# Aminoacyl tRNA synthetases as potential drug targets of the *Panthera* pathogen *Babesia*

**DOI:** 10.1186/s13071-019-3717-z

**Published:** 2019-10-14

**Authors:** Jyoti Chhibber-Goel, Sarthak Joshi, Amit Sharma

**Affiliations:** Molecular Medicine Group, International Centre for Genetic Engineering and Biotechnology, New Delhi, India

**Keywords:** Aminoacyl-tRNA synthetases, *Babesia*, Drug discovery, *Panthera*

## Abstract

**Background:**

A century ago, pantheras were abundant across Asia. Illegal hunting and trading along with loss of habitat have resulted in the designation of *Panthera* as a genus of endangered species. In addition to the onslaught from humans, pantheras are also susceptible to outbreaks of several infectious diseases, including babesiosis. The latter is a hemoprotozoan disease whose causative agents are the eukaryotic parasites of the apicomplexan genus *Babesia*. Babesiosis affects a varied range of animals including humans (*Homo sapiens)*, bovines (e.g. *Bos taurus*), pantheras (e.g. *Panthera tigris*, *P. leo*, *P. pardus*) and equines. *Babesia* spp. are transmitted by the tick vector *Ixodes scapularis* or ticks of domestic animals, namely *Rhipicephalus* (*Boophilus*) *microplus* and *R.* (*B.*) *decoloratus*. At the level of protein translation within these organisms, the conserved aminoacyl tRNA synthetase (aaRS) family offers an opportunity to identify the sequence and structural differences in the host (*Panthera*) and parasites (*Babesia* spp.) in order to exploit these for drug targeting *Babesia* spp.

**Methods:**

Using computational tools we investigated the genomes of *Babesia* spp. and *Panthera tigris* so as to annotate their aaRSs. The sequences were analysed and their subcellular localizations were predicted using Target P1.1, SignalP 3.0, TMHMM v.2.0 and Deeploc 1.0 web servers. Structure-based analysis of the aaRSs from *P. tigris* and its protozoan pathogens *Babesia* spp. was performed using Phyre2 and chimera.

**Results:**

We identified 33 (*B. bovis*), 34 (*B. microti*), 33 (*B. bigemina*) and 33 (*P. tigris*) aaRSs in these respective organisms. Poor sequence identity (~ 20–50%) between aaRSs from *Babesia* spp. and *P. tigris* was observed and this merits future experiments to validate new drug targets against *Babesia* spp.

**Conclusions:**

Overall this work provides a foundation for experimental investigation of druggable aaRSs from *Babesia* sp. in an effort to control Babesiosis in *Panthera*.

## Background

*Panthera* is a genus within the family Felidae, comprising five species: *P. leo*, *P. onca*, *P. uncia, P. pardus* and *P. tigris.* Sadly, *Panthera* spp. are now endangered due to illegal hunting, loss of habitat and trading [1]. Furthermore, *Panthera* spp. have been subjected to outbreaks of babesiosis which is a zoonotic, hemoprotozoan disease caused by tick-borne piroplasmids of the genus *Babesia* [2]. *Babesia* spp. are the second most common haemoparasites of mammals after trypanosomes. They have a wide range of hosts including humans (*Homo sapiens)*, bovines (e.g. *Bos taurus*), pantheras (e.g. *P. tigris*, *P. leo*, *P. pardus*), equines, and a number of other mammal and bird species [3–5]. To date, more than 100 species of *Babesia* have been identified, which may be attributed to the fact that *Babesia* is not strictly host-specific. While babesiosis infection is most commonly asymptomatic, the disease can cause fever, fatigue and haemolytic anaemia that at times can be fatal [5]. Babesiosis is believed to be a major cause of mortality for big cats held in captivity [6–17] (Table 1). Species of *Babesia* infecting *Panthera* spp. are usually *Babesia leo* or *Babesia canis* [12]. A number of effective therapeutics are currently in use which include imidocarb dipropionate, diminazene aceturate, clindamycin, azithromycin and antiparasitic drugs such as atovaquone-azithromycin [14, 18]. The current drug application is often accompanied by intravenous fluids and blood transfusion; these are difficult to administer in the wilderness [14, 18] and therefore developing new and effective therapeutics against babesiosis infection is very important for animal conservation.

**Table 1:** Reported *Babesia* spp. infections in *Panthera* spp

*Panthera* spp.	*Babesia* spp.	Region	Reference
*P. leo*	*B. leo*; *B. vogeli*	Brazil, South America	[6]
	–	Rajiv Gandhi Zoological Park and Wildlife Research Center, Katraj, Pune, India, Asia	[9]
	*B. felis*	Republic of South Africa	[10]
	*B. felis*; *B. lengau*; *B. canis*	Northern Tuli Game Reserve, Botswana, Africa	[11]
	*Babesia* epidemic 1994–2001	Africa	[12]
	–	India, Asia	[14]
	*B. felis*; *B. leo*; novel *Babesia* (similar to *B. lengau*)	Zambia, Africa	[17]
*P. pardus*	–	Nagpur, India, Asia	[7]
	*B. canis*	Kenya, Africa	[8]
	–	Nagpur, India, Asia	[16]
*P. tigris*	–	Rana Pratap Singh Zoo, Sangli, India, Asia	[13]
	–	Bhagwan Birsa Jaivik Udyan, Ranchi, India, Asia	[15]

Being a crucial part of the protein translation machinery, aminoacyl tRNA synthetases (aaRSs) are currently being studied as drug targets for several pathogens [19–28]. The aaRSs catalyse the addition of amino acids to respective tRNAs, and are usually multi-domain proteins with an anti-codon binding domain (ABD) as well as a conserved catalytic domain. Often aaRSs possess editing domains that remove incorrectly charged tRNAs. The 20 aaRSs fall into either Class I or Class II based on their modes of substrate binding and structural folds. Class I aaRSs contain a Rossmann fold which is characterized by KMSKS and HIGH motifs. Class II aaRSs have three motifs and another different β-sheet arrangement [29]. The aaRSs can localise to different subcellular compartments where they are responsible for protein synthesis [30]. More recently, aaRSs have been explored from several eukaryotic pathogens such as *Plasmodium*, *Toxoplasma* and *Leishmania* as potential druggable targets [19–28]. In the present study we used bioinformatics tools to investigate the genomes of *Babesia* spp. (*B. bovis*, *B. microti* and *B. bigemina*) and *P. tigris* and annotated their aaRSs. We identified 33 (*B. bovis*), 34 (*B. microti*), 33 (*B. bigemina*) and 33 (*P. tigris*) aaRSs in these organisms. We have analysed the aaRSs sequences and predicted their cellular localizations. Finally, we determined the percentage sequence identity in aaRSs from *Babesia* spp. with respect to *P. tigris* to identify divergent aaRSs. This work hence provides a resource for launching experimental investigations of druggable *Babesia* aaRSs in an effort to control babesiosis.

## Methods

Open reading frames (ORFs) for the annotated *B. bovis*, *B. microti*, *B. bigemina* and *P. tigris* were downloaded from the National Center for Biotechnology Information (NCBI) database of genomes and computationally translated. All 20 aaRSs were individually used to perform BLAST searches against non-redundant databases available at the NCBI (Additional file 1: Dataset S1). For each, hidden Markov models (HMMs) [31] were generated using homologs identified from BLAST searches. Sequence alignment and modeling software system (SAM) was used to generate multiple sequence alignment (MSA), and the HMMER package was used for building profile HMM for each MSA. Significantly similar matches for each HMM profile were identified within the genomic sequences for the three *Babesia* spp. and for *P. tigris.* Genome sequences were analysed using blastx and four additional aaRSs were identified. Pfam domains were assigned to computationally translated sequences using the ‘Pfam web server for analysis of domains’ [32]. Details on domain functions were also obtained from the Pfam database. Subcellular localizations were predicted using Target P1.1 [33], SignalP 3.0 [34], TMHMM Server v.2.0 [35] and Deeploc 1.0 web server [36]. The presence of secretory signals was detected using Target P1.1 and SignalP, while transmembrane domains were detected using TMHMM. Furthermore, DeepLoc-1.0 was used to predict the subcellular localisations. If a protein was predicted to have a signal peptide and at least one transmembrane domain and predicted to be either mitochondrial or apicoplastic *via* DeepLoc-1.0 it was annotated as an organellar protein. Homology modelling for protein sequences was performed using Phyre2 server [37] and Chimera [38] was used for structure visualization and analyses.

## Results

**Fig. 1:**
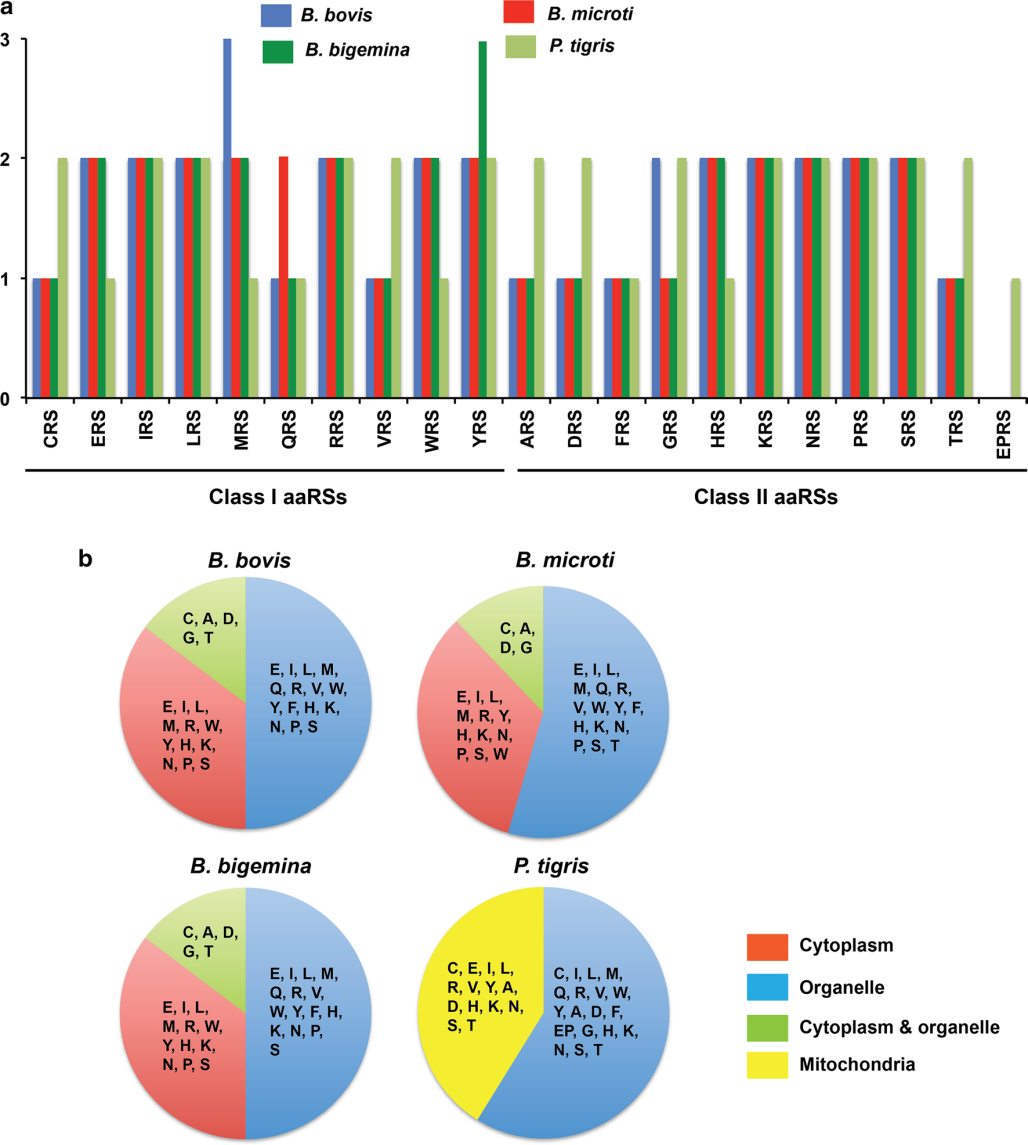
Aminoacyl-tRNA synthetases (aaRSs) and their sites. Number of annotated aaRSs (**a**) and predicted subcellular localisations (**b**) for the studied *Babesia* spp. (*B. bovis*, *B. microti* and *B. bigemina*) and *Panthera tigris*

The *Babesia* spp. that infect *Panthera* spp. are usually *B. leo* or *B. canis* [12]. Since the genomes of *B. leo* and *B. canis* are currently unavailable, we used the open reading frames (ORFs) and genome sequences of *B. bovis*, *B. microti* and *B. bigemina* that are known to infect bovine species and at times *Panthera* spp. as well [39]. Genome sizes for *B. bovis*, *B. microti* and *B. bigemina* are ~ 8.2, ~ 6.3 and ~ 13.8 Mbp, respectively. The aaRSs in the genomes of *B. bovis*, *B. microti*, *B. bigemina* and *P. tigris* were identified *via* HMM-based searches [31]. We annotated aaRSs corresponding to each of the 20 amino acids in these four studied genomes (Tables 2, 3, 4, 5, Additional file 2: Tables S1–S4). Our analyses have annotated 33 aaRSs in *B. bovis*, 34 aaRSs in *B. microti*, 33 aaRSs in *B. bigemina* and 33 aaRSs in *P. tigris* (Fig. 1a).

**Table 2:** Aminoacyl-tRNA synthetase (aaRS) domains in the genome of *B. bovis*

Class	aaRS domain	NCBI ref. seq.	Subcellular localization	Protein length (residues)	Sequence identity (%)
Class 1	CRS	XP_001608890.1	Organelle, cytoplasm	662	42.5
	ERS	XP_001612304.1	Cytoplasm	703	25.3
	ERS	XP_001610985.1	Organelle	601	28.7
	IRS	XP_001611793.1	Cytoplasm	1080	49.4
	IRS	XP_001610095.1	Organelle	1162	27.8
	LRS	XP_001611815.1	Cytoplasm	1098	39.4
	LRS	XP_001609402.1	Organelle	782	30.7
	MRS	XP_001610587.1	Cytoplasm	522	23.4
	MRS	XP_001612148.1	Cytoplasm	385	21.4
	MRS	XP_001608849.1	Organelle	534	25.8
	QRS	XP_001611769.1	Cytoplasm	596	43.4
	RRS	XP_001609801.1	Cytoplasm	581	43.0
	RRS	XP_001609088.1	Organelle	669	22.1
	VRS	XP_001611967.1	Cytoplasm	972	45.3
	WRS	XP_001611386.1	Cytoplasm	587	52.3
	WRS	XP_001612001.1	Organelle	338	20.4
	YRS	XP_001609749.1	Cytoplasm	418	27.4
	YRS	XP_001611016.1	Organelle	605	20.7
Class 2	ARS	XP_001612285.1	Organelle, cytoplasm	978	43.7; 37
	DRS	XP_001609334.1	Organelle, cytoplasm	557	48.9; 28.7
	FRS α	XP_001611853.1	Cytoplasm	448	40.5
	FRS β	XP_001612083.1	Cytoplasm	600	30.2
	FRS	XP_001610235.1	Cytoplasm	448	23.4; 31.8
	GRS	XP_001609027.1	Organelle, cytoplasm	733	44.6
	HRS	XP_001609284.1	Cytoplasm	913	48.5
	HRS	XP_001611649.1	Organelle	548	21.3
	KRS	XP_001609428.1	Cytoplasm	548	49.7
	KRS	XP_001609643.1	Organelle	522	32.9
	NRS	XP_001612247.1	Cytoplasm	605	25.6
	NRS	XP_001610875.1	Organelle	557	31.2
	PRS	XP_001612221.1	Cytoplasm	439	–
	PRS	XP_001609304.1	Organelle	705	–
	SRS	XP_001610648.1	Cytoplasm	502	39.2
	SRS	XP_001609299.1	Organelle	536	20.1
	TRS	XP_001610740.1	Organelle, cytoplasm	736	40.9; 44.1
	I, L, M, V family protein	XP_001611804.1	–	809	–

**Table 3:** Aminoacyl-tRNA synthetase (aaRS) domains in the genome of *B. microti*

Class	aaRS domain	NCBI ref. seq.	Subcellular localization	Protein length (residues)	Sequence identity (%)
Class 1	CRS	XP_021338184.1	Organelle, cytoplasm	657	46.7
	ERS	XP_021337593.1	Cytoplasm	691	25.5
	ERS	XP_021338716.1	Organelle	515	29.6
	IRS	XP_021338677.1	Cytoplasm	1093	48.1
	IRS	XP_021338798.1	Organelle	989	26.8
	LRS	XP_021337733.1	Cytoplasm	1100	39.9
	LRS	XP_012647396.1	Organelle	762	27.7
	MRS	XP_012648167.1	Cytoplasm	708	24.2
	MRS	XP_021338698.1	Organelle	426	20.5
	QRS	XP_012649219.1	Cytoplasm	580	42.9
	QRS	XP_021337505.1	Organelle	384	29.3
	RRS	XP_021338636.1	Cytoplasm	571	40.5
	RRS	XP_021337238.1	Organelle	678	25.6
	VRS	XP_021337780.1	Cytoplasm	987	47.9
	WRS	XP_012649970.1	Cytoplasm	379	18.4
	WRS	XP_021338434.1	Organelle	571	53.7
	YRS	XP_012649345.1	Cytoplasm	550	25.2
	YRS	XP_021337265.1	Organelle	410	26.6
Class 2	ARS	XP_021338305.1	Organelle, cytoplasm	961	44.7; 39.3
	DRS	XP_021337443.1	Organelle, cytoplasm	511	50.5; 28.7
	FRS α	XP_021337722.1	Cytoplasm	499	42.9
	FRS β	XP_021337782.1	Cytoplasm	588	50
	FRS α	XP_012647761.1	Organelle^a^	475	25.7
	GRS	XP_021338402.1	Organelle, cytoplasm	707	44.8
	HRS	XP_012647814.1	Cytoplasm	830	46.3
	HRS	XP_012648429.1	Organelle	359	23.2
	KRS	XP_021337612.1	Cytoplasm	564	56.2
	KRS	XP_021338251.1	Organelle	466	30.1
	NRS	XP_012650212.1	Cytoplasm	538	28.8
	NRS	XP_021338757.1	Organelle	492	35.9
	PRS	XP_021338015.1	Cytoplasm	665	–
	PRS	XP_012650016.2	Organelle	370	–
	SRS	XP_012650114.1	Cytoplasm	448	42.7
	SRS	XP_012647836.1	Organelle	532	30.5
	TRS	XP_021338448.1	Cytoplasm	731	40.9; 44.1
	I, L, M, V family protein	XP_012649205.1	–	987	–

^a^Transmembrane

**Table 4:** Aminoacyl-tRNA synthetase (aaRS) domains in the genome of *B. bigemina*

Class	aaRS domain	NCBI ref. seq.	Sub-cellular localization	Protein length (residues)	Sequence identity (%)
Class 1	CRS	XP_012767680.1	Organelle, cytoplasm	662	40.2
	ERS	XP_012770110.1	Cytoplasm	729	25.4
	ERS	XP_012766722.1	Organelle	607	30.2
	IRS	XP_012769037.1	Cytoplasm	1082	49.9
	IRS	XP_012766327.1﻿	Organelle	1229	29.6
	LRS	XP_012769005.1	Cytoplasm	1120	38.8
	LRS	XP_012770143.1	Organelle	813	30.3
	MRS	XP_012767735.1	Cytoplasm	788	25.8
	MRS	XP_012769850.1	Organelle	506	25.9
	QRS	XP_012769058.1	Cytoplasm	605	42.8
	RRS	XP_012767290.1	Cytoplasm	597	41.3
	RRS	XP_012769919.1	Organelle	753	35.0
	VRS	XP_012768834.1	Cytoplasm	972	45.0
	WRS	XP_012768699.1	Cytoplasm	587	48.2
	WRS	XP_012769504.1﻿	Organelle	399	26.4
	YRS	XP_012767250.1	Cytoplasm	415	28.7
	YRS	XP_012766067.1	Cytoplasm	401	38.2
	YRS	XP_012766758.1	Organelle	601	33.3
Class 2	ARS	XP_012770131.1	Organelle, cytoplasm	984	43.4; 38.1
	DRS	XP_012770238.1	Organelle, cytoplasm	588	51.2; 27.4
	FRS	XP_012768895.1	Cytoplasm	512	47.2; 20.1
	FRS α	XP_012766171.1	Cytoplasm	468	25.6
	FRS β	XP_012769613.1	Cytoplasm	601	27.8
	GRSi	XP_012767840.1	Organelle, cytoplasm	732	45.9
	HRS	XP_012769971.1	Cytoplasm	960	46.5
	HRS	XP_012769231.1	Organelle	524	25.4
	KRS	XP_012768205.1	Cytoplasm	548	51.7
	KRS	XP_012767128.1	Organelle	572	30.1
	NRS	XP_012765953.1	Organelle	610	26.8
	NRS	XP_012766585.1	Cytoplasm	563	32.3
	PRS	XP_012769944.1	Cytoplasm	682	–
	PRS	XP_012765957.1	Organelle	452	–
	SRS	XP_012769786.1	Cytoplasm	506	38.8
	SRS	XP_012769949.1	Organelle	544	28.7
	TRS	XP_012766432.1	Organelle, cytoplasm	734	40.6; 44.4
	I, L, M, V family protein	XP_012769025.1	–	987	–

**Table 5:** Aminoacyl-tRNA synthetase (aaRS) domains in the genome of *P. tigris*

Class	aaRS domain	NCBI ref. seq.	Sub-cellular localization	Protein length (residues)
Class 1	CRS	XP_015397731.1	Cytoplasm	930
	CRS	XP_015400127.1	Mitochondrion	510
	ERS	XP_007084157.1	Mitochondrion	523
	IRS	XP_015399843.1	Cytoplasm	1437
	IRS	XP_007072911.1	Mitochondrion	930
	LRS	XP_007080026.1	Cytoplasm	1176
	LRS	XP_007088460.1	Mitochondrion	903
	MRS	XP_007075037.1	Cytoplasm	908
	QRS	XP_007088554.1	Cytoplasm	775
	RRS	XP_007077700.1	Cytoplasm	660
	RRS	XP_007076978.1	Mitochondrion	578
	VRS	XP_007098881.2	Cytoplasm	1195
	VRS	XP_007090645.1	Mitochondrion	1062
	WRS	XP_007094971.1	Cytoplasm	476
	YRS	XP_007092492.1	Cytoplasm	528
	YRS	XP_007092493.1	Mitochondrion	497
Class 2	ARS	XP_007074943.1	Cytoplasm	968
	ARS	XP_007085183.2	Mitochondrion	983
	DRS	XP_015400574.1	Cytoplasm	551
	DRS	XP_007084072.1	Mitochondrion	653
	EPRS	XP_007072912.1	Cytoplasm	1571
	FRS α	XP_007098251.1	Cytoplasm	470
	FRS β	XP_007073195.1	Cytoplasm	451
	GRS	XP_015396756.1	Cytoplasm	685
	HRS	XP_007077962.1	Cytoplasm	509
	HRS	XP_007077964.1	Mitochondrion	477
	KRS	XP_007083410.1	Cytoplasm	597
	KRS	XP_007083409.1	Mitochondrion	625
	NRS	XP_007080534.1	Cytoplasm	558
	NRS	XP_007079024.1	Mitochondrion	477
	SRS	XP_007076496.1	Cytoplasm	513
	SRS	XP_007097046.1	Mitochondrion	502
	TRS	XP_007097493.1	Cytoplasm	723
	TRS	XP_015395662.1	Mitochondrion	727

The predicted compartmentalisations of all aaRSs were analysed based on the presence of signal sequences using Target P1.1 [33], SignalP 3.0 [34], TMHMM v.2.0 [35] and Deeploc 1.0 web servers [36]. Our analyses predicted subcellular localisation for multiple aaRSs with gene copies in both cytoplasm and in an organelle (apicoplast/mitochondria) for *Babesia* spp. (Fig. 1b, Tables 2, 3, 4). For *P. tigris*, the predicted localisations cover both cytoplasm and mitochondria (Fig. 1b, Table 5). Hereafter, the aaRSs with a predicted subcellular localisation to apicoplast in *Babesia* spp. will be referred to as organellar. We emphasize the need for experimental validation to positively assess the localisations as aaRSs may co-reside in mitochondria [40–42]. In *B. bovis*, the predictions are for 16 aaRSs as cytoplasmic, 12 aaRSs as organellar and 5 aaRSs [cysteinyl-tRNA synthetase (CRS), alanyl-tRNA synthetase (ARS), aspartyl-tRNA synthetase (DRS), glycyl-tRNA synthetase (GRS), threonyl-tRNA synthetase (TRS)] as potential co-localisers between the cytoplasm and organelle (Fig. 1b, Table 2). In *B. microti*, 16 aaRSs are cytoplasmic, 14 aaRSs are organellar and 4 aaRSs (CRS, ARS, DRS, GRS) are possibly dual-localised (Fig. 1b, Table 3). In *B. bigemina*, 16 aaRSs are cytoplasmic, 12 aaRSs are organellar and 5 aaRSs (CRS, ARS, DRS, GRS, TRS) may reside in both the cytoplasm and organelle (Fig. 1b, Table 4). Our HMM profile-based analyses failed to predict a complete set of 20 organellar aaRSs for three aaRSs [glutaminyl-tRNA synthetase (QRS), valyl-tRNA synthetase (VRS), phenylalanyl-tRNA synthetase (FRS)] in *B. bovis*, three aaRSs (VRS, FRS, TRS) in *B. microti* and three aaRSs (QRS, VRS, FRS) in *B. bigemina*. In an attempt to predict the missing organellar aaRSs within the *Babesia* spp., we searched the non-redundant protein sequence databases. Our searches resulted in identification of tRNA synthetases (isoleucine, leucine, methionine and valine; I, L, M and V, respectively) in *B. bovis*, *B. microti* and *B. bigemina* with ~ 55%, 54% and 55% sequence identity, respectively, with, for example, predicted VRS from each of the three species (Tables 2, 3, 4). Searches using Blastx resulted in the identification of four additional aaRSs: methionyl-tRNA synthetase (MRS) (cytoplasmic) in *B. bovis*; QRS (organellar) and tryptophanyl-tRNA synthetase (WRS) (organellar) in *B. microti*; and tyrosyl-tRNA synthetase (YRS) (cytoplasmic) in *B. bigemina.* Based on previous reports, a non-discriminating organellar glutamyl-tRNA synthetase (ERS) is known to convert tRNA^Gln^ into a misacylated Glu-tRNA^Gln^ [43]. The misacylated Glu-tRNA^Gln^ is then converted into Gln-tRNA^Gln^ by an organellar glu-tRNA^Gln^ amidotransferase [43]. While this explains the absence of QRS in the organelles of the three *Babesia* spp., there is a need to identify the missing organellar TRS in *B. microti* to complete its set of 20 aaRSs. A plausible reason for failure to predict a complete set may be poor sequence identity with aaRS homologs that were used for building profile HMMs.

In contrast to *Babesia* spp., for *P. tigris*, 19 aaRSs are present within the cytoplasm including a bi-functional aaRS, namely glutamyl-prolyl-tRNA synthetase (EPRS). In addition, 14 aaRSs are mitochondrial in *P. tigris* (Fig. 1b, Table 5). These observations are supported by previous reports which show that despite the occurrence of protein synthesis in the three distinct compartments in *Plasmodium falciparum*, this apicomplexan does not possess three complete sets of 20 aaRSs for each compartment (i.e. not 60 but has 36 aaRSs) [42]. Furthermore, while cytoplasmic aaRSs supposedly can drive translation in that compartment, several studies have revealed that some aaRSs have the potential to be multi-localized [30]. When a complete set of 20 aaRSs is not available for organellar protein synthesis, as an example, the mitochondria are able to import charged tRNAs, as shown in yeast, *Leishmania*, *Trypanosoma*, *Plasmodium* and *Toxoplasma* [44–48]. The requirement for their own mitochondrial aaRSs in these organisms is therefore bypassed as charged tRNAs are transported between the cellular compartments, thereby compensating for the absence of any aaRS(s) [40–42].

One aaRS enzyme is particularly interesting as it can occur as heterodimers of two different genes: the FRSs. It is well established that FRSs can exist in (αβ)_2_ subunit organization, with exceptions of mitochondrial FRSs from yeast and *Homo sapiens* and possibly others that are monomeric [40]. Our analyses predicted cytoplasmic FRS α and FRS β subunit in all the four studied genomes. A third gene for FRS was predicted in *B. bovis* and *B. bigemina* to localise within the cytoplasm, while the gene for FRS in *B. microti* was predicted to localise within the organelle (Tables 2, 3, 4). Mitochondria of the apicomplexan *P. falciparum* import tRNAs along with an active FRS, and so we analysed the FRSs from *B. bovis*, *B. microti* and *B. bigemina* using Target P1.1 [33] and SignalP 3.0 [34] for the presence of signal peptide and transmembrane domain [35]. The encoded cytoplasmic FRSs from *B. bovis* and *B. bigemina* showed no identifiable organelle targeting sequence or transmembrane domains. However, a transmembrane domain was predicted within the FRS from *B. microti* (Table 3). These predicted FRSs need to be characterised experimentally for validation.

### Domain architectures of aminoacyl tRNA synthetases in *Babesia* spp. and *Panthera tigris*

Based on HMM searches, we identified a total of 33 aaRSs in *B. bovis*, 34 aaRSs in *B. microti*, 33 aaRSs in *B. bigemina* and 33 aaRSs in *P. tigris*. Amongst these, 18 aaRSs belong to Class I and 15 aaRSs to Class II in *B. bovis*; 18 to Class I and 16 Class II in *B. microti*; 18 to Class I and 15 to Class II in *B. bigemina*; and 16 to Class I and 17 to Class II in *P. tigris* (Tables 2, 3, 4, 5). Pfam server predicted catalytic domains for all these aaRSs. Additionally, anticodon-binding domains (ABD), N-terminal domain (NTD) and C-terminal domain (CTD), DALR [aspartate (D), alanine (A), leucine (L), arginine (R)], DHHA [aspartate (D), histidine (H), histidine (H), alanine (A)], SAD (second additional domain), WHEP-TRS (Wh-T) and glutathione S-transferase (GST) domains were predicted.

**Fig. 2:**
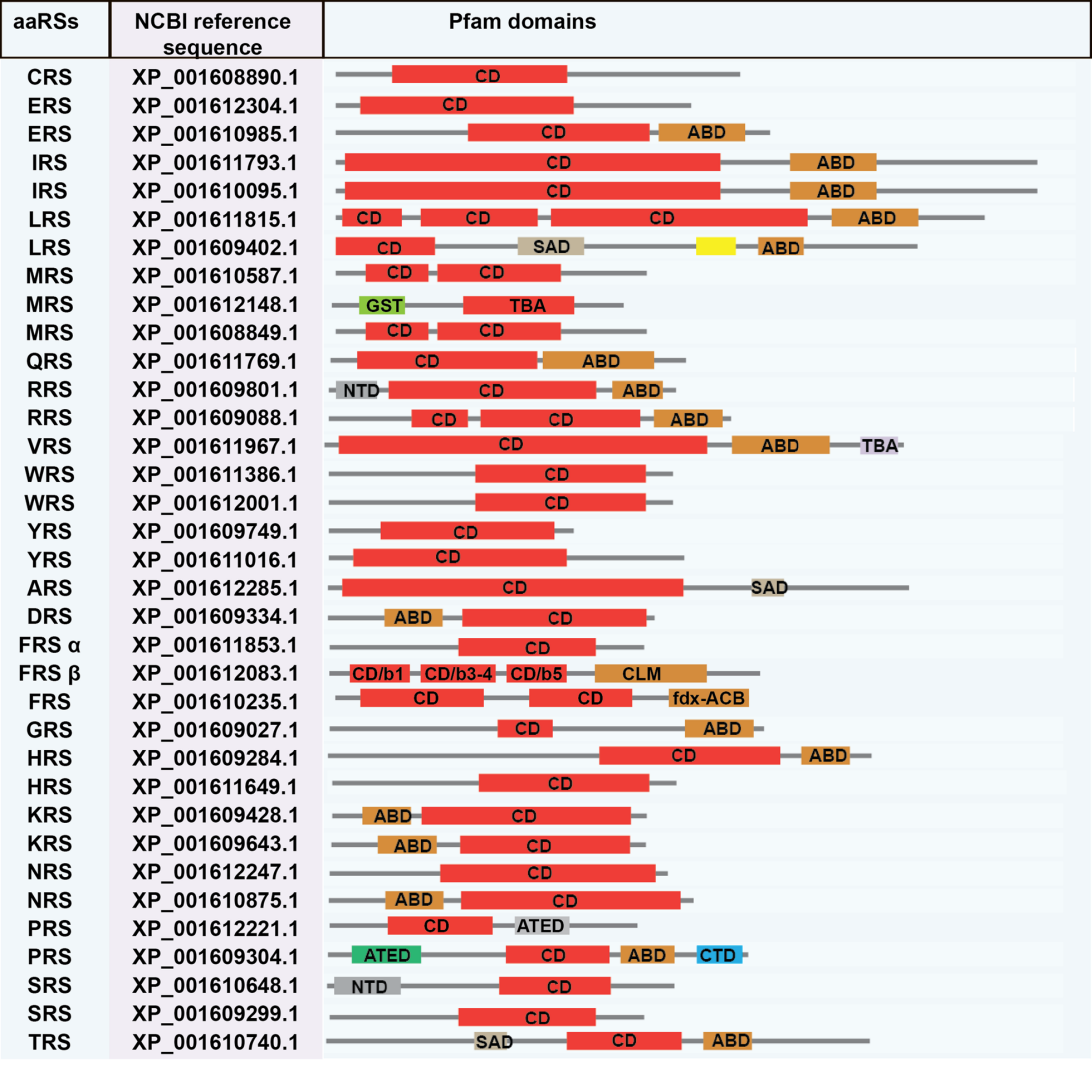
NCBI reference sequence and pfam assigned domains in *B. bovis*. *Abbreviations*: aaRSs, aminoacyl-tRNA synthetase; CD, catalytic domain; ABD, anticodon binding domain; SAD, second additional domain; NTD, N-terminal domain; CTD, C-terminal domain; ATED, aminoacyl tRNA editing domain; TBA, tRNA binding arm; GST, glutathione S-transferase

*Panthera tigris* aaRSs are also predicted to contain DALR and DHHA domains (Fig. 2). DALR is an all alpha-helical anti-codon binding domain named after the characteristic conserved amino acids: aspartate (D), alanine (A), leucine (L) and arginine (R). The DHHA domain is named after conserved amino acids: aspartate (D), histidine (H), histidine (H), alanine (A) in the cytoplasmic version of ARS [49]. Furthermore, in *P. tigris* a GST-like domain was predicted at the N-termini of dual-localised MRS, EPRS and cytoplasmic VRS (Fig. 2). While the functional implications of the GST domains may vary, these seem to be involved in protein assembly and folding [50, 51]. Several studies have reported that GST or GST-like domains play an important role in the formation of complexes between aaRSs and multifunctional factors (p18, p38, p43) [52]. Additionally, Wh-T domain was also predicted in cytoplasmic MRS, WRS, GRS and in the dual-localised HRS/EPRSs (Fig. 2). Wh-T domain has a characteristic helix-turn-helix motif, with consensus lysine and arginine residues. These residues are critical for protein-protein interactions within the multi-synthetase complex [53–56].

**Fig. 3:**
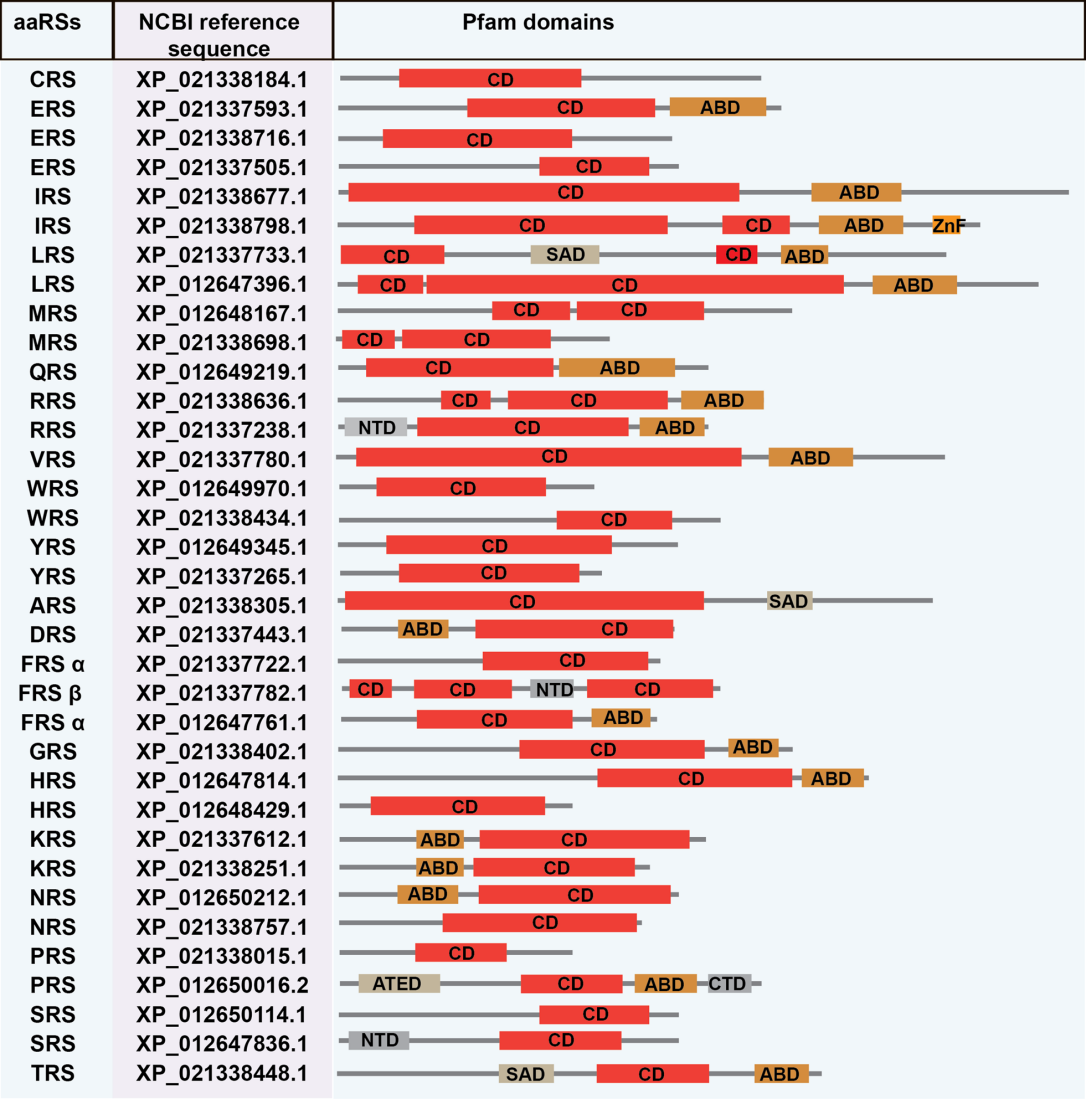
NCBI reference sequence and domain annotations of aaRSs in *B. microti. Abbreviations*: aaRSs, aminoacyl-tRNA synthetases; ABD, anticodon binding domain; SAD second additional domain; NTD, N-terminal domain; CTD, C-terminal domain; ATED, aminoacyl tRNA editing domain; GST, glutathione S-transferase domain

**Fig. 4:**
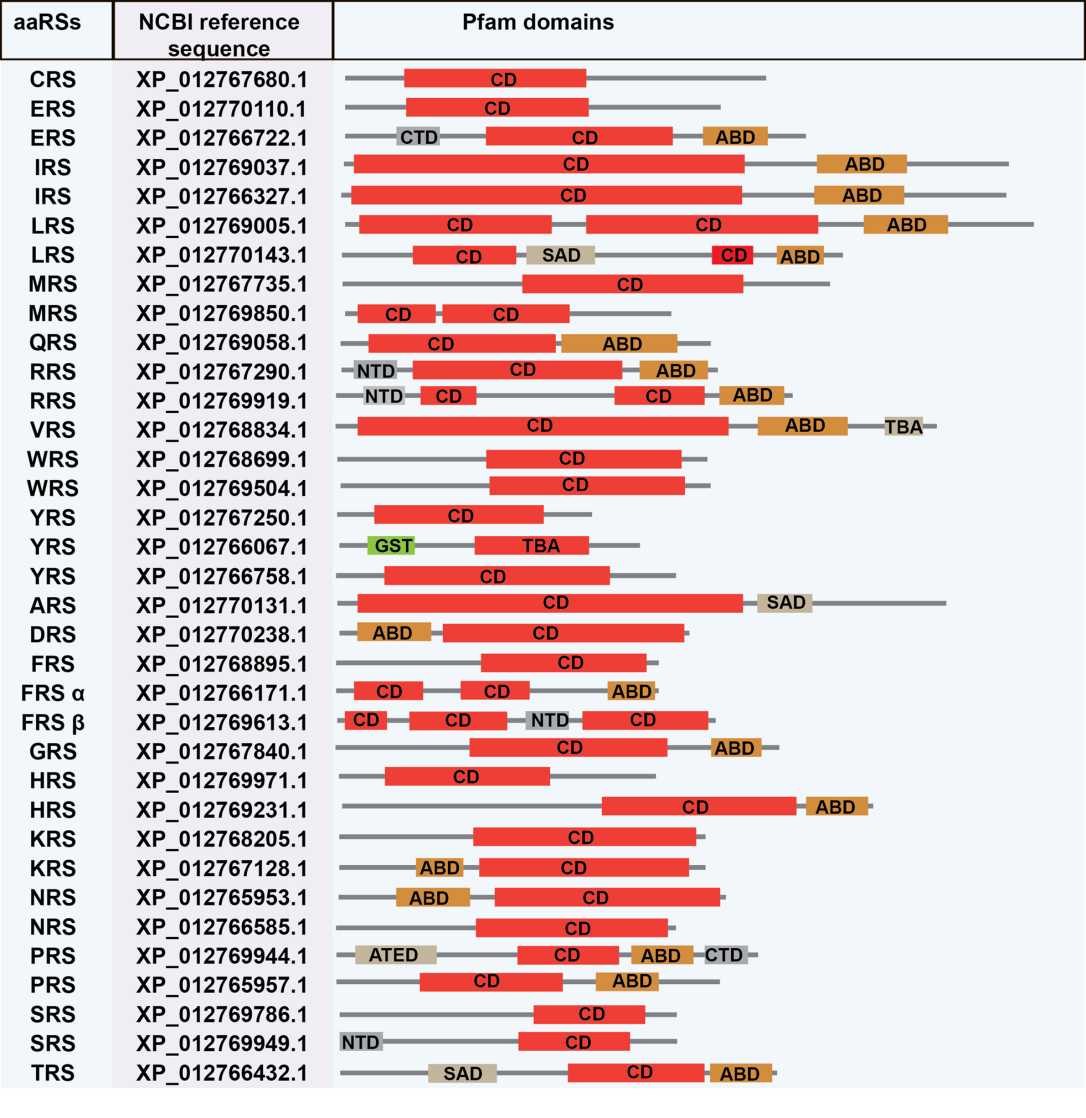
Domain annotations and NCBI reference sequence for aaRSs in *B. bigemina*. *Abbreviations*: aaRSs, aminoacyl-tRNA synthetases; ABD, anticodon binding domain; SAD, second additional domain; NTD, N-terminal domain; CTD, C-terminal domain; ATED, aminoacyl tRNA editing domain; TBA, tRNA binding arm, GST, glutathione S-transferase domain

**Fig. 5:**
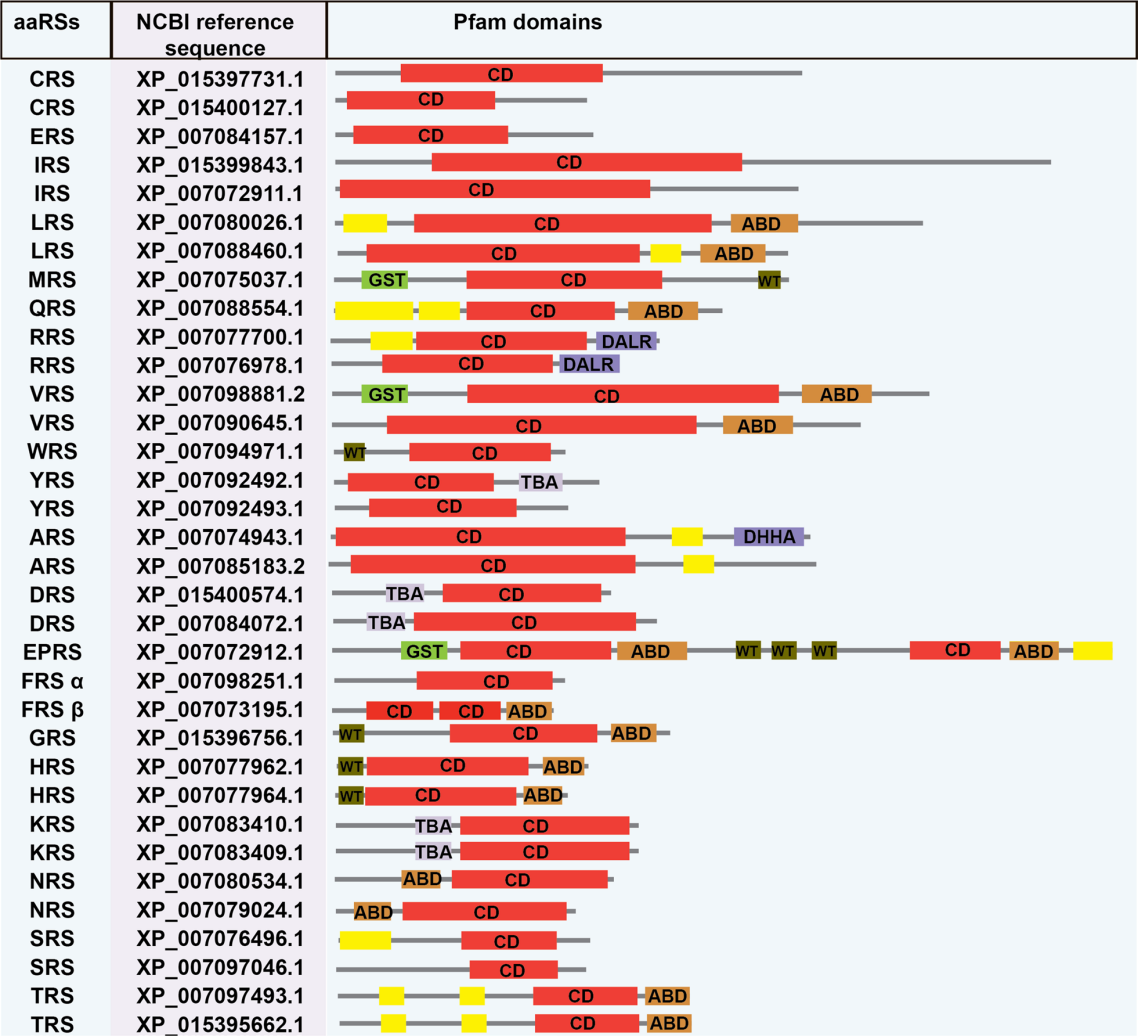
NCBI reference sequence and Pfam assigned domains in *P. tigris*. *Abbreviations*: aaRSs, aminoacyl-tRNA synthetases; ABD, anticodon binding domain; TBA, tRNA-binding arm; NTD, N-terminal domain; CTD, C-terminal domain; ATED, aminoacyl tRNA editing domain; GST, glutathione S-transferase domain; WT, WHEP-TRS domain

We observed no distinct domains amongst the aaRSs from *B. bovis*, *B. microti* and *B. bigemina* (Figs. 3, 4, 5). In *Babesia* spp., RRS and SRS were found to have an additional NTD, while their PRSs have an additional CTD, with no predicted function for either of them (Figs. 3, 4, 5) [57, 58]. Interestingly, for *B. microti*, a ZnF domain was annotated for the organellar IRS. The C-terminal peptide (CP) and C-terminal enzyme-bound zinc participate in aminoacylation of tRNAIle (Fig. 4). These domains were not found for other two species, *B. bovis* and *B. bigemina.* The differences observed within the aaRSs of three *Babesia* spp. and *P. tigris* provide a unique opportunity to exploit some aaRSs as potential drug targets.

We subsequently studied the sequence identities between *Babesia* spp. (*B. bovis*, *B. microti* and *B. bigemina*) and *P. tigris* (Tables 2, 3, 4, 5). While the aaRSs identified in the three *Babesia* spp. share similarities with their homologs in *P. tigris*, our analyses reveal poor identity in the range of ~ 20–50% (Tables 2, 3, 4, 5). The aaRSs with less than 30% identity with the corresponding protein in *P. tigris* include ERS, IRS, MRS, WRS, YRS, HRS, NRS and SRS (Tables 2, 3, 4, 5). Out of eight aaRSs with sequence identity less than 30% between *Babesia* spp. and *P. tigris*, ERS, MRS, YRS and NRS are cytoplasmic. Among the aaRSs with sequence identity less than 30%, IRS and LRS are the targets of drugs currently in use, i.e. IRS (bacterial: muciprocin, available as a 2% topical preparation) and LRS (fungal: tavaborole/AN2690, available as a 5% topical preparation) [19, 24, 59–61].

### Structure-based analysis of cladosporin binding sites in *Babesia* and *Panthera* KRSs

**Fig. 6:**
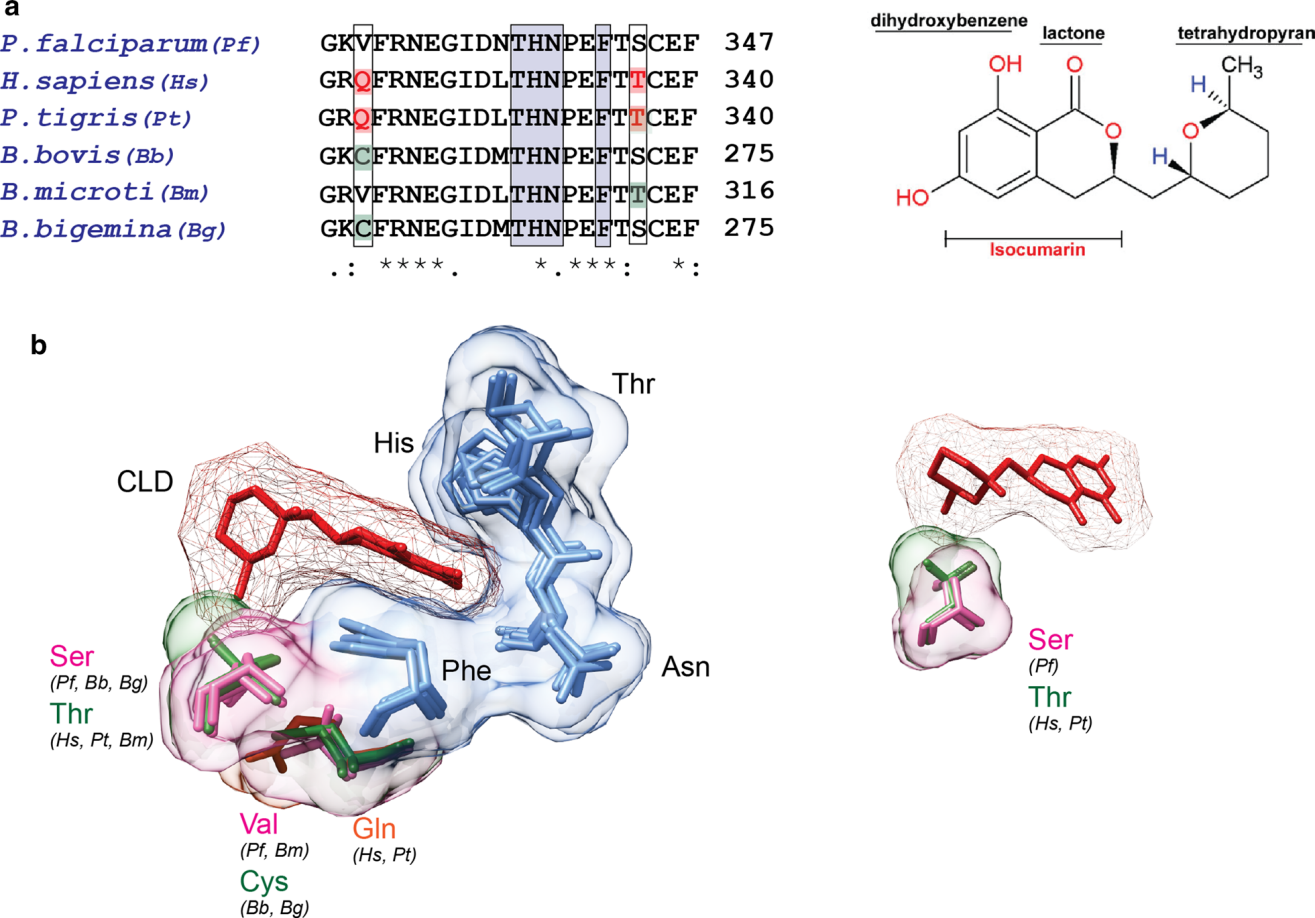
Analysis of CLD binding site in KRSs from *Babesia* spp. and *P. tigris.* **a** Sequence alignment of KRSs. The two key residues are highlighted in a black box and other conserved residues responsible for CLD binding are shown in blue boxes. **b** Structural superimposition of *Hs-*KRS (PDB: 4YCU), *Pf-*KRS (PDB: 4PG3), *P. tigris* and three *Babesia* spp. (*Bb*, *B. bovis*; *Bm*, *B. microti* and *Bg*, *B. bigemina*) (built structure model, this study). The smaller CLD selectivity residues of cysteine-serine (for *Bb*, *Bg*) and valine-threonine (for *Bm*) may accommodate CLD in the binding pocket of *Babesia* KRSs. The bulkier glutamine-threonine (for *Hs*, *H. sapiens*; *Pt*, *P. tigris*) potentially hinder high potency CLD binding. *Abbreviations*: CLD, cladosporin; KRS, lysyl-tRNA synthetase

The *Plasmodium* KRS (for cladosporin, CLD) and PRS (for halofuginone) are currently being studied as potential drug targets [19, 24, 59–61]. We analysed CLD which, along with its analogs, is being investigated as an inhibitor of *P. falciparum*-KRS (*Pf*-KRS) and of various other pathogen KRSs [21, 62]. Several amino acid residues in CLD binding pocket are highly conserved except at two positions near the ATP binding pocket and adjacent to CLD methyl moiety (Fig. 6a). The basis for CLD selectivity has been ascribed to these two key selectivity residues that show clear divergence across species. As proof of concept, we conducted a structural analysis of CLD bound *Homo sapiens* KRS (*Hs*-KRS) (PDB: 4YCU) and *Plasmodium falciparum* KRS (*Pf*-KRS) (PDB: 4PG3) in comparison with the three *Babesia* spp. and *P. tigris* (Fig. 6b). We built a three-dimensional structure model for KRSs using Phyre2 (Protein Homology/AnalogY Recognition Engine)-based protein structure prediction [37]. The two key residues bestowing selectivity to CLD were analysed in *Babesia* spp. and *P. tigris*. In *Pf*-KRS, valine and serine (VS) occupy the two selectivity residue positions thereby providing tight binding. Our analysis here reveals the presence of structurally smaller residues, cysteine and serine (CS), in *B. bovis* and *B. bigemina*, with an exception of *B. microti* that has a smaller valine and a bigger non-favourable threonine (VT) at the site (Fig. 6b). In *Hs*-KRS, these two positions are occupied by bulkier glutamine-threonine (QT) residues that likely hamper CLD binding. The corresponding position in *P. tigris* is also occupied by these bulkier QT residues (Fig. 6b). It is noteworthy that biochemical analysis of recombinant KRSs has previously shown that CLD displays a nanomolar range potency of inhibition (IC50 ~ 40–90 nM) against *Pf*-KRS, which is ~ 500-fold higher when compared with *Hs*-KRS [20, 62, 63]. This suggests potential poor selectivity for *P. tigris* KRS due to the same bulkier residues (QT) at the CLD binding pocket in comparison to the smaller, more favourable residues (CS/VT) in *Babesia* spp. This analysis suggests that small molecule targeting of KRS active site in *Babesia* spp. is an attractive avenue from the perspective of developing anti-infectives.

## Discussion

The treatment of babesiosis presents an emerging challenge. It is a hemoprotozoan disease whose causative agents are apicomplexan *Babesia* spp. *Panthera* spp. have been subjected to outbreaks of babesiosis, caused by *Babesia* spp., the second most common haemoparasites of mammals after trypanosomes, with a worldwide distribution. The current regime of therapy for targeting babesiosis requires the use of antibiotics and antiparasitic drugs. While atovaquone plus azithromycin is used to treat most cases, clindamycin plus quinine is used in more severe cases with chances of relapses [19, 24, 59–61]. Therefore, new drugs with high specificity and low toxicity are desirable. The recent availability of *Babesia* spp. (*B. bovis*, *B. microti* and *B. bigemina*) genomes has paved the way for screening of new chemotherapy targets. In this study, we focused on the housekeeping enzymes aminoacyl-tRNA synthetases that are essential for protein synthesis and cell viability. Lately, parasitic, microbial and fungal aaRSs have been explored for druggability [19–27]. A bacterial IRS inhibitor, mupirocin (marketed as Bactroban), and a fungal LRS inhibitor, 5-fluoro-1,3-dihydro-1-hydroxy-2,1-benzoxaborole (AN2690), have been developed for human use [19, 24, 59–61]. Recent studies on potential anti-malarial compounds like CLD (against KRS) and halofuginone (against PRS) are also promising [19, 20, 22, 24, 59, 64]. Considering this evidence, we have identified *Babesia* aaRSs with poor sequence identity to *Panthera* aaRSs as proteins of interest [65, 66]. As proof-of-concept, we performed a structure-based analysis of *P. tigris* and *Babesia* KRSs and have indicated a potential for selective drug targeting (Fig. 6). Hence, our work here lays a foundation for the future to further investigate and exploit *Babesia* aaRSs as potential targets.

## Conclusions

In the present study, we provide data on genome-wide identification and annotation of aaRSs from *Babesia* spp. and *P. tigris.* Poor sequence identity (~ 20–50%) between pathogen/host aaRS pairs offers a window for specific studies to explore druggability. This detailed genomic cataloguing of aaRSs from pathogenic *Babesia* merits future experiments to validate new drug targets against *Babesia* spp.

## Supplementary information

**Additional file 1: Dataset S1.** Aminoacyl-tRNA synthetase sequences used to generate the HMM profiles.

**Additional file 2: Table S1.** Location of aaRS domains in the *B. bovis* genome. **Table S2.** Location of aaRS domains in the *B. microti* genome. **Table S3.** Location of aaRS domains in the *B. bigemina* genome. **Table S4.** Location of aaRS domains in the *P. tigris* genome.

## Data Availability

Aminoacyl-tRNA synthetase sequences used to generate HMM profiles are provided in Additional file 1: Dataset S1. The datasets analysed during the present study are available in UniProt repository (https://www.uniprot.org) and are included within the article and Additional file 2: Tables S1–S4. The HMM profiles generated are available from the corresponding author upon request.

## Abbreviations

ARS: alanyl-tRNA synthetase
aaRSs: aminoacyl-tRNA synthetase
ABD: anticodon binding domain
ATED: aminoacyl-tRNA editing domain
CRS: cysteinyl-tRNA synthetase
CTD: c-terminal domain
DRS: aspartyl-tRNA synthetase
DALR: aspartate (D), alanine (A), leucine (L), arginine (R)
DHHA: aspartate (D), histidine (H), histidine (H), alanine (A)
EPRS: glutamyl-prolyl-tRNA synthetase
ERS: glutamyl-tRNA synthetase
FRS: phenylalanyl-tRNA synthetase
GRS: glycyl-tRNA synthetase
GST: glutathione S-transferase
HRS: histidyl-tRNA synthetase
HMM: hidden Markov model
IRS: isoleucyl-tRNA synthetase
KRS: lysyl-tRNA synthetase
LRS: leucyl-tRNA synthetase
MRS: methionyl-tRNA synthetase
NRS: asparaginyl-tRNA synthetase
NCBI: National Center for Biotechnology Information
NTD: n-terminal domain
ORF: open reading frame
PRS: prolyl-tRNA synthetase
QRS: glutaminyl-tRNA synthetase
RRS: arginyl-tRNA synthetase
SRS: seryl-tRNA synthetase
SAD: second additional domain
TRS: threonyl-tRNA synthetase
TBA: tRNA-binding domain
VRS: valyl-tRNA synthetase
WRS: tryptophanyl-tRNA synthetase
Wh-T: WHEP-TRS domain
YRS: tyrosyl-tRNA synthetase
