# Supplementary material for: Aminoacyl tRNA synthetases as potential drug targets of the Panthera pathogen Babesia
Source: Parasit Vectors. 2019 Oct 14;12:482. doi: 10.1186/s13071-019-3717-z (PMC6792207; doi:10.1186/s13071-019-3717-z)
Supplement: Supplementary file 2 — Additional file 2: Table S1. Location of aaRS domains in the B. bovis genome. Table S2. Location of aaRS domains in the B. microti genome. Table S3. Location of aaRS domains in the B. bigemina genome. Table S4. Location of aaRS domains in the P. tigris genome. [file 13071_2019_3717_MOESM2_ESM.pdf]

Additional file 2: Table S1. Location of aminoacyl-tRNA synthetase (aaRS) genes in *B. bovis* genome

Class	aaRS Domain	NCBI ref seq	Location on the genome
**Class 1**	CRS	XP_001608890.1	Chromosome: 1; NW_001820854.1 (213,842..216,104)
	ERS	XP_001612304.1	Chromosome: 3; NC_010575.1 (2549536..2551963)
	ERS	XP_001610985.1	Chromosome: 4; NW_001820857.1 (1466874..1469147)
	IRS	XP_001611793.1	Chromosome: 3; NC_010575.1 (1424596..1427947)
	IRS	XP_001610095.1	Chromosome: 2; NC_010574.1 (1311463..1314951)
	LRS	XP_001611815.1	Chromosome: 3; NC_010575.1 (1475915..1479279, complement)
	LRS	XP_001609402.1	Chromosome: 4; NW_001820855.1 (539055..541414)
	MRS	XP_001610587.1	Chromosome: 4; NW_001820857.1 (630862..632643)
	MRS	XP_001612148.1	Chromosome: 3; NC_010575.1 (2200980..2202476, complement)
	MRS	XP_001608849.1	Chromosome: 1; NW_001820854.1 (134046..135650, complement)
	QRS	XP_001611769.1	Chromosome: 3; NC_010575.1 (1372794..1374591)
	RRS	XP_001609801.1	Chromosome: 2; NC_010574.1 (655787..657722)
	RRS	XP_001609088.1	Chromosome: 1; NW_001820854.1 (647,158..649,312)
	VRS	XP_001611967.1	Chromosome: 3; NC_010575.1 (1810529..1813583, complement)
	WRS	XP_001611386.1	Chromosome: 3; NC_010575.1 (592262..594369, complement)
	WRS	XP_001612001.1	Chromosome: 3; NC_010575.1 (1887054..1888531, complement)
	YRS	XP_001609749.1	Chromosome: 2; NC_010574.1 (540163..541419)
	YRS	XP_001611016.1	Chromosome: 4; NW_001820857.1 (1531632..1533484, complement)
**Class 2**	ARS	XP_001612285.1	Chromosome: 3; NC_010575.1 (2501122..2504128)
	DRS	XP_001609334.1	Chromosome: 4; NW_001820855.1 (378504..380540)
	FRS α	XP_001611853.1	Chromosome: 3; NC_010575.1 (1571702..1573116, complement)
	FRS β	XP_001612083.1	Chromosome: 2; NC_010574.1 (1311463..1314951)
	FRS	XP_001610235.1	Chromosome: 2; NC_010574.1 (1604993..1606376)
	GRS	XP_001609027.1	Chromosome: 1; NW_001820854.1 (515758..517968)
	HRS	XP_001609284.1	Chromosome: 4; NW_001820855.1 (275950..278763, complement)
	HRS	XP_001611649.1	Chromosome: 3; NC_010575.1 (1101974..1103755)
	KRS	XP_001609428.1	Chromosome: 4; NW_001820855.1 (591547..593661)
	KRS	XP_001609643.1	Chromosome: 2; NC_010574.1 (293315..295247)
	NRS	XP_001612247.1	Chromosome: 3; NC_010575.1 (2410705..2412560)
	NRS	XP_001610875.1	Chromosome: 4; NW_001820857.1 (1236282..1238192, complement)
	PRS	XP_001612221.1	Chromosome: 3; NC_010575.1 (2351560..2352935, complement)
	PRS	XP_001609304.1	Chromosome: 4; NW_001820855.1 (314665..317009, complement)
	SRS	XP_001610648.1	Chromosome: 4; NW_001820857.1 (748324..749832, complement)
	SRS	XP_001609299.1	Chromosome: 4; NW_001820855.1 (306236..307846)
	TRS	XP_001610740.1	Chromosome: 4; NW_001820857.1 (953999..956347)
I, L, M, V family protein		XP_001611804.1	Chromosome: 3; NC_010575.1 (1443965..1446518, complement)

Additional file 2: Table S2. Location of aminoacyl-tRNA synthetase (aaRS) genes in *B. microti* genome

Class	aaRS Domain	NCBI ref seq	Location on the genome
**Class 1**	CRS	XP_021338184.1	Chromosome: II; NC_027206.1 (826197..828451, complement)
	ERS	XP_021337593.1	Chromosome: IV; NC_034969.1 (34244..36453, complement)
	ERS	XP_021337505.1	Chromosome: I; NC_027205.1 (974269..975592, complement)
	ERS	XP_021338716.1	Chromosome: III; NC_027207.2 (1235559..1237230)
	IRS	XP_021338677.1	Chromosome: III; NC_027207.2 (1068796..1072954, complement)
	IRS	XP_021338798.1	Chromosome: III; NC_027207.2 (1562429..1565398)
	LRS	XP_021337733.1	Chromosome: IV; NC_034969.1 (621518..625071, complement)
	LRS	XP_012647396.1	Chromosome: I; NC_027205.1 (597415..599724, complement)
	MRS	XP_012648167.1	Chromosome: II; NC_027206.1 (722671..724797, complement)
	MRS	XP_021338698.1	Chromosome: III; NC_027207.2 (1151518..1152878)
	QRS	XP_012649219.1	Chromosome: III; NC_027207.2 (1082921..1084707)
	RRS	XP_021338636.1	Chromosome: III; NC_027207.2 (918904..920932)
	RRS	XP_021337238.1	Chromosome: I; NC_027205.1 (32141..34258, complement)
	VRS	XP_021337780.1	Chromosome: IV; NC_034969.1 (816074..819549)
	WRS	XP_012649970.1	Chromosome: IV; NC_034969.1 (763000..764613)
	WRS	XP_021338434.1	Chromosome: III; NC_027207.2 (198738..201162)
	YRS	XP_012649345.1	Chromosome: III; NC_027207.2 (1294030..1295723, complement)
	YRS	XP_021337265.1	Chromosome: I; NC_027205.1 (107774..109612)
**Class 2**	ARS	XP_021338305.1	Chromosome: II; NC_027206.1 (1204560..1208246, complement)
	DRS	XP_021337443.1	Chromosome: I; NC_027205.1 (710638..712484)
	FRS α	XP_021337722.1	Chromosome: IV; NC_034969.1 (564105..565775, complement)
	FRS β	XP_021337782.1	Chromosome: IV; NC_034969.1 (834295..836286, complement)
	FRS α	XP_012647761.1	Chromosome: I; NC_027205.1 (1273511..1274958, complement)
	GRS	XP_021338402.1	Chromosome: III; NC_027207.2 (81321..83465)
	HRS	XP_012647814.1	Chromosome: II; NC_027206.1 (64572..67064)
	HRS	XP_012648429.1	Chromosome: II; NC_027206.1 (1173650..1174852, complement)
	KRS	XP_021337612.1	Chromosome: IV; NC_034969.1 (116666..118638, complement)
	KRS	XP_021338251.1	Chromosome: II; NC_027206.1 (1022160..1023620)
	NRS	XP_012650212.1	Chromosome: IV; NC_034969.1 (1197307..1198923, complement)
	NRS	XP_021338757.1	Chromosome: III; NC_027207.2 (1398075..1399724)
	PRS	XP_021338015.1	Chromosome: II; NC_027206.1 (115969..118564, complement)
	PRS	XP_012650016.2	Chromosome: IV; NC_034969.1 (857011..858287, complement)
	SRS	XP_012650114.1	Chromosome: IV; NC_034969.1 (1028282..1029628)
	SRS	XP_012647836.1	Chromosome: II; NC_027206.1 (107095..108846)
	TRS	XP_021338448.1	Chromosome: III; NC_027207.2 (246629..249114)
	I, L, M, V family protein	XP_012649205.1	Chromosome: III; NC_027207.2 (1055301..1057887, complement)

Additional file 2: Table S3. Location of aminoacyl-tRNA synthetase (aaRS) genes in *B. bigemina* genome

Class	aaRS Domain	NCBI ref seq	Location on the genome
**Class 1**	CRS	XP_012767680.1	Chromosome: II; NC_027217.1 (1455622..1457912)
	ERS	XP_012770110.1	Chromosome: V; NC_027220.1 (37704..40216, complement)
	ERS	XP_012766722.1	Chromosome: I; NC_027216.1 (1853771..1855902)
	IRS	XP_012769037.1	Chromosome: III; NC_027218.1 (1841478..1844843, complement)
	IRS	XP_012766327.1	Chromosome: I; NC_027216.1 (999044..1002912, complement)
	LRS	XP_012769005.1	Chromosome: III; NC_027218.1 (1776022..1779636)
	LRS	XP_012770143.1	Chromosome: V; NC_027220.1 (117175..119616, complement)
	MRS	XP_012767735.1	Chromosome: II; NC_027217.1 (1560112..1562554)
	MRS	XP_012769850.1	Chromosome: IV; NC_027219.1 (357103..358646, complement)
	QRS	XP_012769058.1	Chromosome: III; NC_027218.1 (1888149..1889966, complement)
	RRS	XP_012767290.1	Chromosome: II; NC_027217.1 (632367..634360, complement)
	RRS	XP_012769919.1	Chromosome: IV; NC_027219.1 (493195..495650)
	VRS	XP_012768834.1	Chromosome: III; NC_027218.1 (1384216..1387278)
	WRS	XP_012768699.1	Chromosome: III; NC_027218.1 (1054286..1056372, complement)
	WRS	XP_012769504.1	Chromosome: III; NC_027218.1 (2992002..2993534, complement)
	YRS	XP_012767250.1	Chromosome: II; NC_027217.1 (544432..545768)
	YRS	XP_012766067.1	Chromosome: I; NC_027216.1 (472950..474419)
	YRS	XP_012766758.1	Chromosome: I; NC_027216.1 (1939628..1941496, complement)
**Class 2**	ARS	XP_012770131.1	Chromosome: V; NC_027220.1 (87545..90615, complement)
	DRS	XP_012770238.1	Chromosome: V; NC_027220.1 (308776..310974)
	FRS	XP_012768895.1	Chromosome: III; NC_027218.1 (1528896..1530512, complement)
	FRS α	XP_012766171.1	Chromosome: I; NC_027216.1 (688744..690185, complement)
	FRS β	XP_012769613.1	Chromosome: III; NC_027218.1 (3250943..3253097, complement)
	GRS	XP_012767840.1	Chromosome: II; NC_027217.1 (1809587..1811785)
	HRS	XP_012769971.1	Chromosome: IV; NC_027219.1 (601480..604475, complement)
	HRS	XP_012769231.1	Chromosome: III; NC_027218.1 (2375579..2377369, complement)
	KRS	XP_012768205.1	Chromosome: II; NC_027217.1 (2624165..2626533)
	KRS	XP_012767128.1	Chromosome: II; NC_027217.1 (281041..282759)
	NRS	XP_012765953.1	Chromosome: I; NC_027216.1 (249179..251011)
	NRS	XP_012766585.1	Chromosome: I; NC_027216.1 (1539515..1541539, complement)
	PRS	XP_012769944.1	Chromosome: IV; NC_027219.1 (540336..542572)
	PRS	XP_012765957.1	Chromosome: I; NC_027216.1 (261183..262820)
	SRS	XP_012769786.1	Chromosome: IV; NC_027219.1 (229527..231047)
	SRS	XP_012769949.1	Chromosome: IV; NC_027219.1 (549903..551537, complement)
	TRS	XP_012766432.1	Chromosome: I; NC_027216.1 (1220381..1222800)
I, L, M, V family protein		XP_012769025.1	Chromosome: III; NC_027218.1 (1822370..1825204)

Additional file 2: Table S4. Location of aminoacyl-tRNA synthetase (aaRS) genes in *P. tigris* genome

Class	aaRS Domain	NCBI ref seq	Location on the genome
**Class 1**	CRS	XP_015397731.1	NW_006712098.1 (1078650..1132218, complement)
	CRS	XP_015400127.1	NW_006711693.1 (2578699..2622625)
	ERS	XP_007084157.1	NW_006711882.1 (4118593..4139145)
	IRS	XP_015399843.1	NW_006712473.1 (1506440..1574795)
	IRS	XP_007072911.1	NW_006711278.1 (349712..414421, complement)
	LRS	XP_007080026.1	NW_006711819.1 (32264710..32340767)
	LRS	XP_007088460.1	NW_006711971.1 (4264107..4429196)
	MRS	XP_007075037.1	NW_006711628.1 (144160..157631)
	QRS	XP_007088554.1	NW_006711971.1 (7266925..7273932, complement)
	RRS	XP_007077700.1	NW_006711788.1 (14803963..14828592)
	RRS	XP_007076978.1	NW_006711778.1 (2692426..2769550, complement)
	VRS	XP_007098881.2	NW_006712705.1 (200508..215033)
	VRS	XP_007090645.1	NW_006712025.1 (396853..409507, complement)
	WRS	XP_007094971.1	NW_006712282.1 (4298457..4328773)
	YRS	XP_007092492.1	NW_006712094.1 (2571397..2600068, complement)
	YRS	XP_007092493.1	NW_006712031.1 (479126..499149, complement)
**Class 2**	ARS	XP_007085183.2	NW_006711908.1 (4101073..4113902)
	ARS	XP_007074943.1	NW_006711606.1 (42950..67155, complement)
	DRS	XP_015400574.1	NW_006712715.1 (7346161..7409642, complement)
	DRS	XP_007084072.1	NW_006711880.1 (2964458..2993387, complement)
	EPRS	XP_007072912.1	NW_006711278.1 (452950..524839)
	FRS α	XP_007098251.1	NW_006712602.1 (1559016..1567149)
	FRS β	XP_007073195.1	NW_006711318.1 (6965601..7499787, complement)
	GRS	XP_015396756.1	NW_006712022.1 (1905276..1949920, complement)
	HRS	XP_007077962.1	NW_006711792.1 (4962409..4976212, complement)
	HRS	XP_007077964.1	NW_006711792.1 (4962409..4976212, complement)
	KRS	XP_007083410.1	NW_006711869.1 (3950134..3964758)
	KRS	XP_007083409.1	NW_006711869.1 (3950134..3964758)
	NRS	XP_007080534.1	NW_006711828.1 (4584305..4601802)
	NRS	XP_007079024.1	NW_006711808.1 (2280775..2410379, complement)
	SRS	XP_007076496.1	NW_006711766.1 (5545761..5567409, complement)
	SRS	XP_007097046.1	NW_006712432.1 (16767230..16776766, complement)
	TRS	XP_007097493.1	NW_006712501.1 (1906291..1931672, complement)
	TRS	XP_015395662.1	NW_006711963.1 (728205..744187)
