# Supplementary material for: Aminoacyl tRNA synthetases as potential drug targets of the Panthera pathogen Babesia
Source: Parasit Vectors. 2019 Oct 14;12:482. doi: 10.1186/s13071-019-3717-z (PMC6792207; doi:10.1186/s13071-019-3717-z)
Supplement: Supplementary file 1 — Additional file 1: Dataset S1. Aminoacyl-tRNA synthetase sequences used to generate the HMM profiles. [file 13071_2019_3717_MOESM1_ESM.docx]

### **Additional file 1: Sequences used for Building HMM for Alanyl-tRNA synthetase (ARS)**

>NP_001596.2 alanine--tRNA synthetase, cytoplasmic [Homo sapiens]

MDSTLTASEIRQRFIDFFKRNEHTYVHSSATIPLDDPTLLFANAGMNQFKPIFLNTIDPSHPMAKLSRAANTQKCIRAGG

KHNDLDDVGKDVYHHTFFEMLGSWSFGDYFKELACKMALELLTQEFGIPIERLYVTYFGGDEAAGLEADLECKQIWQNLG

LDDTKILPGNMKDNFWEMGDTGPCGPCSEIHYDRIGGRDAAHLVNQDDPNVLEIWNLVFIQYNREADGILKPLPKKSIDT

GMGLERLVSVLQNKMSNYDTDLFVPYFEAIQKGTGARPYTGKVGAEDADGIDMAYRVLADHARTITVALADGGRPDNTGR

GYVLRRILRRAVRYAHEKLNASRGFFATLVDVVVQSLGDAFPELKKDPDMVKDIINEEEVQFLKTLSRGRRILDRKIQSL

GDSKTIPGDTAWLLYDTYGFPVDLTGLIAEEKGLVVDMDGFEEERKLAQLKSQGKGAGGEDLIMLDIYAIEELRARGLEV

TDDSPKYNYHLDSSGSYVFENTVATVMALRREKMFVEEVSTGQECGVVLDKTCFYAEQGGQIYDEGYLVKVDDSSEDKTE

FTVKNAQVRGGYVLHIGTIYGDLKVGDQVWLFIDEPRRRPIMSNHTATHILNFALRSVLGEADQKGSLVAPDRLRFDFTA

KGAMSTQQIKKAEEIANEMIEAAKAVYTQDCPLAAAKAIQGLRAVFDETYPDPVRVVSIGVPVSELLDDPSGPAGSLTSV

EFCGGTHLRNSSHAGAFVIVTEEAIAKGIRRIVAVTGAEAQKALRKAESLKKCLSVMEAKVKAQTAPNKDVQREIADLGE

ALATAVIPQWQKDELRETLKSLKKVMDDLDRASKADVQKRVLEKTKQFIDSNPNQPLVILEMESGASAKALNEALKLFKM

HSPQTSAMLFTVDNEAGKITCLCQVPQNAANRGLKASEWVQQVSGLMDGKGGGKDVSAQATGKNVGCLQEALQLATSFAQ

LRLGDVKN

>BAD96544.1 alanyl-tRNA synthetase variant, partial [Homo sapiens]

MDSTLTASEIRQRFIDFFKRNEHTYVHSSATIPLDDPTLLFANAGMNQFKPIFLNTIDPSHPMAKLSRAANTQKCIRAGG

KHNDLDDVGKDVYHHTFFEMLGSWSFGDYFKELACKMALELLTQEFGIPIERLYVTYFGGDEAAGLEADLECKQIWQNLG

LDDTKILPGNMKDNFWEMGDTGPCGPCSEIHYDRIGGRDAAHLVNQDDPNVLEIWNLVFIQYNREADGILKPLPKKSIDT

GMGLERLVSVLQNKMSNYDTDLFVPYFEAIQKGTGARPYTGKVGAEDADGIDMAYRVLADHARTITVALADGGRPDNTGR

GYVLRRILRRAVRYAHEKLNASRGFFATLVDVVVQSLGDAFPELKKDPDMVKDIINEEEVQFLKTLSRGRRILDRKIQSL

GDSKTIPGDTAWLLYDTYGFPVDLTGLIAEEKGLVVDMDGFEEERKLAQLKSQGKGAGGEDLIMLDIYAIEELRARGLEV

TDDSPKYNYHLDSSGSYVFENTVATVMALRREKMFVEEVSTGQECGVVLDKTCFYAEQGGQIYDEGYLVKVDDSSEDKTE

FTVKNAQVRGGYVLHIGTIYGDLKVGDQVWLFIDEPRRRPIMSNHTATHILNFALRSVLGEADQKGSLVAPDRLRFDFTA

KGAMSTQQIKKAEEIANEMIEAAKAVYTQDCPLAAAKAIQGLRAVFDETYPDPVRVVSIGVPVSELLDDPSGPAGSLTSV

EFCGGTHLRNSSHAGAFVIVTEEAIAKGIRRIVAVTGAEAQKALRKAESLKKCLSVMEAKVKAQTAPNKDVQREIADLGE

ALATAVIPQWQKDELRETLKSLKKVMDDLDRASKADVQKRVLEKTKQFIDSNPNQPLVILEMESGATAKALNEALKLFKM

HSPQTSAMLFTVDNEAGKITCLCQVPQNAANRGLKASEWVQQVSGLMDGKGGGKDVSAQATGKNVGCLQEALQLATSFAQ

LRLGDVKN

>BAA06808.1 alanyl-tRNA synthetase [Homo sapiens]

MDSTLTASEIRQRFIDFFKRNEHTYVHSSATIPLDDPTLLFANAGMNQFKPIFLNTIDPSHPMAKLSRAANTQKCIRAGG

KQNDLDDVGKDVYHHTFFEMLGSWSFGDYFKELACKMALELLTQEFGIPIERLYVTYFGGDEAAGLEADLECKQIWQNLG

LDDTKILPGNMKDNFWEMGDTGPCGPCSEIHYDRIGGRDAAHLVNQDDPNVLEIWNLVFIQYNREADGILKPLPKKSIDT

GMGLERLVSVLQNKMSNYDTDLFVPYFEAIQKGTGARPYTGKVGAEDADGIDMAYRVLADHARTITVALADGGRPDNTGR

GYVLRRILRRAVRYAHEKLNASRGFFATLVDVVVQSLGDAFPELKKDPDMVKDIINEEEVQFLKTLSRGRRILDRKIQSL

GDSKTIPGDTAWLLYDTYGFPVDLTGLIAEEKGLVVDMDGFEEERKLAQLKSQGKGAGGEDLIMLDIYAIEELRARGLEV

TDDSPKYNYHLDSSGSYVFENTVATVMALRREKMFVEEVSTGQECGVVLDKTCFYAEQGGQIYDEGYLVKVDDSSEDKTE

FTVKNAQVRGGYVLHIGTIYGDLKVGDQVWLFIDEPRRRPIMSNHTATHILNFALRSVLGEADQKGSLVAPDRLRFDFTA

KGAMSTQQIKKAEEIANEMIEAAKAVYTQDCPLAAAKAIQGLRAVFDETYPDPVRVVSIGVPVSELLDDPSGPAGSLTSV

EFCGGTHLRNSSHAGAFVIVTEEAIAKGIRRIVAVTGAEAQKALRKAESLKKCLSVMEAKVKAQTAPNKDVQREIADLGE

ALATAVIPQWQKDELRETLKSLKKVMDDLDRASKADVQKRVLEKTKQFIDSNPNQPLVILEMESGASAKALNEALKLFKM

HSPQTSAMLFTVDNEAGKITCLCQVPQNAANRGLKASEWVQQVSGLMDGKGGGKDVSAQATGKNVGCLQEALQLATSFAQ

LRLGDVKN

>XP_003811107.1 alanine--tRNA synthetase, cytoplasmic [Pan paniscus]

MDSTLTASEIRQRFIDFFKRNEHTYVHSSATIPLDDPTLLFANAGMNQFKPIFLNTIDPSHPMAKLSRAANTQKCIRAGG

KHNDLDDVGKDVYHHTFFEMLGSWSFGDYFKELACKMALELLTQEFGIPIERLYVTYFGGDEAAGLEADLECKQIWQNLG

LDDTKILPGNMKDNFWEMGDTGPCGPCSEIHYDRIGGRDAAHLVNQDDPNVLEIWNLVFIQYNREADGILKPLPKKSIDT

GMGLERLVSVLQNKMSNYDTDLFVPYFEAIQKGTGARPYTGKVGAEDADGIDMAYRVLADHARTITVALADGGRPDNTGR

GYVLRRILRRAVRYAHEKLNAIRGFFATLVDVVVQSLGDAFPELKKDPDMVKDIINEEEVQFLKTLSRGRRILDRKIQSL

GDSKTIPGDTAWLLYDTYGFPVDLTGLIAEEKGLVVDMDGFEEERKLAQLKSQGKGAGGEDLIMLDIYAIEELRARGLEV

TDDSPKYNYHLDSSGSYVFENTVATVMALRREKMFVEEVSTGQECGVVLDKTCFYAEQGGQIYDEGYLVKVDDSSEDKTE

FTVKNAQVRGGYVLHIGTIYGDLKVGDQVWLFIDEPRRRPIMSNHTATHILNFALRSVLGEADQKGSLVAPDRLRFDFTA

KGAMSTQQIKKAEEIANEMIEAAKAVYTQDCPLAAAKAIQGLRAVFDETYPDPVRVVSIGVPVSELLDDPSGPAGSLTSV

EFCGGTHLRNSSHAGAFVIVTEEAIAKGIRRIVAVTGAEAQKALRKAESMKKCLSVMEAKVKAQTAPNKDVQREIADLGE

ALATAVIPQWQKDELRETLKSLKKVMDDLDRASKADVQKRVLEKTKQFIDSNPNQPLVILEMESGASAKALNEALKLFKM

HSPQTSAMLFTVDNEAGKITCLCQVPQNAANRGLKASEWVQQVSGLMDGKGGGKDVSAQATGKNVGCLQEALQLATSFAQ

LRLGDVKN

>XP_016785595.2 alanine--tRNA synthetase, cytoplasmic [Pan troglodytes]

MDSTLTASEIRQRFIDFFKRNEHTYVHSSATIPLDDPTLLFANAGMNQFKPIFLNTIDPSHPMAKLSRAANTQKCIRAGG

KHNDLDDVGKDVYHHTFFEMLGSWSFGDYFKELACKMGLELLTQEFGIPIERLYVTYFGGDEAAGLEADLECKQIWQNLG

LDDTKILPGNMKDNFWEMGDTGPCGPCSEIHYDRIGGRDAAHLVNQDDPNVLEIWNLVFIQYNREADGILKPLPKKSIDT

GMGLERLVSVLQNKMSNYDTDLFVPYFEAIQKGTGARPYTGKVGAEDADGIDMAYRVLADHARTITVALADGGRPDNTGR

GYVLRRILRRAVRYAHEKLNAIRGFFATLVDVVVQSLGDAFPELKKDPDMVKDIINEEEVQFLKTLSRGRRILDRKIQSL

GDSKTIPGDTAWLLYDTYGFPVDLTGLIAEEKGLVVDMDGFEEERKLAQLKSQGKGAGGEDLIMLDIYAIEELRARGLEV

TDDSPKYNYHLDSSGSYVFENTVATVMALRREKMFVEEVSTGQECGVVLDKTCFYAEQGGQIYDEGYLVKVDDSSEDKTE

FTVKNAQVRGGYVLHIGTIYGDLKVGDQVWLFIDEPRRRPIMSNHTATHILNFALRSVLGEADQKGSLVAPDRLRFDFTA

KGAMSTQQIKKAEEIANEMIEAAKAVYTQDCPLAAAKAIQGLRAVFDETYPDPVRVVSIGVPVSELLDDPSGPAGSLTSV

EFCGGTHLRNSSHAGAFVIVTEEAIAKGIRRIVAVTGAEAQKALRKAESMKKCLSVMEAKVKAQTAPNKDVQREIADLGE

ALATAVIPQWQKDELRETLKSLKKVMDDLDRASKADVQKRVLEKTKQFIDSNPNQPLVILEMESGASAKALNEALKLFKM

HSPQTSAMLFTVDNEAGKITCLCQVPQNAANRGLKASEWVQQVSGLMDGKGGGKDVSAQATGKNVGCLQEALQLATSFAQ

LRLGDVKN

>XP_024088780.1 alanine--tRNA synthetase, cytoplasmic isoform X1 [Pongo abelii]

MDSTLTASEIRQRFIDFFKRNEHTYVHSSATIPLDDPTLLFANAGMNQFKPIFLNTIDPSHPMAKLSRAANTQKCIRAGG

KHNDLDDVGKDVYHHTFFEMLGSWSFGDYFKELACKMALELLTQEFGIPIERLYVTYFGGDEAAGLEPDLECKQIWQNLG

LDDTKILPGNMKDNFWEMGDTGPCGPCSEIHYDRIGGRDAAHLVNQDDPNVLEIWNLVFIQYNREADGILKPLPKKSIDT

GMGLERLVSVLQNKMSNYDTDLFVPYFEAIQKGTGARPYTGKVGAEDADGIDMAYRVLADHARTITVALADGGRPDNTGR

GYVLRRILRRAVRYAHEKLNASRGFFATLVDVVVQSLGDAFPELKKDPDMVKDIINEEEVQFLKTLSRGRRILDRKIQSL

GDSKTIPGDTAWLLYDTYGFPVDLTGLIAEEKGLVVDMDGFEEERKLAQLKSQGKGAGGEDLIMLDIYAIEELRARDLEV

TDDSPKYNYHLDSSGSYVFENTVATVMALRREKMFVEEVSTGQECGVVLDKTCFYAEQGGQIYDEGYLVKVDDSSEDKTE

FTVKNAQVRGGYVLHIGTIYGDLKVGDQVWLFIDEPRRRPIMSNHTATHILNFALRSVLGEADQKGSLVAPDRLRFDFTA

KGAMSTQQIKKAEEIANEMIEAAKPVYTQDCSLAAAKAIQGLRAVFDETYPDPVRVVSIGVPVSELLDDPSGPAGSLTSV

EFCGGTHLRNSSHAGAFVIVTEEAIAKGIRRIVAVTGAEAQKALRKAESLKKSLSVMEAKVKAQTAPNKDVQREIADLGE

ALATAVIPQWQKDELRETLKSLKKVMDDLDRASKADVQKRVLEKTKQLIDSNPNQPLVILEMESGASAKALNEALKLFKM

HSPQTAAMLFTVDNEAGKITCLCQVPQNAANRGLKASEWVQQVSGLMDGKGGGKDVSAQATGKNVGCLQEALQLATSFAQ

LRLGDVKN

>XP_011817628.1 PREDICTED: alanine--tRNA synthetase, cytoplasmic [Colobus angolensis palliatus]

MDSTLTASEIRQRFIDFFKRNEHTYVHSSATIPLDDPTLLFANAGMNQFKPIFLNTIDPSHPMAKLSRAANTQKCIRAGG

KHNDLDDVGKDVYHHTFFEMLGSWSFGDYFKELACKMALELLTQEFGISIERLYVTYFGGDEAAGLEPDLECKQIWQNLG

LDDTKILPGNMKDNFWEMGDTGPCGPCSEIHYDRIGGRDAAHLVNQDDPNVLEIWNLVFIQYNREADGILKPLPKKSIDT

GMGLERLVSVLQNKMSNYDTDLFVPYFEAIQKGTGARPYTGKVGAEDADGIDMAYRVLADHARTITVALADGGRPDNTGR

GYVLRRILRRAVRYAHEKLNASRGFFATLVDVVVQSLGDAFPELKKDPDMVKDIINEEEVQFLKTLSRGRRILDRKIQSL

GDSKTIPGDTAWLLYDTYGFPVDLTGLIAEEKGLVVDMDGFEEERKLAQLKSQGKGAGGEDLIMLDIYAIEELRARGLEV

TDDSPKYNYHLDSSGSYVFENTVATVMALRREKMFVEEVSTGQECGVVLDKTCFYAEQGGQIYDEGYLVKVDDSSEDKTE

FTVKNAQVRGGYVLHIGTIYGDLKVGDQVWLFIDEPRRRPIMSNHTATHILNFALRSVLGEADQKGSLVAPDRLRFDFTA

KGAMSTQQIKKAEEIANEMIEAAKPVYTQDCPLAAAKAIQGLRAVFDETYPDPVRVVSIGVPVSELLDDPSGPAGSLTSV

EFCGGTHLRNSSHAGAFVIVTEEAIAKGIRRIVAVTGAEAQKALRKAESLKKSLSVMEAKVKAQTAPNKDVQREIADLGE

ALATAVIPQWQKDELRETLKSLKKVMDDLDRASKADVQKRVLEKTKQLIDSNPNQPLVILEMESGASAKALNEALKLFKT

HSPQTSAMLFTVDNDAGKITCLCQVPQNAANRGLKASEWVQQVSGLMDGKGGGKDVSAQATGKNIGCLQEALQLATSFAQ

LRLGDVKN

>XP_007991840.1 PREDICTED: alanine--tRNA synthetase, cytoplasmic [Chlorocebus sabaeus]

MESTLTASEIRQRFIDFFKRNEHTYVHSSATIPLDDPTLLFANAGMNQFKPIFLNTIDPSHPMAKLSRAANTQKCIRAGG

KHNDLDDVGKDVYHHTFFEMLGSWSFGDYFKELACKMALELLTQEFGIPIERLYVTYFGGDEAAGLEPDLECKQIWQNLG

LDDTKILPGNMKDNFWEMGDTGPCGPCSEIHYDRIGGRDAAHLVNQDDPNVLEIWNLVFIQYNREADGILKPLPKKSIDT

GMGLERLVSVLQNKMSNYDTDLFVPYFEAIQKGTGARPYTGKVGAEDADGIDMAYRVLADHARTITVALADGGRPDNTGR

GYVLRRILRRAVRYAHEKLNASRGFFATLVDVVVQSLGDAFPELKKDPDMVKDIINEEEVQFLKTLSRGRRILDRKIQSL

GDSKTIPGDTAWLLYDTYGFPVDLTGLIAEEKGLVVDMDGFEEERKLAQLKSQGKGAGGEDLIMLDIYAIEELRARGLEV

TDDSPKYNYHLDSSGSYVFENTVATVMALRREKMFVEEVSTGQECGVVLDKTCFYAEQGGQIYDEGYLVKVDDSSEDKTE

FTVKNAQVRGGYVLHIGTIYGDLKVGDQVRLFIDEPRRRPIMSNHTATHILNFALRSVLGEADQKGSLVAPDRLRFDFTA

KGAMSTQQIKKAEEIANEMIEAAKPVYTQDCPLAAAKAIQGLRAVFDETYPDPVRVVSIGVPVSELLDDPSGPAGSLTSV

EFCGGTHLRNSSHAGAFVIVTEEAIAKGIRRIVAVTGAEAQKALRKAESLKKSLSVMEAKVKAQTAPNKDVQREIADLGE

ALATAVIPQWQKDELRETLKSLKKVMDDLDRASKADVQKRVLEKTKQLIDSNPNQPLVILEMESGASAKALNEALKLFKT

HSPQTSAMLFTVDNEAGKITCLCQVPQNAANRGLKASEWVQQVSGLMDGKGGGKDVSAQATGKNIGCLQEALQLATSFAQ

LRLGDVKN

>XP_010381255.1 PREDICTED: alanine--tRNA synthetase, cytoplasmic [Rhinopithecus roxellana]

MDSTLTASEIRQRFIDFFRRNEHTYVHSSATIPLDDPTLLFANAGMNQFKPIFLNTIDPSHPMAKLSRAANTQKCIRAGG

KHNDLDDVGKDVYHHTFFEMLGSWSFGDYFKELACKMALELLTQEFGIPIERLYVTYFGGDEAAGLEPDLECKQIWQNLG

LDDTKILPGNMKDNFWEMGDTGPCGPCSEIHYDRIGGRDAAHLVNQDDPNVLEIWNLVFIQYNREADGILKPLPKKSIDT

GMGLERLVSVLQNKMSNYDTDLFVPYFEAIQKGTGARPYTGKVGAEDADGIDMAYRVLADHARTITVALADGGRPDNTGR

GYVLRRILRRAVRYAHEKLNASRGFFATLVDVVVQSLGDAFPELKKDPDMVKDIINEEEVQFLKTLSRGRRILDRKIQSL

GDSKTIPGDTAWLLYDTYGFPVDLTGLIAEEKGLVVDMDGFEEERKLAQLKSQGKGAGGEDLIMLDIYAIEELRARGLEV

TDDSPKYNYHLDSSGSYVFENTVATVMALRREKMFVEEVSTGQECGVVLDKTCFYAEQGGQLYDEGYLVKVDDSSEDKTE

FTVKNAQVRGGYVLHIGTIYGDLKVGDQVRLFIDEPRRRPIMSNHTATHILNFALRSVLGEADQKGSLVAPDRLRFDFTA

KGAMSTQQIKKAEEIANEMIEAAKPVYTQDCPLAAAKAIQGLRAVFDETYPDPVRVVSIGVPVSELLDDPSGPAGSLTSV

EFCGGTHLRNSSHAGAFVIVTEEAIAKGIRRIVAVTGAEAQKALRKAESLKKSLSVMEAKVKAQTAPNKDVQREIADLGE

ALATAVIPQWQKDEFRETLKSLKKVMDDLDRASKADVQKRVLEKTKQLIDSNPNQPLVILEMESGASAKALNEALKLFKT

HSPQTSAMLFTVDNEAGKITCLCQVPQNAANRGLKASEWVQQVSGLMDGKGGGKDVSAQATGKNIGCLQEALQLATSFAQ

LRLGDVKN

>XP_011892606.1 PREDICTED: alanine--tRNA synthetase, cytoplasmic isoform X1 [Cercocebus atys]

MDSTLTASEIRQRFIDFFKRNEHTYVHSSATIPLDDPTLLFANAGMNQFKPIFLNTIDPSHPMAKLSRAANTQKCIRAGG

KHNDLDDVGKDVYHHTFFEMLGSWSFGDYFKELACKMALELLTQEFGIPIERLYVTYFGGDEAAGLEPDLECKQIWQNLG

LDDTKILPGNMKDNFWEMGDTGPCGPCSEIHYDRIGGRDAAHLVNQDDPNVLEIWNLVFIQYNREADGILKPLPKKSIDT

GMGLERLVSVLQNKMSNYDTDLFVPYFEAIQKGTGARPYTGKVGAEDADGIDMAYRVLADHARTITVALADGGRPDNTGR

GYVLRRILRRAVRYAHEKLNASRGFFATLVDVVVQSLGDAFPELKKDPDMVKDIINEEEVQFLKTLSRGRRILDRKIQSL

GDSKTIPGDTAWLLYDTYGFPVDLTGLIAEEKGLVVDMDGFEEERKLAQLKSQGKGAGGEDLIMLDIYAIEELRARGLEV

TDDSPKYNYHLDFSGSYVFENTVATVMALRREKMFVEEVSTGQECGVVLDKTCFYAEQGGQIYDEGYLVKVDDSSEDKTE

FTVKNAQVRGGYVLHIGTIYGDLKVGDQVRLFIDEPRRRPIMSNHTATHILNFALRSVLGEADQKGSLVAPDRLRFDFTA

KGAMSTQQIKKAEEIANEMIEAAKPVYTQDCPLAAAKAIQGLRAVFDETYPDPVRVVSIGVPVSELLDDPSGPAGSLTSV

EFCGGTHLRNSSHAGAFVIVTEEAIAKGIRRIVAVTGAEAQKALRKAESLKKSLSVMEAKVKAQTAPNKDVQREIADLGE

ALATAVIPQWQKDELRETLKSLKKVMDDLDRASKADVQKRVLEKTKQLIDSNPNQPLVILEMESGASAKALNEALKLFKT

HSPQTSAMLFTVDNEAGKITCLCQVPQNAANRGLKASEWVQQVSGLMDGKGGGKDVSAQATGKNIGCLQEALQLATSFAQ

LRLGDVKN

>BAG61157.1 unnamed protein product [Homo sapiens]

MDSTLTASEIRQRFIDFFKRNEHTYVHSSATIPLDDPTLLFANAGMNQFKPIFLNTIDPSHPMAKLSRAANTQKCIRAGG

KHNDLDDVGKDVYHHTFFEMLGSWSFGDYFKELACKMALELLTQEFGIPIERLYVTYFGGDEAAGLEADLECKQIWQNLG

TYLYSFVRLDDTKILPGNMKDNFWEMGDTGPCGPCSEIHYDRIGGRDAAHLVNQDDPNVLEIWNLVFIQYNREADGILKP

LPKKSIDTGMGLERLVSVLQNKMSNYDTDLFVPYFEAIQKGTGARPYTGKVGAEDADGIDMAYRVLADHARTITVALADG

GRPDNTGRGYVLRRILRRAVRCAHEKLNASRGFFATLVDVVVQSLGDAFPELKKDPDMVKDIINEEEVQFLKTLSRGRRI

LDRKIQSLGDSKTIPGDTAWLLYDTYGFPVDLTGLIAEEKGLVVDMDGFEEERKLAQLKSQGKGAGGEDLIMLDIYAIEE

LRARGLEVTDDSPKYNYHLDSSGSYVFENTVATVMALRREKMFVEEVSTGQECGVVLDKTCFYAEQGGQIYDEGYLVKVD

DSSEDKTEFTVKNAQVRGGYVLHIGTIYGDLKVGDQVWLFIDEPRRRPIMSNHTATHILNFALRSVLGEADQKGSLVAPD

RLRFDFTAKGAMSTQQIKKAEEIANEMIEAAKAVYTQDCPLAAAKAIQGLRAVFDETYPDPVRVVSIGVPVSELLDDPSG

PAGSLTSVEFCGGTHLRNSSHAGAFVIVTEEAIAKGIRRIVAVTGAEAQKTLRKAESLKKCLSVMEAKVKAQTAPNKDVQ

REIADLGEALATAVIPQWQKDELRETLKSLKKVMDDLDRASKADVQKRVLEKTKQFIDSNPNQPLVILEMESGASAKATQ

GPGSPPLGLISSLALNEALKLFKMHSPQTSAMLFTVDNEAGKITCLCQVPQNAANRGLKASEWVQQVSGLMDGKGGGKDV

SAQATGKNVGCLQEALQLATSFAQLRLGDVKN

>XP_023058192.1 alanine--tRNA synthetase, cytoplasmic [Piliocolobus tephrosceles]

MDSTLTASEIRQRFIDFFKRNEHTYVHSSATIPLDDPTLLFANAGMNQFKPIFLNTIDPSHPMAKLSRAANTQKCIRAGG

KHNDLDDVGKDVYHHTFFEMLGSWSFGDYFKELACKMALQLLTQVFGIPIERLYVTYFGGDEAAGLEPDLECKQIWQNLG

LDDTKILPGNMKDNFWEMGDTGPCGPCSEIHYDRIGGRDAAHLVNQDDPNVLEIWNLVFIQYNREADGILKPLPKKSIDT

GMGLERLVSVLQNKMSNYDTDLFVPYFEAIQKGTGARPYTGKVGAEDADGIDMAYRVLADHARTITVALADGGRPDNTGR

GYVLRRILRRAVRYAHEKLNASRGFFATLVDVVVQSLGDAFPELKKDPDMVKDIINEEEVQFLKTLSRGRRILDRKIQSL

GDSKTIPGDTAWLLYDTYGFPVDLTGLIAEEKGLVVDMDGFEEERKLAQLKSQGKGAGGEDLIMLDIYAIEELRARGLEV

TDDSPKYNYHLDSSGSYAFENTVATVMALRREKMFVEEVSTGQECGVVLDKTCFYAEQGGQIYDEGYLVKVDDSSEDKTE

FTVKNAQVRGGYVLHIGTIYGDLKVGDQVRLFIDEPRRRPIMSNHTATHILNFALRSVLGEADQKGSLVAPDRLRFDFTA

KGAMSTQQIKKAEEIANEMIEAAKPVYTQDCPLAAAKAIQGLRAVFDETYPDPVRVVSIGVPVSELLDDPSGPAGSLTSV

EFCGGTHLRNSSHAGAFVIVTEEAIAKGIRRIVAVTGAEAQKALRKAESLKKSLSVMEAKVKAQTAPNKDVQREIADLGE

ALATAVIPQWQKDELRETLKSLKKVMDDLDRASKADVQKRVLEKTKQLIDSNPNQPLVILEMESGASAKALNEALKLFKT

HSPQTSAMLFTVDNEAGKITCLCQVPQNAANRGLKASEWVQQVSGLMDGKGGGKDVSAQATGKNIGCLQEALQLATSFAQ

LRLGDVKN

>NP_001125391.1 alanine--tRNA synthetase, cytoplasmic [Pongo abelii]

MDSTLTASKIRQRFIDFFKRNEHTYIHSSATIPLDDPTLLFANAGMNQFKPIFLNTIDPSHPMAKPSRAANTQKCIRAGG

KHNDLDDVGKDVYHHTFFEMLGSWSFGDYFKELACKMALELLTQEFGIPIERLYVTYFGGDEAAGLEPDLECKQIWQNLG

LDDTKILPGNMKDNFWEMGDTGPCGPCSEIHYDRIGGRDAAHLVNQDDPNVLEIWNLVFIQYNREADGILKPLPKKSIDT

GMGLERLVSVLQNKMSNYDTDLFVPYFEAIQKGTGARPYTGKVGAEDADGIDMAYRVLADHARTITVALADGGRPDNTGR

GYVLRRILRRAVRYAHEKLNASRGFFATLVDVVVQSLGDAFPELKKDPDMVKDIINEEEVQFLKTLSRGRRILDRKIQSL

GDSKTIPGDTAWLLYDTYGFPVDLTGLIAEEKGLVVDMDGFEEERKLAQLKSQGKGAGGEDLIMLDIYAIEELRARDLEV

TDDSPKYNYHLDSSGSYVFENTVATVMALRREKMFVEEVSTGQECGVVLDKTCFYAEQGGQIYDEGYLVKVDDSSEDKTE

FTVKNAQVRGGYVLHIGTIYGDLKVGDQVWLFIDEPRRRPIMSNHTATHILNFALRSVLGEADQKGSLVAPDRLRFDFTA

KGAMSTQQIKKAEEIANEMIEAAKPVYTQDCSLAAAKAIQGLRAVFDETYPDPVRVVSIGVPVSELLDDPSGPAGSLTSV

EFCGGTHLRNSSHAGAFVIVTEEAIAKGIRRIVAVTGAEAQKALRKAGSLKKPLSVMEAKVKAQTAPNKDVQREIADLGE

ALATAVIPQWQKDELRETLKSLKKVMDDLDRASKADVQKRVLEKTKQLIDSNPNQPLVILEMESGASAKALNEALKLFKM

HSPQTAAMLFTVDNEAGKITCLCQVPQNAANRGLKASEWVQQVSGLMDGKGGGKDVSAQATGKNVGCLQEALQLATSFAQ

LRLGDVKN

>XP_005592592.1 PREDICTED: alanine--tRNA synthetase, cytoplasmic [Macaca fascicularis]

MDSTLTASEIRQRFIDFFKRNEHTYIHSSATIPLDDPTLLFANAGMNQFKPIFLNTIDPSHPMAKLSRAANTQKCIRAGG

KHNDLDDVGKDVYHHTFFEMLGSWSFGDYFKELACKMALELLTQEFGIPIGRLYVTYFGGDEAAGLEPDLECKQIWQNLG

LDDTKILPGNMKDNFWEMGDTGPCGPCSEIHYDRIGGRDAAHLVNQDDPNVLEIWNLVFIQYNREADGILKPLPKKSIDT

GMGLERLVSVLQNKMSNYDTDLFVPYFEAIQKGTGARPYTGKVGAEDADGIDMAYRVLADHARTITVALADGGRPDNTGR

GYVLRRILRRAVRYAHEKLNASRGFFATLVDVVVQSLGDAFPELKKDPDMVKDIINEEEVQFLKTLSRGRRILDRKIQSL

GDSKTIPGDTAWLLYDTYGFPVDLTGLIAEEKGLVVDMDGFEEERKLAQLKSQGKGAGGEDLIMLDIYAIEELRARGLEV

TDDSPKYNYHLDFSGSYVFENTVATVMALRREKMFVEEVSTGQECGVVLDKTCFYAEQGGQIYDEGYLVKVDDSSEDKTE

FTVKNAQVRGGYVLHIGTIYGDLKVGDQVRLFIDEPRRRPIMSNHTATHILNFALRSVLGEADQKGSLVAPDRLRFDFTA

KGAMSTQQIKKAEEIANEMIEAAKPVYTQDCPLAAAKAIQGLRAVFDETYPDPVRVVSIGVPVSELLDDPSGPAGSLTSV

EFCGGTHLRNSSHAGAFVIVTEEAIAKGIRRIVAVTGAEAQKALRKAESLKKSLSVMEAKVKAQTAPNKDVQREIADLGE

ALATAVIPQWQKDELRETLKSLKKVMDDLDRASKADVQKRVLEKTKQLIDSNPNQPLVILEMESGASAKALNEALKLFKT

HSPQTSAMLFTVDNEAGKITCLCQVPQNAANRGLKASEWVQQVSGLMDGKGGGKDVSAQATGKNIGCLQEALQLATSFAQ

LRLGDVKN

>XP_003918629.1 alanine--tRNA synthetase, cytoplasmic [Papio anubis]

MDSTLTASEIRLRFIDFFKRNEHTYVHSSATIPLDDPTLLFANAGMNQFKPIFLNTIDPSHPMAKLSRAANTQKCIRAGG

KHNDLDDVGKDVYHHTFFEMLGSWSFGDYFKELACKMALELLTQEFGIPIERLYVTYFGGDEAAGLEPDLECKQIWQNLG

LDDSKILPGNMKDNFWEMGDTGPCGPCSEIHYDRIGGRDAAHLVNQDDPNVLEIWNLVFIQYNREADGILKPLPKKSIDT

GMGLERLVSVLQNKMSNYDTDLFVPYFEAIQKGTGARPYTGKVGAEDADGIDMAYRVLADHARTITVALADGGRPDNTGR

GYVLRRILRRAVRYAHEKLNASRGFFATLVDVVVQSLGDAFPELKKDPDMVKDIINEEEVQFLKTLSRGRRILDRKIQSL

GDSKTIPGDTAWLLYDTYGFPVDLTGLIAEEKGLVVDMDGFEEERKLAQLKSQGKGAGGEDLIMLDIYAIEELRARGLEV

TDDSPKYNYHLDFSGSYVFENTVATVMALRREKMFVEEVSTGQECGVVLDKTCFYAEQGGQIYDEGYLVKVDDSSEDKTE

FTVKNAQVRGGYVLHIGTIYGDLKVGDQVRLFIDEPRRRPIMSNHTATHILNFALRSVLGEADQKGSLVAPDRLRFDFTA

KGAMSTQQIKKAEEIANEMIEAAKPVYTQDCPLAAAKAIQGLRAVFDETYPDPVRVVSIGVPVSELLDDPSGPAGSLTSV

EFCGGTHLRNSSHAGAFVIVTEEAIAKGIRRIVAVTGTEAQKALRKAESLKKSLSVMEAKVKAQTAPNKDVQREIADLGE

ALATAVIPQWQKDELRETLKSLKKVMDDLDRASKADVQKRVLEKTKQLIDSNPNQPLVILEMESGASAKALNEALKLFKT

HSPQTSAMLFTVDNEAGKITCLCQVPQNAANRGLKASEWVQQVSGLMDGKGGGKDVSAQATGKNIGCLQEALQLATSFAQ

LRLGDVKN

>XP_014981998.1 PREDICTED: alanine--tRNA synthetase, cytoplasmic [Macaca mulatta]

MDSTLTASEIRQRFIDFFKRNEHTYIHSSATIPLDDPTLLFANAGMNQFKPIFLNTIDPSHPMAKLSRAANTQKCIRAGG

KHNDLDDVGKDVYHHTFFEMLGSWSFGDYFKELACKMALELLTQEFGIPIGRLYVTYFGGDEAAGLEPDLECKQIWQNLG

LDDTKILPGNMKDNFWEMGDTGPCGPCSEIHYDRIGGRDAAHLVNQDDPNVLEIWNLVFIQYNREADGILKPLPKKSIDT

GMGLERLVSVLQNKMSNYDTDLFVPYFEAIQKGTGARPYTGKVGAEDADGIDMAYRVLADHARTITVALADGGRPDNTGR

GYVLRRILRRAVRYAHEKLNASRGFFATLVDVVVQSLGDAFPELKRDPDMVKDIVNEEEVQFLKTLSRGRRILDRKIQSL

GDSKTIPGDTAWLLYDTYGFPVDLTGLIAEEKGLVVDMDGFEEERKLAQLKSQGKGAGGEDLIMLDIYAIEELRARGLEV

TDDSPKYNYHLDFSGSYVFENTVATVMALRREKMFVEEVSTGQECGVVLDKTCFYAEQGGQIYDEGYLVKVDDSSEDKTE

FTVKNAQVRGGYVLHIGTIYGDLKVGDQVRLFIDEPRRRPIMSNHTATHILNFALRSVLGEADQKGSLVAPDRLRFDFTA

KGAMSTQQIKKAEEIANEMIEAAKPVYTQDCPLAAAKAIQGLRAVFDETYPDPVRVVSIGVPVSELLDDPSGPAGSLTSV

EFCGGTHLRNSSHAGAFVIVTEEAIAKGIRRIVAVTGAEAQKALRKAESLKKSLSVMEAKVKAQTAPNKDVQREIADLGE

ALATAVIPQWQKDELRETLKSLKKVMDDLDRASKADVQKRVLEKTKQLIDSNPNQPLVILEMESGASAKALNEALKLFKT

HSPQTSAMLFTVDNEAGKITCLCQVPQNAANRGLKASEWVQQVSGLMDGKGGGKDVSAQATGKNIGCLQEALQLATSFAQ

LRLGDVKN

>XP_017724215.1 PREDICTED: alanine--tRNA synthetase, cytoplasmic isoform X1 [Rhinopithecus bieti]

MDSTLTASEIRQRFIDFFRRNEHTYVHSSATIPLDDPTLLFANAGMNQFKPIFLNTIDPSHPMAKLSRAANTQKCIRAGG

KHNDLDDVGKDVYHHTFFEMLGSWSFGDYFKELACKMALELLTQEFGIPIERLHVTYFGGDEAAGLEPDLECKQIWQNLG

LDDTKILPGNMKDNFWEMGDTGPCGPCSEIHYDRIGDRDAAHLVNQDDPNVLEIWNLVFIQYNREADGILKPLPKKSIDT

GMGLERLVSVLQNKMSNYDTDLFVPYFEAIQKGTGARPYTGKVGAEDADGIDMAYRVLADHARTITVALADGGRPDNTGR

GYVLRRILRRAVRYAHEKLNASRGFFATLVDVVVQSLGDAFPELKKDPDMVKDIINEEEVQFLKTLSRGRRILDRKIQSL

GDSETIPGDTAWLLYDTYGFPVDLTGLIAEEKGLVVDMDGFEEERKLAQLKSQGKGAGGEDLIMLDIYAIEELRARGLEV

TDDSPKYNYHLDSSGSYVFENTVATVMALRREKMFVEEVSTGQECGVVLDKTCFYAEQGGQLYDEGYLVKVDDSSEDKTE

FTVKNAQVRGGYVLHIGTIYGDLKVGDQVRLFIDEPRRRPIMSNHTATHILNFALRSVLGEADQKGSLVAPDRLRFDFTA

KGAMSTQQIKKAEEIANEMIEAAKPVYTQDCPLAAAKAIQGLRAVFDETYPDPVRVVSIGVPVSELLDDPSGPAGSLTSV

EFCGGTHLRNSSHAGAFVIVTEEAIAKGIRRIVAVTGAEAQKALRKAESLKKSLSVMEAKVKAQTAPNKDVQREIADLGE

ALATAVIPQWQKDEFRETLKSLKKVMDDLDRASKADVQKRVLEKTKQLIDSNPNQPLVILEMESGASAKALNEALKLFKT

HSPQTSAMLFTVDNEAGKITCLCQVPQNAANRGLKASEWVQQVSGLMDGKGGGKDVSAQATGKNIGCLQEALQLATSFAQ

LRLGDVKN

>XP_012497019.1 PREDICTED: alanine--tRNA synthetase, cytoplasmic [Propithecus coquereli]

MDSTLTASEIRQRFIDFFKRNEHTYVHSSATIPLDDPTLLFANAGMNQFKPIFLNTIDPSHPMAKLSRAANTQKCIRAGG

KHNDLDDVGKDVYHHTFFEMLGSWSFGDYFKELACKMALELLTQEFGIPVERLYVTYFGGDEAAGLEPDLECKQIWQNLG

LDDGKILPGNMKDNFWEMGDTGPCGPCSEIHYDRIGGRDAAHLVNQDDPNVLEIWNLVFIQYNRETDGILKPLPKKSIDT

GMGLERLVSVLQNKMSNYDTDLFVPYFEAIQKGTGARPYTGQVGADDADGIDMAYRVLADHARTITVALADGGRPDNTGR

GYVLRRILRRAVRYSHEKLNASRGFFATLVDVVVQSLGDAFPELKKDPDMVKDIINEEEVQFLKTLSRGRRILDRKIQSL

GDSKTIPGDTAWLLYDTYGFPVDLTGLIAEEKGLVVDMDGFEEERKLAQLKSQGKGAGGEDLIMLDIYAIEELRAQGLEV

TDDSPKYNYHSDSSGSYVFENTVATVMALRREKMFVEEVSTGQECGVVLDKTCFYAEQGGQIYDEGYLVKVDDSSEDKTE

FTVKNAQVRGGYVLHIGTVYGYLKVGDQVRLFIDEPRRRPVMSNHTATHILNFALRAVLGEADQKGSLVAPDRLRFDFTA

KGAMSTQQIKKAEEIANEMIEAAKPVYTQDCPLAAAKAIQGLRAVFDETYPDPVRVVSIGVPVSELLDDPSGPAGSLTSV

EFCGGTHLRNSSHAGAFVIVTEEAIAKGIRRIVAVTGAEAQKALRKAESLKKSLSLMEAKVKAQTAPNKDVQREIADLGE

SLATAVIPQWQKDELRETLKSLKKIMDDLDRASKADVQKRVLEKTKQLIDSNPNQPLVILEMESGASAKALNEALKLFKT

HSPQTSAMLFTVDNEAGKITCLCQVPQNAANQGLKASEWVQQVSGLMDGKGGGKDVSAQATGKNVGCLQEALQLATSFAQ

LRLGDVKN

>XP_012627917.1 alanine--tRNA synthetase, cytoplasmic [Microcebus murinus]

MDSTLTASEIRQRFIDFFKRNEHTYVHSSATIPLDDPTLLFANAGMNQFKPIFLNTIDPSHPMAKLSRAANTQKCIRAGG

KHNDLDDVGKDVYHHTFFEMLGSWSFGDYFKELACKMALELLTQEFGIPVERLYVTYFGGDEAAGLEPDLECKQIWQNLG

LDDGKILPGNMKDNFWEMGDTGPCGPCSEIHYDRIGGRDAAHLVNQDDPNVLEIWNLVFIQYNRETDGILKPLPKKSIDT

GMGLERLVSVLQNKMSNYDTDLFVPYFEAIQKGTGARPYTGKVGADDADGIDMAYRVLADHARTITVALADGGRPDNTGR

GYVLRRILRRAVRYSHEKLNASRGFFATLVDVVVQSLGDAFPELKKDPDMVKDIINEEEVQFLKTLSRGRRILDRKIQSL

GDSKTIPGDTAWLLYDTYGFPVDLTGLIAEEKGLVVDMDGFEEERKLAQLKSQGKGAGGEDLIMLDIYAIEELRAKGLEV

TDDSPKYNYHSDSSGSYMFENTVATVMALRREKMFVEEVSTGQECGVVLDKTCFYAEQGGQIYDEGYLVKVDDSSEDKTE

FTVKNAQVRGGYVLHIGTVYGDLKVGDQVRLFIDEPRRRPVMSNHTATHILNFALRSVLGEADQKGSLVAPDRLRFDFTA

KGAMSTQQIKKAEEIANEMIEAAKPVYIQDCPLAAAKAIQGLRAVFDETYPDPVRVVSIGVPVSELLDDPSGPAGSLTSV

EFCGGTHLRNSSHAGAFVIVTEEAIAKGIRRIVAVTGAEAQKALRKAESLNKSLSVMEAKVKAQTAPNKDVQREIADLGE

SLATAVIPQWQKDELRETLKSLKKIMDDLDRASKADVQKRVLERTKQLIDSNPNQPLVILEMESGASAKALNEALKLFKT

HSPQTSAMLFTVDNEAGKITCLCQVPQNAANRGLKASEWVQQVSGLMDGKGGGKDVSAQATGKNVGCLQEALQLATSFAQ

LRLGAVKN

>XP_017371742.1 PREDICTED: alanine--tRNA synthetase, cytoplasmic [Cebus capucinus imitator]

MDSTLTASEIRQRFIEFFKRNEHTYVHSSATIPLDDPTLLFANAGMNQFKPIFLNTIDPSHPMAKLSRAANTQKCIRAGG

KHNDLDDVGKDVYHHTFFEMLGSWSFGDYFKELACKMALELLTQEFGIPIERLYVTYFGGDEAAGLEPDLECKQIWQNLG

LEDTKILPGNMKDNFWEMGDTGPCGPCSEIHYDRIGGRDAAHLVNQDDPNVLEIWNLVFIQYNRETDGILKPLPKKSIDT

GMGLERLVSVLQNKMSNYDTDLFVPYFEAIQKGTGARPYTGKVGAEDADGIDMAYRVLADHARTITVALADGGRPDNTGR

GYVLRRILRRAVRYAHEKLNASRGFFATLVDVVVQSLGDAFPELKKDPDMVKDIINEEEVQFLKTLSRGRRILDRKILSL

GDSKTIPGDTAWLLYDTYGFPVDLTGLIAEEKGLLVDMDGFEEERKLAQLKSQGKGAGGEDLIMLDIYAIEELRAQGLEV

TDDSPKYNYHSDSSGNYVFENTVATVLALRREKMFVEEVSTGQECGVLLDKTCFYAEQGGQICDEGYLVKVDDSSEDKTE

FTVKNAQVRGGYVLHIGTIYGDLKVGDQVWLFIDEPRRRPIMSNHTATHILNFALRSVLGEADQKGSLVAPDRLRFDFTA

KGAMSTQQIKKAEEIANEMIEAAKPVYTQDCPLAAAKAIQGLRAVFDETYPDPVRVVSIGVPVSELLDDPSGPAGSLTSV

EFCGGTHLQNSSHAGAFVIVTEEAIAKGIRRIVAVTGAEAQKALRKAESLKTSLSVMEAKVKAQTAPNKDVQREIADLGE

VLATAVVPQWQKDELRETLKSLKKVMDDLDRASKADVQKRVLEKTKQLIDSNPNQPLVILEMESGASAKALNEALKLFKM

HSPQTSAMLFTVDNEAGKITCLCQVPQNAANRGLKASEWVQQVSGLMDGKGGGKDVSAQATGKNIGCLQEALQLATSFAQ

LRLRDVKN

>XP_002761188.1 PREDICTED: alanine--tRNA synthetase, cytoplasmic [Callithrix jacchus]

MDSTLTASEIRQRFIEFFKRNEHTYVHSSATIPLDDPTLLFANAGMNQFKPIFLNTIDPSHPMAKLSRAANTQKCIRAGG

KHNDLDDVGKDVYHHTFFEMLGSWSFGDYFKELACKMALELLTQEFGIPIERLYVTYFGGDEAAGLEPDLECKQIWQNLG

LEDTKILPGNMKDNFWEMGDTGPCGPCSEIHYDRIGGRDAAHLVNQDDPNVLEIWNLVFIQYNRETDGILKPLPKKSIDT

GMGLERLVSVLQNKMSNYDTDLFVPYFEAIQKGTGARPYTGKVGAEDADGIDMAYRVLADHARTITVALADGGRPDNTGR

GYVLRRILRRAVRYAHEKLNASRGFFATLVDVVVQSLGDAFPELKKDPDMVKDIINEEEVQFLKTLSRGRRILDRKILSL

GDSKTIPGDTAWLLYDTYGFPVDLTGLIAEEKGLLVDMDGFEEERKLAQLKSQGKGAGGEDLIMLDIYAIEELRAQGLEV

TDDSPKYNYHSDSSGNYVFENTVATVLALRREKMFVEEVSTGQECGVVLDKTCFYAEQGGQIYDEGYLVKVDDSNEDKTE

FTVKNAQVRGGYVLHIGTIYGDLKVGDQVRLFIDEPRRRPVMSNHTATHILNFALRSVLGEADQKGSLVAPDRLRFDFTA

KGAMSTQQIKKAEEIANEMIEAAKPVYTQDCPLAAAKAIQGLRAVFDETYPDPVRVVSVGVPVSELLDDPSGPAGSLTSV

EFCGGTHLQNSSHAGAFVIVTEEAIAKGIRRIVAVTGAEAQKALRKAESLKTSLSLMEAKVKAQTAPNKDVQREIADLGE

VLATAVIPQWQKDELRETLKSLKKVMDDLDRASKADVQKRVLEKTKQLIDSNPNQPLVILEMESGASAKALNEALKLFKM

HSPQTSAMLFTVDNEAGKITCLCQVPQNAANRGLKASEWVQQVSGLMDGKGGGKDVSAQATGKNVGCLQEALQLATSFAQ

LHLGDVKN

>XP_021570822.1 alanine--tRNA synthetase, cytoplasmic [Carlito syrichta]

MDSTLTASEIRQRFIDFFKRNEHTYVHSSATIPLDDPTLLFANAGMNQFKPIFLNTIDPSHPMAKLSRAANTQKCIRAGG

KHNDLDDVGKDVYHHTFFEMLGSWSFGDYFKELACKMALELLTQEFGIPVERLYVTYFGGDEAAGLEPDLECKQIWQNLG

LDDTKILPGNMKDNFWEMGDTGPCGPCSEIHYDRIGGRDAAHLVNQDDPNVLEIWNLVFIQYNRETDGVLKPLPKKSIDT

GMGLERLVSVLQNKMSNYDTDLFVPYFEAIQKGTGARPYTGKVGADDADGIDMAYRVLADHARTITVALADGGRPDNTGR

GYVLRRILRRAVRYSHEKLNASRGFFATLVDVVVQSLGDAFPELKKDPDMVKDIINEEEVQFLKTLSRGRRILDRKIQSL

GDGKTIPGDTAWLLYDTYGFPVDLTGLIAEEKGLVVDMDGFEEERKLAQLKSQGKGAGGEDLIMLDIYAIEELRAKGLEV

TDDSPKYNYHLDASGNYVFENTVATVMALRREKMFVEEVSTGQECGVVLDKTCFYAEQGGQIYDEGYLVKVDDSSEDKTE

FTVKNAQVRGGYVLHIGTIYGNLKMGDQVRLFIDEPRRRPVMSNHTATHILNFALRSVLGEADQKGSLVAPDRLRFDFTA

KGAMSTQQIKKAEEIANEMIEAAKPVYTQDCPLAAAKAIQGLRAVFDETYPDPVRVVSIGIPVSELLEDPSGPAGSLTSV

EFCGGTHLRNSSHAGAFVIVTEEAIAKGIRRIVAVTGAEAQKALRKAESLKKLLSVMATKVKAQSVPNKDVQREIADLGE

ALATAVIPQWQKDELRETLKSLKKIMDDLDRASKADVQKRVLERTKQLIDNNPNQPLVILEMESGASAKALNEALKLFKT

HSPQTSAMLFTVDNEAGKITCLCQVPQNAANRGLKASEWVQQVSGLMDGKGGGKDVSAQATGKNVGCLQEALQLATSFAQ

LRLGDVKN

>XP_003939968.1 PREDICTED: alanine--tRNA synthetase, cytoplasmic [Saimiri boliviensis boliviensis]

MDSTLTASEIRQRFIEFFKRNEHTYVHSSATIPLDDPTLLFANAGMNQFKPIFLNTIDPSHPMAKLSRAANTQKCIRAGG

KHNDLDDVGKDVYHHTFFEMLGSWSFGDYFKELACKMALELLTQEFGIPIERLYVTYFGGDEAAGLEPDLECKQIWQNLG

LEDTKILPGNMKDNFWEMGDTGPCGPCSEIHYDRIGGRDAAHLVNQDDPNVLEIWNLVFIQYNRETDGILKPLPKKSIDT

GMGLERLVSVLQNKMSNYDTDLFVPYFEAIQKGTGARPYTGKVGAEDADGIDMAYRVLADHARTITVALADGGRPDNTGR

GYVLRRILRRAVRYAHEKLNASRGFFATLVDVVVRSLGDAFPELKKDPDMVKDIINEEEVQFLKTLSRGRRILDRKILSL

GDSKTIPGDTAWLLYDTYGFPVDLTGLIAEEKGLLVDMDGFEEERKLAQLKSQGKGAGGEDFIMLDIYAIEELRAQGLEV

TDDSPKYNYHSDSSGNYVFENTVATVLALRREKMFVEEVSTGQECGVVLDKTCFYAEQGGQIYDEGYLVKVDDSSEDKTE

FTVKNAQVRGGYVLHIGTIYGDLKVGDQVQLFIDEPRRRPVMSNHTATHILNFALRSVLGEADQKGSLVAPDRLRFDFTA

KGAMSTQQIKRAEEIANEMIEAAKPVYTQDCPLAAAKAIQGLRAVFDETYPDPVRVVSIGVPVSELLDDPSGPAGSLTSV

EFCGGTHLQNSSHAGAFVIVTEEAIAKGIRRIVAVTGAEAQKALRKAESLRTSLSVMEAKVRAQTAPNKDVQREIADLGE

VMATAVIPQWQKDELRETLKSLKKVMDDLDRASKADVQKRVLEKTKQLIDSNPNQPLVILEMESGASAKALNEALKLFKM

HSPQTSAMLFTVDNEAGKITCLCQVPQNAANRGLKASEWVQQVSGLMDGKGGGKDVSAQATGKNVGCLQEALQLATSFAQ

LRLSDVKN

>EHH31841.1 hypothetical protein EGK_12990 [Macaca mulatta]

MDSTLTASEIRQRFIDFFKRNEHTYIHSSATIPLDDPTLLFANAGMNQFKPIFLNTIDPSHPMAKLSRAANTQKCIRAGG

KHNDLDDVGKDVYHHTFFEMLGSWSFGDYFKELACKMALELLTQEFGIPIGRLYVTYFGGDEAAGLEPDLECKQIWQNLG

AYLYSFVRLDDTKILPGNMKDNFWEMGDTGPCGPCSEIHYDRIGGRDAAHLVNQDDPNVLEIWNLVFIQYNREADGILKP

LPKKSIDTGMGLERLVSVLQNKMSNYDTDLFVPYFEAIQKGTGARPYTGKVGAEDADGIDMAYRVLADHARTITVALADG

GRPDNTGRGYVLRRILRRAVRYAHEKLNASRGFFATLVDVVVQSLGDAFPELKKDPDMVKDIVNEEEVQFLKTLSRGRRI

LDRKIQSLGDSKTIPGDTAWLLYDTYGFPVDLTGLIAEEKGLVVDMDGFEEERKLAQLKSQGKGAGGEDLIMLDIYAIEE

LRARGLEVTDDSPKYNYHLDFSGSYVFENTVATVMALRREKMFVEEVSTGQECGVVLDKTCFYAEQGGQIYDEGYLVKVD

DSSEDKTEFTVKNAQVRGGYVLHIGTIYGDLKVGDQVRLFIDEPRRRPIMSNHTATHILNFALRSVLGEADQKGSLVAPD

RLRFDFTAKGAMSTQQIKKAEEIANEMIEAAKPVYTQDCPLAAAKAIQGLRAVFDETYPDPVRVVSIGVPVSELLDDPSG

PAGSLTSVEFCGGTHLRNSSHAGAFVIVTEEAIAKGIRRIVAVTGAEAQKVLRKAESLKKSLSVMEAKVKAQTAPNKDVQ

REIADLGEALATAVIPQWQKDELRETLKSLKKVMDDLDRASKADVQKRVLEKTKQLIDSNPNQPLVILEMESGASAKATQ

GPGPPPLGHISPLALNEALKLFKTHSPQTSAMLFTVDNEAGKITCLCQVPQNAANRGLKASEWVQQVSGLMDGKGGGKDV

SAQATGKNIGCLQEALQLATSFAQLRLGDVKN

>XP_012661601.1 alanine--tRNA synthetase, cytoplasmic [Otolemur garnettii]

MDSTLTASEIRQRFIDFFKRNEHTYVHSSATIPLDDPTLLFANAGMNQFKPIFLNTIDPSHPMAKLSRAANTQKCIRAGG

KHNDLDDVGKDVYHHTFFEMLGSWSFGDYFKELACKMALELLTQEFGIPVERLYVTYFGGDEAAGLEPDLECKQIWQNLG

LDDGKILPGNMKDNFWEMGDTGPCGPCSEIHYDRIGGRDAAHLVNQDDPNVLEIWNLVFIQYNRETDGILKPLPKKSIDT

GMGLERLVSVLQNKMSNYDTDLFVPYFEAIQKGTGARPYTGKVGADDADGIDMAYRVLADHARTITVALADGGRPDNTGR

GYVLRRILRRAVRYSHEKLNASRGFFATLVDVVVQSLGEAFPELKKDPDMVKDIINEEEVQFLKTLSRGRRILDRKIQSL

GETKTIPGDTAWLLYDTYGFPVDLTGLIAEEKGLVVDMDGFEEERKLAQLKSQGKGAGGEDLIMLDIYAIEELRAKGLEV

TDDSPKYNYHSDSSGSYVFENTVATVMALRREKMFVEEVSTGQECGVVLDKTCFYAEQGGQIYDEGYLVKVDDSSEDKTE

FTVKNAQVRGGYVLHIGTIYGDLKVGDQVRLFIDEPRRRPVMSNHTATHILNFALRSVLGEADQKGSLVAPDRLRFDFTA

KGAMSTQQIKKAEEIANEMIEAAKPVYTQDCPLSTAKAIQGLRAVFDETYPDPVRVVSIGVPVSKLLDDPSGPAGSLTSV

EFCGGTHLRNSSHAGAFVIVTEEAIAKGIRRIVAVTGAEAQKALRKAENLKTSLSIMEAKVKVQTAPNKDVQREIADLGE

SLATAVIPQWQKDELRETLKSLKKIMDDLDRASKADVQKRVLEKTKQLIDSNPNQPLVILEMESGASAKALNEALKLFKT

HSPQTSAMLFAVDNEAGKITCLCQVPQNAANRGLKASEWVQQVSGLMDGKGGGKDMSAQATGKNVGCLQEALQLATSFAQ

LRLGDVKN

>XP_014644055.1 PREDICTED: alanine--tRNA synthetase, cytoplasmic [Ceratotherium simum simum]

MDSTLTASEIRQRFIDFFKRNEHTYVHSSATIPLDDPTLLFANAGMNQFKPIFLNTIDPSHPMAKLSRAANTQKCIRAGG

KHNDLDDVGKDVYHHTFFEMLGSWSFGDYFKELACKMALELLTQEFGIPVERLYVTYFGGDEAAGLEPDLECKQIWQNLG

LDDTKILPGNMKDNFWEMGDTGPCGPCSEIHYDRIGGRDAAHLVNQDDPNVLEIWNLVFIQYNRETDGILKPLPKKSIDT

GMGLERLVSVLQNKMSNYDTDLFVPYFEAIQKGTGARPYTGKVGADDADGIDMAYRVLADHARTITVALADGGRPDNTGR

GYVLRRILRRAVRYSHEKLNASRGFFATLVDVVVQSLGDAFPELKKDPDMVKDIINEEEVQFLKTLSRGRRILDRKIQSL

GDSKTIPGDTAWLLYDTYGFPVDLTGLIAEEKGLVVDMDGFEEERKLAQLKSQGKGAGGEDLIMLDIYAIEELREKGLEA

TDDSPKYNYHSDSSGSYVFESAVATVMALRRDKMFVEEVSTGQECGVMLDKTCFYAEQGGQIYDEGYLVKVDDSSEDKTE

FTVKNAQVRGGYILHIGTIYGNLKVGDQVRLFIDEPRRRPVMSNHTATHILNFALRSVLGEADQRGSLVAPDRLRFDFTA

KGAMSTQQIKKAEEIANEMIEAAKPVYTQDCPLAAAKAIQGLRAVFDETYPDPVRVVSIGVPVSELLDDPSGPAGSLTSV

EFCGGTHLQNSSHAGAFVIVSEEAIAKGIRRIVAVTGAEAQKALRKAESLKKSLSVMEAKVKAQTAPNKDVQREIADLGE

ALATAVLPQWQKDEFRDNLKSLKKIMDDLDRASKADVQKRVLEKTKQLIDSNPNQPLVILEMESGASAKALNEALKLFKT

HSPQTSAMLFTVDNEAGRITCLCQVPQNAANRGLKASEWVQQVSGLMDGKGGGKDVSAQATGKNVGCLQEALQLATSFAQ

LRLGDVKN

>XP_005318473.1 alanine--tRNA synthetase, cytoplasmic [Ictidomys tridecemlineatus]

MDSTLTASEIRARFIDFFKRNEHTYVHSSATIPLDDPTLLFANAGMNQFKPIFLNTIDPSHPMAKLSRAANTQKCIRAGG

KHNDLDDVGKDVYHHTFFEMLGSWSFGDYFKELACKMALELLTQEFGIPVERLYVTYFGGDEAAGLEPDLECKQIWQNLG

LDDTKILPGNMKDNFWEMGDTGPCGPCSEIHYDRIGGRDAAHLVNQDDPNVLEIWNLVFIQYNRETDGILKPLPKKSIDT

GMGLERLVSVLQNKMSNYDTDLFVPYFEAIQKGTGARPYTGKVGAEDADGIDMAYRVLADHARTITVALADGGRPDNTGR

GYVLRRILRRAVRYSHEKLNASRGFFATLVDVVVQSLGDAFPELKKDPDMVKDIINEEEVQFLKTLSRGRRILDRKIQSL

GDCKTIPGDTAWLLYDTYGFPVDLTGLIAEEKGMVVDMDGFEEERKLAQLKSQGKGAGGEDLIMLDIYAIEELRAKGLEA

TDDSPKYNYYSDSNGSYVFESAVATVLALRREKMFVEEVSTGQECGVVLDKTCFYAEQGGQIYDEGYLVKVDDCSEDKTE

FTVKNAQVRGGYVLHIGTIYGNLKVGDQVRLFIDEPRRRPVMSNHTATHILNFALRSVLGEADQKGSLVAPDRLRFDFTA

KGAMSTQQIKKAEEIANEMIEAAKPVYTQDCPLAAAKAIQGLRAVFDETYPDPVRVVSIGVPVSELLDDPSGPAGSLTSV

EFCGGTHLRNSSHAGAFVIVTEEAIAKGIRRIVAVTGAEAQKALRKAESLKKSLSVMEAKVKAQTAPNKDVQREIADLGE

ALATAVIPQWQKDELRETLKSLKKIMDDLDRASKADVQKRVLERTKQLIDSNPNQPLVILEMESGASAKALNEALKLFKT

HSPQTSAMLFTVDNEAGKITCLCQVTQNAANRGLKASEWVQQVSGLMDGKGGGKDVSAQATGKNVGCLQEALQLATSFAQ

LRLGDVKN

>XP_015351567.1 PREDICTED: alanine--tRNA synthetase, cytoplasmic [Marmota marmota marmota]

MDSTLTASEIRARFIDFFKRNEHTYVHSSATIPLDDPTLLFANAGMNQFKPIFLNTIDPSHPMAKLSRAANTQKCIRAGG

KHNDLDDVGKDVYHHTFFEMLGSWSFGDYFKELACKMALELLTQEFGIPVERLYVTYFGGDEAAGLEPDLECKQIWQNLG

LDDTKILPGNMKDNFWEMGDTGPCGPCSEIHYDRIGGRDAAHLVNQDDPNVLEIWNLVFIQYNRETDGILKPLPKKSIDT

GMGLERLVSVLQNKMSNYDTDLFVPYFEAIQKGTGARPYTGKVGAEDADGIDMAYRVLADHARTITVALADGGRPDNTGR

GYVLRRILRRAVRYSHEKLNASRGFFATLVDVVVQSLGDAFPELKKDPDMVKDIINEEEVQFLKTLSRGRRILDRKIQSL

GDCKTIPGDTAWLLYDTYGFPVDLTGLIAEEKGMVVDMDGFEEERKLAQLKSQGKGAGGEDLIMLDIYAIEELRATGLEA

TDDSPKYNYYSDSNGSYVFESAVATVLALRREKMFVEEVSTGQECGVVLDKTCFYAEQGGQIYDEGYLVKVDDCSEDKTE

FTVKNAQVRGGYVLHIGTIYGNLKVGDQVRLFIDEPRRRPVMSNHTATHILNFALRSVLGEADQKGSLVAPDRLRFDFTA

KGAMSTQQIKKAEEIANEMIEAAKPVYTQDCPLAAAKAIQGLRAVFDETYPDPVRVVSIGVPVSELLDDPSGPAGSLTSV

EFCGGTHLRNSSHAGAFVIVTEEAIAKGIRRIVAVTGAEAQKALRKAESLKKSLSVMEAKVKAQTAPNKDVQREIADLGE

ALATAVIPQWQKDELRETLKSLKKIMDDLDRASKADVQKRVLERTKQLIDSNPNQPLVILEMESGASAKALNEALKLFKT

HSPQTSAMLFTVDNEAGKITCLCQVTQNAANRGLKASEWVQQVSGLMDGKGGGKDVSAQATGKNVGCLQEALQLATSFAQ

LRLGDVKN

>XP_023493081.1 alanine--tRNA synthetase, cytoplasmic isoform X2 [Equus caballus]

MDSTLTASEIRQRFIDFFKRNEHTYVHSSATIPLDDPTLLFANAGMNQFKPIFLNTIDPSHPMAKLSRAANTQKCIRAGG

KHNDLDDVGKDVYHHTFFEMLGSWSFGDYFKELACKMALELLTQEFGIPIERLYVTYFGGDEAAGLEPDLECKQIWQNLG

LDDTKILPGNMKDNFWEMGDTGPCGPCSEIHYDRIGGRDAAHLVNQDDPNVLEIWNLVFIQYNRETDGVLKPLPKKSIDT

GMGLERLVSVLQNKMSNYDTDLFVPYFEAIQKGTGARPYAGKVGADDADGIDMAYRVLADHARTITVALADGGRPDNTGR

GYVLRRILRRAVRYSHEKLNASRGFFATLVDVVVQSLGDAFPELKKDPDMVKDIINEEEVQFLKTLSRGRRILDRKIQSL

GESKTIPGDTAWLLYDTYGFPVDLTGLIAEEKGLVVDMDGFEEERKLAQLKSQGKGAGGEDLIMLDIYAIEELREKGLEA

TDDSPKYNYYSDSSGSYVFESAVATVMALRRDKMFVEEVSTGQECGVVLDKTCFYAEQGGQIYDEGYLVKVDDSSEDKTE

FTVKNAQVRGGYVLHIGTIYGNLKVGDQVRLFIDEPRRRPVMSNHTATHILNFALRSVLGEADQRGSLVAPDRLRFDFTA

KGAMSTQQIKKAEEIANEMIEAAKPVYTQDCSLAAAKAIQGLRAVFDETYPDPVRVVSIGVPVSELLDDPSGPAGSLTSV

EFCGGTHLQNSSHAGAFVIVSEEAIAKGIRRIVAVTGAEAQKALRKAESLKKSLSVMEGKVKAQTAPNKDVQREIADLGE

ALATAVIPQWQKDEFRENLKSLKKIMDDLDRASKADVQKRVLEKTKQLIDSNPNQPLVILEMESGASAKALNEALKLFKT

HSPQTSAMLFTVDNEAGKITCLCQVPQNAANRGLKASEWVQQVSGLMDGKGGGKDVSAQATGKNVGCLQEALQLATSFAQ

LRLGDVKN

>XP_001501062.4 alanine--tRNA synthetase, cytoplasmic isoform X1 [Equus caballus]

MSRRGKARRGTFYRRALIGRGGASCGRRWRGAGAAAHLREQFCWVTFKMDSTLTASEIRQRFIDFFKRNEHTYVHSSATI

PLDDPTLLFANAGMNQFKPIFLNTIDPSHPMAKLSRAANTQKCIRAGGKHNDLDDVGKDVYHHTFFEMLGSWSFGDYFKE

LACKMALELLTQEFGIPIERLYVTYFGGDEAAGLEPDLECKQIWQNLGLDDTKILPGNMKDNFWEMGDTGPCGPCSEIHY

DRIGGRDAAHLVNQDDPNVLEIWNLVFIQYNRETDGVLKPLPKKSIDTGMGLERLVSVLQNKMSNYDTDLFVPYFEAIQK

GTGARPYAGKVGADDADGIDMAYRVLADHARTITVALADGGRPDNTGRGYVLRRILRRAVRYSHEKLNASRGFFATLVDV

VVQSLGDAFPELKKDPDMVKDIINEEEVQFLKTLSRGRRILDRKIQSLGESKTIPGDTAWLLYDTYGFPVDLTGLIAEEK

GLVVDMDGFEEERKLAQLKSQGKGAGGEDLIMLDIYAIEELREKGLEATDDSPKYNYYSDSSGSYVFESAVATVMALRRD

KMFVEEVSTGQECGVVLDKTCFYAEQGGQIYDEGYLVKVDDSSEDKTEFTVKNAQVRGGYVLHIGTIYGNLKVGDQVRLF

IDEPRRRPVMSNHTATHILNFALRSVLGEADQRGSLVAPDRLRFDFTAKGAMSTQQIKKAEEIANEMIEAAKPVYTQDCS

LAAAKAIQGLRAVFDETYPDPVRVVSIGVPVSELLDDPSGPAGSLTSVEFCGGTHLQNSSHAGAFVIVSEEAIAKGIRRI

VAVTGAEAQKALRKAESLKKSLSVMEGKVKAQTAPNKDVQREIADLGEALATAVIPQWQKDEFRENLKSLKKIMDDLDRA

SKADVQKRVLEKTKQLIDSNPNQPLVILEMESGASAKALNEALKLFKTHSPQTSAMLFTVDNEAGKITCLCQVPQNAANR

GLKASEWVQQVSGLMDGKGGGKDVSAQATGKNVGCLQEALQLATSFAQLRLGDVKN

>XP_014684619.1 PREDICTED: alanine--tRNA synthetase, cytoplasmic isoform X1 [Equus asinus]

MDSTLTASEIRQRFIDFFKRNEHTYVHSSATIPLDDPTLLFANAGMNQFKPIFLNTIDPSHPMAKLSRAANTQKCIRAGG

KHNDLDDVGKDVYHHTFFEMLGSWSFGDYFKELACKMALELLTQEFGIPIERLYVTYFGGDEAAGLEPDLECKQIWQNLG

LDDAKILPGNMKDNFWEMGDTGPCGPCSEIHYDRIGGRDAAHLVNQDDPNVLEIWNLVFIQYNRETDGVLKPLPKKSIDT

GMGLERLVSVLQNKMSNYDTDLFVPYFEAIQKGTGARPYAGKVGADDADGIDMAYRVLADHARTITVALADGGRPDNTGR

GYVLRRILRRAVRYSHEKLNASRGFFATLVDVVVQSLGDAFPELKKDPDMVKDIINEEEVQFLKTLSRGRRILDRKIQSL

GESKTIPGDTAWLLYDTYGFPVDLTGLIAEEKGLVVDMDGFEEERKLAQLKSQGKGAGGEDLIMLDIYAIEELREKGLEA

TDDSPKYNYYSDSSGSYVFESAVATVMALRRDKMFVEEVSTGQECGVVLDKTCFYAEQGGQIYDEGYLVKVDDSSEDKTE

FTVKNAQVRGGYVLHIGTIYGNLKVGDQVRLFIDEPRRRPVMSNHTATHILNFALRSVLGEADQRGSLVAPDRLRFDFTA

KGAMSTQQIKKAEEIANEMIEAAKPVYTQDCSLAAAKAIQGLRAVFDETYPDPVRVVSIGVPVSELLDDPSGPAGSLTSV

EFCGGTHLQNSSHAGAFVIVSEEAIAKGIRRIVAVTGAEAQKALRKAESLKKSLSVMEGKVKAQTAPNKDVQREIADLGE

ALATAVIPQWQKDEFRENLKSLKKIMDDLDRASKADVQKRVLEKTKQLIDSNPNQPLVILEMESGASAKALNEALKLFKT

HSPQTSAMLFTVDNEAGKITCLCQVPQNAANRGLKASEWVQQVSGLMDGKGGGKDVSAQATGKNVGCLQEALQLATSFAQ

LRLGDVKN

>XP_008520581.1 PREDICTED: alanine--tRNA synthetase, cytoplasmic [Equus przewalskii]

MSRRGKARRGTFYRRALIGRGGASCGRRWRGAGAAAHLREQFCWVTFKMDSTLTASEIRQRFIDFFKRNEHTYVHSSATI

PLDDPTLLFANAGMNQFKPIFLNTIDPSHPMAKLSRAANTQKCIRAGGKHNDLDDVGKDVYHHTFFEMLGSWSFGDYFKE

LACKMALELLTQEFGIPIERLYVTYFGGDEAAGLEPDLECKQIWQNLGLDDTKILPGNMKDNFWEMGDTGPCGPCSEIHY

DRIGGRDAAHLVNQDDPNVLEIWNLVFIQYNRETDGVLKPLPKKSIDTGMGLERLVSVLQNKMSNYDTDLFVPYFEAIQK

GTGARPYAGKVGADDADGIDMAYRVLADHARTITVALADGGRPDNTGRGYVLRRILRRAVRYSHEKLNASRGFFATLVDV

VVQSLGDAFPELKKDPDMVKDIINEEEVQFLKTLSRGRRILDRKIQSLGESKTIPGDTAWLLYDTYGFPVDLTGLIAEEK

GLVVDMDGFEEERKLAQLKSQGKGAGGEDLIMLDIYAIEELREKGLEATDDSPKYNYYSDSSGSYVFESAVATVMALRRD

KMFVEEVSTGQECGVVLDKTCFYAEQGGQIYDEGYLVKVDDSSEDKTEFTVKNAQVRGGYVLHIGTIYGNLKVGDQVRLF

IDEPRRRPVMSNHTATHILNFALRSVLGEADQRGSLVAPDRLRFDFTAKGAMSTQQIKKAEEIANEMIEAAKPVYTQDCS

LAAAKAIQGLRAVFDETYPDPVRVVSIGVPVSELLDDPSGPAGSLTSVEFCGGTHLQNSSHAGAFVIVSEEAIAKGIRRI

VAVTGAEAQKALRKAESLKKSLSLMEGKVKAQTAPNKDVQREIADLGEALATAVIPQWQKDEFRENLKSLKKIMDDLDRA

SKADVQKRVLEKTKQLIDSNPNQPLVILEMESGASAKALNEALKLFKTHSPQTSAMLFTVDNEAGKITCLCQVPQNAANR

GLKASEWVQQVSGLMDGKGGGKDVSAQATGKNVGCLQEALQLATSFAQLRLGDVKN

>XP_008255830.1 PREDICTED: alanine--tRNA synthetase, cytoplasmic [Oryctolagus cuniculus]

MDSFLTASEIRQRFIDFFKRNEHTYVHSSGTIPLDDPTLLFANAGMNQFKPIFLNTIDPTHPMAKLSRAANTQKCIRAGG

KHNDLDDVGKDVYHHTFFEMLGSWSFGDYFKELACKMALELLTQEFGIPIERLYVTYFGGDEAAGLEPDLECKQIWQNLG

LDDTKILPGNMKDNFWEMGDTGPCGPCSEIHYDRIGGRDAAHLVNQDDPNVLEIWNLVFIQYNRETDGVLKPLPKKSIDT

GMGLERLVSVLQNKMSNYDTDLFVPYFEAIQKGTGARPYTGKVGAEDVDGIDMAYRVLADHARTITVALADGGRPDNTGR

GYVLRRILRRAVRYSHEKLNASRGFFATLVDVVVQSLGDAFPELKKDPDMVKDIINEEEVQFLKTLSRGRRILDRKIQSL

GDCKTIPGDTAWLLYDTYGFPVDLTGLIAEEKGLVVDMDGFEEERKLAQLKSQGKGAGGEDLIMLDIYAIEELRAKGLEA

TDDSPKYNYQADSSGSYVFESTVATVLALRREKMFVEEVSTGQECGVVLDKTCFYAEQGGQIYDEGYLVKVDDSSEDKTE

FTVKNAQVRGGYVLHIGTVYGSLRVGDQVRLFIDEPRRRPVMSNHTATHILNFALRSVLGEADQKGSLVAPDRLRFDFTA

KGAMSTQQIKKAEEIANEMIAAAKPVYTQDCALAAAKAIQGLRAVFDETYPDPVRVVSIGVPVSELLEDPSGPAGSLTSV

EFCGGTHLRNSSHAGDFVIVTEEAIAKGIRRIVAVTGAEAQKALRKAESLKKSLSVMEAKVKAQTAPNKDVQKEIADLGE

ALAAAVIPQWQKDELRETLKSLKKIMDDLDRASKADVQKRVLEKTKQLIDSNPNQPLVILEMESGASAKALNEALKLFKT

HSPQTSAMLFTVDNEAGKITCLCQVPQNAANRGLKASEWVQQVSGLMDGKGGGKDMSAQATGKNVGCLQEALQLATSFAQ

LRLGDVKN

>XP_007198321.1 PREDICTED: alanine--tRNA synthetase, cytoplasmic isoform X1 [Balaenoptera acutorostrata scammoni]

MDSTLTASEIRQRFIDFFKRNEHTYVHSSATIPLDDPTLLFANAGMNQFKPIFLNTVDPSHPMAKLSRAANTQKCIRAGG

KHNDLDDVGKDVYHHTFFEMLGSWSFGDYFKELACKMALELLTQEFGIPVERLYVTYFGGDEAAGLEPDLECKQIWQNLG

LDDSKILPGNMKDNFWEMGDTGPCGPCSEIHYDRIGGRDAAHLVNQDDPNVLEIWNLVFIQYNRETDGILKPLPKKSIDT

GMGLERLVSVLQNKMSNYDTDLFVPYFEAIQKGTGARPYTGKVGADDADGIDMAYRVLADHARTITVALADGGRPDNTGR

GYVLRRILRRAVRYSHEKLNASRGFFATLADVVVQSLGDAFPELKKDPDMVKDIINEEEVQFLKTLSRGRRILDRKIQSL

GDSKTIPGDTAWLLYDTYGFPVDLTGLIAEEKGLVVDMDGFEEERKLAQLKSQGKGAGGEDLIMLDIYAIEELREKGLEA

TDDSPKYNYHSDSSGSYVFESAVATVMALRRDKMFVEEVSTGQECGVVLDKTCFYAEQGGQIYDEGYLVKVDDSSEDKTE

FTVKNAQVRGGYVLHIGTIYGNLKVGDQVRLFIDEPRRRPVMSNHTATHILNFALRSVLGEADQRGSLVAPDRLRFDFTA

KGAMSTQQIKKAEEIANEMIEAAKPVYTQDCPLAAAKAIQGLRAVFDETYPDPVRVVSIGVPVSELLDDPSGPAGSLTSV

EFCGGTHLQNSSHAGAFVIVSEEAIAKGIRRIVAVTGAEAQKALRKAESLKKSLSVTEAKVKAQTAPNKDVQREIADLGE

ALATAVIPQWQKDEFRENLRSLKKIMDDLDRANKADVQKRVLEKTKQLIDSSPNQPLVILEMESGASAKALNEALKLFKT

HSPETSAMLFTVDNEAGKITCLCQVPQNAANRGLKASEWVQQVSGLMDGKGGGKDVSAQATGKNVGCLQEALQLATSFAQ

LHLGDVKN

>XP_003998291.2 alanine--tRNA synthetase, cytoplasmic [Felis catus]

MDSTLTASEIRQRFIDFFKRNEHTYVHSSATIPLDDPTLLFANAGMNQFKPIFLNTIDPSHPMAKLSRAANTQKCIRAGG

KHNDLDDVGKDVYHHTFFEMLGSWSFGDYFKELACKMALELLTQEFGIPVERLYVTYFGGDEAAGLEPDLECKQIWQNLG

LDDSKILPGNMKDNFWEMGDTGPCGPCSEIHYDRIGGRDAAHLVNQDDPNVLEIWNLVFIQYNREADGILKPLPKKSIDT

GMGLERLVSVLQNKMSNYDTDLFVPYFEAIQKGTGARPYTGKVGADDADGIDMAYRVLADHARTITVALADGGRPDNTGR

GYVLRRILRRAVRYSHEKLNASRGFFATLVDVVVQSLGDAFPELKKDPDMVKDIINEEEVQFLKTLSRGRRILDRKIQSL

GDCKTIPGDTAWLLYDTYGFPVDLTGLIAEEKGLVVDMDGFEEERKLAQLKSQGKGAGAEDLIMLDIYAIEELREKGLEA

TDDSPKYNYHSDSSGSYAFESAVATVMALRRDKMFVEEVSTGQECGVVLDKTCFYAEQGGQIYDEGYLVKVEDSSEDKTE

FTVKNAQVRGGYVLHIGTIYGSLRVGDQVRLFIDEPRRRPIMSNHTATHILNFALRSVLGEADQRGSLVAPDRLRFDFTA

KGAMSTQQIKEAEEIANGMIEAAKPVYTQDCPLAAAKAIQGLRAVFDETYPDPVRVVSIGVPVSELLDDPSGPAGSLTSV

EFCGGTHLQNSSHAGAFVIVSEEAIAKGIRRIVAVTGAEAQKALRKAESLKKSLSVMEAKVKAQTAPNKDVQREIADLGE

ALATVVIPQWQKDEFRENLKSLKKIMDDLDRASKADVQKRVLEKTKQLIDSNPNQPLVILEMENGASAKALNEALKLFKT

HSPQTSAMLFTVDNEAGKITCLCQVPQNAANRGLKASEWVQQVSGLMDGKGGGKDVSAQATGKNVGCLQEALQLATSFAQ

LRLGDVKN

>XP_021488199.1 alanine--tRNA synthetase, cytoplasmic [Meriones unguiculatus]

MDSTLTAGEIRERFINFFKRNEHTYVHSSATIPLDDPTLLFANAGMNQFKPIFLNTIDPSHPMAKLSRAANTQKCIRAGG

KHNDLDDVGKDVYHHTFFEMLGSWSFGDYFKELACKMALELLTQEFGIPVERLYVTYFGGDEAAGLEPDLECRQIWQNLG

LDDAKILPGNMKDNFWEMGDTGPCGPCSEIHYDRIGGRDAAHLVNQDDPNVLEIWNLVFIQYNREADGVLKPLPKKSIDT

GMGLERLVSVLQNKMSNYDTDLFLPYFEAIQKGTGARPYTGKVGAEDVDGIDMAYRVLADHARTITVALADGGRPDNTGR

GYVLRRILRRAVRYSHEKLNASRGFFATLVDVVVQSLGDAFPELKKDPDMVKDIINEEEVQFLKTLSRGRRILDRKIQSL

GDCKTIPGDTAWLLYDTYGFPVDLTGLIAEEKGLVVDMDGFEEERKLAQLKSQGKGAGGEDLIMLDIYAIEELRAKGLEA

TDDSPKYNYHSDSSGSYVFDCTVATVLALRREKMFVEEVVTGQECGVVLDKTCFYAEQGGQIYDEGYLVKVDDSSEDKTE

FTVKNAQVRGGYVLHIGTVYGTLKVGDQVRLFIDEPRRRPVMSNHTATHILNFALRSVLGEADQKGSLVAPDRLRFDFTA

KGAMSTQQIKKAEEIANAMIEAAKPVYTQDCPLAAAKAIQGLRAVFDETYPDPVRVVSIGVPVSELLDDPSGPAGSLTSV

EFCGGTHLQNSSHAGAFVIVTEEAIAKGIRRIVAVTGAEAQKALRKAESLKKSLSTMEAKVKAQTAPNKDVQKEIADLGE

ALATAVIPQWQKDEQRETLKSLKKVMDDLDRASKADVQKRVLEKTKQLIDSNPNQPLVILEMESGASAKALNEALKLFKT

HSPQTSAMLFTVDNEAGKITCLCQVPQNAANRGLKASEWVQQVSGLMDGKGGGKDVSAQATGKNVGCLQEALQLATSFAQ

LRLGDVKN

>XP_004754080.1 PREDICTED: alanine--tRNA synthetase, cytoplasmic [Mustela putorius furo]

MDSTLTASEIRQRFIDFFKRNEHTYVHSSATIPLDDPTLLFANAGMNQFKPIFLNTIDPSHPMAKLSRAANTQKCIRAGG

KHNDLDDVGKDVYHHTFFEMLGSWSFGDYFKELACKMALELLTQEFGIPVERLYVTYFGGDEAAGLEPDLECKQIWQNLG

LDDSKILPGNMKDNFWEMGDTGPCGPCSEIHYDRIGGRDAAHLVNQDDPNVLEIWNLVFIQYNRESDGILKPLPKKSIDT

GMGLERLVSVLQNKMSNYDTDLFVPYFEAIQKGTGARPYSGKVGADDADGIDMAYRVLADHARTITVALADGGRPDNTGR

GYVLRRILRRAVRYSHEKLNASRGFFATLVDVVVQSLGDAFPELKKDPDMVKDIINEEEVQFLKTLSRGRRILDRKIQSL

GDCKTIPGDTAWLLYDTYGFPVDLTGLIAEEKGLVVDMDGFEEERKLAQLKSQGKGAGGEDLIMLDIYAIEELREKGLEA

TDDSPKYNYHSDSSGSYAFESAVATVMALRRDKMFVEEVCTGQECGVVLDKTCFYAEQGGQIYDEGYLVKVEDNSEDKTE

FTVKNAQVRGGYVLHIGTVYGSLRVGDQVRLFIDEPRRRPIMSNHTATHILNFALRSVLGEADQRGSLVAPDRLRFDFTA

KGAMSTQQIKKAEEIANQMIEAAKPVYTQDCPLAAAKAIQGLRAVFDETYPDPVRVVSIGVPVSELLDDPSGPAGSLTSI

EFCGGTHLRNSSHAGTFVIVSEEAIAKGIRRIVAVTGAEAQKALRKAESLKNSLSVMEAKVKAQTAPNKDVQREIADLGE

ALATAVIPQWQKDEFRENLKSLKKIMDDLDRASKADVQKRVLEKTKQLIDSNPNQPLVILEMESGASAKALNEALKLFKT

HSPQTSAMLFTVDNEAGKITCLCQVPQNAANRGLKASEWVQQVSGLMDGKGGGKDVSAQATGKNVGCLQEALELATSFAQ

LRLGDVKN

>XP_006172795.1 PREDICTED: alanine--tRNA synthetase, cytoplasmic [Camelus ferus]

MDSTLTASEIRQRFIDFFKRNEHTYVHSSATIPLDDPTLLFANAGMNQFKPIFLNTIDPSHPMAKLSRAANTQKCIRAGG

KHNDLDDVGKDVYHHTFFEMLGSWSFGDYFKGLACKMALELLTQEFGIPIERLYVTYFGGDEAAGLEPDLECKQIWQNLG

LDDAKILPGNMKDNFWEMGDTGPCGPCSEIHYDRIGGRDAAHLVNQDDPNVLEIWNLVFIQYNRENDGVLKPLPKKSIDT

GMGLERLVSVLQNKMSNYDTDLFVPYFEAIQKGTGARPYTGKVGADDADGIDMAYRVLADHARTITVALADGGRPDNTGR

GYVLRRILRRAVRYSHEKLNASRGFFATLVDVVVQSLGDAFPELKKDPDMVKDIINEEEVQFLKTLSRGRRILDRKIQSL

GDNRTIPGDTAWLLYDTYGFPVDLTGLIAEEKGLVVDMDGFEEERKLAQLKSQGKGASGEDLIMLDIYAIEELRERGLEA

TDDSPKYNYHSDSSGSYAFESAVATVMALRRDKMFVEEVSTGQECGVVLDKTCFYAEQGGQIYDEGYLVKVDDSSEDKTE

FTVKNAQVRGGYVLHIGTVYGSLKVGDQVRLFIDEPRRRPVMSNHTATHILNFALRSVLGEADQRGSLVAPDRLRFDFTA

KGAMSTQQIKKAEEIANEMIEAAKPVYTQDCPLAAAKAIQGLRAVFDETYPDPVRVVSIGVPVSELLDDPSGPAGSLTSV

EFCGGTHLQNSSHAGAFVIVSEEAIAKGIRRIVAVTGAEAQKALRKAESLKNSLSLMEAKVKAQTAPNKDVQREIADLGE

ALATAVIPQWQKDEFRENLRSLKKIMDDLDRASKADVQKRVLEKTKQLIDSNPNQPLVILEMESGASAKALNEALKLFKT

HSPQTSAMLFTVDNEAGKITCLCQVPQNAANRGLKASEWVQQVSGLMDGKGGGKDVSAQATGKNVGCLQEALQLATSFAQ

LHLGDVKN

>XP_006199751.1 PREDICTED: alanine--tRNA synthetase, cytoplasmic [Vicugna pacos]

MDSTLTASEIRQRFIDFFKRNEHTYVHSSATIPLDDPTLLFANAGMNQFKPIFLNTIDPSHPMAKLSRAANTQKCIRAGG

KHNDLDDVGKDVYHHTFFEMLGSWSFGDYFKELACKMALELLTQEFGIPIERLYVTYFGGDEAAGLEPDLECKQIWQNLG

LDDAKILPGNMKDNFWEMGDTGPCGPCSEIHYDRIGGRDAAHLVNQDDPNVLEIWNLVFIQYNRENDGVLKPLPKKSIDT

GMGLERLVSVLQNKMSNYDTDLFVPYFEAIQKGTGARPYTGKVGADDADGIDMAYRVLADHARTITVALADGGRPDNTGR

GYVLRRILRRAVRYSHEKLNASRGFFATLVDVVVQSLGDAFPELKKDPDMVKDIINEEEVQFLKTLSRGRRILDRKIQSL

GDNRTIPGDTAWLLYDTYGFPVDLTGLIAEEKGLVVDMDGFEEERKLAQLKSQGKGASGEDLIMLDIYAIEELRERGLEA

TDDSPKYNYHSDSSGSYAFESAVATVMALRRDKMFVEEVSTGQECGVVLDKTCFYAEQGGQIYDEGYLVKVDDSSEDKTE

FTVKNAQVRGGYVLHIGMVYGSLKVGDQVRLFIDEPRRRPVMSNHTATHILNFALRSVLGEADQRGSLVAPDRLRFDFTA

KGAMSTQQIKKAEEIANEMIEAAKPVYTQDCPLAAAKAIQGLRAVFDETYPDPVRVVSIGVPVSELLDDPSGPAGSLTSV

EFCGGTHLQNSSHAGAFVIVSEEAIAKGIRRIVAVTGAEAQKALRKAESLKNSLSLMEAKVKAQTAPNKDVQREIADLGE

ALATAVIPQWQKDEFRENLRSLKKIMDDLDRASKADVQKRVLEKTKQLIDSNPNQPLVILEMESGASAKALNEALKLFKT

HSPQTSAMLFTVDNEAGKITCLCQVPQNAANRGLKASEWVQQVSGLMDGKGGGKDVSAQATGKNVGCLQEALQLATSFAQ

LHLGDVKN

>XP_004659629.1 PREDICTED: alanine--tRNA synthetase, cytoplasmic [Jaculus jaculus]

MNSTLTASEIRERFISFFKRNEHTYVHSSATIPLDDPTLLFANAGMNQFKPIFLNTIDPSHPMAKLSRAANTQKCIRAGG

KHNDLDDVGKDVYHHTFFEMLGSWSFGDYFKELACKMALELLTQEFGIPIERLYVTYFGGDEAAGLEPDLECKQIWQNLG

VDDTKILPGNMKDNFWEMGDTGPCGPCSEIHYDRIGGRDAAHLVNQDDPNVLEIWNLVFIQYNREPDGLLKPLPKKSIDT

GMGLERLVSVLQNKMSNYDTDLFVPYFDAIQKGTGARPYTGKVGAEDADGIDMAYRVLADHARTITVALADGGRPDNTGR

GYVLRRILRRAVRYSHEKLNASRGFFATLVDVVVQSLGDAFPELKKDPDMVKDIINEEEVQFLKTLSRGRRILDRKIQSL

GDCKTIPGDTAWLLYDTYGFPVDLTGLIAEEKGLVVDMDGFEEERKLAQLKSQGKGAGGEDLIMLDIYAIEELRAKGLEA

TDDSPKYNYHADSSGSYVFENEVATVLALRREKMFVEEVVTGQECGVVLDKTCFYAEQGGQIYDEGYLVKADDSSEDKTE

FTVKNAQVRGGYVLHIGTIYGNLKVGDQVRLFIDETRRRPVMSNHTATHILNFALRSVLGEADQKGSLVAPDRLRFDFTA

KGAMSTQQIKKAEEIANELIEAAKPVYTQDCPLAAAKAIQGLRAVFDETYPDPVRIVSIGVPVSELLDDPSGPAGSLTSV

EFCGGTHLRNSSHAGAFVIVTEEAIAKGIRRIVAVTGAEAQKALRKAESLKKSLSAMEVKVKAQTAPNKDVQREIADLGE

ALATAVIPQWQKDEQRETLKSLKKVMDDLDRASKADVQKRVLEKTKQLIDSNPNQPLVILEMENGASAKALNEALKLFKT

HSPETSAMLFTVDNEAGKITCLCQVPQNAANRGLKASEWVQQVSGLMDGKGGGKDVSAQATGKNVGCLQEALQLATSFAQ

LHLGDVKN

>XP_003417126.1 alanine--tRNA synthetase, cytoplasmic [Loxodonta africana]

MDLTLTASEIRQRFIDFFKRNEHTYVHSSATIPLDDPTLLFANAGMNQFKPIFLNTIDPSHPMAKLSRAANTQKCIRAGG

KHNDLDDVGKDVYHHTFFEMLGSWSFGDYFKELACKMALELLTQEFGIPVERLYVTYFGGDEEAGLEPDLECKQIWQNLG

LDDTKILPGNMKDNFWEMGDTGPCGPCSEIHYDRIGGRDAAHLVNQDDPNVLEIWNLVFIQYNRETDGILKPLPKKSIDT

GMGLERLVSVLQNKMSNYDTDLFVPYFEAIQKGTGARPYTGKVGADDADGIDMAYRVLADHARTITVALADGGRPDNTGR

GYVLRRILRRAVRYSHEKLNANRGFFATLVDVVVQSLGDAFPELKKDPDMVKDIINEEEVQFLKTLSRGRRILDRKIQSL

GDSKTIPGDTAWLLYDTYGFPVDLTGLIAEEKGLAVDMDGFEEERKLAQLKSQGKGAGGEDLIMLDIYAIEELRSKGLEA

TDDSPKYNYHSDSSGSYVFENAVATVMALRRDKTFVEEVSTGQECGVVLDKTCFYAEQGGQIYDEGYLVKVDDNSEDKTE

FTVKNAQVRGGYVLHIGTIYGNLKVGDQVRLFIDEPRRRPVMSNHTATHILNFALRSVLGEADQKGSLVAPDRLRFDFTA

KGAMSTEQIKEAEEISNQMIEAAKPVYTQDCPLAAAKAIQGLRAVFDETYPDPVRVVSIGVPVSELLEDPSGPAGSLTSV

EFCGGTHLQNSSHAGAFVIVSEEAIAKGIRRIVAVTGAEAQKALRKAESLKKSLSVMEAKVKAQTAPNKDVQREIADLGE

ALATAVIPQWQKDELREHLKSLKKIMDDLDRASKADVQKRVLEKTKQLIDSHPNQPLVILEMESGASAKALNEALKLFKT

HSPQTAAMLFTVDNEAGKITCLCQVPQNAANRGLKASEWVQQVSGLMDGKGGGKDVSAQATGKNVGCLQEALQLATSFAQ

LHLGDVKN

>XP_019796718.1 PREDICTED: alanine--tRNA synthetase, cytoplasmic isoform X1 [Tursiops truncatus]

MDSTLTASEIRQRFIDFFKRNEHTYVHSSATIPLDDPTLLFANAGMNQFKPIFLNTVDPSHPMAKLSRAANTQKCIRAGG

KHNDLDDVGKDVYHHTFFEMLGSWSFGDYFKELACKMALELLTQEFGIPVERLYVTYFGGDEAASLEPDLECKQIWHNLG

LDDSKILPGNMKDNFWEMGDTGPCGPCSEIHYDRIGGRDAAHLVNQDDPNVLEIWNLVFIQYNRETDGILKPLPKKSIDT

GMGLERLVSVLQNKMSNYDTDLFVPYFEAIQKGTGARPYTGKVGAEDADGIDMAYRVLADHARTITVALADGGRPDNTGR

GYVLRRILRRAVRYSHEKLNASRGFFATLADVVVQSLGDAFPELKKDPDMVKDIINEEEVQFLKTLSRGRRILDRKIQSL

GDSKTIPGDTAWLLYDTYGFPVDLTGLIAEEKGLVVDMDGFEEERKLAQLKSQGKGAGGEDLIMLDIYAIEELREKGLEA

TDDSPKYNYHSDSSGSYVFESAVATVMALRRDKMFVEEVSTGQECGVVLDKTCFYAEQGGQIYDEGYLVKVDDSSEDKTE

FTVKNAQVRGGYVLHVGTIYGSLKVGDQVRLFIDEPRRRPVMSNHTATHILNFALRSVLGEADQRGSLVAPDRLRFDFTA

KGAMSTQQIKKAEEIANEMIEAAKPVYTQDCPLAAAKAIQGLRAVFDETYPDPVRVVSIGVPVSELLDDPSGPAGSLTSV

EFCGGTHLQNSSHAGAFVIVSEEAIAKGIRRIVAVTGAEAQKALRKAESLKKSLSVAEAKVKAQTAPNKDVQREIADLGE

ALATAVIPQWQKDEFRENLKSLKKIMDDLDRANKADVQKRVLEKTKQLIDSSPNQPLVILEMESGASAKALNEALKLFKT

HSPETSAMLFTVDNEAGKITCLCQVPQNAANRGLKASEWVQQVSGLMDGKGGGKDVSAQATGKNVGCLQEALQLAATFAQ

LHLGDVKN

>XP_012868374.1 PREDICTED: alanine--tRNA synthetase, cytoplasmic [Dipodomys ordii]

MDLPLTAREIRKRFIDFFQRNEHTYVHSSATIPLDDPTLLFANAGMNQFKPIFLNTIDPTHPMAKLSRAANTQKCIRAGG

KHNDLDDVGKDVYHHTFFEMLGSWSFGDYFKELACKMALELLTQEFGIPVERLYVTYFGGDEAAGLEPDLECKQIWQNLG

LDDTKILPGNMKDNFWEMGDTGPCGPCSEIHYDRIGGRDAAHLVNQDDPNVLEIWNLVFIQYNREMDGVLKPLPKKSIDT

GMGLERLVSVLQNKMSNYDTDLFVPYFEAIQKGTGARPYTGKVGAEDVDGIDMAYRVLADHARTITVALADGGRPDNTGR

GYVLRRILRRAVRYSHEKLNASRGFFATLVDVVVQSLGDAFPELKKDPDMVKDIINEEEVQFLKTLSRGRRILDRKIQSL

GDCKTIPGDTAWLLYDTYGFPVDLTGLIAEEKGLVVDMDGFEEERKLAQLKSQGKGAGGEDLIMLDIYAIEELRAKGLEA

TDDSPKYNYHSDSSGSYAFESTVATVLALCREKMFVEEVSTGQECGVVLDKTCFYAEQGGQIYDEGYLVKVDDSSDDKTE

FTVKNAQVRGGYVLHIGTIYGSLKVGDQVRLFLDEPRRRPVMSNHTATHILNFALRSVLGEADQKGSLVAPDRLRFDFTA

KGAMSTQQIKKAEEIANEMIEAAKPVYTQDCPLAAAKAIQGLRAVFDETYPDPVRVVSIGVPVSDLLDDPSGPAGSLTSV

EFCGGTHLQNSSHAGAFVIVTEEAIAKGIRRIVAVTGAEAQKALRKADSLKKSLCVMEAKVKAQTAPNKDVQREIADLGE

ALATAVIPQWQKDELRETLKSLKKVMDDLDRASKADVQKRVLEKTKQLIDSNPNQPLVILEMESGASAKALNEALKLFKT

HSPQTSAMLFTVDNEAGKITCLCQVPQNAANRGLKASEWVQQVSGLMDGKGGGKDVSAQATGKNVGCLQEALQLATSFAQ

LRLGDVKN

>XP_003472166.1 alanine--tRNA synthetase, cytoplasmic [Cavia porcellus]

MSSTLTASEIRERFIDFFKRNEHTYVHSSATIPLDDPTLLFANAGMNQFKPIFLNTIDPSHPLAKLSRAANTQKCIRAGG

KHNDLDDVGKDVYHHTFFEMLGSWSFGDYFKELACKMALELLTQEFGIPVDRLYVTYFGGDEAAGLEPDLECKQIWQNLG

LDDTKILPGNMKDNFWEMGDTGPCGPCSEIHYDRIGGRDAAHLVNQDDPNVLEIWNLVFIQYNRETDGTLKPLPKKSIDT

GMGLERLVSVLQNKMSNYDTDLFVPYFEAIQKGTGARPYTGKVGAEDADGIDMAYRVLADHARTITVALADGGRPDNTGR

GYVLRRILRRAVRYSHEKLNASRGFFATLVDVVVQSLGDAFPELKKDPDMVKDIINEEEVQFLKTLSRGRRILDRKIQSL

GDCKTIPGDTAWLLYDTYGFPVDLTGLIAEEKGLVVDMDGFEEERKLAQLKSHGKGAGGEDLIMLDIYAIEELRAKGLEA

TDDSPKYDYHSDSGGSYVFESAVATVLALRREKMFVEEVSTGQECGVVLDKTCFYAEQGGQIYDEGYLVKVDDSSEDKTE

FTVKNTQVRGGYVLHIGTIYGSLKVGDQVRLFIDEPRRRPIMSNHTATHILNFALRSVLGEADQKGSLVAPDRLRFDFTA

KGAMSTQQIKKAEEIANEMIEAAKPVYTQDCPLAAAKAIQGLRAVFDETYPDPVRVVSIGVPVSELLDDPSGPAGSLTSV

EFCGGTHLQNSSHAGAFVIVTEEAIAKGIRRIVAVTGAEAQKALRKAESLSKSLSAMESTVKAQTAPNKDVQREIADLGE

ALASAVIPQWQKDELRETLKSLKKVMDDLDRASKADVQKRVLERTKQLIDSNPNQPLVILEMESGASAKALNEALKLFKT

HSPETSAMLFTVDNEAGKITCLCQVPQNAANRGLKASEWVQQVSGLMDGKGGGKDMSAQATGKNVGCLQEALQLATSFAQ

LRLGDVKN

>XP_003513729.1 PREDICTED: alanine--tRNA synthetase, cytoplasmic [Cricetulus griseus]

MDSMLTAREIRERFINFFKKNEHTYVHSSATIPLDDPTLLFANAGMNQFKPIFLNTIDPSHPMAKLSRAANTQKCIRAGG

KHNDLDDVGKDVYHHTFFEMLGSWSFGDYFKELACKMALELLTQEFGIPVERLYVTYFGGDEAAGLEPDLECKQIWQNLG

LDDARILPGNMKDNFWEMGDTGPCGPCSEIHYDRIGGRDAAHLVNQDDPNVLEIWNLVFIQYNRETDGLLKPLPKKSIDT

GMGLERLVSVLQNKMSNYDTDLFVPYFEAIQKGTGARPYTGKVGAEDADGIDMAYRVLADHARTITVALADGGRPDNTGR

GYVLRRILRRAVRYSHEKLNASRGFFATLVDVVVQSLGDAFPELKKDPDMVKDIINEEEVQFLKTLSRGRRILDRKIQSL

GDCKTIPGDTAWLLYDTYGFPVDLTGLIAEEKGLVVDMDGFEEERKLAQLKSQGKGAGGEDLIMLDIYAIEELRAKGLEA

TDDSPKYNYHSDSNGSYVFECTVATVLALRREKMFVEEVVTGQECGVVLDKTCFYAEQGGQIYDEGYLVKVDDSSEDKTE

FTVKNTQVRGGYVLHIGTIYGNLKVGDQVRLFIDEPRRRPVMSNHTATHILNFALRSVLGEADQKGSLVAPDRLRFDFTA

KGAMSTQQIKEAEEIVNAMIEAAKPVYTQDCPLAAAKAIQGLRAVFDETYPDPVRVVSIGVPVSELLDDPSGPAGSLTSV

EFCGGTHLRNSSHAGAFVIVTEEAIAKGIRRIVAVTGAEAQKALRKAESLKRSLSVMETKVKAQTAPNKDVQREIADLGE

ALATAVIPQWQKDEQRETLKSLKKVMDDLDRASKADVQKRVLEKTKQLIDSNPNQPLVILEMESGASAKALNEALKLFKT

HSPQTSAMLFTVDNEAGKITCLCQVPQNAANRGLKASEWVQQVSGLMDGKGGGKDVSAQATGKNVGCLQEALQLATSFAQ

LRLGDVKN

>XP_008821199.1 PREDICTED: alanine--tRNA synthetase, cytoplasmic [Nannospalax galili]

MDSTLTAREIRERFISFFKRNEHTYVHSSATIPLDDPTLLFANAGMNQFKPIFLNTIDPSHPMAKLSRAANTQKCIRAGG

KHNDLDDVGKDVYHHTFFEMLGSWSFGDYFKELACKMALELLTQEFGIPVERLYVTYFGGDEAAGLEPDLECKQIWQNLG

LDDAKILPGNMKDNFWEMGDTGPCGPCSEIHYDRIGGRDAAHLVNQDDPNVLEIWNLVFIQYNRETDGVLKPLPKKSIDT

GMGLERLVSVLQNKMSNYDTDLFIPYFEAIQKGTGARPYTGKVGAEDVDGIDMAYRVLADHARTITVALADGGRPDNTGR

GYVLRRILRRAVRYSHEKLNASRGFFATLVDVVVQSLGDAFPELKKDPDMVKDIINEEEVQFLKTLSRGRRILDRKIQSL

GDCKTIPGDTAWLLYDTYGFPVDLTGLIAEEKGLVVDMDGFEEERKLAQLKSQGKGAGGEDLIMLDIYAIEELRATGLEA

TDDSPKYNYHADSSGSYVFESTEATVLALRREKMFVEEVFTGQECGVVLDKTCFYAEQGGQICDEGYLVKVDDSSEDKTE

FTVKNAQVRGGYVLHIGTVYGTLKVGDQVRLFIDEPRRRPIMSNHTATHILNFALRSVLGEADQKGSLVAPDRLRFDFTA

KGAMSTQQIKKAEEIANEVIEAAKPVYTQDCPLAAAKAIQGLRAVFDETYPDPVRVVSIGVPVSKLLDDPSGPAGSLTSV

EFCGGTHLRNSNHAGAFVIVTEEAIAKGIRRIVAVTGAEAQKALRKAESLKKSLSAMEAKVKAQTAPNKDVQREIADLGE

ALATAVIPQWQKDEQRETLKSLKKVMDDLDRASKADVQKRVLEKTKQLIDSNPNQPLVILEMESGASAKALNEALKLFKT

HSPQTSAMLFTVDNEAGKITCLCQVPQNAANQGLKASEWVQQVSGLMDGKGGGKDVSAQATGKNVGCLQEALQLATSFAQ

LRLGDVKN

>XP_022438919.1 alanine--tRNA synthetase, cytoplasmic [Delphinapterus leucas]

MDSTLTASEIRQRFIDFFKRNEHTYVHSSATIPLDDPTLLFANAGMNQFKPIFLNTVDPSHPMAKLSRAANTQKCIRAGG

KHNDLDDVGKDVYHHTFFEMLGSWSFGDYFKELACKMALELLTQEFGIPVERLYVTYFGGDEAAGLEPDLECKQIWHNLG

LDDSKILPGNMKDNFWEMGDTGPCGPCSEIHYDRIGGRDAAHLVNQDDPNVLEIWNLVFIQYNRETDGILKPLPKKSIDT

GMGLERLVSVLQNKMSNYDTDLFVPYFEAIQKGTGARPYTGKVGADDADGIDMAYRVLADHARTVTVALADGGRPDNTGR

GYVLRRILRRAVRYSHEKLNASRGFFATLADVVVQSLGDAFPELKKDPDMVKDIINEEEVQFLKTLSRGRRILDRKIQSL

GDSKTIPGDTAWLLYDTYGFPVDLTGLIAEEKGLVVDMDGFEEERKLAQLKSQGKGAGGEDLIMLDIYAIEELREKGLEA

TDDSPKYNYHSDSSGSYVFESAVATVMALRRDKMFVEEVSTGQECGVVLDKTCFYAEQGGQIYDEGYLVKVDDSSEDKTE

FTVKNAQVRGGYVLHVGTIYGSLKVGDQVRLFIDEPRRRPVMSNHTATHILNFALRSVLGEADQRGSLVAPDRLRFDFTA

KGAMSTQQIKKSEEIANEMIEAAKPVYTQDCPLAAAKAIQGLRAVFDETYPDPVRVVSIGVPVSELLDDPSGPAGSLTSV

EFCGGTHLQNSSHAGAFVIVSEEAIAKGIRRIVAVTGAEAQKALRKAESLKKSLSVAEAKVKAQTAPNKDVQREIADLGE

ALATAVIPQWQKDEFRENLKSLKKIMDDLDRANKADVQKRVLEKTKQLIDSSPNQPLVILEMESGASAKALNEALKLFKT

HSPETSAMLFTVDNEAGKITCLCQVPQNAANRGLKASEWVQQVSGLMDGKGGGKDVSAQATGKNVGCLQEALQLAATFAQ

LHLGDVKN

>XP_020017211.1 alanine--tRNA synthetase, cytoplasmic [Castor canadensis]

MDSTLTAREIRERFIDFFKRNEHTYVHSSATIPLDDPTLLFANAGMNQFKPIFLNTIDPTHPMAKLSRAANTQKCIRAGG

KHNDLDDVGKDVYHHTFFEMLGSWSFGDYFKELACKMALELLTQEFGIPVERLYVTYFGGDEAAGLEPDLECKQIWQNLG

LDDTKILPGNMKDNFWEMGDTGPCGPCSEIHYDRIGGRDAAHLVNQDDPNVLEIWNLVFIQYNRENDGVLKPLPKKSIDT

GMGLERLVSVLQNKMSNYDTDLFVPYFEAIQKGTGARPYTGKVGAEDVDGIDMAYRVLADHARTITVALADGGRPDNTGR

GYVLRRILRRAVRYSHEKLNASKGFFATLVDVVVQSLGDAFPELKKDPDMVKDIINEEEVQFLKTLSRGRRILDRKIQSL

GDCKTIPGDTAWLLYDTYGFPVDLTGLIAEEKGLVVDMDGFEEERKLAQLKSQGKGAGGEDLIMLDIYAIEELRGKCLEA

TDDSPKYNYHSDSSGSYVFESTVATVLALRRDKMFVEEVSTGQECGVVLDKTCFYAEQGGQIYDEGYLVKVDDSSDDKSE

FTVKNAQVRGGYVLHIGTIYGHLKVGDQVRLFIDEPRRRPVMSNHTATHILNFALRSVLGEADQKGSLVAPDRLRFDFTA

KGAMSTQQIKKAEEIANEMIEAAKPVYTQDCSLAAAKAIQGLRAVFDETYPDPVRVVSIGVPVSELLDDPSGPAGSLTSV

EFCGGTHLQNSSHAGAFVIVTEEAIAKGIRRIVAVTGAEAQKALRKAESLKKLLSVMEAKVKAQTAPNKDVQREITDLGE

ALATAVIPQWQKDELRETLKSLKKVMDDLDRASKADVQKRVLEKTKQLIDSNPNQPLVILEMESGASAKALNEALKLFKT

YSPQTSAMLFTVDNEAGKITCLCQVPQNAANRGLKASEWVQQVSGLMDGKGGGKDGSAQATGKNVGCLQEALQLATSFAQ

LRLGDVKN

>XP_006747779.1 PREDICTED: alanine--tRNA synthetase, cytoplasmic [Leptonychotes weddellii]

MDSTLTASEIRQRFIDFFKRNEHTYVHSSATIPLDDPTLLFANAGMNQFKPIFLNTIDPSHPMAKLSRAANTQKCIRAGG

KHNDLDDVGKDVYHHTFFEMLGSWSFGDYFKELACKMALELLTQEFGIPVERLYVTYFGGDEAAGLEPDLECKQIWQNLG

LDDTKILPGNMKDNFWEMGDTGPCGPCSEIHYDRIGGRDAAHLVNQDDPNVLEIWNLVFIQYNREGDGILKPLPKKSIDT

GMGLERLVSVLQNKMSNYDTDLFVPYFEAIQKGTGARPYAGKVGADDADGIDMAYRVLADHARTITVALADGGRPDNTGR

GYVLRRILRRAVRYSHEKLNASRGFFATLVDVVVQSLGDAFPELKKDPDMVKDIINEEEVQFLKTLSRGRRILDRKIQSL

GDCKTIPGDTAWLLYDTYGFPVDLTGLIAEEKGLVVDMDGFEEERKLAQLKSQGKGAGGEDLIMLDIYAIEELREKGLEA

TNDSPKYNYHSDSSGNYAFESAVATVLALRRDKMFVEEVSTGQECGVVLDKTCFYAEQGGQTYDEGYLVKVEDNSEDKTE

FTVKNAQVRGGYVLHIGTIYGSLRVGDQVWLFIDEARRRPIMSNHTATHILNFALRSVLGEADQRGSLVAPDRLRFDFTA

KGAMSTQQIKKAEEIANGMIEAAKPVYTQDCPLAAAKAIQGLRAVFDETYPDPVRVVSIGVPVSELLDDPSGPAGSLTSV

EFCGGTHLRNSSHAGAFVIVSEEAIAKGIRRIVAVTGAEAQKALRKAESLKNTLSVTEAKVKAQTAPNKDVQREIADLGE

ALATAVIPQWQKDEFREDLKSLKKIMDDLDRASKADVQKRVLEKTKQLIDSNPNQPLVILEMESGASAKALNEALKLFKT

HSPQTSAMLFTVDNEAGKITCLCQVPQNAANRGLKASEWVQQVSGLMDGKGGGKDVSAQATGKNVGCLQEALQLATSFAQ

LRLGDVKN

>XP_024591314.1 alanine--tRNA synthetase, cytoplasmic [Neophocaena asiaeorientalis asiaeorientalis]

MDSTLTASEIRQRFIDFFKRNEHTYVHSSATIPLDDPTLLFANAGMNQFKPIFLNTVDPSHPMAKLSRAANTQKCIRAGG

KHNDLDDVGKDVYHHTFFEMLGSWSFGDYFKELACKMALELLTQEFGIPVERLYVTYFGGDEAAGLEPDLECKQIWHNLG

LDDSKILPGNMKDNFWEMGDTGPCGPCSEIHYDRIGGRDAAHLVNQDDPNVLEIWNLVFIQYNRETDGILKPLPKKSIDT

GMGLERLVSVLQNKMSNYDTDLFVPYFEAIQKGTGARPYTGKVGADDADGIDMAYRVLADHARTITVALADGGRPDNTGR

GYVLRRILRRAVRYSHEKLNASRGFFATLADVVVQSLGDAFPELKKDPDMVKDIINEEEVQFLKTLSRGRRILDRKIQSL

GDSKTIPGDTAWLLYDTYGFPVDLTGLIAEEKGLVVDMDGFEEERKLAQLKSQGKGAGGEDLIMLDIYAIEELREKGLEA

TDDSPKYNYHSDSSGSYVFESAVATVMALRRDKMFVEEVSTGQECGVVLDKTCFYAEQGGQIYDEGYLVKVDDSSEDKTE

FTVKNAQVRGGYVLHVGTIYGSLKVGDQVRLFIDEPRRRPVMSNHTATHILNFALRSVLGEADQRGSLVAPDRLRFDFTA

KGAMSTQQIKKAEEIANEMIEAAKPVYTQDCPLAAAKAIQGLRAVFDETYPDPVRVVSIGVPVSELLDDPSGPAGSLTSV

EFCGGTHLQNSSHAGAFVIVSEEAIAKGIRRIVAVTGAEAQKALRKAESLKKSLCVAEAKVKAQTAPNKDVQREIADLGE

ALATAVIPQWQKDEFRENLKSLKKIMDDLDRANKADVQKRVLEKTKQLIDSSPNQPLVILEMESGASAKALNEALKLFKT

HSPETSAMLFTVDNEAGKITCLCQVPQNAANRGLKASEWVQQVSGLMDGKGGGKDVSAQATGKNVGCLQEALQLAATFAQ

LHLGDVKN

>XP_021558694.1 alanine--tRNA synthetase, cytoplasmic [Neomonachus schauinslandi]

MDSTLTASEIRQRFIDFFKRNEHTYVHSSATIPLDDPTLLFANAGMNQFKPIFLNTIDPSHPMAKLSRAANTQKCIRAGG

KHNDLDDVGKDVYHHTFFEMLGSWSFGDYFKELACKMALELLTQEFGIPVERLYVTYFGGDEAAGLEPDLECKQIWQNLG

LDDTKILPGNMKDNFWEMGDTGPCGPCSEIHYDRIGGRDAAHLVNQDDPNVLEIWNLVFIQYNREGDGILKPLPKKSIDT

GMGLERLVSVLQNKMSNYDTDLFVPYFEAIQKGTGARPYAGKVGADDADGIDMAYRVLADHARTITVALADGGRPDNTGR

GYVLRRILRRAVRYSHEKLNASRGFFATLVDVVVQSLGDAFPELKKDPDMVKDIINEEEVQFLKTLSRGRRILDRKIQSL

GDCKTIPGDTAWLLYDTYGFPVDLTGLIAEEKGLVVDMDGFEEERKLAQLKSQGKGAGGEDLIMLDIYAIEELREKGLEA

TNDSPKYNYHSDSSGSYAFESTVAAVLALRRDKMFVEEVSTGQECGVVLDKTCFYAEQGGQTYDEGYLVKVEDNSEDKTE

FTVKNAQVRGGYVLHIGTIYGSLRVGDQVWLFIDEARRRPIMSNHTATHILNFALRSVLGEADQRGSLVAPDRLRFDFTA

KGAMSTQQIKKAEEIANGMIEAAKPVYTQDCPLAAAKAIQGLRAVFDETYPDPVRVVSIGVPVSELLDDPSGPAGSLTSV

EFCGGTHLRNSSHAGAFVIVSEEAIAKGIRRIVAVTGAEAQKALRKAESLKNTLVVTEAKVKAQTAPNKDVQREIADLGE

ALATAVIPQWQKDEFREDLKSLKKIMDDLDRASKADVQKRVLEKTKQLIDSNPNQPLVILEMESGASAKALNEALKLFKT

HSPQTSAMLFTVDNEAGKITCLCQVPQNAANRGLKASEWVQQVSGLMDGKGGGKDVSAQATGKNVGCLQEALQLATSFAQ

LRLGDVKN

>XP_019310850.1 PREDICTED: alanine--tRNA synthetase, cytoplasmic [Panthera pardus]

MDSTLTASEIRQRFIDFFKRNEHTYVHSSATIPLDDPTLLFANAGMNQFKPIFLNTIDPSHPMAKLSRAANTQKCIRAGG

KHNDLDDVGKDVYHHTFFEMLGSWSFGDYFKELACKMALELLTQEFGIPVERLYVTYFGGDEAAGLEPDLECKQIWQNLG

LDDSKILPGNMKDNFWEMGDTGPCGPCSEIHYDRIGGRDAAHLVNQDDPNVLEIWNLVFIQYNRETDGILKPLPKKSIDT

GMGLERLVSVLQNKMSNYDTDLFVPYFEAIQKGTGARPYTGKVGADDADGIDMAYRVLADHARTITVALADGGRPDNTGR

GYVLRRILRRAVRYSHEKLNACRGFFATLVDVVVQSLGDAFPELKKDPDMVKDIINEEEVQFLKTLSRGRRILDRKIQSL

GDCKTIPGDTAWLLYDTYGFPVDLTGLIAEEKGLVVDMDGFEEERKLAQLKSQGKGAGAEDLIMLDIYAIEELREKGLEA

TDDSPKYNYHSDSSGSYAFESAVATVMALRRDKMFVEEVSTGQECGVVLDKTCFYAEQGGQIYDEGYLVKVEDSGEDKTE

FTVKNAQVRGGYVLHIGTIYGSLRVGDQVRLFIDEPRRRPIMSNHTATHILNFALRSVLGEADQRGSLVAPDRLRFDFTA

KGAMSTQQIKEAEEIANGMIEAAKPVYTQDCPLAAAKAIQGLRAVFDETYPDPVRVVSIGVPVSELLDDPSGPAGSLTSV

EFCGGTHLQNSSHAGAFVIVSEEAIAKGIRRIVAVTGAEAQKALRKAESLKKSLSVMEAKVKAQTAPNKDVQREIADLGE

ALATVVIPQWQKDEFRENLKSLKKIMDDLDRASKADVQKRVLEKTKQLIDSNPNQPLVILEMENGASAKALNEALKLFKT

HSPQTSAMLFTVDNEAGKITCLCQVPQNAANRGLKASEWVQQVSGLMDGKGGGKDVSAQATGKNVGCLQEALQLATSFAQ

LRLGDVKN

>XP_005862694.1 PREDICTED: alanine--tRNA synthetase, cytoplasmic [Myotis brandtii]

MDSTLTASEIRQRFIDFFKRNEHTYVHSSATIPLDDPTLLFANAGMNQFKPIFLNTIDPSHPMAKLSRAANTQKCIRAGG

KHNDLDDVGKDVYHHTFFEMLGSWSFGDYFKELACKMALELLTQEFGIPVERLYVTYFGGDEAAGLEPDLECKQIWQNLG

VDDTKILPGNMKDNFWEMGDTGPCGPCSEIHYDRIGGRDAAHLVNQDDPNVLEIWNLVFIQYNRETDGTLKPLPKKSIDT

GMGLERLVSVLQNKMSNYDTDLFVPYFEAIQKGTGARPYTGKVGADDADGIDMAYRVLADHARTITVALADGGRPDNTGR

GYVLRRILRRAVRYSHEKLNASKGFFATLVDVVVQSLGDAFPELKKDPDMVKDIINEEEVQFLKTLSRGRRILDRKIQSL

GDSKTIPGDTAWLLYDTYGFPVDLTGLIAEEKGLVVDMDGFEEERKLAQLKSQGKGAGGEDHIMLDIYAIEELRGKSLEA

TDDSPKYSYLSDSSGSYAFESTVATVMALRRDKMFVEEVSTGQECGVVLDKTCFYAEQGGQIYDEGYLVKVDDSSEDKTE

FTVKNTQVRGGYVLHIGTIYGNLKVGDQVRLFIDEPRRRPVMSNHTATHILNFALRSVLGEADQRGSLVAPDRLRFDFTA

KGAMSTQQIKKAEQIVNEMIEAAKPVYTQDCPLAAAKAIQGLRAVFDETYPDPVRVVSIGVPVSKLLDDPSGPAGSLTSV

EFCGGTHLQNSSHAGAFVIVSEEAIAKGIRRIVAVTGAEAQKALRKAESLKKSLSGMEAKVKAQTSPNKDVQREIADLGE

ALATAVIPQWQKDEFRETLKSLKKIMDDLDRASKADVQKRVLEKTKQLIDSNPNQPLVILEMESGASAKALNEALKLFKT

HSPQTSAMLFTVDNEAGKITCLCQVPQNAANRGLKASEWVQQVSGLMDGKGGGKDVSAQATGKNVGCLQEALQLATSFAQ

LRLGDVKN

>XP_012586153.1 PREDICTED: alanine--tRNA synthetase, cytoplasmic [Condylura cristata]

MDSILTASEIRQRFIDFFKRNEHTYVHSSATIPLDDPTLLFANAGMNQFKPIFLNTIDPSHPMAKLSRAANTQKCIRAGG

KHNDLDDVGKDVYHHTFFEMLGSWSFGDYFKELACKMALELLTQEFGIPVERLYVTYFGGDEAAGLEPDLECKQIWQNLG

LDDTKILPGNMKDNFWEMGDTGPCGPCSEIHYDRIGGRDAAHLVNQDDPNVLEIWNLVFIQYNREADGVLKPLPKKSIDT

GMGLERLVSVLQNKMSNYDTDLFVPYFEAIQKGTGARPYTGKVGAEDADGIDMAYRVLADHARTITVALADGGRPDNTGR

GYVLRRILRRAVRYSHEKLNASRGFFATLVDVVVQSLGDAFPELKKDPDMVKDIINEEEEQFLKTLSRGRRILDRKIQSL

GDSKTIPGDTAWLLYDTYGFPVDLTGLIAEEKGLVVDMDGFEEERKLAQLKSQGKGAGGEDLIMLDIYAIEELRERGLEA

TDDSPKYNYHSDSSGSYAFESAVATVMALRRDKMFVDEVSTGQECGVVLDKTCFYAEQGGQTYDEGYLVKLEDGSEDKTE

FTVKNAQVRGGYVLHIGTVYGNLKVGDQVRLFIDEPRRRPVMSNHTATHILNFALRAVLGEADQRGSLVAPDRLRFDFTA

KGAMSTQQIKKAEEIANEMIEAAKPVYTQDCALAAAKAIQGLRAVFDETYPDPVRVVSIGVPVSTLLDDPSSPAGSLTSV

EFCGGTHLQNSSHAGAFVIVSEEAIAKGIRRIVAVTGAEAQKALRKAESLKKSLSVMETKVKAQTAPNKDVQKEIADLGE

ALATAVIPQWQKDEFRENLKSLKKIMDDLDRASKADVQKRVLEKTKQLIDSNPNQPLVILEMESGASAKALNEALKLFKT

HSPQTSAMLFTVDNEAGKITCLCQVPQNAANRGLKASEWVQQVSGLMDGKGGGKDVSAQATGKNVGCLQEALQLATSFAQ

LRLGDVKN

>XP_019565839.1 PREDICTED: alanine--tRNA synthetase, cytoplasmic [Rhinolophus sinicus]

MDSTLTASEIRQRFIDFFKRNEHTYVHSSATIPLDDPTLLFANAGMNQFKPIFLNTIDPSHPMAKLSRAANTQKCIRAGG

KHNDLDDVGKDVYHHTFFEMLGSWSFGDYFKELACKMALELLTQEFGIPVERLYVTYFGGDEAAGLEPDLECKQIWQDLG

LDETKILPGNMKDNFWEMGDTGPCGPCSEIHYDRIGGRDAAHLVNQDDPNVLEIWNLVFIQYNRETDGILKPLPKKSIDT

GMGLERLVSVLQNKMSNYDTDLFVPYFEAIQKGTGARPYSGKVGADDADGIDMAYRVLADHARTITVALADGGRPDNTGR

GYVLRRILRRAVRYSHEKLNASKGFFATLVDVVVQSLGDAFPELKKDPDMVKDIINEEEVQFLKTLSRGRRILDRKIQSL

GDSKTIPGDTAWLLYDTYGFPVDLTGLIAEEKGLVVDMDGFEEERKMAQLKSQGKGAGGEDHIMLDIYAIEELREKSLKA

TDDSPKYNYHSDSSGSYSFESAVATVLALRRDKMFVGEVSTGQECGVVLDKTCFYAEQGGQIYDEGYLVKVDDSSEDKTE

FTVKNTQVRGGYVLHIGTIYGNLKVGDQVRLFIDEPRRRPVMSNHTATHILNFALRSVLGEADQRGSLVAPDRLRFDFTA

KGAMSSQQIKKAEELANEMIEAAKPVYTQDCPLAAAKAIQGLRAVFDETYPDPVRVVSIGVPVSELLDDPSGPAGSLTSV

EFCGGTHLQNSSHAGAFVIVSEEAIAKGIRRIVAVTGAEAQKALRKAESLKKSLSVMEAKVKAQTAPNKDVQREIADLGE

ALATAVIPQWQKDEFRENLKSLKKIMDDLDRASKADVQKRVLEKTKQLIDSNPNQPLVILEMESGASAKALNEALKLFKT

HSPQTSAMLFTVDNEAGKITCLCQVPQNAANRGLKASEWVQQVSGLMDGKGGGKDVSAQATGKNVGCLQEALQLATSFAQ

LRLGDVKN

>XP_014940475.1 PREDICTED: alanine--tRNA synthetase, cytoplasmic [Acinonyx jubatus]

MDSTLTASEIRQRFIDFFQRNEHTYVHSSATIPLDDPTLLFANAGMNQFKPIFLNTIDPSHPMAKLSRAANTQKCIRAGG

KHNDLDDVGKDVYHHTFFEMLGSWSFGDYFKELACKMALELLTQEFGIPVERLYVTYFGGDEAAGLEPDLECKQIWQNLG

LDDSKILPGNMKDNFWEMGDTGPCGPCSEIHYDRIGGRDAAHLVNQDDPNVLEIWNLVFIQYNRETDGILKPLPKKSIDT

GMGLERLVSVLQNKMSNYDTDLFVPYFEAIQKGTGARPYAGKVGADDADGIDMAYRVLADHARTITVALADGGRPDNTGR

GYVLRRILRRAVRYSHEKLNASRGFFATLVDVVVQSLGDAFPELKKDPDMVKDIINEEEVQFLKTLSRGRRILDRKIQSL

GDCKTIPGDTAWLLYDTYGFPVDLTGLIAEEKGLVVDMDGFEEERKLAQLKSQGKGAGAEDLIMLDIYAIEELREKGLEA

TDDSPKYNYHSDSSGSYAFESAVATVMALRRDKMFVEEVSTGQECGVVLDKTCFYAEQGGQIYDEGYLVKVEDSSEDKTE

FTVKNAQVRGGYVLHIGTIYGSLRVGDQVRLFIDEPRRRPIMSNHTATHILNFALRSVLGEADQRGSLVAPDRLRFDFTA

KGAMSTQQIKEAEEIANGMIEAAKPVYTQDCPLAAAKAIQGLRAVFDETYPDPVRVVSIGVPVSELLDDPSGPAGSLTSV

EFCGGTHLQNSSHAGAFVIVSEEAIAKGIRRIVAVTGAEAQKALRKAESLKKSLSVMEAKVKAQTAPNKDVQREIADLGE

ALATVVIPQWQKDEFRENLKSLKKIMDDLDRASKADVQKRVLEKTKQLIDSNPNQPLVILEMENGASAKALNEALKLFKT

HSPQTSAMLFTVDNEAGKITCLCQVPQNAANRGLKASEWVQQVSGLMDGKGGGKDVSAQATGKNVGCLQEALQLATSFAQ

LHLGDVKN

>XP_006091282.1 alanine--tRNA synthetase, cytoplasmic isoform X1 [Myotis lucifugus]

MDSTLTASEIRQRFIDFFKRNEHTYVHSSATIPLDDPTLLFANAGMNQFKPIFLNTIDPSHPMAKLSRAANTQKCIRAGG

KHNDLDDVGKDVYHHTFFEMLGSWSFGDYFKELACKMALELLTQEFGIPVERLYVTYFGGDEAAGLEPDLECKQIWQNLG

VDDAKILPGNMKDNFWEMGDTGPCGPCSEIHYDRIGGRDAAHLVNQDDPNVLEIWNLVFIQYNRETDGTLKPLPKKSIDT

GMGLERLVSVLQNKMSNYDTDLFVPYFEAIQKGTGARPYTGKVGADDADGIDMAYRVLADHARTITVALADGGRPDNTGR

GYVLRRILRRAVRYSHEKLSASKGFFATLVDVVVQSLGDAFPELKKDPDMVKDIINEEEVQFLKTLSRGRRILDRKIQSL

GDSKTIPGDTAWLLYDTYGFPVDLTGLIAEEKGLVVDMDGFEEERKLAQLKSQGKGAGGEDHIMLDIYAIEELRGKSLEA

TDDSPKYSYLSDSSGSYAFESTVATVMALRRDKMFVEEVSTGQECGVVLDKTCFYAEQGGQIYDEGYLVKVDDSSEDKTE

FTVKNTQVRGGYVLHIGTIYGNLKVGDQVRLFIDEPRRRPVMSNHTATHILNFALRSVLGEADQRGSLVAPDRLRFDFTA

KGAMSTQQIKKAEQIVNEMIEAAKPVYTQDCPLAAAKAIQGLRAVFDETYPDPVRVVSIGVPVSKLLDDPSGPAGSLTSV

EFCGGTHLQNSSHAGAFVIVSEEAIAKGIRRIVAVTGAEAQKALRKAESLKKSLSVMEAKVKAQTSPNKDVQREIADLGE

ALATAVIPQWQKDEFRETLKSLKKIMDDLDRASKADVQKRVLEKTKQLIDSNPNQPLVILEMESGASAKALNEALKLFKT

HSPQTSAMLFTVDNEAGKITCLCQVPQNAANRGLKASEWVQQVSGLMDGKGGGKDVSAQATGKNVGCLQEALQLATSFAQ

LRLGDVKN

>XP_004273308.1 PREDICTED: alanine--tRNA synthetase, cytoplasmic [Orcinus orca]

MDSTLTASEIRQRFIDFFKRNEHTYVHSSATIPLDDPTLLFANAGMNQFKPIFLNTVDPSHPMAKLSRAANTQKCIRAGG

KHNDLDDVGKDVYHHTFFEMLGSWSFGDYFKELACKMALELLTQEFGIPVERLYVTYFGGDEAASLEPDLECKQIWHNLG

LDDSKILPGNMKDNFWEMGDTGPCGPCSEIHYDRIGGRDAAHLVNQDDPNVLEIWNLVFIQYNRETDGILKPLPKKSIDT

GMGLERLVSVLQNKMSNYDTDLFVPYFEAIQKGTGARPYTGKVGAEDADGIDMAYRVLADHARTITVALADGGRPDNTGR

GYVLRRILRRAVRYSHEKLNASRGFFATLADVVVQSLGDAFPELKKDPDMVKDIINEEEVQFLKTLSRGRRILDRKIQSL

GDSKTIPGDTAWLLYDTYGFPVDLTGLIAEEKGLVVDMDGFEEERKLAQLKSQGKGAGGEDLIMLDIYAIEELREKGLEA

TDDSPKYNYHSDSSGSYVFESAVATVMALRRDKMFVEEVSTGQECGVVLDKTCFYAEQGGQIYDEGYLVKVDDGSEDKTE

FTVKNAQVRGGYVLHVGTIYGSLKVGDQVRLFIDEPRRRPVMSNHTATHILNFALRSVLGEADQRGSLVAPDRLRFDFTA

KGAMSTQQIKKAEEIANEMIEAAKPVYTQDCPLAAAKAIQGLRAVFDETYPDPVRVVSIGVPVSELLDDPSGPAGSLTSV

EFCGGTHLQNSSHAGAFVIVSEEAIAKGIRRIVAVTGAEAQKALRKAESLKKSLSVAEAKVKAQTAPNKDVQREIADLGE

ALATAVIPQWQKDEFRENLKSLKKIMDDLDRANKADVQKRVLEKTKQLIDSSPNQPLVILEMESGASAKALNEALKLFKT

HSPETSAMLFTVDNEAGKITCLCQVPQNAANRGLKASEWVQQVSGLMDGKGGGKDVSAQATGKNVGCLQEALQLAATFAH

LHLGDVKN

>XP_008138224.1 PREDICTED: alanine--tRNA synthetase, cytoplasmic [Eptesicus fuscus]

MDSTLTASEIRQRFIDFFKRNEHTYVHSSATIPLDDPTLLFANAGMNQFKPIFLNTIDPSHPMAKLSRAANTQKCIRAGG

KHNDLDDVGKDVYHHTFFEMLGSWSFGDYFKELACKMALELLTQEFGIPVERLYVTYFGGDEAAGLEPDLECKQIWQNLG

VDDAKILPGNMKDNFWEMGDTGPCGPCSEIHYDRVGGRDAAHLVNQDDPNVLEIWNLVFIQYNRETDGTLKPLPKKSIDT

GMGLERLVSVLQNKMSNYDTDLFVPYFEAIQKGTGARPYTGKVGADDADGIDMAYRVLADHARTITVALADGGRPDNTGR

GYVLRRILRRAVRYSHEKLNASKGFFATLVDVVVQSLGDAFPELKKDPDMVKDIINEEEVQFLKTLSRGRRILDRKIQSL

GDSKTIPGDTAWLLYDTYGFPVDLTGLIAEEKGLVVDMDGFEEERKLAQLKSQGKGAGGEDHIMLDIYAIEELRGKSLEA

TDDSPKYSYLSDSSGSYAFESAVATVMALRRDKMFVEEVSTGQECGVVLDKTCFYAEQGGQIYDEGYLVKVDDSSEDKTE

FTVKNTQVRGGYVLHIGTIYGNLKVGDQVRLFIDEPRRRPVMSNHTATHILNFALRSVLGEADQRGSLVAPDRLRFDFTA

KGAMSTQQIKKAEQIVNEMIEAAKPVYTQDCPLAAAKAIQGLRAVFDETYPDPVRVVSIGVPVSKLLDDPSGPAGSLTSV

EFCGGTHLQNSSHAGAFVIVSEEAIAKGIRRIVAVTGAEAQKALRKAESLKKSLSVMEAKVKAQTSPNKDVQREIADLGE

ALATAVIPQWQKDEFRETLKSLKKIMDDLDRASKADVQKRVLEKTKQLIDSNPNQPLVILEMESGASAKALNEALKLFKT

HSPQTSAMLFTVDNEAGKITCLCQVPQNAANRGLKASEWVQQVSGLMDGKGGGKDVSAQATGKNVGCLQEALQLATSFAQ

LRLGDVKN

>XP_006878675.1 PREDICTED: alanine--tRNA synthetase, cytoplasmic [Elephantulus edwardii]

MDSTLTASEIRQRFIDFFKRNEHTYVHSSATIPLDDPTLLFANAGMNQFKPIFLNTIDPSHPMAKLRRAANTQKCIRAGG

KHNDLDDVGKDVYHHTFFEMLGSWSFGDYFKELACKMALELLTQEFGIPVERLYVTYFGGDEAAGLEPDLECKQIWQNLG

LDDAKILPGNMKDNFWEMGDTGPCGPCSEIHYDRIGGRDAAHLVNQDDPNVLEIWNLVFIQYNRENDGVLKPLPKKSIDT

GMGLERLVSVLQNKMSNYDTDLFVPYFEAIQKGTGARPYTGQVGADDADGIDMAYRVLADHARTITVALADGGRPDNTGR

GYVLRRILRRAVRYSHEKLNASRGFFATLVDVVVQSLGDAFPELKKDPDMVKDIINEEEVQFLKTLSRGRRILDRKIQSL

GDSKTIPGDTAWLLYDTYGFPVDLTGLIAEEKGLVVDMDGFEEERKLAQLKSQGKGAGGEDLIMLDIYAIEELRSRGLEA

TDDSPKYNYHSDASGNYVFENAVATVMALRRDKMFVDEVSTGQECGVVLDKTCFYAEQGGQIYDEGYLVKVEDSSEDKTE

FTVKNAQVRGGYVLHIGTIYGHLKVGDQVRLFIDEPRRRPVMSNHTATHILNFALRSVLGEADQRGSLVAPDRLRFDFTA

KGAMSTQQIKKAEEISNQMIEAAKPVYTQDCPLAAAKAIQGLRAVFDETYPDPVRVVSIGVPVSELLEDPSSPAGSLTSV

EFCGGTHLQNSSHAGPFVIVSEEAIAKGIRRIVAVTGAEAQKALRKAESLKKSLSVMEAKVKAQTAPNKDVQREIADLGE

ALAAAVIPQWQKDELREDLKSLKKIMDNLDRASKADVQKRVLEKTKQLIDSHPNQPLVILEMESGASAKALNEALKLFKT

HSPQTAAMLFTVDNEAGKITCLCQVPQNAANRGLKASEWVQQVSGLMDGKGGGKDVSAQATGKNVGCLQEALQLASSFAQ

LRLGDVKN

>XP_007074943.1 PREDICTED: alanine--tRNA synthetase, cytoplasmic [Panthera tigris altaica]

MDSTLTASEIRQRFIDFFKRNEHTYVHSSATIPLDDPTLLFANAGMNQFKPIFLNTIDPSHPMAKLSRAANTQKCIRAGG

KHNDLDDVGKDVYHHTFFEMLGSWSFGDYFKELACKMALELLTQEFGIPVERLYVTYFGGDEAAGLEPDLECKQIWQNLG

LDDSKILPGNMKDNFWEMGDTGPCGPCSEIHYDRIGGRDAAHLVNQDDPNVLEIWNLVFIQYNRETDGILKPLPKKSIDT

GMGLERLVSVLQNKMSNYDTDLFVPYFEAIQKGTGARPYTGKVGADDADGIDMAYRVLADHARTITVALADGGRPDNTGR

GYVLRRILRRAVRYSHEKLNACRGFFATLVDVVVQSLGDAFPELKKDPDMVKDIINEEEVQFLKTLSRGRRILDRKIQSL

GDCKTIPGDTAWLLYDTYGFPVDLTGLIAEEKGLVVDMDGFEEERKLAQLKSQGKGAGAEDLIMLDIYAIEELREKGLEA

TDDSPKYNYHSDSSGSYAFESAVATVMALRRDKMFVEEVSTGQECGVVLDKTCFYAEQGGQIYDEGYLVKVEDSGEDKTE

FTVKNAQVRGGYVLHIGTIYGSLRVGDQVRLFIDEPRRRPIMSNHTATHILNFALRSVLGEADQRGSLVAPDRLRFDFTA

KGAMSTQQIKEAEEIANGMIEAAKPVYTQDCPLAAAKAIQGLRAVFDETYPDPVRVVSIGVPVSELLDDPSGPAGSLTSV

EFCGGTHLQNSSHAGAFVIVSEEAIAKGIRRIVAVTGAEAQKALRKAESLKKSLSVMEAKVKAHTAPNKDVQREIADLGE

ALATVVIPQWQKDEFRENLKSLKKIMDDLDRASKADVQKRVLEKTKQLIDSNPNQPLVILEMENGASAKALNEALKLFKT

HSPQTSAMLFTVDNEAGKITCLCQVPQNAANRGLKASEWVQQVSGLMDGKGGGKDVSAQATGKNVGCLQEALQLATSFAQ

LRLGDVKN

>XP_006860236.1 PREDICTED: alanine--tRNA synthetase, cytoplasmic [Chrysochloris asiatica]

MDSTLTAKEIRQRFIDFFKRNKHTYVHSSATIPLDDPTLLFANAGMNQFKPIFLNTIDPSHPMAKLSRAANTQKCIRAGG

KHNDLDDVGKDVYHHTFFEMLGSWSFGDYFKELACKMALELLTQEFGIPVERLYVTYFGGDDAAGLEPDLECKQIWQNLG

LDDTKILPGNMKDNFWEMGDTGPCGPCSEIHYDRLGGRDAAHLVNQDDPNVLEIWNLVFIQYNRETDGTLKPLPKKSIDT

GMGLERLVSVLQNKMSNYDTDLFVPYFEAIQKGTGARPYTGQVGADDTDGIDMAYRVLADHARTITVALADGGRPDNTGR

GYVLRRILRRAVRYAHEKLNASRGFFATLVDVVVQSLGDAFPELKKDPDMVKDIINEEEVQFLKTLSRGRRILDRKIQSL

GDSKTIPGDTAWLLYDTYGFPVDLTGLIAEEKGLLVDMEGFEEERKLAQLKSQGKGAGGEDLIMLDIYAIEELRSKGLEA

TDDSPKYNYHSDSSGSYVFENTVATVMALRRDKMFVEEVSTGQECGVVLDKTCFYAEQGGQIYDEGYLVKVDDSSEDKTE

FTVKTAQVRGGYVLHVGTIYGNLRVGDQVRLFIDEPRRRPVMSNHTATHILNFALRSVLGEADQRGSLVAPDRLRFDFTA

KGAMSTQQIKKAEEIANQMIEAAKPVYTQDCALAAAKAIQGLRAVFDETYPDPVRVVSIGVPVSELLEDPSSPAGSLTSV

EFCGGTHLQNSSHAGAFVVVSEEAIAKGIRRIVAVTGAEAQKALRKAESLKKSISVMEAKVKAQTAPNKDVQREIADLGE

ALATAVIPQWQKDELREDLKSLKKVMDDLDRASKADVQKRVLEKTKQLIDCHPNQPLVILEMESGASAKALNEALKLFKT

HSPQTAAMLFTVDNEAGKITCLCQVPQNAANRGLKASEWVQQVSGLMDGKGGGKDVSAQATGKNVGCLQEALQLATSFAQ

LRLGDVKN

>XP_536788.2 alanine--tRNA synthetase, cytoplasmic [Canis lupus familiaris]

MDSTLTASEIRQRFIDFFKRNEHTYVHSSATIPLDDPTLLFANAGMNQFKPIFLNTIDPSHPMAKLSRAANTQKCIRAGG

KHNDLDDVGKDVYHHTFFEMLGSWSFGDYFKELACKMALELLTQEFGIPVERLYVTYFGGDEAAGLEPDLECKQIWQNLG

LDDTKILPGNMKDNFWEMGDTGPCGPCSEIHYDRIGGRDAAHLVNQDDPNVLEIWNLVFIQYNRESDGILKPLPKKSIDT

GMGLERLVSVLQNKMSNYDTDLFVPYFEAIQKGTGARPYTGKVGADDADGIDMAYRVLADHARTITVALADGGRPDNTGR

GYVLRRILRRAVRYSHEKLNASRGFFATLVDVVVQSLGDAFPELKKDPDMVKDIINEEEVQFLKTLSRGRRILDRKIQSL

GDCNTIPGDTAWLLYDTYGFPVDLTGLIAEEKGMVVDMDGFEEERKLAQLKSQGKGAGGEDLIMLDIYAIEELREKGLEA

TEDSPKYNYHSDSSGSYTFENVVATVVALRRDKMFVEEVSTGQECGVVLDKTCFYAEQGGQIYDEGYLVKVEDNSEDKME

FTVKNAQVRGGYVLHIGTIYGSLRVGDQVRLFIDEPRRRPIMSNHTATHILNFALRSVLGEADQKGSLVAPDRLRFDFTA

KGAMSTQQIKKAEEIANGMIEAAKPVYTQDCPLAAAKAIQGLRAVFDETYPDPVRVVSIGVPVSELLEDPSGPAGSLTSV

EFCGGTHLQNSSHAGAFVIVSEEAIAKGIRRIVAVTGAEAQKALRKAESLKNSLSVMEAKVKAQTAPNKDVQREIADLGE

VLATAVIPQWQKDEFRENLKSLKKIMDDLDRASKADVQKRVLEKTKQLIDSNPNQPLVILEMESGASAKALNEALKLFKT

HSPETSAMLFTVDNEAGRITCLCQVPQNAANRGLKASEWVQQVSGLMDGKGGGKDVSAQATGKNVGCLQEALQLATSFAQ

LHLGDVKN

>XP_011378935.1 alanine--tRNA synthetase, cytoplasmic isoform X1 [Pteropus vampyrus]

MDSILTASEIRQRFIDFFKRNEHTYVHSSATIPLDDPTLLFANAGMNQFKPIFLNTIDPSHPMAKLSRAANTQKCIRAGG

KHNDLDDVGKDVYHHTFFEMLGSWSFGDYFKELACKMALELLTQEFGIPVERLYVTYFGGDEAAGLEPDLECKQIWQDLG

LDDTKILPGNMKDNFWEMGDTGPCGPCSEIHYDRIGGRDAAHLVNQDDPNVLEIWNLVFIQYNRETDGILKPLPKKSIDT

GMGLERLVSVLQNKMSNYDTDLFVPYFEAIQKGTGARPYTGKVGAEDADGIDMAYRVLADHARTITVALADGGRPDNTGR

GYVLRRILRRAVRYSHEKLNASKGFFATLVDVVVQSLGDAFPELKKDPDMVKDIINEEEVQFLKTLSRGRRILDRKIQSL

GDSKTIPGDTAWLLYDTYGFPVDLTGLIAEEKGLVVDMDGFEEERKMAQLKSQGKGAGGEDHIMLDIYAIEELREKSLEA

TDDSPKYNYHSDSSGSYAFESAVATVMALRRDKTFIEEVSTGQECGVVLDKTCFYAEQGGQIYDEGYLVKVDDSSEDKTE

FTVKNTQVRGGYVLHIGTIYGSLKVGDQVRLFIDEPRRRPVMSNHTATHILNFALRSVLGEADQRGSLVAPDRLRFDFTA

KGAMSTQQIKRVEEIANEMIEAAKPVYTQDCPLAAAKAIQGLRAVFDETYPDPVRVVSIGVPVSELLDDPSGPAGSLTSV

EFCGGTHLQNSSHAGAFVIVSEEAIAKGIRRIVAVTGAEAQKALRKAESLKKSLSVMEAKVKAQTVPNKDVQREIADLGE

ALASAVIPQWQKDEFRENLKSLKKIMDDLDRASKADVQKRVLEKTKQLIDSNPNQPLVILEMESGASAKALNEALKLFKT

HSPQTSAMLFTVDNEAGKITCLCQVSQNAANRGLKASEWVQQVSGLMDGKGGGKDVSAQATGKNVSCLQEALQLATSFAQ

LRLGDVKN

>XP_021075729.1 alanine--tRNA synthetase, cytoplasmic [Mus pahari]

MEATLTAREIRERFINFFKRNEHTYVHSSATIPLDDPTLLFANAGMNQFKPIFLNTIDPSHPMAKLSRAANTQKCIRAGG

KHNDLDDVGKDVYHHTFFEMLGSWSFGDYFKELACKMALELLTQEFGIPVERLYVTYFGGDEAAGLEPDLECKQIWQNLG

LDEARILPGNMKDNFWEMGDTGPCGPCSEIHYDRIGGRDAAHLVNQDDPNVLEIWNLVFIQYNRESDGVLKPLPKKSIDT

GMGLERLVSVLQNKMSNYDTDLFVPYFEAIQKGTGARPYTGKVGAEDADGIDMAYRVLADHARTITVALADGGRPDNTGR

GYVLRRILRRAVRYSHEKLNASRGFFATLVDVVVQSLGDAFPELKKDPDMVKDIINEEEVQFLKTLSRGRRILDRKIQSL

GDCKTIPGDTAWLLYDTYGFPVDLTGLIAEEKGLVVDMDGFEEERRLAQLKSQGKGAGDEDLLMLDIYAIEELRAKGLEA

TDDAPKYNYHSDSSGSYVFECTVATVLALRREKMFVDEVVTGQECGVVLDKTCFYAEQGGQIYDEGYLVKVDDSSEDKTE

FTVKNAQVRGGYVLHIGTIYGNLKVGDQVRLFIDEPRRRPVMSNHTATHILNFALRSVLGEADQKGSLVAPDRLRFDFTA

KGAMSTQQIKKAEEIVNGMIEAAKPVYTQDCPLAAAKAIQGLRAVFDETYPDPVRVVSIGVPVSELLDDPSGPAGSLTSV

EFCGGTHLRNSSHAGAFVIVTEEAIAKGIRRIVAVTGAEAQKALRKSETLKKSLSAMEAKVKAQTAPNKDVQREIADLGE

ALATAVIPQWQKDEQRETLKSLKKVMDDLDRASKADVQKRVLEKTKQLIDSNPNQPLVILEMESGASAKALNEALKLFKT

HSPQTSAMLFTVDNEAGKITCLCQVPQNAANRGLKASEWVQQVSGLMDGKGGGKDMSAQATGKNVGCLQEALQLATSFAQ

LRLGDVKN

>XP_006974156.1 PREDICTED: alanine--tRNA synthetase, cytoplasmic [Peromyscus maniculatus bairdii]

MDSMLTAREIRERFINFFKKNEHTYVHSSATIPLDDPTLLFANAGMNQFKPIFLNTIDPSHPMAKLSRAANTQKCIRAGG

KHNDLDDVGKDVYHHTFFEMLGSWSFGDYFKELACKMALELLTQEFGIPVDRLYVTYFGGDEAAGLEPDLECRQIWQNLG

LDDAKILPGNMKDNFWEMGDTGPCGPCSEIHYDRIGGRDAAHLVNQDDPNVLEIWNLVFIQYNRETDGLLKPLPKKSIDT

GMGLERLVSVLQNKMSNYDTDLFVPYFEAIQKGTGARPYTGKVGAEDADGIDMAYRVLADHARTITVALADGGRPDNTGR

GYVLRRILRRAVRYSHEKLNASRGFFATLVDVVVQSLGDAFPELKKDPDMVKDIINEEEVQFLKTLSRGRRILDRKIQSL

GDCKTIPGDTAWLLYDTYGFPVDLTGLIAEEKGLVVDMDGFEEERKLAQLKSQGKGAGGEDLIMLDIYAIEELRAKGLEA

TDDSPKYNYHADSSDSYVFECTVATVLALRREKMFVEEVVTGQECGVVLDKTCFYAEQGGQIYDEGYLVKIDDSSEDKTE

FTVKNAQVRGGYVLHIGTLYGNLKVGDQVRLFIDEPRRRPVMSNHTATHILNFALRSVLGEADQKGSLVAPDRLRFDFTA

KGAMSTQQIKKAEEIVNAMIAAAKPVYTQNCPLAAAKAIQGLRAVFDETYPDPVRVVSIGVPVLELLEDPSGPAGSLTSV

EFCGGTHLRNSSHAGAFVIVTEEAIAKGIRRIVAVTGAEAQKALRKAESLKKSLSVLEAKVKAQTAPNKDVQREIADLGE

ALATAVIPQWQKDEQRETLKSLKKVMDDLDRASKADVQKRVLEKTKQLIDSNPNQPLVILEMESGASAKALNEALKLFKT

HSPQTSAMLFTVDNEAGKITCLCQVPQNAANRGLKASEWVQQVSGLMDGKGGGKDVSAQATGKNVGCLQEALQLATSFAQ

LRLGDVKN

>NP_001268606.1 alanine--tRNA synthetase, cytoplasmic [Mesocricetus auratus]

MDSPLTAREIRERFINFFRRNEHTYVHSSATIPLDDPTLLFANAGMNQFKPIFLNTIDPSHPLAKLSRAANTQKCIRAGG

KHNDLDDVGKDVYHHTFFEMLGSWSFGDYFKELACKMALELLTQEFAIPVERLYVTYFGGDEAAGLEPDLECKQIWQNLG

LDDAKILPGNMKDNFWEMGDTGPCGPCSEIHYDRIGGRDAAHLVNQDDPNVLEIWNLVFIQYNRETDGLLKPLPKKSIDT

GMGLERLVSVLQNKMSNYDTDLFVPYFEAIQKGTGARPYTGKVGAEDADGIDMAYRVLADHARTITVALADGGRPDNTGR

GYVLRRILRRAVRYSHEKLNASRGFFATLVDVVVQSLGDAFPELKKDPDMVKDIINEEEVQFLKTLSRGRRILDRKIQSL

GDCKTIPGDTAWLLYDTYGFPVDLTGLIAEEKGLVVDMDGFEEERKLAQLKSQGKGAGGEDLIMLDIYAIEELRAKGLEA

TDDSPKYNYHSDSSGAYVFECTVATVLALRREKMFVEEVVTGQECGVVLDKTCFYAEQGGQIYDEGYLVKVDDSSEDKTE

FTVKNAQVRGGYVLHIGTIYGNLKVGDQVRLFIDEPRRRPVMSNHTATHILNFALRSVLGEADQKGSLVAPDRLRFDFTA

KGAMSTQQIKEAEEIVNAMIEAAKPVYTQDCPLAAAKAIQGLRAVFDETYPDPVRVVSIGVPVSELLDDPSGPAGSLTSV

EFCGGTHLQNSSHAGAFVIVTEEAIAKGIRRIVAVTGAEAQKALRKAESLRRSLSIMEAKVKAQTAPNKDVQREIADLGE

ALATAVIPQWQKDEQRETLKSLKKVMDDLDRASKADVQKRVLEKTKQLIDSNPNQPLVILEMESGASAKALNEALKLFKT

HSPQTSAMLFTVDNEAGKITCLCQVPQNAANRGLKASEWVQQVSGLMDGKGGGKDVSAQATGKNVGCLQEALQLATSFAQ

LRLGAVEN

>XP_004842923.1 alanine--tRNA synthetase, cytoplasmic [Heterocephalus glaber]

MNSTLTASEIRERFIDFFKRNEHTYVHSSATIPLDDPTLLFANAGMNQFKPIFLNTIDPSHPLAKLSRAANTQKCIRAGG

KHNDLDDVGKDVYHHTFFEMLGSWSFGDYFKESACKMALELLTQEFGIPVDRLYVTYFGGDEAAGLEPDLECKQIWQTLG

LDDSRILPGNMKDNFWEMGDTGPCGPCSEIHYDRIGGRDASYLVNQDDPNVLEIWNLVFIQYNRETDGTLKPLPKKSIDT

GMGLERLVSVLQNKMSNYDTDLFVPYFEAIQKGTGARPYTGKVGAEDADGIDMAYRVLADHARTITVALADGGRPDNTGR

GYVLRRILRRAVRYSHEKLNASRGFFATLVDVVVQSLGDAFPELKKDPDMVKDIINEEEVQFLKTLSRGRRILDRKIQSL

GDCKTIPGDTAWLLYDTYGFPVDLTGLIAEEKGLVVDMDGFEEERKLAQLKSQGKGAGGEDLIMLDIYAIEELRARGLEA

TDDSPKYNYHSDSSGGYLFESAVATVLALRREKMFVEEVSTGQECGVLLDKTCFYAEQGGQIYDEGYLVKVDDSSEDKTE

FTVKNAQVRGGYVLHIGTIYGNLKVGDQVRLFIDEPRRRPIMSNHTATHILNFALRSVLGEADQKGSLVAPDRLRFDFTA

KGAMSTQQIKKAEETGNEMIKAAKPVYTQDCPLAAAKAIQGLRAVFDETYPDPVRVVSIGVPVSELLDDPSGPAGSLTSV

EFCGGTHLRNSSHAGAFVIVTEEAIAKGIRRIVAVTGAEAQKALRKAESLKKSFSTMESKVKAQTAPNKDVQREIVDLGE

ALATAVIPQWQKDELRETLKSLKKVMDDLDRASKADVQKRVLERTKQLIDSNPNQPLVILEMESGASAKALNEALKLFKT

HSPETSAMLFTVDNEAGKITCLCQVPQNAANRGLKASEWVQQVSGLMDGKGGGKDVSAQATGKNVGCLQEALQLATSFAQ

LHLGDVKN

>XP_004014952.1 PREDICTED: alanine--tRNA synthetase, cytoplasmic [Ovis aries]

MDSTLTAGEIRQRFIDFFKRNEHTYVHSSATIPLDDPTLLFANAGMNQFKPIFLNTIDPSHPMAKLSRAANTQKCIRAGG

KHNDLDDVGKDVYHHTFFEMLGSWSFGDYFKELACKMALELLTQEFGIPVERLYVTYFGGDEAAGLEPDLECKQIWQNLG

LDDSRILPGSMKDNFWEMGDTGPCGPCSEIHYDRVGGRDAAHLVNQDDPNVLEIWNLVFIQYNRETDGILKPLPKKSIDT

GMGLERLVSVLQNKMSNYDTDLFVPYFEAIQKGTGARPYTGKVGAEDADGIDMAYRVLADHARTITVALSDGGRPDNTGR

GYVLRRILRRAVRYSHEKLNASRGFFATLVDVVVQSLGDAFPELKKDPDMVKDIINEEEVQFLKTLSRGRRILDRKIQSL

GDSKTIPGDTAWLLYDTYGFPVDLTALIAEEKGLVVDMDGFDEERKLAQLKSQGKGAGGEDLIMLDIYAIEELREKGLEA

TDDSPKYSYHSDSNGTYVFESAVATVMALRRDKMFVEEVSTGQECGVVLDKTCFYAEQGGQIYDEGYLVKVDDSSEDKTE

FTVKNTQVRGGYVLHIGTLYGSLKVGDQVRLFIDEPRRRPVMSNHTATHILNFALRSVLGEADQRGSLVAPDRLRFDFTA

KGAMSTQQIKRAEEIANEMIEAAKPVYTEDCSLAAAKAIQGLRAVFDETYPDPVRVVSIGVPVSELLDDPSGPAGSLTSV

EFCGGTHLQNSSHAGVFVIVSEEAIAKGIRRIVAVTGAEAQKALRKAESLKKSLSVMEAKVKAQTAPNKDVQREIADLGE

ALATAIIPQWQKDEFRENLKSLKKVMDDLDRASKADVQKRVLEKTKQLIDSSPNQPLVILEVESGASAKALNEALKLFKM

HSPQTSAMLFTVDNEAGRITCLCQVPQNAANRGLKASEWVQQVSGLMDGKGGGKDVSAQATGKNVGCLQEALQLATSFAQ

LRLGDVKN

>XP_016019908.1 PREDICTED: alanine--tRNA synthetase, cytoplasmic [Rousettus aegyptiacus]

MDSTITASEIRQRFIDFFKRNEHTYVHSSATIPLDDPTLLFANAGMNQFKPIFLNTIDPSHPMAKLSRAANTQKCIRAGG

KHNDLDDVGKDVYHHTFFEMLGSWSFGDYFKELACKMALELLTQEFGIPVERLYVTYFGGDEAAGLEPDLECKQIWQDLG

LDDTRILPGNMKDNFWEMGDTGPCGPCSELHYDRIGGRDAAHLVNQDDPNVLEIWNLVFIQYNRETDGILKPLPKKSIDT

GMGLERLVSVLQNKMSNYDTDLFVPYFEAIQKGTGARPYTGKVGAEDADGIDMAYRVLADHARTITVALTDGGRPDNTGR

GYVLRRILRRAVRYSHEKLNASKGFFATLVDVVVQSLGDAFPELKKDPDMVKDIINEEEVQFLKTLSRGRRILDRKIQSL

GDSKTIPGDTAWLLYDTYGFPVDLTGLIAEEKGLVVDMDGFEEERKMAQLKSQGKGAGGEDHIMLDIYAIEELREKSLEA

TDDSPKYNYHSDSSGSYAFESAVATVVALRRDKMFVEEVSTGQECGVVLDKTCFYAEQGGQIYDEGYLVKVDDSSEDKTE

FTVKNTQVRGGYVLHIGTIYGSLKVGDQVRLFIDEPRRRPVMSNHTATHILNFALRSVLGEADQRGSLVAPDRLRFDFTA

KGAMSTQQIKEVEEIANEMIEAAKPVYTQDCPLAAAKAIQGLRAVFDETYPDPVRVVSIGVPVSELLDDPSGPAGSLTSV

EFCGGTHLQNSSHAGAFVIVSEEAIAKGIRRIVAVTGAEAQKALRKAESLKKSLSVMEAKVKAQTMPNKDVQREIADLGE

VLATAVIPQWQKDEFRENLKSLKKIMDDLDRASKADVQKRVLEKTKQLIDSNPNQPLVILEMESGASAKALNEALKLFKT

HSPQTSAMLFTVDNEAGKITCLCQVPQNAANRGLKASEWVQQVSGLMDGKGGGKDVSAQATGKNVGCLQEALQLATSFAQ

LHLGNVKN

>XP_007463008.1 PREDICTED: alanine--tRNA synthetase, cytoplasmic [Lipotes vexillifer]

MENSCVFWNGTAYFPQFRRVIFEMDSTLTASEIRQRFIDFFKRNEHTYVHSSATIPLDDPTLLFANAGMNQFKPIFLNTV

DPSHPMAKLSRAANTQKCIRAGGKHNDLDDVGKDVYHHTFFEMLGSWSFGDYFKELACKMALELLTQEFGIPVERLYVTY

FGGDEAAGLEPDLECKQIWHSLGLDDSKILPGNMKDNFWEMGDTGPCGPCSEIHYDRIGGRDAAHLVNQDDPNVLEIWNL

VFIQYNRETDGILKPLPKKSIDTGMGLERLVSVLQNKMSNYDTDLFVPYFEAIQKGTGARPYTGKVGADDADGIDMAYRV

LADHARTITVALADGGRPDNTGRGYVLRRILRRAVRYSHEKLNASRGFFATLADVVVQSLGDAFPELKKDPDLVKDIINE

EEVQFLKTLSRGRRILDRKIQSLGDSKTIPGDTAWLLYDTYGFPVDLTGLIAEEKGLVVDMDGFEEERKLAQLKSQGKGA

GGEDLIMLDIYAIEELREKGLEATDDSPKYNYHSDSSGSYVFESAVATVMALRRDKMFVEEVSTGQECGVVLDKTCFYAE

QGGQIYDEGYLVKVDDSSEDKTEFTVKNAQVRGGYVLHIGTIYGSLKVGDQVRLFIDEPRRRPVMSNHTATHILNFALRS

VLGEADQRGSLVAPDRLRFDFTAKGAMSTQQIKKAEEIANEMIEAAKPVYTQDCPLAAAKAIQGLRAVFDETYPDPVRVV

SIGVPVSELLDDPSGPAGSLTSVEFCGGTHLQNSSHTGAFVIVSEEAIAKGIRRIVAVTGAEAQKALRKAESLKKSLSVA

EAKVKTQTAPNKDVQREIADLGEALATAVIPQWQKDEFRENLKSLKKIMDDLDRANKADVQKRVLEKTKQLIDSSPNQPL

VILEMESGASAKALNEALKLFKTHSPETSAMLFTVDNEAGKITCLCQVPQNAANRGLKASEWVQQVSGLMDGKGGGKDVS

AQATGKNVGCLQEALQLAATFAQLHLGDVKN

>XP_004393329.1 PREDICTED: alanine--tRNA synthetase, cytoplasmic [Odobenus rosmarus divergens]

MDSTLTASEIRQRFIGFFKRNEHTYVHSSATIPLDDPTLLFANAGMNQFKPIFLNTIDPSHPMAKLSRAANTQKCIRAGG

KHNDLDDVGKDVYHHTFFEMLGSWSFGDYFKELACKMALELLTEEFGIPVERLYVTYFGGDEAAGLEPDLECKQIWQNLG

LDDTKILPGNMKDNFWEMGDTGPCGPCSEIHYDRIGGRDAAHLVNQDDPNVLEIWNLVFIQYNRESDGILKPLPKKSIDT

GMGLERLVSVLQNKMSNYDTDLFVPYFEAIQKGTGARPYAGKVGADDADGIDMAYRVLADHARTITVALADGGRPDNTGR

GYVLRRILRRAVRYSHEKLNASRGFFATLVDVVVQSLGDAFPELKKDPDMVKDIINEEEVQFLKTLSRGRRILDRKIQSL

GDCKTIPGDTAWLLYDTYGFPVDLTGLIAEEKGLVVDMDGFEEERKLAQLKSQGKGAGGEDLIMLDIYAIEELREKGLEA

TDDSPKYNYHSDSGGSYAFESAVATVMALRRDKMFLEEVSTGQECGVVLDKTCFYAEQGGQTYDEGYLVKVEDNSEDKTE

FTVKNVQVRGGYVLHIGTIYGSLRVGDQVRLFIDEPRRRPVMSNHTATHILNFALRSVLGEADQRGSLVAPDRLRFDFTA

KGAMSTQQIKKAEEIANGMIEAAKPVYTQDCPLAAAKAIQGLRAVFDETYPDPVRVVSIGVPVSELLEDPSGPAGSLTSV

EFCGGTHLQNSSHAGAFVIVSEEAIAKGIRRIVAVTGAEAQKALRKAESLKNTLSVMEAKVKAQTAPNKDVQREIADLGE

ALATAVIPQWQKDEFREDLKSLKKIMDDLDRASKADVQKRVLEKTKQLIDSNPNQPLVILEMESGASAKALNEALKLFKT

HSPQTSAMLFTVDNEAGKITCLCQVPQNAANRGLKASEWVQQVSGLMDGKGGGKDVSAQATGKNVGCLQEALQLATSFAQ

LRLGDVKN

>XP_012004081.1 PREDICTED: alanine--tRNA synthetase, cytoplasmic [Ovis aries musimon]

MDSTLTAGEIRQRFIDFFKRNEHTYVHSSATIPLDDPTLLFANAGMNQFKPIFLNTIDPSHPMAKLSRAANTQKCIRAGG

KHNDLDDVGKDVYHHTFFEMLGSWSFGDYFKELACKMALELLTQEFGIPVERLYVTYFGGDEAAGLEPDLECKQIWQNLG

LDDSRILPGSMKDNFWEMGDTGPCGPCSEIHYDRVGGRDAAHLVNQDDPNVLEIWNLVFIQYNRETDGILKPLPKKSIDT

GMGLERLVSVLQNKMSNYDTDLFVPYFEAIQKGTGARPYTGKVGAEDADGIDMAYRVLADHARTITVALSDGGRPDNTGR

GYVLRRILRRAVRYSHEKLNASRGFFATLVDVVVQSLGDAFPELKKDPDMVKDIINEEEVQFLKTLSRGRRILDRKIQSL

GDSKTIPGDTAWLLYDTYGFPVDLTALIAEEKGLVVDMDGFDEERKLAQLKSQGKGAGGEDLIMLDIYAIEELREKGLEA

TDDSPKYSYHSDSNGTYVFESAVATVMALRRDKMFVEEVSTGQECGVVLDKTCFYAEQGGQIYDEGYLVKVDDSSEDKTE

FTVKNTQVRGGYVLHIGTLYGSLKVGDQVRLFIDEPRRRPVMSNHTATHILNFALRSVLGEADQRGSLVAPDRLRFDFTA

KGAMSTQQIKRAEEIANEMIEAAKPVYTEDCSLAAAKAIQGLRAVFDETYPDPVRVVSIGVPVSELLDDPSGPAGSLTSV

EFCGGTHLQNSSHAGVFVIVSEEAIAKGIRRIVAVTGAEAQKALRKAESLKKSLCVMEAKVKAQTAPNKDVQREIADLGE

ALATAIIPQWQKDEFRENLKSLKKVMDDLDRASKADVQKRVLEKTKQLIDSSPNQPLVILEVESGASAKALNEALKLFKM

HSPQTSAMLFTVDNEAGRITCLCQVPQNAANRGLKASEWVQQVSGLMDGKGGGKDVSAQATGKNVGCLQEALQLATSFAQ

LRLGDVKN

>XP_004600586.1 PREDICTED: alanine--tRNA synthetase, cytoplasmic [Sorex araneus]

MDSTLTASEIRQRFIDFFKRNEHTYVHSSATIPLDDPTLLFANAGMNQFKPIFLNTIDPSHPMAKLSRAANTQKCIRAGG

KHNDLDDVGKDVYHHTFFEMLGSWSFGDYFKELACKMALELLTQEFGIPVERLYVTYFGGDEAAGLEPDLECKQIWQNLG

LDDSKILPGNMKDNFWEMGDTGPCGPCSEIHYDRIGGRDAAHLVNQDDPNVLEIWNLVFIQYNREADGVLKPLPKKSIDT

GMGLERLVSVLQNKMSNYDTDLFVPYFEAIQKGTGARPYSGKVGADDVDGIDMAYRVLADHARTITVALADGGRPDNTGR

GYVLRRILRRAVRYSHEKLNASRGFFATLVDVVVQSLGDAFPELKKDPDMVKDIINEEEVQFLKTLSRGRRILDRKIQSL

GDSKTIPGDTAWLLYDTYGFPVDLTGLIAEEKGMLVDMDGFEEERKLAQLKSQGKGAGGEDLIMLDIYAIEELRERGLPA

TDDSPKYNYHSDSSGSYAFESTVATVLALRRDKMFVDEVSTGQECGVVLDKTCFYAEQGGQIYDEGYLVKLEDSSEDKTE

FTVKNTQVRGGYVLHVGTIYGSLQVGDRVRLFIDEPRRRPVMSNHTATHILNFALRSVLGEADQRGSLVAPDRLRFDFTA

KGAMSTEQIKKAEEIANEMIEAAKPVYTQDCPLAAAKAIQGLRAVFDETYPDPVRVVSIGVPVSKLLDDPSGPAGSLTSV

EFCGGTHLQNSSHAGAFVIVNEEAIAKGIRRIVGVTGAEAQKALRKAESLKNALSDMEARVKAQTTPNKDVQREIADLGE

ALATAVIPQWQKDEFREHLKSLKKVMDDLDRASKADVQKRVLEKTKQLIDSNPNQPLVILEMESGASAKALNEALKLFKM

HSPQTSAMLFTVDNEAGKITCLCQVPQNAANRGLKASEWVQQVSGLMDGKGGGKDVSAQATGKNVGCLQEALQLATSFAQ

LRLGDVKN

>XP_016051979.1 PREDICTED: alanine--tRNA synthetase, cytoplasmic [Miniopterus natalensis]

MDSTLTASEIRQRFIDFFKRNGHTYVHSSATIPLDDPTLLFANAGMNQFKPIFLNTIDPSHPMAKLSRAANTQKCIRAGG

KHNDLDDVGKDVYHHTFFEMLGSWSFGDYFKELACKMALELLTQEFGIPVERLYVTYFGGDEAAGLEPDLECKQIWQNLG

MDDSKILPGNMKDNFWEMGDTGPCGPCSEIHYDRIGGRDAAHLVNQDDPNVLEIWNLVFIQYNRETDGILKPLPKKSIDT

GMGLERLVSVLQNKMSNYDTDLFVPYFEAIQKGTGARPYTGKVGADDADGIDMAYRVLADHARTITVALADGGRPDNTGR

GYVLRRILRRAVRYSHEKLNASKGFFATLVDVVVQSLGDAFPELKKDPDMVKDIINEEEVQFLKTLSRGRRILDRKIQSL

GDSKTIPGDTAWLLYDTYGFPVDLTGLIAEEKGLVVDMDGFEEERKLAQLKSQGKGAGGEDHIMLDIYAIEELRGKSLEA

TDDSPKYNYLSDSSGSYAFESTVATVMALRRDKMFVEEVSTGQECGVVLDKTCFYAEQGGQIYDEGYLVKVDDSSEDKTE

LTVKNTQVRGGYVLHIGTVYGNLKVGDRVRLFIDEPRRRPVMSNHTATHILNFALRSVLGEADQRGSLVAPDRLRFDFTA

KGAMSTQQIKKAEQIVNEMIEAAKPVYTQDCPLAAAKAIQGLRAVFDETYPDPVRVVSIGIPVSKLLDDPSGPAGSLTSV

EFCGGTHLQNSSHAGAFVIVSEEAIAKGIRRIVAVTGTEAHKALRKAESLKKSLSVMEAKVKAQTAPNKDVQREIADLGE

ALATAVIPQWQKDEFRENLKSLKKIMDDLDRASKADVQKRVLEKTKQLIDSNPNQPLVILEMESGASAKALNEALKLFKT

HSPQTSAMLFTVDNEAGKITCLCQVPQNAANRGLKASEWVQQVSGLMDGKGGGKDVSAQATGKNVGCLQEALQLATSFAQ

LRLGDVKN

>XP_002930871.1 PREDICTED: alanine--tRNA synthetase, cytoplasmic [Ailuropoda melanoleuca]

MDTTLTASEIRQRFIDFFRRNEHTYVHSSATIPLDDPTLLFANAGMNQFKPIFLNTIDPSHPMAKLSRAANTQKCIRAGG

KHNDLDDVGKDVYHHTFFEMLGSWSFGDYFKELACKMALELLTQEFGIPLERLYVTYFGGDEAAGLEPDLECKQIWQNLG

LDDTKILPGNMKDNFWEMGDTGPCGPCSEIHYDRIGGRDAAHLVNQDDPNVLEIWNLVFIQYNRESDGILKPLPKKSIDT

GMGLERLVSVLQNKMSNYDTDLFVPYFEAIQKGTGARPYSGKVGADDADGIDMAYRVLADHARTITVALADGGRPDNTGR

GYVLRRILRRAVRYSHEKLNASRGFFATLVDVVVQSLGDAFPELKKDPDMVKDIINEEEVQFLKTLSRGRRILDRKIQSL

GDCKTIPGDTAWLLYDTYGFPVDLTGLIAEEKGLVVDMDGFEEERKLAQLKSQGKGAGGDDLIMLDIYAIEELREKGLEA

TDDSPKYNYHSDSSGSYAFESTVATVMALRRDKMFVEEVCTGQECGVVLDKTCFYAEQGGQIYDEGYLVKVEDNSEDKTE

FTVKNAQVRGGYVLHIGTIYGSLRVGDQVRLFIDEPRRRPVMSNHTATHILNFALRSVLGEADQRGSLVAPDRLRFDFTA

KGAMSTQQIKKAEEIANGMIEAAKPVYTQDCPLAAAKAIQGLRAVFDETYPDPVRVVSIGVPVSELLDDPSGPAGSLTSV

EFCGGTHLQNSSHAGAFVLVSEEAIAKGIRRIVAVTGAEAQKALRKAESLKNSLSIMEAKVKAQTVPNKDVQREIADLGE

ALATAVIPQWQKDEFRENLKSLKKIMDDLDRASKADVQKRVLEKTKQLIDSNPNQPLVILEMESGASAKALNEALKLFKT

HSPQTSAMLFTVDNEAGKITCLCQVPQNAANRGLKASEWVQQVSGLMDGKGGGKDVSAQATGKNVGCLQEALQLAASFAQ

LHLGDMKN

>XP_020949450.1 LOW QUALITY PROTEIN: alanine--tRNA synthetase, cytoplasmic [Sus scrofa]

MHSTLTASEIRQRFIDFFKRNEHTYVHSSATIPLDDPTLLFANAGMNQFKPIFLNTIDPSHPMAKLSRAANTQKCIRAGG

KHNDLDDVGKDVYHHTFFEMLGSWSFGDYFKELACKMALELLTQEFGIPVERLYVTYFGGDEAAGLEPDLECKQIWQNLG

LDDSKILPGNMKDNFWEMGDTGPCGPCSEIHYDRIGGRDAAHLVNQDDPNVLEIWNLVFIQYNRETDGVLKPLPKKSIDT

GMGLERLVSVLQNKMSNYDTDLFVPYFEAIQKGTGARPYTGKVGADDADGIDMAYRVLADHARTITVALADGGRPDNTGR

GYVLRRILRRAVRYSHEKLNASRGFFATLVDVVVQSLGDAFPELKKDPDMVKDIINEEEVQFLKTLSRGRRILDRKIQSL

GDSKTIPGDTAWLLYDTYGFPVDLTGLIAEEKGLVVDMDGFEEERKLAQLKSQGKGAGGEDLIMLDIYAIEELRERGLEA

TDDSPKYNYHSDSSGSYVFESAVATVMALRRDRMFVEEVCTGQECGVVLDKTCFYAEQGGQIYDEGYLVKVDDSSEDKTE

FTVKNAQVRGGYVLHIGTIYGSLKVGDQVRLFIDEPRRRPVMSNHTATHILNFALRSVLGEADQRGSLVAPDXLRFDFTA

KGAMSTQQIKKAEELANEMIEAAKPVYAQNCPLAAAKAIQGLRAVFDETYPDPVRVVSIGVPVSELLDDPSGPAGSLTSV

EFCGGTHLQNSSHAGAFVIVSEEAIAKGIRRIVAVTGAEAQKALRKADSLMKSLCAMEAKVKAQTAPNKDVQREIADLGE

ALATAVIPQWQKDEFRENLRSLKKVMDDLDRASKADVQKRVLERTKQLIDSNPNQPLVILEMESGASAKALNEALKLFKT

HSPQTSAMLFTVDNEAGKIICLCQVPQNIANRGLKASEWVQQVSGLMDGKGGGKDVSAQATGKNVGCLQEALQLATSFAQ

LRLEDVKN

>XP_007526734.1 PREDICTED: alanine--tRNA synthetase, cytoplasmic [Erinaceus europaeus]

MDSTLTASEIRRRFIDFFKRNEHTYVHSSATIPLDDPTLLFANAGMNQFKPIFLNTIDPSHPMAKLNRAANTQKCIRAGG

KHNDLDDVGKDVYHHTFFEMLGSWSFGDYFKELACKMALELLTQEFGIPVERLYVTYFGGDEAAGLEPDLECKQIWQNLG

LDDSKILPGNMKDNFWEMGDTGPCGPCSEIHYDRIGGRDAAHLVNQDDPNVLEIWNLVFIQYNRETDGILKPLPKKSIDT

GMGLERLVSVLQNKMSNYDTDLFVPYFEAIQKGTGARPYTGKVGAEDTDGIDMAYRVLADHARTITVALADGGRPDNTGR

GYVLRRILRRAVRYSHEKLNANRGFFATLVDVVVQSLGDAFPELKKDPDMVKDIINEEEVQFLKTLSRGRRILDRKIQSL

GDSKTIPGDTAWLLYDTYGFPVDLTGLIAEEKGMVVDMDGFEEERKLAQLKSQGKGAGGEDHIMLDIYAIEELREKDLEA

TDDSPKYNYHSDSSGSYVFESTLATVMALRRDKMFVEEVSTGQECGVVLDKTCFYAEQGGQTYDEGYLVKVDDSSEDKTE

FTVKNVQVRGGYVLHIGTIYGNLKVGDQVRLFIDEPRRRPVMSNHTATHILNFALRSVLGEADQRGSLVAPDRLRFDFTA

KGAMSSQQIKKAEEIANGMIEAAKPVYTQDCPLAAAKAIQGLRAVFDETYPDPVRVVSIGVPVSKLLDDPSSPAGSLTSV

EFCGGTHLQNSSHAAAFVIVSEEAIAKGIRRIVAVTGAEAQKALRKAESLKKSLSVMEAKVKAQTAPNKDVQKEIADLGE

ALATAVIPQWQKDEFRENLKSLKKIMDDLDRASKADVQKRVLEKTKQLIDSNPNQPLVILEMESGASAKALNEALKLFKT

HSPQTSAMLFTVDNEAGKITCLCQVPQNAANRGLKASEWVQQVSGLMDGKGGGKDVSAQATGKNVGCLQEALQLATSFAQ

LHLGDVQN

>XP_004584081.1 PREDICTED: alanine--tRNA synthetase, cytoplasmic [Ochotona princeps]

MNFVLTASEIRQRFIDFFKRHEHTYVHSSATIPLDDPTLLFANAGMNQFKPIFLNTIDPSHPMAKLSRAANTQKCIRAGG

KHNDLDDVGKDVYHHTFFEMLGSWSFGDYFKELACKMALELLTQEFGIPIERLYVTYFGGDEAAGLEPDLECKQIWQNLG

LDDSRILPGNMKDNFWEMGDTGPCGPCSEIHYDRIGGRDAAHLVNQDDPNVLEIWNLVFIQYNREADGVLKPLPKKSIDT

GMGLERLVSVLQNKMSNYDTDLFMPYFEAIQKGTGARPYTGKVGVDDVDGIDMAYRVLADHARTITVALADGGRPDNTGR

GYVLRRILRRAVRYSHEKLNASRGFFATLVDVVVQSLGDAFPELKKDPDMVKDIINEEEMQFLKTLSRGRRILDRKIQSL

GDCKIIPGDTAWLLYDTYGFPVDLTGLIAEEKGLVVDMDGFEEERKLAQLKSQGKGAGGEDLIMLDVYAIEELRAKGLEA

TDDSPKYNYHADASGSYVFESTVATVLALRREKTFVEEVSTGQECGVVLDRTCFYAEQGGQIYDEGYLVKADDSSEDKTE

FTVKNAQVRGGYVLHIGTVYGSLKVGDQVRLFIDEPRRRPVMSNHTATHILNFALRSVLGEADQKGSLVAPDRLRFDFTA

KGAMSTQQIKKAEEIANEMIEAAKPVYTQDCALAAAKAIQGLRAVFDETYPDPVRVVSIGVPVSELLEDPSGPAGSLTSV

EFCGGTHLRNSSHAGAFVIVTEEAIAKGIRRIVAVTGAEAQKALRKAESLKKSLSLMEAKVKAQTTPNKDVQKEIADLGE

VLATAVIPQWQKDELREMLKSLKKIMDDLDRASKADVQKRVLEKTKQLIDSNPNQPLVILEMESGASAKALNEALKLFKT

HSPQTSAMLFTVDNEAGKITCLCQVPQNAANRGLKASEWVQQVSGLMDGKGGGKDMSAQATGKNVGCLQEALQLATSFAQ

LRLGDVKN

>XP_013210200.1 PREDICTED: alanine--tRNA synthetase, cytoplasmic [Microtus ochrogaster]

MESTLTAREIRERFINFFKRNEHTYVHSSATIPLDDPTLLFANAGMNQFKPIFLNTIDPSHPMAKLSRAANTQKCIRAGG

KHNDLDDVGKDVYHHTFFEMLGSWSFGDYFKELACKMALELLTQEFGIPVERLYVTYFGGDEAAGLEPDLECKQIWQNLG

LDDARILPGNMKDNFWEMGDTGPCGPCSEIHYDRIGGRDAAHLVNQDDPNVLEIWNLVFIQYNRESDGLLKPLPKKSIDT

GMGLERLVSVLQNKMSNYDTDLFVPYFEAIQKGTGARPYTGKVGAEDADGIDMAYRVLADHARTITVALADGGRPDNTGR

GYVLRRILRRAVRYSHEKLNASRGFFATLVDVVVQSLGDAFPELKKDPDMVKDIINEEEVQFLKTLSRGRRILDRKIQSL

GDCMTIPGDTAWLLYDTYGFPVDLTGLIAEEKGLVVDMDGFEEERKLAQLKSQGKGAGGEDLIMLDIYAIEELRAKGLEA

TDDSPKYNYRSDSSGSYEFECTVATVLALRREKMFVEEVVTGQECGVVLDKTCFYAEQGGQIYDEGYLVKVDDSSEDKTE

FTVKNAQVRGGYVLHIGTIYGNLKVGDHVRLFIDEPRRRPVMSNHTATHILNFALRSVLGEADQKGSLVAPDRLRFDFTA

KGAMSTQQIKQAEEIANAMIEAAKPVYTQDCPLAAAKAIQGLRAVFDETYPDPVRVVSIGVPVSELLDDPSGPAGSLTSV

EFCGGTHLRNSSHAGAFVIVTEEAIAKGIRRIVAVTGAEAQKALRKAESLKKSLSDLEAKVKAQTAPNKDVQREIADFGE

ALATAVMPQWQKDEQRETLKSLKKVMDDLDRASKADVQKRVLEKTKQLIDSNPNQPLVILEMESGASAKALNEALKLFKT

HSPQTSAMLFTVDNEAGKITCLCQVPQNAANRGLKASEWVQQVSVLMDGKGGGKDMSAQATGKNVGCLQEALQLATSFAQ

LRLGDVKN

>XP_008570991.1 PREDICTED: alanine--tRNA synthetase, cytoplasmic [Galeopterus variegatus]

MDPTLTASEIRQRFIDFFKRNEHTYVHSSATIPLDDPTLLFANAGMNQFKPIFLNTIDPSHPLAKLSRAANTQKCIRAGG

KHNDLDDVGKDVYHHTFFEMLGSWSFGDYFKELACKMALELLTQEFGIPVERLYVTYFGGDEAAGLEPDLECRQIWQNLG

LDDNKILPGNMKDNFWEMGDTGPCGPCSEIHYDRIGGRDAAHLVNQDDPNVLEIWNLVFIQYNREMDGILKPLPKKSIDT

GMGLERLVSVLQNKMSNYDTDLFVPYFEAIQKGTGARPYTGRVGADDADGIDMAYRVLADHARTITVALADGGRPDNTGR

GYVLRRILRRAVRYSHEKLNASRGFFATLVDVVVQSLGDAFPELKKDPDMVKDIINEEEVQFLKTLSRGRRILDRKIQSL

GDSKTIPGDTAWLLYDTYGFPVDLTGLIAEEKGLVVDMDGFEEERKLAQLKSQGKGAGGEDLIMLDIYAIEELRAKGLQA

TDDSPKYNYRSDSSGNYEFESAVATVLALRRERMFVEEVSTGQECGVVLDKTCFYAEQGGQIYDEGYLVKVDDSSEDKTE

FTVKNAQVRGGYVLHIGAIYGDLRVGDQVRLFIDEPRRRPVMSNHTATHILNFALRSVLGEADQKGSLVAPDRLRFDFTA

KGAMSTQQIKKAEEIANEMIAAGKPVYTQDCPLAAAKAIQGLRAVFDETYPDPVRVVSIGVPVSKLLDDPSGPAGSLTSV

EFCGGTHLQNSSHAGAFVIVTEEAIAKGIRRIVAVTGPEAQKALRKAESLKKALSVTEAKVKAQTAPNKDVQREIADLGE

ALATAIIPQWQKDEFRESLKSLKKVMDDLDRASKADVQKRVLEKTKQLIDSNPNQPLVILEMESGASAKALNEALKLFKT

HSPQTSAMLFTVDNEAGKITCLCQVPQNAANRGLKASEWVQQVSGLMDGKGGGKDMSAQATGKNVDCLQEALQLATSFAQ

LHLGDVKN

>EPY90010.1 alanyl-tRNA synthetase, cytoplasmic [Camelus ferus]

MDSTLTASEIRQRFIDFFKRNEHTYVHSSATIPLDDPTLLFANAGMNQFKPIFLNTIDPSHPMAKLSRAANTQKCIRAGG

KHNDLDDVGKDVYHHTFFEMLGSWSFGDYFKMALELLTQEFGIPIERLYVTYFGGDEAAGLEPDLECKQIWQNLGLDDAK

ILPGNMKDNFWEMGDTGPCGPCSEIHYDRIGGRDAAHLVNQDDPNVLEIWNLVFIQYNRENDGVLKPLPKKSIDTGMGLE

RLVSVLQNKMSNYDTDLFVPYFEAIQKGTGARPYTGKVGADDADGIDMAYRVLADHARTITVALADGGRPDNTGRGYVLR

RILRRAVRYSHEKLNASRGFFATLVDVVVQSLGDAFPELKKDPDMVKDIINEEEVQFLKTLSRGRRILDRKIQSLGDNRT

IPGDTAWLLYDTYGFPVDLTGLIAEEKGLVVDMDGFEEERKLAQLKSQGKGASGEDLIMLDIYAIEELRERGLEATDDSP

KYNYHSDSSGSYAFESAVATVMALRRDKMFVEEVSTGQECGVVLDKTCFYAEQGGQIYDEGYLVKVDDSSEDKTEFTVKN

AQVRGGYVLHIGTVYGSLKVGDQVRLFIDEPRRRPVMSNHTATHILNFALRSVLGEADQRGSLVAPDRLRFDFTAKGAMS

TQQIKKAEEIANEMIEAAKPVYTQDCPLAAAKAIQGLRAVFDETYPDPVRVVSIGVPVSELLDDPSGPAGSLTSVEFCGG

THLQNSSHAGAFVIVSEEAIAKGIRRIVAVTGAEAQKALRKAESLKNSLSLMEAKVKAQTAPNKDVQREIADLGEALATA

VIPQWQKDEFRENLRSLKKIMDDLDRASKADVQKRVLEKTKQLIDSNPNQPLVILEMESGASAKALNEALKLFKTHSPQT

SAMLFTVDNEAGKITCLCQVPQNAANRGLKASEWVQQVSGLMDGKGGGKDVSAQATGKNVGCLQEALQLATSFAQLHLGD

VKN

>XP_022367556.1 alanine--tRNA synthetase, cytoplasmic [Enhydra lutris kenyoni]

MDSTLTAGGIRQRFIDFFRRNGHTYVHSSATIPLDDPTLLFANAGMNQFKPIFLNTIDPSHPMAKLSRAANTQKCIRAGG

KHNDLDDVGKDVYHHTFFEMLGSWSFGDYFKELACKMALELLTQEFGIPVERLYVTYFGGDEAAGLEPDLECKQIWQNLG

LDDTRILPGNMKDNFWEMGDTGPCGPCSEIHYDRIGGRDAAHLVNQDDPNVLEIWNLVFIQYNRESDGILKPLPKKSIDT

GMGLERLVSVLQNKMSNYDTDLFVPYFEAIQKGTGARPYSGKVGADDADGIDMAYRVLADHARTITVALADGGRPDNTGR

GYVLRRILRRAVRYSHEKLNASRGFFATLVDVVVQSLGDAFPELKKDPDMVKDIINEEEVQFLKTLSRGRRILDRKIQSL

GDCKTIPGDTAWLLYDTYGFPVDLTGLIAEEKGLVVDMDGFEEERKLAQLKSQGKGAGGEDLIMLDIYAIEELREKGLEA

TDDSPKYNYHSDSSGSYAFESAVATVMALRRDKMFVEEVCTGQECGVVLDKTCFYAEQGGQIYDEGYLVKVEDNSEDKTE

FTVKNAQVRGGYVLHIGTIYGSLRVGDQVRLFIDEPRRRPIMSNHTATHILNFALRSVLGEADQRGSLVAPDRLRFDFTA

KGAMSTQQIKKAEEIANQMIEAAKPVYTQDCPLAAAKAIQGLRAVFDETYPDPVRVVSIGVPVSELLDDPSGPAGSLTSV

EFCGGTHLQNSSHAGTFVIVSEEAIAKGIRRIVAVTGAEAQKALRKAESLKNSLLVMEAKVKAQTAPNKDVQREIADLGE

ALATAVIPQWQKDEFRENLKSLKKIMDDLDRASKADVQKRVLEKTKQLIDSNPNQPLVILEMESGASAKALNEALKLFKT

HSPQTSAMLFTVDNEAGKITCLCQVPQNAANRGLKASEWVQQVSGLMDGKGGGKDVSAQATGKNVGCLQEALELATSFAQ

LRLGDVKN

>NP_001094545.1 alanine--tRNA synthetase, cytoplasmic [Bos taurus]

MDSTLTAGEIRQRFIDFFKRNEHTYVHSSATIPLDDPTLLFANAGMNQFKPIFLNTIDPSHPMAKLSRAANTQKCIRAGG

KHNDLDDVGKDVYHHTFFEMLGSWSFGDYFKELACKMALELLTQEFGIPVERLYVTYFGGDEAAGLEPDLECKQIWQNLG

LDDSRILPGNMKDNFWEMGDTGPCGPCSEIHYDRIGGRDAAHLVNQDDPNVLEIWNLVFIQYNRETDGILKPLPKKSIDT

GMGLERLVSVLQNKMSNYDTDLFVPYFEAIQKGTGARPYTGRVGAEDADGIDMAYRVLADHARTITVALADGGRPDNTGR

GYVLRRILRRAVRYSHEKLNASRGFFATLVDVVVQSLGDAFPELKKDPDMVKDIINEEEVQFLKTLSRGRRILDRKIQSL

GDSKTIPGDTAWLLYDTYGFPVDLTALIAEEKGLVVDMDGFDEERKLAQLKSQGKGAGGEDLIMLDIYAIEELREKGLEA

TDDSPKYSYHSDSSGTYVFESAVATVMALRRDRMFVEEASTGQECGVVLDKTCFYAEQGGQTYDEGYLVKVDDSSEDKTE

FTVKNTQVRGGYVLHIGTLYGSLKVGDQVRLFIDEPRRRPVMSNHTATHILNFALRSVLGEADQRGSLVAPDRLRFDFTA

KGAMSTQQIKKAEEIANEMIEAAKPVYTEDCSLAAAKAIQGLRAVFDETYPDPVRVVSIGVPVSELLDDPSGPAGSLTSV

EFCGGTHLQNSSHAGAFVIVSEEAIAKGIRRIVAVTGAEAQKALRKAESLKKSLCVVEAKVKAQAAPNKDVQREIADLGE

ALATAIIPQWQKDEFRENLKSLKKVMDDLDRASKADVQKRVLEKTKQLIDSSPNQPLVILEVESGASAKALNEALKLFKM

HSPQTSAMLFTVDNEAGRITCLCQVPQNAANRGLKASEWVQQVSGLMDGKGGGKDVSAQATGKNVGCLQEALQLATSFAQ

LRLGDVKN

>XP_008700570.1 PREDICTED: alanine--tRNA synthetase, cytoplasmic [Ursus maritimus]

MDTTLTASEIRQRFIDFFRRNEHTYVHSSATIPLDDPTLLFANAGMNQFKPIFLNTIDPSHPMAKLSRAANTQKCIRAGG

KHNDLDDVGKDVYHHTFFEMLGSWSFGDYFKELACKMALELLTQEFGIPLERLYVTYFGGDEAAGLEPDLECKQIWQNLG

LDDTKILPGNMKDNFWEMGDTGPCGPCSEIHYDRIGGRDAAHLVNQDDPNVLEIWNLVFIQYNRESDGILKPLPKKSIDT

GMGLERLVSVLQNKMSNYDTDLFVPYFEAIQKGTGARPYSGKVGADDADGIDMAYRVLADHARTITVALADGGRPDNTGR

GYVLRRILRRAVRYSHEKLNASRGFFATLVDVVVQSLGDAFPELKKDPDMVKDIINEEEVQFLKTLSRGRRILDRKIQSL

GDCKTIPGDTAWLLYDTYGFPVDLTGLIAEEKGLVVDMGGFEEERKLAQLKSQGKGAGGDDLIMLDIYAIEELREKGLEA

TDDSPKYNYHSDSSGSYAFESAVATVMALRRDKMFVEEVCTGQECGVVLDKTCFYAEQGGQIYDEGYLVKVEDNSEDKTE

FTVKSAQVRGGYVLHIGTVYGSLRVGDQVRLFIDEPRRRPIMSNHTATHILNFALRSVLGEADQRGSLVAPDRLRFDFTA

KGAMSTQQIKKAEEIANGMIEAAKPVYTQDCPLAAAKAIQGLRAVFDETYPDPVRVVSIGVPVSELLDDPSGPAGSLTSV

EFCGGTHLQNSSHAGAFVIVSEEAIAKGIRRIVAVTGAEAQKALRKAESLKNSLSVMEAKVKAQTVPNKDVQREIADLGE

ALATAVIPQWQKDEFRENLKSLKKIMDDLDRASKADVQKRVLEKTKQLIDSNPNQPLVILEMESGASAKALNEALKLFKT

HSPQTSAMLFTVDNEAGKITCLCQVPQNAANRGLKASEWVQQVSGLMDGKGGGKDVSAQATGKNVGCLQEALQLAASFAQ

LHLGDMKN

>XP_006040799.1 PREDICTED: alanine--tRNA synthetase, cytoplasmic-like [Bubalus bubalis]

MDSTLTAGEIRQRFIDFFKRNEHTYVHSSATIPLDDPTLLFANAGMNQFKPIFLNTIDPSHPMAKLSRAANTQKCIRAGG

KHNDLDDVGKDVYHHTFFEMLGSWSFGDYFKELACKMALELLTQEFGIPVERLYVTYFGGDEAAGLEPDLECKQIWQNLG

LDDSRILPGNMKDNFWEMGDTGPCGPCSEIHYDRIGGRDAAHLVNQDDPNVLEIWNLVFIQYNRETDGILKPLPKKSIDT

GMGLERLVSVLQNKMSNYDTDLFVPYFEAIQKGTGARPYTGKVGAEDADGIDMAYRVLADHARTITVALADGGRPDNTGR

GYVLRRILRRAVRYSHEKLNASRGFFATLVDVVVQSLGDAFPELKKDPDMVKDIINEEEVQFLKTLSRGRRILDRKIQSL

GDSKTIPGDTAWLLYDTYGFPVDLTALIAEEKGLVVDMDGFDEERKLAQLKSQGKGAGGEDLIMLDIYAIEELREKGLEA

TDDSPKYSYHSDSSGTYAFEGAVATVMALRRDKLFVEEASTGQECGVLLDKTCFYAEQGGQIYDEGYLVKADDSSEDKTE

FTVKNTQVRGGYVLHIGTLYGSLKVGDQVRLFIDEPRRRPVMSNHTATHILNFALRSVLGEADQRGSLVAPDRLRFDFTA

KGAMSTQQIKKAEEIANEMIEAAKPVYTEDCSLAAAKAIQGLRAVFDETYPDPVRVVSIGVPVSELLDDPSGPAGSLTSV

EFCGGTHVQNSSHVGAFVIVSEEAIAKGIRRIVAVTGAEAQKALRKAESLKKCLCVVEAKVKAQTAPNKDVQREIADLGE

ALATAIIPQWQKDEFRENLKSLKKVMDDLDRASKADVQKRVLEKTKQLIDSSPNQPLVILEVESGASAKALNEALKLFKM

HSPQTSAMLFTVDNEAGRITCLCQVPQNAANRGLKASEWVQQVSGLMDGKGGGKDVSAQATGKNVGCLQEALQLATSFAQ

LRLGDVKN

>NP_666329.2 alanine--tRNA synthetase, cytoplasmic [Mus musculus]

MDATLTAREIRERFINFFRRNEHTYVHSSATIPLDDPTLLFANAGMNQFKPIFLNTIDPSHPMAKLSRAANTQKCIRAGG

KHNDLDDVGKDVYHHTFFEMLGSWSFGDYFKELACKMALELLTQEFGIPVERLYVTYFGGDEAAGLEPDLECRQIWQNLG

LDEARILPGNMKDNFWEMGDTGPCGPCSEIHYDRIGGRDAAHLVNQDDPNVLEIWNLVFIQYNRESDGVLKPLPKKSIDT

GMGLERLVSVLQNKMSNYDTDLFMPYFEAIQKGTGARPYTGKVGAEDADGIDMAYRVLADHARTITVALADGGRPDNTGR

GYVLRRILRRAVRYSHEKLNASRGFFATLVDVVVQSLGDAFPELKKDPEMVKDIINEEEVQFLKTLSRGRRILDRKIQSL

GDCKTIPGDTAWLLYDTYGFPVDLTGLIAEEKGLVVDMNGFEEERRLAQLKSQGKGAGDEDLIMLDIYAIEELRAKGLEA

TDDSPKYNYQSDSSGSYVFECTVATVLALRREKMFVDEVVTGQECGVVLDKTCFYAEQGGQIYDEGYLVKVDDSSEDKTE

FTVKNAQVRGGYVLHIGTIYGNLKVGDQVRLFIDEPRRRPVMSNHTATHILNFALRSVLGEADQKGSLVAPDRLRFDFTA

KGAMSTQQIKKAEEIVNGMIEAAKPVYTQDCPLAAAKAIQGLRAVFDETYPDPVRVVSIGVPVSELLDDPCGPAGSLTSV

EFCGGTHLRNSSHAGAFVIVTEEAIAKGIRRIVAVTGAEAQKALRKSETLKKSLSAMEAKVKAQTAPNKDVQREIADLGE

ALATAVIPQWQKDEQRETLKSLKKVMDDLDRASKADVQKRVLEKTKQLIDSNPNQPLVILEMESGASAKALNEALKLFKT

HSPQTSAMLFTVDNEAGKITCLCQVPQNAANRGLKASEWVQQVSGLMDGKGGGKDMSAQATGKNVGCLQEALQLATSFAQ

LRLGDVKN

>XP_021025063.1 alanine--tRNA synthetase, cytoplasmic [Mus caroli]

MDATLTAREIREKFINFFRRNEHTYVHSSATIPLDDPTLLFANAGMNQFKPIFLNTIDPSHPMAKLSRAANTQKCIRAGG

KHNDLDDVGKDVYHHTFFEMLGSWSFGDYFKELACKMALELLTQEFGIPVDRLYVTYFGGDEAAGLEPDLECRQIWQNLG

LDEARILPGNMKDNFWEMGDTGPCGPCSEIHYDRIGGRDAAHLVNQDDPNVLEIWNLVFIQYNRESDGVLKPLPKKSIDT

GMGLERLVSVLQNKMSNYDTDLFVPYFEAIQKGTGARPYTGKVGAEDADGIDMAYRVLADHARTITVALADGGRPDNTGR

GYVLRRILRRAVRYSHEKLNASRGFFATLVDVVVQSLGDAFPELKKDPDMVKDIINEEEVQFLKTLSRGRRILDRKIQSL

GDCKTIPGDTAWLLYDTYGFPVDLTGLIAEEKGLVVDMNGFEEERRLAQLKSQGKGAGDEDLIMLDIYAIEELRAKGLEA

TDDSPKYNYQSDSSGSYVFECTVATVLALRREKMFVDEVVTGQECGVVLDKTCFYAEQGGQIYDEGYLVKVDDSSEDKTE

FTVKNAQVRGGYVLHIGTIYGNLKVGDQVRLFIDEPRRRPVMSNHTATHILNFALRSVLGEADQKGSLVAPDRLRFDFTA

KGAMSTQQIKKAEEIVNGMIEAAKPVYTQDCPLAAAKAIQGLRAVFDETYPDPVRVVSIGVPVSELLDDPCGPAGSLTSV

EFCGGTHLRNSSHAGAFVIVTEEAIAKGIRRIVAVTGAEAQKALRKSETLKKSLSAMEAKVKAQTAPNKDVQREIADLGE

ALATAVIPQWQKDEQRETLKSLKKVMDDLDRASKADVQKRVLEKTKQLIDSNPNQPLVILEMESGASAKALNEALKLFKT

HSPQTSAMLFTVDNEAGKITCLCQVPQNAANRGLKASEWVQQVSGLMDGKGGGKDMSAQATGKNVGCLQEALQLATSFAQ

LRLGDVKN

>BAC31927.1 unnamed protein product [Mus musculus]

MDATLTAREIRERFINFFRRNEHTYVHSSATIPLDDPTLLFANAGMNQFKPIFLNTIDPSHPMAKLSRAANTQKCIRAGG

KHNDLDDVGKDVYHHTFFEMLGSWSFGDYFKELACKMALELLTQEFGIPVERLYVTYFGGDEAAGLEPDLECRQIWQNLG

LDEARILPGNMKDNFWEMGDTGPCGPCSEIHYDRIGGRDAAHLVNQDDPNVLEIWNLVFIQYNRESDGVLKPLPKKSIDT

GMGLERLVSVLQNKMSNYDTDLFMPYFEAIQKGTGARPYTGKVGAEDADGIDMAYRVLADHARTITVALADGGRPDNTGR

GYVLRRILRRAVRYSHEKLNASRGFFATLVDVVVQSLGDAFPELKKDPDMVKDIINEEEVQFLKTLSRGRRILDRKIQSL

GDCKTIPGDTAWLLYDTYGFPVDLTGLIAEEKGLVVDMNGFEEERRLAQLKSQGKGAGDEDLIMLDIYAIEELRAKGLEA

TDDSPKYNYQSDSSGSYVFECTVATVLALRREKMFVDEVVTGQECGVVLDKTCFYAEQGGQIYDEGYLVKVDDSSEDKTE

FTVKNAQVRGGYVLHIGTIYGNLKVGDQVRLFIDEPRRRPVMSNHTATHILNFALRSVLGEADQKGSLVTPDRLRFDFTA

KGAMSTQQIKKAEEIVNGMIEAAKPVYTQDCPLAAAKAIQGLRAVFDETYPDPVRVVSIGVPVSELLDDPCGPAGSLTSV

EFCGGTHLRNSSHAGAFVIVTEEAIAKGIRRIVAVTGAEAQKALRKSETLKKSLSAMEAKVKAQTAPNKDVQREIADLGE

ALATAVIPQWQKDEQRETLKSLKKVMDDLDRASKADVQKRVLEKTKQLIDSNPNQPLVILEMESGASAKALNEALKLFKT

HSPQTSAMLFTVDNEAGKITCLCQVPQNAANRGLKASEWVQQVSGLMDGKGGGKDMSAQATGKNVGCLQEALQLATSFAQ

LRLGDVKN

>XP_005980209.1 PREDICTED: alanine--tRNA synthetase, cytoplasmic isoform X1 [Pantholops hodgsonii]

MDSTLTAGEIRQRFIDFFKRNEHTYVHSSATIPLDDPTLLFANAGMNQFKPIFLNTIDPSHPMAKLSRAANTQKCIRAGG

KHNDLDDVGKDVYHHTFFEMLGSWSFGDYFKDLACKMALELLTQEFGIPVERLYVTYFGGDEAAGLEPDVECKQIWQNLG

LDDSRILPGNMKDNFWEMGDTGPCGPCSEIHYDRVGGRDAAHLVNQDDPNVLEIWNLVFIQYNRETDGILKPLPKKSIDT

GMGLERLVSVLQNKMSNYDTDLFVPYFEAIQKGTGARQYTGKVGAEDADGIDMAYRVLADHARTITVALSDGGRPDNTGR

GYVLRRILRRAVRYSHEKLNASRGFFATLVDVVVQSLGDAFPELKKDPDMVKDIINEEEVQFLKTLSRGRRILDRKIQSL

GDSKTIPGDTAWLLYDTYGFPVDLTALIAEEKGLVVDMDGFDEERKLAQLKSQGKGAGGEDLIMLDIYAIEELREKGLEA

TDDSPKYSYHSDSSGTYVFESAVATVMALRRDKMFVEEVSTGQECGVVLDKTCFYAEQGGQIYDEGYLVKVDDSSEDKTE

FTVKNTQVRGGYVLHIGTLYGSLKVGDQVRLFIDEPRRRPVMSNHTATHILNFALRSVLGEADQRGSLVAPDRLRFDFTA

KGAMSTQQIKRAEEIANEMIEAAKPVYTEDCSLAAAKAIQGLRAVFDETYPDPVRVVSIGVPVSELLDDPSGPAGSLTSV

EFCGGTHLQNSSHAGVFVIVSEEAIAKGIRRIVAVTGAEAQKALRKAESLKKSLCVMEAKVKAQTAPNKDVQREIADLGE

ALATASIPQWQKDEFRENLKSLKKVMDDLDRASKADVQKRVLEKTKQLIDSSPNQPLVILEVESGASAKALNEALKLFKM

HSPQTSAMLFTVDNEAGRITCLCQVPQNAANRGLKASEWVQQVSGLMDGKGGGKDVSAQATGKNVGCLQEALQLATSFAQ

LRLGDVKN

>XP_005691846.1 PREDICTED: alanine--tRNA synthetase, cytoplasmic [Capra hircus]

MDSTLTAGEIRQRFIDFFKRNEHTYVHSSATIPLDDPTLLFANAGMNQFKPIFLNTIDPSHPMAKLSRAANTQKCIRAGG

KHNDLDDVGKDVYHHTFFEMLGSWSFGDYFKELACKMALELLTQEFGIPVGRLYVTYFGGDEAAGLEPDLECKQIWQNLG

LDDSRILPGSMKDNFWEMGDTGPCGPCSEIHYDRVGGRDAAHLVNQDDPNVLEIWNLVFIQYNRETDGILKPLPKKSIDT

GMGLERLVSVLQNKMSNYDTDLFVPYFEAIQKGTGARPYTGKVGAEDADGIDMAYRVLADHARTITVALSDGGRPDNTGR

GYVLRRILRRAVRYSHEKLNASRGFFATLVDVVVQSLGDAFPELKKDPDMVKDIINEEEVQFLKTLSRGRRILDRKIQSL

GDSKTIPGDTAWLLYDTYGFPVDLTALIAEEKGLVVDMDGFDEERKLAQLKSQGKGAGGEDLIMLDIYAIEELREKGLEA

TDDSPKYSYHSDSSGTYVFESAVATVMALRRDKMFVEEVSMGQECGVVLDKTCFYAEQGGQIYDEGYLVKVDDSSEDKTE

FTVKNTQVRGGYVLHIGTLYGSLKVGDQVRLFIDEPRRRPVMSNHTATHILNFALRSVLGEADQRGSLVAPDRLRFDFTA

KGAMSTQQIKRAEEIANEMIEAAKPVYTEDCSLAAAKAIQGLRAVFDETYPDPVRVVSIGVPVSELLDDPSGPAGSLTSV

EFCGGTHLQNSSHAGVFVIVSEEAIAKGIRRIVAVTGAEAQKALRKAESLKKSLCVMEAKVKAQTAPNKDVQREIADLGE

ALATAIIPQWQKDEFRENLKSLKKVMDDLDRASKADVQKRVLEKTKQLIDSSPNQPLVILEVESGASAKALNEALKLFKM

HSPQTSAMLFTVDNEAGRITCLCQVPQNAANRGLKASEWVQQVSGLMDGKGGGKDVSAQATGKNVGCLQEALQLATSFAQ

LRLGDVKN

>AAP57355.1 Alanyl-tRNA synthase [Mus musculus]

MDATLTAREIRERFINFFRRNEHTYVHSSATIPLDDPTLLFANAGMNQFKPIFLNTIDPSHPMAKLSRAANTQKCIRAGG

KHNDLDDVGKDVYHHTFFEMLGSWSFGDYFKELACKMALELLTQEFGIPVERLYVTYFGGDEAAGLEPDLECRQIWQNLG

LDEARILPGNMKDNFWEMGDTGPCGPCSEIHYDRIGGRDAAHLVNQDDPNVLEIWNLVFIQYNRESDGVLKPLPKKSIDT

GMGLERLVSVLQNKMSNYDTDLFMPYFEAIQKGTGARPYTGKVGAEDADGIDMAYRVLADHARTITVALADGGRPDNTGR

GYVLRRILRRAVRYSHEKLNASRGFFATLVDVVVQSLGDAFPELKKDPEMVKDIINEEEVQFLKTLSRGRRILDRKIQSL

GDCKTIPGDTAWLLYDTYGFPVDLTGLIAEEKGLVVDMNGFEEERRLAQLKSQGKGAGDEDLIMLDIYAIEELRAKGLEA

TDDSPKYNYQSDSSGSYVFECTVATVLALRREKMFVDEVVTGQECGVVLDKTCFYAEQGGQIYDEGYLVKVDDSSEDKTE

FTVKNAQVRGGYVLHIGTIYGNLKVGDQVRLFIDEPRRRPVMSNHTATHILNFALRSVLGEADQKGSLVAPDRLRFDFTA

KGAMSTQQIKKAEEIVNGMIEAAKPVYTQDCPLAAAKAIQGLRAVFDETYPDPVRVVSIGVPVSELLDDPCGPAGSLTSV

EFCGGTHLRNSSHEGAFVIVTEEAIAKGIRRIVAVTGAEAQKALRKSETLKKSLSAMEAKVKAQTAPNKDVQREIADLGE

ALATAVIPQWQKDEQRETLKSLKKVMDDLDRASKADVQKRVLEKTKQLIDSNPNQPLVILEMESGASAKALNEALKLFKT

HSPQTSAMLFTVDNEAGKITCLCQVPQNAANRGLKASEWVQQVSGLMDGKGGGKDMSAQATGKNVGCLQEALQLATSFAQ

LRLGDVKN

>EPQ05513.1 Alanyl-tRNA synthetase, cytoplasmic [Myotis brandtii]

MRVTFKMDSTLTASEIRQRFIDFFKRNEHTYVHSSATIPLDDPTLLFANAGMNQFKPIFLNTIDPSHPMAKLSRAANTQK

CIRAGGKHNDLDDVGKDVYHHTFFEMLGSWSFGDYFKELACKMALELLTQEFGIPVERLYVTYFGGDEAAGLEPDLECKQ

IWQNLGVDDTKILPGNMKDNFWEMGDTGPCGPCSEIHYDRIGGRDAAHLVNQDDPNVLEIWNLVFIQYNRETDGTLKPLP

KKSIDTGMGLERLVSVLQNKMSNYDTDLFVPYFEAIQKGTGARPYTGKVGADDADGIDMAYRVLADHARTITVALADGGR

PDNTGRGYVLRRILRRAVRYSHEKLNASKGFFATLVDVVVQSLGDAFPELKKDPDMVKDIINEEEVQFLKTLSRGRRILD

RKIQSLGDSKTIPGDTAWLLYDTYGFPVDLTGLIAEEKGLVVDMDGFEEERKLAQLKSQGKGAGGEDHIMLDIYAIEELR

GKSLEATDDSPKYSYLSDSSGSYAFESTVATVMALRRDKMFVEEVSTGQECGVVLDKTCFYAEQGGQIYDEGYLVKVDDS

SEDKTEFTVKNTQVRGGYVLHIGTIYGNLKVGDQVRLFIDEPRRRPVMSNHTATHILNFALRSVLGEADQRGSLVAPDRL

RFDFTAKGAMSTQQIKKAEQIVNEMIEAAKPVYTQDCPLAAAKAIQGLRAVFDETYPDPVRVVSIGVPVSKLLDDPSGPA

GSLTSVEFCGGTHLQNSSHAGAFVIVSEEAIAKGIRRIVAVTGAEAQKALRKAESLKKSLSGMEAKVKAQTSPNKDVQRE

IADLGEALATAVIPQWQKDEFRETLKSLKKIMDDLDRASKADVQKRVLEKTKQLIDSNPNQPLVILEMESGASAKATQGQ

GSRGWVSRHCSALNEALKLFKTHSPQTSAMLFTVDNEAGKITCLCQVPQNAANRGLKASEWVQQVSGLMDGKGGGKDVSA

QATGKNVGCLQEALQLATSFAQLRLGDVKN

>BAE34378.1 unnamed protein product [Mus musculus]

MDATLTAREIRERFINFFRRNEHTYVHSSATIPLDDPTLLFANAGMNQFKPIFLNTIDPSHPMAKLSRAANTQKCIRAGG

KHNDLDDVGKDVYHHTFFEMLGSWSFGDYFKELACKMALELLTQEFGIPVERLYVTYLGGDEAAGLEPDLECRQIWQNLG

LDEARILPGNMKDNFWEMGDTGPCGPCSEIHYDRIGGRDAAHLVNQDDPNVLEIWNLVFIQYNRESDGVLKPLPKKSIDT

GMGLERLVSVLQNKMSNYDTDLFMPYFEAIQKGTGARPYTGKVGAEDADGIDMAYRVLADHARTITVALADGGRPDNTGR

GYVLRRILRRAVRYSHEKLNASRGFFATLVDVVVQSLGDAFPELKKDPDMVKDIINEEEVQFLKTLSRGRRILDRKIQSL

GDCKTIPGDTAWLLYDTYGFPVDLTGLIAEEKGLVVDMNGFEEERRLAQLKSQGKGAGDEDLIMLDIYAIEELRAKGLEA

TDDSPKYNYQSDSSGSYVFECTVATVLALRREKMFVDEVVTGQECGVVLDKTCFYAEQGGQIYDEGYLVKVDDSSEDKTE

FTVKNAQVRGGYVLHIGTIYGNLKVGDQVRLFIDEPRRRPVMSNHTATHILNFALRSVLGEADQKGSLVAPDRLRFDFTA

KGAMSTQQIKKAEEIVNGMIEAAKPVYTQDCPLAAAKAIQGLRAVFDETYPDPVRVVSIGVPVSELLDDPCGPAGSLTSV

EFCGGTHLRNSSHAGAFVIVTEEAIAKGIRRIVAVTGAEAQKALRKSETLKKSLSAMEAKVKAQTAPNKDVQREIADLGE

ALATAVIPQWQKDEQRETLKSLKKVMDDLDRASKADVQKRVLEKTKQLIDSNPNQPLVILEMESGASAKALNEALKLFKT

HSPQTSAMLFTVDNEAGKITCLCQVPQNAANRGLKASEWVQQVSGLMDGKGGGKDMSAQATGKNVGCLQEALQLATSFAQ

LRLGDVKN

>XP_005403528.1 PREDICTED: alanine--tRNA synthetase, cytoplasmic [Chinchilla lanigera]

MSSTLTASEIRERFIDFFKRNEHTYVHSSATIPLDDPTLLFANAGMNQFKPIFLNTIDPSHPLAKLSRAANTQKCIRAGG

KHNDLDDVGKDVYHHTFFEMLGSWSFGDYFKELACKMALELLTEEFGIPVDRLYVTYFGGDEAAGLEPDLECKQIWQNLG

LDGTRILPGNMKDNFWEMGDTGPCGPCSEIHYDRIGDRDAAHLVNQDDPNVLEIWNLVFIQYNRETDGTLKPLPKKSIDT

GMGLERLVSVLQNKMSNYDTDLFVPYFEAIQKGTGARPYAGKVGAEDVDGIDMAYRVLADHARTITVALADGGRPDNTGR

GYVLRRILRRAVRYSHEKLNASRGFFATLVDVVVQSLGDAFPELKKDPDMVKDIINEEEVQFLKTLSRGRRILDRKIQSL

GDCRTIPGDTAWLLYDTYGFPVDLTGLIAEEKGLVVDMDGFEEERKLAQLKSQGKGAGGEDLIMLDIYAIEELRAQGLEA

TDDSPKYNYRSDSSGGYVFESAVATVQALRREKMFVEEVSTGQECGVVLDKTCFYAEQGGQIYDEGYLVKVDDSSEDKTE

FTVKNTQVRGGYVLHIGTIYGSLKVGDQVRLFIDEPRRRPIMSNHTATHILNFALRSVLGEADQKGSLVAPDRLRFDFTA

KGAMSTQQIKKAEGIANEMIEAAKPVYTQDCPLAAAKAIQGLRAVFDETYPDPVRVVSIGVPVSELLDDPSGPAGSLTSV

EFCGGTHLQNSSHAGAFVIVTEEAIAKGIRRIVAVTGAEAQKALRKAESLKKSLSTMESKVKAQTAPNKDVQREIADLSE

VLATAVIPQWQKDELRETLKSLKKVMDDLDRASKADVQKRVLERTKQLINSNPNQPLVILEMESGASAKALNEALKLFKT

HSPETSAMLFTVDNEAGKITCLCQVPQNAANRGLKASEWVQQVSGLMDGKGGGKDVSAQATGKNVGCLQEALQLATSFAQ

LRLGDVKN

>XP_007938543.1 PREDICTED: alanine--tRNA synthetase, cytoplasmic [Orycteropus afer afer]

MDSTLTASEIRQRFIDFFKRNEHTYVHSSATIPLDDPTLLFANAGMNQFKPIFLNTVDPSHPMAKLSRAANTQKCIRAGG

KHNDLDDVGKDVYHHTFFEMLGSWSFGDYFKELACQMALELLTQEFGIPVERLYVTYFGGDEAAGLEPDLECKHIWRNLG

LDDTKILPGNMKDNFWEMGDTGPCGPCSEIHYDRIGGRDAAHLVNQDDPNVLEIWNLVFIQYNRESDGILKSLPKKSIDT

GMGLERLVSVLQNKMSNYDTDLFIPYFEVIQKGTGARPYTGKVGADDTDGIDMAYRVLADHARTITVALADGGRPDNTGR

GYVLRRILRRAVRYSHEKLNASRGFFATLVDVVVQSLGDAFPELKKDPDMVKDIINEEEVQFLKTLSRGRRILDRKIQSL

GDSNTIPGDTAWLLYDTYGFPVDLTGLIAEEKGLVVDMDGFEEERKLAQLKSQGKGAGGEDLIMLDIYAIEELRSTGLAA

TDDSPKYNYHSDPSGSYVFENSVATVMALRRDKMFVEEVSTGQECGVVLDKTCFYAEQGGQIYDEGYLVKVDDSSEDKTE

FTVKNAQVRGGYVLHIGTIYGNLKVGDQVRLFIDEPRRRPIMSNHTATHILNFALRSVLGEADQRGSLVAPDRLRFDFTA

KGAMSTQQIKKAEDIVNQMIEAAKPVYTQDCALAAAKAIQGLRAVFDETYPDPVRVVSIGVPVSELLEDPSGPAGSLTSV

EFCGGTHLQNSSHTGAFVIVSEEAIAKGIRRIVAVTGVEAQKALRKAESLKKSLSVMEAKVKAQIAPNKDVQREIADLSE

ALATAIIPQWQKDELREDLKSLKKIMDDLDRASKADVQKRVLEKTKQLIDSHPNQPLVILEMESGASAKALNEALKLFKT

HSPQTAAMLFTVDNEAGKITCLCQVPQNAANRGLKASEWVQQVSGLMDGKGGGKDVSAQATGKNVGCLQEALQLATSFAQ

LRLGDVKN

>EDL11484.1 alanyl-tRNA synthetase, isoform CRA_a, partial [Mus musculus]

APRALREEPALGGGGAAAAGCRAVPPLAWVRTRSPQEPDLESSFCYSLGALRVVWFLVDLQRTKTLGRSRLLFSRHPELR

FPRRLSEMDATLTAREIRERFINFFRRNEHTYVHSSATIPLDDPTLLFANAGMNQFKPIFLNTIDPSHPMAKLSRAANTQ

KCIRAGGKHNDLDDVGKDVYHHTFFEMLGSWSFGDYFKELACKMALELLTQEFGIPVERLYVTYFGGDEAAGLEPDLECR

QIWQNLGLDEARILPGNMKDNFWEMGDTGPCGPCSEIHYDRIGGRDAAHLVNQDDPNVLEIWNLVFIQYNRESDGVLKPL

PKKSIDTGMGLERLVSVLQNKMSNYDTDLFMPYFEAIQKGTGARPYTGKVGAEDADGIDMAYRVLADHARTITVALADGG

RPDNTGRGYVLRRILRRAVRYSHEKLNASRGFFATLVDVVVQSLGDAFPELKKDPEMVKDIINEEEVQFLKTLSRGRRIL

DRKIQSLGDCKTIPGDTAWLLYDTYGFPVDLTGLIAEEKGLVVDMNGFEEERRLAQLKSQGKGAGDEDLIMLDIYAIEEL

RAKGLEATDDSPKYNYQSDSSGSYVFECTVATVLALRREKMFVDEVVTGQECGVVLDKTCFYAEQGGQIYDEGYLVKVDD

SSEDKTEFTVKNAQVRGGYVLHIGTIYGNLKVGDQVRLFIDEPRRRPVMSNHTATHILNFALRSVLGEADQKGSLVAPDR

LRFDFTAKGAMSTQQIKKAEEIVNGMIEAAKPVYTQDCPLAAAKAIQGLRAVFDETYPDPVRVVSIGVPVSELLDDPCGP

AGSLTSVEFCGGTHLRNSSHAGAFVIVTEEAIAKGIRRIVAVTGAEAQKALRKSETLKKSLSAMEAKVKAQTAPNKDVQR

EIADLGEALATAVIPQWQKDEQRETLKSLKKVMDDLDRASKADVQKRVLEKTKQLIDSNPNQPLVILEMESGASAKALNE

ALKLFKTHSPQTSAMLFTVDNEAGKITCLCQVPQNAANRGLKASEWVQQVSGLMDGKGGGKDMSAQATGKNVGCLQEALQ

LATSFAQLRLGDVKN

>XP_006172228.1 PREDICTED: LOW QUALITY PROTEIN: alanine--tRNA synthetase, cytoplasmic [Tupaia chinensis]

MDSTLTASDIRSRFIDFFKRNEHTYVHSSATIPLDDPTLLFANAGMNQFKPIFLNTIDPSHPMAKLSRAANTQKCIRAGG

KHNDLDDVGKDVYHHTFFEMLGSWSFGDYFKELACKMALELLTEEFGIPVERLYVTYFGGDEAAGLEPDLECKQIWQNLG

LDDSKILPGNMKDNFWEMGDTGPCGPCSEIHFDRIGGRDAAHLVNQDDPNVLEIWNLVFIQYNRETDGILKPLPKKSIDT

GMGLERLVSVLQNKMSNYDTDLFVPYFEAIQKGTGARPYSGKVGADDTDGIDMAYRVLADHARTITXADGGRPDNTGRGY

VLRRILRRAVRYSHEKLNASRGFFATLVDVVVQSLGDAFPELKKDPDMVKDIINEEEVQFLKTLSRGRRILDRKIQSLGD

SKTIPGDTAWLLYDTYGFPVDLTGLIAEEKGLVVDMDGFEEERKLAQLKSQGKGAGGEDLIMLDIYAIEELRAKGLEATD

DSPKYNYLSDSSGNYVFESTVATVLALRREKMFVAEVFTGQECGVVLDRTCFYAEQGGQTYDEGYLVKADDSSEDKTEFT

VKNVQVRGGYVLHVGTIYGHLKVGDQVRLFIDEPRRRPVMSNHTATHILNFALRCVLGEADQKGSLVAPDRLRFDFTAKG

AMSTQQIKKAEEIANEMIEAAKPVYTQDCPLAAAKAIQGLRAVFDETYPDPVRVVSIGVPVSELLDDPSGPAGSLTSVEF

CGGTHLRNSSHAGAFVIVTEEAIAKGIRRIVAVTGAEAQKALRKAESLKKSLSAMEAKVKAQTAPNKDVQREIADLGEAL

ATAVIPQWQKDELREMLKSLKKVMDDLDRASKADVQKRVLERTKQLIDSNPNQPLVILEMENGASAKALNEALKLFKTHS

PQTSAMLFTVDNEAGKITCLCQVPQNAANRGLKASEWVQQVSGLMDGKGGGKDVSAQATGKNVGCLQEALQLATSFAQLR

LGDVKN

>XP_005894383.1 PREDICTED: alanine--tRNA synthetase, cytoplasmic [Bos mutus]

MDSTLTAGEIRQRFIDFFKRNEHTYVHSSATIPLDDPTLLFANAGMNQFKPIFLNTIDPSHPMAKLSRAANTQKCIRAGG

KHNDLDDVGKDVYHHTFFEMLGSWSFGDYFKELACKMALELLTQEFGIPVERLYVTYFGGDEAAGLEPDLECKQIWQNLG

LDDSRILPGNMKDNFWEMGDTGPCGPCSEIHYDRIGGRDAAHLVNQDDPNVLEIWNLVFIQYNRETDGILKPLPKKSIDT

GMGLERLVSVLQNKMSNYDTDLFVPYFEAIQKGTGARPYTGRVGAEDADGIDMAYRVLADHARTITVALADGGRPDNTGR

GYVLRRILRRAVRYSHEKLNASRGFFATLVDVVVQSLGDAFPELKKDPDMVKDIINEEEVQFLKTLSRGRRILDRKIQSL

GDSKTIPGDTAWLLYDTYGFPVDLTALIAEEKGLVVDMDGFDEERKLAQLKSQGKGAGGEDLIMLDIYAIEELREKGLEA

TDDSPKYSYHSDSSGTYMFESAVATVMALRRDRMFVEEASTGQECGVVLDKTCFYAEQGGQTYDEGYLVKVDDSSEDKTE

FTVKNTQVRGGYVLHIGTLYGSLKVGDQVRLFIDEPRRRPVMSNHTATHILNFALRSVLGEADQRGSLVAPDRLRFDFTA

KGAMSTQQIKKAEEIANEMIEAAKPVYTEDCSLAAAKAIQGLRAVFDETYPDPVRVVSIGVPVSELLDDPSGPAGSLTSV

EFCGGTHLQNSSHVGAFVIVSEEAIAKGIRRIVAVTGAEAQKALRKAENLKKSLCVLEAKVKAQAAPNKDVQREIADLGE

ALATAIIPQWQKDEFRENLKSLKKVMDDLDRASKADVQKRVLEKTKQLIDSSPNQPLVILEVESGASAKALNEALKLFKM

HSPQTSAMLFTVDNEAGRITCLCQVPQNAANRGLKASEWVQQVSGLMDGKGGGKDVSAQATGKNVGCLQEALQLATSFAQ

LRLGDVKN

>XP_017533241.1 PREDICTED: alanine--tRNA synthetase, cytoplasmic [Manis javanica]

MDSTLTASEIRQRFIDFFKRNEHTYVHSSATIPLDDPTLLFANAGMNQFKPIFLNTIDPSHPMAKLSRAANTQKCIRAGG

KHNDLDDVGKDVYHHTFFEMLGSWSFGDYFKELACKMALELLTQEFGIPVERLYVTYFGGDEAAGLEPDLECKQIWQNLG

LDDTKILPGNMKDNFWEMGDTGPCGPCSEIHYDRIGGRDAAHLVNQDDPNVLEIWNLVFIQYNRETDGILKPLPKKSIDT

GMGLERLVSVLQNKMSNYDTDLFVPYFEAIQKGTGARPYTGKVGAEDADGIDMAYRVLADHARTITVALADGGRPDNTGR

GYVLRRILRRAVRYSHEKLNASRGFFATLVDVVVQSLGDAFPELKKDPDLVKDIVNEEEVQFLKTLSRGRRILDRKIQSL

GDSRTIPGDTAWLLYDTYGFPVDLTALIAEEKGLVVDMDGFEEERKLAQVKSQGKGAGGEDLIMLDIYAIEELREKGLEA

TDDSPKYNYYSDSSGSYAFESAVATVMALRRDKMFVEEVSTGQECGVVLDKTCFYAEQGGQIYDEGYLVKVDDSSEDKTE

FTVKNAQVRGGYVLHIGTVYGGLRVGDEVRLFIDEPRRRPVMSNHTATHILNFALRSVLGEADQRGSLVAPDRLRFDFTA

KGAMSTQQIKKAEETVNEMIEAARPVYTQDCPLAAAKAIQGLRAVFDETYPDPVRVVSIGVPVSELLDDPSGPAGSLTSV

EFCGGTHLQNSSHAGAFVIVSEEAIAKGIRRIVGVTGAEAQKALRKAESLTKSLSAMEAKVKAQAAPNKDVQREIADLSE

ALATAVIPQWQKDEFRENLRSLKKVMDDLDRASKADVQKRVLEKTKQLIDSNPNQPLVILEMESGASAKALNEALKLFKT

HSPQTSAMLFTVDNEAGKITCLCQVPQNAASRGLKASEWVQQVSGLMDGKGGGKDVSAQATGKNVGCLQEALQLATSFAQ

LHLGAVEN

>EFB26461.1 hypothetical protein PANDA_021488, partial [Ailuropoda melanoleuca]

MDTTLTASEIRQRFIDFFRRNEHTYVHSSATIPLDDPTLLFANAGMNQFKPIFLNTIDPSHPMAKLSRAANTQKCIRAGG

KHNDLDDVGKDVYHHTFFEMLGSWSFGDYFKELACKMALELLTQEFGIPLERLYVTYFGGDEAAGLEPDLECKQIWQNLG

LDDTKILPGNMKDNFWEMGDTGPCGPCSEIHYDRIGGRDAAHLVNQDDPNVLEIWNLVFIQYNRESDGILKPLPKKSIDT

GMGLERLVSVLQNKMSNYDTDLFVPYFEAIQKGTGARPYSGKVGADDADGIDMAYRVLADHARTITVALADGGRPDNTGR

GYVLRRILRRAVRYSHEKLNASRGFFATLVDVVVQSLGDAFPELKKDPDMVKDIINEEEVQFLKTLSRGRRILDRKIQSL

GDCKTIPGDTAWLLYDTYGFPVDLTGLIAEEKGLVVDMDGFEEERKLAQLKSQGKGAGGDDLIMLDIYAIEELREKGLEA

TDDSPKYNYHSDSSGSYAFESTVATVMALRRDKMFVEEVCTGQECGVVLDKTCFYAEQGGQIYDEGYLVKVEDNSEDKTE

FTVKNAQVRGGYVLHIGTIYGSLRVGDQVRLFIDEPRRRPVMSNHTATHILNFALRSVLGEADQRGSLVAPDRLRFDFTA

KGAMSTQQIKKAEEIANGMIEAAKPVYTQDCPLAAAKAIQGLRAVFDETYPDPVRVVSIGVPVSELLDDPSGPAGSLTSV

EFCGGTHLQNSSHAGAFVLVSEEAIAKGIRRIVAVTGAEAQKALRKAESLKNSLSIMEAKVKAQTVPNKDVQREIADLGE

ALATAVIPQWQKDEFRENLKSLKKIMDDLDRASKADVQKRVLEKTKQLIDSNPNQPLVILEMESGASAKATRGGGEGALN

EALKLFKTHSPQTSAMLFTVDNEAGKITCLCQVPQNAANRGLKASEWVQQVSGLMDGKGGGKDVSAQATGKNVGCLQEAL

QLAASFAQLHLGDMKN

>BAE29394.1 unnamed protein product [Mus musculus]

MDATLTAREIRERFINFFRRNEHTYVHSSATIPLDDPTLLFANAGMNQFKPIFLNTIDPSHPMAKLSRAANTQKCIRAGG

KHNDLDDVGKDVYHHTFFEMLGSWSFGDYFKELACKMALELLTQEFGIPVERLYVTYFGGDEAAGLEPDLECRQIWQNLG

LDEARILPGNMRDNFWEMGDTGPCGPCSEIHYDRIGGRDAAHLVNQDDPNVLEIWNLVFIQYNRESDGVLKPLPKKSIDT

GMGLERLVSVLQNKMSNYDTDLFMPYFEAIQKGTGARPYTGKVGAEDADGIDMAYRVLADHARTITVALADGGRPDNTGR

GYVLRRILRRAVRYSHEKLNASRGFFATLVDVVVQSLGDAFPELKKDPEMVKDIINEEEVQFLKTLSRGRRILDRKIQSL

GDCKTIPGDTAWLLYDTYGFPVDLTGLIAEEKGLVVDMNGFEEERRLAQLKSQGKGAGDEDLIMLDIYAIEELRAKGLEA

TDDSPKYNYQSDSSGSYVFECTVATVLALRREKMFVDEVVTGQECGVVLDKTCFYAEQGGQIYDEGYLVKVDDSSEDKTE

FTVKNAQVRGGYVLHIGTIYGNLKVGDQVRLFIDEPRRRPVMSNHTATHILNFALRSVLGEADQKGSLVAPDRLRFDFTA

KGAMSTQQIKKAEEIVNGMIEAAKPVYTQDCPLAAAKAIQGLRAVFDETYPDPVRVVSIGVPVSELLDDPCGPAGSLTSV

EFCGGTHLRNSSHAGAFVIVTEEAIAKGIRRIVAVTGAEAQKALRKSETLKKSLSAMEAKVKAQTAPNKDVQREIADLGE

ALATAVIPQWQKDEQRETLKSLKKVMDDLDRASKAGVQKRVLEKTKQLIDSNPNQPLVILEMESGASAKALNEALKLFKT

HSPQTSAMLFTVDNEAGKITCLCQVPQNAANRGLKASEWVQQVSGLMDGKGGGKDMSAQATGKNVGCLQEALQLATSFAQ

LRLGDVKN

>NP_001093987.1 alanine--tRNA synthetase, cytoplasmic [Rattus norvegicus]

MDSTLTAREIRERFINYFKRNEHTYVHSSATIPLDDPTLLFANAGMNQFKPIFLNTVDPSHPMAKLSRAANTQKCIRAGG

KHNDLDDVGKDVYHHTFFEMLGSWSFGDYFKELACKMALELLTQEFGIPVERLYVTYFGGDEAAGLEPDLECRQIWQNLG

LDEAKILPGNMKDNFWEMGDTGPCGPCSEIHYDRIGGRDAAHLVNQDDPNVLEIWNLVFIQYNRESDGVLKPLPKKSIDT

GMGLERLVSVLQNKMSNYDTDLFVPYFEAIQKGTGARPYTGKVGAEDTDGIDMAYRVLADHARTITVALADGGRPDNTGR

GYVLRRILRRAVRYSHEKLNASRGFFATLVDVVVQSLGDAFPELKKDPDMVKDIINEEEVQFLKTLSRGRRILDRKIQSL

GDCQTIPGDTAWLLYDTYGFPVDLTGLIAEEKGLVVDMDGFEEERKLAQLKSQGKGAGGEDLIMLDIYAIEELRAKGLEA

TDDSPKYNYHSDSSGSYVFECTVATVLALRREKMFVDEVVTGQECGVVLDKTCFYAEQGGQIFDEGYLVKVDDSSEDKTE

FTVKNAQVRGGYVLHIGTIYGNLRVGDQVRLFIDEPRRRPVMSNHTATHILNFALRSVLGDADQKGSLVAPDRLRFDFTA

KGAMSTEQIKKTEEIVNGMIEAAKPVYTLDCPLAAAKAIQGLRAVFDETYPDPVRVVSIGVPVSELLDDPSGPAGSLTSV

EFCGGTHLRNSSHAGAFVIVTEEAIAKGIRRIVAVTGAEAQKALRKSETLKKSLSAMEVKVKAQSAPNKDVQKEIADLGE

VLATAVIPQWQKDEQRETLKSLKKVMDDLDRASKADVQKRVLEKTKQLIDSNPNQPLVILEMESGASAKALNEALKLFKT

HSPQTSAMLFTVDNEAGKITCLCQVPQNAANRGLKASEWVQQVSGLMDGKGGGKDMSAQATGKNVGCLQEALQLATSFAQ

LRLGDVKN

>EHB04674.1 Alanyl-tRNA synthetase, cytoplasmic [Heterocephalus glaber]

MNSTLTASEIRERFIDFFKRNEHTYVHSSATIPLDDPTLLFANAGMNQFKPIFLNTIDPSHPLAKLSRAANTQKCIRAGG

KHNDLDDVGKDVYHHTFFEMLGSWSFGDYFKESACKMALELLTQEFGIPVDRLYVTYFGGDEAAGLEPDLECKQIWQTLG

LDDSRILPGNMKDNFWEMGDTGPCGPCSEIHYDRIGGRDASYLVNQDDPNVLEIWNLVFIQYNRETDGTLKPLPKKSIDT

GMGLERLVSVLQNKMSNYDTDLFVPYFEAIQKGTGARPYTGKVGAEDADGIDMAYRVLADHARTITVALADGGRPDNTGR

GYVLRRILRRAVRYSHEKLNASRGFFATLVDVVVQSLGDAFPELKKDPDMVKDIINEEEVQFLKTLSRGRRILDRKIQSL

GDCKTIPGDTAWLLYDTYGFPVDLTGLIAEEKGLVVDMDGFEEERKLAQLKSQGKGAGGEDLIMLDIYAIEELRARGLEA

TDDSPKYNYHSDSSGGYLFESAVATVLALRREKMFVEEVSTGQECGVLLDKTCFYAEQGGQIYDEGYLVKVDDSSEDKTE

FTVKNAQVRGGYVLHIGTIYGNLKVGDQVRLFIDEPRRRPIMSNHTATHILNFALRSVLGEADQKGSLVAPDRLRFDFTA

KGAMSTQQIKKAEETGNEMIKAAKPVYTQDCPLAAAKAIQGLRAVFDETYPDPVRVVSIGVPVSELLDDPSGPAGSLTSV

EFCGGTHLRNSSHAGAFVIVTEEAIAKGIRRIVAVTGAEAQKALRKAESLKKSFSTMESKVKAQTAPNKDVQREIVDLGE

ALATAVIPQWQKDELRETLKSLKKVMDDLDRASKANVQKRVLERTKQLIDSNPNQPLVILEMESGASAKATRGWGPGTTL

PVTDALNEALKLFKTHSPETSAMLFTVDNEAGKITCLCQVPQNAANRGLKASEWVQQVSGLMDGKGGGKDVSAQATGKNV

GCLQEALQLATSFAQLHLGDVKN

>XP_010642720.1 PREDICTED: alanine--tRNA synthetase, cytoplasmic [Fukomys damarensis]

MNSTLKASEIRERFIDFFKRNAHTYVHSSATIPLDDPTLLFANAGMNQFKPIFLNTIDPSHPLAKLSRAANTQKCIRAGG

KHNDLDDVGKDVYHHTFFEMLGSWSFGDYFKELACKMALELLTQEFGIPVDRLYVTYFGGDEAAGLEPDLECKQIWQNLG

LDDSRILPGNMKDNFWEMGDTGPCGPCSEIHYDRIGGRDAAHLVNQDDPNVLEIWNLVFIQYNRETDSTLKPLPKKSIDT

GMGLERLVSVLQNKMSNYDTDLFVPYFEAIQKGTGARPYTGKVGAEDADGIDMAYRVLADHARTITVALADGGRPDNTGR

GYVLRRILRRAVRYSHEKLNASRGFFATLVDVVVQSLGDAFPELKKDPDLVKDIINEEEVQFLKTLSRGRRILDRKIQSL

GDCKTIPGDTAWLLYDTYGFPVDLTGLIAEEKGLVVDMNGFEEERKLAQLKSQGKGAGGEDLIMLDIYAIEELRAKGLEA

TDDFPKYDYHSDPSGGYVFESAVATVLALRREKMFVEEVSTGQECGVVLDKTCFYAEQGGQIYDEGYLVKVDDSSEDKTE

FTVKNTQVRGGYVLHIGTIYGTLKVGDQVRLFIDEPRRRPIMSNHTATHILNFALRSVLGEADQKGSLVAPDRLRFDFTA

KGAMSTQQIKKAEEMANEMIKAAKPVYTQDCPLAAAKAIQGLRAVFDETYPDPVRVVSIGVPVSQLLDDPSGPAGSLTSV

EFCGGTHLRNSSHAGAFVIVTEEAIAKGIRRIVAVTGAEAQKALRKADSLKKSLSTVESKVKAQTAPNKDMQREIADLGE

ALATAVIPQWQKDELRETLKSLKKVMDDLDRASKADVQKRVLERTKRLIDSNPNQPLVILEMESGASAKALNEALKLFKT

HSPETSAMLFTVDNEAGKITCLCQVPQNAANRGLKASEWVQQVSGLMDGKGGGKDVSAQATGKNVGCLQEALQLATSFAQ

LHLGDVKN

>XP_024411855.1 alanine--tRNA synthetase, cytoplasmic [Desmodus rotundus]

MDSTLTASEIRQRFIDFFKKNEHTYVHSSATIPLDDPTLLFANAGMNQFKPIFLNTIDPSHPMAKLRRAANTQKCIRAGG

KHNDLDDVGKDVYHHTFFEMLGSWSFGDYFKELACKMALELLTQEFGIPVERLYVTYFGGDEAAGLEADLECRQIWQNLG

LDDSKILPGNMKDNFWEMGDTGPCGPCSEVHYDRIGGRDAAHLVNQDDPNVLEIWNLVFIQYNREADGVLKPLPKKSIDT

GMGLERLVSVLQNKMSNYDTDLFVPYFEAIQKGTGARPYTGKVGADDADGIDMAYRVLADHARTITVALADGGRPDNTGR

GYVLRRILRRAVRYSHEKLSASKGFFATLVDVVVQSLGDAFPELKKDPDMVKDIINEEEVQFLKTLSRGRRILDRKIQSL

GDSKTIPGDTAWLLYDTYGFPVDLTGLIAEEKGLAVDMDGFEEERRLAQLKSQGKGAGGEDHLMLDIYAIEELREKSLEA

TDDSPKYSYLSDAGGSYTFESTVATVMALRREKRFVEEVSTGQECGVVLDKTCFYAEQGGQIYDEGYLVKVDDSMEDKTE

FTVKNTQVRGGYVLHVGTIYGHLKVGDQVRLFIDEPRRRPVMSNHTATHILNFALRSVLGEADQRGSLVAPDRLRFDFTA

KGAMSTQQIKKAEKIVNEMIEAAKPVYAQDCPLAAAKAIQGLRAVFDETYPDPVRVISIGVPVSELLDDPSGPAGSLTSV

EFCGGTHLQNSSHAGAFVIVSEEAIAKGIRRIVAVTGAEAQKALRKAESLKKSLCVMEAKVKAQTAPNKDVQREVADLGE

ALATAVIPQWQKDEFRESLKSLKKIMDDLDRASKADVQKRVLEKTKQLIDSNPNQPLVILEMESGASAKALNEALKLFKT

HSPQTAAMLFTVDNEAGKITCLCQVPQNAANRGLKASEWVQQVSGLMDGKGGGKDVSAQATGKNVGCLQEALRLATSFAQ

LRLGDVKN

>XP_020757134.1 alanine--tRNA synthetase, cytoplasmic isoform X2 [Odocoileus virginianus texanus]

MDATLTAGEIRQRFIDFFKRNEHTYVHSSATIPLDDPTLLFANAGMNQFKPIFLNTIDPSHPMAKLSRAANTQKCIRAGG

KHNDLDDVGKDVYHHTFFEMLGSWSFGDYFKELACKMALELLTQEFGIPVERLYVTYFGGDEAAGLEPDLECKQIWQNLG

LDDSRILPGNMKDNFWEMGDTGPCGPCSEIHYDRVGGRDAAHLVNQDDPNVLEIWNLVFIQYNRETDGILKPLPKKSIDT

GMGLERLVSVLQNKMSNYDTDLFVPYFEAIQKGTGARPYTGKVGAEDADGIDMAYRVLADHARTITVALADGGRPDNTGR

GYVLRRILRRAVRYSHEKLNASRGFFATLVDVVVQSLGDAFPELKKDPDMVKDIINEEEVQFLKTLSRGRRILDRKIQSL

GDSKIIPGDTAWLLYDTYGFPVDLTALIAEEKGLVVDMDGFDEERKLAQLKSQGKGAGGEDLIMLDIYAIEELREKGLEA

TDDSPKYSYYSDSSGTYVFESAVATVMALRRDKMFVEEVSTGQECGVVLDKTCFYAEQGGQIYDEGYLVKVDDSSEDKTE

FTVKNTQVRGGYVLHMGTLYGSLQVGDRVRLFIDEPRRRPVMSNHTATHILNFALRSVLGEADQRGSLVAPDRLRFDFTA

KGAMSTQQIRKAEEIANEMIEAAQPVYTEDCALAAAKAIQGLRAVFDETYPDPVRVVSIGVPVSELLDDPSGPAGSLTSV

EFCGGTHLQNSSHAGAFVIVSEEAIAKGIRRIVAVTGAEAHKALRKAESLKKSLCVMEAKVKAQTAPSKDVQREIADLGE

ALATAIIPQWQKDEFRENLKSLKKIMDDLDRASKADVQKRVLEKTKQLIDSSPNQPLVILEVESGASAKALNEALKLFKT

HSPQTSAMLFTVDNEAGKITCLCQVPQNAANRGLKASEWVQQVSGLMDGKGGGKDVSAQATGKNVGCLQEALQLATSFAQ

LHLGDVKN

>XP_020757133.1 alanine--tRNA synthetase, cytoplasmic isoform X1 [Odocoileus virginianus texanus]

MAVQPHATSLSPVCQRSQFPRVIFKMDATLTAGEIRQRFIDFFKRNEHTYVHSSATIPLDDPTLLFANAGMNQFKPIFLN

TIDPSHPMAKLSRAANTQKCIRAGGKHNDLDDVGKDVYHHTFFEMLGSWSFGDYFKELACKMALELLTQEFGIPVERLYV

TYFGGDEAAGLEPDLECKQIWQNLGLDDSRILPGNMKDNFWEMGDTGPCGPCSEIHYDRVGGRDAAHLVNQDDPNVLEIW

NLVFIQYNRETDGILKPLPKKSIDTGMGLERLVSVLQNKMSNYDTDLFVPYFEAIQKGTGARPYTGKVGAEDADGIDMAY

RVLADHARTITVALADGGRPDNTGRGYVLRRILRRAVRYSHEKLNASRGFFATLVDVVVQSLGDAFPELKKDPDMVKDII

NEEEVQFLKTLSRGRRILDRKIQSLGDSKIIPGDTAWLLYDTYGFPVDLTALIAEEKGLVVDMDGFDEERKLAQLKSQGK

GAGGEDLIMLDIYAIEELREKGLEATDDSPKYSYYSDSSGTYVFESAVATVMALRRDKMFVEEVSTGQECGVVLDKTCFY

AEQGGQIYDEGYLVKVDDSSEDKTEFTVKNTQVRGGYVLHMGTLYGSLQVGDRVRLFIDEPRRRPVMSNHTATHILNFAL

RSVLGEADQRGSLVAPDRLRFDFTAKGAMSTQQIRKAEEIANEMIEAAQPVYTEDCALAAAKAIQGLRAVFDETYPDPVR

VVSIGVPVSELLDDPSGPAGSLTSVEFCGGTHLQNSSHAGAFVIVSEEAIAKGIRRIVAVTGAEAHKALRKAESLKKSLC

VMEAKVKAQTAPSKDVQREIADLGEALATAIIPQWQKDEFRENLKSLKKIMDDLDRASKADVQKRVLEKTKQLIDSSPNQ

PLVILEVESGASAKALNEALKLFKTHSPQTSAMLFTVDNEAGKITCLCQVPQNAANRGLKASEWVQQVSGLMDGKGGGKD

VSAQATGKNVGCLQEALQLATSFAQLHLGDVKN

>XP_004704604.1 PREDICTED: alanine--tRNA synthetase, cytoplasmic isoform X1 [Echinops telfairi]

MMDSTLTASEIRQRFIAFFKRNKHTYVHSSATIPLDDPTLLFANAGMNQFKPIFLNTIDPSHPMAKLTRAANTQKCIRAG

GKHNDLDDVGKDVYHHTFFEMLGSWSFGDYFKELACKMALELLTQEFGIPVERLYVTYFGGDEAAGLEPDLECKQIWQNL

GLDDAKILPGNMKDNFWEMGDTGPCGPCSEIHYDRIGGRDAAHLVNQDDPNVLEIWNLVFIQYNREADGILRPLPKKSID

TGMGLERLVSVLQNKMSNYDTDLFVPYFEAIQKGTGARPYTGKVGADDADGIDMAYRVLADHARTITVALADGGRPDNTG

RGYVLRRILRRAVRYSHEKLNASRGFFATLVDVVVQSLGDAFPELKKDPDMVKDIINEEEVQFLKTLSRGRRILDRKIQS

LGDSQTIPGDTAWLLYDTYGFPVDLTGLIAEEKGLAVDMEGFEEERKLAQLKSQGKGAGGEDLIMLDIYAIEELRSRGLE

ATDDSLKYSYHSEPGGSYVFENTVATVMALRRDKMFVDEVATGQECGVVLDKTCFYAEQGGQIYDEGYLVKLDDNSEDRT

EFTVKNAQVRGGYVLHIGTVYGNLRVGDQVRLFIDETRRRPVMSNHTATHILNFALRAVLGEADQKGSLVAPDRLRFDFT

AKGAMSTQQIKEAEEIANQTIEAAKPVYTQDCALAAAKAIQGLRAVFDETYPDPVRVVSIGVPVSELLEDPTSPAGSLTS

VEFCGGTHLQNSSHAGAFVIVSEEAIAKGIRRIVAVTGAEAQKALRKAESMKKSLSVLEAKVKAQTSPNKDVQREIADLG

EALATAVVPQWQKDELREELKSLKKVMDDLDRASKADVQKRVLEKTKQLIDSRPNQPLVILEMESGASAKALNEALKLFK

THSPQTAAMLFTVDNEAGKITCLCQVPQNAANRGLKASEWVQQVSGLMDGKGGGKDVSAQATGKNVGCLQEALQLATSFA

QLRLGDVKN

>XP_004387867.1 alanine--tRNA synthetase, cytoplasmic isoform X1 [Trichechus manatus latirostris]

MDSTLTATEIRQRFIDFFKRNEHTYVHSSATIPLDDPTLLFANAGMNQFKPIFLNTIDPSHPMAKLSRAANTQKCIRAGG

KHNDLDDVGKDVYHHTFFEMLGSWSFGDYFKELACKMALELLTQEFGIPVERLYVTYFGGDEATRLEPDLECKQIWQNLG

LDDTKILPGNMKDNFWEMGDTGPCGPCSEIHYDRIGGRDAAYLVNQDDPNVLEIWNLVFIQYNRETDGILKPLPRKSIDT

GMGLERLVSVLQNKMSNYDTDLFVPYFEAIQKGTGARPYTGKVGADDADGIDMAYRVLADHARTITVALADGGRPDNTGR

GYVLRRILRRAVRYSHEKLNANRGFFATLVDVVIQSLGDAFPELKKDPDMVKDIINEEEVQFLKTLSRGRRILDRKIQSL

GDSKTIPGDTAWLLYDTYGFPVDLTGLIAEEKGLVVDMDSFEEERKLAQLKSQGKGAGGEDLIMLDIYAIEELRSKGLEA

TDDSLKYNYHSDSSGSYVFENAVATVMALRRDKMFVEEVFTGQECGVVLDKTCFYAEQGGQIYDEGYLVKVDDSSEDKAE

FTVKNAQVRGGYVLHIGTIYGNLKVGDRVRLFIDEPRRRPVMSNHTATHILNFALRSVLGEADQRGSLVAPDRLRFDFTA

KGAMSTEQIKEAEEIANQMIEAAKPVYTQDCLLAAAKAIQGLRAVFDETYPDPVRVVSIGVPVSELLEDPSGPAGSLTSV

EFCGGTHLQNSSHAGTFVIVSEEAIAKGIRRIVAITGAEAQKALRKAESLKKSLSVIEAKVKAQTTPNKDVQREIADLGE

ALATAVVPQWQKDELREHLKSLKKIMDDLDRASKADVQKRVLEKTKQLIDSHPNQPLVILEMESGASAKALNEALKLFKT

HSPQTAAMLFTVDSEAGKITCLCQVPQNAANQGLKASEWVQQVSGLMDGKGGGKDASAQATGKNVGCLQEALQLATSFAQ

LHLGDLKN

>KFO24640.1 Alanyl-tRNA synthetase, cytoplasmic [Fukomys damarensis]

MRVPAGCGLGRAQLRISASARTGNYGRLESSGVTFKMNSTLKASEIRERFIDFFKRNAHTYVHSSATIPLDDPTLLFANA

GMNQFKPIFLNTIDPSHPLAKLSRAANTQKCIRAGGKHNDLDDVGKDVYHHTFFEMLGSWSFGDYFKELACKMALELLTQ

EFGIPVDRLYVTYFGGDEAAGLEPDLECKQIWQNLGLDDSRILPGNMKDNFWEMGDTGPCGPCSEIHYDRIGGRDAAHLV

NQDDPNVLEIWNLVFIQYNRETDSTLKPLPKKSIDTGMGLERLVSVLQNKMSNYDTDLFVPYFEAIQKGTGARPYTGKVG

AEDADGIDMAYRVLADHARTITVALADGGRPDNTGRGYVLRRILRRAVRYSHEKLNASRGFFATLVDVVVQSLGDAFPEL

KKDPDLVKDIINEEEVQFLKTLSRGRRILDRKIQSLGDCKTIPGDTAWLLYDTYGFPVDLTGLIAEEKGLVVDMNGFEEE

RKLAQLKSQGKGAGGEDLIMLDIYAIEELRAKGLEATDDFPKYDYHSDPSGGYVFESAVATVLALRREKMFVEEVSTGQE

CGVVLDKTCFYAEQGGQIYDEGYLVKVDDSSEDKTEFTVKNTQVRGGYVLHIGTIYGTLKVGDQVRLFIDEPRRRPIMSN

HTATHILNFALRSVLGEADQKGSLVAPDRLRFDFTAKGAMSTQQIKKAEEMANEMIKAAKPVYTQDCPLAAAKAIQGLRA

VFDETYPDPVRVVSIGVPVSQLLDDPSGPAGSLTSVEFCGGTHLRNSSHAGAFVIVTEEAIAKGIRRIVAVTGAEAQKAL

RKADSLKKSLSTVESKVKAQTAPNKDMQREIADLGEALATAVIPQWQKDELRETLKSLKKVMDDLDRASKADVQKRVLER

TKRLIDSNPNQPLVILEMESGASAKATGGWGPGAALPALNEALKLFKTHSPETSAMLFTVDNEAGKITCLCQVPQNAANR

GLKASEWVQQVSGLMDGKGGGKDVSAQATGKNVGCLQEALQLATSFAQLHLGDVKN

>XP_006755834.1 PREDICTED: alanine--tRNA synthetase, cytoplasmic [Myotis davidii]

MDSTLTASEIRQRFIDFFKRNENTYVHSSATIPLDDPTLLFANAGMNQFKPIFLNTIDPSHPMAKLSRAANTQKCIRAGG

KHNDLDDVGKDVYHHTFFEMLGSWSFGDYFKELACKMALELLTQEFGIPVERLYVTYFGGDEAAGLEPDLECKQIWQNLG

VDDTKILPGNMKDNFWEMGDTGPCGPCSEIHYDRIGGRDAAHLVNQDDPNVLEIWNLVFIQYNRETDGTLKPLPKKSIDT

GMGLERLVSVLQNKMSNYDTDLFVPYFEAIQKGTGARPYTGKVGADDADGIDMAYRVLADHARTITVALADGGRPDNTGR

GYVLRRILRRAVRYSHEKLNASKGFFATLVDVVVQSLGDAFPELKKDPDMVKDIINEEEVQFLKTLSRGRRILDRKIQSL

GDSKTIPGDTAWLLYDTYGFPVDLTGLIAEEKGLVVDMDGFEEERKLAQLKSQGKGAGGEDHIMLDIYAIEELRGKSLEA

TDDSPKYSYLSDSTGSYAFESTVATVMALRRDKMFVEEVSTGQECGVVLDKTCFYAEQGGQIYDEGYLVKVDDSSEDKTE

FTVKNTQVRGGYVLHIGTIYGNLKVGDQVRLFIDEPRRRPVMSNHTATHILNFALRSVLGEADQRGSLVAPDRLRFDFTA

KGAMSTQQIKKAEQIVNEMIEAAKPVYTQDCPLAAAKAIQGLRAVFDETYPDPVRVVSVGVPVSELLDDPSGPAGSLTSV

EFCGGTHLQNSSHAGAFVIVSEEAIAKGIRRIVAVTGAEAQKALRKAESLKKSLSVMEAKVKAQTSPNKDVQREIADLGE

ALATAVIPQWQKDEFRETLKSLKKIMDDLDRASKADVQKRVLEKTKQLIDSNPNQPLVILEMESGASAKALNEALKLFKT

HSPQTSAMLFTVDNEAGKITCLCQVPQNAANRGLKASEWVQQVSGLMDGKGGGKDVSAQATGKNVGCLQEALQLATSFAQ

LRLGDVKN

>XP_023980673.1 alanine--tRNA synthetase, cytoplasmic isoform X2 [Physeter catodon]

MDSTLTASEIRQRFIDFFKRNEHTYVHSSATIPLDDPTLLFANAGMNQFKPIFLNTVDPSHPMAKLSRAANTQKCIRAGG

KHNDLDDVGKDVYHHTFFEMLGSWSFGDYFKELACKMALELLTQEFGIPVERLYVTYFGGDEPAGLEPDLECKQIWHHLG

LDDSRILPGNMKDNFWEMGDTGPCGPCSEIHYDRIGGRDAAHLVNQDDPNVLEIWNLVFIQYNRETDGILKPLPKKSIDT

GMGLERLVSVLQNKMSNYDTDLFVPYFEAIQKGTGARPYTGKVGADDADGIDMAYRVLADHARTITVALADGGRPDNTGR

GYVLRRILRRAVRYSHEKLNASRGFFATLADVVVQSLGDAFPELKKDPDVVKDIINEEEVQFLKTLSRGRRILDRKIQSL

GDSKTIPGDTAWLLYDTYGFPVDLTGLIAEEKGLVVDMDGFEEERKLAQLKSQGKGAGGEDLIMLDIYAIEELREKGLEA

TDDSPKYNYHSDSSGSYVFESAVATVMALRRDKMFVEEVSTGQECGVVLDKTCFYAEQGGQIYDEGYLVKVDDSSEDKTE

FTVKNAQVRGGYVLHTGTVYGSLKVGDQVRLFIDEPRRRPVMSNHTATHILNFALRSVLGEADQRGSLVAPDRLRFDFTA

KGAMSTQQIKKAEEIANETIEAAKPVYTQDCPLAAAKAIQGLRAVFDETYPDPVRVVSVGVPVSELLDDPSGPAGSLTSV

EFCGGTHLQNSSHAGAFVIVSEEAIAKGIRRIVAVTGAEAQKALRKAESLEKSLSVMEAKVKAQTAPNKDVQREIADLGE

ALATAVIPQWQKDEFRENLRSLKKIMDDLDRANKADVQKRVLEKTKQLIDSSPNQPLVILEMESGASAKALNEALKLFKT

HSPETSAMLFTVDNEAGKITCLCQVPQNAANRGLKASEWVQQVSGLMDGKGGGKDVSAQATGKNVGCLQEALQLATSFAQ

LHLGDVKN

>ELK36179.1 Alanyl-tRNA synthetase, cytoplasmic [Myotis davidii]

MFSYAFLSRVTFKMDSTLTASEIRQRFIDFFKRNENTYVHSSATIPLDDPTLLFANAGMNQFKPIFLNTIDPSHPMAKLS

RAANTQKCIRAGGKHNDLDDVGKDVYHHTFFEMLGSWSFGDYFKELACKMALELLTQEFGIPVERLYVTYFGGDEAAGLE

PDLECKQIWQNLGVDDTKILPGNMKDNFWEMGDTGPCGPCSEIHYDRIGGRDAAHLVNQDDPNVLEIWNLVFIQYNRETD

GTLKPLPKKSIDTGMGLERLVSVLQNKMSNYDTDLFVPYFEAIQKGTGARPYTGKVGADDADGIDMAYRVLADHARTITV

ALADGGRPDNTGRGYVLRRILRRAVRYSHEKLNASKGFFATLVDVVVQSLGDAFPELKKDPDMVKDIINEEEVQFLKTLS

RGRRILDRKIQSLGDSKTIPGDTAWLLYDTYGFPVDLTGLIAEEKGLVVDMDGFEEERKLAQLKSQGKGAGGEDHIMLDI

YAIEELRGKSLEATDDSPKYSYLSDSTGSYAFESTVATVMALRRDKMFVEEVSTGQECGVVLDKTCFYAEQGGQIYDEGY

LVKVDDSSEDKTEFTVKNTQVRGGYVLHIGTIYGNLKVGDQVRLFIDEPRRRPVMSNHTATHILNFALRSVLGEADQRGS

LVAPDRLRFDFTAKGAMSTQQIKKAEQIVNEMIEAAKPVYTQDCPLAAAKAIQGLRAVFDETYPDPVRVVSVGVPVSELL

DDPSGPAGSLTSVEFCGGTHLQNSSHAGAFVIVSEEAIAKGIRRIVAVTGAEAQKALRKAESLKKSLSVMEAKVKAQTSP

NKDVQREIADLGEALATAVIPQWQKDEFRETLKSLKKIMDDLDRASKADVQKRVLEKTKQLIDSNPNQPLVILEMESGAS

AKATQGQGSRGWALNEALKLFKTHSPQTSAMLFTVDNEAGKITCLCQVPQNAANRGLKASEWVQQVSGLMDGKGGGKDVS

AQATGKNVGCLQEALQLATSFAQLRLGDVKN

>XP_007102619.2 alanine--tRNA synthetase, cytoplasmic isoform X1 [Physeter catodon]

MENSCVFWNGTAYFPQFRRVIFEMDSTLTASEIRQRFIDFFKRNEHTYVHSSATIPLDDPTLLFANAGMNQFKPIFLNTV

DPSHPMAKLSRAANTQKCIRAGGKHNDLDDVGKDVYHHTFFEMLGSWSFGDYFKELACKMALELLTQEFGIPVERLYVTY

FGGDEPAGLEPDLECKQIWHHLGLDDSRILPGNMKDNFWEMGDTGPCGPCSEIHYDRIGGRDAAHLVNQDDPNVLEIWNL

VFIQYNRETDGILKPLPKKSIDTGMGLERLVSVLQNKMSNYDTDLFVPYFEAIQKGTGARPYTGKVGADDADGIDMAYRV

LADHARTITVALADGGRPDNTGRGYVLRRILRRAVRYSHEKLNASRGFFATLADVVVQSLGDAFPELKKDPDVVKDIINE

EEVQFLKTLSRGRRILDRKIQSLGDSKTIPGDTAWLLYDTYGFPVDLTGLIAEEKGLVVDMDGFEEERKLAQLKSQGKGA

GGEDLIMLDIYAIEELREKGLEATDDSPKYNYHSDSSGSYVFESAVATVMALRRDKMFVEEVSTGQECGVVLDKTCFYAE

QGGQIYDEGYLVKVDDSSEDKTEFTVKNAQVRGGYVLHTGTVYGSLKVGDQVRLFIDEPRRRPVMSNHTATHILNFALRS

VLGEADQRGSLVAPDRLRFDFTAKGAMSTQQIKKAEEIANETIEAAKPVYTQDCPLAAAKAIQGLRAVFDETYPDPVRVV

SVGVPVSELLDDPSGPAGSLTSVEFCGGTHLQNSSHAGAFVIVSEEAIAKGIRRIVAVTGAEAQKALRKAESLEKSLSVM

EAKVKAQTAPNKDVQREIADLGEALATAVIPQWQKDEFRENLRSLKKIMDDLDRANKADVQKRVLEKTKQLIDSSPNQPL

VILEMESGASAKALNEALKLFKTHSPETSAMLFTVDNEAGKITCLCQVPQNAANRGLKASEWVQQVSGLMDGKGGGKDVS

AQATGKNVGCLQEALQLATSFAQLHLGDVKN

>XP_006909907.1 alanine--tRNA synthetase, cytoplasmic [Pteropus alecto]

MDSILTASEIRQRFIDFFKRNEHTYVHSSATIPLDDPTLLFANAGMNQFKPIFLNTIDPSHPMAKLSRAANTQKCIRAGG

KHNDLDDVGKDVYHHTFFEMLGSWSFGDYFKELACKMALELLTQEFGIPLXXXXXXXXAAGLEPDLECKQIWQDLGLDDT

KILPGNMKDNFWEMGDTGPCGPCSEIHYDRIGGRDAAHLVNQDDPNVLEIWNLVFIQYNRETDGILKPLPKKSIDTGMGL

ERLVSVLQNKMSNYDTDLFVPYFEAIQKGTGARPYTGKVGAEDADGIDMAYRVLADHARTITVALADGGRPDNTGRGYVL

RRILRRAVRYSHEKLNASKGFFATLVDVVVQSLGDAFPELKKDPDMVKDIINEEEVQFLKTLSRGRRILDRKIQSLGDSK

TIPGDTAWLLYDTYGFPVDLTGLIAEEKGLLVDMDGFEEERKMAQLKSQGKGAGGEDHIMLDIYAIEELREKSLEATDDS

PKYNYHSDSSGSYAFESAVATVMALRRDKTFIEEVSTGQECGVVLDKTCFYAEQGGQIYDEGYLVKVDDSSEDKTEFTVK

NTQVRGGYVLHIGTIYGSLKVGDQVRLFIDEPRRRPVMSNHTATHILNFALRSVLGEADQRGSLVAPDRLRFDFTAKGAM

STQQIKEVEEIANEMIEAAKPVYTQDCPLAAAKAIQGLRAVFDETYPDPVRVVSIGVPVSELLDDPSGPAGSLTSVEFCG

GTHLQNSSHAGAFVIVSEEAIAKGIRRIVAVTGAEAQKALRKAESLKKSLSVMEAKVKAQTVPNKDVQREIADLGEALAS

AVIPQWQKDEFRENLKSLKKIMDDLDRASKADVQKRVLEKTKQLIDSNPNQPLVILEMESGASAKALNEALKLFKTHSPQ

TSAMLFTVDNEAGKITCLCQVSQNAANQGLKASEWVQQVSGLMDGKGGGKDVSAQATGKNVSCLQEALQLATSFAQLRLG

DVKN

>ELR57374.1 Alanyl-tRNA synthetase, cytoplasmic [Bos mutus]

MDSTLTAGEIRQRFIDFFKRNEHTYVHSSATIPLDDPTLLFANAGMNQFKPIFLNTIDPSHPMAKLSRAANTQKCIRAGG

KHNDLDDVGKDVYHHTFFEMLGSWSFGDYFKELACKMALELLTQEFGIPVERLYVTYFGGDEAAGLEPDLECKQIWQNLG

SVQNPVTGCNWAFLPALCRLDDSRILPGNMKDNFWEMGDTGPCGPCSEIHYDRIGGRDAAHLVNQDDPNVLEIWNLVFIQ

YNRETDGILKPLPKKSIDTGMGLERLVSVLQNKMSNYDTDLFVPYFEAIQKGTGARPYTGRVGAEDADGIDMAYRVLADH

ARTITVALADGGRPDNTGRGYVLRRILRRAVRYSHEKLNASRGFFATLVDVVVQSLGDAFPELKKDPDMVKDIINEEEVQ

FLKTLSRGRRILDRKIQSLGDSKTIPGDTAWLLYDTYGFPVDLTALIAEEKGLVVDMDGFDEERKLAQLKSQGKGAGGED

LIMLDIYAIEELREKGLEATDDSPKYSYHSDSSGTYMFESAVATVMALRRDRMFVEEASTGQECGVVLDKTCFYAEQGGQ

TYDEGYLVKVDDSSEDKTEFTVKNTQVRGGYVLHIGTLYGSLKVGDQVRLFIDEPRRRPVMSNHTATHILNFALRSVLGE

ADQRGSLVAPDRLRFDFTAKGAMSTQQIKKAEEIANEMIEAAKPVYTEDCSLAAAKAIQGLRAVFDETYPDPVRVVSIGV

PVSELLDDPSGPAGSLTSVEFCGGTHLQNSSHVGAFVIVSEEAIAKGIRRIVAVTGAEAQKALRKAENLKKSLCVLEAKV

KAQAAPNKDVQREIADLGEALATAIIPQWQKDEFRENLKSLKKVMDDLDRASKADVQKRVLEKTKQLIDSSPNQPLVILE

VESGASAKASRASRAALCPFSPQALNEALKLFKMHSPQTSAMLFTVDNEAGRITCLCQVPQNAANRGLKASEWVQQVSGL

MDGKGGGKDVSAQATGKNVGCLQEALQLATSFAQLRLGDVKN

>ELK14592.1 Alanyl-tRNA synthetase, cytoplasmic [Pteropus alecto]

MATFGRSRKISESSAALQERLGPGYESTGATAGPWARPHEASGTGPCAGARAGAGAGAGAEAGAEAEAEAEAEAAAWRHG

SRSVGSRTASSGLVEDQDLGKIYSSGFNLPGTGGSAAWRYSTMPLFCGVTFKMDSILTASEIRQRFIDFFKRNEHTYVHS

SATIPLDDPTLLFANAGMNQFKPIFLNTIDPSHPMAKLSRAANTQKCIRAGGKHNDLDDVGKDVYHHTFFEMLGSWSFGD

YFKELACKMALELLTQEFGIPLXXXXXXXXAAGLEPDLECKQIWQDLGLDDTKILPGNMKDNFWEMGDTGPCGPCSEIHY

DRIGGRDAAHLVNQDDPNVLEIWNLVFIQYNRETDGILKPLPKKSIDTGMGLERLVSVLQNKMSNYDTDLFVPYFEAIQK

GTGARPYTGKVGAEDADGIDMAYRVLADHARTITVALADGGRPDNTGRGYVLRRILRRAVRYSHEKLNASKGFFATLVDV

VVQSLGDAFPELKKDPDMVKDIINEEEVQFLKTLSRGRRILDRKIQSLGDSKTIPGDTAWLLYDTYGFPVDLTGLIAEEK

GLLVDMDGFEEERKMAQLKSQGKGAGGEDHIMLDIYAIEELREKSLEATDDSPKYNYHSDSSGSYAFESAVATVMALRRD

KTFIEEVSTGQECGVVLDKTCFYAEQGGQIYDEGYLVKVDDSSEDKTEFTVKNTQVRGGYVLHIGTIYGSLKVGDQVRLF

IDEPRRRPVMSNHTATHILNFALRSVLGEADQRGSLVAPDRLRFDFTAKGAMSTQQIKEVEEIANEMIEAAKPVYTQDCP

LAAAKAIQGLRAVFDETYPDPVRVVSIGVPVSELLDDPSGPAGSLTSVEFCGGTHLQNSSHAGAFVIVSEEAIAKGIRRI

VAVTGAEAQKALRKAESLKKSLSVMEAKVKAQTVPNKDVQREIADLGEALASAVIPQWQKDEFRENLKSLKKIMDDLDRA

SKADVQKRVLEKTKQLIDSNPNQPLVILEMESGASAKAIQGLGFQALLCACLLRIRFLLALNEALKLFKTHSPQTSAMLF

TVDNEAGKITCLCQVSQNAANQGLKASEWVQQVSGLMDGKGGGKDVSAQATGKNVSCLQEALQLATSFAQLRLGDVKN

>XP_001506178.1 PREDICTED: alanine--tRNA synthetase, cytoplasmic [Ornithorhynchus anatinus]

MELSLSASEIRQRFIDFFKRNRHTYVHSSATIPLDDPTLLFANAGMNQFKPIFLNTIDPSHPMAKLSRAANTQKCIRAGG

KHNDLDDVGKDVYHHTFFEMLGSWSFGDYFKELACKMALELLTQEFGIPIERLYVTYFGGNEAAGLDPDLECKQIWQNLG

LDDSKILPGNMKDNFWEMGDTGPCGPCSEIHYDRIGGRDAAHLVNQDDPNVLEIWNLVFIQFNREADGSLKPLPKKSIDT

GMGLERLVSVLQNKMSNYDTDLFVPYFEAIQKGTGARPYMGQVGADDTDGIDMAYRVLADHARTITVALADGGRPDNTGR

GYVLRRILRRAVRYSHEKLNASKGFFATLVDVVVQSLGDAFPELKKDPEMVKDIINEEEVQFLKTLSRGRRILDRKIQSL

GDGKTIPGDTAWLLYDTYGFPVDLTGLIAEEKGLVVDMDGFEEERKLAQLKSQGKGAGGEDLIMLDIYAIEELRARDLEV

TDDSPKYNYRANASGSYEFESPVATVKALRREKTFVDEVSTGQECGVVLDKTCFYAEQGGQIFDEGYLVKDDDGSEDKTE

FTVKNVQVRGGYVLHIGTVYGNLKVGDQVRLFIDEPRRRPVMSNHTATHILNFALRSVLGEADQRGSLVAPDRLRFDFTA

KGAMSTQEIKKTEEIANGMIQAAKPVYAQDCPLAAAKAIQGLRAVFDETYPDPVRVVSIGVPVAELLEDPSSPAGSLTSV

EFCGGTHLRNSGHAGAFVIVSEEAIAKGIRRIVAVTGTEAQKALRKAESLKTALSVLEAKVKAQTAPNKDVQKEIADLGE

TLATAVVPQWHKDELREALKALKKIMDDLDRASKADVQKRVLEKTKQLIESQPNQPLVILEMESGASAKALNEALKLFKT

HSPQTSAMLFTVDNEAGKITCLCQVPQNAANKGLKASEWVQQVSGLMDGKGGGKDVSAQATGKNVGCLPEAVQLATSFAQ

LRLGALKN

>XP_001362507.1 PREDICTED: alanine--tRNA synthetase, cytoplasmic [Monodelphis domestica]

MESTLTASEIRRRFIDFFKRNQHTYVHSSATIPLDDPTLLFANAGMNQFKPIFLNTIDPAHPMAKLNRAANTQKCIRAGG

KHNDLDDVGKDVYHHTFFEMLGSWSFGDYFKELACKMALELLTQEFGIPVDRLYVTYFGGNEAAGLEPDLECKQIWQNLG

LDDSKILPGNMKDNFWEMGDTGPCGPCSEIHYDRIGDRDAAHLVNQDDPNVLEIWNLVFIQFNREADGSLKPLPKKSIDT

GMGLERLVSVLQNKMSNYDTDLFVPYFEAIQKGTGARQYTGCVGADDVDGIDMAYRVLADHARTITVALADGGRPDNTGR

GYVLRRILRRAVRYSHEKLNAKRGFFATLVDVVVQSLGDAFPELKKDPEMVKDIINEEEVQFLKTLSRGRRILDRKIQSL

GDCKTIPGDTAWLLYDTYGFPVDLTGLIAEEKGLVVDMDGFEEERKLAQLKSQGKGAGGEDHIMLDIYAIEELRIKGLQV

TDDSLKYNYQSDSSGTYEFENPVATVKALRREKMFVDEVSTGQECGVVLDKTCFYAEQGGQIYDEGYLVKEDDSSEDKTE

FTVKDAQVRGGYVLHIGTVYGSLRVGDRVRLYIDEPRRRPVMSNHTATHILNFALRSVLGEADQRGSLVAPDRLRFDFTA

KGAMSTQQLRETETIANGMIAAAKPVYAQDCPLAAAKAIQGLRAVFDETYPDPVRVVSIGIPVAKLLDDPSGPAGSLTSV

EFCGGTHLQNSSHAGALVIVSEEAIAKGIRRVVAVTGVEAQKALRKADSLKSSLSELETKVKAQTAPNKDIQREISDFGE

LLATTVVPQWQKDELRENLKALKKVMDDLDRANKADVQKRVLEKTRQLIESHPNQPLVIMEMESGASAKALNEALKLFKT

HSPETSAMLFTVDSEANKIICLCQVPQNAANKGLKASEWVQQVSGLMDGKGGGKDVSAQATGKNVGCLQEALSLATSFAH

LRLGDLKN

>XP_020819319.1 alanine--tRNA synthetase, cytoplasmic [Phascolarctos cinereus]

MESTLTASEVRRRFVDFFRKNQHTYVHSSATIPLDDPTLLFTNAGMNQFKPIFLNTIDPSHPMAKLSRAANTQKCIRAGG

KHNDLDDVGKDVYHHTFFEMLGSWSFGDYFKELACKMALELLTQEFGIPLDKLYVTYFGGNEAAGLEPDLECKQIWQNLG

LDDSKILPGNMKDNFWEMGDTGPCGPCSEVHYDRIGGRDAAHLVNQDDPNVLEIWNLVFIQFNRETDGSLKPLPKKSIDT

GMGLERLVSVLQNKMSNYDTDLFVPYFEAIQKGTGARPYTGCVGADDVDGIDMAYRVLADHARTLTVALADGGRPDNTGR

GYVLRRILRRAVRYSHEKLNARRGFFATLVDVVVQSLGDAFPELKKDPDMVKDIINEEEGQFLKTLSRGRRILDRKIQSL

GDCKTIPGDTAWLLYDTYGFPVDLTGLIAEEKGLVVDMDGFEEERKLAQLKSQGKGAEGENHIMLDIYAIEELRVKGLEA

TDDSLKYNYQSDSSGTYEFETPVATVKALHREKTFVDEVSTGQECGVVLDKTCFYAEQGGQIYDEGFLVKEDDSSEDKTE

FTVKDTQVRGGYVLHVGTVYGSLRVGDRVRLFIDEPRRRPVMSNHTATHILNFALRSVLGEADQRGSLVAPDRLRFDFTA

KSAMSTQQIKETEKIANGMIEAAKPVYAQDCPLAAAKAIQGLRAVFDETYPDPVRVVSIGIPVAKLLDDPSGPAGSLTSV

EFCGGTHLQNSSHAGALVIVSEEAIAKGIRRIVAVTGIEAQKALRKADSLKMSLSELEAKVKAQTAPNKDIQKEISDFGE

LLATTVVPQWQKDELRENLKALKKIMDDLDRASKADVQKRVLERTKQLIERQPNQPLVIMEMESGASAKALNEALKLFKT

HSPETSAMLFTVDNEANKIICLCQVPQNAANRGLKASEWVQQVSGLMDGKGGGKDVSAQATGKNVGCLQEALRLATSFAH

LRLGDLKN

>XP_011756365.2 alanine--tRNA synthetase, cytoplasmic [Macaca nemestrina]

MPGFLFFGVTFKMDSTLTASEIRQRFIDFFKRNEHTYIHSSATIPLDDPTLLFANAGMNQFKPIFLNTIDPSHPMAKLSR

AANTQKCIRAGGKHNDLDDVGKDVYHHTFFEMLGSWSFGDYFKELACKMALELLTQEFGIPIGRLYVTYFGGDEAAGLKP

DLECKQIWQNLGLDDTKILPGNMKDNFWEMGDTGPCGPCSEIHYDRIGGRDAAHLVNQDDPNVLEIWNLVFIQYNREADG

ILKPLPKKSIDTGMGLERLVSVLQNKMSNYDTDLFVPYFEAIQKGTGARPYTGKVGAEDADGIDMAYRVLADHARTITVA

LADGGRPDNTGRGYVLRRILRRAVRYAHEKLNASRGFFATLVDVVVQSLGDAFPELKKDPDMVKDIINEEEVQFLKTLSR

GRRILDRKIQSLGDSKTIPGDTAWLLYDTYGFPVDLTGLIAEEKGLVVDMDGFEEERKLAQLKSQGKGAGGEDLIMLDIY

AIEELRARGLEVTDDSPKYNYHLDFSGSYGMVLCSFHFKTEFTVKNAQVRGGYVLHIGTIYGDLKVGDQVRLFIDEPRRR

PIMSNHTATHILNFALRSVLGEADQKGSLVAPDRLRFDFTAKGAMSTLQIKKAEEIANEMIEAAKPVYTQDCPLAAAKAI

QGLRAVFDETYPDPVRVVSIGVPVSELLDDPSGPAGSLTSVEFCGGTHLRNSSHAGAFVIVTEEAIAKGIRRIVAVTGAE

AQKALRKAESLKKSLSVMEAKVKAQTAPNKDVQREIADLGEALATAVIPQWQKDELRETLKSLKKVMDDLDRASKADVQK

RVLEKTKQLIDSNPNQPLVILEMESGASAKALNEALKLFKTHSPQTSAMLFTVDNEAGKITCLCQVPQNAANRGLKASEW

VQQVSGLMDGKGGGKDVSAQATGKNIGCLQEALQLATSFAQLRLGDVKN

>XP_023350969.1 alanine--tRNA synthetase, cytoplasmic [Sarcophilus harrisii]

MESTLTASEIRQRFIDFFKRNQHTYVHSSATIPLDDPTLLFANAGMNQFKPIFLNTIDPSHPMAKLSRAANTQKCIRAGG

KHNDLDDVGKDVYHHTFFEMLGSWSFGDYFKELACKMALELLTQEFGIPVNRLYVTYFGGNEAAGLEPDLECKQIWQNLG

LEDSKILPGNMKDNFWEMGDTGPCGPCSEIHYDRIGDRDAAHLVNQDDPNVLEIWNLVFIQFNRETDGSLKPLPKKSIDT

GMGLERLVSVLQNKMSNYDTDLFVPYFEAIQKGTGVRQYTGQVGADDVDGIDMAYRVLADHARTLTVALADGGRPDNTGR

GYVLRRILRRAIRYSHEKLNAKRGFFATLVDVVVQSLGDAFPELKKDPDMVKDIINEEEVQFLKTLSRGRRILDRKIQSL

GDCKTIPGDTAWLLYDTYGFPVDLTGLIAEEKGLVVDMNGFEEERKLAQLKSQGKGAGGEDHIMLDIYAIEELRVKGLKV

TDDSLKYNYRSDSSGTYEFENPMATVQALRREKMFVDEVSTGQECGVVLDKTCFYAEQGGQIYDEGFLVKEDDSSEDKTE

FTVKDAQVRGGYVLHIGTVYGSLRVGDRVRLFIDEPRRRPVMSNHTATHILNFALRSVLGEADQRGSLVAPDRLRFDFTA

KAAMSTQQIKETEKIANGMIEAAKPVYAQDCPLAAAKAIQGLRAVFDETYPDPVRVVSIGIPVTKLLDDPSGPAGSLTSV

EFCGGTHLQNSSHAGALVIVSEEAIAKGIRRIVAVTGTEAQKALRKADSLKTSLSELEAKVKAQTAPNKDLQREISDFGE

LLATTVVPQWQKDELRENLKALKKIMDDLDRASKADVQKRVLERTKQLIESQPNQPLVIMEMESGASAKALNEALKLFKT

HSPETSAMLFTVDNEANKIICLCQVPQNAANRGLKASEWVQQVSGLMDGKGGGKDVSAQATGKNVGCLQEALRLATSFAH

LRLGDLKN

>XP_023439353.1 alanine--tRNA synthetase, cytoplasmic [Dasypus novemcinctus]

MDSTLSASEIRQRFIDFFKRNEHTYVHSSATIPLDDPTLLFANAGMNQFKPIFLNTIDPSHPMAKLRRAANTQKCIRAGG

KHNDLDDVGKDVYHHTFFEMLGSWSFGDYFKELACKMALELLTQEFGITIERLYVTYFGGDEAAGLEPDLECKQIWQNLG

LDHTRILPGSMKDNFWEMGDTGPCGPCSEIHYDRIGGRDAAHLVNQDDPNVLEIWNLVFIQYNRETDGILKPLPKKSIDT

GMGLERLVSVLQNKMSNYDTDLFVPYFEAIQQGTGARPYTGKVGADDADGIDMAYRVLADHARTITVALADGGRPDNTGR

GYVLRRILRRAVRYSHEKLNASRGFFATLVDVVVQSLGDAFPELKKDPDMVKDIINEEEVQFLKTLSRGRRILDRKIQSL

GDNKTIPGDTAWLLYDTYGFPVDLTGLIAEEKGLVVDMDGFEEERKLAQETLQFQVNQDKHENKVSDPVFPLLSILHRAV

FENAVATVVALRRDKMFVEEVSTGQECGVVLDKTCFYAEQGGQIYDEGYLVKVDDSSEDKTEFTVKNAQVRGGYVLHIGT

IYGTLKVGDQVRLFIDEPRRRPVMSNHTATHILNFALRSVLGEADQRGSLVAPDRLRFDFTAKGAMSTQQIKKTEEITNK

MIEAAKPVYTQDCPLAAAKAIQGLRAVFDETYPDPVRVVSIGIPVSELLNDPSGPAGSLTSVEFCGGTHLQNSSHAGAFV

IVSEEAIAKGIRRIVAVTGAEAQKALRKAESLKKLLSVMEAKVKAQTLPNKDVQREIADLGESLAAAVVPQWQKDELREH

LKSLKKVMDDLDRASKADIQKRVLEKTKQLIDSHPNQPLVILEMENGASAKALNEALKLFKTHSPQTSAMLFTVDNEAGK

IICLCQVPQNAANRGLKASEWVQQVSDLMDGKGGGKDVSAQATGKNVGCLQEALQLATSFAQLRLGDVKN

>XP_006036357.1 PREDICTED: LOW QUALITY PROTEIN: alanine--tRNA synthetase, cytoplasmic [Alligator sinensis]

MESTLTGSQIRQRFIDFFKENQHTYVHSSSTIPLDDPTLLFANAGMNQFKPIFLNTIDPSHPMAKLNRAANTQKCIRAGG

KHNDLDDVGKDVYHHTFFEMLGSWSFGDYFKALACKLALELLTKEFGIPIERLYVTYFGGNEAAGLEPDLECKQIWLDLG

LAENKILPGSMKDNFWEMGDTGPCGPCSEIHYDRIGNRDASHLVNQDDPNVLEIWNLVFIQFNREADGTLKPLPKKSIDT

GLGLERLVSVLQNKMSNYDTDLFIPYFEAIQKGTGARPYTGQVGAEDMDGIDMAYRVLADHARTITVALSDGGRPDNTGR

GYVLRRILRRAVRYSHEKLNAPKGFFATLVDVVVQSLGDAFPELKKDPDMVKDIINEEEVQFLKTLSRGRRILDRKIQSL

GDSKIIPGDTAWLLYDTYGFPVDLTGLIAEEKGLVVDMEGFEXERRNAQLKSQGKGTGVEDLIMLDIYAIEELRAQGLEV

TDDSPKYSYTSDPSGAYEFENLMATVKAIRREKTFVEEVSTGQECGIVLDKTCFYAEQGGQIYDEGYLVKADDCSEDKME

FTVKNVQVRGGYVLHVGTVYGSLKVGDQVHLYVDEPRRRPVMSNHTATHILNFALRSVLGEADQRGSLVAPDKLRFDFTA

KGAMSTQEIKKTEKIANQMIEEAKTVYAKDCPLAAAKAIQGLRAVFDETYPDPVRVVSIGIPVDDLLADPSGPAGSVTSV

EFCGGTHLQNSSHAGPFVIVSEEAIAKGIRRIVAVTGAEAQKALRKVDSLKKSLSALDAKVKVQTAPNKEVQKEIADLSE

TLATAIIPQWQKDELREALKVLKKVMDDLDRASKADIQKRVLEKTKQFIDSHPNQPLVILEMESGASAKALNESLKLFKT

HSPQTAAMLFAVDNEAGKITCLCQVPQEAANKGLKANQWVQEVSTLIDGKGGGKDVSAQATGKNVGCLQEALKLATDFAE

LRLGELKN

>XP_019391089.1 PREDICTED: alanine--tRNA synthetase, cytoplasmic isoform X1 [Crocodylus porosus]

MELTLTGSQIRQRFIDFFKENRHTYVHSSSTIPLDDPTLLFANAGMNQFKPIFLNTIDPSHPMAKLNRAANTQKCIRAGG

KHNDLDDVGKDVYHHTFFEMLGSWSFGDYFKALACKLALELLTKEFGIPIERLYVTYFGGNEAAGLEPDLECKQIWLDLG

LAENKILPGSMKDNFWEMGDTGPCGPCSEIHYDRIGNRDASHLVNQDDPNVLEIWNLVFIQFNREADGTLKPLPKKSIDT

GMGLERLVSVLQNKMSNYDTDLFIPYFEAIQKGTGARPYTGQVGAEDMDGIDMAYRVLADHARTITVALSDGGRPDNTGR

GYVLRRILRRAVRYSHEKLSAPKGFFATLVDVVVQSLGDAFPELKKDPDMVKDIINEEEVQFLKTLSRGRRILDRKIQSL

GDSKIIPGDTAWLLYDTYGFPVDLTGLIAEEKGLAVDMEGFEEERRNAQLKSQGKGTGVEDLIMLDIYAIEELRAQGLEV

TDDSPKYSYTSDPSGVYEFENLMATVKAIRREKTFVEEVSTGQECGIVLDKTCFYAEQGGQIYDEGYLVKADDYSEDKTE

FTVKNVQVRGGYVLHVGTVYGSLKVGDQVHLYVDEPRRRPVMSNHTATHILNFALRSVLGEADQRGSLVAPDKLRFDFTA

KGAMSTQEIKKTEKIANQMIEEAKTVYAKDCPLAAAKAIQGLRAVFDETYPDPVRVVSIGIPVDDLLADPSGPAGSVTSV

EFCGGTHLQNSSHAGPFVIVSEEAIAKGIRRIVAVTGAEAQKALRKVDSLKKSLSALDAKVKVQTAPNKEVQKEIADLSE

TLATAVIPQWQKDELREALKVLKKVMDDLDRASKADIQKRVLEKTKQFIDSHPNQPLVILEMESGASAKALNESLKLFKT

HSPQTAAMLFAVDNEAGKITCLCQVPQEAANKGLKANQWVQEVSTLMDGKGGGKDVSAQATGKNVGCLQEALKLATDFAE

LHLGELKN

>XP_014454761.1 PREDICTED: alanine--tRNA synthetase, cytoplasmic [Alligator mississippiensis]

MESTLTGSQIRQRFIDFFKENQHTYVHSSSTIPLDDPTLLFANAGMNQFKPIFLNTIDPSHPMAKLNRAANTQKCIRAGG

KHNDLDDVGKDVYHHTFFEMLGSWSFGDYFKALACKLALELLTKEFGIPIERLYVTYFGGNEAAGLEPDLECKQIWLDLG

MAENKILPGSMKDNFWEMGDTGPCGPCSEIHYDRIGNRDASHLVNQDDPNVLEIWNLVFIQFNREADGTLKPLPKKSIDT

GLGLERLVSVLQNKMSNYDTDLFIPYFEAIQKGTGARPYTGQVGAEDMDGIDMAYRVLADHARTITVALSDGGRPDNTGR

GYVLRRILRRAVRYSHEKLNAPKGFFATLVDVVVQSLGDAFPELKKDPDMVKDIINEEEVQFLKTLSRGRRILDRKIQSL

GDSKIIPGDTAWLLYDTYGFPVDLTGLIAEEKGLVVDMEGFEEERRNAQLKSQGKGTGVEDLIMLDIYAIEELRAQGLEV

TDDSPKYSYTSDPSGAYEFENLMGTVKAIRREKTFVEEVSTGQECGIVLDKTCFYAEQGGQIYDEGYLVKADDCSEDKTE

FTVKNVQVRGGYVLHVGTVYGSLKVGDQVHLYVDEPRRRPVMSNHTATHILNFALRSVLGEADQRGSLVAPDKLRFDFTA

KGAMSTQEIKKTEKIANQMIEEAKTVYAKDCPLAAAKAIQGLRAVFDETYPDPVRVVSIGIPVDDLLADPSGPAGSVTSV

EFCGGTHLQNSSHAGPFVIVSEEAIAKGIRRIVAVTGAEAQKALRKVDSLKKSLSALDAKVKVQRAPNKEVQKEIADLSE

TLATAIIPQWQKDELREALKVLKKVMDDLDRASKADIQKRVLEKTKQFIDSHPNQPLVILEMESGASAKALNESLKLFKT

HSPQTAAMLFAVDNEAGKITCLCQVPQEAANKGLKANQWVQEVSTLMDGKGGGKDVSAQATGKNVGCLQEVLKLATDFAE

LCLGELKN

>KYO36992.1 hypothetical protein Y1Q_0000866 [Alligator mississippiensis]

MTAARTNHNGGLVSSRRRGWCEGRREAGCAAAAGTGVRSRAGRGSCRLDRAVLCRPPKMESTLTGSQIRQRFIDFFKENQ

HTYVHSSSTIPLDDPTLLFANAGMNQFKPIFLNTIDPSHPMAKLNRAANTQKCIRAGGKHNDLDDVGKDVYHHTFFEMLG

SWSFGDYFKALACKLALELLTKEFGIPIERLYVTYFGGNEAAGLEPDLECKQIWLDLGMAENKILPGSMKDNFWEMGDTG

PCGPCSEIHYDRIGNRDASHLVNQDDPNVLEIWNLVFIQFNREADGTLKPLPKKSIDTGLGLERLVSVLQNKMSNYDTDL

FIPYFEAIQKGTGARPYTGQVGAEDMDGIDMAYRVLADHARTITVALSDGGRPDNTGRGYVLRRILRRAVRYSHEKLNAP

KGFFATLVDVVVQSLGDAFPELKKDPDMVKDIINEEEVQFLKTLSRGRRILDRKIQSLGDSKIIPGDTAWLLYDTYGFPV

DLTGLIAEEKGLVVDMEGFEEERRNAQLKSQGKGTGVEDLIMLDIYAIEELRAQGLEVTDDSPKYSYTSDPSGAYEFENL

MGTVKAIRREKTFVEEVSTGQECGIVLDKTCFYAEQGGQIYDEGYLVKADDCSEDKTEFTVKNVQVRGGYVLHVGTVYGS

LKVGDQVHLYVDEPRRRPVMSNHTATHILNFALRSVLGEADQRGSLVAPDKLRFDFTAKGAMSTQEIKKTEKIANQMIEE

AKTVYAKDCPLAAAKAIQGLRAVFDETYPDPVRVVSIGIPVDDLLADPSGPAGSVTSVEFCGGTHLQNSSHAGPFVIVSE

EAIAKGIRRIVAVTGAEAQKALRKVDSLKKSLSALDAKVKVQRAPNKEVQKEIADLSETLATAIIPQWQKDELREALKVL

KKVMDDLDRASKADIQKRVLEKTKQFIDSHPNQPLVILEMESGASAKALNESLKLFKTHSPQTAAMLFAVDNEAGKITCL

CQVPQEAANKGLKANQWVQEVSTLMDGKGGGKDVSAQATGKNVGCLQEVLKLATDFAELCLGELKN

>XP_014804137.1 PREDICTED: alanine--tRNA synthetase, cytoplasmic [Calidris pugnax]

MEATLTASQIRQRFIDFFKANKHTYVHSSSTIPLDDPTLLFANAGMNQFKPIFLNTIDPSHPLAKLSRATNTQKCIRAGG

KHNDLDDVGKDVYHHTFFEMLGSWSFGDYFKELACKLALELLTKEFGIPAERLYVTYFGGNEAAGLQPDLECKQIWLNLG

LAEGRILPGNMKDNFWEMGDTGPCGPCSEIHYDRIGDRDASHLVNQDDPNVLEIWNLVFIQFNREADGTLKPLPKKSIDT

GMGLERLVSVLQNKMSNYDTDLFLPYFEAIQKGTGARPYMGQVGAEDADGIDMAYRVLADHARTITLALSDGGRPDNTGR

GYVLRRILRRAVRYSHEKLNAPKGFFATLVDVVVESLGDAFPELKKDPDMVKDIINEEEDQFLKTLSRGRRILDRKIQSL

GDSKTIPGDTAWLLYDTYGFPADLTALIAEEKGLVVDMEGFEEERKNAQLKSQGKGAGGEDLLMLDIYAIEELRARGLEV

TDDSPKYNYASDQSGSYDFGSLVATVKAIRREKKFVEEVSTGQECGIVLDRTCFYAEQGGQIYDEGYMVKDDDSREDKTE

FTVKNVQVRGGYVLHIGTLYGSLKVGDQVHLSIDETRRRPVMSNHTATHILNFALRSVLGEADQRGSLVAPDRLRFDFTA

KGALSTQEIKKVEGIANQMIEEAKTVYAKDCPLAAAKAIQGLRAVFDETYPDPVRVVSIGIPVEELLADPSGPAGSLTSI

EFCGGTHLQNSSHAGPFVIVSEEAIAKGIRRIVAVTGAEARKALRKVDSLRKLLSALEAKVKVQTAPNKDVQKEITDLSE

TLATAVIPQWQKDELREAVKALKKVMDDLDRASKADIQKRVLEKTKQVIESHPNQPLVIMEMEHGASAKALNESLKLFKT

HSPQTAAMLFAVDSEAGRITCLCQVPQETANKGLKASQWVQEVSGLMDGKGGGKDISAQATGKNVGCLQEALKLATDFAQ

LRLGELKN

>XP_010582146.1 PREDICTED: alanine--tRNA synthetase, cytoplasmic [Haliaeetus leucocephalus]

MEATLTASQIRQRFIDFFKENKHTYVHSSSTIPLDDPTLLFANAGMNQFKPIFLNTIDPSHPLAKLNRATNTQKCIRAGG

KHNDLDDVGKDVYHHTFFEMLGSWSFGDYFKELACKLALDLLTKEFGIPAERLYVTYFGGNEAAGLQPDLECKQIWLNLG

LAEGRILPGNMKDNFWEMGDTGPCGPCSEIHYDRIGNRDASHLVNQDDPNVLEIWNLVFIQFNREADGTLKPLPKKSIDT

GMGLERLVSVLQNKMSNYDTDLFLPYFEAIQKGTGARPYMGQVGAEDADGIDMAYRVLADHARTITLALSDGGRPDNTGR

GYVLRRILRRAVRYSHEKLNAPKGFFATLVDVVVQSLGDAFPELKKDPDMVKDIINEEEDQFLKTLSRGRRILDRKIQSL

GDSKTIPGDTAWLLYDTYGFPADLTGLIAEEKGLVVDMEGFEEERKNAQLKSQGKGAGGEDLLMLDIYAIEELRARGLEV

TDDSPKYSYASDPSGTYDFGSLVATVKAIRREKKFVEEVSTGQECGIVLDRTCFYAEQGGQIYDEGYMVKDDDGREDKTE

FTVKNVQVRGGYVLHIGTLYGSLKVGDQVHLSIDETRRRPVMSNHTATHILNFALRSVLGEADQRGSLVAPDRLRFDFTA

KGALSTQEIKKVEGIANQMIEEAKTVYAKDCPLAAAKAIQGLRAVFDETYPDPVRVVSIGIPVEELLADPSGPAGSITSI

EFCGGTHLQNSSHAGPFVIVSEEAIAKGIRRIVAVTGAEARKALRKVDSLKKLLSALEAKVKVQTAPNKDVQKEITDLSE

TLATAVIPQWQKDELREAVKALKKVMDDLDRASKADIQKRVLEKTKQVIESHPNQPLVIMEMENGASAKALNESLKLFKT

YSPQTATMLFTVDNEAGRITCLCQVPQETANKGLKASQWVQEVSGLMDGKGGGKDVSAQATGKNVGCLQEALKLATDFAR

LRLGELKN

>OPJ78066.1 alanine--tRNA synthetase, cytoplasmic [Patagioenas fasciata monilis]

MEATLTASQIRQRFIDFFKENEHTYVHSSSTIPLDDPTLLFANAGMNQFKPIFLNTIDPSHPLAKLNRATNTQKCIRAGG

KHNDLDDVGKDVYHHTFFEMLGSWSFGDYFKELACKLALELLTKEFGIPAERLYVTYFGGNEAAGLQPDLECKQIWLNLG

LAEGRILPGNMKDNFWEMGDTGPCGPCSEIHYDRIGDRDASHLVNQDDPNVLEIWNLVFIQFNREADGTLKPLPKKSIDT

GMGLERLVSVLQNKMSNYDTDLFLPYFEAIQKGTGARPYMGQVGAEDADGIDMAYRVLADHARTITLALSDGGRPDNTGR

GYVLRRILRRAVRYSHEKLNAPKGFFATLVDVVVQSLGDAFPELKKDPDMVKDIINEEEDQFLKTLSRGRRILDRKIQSL

GDSKTIPGDTAWLLYDTYGFPADLTGLIAEEKGLVVDMEGFEEERKNAQLKSQGKGAGGEDLLMLDIYAIEELRSRGLEV

TDDSPKYSYASDPSGAYDFGSLVATVKAIRREKKFVEEVSTGQECGIVLDRTCFYAEQGGQIYDEGYMVKEDDGREDKTE

FTVKNVQVRGGYVLHIGTLYGNLKVGDQVYLSIDETRRRPVMSNHTATHILNFALRSVLGEADQRGSLVAPDRLRFDFTA

KGALSTQEIKKVEGIANQMIEEAKTVYAKDCPLAAAKAIQGLRAVFDETYPDPVRVVSIGIPVEELLADPSGPAGSVTSV

EFCGGTHLQNSSHAGPFVIVSEEAIAKGIRRIVAVTGAEARKALRKVDSLKKLLSALEAKVKVQTAPNKDVQKEITDLSE

TLATAVIPQWQKDELREAVKALKKVMDDLDRASKADIQKRVLEKTKQVIESHPNQPFVIMEMENGASAKALNESLKLFKT

YSPQTATMLFAVDNEAGRITCLCQVPQETANKGLKASQWVQEVSGLMDGKGGGKDVSAQATGKNVGCLQQALKLATDFAT

LRLGELKN

>XP_011832214.1 PREDICTED: alanine--tRNA synthetase, cytoplasmic [Mandrillus leucophaeus]

MPVQAHLDRHLQLFCKTVCISRCALKHFKPIFLNTIDPSHPMAKLSRAANTQKCIRAGGKHNDLDDVGKDVYHHTFFEML

GSWSFGDYFKELACKMALELLTQEFGIPIERLYVTYFGGDEAAGLEPDLECKQIWQNLGLDDTKILPGNMKDNFWEMGDT

GPCGPCSEIHYDRIGGRDAAHLVNQDDPNVLEIWNLVFIQYNREADGILKPLPKKSIDTGMGLERLVSVLQNKMSNYDTD

LFVPYFEAIQKGTGARPYTGKVGAEDADGIDMAYRVLADHARTITVALADGGRPDNTGRGGDAFPELKKDPDMVKDIINE

EEVQFLKTLSRGRRILDRKIQSLGDSKTIPGDTAWLLYDTYGFPVDLTGLIAEEKGLVVDMDGFEEERKLAQLKSQGKGA

GGEDLIMLDIYAIEELRARGLEVTDDSPKYNYHLDFSGSYVFENTVATVMALRREKMFVEEVSTGQECGVVLDKTCFYAE

QGGQIYDEGYLVKVDDSSEDKTEFTVKNAQVRGGYVLHIGTIYGDLKVGDQVRLFIDEPRRRPIMSNHTATHILNFALRS

VLGEADQKGSLVAPDRLRFDFTAKGAMSTQQIKKAEEIANEMIEAAKPVYTQDCPLAAAKAIQGLRAVFDETYPDPVRVV

SIGVPVSELLDDPSGPAGSLTSVEFCGGTHLRNSSHAGAFVIVTEEAIAKGIRRIVAVTGAEAQKALRKAESLKKSLSVM

EAKVKAQTAPNKDVQREIADLGEALATAVIPQWQKDELRETLKSLKKVMDDLDRASKADVQKRVLEKTKQLIDSNPNQPL

VILEMESGASAKALNEALKLFKTHSPQTSAMLFTVDNEAGKITCLCQVPQNAANRGLKASEWVQQVSGLMDGKGGGKDVS

AQATGKNIGCLQEALQLATSFAQLRLGDVKN

>XP_011572150.1 PREDICTED: alanine--tRNA synthetase, cytoplasmic [Aquila chrysaetos canadensis]

MEAMLTASQIRQRFIDFFKENKHTYVHSSSTIPLDDPTLLFANAGMNQFKPIFLNTIDPSHPLAKLNRATNTQKCIRAGG

KHNDLDDVGKDVYHHTFFEMLGSWSFGDYFKELACKLALDLLTKEFGIPAERLYVTYFGGNEAAGLQPDLECKQIWLNLG

LAEGRILPGNMKDNFWEMGDTGPCGPCSEIHYDRIGDRDASHLVNQDDPNVLEIWNLVFIQFNREADGTLKPLPKKSIDT

GMGLERLVSVLQNKMSNYDTDLFLPYFEAIQKGTGARPYMGQVGAEDADGIDMAYRVLADHARTITLALSDGGRPDNTGR

GYVLRRILRRAVRYSHEKLNAPKGFFATLVDVVVQSLGDAFPELKKDPDMVKDIINEEEDQFLKTLSRGRRILDRKIQSL

GDSKTIPGDTAWLLYDTYGFPADLTGLIAEEKGLVVDMEGFEEERKNAQLKSQGKGAGGEDLLMLDIYAIEELRARGLEV

TDDSPKYSYASDPSGTYDFGSLVATVKAIRREKKFVEEVSTGQECGIVLDRTCFYAEQGGQIYDEGYMVKDDDGREDKTE

FTVKNVQVRGGYVLHIGTLYGSLKVGDQVHLSIDETRRRPVMSNHTATHILNFALRSVLGEADQRGSLVAPDRLRFDFTA

KGALSTQEIKKVEGIANQMIEEAKTVYAKDCPLAAAKAIQGLRAVFDETYPDPVRVVSIGIPVEELLADPSGPAGSITSI

EFCGGTHLQNSSHAGPFVIVSEEAIAKGIRRIVAVTGAEARKALRKVDSLKKLLSALEAKVKVQTAPNKDVQKEITDLSE

TLATAVIPQWQKDELREAVKALKKVMDDLDRASKADIQKRVLEKTKQVIESHPNQPLVIMEMENGASAKALNESLKLFKT

YSPQTATMLFTVDNEAGRITCLCQVPQETANKGLKASQWVQEVSGLMDGKGGGKDVSAQATGKNVGCLQEALKLATDFAR

LRLGELKN

>XP_017679687.1 PREDICTED: alanine--tRNA synthetase, cytoplasmic [Lepidothrix coronata]

MEATLTASQIRQRFIDFFKENKHTYVHSSSTIPLDDPTLLFANAGMNQFKPIFLNTIDPSHPLAKLSRATNTQKCIRAGG

KHNDLDDVGKDVYHHTFFEMLGSWSFGDYFKELACKLALELLTKEFGIPAERLYVTYFGGNEAAGLQPDLECKQIWLDLG

LAEGRILPGNMKDNFWEMGDTGPCGPCSEIHYDRIGDRDASHLVNQDDPNVLEIWNLVFIQFNREADGTLKPLPKKSIDT

GMGLERLVSVLQNKMSNYDTDLFLPYFEAIQKGTGARPYQGQVGAEDADGIDMAYRVLADHARTITLALSDGGRPDNTGR

GYVLRRILRRAVRYSHEKLNAPKGFFATLVDVVVQSLGDAFPELKKDPDMVKDIINEEEDQFLKTLSRGRRILDRKIQSL

GDSKIIPGDTAWLLYDTYGFPADLTGLIAEEKGLVVDMEGFEEERKNAQLKSQGKGAGGEDLLMLDIYAIEELRARGLEV

TDDSPKYSYASDPSGTYDFGSLVATVKAIRREKKFVEEVSTGQECGIVLDRTCFYAEQGGQIYDQGYMVKDDDSREDKTE

FTVKNVQVRGGYVLHIGTLYGSLKVGDQVHLSIDEARRRPVMSNHTATHILNFALRSVLGEADQRGSLVAPDRLRFDFTA

KGALSTQEVKKVEGIANQMIEESKTVYAKDCPLAAAKAIQGLRAVFDETYPDPVRVVSIGIPVEELLADPSGPGGSITSV

EFCGGTHLQNSSHAGPFVIVSEEAIAKGIRRIVAVTGAEARKALRKVDSLRKLLSALEAKVKVQTAPNKDVQKEIADLGE

TLATAVIPQWQKDELREAVKALKKVMDDLDRASKADIQKRVLEKTKQVIESHPNQPWVIMEMENGASAKALNESLKLFKT

NSPQTAAMLFAVDNEAGRITCLCQVPQETANKGLKASQWVQEVSGLMDGKGGGKDISAQATGKNVGCLQEALKLATDFAR

LRLGELKN

>XP_017938322.1 PREDICTED: alanine--tRNA synthetase, cytoplasmic [Manacus vitellinus]

MEATLTASQIRQRFIDFFKENKHTYVHSSSTIPLDDPTLLFANAGMNQFKPIFLNTIDPSHPLAKLSRATNTQKCIRAGG

KHNDLDDVGKDVYHHTFFEMLGSWSFGDYFKELACKLALELLTKEFGIPAERLYVTYFGGNEAAGLQPDLECKQIWLDLG

LAEGRILPGNMKDNFWEMGDTGPCGPCSEIHYDRIGDRDASHLVNQDDPNVLEIWNLVFIQFNREADGTLKPLPKKSIDT

GMGLERLVSVLQNKMSNYDTDLFLPYFEAIQKGTGARPYQGQVGAEDADGIDMAYRVLADHARTITLALSDGGRPDNTGR

GYVLRRILRRAVRYSHEKLNAPKGFFATLVDVVVQSLGDAFPELKKDPDMVKDIINEEEDQFLKTLSRGRRILDRKIQSL

GDSKIIPGDTAWLLYDTYGFPADLTGLIAEEKGLVVDMEGFEEERKNAQLKSQGKGAGGEDLLMLDIYAIEELRARGLEV

TDDSPKYSYASDPSGTYDFGSLVATVKAIRREKKFVEEVSTGQECGIVLDRTCFYAEQGGQIYDQGYMVKDDDSREDKTE

FTVKNVQVRGGYVLHIGTLYGSLKVGDQVHLSIDEARRRPVMSNHTATHILNFALRSVLGEADQRGSLVAPDRLRFDFTA

KGALSTQEVKKVEGIANQMIEESKTVYAKDCPLAAAKAIQGLRAVFDETYPDPVRVVSIGIPVEELLADPSGPGGSITSI

EFCGGTHLQNSSHAGPFVIVSEEAIAKGIRRIVAVTGAEARKALRKVDSLRKLLSALEAKVKVQTAPNKDVQKEIADLGE

TLATAVIPQWQKDELREAVKALKKVMDDLDRASKADIQKRVLEKTKQVIESHPNQPWVIMEMENGASAKALNESLKLFKT

NSPQTAAMLFAVDNEAGRITCLCQVPQETANKGLKASQWVQEVSGLMDGKGGGKDISAQATGKNVGCLQEALKLATDFAR

LRLGELKN

>XP_009569488.1 PREDICTED: alanine--tRNA synthetase, cytoplasmic [Cuculus canorus]

MEAPLTASQIRQRFIDFFKENKHTYVHSSSTIPLDDPTLLFANAGMNQFKPIFLNTIDPSHPLAKLTRATNTQKCIRAGG

KHNDLDDVGKDVYHHTFFEMLGSWSFGDYFKELACKLALDLLTKEFGIPAERLYVTYFGGNEAAGLQPDLECKQIWLNLG

LDEGRILPGNMKDNFWEMGDTGPCGPCSEIHYDRIGDRDASHLVNQDDPNVLEIWNLVFIQFNREADGTLKPLPKKSIDT

GMGLERLVSVLQNKMSNYDTDLFLPYFEAIQKGTGARPYMGQVGAEDADGIDMAYRVLADHARTITLALSDGGRPDNTGR

GYVLRRILRRAVRYSHEKLNAPKGFFATLVDVVVQSLGDAFPELKKDPDMVKDIINEEEDQFLKTLSRGRRILDRKIQSL

GDSKTIPGDTAWLLYDTYGFPADLTGLIAEEKGLVVDMEGFEEERRNAQLKSQGKGAGGEDLLMLDIYAIEELRARGLEV

TDDSPKYSYTSDQSGVYEFASLVATVKAIRREKKFVEEVSTGQECGIVLDRTCFYAEQGGQIYDEGYMVKDDDGREDKTE

FTVKNVQVRGGYVLHIGTLYGNLKVGDQVHLSIDETRRRPVMSNHTATHILNFALRSVLGEADQRGSLVAPDRLRFDFTA

KGALSTQEIKKVEGIANQMIEEAKTVYAKDCPLAAAKAIQGLRAVFDETYPDPVRVVSIGIPVEELLTDPCGPGGSITSI

EFCGGTHLQNSSHAGPFVIVSEEAIAKGIRRIVAVTGAEARKALRKVSSLRKLLSTLEAKVKVQTAPNKDVQKEITDLSE

ALATAVIPQWQKDELREAVKALKKVMDDLDRASKADIQKRVLEKTKQVIESHPNQPLVIMEMENGASAKALNESLKLFKT

QSPQTAAMLFAVDNEAGRITCLCQVPQETANKGLKASQWVQEVSGLMDGKGGGKDISAQATGKNVGCLQEALRVATDFAR

LRLGELKN

>XP_009289050.1 PREDICTED: alanine--tRNA synthetase, cytoplasmic [Aptenodytes forsteri]

MEATLTASQIRQRFIDFFKENKHTYVHSSSTIPLDDPTLLFANAGMNQFKPIFLNTIDPSHPLAKLNRATNTQKCIRAGG

KHNDLDDVGKDVYHHTFFEMLGSWSFGDYFKELACKLALDLLTKEFGIPAERLYVTYFGGNEVAGLQPDLECKQIWLNLG

LAEGRILPGNMKDNFWEMGDTGPCGPCSEIHYDRIGDRDASHLVNQDDPNVLEIWNLVFIQFNREADGTLKPLPKKSIDT

GMGLERLVSVLQNKMSNYDTDLFLPYFEAIQKGTGVRPYMGQVGAEDTDGIDMAYRVLADHARTITLALSDGGRPDNTGR

GYVLRRILRRAVRYSHEKLNAPKGFFATLVDVVVQSLGDAFPELKKDPDMVKDIINEEEDQFLKTLSRGRRILDRKIQSL

GDSKTIPGDTAWLLYDTYGFPADLTGLIAEEKGLVVDMEGFEEERKNAQLKSQGKGAGGEDLLMLDIYAIEELRARGLEV

TDDSPKYSYASDQSGAYDFGSLVATVKAIRREKKFVEEVSTGQECGIVLDRTCFYAEQGGQIYDEGYMVKDDDGREDKTE

FTVKNVQVRGGYVLHIGTLYGSLKVGDQVHLSIDETRRRPVMSNHTATHILNFALRSVLGEADQRGSLVAPDRLRFDFTA

KGALSTQEIKKVEGIANQMIEEAKTVYAKDCPLAAAKAIQGLRAVFDETYPDPVRVVSIGIPVEELLADPSGPAGSITSI

EFCGGTHLQNSSHAGPFVIVSEEAIAKGIRRIVAVTGAEARKALRKVDSLRKLLSALEAKVKVQTAPNKDVQKEITDLSE

TLATAVIPQWQKDELREAVKALKKVMDDLDRASKADIQKRVLEKTKQVIESHPNQPLVIMEMENGASAKALNESLKLFKT

YSPQTAAMLFAIDNEAGRITCLCQVPQETANKGLKASQWVQEVSGLMDGKGGGKDVSAQATGKNVGCLQEALNLATDFAR

LRLGELKN

>XP_009468740.1 PREDICTED: alanine--tRNA synthetase, cytoplasmic [Nipponia nippon]

MEATLTASQIRQRFIDFFKENKHTYVHSSSTIPLDDPTLLFANAGMNQFKPIFLNAIDPSHPLAKLNRATNTQKCIRAGG

KHNDLDDVGKDVYHHTFFEMLGSWSFGDYFKELACKLALDLLTKEFGIPAERLYVTYFGGNEAAGLQPDLECKQIWLNLG

LAEGRILPGSMKDNFWEMGDTGPCGPCSEIHYDRIGDRDASHLVNQDDPNVLEIWNLVFIQFNREADGTLKPLPKKSIDT

GMGLERLVSVLQNKMSNYDTDLFLPYFEAIQKGTGARPYMGRVGAEDADGIDMAYRVLADHARTITLALSDGGRPDNTGR

GYVLRRILRRAVRYSHEKLNAPKGFFATLVDVVVQSLGDAFPELKKDPDMVKDIINEEEDQFLKTLSRGRRILDRKIQSL

GDSKTIPGDTAWLLYDTYGFPADLTGLIAEEKGLVVDMEGFEEERKNAQLKSQGKGAGGEDLLMLDIYAIEELRARGLEV

TDDSPKYSYASDQSGAYDFGSLVATVKAIRREKKFVEEVSTGQECGIVLDRTCFYAEQGGQIYDEGYMVKDDDGREDKTE

FTVKNVQVRGGYVLHIGTLYGSLKVGDQVHLSIDETRRRPVMSNHTATHILNFALRSVLGEADQRGSLVAPDRLRFDFTA

KGALSTQEIKKVEGIANQMIEEAKTVYAKDCPLAAAKAIQGLRAVFDETYPDPVRVVSIGIPVEELLADPSGPAGSVTSV

EFCGGTHLQNSSHAGPFVIVSEEAIAKGIRRIVAVTGAEARKALRKVDSLRKLLSALEAKVKVQTAPNKDVQKEITDLSE

TLATAVIPQWQKDELREAVKALKKVMDDLDRASKADIQKRVLEKTKQVIESHPNQPLVIMEMENGASAKALNESLKLFKT

YSPQTATMLFTVDNEAGRITCLCQVPQETATKGLKASQWVQEVSGLMDGKGGGKDVSAQATGKNVGCLQEALKLATDFAR

LRLGELKN

>XP_009507042.1 PREDICTED: alanine--tRNA synthetase, cytoplasmic [Phalacrocorax carbo]

MEATLMASQIRQRFIDFFKENEHTYVHSSSTIPLDDPTLLFANAGMNQFKPIFLNTIDPSHPLAKLNRATNTQKCIRAGG

KHNDLDDVGKDVYHHTFFEMLGSWSFGDYFKELACKLALDLLTKEFGIPVERLYVTYFGGNEAAGLQPDLECKQIWLNLG

LAQGRILPGNMKDNFWEMGDTGPCGPCSEIHYDRIGDRDASHLVNQDDPNVLEIWNLVFIQFNREADGTLKPLPKKSIDT

GMGLERLVSVLQNKMSNYDTDLFLPYFEAIQKGTGARPYMGRVGAEDADGIDMAYRVLADHARTITLALSDGGRPDNTGR

GYVLRRILRRAVRYSHEKLNAPKGFFATLVDVVVQSLGDAFPELKKDPDMVKDIINEEEDQFLKTLSRGRRILDRKIQSL

GDSKTIPGDTAWLLYDTYGFPVDLTGLIAEEKGLVVDMEAFEEERKNAQLKSQGKGAGGEDLLMLDIYAIEELRAQGLEV

TDDSPKYSYASDQSGVYDFGSLVATVKAIRREKKFVEEVSTGQECGIVLDQTCFYAEQGGQIYDKGYMVKDDDGREDKTE

FTVKNVQVRGGYVLHIGTLYGSLKVGDQVHLSIDEARRRPVMSNHTATHILNFALRSVLGEADQRGSLVAPDRLRFDFTA

KGALSTQEIKKVEGIANQMIEEAKTVYARDCPLAAAKAIQGLRAVFDETYPDPVRVVSIGIPVEELLTDPSGPAGSITSV

EFCGGTHLQNSSHAGPFVIVSEEAIAKGIRRIVAVTGAEARKALRKVDSLRKLLSALEAKVKVQTAPNKDVQKEIADLSE

TLATAVIPQWQKDELREAVKALKKVMDDLDRASKADIQKRVLEKTKQVIESHPNQPLVIMEMENGASAKALNESLKLFKA

YSPQTATMLFAVDNEAGRITCLCQVPQETANKGLKASQWVQEVSALMDGKGGGKDVSAQATGKNVGCLQEALKLATDFAR

LRLGELKN

>XP_010401721.1 alanine--tRNA synthetase, cytoplasmic [Corvus cornix cornix]

METSLTASQIRQRFIDFFKENKHTYVHSSSTIPLDDPTLLFANAGMNQFKPIFLNTIDPSHPLAKLSRATNTQKCIRAGG

KHNDLDDVGKDVYHHTFFEMLGSWSFGDYFKELACKLALELLTKEFGIPAERLYVTYFGGNEAAGLQPDLECKQIWLDLG

LAEGRILPGNMKDNFWEMGDTGPCGPCSEIHYDRIGDRDASHLVNQDDPNVLEIWNLVFIQFNREADGTLKPLPKKSIDT

GMGLERLVSVLQNKMSNYDTDLFLPYFEAIQKGTGARPYLGQVGAEDADGIDMAYRVLADHARTITLALSDGGRPDNTGR

GYVLRRILRRAVRYSHEKLNAPKGFFATLVDVVVQSLGDAFPELKKDPDMVKDIINEEEDQFLKTLSRGRRILDRKIQSL

GDSKIIPGDTAWLLYDTYGFPADLTGLIAEEKGLVVDMEGFEEERKNAQLKSQGKGAGGEDLLMLDIYAIEELRARGLEV

TDDSPKYNYASDPSGTYDFGSLVATVKAIRREKKFVEEVSTGQECGIVLDQTCFYAEQGGQIYDQGYMVKDDDSREDKTE

FTVKNVQVRGGYVLHIGTLYGSLKVGDQVHLTIDEARRRPVMSNHTATHILNFALRSVLGEADQRGSLVAPDRLRFDFTA

KGALSTQEIKTVEGIANQMIEESKTVYAKDCPLAAAKAIQGLRAVFDETYPDPVRVVSIGIPVEELLADPSGPGGSITSI

EFCGGTHLQNSSHAGPFVIVSEEAIAKGIRRIVAVTGAEARKALRKVDSLRKLLSALEAKVKVQTAPNKDVQKEITDLGE

TLATAVIPQWQKDELREAVKALKKVMDDLDRASKADIQKRVLEKTKQVIESHPNQPWVIMEMENGASAKALNESLKLFKT

NSPQTAAMLFAVDNEAGRITCLCQVPQETANKGLKASQWVQEVSGLMDGKGGGKDISAQATGKNVGCLQEALKLATDFAR

LRLGELKN

>XP_005152343.1 PREDICTED: alanine--tRNA synthetase, cytoplasmic [Melopsittacus undulatus]

METTLTASQIRQQFLDFFKENKHVYVHSSSTIPLDDPTLLFANAGMNQFKPIFLNTIDPSHPLAKLKRATNTQKCIRAGG

KHNDLDDVGKDVYHHTFFEMLGSWSFGDYFKELACKLALDLLTKEFGIPVERLYVTYFGGNEAAGLQPDLECKQIWLDLG

LAEGRILPGNMKDNFWEMGDTGPCGPCSEIHYDRIGDRDASHLVNQDDPNVLEIWNLVFIQFNRETDGALKPLPKKSIDT

GMGLERLVSVLQNKMSNYDTDLFLPYFEAIQKGTGARPYMGQVGAEDADGIDMAYRVLADHARTVTLALSDGGRPDNTGR

GYVLRRILRRAVRYSHEKLNAPKGFFATLVDVVVQSLGDAFPELKKDPDMVKDIINEEEDQFLKTLSRGRRILDRKIQSL

GDSKIIPGDTAWLLYDTYGFPADLTGLIAEEKGLVVDMEGFEEERKNAQLKSQGKGAGGEDLLMLDIYAIEELRARGLEV

TDDSPKYSYASDPSGTYDFGSLLATVKAIRREKMFVEEVSTGQECGIVLDKTCFYAEQGGQIYDEGYMVKDDNSREDRTE

FTVKNVQVRGGYVLHIGTLYGNLKVGDQVHLSIDETRRRPVMSNHTATHILNFALRSVLGEADQRGSLVAPDRLRFDFTA

KGALSTQEIKKVEEIANQMIEEAKTVYAQDCPLAAAKAIQGLRAVFDETYPDPVRVVSIGIPVEELLADPSGPAGSITSV

EFCGGTHLQNSSHAGPFVIVSEEAIAKGIRRIVAVTGGEARKALRKVDSLRKLLSALEAKVKVQTAPSKDVQKEIVDLSE

TLATAVIPQWQKDELREAVKALKKVMDDLDRASKADIQKRVLEKTKQVIESHPNQPLVIMEMENGASAKALNESLKLFKT

YSPQTATMLFTVDNEAGRITCLCQVPQETANKGLKASQWVQEVSGLMDGKGGGKDVSAQATGKNVGCLREALQLATDFAR

LRLGEQKN

>XP_003209787.1 PREDICTED: alanine--tRNA synthetase, cytoplasmic [Meleagris gallopavo]

MESALTASQIRQRFIDFFKENRHTYVHSSSTIPLDDPTLLFANAGMNQFKPIFLNTIDPSHPLAKLSRATNTQKCIRAGG

KHNDLDDVGKDVYHHTFFEMLGSWSFGDYFKELACKLALDLLTKEFGIPAERLYVTYFGGNEAAGLQPDLECKQIWLDLG

LDEGRILPGNMKDNFWEMGDTGPCGPCSEIHYDRIGDRDASHLVNQDDPNVLEIWNLVFIQFNREADGSLKPLPKKSIDT

GMGLERLVSVLQNKMSNYDTDLFLPYFEAIQKGTGARPYMGQVGAEDADGIDMAYRVLADHARTITLALSDGGRPDNTGR

GYVLRRILRRAVRYSHEKLNAPKGFFATLVDVVVQSLGDAFPELKKDPDMVKDIINEEEDQFLKTLSRGRRILDRKIQSM

GDSKTIPGDTAWLLYDTYGFPVDLTGLIAEEKGLVVDMEGFEEERKNAQLKSQGKGAGGEDLLMLDIYAIEELRARGLEV

TDDSPKYGYTSDPSGTYDFGSLVATVKAIRREKKFVEEVSTGQECGIVLDRTCFYAEQGGQIYDQGYMVKDDDSREDKTE

FTVKNVQVRGGYVLHIGTLYGSLKVGDQVHLSIDETRRRPVMSNHTATHILNFALRSVLGEADQRGSLVAPDRLRFDFTA

KGALSTQEIKKVEGIANQMIEEAKTVYARDCPLAAAKAIQGLRAVFDETYPDPVRVVSIGIPVDELLADPSGPAGSITSI

EFCGGTHLQNSGHAGPFVIVSEEAIAKGIRRIVAVTGAEARKALRKVESLKKLLSALDAKVKVQTAPNKDVQKEITDLSE

MLATAVIPQWQKDELREAVKALKKVMDDLDRASKADIQKRVLEKTKQVIESHPNQPLVIMEMENGASAKALNESLKLFKT

HSPQTATMLFAVDNEAGRITCLCQVPQETAQKGLKANQWVQEVSALMDGKGGGKDISAQATGKNVGCLQEALRLATDFAR

LHLGELKN

>XP_021396809.1 alanine--tRNA synthetase, cytoplasmic [Lonchura striata domestica]

METPLTASQIRQRFIDFFRENKHTYVHSSSTIPLDDPTLLFANAGMNQFKPIFLNTIDPSHPLAKLSRATNTQKCIRAGG

KHNDLDDVGKDVYHHTFFEMLGSWSFGDYFKELACKLALELLTKEFGIPAERLYVTYFGGNEAAGLQPDLECKQIWLDLG

LAEGRILPGNMKDNFWEMGDTGPCGPCSEIHYDRIGDRDASHLVNQDDPNVLEIWNLVFIQFNREADGTLKPLPKKSIDT

GMGLERLVSVLQNKMSNYDTDLFLPYFEAIQKGTGARPYLGQVGAEDADGIDMAYRVLADHARTITLALSDGGRPDNTGR

GYVLRRILRRAVRYSHEKLNAPKGFFATLVDVVVQSLGDAFPELKKDPDMVKDIINEEEDQFLKTLSRGRRILDRKIQSL

GDSKVIPGDTAWLLYDTYGFPADLTGLIAEEKGLVVDMEGFEEERKNAQLKSQGKGAGGEDLLMLDIYAIEELRAQGLEV

TDDSPKYNYASDLSGTYDFGSLVATVKAIRREKKFVEEVSTGQECGIVLDRTCFYAEQGGQIYDQGYMVKDDDSREDKTE

FTVKNVQVRGGYVLHIGTLYGSLKVGDQVHLTIDEARRRPVMSNHTATHILNFALRSVLGEADQRGSLVAPDRLRFDFTA

KGALSTQEIKKVEGIANQMIEESKTVYAKDCPLAEAKAIQGLRAVFDETYPDPVRVVSIGIPVEELLADPSGPGGSITSI

EFCGGTHLQNSSHAGSFVIVSEEAIAKGIRRIVAVTGAEARKALRKVDSLRKLLSALEAKVKVQTAPNKDVQKEITDLGE

ALATAIIPQWQKDELREAVKALKKVMDDLDRASKADIQKRVLEKTKQVIESHPNQPWVIMEMENGASAKALNESLKLFKT

NSPQTAAMLFAVDNEAGRITCLCQVPQETANKGLKASQWVQEVSGLMDGKGGGKDISAQATGKNVGCLQEALKLATDFAR

LRLGELKN

>XP_005309857.1 alanine--tRNA synthetase, cytoplasmic [Chrysemys picta bellii]

METTLSARQIRQKFIDFFKEHQHTYVHSSATIPLDDPTLLFANAGMNQFKPIFLNTLDPSHPMAKLTRAANTQKCIRAGG

KHNDLDDVGKDVYHHTFFEMLGSWSFGDYFKELACKLALDLLTNEFGIPIERLYVTYFGGNEAAGLEPDLECKQIWRDLG

LAESKILPGSMKDNFWEMGDTGPCGPCSEIHYDRIGDRDAAHLVNQDDPNVLEIWNLVFIQFNREADGTLKPLPKKNIDT

GLGLERLVSVLQNKMSNYDTDLFIPYFEAIQKGTGARPYTGKVGAEDVDGIDMAYRVLADHARTITVALADGGRPDNTGR

GYVLRRILRRAVRYSHEKLNAPKGFFASLVDVVVQSLGDAFPELKKDPYMVKDIINEEEVQFLKTLSRGRRILDRKIQSL

GGSETIPGDTAWLLYDTYGFPVDLTGLIAEEKGLTVDMEGFEEERKMAQLKSQGKGDGAEDLIMLDIYAIEELRTRGVEV

TDDSPKYNYTSDPSGVYEFESPVATVQAIRREKRFVEEVSTGQECGIVLDKSCFYAEQGGQIYDEGYLVKDNDSSEDKTE

FTVKNVQIRGGYVLHIGMVYGSLKVGDQVRLYIDEPRRRPIMSNHTATHILNFALRSVLGDADQRGSLVAPDRLRFDFTA

RGAMSTQEIKKTEEIANQMIKEAKTVYAKDCPLAAAKAIQGLRAVFDETYPDPVRVVSIGIPVEDMLADPSSAAGSITSV

EFCGGTHLQNSSHAGPFVIASEEAIAKGIRRIVAITGAEALKALRNADSLMKSLSALDAKVKVQTAPNKDVQKEIADLSE

TLATAVIPQWQKDELREALRALKKVMDDLDRTSKADVQKRVLEKTKQLIDSHPNQPLVVLELESGASAKALNESLKLFKA

YSPQTAAMLFSVDNEAGKITCLCQVPQEAANKGLKANQWVQEVSGLMDGKGGGKDVSAQATGKNVGCLEKALKLATDFAR

LHLGELKN

>XP_013031975.1 PREDICTED: alanine--tRNA synthetase, cytoplasmic [Anser cygnoides domesticus]

MESTLTASQIRQRFIDFFKENQHTYVHSSSTIPLDDPTLLFANAGMNQFKPIFLNTIDPSHPLAKLSRATNTQKCIRAGG

KHNDLDDVGKDVYHHTFFEMLGSWSFGDYFKELACKLALDLLTKEFGIPAERLYVTYFGGNEAAGLQPDLECKQIWLNLG

LPEGRILPGNMKDNFWEMGDTGPCGPCSEIHYDRIGDRDASHLVNQDDPNVLEIWNLVFIQFNREADGTLKPLPKKSIDT

GMGLERLVSVLQNKMSNYDTDLFLPYFEAIQKGTGARPYMGQVGAEDADGIDMAYRVLADHARTITLALSDGGRPDNTGR

GYVLRRILRRAVRYSHEKLNAPKGFFATLVDVVVQSLGDAFPELKKDPDMVKDIINEEEDQFLKTLSRGRRILDRKIQSL

GDSKTIPGDTAWLLYDTYGFPVDLTGLIAEEKGLVVDMEGFEEERKNAQLKSQGKGAGGEDLLMLDIYAIEELRAQGLEV

TDDSPKYGYTSDPSGTYDFGSLVATVKAIRREKKFVEEVSTGQECGIVLDRTCFYAEQGGQIYDQGYMVKDDDSREDKTE

FTVKNVQVRGGYVLHIGTLYGSLKVGDQVLLSIDETRRRPVMSNHTATHILNFALRSVLGEADQRGSLVAPDRLRFDFTA

KGALSTQEIKQVEGIANQMIEEAKTVYAKDCPLAAAKAIQGLRAVFDETYPDPVRVVSIGIPVEELLADPSGPAGSITSI

EFCGGTHLQNSGHAGHFVIVSEEAIAKGIRRIVAVTGAEARKALRKVDSLKKLLSALDAKVKVQTAPNKDVQKEITDLSE

TLATAVIPQWQKDELREAVKALKKVMDDLDRASKADIQKRVLEKTKQVIESHPNQPLVIMEMENGASAKALNESLKLFKT

HSPQTATMLFAVDNEAGRITCLCQVPQETVKKGLKASQWVQEVSGLMDGKGGGKDVSAQATGKNVGCLPEALRLATDFAR

LHLGELKN

>XP_009641635.1 PREDICTED: alanine--tRNA synthetase, cytoplasmic, partial [Egretta garzetta]

MEATLTASQIRQRFIDFFKANKHTYVHSSSTIPLDDPTLLFANAGMNQFKPIFLNTIDPSHPLAKLNRATNTQKCIRAGG

KHNDLDDVGKDVYHHTFFEMLGSWSFGDYFKELACKLALDLLTKEFGIPADRLYVTYFGGNEAAGLQPDLECKQIWLNLG

LAEGRILPGNMKDNFWEMGDTGPCGPCSEIHYDRIGDRDASHLVNQDDPNVLEIWNLVFIQFNREADGTLKPLPKKSIDT

GMGLERLVSVLQNKMSNYDTDLFLPYFEAIQKGTGARPYMGKVGAEDADGIDMAYRVLADHARTITLALSDGGRPDNTGR

GYVLRRILRRAVRYSHEKLNAPKGFFASLVDVVVQSLGDAFPELKKDPDMVKDIINEEEDQFLKTLSRGRRILDRKIQSL

GDSKTIPGDTAWLLYDTYGFPVDLTGLIAEEKGLVVDMEGFEEERKNAQLKSQGKGAGGEDLLMLDIYAIEELRARGLEV

TDDSPKYSYASDQSGAYDFGSLVATVKAIRREKKFVEEVSTGQECGIVLDRTCFYAEQGGQIYDEGYMVKDDDGREDKTE

FTVKNVQVRGGYVLHIGTLYGSLKVGDQVQLSIDEARRGPGMSNHTATHILNFALRSVLGEADQRGSLVAPDRLRFDFTA

KGALSTQEIKKVEGIANQMIEEAKTVYAKDCPLAAAKAIQGLRAVFDETYPDPVRVVSIGIPVEELLADPSGPAGSITSI

EFCGGTHLQNSSHAGPFVIVSEEAIAKGIRRIVAVTGAEARKALRKVDSLRKLLSALEAKVKVQTAPNKDLQKEIADLSE

TLATAVIPQWQKDELREAVKALKKVMDDLDRASKADIQKRVLEKTKQVIESHPNQPLVIMEMENGASAKALNESLKLFKT

HSPQTATMLFAVDNEAGRITCLCQVPQETANKGLKASQWVQEVSGLMDGKGGGKDISAQATGKNVGCLREALKLATDFAR

LRLGEL

>XP_005526340.1 PREDICTED: alanine--tRNA synthetase, cytoplasmic isoform X1 [Pseudopodoces humilis]

METPLTASQIRQRFIDFFKENKHTYVHSSSTIPLDDPTLLFANAGMNQFKPIFLNTIDPSHPLAKLSRATNTQKCIRAGG

KHNDLDDVGKDVYHHTFFEMLGSWSFGDYFKELACKLALELLTKEFGIPAERLYVTYFGGNEAAGLQPDLECKQIWLDLG

LAEGRILPGSMKDNFWEMGDTGPCGPCSEIHYDRIGDRDASHLVNQDDPNVLEIWNLVFIQFNREADGTLKPLPKKSIDT

GMGLERLVSVLQNKMSNYDTDLFLPYFEAIQKGTGARPYLGQVGAEDADGIDMAYRVLADHARTITLALSDGGRPDNTGR

GYVLRRILRRAVRYSHEKLNAPKGFFATLVDVVVQSLGDAFPELKKDPDMVKDIINEEEDQFLKTLSRGRRILDRKIQSL

GDSKVIPGDTAWLLYDTYGFPADLTGLIAEEKGLVVDMEGFEEERKNAQLKSQGKGAGGEDLLMLDIYAIEELRAQGLEV

TDDSPKYNYASDPSGTYDFGSLVATVKAIRREKRFVEEASTGQECGIVLDRTCFYAEQGGQIYDQGYMVKDDDSREDKTE

FTVKNVQVRGGYVLHIGTLYGSLKVGDQVHLTIDEARRRPVMSNHTATHILNFALRSVLGEADQRGSLVAPDRLRFDFTA

KGALSTQEIKKVEGIANQMIEESKTVYAKDCPLAAAKAIQGLRAVFDETYPDPVRVVSIGIPVEELLADPSGPGGSITSI

EFCGGTHLQNSSHAGPFVIVSEEAIAKGIRRIVAVTGAEARKALRKVDSLRKLLSALEAKVKVQTAPNKDVQKEITDLGE

TLATAVIPQWQKDELREAVKALKKVMDDLDRASKADIQKRVLEKTKQVIESHPNQPWVIMEMENGASAKALNESLKLFKT

NSPQTAAMLFAVDNEAGRITCLCQVPQETANKGLKASQWVQEVSGLMDGKGGGKDISAQATGKNVGCLQEALKLATDFAR

LRLGELKN

>XP_005490794.1 PREDICTED: alanine--tRNA synthetase, cytoplasmic [Zonotrichia albicollis]

METPLTASQIRQRFIDFFRENQHTYVHSSSTIPLDDPTLLFANAGMNQFKPIFLNTIDPSHPLAKLSRATNTQKCIRAGG

KHNDLDDVGKDVYHHTFFEMLGSWSFGDYFKELACKLALELLTKEFGIPAERLYVTYFGGNEAAGLQPDLECKQIWLDLG

LAEGRILPGNMKDNFWEMGDTGPCGPCSEIHYDRIGDRDASHLVNQDDPNVLEIWNLVFIQFNREADGTLKPLPKKSIDT

GMGLERLVSVLQNKMSNYDTDLFLPYFEAIQKGTGARPYLGQVGAEDADGIDMAYRVLADHARTITLALSDGGRPDNTGR

GYVLRRILRRAVRYSHEKLNAPKGFFATLVDVVVQSLGDAFPELKKDPDMVKDIINEEEDQFLKTLSRGRRILDRKIQSL

GDSKVIPGDTAWLLYDTYGFPADLTGLIAEEKGLVVDMEGFEEERKNAQLKSQGKGAGGEDLLMLDIYAIEELRARGLEV

TDDSPKYNYASDSSGTYDFGSLVATVKAIRREKTFVEEVSTGQECGIVLDRTCFYAEQGGQIYDQGYMVKDDDSREDKTE

FTVKNVQVRGGYVLHIGTLYGSLKVGDQVHLTIDEARRRPVMSNHTGTHILNFALRSVLGEADQRGSLVAPDRLRFDFTA

KGALSTQEIKKVEGIANQMIEESKTVYAKDCPLAEAKAIQGLRAVFDETYPDPVRVVSIGIPVEELLADPSGPGGSITSI

EFCGGTHLQNSSHAGPFVIVSEEAIAKGIRRIVAVTGAEARKALRKVDSLRKLLSALEAKVKVQTTPNKDVQKEITDLSE

TLATAVIPQWQKDELREAVKALKKVMDDLDRASKADIQKRVLEKTKQVIESHPNQPWVIMEMENGASAKALNESLKLFKT

NSPQTAAMLFAVDNEAGRITCLCQVPQETANKGLKASQWVQEVSGLMDGKGGGKDISAQATGKNVGCLQEALKLATDFAR

LRLGELKN

>XP_010182234.1 PREDICTED: alanine--tRNA synthetase, cytoplasmic [Mesitornis unicolor]

MEAMLTASQIRQRFIDFFKENKHTYVHSSSTIPLDDPTLLFANAGMNQFKPIFLNTVDPSHPLAKLNRATNTQKCIRAGG

KHNDLDDVGKDVYHHTFFEMLGSWSFGDYFKELACKLALDLLTKEFGIPAERLYVTYFGGNEAAGLQPDLECKQIWLNLG

LAEGKILPGNMKDNFWEMGDTGPCGPCSEIHYDRIGDRDASHLVNQDDPNVLEIWNLVFIQFNREADGTLKPLPKKSIDT

GMGLERLVSVLQNKMSNYDTDLFLPYFEAIQKGTGARPYMGQVGAEDADGIDMAYRVLADHARTITLALSDGGRPDNTGR

GYVLRRILRRAVRYSHEKLNAPKGFFATLVDVVVQSLGDAFPELKKDPDMVKDIINEEEDQFLKTLSRGRRILDRKIQSL

GDSKTIPGDTAWLLYDTYGFPADLTGLIAEEKGLVVDMEGFEEERKNAQLKSQGKGAGGEDLLMLDIYAIEELRARGLEV

TDDSPKYSYASDPNGTYDFGSLVATVKAIRRDKKFVEEVSTGQECGIVLDRTCFYAEQGGQIYDEGYMVKDDDGREDKTE

FTVKNVQVRGGYVLHIGTLYGTLKVGDQVHLSIDETRRRPVMSNHTATHILNFALRSVLGEADQRGSLVAPDRLRFDFTA

KGALSTQEIKKVEGITNQMILEAKTVYAKDCPLAAAKAIQGLRAVFDETYPDPVRVVSIGIPVEELLADPSGPAGSITSI

EFCGGTHLQNSSHAGPFVIVSEEAIAKGIRRIVAVTGAEARKALRKVDSLRKLLSALEAKVKVQTAPSKDVQKEITDLSE

TLATAVIPQWQKDELREAVKALKKVMDDLDRASKADIQKRVLEKTKQIIESHPNQPLVIMEMENGASAKALNESLKLFKT

HSPQTATMLFAVDNEAGRITCLCQVPQETANKGLKASQWVQEVSGLMDGKGGGKDVSAQATGKNVGCLREAMQLATDFAR

LRLGELKN

>XP_012425826.1 PREDICTED: alanine--tRNA synthetase, cytoplasmic [Taeniopygia guttata]

METPLTASQIRQRFIDFFRENKHTYVHSSSTIPLDDPTLLFANAGMNQFKPIFLNTIDPSHPLAKLSRATNTQKCIRAGG

KHNDLDDVGKDVYHHTFFEMLGSWSFGDYFKELACKLALELLTKEFGIPAERLYVTYFGGNEAAGLQPDLECKQIWLDLG

LAEGRILPGSMKDNFWEMGDTGPCGPCSEIHYDRIGDRDASHLVNQDDPNVLEIWNLVFIQFNREADGTLKPLPKKSIDT

GMGLERLVSVLQNKMSNYDTDLFLPYFEAIQKGTGARPYLGQVGAEDADGIDMAYRVLADHARTITLALSDGGRPDNTGR

GYVLRRILRRAVRYSHEKLNAPKGFFATLVDVVVQSLGDAFPELKKDPDMVKDIINEEEDQFLKTLSRGRRILDRKIQSL

GDSKVIPGDTAWLLYDTYGFPADLTGLIAEEKGLVVDMEGFEEERKNAQLKSQGKGAGGEDLLMLDIYAIEELRAQGLEV

TDDSPKYNYASDLSGTYDFGSLVATVKAIRREKKFVEEVSTGQECGIVLDRTCFYAEQGGQIYDQGYMVKDDDSREDKTE

FTVKNVQVRGGYVLHIGTLYGSLKVGDQVHLTIDEARRRPVMSNHTATHILNFALRSVLGEADQRGSLVAPDRLRFDFTA

KGALSTQEIKKVEGIANQMIEESKTVYAKDCPLAEAKAIQGLRAVFDETYPDPVRVVSIGIPVEELLADPSGPGGSITSI

EFCGGTHLQNSSHAGSFVIVSEEAIAKGIRRIVAVTGAEARKALRKVDSLRKLLSALEAKVKVQTAPNKDVQKEITDLGE

TLATAIIPQWQKDELREAVKALKKVMDDLDRASKADIQKRVLEKTKQVIESHPNQPWVIMEMENGASAKALNESLKLFKT

NSPQTAAMLFAVDNEAGRITCLCQVPQETANKGLKASQWVQEVSGLMDGKGGGKDISAQATGKNVGCLQEALKLATDFAR

LRLGELKN

>XP_021264356.1 alanine--tRNA synthetase, cytoplasmic [Numida meleagris]

MESTLTASQIRQRFIDFFKENQHTYVHSSSTIPLDDPTLLFANAGMNQFKPIFLNTIDPSHPLAKLSRATNTQKCIRAGG

KHNDLDDVGKDVYHHTFFEMLGSWSFGDYFKELACKLALDLLTKEFGIPADRLYVTYFGGNEAAGLQPDLECKQIWLDLG

LAEGRILPGNMKDNFWEMGDTGPCGPCSEIHYDRIGDRDASHLVNQDDPNVLEIWNLVFIQFNREADGSLKPLPKKSIDT

GMGLERLVSVLQNKMSNYDTDLFLPYFEAIQKGTGARPYMGQVGTEDADGIDMAYRVLADHARTITLALSDGGRPDNTGR

GYVLRRILRRAVRYSHEKLNAPKGFFATLVDVVVQSLGDAFPELKKDPDMVKDIINEEEDQFLKTLSRGRRILDRKIQSM

GDSKTIPGDTAWLLYDTYGFPVDLTGLIAEEKGLVVDMEGFEEERKNAQLKSQGKGAGGEDLLMLDIYAIEELRARGLEV

TDDSPKYGYTSDPSGTYDFGSLVATVKAIRREKKFVEEVSTGQECGIVLDRTCFYAEQGGQIYDQGYMVKDDDSREDKTE

FTVKNVQVRGGYVLHIGTLYGSLKVGDQVHLSIDEMRRRPVMSNHTATHILNFALRSVLGEADQRGSLVAPDRLRFDFTA

KGALSTQEIKKVEGIANQMIEEAKTVYAKDCPLAAAKAIQGLRAVFDETYPDPVRVVSIGIPVEELLADPSGPAGSITSI

EFCGGTHLQNSGHAGPFVIVSEEAIAKGIRRIVAVTGAEARKALRKVESLKKLLSALDAKVKVQTAPNKDVQKEITDLSE

MLATAVIPQWQKDELREAVKALKKVMDDLDRASKADIQKRVLEKTKQVIESHPNQPLVIMEMENGASAKALNESLKLFKT

HSPQTATMLFAVDNEAGRITCLCQVPQETVQKGLKASQWVQEVSALMDGKGGGKDVSAQATGKNVGCLQEALRLATDFAR

LHLGELKN

>XP_015495694.1 PREDICTED: alanine--tRNA synthetase, cytoplasmic [Parus major]

METPLTASQIRQRFIDFFKENKHTYVHSSSTIPLDDPTLLFANAGMNQFKPIFLNTIDPSHPLAKLSRATNTQKCIRAGG

KHNDLDDVGKDVYHHTFFEMLGSWSFGDYFKDIACKLALELLTKEFGIPAERLYVTYFGGNEAAGLQPDLECKQIWLDLG

LAEGRILPGSMKDNFWEMGDTGPCGPCSEIHYDRIGDRDASHLVNQDDPNVLEIWNLVFIQFNREADGTLKPLPKKSIDT

GMGLERLVSVLQNKMSNYDTDLFLPYFEAIQKGTGARPYLGQVGAEDADGIDMAYRVLADHARTITLALSDGGRPDNTGR

GYVLRRILRRAVRYSHEKLNAPKGFFATLVDVVVQSLGDAFPELKKDPDMVKDIINEEEDQFLKTLSRGRRILDRKIQSL

GDSKVIPGDTAWLLYDTYGFPADLTGLIAEEKGLVVDMEGFEEERKNAQLKSQGKGAGGEDLLMLDIYAIEELRAQGLEV

TDDSPKYNYASDPSGTYDFGSLVATVKAIRREKRFVEEASTGQECGIVLDRTCFYAEQGGQIYDQGYMVKDDDSREDKTE

FTVKNVQVRGGYVLHIGTLYGSLKVGDQVHLTIDEARRRPVMSNHTATHILNFALRSVLGEADQRGSLVAPDRLRFDFTA

KGALSTQEIKKVEGIANQMIEESKTVYAKDCPLAAAKAIQGLRAVFDETYPDPVRVVSIGIPVEELLADPSGPGGSITSI

EFCGGTHLQNSSHAGPFVIVSEEAIAKGIRRIVAVTGAEARKALRKVDSLRKLLSALEAKVKVQTAPNKDVQKEITDLGE

TLATAVIPQWQKDELREAVKALKKVMDDLDRASKADIQKRVLEKTKQVIESHPNQPWVIMEMENGASAKALNESLKLFKT

NSPQTAAMLFAVDNEAGRITCLCQVPQETANKGLKASQWVQEVSGLMDGKGGGKDISAQATGKNVGCLQEALKLATDFAR

LRLGELKN

>XP_023800905.1 alanine--tRNA synthetase, cytoplasmic [Cyanistes caeruleus]

METPLTASQIRQRFIDFFKENKHTYVHSSSTIPLDDPTLLFANAGMNQFKPIFLNTIDPSHPLAKLSRATNTQKCIRAGG

KHNDLDDVGKDVYHHTFFEMLGSWSFGDYFKELACKLALELLTKEFGIPAERLYVTYFGGNEAAGLQPDLECKQIWLDLG

LAEGRILPGSMKDNFWEMGDTGPCGPCSEIHYDRIGDRDASHLVNQDDPNVLEIWNLVFIQFNREADGTLKPLPKKSIDT

GMGLERLVSVLQNKMSNYDTDLFLPYFEAIQKGTGARPYLGQVGAEDADGIDMAYRVLADHARTITLALSDGGRPDNTGR

GYVLRRILRRAVRYSHEKLNAPKGFFATLVDVVVQSLGDAFPELKKDPDMVKDIINEEEDQFLKTLSRGRRILDRKIQSL

GDSKVIPGDTAWLLYDTYGFPADLTGLIAEEKGLVVDMEGFEEERKNAQLKSQGKGAGGEDLLMLDIYAIEELRAQGLEV

TDDSPKYSYASDLSGTYDFGSLVATVKAIRREKRFVEEASTGQECGIVLDRTCFYAEQGGQIYDQGYMVKDDDNREDKTE

FTVKNVQVRGGYVLHIGTLYGSLKVGDQVHLTIDEARRRPVMSNHTATHILNFALRSVLGEADQRGSLVAPDRLRFDFTA

KGALSTQEIKKVEGIANQMIEESKTVYAKDCPLAAAKAIQGLRAVFDETYPDPVRVVSIGIPVEELLADPSGPGGSITSI

EFCGGTHLQNSSHAGPFVIVSEEAIAKGIRRIVAVTGAEARKALRKVDSLRKLLSALEAKVKVQTAPNKDVQKEIADLGE

TLATAVIPQWQKDELREAVKALKKVMDDLDRASKADIQKRVLEKTKQVIESHPNQPWVIMEMENGASAKALNESLKLFKT

NSPQTAAMLFAVDNEAGRITCLCQVPQETANKGLKASQWVQEVSGLMDGKGGGKDISAQATGKNVGCLQEALKLATDFAR

LRLGELKN

>XP_005052520.1 PREDICTED: alanine--tRNA synthetase, cytoplasmic [Ficedula albicollis]

METPLTASQIRQRFIDFFRENKHTYVHSSSTIPLDDPTLLFANAGMNQFKPIFLNTIDPSHPLAKLSRATNTQKCIRAGG

KHNDLDDVGKDVYHHTFFEMLGSWSFGDYFKELACKLALELLTKEFGIPAERLYVTYFGGNEAAGLQPDLECKQIWLDLG

LAEGRILPGNMKDNFWEMGDTGPCGPCSEIHYDRIGDRDASHLVNQDDPNVLEIWNLVFIQFNREADGTLKPLPKKSIDT

GMGLERLVSVLQNKMSNYDTDLFLPYFEAIQKGTGARPYLGQVGAEDADGIDMAYRVLADHARTITLALSDGGRPDNTGR

GYVLRRILRRAVRYSHEKLNAPKGFFATLVDVVVQSLGDAFPELKKDPDMVKDIINEEEDQFLKTLSRGRRILDRKIQSL

GDSKVIPGDTAWLLYDTYGFPADLTGLIAEEKGLVVDMEGFEEERKNAQLKSQGKGAGGEDLLMLDIYAIEELRAQGLEA

TDDSPKYNYASDPSGTYDFGSLVATVKAIRRDKKFVEEVSTGQECGIVLDRTCFYAEQGGQIYDQGYMVKDDDSREDKTE

FTVKNVQVRGGYVLHIGTLYGSLKVGDQVHLTIDEARRRPVMSNHTATHILNFALRSVLGEADQRGSLVAPDRLRFDFTA

KGALSTQEIKKVEGIANQMIEESKTVYAKDCPLAAAKAIQGLRAVFDETYPDPVRVVSIGIPVEELLADPSGPGGSITSI

EFCGGTHLQNSSHAGPFVIVSEEAIAKGIRRIVAVTGAEARKALRKVDSLRKLLSALEAKVKVQTAPNKDVQKEITDLSE

TLATAVIPQWQKDELREAVKALKKVMDDLDRASKADIQKRVLEKTKQVIESHPNQPWVIMEMENGASAKALNESLKLFKT

NSPQTAAMLFAVDNEAGRITCLCQVPQETANKGLKASQWVQEVSGLMDGKGGGKDISAQATGKNVGCLQEALKLATDFAR

LRLGELKN

>XP_005422810.1 PREDICTED: alanine--tRNA synthetase, cytoplasmic [Geospiza fortis]

METPLTASQIRQRFIDFFRENQHTYVHSSSTIPLDDPTLLFANAGMNQFKPIFLNTIDPSHPLAKLSRATNTQKCIRAGG

KHNDLDDVGKDVYHHTFFEMLGSWSFGDYFKELACKLALELLTKEFGIPAERLYVTYFGGNEAAGLQPDLECKQIWLDLG

LAEGRILPGSMKDNFWEMGDTGPCGPCSEIHYDRIGDRDASHLVNQDDPNVLEIWNLVFIQFNREADGTLKPLPKKSIDT

GMGLERLVSVLQNKMSNYDTDLFLPYFEAIQKGTGARPYLGQVGAEDADGIDMAYRVLADHARTITLALSDGGRPDNTGR

GYVLRRILRRAVRYSHEKLNAPKGFFATLVDVVVQSLGDAFPELKKDPDMVKDIINEEEDQFLKTLSRGRRILDRKIQSL

GDSKVIPGDTAWLLYDTYGFPADLTGLIAEEKGLVVDMEGFEEERKNAQLKSQGKGAGGEDLLMLDIYAIEELRARGLEV

TDDSPKYSYASDLSGTYDFGSLVATVKAIRREKTFVEEVSTGQECGIVLDRTCFYAEQGGQIYDQGYMVKDDDSREDKTE

FTVKNVQVRGGYVLHIGTLYGSLKVGDQVHLTIDEARRRPVMSNHTATHILNFALRSVLGEADQRGSLVAPDRLRFDFTA

KGALSTQEIKKVEGIANQMIEESKTVYAKDCPLAEAKAIQGLRAVFDETYPDPVRVVSIGIPVEELLADPSGPGGSITSI

EFCGGTHLQNSSHAGPFVIVSEEAIAKGIRRIVAVTGAEARKALRKVDSLRKLLSALEAKVKVQTAPNKDVQKEITDLSE

TLATAIIPQWQKDELREAVKALKKVMDDLDRASKADIQKRVLEKTKQVIESHPNQPWVIMEMENGASAKALNESLKLFKT

NSPQTAAMLFAVDNEAGRITCLCQVPQETANKGLKASQWVQEVSGLMDGKGGGKDVSAQATGKNVGCLQEALKLATDFAR

LRLGELKN

>XP_009088945.1 PREDICTED: alanine--tRNA synthetase, cytoplasmic [Serinus canaria]

METPLTASQIRQRFIDFFRENQHTYVHSSSTIPLDDPTLLFANAGMNQFKPIFLNTIDPSHPLAKLSRATNTQKCIRAGG

KHNDLDDVGKDVYHHTFFEMLGSWSFGDYFKELACKLALELLTKEFGIPAERLYVTYFGGNEAAGLQPDLECKQIWLDLG

LAEGRILPGSMKDNFWEMGDTGPCGPCSEIHYDRIGDRDASHLVNQDDPNVLEIWNLVFIQFNREADGTLKPLPKKSIDT

GMGLERLVSVLQNKMSNYDTDLFLPYFEAIQKGTGARPYLGQVGAEDADGIDMAYRVLADHARTITLALSDGGRPDNTGR

GYVLRRILRRAVRYSHEKLNAPKGFFATLVDVVVQSLGDAFPELKKDPDMVKDIINEEEDQFLKTLSRGRRILDRKIQSL

GDSKVIPGDTAWLLYDTYGFPADLTGLIAEEKGLVVDMEGFEEERKNAQLKSQGKGAGGEDLLMLDIYAIEELRAQGLEV

TDDSPKYNYASDPSGTYDFGNLVATVKAIRREKTFVEEVSTGQECGIVLDRTCFYAEQGGQIYDQGYMVKDDNSREDKTE

FTVKNVQVRGGYVLHIGTLYGSLKVGDQVHLTIDEARRRPVMSNHTATHILNFALRSVLGEADQRGSLVAPDRLRFDFTA

KGALSTQEIKKVEGIANQMIEESKTVYAKDCPLAEAKAIQGLRAVFDETYPDPVRVVSIGIPVEELLADPSGPGGSITSI

EFCGGTHLQNSSHAGPFVIVSEEAIAKGIRRIVAVTGAEARKALRKVDSLRKLLSALEAKVKVQTAPNKDVQKEITDLSE

TLATAIIPQWQKDELREAVKALKKVMDDLDRASKADIQKRVLEKTKQVIESHPNQPWVIMEMENGASAKALNESLKLFKT

NSPQTAAMLFAVDNEAGRITCLCQVPQETANKGLKASQWVQEVSGLMDGKGGGKDISAQATGKNVGCLQEALKLATDFAR

LRLGELKN

>XP_015729141.1 PREDICTED: alanine--tRNA synthetase, cytoplasmic [Coturnix japonica]

MESTLTARQIRQRFIDFFKENQHTYVHSSSTIPLDDPTLLFANAGMNQFKPIFLNTIDPSHPLAKLSRATNTQKCIRAGG

KHNDLDDVGKDVYHHTFFEMLGSWSFGDYFKELACKLALDLLTKEFGIPAERLYVTYFGGNENAGLQPDLECKQIWLDLG

LPEGRILPGNMKDNFWEMGDTGPCGPCSEIHYDRIGDRDASHLVNQDDPNVLEIWNLVFIQFNREADGSLKPLPKKSIDT

GMGLERLVSVLQNKMSNYDTDLFLPYFEAIQKGTGARPYMGQVGAEDADGIDMAYRVLADHARTITLALSDGGRPDNTGR

GYVLRRILRRAVRYSHEKLNAPKGFFATLVDVVVQSLGDAFPELKKDPDMVKDIINEEEDQFLKTLSRGRRILDRKIQSM

GDSKTIPGDTAWLLYDTYGFPVDLTGLIAEEKGLVVDMEGFEEERKNAQLKSQGKGAGGEDLLMLDIYAIEELRARGLEV

TDDSPKYGYTSDPSGTYDFGSLVATVKAIRREKKFVEEVSTGQECGIVLDRTCFYAEQGGQIYDQGYMVKEDDSKEDKTE

FTVKNVQVRGGYVLHIGTLYGSLKVGDQVHLSIDETRRRPVMSNHTATHILNFALRSVLGEADQRGSLVAPDRLRFDFTA

KGALSTKEIKEVESIANQMIEEAKTVYAKDCPLAAAKAIQGLRAVFDETYPDPVRVVSIGIPVEELLADPSGPAGSITSI

EFCGGTHLQNSGHAGPFVIVSEEAIAKGIRRIVAVTGAEARKALRKVESLKKLLSALDAKVKVQTAPNKDVQKEITDLSE

MLATAVIPQWQKDELREAVKALKKVMDDLDRASKADIQKRVLEKTKQVIESHPNQPLVIMEMENGASAKALNESLKLFKT

HSPQTATMLFAVDNEAGRITCLCQVPQETAQKGLKANQWVQEVSALMDGKGGGKDVSAQATGKNVGCLHEALRLATDFAR

LHLGELKN

>KFQ70939.1 Alanine--tRNA synthetase, cytoplasmic, partial [Phaethon lepturus]

MEVTLTASQIRQRFIDFFKENKHTYVHSSSTIPLDDPTLLFANAGMNQFKPIFLNTIDPSHPLAKLNRATNTQKCIRAGG

KHNDLDDVGKDVYHHTFFEMLGSWSFGDYFKELACKLALDLLTKEFGIPAERLYVTYFGGNEAAGLQPDLECKQIWLNLG

LAEGRILPGNMKDNFWEMGDTGPCGPCSEIHYDRIGDRDASHLVNQDDPNVLEIWNLVFIQFNREADGTLKPLPKKSIDT

GMGLERLVSVLQNKMSNYDTDLFLPYFEAIQKGTGARPYMGQVGVEDADGIDMAYRVLADHARTITLALSDGGRPDNTGR

GYVLRRILRRAVRYSHEKLNAPKGFFATLVDVVVQSLGDAFPELKKDPDMVKDIINEEEDQFLKTLSRGRRILDRKIQSL

GDSKTIPGDTAWLLYDTYGFPADLTGLIAEEKGLVVDMEGFEEERKNAQLKSQGKGAGGEDLLMLDIYAIEELRARGLEV

TDDSPKYSYTSDQSGAYDFGSLVATVKAIRRDKRFVEEVSTGQECGIVLDRTCFYAEQGGQIYDEGYMVKDDDGREDKTE

FTVKNVQVRGGYVLHIGTLYGSLKVGDQVHLSVDETRRRPVMSNHTATHILNFALRSVLGEADQRGSLVAPDRLRFDFTA

KGALSTQEIKKVEGIANQMIEEAKTVYAKDCPLAAAKAIQGLRAVFDETYPDPVRVVSIGIPVEELLADPSGPAGSITSI

EFCGGTHLQNSSHTGPFVIVSEEAIAKGIRRIVAVTGAEARKALRKVDSLRKLLSALEAKVKVQTAPNKDVQKEITDLSE

TLATAVIPQWQKDELREAVKALKKVMDDLDRASKADIQKRVLEKTKQVIESHPNQPLVIMEMENGASAKALNESLKLFKT

YSPQTATMLFAVDNEAGRITCLCQVPQETANKGLKASQWVQEVSGLMDGKGGGKDVSAQATGKNVGCLREALQLATDFAR

LRL

>KFO71395.1 Alanine--tRNA synthetase, cytoplasmic [Cuculus canorus]

MEAPLTASQIRQRFIDFFKENKHTYVHSSSTIPLDDPTLLFANAGMNQFKPIFLNTIDPSHPLAKLTRATNTQKCIRAGG

KHNDLDDVGKDVYHHTFFEMLGSWSFGDYFKELACKLALDLLTKEFGIPAERLYVTYFGGNEAAGLQPDLECKQIWLNLG

LDEGRILPGNMKDNFWEMGDTGPCGPCSEIHYDRIGDRDASHLVNQDDPNVLEIWNLVFIQFNREADGTLKPLPKKSIDT

GMGLERLVSVLQNKMSNYDTDLFLPYFEAIQKGTGARPYMGQVGAEDADGIDMAYRVLADHARTITLALSDGGRPDNTGR

GYVLRRILRRAVRYSHEKLNAPKGFFATLVDVVVQSLGDAFPELKKDPDMVKDIINEEEDQFLKTLSRGRRILDRKIQSL

GDSKTIPGDTAWLLYDTYADLTGLIAEEKGLVVDMEGFEEERRNAQLKSQGKGAGGEDLLMLDIYAIEELRARGLEVTDD

SPKYSYTSDQSGVYEFASLVATVKAIRREKKFVEEVSTGQECGIVLDRTCFYAEQGGQIYDEGYMVKDDDGREDKTEFTV

KNVQVRGGYVLHIGTLYGNLKVGDQVHLSIDETRRRPVMSNHTATHILNFALRSVLGEADQRGSLVAPDRLRFDFTAKGA

LSTQEIKKVEGIANQMIEEAKTVYAKDCPLAAAKAIQGLRAVFDETYPDPVRVVSIGIPVEELLTDPCGPGGSITSIEFC

GGTHLQNSSHAGPFVIVSEEAIAKGIRRIVAVTGAEARKALRKVSSLRKLLSTLEAKVKVQTAPNKDVQKEITDLSEALA

TAVIPQWQKDELREAVKALKKVMDDLDRASKADIQKRVLEKTKQVIESHPNQPLVIMEMENGASAKALNESLKLFKTQSP

QTAAMLFAVDNEAGRITCLCQVPQETANKGLKASQWVQEVSGLMDGKGGGKDISAQATGKNVGCLQEALRVATDFARLRL

GELKN

>XP_014736211.1 PREDICTED: alanine--tRNA synthetase, cytoplasmic [Sturnus vulgaris]

METPLTASQIRQRFIDFFRENKHTYVHSSSTIPLDDPTLLFANAGMNQFKPIFLNTIDPSHPLAKLSRATNTQKCIRAGG

KHNDLDDVGKDVYHHTFFEMLGSWSFGDYFKELACKLALELLTKEFGIPAERLYVTYFGGNEAAGLQPDLECKQIWLDLG

LAEGRILPGSMKDNFWEMGDTGPCGPCSEIHYDRIGDRDASHLVNQDDPNVLEIWNLVFIQFNREADGTLKPLPKKSIDT

GMGLERLVSVLQNKMSNYDTDLFLPYFEAIQKGTGARPYLGQVGAEDTDGIDMAYRVLADHARTITLALSDGGRPDNTGR

GYVLRRILRRAVRYSHEKLNAPKGFFATLVDVVVQSLGDAFPELKKDPDMVKDIINEEEDQFLKTLSRGRRILDRKIQSL

GDSKVIPGDTAWLLYDTYGFPADLTGLIAEEKGLVVDMEGFEEERKNAQLKSQGKGAGGEDLLMLDIYAIEELRAQGLEA

TDDSPKYNYASDLSGTYDFGSLVATVKAIRREKKFVEEVSTGQECGIVLDRTCFYAEQGGQIYDQGYMVKDDDSREDKTE

FTVKNVQVRGGYVLHIGTLYGSLKVGDQVHLTIDEARRRPVMSNHTATHILNFALRSVLGEADQRGSLVAPDRLRFDFTA

KGALSTQEIKKVEGIANQMIEESKTVYAKDCPLAAAKAIQGLRAVFDETYPDPVRVVSIGIPVEELLADPSGPGGSITSI

EFCGGTHLQNSSHAGPFVIVSEEAIAKGIRRIVAVTGAEARKALRKVDSLRKLLSALEAKVKVQTAPNKDVQKEITDLSE

TLATAVIPQWQKDELREAVKALKKVMDDLDRASKADIQKRVLEKTRQVIESHPNQPWVIMEMENGASAKALNESLKLFKT

NSPQTAAMLFAVDNEAGRITCLCQVPQETANKGLKASQWVQEVSGLMDGKGGGKDISAQATGKNVGCLQEALKLATDFAR

LRLGELKN

>XP_010279791.1 PREDICTED: alanine--tRNA synthetase, cytoplasmic, partial [Phaethon lepturus]

MEVTLTASQIRQRFIDFFKENKHTYVHSSSTIPLDDPTLLFANAGMNQFKPIFLNTIDPSHPLAKLNRATNTQKCIRAGG

KHNDLDDVGKDVYHHTFFEMLGSWSFGDYFKELACKLALDLLTKEFGIPAERLYVTYFGGNEAAGLQPDLECKQIWLNLG

LAEGRILPGNMKDNFWEMGDTGPCGPCSEIHYDRIGDRDASHLVNQDDPNVLEIWNLVFIQFNREADGTLKPLPKKSIDT

GMGLERLVSVLQNKMSNYDTDLFLPYFEAIQKGTGARPYMGQVGVEDADGIDMAYRVLADHARTITLALSDGGRPDNTGR

GYVLRRILRRAVRYSHEKLNAPKGFFATLVDVVVQSLGDAFPELKKDPDMVKDIINEEEDQFLKTLSRGRRILDRKIQSL

GDSKTIPGDTAWLLYDTYGFPADLTGLIAEEKGLVVDMEGFEEERKNAQLKSQGKGAGGEDLLMLDIYAIEELRARGLEV

TDDSPKYSYTSDQSGAYDFGSLVATVKAIRRDKRFVEEVSTGQECGIVLDRTCFYAEQGGQIYDEGYMVKDDDGREDKTE

FTVKNVQVRGGYVLHIGTLYGSLKVGDQVHLSVDETRRRPVMSNHTATHILNFALRSVLGEADQRGSLVAPDRLRFDFTA

KGALSTQEIKKVEGIANQMIEEAKTVYAKDCPLAAAKAIQGLRAVFDETYPDPVRVVSIGIPVEELLADPSGPAGSITSI

EFCGGTHLQNSSHTGPFVIVSEEAIAKGIRRIVAVTGAEARKALRKVDSLRKLLSALEAKVKVQTAPNKDVQKEITDLSE

TLATAVIPQWQKDELREAVKALKKVMDDLDRASKADIQKRVLEKTKQVIESHPNQPLVIMEMENGASAKALNESLKLFKT

YSPQTATMLFAVDNEAGRITCLCQVPQETANKGLKASQWVQEVSGLMDGKGGGKDVSAQATGKNVGCLREALQLATDFAR

LR

>XP_015280281.1 PREDICTED: alanine--tRNA synthetase, cytoplasmic [Gekko japonicus]

MEALTASQIRQRFINFFVENRHTYVHSSATIPLDDPTLLFANAGMNQFKPIFLNTIDPSHPMAKLTRAANTQKCIRAGGK

HNDLDDVGKDVYHHTFFEMLGSWSFGDYFKELACKFALDLLTKEFGIPIERLYVTYFGGNEAAGLQPDLECKQIWLDLGL

AECRILPGSMKDNFWEMGDTGPCGPCSEIHYDRIGDRDAAHLVNQDDPNVLEIWNLVFIQFNRESDGILKPLPKKSIDTG

MGLERLVSVLQNKMSNYDTDLFVPYFEAIQKGTGCRPYSGKVGEEDADGIDMAYRVLADHARTITVALSDGGRPDNTGRG

YVLRRILRRAVRYAHEKLNARKGFFATLVDVVVESLGGAFPELKKDPDMVKDIINEEEVQFLKTLNRGRRILDRKIQSLG

DNKTIPGDTAWLLYDTYGFPVDLTGLIAEEKGLDVDMEGFEEERKAAQLKSQGKGGGDEDLIMLDIYAIEELRSRGLEVT

DDSVKYNYTSDPSGAYEFDSPVATVKALRRDKKFVEEVSTGQECGVILDQTSFYAEQGGQIYDEGYLVKEDEGSEDKTEF

TVKNVQVRGGYVLHIGTVYGNLKVGDVVRLYVDESRRRPIMSNHTSTHILNFALRAVLGEADQRGSLVAPDRLRFDFTAK

GAMSTKEIKKAEEIANRMIQEAKVVYAKDCPLAAAKAIQGLRAVFDETYPDPVRVVSIGIPVEDMLADPSSAAGAITSVE

FCGGTHLQNSSHAGSFVIVSEEAIAKGIRRIVAVTGAEAQKALRKADSLKKLLSDLEAKVKVQTAPNKEVQREIADLSET

LATAVIPQWQKDELREALKALKKVMDDLDRASKADVQKRVLDKTKQLIETQPNQPLVILEMESGASAKALNESLKLLKTH

SPETAAMLFTVDNEAGKIICLCQVPQEVANKGLKASEWVQQVSGLMDGKGGGKDLSAQATGKNVECLEEALKLATDFAKL

KLGGLKN

>XP_004626056.1 alanine--tRNA synthetase, cytoplasmic [Octodon degus]

MNSTLTASVIWERFIDFFKRNEHTYVHSSATIPLDDPTLLFANAGMNQFKPIFLNTIDPSHPLAKLSRAANTQKCIRAGG

KHNDLDDVGKDVYHHTFFEMLGSWSFGDYFKELACKMALELLTQEFGIPVDRLYVTYFGGDEAAGLEPDLECKQIWQNLG

LDDTKILPGNMKDNFWEMGDTGPCGPCSEIHYDRIGGRDAAHLVNQDDPNVLEIWNLVFIQYNRETDGTLKPLPKKSIDT

GMGLERLVSVLQNKMSNYDTDLFVPYFEAIQKGTGARPYTGKVGAEDADGIDMAYRVLADHARTITVALADGGRPDNTGR

GYVLRRILRRAVRYSHEKLNASRGFFATLVDVVVQSLGDAFPELKKDPDLVKDIINEEEVQFLKTLSRGRRILDRKIQSL

GDCRTIPGDTAWLLYDTYGFPVDLTGLIAEEKGLVVDMDGFEEERKLAQLKSQGKGAGGEDHIMLDIYAIEELRAQGLEA

TDDSPKYSYHSDSSGGYGKTEFTVKNTQVRGGYVLHIGTIYGSLKVGDQVRLFIDEPRRRPIMSNHTATHILNFALRSVL

GEADQKGSLVAPDRLRFDFTAKGAMSTQQIKKAEEIANEMIEAAKPVYTQDCPLAAAKAIQGLRAVFDETYPDPVRVVSI

GVPVSELLDDPSGPAGSLTSVEFCGGTHLRNSSHAGAFVIVTEEAIAKGIRRIVAVTGAEAQKALRKAESLKKSLSTMES

KVNAQTAPNKDVQKEIADLGEALATAVIPQWKKDELRETLKSLKKVMDDLDRASKADLQKRVLERTKQLIDSNPNQPLVI

LEMESGASAKALNEALKLFKTHSPETSAMLFTVDNEAGKITCLCQVPQNAANRGLKASEWVQQVSGLMDGKGGGKDVSAQ

ATGKNVGCLQEALQLATSFAQLHLGDVKN

>XP_013798190.1 PREDICTED: alanine--tRNA synthetase, cytoplasmic [Apteryx australis mantelli]

MEVTLTASQIRQRFIDFFKENKHTYVHSSSTIPLDDPTLLFANAGMNQFKPIFLNTIDPSHPLAKLNRATNTQKCIRAGG

KHNDLDDVGKDVYHHTFFEMLGSWSFGDYFKELACKLALDLLTKEFGIPVERLYVTYFGGNEAAGLQPDLECKQIWLDLG

LAESRILPGNMKDNFWEMGDTGPCGPCSEIHYDRIGDRDASHLVNQDDPNVLEIWNLVFIQFNREADGSLKPLPKKSIDT

GMGLERLVSVLQNKMSNYDTDLFIPYFEAIQKGTGARPYMGQVGADDADGIDMAYRVLADHARTITLALSDGGRPDNTGR

GYVLRRILRRAVRYSHEKLNAPKGFFATLVDVVVQSLGDAFPELKKDPDMVKDIINEEEDQFLKTLSRGRRILDRKIQSL

GDSKSIPGDTAWLLYDTYGFPVDLTGLIAEEKGLVVDMEGFEEERKNAQLKSQGKGAGGEDLLMLDIYAIEELRARGLEV

TDDSPKYSYVSDPSGAYDFGSLVATVKAIRREKKFVEEVSTGQECGIVLDCTCFYAEQGGQIYDEGYMVKDDASGEDKTE

FTVKNVQVRGGYVLHIGTLYGSLKVGDQVHLSIDETRRRSVMSNHTATHILNFALRSVLGEADQRGSLVAPDKLRFDFTA

KGALSTQEIKKVEGIANQIIEEAKTVYAKDCILAAAKAIQGLRAVFDETYPDPVRVVSIGIPVEELLADPSGPAGSVASI

EFCGGTHLQNSSHAGPFVVVSEEAIAKGIRRIVAVTGAEARKALRKVDSLKKLLSDLDAKVKVQTAPNKDVQKEIADLSE

MLATAIIPQWQKDELREAVKALKKVMDDLDRASKANIQKRVLEKTKQVIESHPNQPLVIMEMENGASAKALNESLKLFKT

QSPQTATMLFAVDNEAGRITCLCQVPQETVKKGLKASQWVQEVSTLMDGKGGGKDVSAQATGKNVGCLEEALKLATDFAK

LRLGELKN

>XP_015134617.1 alanine--tRNA synthetase, cytoplasmic isoform X1 [Gallus gallus]

MESTLTASQIRQRFIDFFKENQHTYVHSSSTIPLDDPTLLFANAGMNQFKPIFLNTIDPSHPLAKLSRATNTQKCIRAGG

KHNDLDDVGKDVYHHTFFEMLGSWSFGDYFKELACKLALDLLTKEFGIPAERLYVTYFGGNEAAGLQPDLECKQIWLDLG

LAEGRILPGNMKDNFWEMGDTGPCGPCSEIHYDRIGDRDASHLVNQDDPNVLEIWNLVFIQFNREADGSLKPLPKKSIDT

GMGLERLVSVLQNKMSNYDTDLFLPYFEAIQKGTGARPYMGQVGAEDADGIDMAYRVLADHARTITLALSDGGRPDNTGR

GYVLRRILRRAVRYSHEKLNAPKGFFATLVDVVVQSLGDAFPELKKDPDMVKDIINEEEDQFLKTLSRGRRILDRKIQSM

GDSKTIPGDTAWLLYDTYGFPVDLTGLIAEEKGLVVDMEGFEEERKNAQLKSQGKGAGGEDLLMLDIYAIEELRARGLEV

TDDSPKYGYTSDPSGTYDFGSLVATVKAIRREKKFVEEVSTGQECGIVLDRTCFYAEQGGQIYDQGYMVKDDDSKEDKTE

FTVKNVQVRGGYVLHIGTLYGSLKVGDQVHLSIDETRRRPVMSNHTATHILNFALRSVLGEADQRGSLVAPDRLRFDFTA

KGALSTQEIKKVEGIANQMIEEAKPVYARDCPLAAAKAIQGLRAVFDETYPDPVRVVSIGIPVEELLADPSGPAGSITSI

EFCGGTHLQNSGHAGPFVIVSEEAIAKGIRRIVAVTGAEARKALRKVESLKKVLSALDAKVKVQTAPNKDVQKEITDLSE

MLATAVIPQWQKDELREAVKALKKVMDDLDRASKADIQKRVLEKTKQVIESHPNQPLVIMEMENGASAKALNESLKLFKT

HSPQTATMLFAVDNEAGRITCLCQVPQETVQKGLKANQWVQEVSALMDGKGGGKDGSAQATGKNVGCLQEALRLATDFAR

LHLGELKN

>XP_005512669.1 LOW QUALITY PROTEIN: alanine--tRNA synthetase, cytoplasmic [Columba livia]

MEATLTASQIRQRFIDFFKENEHTYVHSSSTIPLDDPTLLFANAGMNQFKPIFLNTIDPSHPLAKLNRATNTQKCIRAGG

KHNDLDDVGKDVYHHTFFEMLGSWSFGDYFKELACKLALELLTKEFGIPTERLYVTYFGGNEAAGLQPDLECKQIWLNLG

LAESRILPGNMKDNFWEMGDTGPCGPCSEIHYDRIGDRDASHLVNQDDPNVLEIWNLVFIQFNREADGTLKPLPKKSIDT

GMGLERLVSVLQNKMSNYDTDLFLPYFEAIQKGTGARPYMGQVGAEDADGIDMAYRVLADHARTITLALSDGGRPDNTGR

GYVLRRILRRAVRYSHEKLNAPKGFFATLVDVVVQSLGDAFPELKKDPDMVKDIINEEEDQFLKTLSRGRRILDRKIQSL

GDSKTIPGDTAWLLXXXXXXXXXXXXXLIAEEKGLVVDMEGFEEERKNAQLKSQGKGAGGEDLLMLDIYAIEELRSRGLE

VTDDSPKYSYASDSSGAYDFGSLVATVKAIRREKKFVEEVSTGQECGIVLDRTCFYAEQGGQIYDEGYMVKEDDGREDKT

EFTVKNVQVRGGYVLHIGTLYGNLKVGDQVCLSIDETRRRPVMSNHTATHILNFALRSVLGEADQRGSLVAPDRLRFDFT

AKGALSTQEIKKVEGIANQMIEEAKTVYAKDCPLAAAKAIQGLRAVFDETYPDPVRVVSIGIPVEELLADPSGPAGSVTS

VEFCGGTHLQNSSHAGPFVIVSEEAIAKGIRRIVAVTGAEARKALRKVDSLKKLLSALEAKVKVQTAPNKDVQKEITDLS

ETLATAVIPQWQKDELREAVKALKKVMDDLDRASKADIQKRVLEKTKQVIESHPNQPFVIMEMENGASAKALNESLKLFK

TYSPQTATMLFAVDNEAGRITCLCQVPQETANKGLKASQWVQEVSGLMDGKGGGKDVSAQATGKNVGCLQQALKLATDFA

TLRLGELKN

>KQK74856.1 alanine--tRNA synthetase, cytoplasmic-like protein [Amazona aestiva]

MSSEGRGGAEAAEENKHVYVHSSSTIPLDDPTLLFANAGMNQFKPIFLNTIDPSHPLAKLNRATNTQKCIRAGGKHNDLD

DVGKDVYHHTFFEMLGSWSFGDYFKELACKLALDLLTKEFGIPVERLYVTYFGGNEAAGLQPDLECKQIWLDLGLAEGRI

LPGNMKDNFWEMGDTGPCGPCSEIHYDRIGDRDASHLVNQDDPNVLEIWNLVFIQFNREADGALKPLPKKSIDTGMGLER

LVSVLQNKMSNYDTDLFLPYFEAIQKGTGARPYMGQVGAEDADGIDMAYRVLADHARTITLALSDGGRPDNTGRGYVLRR

ILRRAVRYSHEKLNAPKGFFATLVDVVVQSLGDAFPELKKDPDMVKDVINEEEDQFLKTLSRGRRILDRKIQSLGDSKVL

PGDTAWLLYDTYGFPADLTGLIAEEKGLVVDMEGFEEERKNAQLKSQGKGAGGEDLLMLDIYAIEELRARGLEVTDDSPK

YSYASDPSGTYDFGSLVATVKAIRREKMFVEEVSTGQECGIVLDKTCFYAEQGGQIYDEGYMVKDDNSREDKTEFTVKNV

QVRGGYVLHIGTLYGNLKVGDQVHLSIDEMRRRPVMSNHTATHILNFALRSVLGEADQRGSLVAPDRLRFDFTAKGALST

QEIKKIEGIANQMIEEAKTVYTQDCPLAAAKAIQGLRAVFDETYPDPVRVVSIGIPVEELLADPSGPAGSITSVEFCGGT

HLQNSSHAGPFVIVSEEAIAKGIRRIVAVTGGEARKALRKVDSLRKLLSALEAKVKVQTAPNKDVQKEIVDLSEALATAV

IPQWQKDELREAVKALKKVMDDLDRASKADIQKRVLEKTKQVIESHPNQPLVIMEMENGASAKALNESLKLFKTHSPQTA

TMLFTVDNEAGRITCLCQVPQETANKGLKASQWVQEVSGLMDGKGGGKDVSAQATGRNVGCLREALQLATDFARLRLGEQ

KN

>XP_010123495.1 PREDICTED: alanine--tRNA synthetase, cytoplasmic [Chlamydotis macqueenii]

MEATLTASQIRQRFIDFFKENKHTYVHSSSTIPLDDPTLLFANAGMNQFKPIFLNTIDPSHPLAKLNRATNTQKCIRAGG

KHNDLDDVXXXXXXXXXXXTFFEMLGSWSFGDYFKELACKLALDLLTKEFGIPAERLYVTYFGGNEAAGLQPDLECKQIW

LNLGLAEGRILPGNMKDNFWEMGDTGPCGPCSEIHYDRIGDRDASHLVNQDDPNVLEIWNLVFIQFNREADGTLKPLPKK

SIDTGMGLERLVSVLQNKMSNYDTDLFLPYFEAIQKGTGARPYMGRVGAEDADGIDMAYRVLADHARTITLALSDGGRPD

NTGRGYVLRRILRRAVRYSHEKLNAPKGFFATLVDVVVQSLGDAFPELKKDPDMVKDIINEEEDQFLKTLSRGRRILDRK

IQSLGDSKVIPGDTAWLLYDTYGFPAALTGLIAEEKGLVVDMEGFEEERKNAQLKSQGKGAGGEDLLMLDIYAIEELRAR

GLEVTDDSPKYSYTSDQSGAYDFGSIVATVKAIRREKKFVEEVSTGQECGIVLDRTCFYAEQGGQIYDEGYMVKDDDGRE

DKTEFTVKNVQVRGGYVLHIGTLYGNLKVGDQVHLSIDETRRRPVMSNHTATHILNFALRSVLGEADQRGSLVAPDRLRF

DFTAKGALSNQEIKKVEGIANQMIEEAKTVYAKDCPLAAAKAIQGLRAVFDETYPDPVRVVSIGIPVEELLADPSGPAGS

VTSIEFCGGTHLQNSSHAGPFVIVSEEAIAKGIRRIVAVTGAEARKALRKVDSLRKLLSALEAKVKVQTAPNKDVQKEIT

DLSEMLATAVIPQWQKDELREAVKALKKVMDDLDRASKADIQKRVLEKTKQVIESHPNQPLVIMEMENGASAKALNESLK

LFKTHSPQTATMLFAVDNEAGRITCLCQVPQETANKGLKASQWVQEVSGLMDGKGGGKDVSAQATGKNVGCLQEALKLAT

DFARLRLEELKN

>KFO93414.1 Alanine--tRNA synthetase, cytoplasmic, partial [Buceros rhinoceros silvestris]

MEATLTASQIRQRFIDFFKENKHTYVHSSSTIPLDDPTLLFANAGMNQFKPIFLNTIDPSHPLAKLSRATNTQKCIRAGG

KHNDLDDVGKDVYHHTFFEMLGSWSFGDYFKELACKLALDLLTKEFGIPPERLYVTYFGGNEAAGLQPDLECKQIWLNLG

LPEGRILPGNMKDNFWEMGDTGPCGPCSEIHYDRIGDRDASHLVNQDDPNVLEIWNLVFIQFNREADGTLKPLPKKSIDT

GMGLERLVSVLQNKMSNYDTDLFLPYFEAIQKGTGARPYMGQVGAEDADGIDMAYRVLADHARTITLALSDGGRPDNTGR

GYVLRRILRRAVRYSHEKLNAPKGFFATLVDVVVQSLGDAFPELKKDPDMVKDIINEEEDQFLKTLSRGRRILDRKIQSL

GDSKTIPGDTAWLLYDTYGFPADTDLTGLIAEEKGLVVDMEGFEEERKNAQLKSQGKGAGGEDLLMLDIYAIEELRARGL

EVTDDSPKYSYASDPSGTYDFGSFVATVKAIRREKKFVEEVSTGQECGIVLDQTCFYAEQGGQIYDEGYMVKEDDSKEDK

TEFMVKNVQVRGGYVLHIGTLYGSLKVGDQVHLSIDETRRRPVMSNHTATHILNFALRSVLGEADQRGSLVAPDRLRFDF

TAKGALSTQEIKKVEGIANQMIEEAKPVYAKDCPLAAAKAIQGLRAVFDETYPDPVRVVSIGVPVEELLADPSGPAGSLT

SVEFCGGTHLQNSSHAGPFVIVSEEAIAKGIRRIVAVTGAEARKALRKVDSLGKLLTALEAKVKVQTAPNKDVQKEITDL

SEALATAVIPQWQKDELREAVKALKKVMDDLDRASKADIQKRGLEKTKQVIESQPNQPLVIMEMENGASAKALNESLKLF

KTLSPQTAAMLFAVDPEAGRITCLCQVPQETANNGLKASQWVQEVSALMDGKGGGKDVSAQATGKNVGCLQEALRVA

>XP_003225388.1 PREDICTED: alanine--tRNA synthetase, cytoplasmic [Anolis carolinensis]

MEATLTASEIRQRFINFFVENQHTYVHSSATIPLDDPTLLFANAGMNQFKPIFLNTIDPSHPMAKLSRAANTQKCIRAGG

KHNDLDDVGKDVYHHTFFEMLGSWSFGDYFKELACKFALDLLTKEFGIPIERLYVTYFGGNEAAGLQPDLDCKQIWLDLG

LPEDRILPGSMKDNFWEMGDTGPCGPCSEMHYDRIGGRDASHLVNQDDPNVLEIWNLVFIQFNREADGTLKPLPKKSIDT

GMGLERLVSVLQNKMSNYDTDLFVPYFEAIQKGTGCRPYTGQVGEDDADGIDMAYRVLADHARTITVALSDGGRPDNTGR

GYVLRRILRRAVRYAHEKLNAPRGFFATLVDVVVESLGGAFPELKKDPEMVKDIINEEEVQFLKTLNRGRRILDRKIQSL

GDNKVIPGDTAWLLYDTYGFPVDLTGLIAEEKGLKVDMEGFEEERKAAQLKSQGKGGGDEDLIILDIYAIEELRSKGLKA

TDDSLKYNYASDPSGTYEFQSPVATVKALRREKVFVEEVSTGQECGIVLDQTCFYAEQGGQIYDEGYLVKEEDEGSEDKT

EFTVKNVQVRGGYVLHVGTVYGSLKVGDTVRLYIDEPRRRPVMSNHTATHILNFALRAVLGEADQRGSLVAPDRLRFDFT

AKGAMSTQEIKKAEAIVNQMIQEAKVVYAKECALAAAKAIQGLRAVFDETYPDPVRVVSIGIPIEDMLADPSNAAGAITS

VEFCGGTHLQNSSHAGHFVIVSEEAIAKGIRRIVAVTGAEAQKALRKVESLKKLLADLEAKVKVQTAPNKDVQHEIADLG

ESLATAVIPQWQKDELRESLKALKKIMDDLDRACKADIQKRVLDKMKQLIEAHPNQPLVILEMESGASAKALNESLKLLK

THSPETAAMLFTVDNDAGKITCLCQVPQDVVSKGLKASEWVQQVSGLMDGKGGGKDLSAQATGKNTDCLKEALEVATDFA

KLKLGELKN

>KGL75566.1 Alanine--tRNA synthetase, cytoplasmic, partial [Tinamus guttatus]

PLTASEIRRRFIAFFQENQHTYVHSSSTIPLDDPTLLFANAGMNQFKPIFLNTIDPSHPLAKLNRATNTQKCIRAGGKHN

DLDDVGKDVYHHTFFEMLGSWSFGDYFKELACKLALDLLTKEFGIPIERLYVTYFGGNEAAGLQPDLECKQIWLDLGLDE

SRILPGNMKDNFWEMGDTGPCGPCSEIHYDRIGDRDASHLVNQDDPNVLEIWNLVFIQFNREADGSLKPLPKKSIDTGMG

LERLVSVLQNKMSNYDTDLFIPYFEAIQKGTGARPYMGQVGADDADGIDMAYRVLADHARTITLALSDGGRPDNTGRGYV

LRRILRRAVRYSHEKLNAPKGFFATLVDVVVQSLGDAFPELKKDPDMVKDIINEEEDQFLKTLSRGRRILDRKIQSLGDS

KSIPGDTAWLLYDTYGFPVDLTGLIAEEKGLVVDMEGFEEERKNAQLKSQGKGAGGEDLLMLDIYAIEELRARGLEVTDD

SPKYSYISDPSGAYDFGSLVATVRAIRREKKFVEEVSTGQECGLVLDHTCFYAEQGGQIYDEGYMVKDDASGEDKTEFTV

KNVQVRGGYVLHMGTIYGSLKVGDQVHLSIDETRRRSVMSNHTATHILNFALRSVLGEADQRGSLVAPDKLRFDFTAKGA

LSTQEIKKVEGIANQIIGEEKTVYAKDCILAAAKAIQGLRAVFDETYPDPVRVVSIGIPVEELLADPSGPAGSIASIEFC

GGTHLQNSGHAGPFVIVSEEAIAKGIRRIVAVTGAEARKALRKVDSLKKLLADLDAKVKVQTAPNKDVQKEIADLSEMLA

TAVVPQWQKDELREAVKALKKVMDDLDRASKANIQKRVLEKTKQVIESHPNQPLVIMEMENGASAKALNESLKLFKTQSP

QTATMLFAVDNEAGRITCLCQVPQETVQRGLKASQWVQEVSALMDGKGGGKDVSAQATGKNVGCLQEALKLATDFARLRL

GELKN

>XP_009688682.1 PREDICTED: alanine--tRNA synthetase, cytoplasmic [Struthio camelus australis]

MEATLTASQIRQRFIDFFKDNKHTYVHSSSTIPLDDPTLLFANAGMNQFKPIFLNTIDPSHPLAKLNRATNTQKCIRAGG

KHNDLDDVGKDVYHHTFFEMLGSWSFGDYFKELACKLALDLLTKEFGIPVERLYVTYFGGNEAAGLQPDLECKQIWLDLG

LAEGRILPGNMKDNFWEMGDTGPCGPCSEIHYDRIGDRDASHLVNQDDPNVLEIWNLVFIQFNREADGSLKPLPKKSIDT

GMGLERLVSVLQNKMSNYDTDLFIPYFEAIQKGTGARPYKGQVGADDADGIDMAYRVLADHARTITLALSDGGRPDNTGR

GYVLRRILRRAVRYSHEKLNAPKGFFATLVDVVVQSLGDAFPELKKDPDMVKDIINEEEDQFLKTLSRGRRILDRKIQSL

GDSKSIPGDTAWLLYDTYGFPVDLTGLIAEEKGLAVDMEGFEEERKNAQLKSQGKGAGGEDLLMLDIYAIEELRARGLEV

TDDSPKYSYASDPSGVYDFGSLVATVKAIRREKKFVEEVSTGQECGIILDCTCFYAEQGGQIYDEGYMVKDDAGREDKTE

FTVKNVQVRGGYVLHIGTLYGSLKVGDQVHLSIDETRRRSVMSNHTATHILNFALRSVLGEADQRGSLVAPDKLRFDFTA

KGALSTQEIKKVEGIANQIIEEAKTVYAKDCILAAAKAIQGLRAVFDETYPDPVRVVSIGIPVEELLADPSGPAGSITSI

EFCGGTHLQNSSHAGPFVIVSEEAIAKGIRRIVAVSGAEARKALRKVDSLKKLLSDLDAKVKVQTAPNKDVQKEIADLSE

TLATAIIPQWQKDELREAVKALKKVMDDLDRASKANIQKRVLEKTKQVIESHPNQPLVIMEMENGASAKALNESLKLFKT

QSPQTATMLFAVDNEAGRITCLCQVPQETVKKGLKASQWVQEVSALMDGKGGGKDVSAQATGKNVGCLEQALKLATDFAK

LHLGELKN

>XP_010140350.1 PREDICTED: LOW QUALITY PROTEIN: alanine--tRNA synthetase, cytoplasmic, partial [Buceros rhinoceros silvestris]

MEATLTASQIRQRFIDFFKENKHTYVHSSSTIPLDDPTLLFANAGMNQFKPIFLNTIDPSHPLAKLSRATNTQKCIRAGG

KHNDLDDVGKDVYHHTFFEMLGSWSFGDYFKELACKLALDLLTKEFGIPPERLYVTYFGGNEAAGLQPDLECKQIWLNLG

LPEGRILPGNMKDNFWEMGDTGPCGPCSEIHYDRIGDRDASHLVNQDDPNVLEIWNLVFIQFNREADGTLKPLPKKSIDT

GMGLERLVSVLQNKMSNYDTDLFLPYFEAIQKGTGARPYMGQVGAEDADGIDMAYRVLADHARTITLALSDGGRPDNTGR

GYVLRRILRRAVRYSHEKLNAPKGFFATLVDVVVQSLGDAFPELKKDPDMVKDIINEEEDQFLKTLSRGRRILDRKIQSL

GDSKTIPGDTAWLLYDTYGFPGSHPADTDLTGLIAEEKGLVVDMEGFEEERKNAQLKSQGKGAGGEDLLMLDIYAIEELR

ARGLEVTDDSPKYSYASDPSGTYDFGSFVATVKAIRREKKFVEEVSTGQECGIVLDQTCFYAEQGGQIYDEGYMVKEDDS

KEDKTEFMVKNVQVRGGYVLHIGTLYGSLKVGDQVHLSIDETRRRPVMSNHTATHILNFALRSVLGEADQRGSLVAPDRL

RFDFTAKGALSTQEIKKVEGIANQMIEEAKPVYAKDCPLAAAKAIQGLRAVFDETYPDPVRVVSIGVPVEELLADPSGPA

GSLTSVEFCGGTHLQNSSHAGPFVIVSEEAIAKGIRRIVAVTGAEARKALRKVDSLGKLLTALEAKVKVQTAPNKDVQKE

ITDLSEALATAVIPQWQKDELREAVKALKKVMDDLDRASKADIQKRGLEKTKQVIESQPNQPLVIMEMENGASAKALNES

LKLFKTLSPQTAAMLFAVDPEAGRITCLCQVPQETANNGLKASQWVQEVSALMDGKGGGKDVSAQATGKNVGCLQEALR

>XP_010210440.1 PREDICTED: alanine--tRNA synthetase, cytoplasmic [Tinamus guttatus]

MMASTRVVVLDVFRGEWLVSDPGPLTASEIRRRFIAFFQENQHTYVHSSSTIPLDDPTLLFANAGMNQFKPIFLNTIDPS

HPLAKLNRATNTQKCIRAGGKHNDLDDVGKDVYHHTFFEMLGSWSFGDYFKELACKLALDLLTKEFGIPIERLYVTYFGG

NEAAGLQPDLECKQIWLDLGLDESRILPGNMKDNFWEMGDTGPCGPCSEIHYDRIGDRDASHLVNQDDPNVLEIWNLVFI

QFNREADGSLKPLPKKSIDTGMGLERLVSVLQNKMSNYDTDLFIPYFEAIQKGTGARPYMGQVGADDADGIDMAYRVLAD

HARTITLALSDGGRPDNTGRGYVLRRILRRAVRYSHEKLNAPKGFFATLVDVVVQSLGDAFPELKKDPDMVKDIINEEED

QFLKTLSRGRRILDRKIQSLGDSKSIPGDTAWLLYDTYGFPVDLTGLIAEEKGLVVDMEGFEEERKNAQLKSQGKGAGGE

DLLMLDIYAIEELRARGLEVTDDSPKYSYISDPSGAYDFGSLVATVRAIRREKKFVEEVSTGQECGLVLDHTCFYAEQGG

QIYDEGYMVKDDASGEDKTEFTVKNVQVRGGYVLHMGTIYGSLKVGDQVHLSIDETRRRSVMSNHTATHILNFALRSVLG

EADQRGSLVAPDKLRFDFTAKGALSTQEIKKVEGIANQIIGEEKTVYAKDCILAAAKAIQGLRAVFDETYPDPVRVVSIG

IPVEELLADPSGPAGSIASIEFCGGTHLQNSGHAGPFVIVSEEAIAKGIRRIVAVTGAEARKALRKVDSLKKLLADLDAK

VKVQTAPNKDVQKEIADLSEMLATAVVPQWQKDELREAVKALKKVMDDLDRASKANIQKRVLEKTKQVIESHPNQPLVIM

EMENGASAKALNESLKLFKTQSPQTATMLFAVDNEAGRITCLCQVPQETVQRGLKASQWVQEVSALMDGKGGGKDVSAQA

TGKNVGCLQEALKLATDFARLRLGELKN

>KFO63061.1 Alanine--tRNA synthetase, cytoplasmic [Corvus brachyrhynchos]

METSLTASQIRQRFIDFFKENKHTYVHSSSTIPLDDPTLLFANAGMNQFKPIFLNTIDPSHPLAKLSRATNTQKCIRAGG

KHNDLDDVGKDVYHHTFFEMLGSWSFGDYFKELACKLALELLTKEFGIPAERLYVTYFGGNEAAGLQPDLECKQIWLDLG

LAEGRILPGNMKDNFWEMGDTGPCGPCSEIHYDRIGDRDASHLVNQDDPNVLEIWNLVFIQFNREADGTLKPLPKKSIDT

GMGLERLVSVLQNKMSNYDTDLFLPYFEAIQKGTGARPYLGQVGAEDADGIDMAYRVLADHARTITLALSDGGRPDNTGR

GYVLRRILRRAVRYSHEKLNAPKGFFATLVDVVVQSLGDAFPELKKDPDMVKDIINEEEDQFLKTLSRGRRILDRKIQSL

GDSKIIPGDTAWLLYDTYGFPADLTGLIAEEKGLVVDMEGFEEERKNAQLKSQGKGAGGEDLLMLDIYAIEELRARGLEV

TDDSPKYNYASDPSGTYDFGSLVATVKAIRREKKFVEEVSTGQECGIVLDQTCFYAEQGGQIYDQGYMVKDDDSREDKTE

FTVKNVQVRGGYVLHIGTLYGSLKVGDQVHLTIDEARRRPVMSNHTATHILNFALRSVLGEADQRGSLVAPDRLRFDFTA

KGALSTQEIKTVEGIANQMIEESKTVYAKDCPLAAAKAIQGLRAVFDETYPDPVRVVSIGIPVEELLADPSGPGGSITSI

EFCGGTHLQNSSHAGPFVIVSEEAIAKGIRRIVAVTGAEARKALRKVDSLRKLLSALEAKVKVQTAPNKDVQKEITDLGE

TLATAVIPQWQKDELREAVKALKKVMDDLDRASKADIQKRVLEKTKQVIESHPNQPWVIMEMENGASAKALNESLKLFKT

NSPQTAAMLFAVDLPADRWVSFPAQETANKGLKASQWVQEVSGLMDGKGGGKDISAQATGKNVGCLQEALKLATDFARLR

LGELKN

>KFP44259.1 Alanine--tRNA synthetase, cytoplasmic [Chlamydotis macqueenii]

MEATLTASQIRQRFIDFFKENKHTYVHSSSTIPLDDPTLLFANAGMNQFKPIFLNTIDPSHPLAKLNRATNTQKCIRAGG

KHNDLDDMLGSWSFGDYFKELACKLALDLLTKEFGIPAERLYVTYFGGNEAAGLQPDLECKQIWLNLGLAEGRILPGNMK

DNFWEMGDTGPCGPCSEIHYDRIGDRDASHLVNQDDPNVLEIWNLVFIQFNREADGTLKPLPKKSIDTGMGLERLVSVLQ

NKMSNYDTDLFLPYFEAIQKGTGARPYMGRVGAEDADGIDMAYRVLADHARTITLALSDGGRPDNTGRGYVLRRILRRAV

RYSHEKLNAPKGFFATLVDVVVQSLGDAFPELKKDPDMVKDIINEEEDQFLKTLSRGRRILDRKIQSLGDSKVIPGDTAW

LLYDTYGFPAALTGLIAEEKGLVVDMEGFEEERKNAQLKSQGKGAGGEDLLMLDIYAIEELRARGLEVTDDSPKYSYTSD

QSGAYDFGSIVATVKAIRREKKFVEEVSTGQECGIVLDRTCFYAEQGGQIYDEGYMVKDDDGREDKTEFTVKNVQVRGGY

VLHIGTLYGNLKVGDQVHLSIDETRRRPVMSNHTATHILNFALRSVLGEADQRGSLVAPDRLRFDFTAKGALSNQEIKKV

EGIANQMIEEAKTVYAKDCPLAAAKAIQGLRAVFDETYPDPVRVVSIGIPVEELLADPSGPAGSVTSIEFCGGTHLQNSS

HAGPFVIVSEEAIAKGIRRIVAVTGAEARKALRKVDSLRKLLSALEAKVKVQTAPNKDVQKEITDLSEMLATAVIPQWQK

DELREAVKALKKVMDDLDRASKADIQKRVLEKTKQVIESHPNQPLVIMEMENGASAKALNESLKLFKTHSPQTATMLFAV

DNEAGRITCLCQVPQETANKGLKASQWVQEVSGLMDGKGGGKDVSAQATGKNVGCLQEALKLATDFARLRLEELKN

>XP_021528261.1 LOW QUALITY PROTEIN: alanine--tRNA synthetase, cytoplasmic [Aotus nancymaae]

MDLTLTASEIRQRFIEFFKRNEHTYVHSSATIPLDDPTLLFANAGMNQFKPIFLNTIDPSHPMAKLSRAANTQKCIRAGG

KHNDLDDVGKDVYHHTFFEMLGSWSFGDYFKELACKMALELLTQEFGIPIERLYVTYFGGDEAAGLEPDLECKQIWQNLG

LEDTKILPGNMKDNFWEMGDTGPCGPCSEIHYDRIGGRDAAHLVNQDDPNVLEIWNLVFIQYNRWPWGPTPFLLRAVPFL

ELSRGGGTQSFMAVFLAEGPFLHFSDFPSDLWDRDGPGTGARPYTGKVGAEDADGIDMAYRVLADHARTITVALADGGRP

DNTGRGYVLRRILRPSVVYAHEKLNASRGFFATLVDVVVQSLGDAFPELKKDPDMVKDIINEEEVQFLKTLSRGRRILDR

KILSLGDSKTIPGDTAWLLYDTYGFPVDLTGLIAEEKGLLVDMDGFEDERKLAQVKNLEKSNGVASEVQWTIRSXEEFEK

ENAAKPSXESVKRSLTHCPTSVHFPRAVFENTVATVLALRREKMFVEEVSTGQECGVVLDKTCFYAEQGGQIYDEGYLVK

VDDSSDDKTEFTVKNAQVRGGYVLHIGTIYGDLKVGDQVRLFIDEPRRRPVMSNHTATHILNFALRSVLGEADQKGSLVA

PDRLRFDFTAKGAMSTRQIKKAEEIANEMIEAAKPVYTQDCPLAAAKAIQGLRAVFDETYPDPVRVVSIGVPVSELLDDP

SGPAGSLTSVEFCGGTHLQNSSHAGAFVIVTEEAIAKGVRRIVAVTGAEAQKALRKAESLKTSLSVMEAKVKAQTAPNKD

VQREITDLGEVLATAVIPQWQKDELRETLKSLKKVMDDLDRASKADVQKRVLEKTKQLIDSNPNQPLVILEMESGASAKA

LNEALKLFKMHSPQTSAMLFTVDNEAGKITCLCQVPQNAANRGLKASEWVQQVSGLMDGKGGGKDVSAQATGKNVGCLQE

ALQLATSFAQLRLRDVKN

>XP_025021896.1 alanine--tRNA synthetase, cytoplasmic [Python bivittatus]

METLTASEIRQRFINFFVENQHTYVHSSATIPLDDPTLLFANAGMNQFKPIFLNTIDPSHPMAKLTRAVNTQKCIRAGGK

HNDLDDVGKDVYHHTFFEMLGSWSFGDYFKELACKLALDLLTKEFGIPIERLYVTYFGGNEAAGLEPDLDCKQIWLDLGL

AESKILPGSMKDNFWEMGDTGPCGPCSEMHYDRIGGRDASHLVNQDDPNVLEIWNLVFIQFNREADGTLKPLPKKSIDTG

MGLERLVSVLQNKMSNYDTDLFVPYFEAIQKGTGCRPYTGQVGEEDVDGIDMAYRVLADHARTITIALSDGGRPDNTGRG

YVLRRILRRAVRYAHEKLNAPKGFFATLVDVVVQSLGGAFPELTKDPDMVKDIINEEEVQFLKTLNRGRRILDRKIQSLG

DNKIIPGDTAWLLYDTYGFPVDLTGLIAEEKGLKVDMEGFEEERKTAQLKSQGKGGGDEDLIMLDIYAIEDLRCRGLEAT

DDSLKYSYSSDTNGSYEFDSPGATVKALRREKTFVEEVHTGQECGVVLDQTCFYAEQGGQIYDEGYLVKEDDGGEDKTEF

TVKNVQVRGGYVLHVGTVYGSLKVGDSVQLFIDEPRRRPIMSNHTATHILNFALRSVLGEADQRGSLVAPDRLRFDFTAK

GAMSTQEIKKTEEIVNGMIQEAKIVYAKDCPLAAAKAIQGLRAVFDETYPDPVRVVSIGIPVEDMLADPSSAAGAITSVE

FCGGTHLQNSGHAGHFVIVSEEAIAKGIRRIVAVTGAEAQKALRKADGLKKQLADLETKVKVQRTPNKEVQREIADLGEA

LATAVIPQWQKDELREALKALKKVMDDLDRANKADIQKRVLDKMKQLIEAYPNQPLVILEMENGASAKALNESLKLLKTH

SPETAAMLFTVDNEAGKITCLCQVPQEVANKGLKASEWVQQVSVLMDGKGGGKDLSAQATGKNTDCLEEALQVATEFAKL

KLVGLKN

>XP_007066333.1 PREDICTED: LOW QUALITY PROTEIN: alanine--tRNA synthetase, cytoplasmic-like [Chelonia mydas]

MEITLSARQIRQKFLDFFKERQHTYVHSSATIPLDDPTLLFANAGMNQFKPIFLNTLDPSHPMAKLTRAVNTQKCIRAGG

KHNDLDDVGKDVYHHTFFEMLGSWSFGDYFKELACKLALELLTNEFGIPIERLYVTYFGGNKAAGLEPDLECKQIWQDLG

LAQSKILPGSMKDNFWEMGETGPCGPCSEIHYDRIGDRDASHLVNQDDPNVLEIWNLVFIQFNREADGTLKPLPKKNIDT

GLGLERLVSVLQNKMSNYDTDLFIPYFEAIQKGTGARPYTGKVGAEDADGIDMAYRVLADHARTITVALADGGRPDNTGR

GYVLRRILRRAVRYSHEKLNAPKGFFASLVDVVVQLLGDAFPELKKDPFMVKDIINEEEVQFLKTLSRGRRILDRKIQSL

GDSKTIPGDTAWLLYDTYGFPVDLTGLIAEEKGLXXXXXXXERKMAQLKSQGKGDGAEDRIMLDIYAIEELRTRGLEVTD

DSPKYNYTSDPSGAYEFESPAATVQAIRREKRFVEEVSTGQECGIVLDKTCFYAEQGGQIYDEGYLVKDDDSSEDKTEFT

VKNVQIRGGYVLHIGMVYGSLKVGDQVRLYIDEPRRRPIMSNHTATHILNFALRLVLGDADQRGSLVAPDRLRFDFTARG

AMSTQEIKKTEEIANQMIKDAKTVYAKDCPLAAAKAIQGLRAVFDETYPDPVRVVSIGIPVENMLADPSSAAGSITSVEF

CGGTHLQNSSHAGPFVITSEEAIAKGIRRIVAITGAEALKALRNADSLMKSLSALDAKVKVQTAPNKDVQKEIADLSETL

ATAVIPQWRKDELREALRALKKIMDDLDRTSKTDIQKRVLEKTKQLIDGHPNQPLVVLELESGASAKALNESLKLFKAYS

PQTAAMLFSVDNEAGKITCLCQVPQEVANNGLKANQWVQEVSGLMDGKGGGKDVSAQATGKNVGCLEEALKLATDFAKLH

LGELKN

>XP_011892767.1 PREDICTED: alanine--tRNA synthetase, cytoplasmic isoform X2 [Cercocebus atys]

MDSTLTASEIRQRFIDFFKRNEHTYVHSSATIPLDDPTLLFANAGMNQELACKMALELLTQEFGIPIERLYVTYFGGDEA

AGLEPDLECKQIWQNLGLDDTKILPGNMKDNFWEMGDTGPCGPCSEIHYDRIGGRDAAHLVNQDDPNVLEIWNLVFIQYN

READGILKPLPKKSIDTGMGLERLVSVLQNKMSNYDTDLFVPYFEAIQKGTGARPYTGKVGAEDADGIDMAYRVLADHAR

TITVALADGGRPDNTGRGYVLRRILRRAVRYAHEKLNASRGFFATLVDVVVQSLGDAFPELKKDPDMVKDIINEEEVQFL

KTLSRGRRILDRKIQSLGDSKTIPGDTAWLLYDTYGFPVDLTGLIAEEKGLVVDMDGFEEERKLAQLKSQGKGAGGEDLI

MLDIYAIEELRARGLEVTDDSPKYNYHLDFSGSYVFENTVATVMALRREKMFVEEVSTGQECGVVLDKTCFYAEQGGQIY

DEGYLVKVDDSSEDKTEFTVKNAQVRGGYVLHIGTIYGDLKVGDQVRLFIDEPRRRPIMSNHTATHILNFALRSVLGEAD

QKGSLVAPDRLRFDFTAKGAMSTQQIKKAEEIANEMIEAAKPVYTQDCPLAAAKAIQGLRAVFDETYPDPVRVVSIGVPV

SELLDDPSGPAGSLTSVEFCGGTHLRNSSHAGAFVIVTEEAIAKGIRRIVAVTGAEAQKALRKAESLKKSLSVMEAKVKA

QTAPNKDVQREIADLGEALATAVIPQWQKDELRETLKSLKKVMDDLDRASKADVQKRVLEKTKQLIDSNPNQPLVILEME

SGASAKALNEALKLFKTHSPQTSAMLFTVDNEAGKITCLCQVPQNAANRGLKASEWVQQVSGLMDGKGGGKDVSAQATGK

NIGCLQEALQLATSFAQLRLGDVKN

>KYO36993.1 hypothetical protein Y1Q_0000866 [Alligator mississippiensis]

MTAARTNHNGGLVSSRRRGWCEGRREAGCAAAAGTGVRSRAGRGSCRLDRAVLCRPPKMESTLTDDPTLLFANAGMNQFK

PIFLNTIDPSHPMAKLNRAANTQKCIRAGGKHNDLDDVGKDVYHHTFFEMLGSWSFGDYFKALACKLALELLTKEFGIPI

ERLYVTYFGGNEAAGLEPDLECKQIWLDLGMAENKILPGSMKDNFWEMGDTGPCGPCSEIHYDRIGNRDASHLVNQDDPN

VLEIWNLVFIQFNREADGTLKPLPKKSIDTGLGLERLVSVLQNKMSNYDTDLFIPYFEAIQKGTGARPYTGQVGAEDMDG

IDMAYRVLADHARTITVALSDGGRPDNTGRGYVLRRILRRAVRYSHEKLNAPKGFFATLVDVVVQSLGDAFPELKKDPDM

VKDIINEEEVQFLKTLSRGRRILDRKIQSLGDSKIIPGDTAWLLYDTYGFPVDLTGLIAEEKGLVVDMEGFEEERRNAQL

KSQGKGTGVEDLIMLDIYAIEELRAQGLEVTDDSPKYSYTSDPSGAYEFENLMGTVKAIRREKTFVEEVSTGQECGIVLD

KTCFYAEQGGQIYDEGYLVKADDCSEDKTEFTVKNVQVRGGYVLHVGTVYGSLKVGDQVHLYVDEPRRRPVMSNHTATHI

LNFALRSVLGEADQRGSLVAPDKLRFDFTAKGAMSTQEIKKTEKIANQMIEEAKTVYAKDCPLAAAKAIQGLRAVFDETY

PDPVRVVSIGIPVDDLLADPSGPAGSVTSVEFCGGTHLQNSSHAGPFVIVSEEAIAKGIRRIVAVTGAEAQKALRKVDSL

KKSLSALDAKVKVQRAPNKEVQKEIADLSETLATAIIPQWQKDELREALKVLKKVMDDLDRASKADIQKRVLEKTKQFID

SHPNQPLVILEMESGASAKALNESLKLFKTHSPQTAAMLFAVDNEAGKITCLCQVPQEAANKGLKANQWVQEVSTLMDGK

GGGKDVSAQATGKNVGCLQEVLKLATDFAELCLGELKN

>PKK18688.1 hypothetical protein A306_00000067 [Columba livia]

MEATLTASQIRQRFIDFFKENEHTYVHSSSTIPLDDPTLLFANAGMNQFKPIFLNTIDPSHPLAKLNRATNTQKCIRAGG

KHNDLDDVGKDVYHHTFFEMLGSWSFGDYFKELACKLALELLTKEFGIPTERLYVTYFGGNEAAGLQPDLECKQIWLNLG

LAESRILPGNMKDNFWEMGDTGPCGPCSEIHYDRIGDRDASHLVNQDDPNVLEIWNLVFIQFNREADGTLKPLPKKSIDT

GMGLERLVSVLQNKMSNYDTDLFLPYFEAIQKGTGARPYMGQVGAEDADGIDMAYRVLADHARTITLALSDGGRPDNTGR

GYVLRRILRRAVRYSHEKLNAPKGFFATLVDVVVQSLGDAFPELKKDPDMVKDIINEEEDQFLKTLSRGRRILDRKIQSL

GDSKTIPEEKGLVVDMEGFEEERKNAQLKSQGKGAGGEDLLMLDIYAIEELRSRGLEVTDDSPKYSYASDSSGAYDFGSL

VATVKAIRREKKFVEEVSTGQECGIVLDRTCFYAEQGGQIYDEGYMVKEDDGREDKTEFTVKNVQVRGGYVLHIGTLYGN

LKVGDQVCLSIDETRRRPVMSNHTATHILNFALRSVLGEADQRGSLVAPDRLRFDFTAKGALSTQEIKKVEGIANQMIEE

AKTVYAKDCPLAAAKAIQGLRAVFDETYPDPVRVVSIGIPVEELLADPSGPAGSVTSVEFCGGTHLQNSSHAGPFVIVSE

EAIAKGIRRIVAVTGAEARKALRKVDSLKKLLSALEAKVKVQTAPNKDVQKEITDLSETLATAVIPQWQKDELREAVKAL

KKVMDDLDRASKADIQKRVLEKTKQVIESHPNQPFVIMEMENGASAKALNESLKLFKTYSPQTATMLFAVDNEAGRITCL

CQVPQETANKGLKASQWVQEVSGLMDGKGGGKDVSAQATGKNVGCLQQALKLATDFATLRLGELKN

>EHH62253.1 hypothetical protein EGM_20519 [Macaca fascicularis]

MDSTLTASEIRQRFIDFFKRNEHTYIHSSATIPLDDPTLLFANAGMNQFKPIFLNTIDPSHPMAKLSRAANTQKCIRAGG

KHNDLDDVGKDVYHHTFFEMLGSWSFGDYFKELACKMALELLTQEFGIPIGRLYVTYFGGDEAAGLEPDLECKQIWQNLG

YLYSFVRLDDTKILPGNMKDNFWEMGDTGPCGPCSEIHYDRIGGRDAAHLVNQDDPNVLEIWNLVFIQYNREADGILKPL

PKKSIDTGMGLERLVSVLQNKMSNYDTDLFIPYFEAIQKGTGARPYTGKVGAEDADGIDMAYRVLADHARTITVALADGG

RPDNTGYVLRRILRRAVRYAHEKLNASRGFFATLVDVVVQSLGDAFPELKKDPDMVKDIINEEEVQFLKTLSRGRRILDR

KIQSLGDSKTIPGDTAWLLYDTYGFPVDLTGLIAEEKGLVVDMDGFEEERKLAQLKSQGKGAGGEDLIMLDIYAIEELRA

RGLEVTDDSPKYNYHLDFSGSKTEFTVKNAQVRGGYVLHIGTIYGDLKVGDQVRLFIDEPRRRPIMSNHTATHILNFALR

SVLGEADQKGSLVAPDRLRFDFTAKGAMSTQQIKKAEEIANEMIEAAKAVYTQDCPLAAAKAIQGLRAVFDETYPDPVRV

VSIGVPVSELLDDPSGPAGSLTSVEFCGGTHLRNSSHAGAFVIVTEEAIAKGIRRIVAVTGAEAQKALRKAESLKKSLSV

MEAKVKAQTAPNKDVQREIADLGEALATAVIPQWQKDELRETLKSLKKVMDDLDRASKADVQKRVLEKTKQLIDSNPNQP

LVILEMESGASAKATQGPGPPPLGHISPALNEALKLFKTHSPQTSAMLFTVDNEAGKITCLCQVPQNAANRGLKASEWVQ

QVSGLMDGKGGGKDVSAQATGKNIGCLQEALQLATSFAQLRLGDVKN

>XP_020668301.1 alanine--tRNA synthetase, cytoplasmic isoform X1 [Pogona vitticeps]

MEMTLTASEIRQRFIDFFVKNAHTYVHSSATIPLDDPTLLFANAGMNQFKPIFLNTLDPSHPMAKLTRAANTQKCIRAGG

KHNDLDDVGKDVYHHTFFEMLGSWSFGDYFKELACRFALDLLTKEFGIPVERLYVTYFGGNEAAGLHPDLDCKQIWLDLD

LPESRILPGSMKDNFWEMGDTGPCGPCSEIHYDRIGGRDASHLVNQDDPNVLEIWNLVFIQFNREADGTLKPLPKKSIDT

GMGLERLVSILQSKMSNYDTDLFVPYFEAIQKGTGCRPYTGKVGEDDADGIDMAYRVLADHARTITVALSDGGRPDNTGR

GYVLRRILRRAVRYAHEKLSAPKGFFASLVDVVVESLGGAFPELKKDPEMVKDIINEEEVQFLKTLNRGRRILDRKIQSL

GDSKVIPGDTAWLLYDTYGFPVDLTGLIAEEKGLKVDMEGFEEERKTAQLKSQGKGGGDEDLIMLDIYAIEELRSRGLEP

TDDSLKYNYSSDPRGTYKFSSPVATVKALRREKAFVEQVSTGQECGVILDHTCFYAEQGGQIYDEGYLVKEDEGSEDKTE

FTVKNVQVRGGYVLHVGTVYGNLKVGDTVQLYIDEARRRPIMSNHTATHILNFALRAVLGEADQRGSLVAPDRLRFDFTA

KGAMSTEEIRKAEETVNQMIQEAKVVYAKDCPLAAAKAIQGLRAVFDETYPDPVRVISIGIPVEDMLDDPSSPAGAITSV

EFCGGTHLQNSSHAGHFVIVSEEAIAKGIRRIVAVTGVEAQKALRKADIIQKLLTDLEAKVQIQTAPNKDVQREIADLGE

TLATAIIPQWQKDEFREALKALKKIMDDLDRAYKADIQKRVLDKTKRLIEAHPNQPLVILEMESGATAKALNESLKLLKT

HSPETAAMLFAIDNEAEKIICLCQVPQEAVNKGLKASEWVQQVSGLMEGRGGGKNLSAQATGRNTSCLEEALQVAMDFAK

LKLGELKN

>KGL99165.1 Alanine--tRNA synthetase, cytoplasmic [Charadrius vociferus]

MEATLTASQIRQRFIDFFKENKHTYVHSSSTIPLDDPTLLFANAGMNQFKPIFLNTIDPSHPLAKLSRATNTQKCIRAGG

KHNDLDDVGKDVYHHTFFEMLGSWSFGDYFKELACKLALDLLTKEFGIPAERLYVTYFGGNEAAGLQPDLECKQIWLNLG

LAEGRILPGNMKDNFWEMGDTGPCGPCSEIHYDRIGDRDASHLVNQDDPNVLEIWNLVFIQFNREADGTLKPLPKKSIDT

GMGLERLVSVLQNKMSNYDTDLFLPYFEAIQKGTGARPYMGQVGAEDADGIDMAYRVLADHARTITLALSDGGRPDNTGR

GYVLRRILRRAVRYSHEKLNAPKGFFATLVDVVVQSLGDAFPELKKDPDMVKDIINEEEDQFLKTLSRGRRILDRKIQSL

GDSKIIPGKAVTSVWMHQKTFIIWDQKGLVVDMEGFEEERKNAQLKSQGKGAGGEDLLMLDIYAIEELRARGLEVTDDSP

KYSYTSDQSGAYDFGSLVATVKAIRREKKFVEEVSTGQECGIVLDRTCFYAEQGGQIYDEGYMVKDDDGREDKTEFTVKN

VQVRGGYVLHIGTLYGSLKVGDQVHLSIDETRRRPVMSNHTATHILNFALRSVLGEADQRGSLVAPDRLRFDFTAKGALS

TQEIKKVEGIANQMIEEAKTVYAKDCPLAAAKAIQGLRAVFDETYPDPVRVVSIGIPVEELLADPSGPAGSITSIEFCGG

THLQNSGHAGPFVIVSEEAIAKGIRRIVAVTGAEARKALRKVDSLRKLLSALEAKVKVQTAPNKDVQKEITDLSETMATA

VIPQWQKDELREAVKALKKVMDDLDRASKADIQKRVLEKTKQVIESHPNQPLVIMEMENGASAKALNESLKLFKTYSPQT

ATMLFAVDNEAGRITCLCQVPQETANKGLKASQWVQEVSGLMDGKGGGKDVSAQATGKNVGCLQEALKLATDFARLRLGE

LKN

>XP_009890702.1 PREDICTED: alanine--tRNA synthetase, cytoplasmic [Charadrius vociferus]

MEATLTASQIRQRFIDFFKENKHTYVHSSSTIPLDDPTLLFANAGMNQFKPIFLNTIDPSHPLAKLSRATNTQKCIRAGG

KHNDLDDVGKDVYHHTFFEMLGSWSFGDYFKELACKLALDLLTKEFGIPAERLYVTYFGGNEAAGLQPDLECKQIWLNLG

LAEGRILPGNMKDNFWEMGDTGPCGPCSEIHYDRIGDRDASHLVNQDDPNVLEIWNLVFIQFNREADGTLKPLPKKSIDT

GMGLERLVSVLQNKMSNYDTDLFLPYFEAIQKGTGARPYMGQVGAEDADGIDMAYRVLADHARTITLALSDGGRPDNTGR

GYVLRRILRRAVRYSHEKLNAPKGFFATLVDVVVQSLGDAFPELKKDPDMVKDIINEEEDQFLKTLSRGRRILDRKIQSL

GDSKIIPGKAVTSVWMHQKTFIIWDQEEKGLVVDMEGFEEERKNAQLKSQGKGAGGEDLLMLDIYAIEELRARGLEVTDD

SPKYSYTSDQSGAYDFGSLVATVKAIRREKKFVEEVSTGQECGIVLDRTCFYAEQGGQIYDEGYMVKDDDGREDKTEFTV

KNVQVRGGYVLHIGTLYGSLKVGDQVHLSIDETRRRPVMSNHTATHILNFALRSVLGEADQRGSLVAPDRLRFDFTAKGA

LSTQEIKKVEGIANQMIEEAKTVYAKDCPLAAAKAIQGLRAVFDETYPDPVRVVSIGIPVEELLADPSGPAGSITSIEFC

GGTHLQNSGHAGPFVIVSEEAIAKGIRRIVAVTGAEARKALRKVDSLRKLLSALEAKVKVQTAPNKDVQKEITDLSETMA

TAVIPQWQKDELREAVKALKKVMDDLDRASKADIQKRVLEKTKQVIESHPNQPLVIMEMENGASAKALNESLKLFKTYSP

QTATMLFAVDNEAGRITCLCQVPQETANKGLKASQWVQEVSGLMDGKGGGKDVSAQATGKNVGCLQEALKLATDFARLRL

GELKN

>KFW77501.1 Alanine--tRNA synthetase, cytoplasmic [Manacus vitellinus]

MEATLTASQIRQRFIDFFKENKHTYVHSSSTIPLDDPTLLFANAGMNQFKPIFLNTIDPSHPLAKLSRATNTQKCIRAGG

KHNDLDDVGKDVYHHTFFEMLGSWSFGDYFKELACKLALELLTKEXXXXXXXXYFGGNEAAGLQPDLECKQIWLDLGLAE

GRILPGNMKDNFWEMGDTGPCGPCSEIHYDRIGDRDASHLVNQDDPNVLEIWNLVFIQFNREADGTLKPLPKKSIDTGMG

LERLVSVLQNKMSNYDTDLFLPYFEAIQKGTGARPYQGQVGAEDADGIDMAYRVLADHARTITLALSDGGRPDNTGRGYV

LRRILRRAVRYSHEKLNAPKGFFATLVDVVVQSLGDAFPELKKDPDMVKDIINEEEDQFLKTLSRGRRILDRKIQSLGDS

KIIPGDTAWLLYDTYGFPADLTGLIAEEKGLVVDMVEFSGIACHWQLKSQGKGAGGEDLLMLDIYAIEELRARGLEVTDD

SPKYSYASDPSGTYDFGSLVATVKAIRREKKFVEEVSTGQECGIVLDRTCFYAEQGGQIYDQGYMVKDDDSREDKTEFTV

KNVQVRGGYVLHIGTLYGSLKVGDQVHLSIDEARRRPVMSNHTATHILNFALRSVLGEADQRGSLVAPDRLRFDFTAKGA

LSTQEVKKVEGIANQMIEESKTVYAKDCPLAAAKAIQGLRAVFDETYPDPVRVVSIGIPVEELLADPSGPGGSITSIEFC

GGTHLQNSSHAGPFVIVSEEAIAKGIRRIVAVTGAEARKALRKVDSLRKLLSALEAKVKVQTAPNKDVQKEIADLGETLA

TAVIPQWQKDELREAVKALKKVMDDLDRASKADIQKRVLEKTKQVIESHPNQPWVIMEMENGASAKALNESLKLFKTNSP

QTAAMLFAVDNEAGRITCLCQVPQETANKGLKASQWVQEVSGLMDGKGGGKDISAQATGKNVGCLQEALKLATDFARLRL

GELKN

>XP_012360043.1 PREDICTED: LOW QUALITY PROTEIN: alanine--tRNA synthetase, cytoplasmic [Nomascus leucogenys]

MDSTLTASEIRQRFIDFFKRNEHTYVHSSATIPLDDPTLLFANAGMNQFKPIFLNTIDPSHPMAKLSRAANTQKCIRAGG

KHNDLDDVGKDVYHHTFFEMLGSWSFGDYFKELACKMALELLTQEFGIPVERLYVTYFGGDEAAGLEPDLECKQIWQNLG

LDDTKILPGNMKDNFWEMGDTGPCGPCSEIHYDRIGGRDAAHLVNQDDPNVLEIWNLVFIQYNREADGILKPLPKKSIDT

GMGLERLVSVLQNKMSNYDTDLFVPYFEAIQKGTGARPYTGKVGAEDADGIDMAYRVLADHARTITVALADGGRPDNTGR

GYVLRRILRRAVRYAHEKLNASRGFFATLVDVVVQSLGDAFPELKKDPDMVKDIINEEEVQFLKTLSRGRRILDRKIQSL

GDSKNIPGDTAWLLYDTYGFPVDLTGLIAEEKGLVVDMDGFEEERKLAQLKSQGKGAGGEDLIMLDIYAIEELRARGLEV

TDDSPKYNYHLDSSGSYVFETPVATXDALRREKMFVEEVSTGQECGVVLDKTCFYAEQGGQIYDEGYLVKVDDSSEDKTE

FTVKNAQVRGGYVLHIGTIYGDLKVGDQVRLFIDEPKILKNEEVGSGMXQSSRPVYTQDCPLAAAKGIQGLRAVFDETYP

DPVRVVSIGVPVSELLDDPSGPAGSLTSVEFCGGTHLRNSSHAGAFVIVTEEAIAKGIRRIVAVTGAEAQKAPGSTKQGS

RAIVPHLIAGTPWILALDAGLSLLLPALATAVIPQWQKDELRETLKSLKKVMDDLDRASKADVQKRVLEKTKQLIDSNPN

QPLVILEMESGASAKALNEALKLFKTYSPQTSAMLFTVDNEAGKITCLCQVPQNAANRGLKASEWVQQVSGLMDGKGGGK

DVSAQATGKNVGCLQEALQLATSFAQLHLGDVKN

>XP_009999343.1 PREDICTED: LOW QUALITY PROTEIN: alanine--tRNA synthetase, cytoplasmic [Chaetura pelagica]

MAAPLTASQIRQRFIDFFKANKHTYVHSSSTIPLDDPTLLFANAGMNQFKPIFLNTIDPSHPLAKLSRATNTQKCIRAGG

KHNDLDDVGKDVYHHTFFEMLGSWSFGDYFKELACKLALDLLTKEFGIPAERLYVTYFGGNEAAGLQPDLECKQIWLDLG

LAEGRILPGNMKDNFWEMGDTGPCGPCSEIHYDRIGDRDASHLVNQDDPNVLEIWNLVFIQFNREADGTLKPLPKKSIDT

GMGLERLVSVLQNKMSNYDTDLFLPYFEAIQKGTGARPYLGQVGAEDADGIDMAYRVLADHARTITLALSDGGRPDNTGR

GYVLRRILRRAVRYSHEKLNAPKGFFATLVDVVVQSLGDAFPELKKDPDMVKDIINEEEDQFLKTLSRGRRILDRKIQSL

GDSKTIPGDTAWLLYDTYVDMEGFEEERKNAQLKSQGKGAGGEDLLMLDIYAIEELRARGLEVTDDSPKYSYASDQSGAY

DFGSLVATVQAIRREKRFVEEVSTGQECGIVLDQTCFYAEQGGQIYDQGYMVKEDESREDKTEFTVKNVQVRGGYVLHIG

TLYGSLKVGDQVHLSIDESRRRPVMSNHTATHILNFALRSVLGEAEQRGSLVAPDRLRFDFTAKGALSSQEIKKVEGIAN

QMIEEAKPVYAKDCPLAAAKAIQGLRAVFDETYPDPVRVVSIGIPVEDLLADPSGPAGSITSIEFCGGTHLQNSSHAGPF

VIVSEEAIAKGIRRIVAVTGAEARKALRKVDSLRKVLSALEAKVKVQTAPNKDVQKEITDLGETLATAVIPQWQKDELRE

AVKALKKVMDDLDRASKADIQKRVLEKTKQVIESHPNQPLVIMEMENGASAKALNESLKLFKTHSPQTAAMLFAVDTEAG

RITCLCQVPQEAASRGLKANQWVQEVSGLMDGKGGGKDVSAQATGRNVGCLQDALRLATDFARPRLGDDPWSLPDPHLTL

TDPLELPKQLKA

>XP_009328635.1 PREDICTED: LOW QUALITY PROTEIN: alanine--tRNA synthetase, cytoplasmic [Pygoscelis adeliae]

MEATLTASQIRQRFIDFFKENKHTYVHSSSTIPLDDPTLLFANAGMNQFKPIFLNTIDPSHPLAKLNRATNTQKCIRAGG

KHNDLDDVGKDVYHHTFFEMLGSWSFGDYFKELACKLALDLLTKEFGIPAERLYVTYFGGNEAAGLQPDLECKQIWLNLG

LAEGRILPGNMKDNFWEMGDTGPCGPCSEIHYDRIGDRDASHLVNQDDPNVLEIWNLVFIQFNREADGTLKPLPKKSIDT

GMGLERLGSVLQNKMSNYDTDLFLPYFEAIQKGTGARPYMGQVGAEDADGIDMAYRVLADHARTITLALSDGGRPDNTGR

GYVLRRILRRAVRYSHEKLNAPKGFFATLVDVVVQSLGDAFPELKKDPDMVKDIINEEEDQFLKTLSRGRRILDRKIQSL

GDSKTIPGDTAWLLYDTYGFPADLTGLIAEEKGLVVDMEGFEEERKNAQLKSQGKGAGGEDLLMLDIYAIEELRARGLEV

TDDSPKYSYASDQSGAYDFGSLVATVKAIRREKKFVEEVSTGQECGIVLDRTCFYAEQGGQIYDEGYMVKDDDGREDKTE

FTVKNVQVRGGYVLHIGTLYGSLKVGDQVHLSIDETRRRPVMSNHTATHILNFALRSVLGEADQRGSLVAPDRLRFDFTA

KGALSTQEIKKVEGIANQMIEEAKTVYAKDCPLAAAKAIQGLRAVFDETYPDPVRVVSIGIPVEELLADPSGPAGSITSI

EFCGGTHLQNSSHAGPFVIVSEEAIAKGIRRIVAVTGAEARKALRKVDSLRKLLSALEAKVKVQTAPNKDVQKEIVDLGE

MLATAVIPQWQKDELREAVKALKKVMDDLDRASKADIQKRVSPGREXRAEWEDPPRSPWVPRGLVASRGKACCNDFVVLQ

ALNESLKLFKTYSPQTAAMLFAVDNEAGRITCLCQVPQETANKGLKASQWVQEVSGLMDGKGGGKDVSAQATGKNVGCLQ

EALKLATDFARLRLGELKN

>XP_015677165.1 PREDICTED: alanine--tRNA synthetase, cytoplasmic [Protobothrops mucrosquamatus]

METLTACEIRQRFINFFVDNQHTYVHSSATIPLDDPTLLFANAGMNQFKPIFLNTIDPSHPMAKLTRAVNTQKCIRAGGK

HNDLDDVGKDVYHHTFFEMLGSWSFGDYFKELACKLALDLLTKEFGIPVERLYVTYFGGNEAAGLEPDLDCKQIWLNLGL

AESKILPGSMKDNFWEMGDTGPCGPCSEMHYDRIGDRDASHLVNQDDPNVLEIWNLVFIQFNREADGTLKPLPKKSIDTG

MGLERLVSVLQNKMSNYDTDLFMPYFEAIQKGTGCRPYAGRVGEEDVDGIDMAYRVLADHARTITIALSDGGRPDNTGRG

YVLRRILRRAVRYAHEKLNAPKGFFATLVDVVVQSLGGAFPELTKDPDMVKDIINEEEVQFLKTLNRGRRILDRKIQSLG

DNKIIPGDTAWLLYDTYGFPVDLTGLIAEEKGLKVDMEAFEEERKTAQLKSQGKGAGDEDLIMLDIYAIEDLRARDLEPT

DDSLKYNYRSDPEGFYEFNNPGATVKALRREKTFVEEVHTGQECGVVLDQTCFYAEQGGQIYDEGYFVKEDDGAEDKTEF

TVKNVQVRGGYVLHVGTVYGTLKVGDLVRLFIDEPRRRPIMSNHTATHILNFALRSVLGEADQRGSLVAPDRLRFDFTAK

GAMSTQEIKKAEEIVNGMVQEAKIVYAKDCSLAAAKAIQGLRAVFDETYPDPVRVVSIGIPVEDMLNDPSSAAGAVTSVE

FCGGTHLQNSSHAGHFAIVSEEAIAKGIRRIVAVTGAEAQKALRKADGLNKQLADLETKVKIQTTPNKEVQREIADLGEA

LATAIIPQWQKDELREALKALKKIMDDLDRASKADIQKRVLDKMKQLIEAYPNQPLVILEMENGASAKALNESLKLLKTL

SPETAAMLFSVDNEAGKITCLCQVPQEVARQGLKASEWVQQVTVLMDGKGGGKDLSAQATGKNTDCLEEALQVATEFAKL

KLSELKN

>ABI97370.1 AlaRS, partial [Bos taurus]

MDSTLTAGEIRQRFIDFFKRNEHTYVHSSATIPLDDPTLLFANAGMNQFKPIFLNTIDPSHPMAKLSRAANTQKCIRAGG

KHNDLDDVGKDVYHHTFFEMLGSWSFGDYFKELACKMALELLTQEFGIPVERLYVTYFGGDEAAGLEPDLECKQIWQNLG

LDDSRILPGNMKDNFWEMGDTGPCGPCSEIHYDRIGGRDAAHLVNQDDPNVLEIWNLVFIQYNRETDGILKPLPKKSIDT

GMGLERLVSVLQNKMSNYDTDLFVPYFEAIQKGTGARPYTGRVGAEDADGIDMAYRVLADHARTITVALADGGRPDNTGR

GYVLRRILRRAVRYSHEKLNASRGFFATLVDVVVQSLGDAFPELKKDPDMVKDIINEEEVQFLKTLSRGRRILDRKIQSL

GDSKTIPGDTAWLLYDTYGFPVDLTALIAEEKGLVVDMDGFDEERKLAQLKSQGKGAGGEDLIMLDIYAIEELREKGLEA

TDDSPKYSYHSDSSGTYVFESAVATVMALRRDRMFVEEASTGQECGVVLDKTCFYAEQGGQTYDEGYLVKVDDSSEDKTE

FTVKNTQVRGGYVLHIGTLYGSLKVGDQVRLFIDEPRRRPVMSNHTATHILNFALRSVLGEADQRGSLVAPDRLRFDFTA

KGAMSTQQIKKAEEIANEMIEAAKPVYTEDCSLAAAKAIQGLRAVFDETYPDPVRVVSIGVPVSELLDDPSGPAGSLTSV

EFCGGTHLQNSSHAGAFVIVSEEAIAKGIRRIVAVTGAEAQKALRKAESLKKSLCVVEAKVKAQAAPNKDVQREIADLGE

ALATAIIPQWQKDEFRENLKSLKKVMDDLDRASKADVQKRVLEKTKQLIDSSPNQPLVILEVESGASAK

>XP_009072409.1 PREDICTED: alanine--tRNA synthetase, cytoplasmic [Acanthisitta chloris]

MESTLTASEIRQRFIDFFKKNKHTYVHSSSTIPLDDPTLLFANAGMNQFKPIFLNTIDPSHPLAKLSRATNTQKCIRAGG

KHNDLDDVGKDVYHHTFFEMLGSWSFGDYFKELACKLAXXXXXXXXXXXXXPADRLYVTYFGGNEAAGLQPDLECKQIWL

DLGLDEARILPGNMKDNFWEMGDTGPCGPCSEIHYDRIGGRDASHLVNQDDPNVLEIWNLVFIQFNREADGTLKSLPKKS

IDTGMGLERLVSVLQNKMSNYDTDLFLPYFEAIQKGTGARPYLGQVGAEDADGIDMAYRVLADHARTITLALSDGGRPDN

TGRGYVLRRILRRAVRYSHEKLNAPKGFFATLVDVVVQSLGDAFPELKKDPDMVKDIINEEEDQFLKTLSRGRRILDRKI

QSLGDSKIIPVQSQLGGTEPVGQYDTYGFPADLTGLIAEEKGLVVDMDGFEEERKNAQLKSQGKGAGGEDLLMLDIYAIE

ELRAQGLEVTDDSPKYSYASDPSGTYDFGSLVATVKAIRREKKFVEEVSTGQECGIVLDRTCFYAEQGGQIYDQGYMVKD

DDSREDKTEFTVKNVQVRGGYVLHIGTLYGNLKVGDQVHLSIDETRRRPVMSNHTATHILNFALRSVLGEADQRGSLVAP

DRLRFDFTAKGALSTQEIKEVEGIANQMIEESKTVYAKDCPLAAAKAIQGLRAVFDETYPDPVRVVSIGIPVEELLADPS

GPGGSITSIEFCGGTHLQNSSHAGPFVIVSEEAIAKGIRRIVAVTGAEARKALRKVDSLRKLLSALEAKVKIQTAPNKDV

QKEIADLGETLATAVIPQWQKDELREAVKALKKVMDDLDRASKADIQKRVLEKTKQVIESHPNQPWVIMEMENGASAKAL

NESLKLFKTCSPQTAAMLFAVDNEAGRITCLCQVPQETANKGLKASQWVQEVSGXXXXXXGGKDISAQATGKNVGCLQEA

LKLATDFARLRLGELKN

>XP_013152082.1 PREDICTED: alanine--tRNA synthetase, cytoplasmic, partial [Falco peregrinus]

MEALLTASQIRQRFIDFFKENKHTYVHSSSTIPLDDPTLLFANAGMNQFKPIFLNTIDPSHPLAKLNRATNTQKCIRAGG

KHNDLDDVGKDVYHHTFFEMLGSWSFGDYFKELACKLALDLLTKEFGIPVERLYVTYFGGNEAAGLQPDLECKQIWLNLG

LAEGRILPGNMKDNFWEMGDTGPCGPCSEIHYDRIGDRDASHLVNQDDPNVLEIWNLVFIQFNREADGTLKPLPKKSIDT

GMGLERLVSVLQNKMSNYDTDLFLPYFEAIQKGTGARPYMGQVGAEDADGIDMAYRVLADHARTITLALADGGRPDNTGR

GYVLRRILRRAVRYSHEKLNAPKGFFATLVDVVVQSLGDAFPELKKDPDMVKDIINEEEDQFLKTLSRGRRILDRKIQSL

GDSKTIPGDTAWLLYDTYGFPADLTALIAEEKGLVVDMEGSEEEXXHWQLKSQGKGAGGEDLLMLDIYAIEELRARGLEV

TDDSPKYSYSSDPSGTYDFGSLVATVKAIRREKKFVEEVSTGQECGIVLDRTCFYAEQGGQIYDEGYMVKDDDGREDKTE

FTVKNVQVRGGYVLHMGTLYGSLKVGDQVHLSIDETRRRPVMSNHTATHILNFALRSVLGEADQRGSLVAPDRLRFDFTA

KGALSTQEIKKVEGIANQMVEEAKTVYAKDCPLAAAKAIQGLRAVFDETYPDPVRVVSIGIPVEELLADPSGPAGSITSI

EFCGGTHLQNSSHAGPFVIVSEEAIAKGIRRIVAVTGAEARKALRKVDSLGKLLSALEAKVKVQTAPNKDVQKEIMDLSE

TLATAVIPQWQKDELREAVKALKKIMDDLDRASKADIQKRVLEKTKQVIESHPNQPLVIMEMENGASAKALNESLKLFKA

HSPQTATMLFAVDKEAGRITCLCQVPQEAAARGLKASRWVQEVSGLMDGKGGGKDGAAQATGRNVGCLPQALRVAADF

>XP_009702540.1 PREDICTED: alanine--tRNA synthetase, cytoplasmic, partial [Cariama cristata]

MEAMLTASQIRQRFIDFFKENKHTYVHSSSTIPLDDPTLLFANAGMNQFKPIFLNTIDPSHPLAKLTRVTNTQKCIRAGG

KHNDLDDVGKDVYHHTFFEMLGSWSFGDYFKELACKLALDLLTKEFGIPAERLYVTYFGGNEAAGLQPDLECKQIWLNLG

LAEGRILPGNMKDNFWEMGDTGPCGPCSEIHYDRIGDRDASHLVNQDDPNVLEIWNLVFIQFNREADGTLKPLPKKSIDT

GMGLERLVSVLQNKMSNYDTDLFLPYFEAIQKGTGARPYMGQVGAEDTDGIDMAYRVLADHARTITLALSDGGRPDNTGR

GYVLRRILRRAVRYSHEKLNAPKGFFATLVDVVVQSLGDAFPELKKDPDMVKDIINEEEDQFLKTLSRGRRILDRKIQSL

GDSKTIPGDTAWLLYDTYGFPADLTALIAEEKGLVVDMEGFEEERKNAQLKSQGKGAGGEDLLMLDIYAIDELRAQELEV

TDDLPKYSYASDPSGTYDFGSLVATVKAIRREKKFVEEVSTGQECGIVLDRTCFYAEQGGQIYDEGYMVKDDDGKEDKTE

FTVKNVQVRGGYVLHIGTLYGSLKVGDQVHLSIDETRRRSVMSNHTATHILNFALRSVLGEADQRGSLVAPDRLRFDFTA

KGALSTQEIKKVEGIANQMIEEAKTVYAKDCALAAAKAIQGLRAVFDETYPDPVRVVSIGIPVEELLADPSGPAGSITSI

EFCGGTHLQNSSHAGHFVIVSEEAIAKGIRRIVAVTGAEAQKALRKVDSLRKLLSALEAKVKVQTAPNKDVQKEITDLSE

TLATAVIPQWQKDELREAVKALKKIMDDLDRASKSDIQKRVLEKTKQVIESHPNQLLVVMEMENGASAKALNESLKLFKT

YSPQTATMLFAVDNEAGRITCLCQVPQETANKGLKASQWVQEVSGLMDGKGGGKDVSAQAT

>XP_014136270.1 PREDICTED: alanine--tRNA synthetase, cytoplasmic [Falco cherrug]

MEALLTASQIRQRFIDFFKENKHTYVHSSSTIPLDDPTLLFANAGMNQFKPIFLNTIDPSHPLAKLNRATNTQKCIRAGG

KHNDLDDVGKDVYHHTFFEMLGSWSFGDYFKELACKLALDLLTKEFGIPVERLYVTYFGGNEAAGLQPDLECKQIWLNLG

LAEGRILPGNMKDNFWEMGDTGPCGPCSEIHYDRIGDRDASHLVNQDDPNVLEIWNLVFIQFNREADGTLKPLPKKSIDT

GMGLERLVSVLQNKMSNYDTDLFLPYFEAIQKGTGARPYMGQVGAEDADGIDMAYRVLADHARTITLALADGGRPDNTGR

GYVLRRILRRAVRYSHEKLNAPKGFFATLVDVVVQSLGDAFPELKKDPDMVKDIINEEEDQFLKTLSRGRRILDRKIQSL

GDSKTIPGDTAWLLYDTYGFPADLTALIAEEKGLVVDMEGFLKSQGKGAGGEDLLMLDIYAIEELRARGLEVTDDSPKYS

YSSDPSGTYDFGSLVATVKAIRREKKFVEEVSTGQECGIVLDRTCFYAEQGGQIYDEGYMVKDDDGREDKTEFTVKNVQV

RGGYVLHMGTLYGSLKVGDQVHLSIDETRRRPVMSNHTATHILNFALRSVLGEADQRGSLVAPDRLRFDFTAKGALSTQE

IKKVEGIANQMVEEAKTVYAKDCPLAAAKAIQGLRAVFDETYPDPVRVVSIGIPVEELLADPSGPAGSITSIEFCGGTHL

QNSSHAGPFVIVSEEAIAKGIRRIVAVTGAEARKALRKVDSLGKLLSALEAKVKVQTAPNKDLQKEIMDLSETLATAVIP

QWQKDELREAVKALKKIMDDLDRASKADIQKRVLEKTKQVIESHPNQPLVIMEMENGASAKALNESLKLFKAHSPQTATM

LFAVDKEAGRITCLCQVPQEAAARGLKASRWVQEVSGLMDGKGGGKDGAAQATGRNVGCLPQALRVAADFARLRSPEPA

>POI28043.1 hypothetical protein CIB84_008207 [Bambusicola thoracicus]

MESTLTASQIRQRFIDFFKENQHTYVHSSSTIPLDDPTLLFANAGMNQFKPIFLNTIDPSHPLAKLSRATNTQKCIRAGG

KHNDLDDVGKDVYHHTFFEMLGSWSFGDYFKELACKLALDLLTKEFGIPAERLYVTYFGGNEAAGLQPDLECKQIWLDLG

LAEGRILPGNMKDNFWEMGDTGPCGPCSEIHYDRIGDRDASHLVNQDDPNVLEIWNLVFIQFNREADGSLKPLPKKSIDT

GMGLERLVSVLQNKMSNYDTDLFLPYFEAIQKGTGARPYMGQVGAEDADGIDMAYRVLADHARTITLALSDGGRPDNTGR

GSTVIKAMKFGLRGRAVGFFATLVDVVVQSLGDAFPELKKDPDMVKDIINEEEDQFLKTLSRGRRILDRKIQSMGDSKTI

PGDTAWLLYDTYGFPVDLTGLIAEEKGLVVDMEGFEEERKNAQLKSQGKGAGGEDLLMLDIYAIEELRARGLEVTDDSPK

YGYTSDSSGTYDFGSLVATVKAIRREKKFVEEVSTGQECGIVLDRTCFYAEQGGQIYDQGYMVKDDESKEDKTEFTVKNV

QVRGGYVLHIGTLYGSLKVGDQVHLSIDETRRRPVMSNHTATHILNFALRSVLGEADQRGSLVAPDRLRFDFTAKGALST

QEIKKVEGIANQMIEEAKPVYAKDCPLAAAKAIQGLRAVFDETYPDPVRVVSIGIPVEELLDDPSGPAGSITSIEFCGGT

HLQNSGHAGPFVIVSEEAIAKGIRRIVAVTGAEARKALRKVESLKKLLSALDAKVKVQTAPNKDVQKEITDLSEMLATAV

IPQWQKDELRETVKALKKVMDDLDRASKADIQKRVLEKTKQVIESHPNQPLVIMEMENGASAKALNESLKLFKTHSPQTA

TMLFAVDHEAGRITCLCQVPQETVQKGLKANQWVQEVSALMDGKGGGKDGSAQATGKNVGCLQEALRLATDFARLHLGEL

KN

>XP_008503621.1 PREDICTED: alanine--tRNA synthetase, cytoplasmic [Calypte anna]

MESPLTASEIRQRFIDFFKENKHTYVHSSSTIPLDDPTLLFANAGMNQFKPIFLNTIDPSHPFAKLSRATNTQKCIRAGG

KHNDLDDVGKDVYHHTFFEMLGSWSFGDYFKELACKMALDLLTKEFSIPIERLYVTYFGGNEAAGLQPDLECKQIWLNLG

LAEGRILPGNMKDNFWEMGDTGPCGPCSEIHYDRIGDRDASHLVNQDDPNVLEIWNLVFIQFNREADGTLKPLPKKSIDT

GMGLERLVSVLQNKMSNYDTDLFLPYFEAIQKGTGARPYLGQVGAEDADGIDMAYRVLADHARTITLALSDGGRPDNTGR

GYVLRRILRRAVRYSHEKLNAPKGFFATLVDVVVQSLGDAFPELKKDPDMVKDIINEEEDQFLKTLSRGRRILDRKIQSL

GDSKIIPGKAVTSAWVHLKAFIIWDQGLVVDMEGFEEERRNAQLKSQGKGAGGEDLLMLDIYAIEELRAQGLEVTDDSPK

YSYAADQSGAYDFGSLVATVKAIRREKKFVEEASTGQECGIVLDRTCFYAEQGGQIYDQGYMVKDDDSREDRTEFTVKNV

QVRGGYVLHMGTLYGSLKVGDQVHLSIDEARRVPTMSNHTATHVLNFALRSVLGDAEQRGSLVAPDRLRFDFTAKGALSA

QEIKKVEGIANQMIEEAKPVYAKNCPLAAAKAIQGLRAVFEETYPDPVRVVSIGIPVEDLLADPSGPGGSLTSVEFCGGT

HLQNSSHAGPFVIVSEEAIAKGIRRIVAVTGAEARKALRKVDSLRKVLSALEAKVKVQTAPNKDVQKEITDLSETVATAV

IPQWQKDELREAVKALKKVMDDLDRASKADIQKRVLEKTKQVIESHPNQPLVIMEMESGASAKALNESLKLFKTHSPQTA

TMLFAVDNEAGRITCLCQVPQETANKGLKASLWVQEVSGLMDGKGGGKDISAQATGKNVGCLQEALKLATDFARLRLGEL

KN

>KFU92165.1 Alanine--tRNA synthetase, cytoplasmic, partial [Chaetura pelagica]

MAAPLTASQIRQRFIDFFKANKHTYVHSSSTIPLDDPTLLFANAGMNQFKPIFLNTIDPSHPLAKLSRATNTQKCIRAGG

KHNDLDDVGKDVYHHTFFEMLGSWSFGDYFKELACKLALDLLTKEFGIPAERLYVTYFGGNEAAGLQPDLECKQIWLDLG

LAEGRILPGNMKDNFWEMGDTGPCGPCSEIHYDRIGDRDASHLVNQDDPNVLEIWNLVFIQFNREADGTLKPLPKKSIDT

GMGLERLVSVLQNKMSNYDTDLFLPYFEAIQKGTGARPYLGQVGAEDADGIDMAYRVLADHARTITLALSDGGRPDNTGR

GYVLRRILRRAVRYSHEKLNAPKGFFATLVDVVVQSLGDAFPELKKDPDMVKDIINEEEDQFLKTLSRGRRILDRKIQSL

GDSKTIPGKAGTSVWVHQRRADCFAFPLQVTLPGCSMTPIVDMEGFEEERKNAQLKSQGKGAGGEDLLMLDIYAIEELRA

RGLEVTDDSPKYSYASDQSGAYDFGSLVATVQAIRREKRFVEEVSTGQECGIVLDQTCFYAEQGGQIYDQGYMVKEDESR

EDKTEFTVKNVQVRGGYVLHIGTLYGSLKVGDQVHLSIDESRRRPVMSNHTATHILNFALRSVLGEAEQRGSLVAPDRLR

FDFTAKGALSSQEIKKVEGIANQMIEEAKPVYAKDCPLAAAKAIQGLRAVFDETYPDPVRVVSIGIPVEDLLADPSGPAG

SITSIEFCGGTHLQNSSHAGPFVIVSEEAIAKGIRRIVAVTGAEARKALRKVDSLRKVLSALEAKVKVQTAPNKDVQKEI

TDLGETLATAVIPQWQKDELREAVKALKKVMDDLDRASKADIQKRVLEKTKQVIESHPNQPLVIMEMENGASAKALNESL

KLFKTHSPQTAAMLFAVDTEAGRITCLCQVPQEAASRGLKANQWVQEVSGLMDGKGGGKDVSAQATGRNVGCLQDALRLA

TDFARPRLGEL

>KFP67037.1 Alanine--tRNA synthetase, cytoplasmic, partial [Cariama cristata]

MEAMLTASQIRQRFIDFFKENKHTYVHSSSTIPLDDPTLLFANAGMNQFKPIFLNTIDPSHPLAKLTRVTNTQKCIRAGG

KHNDLDDVGKDVYHHTFFEMLGSWSFGDYFKELACKLALDLLTKEFGIPAERLYVTYFGGNEAAGLQPDLECKQIWLNLG

LAEGRILPGNMKDNFWEMGDTGPCGPCSEIHYDRIGDRDASHLVNQDDPNVLEIWNLVFIQFNREADGTLKPLPKKSIDT

GMGLERLVSVLQNKMSNYDTDLFLPYFEAIQKGTGARPYMGQVGAEDTDGIDMAYRVLADHARTITLALSDGGRPDNTGR

GEYVLRRILRRAVRYSHEKLNAPKGFFATLVDVVVQSLGDAFPELKKDPDMVKDIINEEEDQFLKTLSRGRRILDRKIQS

LGDSKTIPGDTAWLLYDTYGFPADLTALIAEEKGLVVDMEGFEEERKNAQLKSQGKGAGGEDLLMLDIYAIDELRAQELE

VTDDLPKYSYASDPSGTYDFGSLVATVKAIRREKKFVEEVSTGQECGIVLDRTCFYAEQGGQIYDEGYMVKDDDGKEDKT

EFTVKNVQVRGGYVLHIGTLYGSLKVGDQVHLSIDETRRRSVMSNHTATHILNFALRSVLGEADQRGSLVAPDRLRFDFT

AKGALSTQEIKKVEGIANQMIEEAKTVYAKDCALAAAKAIQGLRAVFDETYPDPVRVVSIGIPVEELLADPSGPAGSITS

IEFCGGTHLQNSSHAGHFVIVSEEAIAKGIRRIVAVTGAEAQKALRKVDSLRKLLSALEAKVKVQTAPNKDVQKEITDLS

ETLATAVIPQWQKDELREAVKALKKIMDDLDRASKSDIQKRVLEKTKQVIESHPNQLLVVMEMENGASAKALNESLKLFK

TYSPQTATMLFAVDNEAGRITCLCQVPQETANKGLKASQWVQEVSGLMDGKGGGKDVSAQATG

>XP_014431613.1 PREDICTED: alanine--tRNA synthetase, cytoplasmic [Pelodiscus sinensis]

MEETLSARQIRQKFIDFFKAHQHTYVHSSATIPLDDPTLLFANAGMNQFKPIFLNTLDPSHPMAKLTRAANTQKCIRAGG

KHNDLDDVGKDVYHHTFFEMLGSWSFGDYFKELACKLALDLLTNEFGIPIERLYVTYFGGNEAAGLEPDLECKQIWQDLG

LAESRILPGSMKDNFWEMGDTGPCGPCSEIHYDRIGGRNASHLVNQDDPNVLEIWNLVFIQFNREADGTLKPLPKKNIDT

GLGLERLVSVLQNKMSNYDTDLFIPYFEAIQKGTGARPYTGKVGAEDEDGIDMAYRVLADHARTITVALADGGRPDNTGR

GYVLRRILRRAVRYSHEKLSAPKGFFASLVDVVVQSLGDAFPELKKDPAMVKDIINEEEVQFLKTLSRGRRILDRKIQTL

GDSKRIPGDTAWLLYDTYGFPVDLTGLIAEEKGYSTWSGMLVSILLQSPPAPNVLLKPNQPIHKSGIPLAVLRGHFPVPN

ASTCCHSQLKSQGKGAGEEDRLMLDIYAIDELRTRGLEVTDDSPKYNYTSDPSGTYVFESPVATVQAIRWEKRFVEEVST

GQECGIVLDKSCFYAEQGGQIYDEGYLVKDDDSSEDKTEFTVKNVQIRGGYVLHMGTVYGSLKVGDQVRLYIDEPRRRPI

MSNHTATHILNFALRLVLGDADQRGSLVAPDRLRFDFTARGAMSTQEIKKTEEIANQMIQEAKTVYAKDCPLAAAKAIQG

LRAVFDETYPDPVRVVSIGIPVEDMLADPSSAAGSITSVEFCGGTHLQNSSHAGPLAIVSEEAIAKGIRRIVAVTGAEAL

KALRKADSLMTSLSALEAKVKVQTVPNKDVQKEIADLSEVLATAVIPQWRKDELREALRALKKVMDDLDRTSKADIQKRV

LDKTKQLIDSHPNQPLVVLELESGASAKALNESLKLFKAYSPQTAAMLFSVDSEAGKITCLCQVPQELATKGLKANQWVQ

EVTGLMDGKGGGKDVSAQATGKNVDCLEEALKLATDFARLHLGELKN

>OCT84660.1 hypothetical protein XELAEV_18022814mg [Xenopus laevis]

MFKGGDCLAIVVRRSVPTQRECCFGSGSSLLGLSQEPKRFESKSSSALIMKTSMTASEIRQKFIDFFKDNGHTYVHSSAT

VPLDDPTLLFANAGMNQFKPIFLNTIDPSHPMAKLTRAANTQKCIRAGGKHNDLDDVGKDVYHHTFFEMLGSWSFGDYFK

ELACKLALDLLTKEFGIDIERLYVTYFGGNEAAGLEPDLECKQIWLNLGLAESRILPGSMKDNFWEMGDTGPCGPCSEIH

FDRIGGRDAAHLVNMDDPNVLEIWNLVFIQFNRETDGTLKPLPKKSIDTGMGLERLVSVLQNKMSNYDTDLFVPYFEAIQ

KGTGARPYTGKVGDEDVDGIDMAYRVLADHARTITVALADGGRPDNTGRGYVLRRILRRAVRYAHEKLNASKGFFSTLVD

VVVQSLGNAFPELKKDPDMVKDIINDEETQFLKTLNRGRRILDRKIQSLGEHKVIPGDTAWLLYDTYGFPVDLTSLIAEE

RGLTLDMESFEEERKAAQLKSQGKGAGDEDLLMLDIYAIEELRSRGLEPTDDLPKYTYTSNAEGVYEFDNLLATVKAIRR

EKMFVEEASTGQECGLVLDKTCFYAEQGGQTYDEGYMVKEEESSEDKTEFTVRNTQVRGGYVLHVGTLYGNLKVGDQVHL

YIDEPKRRPIMSNHTATHILNFALRSVLGEADQRGSLVAPDRLRFDFTAKGAMTTQEIRKTEEIANDIIKENKVVYALDC

PLATAKAIQGLRAVFDETYPDPVRVVSIGIPVEDLLADPSSPAGSVTSVEFCGGTHLKNSGHAGPFIIVTEEAIAKGIRR

IVAVTGAEAQKALRRQDAIKVSLSDLEKKVKKQTDPNKDVQKEIADLSENLATVVIAQWQKDVLREQLKTLKKTMDDLDR

ASKADVQKRVLEITKQLIEEHPNQPLLVLEMENGASAKALNESLKLLKSQSPQTAAMIFSVDSDAGKITCLCQVPKETAD

KGLKANEWVQQVSELMDGKGGGKDTSAQATGKNVGCLSKVLKLAEDFGKMKLESMKN

>NP_001121342.1 alanyl-tRNA synthetase L homeolog [Xenopus laevis]

MKTSMTASEIRQKFIDFFKDNGHTYVHSSATVPLDDPTLLFANAGMNQFKPIFLNTIDPSHPMAKLTRAANTQKCIRAGG

KHNDLDDVGKDVYHHTFFEMLGSWSFGDYFKELACKLALDLLTKEFGIDIERLYVTYFGGNEAAGLEPDLECKQIWLNLG

LAESRILPGSMKDNFWEMGDTGPCGPCSEIHFDRIGGRDAAHLVNMDDPNVLEIWNLVFIQFNRETDGTLKPLPKKSIDT

GMGLERLVSVLQNKMSNYDTDLFVPYFEAIQKGTGARPYTGKVGDEDVDGIDMAYRVLADHARTITVALADGGRPDNTGR

GYVLRRILRRAVRYAHEKLNASKGFFSTLVDVVVQSLGNAFPELKKDPDMVKDIINDEETQFLKTLNRGRRILDRKIQSL

GEHKVIPGDTAWLLYDTYGFPVDLTSLIAEERGLTLDMESFEEERKAAQLKSQGKGAGDEDLLMLDIYAIEELRSRGLEP

TDDLPKYTYTSNAEGVYEFDNLLATVKAIRREKMFVEEASTGQECGLVLDKTCFYAEQGGQTYDEGYMVKEEESSEDKTE

FTVRNTQVRGGYVLHVGTLYGNLKVGDQVHLYIDEPKRRPIMSNHTATHILNFALRSVLGEADQRGSLVAPDRLRFDFTA

KGAMTTQEIRKTEEIANDIIKENKVVYALDCPLATAKAIQGLRAVFDETYPDPVRVVSIGIPVEDLLADPSSPAGSVTSV

EFCGGTHLKNSGHAGPFIIVTEEAIAKGIRRIVAVTGAEAQKALRRQDAIKVSLSDLEKKVKKQTDPNKDVQKEIADLSE

NLATVVIAQWQKDVLREQLKTLKKTMDDLDRASKADVQKRVLEITKQLIEEHPNQPLLVLEMENGASAKALNESLKLLKS

QSPQTAAMIFSVDSDAGKITCLCQVPKETADKGLKANEWVQQVSELMDGKGGGKDTSAQATGKNVGCLSQVLKLAEDFGK

MKLESMKN

>XP_009931942.1 PREDICTED: alanine--tRNA synthetase, cytoplasmic [Opisthocomus hoazin]

MEATLTASQIRQRFIDFFKENKHTYVHSSSTIPLDDPTLLFANAGMNQFKPIFLNTIDPSHPLAKLSRATNTQKCIRAGG

KHNDLDDVGKDVYHHTFFEMLGSWSFGDYFKELACKLALDLLTKEFGIPAERLYVTYFGGNEASGLQPDLECKQIWLNLG

LAEGRILPGSMKDNFWEMGDTGPCGPCSEIHYDRIGDRDASHLVNQDDPNVLEIWNLVFIQFNREADGTLKPLPKKSIDT

GMGLERLVSVLQNKMSNYDTDLFLPYFEAIQKGTGARPYMGQVGAEDADGTDMAYRVLADHARTITLALSDGGRPDNTGR

GYVLRRILRRAVRYSHEKLNAPKGFFATLVDVVVQSLGDAFPELKKDPDVVKDIINEEEDQFLKTLSRGRRILDRKIQSL

GDSKTIPGDTAWLLYDTYGFPADLTGLIAEEKGLVVDMEGFEEERKNAQLKSQGKGAGGEDLLMLDIYAIEELRAQGLEV

TDDSPKYNYASDQSGAYDFGSLVATVKAIRREKRFVEEVSTGQECGIVLDRTCFYAEQGGQIYDEGYMVKDDDGREDKTE

FTVKNVQVRGGYVLHIGTLYGSLKVGDQVHLSIDEARRRPVMSNHTATHILNFALRSVLGEADQRGSLVAPDRLRFDFTA

KGALSTQEIKEVEGITNQMIEEAKTVYAKDCPLAAAKAIQGLRAVFDETYPDPVRVVSIGIPVEELLADPSGPAGSVTSV

EFCGGTHLQNSSHAGPFVIVSEEAIAKGIRRIVAVTGAEARKALRKVGSLRKLLSALEAKVKVQTAPNKDVQKEIADLSE

TLATAVIPQWQKDELREAVKALKKVMDDLDRASKADIQKRVLEKTKQVIESHPNQPLVIMEMENGASAKALNESLKLFKT

HSPQTAAMLFAVDNEAGRITCLCQVPQETAAKGLKASQWVQEVAGLMDECLSQQQALGAVRQMQKLLAVVEAAHLRGTRG

LRQQLSVLQSRLQRPATTRNETCPQPAVPLNGRMLGRSVRVGHDVHFVCDAGFGLVGSETRSCRHNRTWSGTQPFRRRPA

VGLETMPAAWARLVCKAAGSPPRAISRAAGPAVGLETMPAARASQCERNPCPMESRACRLAATSISFHYLPLQANRTVPH

VLFTMSTTRFVSDSLRFSILGSRGQGVFAVRRSDRQTGELVLTSPIAGPATLEVELEMSEFSRKVLLGKHIFKVTAFVSP

YEF

>KFR09942.1 Alanine--tRNA synthetase, cytoplasmic, partial [Opisthocomus hoazin]

MEATLTASQIRQRFIDFFKENKHTYVHSSSTIPLDDPTLLFANAGMNQFKPIFLNTIDPSHPLAKLSRATNTQKCIRAGG

KHNDLDDVGKDVYHHTFFEMLGSWSFGDYFKELACKLALDLLTKEFGIPAERLYVTYFGGNEASGLQPDLECKQIWLNLG

LAEGRILPGSMKDNFWEMGDTGPCGPCSEIHYDRIGDRDASHLVNQDDPNVLEIWNLVFIQFNREADGTLKPLPKKSIDT

GMGLERLVSVLQNKMSNYDTDLFLPYFEAIQKGTGARPYMGQVGAEDADGTDMAYRVLADHARTITLALSDGGRPDNTGR

GYVLRRILRRAVRYSHEKLNAPKGFFATLVDVVVQSLGDAFPELKKDPDVVKDIINEEEDQFLKTLSRGRRILDRKIQSL

GDSKTIPGDTAWLLYDTYGFPADLTGLIAEEKGLVVDMEGFEEERKNAQLKSQGKGAGGEDLLMLDIYAIEELRAQGLEV

TDDSPKYNYASDQSGAYDFGSLVATVKAIRREKRFVEEVSTGQECGIVLDRTCFYAEQGGQIYDEGYMVKDDDGREDKTE

FTVKNVQVRGGYVLHIGTLYGSLKVGDQVHLSIDEARRRPVMSNHTATHILNFALRSVLGEADQRGSLVAPDRLRFDFTA

KGALSTQEIKEVEGITNQMIEEAKTVYAKDCPLAAAKAIQGLRAVFDETYPDPVRVVSIGIPVEELLADPSGPAGSVTSV

EFCGGTHLQNSSHAGPFVIVSEEAIAKGIRRIVAVTGAEARKALRKVGSLRKLLSALEAKVKVQTAPNKDVQKEIADLSE

TLATAVIPQWQKDELREAVKALKKVMDDLDRASKADIQKRVLEKTKQVIESHPNQPLVIMEMENGASAKALNESLKLFKT

HSPQTAAMLFAVDNEAGRITCLCQVPQETAAKGLKASQWVQEVAGLMD

>XP_002931707.1 PREDICTED: alanine--tRNA synthetase, cytoplasmic isoform X3 [Xenopus tropicalis]

MKTCMTASEIRQKFIDFFKENAHTYVHSSATVPLDDPTLLFANAGMNQFKPIFLNTIDPSHPMAKLNRAANTQKCIRAGG

KHNDLDDVGKDVYHHTFFEMLGSWSFGDYFKELACKLALDLLTKEFGIDIERLYVTYFGGNEAAGLEPDLECKQIWLNLG

LPESRILPGSMKDNFWEMGDTGPCGPCSEIHFDRIGGRDAAHLVNMDDPNVLEIWNLVFIQFNRETDGTLKPLPKKSIDT

GMGLERLVSVLQNKMSNYDTDLFVPYFEAIEKGTGARPYTGKVGAEDVDGIDMAYRVLADHARTITVALADGGRPDNTGR

GYVLRRILRRAVRYAHEKLNASKGFFATLVDVVVQSLGNAFPELKKDPDMVKDIINEEETQFLKTLNRGRRILDRKINSL

GEHKVIPGDTAWLLYDTYGFPVDLTSLIAEERGLTLDLESFEEERKAAQLKSQGKGTGDEDLLMLDIYAIEELRSQGLEP

TDDLPKYKYTSNADGVYEFESLIATVKAIRRDKMFVEEASTGQECGLVLDKTCFYAEQGGQTYDEGYMVKEEESSEDKTE

FTVRNTQVRGGYVLHMGTLYGHLKVGDQVCLYVDEPKRRPIMSNHTATHILNFALRSVLGEADQRGSLVAPDRLRFDFTA

KGAMSTQEIRKTEEIANDIIKENKVVYALDCSLAEAKAIQGLRAVFDETYPDPVRVVSIGIPVEDLLADPSSPAGSITSV

EFCGGTHLQNSGHAGPFIIVTEEAIAKGIRRIVAVTGAEAQKALRRQDALKVNLSDLEKKVKKQNSPNKDVQKEIADLSE

NLATASIAQWQKDVLREQLKTLKKTMDDLDRASKADVQKRVLEITKQLIEEHPNQPLLVLEMENGASAKALNESLKLLKS

QSPHTAAMIFSVDNDAGKITCLCQVPQDTADKGLKANEWVQQVSDLMDGRGGGKDTSAQATGKNVGCLSQVLKLAEDFAK

MKLESMKN

>XP_012817515.1 PREDICTED: alanine--tRNA synthetase, cytoplasmic isoform X2 [Xenopus tropicalis]

MRTLTMKTCMTASEIRQKFIDFFKENAHTYVHSSATVPLDDPTLLFANAGMNQFKPIFLNTIDPSHPMAKLNRAANTQKC

IRAGGKHNDLDDVGKDVYHHTFFEMLGSWSFGDYFKELACKLALDLLTKEFGIDIERLYVTYFGGNEAAGLEPDLECKQI

WLNLGLPESRILPGSMKDNFWEMGDTGPCGPCSEIHFDRIGGRDAAHLVNMDDPNVLEIWNLVFIQFNRETDGTLKPLPK

KSIDTGMGLERLVSVLQNKMSNYDTDLFVPYFEAIEKGTGARPYTGKVGAEDVDGIDMAYRVLADHARTITVALADGGRP

DNTGRGYVLRRILRRAVRYAHEKLNASKGFFATLVDVVVQSLGNAFPELKKDPDMVKDIINEEETQFLKTLNRGRRILDR

KINSLGEHKVIPGDTAWLLYDTYGFPVDLTSLIAEERGLTLDLESFEEERKAAQLKSQGKGTGDEDLLMLDIYAIEELRS

QGLEPTDDLPKYKYTSNADGVYEFESLIATVKAIRRDKMFVEEASTGQECGLVLDKTCFYAEQGGQTYDEGYMVKEEESS

EDKTEFTVRNTQVRGGYVLHMGTLYGHLKVGDQVCLYVDEPKRRPIMSNHTATHILNFALRSVLGEADQRGSLVAPDRLR

FDFTAKGAMSTQEIRKTEEIANDIIKENKVVYALDCSLAEAKAIQGLRAVFDETYPDPVRVVSIGIPVEDLLADPSSPAG

SITSVEFCGGTHLQNSGHAGPFIIVTEEAIAKGIRRIVAVTGAEAQKALRRQDALKVNLSDLEKKVKKQNSPNKDVQKEI

ADLSENLATASIAQWQKDVLREQLKTLKKTMDDLDRASKADVQKRVLEITKQLIEEHPNQPLLVLEMENGASAKALNESL

KLLKSQSPHTAAMIFSVDNDAGKITCLCQVPQDTADKGLKANEWVQQVSDLMDGRGGGKDTSAQATGKNVGCLSQVLKLA

EDFAKMKLESMKN

>KFZ64795.1 Alanine--tRNA synthetase, cytoplasmic, partial [Antrostomus carolinensis]

MEATLTASQIRQRFIDFFKANNHTYVHSSSTIPLDDPTLLFANAGMNQFKPIFLNTIDPSHPLAKLNRATNTQKCIRAGG

KHNDLDDVGKDVYHHTFFEMLGSWSFGDYFKELACKLALELLTKEFGIPAERLYVTYFGGNEAAGLQPDLECKQIWLDLG

LAEGRILPGNMKDNFWEMGDTGPCGPCSEIHYDRIGDRDASHLVNQDDPNVLEIWNLVFIQFNREADGTLKPLPKKSIDT

GMGLERLVSVLQNKMSNYDTDLFLPYFEAIQKGTGARPYMGQVGAEDADGIDMAYRVLADHARTITLALADGGRPDNTGR

GYVLRRILRRAVRYSHEKLNAPKGFFATLVDVVVQSLGDAFPELKKDPDMVKDIINEEEDQFLKTLSRGRRILDRKIQSL

GDSKIIPGDTAWLLYDTYGYLTGLIAEEKGLVVDMEGFEEERKNAQLKSQGKGAGGEDLLMLDIYAIEELRARGLEVTDD

SPKYSYASDPSGTYDFGNLMATVKAIRREKKFVEEVSTGQECGIVLDRTCFYAEQGGQIYDEGYMVKDDDGREDKTEFTV

KNVQVRGGYVLHMGTLYGSLKVGDQVHLSIDESRRRPVMSNHTGTHILNFALRSVLGEADQRGSLVAPDRLRFDFTAKGA

LSTQEIKKVEGIANQMIEEAKTVYAKDCPLAAAKAIQGLRAVFDETYPDPVRVVSIGIPVEELLADPSGPAGSVTSIEFC

GGTHLQNSSHAGSFVIVSEEAIAKGIRRIVAVTGAEARRALRKVESLRKLLSALEAKVKVQTAPNKDVQKEITDLSETLA

TAVIPQWQKDELREAVKALKKVMDDLDRASKADIQKRVLEKTKQVIESHPNQPLVIMEMENGASAKALNESLKLFKTHSP

QTATMLFAVDNEAGRITCLCQVPQETANKGLKASQWVQEVSALMDG

>XP_010172936.1 PREDICTED: alanine--tRNA synthetase, cytoplasmic, partial [Antrostomus carolinensis]

MEATLTASQIRQRFIDFFKANNHTYVHSSSTIPLDDPTLLFANAGMNQFKPIFLNTIDPSHPLAKLNRATNTQKCIRAGG

KHNDLDDVGKDVYHHTFFEMLGSWSFGDYFKELACKLALELLTKEFGIPAERLYVTYFGGNEAAGLQPDLECKQIWLDLG

LAEGRILPGNMKDNFWEMGDTGPCGPCSEIHYDRIGDRDASHLVNQDDPNVLEIWNLVFIQFNREADGTLKPLPKKSIDT

GMGLERLVSVLQNKMSNYDTDLFLPYFEAIQKGTGARPYMGQVGAEDADGIDMAYRVLADHARTITLALADGGRPDNTGR

GYVLRRILRRAVRYSHEKLNAPKGFFATLVDVVVQSLGDAFPELKKDPDMVKDIINEEEDQFLKTLSRGRRILDRKIQSL

GDSKIIPGDTAWLLYDTYGYLTGLIAEEKGLVVDMEGFEEERKNAQLKSQGKGAGGEDLLMLDIYAIEELRARGLEVTDD

SPKYSYASDPSGTYDFGNLMATVKAIRREKKFVEEVSTGQECGIVLDRTCFYAEQGGQIYDEGYMVKDDDGREDKTEFTV

KNVQVRGGYVLHMGTLYGSLKVGDQVHLSIDESRRRPVMSNHTGTHILNFALRSVLGEADQRGSLVAPDRLRFDFTAKGA

LSTQEIKKVEGIANQMIEEAKTVYAKDCPLAAAKAIQGLRAVFDETYPDPVRVVSIGIPVEELLADPSGPAGSVTSIEFC

GGTHLQNSSHAGSFVIVSEEAIAKGIRRIVAVTGAEARRALRKVESLRKLLSALEAKVKVQTAPNKDVQKEITDLSETLA

TAVIPQWQKDELREAVKALKKVMDDLDRASKADIQKRVLEKTKQVIESHPNQPLVIMEMENGASAKALNESLKLFKTHSP

QTATMLFAVDNEAGRITCLCQVPQETANKGLKASQWVQEVSALMD

>XP_017948852.1 PREDICTED: alanine--tRNA synthetase, cytoplasmic isoform X1 [Xenopus tropicalis]

MNFILCAWNGPLLAQGCPDTLSPCDLLLALTMKTCMTASEIRQKFIDFFKENAHTYVHSSATVPLDDPTLLFANAGMNQF

KPIFLNTIDPSHPMAKLNRAANTQKCIRAGGKHNDLDDVGKDVYHHTFFEMLGSWSFGDYFKELACKLALDLLTKEFGID

IERLYVTYFGGNEAAGLEPDLECKQIWLNLGLPESRILPGSMKDNFWEMGDTGPCGPCSEIHFDRIGGRDAAHLVNMDDP

NVLEIWNLVFIQFNRETDGTLKPLPKKSIDTGMGLERLVSVLQNKMSNYDTDLFVPYFEAIEKGTGARPYTGKVGAEDVD

GIDMAYRVLADHARTITVALADGGRPDNTGRGYVLRRILRRAVRYAHEKLNASKGFFATLVDVVVQSLGNAFPELKKDPD

MVKDIINEEETQFLKTLNRGRRILDRKINSLGEHKVIPGDTAWLLYDTYGFPVDLTSLIAEERGLTLDLESFEEERKAAQ

LKSQGKGTGDEDLLMLDIYAIEELRSQGLEPTDDLPKYKYTSNADGVYEFESLIATVKAIRRDKMFVEEASTGQECGLVL

DKTCFYAEQGGQTYDEGYMVKEEESSEDKTEFTVRNTQVRGGYVLHMGTLYGHLKVGDQVCLYVDEPKRRPIMSNHTATH

ILNFALRSVLGEADQRGSLVAPDRLRFDFTAKGAMSTQEIRKTEEIANDIIKENKVVYALDCSLAEAKAIQGLRAVFDET

YPDPVRVVSIGIPVEDLLADPSSPAGSITSVEFCGGTHLQNSGHAGPFIIVTEEAIAKGIRRIVAVTGAEAQKALRRQDA

LKVNLSDLEKKVKKQNSPNKDVQKEIADLSENLATASIAQWQKDVLREQLKTLKKTMDDLDRASKADVQKRVLEITKQLI

EEHPNQPLLVLEMENGASAKALNESLKLLKSQSPHTAAMIFSVDNDAGKITCLCQVPQDTADKGLKANEWVQQVSDLMDG

RGGGKDTSAQATGKNVGCLSQVLKLAEDFAKMKLESMKN

>KFP79842.1 Alanine--tRNA synthetase, cytoplasmic [Acanthisitta chloris]

MESTLTASEIRQRFIDFFKKNKHTYVHSSSTIPLDDPTLLFANAGMNQFKPIFLNTIDPSHPLAKLSRATNTQKCIRAGG

KHNDLDDVGKDVYHHTFFEMLGSWSFGDYFKVKQSWCNSESLAQNYLSLRAEQLYVTYFGGNEAAGLQPDLECKQIWLDL

GLDEARILPGNMKDNFWEMGDTGPCGPCSEIHYDRIGGRDASHLVNQDDPNVLEIWNLVFIQFNREADGTLKSLPKKSID

TGMGLERLVSVLQNKMSNYDTDLFLPYFEAIQKGTGARPYLGQVGAEDADGIDMAYRVLADHARTITLALSDGGRPDNTG

RGYVLRRILRRAVRYSHEKLNAPKGFFATLVDVVVQSLGDAFPELKKDPDMVKDIINEEEDQFLKTLSRGRRILDRKIQS

LGDSKIIPGALTALLFPRRGQYDTYGFPADLTGLIAEEKGLVVDMDGFEEERKNAQLKSQGKGAGGEDLLMLDIYAIEEL

RAQGLEVTDDSPKYSYASDPSGTYDFGSLVATVKAIRREKKFVEEVSTGQECGIVLDRTCFYAEQGGQIYDQGYMVKDDD

SREDKTEFTVKNVQVRGGYVLHIGTLYGNLKVGDQVHLSIDETRRRPVMSNHTATHILNFALRSVLGEADQRGSLVAPDR

LRFDFTAKGALSTQEIKEVEGIANQMIEESKTVYAKDCPLAAAKAIQGLRAVFDETYPDPVRVVSIGIPVEELLADPSGP

GGSITSIEFCGGTHLQNSSHAGPFVIVSEEAIAKGIRRIVAVTGAEARKALRKVDSLRKLLSALEAKVKIQTAPNKDVQK

EIADLGETLATAVIPQWQKDELREAVKALKKVMDDLDRASKADIQKRVLEKTKQVIESHPNQPWVIMEMENGASAKALNE

SLKLFKTCSPQTAAMLFAVDNEAGRITCLCQVPQETANKGLKASQWVQEDISAQATGKNVGCLQEALKLATDFARLRLGE

LKN

>OXB57244.1 hypothetical protein ASZ78_016419 [Callipepla squamata]

MESVLTASQIRQRFIDFFKENQHTYVHSSSTIPLDDPTLLFANAGMNQFKPIFLNTIDPSHPLAKLSRATNTQKCIRAGG

KHNDLDDVGKDVYHHTFFEMLGSWSFGDYFKELACKLALDLLTKEFGIPAERLYVTYFGGNEAAGLQPDLECKQIWLDLG

LAEGRILPGNMKDNFWEMGDTGPCGPCSEIHYDRIGDRDASHLVNQDDPNVLEIWNLVFIQFNREADGSLKPLPKKSIDT

GMGLERLVSVLQNKMSNYDTDLFLPYFEAIQKGTGARPYMGQVGAEDADGIDMAYRVLADHARTITLALSDGGRPDNTGR

GEDCRGQGDAFPELKKDPDMVKDIINEEEDQFLKTLSRGRRILDRKIQSMGDSKTIPGDTAWLLYDTYGFPVDLTGLIAE

EKGLVVDMEGFEEERKNAQLKSQGKGAGGEDLLMLDIYAIEELRARGLEVTDDSPKYSYTSDPSGTYDFGSLVATVKAIR

REKKFVEEVSTGQECGIVLDRTCFYAEQGGQIYDQGYMVKDDDSREDKTEFTVKNVQVRGGYVLHIGTLYGSLKVGDQVH

LTIDEMRRRPVMSNHTATHILNFALRSVLGEADQRGSLVAPDRLRFDFTAKGALSTQEIKKVEGIANQMIEEAKTVYARD

CPLAAAKAIQGLRAVFDETYPDPVRVVSIGIPVEELLADPSGPAGSITSIEFCGGTHLQNSGHAGPFVIVSEEAIAKGIR

RIVAVTGAEARKALRKVESLKKLLSALDAKVKVQTAPNKDMQKEITDLSEMLATAVIPQWQKDELREAVKALKKVMDDLD

RASKADIQKRVLEKTKQVIESHPNQPLVIMEMENGASAKALNESLKLFKTHSPQTAAMLFAVDNEAGRITCLCQVPQETV

QKGLKASQWVQEVSALMDGKGGGKDVSAQATGKNVGCLQEALRLATDFARLHLGELKN

>OXB74037.1 hypothetical protein H355_003100 [Colinus virginianus]

MESVLTASQIRQRFIDFFKENQHTYVHSSSTIPLDDPTLLFANAGMNQFKPIFLNTIDPSHPLAKLSRATNTQKCIRAGG

KHNDLDDVGKDVYHHTFFEMLGSWSFGDYFKELACKLALDLLTKEFGIPAERLYVTYFGGNEAAGLQPDLECKQIWLDLG

LAEGRILPGNMKDNFWEMGDTGPCGPCSEIHYDRIGDRDASHLVNQDDPNVLEIWNLVFIQFNREADGSLKPLPKKSIDT

GMGLERLVSVLQNKMSNYDTDLFLPYFEAIQKGTGARPYMGQVGAEDADGIDMAYRVLADHARTITLALSDGGRPDNTGR

GEDCHGQGDAFPELKKDPDMVKDIINEEEDQFLKTLSRGRRILDRKIQSMGDSKTIPGDTAWLLYDTYGFPVDLTGLIAE

EKGLVVDMEGFEEERKNAQLKSQGKGAGGEDLLMLDIYAIEELRARGLEVTDDSPKYSYTSDPSGTYDFGSLVATVKAIR

REKKFVEEVSTGQECGVVLDRTCFYAEQGGQIYDQGYMVKDDDSREDKTEFTVKNVQVRGGYVLHIGTLYGSLKVGDQVH

LTIDEMRRRPVMSNHTATHILNFALRSVLGEADQRGSLVAPDRLRFDFTAKGALSTQEIKKVEGIANQMIEEAKTVYARD

CPLAAAKAIQGLRAVFDETYPDPVRVVSIGIPVEELLADPSGPAGSITSIEFCGGTHLQNSGHAGPFVIVSEEAIAKGIR

RIVAVTGAEARKALRKVESLKKLLSALDAKVKVQTAPNKDVQKEITDLSEMLATAVIPQWQKDELREAVKALKKVMDDLD

RASKADIQKRVLEKTKQVIESHPNQPLVIMEMENGASAKALNESLKLFKAHSPQTAAMLFAVDNEAGRITCLCQVPQETV

QKGLKASQWVQEVSALMDGKGGGKDVSAQATGKNVGCLQEALRLATDFARLHLGELKN

>XP_007249234.2 alanine--tRNA synthetase, cytoplasmic isoform X1 [Astyanax mexicanus]

MALHLALVCAKAFSCFCQSSVNSPTSMDSSLTAAQIREKFIDFFRRHEHQYVHSSSTIPLDDPTLLFANAGMNQFKPIFL

NTIDPSHPMARLKRAANTQKCIRAGGKHNDLDDVGKDVYHHTFFEMLGSWSFGDYFKHLACEMALELLTKEFGIPIERLY

VTYFGGHEEAGLEPDLECKQIWLDLGMDESRILPGSMKDNFWEMGDTGPCGPCSEIHFDRIGGRDASHLVNMDDPNVLEI

WNLVFIQFNRESETELKPLPKKSIDTGMGLERLVSVLQNKMSNYDTDLFIPYFEAIQKGTGARPYTGKVGAEDTDGIDMA

YRVLADHARTITIALSDGGRPDNTGRGYVLRRILRRAVRYSHEKLGAQKGFFATLVDVVVESLGDAFPELRKDPDMVKDI

INEEEAQFLKTLSRGRRILDRKIQSLGDSKTIPGDTAWLLYDTYGFPLDLTALIAEERGMGVDMAAFEEEKKAAQLKSQG

KGSGDEDHIMLDIYAIEELRNKGIPATDDSPKYRYTSDDNGSYGFEEVVGTVLALRRERTFVDEVTTGQECGVLLDQTSF

YAEQGGQTFDEGYMLRENDSTEDRMEFTVKNTQVRGGYVLHVGTVYGTLKVGDRVTLHIDEARRRPIMSNHTATHILNFA

LRGVLGEADQRGSLVAPDRLRFDFTAKGAMSTKEVHRTEEMACAMIADSKQVYAMEAPLAAAKAIQGLRAVFDETYPDPV

RVVSIGVPVEELLSDPNSPSGSLTSIEFCGGTHLQNSSHAAPFVIVSEEAIAKGIRRIVAVTGAEAQKAQRKADALRQAL

SAMDEKVKAQTAPNKDIQKEIADLTESLGTAVISQWQKDEMRESLKGLKKVMDDLDRASKADVQKRVLDKTKEIIDGNPN

QPLIVMEMESGASAKALNESLKLLKTNSPQTAAMLFTVDNDAGKIICLCQVPQEVANRGLKANEWVQEVCPLLDGKGGGK

DMSAQATGRNTHCIQEALQLANEFARLKLGEN

>XP_007249236.2 alanine--tRNA synthetase, cytoplasmic isoform X2 [Astyanax mexicanus]

MDSSLTAAQIREKFIDFFRRHEHQYVHSSSTIPLDDPTLLFANAGMNQFKPIFLNTIDPSHPMARLKRAANTQKCIRAGG

KHNDLDDVGKDVYHHTFFEMLGSWSFGDYFKHLACEMALELLTKEFGIPIERLYVTYFGGHEEAGLEPDLECKQIWLDLG

MDESRILPGSMKDNFWEMGDTGPCGPCSEIHFDRIGGRDASHLVNMDDPNVLEIWNLVFIQFNRESETELKPLPKKSIDT

GMGLERLVSVLQNKMSNYDTDLFIPYFEAIQKGTGARPYTGKVGAEDTDGIDMAYRVLADHARTITIALSDGGRPDNTGR

GYVLRRILRRAVRYSHEKLGAQKGFFATLVDVVVESLGDAFPELRKDPDMVKDIINEEEAQFLKTLSRGRRILDRKIQSL

GDSKTIPGDTAWLLYDTYGFPLDLTALIAEERGMGVDMAAFEEEKKAAQLKSQGKGSGDEDHIMLDIYAIEELRNKGIPA

TDDSPKYRYTSDDNGSYGFEEVVGTVLALRRERTFVDEVTTGQECGVLLDQTSFYAEQGGQTFDEGYMLRENDSTEDRME

FTVKNTQVRGGYVLHVGTVYGTLKVGDRVTLHIDEARRRPIMSNHTATHILNFALRGVLGEADQRGSLVAPDRLRFDFTA

KGAMSTKEVHRTEEMACAMIADSKQVYAMEAPLAAAKAIQGLRAVFDETYPDPVRVVSIGVPVEELLSDPNSPSGSLTSI

EFCGGTHLQNSSHAAPFVIVSEEAIAKGIRRIVAVTGAEAQKAQRKADALRQALSAMDEKVKAQTAPNKDIQKEIADLTE

SLGTAVISQWQKDEMRESLKGLKKVMDDLDRASKADVQKRVLDKTKEIIDGNPNQPLIVMEMESGASAKALNESLKLLKT

NSPQTAAMLFTVDNDAGKIICLCQVPQEVANRGLKANEWVQEVCPLLDGKGGGKDMSAQATGRNTHCIQEALQLANEFAR

LKLGEN

>XP_017546933.1 PREDICTED: alanine--tRNA synthetase, cytoplasmic isoform X2 [Pygocentrus nattereri]

MDSSLSAAQIREKFIDFFRRYEHQYVHSSSTVPLDDPTLLFANAGMNQFKPIFLNTIDPSHPMARLKRAANTQKCIRAGG

KHNDLDDVGKDVYHHTFFEMLGSWSFGDYFKHLACKMALDLLTQEFGIPIDRLYVTYFGGHAEAGLEPDLECRQIWLDLG

MEESRILPGSMKDNFWEMGDTGPCGPCSELHYDRIGGRDAAHLVNMDDPNVLEIWNLVFIQFNRESETELKPLPKKSIDT

GMGLERLVSVLQNKMSNYDTDLFIPYFEAIQKGTGARPYTGKVGTEDTDGVDMAYRVLADHARTITIALSDGGRPDNTGR

GYVLRRILRRAVRYSHEKLAAQKGFFATLVDVVVESLGDAFPELRKDPDMVKDIINEEEAQFLKTLSRGRRILDRKIQSL

GDSKTIPGDTAWLLYDTYGFPLDLTALIAEERGMGVDMAAFEEEKKAAQLKSQGKGSGDEDHIMLDIYAIEELRNKGVPA

TDDGPKYRYTSDGSGDYVFEPTVATVLALRRERTFVDEVTTGQECGVLLDQTSFYAEQGGQTFDEGYLLRENDSTEDRME

FTVKNTQVRGGYVLHVGTVYGTLKVGDRLTLHIDEARRRPIMSNHTATHILNFALRGVLGEADQRGSLVAPDRLRFDFTA

KGAMSTQEVHRTEEIACAMITDAKQVYAMEAPLAAAKAIQGLRAVFDETYPDPVRVVSIGIPVEELLADPNSPAGSLTSI

EFCGGTHLQNSSHAAPFVIVSEEAIAKGIRRIVAVTGAEAQKAQRKADALKQALSAMGEKVKAQTAPNKDIQKEIADLTE

TLGTAVVSQWQKDEMRESLKGLKKIMDDLDRASKADVQKRVLEKTKEIIESSPNQPLLVMEMESGASAKALNESLKLLKT

QSPQTAAMLFTVDNDAGKIICLCQVPQEAANRGLKASEWVHEVCALLDGKGGGKDMSAQATGRNTHCIQEALQLANEFAR

LKLGEN

>XP_017546924.1 PREDICTED: alanine--tRNA synthetase, cytoplasmic isoform X1 [Pygocentrus nattereri]

MALHFTYFCAKAFSCCQSSVKSPGSMDSSLSAAQIREKFIDFFRRYEHQYVHSSSTVPLDDPTLLFANAGMNQFKPIFLN

TIDPSHPMARLKRAANTQKCIRAGGKHNDLDDVGKDVYHHTFFEMLGSWSFGDYFKHLACKMALDLLTQEFGIPIDRLYV

TYFGGHAEAGLEPDLECRQIWLDLGMEESRILPGSMKDNFWEMGDTGPCGPCSELHYDRIGGRDAAHLVNMDDPNVLEIW

NLVFIQFNRESETELKPLPKKSIDTGMGLERLVSVLQNKMSNYDTDLFIPYFEAIQKGTGARPYTGKVGTEDTDGVDMAY

RVLADHARTITIALSDGGRPDNTGRGYVLRRILRRAVRYSHEKLAAQKGFFATLVDVVVESLGDAFPELRKDPDMVKDII

NEEEAQFLKTLSRGRRILDRKIQSLGDSKTIPGDTAWLLYDTYGFPLDLTALIAEERGMGVDMAAFEEEKKAAQLKSQGK

GSGDEDHIMLDIYAIEELRNKGVPATDDGPKYRYTSDGSGDYVFEPTVATVLALRRERTFVDEVTTGQECGVLLDQTSFY

AEQGGQTFDEGYLLRENDSTEDRMEFTVKNTQVRGGYVLHVGTVYGTLKVGDRLTLHIDEARRRPIMSNHTATHILNFAL

RGVLGEADQRGSLVAPDRLRFDFTAKGAMSTQEVHRTEEIACAMITDAKQVYAMEAPLAAAKAIQGLRAVFDETYPDPVR

VVSIGIPVEELLADPNSPAGSLTSIEFCGGTHLQNSSHAAPFVIVSEEAIAKGIRRIVAVTGAEAQKAQRKADALKQALS

AMGEKVKAQTAPNKDIQKEIADLTETLGTAVVSQWQKDEMRESLKGLKKIMDDLDRASKADVQKRVLEKTKEIIESSPNQ

PLLVMEMESGASAKALNESLKLLKTQSPQTAAMLFTVDNDAGKIICLCQVPQEAANRGLKASEWVHEVCALLDGKGGGKD

MSAQATGRNTHCIQEALQLANEFARLKLGEN

>XP_014110994.1 PREDICTED: alanine--tRNA synthetase, cytoplasmic isoform X2 [Pseudopodoces humilis]

MCTHLLQSPWMTPHCSLPMLFKPIFLNTIDPSHPLAKLSRATNTQKCIRAGGKHNDLDDVGKDVYHHTFFEMLGSWSFGD

YFKELACKLALELLTKEFGIPAERLYVTYFGGNEAAGLQPDLECKQIWLDLGLAEGRILPGSMKDNFWEMGDTGPCGPCS

EIHYDRIGDRDASHLVNQDDPNVLEIWNLVFIQFNREADGTLKPLPKKSIDTGMGLERLVSVLQNKMSNYDTDLFLPYFE

AIQKGTGARPYLGQVGAEDADGIDMAYRVLADHARTITLALSDGGRPDNTGRGYVLRRILRRAVRYSHEKLNAPKGFFAT

LVDVVVQSLGDAFPELKKDPDMVKDIINEEEDQFLKTLSRGRRILDRKIQSLGDSKVIPGDTAWLLYDTYGFPADLTGLI

AEEKGLVVDMEGFEEERKNAQLKSQGKGAGGEDLLMLDIYAIEELRAQGLEVTDDSPKYNYASDPSGTYDFGSLVATVKA

IRREKRFVEEASTGQECGIVLDRTCFYAEQGGQIYDQGYMVKDDDSREDKTEFTVKNVQVRGGYVLHIGTLYGSLKVGDQ

VHLTIDEARRRPVMSNHTATHILNFALRSVLGEADQRGSLVAPDRLRFDFTAKGALSTQEIKKVEGIANQMIEESKTVYA

KDCPLAAAKAIQGLRAVFDETYPDPVRVVSIGIPVEELLADPSGPGGSITSIEFCGGTHLQNSSHAGPFVIVSEEAIAKG

IRRIVAVTGAEARKALRKVDSLRKLLSALEAKVKVQTAPNKDVQKEITDLGETLATAVIPQWQKDELREAVKALKKVMDD

LDRASKADIQKRVLEKTKQVIESHPNQPWVIMEMENGASAKALNESLKLFKTNSPQTAAMLFAVDNEAGRITCLCQVPQE

TANKGLKASQWVQEVSGLMDGKGGGKDISAQATGKNVGCLQEALKLATDFARLRLGELKN

>XP_010309890.1 PREDICTED: alanine--tRNA synthetase, cytoplasmic, partial [Balearica regulorum gibbericeps]

MEATLTASQIRQRFIDFFKENKHTYVHSSSTIPLDDPTLLFANAGMNQFKPIFLNTIDPSHPLAKLNRATNTQKCIRAGG

KHNDLDDVGKDVYHHTFFEMLGSWSFGDYFKELACKLALDLLTKEFGIPAERLYVTYFGGNEAAGLEPDLECKQIWLNLG

LAEGRILPGNMKDNFWEMGDTGPCGPCSEIHYDRIGDRDASHLVNQDDPNVLEIWNLVFIQFNREADGTLKPLPKKSIDT

GMGLERLVSVLQNKMSNYDTDLFLPYFEAIQKGTGARPYTGQVGAEDADGIDMAYRVLADHARTITLALSDGGRPDNTGR

GYVLRRILRRAVRYSHEKLNAPKGFFATLVDVVVQSLGDAFPELKKDPDMVKDIINEEEDQFLKTLSRGRRILDRKIQSL

GDSKTIPGDTAWLLYDTYGFPADLTGLIAEEKGLVVDMEGFEEERKNAQLKSQGKGAGGEDLLMLDIYAIEELRARGLEV

TDDSPKYSYASDPSGTYDFGSLVATVKAIRREKKFVEEVSTGQECGIVLDRTCFYAEQGGQIYDEGYMVKDDDGREDKTE

FTVKNVQVRGGYVLHIGTLYGSLKVGDQVHLCIDETRRRPVMSNHTATHILNFALRSVLGEADQRGSLVAPDRLRFDFTA

KGALSTQEIKKVEGIANEMIEEAKTVYAKDCPLAAAKAIQGLRAVFDETYPDPVRVVSIGIPVEELLADPSGPAGSITSI

EFCGGTHLQNSSHAGPFVIVSEEAIAKGIRRIVAVTGAEARKALRKVDSLRKLLSALEAKVKVQTAPSKDVQKEITDLSE

TLATAVIPQWQKDELREAVKALKKIMDDLDRASKADIQKRVLEKTKQVIESHPNQPLVIMEMENGASAKALNESLKLFKT

YSPQTATMLFAVDNEAGRITCLCQVPQ

>XP_009991186.1 PREDICTED: alanine--tRNA synthetase, cytoplasmic, partial [Tauraco erythrolophus]

MEATLTASQIRQRFIDFFKENKHTYVHSSSTIPLDDPTLLFANAGMNQFKPIFLNTIDPSHPLAKLNRATNTQKCIRAGG

KHNDLDDVGKDVYHHTFFEMLGSWSFGDYFKELACKLALDLLTKEFGIPAERLYVTYFGGNEAAGLQPDLECKQIWLNLG

LAEGRILPGNMKDNFWEMGDTGPCGPCSEIHYDRIGDRDASHLVNQDDPNVLEIWNLVFIQFNREADGTLKPLPKKSIDT

GMGLERLVSVLQNKMSNYDTDLFLPYFEAIQKGTGARPYMGQVGAEDADGIDMAYRVLADHARTITLALSDGGRPDNTGR

GYVLRRILRRAVRYSHEKLNAPKGFFATLVDVVVQSLGDAFPELKKDPDMVKDIINEEEDQFLKTLSRGRRILDRKIQSL

GDSKTIPGDTAWLLYDTYGFSADLTGLIAEEKGLVVDMEGFEEERKNAQLKSQGKGAGGEDLLMLDIYAIEELRARGLEV

TDDSPKYSYASDPSGAYDFGSLVATVKAIRREKKFVEEVSTGQECGIVLDRTCFYAEQGGQIYDEGYMVKDDDGREDKTE

FTVKNVQVRGGYVLHIGTLYGSLKVGDQVHLSIDETRRRPVMSNHTATHILNFALRSVLGEADQRGSLVAPDRLRFDFTA

KGALSTQEIKKVEGIANQMIEEAKTVYAKDCPLAAAKAIQGLRAVFDETYPDPVRVVSIGIPVEELLADPSGPAGSVTSV

EFCGGTHLQNSSHAGPFVIVSEEAIAKGIRRIVAVTGAEARKALRKVDSLRKLLSALEAKVKVQTAPNKDVQKEITDLSE

SLATAVIPQWQKDELREAVKALKKVMDDLDRASKADIQKRVLEKTKQVIESHPNQPLVIMEMENGASAKALNESLKLFKT

HSPQTAAMLFAVDNEAGRITCLCQVPQ

>KFU99162.1 Alanine--tRNA synthetase, cytoplasmic, partial [Tauraco erythrolophus]

MEATLTASQIRQRFIDFFKENKHTYVHSSSTIPLDDPTLLFANAGMNQFKPIFLNTIDPSHPLAKLNRATNTQKCIRAGG

KHNDLDDVGKDVYHHTFFEMLGSWSFGDYFKELACKLALDLLTKEFGIPAERLYVTYFGGNEAAGLQPDLECKQIWLNLG

LAEGRILPGNMKDNFWEMGDTGPCGPCSEIHYDRIGDRDASHLVNQDDPNVLEIWNLVFIQFNREADGTLKPLPKKSIDT

GMGLERLVSVLQNKMSNYDTDLFLPYFEAIQKGTGARPYMGQVGAEDADGIDMAYRVLADHARTITLALSDGGRPDNTGR

GYVLRRILRRAVRYSHEKLNAPKGFFATLVDVVVQSLGDAFPELKKDPDMVKDIINEEEDQFLKTLSRGRRILDRKIQSL

GDSKTIPGDTAWLLYDTYGFSADLTGLIAEEKGLVVDMEGFEEERKNAQLKSQGKGAGGEDLLMLDIYAIEELRARGLEV

TDDSPKYSYASDPSGAYDFGSLVATVKAIRREKKFVEEVSTGQECGIVLDRTCFYAEQGGQIYDEGYMVKDDDGREDKTE

FTVKNVQVRGGYVLHIGTLYGSLKVGDQVHLSIDETRRRPVMSNHTATHILNFALRSVLGEADQRGSLVAPDRLRFDFTA

KGALSTQEIKKVEGIANQMIEEAKTVYAKDCPLAAAKAIQGLRAVFDETYPDPVRVVSIGIPVEELLADPSGPAGSVTSV

EFCGGTHLQNSSHAGPFVIVSEEAIAKGIRRIVAVTGAEARKALRKVDSLRKLLSALEAKVKVQTAPNKDVQKEITDLSE

SLATAVIPQWQKDELREAVKALKKVMDDLDRASKADIQKRVLEKTKQVIESHPNQPLVIMEMENGASAKALNESLKLFKT

HSPQTAAMLFAVDNEAGRITCLCQVPQVT

>XP_005159107.1 alanine--tRNA synthetase, cytoplasmic isoform X1 [Danio rerio]

MDSSLTAAQIREKFIDFFRRHEHQYVHSSATIPLDDPTLLFANAGMNQFKPIFLNTIDPSHPMARLRRAANTQKCIRAGG

KHNDLDDVGKDVYHHTFFEMLGSWSFGDYFKHLACKMALELLTQEFGIPIERLYVTYFGGHADAGLEPDLECKQIWLDLG

MEESRILPGSMKDNFWEMGDTGPCGPCSEIHYDRIGGRDAAHLVNMDDPNVLEIWNLVFIQFNRESETVLKPLPKKSIDT

GMGLERLVSVLQNKMSNYDTDLFVPYFEAIQKGTGARAYTGKVGAEDTDGIDMAYRVLADHARTITIALSDGGRPDNTGR

GYVLRRILRRAVRYSHEKLGAQRGFFASLVDVVVKSLGDAFPELRKDPDMVKDIINEEEAQFLKTLSRGRRILDRKIQSL

GESTTIPGDTAWLLYDTYGFPLDLTALIAEERGMGVDLLAFEEEKKAAQLKSQGKGSGDEDHIMLDIYAIDELRNKGVAA

TDDSPKYKYTSDDNGNYEFEQAVGTVLALRRERMFVDEVITGQECGVLLDKTSFYAEQGGQSFDEGYMLRENDSAEDRME

FTVKNTQVRGGYVLHIGTVYGTLKVGDRLTLHVDEARRRPIMSNHTATHILNYGLRSVLGEADQRGSLVAPDRLRFDFTA

KGAMSTDEVRRTEEIAAAMIADAKGVYALDAPLAAAKAIQGLRAVFDETYPDPVRVVSIGVPVEELLADPNSPAGSLTSI

EFCGGTHLQNSSHAAPFVIVSEEAIAKGIRRIVAVTGAEAQKAQRKADALKLSLDALAEKVKAQSIPNKDVQKEIADMTE

SLGTAVISQWRKDEMRESLKGLKKIMDDLDRASKADVQKRVLEKTKEIIDSNPNQPLIVMEMESGASAKALNESLKMLKT

NSPQTAAMLFTVDNDAGKIICLCQVPQDVANRGLKASEWVQEVCPLLDGKGGGKDMSAQATGRNTQCIQEALQLASEFAR

LKLGEN

>AAI28795.1 Aars protein [Danio rerio]

MDSSLTAAQIREKFIDFFRRHEHQYVHSSATIPLDDPTLLFANAGMNQFKPIFLNTIDPSHPMARLRRAANTQKCIRAGG

KHNDLDDVGKDVYHHTFFEMLGSWSFGDYFKHLACKMALELLTQEFGIPIERLYVTYFGGHADAGLEPDLECKQIWLDLG

MEESRILPGSMKDNFWEMGDTGPCGPCSEIHYDRIGGRDAAHLVNMDDPNVLEIWNLVFIQFNRESETVLKPLPKKSIDT

GMGLERLVSVLQNKMSNYDTDLFVPYFEAIQKGTGARAYTGKVGAEDTDGIDMAYRVLADHARTITIALSDGGRPDNTGR

GYVLRRILRRAVRYSHEKLGAQRGFFASLVDVVVESLGDAFPELRKDPDMVKDIINEEEAQFLKTLSRGRRILDRKIQSL

GDSTTIPGDTAWLLYDTYGFPLDLTALIAEERGMGVDLLAFEEEKKAAQLKSQGKGSGDEDHIMLDIYAIDELRNKGVAA

TDDSPKYKYTSDDNGNYEFEQAVGTVLALRRERMFVDEVITGQECGVLLDKTSFYAEQGGQSFDEGYMLRENDSAEDRME

FTVKNTQVRGGYVLHIGTVYGTLKVGDRLTLHVDEARRRPIMSNHTATHILNYGLRSVLGEADQRGSLVAPDRLRFDFTA

KGAMSTDEVRRTEEIAAAMIADAKGVYALDAPLAAAKAIQGLRAVFDETYPDPVRVVSIGVPVEELLADPNSPAGSLTSI

EFCGGTHLQNSSHAAPFVIVSEEAIAKGIRRIVAVTGAEAQKAQRKADALKLSLDALAEKVKAQSIPNKDVQKEIADMTE

SLGTAVISQWRKDEMRESLKGLKKIMDDLDRASKADVQKRVLEKTKEIIESNPNQPLIVMEMESGASAKALNESLKMLKT

NSPQTAAMLFTVDNDAGKIICLCQVPQDVANRGLKASEWVQEVCPLLDGKGGGKDMSAQATGRNTQCIQEALQLASEFAR

LKLGEN

>NP_001005836.1 alanine--tRNA synthetase, cytoplasmic [Gallus gallus]

MESTLTASQIRQRFIDFFKENQHTYVHSSSTIPLDDPTLLFANAGMNQFKPIFLNTIDPSHPLAKLSRATNTQKCIRAGG

KHNDLDDVGKDVYHHTFFEMLGSWSFGDYFKELACKLALDLLTKEFGIPAERLYVTYFGGNEAAGLQPDLECKQIWLDLG

LAEGRILPGNMKDNFWEMGDTGPCGPCSEIHYDRIGDRDASHLVNQDDPNVLEIWNLVFIQFNREADGSLKPLPKKSIDT

GMGLERLVSVLQNKMSNYDTDLFLPYFEAIQKGTGARPYMGQVGAEDADGIDMAYRVLADHARTITLALSDGGRPDNTGR

GYVLRRILRRAVRYSHEKLNAPKGFFATLVDVVVQSLGDAFPELKKDPDMVKDIINEEEDQFLKTLSRGRRILDRKIQSM

GDSKTIPGDTAWLLYDTYGFPVDLTGLIAEEKGLVVDMEGFEEERKNAQLKSQGKGAGGEDLLMLDIYAIEELRARGLEV

TDDSPKYGYTSDPSGTYDFGSLVATVKAIRREKKFVEEVSTGQECGIVLDRTCFYAEQGGQIYDQGYMVKDDDSKEDKTE

FTVKNVQVRGGYVLHIGTLYGSLKVGDQVHLSIDETRRRPVMSNHTATHILNFALRSVLGEADQRGSLVAPDRLRFDFTA

KGALSTQEIKKVEGIANQMIEEAKPVYARDCPLAAAKAIQGLRAVFDETYPDPVRVVSIGIPVEELLADPSGPAGSITSI

EFCGGTHLQNSGHAGPFVIVSEEAIAKGIRRIVAVTGAEARKALRKVESLKKVLSALDAKVKVQTAPNKDVQKEITDLSE

MLATAVIPQWQKDELREAVKALKKVMDDLDRASKADIQKRVLEKTKQVIESHPNQPLVIMEMENGASAKALNESLKLFKT

HSPQTATMLFAVDNEAGRITCLCQVPRRRCRRA

>NP_001037775.1 alanine--tRNA synthetase, cytoplasmic isoform 1 [Danio rerio]

MALHLTFLCAKAFFCCQSPVKKATSMDSSLTAAQIREKFIDFFRRHEHQYVHSSATIPLDDPTLLFANAGMNQFKPIFLN

TIDPSHPMARLRRAANTQKCIRAGGKHNDLDDVGKDVYHHTFFEMLGSWSFGDYFKHLACKMALELLTQEFGIPIERLYV

TYFGGHADAGLEPDLECKQIWLDLGMEESRILPGSMKDNFWEMGDTGPCGPCSEIHYDRIGGRDAAHLVNMDDPNVLEIW

NLVFIQFNRESETVLKPLPKKSIDTGMGLERLVSVLQNKMSNYDTDLFVPYFEAIQKGTGARAYTGKVGAEDTDGIDMAY

RVLADHARTITIALSDGGRPDNTGRGYVLRRILRRAVRYSHEKLGAQRGFFASLVDVVVKSLGDAFPELRKDPDMVKDII

NEEEAQFLKTLSRGRRILDRKIQSLGESTTIPGDTAWLLYDTYGFPLDLTALIAEERGMGVDLLAFEEEKKAAQLKSQGK

GSGDEDHIMLDIYAIDELRNKGVAATDDSPKYKYTSDDNGNYEFEQAVGTVLALRRERMFVDEVITGQECGVLLDKTSFY

AEQGGQSFDEGYMLRENDSAEDRMEFTVKNTQVRGGYVLHIGTVYGTLKVGDRLTLHVDEARRRPIMSNHTATHILNYGL

RSVLGEADQRGSLVAPDRLRFDFTAKGAMSTDEVRRTEEIAAAMIADAKGVYALDAPLAAAKAIQGLRAVFDETYPDPVR

VVSIGVPVEELLADPNSPAGSLTSIEFCGGTHLQNSSHAAPFVIVSEEAIAKGIRRIVAVTGAEAQKAQRKADALKLSLD

ALAEKVKAQSIPNKDVQKEIADMTESLGTAVISQWRKDEMRESLKGLKKIMDDLDRASKADVQKRVLEKTKEIIDSNPNQ

PLIVMEMESGASAKALNESLKMLKTNSPQTAAMLFTVDNDAGKIICLCQVPQDVANRGLKASEWVQEVCPLLDGKGGGKD

MSAQATGRNTQCIQEALQLASEFARLKLGEN

>KFQ00883.1 Alanine--tRNA synthetase, cytoplasmic, partial [Haliaeetus albicilla]

MEATLTASQIRQRFIDFFKENKHTYVHSSSTIPLDDPTLLFANAGMNQFKPIFLNTIDPSHPLAKLNRATNTQKCIRAGG

KHNDLDDVGKDVYHHTFFEMLGSWSFGDYFKELACKLALDLLTKEFGIPAERLYVTYFGGNEAAGLQPDLECKQIWLNLG

LAEGRILPGNMKDNFWEMGDTGPCGPCSEIHYDRIGNRDASHLVNQDDPNVLEIWNLVFIQFNREADGTLKPLPKKSIDT

GMGLERLVSVLQNKMSNYDTDLFLPYFEAIQKGTGARPYMGQVGAEDADGIDMAYRVLADHARTITLALSDGGRPDNTGR

GYVLRRILRRAVRYSHEKLNAPKGFFATLVDVVVQSLGDAFPELKKDPDMVKDIINEEEDQFLKTLSRGRRILDRKIQSL

GDSKTIPGDTAWLLYDTYGFPADLTGLIAEEKGLVVDMEGFEEERKNAQLKSQGKGAGGEDLLMLDIYAIEELRARGLEV

TDDSPKYSYASDPSGTYDFGSLVATVKAIRREKKFVEEVSTGQECGIVLDRTCFYAEQGGQIYDEGYMVKDDDGREDKTE

FTVKNVQVRGGYVLHIGTLYGSLKVGDQVHLSIDETRRRPVMSNHTATHILNFALRSVLGEADQRGSLVAPDRLRFDFTA

KGALSTQEIKKVEGIANQMIEEAKTVYAKDCPLAAAKAIQGLRAVFDETYPDPVRVVSIGIPVEELLADPSGPAGSITSI

EFCGGTHLQNSSHAGPFVIVSEEAIAKGIRRIVAVTGAEARKALRKVDSLKKLLSALEAKVKVQTAPNKDVQKEITDLSE

TLATAVIPQWQKDELREAVKALKKVMDDLDRASKADIQKRVLEKTKQVIESHTNQPLVIMEMENGASAKALNESLKLFKT

YSPQTATMLFTVDNEAGRITCLCQVPQ

>XP_009913201.1 PREDICTED: alanine--tRNA synthetase, cytoplasmic [Haliaeetus albicilla]

MEATLTASQIRQRFIDFFKENKHTYVHSSSTIPLDDPTLLFANAGMNQFKPIFLNTIDPSHPLAKLNRATNTQKCIRAGG

KHNDLDDVGKDVYHHTFFEMLGSWSFGDYFKELACKLALDLLTKEFGIPAERLYVTYFGGNEAAGLQPDLECKQIWLNLG

LAEGRILPGNMKDNFWEMGDTGPCGPCSEIHYDRIGNRDASHLVNQDDPNVLEIWNLVFIQFNREADGTLKPLPKKSIDT

GMGLERLVSVLQNKMSNYDTDLFLPYFEAIQKGTGARPYMGQVGAEDADGIDMAYRVLADHARTITLALSDGGRPDNTGR

GYVLRRILRRAVRYSHEKLNAPKGFFATLVDVVVQSLGDAFPELKKDPDMVKDIINEEEDQFLKTLSRGRRILDRKIQSL

GDSKTIPGDTAWLLYDTYGFPADLTGLIAEEKGLVVDMEGFEEERKNAQLKSQGKGAGGEDLLMLDIYAIEELRARGLEV

TDDSPKYSYASDPSGTYDFGSLVATVKAIRREKKFVEEVSTGQECGIVLDRTCFYAEQGGQIYDEGYMVKDDDGREDKTE

FTVKNVQVRGGYVLHIGTLYGSLKVGDQVHLSIDETRRRPVMSNHTATHILNFALRSVLGEADQRGSLVAPDRLRFDFTA

KGALSTQEIKKVEGIANQMIEEAKTVYAKDCPLAAAKAIQGLRAVFDETYPDPVRVVSIGIPVEELLADPSGPAGSITSI

EFCGGTHLQNSSHAGPFVIVSEEAIAKGIRRIVAVTGAEARKALRKVDSLKKLLSALEAKVKVQTAPNKDVQKEITDLSE

TLATAVIPQWQKDELREAVKALKKVMDDLDRASKADIQKRVLEKTKQVIESHTNQPLVIMEMENGASAKALNESLKLFKT

YSPQTATMLFTVDNEAGRITCLCQVPQVRLRLCICLSLSIGGGSAASRQ

>XP_024070042.1 alanine--tRNA synthetase, cytoplasmic, partial [Terrapene mexicana triunguis]

METILSARQIRQKFIDFFKEHQHTYVHSSATIPLDDPTLLFANAGMNQFKPIFLNTLDPSHPMAKLTRAANTQKCIRAGG

KHNDLDDVGKDVYHHTFFEMLGSWSFGDYFKELACKLALDLLTNEFGIPIERLYVTYFGGNEAAGLEPDLECKQIWRDLG

LAESKILPGSMKDNFWEMGDTGPCGPCSEIHYDRIGDRDASHLVNQDDPNVLEIWNLVFIQFNREADGTLKPLPKKNIDT

GLGLERLVSVLQNKMSNYDTDLFVPYFEAIQKGTGARPYTGKVGAEDVDGIDMAYRVLADHARTITVALADGGRPDNTGR

GYVLRRILRRAVRYSHEKLNAPKGFFASLVDVVVQSLGDAFPELKKDPYMVKDIINEEEVQFLKTLSRGRRILDRKIQSL

GDSKTIPGDTAWLLYDTYGFPVDLTGLIAEEKGLTVDMEGFEEERKMAQLKSQGKGDGAEDLIMLDIYAIEELRTRGLEV

TDDSPKYNYTSDPSGVYEFESPVATVQAIRREKRFVEEVSTGQECGIVLDKSCFYAEQGGQIYDEGYLVKDNDSSEDKTE

FTVKNVQIRGGYVLHIGMVYGSLKVGDQVRLYIDEPRRRPIMSNHTATHILNFALRSVLGDADQRGSLVAPDRLRFDFTA

RGAMSTQEIKKTEEIANQMIKEAKTVYAKDCPLAAAKAIQGLRAVFDETYPDPVRVVSIGIPVEDMLADPSSAAGSITSI

EFCGGTHLQNSSHAGPFVIASEEAIAKGIRRIVAITGAEALKALRNADSLMKSLSALDAKVKVQTAPNKDVQKEIADLSE

TLATAVIPQWRKDELREALRALKKVMDDLDRTSKADVQKRVLEKTKQLIDSHPNQPLVVLELESGASAKALNESLKLFKA

YSPQTAAMLFSVDNEAGKITCLCQVPQVTMSDITLMRI

>XP_010882298.1 PREDICTED: alanine--tRNA synthetase, cytoplasmic [Esox lucius]

MDSSLTAAQIREKFIDFFRRHEHQYVHSSATIPLDDPTLLFANAGMNQFKPIFLNTIDPSHPMARLKRAANTQKCIRAGG

KHNDLDDVGKDVYHHTFFEMLGSWSFGDYFKHLACTMALELLTKEFGIPIERLYVTYFGGHEEAGLEPDLECKQIWLDLG

MDEARILPGSMKDNFWEMGDTGPCGPCSEIHFDRIGGRDAAHLVNMDDPNVLEIWNLVFIQFNRESETELKPLPKKSIDT

GMGLERLVSVLQNKMSNYDTDLFIPYFQAIQKGTGARDYTGKVGAEDVDGIDMAYRVLADHARTITIALSDGGRPDNTGR

GYVLRRILRRAVRYSHEKLGAQRGFFATLVDVVVESLGNAFPELKKDPDMVKDIINEEEEQFLKTLSRGRRILDRKIQSL

GDSKTIPGDTAWLLYDTYGFPLDLTALIAEERGLVVDVAAFEEEKKAAQIKSQGKGSGDADHIMLDIYAIEDLRNKDIAA

TDDTPKYNYKTDDGGKYEFEQAVGTVLALRRERAFVDEVTTGQECGVLLDQTSFYAEQGGQSFDEGYMLKENDSGDDKTE

FTVKNTQVRGGYVLHIGTVYGTLKVGDRVVLHVDEARRRPIMSNHTATHILNFALRGVLGEADQRGSLVAPDRLRFDFTA

KGAMSTAEVQRTEEIACTMIREAKPVYAMDAPLAAAKAIQGLRAVFDETYPDPVRVVSIGIPVADLLADPSSAAGSLTSI

EFCGGTHLRNSSHAAPFVIVSEEAIAKGIRRIVAVTGAEAQKAQRKADALRQSLSALGEKVKAQTAPNKDVQKEIADMTE

SLGMAVISQWQKDEMREALKGLKKTMDDLDRASKADVQKRVLEKTREMIDSNPNQPLVVMEMESGASAKALNESLKLLKT

QSPQTAAMLFTVDNDAGKIICLCQVPQETANRGLKASEWVQELCPLMDGKGGGKDMSAQATGRNTSCMEEALRMANQFAR

LKLGEN

>XP_010016916.1 PREDICTED: alanine--tRNA synthetase, cytoplasmic, partial [Nestor notabilis]

METTLTASQIRQRFIDFFKENKHTYVHSSSTIPLDDPTLLFANAGMNQFKPIFLNTIDPSHPLAKLNRATNTQKCIRAGG

KHNDLDDVGKDVYHHTFFEMLGSWSFGDYFKELACKLALNLLTKEFGIPVERLYVTYFGGNEAAGLQPDLECKQIWLDLG

LAEGRILPGNMKDNFWEMGDTGPCGPCSEIHYDRIGDRDASHLVNQDDPNVLEIWNLVFIQFNREADGTLKPLPKKSIDT

GMGLERLVSVLQNKMSNYDTDLFLPYFEAIQKGTGARPYMGRVGAEDADGIDMAYRVLADHARTITLALSDGGRPDNSGR

GYVLRRILRRAVRYSHEKLSAPKGFFATLVDVVVQSLGDAFPELKKDPDMVKDIINEEEDQFLKTLIRGRRILDRKIQSL

GDSKTIPGDTAWLLYDTYGFPADLTGLIAEEKGLVVDMEGFEEERKNAQLKSQGKGAGGEDLLMLDIYAIEELRARGLEV

TDDSPKYSYASYPSGTYDFGSLVATVKAIRREKKFVEEVSTGQECGIVLDRTCFYAEQGGQIYDEGYMVKDDDSREDKTE

FTVKNVQVRGGYVLHIGTLYGNLKVGDQVHLSIDETRRRPVMSNHTATHILNFALRSVLGEADQRGSLVAPDRLRFDFTA

KAALSTQEIKKVEGIANQMIEEAKTVYTQDCPLAAAKAIQGLRAVFDETYPDPVRVVSIGIPVEELLADPSGPAGSITSV

EFCGGTHLQNSSHAGPFVIVSEEAIAKGIRRIVAVTGAEARKALRKVDSLRRLLSALEAKVKVQTAPNKDVQKEITDLSE

TLATAVIPQWQKDELREAVKALKKVMDDLDRASKADIQKRVLEKTKQVIESHPNQPLVIMEMENGASAKALNESLKLFKT

YSPQTATMLFTVDNEAGRITCLCQVPQ

>CAQ13591.1 novel protein similar to vertebrate alanyl-tRNA synthetase (AARS) [Danio rerio]

MDSSLTAAQIREKFIDFFRRHEHQYVHSSATIPLDDPTLLFANAGMNQFKPIFLNTIDPSHPMARLRRAANTQKCIRAGG

KHNDLDDVGKDVYHHTFFEMLGSWSFGDYFKHLACKMALELLTQEFGIPIERLYVTYFGGHADAGLEPDLECKQIWLDLG

MEESRILPGSMKDNFWEMGDTGPCGPCSEIHYDRIGGRDAAHLVNMDDPNVLEIWNLVFIQFNRESETVLKPLPKKSIDT

GMGLERLVSVLQNKMSNYDTDLFVPYFEAIQKGTGARAYTGKVGAEDTDGIDMAYRVLADHARTITIALSDGGRPDNTGR

GYVLRRILRRAVRYSHEKLGAQRGFFASLVDVVVESLGDAFPELRKDPDMVKDIINEEEAQFLKTLSRGRRILDRKIQSL

GDSTTIPGDTAWLLYDTYGFPLDLTALIAEERGMGVDLLAFEEEKKAAQLKSQGKGSGDEDHIMLDIYAIDELRNKGVAA

TDDSPKYKYTSDDNGNYEFEQAVGTVLALRRERMFVDEVITGQECGVLLDKTSFYAEQGGQSFDEGYMLRENDSAEDFTV

KNTQVRGGYVLHIGTVYGTLKVGDRLTLHVDEARRRPIMSNHTATHILNYGLRSVLGEADQRGSLVAPDRLRFDFTAKGA

MSTDEVRRTEEIAAAMIADAKGVYALDAPLAAAKAIQGLRAVFDETYPDPVRVVSIGVPVEELLADPNSPAGSLTSIEFC

GGTHLQNSSHAAPFVIVSEEAIAKGIRRIVAVTGAEAQKAQRKADALKLSLDALAEKVKAQSIPNKDVQKEIADMTESLG

TAVISQWRKDEMRESLKGLKKIMDDLDRASKADVQKRVLEKTKEIIDSNPNQPLIVMEMESGASAKALNESLKMLKTNSP

QTAAMLFTVDNDAGKIICLCQVPQDVANRGLKASEWVQEVCPLLDGKGGGKDMSAQATGRNTQCIQEALQLASEFARLKL

GEN

>XP_009575385.1 PREDICTED: alanine--tRNA synthetase, cytoplasmic, partial [Fulmarus glacialis]

MEAMLTASQIRQRFIDFFKENKHTYVHSSSTIPLDDPTLLFANAGMNQFKPIFLNTIDPSHPLAKLNRATNTQKCIRAGG

KHNDLDDVGKDVYHHTFFEMLGSWSFGDYFKELACKLALDLLTKEFGIPAERLYVTYFGGHEAAGLQPDLECKQIWLNLG

LAEGRILPGNMKDNFWEMGDTGPCGPCSEIHYDRIGDRDASHLVNQDDPNVLEIWNLVFIQFNREADGTLKPLPKKSIDT

GMGLERLVSVLQNKMSNYDTDLFLPYFEAIQKGTGARPYMGQVGAEDADGIDMAYRVLADHARTITLALSDGGRPDNTGR

GYVLRRILRRAVRYSHEKLHAPKGFFATLVDVVVQSLGDAFPELKKDPDMVKDIINEEEDQFLKTLSRGRRILDRKIQSL

GDSKTIPGDTAWLLYDTYGFPADLTGLIAEEKGLVVDMEGFEEERKNAQLKSQGKGAGGEDLLMLDIYAIEELRARGLEV

TDDSPKYSYASDQSGAYDFGSLVATVKAIRREKKFVEEVSTGQECGIVLDRTCFYAEQGGQIYDEGYMVKDDDGREDKTE

FTVKNVQVRGGYVLHIGTLYGSLKVGDQVHLFIDETRRRPVMSNHTATHILNFALRSVLGEADQRGSLVAPDRLRFDFTA

KGALSTQEIKKVEGIANQMIEEAKTVYAKDCPLAAAKAIQGLRAVFDETYPDPVRVVSIGIPVEELLADPSGPAGSITSI

EFCGGTHLQNSSHAGPFVIVSEEAIAKGIRRIVAVTGAEARKALRKVDSLRKLLSALEAKVKVQTTPNKDVQKEITDLSE

TLATAVIPQWQKDELREAVKALKKVMDDLDRASKAGIQKRVLEKTKQVIESHPNQPLVIMEMENGASAKALNESLKLFKT

YSPQTATMLFTVDNEAGRITCLCQVPQ

>CAQ13590.1 novel protein similar to vertebrate alanyl-tRNA synthetase (AARS) [Danio rerio]

MALHLTFLCAKAFFCCQSPVKKATSMDSSLTAAQIREKFIDFFRRHEHQYVHSSATIPLDDPTLLFANAGMNQFKPIFLN

TIDPSHPMARLRRAANTQKCIRAGGKHNDLDDVGKDVYHHTFFEMLGSWSFGDYFKHLACKMALELLTQEFGIPIERLYV

TYFGGHADAGLEPDLECKQIWLDLGMEESRILPGSMKDNFWEMGDTGPCGPCSEIHYDRIGGRDAAHLVNMDDPNVLEIW

NLVFIQFNRESETVLKPLPKKSIDTGMGLERLVSVLQNKMSNYDTDLFVPYFEAIQKGTGARAYTGKVGAEDTDGIDMAY

RVLADHARTITIALSDGGRPDNTGRGYVLRRILRRAVRYSHEKLGAQRGFFASLVDVVVESLGDAFPELRKDPDMVKDII

NEEEAQFLKTLSRGRRILDRKIQSLGDSTTIPGDTAWLLYDTYGFPLDLTALIAEERGMGVDLLAFEEEKKAAQLKSQGK

GSGDEDHIMLDIYAIDELRNKGVAATDDSPKYKYTSDDNGNYEFEQAVGTVLALRRERMFVDEVITGQECGVLLDKTSFY

AEQGGQSFDEGYMLRENDSAEDFTVKNTQVRGGYVLHIGTVYGTLKVGDRLTLHVDEARRRPIMSNHTATHILNYGLRSV

LGEADQRGSLVAPDRLRFDFTAKGAMSTDEVRRTEEIAAAMIADAKGVYALDAPLAAAKAIQGLRAVFDETYPDPVRVVS

IGVPVEELLADPNSPAGSLTSIEFCGGTHLQNSSHAAPFVIVSEEAIAKGIRRIVAVTGAEAQKAQRKADALKLSLDALA

EKVKAQSIPNKDVQKEIADMTESLGTAVISQWRKDEMRESLKGLKKIMDDLDRASKADVQKRVLEKTKEIIDSNPNQPLI

VMEMESGASAKALNESLKMLKTNSPQTAAMLFTVDNDAGKIICLCQVPQDVANRGLKASEWVQEVCPLLDGKGGGKDMSA

QATGRNTQCIQEALQLASEFARLKLGEN

>XP_010148838.1 PREDICTED: alanine--tRNA synthetase, cytoplasmic, partial [Eurypyga helias]

MEATLTASQIRQRFIDFFKENKHTYVHSSSTIPLDDPTLLFANAGMNQFKPIFLNTIDPSHPLAKLNRATNTQKCIRAGG

KHNDLDDVGKDVYHHTFFEMLGSWSFGDYFKELACKLALDLLTKEFGIPAERLYVTYFGGNEAAGLQPDLECKQIWLNLG

LAEGRILPGNMKDNFWEMGDTGPCGPCSEIHYDRIGDRDASHLVNQDDPNVLEIWNLVFIQFNREADGTLKPLPKKSIDT

GMGLERLVSVLQNKMSNYDTDLFLPYFEAIQKGTGARPYAGQVGAEDADGIDMAYRVLADHARTITLALSDGGRPDNTGR

GYVLRRILRRAVRYSHEKLNAPKGFFATLVDVVVQSLGDAFPELKKDPDMVKDIINEEEDQFLKTLSRGRRILDRKIQSL

GDSKTIPGDTAWLLYDTYGFPADLTGLIAEEKGLVVDMEGFEEERKNAQLKSQGKGAGGEDLLMLDIYAIEELRARGLEV

TDDSPKYSYASDQSGYYDFGSLVATVKAIRREKKFVEEVSTGQECGIVLDRTCFYAEQGGQIYDEGYMVKDDDSREDKTE

FTVKNVQVRGGYVLHIGTLYGSLKVGDQVHLSIDETRRRPVMSNHTATHILNFALRSVLGEADQRGSLVAPDRLRFDFTA

KGALSTQEIKKVEGIASQMIEEAKPVYAKDCPLAAAKAIQGLRAVFDETYPDPVRVVSIGIPVEELLADPTGPAGSITSV

EFCGGTHLQNSSHAGPFVIVSEEAIAKGIRRIVAVTGAEARKALRKVDSLRKLLSALEAKVKVQTAPNKDVQKEITDLSE

TLATAVIPQWQKDELREAVKALKKVMDDLDRASKADIQKRVXXXXKQVIESHPNQPLVIMEMENGASAKALNESLKLFKT

HSPQTAAMLFAVDNEAGRITCLCQVPQ

>XP_023683025.1 alanine--tRNA synthetase, cytoplasmic [Paramormyrops kingsleyae]

MDTSLTANEIRRKFIEFFERNEHQYVHSSSCIPLDDPTLLFANAGMNQFKPIFLNTIDPSHPMAKLRRAANTQKCIRAGG

KHNDLDDVGKDVYHHTFFEMLGSWSFGDYFKHLACKMALELLTQEFGIPIDRLYVTYFGGNAEAGLEPDLECKQIWLDLG

MEDCRILPGSMKDNFWEMGDTGPCGPCSEMHFDRIGGRDAAHLVNMDDPNVLEIWNLVFIQFNRESETELKPLPKKSIDT

GMGLERLVSVLQNKMSNYDTDLFVPYFEAIQKGTGARPYTGKVGAEDSDGIDMAYRVLADHARTITIALSDGGRPDNTGR

GYVLRRILRRAVRYAHEKLAAHRGFFATLVDVVVESLGDAFPELRKDPDMVKDIINEEEAQFLKTLSRGRRILDRKIQSL

GDSTTIPGDTAWLLYDTYGFPLDLTALIAEERGLGIDHEGFEEEKKAAQLRSQGKGSGDEDHIMLDIYAIEELRNKALQA

TDDSPKYNYTSDDNGNYGFEQTTATVLALRRERAFVNEVFTGQECGVLLDRTCFYAEQGGQTFDEGYILREDDSTEDKME

FTVKNTQVRGGYVLHVGTVYGSLRVGDRVTLHIDEARRRPIMSNHTATHILNFALRGVLGEADQRGSLVASDRLRFDFTA

KAAMSTKEVQRTEEMARTMIADGKPVFAMEAPLAAAKAIQGLRAVFDETYPDPVRVVSIGMPVQELLADPSSAAGSLSSI

EFCGGTHLQNSSHASPFVIVSEEAIAKGIRRIVAVTGTEALKAQRKADALRQSLSALGEKVKSQTAPNKDVQKEIGDLTE

SLGTAVTSQWQKDEMREELKGLKKMMDDLDRASKADVQKRVLEKTKQVIDSNPNQPLLVMEMESGASAKALNESLKLLKS

HSPSTAAMLFTVDNDAGKIICLCQVPQEAANQGLKASEWVQEVCPLLDGKGGGKDTSAQATGRNMHCLQEALELADTFAR

LKLDLQN

>XP_009944489.1 PREDICTED: LOW QUALITY PROTEIN: alanine--tRNA synthetase, cytoplasmic-like, partial [Leptosomus discolor]

MEATLTASQIRQRFIDFFKENKHTYVHSSSTIPLDDPTLLFANAGMNQFKPIFLNTIDPSHPLAKLNRATNTQKCIRAGG

KHNDLDDVGKDVYHHTFFEMLGSWSFGDYFKELACKLALDLLTKEFGXXXXXXXXXXXXXNEAAGLQPDLECKQIWLNLG

LAEGRILPGNMKDNFWEMGDTGPCGPCSEIHYDRIGDRDASHLVNQDDPNVLEIWNLVFIQFNREADGTLKPLPKKSIDT

GMGLERLVSVLQNKMSNYDTDLFLPYFQAIQKGTGARPYMGQVGAEDADGIDMAYRVLADHARTITLALSDGGRPDNTGR

GYVLRRILRRAVRYSHEKLNAPKGFFATLVDVVVQSLGDAFPELKKDPDMVKDIINEEEDQFLKTLSRGRRILDRKIQSL

GDSKTIPGDTAWLLYDTYGFPADLTGLIAEEKGLVVDMEGFEEERKNAQLKSQGKGAGGEDLLMLDIYAIEELRARGLEV

TDDSPKYSYASDPSGTYDFGSLVATVKAIRREKKFVEEVSTGQECGIVLDRTCFYAEQGGQIYDEGYMVKDDDGREDKTE

FTVKNVQVRGGYVLHIGTLYGSLKVGDQVHLSIDETRRRPVMSNHTATHILNFALRSVLGEADQRGSLVAPDRLRFDFTA

KGALSTQEIKKVEGIANQMIEEAKPVYAEDCPLAAAKAIQGLRAVFDETYPDPVRVVSIGIPVEELLADPSGPAGSVTSV

EFCGGTHLQNSSHAGPFVIVSEEAIAKGIRRIVAVTGAEARKALRKVDSLRKLLSALEAKVKVQTAPNKDVQKEITDLSE

TLATAVIPQWQKDELREAVKVLKKVMDDLDRASKADIQKRVLEKTKQVIESHPNQPLVIMEMENGASAKALNESLKLFKT

YSPQTATMLFTVDNEAGRITCLCQVPQETVNKGLKASQWVQEV

>XP_018606970.1 PREDICTED: alanine--tRNA synthetase, cytoplasmic-like [Scleropages formosus]

MDTSLTAAEIREKFIDFFRRHEHQYVHSSSTVPLDDPTLLFANAGMNQFKPIFLNTIDPSHPMARLKRAANTQKCIRAGG

KHNDLDDVGKDVYHHTFFEMLGSWSFGDYFKHLACEMALELLTKEFGIPIERLYVTYFGGDEKAMLEPDLECRQIWLDLG

IEECRILPGSMKDNFWEMGDTGPCGPCSEIHFDRIGGRDAAHLVNMDDPNVLEIWNLVFIQFNRESETVLKPLPKKSIDT

GMGLERLVSVLQNKMSNYDTDLFVPYFEAIQKGTGSRPYTGKVGAEDTDGIDMAYRVLADHARTITIALSDGGRPDNTGR

GYVLRRILRRAVRYAHEKLGAQRGFFASLVDVVVESLGDAFPELKKDPDMVKDIINDEEVQFLKTLSRGRRILDRKIQSL

GDSQTIPGDTAWLLYDTYGFPLDLTALIAEERGLGVNMQGFEEERRAAQLKSQGKGSGDEDHIMLDIYSIEELRNKGLPA

TDDAPKYGYYVSDDGSYELEQAMGTVLALRREREFVEEVTTGQECGVLLDRTSFYAEQGGQTFDEGYMLKENDFAEDKME

FTVKNTQVRGGFVLHVGTVYGTLRVGDCVTLHVDEARRRPIMSNHTATHILNFALRSVLGEADQRGSLVAPDRLRFDFTA

KGAMSTVEVQRTEEMACTMIHEAKSVYAMEVPLAAAKAIQGLRAVFDETYPDPVRVVSIGVPVPDLLADPSGPAASLTSV

EFCGGTHLRNSAHVGPFVIVSEEAIAKGIRRIVAVTGAEAQKAQRKADALRQTLAALGGKVVAQTAPNKDVQKEIADLTE

ALGTAAISQWQKDEMREALKGLKKTMDDLDRASKADVQKRVLEKTKQLIDSNPNQPLIVVEMESGASAKALNESLKLLKA

HSPHTAAMLFAVDNDAGKIICLCQVPQEVVDKGLKASDWVQEVCPVLDGKGGGKDTSAQATGKNTQSIQEALKLAQDFAS

LRLQN

>KFQ06210.1 Alanine--tRNA synthetase, cytoplasmic, partial [Leptosomus discolor]

MEATLTASQIRQRFIDFFKENKHTYVHSSSTIPLDDPTLLFANAGMNQFKPIFLNTIDPSHPLAKLNRATNTQKCIRAGG

KHNDLDDVGKDVYHHTFFEMLGSWSFGDYFKELACKLALDLLTKENEAAGLQPDLECKQIWLNLGLAEGRILPGNMKDNF

WEMGDTGPCGPCSEIHYDRIGDRDASHLVNQDDPNVLEIWNLVFIQFNREADGTLKPLPKKSIDTGMGLERLVSVLQNKM

SNYDTDLFLPYFQAIQKGTGARPYMGQVGAEDADGIDMAYRVLADHARTITLALSDGGRPDNTGRGYVLRRILRRAVRYS

HEKLNAPKGFFATLVDVVVQSLGDAFPELKKDPDMVKDIINEEEDQFLKTLSRGRRILDRKIQSLGDSKTIPGDTAWLLY

DTYGFPADLTGLIAEEKGLVVDMEGFEEERKNAQLKSQGKGAGGEDLLMLDIYAIEELRARGLEVTDDSPKYSYASDPSG

TYDFGSLVATVKAIRREKKFVEEVSTGQECGIVLDRTCFYAEQGGQIYDEGYMVKDDDGREDKTEFTVKNVQVRGGYVLH

IGTLYGSLKVGDQVHLSIDETRRRPVMSNHTATHILNFALRSVLGEADQRGSLVAPDRLRFDFTAKGALSTQEIKKVEGI

ANQMIEEAKPVYAEDCPLAAAKAIQGLRAVFDETYPDPVRVVSIGIPVEELLADPSGPAGSVTSVEFCGGTHLQNSSHAG

PFVIVSEEAIAKGIRRIVAVTGAEARKALRKVDSLRKLLSALEAKVKVQTAPNKDVQKEITDLSETLATAVIPQWQKDEL

REAVKVLKKVMDDLDRASKADIQKRVLEKTKQVIESHPNQPLVIMEMENGASAKALNESLKLFKTYSPQTATMLFTVDNE

AGRITCLCQVPQETVNKGLKASQWVQEVS

>XP_012674239.1 PREDICTED: alanine--tRNA synthetase, cytoplasmic isoform X1 [Clupea harengus]

MGLHLTVLCAKALGCCQVNPSQSRASSPLIMDSSLTAAQIREKFIDFFRRHEHQYVHSSATIPLDDPTLLFANAGMNQFK

PIFLNTIDPSHPMARLRRAANTQKCIRAGGKHNDLDDVGKDVYHHTFFEMLGSWSFGDYFKQLACKMAMELLTQELGIPI

ERLYVTYFGGHAEAGLEPDLECKQIWLDLGMEECRILPGSMKDNFWEMGDTGPCGPCSEIHYDRIGGRDAAHLVNMDDPN

VLEIWNLVFIQFNRESETELKPLPKKSIDTGMGLERLVSVLQNKMSNYDTDLFVPYFQAIQKGTGARPYTGKVGVEDTDG

IDMAYRVLADHARTITIALSDGGRPDNTGRGYVLRRILRRAVRYSHEKLGAQRGFFASLVDVVVESLGDAFPELKKDPDM

VKDIINEEEVQFLKTLSRGRRILDRKIQSLGESTTIPGDTAWLLYDTYGFPLDLTALIAEERGMGVDIEGFEEEKKAAQI

KSQGKGSGDADHLMLDIYAIEELRNKGIAATDDSPKYRYNSDDSGSYDFEQVVSTVLALRRERTFVDEVTTGQECGVVLD

QTSFYAEQGGQTFDEGYMLRENDSAEDKMEFTVKNTQVRGGFVLHVGTVYGTLKVGDRLVLHVDEARRRPIMSNHTATHI

LNFALRGVLGEADQRGSMVAPDRLRFDFTAKGALTTGEVRRTEEIACALIRDNKPVYAMEAPLAAAKAIQGLRAVFDETY

PDPVRVVSIGIPVTDLLSDPDSPAGSLTSIEFCGGTHLQNSGHAGPFIIVSEEAIAKGVRRIVAVTGAEAQKAQRKADAL

RQSLAAMSEKVKAQTAPNKDVQKEIADMTESLGTAVTSQWQKDEMRDTLKGLKKTMDDLDRASKADVQKRVLEKTKELIE

SSPNQPLLVMEIESGASAKALNESLKLLKSSSPQTAAMLFTVDPDAGKIICLCQVPKEVASQGLKASEWVQEVCPLMDGK

GGGKDMSAQATGRNVHCMQEALQLANEFARLKLGEN

>XP_012674241.1 PREDICTED: alanine--tRNA synthetase, cytoplasmic isoform X3 [Clupea harengus]

MDSSLTAAQIREKFIDFFRRHEHQYVHSSATIPLDDPTLLFANAGMNQFKPIFLNTIDPSHPMARLRRAANTQKCIRAGG

KHNDLDDVGKDVYHHTFFEMLGSWSFGDYFKQLACKMAMELLTQELGIPIERLYVTYFGGHAEAGLEPDLECKQIWLDLG

MEECRILPGSMKDNFWEMGDTGPCGPCSEIHYDRIGGRDAAHLVNMDDPNVLEIWNLVFIQFNRESETELKPLPKKSIDT

GMGLERLVSVLQNKMSNYDTDLFVPYFQAIQKGTGARPYTGKVGVEDTDGIDMAYRVLADHARTITIALSDGGRPDNTGR

GYVLRRILRRAVRYSHEKLGAQRGFFASLVDVVVESLGDAFPELKKDPDMVKDIINEEEVQFLKTLSRGRRILDRKIQSL

GESTTIPGDTAWLLYDTYGFPLDLTALIAEERGMGVDIEGFEEEKKAAQIKSQGKGSGDADHLMLDIYAIEELRNKGIAA

TDDSPKYRYNSDDSGSYDFEQVVSTVLALRRERTFVDEVTTGQECGVVLDQTSFYAEQGGQTFDEGYMLRENDSAEDKME

FTVKNTQVRGGFVLHVGTVYGTLKVGDRLVLHVDEARRRPIMSNHTATHILNFALRGVLGEADQRGSMVAPDRLRFDFTA

KGALTTGEVRRTEEIACALIRDNKPVYAMEAPLAAAKAIQGLRAVFDETYPDPVRVVSIGIPVTDLLSDPDSPAGSLTSI

EFCGGTHLQNSGHAGPFIIVSEEAIAKGVRRIVAVTGAEAQKAQRKADALRQSLAAMSEKVKAQTAPNKDVQKEIADMTE

SLGTAVTSQWQKDEMRDTLKGLKKTMDDLDRASKADVQKRVLEKTKELIESSPNQPLLVMEIESGASAKALNESLKLLKS

SSPQTAAMLFTVDPDAGKIICLCQVPKEVASQGLKASEWVQEVCPLMDGKGGGKDMSAQATGRNVHCMQEALQLANEFAR

LKLGEN

>KFV97615.1 Alanine--tRNA synthetase, cytoplasmic, partial [Eurypyga helias]

MEATLTASQIRQRFIDFFKENKHTYVHSSSTIPLDDPTLLFANAGMNQFKPIFLNTIDPSHPLAKLNRATNTQKCIRAGG

KHNDLDDVGKDVYHHTFFEMLGSWSFGDYFKELACKLALDLLTKEFGIPAERLYVTYFGGNEAAGLQPDLECKQIWLNLG

LAEGRILPGNMKDNFWEMGDTGPCGPCSEIHYDRIGDRDASHLVNQDDPNVLEIWNLVFIQFNREADGTLKPLPKKSIDT

GMGLERLVSVLQNKMSNYDTDLFLPYFEAIQKGTGARPYAGQVGAEDADGIDMAYRVLADHARTITLALSDGGRPDNTGR

GYVLRRILRRAVRYSHEKLNAPKGFFATLVDVVVQSLGDAFPELKKDPDMVKDIINEEEDQFLKTLSRGRRILDRKIQSL

GDSKTIPGDTAWLLYDTYGFPADLTGLIAEEKGLVVDMEGFEEERKNAQLKSQGKGAGGEDLLMLDIYAIEELRARGLEV

TDDSPKYSYASDQSGYYDFGSLVATVKAIRREKKFVEEVSTGQECGIVLDRTCFYAEQGGQIYDEGYMVKDDDSREDKTE

FTVKNVQVRGGYVLHIGTLYGSLKVGDQVHLSIDETRRRPVMSNHTATHILNFALRSVLGEADQRGSLVAPDRLRFDFTA

KGALSTQEIKKVEGIASQMIEEAKPVYAKDCPLAAAKAIQGLRAVFDETYPDPVRVVSIGIPVEELLADPTGPAGSITSV

EFCGGTHLQNSSHAGPFVIVSEEAIAKGIRRIVAVTGAEARKALRKVDSLRKLLSALEAKVKVQTAPNKDVQKEITDLSE

TLATAVIPQWQKDELREAVKALKKVMDDLDRASKADIQKRVIESHPNQPLVIMEMENGASAKALNESLKLFKTHSPQTAA

MLFAVDNEAGRITCLCQVPQ

>XP_016086304.1 PREDICTED: alanine--tRNA synthetase, cytoplasmic isoform X2 [Sinocyclocheilus grahami]

MDSSLTAAQIREKFIDFFRRHEHQYVHSSATIPLDDPTLLFANAGMNQFKPIFLNTIDPSHPMARLHRAANTQKCIRAGG

KHNDLDDVGKDVYHHTFFEMLGSWSFGDYFKHLACEMALELLTKEFGIPIGRLYVTYFGGHADAGLEPDLECKQIWLDLG

MEESRILPGSMKDNFWEMGDTGPCGPCSEIHYDRIGGRDAAHLVNMDDPNVLEIWNLVFIQFNRESETVLKPLPKKSIDT

GMGLERLVSVLQNKMSNYDTDLFIPYFEAIQKGTGARAYTGKVGAEDTDGIDMAYRVLADHARTITIALSDGGRPDNTGR

GYVLRRILRRAVRYAHEKLGAQKGFFASLVDVVVESLGDAFPELRKDPDMVKDIINEEEVQFLKTLSRGRRILDRKIQSL

GDSKTIPGDTAWLLYDTYGFPLDLTALIAEERGMGVDMQAFEDEKKAAQLKSQGKGSGDEDHIMLDIYAIEELRNKGVTA

TDDSLKYKYTSNENGNYEFEQAVGTVLALRRERAFVDEATTGQECGVLLDKTSFYAEQGGQSFDEGYMLRENDSAEDRME

FTVKNTQVRGGYALHIGTVYGTLKVGDRLTMHVDEARRRPIMSNHTATHILNFALRSVLGEADQRGSLVAPDRLRFDFTA

KGAMSTDEVRRTEEIASTVIRDAKPVYALDSPLAAAKAIQGLRAVFDETYPDPVRVVSIGVPVEELLADPNSPAGSLTSI

EFCGGTHLQNSGHAAPFVIVSEEAIAKGIRRIVAVTGAEAQKAQRKADALKQELDAMAEKVKAQTSPNKDIQKEIADMTE

ALGTAVTSQWRKDEMRDSLKGLKKIMDDLDRASKADVQKRVLEKTKEIIETNPNQPLIVMEMENGASAKALNESLKLLKT

NSPQTAAMLFAVDNDVGKIICLCQVPQDVANQGLKASEWVQEVCPLLDGKGGGKDMSAQATGRNTHCIQEALQLANEFAR

LKLGEN

>XP_016086303.1 PREDICTED: alanine--tRNA synthetase, cytoplasmic isoform X1 [Sinocyclocheilus grahami]

MALHLTFLCAKAFFCCQSPEKNTTNMDSSLTAAQIREKFIDFFRRHEHQYVHSSATIPLDDPTLLFANAGMNQFKPIFLN

TIDPSHPMARLHRAANTQKCIRAGGKHNDLDDVGKDVYHHTFFEMLGSWSFGDYFKHLACEMALELLTKEFGIPIGRLYV

TYFGGHADAGLEPDLECKQIWLDLGMEESRILPGSMKDNFWEMGDTGPCGPCSEIHYDRIGGRDAAHLVNMDDPNVLEIW

NLVFIQFNRESETVLKPLPKKSIDTGMGLERLVSVLQNKMSNYDTDLFIPYFEAIQKGTGARAYTGKVGAEDTDGIDMAY

RVLADHARTITIALSDGGRPDNTGRGYVLRRILRRAVRYAHEKLGAQKGFFASLVDVVVESLGDAFPELRKDPDMVKDII

NEEEVQFLKTLSRGRRILDRKIQSLGDSKTIPGDTAWLLYDTYGFPLDLTALIAEERGMGVDMQAFEDEKKAAQLKSQGK

GSGDEDHIMLDIYAIEELRNKGVTATDDSLKYKYTSNENGNYEFEQAVGTVLALRRERAFVDEATTGQECGVLLDKTSFY

AEQGGQSFDEGYMLRENDSAEDRMEFTVKNTQVRGGYALHIGTVYGTLKVGDRLTMHVDEARRRPIMSNHTATHILNFAL

RSVLGEADQRGSLVAPDRLRFDFTAKGAMSTDEVRRTEEIASTVIRDAKPVYALDSPLAAAKAIQGLRAVFDETYPDPVR

VVSIGVPVEELLADPNSPAGSLTSIEFCGGTHLQNSGHAAPFVIVSEEAIAKGIRRIVAVTGAEAQKAQRKADALKQELD

AMAEKVKAQTSPNKDIQKEIADMTEALGTAVTSQWRKDEMRDSLKGLKKIMDDLDRASKADVQKRVLEKTKEIIETNPNQ

PLIVMEMENGASAKALNESLKLLKTNSPQTAAMLFAVDNDVGKIICLCQVPQDVANQGLKASEWVQEVCPLLDGKGGGKD

MSAQATGRNTHCIQEALQLANEFARLKLGEN

>KTG42561.1 hypothetical protein cypCar_00013813 [Cyprinus carpio]

MDSSLTTAQIREKFIDFFRRYDHQYVHSSSTIPLDDPTLLFANAGMNQFKPIFLNTIDPSHPMARLRRAANTQKCIRAGG

KHNDLDDVGKDVYHHTFFEMLGSWSFGDYFKQLACKMALELLTQEFGIPIGRLYVTYFGGHADAGLEPDLECKQIWLDLG

MEESRILPGSMKDNFWEMGDTGPCGPCSEIHYDRIGGRDAAHLVNMDDPNVLEIWNLVFIQFNRESETVLKPLPKKSIDT

GMGLERLVSVLQNKMSNYDTDLFIPYFEAIQKGTGARAYTGKVGAEDTDGIDMAYRVLADHARTITIALSDGGRPDNTGR

GYVLRRILRRAVRYSHEKLGAQKGFFASLVDVVVESLGDAFPELRKDPDMVKDIINEEEAQFLKTLSRGRRILDRKIQSL

GDSKTIPGDTAWLLYDTYGFPLDLTALIAEERGMGVDMQAFEDEKKAAQLKSQGKGSGDEEHIMLDIYAIEELRNNGVAA

TDDSPKYKYTSDDNGNYEFEQAVGTVLALRRERAFVDEVTTGQECGVLLDKTSFYAEQGGQSFDEGYMLRENDSAEDRME

FTVKNTQVRGGYVLHIGTVYGTLKVGDRLTLHVDEARRRPIMSNHTATHILNFALRSVLGEADQRGSLVAPDRLRFDFTA

KGAMSTDEVRRTEENASTMICDAKPVYALDAPLAAAKAIQGLRAVFDETYPDPVRVVSIGVPVEELLADPNSPAGSLTSI

EFCGGTHLKNSGHAAPFVIVSEEAIAKGIRRIVAVTGAEAQKAQRKADALKLELDAMAEKVKAQTTPNKDIQKEIADMTE

SLGTAVISQWRKDEMRDSLKGLKKIMDDLDRASKADVQKRVLEKTKEIIESNPNQPLIVMEMENGASAKALNESLKLLKT

NSPQTAAMLFAVDNDAGKIICLCQVPQDVANRGLKASEWVQKVCPLLDGKGGGKDMSAQATGRNTHCIQEALQLANEFAH

LKLVEN

>XP_012674240.1 PREDICTED: alanine--tRNA synthetase, cytoplasmic isoform X2 [Clupea harengus]

MGLHLTVLCAKALGCCQSRASSPLIMDSSLTAAQIREKFIDFFRRHEHQYVHSSATIPLDDPTLLFANAGMNQFKPIFLN

TIDPSHPMARLRRAANTQKCIRAGGKHNDLDDVGKDVYHHTFFEMLGSWSFGDYFKQLACKMAMELLTQELGIPIERLYV

TYFGGHAEAGLEPDLECKQIWLDLGMEECRILPGSMKDNFWEMGDTGPCGPCSEIHYDRIGGRDAAHLVNMDDPNVLEIW

NLVFIQFNRESETELKPLPKKSIDTGMGLERLVSVLQNKMSNYDTDLFVPYFQAIQKGTGARPYTGKVGVEDTDGIDMAY

RVLADHARTITIALSDGGRPDNTGRGYVLRRILRRAVRYSHEKLGAQRGFFASLVDVVVESLGDAFPELKKDPDMVKDII

NEEEVQFLKTLSRGRRILDRKIQSLGESTTIPGDTAWLLYDTYGFPLDLTALIAEERGMGVDIEGFEEEKKAAQIKSQGK

GSGDADHLMLDIYAIEELRNKGIAATDDSPKYRYNSDDSGSYDFEQVVSTVLALRRERTFVDEVTTGQECGVVLDQTSFY

AEQGGQTFDEGYMLRENDSAEDKMEFTVKNTQVRGGFVLHVGTVYGTLKVGDRLVLHVDEARRRPIMSNHTATHILNFAL

RGVLGEADQRGSMVAPDRLRFDFTAKGALTTGEVRRTEEIACALIRDNKPVYAMEAPLAAAKAIQGLRAVFDETYPDPVR

VVSIGIPVTDLLSDPDSPAGSLTSIEFCGGTHLQNSGHAGPFIIVSEEAIAKGVRRIVAVTGAEAQKAQRKADALRQSLA

AMSEKVKAQTAPNKDVQKEIADMTESLGTAVTSQWQKDEMRDTLKGLKKTMDDLDRASKADVQKRVLEKTKELIESSPNQ

PLLVMEIESGASAKALNESLKLLKSSSPQTAAMLFTVDPDAGKIICLCQVPKEVASQGLKASEWVQEVCPLMDGKGGGKD

MSAQATGRNVHCMQEALQLANEFARLKLGEN

>NP_001133550.1 Alanyl-tRNA synthetase, cytoplasmic [Salmo salar]

MDSLLTAAQIREKFIDFFIRHEHQYVHSSATIPLDDPTLLFANAGMNQFKPIFLNTIDPSHPMARLKRAANTQKCIRAGG

KHNDLDDVGKDVYHHTFFEMLGSWSFGDYFKHLACTMALELLTKEFGIPIERLYVTYFGGHEEAGLDPDLECKQIWLDLG

MDEARIIPGSMKDNFWEMGDTGPCGPCSEIHFDRIGGRDAAHLVNMDDPNVLEIWNLVFIQYNRESETELKPLPKKSIDT

GMGLERLVSVLQNKMSNYDTDLFIPYFQAIQKGTGARDYTGKVGAEDVDGIDMAYRVLADHARTITIALSDGGRPDNTGR

GYVLRRILRRAVRYSHEKLGAQKGFFATLVDVVVESLGDAFPELKKDPDMVKDIINEEEEQFLKTLSRGRKILDRKIQSL

GDSKTIPGDTAWLLYDTYGFPLDLTALIAEERGMMVDVAAFEEEKKAAQVKSQGKGSGDEDHIMLDIYAIEDLRNKGIAA

TDDTLKYSYSADDSGKYVFEQAVGTVLALRRERAFVEEVTTGQECGVLLDKTSFYAEQGGQSFDEGYMLREDDSVDDRME

FTVKNTQVRGGYVLHVGTVYGTLKVGDHVILHVDEARRRPIMSNHTATHILNFALREVLGEADQRGSLVAPDRLRFDFTA

KGALSTEEVRRTEEIACTMIRDAKHVYAMDTPLAAAKAIQGLRAVFDETYPDPVRVVSIGIPVADLLADPSSTAGSLTSI

EFCGGTHLRNSGHAAPFVIVSEEAIAKGIRRIVAVTGAEAQKAHRKADALRQSLSALGEKVKVQTAPNKDVQKEIADMTE

FLGMAVISQWQKDKMREALKGLKKTMDDLDRASKADVQKRVLEKTKEMIDSSPNQPLVVMEMESGASAKALNESLKLLKT

QSPQTAAMLFTVDHDAGKIVCLCQVPQDVANRGLKASEWVQELCPLMDGKGGGKDMSAQATGRNTHCIEEALQMANQFAQ

LKLSENE

>XP_012715490.2 alanine--tRNA synthetase, cytoplasmic-like [Fundulus heteroclitus]

MDSSLSAAQIRQRFIDFFVRYEHQYVHSSSTIPLDDPTLLFANAGMNQFKPIFLNTIDPSHPMAKLRRAANTQKCIRAGG

KHNDLDDVGKDVYHHTFFEMLGSWSFGDYFKHLACKMALELLTQEFGIPIDRLYVTYFGGNSDAGLEPDLECKQIWMDLG

VDEARILPGSMKDNFWEMGDTGPCGPCSEIHYDRIGGRDASHLVNMDDPNVLEIWNLVFIQFNRESETELKPLPKKSIDT

GMGLERLVSVLQNKMSNYDTDLFVPYFEAIQKGTGARPYSGKVGSDDADGIDMAYRVLADHARTITISLSDGGRPDNTGR

GYVLRRILRRAVRYSHEKLGAKRGFFASLVDVVVDSLGDAFPELKKDPEMVKDIINEEEAQFLKTLSRGRRILDRKIMSL

GDGKTIPGDTAWLLYDTYGFPLDLTSLIAEEKGMQVDLAAFEEEKKAAQLKSQGKGAGGEDHIMLDIYAIEELRNKQIPA

TDDSPKYRYTSEDNGSYEFQPASATVLALRRDRAFCEEVITGQECGVLLDQTSFYAEQGGQTFDEGYMLREDDSSEDRME

FTVKNTQVRGGYVLHIGTVYGTLRVGDRVTLHVDEARRRPIMSNHTATHILNFALRGVLGEADQRGSLVAPDRLRFDFTA

KGALSTGEVRRTEEIACAMIKDAKPVYAMEAPLAEAKAIQGLRAVFDETYPDPVRVVSIGIPVQELLDDPNSPAGSLTSI

EFCGGTHLQNSSHAAPFVIVSEEAIAKGIRRIVAVTGAEAQKAHRKADALLQSLSALGDKVKQQTAPNKDVQKEIADMTE

SIGTAVISQWQKDEMRETLKSLKKTMDDLDRSYKADIQKRVLEKTKEVIENNPNQPLLIMEMETGASAKALNESLKLLKS

QSPQTAAMLFTVDPDAGKITCLCQVPQDVANRGLKASEWVQELCPLLDGKGGGKDMSAQATGKNTQSLQEALQMANEFAK

LKLGEN

>XP_016320915.1 PREDICTED: alanine--tRNA synthetase, cytoplasmic-like [Sinocyclocheilus anshuiensis]

MDSSLTAAQIREKFIDFFRRYDHQYVHSSSTIPLDDPTLLFANAGMNQFKPIFLNTIDPSHPMARLRRAANTQKCIRAGG

KHNDLDDVGKDVYHHTFFEMLGSWSFGDYFKHLACKMALELLTQEFGIPIGRLYVTYFGGNADAGLEPDLECKQIWLDLG

MEESRILPGSMKDNFWEMGDTGPCGPCSEIHYDRIGGRDAAHLVNMDDPNVLEIWNLVFIQFNRESETVLKPLPKKSIDT

GMGLERLVSVLQNKMSNYDTDLFIPYFEAIQKGTGARAYTGKVGAEDTDGIDMAYRVLADHARTITIALSDGGRPDNTGR

GYVLRRILRRAVRYSHEKLGAQKGFFASLVDVVVESLGDAFPELRKDPDMVKDIINEEEAQFLKTLSRGRRILDRKVQSL

GDSKTIPGDTAWLLYDTYGFPLDLTALIAEERGMGVDMQAFEDEKKAAQLKSQGKGSGDEDHIMLDIYAIEELRNNGVAA

TDDNPKYKYTSDDNGNYEFEQAVGTVLALRRERAFVDEVTTGQECGVLLDKTSFYAEQGGQSFDEGYMLRENDFAEDRME

FTVKNTQVRGGYVLHIGTVYGTLKVGDRLTLHVDEARRRPIMSNHTATHILNFALRSVLGEADQRGSLVAPDRLRFDFTA

KGAMTTDEVRHTEEKASTMIRDAKPVYALDAPLAAAKAIQGLRAVFDETYPDPVRVVSIGIPVEELLADPNSPAGSLTSI

EFCGGTHLKNSGHAAPFVIVSEEAIAKGIRRIVAVTGAEAQKAQRKADALKLELDAMAEKVKAQTTPNKDIQNEIADMTE

SLGTAVISQWRKDEMRDSLKGLKKIMDDLDRASKADVQKRVLEKTKEIIESNPNQPLIVMEMENGASAKALNESLKLLKA

NSPQTAAMLFAVDNDAGKIICLCQVPQDVANQGLKASEWVQKVCPLLDGKGGGKDMSAQATGRNTHCIQEALQLANEFAH

LKLGEN

>XP_023992823.1 alanine--tRNA synthetase, cytoplasmic [Salvelinus alpinus]

MDSLLTAAQIREKFIDFFRRHEHQYVHSSATIPLDDPTLLFANAGMNQFKPIFLNTIDPSHPMARLKRAANTQKCIRAGG

KHNDLDDVGKDVYHHTFFEMLGSWSFGDYFKHLACTMALELLTKEFGIPIERLYVTYFGGHEEAGLDPDLECKQIWLDLG

MDEARIIPGSMKDNFWEMGDTGPCGPCSEIHFDRIGGRDAAHLVNMDDPNVLEIWNLVFIQFNRESETELKPLPKKSIDT

GMGLERLVSVLQNKMSNYDTDLFIPYFQAIQKGTGARDYTGKVGVEDVDGIDMAYRVLADHARTITIALSDGGRPDNTGR

GYVLRRILRRAVRYSHEKLGAQKGFFATLVDVVVESLGDAFPELKKDPDMVKDIINEEEEQFLKTLSRGRKILDRKIQSL

GDSKTIPGDTAWLLYDTYGFPLDLTALIAEERGMMVDVAAFEEEKKAAQVKSQGKGSGDEDHIMLDIYAIEDLRNKGIXA

TDDALKYSYSADDSGKYVFEQAVGTVLALRRERAFVDEVTTGQECGVLLDKTSFYAEQGGQSFDEGYMLREDDSVDDRME

FTVKNTQVRGGYVLHVGTVYGTLKVGDNVILHVDEARRRPIMSNHTATHILNFALREVLGEADQRGSLVAPDRLRFDFTA

KGALSTEEVRRTEEIACTMIRDAXPVYAMDTPLAAAKAIQGLRAVFDETYPDPVRVVSIGIPVADLLADPSSAAGSLTSI

EFCGGTHLRNSGHAAPFVIVSEEAIAKGIRRIVAVTGAEAQKAHRKADALRQSLSALGEKVKVQTAPNKDVQKEIADMTE

FLGMAVISQWQKDKMREALKGLKKTMDDLDRASKADVQKRVLEKTKEMIDSSPNQPLVVMEMESGASAKALNESLKLLKT

QSPQTAAMLFTVDNDAGKIICLCQVPQDVANRGLKASEWVQELCPLMDGKGGGKDMSAQATGRNTHCIEEALQMANQFAQ

LKLSKNE

>XP_016411443.1 PREDICTED: alanine--tRNA synthetase, cytoplasmic-like [Sinocyclocheilus rhinocerous]

MDSSLTAAQIREKFIDFFRRYDHQYVHSSSTIPLDDPTLLFANAGMNQFKPIFLNTIDPSHPMARLRRAANTQKCIRAGG

KHNDLDDVGKDVYHHTFFEMLGSWSFGDYFKHLACKMALELLTQEFGIPIGRLYVTYFGGNADAGLEPDLECKQIWLDLG

MEESRILPGSMKDNFWEMGDTGPCGPCSEIHYDRIGGRDAAHLVNMDDPNVLEIWNLVFIQFNRESETVLKPLPKKSIDT

GMGLERLVSVLQKKMSNYDTDLFIPYFEAIQKGTGARAYTGKVGAEDTDGIDMAYRVLADHARTITIALSDGGRPDNTGR

GYVLRRILRRAVRYSHEKLGARKGFFASLVDVVVESLGDAFPELRKDPDMVKDIINEEEAQFLKTLSRGRRILDRKVQSL

GDSKTIPGDTAWLLYDTYGFPLDLTALIAEERGMGVDMQAFEDEKKTAQLKSQGKGSGDEDHIMLDIYAIEELRNNGVAA

TDDNPKYKYTSDDNGNYEFEQAVGTVLALRRERAFVDEVTTGQECGVLLDKTSFYAEQGGQSFDEGYMLRENDTAEDRME

FTVKNTQVRGGYVLHIGTVYGTLKVGDRLTLHVDEARRRPIMSNHTATHILNFALRSVLGEADQRGSLVAPDRLRFDFTA

KGAMTTDEVRRTEENASTMIRDAKPVYALDAPLAAAKAIQGLRAVFDETYPDPVRVVSIGVPVEELLADPNSPAGSLTSI

EFCGGTHLKNSGHAAPFVIVSEEAIAKGIRRIVAVTGAEAQKAQRKADALKLELDAMAEKVKAQTTPNKDIQKEIADMTE

SLGTAVISQWRKDEMRDFLKGLKKIMDDLDRASKADVQKRVLEKTKEIIESNPNQPLIVMEMENGASAKALNESLKLLKT

NSPQTAAMLFAVDNDAGKIICLCQVPQDVANQGLKASEWVQKVCPLLDGKGGGKDMSAQATGRNTHCIQEALQLANEFAH

LKLGEN

>XP_016338710.1 PREDICTED: alanine--tRNA synthetase, cytoplasmic-like isoform X1 [Sinocyclocheilus anshuiensis]

MALHLTFLCAKAFFCCQSPEKNTTNMDSSLTAAQIREKFIDYFRRHEHQYVHSSATIPLDDPTLLFANAGMNQFKPIFLN

TIDPSHPMARLRRAANTQKCIRAGGKHNDLDDVGKDVYHHTFFEMLGSWSFGDYFKHLACEMALELLTKEFGIPIGRLYV

TYFGGHADAGLEPDLECKQIWLDLGMEESRILPGSMKDNFWEMGDTGPCGPCSEIHYDRIGGRDAAHLVNMDDPNVLEIW

NLVFIQFNRESETVLKPLPKKSIDTGMGLERLVSVLQNKMSNYDTDLFIPYFEAIQKGTGARAYTGKVGAEDTDGIDMAY

RVLADHARTITIALSDGGRPDNTGRGYVLRRILRRAVRYSHEKLGAQKGFFASLVDVVVESLGDAFPELRKDPDMVKDII

NEEEVQFLKTLSRGRRILDRKIQSLGDSKTIPGDTAWLLYDTYGFPLDLTALIAEERGMGVDMQAFEDEKKAAQLKSQGK

GSGDEDHIMLDIYAIEELRNKCVTATDDSLKYKYTSNENGNYEFEQAVGTVLALRRERAFVDEATTGQECGVLLDKTSFY

AEQGGQSFDEGYMLRENDSAEDRMEFTVKNTQVRGGYVLHIGTVYSTLKVGDRLTLHVDEARRRPIMSNHTATHILNFAL

RSVLGEADQRGSLVAPDRLRFDFTAKGAMSTDEVRRTEEIASTVIRDAKPVYALDSPLAAAKAIQGLRAVFDETYPDPVR

VVSIGVPVEELLADPNSPAGSLTSIEFCGGTHLQNSGHAAPFVIVSEEAIAKGIRRIVAVTGAEAQKAQRKADALKQELD

AMAEKVKAQTSPNKDIQKEIADMTEALGTAVTSQWRKDEMRDSLKGLKKIMDDLDRASKADVQKRVLEKTKEIIETNPNQ

PLIVMEMENGASAKALNESLKLLKTNSPQTAAMLFAVDNDVGKIICLCQVPQDVANQGLKASEWVQEVCPLLDGKGGGKD

MSAQATGRNTHCIQEALQLANEFARLKLGEN

>XP_020369373.1 alanine--tRNA synthetase, cytoplasmic isoform X1 [Rhincodon typus]

MDSTMNAGQIRQKFIDFFKCYNHEYVHSSATIPLDDPTLLFANAGMNQFKPIFLNTIDPSHPMAKLTRAANTQKCIRAGG

KHNDLDDVGKDVYHHTFFEMLGSWSFGDYFKALACEMALELLTNEFGIPIERLYVTYFGGNEKAGLEPDLECKQIWLNLG

MAEERILPGSMKDNFWEMGDTGPCGPCSEIHYDRIGGRDAAHLVNMDDPNVLEVWNLVFIQFNREVDGSLKPLAKKSIDT

GMGLERLVSILQQKMSNYDTDLFQPYFKAIQKGTGARPYTGKVGSEDVDGIDMAYRVLADHARTITIALSDGGRPDNTGR

GYVLRRILRRAVRYSHEKLNASKGFFATLVDVVVESLGDAFPELKKDPDMVKDIINDEEEQFLKTLNRGRRILERKIQSL

GENKRIPGDTAWLLYDTYGFPVDLTGLIAEEKGLIVDLEGFENEKKAAQLKSQGKGMGDDDHIMLDVNAIDDLRAKGFEA

TDDLPKYNYTSDENGNYVFESIEATVKTVRREKTFMEEVQTGQDCGLLLDRTCFYAEQGGQIYDEGYLIKVDDSSEDKTE

FTVKNVQVRGGYVLHIGTVYGQLKVGDKVRLFVDEAKRRSVMSNHTSTHMLNFALRSVLGEADQRGSLVAPDRLRFDFTA

KGAMSTQQIKKTEEITCKMIQEAKPIYAKDAALAAAKAIQGLRAVFDETYPDPVRVVSVGIPVEDMLADPSGPAGSITSV

EFCGGTHLQNSRHAGPFVIVSEEAIAKGIRRIVAVTGSEAQKALRKADMLQNFLVQLESKVQIQTSPNKEIQKEIADLTE

MISAAVIPQWQKDELRDKLKSLKKIMDDLDRASKADLLKKVREKTKQFIDTNPNQPLVVMEMEAGAPAKALNEALKLFKT

HSPQTAAMLFAVDIDAGMITCLCQVPQEVVSRGLKASDWVNHILDLLDGRGGGKDMSAQATGKNVTSLDKALKLATEFAE

LKIGDLKN

>XP_016338712.1 PREDICTED: alanine--tRNA synthetase, cytoplasmic-like isoform X2 [Sinocyclocheilus anshuiensis]

MDSSLTAAQIREKFIDYFRRHEHQYVHSSATIPLDDPTLLFANAGMNQFKPIFLNTIDPSHPMARLRRAANTQKCIRAGG

KHNDLDDVGKDVYHHTFFEMLGSWSFGDYFKHLACEMALELLTKEFGIPIGRLYVTYFGGHADAGLEPDLECKQIWLDLG

MEESRILPGSMKDNFWEMGDTGPCGPCSEIHYDRIGGRDAAHLVNMDDPNVLEIWNLVFIQFNRESETVLKPLPKKSIDT

GMGLERLVSVLQNKMSNYDTDLFIPYFEAIQKGTGARAYTGKVGAEDTDGIDMAYRVLADHARTITIALSDGGRPDNTGR

GYVLRRILRRAVRYSHEKLGAQKGFFASLVDVVVESLGDAFPELRKDPDMVKDIINEEEVQFLKTLSRGRRILDRKIQSL

GDSKTIPGDTAWLLYDTYGFPLDLTALIAEERGMGVDMQAFEDEKKAAQLKSQGKGSGDEDHIMLDIYAIEELRNKCVTA

TDDSLKYKYTSNENGNYEFEQAVGTVLALRRERAFVDEATTGQECGVLLDKTSFYAEQGGQSFDEGYMLRENDSAEDRME

FTVKNTQVRGGYVLHIGTVYSTLKVGDRLTLHVDEARRRPIMSNHTATHILNFALRSVLGEADQRGSLVAPDRLRFDFTA

KGAMSTDEVRRTEEIASTVIRDAKPVYALDSPLAAAKAIQGLRAVFDETYPDPVRVVSIGVPVEELLADPNSPAGSLTSI

EFCGGTHLQNSGHAAPFVIVSEEAIAKGIRRIVAVTGAEAQKAQRKADALKQELDAMAEKVKAQTSPNKDIQKEIADMTE

ALGTAVTSQWRKDEMRDSLKGLKKIMDDLDRASKADVQKRVLEKTKEIIETNPNQPLIVMEMENGASAKALNESLKLLKT

NSPQTAAMLFAVDNDVGKIICLCQVPQDVANQGLKASEWVQEVCPLLDGKGGGKDMSAQATGRNTHCIQEALQLANEFAR

LKLGEN

>XP_016125555.1 PREDICTED: alanine--tRNA synthetase, cytoplasmic-like [Sinocyclocheilus grahami]

MDSSLTAVQIREKFIDFFRRYDHQYVHSSSTIPLDDPTLLFANAGMNQFKPIFLNTIDPSHPMARLRRAANTQKCIRAGG

KHNDLDDVGKDVYHHTFFEMLGSWSFGDYFKHLACKMALELLTQEFGIPIGRLYVTYFGGNADAGLEPDLECKQIWLDLG

MEESRILPGSMKDNFWEMGDTGPCGPCSEIHYDRIGSRDAAHLVNMDDPNVLEIWNLVFIQFNRESETVLKPLPKKSIDT

GMGLERLVSVLQNKMSNYDTDLFIPYFEAIQKGTGARAYRGKVGAEDTDGIDMAYRVLADHARTITIALSDGGRPDNTGR

GYVLRRILRRAVRYSHEKLGAQKGFFASLVDVVVESLGDAFPELRKDPDMVKDIINEEEAQFLKTLSRGRRILDRKVQSL

GDSKTIPGDTAWLLYDTYGFPLDLTALIAEERGMGVDMQASEDEKKAAQLKSQGKGSGDEDHIMLDIYAIEELRNNGVAA

TDDNPKYKYTSDDNGNYEFEQAVGTVLALRRERAFVDEVTTGQECGVLLDKTSFYAEQGGQSFDEGYMLRENDSAEDRME

FTVKNTQVRGGYVLHIGTVYGSLKVGDRLTLHVDEARRRPIMSNHTATHILNFALRSVLGEADQRGSLVAPDRLRFDFTA

KGAMTTDEVRHTEENASTMIRDAKPVYALDAPLAAAKAIQGLRAVFDETYPDPVRVVSIGVPVEELLADPNSPAGSLTSI

EFCGGTHLKNSGHAAPFVIVSEEAIAKGIRRIVAVTGAEAQKAQRKADALKLELDAMAEKVKAQTTPNKDIQKEIADMTE

SLGTAVISQWRKDEMRDSLKGLKKIMDDLDRASKADVQKRVLEKTKEIIESNPNQPLIVMEMENGASAKALNESLKLLKT

NSPQTAAMLFAVDNDAGKIICLCQVPQDVANQGLKASEWVQKVCPLLDGKGGGKDMSAQATGRNTHCIQEALQLANEFAH

LKLGEN

>XP_006002660.1 PREDICTED: alanine--tRNA synthetase, cytoplasmic [Latimeria chalumnae]

MMDTMLTASQIRQRFIDFFKKHQHAYVHSSSTIPLDDPTLLFANAGMNQFKPIFLNTIDPSHPMAKLKRAANTQKCIRAG

GKHNDLDDVGKDVYHHTFFEMLGSWSFGDYFKQLACQLALELLTKELGIPLERLYVTYFGGSEEAGLEPDLECKQIWLDL

GMEENRILPGSMKDNFWEMGDTGPCGPCSEMHFDRIGGRDVAHLVNQDDPNVLEVWNLVFIQFNRESDGTLKPLAKKSID

TGMGLERLVSILQNKMSNYDTDLFVPYFDAIQKSTGARPYTGKVGAEDVDGIDMAYRVLADHARTITIALADGGRPDNTG

RGYVLRRILRRAVRYSHEKLNASKGFFATLVDVVVQSLGDAFPELKKDPDLVKDIINEEEAQFLKTLNRGRRILDRKIQS

LGDEKTIPGDTAWLLYDTYGFPVDLTALIAEERGLGIDMQSFEEEKKSAQLKSQGKGAGDEDHLMLDIYAIDDLHSKEIV

PTDDSPKYNYKADNGTYVFEPCVATVKAIRREKMFTEEVLTGQQCGLVLNQTCFYAEQGGQIYDEGYMVIEEDSNEDKTE

FTVKNVQVRGGYVLHVGTVYGKVKVGDRLRLYIDEARRRPIMSNHTSTHILNFALRSVLGEADQRGSLVAPDRLRFDFTA

KGAMSTEEIKKTEEIVSRMIREAKTVYAKDAPLAAAKAIQGLRAVFDETYPDPVRIVSIGIPVEDMLADPSGAAGSVTSV

EFCGGTHLCNSAHASPFVIVSEEAIAKGIRRIVAVTGAEAQKALRKADALSLALLELRAKVKVQTAPNKDIQKELADFNE

MLGTAVIAQWQKDDMREALRVLKKTMDDLDRASKLDVQKRVLEKTKQLIEEKPNQPLVVMEMERGASAKALNESLKLFKM

HSPETATMLFTVDSDAGKITCLCQVPQEVVNKGLKASEWVQHITGLLDGKGGGKDMSAQATGKNTNCTEEALRLAAEFAS

LKLGDLKN

>XP_017321765.1 PREDICTED: alanine--tRNA synthetase, cytoplasmic [Ictalurus punctatus]

MDTSLTAAQIREKFIDFFRRYEHEYVHSSSTIPLDDPTLLFANAGMNQFKPIFLNTIDPSHPMARLKRAANTQKCIRAGG

KHNDLDDVGKDVYHHTFFEMLGSWSFGDYFKQLACKMALELLTQEFGIPLERLYFTYFGGHAEAGLECDNECKQIWLDLG

VAEERILPGNMKDNFWEMGDTGPCGPCSEIHYDRIGGRDASHLVNMDDPNVLEIWNLVFIQFNRESETELKPLPKKSIDT

GMGLERLVSVLQNTMSNYDTDLFIPYFEAIQKGTGARLYTGKVGSEDQDGIDMAYRVLAAHARTITIALSDGGRPDNTGR

GYVLRRILRRAVRYAHEKLGAQKGFFASLVDVVVQSLGDAFPELKKDPDSVKDVINEEEMQFLKTLSRGRRILDRKIQNL

GDSKTIPGDTAWLLYDTYGFPLDLTVLIAEEKGMGIDLGAFEEEKKAAQLKSQGKGAGGEDHIMLDIYAIEELRNKGVPA

TDDTLKYSYTSDDNGNYEFEQAVGTVLALRRERAFVDEVSTGQECGVVLDKTSFYAEQGGQTFDEGYMLRENDSTEDRME

FTVKNTQVRGGYVLHVGTVYGTLKVGDRLTLHVDEARRRPIMSNHTSTHILNFALRSVLGEADQRGSLVAPDRLRFDFTA

KGAMSTEEVRRTEEIASAMIKDAKPVYAKEASLAAAKAIQGLRAVFDETYPDPVRVVSIGIPVEDLLADPNSTAGALTSV

EFCGGTHLQNSGHAAQFVIVSEEAIAKGIRRIVAVTGVPAQKAQRKADALRQSLSAMAEKVKAQTAPNKDVQKEIADLTE

TLGTAVISQWQKDEMRETLKGLKKIMDDLDRSSKADVQKRVLEKTKEIIESNPNQPLLVMEMESGASAKALNESLKLLKS

QSPQTAAMLFAVDNEAGKIICLCQVPQELSNQGLKANEWVQEVCPLLDGKGGGKDMSAQATGRNTQCVQEALKLANKFAQ

LKLGQN

>XP_024235121.1 LOW QUALITY PROTEIN: alanine--tRNA synthetase, cytoplasmic-like [Oncorhynchus tshawytscha]

MDSLLPAAQIREKFIDFFRCHEHQYVHSSATIPLDDPTLLFANAGMNQFKPIFLNTIDPSHPMARLKRAANTQKCIRAGG

KHNDLDDVGKDVYHHTFFEMLGSWSFGDYFKHLACTMALELLTKEFGIPIERLYVTYFGGHEEAGLDPDLECKQIWLDLG

MDEARIIPGSMKDNFWEMGDTGPCGPCSEIHFDRIGGRDAAHLVNMDDPNVLEIWNLVFIQYNRESETELKPLPKKSIDT

GMGLERLVSVLQNKMSNYDTDLFIPYFQAIQKGTGARDYTGKVGVEDVDGIDMAYRVLADHARTITIALSDGGRPDNTGR

GYVLRRILRRAVRYSHEKLGAQKGFFATLVDVVVESLGDAFPELKKDPDMVKDIINEEEEQFLKTLSRGRKILDRKIQSL

GDSKTIPGDTAWLLYDTYGFPLDLTALIAEERGMMVDVAAFEEEKKAAQVKSQGKGSGDEDHIMLDIYAIEDLRNKGIAA

TDDVLKYSYSADDSGKYVFEQAVSTVLALRRERAFVDEVTTGQECGVLLDKTSFYAEQGGQSFDEGYMLREDDSVDDRME

FTVKNTQVRGGYVLHVGTVYGTLKVGDNVILHVDEARRRPIMSNHTATHILNFALREVLGEADQRGSLVAPDRLRFDFTA

KGALSTEEVQRTEEIACTMIRDAKPVYAMDTPLAAAKAIQGLRAVFDETYPDPVRVVSIGIPVADLLADPSSAAGSLTSI

EFCGGTHLRNSGHAAPFVIVSEEAIAKGIRRIVAVTGAEAQKAHRKADALRQSLSALGANVKVQTAPNKDVQKEIADMTE

FLGMAVISQWQKDKMREALKGLKKTMDDLDRASKADVQKRVLEKTKEMIDSSPNQTLVVMEMESGASAKALNESLKLLKT

QSPQTAAMLFTVDNDAGKIICLCQVPQDVANRGLKASEWVQELCPLMDGKGGGKDMSAQATGRNTHCIEEALQMANQFAQ

LKLSENK

>XP_003439495.1 PREDICTED: alanine--tRNA synthetase, cytoplasmic [Oreochromis niloticus]

MDSSLNAAQIRQKFIDFFCRYEHQYVHSSSTIPLDDPTLLFANAGMNQFKPIFLNTIDPSHPMARLHRAANTQKCIRAGG

KHNDLDDVGKDVYHHTFFEMLGSWSFGDYFKELACKMALELLTQEFGIPLERLYVTYFGGNEDAGLEPDLECKQIWMDLG

VDEARILPGSMKDNFWEMGDTGPCGPCSEIHYDRIGGRDASHLVNMDDPNVLEIWNLVFIQFNRESETELKPLPKKSIDT

GMGLERLVSVLQNKMSNYDTDLFIPYFEAIQKGTGARPYTGKVGAEDADGIDMAYRVLADHARTITIALSDGGRPDNTGR

GYVLRRILRRAVRYSHEKLGAQRGFFASLVDVVVDSLGEAFPELKKDPEMVKDIINEEEAQFLKTLSRGRRILDRKIMSL

ADTKTIPGDTAWLLYDTYGFPLDLTSLIAEEKGMEVDLAGFEEEKKAAQLKSQGKGAGDEDHIMLDIYAIEELRNKQIPA

TDDSPKYNYTSDENGNYQFQQASATVLALRRDRAFCDEVTTGQECGVVLDQTSFYAEQGGQTFDEGYLLREDDSTEDRME

FSVKNTQVRGGYVLHVGTVYGTLKVGDRVTLHVDEARRRPIMSNHTATHILNFALRGVLGEADQRGSLVAPDRLRFDFTA

KGALSTDEIRRTEEIACTMIKDAKTVYAMEATLAEAKAIQGLRAVFDETYPDPVRVVSIGIPVPELLADPNSAAGSLTSI

EFCGGTHLQNSGHAAPFVIVSEEAIAKGIRRIVAVTGTEAQKAQRKADALRQSLSALGDKVKQQTSPNKDTQKEIADMTE

SIGTAVISQWQKDEMRETLKGLKKTMDDLDRNYKADIQKRVLEKTKEVIESNPNQPLLVMEMETGASAKALNESLKLLKS

QSPQTAAMLFTVDPDAGKITCLCQVPQDVANRGLKASQWVQELCPLLDGKGGGKDMSAQATGRNTQCLQEALQMANEFAR

LKLGEN

>XP_021425557.1 alanine--tRNA synthetase, cytoplasmic [Oncorhynchus mykiss]

MDSLLPAAQIREKFIDFFRCHEHQYVHSSATIPLDDPTLLFANAGMNQFKPIFLNTIDPSHPMARLKRAANTQKCIRAGG

KHNDLDDVGKDVYHHTFFEMLGSWSFGDYFKHLACTMALELLTKEFGIPIERLYVTYFGGHEEAGLDPDLECKQIWLDLG

MDEARIIPGSMKDNFWEMGDTGPCGPCSEIHFDRIGGRDAAHLVNMDDPNVLEIWNLVFIQYNRESETELKPLPKKSIDT

GMGLERLVSVLQNKMSNYDTDLFIPYFQAIQKGTGARDYTGKVGVEDVDGIDMAYRVLADHARTITIALSDGGRPDNTGR

GYVLRRILRRAVRYSHEKLGAQKGFFATLVDVVVESLGDAFPELKKDPDMVKDIINEEEEQFLKTLSRGRKILDRKIQSL

GDSKTIPGDTAWLLYDTYGFPLDLTALIAEERGMMVDVAAFEEEKKAAQVKSQGKGSGDEDHIMLDIYAIEDLRNKGIAA

TDDVLKYSYSADDSGKYVFEQAVSTVLALRRERAFVDEVTTGQECGVLLDKTSFYAEQGGQSFDEGYMLREDDSVDDRME

FTVKNTQVRGGYVLHVGTVYGTLKVGDNVILHVDEARRRPIMSNHTATHILNFALREVLGEADQRGSLVAPDRLRFDFTA

KGALSTEEVRRTEEIACTMIRDAKPVYAMDTPLAAAKAIQGLRAVFDETYPDPVRVVSIGIPVADLLADPSSTAGSCTSI

EFCGGTHLRNSGHAAPFVIVSEEAIAKGIRRIVAVTGAEAQKAHRKADALRQSLSALGDKVKVQTAPNKDVQKEIADMTE

FLGMAVISQWQKDKMREALKGLKKTMDDLDRASKADVQKRVLEKTKEMIDSSPNQPLVVMEMESGASAKALNESLKLLKT

QSPQTAAMLFTVDNDAGKIICLCQVPQDVANRGLKASEWVQELCPLMDGKGGGKDMSAQATGRNTHCIEEALQMANQFAQ

LKLSENE

>XP_016406040.1 PREDICTED: alanine--tRNA synthetase, cytoplasmic-like isoform X1 [Sinocyclocheilus rhinocerous]

MALHLTFLCAKAFFCCQSPEKNTTSMDSSLTAAQIREKFIDFFRHHEHQYVHSSATVPLDDPTLLFANAGMNQFKPIFLN

TIDPSHPMARLRRAANTQKCIRAGGKHNDLDDVGKDVYHHTFFEMLGSWSFGDYFKHLACEMALELLTKEFGIPIGRLYV

TYFGGHADAGLEPDLECKQIWLDLGMEESRILPGSMKDNFWEMGDTGPCGPCSEIHYDRIGGRDAAHLVNMDDPNVLEIW

NLVFIQFNRESETVLKPLPKKSIDTGMGLERLVSVLQNKMSNYDTDLFIPYFEAIQKGTGARAYTGKVGAEDTDGIDMAY

RVLADHARTITIALSDGGRPDNTGRGYVLRRILRRAVRYSHEKLGAQKGFFASLVDVVVESLGDAFPELRKDPDMVKDII

NEEEAQFLKTLSRGRRILDRKIQSLGDSNTIPGDTAWLLYDTYGFPLDLTALIAEERGMGVDIQAFEDEKKAAQLKSQGK

GSGDEDHIMLDIYAIEELRNKGVTATDDSLKYKYTSNENGNYEFEQAVGTVLALRRERAFVDEATTGQECGVLLDKTSFY

AEQGGQSFDEGYMLRENDSAEDRMEFTVKNTQVRGGYVLHIGTVYGTLKVGDRLTLHVDEARRRPIMSNHTATHILNFAL

RSVLGEADQRGSLVAPDRLRFDFTAKGAMSTDEVRRTEEIASTVIRDAKPVYALDSPLAAAKAIQGLRAVFDETYPDPVR

VVSIGIPVEELLADPNSPAGSLASIEFCGGTHLQNSGHAAPFVIVSEEAIAKGIRRIVAVTGAEAQKAQRKADALKQELD

AMAEKVKAQTSPNKDIQKEIADMTEALGTAVTSQWRKDEMRDSLKGLKKIMDDLDRASKADVQKRVLEKTKEIIETNPNQ

PLIVMEMENGASAKALNESLKLLKTNSPQTAAMLFAVDNDAGKIICLCQVPQDVANQGLKAFEWVQEVCPLLDGKGGGKD

MSAQATGRNTHCIQEALQLANEFARLKLGENEGKREIAL

>XP_016406041.1 PREDICTED: alanine--tRNA synthetase, cytoplasmic-like isoform X2 [Sinocyclocheilus rhinocerous]

MDSSLTAAQIREKFIDFFRHHEHQYVHSSATVPLDDPTLLFANAGMNQFKPIFLNTIDPSHPMARLRRAANTQKCIRAGG

KHNDLDDVGKDVYHHTFFEMLGSWSFGDYFKHLACEMALELLTKEFGIPIGRLYVTYFGGHADAGLEPDLECKQIWLDLG

MEESRILPGSMKDNFWEMGDTGPCGPCSEIHYDRIGGRDAAHLVNMDDPNVLEIWNLVFIQFNRESETVLKPLPKKSIDT

GMGLERLVSVLQNKMSNYDTDLFIPYFEAIQKGTGARAYTGKVGAEDTDGIDMAYRVLADHARTITIALSDGGRPDNTGR

GYVLRRILRRAVRYSHEKLGAQKGFFASLVDVVVESLGDAFPELRKDPDMVKDIINEEEAQFLKTLSRGRRILDRKIQSL

GDSNTIPGDTAWLLYDTYGFPLDLTALIAEERGMGVDIQAFEDEKKAAQLKSQGKGSGDEDHIMLDIYAIEELRNKGVTA

TDDSLKYKYTSNENGNYEFEQAVGTVLALRRERAFVDEATTGQECGVLLDKTSFYAEQGGQSFDEGYMLRENDSAEDRME

FTVKNTQVRGGYVLHIGTVYGTLKVGDRLTLHVDEARRRPIMSNHTATHILNFALRSVLGEADQRGSLVAPDRLRFDFTA

KGAMSTDEVRRTEEIASTVIRDAKPVYALDSPLAAAKAIQGLRAVFDETYPDPVRVVSIGIPVEELLADPNSPAGSLASI

EFCGGTHLQNSGHAAPFVIVSEEAIAKGIRRIVAVTGAEAQKAQRKADALKQELDAMAEKVKAQTSPNKDIQKEIADMTE

ALGTAVTSQWRKDEMRDSLKGLKKIMDDLDRASKADVQKRVLEKTKEIIETNPNQPLIVMEMENGASAKALNESLKLLKT

NSPQTAAMLFAVDNDAGKIICLCQVPQDVANQGLKAFEWVQEVCPLLDGKGGGKDMSAQATGRNTHCIQEALQLANEFAR

LKLGENEGKREIAL

>XP_014852801.1 PREDICTED: alanine--tRNA synthetase, cytoplasmic [Poecilia mexicana]

MDSSLTAAQIRQKFIDFFIRYEHQYVHSSSTIPLDDPTLLFANAGMNQFKPIFLNTIDPSHPMAKLRRAANTQKCIRAGG

KHNDLDDVGKDVYHHTFFEMLGSWSFGDYFKQLACKMALELLTEEFGIPIDRLYVTYFGGNADAGLEPDLECKQIWIDLG

VAEERILPGSMKDNFWEMGDTGPCGPCSEIHYDRIGGRDASHLVNMDDPNVLEIWNLVFIQFNRESETELKPLPKKSIDT

GMGLERLVSVLQNKMSNYDTDLFVPYFEAIQKGTGARPYTGKVGADDVDGIDMAYRVLADHARTITISLSDGGRPDNTGR

GYVLRRILRRAVRYSHEKLGAQRGFFASLVDVVVDSLGDAFPELRKDPEMVKDVINEEETQFLKTLSRGRRILDRKIMSL

GDCKTIPGDTAWLLYDTYGFPLDLTSLIAEEKGMEVNLAAFEEEKKAAQLKSQGKGAGGEDHIMLDIYAIEELRNQQVPA

TDDSPKYRYASEENGTYEFQPTSATVLALRRDRAFCQEVTTGQECGVLLDQTSFYAEQGGQTFDEGYMLREDDSSEDRME

FTVKNTQVRGGYVLHIGTVYGTLKVGDRVTLHIDEARRRPIMSNHTATHILNFALRGVLGEADQRGSLVAPDRLRFDFTA

KGALSTGEIRRTEEIACAMIKDAKPVYAMEAPLAQAKAIQGLRAVFDETYPDPVRVVSIGTPVQELLDDPNSPAGSLTSI

EFCGGTHLQNSGHAAPFVIVSEEAIAKGIRRIVAVTGAEAQKAHRKADALRLSLSALGDKVKQQTSPNKDVQKEIADMTE

SIGTALISQWQKDEMRETLKALKKTMDDLDRSYKADIQKRVLEKTKEEIENNPNQPLLIMEMETGASAKALNESLKLLKS

QSPQTAAMLFTVDPDAGKITCLCQVPQDVANRGLKASQWVQELCPLLDGKGGGKDMSAQATGRNTQCLQEALQMANEFAR

LKLGEN

>XP_005795856.1 alanine--tRNA synthetase, cytoplasmic [Xiphophorus maculatus]

MDSSLSAAQIRQKFIDFFIRYEHQYVHSSSTIPLDDPTLLFANAGMNQFKPIFLNTIDPSHPMAKLRRAANTQKCIRAGG

KHNDLDDVGKDVYHHTFFEMLGSWSFGDYFKQLACKMALELLTEEFGIPIDRLYVTYFGGNADAGLEPDLECKQIWMDLG

VDEARILPGSMKDNFWEMGDTGPCGPCSEIHYDRIGGRDASHLVNMDDPNVLEIWNLVFIQFNRESETELKPLPKKSIDT

GMGLERLVSVLQNKMSNYDTDLFVPYFEAIQKGTGARPYTGKVGADDVDGIDMAYRVLADHARTITISLSDGGRPDNTGR

GYVLRRILRRAVRYSHEKLGAQRGFFASLVDVVVDSLGDAFPELRKDPEMVKDVINEEETQFLKTLSRGRRILDRKIMSL

GDCKTIPGDTAWLLYDTYGFPLDLTSLIAEEKGMEVNLAAFEEEKKAAQLKSQGKGAGGEDHIMLDIYAIEELRNQQVPA

TDDSPKYRYASEENGNYEFRPTSATVLALRRDRAFCQEVTTGQECGVLLDQTSFYAEQGGQTFDEGYMLREDDSSEDRME

FTVKNTQVRGGYVLHIGTVYGTLKVGDRVTLHIDEARRRPIMSNHTATHILNFALRCVLGEADQRGSLVAPDRLRFDFTA

KGALSTGEIRRTEEIACAMIKEAKPVYAMETPLAEAKAIQGLRAVFDETYPDPVRVVSIGIPVQELLDDPNSPAGSLTSI

EFCGGTHLQNSAHAAPFVIVSEEAIAKGIRRIVAVTGAEAQKAHRKADTLHLSLSALGDKVKQQTCPNKDVQKEIADMTE

SIGTALISQWQKDEMRETLKALKKTMDDLDRSYKADIQKRVLEKTKEEIENKPNQPLLIMEMETGASAKALNESLKLLKS

QSPQTAAMLFTVDPDAGKITCLCQVPQDVANRGLKASEWVQELCPLLDGKGGGKDMSAQATGRNTQCLQEALQMANEFAR

LKLGEN

>XP_006797458.1 PREDICTED: alanine--tRNA synthetase, cytoplasmic-like [Neolamprologus brichardi]

MDSSLNAAQIRQKFIDFFCRYEHPYVHSSSTIPLDDPTLLFANAGMNQFKPIFLNTIDPSHPMARLRRAANTQKCIRAGG

KHNDLDDVGKDVYHHTFFEMLGSWSFGDYFKELACKMALELLTQEFGIALERLYVTYFGGNEDAGLEPDLECKQIWMDLG

VDEARILPGSMKDNFWEMGDTGPCGPCSEIHYDRIGGRDASHLVNMDDPDVLEIWNLVFIQFNRESETELKPLPKKSIDT

GMGLERLVSVLQNKMSNYDTDLFIPYFEAIQKGTGARPYTGKVGAEDADGIDMAYRVLADHARTITIALSDGGRPDNTGR

GYVLRRILRRAVRYSHEKLGAQRGFFASLVDVVVDSLGEAFPELKKDPEMVKDIINEEEAQFLKTLSRGRRILDRKIMSL

ADTKTIPGDTAWLLYDTYGFPLDLTSLIAEEKGMEVDLAGFEEEKKAAQLKSQGKGAGDEDHIMLDIYAIEELRNKQIPA

TDDSPKYNYTSDENGNYQFQQTSATVLALRHDRAFCDEVTTGQECGVVLDQTSFYAEQGGQTFDEGYMLREDDSTEDRME

FSVKNTQVRGGYVLHVGTVYGTLKVGDRVTLHVDEARRRPIMSNHTATHILNFALRGVLGEADQRGSLVAPDRLRFDFTA

KGALSTDEIRRSEEIACTMIKDAKPVYAMEATLAEAKAIQGLRAVFDETYPDPVRVVSVGIPVPELLADPNSAAGSLTSI

EFCGGTHLQNSSHAAPFVIVSEEAIAKGIRRIVAVTGTEAQKAQRKADALRQYLSALGDKVKQQTSPNKDIQKEIADMTE

SIGTAVISQWQKDEMRETLKGLKKTMDDLDRNYKADIQKRVLEKTKEVIESNPNQPLLVMEMETGASAKALNESLKLLKS

QSPQTAAMLFTVDPDAGKITCLCQVPQDVANRGLKASQWVQELCPLLDGKGGGKDMSAQATGRNTQCLQEALQMANEFAR

LKLGEN

>XP_008297854.1 PREDICTED: alanine--tRNA synthetase, cytoplasmic [Stegastes partitus]

MDSSLSAAQIREKFIDFFRRYEHQYVHSSSTIPLDDPTLLFANAGMNQFKPIFLNTIDPSHPMARLHRAANTQKCIRAGG

KHNDLDDVGKDVYHHTFFEMLGSWSFGDYFKQLACKMALELLTQEFGIPIDRLYVTYFGGNEDAGLEPDLECKQIWIDLG

MEEARILPGSMKDNFWEMGDTGPCGPCSEIHFDRIGGRDASHLVNMDDPNVLEIWNLVFIQFNRESETELKPLPKKSIDT

GMGLERLVSVLQNKMSNYDTDLFVPYFEAIQKGTGARSYTGKVGAEDTDGIDMAYRVLADHARTITIALSDGGRPDNTGR

GYVLRRILRRAVRYSHEKLGAQRGFFASLVDVVVDSLGEAFPELKKDPEMVKDIINEEEAQFLKTLSRGRRILDRKIMSL

GDSKTIPGDTAWLLYDTYGFPLDLTSLIAEEKGMDVDMAAFEEEKKAAQLKSQGKGAGYEDHIMLDIYAIEELRNKQTPA

TDDAPKYKYTSDENGNYEFQQASATVLALRRDRAFCDEVTTGQECGVVLDQTSFYAEQGGQTFDEGYMLREDDSTEDRME

FTVKNTQVRGGYVLHVGTVYGTLKVGDRVTLHVDEARRRPIMSNHTATHILNFALRGVLGEADQRGSLVAPDRLRFDFTA

KGALSTGEVRRTEEMACAMIRDAKPVYAMDAPLAAAKAIQGLRAVFDETYPDPVRVVSVGIPVQELLDDPTSAAGSLTSI

EFCGGTHLQNSGHAAPFVIVSEEAIAKGIRRIVAVTGTEAQKAQRKADALRQSLSALGDKVKQQTTPNKDIQKEIADMTE

SIGTAVISQWQKDEMRESLKGLKKTMDDLDRNYKADIQKRVLEKTKEMIESSPNQPLLIMEMETGASAKALNESLKLLKS

QSPQTAAMLFTVDPDAGKITCLCQVPQDVANRGLKASEWVQELCPLLDGKGGGKDMSAQATGRNMLCLQEALQIANEFAR

LKLGEN

>XP_023138098.1 alanine--tRNA synthetase, cytoplasmic [Amphiprion ocellaris]

MDSSLSAAQIREKFIDFFKRYEHQYVHSSSTIPLDDPTLLFANAGMNQFKPIFLNTIDPSHPMARLRRAANTQKCIRAGG

KHNDLDDVGKDVYHHTFFEMLGSWSFGDYFKQLACQMALELLTKEFGIPIDRLYVTYFGGNEDAGLEPDLECKQIWIDLG

VEEARILPGSMKDNFWEMGDTGPCGPCSEIHYDRIGGRDASHLVNMDDPNVLEIWNLVFIQFNRESETELKPLPKKSIDT

GMGLERLVSVLQNKMSNYDTDLFVPYFEAIQKGTGARPYTGKVGAEDTDGIDMAYRVLADHARTITIALSDGGRPDNTGR

GYVLRRILRRAVRYSHEKLGAQKGFFASLVDVVVDSLGEAFPELKKDPEMVKDIINEEEAQFLKTLSRGRRILDRKIMSL

GDSKTIPGDTAWLLYDTYGFPLDLTSLIAEEKGMDVDMAAFEEEKKAAQLKSQGKGAGDEDHIMLDIYAIEELRNKQTPA

TDDSPKYKYTSDNNGDYDFQQASATVLALRRDRAFCDEVTTGQECGVLLDQTSFYAEQGGQTFDEGYLLREDDSTEDRME

FTVKNTQVRGGYVLHVGTVYGTLKVGDRVTLHVDEARRRPIMSNHTATHILNFALRGVLGEADQRGSLVAPDRLRFDFTA

KGALSTGEVRRTEEIACAMIRDAKPVYAMEAPLAEAKAIQGLRAVFDETYPDPVRVVSIGIPVQELLDDPTSAAGSLTSI

EFCGGTHLQNSGHAAPFVIVSEEAIAKGIRRIVAVTGTEAQKAQRKADALRQSLSALGDKVKQQTTPNKDIQKEIADMTE

SIGTAVISQWQKDEMRETLKGMKKTMDDLDRNYKADIQKRVLDKTKEMIESSPNQPLLIMEMETGASAKALNESLKLLKA

QSPQTAAMLFTVDTDAGKITCLCQVPQDVANRGLKASEWVQELCPLLDGKGGGKDMSAQATGRNTQCLQEALQMANNFAR

LKLGEN

>XP_007910546.1 PREDICTED: alanine--tRNA synthetase, cytoplasmic [Callorhinchus milii]

MNSTLTGRQIRQQFIDFFKRHNHEYVHSSATIPLDDPTLLFANAGMNQFKPIFLNTIDPSHPMAKLTRAANTQKCIRAGG

KHNDLDDVGKDVYHHTFFEMLGSWSFGDYFKHLACEMAFELLTKEFGIPIERLYVTYFGGDEKAELEPDLECKQIWVNLG

MAEERVLPGSMKDNFWEMGDTGPCGPCSEMHFDRIGGRDAAHLVNMDDPNVLEIWNLVFIQFNRETDGSLKPLAKKSIDT

GLGLERLVSVLQGKMSNYDTDLFVPYFEAIQKRTGSRPYTGKVGAEDVDGIDMAYRVLADHARTITIALADGGRPDNTGR

GYVLRRILRRAVRYSHEKLNAQKGFFASLVDVVVDSLGDAFPELKKDPDMVKDIVNEEEEQFLKTLSRGRRILERKIHSL

TDGQTIPGDTAWLLYDTYGFPVDLTALIAEEKGLRVDLESFEEEKKAAQLKSHGKGTGYEDHIMLDVNAIDELRAKGFEV

TDDSPKYSYRAENNGNYVFTSVEATVKAIRREKTFVDEVTTGQDCGVLLDGTCFYAEQGGQIYDEGYLVKEDDSSDDKTE

FSVKNVQVRGGYILHIGTVYGHLKVGDQVHLFIDEARRRPIMSNHTSTHILNFSLRSVLGEADQRGSLVAPDRLRFDFTA

KGAMSTQEIRQTEEITNRIIQEGKPIYSKDTPLAVAKAIQGLRAVFDETYPDPVRVVSVGIPVEDMLADPSGPAGSVTSV

EFCGGTHLQNAGHVGSFVIVSEEAIAKGIRRIVAVTGPEAQKALRKAEALEKALVALGSKVHGQTAPNKEIQKEIADLTE

MIGTAVIPQWQKDEMREKQKSLKKVMDDLDRASKANTQKKVLDKTKQLIESNPNQPLVVMELEAGASAKVLNEALKLFKT

TSPQTAAMLFAVDSEAGRITCLCQVPQEVASRGLKANEWIGHISGLLDGKGGGKDLSAQATGKNINCLDKALNLATEFAK

LKLGGIKN

>XP_018559078.1 PREDICTED: alanine--tRNA synthetase, cytoplasmic isoform X1 [Lates calcarifer]

MDSSLSAAQIREKFIDFFRRHEHQYVHSSSTIPLDDPTLLFANAGMNQFKPIFLNTIDPSHPMARLRRAANTQKCIRAGG

KHNDLDDVGKDVYHHTFFEMLGSWSFGDYFKQLACKMALELLTEEFGISIDRLYVTYFGGNADAGLEPDLECKQIWIDLG

MDEARILPGSMKDNFWEMGDTGPCGPCSEIHFDRIGGRDAAHLVNMDDPNVLEIWNLVFIQFNRESETELKPLPKKSIDT

GMGLERLVSVLQNKMSNYDTDLFIPYFEAIQKGTGARPYTGKVGAEDADGIDMAYRVLADHARTITIALSDGGRPDNTGR

GYVLRRILRRAVRYSHEKLGAQRGFFASLVDVVVDSLGDAFPELKKDPEMVKDIINEEETQFLKTLSRGRRILDRKIMSL

GDSKTIPGDTAWLLYDTYGFPLDLTSLIAEEKGMGVDMAAFEEEKKAAQLKSQGKGAGDEDHIMLDIYAIEELRNKQIPA

TDDSPKYKYTSEENGNYEFQQASATVLALRRDRAFCDEVTTGQECGVLLDQTSFYAEQGGQTFDEGYMLREDDTTEDRME

FTVKNTQVRGGYVLHVGTVYGTLKVGDRVTLHVDEARRRPIMSNHTATHILNFALRGVLGEADQRGSLVAPDRLRFDFTA

KGALSTGEIRRTEEIACAMIRDAKPVYAMEAPLAEAKAIQGLRAVFDETYPDPVRVVSIGIPVQDLLDDPSSAAGSLTSI

EFCGGTHLQNSGHAAPFVIVSEEAIAKGIRRIVAVTGTEAQKAQRKADALLQSLAALGDKVKQQTAPNKDIQKEIADMTE

SIGTAVISQWQKDEMRETLKGLKKTMDDLDRNYKADIQKRVLEKTKEVIESSPNQTLLIMEMETGASAKALNESLKLLKS

QSPQTAAMLFTVDPDAGKITCLCQVPQDLANRGLKASEWVQELCPLLDGKGGGKDMSAQATGRNTQCLQEALQMANEFAR

LKLGEN

>XP_004564645.1 alanine--tRNA synthetase, cytoplasmic [Maylandia zebra]

MDSSLNAAQIRQKFIDFFCRYEHPYVHSSSTIPLDDPTLLFANAGMNQFKPIFLNTIDPSHPMARLRRAANTQKCIRAGG

KHNDLDDVGKDVYHHTFFEMLGSWSFGDYFKELACKMALELLTQEFGIALERLYVTYFGGNEDAGLEPDLECKQIWMDLG

VDEARILPGSMKDNFWEMGDTGPCGPCSEIHYDRIGGRDASHLVNMDDPDVLEIWNLVFIQFNRESETELKPLPKKSIDT

GMGLERLVSVLQNKMSNYDTDLFIPYFEAIQKGTGARPYTGKVGAEDADGIDMAYRVLADHARTITIALSDGGRPDNTGR

GYVLRRILRRAVRYSHEKLGAQRGFFASLVDVVVDSLGEAFPELKKDPEMVKDIINEEEAQFLKTLSRGRRILDRKIMSL

ADTKTIPGDTAWLLYDTYGFPLDLTSLIAEEKGMEVDLAGFEEEKKAAQLKSQGKGAGDEDHIMLDIYAIEELRNKQIPA

TDDSPKYNYTSDENGNYQFQQTSATVLALRHDRAFCDEVTTGQECGVVLDQTSFYAEQGGQTFDEGYMLREDDSTEDRME

FSVKNTQVRGGYVLHVGTVYGTLKVGDRVTLHVDEARRRPIMSNHTATHILNFALRGVLGEADQRGSLVAPDRLRFDFTA

KGALSTDEIHRTEEIACTMIKDAKPVYAMEATLAEAKAIQGLRAVFDETYPDPVRVVSVGIPVPELLADPNSAAGSLTSI

EFCGGTHLQNSGHAAPFVIVSEEAIAKGIRRIVAVTGTEAQKAQRKADALRQSLSALGDKVKQQTSPNKDIQKEIADMTE

SIGTAVISQWQKDEMRETLKGLKKTMDDLDRNYKADIQKRVLEKTKEVIESNPNQPLLIMEMETGASAKALNESLKLLKS

QSPQTAAMLFTVDPDAGKITCLCQVPQDVANRGLKASQWVQELCPLLDGKGGGKDMSAQATGRNTQCLQEALQMANEFAR

LKLGEN

>XP_022061274.1 alanine--tRNA synthetase, cytoplasmic isoform X1 [Acanthochromis polyacanthus]

MDSSLSAAQIREKFIDFFKRHEHQYVHSSSTIPLDDPTLLFANAGMNQFKPIFLNTIDPSHPMARLHRAANTQKCIRAGG

KHNDLDDVGKDVYHHTFFEMLGSWSFGDYFKQLACQMALELLTKELGIPIDRLYVTYFGGNEDAGLEPDLECKQIWMDLG

VEEARILPGSMKDNFWEMGDTGPCGPCSEIHYDRIGGRDASHLVNMDDPNVLEIWNLVFIQFNRESETELKPLPKKSIDT

GMGLERLVSVLQNKMSNYDTDLFVPYFEAIQKGTGARPYTGKVGAEDTDGIDMAYRVLADHARTITIALSDGGRPDNTGR

GYVLRRILRRAVRYSHEKLGAQRGFFASLVDVVVDSLGEAFPELKKDPEMVKDIINEEEEQFLKTLSRGRRILDRKIMSL

GDSKTIPGDTAWLLYDTYGFPLDLTSLIAEEKGMDVDMAAFEEEKKAAQLKSQGKGAGDEDHIMLDIYAIEELRNKQTPA

TDDSPKYKYTSDDSGSYDFQQASATVLALRRDRAFCDEVTTGQECGVLLDQTSFYAEQGGQTFDEGYLLREDDSTEDRME

FTVKNTQVRGGYVLHVGTVYGTLKVGDRVTLHVDEARRRPIMSNHTATHILNFALRGVLGEADQRGSLVAPDRLRFDFTA

KGALSTGEVRRTEEMACAMIRDAKPVYAMEAPLAEAKAIQGLRAVFDETYPDPVRVVSIGIPVQKLLDDPTSAAGSLTSI

EFCGGTHLQNSGHAAPFVIVSEEAIAKGIRRIVAVTGTEAQKAQRKADALRQSLSALGDKVKQQTTPNKDIQKEIADMTE

SIGTAVISQWQKDEMRETLKGMKKTMDDLDRNYKADIQKRVLEKTKEVIESSPNQPLLIMEMETGASAKALNESLKLLKS

KSPQTAVMLFTVDADAGKITCLCQVPQDVANRGLKASEWVQELCPLLDGKGGGKDMSAQATGRNTQCLQEVLQMANNFAR

LKLGEN

>KTF84216.1 hypothetical protein cypCar_00033421, partial [Cyprinus carpio]

LAHCVFVYIYLQSTEKNTTSMDSSLTAAQIREKFIDFFRRHEHQYVHSSSTIPLDDPTLLFANAGMNQFKPIFLNTIDPS

HPMARLHRAANTQKCIRAGGKHNDLDDVGKDVYHHTFFEMLGSWSFGDYFKHLACKMALELLTKEFGIPIGRLYVTYFGG

HADAGLEPDLECKQIWLDLGMEESRILPGSMKDNFWEMGDTGPCGPCSEIHYDRIGGRDAAHLVNMDDPNVLEIWNLVFI

QFNRESETVLKPLPKKSIDTGMGLERLVSVLQNKMSNYDTDLFIPYFEAIQKGTGARAYTGKVGAEDEDGIDMAYRVLAD

HARTITIALSDGGRPDNTGRGYVLRRILRRAVRYSHEKLGAHKGFFASLVDVVVESLGDAFPELRKDPDMVKDIINEEEE

QFLKTLSRGRRILDRKIQSLGDSKTIPGDTAWLLYDTYGFPLDLTALIAEERGMGVDMQAFEDEKKAAQLKSQGKGSGDE

DHIMLDIYAIEELRNKGVAATDDSPKYKYTSNDNGNYEFEQAVGTVLALRRERAFVDEVTTGQECGVLLDKTSFYAEQGG

QSFDEGYMLRENDSAEDRMEFTVKNTQVRGGYVLHIGTVYGTLKARRRPIMSNHTATHILNFALRSVLGEADQRGSLVAP

DRLRFDFTAKGAMSTDEVRRTEENASTMIRDAKPVYALDAPLAAAKAIQGLRAVFDETYPDPVRVVSIGVPVEELLADPN

SPAGSLTSIEFCGGTHLQNSGHAAPFVIVSEEAIAKGIRRIVAVTGAEAQKAQRKADALKLELDAMAEKVKAQNTPNKDI

QKEIADMTEALGTAVISQWRKDEMRDSLKGLKKIMDDLDRASKADVQKRVLEKTKEIIESNPNKPLIVMEMENGASAKAL

NESLKLLKTNSPQTAAMLFAVDNDAGKIICLCQVPQDVANRGLKASEWVQEVCPLLDGKGGGKDMSAQATGRNTHCIQEA

LQLANEFARLKLGEN

>XP_020459968.1 alanine--tRNA synthetase, cytoplasmic [Monopterus albus]

MDSSLSAAQIREKFIDFFRRYEHQYVHSSATIPLDDPTLLFANAGMNQFKPIFLNTIDPSHPMARLRRAANTQKCIRAGG

KHNDLDDVGKDVYHHTFFEMLGSWSFGDYFKHLACKMALELLTQEFGIPIDRLYVTYFGGDDEAGLEPDLECKQIWIDLG

VEDVRILPGSMKDNFWEMGDTGPCGPCSEIHYDRIGGRDASHLVNMDDPNVLEIWNLVFIQFNRESETVLNPLPKKSIDT

GMGLERLVSVLQNKMSNYDTDLFIPYFEAIQKGTGARPYTGKVGAEDADGIDMAYRVLADHARTITIALSDGGRPDNTGR

GYVLRRILRRAVRYSHEKLGAHRGFFATLVDVVVNSLGDAFPELKKDPEMVKDVINEEEAQFLKTLSRGRRILDRKIMSL

GDSKTIPGDTAWLLYDTYGFPLDLTSLIAEEKGMSVDLAAFEEEKKAAQIKSQGRGAGDQDHIMLDIYAIEELRNKKIPS

TDDSPKYKYTSDENGSYEFQQASATVLALRCDRTFCNEVTTGQECGVVLDQTSFYAEQGGQTFDEGYMLREDDNTEDRME

FTVKNTQVRGGYVLHVGTIYGTLKVGDRVTLHVDEARRRPIMSNHTATHILNFALRKVLGEADQRGSLVAPDRLRFDFTA

KGAMTTEQIRQTEESACDMIRDAKPVYAMEAPLANAKAINGLRAVFDETYPDPVRVVSIGIPVQELLADPSSPAGSLTSI

EFCGGTHLQNSGHAAPFVIVSEEAIAKGIRRIVAVTGTEAQKAQRKAHALHQILSALGDKVKQQTAPNKDIQKEIADMTE

LNGTAVISQWQKDEMRETLKGLKKIMDDLDRNCKADIQKRVLEKTKEVIKSSPNQPLLVMEMETGASPKALNESLKLLKS

NSPQTAVMLFTVDPDAGKITCLCQVPQDVANRGLRASAWVQELCPLLDGKGGGKDLSAQATGRNMQCLQEALQMANEFAR

LKLGEN

>XP_020311999.1 alanine--tRNA synthetase, cytoplasmic [Oncorhynchus kisutch]

MDSLLPAAQIREKFIDFFRCHEHQYVHSSATIPLDDPTLLFANAGMNQFKPIFLNTIDPSHPMARLKRAANTQKCIRAGG

KHNDLDDVGKDVYHHTFFEMLGSWSFGDYFKHLACTMALELLTKEFGIPIERLYVTYFGGHEEAGLDPDLECKQIWLDLG

MDEARIIPGSMKDNFWEMGDTGPCGPCSEIHFDRIGGRDAGHLVNMDDPNVLEIWNLVFIQYNRESETELKPLPKKSIDT

GMGLERLVSVLQNKMSNYDTDLFIPYFQAIQKGTGARDYTGKVGLEDVDGIDMAYRVLADHARTITIALSDGGRPDNTGR

GYVLRRILRRAVRYSHEKLGAQKGFFATLVDVVVESLGDAFPELKKDPDMVKDIINEEEEQFLKTLSRGRKILDRKIQSL

GDSKTIPGDTAWLLYDTYGFPLDLTALIAEERGMMVDVAAFEEEKKAAQVKSQGKGSGDEDHIMLDIYAIEDLRNKGIAA

TDDVLKYSYSADDSGKYVFEQAVSTVLALRRERAFVDEVTTGQECGVLLDKTSFYAEQGGQSFDEGYMLREDDSVDDRME

FTVKNTQVRGGYVLHVGTVYGTLKVGDNVILHVDEARRRPIMSNHTATHILNFALRDVLGEADQRGSLVAPDRLRFDFTA

KGALSTEEVRRTEEIACTMIRDAKPVYAMDTPLAAAKAIQGLRAVFDETYPDPVRVVSIGIPVADLLADPSSTDGFFTSI

EFCGGTHLRNSGHAAPFVIVSEEAIAKGIRRIVAVTGAEAQKAHRKADALRQSLSALGDNVKVQTAPNKDVQKEIADMTE

FLGMAVISQWQKDKMREALKGLKKTMDDLDRASKADVQKRVLEKTKEMIDSSPNQPLVVMEMESGASAKALNESLKLLKT

QSPQTAAMLFTVDNDAGKIICLCQVPQDVANRGLKASEWVQELCPLMDGKGGGKDMSAQATGRNTHCIEEALQMANQFAQ

LKLSENE

>XP_007567599.1 PREDICTED: alanine--tRNA synthetase, cytoplasmic [Poecilia formosa]

MDSSLTAAQIRQKFIDFFIRYEHQYVHSSSTIPLDDPTLLFANAGMNQFKPIFLNTIDPSHPMAKLRRAANTQKCIRAGG

KHNDLDDVGKDVYHHTFFEMLGSWSFGDYFKQLACKMALELLTEEFGIPIDRLYVTYFGGNADAGLEPDLECKQIWIDLG

VAEERILPGSMKDNFWEMGDTGPCGPCSEIHYDRIGGRDASHLVNMDDPNVLEIWNLVFIQFNRESETELKPLPKKSIDT

GMGLERLVSVLQNKMSNYDTDLFVPYFQAIQKGTGARPYTGKVGADDVDGIDMAYRVLADHARTITISLSDGGRPDNTGR

GYVLRRILRRAVRYSHEKLGAQRGFFASLVDVVVDSLGDAFPELRKDPEMVKDVINEEETQFLKTLSRGRRILDRKIMSL

GDCKTIPGDTAWLLYDTYGFPLDLTSLIAEEKGMEVNLAAFEEEKKAAQLKSQGKGAGGEDHIMLDIYAIEELRNQQVPA

TDDSPKYRYASEENGTYEFQPTSATVLALRRDRAFCQEVTTGQECGVLLDQTSFYAEQGGQTFDEGYMLREDDSSEDRME

FTVKNTQVRGGYVLHIGTVYGTLKVGDRVTLHIDEARRRPIMSNHTATHILNFALRGVLGEADQRGSLVAPDRLRFDFTA

KGALSTGEIRRTEEIACAMIKDAKPVYAMEAPLAQAKAIQGLRAVFDETYPDPVRVVSIGTPVQELLDDPNSPAGCLTSI

EFCGGTHLQNSGHAAPFVIVSEEAIAKGIRRIVAVTGAEAQKAHRKADALRLSLSALGDKVKQQTSPNKDVQKEIADMTE

SIGTALISQWQKDEMRETLKALKKTMDDLDRSYKADIQKRVLEKTKEEIENNPNQPLLIMEMETGASAKALNESLKLLKS

QSPQTAAMLFTVDPDAGKITCLCQVPQDVANRGLKASQWVQELCPLLDGKGGGKDMSAQATGRNTQCLQEALQMANEFAR

LKLGEN

>XP_014193174.1 PREDICTED: alanine--tRNA synthetase, cytoplasmic [Haplochromis burtoni]

MDSSLNAAQIRQKFIDFFCRYEHPYVHSSSTIPLDDPTLLFANAGMNQFKPIFLNTIDPSHPMARLRRAANTQKCIRAGG

KHNDLDDVGKDVYHHTFFEMLGSWSFGDYFKELACKMALELLTQEFGIALERLYVTYFGGNEDAGLEPDLECKQIWMDLG

VDEARILPGSMKDNFWEMGDTGPCGPCSEIHYDRIGGRDASHLVNMDDPDVLEIWNLVFIQFNRESETELKPLPKKSIDT

GMGLERLVSVLQNKMSNYDTDLFIPYFEAIQKGTGARPYTGKVGAEDADGIDMAYRVLADHARTITIALSDGGRPDNTGR

GYVLRRILRRAVRYSHEKLGAQRGFFASLVDVVVDSLGEAFPELKKDPEMVKDIINEEEAQFLKTLSRGRRILDRKIMSL

ADTKTIPGDTAWLLYDTYGFPLDLTSLIAEEKGMEVDLAGFEEEKKAAQLKSQGKGAGDEDHIMLDIYAIEELRNKQIPA

TDDSPKYNYTSDENGNYQFQQTSATVLALRHDRAFCDKVTTGQECGVVLDQTSFYAEQGGQTFDEGYMLREDDSTEDRME

FSVKNTQVRGGYVLHVGTVYGTLKVGDRVTLHVDEARRRPIMSNHTATHILNFALRGVLGEADQRGSLVAPDRLRFDFTA

KGALSTDEIHRTEEIACTMIKDAKPVYAMEATLAEAKAIQGLRAVFDETYPDPVRVVSVGIPVPELLADPNSAAGSLTSI

EFCGGTHLQNSGHAAPFVIVSEEAIAKGIRRIVAVTGTEAQKAQRKADALRQSLSALGDKVKQQTSPNKDIQKEIADMTE

SIGTAVISQWQKDEMRETLKGLKKTMDDLDRNYKADIQKRVLEKTKEVIESNPNQPLLIMEMETGASAKALNESLKLLKS

QSPQTAAMLFTVDPDAGKITCLCQVPQDVANRGLKASQWVQELCPLLDGKGGGKDMSAQATGRNTQCLQEALQMANEFAR

LKLGEN

>XP_017596382.1 PREDICTED: alanine--tRNA synthetase, cytoplasmic [Corvus brachyrhynchos]

METSLTASQIRQRFIDFFKENKHTYVHSSSTIPLDDPTLLFANAGMNQFKPIFLNTIDPSHPLAKLSRATNTQKCIRAGG

KHNDLDDVGKDVYHHTFFEMLGSWSFGDYFKELACKLALELLTKEFGIPAERLYVTYFGGNEAAGLQPDLECKQIWLDLG

LAEGRILPGNMKDNFWEMGDTGPCGPCSEIHYDRIGDRDASHLVNQDDPNVLEIWNLVFIQFNREADGTLKPLPKKSIDT

GMGLERLVSVLQNKMSNYDTDLFLPYFEAIQKGTGARPYLGQVGAEDADGIDMAYRVLADHARTITLALSDGGRPDNTGR

GYVLRRILRRAVRYSHEKLNAPKGFFATLVDVVVQSLGDAFPELKKDPDMVKDIINEEEDQFLKTLSRGRRILDRKIQSL

GDSKIIPGDTAWLLYDTYGFPADLTGLIAEEKGLVVDMEGFEEERKNAQLKSQGKGAGGEDLLMLDIYAIEELRARGLEV

TDDSPKYNYASDPSGTYDFGSLVATVKAIRREKKFVEEVSTGQECGIVLDQTCFYAEQGGQIYDQGYMVKDDDSREDKTE

FTVKNVQVRGGYVLHIGTLYGSLKVGDQVHLTIDEARRRPVMSNHTATHILNFALRSVLGEADQRGSLVAPDRLRFDFTA

KGALSTQEIKTVEGIANQMIEESKTVYAKDCPLAAAKAIQGLRAVFDETYPDPVRVVSIGIPVEELLADPSGPGGSITSI

EFCGGTHLQNSSHAGPFVIVSEEAIAKGIRRIVAVTGAEARKALRKVDSLRKLLSALEAKVKVQTAPNKDVQKEITDLGE

TLATAVIPQWQKDELREAVKALKKVMDDLDRASKADIQKRVLEKTKQVIESHPNQPWVIMEMENGASAKALNESLKLFKT

NSPQTAAMLFAVDLPLPEPPGRSCRLQAGAI

>XP_014885987.1 PREDICTED: alanine--tRNA synthetase, cytoplasmic [Poecilia latipinna]

MDSSLTAAQIRQKFIDFFIRYEHQYVHSSSTIPLDDPTLLFANAGMNQFKPIFLNTIDPSHPMAKLRRAANTQKCIRAGG

KHNDLDDVGKDVYHHTFFEMLGSWSFGDYFKQLACKMALELLTEEFGIPIDRLYVTYFGGNADAGLEPDLECKQIWIDLG

VAEERILPGSMKDNFWEMGDTGPCGPCSEIHYDRIGGRDASHLVNMDDPNVLEIWNLVFIQFNRESETELKPLPKKSIDT

GMGLERLVSVLQNKMSNYDTDLFVPYFQAIQKGTGARPYTGKVGADDVDGIDMAYRVLADHARTITISLSDGGRPDNTGR

GYVLRRILRRAVRYSHEKLGAQRGFFASLVDVVVDSLGDAFPELRKDPEMVKDVINEEETQFLKTLSRGRRILDRKIMSL

GDCKTIPGDTAWLLYDTYGFPLDLTSLIAEEKGMEVNLAAFEEEKKAAQLKSQGKGAGGEDHIMLDIYAIEELRNQQVPA

TDDSPKYRYASEENGTYEFQPTSATVLALRRDRTFCQEVTTGQECGVLLDQTSFYAEQGGQTFDEGYMLREDDSSEDRME

FTVKNTQVRGGYVLHIGTVYGTLKVGDRVTLHIDEARRRPIMSNHTATHILNFALRGVLGEADQRGSLVAPDRLRFDFTA

KGALSTGEIRRTEEIACAMIKDAKPVYAMEAPLAQAKAIQGLRAVFDETYPDPVRVVSIGTPVQELLDDPNSPAGCLTSI

EFCGGTHLQNSGHAAPFVIVSEEAIAKGIRRIVAVTGAEAQKAHRKADALCLSLSALGDKVKQQTSPNKDVQKEIADMTE

SIGTALISQWQKDEMRETLKALKKTMDDLDRSYKADIQKRVLEKTKEEIENNPNQPLLIMEMETGASAKALNESLKLLKS

QSPQTAAMLFTVDPDAGKITCLCQVPQDVANRGLKASQWVQELCPLLDGKGGGKDMSAQATGRNTQCLQEALQMANEFAR

LKLGEN

>XP_024137486.1 alanine--tRNA synthetase, cytoplasmic [Oryzias melastigma]

MDSSLSAAQIRQKFIDFFRRYEHQYVHSSSTIPLDDPTLLFANAGMNQFKPIFLNTIDPSHPMAKLRRAANTQKCIRAGG

KHNDLDDVGKDVYHHTFFEMLGSWSFGDYFKQLACKMALELLTQEFGISIDRLYVTYFGGNPDAGLEPDLECKQIWIDLG

VEEARILPGSMKDNFWEMGDTGPCGPCSEIHYDRIGDRDASHLVNMDDPNVLEIWNLVFIQFNRESETELKPLPKKSIDT

GMGLERLVSVLQNKMSNYDTDLFVPYFEAIQKGTGARPYTGKVGAEDVDGIDMAYRVLADHARTITIALSDGGRPDNTGR

GYVLRRILRRAVRYSHEKLGAQRGFFASLVDVVVDSLGDAFPELKKDPDMVKDIINEEEAQFLKTLSRGRRILDRKIMSL

GDCKTIPGDTAWLLYDTYGFPLDLTSLIAEEKGMGVDLAGFEEEKKAAQLKSQGKGAGDEDHIMLDIYAIEELRNKQVPP

TDDSPKYMYTSDENGNYEFQQASATVLALRRDRAFCEEVTTGQECGVVLDQTSFYAEQGGQTYDEGYMLRENDNMDDRTE

FTVKNTQVRGGYVLHIGTVYGTLKVGDRVSLCIDEARRRPIMSNHTATHILNFALRGVLGEADQRGSLVAPDRLRFDFTA

KGAMKTDEIHRAEEIACGLIKDAKPVYAKEASLAEAKAIQGLRAVFDETYPDPVRVVSIGIPVQELLDDPNSPAGSLTSI

EFCGGTHLQNSSHAAPFVIVSEEAIAKGIRRIVAVTGAEAQKAQRKADALRQALCALGDKVKQQTSPNKDVQKEIADMSE

SLGTAVISQWQKDEMRESLKGLKKTMDDLDRSYKADIQKRVLEKTKEVIQSNPNQPLLIMAMETGASAKALNESLKLLKA

QSPQTATMLLTVDPDAGKITCLCQVPQDVAGRGLKASEWVQELCPLLDGKGGGKDTSAQATGKNTQCLEEALQVANEFAR

LKLGEN

>XP_015820628.1 PREDICTED: alanine--tRNA synthetase, cytoplasmic [Nothobranchius furzeri]

MDSTLSAAQIRQKFIDFFVRYEHQYVHSSSTIPLDDPTLLFANAGMNQFKPIFLNTIDPSHPMAKLRRAANTQKCIRAGG

KHNDLDDVGKDVYHHTFFEMLGSWSFGDYFKQLACKMALELLTQEFGISIDRLYVTYFGGNSDAGLEPDLECKQIWMDLG

VEEDRILPGSMKDNFWEMGDTGPCGPCSEIHYDRIGGRDASHLVNMDDPNVLEIWNLVFIQFNRESETELKPLPKKSIDT

GMGLERLVSVLQNKMSNYDTDLFVPYFEAIQKGTGARLYTGKVGADDADGIDMAYRVLADHARTITIALSDGGRPDNTGR

GYVLRRILRRAVRYSHEKLGAQRGFFASLVDVVVESLGEAFPELKKDPEMVKDIINEEESQFLKTLSRGRRILDRKIVSL

GDCKIIPGDTAWLLYDTYGFPLDLTSLIAEEKGMAVDLAAFEEEKKAAQLKSQGKGAGGEDHIMLDIYAIEELRNSQVPA

TDDSPKYRYTSDQNGSYAFQQTSATVLALRRDRAFCQEVTTGQECGVLLDQTSFYAEQGGQTFDEGYMLREDDNMEDRTE

FTVKNTQVRGGYVLHIGTVYGTLKVGDRVTLCVDEARRRPIMSNHTATHILNFALRGVLGEADQRGSLVAPDRLRFDFTA

KGALSTAEIRRAEEIACAMIKDAKPVYAKEAALAEAKAIQGLRAVFDETYPDPVRVVSIGVPVEELLNDPNSPAGSLTSI

EFCGGTHLQNSSHAAPFVIVSEEAIAKGIRRIVAVTGAEAQKAQRKADALRQSLSALGDKVKQQATPNKDVQREIADMTE

SLGTAVIPQWQKDEMRDILKGLKKTMDDLDRTYKADIQKRVLEKTKEVIENSPNQPLLVMEMETGASAKALNESLKLLKS

HSPQTATMLFTVDPDAGRITCLCQVPQEVANRGLKASEWVQELCPLLDGKGGGKDMSAQATGKNTQCLQEALQIANEFAR

LKLGEN

>XP_009818876.1 PREDICTED: alanine--tRNA synthetase, cytoplasmic, partial [Gavia stellata]

MEATLTASQIRQRFIDFFKENKHTYVHSSSTIPLDDPTLLFANAGMNQFKPIFLNTIDPSHPLAKLNRATNTQKCIRAGG

KHNDLDDVGKDVYHHTFFEMLGSWSFGDYFKELACKLALDLLTKEFGIPAERLYVTYFGGNEAAGLQPDLECKQIWLNLG

LAEGRILPGNMKDNFWEMGDTGPCGPCSEIHYDRIGDRDASHLVNQDDPNVLEIWNLVFIQFNREADGTLKPLPKKSIDT

GMGLERLVSVLQNKMSNYDTDLFVPYFEAIQKGTGARPYMGQVGAEDADGIDMAYRVLADHARTITLALSDGGRPDNTGR

GYVLRRILRRAVRYSHEKLNAPKGFFATLVDVVVQSLGDAFPELKKDPDMVKDIINEEEDQFLKTLSRGRRILDRKIQSL

GDSKTIPGDTAWLLYDTYGFPADLTGLIAEEKGLVVDMEGFEEERKNAQLKSQGKGAGGEDLLMLDIYAIEELRARGLEV

TDDSPKYSYASDQSGAYDFGSLVATVKAIRREKKFVEEVSTGQECGIVLDRTCFYAEQGGQIYDEGYMVKDDDGREDKTE

FTVKNVQVRGGYVLHIGTLYGSLKVGDQVHLSIDETRRRPVMSNHTATHILNFALRSVLGEADQRGSLVAPDRLRFDFTA

KGALSTQEIKKVEGIANQMIEEAKTVYAKDCPLAAAKAIQGLRAVFDETYPDPVRVVSIGIPVEELLADPSGPAGSITSI

EFCGGTHLQNSSHAGPFVIVSEEAIAKGIRRIVAVTGAEARKALRKVDSLRKLLSALEAKVKVQTAPNKDVQKEITDLSE

TLATAVIPQWQKDELREAVKALKKVMDDLDRASKADIQKRVLEKTKQVIESHPSNEFVVLQALNESLKLFKTYSPQTATM

LFAVDNEAGRITCLC

>XP_023257338.1 alanine--tRNA synthetase, cytoplasmic [Seriola lalandi dorsalis]

MDSSLSAAQIREKFIDFFRRYEHQYVHSSSTIPLDDPTLLFANAGMNQFKPIFLNTIDPSHPMARLHRAANTQKCIRAGG

KHNDLDDVGKDVYHHTFFEMLGSWSFGDYFKQLACKMALELLTQEFGISIDRLYVTYFGGNADAGLEPDLECKQIWIDLG

MEEARILPGSMKDNFWEMGDTGPCGPCSEIHYDRIGGRDASHLVNMDDPNVLEIWNLVFIQFNRESETELKPLPKKSIDT

GMGLERLVSVLQNKMSNYDTDLFIPYFDAIQKGTGARPYTGKVGAEDADGIDMAYRVLADHARTITIALSDGGRPDNTGR

GYVLRRILRRAVRYSHEKLGAQRGFFASLVDVVVDSLGNAFPELKKDPEMVKDIINEEETQFLKTLSRGRRILDRKIMTL

GDSKTIPGDTAWLLYDTYGFPLDLTSLIAEEKGMAVDMAAFEEEKKAAQLKSQGKGAGDVDHIMLDIYAIEELRNKKIPA

TDDSPKYKYSSDDNGSYDFQQASATVLALRHDRAFCDEVTTGQECGVLLDQTSFYAEQGGQTFDEGFMLREDDSTEDRME

FTVKNTQVRGGYVLHVGTVYGKLKVGDRVTLHVDEARRRPIMSNHTATHILNFALRGVLGEADQRGSLVASDRLRFDFTA

KGALSTGEVRRTEEIACAMIKEAKPVYAMDAPLAEAKAIQGLRAVFDETYPDPVRVVSIGIPVEDLLKDPTSTAGSLTSI

EFCGGTHLQNSGHAAPFVIVSEEAIAKGIRRIVAVTGAEAQKALRKADALRQSLSALGDKVKQQTAPNKDMQKEIADMTE

SIGTAVISQWQKDEMRETLKGLKKIMDDLDRNYKTDIQKRVLEKTKEVIESSPNQALLIMEMETGASAKALNESLKLLKT

QSPQTAAMLFTVDPDAGKITCLCQVPQDVANRGLKASEWVQELCPLLDGKGGGKDMSAQATGRNTQCLQEALQMANEFAR

LKLGEN

>XP_008410788.1 PREDICTED: alanine--tRNA synthetase, cytoplasmic [Poecilia reticulata]

MDSSLSAAQIRQKFIDFFIRYEHQYVHSSSTIPLDDPTLLFANAGMNQFKPIFLNTIDPSHPMAKLRRAANTQKCIRAGG

KHNDLDDVGKDVYHHTFFEMLGSWSFGDYFKQLACKMALELLTEEFGIPIDRLYVTYFGGNADAGLEPDLECKQIWMDLG

VAEERILPGSMKDNFWEMGDTGPCGPCSEIHYDRIGGRDASHLVNMDDPNVLEIWNLVFIQFNRESETELKPLPKKSIDT

GMGLERLVSVLQNKMSNYDTDLFVPYFEAIQKGTGARPYTGKVGADDVDGIDMAYRVLADHARTITISLSDGGRPDNTGR

GYVLRRILRRAVRYSHEKLGAQRGFFASLVDVVVDSLGDAFPELRKDPEMVKDVINEEETQFLKTLSRGRRILDRKIMSL

GDCKTIPGDTAWLLYDTYGFPLDLTSLIAEEKGMEVNLAAFEEEKKAAQLKSQGKGAGGEDHIMLDIYAIEELRNQQVPA

TDDSPKYRYASEENGTYEFQPTSATVLALRRDRAFCQDVTTGQECGVLLDQTSFYAEQGGQTFDEGYMLREDDSSEDRME

FTVKNTQVRGGYVLHIGTVYGTLKVGDRVTLHIDEARRRPIMSNHTATHILNFALRGVLGEADQRGSLVAPDRLRFDFTA

KGALSTGEIRRTEEIACAMIKDAKPVYAMEAPLAQAKAIQGLRAVFDETYPDPVRVVSIGTPVQELLDDPNSPAGSLASI

EFCGGTHLQNSGHAAPFVVVSEEAIAKGIRRIVAVTGAEAQKAHRKADALRLSLSALGDKVKQQTSPNKDVQKEIADMTE

SIGTALISQWQKDEMREALKALKKTMDDLDRSYKADIQKRVLEKTKEEIENNPNQLLLIMEMETGASAKALNESLKLLKS

QSPQTAAMLFTVDPDAGKITCLCQVPQDVANRGLKASEWVQELCPLLDGKGGGKDMSAQATGRNTQCLQEALQMANEFAR

LKLGEN

>XP_020790758.1 alanine--tRNA synthetase, cytoplasmic [Boleophthalmus pectinirostris]

MDTSLSASQIREKFIDFFKRYEHQYVHSSATIPLDDPTLLFANAGMNQFKPIFLNTIDPSHPMARLKRAANTQKCIRAGG

KHNDLDDVGKDVYHHTFFEMLGSWSFGDYFKHLACKMALELLTQEFGISIDRLYVTYFGGSAEAGLEPDLECKQIWIDLG

VAEARILPGNMKDNFWEMGDTGPCGPCSEIHYDRIGGRDASHLVNMDDPNVLEIWNLVFIQFNRESETVLKPLPKKSIDT

GMGLERLVSVLQNKMSNYDTDLFLPYFDAIQKGTGARPYTGKVGAEDEDGIDMAYRVLADHARTITIALSDGGRPDNTGR

GYVLRRILRRAVRYSHEKLGAKRGFFASLVDVVVESLGNAFPELKKDPEMVKDIINEEEAQFLKTLSRGRRILDRKIMSL

ADSKTIPGDTAWLLYDTYGFPLDLTSLIAEEKGMQVDLAAFEEEKKAAQLKSQGKGAGGEDHIMLDIYAIEELRNKNVPA

TDDSPKYKYSSDEKGSYVFEEATATVLALRRDRAFCDEVTTGQECGVLLDQTSFYAEQGGQTFDEGYMLREHDSAEDRME

FTVKNTQVRGGYVLHIGTVYGTLKVGDRVTLHVDEARRRPIMSNHTATHILNFALRGVLGEADQRGSLVASDRLRFDFTA

KGALSTGEVRRTEEIASAMIRDAKPVYAMEAPLAQAKAIQGLRAVFDETYPDPVRVVSIGIPVQDLLEDPNSAAGSLTSI

EFCGGTHLQNSGHAAPFVIVSEEAIAKGIRRIVAVTGSEAQKAQRKADALHQALAALGKTVKQQSSPNKDIQKEIADMTE

SIGTAVISQWQKDEMRETLKGLKKIMDDLDRNYKADIQKRVVEKIKEEIEKNPNQELLIMEMEPGASAKALNESLKLLKT

NSPQTAAMLFTVDPEAGKITCLCQVPQETANRGLKASEWVQELCPLLDGKGGGKDMSAQAAGRNTECLQEALQMANNFAR

LKLGEN

>XP_022605689.1 alanine--tRNA synthetase, cytoplasmic [Seriola dumerili]

MDSSLSAAQIREKFIDFFRRYEHQYVHSSSTIPLDDPTLLFANAGMNQFKPIFLNTIDPSHPMARLHRAANTQKCIRAGG

KHNDLDDVGKDVYHHTFFEMLGSWSFGDYFKQLACKMALELLTQEFGISIDRLYVTYFGGNADAGLEPDLECKQIWIDLG

MEEARILPGSMKDNFWEMGDTGPCGPCSEIHYDRIGGRDASHLVNMDDPNVLEIWNLVFIQFNRESETELKPLPKKSIDT

GMGLERLVSVLQNKMSNYDTDLFIPYFDAIQKGTGARPYTGKVGAEDADGIDMAYRVLADHARTITIALSDGGRPDNTGR

GYVLRRILRRAVRYSHEKLGAQRGFFASLVDVVVDSLGNAFPELKKDPEMVKDIINEEETQFLKTLSRGRRILDRKIMTL

GDSKTIPGDTAWLLYDTYGFPLDLTSLIAEEKGMAVDMAAFEEEKKAAQLKSQGKGAGDVDHIMLDIYAIEELRNKNIPA

TDDSPKYKYSSDDNGSYDFQQASATVLALRHDRAFCDEVTTGQECGVLLDQTSFYAEQGGQTFDEGFMLREDDSTEDRME

FTVKNTQVRGGYVLHVGTVYGKLKVGDRVTLHVDEARRRPIMSNHTATHILNFALRGVLGEADQRGSLVASDRLRFDFTA

KGSLSTGEVRRTEEIACAMIREAKPVYAMDAPLAEAKAIQGLRAVFDETYPDPVRVVSIGIPVEDLLKDPTSTAGSLTSI

EFCGGTHLQNSGHAAPFVIVSEEAIAKGIRRIVAVTGAEAQKALRKADALRQSLSALGDKVKQQTAPNKDMQKEIADMTE

SIGTAVISQWQKDEMRETLKGLKKIMDDLDRNYKTDIQKRVLEKTKEVIESSPNQALLIMEMETGASAKALNESLKLLKT

QSPQTAAMLFTVDPDAGKITCLCQVPQDVANRGLKASEWVQELCPLLDGKGGGKDMSAQATGRNTQCLQEVLQMANEFAR

LKLGEN

>CDQ73562.1 unnamed protein product [Oncorhynchus mykiss]

MDSLLPAAQIREKFIDFFRCHEHQYVHSSATIPLDDPTLLFANAGMNQFKPIFLNTIDPSHPMARLKRAANTQKCIRAGG

KHNDLDDVGKDVYHHTFFEMLGSWSFGDYFKHLACTMALELLTKEFGIPIERLYVTYFGGHEEAGLDPDLECKQIWLDLG

MDEARIIPGSMKDNFWEMGDTGPCGPCSEIHFDRIGGRDAAHLVNMDDPNVLEIWNLVFIQYNRESETELKPLPKKSIDT

GMGLERLVSVLQNKMSNYDTDLFIPYFQAIQKASTTTKHYLPLLSGDYTGKVGVEDVDGIDMAYRVLADHARTITIALSD

GGRPDNTGRGYVLRRILRRAVRYSHEKLGAQKGFFATLVDVVVESLGDAFPELKKDPDMVKDIINEEEEQFLKTLSRGRK

ILDRKIQSLGDSKTIPGDTAWLLYDTYGFPLDLTALIAEERGMMVDVAAFEEEKKAAQVKSQGKGSGDEDHIMLDIYAIE

DLRNKGIAATDDVLKYSYSADDSGKYVFEQAVSTVLALRRERAFVDEVTTGQECGVLLDKTSFYAEQGGQSFDEGYMLRE

DDSVDDRMEFTVKNTQVRGGYVLHVGTVYGTLKVGDNVILHVDEARRRPIMSNHTATHILNFALREVLGEADQRGSLVAP

DRLRFDFTAKGALSTEEVRRTEEIACTMIRDAKPVYAMDTPLAAAKAIQGLRAVFDETYPDPVRVVSIGIPVADLLADPS

STAGSCTSIEFCGGTHLRNSGHAAPFVIVSEEAIAKGIRRIVAVTGAEAQKAHRKADALRQSLSALGDKVKVQTAPNKDV

QKEIADMTEFLGMAVISQWQKDKMREALKGLKKTMDDLDRASKADVQKRVLEKTKEMIDSSPNQPLVVMEMESGASAKAL

NESLKLLKTQSPQTAAMLFTVDNDAGKIICLCQVPQDVANRGLKASEWVQELCPLMDGKGGGKDMSAQATGRNTHCIEEA

LQMANQFAQLKLSENE

>XP_019112731.1 PREDICTED: alanine--tRNA synthetase, cytoplasmic [Larimichthys crocea]

MDSSLSASQIREKFIDFFRRYEHKYVHSSSTIPLDDPTLLFANAGMNQFKPIFLNTIDPSHPMARLHRAANTQKCIRAGG

KHNDLDDVGKDVYHHTFFEMLGSWSFGDYFKQLACKMALELLTQEFGIPIERLYVTYFGGNEDAGLEPDLECKQIWIDLG

MEEARILPGSMKDNFWEMGDTGPCGPCSEMHFDRIGGRDASHLVNMDDPNVLEIWNLVFIQFNRESETELKPLPKKSIDT

GMGLERLVSVLQNKMSNYDTDLFIPYFEAIQKGTGARPYTGKVGAEDADGIDMAYRVLADHARTITIALSDGGRPDNTGR

GYVLRRILRRAVRYSHEKLGAQRGFFASLVDVVVDSLGDAFPELRKDPDMVKDVINEEEVQFLKTLSRGRRILDRKIMSL

GDCKTIPGDTAWLLYDTYGFPLDLTSLIAEEKGMAVDLAAFEEEKKAAQLKSQGKGAGDEDHIMLDIYAIEELRNKNTPA

TDDSPKYKYTSDENGKYEFQQVTATVLALRRDRAFCDEVTTGQECGVVLDQSSFYAEQGGQTFDEGYMLREDDSTEDRME

FTVKNTQVRGGYVLHVGTVYGTLKVGDHVTLHVDEARRRLVMSNHTATHILNFALRGVLGEADQRGSLVAADRLRFDFTA

KGALSTGEVRRTEEIACVLIREAKTVYALDAPLAQAKAIQGLRAVFDETYPDPVRVVSIGIPVEELLKDPDSAAGSLTSI

EFCGGTHLQNSGHAAPFVIVSEEAIAKGIRRIVAVTGTEAQKAQRKADSLHQSLSALGDRVKQQTAPNKDIQKEIADMTE

SIGTAVISQWQKDEMRETLKGLKKIMDDLDRSYKADIQKRVLEKTREVIESNPNQPLLVMEMETGASSKALNESLKLLKS

NSPQTAAMLFTVDPEAGKITCLCQVPQDVVKRGLKASEWVQELCPLLDGKSGGKEMSAQATGRNTQCLEDALQMANEFAR

LKLQEN

>XP_008309287.1 alanine--tRNA synthetase, cytoplasmic [Cynoglossus semilaevis]

MDSSLSAAQIREKFIDFFRQYEHQYVHSSATIPLDDPTLLFANAGMNQFKPIFLNTIDPSHPMARLRRAANTQKCIRAGG

KHNDLDDVGKDVYHHTFFEMLGSWSFGDYFKQLACEMALELLTKEFGISIDRLYVTYFGGSEAAGLEPDLECKQIWIDLG

MDEARVLPGSMKDNFWEMGDTGPCGPCSEIHYDRIGGRDASHLVNMDDPNVLEVWNLVFIQFNRESETELKPLPKKSIDT

GMGLERLVSVLQNKMSNYDTDLFIPYFEAIQKGTGARAYTGKVGAEDSDGIDMAYRVLADHARTITIALSDGGRPDNTGR

GYVLRRILRRAVRYAHEKLGAQKGFFATLVDTVVASLGEAFPELKKDPEMVKDIINEEETQFLKTLSRGRRILDRKIMSL

GESKTIPGDTAWLLYDTYGFPLDLTSLIAEEKGMAVDLAAFEEEKKAAQLKSQGKGAGDADHIMLDIYAIEELRNKNVPA

TDDSPKYKYTSDKNGNYEFQETTATVLALRRERAFCDEVTTGQECGVLLDQTTFYAEQGGQTFDEGFMLREDDNTEDRME

FTVKNTQVRGGYVLHVGTVYGTLKVGDSVVLHVDEARRRLVMSNHTATHILNFALRGVLGEADQRGSLVAPDRLRFDFTA

KGALSTGEIRRTEEMACAMIRDAKPVYAMEAPLAAAKAIQGLRAVFDETYPDPVRVVSVGIPVQDLLDDPNSAAGALNSI

EFCGGTHLQNSGHAAPFVIVSEEAIAKGIRRIVAVTGAEAQKALRKADALNQALSDLGDKVKQQTAPNKDVQKDIADMNE

SIGTAVISQWQKDEMRENLKALKKTMDDLDRNYKADIQKRVLEKTKEVIESSPNQQLLVMEMETGASAKALNESLKLLKS

NSPQTAAMLFTVDPDAGKITCLCQVPQDVANRGLKASEWVQELCPLLDGKGGGKDMSAQATGRNTQCLQEALRMANEFAQ

LKLGEN

>XP_017724218.1 PREDICTED: alanine--tRNA synthetase, cytoplasmic isoform X2 [Rhinopithecus bieti]

MDSTLTASEIRQRFIDFFRRNEHTYVHSSATIPLDDPTLLFANAGMNQFKPIFLNTIDPSHPMAKLSRAANTQKCIRAGG

KHNDLDDVGKDVYHHTFFEMLGSWSFGDYFKELACKMALELLTQEFGIPIERLHVTYFGGDEAAGLEPDLECKQIWQNLG

LDDTKILPGNMKDNFWEMGDTGPCGPCSEIHYDRIGDRDAAHLVNQDDPNVLEIWNLVFIQYNREADGILKPLPKKSIDT

GMGLERLVSVLQNKMSNYDTDLFVPYFEAIQKGTGARPYTGKVGAEDADGIDMAYRVLADHARTITVALADGGRPDNTGR

GYVLRRILRRAVRYAHEKLNASRGFFATLVDVVVQSLGDAFPELKKDPDMVKDIINEEEVQFLKTLSRGRRILDRKIQSL

GDSETIPGDTAWLLYDTYGFPVDLTGLIAEEKGLVVDMDGFEEERKLAQLKSQGKGAGGEDLIMLDIYAIEELRARGLEV

TDDSPKYNYHLDSSGSYVFENTVATVMALRREKMFVEEVSTGQECGVVLDKTCFYAEQGGQLYDEGYLVKVDDSSEDKTE

FTVKNAQVRGGYVLHIGTIYGDLKVGDQVRLFIDEPRRRPIMSNHTATHILNFALRSVLGEADQKGSLVAPDRLRFDFTA

KGAMSTQQIKKAEEIANEMIEAAKPVYTQDCPLAAAKAIQGLRAVFDETYPDPVRVVSIGVPVSELLDDPSGPAGSLTSV

EFCGGTHLRNSSHAGAFVIVTEEAIAKGIRRIVAVTGAEAQKALRKAESLKKSLSVMEAKVKAQTAPNKDVQREIADLGE

PCLFHLPQLLQKGLVSKPEDSFVFLLSFPGH

>KFV46629.1 Alanine--tRNA synthetase, cytoplasmic, partial [Gavia stellata]

MEATLTASQIRQRFIDFFKENKHTYVHSSSTIPLDDPTLLFANAGMNQFKPIFLNTIDPSHPLAKLNRATNTQKCIRAGG

KHNDLDDVGKDVYHHTFFEMLGSWSFGDYFKELACKLALDLLTKEFGIPAERLYVTYFGGNEAAGLQPDLECKQIWLNLG

LAEGRILPGNMKDNFWEMGDTGPCGPCSEIHYDRIGDRDASHLVNQDDPNVLEIWNLVFIQFNREADGTLKPLPKKSIDT

GMGLERLVSVLQNKMSNYDTDLFVPYFEAIQKGTGARPYMGQVGAEDADGIDMAYRVLADHARTITLALSDGGRPDNTGR

GYVLRRILRRAVRYSHEKLNAPKGFFATLVDVVVQSLGDAFPELKKDPDMVKDIINEEEDQFLKTLSRGRRILDRKIQSL

GDSKTIPGDTAWLLYDTYGFPADLTGLIAEEKGLVVDMEGFEEERKNAQLKSQGKGAGGEDLLMLDIYAIEELRARGLEV

TDDSPKYSYASDQSGAYDFGSLVATVKAIRREKKFVEEVSTGQECGIVLDRTCFYAEQGGQIYDEGYMVKDDDGREDKTE

FTVKNVQVRGGYVLHIGTLYGSLKVGDQVHLSIDETRRRPVMSNHTATHILNFALRSVLGEADQRGSLVAPDRLRFDFTA

KGALSTQEIKKVEGIANQMIEEAKTVYAKDCPLAAAKAIQGLRAVFDETYPDPVRVVSIGIPVEELLADPSGPAGSITSI

EFCGGTHLQNSSHAGPFVIVSEEAIAKGIRRIVAVTGAEARKALRKVDSLRKLLSALEAKVKVQTAPNKDVQKEITDLSE

TLATAVIPQWQKDELREAVKALKKVMDDLDRASKADIQKRVLEKTKQVIETGGKVCCNEFVVLQALNESLKLFKTYSPQT

ATMLFAVDNEAGRITCLCQ

>XP_019391090.1 PREDICTED: alanine--tRNA synthetase, cytoplasmic isoform X2 [Crocodylus porosus]

MELTLTGSQIRQRFIDFFKENRHTYVHSSSTIPLDDPTLLFANAGMNQFKPIFLNTIDPSHPMAKLNRAANTQKCIRAGG

KHNDLDDVGKDVYHHTFFEMLGSWSFGDYFKALACKLALELLTKEFGIPIERLYVTYFGGNEAAGLEPDLECKQIWLDLG

LAENKILPGSMKDNFWEMGDTGPCGPCSEIHYDRIGNRDASHLVNQDDPNVLEIWNLVFIQFNREADGTLKPLPKKSIDT

GMGLERLVSVLQNKMSNYDTDLFIPYFEAIQKGTGARPYTGQVGAEDMDGIDMAYRVLADHARTITVALSDGGRPDNTGR

GYVLRRILRRAVRYSHEKLSAPKGFFATLVDVVVQSLGDAFPELKKDPDMVKDIINEEEVQFLKTLSRGRRILDRKIQSL

GDSKIIPGDTAWLLYDTYGFPVDLTGLIAEEKGLAVDMEGFEEERRNAQLKSQGKGTGVEDLIMLDIYAIEELRAQGLEV

TDDSPKYSYTSDPSGVYEFENLMATVKAIRREKTFVEEVSTGQECGIVLDKTCFYAEQGGQIYDEGYLVKADDYSEDKTE

FTVKNVQVRGGYVLHVGTVYGSLKVGDQVHLYVDEPRRRPVMSNHTATHILNFALRSVLGEADQRGSLVAPDKLRFDFTA

KGAMSTQEIKKTEKIANQMIEEAKTVYAKDCPLAAAKAIQGLRAVFDETYPDPVRVVSIGIPVDDLLADPSGPAGSVTSV

EFCGGTHLQNSSHAGPFVIVSEEAIAKGIRRIVAVTGAEAQKALRKVDSLKKSLSALDAKVKVQTAPNKEVQKEIADLSE

TLATAVIPQWQKDELREALKVLKKVMDDLDRASKADIQKRVLEKTKQFIDSHPNQPLVILEMESGASAKALKEIQTQERK

RCPAWGWL

>XP_011474655.1 alanine--tRNA synthetase, cytoplasmic [Oryzias latipes]

MDSSLSAAQIRQKFIDFFLRYEHQYVHSSSTIPLDDPTLLFANAGMNQYKPIFLNTIDPSHPMAKLRRAANTQKCIRAGG

KHNDLDDVGKDVYHHTFFEMLGSWSFGDYFKQLACKMALELLTEEFGISIDRLYVTYFGGNPDAGLEPDLECKQIWMDLG

VEEARILPGSMKDNFWEMGDTGPCGPCSEIHYDRIGGRDASHLVNMDDPNVLEIWNLVFIQFNRESETELKPLPKKSIDT

GMGLERLVSVLQNKMSNYDTDLFVPYFEAIQKGTGARPYSGKVGAEDVDGIDMAYRVLADHARTITIALSDGGRPDNTGR

GYVLRRILRRAVRYSHEKLGAQRGFFASLVDVVVDSLGDAFPELKKDPDMVKDVINEEEAQFLKTLSRGRRILDRKIMSL

GDCKTLPGDTAWLLYDTYGFPLDLTSLIAEEKGMEVDLAGFEEEKKAAQLKSQGKGAGDVDHIMLDIYAIEELRNKHVPP

TDDSPKYVYTSDENGNYEFQQASATVLALRCDRAFCEEVTTGQECGVVLDQTSFYAEQGGQTYDEGYMLRENDNTDDRME

FTVKNTQVRGGYVLHIGTVYGTLRVGDRVSLCIDEARRRPIMSNHTATHILNFALRGVLGEADQRGSLVAPDRLRFDFTA

KGALKTDEIHRTEDIACAMIRDAKPVYAKEASLAEAKAIQGLRAVFDETYPDPVRVVSIGVPVQELLDDPNSPAGSLTSI

EFCGGTHLQNSSHAAPFVIVSEEAIAKGIRRIVAVTGAEAQKAQRKADALRQSLSALGDKVKQQASPNKDIQKEIADMTE

SLGTAVISQWQKDEMRETLKGLKKTMDDLDRSYKADIQKRVLEKTKEVIESNPNQPLLIMEMETGASAKALNESLKLLKA

QSPHTATMLLTVDPDAGKITCLCQVPQEVAGRGLKASEWVQELCPLLDGKGGGKDTSAQATGRNTQCLQEALQMANEFAR

LKLGEN

>KKF31274.1 Alanine--tRNA synthetase, cytoplasmic [Larimichthys crocea]

MDSSLSASQIREKFIDFFRRYEHKYVHSSSTIPLDDPTLLFANAGMNQFKPIFLNTIDPSHPMARLHRAANTQKCIRAGG

KHNDLDDVGKDVYHHTFFEMLGSWSFGDYFKQLACKMALELLTQEFGIPIERLYVTYFGGNEDAGLEPDLECKQIWIDLG

MEEARILPGSMKDNFWEMGDTGPCGPCSEMHFDRIGGRDASHLVNMDDPNVLEIWNLVFIQFNRESETELKPLPKKSIDT

GMGLERLVSVLQNKMSNYDTDLFIPYFEAIQKGTGARPYTGKVGAEDADGIDMAYRVLADHARTITIALSDGGRPDNTGR

GYVLRRILRRAVRYSHEKLGAQRGFFASLVDVVVDSLGDAFPELRKDPDMVKDVINEEEVQFLKTLSRGRRILDRKIMSL

GDCKTIPGDTAWLLYDTYGFPLDLTSLIAEEKGMAVDLAAFEEEKKAAQLKSQGKGAGDEDHIMLDIYAIEELRNKNTPA

TDDSPKYKYTSDENGKYEFQQVTATVLALRRDRAFCDEVTTGQECGVVLDQSSFYAEQGGQTFDEGYMLREDDSTEDRME

FTVKNTQVRGGYVLHVGTVYGTLKVGDHVTLHVDEARRRLVMSNHTATHILNFALRGVLGEADQRGSLVAADRLRFDFTA

KGALSTGEVRRTEEIACVLIREAKTVYALDAPLAQAKAIQGLRAVFDETYPDPVRVVSIGIPVEELLKDPDSAAGSLTSI

EFCGGTHLQNSGHAAPFVIVSEEAIAKGIRRIVAVTGTEAQKAQRKADSLHQSLSALGDRVKQQTAPNKDIQKEIADMTE

SIGTAVISQWQKDEMRETLKGLKKIMDDLDRSYKADIQKRVLEKTREVIESNPNQPLLVMEMETGASSKALNESLKLLKS

NSPQTAAMLFTVDPEAGKITCLCQVPQDVVKRGLKASEWVQELCPLLDGKSGGKEMSAQATGRNTQCLEDALQMANDFLF

GLFSSVIMSVEAAELGYNASSGSGTTLAPLPAHIHPKLLPGGTKTLNAISVL

>XP_017296798.1 alanine--tRNA synthetase, cytoplasmic [Kryptolebias marmoratus]

MDSSLSAAQIRQKFIDFFIRYEHQYVHSSSTIPLDDPTLLFANAGMNQFKPIFLNTIDPSHPMAKLRRAANTQKCIRAGG

KHNDLDDVGKDVYHHTFFEMLGSWSFGDYFKHLACKMALELLTQEFGISIDRLYVTYFGGNPEAGLEPDLECKQIWMDLG

VEEARILPGSMKDNFWEMGDTGPCGPCSEIHYDRIGGRDASHLVNMDDPNVLEIWNLVFIQFNRESETELKPLPKKSIDT

GMGLERLVSVLQNKMSNYDTDLFVPYFEAIQKGTGARSYTGKVGAEDVDGIDMAYRVLADHARTITIALSDGGRPDNTGR

GYVLRRILRRAVRYSHEKLGAQRGFFASLVDVVIESLGDAFPEVKKDPDMVKDIINDEEAQFLKTLSRGRRILDRKIMSL

GDCRTIPGDTAWLLYDTYGFPLDLTSLIAEEKGMEVDLAAFEEEKRAAQLKSQGKGAGGEDHIMLDIYAIEELRNKQIPA

TDDSPKYRYASDQNGNYEFQQASAAVLALRRDRAFCDEVTTGQECGVLLDQTSFYAEQGGQTFDEGYMLRQDDNTEDRME

FTVKNTQVRGGYILHIGTVYGTLKVGDRVTLHLDEARRRPIMSNHTATHILNFALRGVLGEADQRGSLVAPDRLRFDFTA

KGALSTGEIRRTEEIACALIRDAKPVYAMDAPLAEAKAIQGLRAVFDETYPDPVRVVSIGIPVQELLQDPNSPSGSLTSI

EFCGGTHLQNSGHAAPFVIVSEEAIAKGIRRIVAVTGTEAQKAQRKADAVLQSLSALGDKVKQQTAPNKDLQKEIADLTE

ELGAAVISQWQKDEMRDTLKGLKKTMDDLDRSYKADIQKRVLEKTKEVIENNPNQPLLIMEMETGASAKALNESLKLLKS

QSPQTATMLFTVDPEAGKITCLCQVPQEVAKRGLKASEWVQELCPLLDGKGGGKDMSAQATGRNTERLQEALQVANEFAR

LKLGEN

>KPP66654.1 alanine--tRNA synthetase, cytoplasmic-like [Scleropages formosus]

MDTSLTAAEIREKFIDFFRRHEHQYVHSSSTVPLDDPTLLFANAGMNQVNVSNGLGLYLGLGSPRAVIHPLVFSPTMQFK

PIFLNTIDPSHPMARLKRAANTQKCIRAGGKHNDLDDVGKDVYHHTFFEMLGSWSFGDYFKHLACEMALELLTKEFGIPI

ERLYVTYFGGDEKAMLEPDLECRQIWLDLGIEECRILPGSMKDNFWEMGDTGPCGPCSEIHFDRIGGRDAAHLVNMDDPN

VLEIWNLVFIQFNRESETVLKPLPKKSIDTGMGLERLVSVLQNKMSNYDTDLFGTGSRPYTGKVGAEDTDGIDMAYRVLA

DHARTITIALSDGGRPDNTGRGYVLRRILRRAVRYAHEKLGAQRGFFASLVDVVVESLGDAFPELKKDPDMVKDIINDEE

VQFLKTLSRGRRILDRKIQSLGDSQTIPGDTAWLLYDTYGFPLDLTALIAEERGLGVNMQGFEEERRAAQLKSQGKGSGD

EDHIMLDIYSIEELRNKGLPATDDAPKYGYYVSDDGSYELEQAMGTVLALRREREFVEEVTTGQECGVLLDRTSFYAEQG

GQTFDEGYMLKENDFAEDKMEFTVKNTQVRGGFVLHVGTVYGTLRVGDCVTLHVDEARRRPIMSNHTATHILNFALRSVL

GEADQRGSLVAPDRLRFDFTAKGAMSTVEVQRTEEMACTMIHEAKSVYAMEVPLAAAKAIQGLRAVFDETYPDPVRVVSI

GVPVPDLLADPSGPAASLTSVEFCGGTHLRNSAHVGPFVIVSEEAIAKGIRRIVAVTGAEAQKAQRKADALRQTLAALGG

KVVAQTAPNKDVQKEIADLTEALGTAAISQWQKDEMREALKGLKKTMDDLDRASKADVQKRVLEKTKQLIDSNPNQPLIV

VEMESGASAKALNESLKLLKAHSPHTAAMLFAVDNDAGKIICLCQVPQEVVDKGLKASDWVQEVCPVLDGKGGGKDTSAQ

ATGKNTQSIQEALKLAQDFASLRLQN

>XP_013876050.1 PREDICTED: alanine--tRNA synthetase, cytoplasmic [Austrofundulus limnaeus]

MDSSLSAAQIRQKFIDFFIRYEHQYVHSSSTVPLDDPTLLFANAGMNQFKPIFLNTIDPSHPMAKLRRAANTQKCIRAGG

KHNDLDDVGKDVYHHTFFEMLGSWSFGDYFKHLACKMALELLTQEFGISIDRLYVTYFGGNPEAGLEPDLECKQIWMDLG

VDEVRILPGSMKDNFWEMGDTGPCGPCSEIHYDRIGGRDASHLVNMDDPNVLEIWNLVFIQFNRESETELKPLPKKSIDT

GMGLERLVSVLQNKMSNYDTDLFQPYFQAIQKGTGARSYTGKVGPDDVDGIDMAYRVLADHARTITIALSDGGRPDNTGR

GYVLRRILRRAVRYSHEKLGAQRGFFSSLVDVVVHSLGDAFPELKKDPDMVKDIINDEEAQFLKTLSRGRRILDRKILSL

GECRTIPGDTAWLLYDTYGFPLDLTSLIAEEKGLEVDLAAFEEEKKAAQLKSQGKGAGDQDHIMLDIYAIEELRNKQVPT

TDDSPKYRYSSDQNGNYEFQQASATVLALRRDRTFCDQVTTGQECGVLLDQTSFYAEQGGQTFDEGYMLREDDNAEDRME

FSVKNTQVRGGYVLHVGTVYGTLKVGDRVTLHVDEARRRPIMNNHTATHILNFALRGVLGEADQRGSLVAPDRLRFDFTA

KGALSTSEIRRTEEMACTLIREAKPVFAMDAPLAEAKAIQGLRAVFDETYPDPVRVVSIGVPVQELLDDPNSPAGSFTSI

EFCGGTHLQNSGHAAPFVIVSEEAIAKGIRRIVAVTGTEAQKAQRKADGLLQALSSLGDQVKQQTAPNKDLQKEIADLTE

ALGAAVISQWQKDEMRDTLKGLKKTMDDLDRSYKADIQKRVLEKTKEVMEQSPNQPLLIMEMESGASAKALNESLKLLKS

QSPQTATMLFTVDPEAGKITCLCQVPQEVASRGLKASEWVQELCPLMDGKGGGKDLSAQATGRNTQCLQEALQVANDFAR

LKLGEN

>XP_020508728.1 alanine--tRNA synthetase, cytoplasmic [Labrus bergylta]

MDSSMSAAQIRQKFIDFFRRYEHHYVHSSATIPLDDPTLLFANAGMNQFKPIFLNTIDPSHPMAKLRRAANTQKCIRAGG

KHNDLDDVGKDVYHHTFFEMLGSWSFGDYFKHLACEMALELLTKEFGIPIERLYVTYFGGSTEAGLEPDLECKQIWIDLG

MDEARILPGSMKDNFWEMGDTGPCGPCSEIHYDRIGGRDASHLVNMDDPNVLEIWNLVFIQFNRESETELKPLPKKSIDT

GMGLERLVSVLQNKMSNYDTDLFIPYFKAIQKGTGARPYTGKVGAEDADGIDMAYRVLADHARTITIALSDGGRPDNTGR

GYVLRRILRRAVRYSHEKLGAQKGFFASLVDVVVDSLGDAFPELKKDPEMVKEVINEEETQFLKTLSRGRRILDRKIMSL

ADCKTIPGDTAWLLYDTYGFPLDLTSLIAEEKGMMVDLAAFEEEKKAAQLKSQGKGAGDEDHIMLDIYAIEELRNKKIPA

SDDSPKYKYSSDQNGNYAFEQTTATVLALRRDRAFCNEVTTGQQCGVLLDQTTFYAEQGGQTFDEGYMLREDDSTDDRTE

FTVKNTQVRGGFVLHVGTVYGTLKVGDRVTLRVDEARRRPIMSNHTATHILNFALRGVLGEADQRGSLVAADRLRFDFTA

KGALSTGEVRRTEEIACALIRDNKEVYAMDAPLAEAKAIQGLRAVFDETYPDPVRVVSIGIPVEELLKDPESAAGSLTSI

EFCGGTHLRNSGHAAPFVIVSEEAIAKGIRRIVAVTGAEAQKAQRKADSLQQSLSSLGVKVKQQKAPNKDIQKEIADMTE

MIGTAVISQWRKDEMRETLKGLKKIMDDLDRSYKADIQKRVLEKTKEVIASSPNQPLLVMVLESGASAKALNDSLKLLKT

QSPQTAAMLFTIDPEASKIICLCQVPEEVSKRGLKANEWVQEICPLMDGKGGGKNMSAQATGRNTQSLQDALDVANEFAR

LKLGEN

>XP_019743922.1 PREDICTED: alanine--tRNA synthetase, cytoplasmic [Hippocampus comes]

MDSSLSAAQIRQKFIDFFNRYEHQYVHSSSTIPLDDPTLLFANAGMNQFKPIFLNTIDPSHPMARLRRAANTQKCIRAGG

KHNDLDDVGKDVYHHTFFEMLGSWSFGDYFKHLACKMALELLTEEFGISIDRLYVTYFGGNDDAGLEADLECKEIWMSLG

MEESRILPGSMKDNFWEMGDTGPCGPCSEIHYDRIGGRDASHLVNMDDPNVLEVWNLVFIQFNRESETVLKPLPKKSIDT

GMGLERLVSVLQNKMSNYDTDLFIPYFEAIQKGTGARPYTGKVGAEDVDGVDMAYRVLADHARTITIALSDGGRPDNTGR

GYVLRRILRRAVRYSHEKLGAQKGFFASLVDVVVNSLGDAFPELKKDPEIVKDIINEEEAQFLKTLTRGRRILDRKIMSM

GDSKTLPGDTAWLLYDTYGFPLDLTSLIAEEKGMVVDLASFEEEKKAAQLKSQGKGAGDVDHIMLDIYAIEELRNKNIPP

TDDSPKYKYTSNENGIYEFQQTSATVLALRRDRDFCDEVTTGQKCGVLLDQTSFYAEQGGQTFDEGYLLREDDNSEDRME

FSVKNTQVRGGYVLHVGTIYGTLKVGDRVTLHVDEARRRPIMSNHTATHILNFALRGVLGEADQRGSLVASDRLRFDFTA

KGALSTGEVRRTEEIACAMIRDAKPVYAMEASLAEAKAIQGLRAVFDETYPDPVRVVSIGIPVQDLLDDPNSAAGSLTSI

EFCGGTHLQNSAHAAPFVIVSEEAIAKGIRRIVAVTGNEAQKAQRKADTLNQALTALADKVKQQTVPNKDIQKEIADMSE

SIGTAVISQWRKDEMRDTLKALKKTMDDLDRSYKADIQKRVLEKTKEVIESSPNQALLVMAMETGASAKALNECLKVLKA

SSPQTAAMLFAVDPDAGKVICLCEVPQEKANRGLKANEWVQKLCPLMDGKGGGKDMSAQAAGRNTECLQEALQMAKQFAT

LKLGEN

>XP_019796746.1 PREDICTED: alanine--tRNA synthetase, cytoplasmic isoform X2 [Tursiops truncatus]

MDSTLTASEIRQRFIDFFKRNEHTYVHSSATIPLDDPTLLFANAGMNQFKPIFLNTVDPSHPMAKLSRAANTQKCIRAGG

KHNDLDDVGKDVYHHTFFEMLGSWSFGDYFKELACKMALELLTQEFGIPVERLYVTYFGGDEAASLEPDLECKQIWHNLG

LDDSKILPGNMKDNFWEMGDTGPCGPCSEIHYDRIGGRDAAHLVNQDDPNVLEIWNLVFIQYNRETDGILKPLPKKSIDT

GMGLERLVSVLQNKMSNYDTDLFVPYFEAIQKGTGARPYTGKVGAEDADGIDMAYRVLADHARTITVALADGGRPDNTGR

GYVLRRILRRAVRYSHEKLNASRGFFATLADVVVQSLGDAFPELKKDPDMVKDIINEEEVQFLKTLSRGRRILDRKIQSL

GDSKTIPGDTAWLLYDTYGFPVDLTGLIAEEKGLVVDMDGFEEERKLAQLKSQGKGAGGEDLIMLDIYAIEELREKGLEA

TDDSPKYNYHSDSSGSYVFESAVATVMALRRDKMFVEEVSTGQECGVVLDKTCFYAEQGGQIYDEGYLVKVDDSSEDKTE

FTVKNAQVRGGYVLHVGTIYGSLKVGDQVRLFIDEPRRRPVMSNHTATHILNFALRSVLGEADQRGSLVAPDRLRFDFTA

KGAMSTQQIKKAEEIANEMIEAAKPVYTQDCPLAAAKAIQGLRAVFDETYPDPVRVVSIGVPVSELLDDPSGPAGSLTSV

EFCGGTHLQNSSHAGAFVIVSEEAIAKGIRRIVAVTGAEAQKALRKAESLKKSLSVAEAKVKAQTAPNKDVQREIADLGE

PCSLQLMQLLIK

>KFQ89075.1 Alanine--tRNA synthetase, cytoplasmic, partial [Phoenicopterus ruber ruber]

MEATLTASQIRQRFIDFFKENKHTYVHSSSTIPLDDPTLLFANAGMNQFKPIFLNTIDPSHPLAKLSRATNTQKCIRAGG

KHNDLDDVGKDVYHHTFFEMLGSWSFGDYFKELACKLALDLLTKEFGIPTERLYVTYFGGNEAAGLQPDLECKQIWLNLG

LAEGRILPGNMKDNFWEMGDTGPCGPCSEIHYDRIGDRDASHLVNQDDPNVLEIWNLVFIQFNREADGTLKPLPKKSIDT

GMGLERLVSVLQNKMSNYDTDLFLPYFEAIQKGTGARPYTGQVGAEDVDGIDMAYRVLADHARTITLALSDGGRPDNTGR

GYVLRRILRRAVRYSHEKLNAPKGFFATLVDVVVQSLGDAFPELKKDPDMVKDIINEEEDQFLKTLSRGRRILDRKIQSL

GDSKTIPGDTAWLLYDTYGFPADLTGLIAEEKGLVVDMEGFEEERKNAQLKSQGKGAGGEDLLMLDIYAIEELRARGLEV

TDDSPKYSYASDQSGAYDFGSLVATVKAIRREKKFVEEVSTGQECGIVLDRTCFYAEQGGQIYDEGYMVKDDDGREDKTE

FTVKNVQVRGGYVLHIGTLYGSLKVGDQVHLSIDEARRRPVMSNHTATHILNFALRSVLGEADQRGSLVAPDRLRFDFTA

KGALSTQEIKKVEGIANQMIEEAKTVYAKDCPLAAAKAIQGLRAVFDETYPDPVRVVSIGIPVEELLADPSGPAGSITSI

EFCGGTHLQNSSHAGPFVIVSEEAIAKGIRRIVAVTGAEARKALRKVDSLRKLLSALEAKVKVQTAPNKDVQKEITDLSE

TLATAVIPQWQKDELREAVKALKKVMDDLDRASKADIQKRVLEKTRQVIESHPNQPLVIMEMENGASAK

>XP_020668305.1 alanine--tRNA synthetase, cytoplasmic isoform X2 [Pogona vitticeps]

MAKLTRAANTQKCIRAGGKHNDLDDVGKDVYHHTFFEMLGSWSFGDYFKELACRFALDLLTKEFGIPVERLYVTYFGGNE

AAGLHPDLDCKQIWLDLDLPESRILPGSMKDNFWEMGDTGPCGPCSEIHYDRIGGRDASHLVNQDDPNVLEIWNLVFIQF

NREADGTLKPLPKKSIDTGMGLERLVSILQSKMSNYDTDLFVPYFEAIQKGTGCRPYTGKVGEDDADGIDMAYRVLADHA

RTITVALSDGGRPDNTGRGYVLRRILRRAVRYAHEKLSAPKGFFASLVDVVVESLGGAFPELKKDPEMVKDIINEEEVQF

LKTLNRGRRILDRKIQSLGDSKVIPGDTAWLLYDTYGFPVDLTGLIAEEKGLKVDMEGFEEERKTAQLKSQGKGGGDEDL

IMLDIYAIEELRSRGLEPTDDSLKYNYSSDPRGTYKFSSPVATVKALRREKAFVEQVSTGQECGVILDHTCFYAEQGGQI

YDEGYLVKEDEGSEDKTEFTVKNVQVRGGYVLHVGTVYGNLKVGDTVQLYIDEARRRPIMSNHTATHILNFALRAVLGEA

DQRGSLVAPDRLRFDFTAKGAMSTEEIRKAEETVNQMIQEAKVVYAKDCPLAAAKAIQGLRAVFDETYPDPVRVISIGIP

VEDMLDDPSSPAGAITSVEFCGGTHLQNSSHAGHFVIVSEEAIAKGIRRIVAVTGVEAQKALRKADIIQKLLTDLEAKVQ

IQTAPNKDVQREIADLGETLATAIIPQWQKDEFREALKALKKIMDDLDRAYKADIQKRVLDKTKRLIEAHPNQPLVILEM

ESGATAKALNESLKLLKTHSPETAAMLFAIDNEAEKIICLCQVPQEAVNKGLKASEWVQQVSGLMEGRGGGKNLSAQATG

RNTSCLEEALQVAMDFAKLKLGELKN

>XP_003976443.1 PREDICTED: LOW QUALITY PROTEIN: alanine--tRNA synthetase, cytoplasmic [Takifugu rubripes]

MDSTLTAAQIRQKFIDFFLRHEHSYVHSSATVPLDDPTLLFANAGMNQFKPIFLNTIDPSHPMAKLRRAANTQKCIRAGG

KHNDLDDVGKDVYHHTFFEMLGSWSFGDYFKQLACKMALELLTQEFGISIDRLYVTYFGGSEEAGLEADLECKQLWINLG

MDEARILPGSMKDNFWEMGDTGPCGPCSEIHYDRIGGRDASHLVNMDDPNVLEIWNLVFIQFNRESESVLKPLPKKSIDT

GMGLERLVSVLQNKMSNYDTDLFLPYFEAIQQGTGARPYTGKVGAEDVDGIDMAYRVLADHARTLTIALSDGGRPDNTGR

GYVLRRILRRAVRYSHEKLGAQRGFFASLVDVVVKSLGDAFPELRKDPEMVKDVINEEEVQFLKTLSRGRRILDRKIISL

GESKTVPGDTAWLLYDTYGFPLDLTSLIAEEKGLNVDLAAFEEEKKAAQLKSQGKGAGDEDHIMLDIYAIEELRTKGVAA

TDDSLKYRYSSDERGQYEFQPASATVLALRRDRTFCTEVSSGQECGVLLDQTSFYAEQGGQTFDEGFMVREDDSTGDRME

FTVKNTQVRGGYVLHVGTVYGTLKLGDQVTLHVDEARRRLVMSNHTATHILNFALRGVLGEADQRGSLVAPDRLRFDFTA

KGALSTGEVRRTEEIACALIKEAKTVYALDAPLAQAKAIQGLRAVFDETYPDPVRVVSIGIPVEELLKDPDSTAGSLTSI

EFCGGTHLQNSAHAAPFVIVSEEAIAKGIRRIVAVTGGEAQKAQRKAEVLHQALSALSAKVQQQAAPNKDVQKEIADMTE

SVGTAVISQWRKDEMREAVKGLKKVMDDLDRSYKADIQKRVLEKTSEVIQNKPNQPLLIMEMETGASAKALNESLKLLXS

QSPQTAAMLFTVDPDAXXXXXXXXXPQEVASPGLKASEWVQELCPLLDGKGGGKDMSAQATGKNTQGLQEALQVADRFAR

MKLGEN

>XP_010077586.1 PREDICTED: alanine--tRNA synthetase, cytoplasmic, partial [Pterocles gutturalis]

SSQFKPIFLNTIDPSHPLAKLSRATNTQKCIRAGGKHNDLDDVGKDVYHHTFFEMLGSWSFGDYFKELACKLALDLLTKE

FGIPAERLYVTYFGGNEAVGLQPDLECKQIWLDLGLAEGRILPGNMKDNFWEMGDTGPCGPCSEIHYDRIGDRDASHLVN

QDDPNVLEIWNLVFIQFNREADGTLKPLPKKSIDTGMGLERLVSVLQNKMSNYDTDLFVPYFEAIQKGTGARPYAGQVGA

EDADGIDMAYRVLADHARTVTLALSDGGRPDNTGRGYVLRRILRRAVRYSHEKLNAPKCFFATLVDVVVQSLGDAFPELK

KDPDMVKDIINEEEDQFLKTLSRGRRILDRKIQSLGDSKIIPGDTAWLLYDTYGFPADLTGLIAEEKGLVVDMEGFEEER

KNAQLKSQGKGAGGEDLLMLDIYAIEELRARGLEVTDDSPKYSYASDQSGAYDFGSLVATVKAIRREKKFVEEVSTGQEC

GIVLDRTCFYAEQGGQIYDEGYMVKDDDGREDKTEFTVKNVQVRGGYVLHIGTLYGSLKVGDQVHLTIDETRRRPVMSNH

TATHILSFALRSVLGEADQRGSLVAPDRLRFDFTAKGALSTQEIKRVEGIANQMIEEAKPVYAKDCPLAAAKAIQGLRAV

FDETYPDPVRVVSIGIPVEELLADPSGPAGSVTSVEFCGGTHLQNSSHAGPFVIVSEEAIAKGIRRIVAVTGAEARKALR

KVDSLRKLLSALEAKVKVQTVPNKDVQKEITDLSETLAMAVIPQWQKDELREAVKALKKVMDDLDRASKADIQKRVLEKT

KQVIESHPNQPLVIMEMENGASAKALNESLKLFKTHSPETGTMLFAVDNGAAQFTXXXXXAQETANKGLKASQWVQEVSA

LMEGKGGGKDVSAQ

>XP_023379421.1 alanine--tRNA synthetase, cytoplasmic isoform X2 [Pteropus vampyrus]

MDSILTASEIRQRFIDFFKRNEHTYVHSSATIPLDDPTLLFANAGMNQFKPIFLNTIDPSHPMAKLSRAANTQKCIRAGG

KHNDLDDVGKDVYHHTFFEMLGSWSFGDYFKELACKMALELLTQEFGIPVERLYVTYFGGDEAAGLEPDLECKQIWQDLG

LDDTKILPGNMKDNFWEMGDTGPCGPCSEIHYDRIGGRDAAHLVNQDDPNVLEIWNLVFIQYNRETDGILKPLPKKSIDT

GMGLERLVSVLQNKMSNYDTDLFVPYFEAIQKGTGARPYTGKVGAEDADGIDMAYRVLADHARTITVALADGGRPDNTGR

GYVLRRILRRAVRYSHEKLNASKGFFATLVDVVVQSLGDAFPELKKDPDMVKDIINEEEVQFLKTLSRGRRILDRKIQSL

GDSKTIPGDTAWLLYDTYGFPVDLTGLIAEEKGLVVDMDGFEEERKMAQLKSQGKGAGGEDHIMLDIYAIEELREKSLEA

TDDSPKYNYHSDSSGSYAFESAVATVMALRRDKTFIEEVSTGQECGVVLDKTCFYAEQGGQIYDEGYLVKVDDSSEDKTE

FTVKNTQVRGGYVLHIGTIYGSLKVGDQVRLFIDEPRRRPVMSNHTATHILNFALRSVLGEADQRGSLVAPDRLRFDFTA

KGAMSTQQIKRVEEIANEMIEAAKPVYTQDCPLAAAKAIQGLRAVFDETYPDPVRVVSIGVPVSELLDDPSGPAGSLTSV

EFCGGTHLQNSSHAGAFVIVSEEAIAKGIRRIVAVTGAEAQKALRKAESLKKSLSVMEAKVKAQTVPNKDVQREIADLGE

VGVAGRLPSSGPARCWVLGLMNQTLPVLEEPSSPVSQCSGPLD

>XP_018582180.1 PREDICTED: alanine--tRNA synthetase, cytoplasmic-like [Scleropages formosus]

MDTSLTADQIRKKFIKFFQQHDHQYVHSSSTIPLDDPTLLFTNAGMNQFKPIFLGTIDPSHPMARLQRAANTQKCIRAGG

KHNDLDDVGKDVYHHTFFEMLGSWSFGDYFKHLACEMALELLTKEFGIPLERLYFTYFGGHSEAGLEPDLECKQIWLNLG

VEESRILPGSMKDNFWEMGDTGPCGPCSEIHFDRIGGRDVAHLVNMDDPNVLEIWNLVFIQFNRESETELKPLPKKSIDT

GMGLERLVSILQNKMSNYDTDLFVPYFKAIEKGTGARPYAGAVGAEDSDGIDMAYRVLADHARTITVALSDGGRPDSTGR

GYVLRRILRRAVRYAHEKLGAQRGFFATLVDVVVESLGDAFPELRKDPDVAKDIINEEEAQFLKTLSRGRRILDRKIQSL

GDRSTIPGDTAWLLYDTYGFPLDLTALIAEERGLGVDLDAFEEERRAAQVRSQGKGSGDEDRIMLDIYAIEKLRDMGLPA

TDDSPKYRYTSDSDGKYVFEQASGTVLALRRERAFVDEVVTGQECGVLLDRTCFYAEQGGQTFDEGYMLKEEDSAEDKME

FTVKNTQIRGGYVLHVGTVYGTLRVGDRVTLHLDEARRRPIMSNHTATHILNFALREVLGEADQRGSLVAPERLRFDFTA

KAALTTEEVHRTEEIVCAVMRDAKPVHAMEAPLAAAKAIQGLRAVFDETYPDPVRVVSIGIPIPQLLADPSGAAGSLTSI

EFCGGTHLQNSSHAAPFVIVSEEAIAKGIRRITAVTGMKALKAQRKADTLRLCLVTLGEEVRSQSSPNKDIQREIADLNE

SLGTAVISQWRKDKMREALKELKKTMDDLDRASKADVQKRVLEKTKQLIDSNPNQPLLVVEMESGASAKALNEAGKMLKT

HSPKTAAMLFSIDSEADKILCLCVSSEVADQGLKASEWVQEVCPLLDGKGGGKDTSAQATGHNAHCLQEALQLASKFALS

KLKPQN

>KPP75235.1 alanine--tRNA synthetase, cytoplasmic-like, partial [Scleropages formosus]

GPLKMDTSLTADQIRKKFIKFFQQHDHQYVHSSSTIPLDDPTLLFTNAGMNQFKPIFLGTIDPSHPMARLQRAANTQKCI

RAGGKHNDLDDVGKDVYHHTFFEMLGSWSFGDYFKHLACEMALELLTKEFGIPLERLYFTYFGGHSEAGLEPDLECKQIW

LNLGVEESRILPGSMKDNFWEMGDTGPCGPCSEIHFDRIGGRDVAHLVNMDDPNVLEIWNLVFIQFNRESETELKPLPKK

SIDTGMGLERLVSILQNKMSNYDTDLFVPYFKAIEKAGCAFSSCRRHYGTGARPYAGAVGAEDSDGIDMAYRVLADHART

ITVALSDGGRPDSTGRGYVLRRILRRAVRYAHEKLGAQRGFFATLVDVVVESLGDAFPELRKDPDVAKDIINEEEAQFLK

TLSRGRRILDRKIQSLGDRSTIPGDTAWLLYDTYGFPLDLTALIAEERGLGVDLDAFEEERRAAQMRSQGKGSGDEDRIM

LDIYAIEKLRDMGLPATDDSPKYRYTSDSDGKYVFEQASGTVLALRRERAFVDEVVTGQECGVLLDRTCFYAKQGGQTFD

EGYMLKEEDSAEDKKMEFTVKNTQIRGGYVLHVGTVYGTLRVGDRVTLHLDEARRRPIMSNHTATHILNFALREVLGEAD

QRGSLVAPERLRFDFTAKAALTTEEVHRTEEIVCAVMRDAKPVHAMEAPLAAAKAIQGLRAVFDETYPDPVRVVSIGIPI

SQLLADPSGAAGSLTSIEFCGGTHLQNSSHAAPFVIVSEEAIAKGIRRITAVTGMKALKAQRKADTLRLCLVTLGEEVRS

QSSPNKDIQREIADLNESLGTAVISQWRKDKMREALKELKKTMDDLDRASKADVQKRVLEKTKQLIDSNPNQPLLVVEME

SGASAKALNEAGKMLKTHSPKTAAMLFSIDSEADKILCLCQVPQEVADQGLKASEWVQEVCPLLDGKGGGKDTSAQATGH

NAHCLQEALQLASKFALSKLKPQN

>KFV11837.1 Alanine--tRNA synthetase, cytoplasmic, partial [Pterocles gutturalis]

SQFKPIFLNTIDPSHPLAKLSRATNTQKCIRAGGKHNDLDDVGKDVYHHTFFEMLGSWSFGDYFKELACKLALDLLTKEF

GIPAERLYVTYFGGNEAVGLQPDLECKQIWLDLGLAEGRILPGNMKDNFWEMGDTGPCGPCSEIHYDRIGDRDASHLVNQ

DDPNVLEIWNLVFIQFNREADGTLKPLPKKSIDTGMGLERLVSVLQNKMSNYDTDLFVPYFEAIQKGTGARPYAGQVGAE

DADGIDMAYRVLADHARTVTLALSDGGRPDNTGRGYVLRRILRRAVRYSHEKLNAPKCFFATLVDVVVQSLGDAFPELKK

DPDMVKDIINEEEDQFLKTLSRGRRILDRKIQSLGDSKIIPGDTAWLLYDTYGFPADLTGLIAEEKGLVVDMEGFEEERK

NAQLKSQGKGAGGEDLLMLDIYAIEELRARGLEVTDDSPKYSYASDQSGAYDFGSLVATVKAIRREKKFVEEVSTGQECG

IVLDRTCFYAEQGGQIYDEGYMVKDDDGREDKTEFTVKNVQVRGGYVLHIGTLYGSLKVGDQVHLTIDETRRRPVMSNHT

ATHILSFALRSVLGEADQRGSLVAPDRLRFDFTAKGALSTQEIKRVEGIANQMIEEAKPVYAKDCPLAAAKAIQGLRAVF

DETYPDPVRVVSIGIPVEELLADPSGPAGSVTSVEFCGGTHLQNSSHAGPFVIVSEEAIAKGIRRIVAVTGAEARKALRK

VDSLRKLLSALEAKVKVQTVPNKDVQKEITDLSETLAMAVIPQWQKDELREAVKALKKVMDDLDRASKADIQKRVLEKTK

QVIESHPNQPLVIMEMENGASAKALNESLKLFKTHSPETGTMLFAVDNGAAHCLFFFLIFSPSFPAQETANKGLKASQWV

QEVSALMEGKGGGKDVSAQA

>XP_014684620.1 PREDICTED: alanine--tRNA synthetase, cytoplasmic isoform X2 [Equus asinus]

MKDNFWEMGDTGPCGPCSEIHYDRIGGRDAAHLVNQDDPNVLEIWNLVFIQYNRETDGVLKPLPKKSIDTGMGLERLVSV

LQNKMSNYDTDLFVPYFEAIQKGTGARPYAGKVGADDADGIDMAYRVLADHARTITVALADGGRPDNTGRGYVLRRILRR

AVRYSHEKLNASRGFFATLVDVVVQSLGDAFPELKKDPDMVKDIINEEEVQFLKTLSRGRRILDRKIQSLGESKTIPGDT

AWLLYDTYGFPVDLTGLIAEEKGLVVDMDGFEEERKLAQLKSQGKGAGGEDLIMLDIYAIEELREKGLEATDDSPKYNYY

SDSSGSYVFESAVATVMALRRDKMFVEEVSTGQECGVVLDKTCFYAEQGGQIYDEGYLVKVDDSSEDKTEFTVKNAQVRG

GYVLHIGTIYGNLKVGDQVRLFIDEPRRRPVMSNHTATHILNFALRSVLGEADQRGSLVAPDRLRFDFTAKGAMSTQQIK

KAEEIANEMIEAAKPVYTQDCSLAAAKAIQGLRAVFDETYPDPVRVVSIGVPVSELLDDPSGPAGSLTSVEFCGGTHLQN

SSHAGAFVIVSEEAIAKGIRRIVAVTGAEAQKALRKAESLKKSLSVMEGKVKAQTAPNKDVQREIADLGEALATAVIPQW

QKDEFRENLKSLKKIMDDLDRASKADVQKRVLEKTKQLIDSNPNQPLVILEMESGASAKALNEALKLFKTHSPQTSAMLF

TVDNEAGKITCLCQVPQNAANRGLKASEWVQQVSGLMDGKGGGKDVSAQATGKNVGCLQEALQLATSFAQLRLGDVKN

>OBS81396.1 hypothetical protein A6R68_20424, partial [Neotoma lepida]

FPEVTFKMDSVLTAREIRERFINFFKQNKHTYFKPIFLNTIDPSHPMAKLSRAANTQKCIRAGGKHNDLDDVGKDVYHHT

FFEMLGSWSFGDYFKELACKMALELLTQELDDAKILPGNMKDNFWEMGDTGPCGPCSEIHYDRIGGRDAAHLVNQDDPNV

LEIWNLVFIQYNRETDGLLKPLPKKSIDTGMGLERLVSVLQNKMSNYDTDLFVPYFEAIQKGTGARPYTGKVGAEDADGI

DMAYRVLADHARTITVALADGGRPDNTGRGYVLRRILRRAVRYSHEKLNASRGFFATLVDVVVQSLKDPDMVKDIINEEE

VQFLKTLSRGRRILDRKIQSLGDCKTIPGTGNPSEKEWFLASAKVESTLTKLLLLKSQGKGAGGEDLIMLDIYAIEELRA

KGLEATDDSPKYNYHSDSSGSYGRLLFLLLPCSLPLNLRSLKRSVPREKMFVEEVVTGQECGVVLDKTCFYAEQGGQIYD

EGYLVKVDDSSEDKTEFTVKNAQVRGGYVLHIGTIYGNLKVGDQVRLFIDEPRRRPVMSNHTATHILNFALRSVLGEADQ

KGSLVAPDRLRFDFTAKGAMSTQQIKKAEEIVNDMIDAAKPVYTQDCPLAAAKAIQGLRAVFDETYPDPVRVVSIGVPVS

ELLXDPSGPAGSLTSVEFCGGTHLQNSNDAGAFVIVTEEAIAKGIRRIVAVTGAEAQKALRKAESLKKSLSVMEAKVKAQ

TAPNKDVQREIADLGEALATAVIPQWQKDEQRETLKSLKKVMDDLDRASKADVQKRVLEKTKQLIDSNPNQPLALNEALK

LFKTHSPQTSAMLFTVDNEAGKITCLCQNAANRGLKASEWVQQVSGLMDGKGGGKDVSAQATGKNVGCLQEALQLATSFA

QLRLGDVKN

>KFP31779.1 Alanine--tRNA synthetase, cytoplasmic, partial [Colius striatus]

MEVTLTASQIRQRFINFFKANQHTYVHSSSTIPLDDPTLLFANAGMNQFKPIFLNTIDPSHPLAKLSRATNSQKCIRAGG

KHNDLDDVGKDVYHHTFFEMLGSWSFGDYFKELACKLALDLLTKEFGIPVDRLYATYFGGNEAAGLQPDLECKQIWLDLG

LAEGRILPGNMKDNFWEMGDTGPCGPCSEIHYDRIGDRDASHLVNQDDPNVLEIWNLVFIQFNREADGTLKPLPKKSIDT

GMGLERLVSVLQNKMSNYDTDLFLPYFEAIQKGTGARPYLGQVGAEDTDGIDMAYRVLADHARTITLALADGGRPDNTGR

GYVLRRVLRRAVRYSHEKLNAPKGFFATLVDVVVQSLGEAFPELKKDPDMVKDIINEEEDQFLKTLSRGRRILDRKIQSL

GDSKIIPGDTAWLLYDTYGFPVDLTGLIAEEKGLAVDMEAFEEEKRNAQLKSQGKGTGGEELLMLDIYAIEELRARGLEA

TDDSPKYSYTADPSGTYDFGSLVATVKAIRREKHFVEEASTGQECGVVLDQTCFYAEQGGQSYDQGFMVREDDGREDKTE

FTVKNVQVRGGYVLHIGTLYGSLKVGDQVHLSIDEGRRRPIMSNHTATHILNFALRSVLGEAEQRGSLVAPDRLRFDFTS

KGALSTQDIKRVEGIANQMIQEAKPVFAKECPLAAAKAIQGLRAVFDETYPDPVRVVSVGVPVEQLLDGPAGSLTSVEFC

GGTHLQNSSHAGPFVIVSEEAIAKGIRRIVAVTGAEARKALRKVDSLRKLLSALEAKVKVQTAPNKDVQKEITDLSETLA

TAVIPQWQKDELREAVKALKKVMDDLDRASKADLQKRVLEKTKQVIESHPNQPLVIMEMESGASAKALNESLKLFKTHSP

QTATMLFTVDNEAGRIVC

>XP_023611841.1 alanine--tRNA synthetase, cytoplasmic isoform X2 [Myotis lucifugus]

MKDNFWEMGDTGPCGPCSEIHYDRIGGRDAAHLVNQDDPNVLEIWNLVFIQYNRETDGTLKPLPKKSIDTGMGLERLVSV

LQNKMSNYDTDLFVPYFEAIQKGTGARPYTGKVGADDADGIDMAYRVLADHARTITVALADGGRPDNTGRGYVLRRILRR

AVRYSHEKLSASKGFFATLVDVVVQSLGDAFPELKKDPDMVKDIINEEEVQFLKTLSRGRRILDRKIQSLGDSKTIPGDT

AWLLYDTYGFPVDLTGLIAEEKGLVVDMDGFEEERKLAQLKSQGKGAGGEDHIMLDIYAIEELRGKSLEATDDSPKYSYL

SDSSGSYAFESTVATVMALRRDKMFVEEVSTGQECGVVLDKTCFYAEQGGQIYDEGYLVKVDDSSEDKTEFTVKNTQVRG

GYVLHIGTIYGNLKVGDQVRLFIDEPRRRPVMSNHTATHILNFALRSVLGEADQRGSLVAPDRLRFDFTAKGAMSTQQIK

KAEQIVNEMIEAAKPVYTQDCPLAAAKAIQGLRAVFDETYPDPVRVVSIGVPVSKLLDDPSGPAGSLTSVEFCGGTHLQN

SSHAGAFVIVSEEAIAKGIRRIVAVTGAEAQKALRKAESLKKSLSVMEAKVKAQTSPNKDVQREIADLGEALATAVIPQW

QKDEFRETLKSLKKIMDDLDRASKADVQKRVLEKTKQLIDSNPNQPLVILEMESGASAKALNEALKLFKTHSPQTSAMLF

TVDNEAGKITCLCQVPQNAANRGLKASEWVQQVSGLMDGKGGGKDVSAQATGKNVGCLQEALQLATSFAQLRLGDVKN

>XP_010200383.1 PREDICTED: alanine--tRNA synthetase, cytoplasmic, partial [Colius striatus]

MEVTLTASQIRQRFINFFKANQHTYVHSSSTIPLDDPTLLFANAGMNQFKPIFLNTIDPSHPLAKLSRATNSQKCIRAGG

KHNDLDDVGKDVYHHTFFEMLGSWSFGDYFKELACKLALDLLTKEFGIPVDRLYATYFGGNEAAGLQPDLECKQIWLDLG

LAEGRILPGNMKDNFWEMGDTGPCGPCSEIHYDRIGDRDASHLVNQDDPNVLEIWNLVFIQFNREADGTLKPLPKKSIDT

GMGLERLVSVLQNKMSNYDTDLFLPYFEAIQKGTGARPYLGQVGAEDTDGIDMAYRVLADHARTITLALADGGRPDNTGR

GYVLRRVLRRAVRYSHEKLNAPKGFFATLVDVVVQSLGEAFPELKKDPDMVKDIINEEEDQFLKTLSRGRRILDRKIQSL

GDSKIIPGDTAWLLYDTYGFPVDLTGLIAEEKGLAVDMEAFEEEKRNAQLKSQGKGTGGEELLMLDIYAIEELRARGLEA

TDDSPKYSYTADPSGTYDFGSLVATVKAIRREKHFVEEASTGQECGVVLDQTCFYAEQGGQSYDQGFMVREDDGREDKTE

FTVKNVQVRGGYVLHIGTLYGSLKVGDQVHLSIDEGRRRPIMSNHTATHILNFALRSVLGEAEQRGSLVAPDRLRFDFTS

KGALSTQDIKRVEGIANQMIQEAKPVFAKECPLAAAKAIQGLRAVFDETYPDPVRVVSVGVPVEQLLDGPAGSLTSVEFC

GGTHLQNSSHAGPFVIVSEEAIAKGIRRIVAVTGAEARKALRKVDSLRKLLSALEAKVKVQTAPNKDVQKEITDLSETLA

TAVIPQWQKDELREAVKALKKVMDDLDRASKADLQKRVLEKTKQVIESHPNQPLVIMEMESGASAKALNESLKLFKTHSP

QTATMLFTVDNEAGRI

>XP_023597666.1 alanine--tRNA synthetase, cytoplasmic isoform X2 [Trichechus manatus latirostris]

MDSTLTATEIRQRFIDFFKRNEHTYVHSSATIPLDDPTLLFANAGMNQFKPIFLNTIDPSHPMAKLSRAANTQKCIRAGG

KHNDLDDVGKDVYHHTFFEMLGSWSFGDYFKELACKMALELLTQEFGIPVERLYVTYFGGDEATRLEPDLECKQIWQNLG

LDDTKILPGNMKDNFWEMGDTGPCGPCSEIHYDRIGGRDAAYLVNQDDPNVLEIWNLVFIQYNRETDGILKPLPRKSIDT

GMGLERLVSVLQNKMSNYDTDLFVPYFEAIQKGDAFPELKKDPDMVKDIINEEEVQFLKTLSRGRRILDRKIQSLGDSKT

IPGDTAWLLYDTYGFPVDLTGLIAEEKGLVVDMDSFEEERKLAQLKSQGKGAGGEDLIMLDIYAIEELRSKGLEATDDSL

KYNYHSDSSGSYVFENAVATVMALRRDKMFVEEVFTGQECGVVLDKTCFYAEQGGQIYDEGYLVKVDDSSEDKAEFTVKN

AQVRGGYVLHIGTIYGNLKVGDRVRLFIDEPRRRPVMSNHTATHILNFALRSVLGEADQRGSLVAPDRLRFDFTAKGAMS

TEQIKEAEEIANQMIEAAKPVYTQDCLLAAAKAIQGLRAVFDETYPDPVRVVSIGVPVSELLEDPSGPAGSLTSVEFCGG

THLQNSSHAGTFVIVSEEAIAKGIRRIVAITGAEAQKALRKAESLKKSLSVIEAKVKAQTTPNKDVQREIADLGEALATA

VVPQWQKDELREHLKSLKKIMDDLDRASKADVQKRVLEKTKQLIDSHPNQPLVILEMESGASAKALNEALKLFKTHSPQT

AAMLFTVDSEAGKITCLCQVPQNAANQGLKASEWVQQVSGLMDGKGGGKDASAQATGKNVGCLQEALQLATSFAQLHLGD

LKN

>PWA33020.1 hypothetical protein CCH79_00013087, partial [Gambusia affinis]

MDSSLSAAQIRQKFINFFIRYEHQYVHSSSTIPLDDPTLLFANAGMNQFKPIFLNTIDPSHPMAKLRRAANTQKCIRAGG

KHNDLDDVGKDVYHHTFFEMLGSWSFGDYFKQLACKMALELLTEEFGIPIDRLYVTYFGGNADAGLEPDLECKQIWMDLG

VDEARILPGSMKDNFWEMGDTGPCGPCSEIHYDRIGGRDASHLVNMDDPNVLEIWNLVFIQFNRESETELKPLPKKSIDT

GMGLERLVSVLQNKMSNYDTDLFVPYFEAIQKGTGARPYTGKVGADDVDGIDMAYRVLADHARTITISLSDGGRPDNTGR

GYVLRRILRRAVRYSHEKLGAQRGFFASLVDVVVDSLGDAFPELRKDPEMVKDVINEEETQFLKTLSRGRRILDRKIMSL

GDCKTIPGDTAWLLYDTYGFPLDLTSLIAEEKGMAVNLAAFEEEKKAAQLKSQGKGAGGEDHIMLDIYAIEELRNQQVPA

TDDSPKYRYASEENGSYEFRPTSATVLALRRDRAFCQEVTTGQECGVLLDQTSFYAEQGGQTFDEGYMLREDDSSEDRME

FTVKNTQVRGGYVLHIGTVYGTLKVGDRVTLHIDEVENLSFIIRSDFSISAAKNDKHSCFQARRRPIMSNHTATHILNFA

LRSVLGEADQRGSLVAPDRLRFDFTAKGALSTAEIRRTEEIACGMIKDAKPVYAMEAPLAEAKAIQGLRAVFDETYPDPV

RVVSIGIPVQELLDDPNSPAGSLTSIEFCGGTHLQNSGHAAPFVIVSEEAIAKGIRRIVAVTGAEAQKAHRKADALRLSL

SALGDKVKQQTCPNKDVQKEIADMTESIGTALISQWQKDEMRETLKALKKTMDDLDRSYKADIQKRALNESLKLLKSQSP

QTAAMLFTVDPDAGKITCLCQVPQDVANRGLKASEWVQELCPLLDGKGGGKDMSAQATGRNTQCLQEALQLANDQKNLFC

REKCGSTPTAGGKTYFGSSVEESDLCHVLSVGGRNLIWTEGTLLLSSPHRITTSWAAADEEQQPQLKM

>XP_009965055.1 PREDICTED: LOW QUALITY PROTEIN: alanine--tRNA synthetase, cytoplasmic, partial [Tyto alba]

MEGTLTASQIRQRFIDFFKKNKHTYVHSSSTIPLDDPTLLFANAGMNQFKPIFLNTIDPSHPLAKLNRATNTQKCIRAGG

KHNDLDDVGKDVYHHTFFEMLGSWSFGDYFKELACKLALDLLTKEFGIPAERLYVTYFGGNEAAGLQPDLECKQIWLNLG

LAEGRILPGNMKDNFWEMGDTGPCGPCSEIHYDRIGDRDASHLVNQDDPNVLEIWNLVFIQFNREADGTLKPLPKKSIDT

GMGLERLVSVLQNKMSNYDTDLFLPYFEAIQKGTGARPYMGLVGAEDADGIDMAYRVLADHARTITLALSDGGRPDNTGR

GYVLRRILRRAVRYSHEKLNAPKGFFATLVDVVVQSLGDAFPELKKDPDMVKDIINEEEDQFLKTLSRGRRILDRKIQSL

GDSKIIPGDTAWLLYDTYGFPADLTGLIAEEKGLVVDMEGFEEERKNAQLKSQGKGAGGEDLLMLDIYAIEELKAQGLEV

TDDSPKYSYASDPSGTYDFGSLVATVKAIRREKKFVEQVSTGQECGIVLDRTCFYAEQGGQIYDEGYMVKDDDSREDKTE

FTVKNVQVRGGYVLHIGTLYGSLKVGDQVHLSIDETRRRPVMSNHTATHILNFALRSVLGEADQRGSLVAPDRLRFDFTA

KGALSTQEIKKVEGIANQMIEEAKTVYAKDCPLAAAKAIQGLRAVFDETYPDPVRVVSIGIPVEELLADPSGPAGSITSI

EFCGGTHLQNSSHAGPFVIVSEEAIAKGIRRIVAVTGAEARKALRKVDSLRKLLSALEAKVKVQTAPNKDVQKEITDLGE

TLATAVIPQWQKDELREAVKALKKIMDDLDRASKADIQKR

>XP_004704605.1 PREDICTED: alanine--tRNA synthetase, cytoplasmic isoform X2 [Echinops telfairi]

MKDNFWEMGDTGPCGPCSEIHYDRIGGRDAAHLVNQDDPNVLEIWNLVFIQYNREADGILRPLPKKSIDTGMGLERLVSV

LQNKMSNYDTDLFVPYFEAIQKGTGARPYTGKVGADDADGIDMAYRVLADHARTITVALADGGRPDNTGRGYVLRRILRR

AVRYSHEKLNASRGFFATLVDVVVQSLGDAFPELKKDPDMVKDIINEEEVQFLKTLSRGRRILDRKIQSLGDSQTIPGDT

AWLLYDTYGFPVDLTGLIAEEKGLAVDMEGFEEERKLAQLKSQGKGAGGEDLIMLDIYAIEELRSRGLEATDDSLKYSYH

SEPGGSYVFENTVATVMALRRDKMFVDEVATGQECGVVLDKTCFYAEQGGQIYDEGYLVKLDDNSEDRTEFTVKNAQVRG

GYVLHIGTVYGNLRVGDQVRLFIDETRRRPVMSNHTATHILNFALRAVLGEADQKGSLVAPDRLRFDFTAKGAMSTQQIK

EAEEIANQTIEAAKPVYTQDCALAAAKAIQGLRAVFDETYPDPVRVVSIGVPVSELLEDPTSPAGSLTSVEFCGGTHLQN

SSHAGAFVIVSEEAIAKGIRRIVAVTGAEAQKALRKAESMKKSLSVLEAKVKAQTSPNKDVQREIADLGEALATAVVPQW

QKDELREELKSLKKVMDDLDRASKADVQKRVLEKTKQLIDSRPNQPLVILEMESGASAKATRGPGLLPGLGSDISLSPQA

LNEALKLFKTHSPQTAAMLFTVDNEAGKITCLCQVPQNAANRGLKASEWVQQVSGLMDGKGGGKDVSAQATGKNVGCLQE

ALQLATSFAQLRLGDVKN

>XP_018427936.1 PREDICTED: alanine--tRNA synthetase, cytoplasmic, partial [Nanorana parkeri]

MTTPLSASAIRQKFIDFFKQNAHTYVHSSAVVPLDDPTLLFANAGMNQFKPIFLNTIDPSHPMAKLNRAANTQKCIRAGG

KHNDLDDVGKDVYHHTFFEMLGSWSFGDYFKELACKMALDLLTKEFGIDIDRLYVTYFGGNEAAGLEPDLECKQIWLSLG

LPEERILPGSMKDNFWEMGDTGPCGPCSEMHFDRIGGRDAAHLVNMDDPNVLEIWNLVFIQFNRESDGSLKPLPKKSIDT

GMGLERLVSVLQSKMSNYDTDLFVPYFEAIQKGTGARPYSGKVGAEDEDGIDMAYRVLADHARTITVALADGGRPDNTGR

GYVLRRILRRAVRYAHEKLNASKGFFSTLVDVVVQSLGDAFPEIRKDPDMVKDIINEEETQFLKTLTRGRRILDRKIQSL

GEHKIIPGDTAWLLYDTYGFPVDLTGLIAEERGLKVDMEAFEEERKAAQLKSQGKGAGDEDLLMLDIYAIEELRSRGLAP

TDDSPKYNYKSNSEGVYEFEGVVGTVKAIRREKTFVDEVSTGQECGIVLDKTCYYAEQGGQTFDEGYMVREDDASDDKTE

FTVRNTQVRGGYVLHIGTVYGTLKVGDCVHLYVDEARRRPIMSNHTATHILNFALRSVLGEADQRGSLVAPDRLRFDFTA

KGAMTTQEIRQAEEVAAKLIQENKTVYALDCPLAAAKSIQGLRAVFDETYPDPVRVVSVGIPVEDLLADPSGPAGSVTSI

EFCGGTHLQNSGHAGSFVIVTEEAIAKGIRRIVAVTGAEAQKAVRRQEALKSSLSQLEAKVKQQTSPNKDIQKEIADLGE

VRWSHDLPT

>KFQ48816.1 Alanine--tRNA synthetase, cytoplasmic, partial [Pelecanus crispus]

MEATLTASQIRQRFIDFFKENKHTYVHSSSTIPLDDPTLLFANAGMNQFKPIFLNTIDPSHPLAKLNRATNTQKCIRAGG

KHNDLDDVGKDVYHHTFFEMLGSWSFGDYFKELACKLALDLLTKEFGIPAERLYVTYFGGNEAAGLQPDLECKQIWLNLG

LAEGRILPGNMKDNFWEMGDTGPCGPCSEIHYDRIGDRDASHLVNQDDPNVLEIWNLVFIQFNREADGTLKPLPKKSIDT

GMGLERLVSVLQNKMSNYDTDLFLPYFEAIQKGTGARPYMGQVGAEDADGIDMAYRVLADHARTITLALSDGGRPDNTGR

GYVLRRILRRAVRYSHEKLNAPKGFFATLVDVVVQSLGDAFPELKKDPDMVKDIINEEEDQFLKTLSRGRRILDRKIQSL

GDSKTIPGDTAWLLYDTYGFPVDLTGLIAEEKGLVVDMEGFEEERKNAQLKSQGKGAGGEDLLMLDIYAIEELRARGLEV

TDDSPKYSYASDQSGAYDFGSLVATVKAIRREKKFVEEVSTGQECGIVLDRTCFYAEQGGQIYDEGYMVKDDDGREDKTE

FTVKNVQVRGGYVLHIGTLYGSLKVGDQVHLSIDESRRRPVMSNHTATHILNFALRSVLGEADQRGSLVAPDRLRFDFTA

KGALSTQEIKKVEGIANQMIEEAKTVYAKDCPLAAAKAIQGLRAVFDETYPDPVRVVSIGIPVEELLADPSGPAGSITSI

EFCGGTHLQNSSHAGPFVIVSEEAIAKGIRRIVAVTGAEARK

>XP_009491443.1 PREDICTED: alanine--tRNA synthetase, cytoplasmic, partial [Pelecanus crispus]

MEATLTASQIRQRFIDFFKENKHTYVHSSSTIPLDDPTLLFANAGMNQFKPIFLNTIDPSHPLAKLNRATNTQKCIRAGG

KHNDLDDVGKDVYHHTFFEMLGSWSFGDYFKELACKLALDLLTKEFGIPAERLYVTYFGGNEAAGLQPDLECKQIWLNLG

LAEGRILPGNMKDNFWEMGDTGPCGPCSEIHYDRIGDRDASHLVNQDDPNVLEIWNLVFIQFNREADGTLKPLPKKSIDT

GMGLERLVSVLQNKMSNYDTDLFLPYFEAIQKGTGARPYMGQVGAEDADGIDMAYRVLADHARTITLALSDGGRPDNTGR

GYVLRRILRRAVRYSHEKLNAPKGFFATLVDVVVQSLGDAFPELKKDPDMVKDIINEEEDQFLKTLSRGRRILDRKIQSL

GDSKTIPGDTAWLLYDTYGFPVDLTGLIAEEKGLVVDMEGFEEERKNAQLKSQGKGAGGEDLLMLDIYAIEELRARGLEV

TDDSPKYSYASDQSGAYDFGSLVATVKAIRREKKFVEEVSTGQECGIVLDRTCFYAEQGGQIYDEGYMVKDDDGREDKTE

FTVKNVQVRGGYVLHIGTLYGSLKVGDQVHLSIDESRRRPVMSNHTATHILNFALRSVLGEADQRGSLVAPDRLRFDFTA

KGALSTQEIKKVEGIANQMIEEAKTVYAKDCPLAAAKAIQGLRAVFDETYPDPVRVVSIGIPVEELLADPSGPAGSITSI

EFCGGTHLQNSSHAGPFVIVSEEAIAKGIRRIVAVTGAEARKVRGGRRDPVKSHVPRP

>ELW63019.1 Alanyl-tRNA synthetase, cytoplasmic [Tupaia chinensis]

MDSTLTASDIRSRFIDFFKRNEHTYVHSSATIPLDDPTLLFANAGMNQFKPIFLNTIDPSHPMAKLSRAANTQKCIRAGG

KHNDLDDVGKDVYHHTFFEMLGSWSFGDYFKELACKMALELLTEEFGIPVERLYVTYFGGDEAAGLEPDLECKQIWQNLG

LDDSKILPGNMKDNFWEMGDTGPCGPCSEIHFDRIGGRDAAHLVNQDDPNVLEIWNLVFIQYNRETDGILKPLPKKSIDT

GMGLERLVSVLQNKMSNYDTDLFVPYFEAIQKGTGARPYSGKVGADDTDGIDMAYRVLADHARTITGPMAAGLTTRGGGE

YVLRRILRRAVRYSHEKLNASRGFFATLVDVVVQSLGDAFPELKKDPDMVKDIINEEEVQFLKTLSRGRRILDRKIQSLG

DSKTIPGDTAWLLYDTYGFPVDLTGLIAEEKGLVVDMDGFEEERKLAQLKSQGKGAGGEDLIMLDIYAIEELRAKGLEAT

DDSPKYNYLSDSSGNYVFESTVATVLALRREKMFVAEVFTGQECGVVLDRTCFYAEQGGQTYDEGYLVKADDSSEDKTEF

TVKNVQVRGGYVLHVGTIYGHLKVGDQVRLFIDEPRRRPVMSNHTATHILNFALRCVLGEADQKGSLVAPDRLRFDFTAK

GAMSTQQIKKAEEIANEMIEAAKPVYTQDCPLAAAKAIQGLRAVFDETYPDPVRVVSIGVPVSELLDDPSGPAGSLTSVE

FCGGTMVELLRIHMERTWFPPVYTQDCPLAAAKAIQGLRAVFDETYPDPVRVVSIGVPVSELLDDPSGPAGSLTSVEFCG

GTHLRNSSHAGAFVIVTEEAIAKGIRRIVAVTGAEAQKALRKAESLKKSLSAMEAKVKAQTAPNKDVQREIADLGEALAT

AVIPQWQKDELREMLKSLKKVMDDLDRASKADVQKRVLERTKQLIDSNPNQPLVILEMENGASAKAGPAPSALNEALKLF

KTHSPQTSAMLFTVDNEAGKITCLCQVPQNAANRGLKASEWVQQVSGLMDGKGGGKDVSAQATGKNVGCLQEALQLATSF

AQLRLGDVKN

>KFZ55273.1 Alanine--tRNA synthetase, cytoplasmic, partial [Podiceps cristatus]

MQVTLTASQIRQRFIDFFKENQHTYVHSSSTIPLDDPTLLFANAGMNQFKPIFLNTIDPSHPLAKLNRATNTQKCIRAGG

KHNDLDDVGKDVYHHTFFEMLGSWSFGDYFKELACKLALDLLTKEFGIPAERLYVTYFGGNDAAGLQPDLECKQIWLNLG

LAEGRILPGNMKDNFWEMGDTGPCGPCSEIHYDRIGDRDASHLVNQDDPNVLEIWNLVFIQFNREADGTLKPLPKKSIDT

GMGLERLVSVLQNKMSNYDTDLFLPYFEAIQKGTGARPYMGLVGAEDVDGIDMAYRVLADHARTITLALSDGGRPDNTGR

GYVLRRILRRAVRYSHEKLNAPKGFFATLVDVVVQSLGDAFPELKKDPDMVKDIINEEEDQFLKTLSRGRRILDRKIQSL

GDSKIIPGDTAWLLYDTYGFPADLTGLIAEEKGLVVDMEGFEEERKNAQLKSQGKGAGGEDLLMLDIYAIEELRARGLEV

TDDSPKYSYTSDRSGAYDFGNLVATVKAIRREKKFVEEVSTGQECGIVLDRTCFYAEQGGQIYDEGYMVKDDDSREDKTE

FTVKNVQVRGGYVLHIGTLYGSLKVGDRVHLSIDEIRRRPVMSNHTATHILNFALRSVLGEADQRGSLVAPDRLRFDFTA

KGALSTQEIKKVEGIANQMIEEAKPVYAKDCPLAAAKAIQGLRAVFDETYPDPVRVVSIGNPVEELLADPSGPAGSLTSV

EFCGGTHLQNSSHAGPFVIVSEEAIAKGIRRIVAVTGAEARK

>XP_023715038.1 alanine--tRNA synthetase, cytoplasmic isoform X2 [Cryptotermes secundus]

MNTSLTAKEVRKAFIDFFIQKQHEYVHSSSTIPLDDPTLLFANAGMNQFKPIFLGTVDPNSDMAKWVRTVNTQKCIRAGG

KHNDLDDVGKDVYHHTFFEMLGNWSFGDYFKKEICAWAWELLTQVFKLPSDRLYVTYFGGDRAAGLEPDEECREIWINLG

LAPSHILPGSMKDNFWEMGETGPCGPCSELHFDRIGGRNAAHLVNEDDPDVLEIWNLVFIQFNRESDGSLKSLPKKHIDC

GMGLERLVSVIQEKRSNYDTDLFMPLFAAIQKGTGVPPYKGHVGEDDVDGVDMAYRVLADHARTLTIALSDGGHPDNTGR

GYVLRRILRRAVRYATEKLNAKPGFFATLVNTVVEILGDTFPEVKKDPQSVIDLINEEETQFLKTLTRGHNLLNRTIMKL

GNSKTLPGDVAWRLYDTYGFPVDLTQLMSEEKGLTVDMDAYEEAKKQAQILSQGRGGWYDDKIILDIHAITELRDQNVPL

TDDSPKYNYHAKSEDKDAEYEFDGCIAKVLRLRHSKKFVDHVTSGQECGVLLDKTSFYAEQGGQIYDEGFLVKVGDENVE

FSVKNVQIRGGYILHIGTVEGILCEGDEVSLHLDTSRRRLVMNNHTGTHVLNFALRTVLGTEADQKGSLVAPDRLRFDFT

NKGAMSVEQVKATEVHSNAVIDKNEPVYAKEASLAVAKAIQGLRAVFEETYPDPVRVVSIGIPVEKLEEDPYGPAGNNTS

VEFCGGTHLHRAGHVGKFVIASEEAIAKGIRRIVALTGPEASKALKKTELLENKLNKFISLLNSDDSEISNKDLVKKIVE

LTDDVSHATIPYWKKEEMRTNLKHLKKQLDDKDRASKAEVTAKVLEEVKALLAGNPKAPVIVQELKAYSNAKALDGALKQ

VKALSAETSAMFFSVDSEAKKIVCLSAVPKSAVNRGLKANEWVQQVSGLMQGKGGGKPEAAQASGSNVSCLQEALGIAAQ

FANAKLNSSS

>XP_023715037.1 alanine--tRNA synthetase, cytoplasmic isoform X1 [Cryptotermes secundus]

MTRRRKICEAKIMNTSLTAKEVRKAFIDFFIQKQHEYVHSSSTIPLDDPTLLFANAGMNQFKPIFLGTVDPNSDMAKWVR

TVNTQKCIRAGGKHNDLDDVGKDVYHHTFFEMLGNWSFGDYFKKEICAWAWELLTQVFKLPSDRLYVTYFGGDRAAGLEP

DEECREIWINLGLAPSHILPGSMKDNFWEMGETGPCGPCSELHFDRIGGRNAAHLVNEDDPDVLEIWNLVFIQFNRESDG

SLKSLPKKHIDCGMGLERLVSVIQEKRSNYDTDLFMPLFAAIQKGTGVPPYKGHVGEDDVDGVDMAYRVLADHARTLTIA

LSDGGHPDNTGRGYVLRRILRRAVRYATEKLNAKPGFFATLVNTVVEILGDTFPEVKKDPQSVIDLINEEETQFLKTLTR

GHNLLNRTIMKLGNSKTLPGDVAWRLYDTYGFPVDLTQLMSEEKGLTVDMDAYEEAKKQAQILSQGRGGWYDDKIILDIH

AITELRDQNVPLTDDSPKYNYHAKSEDKDAEYEFDGCIAKVLRLRHSKKFVDHVTSGQECGVLLDKTSFYAEQGGQIYDE

GFLVKVGDENVEFSVKNVQIRGGYILHIGTVEGILCEGDEVSLHLDTSRRRLVMNNHTGTHVLNFALRTVLGTEADQKGS

LVAPDRLRFDFTNKGAMSVEQVKATEVHSNAVIDKNEPVYAKEASLAVAKAIQGLRAVFEETYPDPVRVVSIGIPVEKLE

EDPYGPAGNNTSVEFCGGTHLHRAGHVGKFVIASEEAIAKGIRRIVALTGPEASKALKKTELLENKLNKFISLLNSDDSE

ISNKDLVKKIVELTDDVSHATIPYWKKEEMRTNLKHLKKQLDDKDRASKAEVTAKVLEEVKALLAGNPKAPVIVQELKAY

SNAKALDGALKQVKALSAETSAMFFSVDSEAKKIVCLSAVPKSAVNRGLKANEWVQQVSGLMQGKGGGKPEAAQASGSNV

SCLQEALGIAAQFANAKLNSSS

>XP_015239300.1 PREDICTED: alanine--tRNA synthetase, cytoplasmic [Cyprinodon variegatus]

MKDNFWEMGDTGPCGPCSEIHYDRIGGRDASHLVNMDDPNVLEIWNLVFIQFNRESETELKPLPKKSIDTGMGLERLVSV

LQNKMSNYDTDLFIPYFEAIQKGSGARPYTGKVGADDVDGIDMAYRVLADHARTITISLSDGGRPDNTGRGYVLRRILRR

AVRYSHEKLGAPRGFFASLVDVVVASLGDAFPELKKDPEMVKDIINEEEAQFLKTLSRGRRILDRKIMSLGECKTIPGDT

AWLLYDTYGFPLDLTSLIAEEKGMGVDLAAFEEEKKAAQLKSQGKGAGGEDHIMLDIYAIEDLRNKQIPATDDSPKYRYT

SDDNGNYEFQPASATVLALRRDRAFCEEVTTGQECGVLLDQTSFYAEQGGQTFDEGYMLREDDSSEDRMEFTVKNTQVRG

GYVLHIGTVYGTLKVGDRVTLHIDEARRRPIMSNHTATHILNFALRGVLGEADQRGSLVAPDRLRFDFTAKGALSTGEIR

RTEEIACAMIKEAKPVYAMEATLAEAKAIQGLRAVFDETYPDPVRVVSIGIPVQELLDDPNSPAGSLTSIEFCGGTHLQN

SGHAAPFVIVSEEAIAKGIRRIVAVTGAEAQKAARKADALRQSLSALGGKVKQQTTPNKDVQKEIADMNESIGTAVISQW

QKDEMRETLKALKKTMDDLDRSYKADIQKRVLEKTKEVIENNPNQPLLVMEMETGASAKALNESLKLLKNQSPQTAAMLF

TVDPDAGRITCLCQVPQDVANRGLKASEWVQELCPLLDGKGGGKDMSAQATGRNTQCLQEALQMANEFARLKLGEN

>PSN35457.1 Alanine--tRNA synthetase [Blattella germanica]

MNSSLTAKEIRRTFIDFFIQKKKHEYVHSSSTIPLDDPTLLFTNAGMNQFKPIFLGTVDPNSDMAKWVRTVNTQKCIRAG

GKHNDLDDVGKDVYHHTFFEMLGNWSFGDYFKKEICAWAWELLTEVFKLPADRLYVTYFGGDKAAKLEPDEECRQIWINL

GVPPAHVLPGSMKDNFWEMGETGPCGPCSELHFDRIGGRDAAHLVNQDDPDVLEIWNLVFIQFNRETDGSLKPLPKKHID

CGLGLERLVSVIQQKRANYDTDLFMPLFDSIQKGTGAPSYKGRVGEEDVDGMDMAYRVLADHARTLTIALSDGGHPDNTG

RGYVLRRILRRAVRYATEKLNAKPGFFATLVHTVVDLLGDTFPEVKKDPQSVIDIINEEETQFLKTLSRGRNLLNRTITK

LGGSKTVPGDVAWRLYDTYGFPVDLTQLMSEEKGLNVDMVGYEESKKQAQITSQGKGNYYDDKLSLDIHAIAELQKHGIA

VTDDSLKYSYQAKSADKDAEYEFKPCKGKVVALRHSKMFVDSVTSGQECGVVLDKTSFYAEQGGQIYDEGFMVKVGDESV

EFSVRNVQVRGGYILHMGTVEGTLRKGDEVMLHLDTARRRLIMNNHTGTHVLNYALRAVLGTDADQKGSLVAPDRLRFDF

TNKGAMTVDQVKSTEVESNKMINRNEPVYAKESKLPVAKTIQGLRAVFDETYPDPVRVVSIGVPVEQLEADPNGQAGKKT

SVEFCGGTHLQRSGHIGGFVIASEEAIAKGIRRIVALTGPEATKALKKTELLENELNKLIALMKDGQLSSKDIVKKIVEL

TEDVSHAVIPYWKKEELRTTLKGLKKQLDDKDRASKAAVSADVIEDAKAFITANPNIPVVVRELKAFSNSKALDGALKQV

KSLSPETSAMFFSVDPDAKKIVCLAAVPKNAINKGLKANEWVQRVSSLMQGKGGGKPESAQASGTNFSCLKEALKIAEEY

ANSKLGGGNVPASNHSSDVSNGPLIHTYANNIRAYRGLIAAQYSGTSIKTTSTFQMGETNQSQSYLQKFPLGKVPAFEDS

SKGVYLFDGNAIAMYLANDQLKGGDSVLAQAQVLQWLSYADNHIVPYVSEWVYPCLGITPASYKSPVNLQLVKDNVTKIL

NYLNTYLLSRTFLVGQRITLADINVFCSLLPLYEHVLDPALRQPYVNLNRWFDTILNQPQVSAVVGKISLCSAVAQVKIS

SSKQKDAKKKQTPSKDEKKEKSSAKKETEEMDLADEVLAAEPKSKDPFEKFPKGSFDLDDFKRFYSNEDEAKSIPYLWEK

IDTENWSLWFCEYKYPEELRQTFMSCNLISGMFQRLDKLRKHGFASMCLFGENNNSTISGIWLWRGHELAFELSPDLQVD

YESYTWTKLDVKSIKTKELVQQYLSWSGTDKDGRKFKEGKIFK

>AAH71378.1 Aars protein, partial [Danio rerio]

MDSSLTAAQIREKFIDFFRRHEHQYVHSSATIPLDDPTLLFANAGMNQFKPIFLNTIDPSHPMARLRRAANTQKCIRAGG

KHNDLDDVGKDVYHHTFFEMLGSWSFGDYFKHLACKMALELLTQEFGIPIERLYVTYFGGHADAGLEPDLECKQIWLDLG

MEESRILPGSMKDNFWEMGDTGPCGPCSEIHYDRIGGRDAAHLVNMDDPNVLEIWNLVFIQFNRESETVLKPLPKKSIDT

GMGLERLVSVLQNKMSNYDTDLFVPYFEAIQKGTGARAYTGKVGAEDTDGIDMAYRVLADHARTITIALSDGGRPDNTGR

GYVLRRILRRAVRYSHEKLGAQRGFFASLVDVVVESLGDAFPELRKDPDMVKDIINEEEAQFLKTLSRGRRILDRKIQSL

GDSTTIPGDTAWLLYDTYGFPLDLTALIAEERGMGVDLLAFEEEKKAAQLKSQGKGSGDEDHIMLDIYAIDELRNKGVAA

TDDSPKYKYTSDDNGNYEFEQAVGTVLALRRERMFVDEVITGQECGVLLDKTSFYAEQGGQSFDEGYMLRENDSAEDRME

FTVKNTQVRGGYVLHIGTVYGTLKVGDRLTLHVDEARRRPIMSNHTATHILNYGLRSVLGEADQRGSLVAPDRLRFDFTA

KGAMSTDEVRRTEEIAAAMIADAKGVYALDAPLAAAKAIQGLRAVFDETYPDPVRVVSIGVPVEELLADPNSPAGSLTSI

EFCGGTHLQNSSHAAPFVIVSEEAIAKEKKKKKKKKK

>XP_013389030.1 alanine--tRNA synthetase, cytoplasmic [Lingula anatina]

MDQNMSGNDVRKAFIDFFAEKEHLYVHSSSVIPHEDPTLLFANAGMNQFKPIFLGTVDPNSDMSKWVRAANSQKCIRAGG

KHNDLDDVGKDVYHHTFFEMLGNWSFGDYFKKEVCAWAWEFLTERMKLEKDRLYVTYFGGHEPSGLEPDEEAKEIWIKLG

LPADRVLPFDMKDNFWEMGETGPCGPCSEIHYDRIGGRDAKHLVNMDDPDVLEIWNLVFIQYNRENDGSLKLLPSKHIDC

GMGLERLVSVVQQKMSNYDTDMFMPLFDTIQKNTGARPYAGKVGKEDADGVDMAYRVLADHARTLTVALSDGGRPDSVGR

GYVLRRILRRGVRYATEKLGAKPGTFASLVDTVVLLLGDAFPELKKDPEMVKDIINEEEAQFLKTLSRGQKLLMRTIGKL

GDCKTLPGDLAWRLYDTYGFPVDLTQLMVEEKGLDVDMNGYEDSKKQAQLTSQAGGGTVDDQVSLDVHALNELQSQGFPP

TDDLPKYNYTSDESGNYVFESCTAKVKAIRMNKQMVSEVGSGQECGILLDRTCFYAEAGGQTYDEGFITKEGDEEFEFVV

KNVQVRGGYVLHIGSVEGTLKVGDSVKLAIDEGRRRPVMSNHTGTHMLNFALRKVLGEADQRGSLVAPDRLRFDFTAKGS

MTAAQVKEVEEITNGIAKKNEPVYAKDVALPIAKSIQGLRAVFDETYPDPVRVVSVGVPIEDLVADPTGPAGSLTTVEFC

GGTHLQRSGHLGDFVVVSEEAIAKGIRRIVAITGHEAEKALHKAGLLDKDLAALKQKIEQNSKTQQFTQKQLSTEIVRLT

EEASHATIPYWRKDNIRNELKGLKKALDDLDKARKQAVMTEVVEEAKKMIEACPNKPVVIHEFKAGSNAKALDAAMKQYN

SLSPQTAAMFFTVDEDAGKIICMSRVPKEVCTKGLKANEWVQHLSDTIKGKGGGKDVSAQATGTNTSGLSEAMKMAEEFS

TMKLGN

>XP_017773819.1 PREDICTED: alanine--tRNA synthetase, cytoplasmic [Nicrophorus vespilloides]

MNSQLTAKEIRASFIDFFKNKKHEYVHSSSTIPLDDPTLLFANAGMNQYKPIFLGTVDPNSDMSKWVRAVNTQKCIRAGG

KHNDLDDVGKDVYHHTFFEMLGNWSFGDYFKKEICAWAWELLTVVFKLPSDRLYVTYFGGDEKSGLKPDDECKQIWIDLG

IKPSHVLPGNMKDNFWEMGETGPCGPCSELHFDRIGNREVPHLVNMDDPDVLEIWNLVFIQYNRETDGSLKSLPKKHIDC

GLGLERLVSVIQNKRSNYDTDLFGPLFEAIQKGTGAPAYQGRVGDEDVDGVDMAYRVLADHARTLTIALSDGGNPDNTGR

GYVLRRILRRAVRYATEKLNAKPGFFATLVNTVVEILGDTFPEVKKDPQSIMDTINEEEEQFLKTLLRGRNLLNRTIGKL

GDSKTLPGDVAWRLYDTYGFPVDLTQLMAEEKGLNVDMDSYEESKKQAQLASQGKGSGVEDTINLDVHAITDMQNKGIPP

TDDSPKYNYTVSNDLNAEYSFAPCEASIISIRYNKQFENEVSTGQECGIILDKTCFYAEQGGQIYDTGYMVKIGDESVEF

SVKNVQVRGGYIMHIGSVEGTLKVGDKLALNLDKSRRRLVMNNHTGTHVLNYALRKVLGTEADQRGSLVAPDRLRFDFTN

KGALTIDQVKKTELSSNELISKNDEVFAKESNLGVAKTIKGLRAVFEETYPDPVRIVSIGTPVEKLESDPFGPAGENTSI

EFCGGTHLKYAGHIGDFVIASEEAIAKGIRRIVALTGPEAAKALKKNELLEASVKEINNLITADKSGSKSKEYVKKIVEL

TEEISHAIIPYWRKDELRNELKNLKKALDDKERAGKAAVANKVVDEIKQLVTEKPNQPILVKELKAFSNTKALDSALKQV

KTLSPETSAIFFSVDPDSGKIFCLSSVPKSAQEKGLKANEWVASVSPVMGGKGGGKPDSAQASGTNASKLNEILSLAEEF

AKTKLN

>XP_021371285.1 alanine--tRNA synthetase, cytoplasmic-like isoform X2 [Mizuhopecten yessoensis]

MDASLTGNQVRQLFIDYFKEQQHIYVHSSSTIPLDDPTLLFANAGMNQFKPIFLGTVDPNSDMSKYVRVVNSQKCIRAGG

KHNDLDDVGKDVYHHTFFEMLGNWSFGDYFKKDCCAFAWELLVDRIKMPKERLYVTYFGGNEAMGLAPDNDTRDIWINLG

VAVDRVLPFDMGDNFWEMGETGPCGPCTEIHFDRIGGRDAKHLVNMDDPDVLEVWNLVFMQFNRDTDGTLHDLPNKHCDC

GMGLERLVSVMQGKTSNYDTDLFMPIFDAIQKATGARTYTGHVGKEDVDGVDMAYRVLADHARTITIALSDSGRPDNVGR

GYVLRRILRRAVRYATEKLGAKPGMFASLIDTVVELLGDAFPEVTKDPEELKNIINEEEEQFLKTLSRGRRLLERTIGKL

GDTKTLPGEVAWRLYDTYGFPVDLTQLMAEEKGLMVDMDTYEECKKAAQLMSQGSGCTVDDQINLDVHAISDLQKKGLSP

TDDSPKYNYTADEEGNYKFESTMGKVLAIRHSKQFVDEVTSGRECGILLDRTSFYAEQGGQIYDQGFLVKVNEEDTEFQV

RNVQVRGGYVLHIGVIVGTLKVGDTVQLSIDESHRMAVMKNHTGTHMLNFGLRGVLQTADQKGSLVAPDRLRFDFTAKKA

MTPDQVKETEEIVNVMAEKNEEVYAKETSLPVAKSIQGLRAVFDETYPDPVRVLSIGVPVEDLVSDPMGPAASLTSVEFC

GGTHLRRAGHIGKFVIVTEEAIAKGSRRITAVTGAEATRALHRTDLLDKNVKNLAEKFDTCLKTKELSEKELTKMIVQMD

DEITKSVIPYWQRDRLRADLKRLKGKLDDLDKSRKAAITAEVVEQTKKMIEANPNQSFIVHEFKAGSNAKSLDAAMKQYK

TSSPETSAMFFSVDKEASKILCMSCVPKSSVSKGLNAKDWVNEVVPVIQGKCGGKDLAAQATGSNTDGLQEAINMATKFA

QMKLV

>OWF41828.1 Alanine--tRNA synthetase, cytoplasmic [Mizuhopecten yessoensis]

MVGRKHEVKCYQQPSRKKQLKMDASLTGNQVRQLFIDYFKEQQHIYVHSSSTIPLDDPTLLFANAGMNQFKPIFLGTVDP

NSDMSKYVRVVNSQKCIRAGGKHNDLDDVGKDVYHHTFFEMLGNWSFGDYFKKDCCAFAWELLVDRIKMPKERLYVTYFG

GNEAMGLAPDNDTRDIWINLGVAVDRVLPFDMGDNFWEMGETGPCGPCTEIHFDRIGGRDAKHLVNMDDPDVLEVWNLVF

MQFNRDTDGTLHDLPNKHCDCGMGLERLVSVMQGKTSNYDTDLFMPIFDAIQKATGARTYTGHVGKEDVDGVDMAYRVLA

DHARTITIALSDSGRPDNVGRGYVLRRILRRAVRYATEKLGAKPGMFASLIDTVVELLGDAFPEVTKDPEELKNIINEEE

EQFLKTLSRGRRLLERTIGKLGDTKTLPGEVAWRLYDTYGFPVDLTQLMAEEKGLMVDMDTYEECKKAAQLMSQGSGCTV

DDQINLDVHAISDLQKKGLSPTDDSPKYNYTADEEGNYKFESTMGKVLAIRHSKQFVDEVTSGRECGILLDRTSFYAEQG

GQIYDQGFLVKVNEEDTEFQVRNVQVRGGYVLHIGVIVGTLKVGDTVQLSIDESHRMAVMKNHTGTHMLNFGLRGVLQTA

DQKGSLVAPDRLRFDFTAKKAMTPDQVKETEEIVNVMAEKNEEVYAKETSLPVAKSIQGLRAVFDETYPDPVRVLSIGVP

VEDLVSDPMGPAASLTSVEFCGGTHLRRAGHIGKFVIVTEEAIAKGSRRITAVTGAEATRALHRTDLLDKNVKNLAEKFD

TCLKTKELSEKELTKMIVQMDDEITKSVIPYWQRDRLRADLKRLKGKLDDLDKSRKAAITAEVVEQTKKMIEANPNQSFI

VHEFKAGSNAKSLDAAMKQYKTSSPETSAMFFSVDKEASKILCMSCVPKSSVSKGLNAKDWVNEVVPVIQGKCGGKDLAA

QATGSNTDGLQEAINMATKFAQMKLV

>XP_021371284.1 alanine--tRNA synthetase, cytoplasmic-like isoform X1 [Mizuhopecten yessoensis]

MTSSHVLASCIIPDQDDCFCDICNGYQQPSRKKQLKMDASLTGNQVRQLFIDYFKEQQHIYVHSSSTIPLDDPTLLFANA

GMNQFKPIFLGTVDPNSDMSKYVRVVNSQKCIRAGGKHNDLDDVGKDVYHHTFFEMLGNWSFGDYFKKDCCAFAWELLVD

RIKMPKERLYVTYFGGNEAMGLAPDNDTRDIWINLGVAVDRVLPFDMGDNFWEMGETGPCGPCTEIHFDRIGGRDAKHLV

NMDDPDVLEVWNLVFMQFNRDTDGTLHDLPNKHCDCGMGLERLVSVMQGKTSNYDTDLFMPIFDAIQKATGARTYTGHVG

KEDVDGVDMAYRVLADHARTITIALSDSGRPDNVGRGYVLRRILRRAVRYATEKLGAKPGMFASLIDTVVELLGDAFPEV

TKDPEELKNIINEEEEQFLKTLSRGRRLLERTIGKLGDTKTLPGEVAWRLYDTYGFPVDLTQLMAEEKGLMVDMDTYEEC

KKAAQLMSQGSGCTVDDQINLDVHAISDLQKKGLSPTDDSPKYNYTADEEGNYKFESTMGKVLAIRHSKQFVDEVTSGRE

CGILLDRTSFYAEQGGQIYDQGFLVKVNEEDTEFQVRNVQVRGGYVLHIGVIVGTLKVGDTVQLSIDESHRMAVMKNHTG

THMLNFGLRGVLQTADQKGSLVAPDRLRFDFTAKKAMTPDQVKETEEIVNVMAEKNEEVYAKETSLPVAKSIQGLRAVFD

ETYPDPVRVLSIGVPVEDLVSDPMGPAASLTSVEFCGGTHLRRAGHIGKFVIVTEEAIAKGSRRITAVTGAEATRALHRT

DLLDKNVKNLAEKFDTCLKTKELSEKELTKMIVQMDDEITKSVIPYWQRDRLRADLKRLKGKLDDLDKSRKAAITAEVVE

QTKKMIEANPNQSFIVHEFKAGSNAKSLDAAMKQYKTSSPETSAMFFSVDKEASKILCMSCVPKSSVSKGLNAKDWVNEV

VPVIQGKCGGKDLAAQATGSNTDGLQEAINMATKFAQMKLV

>XP_021935281.1 alanine--tRNA synthetase, cytoplasmic isoform X2 [Zootermopsis nevadensis]

MNTSLTAKEIRKAFIDFFIQKKQHEYVHSSSTIPLDDPTLLFANAGMNQFKPIFLGTVDPNSDMAKWLRIVNTQKCIRAG

GKHNDLDDVGKDVYHHTFFEMLGNWSFGDYFKKEICTWAWELLTQVFKLPADRLYVTYFGGDESAGLEPDEECKQIWISL

GLPPSHVLPGNMKDNFWEMGETGPCGPCSELHFDRIGDRDAAYLVNQDDPDVLEIWNLVFIQFNRESDCSLKSLPKRHID

CGLGLERLVSVIQEKRSNYDTDLFLPLFHAIQKGTGVPPYRGRVGSDDDDGVDMAYRVLADHARTLTIALADGGHPDNTG

RGYVLRRILRRAVRYATEKLNAKPGFFATLVNTVVEILGDTFPEVRKDPQSIIDMINDEEAQFLKTLTRGRNLLNRTIVK

LGNSKTVPGDVAWRLYDTYGFPVDLTQLMSEEKGLAVDMDAYEDAKKQAQILSQGKGGWYDDKLNLDIHAINELQEQGVP

RTNDLAKYNYHATSEDKDAEYVFEACSAIVIRLRHSKKFMDCVTSGNECGVLLDRTSFYAEQGGQIYDEGFLVKVGDESV

EFSVKNVQIRGGYILHIGTVEGTLRVGDEVSLHIDTSRRRLVMNNHTGTHVLNFALRSVLGTEADQKGSLVAPDRLRFDF

TNKGAMSVEQVKATEIQSNDLIDRNEPVYAKEASLAVAKTIQGLRAVFEETYPDPVRVVSIGIPVEKLEDDPYGPAGSNT

SIEFCGGTHLHRAGHIGRFVITSEEAIAKGIRRIVALTGPEALKALKKTELLENELNNLISLLKSDDVKISNKDLVKKIV

ELNDDVSHATIPYWKKEDMRSCLRHLKKQLDDKDRASKAAVCADVVEEVKALFAANPKTPIIVQELKAYSNTKALDGALK

QVKALSPETSAMFFSVDSESKKIVCLSAVPKNAVNKGLKANEWVQQVCGLMQGKSGGKPESAQASGSNISCLQEVLSIAT

QFANVKLDTGSRGAEIDGPV

>XP_021935280.1 alanine--tRNA synthetase, cytoplasmic isoform X1 [Zootermopsis nevadensis]

MGENFCEAEIMNTSLTAKEIRKAFIDFFIQKKQHEYVHSSSTIPLDDPTLLFANAGMNQFKPIFLGTVDPNSDMAKWLRI

VNTQKCIRAGGKHNDLDDVGKDVYHHTFFEMLGNWSFGDYFKKEICTWAWELLTQVFKLPADRLYVTYFGGDESAGLEPD

EECKQIWISLGLPPSHVLPGNMKDNFWEMGETGPCGPCSELHFDRIGDRDAAYLVNQDDPDVLEIWNLVFIQFNRESDCS

LKSLPKRHIDCGLGLERLVSVIQEKRSNYDTDLFLPLFHAIQKGTGVPPYRGRVGSDDDDGVDMAYRVLADHARTLTIAL

ADGGHPDNTGRGYVLRRILRRAVRYATEKLNAKPGFFATLVNTVVEILGDTFPEVRKDPQSIIDMINDEEAQFLKTLTRG

RNLLNRTIVKLGNSKTVPGDVAWRLYDTYGFPVDLTQLMSEEKGLAVDMDAYEDAKKQAQILSQGKGGWYDDKLNLDIHA

INELQEQGVPRTNDLAKYNYHATSEDKDAEYVFEACSAIVIRLRHSKKFMDCVTSGNECGVLLDRTSFYAEQGGQIYDEG

FLVKVGDESVEFSVKNVQIRGGYILHIGTVEGTLRVGDEVSLHIDTSRRRLVMNNHTGTHVLNFALRSVLGTEADQKGSL

VAPDRLRFDFTNKGAMSVEQVKATEIQSNDLIDRNEPVYAKEASLAVAKTIQGLRAVFEETYPDPVRVVSIGIPVEKLED

DPYGPAGSNTSIEFCGGTHLHRAGHIGRFVITSEEAIAKGIRRIVALTGPEALKALKKTELLENELNNLISLLKSDDVKI

SNKDLVKKIVELNDDVSHATIPYWKKEDMRSCLRHLKKQLDDKDRASKAAVCADVVEEVKALFAANPKTPIIVQELKAYS

NTKALDGALKQVKALSPETSAMFFSVDSESKKIVCLSAVPKNAVNKGLKANEWVQQVCGLMQGKSGGKPESAQASGSNIS

CLQEVLSIATQFANVKLDTGSRGAEIDGPV

>KRT80774.1 tRNA synthetase [Oryctes borbonicus]

MSTHMTAKEVRDAFINFFKKKDHEYVHSSSTIPLDDPTLLFTNAGMNQFKPIFLGTVDPNSDMAKWKRTVNTQKCIRAGG

KHNDLDDVGKDVYHHTFFEMLGNWSFGDYFKKEICSWAWEFLTEILKLPSERLYVTYFGGDEKQGLEPDDECKQIWLNLG

IKPSHVLPGNMKDNFWEMGDTGPCGPCSELHFDRIGNRDVPHLVNQDDPDVLEIWNLVFIQYNRESGGILKLLPRKHIDC

GLGLERLVSVIQQKRSNYDTDLFGPIFDVIQKGTGAPLYQGRVGNEDTEGIDMAYRVLADHARTLTIALSDGGTPDNTGR

GYVLRRILRRAVRYATEKLHANPRFFASLVYTVVDILGDTFPEVRKDPQGIVDIINEEEEQFLKTLSRGRNLLNRTIGKL

GDSKNVPGDVAWRLYDTYGFPVDLTQLMAEEKGLTVDMSGYEESKKIAQIISQGKGTGVEDTINLDVHAITDLQKRNISP

TDDSPKYNYQADSSSKDYVFDTCEAAVIALRYNKEFVEEVRSGQECGLILDKTSFYAEQGGQIYDTGYIVKAGDESVEFT

VKNVQVRGGYVVHIGNVEGILKVGDKVILQIDTSRRRLVMNNHTGTHVLNYALRKVLSTEADQRGSLVAPDRLRFDFTNK

GAMTVDQVKATETISKEIVARNEEVFAKESSLALAKTINGLRAVFEETYPDPVRIVSIGVPVETLESDPFGPAGTQVSVE

FCGGTHLKYAGHIGDFVIASEEAIAKGIRRIVALTGPEAAKALKKTELLENKLNAIKSSIDDDANGVASRQYVKEIVELT

EEVSRATIPYWKKESIRNSLKNLKKALDDKDRAKKASIANEVVEEMKLFLKENPNQPILVKELNAFSNTKALDSALKQVK

SLSPQTSAMFFSADEENNKIFCLSTVPKEAQEKGLKANEWVLSVASIMGGKGGGKGDSAQASGSNTTCLNQVLDLALQFA

QMKLH

>ETE60198.1 Alanyl-tRNA synthetase, cytoplasmic, partial [Ophiophagus hannah]

MEALTASEIRQRFINFFVDNQHTYVHSSATIPLDDPTLLFANAGMNQELACKLALDLLTKEFGIPIERLYVTYFGGNEAA

GLEPDLDCKQIWLDLGLSESKILPGSMKDNFWEMGDTGPCGPCSEMHYDRIGDRDASHLVNQDDPNVLEIWNLVFIQFNR

EADGTLKPLPKKSIDTGMGLERLVSVLQNKMSNYDTDLFVPYFEAIQKGTGCRPYAGQVGEEDVDGIDMAYRVLADHART

ITIALSDGGRPDNTGRGYVLRRILRRAVRYAHEKLNAPKGFFATLVDVVVQSLGGAFPELTKDPDMVKDIINEEEVQFLK

TLNRGRRILDRKIQSLGDNKIIPGDTAWLLYDTYGFPVDLTGLIAEEKGLRVDMEAFEEERKSAQGGGDEDLIMLDIYAI

EELRARGLEPTDDSPKYNYRSDPEGFYGQECGVVLDQTCFYAEQGGQIYDEGYFKTEFTVKNVQVRGGYVLHVGTVYGSL

KVGDFVRLFIDEPRRRPIMSNHTATHILNFALRSVLGEADQRGSLVAPDRLRFDFTAKGAMSTQEIKKAEEIVNGMIQEA

KVVYTKDCPLAAAKAIQGLRAVFDETYPDPVRVVSIGIPVEDMLNDPSSAAGAITSVEFCGGTKSEGLNKQLADLETKVK

IQTTPNKDVQREIADLGEALATAIIPQWQKDELREALKALKKIMDDLDRASKADIQKRVLDKMKQLIEAYPNQPLALNES

LKLLKTHSPETAAMLFSVDNEAGKITCLCQVPQVSVLMDGKGGGKDLSAQATGKNTDCLEEALQVATEFAKLKLSELKN

>XP_020369375.1 alanine--tRNA synthetase, cytoplasmic isoform X2 [Rhincodon typus]

MDSTMNAGQIRQKFIDFFKCYNHEYVHSSATIPLDDPTLLFANAGMNQFKPIFLNTIDPSHPMAKLTRAANTQKCIRAGG

KHNDLDDVGKDVYHHTFFEMLGSWSFGDYFKALACEMALELLTNEFGIPIERLYVTYFGGNEKAGLEPDLECKQIWLNLG

MAEERILPGSMKDNFWEMGDTGPCGPCSEIHYDRIGGRDAAHLVNMDDPNVLEVWNLVFIQFNREVDGSLKPLAKKSIDT

GMGLERLVSILQQKMSNYDTDLFQPYFKAIQKGTGARPYTGKVGSEDVDGIDMAYRVLADHARTITIALSDGGRPDNTGR

GYVLRRILRRAVRYSHEKLNASKGFFATLVDVVVESLGDAFPELKKDPDMVKDIINDEEEQFLKTLNRGRRILERKIQSL

GENKRIPGDTAWLLYDTYGFPVDLTGLIAEEKGLIVDLEGFENEKKAAQLKSQGKGMGDDDHIMLDVNAIDDLRAKGFEA

TDDLPKYNYTSDENGNYVFESIEATVKTVRREKTFMEEVQTGQDCGLLLDRTCFYAEQGGQIYDEGYLIKVDDSSEDKTE

FTVKNVQVRGGYVLHIGTVYGQLKVGDKVRLFVDEAKRRSVMSNHTSTHMLNFALRSVLGEADQRGSLVAPDRLRFDFTA

KGAMSTQQIKKTEEITCKMIQEAKPIYAKDAALAAAKAIQGLRAVFDETYPDPVRVVSVGIPVEDMLADPSGPAGSITSV

EFCGGTH

>XP_012272687.1 alanine--tRNA synthetase, cytoplasmic [Orussus abietinus]

MDMSMSAKDIRQAYIDFFKSKGHQYVHSSSTIPHDDPTLLFTNAGMNQFKPIFLGTVDPNSDMAKWDRVVNTQKCIRAGG

KHNDLDDVGKDVYHHTFFEMMGNWSFGDYFKNEICTWAWELLTDILKLPSDRLYVTYFGGDAKTNLPPDEECKNIWIQLG

VPASHILPGNMKDNFWEMGETGPCGPCSELHYDRIGGREAAHLVNMDDPDVLEIWNLVFIQYNRESDGSLRPLPKKHIDC

GLGLERLVSVIQNKRANYDTDLFVPLFEAIQKGTGARPYQGNVGTDDNDGIDMAYRVLADHARTLTIALADGGVPDNTGR

GYVLRRILRRAVRYATEKLNAKPGFFGSLIHVVVDLLGDVFPELRKDPQSIVDIINEEEIQFLKTLSRGRNLLNRTIAKL

KSTTVVPGDIAWRLYDTYGFPVDLTQLMTEEIGLSVDMTGYEEAKKQAQIISQGKTGGADDWINLDVHAITELQNKGIKT

TDDTPKYGYSVKSEEKYSEYEFDTCIGTVVALRRAKQFVESVSSGDEVGILLDRTSFYAEQGGQIYDEGFLVKVDDENTE

IRIKNVQVRGGYILHIGTVCEGTLRSGDKVSTNVDTVRRRLIMSNHTATHILNYALRNVLGTEADQKGSLVAPDRLRFDF

TNKGSMSATQVKKTEETANELINNKKKVYAKESSLALARTIQGLRAMFEETYPDPVRIVSVGIPVEDLEKNPLSPAGMKT

SVEFCGGTHLHNANHIGDFVIVNEEAIAKGIRRIVALTGPEASKALKKASLLENQLNQIQTELNADKNGANIKEYVKRIV

ELTEDVSRAVIPYWKKDNIRNSLKSLKKAIDDKERATKNAIVTIVAETAKQMIQDIPGVPLMVEVLKADSNTKALDAAIK

QVRAISPETSVIFFSVDNEAKKIFALSSVPKSAIQKGLKANDWIQKIAILMQGKGGGKPESAQASGPNLSCLQEAITVAK

VFAMSKLKTEAQVAEIPKMCNASSDPNDVLTKEKSRPSGLILLTSAESVTRYRAEIVAQFCQQVLTVEIETENNQKLPIL

QDGSLELRDSNAIAFYLADSSLKGGDNLFDRSQVIQWMSIADNHILPSVFSWALALCSSGKGKNSKMISKDGREDFLAML

KNVNTLLLTKTYFVGDRITLADISMFVSLLPFYEHVFDPDTRKNYVNLNRWFNTILHQPQVKNVVKSFKMCCQRKKI

>XP_015591734.1 alanine--tRNA synthetase, cytoplasmic [Cephus cinctus]

MNSSMTANEIRQAYIDFFKSKGHEYVHSSSTIPHDDPTLLFANAGMNQFKPIFLGTVDPNSDMSKWVRVVNTQKCIRAGG

KHNDLDDVGKDVYHHTFFEMMGNWSFGDYFKKEICTWAWELLTSVLKLPANQLYVTYFGGDKKAGLEPDNECKDIWLSLG

LLPSHVLPGSMKDNFWEMGETGPCGPCSELHFDRIGGREAAHLVNMDDPDVLEIWNLVFIQFNRETDGGLRPLPKKHIDC

GLGLERLISVIQNKRANYDTDLFIPLFEAIQKGTGAPAYQGRVGADDKDGIDMAYRVLADHARTITIALADGGVPDNTGR

GYVLRRILRRAVRYATEKLNAKPGFFSSLVNVVVELLGDVFPEVRKDPQSIIDIINEEEDQFLKTLSRGRNLLNRTIAKL

EKTKIVPGDVAWRLYDTYGFPVDLTQLMTEEKGLTVDMTAYEEAKKQAQILSQGKAGGVDDQINLDVHAITELQSKGIKP

TNDLPKYNYTVKSNDKNAEYQFTPCTSTVIALRRAKQFVDEVSSGEEVGVLLDQTCFYAEQGGQIYDEGFLVKVGDEDTE

IRVKNVQVRGGYVLHIGTVGQGKLKNGDKVHANIDTARRRLIMSNHTGTHALNFALRKVLGGEADQKGSLVAPDRLRFDF

TNKGAMTPLQVKQSEEFTNDLIKRNEMIYAKESNLSVAKTIQGLRAMFEETYPDPVRVVSIGVPVEELEKDPFSPAGTKT

SIEFCGGTHLHYTGHIGDFVIASEEAIAKGIRRIVALTGPEATKALKKTQLLQNQLNQIRNLIDNDKSGANSKDYVKKIV

ELTEDVSHAVIPYWKKDEIRNSLKALKKALDDKERAAKVAVANLVVEKVQKIIQANVGIPILVEVLEAYSNTKALDSALK

QVKTISPETSALFLSVDQDANKIFALSAVPKTAVAKGLKANEWIQAIAPLMKGKGGGKPESAQASGSNVSCLDQALEVAK

TFANSKLGISPEATCLKPAVCSKPKTNKDQSCSKPSSDLILYTKVGNVRGYRAEITAKFSGKIVTVKNDNNKKEYLPILE

IKNVELRDSNAIAFYLANDKLKGKDVFTQSQIIQWISYADNHILPSVFGWVLPSLELGTSKNTGSVKVAKEDLLQSLKHL

NNILLTKTFFVGERITLADISMFTALLPLYEHIFDPETRKKFENVNRWFYTILNQPEVKAIVKDFKVCEKKTKKAA

>XP_009066253.1 hypothetical protein LOTGIDRAFT_197664 [Lottia gigantea]

MADMTGTDVRNLFINFFKEKKHTYVHSSSTIPLDDPTLLFTNAGMNQFKPIFLGVVDPNSDMAKWSRAVNSQKCVRAGGK

HNDLDDVGKDVYHHTFFEMLGNWSFGDYYKKETCTWAWEMLTERLKLDKTRLYVSYFGGKPEAGLEPDNEARDIWLSLGL

PANRVLPFDMKDNFWEMGETGPCGPCSEIHYDRIGGREVPELVNQDDPDVLEIWNLVFIQYNRDADGSLKSLPRKHIDCG

LGLERLVSVIQDKRSNYDTDLFTPIFDVIQKMTGTKAYTGKVGKEDTDGVDMAYRVLADHIRTLTITLSDGGRPDNTGRG

YVLRRILRRAVRYGTEKLNFKPGMFSSLVDTVVTLLGDAFPEVKRDPETVKAILNEEEVQFLKTLTRGHHLLQRTIKKLG

DTNILPGDVAWRLYDTYGFPVDLTTLMAEENGLSIDMGAFEESRKKAQLMSQGAGGSVDDKINLDVHGINHLQSKNIPPT

DDSSKYNYRADTQGNYVFEPASGKVIALRYNKEFVDSVSTGQECGVLLDKTNFYAEQGGQIYDEGFMVKEGEEEVELRIK

NVQVRGGYVLHIGTIEGVLKVGDQVKLSVDEERRCSVMKNHTGTHMLNYGLRCVLNEADQRGSLVAPDRLRFDFTSKGSM

TPAQVKKVEEEVNKMADKNEEVYAKESSLSVAKSIQGLRAVFDEVYPDPVRIVSVGIPVEKLESDPMGPAGSVTSVEFCG

GTHLRRAGHIGSMVIVSEDAISKGIRRIIAVTGSEAQKSHHKVDVLQKSVTELKNILESKSRDITDKELTRRIVQLDDEV

AQSTIAYWQRDGLRSELKSLKKKMDDLDKNKKAAVLNEVVEECKKMIEANPNIKYIVHEFKAGSNAKALDAAMKQYKASS

PQTSAFFVSVDNEANKILCMSCVPKEAASKGLSAKLWIDQVVPAINGKGGGKDVSAQATGTNIGALNEAISLATKFAEMK

LRLIIKT

>XP_022907416.1 alanine--tRNA synthetase, cytoplasmic [Onthophagus taurus]

MNTEMTAKEIRSAFINFFKSKKHEYVHSSSTIPLDDPTLLFTNAGMNQFKPIFLGIADPNSDMAKWTRTVNTQKCIRAGG

KHNDLDDVGKDVYHHTFFEMLGNWSFGDYFKKEICAWAWELLTEVLKLPSERLYVTYFGGDEKSGLEPDLECKQIWLELG

IKPEHIIPGNMKDNFWEMGETGPCGPCSELHFDRIGNRDVPHLVNQDDPDVLEIWNLVFIQFNRESDGSLKLLPKKHIDC

GLGLERLVSVIQNKRSNYDTDLFQPLFEAIQKGTGAPKYQGRVGDEDKDGIDMAYRVLADHARTLTIALSDGGSPDNTGR

GYVLRRILRRAVRYATEKLHAQPKFFASLVNTVVELLGETFPEVKKDPQGIIDVINEEEEQFLKTLSRGRNLLNRTITKL

GGGCNNLPGDVAWRLYDTYGFPVDLTQLMAEEKGLAVDMNGYEESKKQAQLISQGKGIGIEDTINLDVHSITELQQKGVS

PTDDSPKYNYSATSTSFDYTFEPCEAKVIALRFNKQFVDSITTGQEAGILLDKTCFYAEQGGQIYDTGYIVKTDDESVEF

TVKNVQVRGGYIIHIGNIEGVLKVGDKVNLQIDSSRRRLVMNNHTGTHILNYALRKVLGTEADQRGSLVAPDRLRFDFTN

KGALNAEQVKQVELASKEIIATNKEVFAKETALGVAKTINGLRAVFEETYPDPVRVVSVGIPVETLETDPFGPEGTKTSV

EFCGGTHLKYVGHCGDFVISSEEAIAKGIRRIVALTGPEAAKALKKTELLENKLNDIKTTIDKSDFDSKEIVKSIVELTE

EASHSIIPYWKKEEIRNALKTLKKNLDDKERAKKAAIANQVVDEVKLFLKENPNLPIIVKCLNAASNTKALDSALKQVKA

LSPKSAAMFVSVDMDCSKVFSLAAVPKEAQEKGLKANEWIQQVSVLMNGKGGGKADSAQASGTNVAAIEQVLIVAKQYAE

TFLV

>XP_011438498.1 PREDICTED: alanine--tRNA synthetase, cytoplasmic [Crassostrea gigas]

MESKLTGAEIRNLFVDFFKEKEHLYVHSSSVVPLDDPTLLFTNAGMNQFKPIFLEIVDPNSDMAKYVRAANSQKCIRAGG

KHNDLDDVGKDVYHHTFFEMLGNWSFGDYFKTECCGWAWEFLTERVGLPKERLYVTYFGGNEKFNLKPDDETKNIWLNLG

VPAERVLPFGMKDNFWEMGETGPCGPCTEIHYDKVGGRDAKALVNMDDPEVIEIWNLVFIQFNRESETELNELPRKHCDC

GMGLERLVCVMQGKTSNYDTDLFSPYFEFIQKATNSQPYQGRVGKDDVDGVDMAYRVLADHARTLTVALADGGRPDNMGR

GYVLRRILRRAVRYGTEKLNAKPGMFASLVDVVVESLGNAFPELKKDPEMIKDIINEEEAQFLKTLTRGRRLLERTIGKL

GDTTSLPGDIAWRLYDTYGFPVDLTALMAEEKGLAVDMPAYESAKKDAQALSQGTGGTAADLIGLDVHAISDLQNRGVPT

TDDAPKYKYQATENGKYSFEPATGKVIALRRNKEFVEEVSSGQECGVLLDRTSFYAEQGGQIYDTGFLVKENDPDVEFSV

RNVQVRAGYVLHIGVMEGVLRTGDIVQLNIDEERRCSVMKNHTGTHMLNFGLREILKEADQRGSLVAPDRLRFDFSAKGA

MKVSEVKQVEEIVNKMAEKNGEVYDMNVSLAVAKAIQGLRAVFDEVYPDPVRVVSIGVDVNQLVEDPMGPGASTTSVEFC

GGTHLKRAGHIGKMVIVSEEAIAKGIRRIIALTGTEAVKAQHKVDLLEKSLLQLQKDVDEGLKSKSVSEKELTRQIVSFD

DEVSQSLISYWKKDELRSKVKKLKQKMDELDKNRKAAVMTEVVEEAKKMIEASPNQKFVVHEFKADANAKALDAAMKQYK

ALSPETAALFFTVDQSAGKIVCMSCTPKSSIAAGLTANGWINEIVPLIKGKGGGKEASAQATGTNTGALKEAMDIATKFA

GLKLS

>XP_015118346.1 PREDICTED: alanine--tRNA synthetase, cytoplasmic [Diachasma alloeum]

MNRSMTAKEIRQAYIDFFKSKGHEYVHSSSTIPHDDPTLLFANAGMNQFKPIFLGTVDPNSDMSRWVRVVNSQKCIRAGG

KHNDLDDVGKDVYHHTFFEMMGNWSFGDYFKREICTWAWEFLTKVLSLPGDRLYVTYFGGDEGAGLAPDDECKDLWLSLG

IPPSHVLPGSMKDNFWEMGETGPCGPCSELHYDRIGAREAAHLVNMDDPDVLEIWNLVFIQFNRESDGSLRSLPKKHIDC

GLGLERLVSVIQDKRANYDTDLFVPLFEAIQVGTGARPYAGKVGAEDGDGIDMAYRVLADHARTITIALADGGMPDNTGR

GYVLRRILRRAVRYATEKLNAKPGFFGSLVHVVVDLLGEIFPELQKDPQSIVDVINEEESQFLKTLSRGRNLLDKTITKL

EDKTVPGDVAWRLYDTYGFPVDLTQLMAEEKGLRVDMAAYEESKRLAQLASQGKAGGVDDQINLDVHAITELQSSGVPPT

DDSCKYNYSTSAEASAEYTFAPCSATVITLRRQKKFVEEAQPGEEVGVLLDKTSFYAEQGGQIYDEGFLVKSPGGNDAED

SAEVRVTNVQVRGGYVLHIGTVGSGVLRKGDKLQLNIDTSRRRLVMSNHSATHALNYALRRVLGTEADQKGSLVAPDRLR

FDFTNKGAMSAAQVKDTESYTTEMVKDNRKIYAKESKLAVAKTVNGLRAMFEETYPDPVRIVSMGVPVEELEKDPLGPAG

TTTSVEFCGGTHLHYTGHIGDFVIASEEAIAKGIRRIVALTGPEATRALKKAELLQNRLNDIQREINGADCLKNAKESVR

KIVELNDDISHAVIPYWRKDEMRVLLKGLKKTLDDEDRAAKARLANLVVEKAAEIIQNNVGVPVLVEQLEALSNTKALDG

ALKKVKALSPETSALFLSVDEEAKKIVALSAVPKSAIGKGLKANEWIQAISPLMGGKGGGKAESAQASGGNVGALEEALK

IAREFADMKLGA

>XP_015519721.1 PREDICTED: alanine--tRNA synthetase, cytoplasmic [Neodiprion lecontei]

MNSVMTAKEIRQAFIDFFKSKNHEYVHSSATIPHDDPTLLFANAGMNQFKPIFLGTVDPNSDMAKWVRVVNTQKCIRAGG

KHNDLDDVGKDVYHHTFFEMMGNWSFGDYFKKEICAWAWELLTEVFKLPSDRLYVTYFGGDEQSGLKPDEECKQIWLSLG

VPPSHVLPGSMKDNFWEMGETGPCGPCSELHFDRIGGREVPHLVNMDDPDVLEIWNLVFIQFNRESDGSLRSLPKKHIDC

GLGLERLVSVIQGKRANYDTDLFMPLFDMIQKGTGARTYQGKVGSEDTDGIDMAYRVLADHARTITIALADGGMPDNTGR

GYVLRRILRRAVRYATEKLNAKPGFFGSLVNTVVDLLGDVFPEVRKDPQSIIDVINEEEQQFLKTLSRGRNLLNRTMAKL

EATNVVPGDVAWRLYDTYGFPVDLTQLMTEEKGLTIDMKGYEEAKKQAQLLSQGKAGGVDDQINLDVHAITELQNQGVKP

TNDSHKYNYKAKSSDKDAEYEFASCVGTVIALRFAKQFVEQVTSGQEVGVLLDQTNFYAEQGGQIYDEGFLVKVGDESTE

VCIQNVQVRGGYVLHIGTVGEGILKKGDKVNLNVDTARRRLIMNNHTGTHALNYALRTVLGWEADQKGSLVAPDRLRFDF

TNKGAMTVEQVKQTEINTKQLIQRNNPIYAKESGLAIAKTIQGLRAMFEETYPDPVRIISIGIPVEELEKNPLSTAGTKT

SVEFCGGTHLHHAGHIGDFVIASEEAIAKGIRRIVALTGPEATKALKKVELLENELNKIKSIIEADKTGVKSKEHVKAIV

DLTEDVSHAIIPYWKKDEMRNTLKTLKKSIDDKERAAKAAVANLVVQKTADLIKRNVGVAVLVELLEAYSNTKALDSALK

QVKSLSPETSALFFSVDNDAKKIFALSAVPKSAVAKGLKANEWIQAVAPLMQGKGGGKPESAQASGPNFSCLNEALETAK

SFANSKLGIPSGGTELMKKDAYSSNVEGPTLYATSGNIKSYRGQIAARYSGKKITFEFVTKKEKVDVVLKDNSVILRDAN

AIAFYLSNDQLKGAKNVFAESEVIQWMSYADNHILPAVLGWVLPSIDSSTAVNSNKLSKIAQEDCKKVLNSLNIILLTKT

YLVGEKITLADIAVFTSFLPLYEHVFDPESRKPYQNVNRWFLTILHQPEVQEIVKSFKICDKIVKNHGDSKKINN

>XP_966465.1 PREDICTED: alanine--tRNA synthetase, cytoplasmic [Tribolium castaneum]

MDISMSAKDVRNAYINFFKEKNHDYVHSSSTIPLDDPTLLFTNAGMNQFKPIFLGTVDPNSDLAKLTRAVNSQKCIRAGG

KHNDLDDVGKDVYHHTFFEMMGNWSFGDYFKKEICAWAWEFLTKRLHLPGDRLYVTYFGGDKAAGLEPDNECKQIWLGLG

VPESHVLPGSMKDNFWEMGETGPCGPCSELHFDRIGGRDVPELVNQDDPDVLEIWNLVFIQYNRESDGSLKFLPKKHIDC

GLGLERLVSIMQNKRSNYDTDLFLPLFDAIQKGTGAPPYGGKIGDADKDGIDMAYRVLADHARTITIALSDGGNPDNTGR

GYVLRRILRRAVRYATEKLNAKPGFFSTLVNTVVEILGDTFPEVCKDPQSIIDTINEEEEQFLKTLSRGRNLLNRTITKL

GDSKTLPGDVAWRLYDTYGFPVDLTQLMAEEKGLNVDMAVYEESKKLAQIASQGKGSGVEDTINLDVHAITELQNLGVPP

TDDAPKYNYIAGSGINEEYKFELCHSTVIALRHNKQFVEQVHTGQECGILLDKTNFYAEQGGQIYDTGYIVKIGDESVEF

SVKNVQVRGGYVIHIGSLEGTLKKGDKVALHVDSARRRLIMNNHTGTHVLNYALRKVLGTEADQRGSLVAPDRLRFDFTN

KGAMTVDQVKNTELNSKELISRNDQVFAKESNLAVAKTIRGLRAVFEETYPDPVRIVSIGIPVEKLEVDPFGPAGDNTSI

EFCGGTHLKYSGHIGDFVIASEEAIAKGIRRIVALTGPEATKALKRTEYLENCLNDIKFLVETDKNGEKSKELVKKIVQL

TEEVSHAVIPYWKKEEIRNALKTLKKNLDDKERAAKAAVANQVVDQIKEFVKANPNLPILVKELKAFSNTKALDTALKQV

RSLSPSTSALFVTVDPDSNKMFCLASVSKEGVDKGLKANEWVQAVASKLGGKGGGKADSAQASGSNSVAVEEILQLAKQF

AESRI

>XP_018561574.1 alanine--tRNA synthetase, cytoplasmic [Anoplophora glabripennis]

MDTSMSAKDVRNAYINFFKEKEHVYVHSSSTIPLDDPTLLFTNAGMNQYKPIFLGTVDPNSDLSKLLRAVNTQKCIRAGG

KHNDLDDVGKDVYHHTFFEMMGNWSFGDYFKKEVCTWAWEFLTERLKLPSDRLYVTYFGGDEPSGLEPDIECKQIWLDLG

VKPTHVLPGSMKDNFWEMGETGPCGPCSELHFDRIGGREVPELVNQDDPDVLEIWNLVFIQFNRETDGSLKSLPKKHIDC

GLGLERLVSVIQNKRSNYDTDLFTPLFEAIQKGTGAPQYQGRVGDADKDGIDMAYRVLADHARTLTIALSDGGNPDNTGR

GYVLRRILRRAVRYATEKLNAKPGFFSTLVNTVVDILADTFPEVRKDPQGIIDTINEEEEQFLKTLSRGRNLLNRTISKL

GDNNTLPGDVAWRLYDTYGFPVDLTTLMAEEKGLAVDMNAYEDAKKQAQLLSQGKGSGVADTINLDVHAITELQGKGVPP

TDDSYKYNYESTDKVDSDYTFKPCEATVIALRYNKQFVDEVRTGQECGVLLDKTNFYAEQGGQIYDTGYFIKVGDESVEF

SIKNVQVRGGYVIHIGSLEGTLRKGDQVSLYVDTSRRRLIMSNHTGTHVLNYALRKVLGTEADQRGSLVAPDRLRFDFTN

KGALTSEQVKNTELSSKGMISKNEPVYAKEANLAVAKTVRGLRAVFEETYPDPVRIVSIGIPVEQLESDPFGPAGDNTSI

EFCGGTHVQYSGHIGDFVIASEEAIAKGIRRIVALTGPEATKALKKTELLEKRLNEIKTVIELDKEGAKSKENVKKIVEL

TDEVSQAVIPYWKKEEIRNTLKNLKKTLDDKDRAAKAAIANQVVEQIKEFVKENPNLPILVKELKAFNNTKALDTALKQV

RSLSPNTSALFITVDQDSNKVFCLSSVPKDAIGKGLKANEWVQEVAKKLGGKGGGKPDSAQASGSNSVYITDILSLAKKF

AESKLS

>XP_012262517.1 alanine--tRNA synthetase, cytoplasmic isoform X3 [Athalia rosae]

MIRSSNNFAKVKMNTSMTGKQIRQGFIDFFKSKAHEYVHSSSTVPHDDPTLLFANAGMNQFKPIFLGSVDPNSDMAKWVR

VVNTQKCIRAGGKHNDLDDVGKDVYHHTFFEMMGNWSFGDYFKKEICAWAWELLTEVFKLPGDRLYVTYFGGDEKSGLKP

DEECKQIWIELGIKPSHVLPGSMKDNFWEMGETGPCGPCSELHFDRVGGREAAHLVNMDDPDVLEIWNLVFIQFNRETDG

SLRPLPKKHIDCGLGLERLVSVIQGKRANYDTDLFVPLFDAIQKGTGAPAYQGRVGNDDKDGVDMAYRVLADHARTITVA

LADGGMPDNTGRGYVLRRILRRAVRYATEKLNAKPGFFGSLVNTVVELLGDVFPELKKDPQSIIDVINEEEEQFLKTLSR

GRNLLNRTVAKLEATTVVPGDVAWRLYDTYGFPVDLTQLMIEEKGLTIDMKSYEEAKKQAQLLSQGKTGGVDDQINLDVH

AIIELQNKGVKPSDDSHKYNYKAQSSDKNTEYDFSPCTGIVIALRHMKQFVEQVTSGQEVGILLDQTNFYAEQGGQIYDE

GFLVKVGDESTEVRIKNVQVRGGYVLHIGTVGEGTLKVGDKVHLNVDMARRRLIMNNHTGTHALNYALRTVLGTEADQKG

SLVAPDRLRFDFTNKGAMTVEQVKQTEIYTKQLIERNQPIYAKESSLAIAKTIQGLRAMFEETYPDPVRIISIGVPVEEL

EKNPLSPAGTQTSIEFCGGTHLHQAGHIGDFVIASEEAIAKGIRRIVALTGPEATKALKKVEFLENELNKIKSTIDEDKS

GAKSKEHVKAIVELTEDVSHATIPYWKKDELRNILKTLKKSIDDKERTAKAAVATSVVQKATELAQQNIGAPIVVEVLEA

YSNTKALDSALKQIKSISPETSALLFSVDYDSKKIFALSAVPKSAVVKGLKANEWIQTVTLLMQGKGGGKPESAQASGSN

TSCLQEAMKMAIAFASSKLGIPTSNSVASEKNF

>XP_012262513.1 alanine--tRNA synthetase, cytoplasmic isoform X1 [Athalia rosae]

MIRSSNNFAKVKMNTSMTGKQIRQGFIDFFKSKAHEYVHSSSTVPHDDPTLLFANAGMNQFKPIFLGSVDPNSDMAKWVR

VVNTQKCIRAGGKHNDLDDVGKDVYHHTFFEMMGNWSFGDYFKKEICAWAWELLTEVFKLPGDRLYVTYFGGDEKSGLKP

DEECKQIWIELGIKPSHVLPGSMKDNFWEMGETGPCGPCSELHFDRVGGREAAHLVNMDDPDVLEIWNLVFIQFNRETDG

SLRPLPKKHIDCGLGLERLVSVIQGKRANYDTDLFVPLFDAIQKGTGAPAYQGRVGNDDKDGVDMAYRVLADHARTITVA

LADGGMPDNTGRGYVLRRILRRAVRYATEKLNAKPGFFGSLVNTVVELLGDVFPELKKDPQSIIDVINEEEEQFLKTLSR

GRNLLNRTVAKLEATTVVPGDVAWRLYDTYGFPVDLTQLMIEEKGLTIDMKSYEEAKKQAQLLSQGKTGGVDDQINLDVH

AIIELQNKGVKPSDDSHKYNYKAQSSDKNTEYDFSPCTGIVIALRHMKQFVEQVTSGQEVGILLDQTNFYAEQGGQIYDE

GFLVKVGDESTEVRIKNVQVRGGYVLHIGTVGEGTLKVGDKVHLNVDMARRRLIMNNHTGTHALNYALRTVLGTEADQKG

SLVAPDRLRFDFTNKGAMTVEQVKQTEIYTKQLIERNQPIYAKESSLAIAKTIQGLRAMFEETYPDPVRIISIGVPVEEL

EKNPLSPAGTQTSIEFCGGTHLHQAGHIGDFVIASEEAIAKGIRRIVALTGPEATKALKKVEFLENELNKIKSTIDEDKS

GAKSKEHVKAIVELTEDVSHATIPYWKKDELRNILKTLKKSIDDKERTAKAAVATSVVQKATELAQQNIGAPIVVEVLEA

YSNTKALDSALKQIKSISPETSALLFSVDYDSKKIFALSAVPKSAVVKGLKANEWIQTVTLLMQGKGGGKPESAQASGSN

TSCLQEAMKMAIAFASSKLGIPTSNSVASEKNVCTKGSNDPILYGNEGNIKRYRVLIAAKCSGKKINLKPLEGTEKITIR

FEDDNVVLRDGNAIALYLSNDQLKGSKNLYAESEILQWMSYADNHILPSVLGWVGPSAIEDLSKEISPAKEESFKCLKNL

NTVLLNKTYLVGEQITLADIAVFTSLMPLYEHNFDAKSRKPYQNVNRWFLTILNQLEVQSVVQDFKLCG

>OXU16738.1 hypothetical protein TSAR_010539 [Trichomalopsis sarcophagae]

MAGKTAKDIRQAYIDFFKSKDHEYVHSSSTIPHDDPTLLFTNAGMNQFKPIFLGTVDPNSDMAKWVRVVNSQKCIRAGGK

HNDLDDVGKDVYHHTFFEMMGNWSFGDYFKKEICEWAWEFLTQQLNLPADRLYVTYFGGDEKSNLKPDEECKNFWLSLGV

PESHVLPGSMKDNFWEMGETGPCGPCSELHYDRIGGREAAHLVNMDDPDVLEIWNLVFIQYNREQNGELQPLPKKHIDCG

LGLERLVSVIQNKRSNYDTDLFTPLFDAIQKGTGAPAYQGRVGNDDKDGIDMAYRVLADHARTITIALADGGVPDNTGRG

YVIRRILRRAVRYATEKLNAQPGFFATLINVVVELLGDVFPEVKRDPQSIIDIVNEEEVQFLKTLSRGRNLLNRTIAKLE

STKVVPGDVAWRLYDTYGFPVDLTQLMAEEKGLTIDVAAYEEAKKLAIIASQGKAGGVNDQINLDVHAITELQNKGVKTT

DDSPKYNYKVEKTEKYAEYTFAPCTGTVLALRKDKTFVDEVKSGDEVGVLLDQTNFYAEQGGQIYDEGFLVKVDDESTEI

RVTNVQVRGGYVLHIGTVGEGTLKKGDKLYLNVDTSRRRLIMSNHTATHALNHALRSVLGTDADQKGSLVAPDRLRFDFT

NKGAMTAEQVKKTEQFTNLMVKEDKEIYAKESPLAIARTIQGLRAMFGETYPDPVRVVSMGVTVEELEKDPLSPAGAKTS

VEFCGGTHLHRTGHIGDFVIASEEAIAKGIRRIVALTGPEASKALKKAELLQNNLNQIKSSVETVKDSATSKEIVKKIVE

LTDDVSHAVIPYWKKEEMRSELKALKKALDDKERSAKAAVANAVVETAVNIIESNKGAPVLVEVLNAFNNTKALDAALKK

VKAISPETSALFISVDEDEKKIFALSAVPKSAIAKGLKANEWIQSIASLMQGRGGGKPESAQASGPNISCLKEALATAKT

YAYSVLKLEEETKTCVDTSCSKSTSEPSVYANTGSVKGYLVQIAARYAGKCLKVQNHTKNTRENGPIVYKDDTITLNDGN

AIAFYLADDQLKGTDLFAQSEVLQWMSFSDNHILPSVCSWVLPTVGAVKSSKDSTKEARNNVLQILEALNKTLLKKTYLV

GERITLADIAVFVTLSPAYQHTLDLETRKKYQNTNRWFETILQQSNVKTVDQQQQFAHNHHIGSFQCEVSKS

>XP_012262514.1 alanine--tRNA synthetase, cytoplasmic isoform X2 [Athalia rosae]

MNTSMTGKQIRQGFIDFFKSKAHEYVHSSSTVPHDDPTLLFANAGMNQFKPIFLGSVDPNSDMAKWVRVVNTQKCIRAGG

KHNDLDDVGKDVYHHTFFEMMGNWSFGDYFKKEICAWAWELLTEVFKLPGDRLYVTYFGGDEKSGLKPDEECKQIWIELG

IKPSHVLPGSMKDNFWEMGETGPCGPCSELHFDRVGGREAAHLVNMDDPDVLEIWNLVFIQFNRETDGSLRPLPKKHIDC

GLGLERLVSVIQGKRANYDTDLFVPLFDAIQKGTGAPAYQGRVGNDDKDGVDMAYRVLADHARTITVALADGGMPDNTGR

GYVLRRILRRAVRYATEKLNAKPGFFGSLVNTVVELLGDVFPELKKDPQSIIDVINEEEEQFLKTLSRGRNLLNRTVAKL

EATTVVPGDVAWRLYDTYGFPVDLTQLMIEEKGLTIDMKSYEEAKKQAQLLSQGKTGGVDDQINLDVHAIIELQNKGVKP

SDDSHKYNYKAQSSDKNTEYDFSPCTGIVIALRHMKQFVEQVTSGQEVGILLDQTNFYAEQGGQIYDEGFLVKVGDESTE

VRIKNVQVRGGYVLHIGTVGEGTLKVGDKVHLNVDMARRRLIMNNHTGTHALNYALRTVLGTEADQKGSLVAPDRLRFDF

TNKGAMTVEQVKQTEIYTKQLIERNQPIYAKESSLAIAKTIQGLRAMFEETYPDPVRIISIGVPVEELEKNPLSPAGTQT

SIEFCGGTHLHQAGHIGDFVIASEEAIAKGIRRIVALTGPEATKALKKVEFLENELNKIKSTIDEDKSGAKSKEHVKAIV

ELTEDVSHATIPYWKKDELRNILKTLKKSIDDKERTAKAAVATSVVQKATELAQQNIGAPIVVEVLEAYSNTKALDSALK

QIKSISPETSALLFSVDYDSKKIFALSAVPKSAVVKGLKANEWIQTVTLLMQGKGGGKPESAQASGSNTSCLQEAMKMAI

AFASSKLGIPTSNSVASEKNVCTKGSNDPILYGNEGNIKRYRVLIAAKCSGKKINLKPLEGTEKITIRFEDDNVVLRDGN

AIALYLSNDQLKGSKNLYAESEILQWMSYADNHILPSVLGWVGPSAIEDLSKEISPAKEESFKCLKNLNTVLLNKTYLVG

EQITLADIAVFTSLMPLYEHNFDAKSRKPYQNVNRWFLTILNQLEVQSVVQDFKLCG

>XP_011300406.1 PREDICTED: alanine--tRNA synthetase, cytoplasmic [Fopius arisanus]

MSSSMTAKEIRQSYIDFFKSKGHEYVHSSSTIPHDDPTLLFANAGMNQFKPIFLGTVDPNSDMSRWIRVVNSQKCIRAGG

KHNDLDDVGKDVYHHTFFEMMGNWSFGDYFKKEICTWAWEFLTQVLSLPGDRLYVTYFGGDEKSGLAPDNECKELWLSLG

LPESHVLPGSMKDNFWEMGETGPCGPCSELHYDRIGGREAAHLVNMDDPDVLEIWNLVFIQFNREADASLRSLPKKHIDC

GLGLERLVSVIQGKRANYDTDLFVPLFEAIQKGTGARPYSGKVGVEDVDGIDMAYRVLADHARTITIALADGGMPDNTGR

GYVLRRILRRAVRYATEKLSAKPGFFGTLVHVVVELLGDVFPELQKDPQSIIDVINEEETQFLKTLSRGRNLLDKTIAKL

PDKVVPGEVAWRLYDTYGFPVDLTQLMAEEKGLQVDMTAYEESKRLAQIASQGKAGGVDDQINLDVHAITELQNSGVLPT

DDSAKYDYEATADMSSPYTFAPCVGTVIALRRLKKFVDEAHPGEEVGVLLDRTSFYAEQGGQIYDEGFLVKLEGNDLEDS

TEVKVTNVQVRGGYVLHIGTVGSGILRKGDKLQLNIDTPRRRLVMSNHSATHALNYALRRVLGTEADQKGSLVAPDRLRF

DFTNKGALSTNQVHDTETYTNEMVKDNRKIYAKESKLAVAKTVNGLRAMFEETYPDPVRVVSMGVPVEDLEKDPLGPAGT

TTSVEFCGGTHLLYTGHIGDFVIASEDAIAKGIRRIVALTGPEASKALKKAELLQNRLNEIQGRISRGPTNSKEIVREIV

ELSEDVSHAVIPAWKKDEMRLMLKGMKKTLDDEDRAAKARVAGLVVDKAAEIIQNNVGVPVLVEQLEAFSNTKALDGALK

KIKALSPETSAMVLSIDEEAKKIFALSSVSKGAIQKGLTANEWIQAISPMMGGKGGGKAESAQASGSNVAVLEEAIKVAR

NFANLKLGV

>XP_005102630.1 PREDICTED: alanine--tRNA synthetase, cytoplasmic-like [Aplysia californica]

MKSSEVRDAFINFFKEKEHQYVHSSSTIPHDDPTLLFANAGMNQFKPIFLGVVDPNSDMAKYVRVANTQKCVRAGGKHND

LDDVGKDVYHHTFFEMLGNWSFGDYYKKEICTWAYEFLVDRLKLDNSRLYVTYFGGNEKAGLEPDNEARDIWIKLGVDPE

KVLPFDMGDNFWEMGETGPCGPCSEIHYDRIGGRNAKELVNMDDPDVLEIWNLVFIQYNRETDGSLKTLPRKHIDCGMGF

ERMVSVVQDKPSNYDTDVFVPLFEAIQKATGCPDYQGRIGAADKDGVDMAYRVLADHARTITVTLADGGRPDNSGRGYVL

RRILRRAVRYATEKLNAKPGMFASLVDTVVELLGDAFPEVKKDPQEIKEIINEEEAQFLKTLSRGRRLLERSINKVDGKM

LPGDIAWRLYDTYGFPVDLTLLMAEEKGIGVDMESFEEAKNHSLLMSQGVSGTKEDLIGLDIHALNDLNTRKVPPTDDSL

KYSYQADEQGNYVFDAATAKITGLRYNKAFVESVSPGQECGIMLDRTCFYAEQGGQIYDEGFIVKADSEDDEFRVKNVQV

RGGYVLHIGTAEGNFKIGDSVKLGIDEPRRCSVMKNHTATHILNFALRDVLKETDQKGSLVAPDRLRFDFSAKSAMSVKE

VKAAEEIVNNMVSRNELVYARETPLATAKAIQGLRAVFDEVYPDPVRVVSIGVSVEDLEADPTGPAGSVTTVEFCGGTHL

QRAGHIGEFVIVTEEAISKGVRRIIAVTGVEAHKAVHRQEVLSRNVQELKTKVEDNPKTNAFTDRELNKQIISLDDEIAQ

SSISYWMRDSLRGELKGLKKKMDDIDKARKAAVVSGVTEESKKMIEANPNQKFIVHEFKAGSNAKALDAAMKQYKSLSPQ

TSAMFFSVDQDAGKILCMSCVPKETVSTGLNAKAWVDSVVPLIAGKGGGKELSAQATGTNTGALSDALKTALEFAQLKLS

>ELU16117.1 hypothetical protein CAPTEDRAFT_155717 [Capitella teleta]

MDASLTGNQLRQMFVDFFVEKKQHKYWHSSSTIPLDDPTLLFANAGMNQFKPIFQGCVDPNSEMGRLVRVVNSQKCIRAG

GKHNDLDDVGKDVYHHTFFEMLGNWSFGDYFKKETIEWAWELLTEVYKLPKDRMYVSYFGGVPEKGLEPDNEARDIWLNL

GLPPERVMPFGMADNFWQMGDTGPCGPCSEIHFDRIGGGRDAAHLVNEDDPDVLEIWNLVFTQYNMENDGTLKGLPKKNI

DCGMGLERLTSVIQGKPSNYDTDLFKPIFDAIQKATGCRDYTGKVGADDSDGIDMAYRVLADHARTLTIALADGGRPDNT

GRGYVLRRILRRAVRYANEKLNAKPGVIASLVDTVVDILGDAFPEVRKDPDMVKDIINEEEQQFLKTLTRGRRLLERTIN

KLGSGCKMLPGDVAWRLYDTYGFPLDLTQLMVEEKNMTVDLAGYEECKKAAQIKSQGKSGEVDEQINLDVHAISDLQKQG

LPPTDESPKYIYSATVDGEYDFNMCNATVKALRSNKTFVDQVTSGQEVGVVLDKTCFYATQGGQMFDEGFIVKVDDEDTE

LKVHDVQVRGGYVLHVGVVEGTLSVGDNVRLQVDVERRRPIMCNHTGTHMLNFALRQVLGSADQKGSLVAPDKLRFDFTA

KGAMTAKQVKDTEDIVNQVAAKNEEVFTKVSDLAPAKAIQGLRAVFDETYPDPVRVVSLGISVEDLLSDPDGPAGSLTSV

EFCGGTHLTRTGHIGHMAIIAEEAIAKGIRRIVAVTGHEAQKAVHRADVLEKEIQDLKNRMDQATSSKNFSQSELNKEIV

NLTDEVSQSQTPYWRRDELRNRLKPLKKTLDNMDKARKAAVLTEVVDEAKKQVVENPNQPFVVCEFKAGSNAKALDGAMK

QYKALSPKTACFFISVDNENGKIMAMSCVPQEVTKTGLKANEWIGSVQTLMQARGGGKELSAQATGTNTACLQEALGIAT

KYAQTKLGLEVSEAEPPAKNAENIEGIVNTHLTDRSYIEGYMPSQSDCVVFQALSQAPSGNLPNLQRWYKHIKSYGSQIA

SFPGVKKSLEELGFTAGKDEDDFDLFGEDSEEEDAEEAERLKQERIQAYQDRKAKKPALVAKSNIILDVKPWDDETDMKE

MERLVRTVHCDGLLWGAAKLVPLAYGIKKLQICCVVEDDKVSTDFLEESIKEFEDLVQSVDIAAFQKI

>XP_022110497.1 alanine--tRNA synthetase, cytoplasmic-like [Acanthaster planci]

MDATLTSQQVRQIFIDFHVNHPLFKHDFFRSSSTIPLDDPTLLFANAGMNQFKAIFTGTVDPNSDMGRLKRAVNSQKCIR

AGGKHNDLDDVGKDTYHHTFFEMLGTWSFGDYFKKECCQMSWELLTEVYKLPKDRLYVTYFGGSKEDGLEEDVETRQIWI

DLGVPPERVMPFGTKDNFWEMGATGPCGPCTEVHYDRVGGGRDASEYVNMDDPNVVELWNLVFTQFNRESDGSLRLLPKK

NCDTGMGLERIVSVLKSQDSNYDTDLFMPIFEAVQKATGVRPYAGKLAAEDTDGVDMAYRVVADHIRTLTIALSDGGRPD

NIGRGYVLRRILRRGIRYATEKLKAKPGLFASLVSVVVESLGEAFPEVTKDPQLVMDVINEEEAQFLQTLSRGRRVLERT

VNNLGADVKVLPGKAAWLLYDTYGFPIDLTTLIMEEKGMTINMAEYEEEKKKAQILSQGKGSGVDDTISLDVHAINELQE

KRKVPPTNDKVKYNYTSDAAGNYQFESTVGTVIALRRDKQFVEEVQRGDECGVLLDQTCFYAEAGGQIYDEGFMEKADNA

DIEFRVTNVQVKGGYCLHIGKVSAVDLDGKIKIGDLLKLAIDEVRRRPVMNNHTGTHVLNFALRQVLGEADQKGSLVAPD

RLRFDFTAKGAMTTQEVKETERIANEVIGKADKVYAMNTPLAQAKAIQGLRAVFDEVYPDPVRVVSIGIPVEELVADPAG

PGGSMTSVEFCGGTHLQNSGHIGCFVITSEEAIAKGIRRIQAITGEDADKAISRGKAMEVLIEKLAKEIQDNGNANQKLL

LRKVLELGDDVNVAVIQQWKKDELRAQLKDLKKIMDDMDKACKAAQVQAAKESAKQMIAESPNAPYVVARLEAGSSGKAL

DGALKEYKAGSPNTAAMLFSVDADLGKVLCMAQVPKDVAAKGLKANEWVKQVTEVINGRGGGKDLAAQATGDRTAGIDEA

LRLAKEFAQLKLSS

>XP_019871997.1 PREDICTED: LOW QUALITY PROTEIN: alanine--tRNA synthetase, cytoplasmic [Aethina tumida]

MNTQMTANEVRNAYIDFFKEKQHVYVHSSSTIPLDDPTLLFTNAGMNQYKPIFLGTVDPNSDLAKITRAVNSQKCIRAGG

KHNDLDDVGKDVYHHTFFEMMGNWSFGDYFKKEICAWAWELLTDRLKLPAERLYVTYFGGDTAAGLEPDNECKQIWLDLG

VPANHVIPGNMKDNFWEMGETGPCGPCSELHFDRIGGRDVPELVNQDDPDVLEIWNLVFIQFNRETDGSLRPLPKKHIDC

GLGLERLVSVIQNKRSNYDTDLFTPLFEAIQKGTGAPAYQGRVGSDDVDGVDMAYRVLADHARTITIALSDGGNPDNTGR

GYVLRRILRRAVRYATEKLNAKPGFFATLVHTVGQILGDIFPEIRKDPQSIIDTINEEEDQFLKTLSRGRNLLNRTITKL

GDSKTLPGDVAWRLYDTYGFPVDLTQLMAEEKGLNVNMDAYEESKKQAQLASQGKGAGVEDTINLDVHSITELQNKGVPP

TDDSXKYDYEAGSDVNVEYKFKTCEATVIGLRYNKQFVDEVQTGQECGVLLDRTNFYAEQGGQIYDTGYINKVGDESVEF

SVKNVQVRGGYVIHIGSIEGSLRKGDKVILHIDTSRRRLVMNNHTGTHILNYGLRKVLGTDADQRGSLVAPDRLRFDFTN

KGALTAEQVKNVEAASKELISRNNEVFAKESNLGVAKTIRGLRAVFEETYPDPVRIVSVGIPIDKLEADPFGPAGDDTSI

EFCGGTHLRYAGHIGDFVIASEEAIAKGIRRIVALTGPEATKAIKKNELLENRLNEIKSSIDADKDGAKSKEIVKKIVEL

TEEVSHATIPYWKKEEIRNSLKTLKKNLDDKDRAAKAAVANKVVDEIKEFVKANPNLPILVKELKAFNNTKALDTALKQV

RSLAPNTSALFVTVDQDSNKMFCLSSVPKEAIEKGLKANEWVQAVAAKLGGKGGGKPDSAQASGANTADVNDILDLAKKF

ADTKLA

>XP_008938095.1 PREDICTED: alanine--tRNA synthetase, cytoplasmic, partial [Merops nubicus]

MEAPLTASQIRQRFIDFFKENEHTYVHSSSTIPLDDPTLLFANAGMNQFKPIFLNTIDPSHPLAKLSRATNTQKCIRAGG

KHNDLDDVGKDVYHHTFFEMLGSWSFGDYFKELACKLALDLLTKEFGIPAERLYVTYFGGNEAAGLQPDLECKQIWLNLG

LAESRILPGNMKDNFWEMGDTGPCGPCSEIHYDRIGDRDAAHLVNQDDPNVLEIWNLVFIQFNREADGTLKPLPKKSIDT

GMGLERLVSVLQNKMSNYDTDLFLPYFEAIQKGTGARPYMGRVGAEDADGIDMAYRVLADHARTITLALSDGGRPDNTGR

GYVLRRILRRAVRYSHEKLNAPKGFFATLVDVVVQSLGDAFPELKKDPDMVKDIINEEEDQFLKTLSRGRRILDRKIQSL

GDSKTIPGDTAWLLYDTYGFPADLTGLIAEEKGLVVDMEGFEEERKNAQLKSQGKGAGGEDLLMLDIYAIEELRARGLEV

TDDSPKYSYASDLSGTYDFGSLVATVKAIRREKKFVEEVSTGQECGIVLDQTCFYAEQGGQIYDEGYMVKDDDSREDKTE

FTVKNVQVRGGYVLHIGTLYGSLKVGDQVHLSIDETRRRPVMSNHTATHILNFALRSVLGEADQRGSLVAPDRLRFDFTA

KGALSTQEIKRVEGIANQMIEEAKVGVAAT

>XP_003704866.2 PREDICTED: alanine--tRNA synthetase, cytoplasmic isoform X1 [Megachile rotundata]

MTVTMNAKQVRQAYIDFFKSKGHEYVHSSSTIPHDDPTLLFTNAGMNQFKPIFLGTVDPNSDMAKWVRVVNSQKCIRAGG

KHNDLDDVGKDVYHHTFFEMMGNWSFGDYFKKEICTWAWEFLTVKLRLPADRLYVTYFGGDEKNNLDSDEECKAIWLSLG

VSPNHILPGNMKDNFWEMGETGPCGPCSEIHYDRIGNREAAHLVNQDDPDVLEIWNLVFIQFNRESDGSLKSLPKKHIDC

GLGLERLVSVIQNKQANYDTDLFIPLFDAIQKGTGAPPYQGKVGIDDKDGIDMAYRVLADHARTITIALSDGGVPDNTGR

GYVLRRILRRAVRYATEKLNAKPGFFGTLVNVVVEILGDTFPEVTKDPQYIIDTINEEEEQFLKTLSRGRNLLHRTIAKL

ESSNILPGDIAWRLYDTYGFPVDLTQLMTEEKGLKIDMAGYEEAKKQAQLISQNKCGGVDDQINLDVHAITELQNKGIKP

TNDLPKYNYTVTGNKLYEEYEFAPCTGTVIALRRNKTFVDEVSSGEEVGILLDQTNFYAEQGGQIYDEGFLVKTDDEDTE

VRIKNVQVRGGYVLHIGTVGQGTLKKNDKVYLNVDTTRRRFLMSNHTATHVLNYALRKVLGTEVDQKGSLVAPDRLRFDF

TNKGAMTSEQVKRVEEITNSVVKENKKVYAKESSLALAKTIQGLRAMFEETYPDPVRIVSIGIPVEELEKDPLGPAATQT

SVEFCGGTHLHYTKHMEDFIIASEEAIAKGIRRIVALTGPEATKAQKKAAVLQNHLEQLQTTIANDKNGTNSKEYVKKIV

ELTDDVSRATISSWKKDNMRKMLKDLKKMLDDKERSAKAAIANAVVDTIQQIIQQNVGCPVLVETLQAYSNTKALDSALK

KIKAISPETSALLVTVDPDANKIFALSMASKSAINKGLKANEWIQEIAPLMKGKGGGKPESAQASGTNISCLTKLIHTAK

NFANSKLGVISNNEIVTNNYNQSKASQNSVCSKTSKGKLILSGNTGSIKYYRAQIIAQYSDKELVTNECKNDGTTSNSIK

LEGSGFELFDSNAIAFYLATDQLKCSKDTFMFSEVLQWLSYADNHILPAVSGWVIPCIVKNVPNNMKAGIKTSKEDLLSS

LTKLNKLLFTKTYLVGERISLADVAIFTALIPLYEHVFDSNYRIQYPNLTRWFLTILNQPQVTSVLKNFKMCEKAVKN

>KFQ29263.1 Alanine--tRNA synthetase, cytoplasmic, partial [Merops nubicus]

MEAPLTASQIRQRFIDFFKENEHTYVHSSSTIPLDDPTLLFANAGMNQFKPIFLNTIDPSHPLAKLSRATNTQKCIRAGG

KHNDLDDVGKDVYHHTFFEMLGSWSFGDYFKELACKLALDLLTKEFGIPAERLYVTYFGGNEAAGLQPDLECKQIWLNLG

LAESRILPGNMKDNFWEMGDTGPCGPCSEIHYDRIGDRDAAHLVNQDDPNVLEIWNLVFIQFNREADGTLKPLPKKSIDT

GMGLERLVSVLQNKMSNYDTDLFLPYFEAIQKGTGARPYMGRVGAEDADGIDMAYRVLADHARTITLALSDGGRPDNTGR

GYVLRRILRRAVRYSHEKLNAPKGFFATLVDVVVQSLGDAFPELKKDPDMVKDIINEEEDQFLKTLSRGRRILDRKIQSL

GDSKTIPGDTAWLLYDTYGFPADLTGLIAEEKGLVVDMEGFEEERKNAQLKSQGKGAGGEDLLMLDIYAIEELRARGLEV

TDDSPKYSYASDLSGTYDFGSLVATVKAIRREKKFVEEVSTGQECGIVLDQTCFYAEQGGQIYDEGYMVKDDDSREDKTE

FTVKNVQVRGGYVLHIGTLYGSLKVGDQVHLSIDETRRRPVMSNHTATHILNFALRSVLGEADQRGSLVAPDRLRFDFTA

KGALSTQEIKRVEGIANQMIEEAK

>XP_018322304.1 PREDICTED: alanine--tRNA synthetase, cytoplasmic [Agrilus planipennis]

MNTHMTAKEIRNAYIDFFKERGHTYVHSSSTIPLDDPTLLFTNAGMNQFKPIFLGTADPNSDLYKLVRAVNSQKCIRAGG

KHNDLDDVGKDVYHHTFFEMMGNWSFGDYFKKEICNWAWELLTETLKLDPARLYVTYFGGDSSQALEPDNECKQIWLDIG

VKPDHVIPGNMKDNFWEMGETGPCGPCSELHYDRIGGRSVPQLVNQDDPDVLEIWNLVFIQFNRESDGSLKHLPKKHVDC

GLGLERLVSVLQNKRSNYDTDLFVPIFEAIRRNTGAPQYQGRVGEDDVDGTDMAYRVLADHARTLTIALSDGGTPDNTGR

GYVLRRILRRAVRYGTEKLNAKPGVFASLVPVVCDILGDTFPEIQKDPQSVMDIINEEEEQFLKTLSRGRNLLNRTITKI

GENSKCLPGTVAWRLYDTYGFPVDLTLLMVEEKGLSVDMDEYEEAKKQAQIISQNKAGGVEDTVNLDVYAINELQSQKLP

PTDDQAKYNYHSNGDINSEYKFQSCTATVVALRHSKQFVDKISSGQESGIILDKTSFYAEQGGQIYDTGFIVKAGDDSVE

FAVKNVQVRGGYVIHIGTVEGVLCKGDKVELYVDTSRRRLIMNNHTGTHILNYALRAVLQTETDQKGSLVAPDRLRFDFS

NKGALTTEQVKNVELRSKELIDKNGRVYAKESSLAVAKTINGLRAVFDETYPDPVRVVSIGVPVEDLENDPFGPAGMHTS

VEFCGGTHLHYAGHVGDFIIASEDAIAKGIRRIVALTGPEATKALKKTELLENCLNELKSAIDSDNTSNDTKKYVKKIVE

LTNEISQAVIPYWKKDELRNVLKGLKKNLDDKERADKAAIANQVIEDAKKIITENKALVLVEVLKAYSNTKALDSALKQV

KTLTPNTAAMFFSVDNDAGKIFCLSNVPKEAVDKGLKANEWVSQITQLIKGKGGGKAESAQASGTNPAALNEALQLARKF

ALTKLS

>XP_013133760.1 PREDICTED: alanine--tRNA synthetase, cytoplasmic [Papilio polytes]

MDTSMTGNQIRKTFIDFFIGKGHKYVHSSSTIPLDDPTLLFANAGMNQFKPIFLGSVDPNSDMAQYVRVVNTQKCIRAGG

KHNDLDDVGKDVYHHTFFEMMGNWSFGDYFKKEICAWAWELLTEVYKLPANRLYVTYFGGDAASGLEPDLDCKNIWLNLG

LPESHILPGSMKDNFWEMGETGPCGPCSELHYDRIGERNAAHLVNMDDPDVLEIWNLVFIQFNREADGSLKLLPKKHIDC

GLGLERLVSVIQNKRANYDTDFFMPLFKAIETGTKTRPYTGKVGADDVDGIDMAYRVLADHARTLTIALADGGCPDNTGR

GYVLRRILRRAVRYASEKLNGKPGFFGSLVHTVVELLGDVFPEIRKDPESIIQTINEEEIQFLKTLSRGRNLLNRTIEKL

SGSKIIPGDVAWRLYDTYGFPVDLTHLMCEEKGLSIDMEAYEKAKKESQLNSQGKASGQEDLLALDIHAISHLQESGVPV

TDDSPKYGYSPSSNDKDAVYSFAPCTATVLALRWNKEFVNEVQSGQECGLILDQTNFYAEQGGQIFDEGYMVKVGDDSVE

FTVKNVQVKGGYVLHSGKVEGILKVGDKVSLHIDTERRRLVMNNHTGTHILNNVLRKVLGNDSDQRGSLVMPDRLRFDFT

NKGPMTAKQIKDAEDEIKDIIAQNKAVYAKHTNLSDAKKIKGLRAMFDEHYPDPVRVVSVGIPVEELEKNPDGPSGYETS

VEFCGGSHLHQTGHIGDYVIVSEEGIAKGIRRIVALTGPEAVKALNKLSVLENEVNNIANFIKEQGDAVNQKEIVRKIVD

LTNDVSHAQISYWKKEELRNMLKGLKKQLDDKERASKAMIITQIIERSKELCLEEKDSEVIVTELKASGNTKALDGALKQ

VKQMLPNTAAMFFSVDDETNKIYCLAAVPKSLNGKGLLASEWVQSIVGVMDGKGGGKAESAQASGNNPKCIAEAINIAKE

FASSKLK

>XP_013181141.1 PREDICTED: alanine--tRNA synthetase, cytoplasmic [Papilio xuthus]

MDTSMTGNQIRKTFIDFFIGKGHKYVHSSSTIPLDDPTLLFANAGMNQFKPIFLGSVDPNSDMAQYVRVVNTQKCIRAGG

KHNDLDDVGKDVYHHTFFEMMGNWSFGDYFKKEICAWAWELLTEVYKLPADRLYVTYFGGDAASGLEPDLECKNIWLNLG

LPESHILPGSMKDNFWEMGETGPCGPCSELHYDRIGDRNAAHLVNMDDPDVLEIWNLVFIQFNREADSSLKLLPKKHIDC

GLGLERLVSVIQNKRANYDTDFFMPLFKAIETGTKTRPYTGKVGADDVDGIDMAYRVLADHARTLTIALADGGCPDNTGR

GYVLRRILRRAVRYASEKLNGKPGFFGSLVHTVVELLGDVFPEIKKDPDSIIQTINEEEIQFLKTLSRGRNLLNRTIEKL

SGSKIIPGDVAWRLYDTYGFPVDLTHLMCEEKGLSIDMEAYEKAKKESQLNSQGKAAGQEDLLALDIHAISHLQESGVPV

TDDSPKYGYTPSSNDKDAIYSFAPCTATVLALRCNKEFVSEVQSGQECGLILDRTNFYAEQGGQIFDEGYMVKMDDESVE

FTVKNVQVKGGYVLHSGKVEGTLKVGDKVSLHIDTERRRLIMNNHTGTHILNNVLRKVLGNDSDQRGSLVMPDRLRFDFT

NKGPMTVKQIKDAEDEIKNIIAQNKAVYAKHTSLTDAKKIKGLRAMFDEHYPDPVRVVSVGIPVEELEKNPDGPSGYETS

VEFCGGSHLHQTGHIGDYVIVSEEGIAKGIRRIVALTGPEAVKALNKLSVLENEVNSIANFIKEQGDAVNQKEVVRKIVE

LTNDVSHAQISYWKKEELRNLLKGFKKQLDDKERASKAMIITQVIEKTKELCLEEKDSKVIVTELKASGNTKALDGALKQ

VKQMLPNTAAMFFSIDDETSKIYCLAAVPKSLNEKGLLASEWVQSIVSVMGGKGGGKAESAQASGNNPKSITEAIKIAKE

FASSKL

>XP_012239996.1 alanine--tRNA synthetase, cytoplasmic isoform X2 [Bombus impatiens]

MTAMMSAKQIRQAYIDFFKSKNHEYVHSSSTIPHDDPTLLFTNAGMNQFKPIFLGTVDPNSDMAKLMRAVNSQKCIRAGG

KHNDLDDVGKDVYHHTFFEMMGNWSFGDYFKKEICTWAWEFLTVKLKLSADRLYVTYFGGDEKNNLKSDEECKQIWLSLG

VSPSHVLPGNMKDNFWEMGETGPCGPCSEIHYDRIGNREAADLVNQDDPDVLEIWNLVFIQFNRETDGSLKLLPKRHIDC

GLGLERLVSVIQNKRANYDTDLFVPLFDAIQEGTGAPPYRGRVGMEDEDGIDMAYRVLADHARTITIALADGGVPDNTGR

GYVLRRILRRAVRYATEKLNAKPGFFGSLVNVVVNILGDIFPEVKKDPQYIIDVINEEEIQFLKTLSRGRNLLNRTIAKL

GSSNVLPGDVAWRLYDTYGFPVDLTQLMAEEKGLKVDIIGYEEAKKQAQLISQSKSGGVDDQINLDVHAITELQNKGIKP

TNDLPKYNYKVVNNKLYEEYEFVPCTGTVIAVRRAKTFVDEVSSGEEVGILLDQTNFYAEQGGQIYDEGFLVKIDDDDTE

VRIKNVQIRGGYVLHIGTVGQGKLKKGDKVFLNIDTTRRRLIMSNHTATHALNHALRKVLGTEVDQKGSLVAPDRLRFDF

TNKGAMTAEQVRKVEEITSNMIKGNKKIYAKESNLALAKTIQGLRAMFEETYPDPVRVVSIGIPVEDLEKDPLSSAALQT

SVEFCGGTHLHYTEHIGDFVIASEEAIAKGIRRIVALTGPEATKAQKKASILQNHLDQLQATIVADKDGVNIKEHIKEIV

ELTDDVSHATISSWKKDKMRKMLKDLRKALDDKERVAKIAIVNAVVDTIQEIIQQNIGCLVLVEVLQAYSNTKALDSALK

KIKAISPETSALLISVDPDAKKIFALSTVSKSAINKGLKANEWIQEIASLMEGKGGGKSESAQASGTNISCVTKLVHTAK

NFANSKLGIVDIVNENHDQLKSSEKSVCSKTLKKKLVLSSDIGSIKYYRTQIVAKYSNIELTTSQHKNDDTSKCIKLEGN

GVVLSDSNAIAFYLSSDQLRCSSNAFTFSEVLQWLSYADNHILPAVLGWIVPCLSKNVPNNVKTNIKTSKEDVLSSLKKL

NNILLTKTYLVGERISLADIAVFTALMPLYEHVLDPASRKQYTNLNRWFFTILNQSEVASMIKNFKICEKSVN

>XP_003488850.1 alanine--tRNA synthetase, cytoplasmic isoform X1 [Bombus impatiens]

MTAMMSAKQIRQAYIDFFKSKNHEYVHSSSTIPHDDPTLLFTNAGMNQFKPIFLGTVDPNSDMAKLMRAVNSQKCIRAGG

KHNDLDDVGKDVYHHTFFEMMGNWSFGDYFKKEICTWAWEFLTVKLKLSADRLYVTYFGGDEKNNLKSDEECKQIWLSLG

VSPSHVLPGNMKDNFWEMGETGPCGPCSEIHYDRIGNREAADLVNQDDPDVLEIWNLVFIQFNRETDGSLKLLPKRHIDC

GLGLERLVSVIQNKRANYDTDLFVPLFDAIQEGTGAPPYRGRVGMEDEDGIDMAYRVLADHARTITIALADGGVPDNTGR

GYVLRRILRRAVRYATEKLNAKPGFFGSLVNVVVNILGDIFPEVKKDPQYIIDVINEEEIQFLKTLSRGRNLLNRTIAKL

GSSNVLPGDVAWRLYDTYGFPVDLTQLMAEEKGLKVDIIGYEEAKKQAQLISQSKSGGVDDQINLDVHAITELQNKGIKP

TNDLPKYNYKVVNNKLYEEYEFVPCTGTVIAVRRAKTFVDEVSSGEEVGILLDQTNFYAEQGGQIYDEGFLVKIDDDDTE

VRIKNVQIRGGYVLHIGTVGQGKLKKGDKVFLNIDTTRRRLIMSNHTATHALNHALRKVLGTEVDQKGSLVAPDRLRFDF

TNKGAMTAEQVRKVEEITSNMIKGNKKIYAKESNLALAKTIQGLRAMFEETYPDPVRVVSIGIPVEDLEKDPLSSAALQT

SVEFCGGTHLHYTEHIGDFVIASEEAIAKGIRRIVALTGPEATKAQKKASILQNHLDQLQATIVADKDGVNIKEHIKEIV

ELTDDVSHATISSWKKDKMRKMLKDLRKALDDKERVAKIAIVNAVVDTIQEIIQQNIGCLVLVEVLQAYSNTKALDSALK

KIKAISPETSALLISVDPDAKKIFALSTVSKSAINKGLKANEWIQEIASLMEGKGGGKSESAQASGTNISCVTKLVHTAK

NFANSKLGIVEDIVNENHDQLKSSEKSVCSKTLKKKLVLSSDIGSIKYYRTQIVAKYSNIELTTSQHKNDDTSKCIKLEG

NGVVLSDSNAIAFYLSSDQLRCSSNAFTFSEVLQWLSYADNHILPAVLGWIVPCLSKNVPNNVKTNIKTSKEDVLSSLKK

LNNILLTKTYLVGERISLADIAVFTALMPLYEHVLDPASRKQYTNLNRWFFTILNQSEVASMIKNFKICEKSVN

>KOB76888.1 Alanine--tRNA synthetase, cytoplasmic [Operophtera brumata]

MNTSMTAKDVRNAYIDFFKSKGHQYVHSSSTIPLDDPTLLFTNAGMNQFKPIFLGSVDPNSDMAQWVRIVNTQKCIRAGG

KHNDLDDVGKDVYHHTFFEMMGNWSFGDYFKKEICTWAWELLTDVLKLPADRLYVTYFGGDAASGLEPDSECKNFWLSLG

LPESHVLPGNMKDNFWEMGETGPCGPCSELHFDRIGGREAAHLVNMDDPDVLEIWNLVFIQFNRETDGTLKSLPKKHIDC

GLGLERLVSVIQDKRANYDTDLFMPLFKAIEKGSGARPYSGKVGEEDVDGIDMAYRVLADHARTLTIALSDGGCPDNTGR

GYVLRRILRRGVRYATEKLNGKPGFFGSLVHTVVEILGDVFPEITKDPESIIQTINEEETQFLKTLSRGRGLLNRTIEKL

NDSKVIPGDVAWRLYDTYGFPIDLTQLMCEEKGLFVDMDGYEKARKESQLISQGKTAGQEDLLALDIHAISYLQDNGISV

TDDSPKYKYTPSSADKDAVYKFDSCSGTIVAIRADKQFVTEVATGKECGIILDRTNFYAEQGGQIFDEGYMVKSDDESVE

FTVKNVQVKGGYVLHVGKVEGSFKVGDKVSLYIDTERRKLIMNNHTGTHVLNNVLRKVLGNDSDQRGSLVVPDRLRFDFT

NKGPMTVNQIKSAEDQIKDIISENKVVYAKHTSLSDAKKINGLRAMFDEHYPDPVRVVSVGIPVEELEKNPEGASGFDTS

VEFCGGSHLHRTGHIGEYVIVSEEGIAKGIRRIVALTGPEAIKAINKLSVLENDVNNIASFIKDQGDAINHKDITKKIVD

LTNEISQAQISYWKKDELRNMLKNLKKQLDDKERVAKAATINEVIEKAKNLCLESKDAKVIVSELKAYNNTKALDGALKQ

VKQISPTTAAMFFTVDDDSKKIFCLAAVPKNANEKGLLASEWVQSVVNVMGGKGGGKAESAQASGNNPNSLKEALKIAEE

FAVAKLS

>XP_014358431.1 PREDICTED: LOW QUALITY PROTEIN: alanine--tRNA synthetase, cytoplasmic-like [Papilio machaon]

MDTSLTGNQIRKTFIDFFIGKGHKYVHSSSTIPLDDPTLLFANAGMNQFKPIFLGSVDPNSDMAQYVRVVNTQKCIRAGG

KHNDLDDVGKDVYHHTFFEMMGNWSFGDYFKKEICAWAWELLTEVYKLSADRLYVTYFGGDAASGLEPDLECKNIWLNLG

LPESHILPGSMKDNFWEMGETGPCGPCSELHYDRIGDRNAAHLVNMDDPDVLEIWNLVFIQFNREADSSLKPLPKKHIDC

GLGLERLVSVIQNKKANYDTDFFMPLFKAIETGTKTRPYTGKVGADDVDGIDMAYRVLADHARTLTIALADGGCPDNTGR

GYVLRRILRRAVRYASEKLNGKPGFFGSLVHTVVELLGDVFPEIKKDPESIIQTINEEEIQFLKTLSRGRNLLNRTIEKL

SGSKIIPGDVAWRLYDTYGFPVDLTHLMCEEKGLSIDMEAYEKAKKESQLNSQGKAAGQEDLLALDIHAINHLQESGVPV

TDDSPKYSYTPSSNDKDAIYSFAPCTATVLALRWNKEFVSEVQSGQECGLXLDRTNFYAEQGGQIFDEGYMVKIDDDSVE

FTVKNVQVKGGYVLHAGKVEGTLKVGDKVSLHIDTERRRLVMNNHTGTHILNNVLRKVLGNDSDQRGSLVMPDRLRFDFT

NKGPMTVKQIKDAEDEIKNIIAENKAVYAKHTSLADAKKIKGLRAMFDEHYPDPVRVVSVGIPVEELEKNPDGPSGYETS

VEFCGGSHLHQTGHIGDYVIVSEEGIAKGIRRIVALTGPEAVKALNKLSVLENEVNNIANFIKEQGDAVNHKEVVKKIVE

LTNDVSHAQISYWKKEELRNMLKGLKKQLDDKERASKAMIITQVIEKTKELCLEEKDSDVIVTELKALGNTKALDGALKQ

VKQILPNTAAMFFSVDDETNKIYCLAAVPKTLNEKGLLASEWVQSIVSVMGGKGGGKAESAQASGNNPKSITEAIKIAKE

FASSKL

>KPI93859.1 Alanyl-tRNA synthetase, cytoplasmic [Papilio xuthus]

MDTSMTGNQIRKTFIDFFIGKGHKYVHSSSTIPLDDPTLLFANAGMNQFKPIFLGSVDPNSDMAQYVRVVNTQKCIRAGG

KHNDLDDVGKDVYHHTFFEMMGNWSFGDYFKKEICAWAWELLTEVYKLPADRLYVTYFGGDAASGLEPDLECKNIWLNLG

LPESHILPGSMKDNFWEMGETGPCGPCSELHYDRIGDRNAAHLVNMDDPDVLEIWNLVFIQFNREADSSLKLLPKKHIDC

GLGLERLVSVIQNKRANYDTDFFMPLFKAIETGTKTRPYTGKVGADDVDGIDMAYRVLADHARTLTIALADGGCPDNTGR

GYVLRRILRRAVRYASEKLNGKPGFFGSLVHTVVELLGDVFPEIKKDPDSIIQTINEEEIQFLKTLSRGRNLLNRTIEKL

SGSKIIPGDVAWRLYDTYGFPVDLTHLKCEEKGLSIDMEAYEKAKKESQLNSQGKAAGQEDLLALDIHAISHLQESGVPV

TDDSPKYGYTPSSNDKDAIYSFAPCTATVLALRCNKEFVSEVQSGQECGLILDRTNFYAEQGGQIFDEGYMVKMDDDSVE

FTVKNVQVKGGYVLHSGKVEGTLKVGDKVSLHIDTERRRLIMNNHTGTHILNNVLRKVLGNDSDQRGSLVMPDRLRFDFT

NKGPMTVKQIKDAEDEIKNIIVQNKAVYAKHTSLTDAKKIKGLRAMFDEHYPDPVRVVSVGIPVEELEKNPDGPSGYETS

VEFCGGSHLHQTGHIGDYVIVSEEGIAKGIRRIVALTGPEAVKALNKLSVLENEVNSIANFIKEQGDAVNQKEVVRKIVE

LTNDVSHAQISYWKKEELRNLLKGFKKQLDDKERASKAMIITQVIEKTKELCLEEKDSKVIVTELKASGNTKALDGALKQ

VKQMLPNTAAMFFSIDDETSKIYCLAAVPKSLNEKGLLASEWVQSIVSVMGGKGGGKAESAQASGNNPKSITEAIKIAKE

FASSKL

>XP_014472783.1 PREDICTED: alanine--tRNA synthetase, cytoplasmic [Dinoponera quadriceps]

MITTMNAKQIRQAYIDFFKSKGHEYVHSSSTIPHDDPTLLFTNAGMNQFKPIFLGTVDPNSDMAKWVRVVNSQKCIRAGG

KHNDLDDVGKDVYHHTFFEMMGNWSFGDYFKKEICSWAWEFLTEILKLPGDRLYVTYFGGDEKNGLAPDDECKELWLSLG

IPASHVLPGNMKDNFWEMGETGPCGPCSELHYDRIGGREAAHLVNMDDPDVLEIWNLVFIQYNREADGNLKSLPKKHIDC

GLGLERLVSVIQNKRANYDTDLFMPLFAAIEKGTGAPPYQGKIGADDVNGVDMAYRVLADHARTITIALADGGMPDNTGR

GYVLRRILRRAVRYATEKLNAKPGFFGSLINVVVELLGEVFPEVTKDPQSIIDIVNEEETQFLKTLSRGRNLLNRTIAKL

ESNDTVPGDVAWRLYDTYGFPIDLTQLMTEEKGLKIDMAGYEESKKQAQLISQNKAGGVDDQINLDVHSITELQGLGIKP

TDDSPKYNYKITSTTTYKEYEFAPCTGTVIALRRAKTFVDEVSSGEEVGILLDKTNFYAEQGGQIYDEGFLVKVDDEATE

IRVKNVQVRAGYVLHIGTVGEGTLRKGDRVHANVDTARRRLVMANHSATHALNHALRKVLGTEADQKGSLVAPDRLRFDF

TNRGPMTMEQVKNTENITNDMIRDNRKIYAKESNLALAKTIQGLRAMFEETYPDPVRVVSMGVPVEDLEEDPLSPAATQT

SVEFCGGTHLHYTGHIGDFVIASEEAIAKGIRRIVALTGPEAAKALKKATLLQNQLTQLQATIEADKSGAHSKDHVKKIV

ELTEDISHATIPGWRKDKMRGMLKELKKSLDDKERAAKAAIANTVVETAQSMIQSKVGCQVLVEVLEAYSNTKALDSALK

KIRAISPETSALLISIDRDVKKIFALSSVPKSAVSKGLKASEWIQAITPMMQGKGGGKAESAQASGVNVACLNEIVSKAS

EFANQKLGITDNKTASDTFKTSESDDRSFNTECSSKLVLYANVGSTKCYLAQIIAQYSGTCLSVKQVDETSSGSVTLNMG

DITLRNGNAIAFFLSNPQLRRDDDLLASSQVLGWLSYAQNHILPAVTAWVLPTLGVSVSKDMKANSKLSKEDLLRDLKRL

DGMLHMRTYLVGERITLADVSIFTALLPLYEHVLDPHHRKSYTNLNRWFSTILNQPQVTAVVGNFAFCAKTARC

>XP_024873662.1 alanine--tRNA synthetase, cytoplasmic isoform X1 [Temnothorax curvispinosus]

MKSSMNAKQIRQAYIDFFKSKGHEYVHSSSTIPHDDPTLLFTNAGMNQFKPIFLGTVDPNSDMARWVRVVNSQKCIRAGG

KHNDLDDVGKDVYHHTFFEMMGNWSFGDYFKKEICAWAWEFLTDVMKLPVDRLYVTYFGGDDKSGLAPDNECKDLWLSLG

VPASHVLPGNMKDNFWEMGETGPCGPCSELHYDRIGGREAAHLVNMDDPDVLEIWNLVFIQYNRESDGSLKPLPKKHIDC

GLGLERLVSVIQNKRANYDTDLFMPLFAAIEKSTGAPPYQGKVGADDVNGIDMAYRVLADHARTITIALADGGMPDNTGR

GYVLRRILRRAVRYATEKLNAKPGFFGSLVNVVVDLLGEVFPEVKKDPQSIVDVINEEEAQFLKTLSRGRNLLNRTIAKL

ESANTVPGDVAWRLYDTYGFPIDLTQLMTEEKGLKIDMAGYEESKKQAQLISQNKAGGVDDQINLDVHAITELQKQGVKP

TDDSPKYNYKVTNSVKYEEYEFAPCTGTVIALRRAKTFVDQVSSGEEVGILLDRTNFYAEQGGQIYDEGFLVKVDDEATE

IRVKNVQVRAGYVLHIGTVGEGTLRKGDKVHANVDTERRRLVMANHSATHALNYALRKVLGTEADQKGSLVAPDRLRFDF

TNKGPMTGGQLKDTESFTNDMIQANKKIYAKESNLAIAKTIQGLRAMFEETYPDPVRVVSMGIPVENLEKDPLSTAALET

SVEFCGGTHLHYTGHIGDFVIASEDAIAKGIRRIVALTGPEATKALRTARILEKQLSQLQATIETDKSGTNSKEYVKKII

ELTEEISHALIPAWWKDNVRNTLKELKKSLDDKERAVRAAVTTTVVQTAQSIIESKVGCQVLVEVLEAYNNTKALDLALK

KIRAISPETSALLISVDHDVKKIFALSSVPKSAISKGLKANEWIQAIASLMQGKGGGKPESAQASGTNIACLKKIVSKAN

DFANQKLGIIDNKTANNTTRKTSETFRLLNTESSSKLVLYGNTGSTKCYLAQIIAQYSGKCLTIKQVKDNKICSSDIILE

TADVTLHNSNAIAFFLSNSQLRCEDDLFASSQVLQWMSYAQNHILPAVSGWVVPSLGVSVSKDMKANMKISKEDLLCTVR

RLDDILRTRTYLIGERVTLADISVFTALLPLYEYVFDPHYRKQYTNLNRWFSTILNQPQVKCVVKNFAFCEEATRC

>XP_022820652.1 alanine--tRNA synthetase, cytoplasmic [Spodoptera litura]

MDTSMSGNQIRQSFIDFFINKGHKYVHSSSTIPLDDPTLLFANAGMNQFKPIFLGSVDPNSDMAQYVRVVNTQKCIRAGG

KHNDLDDVGKDVYHHTFFEMMGNWSFGDYFKKEICSWAWELLTEVYKLPGDRLYVTYFGGDAASGLEPDLECKEIWLKLG

LPESHILPGSMKDNFWEMGETGPCGPCSELHYDRIGGRDAAHLVNMDDPDVLEIWNLVFIQFNRESDGSLKLLPKKHIDC

GLGLERLVSVIQNKRANYDTDFFMPIFKAIEIGTGARPYTGKVGAEDVDGLDMAYRVLADHARTLTIALSDGGCPDNTGR

GYVLRRILRRAVRYASEKLNAKPGFFGSLVHVVVELLGDVFPEITKDPLSLIQLINEEELQFLKTLTRGRNLLNRTIEKL

NGKVIPGDVAWRLYDTYGFPIDLTQLMCEENGLTIDMEAYEKSKKESQLISQGKAAGQEDLLALDVHAISHLQDTGVPAT

DDSPKYNYVASSSEIEAVYNFSSCIGTVIALRQNKQFVNEVSGQECGVILDKTSFYAEQGGQIYDEGYMVKTDDDSVEFV

VTNVQVKGGYVLHMGKLEGTLKVGDKLSLHIDTERRRLVMNNHTGTHVLNNVLRKVLGNDSDQRGSLVMPDRLRFDFTNK

GPMSLKQIKDTEDQIKEIIYKNKPVYAKHTSLNEAKKIKGLRAMFDEHYPDPVRVVSVGIPVEELEKNPEGPAAYETSVE

FCGGSHLHQTGHIGDYVIVSEEGIAKGIRRIVALTGPEALKAISKSSSLENEVNNIANFIKDQSDNMNQKEILRKIVDLT

NEISQAQISYWKKEELRTMLKNLKKQLDDKERAAKALTITQVAERAKEICTEKKDNKVIVSELKAFGNTKALDGALKQVK

LLCPATAAMFFSVDDETNKIFCLAAVPKNAIEKGLLASEWIQSVVPVIGGKGGGKAESGQASGINFASLEEAMKVAEEFA

NLKLC

>XP_024873663.1 alanine--tRNA synthetase, cytoplasmic isoform X2 [Temnothorax curvispinosus]

MKSSMNAKQIRQAYIDFFKSKGHEYVHSSSTIPHDDPTLLFTNAGMNQFKPIFLGTVDPNSDMARWVRVVNSQKCIRAGG

KHNDLDDVGKDVYHHTFFEMMGNWSFGDYFKKEICAWAWEFLTDVMKLPVDRLYVTYFGGDDKSGLAPDNECKDLWLSLG

VPASHVLPGNMKDNFWEMGETGPCGPCSELHYDRIGGREAAHLVNMDDPDVLEIWNLVFIQYNRESDGSLKPLPKKHIDC

GLGLERLVSVIQNKRANYDTDLFMPLFAAIEKSTGAPPYQGKVGADDVNGIDMAYRVLADHARTITIALADGGMPDNTGR

GYVLRRILRRAVRYATEKLNAKPGFFGSLVNVVVDLLGEVFPEVKKDPQSIVDVINEEEAQFLKTLSRGRNLLNRTIAKL

ESANTVPGDVAWRLYDTYGFPIDLTQLMTEEKGLKIDMAGYEESKKQAQLISQNKAGGVDDQINLDVHAITELQKQGVKP

TDDSPKYNYKVTNSVKYEEYEFAPCTGTVIALRRAKTFVDQVSSGEEVGILLDRTNFYAEQGGQIYDEGFLVKVDDEATE

IRVKNVQVRAGYVLHIGTVGEGTLRKGDKVHANVDTERRRLVMANHSATHALNYALRKVLGTEADQKGSLVAPDRLRFDF

TNKGPMTGGQLKDTESFTNDMIQANKKIYAKESNLAIAKTIQGLRAMFEETYPDPVRVVSMGIPVENLEKDPLSTAALET

SVEFCGGTHLHYTGHIGDFVIASEDAIAKGIRRIVALTGPEATKALRTARILEKQLSQLQATIETDKSGTNSKEYVKKII

ELTEEISHALIPAWWKDNVRNTLKELKKSLDDKERAVRAAVTTTVVQTAQSIIESKVGCQVLVEVLEAYNNTKALDLALK

KIRAISPETSALLISVDHDVKKIFALSSVPKSAISKGLKANEWIQAIASLMQGKGGGKPESAQASGTNIACLKKIVSKAN

DFANQKLGIIATV

>XP_012540755.1 PREDICTED: alanine--tRNA synthetase, cytoplasmic isoform X2 [Monomorium pharaonis]

MNGNQIRQAFIQFFKDKGHEYVHSSSTIPHDDPTLLFTNAGMNQFKPIFLGTVDPNSDMAKWVRAVNSQKCIRAGGKHND

LDDVGKDVYHHTFFEMLGNWSFGDYFKKEICKWAWEFLTEILKLPADRLYVTYFGGDKKSGLASDDECKNLWLSLGIPAS

HVLPGNMKDNFWEMGETGPCGPCSELHYDRIGGREAAHLVNMDDPDVLEIWNLVFIQYNRESDGSLKSLPKKHIDCGLGL

ERLVSVIQNKRANYDTDLFTPLFTAIEKGTGAPPYQGRVGADDVDGVDMAYRVLADHARTITIALADGGMPDNTGRGYVL

RRILRRAVRYATEKLNAKPGFFGTLINVVVSLLGDVFPELKKDPQSIIDTVNEEEAQFLKTLSRGRNLLNRTIAKLESTD

VVPGDVAWRLYDTYGFPIDLTQLMTEEKGLKIDMIGYEESKKQAQLISQNKAGGVDDQINLDIHAITELQNQGVEPTDDS

PKYNYKVISTKLYYEYDFEPCIGTVIALRRAKTFVDEVFSGEEVGILLDTTNFYAEQGGQIYDEGFLVKVDDEATEIRVK

NVQVRAGYILHIGTVGEGTLRKGDKVHTNVDTKRRRFVMANHSATHALNYALRKVLGTEADQKGSLVAPDRLRFDFTNKG

SMTTKQLQDTEVTTQKMIEESKPIYAKESNLVVAKTIRGLRAMFEETYPDPVRVVSMGIPVEKLEEDPLSSAALETSVEF

CGGTHLHNTGHIGDFVIASEDAIAKGIRRIVALTGPEATKAIRTATRLQDRLVELEKTVKSEINPKEYVKKIVELTEEIS

HALIPAWQKTNMRNTLKDVKKTLDDKERKAMAGLANKVIQTAQILIESKCGQEVFVEVLDAKSNTKALDMALKKVRAISP

ETSALLISVDRDVGKIFVLSSVPESAIHKGLKANEWIQAIVPLMDGKGGGKPEAAQASGTNIQCLDEIVSKAKEFAYKKF

GTTEPLYNSTA

>XP_012167104.1 alanine--tRNA synthetase, cytoplasmic isoform X1 [Bombus terrestris]

MTAMMSAKQIRQAYIDFFKSKNHEYVHSSSTIPHDDPTLLFTNAGMNQFKPIFLGTVDPNSDMAKLMRAVNSQKCIRAGG

KHNDLDDVGKDVYHHTFFEMMGNWSFGDYFKKEICTWAWEFLTVKLKLSADRLYVTYFGGDEKNNLKSDEECKQIWLSLG

VSPSHVLPGNMKDNFWEMGETGPCGPCSEIHYDRIGNREAADLVNQDDPDVLEIWNLVFIQFNRETDGSLKLLPKKHIDC

GLGLERLVSVIQNKRANYDTDLFVPLFDAIQEGTGAPPYRGRVGMEDEDGIDMAYRVLADHARTITIALADGGVPDNTGR

GYVLRRILRRAVRYATEKLNAKPGFFGSLVNVVVNILGDIFPEVRKEPQYIIDVINEEEIQFLKTLSRGRNLLNRTIAKL

ESSNVLPGDVAWRLYDTYGFPVDLTQLMAEEKGLKVDIIGYEEAKKQAQLISQSKSGGVDDQINLDVHAITELQNKGIKP

TNDLPKYNYSVVNNKLYEEYEFVPCTGTVIAVRRAKTFVDEVSSGEEVGILLDQTNFYAEQGGQIYDEGFLVKIDDDDTE

VRIKNVQVRGGYVLHIGTVGQGKLKKGDKVFLNIDTTRRRLIMSNHTATHALNHALRKVLGTEVDQKGSLVAPDRLRFDF

TNKGAMTAEQVRKVEEITSNMIKGNKKIYAKESNLALAKTIQGLRAMFEETYPDPVRVVSIGVPVEDLEKDPLSSAALQT

SVEFCGGTHLHCTEHIGDFVIASEEAIAKGIRRIVALTGPEATKAQKKASILQNHLDQLQATIVADKGGINIKEHIKEII

ELTDDVSHATISSWKKDKMRKMLKDLRKALDDKERVAKIAIVNAVVDTIQEIIQQNIGCLVLVEVLQAYSNTKALDSALK

KIKAISPETSALLISVDPDAKKIFALSAVSKSAINKGLKANEWIQEIVSLMEGKGGGKSESAQASGTNISCVTKLVHTAK

NFANSKLGIVEDIVNENHDQLKSSKKSVCSKTLKKKLVLSSDIGSIKYYRTQIVAKYSDIELTTSQHKNDDTSKCIKLEG

NDVVLSDSSAIAFYLSSDQLRCSSNAFTFSEVLQWLSYADNHILPAVLGWIVPYLSKNVSNNVKTNIKTSKEDVLSSLKK

LNNILLTKTYLVGERISLADIAVFTALMPLYEHVLDPAARKQYTNLNRWFFTILNQSEVASIIKNFKICEKSVN

>XP_003397717.1 alanine--tRNA synthetase, cytoplasmic isoform X2 [Bombus terrestris]

MTAMMSAKQIRQAYIDFFKSKNHEYVHSSSTIPHDDPTLLFTNAGMNQFKPIFLGTVDPNSDMAKLMRAVNSQKCIRAGG

KHNDLDDVGKDVYHHTFFEMMGNWSFGDYFKKEICTWAWEFLTVKLKLSADRLYVTYFGGDEKNNLKSDEECKQIWLSLG

VSPSHVLPGNMKDNFWEMGETGPCGPCSEIHYDRIGNREAADLVNQDDPDVLEIWNLVFIQFNRETDGSLKLLPKKHIDC

GLGLERLVSVIQNKRANYDTDLFVPLFDAIQEGTGAPPYRGRVGMEDEDGIDMAYRVLADHARTITIALADGGVPDNTGR

GYVLRRILRRAVRYATEKLNAKPGFFGSLVNVVVNILGDIFPEVRKEPQYIIDVINEEEIQFLKTLSRGRNLLNRTIAKL

ESSNVLPGDVAWRLYDTYGFPVDLTQLMAEEKGLKVDIIGYEEAKKQAQLISQSKSGGVDDQINLDVHAITELQNKGIKP

TNDLPKYNYSVVNNKLYEEYEFVPCTGTVIAVRRAKTFVDEVSSGEEVGILLDQTNFYAEQGGQIYDEGFLVKIDDDDTE

VRIKNVQVRGGYVLHIGTVGQGKLKKGDKVFLNIDTTRRRLIMSNHTATHALNHALRKVLGTEVDQKGSLVAPDRLRFDF

TNKGAMTAEQVRKVEEITSNMIKGNKKIYAKESNLALAKTIQGLRAMFEETYPDPVRVVSIGVPVEDLEKDPLSSAALQT

SVEFCGGTHLHCTEHIGDFVIASEEAIAKGIRRIVALTGPEATKAQKKASILQNHLDQLQATIVADKGGINIKEHIKEII

ELTDDVSHATISSWKKDKMRKMLKDLRKALDDKERVAKIAIVNAVVDTIQEIIQQNIGCLVLVEVLQAYSNTKALDSALK

KIKAISPETSALLISVDPDAKKIFALSAVSKSAINKGLKANEWIQEIVSLMEGKGGGKSESAQASGTNISCVTKLVHTAK

NFANSKLGIVDIVNENHDQLKSSKKSVCSKTLKKKLVLSSDIGSIKYYRTQIVAKYSDIELTTSQHKNDDTSKCIKLEGN

DVVLSDSSAIAFYLSSDQLRCSSNAFTFSEVLQWLSYADNHILPAVLGWIVPYLSKNVSNNVKTNIKTSKEDVLSSLKKL

NNILLTKTYLVGERISLADIAVFTALMPLYEHVLDPAARKQYTNLNRWFFTILNQSEVASIIKNFKICEKSVN

>XP_012540753.1 PREDICTED: alanine--tRNA synthetase, cytoplasmic isoform X1 [Monomorium pharaonis]

MNGNQIRQAFIQFFKDKGHEYVHSSSTIPHDDPTLLFTNAGMNQFKPIFLGTVDPNSDMAKWVRAVNSQKCIRAGGKHND

LDDVGKDVYHHTFFEMLGNWSFGDYFKKEICKWAWEFLTEILKLPADRLYVTYFGGDKKSGLASDDECKNLWLSLGIPAS

HVLPGNMKDNFWEMGETGPCGPCSELHYDRIGGREAAHLVNMDDPDVLEIWNLVFIQYNRESDGSLKSLPKKHIDCGLGL

ERLVSVIQNKRANYDTDLFTPLFTAIEKGTGAPPYQGRVGADDVDGVDMAYRVLADHARTITIALADGGMPDNTGRGYVL

RRILRRAVRYATEKLNAKPGFFGTLINVVVSLLGDVFPELKKDPQSIIDTVNEEEAQFLKTLSRGRNLLNRTIAKLESTD

VVPGDVAWRLYDTYGFPIDLTQLMTEEKGLKIDMIGYEESKKQAQLISQNKAGGVDDQINLDIHAITELQNQGVEPTDDS

PKYNYKVISTKLYYEYDFEPCIGTVIALRRAKTFVDEVFSGEEVGILLDTTNFYAEQGGQIYDEGFLVKVDDEATEIRVK

NVQVRAGYILHIGTVGEGTLRKGDKVHTNVDTKRRRFVMANHSATHALNYALRKVLGTEADQKGSLVAPDRLRFDFTNKG

SMTTKQLQDTEVTTQKMIEESKPIYAKESNLVVAKTIRGLRAMFEETYPDPVRVVSMGIPVEKLEEDPLSSAALETSVEF

CGGTHLHNTGHIGDFVIASEDAIAKGIRRIVALTGPEATKAIRTATRLQDRLVELEKTVKSEINPKEYVKKIVELTEEIS

HALIPAWQKTNMRNTLKDVKKTLDDKERKAMAGLANKVIQTAQILIESKCGQEVFVEVLDAKSNTKALDMALKKVRAISP

ETSALLISVDRDVGKIFVLSSVPESAIHKGLKANEWIQAIVPLMDGKGGGKPEAAQASGTNIQCLDEIVSKAKEFAYKKF

GTTEPLYNCEIGTNKINIKHIKSNMKVTQPCFTLIGNSGSPKCYLAQIVAQYSDKFINFVQIDENVEYPGYVVLLTPDCK

IRDSNAIAFFLSNSQLRRDDDLAASCQVIQWMNYAHNEIAPVVVGWVLPSLFKPYQPKKMKAKMESCREAVIEIVKNMDS

ILHSKTYLIGERISLADLVVFAAFLPMYEYVFDRWYKKEYVNMNRWFSTILNQPQVQRIIETLKI

>XP_023955226.1 alanine--tRNA synthetase, cytoplasmic [Bicyclus anynana]

MDTSMTGSQIRSSFIDFFISKGHKYVHSSSTIPHDDPTLLFTNAGMNQFKPIFLGTIDPNSDMGQYVRVVNTQKCIRAGG

KHNDLDDVGKDVYHHTFFEMMGNWSFGDYFKKEICSWAWELLTDVYKLSGDRLYVTYFGGDEASGLEPDLECKNIWINLG

VPESHILPGSMKDNFWEMGETGPCGPCSELHYDRIGGRNAANLVNMDDPDVLEIWNLVFIQFNREMDGSLKALPKKHIDC

GLGLERLVSVIQNKRANYDTDFFMPIFQAIQNGTKIRPYTGKVGADDTDGIDMAYRVLADHARTLTIALSDGGHPDNTGR

GYVLRRILRRAVRFASEKLNAKPGFFGSLVNTVVKLLGDVFPEITKDPESIIQTINDEEIQFLKTLSRGRNLLNRTIEKL

GDSKTVPGDVAWRMYDTYGFPIDLTQLMCEEKNLAIDMEAYERAKKESQLSSQGKSVGQEDLIALDIHAISHLQEIGMPI

TDDSPKYNYTPSSNEKEAEYEFAPCTGTVLALRRNKEFVNEITSGQEGGVILDRTSFYAEQGGQIFDEGYMVKTDDDSVE

FTVNNVQVKGGYILHTGKVEGTLKVGDKVLLHIDTERRRLVMNNHTGTHVLNNVLRKVLGNDSDQRGSLVMPDRLRFDFT

NKGPMTVKQIKDTEDQIKEIINCNKRVYAKHTSLSAAKKINGLRAMFDEHYPDPVRVVSVGIPVEELENNPEGTAGFDTS

VEFCGGSHLHQTGHIGDYVIVSEEGIAKGIRRIVAFTGPEATKAINKLSILENEVNNIANYVKDQGDNIDNKEIVKKIVD

LTNDVSHAQIAYWKKEELRTMLKNLKKQLDDKERAAKAMTINLVIEKAKEICLQDNSSDVIVSELKAFNNTKALDGALKQ

VKLLQPNAAAMFFSVDDDSNKIFCLAAVPKNLIEKGLLASEWVQAVVPIMAGKGGGKAESAQASGNNPKGLYEAIKIARN

FAENKLS

>XP_017885436.1 PREDICTED: alanine--tRNA synthetase, cytoplasmic-like [Ceratina calcarata]

MNTMMSAKEIRQAYIDFFKSKDHEYVHSSSTIPHDDPTLLFTNAGMNQFKPIFLGVVDPNSDMAKLVRAVNSQKCIRAGG

KHNDLDDVGKDVYHHTFFEMMGNWSFGDYFKKEICTWAWEFLTDKLKLPKDRLYVTYFGGDEKNNLKPDEECKQIWLSLG

LSPSHVLPGSMKDNFWEMGETGPCGPCSEVHYDRIGNREAAHLVNQDDPDVLEIWNLVFIQFNRESDGSLKSLPKKHIDC

GLGLERLVSVIQNKRANYDTDLFVPLFDAIQKGTNAPAYQGRVGAEDKDGIDMAYRVLADHARTITIALADGGVPDNTGR

GYVLRRILRRAVRYATEKLNAKPGFFGSLVNVVVDLLGDVFPELKKDPQYIIDTVNEEEAQFLKTLSRGRNLLNRTIGKL

ESSNELPGDVAWRLYDTYGFPVDLTKLMAEEKGLIVDMNGYEEAKKQAQILSQGKTGGVDDRINLDVHAITELQSKGIKP

TNDLLKYNYKVTNSKMYEEYEFTPCIATVIALRRSKTFVNEVSSGEEVGILLDKTSFYAEQGGQIYDEGFLAKIDDEGTE

VRIKNVQVRGGYVLHIGTVGEGVLKKGDKVTLNVDTTRRRYLMSNHTATHLLNYSLRRILGTDVDQKGSLVAPDRLRFDF

TNKGAVTTEQVRKVEELTSNIVKQNKKVYAKQSNLAVAKTIQGLRAMFEETYPDPVRVVSIGIPVEELERDPLGPAAFET

SVEFCGGTHLHYTGHIGDFIIANEEAIAKGIRRIVALTGPEAAKAQKKASILQNDLDRLKATIESDKNGSNTKDHVKKIV

ELTDDVSRATISSWRKDQMRKMLKDLKKALDDKERAAKTAIANAVVDNVQRIIEQNVGCPVLVQVVQAYSNTKALDSALK

KIKAISPDTSALLVSVDPDAKKIFALAAVSKCAVNKGLKANEWIEEIALLMGGKGGGKSESAQASGSNTSCVAKLVHTAK

NFANSKLGMADKNQNSLLSNESVEFENSDNVMILSGNTRGVKFYRAEIVAKYSGKHVIKQHRDDSDIKLEGKGFQLFDGT

AIAFYLSSDSLKCANDVAKFTDVLQWSIYSDTQILPAVLGWVVPCISKNVSHALKANMKISKEDILCSLNKLNNVLLTRT

YLVGERISVADISLFTSLMPLYEYVLDPASRKQYTNLNRWFFTMLNQPQIAEVIKNFEVCQKTVKC

>XP_014253263.1 alanine--tRNA synthetase, cytoplasmic [Cimex lectularius]

MDSSMSAKEIRNQFINFFVKKKNHEYVHSSPVIPHDDPTLLFANAGMNQFKPIFLGTADPNSAMATWKRAVNTQKCIRAG

GKHNDLDDVGKDVYHHTFFEMLGNWSFGDYFKKEICTWAWELLTEVFHLPKDRLYVTYFGGHKESGLEPDEECRQIWLKL

GIPESHVLPGSMKDNFWEMGETGPCGPCSELHFDRIGNREVPHLVNQDDPDVLEIWNLVFIQYNRETGGSLRPLPKKHID

CGMGFERLVSVIQNKRSNYDTDIFKPLFDAIQKGTNAPPYQGRVGDEDKDGIDMAYRVLADHARTLTIAISDGGTPDNTG

RGYVLRRILRRAVRYAVEKLNAKPGFFSSLVQVVVDLLGDTFSEVKKCPQNVVAIINEEEEQFLKTLSRGRNLLNRTIDK

LGSTNIFPGDIAWRLYDTYGFPVDLTGLMTEEKGLTIDMNAYEESKKHAQLVSQGKDSANANLISLDVHAISELQDSDVP

VTDDSPKYNYKAVAGEGAFAKYKFEPCQGKVLALRKGTQFVQEVKSGDRCGIVLDKTCFYAEQGGQIYDQGFMVKVGDES

VEVSVDNVQLKGGYILHTGKVEGLLKVGDKLNLQIDEVRRRLVMSNHTATHILNHALRAVLGPESDQKGSLVAPDRLRFD

FTNKKAMSPDQVKKTEFMINNISKADKVVYAQSAPLATAKKVVGLRAMFSEVYPDPVRIVSVGEPVERLISSPESKAAIE

TSVEFCGGTHLHRSSHMGDFVITSEEAIAKGIRRIVALTGAEAKKAMDHSIALDNAVNMLSDIASTKSLTGKDLVRAITQ

LNDEISQAVIPYWKKEELRTTLKNMKKKYDDSERAGKTEQIQQVVDSAKQLATQQKGVPFLVAELKAGSNTKALDGALKQ

VKQLSPETSAMFFSVEESSEKIFCLCAVSKSGIEKGLKANEWVNNVSGLMNGKGGGKADSAQASGSNGKCLATALAKSKE

FAAAKLGGGVCSNGGGDAEGLVYTDNCLGSVLVCLTHEYTQAKAGLKKGENLNFNKNGLNLTDSVAIAFYLADTQLKGGD

STLREAQVLQWVWYAENTVRNSSNPSEWMPQLNNYLLTRTFFVGERVTLADLALFTRLLPSYHKNVPLTKYTNLNRWFTT

MCNQKAVMKVVGDLIKSK

>XP_020285640.1 alanine--tRNA synthetase, cytoplasmic isoform X2 [Pseudomyrmex gracilis]

MSTNMKAKEIRQAYIDFFKSKGHEYVHSSSTIPHDDPTLLFTNAGMNQFKPIFLGTVDPNSDMAKWVRVVNSQKCIRAGG

KHNDLDDVGKDVYHHTFFEMMGNWSFGDYFKKEICTWAWEFLTDVLKLSSDRLYVTYFGGDEKNGLAPDIECKELWLSLG

VPASHVLPGNMKDNFWEMGETGPCGPCSELHYDRIGGREAAHLVNMDDPDVLEIWNLVFIQYNRELDGSLKSLPKKHIDC

GLGLERLVSVIQNKRANYDTDLFIPLFAAIEKGTGAPPYQGKVGADDINGIDMAYRVLADHARTITIALADGGMPDNTGR

GYVLRRILRRAVRYATEKLNAKPGFFGSLVNVVVDLLGEVFPEVTKDPQSIIDIVNEEEAQFLKTLSRGRNLLNRTIAKL

ESTDIVPGDVAWRLYDTYGFPIDLTQLMTEERGLKIDMIGYEESKKQAQLISQNKAGGVDDQINLDVHAITELQQQGIGP

TDDSPKYNYKVINSAKYKEYEFGTCIGTVIALRRAKTFVDEVSSGEEVGILLDKTNFYAEQGGQIYDEGFLVKVDDEATE

IRIKNVQVRGGYVLHIGTVGEGTLRKGDKVHTNVDTTRRRLVMANHSATHALNHALRKVLGTEADQKGSLVAPDRLRFDF

TNKGPMTTEQVENTENITNDMIKENKKIYAKESNLALAKTIQGLRAMFEETYPDPVRVVSMGVPVENLEKDPLSTAALET

SVEFCGGTHLHYTGHIGDFVIASEDAIAKGIRRIVALTGPEAAKALKKATLLQNQLTQLQSTIEADKSGTNSKEYVKKIV

ELTEDISHAVISGWRKDKMRAMLKELKKSLDDKERAAKAAIANTVVETAQAIIQSKVGCQVLVEVLEAHSNTKALDSALK

KIRAISPETSALLVSVDRDAKKIFALSSVPKSAVSKGLKANEWIQAITSVMQGKGGGKAESAQASGSNIAGLDEVVNKAT

EFANQKLGIIENDVRSLDMETPSKQNAKLVLHANKWSTKCYLAEITAQYSGKCLTVKQIENDKNNSSDIILETTDVTLSE

SNAIAYFLSNSQLRREDDLIASSQVFQWMIYARNHILPAASTWILSSSDFPVSKGMRPNVKATEDSKADIL

>PCG78534.1 hypothetical protein B5V51_3836 [Heliothis virescens]

MDTSMTGNQIRKAFIDFFISKGHKYVHSSSTIPLDDPTLLFTNAGMNQFKPVFLGSVDPNSDMAQYVRVVNTQKCIRAGG

KHNDLDDVGKDVYHHTFFEMMGNWSFGDYFKKEICGWAWELLTEVYKLSGDRLYVTYFGGDASSGLEPDLECKEIWLKLG

VPESHILPGSMKDNFWEMGETGPCGPCSELHYDRIGGRDAAHLVNMDDPDVLEIWNLVFIQFNRETDGSLKLLPKKHIDC

GLGLERLVSVIQNKRANYDTDFFMPIFKAIEIGTGARPYSGKVGSDDVDGIDMAYRVLADHARTLTIALSDGGCPDNTGR

GYVLRRILRRAVRYASEKLNAKPGFFGSLVHTVVELLGDIFPEITKDPLSLIQLINEEEIQFLKTLSRGRNLLNRTIEKL

NGKVIPGDVAWRLYDTYGFPVDLTQLMCEENGLTIDMDAYEKSKKESQLISQGKVAGQEDLLALDVHAISHLQESGVPAT

DDSPKYNYVASSNEKETVYSFSPCTGTIVALRKDKQFVNEVASGQECGVILDKTNFYAEQGGQIYDEGYMVKVDDDSVEF

MVSNVQVKGGYVLHIGKLEGSLKIGDKLSLHIDTERRRLVMNNHTGTHVLNNVLRKVLGNDSDQRGSLVMPDRLRFDFTN

KGPMSIKQIKETEDQIKEIINKNKPVYAKHTSLNDAKKIKGLRAMFDEQYPDPVRVVSVGIPVEELEKNPDGPAAYETSV

EFCGGSHLHQTGHIGDYVIVSEEGIAKGIRRIVALTGPEAMKAISKSSILENEANNIANYIKDQSDNVNQKEILRKIVDL

TNDISQAQISYWKKEELRTMLKNLKKQLDDKERAAKAVTITQVAEKAKEICVEKKGSPVIVSELKAFGNTKALDGALKQV

KTLCPTTAAMFFSVDDETKKIFCLAAVPKNAIEKGLLASEWIQSVVPVIGGKGGGKPESGQASGNNYTSLNEALKTAEEF

ANLKLN

>XP_022341752.1 alanine--tRNA synthetase, cytoplasmic-like [Crassostrea virginica]

MESKSTGADIRQLFVDFFKEKEHLYVHSSSVVPLDDPTLLFTNAGMNQFKPIFLEIVDPNSDMAKYVRAANSQKCIRAGG

KHNDLDDVGKDVYHHTFFEMLGNWSFGDYFKSECCAWAWEFLTDRVGLPKDRLYVTYFGGNEKFNLSPDEETKNIWLKLG

VPEERVLPFGMKDNFWEMGETGPCGPCTEIHYDKVGGRDAKDLVNMDDPEVIEIWNLVFIQFNRESETELNELPRKHCDC

GMGLERLVCVMQGKTSNYDTDLFTPYFDFIQKATNCQPYQGRVGKEDGDGVDMAYRVLADHARTLTVALADGGRPDNMGR

GYVLRRILRRAVRYGTEKLNAKPGMFASLVDVVVESLGDAFPELKKDPEMIKDIINEEEAQFLKTLNRGRKLLERTIGKL

GDTTSLPGDIAWRLYDTYGFPVDLTALMAEEKGLAVDMLAYKSAMEKAKALSQGMGGTAADQIGLDVHAISDLQGRGVPT

TDDSAKYKYQATEDGKYSFEPATGKVLALRRNKEFVEEVSSGQECGVLLDRTSFYAEQGGQIYDTGFIVKENDPDVEFSV

RNVQVRAGYVLHIGVMEGVLRIGDVVQLNIDEERRCSVMKNHTGTHMLNFGLREILKEADQRGSLVAPERLRFDFSAKGA

MKVSEVKQVEEIVNKMAEKNGEVFDMNVSLAVAKAIQGLRAVFDEVYPDPVRVVSVGVDVKQLVEDPMGPGASMTSVEFC

GGTHLKRAGHIGRMVIVSEEAIAKGIRRILALTGTEAVKAQHKADLLEKLLSQLQKEVDEGLKSKSVSEKELTRKIVSFD

DEVSQSLVSYWKKDELRSKVKKLKQKMDDLDKNRKAAMMTEVVEEAKKMIEASPNQKFVVHEFKADSNAKALDAAMKQYK

ALSPETAALFFTVDQTAGKIVCMSCTPKSSVAAGLTANGWINEIVPLIKGKGGGKEASAQATGTNTGALKEAMEIATKFA

GLKLS

>XP_020285639.1 alanine--tRNA synthetase, cytoplasmic isoform X1 [Pseudomyrmex gracilis]

MSTNMKAKEIRQAYIDFFKSKGHEYVHSSSTIPHDDPTLLFTNAGMNQFKPIFLGTVDPNSDMAKWVRVVNSQKCIRAGG

KHNDLDDVGKDVYHHTFFEMMGNWSFGDYFKKEICTWAWEFLTDVLKLSSDRLYVTYFGGDEKNGLAPDIECKELWLSLG

VPASHVLPGNMKDNFWEMGETGPCGPCSELHYDRIGGREAAHLVNMDDPDVLEIWNLVFIQYNRELDGSLKSLPKKHIDC

GLGLERLVSVIQNKRANYDTDLFIPLFAAIEKGTGAPPYQGKVGADDINGIDMAYRVLADHARTITIALADGGMPDNTGR

GYVLRRILRRAVRYATEKLNAKPGFFGSLVNVVVDLLGEVFPEVTKDPQSIIDIVNEEEAQFLKTLSRGRNLLNRTIAKL

ESTDIVPGDVAWRLYDTYGFPIDLTQLMTEERGLKIDMIGYEESKKQAQLISQNKAGGVDDQINLDVHAITELQQQGIGP

TDDSPKYNYKVINSAKYKEYEFGTCIGTVIALRRAKTFVDEVSSGEEVGILLDKTNFYAEQGGQIYDEGFLVKVDDEATE

IRIKNVQVRGGYVLHIGTVGEGTLRKGDKVHTNVDTTRRRLVMANHSATHALNHALRKVLGTEADQKGSLVAPDRLRFDF

TNKGPMTTEQVENTENITNDMIKENKKIYAKESNLALAKTIQGLRAMFEETYPDPVRVVSMGVPVENLEKDPLSTAALET

SVEFCGGTHLHYTGHIGDFVIASEDAIAKGIRRIVALTGPEAAKALKKATLLQNQLTQLQSTIEADKSGTNSKEYVKKIV

ELTEDISHAVISGWRKDKMRAMLKELKKSLDDKERAAKAAIANTVVETAQAIIQSKVGCQVLVEVLEAHSNTKALDSALK

KIRAISPETSALLVSVDRDAKKIFALSSVPKSAVSKGLKANEWIQAITSVMQGKGGGKAESAQASGSNIAGLDEVVNKAT

EFANQKLGIIDNIIAKNTCKTLENDVRSLDMETPSKQNAKLVLHANKWSTKCYLAEITAQYSGKCLTVKQIENDKNNSSD

IILETTDVTLSESNAIAYFLSNSQLRREDDLIASSQVFQWMIYARNHILPAASTWILSSSDFPVSKGMRPNVKATEDSKA

DIL

>XP_011504370.1 PREDICTED: alanine--tRNA synthetase, cytoplasmic [Ceratosolen solmsi marchali]

MSINLTAKEIRQAYIDFFKSKNHEYVHSSSTIPHNDPTLLFTNAGMNQFKPIFLGTFDPNSDMAKWTRIVNSQKCIRAGG

KHNDLDDVGKDVYHHTFFEMMGNWSFGDYFKKEICSWAWEFLTVVLKLPADQLYVTYFGGDEKTRLEPDLECKNFWLSLG

VPQTHVLPGNMKDNFWEMGETGPCGPCSELHFDRIGGRDASHLVNMDDPDVLEIWNLVFIQYNREKNGNLHLLPKKHIDC

GLGLERLVSVIQNKRSNYDTDLFLPLFNAIQKGTGAPPYRGRVGNDDTDGIDMAYRVLADHARTVTIALADGGVPDNTGR

GYVIRRILRRAVRYATEKLNAQPGFFATLVNDVVELLGDVFPEVKRDPQSTIDIINEEETQFLKTLTRGRNLLNRTIAKL

ESTHIVPGDVAWRLYDTYGFPVDLTQLMAEEKGLTIDVTGYEEAKKLAIIASQNKAGSISDQINLDVHAITELQNNGIKA

TDDSPKYNYEVQSSEKYAEYSFAPCTGTVVALRKNKDFVDQVVSGDEVAIILDQTNFYAEQGGQIYDEGFLVKVDDESTE

IRIKNVQVKGGYVLHIGTVGEGTLKKGDKLYLNIDYSRRRLIMSNHTATHVLNNALREVLGTDSDQKGSLVAPDRLRFDF

SNKGAMTAGQVKKVEELMQEVITADKQIYAKTSPLVIAKGIHGLRAMFGETYPDLVRVVSIGIPVEDLESKPLSDAGMKT

SVEFCGGTHLHRTSHIGDFVIASEEAIAKGIRRIVALTGPEASKALKKSDLLQNHLNLLEDSVKSVKDSADSKNVVKQIV

EHTDDVSHAVISYWKKEKMRDQLKALKKTLDDKERVAKAAVATSVVETVKNIIEANKGTPVLVEILNAYNNTKALDMALK

KVKSISPETSTLFISVDEDEKKIFALSAVPESAVSKGLKANEWIQAIAPLMEGKGGGKPESAQASGSNILCLKEAFATAK

AYAYSALKLDGTS

>KMQ96520.1 alanyl-trna cytoplasmic [Lasius niger]

MEPDAQVPLPLEPIKSNQVEDRRLWNTNLKRKTNSLFVNRYQLLKLVQKYGTIEKFDLLFHRSGPQAGQPRGYAFVTYKT

IQDAKTAKDALHNLKVGAKNIIVRWAHSVTESDMEKPKPKIDIPALAGAKKEDKKISLETAIQAIEAKLKLMKESEEEFE

LNKPLEGSPIIHLYQKAENQKPSTSTQESRGKIWDIGTLINMNAKQIRQAYIDFFKSKGHEYVHSSSTIPHDDPTLLFTN

AGMNQFKPIFLGTVDPNSDMAKWVRVVNSQKCIRAGGKHNDLDDVGKDVYHHTFFEMMGNWSFGDYFKKEICAWAWEFLT

DVLKLPADRLYVTYFGGDEKNGLAPDDECKNLWLSLGIPASHVLPGNMKDNFWEMGETGPCGPCSELHYDRIGGREAAHL

VNMDDPDVLEIWNLVFIQYNRESDGSLKSLPKKHIDCGLGLERLVSVIQNKRANYDTDLFIPLFAAIEKATGAPPYQGKI

GADDVNGIDMAYRVLADHARTITIALADGGMPDNTGRGYVLRRILRRAVRYATEKLNAKPRFFGSLVNVVVDLLGEVFPE

VTKDPQSIIDIVNEEETQFLKTLSRGRNLLNRTIAKLESSDTVPGDVAWRLYDTYGFPIDLTQLMTEEKGLKIDMAGYEE

SKKQAQLISQNKAGGVDDQINLDIHAITELQERGVKPTDDSPKYNYKVISSVKYEEYEFAPCTGTVIALRRAKTFVDQVT

SGQEVGILLDKTNFYAEQGGQIYDEGFLVKVDDEATEIRVKNVQVRAGYVLHIGTVGEGTLRKGDKVHTNVDTARRRLVM

ANHSATHALNYALRKVLGTAADQKGSLVAPDRLRFDFTNKGPMTTEQVKNTENITADMIKKNKKIYAKESNLALAKTIQG

LRAMFEETYPDPVRVISMGIPIDNLEKNPLSTAALETSVEFCGGTHLHYTGHIGDFIIASEEAIAKGIRRIVALTGPEAA

KAFRTATLLQNQLSHLRETIETDKSGTHSKEYVKKIVELTEEITHAVIPGWRKDNMRATLKELKKSLDDKERAAKAAIAN

TVVETAQSIIQSRVGCQVLVEVLEAYSNTKALDSALKKIRVLSPETSALLISVDCDVKKIFALSSVPKSAVSKGLKANEW

IQAIAPVMQGKGGGKSESAQASGTNIACLDEIVSRANEFAKQKLGIIATA

>XP_017755770.1 PREDICTED: alanine--tRNA synthetase, cytoplasmic [Eufriesea mexicana]

MTVIMTAKQIRQAYIEFFKSKNHVYVHSSSTIPHDDPTLLFTNAGMNQFKPIFLGTVDPNSDMAKLVRAVNSQKCIRAGG

KHNDLDDVGKDVYHHTFFEMMGNWSFGDYFKEEICTWAWEFLTIKLKLPTDQLYVTYFGGDEKNNLKPDEECKLIWISLG

LPSSHILPGSMKDNFWEMGETGPCGPCSEIHYDRIGNREAGHLVNQDDPNVLEIWNLVFIQFNREIDGNLKLLPKKHIDC

GLGLERLVSIIQNKHANYDTDLFIPLFDAIQKGTGAPPYQGRVGIEDKDGIDMAYRVLADHARTITIALADGGIPDNTGR

GYVLRRILRRAVRYAIEKLNAKPGFFGSLVNVVIELFCDVFPEVKKDPQHIIDIINEEETQFLKTLTRGRNLLNRTIIKL

EANILPGDVAWRLYDTYGFPVDLTQLMAEEKGLKVDMIGYEEAKKQAQLISQSKSDGVDDQINLDVHAITELQNEGIKPT

NDLQKYNYKITNNKIYEEYEFVPCSGTVIALRHAKTFVDEVSSGEEVGILLDQTNFYAEQGGQIYDEGFLVKIDDEDTEL

CIKNVQVRGGYVLHIGTVGQGKLKKGDKVSLNIDTTRRRLLMSNHTATHALNYALRKVLGTEVDQKGSLVAPDRLRFDFT

NKGAMTMEQIRKVEEITCDMIKKNEKVYAKESNLALAKTIQGLRAMFEETYPDPVRIVSIGIPVEDLEKDPLGPGALQTS

VEFCGGTHLHYTGHIENFVIASEEAIAKGIRRIVALTGPEATKALKKAAVLQNNLDQLQTTILSNKTGVNTKEQVKKIVE

LTDDISHAIISSWKKDKMRKVLKDLKKALDDKERVTKTAISNAVVDTIQQIIQQNVGCPILVEVLQAYSNTKALDSALKK

IKTISPETSALLISVDPEANKIFALSSVSKSAMSKGLKANEWIQEIAPLMEGKGGGKPESAQASGTNTSCVTKLLYNAKN

FANSKLGVTEDNITENHNELRNDKKFVCSKILKNKLILSGNIGSIMCYRTQIVAKYSNTELIISQYKDDDVKIKLEGNDF

KLFDSNAIMLYLSNDELKCSNNLLAFSEVLQWLSYADHHIFPAVIGWVAPCLSKNVPDNMKTNIKASKEDVLSSLRKLND

ILLTKTYFVGERISLADIAVFTSLMPLYEHVLDPIYRKQYTNLNRWFFTILNQSQVVSVIKDFKVCEEALIK

>XP_014789488.1 PREDICTED: alanine--tRNA synthetase, cytoplasmic-like [Octopus bimaculoides]

MKTYLTSSQVRNSFLDFFKEKEHLYVHSSSVIPHDDPTLLFANAGMNQFKPIFLGTVDPSSDMSKWVRTANTQKCIRAGG

KHNDLEDVGKDVYHHTFFEMLGNWSFGDYYKKEICSWAWEYLVHRLGLEKDRLYVTYFGGNPATNLNPDDECKQIWLDLG

LPENHILPFDAKDNFWEMGETGPCGPCSEIHFDRIGGRDASHLVNMDDPNVLEIWNLVFIQFNREADSSLSNLPKKHIDC

GMGFERLVSVLQEKMSNYDTDVFTPIFEAIQKGTDCPVYSGKIGAEDVDGIDMAYRVLADHARTLTVALSDGGRPDNSGR

GYVLRRILRRAVRYATEKLQAKPGFFASLVDTVVAILKSTFPEVAKDPQMVKDIINEEESQFLKTLSRGQRLLQRSIAKL

DGKKSLPGDIAWRLYDTYGFPIDLTQLIVDEHGLVVDMDAYEEARKASLLASQGGGGTATQMVSLDVHALNELQTKGVPL

TDDLPKYKYTADENGNYVFETNTGSIVALRISKDFVQKVPAGVECGVLLDSTNFYAEQGGQIYDEGFMTKQNEEEGFLVK

DVQVYGGYVLHVGVSEGVLEVGDILTQTIFQSRREAIMKNHTGTHMLNFGLRQVLNEADQKGSLVAPDRLRFDFTAKGAM

TVEQVKKTEECVNKLATEKQLVYAKEAALPVAKSIKGLRAMFDEVYPDPVRIVSIGIPVEDLVKDPLGSGGNTNSVEFCG

GTHLKSSDHIGGMVIVSEEAIAKGIRRIIAVTGNEAEKALHRASVFEKDVEELFNKVNSPDCNDRQVSKDIIHVDESITQ

SGISHWKRDQLRAKLKEIKKVLDNRDKARKAAILNEVVEETKQMIAKNPDQHVIIHQFKPGSNAKALDSAVKQYKSLSPQ

TAVLFFAVDAGKLLCMSYVPKELTKTLKANEWIGSVTECINGKGGGKDVSAQATGTNVHAVEEAMKLAQQFADLKLS

>XP_014661746.1 PREDICTED: alanine--tRNA synthetase, cytoplasmic-like [Priapulus caudatus]

MDKTLTGSKIRQIFIDFFREKYQHTYVHSSAVVPLDDPTLLFTNAGMNQFKPIFLGIVDPKSKLARLKRAVNSQKCIRAG

GKHNDLDDVGKDVYHHTFFEMLGNWSFGDYFKKEVCAMAWELLVDVYKLDKERMYVTYFGGDTKAQLEPDLECKDIWLKL

GVPADHVLPGNMKDNFWEMGDTGPCGPCSEIHYDRIGGRNAAHLVNMDVPEVLEIWNLVFIQFNRDSSGCLSVLPSKHID

CGMGLERLVSVIQDKQSNYDTDLFRALFDAIEAGSGARPYTGKVGADDKDGCDMAYRVIADHMRTLTISLSDGGTPDNTG

RGYVLRRILRRAVRYANEKLKAKPGFLASLIDVVVELLGDAFPELSRDPEAIKDVINDEETQFLKTLARGEKMFYRTVAR

LGNDASVLPGDVAWRLYDTYGFPVDLTELMAEEHGFRVDMTEYEDAKKQAQLVSQGPGGGVDDGINLDVHAISDLQQQGV

SPTNDSLKYSYTYEGENPKEATYKFESCVGKVIALRYNKQFVGSVSNQECGILLDRTSFYAEQGGQIYDEGFLVKLGDEE

TEFCVKNVQVRGGYILHIGSLEGTVKVGDEMKVQIDEGRRKLVMNNHTGTHILNFALRQVLGEADQKGSLVAPDRLRFDF

SAKGAMKAAQVKQAEEIAKQLISKNEEVYAKDSPLALAKAIQGLRAVFDETYPDPVRVVSIGIPIEDMQADPTSPAGSVT

SVEFCGGTHLRRSGHVGDYVIASEEAIAKGIRRIVCLTGPEATRALHRATLLNQQFAQLQEQVTARCETKEQQKAASRKI

VDLGDDISQSVIQAWKKDEMRVSLKGLKKKLDDLDKAAKAAMLQRVVEEAKQMIAAKPEQMVVVHRFNAGAVGKALDGAI

QQYKALSPKTAAIFFSTDEDTDKVLCMCCVPKELVTKGLKANEWVGHVSGVLNGKGGGKEAAAQATGSNIESVDIAIKMA

EEFALLKLEN

>XP_020285641.1 alanine--tRNA synthetase, cytoplasmic isoform X3 [Pseudomyrmex gracilis]

MSTNMKAKEIRQAYIDFFKSKGHEYVHSSSTIPHDDPTLLFTNAGMNQFKPIFLGTVDPNSDMAKWVRVVNSQKCIRAGG

KHNDLDDVGKDVYHHTFFEMMGNWSFGDYFKKEICTWAWEFLTDVLKLSSDRLYVTYFGGDEKNGLAPDIECKELWLSLG

VPASHVLPGNMKDNFWEMGETGPCGPCSELHYDRIGGREAAHLVNMDDPDVLEIWNLVFIQYNRELDGSLKSLPKKHIDC

GLGLERLVSVIQNKRANYDTDLFIPLFAAIEKGTGAPPYQGKVGADDINGIDMAYRVLADHARTITIALADGGMPDNTGR

GYVLRRILRRAVRYATEKLNAKPGFFGSLVNVVVDLLGEVFPEVTKDPQSIIDIVNEEEAQFLKTLSRGRNLLNRTIAKL

ESTDIVPGDVAWRLYDTYGFPIDLTQLMTEERGLKIDMIGYEESKKQAQLISQNKAGGVDDQINLDVHAITELQQQGIGP

TDDSPKYNYKVINSAKYKEYEFGTCIGTVIALRRAKTFVDEVSSGEEVGILLDKTNFYAEQGGQIYDEGFLVKVDDEATE

IRIKNVQVRGGYVLHIGTVGEGTLRKGDKVHTNVDTTRRRLVMANHSATHALNHALRKVLGTEADQKGSLVAPDRLRFDF

TNKGPMTTEQVENTENITNDMIKENKKIYAKESNLALAKTIQGLRAMFEETYPDPVRVVSMGVPVENLEKDPLSTAALET

SVEFCGGTHLHYTGHIGDFVIASEDAIAKGIRRIVALTGPEAAKALKKATLLQNQLTQLQSTIEADKSGTNSKEYVKKIV

ELTEDISHAVISGWRKDKMRAMLKELKKSLDDKERAAKAAIANTVVETAQAIIQSKVGCQVLVEVLEAHSNTKALDSALK

KIRAISPETSALLVSVDRDAKKIFALSSVPKSAVSKGLKANEWIQAITSVMQGKGGGKAESAQASGSNIAGLDEVVNKAT

EFANQKLGIIAAA

>OWR44916.1 alanine-tRNA synthetase cytoplasmic [Danaus plexippus plexippus]

MNNNMSGSEIRKTFIDFFINKGHKYVHSSSTIPLDDPTLLFANAGMNQFKPIFLGSVDPNSDMAQYVRVVNTQKCIRAGG

KHNDLDDVGKDVYHHTFFEMLGNWSFGDYFKKEICSWAWELLTEVYKLPADRLYITYFGGDKASGLEPDLECKNIWLSLG

VPESHILPGSMKDNFWEMGETGPCGPCSELHYDRIGGRDAAHLVNMDDPDVLEIWNLVFIQFNRESDGSLKLLPKKHIDC

GLGLERLVSVIQNKKANYDTDFFMPIFKAIENGTKVRPYTGHVGAEDTDGIDMAYRVLADHARTLTIALADGGYPDNTGR

GYVLRRILRRAVRFASEKLNAKPGFFSSLVNTVVELLGDVFPEIKKDPESIIQIINEEEIQFLKTLTRGRNLLNRTIEKL

GDSKIIPGDVAWRMYDTYGFPIDLTQLMCEEKGLNIDMDAYEKAKKESQLSSQGKTAGQEDLLALDIHAISHLQETGVNT

TDDSPKYNYTPSSTDKDAEYKFAPCEGVITALRRNKQFVDEVSSGQECGIILDRTNFYAEQGGQIFDEGYMVKLDDESVE

FTVKSVQVKGGYVLHIGKVEGTLKVGDRVSLHIDTERRRLVMNNHTGTHILNNVLRKVLGNDSDQRGSLVMPDRLRFDFT

NKGPMTVKQIKDAEDQIKEIINKNKVVYAKHTSLSEAKKIKGLRAMFDEQYPDPVRVVSVGIPVEDLENNPDGLSGFETS

VEFCGGSHLHQTGHIGDYVIVSEEGIAKGIRRIVALTGPEAIKALNKMSSLENEVNAIANLIKEQGENVNQKEVVKNIVD

LTNDVSHAQIAYWKKEELRTLLKNLKKQLDDKERAAKAMTANIVIEKAKELCLKDENAIVIVEELKAYNNTKALDGALKQ

VKQMLPNTAAMFFSVDDDSNKIFCLAAVPKILNEKGLLASEWVQSVVPVMGGKGGGKAESAQASGNSPNKLQDAIKIGRD

FANSKLS

>XP_008545550.1 PREDICTED: alanine--tRNA synthetase, cytoplasmic [Microplitis demolitor]

MGESMTAKQIRQAYIDFFKEKGHQYVHSSSTIPHDDPTLLFANAGMNQFKPIFLGTVDPNSDMAKWVRVVNSQKCIRAGG

KHNDLDDVGKDVYHHTFFEMMGNWSFGDYFKKEICTWAWEFLTQVMKLPADRLYVTYFGGDEKANLAADEECKQLWLSVG

VPPSHILPGSMKDNFWEMGETGPCGPCSELHFDRIGGREAAHLVNMDDPDVLEIWNLVFIQFNRESDGSLRNLPKKHIDC

GLGLERLVSVIQNKRANYDTDLFVPLFDAIQSGTGARPYTGKVGDEDKDGIDMAYRVLADHARTITVALADGGMPDNTGR

GYVLRRILRRAVRYATEKLNAKPGFFGSLVNVVVNLLGDTFPELKKDSQSIIDVINEEETQFLKTLSRGRNLLDRTIAKL

ENAKVVPGDVAWRLYDTYGFPVDLTQLMTEEKGMCVDMAGYEEAKKAAQLASQSKAGGVDDQINLDVHAITELQKMSIPP

TDDSYKYNYSSTDEIDAEYKFEPCVGTVIAMRRAKKFVDEALPGEEVGILLDKTCFYAEQGGQIYDEGFLVKESGNDDDS

AEVRVTNVQVRGGYVLHIGTVGIGVLKKGDKLNLKIDTARRRLIMSNHSATHALNFALRKVLGTEADQKGSLVAPDRLRF

DFTNKGAMSTNDIKQVEEFTMELVEDNRKIFARESKLGLAKTIQGLRAMFEETYPDPVRIVSMGVPIDELEKNPLGPGAM

VTSVEFCGGTHLHYTGHIKNFVIASEEAIAKGIRRIVGLTGPEAAKALNKAEILQNRLNTIEKNMADDKEFKYTKEHTKN

ILDLLNDVSQATIAAWKKDTLRNTLNGMKKTLDDRERQAKAAVATSVLEKATLIIEDKIGTPVMVEEFQAYNNTKALDAA

LKKVRTLSPGTSALFFTIDEDTKKIFALASVPKDAVQKGLTANEWIQAIVPLMGGKGGGKAEAAQASGPNYSCLKEVIKL

AKEFASSKLGN

>XP_015436360.1 PREDICTED: alanine--tRNA synthetase, cytoplasmic [Dufourea novaeangliae]

MDSYKNAKNVRQAYIDFFKSKGHEYVHSSSTIPHDDPTLLFTNAGMNQFKPIFLGTVDPNSDMAKYVRVVNSQKCIRAGG

KHNDLDDVGKDVYHHTFFEMMGNWSFGDYFKKEICTWAWEFLTVKLKLPSDRLYVTYFGGDEKNNLKPDEECKEIWLSLG

VPPTHVLPGSMKDNFWEMGETGPCGPCSELHYDRIGNREVAHLVNQDDPDVLEIWNLVFIQFNRQSDGSLKSLPKKHIDC

GLGLERLVSVIQNKRANYDTDLFMPLFEAIQKGTGAPPYQGKVGTDDKDGIDMAYRVLADHARTLTISLADGGVPDNTGR

GYVLRRILRRAVRYATEKLNAKPGFFGTLVNVVVQILGDIFPEVTKDPQYIIDIINEEEAQFLKTLSRGRNLLNRTVAKL

ESSTIVPGDVAWRLYDTYGFPVDLTQLMTEEKGLKVDMIGYEEAKKQAQLISQSKSGGVDDQINLDVHAITELQNKGVKP

TIDLPKYNYTVTGDKMCEEYEFAPCIGTVIALRHAKTFVDEVLSGEEVGILLDQTNFYAEQGGQIYDEGFLVKTDDEETE

VRIKNVQVRGGYVLHIGTVGQGILKKDDKVYLNIDTTRRRLVMSNHTATHALNYALRKVLGTEVDQKGSLVAPDRLRFDF

TNKGAMTSEQVKQVEEITNTIVKEKKKVFAKESSLALAKTIQGLRAMFEETYPDPVRVVSIGVPVEDLENDPLGPYAVQT

SVEFCGGTHLHCTDHIGDFVIASEEAIAKGIRRIVALTGPEATKALKKAKVLQTQLDQLQMTVAIDKNCVNSKEHVKRIV

ELTDDISRVTIPSWRKDKMRKMLKDLKKTLDDKERVAKAAIANTVVETIQQIIQQNIGCPVLVEVCQAYSNTKALDSALK

KIKAVSPETSALLVSVDPDAKKIFALSATSKSGVNKGLKANEWIQEIAPLMGGKGGGKPESAQASGTNVSCLNKLIHTAK

NFANLKLGITDSVRNDILNNQPEVVEHLVCSKISENKLTLFGNVGSVKCYRAQIAAKYSGREIILVQYKTNGGISRDIRL

EGNGFELFDSSAITFYLANDQLRCSSDSYAFSQVLQWISYTDNHILPAMAGWVFPCISENVSSNMKTIIKTSKEDVLSSL

KKLNNILLAKTYLVGERISLADIAVFTTLIPLYQYVFDPECRKQYTNLNRWFHTILNQQEITCVLKNFKLCERAVKC

>XP_011135978.1 PREDICTED: alanine--tRNA synthetase, cytoplasmic isoform X1 [Harpegnathos saltator]

MLTTMSGKQIRQAYIDFFKNKGHEYVHSSSTIPHDDPTLLFTNAGMNQFKPIFLGTVDPNSDMAKWVRVVNSQKCIRAGG

KHNDLDDVGKDVYHHTFFEMMGNWSFGDYFKKEICSWAWEFLTETLKLPADRLYVTYFGGDEKNGLAPDNECKELWLSLG

IPASHVLPGNMKDNFWEMGETGPCGPCSELHYDRIGGREAAHLVNMDDPDVLEIWNLVFIQYNREADGNLKPLPKKHIDC

GLGLERLVSVIQNKRANYDTDLFMPLFAAIEKGTGAPPYQGKIGTDDVNRVDMAYRVLADHARTITIALADGGIPDNTGR

GYVLRRILRRAVRYATEKLNAKPGFFGSLVNVVVELLGEVFPEVTKDPQSIIDIVNEEETQFLKTLSRGRNLLNRTIAKL

ESNDTVPGDVAWRLYDTYGFPIDLTQLMTEEKGLKIDMAGYEESKKQAQLISQNKVGGVDDQINLDVHSITELQERGIKP

TDDSPKYNYKITSTTTYKEYEFAPCIGTVIELRRAKTFVDQVSSGEEVGILLDRTNFYAEQGGQIYDEGFLVKVDDEATE

IRIKNVQVRAGYVLHIGTVGEGILKKGDQVHANVDTTRRRLVMANHSATHALNHALRKVLGTEADQKGSLVAPDRLRFDF

TNKGPMTTEQVKNAENITNDMIKDNKKIYAKESNLALAKTIQGLRAMFEETYPDPVRVVSMGVPIEELEKNPLSPVATQT

SVEFCGGTHLHYTGHIGDFIIASEEAIAKGIRRIVALTGPEAAKALKKATLLQNQLTQLQTTIEADKCGANSKEYVKKIV

ELTEDISHAVIPGWRKDEMRGMLKKLKKSLDDKDRAAKAAIANMVVETAQSIIQSKVGCQVLVEVLEAYSNTKALDSALK

KIRAVSPETSALLISVDQDVKKIFALSSVPKSAVSKGLKANEWIQAIAPMMQGKGGGKSESAQASGADITCLNEIVSKAN

EFANQKLGIMNNKTANDIFKTSESDDRSLNMESSSKLVLYANIGSINYYLAQIIAQYSGKCLSVKQVDNDKMNSNRMMLE

TGDVMLHNGNAIAFFLSNSQLRCDDDSFASSQVLQWMSYSQNHVLSAVTTWILPTLGVCMSKDMKANSKLSKESLLRDLK

ILNSMLHTRTYLVGERITLADISVFTVLLPLYEHVFDLNHRKSYTNLNRWFSTILNQPQVRSVIKNFAFCMKVARY

>XP_022123105.1 alanine--tRNA synthetase, cytoplasmic isoform X2 [Pieris rapae]

MNTSMTGSEVRQAYIDFFISKGHKYVHSASTIPLDDPTLLFANAGMNQFKPIFLGSVDPNSDMANYVRVVNTQKCIRAGG

KHNDLDDVGKDVYHHTFFEMMGNWSFGDYFKKEICSWAWELLTEVLKLPGDRLYVTYFGGDKSAGLEPDLECKDIWLKLG

LPESHIIPGSMKDNFWEMGETGPCGPCSELHYDRIGGREAAHLVNMDDPDVLEIWNLVFIQFNREKNGELKLLPKKHIDC

GLGLERLVSVIQNKRANYDTDLFMPLFKAIENGTKVRPYSGKVGEEDTDGIDMAYRVLADHARTLTIALSDGGYPDNTGR

GYVLRRILRRAVRYASEKLNAKPGFFATLVRTVVEILGDVFPEIKKNPEAVMQIINEEELQFLKTLTRGRNLLNRTIEKL

NDSRVVPGDVAWRMYDTYGFPIDLTCLMCEEKGLTIDMDAYEKAKKESQLSSQGKTAGQDDLLALDIHAISYLKDTGISP

TNDSLKYNYVPTTTEAKAIYEFKPCSGHVVALRKNKEFVDEVMSGDECGILLDQTNFYAEQGGQIYDEGYMVKVGDDSVE

FSVKNVQVKGGYVLHIGKVEGILRVGDTLSLHIDTERRRLIMNNHTGTHVLNNVLRKVLGNDADQRGSLVMPDRLRFDFT

NKGPMTTKQIKEAEDQVKAIINNNKKVYAKYTKLSDAKKINGLRAMFDEQYPDPVRVVSVGIPVEDLEENPEGPNGFETS

IEFCGGSHLHQTGHIGDYVIVSEEGIAKGIRRIVALTGPEALKAINKMSILENEVNDITNQIQEQGSVLNQKEAVKKIVE

LTNEISQAQISYWKKEEMRTMLKNLKKQLDDKERASKTATINLVAEKAKEMCLNEKSSVIVAELKAYGNTKALDGALKQV

KQILPNTAAMFFSVDDDTNKIYCLAVVPKNLVEQGLLASEWVQSVVGIMNGKGGGKAESAQASGSNVSCLNDAIKAARDF

ATTKVSDS

>XP_011269528.1 PREDICTED: alanine--tRNA synthetase, cytoplasmic [Camponotus floridanus]

MNAKQIRQAYIDFFKSKGHEYVHSSSTIPHDDPTLLFTNAGMNQFKPIFLGTVDPNSDMAKWVRVVNSQKCIRAGGKHND

LDDVGKDVYHHTFFEMMGNWSFGDYFKKEICAWAWEFLTDVLKLPADRLYVTYFGGDEKNGLAPDDECKDLWLSLGIPAS

HVLPGNMKDNFWEMGETGPCGPCSELHYDRIGGREAAHLVNMDDPDVLEIWNLVFIQYNRESDGSLKSLPRKHIDCGLGL

ERLVSVIQNKRANYDTDLFMPLFAAIEKATGAPSYQGKVGAEDVDGIDMAYRVLADHARTITIALADGGMPDNTGRGYVL

RRILRRAVRYATEKLNAKPGFFGSLVNVVVDLLGEVFPEVTKDPQNIIDIVNEEETQFLKTLSRGRNLLNRTIAKLESSD

TVPGDVAWRLYDTYGFPVDLTQLMTEEKGLKIDIAGYEESKKQAQLISQNKAGGIDDQINLDIHAITEMQEHGIKPTDDS

PKYDYKVISSVKYEEYEFAPCIGIVIALRRAKTFVDQVTSGEEVGILLDKTNFYAEQGGQIYDEGFLVKVDDEATEIRVK

NVQVRAGYVLHIGTVGEGTLRKGDKVYTNVDTARRRLVMANHSATHALNYALRKVLGTGADQKGSLVAPDRLRFDFTNKG

PMTTEQVKNTENITADMVKENKKIYAKESNLALAKTIQGLRAMFEETYPDPVRVISMGIPVDDLEKNPLSTAALETSVEF

CGGTHLHYTGHIGDFIIASEEAIAKGIRRIVALTGPEAAKAFRTATLLQNQLSHLRETIETDNSGTNSKEYVKKIVELTE

EIMHAVIPSWRKDNMRATLKELKKSLDDKERAAKAAVANTVVETIQSIIQSRVGCQVLVEVLEAYSNTKALDSALKKIRV

LSPETSALLISIDRDVKKIFALSSVPKSAVSKGLKANEWIQVIAPAMQGKGGGKPESAQASGTNIACLNEIISRANEFAN

QKLGIIDNNTANNNKIIENDNRCDVDSSKLILYANIGSAKCYLAQIIARYSNKCLTIKQIENEKLYSSNIILKAADFTLY

DSNAIAFFLSNSQLRREDDLFASSKVLQWTNYAQNHILPAVSGWVLPSLDISVSKEMKTNAKVSKEDVLFALRTLDNTLH

IRTYLIGERITLADISVFVTLLPLYEYVLDPHHRKQYINLNRWFSTILNQPQVKCVVENFTFCTKCNFNY

>XP_022123104.1 alanine--tRNA synthetase, cytoplasmic isoform X1 [Pieris rapae]

MQLYCHNLQLCQVSATFKLLSSLIQYNSQKIEVSLFTMNTSMTGSEVRQAYIDFFISKGHKYVHSASTIPLDDPTLLFAN

AGMNQFKPIFLGSVDPNSDMANYVRVVNTQKCIRAGGKHNDLDDVGKDVYHHTFFEMMGNWSFGDYFKKEICSWAWELLT

EVLKLPGDRLYVTYFGGDKSAGLEPDLECKDIWLKLGLPESHIIPGSMKDNFWEMGETGPCGPCSELHYDRIGGREAAHL

VNMDDPDVLEIWNLVFIQFNREKNGELKLLPKKHIDCGLGLERLVSVIQNKRANYDTDLFMPLFKAIENGTKVRPYSGKV

GEEDTDGIDMAYRVLADHARTLTIALSDGGYPDNTGRGYVLRRILRRAVRYASEKLNAKPGFFATLVRTVVEILGDVFPE

IKKNPEAVMQIINEEELQFLKTLTRGRNLLNRTIEKLNDSRVVPGDVAWRMYDTYGFPIDLTCLMCEEKGLTIDMDAYEK

AKKESQLSSQGKTAGQDDLLALDIHAISYLKDTGISPTNDSLKYNYVPTTTEAKAIYEFKPCSGHVVALRKNKEFVDEVM

SGDECGILLDQTNFYAEQGGQIYDEGYMVKVGDDSVEFSVKNVQVKGGYVLHIGKVEGILRVGDTLSLHIDTERRRLIMN

NHTGTHVLNNVLRKVLGNDADQRGSLVMPDRLRFDFTNKGPMTTKQIKEAEDQVKAIINNNKKVYAKYTKLSDAKKINGL

RAMFDEQYPDPVRVVSVGIPVEDLEENPEGPNGFETSIEFCGGSHLHQTGHIGDYVIVSEEGIAKGIRRIVALTGPEALK

AINKMSILENEVNDITNQIQEQGSVLNQKEAVKKIVELTNEISQAQISYWKKEEMRTMLKNLKKQLDDKERASKTATINL

VAEKAKEMCLNEKSSVIVAELKAYGNTKALDGALKQVKQILPNTAAMFFSVDDDTNKIYCLAVVPKNLVEQGLLASEWVQ

SVVGIMNGKGGGKAESAQASGSNVSCLNDAIKAARDFATTKVSDS

>XP_018978024.1 PREDICTED: alanine--tRNA synthetase, cytoplasmic [Cyprinus carpio]

MAPPAEELHFSEEAGFLMPDQVESETVLKPLPKKSIDTGMGLERLVSVLQNKMSNYDTDLFIPYFEAIQKGTGARAYTGK

VGAEDADGIDMAYRVLADHARTITIALSDGGRPDNTGRGYVLRRILRRAVRYSHEKLGAQKGFFASLVDVVVESLGDAFP

ELRKDPDMVKDIINEEEEQFLKTLSRGRRILDRKIQSLGDSKTIPGDTAWLLYDTYGFPLDLTALIAEERGMGVDMQAFE

DEKKAAQLKSQGKGSGDEDHIMLDIYAIEELRNKGVAATDDSPKYKYTSNDNGNYEFEQAVGTVLALRRERAFVDEVTTG

QECGVLLDKTSFYAEQGGQSFDEGYMLRENDSAEDRMEFTVKNTQVRGGYVLHIGTVYGTLKVGDRLTLHVDEARRRPIM

SNHTATHILNFALRSVLGEADQRGSLVAPDRLRFDFTAKGAMSTDEVRRTEENASTMIRDAKPVYALDAPLAAAKAIQGL

RAVFDETYPDPVRVVSIGVPVEELLADPNSPAGSLTSIEFCGGTHLQNSGHAAPFVIVSEEAIAKGIRRIVAVTGAEAQK

AQRKADALKLELDAMAEKVKSQNTPNKDIQKEIADMTEALGTAVISQWRKDEMRDSLKGLKKIMDDLDRASKADVQKRVL

EKTKEIIESNPNKPLIVMEMENGASAKALNESLKLLKTNSPQTAAMLFAVDNDAGKIICLCQVPQDVANRGLKASEWVQE

VCPLLDGKGGGKDMSAQATGRNTHCIQEALQLANEFARLKLGEN

>XP_017797309.1 PREDICTED: alanine--tRNA synthetase, cytoplasmic [Habropoda laboriosa]

MMNAKQIRQAYIDFFKSKNHEYVHSSSTIPHDDPTLLFTNAGMNQFKPIFLGTVDPNSDMAKLVRVVNSQKCIRAGGKHN

DLDDVGKDVYHHTFFEMMGNWSFGDYFKKEICTWAWEFLTIKLKLPKDQLYVTYFGGDEKNNLKPDEECKQIWLDLGLST

SHVLPGSMKDNFWEMGETGPCGPCSEIHYDRIGNRNAAHLVNQDDPDVLEIWNLVFIQFNRESDGSLKPLPKKHIDCGLG

LERLVSVIQNKRANYDTDLFVPLFDAIQKGTGAPPYQGKIGTDDKDGIDMAYRVLADHARTITIALADGGVPDNTGRGYV

LRRILRRAVRYATEKLNAKPGFFGSLVNVVVDLLGDIFPEVRKDPQYIIDIVNEEEVQFLKTLLRGRNLLNRAIVKLESS

NILPGDVAWRLYDTYGFPVDLTQLMAEEKGLKVDMIGYEVAKKQAQIISQSKSGGVDDQINLDVHAITELKNKGIKPTND

LPKYNYTVTNNKIYEEYEFAPCTGTVIALRQAKTFVDNISSGEEVGILLDQTNFYAEQGGQIYDEGFLVKIDDEDTEVRI

KNVQVRGGYVLHIGTVGQGILKKGDKVSLNIDTTRRRFLMSNHTATHALNYALRKVLGTEVDQKGSLVAPDRLRFDFTNK

GAMTAEQIQKTEEITRNMIKENKKVYAKESNLALAKTIQGLRAMFEETYPDPVRIVSIGIPIEDLEKNPLGPGALQTSVE

FCGGTHLHYTEHIEDFAIASEEAIAKGIRRIVALTGPEATKAQKKAAILQNYLDQLQTTIAADKSGANTKEHVKKIVDVT

DDVSRATISSWKKDKMRKILKDLKKALDDKERAAKTAIANAVVDTIQQIIQQNIGCPVMVEVLKAYNNTKALDSALKKIK

AVSPDTSALLISVDPDANKIFALSTVSKSAINKGLKANEWIQKIAPLMGGKGGGKPESAQASGTNISCVPKLVHDAKNFA

NSKLGVVENIVIENHTQFKNNEESVNFKSSKNKLVLSGNAGSIKYYRAQIIAKYSSKDLITSQHKNSDIITKSIKLEGDG

FELSDSSAIMLYLSNDQLRFSSDPFTFSEILQWLSYADNHILPAVFGWVVPCLSENIPNNMKTNIKTSKEDVLSSLKKLN

NILLTKTYLVRERISLADIAVFTALLPLYEHVLDPACRKQYTNLNRWFFTVLNQPQVVSVIKKFEVCEKARKH

>KOC69822.1 Alanine--tRNA synthetase, cytoplasmic [Habropoda laboriosa]

MNAKQIRQAYIDFFKSKNHEYVHSSSTIPHDDPTLLFTNAGMNQFKPIFLGTVDPNSDMAKLVRVVNSQKCIRAGGKHND

LDDVGKDVYHHTFFEMMGNWSFGDYFKKEICTWAWEFLTIKLKLPKDQLYVTYFGGDEKNNLKPDEECKQIWLDLGLSTS

HVLPGSMKDNFWEMGETGPCGPCSEIHYDRIGNRNAAHLVNQDDPDVLEIWNLVFIQFNRESDGSLKPLPKKHIDCGLGL

ERLVSVIQNKRANYDTDLFVPLFDAIQKGTGAPPYQGKIGTDDKDGIDMAYRVLADHARTITIALADGGVPDNTGRGYVL

RRILRRAVRYATEKLNAKPGFFGSLVNVVVDLLGDIFPEVRKDPQYIIDIVNEEEVQFLKTLLRGRNLLNRAIVKLESSN

ILPGDVAWRLYDTYGFPVDLTQLMAEEKGLKVDMIGYEVAKKQAQIISQSKSGGVDDQINLDVHAITELKNKGIKPTNDL

PKYNYTVTNNKIYEEYEFAPCTGTVIALRQAKTFVDNISSGEEVGILLDQTNFYAEQGGQIYDEGFLVKIDDEDTEVRIK

NVQVRGGYVLHIGTVGQGILKKGDKVSLNIDTTRRRFLMSNHTATHALNYALRKVLGTEVDQKGSLVAPDRLRFDFTNKG

AMTAEQIQKTEEITRNMIKENKKVYAKESNLALAKTIQGLRAMFEETYPDPVRIVSIGIPIEDLEKNPLGPGALQTSVEF

CGGTHLHYTEHIEDFAIASEEAIAKGIRRIVALTGPEATKAQKKAAILQNYLDQLQTTIAADKSGANTKEHVKKIVDVTD

DVSRATISSWKKDKMRKILKDLKKALDDKERAAKTAIANAVVDTIQQIIQQNIGCPVMVEVLKAYNNTKALDSALKKIKA

VSPDTSALLISVDPDANKIFALSTVSKSAINKGLKANEWIQKIAPLMGGKGGGKPESAQASGTNISCVPKLVHDAKNFAN

SKLGVVENIVIENHTQFKNNEESVNFKSSKNKLVLSGNAGSIKYYRAQIIAKYSSKDLITSQHKNSDIITKSIKLEGDGF

ELSDSSAIMLYLSNDQLRFSSDPFTFSEILQWLSYADNHILPAVFGWVVPCLSENIPNNMKTNIKTSKEDVLSSLKKLNN

ILLTKTYLVRERISLADIAVFTALLPLYEHVLDPACRKQYTNLNRWFFTVLNQPQVVSVIKKFEVCEKARKH

>XP_019696023.1 PREDICTED: alanine--tRNA synthetase, cytoplasmic isoform X2 [Harpegnathos saltator]

MLTTMSGKQIRQAYIDFFKNKGHEYVHSSSTIPHDDPTLLFTNAGMNQFKPIFLGTVDPNSDMAKWVRVVNSQKCIRAGG

KHNDLDDVGKDVYHHTFFEMMGNWSFGDYFKKEICSWAWEFLTETLKLPADRLYVTYFGGDEKNGLAPDNECKELWLSLG

IPASHVLPGNMKDNFWEMGETGPCGPCSELHYDRIGGREAAHLVNMDDPDVLEIWNLVFIQYNREADGNLKPLPKKHIDC

GLGLERLVSVIQNKRANYDTDLFMPLFAAIEKGTGAPPYQGKIGTDDVNRVDMAYRVLADHARTITIALADGGIPDNTGR

GYVLRRILRRAVRYATEKLNAKPGFFGSLVNVVVELLGEVFPEVTKDPQSIIDIVNEEETQFLKTLSRGRNLLNRTIAKL

ESNDTVPGDVAWRLYDTYGFPIDLTQLMTEEKGLKIDMAGYEESKKQAQLISQNKVGGVDDQINLDVHSITELQERGIKP

TDDSPKYNYKITSTTTYKEYEFAPCIGTVIELRRAKTFVDQVSSGEEVGILLDRTNFYAEQGGQIYDEGFLVKVDDEATE

IRIKNVQVRAGYVLHIGTVGEGILKKGDQVHANVDTTRRRLVMANHSATHALNHALRKVLGTEADQKGSLVAPDRLRFDF

TNKGPMTTEQVKNAENITNDMIKDNKKIYAKESNLALAKTIQGLRAMFEETYPDPVRVVSMGVPIEELEKNPLSPVATQT

SVEFCGGTHLHYTGHIGDFIIASEEAIAKGIRRIVALTGPEAAKALKKATLLQNQLTQLQTTIEADKCGANSKEYVKKIV

ELTEDISHAVIPGWRKDEMRGMLKKLKKSLDDKDRAAKAAIANMVVETAQSIIQSKVGCQVLVEVLEAYSNTKALDSALK

KIRAVSPETSALLISVDQDVKKIFALSSVPKSAVSKGLKANEWIQAIAPMMQGKGGGKSESAQASGADITCLNEIVSKAN

EFANQKLGIMNNKTANDIFKTSESDDRSLNMESSSKLVLYANIGSINYYLAQIIAQYSGKCLSVKQVDNDKMNSNRMMLE

TGDVMLHNGNAIAFFLSNSQLRCDDDSFASSQVLQWMSYSQNHVLSAVTTWILPTLGERESLLRTYQFLPCCYLYMSTCS

I

>XP_014234728.1 alanine--tRNA synthetase, cytoplasmic [Trichogramma pretiosum]

MSQKTAKEIRQAYIDFFKSKKHEYVHSSSTIPHDDPTLLFTNAGMNQFKPIFLGTVDPNSDMAKWERVVNSQKCIRAGGK

HNDLDDVGKDVYHHTFFEMMGNWSFGDYFKTEICTWAWEFLTDTLKLPPSQLYVTYFGGDEKSNLKPDEECKNMWLSLGV

PESHVLPGSMKDNFWEMGETGPCGPCSELHFDRIGGREAAHLVNMDDPDVLEIWNLVFIQFNREKNGELHPLPKKHIDCG

LGLERLVSVIQNKRSNYDTDLFLPLFDAIQKGTNATPYQGRVGADDVDGIDMAYRVLADHARTITIALADGGVPDNTGRG

YVLRRILRRAVRYATEKLNAKPGFFATLIDVVVELLGDVFPEVTKDPQSIKDIVNEEEAQFLKTLTRGRNLLNRTIAKLN

TDKVVPGDVAWRLYDTYGFPVDLTQLMAEEKGLTIDFAGYEEAKKLAILVSQNKAGGASDLINLDVHAITELQNSGIKTT

DDSPKYNYQVVSKDSYAEYSFAPCIGTVLALRKDKQFVDEVKSGDEVGILLDQTNFYAEQGGQIYDEGFLVKVDDESTEI

CVKNVQVKGGYILHIGTVGEGTLKKGDKLHLNVDFSRRRLIMSNHTATHVLNCALRKVLGPESDQKGSLVAPDRLRFDFT

NKGAMTADQVKKAEEFIVEVVRADKQVYAKESSLAVAKSINGLRAMFGETYPDPVRVVCAGVPIEELEKNPNGTAGLETS

VEFCGGTHLHRTSHIGDFAIVNEEAIAKGIRRIVALTGPEANKALKKAELLQNDLNKLKSKVDDAKDSAATKEIVKKIVE

LTDDVSHATISYWKKEAMRSELKALKKKLDDKERAAKAAVAAEVVETATKLIEAKKGAKVLVEELKAFNNTKALDQALKK

VKAISPETSALFITVDHDEGKIFALSAVPKSAIGEGLKANEWIQAIAPMMNGRGGGKPESAQASGTNVSCLKEALNAAQV

YADTVLLKLQEQNTSVPNDPSCVKKNIKGTLYADNGNVKSYIIQILARYVRRSIKVYKHTKDTEKKGSIFYEDEDVSLFD

GTAIAVYTANSAFKGKTDLEQSHVLQWLNIANNHILPSVYGCIPSTIGAKSEISKDAAKADLLILMNMLNTTLQASKFLM

KSKINLADIYVFGTLIPAYQYVFDSEIRKKYKHVDKWFETIMQEEYVKAVIGDFKYHAEK

>XP_014210304.1 LOW QUALITY PROTEIN: alanine--tRNA synthetase, cytoplasmic [Copidosoma floridanum]

MTVNKTAKEIRQAYIDFFKSKDHEYVHSSSTVPHDDPTLLFTNAGMNQFKPIFLGTVDPNSDMAKWVRVVNSQKCIRAGG

KHNDLDDVGKDVYHHTFFEMMGNWSFXDYFKKQICIWAWEFLTDIPKLPXNQLYVTYFGRNKKANLKPDDECKDLWLSLS

IPDSNVLPSYVKDNFWKMGETGPCGPCSELHFDRIGGRDAAHLVNMDDPDVLEIWNLVFIQFNRERSGELHPLPKKHIDC

GLGLERLVSVIQNKRSNYDTDLFLPLFEAIHKGTNAPVYQGRVGSDDKDGIDMAYRVLADHARTITIALADGGVPDNTGR

GYVVRRILRRAVRYATEKLNAKPGFFATLVNVVVELLGDIFPEVKRDPQSIIDVINEEETQFLKTLTRGRNLLNRTIAKL

ESTQIVPGDVAWRLYDTYGFPVDLTQLMAEEKGLTIDVVGYEEAKKQAIIASQGKAGGVNDQINLDVHAITELQNRGIKT

TDDSYKYNYKSESSEKYAEYSFSPCIGTVVALRKDKKFVDEVKSGDEVGVLLDQTNFYAEQGGQIYDEGFLVKIDDESTE

VCVKNVQVRGGYVLHIGTVGEGTLRKGDKLYLNVDNSRRRLVMSNHTATHALNHALRCVLGTEADQKGSLVAPDRLRFDF

TNKGAMTTEQVKKTEQYTNAIVIADKVVYAKESPLAVARNIQGLRAMFGETYPDPVRVVSIGVPVEDLEKDPLSEAGTKT

SVEFCGGTHLHRTGHIGDFVIASEEAIAKGIRRIVALTGPEASKALKKSELLQNHLNNLKSDVDNVKDSTASKAIVKKIV

ELTDDVSHAVIPYWKKEDMRNELKALKKALDDKERAAKAAVANNVVEFAKDLIEAQKGIAVLVEKLNAYNNTKALDMALK

KVKAISPETSALFITVDEDDNKIFALSAVPKSAITKGLKANEWIQAIAGLMQGRGGGKPESAQASGSNISCLNEALETAK

SYALEKLEGVKSSKDSICTKSSKELVFHCNEGSVKSHLVQIVCRYAGKCLKLNNHTKDSEMKGSVYFNDGKVELIDGNAI

AFYLANAQFKGNNVLEKSQVLQWMSFSDNHILPSVYGWVLPSLKNSSIKTSKDSIKNSKDTLLQIMESLNEVLSGNTYLV

SGKMTLADVSVFVSLSPAYQHVFNSEVRKNYRNVNKWFETVLQQSNVKCLMSNFKYCEKQ

>XP_011881569.1 PREDICTED: alanine--tRNA synthetase, cytoplasmic isoform X4 [Vollenhovia emeryi]

MESSMNAKQIRQAYIDFFKSKGHEYVHSSSTIPHDDPTLLFTNAGMNQFKPIFLGTADPNSDMARWVRVVNSQKCIRAGG

KHNDLDDVGKDVYHHTFFEMMGNWSFGDYFKKEICTWAWEFLTDVLKLPADRLYVTYFGGDEKCGLAPDDECKNLWLSLG

VPASHVLPGNMKDNFWEMGETGPCGPCSELHYDRIGGREAAHLVNMDDPDVLEIWNLVFIQYNRESDGSLKSLPKKHIDC

GLGLERLVSVIQNKRANYDTDLFVPLFAAIEKGTGAPPYQGKIGADDVNGIDMAYRVLADHARTITIALADGGMPDNTGR

GYVLRRILRRAVRYATEKLNAKQGFFGSLVNVVVDLLGEVFPEVKKDPQSIIEVINEEEAQFLKTLSRGRNLLNRTVAKL

ESTDTVPGDVAWRLYDTYGFPIDLTQLMTEEKGLKIDMAGYEESKKQAQLISQNKAGGVDDQINLDVHAITELQKQGVKP

TDDSPKYNYKVTNSVKYEEYEFAPCIGTVIALRRAKTFVDKVSSGEEVGILLDKTNFYAEQGGQIYDEGFLVKVDDEATE

IRVKNVQVRAGYVLHIGTVGEGTLRKGDKVHANVDTERRRLVMANHSATHALNHALRKVLGTEADQKGSLVAPDRLRFDF

TNKGPMTGGQLKDTELFTNDMIQANKKIYAKESNLAIAKTIQGLRAMFEETYPDPVRIVSMGVPIENLEKNPLSTAALET

SVEFCGGTHLHYTSHIGDFVIASEDAIAKGIRRIVALTGPEATKALRTMRILENQIHQIQVTIEADKSGTNSKEYVKKIV

ELTDEISHALVPVWWKDNVRNTLRELKKSLDDKERATKAAVISTVVKTAQTIIESKVGCQVLVEVLEAYNNTKALDLALK

KIRAISPETSALLISVDRDVKKIFALASVSKSAISKGLKANEWIQAIASLMQGKGGGKPESAQASGTNVAGLNETVSKAN

EFANQKLGIIDNNTANTTRKTSETIQSHNTGSSSKLVLYGNNGSTKCYLAQIIAQYSGKCLTVKQVGDEHKICSSDIMLE

TADVTLHNINAIAFFLSNSQLRREDDLFASSQVLQWMSYVQNHILPAVSGWVLPSLGVSVSKDMKANAKISKEDLLCTMK

RMDEILRTRTYLIGERITLADVSVFTALLPLYEHVLDPHYRKQCTNLNRWFSTILNQPQVKCVVKNFTFCAEATRC

>OAD58004.1 Alanine--tRNA synthetase, cytoplasmic [Eufriesea mexicana]

MTAKQIRQAYIEFFKSKNHVYVHSSSTIPHDDPTLLFTNAGMNQFKPIFLGTVDPNSDMAKLVRAVNSQKCIRAGGKHND

LDDVGKDVYHHTFFEMMGNWSFGDYFKEEICTWAWEFLTIKLKLPTDQLYVTYFGGDEKNNLKPDEECKLIWISLGLPSS

HILPGSMKDNFWEMGETGPCGPCSEIHYDRIGNREAGHLVNQDDPNVLEIWNLVFIQFNREIDGNLKLLPKKHIDCGLGL

ERLVSIIQNKHANYDTDLFIPLFDAIQKGTGAPPYQGRVGIEDKDGIDMAYRVLADHARTITIALADGGIPDNTGRGYVL

RRILRRAVRYAIEKLNAKPGFFGSLVNVVIELFCDVFPEVKKDPQHIIDIINEEETQFLKTLTRGRNLLNRTIIKLEANI

LPGDVAWRLYDTYGFPVDLTQLMAEEKGLKVDMIGYEEAKKQAQLISQSKSDGVDDQINLDVHAITELQNEGIKPTNDLQ

KYNYKITNNKIYEEYEFVPCSGTVIALRHAKTFVDEVSSGEEVGILLDQTNFYAEQGGQIYDEGFLVKIDDEDTELCIKN

VQVRGGYVLHIGTVGQGKLKKGDKVSLNIDTTRRRLLMSNHTATHALNYALRKVLGTEVDQKGSLVAPDRLRFDFTNKAM

TMEQIRKVEEITCDMIKKNEKVYAKESNLALAKTIQGLRAMFEETYPDPVRIVSIGIPVEDLEKDPLGPGALQTSVEFCG

GTHLHYTGHIENFVIASEEAIAKGIRRIVALTGPEATKALKKAAVLQNNLDQLQTTILSNKTGVNTKEQVKKIVELTDDI

SHAIISSWKKDKMRKVLKDLKKALDDKERVTKTAISNAVVDTIQQIIQQNVGCPILVEVLQAYSNTKALDSALKKIKTIS

PETSALLISVDPEANKIFALSSVSKSAMSKGLKANEWIQEIAPLMEGKGGGKPESAQASGTNTSCVTKLLYNAKNFANSK

LGVTGNIGSIMCYRTQIVAKYSNTELIISQYKDDDVKIKLEGNDFKLFDSNAIMLYLSNDELKCSNNLLAFSEVLQWLSY

ADHHIFPAVIGWVAPCLSKNVPDNMKTNIKASKEDVLSSLRKLNDILLTKTYFVGERISLADIAVFTSLMPLYEHVLDPI

YRKQYTNLNRWFFTILNQSQVVSVIKDFKNNIN

>XP_011881568.1 PREDICTED: alanine--tRNA synthetase, cytoplasmic isoform X3 [Vollenhovia emeryi]

MASHAAEHQPEDTTMESSMNAKQIRQAYIDFFKSKGHEYVHSSSTIPHDDPTLLFTNAGMNQFKPIFLGTADPNSDMARW

VRVVNSQKCIRAGGKHNDLDDVGKDVYHHTFFEMMGNWSFGDYFKKEICTWAWEFLTDVLKLPADRLYVTYFGGDEKCGL

APDDECKNLWLSLGVPASHVLPGNMKDNFWEMGETGPCGPCSELHYDRIGGREAAHLVNMDDPDVLEIWNLVFIQYNRES

DGSLKSLPKKHIDCGLGLERLVSVIQNKRANYDTDLFVPLFAAIEKGTGAPPYQGKIGADDVNGIDMAYRVLADHARTIT

IALADGGMPDNTGRGYVLRRILRRAVRYATEKLNAKQGFFGSLVNVVVDLLGEVFPEVKKDPQSIIEVINEEEAQFLKTL

SRGRNLLNRTVAKLESTDTVPGDVAWRLYDTYGFPIDLTQLMTEEKGLKIDMAGYEESKKQAQLISQNKAGGVDDQINLD

VHAITELQKQGVKPTDDSPKYNYKVTNSVKYEEYEFAPCIGTVIALRRAKTFVDKVSSGEEVGILLDKTNFYAEQGGQIY

DEGFLVKVDDEATEIRVKNVQVRAGYVLHIGTVGEGTLRKGDKVHANVDTERRRLVMANHSATHALNHALRKVLGTEADQ

KGSLVAPDRLRFDFTNKGPMTGGQLKDTELFTNDMIQANKKIYAKESNLAIAKTIQGLRAMFEETYPDPVRIVSMGVPIE

NLEKNPLSTAALETSVEFCGGTHLHYTSHIGDFVIASEDAIAKGIRRIVALTGPEATKALRTMRILENQIHQIQVTIEAD

KSGTNSKEYVKKIVELTDEISHALVPVWWKDNVRNTLRELKKSLDDKERATKAAVISTVVKTAQTIIESKVGCQVLVEVL

EAYNNTKALDLALKKIRAISPETSALLISVDRDVKKIFALASVSKSAISKGLKANEWIQAIASLMQGKGGGKPESAQASG

TNVAGLNETVSKANEFANQKLGIIDNNTANTTRKTSETIQSHNTGSSSKLVLYGNNGSTKCYLAQIIAQYSGKCLTVKQV

GDEHKICSSDIMLETADVTLHNINAIAFFLSNSQLRREDDLFASSQVLQWMSYVQNHILPAVSGWVLPSLGVSVSKDMKA

NAKISKEDLLCTMKRMDEILRTRTYLIGERITLADVSVFTALLPLYEHVLDPHYRKQCTNLNRWFSTILNQPQVKCVVKN

FTFCAEATRC

>EFN86804.1 Alanyl-tRNA synthetase, cytoplasmic [Harpegnathos saltator]

MLTTMSGKQIRQAYIDFFKNKGHEYVHSSSTIPHDDPTLLFTNAGMNQFKPIFLGTVDPNSDMAKWVRVVNSQKCIRAGG

KHNDLDDVGKDVYHHTFFEMMGNWSFGDYFKKEICSWAWEFLTETLKLPADRLYVTYFGGDEKNGLAPDNECKELWLSLG

IPASHVLPGNMKDNFWEMGETGPCGPCSELHYDRIGGREAAHLVNMDDPDVLEIWNLVFIQYNREADGNLKPLPKKHIDC

GLGLERLVSVIQNKRANYDTDLFMPLFAAIEKGTGAPPYQGKIGTDDVNRVDMAYRVLADHARTITIALADGGIPDNTGR

GYVLRRILRRAVRYATEKLNAKPGFFGSLVNVVVELLGEVFPEVTKDPQSIIDIVNEEETQFLKTLSRGRNLLNRTIAKL

ESNDTVPGDVAWRLYDTYGFPIDLTQLMTEEKGLKIDMAGYEESKKQAQLISQNKVGGVDDQINLDVHSITELQERGIKP

TDDSPKYNYKITSTTTYKEYEFAPCIGTVIELRRAKTFVDQVSSGEEVGILLDRTNFYAEQGGQIYDEGFLVKVDDEATE

IRIKNVQVRAGYVLHIGTVGEGILKKGDQVHANVDTTRRRLVMANHSATHALNHALRKVLGTEADQKGSLVAPDRLRFDF

TNKGPMTTEQVKNAENITNDMIKDNKKIYAKESNLALAKTIQGLRAMFEETYPDPVRVVSMGVPIEELEKNPLSPVATQT

SVEFCGGTHLHYTGHIGDFIIASEEAIAKGIRRIVALTGPEAAKALKKATLLQNQLTQLQTTIEADKCGANSKEYVKKIV

ELTEDISHAVIPGWRKDEMRGMLKKLKKSLDDKDRAAKAAIANMVVETAQSIIQSKVGCQVLVEVLEAYSNTKALDSALK

KIRAVSPETSALLISVDQDVKKIFALSSVPKSAVSKGLKANEWIQAIAPMMQGKGGGKSESAQASGADITCLNEIVSKAN

EFANQKLGIMSE

>XP_019696024.1 PREDICTED: alanine--tRNA synthetase, cytoplasmic isoform X3 [Harpegnathos saltator]

MLTTMSGKQIRQAYIDFFKNKGHEYVHSSSTIPHDDPTLLFTNAGMNQFKPIFLGTVDPNSDMAKWVRVVNSQKCIRAGG

KHNDLDDVGKDVYHHTFFEMMGNWSFGDYFKKEICSWAWEFLTETLKLPADRLYVTYFGGDEKNGLAPDNECKELWLSLG

IPASHVLPGNMKDNFWEMGETGPCGPCSELHYDRIGGREAAHLVNMDDPDVLEIWNLVFIQYNREADGNLKPLPKKHIDC

GLGLERLVSVIQNKRANYDTDLFMPLFAAIEKGTGAPPYQGKIGTDDVNRVDMAYRVLADHARTITIALADGGIPDNTGR

GYVLRRILRRAVRYATEKLNAKPGFFGSLVNVVVELLGEVFPEVTKDPQSIIDIVNEEETQFLKTLSRGRNLLNRTIAKL

ESNDTVPGDVAWRLYDTYGFPIDLTQLMTEEKGLKIDMAGYEESKKQAQLISQNKVGGVDDQINLDVHSITELQERGIKP

TDDSPKYNYKITSTTTYKEYEFAPCIGTVIELRRAKTFVDQVSSGEEVGILLDRTNFYAEQGGQIYDEGFLVKVDDEATE

IRIKNVQVRAGYVLHIGTVGEGILKKGDQVHANVDTTRRRLVMANHSATHALNHALRKVLGTEADQKGSLVAPDRLRFDF

TNKGPMTTEQVKNAENITNDMIKDNKKIYAKESNLALAKTIQGLRAMFEETYPDPVRVVSMGVPIEELEKNPLSPVATQT

SVEFCGGTHLHYTGHIGDFIIASEEAIAKGIRRIVALTGPEAAKALKKATLLQNQLTQLQTTIEADKCGANSKEYVKKIV

ELTEDISHAVIPGWRKDEMRGMLKKLKKSLDDKDRAAKAAIANMVVETAQSIIQSKVGCQVLVEVLEAYSNTKALDSALK

KIRAVSPETSALLISVDQDVKKIFALSSVPKSAVSKGLKANEWIQAIAPMMQGKGGGKSESAQASGADITCLNEIVSKAN

EFANQKLGIMITL

>EFN72738.1 Alanyl-tRNA synthetase, cytoplasmic [Camponotus floridanus]

MNAKQIRQAYIDFFKSKGHEYVHSSSTIPHDDPTLLFTNAGMNQFKPIFLGTVDPNSDMAKWVRVVNSQKCIRAGGKHND

LDDVGKDVYHHTFFEMMGNWSFGDYFKKEICAWAWEFLTDVLKLPADRLYVTYFGGDEKNGLAPDDECKDLWLSLGIPAS

HVLPGNMKDNFWEMGETGPCGPCSELHYDRIGGREAAHLVNMDDPDVLEIWNLVFIQYNRESDGSLKSLPRKHIDCGLGL

ERLVSVIQNKRANYDTDLFMPLFAAIEKATGAPSYQGKVGAEDVDGIDMAYRVLADHARTITIALADGGMPDNTGRGYVL

RRILRRAVRYATEKLNAKPGFFGSLVNVVVDLLGEVFPEVTKDPQNIIDIVNEEETQFLKTLSRGRNLLNRTIAKLESSD

TVPGDVAWRLYDTYGFPVDLTQLMTEEKGLKIDIAGYEESKKQAQLISQNKAGGIDDQINLDIHAITEMQEHGIKPTDDS

PKYDYKVISSVKYEEYEFAPCIGIVIALRRAKTFVDQVTSGEEVGILLDKTNFYAEQGGQIYDEGFLVKVDDEATEIRVK

NVQVRAGYVLHIGTVGEGTLRKGDKVYTNVDTARRRLVMANHSATHALNYALRKVLGTGADQKGSLVAPDRLRFDFTNKG

PMTTEQVKNTENITADMVKENKKIYAKESNLALAKTIQGLRAMFEETYPDPVRVISMGIPVDDLEKNPLSTAALETSVEF

CGGTHLHYTGHIGDFIIASEEAIAKGIRRIVALTGPEAAKAFRTATLLQNQLSHLRETIETDNSGTNSKEYVKKIVELTE

EIMHAVIPSWRKDNMRATLKELKKSLDDKERAAKAAVANTVVETIQSIIQSRVGCQVLVEVLEAYSNTKALDSALKKIRV

LSPETSALLISIDRDVKKIFALSSVPKSAVSKGLKANEWIQVIAPAMQGKGGGKPESAQASGTNIACLNEIISRANEFAN

QKLGIIGK

>XP_011881567.1 PREDICTED: alanine--tRNA synthetase, cytoplasmic isoform X2 [Vollenhovia emeryi]

MLLCTLGSFDTWNLITAERASVRPVSFPEILPKGTESNREDTLNNLIKAYRHSQARARARALRASLPLSFPSRPPCQLCR

LDFRVSPDEPEDTTMESSMNAKQIRQAYIDFFKSKGHEYVHSSSTIPHDDPTLLFTNAGMNQFKPIFLGTADPNSDMARW

VRVVNSQKCIRAGGKHNDLDDVGKDVYHHTFFEMMGNWSFGDYFKKEICTWAWEFLTDVLKLPADRLYVTYFGGDEKCGL

APDDECKNLWLSLGVPASHVLPGNMKDNFWEMGETGPCGPCSELHYDRIGGREAAHLVNMDDPDVLEIWNLVFIQYNRES

DGSLKSLPKKHIDCGLGLERLVSVIQNKRANYDTDLFVPLFAAIEKGTGAPPYQGKIGADDVNGIDMAYRVLADHARTIT

IALADGGMPDNTGRGYVLRRILRRAVRYATEKLNAKQGFFGSLVNVVVDLLGEVFPEVKKDPQSIIEVINEEEAQFLKTL

SRGRNLLNRTVAKLESTDTVPGDVAWRLYDTYGFPIDLTQLMTEEKGLKIDMAGYEESKKQAQLISQNKAGGVDDQINLD

VHAITELQKQGVKPTDDSPKYNYKVTNSVKYEEYEFAPCIGTVIALRRAKTFVDKVSSGEEVGILLDKTNFYAEQGGQIY

DEGFLVKVDDEATEIRVKNVQVRAGYVLHIGTVGEGTLRKGDKVHANVDTERRRLVMANHSATHALNHALRKVLGTEADQ

KGSLVAPDRLRFDFTNKGPMTGGQLKDTELFTNDMIQANKKIYAKESNLAIAKTIQGLRAMFEETYPDPVRIVSMGVPIE

NLEKNPLSTAALETSVEFCGGTHLHYTSHIGDFVIASEDAIAKGIRRIVALTGPEATKALRTMRILENQIHQIQVTIEAD

KSGTNSKEYVKKIVELTDEISHALVPVWWKDNVRNTLRELKKSLDDKERATKAAVISTVVKTAQTIIESKVGCQVLVEVL

EAYNNTKALDLALKKIRAISPETSALLISVDRDVKKIFALASVSKSAISKGLKANEWIQAIASLMQGKGGGKPESAQASG

TNVAGLNETVSKANEFANQKLGIIDNNTANTTRKTSETIQSHNTGSSSKLVLYGNNGSTKCYLAQIIAQYSGKCLTVKQV

GDEHKICSSDIMLETADVTLHNINAIAFFLSNSQLRREDDLFASSQVLQWMSYVQNHILPAVSGWVLPSLGVSVSKDMKA

NAKISKEDLLCTMKRMDEILRTRTYLIGERITLADVSVFTALLPLYEHVLDPHYRKQCTNLNRWFSTILNQPQVKCVVKN

FTFCAEATRC

>XP_011881566.1 PREDICTED: alanine--tRNA synthetase, cytoplasmic isoform X1 [Vollenhovia emeryi]

MLLCTLGSFDTWNLITAERASVRPVSFPEILPKGTESNREDTLNNLIKAYRHSQARARARALRASLPLSFPSRPPCQLCR

LDFRVSPDGKSRGSVATLFRPLVEISVRGMYLHRGNYYIVEGTARSASRTMSRRGSPARLTQPRPYGESRRGAPLQKPED

TTMESSMNAKQIRQAYIDFFKSKGHEYVHSSSTIPHDDPTLLFTNAGMNQFKPIFLGTADPNSDMARWVRVVNSQKCIRA

GGKHNDLDDVGKDVYHHTFFEMMGNWSFGDYFKKEICTWAWEFLTDVLKLPADRLYVTYFGGDEKCGLAPDDECKNLWLS

LGVPASHVLPGNMKDNFWEMGETGPCGPCSELHYDRIGGREAAHLVNMDDPDVLEIWNLVFIQYNRESDGSLKSLPKKHI

DCGLGLERLVSVIQNKRANYDTDLFVPLFAAIEKGTGAPPYQGKIGADDVNGIDMAYRVLADHARTITIALADGGMPDNT

GRGYVLRRILRRAVRYATEKLNAKQGFFGSLVNVVVDLLGEVFPEVKKDPQSIIEVINEEEAQFLKTLSRGRNLLNRTVA

KLESTDTVPGDVAWRLYDTYGFPIDLTQLMTEEKGLKIDMAGYEESKKQAQLISQNKAGGVDDQINLDVHAITELQKQGV

KPTDDSPKYNYKVTNSVKYEEYEFAPCIGTVIALRRAKTFVDKVSSGEEVGILLDKTNFYAEQGGQIYDEGFLVKVDDEA

TEIRVKNVQVRAGYVLHIGTVGEGTLRKGDKVHANVDTERRRLVMANHSATHALNHALRKVLGTEADQKGSLVAPDRLRF

DFTNKGPMTGGQLKDTELFTNDMIQANKKIYAKESNLAIAKTIQGLRAMFEETYPDPVRIVSMGVPIENLEKNPLSTAAL

ETSVEFCGGTHLHYTSHIGDFVIASEDAIAKGIRRIVALTGPEATKALRTMRILENQIHQIQVTIEADKSGTNSKEYVKK

IVELTDEISHALVPVWWKDNVRNTLRELKKSLDDKERATKAAVISTVVKTAQTIIESKVGCQVLVEVLEAYNNTKALDLA

LKKIRAISPETSALLISVDRDVKKIFALASVSKSAISKGLKANEWIQAIASLMQGKGGGKPESAQASGTNVAGLNETVSK

ANEFANQKLGIIDNNTANTTRKTSETIQSHNTGSSSKLVLYGNNGSTKCYLAQIIAQYSGKCLTVKQVGDEHKICSSDIM

LETADVTLHNINAIAFFLSNSQLRREDDLFASSQVLQWMSYVQNHILPAVSGWVLPSLGVSVSKDMKANAKISKEDLLCT

MKRMDEILRTRTYLIGERITLADVSVFTALLPLYEHVLDPHYRKQCTNLNRWFSTILNQPQVKCVVKNFTFCAEATRC

>XP_013200543.1 PREDICTED: alanine--tRNA synthetase, cytoplasmic [Amyelois transitella]

MDSSMTGSQVRKTFIDFFISKGHKYVHSSSTIPLDDPTLLFANAGMNQFKPIFLGTIDPNSDMAHYVRVVNTQKCIRAGG

KHNDLDDVGKDVYHHTFFEMLGNWSFGDYFKKEICTWAWELLTEVYKLPGERLYVTYFGGDSASGLEPDFECKNIWLALG

VPESHILPGSMKDNFWEMGETGPCGPCSELHYDRIGGREAAHLVNMDDPDVLEIWNLVFIQFNRESDGSLKLLPKKHIDC

GLGFERLVSVIQNKRANYDTDIFMPLFKAIEFGTGVRPYSAKVGKEDLDGIDMAYRVLADHARTLTIALSDGGYPDNTGR

GYVLRRILRRGVRYASEKLNAKPGFFASLVHTVVEMLGDVFPEIKKDPESIIHIINEEEVQFLKTLTRGRNLLYRTIEKL

DKSKILPGDVAWRLYDTYGFPIDLTQLMCEEKGLSIDMEAYEKAKKESQIASQGKSAGKEDLLALDIHAITYLQDSHIPV

TDDSPKYDYVASSQDKESTYDFNTCTATILALRRNKEFVHEVFSGQECGIILDKTSFYAEQGGQIYDEGYMVKVDDDTTE

FTVNNVQVKAGYVLHTGKVEGTLKVGDTLSLHIDTVRRRLVMNNHTGTHILNNVLRKVLGSDSDQRGSLVMPDRLRFDFT

NKGPMTIQQIKDTENQIKEIINSNKIVYAKLTNLAEAKKINGLRAMFDEHYPDPVRVVSVGVPVEELEKNPDAATGYATS

VEFCGGSHLHHTGHIGDYVIVSEEGIAKGIRRIVALTGPEAQKAINKQNILENEVNNLANLIKEQGNNIIQKDILKNIID

LTNDISQAQISYWKKDEFRTTLNNLKKKLSDKERAAKAAVVNEVIEKAKDLCIQQKNSDVIVAELKAFGNTKALDSALKQ

VKQLCPNTAAMFFSVDNETKKIYCLAAVPKNIIEKGLLASDWVNSLTNVMGGRGGGKPESAQASGCNYNSLDEAMKKAQE

FAKSKLS

>XP_012235135.1 PREDICTED: alanine--tRNA synthetase, cytoplasmic isoform X1 [Linepithema humile]

MLTSMNAKQIRQAYIDFFKSKGHQYVHSSSTIPHDDPTLLFTNAGMNQFKPIFLGTVDPNSDMAKWVRVVNSQKCIRAGG

KHNDLDDVGKDVYHHTFFEMMGNWSFGDYFKKEICTWAWEFLTDVLKLPADRLYVTYFGGDEKNGLAPDDECKEIWLSLG

VPASHVLPGSMKDNFWEMGETGPCGPCSELHYDRIGGREAAHLVNMDDPDVLEIWNLVFIQYNRELDGSLKSLPKKHIDC

GLGLERLVSVIQNKRANYDTDLFMPLFVAIKKGTGAPPYQGKVGADDVNGIDMAYRVLADHARTITIALADGGMPDNTGR

GYVLRRILRRAVRYATEKLNAKPGFFGSLINVVVDLLGEVFPEVTKDPQSIIDIVNEEEAQFLKTLSRGRNLLNRTIAKL

ESADTVPGDVAWRLYDTYGFPIDLTQLMTEEKGLKIDMMGYEESKKQAQLISQSKAGGIDDQINLDIHAITELQEQGVKP

TDDSLKYDYKVRNSVMYKEYEFGACTGTVIALRRAKTFVDAVSSGDEVGILLDRTNFYAEQGGQIYDEGFLVKVDDEATE

IRIKNVQVRAGYVLHIGTVGEGTLRKGDKVHTNVDTARRRFIMANHSATHALNHALRKVLGTEADQKGSLVAPDRLRFDF

TNKGPMTTEQVKNTENITNDIIKRNKTIYAKESNLALAKTIQGLRAMFEETYPDPVRVVSMGIPVEDLEKDPLSAAALET

SVEFCGGTHLHYTGHIGDFIIASEEAIAKGIRRIVALSGPEATKALKKATLLQNQLIQLQATIEADKSGKKSKEYVKNIV

ELTEDISHAIIPGWRKDCMRNMLKELKKSLDDKERAAKAAIANTVVETAQSIIESKLGCQVLVEVLEAYSNTKALDTALK

KIRAVSPETSALLISVDRDVKKIFALSSVPKSAVSKGLKANEWIQAIALVMCGKGGGKAESAQASGTNIACVNEIVRKAS

EFANEKLGITDNSTANNSPKTSENDIQSLNVGSSSKLVLYANIGSTKCYLAQIVAQYSGKSLTLKQVKSGKINSGDVMLE

TADVTLHNSNAIAFFLSNSQLRREDDLLASSQILQWTSYAQNHILPAVSGWVLSSLGISAFKDMKANAKASKEDLLGALK

KLDDILCTKTYLIGERITLADIFVFTALMPLYEHVFDPHLRKPYTSLNRWFSTILNQPQVKCVVKNFTFCTKAA

>XP_011881570.1 PREDICTED: alanine--tRNA synthetase, cytoplasmic isoform X5 [Vollenhovia emeryi]

MLLCTLGSFDTWNLITAERASVRPVSFPEILPKGTESNREDTLNNLIKAYRHSQARARARALRASLPLSFPSRPPCQLCR

LDFRVSPDGKSRGSVATLFRPLVEISVRGMYLHRGNYYIVEGTARSASRTMSRRGSPARLTQPRPYGESRRGAPLQKPED

TTMESSMNAKQIRQAYIDFFKSKGHEYVHSSSTIPHDDPTLLFTNAGMNQFKPIFLGTADPNSDMARWVRVVNSQKCIRA

GGKHNDLDDVGKDVYHHTFFEMMGNWSFGDYFKKEICTWAWEFLTDVLKLPADRLYVTYFGGDEKCGLAPDDECKNLWLS

LGVPASHVLPGNMKDNFWEMGETGPCGPCSELHYDRIGGREAAHLVNMDDPDVLEIWNLVFIQYNRESDGSLKSLPKKHI

DCGLGLERLVSVIQNKRANYDTDLFVPLFAAIEKGTGAPPYQGKIGADDVNGIDMAYRVLADHARTITIALADGGMPDNT

GRGYVLRRILRRAVRYATEKLNAKQGFFGSLVNVVVDLLGEVFPEVKKDPQSIIEVINEEEAQFLKTLSRGRNLLNRTVA

KLESTDTVPGDVAWRLYDTYGFPIDLTQLMTEEKGLKIDMAGYEESKKQAQLISQNKAGGVDDQINLDVHAITELQKQGV

KPTDDSPKYNYKVTNSVKYEEYEFAPCIGTVIALRRAKTFVDKVSSGEEVGILLDKTNFYAEQGGQIYDEGFLVKVDDEA

TEIRVKNVQVRAGYVLHIGTVGEGTLRKGDKVHANVDTERRRLVMANHSATHALNHALRKVLGTEADQKGSLVAPDRLRF

DFTNKGPMTGGQLKDTELFTNDMIQANKKIYAKESNLAIAKTIQGLRAMFEETYPDPVRIVSMGVPIENLEKNPLSTAAL

ETSVEFCGGTHLHYTSHIGDFVIASEDAIAKGIRRIVALTGPEATKALRTMRILENQIHQIQVTIEADKSGTNSKEYVKK

IVELTDEISHALVPVWWKDNVRNTLRELKKSLDDKERATKAAVISTVVKTAQTIIESKVGCQVLVEVLEAYNNTKALDLA

LKKIRAISPETSALLISVDRDVKKIFALASVSKSAISKGLKANEWIQAIASLMQGKGGGKPESAQASGTNVAGLNETVSK

ANEFANQKLGIIAPA

>XP_021189227.1 alanine--tRNA synthetase, cytoplasmic [Helicoverpa armigera]

MDTSMTGNQIRKAFIDFFISKGHKYVHSSSTIPLDDPTLLFTNAGMNQFKPIFLGSVDPNSDMAQYVRVVNTQKCIRAGG

KHNDLDDVGKDVYHHTFFEMMGNWSFGDYFKKEICAWAWELLTEVYKLPGDRLYVTYFGGDSSSGLEPDLECKEIWLKLG

VPESHILPGSMKDNFWEMGETGPCGPCSELHYDRIGGRDAAHLVNMDDPDVLEIWNLVFIQFNRETDGSLKLLPKKHIDC

GLGLERLVSVIQNKRANYDTDFFMPIFKAIEVGTGARPYSGKVGSDDVDGIDMAYRVLADHARTLTIALSDGGVPDNTGR

GYVLRRILRRAVRYASEKLNAKPGFFGSLVHTVVELLGDIFPEITKDPLSLIQLINEEEIQFLKTLSRGRNLLNRTIEKL

NGKVIPGDVAWRLYDTYGFPVDLTQLMCEENGLTIDMDAYEKSKKESQLISQGKVAGQEDLLALDVHAISHLQEVGIPTT

DDSPKYNYVASSTEKETIYTFSPCTGTIVALRKDKQFVNEVSSGQECGVILDRTNFYAEQGGQIYDEGYMVKVDDDSVEF

MVKNVQVKGGYVLHVGKLEGSLKIGDKLSLHIDTERRRLVMNNHTGTHVLNNVLRKVLGNDSDQRGSLVMPDRLRFDFTN

KGPMSIKQIKETEDQIKDIINKNKPVYAKHTSLNDAKKIKGLRAMFDEQYPDPVRVVSVGVPVEELEKNPEGPAAYETSV

EFCGGSHLHQTGHIGDYVIVSEEGIAKGIRRIVALTGPEAMKAISKSSILENEANNIANFIKDQNDIVNQKEILRKIVDL

TNDISQAQISYWKKEELRTMLKNLKKQLDDKERAAKAVTITQVAEKAKEICEEKKGSQVIVSELKAFGNTKALDGALKQV

KTLSPTTAAMFFSVDDETKKIFCLAAVPKNAIEKGLLASEWIQSVVPVIGGKGGGKPESGQASGSNYTSLNEALRVAEEF

ANLKLS

>XP_012235139.1 PREDICTED: alanine--tRNA synthetase, cytoplasmic isoform X2 [Linepithema humile]

MLTSMNAKQIRQAYIDFFKSKGHQYVHSSSTIPHDDPTLLFTNAGMNQFKPIFLGTVDPNSDMAKWVRVVNSQKCIRAGG

KHNDLDDVGKDVYHHTFFEMMGNWSFGDYFKKEICTWAWEFLTDVLKLPADRLYVTYFGGDEKNGLAPDDECKEIWLSLG

VPASHVLPGSMKDNFWEMGETGPCGPCSELHYDRIGGREAAHLVNMDDPDVLEIWNLVFIQYNRELDGSLKSLPKKHIDC

GLGLERLVSVIQNKRANYDTDLFMPLFVAIKKGTGAPPYQGKVGADDVNGIDMAYRVLADHARTITIALADGGMPDNTGR

GYVLRRILRRAVRYATEKLNAKPGFFGSLINVVVDLLGEVFPEVTKDPQSIIDIVNEEEAQFLKTLSRGRNLLNRTIAKL

ESADTVPGDVAWRLYDTYGFPIDLTQLMTEEKGLKIDMMGYEESKKQAQLISQSKAGGIDDQINLDIHAITELQEQGVKP

TDDSLKYDYKVRNSVMYKEYEFGACTGTVIALRRAKTFVDAVSSGDEVGILLDRTNFYAEQGGQIYDEGFLVKVDDEATE

IRIKNVQVRAGYVLHIGTVGEGTLRKGDKVHTNVDTARRRFIMANHSATHALNHALRKVLGTEADQKGSLVAPDRLRFDF

TNKGPMTTEQVKNTENITNDIIKRNKTIYAKESNLALAKTIQGLRAMFEETYPDPVRVVSMGIPVEDLEKDPLSAAALET

SVEFCGGTHLHYTGHIGDFIIASEEAIAKGIRRIVALSGPEATKALKKATLLQNQLIQLQATIEADKSGKKSKEYVKNIV

ELTEDISHAIIPGWRKDCMRNMLKELKKSLDDKERAAKAAIANTVVETAQSIIESKLGCQVLVEVLEAYSNTKALDTALK

KIRAVSPETSALLISVDRDVKKIFALSSVPKSAVSKGLKANEWIQAIALVMCGKGGGKAESAQASGTNIACVNEIVRKAS

EFANEKLGITATA

>XP_011170503.1 PREDICTED: alanine--tRNA synthetase, cytoplasmic isoform X1 [Solenopsis invicta]

MKSSMNAKQIRQAYIDFFKSKGHEYVHSSSTIPHDDPSLLFTNAGMNQFKPIFLGTVDPNSDMAKWVRVVNSQKCIRAGG

KHNDLDDVGKDVYHHTFFEMMGNWSFGDYFKKEICAWAWEFLTDVLKLPADRLYVTYFGGDMKSGLAPDDECKDLWLSLG

IPASHVLPGNMKDNFWEMGETGPCGPCSELHYDRIGGREAAHLVNMDDPDVLEIWNLVFIQYNREPDGSLKPLPKKHIDC

GLGLERLISVIQDKRANYDTDLFMPLFDAIEKGTGAPPYKGKIGADDVDGIDMAYRVLADHARTITIALADGGMPDNTGR

GYVLRRILRRAVRYATEKLNAKPGFFGSLVNVVVELLGDVFPEVKRDPQSIIEIINEEEIQFLKTLSRGRNLLNRTIAKL

ESTNVVPGDVAWRLYDTYGFPIDLTQLMTEEKGLKIDMTGYEEAKKQAQLISQNKAGGVDDQINLDVHAITELQNQGVKP

TDDSPKYDYKVTNTVQYNEYDFAPCTGTVIALRRAKAFVDEVSSGEEVGILLDRTNFYAEQGGQIFDEGFLVKVDDEATE

IRVKNVQVRAGYVLHIGTVGEGKLRKGDKVHTNVDTKRRRFVMANHSATHALNYALRKVLGTEADQKGSLVAPDRLRFDF

TNKGPMTTKQLLDTEVATQEMIKDNKQIYTKESSLAIAKTIQGLRAMFEETYPDPVRVVSMGVSVEKLESDPLSKAALET

SVEFCGGTHLHYTAHIGDFVIASEDAIAKGIRRIVALTGPEATKALRTSTLLQQQILALKEMIANSEINVKECVRKITDL

SEEISHAVIPAWLKDNMRNTLKDLKKSLDDKERAAKAAVATSVVQTAQSIIESNPECKVLVEVMEAYSNTKALDLALKKI

KAISQETSAMLISVDHNVNKIYALSSVSKSGIDEGLKANEWVQAIVPLMKGKGGGKAESAQASGTNIACLNEIVSKAKEF

ANQKLGINNTANNICKTSKISRLPNTGASEFVLYGNLGSAKCWLARIIAAYSGKSLLVKQIKNAEAYPGYVKLETTGATL

RDSNAIAFFLSNSQLRRDDDLSASCQVLQWMSYAENHITPLVVDWVLAELGVSKMPRNVKADMNIFSKMDIFYALRRMEI

VLRPRTYLVGDRISLADLVLFTAFIPMYEHVFDSQFRKQYVNMNRWFFTILNQPQVKRVLITFKFCDYFKEIKAQYR

>XP_019864269.1 PREDICTED: alanine--tRNA synthetase, cytoplasmic-like [Amphimedon queenslandica]

MDSTLTAKDIRRMFIDFFRSKDHTFVPSSSTIPVDDPTLLFTNAGMNQFKPIFQGTVDPSSDFATLSRAANSQKCIRAGG

KHNDLDDVGKDTYHHTFFEMMGNWSFGDYFKEEAIKMAWSFLTESLKLPKDRLYVTYFGGDEKLKLPADDECKEIWLNTG

LPPERVLPFSVKDNFWEMGEVGPCGPCSEIHYDRIGGRFVADRVNMDDPNVLELWNVVFMQFNKESDGSIKPLPKNNVDT

GLGLERIVSVVQGKFSNYDTDLFLPIFDAIEAKTGSRSYTGKIGAEDVDGVDMAYRVVADHIRTLTIAISDGGKPDATGR

GYVLRRILRRGVRYITEKLNAKPGTFASLVPTVLDVLGEAFPEITKDPEMVMDTINEEEKQFLKTLSHGKRMFERKLRKI

ENNLLPGDVAWRLYDTYGFPVDLTQLMAEERQVTVDMEGYEKAKAMAQEKARGTGREIDESLQLDVHAISHLQSKGVPPT

NDSSKYDYSSDESGNYKFQSCEGSVIALRYNKEFVDEVSSGQVVGVLTDKTCFYAEQGGQIYDEGYIVKIGEEETEFIVR

DVQVRGGYVLHVGSIEGTLRVGDKVNMTIDGDRRSDVMKNHTGTHVLNFALRRVIGEADQKGSLVAPDRLRFDFSAKGAM

KTDQIKKCEEVALEVIKASHPVYAQETPLPVAKEIQGLRAVFDEVYPDPVRVLSIGVPVDKLVEDPSGPWAINYSVEFCG

GTHLRSSGHIQSFAIVTEEAIAKGIRRIVALTGMEAVKAHKRAAVIQERVDALCSTVLNGLSTKSMAPKEANKLIVKEGD

ALSSAVISQWIKDELREKLNTAKKSADEADKAAKELLTQKCIERAQELIGQFDKASPPSFVVEIFEAEGLSKALDNALKQ

FKSLAPTVAALLLSVDHDNHKILCLSNVPKNAIAKGLKANEWVQAVSVTIGGKGGGRDNTAQATGTNVGKLSDAVEVAKQ

FATKFF

>XP_011170505.1 PREDICTED: alanine--tRNA synthetase, cytoplasmic isoform X2 [Solenopsis invicta]

MKSSMNAKQIRQAYIDFFKSKGHEYVHSSSTIPHDDPSLLFTNAGMNQFKPIFLGTVDPNSDMAKWVRVVNSQKCIRAGG

KHNDLDDVGKDVYHHTFFEMMGNWSFGDYFKKEICAWAWEFLTDVLKLPADRLYVTYFGGDMKSGLAPDDECKDLWLSLG

IPASHVLPGNMKDNFWEMGETGPCGPCSELHYDRIGGREAAHLVNMDDPDVLEIWNLVFIQYNREPDGSLKPLPKKHIDC

GLGLERLISVIQDKRANYDTDLFMPLFDAIEKGTGAPPYKGKIGADDVDGIDMAYRVLADHARTITIALADGGMPDNTGR

GYVLRRILRRAVRYATEKLNAKPGFFGSLVNVVVELLGDVFPEVKRDPQSIIEIINEEEIQFLKTLSRGRNLLNRTIAKL

ESTNVVPGDVAWRLYDTYGFPIDLTQLMTEEKGLKIDMTGYEEAKKQAQLISQNKAGGVDDQINLDVHAITELQNQGVKP

TDDSPKYDYKVTNTVQYNEYDFAPCTGTVIALRRAKAFVDEVSSGEEVGILLDRTNFYAEQGGQIFDEGFLVKVDDEATE

IRVKNVQVRAGYVLHIGTVGEGKLRKGDKVHTNVDTKRRRFVMANHSATHALNYALRKVLGTEADQKGSLVAPDRLRFDF

TNKGPMTTKQLLDTEVATQEMIKDNKQIYTKESSLAIAKTIQGLRAMFEETYPDPVRVVSMGVSVEKLESDPLSKAALET

SVEFCGGTHLHYTAHIGDFVIASEDAIAKGIRRIVALTGPEATKALRTSTLLQQQILALKEMIANSEINVKECVRKITDL

SEEISHAVIPAWLKDNMRNTLKDLKKSLDDKERAAKAAVATSVVQTAQSIIESNPECKVLVEVMEAYSNTKALDLALKKI

KAISQETSAMLISVDHNVNKIYALSSVSKSGIDEGLKANEWVQAIVPLMKGKGGGKAESAQASGTNIACLNEIVSKAKEF

ANQKLGITTA

>XP_011636339.1 PREDICTED: alanine--tRNA synthetase, cytoplasmic [Pogonomyrmex barbatus]

MTSSVNAKQIRQAYIDFFKGKGHEYVHSSSTIPHDDPTLLFTNAGMNQFKPIFLGTVDPNSDMAKWIRVVNSQKCIRAGG

KHNDLDDVGKDVYHHTFFEMMGNWSFGDYFKKEICAWAWEFLTEVLKLSADRLYVTYFGGDEKNGLAPDDECKNLWLSLG

IPASHVLPGSMKDNFWEMGETGPCGPCSELHYDRIGDREAAHLVNMDDPDVLEIWNLVFIQYNRESDGSLKSLPKKHIDC

GLGLERLVSVIQNKRANYDTDLFVPLFAAIEKGTGAPPYQGRIGADDINGIDMAYRVLADHARTITIALADGGMPDNTGR

GYVLRRILRRAVRYATEKLNAKPGFFASLVNVVVDLLGEVFPEVKKDPQGIIEIINEEEAQFLKTLSRGRNLLNRTIAKL

ESTDIVPGDVAWRLYDTYGFPVDLTQLMTEEKGLKIDMAGYEESKKQAQLISQNKAGGVNDQINLDIHAITELQKQGVKP

TDDSPKYNYKVTNSVKYEEYEFAPCIGTVIALRRAKTFVDKVSSGEEVGILLDRTNFYAEQGGQIFDEGFLVKVDDETTE

IRVKNVQIRAGYVLHIGTVGEGTLKKGDKVHANVDTERRRFVMANHSATHALNHALRKVLGTEADQKGSLVAPERLRFDF

TNKGPMTGEQLKNTETITNGMIKENKKIYAKESSLAVAKTIQGLRAMFEETYPDPVRVVSMGVPVENLEKDPLSKAALET

SVEFCGGTHLHYTGHIGDFVIASEDAIAKGIRRIVALTGPEATKAIRTATILQKQLSQLQATIEADKSGMNSKEYVKKIV

ELTEEISHALIPAWQKDNMRNMLRELKKSLDDKERAAKAAVANTVVKTAQSIIESKIGCEVLVLMLEAYSNTKALDSALK

KIRAISPETSALLLSVDRDVKKIFALSSVPKSAVSKGLKANEWIQAIAGLMQGKGGGKPESAQASGTDITRVNEILSKAN

EFANQKLGISYNNTVNNACKTSETDIRLFNTGSQSKLVLYGNIGSTKCYLAQIIAQYSGKCLTVKQIENDKLCSSGIILE

TADVTLYGSDAIAFFLSNSQLRRDDDLFASSQVLQWMYYARNHILPAVSGWVLPSFSNSVSKDTKANIKISKEELLCALK

QMNNVLCTKTYLVGERITLADISVFTALLPLYEHVFDSHYRKLYSNLNRWFSTILNQPQVKCIVKNFTFL

>XP_019759480.1 PREDICTED: alanine--tRNA synthetase, cytoplasmic isoform X2 [Dendroctonus ponderosae]

MSISNMTAKEVRSAYIDFFKEKDHVYHHSSSTIPLDDPTLLFANAGMNQFKPIFLGTVDPNSDLSKLVRAVNTQKCIRAG

GKHNDLDDVGKDVYHHTFFEMMGNWSFGDYFKKEICTWAWEFLTVKLSLPADRIYVTYFGGDPKSGLEPDNECKEIWLKL

GVAPSHVIPGSMKDNFWEMGETGPCGPCSELHFDRIGGRSVPELVNMDDPDVLEIWNLVFIQYNREPDSTLKPLPKKHID

CGLGLERLVSVIQNKRSNYDTDLFMPLFEAIHRGTGAAPYQGRVGDDDKDGVDMAYRVLADHARTLTIALADGGNPDNTG

RGYVLRRILRRAVRYATEKLNAKPGFFSTLVNTVVEILGDTFPEVKKDPQYIIETINEEEAQFLKTLSRGRNLLNRTITK

LGESKELPGDVAWRLYDTYGFPVDLTSLMAEEKGLNVNMEAYEEAKKQAQIISQGKGTGVADTINLDVHAITELQNRQIP

PTNDSPKYDYEANQNVDDEYKFGACEASVIGLRYNKQFVEEVTSGQECGVLLDSTNFYAEQGGQIYDTGFLVKLNDDSVE

FSVKNVQVRGGYIIHIGNIEGVLRVGDKVSLHIDADRRRLIMSNHTGTHILNYALRKVLGTDADQRGSLVAPDRLRFDFT

NKGAMTADQVKKAEQSAKDLISKNAKVYAKDSSLATAKTIRGLRAVFEETYPDPVRIVSVGIPVEDLENDPFGPAGDSTS

IEFCGGTHVKYAGHIGDFVISNEEAIAKGIRRIVALTGPEATKALKRNEVLENRLNEIKSSINADKDGAKSKEHVRIIVE

LTDEVSQAIIPYWKKEEIRNNLKNLKKNLDDKDRAAKAAIANKVVDEIKEFVKENPNLPILVKELKAFNNTKALDSALKQ

VKTLSPATAALFVTVDSDSNKMFCLSSVPKEAIEKGLKANEWVQSVAQKIGGKGGGKPDSAQASGANSVSVDEILELAKK

FADSKLL

>XP_011689776.1 PREDICTED: alanine--tRNA synthetase, cytoplasmic isoform X2 [Wasmannia auropunctata]

MKGTMNAKEIRQAYIDFFKSKGHEYVHSSSTIPHDDPTLLFTNAGMNQFKPIFLGTVDPNSDMAKWVRVVNSQKCIRAGG

KHNDLDDVGKDVYHHTFFEMMGNWSFGDYFKKEICTWAWEFLTEVLKLPGDRLYVTYFGGDEKSGLAPDDECKNLWLSLG

IPASHVLPGSMKDNFWEMGETGPCGPCSELHYDRIGGRDAAHLVNMDDPDVLEIWNLVFIQYNRESDGSLKSLPKKHIDC

GLGLERLVSVIQNKRANYDTDLFMPLFAAIEKGTGAPSYQGKIGADDVDGIDMAYRVLADHARTITIALADGGVPDNTGR

GYVLRRILRRAVRYATEKLNAKPGFFGTLVNVVVDLLGDVFPEVKKDPQSIIEVINEEETQFLKTLSRGRNLLNRTITKL

ESTDTVPGDVAWRLYDTYGFPIDLTQLMTEEKGLKIDMAGYEESKKQAQLISQNKAGGVDDQINLDVHAITELQKQGVKP

TDDSPKYNYKVTNDVKCKEYEFASCTGTVIALRRAKTFVDEVSSGEEVGILLDRTNFYAEQGGQIYDEGFLVKVDDEATE

IRVKNVQVRAGYVLHIGTVGEGTLRKGDKVHANVDTERRRFVMANHSATHALNYALRKVLGTEADQKGSLVAPDRLRFDF

TNKGPMTEKQLELTEDFTVKMIKENKKIYAKESNLAIAKAIQGLRAMFEETYPDPVRVISMGVPIENLEKNPLSTAALET

SVEFCGGTHLHYTGHIGDFIIASEDAIAKGIRRIVALTGPEATKAIRTMRMLQERLAQIKATIEADKSGANSKEHVRKIV

ELSDEISHALISAHSKDNMRNALKELKRSLDDKERAAKAAVATTVVKTAQSIIESKVGCEVLIEVLEAYSNTKALDSALK

KIRAISPETSALLISVDRDVNKIFALSSVPKSAVSKGLKANEWIQAIASLMQGKGGGKPESAQASGSNITCLNEVVSKAN

EFAYQKLGIATTA

>EFX88718.1 hypothetical protein DAPPUDRAFT_191289 [Daphnia pulex]

MDTSLSAAKIRQMYMDFFIEKKSHIYVHSSSTIPKDDPTLLFANAGMNQFKPLFLGTVDPNSDMSKWIRVVNTQKCIRAG

GKHNDLDDVGKDVYHHTFFEMLGNWSFGDYFKKEVCTWAWELLTDVFQLPKDRLYVTYFGGNENAGLQPDEECRQIWLSL

GLPAERILPGSMKDNFWEMGDVGPCGPCSEIHFDRIGGRDAAHLVNMDDPDVLEIWNLVFMTFNRETDSSLKPLPRKHID

CGMGFERLVSVIQDKRSNYDTDIFQPIFEAIQKGTNAPAYQGKVGAEDVNGVDMAYRVLADHARTLTIALSDGGRPDNVG

RGYVLRRVLRRGVRYATEKLNAKPGFFASLVPTVCQVLGPTFPEVNRDPQLVIDIINDEETQFLKTLTRGRDLLERTISK

LNGQTTLPGDVAWRLYDTYGFPVDLTQLMSEEKGLSIDMVAYEEAKKQAQLLSQGKGSGVDDTIAFDVHAITELQNKGVP

PTDDSFKYNYKSVSDAVDSPYEFDVCTSKVIAIRFQKAFHEEVSSGVECGILLDRTSFYAESGGQAYDEGFITKIGDEGT

EFTVKNVQVRGGYVIHLGNVEGTLRVGDEVKLSIDQSRRRLIMSNHTGTHVLNYALRQVVGNEADQRGSLVAPDRMRFDF

TNKAAMTVDQVKQTERIANEMIRKNQEVYAKESSLVVAKAVQGLRAVFEETYPDPVRVVSIGIPVEQLEADPANPAGNST

SIEFCGGTHVKRSGHIGDFVISAEEAIAKGIRRIVAITGPEASKALKKADLLQKEVDAVSAKVDAFVSQNDKTLSVKELS

RIIVDLSDDVSQANIAYWKKDDLRNILKGLKKRADDVERAIKAAVVNDVAEAAKKLVAEQVNTPFIVHEFNAFSNSKALD

GALKQVKSLSPETAAIFFSVDAEANKIVVLAAAPKGANDRGLKANEWVADVSALLDGKGGGSAGSAQATGNNPAGLAEAM

KKAKAFAQSKLGLDAEISASTTKLGLV

>XP_019759479.1 PREDICTED: alanine--tRNA synthetase, cytoplasmic isoform X1 [Dendroctonus ponderosae]

MSTPHMGTTCEGVKAENCHLIDEKILFKNTQKTIIRAIVKMSISNMTAKEVRSAYIDFFKEKDHVYHHSSSTIPLDDPTL

LFANAGMNQFKPIFLGTVDPNSDLSKLVRAVNTQKCIRAGGKHNDLDDVGKDVYHHTFFEMMGNWSFGDYFKKEICTWAW

EFLTVKLSLPADRIYVTYFGGDPKSGLEPDNECKEIWLKLGVAPSHVIPGSMKDNFWEMGETGPCGPCSELHFDRIGGRS

VPELVNMDDPDVLEIWNLVFIQYNREPDSTLKPLPKKHIDCGLGLERLVSVIQNKRSNYDTDLFMPLFEAIHRGTGAAPY

QGRVGDDDKDGVDMAYRVLADHARTLTIALADGGNPDNTGRGYVLRRILRRAVRYATEKLNAKPGFFSTLVNTVVEILGD

TFPEVKKDPQYIIETINEEEAQFLKTLSRGRNLLNRTITKLGESKELPGDVAWRLYDTYGFPVDLTSLMAEEKGLNVNME

AYEEAKKQAQIISQGKGTGVADTINLDVHAITELQNRQIPPTNDSPKYDYEANQNVDDEYKFGACEASVIGLRYNKQFVE

EVTSGQECGVLLDSTNFYAEQGGQIYDTGFLVKLNDDSVEFSVKNVQVRGGYIIHIGNIEGVLRVGDKVSLHIDADRRRL

IMSNHTGTHILNYALRKVLGTDADQRGSLVAPDRLRFDFTNKGAMTADQVKKAEQSAKDLISKNAKVYAKDSSLATAKTI

RGLRAVFEETYPDPVRIVSVGIPVEDLENDPFGPAGDSTSIEFCGGTHVKYAGHIGDFVISNEEAIAKGIRRIVALTGPE

ATKALKRNEVLENRLNEIKSSINADKDGAKSKEHVRIIVELTDEVSQAIIPYWKKEEIRNNLKNLKKNLDDKDRAAKAAI

ANKVVDEIKEFVKENPNLPILVKELKAFNNTKALDSALKQVKTLSPATAALFVTVDSDSNKMFCLSSVPKEAIEKGLKAN

EWVQSVAQKIGGKGGGKPDSAQASGANSVSVDEILELAKKFADSKLL

>XP_015178787.1 PREDICTED: alanine--tRNA synthetase, cytoplasmic [Polistes dominula]

MSQSMTAKEIRQSYIDFFKSKGHEYVHSSSTIPHDDPTLLFTNAGMNQFKPIFLGTVDPNSDMAKWVRVVNSQKCIRAGG

KHNDLDDVGKDVYHHTFFEMMGNWSFGDYFKKEICSWAWEYLTVNLKLSSDRLYITYFGGDEKSGLDPDNECKDIWLSLG

VPASHVLPGNMKDNFWEMGETGPCGPCSEIHYDRIGNREAAHLVNMDDPDVLEVWNLVFIQYNRESDGILKPLPKKHIDC

GLGLERLVSVIQNKRANYDTDLFMPLFEAIQKGTGVSPYQGRVGNADKDGIDMAYRVLADHARTITIALADGGMPDNTGR

GYVLRRILRRAVRYATEKLNAKPGFFASLVNVVVELLGDVFPEVKKDPQSIIDIINEEETQFLKTLSRGRNLLNRTISKL

ESSKIVPGNVAWRLYDTYGFPVDLTQLMTEEKGLKVDMVAYEEAKKQAQLISQSKVGGVDDQINLDVHAITELQNTGIPM

TNDLPKYNYNVKGKDPYDEYSFQSCTSTVIALRHAKTFVEEVYSGQEVGILLDKTNFYAEQGGQIYDEGFLVKVDDEETE

IRITNVQVRGGYVLHIGTVGEGVLKKGDKVHLNVDTTRRRLIMSNHTATHVLNHALRQVLGLEADQKGSLVAPDKLRFDF

TNKGAMTTDQVKKTEKITNDIIKQKKNIFAKESNLALAKTIKGLRAMFEETYPDPVRIVSVGIPIEDLEKDPLNDAALKT

SVEFCGGTHLHNTEHIGDFVISSEEAIAKGIRRIVALTGPEAKKALKKAALLEDKLNQLKNTIDTDKSSIHSKEYVKQIV

ELNEDVSHATIPSWKKDKMRGMLKELKKSLDDKERLAKAAVANAVVESVQEMIQSNIGCAVFVEVLKAFSNTKALDSALK

KIKSLSPNTSALLISVDEDAKKIFVLSTVPKSAISKGLKANEWIQEIAPLLGGKGGGKAESAQASGPNISSLYKIIQKAK

EFANSKLGIVNEKLTIQDPTFNSTTNVKNTNSVDATKSQKLTIFGNKNSIKSYKVQIVAQYCNQNLQIKYVEDDKNSKTD

MILEIGDIQISDSSAIAYYLSDPKLKLEDDLFAFCEILQWISYTNNHLLPAVTGWILSSCQITIPKNIKFDWKTSKEDAS

NSLKKLNNTLLKKTYFVNERISLADVTIFSVLLPLYEHILTPELRKQYTNLNRWFSTILNQPEVKAVVKDFHFCTKTQ

>XP_011689775.1 PREDICTED: alanine--tRNA synthetase, cytoplasmic isoform X1 [Wasmannia auropunctata]

MKGTMNAKEIRQAYIDFFKSKGHEYVHSSSTIPHDDPTLLFTNAGMNQFKPIFLGTVDPNSDMAKWVRVVNSQKCIRAGG

KHNDLDDVGKDVYHHTFFEMMGNWSFGDYFKKEICTWAWEFLTEVLKLPGDRLYVTYFGGDEKSGLAPDDECKNLWLSLG

IPASHVLPGSMKDNFWEMGETGPCGPCSELHYDRIGGRDAAHLVNMDDPDVLEIWNLVFIQYNRESDGSLKSLPKKHIDC

GLGLERLVSVIQNKRANYDTDLFMPLFAAIEKGTGAPSYQGKIGADDVDGIDMAYRVLADHARTITIALADGGVPDNTGR

GYVLRRILRRAVRYATEKLNAKPGFFGTLVNVVVDLLGDVFPEVKKDPQSIIEVINEEETQFLKTLSRGRNLLNRTITKL

ESTDTVPGDVAWRLYDTYGFPIDLTQLMTEEKGLKIDMAGYEESKKQAQLISQNKAGGVDDQINLDVHAITELQKQGVKP

TDDSPKYNYKVTNDVKCKEYEFASCTGTVIALRRAKTFVDEVSSGEEVGILLDRTNFYAEQGGQIYDEGFLVKVDDEATE

IRVKNVQVRAGYVLHIGTVGEGTLRKGDKVHANVDTERRRFVMANHSATHALNYALRKVLGTEADQKGSLVAPDRLRFDF

TNKGPMTEKQLELTEDFTVKMIKENKKIYAKESNLAIAKAIQGLRAMFEETYPDPVRVISMGVPIENLEKNPLSTAALET

SVEFCGGTHLHYTGHIGDFIIASEDAIAKGIRRIVALTGPEATKAIRTMRMLQERLAQIKATIEADKSGANSKEHVRKIV

ELSDEISHALISAHSKDNMRNALKELKRSLDDKERAAKAAVATTVVKTAQSIIESKVGCEVLIEVLEAYSNTKALDSALK

KIRAISPETSALLISVDRDVNKIFALSSVPKSAVSKGLKANEWIQAIASLMQGKGGGKPESAQASGSNITCLNEVVSKAN

EFAYQKLGIANHNTAKGACKPSETIQLLNAAGPSLNLVLYGNIGSTKCCLAQIIAQYSGKRLTVKQVDDKIYPSDVALET

ADVTLHHSNAIAFFLSNSQLRRDDDLFASSQVLQWMSHVQNHISPVVSGCALLSLGVSVPDAKAEENRTVKVADLLHVLR

GLDDVLRTRTYLIGERITLADISLFTALLPLYEYVFDYRKKRTNRKQHGFNISYKKRYTNLNRWFSTILNQPQVKCVVEN

FAFSS

>XP_011556212.1 PREDICTED: alanine--tRNA synthetase, cytoplasmic [Plutella xylostella]

MDTSMTGNQIRKTFLDFFIEKKGHKYVHSSSTIPLDDPTLLFANAGMNQFKPIFLGTVDPNSDMANYVRAVNTQKCIRAG

GKHNDLDDVGKDVYHHTFFEMLGNWSFGDYFKKEICSWAWELLTEVYKLPANRLYVTYFGGDAASGLEPDLECKNIWLSL

GLSESHIIPGNMKDNFWEMGETGPCGPCSELHYDRIGGREAAHLVNMDDPDVLEIWNLVFIQFNRESDGSLKLLPKKHID

CGLGLERLVSVIQDKRANYDTDFFTPLFQAIEKGTGIRPYSGKVGADDVDGIDMAYRVLADHARTLTIALSDGGYPDNTG

RGYVLRRVLRRAVRYATEKLNGKPGLFGSLVHTVVDLLGDVFPEIRKDPEGVIQIINEEEIQFLKTLTRGRNLLNRTIEK

LNESKVVPGDVAWRLYDTYGFPIDLTQLMCEEKGLTIDMVAYEKAKKESLLASQGKTAGQEDLLALDIHALSDLQQRGIP

VTDDSLKYNYKSSTAKDAKYEFSPSTGTVVALRSNKQFVNEVSSGMECGLILDKTSFYAEQGGQIYDEGYMVKVGDDSVE

FTVKNVQVKGGYVLHTGKVEGTLKVGDTLSLHIDTERRRLIMNNHTGTHILNNVLRKVLGNDSDQRGSLVMPDRLRFDFT

NKGPMTTKQIKDAEDEIRKIIENNKTVYAKHTSLTLAKNINGLRAMFDEQYPDPVRVVCVGVPIEELEKNPEKPTGFETS

VEFCGGSHLHQTGHIGDYVIVSEEGIAKGIRRIVALTGPEATKAVNRLSVLENEVNNIANLIKEKGDANNKEIVKSIVEL

TNEISQAQISYWKKDELRTMLKNLKKQLDDKERAAKALVINEITEKAKQMCLEQKDAEIIVAELKAYGNTKALDGALKQV

KQHCPTTAAMFFTVDDETNKIYCLAAVPKNAIEKGLLASEWVQSVNAILGGKGGGKAESAQASGSNPKAIEEAIAAAKKY

AVSKLS

>XP_022790035.1 alanine--tRNA synthetase, cytoplasmic-like [Stylophora pistillata]

MVFRRCSRLNRFVSSLNSIILRNVSKRTLSTSIKMEPSLTAKDLRKMFVDFFVDKNEHTFVPSSSTIPHDDPTLLFANAG

MNQYKPIFLGTVDPNSDLSKLKRAVNSQKCIRAGGKHNDLDDVGKDVYHHTFFEMLGNWSFGDYFKKGAIEFAWELLTVK

YGMPKDRLYVTYFGGAEGLESDEDTKQFWLDMGLPPDRVLPFGMKDNFWEMGDTGPCGPCTEIHFDRIGGRDAAYLVNMD

DPTVLEVWNLVFIQFNRETDGSLKSLPNKHVDTGMGLERLTSITQGKMSNYDTDLFQPFFEAIHKGTGVRPYTGLVGAED

KDGVDMAYRVVADHVRTLTMAITDGGKPDNTGRGYVLRRILRRCVRFGTEKLNMPPGFIASLVEVAVETLGDFFPEVKKD

PQQVKDVINEEETMFLKTLARGRRLFDSTVSQLTDNFIPGDVAWRLYDTYGFPVDLTQLMAEERGLTVDMNVYEESKNKA

QEIARGKGSGSDDAITLDVHAINKLQKDLNLEPTNEKSKYIYTSDKEGNYVFEPVIGTVKAIIFEKEFVNEVISGSHCGV

VLDRTCFYAEQGGQIYDQGFMVKEGDDEVEFSVTNVQVHGGYVLHVGAVEGTLRVGDEMKCQIDEQRRRLTMNNHTATHV

LNFALRKHLGDADQRGSLVAPDKLRFDFTAKGAMTTEQIKNTELTSQDIVSRSLDVFAKESSLATAKEIQGLRAIFEEVY

PDPVRVLSIGASFEELQDDPQAGYKYSVEFCGGTHLQNTGHMKSFVLVSEEAISKGIRRIVALTGPEALRAEKKCHSLEE

RVENVQNLVDEKMKDGGLNIKETSQEITRISEDISAAVISAWRKDEMRSKLKIVKKLVDDADKAKKTILQQQAIDKAKGI

VEADRNRSFVVDVFDAGSNSKILDAAMKQFKSLAPKMAALLFSVDEENSKIICLAQVPKEAIARGLKADEWVKSVSELIG

GKCGGKDLSAQGSGPNVGCVKKAVETATNFAQQKL

>XP_018913251.1 PREDICTED: alanine--tRNA synthetase, cytoplasmic isoform X1 [Bemisia tabaci]

MASAASANHVRQRFMDFFILKKNHEYVHSSSVIPLDDPTLLFANAGMNQFKPIFLGVADPNSDVAKWKRVVNTQKCIRAG

GKHNDLDDVGKDVYHHTFFEMLGNWSFGDYFKKEVCAWAWEFLTEEFKLPKEQLYVTYFGGDPKSGLDPDLECKEIWLKL

GLPDTHVIPGSMKDNFWEMGETGPCGPCSELHFDRIGNRDVPHLVNQDDPDVLEIWNLVFIQFNREADSSLKPLPKKHID

CGMGFERLVSVIQNKRSNYDTDVFVPLFDAIQKGTGAPPYSGKVGAEDTDGIDMAYRVLADHARTLTIALADGGFPDNTG

RGYVLRRILRRAVRYATEKLNANPGFFSSLVHTVVDLLGGAFPEVNKDPQSIIDVINEEEEQFLKTLSRGRGLLNREISK

LKGSKVLPGDVAWRLYDTYGFPVDLTQLMAEEKGLSVDLPGYEEAKKHAQVLSQGKSGGIEDCISLDVHAITELQNRGIP

VTDDSPKYDYSTDPNPTSPYVFSRCSGKVLALRCNKNFVDEIHSGQKCGILSDRTNFYAEQGGQIFDEGFIIKSDDKDVE

VNVYNVQVKGGYVLHYGTVEGTLKVGDSIELSIDTKRRRKVMSNHTGTHVLNHSLRHVLGQTDQKGSLVVPERLRFDFSN

KGAMTVDQIRAAEQETQAVIKGNKKVYAKHIDLTNAKKINGLRAMFDEVYPDPVRVVCVGVPIEELESNPESDAGMKTSV

EFCGGTHVHYAGDIGDFVITNEEAIAKGVRRIIALSGAEATKAMKKAELLEKELNVLKNKVDHVGSDVKSTNKEILDLTD

DINQALISYCKKDELRKIIGCMKKKIDDADKKAKAAIVSIIVNEAKELIEEKPNEPVIVAEFNAESNTKALNSALQQVKS

LSPETSAMFFTFDDTTKRLYYLCFVPKDSIAKGLKANEWIQAVSQEINGKGGGNMETAQGSGVTSLDAVAKAVNLANSFA

NLKLGL

>XP_014616196.1 PREDICTED: alanine--tRNA synthetase, cytoplasmic isoform X2 [Polistes canadensis]

MQKIYELQGAEKGAKIKMSQLMTAKEIRQSYIDFFKSKGHEYVHSSSTIPHDDPTLLFTNAGMNQFKPIFLGTVDPNSDM

AKWVRVVNSQKCIRAGGKHNDLDDVGKDVYHHTFFEMMGNWSFGDYFKKEICSWAWEYLTVNLKLSSDRLYITYFGGDEK

SGLDPDNECKDIWLSLGVPASHVLPGSMKDNFWEMGETGPCGPCSEIHYDRIGSREAAHLVNMDDPDVLEVWNLVFIQYN

RESDGILKPLPKKHIDCGLGLERLVSVIQNKKANYDTDLFMPLFETIQKGTGVPPYQGRVGNADKDGIDMAYRVLADHAR

TITIALADGGMPDNTGRGYVLRRILRRAVRYATEKLNAKPGFFASLVNVVVELLGDVFPEVKKDPQSIIDIINEEETQFL

KTLSRGRNLLNRTISKLESSKVVPGNVAWRLYDTYGFPVDLTQLMTEEKGLKVDMVAYEEAKKQAQLISQNKVGGVDDQI

NLDVHAITELQNTGVPMTNDLPKYDYNVKGKDPYEEYSFQTCTSTVIALRHAKTFVEEVHSGEEVGILLDKTNFYAEQGG

QIYDEGFLVKVDDEETEIRITNVQVRGGYVLHIGTVGEGVLKKGDKVHLNVDTTRRRLIMSNHTATHVLNYALRQVLGLE

ADQKGSLVAPDKLRFDFTNKGAMTTDQVKKTEEITNDIVKQKKNIFAKESNLALAKTIKGLRAMFEETYPDPVRIVSVGI

PIEDLEKDPLNNAALKTSVEFCGGTHLHNTGHIGDFVISNEEAIAKGIRRIIALTGPEAKKAIKKAALLEDKLNQLKNTI

EEDTDKNSTYSKEYVRQIVELNEDISHATIHSWKKDNMRVMLKELKKSLDDKERLAKAAVANAVVESVQEIIQSNIGCAV

FVEVLKAFSNTKALDSALKKIKSLSPNTSALLISVDEDAKKIFVLTTVPKSAISKGLKANEWVQEIAPLLGGKGGGKAES

AQASGPNISSLHKIIQKAKEFANSKLGIVNEKSTIQDLPSNSTANVKNTNSVDAKKFQKLTIFGNKNSIKSYKAQIVAQY

CNKNLQIKYVEDDKNSKTDMILEIGDIQISDNSAIAYYLSDPKLKLEDDLFAFCEILQWISYTNNHLLPAVTGWILSSSQ

ITLPKNIKFDWKTSKEDASNSLKKLNNILLKKTYFVNERISLADVTIFSVLLPLYEHVLTPELRKQYTNLNRWFSTILNQ

PEVKAVVKDFHFCTKTQKAEK

>XP_014616195.1 PREDICTED: alanine--tRNA synthetase, cytoplasmic isoform X1 [Polistes canadensis]

MQKIYELQGRAEKGAKIKMSQLMTAKEIRQSYIDFFKSKGHEYVHSSSTIPHDDPTLLFTNAGMNQFKPIFLGTVDPNSD

MAKWVRVVNSQKCIRAGGKHNDLDDVGKDVYHHTFFEMMGNWSFGDYFKKEICSWAWEYLTVNLKLSSDRLYITYFGGDE

KSGLDPDNECKDIWLSLGVPASHVLPGSMKDNFWEMGETGPCGPCSEIHYDRIGSREAAHLVNMDDPDVLEVWNLVFIQY

NRESDGILKPLPKKHIDCGLGLERLVSVIQNKKANYDTDLFMPLFETIQKGTGVPPYQGRVGNADKDGIDMAYRVLADHA

RTITIALADGGMPDNTGRGYVLRRILRRAVRYATEKLNAKPGFFASLVNVVVELLGDVFPEVKKDPQSIIDIINEEETQF

LKTLSRGRNLLNRTISKLESSKVVPGNVAWRLYDTYGFPVDLTQLMTEEKGLKVDMVAYEEAKKQAQLISQNKVGGVDDQ

INLDVHAITELQNTGVPMTNDLPKYDYNVKGKDPYEEYSFQTCTSTVIALRHAKTFVEEVHSGEEVGILLDKTNFYAEQG

GQIYDEGFLVKVDDEETEIRITNVQVRGGYVLHIGTVGEGVLKKGDKVHLNVDTTRRRLIMSNHTATHVLNYALRQVLGL

EADQKGSLVAPDKLRFDFTNKGAMTTDQVKKTEEITNDIVKQKKNIFAKESNLALAKTIKGLRAMFEETYPDPVRIVSVG

IPIEDLEKDPLNNAALKTSVEFCGGTHLHNTGHIGDFVISNEEAIAKGIRRIIALTGPEAKKAIKKAALLEDKLNQLKNT

IEEDTDKNSTYSKEYVRQIVELNEDISHATIHSWKKDNMRVMLKELKKSLDDKERLAKAAVANAVVESVQEIIQSNIGCA

VFVEVLKAFSNTKALDSALKKIKSLSPNTSALLISVDEDAKKIFVLTTVPKSAISKGLKANEWVQEIAPLLGGKGGGKAE

SAQASGPNISSLHKIIQKAKEFANSKLGIVNEKSTIQDLPSNSTANVKNTNSVDAKKFQKLTIFGNKNSIKSYKAQIVAQ

YCNKNLQIKYVEDDKNSKTDMILEIGDIQISDNSAIAYYLSDPKLKLEDDLFAFCEILQWISYTNNHLLPAVTGWILSSS

QITLPKNIKFDWKTSKEDASNSLKKLNNILLKKTYFVNERISLADVTIFSVLLPLYEHVLTPELRKQYTNLNRWFSTILN

QPEVKAVVKDFHFCTKTQKAEK

>XP_014616197.1 PREDICTED: alanine--tRNA synthetase, cytoplasmic isoform X3 [Polistes canadensis]

MSQLMTAKEIRQSYIDFFKSKGHEYVHSSSTIPHDDPTLLFTNAGMNQFKPIFLGTVDPNSDMAKWVRVVNSQKCIRAGG

KHNDLDDVGKDVYHHTFFEMMGNWSFGDYFKKEICSWAWEYLTVNLKLSSDRLYITYFGGDEKSGLDPDNECKDIWLSLG

VPASHVLPGSMKDNFWEMGETGPCGPCSEIHYDRIGSREAAHLVNMDDPDVLEVWNLVFIQYNRESDGILKPLPKKHIDC

GLGLERLVSVIQNKKANYDTDLFMPLFETIQKGTGVPPYQGRVGNADKDGIDMAYRVLADHARTITIALADGGMPDNTGR

GYVLRRILRRAVRYATEKLNAKPGFFASLVNVVVELLGDVFPEVKKDPQSIIDIINEEETQFLKTLSRGRNLLNRTISKL

ESSKVVPGNVAWRLYDTYGFPVDLTQLMTEEKGLKVDMVAYEEAKKQAQLISQNKVGGVDDQINLDVHAITELQNTGVPM

TNDLPKYDYNVKGKDPYEEYSFQTCTSTVIALRHAKTFVEEVHSGEEVGILLDKTNFYAEQGGQIYDEGFLVKVDDEETE

IRITNVQVRGGYVLHIGTVGEGVLKKGDKVHLNVDTTRRRLIMSNHTATHVLNYALRQVLGLEADQKGSLVAPDKLRFDF

TNKGAMTTDQVKKTEEITNDIVKQKKNIFAKESNLALAKTIKGLRAMFEETYPDPVRIVSVGIPIEDLEKDPLNNAALKT

SVEFCGGTHLHNTGHIGDFVISNEEAIAKGIRRIIALTGPEAKKAIKKAALLEDKLNQLKNTIEEDTDKNSTYSKEYVRQ

IVELNEDISHATIHSWKKDNMRVMLKELKKSLDDKERLAKAAVANAVVESVQEIIQSNIGCAVFVEVLKAFSNTKALDSA

LKKIKSLSPNTSALLISVDEDAKKIFVLTTVPKSAISKGLKANEWVQEIAPLLGGKGGGKAESAQASGPNISSLHKIIQK

AKEFANSKLGIVNEKSTIQDLPSNSTANVKNTNSVDAKKFQKLTIFGNKNSIKSYKAQIVAQYCNKNLQIKYVEDDKNSK

TDMILEIGDIQISDNSAIAYYLSDPKLKLEDDLFAFCEILQWISYTNNHLLPAVTGWILSSSQITLPKNIKFDWKTSKED

ASNSLKKLNNILLKKTYFVNERISLADVTIFSVLLPLYEHVLTPELRKQYTNLNRWFSTILNQPEVKAVVKDFHFCTKTQ

KAEK

>XP_011061293.1 PREDICTED: alanine--tRNA synthetase, cytoplasmic isoform X2 [Acromyrmex echinatior]

MPRIIEMQLQQTANNQDNDGSSLNSASGIMSSASNTDHSNNMNAKQIRQAYIDFFKSKGHEYVHSSSTIPHDDPTLLFTN

AGMNQFKPIFLGTVDPNSDMAKWVRVVNSQKCIRAGGKHNDLDDVGKDVYHHTFFEMMGNWSFGDYFKNEICAWAWEFLT

DVLKLPADRLYVTYFGGDEKSGLAPDDECKNLWLSLGVPASHVLPGNMKDNFWEMGETGPCGPCSELHYDRIGGREAAHL

VNMDDPDVLEIWNLVFIQYNRELNGSLKSLPKKHIDCGLGLERLVSVIQNKRANYDTDVFMPLFAAIEEGTGAPPYQGKV

GADDVNGIDMAYRVLADHARTLTIALADGGVPDNTGRGYVLRRILRRAVRYATEKLNAKQGFFGSLVNVVVDLLGDVFPE

VRKDPQSIIEIINEEESQFLKTLSRGRNLLNRTIAKLESADTVPGDVAWRLYDTYGFPIDLTQLMTEEKGLKIDIAGYEE

SKKQAQLISQNKAGGVDDQINFDVHAITELQKQGVKPTDDSPKYDYKVTTEIKYKEYEFAPCTGTVIALRRAKTFVDEVS

SGEEVGILLDRTNFYAEQGGQIYDEGFLVKVDDETTEIRIKNVQIRAGYVLHIGTVGEGTLRKGDKVHTNVDTERRRFVM

ANHSATHVLNYALRKVLGTKADQKGSLVAPDRLRFDFTNKGPMTGGQLQDTEQLANNVITENEKIYAKESNLAIAKTIQG

LRAMFEETYPDPVRVVSVGIPVENLEKDPLSTAALETSVEFCGGTHLHYTSHIGNFTIVSEDAIAKGIRRIVALTGPEAA

KALRTGMLLQRQLSQFQASIEDDKSETNSKEYVREIVELTEEISHALIPAYWKDHMRNTLKNLKKSLDDKERAAKATVAT

TVVQTAQSIIESKVGCQVLVEVLEAYSNTKALDQALKKIRAISPETSALLISIDHDVKKIFALSSVPKSAVSKGLKANEW

IQAITSLMQGKGGGKSESAQASGSNIVCLNEIVSKANEFADQKLGITNKITTNNTHKISETFRLLNTGSSLKLVLYGNIG

NIKCYLAQIIAQYSGKCLIIKQIDNRIRSSDIILETANVTLHDSNAIAFFLSNSQLRRDDDLFACSQVLQWMSYVQNHIL

PMVCGWVLPSLGSVSEDTKINIKISKADLLCALRKLDDVMHTRTYLIGERITLADISLFTALLPLYEYVFDPHYRGQYSN

ITRWFSTILNQPQVKCVVKNFSFCTESM

>XP_011061291.1 PREDICTED: alanine--tRNA synthetase, cytoplasmic isoform X1 [Acromyrmex echinatior]

MPRIIEMQLQQTANNQDNDGSSLNSASGIMSSASNTDHSNNMNAKQIRQAYIDFFKSKGHEYVHSSSTIPHDDPTLLFTN

AGMNQFKPIFLGTVDPNSDMAKWVRVVNSQKCIRAGGKHNDLDDVGKDVYHHTFFEMMGNWSFGDYFKNEICAWAWEFLT

DVLKLPADRLYVTYFGGDEKSGLAPDDECKNLWLSLGVPASHVLPGNMKDNFWEMGETGPCGPCSELHYDRIGGREAAHL

VNMDDPDVLEIWNLVFIQYNRELNGSLKSLPKKHIDCGLGLERLVSVIQNKRANYDTDVFMPLFAAIEEGTGAPPYQGKV

GADDVNGIDMAYRVLADHARTLTIALADGGVPDNTGRGYVLRRILRRAVRYATEKLNAKQGFFGSLVNVVVDLLGDVFPE

VRKDPQSIIEIINEEESQFLKTLSRGRNLLNRTIAKLESADTVPGDVAWRLYDTYGFPIDLTQLMTEEKGLKIDIAGYEE

SKKQAQLISQNKAGGVDDQINFDVHAITELQKQGVKPTDDSPKYDYKVTTEIKYKEYEFAPCTGTVIALRRAKTFVDEVS

SGEEVGILLDRTNFYAEQGGQIYDEGFLVKVDDETTEIRIKNVQIRAGYVLHIGTVGEGTLRKGDKVHTNVDTERRRFVM

ANHSATHVLNYALRKVLGTKADQKGSLVAPDRLRFDFTNKGPMTGGQLQDTEQLANNVITENEKIYAKESNLAIAKTIQG

LRAMFEETYPDPVRVVSVGIPVENLEKDPLSTAALETSVEFCGGTHLHYTSHIGNFTIVSEDAIAKGIRRIVALTGPEAA

KALRTGMLLQRQLSQFQASIEDDKSETNSKEYVREIVELTEEISHALIPAYWKDHMRNTLKNLKKSLDDKERAAKATVAT

TVVQTAQSIIESKVGCQVLVEVLEAYSNTKALDQALKKIRAISPETSALLISIDHDVKKIFALSSVPKSAVSKGLKANEW

IQAITSLMQGKGGGKSESAQASGSNIVCLNEIVSKANEFADQKLGITTTA

>XP_018360120.1 PREDICTED: alanine--tRNA synthetase, cytoplasmic isoform X1 [Trachymyrmex cornetzi]

MPGFIEMQLQQTANNQDNDGLSPTSSGTMCSASNTDHSNDTNAKQIRQTYIDFFKSKGHEYVHSSSTIPHDDPTLLFTNA

GMNQFKPIFLETVDPNSDMAKWVRVVNSQKCIRAGGKHNDLDDVGKDVYHHTFFEMMGNWSFGDYFKKEICAWAWEFLTD

VLKLPADRLYVTYFGGDEKSGLAPDDECKNLWLSLGVPVSHVLSGNMKDNFWEMGETGPCGPCSELHYDRIGGREAAHLV

NMDDPDVLEIWNLVFIQYNRELNGSLKSLPKKHIDCGLGLERLVSVIQNKRANYDTDLFMPLFAAIEEGTGAPPYQGKVG

ADDVNGIDMAYRVLADHARTLTIALADGGVPDNTGRGYVLRRILRRAVRYATEKLNAKQGFFGSLVNVVVDLLGDVFPEV

RKDPQSIIEIINEEESQFLKTLSRGRNLLNRTIAKLESADTVPGDVAWRLYDTYGFPIDLTQLMTEEKGLKIDMAGYEES

KKQAQLISQNKAGGVDDQINLDVHAITELQKQGVKLTDDSPKYDYKVTTEIKYKEYEFAPCTGTVIALRRDKTFVDEVSS

SEEIGILLDRTNFYAEQGGQIYDEGFLVKVDDETTEIRIKNVQVRAGYVLHIGTVGEGTLRKGDKVHTNVDTERRRFVMA

NHSATHVLNYALRKVLGTKADQKGSLVAPDRLRFDFTNKGPMTGGQLQDTEQLANNMIMEKKKIYAKESNLAIAKTIQGL

RAMFEETYPDPVRVVSVGIPVENLEKDPLSTAALETSVEFCGGTHLHYTSHIDNFTIVSEDAIAKGIRRIVALTGPEAAK

ALRTGMLLQRQLFQFQATIEDDQSGTNSKEYVREIVELTEEILHALIPAYWKDYMRNTLKNLKKSLDDKERAAKATVATT

VVQTAQSIIESKVGCQVLVEVLEAYSNTKALDQALKKIRAISPETSALLISVDHDVKKIFALSSVPKSAVSKGLKANEWI

QAITSLMQGKGGGKSESAQASGSNIVCLNEIVSKANEFADQKLGITNKIITNNIYKTSETFRLLNMESSLKLVLYGNVGS

IKCYLAQIIAQYSAKRLTIKQVDNKIHSSDIMLETANVTLHDSNAIAFFLSNSQLRRDDDLFACSQVLQWMSYVQNHILP

MVSGWVLPSLVVSVSEDTKLNIKISKVDLLCALRKLDDIMHTRTYLIGERITLADISLFTAMLPLYEYVFNSHYRGQYSN

ITRWFSTILNQPQVKCVVKNFSFCMKAM

>XP_017097757.1 PREDICTED: alanine--tRNA synthetase, cytoplasmic [Drosophila bipectinata]

MKFLTAKEVRDAYLDFFKEQKHIYVHSSSTIPLDDPTLLFANAGMNQFKPIFLGTADPKGEMAKWVRVANTQKCIRAGGK

HNDLDDVGKDVYHHTFFEMLGNWSFGDYFKKEICSWAWEFLTERLGLPKDRLYVTYFGGDKASGLEPDLECKQLWLDLGL

KPEHILPGSMKDNFWEMGETGPCGPCSELHFDRIGGRSVPELVNQDDPDVLEIWNLVFIQFNRESDGSLKPLPKKHIDCG

MGFERLVSVIQNKRSNYDTDLFVPLFEAIQAGTGAPAYQGRVGADDVDGIDMAYRVLADHARTITIALADGGTPDNTGRG

YVLRRILRRAVRYATEKLNAKPGFFASLVNTVVDLLGDAFPEVKKDPQHIIDIINEEELQFLKTLTRGRNLLNRTIEKLG

SQTTIPGDVAWRLYDTYGFPVDLTQLMAEEKSLQIDMEGYEAAKHNSYILSQGKGASKIEEINLDVHAISQLQEQGVPPT

NDSFKYKYEAVSDERDSAYNYGECTSKIVALRFENQFVNEITSGQKAGIVLDKTNFYAESGGQIYDQGALVKVKDEANEF

LVDRVYNRGGYILHIGVVEGTLKVGDELQLHIDVERRWLTMKNHSATHALNHCLLQVLGKDTEQKGSLVVPEKLRFDFNS

KAAMTIEQVAQTENLTKEMVYNNVPIYAKESKLALAKKIRGLRSVFDEVYPDPVRVISFGVPVDELEQNPDSEAGEKTSV

EFCGGTHLRRSGHIMDFVISSEEAIAKGIRRIVALTGPEALKALKKAEAFEQEIVKLKATINADTSGKDSKSHVKEIVEL

TEQISHATIPYVKKDEMRNLLKGLKKTLDDKERALRAAVSVTVVEKAKELCEKNPDATVLVKELEAFNNTKALDAALKQV

RTQLPDAAAMFFSVDADSKKIFCLSSVPKSAVEKGLKANEWVQHVSATLGGKGGGKPESAQASGTNYEKVDEIVELATKF

AQAKLA

>XP_018360122.1 PREDICTED: alanine--tRNA synthetase, cytoplasmic isoform X2 [Trachymyrmex cornetzi]

MPGFIEMQLQQTANNQDNDGLSPTSSGTMCSASNTDHSNDTNAKQIRQTYIDFFKSKGHEYVHSSSTIPHDDPTLLFTNA

GMNQFKPIFLETVDPNSDMAKWVRVVNSQKCIRAGGKHNDLDDVGKDVYHHTFFEMMGNWSFGDYFKKEICAWAWEFLTD

VLKLPADRLYVTYFGGDEKSGLAPDDECKNLWLSLGVPVSHVLSGNMKDNFWEMGETGPCGPCSELHYDRIGGREAAHLV

NMDDPDVLEIWNLVFIQYNRELNGSLKSLPKKHIDCGLGLERLVSVIQNKRANYDTDLFMPLFAAIEEGTGAPPYQGKVG

ADDVNGIDMAYRVLADHARTLTIALADGGVPDNTGRGYVLRRILRRAVRYATEKLNAKQGFFGSLVNVVVDLLGDVFPEV

RKDPQSIIEIINEEESQFLKTLSRGRNLLNRTIAKLESADTVPGDVAWRLYDTYGFPIDLTQLMTEEKGLKIDMAGYEES

KKQAQLISQNKAGGVDDQINLDVHAITELQKQGVKLTDDSPKYDYKVTTEIKYKEYEFAPCTGTVIALRRDKTFVDEVSS

SEEIGILLDRTNFYAEQGGQIYDEGFLVKVDDETTEIRIKNVQVRAGYVLHIGTVGEGTLRKGDKVHTNVDTERRRFVMA

NHSATHVLNYALRKVLGTKADQKGSLVAPDRLRFDFTNKGPMTGGQLQDTEQLANNMIMEKKKIYAKESNLAIAKTIQGL

RAMFEETYPDPVRVVSVGIPVENLEKDPLSTAALETSVEFCGGTHLHYTSHIDNFTIVSEDAIAKGIRRIVALTGPEAAK

ALRTGMLLQRQLFQFQATIEDDQSGTNSKEYVREIVELTEEILHALIPAYWKDYMRNTLKNLKKSLDDKERAAKATVATT

VVQTAQSIIESKVGCQVLVEVLEAYSNTKALDQALKKIRAISPETSALLISVDHDVKKIFALSSVPKSAVSKGLKANEWI

QAITSLMQGKGGGKSESAQASGSNIVCLNEIVSKANEFADQKLGITKTFRLLNMESSLKLVLYGNVGSIKCYLAQIIAQY

SAKRLTIKQVDNKIHSSDIMLETANVTLHDSNAIAFFLSNSQLRRDDDLFACSQVLQWMSYVQNHILPMVSGWVLPSLVV

SVSEDTKLNIKISKVDLLCALRKLDDIMHTRTYLIGERITLADISLFTAMLPLYEYVFNSHYRGQYSNITRWFSTILNQP

QVKCVVKNFSFCMKAM

>XP_018360123.1 PREDICTED: alanine--tRNA synthetase, cytoplasmic isoform X3 [Trachymyrmex cornetzi]

MPGFIEMQLQQTANNQDNDGLSPTSSGTMCSASNTDHSNDTNAKQIRQTYIDFFKSKGHEYVHSSSTIPHDDPTLLFTNA

GMNQFKPIFLETVDPNSDMAKWVRVVNSQKCIRAGGKHNDLDDVGKDVYHHTFFEMMGNWSFGDYFKKEICAWAWEFLTD

VLKLPADRLYVTYFGGDEKSGLAPDDECKNLWLSLGVPVSHVLSGNMKDNFWEMGETGPCGPCSELHYDRIGGREAAHLV

NMDDPDVLEIWNLVFIQYNRELNGSLKSLPKKHIDCGLGLERLVSVIQNKRANYDTDLFMPLFAAIEEGTGAPPYQGKVG

ADDVNGIDMAYRVLADHARTLTIALADGGVPDNTGRGYVLRRILRRAVRYATEKLNAKQGFFGSLVNVVVDLLGDVFPEV

RKDPQSIIEIINEEESQFLKTLSRGRNLLNRTIAKLESADTVPGDVAWRLYDTYGFPIDLTQLMTEEKGLKIDMAGYEES

KKQAQLISQNKAGGVDDQINLDVHAITELQKQGVKLTDDSPKYDYKVTTEIKYKEYEFAPCTGTVIALRRDKTFVDEVSS

SEEIGILLDRTNFYAEQGGQIYDEGFLVKVDDETTEIRIKNVQVRAGYVLHIGTVGEGTLRKGDKVHTNVDTERRRFVMA

NHSATHVLNYALRKVLGTKADQKGSLVAPDRLRFDFTNKGPMTGGQLQDTEQLANNMIMEKKKIYAKESNLAIAKTIQGL

RAMFEETYPDPVRVVSVGIPVENLEKDPLSTAALETSVEFCGGTHLHYTSHIDNFTIVSEDAIAKGIRRIVALTGPEAAK

ALRTGMLLQRQLFQFQATIEDDQSGTNSKEYVREIVELTEEILHALIPAYWKDYMRNTLKNLKKSLDDKERAAKATVATT

VVQTAQSIIESKVGCQVLVEVLEAYSNTKALDQALKKIRAISPETSALLISVDHDVKKIFALSSVPKSAVSKGLKANEWI

QAITSLMQGKGGGKSESAQASGSNIVCLNEIVSKANEFADQKLGITTTA

>XP_012055195.1 PREDICTED: alanine--tRNA synthetase, cytoplasmic [Atta cephalotes]

MPRIIKMQLQQTANNQDNDNGSSPNSSGTMSSAPNTDHSNNMNAKQIRQAYIDFFKSKGHEYVHSSSTIPHDDPTLLFTN

AGMNQFKPIFLGTVDPNSDMAKWIRVVNSQKCIRAGGKHNDLDDVGKDVYHHTFFEMMGNWSFGDYFKNEICAWAWEFLT

DMLKLPADRLYVTYFGGDEKSGLAPDDECKNLWLSLGVPASHVLPGNMKDNFWEMGETGPCGPCSELHYDRIGGREAAHL

VNMDDPDVLEIWNLVFIQYNRELNGSLKSLPKKHIDCGLGLERLVSVIQNKRANYDTDLFMPLFAAIEEGTDAPPYQGKV

GADDVNGIDMAYRVLADHARTLTIALADGGVPDNTGRGYVLRRILRRAVRYATEKLNAKQGFFGSLVNVVVDLLGDVFSE

VRKDPQSIIEIINEEESQFLKTLSRGRNLLNRTIAKLESADTVPGDVAWRLYDTYGFPIDLTQLMTEEKGLKIDMAGYEE

SKKQAQLISQNKAGGVDDQINLDVHAITELQKQGIKPTDDSPKYDYKVTAEIKYKEYEFAPCTGTVIALRRAKTFVDEVS

SGEEVGILLDRTNFYAEQGGQIYDEGFLVKVNDETTEIRIKNVQIRAGYVLHIGTVGEGTLRKRDKVHTNVDTERRRFVM

ANHSATHVLNYALRKVLGTKADQKGSLVAPDRLRFDFTNKGPMTGGQLQDTEQLANNMITENEKIYAKESNLAIAKTIQG

LRAMFEETYPDPVRVVSVGIPVENLEKDPLSTAALETSVEFCGGTHLHYTSHIGNFTIISEDAIAKGIRRIVALTGPEAA

KALRTGMLLQRQLSQFQATIEDDKSEMNSKEYVREIVELTEEISHALIPAYWKDHMRNTLKNLKKSLDDKERAAKATVAT

TVIQTAQSIIESKVGCQVLVEVLEAYSNTKALDQALKKIRAISPETSALLISIDHDVKKIFALSSVPKSAVSKGLKANEW

IQAITSLMQGKGGGKSESAQASGSNIVCLNEIVSKANEFADQKLGITNKITTNNTHKISETFQLLNTESSLKLVLYGNIG

NIKCYLAQIIAEYSGKCLTIKQIDNRIRSSDIMLETANITLHDSNAIAFFLSNSQLRRDDDLFACSQVLQWMSYVQNHIL

PMVYGWVLPSLVSVSEDAKINIKISKADLLCALRKLDDIMHTRTYLIGERITLADISLFTALLPLYEYVFDPHYRGHYSN

ITRWFSTILNQPQVKCVVKNFSFCTEAI

>XP_001961945.1 uncharacterized protein Dana_GF14684, isoform A [Drosophila ananassae]

MKFLTAKEVRDAYLDFFKEQKHIYVHSSSTIPLDDPTLLFANAGMNQFKPIFLGTADPNSEMAKWVRVANTQKCIRAGGK

HNDLDDVGKDVYHHTFFEMLGNWSFGDYFKKEICSWAWQFLTERLGLPKDRLYVTYFGGDKASGLEPDLECKQLWLDLGL

KPEHILPGSMKDNFWEMGETGPCGPCSELHFDRIGGRSVPELVNMDDPDVLEIWNLVFIQFNRESDGSLKPLPKKHIDCG

MGFERLVSVIQNKRSNYDTDLFVPLFEAIQAGTGAPAYQGRVGTDDVDGIDMAYRVLADHARTITIALADGGTPDNTGRG

YVLRRILRRAVRYATEKLNAKPGFFASLVNTVVDLLGDAFPEVKKDPQHIIDIINEEELQFLKTLTRGRNLLNRTIEKLG

SQATIPGDVAWRLYDTYGFPVDLTQLMAEEKSLQIDMEGYEAAKHNSYILSQGKGASKIEEINLDVHAISQLQEQGVAPT

NDSFKYKYEAVSDERDSAYNYGECTSKIVALRFENQFVNEITSGQKAGIVLDKTNFYAESGGQIYDQGALVKVNDEANEF

LVDRVYNRGGYILHIGVVEGTLKVGDELQLHIDVERRWLTMKNHSATHALNHCLLQVLGKDTEQKGSLVVPEKLRFDFNS

KGAMTIEQVAKTENLTKEMVYNNVPIYAKESKLALAKKIRGLRSVFDEVYPDPVRVISFGVPVDELEQNPDSEAGEKTSV

EFCGGTHLRRSGHIMDFVISSEEAIAKGIRRIVALTGPEALKALKKSEAFEQEIVKLKATIDADTSGKDSKSHVKEIVEL

TEQISHATIPYVKKDEMRNLLKGLKKTLDDKERALRAAVSTTVVEKAKELCEKNPDAIVLVEQLEAFNNTKALDAALKQV

RSQLPNAAAMFFSVDADSKKIFCLSSVPKSAVEKGLKANEWVQHVSATLGGKGGGKPESAQASGTNYEKVDEIVELATKF

AQTKLA

>XP_018345558.1 PREDICTED: alanine--tRNA synthetase, cytoplasmic isoform X3 [Trachymyrmex septentrionalis]

MQLQQTANNQDNDDGSSPNSSGTMSNASNTNHSNNMNAKQIRQAYIDFFKSKGHEYVHSNSTIPHDDPTLLFTNAGMNQF

KPIFLGTVDPNSDMAKWVRVVNSQKCIRAGGKHNDLDDVGKDVYHHTFFEMMGNWSFGDYFKNEICAWAWEFLTDVLKLP

ADRLYVTYFGGDEKSGLAPDDECKNFWLSLGVPTSHVLPGNMKDNFWEMGETGPCGPCSELHYDRIGGREAAHLVNMDDP

DVLEIWNLVFIQYNRELNGSLKSLPKKHIDCGLGLERLVSVIQNKRANYDTDLFMPLFAAIEEGTGAPPYQGKVGADDIN

GIDMAYRVLADHARTLTIALADGGVPDNTGRGYVLRRILRRAVRYATEKLNAKQGFFGSLVNVVVDLLGDVFPEVRKDPQ

SIIEIINEEESQFLKTLSRGRNLLNRTIAKLESADTVPGDVAWRLYDTYGFPIDLTQLMTEEKGLKIDMAGYEEAKKQAQ

LISQNKAGGVDDQINLDVHAITELQKQGVKPTDDSPKYDYKVTTEIKYKEYEFVPCTGTVIALRRAKTFVDEVSSGEEVG

ILLDRTNFYAEQGGQIYDEGFLVKVDDETTEIRIKNVQVRAGYVLHIGTVGEGTLRKGDKVHTNVDTERRRFVMANHSAT

HVLNCALRKVLGTKADQKGSLVAPDRLRFDFTNKGPMTGGQLQDTEQLANNMIIENEKIYAKESNLAIAKTIQGLRAMFE

ETYPDPVRVVSVGIPVENLEKDPLSTAALETSVEFCGGTHLHYTSHIGNFTIISEDAIAKGIRRIVALTGPEAAKALRTG

MLLQRQLSQFQATIEDDQSGTNSKEYVREIVELTEEISHALIPAYWKDCMRNTLKNLKKSLDDKERAAKATVATTVVQTA

QSIIESKVGCQVLVEVLEAYSNTKALDQALKKIRAISPETSALLISVDHDVKKIFALSSVPKSAVSKGLKANEWIQAITS

LMQGKGGGKSESAQASGSNIVRLNEIVSKANEFADQKLGITTTA

>XP_018345556.1 PREDICTED: alanine--tRNA synthetase, cytoplasmic isoform X2 [Trachymyrmex septentrionalis]

MQLQQTANNQDNDDGSSPNSSGTMSNASNTNHSNNMNAKQIRQAYIDFFKSKGHEYVHSNSTIPHDDPTLLFTNAGMNQF

KPIFLGTVDPNSDMAKWVRVVNSQKCIRAGGKHNDLDDVGKDVYHHTFFEMMGNWSFGDYFKNEICAWAWEFLTDVLKLP

ADRLYVTYFGGDEKSGLAPDDECKNFWLSLGVPTSHVLPGNMKDNFWEMGETGPCGPCSELHYDRIGGREAAHLVNMDDP

DVLEIWNLVFIQYNRELNGSLKSLPKKHIDCGLGLERLVSVIQNKRANYDTDLFMPLFAAIEEGTGAPPYQGKVGADDIN

GIDMAYRVLADHARTLTIALADGGVPDNTGRGYVLRRILRRAVRYATEKLNAKQGFFGSLVNVVVDLLGDVFPEVRKDPQ

SIIEIINEEESQFLKTLSRGRNLLNRTIAKLESADTVPGDVAWRLYDTYGFPIDLTQLMTEEKGLKIDMAGYEEAKKQAQ

LISQNKAGGVDDQINLDVHAITELQKQGVKPTDDSPKYDYKVTTEIKYKEYEFVPCTGTVIALRRAKTFVDEVSSGEEVG

ILLDRTNFYAEQGGQIYDEGFLVKVDDETTEIRIKNVQVRAGYVLHIGTVGEGTLRKGDKVHTNVDTERRRFVMANHSAT

HVLNCALRKVLGTKADQKGSLVAPDRLRFDFTNKGPMTGGQLQDTEQLANNMIIENEKIYAKESNLAIAKTIQGLRAMFE

ETYPDPVRVVSVGIPVENLEKDPLSTAALETSVEFCGGTHLHYTSHIGNFTIISEDAIAKGIRRIVALTGPEAAKALRTG

MLLQRQLSQFQATIEDDQSGTNSKEYVREIVELTEEISHALIPAYWKDCMRNTLKNLKKSLDDKERAAKATVATTVVQTA

QSIIESKVGCQVLVEVLEAYSNTKALDQALKKIRAISPETSALLISVDHDVKKIFALSSVPKSAVSKGLKANEWIQAITS

LMQGKGGGKSESAQASGSNIVRLNEIVSKANEFADQKLGITKTFRLLNTGSSLKLVLYGNIGNIKCYLAQIIAQYSGKCL

TIKQVDNRILSSDIMLETANVTLHDSNAIAFFLSNSQLRRDDDLFACSQVLQWMSYVQNHILPMVYGWVLPSLVSVSKDT

KINIKISKADLLCALRKLDDVMHTRTYLIGERITLADISFFTALLPLYEYVFDPHYREQYSNITRWFSTILNQPQVKCVI

KNFSFCTEAM

>XP_018345555.1 PREDICTED: alanine--tRNA synthetase, cytoplasmic isoform X1 [Trachymyrmex septentrionalis]

MQLQQTANNQDNDDGSSPNSSGTMSNASNTNHSNNMNAKQIRQAYIDFFKSKGHEYVHSNSTIPHDDPTLLFTNAGMNQF

KPIFLGTVDPNSDMAKWVRVVNSQKCIRAGGKHNDLDDVGKDVYHHTFFEMMGNWSFGDYFKNEICAWAWEFLTDVLKLP

ADRLYVTYFGGDEKSGLAPDDECKNFWLSLGVPTSHVLPGNMKDNFWEMGETGPCGPCSELHYDRIGGREAAHLVNMDDP

DVLEIWNLVFIQYNRELNGSLKSLPKKHIDCGLGLERLVSVIQNKRANYDTDLFMPLFAAIEEGTGAPPYQGKVGADDIN

GIDMAYRVLADHARTLTIALADGGVPDNTGRGYVLRRILRRAVRYATEKLNAKQGFFGSLVNVVVDLLGDVFPEVRKDPQ

SIIEIINEEESQFLKTLSRGRNLLNRTIAKLESADTVPGDVAWRLYDTYGFPIDLTQLMTEEKGLKIDMAGYEEAKKQAQ

LISQNKAGGVDDQINLDVHAITELQKQGVKPTDDSPKYDYKVTTEIKYKEYEFVPCTGTVIALRRAKTFVDEVSSGEEVG

ILLDRTNFYAEQGGQIYDEGFLVKVDDETTEIRIKNVQVRAGYVLHIGTVGEGTLRKGDKVHTNVDTERRRFVMANHSAT

HVLNCALRKVLGTKADQKGSLVAPDRLRFDFTNKGPMTGGQLQDTEQLANNMIIENEKIYAKESNLAIAKTIQGLRAMFE

ETYPDPVRVVSVGIPVENLEKDPLSTAALETSVEFCGGTHLHYTSHIGNFTIISEDAIAKGIRRIVALTGPEAAKALRTG

MLLQRQLSQFQATIEDDQSGTNSKEYVREIVELTEEISHALIPAYWKDCMRNTLKNLKKSLDDKERAAKATVATTVVQTA

QSIIESKVGCQVLVEVLEAYSNTKALDQALKKIRAISPETSALLISVDHDVKKIFALSSVPKSAVSKGLKANEWIQAITS

LMQGKGGGKSESAQASGSNIVRLNEIVSKANEFADQKLGITNKIITNNTHKISETFRLLNTGSSLKLVLYGNIGNIKCYL

AQIIAQYSGKCLTIKQVDNRILSSDIMLETANVTLHDSNAIAFFLSNSQLRRDDDLFACSQVLQWMSYVQNHILPMVYGW

VLPSLVSVSKDTKINIKISKADLLCALRKLDDVMHTRTYLIGERITLADISFFTALLPLYEYVFDPHYREQYSNITRWFS

TILNQPQVKCVIKNFSFCTEAM

>KOX71748.1 Alanine--tRNA synthetase, cytoplasmic [Melipona quadrifasciata]

MSAEQIRQAYIDFFKSKNHEYVHSSSTIPHDDPTLLFTNAGMNQFKPIFLGIVDPNSDLGKLVRAVNSQKCIRAGGKHND

LDDVGKDVYHHTFFEMMGNWSFGDYFKKEICTWAWEFLTVKLKLPADRLYVTYFGGEEKNNLKPDEECKEIWLSLGVAPN

HVLPGNMKDNFWEMGETGPCGPCSEIHYDRIGNREAAHLVNQDDPDVLEIWNLVFIQFNRETDGSLKLLPKKHVDCGLGL

ERLVSVIQNKKANYDTDLFVPLFDAIQKGTGAPPYQGRIGMEDKDGIDMAYRVLADHARTITIALADGGVPDNTGRGRIL

RRAVRYATEKLNAKPGFFGSLVNVVVDLLGNTFPEVTKDPQYIIDIINEEETQFLKTLSRGRNLLNRTITKLESSNILPG

DVAWRLYDTYGFPVDLTQLMAEEKGLKVDIIGYEEAKKQAQLISQSKSGGVDDQINLDVHAITDLQNKGIKPTNDLPKYN

YNVINNNLYEEYEFVPCSGTVITLRSAKTFVDEISSGEEVGILLDQTNFYAEQGGQIYDEGFLVKINDEDTEVRIKNVQV

RGGYVLHIGTVGQGKLRKGDKVSLNIDITRRRFIMSNHTATHVLNYALRKVLGAEVDQKGSLVAPDRLRFDFTNKEYNIH

FKTFLELIYSKYLFIGAMTAEQIRKVEEIVNDMIKENRKVYTKESNLALAKTIQGLRAMFEETYPDPVRIVSLGIQVEDL

EKDPLGPGALQTSVEFCGGTHLHYTEHIGDFVITSEEAIAKGIRRIVALTGPEATKAQKKASVLESHLDRLQAIIVADTS

GVNIKEHIKKIIELTDDISHAVISSWKKDKMRKMLKDLKKTLDDKERAAKTAIANAVVDTIQQIIQQDIGRLVLVEVLQA

YSNTKALDSALKKIKAISPETSALLISVDLDAKKIFALCAVSKPAVNKGLKANEWIQEIVPLMEGKGGGKTESAQASGTN

ISCVTKLIYTAKNFANSKLGIAEDSVNENHEQLKNNEKSICSENSKNKLVLSSDIGSVKYYRTQIVAKYSDIELITLQYK

NDDTSKSIKLEGNGFELFDSNAIAFYLSNNQLKCSSNPFAFSEVLQWLSYADNHILPAVLGWIVPCFSKNVSNDIKTNIK

TSKEDVLSSLKKLNNILLTKTYLVGERVSLADIAVFTALMPLYEYVLDSMSRKQYTNLNRWFFTILNQSEVASAIKKFKV

CEKAVN

>XP_395392.3 PREDICTED: alanine--tRNA synthetase, cytoplasmic isoform X1 [Apis mellifera]

MTIMMTAKQIRQAYIDFFKSKGHEYVHSSSTIPHDDPTLLFTNAGMNQFKPIFLGIVDPNSDMAKLVRAVNSQKCIRAGG

KHNDLDDVGKDVYHHTFFEMMGNWSFGDYFKKEICTWAWEFLTVKLKLSSDRLYVTYFGGDEKNNLKPDEECKQIWLSLG

LSPTHVLPGNMKDNFWEMGETGPCGPSSEIHYDRIGNREAAHLVNQDDPDVLEIWNLVFIQFNRENDGNLKLLPKKHIDC

GLGLERLVSIIQNKSANYDTDLFMPLFDAIQKGTKAPSYQGKVGKEDINGIDMAYRVLADHARTITIALADGGVPDNTGR

GYVLRRILRRAVRYAIEKLNAKPGFFGSLVNVVVDLLGDVFPEVTKDPQHIIDIINEEETQFLKTLSRGRNLLNRTIVKL

ESSNILPGDVAWRLYDTYGFPFDLTQLMAEEKGLKVDVIGYEEAKKEAQLISQNKSGEVDDQINLDIHAITELQNEKIKP

TNDIPKYNYKVISNKIYEEYEFAPCFSTIIALRRAKTFVDEVSSGEEVGILLDQTNFYAEQGGQIYDEGFLTKIDDEDSE

VHIKNVQVRGGYILHIGIVSQGKLKKNDKVFSNIDTTRRRLLMSNHTATHVLNYALREILGTEVDQKGSLVAPDRLRFDF

TNKGAMTTEQIRKVEEITNNIINENKMVYAKECNLGLAKTIQGLRAMFEETYPDPVRVVSIGIPVEDLEKNPLSSDALKT

SVEFCGGTHLHYTGHIKNFIIVSEEAIAKGIRRIVALTGPEAIKAQKKTSMLQNYLDELQITIEADKNGINTKEHIKKII

ELTDDVSHAIISSWKKDEIRKMLKDLKKILDDKERAIKTAITNVVLDNIQQIIQQNIGCLVLVEILQAYSNTKALDSALK

KIRTISPETSALLISIDPDAKKIFALSSVSKFAINKGLKANEWIQEIVPLMEGKGGGKSESAQASGTNISCVTKLIYTAK

NFANSKLELAENINENHDQLSNEKFIYFKTLKDKLVLSGDIGSIKYYCIQIVAKYNNIELITVPHKNNIFKGIKLQGNDF

ELFDSNAIAFYLSTDQFKCSNNPFTFSEVLQWLSYADNHILPAVLGWVVPYLSKNIPNNIKTNIKTSKEDILSSLQKLNN

ILLTKTYLVGERISLADISVFTTLMPLYEYVLDSEYRKQYTNLNRWFFTILNQSQVISVIKKFNICEKVKIVKY

>XP_003697700.1 PREDICTED: alanine--tRNA synthetase, cytoplasmic isoform X1 [Apis florea]

MTIMMTAKQIRQAYIDFFKNKGHEYVHSSSTIPHDDPTLLFTNAGMNQFKPIFLGIVDPNSDMAKLVRAVNSQKCIRAGG

KHNDLDDVGKDVYHHTFFEMMGNWSFGDYFKKEICTWAWEFLTVKLKLSSDRLYVTYFGGDEKNNLKPDEECKQIWLSLG

LSPTHILPGNMKDNFWEMGETGPCGPSSEIHYDRIGNREAAHLVNQDDPDVLEIWNLVFIQFNRENNGNLKLLPKKHIDC

GLGLERLVSIIQNKSANYDTDLFMPLFDAIQKGTRAPSYQGKVGKEDKDGIDMAYRVLADHARTITIALADGGIPDNTGR

GYVLRRILRRAVRYAIEKLNAKPGFFGSLVNVVVDLLGDVFPEVTKDPQHIIDIINEEETQFLKTLSRGRNLLNRTIVKL

ESSNVLPGDVAWRLYDTYGFPFDLTQLMVEEKGLKVDIIGYEEAKKQAQLISQNKSGEVDDQINLDVHAITELQNKKIKP

TNDIPKYNYKVINNKICEEYEFAPCFSTVIALRRAKTFVNEVSSGEEVGILLDQTNFYAEQGGQMYDEGFLTKIDDEDTE

VHIKNVQVRGGYVLHIGIVSQGKLKKNDKVFSNIDTTRRRLLMSNHTATHALNYALREILGTEVDQKGSLVAPDRLRFDF

TNKGAMTTEQIRKVEEITNNIINENKMVYAKECNLGLAKTIQGLRAMFEETYPDPVRVVSIGIPVEDLEKNPLSSGALKT

SVEFCGGTHLHYTGHIRNFVIVSEEAIAKGIRRIVALTGPEAVKAQKKTSMLQNYLDELQITIEADKDSINTKEHVKKIV

ELTDDVSHAIISSWKKDKIRKMLKDLKKILDDKERAIKTAIANIVLDNIQQIIQQNIGCLVLVEVLQAYSNTKALDSALK

KIRTISPETSALLISIDPDAKKIFALSSVSKSAISKGLKANEWIQEIVPFMEGKGGGKSESAQASGTNISCITKLIYTAK

NFANSKLGLAENINEDHDQLKGDEKFIYFKTLKDKLILSGDSGSIKYYRIQIVAKYNNIELITMPHKNNIFKGIKLQGND

FELFDSNAIAFYLSTDQFKCSNNSFTFSEVLQWLSYADNHILPAVLGWVVPYLSKNIPNNIKTNIKTSKEDVLSSLQKLN

NILLTKTYLVGERISLADITVFTTLMPLYEYVLDSEYRKQFTNLNRWFFTILNQSQVTSVIKKFNICEKVKY

>XP_023323002.1 alanine--tRNA synthetase, cytoplasmic-like isoform X2 [Eurytemora affinis]

MDTSLDAKAIRKIFLDFFIEKKTHIYVHSSSTIPHDDPTLLFANAGMNQFKPVFVGTADPNSDMAKWTRVVNTQKCIRAG

GKHNDLDDVGKDVYHHTFFEMLGNWSFGDYFKKEICTWSWELLTEVFKLDPARLYVTYFGGDEASGLQPDLEAKQLWLDL

GVGESRILPGNMKDNFWEMGDTGPCGPCSEIHYDRIGGGRDAAHLVNMDDPDVLEIWNLVFMQYNREQDKSLKQLPNKHI

DCGMGFERLVSVVQDKRSNYDTDLFLPFFQHIQAGTGVRAYAGRVGAEDTDGIDMAYRVLADHARTLTIALSDGGRPDNV

GRGYVLRRILRRGVRYASEKLGAKPGFFASLVHTVVQVLGETFPEVKKDPQTVIDIINEEETQFLKTLSRGQKLLDRTIS

KLEGAKELPGDIAWRLYDTYGFPVDLTQLMCEERGLQVDMNSYESCKAAAQLASQGKGAGAEDTISLDVHSINELKEKSF

QPTDDSFKYAYEAESSSKDSRYKFHACTAKILGIRHSKQFVDSVSSGQECGVLLDRTCFYAEQGGQIYDEGFLVNGENEM

KVNNVQVRGGYVLHMGTVEGTLKLGDTVELSVDEERRKNVMNNHTGTHILNFALRQVLSSDADQKGSLVAPDKLRFDFTS

KAAMSPAQVKNVEEIVNNVINKNQEIYAKEASLAVAKTIQGLRAVFDETYPDPVRVVSVGVPVVDLEKDPNNPAGTQTSV

EFCGGTHLRRAGHIEHMVIASEEAIAKGIRRIVALTGPEAKKALAKEKLLLGELEKVKTKVESKDMGMKEKVRLITELND

DISAATISYHKKDEMRGELKNVKKRIDDEDRNRKAAVMGDVVEAAKAILTGNPGLPYLVCELQAYANNKAIDGALKQVKL

LSSATPTIFFSGDEDTGKILCMAYSSKEAITSGLKANEWCGSVQTLINGKGGGRPDNAQASGTNVSALNAAMEAAHSFAM

LKLGVERVNLAADATGPAGEKSISASVSAPAGSAVLLAPPGCCGGVLVQVASKYAGQTIAVQSGQAFTFKSGAGSFTSPV

STALYLCSNNKTLIGADLAQQSMVLQYCLYAENDLKHHVSGWVNPTQGVVEGCNPGTVARSKEAVLHHLQVLDNILVAWT

YLLQI

>XP_018398048.1 PREDICTED: alanine--tRNA synthetase, cytoplasmic isoform X3 [Cyphomyrmex costatus]

MSDIIEMPLQQTANNQNNDDGLSVSDTISCALNTNNSNNMNAKQIRQAYIDFFKSKGHEYVHSSSTIPHDDPTLLFTNAG

MNQFKPIFLGTVDPNSDMAKWVRVVNSQKCIRAGGKHNDLDDVGKDVYHHTFFEMMGNWSFGDYFKKEICTWAWEFLTDV

LKLSADRLYVTYFGGDKKNGLAPDDECKNLWLSLGVPASHVLPGNMKDNFWEMGETGPCGPCSELHYDRIGDREAAHLVN

MDDPDVLEIWNLVFIQYNRESDGSLKSLPKKHIDCGLGLERLVSVIQNKRANYDTDLFMPLFTAIEKGTGAPSYQGKVGA

DDVNGIDMAYRVLADHARTLTIALADGGVPDNTGRGYVLRRILRRAVRYAIEKLNAKPGFFGTLVNVVVDLLGDVFPEVK

KDPQSIIETINEEESQFLKTLSRGRNLLNRTIAKLESADTVPGDVAWRLYDTYGFPIDLTQLMTEEKGLKIDMAGYEESK

KQAQLISQGKAGGVDDQINLDVHAITELQNQGVKPTDDSPKYNYKVTNKTKYKEYEFAPCTGTVIALRRAKTFVNEVSSG

EVVGILLDRTNFYAEQGGQIYDEGFLVKVNDETTEIRIKNVQVRAGYVLHIGIVGEGKLKKGDKVRTNVDTERRRLVMAN

HSATHVLNYALRKVLGTEADQKGSLVTPDRLRFDFTNKGPMTGQQLKETELSVIAIIRKNEKIYAKESNLATAKTIQGLR

AMFEETYPDPVRVVSVGIPVENLEKDPLSTAALETSVEFCGGTHLHYTSHIGDFVIASEDAIAKGIRRIVALTGPEARKA

IRTAGRFGSDITMSECQYQMNDMNSKEYVRKIVELTEEISQALIPAYRKDDLRNHLKEMKKSLDDKERAAKAAVATTVVQ

TAQSIIESKVGCQVLVEVLEAYSNTKALDSALKKIRAISPETSALLISVDHDVKKIFALSSVPKSAISKGLKANEWIQAI

TSLMQGKGGGKSESAQASGLNVACLNEIVSKANEFADQKLGIINKITTNNTCKTSETFRLLNTGSSLKLVLYGNIGSIKC

YLAQIISQYSGKHLTIKQVDNKIHFSDIMLETTNVTLHDSNAIAFFLSNSQLRRDDDLFACSQVLQWMSYVQNHILPMVS

GWVLSSLKVYVSGNTRMNVKTSKVDLFYALRRLDNILHTKTYLIDERITLADISVFTALLPLYEHVFNPRYREQYVNVTK

WFSTILNQPQVKCVVKNFTFCMEAT

>XP_002057564.1 uncharacterized protein Dvir_GJ18196 [Drosophila virilis]

MKFLTAKEVRDTYLNFFKEKQHIYVHSSSTIPLDDPTLLFANAGMNQFKPIFLGTADPNSEMAKWVRVANTQKCIRAGGK

HNDLDDVGKDVYHHTFFEMLGNWSFGDYFKKEICAWAWEFLTVRLNLPKDRLYVTYFGGDVASGLEPDLECKQMWLDLGL

KPEHILPGSMKDNFWEMGETGPCGPCSELHFDRIGGRSVPELVNQDDPDVLEIWNLVFIQYNRESDGSLKPLPKKHIDCG

MGFERLVSVIQNKRSNYDTDLFVPLFDAIQAGTGAPAYQGRVGADDADGIDMAYRVLADHARTITIALADGGNPDNTGRG

YVLRRILRRAVRYATEKLNAKPGFFATLVNTVVELLGDAFPEVKKDPQQIMDIINEEELQFLKTLTRGRNLLNRTIEKLG

ANQTTIPGDVAWRLYDTYGFPVDLTQLMAEEKSLQIDMAGYDAAKQNSYILSQGKGASKIEEINLDVHAISQLQEQGVPP

TNDSFKYKYEAVSNERDSAYNYSECTSKIVALRYENKFVQEISSGQKAGIVLDKTNFYAESGGQIYDQGALVKLNDEANE

FLVDRVYNRGGYILHIGVVEGTLAVGDEVQLHIDAERRWLTMKNHSATHALNHCLLQVLGKDTEQKGSLVVPEKLRFDFN

SKAAMTIEQVAKTEQLTRDMVYKNMPIYAKESQLALAKRIRGLRSVFDEVYPDPVRVISFGVPVDQLEAQPDAEAGEQTS

VEFCGGTHLRNSGHIMDFVISSEEAIAKGIRRIVALTGPEALKALKKSEAFQQEISKLQATIEADKTGKDSKLYVKQIVE

LTEQISHATIPYVKKDEMRNLLKSLKKTLDDKERALRAAVSVTVVERAKALCEANPQANVLVQQLEAYNNTKALDAALKQ

VRSQLPDAAAMFVSVDADSKKIFCLCSVPKSAVEKGLKANEWVQHVSPVLGGKGGGKPESAQASGSNFERVDEIVQLARK

FAQAKLN

>XP_019547812.1 PREDICTED: alanine--tRNA synthetase, cytoplasmic [Aedes albopictus]

MDTSLTAKQIRNAFLDFFKEKEHLYVHSSSTIPLDDPTLLFANAGMNQFKPIFLGTADPNSEMAKWVRTANTQKCIRAGG

KHNDLDDVGKDVYHHTFFEMLGNWSFGDYFKKEICTWAWELLTDRLKLPKERLYVTYFGGHPESGLEPDLECREIWLNLG

VKEEHILPGSMKDNFWEMGETGPCGPCSELHFDRIGGRSVPELVNMDDPDVLEIWNLVFIQFNREQDGSLKLLPKKHIDC

GMGFERLVSVIQDKRSNYDTDVFVPLFEAIQKGTGAPSYQGRVGADDVDGVDMAYRVLADHARTITIALADGGFPDNTGR

GYVLRRILRRAVRYATEKLNAKPGFFATLVDTVVFLLGDTFPEVKKDPQSIKNIINEEEQQFLKTLTRGRNLLNRTIAKL

GDSKVIPGDVAWRLYDTYGFPIDLTQLMAEEKNMTIDMEGYEKAKHESYILSQGKEKSKAATVDLDVHAISELQEKKVPT

TDDSFKYRYKADVDPMAEYKFEPCSGNVLALRSNNTFVEEVQAGQECAVILDKTNFYAESGGQIYDQGYLVKVNDESTEF

NVNLVYNRGGYIIHIGVVEGILRVGDEVACHMDILRRQLTMKNHSATHALNHSLLKVLGQDTDQRGSLVVPEKLRFDFTN

KSAMTVEQVAESERLTREVVKKNMQIYAKESKLSVAKSIKGLRSVFDEVYPDPVRVISFGVPVENLEANPEGEEGVETSV

EFCGGTHLHQSGHMWDFVITTEEAIAKGIRRIVALTGPEALKALKKTELLENELNALKSTIEADKTGKDSKEHVKKIVEL

NEDVSQAVIPYVKKDEIRNVLKSLKKTLDDKERAARAAVSTTVVEKAKELSEANKEALFLVHRMEAYNNTKALDSALKEV

RKINPEQSALFVSSDPDSKKIFCLASVPKSAVEKGLKANEWISHISPSMGGKGGGKPESAQASGGNFDKVEEILELARQF

AASKLS

>XP_023323001.1 alanine--tRNA synthetase, cytoplasmic-like isoform X1 [Eurytemora affinis]

MDTSLDAKAIRKIFLDFFIEKKTHIYVHSSSTIPHDDPTLLFANAGMNQFKPVFVGTADPNSDMAKWTRVVNTQKCIRAG

GKHNDLDDVGKDVYHHTFFEMLGNWSFGDYFKKEICTWSWELLTEVFKLDPARLYVTYFGGDEASGLQPDLEAKQLWLDL

GVGESRILPGNMKDNFWEMGDTGPCGPCSEIHYDRIGGGRDAAHLVNMDDPDVLEIWNLVFMQYNREQDKSLKQLPNKHI

DCGMGFERLVSVVQDKRSNYDTDLFLPFFQHIQAGTGVRAYAGRVGAEDTDGIDMAYRVLADHARTLTIALSDGGRPDNV

GRGYVLRRILRRGVRYASEKLGAKPGFFASLVHTVVQVLGETFPEVKKDPQTVIDIINEEETQFLKTLSRGQKLLDRTIS

KLEGAKELPGDIAWRLYDTYGFPVDLTQLMCEERGLQVDMNSYESCKAAAQLASQGKGAGAEDTISLDVHSINELKEKSF

QPTDDSFKYAYEAESSSKDSRYKFHACTAKILGIRHSKQFVDSVSSGQECGVLLDRTCFYAEQGGQIYDEGFLVNGENEM

KVNNVQVRGGYVLHMGTVEGTLKLGDTVELSVDEERRKNVMNNHTGTHILNFALRQVLSSDADQKGSLVAPDKLRFDFTS

KAAMSPAQVKNVEEIVNNVINKNQEIYAKEASLAVAKTIQGLRAVFDETYPDPVRVVSVGVPVVDLEKDPNNPAGTQTSV

EFCGGTHLRRAGHIEHMVIASEEAIAKGIRRIVALTGPEAKKALAKEKLLLGELEKVKTKVESKDMGMKEKVRLITELND

DISAATISYHKKDEMRGELKNVKKRIDDEDRNRKAAVMGDVVEAAKAILTGNPGLPYLVCELQAYANNKAIDGALKQVKL

LSSATPTIFFSGDEDTGKILCMAYSSKEAITSGLKANEWCGSVQTLINGKGGGRPDNAQASGTNVSALNAAMEAAHSFAM

LKLGVERVNLAADATGPAGEKSISASVSAPAGSAVLLAPPGCCGGVLVQVASKYAGQTIAVQSGQAFTFKSGAGSFTSPV

STALYLCSNNKTLIGADLAQQSMVLQYCLYAENDLKHHVSGWVNPTQGVVEGCNPGTVARSKEAVLHHLQVLDNILVART

YLVGERVSLADLATCTTLLPAFRHVLDGGVRNKLRHVTRWFNTIIHQDNFKQIAGEISLCAQEAQIVQGAGKKEKEGKKE

KKEAKKEVEKPKPKKEEKEEGPEEPMPEKKKDPLDALPKGTFDMEDWKRFYSNNDEEKSCEYFWEKFDPENYSIWRGDYR

YNDELSQVFMSCNLMGGMFQRLEKMNKNAFASAMLFGENNNSSIAAIWVWKGQNLAFELSEDWQIDYASYQWSKLDPKSA

ETKTMVNQFWKWEGVDAKGRKFNQGKIFK

>KXJ76147.1 hypothetical protein RP20_CCG010191 [Aedes albopictus]

MDTSLTAKQIRNAFLDFFKEKEHLYVHSSSTIPLDDPTLLFANAGMNQFKPIFLGTADPNSEMAKWVRTANTQKCIRAGG

KHNDLDDVGKDVYHHTFFEMLGNWSFGDYFKKEICTWAWELLTDRLKLPKERLYVTYFGGHPESGLEPDLECREIWLNLG

VKEEHILPGSMKDNFWEMGETGPCGPCSELHFDRIGGRSVPELVNMDDPDVLEIWNLVFIQFNREQDGSLKLLPKKHIDC

GMGFERLVSVIQDKRSNYDTDVFVPLFEAIQKGTGAPSYQGRVGADDVDGVDMAYRVLADHARTITIALADGGFPDNTGR

GYVLRRILRRAVRYATEKLNAKPGFFATLVDTVVFLLGDTFPEVKKDPQSIKNIINEEEQQFLKTLTRGRNLLNRTIAKL

GDSKVIPGDVAWRLYDTYGFPIDLTQLMAEEKNMTIDMEGYEKAKHESYILSQGKEKSKAATVDLDVHAISELQEKKVPT

TDDSFKYRYKADVDPMAEYKFEPCNGKVLALRSNNTFVEEVQAGQECAVILDKTNFYAESGGQIYDQGYLVKVNDESTEF

NVNLVYNRGGYIIHIGVVEGILRVGDEVACHMDILRRQLTMKNHSATHALNHSLLKVLGQDTDQRGSLVVPEKLRFDFTN

KSAMTVEQVAESERLTREVVKKNMQIYAKESKLSVAKSIKGLRSVFDEVYPDPVRVISFGVPVENLEANPEGEEGVETSV

EFCGGTHLHQSGHMWDFVITTEEAIAKGIRRIVALTGPEALKALKKTELLENELNALKSTIEADKTGKDSKEHVKKIVEL

NEDVSQAVIPYVKKDEIRNVLKSLKKTLDDKERAARAAVSTTVVEKAKELSEANKEALFLVHRMEAYNNTKALDSALKEV

RKINPEQSALFVSSDPDSKKIFCLASVPKSAVEKGLKANEWISHISPSMGGKGGGKPESAQASGGNFDKVEEILELARQF

AASKLS

>XP_001658296.1 alanine--tRNA synthetase, cytoplasmic [Aedes aegypti]

MDTSLTAKQIRNAFLDFFKEKEHLYVHSSSTIPLDDPTLLFANAGMNQFKPIFLGTADPNSDMAKWVRTANTQKCIRAGG

KHNDLDDVGKDVYHHTFFEMLGNWSFGDYFKKEICTWAWELLTDRLKLPKDRLYVTYFGGHPESGLEPDLECREIWIKLG

VKEEHILPGSMKDNFWEMGETGPCGPCSELHFDRIGGRSVPELVNMDDPDVLEIWNLVFIQFNREQDGSLKLLPKKHIDC

GMGFERLVSVIQDKRSNYDTDVFVPLFEAIQKGTGAPSYQGRVGADDVDGVDMAYRVLADHARTITIALADGGFPDNTGR

GYVLRRILRRAVRYATEKLNAKPGFFATLVDTVVFLLGDTFPEVKKDPQNIKNIINEEEQQFLKTLTRGRNLLNRTIAKL

GDSKVIPGDVAWRLYDTYGFPIDLTQLMAEEKNMTIDMEGYEKAKHESYILSQGKEKSKAATVDLDVHAISELQEKKVPT

TDDSFKYRYKADVDPMAEYKFEPCNGKVLALRANNIFVDEVQAGQECAVILDKTNFYAESGGQIYDQGYLVKVGDESTEF

NVNLVYNRGGYIIHIGVVEGILRVGDEVACHMDTLRRQLTMKNHSATHALNHSLLKVLGLDTDQRGSLVVPEKLRFDFTN

KSAMTVEQVAESERLTREVVKKNMQIYAKESKLSVAKSIKGLRSVFDEVYPDPVRVISFGVPVENLEANPEGEEGVETSV

EFCGGTHLHQSGHMWDFVITTEEAIAKGIRRIVALTGPEALKALKKTELLENELNALKSTIEADKTGKDSKELVKKIVEL

NEDVSQAVIPYVKKDEIRNVLKSLKKTLDDKERAARAAVSTTVVEKAKELSESNKDAPFLVHRMEAYNNTKALDSALKEV

RKINPEQSALFVSSDPDTKKIFCLASVPKSAVEKGLKANEWISHISPSMGGKGGGKPESAQASGANFDKVEEILELARQF

AASKLS

>KPJ15176.1 Alanyl-tRNA synthetase, cytoplasmic [Papilio machaon]

MDTSLTGNQIRKTFIDFFIGKGHKYVHSSSTIPLDDPTLLFANAGMNQFKPIFLGSVDPNSDMAQYVRVVNTQKCIRAGG

KHNDLDDVGKDVYHHTFFEMMGNWSFGDYFKKEICAWAWELLTEVYKLSADRLYVTYFGGDAASGLEPDLECKNIWLNLG

LPESHILPGSMKDNFWEMGETGPCGPCSELHYDRIGDRNAAHLVNMDDPDVLEIWNLVFIQFNREADSSLKPLPKKHIDC

GLGLERLVSVIQNKKANYDTDFFMPLFKAIETGTKTRPYTGKVGADDVDGIDMAYRVLADHARTLTIALADGGCPDNTGR

GYVLRRILRRAVRYASEKLNGKPGFFGSLVHTVVELLGDVFPEIKKDPESIIQTINEEEIQFLKTLSRGRNLLNRTIEKL

SGSKIIPGDVAWRLYDTYGFPVDLTHLMCEEKGLSIDMEAYEKAKKESQLNSQGKAAGQEDLLALDIHAINHLQESGVPV

TDDSPKYSYTPSSNDKDAIYSFAPCTATVLALRWNKEFGGQIFDEGYMVKIDDDSVEFTVKNVQVKGGYVLHAGKVEGTL

KVGDKVSLHIDTERRRLVMNNHTGTHILNNVLRKVLGNDSDQRGSLVMPDRLRFDFTNKGPMTVKQIKDAEDEIKNIIAE

NKAVYAKHTSLADAKKIKGLRAMFDEHYPDPVRVVSVGIPVEELEKNPDGPSGYETSVEFCGGSHLHQTGHIGDYVIVSE

EGIAKGIRRIVALTGPEAVKALNKLSVLENEVNNIANFIKEQGDAVNHKEVVKKIVELTNDVSHAQISYWKKEELRNMLK

GLKKQLDDKERASKAMIITQVIEKTKELCLEEKDSDVIVTELKALGNTKALDGALKQVKQILPNTAAMFFSVDDETNKIY

CLAAVPKTLNEKGLLASEWVQSIVSVMGGKGGGKAESAQASGNNPKSITEAIKIAKEFASSKL

>NP_001037452.1 alanine--tRNA synthetase, cytoplasmic [Bombyx mori]

MDTSMTGNEIRKTFIDFFIKKGHKYVHSSSTIPLDDPTLLFANAGMNQFKPIFLGSVDPNSDMAQYIRVVNTQKCIRAGG

KHNDLDDVGKDVYHHTFFEMMGNWSFGDYFKKEICAWAWELLTDVFKLSRERLYVTYFEGDPSSGLEPDLECRNIWLNLG

VPEAHILPGSMKDNFWEMGETGPCGPCSELHYDRIGDREAAHLVNMDDPDVLEIWNLVFIQFNRETDGSLKLLPTKHIDC

GLGLERLVSVIQNKRANYDTDFFMPIFKAIENATGVRPYSGKVGVDDVDGIDMAYRVLADHARTLTIALSDGGCPDNTGR

GYVLRRILRRAVRYASEKLNAKPGFFGSLVYTVVELLGDVFPEIKKDPDSIVHVINEEEVQFLKTLLRGRNLLYRTIEKL

NNSKTLPGDVAWRLYDTYGFPIDLTQLMCEEKGLNVDMEGYEKSRKESQLVSQGKAAGQEDLIALDVHAISHLQDTGIPA

TDDSPKYNYLPSSTDKDALYTFAPCTAKIVALRKNKEFVSEISSGQECGVILDRTSFYAEQGGQIFDEGYMVKIDDETVE

FTVKNVQVKGGYVLHAGKVEGILKVGDTLSLHIDTERRRLVMNNHTGTHVLNNVLRKVLGNDSDQRGSLVMPDRLRFDFT

NKGPMTIKQIKDTENEIKEIIAKNKTVYANYTSLSEAKKINGLRAMFDEHYPDPVRVVSVGVPVEDLIKNPDAPTGFETS

VEFCGGSHLHRTSHIGEYVIVSEEGIAKGIRRIVAVTGPEAIKAINKLSVLENEVNNVANFIKEQNESISHKEVSKKIVD

LTNEISQAQISYWKKDELRNMLKNLKKQLDDKERAEKAIIITQVTEKAKELCLERKESKYIVSELKAFGNTKALDGALKQ

VKQFCPNSAAMFFSVDKDADKIYCLAAVPKSDVEKGLLASEWVQSVVDIIGGKGGGKAESAQASGNNPNSLNEAIQIANE

YAKSKLN

>XP_017022471.1 PREDICTED: alanine--tRNA synthetase, cytoplasmic [Drosophila kikkawai]

MKFLTAKEVRDAYLDFFKEQKHIYVHSSSTIPLDDPTLLFANAGMNQFKPIFLGTADPNSEMSKWVRVANTQKCIRAGGK

HNDLDDVGKDVYHHTFFEMLGNWSFGDYFKKEICSWAWQFLTERLGLPKDRLYVTYFGGDEASGLEPDLECKQLWLDLGL

KPEHILPGSMKDNFWEMGETGPCGPCSELHFDRIGGRSVPELVNMDDPDVLEIWNLVFIQYNRESDGSLKQLPKKHIDCG

MGFERLVSVIQNKRSNYDTDLFVPLFDAIQAGTGAPAYQGRVGADDSDGIDMAYRVLADHARTITIALADGGTPDNTGRG

YVLRRILRRAVRYATEKLNAKPGFFATLVNTVVDLLGDAFPEVKKDPQHIIDIINEEELQFLKTLTRGRNLLNRTIEKLG

TATIIPGDVAWRLYDTYGFPVDLTQLMAEEKSLQIDMAGYEAAKQNSYVLSQGKGASKIEEINLDVHAISQLQEQNVPPT

DDSYKYKYEAVSAERDSAYNYGDCTSKIVALRFENQFVNEISNGQKAGIVLDKTNFYAESGGQIYDQGALVKVNDEANEF

LVDRVYNRGGYILHIGVVEGTLKVGDELQLHIDVERRWLTMKNHSATHALNHCLLQVLGKDTEQKGSLVVPEKLRFDFNS

KAAMTIQQVAKTEQLTKEMVYKNVPIYAKESKLAVAKQIRGLRSVFDEVYPDPVRVISFGVPVDELEQNPDSEAGEQTSV

EFCGGTHLRRSGHIMDFVISSEEAIAKGIRRIVALTGPEALKALKKSEGFEQEIVRLKATIDADQSGKDSKSHVKEIVEL

TEQISHATIPYVKKDDMRNLLKGLKKTLDDKERALRAAVSVTVVERAKALCEANPKAIVLVEQLEAFNNTKALDAALKQV

RTQLPEAAAMFFSVDVDSKKIFCLSSVPKSAVEKGLKANEWVQHVSATLGGKGGGKPESAQASGTNYEKVDEILQMANKF

AQAKLA

>XP_002002386.1 uncharacterized protein Dmoj_GI12987 [Drosophila mojavensis]

MKFLTAKEVRDAYLNFFKEQKHIYVHSSSTIPLDDPTLLFANAGMNQFKPIFLGIADPNSEMSKWVRVVNTQKCIRAGGK

HNDLDDVGKDVYHHTFFEMLGNWSFGDYFKKEICSWAWEFLTDRLNLPKDRLYVTYFGGDAANGLEPDLECKQMWLDLGL

KPEHILPGSMKDNFWEMGETGPCGPCSELHFDRIGGRSVPELVNMDDPDVLEIWNLVFIQYNRESDGSLKQLPKKHIDCG

MGFERLVSVIQNKRSNYDTDLFVPLFDAIQAGTGAPAYQGRVGADDADGIDMAYRVLADHARTITIALADGGNPDNTGRG

YVLRRILRRAVRYATEKLNAKPGFFATLVTTVVELLGDAFPEVKKDPQHIMDIINEEEQQFLKTLSRGRNLLNRTIEKLG

ADQKTIPGDVAWRLYDTYGFPVDLTQLMAEEKSLQIDMDGYDAAKHNSYILSQGKGASKIDEINLDVHAISELQEKGVPP

TNDSFKYIYEAESNERDSAYKFSDCTSKIVALRYENKFVQEITSGQKAGIVLDKTNFYAESGGQIFDQGALVKLNDEANE

FLVDRVYNRGGYILHIGVVEGTLAVGDEVQLHIDVERRWLTMKNHSATHALNHCLLQVLGKDTEQKGSLVVPEKLRFDFN

SKAAMTIEQVAKTEQLTRDMVYKEMPIYAKESQLALAKRIRGLRSVFDEVYPDPVRVISFGVPVDQLESQPDSEAGEQTS

VEFCGGTHLRNSGHIMDFVISSEEAIAKGIRRIVALTGPEALKALKKSEGFEKEISTLKATIEADKTGKDSKLYVKQIVE

LTEQISHATIPYVKKDEMRNLLKSLKKTLDDKERALRAAVSVTVVEQAKALCEANPKANVLVQQLEAYNNTKALDAALKQ

VRSQLPDAAAMFVSVDADSKKVFCLCSVPKSAVEKGLKANEWVQHVSPVLGGKGGGKPESAQASGSNFERVDEIVQLASK

FAQAKLN

>NP_523511.2 Alanyl-tRNA synthetase, isoform A [Drosophila melanogaster]

MKLLTAKEVRNAYLDFFKEQKHIYVHSSSTIPLDDPTLLFANAGMNQFKPIFLGTADPNSEMSKWVRVANTQKCIRAGGK

HNDLDDVGKDVYHHTFFEMLGNWSFGDYFKKEICSWAWEFLTQRLALPKDRLYVTYFGGDAASGLEPDLECKQMWLDLGL

KPEHILPGSMKDNFWEMGETGPCGPCSELHFDRIGGRSVPELVNMDDPDVLEIWNLVFIQYNRESDGSLKQLPKKHIDCG

MGFERLVSVIQNKRSNYDTDLFVPLFDAIQAGTGAPPYQGRVGADDVDGIDMAYRVLADHARTITIALADGGTPDNTGRG

YVLRRILRRAVRYATEKLNAKPGFFATLVNTVVDLLGDAFPEVKKDPQHIIDIINEEELQFLKTLTRGRNLLNRTIEKLG

NQTTIPGDVAWRLYDTYGFPVDLTQLMAEEKSLKIDMDGYEAAKHNSYVLSQGKGASKIEEINLDVHAISQLQEQGVPPT

NDTFKYKYEAVSDERDSAYNYGVCNSKIVALRFENQFVNEITSGQKAGIVLDKTNFYAESGGQIYDQGALVKVNDEANEF

LVDRVYNRGGYILHIGVVEGTLKVGDELELHIDVERRWLTMKNHSATHALNHCLLQVLGKDTEQKGSLVVPEKLRFDFNS

KAAMTIEQVSKTEQLTKEMVYKNVPIYAKESKLALAKKIRGLRSVFDEVYPDPVRVISFGVPVDELEQNPDSEAGEQTSV

EFCGGTHLRRSGHIMDFVISSEEAIAKGIRRIVALTGPEALKALKKSEAFEQEIVRLKATIDNDKSGKDSKSHVKEIVEL

TEQISHATIPYVKKDEMRNLLKGLKKTLDDKERALRAAVSVTVVERAKTLCEANPNATVLVEQLEAFNNTKALDAALKQV

RSQLPDAAAMFLSVDADSKKIFCLSSVPKSAVEKGLKANEWVQHVSATLGGKGGGKPESAQASGTNYEKVDEIVQLASKF

AQSKLS

>AAL39400.1 GM03058p [Drosophila melanogaster]

MKLLTAKEVRNAYLDFFKEQKHIYVHSSSTIPLDDPTLLFANAGMNQFKPIFLGTADPNSEMSKWVRVANTQKCIRAGGK

HNDLDDVGKDVYHHTFFEMLGNWSFGDYFKKEICSWAWEFLTQRLALPKDRLYVTYFGGDAASGLEPDLECKQMWLDLGL

KPEHILPGSMKDNFWEMGETGPCGPCSELHFDRIGGRSVPELVNMDDPDVLEIWNLVFIQYNRESDGSLKQLPKKHIDCG

MGFERLVSVIQNKRSNYDTDLFVPLFDAIQAGTGAPPYQGRVGADDVDGIDMAYRVLADHARTITIALADGGTPDNTGRG

YVLRRILRRAVRYATEKLNAKPGFFATLVNTVVDLLGDAFPEVKKDPQHIIDIINEEELQFLKTLTRGRNLLNRTIEKLG

NQTTIPGDVAWRLYDTYGFPVDLTQLMAEEKSLKIDMDGYEAAKHNSYVLSQGKGASKIEEINLDVHAISQLQEQGVPPT

NDTFKYKYDAVSDERDSAYNYGVCNSKIVALRFENQFVNEITSGQKAGIVLDKTNFYAESGGQIYDQGALVKVNDEANEF

LVDRVYNRGGYILHIGVVEGTLKVGDELELHIDVERRWLTMKNHSATHALNHCLLQVLGKDTEQKGSLVVPEKLRFDFNS

KAAMTIEQVSKTEQLTKEMVYKNVPIYAKESKLALAKKIRGLRSVFDEVYPDPVRVISFGVPVDELEQNPDSEAGEQTSV

EFCGGTHLRRSGHIMDFVISSEEAIAKGIRRIVALTGPEALKALKKSEAFEQEIVRLKATIDNDKSGKDSKSHVKEIVEL

TEQISHATIPYVKKDEMRNLLKGLKKTLDDKERALRAAVSVTVVERAKALCEANPNATVLVEQLEAFNNTKALDAALKQV

RSQLPDAAAMFLSVDADSKKIFCLSSVPKSAVEKGLKANEWVQHVSATLGGKGGGKPESAQASGTNYEKVDEIVQLASKF

AQSKLS

>XP_017859017.1 PREDICTED: alanine--tRNA synthetase, cytoplasmic [Drosophila arizonae]

MKFLTAKEVRDAYLNFFKEQKHIYVHSSSTIPLDDPTLLFANAGMNQFKPIFLGIADPNSEMSKWVRVVNTQKCIRAGGK

HNDLDDVGKDVYHHTFFEMLGNWSFGDYFKKEICSWAWEFLTDRLNLPKDRLYVTYFGGDAASGLEPDLECKQMWLDLGL

KPEHILPGSMKDNFWEMGETGPCGPCSELHFDRIGGRSVPELVNMDDPDVLEIWNLVFIQYNRESDGSLKQLPKKHIDCG

MGFERLVSVIQNKRSNYDTDLFVPLFDAIQAGTGAPAYQGRVGADDADGIDMAYRVLADHARTITIALADGGNPDNTGRG

YVLRRILRRAVRYATEKLNAKPGFFATLVTTVVELLGDAFPEVKKDPQHIMDIINEEEQQFLKTLSRGRNLLNRTIEKLG

ADQKTIPGDVAWRLYDTYGFPVDLTQLMAEEKSLQIDMAGYDAAKHNSYILSQGKGASKIDEINLDVHAISELQDKGVPP

TNDSFKYIYEAESNERDSAYKFSDCTSKIVALRYENKFVQEITSGQKAGIVLDKTNFYAESGGQIFDQGALVKLNDEANE

FLVDRVYNRGGYILHIGVVEGTLAVGDEVQLHIDVERRWLTMKNHSATHALNHCLLQVLGKDTEQKGSLVVPEKLRFDFN

SKAAMTIEQVAKTEQLTRDMVYKEMPIYAKESQLALAKRIRGLRSVFDEVYPDPVRVISFGVPVDQLESQPDSEAGEQTS

VEFCGGTHLRNSGHIMDFVISSEEAIAKGIRRIVALTGPEALKALKKSEGFEKEISTLKATIEADKTGKDSKLYVKQIVE

LTEQISHATIPYVKKDEMRNLLKSLKKTLDDKERALRAAVSVTVVEQAKALCEANPKANVLVQQLEAYNNTKALDAALKQ

VRSQLPDAAAMFVSVDADSKKIFCLCSVPKSAVEKGLKANEWVQHVSPVLGGKGGGKPESAQASGSNFERVDEIVQLASK

FAQAKLN

>XP_002088795.1 uncharacterized protein Dyak_GE11031 [Drosophila yakuba]

MKLLTAKEVRNAYLDFFKEQKHIYVHSSSTIPLDDPTLLFANAGMNQFKPIFLGTADPNSEMSKWVRVANTQKCIRAGGK

HNDLDDVGKDVYHHTFFEMLGNWSFGDYFKKEICSWAWEFLTQRLSLPKDRLYVTYFGGDEASGLEPDLECKQMWLDLGL

KPEHILPGSMKDNFWEMGETGPCGPCSELHFDRIGGRSVPELVNMDDPDVLEIWNLVFIQYNRESDGSLKQLPKKHIDCG

MGFERLVSVIQNKRSNYDTDLFVPLFDAIQAGTGAPAYQGRVGADDVDGIDMAYRVLADHARTITIALADGGTPDNTGRG

YVLRRILRRAVRYATEKLNAKPGFFATLVNTVVDLLGDAFPEVKKDPQHIIDIINEEELQFLKTLTRGRNLLNRTIEKLG

NQTTIPGDVAWRLYDTYGFPVDLTQLMAEEKSLKIDMDGYEAAKHNSYVLSQGKGASKIEEINLDVHAISQLQEQGVPPT

NDTFKYKYEAVSDERDSAYNYGVCNSKIVALRFENQFVNEITSGQKAGIVLDKTNFYAESGGQIYDQGALVKVNDEANEF

LVDRVYNRGGYILHIGVVEGTLKVGDELELHIDVERRWLTMKNHSATHALNHCLLQVLGKDTEQKGSLVVPEKLRFDFNS

KGAMTIEQVSKTEQLTKEMVYKNVPIYAKESKLALAKKIRGLRSVFDEVYPDPVRVISFGVSVDELEQNPDSEAGEQTSV

EFCGGTHLRRSGHIMDFVISSEEAIAKGIRRIVALTGPEALKALKKSEAFEQEIIRLKATIDTDKSGKDSKSHVKEIVEL

TEQISHATIPYVKKDEMRNLLKGLKKTLDDKERALRAAVSVTVVERAKALCEANPNATVLVEQLEAFNNTKALDAALKQV

RSQLPDAAAMFLSVDADSKKIFCLSSVPKSAVEKGLKANEWVQHVSATLGGKGGGKPESAQASGTNFEKVDEIVQLASKF

AQSKLS

>XP_016024174.1 uncharacterized protein Dsimw501_GD22412, isoform B [Drosophila simulans]

MKLLTAKEVRNAYLDFFKEQKHIYVHSSSTIPLDDPTLLFANAGMNQFKPIFLGTADPNSEMSKWVRVANTQKCIRAGGK

HNDLDDVGKDVYHHTFFEMLGNWSFGDYFKKEICSWAWEFLTQRLALPKDRLYVTYFGGDAASGLEPDLECKQMWLDLGL

KPEHILPGSMKDNFWEMGETGPCGPCSELHFDRIGGRSVPELVNMDDPDVLEIWNLVFIQYNRESDGSLKQLPKKHIDCG

MGFERLVSVIQNKRSNYDTDLFVPLFDAIQAGTGAPPYQGRVGADDVDGIDMAYRVLADHARTITIALADGGTPDNTGRG

YVLRRILRRAVRYATEKLNAKPGFFATLVNTVVDLLGDAFPEVKKDPQHIIDIINEEELQFLKTLTRGRNLLNRTIEKLG

NQTTIPGDVAWRLYDTYGFPVDLTQLMAEEKSLKIDMDGYEAAKHNSYVLSQGKGASKIEEINLDVHAISQLQEQGVPPT

NDTFKYKYEAVSDERDSAYNYGVCNSKIVALRFENQFVNEITSGQKAGIVLDKTNFYAESGGQIYDQGALVKVNDEANEF

LVDRVYNRGGYILHIGVVEGTLKVGDELELHIDVERRWLTMKNHSATHALNHCLLQVLGKDTEQKGSLVVPEKLRFDFNS

KAAMTIEQVSKTEQLTKEMVYKNVPIYAKESKLALAKKIRGLRSVFDEVYPDPVRVISFGVSVDELEQNPDSEAGEKTSV

EFCGGTHLRRSGHIMDFVISSEEAIAKGIRRIVALTGPEALKALKKSEAFEQEIVRLKATIDNDKSGKDSKSHVKEIVEL

TEQISHATIPYVKKDEMRNLLKGLKKTLDDKERALRAAVSVTVVERAKALCEANPNATVLVEQLEAFNNTKALDAALKQV

RSQLPDAAAMFLSVDADSKKIFCLSSVPKSAVEKGLKANEWVQHVSATLGGKGGGKPESAQASGTNYEKVDEIVQLASKF

AQSKLS

>XP_002036208.1 GM12994 [Drosophila sechellia]

MKLLTAKEVRNAYMDFFKEQKHIYVHSSSTIPLDDPTLLFANAGMNQFKPIFLGTADPNSEMSKWVRVANTQKCIRAGGK

HNDLDDVGKDVYHHTFFEMLGNWSFGDYFKKEICSWAWEFLTQRLALPKDRLYVTYFGGDAASGLEPDLECKQMWLDLGL

KPEHILPGSMKDNFWEMGETGPCGPCSELHFDRIGGRSVPELVNMDDPDVLEIWNLVFIQYNRESDGSLKQLPKKHIDCG

MGFERLVSVIQNKRSNYDTDLFVPLFDAIQAGTGAPPYQGRVGADDVDGIDMAYRVLADHARTITIALADGGTPDNTGRG

YVLRRILRRAVRYATEKLNAKPGFFATLVNTVVDLLGDAFPEVKKDPQHIIDIINEEELQFLKTLTRGRNLLNRTIEKLG

NQTTIPGDVAWRLYDTYGFPVDLTQLMAEEKSLKIDMDGYEAAKHNSYVLSQGKGASKIEEINLDVHAISQLQEQGVPPT

NDTFKYKYEAVSDERDSAYNYGVCSSKIVALRFENQFVNEITSGQKAGIVLDKTNFYAESGGQIYDQGALVKVNDEANEF

LVDRVYNRGGYILHIGVVEGTLKVGDELELHIDVERRWLTMKNHSATHALNHCLLQVLGKDTEQKGSLVVPEKLRFDFNS

KAAMTIEQVSKTEQLTKEMVYKNVPIYAKESKLALAKKIRGLRSVFDEVYPDPVRVISFGVSVDELEQNPDSEAGEQTSV

EFCGGTHLRRSGHIMDFVISSEEAIAKGIRRIVALTGPEALKALQKSETFEQEIVRLKATIENDKSGKDSKSHVKEIVEL

TEQISHATIPYVKKDEMRNLLKGLKKTLDDKERALRAAVSVTVVERAKALCEANPNATVLVEQLEAFNNTKALDAALKQV

RSQLPDAAAMFLSVDADSKKIFCLSSVPKSAVEKGLKANEWVQHVSATLGGKGGGKPESAQASGTNYEKVDEIVQLASKF

AQSKLS

>XP_015774801.1 PREDICTED: alanine--tRNA synthetase, cytoplasmic-like [Acropora digitifera]

MVVHIFRCFQEVRVNSLFSLCVFRKISKVRLLSSSVMDPNLSAKDLRQMFVDFFVEKHEHTFVPSSSTIPHEDPTLLFAN

AGMNQFKSIFLGSVDPNSDFFKLKRAANSQKCIRAGGKHNDLDDVGKDVYHHTFFEMLGNWSFGNYFKKEAIEFAWELLT

VKYGMPKDRLYVTYFGGEEGLDSDEETRQLWISMGLAPERVLPFGMKDNFWEMGETGPCGPCSEIHYDRIGGRDAAFLVN

MDDPTVLEVWNLVFIQFNREADGSLKTLPSKHVDTGMGLERLTSITQGKLSNYDTDLFQPFFEAIHKGTGVRRYQGLVGP

EDRDGIDMAYRVVADHIRTLTMAITDGGKPDNTGRGYVLRRILRRCVRFATEKLNCPPGFVSSLVLVAVDSLGEFFPEVT

KDPQQVMDTINEEEAQFLKTLARGRRLFDRTVSKLTDKLIPGDVAWRLYDTYGFPVDLTQLMAEERGLTVDMATYEESKR

KAQEIARGKGSGNDEGITLDVHAINKLQKEFNLAPTDDKVKYKYTSDEDGNYVFEPVVGTVKAIIFQKEFVNEVTSGSHC

GVLLDKTCFYAEQGGQIYDQGFMTKEDDEEVEFSVSNVQVHGGYVLHVGAIEGTLKVGDMLKCQIDEQRRRVVMNNHTAT

HVLNFSLRKELGEADQRGSLVAPDRLRFDFTAKGAMTTEQIKNTELTSQDIVSKNMAVFAKEVPLALAKDIQGLRAIFEE

VYPDPVRVLSIGSPFDDLQEDPQAGYKYSVEFCGGTHLQRTGHMKTFVLVSEEAISKGIRRIVALTGSEALKAEKKCNLL

ETKVEDIQSRVQDKINEGGLKIKETSQEIARITEDISGSVISAWRKDEMRTKLKAVKKLVDDAEKEKKNAMMQQAMDKAK

AIIEADGNRSFVVDVFDAGSNQKILDAALKQFKTLAPTTATLLFSVDEENSKIICLAQVPKEAIQQGLKADEWVKSVSDL

LGGKCGGKDVSAQGSGPNVGCIDKAVEIASKFAEQKL

>KFB53790.1 AGAP009701-PA-like protein [Anopheles sinensis]

MDTSLTASQIRSIFLDFFKEKEHLYVHSSSVIPLDDPTLLFANAGMNQFKPIFLGTVDPNSDMARWVRTANTQKCIRAGG

KHNDLDDVGKDVYHHTFFEMLGNWSFGDYFKKEICTWAWELLTERLKLPKERLYVTYFGGHPESGLEPDLECREIWLKLG

VKEEHILPGSMKDNFWEMGETGPCGPCSELHFDRIGGRSVPELVNMDDPDVLEIWNLVFIQYNREQDSSLKLLPKKHIDC

GMGFERLVSVIQDKRSNYDTDVFVPLFDAIQKGTGAPAYQGRVGDADVDGVDMAYRVLADHARTITIALADGGFPDNTGR

GYVLRRILRRAVRYATEKLSAKPGFFATLVDTVVLLLGDTFPEVRKDPQHIKNVINEEEQQFLKTLSRGRNLLNRTIAKL

GNSKVIPGDVAWRLYDTYGFPVDLTQLMAEEKQMTIDMEGYEKAKHESYIISQGKEKSKTATIDLDVHAISELQERKVPT

TDDSFKYRYKAQSTDPLAEYVFEPCTGKIVALRHNNAFVEEVLAGQECGVLLDRTNFYAESGGQIYDQGYLVKVNDESSE

FNVTLVYNRGGYVLHIGVVEGTLRVGDEVHCHMDTGRRQLTMKNHSATHALNHSLLKVLGQDTDQRGSLVVPEKLRFDFT

NKAAMTIEQVAEAERLTREVVRKNVPVYAKEANLAVAKTINGLRSVFDEVYPDPVRVISFGVPVEQLEADPTGEAGVLNS

VEFCGGTHLHQSGHMVDFVITTEEAIAKGIRRIVALTGPEALKALKKTELLEQELATLKATIEADATGKEAKEHVKKIVE

LTEDVSQATIPYVKKDELRTVLKGLKKTLDDKERVARAAVASGVVEKAKELSQAHRDAPFLVQRLEAYSNTKALDSALKE

VRKVNPEQSALFLSVDEDTKKIFCLASVPKSAVEKGLKANEWISHIAPVMGGKGGGKPESAQASGGNYDKADEILELARL

FAVSKLE

>XP_017060606.1 PREDICTED: alanine--tRNA synthetase, cytoplasmic [Drosophila ficusphila]

MKFLSAKEVRDAYLGFFKEQKHIYVHSSSTIPLDDPTLLFANAGMNQFKPIFLGTADPNSEMSKWVRVANTQKCIRAGGK

HNDLDDVGKDVYHHTFFEMLGNWSFGDYFKKEICSWAWEFLTVRLGLPKERLYVTYFGGDEASGLEPDLECKQLWLDLGL

KPEHILPGSMKDNFWEMGETGPCGPCSELHFDRIGGRSVPELVNMDDPDVLEIWNLVFIQYNRESDGSLKPLPKKHIDCG

MGFERLVSVIQNKRSNYDTDLFVPLFEAIQAGTGAPAYQGRVGADDVDGIDMAYRVLADHARTITIALADGGTPDNTGRG

YVLRRILRRAVRYATEKLNAKPGFFGTLVNTVVDLLGDAFPEVKKDPQHIIDIINEEELQFLKTLTRGRNLLNRTIEKLG

NQTTIPGDVAWRLYDTYGFPVDLTQLMAEEKSLQIDMDGYEAAKQNSYILSQGKGASKIEEINLDVHAISQLQEQGVPPT

NDSFKYKYEAVSDERDSAYNYGVCNSKIVALRFENQFVNEISSGQKAGIVLDNTNFYAESGGQIYDQGALVKVNDEANEF

LVDRVYNRGGYILHIGVVEGTLKVGDELELHIDVERRWLTMKNHSATHALNHCLLQVLGKDTEQKGSLVVPEKLRFDFNS

KAAMTIEQVSKTEQLTKEMVYKNVPIYAKESKLALAKKIRGLRSVFDEVYPDPVRVISFGVSVDELEQNPDSEAGEQTSV

EFCGGTHLRRSGHIMDFVISSEEAIAKGIRRIVALTGPEALKALKKSEGFEQEIVRLKATIDADQSGKDSKSHVKEIVEL

TEQISHATIPYVKKDEMRNLLKGLKKTLDDKERALRAAVSVTVVERAKALCEANPKATVLVEQLEAFNNTKALDAALKQV

RSQLPDAAAMFLSVDVDSKKIFCLSSVPKSAVEKGLKANEWVQHVSATLGGKGGGKPESAQASGTNYEKVDEIVQLASKF

AQSKLS

>XP_013926747.1 PREDICTED: alanine--tRNA synthetase, cytoplasmic-like, partial [Thamnophis sirtalis]

METLTASEIRQRFINFFVDHQHTYVHSSATIPLDDPTLLFANAGMNQFKPIFLNTLDPSHPMAKLTRAVNTQKCIRAGGK

HNDLDDVGKDVYHHTFFEMLGSWSFGDYFKELACKLALDLLTKEFGIPIERLYVTYFGGNEAAGLEPDLDCKQIWLDLGL

AESKILPGSMKDNFWEMGDTGPCGPCSEMHYDRIGDRDASHLVNQDDPNVLEIWNLVFIQFNRWYVLRRILRRAVRYAHE

KLSAPKGFFATLVDVVVQSLGTGCRPYSGQVGEEDVDGIDMAYRVLADHARTITIALSDGGRPDNTGPSRDLTMETLTAS

EIRQRFINFFVDHQHTYVHSSATIPLDDPTLLFANAGMNQRGASFNTDTLNHPYSVLQKDGELCDTAWLLYDTYGFPVDL

TGLIAEEKGLRVDMEAFEEERKTAQLKSQGKGGGDEDLIMLDIYAIEDLRARALEPTDDSPKYNYRSDSDGFYEFGTPGA

TVKAIRRDKTFVDEVHTGQECGIVLDQTCFYAEQGGQIYDEGYLVKEDGAEDRTEFTVKNVQVRGGYVLHVGTVYGSLKV

GDFVGLFIDEPRRRPIMSNHTATHILNFALRSVLGEADQRGSLVAPDRLRFDFTAKGAMSTQEIKKTEEIVNGMIQDAKV

VYAKECPLAAAKAIQGLRAVFDETYPDPVRVVSIGIPVDDMLNDPSSAAGAVTSVEFCGGTHLQNSSHAGHFVIVSEEAI

AKGIRRIVAVTGAEAQKALRKSEGLIKQLADLETKVKIQTTPNKDVQREIADLGEALATAIIPQWQKDELREALKALKKI

M

>XP_002125465.1 PREDICTED: alanine--tRNA synthetase, cytoplasmic [Ciona intestinalis]

MDASLNSKQLRQMFLSFFQEKYAHTYIHSSSTIPLDDPTLLFANAGMNQFKSIFLGTISPADPLSKCKRVVNSQKCIRAG

GKHNDLDDVGKDTYHHTFFEMLGCWSFGDYFKKDAINWAWELLTEVLKIPKDRLYATYFEGSEEYNVPVDTEVQNLWLQH

LPADHILPGNMKDNFWEMGDVGPCGPCTEIHFDRIGGRNAAHLVNMDDPEVLEVWNLVFMQFNREKDGKLKLLAKQHVDT

GMGLERLVSVIQGKLSNYDTDMFTPLFKAIQLGTGAREYQGKVGKDDVDGVDMAYRVLADHIRTLTIALSDGGRPDNVGR

GYVLRRILRRATRFAVEKLNAKPRFFSSLVHVVVDILGEAFPEIIRDPQMVIDIIDEEEEQFLKTLNRGRKILNQKIKSL

DADAKVFPGETAWLLYDTYGFPFDLTEIMAEESGLTVDCESFEKAKAQARITSKGFAQDVADTICLDVHMLTDLKEKKFP

PTDDSFKYQYSSNDNGEYSFPACTGVVKAILQNKTFVTSVSSGAECGILLDKTCFYAEQGGQLFDLGYFKKQEDEFTEFT

VKNCQVQGGYVLHVGTVAGTLEVGDSVELFIDEQRRQSLMKNHSCTHILNFGLRCVLGEADQRGSLVAPNRLRFDFTAKK

AMTSQQVKETEDVCNQIVNKDEVIYAKPVPLDQAKAIHGLRAIFDETYPDPVRVLSVGVSVDSLVADPNFDAAMNTSVEL

CGGTHLLRSGHADKFIIISEEAISKGVRRIVAVTGQEALKAERKANLLAKSFETIKSEVEKGLVNNKENYKTFSKKCTDF

IELVSQASISQWRREEMRGFASSLKKKVDDVDRVMKENATKLALAQTKESIQQDPNATFVVTELKTNGNAKSLNEALKIY

KSESPDTSVMLFSIDKEANKIVCLSQVPASANDKGLTANGWVQSIATTLNGKGGGKSTSAQAVGRNPDGLNEAIQLAKEF

AKVKLAS

>XP_001970293.1 uncharacterized protein Dere_GG23450 [Drosophila erecta]

MKLLTAKEVRNAYLDFFKEQKHIYVHSSSTIPLDDPTLLFANAGMNQFKPIFLGTADPNSEMSKWVRVANTQKCIRAGGK

HNDLDDVGKDVYHHTFFEMLGNWSFGDYFKKEICSWAWEFLTKRLALPKDRLYVTYFGGDEASGLEPDLECKQMWLDLGL

KPEHILPGSMKDNFWEMGETGPCGPCSELHFDRIGGRSVPELVNMDDPDVLEIWNLVFIQYNRESDGSLKQLPKKHIDCG

MGFERLVSVIQNKRSNYDTDLFVPLFDAIQAGTGAPAYQGRVGPDDVDGIDMAYRVLADHARTITIALADGGTPDNTGRG

YVLRRILRRAVRYATEKLNAKPGFFATLVNTVVELLGDAFPEVKKDPQHIIDIINEEELQFLKTLTRGRNLLNRTIEKLG

NQTTIPGDVAWRLYDTYGFPVDLTQLMAEEKSLKIDMDGYEAAKHNSYVLSQGKGASKIEEINLDVHAISQLQEQGVPPT

NDTFKYKYEAVSDERDSAYNYGVCNSKIVALRFENQFVNEITSGQKAGIVLDKTNFYAESGGQIYDQGALVKVNDEANEF

LVDRVYNRGGYILHIGIVEGTLKVGDELELHIDVERRWLTMKNHSATHALNHCLLQVLGKDTDQKGSLVVPEKLRFDFNS

KAAMTIEQVAKTEQLTKEMVYKNVPIYAKESKLALAKKIRGLRSVFDEVYPDPVRVISFGVSVDDLEQNPDSEAGEQTSV

EFCGGTHLRRSGHIMDFVISSEEAIAKGIRRIVALTGPEALKALKKSEAFEQEIVRLKATIDTDKSGKDSKSHVKEIVEL

TEQISHATIPYVKKDEMRNLLKGLKKTLDDKERALRAAVSVTVVERAKALCEANPNATVLVEQLEAFNNTKALDAALKQV

RSQLPDAAAMFLSVDADSKKIFCLSSVPKSAVEKGLKANEWVQHVSATLGGKGGGKPESAQASGTNFEKVDEIVQLASKF

AQSKLS

>CRL01519.1 CLUMA_CG014203, isoform A [Clunio marinus]

MDTSLTSNQIRQMFIDFFKEHEHIYVHSSSTIPLDDPTLLFTNAGMNQFKPIFLGTADPNSEMSKWVRTANTQKCIRAGG

KHNDLDDVGKDVYHHTFFEMLGNWSFGDYFKKEICTWAWEFLTNRLKLPKERLYVTYFGGDKSAGLEPDYECQEIWTSLG

VLPAHILPGSMKDNFWEMGETGPCGPCSELHFDRIGGRSVPEKVNQDDPDVLEIWNLVFIQFNREADSSLKPLPRKHIDC

GMGFERLVSVIQNKRSNYDTDIFMPLFDAIQKGTGARTYQGRVGVEDVDGIDMAYRVLADHARTITIALADGGFPDNTGR

GYVLRRILRRAVRYGTEKLNAKPGFFATLVDTVVGLLGDTFPELKKDPLHIVNIVNEEEQQFLKTLTRGRNLLNRTIAKL

GSGTKILPGDVAWRLYDTYGFPIDLTKLMVEEKDLTIDMETYEKAKQESYIISQGKAADKIDTINLDVHALSELQDKNIP

TTNDSFKYRYKAASIEPTAEYIFEGCNGKILAIRYNSQFVDEVKSGQECGLILDQTNFYAESGGQIYDQGYFVKVQDEST

EFSVRLVYNKGGYILHIGVVEGVLRVGDEVQIHLDTIRRQLTMKNHSATHALNHSLLKVLGNDTDQRGSLVVPEKLRFDF

TNKNAMTIEQVAAAEKLTQEVVQKNVKIFSKEADLKVAKAINGLRSVFDEVYPDPVRVIAFGVPVEELEKNPKGDSGVIN

SVEFCGGTHLHQSGHMVDFIITSEEAIAKGIRRIVALTGPEAEKARKKTNLLESELKNLQLKIEADKNCENSKELVKEIV

ELTDDISHAVIPYVKKDEMRNTLKLMKKTLDDKDRAKAAAVSNVVIDQTKQLCQDNPDAKFLVHRFEAFNNTKALDSALK

QVRTINPDTSALFVSSDPDSKKIFCLASVPKSGIDKGLKANEWIQQLSSVIGGKGGGKPESAQASGTNYEKVDEVLELAK

KFASSKLN

>XP_017004808.1 PREDICTED: alanine--tRNA synthetase, cytoplasmic [Drosophila takahashii]

MKFLTAKEVRDAYLGFFKEQQHIYVHSSSTIPLDDPTLLFANAGMNQFKPIFLGTADPNSEMSKWVRVANTQKCIRAGGK

HNDLDDVGKDVYHHTFFEMLGNWSFGDYFKKEICSWAWEFLTQRLSLPKDRLYVTYFGGDEASGLEPDLECKQLWLDLGL

KPEHILPGSMKDNFWEMGETGPCGPCSELHFDRIGGRSVPELVNMDDPDVLEIWNLVFIQYNRESDGSLKQLPRKHIDCG

MGFERLVSVIQNKRSNYDTDLFVPLFEAIQAGTGAPAYQGRVGADDVDGIDMAYRVLADHARTITIALADGGTPDNTGRG

YVLRRILRRAVRYATEKLNAKPGFFATLVNTVVDLLGDAFPEVKKDPQHIIDIINEEELQFLKTLTRGRNLLNRTIEKLG

NQTIIPGDVAWRLYDTYGFPVDLTQLMAEEKSLKIDMEGYEAAKQNSYVLSQGKGASKIEEINLDVHAISQLQDQGVPPT

NDSFKYKYEAVSEERDSAYNYGVCNSKIVALRFENQFVNEISSGQKAGIVLDNTNFYAESGGQIYDQGALVKVNDEANEF

LVDRVYNRGGYILHIGVVEGTLKVGDELELHIDVERRWLTMKNHSATHALNHCLLQVLGKDTEQKGSLVVPEKLRFDFNS

KGAMTIEQVAKTEQLTKEMVYKNVPIYAKESQLALAKKIRGLRSVFDEVYPDPVRVISFGVSVDELEQNPDSEAGEQTSV

EFCGGTHLRRSGHIMDFVISSEEAIAKGIRRIVALTGPEALKALKKSEAFEQEIIQLKATIDADQSGKDSKSHVKEIVDL

TEQISHATIPYVKKDEMRNLLKGLKKTLDDKERALRAAVSVTVVERAKALCEANPEATVLVEQLEAFNNTKALDAALKQV

RSQLPDAAAMFLSVDVDSKKIFCLSSVPKSAVEKGLKANEWVQHVSATLGGKGGGKPESAQASGTNYEKVDEIVQLASKF

AQSKLS

>XP_023170252.1 alanine--tRNA synthetase, cytoplasmic [Drosophila hydei]

MKFLTAKEVRDAYLNFFKEQKHIYVHSSSTIPLDDPTLLFANAGMNQFKPIFLGIADPNSEMSKWVRAVNTQKCIRAGGK

HNDLDDVGKDVYHHTFFEMLGNWSFGDYFKKEICSWAWEFLTNRLNLPKDRLYVTYFGGDAASGLEPDLECKQMWLDLGL

KPEHILPGSMKDNFWEMGETGPCGPCSELHFDRIGGRSVPELVNMDDPDVLEIWNLVFIQYNRESDGTLKQLPKKHIDCG

MGFERLVSVIQNKRSNYDTDLFVPLFDAIQAGTGAPAYQGRVGADDVDGIDMAYRVLADHARTITIALADGGNPDNTGRG

YVLRRILRRAVRYATEKLNAKPGFFATLVTTVVDLLGDAFPEVKKDPQHIMDIINEEEQQFLKTLSRGRNLLNRTIEKLG

ADQTTIPGDVAWRLYDTYGFPVDLTQLMAEEKSLQIDMAGYEAAKHNSYILSQGKGASKIDEINLDVHAISELQEKGVPP

TNDSFKYVYEAVSNDRDSAYKYSDCTSKIIALRYENKFVQEISSGQKAGIVLDKTNFYAESGGQIFDQGALVKLNDEANE

FLVDRVYNRGGYILHIGVVEGNLAVGDEVQLHIDVERRWLTMKNHSATHALNHCLLQVLGKDTEQKGSLVVPEKLRFDFN

SKAAMTIEQVAKTEQLTRDMVYKNMPIYAKESQLALAKRIRGLRSVFDEVYPDPVRVISFGVPVDQLESQPEAEAGEQTS

VEFCGGTHLRNSGHIMDFVISSEEAIAKGIRRIVALTGPEALKALKKSEGFEKEINKLKATIEDDKTGKDSKLYVKQIVE

LTEQISHATIPYVKKDEMRNLLKSLKKTLDDKERALRAAVSVTVVEQAKALCEANPKANVLVQQLEAYNNTKALDAALKQ

VRSQLPDAAAMFVSVDADSKKIFCLCSVPKSAVEKGLKANEWVQHVSPVLGGKGGGKPESAQASGGNFERVDEIVQLASK

FAQAKLN

>XP_002111584.1 hypothetical protein TRIADDRAFT_23916 [Trichoplax adhaerens]

MDPNLTSEQIRQMFIDFFVEKYNHLFVPSSSTVPHDDPTLLFANAGMNQFKPIYLGTVDPKSEMATYRRVVNSQKCIRAG

GKHNDLDDVGKDTYHHTFFEMLGNWSFGDFFKKESIDWAWELLVDVYKMPAERMYVTYFGGDEKSGLQPDYETKNIWLAK

GIAPERVLPFGLQDNFWEMGDTGPCGPCTEIHYDRIGGRDAAYLVNMDDPEVLEVWNLVFTQFNREADKSLRPLPRKYVD

TGAGLERVTSVIQGVMSNYDTDLFKPLFDKIRSLTGAREYRGLLGDADVDGIDMAYRVLADHARTLTIAISDGGKPDSTG

RGYVLRRILRRAVRYATEKLDAKPGMFAQLVNVAVEKLGGAFPEVTKDPQMVIDIINEEEAQFLKTLSRGRRMFEREVLK

AKDGVLPGFVAWRLYDTYGFPVDLTHIMAEEKGLSIDMDGYEKAKQDSQELSRGKDTGVDDTVALGIHAIKDLEDLAIPL

TDTSPKYNYESDGNGGYTIHGIAAKISALRYEKSFVNEVLTGSRCGIILDKSCFYAEQGGQIYDLGFIVNSDDEDTEFKV

TDVQIMGGYVLHVGITEGTFKVGDTVQLQVDSERRRTVMNNHTATHILNFALREQLGEADQRGSLVAPDRLRFDFTAKGA

MTTQQIKATEEIVGNIIEQKQQVFAKDATLAKASAIQGLRQVFAETYPDPVRIVSVGVSVDDLLEDPTGPSGLKTSVEFC

GGTHLKNSGDMEAFVISSEEPIAKGIRRIIALTGSDAKKKVTKLQESLSELMKLGRADQTSLKAKKRQITELNDSIAAVT

LQQWRKDELRETLKKVKKTIIDIEKAADSKRSQEVVEKAKEIASQSKDLSAVVEVLEAGLSSKVLDSALKQFHSIAPNTA

AMLFSVDSEGRRVTCLCQVPEAIMKKGLSASDWVKSVMNVLEGKGGGKPTSAQGSGTNVAGLNNALNLAKEFAKMKLDS

>EFZ10997.1 hypothetical protein SINV_01057, partial [Solenopsis invicta]

MKSSMNAKQIRQAYIDFFKSKGHEYVHSSSTIPHDDPSLLFTNAGMNQFKPIFLGTVDPNSDMAKWVRVVNSQKCIRAGG

KHNDLDDVGKDVYHHTFFEMMGNWSFGDYFKKEICAWAWEFLTDVLKLPADRLYVTYFGGDMKSGLAPDDECKDLWLSLG

IPASHVLPGNMKDNFWEMGETGPCGPCSELHYDRIGGREAAHLVNMDDPDVLEIWNLVFIQYNREPDGSLKPLPKKHIDC

GLGLERLISVIQDKRANYDTDLFMPLFDAIEKGTGAPPYKGKIGADDVDGIDMAYRVLADHARTITIALADGGMPDNTGR

GYVLRRILRRAVRYATEKLNAKPGFFGSLVNVVVELLGDVFPEVKRDPQSIIEIINEEEIQFLKTLSRGRNLLNRTIAKL

ESTNVVPGDVAWRLYDTYGFPIDLTQLMTEEKGLKIDMTGYEEAKKQAQLISQNKAGGVDDQINLDVHAITELQNQGVKP

TDDSPKYDYKVTNTVQYNEYDFAPCTGTVIALRRAKAFVDEVSSGEEVGILLDRTNFYAEQGGQIFDEGFLVKVDDEATE

IRVKNVQVRAGYVLHIGTVGEGKLRKGDKVHTNVDTKRRRFVMANHSATHALNYALRKVLGTEADQKGSLVAPDRLRFDF

TNKGPMTTKQLLDTEVATQEMIKDNKQIYTKESSLAIAKTIQGLRAMFEETYPDPVRVVSMGVSVEKLESDPLSKAALET

SVEFCGGTHLHYTAHIGDFVIASEDAIAKGIRRIVALTGPEATKALRTSTLLQQQILALKEMIANSEINVKECDNMRNTL

KDLKKSLDDKERAAKAAVATSVVQTAQSIIESNPECKVLVEVMEAYSNTKALDLALKKIKAISQETSAMLISVDHNVNKI

YALSSVSKSGIDEGLKANEWVQAIVPLMKGKGGGKAESAQASGTNIACLNEIVSKAKEFANQKLGIKLCYPS

>XP_002018701.1 GL25810 [Drosophila persimilis]

MKFLTAKEVRDAYLGFFKEQKHIYVHSSSTIPLDDPTLLFANAGMNQFKPIFLGTADPNSEMSKWVRVCNTQKCIRAGGK

HNDLDDVGKDVYHHTFFEMLGNWSFGDYFKKEICTWAWEFLTNRLHLPKDRLYVTYFGGDEASGLEPDLECKQLWLDLGL

KPEHILPGSMKDNFWEMGETGPCGPCSELHFDRIGGRSVPELVNMDDPDVLEIWNLVFIQYNRESDGSLKQLPKKHIDCG

MGFERLVSVIQNKRSNYDTDLFVPLFEAIQAGTGAPAYQGRVGADDVDGIDMAYRVLADHARTITIALADGGTPDNTGRG

YVLRRILRRAVRYATEKLNAKPGFFGTLVNTVVDLLGDAFPEVKKDPQHIIDIINEEELQFLKTLTRGRNLLNRTIEKLG

SQSIIPGDVAWRLYDTYGFPVDLTQLMAEEKSLEIDMAGYEAAKQNSYVLSQGKGASKIEEINLDVHAISQLQEQGVPPT

NDSFKYKYEAVSEERDSAYNYQECTSKIVALRFENQFVNEISNGQKAGIVLDKTNFYAESGGQIFDQGALVKLNDEANEF

LVDRVYNRGGYILHIGVVEGTLKVGDELQLHIDEERRWLTMKNHSATHALNHCLLQVLGKDTEQKGSLVVPEKLRFDFNT

KAAMTIEQVAKTEQLTKDMVYKNVPIYAKESKLALAKRIRGLRSVFDEVYPDPVRVISFGVPVDELEQNPDAEAGEQTSV

EFCGGTHLRRSGHIMDFVISSEEAIAKGIRRIVALTGPEALKAIKKSEAFEQEIVKLKATIDADKSGKDSKSHVKEIVEL

TEQISHATVPYVKKDEMRNLLKGLKKTLDDKERSLRAAVSVNVVERAKELCEANPGANVLVQQLEAFNNTKALDAALKQV

RTQLPDAAAMFFSVDADSKKIFCLSSVPKSAVEKGLKANEWVQHVSPTLGGKGGGKPESAQASGSNYDRVEEIIQLATKF

AQSKLA

>XP_022208191.1 alanine--tRNA synthetase, cytoplasmic [Drosophila obscura]

MKYLTAKEVRDAYLGFFKEQKHIYVHSSSTIPLDDPTLLFANAGMNQFKPIFLGTADPNSEMSKWVRVCNTQKCIRAGGK

HNDLDDVGKDVYHHTFFEMLGNWSFGDYFKKEICTWAWEFLTDRLNLPKDRLYVTYFGGDKASGLEPDLECKQLWLDLGL

KPEHILPGSMKDNFWEMGETGPCGPCSELHFDRIGGRSVPELVNMDDPDVLEIWNLVFIQYNRESDGSLKQLPKKHIDCG

MGYERLVSVIQNKRSNYDTDLFVPLFEAIQAGTGAPAYQGRVGADDVDGIDMAYRVLADHARTITIALADGGTPDNTGRG

YVLRRILRRAVRYATEKLNAKPGFFGTLVNTVVDLLGDAFPEVKRDPQHIIDIINEEELQFLKTLTRGRNLLNRTIEKLG

SQSIIPGDVAWRLYDTYGFPVDLTQLMAEEKSLEIDMAGYEAAKQNSYVLSQGKGASKIDEINLDVHAISQLQEQGVPPT

NDSFKYKYEAVSEERDSAYNYQECTSKIVALRYENQFVNEISNGQKAGIVLDKTNFYAESGGQIFDQGAFVKLSDEANEF

LVDRVYNRGGYILHIGVVEGTLKVGDELQLHIDEERRWLTMKNHSATHALNHCLLQVLGKDTEQKGSLVVPEKLRFDFNT

KAAMTIEQVAKTEQLTKEMVYKNVPIYAKESKLALAKRIRGLRSVFDEVYPDPVRVISFGVPVDELEQNPDAEAGEQTSV

EFCGGTHLRRSGHIIDFVISSEEAIAKGIRRIVALTGPEALKAIKKSEAFEQEIVKLKATIDADKSGKDSKAHVKEIVEL

TEQISHATVPYVKKDEMRNLLKGLKKTLDDKERTLRAAVSVNVVERAKELCEANPGANLLVQQLEAFNNTKALDAALKQV

RTQLPDAAAMFFSVDADSKKIFCLSSVPKSAVEKGLKANEWVQHVSPTLGGKGGGKPESAQASGSNYERIEEILQLATKF

AQSKLA

>XP_020807847.1 alanine--tRNA synthetase, cytoplasmic [Drosophila serrata]

MKFLTAKEVRDAYLGFFKEQKHIYVHSSSTIPLDDPTLLFANAGMNQFKPIFLGTADPNSEMSKWVRVANTQKCIRAGGK

HNDLDDVGKDVYHHTFFEMLGNWSFGDYFKKEICSWAWQFLTERLGLPKDRLYVTYFGGDEASGLEPDLECKQLWLDLGL

KPEHILPGSMKDNFWEMGETGPCGPCSELHFDRIGGRSVPELVNMDDPDVLEIWNLVFIQYNRESDGSLKQLPKKHIDCG

MGFERLVSVIQNKRSNYDTDLFVPLFDAIQAGTGAPAYQGRVGADDSDGIDMAYRVLADHARTITIALADGGTPDNTGRG

YVLRRILRRAVRYATEKLNAKPGFFATLVNTVVDLLGDAFPEVKKDPQHIIDIINEEELQFLKTLTRGRNLLNRTIEKLG

TATIIPGDVAWRLYDTYGFPVDLTQLMAEEKSLQIDMAGYEAAKQNSYVLSQGKGASKIEEINLDVHAISQLQEQNVPPT

DDSFKYKYEAVSEERDSAYNYGECTSKIVALRFENQFVNEISNGQKAGIVLDKTNFYAESGGQIYDQGALVKVNDEANEF

LVDRVYNRGGYILHIGVVEGTLKVGDELHLHIDVERRWLTMKNHSATHALNHCLLQVLGKDTEQKGSLVVPEKLRFDFNS

KAAMTIQQVAKTEQLTKDMVYKNVPIYAKESKLAVAKQIRGLRSVFDEVYPDPVRVISFGVPVDELEQNPDSEAGEQTSV

EFCGGTHLRRSGHIMDFVISSEEAIAKGIRRIVALTGPEALKALKKSEVFEQEIIRLKATIDADQSGKDSKSHVKEIVEL

TEQISHATIPYVKKDDMRNLLKGLKKTLDDKERALRAAVSVTVVERAKALCEANPKATVLVEQLEAFNNTKALDAALKQV

RTQLPEAAAMFFSVDVDSKKIFCLSSVPKSAVEKGLKANEWVQHVSPTLGGKGGGKPESAQASGTNYEKVDEIVQMANKF

AQAKLA

>XP_017848622.1 PREDICTED: alanine--tRNA synthetase, cytoplasmic [Drosophila busckii]

MKFLTAKEVRDAYLNFFKEQKHIYVHSSSTIPMDDPTLLFANAGMNQFKPIFLGTVDPSSEMAKWVRVANTQKCIRAGGK

HNDLDDVGKDVYHHTFFEMLGNWSFGDYFKKEICSWAWEFLTVRLGLPKDRLYVTYFGGDAASGLEPDLECKQMWLDLGL

KPEHILPGSMKDNFWEMGETGPCGPCSELHFDRIGGRSVPELVNMDDPDVLEIWNLVFIQYNREADSSLKQLPKKHIDCG

MGFERLVSVIQNKRSNYDTDLFVPLFEAIQAGTNAPAYQGRVGADDVDGIDMAYRVLADHARTITIALADGGTPDNTGRG

YVLRRILRRAVRYATEKLNAKPGFFASLVNTVVELLGDAFPEVKKDPQHIMDIINEEELQFLKTLTRGRNLLNRTIEKLG

ANQTTIPGDVAWRLYDTYGFPVDLTQLMAEEKSMQIDMAGYDAAKQNSYILSQGKGASKIEEINLDVHAISQLQEQGVPP

SNDSFKYKYEAESDEHNSPYNFSECTSKIVALRFENQFVNEISSGQKAGIVLDKTNFYAESGGQIYDQGALVKVNDEANE

FLVDRVYNRGGYILHIGVVEGHLSVGDEVQLHIDVERRWLTMKNHSATHALNHCLLQVLGKDTEQKGSLVVPEKLRFDFN

CKAAMTIEQVAKTEQLTRDMVYKNMPIYAKESKLAMAKRIRGLRSVFDEVYPDPVRVISFGVPVDQLEEQPDAEAGEQTS

VEFCGGTHLRRSGHIMDFVISSEEAIAKGIRRIVALTGPEALKALKKSEAFEQEIAKLQATIEADKAGKDSKLYVKQIVE

LTEQISHATIPYVKKDEMRNLLKSLKKTLDDKERALRAAVSVTVVERAKALCDADPKATVLVQQLEAYNNTKALDAALKQ

VRSQLPDAAAMFISVDADSKKIFCLCSVPKTAVEKGLKANEWVAHVSPALGGKGGGKPESAQASGSNYERVDEIVQLACQ

FAKTKLA

>XP_016989274.1 PREDICTED: alanine--tRNA synthetase, cytoplasmic [Drosophila rhopaloa]

MKFLTAKEVRDAYLGFFKEQKHIYVHSSSTIPLDDPTLLFANAGMNQFKPIFLGTADPNSEMSKWVRVANTQKCIRAGGK

HNDLDDVGKDVYHHTFFEMLGNWSFGDYFKKEICSWAWEFLTKRLSLPKDRLYVTYFGGDEASGLEPDLECKQMWLDLGL

KPEHILPGSMKDNFWEMGETGPCGPCSELHFDRIGGRSVPELVNMDDPDVLEIWNLVFIQYNRESDGSLKQLPRKHIDCG

MGFERLVSVIQNKRSNYDTDLFVPLFEAIQAGTGAPAYQGRVGADDVDGIDMAYRVLADHARTITIALADGGTPDNTGRG

YVLRRILRRAVRYATEKLNAKPGFFATLVNTVVDLLGDAFPEVKKDPQHIIDIINEEELQFLKTLTRGRNLLNRTIEKLG

NQTTIPGDVAWRLYDTYGFPVDLTQLMAEEKSLQIDMDGYEAAKQNSYVLSQGKGASKIEEINLDVHAISQLQDQGVPPT

NDTFKYKYEAVSEERDSAYNYGVCNSKIVALRFENQFVNEISSGQKAGIVLDKTNFYAESGGQIYDQGALVKVNEEANEF

LVDRVYNRGGYILHIGVVEGTLKVGDELELHIDVERRWLTMKNHSATHALNHCLLQVLGKDTEQKGSLVVPEKLRFDFNS

KGAMTIEQVAKTEQLTKDMVYKNVPIYAKESQLALAKKIRGLRSVFDEVYPDPVRVISFGVSVDELEQNPDSEAGEQTSV

EFCGGTHLRRSGHIMDFVISSEEAIAKGIRRIVALTGPEALKALKKSEAFEQEIIRLKATIDADQSGKDSKSHVKEIVEL

TEQISHATIPYVKKDEMRNLLKGLKKTLDDKERALRAAVSVTVVERAKALCEANPKATVLVEQLEAFNNTKALDAALKQV

RSQLPDAAAMFLSVDVDSKKIFCLSSVPKSAVEKGLKANEWVQHVSATLGGKGGGKPESAQASGTNYEKADEIVQLASKF

AQSKLS

>XP_001357433.1 uncharacterized protein Dpse_GA12250, isoform A [Drosophila pseudoobscura pseudoobscura]

MKFLTAKEVRDAYLGFFKEQKHIYVHSSSTIPLDDPTLLFANAGMNQYKPIFLGTADPNSEMSKWVRVCNTQKCIRAGGK

HNDLDDVGKDVYHHTFFEMLGNWSFGDYFKKEICTWAWEFLTNRLHLPKDRLYVTYFGGDEASGLEPDLECKQLWLDLGL

KPEHILPGSMKDNFWEMGETGPCGPCSELHFDRIGGRSVPELVNMDDPDVLEIWNLVFIQYNRESDGSLKQLPKKHIDCG

MGFERLVSVIQNKRSNYDTDLFVPLFEAIQAGTGAPAYQGRVGADDVDGIDMAYRVLADHARTITIALADGGTPDNTGRG

YVLRRILRRAVRYATEKLNAKPGFFGTLVNTVVDLLGDAFPEVKKDPQHIIDIINEEELQFLKTLTRGRNLLNRTIEKLG

SQSIIPGDVAWRLYDTYGFPVDLTQLMAEEKSLEIDMAGYEAAKQNSYVLSQGKGASKIEEINLDVHAISQLQEQGVPPT

NDSFKYKYEAVSEERDSAYNYQECTSKIVALRFENQFVNEISNGQKAGIVLDKTNFYAESGGQIFDQGALVKLNDEANEF

LVDRVYNRGGYILHIGVVEGTLKVGDELQLHIDEERRWLTMKNHSATHALNHCLLQVLGKDTEQKGSLVVPEKLRFDFNT

KAAMTIEQVAKTEQLTKDMVYKNVPIYAKESKLALAKRIRGLRSVFDEVYPDPVRVISFGVPVDELEQNPDAEAGEQTSV

EFCGGTHLRRSGHIMDFVISSEEAIAKGIRRIVALTGPEALKAIKKSEAFEQEIVKLKATIDADKSGKDSKSHVKEIVEL

TEQISHATVPYVKKDEMRNLLKGLKKTLDDKERSLRAAVSVNVVERAKELCEANPGANVLVQQLEAFNNTKALDAALKQV

RTQLPDAAAMFFSVDADSKKIFCLSSVPKSAVEKGLKANEWVQHVSPTLGGKGGGKPESAQASGSNYDRVEEIIQLATKF

AQSKLA

>XP_017068517.1 PREDICTED: alanine--tRNA synthetase, cytoplasmic [Drosophila eugracilis]

MKFLTAKEVRDAYLGFFKEQKHIYVHSSSTIPLDDPTLLFANAGMNQFKPIFQGTADPNSEMAKWVRVANTQKCIRAGGK

HNDLDDVGKDVYHHTFFEMLGNWSFGDYFKKEICSWAWEFLTQRLNLPKDRLYVTYFGGDEASGLEPDLECKQMWLDLGL

KPEHILPGSMKDNFWEMGETGPCGPCSELHFDRIGGRSVPELVNMDDPDVLEIWNLVFIQYNRESDGSLKQLPKKHIDCG

MGFERLVSVIQNKRSNYDTDLFVPLFEAIQAGTGAPAYQGRVGADDVDGIDMAYRVLADHARTITIALADGGTPDNTGRG

YVLRRILRRAVRYATEKLNAKPGFFATLVNTVVDLLGDAFPEVKKDPQHIIDIINEEELQFLKTLTRGRNLLNRTIEKLG

NQTTIPGDVAWRLYDTYGFPVDLTQLMAEEKSLKIDMDGYEAAKQNSYVLSQGKGASKIEEINLDVHAISQLQEQGVPPT

NDSFKYKYEAVSEERDSAYNYGVCNSKIVALRFENQFVNEISSGQKAGIVLDNTNFYAESGGQIYDQGALVKVNDEANEF

LVDRVYNRGGYILHIGVVEGTLKVGDELELHIDIERRWLTMKNHSATHALNHCLLQVLGKDTEQKGSLVVPEKLRFDFNS

KAAMTIEQVAKTEQLTKDMVYKNVPIYAKESKLALAKKIRGLRSVFDEVYPDPVRVISFGVSVDELEQNPDSEAGEKTSV

EFCGGTHLRRSGHIMDFVISSEEAIAKGIRRIVALTGPEALKALKKSEAFEQEIVRLKATIDTDQSGKDSKSHVKEIVEL

TEQISHATIPYVKKDEMRNLLKGLKKTLDDKERAMRAAVSVTVVERAKALCEANPKATVLVEQLEAFNNTKALDAALKQV

RSQLPDAAAMFLSVDVDSKKIFCLSSVPKSAVEKGLKANEWVQHVSATLGGKGGGKPESAQASGTNYEKVDEILQLANKF

AQSKLS

>XP_017132354.1 PREDICTED: alanine--tRNA synthetase, cytoplasmic [Drosophila elegans]

MKYLTAKEVRDAYLGFFKEQKHIYVHSSSTIPLDDPTLLFANAGMNQFKPIFLGTADPNSEMSKWVRVANTQKCIRAGGK

HNDLDDVGKDVYHHTFFEMLGNWSFGDYFKKEICSWAWEFLTQRLSLPKDRLYVTYFGGDEASGLEPDLECKQLWLDLGL

KPEHILPGSMKDNFWEMGETGPCGPCSELHFDRIGGRSVPELVNMDDPDVLEIWNLVFIQFNRESDGSLKQLPRKHIDCG

MGFERLVSVIQNKRSNYDTDLFVPLFEAIQAGTGAPAYQGRVGADDVDGIDMAYRVLADHARTITIALADGGTPDNTGRG

YVLRRILRRAVRYATEKLNAKPGFFATLVNTVVDLLGDAFPEVKKDPQHIIDIINEEELQFLKTLTRGRNLLNRTIEKLG

NQTIIPGDVAWRLYDTYGFPVDLTQLMAEEKSLQIDMDGYEAAKQNSYVLSQGKGASKIDEINLDVHGISQLQEQGVPPT

NDSFKYKYEAVSEERDSAYNYGVCNSKIVALRFENQFVNEISSGQKAGIVLDKTNFYAESGGQIYDQGALVKVNDEANEF

LVDRVYNRGGYILHIGVVEGTLKVGDELELHIDVERRWLTMKNHSATHALNHCLLQVLGKDTEQKGSLVVPEKLRFDFNS

KAAMTIEQVAKTEQLTKDMVYKNVPIYAKESQLALAKKIRGLRSVFDEVYPDPVRVISFGVSVDELEQNPDSEAGEQTSV

EFCGGTHLRRSGHIMDFVISSEEAIAKGIRRIVALTGPEAMKALKKSEAFEQEIIRLKATIDADKSGKDSKSHVKEIVEL

TEQISHATIPYVKKDEMRNLLKGLKKTLDDKERALRAAVSVTVVERAKALCEANPKATVLVEQLEAFNNTKALDAALKQV

RSQLPDAAAMFLSVDADSKKIFCLSSVPKSAVEKGLKANEWVQHVSATLGGKGGGKPESAQASGTNYEKVDEIVELASQF

AQSKLS

>XP_016940925.1 PREDICTED: alanine--tRNA synthetase, cytoplasmic [Drosophila suzukii]

MKFLTAKEVRDAYLGFFKEQKHIYVHSSSTIPLDDPTLLFANAGMNQFKPIFLGTADPNSEMSKWVRVANTQKCIRAGGK

HNDLDDVGKDVYHHTFFEMLGNWSFGDYFKKEICSWAWEFLTQRLNLPKDRLYVTYFGGDEASGLEPDLECKQLWLDLGL

KPEHILPGSMKDNFWEMGETGPCGPCSELHFDRIGGRSVPELVNMDDPDVLEIWNLVFIQYNRESDGSLKQLPRKHIDCG

MGFERLVSVIQNKRSNYDTDLFVPLFEAIQAGTGAPAYQGRVGADDVDGIDMAYRVLADHARTITIALADGGTPDNTGRG

YVLRRILRRAVRYATEKLNAKPGFFATLVNTVVDLLGDAFPEVKKDPQHIIDIINEEELQFLKTLTRGRNLLNRTIEKLG

NQSTIPGDVAWRLYDTYGFPVDLTQLMAEEKSLKIDMDGYEAAKQNSYVLSQGKGASKIEEINLDVHAISELQEKGVPPT

NDTFKYKYEAVSDERDSAYNYGVCNSKIVALRFENQFVNEISSGQKAGIVLDKTNFYAESGGQIYDQGALVKVNDEANEF

LVDRVYNRGGYILHIGVVEGTLKVGDELELHIDVERRWLTSKNHSATHALNHCLLQVLGKDTEQKGSLVVPEKLRFDFNS

KAAMTIEQVAKTEQLTKEMVYKNVPIYAKESKLALAKKIRGLRSVFDEVYPDPVRVISFGVSVDELEQSPDSEAGEQTSV

EFCGGTHLRRSGHIMDFVISSEEAIAKGIRRIVALTGPEALKALKKSEAFEQEIIRLKATIDADQSGKDSKSHVKEIVEL

TEQISHATIPYVKKDEMRNLLKGLKKTLDDKERALRAAVSVTVVERAKALCEANPQATVLVEQLEAFNNTKALDAALKQV

RSQLPDAAAMFLSVDADSKKIFCLSSVPKSAVEKGLKANEWVQHVSATLGGKGGGKPESAQASGTNYEKVDEIVLLASKF

AQSKLS

>XP_001847766.1 alanyl-tRNA synthetase [Culex quinquefasciatus]

MDTSLTAKQIRNIFLDFFKEKEHLYVHSSSTIPLDDPTLLFANAGMNQFKPIFLGTADPNSEMSKWVRTANTQKCIRAGG

KHNDLDDVGKDVYHHTFFEMLGNWSFGDYFKKEICTWAWELLTDRLKLPSDRLYVTYFGGHPESGLEPDLECRDIWLKLG

VKEEHILPGSMKDNFWEMGETGPCGPCSELHFDRIGGRSVPELVNMDDPDVLEIWNLVFIQFNREQDGSLKLLPKKHIDC

GMGYERLVSVIQDKRSNYDTDVFVPLFQAIQKGTGAPDYQGRVGAEDVDGVDMAYRVLADHARTITIALADGGFPDNTGR

GYVLRRILRRAVRYASEKLNGKPGFFATLVDTVVELLGDTFPEVKKDPQHIKNIINEEEQQFLKTLSRGRNLLNRTIAKL

GDSKVIPGDVAWRLYDTYGFPVDLTGLMAEEKNMTIDMEGYEKAKHQSYIISQGKEKSKTATVDLDVHAISELQEKKVPT

TNDTFKYHYKAESTDPLAAYVFEPCTGKVLALRYNNAFVDEVQAGQECAVILDKTNFYAESGGQIYDQGYLLKVNDESTE

FNVNLVYNRGGYVLHIGVVEGVLRVGDEVACHMDVVRRQLTMKNHSATHALNHSLLKVLGLDTDQRGSLVVPEKLRFDFT

NKAAMTIEQVAEAERLTREVVKKNMQVYAKESKLASAKAIKGLRSVFDEVYPDPVRVISFGVPVEDLEANPEGVAGTETS

VEFCGGTHLHQSGHMVDFVITTEEAIAKGIRRIVALTGPEAIKALKKTELLEGELNALKATIDADKTGADSREHVKKIVE

LNEDVSQAVIPYVKKDEIRNQLKSLKKTLDDKERAARAAVATTVVEAAKELCAANKDAPFIVHRLEAFSNTKALDSALKE

VRKLNVEQSALFVSSDPDTKKIFCLASVPKSAVEKGLKANEWISHISPSMGGKGGGKPESAQASGGAFDKVEEILELARA

FAASKLG

>XP_017152282.1 PREDICTED: alanine--tRNA synthetase, cytoplasmic [Drosophila miranda]

MKFLTAKEVRDAYLGFFKEQKHIYVHSSSTIPLDDPTLLFANAGMNQFKPIFLGTADPNSEMSKWVRVCNTQKCIRAGGK

HNDLDDVGKDVYHHTFFEMLGNWSFGDYFKKEICTWAWEFLTNRLHLPKDRLYVTYFGGDEASGLEPDLECKQLWLDLGL

KPEHILPGSMKDNFWEMGETGPCGPCSELHFDRIGGRSVPELVNMDDPDVLEIWNLVFIQYNRESDGSLKQLPKKHIDCG

MGFERLVSVIQNKRSNYDTDLFVPLFEAIQAGTGAPAYQGRVGADDVDGIDMAYRVLADHARTITIALADGGTPDNTGRG

YVLRRILRRAVRYATEKLNAKPGFFGTLVNTVVDLLGDAFPEVKKDPQHIIDIINEEELQFLKTLKRGRNLLNRTIEKLG

SQSIIPGDVAWRLYDTYGFPVDLTQLMAEEKSLEIDMVGYEAAKQNSYVLSQGKGASKIEEINLDVHAISQLQEQGVPPT

NDSFKYKYEAVSEERDSAYNYQECTSKIVALRFENQFVNEISNGQKAGIVLDKTNFYAESGGQIFDQGALVKLNDEANEF

LVDRVYNRGGYILHIGVVEGTLKVSDELQLHIDEDRRWLTMKNHSATHALNHCLLQVLGKDTEQKGSLVVPEKLRFDFNT

KAAMTIEQVAKTEQLTKDMVYKNVPIYAKESKLALAKRIRGLRSVFDEVYPDPVRVISFGVPVDELEQNPDAEAGEQTSV

EFCGGTHLRRSGHIMDFVISSEEAIAKGIRRIVALTGPEALKAIKKSEAFEQEIVKLKATIDADGSGKDSKSHVKEIVEL

TEQISHATVPYVKKDEMRNLLKGLKKTLDDKERSLRAAVSVNVVERAKELCEANPGANVLVQQLEAFNNTKALDAALKQV

RSQLPDAAAMFFSVDADSKKIFCLSSVPKSAVEKGLKANEWVQHVSPTLGGKGGGKPESAQASGSNYERVEEIIQLATKF

AQSKLA

>XP_002067163.1 alanine--tRNA synthetase, cytoplasmic [Drosophila willistoni]

MQFMTAKMVRDAYLGFFKEKKHIYVHSSSTIPLDDPTLLFANAGMNQFKPIFLGIADPNSEMSKWVRVANTQKCIRAGGK

HNDLDDVGKDVYHHTFFEMLGNWSFGDYFKKEICTWAWEFLTDRLKLPKDRLYVTYFGGDEASGLEPDLECKQLWLDLGI

KPEHVLPGSMKDNFWEMGETGPCGPCSELHFDRIGGRSVPELVNQDDPDVLEIWNLVFIQYNRESDGSLKPLPKKHIDCG

MGFERLVSVIQNKRSNYDTDLFQPLFEHIQSSTGAPAYQGRVGADDSDGIDMAYRVLADHARTITIALADGGTPDNTGRG

YVLRRILRRAVRYATEKLKAKPGMFASLVNTVVELLGDAFPEVKKDPQHIIDIINEEEQQFLKTLTRGRNLLNRTIEKLG

ADQKIIPGDVAWRLYDTYGFPVDLTQLMAEEKSLEIDMAGYEAAKQNSYILSQGKGSSKIEEINLDVHAISQLQDQGVPT

TNDSFKYKYEAESEERDSPYNYETCSSKILAIRFENQFVQSISNGQKAGIVLDNTNFYAESGGQIYDQGALVKANDEANE

FLVDRVYNRGGYILHIGVVEGTLAVGDDLQLHIDVTRRWLTMKNHSATHALNHCLLQVLGKDTEQKGSLVVPEKLRFDFN

TKGAMTIEQVAKTEELTRQMVYKNVPIYAKESKLAVAKSIRGLRSVFDEVYPDPVRVISFGVPVEQLEEQPNGEAGDNTS

VEFCGGTHLRRSGHIMDFVISSEEAIAKGIRRIVALTGPEAIKAIKKSEQFEQEIVTLKATIEADTTGKDSKQHVKQIVE

LTEQISHATIPYVKKDEMRNLLKSLKKTLDDKERALRAAVSVTVVERAKALCEENPTATVLVQQLEAYNNTKALDAALKQ

VRSQLPDTAAMFFSVDVDSKKIFCLSSVPKSAVEKGLKANEWVQHLSQSLGGKGGGKPESAQASGTNYESVDEILQLATK

FAQTKLA

>XP_006613091.1 PREDICTED: alanine--tRNA synthetase, cytoplasmic-like isoform X1 [Apis dorsata]

MTTMMTAKQIRQAYIDFFKNKGHEYVHSSSTIPHDDPTLLFTNAGMNQFKPIFLGIVDPNSDMAKLVRVVNSQKCIRAGG

KHNDLDDVGKDVYHHTFFEMMGNWSFGDYFKKEICTWAWEFLTVKLKLSSDRLYVTYFGGDEKNNLKPDEECKQIWLSLG

LSPTHILPGNMKDNFWEMGETGPCGPSSEIHYDRIGNREAAHLVNQDNPDVLEIWNLVFIQFNRENDGNLKLLPKKHIDC

GLGLERLVSIIQNKSANYDTDLFMPLFDAIQKGTKAPSYQGKVGKEDKNGIDMAYRVLADHARTITIALADGGIPDNTGR

GYVLRRILRRAVRYAIEKLNAKPGFFGSLVNVVVDLLGDVFPEVTKDPQHIIDIINEEETQFLKTLSRGRNLLNRTIVKL

ESSNILPGDVAWRLYDTYGFPFDLTQLMVEEKGLKVDIIGYEEAKKQAQLISQNKSGEVDDQINLDVHAITELQNKKIKP

TNDIPKYNYKVINNKIYEEYEFAPCFSTVIALRRAKTFVDEVSSGEEVGILLDQTNFYAEQGGQIYDEGFLTKIDDEDTE

IHVKNVQVRGGYVLHIGIVIQGKLKKNDKVFSNIDITRRRLLMSNHTATHVLNYALREILGTEVDQKGSLVAPDRLRFDF

TNKGAMITEQIRKVEEITNNIINENKIVYAKECNLGLAKTIQGLRAMFEETYPDPVRVVSIGIPVEDLEKNPLSSDALKT

SVEFCGGTHLHYTGHIRNFIIVSEEAIAKGIRRIVALTGPEAMKAQKKTSILQNYLDELQISIEADKNGINTKEHIKKIV

ELTDDVSHAIISSWKKDKIRKILKDLKKLLDDKERAIKTAIANVVLDNIQQIIQQNIGCLVLVEVLQAYSNTKALDSALK

KIRTISPETSALLISIDPDAKKIFALSSVSKSAINKGLKANEWIQEIVPLMEGKGGGKSESAQASGTNISCVTRLIYTAK

NFANSKLELAENISENHDQLKGDEKFIYFKTLKDKLVLSGDIGSIKYYRIQIVAKYNNIELITHKNNIFKGIKLQGNDFE

LFDSNAIAFYLSTDQFKCSNNPFTFSEVLQWLSYADNHILPAVLGWVVPCLSKNIPNNIKTNIKISKEDVLSSLQKLNNI

LLTKTYLVGERISLADISVFTTLMPLYEYVLDSEYRKQYTNLNRWFFTILNQSQVISVIKKFNICEKVKIVKY

>XP_001988798.1 GH11356 [Drosophila grimshawi]

MKLMTAKEVRNAYLDFFKEQKHIYVHSSSTIPLDDPTLLFTNAGMNQFKPIFLGTADPNSEMSKWVRTVNTQKCIRAGGK

HNDLDDVGKDVYHHTFFEMLGNWSFGDYFKKEICSWAWEFLTERLRLPKDRLYVTYFGGDAASGLQPDLECKQFWMDLGL

KPEHILPGSMKDNFWEMGETGPCGPCSELHFDRIGGRSVPELVNQDDPDVLEIWNLVFIQFNRESDGSLKPLPKKHIDCG

MGFERLVSVIQNKRSNYDTDLFVPLFDAIQAGTGAPAYQGRVGADDVDGIDMAYRVLADHARTITIALADGGNPDNTGRG

YVLRRILRRAVRYATEKLNAKPGFFASLVSTVVDLLGDAFPEVKKDPQHIMDIINEEELQFLKTLTRGRNLLNRTIEKLG

TSQTTIPGDVAWRLYDTYGFPVDLTQLMAEEKSLQIDMAGYEAAKQNSYILSQGKGASKIDEITLDVHAISELQEKGLPS

TDDSFKYNYEAVSVERDAAYNFSECTSKIVALRFENKFVQEISSGQKAGIVLDKTNFYAESGGQIYDQGVLVKLNDEANE

FLVDRVYNRGGYILHIGVVEGNLAVGDELQLHIDVERRWLTMKNHSATHALNHCLLQVLGKDTEQKGSLVVPEKLRFDFN

CKAAMTIDQVARTEKLTRDMVYQNMPIYAKESQLALAKRIRGLRSVFDEVYPDPVRVISFGVPVDRLESQPDAETGEQTS

VEFCGGTHLRNSGHIMDFVISSEEAIAKGIRRIVALTGPEALKAIKKSEAFEQEISQLQATIEADQSGKESKMYVKLIVE

LTEQISHATIPHVKKDEMRNLLKLLKKSLDDKERALRAAVSVTVVERTKALCEAEPQAKVLVHQLEAYNNTKALDAALKQ

VRLQLPEAAAMFVSVDADSKKIFCLCSVPKAAVEKGLKANEWVQHVSSILGGKGGGKAESAQASGSNFERVDEIVQLANQ

FAQAKLN

>PBC27482.1 Alanyl-tRNA synthetase, cytoplasmic [Apis cerana cerana]

MTTMMTAKEIRQAYIDFFKSKGHEYVHSSSTIPHDDPTLLFTNAGMNQFKPIFLGIVDPNSDMAKLVRVVNSQKCIRAGG

KHNDLDDVGKDVYHHTFFEMMGNWSFGDYFKKEICTWAWEFLTVKLKLSSDRLYVTYFGGDEKNNLKPDEECKQIWLSLG

LSPTHILPGNMKDNFWEMGETGPCGPSSEIHYDRIGNREATHLVNQDDPDVLEIWNLVFIQFNRENDGNLKLLPKKHIDC

GLGLERLVSIIQNKSANYDTDLFMPLFDAIQKGTKAPSYQGKVGKEDKNGIDMAYRVLADHARTITIALADGGVPDNTGR

GYVLRRILRRAVRYAIEKLNAKPGFFGSLVNVVVDLLGDVFPEVTKDPQHIIDIINEEEIQFLKTLSRGRNLLNRTIVKL

ESSNILPGDVAWRLYDTYGFPFDLTQLMAEEKGLKVDIIGYEKAKKQAQLISQNKSGEVDDQINLDVHAITELQNKKIKP

TNDIPKYNYKVINTKIYEEYEFAPCFSTIIALRRAKTFVDEVSSGEEVGILLDQTNFYAEQGGQIYDEGFLTKIDDEDSE

VHIKNVQVRGGYILHIGIVSQGKLKKNDKVFSNIDTTRRRLLMSNHTATHVLNYALREILGTEVDQKGSLVAPDRLRFDF

TNKGAMTTEQIRKVEEITNNIINENKMVYAKECNLGIAKTIQGLRAMFEETYPDPVRVISIGIPVEDLEKNPLSSDALKT

SVEFCGGTHLHYTGHIRNFIIVSEEAIAKGIRRIVALTGPEAMKAQKKTSMLENYLNELQITIEADKNGINTKEHVKKII

ELTDDVSHAIISSWKKDKIRKMLKDLKKILDDKERAIKTAIANVVLDNIQQIIQQNIGCLVLVEVLQAYSNTKALDSALK

KIRTISPETSALLISIDPDAKKIFALSSVSKYAISKGLKANEWIQEIVPLMEGKGGGKSESAQASGTNISCVTRLIYTAK

NFANSKLELSENINENHDQLKRDEKFIYFKTLKDKLVLSGDIGSIKYYRIQIVAKYNNIELITVPHKNNIFKGIKLQGND

FELFDSNAIAFYLSTDQFKCSNNSFTFSEVLQWLSYADNHILPAVLGWVVPYLSKNIPNNIKTNIKTSKEDVLSSLQKLN

NILLTKTYLVGERISLADISVFTTLMPLYEYVLDSEYRKQYTNLNRWFFTILNQSQFL

>XP_016967520.1 PREDICTED: alanine--tRNA synthetase, cytoplasmic [Drosophila biarmipes]

MKFLTAKEVRDAYLGFFKEQKHIYVHSSSTIPLDDPTLLFANAGMNQFKPIFLGTADPNSEMSKWVRVANTQKCIRAGGK

HNDLDDVGKDVYHHTFFEMLGNWSFGDYFKKEICSWAWEFLTQRLNLPKDRLYVTYFGGDEASGLEPDLECKQLWLDLGL

KPEHILPGSMKDNFWEMGETGPCGPCSELHFDRIGGRSVPELVNMDDPDVLEIWNLVFIQYNRESDGSLKQLPRKHIDCG

MGFERLVSVIQNKRSNYDTDLFVPLFEAIQAGTGAPAYQGRVGADDVDGIDMAYRVLADHARTITIALADGGTPDNTGRG

YVLRRILRRAVRYATEKLNAKPGFFATLVNTVVDLLGDAFPEVKKDPQHIIDIINEEELQFLKTLTRGRNLLNRTIEKLG

NQTTIPGDVAWRLYDTYGFPVDLTQLMAEEKSLKIDMDGYEAAKQNSYVLSQGKGASKIEEINLDVHAISELQEKGVPPT

NDTFKYKYEAVSEERDSAYNYGVCNSKIVALRFENQFVNEISSGQKAGIVLDKTNFYAESGGQIYDQGALVKVNDEANEF

LVDRVYNRGGYILHIGVVEGTLKVGDELELHIDVERRWLTMKNHSATHALNHCLLQVLGKDTEQKGSLVVPEKLRFDFNS

KAAMTIEQVSKTEQLTKEMVYKNVPIYAKESKLALAKKIRGLRSVFDEVYPDPVRVISFGVSVDELEQNPDSEAGEQTSV

EFCGGTHLRRSGHIMDFVISSEEAIAKGIRRIVALTGPEALKALKKSEAFEQEIVRLKATIDADKSGKDSKSHVKEIVEL

TEQISHATIPYVKKDEMRNLLKGLKKTLDDKERALRAAVSVTVVERAKALCEANPQATVLVEQLEAFNNTKALDAALKQV

RSQLPDAAAMFLSVDADSKKIFCLSSVPKSAVEKGLKANEWVQHVSAALGGKGGGKPESAQASGTNYEKVDEIVQLASKF

AQSKLS

>XP_016922418.1 PREDICTED: alanine--tRNA synthetase, cytoplasmic [Apis cerana]

MTTMMTAKEIRQAYIDFFKSKGHEYVHSSSTIPHDDPTLLFTNAGMNQFKPIFLGIVDPNSDMAKLVRVVNSQKCIRAGG

KHNDLDDVGKDVYHHTFFEMMGNWSFGDYFKKEICTWAWEFLTVKLKLSSDRLYVTYFGGDEKNNLKPDEECKQIWLSLG

LSPTHILPGNMKDNFWEMGETGPCGPSSEIHYDRIGNREATHLVNQDDPDVLEIWNLVFIQFNRENDGNLKLLPKKHIDC

GLGLERLVSIIQNKSANYDTDLFMPLFDAIQKGTKAPSYQGKVGKEDKNGIDMAYRVLADHARTITIALADGGVPDNTGR

GYVLRRILRRAVRYAIEKLNAKPGFFGSLVNVVVDLLGDVFPEVTKDPQHIIDIINEEEIQFLKTLSRGRNLLNRTIVKL

ESSNILPGDVAWRLYDTYGFPFDLTQLMAEEKGLKVDIIGYEKAKKQAQLISQNKSGEVDDQINLDVHAITELQNKKIKP

TNDIPKYNYKVINTKIYEEYEFAPCFSTIIALRRAKTFVDEVSSGEEVGILLDQTNFYAEQGGQIYDEGFLTKIDDEDSE

VHIKNVQVRGGYILHIGIVSQGKLKKNDKVFSNIDTTRRRLLMSNHTATHVLNYALREILGTEVDQKGSLVAPDRLRFDF

TNKGAMTTEQIRKVEEITNNIINENKMVYAKECNLGIAKTIQGLRAMFEETYPDPVRVISIGIPVEDLEKNPLSSDALKT

SVEFCGGTHLHYTGHIRNFIIVSEEAIAKGIRRIVALTGPEAMKAQKKTSMLENYLNELQITIEADKNGINTKEHVKKII

ELTDDVSHAIISSWKKDKIRKMLKDLKKILDDKERAIKTAIANVVLDNIQQIIQQNIGCLVLVEVLQAYSNTKALDSALK

KIRTISPETSALLISIDPDAKKIFALSSVSKYAISKGLKANEWIQEIVPLMEGKGGGKSESAQASGTNISCVTRLIYTAK

NFANSKLELSENINENHDQLKRDEKFIYFKTLKDKLVLSGDIGSIKYYRIQIVAKYNNIELITVPHKNNIFKGIKLQGND

FELFDSNAIAFYLSTDQFKCSNNSFTFSEVLQWLSYADNHILPAVLGWVVPYLSKNIPNNIKTNIKTSKEDVLSSLQKLN

NILLTKTYLVGERISLADISVFTTLMPLYEYVLDSEYRKQYTNLNRWFFTILNQSQVISVIKKFNICEKVKIVKY

>XP_008211711.2 PREDICTED: LOW QUALITY PROTEIN: alanine--tRNA synthetase, cytoplasmic [Nasonia vitripennis]

MAGKTAKDIRQAYIDFFKSKDHEYVHSSSTIPHDDPTLLFTNAGMNQFKPIFLGTVDPNSDMAKWVRVVNSQKCIRAGGK

HNDLDDVGKDVYHHTFFEMMGNWSFGDYFKKEICEWAWEFLTQKLNLPADRLYVTYFGGDEKSNLKPDEECKNFWLSLGV

PESHVLPGSMKDNFWEMGETGPCGPCSELHYDRIGGREAAHLVNMDDPDVLEIWNLVFIQYNREQNGELQPLPKKHIDCG

LGLERLVSVIQNKRSNYDTDLFTPLFEAIQKGTGAPAYQGRVGNDDKDGIDMAYRVLADHARTITIALADGGVPDNTGRG

YVIRRILRRAVRYATEKLNAQPGFFATLINVVVELLGDVFPEVKRDPQSIIDIVNEEEVQFLKTLSRGRNLLNRTIAKLE

STKVVPGDVAWRLYDTYGFPVDLTQLMAEEKGLTIDVAAYEEAKKLAIIASQGKAGGVNDQINLDVHAITELQNKGVKTT

DDSPKYNYKVAKTEKYAEYTFAPCTGTVLALRKDKTFVDEVKSGDEVGVLLDQTNFYAEQGGQIYDEGFLVKVDDESTEI

RVTNVQVRGGYVLHIGTVGEGTLKKGDKLYLNVDTSRRRLIMSNHTATHALNHALRSVLGTDKESPLAIARTIQGLRAMF

GETYPDPVRVVSMGVTVEELEKDPLSPAGAKTSVEFCGGTHLHRTGHIGDFVIASEEAIAKGIRRIVALTGPEASKALKK

AELLQNNLNQIKSSVETVKDSATSKEIVKKIVELTDDVSHAVIPYWKKEEMRSELKALKKALDDKERSAKAAVANAVVET

AVNIIESNKGAPVLVEVLNAFNNTKALDAALKKVKAISPETSALFISVDEDEKKIFALSAVPKSAIAKGLKANEWIQAIA

SMMQGRGGGKPESAQASGPNISCLKEALATAKTYAYSVLKLEEETKTSVDTSCSKSTSELSVYANTGSVKGYLVQIAARY

AGKCLKVQNHTKNTRESGPIVYKDDTITLNDGNAIAFYLADDQLKGTDLFAQSEVLQWMSFSDNHILPSVCSWVLPTIGA

VKSSKDSTKEARNNVLQILEALNKTLLKKTYLVGERITLADIAVFITLSPAYQHTLDLETRKKYQNTNRWFETILQQSNV

KTVFEDFKYCQKK

>XP_002426803.1 Alanyl-tRNA synthetase, putative [Pediculus humanus corporis]

MPPLSSKEVRQTFIDFFIKKKQHQYVHSSSTIPLDDPTLLFANAGMNQFKPIFLGTADPNSDMAKWVRVVNTQKCIRAGG

KHNDLDDVGKDVYHHTFFEMLGNWSFGDYFKKEICAWAWELLTTVYHISPDRLYVTYFGGDSASGLEPDSECKKIWIDLG

VPESHVLPGNMKDNFWEMGETGPCGPCSELHYDRIGNRNASHLVNQDDPDVLEIWNLVFIQFNRETDGSLKTLPKRHIDC

GMGFERLVSVIQNKRSNYDTDIFTPLFDAIQKGTGSPPYKGKVGAEDVDGIDMAYRVLADHARTLTIALADGGRPDNVGR

GYVLRRILRRAIRYGTEKLNTKPKFFSSLVKTVCDTLGDTFSEIRRDPDTIMEIINEEEEQFLKTLSRGRNLLNRTIAKI

GKSKVLPGDVAWRLYDTYGFPVDLTQLMAEEKGLSVDMTGYEEARKQAQLLSQGKEGKVDDGIALDVHAITELQNLKIPP

TDDSPKYNYKAKQQDKNAEYEFQKCEAKITAIRHKKKFVDSINSGEKCGILLDRTLFYAEQGGQIFDEGYMIKKNDENVE

FSIYNVQVKAGYVLHFGTTEGVFKKGDTVVLHLDTARRRLVMNNHTGTHILNFALREVLGSGCEQKGSLVAPDRLRFDFT

NKSAMTPDQIKAAEDSVKRVIAKNCEVFAKNTPLAVAKTVNGVRAMFDEHYPDPVRIVSVGAAVEDLVANPSSSLGMSVP

AEFCGGTHLHRSGHVGDFVISSEEAIAKGIRRIVALTGPEASKALKKAAILENEVNEIKQKMNDSSAQKMIPKKIISLTD

DISHAVIPYWKKEELRNALKVLKKQLDDKERANKAALAAGVVEQAKKIIEANLNKPFIVAEVKAFSNNKAVDTALKQVKA

LSPNTAAMFFSVDPDANKIFYLASVPKSANSQGLSAGDWVNHVSPLLKGKGGGKAESAQGSGSNFSALNEAMDAARKFAS

SKLGEGSQVVVGTPSSGKLFISF

>XP_022668440.1 alanine--tRNA synthetase, cytoplasmic-like isoform X1 [Varroa destructor]

MMSTAVSTSRPDVLKIFVKTLTGRTITLEVEPSETIATVKEKICDKLLTETKRMSSAKEIRSKFIAFFRDKYAHTEVPSS

CVIPHDDPTLLFTNAGMNQFKPIFLGTVDPSTEAAKWKRTVDTQKCIRAGGKHNDLDDVGKDVYHHTFFEMLGNWSFGDY

FKKEICAWAWELLTKEYGLAEDRLYVTYFGGNKDADLEPDEECKNIWKALGLPENRILPGNMKDNFWEMGETGPCGPCSE

IHYDRLGGRNAAHLVNMDDPDVLEIWNLVFIQFNRESDGKLKPLPKKHVDTGMGLERLVSVIQNKRANYDTDLFVPIFEA

IQKATGVRAYTGKVGDDDTDGIDMAYRVLADHARTLSIALSDGGHPDNVGRGYVLRRILRRAVRYATEKMGAQPGMFAGL

VPVVVEILGDVFPELRKDPEGIMEIINEEEAQFLKTLSRGRRLLEKSISKLERKALLPGEVAWRLYDTYGFPVDLTQLMV

EEKGIFVDMVAYEKAKQAAQLASQGGGDRADDTSKLDVHAIAELQDQKKVPATDDSPKYNYSSKYMGREALYEFESCKGK

VVAIRMNKQFVDEAHSGSLCGLILDRTSFYAEQGGQIYDTGFMQAVCADDDVEFAVNEVHLQGGYVVHIGRIVEGTVKVG

DELRLLIDEMRRLSIMRNHTGTHVLNYALRRVLSADADQRGSLVAPERMRFDFTSNRPMTAAEIRQAEEAASELIWKNGN

VFAKDSSLAQAKAVKGLRAVFNETYPDPVRVVSIGVPVDELLANPDRGDALNTSVEFCGGTHLVNVSHIADFVISQEEAI

AKGIRRVVALTGLEAAKAVKRAELFEAKVNCIKEDLSKNKSLAKRIAELSEEISQSTISCWRKDDLRNELKKVKKVLDDQ

DKQNKAAAQMEALEEIKRELTDRGRAPQFLVKQLQVGNNAKALDIVLKQIRALAPSAAAMLFSVDEERGRLICLCGVPKQ

VVADKGLSAVEWVRQVAEVIGGKGGGKDESAQASGTNPHALNRAMELAKEFARLKFV

>XP_022668441.1 alanine--tRNA synthetase, cytoplasmic-like isoform X2 [Varroa destructor]

MQIFVKTLTGRTITLEVEPSETIATVKEKICDKLLTETKRMSSAKEIRSKFIAFFRDKYAHTEVPSSCVIPHDDPTLLFT

NAGMNQFKPIFLGTVDPSTEAAKWKRTVDTQKCIRAGGKHNDLDDVGKDVYHHTFFEMLGNWSFGDYFKKEICAWAWELL

TKEYGLAEDRLYVTYFGGNKDADLEPDEECKNIWKALGLPENRILPGNMKDNFWEMGETGPCGPCSEIHYDRLGGRNAAH

LVNMDDPDVLEIWNLVFIQFNRESDGKLKPLPKKHVDTGMGLERLVSVIQNKRANYDTDLFVPIFEAIQKATGVRAYTGK

VGDDDTDGIDMAYRVLADHARTLSIALSDGGHPDNVGRGYVLRRILRRAVRYATEKMGAQPGMFAGLVPVVVEILGDVFP

ELRKDPEGIMEIINEEEAQFLKTLSRGRRLLEKSISKLERKALLPGEVAWRLYDTYGFPVDLTQLMVEEKGIFVDMVAYE

KAKQAAQLASQGGGDRADDTSKLDVHAIAELQDQKKVPATDDSPKYNYSSKYMGREALYEFESCKGKVVAIRMNKQFVDE

AHSGSLCGLILDRTSFYAEQGGQIYDTGFMQAVCADDDVEFAVNEVHLQGGYVVHIGRIVEGTVKVGDELRLLIDEMRRL

SIMRNHTGTHVLNYALRRVLSADADQRGSLVAPERMRFDFTSNRPMTAAEIRQAEEAASELIWKNGNVFAKDSSLAQAKA

VKGLRAVFNETYPDPVRVVSIGVPVDELLANPDRGDALNTSVEFCGGTHLVNVSHIADFVISQEEAIAKGIRRVVALTGL

EAAKAVKRAELFEAKVNCIKEDLSKNKSLAKRIAELSEEISQSTISCWRKDDLRNELKKVKKVLDDQDKQNKAAAQMEAL

EEIKRELTDRGRAPQFLVKQLQVGNNAKALDIVLKQIRALAPSAAAMLFSVDEERGRLICLCGVPKQVVADKGLSAVEWV

RQVAEVIGGKGGGKDESAQASGTNPHALNRAMELAKEFARLKFV

>XP_019646963.1 PREDICTED: alanine--tRNA synthetase, cytoplasmic-like [Branchiostoma belcheri]

MTGDLMPQLNLTGEQVRDMFIKYFEEKHEHLEVHSSSTIPLDDPTLLFANAGMNQFKPIFQGTVDPSNPMAKYRRVVNSQ

KCIRAGGKHNDLDDVGKDVYHHTFFEMLGNWSFGDYFKKEACAWAWDLLVNGYQLEKERMYVTYFGGNKDQGLEPDLECR

QIWLDLGVAEDRILPGSMKDNFWEMGEVGPCGPCSEIHYDRVGGRDASHLVNMDVPEVLEIWNLVFMQNNREPDGSITNL

PKKNIDCGMGLERILSVVQGVLSNYDTDLFTPFFAAIQKATGVRPYTGKVGSDDADGIDMAYRVLADHARTLTISMSDGG

KPDNVGRGYVLRRILRRGVRYATEKLNAPRGFFASLVDVAVQTLGSAFPEITKDPQMVKDIINEEEEQFLKTLSRGRRVL

EKRITQLGDSKVVPGETAWLLYDTYGFPVDLTSLMAEEKGLTLDCAAYEECRKQAQIRSQGKGAGVDDSVNLDVHAINEL

QEKGFPPTDDSPKYQYSHDDNGNYVFQPCTAAVVSLRRDKKFVQEVTTGQECGVLLDRTNFYAEQGGQIYDEGYLVKEDD

EETEFTVKNCQVRGGYVMHIGTLVGTLKVGDKVKLFVDEARRRPIMSNHTSTHVLNFALRQVLGEADQRGSLVAPDRLRF

DFSAKGAMTTAQIKAAENICNEIIQKNEAVFAKEAALAQAKAIQGLRAVFDETYPDPVRVLSIGISVEDMLSDPTSPAGT

ITSVEFCGGTHLQRVGHAGDLVIVTEEAIAKGIRRIVALTGHPAQQALRKAKQLQAERGGESGGGRRTTWR

>XP_018310344.1 PREDICTED: alanine--tRNA synthetase, cytoplasmic isoform X1 [Trachymyrmex zeteki]

MSSIKDLSGIVKTQLQWTANNQDDDDDSSVSNNSSTMSSVWNIDCSNNTNANQIRQTYINFFRHKEHEYVHSSSTIPHDD

PTLLFTNAGMNQFKPIFLGTVDPNSDMAKWVRVVNSQKCIRAGGKHNDLDDVGKDVYHHTFFEMMGNWSFGDYFKKEICA

WAWEFLTKVLKLPADRLYVTYFGGDEKSGLAPDDECKNLWLSLGVPASHVLPGNLKDNFWEMGETGPCGPCSELHYDRIG

DREAAHLVNMDDPDVLEIWNLVFIQYNRELDGLKLLPKKHVDCGLGLERLVSVIQNKRANYDTDLFMPLFAAIEKGTGAP

PYQGKVGADDVNGIDMAYRVLADHARTLTIALADGGVPDNTGRGYVLRRILRRAIRYATEKLNAKQGFFGSLVNVVVDLL

GDVFPEIRKDPQSIIEIINEEESQFLKTLSRGRNLLNRTIAKLESANIVPGDVAWRLYDTYGFPIDLTQLMTEEKGLKID

MAGYEESKKQAQLISQNKAGGVDDQINLDVHAITELQKQGVKPTDDTPKYDYKVTNEIKYKEYEFASCIGTVIALRRAKT

FVNEVSSGEEVGILLDKTNFYAEQGGQIYDEGFLVKVDDETTEIRIKNVQVRAGYVLHIGIVGEGTLRKGDKVHANVDTE

RRRFVMANHSATHVLNYALRKVLGTEVDQKGSLVAPDRLRFDFTNKGPMTGQQLQETELVAITMIMKNEKIYAKESNLAI

AKTIQGLRAMFEETYPDPVRVVSVGIPVENLEKDPLSTAALETSVEFCGGTHLHYTSHIGYLTIVSEDAIAKGIRRIVAL

TGPEARKAIRTAKILQEQLTEFEAAVQNKKKFEMKSKECMRKIVELTKEISHSLIPAYWKDYMRNTLKELKKSHDDNERA

ANAAVVTTVVQTAQSIIESKIGCKVLVEVLEAYSNTKALDQALKKIRDLSPGTSALLISVDHDVKKIFALSSVPKSAVYE

GLKANEWIQAIAPLMQGKGGGKYESAQASGSNIACLNEIVSKANEFANQKLGITNKITTNNTHKTSETFRLLNTGSSLKL

ILYGNVGSIKCYLAQIIAQYSGKCLTIKQVDNKIHSSDIMLETANVTLHDSNAIAFFLSNSQLRRDDDLFASSTVLQWMS

YVQNHILPMVSGWVLPSLDVSVLEDTKINIKISKADLLCALRRLDNIMHTRTYLIDERITLADISLFTALLPLYEYVFDP

HYRGQYSNITRWFSMILNQPQVKCVVKNFSFCTEAT

>AAF05593.1 alanyl-tRNA synthetase [Drosophila melanogaster]

MKLLTAKEVRNAYLDFFKEQKHIYVHSSSTIPLDDPTLLFANAGMNQFKPIFLGTADPNSEMSKWVRVANTQKCIRAGGK

HNDLDDVGKDVYHHTFFEMLGNWSFGDYFKKEICSWAWEFLTQRLALPKDRLYVTYFGGDAASGLEPDLECKQMWLDLGL

KPEHILPGSMKDNFWEMGETGPCGPCSELHFDRIGGRSVPELVNMDDPDVLEIWNLVFIQYNRESDGSLKQLPKKHIDCG

MGFERLVSVIQNKRSNYDTDLFVPLSMPSKRALVPQPYQGRVGADDVDGIDMAYRVLADHARTITIALADGGTPDNTGRG

YVLRRILRRAVRYATEKLNAKPGFFATLVNTVVDLLGDAFPEVKKDPQHIIDIINEEELQFLKTLTRGRNLLNRTIEKLG

NQTTIPGDVAWRLYDTYGFPVDLTQLMAEEKSLKIDMDGYEAAKHNSYVLSQGKGASKIEEINLDVHAISQLQEQGVPPT

NDTFKYKYEAVSDERDSAYNYGVCNSKIVALRFENQFVNEITSGQKAGIVLDKTNFYAESGGQIYDQGALVKVNDEANEF

LVDRVYNRGGYILHIGVVEGTLKVGDELELHIDVERRWLTMKNHSATHALNHCLLQVLGKDTEQKGSLVVPEKLRFDFNS

KAAMPSSRCPRRSSLTKEMVYKNVPIYAKESKLALAKKIRGLRSVFDEVYPDPVRVISFGVPVDELEQNPDSEAGEQTSV

EFCGGTHLRRSGHIMDFVISSEEAIAKGIRRIVALTGPEALKALKKSEAFEQEIVRLKATIDNDKSGKDSKSHVKEIVEL

TEQISHATIPYVKKDEMRNLLKGLKKTLDDKERALRAAVSVTVVERAKTLCEANPNATVLVEQLEAFNNTKALDAALKQV

RSQLPDAAAMFLSVDADSKKIFCLSSVPKSAVEKGLKANEWVQHVSATLGGKGGGKPESAQASGTNYEKVDEIVQLASKF

AQSKLS

>XP_022668442.1 alanine--tRNA synthetase, cytoplasmic-like isoform X3 [Varroa destructor]

MSSAKEIRSKFIAFFRDKYAHTEVPSSCVIPHDDPTLLFTNAGMNQFKPIFLGTVDPSTEAAKWKRTVDTQKCIRAGGKH

NDLDDVGKDVYHHTFFEMLGNWSFGDYFKKEICAWAWELLTKEYGLAEDRLYVTYFGGNKDADLEPDEECKNIWKALGLP

ENRILPGNMKDNFWEMGETGPCGPCSEIHYDRLGGRNAAHLVNMDDPDVLEIWNLVFIQFNRESDGKLKPLPKKHVDTGM

GLERLVSVIQNKRANYDTDLFVPIFEAIQKATGVRAYTGKVGDDDTDGIDMAYRVLADHARTLSIALSDGGHPDNVGRGY

VLRRILRRAVRYATEKMGAQPGMFAGLVPVVVEILGDVFPELRKDPEGIMEIINEEEAQFLKTLSRGRRLLEKSISKLER

KALLPGEVAWRLYDTYGFPVDLTQLMVEEKGIFVDMVAYEKAKQAAQLASQGGGDRADDTSKLDVHAIAELQDQKKVPAT

DDSPKYNYSSKYMGREALYEFESCKGKVVAIRMNKQFVDEAHSGSLCGLILDRTSFYAEQGGQIYDTGFMQAVCADDDVE

FAVNEVHLQGGYVVHIGRIVEGTVKVGDELRLLIDEMRRLSIMRNHTGTHVLNYALRRVLSADADQRGSLVAPERMRFDF

TSNRPMTAAEIRQAEEAASELIWKNGNVFAKDSSLAQAKAVKGLRAVFNETYPDPVRVVSIGVPVDELLANPDRGDALNT

SVEFCGGTHLVNVSHIADFVISQEEAIAKGIRRVVALTGLEAAKAVKRAELFEAKVNCIKEDLSKNKSLAKRIAELSEEI

SQSTISCWRKDDLRNELKKVKKVLDDQDKQNKAAAQMEALEEIKRELTDRGRAPQFLVKQLQVGNNAKALDIVLKQIRAL

APSAAAMLFSVDEERGRLICLCGVPKQVVADKGLSAVEWVRQVAEVIGGKGGGKDESAQASGTNPHALNRAMELAKEFAR

LKFV

>XP_023304810.1 alanine--tRNA synthetase, cytoplasmic [Lucilia cuprina]

MKSMSAAEVRNCYLDFFKEKGHIYVHSSSTIPLDDPTLLFANAGMNQFKPIFLGTADPNSDMSKWKRVANTQKCIRAGGK

HNDLDDVGKDVYHHTFFEMLGNWSFGDYFKKEICSWAWEFLTERLGLPKDRLYVTYFGGDESSGLEPDLECKQLWLDLGL

KPEHVLPGNMKDNFWEMGETGPCGPCSELHFDRIGERSVPELVNMDDPDVLEIWNLVFIQFNREQDGSLKPLPKKHIDCG

MGFERLVSVIQDKRSNYDTDIFMPLFEAIESGTGCQPYKGRIGEEDVGGIDMAYRVLADHARTITIALADGGTPDNTGRG

YVLRRILRRAVRYATEKLNAKPGFFATLVHTVVKLLGDAFPEVAKDPQHIIDVINEEEQQFLKTLTRGRNLLNRTIAKLG

DNNIIPGDVTWRLYDTYGFPVDLTQLMAEERGLKIDMDGYEKSKHNSYILSQGKSANKIDEINLDVHAISELKEKQIPVT

DDSFKYKYESVSDDRESEYIFAPCSAKIVAIRYENTFVENIASGQKAGLVLDKTNFYAESGGQIFDQGVLVKIDDEANEF

LIDRVYNRGGYILHIGVVEGNLSVGDEVNLHIDTVRRSLTMKNHSATHALNHCLLQVLGKDTEQKGSLVVPEKLRFDFNY

KSGMTIEQVAKTEQLLKDIVYKNVPIYAKESKLSVAKSIRGLRSVFDEVYPDPVRVISFGVPVEELEQNPDSDVGEKTSV

EFCGGTHLRRSGHIVDVVISSEEAIAKGIRRIVALTGPEALKAIKKEELFAKEIMKLSEKITNDSKNSKFYVKQIVDLSE

EIAHANISHVKKDEMRNCLKNLKKKLDDKERALKNAVAVTVVEKAKELCISNPNIPIIVELLEAYNNTKALDAALKQVRS

LNPDTSAMFVSVDADSKKIFCLTSVPKSAIEKGLKANEWVQHISVAINGKGGGKPESAQASGSNYENVTEVLKMANEFAS

LKLNK

>XP_018310345.1 PREDICTED: alanine--tRNA synthetase, cytoplasmic isoform X2 [Trachymyrmex zeteki]

MSSIKDLSGIVKTQLQWTANNQDDDDDSSVSNNSSTMSSVWNIDCSNNTNANQIRQTYINFFRHKEHEYVHSSSTIPHDD

PTLLFTNAGMNQFKPIFLGTVDPNSDMAKWVRVVNSQKCIRAGGKHNDLDDVGKDVYHHTFFEMMGNWSFGDYFKKEICA

WAWEFLTKVLKLPADRLYVTYFGGDEKSGLAPDDECKNLWLSLGVPASHVLPGNLKDNFWEMGETGPCGPCSELHYDRIG

DREAAHLVNMDDPDVLEIWNLVFIQYNRELDGLKLLPKKHVDCGLGLERLVSVIQNKRANYDTDLFMPLFAAIEKGTGAP

PYQGKVGADDVNGIDMAYRVLADHARTLTIALADGGVPDNTGRGYVLRRILRRAIRYATEKLNAKQGFFGSLVNVVVDLL

GDVFPEIRKDPQSIIEIINEEESQFLKTLSRGRNLLNRTIAKLESANIVPGDVAWRLYDTYGFPIDLTQLMTEEKGLKID

MAGYEESKKQAQLISQNKAGGVDDQINLDVHAITELQKQGVKPTDDTPKYDYKVTNEIKYKEYEFASCIGTVIALRRAKT

FVNEVSSGEEVGILLDKTNFYAEQGGQIYDEGFLVKVDDETTEIRIKNVQVRAGYVLHIGIVGEGTLRKGDKVHANVDTE

RRRFVMANHSATHVLNYALRKVLGTEVDQKGSLVAPDRLRFDFTNKGPMTGQQLQETELVAITMIMKNEKIYAKESNLAI

AKTIQGLRAMFEETYPDPVRVVSVGIPVENLEKDPLSTAALETSVEFCGGTHLHYTSHIGYLTIVSEDAIAKGIRRIVAL

TGPEARKAIRTAKILQEQLTEFEAAVQNKKKFEMKSKECMRKIVELTKEISHSLIPAYWKDYMRNTLKELKKSHDDNERA

ANAAVVTTVVQTAQSIIESKIGCKVLVEVLEAYSNTKALDQALKKIRDLSPGTSALLISVDHDVKKIFALSSVPKSAVYE

GLKANEWIQAIAPLMQGKGGGKYESAQASGSNIACLNEIVSKANEFANQKLGITTTA

>XP_018044471.1 PREDICTED: alanine--tRNA synthetase, cytoplasmic [Atta colombica]

MPRIIKMQLQQTANNQDNDNGSSPNSSGTMSSALNTDHSNNMNAKQIRQAYIDFFKSKGHEYVHSSSTIPHDDPTLLFTN

AGMNQFKPIFLGTVDPNSDMAKWIRVVNSQKCIRAGGKHNDLDDVGKDVYHHTFFEMMGNWSFGDYFKNEICAWAWEFLT

DMLKLPADRLYVTYFGGDEKSGLAPDDECKNLWLSLGVPASHVLPGNMKDNFWEMGETGPCGPCSELHYDRIGDREAAHL

VNMDDPDVLEIWNLVFIQYNRELNGSLKSLPKKHIDCGLGLERLVSVIQNKRANYDTDLFMPLFAAIEEGTDAPPYQGKV

GADDVNGIDMAYRVLADHVRTLTIALADGGVPDNTGRGYVLRRILRRAVRYATEKLNAKQGFFGSLVNVVVDLLGDVFPE

VRKDPQSIIEIINEEESQFLKTLSRGRNLLNRTIAKLESADTVPGDVAWRLYDTYGFPIDLTQLMTELISQNKAGGVDDQ

INLDVHAITELQKQGIKPTDDSPKYDYKVTAEIKYKEYEFAPCTGTVIALRRAKTFVDEVSSGEEVGILLDRTNFYAEQG

GQIYDEGFLVKVNDETTEIRIKNVQIRAGYVLHIGTVGEGTLRKRDKVHTNVDTERRRFVMANHSATHVLNYALRKILGT

KADQKGSLVAPDRLRFDFTNKGPMTGGQLQDTEQLANNMITENEKIYAKESNLAIAKTIQGLRAMFEETYPDPVRVVSVG

IPVENLEKDPLSTAALETSVEFCGGTHLHYTSHIGNFTIISEDAIAKGIRRIVALTGPEAAKALRTGMLLQRQLSQFQAT

IEDDKSEMNSKEYVREIVELTEEISHALIPAYWKDHMRNTLKNLKKSLDDKERAAKATVATTVIQTAQSIIESKVGCQVL

VEVLEAYSNTKALDQALKKIRAISPETSALLISIDHDVKKIFALSSVPKSAVSKGLKANEWIQAITSLMQGKGGGKSESA

QASGSNIVCLNEIVSKANEFADQKLGIAKTFQLLNTESSLKLVLYGNIGNIKCYLAQIIAQYSGKCLTIKQIDNRIHSSD

IMLETANITLHDSNAIAFFLSNSQLRRDDDLFACSQVLQWMSYVQNHILPMVYGWVLPSLVSVSEDAKINIKISKADLLC

ALRKLDDIMHTRTYLIGERITLADISLFTALLPLYEYMFDPHYRGHYSNITRWFSTILNQPQVKCVVKNFSFCTEAI

>XP_011348262.1 PREDICTED: alanine--tRNA synthetase, cytoplasmic isoform X1 [Ooceraea biroi]

MNSDRSVKMSATELEGRSKKRMSAKEIRQAYIDFFQSKGHEYVHSSSTIPHDDPTLLFTNAGMNQFKPMFLGTVDPNSDM

AKLTRVVNSQKCIRAGGKHNDLDDVGKDVYHHTFFEMMGNWSFGDYFKKEICAWAWEFLTGVLELPADRLYVTYFGGDEK

SNLEPDNECKELWLSLGLPASHVIPGSMKDNFWEMGETGPCGPCSELHYDRIGGREAAHLVNMDDPDVLEIWNLVFIQYN

RESDGSLKTLPKKHIDCGLGLERLVSVIQNKRSNYDTDLFARLFATIQKNTGARPYQGKVGAEDVDGIDMAYRVLADHAR

TITIALADGGMPDNTGRGYVIRRILRRAVRYATEKLNAKPQFFGNLVYVVVKLLGNTFPELKKDPQTTIDIVNEEETQFL

KTLSRGRNLLNRTIEKLESTDIVPGDVAWRLYDTYGFPVDLTQLMAEEKGLKIDMAAYEEAKKRAQLISQNKAGGVDDQI

NLDVHAITELQELDIKPTDDSPKYDSRSAYLSRYGTEELYKEYEFKPCTGTVIALRRARTFVDEVSSGEGEVGILLDTTN

FYAEQGGQIYDEGFLVKVDDEATEVRITNVQVRAGYVLHIGTVGEGTLRKGDKVRTNIDTVRRRLIMGNHSATHVLNYAL

RKMYGPEVDQKGSLVAPDRLRFDFSKKGPMTAEQVRLTENIVHDLVKKAEVIFRLECNLALAKSIHGVRAMFEETYPDPV

RVVSIGVPVEVLQKDPSSSAGFENSVEFCGGTHLHTTDHIGDFVIISEEAIAKGIRRIVALTGLPATKAILRAQSMCKVV

YDLQDQVKPDRCRRDTKDLMKRITELLNDVSRATISTWQKFILRDTLNMYKNALEREASMRRGSIMMRVKEDVAQLLRAM

AGSPVLVEKIDASNHTKAVDFALKKIRELSPVTSALLLTYDNEEKKVFALCSVPKSAIMKGLKANEWIQAFVPMINGKGG

GKAESAQASGTIECSDQQFEEIIKKTYEYANEKLGLPLNNMCKKIHIDLETRLESQLVLHAYVGSTKYYLAQIISQYTGF

RLNFKQIHYQEQPHSYDIKLETPEITLHDGSAIAFYLANSDLRCEDDFFASSQVLQWMSYAQNHILPAVTATVLPLIALF

AANDTKSNMNVSKVELTDALERLDAILRTKTYLVRERITLADLFIFSTLLPLYEFAFDPNHREPYKNLNRWFLTILNEPK

VQEVLKDFKLCLKAENIHDFLLFTGFIA

>XP_011348263.1 PREDICTED: alanine--tRNA synthetase, cytoplasmic isoform X2 [Ooceraea biroi]

MNSDRSVKMSATELEGRSKKRMSAKEIRQAYIDFFQSKGHEYVHSSSTIPHDDPTLLFTNAGMNQFKPMFLGTVDPNSDM

AKLTRVVNSQKCIRAGGKHNDLDDVGKDVYHHTFFEMMGNWSFGDYFKKEICAWAWEFLTGVLELPADRLYVTYFGGDEK

SNLEPDNECKELWLSLGLPASHVIPGSMKDNFWEMGETGPCGPCSELHYDRIGGREAAHLVNMDDPDVLEIWNLVFIQYN

RESDGSLKTLPKKHIDCGLGLERLVSVIQNKRSNYDTDLFARLFATIQKNTGARPYQGKVGAEDVDGIDMAYRVLADHAR

TITIALADGGMPDNTGRGYVIRRILRRAVRYATEKLNAKPQFFGNLVYVVVKLLGNTFPELKKDPQTTIDIVNEEETQFL

KTLSRGRNLLNRTIEKLESTDIVPGDVAWRLYDTYGFPVDLTQLMAEEKGLKIDMAAYEEAKKRAQLISQNKAGGVDDQI

NLDVHAITELQELDIKPTDDSPKYDSRSAYLSRYGTEELYKEYEFKPCTGTVIALRRARTFVDEVSSGEGEVGILLDTTN

FYAEQGGQIYDEGFLVKVDDEATEVRITNVQVRAGYVLHIGTVGEGTLRKGDKVRTNIDTVRRRLIMGNHSATHVLNYAL

RKMYGPEVDQKGSLVAPDRLRFDFSKKGPMTAEQVRLTENIVHDLVKKAEVIFRLECNLALAKSIHGVRAMFEETYPDPV

RVVSIGVPVEVLQKDPSSSAGFENSVEFCGGTHLHTTDHIGDFVIISEEAIAKGIRRIVALTGLPATKAILRAQSMCKVV

YDLQDQVKPDRCRRDTKDLMKRITELLNDVSRATISTWQKFILRDTLNMYKNALEREASMRRGSIMMRVKEDVAQLLRAM

AGSPVLVEKIDASNHTKAVDFALKKIRELSPVTSALLLTYDNEEKKVFALCSVPKSAIMKGLKANEWIQAFVPMINGKGG

GKAESAQASGTIECSDQQFEEIIKKTYEYANEKLGLPLNNMCKKIHIDLETRLESQLVLHAYVGSTKYYLAQIISQYTGF

RLNFKQIHYQEQPHSYDIKLETPEITLHDGSAIAFYLANSDLRCEDDFFASSQVLQWMSYAQNHILPAVTATVLPLIALF

AANDTKSNMNVSKVELTDALERLDAILRTKTYLVRERITLADLFIFSTLLPLYEFAFDPNHREPYKNLNRWFLTILNEPK

VQEVLKDFKLCLKAENIHGKTPFRSPQ

>XP_018010833.1 PREDICTED: alanine--tRNA synthetase, cytoplasmic-like [Hyalella azteca]

MLLKEVRSTFVEYFQKKHAHVYVHSSPTIPHDDPTLLFANAGMNQFKPIFLGNVDPSSDMARLKRAVNSQKCIRAGGKHN

DLDDVGKDLYHHTFFEMLGNWSFGDYFKDEICSWSWDLLTNVYKLPKDRLYVTYFGGEPSQQLEPDLECRQKWLDLGVPA

EHILPGSMKDNFWEMGDTGPCGPCSEIHFDRIGGRDASSLVNQDDPDVLEIWNLVFIQFNRESNGQLKSLPKKHIDCGLG

LERLASIMQDKRSNYDTDAFLPIFVKIQELTGARPYTGMIGKADSDCVDMAYRVLADHARTLTVALSDGGMPDNVGRGYV

LRRILRRAVRFASEKLGAKPGVFAELVDVVVDLLQDAFPELAKDSSYVKDVINEEERQFLKTLERGRKLLHRTVGKLGVS

KLIPGDVAWRLYDTYGFPVDLTQLMAEENDLNVDMDAFETCKKEAQERSRGEAKDRDDSYRLDVHAIEELKSRGIPFTDD

SQKYDYKAGNSPDADYSFGSCLGKIVAIRMNGHFVEEVKSGDRCGIVTDRTCFYAESGGQLSDEGFMVKNDDDSTEFKVE

NAEIKGGFVCHVGVVEGTLKIGDEVAITFDGVRRKLLMNNHTGTHILNFALRQVLSADADQKGSLVAPDRLRFDFSSKGA

MTVEQVKRAEQIAVEMVKSNSQVYAKTSSLAVAKSIVGLRAVFGEVYPDPVRVVSVGIPVEDLEKDPQSPAGNKTSIEFC

GGTHLLRSGHVGHFVIAVEEAIAKGIRRIVALTGPEAQKAINRGALLQNKVNQLTQTLSNDSLSYKESVKLITDLMEDIN

QAQIQYWLRDELRNALDSAKKVLGDADRARKMAVSKDAIRFVKELKAANPDVPYVVVELKAFAQNKVLNDALKEVKNGPP

SLFISADEDTGKILAMAAVPKDVVQKGLAADQWVKNLTELLDGKGGGRAENAQLSGTNTSALHKALRLSHEFAQQKLSCP

PVHLKTDELLTTSATNVLEKPPKCCQKREETKVAVKTQAATDTKYDLVLHGADNIQAIFVALAAGFSGKTLTFADNSAKR

VSLVTSEGRLEGALASASFVAPADMCGKDPVHKAQVLSWMITAESELLPLLAAGKLVSQPKPGAGAAAQQMLERLNRYLR

DHTFLVGERLSMADVSVFASLLNAYRDQRLRPSMDKLTSLTRWFNTVLHQPPVQTCLSCMNMTL

>XP_011348264.1 PREDICTED: alanine--tRNA synthetase, cytoplasmic isoform X3 [Ooceraea biroi]

MNSDRSVKMSATELEGRSKKRMSAKEIRQAYIDFFQSKGHEYVHSSSTIPHDDPTLLFTNAGMNQFKPMFLGTVDPNSDM

AKLTRVVNSQKCIRAGGKHNDLDDVGKDVYHHTFFEMMGNWSFGDYFKKEICAWAWEFLTGVLELPADRLYVTYFGGDEK

SNLEPDNECKELWLSLGLPASHVIPGSMKDNFWEMGETGPCGPCSELHYDRIGGREAAHLVNMDDPDVLEIWNLVFIQYN

RESDGSLKTLPKKHIDCGLGLERLVSVIQNKRSNYDTDLFARLFATIQKNTGARPYQGKVGAEDVDGIDMAYRVLADHAR

TITIALADGGMPDNTGRGYVIRRILRRAVRYATEKLNAKPQFFGNLVYVVVKLLGNTFPELKKDPQTTIDIVNEEETQFL

KTLSRGRNLLNRTIEKLESTDIVPGDVAWRLYDTYGFPVDLTQLMAEEKGLKIDMAAYEEAKKRAQLISQNKAGGVDDQI

NLDVHAITELQELDIKPTDDSPKYDSRSAYLSRYGTEELYKEYEFKPCTGTVIALRRARTFVDEVSSGEGEVGILLDTTN

FYAEQGGQIYDEGFLVKVDDEATEVRITNVQVRAGYVLHIGTVGEGTLRKGDKVRTNIDTVRRRLIMGNHSATHVLNYAL

RKMYGPEVDQKGSLVAPDRLRFDFSKKGPMTAEQVRLTENIVHDLVKKAEVIFRLECNLALAKSIHGVRAMFEETYPDPV

RVVSIGVPVEVLQKDPSSSAGFENSVEFCGGTHLHTTDHIGDFVIISEEAIAKGIRRIVALTGLPATKAILRAQSMCKVV

YDLQDQVKPDRCRRDTKDLMKRITELLNDVSRATISTWQKFILRDTLNMYKNALEREASMRRGSIMMRVKEDVAQLLRAM

AGSPVLVEKIDASNHTKAVDFALKKIRELSPVTSALLLTYDNEEKKVFALCSVPKSAIMKGLKANEWIQAFVPMINGKGG

GKAESAQASGTIECSDQQFEEIIKKTYEYANEKLGLPPKKRKTPDKNSSNMKYFRKM

>XP_009021139.1 hypothetical protein HELRODRAFT_100946 [Helobdella robusta]

MDSSMTSKDVRDGFIEFFKEKKHTYVHSSSVIPLDDPTLLFTNAGMNQFKPIFLGTADPKSEMGRLVRAVNTQKCIRAGG

KHNDLDDVGKDVYHHTFFEMLGNWSFGEYFKKEVTEWAWELLTVRWKMPKDRLYVTYFGGDRERNLQPDLEARDCWIKLG

LPPERVMPFGMKENFWMMGETGPCGPCSEIHFDRIGGRNAAHLVNMDVPDVLEIWNLVFITYNMESDGSLKDLPKKNIDC

GMGLERVVSVIQGKGSNYDTDLFQPLFQAINNTMQCRKYTGKVGEEDKDGVDMAYRVVADHVRTLTVALSDGGRPDNVGR

GYVLRRILRRAIRYATEKLNAKPGQLASLVDAAIFILGGAFPEIKKDPDTVKDIINEEEAQFLKTLSRGRRLLERTIDKL

GGAKILPGDIAWRLYDTYGFPVDLTQLMVEERKMKIDHVGYEEAKKKAQLLSQGGGATVDEEISLDVHSINELKSTNQAI

TDDSPKFEYTADDKGNYVFQSCQSKIMAIRKGKKFVNEVTPGEECGILLDRTNYYAEQGGQIYDEGYIVRCDDESNEIKV

KNVQVYAGYILHVGNVEGHFKVGDVVKCFIDEDRRKGIMKNHTGTHVLNFSLRKVLGSTDQKGSLVAPDRMRFDFTAKEA

LKPSEVESVEKISNTFTRGGRTVYSKVVPLAQAKAIQGLRAVFDETYPDPVKVVSIGTPIEELLKDPNGRHGYETSVEFC

GGTHLVRSDDIGSLVIVSEEAIAKGQRRIVAVTGTDAKKALHRSELLEKTYEDLKEKVDGSQSSDVSVQKALVREIVELT

NEVNQAVIQSWKKDNMRNNLSSLKKILDDVERKKKAAISNEVLSEVLRCTVQEDWCSVYVVREFKALSDAKALDGALKQY

KTNLPNTSCMLFSVDFENGKILCMAAAAQDHIKQGLKANEWIGSIQELIKGKGGGKDMNAQATGTQPDQLNEAMKQASRY

AVSKLQLLQMDNVKPISDSGTLQKVQPIIEAHLSNRSYIASHRPSQNDSVVFRALESAILCPEKFPNLCRWRKHLSSFGL

QVYKLPGVCKSFDEIIASIQSMPGGDN

>XP_014270816.1 alanine--tRNA synthetase, cytoplasmic isoform X2 [Halyomorpha halys]

MDSKTIRSKFIDFFVKEKQHKYVHSSPVVPHDDPTLLFTNAGMNQFKAIFLGIADPNSEMATWIRATNTQKCIRAGGKHN

DLDDVGKDVYHHTFFEMLGNWSFGDYFKKEICAWAWELLTEVFALPKERLYVTYFGGDPDSGLQPDEECRQIWLNLGLPE

SHVIPGSMKDNFWEMGETGPCGPCSELHFDRIGGREVPHLVNQDDPDVLEIWNLVFIQFNRENDGSLKVLPRKHIDCGMG

FERLVSVIQNKRSNYDTDIFQPLFAAIQKGTGSPPYEGKVGKEDIDGRDMAYRVLADHARTLTISIADGGVPDNTGRGYV

LRRILRRAVRYGTEKLNAKPGFFASLVHVVIDLLGETFPELKKDPQHIIDTINEEEEQFLKTLTRGRNLLNRTISKLEGK

TVIPGDIAWRLYDTYGFPLDLTRLMAEEKGLTVNMEKYEEARKQSQLASQGKTSGVVERISLDVHALSELQKKNIPQTDD

SYKYHYKVSEKNKQKFADYEFGTSQGKILAIRTESGFVDSVVSGNLCGIILDKTCFYAEQGGQIYDEGYISKIDDEASEV

FVKNVQVQAGYILHIGVVEGHFSIGDKVICNIDEKRRRLIMSNHTATHILNHALRHVLGPDTDQKGSLVAPDRLRFDFTC

KSSVKLDELKKIENEVNRIVNDDDEVFAACATLEDAKKITGLRAMFSEAYPDPVRVVSVGVPVHHLIDNPQNDSGLKTSV

EFCGGTHLKRSSHVGDFIITNEEAIAKGIRRLIAVTGPEAEKAQQRVKALESSVLILKNIIEKNMIDGKEMLKSIVEHDN

DISKAPISYWKKDELRNTLKNLKKVIDSAERAGKTEQTNKVIEEAKNLMNTKKGTPFLVATLNAGSNSKALDSALKQVKN

ISPETAAMFFSVEQSANRIICLCTVPKVCVEKGLKGNEWVQSISSTIGGKGGGKAESAQASGSNVDKLPEVLKIAEDFAK

LKLSVSGS

>XP_014270814.1 alanine--tRNA synthetase, cytoplasmic isoform X1 [Halyomorpha halys]

MDSKTIRSKFIDFFVKEKQHKYVHSSPVVPHDDPTLLFTNAGMNQFKAIFLGIADPNSEMATWIRATNTQKCIRAGGKHN

DLDDVGKDVYHHTFFEMLGNWSFGDYFKKEICAWAWELLTEVFALPKERLYVTYFGGDPDSGLQPDEECRQIWLNLGLPE

SHVIPGSMKDNFWEMGETGPCGPCSELHFDRIGGREVPHLVNQDDPDVLEIWNLVFIQFNRENDGSLKVLPRKHIDCGMG

FERLVSVIQNKRSNYDTDIFQPLFAAIQKGTGSPPYEGKVGKEDIDGRDMAYRVLADHARTLTISIADGGVPDNTGRGYV

LRRILRRAVRYGTEKLNAKPGFFASLVHVVIDLLGETFPELKKDPQHIIDTINEEEEQFLKTLTRGRNLLNRTISKLEGK

TVIPGDIAWRLYDTYGFPLDLTRLMAEEKGLTVNMEKYEEARKQSQLASQGKTSGVVERISLDVHALSELQKKNIPQTDD

SYKYHYKVSEKNKQKFADYEFGTSQGKILAIRTESGFVDSVVSGNLCGIILDKTCFYAEQGGQIYDEGYISKIDDEASEV

FVKNVQVQAGYILHIGVVEGHFSIGDKVICNIDEKRRRLIMSNHTATHILNHALRHVLGPDTDQKGSLVAPDRLRFDFTC

KSSVKLDELKKIENEVNRIVNDDDEVFAACATLEDAKKITGLRAMFSEAYPDPVRVVSVGVPVHHLIDNPQNDSGLKTSV

EFCGGTHLKRSSHVGDFIITNEEAIAKGIRRLIAVTGPEAEKAQQRVKALESSVLILKNIIEKNMIDGKEMLKSIVEHDN

DISKAPISYWKKDELRNTLKNLKKVIDSAERAGKTEQTNKVIEEAKNLMNTKKGTPFLVATLNAGSNSKALDSALKQVKN

ISPETAAMFFSVEQSANRIICLCTVPKVCVEKGLKGNEWVQSISSTIGGKGGGKAESAQASGSNVDKLPEVLKIAEDFAK

LKLSGSSEELVYQAGSVSSTLILIIAEYSGVSLPVRKGPTTLLNYKGVVLSQISSIIFALAPPSLKANNDPFILASILQW

IEFSDTLFKEIYSILVNKSPSNKNKNITLLPYIEYLNRYLSNYTFVVGDRLSIADFYLFCYILPVSEMIFSDEKQYGLLI

NLKRWFITIKNQSPVLKVLRE

>XP_020911043.1 alanine--tRNA synthetase, cytoplasmic-like [Exaiptasia pallida]

MDTTLTSKQVRQMFVDFFVKKYEHVFVPSSSTIPLDDPTLLFANAGMNQYKSIFLETVDPNSDMAKLKRATNSQKCIRAG

GKHNDLDDVGKDVYHHTFFEMLGSWSFGNYFKQEAIDWAWELLTEVYKMPKERLYVTYFGGYGDIEPDEETKNCWLSKGI

HPSRVLPFGMKDNFWEMGDTGPCGPCTEIHFDRIGDRDAKDLVNMDDPTVLEVWNLVFIQFNRENGGNLKPLPNKHVDTG

MGLERLVSIIQGKMSNYDTDIFTPFFDKIHQSTGIRPYAGKVGVEDADGIDMAYRVVADHIRTLSIAISDGGKPDNVGRG

YVLKRVLRRCIRYATEKLNAPPGFVSSLVYVVIETLGDFFPELKKDPQLVIDVINEEETQFLKTLARGRRLFDRTASKIN

DKCIPGDVAWRLYDTYGFPVDLTQLMAEERGLTVDMEKYEESKRRAQEIAKGKGVSSDEAITLDVHMIDKLKKELSLPPT

NDSAKYNYKSDNQGDYDFLPTTGIVKALLYQKEFVQEVPGGVHCGVVMDKTGFYAEQGGQIYDQGFLVKEGDEEVEFKVM

DVQVHGGYVLHVGALEGALRVGDKMKSVIDEERRRHCMNNHTATHVLNFALRKTLTGDSDQRGSLVAPDRLRFDFTSKGA

MTTEQIKETELISQEIINQDLEVFAKEVPLAVAKDIAGLRAIFDEVYPDPVRVVSIGSSFEELQNNPQAGYKYSVEFCGG

THLQRSSHMKAFVLVTEEAISKGIRRIVAITGPEALRAQKKCQLLEDKVQNXKTDVETKLSSKSLNVKESSQEINRLNED

IDGAIISAWKKDELRSVLKQVKKLVTDAEKELKAAAMQIAIEKAKSLIEQKPDMAICVDLFDAGSNTKILDSALKQFKSL

SPKTAAMLFSVDSGNNKAICMAIVPKDVVERGLKADEWAKAISEVLGGKSGGKPVSAQASGPNVKDINKAMEVAEQFAKL

KLQN

>XP_011202499.1 PREDICTED: alanine--tRNA synthetase, cytoplasmic [Bactrocera dorsalis]

MKVLTANEIREAFLGFFKEKDHLYVHSSSTIPLDDPTLLFANAGMNQFKPIFLGTADPNSEMAKWVRTTNTQKCIRAGGK

HNDLDDVGKDVYHHTFFEMLGNWSFGDYFKKEICTWAWEFLTERMKLPKDRLYVTYFGGDKASGLEPDLECKQIWLDLGL

CPEHILPGSMKDNFWEMGETGPCGPCSELHFDRIGGRSVPELVNVDDPDVLEIWNLVFIQYNRESDGSLKPLPKKHIDCG

MGFERLVSVIQNKRSNYDTDLFVPLFKAIQEGTGAPEYAGRVGAEDVEGIDMAYRVLADHARTITIALADGGTPDNTGRG

YVLRRILRRAVRYATEKLSAKPGFFGSLVHTVVDLLGDAFPEVRKNPQQIIDIINEEEAQFLKTLSRGRDLFNRTVAKLS

NQNVIPGDLAWKLYDTYGFPVDLTQLMAEEKNLEINMEEYEQAKQAAYIMSQGKSTTKADEIDLDVHAISELQSKSIQIT

NDSFKYKYEAESENLDSPYKFTSCTAKILALRFGNQFVHKIEAGSKAGIILDQTNFYAESGGQIYDKGLFVKLNNVDDKF

IVDAVYNRGGYILHIGVADGEFTVGDEIDLKIDTERRWLTMKNHSATHALNHSLLQVLGKETDQKGSLVVPDKLRFDFSS

KSAMTIEQVAKAELLTRDIVYKNVPIYAKETKLANAKKIRGLRSVFDEVYPDPVRVISFGVNVEELENNLDSDAGEKTSV

EFCGGTHLQRSGHMMEFVITSEEAIAKGIRRIVALTGPEAVKALEKYDQLHKQIQTLKTTIEDDKDGKSSKNYVRQIVEL

TEEVSQAIIPYVKKDEMRNLLKQLKKTLDDKERTLKAAVSVTVIEKTKEICQENPKATLLVKQFEAYNNTKALDGALKQV

RALCPDAAALFLSVDPDSKKIFCLASVPKSAVNKGLKANEWVQQLSEVIGGKGGGKPESAQASGTNYENVDEVIRVATEF

AKLKVGA

>XP_012144271.1 PREDICTED: alanine--tRNA synthetase, cytoplasmic isoform X2 [Megachile rotundata]

MAKWVRVVNSQKCIRAGGKHNDLDDVGKDVYHHTFFEMMGNWSFGDYFKKEICTWAWEFLTVKLRLPADRLYVTYFGGDE

KNNLDSDEECKAIWLSLGVSPNHILPGNMKDNFWEMGETGPCGPCSEIHYDRIGNREAAHLVNQDDPDVLEIWNLVFIQF

NRESDGSLKSLPKKHIDCGLGLERLVSVIQNKQANYDTDLFIPLFDAIQKGTGAPPYQGKVGIDDKDGIDMAYRVLADHA

RTITIALSDGGVPDNTGRGYVLRRILRRAVRYATEKLNAKPGFFGTLVNVVVEILGDTFPEVTKDPQYIIDTINEEEEQF

LKTLSRGRNLLHRTIAKLESSNILPGDIAWRLYDTYGFPVDLTQLMTEEKGLKIDMAGYEEAKKQAQLISQNKCGGVDDQ

INLDVHAITELQNKGIKPTNDLPKYNYTVTGNKLYEEYEFAPCTGTVIALRRNKTFVDEVSSGEEVGILLDQTNFYAEQG

GQIYDEGFLVKTDDEDTEVRIKNVQVRGGYVLHIGTVGQGTLKKNDKVYLNVDTTRRRFLMSNHTATHVLNYALRKVLGT

EVDQKGSLVAPDRLRFDFTNKGAMTSEQVKRVEEITNSVVKENKKVYAKESSLALAKTIQGLRAMFEETYPDPVRIVSIG

IPVEELEKDPLGPAATQTSVEFCGGTHLHYTKHMEDFIIASEEAIAKGIRRIVALTGPEATKAQKKAAVLQNHLEQLQTT

IANDKNGTNSKEYVKKIVELTDDVSRATISSWKKDNMRKMLKDLKKMLDDKERSAKAAIANAVVDTIQQIIQQNVGCPVL

VETLQAYSNTKALDSALKKIKAISPETSALLVTVDPDANKIFALSMASKSAINKGLKANEWIQEIAPLMKGKGGGKPESA

QASGTNISCLTKLIHTAKNFANSKLGVISNNEIVTNNYNQSKASQNSVCSKTSKGKLILSGNTGSIKYYRAQIIAQYSDK

ELVTNECKNDGTTSNSIKLEGSGFELFDSNAIAFYLATDQLKCSKDTFMFSEVLQWLSYADNHILPAVSGWVIPCIVKNV

PNNMKAGIKTSKEDLLSSLTKLNKLLFTKTYLVGERISLADVAIFTALIPLYEHVFDSNYRIQYPNLTRWFLTILNQPQV

TSVLKNFKMCEKAVKN

>XP_013109973.1 PREDICTED: alanine--tRNA synthetase, cytoplasmic [Stomoxys calcitrans]

MHFLTAAEVRMAYLDFFKEKEHVYVHSSSTIPLDDPTLLFANAGMNQFKPIFLGTADPNSDMSKWLRVVNTQKCIRAGGK

HNDLDDVGKDVYHHTFFEMLGNWSFGDYFKKEICHWAWEFLTDRLGLPKERLYVTYFGGDEANGLAPDLECKQIWIELGL

MPEHIIPGNMKDNFWEMGETGPCGPCSELHFDRIGGRSVPELVNMDDPDVLEIWNLVFIQFNRDQDGSLKQLPKKHIDCG

MGFERLVSVIQNKRSNYDTDIFVPLFDAIQAGTGCPPYKGNVGADDVSGIDMAYRVLADHVRTLTVALADGGVPDNTGRG

YVLRRILRRAVRYGTEKLNAKPGLVGSLVFTAVDLLGDAFPEVKRDPQHIIDIINEEEQQFLKTLTRGRNLFNRTIAKLS

NDKLIPGEVAWRLYDTYGFPIDLTQLMAEERGFGIDMVGYEAAKRNAYILSQGKGINKVDEINLDVHAIAQLREKQIAVT

DDSFKYQYESVSNDPQSEYKFSPCKGTIVAIRFNNEFVDELSSGQKAGVVLDMTNFYAESGGQIYDQGVLTKIEDEENEF

IVDFVYNRGGYILHIGTVEGRISVGDIMNLHIDTERRSLVMKNHSATHALNHSLLQVLGKDTDQKGSLVVPEKLRFDFSN

KSGMTIEQIAKTEQLTKDIIKKNIPIFAKESQLKVAKTIRGLRSVFDEVYPDPVRVISFGVPVEELEQNPESDAGEKTSV

EFCGGTHLKRSGHMIEFVISSEEAISKGIRRIIALTGPEAIKAINKAKLYQNEIEKLSKDIDNNFDEKSSKHIVKKIVDL

SEEIARASIPHVQKDEMRTALKNLKKILDDKERALKNIVSVKVVEKAKELCLSNSNAMFIVEHLEAYNNTKALDAALKQV

RLLNPDTSAMFISVDANIKKIFCLSSVPKSAIEKGLKANEWVQHVSGALNGKGGGKPESAQASGTNYDNVQEVLDLAKEF

AKSKLQI

>XP_005190250.2 PREDICTED: alanine--tRNA synthetase, cytoplasmic [Musca domestica]

MHFLTAAEVRQAYLDFFKEKNHIYVHSSSTIPLDDPTLLFANAGMNQFKPIFLGTADPNSDMSKWLRVANSQKCIRAGGK

HNDLDDVGKDVYHHTFFEMLGNWSFGDYFKKEICAWAWEFLTERLGLDKDRLYVTYFGGDETSGLEPDLECKQIWMNLGL

KAEHIIPGNMKDNFWEMGETGPCGPCSELHFDRIGGRSVPELVNMDDPDVLEIWNLVFIQFNREQDGSLKPLPKKHIDCG

MGFERLVSVIQNKRSNYDTDIFVPLFDAIHVGTGCAPYGGKVGAEDVTGVDMAYRVLADHARTLTIALADGGVPDNSGRG

YVLRRILRRAVRYATEKLNAKPGFFATLVFTVIDLLGDAFPEVKKDPQHIVDIINEEEQQFLKTLSRGRNLFNRTVAKLG

DEKIIPGDVTWRLYDTYGFPVDLTQLMAEERGLQIDMEGYESAKHNSYILSQGKGANKVDEINLDVHAISQLKDKGIPVT

DDSFKYSYDSVSDDPQSEYKFNSCTGKIVAIRYNNEFVQKVYSGQKAGIVLDKTNFYAESGGQIYDQGVLVKVDEEENEF

MVDCVYNRGGYILHIGIVEGCLAVGNTLNLQIDTIRRSLTMKNHSATHALNHSLLQVLGKDTDQKGSLVVPEKLRFDFSC

KSAMTTEQIAKTEQLTKDLVYQNVPIYAKESKLHVAKTIRGLRSVFDEVYPDPVRVISFGIPVEELEQNPERDAGEKTSV

EFCGGTHLKRSGHIVDFVISSEEAISKGIRRIVALTGPEALKAISKSNLFQAEIEKLNKAIEEDSHGKNSKNIVKQIVEI

NEEISRATIPHVKKDEMRNNLKNLKKKLDDKERALKNLVALQVVERVKELCTSKPNAPYMVEQLDAYNNTKALDAALKQV

RSLCPDTSAMFLSVDVDSKKIFCLSSVPKTAVEKGLKANEWVQYISSVLNGKGGGKPESAQASGSNYENIVDIIKVAEEF

AKTKLEA

>XP_318757.4 AGAP009701-PA [Anopheles gambiae str. PEST]

MDTSLTASQIRSIFLDFFKEKEHLYVHSSSVIPLDDPTLLFANAGMNQFKPIFLGTVDPNSDMAKWVRTANTQKCIRAGG

KHNDLDDVGKDVYHHTFFEMLGNWSFGDYFKKEICTWAWELLTERLKLPKERLYVTYFGGHPESGLEPDLECREIWLKLG

VKEEHILPGSMKDNFWEMGETGPCGPCSELHFDRIGGRSVPELVNMDDPDVLEIWNLVFIQYNREQDSSLKLLPKKHIDC

GMGFERLVSVIQDKRSNYDTDVFMPLFDAIQKGTGAAAYQGRVGADDSDGVDMAYRVLADHARTITIALADGGFPDNTGR

GYVLRRILRRAVRYATEKLNAKPGFFATLVDTVVQLLGETFPEVRKDPQHIKNVINEEEQQFLKTLTRGRNLLNRTIAKL

GNSKVIPGDVAWRLYDTYGFPIDLTLLMAEEKQMTIDMEGYEKAKHESYIISQGKEKSKTATIDLDVHGISELQERKVPA

TDDSFKYRYKAESIDPLAQYVFEPCTGKIVALRFNNAFVEEVQAGQECGVILDRTNFYAESGGQIYDQGFLVKVNDESSE

FNVSLVYNRGGYVLHIGVVEGTLRVGDEVHCHMDVVRRQLTMKNHSATHALNHSLLKVLGQDTDQRGSLVVPEKLRFDFT

NKSAMTIEQVAEAERLTREVVKRNVQVYAKEANLAVAKTIRGLRSVFDEVYPDPVRVISFGVPVEQLEADPTGEAGVINS

VEFCGGTHLHQSGHMVDFVITTEEAIAKGIRRIVALTGPEALKALKKTELLEQELSKLRATIDGDKEGREAKEHVKKIIE

LTDDVSQATIPYVKKDELRTVLKGLKKALDDKERVARAAVASGVVEKAKELSQAHKDAPFLVQRLEALNNTKALDSALKE

VRKVNPEQSALFLSGLKANEWISHIAPVMGGKGGGKPESAQASGGNFDKADEILELARLFAASKLE

>XP_015786533.1 alanine--tRNA synthetase, cytoplasmic [Tetranychus urticae]

MDCNQVRQKFIDFFMKKYNHEFVPSSSVIPLDDPTLLFANAGMNQYKPIFLGTVDPNSDQAKYKRTCNSQKCIRAGGKHN

DLDDVGKDVYHHTFFEMLGNWSFGDYFKKEICKWAWELLTEEYGLDKDRLYVTYFGGAPQLGLDPDEECRSIWVDDVGVD

PSKVIPGSMKDNFWEMGDTGPCGPCSEIHFDRIGGRNASDLVNMDDPDVLEIWNLVFIQYNREQDSSLKPLPKQHVDTGM

GFERITSVIQNKRSNYDTDIFVPIFETVSQMTGCRPYTGKVGPEDTDGIDMAYRVIADHARTLTVALSDGGRPDNVGRGY

VLRRILRRAVRYGTEKLGAKPGMFASLVPVVVQILGDFFPEMKKDPQTVMDIINEEETQFLKTLSRGRRLLERTIAKLGD

SKILPGDIAWRLYDTYGFPLDLTHLMVEEKNLSIDMAGYEAAKAKAQASSHSKTSSQQSPIELNVHSIAELKDQLKVPLT

NDEPKYDYKANSDEPNADYIFTPCSGRILALRKDNAFVDSVDSGIECGVILDKTSFYAEAGGQIFDTGFMTKESLGGDEE

DSEFEVRDVKCQGGYIVHLGNVASGVLKVGDVMKLQIDQQRRKYIMSNHTGTHVLNYALRSVLKAESDQRGSLVAPDRLR

FDFTNKGPMSPSQVKETEQVSQKVIAKNEIVYAADASLPKAKEIKGLRAVFDEAYPDPVRVVSIGVPIDTLLNNPDSDSG

LETSVEFCGGTHLKRSGHIGDFVISSEEAISKGIRRIIALTGPEASRAIKKSALFQNEAEIIAEKVNSVTNNANEQKKIV

KRIVELIEEVSQSNISYWKKDELRNYLNASKKKLDDADRASKAANLNQVTEQVKILAESLKDAPFVVKALEAGSNAKALD

AAVKQIKSISPKTAAMFISCDDKKIICLASVPSETVSTSGLKANEWVQQVSPIINGKGGGKPESAQASGTNVNAIDAAVE

AAEKFAKMKLN

>ERL91554.1 hypothetical protein D910_08884 [Dendroctonus ponderosae]

MSISNMTAKEVRSAYIDFFKEKDHVYHHSSSTIPLDDPTLLFANAGMNQFKPIFLGTVDPNSDLSKLVRAVNTQKCIRAG

GKHNDLDDVGKDVYHHTFFEMMGNWSFGDYFKKEICTWAWEFLTVKLSLPADRIYVTYFGGDPKSGLEPDNECKEIWLKL

GVAPSHVIPGSMKDNFWEMGETGPCGPCSELHFDRIGGRSVPELVNMDDPDVLEIWNLVFIQYNREPDSTLKPLPKKHID

CGLGLERLVSVIQNKRSNYDTDLFMPFFEAIHRGTGAAPYQGRVGDDDKDGVDMAYRVLADHARTLTIALADGGNPDNTG

RGYVLRRILRRAVRYATEKLNAKPGFFSTLVNTVVEILGDTFPEVKKDPQYIIETINEEEAQFLKTLSRGRNLLNRTITK

LGESKELPGDVAWRLYDTYGFPVDLTSLMAEEKGLNVNMEAYEEAKKQAQIISQGKGTGVADTINLDVHAITELQNRQIP

PTNDSPKYDYEANQNVDDEYKFGACEASVIGLRYNKQFVEEVTSGQECGVLLDSTNFYAEQGGQIYDTGFLVKLNDDSVE

FSVKNVQVRGGYIIHIGNIEGVLRVGDKVSLHIDADRRRLIMSNHTGTHILNYALRKVLGTDADQRGSLVAPDRLRFDFT

NKGAMTADQVKKAEQSAKDLISKNAKVYAKDSSLATAKTIRGLRAVFEETYPDPVRIVSVGHIGDFVISNEEAIAKGIRR

IVALTGPEATKALKRNEVLENRLNEIKSSINADKDGAKSKEHVRIIVELTDEVSQAIIPYWKKEEIRNNLKNLKKNLDDK

DRAAKAAIANKVVDEIKEFVKENPNLPILVKELKAFNNTKALDSALKQVKTLSPATAALFVTVDSDSNKMFCLSSVPKEA

IEKGLKANEWVQSVAQKIGGKGGGKPDSAQASGANSVSVDEILELAKKFADSKLL

>XP_011189366.1 PREDICTED: alanine--tRNA synthetase, cytoplasmic [Zeugodacus cucurbitae]

MATLTANEIRDAFLGFFKEKNHLYVHSSSTIPLDDPTLLFANAGMNQFKPIFLGTADPNSEMAKWIRTTNTQKCIRAGGK

HNDLDDVGKDVYHHTFFEMLGNWSFGDYFKKEICTWAWEFLTERMKLPKDRLYVTYFGGYKAAGLEPDLECKQIWLDLGL

RPEHILPGSMKDNFWEMGETGPCGPCSELHFDRIGGRSVPELVNVDDPDVLEIWNLVFIQYNRESDGSLKPLPKKHIDCG

MGFERLVSVIQNKRSNYDTDLFIPLFKAIQEGTGAPEYAGRVGAEDVDGTDMAYRVLADHARTITIALADGGTPDNTGRG

YVLRRILRRAVRYATEKLNAKPGFFASLVHTVVDLLGDAFPEVRKNPQQIIDIINEEEAQFLKTLSRGRDLFNRTVAKLG

NQNVIPGDLAWKLYDTYGFPVDLTQLMAEEKNLQINMEEYEQAKQAAYIMSQGKSTTKADEIDLDVHAISELQSKSTPIT

NDSFKYKYQAESENLDSPYKFTPCSAKILALRFENQFVNKIEAGSKAGIILDQTNFYAESGGQIFDKGLFNKPNNVDDKF

IVEAVYNRGGYILHIGVSDGEFTVGDEIDLSIDIERRWLTMKNHSATHALNHSLLQVLGKETDQKGSLVVPDKLRFDFSS

KSAMTIEQVAKAEQFTRDIVYKNVPIYAKETKLANAKKIRGLRSVFDEVYPDPVRVISFGVPVEELESNLDSDAGEKTSV

EFCGGTHLQRSGHMMEFVITSEEAIAKGIRRIVALTGPEAVKALEKYNQLHKQIQTLKTTIEVDKDGKNSKNYVKQIVEL

TEEVSQAIIPYVKKDEMRNLLKQLKKELDDKERTLKAAVAVTVIEKTKEICQENPKATLLVKQFEAYNNTKALDGALKQV

RALCPDAAALFLSVDVDSKKIFCLASVPKSAVEKGLKANEWVQQLCEVIGGKGGGKPESAQASGTNYENVDEVIRLATEF

AKIKVGA

>ENN77299.1 hypothetical protein YQE_06125, partial [Dendroctonus ponderosae]

MSISNMTAKEVRSAYIDFFKEKDHVYHHSSSTIPLDDPTLLFANAGMNQFKPIFLGTVDPNSDLSKLVRAVNTQKCIRAG

GKHNDLDDVGKDVYHHTFFEMMGNWSFGDYFKKEICTWAWEFLTVKLSLPADRIYVTYFGGDPKSGLEPDNECKEIWLKL

GVAPSHVIPGSMKDNFWEMGETGPCGPCSELHFDRIGGRSVPELVNMDDPDVLEIWNLVFIQYNREPDSTLKPLPKKHID

CGLGLERLVSVIQNKRSNYDTDLFMPLFEAIHRGTGAAPYQGRVGDDDKDGVDMAYRVLADHARTLTIALADGGNPDNTG

RGYVLRRILRRAVRYATEKLNAKPGFFSTLVNTVVEILGDTFPEVKKDPQYIIETINEEEAQFLKTLSRGRNLLNRTITK

LGESKELPGDVAWRLYDTYGFPVDLTSLMAEEKGLNVNMEAYEEAKKQAQIISQGKGTGVADTINLDVHAITELQNRQIP

PTNDSPKYDYEANQNVDDEYKFGACEASVIGLRYNKQFVEEVTSGQECGVLLDSTNFYAEQGGQIYDTGFLVKLNDDSVE

FSVKNVQVRGGYIIHIGNIEGVLRVGDKVSLHIDADRRRLIMSNHTGTHILNYALRKVLGTDADQRGSLVAPDRLRFDFT

NKGAMTADQVKKAEQSAKDLISKNAKVYAKDSSLATAKTIRGLRAVFEETYPDPVRIVSVGHIGDFVISNEEAIAKGIRR

IVALTGPEATKALKRNEVLENRLNEIKSSINADKDGAKSKEHVRIIVELTDEVSQAIIPYWKKEEIRNNLKNLKKNLDDK

DRAAKAAIANKVVDEIKEFVKENPNLPILVKELKAFNNTKALDSALKQVKTLSPATAALFVTVDSDSNKMFCLSSVPKEA

IEKGLKANEWVQSVAQKIGGKGGGKPDSAQASGANSVSVDEILELAKKFADSKLL

>XP_018795074.1 PREDICTED: alanine--tRNA synthetase, cytoplasmic [Bactrocera latifrons]

MKVLTANEIREAFLGFFKEKDHLYVHSSSTIPLDDPTLLFANAGMNQFKPIFLGTADPNSEMVKWVRTTNTQKCIRAGGK

HNDLDDVGKDVYHHTFFEMLGNWSFGDYFKKEICTWAWEFLTERMKLPKDRLYVTYFGGDKASGLEPDLECKQIWLDLGL

CPEHILPGSMKDNFWEMGETGPCGPCSELHFDRIGGRSVPELVNVDDPDVLEIWNLVFIQYNRESDGSLKALPKKHIDCG

MGFERLVSVIQNKRSNYDTDLFVPLFKAIQEGTGAPEYAGRVGAEDVEGIDMAYRVLADHARTITIALADGGTPDNTGRG

YVLRRILRRAVRYATEKLSAKPGFFGSLVHTVVDLLGDAFPEVRKNPQQIIDIINEEEAQFLKTLSRGRDLFNRTVAKLS

NQKVIPGDLAWKLYDTYGFPVDLTQLMAEEKNLEINMEEYEQAKKAAYIMSQGKSTTKADEIDLDVHAISELQSKAIQIT

NDSFKYKYEAESENLDSPYKFTSCTSKILALRFGNQFVHKIEAGSKAGIIVDQTNFYAESGGQIYDKGLFVKLNNVDDKF

IVDAVYNRGGYILHIGVADGEFTVGDEIDLKIDTERRWLTMKNHSATHALNHSLLQVLGKETDQKGSLVVPDKLRFDFSS

KSAMTIEQVAKTELLTRDIVYKNVPIYAKETKLANAKKIRGLRSVFDEVYPDPVRVISFGVNVEELENNLDSDAGEKTSV

EFCGGTHLQRSGHIMEFVITSEEAIAKGIRRIVALTGPEALKALEKYDQLHKQIQTLKNTIEDDKDAKNSKNYVKQIVEL

TEEVSQAIIPYVKKDEMRNLLKQLKKTLDDKERTVKAAVSVTVIEKTKEICQENPKATLLVKQFEAYNNTKALDGALKQV

RALCPDAAALFLSVDPDSKKIFCLASVPKSAVNKGLKANEWVQHLSEVIGGKGGGKPESAQASGTNYENVDEVIRVATEF

AKLKVGAQ

>ETN61283.1 alanyl-tRNA synthetase [Anopheles darlingi]

MVAASTMDTSLTASQIRKIFLDFFAEKKHLYVHSSSVIPLDDPTLLFANAGMNQFKPIFLGTVDPSSEMARWVRTANTQK

CIRAGGKHNDLDDVGKDVYHHTFFEMLGNWSFGDYFKREICTWAWELLTERLRLPKDRLYVTYFGGHPESGLEPDLECRS

IWIELGIPEAHVLPGSMKDNFWEMGETGPCGPCSELHFDRIGGGRSVPELVNQDDPDVLEIWNLVFIQYNREQDGSLKLL

PKKHIDCGMGFERLVSVIQDKRSNYDTDVFVPLFDAIRAGTGAPPYGGRVGAEDTDGVDMAYRVLADHARTITIALADGG

FPDNTGRGYVLRRILRRAVRYATEKLNAKPGFFASLVDTVVELLGDTFPEVRKDPQHIKNVIGEEEQQFLKTLTRGRNLL

NRTIAKLGDSKVIPGDVAWRLYDTYGFPVDLTQLMAEEKEMSIDMEGYERAKHESYLISQGKEKSKTATIDLDVHAISEL

QAKQLAPTDDSTKYRYRAESTDPQAQYVFEGCRGRIVALRFGGAFVEEVTAGQECGVILDKTNFYAESGGQIYDQGYLVK

VGDESSEFNVSLVYNRGGYVLHIGVVEGTLRVGDEVDCHLDTVRRQLTMKNHSATHALNHSLLKVLGQDTDQRGSLVVPE

KLRFDFTNKAAMTIEQVGEAERLTREVVRKNVQVFAKETNLAVAKSIRGLRSVFDEVYPDPVRVISFGVPVEQLAADPTS

DAGVLNSVEFCGGTHLHQSGHMVDFVITSEEAIAKGIRRIVALTGPEALKALQKTESLEQEVRQLREKIEASGTVTTGAK

EFVRRIVELTEDVSQAVIPYVKKDELRTVLKNLKKTLDDRERAAKAAVSVEVIVRAKELSEANRDAPVLVHRLEAFSNTK

ALDSALKEVRKINPEQSALFVSVDEDAGKIFCLASVPKGAVEKGTEGERVDLARGPTDGWQGRRKTGIGTSFRWQLAEGR

RNSRACSQLCQHQAGLNAQG

>ODN05318.1 Alanine--tRNA synthetase, cytoplasmic [Orchesella cincta]

MDSSLTTAAIRSSFIDFFKSKQHIYVHSSSTIPLDDPTLLFANAGMNQFKPIFLGSVDPNSDMAKWVRVANTQKCIRAGE

GNLRMVLGVAYTVMKIPGDRLYVTYFGGDEKAGLEPDLECKEFWLKLGVQEEHILPGNMKDNFWEMGDTGPCGPCSEIHF

DRIGGRNAADLVNMDDPDVLEVWNLVFIQFNRESDGSLKSLPRKHIDCGMGLERVVSVIQQKRSNYDTDLFVPIFEAIQK

ATGARTYTGKVGAEDTDGIDMAYRVVADHVRTLTIALSDGGRPDNTGRGYVLRRILRRGIRYAIEKLGGKPGTFANLVDV

VVQILGDTFPEVRKDPHSIMDVINEEEAQFLKTLNRGRSLLERTVSKLSPTEKTLPGDVAWRLYDTYGFPVDLTQLMCEE

RSLSIDMNAYEEAKKRAQLISQAKGAGEDDTLALDVHAINELQSKGVPPTDDLLKYNYSAIDASSYEFPTLNAKVVAIRY

QKQFVDQVSSGQQAALILDKTNFYAEQGGQIFDEGFMTSLSKEDTEFLVKNSQVRGGYVLHIGNVEGTISVGDEMKLQVD

GARRKQIMNNHTGTHILNFALRSVLKEEPDQKGSLVAPDRMRFDFTNKNAMTVQQVKDAERIAQDMVNSDVEVYAKESAL

GQAKTIKGLRAVFNEAYPDPVRVVSIGVPVEELQNDPTSPKGLNTSVEFCGGTHLLRSGHIGDFVISSEEAIAKGIRRIV

ALTGPEASKALKKAALLEQAVTNLKNSASTTPPKEIQRKITELGDEISASQIPYWRKDDLRNSIKELKKSLDDLDKANKA

QQMTQIVEDVKQLLTENKFENGYIVKLLNAQSNTKALDAALKQVKALSPNTSAIFFSVDEAAGKVFCLSNCSGEAVGKGL

KANEWVGQVAKILDGKGGGKPESAQATGKNVSGLNEALKVAEEFAKLKMSG

>KYQ50613.1 Alanyl-tRNA synthetase, cytoplasmic [Trachymyrmex zeteki]

MSSIKDLSGIVKTQLQWTANNQDDDDDSSVSNNSSTMSSVWNIDCSNNTNANQIRQTYINFFRHKEHEYVHSSSTIPHDD

PTLLFTNAGMNQFKPIFLGTVDPNSDMAKWVRVVNSQKCIRAGGKHNDLDDVGKDVYHHTFFEMMGNWSFGDYFKKEICA

WAWEFLTKVLKLPADRLYVTYFGGDEKSGLAPDDECKNLWLSLGVPASHVLPGNLKDNFWEMGETGPCGPCSELHYDRIG

DREAAHLVNMDDPDVLEIWNLVFIQYNRELDGLKLLPKKHVDCGLGLERLVSVIQNKRANYDTDLFMPLFAAIEKGTGAP

PYQGKVGADDVNGIDMAYRVLADHARTLTIALADGGVPDNTGRGYVLRRILRRAIRYATEKLNAKQGFFGSLVNVVVDLL

GDVFPEIRKDPQSIIEIINEEESQFLKTLSRGRNLLNRTIAKLESANIVPGDVAWRLYDTYGFPIDLTQLMTEEKGLKID

MAGYEESKKQAQLISQNKAGGVDDQINLDVHAITELQKQGVKPTDDTPKYDYKVTNEIKYKEYEFASCIGTVIALRRAKT

FVNEVSSGEEVGILLDKTNFYAEQGGQIYDEGFLVKVDDETTEIRIKNVQVRAGYVLHIGIVGEGTLRKGDKVHANVDTE

RRRFVMANHSATHVLNYALRKVLGTEVDQKGSLVAPDRLRFDFTNKGPMTGQQLQETELVAITMIMKNEKIYAKESNLAI

AKTIQGLRAMFEETYPDPVRVVSVGIPVENLEKDPLSTAALETSVEFCGGTHLHYTSHIGYLTIVSEDAIAKGIRRIVAL

TGPEARKAIRTAKILQEQLTEFEAAVQNKKKFEMKSKECMRKIDYMRNTLKELKKSHDDNERAANAAVVTTVVQTAQSII

ESKIGCKVLVEVLEAYSNTKALDQALKKIRDLSPGTSALLISVDHDVKKIFALSSVPKSAVYEGLKANEWIQAIAPLMQG

KGGGKYESAQASGSNIACLNEIVSKANEFANQKLGITSKYRNIIV

>XP_020716408.1 alanine--tRNA synthetase, cytoplasmic [Ceratitis capitata]

MKVPTAKEIRDAFLGFFKEKGHLYVHSSSTIPLDDPTLLFANAGMNQFKPIFLGTADPNSEMAKWVRTANTQKCIRAGGK

HNDLDDVGKDVYHHTFFEMLGNWSFGDYFKIEICTWAWEFLTERMKLPKDRLYVTYFGGDKDAGLEPDLECKQIWLDLGL

RPEHILPGSMKDNFWEMGETGPCGPCSELHFDRIGGRSVPELVNVDDPDVLEIWNLVFIQYNRESDGSLKPLPKKHIDCG

MGFERLVSVVQNKRSNYDTDLFVPLFKAIQEGTGAPEYQGRVGADDADGIDMAYRVLADHARTITIALADGGTPDNTGRG

YVLRRILRRAVRYATEKLNAKPGFFGSLVHTVVSLLGDAFPEVAKNPQQIIDIINEEEAQFLKTLSRGRDLFNRTVAKLG

NQKIIPGDLAWKLYDTYGFPVDLTQLMAEEKNLQINMEEYEEAKQAAYIMSQGKSTAKEDAIDIGVHAISELQTKSIPIT

NDTFKYKYQAESEKFDSPYNFGSCKATILALRFGNEFVNKIEAGSKAGIILDQTNFYAESGGQIYDKGFFAKSKSSDEKF

FVETVYNRAGYILHVGVADGEFSVGDEIELNIDIERRWLTMKNHSATHALNHSLLQVLGKETDQKGSLVVPDKLRFDFSS

KSAMTIEQVAKAEQLTRDVVYKNVPIYAKETKLANAKKIRGLRSVFDEVYPDPVRVISFGVPVEELESNIDSDAGEKTSV

EFCGGTHLQRSGHMMEFVITSEEAIAKGIRRIVALTGPEAVKALEKSNELQKQIQALKSTIEVDKDGKNSKNYVKQIVEL

SEEVSQAVIPYVKKDEMRNLLKQLKKTLDDKERTLKAAVSVTVIEKTKEICQENPQATLLVKQFEAFNNTKALDGALKQV

RALCPEAAALFLSVDVDTKKIFCLASVPKSAVGKGLKANEWVQHLSEIIGGKGGGKPESAQASGSNYENADEVIRLATEF

AKTKLGA

>EDX04231.1 GD22412 [Drosophila simulans]

MKLLTAKEVRNAYLDFFKEQKHIYVHSSSTIPLDDPTLLFANAGMNQFKPIFLGTADPNSEMSKWVRVANTQKCIRAGGK

HNDLDDVGKDVYHHTFFEMLGNWSFGDYFKKEICSWAWEFLTQRLALPKDRLYVTYFGGDAASGLEPDLECKQMWLDLGL

KPEHILPGSMKDNFWEMGETGPCGPCSELHFDRIGGRSVPELVNMDDPDVLEIWNLVFIQYNRESDGSLKQLPKKHIDCG

MGFERLVSVIQNKRSNYDTDLFVPLFDAIQAGTGAPPYQGRVGADDVDGIDMAYRVLADHARTITIALADGGTPDNTGRG

YVLRRILRRAVRYATEKLNAKPGFFATLVNTVVDLLGDAFPEVKKDPQHIIDIINEEELQFLKTLTRGRNLLNRTIEKLG

NQTTIPGDVAWRLYDTYGFPVDLTQLMAEEKSLKIDMDGYEAAKHNSYVLSQGKGASKIEEINLDVHAISQLQEQGVPPT

NDTFKYKYEAVSDERDSAYNYGVCNSKIVALRFENQFVNEITSGQKAGIVLDKTNFYAESGGQIYDQGALVKVNDEANEF

LVDRVYNRGGYILHIGVVEGTLKVGDELELHIDVERRWLTMKNHSATHALNHCLLQVLGKDTEQKGSLVVPEKLRFDFNS

KAAMTIEQVSKTEQLTKEMVYKNVPIYAKESKLALAKKIRGLRSVFDEVYPDPVRPANKPPSSFAAAPTLRRSGHIMDFV

ISSEEAIAKGIRRIVALTGPEALKALKKSEAFEQEIVRLKATIDNDKSGKDSKSHVKEIVELTEQISHATIPYVKKDEMR

NLLKGLKKTLDDKERALRAAVSVTVVERAKALCEANPNATVLVEQLEAFNNTKALDAALKQVRSQLPDAAAMFLSVDADS

KKIFCLSSVPKSAVEKGLKANEWVQHVSATLGGKGGGKPESAQASGTNYEKVDEIVQLASKFAQSKLS

>XP_023216754.1 LOW QUALITY PROTEIN: alanine--tRNA synthetase, cytoplasmic-like [Centruroides sculpturatus]

METKLSAKEIRQKFMNFFIERYDHQYVHSSSVIPHEDPTLLFTNAGMNQFKPIFLGTVDPNSDAAKYVRTVNTQKCIRAG

GKHNDLDDVGKDVYHHTFFEMLGNWSFGDFFKREICHWSWELLTEVYGLDSNRLYVTYFGGCADLGLEPDLECRDIWKEV

GVPEDRILPFGMKDNFWEMGETGPCGPCSEIHFDRIGGRDAKHLVNADVPDVLEIWNLVFIQFNRESDGSLKLLPKKHVD

TGMGLERLVSVIQEKPSNYDTDLFLPLFDAIQKATNVRPYEGRVGKDDTDGIDMAYRVLADHARTLTIALADGGRPDNTG

RGYVLRRILRRAVRYATEKLNGKPGTFASLVPVVVELLVNFYFYXLLNEKEEEKVKIILYERYLXFNNLICLLIKXIIIN

IYIXGDIAWRLYDTYGFPIDLTQLMIEEKGLTIDMTGYEVAKKQAQLASQGKVEGQEDNIKLDVHAISELQQKGISPTYD

LPKYSYVALYDDKNSKYEFEGCTGEVVALRKNKMFVDEVNNGDECGVILNRTCFYGEAGGQIYDIGFMVKESDEDVEFNV

KEVHIQGGYVVHIGNIGGKLAVGNQVKLFIDEERRKLVMNNHTGTHILNFALRKVLSSDADQRGSLVAPDRLRFDFTNKG

AMTVNQIQETEMVANEVINKNQEVYAKECPLPLAKAIQGLRAVFNETYPDPVRVVSVGIPVDDLIADPCGPGGSTTSVEF

CGGTHLKRSGHIGDFVIASEEAIAKGIRRIVALTGPEASKAIRKAEIYEQDVEKIQKLVNDKNRKISGKEIVKKIVELTE

DISKSNISYWKKEEMRNNLKNLKKQLDDMERAQKAAILQEVVEETKNIIQSNSKLKFMVHELKAFGNAKALDAALKQVKS

LSPETSAMFFSVDRETGKIICLSSVPKEAVARGLKANEWIQQITGIIHGKGGGKDESAQATGCKPSALLEAMDVANQFAR

LKLGQDELLL

>OTF70272.1 alanine-tRNA synthetase, cytoplasmic-like protein [Euroglyphus maynei]

MSLKSKSYRDVRQAFIDFFKDKYEHFFVRSSSVIPHEDPTLLFANAGMNQFKPIFLGTVDPNSDMSKYKRVVNSQKCIRA

GGKHNDLDDVGKDSYHHTFFEMLGNWSFGDFFKREICQWSFELLTKVYGLEKDRIYVTYFGGNKAANLEPDEEARKIWTE

IGIDPEKILPFDMKDNFWEMGETGPCGPCSEIHYDRIGGRNASSLVNMDDPNVIEIWNLVFIQFNRENDGSLVSLPKKHV

DTGMGFERIVSVIQNKTSNYDTDIFEPLFKAIQENTGAPNYTGKLADDDINGMDMAYRVVADHVRTLTIALSDGGRPDNV

GRGYVLRRILRRAVRYIEKLGGKPGSFGSLVPVVVQTLGDFFPELNKDPQLVMDIINEEELQFLKTLSRGKRLLKTAIEK

LEGSKVLPGDIAWKLYDTYGFPIDLTRLMVEEHGLCVDMEGFEKAKAQAQLISQAKISQLDTKVELDVHAIAELRDQLKI

PLSNDLPKYNYNSLTDKKDSVYHFESCQSQVLAIRKDKSFVQEVSDTDLIGIILDSTNFYAESGGQEHDIGYMIKENDDQ

NIEIEFKVVQVKSFGGYVVHIGYLMQESSGQNKLRVGDVLRLEIDQERRKLIMNNHTGTHVLNYALRAVLKETDQKGSLV

AADKLRFDFTNKSAMTSAQVKEAESIAREMIDRNEIVYAKESPLSMAKAVKGLRAVFDETYPDPVRVVSIGVPVDDLLKD

PSSPAGMNTSVEFCGGTHLKRSGHIGDFVITSEEAIAKGIRRIVCVTGPEATRSMNKLAELENDAFELGIYINANQSKFS

TELKNMVKKVLDLQQKVSASDISCWKKEELRNKLNQLKKKLDDYERSSKASQANEVIKVAKKLADDNPKSEFIISSLDAG

SNAKALDQAVKAARAINPNLAVLFFSIDSDVNKIICIANVSKETAEKGLAANEWVQNITTLIQGKGGGKQESAQATGTNI

EALEEAIRLSNEFARLKLGN

>XP_002587517.1 hypothetical protein BRAFLDRAFT_237056 [Branchiostoma floridae]

MPQLNLTGDQVRDMFIKYFEEKHEHLEVHSSSTIPLDDPTLLFANAGMNQFKPIFQGTVDPSHPMAKYRRVVNSQKCIRA

GGKHNDLDDVGKDVYHHTFFEMLGNWSFGDYFKKEACSWAWDLLVNGYKLEVERMYVTYFGGNEAQGLQPDLECRQIWLD

LGVAEDRILPGSMKDNFWEMGEVGPCGPCSEIHYDRVGGRDASHLVNMDVPEVLEIWNLVFMQNNREPDGSITNLPKKNI

DCGMGLERILSVVQGVLSNYDTDLFTPFFDAIQKATGVRPYTGKVGADDADGIDMAYRVLADHARTLTISMSDGGKPDNV

GRGYVLRRILRRGVRYATEKLNAPRGFFASLVDVAVQTLGSAFPEITKDPQMVKDIINEEEEQFLKTLSRGRRVLEKRIT

QLGDSKVVPGETAWLLYDTYGFPVDLTSLMAEEKGLTLDCAAYEECKKQAQIRSQGKGAGVDDSVNLDVHAINELQEKGS

PPTDDSPKYQYSHDDNGNYVFQPCTATVVSLRRDKQFVQEVTQGQECGVLLDRTNFYAEQGGQIYDEGYLVKEEDEVKRT

AETEFTVKNCQVRGGYVMHIGTLVGTLNVGDKVKLFVDEARRRPIMSNHTSTHVLNFALRQVLGEADQRGSLVAPDRLRF

DFSAKGAMTTAQIKEAEKICNEIIKKNEQVYAKEAALAQAKAIQGLRAVFDETYPDPVRVLSIGISVEDMLSDPTSPAGT

VTSVEFCGGTHLQRVGHAGDLVIVTEEAIAK

>XP_014099419.1 PREDICTED: alanine--tRNA synthetase, cytoplasmic [Bactrocera oleae]

MKVLTANEIREAFLGFFKEKDHLYVHSSSTIPLDDPTLLFANAGMNQFKPIFLGTADPNSEMAKWVRTTNTQKCIRAGGK

HNDLDDVGKDVYHHTFFEMLGNWSFGDYFKKEICTWAWEFLTERMKLPKDRLYVTYFGGDKAAGLAPDLECKQIWLDLGL

CPEHILPGSMKDNFWEMGETGPCGPCSELHFDRIGGRSVPELVNVDDPDVLEIWNLVFIQYNREADGSLKPLPKKHIDCG

MGFERLVSVIQNKRSNYDTDLFVPLFNAIQEGTGAPEYAGRVGAEDVEGIDMAYRVLADHARTITIALADGGTPDNTGRG

YVLRRILRRAVRYATEKLNAKPGFFGSLVHTVVDLLGDAFPEVRKNPQQIIDIINEEEAQFLKTLSRGRDLFNRTVAKLS

NQNVIPGDLAWKLYDTYGFPVDLTQLMAEEKNLQINMEEYEQAKQAAYIMSQGKSTTKTDEIDLDVHAISELQFKSIKIT

NDSFKYKYEAESENLASPYKFNSCTAKIVALRFGNQFVNKIEAGSKAGVILDQTNFYAESGGQIYDKGLFVKPNNADDKF

IVDAVYNRGGYILHIGVADGEFTIGDEIELKIDIERRWLTMKNHSATHALNHSLLQVLGKETDQKGSLVVPDKLRFDFSS

KSAMTIEQVAKTELLTRDIVYKNVPIYAKETKLANAKKIRGLRSVFDEVYPDPVRVISFGVPVEELENNLDSDAGEKTSV

EFCGGTHLQRSGHMMEFVITSEEAIAKGIRRIVALTGPEAVKALEKYDQLHKKIQRLKTTIEDDKDGKNSKNYVRQIVEL

TEEVSQAIIPYVKKDEMRNLLKQFKKTLDDKERILKAAVSVTVIEKTKEICQENPKATLLVKQFEAYNNTKALDGALKQV

RALCPDAAALFLSVDPDSKKIFCLASVPKSAVDKGLKANEWVQQLCEIIGGKGGGKPESAQASGTNYEKVDEVIRVATEF

AKLKVGA

>OQV23471.1 Alanine--tRNA synthetase, cytoplasmic [Hypsibius dujardini]

MPEITTAKDVRQAFIDFFVGKQHTYWHSSSVVPLDDPTLLFTNAGMNQYKPIFLGTVAPNSDMSRLVRAVNSQKCIRAGG

KHNDLDDVGKDVYHHTFFEMLGTWSFGDYFKKEAAAWAWEFLTSYLGLDKDRLYVTYFGGKEELGLKPDEECRDIWLGLG

LPAHRVIPFPMKENFWEMGETGPCGPCTEIHFDRIGNRDASALVNQDDPDVLEIWNLVFIQFNREQDGSLTSLPRKHIDT

GMGFERLVSVCQNKRSNYDTDVFVPIFSAIERGTNSRPYTGKIGADDKDKMDMAYRVVADHIRTLVIALSDGGRPDNVGR

GYVLRRILRRAIRYSSEKLNAKPGFLSSLVPQVIELLGDAFPEVKKDPQSVMHIIDEEETQFLRTLIRGRRLLERTIAQM

GDSKTLPGETAWRLYDTYGFPIDLTQLMADERDLAVDMVGYEEAKKRSHLLSQAQSAVGIAKLDLDVHAIGALKDRGVPV

TDERPKYDYKSTQDGQPADDRYHFAACSGTILALRVEKGFVEDVHGGECGIILDRSNFYAEQGGQIFDEGYMTLANNEEV

EFQVTNVQVRGGYIIHTGKLDGALKVGDKLNLFIDETRRKLIMNNHTGTHILNFALRKVLAGKEADQRGSLVAPDRLRFD

FACNAAMTIPEVKETEAITQQVVQKNEEVYAKYASLADAKHIQGLRAIFDETYPDPVRIISVGVPVDELLLKPNDGLGFN

TSVEFCGGTHLLRSGHIGDFVIVSEEAIAKGIRRIVALTGPEARNAIQRSDAFQAKVDHVKQTLKSGQYVHKTLLKELND

AAEEVAQAVIPAWRKDALRTELNTAKKEIDSLDKAAKAAKAALVNEKAKEIVERDPGQKIFVLQLDAGGNAKALDGAVKV

MRQVAPDAAILLFTVDEDEGKVLVLASVTDGIVAKGLKASEWIQQVSVAINGKGGGKDLNAQAVGTNVRGVAEGLELAKK

FAEGKL

>KYM92893.1 Alanyl-tRNA synthetase, cytoplasmic [Atta colombica]

MQLQQTANNQDNDNGSSPNSSGTMSSALNTDHSNNMNAKQIRQAYIDFFKSKGHEYVHSSSTIPHDDPTLLFTNAGMNQF

KPIFLGTVDPNSDMAKWIRVVNSQKCIRAGGKHNDLDDVGKDVYHHTFFEMMGNWSFGDYFKNEICAWAWEFLTDMLKLP

ADRLYVTYFGGDEKSGLAPDDECKNLWLSLGVPASHVLPGNMKDNFWEMGETGPCGPCSELHYDRIGDREAAHLVNMDDP

DVLEIWNLVFIQYNRELNGSLKSLPKKHIDCGLGLERLVSVIQNKRANYDTDLFMPLFAAIEEGTDAPPYQGKVGADDVN

GIDMAYRVLADHVRTLTIALADGGVPDNTGRGYVLRRILRRAVRYATEKLNAKQGFFGSLVNVVVDLLGDVFPEVRKDPQ

SIIEIINEEESQFLKTLSRGRNLLNRTIAKLESADTVPGDVAWRLYDTYGFPIDLTHAKILFFTLIFFINQLISQNKAGG

VDDQINLDVHAITELQKQGIKPTDDSPKYDYKVTAEIKYKEYEFAPCTGTVIALRRAKTFVDEVSSGEEVGILLDRTNFY

AEQGGQIYDEGFLVKVNDETTEIRIKNVQIRAGYVLHIGTVGEGTLRKRDKVHTNVDTERRRFVMANHSATHVLNYALRK

ILGTKADQKGSLVAPDRLRFDFTNKGPMTGGQLQDTEQLANNMITENEKIYAKESNLAIAKTIQGLRAMFEETYPDPVRV

VSVGIPVENLEKDPLSTAALETSVEFCGGTHLHYTSHIGNFTIISEDAIAKGIRRIVALTGPEAAKALRTGMLLQRQLSQ

FQATIEDDKSEMNSKEYDHMRNTLKNLKKSLDDKERAAKATVATTVIQTAQSIIESKVGCQVLVEVLEAYSNTKALDQAL

KKIRAISPETSALLISIDHDVKKIFALSSVPKSAVSKGLKANEWIQAITSLMQGKGGGKSESAQASGSNIVCLNEIVSKA

NEFADQKLGIASKYKNITVIRYCNFTVALC

>XP_017484302.1 PREDICTED: alanine--tRNA synthetase, cytoplasmic-like [Rhagoletis zephyria]

MAQSKGFKEIRQDFINFFQDKYAHQFVRSSAVIPLDDPTLLFANAGMNQFKPIFLGTVDPNSDMAKWKRAVNSQKCIRAG

GKHNDLDDVGKDVYHHTFFEMLGNWSFGDFFKKEICKWSFELLTGVYGLNKDRIYVTYFGGNKAANLDPDEECRQIWVDV

GIDPARILPFDMKDNFWEMGETGPCGPCSEIHYDRIGGRDASALVNMDDPDVIEIWNLVFIQFNRENDGSLRQLPSKHVD

TGMGFERLVSVIQDKNSNYDTDVFVPLFNAIQEKTGARLYTGKVGADDTDGVDMAYRVVADHARTLTIALSDGGRPDNVG

RGYVLRRILRRAIRFIEKLGGKPGALSSLVPVVVGLLGDTFPELNKDPELVMDIINDEEQQFLKTLSRGKRLLERTIEKL

TDSKVLPGEIAWKLYDTYGFPFDLTQLMSEEQGLTVDIEGYEKAKVAAQLLSQAKISQLNTTVDLDVHAISELRDQKVPI

TDDSAKYNYSSLSDKKDSVYRFESCQGTVLAIRKDKAFVQQVTDTELIGLILDATNFYAEQGGQEHDTGFMIKGESGAEV

EFQVLQVKVYGGYIMHIGHLIGNSTEGDQALSLKVGDLLRLEINQERRKLIMNNHTGTHVLNYALRAVLNETDQKGSLVA

ADKLRFDFTCKSAMTSAQIKNAEQIARELIDKNEEVYAKDSPLSAAKAVKGLRAVFDETYPDPVRIVSIGVPVEDLLSDP

SSPAGMNTSVEFCGGTHLKRCGHIGDFVITSEEAIAKGIRRILCVTGPEASRSISNLAKLENSVNELKVVIEANKSQFDT

LAKELVKKIIELQDEVNKQDISYWKKDELRSSLNSLKKQIDDHERAIKAAQITEVGNVGKQLATEQMGAKFMVEVLNAGW

NAKALDIALKNVRTVSPSTAALFFSVDEEAKKIICLSSVPKETKEKGLKANEWINNITALINGKGGGKDESAQAMGSNVS

ALDEAVKLSREFAALKLGN

>PAA73021.1 hypothetical protein BOX15_Mlig019934g1 [Macrostomum lignano]

MTQYTAASVRQSFIDFFAQKHKHTYVHSSSTIPYDDPTLLFTNAGMNQYKPIFLGTADPNSDMAKWTRAVNSQKCIRAGG

KHNDLDDVGKDVYHHTFFEMLGNWSFGDYFKLEACTWAWDLLTNVWGLPRDRLYVTYFGGNEAQGLPSDEEARDIWISLG

LPPDRVLPFGMKDNFWEMGETGPCGPCSEIHFDRIGGGRNAADLVNMDDPDVLEIWNLVFIQFNREPDGNLRLLPNKHID

TGMGMERILSVLQGHRSNYDTDAFQPLFAAIEKFTGSRPYSGRVGAEDTDGVDMAYRVLADHARTLTIALSDGGRPDNAG

RGYVLRRILRRGVRYAVEKLRSPPGLLEALVDVVCQSLGSAFPELNRDPDTVREIIAEEEAQFLKTLRRGQRVLERTLAK

VPAGQPVPGDVVWRLYDTYGFPTDLTKLMAEERGLAIDETGFEAAKEASLLASQAAGGGVGVSVDLDVHALAELKDGMAI

AQTDDSPKLTYTTDSDGVYAYPNRISATIKAIRYRQAFVDSVPSGVECGFVLDRTCFYAEQGGQIFDEGVMTKTGEGAGD

DAAEFMVKNCQVRGGYVIHVGTTEGEFKVGDELILSVDMQRRKPVMQNHTGTHVLNYALRQVLGVDADQKGSLVAPDRLR

FDFACKAAMTQEQIGQVENIVNEVVSKRQPVYAAMASLAEAKAVQGLRAVFGEVYPDPVRVVSVGVEVDKLLADPNGPAG

SSTSVEFCGGTHLHNAGHIGQMAIISEEAIAKGIRRIVAVTGTEASRARHSADLLEADVTSLEGLASACTDVAEARGLAR

RIVDLSTLVAQSTTPSVRREAMRRRLADAKRRLDDLDRSKKAAQANQMMEEAKQLSAKAASGGGASPPAFLVHAFDSEGG

AKAVNTALKQLSKDCPETAFLCLSVDAAAGKVVCLAQAPKSAIDRGLKANEWIGHVSPAIDGRGGGRDNAAQTAGGKVDG

IAEAKKLAVEFAKAKLG

>XP_002160302.2 PREDICTED: alanine--tRNA synthetase, cytoplasmic [Hydra vulgaris]

MLLGFVFQYKYFVSNITAKYLLRSFSIKTFQSLRCSKEMTTKTAKQVRQDFIDFFVKKYDHIFVPSSSTIPHEDPTLLFA

NAGMNQFKSLFLGTADPNSDFAKLKRVVNSQKCIRAGGKHNDLDDVGKDVYHHTFFEMMGNWSFGDFFKKEIIAWSWELL

TDVWGLEKERFYVTYFGGADGVPADEEAKQFWLELGLPPNHVLPFGMKENFWEMGETGPCGPCSELHYDRIGGRDASNLV

NMDVPDVLEVWNLVFIQFNREVDGQLVPLPNQHVDTGLGLERIISVLQNKMSNYDTDVFTPYFDAIHQATGIKPYLGLVG

PDDKDGVDMAYRVIADHIRTLTVALSDGGRPDNVGRGYVLRRILRRAVRFATEKLNCKPGVFASLVDVVCTSLGSVYPNI

LKDPQTIKDIINEEESQFLKTLARGRRLFNRTADKTTDGIIAGDLAWRLYDTYGFPSDLTILMAEERNLKVDMVGYEVLK

AKAQEMARARGSEVEDIYSLDVHAIDELKTKGFVQTDESPKYNYEKDEHGNYVFQPTEAIVTALRVNKQFVDEVTNGKCG

VLLDRTSFYAEQGGQLFDTGYINLGEEVEFAVNDVQIHGGYVLHVGNLVGTLKVGDKVTCSIDESRRRSLMNNHTATHLL

NFALRKVLVDDADQRGSLVAPDKLRFDFTAKGALSSEQVKMTEDITREIIKQDQKVFASASSLAVAKNIQGLRAIFDEVY

PDPVRVISIGYDLKDLINDPQAGYKTSVEFCGGTHLKSTGDVVEFAIVSEEAISKGIRRIVSLTGSDALKAHKRANQLAS

TLNEFSEYVEVQKNNNTLNMRQLSQQIAKITEEISESLIPAWCKHELREKLKSIKKHLDDTERMEKASRQNRVIEKASEI

ATLHLNEPFIVQRMEDGCNAKALDAALKSVKAVAPNLSAMLFSVDTETNTVICLCQVPQNVVDNTSLKANEWVKEVSTVI

GGKGGGKPISAQGSGDKMQEIDKAVEVAKSFASLKLQ

>XP_018495959.1 PREDICTED: alanine--tRNA synthetase, cytoplasmic [Galendromus occidentalis]

MTSCKEVRTRFIEFFQKKYQHTFVPSSCVIPHDDPTLLFANAGMNQYKPIFLGTVDPNTEAAKWKRTVNSQKCIRAGGKH

NDLDDVGKDVYHHTFFEMLGNWSFGDFFKKEICAWAWEFLTKEFGLAEDRLYVTYFGGNPEAGLEPDNECKGIWLALGVP

EDHILPGSMKDNFWEMGETGPCGPCSEIHFDRIGGRNAAHLVNMDDPDVLEIWNLVFIQYNREADSSLRPLPKQHVDTGM

GFERLVSVIQNKRSNYDTDVFVPIFDAIQKSTGVRAYTGKVGAEDVDGIDMAYRVLADHARTLTIALSDGGRPDNVGRGY

VLRRILRRAIRYATEKMNAKPGMFAGLVPVVVGLLGDAFPELKKDPDTVMEIINEEETQFLKTLSRGRRLLTKAVSKIEK

GGLLPGEVAWRLYDTYGFPVDLTQLMVEESGIFVDSAGYEKAKQAAILASQGGADKADDSSKLDVHAIAELQDKQKLPAT

DDSPKYVYTAEGTGKSAKYTFQPCTGKIVAIRSKKQFVNGVNAGDLCGIVLDRTNFYAEQGGQIYDTGFLQHVENEDVEF

AVTEVHLQGGFVVHIGRLAAGTLKVGDELKLQIDETRRVAVMQNHTGTHVLNYALRKVLSADADQRGSLVAPERMRFDFA

SNRPMTAQEVKQAEEAAVELVSRNGPVYAKDSSLSQAKAVKGLRAVFNETYPDPVRVVSIGVSVEDLLANPDRGDALNTS

VEFCGGTHLQNAGHIGDFVIAQEEAIAKGIRRIMALTGTEAAKATKRAEHLESEVNTLKSLVGKDKSLVKRITELSDEIS

QSSISYWRKDDLRSELKKIKKVIDDQDKQNKAAAQMEALEEIKRVLGDAPPYLIRKLQVGGNGKALDAVMKQIRTLSPNA

SAMLFSIDEEKKRLVCLSSVPKQVVADKGLSASEWVRHIADVIGGKGGGKDESAQASGTNPEALSEAMNMAEEFAKLKLN

>NP_491281.1 Alanine--tRNA synthetase, cytoplasmic [Caenorhabditis elegans]

MKHLTASEVRSTFINFFREKKEHTYVHSSSVIPHDDPTLLFANAGMNQFKPLFLGIADPNSDLAKLKRAVNTQKCIRAGG

KHNDLDDVGKDVYHHTYFEMLGNWSFGDYFKKEIITWAWELLTTVYGIPAERLYVSVFGGDEANGVPADSEARDIWRSVG

VPDERILNFGMKDNFWEMGDVGPCGPCSEIHYDRIGNRDASHLVNADDPMVVEIWNLVFIQFNREEGGVLKPLPAKHIDC

GLGLERLIAVMQDKTSNYDTDIFQPIFEAIHKGSGVRAYTGFIGDEDKDGVDMAYRVVADHVRTLTIALSDGGRPDNSGR

GYVLRRILRRGVRYASEKLNAQPGFFASLVPVVISILGETFPELSRDPVTVMDIINDEEKQFLKTLSRGRVLFQRAVQSL

PEGTMTFPGDVSWRLYDTYGFPADLTQLMAEEKGLSVDNTAFEEARRKAIETSSAGTGKFRDTLDLDVHALAELQQKGVP

TTDDSPKYAYTFTGEGSDAVYKFEPCVGKILAIRRDGKFVDQLAAGEEGAILLDRTNFYAEQGGQIYDVGVLTKVNDESN

EFNVSNCQVRGGYIVLVGSAEGSFSVGDQVNERFDEDRKQLIMKNHTGTHVLNYALRKVLADSDQKGSLVAPDRMRFDFT

NKAGMTVQQVKKAEEYAQQLIDTKGQVYAKNSPLGEAKKVKGLRAMFDETYPDPVRVVAVGTPVEQLLQNPDAEEGQNTT

VEFCGGTHLQNVSHIGRIVIASEEAIAKGIRRIVALTGPEAERAIARADRLTARLEEESKHADKKDELLANKDKFKALQK

KIQEIVDEANGAQLPYWRKDSIREKAKAIQKTLDGYTKAQQAAVAEKVLGEAKELAAVAEQPTVLVHVFAANANSKAIDN

ALKLLKDTKAVMAFSVNEDSGKVLCLAKVDKSLVSNGLKANEWVNEVCTVLGGKGGGKDANAQLTGENVDKLDAAVELAQ

KFALAAIN

>XP_018653769.1 putative alanyl-tRNA synthetase [Schistosoma mansoni]

MNNKITAKELRKQFFEFFESKGHVYVHSSSTIPHNDPTLLFANAGMNQYKPIFQGTVDPNSELGRLKRSYNSQKCIRAGG

KHNDLDDVGRDVYHHTFFEMLGNWSFGDYFKKEACEWAWELLTEVWKIPSERLYVTYFGGDISNGLPADEEARDIWISIG

LPKDHVLPFGMKENFWEMGETGPCGPCSEIHYDRMGDRDAAHLVNQDDPDVLEIWNLVFIQFNREEGGSLKPLPAKHIDT

GMGFERVLSVLQNKRSNYDTDLFVPLFKTIEAGTNTRPYTGKVGVEDTDKIDMAYRVLADHARVLTIALGDGGRAQNTGR

GYVIRRILRRAVRFAVEVLNAKPGFLATLVDIVIETLGDAFPEITRDPDTIKELINEEEEQFLVTLKRGRRFFDRVIKDL

KASGSNNTTLSGEVAWGLYETYGFPLDLTQILAEEHHLKIDLNGYEKAKEEAQIRSQSRKCTGGSIIDLTVHALAELKSK

NISVTDDSEKFVYTSDLNGNYVFPDSEATVLAILYENKFVESVDLSSQMCGIILNKTIFYAESGGQLYDHGFITSLTDEV

TEFSVSDVQCRGGYILHIGNLHGKLIVGNRVLLSLDTVRRTALMRNHTGTHVLNFALRELVDESEQKGSLVAPDRLRFDF

TAKRGMTRDELAKAEEICDTMISKRLNVYSSNVSLSYAKTIQGVRAVFGEAYPDPVRVVSIGVPVTSLVADPETGYGKTT

SVEFCGGTHVLNTKHIGVLVIVSEEAISKGVRRIIALTGHEAERAQKEALRLDNEVNEFIKLVNQSISLHQNHNNNITDD

FNINRQMSNLSEHVSRAVISQHHRENLREKLFKAKKLLDARDKASKTAITSKVLEEARLLGESLANSPSNYIIHVFDAQS

NTKIMNSAIKEIERICPNKAVMVISSDLTSKKLTVLCQVPKVLISRGLKANEWVTHISKTMDGKGGGKENSAQAVGSRID

TLDEVIHLADIFANTKLTNN

>OZG17539.1 hypothetical protein FL83_08256, partial [Caenorhabditis latens]

MKHLTASEVRSTFINFFKEKKEHTYVHSSSVIPHDDPTLLFANAGMNQFKPLFLGIADPNSDLAKLKRAVNTQKCIRAGG

KHNDLDDVGKDVYHHTYFEMLGNWSFGDYFKKEIITWAWELLTTVYGIPAERLYVSVFGGDEATGVPADSEAREIWRAVG

VPDERILNFGMKDNFWEMGDVGPCGPCSEIHYDRIGNRDASHLVNADDPMVVEIWNLVFIQFNREEGGSLKPLPAKHIDC

GLGLERLIAVMQDKTSNYDTDIFQPIFQAINKGSGVRAYTGHIGDDDKDGVDMAYRVVADHIRTLTIALSDGGRPDNSGR

GYVLRRILRRGVRYATEKLNAQPGFFASLVPVVISILGETFPELSRDPETVMDIINDEEKQFLKTLSRGRVLFQRAVDSL

PTGTKTFPGDVAWRLYDTYGFPADLTQLMAEEKGLSVDLTAFEEARKKAIETSSAGTGKFRDTLDLDVHALAELQQKGVP

TTDDTPKYAYSFTGEGADAVYKFEPCVGKILAIRRDGKFVDELVAGEEGAILLDRTNFYAEQGGQIYDVGVLTKVNDESN

EFNVSNCQVRGGYIVLVGSSEGNFKVGDDVNQRFDEDRKQLIMKNHTGTHVLNYALRKVLADSDQKGSLVAPDRMRFDFT

NKAAMTSEQVKQAEEYAQQLINTKGQVYAKNSPLADAKRINGLRAMFDETYPDPVRVVAVGTPVEQLLQNPDAPEGQNTT

VEFCGGTHLQNVSHIGRIVISSEEAIAKGIRRIVALTGPEADRAIARADRLEARLVEESKHAKNQELLANKDKFKELQKK

IQEIVDEANGALLPSWRKDSLRKKAKDVQKDMDGYSKAQQAAVAEKVLGEAKELAAASEQPAVLVHVFTSNANSKAIDNA

LKLLKDTKAVMAFSVNEDTGKVLCLAKVDKAMSSLKANEWVNEVCTVLGGKGGGKDANAQLTGENVDKLDAAVELAQKFA

LAAIN

>GAV04904.1 hypothetical protein RvY_15107 [Ramazzottius varieornatus]

MPEIKNAKDVRQAFVDFFVGKAHTYWHSSSVIPLDDPTLLFTNAGMNQFKPIFLGTVAPNSDMSKLVRACNSQKCIRAGG

KHNDLDDVGKDVYHHTFFEMLGTWSFGDYFKKEACTWAWEFLTEYIRLPKDRLYVTYFEGKTELGLEPDVECRDVWLKLG

LPENRVISSSMKDNFWEMGETGPCGPCTEIHFDRIGNRDAAALVNQDDPDVLEIWNLVFIQFNREPDGSLTSLPNKHIDT

GMGFERLVSVVQNKRSNYDTDIFVPIFDAIEKGTGSRPYTGKVGAEDTDKMDMAYRVVADHIRTLVIALSDGGRPDNVGR

GYVLRRILRRAIRYSSEKLNAKPGFLSSLVRQVVETLGSAFPEVRKDPSSVKKIIDEEEVQFLRTLTRGRRLLERTIAQM

GDGKTLPGDVAWRLYDTYGFPVDLTQLMADEHGLTVNMEAYEEAKKKAVIASQAQSVASIAKLDLDVHAIGHLKDRYVPP

TDDTPKYAYKAEQNGRPAEDKYSFEPCAGTVLALRVEKGFVEEAATGECGVVLDRSSFYAEQGGQIFDEGYMVLEGSEEV

EFQVKNVQVRGGYVLHTGHLDGVLKVGDKLKLFIDENRRKLVMNNHTGTHILNFALREVLSQSADQRGSLVAPDRLRFDF

AFGSGMKVDEVKKTEAIVQDLISKNEEVFAQYASLGEAKRIKGLRAIFDETYPDPVRIVSVGKPVEELLEQPENGEGYRH

SVEFCGGTHLIRSGHIGDFVIVTEEAIAKGIRRIVALTGPEAKTALGRSDRYKEKVERIKNSVVGDSSGAQQKVITKELN

DLFEEINQAVMAVWQKDRLRNELTALKKELDNKDKERKAAKVLAVNEKMKEMVASGDGRKVFVALVDVGSNSKALDAVVK

VMRGVPGGEDAAIMLFSVDDDEGKCCVLSSVTDSLVKDKGFKAIEWVLQVSGVINGKGGGKELNAQAVGSNVGKVNEAVE

LARKFAEMKLGTL

>XP_017472431.1 PREDICTED: alanine--tRNA synthetase, cytoplasmic [Rhagoletis zephyria]

MKHLTAKEIRDAFLGFFKEKDHVYVHSSSTIPLDDPTLLFANAGMNQFKPIFLGTADPNSEMAKWVRVTNTQKCIRAGGK

HNDLDDVGKDVYHHTFFEMLGNWSFGDYFKKEICSWAWEFLTERMKLPKDRLYVTYFGGDKASGLEPDLECKQIWLDLGL

RPEHILPGSMKDNFWEMGETGPCGPCSELHFDRIGGRSVPELVNVDDPDVLEIWNLVFIQYNREPDGSLKPLPKKHIDCG

LGFERLVSVIQNKRSNYDTDLFVPLFKAIQEGTGASEYAGRVGADDADGIDMAYRVLADHARTITIALADGGTPDSTGRG

YVLRRILRRAVRYATEKLIAKPGFFASLVHTVVDLLGDAFPEVKKSPQQIIEIINEEEAQFLKTLSRGRDLFNRTVAKLG

NRNIIPGDLAWKLYDTYGFPVDLTQLMAEEKNLKINMEEYEQAKQAAYIMSQGKSATKAEEIHLDVHAISELQSRSIPIT

NDSPKYKYQAESDKFDSPYIFSSCKAKIFALRFGNQFVNKIEAGSKAGIILDQTNFYAESGGQIYDKGSFNKSNSGVDKF

FVEAVFNRGGYILHIGVADGEFAVGDEIELKINIERRWLTMKNHSATHALNHSLLQVLGKETDQKGSLVVPDKLRFDFSS

KSAMSIEQIAKAEQLTRDIVYQNMPIFAKETKLAKAKKIRGLRSVFDEVYPDPVRVISFGVPVEELENNPDSGAGEKTSV

EFCGGTHLQRSGHMMEFVITSEEAIAKGIRRIVALTGPDAVKALEKNNELHQKIQALKSTIEADEDGKNSKSYVKQIVEF

TEEVSQAIIPYIKKDQMRNLLKQLKKTLDDKERTMKAAVSVTVIEKTKEICEANPQATLLVKQFEAFNNTKALDGALKQV

RSLCPDAAALFLSVDEDSKKVFCLASVPKSAVDKGLKANEWVQQLSEIIGGKGGGKAESAQASGSRYENVDEVIRTATEF

AKNKIGS

>XP_009175000.1 hypothetical protein T265_10380 [Opisthorchis viverrini]

MASQVTAERVRQQFIEFFKEKGHIYVHSSSTIPHNDPSLLFTNAGMNQGTVDPNTEMAGWKRAVNTQKCIRAGGKHNDLD

DVGRDVYHHTFFEMLGNWSFGDYFKKEACVWAWQLLTEVWKIPKERLYVTYFGGTGSTGLPPDEEARDIWLSIGLPKERV

LPFGMKDNFWEMGETGPCGPCSEIHYDRIGGRDAAGLVNMDDPDVLEIWNLVFIQFNREEDGSLRPLPAKHIDTGLGFER

ILSVLQGKRSNYDTDLFQPLFAAIQSGTGAPPYSGRVGAEDVDGKDMAYRVLADHARVLTIALSDGGRAQNTGRGYVLRR

ILRRAVRYAVEVLGAPPGFFATLVDVVVTSLKDAFPELANDPYTVKELINEEEQQFLTTLKRGQRLLSRTISDIKRTQGA

AGTGVLPGTVAWRLYDTYGFPLDLTQLMAEEHNLKLDLAGYEKAKELAQARSQTGAAGAGAIVDLDVHAIAELRKRGIPG

TDDTEKYNYQADAQGNYAFPEIEATILSIRQESMFVPDVSQSGQLCGFILDKTVFYAEAGGQLYDTGFITKISDDACELC

VTDVQYRGGYVLHIGRLEGHFAIGDKVRVSIDGVRRSGLMRNHTGTHVLNFALRKVLGEADQKGSLVAPDRLRFDFTAKR

GMSRDELRDAEEVAHTMLESNAAVYAANVSLPIGKSIQGLRAVFGEVYPDPVRVVSVAVPVDKLVADPTGPAGNTTSVEF

CGGTHVHNVKHIGSLVIVSEEAIAKGIRRIVALTGHEGERAQREAQELEHEVSRLSGAIHMASSLALANGDKPVDKNKNI

EFRELTQKLASLSERLAQANISQHVRDRLRADLGLAKKKLDDRDKNAKASITTQVLEEARKLVESCCSNKQDGFGSNYVV

HVFQDEANAKAINSALKILERGCPDIAVMAISSDPVGKKLLCLSQVPKNLIDRGLKANEWVASVSSLIDGKGGGKESSAQ

AVGSRVDCLDEVVRLANQFAKAKLCSS

>XP_001628752.1 predicted protein [Nematostella vectensis]

MDSSLTAKDIRRKFIDFFVEKHNHTFVPSSSVIPHEDPTLLFANAGMNQYKSIFLGTVDPNSDMSKLKRAVNSQKCIRAG

GKHNDLEDVGKDVYHHTFFEMLGNWSFGDFFKKGAIDFAWEFLTEVLKVPKERLYVTYFGGCDGVECDNETKDYWIGKGL

DPSRVLPFGMSDNFWEMGDTGPCGPCSEIHYDRIGGRDAKALVNMDDPTVLEIWNLVFIQFNRENDGTLKTLPSQHVDTG

MGLERLVSIIQGKMSNYDTDLFTPFFDKIHQATGVQPYGGQVGLADQNGIDMAYRVVADHIRTLTVAMSDGGKPDNVGRG

YVLRRILRRCIRYSIEKLNASPGFTASLVSVVIDTLGEFFPELNKDPQQVMDVINEEEVQFLKTLARGRRLFERTASKIT

DGTIPGDVAWRLYDTYGFPVDLTELMAEERGLRVDMEKYEEAKRRAQEIARGKDSASDDAITLDVHLLDKLQNVMNLPPT

IDLPKYSYSSDALGSYVFQPVSGTIKALLHEKEFVNEVPGGEHCGIILDQTSFYAEQGGQIYDEGFMTKEGDEDVEFRVI

NVQVHGGYVLHVGALEGAIRVGDTMNCLIDEDRRRQCMNNHTATHILNFALRRTLGEADQRGSLVAPNRLRFDFTAKGAM

TPEQVKSTELFSKEIVTSDLPVFAKEVALAQAKDVQGLRAIFDEVYPDPVRVVSIGASFDELQPNPQAGYKYSIEFCGGT

HLQRSSHMETFVLVSEEAISKGIRRIVALTGPEGLKAEKHCEALEARVEKIRKEVEAESKNLRVKEAGQEITRLIEDINA

AVISAWKKEEMRQTLKKVKKIVDNASKAMQAALMTSAADKAKAMITAEPDRTVVVELFDAGSNQKVLDAAMKQFKNLSPL

TAVLLLSVDQENKKIICLSQVPKDVTKKGLKADEWTGAIADILGGKSGGKEMSAQASGPNVGSIDKALSEAKQFANMKLQ

N

>POM29224.1 hypothetical protein FL81_12959 [Caenorhabditis remanei]

MKHLTASEVRSTFINFFKEKKEHTYVHSSSVIPHDDPTLLFANAGMNQFKPLFLGIADPNSDLAKLKRAVNTQKCIRAGG

KHNDLDDVGKDVYHHTYFEMLGNWSFGDYFKKEIITWAWELLTTVYGIPAERLYVSVFGGDEATGVPADSEARKIWRSVG

VPDERILNFGMKDNFWEMGDVGPCGPCSEIHYDRIGNRDASHLVNADDPMVVEIWNLVFIQFNREEGGSLKPLPAKHIDC

GLGLERLIAVMQDKTSNYDTDIFQPIFQAINKGSGVRAYTGHIGDDDKDGIDMAYRVVADHIRTLTIALSDGGRPDNSGR

GYVLRRILRRGVRYATEKLNAQPGFFASLVPVVISILGETFPELSRDPETVMDIINDEEKQFLKTLSRGRVLFQRAVDSL

PSGTKTFPGDVAWRLYDTYGFPADLTQLMAEEKGLSVDLTAFEEARKKAIETSSAGTGKFRDTLDLDVHALAELQQKGVP

TTDDTPKYAYSFTGEGADAVYKFEPCVGKILAIRRDGKFVDELVAGEEGAILLDRTNFYAEQGGQIYDVGVLTKVNDESN

EFNVSNCQVRGGYIVLVGSSEGNFKIGDDVNQRFDEDRKQLIMKNHTGTHVLNYALRKVLADSDQKGSLVAPDRMRFDFT

NKAAMTSEQVKQAEEYAQQLINTKGQVYAKNSPLADAKKINGLRAMFDETYPDPVRVVAVGTPVEQLLQNPDAPEGQNTT

VEFCGGTHLQNVSHIGRIVISSEEAIAKGIRRIVALTGPEADRAIARADRLEARLVEESKHAKNQELLANKDKFKELQKK

IQEIVDEANGALLPSWRKDSLRKKAKDVQKDMDGYSKAQQAAVAEKVLGEAKELAAASEQPAVLVHVFTSNANSKAIDNA

LKLLKDTKAVMAFSVNEDTGKVLCLAKVDKSLVSSLKANEWVNEVCTVLGGKGGGKDANAQLTGENVDKLDAAVELAQKF

ALAAIN

>XP_002646367.1 C. briggsae CBR-ARS-2 protein [Caenorhabditis briggsae]

MKHLTASEVRSTFIEFFREKKNHTYVHSSSVIPHDDPTLLFANAGMNQFKPLFLGIADPNSDLAKLKRAVNTQKCIRAGG

KHNDLDDVGKDVYHHTYFEMLGNWSFGDYFKKEIITWAWELLTTVYGIPAERLYVSVFGGDEANGVPADSEAREIWRSVG

VPEERILNFGMKDNFWEMGDVGPCGPCSEIHYDRIGNRDASHLVNADDPMVVEIWNLVFIQFNREEGGALKSLPAKHIDC

GLGLERLIAVMQDKTSNYDTDIFQPIFEEITKKSGVRAYTGHIGDEDKDGVDMAYRVVADHIRTLTIALSDGGRPDNSGR

GYVLRRILRRGVRYATEKLNAQPGFFAALVPVVISILGDTFPELKRDPVTVMDIINDEEKQFLKTLSRGRVLFQRAVDSL

PAGTKTFPGDIAWRLYDTYGFPADLTQLMAEEKGLSVDETAFEDARKKAIETSSAGAGKFRDTLDLDVHALAELQQKGIP

TTDDSPKYAYSFTGEGSEAVYKFEPCVGKILAIRRDGKFVDQLVAGEEGAVLLDRTNFYAEQGGQIYDVGVLSKSGDESN

EFNVSNCQVRGGYIVLVGSAEGSFSVGDNVNQRFDEDRKQLIMKNHTGTHVLNYALRKVLADSDQKGSLVAPDRMRFDFT

NKAAMTPEQVKQAEEYAQQLINAKGQVYAKNSPLEDAKKINGLRAMFDETYPDPVRVVAVGTPVEQLLQNPEAEAGQNTT

VEFCGGTHLQNVSHIGRIVISSEEAIAKGIRRIVALTGPEADRAIARAERLEARLVDESKHAKNQEILSNKDKFKELQKK

IQEIVDEANGAQLPYWRKDALRKKAKDVQKDMDGYSKAQQAAVAEKVLGEAKELAAAAEQPPVLVHVFASNANSKAIDNA

LKLLKDTKAVMAFSVNEDTGKVLCLAKVDKSLVSNGLKANEWVNEVCTILGGKGGGKEANAQLTGENVDKLDDAVELAQK

FALAAIN

>XP_006561680.1 PREDICTED: alanine--tRNA synthetase, cytoplasmic isoform X2 [Apis mellifera]

MAKLVRAVNSQKCIRAGGKHNDLDDVGKDVYHHTFFEMMGNWSFGDYFKKEICTWAWEFLTVKLKLSSDRLYVTYFGGDE

KNNLKPDEECKQIWLSLGLSPTHVLPGNMKDNFWEMGETGPCGPSSEIHYDRIGNREAAHLVNQDDPDVLEIWNLVFIQF

NRENDGNLKLLPKKHIDCGLGLERLVSIIQNKSANYDTDLFMPLFDAIQKGTKAPSYQGKVGKEDINGIDMAYRVLADHA

RTITIALADGGVPDNTGRGYVLRRILRRAVRYAIEKLNAKPGFFGSLVNVVVDLLGDVFPEVTKDPQHIIDIINEEETQF

LKTLSRGRNLLNRTIVKLESSNILPGDVAWRLYDTYGFPFDLTQLMAEEKGLKVDVIGYEEAKKEAQLISQNKSGEVDDQ

INLDIHAITELQNEKIKPTNDIPKYNYKVISNKIYEEYEFAPCFSTIIALRRAKTFVDEVSSGEEVGILLDQTNFYAEQG

GQIYDEGFLTKIDDEDSEVHIKNVQVRGGYILHIGIVSQGKLKKNDKVFSNIDTTRRRLLMSNHTATHVLNYALREILGT

EVDQKGSLVAPDRLRFDFTNKGAMTTEQIRKVEEITNNIINENKMVYAKECNLGLAKTIQGLRAMFEETYPDPVRVVSIG

IPVEDLEKNPLSSDALKTSVEFCGGTHLHYTGHIKNFIIVSEEAIAKGIRRIVALTGPEAIKAQKKTSMLQNYLDELQIT

IEADKNGINTKEHIKKIIELTDDVSHAIISSWKKDEIRKMLKDLKKILDDKERAIKTAITNVVLDNIQQIIQQNIGCLVL

VEILQAYSNTKALDSALKKIRTISPETSALLISIDPDAKKIFALSSVSKFAINKGLKANEWIQEIVPLMEGKGGGKSESA

QASGTNISCVTKLIYTAKNFANSKLELAENINENHDQLSNEKFIYFKTLKDKLVLSGDIGSIKYYCIQIVAKYNNIELIT

VPHKNNIFKGIKLQGNDFELFDSNAIAFYLSTDQFKCSNNPFTFSEVLQWLSYADNHILPAVLGWVVPYLSKNIPNNIKT

NIKTSKEDILSSLQKLNNILLTKTYLVGERISLADISVFTTLMPLYEYVLDSEYRKQYTNLNRWFFTILNQSQVISVIKK

FNICEKVKIVKY

>OZF74787.1 hypothetical protein FL82_12780, partial [Caenorhabditis remanei]

MKHLTASEVRSTFINFFKEKKEHTYVHSSSVIPHDDPTLLFANAGMNQFKPLFLGIADPNSDLAKLKRAVNTQKCIRAGG

KHNDLDDVGKDVYHHTYFEMLGNWSFGDYFKKEIITWAWELLTTVYGIPAERLYVSVFGGDEATGVPADSDAREIWRSVG

VPDERILNFGMKDNFWEMGDVGPCGPCSEIHYDRIGNRDASHLVNADDPMVVEIWNLVFIQFNREEGGSLKPLPAKHIDC

GLGLERLIAVMQDKTSNYDTDIFQPIFQAINKGSGVRAYTGHIGDDDKDGIDMAYRVVADHIRTLTIALSDGGRPDNSGR

GYVLRRILRRGVRYATEKLNAQPGFFASLVPVVISILGETFPELSRDPETVMDIINDEEKQFLKTLSRGRVLXQRAVDSL

PAGTKTFPGDVAWRLYDTYGFPADLTQLMAEEKGLSVDLTAFEEARKKAIETSSAGTGKFRDTLDLDVHALAELQQKGVP

TTDDTPKYAYSFTGEGADAVYKFEPCVGKILAIRRDGKFVDELVAGEEGAILLDRTNFYAEQGGQIYDVGVLTKVNDESN

EFNVSNCQVRGGYIVLVGSSEGNFKIGDDVNQRFDEDRKQLIMKNHTGTHVLNYALRKVLADSDQKGSLVAPDRMRFDFT

NKAAMTSEQVKQAEEYAQQLINTKGQVYAKHSPLADAKKINGLRAMFDETYPDPVRVVAVGTPVEQLLQNPDAPEGQNTT

VEFCGGTHLQNVSHIGRIVISSEEAIAKGIRRIVALTGPEADRAIARADRLEARLVEESKHAKNQELLANKDKFKELQKK

IQEIVDEANGALLPSWRKDSLRKKAKDVQKDMDGYSKAQQAAVAEKVLGEAKELAAASEQPAVLVHVFTSNANSKAIDNA

LKLLKDTKAVMAFSVNEDTGKVLCLAKVDKSLVSSLKANEWVNEVCTVLGGKGGGKDANAQLTGENVDKLDAAVELAQKF

ALAAIN

>XP_012347711.1 PREDICTED: alanine--tRNA synthetase, cytoplasmic isoform X2 [Apis florea]

MAKLVRAVNSQKCIRAGGKHNDLDDVGKDVYHHTFFEMMGNWSFGDYFKKEICTWAWEFLTVKLKLSSDRLYVTYFGGDE

KNNLKPDEECKQIWLSLGLSPTHILPGNMKDNFWEMGETGPCGPSSEIHYDRIGNREAAHLVNQDDPDVLEIWNLVFIQF

NRENNGNLKLLPKKHIDCGLGLERLVSIIQNKSANYDTDLFMPLFDAIQKGTRAPSYQGKVGKEDKDGIDMAYRVLADHA

RTITIALADGGIPDNTGRGYVLRRILRRAVRYAIEKLNAKPGFFGSLVNVVVDLLGDVFPEVTKDPQHIIDIINEEETQF

LKTLSRGRNLLNRTIVKLESSNVLPGDVAWRLYDTYGFPFDLTQLMVEEKGLKVDIIGYEEAKKQAQLISQNKSGEVDDQ

INLDVHAITELQNKKIKPTNDIPKYNYKVINNKICEEYEFAPCFSTVIALRRAKTFVNEVSSGEEVGILLDQTNFYAEQG

GQMYDEGFLTKIDDEDTEVHIKNVQVRGGYVLHIGIVSQGKLKKNDKVFSNIDTTRRRLLMSNHTATHALNYALREILGT

EVDQKGSLVAPDRLRFDFTNKGAMTTEQIRKVEEITNNIINENKMVYAKECNLGLAKTIQGLRAMFEETYPDPVRVVSIG

IPVEDLEKNPLSSGALKTSVEFCGGTHLHYTGHIRNFVIVSEEAIAKGIRRIVALTGPEAVKAQKKTSMLQNYLDELQIT

IEADKDSINTKEHVKKIVELTDDVSHAIISSWKKDKIRKMLKDLKKILDDKERAIKTAIANIVLDNIQQIIQQNIGCLVL

VEVLQAYSNTKALDSALKKIRTISPETSALLISIDPDAKKIFALSSVSKSAISKGLKANEWIQEIVPFMEGKGGGKSESA

QASGTNISCITKLIYTAKNFANSKLGLAENINEDHDQLKGDEKFIYFKTLKDKLILSGDSGSIKYYRIQIVAKYNNIELI

TMPHKNNIFKGIKLQGNDFELFDSNAIAFYLSTDQFKCSNNSFTFSEVLQWLSYADNHILPAVLGWVVPYLSKNIPNNIK

TNIKTSKEDVLSSLQKLNNILLTKTYLVGERISLADITVFTTLMPLYEYVLDSEYRKQFTNLNRWFFTILNQSQVTSVIK

KFNICEKVKY

>EYB99703.1 hypothetical protein Y032_0120g894 [Ancylostoma ceylanicum]

MKQLTANEVRSTFIEFFKQKKAHTYVHSSSVIPHDDPTLLFANAGMNQFKPLFLGIADPNSDLAKLKRAVNTQKCIRAGG

KHNDLDDVGKDVYHHTFFEMLGNWSFGDYFKKEIITWAWELLTEVYGIPAERLYVSYFGGDEKAGVPADEEARSIWLSLG

LPENRILPFGMKDNFWEMGDVGPCGPCSEIHYDRVGGRDASHLVNADDPMVVEIWNLVFIQFNREEGGVLRPLPAKHIDC

GLGLERLIAVIQEKTSNYDTDIFQPIFKAIQQGTGIREYTGKVGADDVDGVDMAYRVVADHVRTLTIALSDGGRPDNTGR

GYVLRRILRRGVRYATEKLNAHPGFFASLVPVVIDILGDTFPELRRDPETVQDIINDEEKQFLKTLSRGRVLFQRAVEGL

TADQKTFPGDVAWRLYDTYGFPADLTQLMAEEKGLTVDQNVFEECRKKAVELSAAGAGKFRDTLDLDVHALAELQKRGIP

PTDDSFKYNYQADGAKDGSVKYTFSSCTGKILAIRSDGKFVDSIESGVEGAILLDKTNFYAEQGGQIYDTGVLTKVDGDG

SEFVVSNCQVRGGYIVLVGSANGKFSVGDDVLQNFDEDRRSLIMKNHTGTHVLNHALRCVLADSDQKGSLVAPDRLRFDF

TNKQAMTVKQVKETEEIVQSVINTREPVYAKEAPLAQAREISGLRAMFDENYPDPVRVVCVGVPVEQLLQDPKSGAGLKT

TVEFCGGTHLHNVGHIGHFVISSEEAIAKGIRRIVALTGPEADRAIQRADRLAARAQAISDAINANKDIALDSEKFKAAA

KRIQELIDEINGAQLPYWRKDAIREQCKAAQRTLDGYKKLADAAIAEKVLSEAKELAQGDQQDVIVHVFSSGASSKVLDN

ALKQLKSTKAVMGFSVNEESGKVLVLAKVDKSLVDGGLKANLWVNEVCTILGGRGGGKDANAQATGENIDRLDEAVALAR

KFAQTTIA

>KYN00264.1 Alanyl-tRNA synthetase, cytoplasmic [Cyphomyrmex costatus]

MAKWVRVVNSQKCIRAGGKHNDLDDVGKDVYHHTFFEMMGNWSFGDYFKKEICTWAWEFLTDVLKLSADRLYVTYFGGDK

KNGLAPDDECKNLWLSLGVPASHVLPGNMKDNFWEMGETGPCGPCSELHYDRIGDREAAHLVNMDDPDVLEIWNLVFIQY

NRESDGSLKSLPKKHIDCGLGLERLVSVIQNKRANYDTDLFMPLFTAIEKGTGAPSYQGKVGADDVNGIDMAYRVLADHA

RTLTIALADGGVPDNTGRGYVLRRILRRAVRYAIEKLNAKPGFFGTLVNVVVDLLGDVFPEVKKDPQSIIETINEEESQF

LKTLSRGRNLLNRTIAKLESADTVPGDVAWRLYDTYGFPIDLTQLMTEEKGLKIDMAGYEESKKQAQLISQGKAGGVDDQ

INLDVHAITELQNQGVKPTDDSPKYNYKVTNKTKYKEYEFAPCTGTVIALRRAKTFVNEVSSGEVVGILLDRTNFYAEQG

GQIYDEGFLVKVNDETTEIRIKNVQVRAGYVLHIGIVGEGKLKKGDKVRTNVDTERRRLVMANHSATHVLNYALRKVLGT

EADQKGSLVTPDRLRFDFTNKGPMTGQQLKETELSVIAIIRKNEKIYAKESNLATAKTIQGLRAMFEETYPDPVRVVSVG

IPVENLEKDPLSTAALETSVEFCGGTHLHYTSHIGDFVIASEDAIAKGIRRIVALTGPEARKAIRTAGRFGSDITMSECQ

YQMNDMNSKEYVRKIVELTEEISQALIPAYRKDDLRNHLKEMKKSLDDKERAAKAAVATTVVQTAQSIIESKVGCQVLVE

VLEAYSNTKALDSALKKIRAISPETSALLISVDHDVKKIFALSSVPKSAISKGLKANEWIQAITSLMQGKGGGKSESAQA

SGLNVACLNEIVSKANEFADQKLGIITTA

>PIC51453.1 hypothetical protein B9Z55_001962 [Caenorhabditis nigoni]

MKHLTASEVRSTFIEFFREKKNHTYVHSSSVIPHDDPTLLFANAGMNQFKPLFLGIADPNSDLAKLKRAVNTQKCIRAGG

KHNDLDDVGKDVYHHTYFEMLGNWSFGDYFKKEIITWAWELLTTVYGIPAERLYVSVFGGDEANGVPADSEAREIWRSVG

VPEERILNFGMKDNFWEMGDVGPCGPCSEIHYDRIGNRDASHLVNADDPMVVEIWNLVFIQFNREEGGALKSLPAKHIDC

GLGLERLIAVMQDKTSNYDTDIFQPIFGEITKKSGVRAYTGKIGDEDKDGVDMAYRVVADHIRTLTIALSDGGRPDNSGR

GYVLRRILRRGVRYATEKLNAQPGFFAALVPVVISILGDTFPELKRDPVTVMDIINDEEKQFLKTLSRGRVLFQRAVDSL

PAGTKTFPGDIAWRLYDTYGFPADLTQLMAEEKGLSVDETAFEDARKKAIETSSAGAGKFRDTLDLDVHALAELQQKGIP

TTDDSPKYAYSFTGEGSEAVYKFEPCVGKILAIRRDGKFVDQLVAGEEGAVLLDRTNFYAEQGGQIYDVGVLSKSGDESN

EFNVSNCQVRGGYIVLVGSAEGSFSVGDNVNQRFDEDRKQLIMKNHTGTHVLNYALRKVLADSDQKGSLVAPDRMRFDFT

NKAAMTPEQVKQAEEYAQQLIDAKGQVYAKNSPLEDAKKISGLRAMFDETYPDPVRVVAVGTPVEQLLQNPEAEAGKNTT

VEFCGGTHLQNVSHIGRIVISSEEAIAKGIRRIVALTGPEADRAIARADRLEARLVDESKHAKNQEILSNKDKFKELQKK

IQEIVDEANGAQLPYWRKDALRKKAKDVQKDMDGYSKAQQAAVAEKVLGEAKELAAAAEQPAVLVHVFASNANSKAIDNA

LKLLKDTKAVMAFSVNEDTGKVLCLAKVDKSLVSNGLKANEWVNEVCAILGGKGGGKEANAQLTGENVDKLDAAVELAQK

FALAAIN

>PIC51454.1 hypothetical protein B9Z55_001962 [Caenorhabditis nigoni]

MKIVNCAEGAAASRLVYNTLDASVCPDDDLWTFFLKNENNKYRLQSNLFWGTMKHLTASEVRSTFIEFFREKKNHTYVHS

SSVIPHDDPTLLFANAGMNQFKPLFLGIADPNSDLAKLKRAVNTQKCIRAGGKHNDLDDVGKDVYHHTYFEMLGNWSFGD

YFKKEIITWAWELLTTVYGIPAERLYVSVFGGDEANGVPADSEAREIWRSVGVPEERILNFGMKDNFWEMGDVGPCGPCS

EIHYDRIGNRDASHLVNADDPMVVEIWNLVFIQFNREEGGALKSLPAKHIDCGLGLERLIAVMQDKTSNYDTDIFQPIFG

EITKKSGVRAYTGKIGDEDKDGVDMAYRVVADHIRTLTIALSDGGRPDNSGRGYVLRRILRRGVRYATEKLNAQPGFFAA

LVPVVISILGDTFPELKRDPVTVMDIINDEEKQFLKTLSRGRVLFQRAVDSLPAGTKTFPGDIAWRLYDTYGFPADLTQL

MAEEKGLSVDETAFEDARKKAIETSSAGAGKFRDTLDLDVHALAELQQKGIPTTDDSPKYAYSFTGEGSEAVYKFEPCVG

KILAIRRDGKFVDQLVAGEEGAVLLDRTNFYAEQGGQIYDVGVLSKSGDESNEFNVSNCQVRGGYIVLVGSAEGSFSVGD

NVNQRFDEDRKQLIMKNHTGTHVLNYALRKVLADSDQKGSLVAPDRMRFDFTNKAAMTPEQVKQAEEYAQQLIDAKGQVY

AKNSPLEDAKKISGLRAMFDETYPDPVRVVAVGTPVEQLLQNPEAEAGKNTTVEFCGGTHLQNVSHIGRIVISSEEAIAK

GIRRIVALTGPEADRAIARADRLEARLVDESKHAKNQEILSNKDKFKELQKKIQEIVDEANGAQLPYWRKDALRKKAKDV

QKDMDGYSKAQQAAVAEKVLGEAKELAAAAEQPAVLVHVFASNANSKAIDNALKLLKDTKAVMAFSVNEDTGKVLCLAKV

DKSLVSNGLKANEWVNEVCAILGGKGGGKEANAQLTGENVDKLDAAVELAQKFALAAIN

>XP_003104792.1 CRE-AARS-2 protein [Caenorhabditis remanei]

MKHLTASEVRSTFINFFKEKKEHTYVHSSSVIPHDDPTLLFANAGMNQFKPLFLGIADPNSDLAKLKRAVNTQKCIRAGG

KHNDLDDVGKDVYHHTYFEMLGNWSFGDYFKKEIITWAWELLTTVYGIPSERLYVSVFGGDEATGVPADSEAREIWRSVG

VPDERILNFGMKDNFWEMGDVGPCGPCSEIHYDRIGNRDASHLVNADDPMVVEIWNLVFIQFNREEGGSLKTLPAKHIDC

GLGLERLIAVMQDKTSNYDTDIFQPIFQAINKGSGVRAYTGHIGDDDKDGIDMAYRVVADHIRTLTIALSDGGRPDNSGR

GYVLRRILRRGVRYATEKLNAQPGFFASLVPVVISILGETFPELSRDPETVMDIINDEEKQFLKTLSRGRVLFQRAVDSL

PAGTKTFPGDVAWRLYDTYGFPADLTQLMAEEKGLSVDLTAFEEARKKAIETSSAGTGKFRDTLDLDVHALADLQQKGVP

TTDDTPKYAYSFTGEGADAVYKFEPCVGKILAIRRDGKFVDELVAGEEGAILLDRTNFYAEQGGQIYDVGVLTKVNDESN

EFNVSNCQVRGGYIVLVGSSEGNFKIGDDVNQRFDEDRKQLIMKNHTGTHVLNYALRKVLADSDQKGSLVAPDRMRFDFT

NKAAMTSEQVKQAEEYAQQLINTKGQVYAKNSPLADAKKINGLRAMFDETYPDPVRVVAVGTSVEQLLQNPDAPEGQNTT

VEFCGGTHLQNVSHIGRIVISSEEAIAKGIRRIVALTGPEADRAIARADRLEARLVEESKHAKNQELLANKDKFKELQKK

IQEIVDEANGALLPSWRKDSLRKKAKDVQKDMDGYSKAQQAAVAEKVLGEAKELAAASEQPAVLVHVFTSNANSKAIDNA

LKLLKDTKAVMAFSVNEDTGKVLCLAKVDKSLVSSLKANEWVNEVCTVLGGKGGGKDANAQLTGENVDKLDAAVELAQKF

ALAAIN

>XP_001950431.1 PREDICTED: alanine--tRNA synthetase, cytoplasmic [Acyrthosiphon pisum]

MVPSAKEIRDRYIKFFIDKKGHEYVYSSSVVPVDDPTLLFANAGMNQFKPIFLGTVAPSSDMAKWKRAVNTQKCIRAGGK

HNDLDDVGKDVYHHTFFEMLGNWSFGDYFKTEICQWAWEFLTQEFKLSPDRLYVTYFGGDKKSGLEPDLECKNIWLKLGL

AEDHIIPGNMKDNFWEMGETGPCGPCSEIHYDRIGNRLAADLVNQDDPDVLEIWNLVFMQYNRNQEGTLHNLPKKHIDCG

MGFERLVSVIQDKRSNYDTDLFQPLFVAIQNGTNAPPYEGKVGADDIDQKDMAYRVLADHARTLTIALSDGGSPDNTGRG

YVLRRILRRAVRYATEKLNGKPGFFATLVNVVVDLLGDTFPEITKDPQQIIEIINEEEEQFLRTLSRGRNLLNRTILKLT

DKVIPGEVAWKLYDTYGFPVDLTYLMVEEKGFTIDMEAYEEAKKKAQLISQNKVGGDKEVISLDVHALADLQTKGISTTD

DSPKYNYKAVSDDADAEYTFETCTSTVLSIRLINQFVNEVDTFQECGIILDKTCFYAEQGGQIYDQGSMVKDEFTEFYVN

NVQLRGGYILHVGILESGSLKVGDKVVLKIDEKRRRFVMNNHTATHTLNHTLRVVLGDNLNQKGSLVAPDKLRFDFNNKA

ALTGDQVAKVEKLTNEVIKRNDTVYAKIAPLNDAKRIKGLRAMFDETYPDPVRVVSVGVTVDKLLTDPEGNSGLETSVEF

CGGTHLHKTGHMECFTILSEEAISKGIRRIVAVTGPEATKAYNRGEELKKDVMKLDSKDVNLSKAIIDTQDKINKSVIPY

WVKEELRSVLSGLKKGIAEEERKQLVAQASEVAKSIKTKICSESDKTVFIYKLDVGSNTKVMDAVLKLLKADMPNISIML

LSEDTEGKRIYCQCVVSKDLNKKGLMANKWVTKIASCINGKGGGKADTAQASGCNETGVEQLISIGEQFASLSIS

>XP_015374465.1 PREDICTED: alanine--tRNA synthetase, cytoplasmic [Diuraphis noxia]

MVPSAKEIRDRYIKFFIEKKGHEYVYSSSVVPVDDPTLLFANAGMNQFKPIFLGTVAPSSDMAKLKRAVNTQKCIRAGGK

HNDLDDVGKDVYHHTFFEMLGNWSFGDYFKTEICQWAWEFLTQEFKLSPDRLYVTYFGGDKKSGLEPDLECKNIWLKLGL

EENHIIXGNMKDNFWEMGETGPCGPCSEIHYDRIGNRLAANLVNQDDPDVLEIWNLVFMQYNRNQEGTLHNLPKKHIDCG

MGFERLVSVIQDKRSNYDTDLFQPLFEAIQNGTNAPPYEGKVGADDLDQKDMAYRVLADHARTLTIALSDGGSPDNTGRG

YVLRRILRRAVRYATEKLNGKPGFFATLVNVVVDLLGDTFPEITKDPQQIIEIINEEEEQFLRTLSRGRNLLNRTILKLT

DKVIPGEVAWKLYDTYGFPVDLTYLMVEEKGFSIDMEAYEEAKKKAQLISQNKVGGDKEVISLDVHALADLQTKGISATN

DSPKYNYKAISDDPDTEYTFETCTSTILSIRLINQFVNEVDTFQECGIILDRTCFYAEQGGQIYDQGSMVKDEFTEFYVN

NVQLRGGYILHVGILETGSLKVGDKVVLKIDEKRRRFVMNNHTATHTLNHTLRVVLGDNLNQKGSLVAPEKLRFDFNNKS

ALTGDQVAKVEKLTNDVIKRNDTVYAKVAPLNDAKRIKGLRAMFDETYPDPVRVVSVGVTVDKLLTDPEGNSGLETSVEF

CGGTHLHKTGHMECFTILSEEAISKGIRRIVAVTGPEATKAYNRGEELKKDVMKLDSTNVNLSKAIIDTQDKINKSVIPY

WIKEELRSVLSGLKKGIAEEERKQLAALSSELVKSIKTKICSESDKSVFIYKIDVGSNTKVMDSVLKLLKADMPNVAIML

FSEDTEGKRMYCQCVVSKDLNKKGLMANKWVNQIAICSNGKGGGKPDIAQASGPNTTDIEQLISIGEQFASLSIS

>XP_022176230.1 alanine--tRNA synthetase, cytoplasmic [Myzus persicae]

MVPSAKEIRDRYIKFFIDKKGHEYVYSSSVVPVDDPTLLFANAGMNQFKPIFLGTVAPSSDMAKWKRAVNTQKCIRAGGK

HNDLDDVGKDVYHHTFFEMLGNWSFGDYFKTEICQWAWEFLTQEFNLSPDRLYVTYFGGDKKSGLEPDLECKNIWLKLGL

AENHIIPGNMKDNFWEMGETGPCGPCSEIHYDRIGNRLAADLVNQDDPDVLEIWNLVFMQFNRNQEGTLHNLPKKHIDCG

MGFERLVSVIQDKRSNYDTDLFQPLFVAIQNGTNAPPYEGKVGADDIDQKDMAYRVLADHARTLTIALSDGGSPDNTGRG

YVLRRILRRAVRYATEKLNGKPGFFATLVNVVVDLLGDTFPEITKDPQQIIEIINEEEEQFLRTLSRGRNLLNRTILKLT

DKVIPGEVAWKLYDTYGFPVDLTYLMVEEKGFTIDMEAYEEAKKKAQLISQNKVGGDKEVISLDVHALADLQTKGISTTD

DSPKYNYKAVSDNPDAEYTFETCTSTVLSIRLINQFVNEVDTFQECGIILDKTCFYAEQGGQIYDQGSMVKDEFTEFYVN

NVQLRGGYILHVGILESGSLKVGDKVVLKIDEKRRRFVMNNHTATHTLNHTLRVVLGDNLNQKGSLVAPDKLRFDFNNKA

ALTGDQVAKVEKLTNDVIKRNDTVYAKIAPLNDAKRIKGLRAMFDETYPDPVRVVSVGVAVDKLLTDPEGNSGLETSVEF

CGGTHLHKTGHMECFTILSEEAISKGIRRIVAVTGPEATKAYNRGEELKKDVMKLDSKDANLSKAIIDTQDKINKSVIPY

WVKEELRSVLSGLKKGIAEEERKQLAALASEVTKSLKTKICSESDKSIFIYKLDVGSNTKVMDAVLKLLKADMPNISIML

LSEDIESKRIYCQCVVSKELNKKGLMANKWVTKIASCINGKGGGKADTAQASGCNETGVEQLISIGEQFASLSIS

>EGT52396.1 hypothetical protein CAEBREN_18885 [Caenorhabditis brenneri]

MKQLTASEVRSTFINFFREKKEHTYVHSSSVIPHDDPTLLFANAGMNQFKPLFLGIADPNSDLAKLKRAVNTQKCIRAGG

KHNDLDDVGKDVYHHTYFEMLGNWSFGDYFKKEIITWAWELLTTVYGIPAERLYVSVFGGDEANGVPADTEARDIWRSVG

VPDERILNFGMKDNFWEMGDVGPCGPCSEIHYDRIGNRDASHLVNADDPMVVEIWNLVFIQFNREEGGALKPLPAKHIDC

GLGLERLIAVMQDKTSNYDTDIFQPIFEAIHKGSGVRKYTGLIGDEDKDGVDMAYRVVGDHIRTLTIALSDGGRPDNSGR

GYVLRRILRRGIRYATEKLNAQPGFFASLVPVVISILGETFPELSRDPETVMDIINDEEKQFLKTLSRGRVLFQKAVDSL

PAGTKTFPGDIAWRLYDTYGFPADLTQLMAEEKGLSVDISAFDEARKKAVETSSAGAGKFRDTLDLDVHALAELQQKGVP

TTDDTPKYAYSFTGEGAEAVYKFEPCIGKILAIRRDGKFVDQLVAGEEGALLLDRTNFYAEQGGQIYDVGVLTKVNDESN

EFNVNNCQVRGGYIVLVGSAEGSFAVGDEVNQRFDEDRKQLIMKNHTGTHVLNYALRKVLSDSDQKGSLVAPDRMRFDFT

NKAAMTSDQVKQAEEYAQQLIDTKGQVYAKNSPLSDAKRINGLRAMFDETYPDPVRVVAVGTPVEQLLQNPEAEEGQNTT

VEFCGGTHLQNVSHIGRIVISSEEAIAKGIRRIVALTGPEAERAIARADRLEARLLEESKQAKDQTLLANKDKFKELQKK

IQEIVDEANGAQLPSWRKDALRKKAKDIQKDMDGYSKAQQAAVAEKVLGEAKELAAASEQPAVLVHVFTSNANSKAIDNA

LKLLKDTKAVMAFSVNEDTGKVLCLAKVDKSLVSNLKANEWINEVCTVLGGKGGGKDANAQLTGENVDKLDAAVELAQKF

ALAAIN

>CBY23722.1 unnamed protein product [Oikopleura dioica]

MNTNITANEVRQQFLDFFKELEHPYVHSSSTIPHDDPTLLFANAGMNQFKSIFVGTIDPSSPMAKLKSAVNSQKCIRAGG

KHNDLDDVGKDSYHHTFFEMLGSWSFGDYFKKEAIQWSFELLNKVWGIPADRLYATYFEGNPEEGLEPDNEAREEWLKYL

PADQILPGNAKDNFWEMGDTGPCGPCSELHYDRIGGRNASSLVNMDDPDVLEIWNLVFMQYDRQADKSLISLPAKSVDTG

MGLERVVSVLQDKRANYDTDMFSPYFKKIEDLTGTRAYTGLFGAEDKDGVDMAYRVLADHARTLSIALADGGRPDNVGRG

YVLRRILRRAIRYGHEKLNMKVDDFVALVDVVVESLGDAFPELKKDPESTKEILKEEWIQFMKTLDRGQKILERKIKQLN

LTDRLPGDVAWLLYDTYGFPLDLTTLIAEEHNLTLSCEEFEEERQKAIERSKGADGVATTDDSHKQNYEANSAGEYTFNS

IKAKVVALRFNKEFHQECESCEVGIVLDKTNFYAEQGGQIYDLGYFSRENDDDFEFNVKNVQVRAGYVLHVGTLVGKLSV

GDEINLNIDTERRVNIMPNHTGTHVLNFGLRCVLGEGEADQKGSLVAPERLRFDFTAGGALKPAQIKQVEEICNEVITRG

GAVHCQVSPLAQSKAIQGLRAMFDETYPDPVRVVSIGVPVNELLENPTSGAADKTSVEFCGGTHLRDISHARKLIITAEE

AISKGIRRVIAVTGAEAEKADRQAQRLAKANQLKEKDVNELKAEVKEAIKAKNTAEANQRAISLGKTIEVAPISAWKRAE

LVDAVKKSKMEIVQYEQKVEEYGIQTEKRIDAGDVAEPEVLLLDAGSNNKVLNEILKLYAKKSPKSHVLLFSADHTAKKV

LCLAQTPKGSTLKANEWVSSISPLIDGRGGGKDVQAQASGKNVSGLKGALAKAKEFAAANL

>CBY38956.1 unnamed protein product [Oikopleura dioica]

MNTNITANEVRQQFLDFFKEREHPYVHSSSTIPHDDPTLLFANAGMNQFKSIFVGTIDPSSPMAKLKSAVNSQKCIRAGG

KHNDLDDVGKDSYHHTFFEMLGSWSFGDYFKKEAIQWSFDLLNKVWGIPADRLYATYFEGNPEEGLEPDNEAREEWLKYL

PADQILPGNAKDNFWEMGDTGPCGPCSELHYDRIGGRNASSLVNMDDPDVLEIWNLVFMQYDRQADKSLISLPAKSVDTG

MGLERVVSVLQDKRANYDTDMFSPYFKKIEDLTGTRAYTGLFGAEDKDGVDMAYRVLADHARTLSIALADGGRPDNVGRG

YVLRRILRRAIRYGHEKLKMKVDDFVALVDVVVESLGDAFPELKKDPESTKEILKEEWIQFMKTLDRGQKILERKIKQLN

LTDRLPGDVAWLLYDTYGFPLDLTTLIAEEHNLTLSCEEFEEERQKAIERSKGADGVATTDDSHKQNYEANSAGEYTFNS

IKAKVVALRFNKEFHQECESCEVGIVLDKTNFYAEQGGQIYDLGYFSRENDDDFEFNVKNVQVRAGYVLHVGTLVGKLSV

GDEINLNIDTERRVNIMPNHTGTHVLNFGLRCVLGEGEADQKGSLVAPERLRFDFTAGGALKPAQIKQVEEICNEVITRG

GAVHCQVSPLAQSKAIQGLRAMFDETYPDPVRVVSIGVPVNELLENPTSGAADKTSVEFCGGTHLRDISHARKLIITAEE

AISKGIRRMIAVTGAEAEKADRQAQRLEKDVNELKAEVKEAIKAKNTAEANQRAISLGKTIEVAPISAWKRAELVDAVKK

SKMEIVQYEQKVEEYEIQTEKRIDAGDVAEPEVLLLDAGSNNKVLNEILKLYAKKSPKSHVLLFSADHTAKKVLCLAQTP

KGSTLKANEWVSSISPLIDGRGGGKDVQAQASGKNVSELKGALAKAKEFAAANL

>XP_006613092.1 PREDICTED: alanine--tRNA synthetase, cytoplasmic-like isoform X2 [Apis dorsata]

MAKLVRVVNSQKCIRAGGKHNDLDDVGKDVYHHTFFEMMGNWSFGDYFKKEICTWAWEFLTVKLKLSSDRLYVTYFGGDE

KNNLKPDEECKQIWLSLGLSPTHILPGNMKDNFWEMGETGPCGPSSEIHYDRIGNREAAHLVNQDNPDVLEIWNLVFIQF

NRENDGNLKLLPKKHIDCGLGLERLVSIIQNKSANYDTDLFMPLFDAIQKGTKAPSYQGKVGKEDKNGIDMAYRVLADHA

RTITIALADGGIPDNTGRGYVLRRILRRAVRYAIEKLNAKPGFFGSLVNVVVDLLGDVFPEVTKDPQHIIDIINEEETQF

LKTLSRGRNLLNRTIVKLESSNILPGDVAWRLYDTYGFPFDLTQLMVEEKGLKVDIIGYEEAKKQAQLISQNKSGEVDDQ

INLDVHAITELQNKKIKPTNDIPKYNYKVINNKIYEEYEFAPCFSTVIALRRAKTFVDEVSSGEEVGILLDQTNFYAEQG

GQIYDEGFLTKIDDEDTEIHVKNVQVRGGYVLHIGIVIQGKLKKNDKVFSNIDITRRRLLMSNHTATHVLNYALREILGT

EVDQKGSLVAPDRLRFDFTNKGAMITEQIRKVEEITNNIINENKIVYAKECNLGLAKTIQGLRAMFEETYPDPVRVVSIG

IPVEDLEKNPLSSDALKTSVEFCGGTHLHYTGHIRNFIIVSEEAIAKGIRRIVALTGPEAMKAQKKTSILQNYLDELQIS

IEADKNGINTKEHIKKIVELTDDVSHAIISSWKKDKIRKILKDLKKLLDDKERAIKTAIANVVLDNIQQIIQQNIGCLVL

VEVLQAYSNTKALDSALKKIRTISPETSALLISIDPDAKKIFALSSVSKSAINKGLKANEWIQEIVPLMEGKGGGKSESA

QASGTNISCVTRLIYTAKNFANSKLELAENISENHDQLKGDEKFIYFKTLKDKLVLSGDIGSIKYYRIQIVAKYNNIELI

THKNNIFKGIKLQGNDFELFDSNAIAFYLSTDQFKCSNNPFTFSEVLQWLSYADNHILPAVLGWVVPCLSKNIPNNIKTN

IKISKEDVLSSLQKLNNILLTKTYLVGERISLADISVFTTLMPLYEYVLDSEYRKQYTNLNRWFFTILNQSQVISVIKKF

NICEKVKIVKY

>PDM66516.1 aars-2 [Pristionchus pacificus]

MKNLTANEVRAAFIEFFQKNEHKYVHSSSVIPHEDPTLLFANAGMNQFKPAFLGVADPSSDLAKLKRAVNTQKCIRAGGK

HNDLDDVGKDVYHHTFFEMLGNWSFGDYFKKEVCSWAWELLTKELEIPAERLYVSYFGGDEASGLEPDNECRQIWLDLGL

PSNRILPFSMKDNFWEMGDVGPCGPCSEIHYDRVGNRDASHLVNADDPMVVEIWNLVFMQFNREEGGSLRPLPHKHIDCG

LGFERLVAVLQNKTSNYDTDVFVPIFNAITNGTGARAYTGHVGEEDRDGIDMAYRVVADHIRTLTIALSDGGRPDNTGRG

YVLRRILRRGVRYATEKLGAKAGFFASLVPVVVELLGGVFPELTKDPETVQDIINDEETQFLKTLNRGRILFTRAVSELP

LGEKIFPGAIAWRLYDTYGFPADLTQLMAEEKGLNVDSDAFEECRKKAIELSSAGAGKLRDTVDLDVHAIAELQSQGVSA

TDDKPKYDYTAEESNEMTSKYAFNSCTGKIVAIRKEGAFVEEINDGEEGVIIVDKTNFYAEQGGQIYDIGVLTKEGSEDS

HFNVTNVQIRGGYVVMVGCQEGTLKIGDAVVQKFDEDRRWLIMKNHTGTHILNHALRKVIGDVEQKGSLVAPDRLRFDFT

AKGALTSDQVREVESICQQLADAKGKVYAKESKLEEAKAINGLRAVFDENYPDPVRVVSVGISVDDLLSNPDGDAAHNTA

VEFCGGTHLHNSGHIGRLVITSEEAIAKGIRRIVGVTGPEADRAIHRADRIEGRVQELFSEVVSEKNLAENKEKFNEKSK

KANDLVDEINSLLLPSWRKDAIREKAKTTQKQLDSYDKAAKAAVADKIVAEAKALCEKEDEMVVHVFSPGANSKALDNAL

KQLKKAKAVMAFSVNEDNGKVLVLAKVDKSLVDKLKASSWVNEVCTVLGGRGGGKDGQAQATGEDSSRIEEAKQIAQRFA

ENAIA

>EPB76393.1 alanine--tRNA synthetase [Ancylostoma ceylanicum]

MKQLTANEVRSTFIEFFKQKKAHTYVHSSSVIPHDDPTLLFANAGMNQFKPLFLGIADPNSDLAKLKRAVNTQKCIRAGG

KHNDLDDVGKDVYHHTFFEMLGNWSFGDYFKKEIITWAWELLTEVYGIPAERLYVSYFGGDEKAGVPADEEARSIWLSLG

LPENRILPFGMKDNFWEMGDVGPCGPCSEIHYDRVGGRDASHLVNADDPMVVEIWNLVFIQFNREEGGVLRPLPAKHIDC

GLGLERLIAVIQEKTSNYDTDIFQPIFKAIQQGTGIREYTGKVGADDVDGVDMAYRVVADHVRTLTIALSDGGRPDNTGR

GYVLRRILRRGVRYATEKLNAHPGFFASLVPVVIDILGDTFPELRRDPETVQDIINDEEKQFLKTLSRGRVLFQRAVEGL

TADQKTFPGDVAWRLYDTYGFPADLTQLMAEEKGLTVDQNVFEECRKKAVELSAAGAGKFRDTLDLDVHALAELQKRGIP

PTDDSFKYNYQADGAKDGSVKYTFSSCTGKILAIRSDGKFVDSIESGVEGAILLDKTNFYAEQGGQIYDTGVLTKVDGDG

SEFVVSNCQVRGGYIVLVGSANGKFSVGDDVLQNFDEDRRSLIMKNHTGTHVLNHALRCVLADSDQKGSLVAPDRLRFDF

TNKQAMTVKQVKETEEIVQSVINTREPVYAKEAPLAQAREISGLRAMFDENYPDPVRVVCVGVPVEQLLQDPKSGAGLKT

TVEFCGGTHLHNVGHIGHFVISSEEAIAKGIRRIVALTGPEADRAIQRADRLAARAQAISDAINANKDIALDSEKFKAAA

KRIQELIDEINGAQLPYWRKDAIREQCKAAQRTLDGYKKLADAAIAEKVLSEAKELAQGDQQDVIVHVFSSGASSKVLDN

ALKQLKSTKAVMGFSVNEESGKVLVLAKVDKSLVDGGLKANLWVNEVCTILGGRGGGKDANAQVMSSMLMFPRD

>EGT32559.1 hypothetical protein CAEBREN_22812 [Caenorhabditis brenneri]

MKQLTASEVRSTFINFFREKKEHTYVHSSSVIPHDDPTLLFANAGMNQFKPLFLGIADPNSDLAKLKRAVNTQKCIRAGG

KHNDLDDVGKDVYHHTYFEMLGNWSFGDYFKKEIITWAWELLTTVYGIPAERLYVSVFGGDEANGVPADTEARDIWRSVG

VPDERILNFGMKDNFWEMGDVGPCGPCSEIHYDRIGNRDASHLVNADDPMVVEIWNLVFIQFNREEGGALKPLPAKHIDC

GLGLERLIAVMQDKTSNYDTDIFQPIFEAIHKGSGVRKYTGLIGDEDKDGVDMAYRVVGDHIRTLTIALSDGGRPDNSGR

GYVLRRILRRGIRYATEKLNAQPGFFASLVPVVISILGETFPELSRDPETVMDIINDEEKQFLKTLSRGRVLFQKAVDSL

PAGTKTFPGDIAWRLYDTYGFPADLTQLMAEEKGLSVDISAFDEARKKAVETSSAGAGKFRDTLDLDVHALAELQQKGVP

TTDDTPKYAYSFTGEGAEAVYSKFFHVSKIGSITFSILEFEPCIGKILAIRRDGKFVDQLVAGEEGALLLDRTNFYAEQG

GQIYDVGVLTKVNDESNEFNVNNCQVRGGYIVLVGSAEGSFAVGDEVNQRFDEDRKQLIMKNHTGTHVLNYALRKILSDS

DQKGSLVAPDRMRFDFTNKAAMTPDQVKQAEEYAQQLIDTKGQVYAKNSPLADAKRINGLRAMFDETYPDPVRVVAVGTP

VEQLLQNPEAEEGQNTTVEFCGGTHLQNVSHIGRIVISSEEAIAKGIRRIVALTGPEAERAIARADRLEARLLEESKQAK

DQTLLANKDKFKELQKKIQEIVDEANGAQLPSWRKDALRKKAKDIQKYMDGYSKAQQAAVAEKVLGEAKELAAASEQPAV

LVHVFTSNANSKAIDNALKLLKDTKAVMAFSVNEDTGKVLCLAKVDKSLVSNLKANEWINEVCTVLGGKGGGKDANAQLT

GENVDKLDAAVELAQKFALAAIN

>XP_003137715.1 alanyl-tRNA synthetase [Loa loa]

MKALTCDEIRATFINFFKDREHTYVHSSSVIPFDDPTLLFANAGMNQYKPIFLGTVEPSSEMAKLKRAVNTQKCIRAGGK

HNDLDDVGKDVYHHTFFEMLGNWSFGDYFKLEVCNWAWELLTKVLGIPGERLYVSYFGGDDKTGLDADEECRQIWLNIGV

PPSHILSFGMKDNFWEMGDVGPCGPCSEIHYDRIGDRDASHLVNADDPMVVEIWNLVFIQFNREEDGSLKQLPHKHIDCG

LGFERLVAVLQNKLSNYDTDVFTPLFSAIHNGTKVRPYSGKVGADDVDGIDMAYRVVADHIRTLCIALSDGGRPDNTGRG

YVLRRILRRGIRYANEKLSATPGFLSSLVPTVVNLLGKTFPELHKDPQTVMDIINDEEKQFLKTLNKGRTLFQKAATRLQ

ERNILPGNVAWRLYDTYGFPLDLTQLMAEERGLLIDFEAFENCRQQAAEISAARANKMRDAIDLDVSAISELKNKGIPVT

DDSPKYKYYPLPPENQQTMYNFEKCEGIILALRKDKIFVDTLNSEDFGAIILDRTNFYAEQGGQLFDTGVLREVDKGSEF

IVSNVQIRGGYVVLVGTVEGELSVGDKVIQAIDEERRNLIMKNHTGTHVLNFALRKVVGEVEQKGSLVAPDRLRFDLTSK

QPLTYEQIKIVEEESQMLIDTNATVFAKNAPLAEARQINGLRAVFEEAYPDPVRIVSVGVPIEELLKDKDGDLAVNTAVE

FCGGTHLHNVGHIGKLVITVEEAIAKGIRRIIALTGPEAFRAIHRADRIEQRVEEAHNEIATDRIVTLDKTQFKTASKRI

HDLFEEINQSQLPYCRKESIRKKAKALQKLLDMRDREAKAAVTDKVQQEARELDASLNGDTLYTVHIFSKGANGKVLDGA

LKSIKKSHAVMGFSINDDIGKVVVVARVSKDVANKGLQASEWISKVCRVLDGRGGGTVIQAQATGNNTELVEEAAQIALK

FAEVTLS

>XP_013295859.1 alanine--tRNA synthetase [Necator americanus]

MASSITRAERSGNIFYRITGLIRSGQLAWAERPLWYDVYIAHPPLEAHDWNTKHPKYDEPVRKIFYSEDIVRAAFYKKYR

GGVINLEHGRESLAQQFINECEAVKSEQEGKENLTHDELFRRTEQRIKDLGVELKINEVFLNFLTIHCCSEFSLMYLSFV

RSTFIEYFKQKRAHTYMHSSSVIPHDDPTLLFANAGMNQFKPLFLGVADPNSDLAKLKRAVNTQKCIRAGGKHNDLDDVG

KDVYHHTFFEMLGNWSFGDYFKKEIITWAWELLTEVYGIPADRLYVSYFGGDDSAGVPADEEAKSIWLSLGMPENRILPF

GMKDNFWEMGDVGPCGPCSEIHYDRVGGRDASHLVNADDPMVVEIWNLVFIQFNREESGVLRPLPAKHIDCGLGLERLIA

VIQNKTSNYDTDVFQPIFKAIQEGTGVREYTGNVGADDTDGVDMAYRVVADHIRTLTIALADGGRPDNTGRGYVLRRVLR

RGVRYATEKLNAQPEFFASLVPVVINILGDTFPELRRDPETIQDIINDEEKQFLKTLNRGRVLFQRAVAGLTADQKTFPG

DVAWRLYDTYGFPADLTQLMAEEKGLTIDQVVFEECKKKAVEMSAAGAGKFRDTLDLDVHALAELQKRGIPPTDDSSKYN

YHADGATDGSAKYKFSYCTGKILAIRSEGKFVDSIGSGVEGAILLDKTNFYAEQGGQIYDTGVLCKVDEEGSEFVVSNCQ

VRGGYIVLVGSANGNLSVGDNVIQNFDEDRRSLIMKNHTGTHVLNHALRCVLADSDQKGSLVAPDRLRFDFTNKQAMTVK

QVKETEVIAQAIINTRQPVYAREAPLAQAREISGLRAMFDENYPDPVRVVSVGVPVEQLLNDPKSGAGLKTTVEFCGGTH

LHNVGHIGSLVISSEEAIAKGIRRIVALTGPEAERAINRADRLASRAKALCDEICADKGIALDSEKFKATAKRIQELIDE

INGAQLPYWRKDAMREQCKTAQKTLDGYKKLADAAVAEKVLSEAKELAQNDQKEVIVHVFSAGASSKVLDNALKQLKTTK

AVMGFSVNEESGKVLVLAKVDKSLVEIGLKANLWVNEVCTILGGRGGGKDANAQATGENINRLEEAVALARKFAQMTIA

>XP_001899825.1 Alanyl-tRNA synthetase [Brugia malayi]

MKALTCDEVRAAFINFFKDREHTYVHSSSVIPFDDPTLLFANAGMNQYKPIFLGTVEPSSEMAKLKRAVNTQKCIRAGGK

HNDLDDVGKDVYHHTFFEMLGNWSFGDYFKLEVCNWAWELLTKVLGIPGERLYVSYFGGDEKTGLDADEECRQIWLNIGV

PPSHILSFGMKDNFWEMGDVGPCGPCSEIHYDRVGNRDASHLVNADDPMVVEIWNLVFMQFNREEDGSLKQLPRKHIDCG

LGFERLVAVLQNKLSNYDTDIFTPLFXAIHNGTKVRPYSGKVGADDVDGIDMAYRVIADHIRTLSIALSDGGRPDNTGRG

YVLRRILRRGVRYANEKLSAPPGFLSSLVPTVVNLLGKTFPELHKDPQAVMDIINDEEKQFLKTLSRGRTLFQKAVAGLQ

GHNILPGDVAWRLYDTYGFPLDLTQLMAEERGLLVDFEVFENCRQKAAGISAAHANKMRDTIDLDVNAISELKNKGIPAT

DDSPKYKYYALSSEDQQTLYNFEKCEGIILALRKDKIFLDTLNSGDFGALILDRTNFYAEQGGQIFDTGVLTEVDKGSEF

IVSNVQIRGGYVVLVGTVEGQLCVGDKVIQAIDEDRRNLIMKNHTGTHVLNFALRKVIGEVEQKGSLVAPDRLRFDLTAK

QPLTYEQIRMVEKESQMLIDTNATVFAKNVPLAEARQINGLRAAYPDPVRIVSVGIPIEELLRNKDSDLAVNTAVEFCGG

THLHNVGHIGKLVITVEEAIAKGIRRIIALTGPEAFRAIHRADRLEQRVEDAHHKITTDHMVNLDKAQFKAATKRVLELF

EEINQSQLPYCRKESIRKKAKALQKLLDMHDREAKAVLADKVRCEASELDSSLKDGTLFTVHVFSKGANGKVLDSALKSI

TKSHAIMGFSVSDDGKVVAVARVSKDVASKGLQASEWISRVCQVLDGRGGGTITQAQATGNNAELVEEAAQIALKFAEVI

LS

>PAV57350.1 hypothetical protein WR25_00301 [Diploscapter pachys]

MKFMSSDEVRAAFIDYFRTKQGHTYVHSSSVIPHDDPTLLFANAGMNQFKPLFLGIADPNSDLAKLKRAVNTQKCIRAGG

KHNDLDDVGKDVYHHTYFEMLGNWSFGDYFKKEIITWAWELLTQVYGIPAERLYVSVFGGDEANGVPADEEARQIWKSVG

VPDSRILNFGMKDNFWEMGDVGPCGPCSEIHYDRIGGRDASDLVNADDPMVVEIWNLVFIQFNREEGGKLRSLPAKHIDC

GLGLERLIAVMQGKSSNYDTDMFTPIFKAIQEKTGIREYSGKVGAEDVDGIDMAYRVVGDHIRTLTIALSDGGRPDNVGR

GYVLRRILRRGVRYATEKLNAKSGVFASLVPVVVEILGKTFPEITKDPETVMEIINDEEKQFLKTLSRGKVLFQKSINQL

PKDAKVFPGDVAFRLYDTYGFPPDLTQLMAEEHGLQVDLVRFEEEKKKAIELSAAGAGKFRDTLDMDVHSLAELQKRGIA

TTDDKPKYNYKADGPNDLSAQYNFQTCTGKIVALRHEGKFVDEIATGQEAVVLLDKTNFYAEQGGQIYDTGVLTKVGDDS

TEFNVFNCQARAGYVILVGSVEGNLKVGDEVVQRFDEDRRALIMKNHTGTHVLNYALRKVLVDSDQKGSLVAPDKLRFDF

TYKQAMNPKQVKEAEEICQTVIETRKDVFAKETPLSQAKEINGLRAMFDEAYPDPVRVVCVGTPVEELLKDPQSELGLNT

TVEFCGGTHLHNVGHIGRLVITTEEAIAKGIRRIVAVTGQEAEKTIQRADRLQARLQELVKSIESDKEMASDKAKFKQVS

KSVTDFLDEINSSQLPYWRKDLLREQAKAAGKTLFNYSVALEKVIAEKIVTEAKELAEKSGDVIVHVFSPGASVKTLDNA

FKQLKDAKAVMAFSVNEENGKVLIVAKVDKTLIEKNFKAPAWINEVCKVLGGKGGGKDDRAQANGENTEKLDEAVELAKK

FAETVIA

>XP_022205776.1 alanine--tRNA synthetase, cytoplasmic [Nilaparvata lugens]

MAMTAQEVRQSFLDFFTKTKAHTYVHSSSVIPHDDPTLLFANAGMNQFKPIFLGTVDPSSEMGKMVRAANTQKCIRAGGK

HNDLDDVGKDVYHHTFFEMLGNWSFGDYFKREICTWAWELLTAVYKLPADRLYVTYFGGSEKAGLQPDLEAKQIWLELGV

TESHILPGNMKDNFWEMGETGPCGPCSEIHYDRVGGRNAASLVNQDDPDVLEIWNLVFIQFNREADSSLKLLPKKHIDCG

LGLERLVSVIQGKRSNYDTDLFLPLFAAIQQGTGAREYSGKVGAEDVDGMDMAYRVLADHARTLTIALADGGIPDNTGRG

YVLRRILRRAVRYATEKLNAQPGFFGSLVHTVVKILSSTFPEIAKDPQRIIDIINEEEQQFLKTLSNGRNLLNQTLAKMG

DAKVLPGDVAWRLYDTYGFPLDLTQLMMEEKGCQVDMQGYEQARLHALALSQGKSAGAASGVSLDVHSIDELQAKGIAPT

DDSPKYNYKAKPQDGFAPYEFEGCEAIVKAIRVEKQFVSSVGPDTDCGLLLDCTNFYAEQGGQQYDQGFIFPIGGGSVEM

RVNSVEVRAGFILHVGRTTAADEEYSGDLCVGDKVSLRIDEQRRRLIMSNHTATHALNHTLRTVLGPDCNQRGSLVAPDR

LRFDFANRSALSIDQVQKVEQETREMIDRNEDVFADVVPLAGAKPIVGLRAMFDETYPDPVRVVAIGVPIDELVADPNSG

RGLTTSVEFCGGTHLHKAGHIGDFVIVSEEAIAKGIRRIVALTGPEASKALKLEKSLEKRVVEICSESNENSKDTVKQIT

ALTEEISHALISSSKKDELRSRLASVKRELDEAEKRAAQRLAAAAVDQAKQLANEAADAKRSFVVAKLDAGSNAKI

>CDJ87289.1 Alanyl-tRNA synthetase and Threonyl alanyl tRNA synthetase and Phosphoesterase domain containing protein [Haemonchus contortus]

MIGDNRRLEPYSSRSTVEMKQLTANEVRSTFIEFFKQKKAHTYVHSSSVIPHDDPTLLFANAGMNQFKPLFLGIADPNSD

LAKLKRAVNTQKCIRAGGKHNDLDDVGKDVYHHTFFEMLGNWSFGDYFKKEVITWAWELLTEVYEIPADRLYVSYFGGDT

KSGIPADEEARSIWLSLGLPDSRILPFGMKDNFWEMGDVGPCGPCSEIHFDRIGGRDASHLVNADDPMVVEIWNLVFIQF

NREESGDLRPLPAKHIDCGLGLERLIAVIQGKTSNYDTDMFQPIFKAIQEGTGTRPYTGKVGADDIDGIDMAYRVVADHI

RTLTIALSDGGRPDNTGRGYVLRRVLRRGVRYATEKLNAQPEFFASLVPVAIEILGDTFPELRRDPETVRDIINDEEQQF

LKTLVRGRRLFQRAVAGLGADEKTFPGDVAWRLYDTYGFPADLTQLMAEEKGLTVDQKAFEECRKKAVELSAAGAGKFRD

TLDLNVHAIAELQKQGIPTTDDSFKYRYKADGPKDGTAKYTFETCTGKILAIRHDGKFVDSIEAGVEGALILDKTNFYAE

QGGQIYDTGVLTATADEGTEFIVTNCQVRGGYVVLVGSTNGVLRVGDSVEQMFDGERRALIMKNHTATHVLNHALRCVLT

DSDQKGSLVAPDRLRFDFTNKQAMTVKQVKEAEEIVQSIINTREPVYAKEAPLPQAREISGLRAMFDENYPDPVRVVCVG

VPVEELLANPKSGAGLKTTVEFCGGTHLHNVGHIGHMVISSEEAIAKGIRRIVALSGPEAERAIHRADRLAARVKAISEE

ISANKNIAMDSEKFKAKSKQVQELIDEVNGAQLPYWRKDEIRDQGKAAQRTLDGYKKQADAAVADKVLSEAKELAQNSNE

DVVVHVFAPGASSKALDNALKQLKSTKAVMGFSVNEDSGKVLVLAKVDKSLVDGGLKANLWVNEVCTVLGGRGGGKDANA

QATCENVERLDEAVELALKFAQTTIA

>XP_024507138.1 Alanine--tRNA synthetase, mitochondrial [Strongyloides ratti]

MSGNDVRRTFIEFFKEKEHIYVHSSSVIPHDDPTLLFANAGMNQFKPIFQGVVDPNSDMGKWKRAVNTQKCIRAGGKHND

LDDVGKDVYHHTFFEMLGNWSFGDYFKKEVCEWAWELLTKVYEIPPERLYVSYFGGNEEYGLEPDNECKNIWLNIGVPEN

RIIPFGMKDNFWEMGEVGPCGPCSEIHYDRIGGRDASSLVNADDPMVVEIWNLVFMQYNREEGGSLKPLPNKHIDCGLGL

ERLIAVMQDKVSNYDTDVFQPIFKAIEEGTGARKYTGLVGDDDKDCVDMAYRVVADHIRTLVIAIADGGVPDNVGRGYVL

RRVLRRAVRYATEKLGAKPGFFSSLVDVVITLLGETFPELNKDVETIKDIINDEEIQFLKTLNRGRVLFQKAVKALPEGE

KCFPGDIAWRLYDTYGFPIDLTQLMAEECKLQINMDQYNDSKKKAIEMSAGGVGKFKESLDINVHAIAELQSKGILPTDD

SPKYLYTDNGCFGLDGKYNFEKCTGKIVGIRKNGEFVDSLTSGEEGVLILDRTWFYAEQGGQIYDTGVFVKVGDKDTEFS

VKNCQVRGGYIIFVGEAEGNLKIGDELEQNVDEDRRSLIMKNHTGTHVLNYAINRVIPGIDQKGSLVAPDRLRFDFTAKG

ALTTKQIKEIEDIAQSLINTKKPVYAKQAPLAEAKKVKGLRAVFDENYPDPVRVVSVGTPIETLLANPDSDIGLNTTVEF

CGGTHLKNVSHIGSLVITTEEAIAKGIRRIVALTGPEAEKAIHRGERIEKRVNELKVLVEKDENIVNDKKKFKELTTSTN

ELLLEINGMILPYCKKESVRETTKNIQKLLDKYNKNAQNAAAEKIVAEAKKLVETIDDSTKYLVHVFPEGANGKILDSAL

KQIKKPSAVMAFSVNNEIGKIQVICKVEKNLVEKGFKAQNWINKVCEVIDGKGGGKDTQAQATGNCLEKLNEAVSVAKEF

ASISLN

>OZC07121.1 alanine--tRNA synthetase [Onchocerca flexuosa]

MKVLSCDEVRAAFINFFKDREHTYVHSSSVIPFDDPTLLFANAGMNQYKPIFLGTIEPSSEMAKLKRAVNTQKCIRAGGK

HNDLDDVGKDVYHHTFFEMLGNWSFGDYFKLEVCNWAWELLTKVFGIPGERLYVSYFGGDEKTGLAADEECRQIWLNIGV

PPSHILSFGMKDNFWEMGSVGPCGPCSEIHYDRIGNRDASHLVNADDPMEDGSLKQLPRKHIDCGLGFERLVAVLQNKLS

NYDTDIFTPLFDAIHTGTKIRPYSGKIGVDDVDGIDMAYRVVADHIRTLSIALSDGGRPDNTGRGYVLRRILRRAVRYAN

EKLLAPPGFLSSLVPTVVNLLGKTFPELHKDPQTVMDIINDEEKQFLKTLNRGRRLFQKAVASLQGRNILSGDVAWRLYD

TYGFPLDLTQLMAEERGLVIDLELFENCRQEAAELSAAHINKMRGTIDLDVNAISELKNKGIPATDDSPKYRYHALPIED

QQTMYSFEECEGVILALRKDKMFVDTLNSGDFGALILDRTNFYAEQGGQIFDTGVLTKVDEGSEFIVSNVQIRGGYVVLV

GSAEGELSVGDKVIQIIDEERRNLIMKNHTGTHVLNFALRKIIGEIEQKGSLVAPDRLRFDFTSKQSLTYEQIKMVEEES

QKLIDTNATVFAKNAPLAEARQINGLRAVFEEAYPDPVRIVSVGVPIEELLKNMDSDLAVSTAVEFCGGTHLHNVGHIGK

LVITAEEAIAKGIRRIIALTGPEASRAIHRANRLEQRVEETHDELATDHIITLDKIQFKAATKRVLELIEEVNQSQLPYC

RKENIRKKAKALQKLLDTRDREAKAVLAEKVQREASELDASLNGDTVFTVHIFSKGANGKVLDGALKLIKKSHAVMGFSI

NDDIGKVVVVARVSKDVASKGLQASEWISQVCKVLDGRGGGTVTQAQATGNNIDSVEEAAEVALKFAKITLS

>XP_021124384.1 LOW QUALITY PROTEIN: alanine--tRNA synthetase, cytoplasmic-like [Anas platyrhynchos]

MESTLTASQIRQRFIDFFKENQHTYVHSSSTIPLDDPTLLFANAGMNQFKPIFLNTIDPSHPLAKLSRATNTQKCIRAGG

KHNDLDDVGKDVYHHTFFEMLGSWSFGDYFKELACKLALDLLTKEFGIPAERLYVTYFGGNEAAGLQPDLECKQIWLNLG

LPEGRILPGNMKDNFWEMGDTGPCGPCSEIHYDRIGDRDASHLVNQDDPNVLEIWNLVFIQFNREADGTLKPLPKKSIDT

GMGLERLVSVLQNKMSNYDTDLFLPYFEAIQKGTGARPYMGQVGAEDADGIDMAYRVLADHARTITXLSDGGRPDNTGRG

YVLRRILRRAVRYSHEKLNAPKGFFATLVDVVVQSLGDAFPELKKDPDMVKDIINEEEDQFLKTLSRGRRILDRKIQSLG

DSKTIPGDTAWLLYDTYGFPVDLTGLIAEEKGLVVDMEGFEEERKNAQLKSQGKGAGGEDLLMLDIYAIEELRAQGLEVT

DDSPKYSYTSDPSGTYDFGSLVATVKAIRREKKFVEEVSTGQECGIVLDRTCFYAEQGGQIYDQGYMVKDDDSREDVSAL

RKVDSLKKLLSALDAKVKVQTAPNKDVQKEITDLSETLATAVIPQWQKDELREAVKALKKVMDDLDRASKADIQKRVLEK

TKQVIESHPNQPLVIMEMENGASAKALNESLKLFKTHSPQTATMLFAVDNEAGRITCLCQVPQVTPRKGLKASQWVQEVS

GLMDGKGGGKDVSAQATGKNVGCLPEALRLATDFARLHLGELKN

>XP_012795597.1 Alanine--tRNA synthetase, cytoplasmic, partial [Schistosoma haematobium]

GTVDPNSELGRLKRAYNSQKCIRAGGKHNDLDDVGRDVYHHTFFEMLGNWSFGDYFKKEACEWAWELLTEVWKIPSERLY

VTYFGGDISNGLPADEEARDIWISIGLPKDHVLPFGMKENFWEMGETGPCGPCSEIHYDRVGDRAAAHLVNQDDPDVLEI

WNLVFIQFNREEGGSLKPLPAKHIDTGMGFERVLSVLQNKRSNYDTDLFVPLFKTIEAGTNTRPYTGKVGVEDTDKIDMA

YRVLADHARVLTIALGDGGRAQNTGRGYVIRRILRRAVRFAIEVLNAKPGFLATLVDVVIETLGNAFPEVTRDPDTIKEL

INEEEEQFLVTLKRGRRFLDRVIKDLKVSGSNSTTLSGEVAWGLYETYGFPLDLTQILAEEHHLKIDLNGYEKAKEEAQI

RSQSRKCTGGSIVDLDVHALAELKSKNISVTDDSDKFVYTSDLNGNYVFPDSEATVLAIRYENKFVESVDSSSQMCGIIL

NKTIFYAESGGQLYDHGFITSLTDEVTEFSILDIQCRGGYILHIGTLHGKLNVGSRVLLSLDTVRRTALMRNHTGTHVLN

FALRELVDESEQKGSLVAPDRLRFDFTAKRGMTRDELAKAEEICDTMISKRLNVYSSNVSLSYAKTIQGVRAVFGEAYPD

PVRVVSIGVPVTSLVADPERGYGKTTSVEFCGGTHVLNTKHIGVLVIVSEEAISKGVRRIIALTGHEAERAQKEALRLDN

EVNELIQFVNKSISLSQNNNVTDDFNINRQISNLSELVSRAVISQHHRENLREKLFEAKKLLDARDKASRTATTSKVLEE

ARLLGETLSNSPSNYIIHVFDAQSNTKIMNSAIKEIERICPNKAVMVISSDLISKKLTVLCQVPKVLVSHGLKANEWVTH

ISKTMDGKGGGKESSAQAVGSRIDTLEEVIRLADIFASTKLTNN

>PFX26004.1 Alanine--tRNA synthetase, cytoplasmic [Stylophora pistillata]

MEPSLTAKDLRKMFVDFFVDKNEHTFVPSSSTIPHDDPTLLFANAGMNQYKPIFLGTVDPNSDLSKLKRAVNSQKCIRAG

GKHNDLDDVGKDVYHHTFFEMLGNWSFGDYFKKGAIEFAWELLTVKYGMPKDRLYVTYFGGAEGLESDEDTKQFWLDMGL

PPDRVLPFGMKDNFWEMGDTGPCGPCTEIHFDRIGGRDAAYLVNMDDPTVLEVWNLVFIQFNRETDGSLKSLPNKHVDTG

MGLERLTSITQGKMSNYDTDLFQPFFEAIHKGTGVRPYTGLVGAEDKDGVDMAYRVVADHVRTLTMAITDGGKPDNTGRG

YVLRRILRRCVRFGTEKLNMPPGFIASLVEVAVETLGDFFPEVKKDPQQVKDVINEEETMFLKTLARGRRLFDSTVSQLT

DNFIPGDVAWRLYDTYGFPVDLTQLMAEERGLTVDMNVYEESKNKAQEIARGKGSGSDDAITLDVHAINKLQKDLNLEPT

NEKSKYIYTSDKEGNYVFEPVIGTVKAIIFEKEFVNEVISGSHCGVVLDRTCFYAEQGGQIYDQGFMVKEGDDEVEFSVT

NVQVHGGYVLHVGAVEGTLRVGDEMKCQIDEQRRRLTMNNHTATHVLNFALRKHLGDADQRGSLVAPDKLRFDFTAKGAM

TTEQIKNTELTSQDIVSRSLDVFAKESSLATAKEIQGLRAIFEEVYPDPVRVLSIGASFEELQDDPQAGYKYSVEFCGGT

HLQNTGHMKSFVLVSEEAISKGIRRIVALTGPEALRAEKKCHSLEERVENVQNLVDEKMKDGGLNIKETSQEITRISEVR

NNPFLMFCISTEINFLPPAFRLLCYVYCNRKKIQVYRELR

>XP_013083042.1 PREDICTED: LOW QUALITY PROTEIN: alanine--tRNA synthetase, cytoplasmic-like [Biomphalaria glabrata]

MMNANEVRDSFIDFFKEKEHKYVHSSSTIPLDDPTLLFANAGMNQFKPIFLGIADPNSDMGKYVRVVNTQKCVRAGGKHN

DLDDVGKDVYHHTFFEMLGNWSFGDYYKKEVCTWAYEYLVDRLQLDKSRLYVTYFGGNLSAGLEPDNEARDIWTRLGVDP

EKILPFGMKDNFWEMGETGPCGPCSEIHFDRIGGRNAKDKVNMDDPDVLEIWNLVFIQYNRETDGSLKLLPRKHIDCGMG

FERMVSVVQNKRSNYDTDLFQPIFRAIEQATGARPYQGRVGAEDTDGIDMAYRVLADHARTLTITLADGGRPDNSGRGYV

LRRILRRAVRYATEKLNAKPGMFSSLVDTVVQLLVSILERSIKKMGDTKTLPGDIAWRLYDTYGFPVDLTLLMVEEKGMN

IDMPAFEEAKAHSLLMSQSTTGVKEDLISLDVHSLTELKDRGVPPTDDLRKYAYDADEKGDYKFEPTTAKIIGLRYNKQF

VETVTSGQECGILLDQTSFYSEQGGQTYDEGFIVKDGGEDDEFRVKNVQLRGGYVLHIGSVEGVFKVGDTVRLLIDEPRR

CAIMKNHTATHVLNFALRDVLKETDQKGSLVAPDRLRFDFSAKGAMTVQEVKTAEEIVKNIVAKNEVVYARETPLAVAKT

IQGLRAVFDEVYPDPVRVVSIGIPVEDLESDPNGPGGNITTVEFCGGTHLKKAGHIGDFVIVTEEAISKGVRRIIAVTGS

EAHKAVHKQELLKKNVKELTLKIENNSKTQEFTDRELNKEIITLDEEIAQSSISYWKRDALKSKCYLIYNSSFSCLFCLQ

ETVSKGLNAKEWVDKVVPIINGKGGGKDLAAQATGTNIKGLQEALNVATEFAQLKLS

>GAA28662.2 alanyl-tRNA synthetase [Clonorchis sinensis]

MAGWKRAVNTQKCIRAGGKHNDLDDVGRDVYHHTFFEMLGNWSFGDYFKKEACVWAWQLLTEVWKIPKERLYVTYFGGTE

STGLPPDEEARDIWLSIGLPKERVLPFGMKDNFWEMGETGPCGPCSEIHYDRIGGRDAASLVNMDDPDVLEIWNLVFIQF

NREEDGSLRPLPAKHIDTGLGFERILSVLQGKRSNYDTDLFQPLFAAIQSGTGAPPYSGRVGAEDVDGKDMAYRVLADHA

RVLTIALSDGGRAQNTGRGYVLRRILRRAVRYAVEVLGAPPGFFATLVDVVVTSLKDAFPELANDPYTVKELINEEEQQF

LTTLKRGQRLLSRTISDIKRTQGAAGTGVLPGTVAWRLYDTYGFPLDLTQLMAEEHNLKLDLAGYEKAKELAQARSQTGA

AGAGAIVDLDVHAIAELRKQGIPGTDDTEKYNYQADAQGNYAFPEIEATILSIRQESMFVPDVSQSGQLCGFILDKTIFY

AEAGGQLYDTGFITKISDDACELCVTDVQYRGGYVLHIGRLEGHFAIGDKVRMCIDGVRRSGLMRNHTGTHVLNFALRKV

LGEADQKGSLVAPDRLRFDFTAKRGMSRDEIRDAEEVARTVLESNAPVYAANVSLPVGKSIQGLRAVFGEVYPDPVRVVS

VAVPVDKLVADPTGPAGNTTSVEFCGGTHVHNVKHIGSLVIVSEEAIAKGIRRIVALTGHEGERAQREAQELEHEVSHLS

GAIHMASSLTLANGDKPVDKDKNVEFRELTQKLASLSERLAQANISQHVRDRLRADLGLAKKKLDDRDKNAKASITTQVL

EEARKLVESCCSNKQDGFGGNYMVHVFQDEANAKAINSALKILERGCPDIAVMAISSDPVGKKLLCLSQVPKNLIDRGLK

ANEWVASVSSLINGKGGGKESSAQAVGSRVDCLDEVVRLANQFAKAKLCNS

>KRZ09281.1 Alanine--tRNA synthetase, cytoplasmic, partial [Trichinella zimbabwensis]

LYTASCCVINNLKRTKMDILLTANAIRSTFINYFLEKRNHQYVHSSSVIPKGDPSLLFTNAGMNQFKPIFLGTADPNSQM

AAWKRVVNSQKCIRAGGKHNDLDDVGKDVYHHTFFEMLGNWSFGDYFKKEICAWAWEVLTKEFKIPKEQLYVTYFGGSEK

YRLQPDVECRQIWLDIGLPSEKVLPFGMKDNFWEMGDIGPCGPCSEIHFDRIGNRNAANLVNKDDPDVLEIWNLVFITFN

RDKDGSLHPLPKRHIDCGLGFERLVSVIQGKKSNYDTDLFVPIFNRIAETANVRPYMGKVGEEDEQQIDMAYRVVADHIR

TLTVALSDGGRPDNCGRGYVLRRILRRGVRYATEKLNARSGFFANLVPVVVETLGDTFPELKENPDLVMEIINEEESLFL

KTLSRGSRVLRQEIQKSPKEKLIPGFVAWRLYASYGFPVDLTQLMAEESGRKVDMEEYEQCRQQAQISSSSKFSQQSDDI

DFNINTISCLLDMKVPLTDDKPKYAYRLSENKNDNYEFDELKCEVLALRKNGEFVSEVHSGDSCCLICDKTVFYAESGGQ

IYDEGYMNNDKSEFHVRNVQVRSGYVIHFGDVMGTIILGSKLSQNIDQKRRMQVMKNHTATHLLNFALREILGQVEQKGS

LVLPEKLRFDFTSKAPLTVEQLMRVEQIINDIVKSDVEIYMQEVPLGVAKTIAGLRSTTDESYPDPVRVVSVGITADTLL

NDPNGSGAKLASVEFCGGTHLLRTGHVGSFVIASEEAVARGVRRIVALTSHQAEQAVQKLEYFVKKIETVMGLIRNSLRT

GELEYKMLNREIATCIEEMKSSQIPAWRREHLVRKLTQVKATMDDFDKSERLKFAQLITDEARNLQIPVDGIVVHEFPQN

ATTKALDSALKQLKCKAGIVFSQDSKSKKVVCLANVNRECVDKGLTAKAWVDRLCCVAGGYGGGKETTAQASLGNGLTMD

QIKIEAENFAKLRLSFPK

>XP_021953644.1 alanine--tRNA synthetase, cytoplasmic-like [Folsomia candida]

MDTPTIRSSFINFFTSKHSHSYVHSSSVIPHDDPTLLFANAGMNQFKPVFLGTVDPGSDMAKLTRVVNTQKCIRAGGKHN

DLEDVGKDVYHHTFFEMLGNWSFGDYFKKEICTWSWEWLTETLALPKDRLYVTYFGGDEKAGLEPDEECRGIWEALGLPP

THILPGSMKDNFWEMGDTGPCGPCSEIHFDRIGDRDASHLVNMDDPDVLEVWNLVFIQFNREADTSLRPLPKKHIDCGMG

LERIVSVVQGHRSNYDTDLFTPIFEEIKKWSPTTPTYHGRIAPEDTQGTDMAYRVVADHIRTLTIALADGGRPDSTGRGY

VLRRILRRGIRYSIEKLGAKRGDFAGLVGVVCQILGGTFPELLKSPTTTMTIINEEEAQFMRTLTRGRALLERTIGKLEF

SKFLPGDVAWRLYDTYGFPLDLTQLMCEEKSLSVDLEGYEEAKKRAVMISQGKMGGDEGVGLGLDVHSIGELQGRGVQRT

EDQGKYLYQVGEGGGEEYDFPPHLATLLAIRQPSTFTPVAPQSSTPTILILSSTSFYADAGGQIHDEGFITSTTSDAEFI

VTNTQSRGGYICHYGTVTEGEFTVGDVVELHLDGNRRKQIMNNHTGTHVLNFALRGVLEAEPEQKGSHVGPDRLRFDFSS

KAALTSKQVKDAESICNAVVAQDLVVYAKESSLASAKAIKGLRAVFNEAYPDPVRVVSIGVDVEELERDPSGGKGVDFSV

EFCGGTHLLRSSHMGSFVIASEEAIAKGIRRIVGLTGPEAARAENKANLMIQQVAEIKKGPKADWSKKIVEMGEELNQAV

ISQWKKDELRAQLKELRKILDDEDKAAKAQVVLSVVEQVKGIMVEEEGREFYVKILQSGASAKALDAGLRQVKAASPWKP

ALFISVESPTKVYVMAQVPPAAVEKGLKANEWVGQVAELLQGKGGGKAESAQVQGSSAMNVEKCIEICSEFAKLKLK

>KRX17412.1 Alanine--tRNA synthetase, cytoplasmic [Trichinella nelsoni]

MLYLKTRMTVLLYTTCCCVINNLKRSKMDMLLTANAIRQTFINYFTEKRNHQYVHSSSVIPKGDPSLLFTNAGMNQFKPI

FLGTADPNSQMASWKRVVNSQKCIRAGGKHNDLDDVGKDVYHHTFFEMLGNWSFGDYFKKEICAWAWEVLTKEFKIPKEQ

LYVTYFGGSEKCRLQPDVECRQIWLDIGLPSERVLPFGMKDNFWEMGDIGPCGPCSEIHYDRIGNRNAANLINKGDPDVL

EIWNLVFITYNRDKDGSLHPLPKRHIDCGLGFERLVSVIQGKKSNYDTDLFVPIFNRIAETAKVRPYMGKVGKEDEQQID

MAYRVVADHIRTLTVALSDGGRPDNCGRGYVLRRILRRGVRYATEKLNAPSGFFANLVPVVVETLGDTFPELKEDPNSVM

EIINEEESLFLKTLSRGSRVLRQEIEKASKDKLIPGVVAWRLYASYGFPVDLTQLMAEESGYKVNMEEYEECRQQAQMSS

SSKFSQPNDVVDFNINAVNYLLNGKVPLTDDKPKYAYRLIEDGDDDYEFDEVKCEVLALRKNGEFVSEVCSGDSCCLICD

KTAFYAESGGQIYDEGSMEGDKSEFRVRNVQARSGYVIHFGEVMGTITRGSKLFQNVDQKRRVQVMKNHTATHLLNFALR

EVLGQVEQKGSLVLTDRLRFDFTCKAPLTVEQLMRIEQIVADIVNSDVEIYMQEVPLALAKTIAGLRSTADETYPDPVRV

VSVGTPVDALLKDPDGPGAKLASVEFCGGTHLLRTGHLGDFVIASEEAVARGIRRIVALTCREAEQAIQKLEHFVNKVEE

VIDLIRSSVLSGELDYSFLNREISGCMEEMKASQIPAWRREHLVRKLAQVKATLDDFEKSERMKFVQMITDEAKNLQIPE

NGIVVHEFPPNATAKALDSALKQLKCRASIVFSQDSKNKRVVCLANVNRECVDKGLTAKAWVDRLCCVAGGYGGGKETTA

QASLGTGLPVDKIKTEAEKFAKCKLSRR

>EJW87808.1 alanyl-tRNA synthetase [Wuchereria bancrofti]

MKALTCDEVRAAFINFFKDREHTYVHSSSVIPFDDPTLLFANAGMNQYKPIFLGTVEPSSEMAKLKRAVNTQKCIRAGGK

HNDLDDVGKDVYHHTFFEMLGNWSFGDYFKLEVCNWAWELLTKVLGIPGERLYVSYFGGDEKAGLDADEECRQIWLNIGV

PSSHILSFGMKDNFWEMGDVGPCGPCSEIHYDRVGNRDASHLVNADDPMVVEIWNLVFMQFNREEDGSLKQLPRKHIDCG

LGFERLVAVLQNKLSNYDTDIFTPLFSAIHNGTKVRPYSGKVGADDVDGIDMAYRVVADHIRTLSIALSDGGRPDNTGRG

YVLRRILRRGIRYANEKLSAPPGFLSSLVPTVVNLLGKTFPELHKDPQAVMDIINDEEKQFLKTLSRGRTLFQKAVAGLQ

GHNILPGDVAWRLYDTYGFPLDLTQLMAEERGLVVDFEVFENCRQQAAGISAAHANKMRDTIDLDVNAISELKNKGIPAT

DDSPKYKYHALSSEDEQTLYNFEKCEGIILALRKDKIFLDTLNSGDFGALILDRTNFYAEQGGQIFDTGVLTEVDKGSEF

IVSNVQIRGGYVVLVGTVEGELSVGDRVIQAIDEDRRNLIMKNHTGTHVLNFALRKVIGEVEQKGSLVAPDRLRFDLTAK

QPLTSEQIRMVEEESQMLIDTNARVFAENAPLAEARQINGLRAAYPDPVRIVSVGVPIEELLRNKDSDLAVNTAVEFCGG

THLHNVGHIGKLVITVEEAIAKGIRRIIALTGPEASRAIHRADRLEQRVEDTHNKIATDYIVTVDKAQFKAATKRVLELF

EVQREASELDASLKDGTLFTVHVFSKGANGKVLDSALKSIRKSHATMGFSVNDDGKVVAVARVSKDVASKGLQASEWISR

VCKVLDGRGGGTITQAQATGNNAELVEEAAQIALKFAEVTLS

>KRZ73423.1 Alanine--tRNA synthetase, cytoplasmic, partial [Trichinella papuae]

LYTASCCVINNLKRKNIFYFRTKMDILLTANAIRSTFINYFLEKRNHQYVHSSSVIPKGDPSLLFTNAGMNQFKPIFLGT

ADPNSQMAAWKRVVNSQKCIRAGGKHNDLDDVGKDVYHHTFFEMLGNWSFGDYFKKEMCAWAWEVLTKEFKIPKEQLYVT

YFGGSEKYRLQPDVECRQIWLDIGLPSEKVLPFGMKDNFWEMGDIGPCGPCSEIHFDRIGNRNAANLVNKDDPDVLEIWN

LVFITFNRDKDGSLHPLPKRHIDCGLGFERLVSVIQGKKSNYDTDLFVPIFNRIAETANVRPYMGKVGEEDEEQIDMAYR

VVADHIRTLTVALSDGGRPDNCGRGYVLRRILRRGVRYATEKLNARSGFFANLVPVVVETLGDTFPELKENPDLVMEIIN

EEESLFLKTLSRGSRVLRQEIQKSPKEKLIPGFVAWRLYASYGFPVDLTQLMAEESGRKVDMEEYEQCRQQAQISSSSKF

SQQSDDIDFNINTISCLLDMKVPLTDDKPKYAYRLSENKNDNYEFDELKCEVLALRKNGEFVSEVHSGDSCCIICDKTAF

YAESGGQIYDEGYMNNDKSEFHVRNVQVRSGYVIHFGDVMGTIILGSKLSQNIDQKRRMQVMKNHTATHLLNFALREILS

QVEQKGSLVLPEKLRFDFTSKAPLTVEQLMRVEEIINDIVKSDVEIYMQEVPLGVAKTIAGLRSTTDESYPDPVRVVSVG

ITADTLLNDPNGSGAKLASVEFCGGTHLLRTGHIGSFVIASEEAVARGVRRIVALTSHQAEQAVQKLEYFVKKIETVMGL

IRNSLRTGELEYKMLNREIATCIEEMKSSQIPAWRREHLVRKLTQVKATMDDFDKSERLKFAQLITDEARNLQIPVDGIV

VHEFPQNATTKALDSALKQLKCKAGIVFSQDSKSKKVVCLANVNRECVDKGLTAKAWVDRLCCVAGGYGGGKETTAQASL

GNGLTMDQIKIEAEKFAKLRLSFPK

>KHJ45035.1 alanine--tRNA synthetase [Trichuris suis]

MKAEEVRQKFLDFFSKKYGHTYYHSSPVIPHDDPTLLFANAGMNQFKPIFLGTVNPNSPLAKLKRAVNTQKCIRAGGKHN

DLDDVGKDVYHHTFFEMLGNWSFGDYYKEEVCAWAWELLTECFMLEKDRLYVTYFGGNPSMNLGPDLECRDIWLKLGIPD

DRILPFGMKDNFWEMGEIGPCGPCSEIHYDRIAGRNAASLVNQDDPSVIEIWNLVFITYNRETNGSLQPLPTRHIDCGLG

FERLVSVIQGVSSNYDTDLFLPIFSYIASATGCRPYSSKVGTEDVDQVDMAYRVVADHIRALTVALSDGGRPDSTGRGYV

LRRILRRGVRFATEKLNAPPGLFASLVPQVVAILGTTFPEMTKDPDAVMAIINEEEMQFLKTLSRGGKLLDRTIDKVGPG

GLIQGDVAWRLYDTYGFPVDLTELIAEERGCTVDMAAYEACRLAAQKASQVKLSCKGGLEFSASVLKELNDLGIPTTDDS

WKYKYSVADSALDDYVLETCVCHVLAIRMNGKFVSQVTSGDDCVLICDRTSFYAENGGQIYDEGFMEKANAQVEAEFCVT

NVQARAGYVLHSGKVEGMISVGDKLTQRVDEERRRLVMKNHTATHLLNYALREVVKESFQKGSLVAPDRLRFDFSASSAL

TNDQIKRVEEIVLKLVTENAQVYMSEVPLSAGKALPGLRAEFQETYPDPVRVVSVGYPVNTLLADPSLSMQTSVEFCGGT

HLHRVGHIDDFVIISEEALARGIRRIVALTGPAARRARQRCAHFNERLRSIEQSRPGEDVVPDRFYKAKKSKIVALIEEV

SSSQISCWERESLCEQLQALKKEIEATNKAAQALLASKVLEKAKILSESSEDIVVHTFDSNASAKILDSALKKITSAKAA

MVFSVDEENSKVLCMACVKKQYAENGQLNAKDWTSYLCSTFGGKSGGKDVNSQALLDEPSRLDDIVKAAEDFARLRIGPL

N

>XP_018398046.1 PREDICTED: alanine--tRNA synthetase, cytoplasmic isoform X1 [Cyphomyrmex costatus]

MMGNWSFGDYFKKEICTWAWEFLTDVLKLSADRLYVTYFGGDKKNGLAPDDECKNLWLSLGVPASHVLPGNMKDNFWEMG

ETGPCGPCSELHYDRIGDREAAHLVNMDDPDVLEIWNLVFIQYNRESDGSLKSLPKKHIDCGLGLERLVSVIQNKRANYD

TDLFMPLFTAIEKGTGAPSYQGKVGADDVNGIDMAYRVLADHARTLTIALADGGVPDNTGRGYVLRRILRRAVRYAIEKL

NAKPGFFGTLVNVVVDLLGDVFPEVKKDPQSIIETINEEESQFLKTLSRGRNLLNRTIAKLESADTVPGDVAWRLYDTYG

FPIDLTQLMTEEKGLKIDMAGYEESKKQAQLISQGKAGGVDDQINLDVHAITELQNQGVKPTDDSPKYNYKVTNKTKYKE

YEFAPCTGTVIALRRAKTFVNEVSSGEVVGILLDRTNFYAEQGGQIYDEGFLVKVNDETTEIRIKNVQVRAGYVLHIGIV

GEGKLKKGDKVRTNVDTERRRLVMANHSATHVLNYALRKVLGTEADQKGSLVTPDRLRFDFTNKGPMTGQQLKETELSVI

AIIRKNEKIYAKESNLATAKTIQGLRAMFEETYPDPVRVVSVGIPVENLEKDPLSTAALETSVEFCGGTHLHYTSHIGDF

VIASEDAIAKGIRRIVALTGPEARKAIRTAGRFGSDITMSECQYQMNDMNSKEYVRKIVELTEEISQALIPAYRKDDLRN

HLKEMKKSLDDKERAAKAAVATTVVQTAQSIIESKVGCQVLVEVLEAYSNTKALDSALKKIRAISPETSALLISVDHDVK

KIFALSSVPKSAISKGLKANEWIQAITSLMQGKGGGKSESAQASGLNVACLNEIVSKANEFADQKLGIITTA

>PAA52127.1 hypothetical protein BOX15_Mlig016942g3 [Macrostomum lignano]

MTQYTAASVRQSFIDFFAQKHKHTYVHSSSTIPYDDPTLLFTNAGMNQYKPIFLGTADPNSDMAKWTRAVNSQKCIRAGG

KHNDLDDVGKDVYHHTFFEMLGNWSFGDYFKLEACTWAWDLLTNVWGLPRDRLYVTYFGGNEAQGLPSDEEARDIWISLG

LPPDRVLPFGMKDNFWEMGETGPCGPCSEIHFDRIGGGRNAADLVNMDDPDVLEIWNLVFIQFNREPDGNLRLLPNKHID

TGMGMERILSVLQGHRSNYDTDAFQPLFAAIEKFTGSRPYSGRVGAEDADGVDMAYRVLADHARTLTIALSDGGRPDNAG

RGYVLRRILRRGVRYAVEKLRSPPGLLEALVDVVCQSLGSAFPELNRDPDTVREIIAEEEAQFLKTLRRGQRVLERTLAK

VPAGQPVPGDVVWRLYDTYGFPTDLTKLMAEERGLAIDETGFEAAKEASLLASQAAGGGVGVSVDLDVHALAELKDGMAI

AQTDDSPKLTYTTDSDGVYAYPNRISATIKAIRYRQAFVDSVPSGVECGFVLDRTCFYAEQGGQIFDEGVMTKTGEGAGD

DAAEFMVKNCQVRGGYVIHVGTTEGEFKVGDELILSVDMQRRKPVMQNHTGTHVLNYALRQVLGVDADQKGSLVAPDRLR

FDFACKAAMTQEQIGQVENIVNEVVSKRQPVYAAMASLAEAKAVQGLRAVFGEVYPDPVRVVSVGVEVDKLLADPNGPAG

SSTSVEFCGGTHLHNAGHIGQMAIISEEAIAKGIRRIVAVTGTEASRARHSADLLEADVTSLEGLASACTDVAEARGLAR

RIVDLSTLVAQSTTPSVRREAMRRRLADAKRRLDDLDRSGLPSPGSQIRNRSRPES

>XP_018398047.1 PREDICTED: alanine--tRNA synthetase, cytoplasmic isoform X2 [Cyphomyrmex costatus]

MMGNWSFGDYFKKEICTWAWEFLTDVLKLSADRLYVTYFGGDKKNGLAPDDECKNLWLSLGVPASHVLPGNMKDNFWEMG

ETGPCGPCSELHYDRIGDREAAHLVNMDDPDVLEIWNLVFIQYNRESDGSLKSLPKKHIDCGLGLERLVSVIQNKRANYD

TDLFMPLFTAIEKGTGAPSYQGKVGADDVNGIDMAYRVLADHARTLTIALADGGVPDNTGRGYVLRRILRRAVRYAIEKL

NAKPGFFGTLVNVVVDLLGDVFPEVKKDPQSIIETINEEESQFLKTLSRGRNLLNRTIAKLESADTVPGDVAWRLYDTYG

FPIDLTQLMTEEKGLKIDMAGYEESKKQAQLISQGKAGGVDDQINLDVHAITELQNQGVKPTDDSPKYNYKVTNKTKYKE

YEFAPCTGTVIALRRAKTFVNEVSSGEVVGILLDRTNFYAEQGGQIYDEGFLVKVNDETTEIRIKNVQVRAGYVLHIGIV

GEGKLKKGDKVRTNVDTERRRLVMANHSATHVLNYALRKVLGTEADQKGSLVTPDRLRFDFTNKGPMTGQQLKETELSVI

AIIRKNEKIYAKESNLATAKTIQGLRAMFEETYPDPVRVVSVGIPVENLEKDPLSTAALETSVEFCGGTHLHYTSHIGDF

VIASEDAIAKGIRRIVALTGPEARKAIRTAGRFGSDITMSECQYQMNDMNSKEYVRKIVELTEEISQALIPAYRKDDLRN

HLKEMKKSLDDKERAAKAAVATTVVQTAQSIIESKVGCQVLVEVLEAYSNTKALDSALKKIRAISPETSALLISVDHDVK

KIFALSSVPKSAISKGLKANEWIQAITSLMQGKGGGKSESAQASGLNVACLNEIVSKANEFADQKLGIIRE

>PAA89152.1 hypothetical protein BOX15_Mlig018098g1 [Macrostomum lignano]

MTQYTAASVRQSFIDFFAQKHKHTYVHSSSTIPYDDPTLLFTNAGMNQYKPIFLGTADPNSDMAKWTRAVNSQKCIRAGG

KHNDLDDVGKDVYHHTFFEMLGNWSFGDYFKLEACTWAWDLLTNVWGLPRDRLYVTYFGGNEAQGLPSDEEARDIWISLG

LPPDRVLPFGMKDNFWEMGETGPCGPCSEIHFDRIGGGRNAADLVNMDDPDVLEIWNLVFIQFNREPDGNLRLLPNKHID

TGMGMERILSVLQGHRSNYDTDAFQPLFAAIEKFTGSRPYSGRVGAEDTDGVDMAYRVLADHARTLTIALSDGGRPDNAG

RGYVLRRILRRGVRYAVEKLRSPPGLLEALVDVVCQSLGSAFPELNRDPDTVREIIAEEEAQFLKTLRRGQRVLERTLAK

VPAGQPVPGDVVWRLYDTYGFPTDLTKLMAEERGLAIDETGFEAAKEASLLASQAAGGGVGVSVDLDVHALAELKDGMAI

AQTDDSPKLTYTTDSDGVYAYPNRISATIKAIRYRQAFVDSVPSGVECGFVLDRTCFYAEQGGQIFDEGVMTKTGEGAGD

DAAEFMVKNCQVRGGYVIHVGTTEGEFKVGDELILSVDMQRRKPVMQNHTGTHVLNYALRQVLGVDADQKGSLVAPDRLR

FDFACKAAMTQEQIGQVENIVNEVVSKRQPVYAAMASLAEAKAVQGLRAVFGEVYPDPVRVVSVGVEVDKLLADPNGPAG

SSTSVEFCGGTHLHNAGHIGQMAIISEEAIAKGIRRIVAVTGTEASRARHSADLLEADVTSLEGLASACTDVAEARGLAR

RIVDLSTLVAQSTTPSVRREAMRRRLADAKRRLDDLDRSGLPSPGSQIRNRSRPES

>KRX17413.1 Alanine--tRNA synthetase, cytoplasmic [Trichinella nelsoni]

MLYLKTRMTVLLYTTCCCVINNLKRSKMDMLLTANAIRQTFINYFTEKRNHQYVHSSSVIPKGDPSLLFTNAGMNQFKPI

FLGTADPNSQMASWKRVVNSQKCIRAGGKHNDLDDVGKDVYHHTFFEMLGNWSFGDYFKKEICAWAWEVLTKEFKIPKEQ

LYVTYFGGSEKCRLQPDVECRQIWLDIGLPSERVLPFGMKDNFWEMGDIGPCGPCSEIHYDRIGNRNAANLINKGDPDVL

EIWNLVFITYNRDKDGSLHPLPKRHIDCGLGFERLVSVIQGKKSNYDTDLFVPIFNRIAETAKVRPYMGKVGKEDEQQID

MAYRVVADHIRTLTVALSDGGRPDNCGRGYVLRRILRRGVRYATEKLNAPSGFFANLVPVVVETLVEDVHIVIGDTFPEL

KEDPNSVMEIINEEESLFLKTLSRGSRVLRQEIEKASKDKLIPGVVAWRLYASYGFPVDLTQLMAEESGYKVNMEEYEEC

RQQAQMSSSSKFSQPNDVVDFNINAVNYLLNGKVPLTDDKPKYAYRLIEDGDDDYEFDEVKCEVLALRKNGEFVSEVCSG

DSCCLICDKTAFYAESGGQIYDEGSMEGDKSEFRVRNVQARSGYVIHFGEVMGTITRGSKLFQNVDQKRRVQVMKNHTAT

HLLNFALREVLGQVEQKGSLVLTDRLRFDFTCKAPLTVEQLMRIEQIVADIVNSDVEIYMQEVPLALAKTIAGLRSTADE

TYPDPVRVVSVGTPVDALLKDPDGPGAKLASVEFCGGTHLLRTGHLGDFVIASEEAVARGIRRIVALTCREAEQAIQKLE

HFVNKVEEVIDLIRSSVLSGELDYSFLNREISGCMEEMKASQIPAWRREHLVRKLAQVKATLDDFEKSERMKFVQMITDE

AKNLQIPENGIVVHEFPPNATAKALDSALKQLKCRASIVFSQDSKNKRVVCLANVNRECVDKGLTAKAWVDRLCCVAGGY

GGGKETTAQASLGTGLPVDKIKTEAEKFAKCKLSRR

>KFD48021.1 hypothetical protein M513_11103 [Trichuris suis]

MPAERAGIGKARERHSFAIEAGERSNKRREKTYPLTTAFVEKRSCLLAEFVFNGGCYDNRILYNQQSVETIEVCIGINHE

KSPSSGVAVEFFYVKIAVDWTDNWASITAFMLSLMSVIKLRNCPPSEWDKLSLQQLFCDVGSNAQKKQGSFEFTNIPPKI

LDALKEAVMEQLSELEESITKLVSVLYPDFASRSVANGDKNSALDFVINYLRNGYHVKSDQLYVEQLRELLLQFDTVSRR

REESSSGSCRVDAYIVSLSAESLFLRKEAEFTLAEAEAKKRSLYESDVCEAAMAILQKELDELEKQAADLEKQIADKKRQ

VKIYKLLAQQPSYQQLFTQYKLLKEELELYKFVIENCCSGQNTFLGCFDPRMKAEEVRQKFLDFFSKKYGHTYYHSSPVI

PHDDPTLLFANAGMNQFKPIFLGTVNPNSPLAKLKRAVNTQKCIRAGGKHNDLDDVGKDVYHHTFFEMLGNWSFGDYYKE

EVCAWAWELLTECFMLEKDRLYVTYFGGNPSMNLGPDLECRDIWLKLGIPDDRILPFGMKDNFWEMGEIGPCGPCSEIHY

DRIAGRNAASLVNQDDPSVIEIWNLVFITYNRETNGSLQPLPTRHIDCGLGFERLVSVIQGVSSNYDTDLFLPIFSYIAS

ATGCRPYSSKVGTEDVDQVDMAYRVVADHIRALTVALSDGGRPDSTGRGYVLRRILRRGVRFATEKLNAPPGLFASLVPQ

VVAILGTTFPEMTKDPDAVMAIINEEEMQFLKTLSRGGKLLDRTIDKVGPGGLIQGEIYGDVAWRLYDTYGFPVDLTELI

AEERGCTVDMAAYEACRLAAQKASQVKLSCKGGLEFSASVLKELNDLGIPTTDDSWKYKYSVADSALDDYVLETCVCHVL

AIRMNGKFVSQVTSGDDCVLICDRTSFYAENGGQIYDEGFMEKANAQVEAEFCVTNVQARAGYVLHSGKVEGMISVGDKL

TQRVDEERRRLVMKNHTATHLLNYALREVVKESFQKGSLVAPDRLRFDFSASSALTNDQIKRVEEIVLKLVTENAQVYMS

EVPLSAGKALPGLRAEFQETYPDPVRVVSVGYPVNTLLADPSLSMQTSVEFCGGTHLHRVGHIDDFVIISEEALARGIRR

IVALTGPAARRARQRCAHFNERLRSIEQSRPGEDVVPDRFYKAKKSKIVALIEEVSSSQISCWERESLCEQLQALKKEIE

ATNRAAQALLASKVLEKAKILSESSEDIVVHTFDSNASAKILDSALKKITSAKAAMVFSVDEENSKVLCMACVKKQYAEN

GQLNAKDWTSYLCSTFGGKSGGKDVNSQALLDEPSRLDDILDLNNSSSAESASSHEAQCFAVLPHMLSPHYCIKAGEPCS

WVISDANKSVELPWRHFVRTPALEILDLKNAKLSCSCANMWLAFWDTILNPVAAIESLPFKMDVLLWFLTDVNRLCESDC

PKTVRDVYGLPHRVHFANHVWSHCLEKQQEDKKQAVYAHFKQICGVGSLNMNAKQVVFSHNKTDDVVICNIQNETAGNVS

HKQCPKFTVTSFLEKPQKPDTFTWCMNETTAVLSVNNVKPNSSGWIICFGNGYLPQAYAIPLLVITPPVFIQFESNRVSL

RELHIKFLLHSYPNVDLRLFIWEADIKGWREVSLDEEYVSRKGRRGALWFSRALRSLASTTTEGEIQITDMDTVCRTDEY

NFTACNSIGCAWKAFLVKPDDQNVLCIANADSPVGREKIGSAIFTFKSEPGTSGPTTKIQNESTFQSSSGSAEYGQISMI

MPFYVVLVILLLTALVVVCRQRVEAKQKAKLLKNHPTTSETQDTNSNRLDYAKRPMISSEDHVL

>XP_022255364.1 alanine--tRNA synthetase, cytoplasmic [Limulus polyphemus]

MEATLTSKEIRQMFMDFFVQKYEHFYVHSSPVIPHDDPTLLFTNAGMNQFKPIFLGVVDPNSDSAKYKRVVDTQKCIRAG

GKHNDLDDVGKDVYHHTFFEMLGNWSFGDFFKREICNWSWELLTKVYKLPKDRMYVTYFGGAPEYGLEPDFECRDIWKEV

GLPDDRILPFGMKDNFWEMGETGPCGPCSEIHYDRIGDRDAKHLVNADVPDVLEIWNLVFIQFNRENDGTLRMLPKKHID

TGMGLERLVSVIQQKSSNYDTDLFVPIFEAIQKVTGTRPYQGKVGSDDEDGIDMAYRVLADHVRTLTVSLSDGGRPDNTG

RGYVLRRILRRGVRFATEKLNAKPGVFAGLVPVVIDILGDVFPELRKDPQMVMDIINEEESQFLKTLTRGHRLLERTINK

LGNVKTLPGDVAWRLYDTYGFPVDLTQLMVEEKGLGIDLDGYEECKKKAQLASQVRGEGQDDSVKLDVHAISELQQKQVP

TTNDLPKYNYSAVSDEKNAQYKFEECSGTVLALRCNKTFVNEVKNGQECGVICDQTCFYAESGGQIYDTGFLVKEGDEDT

EVLVKDVQLQGGYIVHMGTVCGNIRVGDQLKMFIDEERRKLVMNNHTGTHILNFALRQVISSEADQRGSLVAPDRLRFDF

TNKGAMKVSQVQETETIANNMINKNEPVYAKDSSLPQAKAIQGLHAVFNETYPDPVRVVSIGIPVEELLSDPTGPAGSVT

SVEFCGGT

>KRY12513.1 Alanine--tRNA synthetase, cytoplasmic [Trichinella patagoniensis]

MTVLLYTTSCCVINNLKRSKMDMLLTANAIRRTFINYFTEKRNHQYVHSSSVIPKGDPSLLFTNAGMNQFKPIFLGTADP

NSQMASWKRVVNSQKCIRAGGKHNDLDDVGKDVYHHTFFEMLGNWSFGDYFKKEICAWAWEVLTKEFKIPKEQLYVTYFG

GSEKCRLQPDVECRQIWLEIGLPSERVLPFGMKDNFWEMGDIGPCGPCSEIHYDRIGNRNAANLINKNDPDVLEIWNLVF

ITYNRDKDGSLHPLPKRHIDCGLGFERLVSVIQGKKSNYDTDLFVPIFNRIAETAKVRPYMGKVGEEDEQQIDMAYRVVA

DHIRTLTVALSDGGRPDNCGRGYVLRRILRRGVRYATEKLNAPSGFFANLVPVVVETLVEDVHIVIGDMFPELKEDPNSV

MEIINEEESLFLKTLSRGSRVLRQEIEKASKDKLIPGVVAWRLYASYGFPVDLTQLMAEESGYKVNMEEYEECRQQAQMS

SSSKFSQANDVVDFNINAVNYLLNGKVPLTDDKPKYAYRLTENGDDDYEFDEVKCEVLALRRNGEFVSEVCSGDSCCLIC

DKTAFYAESGGQIYDEGSMEGERCEFRVRNVQARSGYVIHFGEVMGTITRGSKLFQNVDQKRRVQVMKNHTATHLLNFAL

REVLGQVEQKGSLVLMDRLRFDFTCKAALTVEQLMRIEQIVASIVNSDVEIYMQEVPLGVAKTIAGLRSTADETYPDPVR

VVSVGTPVDALLKDPDGPGAKLASVEFCGGTHLLRTGHLGDFVIASEEAVARGIRRIVALTCHEAEQAVQKLEYFVKRVE

EVMDLIRSSVLSGELDYSFLNREISGCLEEMKASQIPAWRREHLVRKLAQVKATLDDFEKSERMKFVQMITDEAKNLQIP

DDGIVVHEFPPNATAKALDSALKQLKCRACIVFSQDSKNKRVVCLANVNRECVDKGLTAKAWVDRLCCIAGGYGGGKETT

AQASLGTGLPVDKIKTEAEKFAKCKLSKR

>KRY33650.1 Alanine--tRNA synthetase, cytoplasmic [Trichinella spiralis]

MTVLLYTTSCCVINNVKCTCFLQFIIRQFHYKKFVFYIRSKMDMLLTANAIRQTFINYFTEKRNHQYVHSSSVIPKGDPS

LLFTNAGMNQFKPIFLGTADPNSQMASWKRVVNSQKCIRAGGKHNDLDDVGKDVYHHTFFEMLGNWSFGDYFKKDICAWA

WEVLTKEFKIPKEQLYVTYFGGSEKCRLQPDVECRQIWLDMGLPSERVLPFGMKDNFWEMGDIGPCGPCSEIHYDRIGNR

NAANLINKDDPDVLEIWNLVFITYNRDKDGSLHPLPKRHIDCGLGFERLVSVIQGKKSNYDTDLFVPIFNRIAETAKVRP

YMGKVGEEDEQQIDMAYRVVADHIRTLTVALSDGGRPDNCGRGYVLRRILRRGVRYATEKLNAPSGFFANLVPVVVETLV

EDVHIVIGETFPELKEDPNSVMEIINEEESLFLKTLSRGSRVLRQEIEKASKDKLIPGVVAWRLYASYGFPVDLTQLMAE

ESGYKVNMEEYEECRQQAQMSSSSKFSQLNDVVDFNINAVNYLLNGKVPLTDDKPKYAYRLTENGDDDYEFDEVKCEVLA

LRKNGEFVSEVCSGDSCCLICDKTAFYAESGGQIYDEGSMEGDKSEFRVRNVQARGGYIVHFGEVMGTITRGSKLFQNVD

QKRRVQVMKNHTATHLLNFALREVLGQVEQKGSLVLTDRLRFDFTCKAPLTVEQLMKIEQIVANIVNSDVEIYMQEVPLG

VAKTIAGLRSTADETYPDPVRVVSVGTPVDALLKDPDGPGAKLASVEFCGGTHLLRTGHLGDFVIASEEAVARGIRRIVA

LTCHEAEQAVQKLEYFVNKVEEVIDLIRSSVLSGELDYSFLNRKISGCLEEMKASQIPAWRREHLVRKLAQVKATLDDFE

KGERMKFVQMITDEAKNLQIPDDGIVVHEFPPNATAKALDTALKQLKCRAGIVFSQDSKNKRVVCLAIVNRECVDKGLTA

KAWVDRLCCVAGGYGGGKETTAQASLGTGLPVDKIKTEAEKFAKCKLSRR

>KRX69889.1 Alanine--tRNA synthetase, cytoplasmic [Trichinella sp. T6]

MTVLLYTTSCCVINNLKRSKMDMLLTANAIRRTFINYFTEKRNHQYVHSSSVIPKGDPSLLFTNAGMNQFKPIFLGTADP

NSQMASWKRVVNSQKCIRAGGKHNDLDDVGKDVYHHTFFEMLGNWSFGDYFKKEICAWAWEVLTKEFKIPKEQLYVTYFG

GSEKYRLQPDVECRQIWLEIGLPSERVLPFGMKDNFWEMGDIGPCGPCSEIHYDRIGNRNAANLINKNDPDVLEIWNLVF

ITYNRDKDGSLHPLPKRHIDCGLGFERLVSVIQGKKSNYDTDLFVPIFNRIAETAKVRPYMGKVGKEDEQQIDMAYRVVA

DHIRTLTVALSDGGRPDNCGRGYVLRRILRRGVRYATEKLNAPSGFFANLVPVVVETLGDTFPELKEDPNSVMEIINEEE

SLFLKTLSRGSRVLRQEIEKASKDKLIPGVVAWRLYASYGFPVDLTQLMAEESGYKVNMEEYEECRQQAQMSSSSKFSQA

NDVVDFNINAVNYLLNGKVPLTDDKFKYAYRLTENGDDDYEFDEVKCEVLALRRNGEFVSEVCSGDSCCLICDKTAFYAE

SGGQIYDEGSMEGEKCEFRVRNVQARSGYVIHFGEVMGTITRGSKLLQNVDQKRRVQVMKNHTATHLLNFALREVLGQVE

QKGSLVLMDRLRFDFTCKAPLTVEQLVRIEQIVANIVNSDVEIYMQEVPLGVAKTIAGLRSSADETYPDPVRVVSVGTPV

DALLKDPDGPGAKLASVEFCGGTHLLRTGHLGDFVIASEEAVARGIRRIVALTCHEAEQAVQKLEYFVKRVEEVMDLIRS

SVLSGELDYSFLNREISGCLEEMKASQIPAWRREHLVRKLAQVKATLDDFEKSERMKFVQMITDEAKNLQIPDDGIVVHE

FPPNATAKALDSALKQLKCRAGIVFSQDSKNKRVVCLANVNRECVDKGLTAKAWVDRLCCIAGGYGGGKETTAQASLGTG

LPVDKIKTEAEKFAKCKLSKR

>KRZ73422.1 Alanine--tRNA synthetase, cytoplasmic, partial [Trichinella papuae]

LYTASCCVINNLKRTKMDILLTANAIRSTFINYFLEKRNHQYVHSSSVIPKGDPSLLFTNAGMNQFKPIFLGTADPNSQM

AAWKRVVNSQKCIRAGGKHNDLDDVGKDVYHHTFFEMLGNWSFGDYFKKEMCAWAWEVLTKEFKIPKEQLYVTYFGGSEK

YRLQPDVECRQIWLDIGLPSEKVLPFGMKDNFWEMGDIGPCGPCSEIHFDRIGNRNAANLVNKDDPDVLEIWNLVFITFN

RDKDGSLHPLPKRHIDCGLGFERLVSVIQGKKSNYDTDLFVPIFNRIAETANVRPYMGKVGEEDEEQIDMAYRVVADHIR

TLTVALSDGGRPDNCGRGYVLRRILRRGVRYATEKLNARSGFFANLVPVVVETLGDTFPELKENPDLVMEIINEEESLFL

KTLSRGSRVLRQEIQKSPKEKLIPGFVAWRLYASYGFPVDLTQLMAEESGRKVDMEEYEQCRQQAQISSSSKFSQQSDDI

DFNINTISCLLDMKVPLTDDKPKYAYRLSENKNDNYEFDELKCEVLALRKNGEFVSEVHSGDSCCIICDKTAFYAESGGQ

IYDEGYMNNDKSEFHVRNVQVRSGYVIHFGDVMGTIILGSKLSQNIDQKRRMQVMKNHTATHLLNFALREILSQVEQKGS

LVLPEKLRFDFTSKAPLTVEQLMRVEEIINDIVKSDVEIYMQEVPLGVAKTIAGLRSTTDESYPDPVRVVSVGITADTLL

NDPNGSGAKLASVEFCGGTHLLRTGHIGSFVIASEEAVARGVRRIVALTSHQAEQAVQKLEYFVKKIETVMGLIRNSLRT

GELEYKMLNREIATCIEEMKSSQIPAWRREHLVRKLTQVKATMDDFDKSERLKFAQLITDEARNLQIPVDGIVVHEFPQN

ATTKVCIEPATDQFHIQILAYYFQALDSALKQLKCKAGIVFSQDSKSKKVVCLANVNRECVDKGLTAKAWVDRLCCVAGG

YGGGKETTAQASLGNGLTMDQIKIEAEKFAKLRLSFPK

>KRX52312.1 Alanine--tRNA synthetase, cytoplasmic [Trichinella sp. T9]

MTVLLYTTSCCVINNLKRSKMDMLLTANAIRRTFINYFTEKRNHQYVHSSSVIPKGDPSLLFTNAGMNQFKPIFLGTADP

NSQMASWKRVVNSQKCIRAGGKHNDLDDVGKDVYHHTFFEMLGNWSFGDYFKKEICAWAWEVLTKEFKIPKEQLYVTYFG

GSEKCRLQPDVECRQIWLEIGLPSERVLPFGMKDNFWEMGDIGPCGPCSEIHYDRIGNRNAANLINKNDPDVLEIWNLVF

ITYNRDKDGSLHPLPKRHIDCGLGFERLVSVIQGKKSNYDTDLFVPIFNRIAETAKVRPYMGKVGKEDEQQIDMAYRVVA

DHIRTLTVALSDGGRPDNCGRGYVLRRILRRGVRYATEKLNAPSGFFANLVPVVVETLGDMFPELKEDPNSVMEIINEEE

SLFLKTLSRGSRVLRQEIEKASKDKLIPGVVAWRLYASYGFPVDLTQLMAEESGYKVNMEEYEECRQQAQMSSSSKFSQA

NDVVDFNINAVNYLLNGKVPLTDDKPKYAYRLTENGDDDYEFDEVKCEVLALRRNGEFVSEVCSGDSCCLICDKTAFYAE

SGGQIYDEGSMEGEKCEFRVRNVQARSGYVIHFGEVMGTITRGSKLFQNVDQKRRVQVMKNHTATHLLNFALREVLGQVE

QKGSLVLMDRLRFDFTCKAPLTVEQLVRIEQIVANIVNSDVEIYMQEVPLGVAKTIAGLRLTADEVIRRRSTVQFFYTAL

PLQTYPDPVRVVSVGTPVDALLKDPDGPGAKLASVEFCGGTHLLRTGHLGDFVIASEEAVARGIRRIVALTCHEAEQAVQ

KLEYFVKRVEEVMDLIRASVLSGELDYSFLNREISGCSEEMKASQIPAWRREHLVRKLAQVKATLDDFEKSERMKFVQMI

TDEAKNLQIPDDGIVVHEFPPNATAKALDSALKQLKCRACIVFSQDSKNKRVVCLANVNRECVDKGLTAKAWVDRLCCIA

GGYGGGKETTAQASLGTGLPVDKIKTEAEKFAKCKLSKR

>CDQ01942.1 BMA-AARS-2, isoform h [Brugia malayi]

MKALTCDEVRAAFINFFKDREHTYVHSSSVIPFDDPTLLFANAGMNQPIFLGTVEPSSEMAKLKRAVNTQKCIRAGGKHN

DLDDVGKDVYHHTFFEMLGNWSFGDYFKLEVCNWAWELLTKVLGIPGERLYVSYFGGDEKTGLDADEECRQIWLNIGVPP

SHILSFGMKDNFWEMGDVGPCGPCSEIHYDRVGNRDASHLVNADDPMVVEIWNLVFMQFNREEDGSLKQLPRKHIDCGLG

FERLVAVLQNKLSNYDTDIFTPLFNAIHNGTKVRPYSGKVGADDVDGIDMAYRVIADHIRTLSIALSDGGRPDNTGRGYV

LRRILRRGVRYANEKLSAPPGFLSSLVPTVVNLLGKTFPELHKDPQAVMDIINDEEKQFLKTLSRGRTLFQKAVAGLQGH

NILPGDVAWRLYDTYGFPLDLTQLMAEERGLLVDFEVFENCRQKAAGISAAHANKMRDTIDLDVNAISELKNKGIPATDD

SPKYKYYALSSEDQQTLYNFEKCEGIILALRKDKIFLDTLNSGDFGALILDRTNFYAEQGGQIFDTGVLTEVDKGSEFIV

SNVQIRGGYVVLVGTVEGQLCVGDKVIQAIDEDRRNLIMKNHTGTHVLNFALRKVIGEVEQKGSLVAPDRLRFDLTAKQP

LTYEQAYPDPVRIVSVGIPIEELLRNKDSDLAVNTAVEFCGGTHLHNVGHIGKLVITVEEAIAKGIRRIIALTGPEAFRA

IHRADRLEQRVEDAHHKITTDHMVNLDKAQFKAATKRVLELFEEINQSQLPYCRKESIRKKAKALQKLLDMHDREAKAVL

ADKVRCEASELDSSLKDGTLFTVHVFSKGANGKVLDSALKSITKSHAIMGFSVSDDGKVVAVARVSKDVASKGLQASEWI

SRVCQVLDGRGGGTITQAQATGNNAELVEEAAQIALKFAEVILS

>KRY47973.1 Alanine--tRNA synthetase, cytoplasmic [Trichinella britovi]

MTVLLYTTSCCVINNLKRSKMDMLLTANAIRRTFINYFTEKRNHQYVHSSSVIPKGDPSLLFTNAGMNQFKPIFLGTADP

NSQMASWKRVVNSQKCIRAGGKHNDLDDVGKDVYHHTFFEMLGNWSFGDYFKKEICAWAWEVLTKEFKIPKEQLYVTYFG

GSEKCRLQPDVECRQIWLEIGLPSERVLPFGMKDNFWEMGDIGPCGPCSEIHYDRIGNRNAANLINKNDPDVLEIWNLVF

ITYNRDKDGSLHPLPKRHIDCGLGFERLVSVIQGKKSNYDTDLFVPIFNRIAETAKVRPYMGKVGKEDEQQIDMAYRVVA

DHIRTLTVALSDGGRPDNCGRGYVLRRILRRGVRYATEKLNAPSGFFANLVPVVVETLGDMFPELKEDPNSVMEIINEEE

SLFLKTLSRGSRVLRQEIEKASKDKLIPGVVAWRLYASYGFPVDLTQLMAEESGYKVNMEEYEECRQQAQMSSSSKFSQA

NDVVDFNINAVNYLLNGKVPLTDDKPKYAYRLTENGDDDYEFDEVKCEVLALRRNGEFVSEVCSGDSCCLICDKTAFYAE

SGGQIYDEGSMEGEKCEFRVRNVQARSGYVIHFGEVMGTITRGSKLFQNVDQKRRVQVMKNHTATHLLNFALREVLGQVE

QKGSLVLMDRLRFDFTCKAPLTVEQLMRIEQIVANIVNSDVEIYMQEVPLGVAKTIAGLRLTADEVIRRRSTVQFFYTAL

HLQTYPDPVRVVSVGTPVDALLKDPDGPGAKLASVEFCGGTHLLRTGHLGDFVIASEEAVARGIRRIVALTCHEAEQAVQ

KLEYFVKRVEDVMDLIRASVLSGELDYSFLNREISGCLEEMKASQIPAWRREHLVRKLAQVKATLDDFEKSERMKFVQMI

TDEAKNLQIPDDGIVVHEFPPNATAKALDSALKQLKCRACIVFSQDSKNKRVVCLANVNRECVDKGLTAKAWVDRLCCIA

GGYGGGKETTAQASLGTGLPVDKIKTEAEKFAKCKLSKR

>KRX17411.1 Alanine--tRNA synthetase, cytoplasmic [Trichinella nelsoni]

MLYLKTRMTVLLYTTCCCVINNLKRSKMDMLLTANAIRQTFINYFTEKRNHQYVHSSSVIPKGDPSLLFTNAGMNQFKPI

FLGTADPNSQMASWKRVVNSQKCIRAGGKHNDLDDVGKDVYHHTFFEMLGNWSFGDYFKKEICAWAWEVLTKEFKIPKEQ

LYVTYFGGSEKCRLQPDVECRQIWLDIGLPSERVLPFGMKDNFWEMGDIGPCGPCSEIHYDRIGNRNAANLINKGDPDVL

EIWNLVFITYNRDKDGSLHPLPKRHIDCGLGFERLVSVIQGKKSNYDTDLFVPIFNRIAETAKVRPYMGKVGKEDEQQID

MAYRVVADHIRTLTVALSDGGRPDNCGRGYVLRRILRRGVRYATEKLNAPSGFFANLVPVVVETLVEDVHIGDTFPELKE

DPNSVMEIINEEESLFLKTLSRGSRVLRQEIEKASKDKLIPGVVAWRLYASYGFPVDLTQLMAEESGYKVNMEEYEECRQ

QAQMSSSSKFSQPNDVVDFNINAVNYLLNGKVPLTDDKPKYAYRLIEDGDDDYEFDEVKCEVLALRKNGEFVSEVCSGDS

CCLICDKTAFYAESGGQIYDEGSMEGDKSEFRVRNVQARSGYVIHFGEVMGTITRGSKLFQNVDQKRRVQVMKNHTATHL

LNFALREVLGQVEQKGSLVLTDRLRFDFTCKAPLTVEQLMRIEQIVADIVNSDVEIYMQEVPLALAKTIAGLRSTADETY

PDPVRVVSVGTPVDALLKDPDGPGAKLASVEFCGGTHLLRTGHLGDFVIASEEAVARGIRRIVALTCREAEQAIQKLEHF

VNKVEEVIDLIRSSVLSGELDYSFLNREISGCMEVGWLCVVAFQEMKASQIPAWRREHLVRKLAQVKATLDDFEKSERMK

FVQMITDEAKNLQIPENGIVVHEFPPNATAKALDSALKQLKCRASIVFSQDSKNKRVVCLANVNRECVDKGLTAKAWVDR

LCCVAGGYGGGKETTAQASLGTGLPVDKIKTEAEKFAKCKLSRR

>KRY12514.1 Alanine--tRNA synthetase, cytoplasmic [Trichinella patagoniensis]

MTVLLYTTSCCVINNLKRSKMDMLLTANAIRRTFINYFTEKRNHQYVHSSSVIPKGDPSLLFTNAGMNQFKPIFLGTADP

NSQMASWKRVVNSQKCIRAGGKHNDLDDVGKDVYHHTFFEMLGNWSFGDYFKKEICAWAWEVLTKEFKIPKEQLYVTYFG

GSEKCRLQPDVECRQIWLEIGLPSERVLPFGMKDNFWEMGDIGPCGPCSEIHYDRIGNRNAANLINKNDPDVLEIWNLVF

ITYNRDKDGSLHPLPKRHIDCGLGFERLVSVIQGKKSNYDTDLFVPIFNRIAETAKVRPYMGKVGEEDEQQIDMAYRVVA

DHIRTLTVALSDGGRPDNCGRGYVLRRILRRGVRYATEKLNAPSGFFANLVPVVVETLVEDVHIVIGDMFPELKEDPNSV

MEIINEEESLFLKTLSRGSRVLRQEIEKASKDKLIPGVVAWRLYASYGFPVDLTQLMAEESGYKVNMEEYEECRQQAQMS

SSSKFSQANDVVDFNINAVNYLLNGKVPLTDDKPKYAYRLTENGDDDYEFDEVKCEVLALRRNGEFVSEVCSGDSCCLIC

DKTAFYAESGGQIYDEGSMEGERCEFRVRNVQARSGYVIHFGEVMGTITRGSKLFQNVDQKRRVQVMKNHTATHLLNFAL

REVLGQVEQKGSLVLMDRLRFDFTCKAALTVEQLMRIEQIVASIVNSDVEIYMQEVPLGVAKTIAGLRSTADETYPDPVR

VVSVGTPVDALLKDPDGPGAKLASVEFCGGTHLLRTGHLGDFVIASEEAVARGIRRIVALTCHEAEQAVQKLEYFVKRVE

EVMDLIRSSVLSGELDYSFLNREISGCLEVGWLCVEMKASQIPAWRREHLVRKLAQVKATLDDFEKSERMKFVQMITDEA

KNLQIPDDGIVVHEFPPNATAKALDSALKQLKCRACIVFSQDSKNKRVVCLANVNRECVDKGLTAKAWVDRLCCIAGGYG

GGKETTAQASLGTGLPVDKIKTEAEKFAKCKLSKR

>KRY83701.1 Alanine--tRNA synthetase, cytoplasmic, partial [Trichinella pseudospiralis]

LYTASYCIINNLKRTKMDILLTANAIRSTFINYFLEKRNHEYVHSSSVIPKGDPSLLFTNAGMNQFKPIFLGTADPNSQM

ADWKRVVNSQKCIRAGGKHNDLDDVGKDVYHHTFFEMLGNWSFGDYFKKEICAWAWELLTKQFKIPQEQLYVTYFGGSEK

YRLQPDVECRQIWLDIGLPSEKVLPFGMKDNFWEMGDIGPCGPCSEIHFDRIGNRNAANLVNKDDPDVLEIWNLVFITFD

RDKDGSLHPLPKRHVDCGLGFERLVSVIQGKKSNYDTDLFVPIFNRIAETTNVRPYMGKVGEEDEQQIDMAYRVVADHIR

TLTVALSDGGRPDNCGRGYVLRRILRRGVRYATEKLNARPGFFANLVPVVVEILGDTFPELKDDPDLVMEIINEEESLFL

KTLSRGSRVLRQEIQKTPKENLIPGFVAWRLYASYGFPVDLTQLIAEESGRKVNMEEYEQCRQQAQISSSSKFSQQSDDI

NFNINAISCLLDMGVPLTDDKPKFVYRLSENENDNYEFDELECEVLALRKNGEFVSEVRTGDFCCLICDKTAFYAESGGQ

IYDEGSMSNDKCEFHVRNVQVRSGYVIHFGDVLGTITLGSKLSQNIDQKRRMQVMKNHTATHLLNFALREIMSEVEQKGS

LVLPEKLRFDFTRRTPFELEQLMRIQEIIEDVIKSDAEIFMQEVPLDVAKTIAGLRFMADESYPDPVRVVSIGIPVDALL

DNANGIDGNLTSVEFCGGTHLLRAGHVGKFVIASEDAISRGVRRIIALTGHQAEQAVQKLDYFVTKIETMMDVIRETLRA

GEWKDKMLNREIAACMKEMKYSQIPAWRRTLLMRKLLQMKETMDDFEKSQKLKFVQLITDEARNLQIPDDGIVVHEFPQN

ATTKALDSALKQLKCKAGIVFSKNPAINKVVCLANVSKECVDNGLTAKEWVDHLCRVAGGYGGGKDTTAQASLGEELTVD

QIKIEAENFAKLKLSLPK

>KRY33651.1 Alanine--tRNA synthetase, cytoplasmic [Trichinella spiralis]

MTVLLYTTSCCVINNVKCTCFLQFIIRQFHYKKFVFYIRSKMDMLLTANAIRQTFINYFTEKRNHQYVHSSSVIPKGDPS

LLFTNAGMNQFKPIFLGTADPNSQMASWKRVVNSQKCIRAGGKHNDLDDVGKDVYHHTFFEMLGNWSFGDYFKKDICAWA

WEVLTKEFKIPKEQLYVTYFGGSEKCRLQPDVECRQIWLDMGLPSERVLPFGMKDNFWEMGDIGPCGPCSEIHYDRIGNR

NAANLINKDDPDVLEIWNLVFITYNRDKDGSLHPLPKRHIDCGLGFERLVSVIQGKKSNYDTDLFVPIFNRIAETAKVRP

YMGKVGEEDEQQIDMAYRVVADHIRTLTVALSDGGRPDNCGRGYVLRRILRRGVRYATEKLNAPSGFFANLVPVVVETLV

EDVHIVIGETFPELKEDPNSVMEIINEEESLFLKTLSRGSRVLRQEIEKASKDKLIPGVVAWRLYASYGFPVDLTQLMAE

ESGYKVNMEEYEECRQQAQMSSSSKFSQLNDVVDFNINAVNYLLNGKVPLTDDKPKYAYRLTENGDDDYEFDEVKCEVLA

LRKNGEFVSEVCSGDSCCLICDKTAFYAESGGQIYDEGSMEGDKSEFRVRNVQARGGYIVHFGEVMGTITRGSKLFQNVD

QKRRVQVMKNHTATHLLNFALREVLGQVEQKGSLVLTDRLRFDFTCKAPLTVEQLMKIEQIVANIVNSDVEIYMQEVPLG

VAKTIAGLRSTADETYPDPVRVVSVGTPVDALLKDPDGPGAKLASVEFCGGTHLLRTGHLGDFVIASEEAVARGIRRIVA

LTCHEAEQAVQKLEYFVNKVEEVIDLIRSSVLSGELDYSFLNRKISGCLEVGWLCVEMKASQIPAWRREHLVRKLAQVKA

TLDDFEKGERMKFVQMITDEAKNLQIPDDGIVVHEFPPNATAKALDTALKQLKCRAGIVFSQDSKNKRVVCLAIVNRECV

DKGLTAKAWVDRLCCVAGGYGGGKETTAQASLGTGLPVDKIKTEAEKFAKCKLSRR

>KRX69886.1 Alanine--tRNA synthetase, cytoplasmic [Trichinella sp. T6]

MTVLLYTTSCCVINNLKRSKMDMLLTANAIRRTFINYFTEKRNHQYVHSSSVIPKGDPSLLFTNAGMNQFKPIFLGTADP

NSQMASWKRVVNSQKCIRAGGKHNDLDDVGKDVYHHTFFEMLGNWSFGDYFKKEICAWAWEVLTKEFKIPKEQLYVTYFG

GSEKYRLQPDVECRQIWLEIGLPSERVLPFGMKDNFWEMGDIGPCGPCSEIHYDRIGNRNAANLINKNDPDVLEIWNLVF

ITYNRDKDGSLHPLPKRHIDCGLGFERLVSVIQGKKSNYDTDLFVPIFNRIAETAKVRPYMGKVGKEDEQQIDMAYRVVA

DHIRTLTVALSDGGRPDNCGRGYVLRRILRRGVRYATEKLNAPSGFFANLVPVVVETLVEDVHIVIGDTFPELKEDPNSV

MEIINEEESLFLKTLSRGSRVLRQEIEKASKDKLIPGVVAWRLYASYGFPVDLTQLMAEESGYKVNMEEYEECRQQAQMS

SSSKFSQANDVVDFNINAVNYLLNGKVPLTDDKFKYAYRLTENGDDDYEFDEVKCEVLALRRNGEFVSEVCSGDSCCLIC

DKTAFYAESGGQIYDEGSMEGEKCEFRVRNVQARSGYVIHFGEVMGTITRGSKLLQNVDQKRRVQVMKNHTATHLLNFAL

REVLGQVEQKGSLVLMDRLRFDFTCKAPLTVEQLVRIEQIVANIVNSDVEIYMQEVPLGVAKTIAGLRSSADETYPDPVR

VVSVGTPVDALLKDPDGPGAKLASVEFCGGTHLLRTGHLGDFVIASEEAVARGIRRIVALTCHEAEQAVQKLEYFVKRVE

EVMDLIRSSVLSGELDYSFLNREISGCLEEMKASQIPAWRREHLVRKLAQVKATLDDFEKSERMKFVQMITDEAKNLQIP

DDGIVVHEFPPNATAKALDSALKQLKCRAGIVFSQDSKNKRVVCLANVNRECVDKGLTAKAWVDRLCCIAGGYGGGKETT

AQASLGTGLPVDKIKTEAEKFAKCKLSKR

>KRZ49832.1 Alanine--tRNA synthetase, cytoplasmic [Trichinella nativa]

MFSTIHYLLIALQKIFFYIRSKMDMLLTANAIRRTFINYFTEKRNHQYVHSSSVIPKGDPSLLFTNAGMNQFKPIFLGTA

DPNSQMASWKRVVNSQKCIRAGGKHNDLDDVGKDVYHHTFFEMLGNWSFGDYFKKEICAWAWEVLTKEFKIPKEQLYVTY

FGGSEKCRLQPDVECRQIWLEIGLPSERVLPFGMKDNFWEMGDIGPCGPCSEIHYDRIGNRNAANLINKNDPDVLEIWNL

VFITYNRDKDGSLHPLPKRHIDCGLGFERLVSVIQGKKSNYDTDLFVPIFNRIAETAKVRPYMGKVGKEDEQQIDMAYRV

VADHIRTLTVALSDGGRPDNCGRGYVLRRILRRGVRYATEKLNAPSGFFANLVPVVVETLGDTFPELKEDPNSVMEIINE

EESLFLKTLSRGSRVLRQEIEKASKDKLIPGVVAWRLYASYGFPVDLTQLMAEESGYKVNMEEYEECRQQAQMSSSSKFS

QANDVVDFNINAVNYLLNGKVPLTDDKFKYAYRITENGDDDYEFDEVKCEVLALRRNGEFVSEVCSGDSCCLICDKTAFY

AESGGQIYDEGSMEGEKCEFRVRNVQARSGYVIHFGEVMGTITLGSKLLQNVDQKRRVQVMKNHTATHLLNFALREVLGQ

VEQKGSLVLMDRLRFDFTCKAPLTVEQLVRIEQIVANIVNSDVEIYMQEVPLGVAKTIAGLRSSADEVIRRRSTVQFFYT

ALHLQTYPDPVRVVSVGTPVDALLKDPDGPGAKLASVEFCGGTHLLRTGHLGDFVIASEEAVARGIRRIVALTCHEAEQA

VQKLEYFVKRVEEVMDLIRSSVLSGELDYSFLNREISGCLEEMKASQIPAWRREHLVRKLAQVKATLDDFEKSERMKFVQ

MITDEAKNLQIPDDGIVVHEFPPNATAKALDSALKQLKCRAGIVFSQDSKNKRVVCLANVNRECVDKGLTAKAWVDRLCC

IAGGYGGGKETTAQASLGTGLPVDKIKTEAEKFAKCKLSKR

>KRX42767.1 Alanine--tRNA synthetase, cytoplasmic [Trichinella murrelli]

MTVLLYTTSCCVINNLKRSKMDMLLTANAIRRTFINYFTEKRNHQYVHSSSVIPKGDPSLLFTNAGMNQFKPIFLGTADP

NSQMASWKRVVNSQKCIRAGGKHNDLDDVGKDVYHHTFFEMLGNWSFGDYFKKEICAWAWEVLTKEFKIPKEQLYVTYFG

GSEKCRLQPDVECRQIWLEIGLPSERVLPFGMKDNFWEMGDIGPCGPCSEIHYDRIGNRNAANLINKNDPDVLEIWNLVF

ITYNRDKDGSLHPLPKRHIDCGLGFERLVSVIQGKKSNYDTDLFVPIFNRIAETAKVRPYMGKVGKEDEQQIDMAYRVVA

DHIRTLTVALSDGGRPDNCGRGYVLRRILRRGVRYATEKLNAPSGFFANLVPVVVETLVEDVHIGDMFPELKEDPNSVME

IINEEESLFLKTLSRGSRVLRQEIEKASKDKLIPGVVAWRLYASYGFPVDLTQLMAEESGYKVNMEEYEECRQQAQMSSS

SKFSQANDVVDFNINAVNYLLNGKVPLTDDKPKYAYRLTENGDDDYEFDEVKCEVLALRRNGEFVSEVCSGDSCCLICDK

TAFYAESGGQIYDEGSMEGEKCEFRVRNVQARSGYVIHFGEVMGTITRGSKLFQNVDQKRRVQVMKNHTATHLLNFALRE

VLGQVEQKGSLVLMDRLRFDFTCKAPLTVEQLMRIEQIVANIVNSDVEIYMQEVPLGVAKTIAGLRSTADEVIRRRSTVQ

FFYTALHLQTYPDPVRVVSVGTPVDALLKDPDGPGAKLASVEFCGGTHLLRTGHLGDFVIASEEAVARGIRRIVALTCHE

AEQAVQKLEYFVKRVEEVMDLIRASVLSGELDYSFLNREISGCLEEMKASQIPAWRREHLVRKLAQVKATLDDFEKSERM

KFVQMITDEAKNLQIPDDGIVVHEFPPNATAKALDSALKQLKCRACIVFSQDSKNKRVVCLANVNRECVDKGLTAKAWVD

RLCCIAGGYGGGKETTAQASLGTGLPVDKIKTEAEKFAKCKLSKR

>KRX52313.1 Alanine--tRNA synthetase, cytoplasmic [Trichinella sp. T9]

MTVLLYTTSCCVINNLKRSKMDMLLTANAIRRTFINYFTEKRNHQYVHSSSVIPKGDPSLLFTNAGMNQFKPIFLGTADP

NSQMASWKRVVNSQKCIRAGGKHNDLDDVGKDVYHHTFFEMLGNWSFGDYFKKEICAWAWEVLTKEFKIPKEQLYVTYFG

GSEKCRLQPDVECRQIWLEIGLPSERVLPFGMKDNFWEMGDIGPCGPCSEIHYDRIGNRNAANLINKNDPDVLEIWNLVF

ITYNRDKDGSLHPLPKRHIDCGLGFERLVSVIQGKKSNYDTDLFVPIFNRIAETAKVRPYMGKVGKEDEQQIDMAYRVVA

DHIRTLTVALSDGGRPDNCGRGYVLRRILRRGVRYATEKLNAPSGFFANLVPVVVETLVEDVHIVIGDMFPELKEDPNSV

MEIINEEESLFLKTLSRGSRVLRQEIEKASKDKLIPGVVAWRLYASYGFPVDLTQLMAEESGYKVNMEEYEECRQQAQMS

SSSKFSQANDVVDFNINAVNYLLNGKVPLTDDKPKYAYRLTENGDDDYEFDEVKCEVLALRRNGEFVSEVCSGDSCCLIC

DKTAFYAESGGQIYDEGSMEGEKCEFRVRNVQARSGYVIHFGEVMGTITRGSKLFQNVDQKRRVQVMKNHTATHLLNFAL

REVLGQVEQKGSLVLMDRLRFDFTCKAPLTVEQLVRIEQIVANIVNSDVEIYMQEVPLGVAKTIAGLRLTADEVIRRRST

VQFFYTALPLQTYPDPVRVVSVGTPVDALLKDPDGPGAKLASVEFCGGTHLLRTGHLGDFVIASEEAVARGIRRIVALTC

HEAEQAVQKLEYFVKRVEEVMDLIRASVLSGELDYSFLNREISGCSEEMKASQIPAWRREHLVRKLAQVKATLDDFEKSE

RMKFVQMITDEAKNLQIPDDGIVVHEFPPNATAKALDSALKQLKCRACIVFSQDSKNKRVVCLANVNRECVDKGLTAKAW

VDRLCCIAGGYGGGKETTAQASLGTGLPVDKIKTEAEKFAKCKLSKR

>KRX69890.1 Alanine--tRNA synthetase, cytoplasmic [Trichinella sp. T6]

MTVLLYTTSCCVINNLKRSKMDMLLTANAIRRTFINYFTEKRNHQYVHSSSVIPKGDPSLLFTNAGMNQFKPIFLGTADP

NSQMASWKRVVNSQKCIRAGGKHNDLDDVGKDVYHHTFFEMLGNWSFGDYFKKEICAWAWEVLTKEFKIPKEQLYVTYFG

GSEKYRLQPDVECRQIWLEIGLPSERVLPFGMKDNFWEMGDIGPCGPCSEIHYDRIGNRNAANLINKNDPDVLEIWNLVF

ITYNRDKDGSLHPLPKRHIDCGLGFERLVSVIQGKKSNYDTDLFVPIFNRIAETAKVRPYMGKVGKEDEQQIDMAYRVVA

DHIRTLTVALSDGGRPDNCGRGYVLRRILRRGVRYATEKLNAPSGFFANLVPVVVETLGDTFPELKEDPNSVMEIINEEE

SLFLKTLSRGSRVLRQEIEKASKDKLIPGVVAWRLYASYGFPVDLTQLMAEESGYKVNMEEYEECRQQAQMSSSSKFSQA

NDVVDFNINAVNYLLNGKVPLTDDKFKYAYRLTENGDDDYEFDEVKCEVLALRRNGEFVSEVCSGDSCCLICDKTAFYAE

SGGQIYDEGSMEGEKCEFRVRNVQARSGYVIHFGEVMGTITRGSKLLQNVDQKRRVQVMKNHTATHLLNFALREVLGQVE

QKGSLVLMDRLRFDFTCKAPLTVEQLVRIEQIVANIVNSDVEIYMQEVPLGVAKTIAGLRSSADEVIRRRSTVQFFYTAL

HLQTYPDPVRVVSVGTPVDALLKDPDGPGAKLASVEFCGGTHLLRTGHLGDFVIASEEAVARGIRRIVALTCHEAEQAVQ

KLEYFVKRVEEVMDLIRSSVLSGELDYSFLNREISGCLEEMKASQIPAWRREHLVRKLAQVKATLDDFEKSERMKFVQMI

TDEAKNLQIPDDGIVVHEFPPNATAKALDSALKQLKCRAGIVFSQDSKNKRVVCLANVNRECVDKGLTAKAWVDRLCCIA

GGYGGGKETTAQASLGTGLPVDKIKTEAEKFAKCKLSKR

>KRY72747.1 Alanine--tRNA synthetase, cytoplasmic, partial [Trichinella pseudospiralis]

LYTASYCIINNLKRYIAFLTVLLFSLIFKHVIICALHYRKNIFYFRTKMDILLTANAIRSTFINYFLEKRNHEYVHSSSV

IPKGDPSLLFTNAGMNQFKPIFLGTADPNSQMADWKRVVNSQKCIRAGGKHNDLDDVGKDVYHHTFFEMLGNWSFGDYFK

KEICAWAWELLTKQFKIPQEQLYVTYFGGSEKYRLQPDVECRQIWLDIGLPSEKVLPFGMKDNFWEMGDIGPCGPCSEIH

FDRIGNRNAANLVNKDDPDVLEIWNLVFITFDRDKDGSLHPLPKRHVDCGLGFERLVSVIQGKKSNYDTDLFVPIFNRIA

ETTNVRPYMGKVGEEDEQQIDMAYRVVADHIRTLTVALSDGGRPDNCGRGYVLRRILRRGVRYATEKLNARPGFFANLVP

VVVEILGDTFPELKDDPDLVMEIINEEESLFLKTLSRGSRVLRQEIQKTPKENLIPGFVAWRLYASYGFPVDLTQLIAEE

SGRKVNMEEYEQCRQQAQISSSSKFSQQSDDINFNINAISCLLDMGVPLTDDKPKFVYRLSENENDNYEFDELECEVLAL

RKNGEFVSEVRTGDFCCLICDKTAFYAESGGQIYDEGSMSNDKCEFHVRNVQVRSGYVIHFGDVLGTITLGSKLSQNIDQ

KRRMQVMKNHTATHLLNFALREIMSEVEQKGSLVLPEKLRFDFTRRTPFELEQLMRIQEIIEDVIKSDAEIFMQEVPLDV

AKTIAGLRFMADESYPDPVRVVSIGIPVDALLDNANRIDGNLTSVEFCGGTHLLRAGHVGKFVIASEDAISRGVRRIIAL

TGHQAEQAVQKLDYFVTKIETMMDVIRETLRAGEWKDKMLNREISACMKEMKYSQIPAWRRTLLMRKLLQMKETMDDFEK

SQKLKFVQLITDEARNLQIPDDGIVVHEFPQNATTKALDSALKQLKCKAGIVFSKNPAINKVVCLANVSKECVDNGLTAK

EWVDHLCRVAGGYGGGKDTTAQASLGEELTVDQIKIEAENFAKLKLSLPK

>KRY72749.1 Alanine--tRNA synthetase, cytoplasmic, partial [Trichinella pseudospiralis]

LYTASYCIINNLKRTKMDILLTANAIRSTFINYFLEKRNHEYVHSSSVIPKGDPSLLFTNAGMNQFKPIFLGTADPNSQM

ADWKRVVNSQKCIRAGGKHNDLDDVGKDVYHHTFFEMLGNWSFGDYFKKEICAWAWELLTKQFKIPQEQLYVTYFGGSEK

YRLQPDVECRQIWLDIGLPSEKVLPFGMKDNFWEMGDIGPCGPCSEIHFDRIGNRNAANLVNKDDPDVLEIWNLVFITFD

RDKDGSLHPLPKRHVDCGLGFERLVSVIQGKKSNYDTDLFVPIFNRIAETTNVRPYMGKVGEEDEQQIDMAYRVVADHIR

TLTVALSDGGRPDNCGRGYVLRRILRRGVRYATEKLNARPGFFANLVPVVVEILGDTFPELKDDPDLVMEIINEEESLFL

KTLSRGSRVLRQEIQKTPKENLIPGFVAWRLYASYGFPVDLTQLIAEESGRKVNMEEYEQCRQQAQISSSSKFSQQSDDI

NFNINAISCLLDMGVPLTDDKPKFVYRLSENENDNYEFDELECEVLALRKNGEFVSEVRTGDFCCLICDKTAFYAESGGQ

IYDEGSMSNDKCEFHVRNVQVRSGYVIHFGDVLGTITLGSKLSQNIDQKRRMQVMKNHTATHLLNFALREIMSEVEQKGS

LVLPEKLRFDFTRRTPFELEQLMRIQEIIEDVIKSDAEIFMQEVPLDVAKTIAGLRFMADESYPDPVRVVSIGIPVDALL

DNANRIDGNLTSVEFCGGTHLLRAGHVGKFVIASEDAISRGVRRIIALTGHQAEQAVQKLDYFVTKIETMMDVIRETLRA

GEWKDKMLNREISACMKEMKYSQIPAWRRTLLMRKLLQMKETMDDFEKSQKLKFVQLITDEARNLQIPDDGIVVHEFPQN

ATTKALDSALKQLKCKAGIVFSKNPAINKVVCLANVSKECVDNGLTAKEWVDHLCRVAGGYGGGKDTTAQASLGEELTVD

QIKIEAENFAKLKLSLPK

>KRZ35515.1 Alanine--tRNA synthetase, cytoplasmic, partial [Trichinella pseudospiralis]

LYTASYCIINNLKRYIAFLTVLLFSLIFKHVIICALHYRKNIFYFRTKMDILLTANAIRSTFINYFLEKRNHEYVHSSSV

IPKGDPSLLFTNAGMNQFKPIFLGTADPNSQMADWKRVVNSQKCIRAGGKHNDLDDVGKDVYHHTFFEMLGNWSFGDYFK

KEICAWAWELLTKQFKIPQEQLYVTYFGGSEKYRLQPDVECRQIWLDIGLPSEKVLPFGMKDNFWEMGDIGPCGPCSEIH

FDRIGNRNAANLVNKDDPDVLEIWNLVFITFDRDKDGSLHPLPKRHVDCGLGFERLVSVIQGKKSNYDTDLFVPIFNRIA

ETTNVRPYMGKVGEEDEQQIDMAYRVVADHIRTLTVALSDGGRPDNCGRGYVLRRILRRGVRYATEKLNARPGFFANLVP

VVVEILGDTFPELKDDPNLVMEIINEEESLFLKTLSRGSRVLRQEIQKTPKENLIPGFVAWRLYASYGFPVDLTQLIAEE

SGRKVNMEEYEQCRQQAQISSSSKFSQQSDDINFNINAISCLLDMGVPLTDDKPKFVYRLSENENDNYEFDELECEVLAL

RKNGEFVSEVRTGDFCCLICDKTAFYAESGGQIYDEGSMSNDKCEFHVRNVQVRSGYVIHFGDVLGTITLGSKLSQNIDQ

KRRMQVMKNHTATHLLNFALREIMSEVEQKGSLVLPEKLRFDFTRRTPFELEQLMRIQEIIEDVIKSDAEIFMQEVPLDV

AKTIAGLRFMADESYPDPVRVVSIGIPVDALLDNANRIDGNLTSVEFCGGTHLLRAGHVGKFVIASEDAISRGVRRIIAL

TGHQAEQAVQKLDYFVTKIETMMDVIRETLRAGEWKDKMLNREISACMKEMKYSQIPAWRRTLLMRKLLQMKETMDDFEK

SQKLKFVQLITDEARNLQIPDDGIVVHEFPQNATTKALDSALKQLKCKAGIVFSKNPAINKVVCLANVSKECVDNGLTAK

EWVDHLCRVAGGYGGGKDTTAQASLGEELTVDQIKIEAENFAKLKLSLPK

>KRX69885.1 Alanine--tRNA synthetase, cytoplasmic [Trichinella sp. T6]

MTVLLYTTSCCVINNLKRSKMDMLLTANAIRRTFINYFTEKRNHQYVHSSSVIPKGDPSLLFTNAGMNQFKPIFLGTADP

NSQMASWKRVVNSQKCIRAGGKHNDLDDVGKDVYHHTFFEMLGNWSFGDYFKKEICAWAWEVLTKEFKIPKEQLYVTYFG

GSEKYRLQPDVECRQIWLEIGLPSERVLPFGMKDNFWEMGDIGPCGPCSEIHYDRIGNRNAANLINKNDPDVLEIWNLVF

ITYNRDKDGSLHPLPKRHIDCGLGFERLVSVIQGKKSNYDTDLFVPIFNRIAETAKVRPYMGKVGKEDEQQIDMAYRVVA

DHIRTLTVALSDGGRPDNCGRGYVLRRILRRGVRYATEKLNAPSGFFANLVPVVVETLVEDVHIVIGDTFPELKEDPNSV

MEIINEEESLFLKTLSRGSRVLRQEIEKASKDKLIPGVVAWRLYASYGFPVDLTQLMAEESGYKVNMEEYEECRQQAQMS

SSSKFSQANDVVDFNINAVNYLLNGKVPLTDDKFKYAYRLTENGDDDYEFDEVKCEVLALRRNGEFVSEVCSGDSCCLIC

DKTAFYAESGGQIYDEGSMEGEKCEFRVRNVQARSGYVIHFGEVMGTITRGSKLLQNVDQKRRVQVMKNHTATHLLNFAL

REVLGQVEQKGSLVLMDRLRFDFTCKAPLTVEQLVRIEQIVANIVNSDVEIYMQEVPLGVAKTIAGLRSSADETYPDPVR

VVSVGTPVDALLKDPDGPGAKLASVEFCGGTHLLRTGHLGDFVIASEEAVARGIRRIVALTCHEAEQAVQKLEYFVKRVE

EVMDLIRSSVLSGELDYSFLNREISGCLEVGWLCVEMKASQIPAWRREHLVRKLAQVKATLDDFEKSERMKFVQMITDEA

KNLQIPDDGIVVHEFPPNATAKALDSALKQLKCRAGIVFSQDSKNKRVVCLANVNRECVDKGLTAKAWVDRLCCIAGGYG

GGKETTAQASLGTGLPVDKIKTEAEKFAKCKLSKR

>KRX52310.1 Alanine--tRNA synthetase, cytoplasmic [Trichinella sp. T9]

MTVLLYTTSCCVINNLKRSKMDMLLTANAIRRTFINYFTEKRNHQYVHSSSVIPKGDPSLLFTNAGMNQFKPIFLGTADP

NSQMASWKRVVNSQKCIRAGGKHNDLDDVGKDVYHHTFFEMLGNWSFGDYFKKEICAWAWEVLTKEFKIPKEQLYVTYFG

GSEKCRLQPDVECRQIWLEIGLPSERVLPFGMKDNFWEMGDIGPCGPCSEIHYDRIGNRNAANLINKNDPDVLEIWNLVF

ITYNRDKDGSLHPLPKRHIDCGLGFERLVSVIQGKKSNYDTDLFVPIFNRIAETAKVRPYMGKVGKEDEQQIDMAYRVVA

DHIRTLTVALSDGGRPDNCGRGYVLRRILRRGVRYATEKLNAPSGFFANLVPVVVETLGDMFPELKEDPNSVMEIINEEE

SLFLKTLSRGSRVLRQEIEKASKDKLIPGVVAWRLYASYGFPVDLTQLMAEESGYKVNMEEYEECRQQAQMSSSSKFSQA

NDVVDFNINAVNYLLNGKVPLTDDKPKYAYRLTENGDDDYEFDEVKCEVLALRRNGEFVSEVCSGDSCCLICDKTAFYAE

SGGQIYDEGSMEGEKCEFRVRNVQARSGYVIHFGEVMALVSIVLLHIIYYVVYLFVCLFVSKKRRVQVMKNHTATHLLNF

ALREVLGQVEQKGSLVLMDRLRFDFTCKAPLTVEQLVRIEQIVANIVNSDVEIYMQEVPLGVAKTIAGLRLTADEVIRRR

STVQFFYTALPLQTYPDPVRVVSVGTPVDALLKDPDGPGAKLASVEFCGGTHLLRTGHLGDFVIASEEAVARGIRRIVAL

TCHEAEQAVQKLEYFVKRVEEVMDLIRASVLSGELDYSFLNREISGCSEEMKASQIPAWRREHLVRKLAQVKATLDDFEK

SERMKFVQMITDEAKNLQIPDDGIVVHEFPPNATAKALDSALKQLKCRACIVFSQDSKNKRVVCLANVNRECVDKGLTAK

AWVDRLCCIAGGYGGGKETTAQASLGTGLPVDKIKTEAEKFAKCKLSKR

>KRZ88819.1 Alanine--tRNA synthetase, cytoplasmic [Trichinella sp. T8]

MTVLLYTTSCCVINNLKRSKMDMLLTANAIRRTFINYFTEKRNHRYVHSSSVIPKGDPSLLFTNAGMNQFKPIFLGTADP

NSQMASWMRVVNSQKCIRAGGKHNDLDDVGKDVYHHTFFEMLGNWSFGDYFKKEICAWAWEVLTKEFKIPKEQLYVTYFG

GSEKCRLQPDVECRQIWLEIGLPSERVLPFGMKDNFWEMGDIGPCGPCSEIHYVRIGNRNAANLINKNDPDVLEIWNLVF

ITYNRDKDGSLHPLPKRHIDCGLGFERLVSVIQGKKSNYDTDLFVPIFNRIAETAKVRPYMGKVGKEDEQQIDMAYRVVA

DHIRTLTVALSDGGRPDNCGRGYVLRRILRRGVRYATEKLNAPSGFFANLVPVVVETLVEDVHIVIGDMFPELKEDPNSV

MEIINEEESLFLKTLSRGSRVLRQEIEKASKDKLIPGVVAWRLYASYGFPVDLTQLMAEESGYKVNMEEYEECRQQAQMS

SSSKFSQANDVVDFNINAVNYLLNGKVPLTDDKPKYAYRLTENGDDDYEFDEVKCEVLALRRNGEFVSEVCSGDSCSLIC

DKTAFYAESGGQIYDEGSMEGEKCEFRVRNVQARSGYVIHFGEVMGTITRGSKLFQNVDQKRRVQVMKNHTATHLLNFAL

REVLGQVEQKGSLVLMDRLRFDFTCKAPLTVEQLMRIEQIVANIVNSDVEIYMQEVPLGVAKTIAGLRSTADEVIRRRST

VQFFYTALHLQTYPDPVRVVSVGTPVDALLKDPDGPGAKLASVEFCGGTHLLRTGHLGDFVIASEEAVARGIRRIVALTC

HEAEQAVQKLEYFVKRVEEVMDLIRASVLGGELDYSFLNREISGCLEEMKASQIPAWRREHLVRKLAQVKATLDDFEKSE

RMKFVQMITDEAKNLQIPDDGIVVHEFPPNATAKALDSALKQLKCRACIVFSQDSKNKRVVCLANVNRECVDKGLTAKAW

VDRLCCIAGGYGGGKETTAQASLGTGLPVDKIKTEAEKFAKCKLSKR

>KRZ23207.1 Alanine--tRNA synthetase, cytoplasmic [Trichinella pseudospiralis]

MDILLTANAIRSTFINYFLEKRNHEYVHSSSVIPKGDPSLLFTNAGMNQFKPIFLGTADPNSQMADWKRVVNSQKCIRAG

GKHNDLDDVGKDVYHHTFFEMLGNWSFGDYFKKEICAWAWELLTKQFKIPQEQLYVTYFGGSEKYRLQPDVECRQIWLDI

GLPSEKVLPFGMKDNFWEMGDIGPCGPCSEIHFDRIGNRNAANLVNKDDPDVLEIWNLVFITFDRDKDGSLHPLPKRHVD

CGLGFERLVSVIQGKKSNYDTDLFVPIFNRIAETTNVRPYMGKVGEEDEQQIDMAYRVVADHIRTLTVALSDGGRPDNCG

RGYVLRRILRRGVRYATEKLNARPGFFANLVPVVVEILGDTFPELKDDPNLVMEIINEEESLFLKTLSRGSRVLRQEIQK

TPKENLIPGFVAWRLYASYGFPVDLTQLIAEESGRKVNMEEYEQCRQQAQISSSSKFSQQSDDINFNINAISCLLDMGVP

LTDDKPKFVYRLSENENDNYEFDELECEVLALRKNGEFVSEVRTGDFCCLICDKTAFYAESGGQIYDEGSMSNDKCEFHV

RNVQVRSGYVIHFGDVLGTITLGSKLSQNIDQKRRMQVMKNHTATHLLNFALREIMSEVEQKGSLVLPEKLRFDFTRRTP

FELEQLMRIQEIIEDVIKSDAEIFMQEVPLDVAKTIAGLRFMADESYPDPVRVVSIGIPVDALLDNANRIDGNLTSVEFC

GGTHLLRAGHVGKFVIASEDAISRGVRRIIALTGHQAEQAVQKLDYFVTKIETMMDVIRETLRAGEWKDKMLNREISACM

KEMKYSQIPAWRRTLLMRKLLQMKETMDDFEKSQKLKFVQLITDEARNLQIPDDGIVVHEFPQNATTKALDSALKQLKCK

AGIVFSKNPAINKVVCLANVSKECVDNGLTAKEWVDHLCRVAGGYGGGKDTTAQASLGEELTVDQIKIEAENFAKLKLSL

PK

>XP_003372464.1 putative alanine--tRNA synthetase [Trichinella spiralis]

MDMLLTANAIRQTFINYFTEKRNHQYVHSSSVIPKGDPSLLFTNAGMNQFKPIFLGTADPNSQMASWKRVVNSQKCIRAG

GKHNDLDDVGKDVYHHTFFEMLGNWSFGDYFKKDICAWAWEVLTKEFKIPKEQLYVTYFGGSEKCRLQPDVECRQIWLDM

GLPSERVLPFGMKDNFWEMGDIGPCGPCSEIHYDRIGNRNAANLINKDDPDVLEIWNLVFITYNRDKDGSLHPLPKRHID

CGLGFERLVSVIQGKKSNYDTDLFVPIFNRIAETAKVRPYMGKVGEEDEQQIDMAYRVVADHIRTLTVALSDGGRPDNCG

RGYVLRRILRRGVRYATEKLNAPSGFFANLVPVVVETLVEDGETFPELKEDPNSVMEIINEEESLFLKTLSRGSRVLRQE

IEKASKDKLIPGVVAWRLYASYGFPVDLTQLMAEESGYKVNMEEYEECRQQAQMSSSSKFSQLNDVVDFNINAVNYLLNG

KVPLTDDKPKYAYRLTENGDDDYEFDEVKCEVLALRKNGEFVSEVCSGDSCCLICDKTAFYAESGGQIYDEGSMEGDKSE

FRVRNVQARGGYIVHFGEVMGTITRGSKLFQNVDQKRRVQVMKNHTATHLLNFALREVLGQVEQKGSLVLTDRLRFDFTC

KAPLTVEQLMKIEQIVANIVNSDVEIYMQEVPLGVAKTIAGLRSTADETYPDPVRVVSVGTPVDALLKDPDGPGAKLASV

EFCGGTHLLRTGHLGDFVIASEEAVARGIRRIVALTCHEAEQAVQKLEYFVNKVEEVIDLIRSSVLSGELDYSFLNRKIS

GCLEVGWLCVEMKASQIPAWRREHLVRKLAQVKATLDDFEKGERMKFVQMITDEAKNLQIPDDGIVVHEFPPNATAKALD

TALKQLKCRAGIVFSQDSKNKRVECVDKGLTAKAWVDRLCCVAGGYGGGKETTAQASLGTGLPVDKIKTEAEKFAKCKLS

RR

>KRX42768.1 Alanine--tRNA synthetase, cytoplasmic [Trichinella murrelli]

MTVLLYTTSCCVINNLKRSKMDMLLTANAIRRTFINYFTEKRNHQYVHSSSVIPKGDPSLLFTNAGMNQFKPIFLGTADP

NSQMASWKRVVNSQKCIRAGGKHNDLDDVGKDVYHHTFFEMLGNWSFGDYFKKEICAWAWEVLTKEFKIPKEQLYVTYFG

GSEKCRLQPDVECRQIWLEIGLPSERVLPFGMKDNFWEMGDIGPCGPCSEIHYDRIGNRNAANLINKNDPDVLEIWNLVF

ITYNRDKDGSLHPLPKRHIDCGLGFERLVSVIQGKKSNYDTDLFVPIFNRIAETAKVRPYMGKVGKEDEQQIDMAYRVVA

DHIRTLTVALSDGGRPDNCGRGYVLRRILRRGVRYATEKLNAPSGFFANLVPVVVETLVEDVHIVIGDMFPELKEDPNSV

MEIINEEESLFLKTLSRGSRVLRQEIEKASKDKLIPGVVAWRLYASYGFPVDLTQLMAEESGYKVNMEEYEECRQQAQMS

SSSKFSQANDVVDFNINAVNYLLNGKVPLTDDKPKYAYRLTENGDDDYEFDEVKCEVLALRRNGEFVSEVCSGDSCCLIC

DKTAFYAESGGQIYDEGSMEGEKCEFRVRNVQARSGYVIHFGEVMGTITRGSKLFQNVDQKRRVQVMKNHTATHLLNFAL

REVLGQVEQKGSLVLMDRLRFDFTCKAPLTVEQLMRIEQIVANIVNSDVEIYMQEVPLGVAKTIAGLRSTADEVIRRRST

VQFFYTALHLQTYPDPVRVVSVGTPVDALLKDPDGPGAKLASVEFCGGTHLLRTGHLGDFVIASEEAVARGIRRIVALTC

HEAEQAVQKLEYFVKRVEEVMDLIRASVLSGELDYSFLNREISGCLEVGWLCVEMKASQIPAWRREHLVRKLAQVKATLD

DFEKSERMKFVQMITDEAKNLQIPDDGIVVHEFPPNATAKALDSALKQLKCRACIVFSQDSKNKRVVCLANVNRECVDKG

LTAKAWVDRLCCIAGGYGGGKETTAQASLGTGLPVDKIKTEAEKFAKCKLSKR

>KRY12512.1 Alanine--tRNA synthetase, cytoplasmic [Trichinella patagoniensis]

MTVLLYTTSCCVINNLKRSKMDMLLTANAIRRTFINYFTEKRNHQYVHSSSVIPKGDPSLLFTNAGMNQFKPIFLGTADP

NSQMASWKRVVNSQKCIRAGGKHNDLDDVGKDVYHHTFFEMLGNWSFGDYFKKEICAWAWEVLTKEFKIPKEQLYVTYFG

GSEKCRLQPDVECRQIWLEIGLPSERVLPFGMKDNFWEMGDIGPCGPCSEIHYDRIGNRNAANLINKNDPDVLEIWNLVF

ITYNRDKDGSLHPLPKRHIDCGLGFERLVSVIQGKKSNYDTDLFVPIFNRIAETAKVRPYMGKVGEEDEQQIDMAYRVVA

DHIRTLTVALSDGGRPDNCGRGYVLRRILRRGVRYATEKLNAPSGFFANLVPVVVETLVEDVHIVIGDMFPELKEDPNSV

MEIINEEESLFLKTLSRGSRVLRQEIEKASKDKLIPGVVAWRLYASYGFPVDLTQLMAEESGYKVNMEEYEECRQQAQMS

SSSKFSQANDVVDFNINAVNYLLNGKVPLTDDKPKYAYRLTENGDDDYEFDEVKCEVLALRRNGEFVSEVCSGDSCCLIC

DKTAFYAESGGQIYDEGSMEGERCEFRVRNVQARSGYVIHFGEVMGTITRGSKLFQNVDQKRRVQVMKNHTATHLLNFAL

REVLGQVEQKGSLVLMDRLRFDFTCKAALTVEQLMRIEQIVASIVNSDVEIYMQEVPLGVAKTIAGLRSTADEVIRRRST

VQFFYTALHLQTYPDPVRVVSVGTPVDALLKDPDGPGAKLASVEFCGGTHLLRTGHLGDFVIASEEAVARGIRRIVALTC

HEAEQAVQKLEYFVKRVEEVMDLIRSSVLSGELDYSFLNREISGCLEVGWLCVEMKASQIPAWRREHLVRKLAQVKATLD

DFEKSERMKFVQMITDEAKNLQIPDDGIVVHEFPPNATAKALDSALKQLKCRACIVFSQDSKNKRVVCLANVNRECVDKG

LTAKAWVDRLCCIAGGYGGGKETTAQASLGTGLPVDKIKTEAEKFAKCKLSKR

>KRZ49830.1 Alanine--tRNA synthetase, cytoplasmic [Trichinella nativa]

MFSTIHYLLIALQKIFFYIRSKMDMLLTANAIRRTFINYFTEKRNHQYVHSSSVIPKGDPSLLFTNAGMNQFKPIFLGTA

DPNSQMASWKRVVNSQKCIRAGGKHNDLDDVGKDVYHHTFFEMLGNWSFGDYFKKEICAWAWEVLTKEFKIPKEQLYVTY

FGGSEKCRLQPDVECRQIWLEIGLPSERVLPFGMKDNFWEMGDIGPCGPCSEIHYDRIGNRNAANLINKNDPDVLEIWNL

VFITYNRDKDGSLHPLPKRHIDCGLGFERLVSVIQGKKSNYDTDLFVPIFNRIAETAKVRPYMGKVGKEDEQQIDMAYRV

VADHIRTLTVALSDGGRPDNCGRGYVLRRILRRGVRYATEKLNAPSGFFANLVPVVVETLGDTFPELKEDPNSVMEIINE

EESLFLKTLSRGSRVLRQEIEKASKDKLIPGVVAWRLYASYGFPVDLTQLMAEESGYKVNMEEYEECRQQAQMSSSSKFS

QANDVVDFNINAVNYLLNGKVPLTDDKFKYAYRITENGDDDYEFDEVKCEVLALRRNGEFVSEVCSGDSCCLICDKTAFY

AESGGQIYDEGSMEGEKCEFRVRNVQARSGYVIHFGEVMGTITLGSKLLQNVDQKRRVQVMKNHTATHLLNFALREVLGQ

VEQKGSLVLMDRLRFDFTCKAPLTVEQLVRIEQIVANIVNSDVEIYMQEVPLGVAKTIAGLRSSADEVIRRRSTVQFFYT

ALHLQTYPDPVRVVSVGTPVDALLKDPDGPGAKLASVEFCGGTHLLRTGHLGDFVIASEEAVARGIRRIVALTCHEAEQA

VQKLEYFVKRVEEVMDLIRSSVLSGELDYSFLNREISGCLEVGWLCVEMKASQIPAWRREHLVRKLAQVKATLDDFEKSE

RMKFVQMITDEAKNLQIPDDGIVVHEFPPNATAKALDSALKQLKCRAGIVFSQDSKNKRVVCLANVNRECVDKGLTAKAW

VDRLCCIAGGYGGGKETTAQASLGTGLPVDKIKTEAEKFAKCKLSKR

>KRZ49833.1 Alanine--tRNA synthetase, cytoplasmic [Trichinella nativa]

MFSTIHYLLIALQKIFFYIRSKMDMLLTANAIRRTFINYFTEKRNHQYVHSSSVIPKGDPSLLFTNAGMNQFKPIFLGTA

DPNSQMASWKRVVNSQKCIRAGGKHNDLDDVGKDVYHHTFFEMLGNWSFGDYFKKEICAWAWEVLTKEFKIPKEQLYVTY

FGGSEKCRLQPDVECRQIWLEIGLPSERVLPFGMKDNFWEMGDIGPCGPCSEIHYDRIGNRNAANLINKNDPDVLEIWNL

VFITYNRDKDGSLHPLPKRHIDCGLGFERLVSVIQGKKSNYDTDLFVPIFNRIAETAKVRPYMGKVGKEDEQQIDMAYRV

VADHIRTLTVALSDGGRPDNCGRGYVLRRILRRGVRYATEKLNAPSGFFANLVPVVVETLVEDVHIVIGDTFPELKEDPN

SVMEIINEEESLFLKTLSRGSRVLRQEIEKASKDKLIPGVVAWRLYASYGFPVDLTQLMAEESGYKVNMEEYEECRQQAQ

MSSSSKFSQANDVVDFNINAVNYLLNGKVPLTDDKFKYAYRITENGDDDYEFDEVKCEVLALRRNGEFVSEVCSGDSCCL

ICDKTAFYAESGGQIYDEGSMEGEKCEFRVRNVQARSGYVIHFGEVMGTITLGSKLLQNVDQKRRVQVMKNHTATHLLNF

ALREVLGQVEQKGSLVLMDRLRFDFTCKAPLTVEQLVRIEQIVANIVNSDVEIYMQEVPLGVAKTIAGLRSSADEVIRRR

STVQFFYTALHLQTYPDPVRVVSVGTPVDALLKDPDGPGAKLASVEFCGGTHLLRTGHLGDFVIASEEAVARGIRRIVAL

TCHEAEQAVQKLEYFVKRVEEVMDLIRSSVLSGELDYSFLNREISGCLEEMKASQIPAWRREHLVRKLAQVKATLDDFEK

SERMKFVQMITDEAKNLQIPDDGIVVHEFPPNATAKALDSALKQLKCRAGIVFSQDSKNKRVVCLANVNRECVDKGLTAK

AWVDRLCCIAGGYGGGKETTAQASLGTGLPVDKIKTEAEKFAKCKLSKR

>KRX69887.1 Alanine--tRNA synthetase, cytoplasmic [Trichinella sp. T6]

MTVLLYTTSCCVINNLKRSKMDMLLTANAIRRTFINYFTEKRNHQYVHSSSVIPKGDPSLLFTNAGMNQFKPIFLGTADP

NSQMASWKRVVNSQKCIRAGGKHNDLDDVGKDVYHHTFFEMLGNWSFGDYFKKEICAWAWEVLTKEFKIPKEQLYVTYFG

GSEKYRLQPDVECRQIWLEIGLPSERVLPFGMKDNFWEMGDIGPCGPCSEIHYDRIGNRNAANLINKNDPDVLEIWNLVF

ITYNRDKDGSLHPLPKRHIDCGLGFERLVSVIQGKKSNYDTDLFVPIFNRIAETAKVRPYMGKVGKEDEQQIDMAYRVVA

DHIRTLTVALSDGGRPDNCGRGYVLRRILRRGVRYATEKLNAPSGFFANLVPVVVETLVEDVHIVIGDTFPELKEDPNSV

MEIINEEESLFLKTLSRGSRVLRQEIEKASKDKLIPGVVAWRLYASYGFPVDLTQLMAEESGYKVNMEEYEECRQQAQMS

SSSKFSQANDVVDFNINAVNYLLNGKVPLTDDKFKYAYRLTENGDDDYEFDEVKCEVLALRRNGEFVSEVCSGDSCCLIC

DKTAFYAESGGQIYDEGSMEGEKCEFRVRNVQARSGYVIHFGEVMGTITRGSKLLQNVDQKRRVQVMKNHTATHLLNFAL

REVLGQVEQKGSLVLMDRLRFDFTCKAPLTVEQLVRIEQIVANIVNSDVEIYMQEVPLGVAKTIAGLRSSADEVIRRRST

VQFFYTALHLQTYPDPVRVVSVGTPVDALLKDPDGPGAKLASVEFCGGTHLLRTGHLGDFVIASEEAVARGIRRIVALTC

HEAEQAVQKLEYFVKRVEEVMDLIRSSVLSGELDYSFLNREISGCLEEMKASQIPAWRREHLVRKLAQVKATLDDFEKSE

RMKFVQMITDEAKNLQIPDDGIVVHEFPPNATAKALDSALKQLKCRAGIVFSQDSKNKRVVCLANVNRECVDKGLTAKAW

VDRLCCIAGGYGGGKETTAQASLGTGLPVDKIKTEAEKFAKCKLSKR

>KJH51087.1 alanine--tRNA synthetase [Dictyocaulus viviparus]

MASSFTRAERSGNIFYRVTGLIRSGQLTWSGRPLWYDVYIAHPPLQAHDWNVKHAKYDEPVRKIFYEEDIIRAAFYKKYR

GGVINIESSRESVSQQFIKEYNIIKQKNEGKDDLTEDEIFKMTEERMANIGYSNKTSMKYLTANEVRSTFIDFFKQKKGH

SYVHSSSVIPHDDPTLLFANAGMNQFKPLFLGTADPNSDLAKLKRAVNTQKCIRAGGKHNDLDDVGKDVYHHTFFEMLGN

WSFGDYFKKEVITWAWELLTEIYCIPGERLYVSYFGGDKNAGVPADNETKNIWLSLGHDNFWEMGDVGPCGPCSEIHYDR

VGGRNAAHLVNADDPMVVEIWNLVFIQCNREADGTLRYLPAKHIDCGLGLERLIAVIQGKVSNYDTDIFQPIFKAIQEGT

GIREYTGKVGVDDVDGVDMAYRVVADHIRTLTVAISDGGRPDNTGRGYVLRRILRRGVRYAAEKLRAKPGFFASLVPVVV

GLLKETFPELIHDPETVQDIINDEEKQFLKTLSRGRTLFHRAISELEADKKTFPGDVAWRLYDTYGFPADLTQLMAEENG

LSVDQIAFEECRRKAVVRHYEVSAAGVGKFRDSVDLDVHAIAELQERGVPTTDDSFKYNYTADNNTEGNVLYTFSTCIGK

IVAIRSGGRFVDSIEHGVEGSLLLDKTNFYAEQGGQIYDTGVVSKLNDEGTEFVVSNCQVRGGYVTLIGSTNRKLSVGDS

VKQMFDEDRRLLIMKNHTGTHVLNHALRCVLTDSDQKGSLVAPDRLRFDFTNKQAMTVQQVRRAEEIVQAVINTSQPVFA

KENYPDPVRVVSVGVPVDQLLDEPESEVGLKTSVEFCGGTHLHNVGHIGFFVISSEEAIAKGIRRIVALTGPEAKRAIHR

AERLAARVQAIADSVDVYSEVDMGLGKLRDISKRVQELIDVWSFLCCVRFVFSLWNKIKIIYFFKEVNGALLPYWQKDKI

REQGKAIQKTLEGYKKMADAVVAEKIMSEAKQLAQNNKEDVVVHVFSSGASSKALDNALKQFKNSKAVMGFSANEENGKV

LVLAKVDKSLIDEGLKANDWINEVCLVLGGRGGGKDANAQATGENISRLEEAVSLARKFAQSTIS

>KRZ88818.1 Alanine--tRNA synthetase, cytoplasmic [Trichinella sp. T8]

MTVLLYTTSCCVINNLKRSKMDMLLTANAIRRTFINYFTEKRNHRYVHSSSVIPKGDPSLLFTNAGMNQFKPIFLGTADP

NSQMASWMRVVNSQKCIRAGGKHNDLDDVGKDVYHHTFFEMLGNWSFGDYFKKEICAWAWEVLTKEFKIPKEQLYVTYFG

GSEKCRLQPDVECRQIWLEIGLPSERVLPFGMKDNFWEMGDIGPCGPCSEIHYVRIGNRNAANLINKNDPDVLEIWNLVF

ITYNRDKDGSLHPLPKRHIDCGLGFERLVSVIQGKKSNYDTDLFVPIFNRIAETAKVRPYMGKVGKEDEQQIDMAYRVVA

DHIRTLTVALSDGGRPDNCGRGYVLRRILRRGVRYATEKLNAPSGFFANLVPVVVETLVEDVHIVIGDMFPELKEDPNSV

MEIINEEESLFLKTLSRGSRVLRQEIEKASKDKLIPGVVAWRLYASYGFPVDLTQLMAEESGYKVNMEEYEECRQQAQMS

SSSKFSQANDVVDFNINAVNYLLNGKVPLTDDKPKYAYRLTENGDDDYEFDEVKCEVLALRRNGEFVSEVCSGDSCSLIC

DKTAFYAESGGQIYDEGSMEGEKCEFRVRNVQARSGYVIHFGEVMGTITRGSKLFQNVDQKRRVQVMKNHTATHLLNFAL

REVLGQVEQKGSLVLMDRLRFDFTCKAPLTVEQLMRIEQIVANIVNSDVEIYMQEVPLGVAKTIAGLRSTADEVIRRRST

VQFFYTALHLQTYPDPVRVVSVGTPVDALLKDPDGPGAKLASVEFCGGTHLLRTGHLGDFVIASEEAVARGIRRIVALTC

HEAEQAVQKLEYFVKRVEEVMDLIRASVLGGELDYSFLNREISGCLEVGWLCVEMKASQIPAWRREHLVRKLAQVKATLD

DFEKSERMKFVQMITDEAKNLQIPDDGIVVHEFPPNATAKALDSALKQLKCRACIVFSQDSKNKRVVCLANVNRECVDKG

LTAKAWVDRLCCIAGGYGGGKETTAQASLGTGLPVDKIKTEAEKFAKCKLSKR

>KRZ35516.1 Alanine--tRNA synthetase, cytoplasmic, partial [Trichinella pseudospiralis]

LYTASYCIINNLKRYIAFLTVLLFSLIFKHVIICALHYRKNIFYFRTKMDILLTANAIRSTFINYFLEKRNHEYVHSSSV

IPKGDPSLLFTNAGMNQFKPIFLGTADPNSQMADWKRVVNSQKCIRAGGKHNDLDDVGKDVYHHTFFEMLGNWSFGDYFK

KEICAWAWELLTKQFKIPQEQLYVTYFGGSEKYRLQPDVECRQIWLDIGLPSEKVLPFGMKDNFWEMGDIGPCGPCSEIH

FDRIGNRNAANLVNKDDPDVLEIWNLVFITFDRDKDGSLHPLPKRHVDCGLGFERLVSVIQGKKSNYDTDLFVPIFNRIA

ETTNVRPYMGKVGEEDEQQIDMAYRVVADHIRTLTVALSDGGRPDNCGRGYVLRRILRRGVRYATEKLNARPGFFANLVP

VVVEILGDTFPELKDDPNLVMEIINEEESLFLKTLSRGSRVLRQEIQKTPKENLIPGFVAWRLYASYGFPVDLTQLIAEE

SGRKVNMEEYEQCRQQAQISSSSKFSQQSDDINFNINAISCLLDMGVPLTDDKPKFVYRLSENENDNYEFDELECEVLAL

RKNGEFVSEVRTGDFCCLICDKTAFYAESGGQIYDEGSMSNDKCEFHVRNVQVRSGYVIHFGDVLGTITLGSKLSQNIDQ

VMKNHTATHLLNFALREIMSEVEQKGSLVLPEKLRFDFTRRTPFELEQLMRIQEIIEDVIKSDAEIFMQEVPLDVAKTIA

GLRFMADESYPDPVRVVSIGIPVDALLDNANRIDGNLTSVEFCGGTHLLRAGHVGKFVIASEDAISRGVRRIIALTGHQA

EQAVQKLDYFVTKIETMMDVIRETLRAGEWKDKMLNREISACMKEMKYSQIPAWRRTLLMRKLLQMKETMDDFEKSQKLK

FVQLITDEARNLQIPDDGIVVHEFPQNATTKALDSALKQLKCKAGIVFSKNPAINKVVCLANVSKECVDNGLTAKEWVDH

LCRVAGGYGGGKDTTAQASLGEELTVDQIKIEAENFAKLKLSLPK

>KRZ49831.1 Alanine--tRNA synthetase, cytoplasmic [Trichinella nativa]

MFSTIHYLLIALQKIFFYIRSKMDMLLTANAIRRTFINYFTEKRNHQYVHSSSVIPKGDPSLLFTNAGMNQFKPIFLGTA

DPNSQMASWKRVVNSQKCIRAGGKHNDLDDVGKDVYHHTFFEMLGNWSFGDYFKKEICAWAWEVLTKEFKIPKEQLYVTY

FGGSEKCRLQPDVECRQIWLEIGLPSERVLPFGMKDNFWEMGDIGPCGPCSEIHYDRIGNRNAANLINKNDPDVLEIWNL

VFITYNRDKDGSLHPLPKRHIDCGLGFERLVSVIQGKKSNYDTDLFVPIFNRIAETAKVRPYMGKVGKEDEQQIDMAYRV

VADHIRTLTVALSDGGRPDNCGRGYVLRRILRRGVRYATEKLNAPSGFFANLVPVVVETLVEDVHIVIGDTFPELKEDPN

SVMEIINEEESLFLKTLSRGSRVLRQEIEKASKDKLIPGVVAWRLYASYGFPVDLTQLMAEESGYKVNMEEYEECRQQAQ

MSSSSKFSQANDVVDFNINAVNYLLNGKVPLTDDKFKYAYRITENGDDDYEFDEVKCEVLALRRNGEFVSEVCSGDSCCL

ICDKTAFYAESGGQIYDEGSMEGEKCEFRVRNVQARSGYVIHFGEVMGTITLGSKLLQNVDQKRRVQVMKNHTATHLLNF

ALREVLGQVEQKGSLVLMDRLRFDFTCKAPLTVEQLVRIEQIVANIVNSDVEIYMQEVPLGVAKTIAGLRSSADEVIRRR

STVQFFYTALHLQTYPDPVRVVSVGTPVDALLKDPDGPGAKLASVEFCGGTHLLRTGHLGDFVIASEEAVARGIRRIVAL

TCHEAEQAVQKLEYFVKRVEEVMDLIRSSVLSGELDYSFLNREISGCLEVGWLCVEMKASQIPAWRREHLVRKLAQVKAT

LDDFEKSERMKFVQMITDEAKNLQIPDDGIVVHEFPPNATAKALDSALKQLKCRAGIVFSQDSKNKRVVCLANVNRECVD

KGLTAKAWVDRLCCIAGGYGGGKETTAQASLGTGLPVDKIKTEAEKFAKCKLSKR

>KRZ35517.1 Alanine--tRNA synthetase, cytoplasmic, partial [Trichinella pseudospiralis]

LYTASYCIINNLKRYIAFLTVLLFSLIFKHVIICALHYRKNIFYFRTKMDILLTANAIRSTFINYFLEKRNHEYVHSSSV

IPKGDPSLLFTNAGMNQFKPIFLGTADPNSQMADWKRVVNSQKCIRAGGKHNDLDDVGKDVYHHTFFEMLGNWSFGDYFK

KEICAWAWELLTKQFKIPQEQLYVTYFGGSEKYRLQPDVECRQIWLDIGLPSEKVLPFGMKDNFWEMGDIGPCGPCSEIH

FDRIGNRNAANLVNKDDPDVLEIWNLVFITFDRDKDGSLHPLPKRHVDCGLGFERLVSVIQGKKSNYDTDLFVPIFNRIA

ETTNVRPYMGKVGEEDEQQIDMAYRVVADHIRTLTVALSDGGRPDNCGRGYVLRRILRRGVRYATEKLNARPGFFANLVP

VVVEILGDTFPELKDDPNLVMEIINEEESLFLKTLSRGSRVLRQEIQKTPKENLIPGFVAWRLYASYGFPVDLTQLIAEE

SGRKVNMEEYEQCRQQAQISSSSKFSQQSDDINFNINAISCLLDMGVPLTDDKPKFVYRLSENENDNYEFDELECEVLAL

RKNGEFVSEVRTGDFCCLICDKTAFYAESGGQIYDEGSMSNDKCEFHVRNVQVRSGYVIHFGDVLGTITLGSKLSQNIDQ

KRRMQVMKNHTATHLLNFALREIMSEVEQKGSLVLPEKLRFDFTRRTPFELEQLMRIQEIIEDVIKSDAEIFMQEVPLDV

AKTIAGLRFMADESYPDPVRVVSIGIPVDALLDNANRIDGNLTSVEFCGGTHLLRAGHVGKFVIASEDAISRGVRRIIAL

TGHQAEQAVQKLDYFVTKIETMMDVIRETLRAGEWKDKMLNREISACMKVAWRRTLLMRKLLQMKETMDDFEKSQKLKFV

QLITDEARNLQIPDDGIVVHEFPQNATTKALDSALKQLKCKAGIVFSKNPAINKVVCLANVSKECVDNGLTAKEWVDHLC

RVAGGYGGGKDTTAQASLGEELTVDQIKIEAENFAKLKLSLPK

>KRZ23210.1 Alanine--tRNA synthetase, cytoplasmic [Trichinella pseudospiralis]

MDILLTANAIRSTFINYFLEKRNHEYVHSSSVIPKGDPSLLFTNAGMNQFKPIFLGTADPNSQMADWKRVVNSQKCIRAG

GKHNDLDDVGKDVYHHTFFEMLGNWSFGDYFKKEICAWAWELLTKQFKIPQEQLYVTYFGGSEKYRLQPDVECRQIWLDI

GLPSEKVLPFGMKDNFWEMGDIGPCGPCSEIHFDRIGNRNAANLVNKDDPDVLEIWNLVFITFDRFYLRFIIDYHDFYYM

ILFRDKDGSLHPLPKRHVDCGLGFERLVSVIQGKKSNYDTDLFVPIFNRIAETTNVRPYMGKVGEEDEQQIDMAYRVVAD

HIRTLTVALSDGGRPDNCGRGYVLRRILRRGVRYATEKLNARPGFFANLVPVVVEILGDTFPELKDDPNLVMEIINEEES

LFLKTLSRGSRVLRQEIQKTPKENLIPGFVAWRLYASYGFPVDLTQLIAEESGRKVNMEEYEQCRQQAQISSSSKFSQQS

DDINFNINAISCLLDMGVPLTDDKPKFVYRLSENENDNYEFDELECEVLALRKNGEFVSEVRTGDFCCLICDKTAFYAES

GGQIYDEGSMSNDKCEFHVRNVQVRSGYVIHFGDVLGTITLGSKLSQNIDQKRRMQVMKNHTATHLLNFALREIMSEVEQ

KGSLVLPEKLRFDFTRRTPFELEQLMRIQEIIEDVIKSDAEIFMQEVPLDVAKTIAGLRFMADESYPDPVRVVSIGIPVD

ALLDNANRIDGNLTSVEFCGGTHLLRAGHVGKFVIASEDAISRGVRRIIALTGHQAEQAVQKLDYFVTKIETMMDVIRET

LRAGEWKDKMLNREISACMKEMKYSQIPAWRRTLLMRKLLQMKETMDDFEKSQKLKFVQLITDEARNLQIPDDGIVVHEF

PQNATTKALDSALKQLKCKAGIVFSKNPAINKVVCLANVSKECVDNGLTAKEWVDHLCRVAGGYGGGKDTTAQASLGEEL

TVDQIKIEAENFAKLKLSLPK

>CDS37141.1 alanyl tRNA synthetase cytoplasmic [Echinococcus multilocularis]

MENGCDAADIRSQFIEFFKSKGHKYVHSSSVIPRNDPTLLFTNAGMNQWKPIIQGTVDPNTEMATYKRVVNSQKCIRAGG

KHNDLDDVGRDVYHHTFFEMLGNWSFGDYFKREACTWAWELLTKVWKLPEDRFYITYFGGNKKTELESDEEVRQIWIDLG

IPSERILPFGMKDNFWEMGETGPCGPCSEIHYDRIGGRDASKYVNMDDPDVLEIWNLVFMQYNREEDGSLRPLPAKHVDT

GMGLERIVSVLQNRRSNYDTNLFLPYFEIIHKATGVRPYAGKLGEEDEGNLDFAYRVLADHARNLTIALSDGGQISNQGR

GYVLRRILRRAIRYSIEVIGAKPGFFSSLVDVVVASLGDAFPEVKRDPATVKELINEEEQQFLLTLRRGQRLLQREVERL

KKSRVTTLPGAVAWRLYDTYGFPLDLTQVMTSEVGIEVDVEGYEKAKEEALLKSQGTFGTDVFGIDLDVHDIATLQNREV

PLTDDSLKYEYRYNSKRHKYVFSEYMASILAIRTAEGFVESVEGAGTVCGLVLDRTIFYAEAGGQAEDHGFIVSEGNDAN

ELSVCGVRNKGGFVLHLGRLEGSLSRGENVRMGLDTVRRVGLMRNHTATHVLNFALRRLLGEADQKGSLVAPDRLRFDFS

TKHPLTKKQVREIEVISQKMIGSAQPIYAKDVHLADAKAIQGLRAVFGEVYPDPVRVVSVGVPVEKLIAFKSSDKAAKTS

VELCGGTHVTNVAHIGKFVIVSEEAVAKGIRRIVAVTGPEGERAEKQAKHLRAEVAKHIGAIKRAISNKQVDLRTCRQLA

AKLSAVAESVDAAQIGVSPREAMRAAINVAKKRLDDFDKAYKAEMASRVMEEAKNLCHGHTGDFIVHVFNAGSNAKVLNT

ALKEIEKALPSTAVMALSLDAEDGKVLCLTQVPKALVTRGLKANSWVGCVSSLISGKGGGRETSAQASGSSNGVSVAQVV

AAATEFVKANLK

>XP_019967638.1 PREDICTED: alanine--tRNA synthetase, cytoplasmic-like [Paralichthys olivaceus]

MCSYVLRRILRRAVRYSHEKLGAKKGFFASLVDVVVTSLGNAFPELKKDPEMVKDVINEEEEQFLKTLSRGRRILDRKIM

SLGESKTIPGDTAWLLYDTYGFPLDLTSLIAEEKGMAVDLAAFEEVKKAAQLKSQGKGAGDEDHIMLDIYAIDELRNKKI

PATDDSLKYKYTSDDSGNYEFQQASATVLALRRDRAFCDEVTTGQECGVLLDQTSFYAEQGGQTFDEGFMLREDDNAEDR

MEFTVKNTQVRGGYVLHVGTVYGTLKVGDSVVLHVDEARRKPIMSNHTATHILNFALRGVLGEADQRGSLVAADRLRFDF

TAKGALSTGEVRRTEEIACAMIRDAKPVYAMDTPLAEAKAIQGLRAVFDETYPDPVRVVSIGIPVQDLLDEPTSAAGSLT

SIEFCGGTHLQNSGHAAPFVIVSEEAIAKGIRRIVAVTGAEAQKAQRKADALLQSLSALGDKVKQQTSPNKDVQKEIADM

TESIGTAVISQWQKDEMRETLKGLKKTMDDLDRNYKADIQKRVLEKTKEVIESSPNQPLLIMEMETGASAKALNESLKLL

KSNSPQTAAMLFTVDPEAGRITCLCQVPQDVANRGLKASEWVQEVCPLLEGKGGGKDMSAQATGRNTQCLQEALQMANEF

ARLKLGEN

>XP_024353978.1 Alanyl-tRNA synthetase, cytoplasmic [Echinococcus granulosus]

MENGCDAADIRSQFIEFFKSKGHKYVHSSSVIPRNDPTLLFTNAGMNQWKPIIQGTVDPNTEMATYKRVVNSQKCIRAGG

KHNDLDDVGRDVYHHTFFEMLGNWSFGDYFKREACTWAWELLTKVWKLPEDRFYITYFGGDKKTKLESDEEVRQIWIDLG

IPSERILPFGMKDNFWEMGETGPCGPCSEIHYDRIGGRDASKYVNMGDPDVLEIWNLVFMQYNREEDGSLRPLPAKHVDT

GMGLERIVSVLQNRRSNYDTNLFLPYFEIIHKATGVRPYAGKLGEEDEGNLDFAYRVLADHARNLTIALSDGGQISNQGR

GYVLRRILRRAIRYSIEVIGAKPGFFSSLVDVVVASLGDAFPEVKRDPAAVKALINEEEQQFLLTLRRGQRLLQREVERL

KKSRVTTLPGAVAWRLYDTYGFPLDLTQVMTSEVGIEVDVEGYEKAKEEALLKSQGTFGTDVFGIDLDVHDIAALQNREV

PLTDDSLKYEYRYNSKRHKYVFSEYMASILAIRTAEGFVESVEGAGTVCGLVLDRTIFYAEAGGQAEDHGFIVSEGNDAN

ELSVFGVRNKGGFVLHLGRLEGSLSRGENVRMGLDTVRRVGLMRNHTATHVLNFALRRLLGEADQKGSLVAPDRLRFDFS

TKHPLTKKQVRETEVISQKMIGSAQPIYAKDVHLADAKAIQGLRAVFGEVYPDPVRVVSVGVPVEKLIAFKSSDKAAKTS

VELCGGTHVTNVAHIGKFVIVSEEAIAKGIRRIVAVTGPEGERAEKQAKHLRAEVAKHTGAIERAISNKQVDLRTCRQLA

AKLSAVAESVDAAQIGVSPREAMRAAINVAKKRLDDFDKAFKAEMASRVMEEAKNLCHGHTGDFIVHVFNAGSNAKVLNT

ALKEIEKALPSTAVMALSLDAEDGEVLCLTQVPKALVTRGLKANSWVGCVSSLISGKGGGRETSAQASGSSNGVSVAQVV

AAATEFVKANLK

>XP_019501956.1 PREDICTED: alanine--tRNA synthetase, cytoplasmic, partial [Hipposideros armiger]

SNYAYFSYRQLKSQGKGAGGEDHIMLDIYAIEELREKSMEATDDSPKYNYHWDSSGSYAFESAVATVMALRRDKMFVQEV

STGQECGVVLDKTCFYAEQGGQIYDEGYLVKVDDSSEDKTEFTVKNTQVRGGYVLHIGTIYGNLKVGDQVRLFIDEPRRR

PVMSNHTATHILNFALRSVLGEADQRGSLVAPDRLRFDFTAKGAMTTQQIKKAEEIVNEMIEAAKPVYTQDCPLAAAKAI

QGLRAVFDETYPDPVRVVSIGVPVSELLDDPSGPAGSLTSVEFCGGTHLQNSSHAGAFVIVNEEAIAKGIRRIVAVTGAE

AQKALRKAESLKKSLSVMEAKVKAQTAPNKDVQKEIADLGEALATAVIPQWQKDEFRENLKSLKKIMDDLDRASKADVQK

RVLEKTKQLIDSNPNQPLVILEMESGASAKALNEALKLFKTHSPQTSAMLFTVDNEAGKITCLCQVPQNAANRGLKASEW

VQQVSGLMDGKGGGKDVSAQATGKNVGCLQEALQLATSFAQLRLGDVKN

>ORX99184.1 alanyl-tRNA synthetase [Basidiobolus meristosporus CBS 931.73]

MPSDNIEWSASKVRSSYIEFFQNYGHKFVPSSSTIPHDDPTLLFANAGMNQYKPIFLGTVDPASDFAKLTRAVNTQKCIR

AGGKHNDLEDVGKDNYHHTFFEMLGNWSFGDYFKETAIHMAWELLTKVYNLPVDRLYVTYFGGDKEQGLEADLEARDIWL

ATGINPAHVLPFGCKENFWEMGDQGPCGPCSEIHFDRIGGRDAASLVNLDDPTVLEIWNLVFIQYNRESDKSLRPLPNKH

VDTGMGLERVVSVLQNKMSNYDTDVFTPIFEAIQQLTGARPYTGKLGAEDVDGIDMAYRVIADHIRTLTFAISDGGVPSN

EGRGYVLRRILRRGARYARRKFNVQIGTFFSSLIDVVIREMGEAFPEITKRVDYVKEILNEEEESFSKTLDRGERLFETY

LKKAQESNSKFLSGADVWRLYDTYGFPVDLTRLMAEENGLEVDEEEFLKQEAAAKAASKSSRGVTSSGEVIALDVHAISE

VEKNPDVPKTDDSYKYATDDITATVKAIYHNKAFGNEMSTAEQSFGVILDKTNFYSEQGGQEYDLGTIVIDGQAEFVVES

VQVFGGYVLHIGYLKYGKLAVGDEVLCSFDKVRRWSLRNNHTGTHILNFALRKVLDDTVDQKGSLVAQEKLRFDFSYKNA

ITPKQITEIEEICNDFINKNMKVYYKDVPLHVAKAINGLRAVFGEVYPDPVRVVSVGYDVNDIIEDVANPKWSETSIEFC

GGTHVAQTGDIKSFVIVEESAISKGIRRIVAVTGKEAQQAHELANQFEVKLEGLKKLDGAELDTALKVVSKELDTLAISL

GRKASFRETFSAIKKQFDDADKAQKAAQVKQAVDTVKECIEENPDKEFIVKVLDVGSNGKALSSAIQHVKGLKTKAAYLI

SVDAAGGRVAHQCIVPKSLVGKGLKAIEWANVVSEKLGGKKGGKDESAQGQGTNVDSVDEAVSIAEEFARMKLNA

>PRD26757.1 Alanine--tRNA synthetase- cytoplasmic [Nephila clavipes]

MLHCHILISQFFIQREICHWSWELLTEVFKLDGERLYVTYFGGNAKLGLEPDNECKEIWKEVGLPEKRILPFGMKDNFWE

MGDTGPCGPCSEIHYDRIGGRDASKLVNADVPDVLEIWNLVFIQFNRELDGSLKPLPKKHVDTGMGLERLVSVIQDKSSN

YDTDLFVPIFEAIQSSTSAPPYTGKVGADDVTGIDMAYRVLADHARTLTVALADGGRPDSTGRGYVLRRILRRAVRYASE

KLGAKPGVFANLVPVVVNILGEIFPELSKDPQTIMEIINDEEAQFLKTLNRGRVVLERAMKKLESNILPGSIAWRLHDTY

GFPIDLTYLMVEEKGMYIDLMGYEAAKREAQLLSQAKSEGVIDDLKLDVHAISELQEQKVPPTDDMPKYAYQFESCTATI

LALRFQKEFVDQVGGLQNCGIILDRTSFYAESGGQMCDVGLMVKENDEDTEVSIKEVQLQGGYVVHIGTTCNDLRVGDKM

KLLINEERRKLIMNNHTGTHVLNFALRKALATDCDQKGSLVAPDRLRFDFTNKGAMSVAQVKEAEMVANELISKNEEVYA

KEAPLPNAKAVQGLRAVFGEVYPDPVRVVSIGISVEDLLSDPTGPGGTVTSVEFCGGTHLKRSGHIGDFIIASEEAIAKG

IRRVVALTGPEATKAIKRSELFQKEVENIKNLIDDKNRNITEKEIVKKIVELTEEISQSNISYWKKDEMRNNLKNLKKKL

DDLDRANKAAIVQTITEEAKKLFEETSKDKNIQYVVHEFKAGFNAKALDAALKQAKLLSPDTAVMLFTKDTENEKILCMS

SVPQSVTSKGLKAYEWVQQVTEVIGGKGGGKPESAQATGTNTTAVSEAMNLAKKFAELKLS

>CDJ84213.1 Alanyl-tRNA synthetase and Threonyl alanyl tRNA synthetase and Phosphoesterase domain containing protein [Haemonchus contortus]

MIGDNRLEPYSSRSTVEMKQLTANEVRSTFIEFFKQKKAHTYVHSSSVIPHDDPTLLFANAGMNQFKPLFLGIADPNSDL

AKLKRAVNTQKCIRAGGKHNDLDDVGKDVYHHTFFEMLGNWSFGDYFKKEVITWAWELLTEVYEIPADRLYVSYFGGDTK

SGIPADEEARSIWLSLGLPDSRILPFGMKDNFWEMGDVGPCGPCSEIHFDRIGGRDASHLVNADDPMVVEIWNLVFIQFN

REESGKTSNYDTDMFQPIFKAIQEGTGTRPYTGKVGADDIDGIDMAYRVVADHIRTLTIALSDGGRPDNTGRGYVLRRVL

RRGVRYATEKLNAQPEFFASLVPVAIEILGDTFPELRRDPETVRDIINDEEQQFLKTLVRGRRLFQRAVAGLGADEKTFP

GDVAWRLYDTYGFPADLTQLMAEEKGLTVDQKAFEECRKKAVELSAAGAGKFRDTLDLNVHAIAELQKQGIPTTDDSFKY

RYKADGPKDGTAKYTFETCTGKILAIRHDGKFVDSIEAGVEGALILDKTNFYAEQVRGGYVVLVGSTNGVLRVGDSVEQM

FDGERRALIMKNHTATHVLNHALRCVLTDSDQKGSLVAPDRLRFDFTNKQAMTVKQVKEAEEIVQSIINTREPVYAKEAP

LPQAREISGLRAMFDENYPDPVRVVCVGVPVEELLANPKSGAGLKTTVEFCGGTHLHNVGHIGHMVISSEEAIAKGIRRI

VALSGPEAERAIHRADRLAARVKAISEEISANKNIAMDSEKFKAKSKQVQELIDEVNGAQLPYWRKDEIRDQGKAAQRTL

DGYKKQADAAVADKVLSEAKELAQNSNEDVVVHVFAPGASSKALDNALKQLKSTKAVMGFSVNEDSGKVLVLAKVDKSLV

DGGLKANLWVNEVCTVLGGRGGGKDANAQATCENVERLDEAVELALKFAQTTIA

>XP_002734240.1 PREDICTED: alanine--tRNA synthetase, cytoplasmic-like [Saccoglossus kowalevskii]

MSINFKQSSVLSRQLCFCLKNASIAPNVVNRCFHRTHVDCRSTTSTKSNILTSGEIRRKFIDYFKEKQSHLFVPSSSVIP

REDKTLLFTNAGMNQFKSIFQGAVDPSSPIVKYRRVVNSQKCIRAGGKHNDLDDVGKDVYHHTFFEMLGNWSFGDYFKKE

TCEMAWDLLINVYGLSSNRLYVTYFGGNQQLGLSPDIECRNIWKNIGVAEDRILPYGMNENFWEMGTIGPCGMCSEIHYD

RIGNRNATSLINKDDPSVLELWNLVFIQYNREEDGCLSKLPQHNIDTGMGLERLVSIIQGVDSNYDTDLFQPLIQEIQKC

TSMPPYQGLVGDSDNENIDMAYRVLADHIRTLTIAITDGARPGNFKQSYIIKRLIRRAVRYATEKLNASPGQFASLVPIV

VDTLGDAYPELLTNPQQVIDVINREEDLFLKQLKKGRKFLDKKISKLKEPRILPGDIAWKMYDGFGFPIDLTILMCNEKG

VSVDMEGYEEVKEKTHQITSQGGIKNQLEYGFDTNALQQLKHQNISTTDDSYKYSYECDDDGNYEFEVITAKITSLRVGG

QFVESVEPDQSCDVILHQTCFYAEQGGQLWDDGYFTKLGDEAQQIVFEVDNVQIYGGYVVHSGTTKKTLSVGEQLKLHIN

EERRLGLMRNHTAAHILNWSLRHLHGDGIEQRGSLVAPDRLRFDFTYEDSLTSLQVKEIQDQVNEVIEQYTPVDSQEVEL

DEAKQIEGLRAVFGEVYPDPVQVVSVGIPVTGLLTNPFGPGGFMSPVELCGGTHVHMTEHIGKLVIIDEGKVSSGVRRIT

AITGSDGWEADRKADDLLVKVSELKKQIEESIENLTPSLRVLFDEANNLLEVVGASPIPLWRSRELKEELFKLKEDIKSF

DKKRKKMAVKEAISQAADMANDRLDRPFIVKEIHTGSNTKALMQIMDIFKNTTPSSAILLYGVDKDDCTITCVCYTPKHA

IEQGLKANGWLQHVNSLVDGKVGGQDTMGRLQAKNVNLEYLGKLKEIAEQYAKSIL

>XP_021881201.1 alanyl-tRNA synthetase [Lobosporangium transversale]

MTFRPDYWTANKVRETFISFFRDQHQHTFVPSSSTIPHDDPTLLFANAGMNQYKPIFLGTIDPNTEFGKLTRAVNSQKCI

RAGGKHNDLEDVGKDTYHHTYFEMLGNWSFGDYFKKEAIDMAWTLLTEVYGLPKDRLYVTYFGGDEKQGLSPDLEAKELW

LQVGVPENRILPGSAKDNFWEMGEQGPCGPCSEIHFDRIGGRDVAHLVNEDDPNVIEVWNLVFMSFNRESDGSLRPLPNK

HIDTGMGFERLVSILQNKSSNYDTDVFQPIFRAIQNVTGARPYTGLLGAEDKDGIDMAYRVIADHVRTLTFSISDGGVPS

NEGRGYVLRRILRRGSRYARRKFGVQIGSFFSSLVDTIVSEFGDSFPEIRSRIDDVKEILNEEEEAFSRTLDRGEKLFDG

YLQKAIDAKATYLSGADVWRLYDTYGFPVDLTRLMAEENGISVNEEEFSKQQEAAKALSRAGKGSGAGAKEVVAFDVHDI

ASVEAKETVPKTDDSFKYQSGNVNAHIKLIYRNHEFPEDTSAIEDQKSFGILLDRTNMYAEQGGQEYDTGSITVDGKAEF

VVENVQVYGGYVLHTGYLKYGTLHVGDEVVVQFDELRRWPIRNNHTGTHILNFALREILGNIVDQKGSLVAPEKLRFDFT

SKNGMTPQQIHEADLMASDFIKRNLVVYSQEVPLNIARQITGLRAVFGEVYPDPVRVVSIGYDVNEILKDVTNEKWAKTS

VEFCGGTHVAKTADIKHFVIIEESGIAKGIRRIVAVTGEEAYQAERVANEFEQKLNHVGSGALSSSALEQQLKVLAKELD

EIVISAYRKSIFKERFAGIKKAFDDADKARKAKQVKDAVEQCKQHFETDPKSKVFVALLDVGSNAKATAAVVTHVKAQLK

DKAAYVFSVDETNGKVSHACVVGQDILNSGNDLKATEWADVVVKIVGGRKGGKDDSAQGAGEHADKVQEAIDEARKYAES

KLN

>OZJ04024.1 hypothetical protein BZG36_03767 [Bifiguratus adelaidae]

MSDSEDDVPLAKRAFNVAAGSNKGTPALSNGGDTSDNDSDMPLSARIKSKETIADDDSDEDMPLAKRLPLKMETTSESEV

NGSANSKRRAQESDSDSDEPLSKRVPQPPKRKKTGAENGSVHVKKEDMKAKKATKPQEASQAHSRELSIKKEIPEQKPSK

SKKTQKEEEDEEHFKWWEQQENLDSDGSTKWETLEHAGVLFPPEYQPHGVKMKYDGKPITLTPEAEEVASFFAALLGSDH

AQNPTFCKNFFADWQQILRKSPQNPVITDFKKCDFTPIYEYFEKEKGRKKQMTKQEKQEIKEAKAKQEEKFAYAIVDGRK

EKVGNFRIEPPGLFRGRGEHPKTGCLKTRVKPEQITLNLGENAPIPAPPPGHKWGGIVHDQTATWLATWKENVNGSIKYV

FLAATSMWKGQSDMQKFEKARELKKHVAKIRRDYTAELKDKVMATRQRATALYLIDRLALRAGNEKGDEEADTVGCCSLR

CEHVTLEPPNVVIFDFLGKDSIRYHNSVEVDTQVFKNLKIFKKEPKTEEDMLFDRLTTTTLNKHLSSYMKGLTAKVFRTY

NASYTFQEQLKATNPDDTVAEKLLTYNRANRAVAILCNHQRAVSKGHAGQMLKIEDKVKALKYQRMKLRKMILAIDPKLK

KREDLTEVDPDLTDEWIIEHEKQNLEKEKQKAEAKFAKDNEKRIETGETLLSEKDLKLIIKDIEAKYKDLEEERKSGKVE

PKKGATVEKLEAQIEKIEERIKNVELQMVDKEENKETALTTSKINYIDPRISAAWCHKWKLASRVRSEFINFFERNGHTF

VPSSSTIPHDDPTLLFANAGMNQYKPIFLGVADPNSEFGKLKRAANSQKCIRAGGKHNDLEDVGKDVYHHTFFEMLGNWS

FGDYFKEGAIKYAWELLTEVYGMEKDRLYVTYFEGDPKQGLEPDLEAKQLWLNIGVPEDHLVKGNAKDNFWEMGETGPCG

PCSEIHYDRIGGRNASSLVNQDDPNVLEIWNLVFMQFNREADGKLRPLPNKHIDTGMGLERVVSILQGKMSNYDTDVFTP

IFSKIQELTGVRPYQGLLGAEDVDGLDMAYRVVADHIRTLTFAISDGGVPSNEGRGYVLRRILRRGARYARKKFNVQIGH

FFSSLVDVLVETMGEAFPEISKKVVDVKDILDEEEESFSRTLDRGEKLFDSFLTKTKESGSKTLPGADVWRLYDTYGFPV

DLTRVMAEENGVEVNEQEFYAEQQNAKALSRKARGTGAGQAVVALDVHDIAHVDQDASVPKTDDSAKYLAGNINAQVKTI

YSASKFYTSTDELPNDVSFGILLDKTNFYAEQGGQEYDQGNLISPNGQSEFVVENCQVFGGYVLHIGYMKYGQFRLDDEV

VASYDELRRRPLRNNHTATHILNFALREVLRDAIDQKGSLVAPEKLRFDFNYKLHEIESICNTFVLKNHRVYSKEVPLAV

ARAIHGLRAVFGEVYPDPVRVVSIGVDVQDLEADVSNPEWRKTSVEFCGGTHVTKTGDIKVITLIEEGGIAKGIRRIVAI

SGEEAQAANKVAQDFELDLARLQKLKGDELAAELKTVGTKLDGLTISAAKKAAFRETFQGIKKAFDEEDKANRTKQAKEV

IDAVKAYFETNPDSPYMVQKLNVGSNTKAITGAIAHIKTQYQDKAAYLLSVDNDAGRVAHHCIVPKVFVAKGLSASEWAG

VVSARVGGKKGGKDEAAQGSGDRPDQADEALEAAKAFAQLKLQ

>CDS33215.1 alanyl tRNA synthetase cytoplasmic [Hymenolepis microstoma]

MERKVDAATVRSQFIEFFKSKGHKYVHSSSVIPHNDPTLLFANAGMNQWKPIFQATVDPNTEMATYKRVVNSQKCIRAGG

KHNDLEDVGRDVYHHTFFEMLGNWSFGDYFKKEACEWAWELLTQVWKLPKDRFYVTYFGGDKKLGLECDGEARQVWLDLG

IPASRILPFGMKDNFWEMGETGPCGPCSEIHFDRIGGRDASQLVNMGDPDVLEVWNLVFIQYNREEGGNLRHLPAKHVDT

GMGLERIVSVLQGKRSNYDTDLFVPYFEAIHRATGVRPYAGKIAEADKDNIDLSYRVLADHARNLTIALSDGGQISNTGR

GYVLRRILRRAIRYSIEVLGAKPGFFSSLVDTVVASLGDAFPEVKKDPASVKELINAEEQQFLLTLRRGQKLLQREVERI

KDAKQATLSGSVAWRLYDTYGFPLDLTQIMAEELGVQVDVDGYEKAKEEALLKSQGTASAGVADVDLDVHDIASLQAKGT

PLTDDSLKYEYKYSKKKRIYEFAECTAKILAIRIGDGFVDKVDGTGSVIGLVLDRTVLYAEAGGQSEDHGFIFSEENEAN

ELEVIGVRNKGGFVLHLCRFEEGSLRKGEKVRVSLDTVRRVGLMRNHTATHVLNFALRRVLTGQDADQKGSLVAPDRLRF

DFSSKLALTKREVQAVEKASQRMVSSGQPIYAEDVPLVDAKAINGLRAVFGEVYPDPVRVVSVGVPVEKLISEKDGDKAT

RTSVELCGGTHVTNVSHIGKFVIISEEAVAKGIRRIVALTGPEGERAEKLANQLQQEVTTYVDAILQATSKKSIDMAVLR

PLSVRFTSVLESVDSAQIGVWPRETMRSALASVKSRLDELDKATKAATAGLVMEEAKKLCQSAPGDFIVHVFNAGSNAKA

LNSALKEMEKSLPSTAVMALSLDAESNRLLCLAQVPKALLARGLKANLWAGCVSALINGKGGGKDTSAQASGSPNGVSMD

QVVRAAKDFVKANLK

>XP_023990386.1 LOW QUALITY PROTEIN: alanine--tRNA synthetase, cytoplasmic [Salvelinus alpinus]

MKIREKFIDFFRLYDHQYVHSSATIPLDDLTLLFANAGMNQFKPVFLNTIDPSHPMARLKHAANTQKCICAGGKHNDLDD

LGKNVYHHTFFEMLGSWSFGDYFKHLACTMALELLTKEFGIPIECLYVTYFGGHEEAGLEPDLXCKQIWLDLGXPSARIL

PGSMKDNFWEMXDTGPCGPCSEIHFDRIGGXDATCLVHMDDPNVLEIWNLVFTQFNRESETELNPLPMKXVDTGMGLERL

VSVLQNNMSNYDTDLFIPYFQAIMKGTGARDYTGTVGAEDVNGIDIAYCVLADHARTITIALSDEGRPDNTGRGCPFNCL

ALCCSHEKLGTHRGFFATLVDVVVECLGDDVPELKKDHDMVKDIVNEEEEQFLKTLSRGRKILDRKIQSLGDSKTIPGDT

AWLLYNTYGFPMDLTALIAEERGMMVDVTEGDDGFLLHVPGKGSGDEDHIMLDIYATEDLRNKGIAATDDSPKYSYSADD

SGKYEFEQAVGRVLALRRECAFVDEVMTGQECGVLLDHTSFYAEQGGQSFNGGYMLRKDDSGDDRMEFTVKNTQVRGGYV

QLVGTVYGTLEVGNCVILHVYEAGHRPIKSNHTATHILNFTLRXVLGEADQRGSLVAPDRLHFDFTAKGALRTGAVRRTE

KIACAMLPRPGTPLSLTLFWTSALLERSRSSMVFFSPFKASLISSFSHWEITAMPRDCNNTPLVVMEMENGVSAKALNES

LKLLKTQSPQTAAMLFTVDNEAGKIICLCQVPQDVANRGLKASECVQELSPLMDGKGGGKDMSAQGTGRNTHCIAKTPQM

ANQFACLKLGEN

>AAH58620.1 Aars protein, partial [Mus musculus]

LIMLDIYAIEELRAKGLEATDDSPKYNYQSDSSGSYVFECTVATVLALRREKMFVDEVVTGQECGVVLDKTCFYAEQGGQ

IYDEGYLVKVDDSSEDKTEFTVKNAQVRGGYVLHIGTIYGNLKVGDQVRLFIDEPRRRPVMSNHTATHILNFALRSVLGE

ADQKGSLVAPDRLRFDFTAKGAMSTQQIKKAEEIVNGMIEAAKPVYTQDCPLAAAKAIQGLRAVFDETYPDPVRVVSIGV

PVSELLDDPCGPAGSLTSVEFCGGTHLRNSSHAGAFVIVTEEAIAKGIRRIVAVTGAEAQKALRKSETLKKSLSAMEAKV

KAQTAPNKDVQREIADLGEALATAVIPQWQKDEQRETLKSLKKVMDDLDRASKADVQKRVLEKTKQLIDSNPNQPLVILE

MESGASAKALNEALKLFKTHSPQTSAMLFTVDNEAGKITCLCQVPQNAANRGLKASEWVQQVSGLMDGKGGGKDMSAQAT

GKNVGCLQEALQLATSFAQLRLGDVKN

>PKC07439.1 alanyl-tRNA synthetase [Rhizophagus irregularis]

MAQTGTNSNINWPASKVRSTFISYFESKGHKYIPSSSTIPFEDPTLLFANSGMAQFKSIFLATVDPSSDFGKLKRATNSQ

KCIRAGGKHNDLEDVGKDVYHHTFFEMLGNWSFGDYFKKEAIIMAWELLTEVYGLPKERLYVTYFEGDEKLGLPADLEAK

QLWLDVGVEEKMLIRGNSKDNFWEMGETGPCGPCSEIHFDRIGDRDASHLVNKDDPDVLEIWNLVFMEFNRESDGSLRPL

PNKNIDTGMGLERVVSCLQSKLSNYDTDLFLPVFNKIQDLTGARPYSGKVGTDDIDGIDMAYRVIADHVRTLTFAIADGG

VPSNEGRGYVLRRILRRGARYARKKFNVQIGHFFSNLVDTVVNEMGEVFPEITQRVNDIKEILDEEEESFSRTLDRGEKL

FDTYLQKAKQSNAEVLPGSYVWRLYDTYGFPVDLTRLMAEENGLKVDEQEFLKEQEKAKTLSRNARASGGDSQEVVALDM

HDLAKIEKEKFVSITDDSFKYLSGNIDATVKAILYNHTFSQSTSDIPPGKNFGILLDRTNFYSEQGGQQYDTGSITIDGQ

AEFVVENVQMFGGYVLHIGYLKYGNLNVNDEIIANYDELRRWPLRNNHTATHILNYALREVLGNNIDQKGSLVAPEKLRF

DFSYKKSVTNNELVKIESISNKFIKKNQKVYSKDVPLPVAKAIHGLRAVFGEVYPDPVRVVSIGYDVEDIVKDVSSPKWG

ETSIEFCGGTHVAKTGDIKHFTILEESGIAKGIRRIVAVTGEEAQQAQRISEEFQTKIDSISKLKGVELDNALKSITKEL

DASNISALKKSEFREKIQGLKKAFAEADKAQKAAQTKEAVDAINTYFNQNPDIPYLVKKLDVETKAIANAIAHVKTNLKD

KALYIFSVDESSGRITHSCCVGKILTEKGLKASEWAKTVSETVGGKSGGKDDSAQGSGSNIDKLDEAIKVAEQFAKLQVA

S

>PKK72312.1 alanyl-tRNA synthetase [Rhizophagus irregularis]

MAQTGTNSNINWPASKVRSTFISYFESKGHKYIPSSSTIPFEDPTLLFANSGMAQFKSIFLATVDPSSDFGKLKRATNSQ

KCIRAGGKHNDLEDVGKDVYHHTFFEMLGNWSFGDYFKKEAIIMAWELLTVVYGLPKERLYVTYFEGDEKLGLPADLEAK

QLWLDVGVEEKMLIRGNSKDNFWEMGETGPCGPCSEIHFDRIGDRDASHLVNKDDPDVLEIWNLVFMEFNRESDGSLRPL

PNKNIDTGMGLERVVSCLQSKLSNYDTDLFLPVFNKIQDLTGARPYSGKVGTDDIDGIDMAYRVIADHVRTLTFAIADGG

VPSNEGRGYVLRRILRRGARYARKKFNVQIGHFFSNLVDTVVNEMGEVFPEITQRVNDIKEILDEEEESFSRTLDRGEKL

FDTYLQKAKQSNAEVLPGSYVWRLYDTYGFPVDLTRLMAEENGLKVDEQEFLKEQEKAKTLSRNARASGGDSQEVVALDM

HDLAKIEKEKFVSITDDSFKYLSGNIDATVKAILYNHTFSQSTSDIPPGKNFGILLDRTNFYSEQGGQQYDTGSITIDGQ

AEFVVENVQMFGGYVLHIGYLKYGNLNVNDEIIANYDELRRWPLRNNHTATHILNYALREVLGNNIDQKGSLVAPEKLRF

DFSYKKSVTNNELVKIESISNKFIKKNQKVYSKDVPLPVAKAIHGLRAVFGEVYPDPVRVVSIGYDVEDIVKDVSSPKWG

ETSIEFCGGTHVAKTGDIKHFTILEESGIAKGIRRIVAVTGEEAQQAQRISEEFQTKVDSISKLKGVELDNALKSITKEL

DASNISALKKSEFREKIQGLKKAFAEADKAQKAAQTKEAVDAINTYFNQNPDIPYLVKKLDVETKAITNAIAHVKTNLKD

KALYIFSVDESSGRITHSCCVGRILTEKGLKASEWAKTVSETVGGKSGGKDDSAQGSGSNIDKLDEAIKVAEQFAKLQVA

S

>EXX57272.1 alanine--tRNA synthetase [Rhizophagus irregularis DAOM 197198w]

MAQTGTNSNINWPASKVRSTFISYFESKGHKYIPSSSTIPFEDPTLLFANSGMAQFKSIFLATVDPSSDFGKLKRATNSQ

KCIRAGGKHNDLEDVGKDVYHHTFFEMLGNWSFGDYFKKEAIIMAWELLTEVYGLPKERLYVTYFEGDEKLGLPADLEAK

QLWLDVGVEEKMLIRGNSKDNFWEMGETGPCGPCSEIHFDRIGDRDASHLVNKDDPDVLEIWNLVFMEFNRESDGSLRPL

PNKNIDTGMGLERVVSCLQSKLSNYDTDLFLPVFNKIQDLTGARPYSGKVGTDDIDGIDMAYRVIADHVRTLTFAIADGG

VPSNEGRGYVLRRILRRGARYARKKFNVQIGHFFSNLVDTVVNEMGEVFPEITQRVNDIKEILDEEEESFSRTLDRGEKL

FDTYLQKAKQSNAEVLPGSYVWRLYDTYGFPVDLTRLMAEENGLKVDEQEFLKEQEKAKTLSRNARASGGDSQEVVALDM

HDLAKIEKEKIVSITDDSFKYLSGNIDATVKAILYNHTFSRSTSDIPPGKNFGILLDRTNFYSEQGGQQYDTGSITIDGQ

AEFVVENVQMFGGYVLHIGYLKYGNLNVNDEIIANYDELRRWPLRNNHTATHILNYALREVLGNNIDQKGSLVAPEKLRF

DFSYKKSVTNNELVKIESISNKFIKKNQKVYSKDVPLPVAKAIHGLRAVFGEVYPDPVRVVSIGYDVEDIVKDVSSPKWG

ETSIEFCGGTHVAKTGDIKHFTILEESGIAKGIRRIVAVTGEEAQQAQRISEEFQTKIDSISKLKGVELDNALKSITKEL

DASNISALKKSEFREKIQGLKKAFAEADKAQKAAQTKEAVDAINTYFNQNPDIPYLVKKLDVETKAIANAIAHVKTNLKD

KALYIFSVDESSGRITHSCCVGKILTEKGLKASEWAKTVSETVGGKSGGKDDSAQGSGSNIDKLDEAIKVAEQFAKLQVA

S

>OON05404.1 alanine-tRNA synthetase [Batrachochytrium salamandrivorans]

MATDWPLPKVRSTFVDYFKANGHTHVVSSATIPHDDPTLLFANSGMNQFKPIFLGTVDPNSDLARLSRAANSQKCIRAGG

KHNDLEDVGRDTYHHTFFEMLGNWSFGDYFKTEAITMAWDLLTKVYGLPADRLYITYFGGDEALGLQPDLEAKELWLSMG

VPEDHILPGSAKDNFWEMGDQGPCGPCSEIHFDRIGGRNASHLVNMDDPDVLEIWNLVFMQYNREADRSLKSLPNKHIDT

GMGLERLVSILQDKRSNYDTDVFTGIFATIQKLTGAREYRGLLGDADTDGIDTAYRVIADHVRTLTFSISDGGIPSNEGR

GYVLRRILRRGARYARKKFNVPIGSFFSTLVDTVVAEMGEAFPEITLRVNDLKEILDEEECSFAKTLDRGERLFMEYLAK

TKASGEKSIRGADAWQLYDTYGFPVDLTRLMAEESGFTVNEAEFEEEQKIAKERSRGRGEKGTSAVIALDIHAIGELEAN

PDIQPTVDKFKYESDNINANVKALFVDGKFIKSVASSNVQGRFGVILDKTNFYAEQGGQQYDTGSLTIDGEFEFAVEDVQ

VFGGYVLHIGYLKYGSISLDDTLVCSYDELRRWPLRNNHTATHVLNYGLREVIGTIVDQKGSLVTPEKLRFDYSCKNAPS

LEELAKVEEITNAFISKNDTIYTTEVPLALAKSINGLRAVFGEVYPDPVRVVSIGFSIEEMLQDPGNEKWAATSIEFCGG

THVQKTGDIKRFVIIEDSSLAKGIRRVVGVTGDEALKFQKLADEFGMKLERLRQLNGALFESTMKETGKTLDAAVLPVLR

KYKLKETFAAIKKDFDDRDKARKARESKDAIDIVKAFFDENPEKDVFVSVINHSGNSKALSQAIAHVRTLKDKAAMLIAV

DPEGGKVAHQCIVPQCLLDKGLKATEWSEAVSSKVGGKKGGSETAAQGAGDRVEQVQEAIDLATAFAEKLSI

>PKY45116.1 alanyl-tRNA synthetase [Rhizophagus irregularis]

MAQTGTNSNMNWPASKVRSTFISYFESKGHKYIPSSSTIPFEDPTLLFANSGMAQFKSIFLATVDPSSDFGKLKRATNSQ

KCIRAGGKHNDLEDVGKDVYHHTFFEMLGNWSFGDYFKKEAIIMAWELLTEVYGLPKERLYVTYFEGDEKLGLPADLEAK

QLWLDVGVEEKMLIRGNSKDNFWEMGETGPCGPCSEIHFDRIGDRDASHLVNKDDPDVLEIWNLVFMELNRESDGSLRPL

PNKNIDTGMGLERVVSCLQSKLSNYDTDLFLPVFNKIQDLTGVRPYSGKVGTDDIDGIDMAYRVIADHVRTLTFAIADGG

VPSNEGRGYVLRRILRRGARYARKKFNVQIGHFFSNLVDTVVNEMGEVFPEITQRVNDIKEILDEEEESFSRTLDRGEKL

FDTYLQKAKQSNAEVLPGSYVWRLYDTYGFPVDLTRLMAEENGLKVDEQEFLKEQEKAKTLSRNARASGGDSQEVVALDM

HDLAKIEKEKFVSITDDSFKYLSGNINATVKAILYNHTFSQSTSDIPPGKNFGILLDRTNFYSEQGGQQYDTGSITIDGQ

AEFVVENVQMFGGYVLHIGYLKYGDLNVNDEIIANYDELRRWPLRNNHTATHILNYALREVLGNNIDQKGSLVAPEKLRF

DFSYKKSVTNNELVKIESISNKFIKKNQKVYSKDVPLPVAKAIHGLRAVFGEVYPDPVRVVSIGYDVEDIVKDVSSPKWG

ETSIEFCGGTHVAKTGDIKHFTILEESGIAKGVRRIVAVTGEEAQQAQRISEEFQTKIDSISKLKGVELDNALKSITKEL

DASNISALKKSEFREKIQGLKKAFAEADKAQKAAQTKEAVDAINTYFNQNPDIPYLVKKLDVETKAIANAIAHVKTNLKD

KALYIFSVDESSGRITHSCCVGKILTEKGLKASEWAKTVSETVGGKSGGKDDSAQGSGSNIDKLDEAIKVAEQFAKLQVA

S

>KXS15150.1 hypothetical protein M427DRAFT_123846 [Gonapodya prolifera JEL478]

MSADKGGFNEWPVQRVRQAFVDYFTERGHTNYLSSPTIPHDDPTLLFANSGMAQFKPIFLGTVDPNSPLAKLKRAANTQK

CIRAGGKHNDLEDVGKDVYHHTFFEMLGNWSFGDYFKKEAIAFAWDLLTNVYKLPKDRLYITYFGGDSKTGLGPDLEAKQ

LWLDIGVPAAHVIPFGSKENFWEMGETGPCGPCSEIHFDRIGNRDASHLVNLDDPNVLEIWNLVFMQYNREPDQTLRPLP

NKHIDTGMGLERVTSVLQHKMSNYDTDVFTGIFTTIQQISGARHYTGKVGADDVDGVDMAYRVVADHVRTLTFAISDGGV

PSNEGRGYVLRRILRRGARYARRKLGVSIGSFFSRLVDTVVSEMGDAFPEIHKRVDDLKEILDEEERSFAKTLDRGERLF

EGYVAKAKNNTISGADAWRLYDTYGFPVDLTRLMAEERGMTVDESGFLDEQAAAREKSKARTGAGDAGAVALDVHALGDL

ERNPEVSKTDDVAKYSSGDISATVKAIYQKGGFVTTVDSSFQGNFGVILDKTNFYAESGGQEYDTGSLSIDEVADFAVEN

AQVYGGYVLHVGYLKFGKLSVGDTVTASYDELRRWPLRNNHTSTHLLNFALRNVLGENIDQKGSLVAPEKFRFDFSYKTG

LTAKQLEEVENIVNTFVRASYPLYSKDVPLAVAKQINGLRAVFGEVYPDPVRVVAVRFPIEDILADLTNPRWAETSIEFC

GGTHVKNTGDIKACVLVEEGSIAKGIRRIVAVTGEEAFALQRNAADLKPRIDALKSLEGSDLEHGLKVISKDLDATPLPL

IARNSLREQHAAIKKSFDDADKARKAILVKETVEFVKTHFADPAQSILVKVFEVGSEAKALSQAVAHIKTTKGKAALLLS

VDHDSGKVFYHSAVAPDVAKGFKASEWIQVVNQHLEGKSGGKDEAAQGAGINNGNLESAVNAAHAFATEALAKSAVQPSQ

AKSN

>XP_001743509.1 hypothetical protein [Monosiga brevicollis MX1]

MAQEQDWNAAKVRQTYIDFFVEKKEHVFWPSSSSVPHDDPTLLFCNAGMNQFKKVFQGTVDPNSDMAKVSRTVNSQKCIR

AGGKHNDLDDVGKDTYHHTFFEMLGNWSFGDYFKAEAIQWSFELLTEVYGLSADRLYATYFEGDTEQNIPEDVEAKELWL

KLLPADHVLPGNKKDNFWEMGATGPCGPCSELHYDRIGGRNAASLVNQDDPDVLEIWNLVFMQFNREPDASLRLLPARHI

DTGMGLERLVSVLQDKRSNYDTDVFQPYFDVIQQVCGCRPYAGKVGKEDDGIDTAYRVLADHIRTLTISISDGGEPDNTG

RGYVLRLIIRRAVRFAEDYLKAPEYFFSSLVPTVVEQLGDAFPEIKRDPEHVIEVLKAEEKQFRRTLNRGRRLFQRAADK

ATDGVIDGHVAWQLWGTYGFPVDLTKVMAEERGLTVDEKTFEAAREEAVKVSQMGKKMAIEGIDLDVHDLDKLKADGIAA

TDDSFKYDYGLVGDNSYEFKDLEAKVVALRTESGFVDSLDGSDDSVNVGVVLDRTNFYAEQGGQTYDTGFFRDANGELDF

TVMDVQKKGPYCLHIGVLSNGTLKVGDTLTLSFDVERRRPCMSNHTATHVLNFALRKVLGEADQKGSLVEPERLRFDFTA

KKGMTIEQIQQTEKLVNEVVNAKKPVDAMDAPLASAREINGLRAMFGETYPDPVRVVSVGAIVKDVLDDPVSEVALANSI

EFCGGTHVKNSGDIVQFAILNEEAISKGIRRIVAVTGSEARKAQTEANRLDAEVRDDLTMKEVVALDTEVSQAAIPATRK

DAIRSRLKALHKKHSDVLKERRKAMAGNAEKRAKELLEEEAKPVVVEVLEVGANGKAIGEALKVLKVGRPDTAFMFFGVE

EGGVAFNCMVPEALVAKGLKAGDWVSEVNKVLGGRGGGRDLVAQGSADQDGKVSATFCPYRQWPNLLCVLTLTLACPLFV

ARSRLPLQRPRRMLLNSRSKFSACSTAQLARAASQRLSAS

>KRX94120.1 Alanine--tRNA synthetase, cytoplasmic, partial [Trichinella pseudospiralis]

LHYRKNIFYFRTKMDILLTANAIRSTFINYFLEKRNHEYVHSSSVIPKGDPSLLFTNAGMNQFKPIFLGTADPNSQMADW

KRVVNSQKCIRAGGKHNDLDDVGKDVYHHTFFEMLGNWSFGDYFKKEICAWAWELLTKQFKIPQEQLYVTYFGGSEKYRL

QPDVECRQIWLDIGLPSEKVLPFGMKDNFWEMGDIGPCGPCSEIHFDRIGNRNAANLKYGTLCSLLSIDKDGSLHPLPKR

HVDCGLGFERLVSVIQGKKSNYDTDLFVPIFNRIAETTNVRPYMGKVGEEDEQQIDMAYRVVADHIRTLTVALSDGGRPD

NCGRGYVLRRILRRGVRYATEKLNARPGFFANLVPVVVEILGDTFPELKDDPDLVMEIINEEESLFLKTLSRGSRVLRQE

IQKTPKENLIPGFVAWRLYASYGFPVDLTQLIAEESGRKVNMEEYEQCRQQAQISSSSKFSQQSDDINFNINAISCLLDM

GVPLTDDKPKFVYRLSENENDNYEFDELECEVLALRKNGEFVSEVRTGDFCCLICDKTAFYAESGGQIYDEGSMSNDKCE

FHVRNVQVRSGYVIHFGDVLGTITLGSKLSQNIDQKRRMQVMKNHTATHLLNFALREIMSEVEQKGSLVLPEKLRFDFTR

RTPFELEQLMRIQEIIEDVIKSDAEIFMQEVPLDVAKTIAGLRFMADESYPDPVRVVSIGIPVDALLDNANRIDGNLTSV

EFCGGTHLLRAGHVGKFVIASEDAISRGVRRIIALTGHQAEQAVQKLDYFVTKIETMMDVIRETLRAGEWKDKMLNREIS

ACMKEMKYSQIPAWRRTLLMRKLLQMKETMDDFEKSQKLKFVQLITDEARNLQIPDDGIVVHEFPQNATTKALDSALKQL

KCKAGIVFSKNPAINKVVCLANVSKECVDNGLTAKEWVDHLCRVAGGYGGGKDTTAQASLGEELTVDQIKIEAENFAKLK

LSLPK

>OAQ24165.1 alanyl-tRNA synthetase [Mortierella elongata AG-77]

MTFQPEHWTAVRVRETFISYFRDQFGHTFVPSSSTIPHDDPTLLFANAGMNQYKPIFLGTIDPNTEFGKLTRAVNSQKCI

RAGGKHNDLEDVGKDTYHHTYFEMLGNWSFGDYFKKEAIDMGWKLLTEVYGLSKDRLYVTYFGGDEKQGLAPDLEAKELW

LKVGVPENRILPGSAKDNFWEMGEQGPCGPCSEIHYDRIGGRDVAHLVNEDDPNVIEVWNLVFMAFNREADGSLRPLPNK

HIDTGMGFERLVSILQDKPSNYDTDVFQPIFAAIQEVTGARAYTGLLGAEDKDGIDMAYRVIADHVRTLTFAISDGGVPS

NEGRGYVLRRILRRGSRYARRKFGVQIGTFFSSLVDTVLREFGDAFPEIRNRVDDVKEILNEEEEAFSRTLDRGEKLFDG

YLQKALEAKATHLSGADVWRLYDTYGFPVDLTRLMAEENGISVDEEEFSKQQEAAKALSRAGKGSGAGAGETVAFDVHDI

AAVEAKGTVPKTDDSHKYKSGNINAHIKLIYRNHEFPQDTSSIEEQKSFGILLDRTNMYAEQGGQEYDTGSITVDGKAEF

VVENVQVYGGYVLHTGYLKYGTLAVNDEVVVQFDELRRWPLRNNHTGTHVLNYALREILGNVVDQKGSLVAPEKLRFDFT

SKNGMTPQQIHETDLIVSDFIKRNLAVYSQEVPLAVAKSINGLRAVFGEVYPDPVRVVSIGYDVNEILKDVSNEKWTKTS

IEFCGGTHVAKTSDIKQFVIIEETGIAKGIRRIVAVTGEEAHQAERVANEFEQKLNHVGSGALSSNELESQLKVLSKELD

EIVISAYRKSVLKERFAGIKKAFDDADKARKAKQVKDSVDQVKNYFEKNPESKVFVALLDVGSNAKATGAVVTHVKTNLK

DKAAYVFSVDETNGKVSHACVVGQEILSGSSGLKATEWADVVVKVIGGRKGGKDDSAQGAGEHAEKVQEALDLAKQFAEL

KLN

>KFH69574.1 alanyl-tRNA synthetase [Mortierella verticillata NRRL 6337]

MTSNPEQWTAVKVRDTFISYFRDQFGHTFVPSSSTIPHDDPTLLFANAGMNQYKPIFLGTIDPNSEFGKLTRAVNSQKCI

RAGGKHNDLDDVGKDTYHHTYFEMLGNWSFGDYFKKEAIDMGWKLLTEVYGLSKDRLYVTYFGGDEKQGLAPDLEARELW

IKVGVPENRILPGSAKDNFWEMGEQGPCGPCSEIHYDRIGGRDVAHLVNEDDPNVIEVWNLVFMAFNREADGSLRPLPNK

HIDTGMGYERLVSILQDKPSNYDTDVFQPIFKRIQEVTGVRPYTGLLGAEDADGIDMAYRVIADHVRTLTFSISDGGVPS

NEGRGYVLRRILRRGCRYARRKLGVQIGTFFSSLVDTVLAEFGDAFPEIRLRVDDVKEILNEEEEAFSRTLDRGEKLFDG

YLQKALQAKATHLSGADVWRLYDTYGFPVDLTRLMAEENGILVDEEEFSKQQEAAKALSRAGKGTGAGAGEVVAFDVHDI

AAVEAKGTVPKTDDSAKYLNGNINAHIKLIYRNHEFPENTTGIEEQKSFGILLDRTNMYAEQGGQEYDTGSITVDGKAEF

VVENVQVYGGYVLHTGYLKYGTLQVNDEVVVQFDELRRWPLRNNHTGTHVLNYALREILGTVVDQKGSLVAPEKLRFDFT

SKVGMTPQQINEADLIVSDFIKRNLSVYSQEVPLSVAKEINGLRAVFGEVYPDPVRVVSIGFDVNEILKDPSNEKWTKTS

IEFCGGTHVAKTADIKQFVIIEETGIAKGIRRIVAVTGEEAHQAERVANDFEEKLNHIGSGVLSSSELEQQLKVLAKELD

DIVISAYRKSVFRERFADIKKKFDDADKARKAKQVKDAVDECKNYFEANPESKVFVSLLDVGSNTKATGAVVTHVKTQLK

DKAAYVFSVDHSSGKVSHACVVGQELLNGGKLKATEWADVVVKVIGGRKGGKDDSAQGAGEHVEKVQEALDLAKQFAELK

LN

>XP_004988665.1 alanyl-tRNA synthetase [Salpingoeca rosetta]

MSETTTTWTADKVRGKFISYFEERGHKFWPSSSTIPFNDPTLLFANAGMNQYKAKFLGTDSKDTDLGKLTRAVNSQKCIR

AGGKHNDLDDVGKDVYHHTFFEMLGNWSFGDYWKEEAIDFSWDLLTNVFQLDPNRLYITYFEGDSVVPADEETRSLWMKH

VPPERILPFDRKDNFWEMGETGPCGPCTEIHYDRIGGRDASHLVNMDDPDVLEIWNLVFMQYNNEGNGVLKTLPAQHVDT

GMGLERILSVLQNKTSNYDTDLFLPIFAAIKEKCGCREYTGKVNEADTDMVDTAYRVVADHIRTLCIAIADGGRPDKDGR

GYILRLVLRRAIRFCEEYLGAPEYFFASLVPVVVDVLGDAFPELRKDPEETIAVLKEEERQFRRTLSRGRRYFKRAVAAV

GEDGVIDGDTAWRLWDTYGFPIHLTIVMAEERGLKVDMDGFEAAKARAVEISRAGGAAGETNLDLDVNTLDELKKKGVPG

TDDAAKYAYTRDENGSYSFSDAEGTVLALLTEEGGLVDRVSVSEDDEELPVGVVLDKTNFYAEQGGQIYDTGRFESTTGA

PLELDVFNVQKRGAYCLHVATLAQGELKVGDKLTLKFDHDRRRPCMSNHTATHVLNYALRSVLGEADQKGSLVEPERLRF

DFTAKKGMTPEQLKQTEEVVNKIVNANYTVYDKDTPLAQARAINGLRAMFGETYPDPVRVLSVGASIDDMVADPAASYAV

DYSVEFCGGTHVKNSGDIGPFTILSEEAVAKGIRRIVAVTGQEALKAHRAADAYERRIRDDLDAKEVTSLDAELGTATIP

AVRKEEMRARLKAMSKKHAEARLAERKAKAAHAKTRANELIEEKPEGFVVETLDVGANAKAIGEALKLLKKKLPDVAFMF

FGAEEGAVAYTCQVPKAMVDEKGIKASEWIKRVNEVLGGRGGGRDLVAQGTADDSTKLTLAQEAARAYIEERLSA

>OAJ39725.1 alanine-tRNA synthetase [Batrachochytrium dendrobatidis JEL423]

MEPVVVQHFNRIVALNPDMSLPVVAIKALIEFSKKCEASTLAELFDKLDAASGILKAIPSHTISMVAGCDLFMRLAKDTP

SAIQAFEDIKQDLFRRSDVLVSSCIESRKKAANHGSKFIRDDTVILVHSYSRVVMSMLTNAPNKALIKVLVTEAKPSDSG

KRAVEELRKANIQAALISDSAVGHIIERVDMVLVGAEVVVQNGGLINQIGTYQIALVAKAANKPFYVVAESFKFARLFPL

NQDDLPISSPMYFTDEEDQGECRLSNPDIDYTPPTHINLIFTDVGVLTPSGVSEQLSSQHTLKMGQHAKVFLRMKPLSSQ

RYTFNDFSWSVDTSTRTITVPDENKNERKKTFQFDHILDVSESVETLFQEAILPQVKICLNGFNVSLISVGASKSGKSYA

LEGAMGKNKKMPAWVRDLLNPQRDGLQVDTSSATGVAVTGLSTRRISDVETCMDAIERPTKSKFTVVDFCALDHFFTDPT

QLLMKEGTNVGNSIIACHKTIKQVALQKKIAPEAEQTPITRLLLEEFGGNCYTNFLFSMDYLEAKAMNLNLLNFSELLRK

IETYPICNDETFFHLVSLVQERAGHIGKRAALVEIPTNQKQLLVDLNERINVVIEQNTTMQKELILKERLLLDTQKTQLD

LEIEQNLQLEAAENKISDLLDRLAFYEKAENNLNSTCSIQDQKTVNEKLYAQVLQLNVQVAQLEAIIQQTSKDLQASLLK

NEELGLEMIALINQKEDLTIKSLNETYENGTLIRHCIDTKELGSLNNLATIKELESSIKDRLYKSIKTHPVLVDEGRMEI

EMLVEGIANATQKYEQILQRQNEYYKTLTPEDGLAELRKQVKEFVLNTQSDLERERATLLSRCLMVLRVETKRVLFKSYQ

AVVFGLSQTPCAFHPLSSLASASILNSSMATKWPLSKVRKTFIDYFEQHGHTHVVSSSTIPHGDPTLMFANSGMNQFKPI

FLGTVDPNSDLARLSRAANSQKCIRAGGKHNDLEDVGRDTYHHTFFEMLGNWSFGNYFKTEAIEMAWEILTKVYGLPKDR

LYITYFGGDKSLGLESDIEAKHLWRNIGVEEDHIIPGSVKDNFWEMGDQGPCGPCSEIHFDRIGGRNAADLVNMDDPDVL

EIWNLVFMQYNREVDRSLKLLPNKHIDTGMGLERLVSILQDKRSNYDTDVFTGIFSAIQKLTGVREYQGRLGEADQDGID

TAYRVIADHVRTLTFAISDGGVPSNEGRGYVLRRILRRGARYARKKFNVPIGCFFSSLVDTVVTEMGDAFPEITERVDDL

KEILDEEERSFSKTLDRGERLFGEYLAKTKASGGKVISGADAWRLYDTYGFPVDLTRLMAEENGLTVDEVEFEQEQKIAK

ERSRGRNEKETGIVVSLDIHAIGELEANPNVQPTDDQYKYGFDDISSKVKALFVNGKFVESVSSTNHQGRFGVILDKTNF

YAEQGGQQFDTGSLTVDGSLEFAIEDVQVFGGYVLHIGYLKYGSLSLDDSIMCTYDELRRWPLRNNHTATHVLNYGLRSV

IGTIVDQKGSLVSPEKLRFDYSCKNSPSIAELAKVEKITNSFIAKNDIIYTSEVSLALAKSINGLRAVFGEVYPDPVRVV

SIGFPIDEMLKDPSNAKWAETSIEFCGGTHVQRTGDIKRFVIIEDSALAKGIRRIVGVTGDEALKYQKLADEFEIKLEQL

RRIKGALFETTLKEIGKTLDSSVLPVLRKHSLKETFSAIKKDFDDKDKARKALESKEAVDTVKSFFEANPEKTVFVSVIN

QSGNTKALAQAIAHVRTLKDKAAMLIAVDPETSKVAHQCIVPQILIDRGLKATEWADAVASKVGGKKGGSESAAQGAGDR

AASVQEAVDAATAFIAKLTI

>KXN74111.1 hypothetical protein CONCODRAFT_35003 [Conidiobolus coronatus NRRL 28638]

MSNELEWSVTKVRQTYFDFFEKNGHTFVPSSSTIPHDDPTLLFANAGMNQYKPIFLGTCDSASEFGKLKRAYNSQKCIRA

GGKHNDLDDVGKDVYHHTFFEMLGNWSFGDYFKKEAISMAWELLTEVYKLPKERLYVTYYGGDADKGLEPDNEARDYWLA

QGLDASKVLPFGNKENFWEMGDQGPCGPCSEIHFDRIGGRDAAHLVNLDDPDVLEIWNLVFIQFNREADGSLRPLPNKHV

DTGMGLERLVSVLQNKSSNYDTDVFVPIFEAIQKLTGARPYTGKVGAEDADGIDMAYRVVADHVRTLLIAISDSGVPSNE

GRGYVLRRILRRGCRYARRKFQVQIGSFFSSLLPTVVSILGEAYPEITKKLEDVKEILNDEEVSFNRTLDRGERLFEQCL

TNAKANNVNEISGQDAWRLYDTFGFPLDLTTLMAEEAGFNVNMDEFNKQQEAAKELSRKGRSGTGAQGENLVLSVHDIAL

VEKDESIPKTDDIYKFTESSIDATVKGIFFDGKLHSSAESSGSLIGVLLDKTNFYAESGGQEYDTGSIFVDGKVEFAVEN

VQVYAGYVLHTGSLKYGTLSVGDQVVASYDESRRAPLRSNHTATHVLNYALREVMPGEVVDQKGSLVAPEKLRFDFTSKG

ALTAEQLSGAQKICNDIVKQDLPVYYKDVSLTVAKSIYGLRAVFGEVYPDPVRVVSIGADIDAMIADASNTEWAKYSVEF

CGGTHVPRTSEIGSILLIEESAVSKGIRRIIAVTGKEAKQAQKLADEFKVKLDALNSLKGAELDATLKTIVKELDALTIS

AVDKTEFRAIFADNKKRFDDEDKANKAAQVKIALEQTKCALERNPDQHFIVEVLEVGSNSKAIQGAVKHVQSLKDKAALF

ISPDRDNGRVSYQCVVPKTLTAQGIKAIDWVTVVSDLVGGKKGGKDESAQGAGTEVEKVSEAAKLAEEFITEKLSKLSL

>KZS15556.1 Alanyl-tRNA synthetase [Daphnia magna]

MKDNFWEMGDVGPCGPCSEIHFDRIGGRDAAHLVNMDDPDVLEIWNLVFMTFNRESDGSLKPLPRKHIDCGMGFERLVSV

IQNKRSNYDTDIFQPIFEAIQKGTNAPAYQGKVGSEDVNGVDMAYRVLADHARTLTIALSDGGRPDNVGRGYVLRRVLRR

GVRYATEKLNAKPGFFASLVPTVCQVLGLTFPEVNRDPQLVIDIINDEETQFLKTLTRGRDLLERTISKLNGQTTLPGDV

AWRLYDTYGFPVDLTQLMSEEKGLSIDMAAYEEAKKQAQLLSQGKGSGVDDTIAFDVHAITELQNKGVPPTDDSFKYNYK

AVSDAIDSPYEFDVCTSKVIAIRFQKDFHEEVSSGVECGILLDRTSFYAESGGQAYDEGFITKVDGEETEFTVKNVQVRG

GYVIHLGNVEGTLRVGDEVKLSIDQSRRRLIMSNHTGTHVLNYALRQVVGMEADQRGSLVAPDRMRFDFTNKAAMTSEQV

KQTERIANEMISKNEEVYAKESALVVAKAVQGLRAVFEETYPDPVRVVSIGIPVEQLEADPSNPAGNSTSIEFCGGTHVK

RSGHIGDFVISSEEAIAKGIRRIVAITGPEASKALKKAELLQKEVDALSEKVDAFVSQKDKTLTVKELSRIIVDLSDDVS

QANIAYWKKDDLRNLLKGLKKRADDVERAIKAAVVNDVADAAKKLIGERVNTPYIVHEFNAFSNSKALDGALKQVKSLSP

ETAAIFFSVDAEANKVVVLAAAPKGANDRGLKANEWVADISGLLDGKGGGSAGSAQATGNNPAGLAEAMKKATVFAQSKL

GLVSEIAASTAKLGAEPVDGPTLFSTSGSVRTNIALIASRYANTKLNVVTEVLDLPSSTFISNKFPALSTGDVHVSGLAA

VSVYLAPKSLKGNSLFEEAQILQWINLAEHELLPAVLVFLDASFNAKPVRNRARQEIQHYLEILNKILLTHTYLVGETVT

LADIAVVCTLAPVFQLVMEGPSSSKSTNVLRWFNTITQQPIVKHVVGDAM

>OUC47559.1 alanine--tRNA synthetase [Trichinella nativa]

MFSTIHYLPISLQKIFFYIRSKMDMLLTANAIRRTFINYFTEKRNHQYVHSSSVIPKGDPSLLFTNAGMNQFKPIFLGTA

DPNSQMASWKRVVNSQKCIRAGGKHNDLDDVGKDVYHHTFFEMLGNWSFGDYFKKEICAWAWEVLTKEFKIPKEQLYVTY

FGGSEKYRLQPDVECRQIWLEIGLPSERVLPFGMKDNFWEMGDIGPCGPCSEIHYDRIGNRNAANLINKNDPDVLEIWNL

VFITYNRDKDGSLHPLPKRHIDCGLGFERLVSVIQGKKSNYDTDLFVPIFNRIAETAKVRPYMGKVGKEDEQQIDMAYRV

VADHIRTLTVALSDGGRPDNCGRGYVLRRILRRGVRYATEKLNAPSGFFANLVPVVVETLVEDVHIVIGDTFPELKEDPN

SVMEIINEEESLFLKTLSRGSRVLRQEIEKASKDKLIPGVVAWRLYASYGFPVDLTQLMAEESGYKVNMEEYEECRQQAQ

MSSSSKFSQANDVVDFNINAVNYLLNGKVPLTDDKFKYAYRITENGDDDYEFDEVKCEVLALRKNGEFVSEVCSGDSCCL

ICDKTAFYAESGGQIYDEGSMEGEKCEFRVRNVQARSGYVIHFGEVMGTITLGSKLLQNVDQKRRVQVMKNHTATHLLNF

ALREVLGQVEQKGSLVLMDRLRFDFTCKAPLTVEQLVRIEQIVANIVNSDVEIYMQEVPLGVAKTIAGLRSSADETYPDP

VRVVSVGTPVDALLKDPDGPGAKLASVEFCGGTHLLRTGHLGDFVIASEEAVARGIRRIVALTCHEAEQAVQKLEYFVNK

VEEVMDLIRSSVLSGELDYSFLNREISGCLEVGWLCVEMKASQIPAWRREHLVRKLAQVKATLDDFEKSERMKFVQMVF

>CDW58118.1 alanine tRNA synthetase, cytoplasmic [Trichuris trichiura]

MKAEEVRQKFLDFFSKKYGHTYYHSSPVIPHDDPTLLFANAGMNQFKPIFLGTVNPNSPLAKLKRVVNTQKCIRAGGKHN

DLDDVGKDVYHHTFFEMLGNWSFGDYYKEEICAWAWELLTECFMLEKDRLYVTYFGGNPSMNLGPDLECRDIWLKIGIPN

NRILPFGMKDNFWEMGEIGPCGPCSEIHYDRVAGRNAASLVNQDDPSVIEIWNLVFITETNGSLQLLPTRHIDCGLGFER

LVSVIQGVSSNYDTDLFLPIFSYISSVTGCRPYSGKVGTEDVGQVDMAYRVVADHIRALTVALSDGGRPDSTGRGYVLRR

ILRRGVRFATEKLNAPPGLFASLVPQVVAILGTTFPEMAKDPDAVMAIINEEEMQFLKTLSRGGKLLDRAIDKVSPGGHI

QGDIAWRLYDTYGFPVDLTELIAEERGCTVDMVAYEACRLAAQKASQVKLSCKNGLEFSAPVLKELNDLGIPTTDDSWKY

RYSVADVALNNYVLETCVCRVLAIRMNGKFINQVTSGDDCVLICDRTSFYAENGGQIYDEGFMEKGDAQVEAEFCVTNVQ

ARAGYVLHSGKVEGIITVGDKLTQRVDEQIGPDGSSFQERRRLVMKNHTATHLLNFALREIVKESVQKGSLVAPDRLRFD

FSASSGLTNDQIKCVEEIILKLVAENAPVYMSEMPLSAGKTLPGLRAEFQETYPDPVRVVSVGYPIDKLLAVPSLSMQTS

VEFCGGTHLHRVGHIDDFVIICEEALARGIRRIVALTGPAARRARQRCAHFNERLRSIEQSRPGGEVVEDRFYKIKKSKI

VALIEEVSSSQISCWERESLCERLQALKKEVEATNKAAQALLASKILDSALKKIKSAKAAMVFSVDEENSKVLCMACVKK

QYAENGQLNAKDWTSYLCSMFGGKSGGKDVNSQALLDEPSRLDDIVKAAEDFAKLKIGPVNR

>SAM04223.1 hypothetical protein [Absidia glauca]

MTDVWSAKKVRASFIEFFEKNGHTFYASSPTVPHDDPTLLFANAGMNQYKPIFLGTVDPSSPLYNLKRATNSQKCIRAGG

KHNDLDDVGKDTYHHTFFEMLGNWSFGDYFKVDATRFAWEYLTKVLQLPEDRLYVSYFGGDEKLGLEPDLEAKKIWMDLG

VEEKRLLPGDSKDNFWEMGETGPCGPCSEIHYDRIGGRDAAHLVNMDDPNVLEIWNVVFIQFNREQGGKLRPLPNKHIDT

GLGLERLVSILQNKYSNYDTDVFTPIFDKIKELTGARDYAGKLGDEDVDGVDTAYRVVADHIRTLTFAITDGGVPSNEGR

GYVLRRILRRGARYVRKYFDTPIGTFFSSLVDTLVEQMGDAFPELLTKVAEVKAILDEEEVAFAKTLDRGEKLFENYLAA

AKERGSNTLSGTDVWRLYDTYGFPVDLTRVMAEENGMTVDEKEFEASQEAAKELSRRGKAGSNGEEVVALDVHDLAEVGK

DEKVPKTDDSFKYAGGIVQGLIKSIYYQHKFLADTKSIPEGASFGILTDKTNFYAESGGQEYDTGSIITLDGSTEFVVKD

CQVYGGYVLHVGHLKYGQLKYDDQVELSFDDLRRFPLKNNHTSTHILNFALRQVLGESIDQRGSLVAPEKLRFDFSHKSP

VNPKQLAEVESICNDFVIKNSPVYSKEVPLPVAKAIHGLRAVFGETYPDPVRVVSIGYDVDEVTADVSNPKWATTSVEFC

GGTHVTKTGDIKLFAILEESGIAKGIRRIIAVTGEEALKADRTAQDFAKRLDRLEQLTDKNSVETELKNMARDLDTMAIS

VVAKAEFRERYVKRKKAHDELEKVRQKEQAKEVTDAVKKYFEEHPESKYVVLSLDVGNNNKALLAATTYAKTNLKDKAIY

LLSSDAASGRVAHNCIVGKNLITDYGFKASDWAGVVADKVGGKKGGKDDSAQGSGDNIAGLDEAVKMAQEFAKLKIGA

>CEI92968.1 Putative Alanyl-tRNA synthetase [Rhizopus microsporus]

MTEQEWPVNKVRSTFIKFFEENGHTFVPSSSTIPHDDPTLLFANAGMNQFKPMFLGTLDPNSPLANVKRACNSQKCIRAG

GKHNDLDDVGKDTYHHTFFEMLGNWSFGDYFKKEATKLAWEFLTKVIRLPEDRLYVTYFGGDEKMGLPADLEAKQLWLDL

GVDEKRLIPGNVKDNFWEMGETGPCGPCSELHFDRIGNRDAASLVNMDDPNVIEIWNLVFMQFNREADGTLRPLPNQHID

TGLGLERLVSILQNKYSNYDTDVFTPMFDRIKEITGVRSYTGKLGDEDVDGIDTAYRVVADHIRTLTFAIADGGVPSNEG

RGYVLRRILRRGARYVRKYFNVPIGSFFSSLVDTLIAQMHEGYPELAKKANDIKVILDEEEAAFAKTLDRGEKLFEAYMN

KTKESGSNVMSGDDVWRLYDTYGFPVDLTRIMAEENGLQVNEAQFEAAQEAAKNKSRKARGGALGQDTVALDVHDLGALD

NDSSVPKTDDSAKYSRGNIQAKVKAIFFDHKFFKNTSEIPDERNFGLLLDKTNFYAESGGQEYDIGSIITLDGNTEFVVE

DCQLYGGYVLHVGHLKYGQLSVDEDVEVSYDEDRRSPLRHNHTATHILNFALREVLGEDIDQKGSLVAPEKFRFDFSYKS

PVNAKQLEQVENICNEFIAKNQQVYSQEVPLAVAKAIHGLRAVFGETYPDPVRVVSIGFDIDEIVQDVSNPKWKTSSVEF

CGGTHVAKTGDIKHFVIIEESGIAKGIRRIVALTGEAAVQAQREADTFVTKLDRLEKLSGSQLEAELKVVSKELDNAVIS

SVSKAAFRERFAQIKKAFDNAEKVRKAEQVKEATEKVKKYFEENPDAPYYIAELHVGNNSKALSGAINYAKTSLKDKAVY

VFSVDRDSGRVAHNCIVGKVYIEKGLKASEWANVVCEKIGGKKGGKDDAAQGSGDNIDGLPDALKAAEEFAKLKIN

>XP_023461574.1 alanyl-tRNA synthetase [Rhizopus microsporus ATCC 52813]

MTEQEWPVNKVRSTFIKFFEENGHTFVPSSSTIPHDDPTLLFANAGMNQFKPMFLGTLDPNSPLANVKRACNSQKCIRAG

GKHNDLDDVGKDTYHHTFFEMLGNWSFGDYFKKEATKLAWEFLTKVISLPEDRLYVTYFGGDEKMGLPADLEAKQLWLDL

GVDEKRLIPGNVKDNFWEMGETGPCGPCSELHFDRIGNRDAASLVNMDDPNVIEIWNLVFMQFNREADGTLRPLPNQHID

TGLGLERLVSILQNKYSNYDTDVFTPMFDRIKEFTGVRSYTGKLGDEDVDGIDTAYRVVADHIRTLTFAIADGGVPSNEG

RGYVLRRILRRGARYVRKYFNAPIGSFFSSLVDTLIAQMHEGYPELAKKANDIKAILDEEEAAFAKTLDRGEKLFEAYMN

KTKESGSNVMSGDDVWRLYDTYGFPVDLTRIMAEENGLQVNEAQFEAAQEAAKNKSRKARGGALGQDTVALDVHDLGALD

NDSSVPKTDDSAKYSRGNIQAKVKAIFFDHKFLKNTSEIPDERNFGLLLDKTNFYAESGGQEYDIGSIITLDGNTEFVVE

DCQLYGGYVLHVGHLKYGQLSVDEDVEVSYDEDRRSPLRHNHTATHILNFALREVLSEDIDQKGSLVAPEKFRFDFSYKS

PVNVKQLEQVENICNEFIAKNQQVYSQEVPLAVAKAIHGLRAVFGETYPDPVRVVSIGFDIDEIVQDVSNPRWKTSSVEF

CGGTHVAKTGDIKHFVIIEESGIAKGIRRIVALTGEAAVQAQREADTFVTKLDRLEKLSGSQLEAELKVVSKELDIAVIS

SVSKAAFRERFAQIKKAFDNAEKVRKAEQVKEATEKVKKYFEENPDAPYYIAELHVGNNSKALSGAINYAKTSLKDKAVY

VFSADRDSGRVAHNCIVGKVFIEKGLKASEWANIVCEKIGGKKGGKDDAAQGSGDNIDGLPDALKAAEEFAKLKIN

>ORX62144.1 alanyl-tRNA synthetase [Hesseltinella vesiculosa]

MSDTWTVKKVRSTFIDYYKSLDHTFCPSSPTVPVDDPTLLFANAGMNQYKSIFLGTVEPTSPLYSVKRATNSQKCIRAGG

KHNDLDDVGKDTYHHTFFEMLGNWSFGDYFKAEAAKYSWDFLTNVLGLEKSRLYVTYFGGDEKSGMEPDLEAKQIWLDLG

VEEKRLLPGNVKDNFWEMGETGPCGPCSEIHYDRIGGRDAAHLVNMDDPNVLEVWNIVFIQFNREQGGKLRPLPAKHIDT

GMGLERLVSVLQDKPSNYDTDVFMPLFDKIKELTGARDYAGKLGKEDADGIDTAYRVVADHIRTLTFAITDGGVPSNEGR

GYVLRRILRRGCRYVRKYFNVPIGHFFSSLVDTLVDHMGDAFPELHTKVEDVKAILDEEEVAFAKTLDRGEKLFEEYMSA

TKERGSTILSGNDVWRLYDTYGFPTDLTQVMAEENGFTIDEKEFEAAQEAAKELSRRRRGGNEGEEAVALDVHDLAELGK

NDQVPTTDDSFKYAGSNIQSSIKSIYYQHKFLNNTSDIPEGARFGLLTDKTNFYAESGGQEYDTGSIISLDGKNEFAVTD

CQVYGGYVLHVGHLKYGQLNLNDQVELSFDDLRRFPLKNNHTSTHILNFALRKVLGDNVDQRGSLVAPEKLRFDFAYKTA

VTPKQLAEVEKICNEFVTNNSPVYSKEVPLPVAKAIHGLRAVFGETYPDPVRVVSIGYDVDEVTADVSNPKWATTSVEFC

GGTHVAKTGDIKLFCLLEEGGIAKGIRRIIAVTGEEAMRAQQTAQQFSQRLDRLENLKGAELDAEIKNVGRDLDTLTVSV

VAKAEFRDRYAKIRKAFDELEKARQKEQAKQVTDSIKDYFEQNPDAKFVVLSLAVGNNSKALVAAITYAKSNLKDKAVYL

MSADASSGRVAHNCFVGKDLIQQGLKASDWAGVVAEKIGGKKGGKDDAAQGSGDNVAGVDEAVQVAIDFAKLKVSN

>ORX66640.1 alanyl-tRNA synthetase [Linderina pennispora]

MTSTEWTAKHVRETFIGFYEQHGHTFVPSSSTVPHDDPTLLFANAGMNQYKPIFQGTVDPASDFAKLHRACNSQKCIRAG

GKHNDLDDVGKDVYHHTFFEMLGNWSFGDYFKKEAIGYAWELLTKVYRLDPERLYVTYFGGSEADGLPVDTEAKQYWLDL

GVPESRVLPFGSKENFWEMGEVGPCGPCSEIHYDRIGGRDAASLVNLDDPDVLEIWNLVFMEFNRESDGSLRSLPRKHID

TGMGMERLVSVLQGKRSNYDTDVFMPLFAAIQELTGARPYTGNVGADDTDGIDMAYRVIADHIRTLTFAIADGGVPSNEG

RGYVLRRILRRGARYARRKFNVELGSFFARLVDTVVENMGDMFPEITKSTDLIKEILCEEEESFARTLDRGERLFEQTVA

KMGGSKTMPGASVWRLYDTYGFPVDLTRIMAEESGLVVDEAEFEREKQKARDLSKRKKGSSGSGQGIALDVHAIGELNAR

GVAKTDDSPKYSSRSVEAKVLAVFTGNGFADAEERSLATHPEDAPVVGVVLDRTAFYAEQGGQEYDTGSLVSSDLSSEFT

VEDVQVYGGYVLHTGFLKYGALRVGDSVEAQYDVLRRLPIRNNHSATHVLNFALRQVLRAEEPDQRGSLVAPDRLRFDFA

CKAAISAEHVRDIEALTNQIIKQNLKVYAQEVPLDMARQISGLRAVFGEVYPDPVRVVSIGADLDQVLKNPENAEWENYS

IEFCGGTHVAGTADMKVFVITEESAIAKGVRRIVAVTGPEASKAQLLYQSLQAEVAKLKQLSGAELDHEIKRLAKELDAA

AIGVYDKHVLRSEFDAIRAAHVEAEKLAKAQAAKQAVELVQKLIEESPEQDVFVVELPAAGKAMSQVATFVKGLKSKAVY

LLAVDGARVAHQCVVPKTLVQRGLKAGEWADAVSKIVGGKKGGKDESAQGSGTETARVAEAVAAAEEFAKTHLN

>CEG71324.1 Putative Alanyl-tRNA synthetase [Rhizopus microsporus]

MTEQEWPVNKVRSTFIKFFEENGHTFVPSSSTIPHDDPTLLFANAGMNQFKPMFLGTLDPNSPLANVKRACNSQKCIRAG

GKHNDLDDVGKDTYHHTFFEMLGNWSFGDYFKKEATKLAWEFLTKVISLPKDRLYVTYFGGDEKMGLPADLEAKQLWLDL

GVDEKRLIPGNVKDNFWEMGETGPCGPCSELHFDRIGNRDAACLVNMDDPNVIEIWNLVFMQFNREVDGTLRPLPNQHID

TGLGLERLVSILQNKYSNYDTDVFTPMFDRIKETTGVRSYTGKLGDEDVDGIDTAYRVVADHIRTLTFAIADGGVPSNEG

RGYVLRRILRRGARYVRKYFNVPIGSFFSSLVDTLIAQMHEGYPELAKKANDIKAILDEEEAAFAKTLDRGEKLFEAYMN

KTKESGSNVMSGDDVWRLYDTYGFPVDLTRIMAEENGLQVNEAQFEAAQEAAKNKSRKARGGALGQDTVALDVHDLGALD

NDSSVPKTDDSAKYSRGNIQAKVKAIFFDHKFLKNTSEIPDERNFGLLLDKTNFYAESGGQEYDIGSIITLDGNTEFVVE

DCQLYGGYVLHVGHLKYGQLSVDEDVEVSYDEDRRSPLRHNHTATHILNFALREVLSEDIDQKGSLVAPEKFRFDFSYKS

PVNVKQLEQVENICNEFIAKNQQVYSQEVPLAVAKAIHGLRAVFGETYPDPVRVVSIGFDIDEIVQDVSNPKWKTSSVEF

CGGTHVAKTGDIKHFVIIEESGIAKGIRRIVALTGEAAVQAQREADTFVTKLDRLEKLSGSQLEAELKVVSKELDNAVIS

SVSKAAFRERFAQIKKAFDNAEKVRKAEQVKEATEKVKKYFEENPDAPYYIAELHVGNNSKALSGAINYAKTSLKDKAVY

VFSADRDSGRVAHNCIVGKVYIEKGLKASEWANVVCEKIGGKKGGKDDAAQGSGDNIDGLPDALKAAEEFAKLKIN

>KRX94122.1 Alanine--tRNA synthetase, cytoplasmic, partial [Trichinella pseudospiralis]

LHYRKNIFYFRTKMDILLTANAIRSTFINYFLEKRNHEYVHSSSVIPKGDPSLLFTNAGMNQFKPIFLGTADPNSQMADW

KRVVNSQKCIRAGGKHNDLDDVGKDVYHHTFFEMLGNWSFGDYFKKEICAWAWELLTKQFKIPQEQLYVTYFGGSEKYRL

QPDVECRQIWLDIGLPSEKVLPFGMKDNFWEMGDIGPCGPCSEIHFDRIGNRNAANLKYGTLCSLLSIDKDGSLHPLPKR

HVDCGLGFERLVSVIQGKKSNYDTDLFVPIFNRIAETTNVRPYMGKVGEEDEQQIDMAYRVVADHIRTLTVALSDGGRPD

NCGRGYVLRRILRRGVRYATEKLNARPGFFANLVPVVVEILGDTFPELKDDPDLVMEIINEEESLFLKTLSRGSRVLRQE

IQKTPKENLIPGFVAWRLYASYGFPVDLTQLIAEESGRKVNMEEYEQCRQQAQISSSSKFSQQSDDINFNINAISCLLDM

GVPLTDDKPKFVYRLSENENDNYEFDELECEVLALRKNGEFVSEVRTGDFCCLICDKTAFYAESGGQIYDEGSMSNDKCE

FHVRNVQVRSGYVIHFGDVLGTITLGSKLSQNIDQKRRMQVMKNHTATHLLNFALREIMSEVEQKGSLVLPEKLRFDFTR

RTPFELEQLMRIQEIIEDVIKSDAEIFMQEVPLDVAKTIAGLRFMADESYPDPVRVVSIGIPVDALLDNANRIDGNLTSV

EFCGGTHLLRAGHVGKFVIASEDAISRGVRRIIALTGHQAEQAVQKLDYFVTKIETMMDVIRETLRAGEWKDKMLNREIS

ACMKEMKYSQIPAWRRTLLMRKLLQMKETMDDFEKSQKLKFVQLITDEARNLQIPDDGIVVHEFPQNATTKALDSALKQL

KCKAGIVFSKNPAINKECVDNGLTAKEWVDHLCRVAGGYGGGKDTTAQASLGEELTVDQIKIEAENFAKLKLSLPK

>PIA12779.1 hypothetical protein COEREDRAFT_12203 [Coemansia reversa NRRL 1564]

MTDIEWGMRKVRDTFMEFFKSKGHVFVPSSSTIPHDDPTLLFANAGMNQYKPIFQGTVDPASDFAKLTRAYNSQKCIRAG

GKHNDLDDVGKDVYHHTFFEMLGNWSFGDYFKKEAIEFSWELLTKVYGLDSKRLYVTYFEGSTADGLPVDKDARQFWLDL

GLPEERVLPFSSKDNFWEMGDVGPCGPSVEIHYDRIGGRDAGPLVNMDDPDVVEIWNLVFIEFNREVGGKLRPLPNKHID

TGLGFERVVSVLQDKRSNYDTDVFMPLIDKIQSVTNARPYTGNVGADDTDGVDMGYRVIADHIRTLTFAIADGGVPSNEG

RGYVLRRILRRGARYARRKFNVELGSFFAGLVDTVVEQMGDMFPEISKNTDLIKEILCEEELSFARTLDRGERLFEQTVA

KMPEGSTVIPGASVWRLYDTFGFPVDLTRIMAEERGLTVDEDEFNKEQEQSRAMSRQKKGGAGAANNVVLDVHALAQLNA

KGVATTDDEPKYYARTLDAKVLAVFDGKEFVNSEQRSISTHPDSVPVVGVLLDRTNMYAEQGGQEYDTGSLITLDDSSEF

TVENVQVYGGYVLHTGFLKYGELKVGDVVEAQYDVLRRLPIRSNHTATHALNFALRKVLHSDQPDQRGSLVAPDRLRFDF

SYKQPVTAEEVRDVDLLCNQMISENMKVYAREVPLAEAKQIYGLRAVFGEVYPDPVRVVSIGADIDQVVKSPGEHRWADY

SIEFCGGTHVGSTSDLKVFVTVEESGVAKGIRRIVAVTGEEALKAQLLHRSLAAEIEKLHKLLVPPQKPEQQQLLESELK

RLSKELDAAVIGVYEKYQLRQEFDGIKRQYNEADKAAKALVAKQISDQVQSAIEQNPEQEVFVFKFNAAGKAVAQMATFV

KGLKNKAAYFISAEESSGRVAHQCVVGKPLVARGLKANEWAEAVSAVVGGKKGGKEESAQGSGTEIARVDEAVRVAEDFA

KSHLS

>ORZ13205.1 alanyl-tRNA synthetase [Absidia repens]

MSEEVWSAKKVRSSFIEFFGKHGHTFYPSSPAVPHDDPTLLFANAGMNQYKPIFLGTVDPSSPLYSLKRATNSQKCIRAG

GKHNDLDDVGKDTYHHTFFEMLGNWSFGDYFKVDATRFAWEYLTKVLKLPEDRLYVSYFGGDEKLGLEPDLEAKQIWMDL

GVEEKRLLPGDSKDNFWEMGETGPCGPCSEIHYDRIGGRDAAHLVNMDDPNVLEIWNVVFIQFNREQGGKLRPLPNKHID

TGLGLERLVSILQNKYSNYDTDVFTPLFDKIKELTGARDYAGKLGDEDADGVDTAYRVVADHIRTLTFAITDGGVPSNEG

RGYVLRRILRRGARYVRKYFNTPIGNFFSSLVDTLVEQMGEAFPELLTKVDEVKAILDEEEVAFAKTLDRGEKLFENYLA

AAKERGSKTLSGADVWRLYDTYGFPVDLTRVMAEENGVTVDEKEFEVSQEAAKELSRRGKAGGNGDEAVALDVHDLAEVG

KNDKVPKTDDSFKYAGGNVQGLIKSVYYQHKFLEDTKDIPEGANFGILTDKTNFYAESGGQEYDTGSIITLDGSTEFVVK

DCQVYGGYVLHVGHLKYGQLKYDDQVVLSFDDLRRFPLKNNHTSTHILNFALRQVLGETVDQRGSLVAPEKLRFDFSHKS

PVNSKQLAEVESICNDFVIKNSPVHSKEVPLPVAKAIHGLRAVFGETYPDPVRVVSIGYDVDEVTADVSNPKWATTSVEF

CGGTHVAKTGDIKLFAILEESGIAKGIRRIIAVTGEEALKADRTAKEFVKRLERLETMTDKSQVEVELKNVARDLDALTV

SVVIKAEFRERYSKKKKAHDELEKVRQKEQAKEVTDAVKKYFEDNADAKYVVLSLDVGNNNKALLAATAYAKTNLKDKAI

YLLSGDASSGRVAHNCIVGKNLITDYGLKASEWAGVVADKVGGKKGGKDDSAQGSGDNVSGLDEAVKMADEFAKLKVGA

>PKY22414.1 alanyl-tRNA synthetase [Rhizophagus irregularis]

MAQTGTNSNINWPASKVRSTFISYFESKGHKYIPSSSTIPFEDPTLLFANSGMAQFKSIFLATVDPSSDFGKLKRATNSQ

KCIRAGGKHNDLEDVGKDVYHHTFFEMLGNWSFGDYFKKEAIIMAWELLTEVYGLPKERLYVTYFEGDEKLGLPADLEAK

QLWLDVGVEEKMLIRGNSKDNFWEMGETGPCGPCSEIHFDRIGDRDASHLVNKDDPDVLEIWNLVFMEFNRESDGSLRPL

PNKNIDTGMGLERVVSCLQSKLSNYDTDLFLPVFNKIQDLTGARPYSGKVGTDDIDGIDMAYRVIADHVRTLTFAIADGG

VPSNEGRGYVLRRILRRGARYARKKFNVQIGHFFSNLVDTVVNEMGEVFPEITQRVNDIKEILDEEEESFSRTLDRGEKL

FDTYLQKAKQSNAEVLPGSYVWRLYDTYGFPVDLTRLMAEENGLKVDEQEFLKEQEKAKTLSRNARASGGDSQEVVALDM

HDLAKIEKEKFVSITDDSFKYLSGNIDATVKAILYNHTFSQSTSDIPPGKNFGILLDRTNFYSEQGGQQYDTGSITIDGQ

AEFVVENVQMFGGYVLHIGYLKYGNLNVNDEIIANYDELRRWPLRNNHTATHILNYALREVLGNNIDQKGSLVAPEKLRF

DFSYKKIYSKDVPLPVAKAIHGLRAVFGEVYPDPVRVVSIGYDVEDIVKDVSSPKWGETSIEFCGGTHVAKTGDIKHFTI

LEESGIAKGIRRIVAVTGEEAQQAQRISEEFQTKIDSISKLKGVELDNALKSITKELDASNISALKKSEFREKIQGLKKA

FAEADKAQKAAQTKEAVDAINTYFNQNPDIPYLVKKLDVETKAIANAIAHVKTNLKDKALYIFSVDESSGRITHSCCVGK

ILTEKGLKASEWAKTVSETVGGKSGGKDDSAQGSGSNIDKLDEAIKVAEQFAKLQVAS

>XP_010765240.1 PREDICTED: LOW QUALITY PROTEIN: alanine--tRNA synthetase, cytoplasmic, partial [Notothenia coriiceps]

YVLRRILRRAVRYSHEKLGAQKGFFATLVDVVVDSLGNAFPELKKDPEVVKDIYXRGGGCTFLQNPSAGGRRILDRKIMS

LGENKTIPGDTAWLLYDTYGFPLDLTSLIAEEKGMAVDVASFEEEKKAAQIKSQGKGAGDVDHIMLDIYAIEDLRNKNIP

ATDDSSKYKYTSEENGNYNFEQASATVQALRCDRAFCDEVTTGQECGVLLDSTSFYAEQGGQTFDEGYMVREDDSTEDRM

EFTVKNTQVRGGYVLHLGTVYGTLKVGDRVTLRLDEARRRPIMSNHTATHILNFALRGVLGEADQRGSSVAPDRLRFDFT

AKGALSTGEVRRTEEIACAIIKDAKPVYAMDAPLAKAKAIQGLRAVFDETYPDPVRVVSIGIPVEELLENPESTAGALTS

IEFCGGTHLRNSGHAAPFVIVSEEAIAKGIRRIVAVTGTEAQKAQRKADSLLQSLSALGEKVKQQTDPNKDIQKDIADMT

ELIGTAVISQWKKDEMRESLKGLKKTMDDLDRNYKADIQKRVLEKTKEVIESSPNQALLVMEMETGASAKALNESLKLLK

SQSPQTAAMLFTVDPEAGKITCLCQVPQDVAKRGLKASEWVQELCPLLDGKGGGKDMSAQATGKNTQCMQEALQMANQFA

QLKLGEN

>XP_018913253.1 PREDICTED: alanine--tRNA synthetase, cytoplasmic isoform X3 [Bemisia tabaci]

MKDNFWEMGETGPCGPCSELHFDRIGNRDVPHLVNQDDPDVLEIWNLVFIQFNREADSSLKPLPKKHIDCGMGFERLVSV

IQNKRSNYDTDVFVPLFDAIQKGTGAPPYSGKVGAEDTDGIDMAYRVLADHARTLTIALADGGFPDNTGRGYVLRRILRR

AVRYATEKLNANPGFFSSLVHTVVDLLGGAFPEVNKDPQSIIDVINEEEEQFLKTLSRGRGLLNREISKLKGSKVLPGDV

AWRLYDTYGFPVDLTQLMAEEKGLSVDLPGYEEAKKHAQVLSQGKSGGIEDCISLDVHAITELQNRGIPVTDDSPKYDYS

TDPNPTSPYVFSRCSGKVLALRCNKNFVDEIHSGQKCGILSDRTNFYAEQGGQIFDEGFIIKSDDKDVEVNVYNVQVKGG

YVLHYGTVEGTLKVGDSIELSIDTKRRRKVMSNHTGTHVLNHSLRHVLGQTDQKGSLVVPERLRFDFSNKGAMTVDQIRA

AEQETQAVIKGNKKVYAKHIDLTNAKKINGLRAMFDEVYPDPVRVVCVGVPIEELESNPESDAGMKTSVEFCGGTHVHYA

GDIGDFVITNEEAIAKGVRRIIALSGAEATKAMKKAELLEKELNVLKNKVDHVGSDVKSTNKEILDLTDDINQALISYCK

KDELRKIIGCMKKKIDDADKKAKAAIVSIIVNEAKELIEEKPNEPVIVAEFNAESNTKALNSALQQVKSLSPETSAMFFT

FDDTTKRLYYLCFVPKDSIAKGLKANEWIQAVSQEINGKGGGNMETAQGSGVTSLDAVAKAVNLANSFANLKLGL

>XP_006676752.1 hypothetical protein BATDEDRAFT_32863 [Batrachochytrium dendrobatidis JAM81]

MATKWPLSKVRKTFIDYFEQHGHTHVVSSSTIPHGDPTLMFANSGMNQFKPIFLGTVDPNSDLARLSRAANSQKCIRAGG

KHNDLEDVGRDTYHHTFFEMLGNWSFGNYFKTEAIEMAWEILTKVYGLPKDRLYITYFGGDKSLGLESDIEAKHLWRNIG

VEEDHIIPGSVKDNFWEMGDQGPCGPCSEIHFDRIGGRNAADLVNMDDPDVLEIWNLVFMQYNREVDRSLKLLPNKHIDT

GMGLERLVSILQDKRSNYDTDVFTGIFSAIQKLTGVREYQGRLGEADQDGIDTAYRVIADHVRTLTFAISDGGVPSNEGR

GYVLRRILRRGARYARKKFNVPIGCFFSSLVDTVVTEMGDAFPEITERVDDLKEILDEEERSFSKTLDRGERLFGEYLAK

TKASGGKVISGADAWRLYDTYGFPVDLTRLMAEENGLTVDEVEFEQEQKIAKERSRGRNEKETGIVVSLDIHAIGELEAN

PNVQPTDDQYKYGFDDISSKVKALFVNGKFVESVSSTNHQGRFGVILDKTNFYAEQGGQQFDTGSLTVDGSLEFAIEDVQ

VFGGYVLHIGYLKYGSLSLDDSIMCTYDELRRWPLRNNHTATHVLNYGLRSVIGTIVDQKGSLVSPEKLRFDYSCKVNKC

FEYNIYSRRKIGLTAELAKVEKITNSFIAKNDIIYTSEVSLALAKSINGLRAVFGEVYPDPVRVVSIGFPIDEMLKDPSN

AKWAETSIEFCGGTHVQRTGDIKRFVIIEDSALAKGIRRIVGVTGDEALKYQKLADEFEIKLEQLRRIKGALFETTLKEI

GKTLDSSVLPVLRKHSLKETFSAIKKDFDDKDKARKALESKEAVDTVKSFFEANPEKTVFVSVINQSGNTKALAQAIAHV

RTLKDKAAMLIAVDPETSKVAHQCIVPQILIDRGLKATEWADAVASKVGGKKGGSESAAQGAGDRAASVQEAVDAATAFI

AKLTI

>XP_016607026.1 alanine-tRNA synthetase [Spizellomyces punctatus DAOM BR117]

MHGICYRLLQINTSFPRSRYTCSVIQRNVSSSLFCGFFIRSSHSATKMTVDLEWPVSRVRQTYIEFFTQKHQHTFVPSSA

TIPHDDPTLLFANSGMNQFKPIFLGTVDRNSDLGKLKRAANTQKCIRAGGKHNDLEDVGKDTYHHTFFEMLGNWSFGDYF

KKEAIAMAWDLLTNVYKLPSDRLYVTYFGGDEKMRLAPDEEARKLWLDIGVPEDHILPGDSKDNFWEMGDQGPCGPCSEI

HFDRIGGRNASHLVNQDDPDVLEIWNLVFMQFNREPDRSLKPLPNKHIDTGMGLERLVSILQNKRSNYDTDVFAGIFAAI

QKLTGARPYAGRVGPDDVDGIDTAYRVVADHIRTLAFAISDGGVPSNEGRGYVLRRIIRRAARYVRNKFNTPIDGFFSRL

VDTVTAEMGEAFPEITKRIDDLKEILDEEERSFAKTLDRGEKLFGEYLAKARSENASTLNGADVWRLYDTYGFPVDLTRL

MAEESGLQVDEMEFERAQQAAKERSKATGSKDKGDVVALNIHDLGELEKNPDVPRTDDRYKYGTDDIQATVKAIYADGKF

VKSVASESFTRRFGILLDKTNFYAEQGGQQYDTGSITVDGQAEFAVDDVQPFGGYVLHIGYLKYGSLSVGTEVVCSYDEL

RRWPIRNNHTATHILNFALKKTLGDVVDQKGSLVAPEKLRFDFSAKNAPTIEQLTQIEDISNEFIKKNHKMFTKDVPLHL

GKSINGLRAVFGETYPDPVRVVSIGFDVDEMLKDSANPKWSETSVEFCGGTHVKQTGDIKRFVILEESSISKGIRRIVAV

TGEEAFEVQRLADEFDRKIADLKGLQGSALETGLKTTGKDLDEAALPVLRKHKLRQTFAAIKKEFDDQDKARKAKEMKEA

AERVKTYFDQNPDVPFLVEVLPYGGNTKALAKAIEAVKGLKTKAAMLLTVDAESSKVSYQCIVPKEYVDKGLKATDWADV

VSQKVGGRKGGKDESAAGAGTDLAGVEEAVSLSLEFARLRLSA

>KRX94121.1 Alanine--tRNA synthetase, cytoplasmic, partial [Trichinella pseudospiralis]

LHYRKNIFYFRTKMDILLTANAIRSTFINYFLEKRNHEYVHSSSVIPKGDPSLLFTNAGMNQFKPIFLGTADPNSQMADW

KRVVNSQKCIRAGGKHNDLDDVGKDVYHHTFFEMLGNWSFGDYFKKEICAWAWELLTKQFKIPQEQLYVTYFGGSEKYRL

QPDVECRQIWLDIGLPSEKVLPFGMKDNFWEMGDIGPCGPCSEIHFDRIGNRNAANLKYGTLCSLLSIDKDGSLHPLPKR

HVDCGLGFERLVSVIQGKKSNYDTDLFVPIFNRIAETTNVRPYMGKVGEEDEQQIDMAYRVVADHIRTLTVALSDGGRPD

NCGRGYVLRRILRRGVRYATEKLNARPGFFANLVPVVVEILGDTFPELKDDPDLVMEIINEEESLFLKTLSRGSRVLRQE

IQKTPKENLIPGFVAWRLYASYGFPVDLTQLIAEESGRKVNMEEYEQCRQQAQISSSSKFSQQSDDINFNINAISCLLDM

GVPLTDDKPKFVYRLSENENDNYEFDELECEVLALRKNGEFVSEVRTGDFCCLICDKTAFYAESGGQIYDEGSMSNDKCE

FHVRNVQVRSGYVIHFGDVLGTITLGSKLSQNIDQKRRMQVMKNHTATHLLNFALREIMSEVEQKGSLVLPEKLRFDFTR

RTPFELEQLMRIQEIIEDVIKSDAEIFMQEVPLDVAKTIAGLRFMADEVISRRALQYNHDTSYPDPVRVVSIGIPVDALL

DNANRIDGNLTSVEFCGGTHLLRAGHVGKFVIASEDAISRGVRRIIALTGHQAEQAVQKLDYFVTKIETMMDVIRETLRA

GEWKDKMLNREISACMKEMKYSQIPAWRRTLLMRKLLQMKETMDDFEKSQKLKFVQLMKREICKYPTMVLWCMNFRRMPQ

PKFAFNNNRSVLHSDSFQALDSALKQLKCKAGIVFSKNPAINKVVCLANVSKECVDNGLTAKEWVDHLCRVAGGYGGGKD

TTAQASLGEELTVDQIKIEAENFAKLKLSLPK

>4XEM_A Chain A, Crystal Structure Of Wild Type Human Alars Catalytic Domain

MGSSHHHHHHSSGLVPRGSHMDSTLTASEIRQRFIDFFKRNEHTYVHSSATIPLDDPTLLFANAGMNQFKPIFLNTIDPS

HPMAKLSRAANTQKCIRAGGKHNDLDDVGKDVYHHTFFEMLGSWSFGDYFKELACKMALELLTQEFGIPIERLYVTYFGG

DEAAGLEADLECKQIWQNLGLDDTKILPGNMKDNFWEMGDTGPCGPCSEIHYDRIGGRDAAHLVNQDDPNVLEIWNLVFI

QYNREADGILKPLPKKSIDTGMGLERLVSVLQNKMSNYDTDLFVPYFEAIQKGTGARPYTGKVGAEDADGIDMAYRVLAD

HARTITVALADGGRPDNTGRGYVLRRILRRAVRYAHEKLNASRGFFATLVDVVVQSLGDAFPELKKDPDMVKDIINEEEV

QFLKTLSRGRRILDRKIQSLGDSKTIPGDTAWLLYDTYGFPVDLTGLIAEEKGLVVDMDGFEEERKLAQLKSQGK

>CDH55480.1 alanyl-trna synthetase [Lichtheimia corymbifera JMRC:FSU:9682]

MTQQQQLEWPVNRVRSAFIDYFIKENDHTFVPSSSTIPHDDPTLLFANAGMNQYKSVFLGTVDPNSPLAGLKSATNSQKC

IRAGGKHNDLDDVGKDTYHHTFFEMLGNWSFGDYFKTGAAKMCWTFLTEVLKLPSDRLYVTYFGGDEKLGLEADLEAKQI

WMDIGVAEDHLLPGDVKDNFWEMGETGPCGPCSEVHYDRIGGRNASSLVNMDDPNVIEIWNVVFIQFNREADGKLRPLPN

KHIDTGLGLERLVSIMQNVYSNYDTDVFAPLFAKIKEITGARSYEGKLGAEDVDGVDTAYRVVADHIRTLTFAISDGGVP

SNEGRGYVLRRILRRGARYARKYFNYPIGNFFSQLVDTLVDQMGHHFPELTKMSAEVKSILDEEEVAFAKTLDRGEKLFE

QYRIKAQEAGNKTLSGNDVWRLYDTYGFPVDLTRVMAEENGLTVDEKEFEAAQEAAKNASRKAKGGNAGEDVVALDVHDL

GALEKMDAVPTTDDSFKYLAGNVQGKIKAIVFEHKFLDNTEEIPKDRRFGILTDKTNFYAESGGQEYDTGSIITLDGETE

FAVQDCQSFGGYVLHIGYLKYGQLKIDSDVELSFDALRRHPLKNNHTGTHILNFALRKVLGENVDQKGSLVAPEKLRFDF

SFKSALTTPQLAETEKICNEMISKNDPVYSKEVPLPVAKAIHGLRAVFGETYPDPVRVVSVGYDVDEVTSDVSNPKWATT

SIEFCGGTHVAKTGDIKRFAILEESGIAKGIRRIVAVTGEEAQAADRLAKSFESRLDELEKLGAGPELDTALKTVAKDLD

SIAISTVTKAEFRERYNKVKKAAVNAGKALAAQQLKEAQDAVKNHFEQNPEAKFLVTVLNVGNNSKALAGAIAYTKSNHK

DKAVYVFSADQTSGRVSHNCIVSKDLVAQGLKASEWADVVAAKVGGKKGGKDEAAQGSGDKLDGLDEAIKAAEEFAKLKV

SA

>OBZ91910.1 Alanine--tRNA synthetase [Choanephora cucurbitarum]

MTDWPVNKVRSTFIKYFEDNQHTFVPSSSTVPHDDPTLLFANAGMNQYKPLFLGTVDPNSPMASLKRACNSQKCIRAGGK

HNDLDDVGKDTYHHTFFEMLGNWSFGDYFKREATKFGWEFLTKVINLPEDRLYVTYFGGDEKQGLPADLEAKQMWIDLGV

DEKRLIPGNVKDNFWEMGETGPCGPCSEVHFDRIGGRDAAHLVNMDDPNVIEIWNIVFIQFNREQDGSLRTLPSQHIDTG

LGLERLVSILQNKYSNYDTDCFTPIFDKIKELTGARDYTGLLGEEDKDGIDTAYRVVGDHIRTLTFAISDGGVPSNEGRG

YVLRRILRRGARYVRKYFNVPIGSFFSSLVDTLVEQMTEAYPELAKKADDVKAILDEEEVAFSKTLDRGEKLFEAYMKKT

KESGSTVLSGDDVWRLYDTYGFPVDLTRIMAEENGLEVNEAQFEAAQEAAKNKSRKVKGGDNGQGVVALDVHDLGALDNN

DAIPKTDDKYKYLPGNIQGRIKGIFFEHKFLNSTTEIPEDRHFGILTDKSNFYAESGGQEYDTGSIITLDGSTEFAVRDC

QVYGGYVLHVGHLKYGQLSVEDEVELSYDDVRRFPLRNNHTSTHILNFALREVLGEDIDQKGSLVSPEKLRFDFSYKSPV

NATQLEKVEKICNEFIGKNLKVYSKEVPLAVAKAIHGLRAVFGETYPDPVRVVSVGFDIDEVTQDVSNPKWKTSSVEFCG

GTHVAKTSDIKHFAIIEETGIAKGIRRIVAVTGDEAIAAQREAQTFVAKLDQLEKLSGSELEGALKAVAKELDAAVISSV

DKAAFRDRFAQIKKAFDNAEKARKAEQVKEASEAVKKYFEQNPEAPFFIAELPMGNNTKALGGAITYAKTNLKDKAVYVF

SGDKETGRVAHNCIVGKNYIEKGLKASEWAAVVCEKIGGKKGGKDDAAQGSGDKIEGLSDALKAAEEFAKLKINA

>4XEO_A Chain A, Crystal Structure of human AlaRS catalytic domain with R329H mutation

MGSSHHHHHHSSGLVPRGSHMDSTLTASEIRQRFIDFFKRNEHTYVHSSATIPLDDPTLLFANAGMNQFKPIFLNTIDPS

HPMAKLSRAANTQKCIRAGGKHNDLDDVGKDVYHHTFFEMLGSWSFGDYFKELACKMALELLTQEFGIPIERLYVTYFGG

DEAAGLEADLECKQIWQNLGLDDTKILPGNMKDNFWEMGDTGPCGPCSEIHYDRIGGRDAAHLVNQDDPNVLEIWNLVFI

QYNREADGILKPLPKKSIDTGMGLERLVSVLQNKMSNYDTDLFVPYFEAIQKGTGARPYTGKVGAEDADGIDMAYRVLAD

HARTITVALADGGRPDNTGRGYVLRRILHRAVRYAHEKLNASRGFFATLVDVVVQSLGDAFPELKKDPDMVKDIINEEEV

QFLKTLSRGRRILDRKIQSLGDSKTIPGDTAWLLYDTYGFPVDLTGLIAEEKGLVVDMDGFEEERKLAQLKSQGK

>EIE82853.1 alanyl-tRNA synthetase [Rhizopus delemar RA 99-880]

MTEWPANKVRSTFVKFFEDNGHVFVPSSSTIPHDDPTLLFANAGMNQFKPMFLGTLDPNSPLANVKRACNSQKCIRAGGK

HNDLDDVGKDTYHHTFFEMLGNWSFGDYFKKEATKFGWEFLTKVINLPEDRLYVTYFGGDEKMGLPADLEAKQLWMDLGV

EEKRLIPGNVKDNFWEMGETGPCGPCSEIHYDRIGGRDAASLVNMDDPNVLEIWNLVFMQFNREADGSLRPLPNQHIDTG

LGLERLVSILQNKYSNYDTDVFTPMFDKIKELTGARDYTGKLGEEDVDGVDTAYRVIGDHIRTLTFAISDGGVPSNEGRG

YVLRRILRRGARYARKYFNVAIGSFFSSLVDTLISQMHEAYPELGKKANDIKAILDEEEVAFSKTLDRGEKLFEAYMKKT

KESGSTVLSGDDVWRLYDTYGFPVDLTRIMAEENGLEVNEAQFEAAQEAAKNKSRKVKGGDLGQGVVALDVHDLGALDND

DCVPKTDDSAKYLPGNIQGKVKAIFFEHKFLKSTTEIPEDRNFGLLLDKTNFYAESGGQEFDIGSIITLDGNSEFAVQDC

QLYGGYVLHVGHLKYGQLSIDEDVEVSYDDTRRSPLRNNHTATHILNFALREVLQDDIDQKGSLVAPEKFRFDFSYKSPV

NTKQLAEIENITNDFINKNHQVYSKEVPLAVAKAIHGLRAVFGETYPDPVRVVSIGFDVDEITKDVSNPLWKTSSVEFCG

GTHVAKTGDIKHFVIIEESGIAKGIRRIVALTGEAAVKAQREADTFVTKLERLEKLSGSQLEAELKVVSKEIDNAVISSV

SKATFRDRFAQIKKAFDNAEKVRKAEQVKEATEKIKAYFEENPEASYYIAELQVGNNSKALGGAINYAKANLKDKAVYVF

SADKESGRVAHNCIVGKVFIEKGLKASEWANVVCEKIGGKKGGKDDAAQGSGDKIDGLPDALKAAEEFAKLKISA

>ORY23740.1 hypothetical protein LY90DRAFT_706785 [Neocallimastix californiae]

MSEEVYWTTKRVREQFIKFFEGHEHTYVPSSSTIPHDDPTLLFANAGMNQYKPIFLGVVDPNSDMAKLKRACNSQKCIRA

GGKHNDLDDVGKDVYHHTFFEMLGNWSFGDYFKEGAIKMAWELLTEVYKLPADRLYITYFGGDEKLGLEADLECRQIWLD

LGIPADRLLPFGSKDNFWEMGDQGPCGPCCELHYDRKGGRNAASLVNMDDPEVIEIWNLVFMQYNREADGSLRPLPSKHI

DTGMGLERVVSVLQNKMSNYDTDAFTGLFNAIQKLTGAAPYTGKVGAEDVNGMDTAYRVIADHTRTLTFAISDGGVPSNE

GRGYVLRRILRRGARFARRKFGVQIGNFFSQLVDVVISEMGDTYPEITKNVADLKEILDEEEKSFARTLDRGEKLFDACI

AKTKEQGSKVIKGADVWRLYDTYGFPVDLTTLMAEENGMTINQQEFLAAQEEARERSKACKNGNKAEVVTLDVHAIGALD

KNDAVPKTDDSAKYERGAITANIKALYVDGKFVDNVDEGYAKNFGVILDKTNMYAEQGGQEYDFGTITIDGKADFAVESV

QTYGGYVLHVGYLKYGSFAVNDEIVAAYDEDRRDLIQNNHTGTHILNFALREIIGDTVDQKGSLVNAEKLRFDFTCKNAI

PASDIEKVENICNDFIKRDLKVYTQEVSTAIGKQINGIRAVFGEVYPDPVRVVSIGFDVAEVIKDVSNEKWRTTSMEFCG

GTHVNSTGEIKLLVVIEEASIAKGIRRITALTADEAAKVIENAKLFAPKIEALKTLKGAELENTLKTFLKELDGSVLPFV

AKQKYRDEYNAIKKAFDDSEKARKAAQIKEVTEFVKTEVEKNPEAKYFVLQLNAGSNSKAVAGAVNYIKGLKNKAALFIS

VDEAAGRVSHQCIVPKEFTKAGFKAIDWASVVSEKVGGKKGGKDVSAQGSGTEVAAVDEALKLADEFAKLKVASL

>CDR44690.1 CYFA0S15e01530g1_1 [Cyberlindnera fabianii]

MSTPTAEWTAKKVRQTFFDYFQERGHTFVPSSSVIPYDDPTLLFANAGMNQYKPIFLGTVDPSSDFYNLKRAVNSQKCIR

AGGKHNDLEDVGRDSYHHTFFEMLGNWSFGDYFKKEAIEYSWELLTKVYGLDPDRLYVTYFGGDEAAGLEPDLEAKELWK

SVGVDPSHILPGSVKDNFWEMGDQGPCGPCSEVHYDRIGGRNAAHLVNEDDPNVLEIWNNVFMQYNRESDRSLKPLPAKH

IDTGLGFERLVSILQQVYSNYDTDVFQPLFKRIQEITGAREYTGKFGAEDVDGIDTAYRVLADHVRTLTFALADGGVPNN

DGRGYVLRRILRRGARYARKYFNYPIGDFFSKLAPTLIEQVKDVFPELEKNTDSTFEILNEEEISFSRTLDRGEKLFQQY

AEAAKKSSNEVLNGKDVWRLYDTYGFPVDLTQLMAEEAGLKIDEEGFEKARVESYEASKAGGKSKGASIIKLDVHSLGKL

SEDGVPKTDDQFKYATENIEAKILVIHDGENFVDEITDITKEYGIILDKTPFYAEQGGQEYDTGKIVIDGESEFNVENTQ

LYAGYVFHTGRLAEGKLKVGDKVIAGFDELRRWPIRNNHTGTHILNLALRNVLGDDINQRGSLVAPEKLRFDFSHKQGLT

LDEVAKVEKICNDQIKKSQTVYVKEVALDTAKSIYSVRAVFGETYPDPVRVVSIGVPVEELLKAPDNKEWHNISIEFCGG

THVAKTSDIKDFVITEESGIAKGIRRVVAVTGSEAHEVQRIASEFEHELLNAGKLEFSPLKEKKLKELGVRLGQLNISIL

TKNSLKETFNKFDKSVKDELKARQKAESKKTLDTVKEHFEANADSEFLVAHIDISANAKSITEAINYLKKEKPQKSIYLF

TGNADKVAHGCYISNEAIGKGVDASELAKKASTHIGGKAGGKGNVVQGMGDKLAGIDEALKELETQLKEKLTI

>ORY98177.1 alanyl-tRNA synthetase [Syncephalastrum racemosum]

MAQELEWPVSKVRSTFLEFFKERDHTFVPSSSSIPHDDPTLLFANAGMNQYKSIFLGTVDPNSPLANVSRATNSQKCIRA

GGKHNDLDDVGRDTYHHTFFEMLGNWSFGDYFKAGAAEYSWNFLTKVLGLSADRLYVTYFGGDEKLGLEPDLEAKKIWLD

LGVEEKRLIAGNVKDNFWEMGETGPCGPSSEIHYDRIGGRDAASLVNMDDPNVLEIWNVVFIQFNRENDGKLRPLPNKHI

DTGLGLERLVSIMQNVYSNYDTDVFQPLFARVKEITGARDYTGKLGAEDVDGVDTAYRVVADHIRTLTFAITDGGVPSNE

GRGYVLRRILRRGARYVRKYFNYPIGSFFSGLVDTLVGQMAEAYPELLTKVDDVKAILDEEEAAFAKTLDRGEKLFESYK

NKTKEAGQTTLAGADVWRLYDTYGFPVDLTRIMAEEHGMTVDEKGFEEAQEAAKNLSRRTKGGNAGDDVVALDVHDLAAL

DKNEAVPNTDDSFKYLSGNVQAHIKSIFFEHKFLDDSSSIPEGRKFGLLLDKTNFYAESGGQQFDTGSIITLDSENEFAV

EDCQSFGGYVLHIGHLKYGSLKVNDDVEVSFDELRRRPLRNNHTATHILNYALREVLGGNIDQKGSLVAPTKLRFDFTHK

APVTTAELDKVEQICNEFIAKNSKVYSKEVPLPVAKAIHGLRAVFGETYPDPVRVVSIGFDVDEVTQDVSNPNWATSSVE

FCGGTHVAKTGDIKHFAVLEETGIAKGIRRIVAVTGDEAMIADREAKAFGAKMDRLEKMSGPALEVELKTIAKEIDALVI

SAVTKAAYRDRYNAVKKSFDNAEKARKAEEVKQATEAVKAYFAQNPDAVYMIQTLDVGNNGKALTSAINYVKSNLKDKAV

YLFSADAQSGRVSHNCIVSKDLVAKGLKASDWAGLVAEKVGGKKGGKDEAAQGSGDKIDGLDEALKAAEEFAKLKIAA

>ONH66731.1 Alanine--tRNA synthetase [Cyberlindnera fabianii]

MSTPTAEWTAKKVRQTFFDYFQERGHTFVPSSSVIPYDDPTLLFANAGMNQYKPIFLGTVDPSSDFYNLKRAVNSQKCIR

AGGKHNDLEDVGRDSYHHTFFEMLGNWSFGDYFKKDAIEYSWELLTKVYGLDPDRLYVTYFGGDEAAGLEPDLEAKELWK

SVGVDPSHILPGSVKDNFWEMGDQGPCGPCSEVHYDRIGGRNAAHLVNEDDPNVLEIWNNVFMQYNRESDRSLKPLPAKH

IDTGLGFERLVSILQQVYSNYDTDVFQPLFKRIQEITGAREYTGKFGAEDVDGIDTAYRVLADHVRTLTFALADGGVPNN

DGRGYVLRRILRRGARYARKYFNYPIGDFFSKLAPTLIEQVKDVFPELEKNTDSTFEILNEEEISFSRTLDRGEKLFQQY

AEAAKKSSNEVLNGKDVWRLYDTYGFPVDLTQLMAEEAGLKIDEEGFEKARVESYEASKAGGKSKGASIIKLDVHSLGKL

SEDGVPKTDDQFKYATENIEAKILVIHDGENFVDEITDITKEYGIILDKTPFYAEQGGQEYDTGKIVIDGESEFNVENTQ

LYAGYVFHTGRLAEGKLKVGDKVIAGFDELRRWPIRNNHTGTHILNLALRNVLGDDINQRGSLVAPEKLRFDFSHKQGLT

LDEVAKVEKICNDQIKKSQTVYAKEVALDTAKSIYSVRAVFGETYPDPVRVVSIGVPVEELLKAPDNKEWHNISIEFCGG

THVAKTSDIKDFVITEESGIAKGIRRVVAVTGSEAHEVQRIASDFEHELLNAGKLEFSPLKEKKLKELGVRLGQLNISIL

TKNSLKETFNKFDKSVKDELKARQKAESKKTLDTVKEHFEANADSEFLVAHIDISANAKSITEAINYLKKEKPQKSIYLF

TGNADKVAHGCYISNEAIGKGVDASELAKKASTHIGGKAGGKGNVVQGMGDKLAGIDEALKELETQLKEKLTI

>GAV53683.1 hypothetical protein ZYGR_0AK01850 [Zygosaccharomyces rouxii]

MTIGDREKWTATNVRKAFLDFFKSKDHKFVASSPVVPYDDPTLLFANAGMNQYKPIFLGTVDPSSDFYHLKRAYNSQKCI

RAGGKHNDLEDVGKDSYHHTFFEMLGNWSFGDYFKEGAISYAWELLTKVYGLPADRLYVTYFEGDPKNGLEADTEALELW

KSVGVDESHILPGNAKDNFWEMGDQGPCGPCSEIHFDRIGGRNAASLVNMDDPDVLEVWNLVFMQFNREQDGSLKPLPAK

HIDTGMGFERVVSILQNYRSNYDTDVFTPLFARIQEVTGVRPYAGKFGAEDADGVDTAYRVLADHVRTLVFAIADGGVPN

NEGRGYVLRRILRRGARFARKYMNYPIGDFFSKLVPTLISQVQDIFPEVAKDPSYLFEILNEEEVSFAKTLDRGERLFEK

YADAAIKSGKKTLDGLQVWRLYDTYGFPVDLTELMAEEKGLTIDGPGFEKAKQESYEASKRGGKKDGADLLKLNVHDISE

LNSENVPKTNDEYKYGMENVDSTILKLHDGTKFVNEISEPGKKYGVILDKTCFYAEQGGQEYDTGKLVIDDVAEFNVDNV

QLYNGFVFHTGSLVEGKLSVGNKIIASYDELRRFPIKNNHTGTHILNFALREVLGNDIEQKGSLVAAEKLRFDFSHKKGL

AVDEIIKVEDICNKQIKENMDVYYKDVPLTDAKSIYGVRAVFGETYPDPVRVVSVGKSVDDLLANPSNEEWSKYSIEFCG

GTHVVKTGDIKVFVILEEGGIAKGIRRIVAVTGSEAFEAQRIANEYEKELDAADRLPFSPLKEKKLKELSVKLGQLSISV

VSKQELKTKFTKIEKVVKDEIKTRTKEETKKTLDEVKEYFSKNENSQFFAKFIDIPTNAKAITEAVNYVKSSAKEKSIYL

FTGNDPEGKIAHGCYISDAALSKGIDGSAVAKQVSGIIGGKAGGRGNVFQGMGDKPTAALDAVAEVEKLFKEKLQL

>XP_018867113.1 PREDICTED: alanine--tRNA synthetase, cytoplasmic [Gorilla gorilla gorilla]

MDSTLTASEIRQRFIDFFKRNEHTYVHSSATIPLDDPTLLFANAGMNQFKPIFLNTIDPSHPMAKLSRAANTQKCIRAGG

KHNDLDDVGKDVYHHTFFEMLGSWSFGDYFKELACKMALELLTQEFGIPIERLYVTYFGGDEAAGLEADLECKQIWQNLG

LDDTKILPGNMKDNFWEMGDTGPCGPCSEIHYDRIGGRDAAHLVNQDDPNVLEIWNLVFIQYNREADGILKPLPKKSIDT

GMGLERLVSVLQNKMSNYDTDLFVPYFEAIQKGTGARPYTGKVGAEDADGIDMAYRVLADHARTITVALADGGRPDNTGR

GYVLRRILRRAVRYAHEKLNASRGFFATLVDVVVQSLGDAFPELKKDPDMVKDIINEEEVQFLKTLSRGRRILDRKIQSL

GDSKTIPGRYQFLPFSILAAEHSSIELLLVQTGIYTVFENTVATVMALRREKMFVEEVSTGQECGVVLDKTCFYAEQGGQ

IYDEGYLVKVDDSSEDKTEFTVKNAQVRAGYVLHIGTIYGDLKVGDQVWLFIDEPRRRPIMSNHTATHILNFALRSVLGE

ADQKGSLVAPDRLRFDFTAKGAMSTQQIKKAEEIANEMIEAAKAVYTQDCPLAAAKAIQGLRAVFDETYPDPVRVVSIGV

PVSELLDDPSGPAGSLTSVEFCGGTHLRNSSHAGAFVIVTEEAIAKGIRRIVAVTGAEAQKALRKAESLKKCLSVMEAKV

KAQTAPNKDVQREIADLGEALATAVIPQWQKDELRETLKSLKKVMDDLDRASKADVQKRVLEKTKQLIDSNPNQPLVILE

MESGASAKALNEALKLFKMHSPQTSAMLFTVDNEAGKITCLCQVPQNAANRGLKASEWVQQVSGLMDGKGGGKDVSAQAT

GKNVGCLQEALQLATSFAQLRLGDVKN

>XP_002496623.1 uncharacterized protein ZYRO0D04378g [Zygosaccharomyces rouxii]

MTIGDREKWTATNVRKAFLDFFKSKDHKFVASSPVVPYDDPTLLFANAGMNQYKPIFLGTVDPSSDFYHLKRAYNSQKCI

RAGGKHNDLEDVGKDSYHHTFFEMLGNWSFGDYFKQGAISYAWELLTKVYGLPADRLYVTYFEGDPKNGLEADMEAFELW

KSVGVDESHILPGNAEDNFWEMGDQGPCGPCSEIHFDRIGGRNAASLVNMDDPDVLEVWNLVFMQFNREQDGSLKPLPAK

HIDTGMGFERVVSILQDYRSNYDTDVFTPLFARIHEVTGVRPYAGKFGAEDTDGIDTAYRVLADHVRTLVFAIADGGVPN

NEGRGYVLRRILRRGARFARKYMNYPIGDFFSRLVPTLISQVQDIFPEVAKDPSYLFEILNEEEVSFAKTLDRGERLFEK

YADAATSSGKKTLDGLQVWRLYDTYGFPVDLTELMAEEKGLTIDGPGFEKAKQESYEASKRGGKKDGAEVLKLNVHEISE

LNGENVPKTKDEYKYGMENVDSTILKLHDGTKFVNEISEPGKKYGVILDKTCFYAEQGGQEYDTGKLVIDDVAEFNVENV

QLYNGFVFHTGSLLEGKLSVGNKIIASYDELRRFPIKNNHTGTHILNFALREVLGNDIEQKGSLVAPEKLRFDFSHKKGM

AADEILKVEDICNKQIKENMSVYYKDVPLTDAKSIYGVRAVFGETYPDPVRVVSVGKSVDDLLANPSNEEWSKYSVEFCG

GTHVVKTGDIKVFVILEESGIAKGIRRIVAVTGSEAYEAQRIANEFDKELNAVDRLPFSPLKEKKLKELSVKLGQLSISV

VSKQELKTKFAKIEKVVKDEIKTRAKQESKKTLDEVKEYFAKNENSQFLAKFVDIPTNAKAITEAVNYVKSSAKDKSIYL

FTGNDPEGKIAHGCYISDAALSKGIDGSTVAKQVSGIIGGKAGGRGNVFQGMGDKPTAAGDAVAEVEKLFKEKLQL

>ORX43782.1 hypothetical protein BCR36DRAFT_335506 [Piromyces finnis]

MSEQIYWSTKKVREEFIKFFEKYEHTFVPSSSTIPHDDPTLLFANAGMNQYKPIFLGVVDPNSDMAKLKRACNSQKCIRA

GGKHNDLDDVGKDVYHHTFFEMLGNWSFGDYFKEGAIKMAWELLTEVYKLPKDRLYMTYFGGDEKLGLEADLEARQIWLD

LGIPADRLLPFGSKDNFWEMGDQGPCGPCCEIHFDRRGGRDASALVNQDDPEVLEIWNLVFMQYNREADGSLRPLPNKHI

DTGMGLERVVSVLQNKMSNYDTDAFSGLFSAIQKLTGAAPYTGKVGAEDVNGMDTAYRVIADHTRTLTFSISDGGIPSNE

GRGYVLRRILRRGARYARKKFNVQIGNFFSQLVDVVISEMGDVYPEITKNVDELKEILDEEEKSFSRTLDRGEKLFDACI

TKTKEQGSTQIKGADVWRLYDTYGFPVDLTTLMAEENGMTINQEEFLAAQEEARERSKACNNGNKADAVTLDVHAIGDLD

KNDAVPKTNDSAKYERGAINASVKALFVDGKFVDSVDESFTKNFGVILDKTNMYAEQGGQEYDFGTITIDGKTDFAVESV

QTYGGYVLHVGYLKYGAFSLNDEVVATYDEDRRALIQNNHTGTHILNFALREVVGDSVDQKGSLVNTEKLRFDFSCKNAI

PAEDVEKIEAICNDFIKRDLKVYTKEVETSVGKQIYGIRAVFGEVYPDPVRVVSIGFDVEEIIKDVSNTKWRTTSMEFCG

GTHVNTTGEIKQLVIIEETSIAKGIRRITALTADEAAKVIENAKAFAPKVEALKALKGAELENSLKVFLKELDSSVLPFV

AKQKFRDEYNAIKKAFDDSEKARKAAQIKEVTEFVKTEVEKNPEAKYIVLRLEAGNNTKAVAGAVNYIKGLKNKAALFIS

VDEAAGRVSHQCIVPKEFTKAGFKAIDWATVVSDKVGGKKGGKDVSAQGSGTEISAVDEALKLADEFAKLKIASL

>XP_018287813.1 hypothetical protein PHYBLDRAFT_188133 [Phycomyces blakesleeanus NRRL 1555(-)]

MTEQVEWPANKVRSAFISYFEKNSHVFYPSSSTVPHDDPTLLFANAGMNQYKSVFLGTVDPNSPLATIRRAVNSQKCIRA

GGKHNDLDDVGKDTYHHTFFEMLGNWSFGDYFKKEAIRLGWEFLTKELGLPKERLYVTYFGGDAKQGLEPDLEAKQMWLD

IGVDEERLLPGSAKDNFWEMGETGPCGPCSEIHYDRIGGRNASSLVNEDDPNVLEIWNLVFIQFNRENDGSLRLLPNKHI

DTGMGLERLVSIMQNKYSNYDTNIFMPIFAKIQELTGARTYSGKLGAEDVDGIDTAYRVIADHIRTLTFAITDGGVPSNE

GRGYVLRRILRRGARYVRKYFNVPIGTFFSSLVDTLVAEMSSAFPELTTKVTEVKEILDEEEVAFAKTLDRGEKLFENYL

AKTKATGSKTLPGADVWRLYDTYGFPVDLTRIMAEENGMTVDEKEFEIAQETAKNLSRKVKGGNDGDDVVALDVHDIAAL

DANNAVPKTDDSYKYSDSIIQARVKAIFYKRAFYPDSSAVPEGRNFGILTDKTNFYAESGGQEYDTGSIISLDGETEFVV

EDCQVYGGYVLHVGHLKYGKLSLDTEVEVSYDETRRHTLRNYHTSTHILNFALREVLGGEIDQKGSLVSQEKLRFDFSFK

SALTTKQLAEIEKMCSSFITKNTKVYSREVPLPVAKAIHGLRAVFGETYPDPVRVVSIGFDVEDITKDVANPKWKTTSIE

FCGGTHVVKTGDIKSFAILEESGIAKGIRRIVALTGDEALAADRAAKEFVTKLDKLEKLSGADLDAALKVVSKELDALTI

SAVTKAEYRERFNKTKKTFDDSEKARKAEQVKEATEAVKAYFVSNPKATYLVTSLNVGNNSKALAGAINYAKASLKDKAV

YVLSADKDSGRVAHNCIVGKDLIAKGLKASDWAGVVSEKVGGKKGGKEDSAQGSGDKIDGMADALKAAEEFAKLKIGA

>XP_011276651.1 alanyl-tRNA synthetase [Wickerhamomyces ciferrii]

MKPSNYFKERGHTFVPSSSVVPYDDPTLLFANAGMNQYKPIFLGTVDPSSDFHNLKRAVNSQKCIRAGGKHNDLEDVGRD

SYHHTFFEMLGNWSFGDYFKKEAIQYSWELLTKVYGLPADRLYVTYFGGDEASGLEPDLEAKQLWKDVGVADDHILPGSV

KDNFWEMGDQGPCGPCSEVHYDRIGGRNAAHLVNEDDPNVLEIWNNVFMQFNREADRSLKPLPSKHIDTGLGFERLVSIL

QNVYSNYDTDVFQPLFKKIQEITGAREYTGKFGDEDVDGIDTAYRVLADHVRTLTFAIADGGVPNNEGRGYVLRRILRRG

ARYARKYFNYPIGNFFSQLSPTLIDQVKDIFPELTKDPSTIFEILDEEEASFSKTLDRGEKLFQQYATAASKSDKTLNGK

DVWRLYDTYGFPVDLTTLMAEEAGLKIDNEGFEKARIESYEASKAGGKSKGVELIKLDVHALGKLSEDALPKTDDSSKFG

FENIEAKILAIYDGKDFVEEITDITKQYGIILDKTPFYAEQGGQEYDTGKIVIDGSSEFNVENTQQYAGYVFHTGRLAEG

QLKINDKVIAGYDELRRWPIRNNHTATHILNLALREVLGDEVNQKGSLVAQEKLRFDFSNKQGLTLDQVAKVEQISNEEI

KKSQVVYSKEVALDVAKGIYAVRAVFGETYPDPVRVVSIGQPVEDLLNDPQNPKWHGASIEFCGGTHVAKTSDIKDFVIV

EESGIAKGIRRIIAVTGTEAHEVQRIAQEFEHELINAGKLEFSPLKEKKLKELGVKLGQLNISILTKNELKNKFAAIDKS

VKDELKTRQKAESKKTLDAVKEYFQSNEKSEFFVTHVDIGSNAKAITEAINYVKSKLTGKSLYVFTGNEDKVAHGCYISD

EAIAKGIVAKEVAEQATKHIGGKAGGKGNVSQGMGDKVSGVDSAVADIQKLLAEKLNI

>CCE82501.1 Piso0_002228 [Millerozyma farinosa CBS 7064]

MIKLFVHKKAMSTTSNNWTASKVRSTFLDFFKNKNHTFVPSSSVVPHNDPSLLFANAGMNQYKPIFLGTVDPGSEFATLK

RAVNSQKCIRAGGKHNDLEDVGRDSYHHTFFEMLGNWSFGDYFKKDAIAWAWELLTEEYKLEKDRLYVTYFEGDEKNGLE

PDLEAKELWSKVGVPDDHILPGNAKDNFWEMGDQGPCGPCSEIHYDRIGGRNAASLVNEDDPDVIEVWNIVFIQYNREAD

SSLRPLPAKHIDTGMGFERLVSILQNKSSNYDTDVFTPIFNKIREVTGVRPYTGKFGSEDKDGIDTAYRVIADHARTLTF

AISDGGVPNNEGRGYVLRRILRRGARYVRKYMNYPIGSFFQQIISVIIERNSDIFPEISKYQEELKEILNEEELSFAKTL

DRGEKLFEQYAIIASKTPEQTLSGRDVWRLYDTYGFPVDLTRLMAEEAGTKIDEEGFEKARLESREASKSNTSKSGNQPI

KLDVHALSDLDNKQIPKTDDSYKYGLDNVKAKIVEIFDGSSFVDTIDSTEKEYGILLDKTAFYAEQGGQEYDTGNLVIDG

SAEFNVTNVQVYGGYVLHTGTMVEGILKVGNEVIATYNELRRWPIRNNHTGTHVLNFALREVLGDSVDQKGSLVAPEKLR

FDFSHKKAVSTEELRKIEDISNHYIKEDKKVFAKDVSLSEARNINGLRAVFGETYPDPVRVVSIGVPVEDLLKDPSNSQW

HKYSIEFCGGTHVSKTAEIKYLVIIEESGIAKGIRRIVAVTGNEAYEVQRIAADFENELKKASQLPFGEEKQQRVKEIGV

KLKTLSISVLEKQKLNDLFNSLDKSIKDELKSQQKIESKKVLDVVNNWLASENAKPYYVDYIPINANAKAITEAINLLKK

KHNEKSLYLITGSDKVAHGCYISDDAISRGVDITGLASLVSSKVGGKAGGKGNVIQGMGDNAAGVQEAIAQLEKAFAEKL

>5KNN_A Chain A, Evolutionary Gain Of Alanine Mischarging To Non-cognate Trnas With A G4:u69 Base Pair

TLTASEIRQRFIDFFKRNEHTYVHSSATIPLDDPTLLFANAGMNQFKPIFLNTIDPSHPMAKLSRAANTQKCIRAGGKHN

DLDDVGKDVYHHTFFEMLGSWSFGDYFKELACKMALELLTQEFGIPIERLYVTYFGGDEAAGLEADLECKQIWQNLGLDD

TKILPGNMKDNFWEMGDTGPCGPCSEIHYDRIGGRDAAHLVNQDDPNVLEIWNLVFIQYNREADGILKPLPKKSIDTGMG

LERLVSVLQNKMSNYDTDLFVPYFEAIQKGTGARPYTGKVGAEDADGIDMAYRVLADHARTITVALADGGRPDNTGRGYV

LRRILRRAVRYAHEKLNASRGFFATLVDVVVQSLGDAFPELKKDPDMVKDIINEEEVQFLKTLSRGRRILDRKIQSLGDS

KTIPGDTAWLLYDTYGFPVDLTGLIAEEKGLVVDMDGFEEERKLAQLKSQ

>OAD08861.1 hypothetical protein MUCCIDRAFT_105829 [Mucor circinelloides f. lusitanicus CBS 277.49]

MAGQTEWPANKVRATFIKYFEDNGHVFVPSSSTIPHDDPTLLFANAGMNQYKPIFLGTLDPNSPLANVKRACNSQKCIRA

GGKHNDLDDVGRDTYHHTFFEMLGNWSFGDYFKLEATKFGWEFLTKVISLPEDRLYVTYFGGDEKMGLPADLEAKQMWMD

LGVEEKRLIPGSVKDNFWEMGETGPCGPCSEIHYDRIGGRDAAHLVNMDDPNVLEIWNLVFMQFNREIDGSLRPLPNQHI

DTGLGLERLVSILQNKYSNYDTDCFTPLFDKIKELTGARDYTGLLGEEDKDGIDTAYRVVGDHIRTLTFAISDGGVPSNE

GRGYVLRRILRRGARYVRKYFNVPIGTFFSSLVDTLLEQMHEAYPELAKKAADVKAILDEEEVAFSKTLDRGEKLFELYM

NKTKESGSSVLSGDDVWRLYDTYGFPVDLTRIMAEENGLEVNEAQFEAAQEAAKSKSRKVKGGDNGQGVVALDVHDLGAL

DNNADVPKTDDSHKYLPGNVQGDIKAIFFEHKFLDNTADIPEDRNFGLLVDKTNFYAESGGQEYDTGSIITLDGNTEFVV

EDCQLYGGYVLHVGHLKYGQLKLNDKVELSYDDNRRYPLRNNHSATHILNFALREVLKEDIDQKGSLVSPEKLRFDFSFK

NPVNVKQLAEVEKICDDFIAKNLKVYSKEVPLAVAKAIHGLRAVFGETYPDPVRVVSIGFDVDEITNDVSNPKWGTTSIE

FCGGTHVAKTSDIKYFVILEESGIAKGVRRIVAVTGDEAAAAHRNADEFVGKLDRLEKLSGAELEAELKVINKELDLAVI

SAVSKAQFRERFTQIKKVFDNAEKARKAEQVKEASDAVKKYFEANPDAQYLVTELPVGNNSKALGGAINYAKANLKDKAV

YVLSGDKASGRVAHNCIVGKNLIEKGFKASDWATVVCEKVGGKKGGKDDAAQGSGDKVDALSDALKAAEEFAKLKISA

>EPB88726.1 alanyl-tRNA synthetase [Mucor circinelloides f. circinelloides 1006PhL]

MAGQTEWPANKVRSTFVKYFEDNGHVFVPSSSTIPHDDPTLLFANAGMNQYKPIFLGTLDPNSPLANVKRACNSQKCIRA

GGKHNDLDDVGRDTYHHTFFEMLGNWSFGDYFKLEATKFGWEFLTKVINLPEDRLYVTYFGGDEKMGLPADLEAKQMWMD

LGVEEKRLIPGSVKDNFWEMGETGPCGPCSEIHYDRIGGRDAAHLVNMDDPNVLEIWNLVFMQFNREIDGSLRPLPNQHI

DTGLGLERLVSILQNKYSNYDTDCFTPLFDKIKELTGARDYTGLLGEEDKDGIDTAYRVVGDHIRTLTFAISDGGVPSNE

GRGYVLRRILRRGARYVRKYFNVPIGTFFSSLVDTLLEQMHEAYPELAKKADDVKAILNEEEVAFSKTLDRGEKLFEAYM

NKTKESGSSVLSGDDVWRLYDTYGFPVDLTRIMAEENGLEVNEAQFEAAQEAAKNKSRKVKGGDNGQGVVALDVHDLGAL

DNNSAVPKTDDSHKYLPGNVQGEIKAIFFEHKFLDNTADIPEDRNFGLLVDKTNFYAESGGQEYDTGSIITLDGNTEFVV

EDCQLYGGYVLHVGHLKYGQLKIDDKVELSYDDNRRYPLRNNHSATHILNFALREVLKEDIDQKGSLVSPEKFRFDFSFK

NPVNVKQLAEVEKICDDFIAKNLKVYSKEVPLAVAKAIHGLRAVFGETYPDPVRVVSIGFDVDEITNDVSNPKWGTTSIE

FCGGTHVAKTSDIKYFVILEESGIAKGIRRIVAVTGDEAAAAHRNADEFVGKLDKLERLSGAELEAELKVVSKELDLAVI

SAVSKAHFRERFAQIKKAFDNAEKARKAEQIKEASDAVKKYFDANPDAQYLVTELPVGNNSKALGGAINYAKANLKDKAV

YVLSADKASGRVAHNCIVGKSLIEKGFKASDWATVVCEKVGGKKGGKDDAAQGSGDKVDGLSDALKAAEEFAKLKISA

>XP_019039224.1 hypothetical protein WICANDRAFT_42444 [Wickerhamomyces anomalus NRRL Y-366-8]

MSTQTQQEWTAQKVRNTFLDYFKERGHTFVRSSSVIPFEDPTLLFANAGMNQYKPIFLGTVDPASDYYNLKRAVNSQKCI

RAGGKHNDLEDVGRDSYHHTFFEMLGNWSFGDYFKKDAIKYSWELLTKVYGLDPDRLYVTYFGGDEASGLEPDLEAKQLW

SDVGVADDHILPGSVKDNFWEMGDQGPCGPCSEVHYDRIGGRNAAHLVNEDDPNVLEIWNNVFMQFNREADRSLRPLPSK

HIDTGLGFERLVSILQGVYSNYDTDVFQPLFKKIQEITGAREYTGKFGDEDKDGVDTAYRVLADHVRTLTFAIADGGVPN

NEGRGYVLRRILRRGARYVRKYFNYPIGNFFSQLAPTLIEQVKDIFPEIAENTARTFEILDEEEASFSRTLDRGEKLFEQ

YAGAASKGDKTLNGKDVWRLYDTYGFPVDLTTLMAEEAGLKIDNEGFEKARIASYEASKAGGKSKGSNLVKLDVHALGKL

SEQSLPKTDDSFKFGLENVEAKILAIYDGKDFVDEITDITKQYGIILDKTPFYAEQGGQEYDTGKIVIDGSSEFNVENTQ

QYAGYVFHTGRLAEGKLKVDDSVIAGYDELRRWPIRNNHTGTHILNFALREVLGDEINQKGSLVASEKLRFDFSHKQGLT

LDEVAKVEKISNAEIQKAQLVYSKEVPLKTAQSIYAVRAVFGETYPDPVRVVSIGHPVDDLLSDPENPKWHGISIEFCGG

THVAKTSDIKDFVITEESGIAKGIRRIIAVTGNEAHEVQRVASEFEHELEQVGKLEFSPMKEKKLKELGVKLGQLNISIL

TKNELKAKFTKLDKAIKDELKARQKIESKKTLDAVKDHFAANEKSNFLVTHVDIGSNAKAITEAINFIKSKNPEKSIYIF

TGNQDKVAHGCYVSNSAIEKGVNATDLAKQASQFIGGKAGGKGNVVQGMGDKVDGVEDAVKEVEKLLSEKLII

>CCE81569.1 Piso0_002228 [Millerozyma farinosa CBS 7064]

MIKLFVHKKAMSTTSNNWTASKVRSTFLDFFKNKNHTFVPSSSVVPHNDPSLLFANAGMNQYKPIFLGTVDPSSEFATLK

RAVNSQKCIRAGGKHNDLEDVGRDSYHHTFFEMLGNWSFGDYFKKDAIAWAWKLLTEEYKLEKDRLYVTYFEGDEKNGLE

PDLEAKELWLKVGVPEDHILPGNAKDNFWEMGDQGPCGPCSEIHYDRIGGRNAASLVNEDDPDVIEVWNIVFIQYNREAD

SSLRPLPAKHIDTGMGFERLVSILQNKSSNYDTDVFTPIFNKIREVTGVRPYTGKFGSEDKDGIDTAYRVIADHARTLTF

AISDGGVPNNEGRGYVLRRILRRGARYVRKYMNYPIGSFFQQIIPVIIERNSDIFPEISKYQEELKEILNEEELSFAKTL

DRGEKLFEQYAIIASKTPEQTLSGRDVWRLYDTYGFPVDLTRLMAEEAGTKIDEEGFEKARLESREASKSNNSKSGNQPM

KLDVHALSDLDNKQIPKTDDSYKYGLDNVKAKIVEIFDGSGFVDTIDSTEKEYGILLDKTAFYAEQGGQEYDTGKLVIDG

SAEFNVTNVQVYGGYVLHTGTLVEGSLKVGNEVIATYNELRRWPIRNNHTGTHVLNFALREVLGDSVDQKGSLVAPEKLR

FDFSHKKAVTTEELNKIEDISNHYIKEDKKVFAKDVSLSEARSINGLRAVFGETYPDPVRVVSIGVPVEDLLKDPSNSQW

HKYSIEFCGGTHVAKTAEIKHLVIIEESGIAKGIRRIVAVTGNEAYEVQRVAADFEDELKKASELPFGEVKQQKAKELGV

KLKTLSISVLEKQKLNELFNSLDKSIKDEIKSQQKLESKKVLDAVNDWLASENAKPYYVDYIPISANAKAITEAINLLKK

KHNEKSLYLITGSDKVAHGCYVSDDAISKGIDVTGLASLVSSKVGGKAGGKGNVVQGMGDNAAGVQEAIAELEKAFVEKL

>XP_001643652.1 hypothetical protein Kpol_1040p7 [Vanderwaltozyma polyspora DSM 70294]

MTIGDTDRWTAAVVRNTFLDYFKSKEHKFVRSSPVVPFDDPTLLFANAGMNQYKPIFLGTVDPSSEFYTLKRAYNSQKCI

RAGGKHNDLEDVGRDSYHHTFFEMLGNWSFGDYFKKEAVEYSWELLTQVYSIPADRLYVTYFEGDEKQGLEPDHEARELW

RSVGVPDDHILPGNAKDNFWEMGEQGPCGPCSEIHYDRIGGRNAAHLVNQDDPDVLEVWNLVFMQYNREQDRSLKSLPAK

HIDTGMGFERLVSVLQDVRSNYDTDVFQPLFKAIQEIANVRPYTGKFGAEDVDGIDTAYRVLADHVRTLTFALADGGVPN

NEGRGYVLRRILRRGARYARKYMNYPIGNFFSRLMPTLIEQVKDIFPEVAKDPSFVAEILDEEEASFSKTLDRGEKLFEK

YAAAASQTESKTLDGKQVWRLYDTYGFPVDLTELMAEEQGLKIDGPGFEKAKQESYEASKRGGKKDNSDLLKLNVHELSQ

LNEEKIAKSDDSSKYGFDNVDGTILKLYDGEQFVDEITEPGKKYGIILDNTCFYAEQGGQEYDTGKIIIDDVAEFNVENV

QLYNGYVFHTGSLVEGKLSVGDKIIAAYDELRRHPIKNNHTGTHILNYALKETLGNDVDQKGSLVAPEKLRFDFSHKKAL

SLDELRNVEEVCNQQIKDNMAVYYKEVPLDLAKTISSVRAVFGETYPDPVRVVSVGKPIDEILANPSNEEWNKYSIEFCG

GTHVSKTGDIKYFIILEESGIAKGIRRIVAVSGTDAFEAQRIANEYESELESTEKLPFSPLKEKKLKELGVQLGQLSISV

IKKNELKEKFTKIEKSVKDEVKSRAKLETKQVLDEVKEHFTENTESLFLVKHIDIPPNAKAITEAIAHIKTKNKDKTIYL

LTGNDPEGRVAHGCFISDVAISKGVDGAALAKKVSASIGGKAGGKGNVFQGMGDKHIGIEEAVAAIEAEFNQFLTM

>XP_022464561.1 hypothetical protein KNAG_0E00470 [Kazachstania naganishii CBS 8797]

MTIGDKEKWTAKNVRNTFMDYFKQREHTFVHSSPVVPFDDPTLLFANAGMNQYKPIFQGTVDPSSDFYTLKRAYNSQKCV

RAGGKHNDLEDVGKDSYHHTFFEMLGNWSFGDYFKKEAIQFAWELLTVVYGIPSDRLYVTYFEGDSKLGLQPDEEARQLW

RDVGVLDDHILPGNAKDNFWEMGEQGPCGPCSEVHYDRIGGRNAASLVNMDDPDVLEVWNLVFMQFNREQDGSLKSLPAK

HIDTGMGFERLVSVLQDVRSNYDTDVFIPLFKRIQEITNARPYAGKFSEEDADGVDTAYRVLADHVRTLMFALADGGVPN

NEGRGYVLRRILRRGARYARKYMNYPIGNFFSTLAPTLIEQVKDIFPELAKDPSFLFEILDEEEASFAKTLDRGERLFEK

YAATAAENSTKTLDGKQVWRLYDTYGFPVDLTELMAEEKGLKIDGPGFEKAKQESYEASKRGGKKDDTSLIKLNVHELSE

LNNENVPKTDDSAKYGFENIDAKILKLHDGTAFVDEITETDKKYGMILDKSCFYAEQGGQEYDTGKIVIDDVAEFNVENV

QLYNGFVFHTGSLKEGKLSVGDKVIASYDELRRFPIKNNHTGTHILNFALRQVIGKEVDQKGSLVAPEKLRFDFSHKKAL

SLDEVRSVENVCNEQIKESLSVYYKDVPLEQAKAISSVRAVFGETYPDPVRVVSIGKPVDELLADPANEEWNKYSVEFCG

GTHVVKTSDIKFLVILEENGIAKGIRRIVAVTGTEAFEAQRVAQEFDSQLVATEKMFFSAEKEKKLKELGVQLGQLSVSV

IDKHELKEKFNKIDKAVRDEVKSRAKKEIKQTLDDVKSYFTENGESTFLVKHISIPVNAKAITESVNYLKSSDKDKSIYL

FTGNDDNGKIAHGCYISDAAIAKGVDGSALAKQVSGIIGGKAGGKGNVFQGMGDKPSDVAKAIAEVEQALKTKLSL

>XP_011270692.1 alanyl-tRNA synthetase [Capsaspora owczarzaki ATCC 30864]

MSVDWTTAPASAVRQQFVDFFVSKGHEFVPSSSVIPHDDPTLLFANAGMNQFKPAFLGTADPNSRMAKYRRTANSQKCIR

AGGKHNDLEDVGKDVYHHTFFEMLGNWSFGDYFKKEAISWAWELLTVVYKLDPNRLYVTYFGGNEKAGLEADLEARDIWL

SVGLPAERVLPYGMKENFWEMGETGPCGPCSEIHFDRIGGGRDAAALVNADDPDVLEIWNLVFIQFNREVDQSLRKLPRC

HVDTGMGLERIVSVLQNKRSNYDTDVFVPYFTAIQAATGIRAYTGKVGADDTDGLDMAYRVVADHIRTLTIALADGGAPG

PTGRDYVLRRVLRRAVRYCAEKLGAKPGFFASLVDVVGTSLGGFFPELVRSPQDIKDIINEEEAQFRKTLDRGKRLFERA

AAASKNGVIEGLVAWRLYDTYGFPLDLTELMAAELKLTIDKPGFKIAEADAKEASKGKVDEAEETVVLDVHNISALKDKG

VPATDDSPKFNYRSENDKYTFESAVGSIVAIALGKELVSTVDFESLKGAKVGIVLDKTNFYAEQGGQIFDTGFATRVDNE

ASEFAIESVKVYGPYVLHVGRLQGGSLSIGDKLTLSIDDIRRLPIMANHTTTHMLNFALREVLGNSVDQKGSLVRPDNFR

FDFSHPKAVSTEELRKIEKIVNDIIEKNETVYRVPTALHLAKEVAGLRAVFDEVYPDPVRVVSVGVPVEKLLADPASASW

KGYSIEFCGGTHIDRTRDARRFAIFREEGIAKGIRRISGITGKEAIEAYKTADKLEAKVKQVSEIPIGQLGRELPSLIES

VDTAVIPSFRKDEIKAAIAAISKRVSDHEKAQQAEQTKIATAAVKERIDAGAPFVVAFVDINGNRKAMSAGLSAAKDAAG

LLVSKDGDSIIAVASVPKALVGKIKAGEWVTEAVKSLSNTKTGGADTVGQASGTGSDHAAVIDAATKFAAARLQ

>ORX87486.1 hypothetical protein BCR32DRAFT_289308 [Anaeromyces robustus]

MSEEVYWTTKRVREEFIKFFQKYDHTYVPSSSTIPHDDPTLLFANAGMNQYKPIFLGVVDPNSDMAKLKRACNSQKCIRA

GGKHNDLDDVGKDVYHHTFFEMLGNWSFGDYFKEGAIKMAWELLTEVYKLPKDRLYITYFGGDEKLGLKADLECRQIWLD

LGVPAERLLPFGSKDNFWEMGDQGPCGPCCELHYDRKGGRNAADLVNMDDPEVIEIWNLVFMQYNREADGSLRPLPNKHI

DTGMGLERVVSVLQNKMSNYDTDAFTGLFSAIQKLTNAAPYTGKVGAEDVNGMDTAYRVIADHTRTLTFAISDGGVPSNE

GRGYVLRRILRRGARFARRKFNVQIGNFFSQLVDVVISEMGDVYPEITKNVDDLKEILNEEEKSFARTLDRGEKLFDACI

AKTKEQGSTQIKGADVWRLYDTYGFPVDLTTLMAEENGMTINQEEFLAAQEEARERSKACNNGNKAETVTLDVHAIGALD

NNDAVPKTDDAAKYERGAINANIKALYVDGKFVDNVDESFTKNFGVILDKTNMYAEQGGQEYDFGTITIDGKADFAVESV

QTYGGYVLHVGYLKYGSFALNDEVVAAYDEDRRSLIQNNHTGTHILNYALREVVGDTVDQKGSLVNAEKLRFDFSCKNAI

PVEDVEKIEAICADFINRDLKVYTQEVQTSIGKQIYGIRAVFGEVYPDPVRVVSIGFDVAEVIKDVSNPKWRTTSMEFCG

GTHVNTTGEIKQLVIIEETSIAKGIRRITALTADEAAKVVDNAKAFAPKLETLKSLKGAELEEYLKTFLKELDASVLPFV

AKQKYREEYNAVKKAFDDSEKARKAAQIKEVTEFVKTEVEKNPEAKYVVLRLNAGNNSKAVAGAINYIKGLKNKAALFIS

VDEASGRVSHQCIVPKEFTKSGFKAIDWASVVADKVGGKKGGKDVSAQGSGTEVSAVDEALKLADEFAKLKIASL

>CCA39700.1 Alanine-tRNA synthetase [Komagataella phaffii CBS 7435]

MSTLASKEWTANKVRSTFFDFFKSKDHTYVPSSSVIPYDDPTLLFTNAGMNQYKPIFLGTVDPSSDFATLKRATNSQKCI

RAGGKHNDLEDVGRDSYHHTFFEMLGNWSFGDYFKKEAIGYGWELLTKVYGLEKERLYVSYFGGDAGLGLEPDLEAKEYW

KALGVAEDHILPGSVEDNFWEMGDQGPCGPCSEIHYDRIGGRNAASLVNQDDPDVLEIWNIVFIQFNREADGSLKPLPSK

HIDTGMGFERLVSILQNVRSNYDTDVFDPLFTKIQEITGVRPYSGHFGKEDMDGIDTAYRVLADHVRTLTFSLADGGVPN

NEGRGYVLRRILRRGARYARKYMNYPIGKFFSQLAPALIEQVKETFPEVDKNFDTIAEILDEEEASFSRTLDRGERLFEQ

YAIIASKTPEQTLAGRDVWRLYDTYGFPVDLTRLMAEETGLKIDDEGFEIAKEASREASKGNGKTQGKELVKLDVHVLGE

LEKNSEVPKTDDSYKYGTKNIEASIVAIYNGKELIQSTSEVADDQQLGILLDKTPFYAEQGGQIYDTGKIVIDGVAEFNV

ENVQSYAGYVLHTGYVEEGVLSVSDKIIAGYDELRRWPIKNNHTGTHILNFALRDVLGDDVEQKGSLVAPEKLRFDFSHK

KALTLSELQKVEDISNEVIKKNVPVFASDVTLAVGKEINGLRAVFGETYPDPVRVVSIGVPVEELVKNPSNAEWHNASIE

FCGGTHVAKTGDIKDFVIIEESGIAKGIRRIVAVTSTEAHTVQRIAREKNDQLDAIAKMSFGEAKEKKLKEFNVALGKLQ

ISVLAKNDLKTKFTNIEKVVKDELKVRQKAESKKTLDTVKDYFNEHEDSPFFVAHVPVSSNAKLLTEAFSYIKKEIPDKS

LYLIVGNESEPKIAQGCYVSDNALSKGVVASEVTNVASEFIGGKAGGKGNICQGVGTNPEGINKAIEAVTKILKEKLSI

>PIS56237.1 alanine-tRNA synthetase [[Candida] auris]

MLKTLTRKHTMTTWTASTVRQTFLDFFKSKQHTYVPSSSVVPHNDPTLLFANAGMNQYKPIFLGTVDPTSDFASLKRAAN

SQKCIRAGGKHNDLDDVGKDSYHHTFFEMLGNWSFGDYFKKEAIEWSWELLTGIYKLEKDRLYVTYFEGDEKNGLKPDLE

AKQYWLDVGVAEDHILPGNATDNFWEMGDQGPCGPCSEIHYDRIGGRNAASLVNMDDPNVLEVWNIVFIQYNREADSSLR

PLPNKHIDTGMGFERLVSILQNKFSNYDTDVFTPIFEKIREITGVRPYTGKFGADDVDGIDTAYRVIADHVRTLTFAITD

GGVPNNEGRGYVLRRILRRGSRYVRKYMNYPIGSFFQQLVDVVIEQNSKIFPEIAHNLAELKEILVEEEVSFAKTLDRGE

KLFEQYAIIASKTPEQTLSGKDVWRLYDTYGFPVDLTRLMAEEAGLKIDEVAFEKAKQESKEASKASGSKSGAELIKLDV

HALSELDGNDAVPKTDDHYKYGLENTKSKIVALYDGSKFTDSISEAGQQYGILLNKTPFYAEQGGQEYDTGSLVIDGSAE

FNVTNVQSYAGYVLHTGNLVEGTLKVGDELIATYDELRRWPIRNNHTGTHVLNFALREVLGDSVDQKGSLVAAEKLRFDF

SNKQALTTKELEDVERICNENIKANMEVYAEDVSLATAKQINGLRAVFGEVYPDPVRVVSIGKPVSELLKDPENKEWHKY

SIELCGGTHVAKTADIKDFVIIEESGIAKGIRRIVAVTGHDAHRVQQIAKDFDAELDHASNLPFGPDKEQQAKELGVTLK

KLSISVLDKQKLNDKFSKLDKSIKDQLKAKQKEESKKTLEVINEWLKDEEKSKAPFLVAHVPISANAKAITEAFNLIKKQ

AKEKCLYMITGTSEKVAHGCYISDEAISKGVDASQVAQAAIAYIGGKAGGKGNIVQGMGDKPEGIDEALKAVKKLLEEKL

Q

>XP_002492690.1 Cytoplasmic alanyl-tRNA synthetase [Komagataella phaffii GS115]

MLKFPHKGISSIKSKPRIGIIAMSTLASKEWTANKVRSTFFDFFKSKDHTYVPSSSVIPYDDPTLLFTNAGMNQYKPIFL

GTVDPSSDFATLKRATNSQKCIRAGGKHNDLEDVGRDSYHHTFFEMLGNWSFGDYFKKEAIGYGWELLTKVYGLEKERLY

VSYFGGDAGLGLEPDLEAKEYWKALGVAEDHILPGSVEDNFWEMGDQGPCGPCSEIHYDRIGGRNAASLVNQDDPDVLEI

WNIVFIQFNREADGSLKPLPSKHIDTGMGFERLVSILQNVRSNYDTDVFDPLFTKIQEITGVRPYSGHFGKEDMDGIDTA

YRVLADHVRTLTFSLADGGVPNNEGRGYVLRRILRRGARYARKYMNYPIGKFFSQLAPALIEQVKETFPEVDKNFDTIAE

ILDEEEASFSRTLDRGERLFEQYAIIASKTPEQTLAGRDVWRLYDTYGFPVDLTRLMAEETGLKIDDEGFEIAKEASREA

SKGNGKTQGKELVKLDVHVLGELEKNSEVPKTDDSYKYGTKNIEASIVAIYNGKELIQSTSEVADDQQLGILLDKTPFYA

EQGGQIYDTGKIVIDGVAEFNVENVQSYAGYVLHTGYVEEGVLSVSDKIIAGYDELRRWPIKNNHTGTHILNFALRDVLG

DDVEQKGSLVAPEKLRFDFSHKKALTLSELQKVEDISNEVIKKNVPVFASDVTLAVGKEINGLRAVFGETYPDPVRVVSI

GVPVEELVKNPSNAEWHNASIEFCGGTHVAKTGDIKDFVIIEESGIAKGIRRIVAVTSTEAHTVQRIAREKNDQLDAIAK

MSFGEAKEKKLKEFNVALGKLQISVLAKNDLKTKFTNIEKVVKDELKVRQKAESKKTLDTVKDYFNEHEDSPFFVAHVPV

SSNAKLLTEAFSYIKKEIPDKSLYLIVGNESEPKIAQGCYVSDNALSKGVVASEVTNVASEFIGGKAGGKGNICQGVGTN

PEGINKAIEAVTKILKEKLSI

>BAP72231.1 alanyl-tRNA synthetase [Kluyveromyces marxianus]

MTIGDKAKWTATNVRSTFLDYFKSKGHTFVPSSPVIPYDDPTLLFANAGMNQYKPIFLGTVDPSSDFYKLKRATNSQKCI

RAGGKHNDLEDVGRDSYHHTFFEMLGNWSFGDYFKKEAIEYSWELLTKVYGIPGDRLYVTYFEGDEKLGLEPDFEARDLW

KAVGVADDHILPGNAKDNFWEMGEQGPCGPCSEVHYDRIGGRNASSLVNQDDPDVLEIWNNVFIQFNREQDGSLRPLPAK

HVDTGMGFERLVSVLHDVRSNYDTDVFEPLFKRIQEITGVRPYSGKFGAEDVDGIDTAYRVLADHVRTLTFALADGGVPN

NEGRGYVLRRILRRGARYARKYMNYPIGNFFSQLSVTLIEQVKDMFPEVAKDPSYLFEILDEEEASFAKTLDRGEKLFEK

YAEETMKKSDKTLDGKQVWRLYDTYGFPVDLTELMAEEQGLKIDTEGFEKAKKESYEASKQGGKKGTSNLIKLNVHELSQ

LNESNVPKTDDSFKYGTENIEATILRIHDGEAFVDSMTEVGKQYGIVLDKTCFYAEQGGQEYDTGKLVIDGQAEFNVENV

QVYNGYVFHTGILGDGELSTGDKIIASYEELRRHPLRNNHTGTHILNFALKEVLGNDVDQKGSLVAPEKLRFDFSHKRAL

SYEELVDVEKITNEKIKENLTVYYKDVALDLAKTISSVRAVFGETYPDPVRVVSVGKPVDEILENPSSEEWSKYSVEFCG

GTHVSKTGDIKEFVIVEESGIAKGIRRIVAVSGPEAYEVQRVAVEFDGELDAVDKLPFSALKAQKVKELGVRLGQLNISV

ISKTDLKEKFNKIEKEVKDEMKVRAKKEIKQTVDDVKQYFEENKDASFYVKHIDIPTNAKAITEAINHIKTNLKDKSIYL

FTGNDANGKVAHGCYISDAAISKGVDGSSLAKKVSANIGGKAGGKGNVFQGMGDKPSSIDSAISEVSDLLKEKLTI

>CCE39704.1 hypothetical protein CPAR2_601240 [Candida parapsilosis]

MSTTSTINTTEKWTAPKVRSTFLDYFKDKRQHTYVPSSSVVPHNDPTLLFANAGMNQYKPIFLGTVDPASDFAKLKRAAN

SQKCIRAGGKHNDLEDVGRDSYHHTFFEMLGNWSFGDYFKKEAIAWSWELLTDVYGLEKDRLYVTYFGGDEKQGLEADLE

AKKFWLDVGVAEDHILPGDAKDNFWEMGDQGPCGPCSEIHYDRIGGRNAASLVNMDDPNVLEVWNVVFIQYNREADGSLR

PLPNKHIDTGMGFERLVSILQNKSSNYDTDVFLPIFDKIREITGVRPYTGKFGADDKDGIDTAYRVIADHVRTLTFAICD

GGVPNNEGRGYVLRRVLRRGSRYVRKYMNYPIGSFFPQLVDIVIQQNKEIFPEIESGSQDLKEILNEEELSFAKTLDRGE

KLFEQYAIIASKTPEQTLSGKDVWRLYDTYGFPVDLTRLMAEEAGLKIDEKGFEVAKEESREASKGGANKNASSLIKLDV

HALSELEGKVAKTDDDAKYGLDNIKAKVLAIYDGSKFVDSIQQNEEGQQYGILLDKTPFYAEQGGQEYDTGKLVIDGKTE

FNVENVQVYAGYVLHTGFVLEGKLSVGDDVIAAYDELRRWPIRNNHTGTHVLNYALREVLGDDVDQKGSLVAKEKLRFDF

SHKQALTPKELEKVEGISNQYIRDNKQVYYKDVPLTEAKAICGLRAVFGETYPDPVRVVSIGVPVDELLKDPTNEEWRSI

SIEFCGGTHVAKTSEIKDLVIIEESGIAKGIRRIVAVTGHDAHAVQRVAKEYEQELDHANELPNGPIKENKVKELGVALK

KLSISVLDKQKLTEKFNKVDKSIKDHQKSIQKEESKKTLDVVKKWLDEGDDNNKSEFLVAHIPINANAKAITEAFNLVKK

QDKTKSLYLLTGQNDKVAHGCYVSDEAIAKGVDASSLAKLVSGKIGGKAGGKGNIVQGMGDKPSGINEAIEEVTKSFKEK

L

>SGZ55244.1 CIC11C00000005342 [[Candida] intermedia]

MLKNLARKQIMTTSSHPWSASKVRTTFLDYFKDREHSYVPSSSVVPHNDPTLLFANAGMNQYKPIFLGTVDPTSDFASLK

RAVNSQKCIRAGGKHNDLEDVGRDSYHHTFFEMLGNWSFGDYFKKEAIGWSWDLLTNVYGLQKDRLYVTYFEGDEKNGLE

PDLEAKQFWLDVGVAEDHILPGNAKDNFWEMGDQGPCGPCSEVHYDRIGGRNAASLVNMDDPNVLEIWNIVFIQYNREPD

SSLKSLPSKHIDTGMGFERLVSVLQNKSSNYDTDVFQPIFEKIREITGVRPYTGRFGAEDVDGIDTAYRVIADHVRTLTF

AITDGGVPNNEGRGYVLRRILRRGSRYVRKYMNYPIGSFFQQLVDVVIDQNSSIFPEIVKNSRELKEILVEEEVSFAKTL

DRGEKLFEQYAIIASKTPEQTLSGKDVWRLYDTYGFPVDLTRLMAEEAGLKIDEAAFEAAKEESREASKATGSKNAANLI

KLDVHALSELDNNSAVTKTDDSFKYGLENTKSTIVALYDGSKFVDSISEAGAQYGIILDKTPFYAEQGGQEYDTGSLVID

GTAEFSVSNVQQYGGYVLHTGTLTEGTLKVSDDIIATYDELRRWPIRNNHTGTHVLNLALREVLGDGIDQKGSLVAPEKL

RFDFSHKQALAPKELEQVEKISNEVIKANKQVYAQDVSLAEAKGINGVRAVFGETYPDPVRVVSIGVPVDELLRSPENQE

WSNVSIEFCGGTHVAKTAEIKDLVIIEESGIAKGIRRIVAVTGHEAHEVQKIAEEFGVKLDNADKLPFGADKEQKTKELG

VALKKLSISVLDKQKLNEKFTKLDKAIKDNLKAKQKEESKKTLDAVNEWLKDEEKSKAAFFVAHVPIGANAKAITEAINL

IKKQFKEKSLYLVTGTTDKVAHGCYISDEAISKGVDANELASTVASHIGGKAGGKGNVVQGMGDKPSGIGAAVEAVTKLL

SEKL

>XP_022676577.1 alanyl-tRNA synthetase [Kluyveromyces marxianus DMKU3-1042]

MTIGDKAKWTATNVRSTFLDYFKSKGHTFVPSSPVIPYDDPTLLFANAGMNQYKPIFLGTVDPSSDFYKLKRATNSQKCI

RAGGKHNDLEDVGRDSYHHTFFEMLGNWSFGDYFKKEAIEYSWELLTKVYGIPGDRLYVTYFEGDEKLGLEPDFEARDLW

KAVGVADDHILPGNAKDNFWEMGEQGPCGPCSEVHYDRIGGRNASSLVNQDDPDVLEIWNNVFIQFNREQDGSLRPLPAK

HVDTGMGFERLVSVLHDVRSNYDTDVFEPLFKRIQEITGVRPYSGKFGAEDVDGIDTAYRVLADHVRTLTFALADGGVPN

NEGRGYVLRRILRRGARYARKYMNYPIGNFFSQLSVTLIEQVKDMFPEVAKDPSYLFEILDEEEASFAKTLDRGEKLFEK

YAEETMKKSDKTLDGKQVWRLYDTYGFPVDLTELMAEEQGLKIDTEGFEKAKKESYEASKQGGKKGTSNLIKLNVHELSQ

LNESNVPKTDDSFKYGTENIEATILRIHDGEAFVDSMTEVGKQYGIVLDKTCFYAEQGGQEYDTGKIVIDGQAEFNVENV

QVYNGYVFHTGILGDGELSTGDKIIASYEELRRHPLRNNHTGTHILNFALKEVLGNDVDQKGSLVAPEKLRFDFSHKRAL

SYEELVDVEKITNEKIKENLTVYYKDVALDLAKTISSVRAVFGETYPDPVRVVSVGKPVDEILENPSSEEWSKYSVEFCG

GTHVSKTGDIKEFVIVEESGIAKGIRRIVAVSGPEAYEVQRVAVEFDGELDAVDKLPFSALKAQKVKELGVRLGQLNISV

ISKTDLKEKFNKIEKEVKDEMKVRAKKEIKQTVDDVKQYFEENKDASFYVKHIDIPTNAKAITEAINHIKTNLKDKSIYL

FTGNDANGKVAHGCYISDAAISKGVDGSSLAKKVSANIGGKAGGKGNVFQGMGDKPSSIDSAISEVSDLLKEKLTI

>PIS56585.1 alanine-tRNA synthetase [[Candida] auris]

MTTWTASTVRQTFLDFFKSKQHTYVPSSSVVPHNDPTLLFANAGMNQYKPIFLGTVDPTSDFASLKRAANSQKCIRAGGK

HNDLDDVGKDSYHHTFFEMLGNWSFGDYFKKEAIEWSWELLTGIYKLEKDRLYVTYFEGDEKNGLKPDLEAKQYWLDVGV

AEDHILPGNATDNFWEMGDQGPCGPCSEIHYDRIGGRNAASLVNMDDPNVLEVWNIVFIQYNREADSSLRPLPNKHIDTG

MGFERLVSILQNKFSNYDTDVFTPIFEKIREITGVRPYTGKFGADDVDGIDTAYRVIADHVRTLTFAITDGGVPNNEGRG

YVLRRILRRGSRYVRKYMNYPIGSFFQQLVDVVIEQNSKIFPEIAHNLAELKEILVEEEVSFAKTLDRGEKLFEQYAIIA

SKTPEQTLSGKDVWRLYDTYGFPVDLTRLMAEEAGLKIDEVAFEKAKQESKEASKASGSKSGAELIKLDVHALSELDGND

AVPKTDDHYKYGLENTKSKIVALYDGSKFTDSISEAGQQYGILLNKTPFYAEQGGQEYDTGSLVIDGSAEFNVTNVQSYA

GYVLHTGNLVEGTLKVGDELIATYDELRRWPIRNNHTGTHVLNFALREVLGDSVDQKGSLVAAEKLRFDFSNKQALTTKE

LEDVERICNENIKANMEVYAEDVSLATAKQINGLRAVFGEVYPDPVRVVSIGKPVSELLKDPENKEWHKYSIELCGGTHV

AKTADIKDLVIIEESGIAKGIRRIVAVTGHDAHRVQQIAKDFDAELDHASNLPFGPDKEQQAKELGVTLKKLSISVLDKQ

KLNDKFSKLDKSIKDQLKAKQKEESKKTLEVINEWLKDEEKSKAPFLVAHVPISANAKAITEAFNLIKKQAKEKCLYMIT

GTSEKVAHGCYISDEAISKGVDASQVAQAAIAYIGGKAGGKGNIVQGMGDKPEGIDEALKAVKKLLEEKLQ

>SGZ55103.1 CIC11C00000002046 [[Candida] intermedia]

MLKNLARKQIMTTSSHPWSASKVRTTFLDYFKDREHSYVPSSSVVPHNDPTLLFANAGMNQYKPIFLGTVDPTSDFASLK

RAVNSQKCIRAGGKHNDLEDVGRDSYHHTFFEMLGNWSFGDYFKKEAIGWSWDLLTNVYGLQKDRLYVTYFEGDEKNGLE

PDLEAKQFWLDVGVAEDHILPGNAKDNFWEMGDQGPCGPCSEVHYDRIGGRNAASLVNMDDPNVLEIWNIVFIQYNREPD

SSLKSLPSKHIDTGMGFERLVSVLQNKSSNYDTDVFQPIFEKIREITGVRPYTGRFGAEDVDGIDTAYRVIADHVRTLTF

AITDGGVPNNEGRGYVLRRILRRGSRYVRKYMNYPIGSFFQQLVDVVIDQNSSIFPEIVKNSQELKEILVEEEVSFAKTL

DRGEKLFEQYAIIASKTPEQTLSGKDVWRLYDTYGFPVDLTRLMAEEAGLKIDEAAFEAAKEESREASKATGSKNAANLI

KLDVHALSELDNNSAVTKTDDSFKYGLENTKSTIVALYDGSKFVDSISEAGAQYGIILDKTPFYAEQGGQEYDTGSLVID

GTAEFSVSNVQQYGGYVLHTGTLTEGILKVSDDIIATYDELRRWPIRNNHTGTHVLNLALREVLGDGIDQKGSLVAPEKL

RFDFSHKQALAPKELEQVEKISNEVIKANKQVYAQDVSLAEAKGINGVRAVFGETYPDPVRVVSIGVPVDELLRSPENQE

WSNVSIEFCGGTHVAKTAEIKDLVIIEESGIAKGIRRIVAVTGHEAHEVQKIAEEFGVKLDNADKLPFGADKEQKTKELG

VALKKLSISVLDKQKLNEKFTKLDKAIKDNLKAKQKEESKKTLDAVNEWLKDEEKSKAAFFVAHVPIGANAKAITEAINL

IKKQFKEKSLYLVTGTTDKVAHGCYISDEAISKGVDANELASTVASHIGGKAGGKGNVVQGMGDKPSGIGAAVEAVTKLL

SEKL

>CUS20800.1 LAQU0S01e14928g1_1 [Lachancea quebecensis]

MTIGDKQKWTASNVRSTFMEFFKQREHTFVASSPVIPYDDPTLLFANAGMNQYKPIFLGTVDPSSDYYSLKRATNSQKCI

RAGGKHNDLEDVGRDSYHHTFFEMLGNWSFGDYFKKDAIQYSWELLTKVYGIPSERLYVTYFEGDEKQGLEPDLEAYDLW

KSVGVAEDHILPGNAKDNFWEMGDQGPCGPCSEIHYDRIGGRNAASLVNMDDPNVLEVWNIVFIQYNREQNGSLKSLPSK

HIDTGMGFERLVSVLQDVKSNYDTDVFQPLFARIQEITGARPYEGKFGAEDADGIDTAYRVLADHVRTLTFALADGGVPN

NEGRGYVLRRILRRGARYARKYMNYPIGNFFSSLAPTLIEQSKAMFPEINRDPSYLFEILDEEEASFAKTLDRGEKLFEK

YAQAAAQTKDKTLDGLQVWRLYDTYGFPVDLTELMAEEKGLKIDGPGFEKAKQESYEASKKGGKKSASDLIKLEVHELST

LNSENVPKTDDSSKYESANIEATVLRIFDGENFVDEISEVGKQYGVILDSTCFYAEQGGQEYDVGTLVIDDVAEFNVENV

QLYNGYVFHTGQLGEGKLSVGDKVIASFDELRRFPIKNNHTGTHILNFALKEVLGNDVDQKGSLVAPEKLRFDFSHKKAL

ALDELSKVEKISNEQIKANLPVYYKNVPLDLARKISSVRAVFGETYPDPVRVVSVGKPVDDMLADPASEEWQKYSVEFCG

GTHVSKTGDIKEFVIIEESGIAKGTRRIVAVSGSEAYEVQRIATKFDSDLTAAEKMPFSPLKEKKLKELGVALGQLSISV

VSKQELKNKFTKIEKVVKDEIKTRAKKEIKQTVDEVKNYFAENQDASFLVKFINIPTNAKAITEAVNHMKTSAKDKSIYL

FTGEDRVAHGCYISDAALEKGIDGRALLNTVSNNIGGKAGGKGNVFQGVGDKPEGIDAAVKEVENLFREKLDITK

>XP_018172104.1 hypothetical protein QG37_00639 [[Candida] auris]

MLKTLTRKHTMTTWTASTVRQTFLDFFKSKQHTYVPSSSVVPHNDPTLLFANAGMNQYKPIFLGTVDPTSDFASLKRAAN

SQKCIRAGGKHNDLDDVGKDSYHHTFFEMLGNWSFGDYFKKEAIEWSWELLTGIYKLEKDRLYVTYFEGDEKNGLKPDLE

AKQYWLDVGVAEDHILPGNATDNFWEMGDQGPCGPCSEIHYDRIGGRNAASLVNMDDPNVLEVWNIVFIQYNREADSSLR

PLPNKHIDTGMGFERLVSILQNKFSNYDTDVFTPIFEKIREITGVRPYTGKFGADDVDGIDTAYRVIADHVRTLTFAITD

GGVPNNEGRGYVLRRILRRGSRYVRKYMNYPIGSFFQQLVDVVIEQNSKIFPEIAHNLAELKEILVEEEVSFAKTLDRGE

KLFEQYAIIASKTPEQTLSGKDVWRLYDTYGFPVDLTRLMAEEAGLKIDEVAFEKAKQESKEASKASGSKSGAELIKLDV

HALSELDGNDAVPKTDDHYKYGLENTKSKIVALYDGSKFTDSISEAGQQYGILLNKTPFYAEQGGQEYDTGSLVIDGSAE

FNVTNVQSYAGYVLHTGNLVEGTLKVGDELIATYDELRRWPIRNNHTGTHVLNFALREVLGDSVDQKGSLVAAEKLRFDF

SNKQALTTKELEDVERICNENIKANMEVYAEDVSLATAKQINGLRAVFGEVYPDPVRVVSIGKPVSELLKDPENKEWHKY

SIELCGGTHVAKTADIKDLVIIEESGIAKGIRRIVAVTGHDAHRVQQIAKDFDAELDHASNLPFGPDKEQQAKELGVTLK

KLSISVLDKQKLNDKFSKLDKSIKDQLKAKQKEESKKTLEVINEWLKDEEKSKAPFLVAHVPISANAKAITEAFNLIKKQ

AKEKCLYMITGTSEKVAHGCYISDEAISKGVDASQVAQAAIAYIGGKAGGKGNIVQGMGDKPEGIDEALKAVKKLLEEKL

Q

>EJT44225.1 ALA1-like protein [Saccharomyces kudriavzevii IFO 1802]

MTIGDKQKWTAANVRNTFLDYFKSKEHKFVKSSPVVPFDDPTLLFANAGMNQYKPIFLGTVDPASDFYTLKRAYNSQKCI

RAGGKHNDLEDVGKDSYHHTFFEMLGNWSFGDYFKIEAVTYSWTLLTEVYGIPKDRLYVTYFEGDEKLGLEPDIEARELW

RNAGVPDDHILPGNAKDNFWEMGDQGPCGPCSEIHYDRIGGRNAASLVNMDDPDVLEVWNLVFIQFNREQDGSLKSLPAK

HIDTGMGFERLVSVLQDVRSNYDTDVFTPLFERIQEITSVKPYSGKFGEDDSDGVDTAYRVLADHVRTLTFALADGGVPN

NEGRGYVLRRILRRGARYARKYMNYPIGNFFSTLAPTLIEQVQDIFPELAKDPAFLFEILDEEEASFAKTLDRGERLFEK

YASAASKTESRTLDGKQVWRLYDTYGFPVDLTELMAEEQGLKIDGPGFEKAKQESYEASKRGGKKDQSDLIKLNVHELSE

LNDANVPKTNDEFKYGSANVESTILKLHDGTKFVNEITEPGRKYGIILDKTCFYAEQGGQEYDTGKIVIDDAAEFNVENV

QLYNGFVFHTGSLEEGKLSVGDKIIASFDELRRFPIKNNHTGTHILNFALKETLGNDIDQKGSLVASEKLRFDFSYKKAV

TGDELKNIEDTCNQQIKENLQVFYKEIPLELAKSIDGVRAVFGETYPDPVRVVSVGKPIEELLANPANEEWTKYSIEFCG

GTHVNKTGDIKYFVILEESGIAKGIRRIVAVTGTEAFEAQRLAERFAADLDAADKLPFSPVKEKKLKELGVKLGQLSISV

ITKNELKQKFNKIEKAVKDEVKSRAKKENKQTLDEVKTFFETNENTPYFVKFIDISPNAKAITEAINYMKSNDSVKDKSI

YLLAGNDPEGRVAHGCYISNVALAKGVDGSELAKKVSSVIGGKAGGKGNVFQGMGDKPAAIQNAVDDLESLFKEKLSI

>XP_002552804.1 KLTH0D01848p [Lachancea thermotolerans CBS 6340]

MTIGDRQKWTASNVRSTFMEFFKQREHTFVASSPVIPYDDPTLLFANAGMNQYKPIFLGTVDPSSDYYSLKRAMNSQKCI

RAGGKHNDLEDVGRDSYHHTFFEMLGNWSFGDYFKKDAIQYSWELLTKVYGIPSERLYVTYFEGDQKQGLEPDLEAFELW

KSVGVSEDHILPGNAKDNFWEMGDQGPCGPCSEIHYDRIGGRNAASLVNMDDPNVLEVWNIVFIQYNREQNGTLKSLPSK

HIDTGMGFERLVSVLQDVKSNYDTDVFQPLFARIQEITGARPYEGKFGAEDADGIDTAYRVLADHVRTLTFALADGGVPN

NEGRGYVLRRILRRGARYARKYMNYPIGNFFSSLAPTLIDQSKAMFPEINRDPSYLFEILDEEEASFAKTLDRGEKLFEK

YAQAAAQTKDKTLDGLQVWRLYDTYGFPVDLTELMAEEKGLKIDGPGFEKAKQESYEASKKGGKKSASDLIKLEVHELST

LNSENVPKTDDSPKYESANIEAIVLRIFDGENFVDEISEVGKQYGVILDSTCFYAEQGGQEYDMGTLVIDDVAEFSVENV

QLYNGYVFHTGQLGEGKLSVGDKVIASFDELRRFPIKNNHTGTHILNFALKEVLGNDVDQKGSLVAPEKLRFDFSHKKAV

ALDELKEVERICNEQIKANLPVYYKNVPLDLARKISSVRAVFGETYPDPVRVVSVGKPVDDMLVDPTSEEWQKYSVEFCG

GTHVSKTGDIKEFVIIEESGIAKGTRRIVAVSGSEAYEVQRIANKFDSDLIAAEKMPFSPLKEKKLKELGVALGQLSISV

VTKQELKDKFAKIEKVVKDEIKTRAKKEIKQTVDEVKNYFAENQEASFLVKYINIPTNAKAITEAVNHMKTSAKDKSIYL

FTGDDRVAHGCYISDVALEKGIDGRALLNTVSSNIGGKAGGKGNVFQGVGDKPEGIETAVKEVENLFREKLNITK

>PNF25967.1 Alanine--tRNA synthetase, cytoplasmic [Cryptotermes secundus]

MGLERLVSVIQEKRSNYDTDLFMPLFAAIQKGTGVPPYKGHVGEDDVDGVDMAYRVLADHARTLTIALSDGGHPDNTGRG

YVLRRILRRAVRYATEKLNAKPGFFATLVNTVVEILGDTFPEVKKDPQSVIDLINEEETQFLKTLTRGHNLLNRTIMKLG

NSKTLPGDVAWRLYDTYGFPVDLTQLMSEEKGLTVDMDAYEEAKKQAQILSQGRGGWYDDKIILDIHAITELRDQNVPLT

DDSPKYNYHAKSEDKDAEYEFDGCIAKVLRLRHSKKFVDHVTSGQECGVLLDKTSFYAEQGGQIYDEGFLVKVGDENVEF

SVKNVQIRGGYILHIGTVEGILCEGDEVSLHLDTSRRRLVMNNHTGTHVLNFALRTVLGTEADQKGSLVAPDRLRFDFTN

KGAMSVEQVKATEVHSNAVIDKNEPVYAKEASLAVAKAIQGLRAVFEETYPDPVRVVSIGIPVEKLEEDPYGPAGNNTSV

EFCGGTHLHRAGHVGKFVIASEEAIAKGIRRIVALTGPEASKALKKTELLENKLNKFISLLNSDDSEISNKDLVKKIVEL

TDDVSHATIPYWKKEEMRTNLKHLKKQLDDKDRASKAEVTAKVLEEVKALLAGNPKAPVIVQELKAYSNAKALDGALKQV

KALSAETSAMFFSVDSEAKKIVCLSAVPKSAVNRGLKANEWVQQVSGLMQGKGGGKPEAAQASGSNVSCLQEALGIAAQF

ANAKLNSSS

>SJM84235.1 alanine--tRNA synthetase [Zygosaccharomyces bailii]

MTMGNTEKWTAKNVRDTYLNYFKSKGHTFVPSSPVVPYDDPTLLFANAGMNQYKPIFLGTVDPSSDFYNLKRAHNSQKCI

RAGGKHNDLEDVGKDSYHHTFFEMLGNWSFGDYFKKDAIAYAWDLLTKVYGLPEDRLYVTYFEGDAEQGLEPDTEALELW

KSVGVDESHILRGNAKDNFWEMGDQGPCGPCSEIHFDRIGGRNAASLVNMDDPDVLEVWNLVFMQFNREQGGILKPLPAK

HIDTGMGFERLVSILQNCRSNYDTDVFTPLFARIQEITGARPYSGKFGAEDVDGVDTAYRVLADHVRTLTFALADGGVPN

NEGRGYVLRRILRRGARYARKYMNYPIGDFFSKLVPTLISQVEDIFPEVAKDPSYLFEILDEEEVSFAKTLDRGERLFEK

YADAASKSGSKTLDGLQVWRLYDTYGFPVDLTELMAEEKGLTIDGPGFEKAKKESYEASKRGGKKDAANVLKLDVHGISQ

LNNDKVPKTEDVYKYGMDNVDATILKLYDGSNFVDEISEPGQKYGVILDKTCFYAEQGGQEYDTGKLVIDDAAEFNVDNV

QLYNGFVFHTGSLEEGKLSVGDKVIASYDELRRFPIKNNHTGTHILNFALREILGDDIDQKGSLVAPEKLRFDFSHKKAM

TVEEIVKVENICNKQIKDNMAVYYKEVPLEKAKSIYGVRAVFGETYPDPVRVVSVGRSVDDLLSNPSNEEWHKYSVEFCG

GTHVTKTGDIKVFVILEENGIAKGIRRIVAVTGSEAFEAQRLADEFSKELDAANKLPFSPLKEKKLKELGVRLGQLSIAV

ASKQELKNKFAKIEKGVKDEIKARVKQETKKTLDEVKEYFAQNETAPFLVKFIDIPTNAKAITEAVNYMKSSAKDKSIYL

FTGNDPEGKIAHGCYISDIAISKGIDGSSIAKQVSGIVGGKAGGKGNVFQGMGDKPTAAIDAVTELEKLFNEKLHI

>AJU00089.1 Ala1p [Saccharomyces cerevisiae YJM1385]

MTIGDKQKWTATNVRNTFLDYFKSKEHKFVKSSPVVPFDDPTLLFANAGMNQYKPIFLGTVDPASDFYTLKRAYNSQKCI

RAGGKHNDLEDVGKDSYHHTFFEMLGNWSFGDYFKKEAITYSWTLLTEVYGIPKDRLYVTYFEGDEKLGLEPDTEARELW

KNVGVPDDHILPGNAKDNFWEMGDQGPCGPCSEIHYDRIGGRNAASLVNMDDPDVLEVWNLVFIQFNREQDGSLKPLPAK

HIDTGMGFERLVSVLQDVRSNYDTDVFTPLFERIQEITSVRPYSGNFGENDKDGIDTAYRVLADHVRTLTFALADGGVPN

NEGRGYVLRRILRRGARYARKYMNYPIGNFFSTLAPTLISQVQDIFPELSKDPAFLFEILDEEEASFAKTLDRGERLFEK

YASAASKTESKTLDGKQVWRLYDTYGFPVDLTELMAEEQGLKIDGPGFEKAKQESYEASKRGGKKDQSDLIKLNVHELSE

LNDAKVPKTNDEFKYGSANVEGTILKLHDGTNFVDEITEPGKKYGIILDKTCFYAEQGGQEYDTGKIVIDDAAEFNVENV

QLYNGFVFHTGSLEEGKLSVGDKIIASFDELRRFPIKNNHTGTHILNFALKETLGNDVDQKGSLVAPEKLRFDFSHKKAV

SNEELKKVEDICNEQIKENLQVFYKEIPLDLAKSIDGVRAVFGETYPDPVRVVSVGKPIEELLANPANEEWTKYSIEFCG

GTHVNKTGDIKYFVILEESGIAKGIRRIVAVTGTEAFEAQRLAEQFATDLDAADKLPFSPIKEKKLKELGVKLGQLSISV

ITKNELKQKFNKIEKAVKDEVKSRAKKENKQTLDEVKTFFETNENAPYLVKFIDISPNAKAITEAINYMKSNDAVKDKSI

YLLAGNDPEGRVAHGCYISNAALAKGIDGSALAKKVSSIIGGKAGGKGNVFQGMGDKPAAIKDAVDDLESLCKEKLSI

>AJT97628.1 Ala1p [Saccharomyces cerevisiae YJM1342]

MTIGDKQKWTATNVRNTFLDYFKSKEHKFVKSSPVVPFDDPTLLFANAGMNQYKPIFLGTVDPASDFYTLKRAYNSQKCI

RAGGKHNDLEDVGKDSYHHTFFEMLGNWSFGDYFKKEAITYSWTLLTEVYGIPKDRLYVTYFEGDEKLGLEPDTEARELW

KNVGVPDDHILPGNAKDNFWEMGDQGPCGPCSEIHYDRIGGRNAASLVNMDDPDVLEVWNLVFIQFNREQDGSLKPLPAK

HIDTGMGFERLVSVLQDVRSNYDTDVFTPLFERIQEITSVRPYSGNFGENDKDGIDTAYRVLADHVRTLTFALADGGVPN

NEGRGYVLRRILRRGARYARKYMNYPIGNFFSTLAPTLISQVQDIFPELAKDPAFIFEILDEEEASFAKTLDRGERLFEK

YASAASKTESKTLDGKQVWRLYDTYGFPVDLTELMAEEQGLKIDGPGFEKAKQESYEASKRGGKKDQSDLIKLNVHELSE

LNDAKVPKTNDEFKYGSANVEGTILKLHDGTNFVDEITEPGKKYGIILDKTCFYAEQGGQEYDTGKIVIDDAAEFNVENV

QLYNGFVFHTGSLEEGKLSVGDKIIASFDELRRFPIKNNHTGTHILNFALKETLGNDVDQKGSLVAPEKLRFDFSHKKAV

SNEELKKVEDICNEQIKENLQVFYKEIPLDLAKSIDGVRAVFGETYPDPVRVVSVGKPIEELLANPANEEWTKYSIEFCG

GTHVNKTGDIKYFVILEESGIAKGIRRIVAVTGTEAFEAQRLAEQFAADLDAADKLPFSPIKEKKLKELGVKLGQLSISV

ITKNELKQKFNKIEKAVKDEVKSRAKKENKQTLDEVKTFFETNENAPYLVKFIDISPNAKAITEAINYMKSNDAVKDKSI

YLLAGNDPEGRVAHGCYISNAALAKGIDGSALAKKVSSIIGGKAGGKGNVFQGMGDKPAAIKDAVDDLESLFKEKLSI

>AJT75159.1 Ala1p [Saccharomyces cerevisiae YJM326]

MTIGDKQKWTATNVRNTFLDYFKSKEHKFVKSSPVVPFDDPTLLFANAGMNQYKPIFLGTVDPASDFYTLKRAYNSQKCI

RAGGKHNDLEDVGKDSYHHTFFEMLGNWSFGDYFKKEAITYSWTLLTEVYGIPKDRLYVTYFEGDEKLGLEPDTEARELW

KNVGVPDDHILPGNAKDNFWEMGDQGPCGPCSEIHYDRIGGRNAASLVNMDDPDVLEVWNLVFIQFNREQDGSLKPLPAK

HIDTGMGFERLVSVLQDVRSNYDTDVFTPLFERIQEITSVRPYSGNFGENDKDGIDTAYRVLADHVRTLTFALADGGVPN

NEGRGYVLRRILRRGARYARKYMNYPIGNFFSTLAPTLISQVQDIFPELAKDPAFLFEILDEEEASFAKTLDRGERLFEK

YASAASKTESKTLDGKQVWRLYDTYGFPVDLTELMAEEQGLKIDGPGFEKAKQESYEASKRGGKKDQSDLIKLNVHELSE

LNDAKVPKTNDEFKYGSANVEGTILKLHDGTNFVDEITEPGKKYGIILDKTCFYAEQGGQEYDTGKIVIDDAAEFNVENV

QLYNGFVFHTGSLEEGKLSVGDKIIASFDELRRFPIKNNHTGTHILNFALKETLGNDVDQKGSLVAPEKLRFDFSHKKAV

SNEELKKVEDICNEQIKENLQVFYKEIPLDLAKSIDGVRAVFGETYPDPVRVVSVGKPIEELLANPANEEWTKYSIEFCG

GTHVNKTGDIKYFVILEESGIAKGIRRIVAVTGTEAFEAQRLAEQFAADLDAADKLPFSPIKEKKLKELGVKLGQLSISV

ITKNELKQKFNKIEKAVKDEVKSRAKKENKQTLDEVKTFFETNENAPYLVKFIDISPNAKAITEAINYMKSNDAVKDKSI

YLLAGNDSEGRVAHGCYISNTALAKGIDGSALAKKVSSIIGGKAGGKGNVFQGMGDKPAAIKDAVDDLENLFKEKLSI

>P40825.3 RecName: Full=Alanine--tRNA synthetase, mitochondrial; AltName: Full=Alanyl-tRNA synthetase; Short=AlaRS; Flags: Precursor

MTSTTGLRNLTLSFKKQLTTSTRTIMTIGDKQKWTATNVRNTFLDYFKSKEHKFVKSSPVVPFDDPTLLFANAGMNQYKP

IFLGTVDPASDFYTLKRAYNSQKCIRAGGKHNDLEDVGKDSYHHTFFEMLGNWSFGDYFKKEAITYSWTLLTEVYGIPKD

RLYVTYFEGDEKLGLEPDTEARELWKNVGVPDDHILPGNAKDNFWEMGDQGPCGPCSEIHYDRIGGRNAASLVNMDDPDV

LEVWNLVFIQFNREQDGSLKPLPAKHIDTGMGFERLVSVLQDVRSNYDTDVFTPLFERIQEITSVRPYSGNFGENDKDGI

DTAYRVLADHVRTLTFALADGGVPNNEGRGYVLRRILRRGARYARKYMNYPIGNFFSTLAPTLISQVQDIFPELAKDPAF

LFEILDEEEASFAKTLDRGERLFEKYASAASKTESKTLDGKQVWRLYDTYGFPVDLTELMAEEQGLKIDGPGFEKAKQES

YEASKRGGKKDQSDLIKLNVHELSELNDAKVPKTNDEFKYGSANVEGTILKLHDGTNFVDEITEPGKKYGIILDKTCFYA

EQGGQEYDTGKIVIDDAAEFNVENVQLYNGFVFHTGSLEEGKLSVGDKIIASFDELRRFPIKNNHTGTHILNFALKETLG

NDVDQKGSLVAPEKLRFDFSHKKAVSNEELKKVEDICNEQIKENLQVFYKEIPLDLAKSIDGVRAVFGETYPDPVRVVSV

GKPIEELLANPANEEWTKYSIEFCGGTHVNKTGDIKYFVILEESGIAKGIRRIVAVTGTEAFEAQRLAEQFAADLDAADK

LPFSPIKEKKLKELGVKLGQLSISVITKNELKQKFNKIEKAVKDEVKSRAKKENKQTLDEVKTFFETNENAPYLVKFIDI

SPNAKAITEAINYMKSNDSVKDKSIYLLAGNDPEGRVAHGCYISNAALAKGIDGSALAKKVSSIIGGKAGGKGNVFQGMG

DKPAAIKDAVDDLESLFKEKLSI

>PSK76608.1 alanine-tRNA synthetase [[Candida] auris]

MTTWTASTVRQTFLDFFKSKQHTYVPSSSVVPHNDPTLLFANAGMNQYKPIFLGTVDPTSDFASLKRAANSQKCIRAGGK

HNDLDDVGKDSYHHTFFEMLGNWSFGDYFKKEAIEWSWELLTGIYKLEKDRLYVTYFEGDEKNGLKPDLEAKQYWLDVGV

AEDHILPGNATDNFWEMGDQGPCGPCSEIHYDRIGGRNAASLVNMDDPNVLEVWNIVFIQYNREADSSLRPLPNKHIDTG

MGFERLVSILQNKFSNYDTDVFTPIFEKIREITGVRPYTGKFGADDVDGIDTAYRVIADHVRTLTFAITDGGVPNNEGRG

YVLRRILRRGSRYVRKYMNYPIGSFFQQLVDVVIEQNSKIFPEIAHNLTELKEILVEEEVSFAKTLDRGEKLFEQYAIIA

SKTPEQTLSGKDVWRLYDTYGFPVDLTRLMAEEAGLKIDEVAFEKAKQESKEASKASGSKNGAELIKLDVHALSELDGND

AVPKTDDHYKYGLENTKSKIVALYDGSKFTDSISEAGQQYGILLNKTPFYAEQGGQEYDTGSLVIDGSAEFNVTNVQSYA

GYVLHTGNLVEGTLKVGDELIATYDELRRWPIRNNHTGTHVLNFALREVLGDSVDQKGSLVAAEKLRFDFSNKQALTTKE

LEDVERICNENIKANMEVYAEDVSLATAKQINGLRAVFGEVYPDPVRVVSIGKPVSELLKDPENKEWHKYSIELCGGTHV

AKTADIKDLVIIEESGIAKGIRRIVAVTGHDAHRVQQIAKDFDAELDHASNLPFGPDKEQQAKELGVTLKKLSISVLDKQ

KLNDKFSKLDKSIKDQLKAKQKEESKKTLEVINEWLKDEEKSKAPFLVAHVPISANAKAITEAFNLIKKQAKEKCLYMIT

GTSEKVAHGCYISDEAISRGVDANQVAQAAIAHIGGKAGGKGNIVQGMGDKPEGIDEALKAVKKLLEEKLQ

>SMN20736.1 similar to Saccharomyces cerevisiae YOR335C ALA1 Cytoplasmic and mitochondrial alanyl-tRNA synthetase, required for protein synthesis [Kazachstania saulgeensis]

MSSSLGDKQKWTATNVRNTFLDYFKSKDHKFVASSPVVPFDDPTLLFANAGMNQYKPIFLGTVDPSSDFYTLKRAYNSQK

CIRAGGKHNDLEDVGKDSYHHTFFEMLGNWSFGDYFKAEAVDFAWTLLTEVYGIPKDRLYVTYFEGDAKQGLEPDIEALE

LWKNAGVPEDHILPGNAKDNFWEMGDQGPCGPCSEIHYDRIGGRNASSLVNQDDPDVLEVWNLVFMQYNREADGSLKSLP

SKHIDTGMGFERLVSVLQDVRSNYDTDVFQPLFTRIQEITQVRPYQGKFGEEDTDNIDTAYRVLADHVRTLTFALSDGGV

PNNEGRGYVLRRILRRGARYARKYMNYPIGNFFSTLAPTLIDQVKDIFPEVSKDPSFLFEILDEEEASFAKTLDRGERLF

EKYAKAAAETESKTLDGKQVWRLYDTYGFPVDLTELMAEEQGLKIDSVGFEKAKQESYEASKRGGKKGNDDLIKLNVHEL

NELNQNNVAKTDDHFKYGFDDVESKILKLHDGEKFVNEITESGKKYGVLLDKTCFYAEQGGQEYDTGKIVIDDVAEFNVD

NVQLYNGFVFHTGTLEEGKLSVGDSIIASYDELRRFPIKNNHTGTHILNFALKDVLGNDVDQKGSLVAPEKLRFDFSHKK

ALSMDELRKVEEICNQQIKDNLPVYYKDVPLELAKSISSVRAVFGETYPDPVRVVSIGKPVDDLLKDPTNAEWENFSVEF

CGGTHVSKTSDIKYYIILEENGIAKGIRRIVAVTGSEAFEAQRIADEFDAELIAAEKLPFSPLKEKKLKELGVRLGQLSI

SVISKHELKEKFTKIEKVVKDEVKTRAKAETKQTLDEVKAYFTENPEAKYFVKFIDIPTNAKAITEAVNYMKTSAKDKSM

YLFTGNDPEGRLAHGCYISDDALTTGVEGPALAKEVSNIIGGKAGGKGNVFQGMGDKPTEVNKAVEEVTKLLSEKL

>XP_003870578.1 Ala1 alanyl-tRNA synthetase [Candida orthopsilosis Co 90-125]

MIKRTFIRAMSTTSTINNTEKWTAPKVRSTFLDFFKDKKQHTYVPSSSVVPHNDPTLLFANAGMNQYKPIFLGTVDPASD

FAKLKRAANSQKCIRAGGKHNDLEDVGRDSYHHTFFEMLGNWSFGDYFKKEAIAWSWELLTEVYGLEKDRLYVTYFGGDD

KQGLEADLEAKQFWLDVGVAEDHILPGDAKDNFWEMGDQGPCGPCSEIHYDRIGGRNAASLVNMDDPNVLEVWNVVFIQY

NREADGSLRSLPNKHIDTGMGFERLVSILQNKSSNYDTDVFLPIFEKIREITGVRPYTGKFGTDDKDGIDTAYRVIADHV

RTLTFAICDGGVPNNEGRGYVLRRVLRRGSRYVRKYMNYPIGSFFPQLVDIVIEQNKEIFPEIESGSQDLKEILNEEELS

FAKTLDRGEKLFEQYAIIASKTPEQTLSGKDVWRLYDTYGFPVDLTRLMAEEAGLKIDEKGFEVAKEESREASKGGASKN

ASSLIKLDVHALSELEGKVVKTNDSAKYGLDNIKASVLAIYDGGKFVDSIQQDNEGKQYGILLDKTPFYAEQGGQEYDTG

KLVIDGKTEFNVENVQVYAGYVLHTGFVLEGKLSVGDEVIAAYDELRRWPIRNNHTGTHVLNYALREVLGDDVDQKGSLV

AKEKLRFDFSHKQALTPKELEKVEDKSNQYIRDNKQVYYKDVPLAEAKAINGLRAVFGETYPDPVRVVSIGVPVDDLLKD

PTNEEWRSLSIEFCGGTHVAKTSEIKDLVIIEESGIAKGIRRIVAVTGHDAHAVQRIAEEFGDELDHANALPNGPIKENK

VKELGVSLKKLSISVLDKQKLTEKFNKVDKSIKDHQKSIQKEESKKTLDVVKKWLDDGDNKSEFLVAHIPINANAKAITE

AFNLVKKQDKTKSLYLLTGQNDKVAHGCYVSDEAIGKGVDASSLAKLVSDKIGGKAGGKGNIVQGMGDKPSGINDAIEEV

TKAFKEKL

>GAA26640.1 K7_Ala1p [Saccharomyces cerevisiae Kyokai no. 7]

MTIGDKQKWTATNVRNTFLDYFKSKEHKFVKSSPVVPFDDPTLLFANAGMNQYKPIFLGTVDPASDFYTLKRAYNSQKCI

RAGGKHNDLEDVGKDSYHHTFFEMLGNWSFGDYFKKEAITYSWTLLTEVYGIPKDRLYVTYFEGDEKLGLEPDTEARELW

KNVGVPDDHILPGNAKDNFWEMGDQGPCGPCSEIHYDRIGGRNAASLVNMDDPDVLEVWNLVFIQFNREQDGSLKPLPAK

HIDTGMGFERLVSVLQDVRSNYDTDVFTPLFERIQEITSVRPYSGNFGENDKDGIDTAYRVLADHVRTLTFALADGGVPN

NEGRGYVLRRILRRGARYARKYMNYPIGNFFSTLAPTLISQVQDIFPELAKDPAFLFEILDEEEASFAKTLDRGERLFEK

YASAASKTESKTLDGKQVWRLYDTYGFPVDLTELMAEEQGLKIDGPGFEKAKQESYEASKRGGKKDQSDLIKLNVHELSE

LNDAKVPKTNDEFKYGSANVEGTILKLHDGTNFVDEITEPGKKYGIILDKTCFYAEQGGQEYDTGKIVIDDAAEFNVENV

QLYNGFVFHTGSLEEGKLSVGDKIIASFDELRRFPIKNNHTGTHILNFALKETLGNDVDQKGSLVAPEKLRFDFSHKKAV

SNEELKKVEDICNEQIKENLQVFYKEIPLDLAKSIDGVRAVFGETYPDPVRVVSVGKPIEELLANPANEEWTKYSIEFCG

GTHVNKTGDIKYFVILEESGIAKGIRRIVAVTGTEAFEAQRLAEQFGTDLDAADKLPFSPIKEKKLKELGVKLGQLSISV

ITKNELKQKFNKIEKAVKDEVKSRAKKENKQTLDEVKTFFETNENAPYLVKFIDISPNAKAITEAINYMKSNDSVKDKSI

YLLAGNDPEGRVAHGCYISNAALAKGIDGSALAKKVSSIIGGKAGGKGNVFQGMGDKPAAIKDAVDDLESLFKEKLSI

>NP_014980.3 alanine--tRNA synthetase [Saccharomyces cerevisiae S288C]

MTIGDKQKWTATNVRNTFLDYFKSKEHKFVKSSPVVPFDDPTLLFANAGMNQYKPIFLGTVDPASDFYTLKRAYNSQKCI

RAGGKHNDLEDVGKDSYHHTFFEMLGNWSFGDYFKKEAITYSWTLLTEVYGIPKDRLYVTYFEGDEKLGLEPDTEARELW

KNVGVPDDHILPGNAKDNFWEMGDQGPCGPCSEIHYDRIGGRNAASLVNMDDPDVLEVWNLVFIQFNREQDGSLKPLPAK

HIDTGMGFERLVSVLQDVRSNYDTDVFTPLFERIQEITSVRPYSGNFGENDKDGIDTAYRVLADHVRTLTFALADGGVPN

NEGRGYVLRRILRRGARYARKYMNYPIGNFFSTLAPTLISQVQDIFPELAKDPAFLFEILDEEEASFAKTLDRGERLFEK

YASAASKTESKTLDGKQVWRLYDTYGFPVDLTELMAEEQGLKIDGPGFEKAKQESYEASKRGGKKDQSDLIKLNVHELSE

LNDAKVPKTNDEFKYGSANVEGTILKLHDGTNFVDEITEPGKKYGIILDKTCFYAEQGGQEYDTGKIVIDDAAEFNVENV

QLYNGFVFHTGSLEEGKLSVGDKIIASFDELRRFPIKNNHTGTHILNFALKETLGNDVDQKGSLVAPEKLRFDFSHKKAV

SNEELKKVEDICNEQIKENLQVFYKEIPLDLAKSIDGVRAVFGETYPDPVRVVSVGKPIEELLANPANEEWTKYSIEFCG

GTHVNKTGDIKYFVILEESGIAKGIRRIVAVTGTEAFEAQRLAEQFAADLDAADKLPFSPIKEKKLKELGVKLGQLSISV

ITKNELKQKFNKIEKAVKDEVKSRAKKENKQTLDEVKTFFETNENAPYLVKFIDISPNAKAITEAINYMKSNDSVKDKSI

YLLAGNDPEGRVAHGCYISNAALAKGIDGSALAKKVSSIIGGKAGGKGNVFQGMGDKPAAIKDAVDDLESLFKEKLSI

>AJU01059.1 Ala1p [Saccharomyces cerevisiae YJM1387]

MTIGDKQKWTATNVRNTFLDYFKSKEHKFVKSSPVVPFDDPTLLFANAGMNQYKPIFLGTVDPASDFYTLKRAYNSQKCI

RAGGKHNDLEDVGKDSYHHTFFEMLGNWSFGDYFKKEAITYSWTLLTEVYGIPKDRLYVTYFEGDEKLGLEPDTEARELW

KNVGVPDDHILPGNAKDNFWEMGDQGPCGPCSEIHYDRIGGRNAASLVNMDDPDVLEVWNLVFIQFNREQDGSLKPLPAK

HIDTGMGFERLVSVLQDVRSNYDTDVFTPLFERIQEITSVRPYSGNFGENDKDGIDTAYRVLADHVRTLTFALADGGVPN

NEGRGYVLRRILRRGARYARKYMNYPIGNFFSTLAPTLISQVQDIFPELAKDPAFLFEILDEEEASFAKTLDRGERLFEK

YASAASKTESKTLDGKQVWRLYDTYGFPVDLTELMAEEQGLKIDGPGFEKAKQESYEASKRGGKKDQSDLIKLNVHELSE

LNDAKVPKTNDEFKYGSANVEGTILKLHDGTNFVDEITEPGKKYGIILDKTCFYAEQGGQEYDTGKIVIDDAAEFNVENV

QLYNGFVFHTGSLEEGKLSVGDKIIASFDELRRFPIKNNHTGTHILNFALKETLGNDVDQKGSLVAPEKLRFDFSHKKAV

SNEELKKVEDICNEQIKENLQVFYKEIPLDLAKSIDGVRAVFGETYPDPVRVVSVGKPIEELLANPANEEWAKYSIEFCG

GTHVNKTGDIKYFVILEESGIAKGIRRIVAVTGTEAFEAQRLAEQFGTDLDAADKLPFSPIKEKKLKELGVKLGQLSISV

ITKNELKQKFNKIEKAVKDEVKSRAKKENKQTLDEVKTFFETNENAPYLVKFIDISPNAKAITEAINYMKSNDSVKDKSI

YLLAGNDPEGRVAHGCYISNAALAKGIDGSALAKKVSSIIGGKAGGKGNVFQGMGDKPAAIKDAVDDLESLFKEKLSI

>AJU00572.1 Ala1p [Saccharomyces cerevisiae YJM1386]

MTIGDKQKWTATNVRNTFLDYFKSKEHKFVKSSPVVPFDDPTLLFANAGMNQYKPIFLGTVDPASDFYTLKRAYNSQKCI

RAGGKHNDLEDVGKDSYHHTFFEMLGNWSFGDYFKKEAITYSWTLLTEVYGIPKDRLYVTYFEGDEKLGLEPDTEARELW

KNVGVPDDHILPGNAKDNFWEMGDQGPCGPCSEIHYDRIGGRNAASLVNMDDPDVLEVWNLVFIQFNREQDGSLKPLPAK

HIDTGMGFERLVSVLQDVRSNYDTDVFTPLFERIQEITSVRPYSGNFGENDKDGIDTAYRVLADHVRTLTFALADGGVPN

NEGRGYVLRRILRRGARYARKYMNYPIGNFFSTLAPTLISQVQDIFPELAKDPAFLFEILDEEEASFAKTLDRGERLFEK

YASAASKTESKTLDGKQVWRLYDTYGFPVDLTELMAEEQGLKIDGPGFEKAKQESYEASKRGGKKDQSDLIKLNVHELSE

LNDAKVPKTNDEFKYGSANVEGTILKLHDGTNFVDEITEPGKKYGIILDKTCFYAEQGGQEYDTGKIVIDDAAEFNVENV

QLYNGFVFHTGSLEEGKLSVGDKIIASFDELRRFPIKNNHTGTHILNFALKETLGNDVDQKGSLVAPEKLRFDFSHKKAV

SNEELKKVEDICNEQIKENLQVFYKEIPLDLAKSIDGVRAVFGETYPDPVRVVSVGKPIEELLANPANEECTKYSIEFCG

GTHVNKTGDIKYFVILEESGIAKGIRRIVAVTGTEAFEAQRLAEQFATDLDAADKLPFSPIKEKKLKELGVKLGQLSISV

ITKNELKQKFNKIEKAVKDEVKSRAKKENKQTLDEVKTFFETNENAPYLVKFIDISPNAKAITEAINYMKSNDAVKDKSI

YLLAGNDPEGRVAHGCYISNAALAKGIDGSALAKKVSSIIGGKAGGKGNVFQGMGDKPAAIKDAVDDLESLFKEKLSI

>AJT72237.1 Ala1p [Saccharomyces cerevisiae YJM195]

MTIGDKQKWTATNVRNTFLDYFKSKEHKFVKSSPVVPFDDPTLLFANAGMNQYKPIFLGTVDPASDFYTLKRAYNSQKCI

RAGGKHNDLEDVGKDSYHHTFFEMLGNWSFGDYFKKEAITYSWTLLTEVYGIPKDRLYVTYFEGDEKLGLEPDTEARELW

KNVGVPDDHILPGNAKDNFWEMGDQGPCGPCSEIHYDRIGGRNAASLVNMDDPDVLEVWNLVFIQFNREQDGSLKPLPAK

HIDTGMGFERLVSVLQDVRSNYDTDVFTPLFERIQEITSVRPYSGNFGENDKDGIDTAYRVLADHVRTLTFALADGGVPN

NEGRGYVLRRILRRGARYARKYMNYPIGNFFSTLAPTLISQVQDIFPELAKDPAFLFEILDEEEASFAKTLDRGERLFEK

YASAASKTESKTLDGKQVWRLYDTYGFPVDLTELMAEEQGLKIDGPGFEKAKQESYEASKRGGKKDQSDLIKLNVHELSE

LNDAKVPKTNDEFKYGSANVEGTILKLHDGTNFVDEITEPGKKYGIILDKTCFYAEQGGQEYDTGKIVIDDAAEFNVENV

QLYNGFVFHTGSLEEGKLSVGDKIIASFDELRRFPIKNNHTGTHILNFALKETLGNDVDQKGSLVAPEKLRFDFSHKKAV

SNEELKKVEDICNEQIKENLQVFYKEIPLDLAKSIDGVRAVFGETYPDPVRVVSVGKPIEELLANPANEEWTKYSIEFCG

GTHVNKTGDIKYFVILEESGIAKGIRRIVAVTGTEAFEAQRLAEQFATDLDAADKLPFSPIKEKKLKELGVKLGQLSISV

ITKNELKQKFNKIEKAVKDEVKSRAKKENKQTLDEVKTFFETNENAPYLVKFIDISPNAKAITEAINYMKSNDAVKDKSI

YLLAGNDPEGRVAHGCYISNAALAKGIDGSALAKKVSSIIGGKAGGKGNVFQGMGDKPAAIKDAVDDLESLFKEKLSI

>EDV10897.1 alanyl-tRNA synthetase [Saccharomyces cerevisiae RM11-1a]

MTIGDKQKWTATNVRNTFLDYFKSKEHKFVKSSPVVPFDDPTLLFANAGMNQYKPIFLGTVDPASDFYTLKRAYNSQKCI

RAGGKHNDLEDVGKDSYHHTFFEMLGNWSFGDYFKKEAITYSWTLLTEVYGIPKDRLYVTYFEGDEKLGLEPDTEARELW

KNVGVPDDHILPGNAKDNFWEMGDQGPCGPCSEIHYDRIGGRNAASLVNMDDPDVLEVWNLVFIQFNREQDGSLKPLPAK

HIDTGMGFERLVSVLQDVRSNYDTDVFTPLFERIQEITSVRPYSGNFGENDKDGIDTAYRVLADHVRTLTFALADGGVPN

NEGRGYVLRRILRRGARYARKYMNYPIGNFFSTLAPTLISQVQDIFPELAKDPAFLFEILDEEEASFAKTLDRGERLFEK

YASAASKTESKTLDGKQVWRLYDTYGFPVDLTELMAEEQGLKIDGPGFEKAKQESYEASKRGGKKDQSDLIKLNVHELSE

LNDAKVPKTNDEFKYGSANVEGTILKLHDGTNFVDEITEPGKKYGIILDKTCFYAEQGGQEYDTGKIVIDDAAEFNVENV

QLYNGFVFHTGSLEEGKLSVGDKIIASFDELRRFPIKNNHTGTHILNFALKETLGNDVDQKGSLVAPEKLRFDFSHKKAV

SNEELKKVEDICNEQIKENLQVFYKEIPLDLAKSIDGVRAVFGETYPDPVRVVSVGKPIEELLANPANEEWTKYSIEFCG

GTHVNKTGDIKYFVILEESGIAKGIRRIVAVTGTEAFEAQRLAEQFAADLDAADKLPFSPIKEKKLKELGVKLGQLSISV

ITKNELKQKFNKIEKAVKDEVKSRAKKENKQTLDEVKTFFETNENAPYLVKFIDISPNAKAITEAINYMKSNDAVKDKSI

YLLAGNDPEGRVAHGCYISNAALAKGIDGSALAKKVSSIIGGKAGGKGNVFQGMGDKPAAIKDAVDDLESLFKEKLSI

>EEU07514.1 Ala1p [Saccharomyces cerevisiae JAY291]

MTIGDKQKWTATNVRNTFLDYFKSKEHKFVKSSPVVPFDDPTLLFANAGMNQYKPIFLGTVDPASDFYTLKRAYNSQKCI

RAGGKHNDLEDVGKDSYHHTFFEMLGNWSFGDYFKKEAITYSWTLLTEVYGIPKDRLYVTYFEGDEKLGLEPDTEARELW

KNVGVPDDHILPGNAKDNFWEMGDQGPCGPCSEIHYDRIGGRNAASLVNMDDPDVLEVWNLVFIQFNREQDGSLKPLPAK

HIDTGMGFERLVSVLQDVRSNYDTDVFTPLFERIQEITSVRPYSGNFGENDKDGIDTAYRVLADHVRTLTFALADGGVPN

NEGRGYVLRRILRRGARYARKYMNYPIGNFFSTLAPTLISQVQDIFPELAKDPAFLFEILDEEEASFAKTLDRGERLFEK

YASAASKTESKTLDGKQVWRLYDTYGFPVDLTELMAEEQGLKIDGPGFEKAKQESYEASKRGGKKDQSDLIKLNVHELSE

LNDAKVPKTNDEFKYGSANVEGTILKLHDGTNFVDEITEPGKKYGIILDKTCFYAEQGGQEYDTGKIVIDDAAEFNVENV

QLYNGFVFHTGSLEEGKLSVGDKIIASFDELRRFPIKNNHTGTHILNFALKETLGNDVDQKGSLVAPEKLRFDFSHKKAV

SNEELKKVEDICNEQIKENLQVFYKEIPLDLAKSIDGVRAVFGETYPDPVRVVSVGKPIEELLANPANEEWTKYSIEFCG

GTHVNKTGDIKYFVILEESGIAKGIRRIVAVTGTEAFEAQRLAEQFAADLDAADKLPFSPIKEKKLKELGVKLGQLSISV

ITKNELKQKFNKIEKAVKDEVKSRAKKENKQTLDEVKTFFETNENAPYLVKFIDISPNAKAITEAINYIKSNDAVKDKSI

YLLAGNDPEGRVAHGCYISNAALAKGIDGSALAKKVSSIIGGKAGGKGNVFQGMGDKPAAIKDAVDDLESLFKEKLSI

>EDN63655.1 alanyl-tRNA synthetase [Saccharomyces cerevisiae YJM789]

MTIGDKQKWTATNVRNTFLDYFKSKEHKFVKSSPVVPFDDPTLLFANAGMNQYKPIFLGTVDPASDFYTLKRAYNSQKCI

RAGGKHNDLEDVGKDSYHHTFFEMLGNWSFGDYFKKEAITYSWTLLTEVYGIPKDRLYVTYFEGDENLGLEPDTEARELW

KNVGVPDDHILPGNAKDNFWEMGDQGPCGPCSEIHYDRIGGRNAASLVNMDDPDVLEVWNLVFIQFNREQDGSLKPLPAK

HIDTGMGFERLVSVLQDVRSNYDTDVFTPLFERIQEITSVRPYSGNFGENDKDGIDTAYRVLADHVRTLTFALADGGVPN

NEGRGYVLRRILRRGARYARKYMNYPIGNFFSTLAPTLISQVQDIFPELAKDPAFLFEILDEEEASFAKTLDRGERLFEK

YASAASKTESKTLDGKQVWRLYDTYGFPVDLTELMAEEQGLKIDGPGFEKAKQESYEASKRGGKKDQSDLIKLNVHELSE

LNDAKVPKTNDEFKYGSANVEGTILKLHDGTNFVDEITEPGKKYGIILDKTCFYAEQGGQEYDTGKIVIDDAAEFNVENV

QLYNGFVFHTGSLEEGKLSVGDKIIASFDELRRFPIKNNHTGTHILNFALKETLGNDVDQKGSLVAPEKLRFDFSHKKAV

SNEELKKVEDICNEQIKENLQVFYKEIPLDLAKSIDGVRAVFGETYPDPVRVVSVGKPIEELLANPANEEWTKYSIEFCG

GTHVNKTGDIKYFVILEESGIAKGIRRIVAVTGTEAFEAQRLAEQFAADLDAADKLPFSPIKEKKLKELGVKLGQLSISV

ITKNELKQKFNKIEKAVKDEVKSRAKKENKQTLDEVKTFFETNENAPYLVKFIDISPNAKAITEAINYMKSNDAVKDKSI

YLLAGNDPEGRVAHGCYISNAALAKGIDGSALAKKVSSIIGGKAGGKGNVFQGMGDKPAAIKDAVDDLESLFKEKLSI

>EGA76661.1 Ala1p [Saccharomyces cerevisiae Vin13]

MTIGDKQKWTATNVRNTFLDYFKSKEHKFVKSSPVVPFDDPTLLFANAGMNQYKPIFLGTVDPASDFYTLKRAYNSQKCI

RAGGKHNDLEDVGKDSYHHTFFEMLGNWSFGDYFKKEAITYSWTLLTEVYGIPKDRLYVTYFEGDEKLGLEPDTEARELW

KNVGVPDDHILPGNAKDNFWEMGDQGPCGPCSEIHYDRIGGRNAASLVNMDDPDVLEVWNLVFIQFNREQDGSLKPLPAK

HIDTGMGFERLVSVLQDVRSNYDTDVFTPLFERIQEITSVRPYSGNFGENDKDGIDTAYRVLADHVRTLTFALADGGVPN

NEGRGYVLRRILRRGARYARKYMNYPIGNFFSTLAPTLISQVQDIFPELAKDPAFLFEILDEEEASFAKTLDRGERLFEK

YASAASKTESKTLDGKQVWRLYDTYGFPVDLTELMAEEQGLKIDGPGFEKAKQESYEASKRGGKKDQSDLIKLNVHELSE

LNDAKVPKTNDEFKYGSANVEGTILKLHDGTNFVDEITEPGKXYGIILDKTCFYAEQGGQEYDTGKIVIDDAAEFNVENV

QLYNGFVFHTGSLEEGKLSVGDKIIASFDELRRFPIKNNHTGTHILNFALKETLGNDVDQKGSLVAPEKLRFDFSHKKAV

SNEELKKVEDICNEQIKENLQVFYKEIPLDLAKSIDGVRAVFGETYPDPVRVVSVGKPIEELLANPANEEWTKYSIEFCG

GTHVNKTGDIKYFVILEESGIAKGIRRIVAVTGTEAFEAQRLAEQFAADLDAADKLPFSPIKEKKLKELGVKLGQLSISV

ITKNELKQKFNKIEKAVKDEVKSRAKKENKQTLDEVKTFFETNENAPYLVKFIDISPNAKAITEAINYMKSNDAVKDKSI

YLLAGNDPEGRVAHGCYISNAALAKGIDGSALAKKVSSIIGGKAGGKGNVFQGMGDKPAAIKDAVDDLESLFKEKLSI

>PSK47347.1 alanine-tRNA synthetase [[Candida] auris]

MTTWTASTVRQTFLDFFKSKQHTYVPSSSVVPHNDPTLLFANAGMNQYKPIFLGTVDPTSDFASLKRAANSQKCIRAGGK

HNDLDDVGKDSYHHTFFEMLGNWSFGDYFKKEAIEWSWELLTGIYKLEKDRLYVTYFEGDEKNGLKPDLEAKQYWLDVGV

AEDHILPGNATDNFWEMGDQGPCGPCSEIHYDRIGGRNAASLVNMDDPNVLEVWNIVFIQYNREADSSLRPLPNKHIDTG

MGFERLVSILQNKFSNYDTDVFTPIFEKIREITGVRPYTGKFGADDVDGIDTAYRVIADHVRTLTFAITDGGVPNNEGRG

YVLRRILRRGSRYVRKYMNYPIGSFFQQLVDVVIEQNSKIFPEIAHNLAELKEILVEEEVSFAKTLDRGEKLFEQYAIIA

SKTPEQTLSGKDVWRLYDTYGFPVDLTRLMAEEAGLKIDEVAFEKAKQESKEASKASGSKSGAELIKLDVHALSELDGND

AVPKTDDHYKYGLENTKSKIVALYDGSKFTDSISEAGQQYGILLNKTPFYAERGGQEYDTGSLVIDGSAEFNVTNVQSYA

GYVLHTGNLVEGTLKVGDELIATYDELRRWPIRNNHTGTHVLNFALREVLGDSVDQKGSLVAAEKLRFDFSNKQALTTKE

LEDVERICNENIKANMEVYAEDVSLATAKQINGLRAVFGEVYPDPVRVVSIGKPVSELLKDPENKEWHKYSIELCGGTHV

AKTADIKDLVIIEESGIAKGIRRIVAVTGHDAHRVQQIAKDFDAELDHASNLPFGPDKEQQAKELGVTLKKLSISVLDKQ

KLNDKFSKLDKSIKDQLKAKQKEESKKTLEVINEWLKDEEKSKAPFLVAHVPISANAKAITEAFNLIKKQAKEKCLYMIT

GTSEKVAHGCYISDEAISKGVDASQVAQAAIAYIGGKAGGKGNIVQGMGDKPEGIDEALKAVKKLLEEKLQ

>EWH16115.1 Ala1p [Saccharomyces cerevisiae P283]

MTIGDKQKWTATNVRNTFLDYFKSKEHKFVKSSPVVPFDDPTLLFANAGMNQYKPIFLGTVDPASDFYTLKRAYNSQKCI

RAGGKHNDLEDVGKDSYHHTFFEMLGNWSFGDYFKKEAITYSWTLLTEVYGIPKDRLYVTYFEGDEKLGLEPDTEARELW

KNVGVPDDHILPGNAKDNFWEMGDQGPCGPCSEIHYDRIGGRNAASLVNMDDPDVLEVWNLVFIQFNREQDGSLKPLPAK

HIDTGMGFERLVSVLQDVRSNYDTDVFTPLFERIQEITSVRPYSGNFGENDKDGIDTAYRVLADHVRTLTFALADGGVPN

NEGRGYVLRRILRRGARYARKYMNYPIGNFFSTLAPTLISQVQDIFPELAKDPAFLFEILDEEEASFAKTLDRGERLFEK

YASAASKTESKTLDGKQVWRLYDTYGFPVDLTELMAEEQGLKIDGPGFEKAKQESYEASKRGGKKDQSDLIKLNVHELSE

LNDAKVPKTNDEFKYGSANVEGTILKLHDGTNFVDEITEPGKKYGIILDKTCFYAEQGGQEYDTGKIVIDDAAEFNVENV

QLYNGFVFHTGSLEEGKLSVGDKIIASFDELRRFPIKNNHTGTHILNFALKETLGNDVDQKGSLVAPEKLRFDFSHKKAV

SNEELKKVEDICNEQIKENLQVFYKEIPLDLAKSIDGVRAVFGETYPDPVRVVSVGKPIEELLANPANEEWTKYSIEFCG

GTHVNKTGDIKYFVILEESGIAKGIRRIVAVTGTEAFEAQRLAEQFAADLDAADKLPFSPIKEKKLKELGVKLGQLSISV

ITKNELKQKFNKIEKAVKDEVKSRAKKENKQTLDEVKTFFETNENAPYLVKFIDISPNAKAITEAINYMKSNDAVKDKSI

YLLAGNDPEGRVAHGCYISNAALAKGIDGSALAKKVSSIIGGKAGGKGNVFQGMGDKPAAIKDAVDDLESLFKVEAFHLR

S

>AJU08879.1 Ala1p [Saccharomyces cerevisiae YJM1447]

MTIGDKQKWTATNVRNTFLDYFKSKEHKFVKSSPVVPFDDPTLLFANAGMNQYKPIFLGTVDPASGFYTLKRAYNSQKCI

RAGGKHNDLEDVGKDSYHHTFFEMLGNWSFGDYFKKEAITYSWTLLTEVYGIPKDRLYVTYFEGDEKLGLEPDTEARELW

KNLGVPDDHILPGNAKDNFWEMGDQGPCGPCSEIHYDRIGGRNAASLVNMDDPDVLEVWNLVFIQFNREQDGSLKPLPAK

HIDTGMGFERLVSVLQDVRSNYDTDVFTPLFERIQEITSVRPYSGNFGENDKDGIDTAYRVLADHVRTLTFALADGGVPN

NEGRGYVLRRILRRGARYARKYMNYPIGNFFSTLAPTLISQVQDIFPELAKDPAFLFEILDEEEASFAKTLDRGERLFEK

YASAASKTESKTLDGKQVWRLYDTYGFPVDLTELMAEEQGLKIDGPGFEKAKQESYEASKRGGKNDQSDLIKLNVHELSE

LNDAKVPKTNDEFKYGSANVEGTILKLHDGTNFVDEITEPGKKYGIILDKTCFYAEQGGQEYDTGKIVIDDAAEFNVENV

QLYNGFVFHTGSLEEGKLSVSDKIIASFDELRRFPIKNNHTGTHILNFALKETLGNDVDQKGSLVAPEKLRFDFSHKKAV

SNEELKKVEDICNEQIKENLQVFYKEIPLDLAKSIDGVRAVFGETYPDPVRVVSVGKPIEELLANPANEEWTKYSIEFCG

GTHVNKTGDIKYFVILEESGIAKGIRRIVAVTGTEAFEAQRLAEQFAADLDAADKLPFSPIKEKKLKELGVKLGQLSISV

ITKNELKQKFNKIEKAVKDEVKSRAKKENKQTLDEVKTFFETNENAPYLVKFIDISPNAKAITEAINYMKSNDAVKDKSI

YLLAGNDPEGRVAHGCYISNAALAKGIDGSALAKKVSSIIGGKAGGKGNVFQGMGDKPAAIKDAVDDLESLFKEKLSI

>AJT87923.1 Ala1p [Saccharomyces cerevisiae YJM1129]

MTIGDKQKWTATNVRNTFLDYFKSKEHKFVKSSPVVPFDDPTLLFANAGMNQYKPIFLGTVDPASDFYTLKRAYNSQKCI

RAGGKHNDLEDVGKDSYHHTFFEMLGNWSFGDYFKKEAITYSWTLLTEVYGIPKDRLYVTYFEGDEKLGLEPDTEARELW

KNVGVPDDHILPGNAKDNFWEMGDQGPCGPCSEIHYDRIGGRNAASLVNMDDPDVLEVWNLVFIQFNREQDGSLKPLPAK

HIDTGMGFERLVSVLQDVRSNYDTDVFTPLFERIQEITSVRPYSGNFGENDKDGIDTAYRVLADHVRTLTFALADGGVPN

NEGRGYVLRRILRRGARYARKYMNYPIGNFFSTLAPTLISQVQDIFPELAKDPAFLFEILDEEEASFAKTLDRGERLFEK

YASAASKTESKTLDGKQVWRLYDTYGFPVDLTELMAEEQGLKIDGPGFEKAKQESYEASKRGGKKDQSDLIKLNVHELSE

LNDAKVPKTNDEFKYGSANVEGTILKLHDGTNFVDEITEPGKKYGIILDKTCFYAEQGGQEYDTGKIVIDDAAEFNVENV

QLYNGFVFHTGSLEEGKLSVGDKIIASFDELRRFPIKNNHTGTHILNFALKETLGNDVDQKGSLVAPEKLRFDFSHKKAV

SNEELKKVEDICNEQIKENLQVFYKEIPLDLAKSIDGVRAVFGETYPDPVRVVSVGKPIEELLANPANEEWTKYSIEFCG

GTHVNKTGDIKYFVILEESGIAKGIRRIVAVTGTEAFEAQRLAEQFAADLDSADKLPFSPIKEKKLKELGVKLGQLSISV

ITKNELKQKFNKIEKAVKDEVKSRAKKENKQTLDEVKTFFETNENAPYLVKFIDISPNAKAITEAINYMKSNDAVKDKSI

YLLAGNDPEGRVAHGCYISNAALAKGIDGSALAKKVSSIIGGKAGGKGNVFQGMGDKPAAIKDAVDDLESLFKEKLSI

>AJT78594.1 Ala1p [Saccharomyces cerevisiae YJM541]

MTIGDKQKWTATNVRNTFLDYFKSKEHKFVKSSPVVPFDDPTLLFANAGMNQYKPIFLGTVDPASDFYTLKRAYNSQKCI

RAGGKHNDLEDVGKDSYHHTFFEMLGNWSFGDYFKKEAITYSWTLLTEVYGIPKDRLYVTYFEGDEKLGLEPDTEARELW

KNVGVPDDHILPGNAKDNFWEMGDQGPCGPCSEIHYDRIGGRNAASLVNMDDPDVLEVWNLVFIQFNREQDGSLKPLPAK

HIDTGMGFERLVSVLQDVRSNYDTDVFTPLFERIQEITSVRPYSGNFGENDKDGIDTAYRVLADHVRTLTFALADGGVPN

NEGRGYVLRRILRRGARYARKYMNYPIGNFFSTLAPTLISQVQDIFPELAKDPAFLFEILDEEEASFAKTLDRGERLFEK

YASAASKTESKTLDGKQVWRLYDTYGFPVDLTELMAEEQGLKIDGPGFEKAKQESYEASKRGGKKDQSDLIKLNVHELSE

LNDAKVPKTNDEFKYGSANVEGTILKLHDGTNFVDEITEPGKKYGIILDKTCFYAEQGGQEYDTGKIVIDDAAEFNVENV

QLYNGFVFHTGSLEEGKLSVGDKIIASFDELRRFPIKNNHTGTHILNFALKETLGNDVDQKGSLVAPEKLRFDFSHKKAV

SNEELKKVEDICNEQIKENLQVFYKEIPLDLAKSIDGVRAVFGETYPDPVRVVSVGKPIEELLANPANEEWTKYSIEFCG

GTHVNKTGDIKYFVILEESGIAKGIRRIVAVTGTEAFEAQRLAEQFAADLDSADKLPFSPVKEKKLKELGVKLGQLSISV

ITKNELKQKFNKIEKAVKDEVKSRAKKENKQTLDEVKTFFETNENAPYLVKFIDISPNAKAITEAINYMKSNDAVKDKSI

YLLAGNDPEGRVAHGCYISNAALAKGIDGSALAKKVSSIIGGKAGGKGNVFQGMGDKPAAIKDAVDDLESLFKEKLSI

>AFR32146.1 alanyl-tRNA synthetase [Galactomyces candidus]

MIRPRIFFPRLYSQQFRSIMTSKLTPENWPASKVRNTFLDYFKSNEHTFVPSSSVIPYDDPTLLFANAGMNQYKPIFLGT

VDPSSPLFKLKRAANSQKCIRAGGKHNDLEDVGRDSYHHTFFEMLGNWSFGDYFKKEAIAFGWELLTKVYGIPADRLYVS

YFGGEEKLGLEPDLEAKQIWLDLGVPEDHILPGDSADNFWEMGDQGPCGPCSEIHYDRIGGRNASSLVNMDDPNVLEIWN

IVFIQYNREANGELRPLPNKHIDTGMGFERLVSVLQNKLSNYDTDVFTPLFAKIQELTGAREYTGKFGKEDVDGIDTAYR

VVADHVRTLTFALADGGVPNNDGRGYVLRRILRRGARYVRKKFDVPIGNFFSRLAPTLIEQVSDIFPEVTKKTDDIFEIL

NEEEESFSRTLDRGEKLFEQYAAAAIANKQTTLNGKDVWRLYDTYGFPVDLTRLMAEEAGLEIDEQEFEKAKLESKEASK

GGKKSEGKTIVKLDVHSLQELADKKVPVTDDNAKYIPGDIESSVLAIYANNTFVENTEGLEKDLFFGIVLDKTNFYAEQG

GQEYDTGKFVIDGETEFEVTNVQVYAGYVLHTGYLKYGNLKVGSKVNAGFDELRRWPIRNNHTGTHVLNYALREVLGDSI

DQKGSLVAPEKMRFDFSHKAALSLKEVEAVENISNEYIKKNLSVYAKDVPLEQATEINGLRAVFGEKYPDPVRVVSVGVS

IDDLLADPTNSEWQKYSIEFCGGTHVGKTGEIKELVIIEESGIAKGIRRIVAVTSTDAYEVQKLAKAFDERLDHLSAMPI

GAEKEALAKTIGVELKKLTVAVLAKNELQTKFAAIQKKISDDAKAKHKQEAKIVLDAVTEIFDAENSPEYFIGRFDNISG

NSKALTETINFIKTKKADKSVYLFAGDVSAGKVAHGCYISKEAEAKGITASDLGALAAQSVGGRSGGKGQTVQGAGVKPE

GIDEALEKLSALLKEKLTV

>OVF07805.1 putative alanine--tRNA synthetase [Clavispora lusitaniae]

MTTSSHPWSASTVRSTFLDYFKNKSHTYVPSSSVVPHNDPTLLFANAGMNQYKPIFLGTVDPTSEFATLKRAVNSQKCIR

AGGKHNDLEDVGRDSYHHTFFEMLGNWSFGDYFKKEAIAYSWELLTEVYKLDKDRLYVTYFEGDEKNGLEPDLEAKEYWL

SVGVAPDHILPGDAHDNFWEMGDQGPCGPCSEIHYDRIGGRNAASLVNQDDPNVLEVWNIVFIQYNREADSSLKPLPSKH

IDTGMGFERLVSVLQDKSSNYDTDVFQPIFEEIKNITGVRPYTGKFGAEDVDGIDTAYRVIADHVRTLTFAISDGGVPNN

EGRGYVLRRILRRGARYVRKYMNCPIGSFFQQLVDVVIENNSSIFPEIAKNKDMLKEILMEEEVSFAKTLDRGEKLFEQY

AIIASKTPEQTLSGKDVWRLYDTYGFPVDLTRLMAEEAGLKIDEEAFEKAKEESREASKATNTKGGATLIKLDVHALSEL

DNNESITKTNDVYKYGLDNIESKIVAIYDGSKFVDTISEAGQQFGIILDRTPFYAEQGGQEYDTGSLVIDGSAEFAVTNV

QSYGSYILHTGTMVEGTLKLNDNVIATYDELRRWPIRNNHTGTHVLNFALKEVLGDGIDQKGSLVAPEKLRFDFSHKQAL

TPKELEQVENISSEIIKSNKQVYAEEVELTKAKQIAGVRAVFGETYPDPVRVVSIGVPVADLLKDPTNQDWTKLSIEFCG

GTHVAKTGDIKDFVIIEESGIAKGIRRIVAVTGHEAQEVQRIAREFSASLDSADKLSFGPEKEQKAKELGVALKQLSISV

LDKQKLNEKFTKLDKSIKDHLKAKQKEESKKTLEAVNGWLKDEEKSKTAFFVAHVPISANAKAITEAINLIKKQHKDKSL

YLLTGTSEKVAHGCYISDEAISKGIDASELAKAVSAHIGGKAGGKGNVVQGMGDSPEGIDAAVETVTKLLAEKL

>AJT74669.1 Ala1p [Saccharomyces cerevisiae YJM320]

MTIGDKQKWTATNVRNTFLDYFKSKEHKFVKSSPVVPFDDPTLLFANAGMNQYKPIFLGTVDPASDFYTLKRAYNSQKCI

RAGGKHNDLEDVGKDSYHHTFFEMLGNWSFGDYFKKEAITYSWTLLTEVYGIPKDRLYVTYFEGDEKLGLEPDTEARELW

KNVGVPDDHILPGNAKDNFWEMGDQGPCGPCSEIHYDRIGGRNAASLVNMDDPDVLEVWNLVFIQFNREQDGSLKPLPAK

HIDTGMGFERLVSVLQDVRSNYDTDVFTPLFERIQEITSVRPYSGNFGENDKDGIDTAYRVLADHVRTLTFALADGGVPN

NEGRGYVLRRILRRGARYARKYMNYPIGNFFSTLAPTLISQVQDIFPELAKDPAFLFEILDEEEASFAKTLDRGERLFEK

YASAASKTESKTLDGKQVWRLYDTYGFPVDLTELMAEEQGLKIDGPGFEKAKQESYEASKRGGKKDQSDLIKLNVHELSE

LNDAKVPKTNDEFKYGSANVEGTILKLHDGTNFVDEITEPGKKYGIILDKTCFYAEQGGQEYDTGKIVIDDAAEFNVENV

QLYNGFVFHTGSLEEGKLSVGDKIIASFDELRRFPIKNNHTGTHILNFALKETLGNDVDQKGSLVAPEKLRFDFSHKKAV

SNEELKKVEDICNEQIKENLQVFYKEIPLDLAKSIDGVRAVFGETYPDPVRVVSVGKPIEELLANPANEEWTKYSIEFCG

GTHVNKTGDIKYFVILEESGIAKGIRRIVAVTGTEAFEAQRLAEQFAADLDAADKLPFSPIKEKKLKELGVKLGQLSISV

ITKNELKQKFNIIEKAVKDEVKSRAKKENKQTLDEVKTFFETNENAPYLVKFIDISPNAKAITEAINYMKSNDAVKDKSI

YLLAGNDPEGRVAHGCYISNAALAKGIDGSALAKKVSSIIGGKAGGKGNVFQGMGDKPAAIKDAVDDLESLFKEKLSI

>AJT73685.1 Ala1p [Saccharomyces cerevisiae YJM270]

MTIGDKQKWTATNVRNTFLDYFKSKEHKFVKSSPVVPFDDPTLLFANAGMNQYKPIFLGTVDPASDFYTLKRAYNSQKCI

RAGGKHNDLEDVGKDSYHHTFFEMLGNWSFGDYFKKEAITYSWTLLTEVYGIPKDRLYVTYFEGDEKLGLEPDTEARELW

KNVGVPDDHILPGNAKDNFWEMGDQGPCGPCSEIHYDRIGGRNAASLVNMDDPDVLEVWNLVFIQFNREQDGSLKPLPAK

HIDTGMGFERLVSVLQDVRSNYDTDVFTPLFERIQEITSVRPYSGNFGENDKDGIDTAYRVLADHVRTLTFALADGGVPN

NEGRGYVLRRILRRGARYARKYMNYPIGNFFSTLAPTLISQVQDIFPELAKDPAFLFEILDEEEASFAKTLDRGERLFEK

YASAASKTESKTLDGKQVWRLYDTYGFPVDLTELMAEEQGLKIDGPGFEKAKQESYEASKRGGKKDQSDLIKLNVHELSE

LNDAKVPKTNDEFKYGSANVEGTILKLHDGTNFVDEITEPGKMYGIILDKTCFYAEQGGQEYDTGKIVIDDAAEFNVENV

QLYNGFVFHTGSLEEGKLSVGDKIIASFDELRRFPIKNNHTGTHILNFALKETLGNDVDQKGSLVAPEKLRFDFSHKKAV

SNEELKKVEDICNEQIKENLQVFYKEIPLDLAKSIDGVRAVFGETYPDPVRVVSVGKPIEELLANPANEEWTKYSIEFCG

GTHVNKTGDIKYFVILEESGIAKGIRRIVAVTGTEAFEAQRLAEQFAADLDAADKLPFSPIKEKKLKELGVKLGQLSISV

ITKNELKQKFNKIEKAVKDEVKSRAKKENKQTLDEVKTFFETNENAPYLVKFIDISPNAKAITEAINYMKSNDAVKDKSI

YLLAGNDPEGRVAHGCYISNAALAKGIDGSALAKKVSSIIGGKAGGKGNVFQGMGDKPAAIKDAVDDLESLFKEKLSI

>GAN03494.1 alanyl-tRNA synthetase [Mucor ambiguus]

MAGQSEWPANKVRATFIKYFEDNGHVFVPSSSTIPHDDPTLLFANAGMNQYKPIFLGTLDPNSPLANVKRACNSQKCIRA

GGKHNDLDDVGRDTYHHTFFEMLGNWSFGDYFKLEATKFGWEFLTKVINLPEDRLYVTYFGGDEKMGLPADLEAKQMWMD

LGVEEKRLIPGSVQDNFWEMGETGPCGPCSEIHYDRIGDRDAAHLVNMDDPNVLEIWNLVFMQFNREIDGSLRSLPNQHI

DTGLGLERLVSILQNKYSNYDTDCFTPLFDKIKELTGARDYTGLLGEEDKDGIDTAYRVVGDHIRTLTFAISDGGVPSNE

GRGYVLRRILRRGARYVRKYFNVSIGTFFSSLVDTLLEQMHEAYPELAKKAADVKAILDEEEVAFSKTLDRGEKLFELYM

NKTKESGSSVMSGDDVWRLYDTYGFPVDLTRIMAEENGLQVNEAQFEAAQEAAKNKSRKVKGGDNGQGVVALDVHDLGAL

DNNAAVPKTDDSYKYLPGNVQGDIKAIFFEHKFLDTTADIPEDRNFGLLVDKTNFYAESGGQEYDTGSIITLDGNTEFVV

EDCQLYGGYVLHVGHLKYGQLKLDDKVELSYDDNRRYPLRNNHSATHILNFALREALKEDIDQKGSLVSPEKLRFDFSFK

NPVNVKQLAEVEKICDDFIAKNLKVYSKEVPLAVAKAIHGLRAVFGETYPDPVRVVSIGYDVDEITNDVSNPKWGTTSIE

FCGGTHVAKTSDIKYFVILEESGIAKGIRRIVAVTGDEAAAAHRNAEEFVGKLDRLEKLSGAELEAELKVVNKELDLAVI

SAVSKAHFRERFTQIKKVFDNAEKVRKAEQIKEASDAVKKYFEANPDAQYLVTELPVGNNSKALGGAINYAKANLKDKAV

YVLSGDKASGRVAHNCIVGKNLIEKGFKASDWATVVCEKVGGKKGGKDDAAQGSGDKVDALSDALKAAEEFAKLKISA

>EHN00072.1 Ala1p [Saccharomyces cerevisiae x Saccharomyces kudriavzevii VIN7]

MTIGDKQKWTAANVRNTFLDYFKSKEHKFVKSSPVVPFDDPTLLFANAGMNQYKPIFLGTVDPASDFYTLKRAYNSQKCI

RAGGKHNDLEDVGKDSYHHTFFEMLGNWSFGDYFKIEAVTYSWTLLTEVYGIPKDRLYVTYFEGDEKLGLEPDIEARELW

RNAGVPDDHILPGNAKDNFWEMGDQGPCGPCSEIHYDRIGGRNAASLVNMDDPDVLEVWNLVFIQFNREQDGSLKSLPAK

HIDTGMGFERLVSVLQDVRSNYDTDVFTPLFERIQEITSVKPYSGKFGEDDSDGVDTAYRVLADHVRTLTFALADGGVPN

NEGRGYVLRRILRRGARYARKYMNYPIGNFFSTLAPTLIEQVQDIFPELAKDPAFLFEILDEEEASFAKTLDRGERLFEK

YASAASKTESRTLDGKQVWRLYDTYGFPVDLTELMAEEQGLKIDGPGFEKAKQESYEASKRGGKKDQSDLIKLNVHELSE

LNDANVPKTNDEFKYGSANVESTILKLHDGTKFVNEITEPGRKYGIILDKTCFYAEQGGQEYDTGKIVIDDAAEFNVENV

QLYNGFVFHTGSLEEGKLSVGDKIIASFDELRRFPIKNNHTGTHILNFALKETLGNDIDQKGSLVASEKLRFDFSYKKAV

TGDELKNIEDTCNQQIKENLQVFYKEIPLELAKSIDGVRAVFGETYPDPVRVVSVGKPIEELLANSANEEWTKYSIEFCG

GTHVNKTGDIKYFVILEESGIAKGIRRIVAVTGTEAFEAQRLAERFAADLDAADKLPFSPVKEKKLKELGVKLGQLSISV

ITKNELKQKFNKIEKAVKDEVKSRAKKENKQTLDEVKTFFETNENTPYFVKFIDISPNAKAITEAINYMKSNDSVKDKSI

YLLAGNDPEGRVAHGCYISNVALAKGVDGSELAKKVSSVIGGKAGGKGNVFQGMGDKPAAIQNAVDDLESLFKEKLSI

>AJP41837.1 Ala1p [Saccharomyces cerevisiae YJM1078]

MTIGDKQKWTATNVRNTFLDYFKSKEHKFVKSSPVVPFDDPTLLFANAGMNQYKPIFLGTVDPASDFYTLKRAYNSQKCI

RAGGKHNDLEDVGKDSYHHTFFEMLGNWSFGDYFKKEAITYSWTLLTEVYGIPKDRLYVTYFEGDEKLGLEPDTEARELW

KNVGVPDDHILPGNAKDNFWEMGDQGPCGPCSEIHYDRIGGRNAASLVNMDDPDVLEVWNLVFIQFNREQDGSLKPLPAK

HIDTGMGFERLVSVLQDVRSNYDTDVFTPLFERIQEITSVRPYSGNFGENDKDGIDTAYRVLADHVRTLTFALADGGVPN

NEGRGYVLRRILRRGARYARKYMNYPIGNFFSTLAPTLISQVQDIFPELAKDPAFLFEILDEEEASFAKTLDRGERLFEK

YASDASKTESKTLDGKQVWRLYDTYGFPVDLTELMAEEQGLKIDGPGFEKAKQESYEASKRGGKKDQSDLIKLNVHELSE

LNDAKVPKTNDEFKYGSANVEGTILKLHDGTNFVDEITEPGKKYGIILDKTCFYAEQGGQEYDTGKIVIDDAAEFNVENV

QLYNGFVFHTGSLEEGKLSVGDKIIASFDELRRFPIKNNHTGTHILNFALKETLGNDVDQKGSLVAPEKLRFDFSHKKAV

SNEELKKVEDICNEQIKENLQVFYKEIPLDLAKSIDGVRAVFGETYPDPVRLVSVGKPIEELLANPANEEWTKYSIEFCG

GTHVNKTGDIKYFVILEESGIAKGIRRIVAVTGTEAFEAQRLAEQFAADLDAADKLPFSPIKEKKLKELGVKLGQLSISV

ITKNELKQKFNKIEKAVKDEVKSRAKKENKQTLDEVKTFFETNENAPYLVKFIDISPNAKAITEAINYMKSNDAVKDKSI

YLLAGNDPEGRVAHGCYISNAALAKGIDGSALAKKVSSIIGGKAGGKGNVFQGMGDKPAAIKDAVDDLESLFKEKLSI

>AJT94197.1 Ala1p [Saccharomyces cerevisiae YJM1307]

MTIGDKQKWTATNVRNTFLDYFKSKEHKFVKSSPVVPFDDPTLLFANAGMNQYKPIFLGTVDPASDFYTLKRAYNSQKCI

RAGGKHNDLEDVGKDSYHHTFFEMLGNWSFGDYFKKEAITYSWTLLTEVYGIPKDRLYVTYFEGDEKLGLEPDTEARELW

KNVGVPDDHILPGNAKDNFWEMGDQGPCGPCSEIHYDRIGGRNAASLVNMDDPDVLEVWNLVFIQFNREQDGSLKPLPAK

HIDTGMGFERLVSVLQDVRSNYDTDVFTPLFERIQEITSVRPYSGNFGENDKDGIDTAYRVLADHVRTLTFALADGGVPN

NEGRGYVLRRILRRGARYARKYMNYPIGNFFSTLAPTLISQVQDIFPELAKDPAFLFEILDEEEASFAKTLDRGERLFEK

YASAASKTESKTLDGKQVWRLYDTYGFPVDLTELMAEEQGLKIDGPGFEKAKQESYEASKRGGKKDQSDLIKLNVHELSE

LNDAKVPKTNDEFKYGSANVEGTILKLHDGTNFVDEITEPGKKYGIILDKTCFYAEQGGQEYDTGKIVIDDAAEFNVENV

QLYNGFVFHTGSLEEGKLSVGDKIIASFDELRRFPIKNNHTGTHILNFALKETLGNDVDQKGSLVAPEKLRFDFSHKKAV

SNEELKKVEDICNEQIKENLQVFYKEIPLDLAKSIDSVRAVFGETYPDPVRVVSVGQPIEELLANPANEEWTKYSIEFCG

GTHVNKTGDIKYFVILEESGIAKGIRRIVAVTGTEAFEAQRLAEQFAADLDAADKLPFSPIKEKKLKELGVKLGQLSISV

ITKNELKQKFNKIEKAVKDEVKSRAKKENKQTLDEVKTFFETNENAPYLVKFIDISPNAKAITEAINYMKSNDAVKDKSI

YLLAGNDPEGRVAHGCYISNAALAKGIDGSALAKKVSSIIGGKAGGKGNVFQGMGDKPAAIKDAVDDLESLFKEKLSI

>AFR32145.1 alanyl-tRNA synthetase, partial [Galactomyces candidus]

PRLYSQQFRSIMTSKLTPENWPASKVRNTFLDYFKSKEHTFVPSSSVIPYDDPTLLFANAGMNQYKPIFLGTVDPSSPLF

KLKRAANSQKCIRAGGKHNDLEDVGRDSYHHTFFEMLGNWSFGDYFKKEAIAFGWELLTKVYGIPADRLYVSYFGGEEKL

GLEPDLEAKQIWLDLGVPEDHILPGDSADNFWEMGDQGPCGPCSEIHYDRIGGRNASSLVNMDDPNVLEIWNIVFIQYNR

EANGELRPLPNKHIDTGMGFERLVSVLQNKLSNYDTDVFTPLFAKIQELTGAREYTGKFGKEDVDGIDTAYRVVADHVRT

LTFALADGGVPNNDGRGYVLRRILRRGARYVRKKFDVPIGNFFSRLAPTLIEQVSDIFPEVTKKTDDIFEILNEEEESFS

RTLDRGEKLFEQYAAAAIANKQTTLNGKDVWRLYDTYGFPVDLTRLMAEEAGLEIDEQEFEKAKLESKEASKGGKKSEGK

TIVKLDVHSLQELADKKVPVTDDNAKYIPGDIESSVLAIYANNTFVENTEGLEKDLFFGIVLDKTNFYAEQGGQEYDTGK

FVIDGETEFEVTNVQVYAGYVLHTGYLKYGNLKVGSKVNAGFDELRRWPIRNNHTGTHVLNYALREVLGDSIDQKGSLVA

PEKMRFDFSHKAALSLKEVEAVENISNEYIKKNLSVYAKDVPLEQATEINGLRAVFGEKYPDPVRVVSVGVSIDDLLADP

TNSEWQKYSIEFCGGTHVGKTGEIKELVIIEESGIAKGIRRIVAVTSTDAYEVQKLAKAFDERLDHLSAMPIGAEKEALA

KTIGVELKKLTVAVLAKNELQTKFAAIQKKISDDAKAKHKQEAKIVLDAVTEIFDAENSPEYFIGRFDNISGNSKALTET

INFIKTKKADKSVYLFAGDVSAGKVAHGCYISKEAEAKGITASDLGALAAQSVGGRSGGKGQTVQGAGVKPEGIDEALEK

LSALLKEKLTV

>XP_003670443.1 hypothetical protein NDAI_0E03830 [Naumovozyma dairenensis CBS 421]

MTIGDKQKWTAVQVRNTFLDYFKSKNHKFIPSSPVVPFDDPTLLFANAGMNQYKPIFLGTVDPSSDFYTLKRAYNSQKCV

RAGGKHNDLEDVGKDSYHHTFFEMLGNWSFGDYFKTEAINYAWDLLTNVYGIPKDRLYVTYFEGDAKQGLEPDTEAQELW

KKVGVPEDHILPGNAKDNFWEMGDQGPCGPCSEIHYDRIGGRNAAQLVNQDDPDVLEVWNLVFIQFNRESNGELKSLPAK

HIDTGMGFERLVSVLQDVRSNYDTDVFQPLFKRIQEITGARDYTGKFGDEDKDNIDTAYRVLADHVRTLMFALADGGVPN

NEGRGYVLRRILRRGARYARKYMNYPIGNFFSTLAPTLIDQVKHIFPEIAKDPSFLFEILDEEEASFAKTLDRGERLFEK

YAAAASQTESKTLDGKQVWRLYDTYGFPVDLTELMAEEQNLKIDGPGFEKAKQESYEASKRGGKKDNGDLIKLDVHQLSE

LNDNNVPKTDDNAKYGTENIESTILRIHDGTNFVDSITEVGKKYGIILDKTCFYAEQGGQEYDTGKIVIDDAAEFNVENV

QLYNGFVFHTGSLEEGKLSVGDKIIASYDELRRFPIKNNHTGTHILNLALKETLGADVDQKGSLVNPEKLRFDFSHKKAL

TTDELRKVEDISNEQIKENLIVYYKDVPLEQAKSINNVRAVFGETYPDPVRVVCVGKPIDELLADPTNEEWNKYSIEFCG

GTHVAKTGDIKYFVVLEESGIAKGIRRIVAVTGSEAFEAQRIANEFASELETAEKLPFSPLKEKKLKELGVKLGQLSISV

INKHELKEKFTAIEKVVKDEVKSRAKKEAKQTLDEVKTYFTENADAPYFVKFIDIPVNAKAITEAINYIKSSAKDKSIYL

LTGNDSNGKIAHGCYISDAALAKGVDGAVLAKQVSNVIGGKAGGKGNVFQGMGDKPDAATEAVSEIEKLLREKLTI

>CDO55862.1 similar to Saccharomyces cerevisiae YOR335C ALA1 Cytoplasmic and mitochondrial alanyl-tRNA synthetase [Galactomyces candidum]

MTSKLTPENWPASKVRNTFLDYFKSKEHTFVPSSSVIPYDDPTLLFANAGMNQYKPIFLGTVDPSSPLFKLKRAANSQKC

IRAGGKHNDLEDVGRDSYHHTFFEMLGNWSFGDYFKKEAIAFGWELLTKVYGIPADRLYVSYFGGEEKLGLEPDLEAKQI

WLDLGVPEDHILPGDSADNFWEMGDQGPCGPCSEIHYDRIGGRNASSLVNMDDPNVLEIWNIVFIQYNREANGELRPLPN

KHIDTGMGFERLVSVLQNKLSNYDTDVFTPLFAKIQELTGAREYTGKFGKEDVDGIDTAYRVVADHVRTLTFALADGGVP

NNDGRGYVLRRILRRGARYVRKKFDVPIGNFFSRLAPTLIEQVSDIFPEVTKKTDDIFEILNEEEESFSRTLDRGEKLFE

QYAAAAIANKQTTLNGKDVWRLYDTYGFPVDLTRLMAEEAGLEIDEQEFEKAKLESKEASKGGKKSEGKTIVKLDVHSLQ

ELADKKVPVTDDNAKYIPGDIESSVLAIYANNTFVENTEGLEKDLFFGIVLDKTNFYAEQGGQEYDTGKFVIDGETEFEV

TNVQVYAGYVLHTGYLKYGNLKVGSKVNAGFDELRRWPIRNNHTGTHVLNYALREVLGDSIDQKGSLVAPEKMRFDFSHK

AALSLKEVEAVENISNEYIKKNLSVYAKDVPLEQATEINGLRAVFGEKYPDPVRVVSVGVSIDDLLADPTNSEWQKYSIE

FCGGTHVGKTGEIKELVIIEESGIAKGIRRIVAVTSTDAYEVQKLAKAFDERLDHLSAMPIGAEKEALAKTIGVELKKLT

VAVLAKNELQTKFAAIQKKISDDAKAKHKQEAKIVLDAVTEIFDAENSPEYFIGRFDNISGNSKALTETINFIKTKKADK

SVYLFAGDVSAGKVAHGCYISKEAEAKGITASDLGALAAQSVGGRSGGKGQTVQGAGVKPEGIDEALEKLSALLKEKLTV

>KOH47664.1 SYC1p Subunit of the APT subcomplex of cleavage and polyadenylation factor [Saccharomyces sp. 'boulardii']

MTIGDKQKWTATNVRNTFLDYFKSKEHKFVKSSPVVPFDDPTLLFANAGMNQYKPIFLGTVDPASDFYTLKRAYNSQKCI

RAGGKHNDLEDVGKDSYHHTFFEMLGNWSFGDYFKKEAITYSWTLLTEVYGIPKDRLYVTYFEGDEKLGLEPDTEARELW

KNVGVPDDHILPGNAKDNFWEMGDQGPCGPCSEIHYDRIGGRNAASLVNMDDPDVLEVWNLVFIQFNREQDGSLKPLPAK

HIDTGMGFERLVSVLQDVRSNYDTDVFTPLFERIQEITSVRPYSGNFGENDKDGIDTAYRVLADHVRTLTFALADGGVPN

NEGRGYVLRRILRRGARYARKYMNYPIGNFFSTLAPTLISQVQDIFPELAKDPAFLFEILDEEEASFAKTLDRGERLFEK

YASAASKTESKTLDGKQVWRLYDTYGFPVDLTELMAEEQGLKIDGPGFEKAKQESYEASKRGGKKDQSDLIKLNVHELSE

LNDAKVPKTNDEFKYGSANVEGTILKLHDGTNFVDEITEPGKKYGIILDKTCFYAEQGGQEYDTGKIVIDDAAEFNVENV

QLYNGFVFHTGSLEEGKLSVGDKIIASFDELRRFPIKNNHTGTHILNFALKETLGNDVDQKGSLVAPEKLRFDFSHKKAV

SNEELKKVEDICNEQIKENLQVFYKEIPLDLAKSIDGVRAVFGETYPDPVRVVSVGKPIEKLLANPANEEWTKYSIEFCG

GTHVNKTGDIKYFVILEESGIAKGIRRIVAVTGTEAFEAQRLAEQFAADLDAADKLPFSPIKEKKLKELGVKLGQLSISV

ITKNELKQKFNKIEKAVKDEVKSRAKKENKQTLDEVKTFFETNENAPYLVKFIDISPNAKAITEAINYMKSNDAVKDKSI

YLLAGNDPEGRVAHGCYISNAALAKGIDGSALAKKVSSIIGGKAGGKGNVFQGMGDKPAAIKDAVDDLESLFKEKLSI

>AJT76134.1 Ala1p [Saccharomyces cerevisiae YJM450]

MTIGDKQKWTATNVRNTFLDYFKSKEHKFVKSSPVVPFDDPTLLFANAGMNQYKPIFLGTVDPASDFYTLKRAYNSQKCI

RAGGKHNDLEDVGKDSYHHTFFEMLGNWSFGDYFKKEAITYSWTLLTEVYGIPKDRLYVTYFEGDEKLGLEPDTEARELW

KNVGVPDDHILPGNAKDNFWEMGDQGPCGPCSEIHYDRIGGRNAASLVNMDDPDVLEVWNLVFIQFNREQDGSLKPLPAK

HIDTGMGFERLVSVLQDVRSNYDTDVFTPLFERIQEITSVRPYSGNFGENDKDGIDTAYRVLADHVRTLTFALADGGVPN

NEGRGYVLRRILRRGARYARKYMNYPIGNFFSTLAPTLISQVQDIFPELAKDPAFLFEILDEEEASFAKTLDRGERLFEK

YASAASKTESKTLDGKQVWRLYDTYGFPVDLTELMAEEQGLKIDGPGFEKAKQESYEASKRGGKKDQSDLIKLNVHELSE

LNDAKVPKTNDEFKYGSANVEGTILKLHDGTNFVDEITEPGKKYGIILDKTCFYAEQGGQEYDTGKIVIDDAAEFNVENV

QLYNGFVFHTGSLEEGKLSVGDKIIASFDELRRFPIKNNHTGTHILNFALKETLGNDVDQKGSLVAPEKLRFDFSHKKAV

SNEELKKVEDICNEQIKENLQVFYKEIPLDLAKSIDSVRAVFGETYPDPVRVVSVGQPIEELLANPANEEWTKYSIEFCG

GTHVNKTGDIKYFVILEESGIAKGIRRIVAVTGTEAFEAQRLAEQFAADLDAADKLPFSPIKEKKLKELGVKLGQLSISV

ITKNELKQKFNKIEKAVKDEVKSRAKKENKQTLDEVKTFFETNENAPYLVKFIDISPNAKAITEAINYMKSNDAVKDKSI

YLLAGNDPEGRVAHGCYISNAALAKGIDGSALAKKVSSIIGGKAGGKGNVFQGMGDKPAAIKDAVDDLENLFKEKLSI

>AAC49007.1 cytoplasmic alanyl-tRNA synthetase [Saccharomyces cerevisiae]

MTIGDKQKWTATNVRNTFLDYFKSKEHKFVKSSPVVPFDDPTLLFANAGMNQYKPIFLGTVDPASDFYTLKRAYNSQKCI

RAGGKHNDLEDVGKDSYHHTFFEMLGNWSFGDYFKKEAITYSWTLLTEVYGIPKDSLYVTYFEGDEKLGLEPDTEARELW

KNVGVPDDHILPGNAKDNFWEMGDQGPCGPCSEIHYDRIGGRNAASLVNMDDPDVLEVWNLVFIQFNREQDGSLKPLPAK

HIDTGMGFERLVSVLQDVRSNYDTDVFTPLFERIQEITSVRPYSGNFGENDKDGIDTAYRVLADHVRTLTFALADGGVPN

NEGRGYVLRRILRRGARYARKYMNYPIGNFFSTLAPTLISQVQDIFPELAKDPAFLFEILDEEEASFAKTLDRGERLFEK

YASAASKTESKTLDGKQVWRLYDTYGFPVDLTELMAEEQGLKIDGPGFEKAKQESYEASKRGGKRTKSDLIKLNVHELSE

LNDAKVPKTNDEFKYGSANVEGTILKLHDGTNFVDEITEPGKKYGIILDKTCFYAEQGGQEYDTGKIVIDDAAEFNVENV

QLYNGFVFHTGSLEEGKLSVGDKIIASFDELRRFPIKNNHTGTHILNFALKETLGNDVDQKGSLVAPEKLRFDFSHKKAV

SNEELKKVEDICNEQIKENLQVFYKEIPLDLAKSIDGVRAVFGETYPDPVRVVSVGKPIEELLANPANEEWTKYSIEFCG

GTHVNKTGDIKYFVILEESGIAKGIRRIVAVTGTEAFEAQRLAEQFAADLDAADKLPFSPIKEKKLKELGVKLGQLSISV

ITKNELKQKFNKIEKAVKDEVKSRAKKENKQTLDEVKTFLQTNENAPYLVKFIDISPNAKAITEAINYMKSNDSVKDKSI

YLLAGNDPEGRVAHGCYISNAALAKGIDGSALAKKVSSIIGGKAGGKGNVFQGMGDKPAAIKDAVDDLESLFKEKLSI

>XP_020071552.1 hypothetical protein CYBJADRAFT_172487 [Cyberlindnera jadinii NRRL Y-1542]

MSQSTEWPAQKVRNTFLDYFKSKGHTFVPSSSVIPYDDPTLLFANAGMNQYKPIFLGTVDPASEFYSLKRAMNSQKCIRA

GGKHNDLEDVGRDSYHHTFFEMLGNWSFGDYFKKEAIEYSWELLTKVYGLKEDRLYVTYFGGDEASGLLPDLEAKKLWED

VGVDPSHILPGSVKDNFWEMGEQGPCGPCSEVHYDRIGGRNAAELVNEDDPNVIEIWNNVFMQFNREADGTLRPLPAKHI

DTGLGFERLVSILQNCSSNYDTDVFQPLFKKIQEMTGARDYTGKFGAEDVDGIDTAYRVLADHVRTLTFALADGGVPNNE

GRGYVLRRILRRGARYARKYFNYPIGNFFSTLAPTLIDQVQDIFPELTKNTSNTFEILDEEEASFARTLDRGEKLFEQYA

IAAQKGTKILNGKDVWRLYDTYGFPVDLTQLMALEAGLKIDEAGFEKARIESYEASKAGGKSKGSDLIKLDVHALAKLNE

EKLDRTDDHYKYGTENVEAKILKIYDGQEFVDEISDITKQYGIILDKTPFYAEQGGQEYDTGKIVIDGASEFNVENTQQY

NGYVFHTGRLAEGSLKVGDAVIAGYEELRRWPLRNNHTGTHILNLALREVLGTEVHQKGSLVAPEKLRFDFSHKQGLTLD

EVSKIEAICNEEIKKAQIVYAKDVPLEQAKGITSVRAVFGETYPDPVRVVSIGVPIDDLLSSPTDEKWMTVSIEFCGGTH

VAKTSDIKDLVLTEESGIAKGIRRIVAVTGNEAHDVQRIASEFSKDLESAGKLEFGPLKEKKLKELGVKLGQLNISILSK

SELKNRFNVFDKEIKDENKARQKAESKKTLDTVKDYFAENESSAFLVKHVDISANAKSITEAINFIKSKNPDKSIYIFTG

NADKVAHGCYISDKAIEKGVNASELAKHASEFIGGKAGGKGNVVQGMGDKLDGIPNAVDELEKLLKEKLSI

>ANZ74405.1 BA75_00493T0 [Komagataella pastoris]

MLKFPHKGISSIKSKTRIGIIAMSTLASKEWTANKVRSTFFDFFKGKDHTYVPSSSVIPYDDPTLLFTNAGMNQYKPIFL

GTVDPSSDFATLKRATNSQKCIRAGGKHNDLEDVGRDSYHHTFFEMLGNWSFGDYFKKEAIGYGWELLTKVYGLDKERLY

VSYFGGDADLGLEPDLEAKEYWKALGVAEDHILPGSVADNFWEMGDQGPCGPCSEIHYDRIGGRNAASLVNQDDPDVLEI

WNIVFIQFNREADGSLRPLPSKHIDTGMGFERLVSILQNVRSNYDTDVFDPLFTKIQEITGVRPYSGHFGKDDMDGIDTA

YRVLADHVRTLTFSLADGGVPNNEGRGYVLRRILRRGARYARKYMNYPIGKFFSQLAPALIEQVKETFPEVDKNFDTIAE

ILDEEEASFARTLDRGERLFEQYAIIASKTPEQTLAGRDVWRLYDTYGFPVDLTRLMAEETGLKIDDEGFEIAKEASREA

SKGNGKTQGKELVKLDVHVLGELEKNSEVPKTDDSYKYGTKNIEASIVAIYNGKDLIQSTSTVAENQQLGILLDKTPFYA

EQGGQIYDTGKIVIDGVAEFNVENVQSYAGYVLHTGYVEEGVLSVSDKIIAGYDELRRWPIKNNHTGTHILNFALRDVLG

DDVEQKGSLVAPEKLRFDFSHKKALTLSELRKVEDISNEIIKKNVPVFASDVTLAVGKEINGLRAVFGETYPDPVRVVSI

GVPVEDLVKNPSNGEWHNASIEFCGGTHVAKTGDIKDFVIIEESGIAKGIRRIVAVTSTEAHTVQRIAREKNDQLDAISK

MSFGEAKEKKLKEFNVALGKLQISVLAKNDLKTKFTNIEKVVKDELKARQKAESKKTLDTVKDYFNEHKDSPFFVAHVPV

SSNAKLLTEAFSYVKKEIPDKSLYLIVGNESEPKIAQGCYVSENAISKGVVASEVTGVASEFIGGKAGGKGNICQGVGTN

PEGINKAIEAVTKMFKEKVNI

>AFR32153.1 alanyl-tRNA synthetase [Galactomyces candidus]

MIRPRIFFPRLYSQQFRSIMTSKLTPENWPASKVRNTFLDYFKSKEHTFVPSSSVIPYDDPTLLFANAGMNQYKPIFLGT

VDPSSPLFKLKRAANSQKCIRAGGKHNDLEDVGRDSYHHTFFEMLGNWSFGDYFKKEAIAFGWELLTKVYGIPADRLYVS

YFGGEEKLGLEPDLEAKQIWLDLGVPEDHILPGDSADNFWEMGDQGPCGPCSEIHYDRIGGRNASSLVNMDDPNVLEIWN

IVFIQYNREANGELRPLPNKHIDTGMGFERLVSVLQNKLSNYDTDVFTPLFAKIQELTGAREYTGKFGKEDVDGIDTAYR

VVADHVRTLTFALADGGVPNNDGRGYVLRRILRRGARYVRKKFDVPIGNFFSRLAPTLIEQVSDIFPEVTKKTDDIFEIL

NEEEESFSRTLDRGEKLFEQYAAAAIANKQTTLNGKDVWRLYDTYGFPVDLTRLMAEEAGLEIDEQEFEKAKLESKEASK

GGKKSEGKTIVKLDVHSLQELADKKVPVTDDNAKYIPGDIESSVLAIYANNTFVENTEGLEKDLFFGIVLDKTNFYAEQG

GQEYDTGKFVIDGETEFEVTNVQVYAGYVLHTGYLKYGNLKVGSKVNAGFDELRRWPIRNNHTGTHVLNYALREVLGDSI

DQKGSLVAPEKMRFDFSHKAALSLKEVEAVENISNEYIKKNLSVYAKDVPLEQATEINGLRAVFGEKYPDPVRVVSVGVS

IDDLLADPTNSEWQKYSIEFCGGTHVGKTGEIKELVIIEESGIAKGIRRIVAVTSTDAYEVQKLAKAFDERLDHLSAMPI

GAEKEALAKTIGVELKKLTVAVLAKNELQTKFAAIQKKISDDAKAKHKQEAKIVLDAVTEIFDAENSPEYFIGRFDNISG

NSKALTETINFIKTKKADKSVYLFAGDVSAGKVAHGCYISKEAEAKGITASDLGALAAQSVGGRSGGKGQTVQGAGVKPE

GIDEALEKLSALLKEKLTV

>AJU04002.1 Ala1p [Saccharomyces cerevisiae YJM1402]

MTIGDKQKWTATNVRNTFLDYFKSKEHKFVKSSPVVPFDDPTLLFANAGMNQYKPIFLGTVDPASDFYTLKRAYNSQKCI

RAGGKHNDLEDVGKDSYHHTFFEMLGNWSFGDYFKKEAITYSWTLLTEVYGIPKDRLYVTYFEGDEKLGLEPDTEARELW

KNVGVPDDHILPGNAKDNFWEMGDQGPCGPCSEIHYDRIGGRNAASLVNMDDPDVLEVWNLVFIQFNREQDGSLKPLPAK

HIDTGMGFERLVSVLQDVRSNYDTDVFTPLFERIQEITSVRPYSGNFGENDKDGIDTAYRVLADHVRTLTFALADGGVPN

NEGRGYVLRRILRRGARYARKYMNYPIGNFFSTLAPTLISQVQDIFPELAKDPAFLFEILDEEEASFAKTLDRGERLFEK

YASAASKTESKTLDGKQVWRLYDTYGFPVDLTELMAEEQGLKIDGPGFEKAKQESYEASKRGGKKDQSDLIKLNVHELSE

LNDAKVPKTNDEFKYGSANVEGTILKLHDGTNFVDEITEPGKKYGIILDKTCFYAEQGGQEYDTGKIVIDDAAEFNVENV

QLYNGFVFHTGSLEEGKLSVGDKIIASFDELRRFPIKNNHTGTHILNFALKETLGNDVDQKGSLVAPEKLRFDFSHKKAV

SNEELKKVEDICNEQIKENLQVFYKEISLDLAKSIDGVRAVFGETYPDPVRVVSVGKPIEELLANPANEEWTKYSIEFCG

GTHVNKTGDIKYFVILEESGIAKGIRRIVAVTGTEAFEAQRLAEQFAADLDAADKLPFSPIKEKKLKELGVKLGQLSISV

ITKNELKQKFNKIEKAVKDEVKSRAKKENKQTLDEVKTFFETNENAPYLVKFIDISPNAKAITEAINYMKSNDAVKDKSI

YLLAGNDPEGRVAHGCYISNAALAKGIDGSALAKKVSSIIGGKAGGKGNVFQGMGDKPAAIKDAVDDLESLFKEKLSI

>CDF90599.1 ZYBA0S07-04786g1_1 [Zygosaccharomyces bailii CLIB 213]

MTMGNTEKWTAKNVRDTYLNYFKSKGHTFVPSSPVVPYDDPTLLFANAGMNQYKPIFLGTVDPSSDFYNLKRAHNSQKCI

RAGGKHNDLEDVGKDSYHHTFFEMLGNWSFGDYFKKDAIAYAWDLLTKVYGLPEDRLYVTYFEGDAEQGLEPDTEALELW

KSVGVDESHILRGNAKDNFWEMGDQGPCGPCSEIHFDRIGGRNAASLVNMDDPDVLEVWNLVFMQFNREQGGILKPLPAK

HIDTGMGFERLVSILQNCRSNYDTDVFTPLFARIQEITGARPYSGKFGAEDVDGVDTAYRVLADHVRTLTFALADGGVPN

NEGRGYVLRRILRRGARYARKYMNYPIGDFFSKLVPTLISQVEDIFPEVAKDPSYLFEILDEEEVSFAKTLDRGERLFEK

YADAASKSGSKTLDGLQVWRLYDTYGFPVDLTELMAEEKGLTIDGPGFEKAKKESYEASKRGGKKDAANVLKLDVHGISQ

LNNDKVPKTEDVYKYGMDNVDATILKLYDGSNFVDEISEPGQKYGVILDKTCFYAEQGGQEYDTGKLVIDDAAEFNVDNV

QLYNGFVFHTGSLEEGKLSVGDKVIASYDELRRFPIKNNHTGTHILNFALREILGDDIDQKGSLVAPEKLRFDFSHKKAM

TVEEIVKVENICNKQIKDNMAVYYKEVPLEKAKSIYGVRAVFGETYPDPVRVVSVGRSVDDLLSNPSNEEWHKYSVEFCG

GTHVTKTGDIKVFVILEENGIAKGIRRIVAVTGSEAFEAQRLADEFSKELDAANKLPFSPLKEKKLKELGVRLGQLSIAV

ASKQELKTKFAKIEKGVKDEIKARVKQETKKTLDEVKEYFAQNETASFLVKFIDIPTNAKAITEAVNYMKSSAKDKSIYL

FTGNDPEGKIAHGCYISDIAISKGIDGSSIAKQVSGIVGGKAGGKGNVFQGMGDKPTAAIDAVTELEKLFNEKLHI

>XP_002615762.1 hypothetical protein CLUG_04644 [Clavispora lusitaniae ATCC 42720]

MLKSLARKHNMTTSSHPWSASTVRSTFLDYFKNKSHTYVPSSSVVPHNDPTLLFANAGMNQYKPIFLGTVDPTSEFATLK

RAVNSQKCIRAGGKHNDLEDVGRDSYHHTFFEMLGNWSFGDYFKKEAIAYSWELLTEVYKLDKDRLYVTYFEGDEKNGLE

PDLEAKEYWLSVGVAPDHILPGDAHDNFWEMGDQGPCGPCSEIHYDRIGGRNAASLVNQDDPNVLEVWNIVFIQYNREAD

SSLKPLPSKHIDTGMGFERLVSVLQDKSSNYDTDVFQPIFEEIRNITGVRPYTGKFGAEDVDGIDTAYRVIADHVRTLTF

AISDGGVPNNEGRGYVLRRILRRGARYVRKYMNCPIGSFFQQLVDVVIENNSSIFPEIAKNKDMLKEILMEEEVSFAKTL

DRGEKLFEQYAIIASKTPEQTLSGKDVWRLYDTYGFPVDLTRLMAEEAGLKIDEEAFEKAKEESREASKATNTKGGATLI

KLDVHALSELDNNESITKTNDVYKYGLDNIESKIVAIYDGSKFVDTISEAGQQFGIILDRTPFYAEQGGQEYDTGSLVID

GSAEFAVTNVQSYGSYILHTGTMVEGTLKLNDNVIATYDELRRWPIRNNHTGTHVLNFALKEVLGDGIDQKGSLVAPEKL

RFDFSHKQALTPKELEQVENISSEIIKSNKQVYAEEVELTKAKQIAGVRAVFGETYPDPVRVVSIGVAVADLLKDPTNQD

WTKLSIEFCGGTHVAKTGDIKDFVIIEESGIAKGIRRIVAVTGHEAQEVQRIAREFSASLDSADKLSFGPEKEQRAKELG

VALKQLSISVLDKQKLNEKFTKLDKSIKDHLKAKQKEESKKTLEAVNGWLKDEEKSKTAFFVAHVPISANAKAITEAINL

IKKQHKDKSLYLLTGTSEKVAHGCYISDEAISKGIDASELAKAVSAHIGGKAGGKGNVVQGMGDSPEGIDAAVETVTKLL

AEKL

>XP_002421135.1 alanyl-tRNA synthetase, putative [Candida dubliniensis CD36]

MIKTLFRRMSSNTTIPTPNGSNHWTASKVRSTFLDYFKQQQHTYVPSSSVVPHNDPTLLFANAGMNQYKPIFLGTVDPAS

DFASLKRAANSQKCIRAGGKHNDLEDVGRDSYHHTFFEMLGNWSFGDYFKKEAIDYSWELLTKVYGLKEDRLYVTYFGGD

EKQGLEPDLEAKNFWLKVGVPEDHILPGSVKDNFWEMGDQGPCGPCSEIHYDRIGGRNASDLVNMDDPNVIEVWNVVFIQ

YNREADGNLRTLPNKHIDTGMGFERLVSILQNKFSNYDTDVFLPIFDKIREITGVRPYTGKFGNEDKDGIDTAYRVIADH

VRTLTFAICDGGVPNNEGRGYVLRRILRRGSRYVRKYMNYPIGGFFQQLVDVVIEQNKEIFPEISSGAQDLKEILNEEEL

SFAKTLDRGEKLFEQYAIIASKTPEQTLSGKDVWRLYDTYGFPVDLTRLMAEEAGLKIDEDGFERAKEESREASKGSGTK

DGKTLVKLDVHALSELDQNDTIPKTNDEFKYGLENVKAKVVGIYDGSKFVESIEDPSIQYGILLDKTPFYAEQGGQEYDT

GKLVIDGKSEFNVANVQVYAGYVLHTGNIVDGKLNVGDEIIATYDELRRWPIRNNHTGTHILNFALREVLGDGVDQKGSL

VAPEKLRFDFSYKQAVTPKELEKIEAISNKYIKNNDKVYYKDVSLTKAKEINGLRAVFGETYPDPVRVVSIGVSVDDLLA

DPTNAKWHEISIEFCGGTHVAKTGDIKDLVIVEESGIAKGIRRIVAVTGHDAHHVQKVANEFEQEIDAAASLPFGVVKES

KSKELGVALKKLSISVLDKQRLTEKFNKLDKSIKDNLKARQKEETKKTLDVVNNWLNDKENVSSFLVSHVPITANAKAIT

EAINLIKKQDKSKSIYLLTGETDKVAHGCYVSDEAIAKGINANELAKSVSENIGGKAGGKGNIVQGMGDKPEGINTAIEE

VTKLFKEKL

>CDH13133.1 probable ALA1-Alanyl-tRNA synthetase,cytosolic [Zygosaccharomyces bailii ISA1307]

MGNTEKWTAKNVRDTYLNYFKSKGHTFVPSSPVVPYDDPTLLFANAGMNQYKPIFLGTVDPSSDFYNLKRAHNSQKCIRA

GGKHNDLEDVGKDSYHHTFFEMLGNWSFGDYFKKDAIAYAWDLLTKVYGLPEDRLYVTYFEGDAEQGLEPDTEALELWKS

VGVDESHILRGNAKDNFWEMGDQGPCGPCSEIHFDRIGGRNAASLVNMDDPDVLEVWNLVFMQFNREQGGILRPLPAKHI

DTGMGFERLVSILQNRRSNYDTDVFTPLFARIQEITGARPYSGKFGAEDVDGVDTAYRVLADHVRTLTFALTDGGVPNNE

GRGYVLRRILRRGARYARKYMNYPIGDFFSKLVPTLISQVEDIFPEVAKDPSYLFEILDEEEVSFAKTLDRGERLFEKYA

DAASKSGSKTLDGLQVWRLYDTYGFPVDLTELMAEEKGLTIDGPGFEKAKKESYEASKRGGKKDAANVLKLDVHGISQLN

NDKVPKTEDVYKYGMDNVDATILKLHDGSDFVDEISEPGQKYGVILDKTCFYAEQGGQEYDTGKLVIDDAAEFNVDNVQL

YNGFVFHTGSLEEGKLSVGDKVIASYDELRRFPIKNNHTGTHILNFALREVLGDDIDQKGSLVAPEKLRFDFSHKKAMTV

DEIVKVESICNKQIKDNMAVYYKEVPLEKAKSIYGVRAVFGETYPDPVRVVSVGRSVEDLLSNPSNEEWHKYSVEFCGGT

HVAKTGDIKVFVILEENGIAKGTRRIVAVTGSKAFEAQRFADEFFKELDAASKLPFSPLKEKKLKELGVRLGQLSIAVAS

KQELKNKFAKIEKGVKDEIKARVKQETKKTLDDVKEYFAQNESAPFLIKFVDIPTNAKAITEAVNYMKSSAKDKSIYLFT

GNDPEGKIAHGCYISDVAISKGIDGSSIAKQVSGIVGGKAGGKGNVFQGMGDKPTAAVDAVTELEKLFNEKLQI

>XP_003676658.1 hypothetical protein NCAS_0E02290 [Naumovozyma castellii CBS 4309]

MTIGDKQKWTAQNVRNTFLDYFKSKEHTFVPSSPVVPFDDPTLLFANAGMNQYKPIFLGTVDPSSDFYTLKRAYNSQKCV

RAGGKHNDLEDVGKDSYHHTFFEMLGNWSFGDYFKKEAIHYAWELLTVVYGIPADRLYVTYFEGDAKQGLEPDSEAQDLW

RKVGVPEDHILPGNAKDNFWEMGDQGPCGPCSEIHYDRIGGRNASSLVNMDDPDVLEVWNLVFIQFNRESDGSLKSLPAK

HIDTGMGFERLVSVLQDVRSNYDTDVFQPLFKRIQEITNARDYTGKFGAEDKDNIDTAYRVLADHVRTLTFALADGGVPN

NEGRGYVLRRILRRGARYARKYMNYPIGDFFSKLAPTLIEQVKDIFPEVAKDPAFLFEILDEEEASFAKTLDRGERLFEK

YAATAAETQSKTLDGKQVWRLYDTYGFPVDLTELMAEEQGLKIDGPGFEKAKQESYEASKRGGKKDQSDLIKLNVHELSE

LNDNNVAKTDDSAKYGFENIESTILKLHDGTKFVDEITESGKKYGVLLDKTCFYAEQGGQEYDTGKLVIDDVAEFNVENV

QLYNGFVFHTGSLEEGKLSVGDKIIASYDELRRFPIKNNHTGTHILNFALLETLGNEVDQKGSLVAPEKLRFDFSHKKAL

AMDELRKVEEICNQQIKENFPVYYKEVPLDLAKSIFNVRAVFGETYPDPVRVVCVGKPVEELLKNPTSEEWGNYSIEFCG

GTHVVKTGDIKYFVILEESGIAKGIRRIVAVTGSDAFEAQRIANEFSTELDAAEKLPFSPLKEKKLKELGVSLGQLSISV

ISKHELKERFTAIEKVVKDEVKSRAKKEAKQTVDEVKNYFAENENSPYFVKFINIPVNAKAITEAINYVKSSAKGKSIYL

LTGNDSNGKIAHGCYISDEALAKGVDGAAIAKQVSSIIGGKAGGKGNVFQGMGDKPAAAEQAVSEIETLLKEKLTI

>AQZ09156.1 ALA1 (YOR335C) [Zygosaccharomyces parabailii]

MTMGNTEKWTAKNVRDTYLNYFKSKGHTFVPSSPVVPYDDPTLLFANAGMNQYKPIFLGTVDPSSDFYNLKRAHNSQKCI

RAGGKHNDLEDVGKDSYHHTFFEMLGNWSFGDYFKKDAIAYAWDLLTKVYGLPEDRLYVTYFEGDAEQGLEPDTEALELW

KSVGVDESHILRGNAKDNFWEMGDQGPCGPCSEIHFDRIGGRNAASLVNMDDPDVLEVWNLVFMQFNREQGGILRPLPAK

HIDTGMGFERLVSILQNRRSNYDTDVFTPLFARIQEITGARPYSGKFGAEDVDGVDTAYRVLADHVRTLTFALTDGGVPN

NEGRGYVLRRILRRGARYARKYMNYPIGDFFSKLVPTLISQVEDIFPEVAKDPSYLFEILDEEEVSFAKTLDRGERLFEK

YADAASKSGSKTLDGLQVWRLYDTYGFPVDLTELMAEEKGLTIDGPGFEKAKKESYEASKRGGKKDAANVLKLDVHGISQ

LNNDKVPKTEDVYKYGMDNVDATILKLHDGSDFVDEISEPGQKYGVILDKTCFYAEQGGQEYDTGKLVIDDAAEFNVDNV

QLYNGFVFHTGSLEEGKLSVGDKVIASYDELRRFPIKNNHTGTHILNFALREVLGDDIDQKGSLVAPEKLRFDFSHKKAM

TVDEIVKVESICNKQIKDNMAVYYKEVPLEKAKSIYGVRAVFGETYPDPVRVVSVGRSVEDLLSNPSNEEWHKYSVEFCG

GTHVAKTGDIKVFVILEENGIAKGTRRIVAVTGSKAFEAQRFADEFFKELDAASKLPFSPLKEKKLKELGVRLGQLSIAV

ASKQELKNKFAKIEKGVKDEIKARVKQETKKTLDDVKEYFAQNESAPFLIKFVDIPTNAKAITEAVNYMKSSAKDKSIYL

FTGNDPEGKIAHGCYISDVAISKGIDGSSIAKQVSGIVGGKAGGKGNVFQGMGDKPTAAVDAVTELEKLFNEKLQI

>CEP23010.1 K01872 alanyl-tRNA synthetase [Cyberlindnera jadinii]

MSQSTEWPAQKVRNTFLDYFKSKGHTFVPSSSVIPYDDPTLLFANAGMNQYKPIFLGTVDPASDFYSLKRAMNSQKCIRA

GGKHNDLEDVGRDSYHHTFFEMLGNWSFGDYFKKEAIEYSWELLTKVYGLKEDRLYVTYFGGDEASGLLPDLEAKKLWED

VGVDPSHILPGSVKDNFWEMGEQGPCGPCSEVHYDRIGGRNAAELVNEDDPNVIEIWNNVFMQFNREADGTLRPLPAKHI

DTGLGFERLVSILQNCSSNYDTDVFQPLFKKIQEMTGARDYTGKFGAEDVDGIDTAYRVLADHVRTLTFALADGGVPNNE

GRGYVLRRILRRGARYARKYFNYPIGNFFSTLAPTLIDQVQDIFPELTKNTSNTFEILDEEEASFARTLDRGEKLFEQYA

IAAQKGTKILNGKDVWRLYDTYGFPVDLTQLMALEAGLKIDEAGFEKARIESYEASKAGGKSKGSDLIKLDVHALAKLNE

EKLDRTDDHYKYGTENVEAKILKIYDGQEFVDEISDITKQYGIILDKTPFYAEQGGQEYDTGKIVIDGASEFNVENTQQY

NGYVFHTGRLAEGSLKVGDAVIAGYEELRRWPLRNNHTGTHILNLALREVLGTEVHQKGSLVAPEKLRFDFSHKQGLTLD

EVSKIEAICNQEIKKAQIVYAKDVPLEQAKGITSVRAVFGETYPDPVRVVSIGVPIDDLLSSPTDEKWMTVSIEFCGGTH

VAKTSDIKDLVLTEESGIAKGIRRIVAVTGNEAHDVQRIASEFSKDLESAGKLEFGPLKEKKLKELGVKLGQLNISILSK

SELKNRFNVFDKEIKDENKARQKAESKKTLDTVKDYFAENESSAFLVKHVDISANAKSITEAINFIKSKNPDKSIYIFTG

NADKVAHGCYISDKAIEKGVNASELAKHASEFIGGKAGGKGNVVQGMGDKLDGIPNAVDELEKLLKEKLSI

>ORY48539.1 hypothetical protein BCR33DRAFT_803846 [Rhizoclosmatium globosum]

MPEETQWPLNKVRSTFVDYFVQKKGHTHVPSSSTIPVNDPTLLFANSGMNQFKPVFLGTVDPNSDMSKWKRAANSQKCIR

AGGKHNDLEDVGKDVYHHTFFEMLGNWSFGDYFKKEAIHMAWELLTEVYKMPKDRLYITYFGGDKDLGIGPDEEAKQLWL

ELGLDPKHVIPFGPKENFWEMGDQGPCGPCSEIHFDRIGGRDASHLVNLDDPDVLEIWNLVFMQYNREPDRSLRPLPNKH

IDTGAGLERVTSVLQNKRSNYDTDAFMGIFKKIQDMTGARPYQGLVGADDKDGIDTAYRVVADHVRTLTFAISDGGVPSN

EGRGYVLRRILRRGARYVRRRFNAPIGNFFSTLVDTVVAEMGDAFPEIRRKVVDLKQILNEEEQSFAKTLDRGEKLFEGC

IQTAKDRGTKVLDGADVWKLYDTYGFPVDLTRLMAEEIGFSVDEAQFLEEQLKAKEKSKGGKAGDKGENVGLDIHAIGEL

EKNADVPPTDDSHKYGTFTIKATVKAIYKAPTFVKNVARKDFSGNFGIVLDKTNFYAESGGQQYDTGILKIGETEFVVDD

VQVYGSYVLHIGSLSSGSIKIGDVVDATYDEDRRLPMANNHTSTHILNYGLLKTLGDGVDQKGSLVTPEKLRFDFSQKNA

PTTEQLKEIEATCNLIISNNVQVYSREVPLAQAKAINGLRAVFGEVYPDPVRVVSVGFNLDEVLADPSNEKWASSSIEFC

GGTHVPRTGLIKKLVILEESSISKGIRRIIAATGDEAKAAINLAAEFKYKLIMARSLNGSSLEGALKSLGKELDEIVIPV

VTKAEFRKEFNDLKKAFDDADKLRKAQEVKEAVEKVKAVFDGDKAPSFAVINLEVGSNSKAVSSAAVHVKGLNNKSALFI

SVDEEENKVVYQAVVAKEHIAKGLKANEWAAVVGNTVGGKSGGKDDSAQGAGSDPTRAEEAIKLAQSHAKKLL

>SCU84598.1 LANO_0C01838g1_1 [Lachancea nothofagi CBS 11611]

MTVGDKQKWTASNVRSTFLDYFKEREHKFVASSPVVPYDDPTLLFANAGMNQYKPIFLGTVDPSSDFYTLKSAVNSQKCI

RAGGKHNDLEDVGRDSYHHTFFEMLGNWSFGDYFKKEAIEYAWDLLTKVYGIPADRLYVTYFEGDAKQGLEPDTEARDLW

LKVGVLEDHILTGNAQDNFWEMGDQGPCGPCSEIHYDRIGGRNAASLVNMDDPDVLEIWNIVFIQYNREQNGKLRPLPAK

HIDTGMGFERLVSVLQDVRSNYDTDVFQPLFSRIQEITGARPYTGNFGADDVDGIDTAYRVLADHVRTLMFALADGGVPN

NEGRGYVLRRILRRGARYARKYMNYPIGNFFSNLAPTLIEQTKSMFPEIAKDPSYLFEILNEEEASFAKTLDRGEKLFEK

YAQTASQTQSKTLDGLQVWRLYDTYGFPVDLTELMAEEQGLKIDGPGFEKAKQESYEASKKGGKKSASDLIKLDVHEIST

LNSENVPKTDDSPKYNSANIEATVLKIYDGESFVSHVSEVGKQYGVILDTTCFYAEQGGQEYDMGNLVIDGAVEFKVENA

QLYNGYVFHTGSLAEGKLSVGDKIIASFDELRRFPIKNNHTGTHILNFALKEVLGGEVDQKGSLVAPEKLRFDFSHKKGL

SLNELQKVESICNDQIKADLTVYYKNVPLELAQKISSVRAVFGETYPDPVRVVSVGMPIDDLLANPTSEEWQKFSVEFCG

GTHVAKTSDIKEFIIIEESGIAKGIRRIVAVSGTEAHEVQRTANSFDADLAAAEKLPFSPLKEKRLKELGVKLGQLTISV

VAKDALKTKFSKIDKVVKDEVKSRAKKEIKQTVDEVKNFFAENESAQFFVKYVDIPTNAKAITEAVNHMKTSAKDKSIYL

LNGNDKVAHGCYISDVAIEKGIDGRELLEAISRNIGGKAGGKANVFQGVGDKPQGIENAVTEVEQLFMQKLSI

>CDH15032.1 probable ALA1-Alanyl-tRNA synthetase,cytosolic [Zygosaccharomyces bailii ISA1307]

MTMGNTEKWTAKNVRDTYLNYFKSKGHTFVPSSPVVPYDDPTLLFANAGMNQYKPIFLGTVDPSSDFYNLKRAHNSQKCI

RAGGKHNDLEDVGKDSYHHTFFEMLGNWSFGDYFKKDAIAYAWDLLTKVYGLPEDRLYVTYFEGDAEQGLEPDTEALELW

KSVGVDESHILRGNAKDNFWEMGDQGPCGPCSEIHFDRIGGRNAASLVNMDDPDVLEVWNLVFMQFNREQGGILKPLPAK

HIDTGMGFERLVSILQNCRSNYDTDVFTPLFARIQEITGARPYSGKFGAEDVDGVDTAYRVLADHVRTLTFALADGGVPN

NEGRGYVLRRILRRGARYARKYMNYPIGDFFSKLVPTLISQVEDIFPEVAKDPSYLFEILDEEEVSFAKTLDRGERLFEK

YADAASKSGSKTLDGLQVWRLYDTYGFPVDLTELMAEEKGLTIDGPGFEKAKKESYEASKRGGKKDAANVLKLDVQGISQ

LNNDKVPKTEDVYKYGMDNVDATILKLYDGSNFVDEISEPGQKYGVILDKTCFYAEQGGQEYDTGKLVIDDAAEFNVDNV

QLYNGFVFHTGSLEEGKLSVGDKVIASYDELRRFPIKNNHTGTHILNFALREILGDDIDQKGSLVAPEKLRFDFSHKKAM

TVEEIVKVENICNKQIKDNMAVYYKEVPLEKAKSIYGVRAVFGETYPDPVRVVSVGRSVDDLLSNPSNEEWHKYSVEFCG

GTHVTKTGDIKVFVILEENGIAKGIRRIVVVTGSEAFEAQRLADEFSKELDAANKLPFSPLKEKKLKELGVRLGQLSIAV

ASKQELKTKFAKIEKGVKDEIKARVKQETKKTLDEVKEYFAQNETASFLVKFIDIPTNAKAITEAVNYMKSSAKDKSIYL

FTGNDPEGKIAHGCYISDIAISKGIDGSSIAKQVSGIVGGKAGGKGNVFQGMGDKPTAAIDAVTELEKLFNEKLHI

>XP_003957527.1 hypothetical protein KAFR_0E02390 [Kazachstania africana CBS 2517]

MTIGDKQKWTATNVRNTFLNYFNSKEHKFIKSSPVVPFDDPTLLFANAGMNQYKPIFLGTVDPASEFYTLKRAYNSQKCV

RAGGKHNDLEDVGKDSYHHTFFEMLGNWSFGDYFKTDAIHYAWDLLTNVYGIPKDRLYVTYFEGDAKQGLEADEEARQLW

RDVGVPEDHILPGNAKDNFWEMGDQGPCGPCSEIHYDRIGGRNAASLVNQDDPDVLEVWNLVFIQYNRESDGSLKSLPAK

HIDTGMGFERLVSVLQDVRSNYDTDVFQPLFAKIQEITGARSYTGKFGDEDKDNIDTAYRVLADHVRTLTFALADGGVPN

NEGRGYVLRRILRRGARYARKYMNYPIGNFFSTLAPTLIEQVKDIFPELTKDPSFLFEILDEEEASFAKTLDRGERLFEK

YATTASQTTSKTLDGKQVWRLYDTYGFPVDLTELMAEEQGLKIDGPGFEKAKQESYEASKRGGKKGGSDLIKLNVHELNE

LNEENVAKTDDKAKYGTENIEGTILKLHDGSNFVNEISEPGKKYGIILDKTCFYAEQGGQEYDTGKIVIDDVAEFNVENV

QLYNGFVFHTGSLEEGKLSVGDKIIAAYDELRRFPIKNNHTGTHILNLALKETLGNDVDQKGSLVAPEKLRFDFSHKKAL

TIDELRDVEKISNQQIKDNLTVYYKDVPLDLAKQISSVRAVFGETYPDPVRVVSVGVPVDDLLANPTSEEWHKYSVEFCG

GTHVNKTSDIKYFVVLEESGIAKGIRRIVAVTGTEAFEAQRIADEFAEALTRAEQLPFSPIKEKKLKELGVQLGQLSISV

ITKHELKEKFNKADKLVKDEVKSRAKKEIKQTVDEVKNYFAENQDAKYFVKFIDIPTNAKAITEAVNFMKTSAKDKSMYL

FTGNDPEGKIAHGCYISDDALSQGIEGQTLIKQVSSIVGGKAGGKGNVFQGMGDKSVEVNKAVVELDTLFKEKLSI

>PPJ57623.1 hypothetical protein CBER1_04771 [Cercospora berteroae]

MRSFSTLLRFFKPAPGISRISATKATTAHTPPPRVLHHYSFRRMASTTGQEPEWSAVQVRKTFIEYFEKNGHKFVPSSSV

VPLSDPTLLFTNAGMNQYKAIFLGTVDRDSDFAKLKRAANSQKCIRAGGKHNDLDDVGKDSYHHTFFEMLGNWSFGDYFK

EEAIKFSWELLTKVYGLETDRLYVTYFEGKADAGLEPDHEARDFWLKQGVPEDHILTGDMKDNFWEMGDQGPCGPCSEIH

YDRIGGRNASDLVNQDDPNVIEIWNNVFIQYNREPDKSLKSLPSKHIDTGMGFERLVSILQKKSSNYDTDVFTPLFARIQ

EVTGARPYQGKFGAEDADGIDTAYRVVADHLRLLTFAISDGGAPNNVGRGYVVRRVLRRGARYARKYFNTNIGNFFSKLV

PTLVEQMGDMFPEIKKKQEEVKEILDEEEVSFAKTLDRGESMFEKYAQNCKANGTKTLAGADVWRLYDTYGFPVDLTKLM

CEERGLEIIDEDVAEAQEKARLASMGAKKEGATLVKLDVHDISTLENMPDVSKTDDSPKFQRENIKATIKAIYHGRKFLQ

STEEIPEGEQFGVILDRTNFYAEQGGQENDTGRILIDGVAEIKVENVQQYAGYVMHTGYVGYGQLKVGDEVLAEYDELRR

QPIRNNHTGTHVLNYALREVLGDDVNQKGSLVAPEKLRFDFSHKTGVTDEELAKIEEVSTNYIRQNCEVYAKDVPLATAR

QINGVRAVFGETYPDPVRVVSVGISVEDLLENPSDPKWNDISIEFCGGTHVKNTTEIKDLVVIEESGIAKGIRRIIAVTG

QEAHKVQAEAETWDKEVSALERLPYGPDKEAKTKQLTVDLSKLEISALQKTSLRNRVAAVAKANLDQQKTAQKAENKKVL

DAVTDHFAKNADSKTLVLSLPISAGSKAIQETIKVVQNKQKEKTVYLVGKTDDGKVVHGCHVSADAQAKGADASKWSGEV

AALVGGKAGGKGPTSLGQGTDASKVDEAVEAARKYLESLSM

>EMG50125.1 Alanyl-tRNA synthetase, putative, partial [Candida maltosa Xu316]

MSTTTTNTSHPWTASKVRSTFLDFFKQKQHTYVPSSSVVPHNDPTLLFANAGMNQYKPIFLGTVDPASDFATLKRAANSQ

KCIRAGGKHNDLEDVGRDSYHHTFFEMLGNWSFGDYFKKEAIDYSWELLTKVYGLQEDRLYVTYFGGDEAQGLEPDLEAK

DFWLKVGVPEDHILPGSVKDNFWEMGDQGPCGPCSEIHYDRIGGRNASDLVNMDDPNVIEVWNVVFIQFNREADGNLRSL

PSKHIDTGMGFERLVSILQDKSSNYDTDVFLPIFEKIREITGVRPYTGKFASEDKDGIDTAYRVIADHVRTLTFAICDGG

VPNNEGRGYVLRRILRRGSRYVRKYMNYPIGGFFQQLVDVVIEQNKEIFPEIVTGAQDLKEILNEEELSFAKTLDRGEKL

FEQYAIIASKTPEQTLSGKDVWRLYDTYGFPVDLTRLMAEEAGLKIDEPGFERAKEESREASKGSGTKDGKQLVKLDVHA

LSELDKNDAIPKTDDEYKYGTENVKATVVGIFDGSKFVDSIEEPSTQYGILLDKTPFYAEQGGQEYDTGKIVIDGKSEFN

VTNVQVYAGYVLHTGNVVEGKLSVGDEIIATYDELRRWPIRNNHTGTHILNYALREVLGDGVDQKGSLVAPEKLRFDFSH

KQAVSPKELEKVEAISNKYIKDNQKVFYKDVSLAQAKEINGLRAVFGETYPDPVRVVSIGVSVDDLLADPTNSKWHEVSI

EFCGGTHVAKTSDIKDLVITEESGIAKGIRRIVAVTGHDAHHVQKIAKEFEQEIEHASKLPLGPVKEAKAKELGVALKKL

AISVLDKQRLTEQFNKLDKAIKDNLKAKQKEESKKTLEVVNNWLNDKENSTPFLVAHIPIDANAKALTEAFNLVKKQDKT

RSIYLLTGEKEKVAHGCYLSDEAIAKGLNVGDLTAIVSENVGGKAGGKGNTAQ

>XP_006687556.1 hypothetical protein CANTEDRAFT_109802 [[Candida] tenuis ATCC 10573]

MSTNNNIEWSASKVRTTFLDFFKDKKHTFVPSSSVVPHNDPTLLFANAGMNQYKPIFLGTVDPSSDFANLKRAANSQKCI

RAGGKHNDLEDVGRDSYHHTFFEMLGNWSFGDYFKKEAIGFSWNLLVGTYGLDKDRLYVSYFGGDEKQGLDPDLDAKQHW

LNIGVAEDHILPGDAKDNFWEMGDQGPCGPCSEIHYDRIGGRNAAHLVNMDDPNVLEIWNIVFIQYNREPDSTLRSLPAK

HIDTGMGFERLVSILQDKSSNYDTDVFTPIFDKIREITGVRPYTGKFGTEDKDGIDTAYRVIADHVRTLTFAITDGGVPN

NEGRGYVLRRILRRGSRYVRKYMNYPIGTFFQQLAPVVIEQNKEIFPEIAHKSQDLMEILNEEELSFAKTLDRGEKLFEQ

YSIIASKTPEQTLSGKDVWRLYDTYGFPVDLTRLMAEEAGLSIDEVGFEKAKEESREASKGRNSKSGASLVKLDVHALSE

LDSNDSIPKTDDSYKYGLDNIKAKVVAIYDGAGFVEVVSDTTSKQYGILLDRTPFYAEQGGQEYDTGKFVIDGQAEFNVE

NVQVYAGYVLHTGNLVEGSIKVGDEVIAGYDELRRWPIRNNHTGTHVLNYALREVLGNSVDQKGSLVAPEKLRFDFSHKQ

ALKPEEVSKVEDISNEYIKANKQVYAKEVSLADAKQIYGLRAVFGETYPDPVRVVSIGKSVDDLLSNPDNKDWYHFSVEF

CGGTHVTKTGDIKDLVIIEESGIAKGIRRIVAVTGTEAHRVQQIAHDNEKELHEINSLPFGPAKESRAKDFGVNLKKLSI

SVTEKQKLNEKFNKLDKAIKDNLKAIQKEETKKVMDVVNEWLQDKEAKNFLVSHVPINSNAKAIAEGLNLIKKSHPEKSI

YLFTGKDKVAHGCYISNDALEKGINVTDLGVAVSSNIGGKAGGKGNTIQGMGDNVGGVDKAIEELTALLSSKL

>PVU99863.1 hypothetical protein BB559_000336 [Furculomyces boomerangus]

MDSPPSTTEKWTANAVRTAFIDYFKNKDHEYVPSSSVVPHEDPTLLFANAGMNQFKPLFLGTSDPNTDLGKLKRACNTQK

CIRAGGKHNDLEDVGKDVYHHTFFEMLGNWSFGDYFKREAIQHAWVLLTEVYGLEKERLYITYFGGDSQQGLEADEEAKN

LWLELGVPQNRIIPFGTKDNFWEMGEVGPCGPCSEIHYDRIGGRDASHLVNYDDPDVLEIWNLVFMQYNRESKTELKLLP

NKHIDTGMGFERLVSVLQNKPSNYDTDVFVPIFLAIQEKSGARPYTGKVGDEDVDGIDMAYRVIADHTRTLLFSISDGAV

PSNEGRGYVVRRILRRGARYARKKFGVELGNFFPSLVDVVVAQMAHEFPSISKKVEYVKQILQDEEKSFSRTLDRGEKLF

ESTLSKMKLSNQKIVPGAEVWRLYDTFGFPVDLTRIMAEESSVTIDEQEFEREQARSKELSRRKKNAGANAGSVTLDVHA

IAELDSKGNIPKTDDIYKFNEPEITAKVVALYSNKTFVQSEDSSEEKFNENALTIGVLLDKTNFYAEAGGQEWDTGYLLS

EDRQTEFSVTQVKNYGGYILHVGTLKSGVIKVGDTMQCSYDELRRTPIRSNHSATHILNFSLRKILNSNEVDQRGSLVAP

DKLRFDYSWGGQITPEQIKEIQDHTLSVIKDDLTVYSKEINLQDAKAIYGLRAVFGETYPNPVRVISIGANLGDVLKDHS

NPDWYNYSIELCGGTHVNKTSEIGNFIVTEESSISKGIRRIVAVTGEDAAKVQIESERFSEVLNDLKKLKGGQLEAGIKK

VGKDLDNLYTGVWLKHLLRQEYNQIRKNYIEADKEAKLAASKFVTNEVISSIEKNPDSDYHVLVINFDESSSTNSKALAN

AITHAKSLNTKAVYLISPDKTTKKVAHLCFVPQSLISKGLSAKKWASTVSEVVGGKSGGKDDSAQGSGINVDSVSEAEKV

ALEFAKISLS

>XP_020074944.1 hypothetical protein HYPBUDRAFT_157945 [Hyphopichia burtonii NRRL Y-1933]

MIRTLFSKRTMSTSTNQWTAPKVRSTFLEFFKEHNHKYVPSSSVVPHNDPSLLFANAGMNQYKPIFLGTVDPSSDFAGLK

RAANSQKCIRAGGKHNDLDDVGKDSYHHTFFEMLGNWSFGDYFKKEAIQWSWDLLTKVYKLESDRLYVTYFEGDAKNGLE

PDLEAKNFWLDVGVAEDHILPGNAKDNFWEMGDQGPCGPCSEVHYDRIGGRNAASLVNMDDPNVLEIWNIVFIQYNREAD

TSLRSLPAKHIDTGMGFERLVSILQNKSSNYDTDVFTPIFNKIREITGVRPYTGKFGSDDVDGIDTAYRVIADHVRTLTF

AISDGGVPNNEGRGYVLRRILRRGARYVRKYMAYPIGTFFPQLVDIVIEQNSEIFPEISKKSQDLKEILHEEEVSFSKTL

DRGEKLFEQYAIIASKTPEQTLSGKDVWRLYDTYGFPVDLTRLMAEEAGLNIDEVGFEKAKEESRLASKATGNKTGVELI

KLDVHALSKLENEVPKTNDIYKYGLENTKSKILAIYDGSEFIESISDSKKQVGILLDKTPFYAEQGGQEYDTGTLVIDGS

VEFNVSNVQVYGGYVLHTGHLVEGSLKLQDEVIATYDELRRWPIRNNHTGTHVLNYALREVLGDGVDQKGSLVAPEKLRF

DFSHKQGLKPEELVKVEDISNEYIKSNKEVFSKEVNLLTAKEINGLRAVFGETYPDPVRVVSIGAPVEELIKNPSNSDWS

KLSIEFCGGTHVAKTGEIKDLIIIEESGIAKGIRRIVAVTGNEAHEVQRIAKEFGEEISKAESLPFGSEKETKAKELGVA

LKKLSISVISKAKLTESFNKLDKSIKDQLKSKQKEELKKTLDAVNKWLKDEESKHEFFVSHIPISANAKAITEAINLIKK

QHKEKSLYLITGGASDAKVAHGCYVSDEAIAKGLDAQDLAKAVSAQIGGKAGGKGNVVQGMGDKPEGINQAIDEVTKLFG

EKL

>KGQ88605.1 alanyl-tRNA synthetase [Candida albicans GC75]

MIKTLLRRMSSNTTIPTPNGSNHWTASKVRSTFLDYFKKQQHTYVPSSSVVPHNDPTLLFANAGMNQYKPIFLGTVDPAS

DFASLKRAANSQKCIRAGGKHNDLEDVGRDSYHHTFFEMLGNWSFGDYFKKEAIDYSWELLTKVYGLQEDRLYVTYFGGD

EKQGLEPDLEAKNFWLKVGVPEDHILPGSVEDNFWEMGDQGPCGPCSEIHYDRIGGRNASALVNMDDPNVLEVWNVVFIQ

YNREADGNLRTLPNKHIDTGMGFERLVSILQNKYSNYDTDVFLPIFDKIREITGVRPYTGKFGNEDKDGIDTAYRVIADH

VRTLTFAICDGGVPNNEGRGYVLRRILRRGSRYVRKYMNYPIGGFFQQLVDVVIEQNKEIFPEISSGAQDLKEILNEEEL

SFAKTLDRGEKLFEQYAIIASKTPEQTLSGKDVWRLYDTYGFPVDLTRLLAEEAGLKIDEEGFERAKEESREASKGSGTK

DGKTLVKLDVHALSELDQNDAIPKTNDEFKYGLENVKAKVVGIYDGSKFVDSIEDPSIQYGILLDKTPFYAEQGGQEYDT

GKLVIDGKSEFNVANVQVYAGYVLHTGNIVDGKLNVGDEIIATYDELRRWPIRNNHTGTHILNFALREVLGDGVDQKGSL

VAPEKLRFDFSHKQAVTAKELEKIEAISNKYIKNNDKVYYKDVSLTKAKEINGLRAVFGETYPDPVRVVSIGVSVDDLLA

DPTNTKWHEISIEFCGGTHVAKTGDIKDLVIIEESGIAKGIRRIVAVTGHDAHHVQKVANEFEQEIDNASSLPFGVAKES

KSKELGVALKKLSISVLDKQRLTEKFNKLDKSIKDNLKAKQKEETKKTLDVVNNWLNDKENASSFLVAHVPITANAKAIT

EAINLIKKQDKTKSIYLLTGETDKVAHGCYVSDEAIVKGINANELAKAVSENIGGKAGGKGNIVQGMGDKPQGINTAIEE

VTKLFKEKL

>SCU96317.1 LAMI_0F06128g1_1 [Lachancea mirantina]

MTIGDKQKWTAQKVRSTFLDYFKERNHRFVPSSPVIPFDDPTLLFANAGMNQYKPIFLGTVDPSSDFYGLKRAVNSQKCI

RAGGKHNDLEDVGRDSYHHTFFEMLGNWSFGDYFKKEAIEYSWELLTKVYGISADNLYVTYFEGDEKLGLQPDIEARDLW

KAVGVAEDHILPGNAKDNFWEMGDQGPCGPCSEVHYDRIGGRNAASLVNMDDPDVLEIWNNVFIQFNREQDGSLKPLPAK

HVDTGMGFERLVSVLQDVRSNYDTDVFQPLFARIQEMTGCRPYSGKFGTEDVDGIDTAYRVLADHVRTLTFALADGGVPN

NEGRGYVLRRVLRRGARYARKYMNYPIGNFFSSLAPTLIDQAKGMFPELAKDPSFLFEILDEEEASFSKTLDRGERLFEK

YAEEASKTSSKTLDGKQVWRLYDTYGFPVDLTELMAEELGLKIDGPGFEKAKQESYEASKRGGKKGASDLIKLNVHDLSN

LNSDNVPKTDDSHKYNAANVKAKILRLHDGNGFVEKINEVGKQYGIILDQTCFYAEQGGQEFDTGSIVIDGETEFKVENV

QLYNGFVFHTGALDEGSLSVGDGIIATFDELRRFPIKNNHTGTHILNFALKEVLGSDVDQKGSLVAPEKLRFDFSHKKAL

SNDEIKKVEDISNDQVKANHVVYYKSVPLDLAKSICSVRAVFGETYPDPVRVVSVGRPVDDLIADPQNEEWQKYSVEFCG

GTHVAKTGDIKEFIIIDESGIAKGTRRIVAVSGSAAHEAQRVARKFGDDLVATEKLPFSPLKEKKLKELGVQLGQLPISV

VAKDELKKKFTKIEKSVKDEVKTRAKKEIKQTIDEVKQFFADNEQSSYLVKHVNIPANAKAITEAVNHIKSSSNEKSIYL

FVGNDPSGKVAHGGFISDAALQKGLDGPRLLKLVSAQVGGKAGGKGNVFQGMGDKLDGVDPAVKELKNAFAQKLSID

>EEQ46641.1 alanyl-tRNA synthetase [Candida albicans WO-1]

MIKTLLRRMSSNTTIPTPNGSNHWTASKVRSTFLDYFKKQQHTYVPSSSVVPHNDPTLLFANAGMNQYKPIFLGTVDPAS

DFASLKRAANSQKCIRAGGKHNDLEDVGRDSYHHTFFEMLGNWSFGDYFKKEAIDYSWELLTKVYGLQEDRLYVTYFGGD

EKQGLEPDLEAKNFWLKVGVPEDHILPGSVEDNFWEMGDQGPCGPCSEIHYDRIGGRNASALVNMDDPNVLEVWNVVFIQ

YNREADGNLRTLPNKHIDTGMGFERLVSILQNKYSNYDTDVFLPIFDKIREITGVRPYTGKFGNEDKDGIDTAYRVIADH

VRTLTFAICDGGVPNNEGRGYVLRRILRRGSRYVRKYMNYPIGGFFQQLVDVVIEQNKEIFPEISSGAQDLKEILNEEEL

SFAKTLDRGEKLFEQYAIIASKTPEQTLSGKDVWRLYDTYGFPVDLTRLMAEEAGLKIDEEGFERAKEESREASKGSGTK

DGKTLVKLDVHALSELDQNDAIPKTNDEFKYGLENVKAKVVGIYDGSKFVDSIEDPSIQYGILLDKTPFYAEQGGQEYDT

GKLVIDGKSEFNVANVQVYAGYVLHTGNIVDGKLNVGDEIIATYDELRRWPIRNNHTGTHILNFALREVLGDGVDQKGSL

VAPEKLRFDFSHKQAVTAKELEKIEAISNKYIKNNDKVYYKDVSLTKAKEINGLRAVFGETYPDPVRVVSIGVSVDDLLA

DPTNTKWHEISIEFCGGTHVAKTGDIKDLVIIEESGIAKGIRRIVAVTGHDAHHVQKVANEFEQEIDNASSLPFGVAKES

KSKELGVALKKLSISVLDKQRLTEKFNKLDKSIKDNLKAKQKEETKKTLDVVNNWLNDKENASSFLVAHVPITANAKAIT

EAINLIKKQDKTKSIYLLTGETDKVAHGCYVSDEAIVKGINANELAKAVSENIGGKAGGKGNIVQGMGDKPQGINTAIEE

VTKLFKEKL

>KGR08732.1 alanyl-tRNA synthetase [Candida albicans P57072]

MIKTLLRRMSSNTTIPTPNGSNHWTASKVRSTFLDYFKKQQHTYVPSSSVVPHNDPTLLFANAGMNQYKPIFLGTVDPAS

DFASLKRAANSQKCIRAGGKHNDLEDVGRDSYHHTFFEMLGNWSFGDYFKKEAIDYSWELLTKVYGLQEDRLYVTYFGGD

EKQGLEPDLEAKNFWLKVGVPEDHILPGSVEDNFWEMGDQGPCGPCSEIHYDRIGGRNASALVNMDDPNVLEVWNVVFIQ

YNREADGNLRTLPNKHIDTGMGFERLVSILQNKYSNYDTDVFLPIFDKIREITGVRPYTGKFGNEDKDGIDTAYRVIADH

VRTLTFAICDGGVPNNEGRGYVLRRILRRGSRYVRKYMNYPIGGFFQQLVDVVIEQNKEIFPEISSGAQDLKEILNEEEL

SFAKTLDRGEKLFEQYAIIASKTPEQTLSGKDVWRLYDTYGFPVDLTRLMAEEAGLKIDEEGFERAKEESREASKGSGTK

DGKTLVKLDVHALSELDQNDAIPKTNDEFKYGLENVKAKVVGIYDGSKFVDSIEDPSIQYGILLDKTPFYAEQGGQEYDT

GKLVIDGKSEFNVANVQVYAGYVLHTGNIVDGKLNVGDEIIATYDELRRWPIRNNHTGTHILNFALREVLGDGVDQKGSL

VAPEKLRFDFSHKQAVTAKELEKIEAISNKYIKNNDKVYYKDVSLTKAKEINGLRAVFGETYPDPVRVVSIGVSVDDLLA

DPTNTKWHEISIEFCGGTHVAKTGDIKDLVIIEESGIAKGIRRIVAVTGHDAHHVQKVANEFEQEIDNASSLPFGVAKES

KSKELGVALKKLSISVLDKQRLTEKFNKLDKSIKDNLKAKQKEETKKTLDVVNNWLNDKENSSSFLVAHVPITANAKAIT

EAINLIKKQDKTKSIYLLTGETDKVAHGCYVSDEAIVKGINANELAKAVSENIGGKAGGKGNIVQGMGDKPQGINTAIEE

VTKLFKEKL

>XP_718082.1 alanine--tRNA synthetase [Candida albicans SC5314]

MIKTLLRRMSSNTTIPTPNGSNHWTASKVRSTFLDYFKKQQHTYVPSSSVVPHNDPTLLFANAGMNQYKPIFLGTVDPAS

DFASLKRAANSQKCIRAGGKHNDLEDVGRDSYHHTFFEMLGNWSFGDYFKKEAIDYSWELLTKVYGLQEDRLYVTYFGGD

EKQGLEPDLEAKNFWLKVGVPEDHILPGSVEDNFWEMGDQGPCGPCSEIHYDRIGGRNASALVNMDDPNVLEVWNVVFIQ

YNREADGNLRTLPNKHIDTGMGFERLVSILQNKYSNYDTDVFLPIFDKIREITGVRPYTGKFGNEDKDGIDTAYRVIADH

VRTLTFAICDGGVPNNEGRGYVLRRILRRGSRYVRKYMNYPIGGFFQQLVDVVIEQNKEIFPEISSGAQDLKEILNEEEL

SFAKTLDRGEKLFEQYAIIASKTPEQTLSGKDVWRLYDTYGFPVDLTRLMAEEAGLKIDEEGFERAKEESREASKGSGTK

DGKTLVKLDVHALSELDQNDAIPKTNDEFKYGLENVKAKVVGIYDGSKFVDSIEDPSIQYGILLDKTPFYAEQGGQEYDT

GKLVIDGKSEFNVANVQVYAGYVLHTGNIVDGKLNVGDEIIATYDELRRWPIRNNHTGTHILNFALREVLGDGVDQKGSL

VAPEKLRFDFSHKQAVTAKELEKIEAISNKYIKNNDKVYYKDVSLTKAKEINGLRAVFGETYPDPVRVVSIGVSVDDLLA

DPTNTKWHEISIEFCGGTHVAKTGDIKDLVIIEESGIAKGIRRIVAVTGHDAHHVQKVANEFEQEIDNASSLPFGVAKES

KSKELGVALKKLSISVLDKQRLTEKFNKLDKSIKDNLKAKQKEETKKTLDVVNNWLNDKENASSFLVAHVPITANAKAIT

EAINLIKKQDKTKSIYLLTGETDKVAHGCYVSDEAIAKGINANELAKAVSENIGGKAGGKGNIVQGMGDKPQGINTAIEE

VTKLFKEKL

>XP_023452737.1 Alanine--tRNA synthetase [Cercospora beticola]

MRSFSTLLRFFKPAPGISRNSATKATTARTPPPRVLHHYSFRRMASTTGQEPEWSAVKVRKTFIEYFEKNGHTFVPSSSV

VPLSDHTLLFTNAGMNQYKAIFLGTVDRDSDFAKLKRAANSQKCIRAGGKHNDLDDVGKDSYHHTFFEMLGNWSFGDYFK

EDAIKFSWELLTKVYGLETDRLYVTYFEGKADAGLEPDHEARDFWLKQGVPEDHILTGDMKDNFWEMGDQGPCGPCSEIH

YDRIGGRNASDLVNQDDPNVIEIWNNVFIQYNREPDKSLKSLPSKHIDTGMGFERLVSILQKKSSNYDTDVFTPLFARIQ

EVTGARPYQGKFGAEDADGIDTAYRVVADHLRLLTFAISDGGVPNNVGRGYVVRRVLRRGARYARKYFNTNIGNFFSKLV

PTLVEQMGDMFPEIKKKQEEVKEILDEEEVSFAKTLDRGESMFEKYAQNCKANGTKTLAGADVWRLYDTYGFPVDLTKLM

CEERELEIIDEDVAEAQEKARLASMGAKKEGATLVKLDVHDISTLENMPDVSKTDDSPKFQRENIKATIKAIYHGRKFLQ

STAEIPEGEQFGVILDRTNFYAEQGGQENDTGRILIDGVAEIKVENVQQYAGYVMHTGYVGYGQLKVGDEVLAEYDELRR

QPIRNNHTGTHVLNYALREVLGDDVNQKGSLVAPEKLRFDFSHKAGVTDEELTKIEEVSTNYIRQNCEVYAKDVPLATAR

QINGVRAVFGETYPDPVRVVSVGISVEDLLENPSDPKWNDISIEFCGGTHVKNTTEIKDLVVIEESGIAKGIRRIIAVTG

QEAHKVQAEAETWDKEVSALERLPYGPDKEAKTKQLTVDLSKLEISALQKTSLRNRVAAVAKANLDQQKAAQKAENKKVL

DAVTDHFTNNADSKTLVLSLPISAGSKAIQETIKVVQNKQKEKTVYLVGKTDDGKVVHGCHVSSDAQAKGADASKWSGEV

AALVGGKAGGKGPTSLGQGTDASKVDEAVEAARKYLESLSL

>XP_003683463.1 hypothetical protein TDEL_0H03930 [Torulaspora delbrueckii]

MTIGDKQRWTAKNVRNTFLDYFKSKEHRFVPSSPVIPYDDPTLLFANAGMNQYKPIFLGTVDPASDFYTMKRAYNSQKCI

RAGGKHNDLEDVGRDSYHHTFFEMLGNWSFGDYFKKEAINYSWELLTKVYGIPADRLYVTYFEGDSKQGLEPDVEALELW

KSVGVPEDHILPGNAKDNFWEMGDQGPCGPCSEIHYDRIGGRNAASLVNQDDPDVLEVWNLVFIQFNREQDGSLKSLPAK

HIDTGMGFERLVSVLQDCRSNYDTDVFMPLFASIQELTGARPYAGKFGTEDADGVDTAYRVLADHVRTLTFALADGGVPN

NEGRGYVLRRILRRGARYARKYMNYPIGNFFSRLAPTLIDQVQEMFPEVAKDPSFLFEILDEEEASFAKTLDRGERLFEK

YADAAAQSGNKTLDGLQVWRLYDTYGFPVDLTELMAEEKGLTIDGPGFEKAKQESYEASKRGGKKDNSSLLKLNVHELSQ

LNEEHVPKTDDSAKYGTQDVDATILRLFDGSQFVNEITEPGKKYGIILDKTCFYAEQGGQEYDMGKIVIDGSAEFDVDNV

QLYNGFVFHTGTLLEGKLSINDKIIASYDELRRFPIRNNHTGTHILNYGLLETLGTEVDQKGSLVAPEKLRFDFSHKKAL

TVDELRKVENISNEQIKQNLSVYYKDVPLDLARSIYNVRAVFGETYPDPVRVVSVGRPVDELLENPASEEWGKYSIEFCG

GTHVAKTSDIREFVILEESGIAKGIRRIVAVTGSEAHEAQRIAKQYESELLAAEKLPFSPLKEKKLKELGVQLGQLSISV

IRKQELREHFNKIDKAVKDEVKSRSKKEAKQTLDEVKEYFAANESSPYFVKFINIPTNAKAITEAINYMKSSAKDKSIYL

FTGNDPEGKVAHGCYISDDALSKGIDGPALGKIVSGAVGGKAGGKGNVFQGMGDKPAGVENATTELESLFKEKLVI

>XP_001248510.1 alanine-tRNA synthetase [Coccidioides immitis RS]

MLSGRSSLGVLANRRAAGSTRKFFSPSTTFHPAAAAIAIPLSSRNKFVPLRPYPFTLLKMASSTSTPSQNEVEWPAIKVR

DTFLDFFKKNGHTFVPSSSVVPLSDPTLLFANAGMNQYKSIFLGTVDPNSDFAQLKRAHNSQKCIRAGGKHNDLDDVGKD

SYHHTFFEMLGNWSFGDYFKKEAIEYSWELLTKVYGLEPDRLYVTYFEGNKDAGIEPDLETKDLWLSVGVKEDHILSGNM

KDNFWEMGDQGPCGPCSEVHYDRIGGRNAAHLVNMDDPNVLEIWNNVFIQYNRESESILRPLPNKHVDTGMGYERLVSVL

QKKYSNYDTDVFTPLFDAIREITGVRPYSGKFGEEDVDGIDTAYRVVADHVRTLTFAISDGAPPNNEGRGYVVRRVLRRG

ARYARKYLKVEIGNFFSKIVPTLVDQMGGMFPEIKKKQTDVMEILDEEEISFAKTLDRGERQFEIYAQQAKDSGSNKLHG

ADVWRLYDTFGFPVDLTQLMAEERGLSIDNVEFEEARLKAKEASKGQAKAASDLLKLSVHDLSKLEKDKVPKTNDDAKFG

RGNILSQIVAIYHDKEFVDSTSGIPEGDQIGIILDKTNFYAEQGGQEYDTGKIIIDGKAELDVRNVQMYGGYVFHTGFIK

YGDLSVGDSVISEYDELRRWPIRNNHTGTHILNFALREVLGNGIDQKGSLVAAEKLRFDFSHKSGVSDSDLEKIEEKCTE

YIRQNCAVYSKDVPLSVAREITGVRAVFGETYPDPVRVVSVGVELEEILKDVKDPRWKGVSIEFCGGTHVQKTGDIKDLV

ILEEGGIAKGIRRIIAVTGEDAHEVQRIAKEFGDRLNRFEKMEIGPKKEQEAKLIQADLNQLSISAVEKARFREQFARIH

KQVLEAQKALQKQEIKAALDAINSHFEKPENKDSSHLIVRLPTSANAKAISESINHAKSKLKDKSIYVFAADAEQGRVAH

GCYVSSSLSAQGASASNWSGVVSGIVGGKAGGKAPTSIGNGTEVDRVDEAIEAATKYLEQFQL

>KHC63511.1 alanyl-tRNA synthetase [Candida albicans P75016]

MIKTLLRRMSSNTTIPTPNGSNHWTASKVRSTFLDYFKKQQHTYVPSSSVVPHNDPTLLFANAGMNQYKPIFLGTVDPAS

DFASLKRAANSQKCIRAGGKHNDLEDVGRDSYHHTFFEMLGNWSFGDYFKKEAIDYSWELLTKVYGLQEDRLYVTYFGGD

EKQGLEPDLEAKNFWLKVGVPEDHILPGSVEDNFWEMGDQGPCGPCSEIHYDRIGGRNASALVNMDDPNVLEVWNVVFIQ

YNREADGNLRTLPNKHIDTGMGFERLVSILQNKYSNYDTDVFLPIFDKIREITGVRPYTGKFGNEDKDGIDTAYRVIADH

VRTLTFAICDGGVPNNEGRGYVLRRILRRGSRYVRKYMNYPIGGFFQQLVDVVIEQNKEIFPEISSGAQDLKEILNEEEL

SFAKTLDRGEKLFEQYAIIASKTPEQTLSGKDVWRLYDTYGFPVDLTRLMAEEAGLKIDEEGFERAKEESREASKGSGTK

DGKTLVKLDVHALSELDQNDAIPKTNDEFKYGLENVKAKVVGIYDGSKFVDSIEDPSIQYGILLDKTPFYAEQGGQEYDT

GKLVIDGKSEFNVANVQVYAGYVLHTGNIVDGKLNVGDEIIATYDELRRWPIRNNHTGTHILNFALREVFGDGVDQKGSL

VAPEKLRFDFSHKQAVTAKELEKIEAISNKYIKNNDKVYYKDVSLTKAKEINGLRAVFGETYPDPVRVVSIGVSVDDLLA

DPTNTKWHEISIEFCGGTHVAKTGDIKDLVIIEESGIAKGIRRIVAVTGHDAHHVQKVANEFEQEIDNASSLPFGVAKES

KSKELGVALKKLSISVLDKQRLTEKFNKLDKSIKDNLKAKQKEETKKTLDVVNNWLNDKENASSFLVAHVPITANAKAIT

EAINLIKKQDKTKSIYLLTGETDKVAHGCYVSDEAIAKGINANELAKAVSENIGGKAGGKGNIVQGMGDKPQGINTAIEE

VTKLFKEKL

>KHC33201.1 alanyl-tRNA synthetase [Candida albicans Ca6]

MIKTLLRRMSSNTTIPTPNGSNHWTASKVRSTFLDYFKKQQHTYVPSSSVVPHNDPTLLFANAGMNQYKPIFLGTVDPAS

DFASLKRAANSQKCIRAGGKHNDLEDVGRDSYHHTFFEMLGNWSFGDYFKKEAIDYSWELLTKVYGLQEDRLYVTYFGGD

EKQGLEPDLEAKNFWLKVGVPEDHILPGSVEDNFWEMGDQGPCGPCSEIHYDRIGGRNASALVNMDDPNVLEVWNVVFIQ

YNREADGNLRTLPNKHIDTGMGFERLVSILQNKYSNYDTDVFLPIFDKIREITGVRPYTGKFGNEDKDGIDTAYRVIADH

VRTLTFAICDGGVPNNEGRGYVLRRILRRGSRYVRKYMNYPIGGFFQQLVDVVIEQNKEIFPEISSGAQDLKEILNEEEL

SFAKTLDRGEKLFEQYAIIASKTPEQTLSGKDVWRLYDTYGFPVDLTRLLAEEAGLKIDEEGFERAKEESREASKGSGTK

DGKTLVKLDVHALSELDQNDAIPKTNDEFKYGLENVKAKVVGIYDGSKFVDSIEDPSIQYGILLDKTPFYAEQGGQEYDT

GKLVIDGKSEFNVANVQVYAGYVLHTGNIVDGKLNVGDEIIATYDELRRWPIRNNHTGTHILNFALREVFGDGVDQKGSL

VAPEKLRFDFSHKQAVTAKELEKIEAISNKYIKNNDKVYYKDVSLTKAKEINGLRAVFGETYPDPVRVVSIGVSVDDLLA

DPTNTKWHEISIEFCGGTHVAKTGDIKDLVIIEESGIAKGIRRIVAVTGHDAHHVQKVANEFEQEIDNASSLPFGVAKES

KSKELGVALKKLSISVLDKQRLTEKFNKLDKSIKDNLKAKQKEETKKTLDVVNNWLNDKENASSFLVAHVPITANAKAIT

EAINLIKKQDKTKSIYLLTGETDKVAHGCYVSDEAIVKGINANELAKAVSENIGGKAGGKGNIVQGMGDKPQGINTAIEE

VTKLFKEKL

>KGU04317.1 alanyl-tRNA synthetase [Candida albicans P87]

MIKTLLRRMSSNTTIPTPNGSNHWTASKVRSTFLDYFKKQQHTYVPSSSVVPHNDPTLLFANAGMNQYKPIFLGTVDPAS

DFASLKRAANSQKCIRAGGKHNDLEDVGRDSYHHTFFEMLGNWSFGDYFKKEAIDYSWELLTKVYGLQEDRLYVTYFGGD

EKQGLEPDLEAKNFWLKVGVPEDHILPGSVEDNFWEMGDQGPCGPCSEIHYDRIGGRNASALVNMDDPNVLEVWNVVFIQ

YNREADGNLRTLPNKHIDTGMGFERLVSILQNKYSNYDTDVFLPIFDKIREITGVRPYTGKFGNEDKDGIDTAYRVIADH

VRTLTFAICDGGVPNNEGRGYVLRRILRRGSRYVRKYMNYPIGGFFQQLVDVVIEQNKEIFPEISSGAQDLKEILNEEEL

SFAKTLDRGEKLFEQYAIIASKTPEQTLSGKDVWRLYDTYGFPVDLTRLMAEEAGLKIDEEGFERAKEESREASKGSGTK

DGKTLVKLDVHALSELDQNDAIPKTNDEFKYGLENVKAKVVGIYDGSKFVDSIEDPSIQYGILLDKTPFYAEQGGQEYDT

GKLVIDGKSEFNVANVQVYAGYVLHTGNIVDGKLNVGDEIIATYDELRRWPIRNNHTGTHILNFALREVFGDGVDQKGSL

VAPEKLRFDFSHKQAVTAKELEKIEAISNKYIKNNDKVYYKDVSLTKAKEINGLRAVFGETYPDPVRVVSIGVSVDDLLA

DPTNTKWHEISIEFCGGTHVAKTGDIKDLVIIEESGIAKGIRRIVAVTGHDAHHVQKVANEFEQEIDNASSLPFGVAKES

KSKELGVALKKLSISVLDKQRLTEKFNKLDKSIKDNLKAKQKEETKKTLDVVNNWLNDKENASSFLVAHVPITANAKAIT

EAINLIKKQDKTKSIYLLTGETDKVAHGCYVSDEAIVKGINANELAKAVSENIGGKAGGKGNIVQGMGDKPQGINTAIEE

VTKLFKEKL

>ORZ31772.1 putative alanyl-tRNA synthetase [Catenaria anguillulae PL171]

MTTNTQQQQWTAAKVRSTFIDYFKSLPGGNDHIHVPSSKTIPHDDPTLLFANAGMNQFKPIFLGTADPNSPMAKWQRAAN

SQKCIRAGGKHNDLDDVGKDTYHHTFFEMLGNWSFGNYFKKEAIQWAWTLLTQVYGLDKNRLYVTYFGGDEKQGLEPDTE

ARDLWIQMGLPKDRVLPYGCKENFWEMGDQGPCGPCSEIHYDLLDTGRFVPALVNADDPTLIEIWNLVFIQFNREADGSL

RQLPAKHVDTGMGFERLVCALQGQLSNYDTDVFSPIFARIQEVTGARPYAGKLGKDDADGVDMAYRVVADHIRTLTVSIS

DGGVPSNDGRGYVLRRILRRGVRYARSGFNVNIGTFFSSLVDVVAASLGDAFPEVRAHVETVKQILDEEEAAFAKTLDRG

IKLFESIVAKGGDAAKTISGADAWKLYDTFGFPIDLTRLMAEEKGLQVDDAGFEAEQAKAKELSRRRKDAQGEQVIKLDV

HAIKAAEAAGVAKTDDSFKYNTATLEGANVIAVYKNGEWILDNKQVGVSEGEAAGILLDRTNFYAESGGQEYDTGSLAVD

GELEFAVENVQVYAGYVLHTGLLKYGTLSVGQSVTAAFDALRRWPLRNNHTATHVLNHALRATLGDGVDQKGSLVAPTKL

RFDFSAKAPLAHKDLVKIEAEAADTIKRALPVYAKEVPLAQAKAINGLRAVFGEVYPDPVRVVSIGVAVEDLVSDPNNAK

WPEYSIELCGGTHAGTSLELGSFAVMEESAIAKGVRRIVAVTGPEAIAAHDEAASFVQELNKLSGLSGFALGEAVKEAAK

EVDLRVMPAAAKMQAREVVGAHKKRFDEWDKAQKAAKATAAVEEVKAYFAGDEAKNKPYLVRVLDVGGNNKAVQSAIAAV

KATGGEGKAALFITVAEGAKVSYAAYVDKSLHGKVTATQWAEVVAKAVDGKMGGKEEMAQGAGSKVEAVDEAVRLAEEFA

KVKLA

>KMP09839.1 alanyl-tRNA methyltransferase synthetase [Coccidioides immitis RMSCC 2394]

MLSGRSSLGVLANRRAAGSTRKFFSPSTTFHPAAAAIAIPLSSRNKFVPLRPYPFTLLKMASSTSTPSQNEVEWPAIKVR

DTFLDFFKKNGHTFVPSSSVVPLSDPTLLFANAGMNQYKSIFLGTVDPNSDFAQLKRAHNSQKCIRAGGKHNDLDDVGKD

SYHHTFFEMLGNWSFGDYFKKEAIEYSWELLTKVYGLEPDRLYVTYFEGNKDAGIEPDLETKDLWLSVGVKEDHILSGNM

KDNFWEMGDQGPCGPCSEVHYDRIGGRNAAHLVNMDDPNVLEIWNNVFIQYNRESESILRPLPNKHVDTGMGYERLVSVL

QKKYSNYDTDVFTPLFDAIREITGVRPYSGKFGEEDVDGIDTAYRVVADHVRTLTFAISDGAPPNNEGRGYVVRRVLRRG

ARYARKYLKVEIGNFFSKIVPTLVDQMGGMFPEIKKKQTDVMEILDEEEISFAKTLDRGERQFEIYAQQAKDSGSNKLHG

ADVWRLYDTFGFPVDLTQLMAEERGLSIDNVEFEEARLKAKEASKGQAKAASDLLKLSVHDLSKLEKDKVPKTNDDAKFG

RGNILSQIVAIYHDKEFVDSTSGIPEGDQIGIILDKTNFYAEQGGQEYDTGKIIIDGKAELDVRNVQMYGGYVFHTGFIK

YGDLSVGDSVISEYDELRRWPIRNNHTGTHILNFALREVLGNGIDQKGSLVAAEKLRFDFSHKSGVSDSDLEKIEEKCTE

YIRQNCAVYSKDVPLSVAREITGVRAVFGETYPDPVRVVSVGVELEEILKDVKDPRWKGVSIEFCGGTHVQKTGDIKDLV

ILEEGGIAKGIRRIIAVTGEDAHEVQRIAKEFGDRLNRFEKMEIGPKKEQEAKLIQADLNQLSISAVEKARFREQFARIH

KQVLEAQKSLQKQEIKAALDAINSHFEKPENKDSSHLIVRLPTSANAKAISESINHAKSKLKDKSIYVFAADAEQGRVAH

GCYVSSSLSAQGASASNWSGVVSGIVGGKAGGKAPTSIGNGTEVDRVDEAIEAATKYLEQFQL

>XP_455190.1 uncharacterized protein KLLA0_F02431g [Kluyveromyces lactis]

MTIGDKSKWTAANVRSTFLDYFKSKGHTFVPSSPVIPFDDPTLLFANAGMNQYKPIFLGTVDPSSDFYKLKRATNSQKCI

RAGGKHNDLEDVGRDSYHHTFFEMLGNWSFGDYFKKEAIEYSWELLTKVYGIPGDRLYVTYFEGDEKLGLEPDFEARDLW

KSVGVKDDHILPGNAKDNFWEMGEQGPCGPCSEVHYDRIGGRNAAHLVNQDDPDVLEIWNNVFIQFNREQDRSLRALPAK

HVDTGMGFERLVSVLHDVRSNYDTDVFEPLFNRIQEITGVRSYTGKFGAEDTDGIDTAYRVLADHVRTLTFALADGGVPN

NEGRGYVLRRILRRGARYARKYMNYPIGNFFSQLSVTLIEQVKDMFPEVARDPSYLFEILDEEEASFAKTLDRGEKLFEK

YAEDALKKADKTLDGKQVWRLYDTYGFPVDLTELMAEEQGLKIDTIGFEKAKQESYEASKKGGKKGASDLIRLNVHELSQ

LNGDNVAKTDDSSKYGTENVEATILRIYDGEKFVDSMTEIGKQYGVILDKTCFYAEQGGQEFDTGKLVIDGEAEFNVDNV

QAYNGYVFHTGSLGDGKLSIGDSIIASYEELRRHPLRNNHTGTHILNFALKEVLGNDVDQKGSLVAPEKLRFDFSHKRAL

SYEELVEVEKLSNEKIRDNLTVYYKEVALDLAKTISSVRAVFGETYPDPVRVVSIGKAVEEVLENPTSEEWQKYSIEFCG

GTHVAKTSDIKEFVIVEESGIAKGIRRIVAVSGPEAYEVQRIATEFDGELTAVEKLPFSPLKAQKLKELGVRLGQLTISV

ITKTDLKDKFNKIEKEVKDELKSRAKKEIKQTLDDVKHHFDANKESSFYVKHIDIPTNAKAITEAINHIKTNSKDKSIYL

FTCNEPDGKVAHGCYISDAAIAQGVDGSSLAKTVSSSIGGKAGGKGNVFQGMGDKPNGIDSAVAEVEALLKERLTI

>PVH23048.1 alanine-tRNA synthetase [[Candida] haemulonis]

MTSWPASTVRQTFLDFFKGKQHSFVPSSSVVPHNDPTLLFANAGMNQYKPIFLGTVDPTTDFGSLKRAANSQKCIRAGGK

HNDLDDVGKDSYHHTFFEMLGNWSFGDYFKKEAIEWSWELLTEVYKLEKDRLYVTYFEGDEKNGLKPDLEAKQFWLDVGV

AEDHILPGDATDNFWEMGDQGPCGPCSEIHYDRIGGRNASSLVNQDDPNVLEVWNVVFIQYNREADASLRPLPNKHIDTG

MGFERLVSILQNKFSNYDTDVFSPLFEKIREITGVRPYTGKFGADDVDGIDTAYRVIADHVRTLTFAITDGGVPNNEGRG

YVLRRILRRGSRYVRKYMNYPIGSFFQQLVDVVIQENSQIFPEIASHKDELKEILVEEELSFAKTLDRGEKLFEQYAIIA

SKTPEQTLSGKDVWRLYDTYGFPVDLTRLMAEEAGLKIDEVAFEKAKEESREASKASGSKTGADLIKLDVHALSELEGND

AVPKTDDHYKFGLENTKSKIVAFYDGSAFTDSIDNAGQQYGILLNKTPFYAEQGGQEYDTGSLVIDGSAEFNVTNVQSYA

GYVLHTGNLVEGTLKVGDEVIATYDELRRWPIRNNHTGTHVLNFALREVLGDNVDQKGSLVAAEKLRFDFSNKQALTIKE

VEQVEKISNENIKANQEVFAEDVSLATAKSINGLRAVFGEVYPDPVRVVSIGVPVSKLLNDPENKEWHKYSIELCGGTHV

AKTAEIKDLVIIEESGIAKGTRRIVAVTGHDAHRVQQVAKDFDADLDAAANLPFGADKEQKAKELGVALKKLSISVVDKS

KLNEKFTKLDKSIKDNLKAKQKEESKKTLNVVNEWLKDEEKSKAPFLVAHVPISANAKAITEAFNLIKKEAKEKSLYLIT

GTSDKVAHGCYISDEAISKGVDANEIAKAAIAHIGGKAGGKGNIVQGMGDKPDGVNEALEAVKKALTEKLT

>OEJ85664.1 Alanine--tRNA synthetase, mitochondrial [Hanseniaspora osmophila]

MTVGNKELWTAPHVRDTFLKYFIEKKGHKYVKSSPVIPYDDPTLLFANAGMNQYKPIFLGTVDPQSDFASLKRACNSQKC

IRAGGKHNDLEDVGKDSYHHTFFEMLGNWSFGDYFKKEAIDYSWDLLTNVYGIPKDRLYVTYFEGDEKNGLEADLEAKQY

WKNVGVEEDHILPGNAKDNFWEMGDQGPCGPCSEIHYDRIGGRNAAHLVNMDDPDVLEIWNNVFIQYNREQGGSLKSLPA

KHVDTGMGFERLVSVLQDVRSNYDTDVFQPLFVKIQEITGCRPYSGKFGDEDKDGIDTAYRVLSDHVRTLIFALSDGGVP

NNEGRGYVLRRILRRGARYVRKYMNYPIGDFFSKLAPTVIDQVKDIFPEVNKDPSYLFEILNEEELSFAKTLDRGEKLFE

KYAEVASKSANKTLNGKEVWRLYDTYGFPVDLTRLMAEEQSLKIDEEGFEKAKLESYEASKKXNKKNQGTLIKLSVHDLS

KLNEDSVSKTNDEFKYGLENIEANILAIHDGENFVDEITESGKTYGIILDKTCFYAEQGGQEYDTGKIVVDGVSEFNVEN

VQLYNGYVFHTGVLQEGKLSKNEKVIASYDELRRFPLRNNHTGTHILNFALKETLGADVDQKGSLVAPEKLRFDFSHKKA

LSFEELEKVEGICNKLIKENMTVYYKEVPLELAQSIISVRAVFGETYPDPVRVVSVGKPVEDLLADPKNEEWNNYSVEFC

GGTHVAKTGDIKEFVIIEESGIAKGIRRMVAVSGSEAYEVQRVAAEFNKQLEHVESLPFGPAKEKKIKELSVELGQLSIS

VITKNNLKEKFNKIEKEVKDEIKTRAKAEIKQTVDEVTQFFEQNEDAAFLVKHVEIPTNAKAITEAINYIKNNKKEKSLY

LLTGNDASGRVAHGCYISNEALTKSVDGSALAKAVSASIGGKAGGKGNVFQGMGDQYAGINAAVEEVTKLLEEKL

>CEP13586.1 hypothetical protein [Parasitella parasitica]

MMAVQTEWPANKVRSTFIKYFEDNGHVFVPSSSTIPHDDPTLLFANAGMNQYKPIFLGTVDPNSPLANVKRACNSQKCIR

AGGKHNDLDDVGRDTYHHTFFEMLGNWSFGDYFKLEATKFGWEFLTKVIKLPEDRLYVTYFGGDEKMGLPADLEAKQMWM

DLGVEEKRLIPGSVKDNFWEMGETGPCGPCSEIHYDRIGGRDAAHLVNMDDPNVLEIWNLVFMQFNREQDGSLRPLPNQH

IDTGLGLERLVSILQNKYSNYDTDCFTPLFDKVKELTGVREYTGLLGKEDTDGIDTAYRVVGDHIRTLTFAISDGGVPSN

EGRGYVLRRILRRGARYVRKYFNIPIGSFFSSLVDTLIAQMHEAYPELAKKAEDVKAILDEEEAAFSKTLDRGEKLFEAY

MNKTKESGSTELSGDDVWRLYDTYGFPVDLTRIMAEENGLEVNEKQFEAAKEAAKEKSRKVKRNVNDQDVVALDVHDLGA

LENNDAVPKTDDSHKYLAGNVQGEIKAIFYEHKFLDNTADIPEDRNFGLLVDKTNFYAESGGQEYDVGSIITLDANTEFV

VEDCQLYGGYVLHVGHLKYGQLKLNDKVEISYDDLRRFPLRNNHSATHILNFALREVLKEDIDQKGSLVAPEKLRFDFSY

KTPVTVDQLAKVENICNDFIAKNLKVYSKEVPLAVAKAIHGLRAVFGETYPDPVRVVSIGFDVDEVTADVSNPKWKTTSI

EFCGGTHVAKTSDIKYFAILEESGIAKGIRRIVAVTGDEAAAAQREAQEVTAKLDKLEKLSGAQLEAELKVVSKELDTSK

ISVVTKTELRERFATIKKKFDDAEKSRKSKQAEEAINSVKEYFTANPDAQYYVAELPVDGNSKALIGITNYAKTNLNDKA

ICVLSSDESTGRVAHSCFVGKIYIEKGLKASEWAGIVSEKVGGKKGGKDDAAQGSGDKITGLPDALKAAEEFAKLKISA

>KGQ84359.1 alanyl-tRNA synthetase [Candida albicans P94015]

MIKTLLRRMSSNTTIPTPNGSNHWTASKVRSTFLDYFKKQQHTYVPSSSVVPHNDPTLLFANAGMNQYKPIFLGTVDPAS

DFASLKRAANSQKCIRAGGKHNDLEDVGRDSYHHTFFEMLGNWSFGDYFKKEAIDYSWELLTKVYGLQEDRLYVTYFGGD

EKQGLEPDLEAKNFWLKVGVPEDHILPGSVEDNFWEMGDQGPCGPCSEIHYDRIGGRNASALVNMDDPNVLEVWNVVFIQ

YNREADGNLRTLPNKHIDTGMGFERLVSILQNKYSNYDTDVFLPIFDKIREITGVRPYTGKFGNEDKDGIDTAYRVIADH

VRTLTFAICDGGVPNNEGRGYVLRRILRRGSRYVRKYMNYPIGGFFQQLVDVVIEQNKEIFPEISSGAQDLKEILNEEEL

SFAKTLDRGEKLFEQYAIIASKTPEQTLSGKDVWRLYDTYGFPVDLTRLMAEEAGLKIDEEGFERAKEESREASKGSGTK

DGKTLVKLDVHALSELDQNDAIPKTNDEFKYGLENVKAKVVGIYDGSKFVDSIEDPSIQYGILLDKTPFYAEQGGQEYDT

GKLVIDGKSEFNVANVQVYAGYVLHTGNIVDGKLNVGDEIIATYDELRRWPIRNNHTGTHILNFALREVLGDGVDQKGSL

VAPEKLRFDFSHKQAVTAKELEKIEAISNKYIKNNDKVYYKDVSLTKAKEINGLRAVFGETYPDPVRVVSIGVSVDDLLA

DPTNTKWHEISIEFCGGTHVAKTGDIKDLVIIEESGIAKGIRRIVAVTGHDAHHVQKVANEFEQEIDNASSLPFGVAKES

KSKELGVALKKLSISVLDKQRLTKKFNKLDKSIKDNLKAKQKEETKKTLDVVNNWLNDKENASSFLVAHVPITANAKAIT

EAINLIKKQDKTKSIYLLTGETDKVAHGCYVSDEAIAKGINANELAKAVSENIGGKAGGKGNIVQGMGDKPQGINTAIEE

VTKLFKEKL

>PKS02820.1 Alanine--tRNA synthetase [Cercospora zeina]

MRSFSTLLRFSRPAPGISRTSAAKATTAPTPPPRVLHHYYLRTMASTTGQEPEWSAVKVRKTFIEYFEKNGHAFVPSSSV

VPLSDPTLLFTNAGMNQYKSIFLGTVDRDSDFAKLKRAANSQKCIRAGGKHNDLDDVGKDSYHHTFFEMLGNWSFGDYFK

EDAIKFSWELLTKVYGLEPDRLYVTYFEGKPDAGLEPDIEARDLWIQQGVPEDHILTGDMKDNFWEMGDQGPCGPCSEIH

YDRIGGRNASDLVNQDDPNVIEIWNNVFIQYNREPDKSLKSLPSKHIDTGMGYERLVSILQRKSSNYDTDVFMPLFARIQ

EVTGARPYQGKFGAEDADGIDTAYRVVADHLRLLTFAISDGGVPNNVGRGYVVRRVLRRGARYARKYFNTNIGNFFSKLV

PTLVEQMGDMFPEIKKKQEEVKEILDEEEVSFAKTLDRGESMFEKYAQNCKASGTKTLAGADVWRLYDTYGFPVDLTKLM

CEERGLQIIDEDVAEAQEKARLASMGAKKEGATLVKLDVHDISTLENMADVSKTDDSPKFQRENIKATIKAIYYGRKFLQ

STEEIPEGEQFGVILDRTNFYAEQGGQENDTGRILIDGIAEIKVDNVQQYAGYVMHTGYVGYGQLKVGDEVLAEYDELRR

QPIRNNHTGTHILNYALREVLGDDVNQKGSLVAPEKLRFDFSHKTGVTDEELAKIEEVSTNYIRQNCEVYAKDVPLATAR

QINGVRAVFGETYPDPVRVVSVGMSVEDLLENPSDPKWNDISIEFCGGTHVKSTTEIKDLVVIEESGIAKGIRRITAVTG

QEAHKVQAEAETWDKKVSALERLPYGADKEAKTKQLTVDLSKLEISALQKTSLRNRVAAVAKANLDQQKAAQKTENKKVL

DAVTDHFANNADSKTLVLSLPVSAGSKAIQETIKVVQNKQKEKTVYLIGKTDDGKVVHGCHVSADAQAMGADASRWSGEV

ATLVGGKAGGKGPTSLGQGTDASKVDEAVEAARKYLEKLSL

>XP_007377582.1 alanyl-tRNA synthetase, cytoplasmic [Spathaspora passalidarum NRRL Y-27907]

MSTNTTNSVVANEWTASKVRSTFLDFFTKKKGHTYVPSSSVVPHNDPTLLFANAGMNQYKPIFLGTVDPASDFASLKRAA

NSQKCIRAGGKHNDLEDVGRDSYHHTFFEMLGNWSFGDYFKKEAIDWSWELLTQVYGLDKDRLYVTYFGGDEKQGLQPDL

EAKQFWLNVGVAEDHILPGSAKDNFWEMGDQGPCGPCSEIHYDRIGGGRNAADLVNMDDPNVLEVWNVVFIQYNREPDSS

LRTLPNKHIDTGMGFERLVSILQDKSSNYDTDVFGPIFDKITQITGVRPYAGRFGAEDKDGIDTAYRVIADHVRTLTFAI

CDGGVPNNEGRGYVLRRILRRGSRYVRKYMNYPIGSFFQQLVDVVIEQNKEIFPEIVHGASDLKEILNEEEVSFAKTLDR

GEKLFEQYAIIASKTPEQTLSGKDVWRLYDTYGFPVDLTRLMAEEAGLKIDEPGFERAKEESREASKGTGTKNGTTLIKL

DVHALSSLETELVPKTDDEYKYGTENIEAKIVSIYDGAEFVTKINDSTKQYGILLDKTPFYAEQGGQEYDTGKLVIDGKS

EFTVDNVQVYGGYVLHTGTITEGELSVGDSIIASYDELRRWPIRNNHTGTHVLNYALREVLGDSVDQKGSLVAPEKLRFD

FSHKQALSTAELEKVEAISNKYIKDNKTVYYQDVSLSQAREINGLRAVFGETYPDPVRVVSIGVPVDTLLSNPKNPEWHE

VSIEFCGGTHVGETGEIKDLVVIEESGIAKGIRRIVAVTGHDARNVQRLASEFDQELDLADKLPFGQDKEARAKELGVRL

KQLSISVLEKQKLNAKFNKLDKSIKDNLKAIQKEESKKTLDVVKQWLDQEKPADFLVSHVPINANAKAITEAFNLIKKQD

KTKSVYLITGTSGKVAHGCYISDEAIAKGVDATVLAKAVSDKIGGKAGGKGNIVQGMGDKPEGINTAVEEVTKLLAEKL

>XP_003070819.1 Alanyl-tRNA synthetase, cytoplasmic, putative [Coccidioides posadasii C735 delta SOWgp]

MLSGRSSLGVLANRRAAGSTRKFFSPSTTFHPAAAAIAIPLFSRNKFVPLRPYPFTLLKMASSTSNPSQNEVEWPAIKVR

DTFLDFFKKNGHTFVPSSSVVPLSDPTLLFANAGMNQYKSIFLGTVDPNSDFAQLKRAHNSQKCIRAGGKHNDLDDVGKD

SYHHTFFEMLGNWSFGDYFKKEAIEYSWELLTKVYGLEPDRLYVTYFEGNKDAGIEPDLETKDLWLSVGVKEDHILSGNM

KDNFWEMGDQGPCGPCSEVHYDRIGGRNAAHLVNMDDPNVLEIWNNVFIQYNRESESILRPLPNKHVDTGMGYERLVSVL

QKKYSNYDTDVFTPLFDAIREITGVRPYSGKFGEEDLDGIDTAYRVVADHVRTLTFAISDGAPPNNEGRGYVVRRVLRRG

ARYARKYLKVEIGNFFSKIVPTLVDQMGGMFPEIKKKQTDVMEILDEEEISFAKTLDRGERQFEIYAQQAKDSGSNKLHG

ADVWRLYDTFGFPVDLTQLMAEERGLSIDNVEFEEARLKAKEASKGQAKAASDLLKLSVHDLSKLEKDKVPKTNDDAKFG

RGNILSQIVAVYHDKEFVDSTSGIPEGDQIGIILDKTNFYAEQGGQEYDTGKIIIDGKAELDVRNVQMYGGYVFHTGFIK

YGNLSVGDSVISEYDELRRWPIRNNHTGTHILNFALREVLGNGIDQKGSLVAAEKLRFDFSHKSGVSDSDLEKIEEKCTE

YIRQNCAVYSKDVPLSVAREITGVRAVFGETYPDPVRVVSVGVELEEILKDVKDPRWKGVSIEFCGGTHVQKTGDIKDLV

ILEEGGIAKGIRRIIAVTGEDAHEVQRIAKEFGDRLNRFEKMEIGPKKEQEAKLIQADLNQLSISAVEKARFREQFARIH

KQVLEAQKALQKQEIKAALDAINSHFEKPENKDSSHLIVRLPTSANAKAISESINHAKSKLKDKSIYVFAADAEQGRVAH

GCYVSSSLSAQGASASNWSGVVSGIVGGKAGGKAPTSIGNGTEVDRVDEAIEAATKYLEQFQL

>ODV83506.1 hypothetical protein CANARDRAFT_202947 [[Candida] arabinofermentans NRRL YB-2248]

MSSASKLEQWPASKVRSVFFDYFKENKHTFVPSSSVIPHDDPTLLFANAGMNQYKPIFLGTVDPTSPFATLKRAVNSQKC

IRAGGKHNDLEDVGRDSYHHTFFEMLGNWSFGDYFKKEAIEMSWKLLTKVYGLDPDRLYVTYFEGDSKLGLEPDLEAKEY

WKSVGVADDHILPGSAKDNFWEMGDQGPCGPCSEVHYDRIGGRNAAAMVNEDDPDVLEIWNNVFIQYNREADGSLRSLPA

KHVDTGMGFERLVSILQDVRSNYDTDVFQPLFAKIQEITGVRPYSGKFGKDDMDGIDTAYRVLADHVRTLTFALADGGVP

NSDGRGYVLRRILRRGARYVRKYMNYPIGKFFSQLSPTLIEQVKDVFPELAKDPSSIFEILDEEEASFARTLDRGEKLFE

QYAIIASKTPEQTLAGKDVWRLYDTYGFPVDLTRLMAEEAGLKIDDEAFEKARIASREASKGNGSKSGKELLKLDVHALG

SLDNNSAVPKTDDSYKYGTEDIKTKIVAIYSGKEFYESTKNIKEGEQIGLILEKTPFYAEQGGQEYDTGKIVIDGSAEFN

VENVQTYAGYVLHTGFITEGELSVGDDVIAGYDELRRWPLRNNHTGTHILNLALREILGNGVDQKGSLVAAEKLRFDFSH

KAGVSLKELEKIEKICTDTIKAALPVYSKPVTLETATAINGLRAVFGETYPDPVRVVSVGAPVDDLLKDPSNPKWIDYSV

EFCGGTHVSKTSDIKDLVIVEESGIAKGIRRIVAVTGLDAHEVQRIAKEFDDFLDQTEKLPFGDEKEKRLKEIGTKLGQA

SISTLQKAALKEKFTKIEKTVKDELKVRQKADTKKALDTVKEFFAENTEAPYLVKYVDIAANAKVITEAINLVKKENKSK

SLFLITGLKTDAKIAQGVYISEEHLSKVPATEVAELASTFIGGKAGGKGNVCQGMGTEVGGIDAAIAAVESLLKEKLQI

>EFW22234.1 alanyl-tRNA synthetase [Coccidioides posadasii str. Silveira]

MASSTSNPSQNEVEWPAIKVRDTFLDFFKKNGHTFVPSSSVVPLSDPTLLFANAGMNQYKSIFLGTVDPNSDFAQLKRAH

NSQKCIRAGGKHNDLDDVGKDSYHHTFFEMLGNWSFGDYFKKEAIEYSWELLTKVYGLEPDRLYVTYFEGNKDAGIEPDL

ETKDLWLSVGVKEDHILSGNMKDNFWEMGDQGPCGPCSEVHYDRIGGRNAAHLVNMDDPNVLEIWNNVFIQYNRESESIL

RPLPNKHVDTGMGYERLVSVLQKKYSNYDTDVFTPLFDAIREITGVRPYSGKFGEEDLDGIDTAYRVVADHVRTLTFAIS

DGAPPNNEGRGYVVRRVLRRGARYARKYLKVEIGNFFSKIVPTLVDQMGGMFPEIKKKQTDVMEILDEEEISFAKTLDRG

ERQFEIYAQQAKDSGSNKLHGADVWRLYDTFGFPVDLTQLMAEERGLSIDNVEFEEARLKAKEASKGQAKAASDLLKLSV

HDLSKLEKDKVPKTNDDAKFGRGNILSQIVAVYHDKEFVDSTSGIPEGDQIGIILDKTNFYAEQGGQEYDTGKIIIDGKA

ELDVRNVQMYGGYVFHTGFIKYGNLSVGDSVISEYDELRRWPIRNNHTGTHILNFALREVLGNGIDQKGSLVAAEKLRFD

FSHKSGVSDSDLEKIEEKCTEYIRQNCAVYSKDVPLSVAREITGVRAVFGETYPDPVRVVSVGVELEEILKDVKDPRWKG

VSIEFCGGTHVQKTGDIKDLVILEEGGIAKGIRRIIAVTGEDAHEVQRIAKEFGDRLNRFEKMEIGPKKEQEAKLIQADL

NQLSISAVEKARFREQFARIHKQVLEAQKALQKQEIKAALDAINSHFEKPENKDSSHLIVRLPTSANAKAISESINHAKS

KLKDKSIYVFAADAEQGRVAHGCYVSSSLSAQGASASNWSGVVSGIVGGKAGGKAPTSIGNGTEVDRVDEAIEAATKYLE

QFQL

>SCW01388.1 LAFE_0D11474g1_1 [Lachancea fermentati]

MTIGDKQKWTAKTVRSTFLDYFKERGHKFVPSSPVIPFDDPTLLFANAGMNQYKPIFLGTVDPSSDFYTLKRAVNSQKCI

RAGGKHNDLEDVGRDSYHHTFFEMLGNWSFGDYFKKEAIHYSWELLTKVYGIPEDRLYVTYFGGDEKLGLAPDLEARELW

KAAGVPDDHILPGDAKDNFWEMGDQGPCGPCSEIHYDRIGGRNAASLVNMDDPDVLEIWNNVFIQFNREQDGSLKTLPAQ

HIDTGMGFERLVSVLHDVRSNYDTDVFIPLFERIQAITGVRPYAGKFGGDDADGIDTAYRVLADHVRTLTFALADGGVPN

NEGRGYVLRRILRRGARYARKYMNYPIGNFFSQLAPTLIDQVQDMFPEVAKDPSYLYEILDEEEASFAKTLDRGEKLFEK

YAEAASKTESKTLDGKQVWRLYDTYGFPVDLTELMAEELNLKIDGPGFEKAKQESYEASKRGGKKGTSDLIKLNVHELST

LNTENVPKTNDSAKYESANIEATILKIYDGEKFVSEMTEEGKQYGIILDKTCFYAEQGGQEYDVGKIVIDGAAEFNVENV

QLYNGYVFHTGLLGEGKLSVGDKTIASYDELRRFPIRNNHTGTHILNFALMTVLGDDVDQKGSLVAPEKLRFDFSHKRAL

TMDELKKVEDISNGQIKKNMPVYYKNVPLDLAKTISSVRAVFGETYPDPVRVVSVGMPVDDLLANPSNEEWKKYSVEFCG

GTHVAKTGDIKEFVIIEESGIAKGIRRIVAVSGSEAHEVQRVASEFDSELDAVEKMPFSLLKEKKIKELGVKLNELTISV

VTKSALREKFAGIEKSVKDEVKSRAKKEIKQTLDEVKQYFAEHEDAQYFVKFIDIPTNAKAITEAVNHMKSSAKDKSIYL

FTGNDSSGRVAHGCYISDAALKKGVDGPQLVKLVSSSIGGKAGGKGNVFQGMGDKPQGIESAVDEVQRLFSEKVTL

>XP_007266315.1 hypothetical protein FOMMEDRAFT_167809 [Fomitiporia mediterranea MF3/22]

MAYNPYKGDWSSRKVRQTFFDYFKERGHTYVQSSSTIPYEDPTLLFANAGMNQFKAIFLGTVDPNSEMAKWKRVFNSQKC

IRAGGKHNDLDDVGKDSYHHTFFEMLGNWSFGDYFKKDAITFSWQLLTEVYGLPKDRLYVTYFEGDPKNGLEPDDEAREY

WKAQGLPDSHILTGNAKDNFWEMGATGPCGPCSEIHYDRIGGRNAAHLVNQDDPNVLEIWNNVFIQFNRESDGSLRPLPA

KHVDTGMGFERLVSVIQDRSSNYDTDIFEPLFKRIHELTGVRPYEGRFGKDDEDGVDMAYRVVADHVRTLSFALADGGVP

NNVGRGYVLRRILRRGARYARKKLGVQIGSFFSSLVPTVVEQLGDVFPELHKRTDEIKEILDEEEESFARTLDRGEKLFE

QYASKALQSPSKTLNGEDVWRLYDTFGFPVDLTLLMAEELGLGIDQEGFDKAQAKSKEASKGGVKKGDKDLVKLDVHDIA

SLDKNDTVPKTNDDAKFNLGNITATVKAIYHDKSFVNHTGAISEDTTFGIILDKTNFYAEAGGQEYDTGSITIDGVAEFE

VTNVQSYSGYVLHTGILKYGRLSVGDEVIATYDELRRWPLRNNHTATHILNFSLREVLGDHIDQKGSLVAPTKLRFDFSH

KSQISPTELQKIEDMSIDWIKRDVKVFSKELDLKSGYKIPGLRAVFGEAYPDPVRVVTLEYDVEEIAKDLENSKWRKTSI

EFCGGTHVAKTGDIKGFVIVEESGIAKGIRRVTAVTGHEAADVTRQAKTFTATLDKIEKASGKEKDVMLKTFQVELGQAD

ISVLKKNELRDRLAAIRKAFDKQTKEREQALQKAEIEKLTEFFKANPNANAYFAILDVEGNAKIIGSVITQARKLSKAAY

LFSIDTENNKVTHANHVPEAFRRGEDFNAKTWSAGVVEVIGGKAGGKDEGCQGVGVNTNKVQEALDVAKQLFSKVG

>SCU82138.1 LAFA_0C09274g1_1 [Lachancea sp. CBS 6924]

MTVGDKQKWTATNVRSTFLEYFGERNHKFVASSPVVPYDDPTLLFANAGMNQYKPIFLGTVDPSSDFYNLKSAMNSQKCI

RAGGKHNDLEDVGRDSYHHTFFEMLGNWSFGDYFKKEAISYAWELLTKVYGIPADRLYVTYFEGDAKQGLEADTEARDFW

RQVGVLDDHILTGNAKDNFWEMGEQGPCGPCSEIHYDRIGGRNASSLVNMDDPDVLEIWNIVFIQYNREQNGKLRPLPAK

HIDTGMGFERLVSVLQDVRSNYDTDVFQPLFARIQEITGARAYAGKFGAEDADGVDTAYRVLADHVRTLMFALADGGVPN

NEGRGYVLRRILRRGARYARKYLNYPIGNFFSTLAPTLIEQTKTMFPEIAKDPSYLFEILDEEEASFAKTLDRGEKLFEK

YAQAALETEAKTLDGLQVWRLYDTYGFPVDLTELMAEEQGLKIDGPGFEKAKQESYEASKKGGKKGVAELIKLDVHGLST

LNSEGIAKTDDSPKYTSANVEATILKIYDGENFVSSTGEVGSQYGVILDQTCFYAEQGGQEYDVGTLVIDGSTEFKVENV

QLYNGYVFHTGFVAEGELKVGDKIIASFDELRRFPIKNNHTGTHILNFALKEVLGGDIDQKGSLVAPEKLRFDFSHKKAL

TLDEVKKVEDLCGQQIKANLQVYYKNVPLQLAQQISSVRAVFGESYPDPVRVVSVGMPVDELLSNPANEEWQKYSVEFCG

GTHVNKTGDIKDFVITEESGIAKGIRRIVAVSGTEAHEVQRIASSFDAELEAAEKLPFSPLKEKKLKELGVSLGQLNISV

LAKNDLKTKFAKVEKAVKDELRTRAKKETKQTVDEVKNFFAENESAQFFVKYVDIPTNAKAITEAVNYMKTSAKDKTIYL

LNGSEKVAHGCYISDNALGKGVIGRDLLDRISRNIGGKAGGKANVFQGVGDKPDGIENAVKEVSELLQEKLSI

>GAO49636.1 hypothetical protein G7K_3785-t1 [Saitoella complicata NRRL Y-17804]

MSIWPRPLLQLNFRPVSVGIRCTSRFSRLLASRQVLRYSHSHNNANDRMAESQEWPSRKVRTTFLEYFKERGHTYVPSSS

VVPHDDPTLLFANAGMNQFKPIFLGTVDPTTEAAKWKRAVNSQKCIRAGGKHNDLEDVGRDSYHHTMFEMLGNWSFGDYF

KKEAIEYSWELLTKTFGLPADRIYVTYFEGSEKAGLGPDLETKNLWLSVGVAEDHILPGNMKDNFWEMGDQGPCGPCSEL

HFDRIGGRNAAHLVNMDDPNVLEIWNNVFMQYNREADGTLRTLPAQHVDTGMGFERLVSVLQNKMSNYDTDVFTPLFDKI

QELTGARPYGGAYGEEDVDGIDTAYRVVADHVRTLTFAICDGGVPNNEGRGYVLRRILRRGARYIRKKFNVPIGSFFSRL

APVLVDQMSDIFPELNNKMGDVKEILDEEEESFSRTLDRGEKMFEQYLKAAQAKGAKQLEGADVWRLYETYGFPVDLTRL

MAEENGLGVDEEGFEKAQEDSKAIARASRNAGGKGAELLKLDVHVLGKLAEMDEVPKTDDKYKYELGNINAQVKAIVLSN

KDFAQTTEGSREGEQIGILLDKTNFYAEQGGQEFDTGKIIIDGQAEFEVDNTQVYAGYVLHTGYIKYGSLKVNDEVICEY

DELRRWPIRNNHTGTHILNYALREVIGDNVHQRGSLVAPEKLRFDFTHKTGLTIPELAKVEELSNIAVKDNVKVYAKDVK

LDLAKEIAGVRAVFGETYPDPVRVVSVGVSIEDILADPKNERWEKTSIEFCGGTHVAKTGDIKDFVIVEESGIAKGIRRI

IAVTGEEAHRVSREANEFSARLDTLDKMALGPQKEAEVKSTSVELNNLSISAVRKQEFRDRFAKISKEVADAEKAKKNAE

SKETVEAVKKYIEENKEAQFMVAVVPNVSANPKALQEAIKHVSKNAKDKSVYLLAADAAADGKVAHACIVSKDAAGKGVN

AQEWSGAVSKIVGGRAGGKPDSCQGVGTEVSKVDEAVAAAEEYLKNLKL

>XP_445923.1 uncharacterized protein CAGL0E05390g [[Candida] glabrata]

MTIGDKQKWTAHNVRKTFMDYFEEKNHKYVKSSPVVPFDDPTLLFTNAGMNQYKPIFLGTVDPSSDFYTLKRAYNSQKCI

RAGGKHNDLEDVGKDSYHHTFFEMLGNWSFGDYFKKEAIDYAWKLLTEVYGIPADRLYVTYFEGDAKQGLEPDTEALDLW

KKVGVPEDHILPGNAKDNFWEMGDQGPCGPCSEIHYDRIGGRNAASLVNQDDPDVLEVWNLVFMQFNREQDGSLKSLPAK

HIDTGMGFERLVSVLQDVRSNYDTDVFMPLFARIQEITGARPYAGKFGDEDKDNIDTTYRVLADHVRTLVFALADGGVPN

NEGRGYVLRRILRRGARFARKYMNYPIGNFFSSLAPTLIDQVKHIFPEVAKDPSFLFEILDEEESSFSKTLDRGERIFEK

YAAAASQTESKTLDGKQVWRLYDTYGFPVDLTELMAEEQNLKIDGPGFEKAKQESYEASKRGGQKGGDDLIKLNVHSIAE

LNSENVPKTDDSFKYGNENVEGKILKLHDGEKFVTEISEPGKKYGIILDKTCFYAEQGGQEYDTGKIVIDDKAEFSVENV

QLYNGFVFHTGTLSEGKLSVDDVIIASYDELRRFPIKNNHTGTHILNYALKETLGNDIDQKGSLVAPEKLRFDFSHKKAL

TIEEVTKVENICNEQIKNNYNVYYKDVPLDLAKSIYSVRAVFGETYPDPVRVVSVGKPVDDLLANPSNEEWGKYSIEFCG

GTHVKTTGDIKYFVILEESGIAKGIRRIVAVTGTEAFEAQRVADQFAADLEAADKLPFSPLKEKKLKELGVKLGQLSISV

ISKNDLKTKFTKIEKVVKDEVKSRAKKEAKLVLDEVKGHFTENPESQFFVKFIDVPANAKAITEAVNYMKTSAKDKSMYL

ITGNDPEGRVAHGCFISDVALEKGIAGQELAGKVSGIIGGKAGGKGNVFQGVGDKPAAINDAINEITTAFKEKLSI

>XP_019022100.1 alanyl-tRNA synthetase [Saitoella complicata NRRL Y-17804]

MAESQEWPSRKVRTTFLEYFKERGHTYVPSSSVVPHDDPTLLFANAGMNQFKPIFLGTVDPTTEAAKWKRAVNSQKCIRA

GGKHNDLEDVGRDSYHHTMFEMLGNWSFGDYFKKEAIEYSWELLTKTFGLPADRIYVTYFEGSEKAGLGPDLETKNLWLS

VGVAEDHILPGNMKDNFWEMGDQGPCGPCSELHFDRIGGRNAAHLVNMDDPNVLEIWNNVFMQYNREADGTLRTLPAQHV

DTGMGFERLVSVLQNKMSNYDTDVFTPLFDKIQELTGARPYGGAYGEEDVDGIDTAYRVVADHVRTLTFAICDGGVPNNE

GRGYVLRRILRRGARYIRKKFNVPIGSFFSRLAPVLVDQMSDIFPELNNKMGDVKEILDEEEESFSRTLDRGEKMFEQYL

KAAQAKGAKQLEGADVWRLYETYGFPVDLTRLMAEENGLGVDEEGFEKAQEDSKAIARASRNAGGKGAELLKLDVHVLGK

LAEMDEVPKTDDKYKYELGNINAQVKAIVLSNKDFAQTTEGSREGEQIGILLDKTNFYAEQGGQEFDTGKIIIDGQAEFE

VDNTQVYAGYVLHTGYIKYGSLKVNDEVICEYDELRRWPIRNNHTGTHILNYALREVIGDNVHQRGSLVAPEKLRFDFTH

KTGLTIPELAKVEELSNIAVKDNVKVYAKDVKLDLAKEIAGVRAVFGETYPDPVRVVSVGVSIEDILADPKNERWEKTSI

EFCGGTHVAKTGDIKDFVIVEESGIAKGIRRIIAVTGEEAHRVSREANEFSARLDTLDKMALGPQKEAEVKSTSVELNNL

SISAVRKQEFRDRFAKISKEVADAEKAKKNAESKETVEAVKKYIEENKEAQFMVAVVPNVSANPKALQEAIKHVSKNAKD

KSVYLLAADAAADGKVAHACIVSKDAAGKGVNAQEWSGAVSKIVGGRAGGKPDSCQGVGTEVSKVDEAVAAAEEYLKNLK

L

>KNE56176.1 alanine-tRNA synthetase [Allomyces macrogynus ATCC 38327]

MPPKSKKSPQKQAPAAAAPKAAAAPAPTKAAPAAAAPAPKAAAPAPAPVAKAAPAAAPKAAVVAAPKEATPAPAAAPAWS

APLVRETFIKFFQDKHQHAFVPSSKTIPHDDPTLLFANAGMNQFKPVFLGTVDPSAPMAKLTRAANSQKCIRAGGKHNDL

DDVGKDTYHHTFFEMLGNWSFGNYFKKEAIEWAWDLLTNIFKLDKQRLYVTYFGGDAKQGLAADLEARDLWIKIGVPADR

VIPYGAKENFWEMGDQGPCGPCSEIHYDLIGGRFVPALVNADDPTLIEIWNLVFMQFNREADGSLRPLPAKHVDTGMGFE

RLVCALQAKMSNYDTDVFTPIFSRIQEVAKVRPYEGKLGAADADGIDMAYRVVADHIRTLTVAISDGGMPSNEGRGYVLR

RILRRGVRYARNAFKVPVGTFFSSLVDTVAEHMGAFFPELREHVETVKSVLDEEEAAFAKTLDRGLKLFDGILAKAGNAK

TISGADAWRLYDTFGFPVDLTRLMAEEKGMTVDEAAFEAEQAKAKEQSKKRKDQAGDKAIKMDVHAIKEAETLGFPPTDD

SYKYTAATLEANIAAIFHPHQEKKGASSHWVKSVSEGETVGILLNRTNFYAESGGQEYDTGSLVVDGEAEFVVENVQVYG

GYVLHVGTLKYGTLTVDQEVVAAYDELRRWPLRNNHTATHVLNYALRKVLGDGIDQKGSLVSAGKLRFDFSYRSAVTADQ

LRAIEKECSAVIAKALTVFAKDIPLAQAKSIHSLRAVFGEVYPDPVRVVSIGVAVEELLKNPNNAAWMDLSIELCGGTHA

PTTKELAAFVVMEESAIAKGVRRIVAVTGHEAAEAAKEGKAVLAEIAQLKSLSGHALGEAVKNATKDLEARTMSALDKAA

ARDLLASLKKQFDDWDKAQKAAKAAAATDEVKAYFADENAKPFLVRSLDVGGNGKALTSAINAVKSLPGKAALLLAVDGD

KVMYHVYVDKTLHGKITALQWADIVAQKVGGKKGGRDESAQGAGTNVNAVDEAVKLATEFAAMKLK

>KNE54528.1 alanine-tRNA synthetase [Allomyces macrogynus ATCC 38327]

MPPKSKKSPQKQAPAAAAPKAAAAPAPAKAAPAAAAPAPKAAAPTPAPVAKAAPAAAPKAAVVAAPKEATPAPVSPPAWS

APLVRETFIKFFQDKHQHAFVPSSKTIPHDDPTLLFANAGMNQFKPVFLGTVDPSAPMAKLTRAANSQKCIRAGGKHNDL

DDVGKDTYHHTFFEMLGNWSFGNYFKKEAIEWAWDLLTNIFKLDKQRLYVTYFGGDTKQGLAADLEARDLWIKIGVPADR

VIPYGAKENFWEMGDQGPCGPCSEIHYDLIGGRFVPALVNADDPTLIEIWNLVFMQFNREADGSLRPLPAKHVDTGMGFE

RLVCALQAKMSNYDTDVFTPIFSRIQEVAKVRPYEGKLGAADVDGIDMAYRVVADHIRTLTVAISDGGMPSNEGRGYVLR

RILRRGVRYARNAFKVPVGTFFSSLVDTVAEHMGAFFPELREHVETVKSVLDEEEAAFAKTLDRGLKLFDGILAKAGNAK

TISGADAWRLYDTFGFPVDLTRLMAEEKGMTVDEAAFEAEQAKAKEQSKKRKDQAGDKAIKMDVHAIKEAETLGFPPTDD

SYKYTAATLEANIAAIFHPHQEKKGASSHWVKSVSEGETVGILLNRTNFYAESGGQEYDTGSLVVDGEAEFVVENVQVYG

GYVLHVGTLKYGTLTVDQEVVAAYDELRRWPLRNNHTATHVLNYALRKVLGDGIDQKGSLVSAGKLRFDFSYRSAVTADQ

LRAIEKECSAVIAKALTVFAKDIPLAQAKSIHSLRAVFGEVYPDPVRVVSIGVAVEELLKNPNNAAWMDLSIELCGGTHA

PTTKELAAFVVMEESAIAKGVRRIVAVTGHEAAEAAKEGKAVLAEIAQLKSLSGHALGEAVKNATKDLEARTMSALDKAA

ARDLLASLKKQFDDWDKAQKAAKAAAATDEVKAYFADENAKPFLVRSLDVGGNGKALTSAINAVKSLPGKAALLMAVDGD

KVMYHVYVDKTLHGKITALQWADIVAQKVGGKKGGRDESAQGAGTNVNAVDEAVKLATEFAAMKLQ

>OWB81931.1 hypothetical protein B5S33_g551 [[Candida] boidinii]

MNSFGSKLYKLNIINNSNLHSNPFLKKIPFNNISNTINTTIIFNQRMSSTTATNNINSSSLTKEQIEWPASKVRNTFLEY

FKENGHTFVPSSSVVPHDDPTLLFANAGMNQYKPIFLGTVDPSSPLATLKRAVNSQKCIRAGGKHNDLEDVGRDSYHHTF

FEMLGNWSFGDYFKKEAIAFSWNLLTKVYALPKDRLYVTYFGGDEKLGLEPDLEAKEYWQQIGVDDDHILPGSAKDNFWE

MGDQGPCGPCSEIHYDRIGGRNASSLVNEDDPNVLEIWNNVFIQFNREADGSLKSLPAKHIDTGMGFERVVSILQNVYSN

YDTDVFEPLFSKIQEITGVRPYSGKFGKDDLDGIDTAYRVLADHVRTLSFAIADGGVPNNEGRGYVLRRILRRGARYARK

YMNYPIGKFFSQLAPTLIEQVKDIFPELSKDPSTIYEILDEEEASFARTLDRGEKLFEQYAIVASKTPEQTLSGKDVWRL

YDTYGFPVDLTRLMAEEAGLKIDEAGFEKAKLAAYEASKGNGKKAGKELVKLDVHALGYLDSNDSIPKTDDSFKYGTKDI

NSKIVAIYNGKEFIDSTDSLEEPTKQQIGILLDKSPFYAEQGGQEFDTGKIVIDGVSEFNVENVQAYAGYVLHTGFLVEG

SLKVNDSVIAGYDELRRWPLRNNHTGTHILNLALKKTLGDGIDQKGSLVAAEKLRFDFSHKSGISLDDLKKIEDICNKQI

EDNLQVFSKPVKLELAKEIKGLRAVFGETYPDPVRVVSVGIPVEDLLSNPSNEEWENYSIEFCGGTHVAKTNDIKKFVIT

EENGIAKGIRRIVALSGTEAFEAQRIANEFNDLLDSIDKLPFGDDKERKLKENGTKLSQLNVSLLDKDQLKNKFNKIDKE

VKNEIKIRQKADSKKTLDTVKNYFIENKDSEFLVAHVPINANAKVITEAINFLKKENKDKSLFLITGKQDESKIAQGVYI

SDAAIAKGLDAAEIANVASKFIGGKAGGKGNVCQGMGTESAGINDAIDAVEKLLKEKLSI

>KTB10709.1 Alanine--tRNA synthetase, mitochondrial [[Candida] glabrata]

MTIGDKQKWTAHNVRKTFMDYFEEKNHKYVKSSPVVPFDDPTLLFTNAGMNQYKPIFLGTVDPSSDFYTLKRAYNSQKCI

RAGGKHNDLEDVGKDSYHHTFFEMLGNWSFGDYFKKEAIDYAWKLLTEVYGIPADRLYVTYFEGDAKQGLEPDTEALDLW

KKVGVPEDHILPGNAKDNFWEMGDQGPCGPCSEIHYDRIGGRNAASLVNQDDPDVLEVWNLVFMQFNREQDGSLKSLPAK

HIDTGMGFERLVSVLQDVRSNYDTDVFMPLFARIQEITGARPYAGKFGDEDKDNIDTTYRVLADHVRTLVFALADGGVPN

NEGRGYVLRRILRRGARFARKYMNYPIGNFFSSLAPTLIDQVKHIFPEVAKDPSFLFEILDEEESSFSKTLDRGERLFEK

YAAAASQTESKTLDGKQVWRLYDTYGFPVDLTELMAEEQNLKIDGPGFEKAKQESYEASKRGGQKGGDDLIKLNVHSIAE

LNSENVPKTDDSFKYGNENVEGKILKLHDGEKFVTEISEPGKKYGIILDKTCFYAEQGGQEYDTGKIVIDDKAEFSVENV

QLYNGFVFHTGTLSEGKLSVDDVIIASYDELRRFPIKNNHTGTHILNYALKETLGNDIDQKGSLVAPEKLRFDFSHKKAL

TIEEVTKVENICNEQIKNNYNVYYKDVPLDLAKSIYSVRAVFGETYPDPVRVVSVGKPVDDLLANPSNEEWGKYSIEFCG

GTHVKTTGDIKYFVILEESGIAKGIRRIVAVTGTEAFEAQRVADQFAADLEAADKLPFSPLKEKKLKELGVRLGQLSISV

ISKNDLKTKFTKIEKVVKDEVKSRAKKEAKLVLDEVKGHFTENPESQFFVKFIDVPANAKAITEAVNYMKTSAKDKSMYL

ITGNDPEGRVAHGCFISDVALEKGIAGQELAGKVSGIIGGKAGGKGNVFQGVGDKPAAINDAINEITTAFKEKLSI

>KTA99939.1 Alanine--tRNA synthetase, mitochondrial [[Candida] glabrata]

MTIGDKQKWTAHNVRKTFMDYFEEKNHKYVKSSPVVPFDDPTLLFTNAGMNQYKPIFLGTVDPSSDFYTLKRAYNSQKCI

RAGGKHNDLEDVGKDSYHHTFFEMLGNWSFGDYFKKEAIDYAWKLLTEVYGIPADRLYVTYFEGDAKQGLEPDTEALDLW

KKVGVPEDHILPGNAKDNFWEMGDQGPCGPCSEIHYDRIGGRNAASLVNQDDPDVLEVWNLVFMQFNREQDGSLKSLPAK

HIDTGMGFERLVSVLQDVRSNYDTDVFMPLFARIQEITGARPYAGKFGDEDKDNIDTTYRVLADHVRTLVFALADGGVPN

NEGRGYVLRRILRRGARFARKYMNYPIGNFFSSLAPTLIDQVKHIFPEVAKDPSFLFEILDEEESSFSKTLDRGERLFEK

YAAAASQTESKTLDGKQVWRLYDTYGFPVDLTELMAEEQNLKIDGPGFEKAKQESYEASKRGGQKGGDDLIKLNVHSIAE

LNSENVPKTDDSFKYGNENVEGKILKLHDGEKFVTEISEPGKKYGIILDKTCFYAEQGGQEYDTGKIVIDDKAEFSVENV

QLYNGFVFHTGTLSEGKLSVDDVIIASYDELRRFPIKNNHTGTHILNYALKETLGNDIDQKGSLVAPEKLRFDFSHKKAL

TIEEVTKVENICNEQIKNNYNVYYKDVPLDLAKSIYSVRAVFGETYPDPVRVVSVGKPVDDLLANPSNEEWGKYSIEFCG

GTHVKTTGDIKYFVILEESGIAKGIRRIVAVTGTEAFEAQRVADQFAADLEAADKLPFSPLKEKKLKELGVKLGQLSISV

ISKNDLKTKFTKIEKVVKDEVKSRAKKEAKFVLDEVKGHFTENPESQFFVKFIDVPANAKAITEAVNYMKTSAKDKSMYL

ITGNDPEGRVAHGCFISDVALEKGIAGQELAGKVSGIIGGKAGGKGNVFQGVGDKPAAINDAINEITTAFKEKLSI

>SLM16879.1 alanine--tRNA synthetase [[Candida] glabrata]

MTIGDKQKWTAHNVRKTFMDYFEEKNHKYVKSSPVVPFDDPTLLFTNAGMNQYKPIFLGTVDPSSDFYTLKRAYNSQKCI

RAGGKHNDLEDVGKDSYHHTFFEMLGNWSFGDYFKKEAIDYAWKLLTEVYGIPADRLYVTYFEGDAKQGLEPDTEALDLW

KKVGVPEDHILPGNAKDNFWEMGDQGPCGPCSEIHYDRIGGRNAASLVNQDDPDVLEVWNLVFMQFNREQDGSLKSLPAK

HIDTGMGFERLVSVLQDVRSNYDTDVFMPLFARIQEITGARPYAGKFGDEDKDNIDTTYRVLADHVRTLVFALADGGVPN

NEGRGYVLRRILRRGARFARKYMNYPIGNFFSSLAPTLIDQVKHIFPEVAKDPSFLFEILDEEESSFSKTLDRGERLFEK

YAAAASQTESKTLDGKQVWRLYDTYGFPVDLTELMAEEQNLKIDGPGFEKAKQESYEASKRGGQKGGDDLIKLNVHSIAE

LNSENVPKTDDSFKYGNENVEGKILKLHDGEKFVTEISEPGKKYGIILDKTCFYAEQGGQEYDTGKIVIDDKAEFSVENV

QLYNGFVFHTGTLSEGKLSVDDVIIASYDELRRFPIKNNHTGTHILNYALKETLGNDIDQKGSLVAPEKLRFDFSHKKAL

TIEEVTKVENICNEQIKNNYNVYYKDVPLDLAKSIYSVRAVFGETYPDPVRVVSVGKPVDDLLANPSNEEWGKYSIEFCG

GTHVKTTGDIKYFVILEESGIAKGIRRIVAVTGTEAFEAQRVADQFAADLEAADKLPFSPLKEKKLKELGVKLGQLSISV

ISKNDLKTKFTKIEKVVKDEVKSRAKKEAKLVLDEVKGHFTENPESQFFVKFIDVPANAKAITEAVNYMKTSAKDKSMYL

ITGNDPEGRVAHGCFISDVALEKGIAGQELAGKVSGIIGGKAGGKGNVFQGVGDKPVAINDAINEITTAFKEKLSI

>XP_024367679.1 alanine--tRNA synthetase-like [Physcomitrella patens]

MAPTVVDDLVEAAQSVYLTGGSDKMDSSFAWPANRVRETFNSFFESKNHTFVPSSPVVPVDDPTLLFANAGMNQFKPIFL

GTVDPRSARGNLKRACNTQKCIRAGGKHNDLDDVGKDTYHHTFFEMLGNWSFGDYFKKEAIAWAWELLTSVYKLPKDRLY

ATYFGGDENLGLAADDEARDIWRGLLPESHVLPFGCKDNFWEMGDSGPCGPCSEIHFDRIGGRDASSLVNMDDPNCLEIW

NLVFIQFNREQDGSLKVLPAQHVDTGLGFERVTSVLQEKMSNYDTDLFTPIFGAIQKATGAREYSGKVGKDDTDNVDMAY

RVVADHIRTLSFAIADGSRPGNEGREYVLRRMLRRAVRYGREVLKAQEGFFSGLVDIVVEVMGEAFPELKKNQAKIKEII

SEEESSFSRTLVKGIEKFKKAAAEVKDGVLGAQDAFILWDTFGFPIDLTELMAEEMGLKVDKEGFNRTMEEAREKARGAR

GKAGGKALVMEAEATSQLQKLGILPTDDKPKYIWHKNPTVTVKAIFTSSGFEESTSDEDVGIVLDTTSYYAEQGGQIYDT

GVLEGPNGAFTVREVQIYGGYVLHIGSINNGGKISVGDKVTSQVDYARRAVVAPNHTCTHLLNYALKEVLGDHIDQKGSL

VAPDKLRFDFSHGKPIDSKDLGEIEAIVAQQIRDGMTVYAKEASLAEAKRIMGLRAVFGEVYPDPVRVVAIGRPVEDLLA

DPENPNWAKLSTEFCGGTHLSNTKEAEAFALVSEEGIAKGVRRVTALTMGAAKEAIHQSEVFSSQIDKASKLEGPALEKE

VASLKNSLENVVIPAAKKAEFRTRLGELQERIRKAQKAAAGSNLQTALNGAVSSADTAVAQNQPFCVLRIDVGLDTNAVR

EAVTLVMEKHKDLAVMIFSVDEQKNKVLVYAGVPDSAAKQGLKVLDWLRGSLAPVNGKGGGGKNGLAQGQGGNAAGIDEA

LTVAKDFATKVFGA

>SCU80107.1 LAME_0B01640g1_1 [Lachancea meyersii CBS 8951]

MTVGDKQKWTASNVRSTFLDFFAERSHKFVPSSPVVPYDDPTLLFANAGMNQYKPIFLGTVDPSSDFYTLKCAMNSQKCI

RAGGKHNDLEDVGRDSYHHTFFEMLGNWSFGDYFKKEAIAYAWELLTKVYGIPADRLYVTYFEGDAKQGLEADTEARDFW

RQVGVLDDHILTGDAKDNFWEMGDQGPCGPCSEIHYDRIGGRNASSLVNMDDPDVLEIWNIVFIQYNREQNGKLRPLPAK

HIDTGMGFERLVSVLQDVRSNYDTDVFQPLFSRIQEITGARAYSGKFGSEDVDGIDTAYRVLADHVRTLMFALADGGVPN

NEGRGYVLRRILRRGARYARKYLNYPIGNFFSTLAPTLIEQTKAMFPEIAKDPSYLFEILDEEEASFAKTLDRGEKLFEK

YAQAALQTESKTLDGLQVWRLYDTYGFPVDLTELMAEEQGLKIDGPGFEKAKQESYEASKKGGKKGASDLIKLDVHGLST

LNSENVPKTDDSTKYTSANVEATVLKIYDGEIFASGISEVGSQYGVILDQTCFYAEQGGQEYDVGTLVIDGSTEFKVENV

QLYNGYVFHTGFLAEGQLNVGDKIIASFDELRRFPIKNNHTGTHILNFALKEVLGGEVDQKGSLVAPEKLRFDFSHKKAL

SLEEVEKVENTCNKQIKANLPVYYKNVPLQLAQQISSVRAVFGETYPDPVRVVSVGKPVDDLLANPSSDEWQNYSVEFCG

GTHVNKTGDIKEFVITEESGIAKGIRRIVAVSGTEAHEVQRIALAFDAELTAAEKMSFSPLKEKKLKELGVQLGQLNISV

LSKNDLKNKFAKVEKAVKDELRSRAKKEIKQTVDEVKNFFAENESAQFLVKYVDIPTNAKAITEAVNYIKTSAKDKSIYL

LNGSEKVAHGCYISDSALEKGVVGRDLLDAISRNIGGKAGGKANVFQGVGDKPEGIENAVKEVSQLLQEKLSI

>OXB44458.1 hypothetical protein B1J91_E05390g [[Candida] glabrata]

MTIGDKQKWTAHNVRKTFMDYFEEKNHKYVKSSPVVPFDDPTLLFTNAGMNQYKPIFLGTVDPSSDFYTLKRAYNSQKCI

RAGGKHNDLEDVGKDSYHHTFFEMLGNWSFGDYFKKEAIDYAWKLLTEVYGIPADRLYVTYFEGDAKQGLEPDTEALDLW

KKVGVPEDHILPGNAKDNFWEMGDQGPCGPCSEIHYDRIGGRNAASLVNQDDPDVLEVWNLVFMQFNREQDGSLKSLPAK

HIDTGMGFERLVSVLQDVRSNYDTDVFMPLFARIQEITGARPYAGKFGDEDKDNIDTTYRVLADHVRTLVFALADGGVPN

NEGRGYVLRRILRRGARFARKYMNYPIGNFFSSLAPTLIDQVKHIFPEVAKDPSFLFEILDEEESSFSKTLDRGERLFEK

YAAAASQTESKTLDGKQVWRLYDTYGFPVDLTELMAEEQNLKIDGPGFEKAKQESYEASKRGGQKGGDDLIKLNVHSIAE

LNSENVPKTDDNFKYGNENVEGKILKLHDGEKFVTEISEPGKKYGIILDKTCFYAEQGGQEYDTGKIVIDDKAEFSVENV

QLYNGFVFHTGTLSEGKLSVDDVIIASYDELRRFPIKNNHTGTHILNYALKETLGNDIDQKGSLVAPEKLRFDFSHKKAL

TIEEVTKVENICNEQIKNNYNVYYKDVPLDLAKSIYSVRAVFGETYPDPVRVVSVGKPVDDLLANPSNEEWGKYSIEFCG

GTHVKTTGDIKYFVILEESGIAKGIRRIVAVTGTEAFEAQRVADQFAADLEAADKLPFSPLKEKKLKELGVRLGQLSISV

ISKNDLKTKFTKIEKVVKDEVKSRAKKEAKLVLDEVKGHFTENPESQFFVKFIDVPANAKAITEAVNYMKTSAKDKSMYL

ITGNDPEGRVAHGCFISDVALEKGIAGQELAGKVSGIIGGKAGGKGNVFQGVGDKPAAINDAINEITTAFKEKLSI

>XP_003686912.1 hypothetical protein TPHA_0H02750 [Tetrapisispora phaffii CBS 4417]

MTVGDKSKWMAKNVRQTFFDYFKSKGHTYVPSSSVVPFDDPTLLFTNAGMNQYKPIFLGTVDPSSDFATLKRAYNSQKCI

RAGGKHNDLEDVGRDSYHHTFFEMLGNWSFGDYFKKEAIAYAWELLTVVYGLPADRLYVTYFEGDAQQGLEPDLEAKDLW

LAVGVSEDHIIPGNSHDNFWEMGEQGPCGPCSEMHYDRIGGRNAAHLVNMDDPDVLEIWNLVFMQYNREQDRSLKSLPAK

HIDTGMGFERLVSILQDVRSNYDTDVFQPLFVKIQEITGVRPYSGKFGAEDVDGIDTAYRVLADHVRTLTFAITDGGIPN

NEGRGYVLRRILRRGARYARKYMNYPIGNFFSTLAPTLIEQVKEIFPQVTKDPAFLFEILDEEEASFAKTLDRGEKLFEK

YAEAAAKSQTKILDGLQVWRLFDTYGFPVDLTELMAEEQGLKIDEEGFEKAKQESYEASKRGGKKDNFDMLKLNVHELSQ

LNELKVPKTDDSAKYISTDAESVILSLYDGETFVDSISEAGKKYGVILDKTSFYAEQGGQEYDTGKIIIDGEAEFQVDNV

QLYNGYVFHTGTLIEGTLNKGDKTISSHDEERRLPIKNNHTGTHILNLALRQTLGNDVDQKGSLVAPEKLRFDFSHKKAL

SLDELRKVEEIANDQIKKNLPVYYKDVPLELAKGISSVRAVFGETYPDPVRVVSVGKTVEDLLADPTNEEWNKYSVEFCG

GTHVPKTGDIKFFVILEESGIAKGIRRIVAVSGAEAFEVQRIANEFDEELNKTEQLPFSQAKEKKLKELGVQLNQLSISV

IRKNELKEKFTKIEKVVKDEVKARAKVETKQVVDAVKEHFENNKDSLYFAQFIDIPANAKAITEAINHIKSKSKDKSIYL

LTGNDPEGRVAHGCYISDESLASGLDGAALAKQVSQLIGGKAGGKGNVFQGMGDKPEAIEEAVKTIEIAFNEKLSI

>EJS41521.1 ala1p [Saccharomyces arboricola H-6]

MTIGDKQKWTAANVRNTFLDYFKCKEHKFVKSSPVVPFDDPTLLFANAGMNQYKPIFLGTVDPASDFYTLKRAYNSQKCI

RAGGKHNDLEDVGKDSYHHTFFEMLGNWSFGDYFKKEAITYSWTLLTEVYGIPKNRLYVTYFEGDEKLGLEPDTEARELW

INVGVPEDHILPGNAKDNFWEMGDQGPCGPCSEIHYDRIGGRNASSLVNMDDPDVLEVWNLVFIQFNREQDGSLKSLPAK

HIDTGMGFERLVSVLQDVRSNYDTDVFTPLFERIQEITSARPYSGNFGEDDKDGIDTAYRVLADHVRTLTFALADGGVPN

NEGRGYVLRRILRRGARYARKYMNYPIGNFFSTLSPTLIAQVQDIFPELAKDPAFLFEILDEEEASFAKTLDRGERLFEK

YAAAASKTESKTLDGKQVWRLYDTYGFPVDLTELMAEEQGLKIDGPGFEKAKQESYEASKRGGKKDQSDLIKLNVHELSD

LNDAKVPKTNDEFKYGSANVESTILKLHDGTNFVEEITEQGKKYGVILDKTCFYAEQGGQEYDTGKIVIDDVAEFNVENV

QLYNGFVFHTGSLEEGKLSVGDKIIASFDELRRFPIKNNHTGTHILNLALKETLGNDIDQKGSLVAPEKLRFDFSHKKAV

TGDELKKIEDICNKQIKDNLQVFYKEIPLDLAKSIDGVRAVFGETYPDPVRVVSVGKPIDELLANPANEEWNNYSIEFCG

GTHVNKTGDIKFFVVLEESGIAKGIRRIVAVTGTEAFEAQRLADQFATDLDAADKLPFSPIKEKKLKELGVKLGQLSISV

ITKNELKQKFNKIEKAVKDEVKSRAKKENKQTLDEVKTFFETNEDAPYLVKFIDISPNAKAITEAINYMKSNDSVKDKSI

YLLAGNDPEGRVAHGCYISNAALTKGVDGSELAKKVSSIIGGKAGGKGNVFQGMGDKPAAIKNAVDDLESSFKEKLSI

>XP_018191610.1 hypothetical protein L228DRAFT_242453 [Xylona heveae TC161]

MPSIIEAEPEWSAVRVRAAFLEFFKKKGHTFVPSSSVVPLSDPTLLFTNAGMNQYKSIFLGTVDPQSDFAQLKRAVNSQK

CIRAGGKHNDLDDVGKDSYHHTFFEMLGNWSFGDYFKKEAIEYSWDLLTKVFGIAPDRLYVTYFEGYEAGGLEPDLEAKE

FWKSVGVPEDHILPGNMKDNFWEMGDQGPCGPCSEVHYDRIGGRNAAHLVNEDDPNVLEIWNNVFIQYNREADKSLRPLP

NKHVDTGMGFERLVSVLQNKESNYDTDVFSPLFKKIQEITGAREYRGKFGSEDPDGIDTAYRVCADHVRTLTFAISDGGI

PNNEGRGYVVRRVLRRGARYARKYFETDIGNFFSRIVPTLVEQMGGMFPEIAKKEKDVKEILDEEEESFSKTLDRGERMF

EHYAQQCKIHDSKKLHGADVWRLYDTFGFPVDLTRLMAEERGLDIDEKEFQEAQAKAKEASKGEKKAVADLVKFDVHDLG

NLDKMHEVPKTDDSAKFAKGNITSQIKAIFHSKKFLNSTSEIPEGEQLGVLLDRTNFYAEQGGQEYDTGKILIDGHAELE

VQNVQSYAGYVLHTGYLKYGNLTVGNEVICEYDELRRWPIRNNHTGTHILNYALREVLGDAIDQKGSLVAAEKLRFDFSH

KSSISDAELARIEDICSDYIERNDTVYAKDVPLATAREIQGVRAVFGETYPDPVRVVSVGVDIETLLHNIKNPDWEHVSV

EFCGGTHVQKTGDIKRLVILEESGIAKGIRRIIAVTGEEAYEVQRIANEFSDRLDHLTKMPHSSEKEQAVKQTQTELNQL

SISAITKASFRDRFAKISKQVLDEQKAKQKAEAKEAVDTITKYFADEKNKDKKHLVIKLPISANSKALGEALNFVKSKNS

DKTVYLFAADSTESKVVHGCHVSSDASSKGASANEWASAVSGVIGGKAGGKGATCIGNGTQADKVDEALSKAEEYLSKLS

I

>XP_020062456.1 alanyl-tRNA synthetase, cytoplasmic [Suhomyces tanzawaensis NRRL Y-17324]

MSTSHSDWSASKVRSTFLEFFKTKKNHTFVPSSSVVPHNDPTLLFANAGMNQYKPIFLGTVDPLSDFATLKRSVNSQKCI

RAGGKHNDLEDVGRDSYHHTFFEMLGNWSFGDYFKKEAISWSWELLTEVYKLDKDRLYVTYFGGDEKQGLEPDLEAKNFW

SAVGVADDHILPGDAKDNFWEMGDQGPCGPCSEIHYDRIGGRNAAHLVNMDDPNVLEIWNIVFIQYNREADASLRSLPAK

HIDTGMGFERLVSILQNKYSNYDTDVFLPIFDKIREITGVRPYTGKFGAEDVDGIDTAYRVIADHVRTLTFAICDGGVPN

NEGRGYVLRRILRRGSRYVRKYMNYPIGTFFQQLVDVVIEQNKEIFPEIVKGAQDLKEILNEEELSFAKTLDRGEKLFEQ

YAIIASKTPEQTLSGKDVWRLYDTYGFPVDLTRLMAEEAGLMIDEPGFEKAKEQSREASKATGNKDTATLVKLDVHSLSE

LDSNDDVPKTDDSYKYGLENIKANIVAIYDGSAFVSSISDSTKQYGILLDKSPFYAEQGGQEYDTGKLVIDGKAEFNVAN

VQVYAGYVLHTGSLTEGTLSVKDEVIAAYDELRRWPIRNNHTGTHVLNYALREVLGDSVDQKGSLVAPEKLRFDFSHKQA

LKPEELAKVENISNEYIKADKAVYSKEVSLAAAKEIYGLRAVFGETYPDPVRVVSIGAPVDELVANPKNEDWNKLSIEFC

GGTHVAKTGEIKDLVVIEESGIAKGIRRIVAVTGNEAHEVQRIAKDFDTELNHANGLPFGPAKEAKSKELGVALKKLSIS

VLDKQKLTEKFNKLDKAIKDELKTKQKEESKKTLEVVNEWLAKEDAGEYLVSHIPINANAKAITEAINLIKKSHKTKSIY

LITGATDKVAHGCYVSDEAIAKGIDTTALGQAVSTKIGGKAGGKGNIVQGMGDKPEGIQAAIDEVAKLFNEKL

>OWB61758.1 hypothetical protein B5S29_g2661 [[Candida] boidinii]

MNLFGSKLLKLNIINKSNFHLKPFLKKIPFNNNLNTINIIIFNQKMSSSSNNTSSLTKEQIEWPASKVRNTFLEYFKENG

HTFVPSSSVVPHDDPTLLFANAGMNQYKPIFLGTVDPSSPLATLKRAVNSQKCIRAGGKHNDLEDVGRDSYHHTFFEMLG

NWSFGDYFKKEAIAFSWNLLTKVYALPKDRLYVTYFGGDEKLGLEPDLEAKEYWQQIGVDDDHILPGSAKDNFWEMGDQG

PCGPCSEIHYDRIGGRNASSLVNEDDPNVLEIWNNVFIQFNRESDGSLKSLPAKHIDTGMGFERVVSILQNVYSNYDTDV

FEPLFSKIQEITGVRPYSGKFGKDDIDGIDTAYRVLADHVRTLSFAIADGGVPNNEGRGYVLRRILRRGARYVRKYMNYP

IGKFFSQLAPTLIEQVKDIFPELSKDPTTIYEILDEEEASFARTLDRGEKLFEQYAIVASKTPEQTLSGKDVWRLYDTYG

FPVDLTRLMAEEAGLKIDEEGFEKAKLAAYEASKGNGKKAGKELVKLDVHALGYLDSNDSIPKTDDSFKYGTKDINSKIV

AIYNGKEFIDSTDSLEEPTKQQIGILLDNSPFYAEQGGQEFDTGKIVIDGVSEFNVENVQVYAGYVLHTGFLVEGSLKVN

DSVIAGYDELRRWPLRNNHTGTHILNLALRKTLGDGIDQKGSLVAAEKLRFDFSHKSGISLDDLKKIEDICNKQIEDNLQ

VYSKPVELELAKEIKGLRAVFGETYPDPVRVVSVGIPVEDLLSNPSNEEWENYSIEFCGGTHVAKTNDIKKFVITEENGI

AKGIRRIVALSGTEAFEAQRIANEFNSLLDSIDKLPFGDEKEKRLKENGTKLSQLNVSLLDKDQLKNKFNKIDKEVKNEI

KIRQKADSKKTLDTVKNYFIENKDSEFLVTHVPINANAKVITEAINFLKKENKDKSLFLITGKQDESKIAQGVYISDVAI

AKGLDAAEIANVASKFIGGKAGGKGNVCQGMGTESAGINDAIDAVEKLLKEKLSI

>OWB58433.1 hypothetical protein B5S28_g4464 [[Candida] boidinii]

MNLFGSKLLKLNIINKSNFHLKPFLKKIPFNNNLNTINIIIFNQKMSSSSNNTSSLTKEQIEWPASKVRNTFLEYFKENG

HTFVPSSSVVPHDDPTLLFANAGMNQYKPIFLGTVDPSSPLATLKRAVNSQKCIRAGGKHNDLEDVGRDSYHHTFFEMLG

NWSFGDYFKKEAIAFSWNLLTKVYALPKDRLYVTYFGGDEKLGLEPDLEAKEYWQQIGVDDDHILPGSAKDNFWEMGDQG

PCGPCSEIHYDRIGGRNASSLVNEDDPNVLEIWNNVFIQFNRESDGSLKSLPAKHIDTGMGFERVVSILQNVYSNYDTDV

FEPLFSKIQEITGVRPYSGKFGKDDIDGIDTAYRVLADHVRTLSFAIADGGVPNNEGRGYVLRRILRRGARYVRKYMNYP

IGKFFSQLAPTLIEQVKDIFPELSKDPSTIYEILDEEEASFARTLDRGEKLFEQYAIVASKTPEQTLSGKDVWRLYDTYG

FPVDLTRLMAEEAGLKIDEEGFEKAKLAAYEASKGNGKKAGKELVKLDVHALGYLDSNDSIPKTDDSFKYGTKDINSKIV

AIYNGKEFIDSTDSLEEPTKQQIGILLDNSPFYAEQGGQEFDTGKIVIDGVSEFNVENVQVYAGYVLHTGFLVEGSLKVN

DSVIAGYDELRRWPLRNNHTGTHILNLALRKTLGDGIDQKGSLVAAEKLRFDFSHKSGISLDDLKKIEDICNKQIEDNLQ

VYSKPVELELAKEIKGLRAVFGETYPDPVRVVSVGIPVEDLLSNPSNEEWENYSIEFCGGTHVAKTNDIKKFVITEENGI

AKGIRRIVALSGTEAFEAQRIANEFNSLLDSIDKLPFGDEKEKRLKENGTKLSQLNVSLLDKDQLKNKFNKIDKEVKNEI

KIRQKADSKKTLDTVKNYFIENKDSEFLVTHVPINANAKVITEAINFLKKENKDKSLFLITGKQDESKIAQGVYISDVAI

AKGLDAAEIANVASKFIGGKAGGKGNVCQGMGTESAGINDAIDAVEKLLKEKLSI

>ODH13392.1 alanine-tRNA synthetase [Paracoccidioides brasiliensis]

MTSTQRVEQPEWPALRVRNAFLDFFKDNGHTFVPSSSVVPLSDPTLLFANAGMNQYKAIFLGTVEPNSDFAQLKRAHNTQ

KCIRAGGKHNDLDDVGKDSYHHTFFEMLGNWSFGDYFKKEAIRYSWDLLTKVYGLDPDRLYVTYFEGNPDAGIEPDLEAR

DLWLSVGVAEDHLLSGNMKDNFWEMGEQGPCGPCSEIHYDRIGGRNASHLVNQDDPNVLEIWNNVFIQYNREQDKSLRPL

PNKHVDTGMGYERLVSILQNKSSNYDTDVFTPLFEKIREITGSRPYTGKFHEEDVDGVDTAYRVVADHIRTLTFAISDGA

VPNNEGRGYVVRRVLRRGARYARKYLNVEIGGFFSQIVPTLVEQMGDMFPEIKRKATDVMEILDEEEISFAKTLDRGERL

FEEYALQAKQKGLDRLHGADVWRLYDTFGFPVDLTQLMAEERGLKIDNGEFEEARLRAKEASKGQGKLTKDVVKLTVHDL

GLLETMDDVPKTDDSAKFGRGNITSQIKAIYHNKKFVDSSKDIPEGEQFGIILDRTNFYAEQGGQEYDTGKIIIDGKAEL

AVEDVQVYGGYVLHTGFMKYGSFNVNDSVIAEYDELRRWPIRNNHTGTHILNFALRKVLGDGVEQKGSLVAAEKLRFDFS

HKSAVSDSDLEEIERISTDYIRQNCAVYGKDVPLSIAREITGVRAVFGETYPDPVRVVSVGVELEEILKDVHDPRWKEIS

IEFCGGTHVQKTGDIKDLVILEESGIAKGIRRIIAVTGEEAHEVQRIAKEFGERLKRFEKMELGPKKEMEAKLLQVDLNQ

LTISAVEKAQFREKFTQIHKKVLEGQKAAQKLESKKALDAIMGYFQAPENKDAPHLVLQLPISANAKAVSDSLNFVKTKM

QDKSLYVFAADKEAGKIVHGCHVAENLSQQGASPNDWANSVANVVGGRAGGKGPISIGNGTDAEKLDDAIKAATEYLEKF

KL

>KIM31429.1 hypothetical protein M408DRAFT_327641 [Serendipita vermifera MAFF 305830]

MSSAPYEGPWTASKVRETYIDFFKSKGHTFWPSSSTIPYEDPTLLFANAGMNQYKAVFLGTVDPNSDMSKLKRAVNSQKC

IRAGGKHNDLEDVGRDSYHHTFFEMLGNWSFGDYFKREALSWAWELLTEVYKLPADRLYVSYFEGDPKQGLLPDEETKQI

WLDMGVPEDRIVKGNAKDNFWEMGATGPCGPCSEIHYDRIGGRNAAHIVNADDPNVVEIWNNVFIQFNRENDGSLKTLPA

KHVDTGMGFERLVSAIQDRLSNYDTDVFRPIFERIQEVSGVRPYSGKFGAEDVDGIDTAYRVIADHARTLTFALSDGGIP

SNIGRGYVLRRILRRGARYARKKLNVPIGSFFSSISPVLVEQMGGFFPELKKTPEEVKELLDEEEISFAKTLDRGEKLFD

NYVAQTKERGGTVLDGKDVWRLYDTYGFPVDLTRLMAEELGLTVDEEAFAAAQAHSKEASKGAAKVDAKDTVKLDVHDIG

ALEANSSVAVTDDNAKYTSGSISATIKAIYHDKTFPSSTSNIPNGASFGIILDKTCFYAESGGQEYDTGSIALDMKDREA

KFEVVNCQSFKGYVLHIGQVEEGQLDVGDEVIVTYDPDRRRPIRSNHTATHILNYALRETLGDHVDQKGSLVAPSKLRFD

FSHKAPVKPEELQKIEAICLEWIKKDVPVFSKELDLKSARVIPGLRAVFGETYPDPVRVVTLEYDVEEIAKDLENPKWRG

TSVEFCGGTHVAKTGDIKEFTIMEESGIAKGIRRLIAVTGPEAREAMAVAASFESRFASVQSMTGKDKDEGFKTLTLELN

QADMPAILKTDLRDRVAAKRKVWDAEVKARETAATKAATESVAKYFQENPNAMGYFAQASVGSNVKAVQTILNQGKSLGK

AVYILSVDEHAGKVTHGNYLPAELAGEGFDARSWATSVAEVLGGKVGGKPDTAQGVGTDISKVEEGVKVAQEVFTKATQK

>XP_017988050.1 HEL227Cp [Eremothecium sinecaudum]

MTIGDKEKWTATNVRNTFLNYFKERGHKFVPSSSVIPYDDPTLLFANAGMNQYKPIFLGTVDPASDFYTLKRAVNSQKCI

RAGGKHNDLEDVGRDSYHHTFFEMLGNWSFGDYFKKEAILYSWELLTKVYGIPEDRLYVTYFGGDEGLGLGPDLEARDLW

LEVGVRQDRILPGDIKDNFWEMGDQGPCGPCSEIHYDRIGGRNASDLVNQDDPDVLEIWNNVFMQFNREQDGSLKPLPAK

HVDTGMGFERLVSVLQDVRSNYDTDVFQPLFERIQEITGVRPYEGKFGAEDKDGIDTAYRVLADHVRTSTFALADGGVPN

NEGRGYVLRRILRRGARYARKYMNYPIGNFFSQLAPTLIEQVKDVFPEVAKNTSYLFDILDEEEASFAKTLDRGEKLFEK

YAEAAALTESKTLDGKQVWRLYDTYGFPVDLTELMAEEKGLKIDSVGFEKAKLESYEASKKGGRKGGDDMIKLNVHDISK

LNEDAISRTDDSAKYGTKDIEGKILCIHDGEGFVQEMNEIGKQYGIILDKTCFYAEQGGQEYDVGKIVIDGVSEFNVENV

QLYNGYVFHTGVLADGKLAVNDSVIASYDELRRYPIRNNHTGTHILNFALREVLGDNIDQRGSLVNPEKLRFDFSHKKPL

SYEELAEVENICNRMIKDNLNVYYKDVPLDMAKSIYSVRAVFGETYPDPVRVVSVGMPVEDMLENPSNEKWASYSIEFCG

GTHVAKTGDIKEFVITEESGIAKGIRRIVAVSNTEAYQVQRVATEFEAELSACEKLPFSQYKEKKVKELGVRLSELDISV

ITKNELRMTFNKMEKAVKDEVKLRAKKETKQTLDEVKNHFAENAESDFFVKFIDIPANAKAITEAIQYLKNSAKDKSIYL

ITGNDPTGKVAHGCFISNDAISKGLDGSALARAVAGKIGGKAGGKDNVFQGMGDKPAGIEAALSEIEAAFKEKLTI

>XP_010755650.1 alanyl-tRNA synthetase [Paracoccidioides brasiliensis Pb18]

MGVGGERVSGVVLHQGLSLTRYCSRSVCRNLVEVVGVNVGGSVPVPLPPSRRFFAWHFFSPAITNTFSTTPLFFRRHNST

LPRSQAAFNMTSTQMVEQPEWPALRVRNAFLDFFKDNGHTFVPSSSVVPLSDPTLLFANAGMNQYKAIFLGTVEPNSDFA

QLKRAHNTQKCIRAGGKHNDLDDVGKDSYHHTFFEMLGNWSFGDYFKKEAIRYSWDLLTKVYGLDPDRLYVTYFEGNPDA

GIEPDLEARDLWLSVGVAEDHLLSGNMKDNFWEMGEQGPCGPCSEIHYDRIGGRNASHLVNQDDPNVLEIWNNVFIQYNR

EQDKSLRPLPNKHVDTGMGYERLVSILQNKSSNYDTDVFTPLFEKIREITGSRPYTGKFHEEDVDGVDTAYRVVADHIRT

LTFAISDGAVPNNEGRGYVVRRVLRRGARYARKYLNVEIGGFFSQIVPTLVEQMGDMFPEIKRKATDVMEILDEEEISFA

KTLDRGERLFEEYALQAKQKGLDRLHGADVWRLYDTFGFPVDLTQLMAEERGLKIDNGEFEEARLRAKEASKGQGKQTKD

VVKLTVHDLGLLETMDDVPKTDDSAKFGRGNITSQIKAIYHNKKFVDSSKDIPEGEQFGIILDRTNFYAEQGGQEYDTGK

IIIDGKAELAVEDVQVYGGYVLHTGFMKYGSFNINDSVIAEYDELRRWPIRNNHTGTHILNFALRKVLGDGVEQKGSLVA

AEKLRFDFSHKSAVSDSDLEEIERISTDYIRQNCAVYGKDVPLSIAREITGVRAVFGETYPDPVRVVSVGVELEEILKDV

HDPRWKEISIEFCGGTHVQKTGDIKDLVILEESGIAKGIRRIIAVTGEEAHEVQRIAKEFGERLKRFEKMELGPKKEMEA

KLLQVDLNQLTISAVEKAQFREKFTQIHKKVLEGQKAAQKLESKKALDAIMGYFQAPENKDAPHLVLQLPISANAKAVSD

SLNFVKTKMQDKSLYVFAADKEAGKIVHGCHVAENLSQQGASPNDWANSVANVVGGRAGGKGPISIGNGTDAEKLDDAIK

AATEYLEKFKL

>ODH46028.1 alanine-tRNA synthetase [Paracoccidioides brasiliensis]

MGVGGERVSGVVLHQGLSLTRYCSRSVCRNLVEVVGVNVGGSVPVPLPPSRRFFAWHFFSPAITNTFSTTPLFFRRHNST

LPRSQAAFNMTSTQRVEQPEWPALRVRNAFLDFFKDNGHTFVPSSSVVPLSDPTLLFANAGMNQYKAIFLGTVEPNSDFA

QLKRAHNTQKCIRAGGKHNDLDDVGKDSYHHTFFEMLGNWSFGDYFKKEAIRYSWDLLTKVYGLDPDRLYVTYFEGNPDA

GIEPDLEARDLWLSVGVAEDHLLSGNMKDNFWEMGEQGPCGPCSEIHYDRIGGRNASHLVNQDDPNVLEIWNNVFIQYNR

EQDKSLRPLPNKHVDTGMGYERLVSILQNKSSNYDTDVFTPLFEKIREITGSRPYTGKFHEEDVDGVDTAYRVVADHIRT

LTFAISDGAVPNNEGRGYVVRRVLRRGARYARKYLNVEIGGFFSQIVPTLVEQMGDMFPEIKRKATDVMEILDEEEISFA

KTLDRGERLFEEYALQAKHKGLDRLHGADVWRLYDTFGFPVDLTQLMAEERGLKIDNGEFEEARLRAKEASKGQGKLTKD

VVKLTVHDLGLLETMDDVPKTDDSAKFGRGNITSQIKAIYHNKKFVDSSKDIPEGEQFGIILDRTNFYAEQGGQEYDTGK

IIIDGKAELAVEDVQVYGGYVLHTGFMKYGSFNVNDSVIAEYDELRRWPIRNNHTGTHILNFALRKVLGDGVEQKGSLVA

AEKLRFDFSHKSAVSDSDLEEIERISTDYIRQNCAVYGKDVPLSIAREITGVRAVFGETYPDPVRVVSVGVELEEILKDV

HDPRWKEISIEFCGGTHVQKTGDIKDLVILEESGIAKGIRRIIAVTGEEAHEVQRIAKEFGERLKRFEKMELGPKKEMEA

KLLQVDLNQLTISAVEKAQFREKFTQIHKKVLEGQKAAQKLESKKALDAIMGYFQAPENKDAPHLVLQLPISANAKAVSD

SLNFVKTKMQDKSLYVFAADKEAGKIVHGCHVAENLSQQGASPNDWANSVANVVGGRAGGKGPISIGNGTDAEKLDDAIK

AATEYLEKFKL

>KZZ88409.1 alanyl-tRNA synthetase [Ascosphaera apis ARSEF 7405]

MSSVAAGQEAATQWPAKKVRDTFLDYFKQNGHTFVPSSSVVPLSDPTLLFANAGMNQYKSIFLGTVDPNSDFAHLKRANN

SQKCIRAGGKHNDLDDVGKDSYHHTFFEMLGNWSFGDYFKKEAIHYSWELLTKVYGLDPDRLYVTYFEGKPDAGLEPDLE

SKQYWLDAGVAEDHILTGDMKDNFWEMGDQGPCGPCSEIHYDRIGGRNASSLVNQDDPNVLEIWNNVFIQYNRESDGSLK

PLPARHVDTGMGYERLVSVLQNKSSNYDTDVFTPLFDRIQEITGARAYTGKFGVEDADGVDTAYRVVADHVRTLSFAIAD

GAPPNNEGRGYVVRRVLRRGARYARKYLGVDIGNFFSKIVPTLVDQMGDMFPELKRKASDICEILNEEELSFAKTLDRGE

KLFENYADQAKKNGLTKLHGADVWRLYDTYGFPVDLTQLMCEERGLTIDNNEFEEARLKAREASKGQGKVAGETVKLSVH

DIGELDAREDIQKTDDSAKYGRDDIDATVKAIYYNGQFINDTTSVPENARVGVVLDRTNFYAEQGGQEYDTGHIMIDEKV

ELEVLDVQQYGGYVLHVGYLKYGVLTTGDKVICEYDELRRWPIRNNHTGTHILNFALREVLGDGVEQKGSLVAAEKLRFD

FSHKSAISDKDLDTVENKCTDYIRQNCSVYSQEVPLSIARNIAGVRAVFGETYPDPVRVVSVGVELEEILKDVSDERWKD

ISIEFCGGTHVAKTGDIKELIILEESGIAKGIRRIIAVTGEDALRAQIVAKDFEKRLDAFEKMENGPAKEQEAKVMQTDL

NQLTISAVTKSKFRERFTAAHKKLMDAQKAIQKAESKKALDTITAYFKDEQNKDKPYLVVKLPIGANAKAVSDSLNHMKS

KVPEKSLYVFAADDSKVAHGCYVAKNLSEQGASANDWAGAVSKIVGGRSGGKAPTSIGNGPEASKVDDALEAATEYLKNF

KL

>XP_002796769.2 alanyl-tRNA synthetase [Paracoccidioides lutzii Pb01]

MGVGGERVSGVVLHQGLSLTRYCSRSVYCNLVEVVGVNVGGSVPVPLPPSRRFFAWHLFSPAITNTLSTTPLFFRRHNST

LPRSQAAPNMTSTQRVEQPEWPALRVRNAFLDFFKDNGHTFVPSSSVVPLSDPTLLFANAGMNQYKAIFLGTVEPNSDFA

QLKRAHNTQKCIRAGGKHNDLDDVGKDSYHHTFFEMLGNWSFGDYFKKEAIRYSWDLLTKVYGLDPDRLYVTYFEGNPDA

GIEPDLEARDLWLSVGVAEDHLLSGNMKDNFWEMGEQGPCGPCSEIHYDRIGGRNASHLVNQDDPNVLEIWNNVFIQYNR

EQDKSLRSLPNKHIDTGMGYERLVSILQNKSSNYDTDVFTPLFEKIREITGSRPYTGKFHEEDVDGVDTAYRVVADHIRT

LTFAISDGAVPNNEGRGYVVRRVLRRGARYARKYLNVEIGGFFSQIVPTLVEQMGDMFPEIKRKATDVMEILDEEEISFA

KTLDRGERLFEEYALQAKQKGLDRLHGADVWRLYDTFGFPVDLTQLMAEERGLKIDNGEFEEARLRAKEASKGQGKLTKD

VVKLTVHDLGLLETMDDVPKTDDSAKFGRGNITSQIKAIYHNKKFVNSSKDIPEGEQFGIILDRTNFYAEQGGQEYDTGK

IIIDGKAELTVEDVQVYGGYVLHTGFMKYGSFNVNDSVIAEYDELRRWPIRNNHTGTHILNFALRKVLGDGVEQKGSLVA

AEKLRFDFSHKSAVSDSDLEEIERISTDYIRQNCAVYGKDVPLSIAREITGVRAVFGETYPDPVRVVSVGVELEEILKDV

HDPRWKEISIEFCGGTHVQKTGDIKDLVILEESGIAKGIRRIIAVTGEEAHEVQRIAKEFGERLKRFEKMELGPKKEMEA

KLLQVDLNQLTISAVEKAQFREQFTQIHKKVLEGQKAAQKLESKKALDAIMGYFQAPENKDAPHLVLQLPISANAKAVSD

SLNFVKTKMQDKSLYVFAADKEAGKIVHGCHVAENLSQQGASPNDWANSVANVVGGRAGGKAPISIGNGTDADKLDEAIK

AATEYLEKFKL

>PJF18581.1 hypothetical protein PSACC_01613 [Paramicrosporidium saccamoebae]

MIVGLPIMNGYFLTRFTFSVISIKTCYEINVELSSELELSNRRPTASTRTLASLLFRRTARRLMSFSDKSRVESLTPFPS

SVPFTDARRLEYWTAQKTRQTFLDYFVEACGHSFVPSSATIPHDDPTLLFANSGMAQFKTIFQGVIDPAAPFAKLKRAAN

SQKCIRAGGKHNDLEDVGKDVYHHTFFEMLGNWSFGDYFKKEAISMAWELLTTVYGLPKERLYVTYFGGDERCNLPADLE

AKQLWMEAGVAEERVLPFGMKENFWEMGESGPCGPCSEIHFDRIGGRDASAMVNMDDPDVLEIWNLVFMQFNRELDGSLR

SLPNKHVDTGMGFERVLSVLQNKRSNYDTDVFMPIFAEIEQRSGCRPYSGKVGRSEDVDGIDMAYRVIADHIRTLTFSIS

DGGCPSNDGRGYVLRRILRRAVRYSHDKLKASPGFFASLVDIVVERFGDAFPEIKKSPELVKELLLEEETQFRKTLERGL

VQFGKFSKTAKDGVLSGSDVWRLYDTYGFPVDLTRLMAEEANLRIDEDGFLVAQSTAKELSRAGKHSDSDNPLNKVTIDV

HLASDLEKKHTTPITDDSFKYTDSIVEAKIVGLISEGEVVSSLPSTSDVKYFGVLLDRTNFYAESGGQVYDEGVITGADG

QMEFAVEAVQSYGGYVLHVGYLKYGGISVGENVIATFDETRRRPLRHNHTATHLLNYAIGNVMTEQNPDQKGSLVAPDRL

RFDFNSTKAPSSEQVEQIESIVNGFIEKNFEVSTMPLPLEQAMALPGLRAVFGETYPDPVRVVCVGAPLSQVLANPTDDR

WKDVSVELCGGTHVKRSSDIKKFLILTESAIAKGIRRIVAVTGEEAVKAQQLSRELDGRLEAISRKSIMDEEAIKSLSKD

VDEAAISLLEKNRLRDGVAAVRQKMVDADKAVRLTQSRLALDTVAPFLESTAHPVVVKRIDVGDNGKALLDALNALKKAG

HSGLLYSVDQQSGKIHYYSVVAEQHKSLDASEWAKVFGEPLEGRSGGRALAAQGSAPMRDCADAERLALEFASIKVRD

>XP_007929747.1 hypothetical protein MYCFIDRAFT_57749 [Pseudocercospora fijiensis CIRAD86]

MASTAGQEPEWTAVKVRKTFIDYFGKNGHTFVPSGSVVPLADPTLLFTNAGMNQYKSIFLGTVDPDSDFAKLKRAANSQK

CIRAGGKHNDLDDVGKDSYHHTFFEMLGNWSFGDYFKKEAIHFSWELLTKVYGLSPDRLYVTYFEGKKDAGLEPDTEARE

FWRAEGVPDDHILTGDMKDNFWEMGDQGPCGPCSEIHYDRIGGRNAADLVNKDDPNVIEIWNNVFIQYNREPDKSLRPLP

AKHIDTGMGFERLVSILQNKYSNYDTDVFTPIFARIREITGAREYSGKFGDEDVDGIDTAYRVVADHVRLLTFAISDGGI

PNNVGRGYVVRRVLRRGARYARKYFNTDIGSFFSKIVPTLVEQMGDMFPEIKKKQAEVIEILDEEELSFAKTLDRGENMF

EKYAQKCKANNSKVLEGDDVWRLYDTYGFPVDLTRLMAEERGLDIIDEDVAKAQEKAREASKGEKKAGTDLVKLDVHDIN

ALEQMADVPKTDDSPKFQRDNITATIKAIYHGRKFLQSTEEVPKGEQFGVFLDRTNFYAEQGGQENDTGRILIDGEAEID

VHNVQLYGGYVMHTGYMNYGTLKVGDQVLSEYDELRRQPIRNNHTGTHILNFALRETLGDDVNQKGSLVAPEKLRFDFSH

KQGCSDAELEKIEELSTGYIRQNSEVYAKDVPLATAKQIEGVRAVFGETYPDPVRVVSIGVPVEDLLEDPKRSDWRNVSV

EFCGGTHVKNTTEIKDLIVLEESGIAKGIRRIVAVTGQTAHRIQLLGEEWNKKVAHIEGMKYGPEKEGAVKEAQHDLGKL

EMAALTKTKLRERVAKVVKENLDHQKAAQKAENKKLLDAVNEHFDKNKDSNTLVLSFPVSAGSKAIQETMKVIGNKQKDK

TVYLLGKTEDGKVVHGCHVSSDAQGKGADAAKWSNEVAGLVGGKAGGKGATSLGQGTNADKVDDAVEVARKYLEGLSI

>OJD19759.1 alanyl-tRNA synthetase [Emergomyces pasteuriana Ep9510]

MGGGGERVSSVVLRRGLSLPGYRSRVVRSGGAGGAGAGNIPSPPSRRFFASSPALTTNTSTSTSTTATPRARFHHHGLAQ

TAFKMASTQSVEQPEWSALRVRDAFLDFFKENGHTFVPSSSVVPLSDPTLLFTNAGMNQYKSIFLGTVDPNSDFATLKRA

HNSQKCIRAGGKHNDLDDVGKDSYHHTFFEMLGNWSFGDYFKKEAISYSWNLLTKVYGLDADRLYVTYFEGNPDAGIGPD

LEAKELWLSVGVAEDHLLSGNMKDNFWEMGEQGPCGPCSEIHYDRIGGRNASHLVNQDDPNVLEIWNNVFIQYNREPDRS

LRPLPNKHIDTGMGYERLVSILQNKSSNYDTDVFTPLFEKISEITGCRPYTGKFHEEDTDGIDTAYRVVADHIRTLTFAI

SDGASPNNEGRGYVVRRVLRRGARYARKYLNVEIGGFFSQIVPTLVEQMGDMFPEIKRKQTDVMEILDEEEISFAKTLDR

GERLFEEYALQAKQKGLGKLHGADVWRLYDTFGFPVDLTQLMAEERGLAIDNNEFEEARLKAKEASRGKGKLTADAVKLT

VHDLGRLETMDDVPKTNDDAKFGRGNITSQIKAIYHNKNFVDSTKDIPEGEQFGIILDRTNFYAEQGGQENDTGKIIIDG

KAELAVEDVQVYGGYILHTGFLKYGSFNINDAVIAEYDELRRWPIRNNHTGTHILNFALRQVLGDGVEQKGSLVAAEKLR

FDFSHKSAVSDSDLEKIERISTDYIRQNCAVYAKDVPLSTAREISGVRAVFGETYPDPVRVVSVGVELEEILQNVHDPRW

KDISIEFCGGTHVQKTGDIKDLIILEESGIAKGIRRIIAVTGEEAHEAQRIAKEFGERLKRFEKMELGPQKEIEAKLLQV

DLNQLTISAVEKARFREQFTQTHKKLLEGQKAAQKLESKKALDAITGYFQAPENKDAPHLVLQLPISANPKAVSDSLNFV

KTKMQDKSIYVFAADKEAGKVVHGCHVAQNLSEQGATPGDWATTVSNIVGGRAGGKAPISIGNGTEAAKLDEAIKAATEY

LGKFKL

>KII92325.1 hypothetical protein PLICRDRAFT_37088 [Plicaturopsis crispa FD-325 SS-3]

MAASSWTAHRVRQEFFDYFNAKNHTFVASSPTIPYDDPTLLFANAGMNQYKSIFLGTVDPHSDMARLKRAYNSQKCIRAG

GKHNDLDDVGKDSYHHTFFEMLGNWSFGDYFKKEAIEYSWDLLTNVYGLSKDRLYVTYFEGDTKGGLEPDLEARQYWINQ

GVAEDHILPGNAKDNFWEMGATGPCGPCSEIHYDRIGGRNAAHLVNQDDPDVLEIWNNVFIQFNREENGSLKSLPSKHVD

TGMGFERLVSVVQDKRSNYDTDVFSPLFAKVQELTGVRPYGGKFGEEDQDGIDTAYRVVADHVRTLTFALSDGGVPNNVG

RGYVLRRILRRGSRYARKKLGVTIGSFFSSLMPVVVQEMGDVFPELTKRGAEIKEILDEEELSFSRTLDRGEKLFDQYAA

RAKQQGLKELSGKDVWRLYDTYGFPEDLTRLMAEELGLGVNDKEFEEAQLASKEASKATQKKGTSDVVKLDVHDIAVLDN

GAVPKTDDSPKFDLGNIDAHIIAIYYNRSFVQSTSEIPADANFGLILDRTNFYAESGGQEYDTGSIVIDGAADFEVTNVQ

TYRGYVLHAGHLKYGSLAVGNEVLATYDELRRWPLRNNHTATHILNFSLREVLGDHIDQKGSLVAPTKLRFDFSHKAQIP

PSELAKIESLSVDWIKKNVKVFSKELDLKVAQQIPGLRAVFGETYPDPVRVVTLEFDVEEISKDITNPKWRGTSVEFCGG

THVAKTGDIKDFVITEESGIAKGIRRITAVTGHEAHEVSRAASILKSKLEYLEKLGESKEKDAGLKALSVELGQADISVV

IKSELRDRLAVVRKAFDKIIKDKEAAANKEAVDSVTAYFKENPESEAFFATLDVSGNAKILKNVVAQASKVQKAVYVFSV

DHEGGKVAHVNYVPPSLRPKLDARTWASTVSDILSGKAGGKEDSVQGSGTNVERVDEALDAAKKYFAKQIAA

>PVH15373.1 alanine-tRNA synthetase [[Candida] duobushaemulonis]

MTTWPASTVRQTFLDFFKGKQHTFVPSSSVVPHNDPSLLFANAGMNQYKPIFLGTVDTSSDFGSLKRAANSQKCIRAGGK

HNDLDDVGKDSYHHTFFEMLGNWSFGDYFKKEAIEWSWELLTEVFKLEKDRLYVTYFEGDEKNGLQPDLEAKQFWLDVGV

AEDHILPGDASDNFWEMGDQGPCGPCSEIHYDRIGGRNAAHLVNMDDPNVLEVWNVVFIQYNREADSSLRPLPNKHIDTG

MGFERLVSILQSKFSNYDTDVFSPLFEKIRGITGVRPYTGKFGSDDVDGIDTAYRVIADHVRTLTFAITDGGVPNNEGRG

YVLRRILRRGSRYVRKYMNYPIGHFFEQLVDVVIEENSQIFPEIASHKDELKEILVEEELSFAKTLDRGEKLFEQYAIIA

SKTPEQTLSGKDVWRLYDTYGFPVDLTRLMAEEAGLKIDEEAFEKAKEESREASKASGSKTGADLIKLDVHALSELDGND

AVPKTDDHYKFGLENTKSKILAFYDGSSFVDSIDKTGQQFGILLDKTPFYAEQGGQEFDTGSLVIDGSAEFHVDNVQSYA

GYVLHTGTLSEGTLKVGDKVIATYDELRRWPIRNNHTGTHVLNFALREVLGDSVDQKGSLVAAEKLRFDFSNKQALTLKE

VEEVERISNENIKANMEVFAEDVSLTVAKSINGLRAVFGEVYPDPVRVVSIGVPVSTLVSEPENKEWHKYSIELCGGTHV

AKTSEIKDLVIIEESGIAKGTRRIVAVTGHDAHRVQQIARDFGAELDAAANLPFGPDKEQKAKELGVALKKLSISVVDKS

KLNEKFTKLDKSIKDNLKAKQKEESKKTLAVVKDWLKDEEKSKAAFLVAHVPISANAKAITEAFNLIKKENKDKSLYLIT

GTDDKVAHGCYISDEAISKGVDANEVAKVAIAHIGGKAGGRGNIVQGMGDKPEGVEEAVEAVKKALTEKLA

>KKZ62953.1 alanyl-tRNA synthetase [Emmonsia crescens UAMH 3008]

MASTQNVEQPEWSALRVRNAFLDFFKDNGHTFVPSSSVVPLSDPTLLFTNAGMNQYKSIFLGTVDPNSEFATLKRAHNSQ

KCIRAGGKHNDLDDVGKDSYHHTFFEMLGNWSFGDYFKKEAISYSWDLLTKVYGLDPDRLYVTYFEGNSDAGIGPDLEAK

ELWLSVGVAEDHLLSGNMQDNFWEMGEQGPCGPCSEIHYDRIGGRNASHLVNQDDPNVLEIWNNVFIQYNRESDRSLRPL

PNKHVDTGMGYERLVSILQNKSSNYDTDVFTPLFDKIREVTGCRPYTGKFHEEDTDGIDTAYRVVADHVRTLTFAISDGA

SPNNEGRGYVVRRVLRRGSRYARKYLNVEIGGFFSKIVPTLVEQMGDMFPEIKRKQTDVMEILDEEEISFAKTLDRGERL

FEEYALQAKQKGLGKLHGADVWRLYDTFGFPVDLTQLMAEERGLTIDNVEFEEARLKAKEASKGQGKLTTDVVKLTVHDL

GRLETMDDVPKTNDDAKFGRGNITSQIKAIYHNRKFVDSTKEIPEGEQFGIILDRTNFYAEQGGQENDTGKIIIDGKAEL

AVEDVQVYGGYILHTGFMKYGSFNINDSVIAEYDELRRWPIRNNHTGTHILNFALRQVLGDGVEQKGSLVAAEKLRFDFS

HKSAVSDSDIEKIERISTDYIRQNCAVYAKDVPLSTAREISGVRAVFGETYPDPVRVVSVGVELEEILQNVHDPRWKDIS

IEFCGGTHVQKTGDIKDLVILEESGIAKGIRRIIAVTGEEAHEVQRIAKEFGERLRRFEKMELGPQKEIEAKLLQVDLNQ

LTISAVEKARFRELFTQIHKKVLEGQKAAQKLESKKSLDAITEYFQAPENKDAPHLVLQLPISANPKAVSDSLNFIKTKM

QDKSIYVFAADKEAGKVVHGCHVAQNLSAQGATPNDWANTVSSIVGGRAGGKAPICIGNGTEAEKLDEAIKAATEYLEKF

KL

>EEH21600.2 alanyl-tRNA synthetase [Paracoccidioides brasiliensis Pb03]

MGVGGERVSGVVLHQGLSLTRYCSRSVCRNLVEVVGVNVGGSVPVPLPPSRRFFAWHFFSPAITNTFSTTPLFFRRHNST

LPRSQAAFNMTSTQRVEQPEWPALRVRNAFLDFFKDNGHTFVPSSSVVPLSDPTLLFANAGMNQYKAIFLGTVEPNSDFA

QLKRAHNTQKCIRAGGKHNDLDDVGKDSYHHTFFEMLGNWSFGDYFKKEAIRYSWDLLTKVYGLDPDRLYVTYFEGNPDA

GIEPDLEARDLWLSVGVAEDHLLSGNMKDNFWEMGEQGPCGPCSEIHYDRIGGRNASHLVNQDDPNVLEIWNNVFIQYNR

EQDKSLRPLPNKHVDTGMGYERLVSILQNKTSNYDTDVFTPLFEKIREITGSRPYTGKFHEEDVDGVDTAYRVVADHIRT

LTFAISDGAVPNNEGRGYVVRRVLRRGARYARKYLNVEIGGFFSQIVPTLVEQMGDMFPEIKRKATDVMEILDEEEISFA

KTLDRGERLFEEYALQAKQKGLDRLHGADVWRLYDTFGFPVDLTQLMAEERGLKIDNGEFEEARLRAKEASKGQGKLTKD

VVKLTVHDLGLLETMDDVPKTDDSAKFGRGNITSQIKAIYHNKKFVDSSKGIPEGEQFGIILDRTNFYAEQGGQEYDTGK

IIIDGKAELAVEDVQVYGGYVLHTGFMKYGSFNVNDSVIAEYDELRRWPIRNNHTGTHILNFALRKVLGDGVEQKGSLVA

AEKLRFDFSHKSAVSDSDLEEIERISTDYIRQNCAVYGKDVPLSIAREITGVRAVFGETYPDPVRVVSVGVELEEILKDV

HDPRWKEISIEFCGGTHVQKTGDIKDLVILEESGIAKGIRRIIAVTGEEAHEVQRIAKEFGERLKRFEKMELGPKKEMEA

KLLQVDLNQLTISSVEKAQFREKFTQIHKKVLEGQKAAQKLESKKALDAIMGYFQAPENNDAPHLVLQLPISANAKAVSD

SLNFVKTKMQDKSLYVFAADKEAGKIVHGCHVAENLSQQGASPNDWANSVANVVGGRAGGKGPISIGNGTDAEKLDDAIK

AATEYLEKFKL

>OQN95818.1 Alanine--tRNA synthetase [Rachicladosporium antarcticum]

MGSQAQNEVQWTAQKVRSTFLEFFEKNGHTIVPSGSVVPLSDPTLLFTNAGMNQFKSIFLGTVDPDSDFAKLKRAVDSQK

CIRAGGKHNDLDDVGKDSYHHTFFEMLGNWSFGDYFKKEAIAFSWELLTKVYGLEPDRMYVTYFEGKADVGLEPDLEAKE

LWKSVGVADDHILPGDMKDNFWEMGDQGPCGPCSEIHYDRIGGRNAAHLVNQDDPNVLEIWNNVFIQYNREPDKSLKPLP

SKHVDTGMGFERLVSVLQDKLSNYDTDVFTPLFKAVQDITGAREYSGKFGEEDVDGIDTAYRVVADHVRLLTFAISDGGV

PNNVGRGYVVRRVLRRGARYARKYFKTEIGGFFSKLVPTLVNQMGDMFPEIKKKELDVKEILDEEELSFAKTLDRGEVMF

EKYAEQCKQRDSKELSGADVWRLYDTYGFPVDLTKLMAEERGLSIDDDAVHEAQEKAREASKGEKKSAAELVKFSVHDIN

TIENMEDVPKTDDSSKFLRENVNGTVKAIFHGGKFLQSTADAPEGEQLGIVLDKTCFYAEQGGQEFDMGRILIDGEAEID

VQNVQVYAGYVLHTGFMSYGALKTGDNVLCQYDELRRQPIRNNHTGTHILNFALREVLGEDVNQRGSLVAPEKLRFDFSH

KAGCSDAELEKIEEISTSYIRQNSEVYSTDVPLSTARNIEGVRAVFGETYPDPVRVVSIGVPVEELLEDTTRKEWRSVSV

EFCGGTHVKNTSEIKDLVVVEESGIAKGIRRIIAFTGAAAHEVQRIAGEWDEKFTRLEAQAYTPEKEARTKEYQHALNSL

EISALAKTKLRDRVSKIVKANMEHQKTAQKAENKALTDAVTAHFTQHPDSKSFVTSHPVSAGSKAIQETLKLVSSKQKDK

NVYIVGASADGKVVHGCFVSSDAQAKGADAGKWSTTVAGIVGGKAGGKGATSFGQGTEAEKVDEGVEAARKYLEGLGL

>XP_018709567.1 hypothetical protein METBIDRAFT_33653 [Metschnikowia bicuspidata var. bicuspidata NRRL YB-4993]

MIMPLQDKAALPRVYGCAGRTWRREVRKKLSRARPPLPMLKTIARKPAMSSNPWSAAKVRSTFLDYFKERSHTYVPSSSV

VPHNDPTLLFANAGMNQYKPIFLGTVDPSSDFAKLKSAANSQKCIRAGGKHNDLDDVGKDSYHHTFFEMLGNWSFGDYFK

KEAIQYSWGLLTDVYGLDGDRMYVTYFEGDEKNGLQPDLEAKQFWLDVGVPEDHILPGNAADNFWEMGDQGPCGPCSEIH

YDRIGGRNASALVNQDDPNVLEIWNVVFMQYNREPDSSLRPLPNKHIDTGMGFERLVSILQDKSSNYDTDVFTPIFDKIK

EITGVRPYAGRFGDDDIDGIDTAYRVIADHVRTLTFAISDGGVPNNEGRGYVLRRILRRGARYVRKYMNYPIGSFFQQLV

DVVIEQNSSIFPEIAKNSTELKEILVEEEVSFAKTLERGEKLFEKYAIICSKTPEQTLPGNIVWRLYDTYGFPVDLTRLM

AEEAGLKIDEEGFEQAKEASREASKGLNSKSGSSMIKLDVHALSELDKNDAVPKTDDSFKYGLDNTKSKIVALYDGSKFI

DSISESGEQYGILLDKTPFYAEQGGQEYDTGSLVIDGVSEFNVRNVQQYAGYVLHTGFLVEGTLNVSDEIIASYDELRRW

PIRNNHTGTHVLNLALREVLGDGVDQKGSLVASEKLRFDFSHKQALSQKELEQVEKISNENIAANKQVFYKDVSLAAAKE

INGLRAVFGETYPDPVRVVSIGVSVEDLLSNPKNEDWHGLSIEFCGGTHVAKTGEIKDLVIIEESGIAKGIRRIVAVTGQ

EAHEVQRIARDYEESLTAADKLPFGSEKEKRTKELGVGLKQLSISVLGKSKLNDKFNKIDKSIKDNLKAKQREESKKTLE

VVKQWFDDEEKSKAPFFVAHVQIEANAKAITEAINLVKKQDKTKCLYLITGVEGKVAHGCYISDEAIAKGLNANELASVV

SSHVGGKAGGKGNVVQGMGDKPEGIPQAVEDLSKLLAEKL

>OCF41937.1 alanine-tRNA synthetase [Kwoniella heveanensis CBS 569]

MSMANSAPKVPDTAPAHWPSPTEWPAAKVRQTFIDYFVKQPGFEHTFWPSSGCIPFDDDTLLFANAGMNQYKPLFLGTAD

PKSDLSKLLRAVNSQKCIRAGGKHNDLDDVGKDTYHHTFFEMLGNWSFGNYFKVGACTMAWDLLTRVYGLPKDRLYVTYF

EGDAKQGLEPDTEAIQIWKDLGVPEHHILPGNAKDNFWEMGATGPCGPCSEIHFDRIGGREAAELVNADDPNVIEIWNNV

FMQYNREQNGELRPLPAKHIDTGMGFERLVSILQNVPSNYDTDVFTPIFAKIQELTGARPYEGKLGEEDADGVDTAYRVV

ADHIRTLTIAISDGGVPDKDGRGYVLRRILRRGVRYASNKLNVKIGSFFSSLVPTVVESLGDIFPEIRKKIPELVEILNE

EEASFARTLTRGEALFTKYAESAKGEKRTVLAGKDIWRLYDTYGFPVDLTQIMAEERGLQIDQDAFEKARLESLEASKAG

GKDKATAGVKLDVHDLAALEANDAVPKTDDSSKYQLDDIKATVKSIYHASKFYTSTSELPAHAPFGVLLDKTNFYAESGG

QEYDTGVIAIDGQAEFKVEDVQVFNGYVLHIGQMEEGEIKVGDEVICTYDELRRWPIRNNHTGTHILNFALREVLGDHID

QKGSLVAPTKLRFDFSHGKSIGTPEITKIEAISNDWIKKAAPVYAKEMSLAEAYKIPGLRAVFGEAYPDPVRVVSLGFPL

EEIAQNVEDNKWRQTSIEFCGGTHVAKTDDIKDFVIVEETSIAKGIRRIVAVTGHEAHEVSRKAGDFERRLDRIAQLEGK

EKETALKPYLVELGQSGISIIKKDALKKRFEKMQSELVAAAKAKTAADSKVIQEEIKAFFKANPNDNVYAGSFDVAAGNA

KTLAAATSTAKSLSKAVYVFSVDPETGKVAHTNYLPKEVLDRKVIDAKTWLGEVSKVIGGKGGGRDDSATGVGSEVSKID

EAIAVAKSFYKSKVEA

>PGH08001.1 alanine-tRNA synthetase [Emmonsia parva]

MRGVGGERVARGVVLHRGLSYSRPRARAGAGVAGSCAGIGAAGVAAGVFSGPSPPSRRFFLFLARRRFRSPAICNTSTST

PSPAATLSARSHQHGLAHSAFKMASAQTVEQPEWSALRVRNAFLDFFKDNGHTFVPSSSVVPLSDPTLLFANAGMNQYKS

IFLGTVDPNSDFATLKRAHNSQKCIRAGGKHNDLDDVGKDSYHHTFFEMLGNWSFGDYFKKEAISYSWDLLTKVYGLDPD

RLYVTYFEGNPDAGIGPDLEAKELWMSVGVAEDHLLSGNMQDNFWEMGEQGPCGPCSEIHYDRIGGRNAAHLVNQDDPNV

LEIWNNVFIQYNREPDRSLRPLPNKHIDTGMGYERLVSILQNKSSNYDTDVFSPLFEKISEITGCRPYAGKFGDDDADGI

DTAYRVVADHVRTLTFAISDGASPNNEGRGYVVRRVLRRGARYARKYLNVEIGNFFSKIVPTLVDQMGDMFPEIRRKQTD

VMEILDEEEISFAKTLDRGERLFEEYALKAKQTGLGKLHGADVWRLYDTFGFPVDLTQLMAEERGLTIDNTEFEEARLKA

KEASKGQGKQTADVVKLTVHDLGLLETMDNVPKTNDDAKFGRGNITSQIKAIYYNKQFVDSTKDIPEGEQFGIILDRTNF

YAEQGGQENDTGKIIIDGKAELAVEDVQVYGGYILHTGFMKYGSFNVNDSVIAEYDELRRWPIRNNHTGTHILNFALRQV

LGEGVEQKGSLVAAEKLRFDFSHKSAVSDSDLEKIERISTDYIRQNCAVYAKDVPLSTAREISGVRAVFGETYPDPVRVV

SVGVELEEILQNVHDPRWKDISIEFCGGTHVQKTGDIKDLVILEENGIAKGIRRIIAVTGEDAHEVQRIAKEFGERLQRF

EKMELGPQKEMEAKLLQVDLNQLTISAVEKVRFREQFTQIHKKVVEGQKAAQKLESKKALDAITGYFEAPENKDAPHLVL

QLPISANPKAVSDSLNFVKTKMPDKSVYVFAADKGAGKVVHGCHVAQSLSAQGATPNEWANTVSSIVGGRAGGKAPVAIG

NGTEAEKLDDALKAATEYLEKFKL

>KRX52311.1 Alanine--tRNA synthetase, cytoplasmic [Trichinella sp. T9]

MTVLLYTTSCCVINNLKRSKMDMLLTANAIRRTFINYFTEKRNHQYVHSSSVIPKGDPSLLFTNAGMNQFKPIFLGTADP

NSQMASWKRVVNSQKCIRAGGKHNDLDDVGKDVYHHTFFEMLGNWSFGDYFKKEICAWAWEVLTKEFKIPKEQLYVTYFG

GSEKCRLQPDVECRQIWLEIGLPSERVLPFGMKDNFWEMGDIGPCGPCSEIHYDRIGNRNAANLINKNDPDVLEIWNLVF

ITYNRDKDGSLHPLPKRHIDCGLGFERLVSVIQGKKSNYDTDLFVPIFNRIAETAKVRPYMGKVGKEDEQQIDMAYRVVA

DHIRTLTVALSDGGRPDNCGRGYVLRRILRRGVRYATEKLNAPSGFFANLVPVVVETLGDMFPELKEDPNSVMEIINEEE

SLFLKTLSRGSRVLRQEIEKASKDKLIPGVVAWRLYASYGFPVDLTQLMAEESGYKVNMEEYEECRQQAQMSSSSKFSQA

NDVVDFNINAVNYLLNGKVPLTDDKPKYAYRLTENGDDDYEFDEVKCEVLALRRNGEFVSEVCSGDSCCLICDKTAFYAE

SGGQIYDEGSMEGEKCEFRVRNVQARSGYVIHFGEVMGTITRGSKLFQNVDQKRRVQVMKNHTATHLLNFALREVLGQVE

QKGSLVLMDRLRFDFTCKAPLTVEQLVRIEQIVANIVNSDVEIYMQEVPLGVAKTIAGLRLTADEVIRRRSTVQFFYTAL

PLQTYPDPVRVVSVGTPVDALLKDPDGPGAKLASVEFCGGT

>GAV27197.1 Alanine--tRNA synthetase [Pichia membranifaciens]

MHALRSLQLSPLASLKPVNSLRADFHPASRAFSSTMSIYTNPATNPKVQEWPAAKVRDTFLDFFKQKQHTFVPSSSVVPH

DDPTLLFANAGMNQYKPIFLGTVDPSSSFSTLKRAVNSQKCIRAGGKHNDLEDVGRDSYHHTFFEMLGNWSFGDYFKKEA

IQYSWELLTQVYGIPADRLYVTYFEGDEKAGLEPDLEAKKLWTAIGVPDDHILPGNAKDNFWEMGDQGPCGPCSEIHYDR

IGGRNAAALVNEDDPDVLEIWNNVFIQFNREPDGSLKPLPAKHIDTGLGFERLVSLLQDVRSNYDTDVFEPLFLKIQEIT

GVRPYSGKFGKDDVDGIDTAYRVLADHVRTLTFALADGGVPNNEGRGYVLRRILRRGARYTRKYMNYPIGNFFSQLAPTL

IEQDEHIFPELAKDPFSIFEILDEEEASFARTLDRGEKLFEQYALIASKTPEQTLSGKDVWRLYDTYGFPVDLTRLMAEE

AGLKIDEEGFEKARLASKEASKGGASKSAKALLKLDVHALALLGEQKVPTTDDSAKYDTKDINSQVLAIYTEGGFVDSIT

TESSKDKQIGIILDKTPFYAEQGGQEYDTGRIVIDGKSEFHVENVQVYAGYVLHTGFLDEGELKVNDIVISGYDELRRWP

LRNNHTGTHILNFALRNVIGEGVEQKGSLVASEKLRFDFSHKKAVELKELEQVERIVNKIIQDDLKVYAKPVALETAKSI

NGLRAVFGESYPDPVRVVSVGIPVEDLLADPSNEKWKEYSIEFCGGTHVAKTDDIKEFVIIEESGIAKGIRRIVAVSGSE

AREVQRKASEFSKILDDVNKLPYGEIKERKVKDLGTELGKLSISVVEKAALKAKFVAIEKSVKDETKLRVKQDTTKVMDA

VKSFFAAPDHKSVCAVHVSAGANAKIISEAINYMKKEQKDSCLYIVLAEAEGKAAQGVYLSPAVQEKVPAVEVATKGAAF

IGGRAGGKGNTCQGMGDKAEGADAAVEEVKSLLEKLAI

>XP_022459196.1 uncharacterized protein KUCA_T00003177001 [Kuraishia capsulata CBS 1993]

MNILPWKRIVYSKRRISCSLLPLPLQPFRTMSAINSTEDWTAAKVRSTFLKFFESRGHTFVPSSSVIPYDDPTLLFANAG

MNQYKPIFLGTVDPSSDFATLKRAVNSQKCIRAGGKHNDLEDVGKDSYHHTFFEMLGNWSFGDYFKKEAIQWSWELLTVV

YGIPADRLYVTYFEGDAKNGLEPDLEAKDLWKSVGVPDDHILPGNAKDNFWEMGDQGPCGPCSEVHYDRIGGRNAAALVN

MDDPDVLEIWNNVFIQFNREPDSSLKPLPAKHVDTGMGFERLVSVLQDVRSNYDTDVFQPLFQRIHEITGVRPYSGHFGK

EDLDGIDTAYRVLADHVRTLTFALADGGVPNNEGRGYVLRRILRRGSRYVRKYMNYPIGKFFSQLAPTLIEQVKDIFPEV

AKDPSTIFEILDEEEASFARTLDRGEKLFEQYAIIASKTPEQTLSGKDVWRLYDTYGFPVDLTRLMAEEAGLKIDEEGFE

KAKLASWEASKGAGKTAGKALLKFDVHALGKLDSDGTVPKTDDSPKYGLENVKAEIVAIYNGEFLDSTEGIAEGEQIGIL

LDKTPFYAEQGGQEFDTGKIVIDGVAEFNVENVQQYAGYVLHTGYVVEGSFAVKSSVIAGYDELRRWPIRNNHTGTHILN

FALREVLGDGVDQKGSLVSAEKLRFDFSHKAALSLKEIERVENICSEQIKSNVQVFSKPVELDTAKKISGLRAVFGEVYP

DPVRVVSIGVPVETLLANPESEEWNKYSIEFCGGTHVAKTGDIKEFVIIEESGIAKGIRRIVAVSGTEAHAVQRIAKSFD

EELDAISKLTFGAEKEKKLKEANVALGKLSVQVVAKNALKEKFAKIDKSVKDEQKAIQKAESKKTLDAVKNFFTENAEAP

FFAAHVDISPNAKVITEAINFLKKENKDKSLFLITGKESEARVAQGVYISDAAGAKGLDATAVAQLAAKFIGGKAGGKGN

VCQGMGDDVKGINEAVEAVSSLLKEKLSI

>XP_022631038.1 uncharacterized protein LALA0_S14e00408g [Lachancea lanzarotensis]

MTVGDKQKWTATNVRSTFLDFFSQRSHKFVASSPVVPYDDPTLLFANAGMNQYKPIFLGTVDPSSDFYNLKCAMNSQKCI

RAGGKHNDLEDVGRDSYHHTFFEMLGNWSFGDYFKKEAIAYAWELLTKVYEIPADRLYVTYFEGDAKQGLEADTEARDFW

RQVGVLDDHILTGDTKDNFWEMGEQGPCGPCSEIHYDRIGGRNASSLVNMDDPDVLEIWNIVFIQYNREQNGKLRPLPAK

HIDTGMGFERLVSVLQDVRSNYDTDVFQPLFARIQEITGARGYAGKFGAEDVDGVDTAYRVLADHVRTLMFALADGGVPN

NEGRGYVLRRILRRGARYARKYLNYPIGNFFSTLAPTLIEQTKSMFPEIAKDPSYLFEILDEEEASFAKTLDRGEKLFEK

YAQTALETEAKTLDGLQVWRLYDTYGFPVDLTELMAEEQGLKIDGPGFEKAKQESYEASKKGGKKSASDLIKLDVHGLST

LNSESVAKTDDSPKYTSANVEATVLKIYDGENFVSSTGEVGNQYGVILDQTCFYAEQGGQEYDVGTLVIDGSTEFKVENV

QLYNGYVFHTGFVAEGELKVGDKIIASFDELRRFPIKNNHTGTHILNFALKEVLGGEVDQKGSLVSPEKLRFDFSHKKAL

TLDEVKKVEDVCSQQIKANLQVYFKNVPLHLAQQISSVRAVFGESYPDPVRVVSVGKPVDDLLTNPTSEEWQKYSVEFCG

GTHVNKTGDIKEFVITEESGIAKGIRRIVAVSGTEAHEVQRIASSFDAELEAADKLPFSPLKEKKLKELGVNLGQLNISV

LAKNDLKTKFAKVEKAVKDELRTRAKKETKQTVDEVKNFFAENESAQFFVKYIDIPTNAKAITEAVTYMKTSAQDKTIYL

LNGSEKVAHGCYISDKALEKGVVGRDLLDRISRNIGGKAGGKANVFQGVGDKPDGIENAVKEVSELLQEKLSI

>XP_002628910.1 alanyl-tRNA synthetase [Blastomyces gilchristii SLH14081]

MASTQTVEQPEWSALRVRNAFLDFFKENGHTFVPSSSVVPLSDPTLLFTNAGMNQYKSIFLGTVDPNSDFATLKRAHNSQ

KCIRAGGKHNDLDDVGKDSYHHTFFEMLGNWSFGDYFKKEAISYSWDLLTKVYGLEPDRLYVTYFEGNPDAGIGPDLEAK

ELWLSVGVAEDHLLSGNMKDNFWEMGEQGPCGPCSEIHYDRIGGRNAAHLVNQDDPNVLEIWNNVFIQYNREPDRSLRPL

PNRHIDTGMGYERLVSILQNKSSNYDTDVFTPLFDKIREITGCRPYTGKFGEEDTDGIDTAYRVVADHIRTLTFAISDGA

SPNNEGRGYVVRRVLRRGARYARKYLNVEIGSFFSKIVPTLVEQMGDMFPEIRRKQTDVMEILDEEEISFAKTLDRGERL

FEEYALQAKQKGLGNLHGADVWRLYDTFGFPVDLTQLMAEERGLTIDNAEFEEARLKAKEASKGQGKQTADVVKLTVHDL

GLLETMDNVPKTNDDAKFGRGNITSQIKAIYHNKKFVDSTKDIPEGEQFGIILDRTNFYAEQGGQENDTGKIIIDGKAEL

AVEDVQVYGGYILHTGFMKYGSFNVNDSVIAEYDELRRWPIRNNHTGTHILNFALRQVLGEGVEQKGSLVAAEKLRFDFS

HKSAVSDSDLEKIERISTDYIRQNCAVYAKDVPLSTAREISGVRAVFGETYPDPVRVVSVGVELEEILQNVHDPRWKDIS

IEFCGGTHVQKTGDIKDLVILEENGIAKGIRRIIAVTGEEAHEVQRIAKEFGERLQRFDKMELGPQKEMEAKLLQVDLNQ

LTISAVEKVRFRERFTQIHKKVVEGQKAAQKLESKKALDAITGYFETPENKDAPHLVLQLPISANPKAVSDSLNFVKTKM

QNKSVYVFAADKGAGKVVHGCHVAQSLSAQGATPNEWANTVSSIVGGRAGGKAPVAIGNGTEAEKLEDAIKAATEYLEKF

KL

>EIF48501.1 cytoplasmic alanyl-trna synthetase [Brettanomyces bruxellensis AWRI1499]

MSFKLARLFVAPLERLRLFHFRKMSTTNXNGQPXXSSAXNAENWPASKVRDTFLNFFKERGHTFVPSSSVIPHDDPTLLF

ANAGMNQYKPIFLGTIDPSSPLAHIKRATNSQKCIRAGGKHNDLEDVGRDSYHHTFFEMLGNWSFGDYFKEGAVGFSWEL

LTKVYKLDPSRLYVTYFGGDKKAGLQPDLETXKIWENIGVSESHILPGSAKDNFWEMGDQGPCGPCSEIHYDRIGGRNAA

DLVNKDDPDVLEIWNNVFIQFNREQDGSLKPLPQKHIDTGMGFERLVSILQNVRSNYDTDVFRPLFAKIQEITGXRXYTG

KFGKEDVDGIDTAYRVLADHVRTLTFAIADGGVPNNEGRGYVLRRILRRGARYARKYMNYPIGKFFSSLAPTLIEQVKNI

FPELTKDPQTIYEIMDEEEASFARTLDRGEKLFESYAEKASKTSEKMLSGKDVWRLYDTYGFPVDLTRLMAEEAXLKIDE

EGFEKAKLASWKASKSGNSKGGKKLLKLDVHALGDLQSNDKISQTDDQFKYTSNDVEADIQAIYVDQKFVNSTEGLKEGZ

QIGIILSKTPFYAEQGGQVXDTGKLVIDGQAEFNVENVQTYAGYVLHTGYIEEGSLKVGESVIASYDETRRESLRHNHTG

THILNFALRSVLGDSVDQKGSLVAPDKLRFDFSFKKALKLDQIKKTEEIVNKIIKDDVKVYTKSVPLEEAKKINGLRAVF

GESYPDPVRVVSVGNSVDELIADPSNEXWLNXPIEFCGGTHVSRSSDIKFLVITEEGGIAKGIRRIXALTGEEALKTKTV

ANEFEAVLDNIEKMPFGNXKQKALKANTTKLGKLIVSILDKESLKSKFNKIDKDVKNELKKRQKAATKKAEEEISQYFEE

HPDSQFVALHIDTGSNARVITEAFNYLKKNFKGKSLFIIIGTSEDQRVAQGVYISDEHLKSLASSDVAKVASEYIGGKAG

GRGNICQGMGDKPDGINKAVDAVTKLFEEKLKL

>OTA33780.1 Alanine--tRNA synthetase [Hortaea werneckii EXF-2000]

MRRVWSFPQTLSFALWKRQPPRRIRPHDLRRCWYSLQTRSYVNMGSTPGETPQWPAPKVRSAFIDYFKEKGHTFVPSSSV

VPLDDPTLLFTNAGMNQYKSIFLGTVDPNSDFSKLKRAVNSQKCIRAGGKHNDLDDVGKDSYHHTFFEMLGNWSFGDYFK

QEAIAYSWELLTKVFGLQPDRLYVTYFEGKADAGLEPDLEAKEFWKSVGVAEDHILPGNMKDNFWEMGDQGPCGPCSEVH

YDRIGNRNAAYLVNQDDPNVLEIWNNVFIQYNREPDRTLKPLPNKHVDTGLGYERLVSVLQNVYSNYDTDVFTPLFKRIQ

EITGAREYRGKFAEEDPDGIDTAYRVIADHIRTLTFAISDGGVPNNVGRGYVVRRILRRGARYARKYFNVEIGNFFEKLL

PTLVEQMGDMFPEIKKKQSDVAEILNEEELSFAKTLDRGEAMFEKIAQNCKVKDIKELAGTDVWRLYDTYGFPVDLTSIM

AEERGLTIDENAVAEAQEKAREASKGEKKAGGDLIKLNVHDINALEKMADVPKTDDSAKFQRDDIHSVIKAIYHSGNFVD

ATSAAPEGEQIGLILDRTNFYAEQGGQEYDTGRILIDGEAELDVQNVQGYGGYVLHTGYMKYGSLKKGDEVRCEYEELRR

APIRNNHTGTHILNFALREVLGEEVNQRGSLVAPEKLRFDFSHKTGCSEAELEKIEDLCTGYIRQNSEVYATDVPLATAK

SIEGVRAVFGETYPDPVRLVSIGVPVEDLLENVKRAEWRNVSVEFCGGTHVKNTTEIKDLVILEESGIAKGIRRIVAVTG

STAHEVQRQVAEWEKRVQQLEGMQYSAEKESKTKEWQHELGKVEISAVGKSKLRERFAKVTKDNLDHQKAAQKVENKKVL

DAVNEYFEKNKDDKTAVLTLPLSAGSKAVQEAIKTISSKNKDKTVYILGAAEDKVMHGCHVSSDAQGKGADASKWANSVA

SLVGGKAGGKGATSIGQGTEVSKVDEALEAARKYLQDLSL

>XP_018212815.1 hypothetical protein OGAPODRAFT_15056 [Ogataea polymorpha]

MSQQWTATKVRNTFLDYFKERGHTFVSSSSVIPHDDPTLLFANAGMNQYKPIFLGTVDPSSSFASLKRAVNSQKCIRAGG

KHNDLEDVGRDSYHHTFFEMLGNWSFGDYFKKEAIQYSWDLLTNVYGLNKDRLYVTYFEGDAANGLEPDLEAKQYWKDVG

VADDHILPGNAKDNFWEMGDQGPCGPCSEIHYDRIGGRNASSLVNQDDPDVLEIWNNVFIQFNREQDGSLKPLPAKHVDT

GMGFERLVSVLQNVRSNYDTDVFQPLFSKIQEITGVRPYSGKFGKDDLDGIDTAYRVLADHVRTLTFALADGGVPNNEGR

GYVLRRILRRGARYVRKYMNYPIGKFFSQLAPTLIEQVKDIFPEVATNTDTIFEILDEEEASFARTLDRGEKLFEQYAII

ASKTPEQTLAGRDVWRLYDTYGFPVDLTRLMAEEAGLNIDEEGFEKARLASLEASKAGGSKSGKTLLKLDVHILGALDSN

KDIPVTDDSYKYGNQDIKTKIVGIYNGKELLGSTEGTEAGQQLGVLLEKTPFYAEQGGQEYDTGKIVIDGVAEFEVENVQ

SYAGYVLHTGHVVEGKFSVGDQVIAGYDELRRWPIRNNHTGTHILNLALREILGDGVDQKGSLVAAEKLRFDFSNKAAVQ

LEQLEKIESICNKIIKDSMPVYSKPVALSIAKSIYGLRAVFGETYPDPVRVVSIGIPVEELLAKPDNKEWYDYSVEFCGG

THVAKTSDIKELVIVEESGIAKGIRRIVAVTGTEAHEVQRIAKDFDEQLDAIEKMPFGEAKEKKLKETSTKLGQLTISVL

QKAQLKDKFAKIEKAVKDEVKARQKADTKKALDAVKEFFSEHKDAPFLVKHIDISPNAKIITEAINMIKKENKEKSLFLI

TGQKDDARVAQGVYISDEHLGKVQATEVANVAASYIGGKAGGKGNVCQGMGTEAAQIDKAISAVEKLLESKLIM

>XP_013932997.1 Alanine--tRNA synthetase [Ogataea parapolymorpha DL-1]

MNICCFQRHTLKPRATQQIRKMSQQWTATKVRNTFLDYFKDRGHTFVSSSSVIPHDDPTLLFANAGMNQYKPIFLGTVDP

SSSFASLKRAVNSQKCIRAGGKHNDLEDVGRDSYHHTFFEMLGNWSFGDYFKKEAIQYSWDLLTNVYGLNKDRLYVTYFE

GDAANGLEPDLEAKQYWKDVGVADDHILPGNAKDNFWEMGDQGPCGPCSEIHYDRIGGRNAASLVNQDDPDVLEIWNNVF

IQFNREQDGSLKPLPAKHVDTGMGFERLVSVLQNVRSNYDTDVFQPLFSKIQEITGVRPYSGKFGKDDLDGIDTAYRVLA

DHVRTLTFALADGGVPNNEGRGYVLRRILRRGARYVRKYMNYPIGKFFSQLAPTLIEQVKDIFPEVATNTDTIFEILDEE

EASFARTLDRGEKLFEQYAIIASKTPEQTLAGRDVWRLYDTYGFPVDLTRLMAEEAGLNIDEEGFEKARLASLEASKAGG

SKSGKTLLKLDVHILGALDSNKDIPVTDDSYKYGNQDIKTKIVGIYNGKELLGSTEGTEAGQQLGVLLEKTPFYAEQGGQ

EYDTGKIVIDGVAEFEVENVQSYAGYVLHTGHVVEGKFSVGDQVIAGYDELRRWPIRNNHTGTHILNLALREILGDGVDQ

KGSLVAAEKLRFDFSNKAAVTLEQLEKIEAICNKIIKDSMPVYSKPVALNVAKSIYGLRAVFGETYPDPVRVVSIGVPVE

ELLAKPDNKEWYDYSVEFCGGTHVAKTSDIKELVIVEESGIAKGIRRIVAVTGTEAHEVQRIAKEFDEQLDAIEKMPFGE

AKEKKLKETSTKLGQLSISVLQKAQLKDKFAKIDKAVKDEVKARQKADTKKALDAVKEFFAEHEDAPFLVKHIDISANAK

IITETINMIKKENKEKSLFLITGQKDDARVAQGVYISDEHLGKVQATEVANVAASYIGGKAGGKGNVCQGMGTEAAQIDK

AISAVEKLLESKLIM

>OAT03431.1 alanyl-tRNA synthetase [Blastomyces gilchristii SLH14081]

MRGVGGERVASGVVLRRGLGFSRLRARAGAGAGAGAGAGVAGIGAAGICVGGAPRGVCGVGPSPPSTRFFARRRFPSPAI

YNSSTSTPSPVATLPARFHHHGLAHSAFKMASTQTVEQPEWSALRVRNAFLDFFKENGHTFVPSSSVVPLSDPTLLFTNA

GMNQYKSIFLGTVDPNSDFATLKRAHNSQKCIRAGGKHNDLDDVGKDSYHHTFFEMLGNWSFGDYFKKEAISYSWDLLTK

VYGLEPDRLYVTYFEGNPDAGIGPDLEAKELWLSVGVAEDHLLSGNMKDNFWEMGEQGPCGPCSEIHYDRIGGRNAAHLV

NQDDPNVLEIWNNVFIQYNREPDRSLRPLPNRHIDTGMGYERLVSILQNKSSNYDTDVFTPLFDKIREITGCRPYTGKFG

EEDTDGIDTAYRVVADHIRTLTFAISDGASPNNEGRGYVVRRVLRRGARYARKYLNVEIGSFFSKIVPTLVEQMGDMFPE

IRRKQTDVMEILDEEEISFAKTLDRGERLFEEYALQAKQKGLGNLHGADVWRLYDTFGFPVDLTQLMAEERGLTIDNAEF

EEARLKAKEASKGQGKQTADVVKLTVHDLGLLETMDNVPKTNDDAKFGRGNITSQIKAIYHNKKFVDSTKDIPEGEQFGI

ILDRTNFYAEQGGQENDTGKIIIDGKAELAVEDVQVYGGYILHTGFMKYGSFNVNDSVIAEYDELRRWPIRNNHTGTHIL

NFALRQVLGEGVEQKGSLVAAEKLRFDFSHKSAVSDSDLEKIERISTDYIRQNCAVYAKDVPLSTAREISGVRAVFGETY

PDPVRVVSVGVELEEILQNVHDPRWKDISIEFCGGTHVQKTGDIKDLVILEENGIAKGIRRIIAVTGEEAHEVQRIAKEF

GERLQRFDKMELGPQKEMEAKLLQVDLNQLTISAVEKVRFRERFTQIHKKVVEGQKAAQKLESKKALDAITGYFETPENK

DAPHLVLQLPISANPKAVSDSLNFVKTKMQNKSVYVFAADKGAGKVVHGCHVAQSLSAQGATPNEWANTVSSIVGGRAGG

KAPVAIGNGTEAEKLEDAIKAATEYLEKFKL

>KDQ21195.1 hypothetical protein BOTBODRAFT_25615 [Botryobasidium botryosum FD-172 SS1]

MSTTTTPEWPAAKVRQVFIDFFKNKGHTFVPSSSTIPYEDPTLLFANAGMNQYKSIFLGTVDPNSDFAKLKRAVNSQKCI

RAGGKHNDLEDVGKDTYHHTMFEMLGNWSFGDYFKKEAILWAWELLTEVYKLPKDRLYVTYFEGDAKQGLSPDLEAKQLW

LDVGVPEDHILPGNAKDNFWEMGATGPCGPCSEIHYDRIGGRNAGHLVNQDDPNVLEIWNNVFMEFNREDDGSLRPLPAR

HIDTGMGFERLVSALQDKYSNYDTDVFLPLFAKIQELTGARAYTGKLGAEDVDGIDTAYRVVADHVRTLTFALADGGVPS

NVGRGYVLRRILRRGARYVRKKFDVPIGSFFSSLMPAVIDQLGGFFPELNKRTEDIKEILNEEEESFSRTLDRGEKLFER

YATRAKEAGATQLSGTDVWRLYDTYGFPVDLTRLMAEELGLGVNDKEFEAAQAHSKEASKATKKTGDKDVVKLDVHDIAA

LEKNDNVPKTDDSHKFALGNVTARLKGIYHSKAFFTSTKEVPQDATFGLLLDRTSFYAESGGQEYDTGSIVIDDVAEFEV

ENVQVFNGYVLHIGHMKYGQLALEDEVVASYDELRRWPLRNNHTATHILNYALREVLGDHVDQKGSLVAPSKLRFDFSHK

AQITLPELTKIEEISNEWIRRNVKVYSKELNLEVARKIPGLRAVFGEVYPDPVRVVTLEYDVEDIAKDVENPKWRNTSVE

FCGGTHVAKTGDIKDFVITEESGIAKGIRRLVAVTGAEAHDVTREASTFESRLSQVERLTGKEKDAGLKTLTADILGAEI

SVLRKADLKERLTKVRKAFDAENKAVEVALTKAATEGLTKHFTENPNSNSYISHVDVEGSPKILQSLVAQARTLQKAVFL

FSVDSGAGKVAYLNYLPKTSISKEFNAKSWATSVADVLGGKGGGKDDSAQGVGTNIDKVSEAIQKAEAAYKANVSS

>XP_002173036.1 cytoplasmic alanine-tRNA synthetase Ala1 [Schizosaccharomyces japonicus yFS275]

MSTTETLEWPANKVRSTFIDYFKEHGHTFVPSSSVIPFEDPTLLFANAGMNQYKPIFLGTVDPSSSFAKLKRACNSQKCI

RAGGKHNDLEDVGKDNYHHTMFEMLGNWSFGDYFKKEAIPLAWKLLTEVFKLNPDQLYVTYFGGHEESGLGPDLEAKNIW

LEMGIPPERVIPGNLKDNFWEMGEQGPCGPCSEIHYDRIGGRSVPELVNQDDPNVLEIWNIVFMEFNREKDGSLRPLPNK

HIDTGMGFERLVSVVQNKSSNYDTDLFAPLFQKIQELTGARPYEGRMGEDDVDGIDTAYRVVADHVRTLTFAISDGGVPN

NEGRGYVLRRILRRGARYVRKKFNTPIGDFFSRLALVVAEQMGEFFPEVRSKLEDVKEILDEEEESFSRTLDRGEKMFEQ

YAAAAQKTESKMLKGMDVWRLYETYGFPVDLTHLMAEEAGLKIDVEGFNAAQAHSKEISRRAAKAGGDSATMVTLDIHAL

GALAKMPEVPKTDDHFKHESGTINATVKALYHPSGFQTSTEKFEAGENIGILLDRTNFYAEQGGQEYDTGRITIDGKLEF

RVTNVQVYGGYVLHTGFCEYGSLNVGDEVICEYDELRRRPLMNNHTVTHILNFALREVLGEGVDQRGSLVSPEKLRFDFS

HKAALSIKELERVENICNQKIAENLRVYAKELPLAISKEIAGLRAVFGEVYPDPVRVVCVGADIDEIIKDPKNEQWRQYS

IEFCGGTHLPKTGEIKDFVILEESGIAKGIRRIVAVTAADANQVCRIANEFDQRLDRLAAMPHGPEKEAEIKLVTVDLSQ

LSVSALRKHAMKEKFTKITKQFQDQVKAANAKEQKDAVKAVADYFEEHPESKFFVSKLNVSPNPKVLNFAISYAKKNLAG

KSLYLLASNGAQPDKVAHACLVSQEALPKVTAQEWSQKVSHVVGGRCGGKKDSCQGVGTNADKIDQAIEEAIEFFQGKLT

L

>EQL37596.1 alanyl-tRNA synthetase [Blastomyces dermatitidis ATCC 26199]

MRGVGGERVASGVVLRRGLGFSRLRARAGAGAGAGAGAGVAGIGAAGICVGGAPRGVSGVGPSPPSTRFFARRRFPSPAI

CNSSTSTPSPVATLPARFHHHGLAHSAFKMASAQTVEQPEWSALRVRNAFLDFFKENGHTFVPSSSVVPLSDPTLLFTNA

GMNQYKSIFLGTVDPNSDFATLKRAHNSQKCIRAGGKHNDLDDVGKDSYHHTFFEMLGNWSFGDYFKKEAISYSWDLLTK

VYGLEPDRLYVTYFEGNPDAGIGPDLEAKELWLSVGVAEDHLLSGNMKDNFWEMGEQGPCGPCSEIHYDRIGGRNAAHLV

NQDDPNVLEIWNNVFIQYNREPDRSLRPLPNRHIDTGMGYERLVSILQNKSSNYDTDVFTPLFDKIREITGCRPYTGKFG

EEDTDGIDTAYRVVADHIRTLTFAISDGASPNNEGRGYVVRRVLRRGARYARKYLNVEIGSFFSKIVPTLVEQMGDMFPE

IRRKQTDVMEILDEEEISFAKTLDRGERLFEEYALQAKQKGLGNLHGADVWRLYDTFGFPVDLTQLMAEERGLTIDNAEF

EEARLKAKEASKGQGKQTADVVKLTVHDLGLLETMDNVPKTNDDAKFGRGNITSQIKAIYHNKKFVDSTKDIPEGEQFGI

ILDRTNFYAEQGGQENDTGKIIIDGKAELAVEDVQVYGGYILHTGFMKYGSFNVNDSVIAEYDELRRWPIRNNHTGTHIL

NFALRQVLGEGVEQKGSLVAAEKLRFDFSHKSAVSDSDLEKIERISTDYIRQNCAVYAKDVPLSTAREISGVRAVFGETY

PDPVRVVSVGVELEEILQNVHDPRWKDISIEFCGGTHVQKTGDIKDLVILEENGIAKGIRRIIAVTGEEAHEVQRIAKEF

GERLQRFDKMELGPQKEMEAKLLQVDLNQLTISAVEKVRFRERFTQIHKKVVEGQKAAQKLESKKALDAITGYFETPENK

DAPHLVLQLPISANPKAVSDSLNFVKTKMQNKSVYVFAADKGAGKVVHGCHVAQSLSAQGATPNEWANTVSSIVGGRAGG

KAPVAIGNGTEAEKLKDAIKAATEYLEKFKL

>OMJ23467.1 Alanine-tRNA synthetase [Smittium culicis]

MSSQDICPEWPADKVRSTFIEFFCSKMHVNHLSSSVVPHDDPTLLFANAGMNQFKPIFLGTVDPNSDMSKLKRATNSQKC

IRAGGKHNDLDDVGKDVYHHTFFEMLGNWSFGDYFKKDAISFAWELLTKVYRLSEDRLYVTYFGGDSSQNLPEDIEAKQL

WIDIGVPESRILPFGTKDNFWEMGEVGPCGPCSEIHYDRIGNRDAAHLVNLDDPDVLEIWNLVFMQYNRESQTELKPLPN

SHIDTGMGFERLVSVLQNKSSNYDTDVFVPIFKAIQEKSGVREYTGKVGPEDVDGIDMAYRVIADHVRTLLFAITDGAVP

SNEGRGYVVRRILRRGARYARKKMQVELGNFFPSLLDVVVQQMSPTFPEILKKVDFIKQTLQSEEISFARTLDRGEKLFD

ATLNKMRTSGQTTVPGAEVWKLYDTFGFPVDLTRVMADENSVFIDEAEFEAEKNKSKELSKKKKGNDSSSETVLLDVHTI

SHLETSLKIQKTDDSFKFSKSDITSSILSIYSNKGFVQTQEASSDGSANTGQLFGILLDKTNFYAEAGGQEWDTGYLLSQ

TQDSQFVVTQVKNFGGYVLHVGYLKNGSLSIGESVLCCYDQDRRRSLCSNHTSTHILNHSLRNVLVSDELDQRGSLVASD

KLRFDFSFNGQISADQIKEIELKCKKVIDDDLEVFSSDVPLQNSKEINGLRAVFGETYPNPVRVVSIGKSVDELVNDPHN

ESWVNYSIELCGGTHVKSTKEISDFIITEEASISKGIRRIVAVTGPESEQVLNSYNIAKQMVDKLSTLSGSQLESEIKVV

GKFLDNVNAGVYNKHVLRTKFNQIRSDFIEADKAAKALATKFAIDQVNIAIESNPDAKYHVINLQFDNSLISNSGKALSN

AIAHVKSLNSKAVYLISPDATLGKVAHLCYVPKDLINSGLHAKQWAESVSSVIGGKCGGKNESAQGSGTNVQSVSDAERV

ANDFAKLNIK

>CDO55361.1 similar to Saccharomyces cerevisiae YOR335C ALA1 Cytoplasmic and mitochondrial alanyl-tRNA synthetase [Galactomyces candidum]

MTVTELTPENWSASKVRQTFLEFFKQKEHNYIPSSAVIPYDDPSLLFTNAGMNQFKPIFLGTVDPTSSLYNLRRAANSQK

CIRAGGKHNDLEDVGKDSYHHTFFEMLGNWSFGDYFKKEAIAWAWELLTQVYGLDPNRLYVTYFEGDLKNGLEPDLEAKQ

IWLNTGVPEDHIIPGNAKDNFWEMGDQGPCGPCSEIHYDRIGGGRNASSLVNMDDPNVLEIWNIVFIQFNREASGELKSL

PAKHIDTGMGFERLVSILQDKSSNYDTDVFTPLFEKIQQLTGAKKYTGKFGKEDIDGIDTAYRVLADHVRTLTFAISDGG

IPNNDGRGYVLRRILRRGTRYLRKYFNVPIGEFFSRLAPTLISQVSDIFPELEKAPRDVFEILNEEEESFSRTLDRGEKL

FQQYARNAIENNINTLNGKDVWRLYDTYGFPADLTRLMAEEAGLDIDEDDFEKARIASKEASKGKKQTENKAKVKLDVHT

LQELSSNNIPITDDKYKYQSSNIEASVLAIFVDNKFVDSTKSISDNTIFGIILDKTNLYAEQGGQEYDTGKLVIDGEAEF

EVTEVQAYGGYVLHIGFIDYGSLNVGSPVNVGFDELRRWPIRNNHTGTHVLNFALREVFGDSVDQKGSLVAPEKLRFDFS

QKTAPTLDEVKKIEKLSNEKIRKNLLVYSQDVPFAKASEISGLRAVFGEKYPDPVRVVSIGVPVEELLKDPTSPKWHEYS

IEFCGGTHVAKTGDIKDLVIIEESGIAKGIRRIVAVTGITAHEVQNRAIEFEKKLDHLESLPTSESKENLSKQLGIELKQ

ITVSVLSKNKLQQKYTMIQKKIVDEAKIKHKAEVRKAINLISSYFGGDETGLTPSYFVGEIDGISANPKVLTEVVNHFRT

KLPIKSVYLFAKDDIKVVHGSFVSPEASGITAQELGDIARNIVGGKSGGKGPTVQGVGDKPDSVEEAIQKLAAVLKEKVT

I

>GAD98516.1 alanyl-tRNA synthetase, cytoplasmic [Byssochlamys spectabilis No. 5]

MASTLSRPEWTASQVRETFLDYFKKNGHTFVPSSSVVPLSDPTLLFTNAGMNQFKSIFLGTVDPQSDFAQLKRAVNSQKC

IRAGGKHNDLDDVGKDSYHHTFFEMLGNWSFGDYFKKDAIRYSWELLTKVYGLSPDRLYVTYFEGNEAGGLEPDLEAKEL

WRSVGVADDHILPGNMKDNFWEMGDQGPCGPCSEIHYDRVGGRNAAHLVNQDDPDVLEIWNNVFIQYNREPDRSLRSLPN

KHVDTGMGYERLVSVLQDKSSNYDTDVFLPLFKKIQEITGARDYQGRFGAEDPDGIDTAYRVIADHVRTLTIAITDGAAP

NNEGRGYVIRRVLRRGARFARKYFQVEIGSFFSKIVPTVVEQMGDMFPELKKKQADVMEILDEEEVSFAKTLDRGERQFE

NYAQQAKVKGLDKLHGADVWRLYDTFGFPVDLTQIMAEERGLRIDDAEFEEARLKAKEASRGEKKAAAETVKLDVHDLGK

LEKMNDVPKTDDNAKFEKGNINAQVKAIYHAKKFVSSTDEIPEGEQLGIILDRTNFYAEQGGQENDTGKIIIDGKAELEV

GDVQSYAGYVLHTGSMKYGSFSLGDTVICEYDELRRWPIRNNHTGTHILNFALRAILGDGVEQRGSLVAAEKLRFDFSHK

SAISDSDLAKIEDKSTEYIRQNCAVYSKEVPLATAREISGVRAVFGETYPDPVRVVSVGVELEEILKNVKDPRWEEVSIE

FCGGTHVQKTGDIKDLIILEESGIAKGIRRIIAVTGEEAHQVQRVAQEFEKRVDLLDKMAMGPKKEQEAKQIQVELSQLS

ISAVHKAKLRERFNQINKQVIEGQKAQQKLESKKALETITTYFQDPENKDKTSLVVRLPISANAKAVSESLNYVKSKLSD

KAVYVLAADSEQGRVSHGCYVPKNLGDQGATPNEWAGVVAGAVGGKAGGKAPTSIGSGTNVDKVDEAIAQATEYLQKFKL

>XP_016759050.1 alanyl-tRNA synthetase [Sphaerulina musiva SO2202]

MRSFHRQLVSGLLHFRISRNATKTTTTTTTTITSTYTAPIRVIFPQIRRMASTTGHEPEWSAVKVRKTFIEYFEKNGHTF

VPSGSVVPLSDPTLLFTNAGMNQFKAIFLGTVDRDSDFAKLKRAANSQKCIRAGGKHNDLDDVGKDSYHHTFFEMLGNWS

FGDYFKEEAIQLSWQLLTKVYGLQPDRLYVTYFEGKADAGLDPDTEARDFWLAQGVPEDHILTGDMKDNFWEMGDQGPCG

PCSEIHYDRIGGRNASHLVNQDDPNVIEIWNNVFIQYNREPDKSLKSLPNKHVDTGMGFERLVSILQNKPSNYDTDVFTP

LFARIQEITGAREYQGKFGEEDADGVDTAYRVVADHVRLLTFAISDGGVPNNVGRGYVVRRVLRRGARYARKYFNTDIGS

FFSKLVPTLVEQMGDMFPEIKKKQDEVKEILDEEEVSFAKTLDRGESMFEKYAQNCKSSGTKTLAGADVWRLYDTYGFPV

DLTRLMCDERGLEIIDKDVAEAQEKAREASKGEKKAGATLVKLDVHDISTLENMPDVPKTDDTPKFQRDNIKATIKAIYH

DRKFIKSTSEIPEGEQFGVILDKTNFYAEQGGQENDTGRILIDGEAEIKVDNVQQYAGYVMHTGYVEYGSLKVGDEVLSE

YDELRRQPIRNNHTGTHILNYALREVLGDDVNQKGSLVAPEKLRFDFSHKAGVTDEELAKIEEVSTNYIRQNFEVFSKDV

PLATARQINGVRAVFGETYPDPVRVVSVGMAVEDLLENPSDPKWNDISIEFCGGTHVKSTTEIKDLVVVEESGIAKGIRR

IVAVTGAEAHRVQAIAQEWEKQVSALEKLPYGPDKEAKTKQLTTDLSKLDISALQKTSLRNRVAAVAKANLDQQKTAQKA

ESKKVLDEVTNHFVSNAGSTTLVLAVPVSAGGKSIQETIKVVQTKQKDKTVYLLGKTDDGKVIHGCHVSADAQAKGADAA

KWSSEVSSLVGGKAGGKGATSLGQGTDATKVGEAVEAAQKYLANLSL

>PGH33736.1 alanine-tRNA synthetase [Emmonsia crescens]

MRVGGQRVSGVVLRRGLSLPGYGSRAVCHSGVGGAGAGAGAGAGGGLNVPSPPSRRFFARRFPFPAITNTSTSTSTTATL

TARFHHHGLAQSAFKMASTQNVEQPEWSALRVRNAFLDFFKDNGHTFVPSSSVVPLSDPTLLFTNAGMNQYKSIFLGTVD

PNSEFATLKRAHNSQKCIRAGGKHNDLDDVGKDSYHHTFFEMLGNWSFGDYFKKEAISYSWDLLTKVYGLDPDRLYVTYF

EGNSDAGIGPDLEAKELWLSVGVAEDHLLSGNMQDNFWEMGEQGPCGPCSEIHYDRIGGRNASHLVNQDDPNVLEIWNNV

FIQYNREPDRSLRPLPNKHVDTGMGYERLVSILQNKSSNYDTDVFTPLFDKIREVTGCRPYTGKFHEEDTDGIDTAYRVV

ADHVRTLTFAISDGASPNNEGRGYVVRRVLRRGSRYARKYLNVEIGGFFSKIVPTLVEQMGDMFPEIKRKQTDVMEILDE

EEISFAKTLDRGERLFEEYALQAKQKGLGKLHGADVWRLYDTFGFPVDLTQLMAEERGLTIDNVEFEEARLKAKEASRGQ

GKLTTDVVKLTVHDLGRLETMDDVPKTNDDAKFGRGNITSQIKAIYHNKKFVDSTKDIPEGEQFGIILDRTNFYAEQGGQ

ENDTGKIIIDGKAELVVEDVQVYGGYILHTGFMKYGSFNINNSVIAEYDELRRWPIRNNHTGTHILNFALRQVLGDGVEQ

KGSLVAAEKLRFDFSHKSAVSDSDIEKIERISTDYIRQNCAVYAKDVPLSTAREISGVRAVFGETYPDPVRVVSVGVELE

EILQNVHDPRWKDISIEFCGGTHVQKTGDIKDLVILEESGIAKGIRRIIAVTGEEAHEVQRIAKEFGERLRRFEKMELGP

QKEIEAKLLQVDLNQLTISAVEKARFRELFTQIHKKVLEGQKAAQKLESKKALDAITEYFQAPENKDAPHLVLQLPISAN

PKAVSDSLNFIKTKMQDKSIYVFAADKDAGKVVHGCHVAQNLSAQGATPNDWANTVSSIVGGRAGGKAPISIGNGTEAEK

LDEAIKAATEYLGKFKL

>CDO95315.1 unnamed protein product [Kluyveromyces dobzhanskii CBS 2104]

MTIGDKAKWTATNVRTTFLDFFKSKGHTFVPSSPVIPYEDPTLLFANAGMNQYKPIFLGTVDPSSDFYKLKRATNSQKCI

RAGGKHNDLEDVGRDSYHHTFFEMLGNWSFGDYFKKEAIEYSWELLTKVYGIPGDRLYVTYFEGDESLGLEPDFEARDLW

KAVGVTDDHILPGNAKDNFWEMGEQGPCGPCSEVHYDRIGGRNAAHLVNQDDPDVLEIWNNVFIQFNREQDRSLRSLPAK

HVDTGMGFERLVSVLHDVRSNYDTDVFEPLFKRIQEITQVRPYTGKFGAEDTDGIDTAYRVLADHVRTLTFALADGGVPN

NEGRGYVLRRILRRGARYVRKYMNYPIGNFFSQLSITLIDQVKVMFPEVAKDPSYLFEILDEEEASFSKTLDRGEKLFEK

YAEQAVKEAHKTLDGTQVWRLYDTYGFPVDLTELMAEEQGLKIDTVGFEQAKQESYEASKQGGKKGASGLLKLNVHELSQ

LNEDNVPKTDDSSKYGTENIEATILKIYDGEKFVTSMTEIGKQYGVVLDSTCFYAEQGGQEYDTGKLVIDGEAEFKVDNV

QVYNGYVFHTGSLGDGKLCVGNSVIASYEELRRHPLRNNHTGTHILNFALKEVLGNTVDQKGSLVSPEKLRFDFSHKRAL

SYDELVEVEKISNEKVKENLTVFYKDVALDLAKTISSVRAVFGETYPDPVRVVSVGKAVDEILQNPSSEEWNKYSIEFCG

GTHVAKTGDIKEFVIVEESGIAKGIRRIVAVSGPEAYEVQRIALKFEDELTAVDSLPFSALKAQKLKDLGVRLGQLNISV

TSKTDLKDKFNKIEKEVKDEVKSRAKKEIKQTVDDVKQHFDEHKESLYYVKYIDIPPNAKAITEAINHIKSNLKDKSIYL

FTGNEEAGKVAHGCYISEAAISKGVDGSSLAKTVSSSIGGKAGGKGNVFQGMGDKPTNVDSAVAEVEVLLREKLTI

>PVF97475.1 hypothetical protein CPB86DRAFT_807243 [Serendipita vermifera 'subsp. bescii']

MSSTPYQGPWTAPKIRQTYIDFFKSKGHTFWPSSSTIPFEDPTLLFANAGMNQYKSVFLGTVDPNSDMSKLKRAVNSQKC

IRAGGKHNDLEDVGKDSYHHTFFEMLGNWSFGDYFKREALTWAWELLTEVYKLPVDRLYVSYFEGDPKQGLAPDEETRQI

WLDIGVPEERIVKGNAKDNFWEMGPTGPCGPCSEIHYDRIGGRDAAKIVNADDPTVVEIWNNVFIQFNREEDGSLKSLPA

KHVDTGMGFERLVSAIQDVLSNYDTDIFQPIFNRIQEVSGVRPYTGKFGAEDVDGIDTAYRVIADHVRTLTFALSDGGVP

SNIGRGYVLRRILRRGARYARKKLGAPIGSFFSSVSPVLVEQLGDFFPELKRSPEEVKKLLDEEEVSFARTLDRGERLFD

SYVAKIQGTGSTVLDGKDVWRLYDTYGFPVDLTRLMAEELNLTVDEEAFKAAQAQSKEASKGGAKTSVKDTVKLDVHDIG

ALEKNTEVPTTNDSFKFDSSSISATIKAIYYEKSFPSSTANIPTGASFGIILDKTCFYAEAGGQEYDTGVIALDMKDREA

KFEVINCQSFKGYVLHIGQLDEGELHVGDEIIVSYDADRRRPIRSNHTATHILNYALREVLGDHIDQKGSLVAPSKLRFD

FSHKAPVSLPELKKIEDICLEWIRKDVPVFSKDLDLKSARLIPGLRAVFGETYPDPVRVVSLEYDVDEISRDLENPKWRG

TSVEFCGGTHVAKTGDIKEFTIMEESGIAKGIRRIIAVTGPESRAAMAALAQFELRFTGVQGMTGKEKDEGFKTLTLDLN

QADLPALLKADLKDRLAAKRKAWDSEVKAREAAATKEATESVAKYFAENPDAQVYCGQVEIGSNIKALQSIINQAKTLNK

AVYVLSIDSQSGKVTHGNYLPPSLAADGFDARTWASGVSEIVGGRAGGKPDTAQGVGTDASKVADAVTVANELFAKATQK

>OQO19478.1 Alanine--tRNA synthetase [Rachicladosporium sp. CCFEE 5018]

MGSQAQDEVQWTAQKVRSTFLEFFEKNGHTIVPSGSVVPLSDPTLLFTNAGMNQFKSIFLGTVDPDSDFAKLKRAVDSQK

CIRAGGKHNDLDDVGKDSYHHTFFEMLGNWSFGDYFKKEAIAFSWELLTKVYGLEPDRMYVTYFEGKADVALEPDLEAKE

LWKSVGVADDHILPGDMKDNFWEMGDQGPCGPCSEIHYDRIGGRNAAHLVNQDDPNVLEIWNNVFIQYNREPDKSLKPLP

SKHVDTGMGFERLVSVLQDKLSNYDTDVFTPLFKAVQEITGAREYSGKFGEDDVDGIDTAYRVVADHVRLLTFAISDGGV

PNNVGRGYVVRRVLRRGARYARKYFKTEIGGFFSKLVPTLVNQMGDMFPEIKKKELDVKEILDEEELSFAKTLDRGEVMF

EKYAEQCKQRDSKELSGADVWRLYDTYGFPVDLTKLMAEERGLSIDDDAVHEAQEKAREASKGEKKSAAELVKFSVHDIN

TIENMEDVPKTDDSSKFLRENVSGTVKAIFHGGKFLQSTADAPEGEQLGIVLDKTCFYAEQGGQEFDMGRILIDGEAEID

VQNVQVYAGYVLHTGFMSYGALKTGDNVLCQYDELRRQPIRNNHTGTHILNFALREVLGEDVNQRGSLVAPEKLRFDFSH

KAGCSDAELEKIEEISTSYIRQNSEVYSTDVPLATARNIEGVRAVFGETYPDPVRVVSIGVPVEELLEDTTRKEWRSVSV

EFCGGTHVKNTSEIKDLVVVEESGIAKGIRRIIAFTGAAAHEVQRIAGEWDEKFTKLESQAYTPEKEVRTKEYQHALNSL

EISALAKTKLRDRVSKIVKANMEHQKTAQKAENKALTDAVTAHFTQHPDAKSFVTSHPVSAGSKAIQETLKLVSSKQKDK

NVYIVGASADGKVVHGCFVSSDAQAKGADAGKWSTTVAGVVGGKAGGKGATSFGQGTEAEKVDEGVEAARKYLEGLRL

>OJT03379.1 Alanine--tRNA synthetase [Trametes pubescens]

MASSTHTDWSASKVREEFFNYFKSREHTFVPSSSTIPYDDPTLLFANAGMNQYKGIFLGTVDPQSELARLKRAFNSQKCI

RAGGKHNDLEDVGKDSYHHTFFEMLGNWSFGDYFKKEAIEYSWELLTEVYGLPKDQLYVTYFEGDPKAGLEPDLEAKDWW

LKTGVAEDHIIPGNAKDNFWEMGATGPCGPCSEIHYDRIGGRNAASHVNQDDPDVLEIWNNVFIQFNREDDGSLKPLPAK

HVDTGMGFERLVSVVQNKRSNYDTDVFQPIFARIQEVTGVRPYGGKFGDEDENGIDTAYRVIADHVRTLTFALSDGGVPN

NVGRGYVLRRILRRGARFARKKLGVQIGAFFSSLVPVVVEQMGAVFPEITQKSAEIKEILDEEEESFSRTLDRGEKLFEQ

YAQKTLQSGLKELSGKDVWRLYDTYGFPVDLTRLMAEELGLSINEAEFEAAQAHSKEASKGGVKKGTGEAVALDVHDIAA

LEKNDGVPKTDDSFKFQQGNITAKVVAIYSKKAFLKSTEEIPEDASFGLLLDRTSFYAESGGQEYDTGSIVIDGVAEFEV

TNVQVYNGYVLHIGHMKYGKLNIGDEIVSSYDELRRWPLRNNHTATHILNYSLREVLGDHIDQKGSLVAPTKLRFDFSHK

AQVSTSELTKIESISQDFIKRNVGVFGKELPLHVAQKINSLRAVFGESYPDPVRVVSLEFDVDDIAKDLENPKWRKTSIE

LCGGTHVARTGEIKDFVVTEESGIAKGIRRVVAVTGHEAAEARRLGESLTAQLNRIEAMPDAEKDAALKAYTVHIGQVDI

ALLKKNELRDRLASVRKAFDKHLKDKEAAANKASVDALQQYFMDKPGAPAYFAALDVNANAKVMQGVVLQAKKLGKSIYI

FSADQSAGKVAHVNAVSEEAKGMGLDGREWASAVVEILGGKAGGKPDGAQGVGAEVSKVQDALAAAQKFFESKTGLSV

>OCF35527.1 alanine-tRNA synthetase [Kwoniella heveanensis BCC8398]

MSMANSAPKVPDTAPAHWPSPTEWPAAKVRQTFIDYFVKQPGFEHTFWPSSGCIPFDDDTLLFANAGMNQYKPLFLGTAD

PKSDLSKLLRAVNSQKCIRAGGKHNDLDDVGKDTYHHTFFEMLGNWSFGNYFKAGACTMAWDLLTRVYGLPKDRLYVTYF

EGDAKQGLEPDTEAIQIWKDLGVPEHHILPGNAKDNFWEMGATGPCGPCSEIHFDRIGGREAAELVNADDPNVIEIWNNV

FMQYNREQNGELRPLPAKHIDTGMGFERLVSILQNVPSNYDTDVFTPIFAKIQELTGARPYEGKLGEEDADGVDTAYRVV

ADHIRTLTIAISDGGVPDKDGRGYVLRRILRRGVRYASNKLNVKIGSFFSSLVPTVVESLGDIFPEIRKKIPELVEILNE

EEASFARTLTRGEALFTKYAETAKAEKRTVLAGKDIWRLYDTYGFPVDLTQIMAEERGLQIDQDAFEEARLESLEASKAG

GKDKATAGVKLDVHDLAALEANDAVPKTDDSSKYQLDDIKATVKSIYHASKFYTSTSELPAHAPFGVLLDKTNFYAESGG

QEYDTGVIAIDGQAEFKVEDVQVFNGYVLHIGQMEEGEIKVGDEVICTYDELRRWPIRNNHTGTHILNFALREVLGDHID

QKGSLVAPTKLRFDFSHGKSIGTPEITKIEAISNDWIKKAAPVYAKEMSLAEAYKIPGLRAVFGEAYPDPVRVVSLGFPL

EEIAQNVEDNKWRQTSIEFCGGTHVAKTDDIKDFVIVEETSIAKGIRRIVAVTGHEAHEVSRKAGDFERRLDRIAQLEGK

EKETALKPYLVELGQSGISIIKKDALKKRFEKMQSELVAAAKAKTAADSKVIQEEIKAFFKANPNDNVYAGSFDVAAGNA

KTLAAATTTAKSLSKAVYVFSVDPETGKVAHTNYLPKEVLDRKVIDAKTWLGEVSKVIGGKGGGRDDSATGVGSEVSKID

EAIAVAKSFYKSKVEA

>EKG22385.1 Alanyl-tRNA synthetase class IIc [Macrophomina phaseolina MS6]

MASSTVDQQTEWSAQRVRDTFLDYFKKNGHTFVPSSSVVPLSDPTLLFTNAGMNQYKSIFLGTVDPNSDFATLKRAVNSQ

KCIRAGGKHNDLDDVGKDSYHHTFFEMLGNWSFGDYFKKEAIEYSWELLTKVYGLDPDRLYVTYFEGDQSLGLEADEEAR

NYWKAVGVAEDHILKGNAKDNFWEMGDQGPCGPCSEVHYDRIGGRNAAHLVNQDDPNVLEIWNNVFIQYNREPDRSLRSL

PNKHVDTGMGYERLVSVLQNKMSNYDTDVFTPLFKRIQEITGVREYRGKFGEEDADGIDTAYRVVADHVRLLTFAISDGA

IPDAVGRGYVVRRVLRRGARYARKYLQTDIGSFFSKIVPTLVEQVGDMFPEIKKREQDVKEILDEEEQAFAKSLDRGEVM

FDRYAKQVEASGSKKIPGKDVWRLYDTYGFPEDLTLIMAEERGLTIDDAEVREAQAIAREASKGAKKAGSNIVKLDVHAL

GQLQDMKVPTTDDSFKFAKENITAKVKAIYHPEKKFIKSTSEIGEGEQIGVVLDKTNFYAEQGGQEYDTGKILIEGANEA

AFDVQNVQVSNGFVLHTGYLSYGALSVEDEVICEYDELRRNPIRNNHTGTHVLNYALREVLGGDIEQKGSLVAPEKLRFD

FSHKQGITEAEIQKIETISNGYIRDNRTVYIEPVAVAEAHKIRGVRAVFGEKYPDPVRVVSIGVPVSELLKDPENEAWAK

LSIEFCGGTHVASTGEIKELVVVEESGIAKGIRRIIAVTGQGAYDVQRVAADFSKELERLEKMAFGPEKEALAKRIQNEL

NELSVSLLDKAKMRKRFEAVSKSVLAEQKKQSKAVSTKVQETVTNYFAANKDAKFLVQKLDYNAHTSKAITEALKHVSTK

QKDKTVYLIAGDAQEGKVAHGCYMGDHFETQGASAGDWSKAVSELVGGKAGGKGKTAIGQGTNPDKLDQALDQATKYLEK

FQI

>EGE78320.1 alanyl-tRNA synthetase [Blastomyces dermatitidis ATCC 18188]

MRGVGGERVASGVVLRRGLGFSRLRARAGAGAGAGAGAGVAGIGAAGICVGGAPRGVSGVGPSPPSTRFFARRRFPSPAI

CNSSTSTPSPVATLPARFHHHGLAHSAFKMASAQTVEQPEWSALRVRNAFLDFFKENGHTFVPSSSVVPLSDPTLLFTNA

GMNQYKSIFLGTVDPNSDFATLKRAHNSQKCIRAGGKHNDLDDVGKDSYHHTFFEMLGNWSFGDYFKKEAISYSWDLLTK

VYGLEPDRLYVTYFEGNPDAGIGPDLEAKELWLSVGVAEDHLLSGNMKDNFWEMGEQGPCGPCSEIHYDRIGGRNAAHLV

NQDDPNVLEIWNNVFIQYNREPDRSLRPLPNRHIDTGMGYERLVSILQNKSSNYDTDVFTPLFDKIREITGCRPYTGKFG

EEDTDGIDTAYRVVADHIRTLTFAISDGASPNNEGRGYVVRRVLRRGARYARKYLNVEIGSFFSKIVPTLVEQMGDMFPE

IRRKQTDVMEILDEEEISFAKTLDRGERLFEEYALQAKQKGLGNLHGADVWRLYDTFGFPVDLTQLMAEERGLTIDNAEF

EEARLKAKEASKGQGKQTADVVKLTVHDLGLLETMNNVPKTNDDAKFGRGNITSQIKAIYHNKKFVDSTKDIPEGEQFGI

ILDRTNFYAEQGGQENDTGKIIIDGKAELAVEDVQVYGGYILHTGFMKYGSFNVNDSVIAEYDELRRWPIRNNHTGTHIL

NFALRQVLGEGVEQKGSLVAAEKLRFDFSHKSAVSDSDLEKIERISTDYIRQNCAVYAKDVPLSTAREISGVRAVFGETY

PDPVRVVSVGVELEEILQNVHDPRWKDISIEFCGGTHVQKTGDIKDLVILEENGIAKGIRRIIAVTGEEAHEVQRIAKEF

GERLQRFDKMELGPQKEMEAKLLQVDLNQLTISAVEKVRFRERFTQIHKKVVEGQKAAQKLESKKALDAITGYFETPENK

DAPHLVLQLPISANPKAVSDSLNFVKTKMHNKSVYVFAADKGAGKVVHGCHVAQSLSAQGATPNEWANTVSSIVGGRAGG

KAPVAIGNGTEAEKLEDAIKAATEYLEKFKLCIFFFSFLLSLLLFSSV

>KIL68962.1 hypothetical protein M378DRAFT_71103 [Amanita muscaria Koide BX008]

MSSLSWSSVRVRQEFFDYFRSRNHTHVPSSSTIPYEDPTLLFANAGMNQFKSIFLGTVDPNSDLAKLKRAFNSQKCIRAG

GKHNDLEDVGKDSYHHTFFEMLGNWSFGDYFKKDAIAYSWELLTEVYGLPKEQLYVTYFEGDSKNGLEPDLEAKQFWLNQ

GVAEDHMLPGNTKDNFWEMGATGPCGPCSEIHFDRIGGRNAAHLVNKDDPDVLEIWNNVFIQFNREDDGSLKPLPAKHVD

TGMGFERLVSVIQNKRSNYDTDVFIPIFDKIHQLTGVRPYQGLFGDDDKDGIDMAYRVVADHVRTLTFALSDGGIPDKDG

RGYVLRRILRRGARYARKKLGVTIGSFFSSLVPVVIEQMGDVFPEIGRKVDAIKEVLDEEEESFSRTLDRGEKLFEEFAS

RAQTSGTRILNGKDVWRLYDTFGFPVDLTRLMAEELKLRINEEEFEEAQIQSREASKGLAKKGDVDIVKLDVHDIAFLDK

NEDVPKTDDSAKFNLGNTNATVKAIYHNRTFLDSSSSIPQGSAFGIILNRTNFYAEAGGQEYDTGSIVIDGVADFQVSNV

QVYNGYVLHTGQLKYGQLQVGNDVVSSYDELRRWPLRNNHTATHILNFCLRDVLGDHIDQKGSLVAPEKLRFDFSHKAQI

SPEELSAIESQSVQWIRKNVPVFSKELSLQEAYKIAGVRAVFGEAYPDPVRVVTLEYDVDEIAKDVENSKWRQTSIEFCG

GTHVSKTSDIKDFVVTEESGIAKGIRRIVAVTGHEAQEVTRLAQRFTERLDRADSLSGKEKDLALRSLTVELGQANISIL

KKAELKERLASIRKAFDKQVKEIEAAANKKAVEDVSNYFKDNKEAEHYIALLDVNGNPKILQNVMLQAKKLSKAAYLFSV

DAEAGKVAHVNYIPPQYKAKGADARVWATKITEIVGGKAGGKEDSAQGVGANVDKVADAIDVAEQYLTSF

>EOD50240.1 putative alanyl-trna synthetase protein [Neofusicoccum parvum UCRNP2]

MASLVDQQAEWPAQRVRQTFLDFFKKNGHTFVPSSSVVPLSDPTLLFTNAGMNQYKSIFLGTVDPNSDFATLKRAVNSQK

CIRAGGKHNDLDDVGKDSYHHTFFEMLGNWSFGDYFKKEAIEYSWELLTKVYGLDPDRLYVTYFEGDKSLGLEPDDEARK

YWKAVGVADDHILTGNVKDNFWEMGDQGPCGPCSEIHYDRIGGRNAAHLVNMDDPNVLEIWNNVFIQYNREPDRSLRPLP

NKHVDTGLGYERLVSVLQNKMSNYDTDVFTPLFKTIQEITGVREYQGKFGDEDTDGIDTAYRVVADHVRLLTFAISDGAI

PDAVGRGYVVRRVLRRGARYARKYLNTDIGNFFSKIVPKLVEQVGDMFPEIKKKEQDVKEILDEEEQAFAKSLDRGEVMF

DRYAKQVEASGSKKIPGKDVWRLYDTYGFPEDLTLIMAEERGLTIDDAEVREAQAIAKEASKGAKKAGANVVKLDVHALG

QLQDMKVPTTDDSFKFARDNITAKIKAIFHPEKKFIKSTSEVGEGEQIGLVLDRTNFYAEQGGQEFDTGKILIEGANEAA

FDVQNVQVSNGFVLHTGYLSYGALSLEDEVIAEYDELRRNPIRSNHTGTHVLNYALREVLGWDIDQKGSLVAPEKLRFDF

SHKQGVTEAEIAKIETIANGYIKDNRTVYIEPVAVAEAHKIRGVRAIFGEKYPDPVRVVSIGTPVSDILKDPENEKWEKL

SIEFCGGTHVASTGEIKELVVVEESGIAKGIRRIIAVTGQGAYDVQRVAADFSKELESLEKMAFSPEKEVRAKKSQNELN

ELSISVIDKEKLRKRLGAVTKSILDEQKKQAKVINKKVLDTVTNYFSENKDAKFLVQKLDYNAHTSKAIAEAIKHVSSKQ

KDKTVYLMAGDAQDGKVVHGCYMGDHFETQGASAGEWSKSVSGVIGGKAGGKGKTAIGQGTSPDKLDEGLEQATKYLEKF

QI

>OMJ15551.1 Alanine-tRNA synthetase [Smittium culicis]

MSSQDTSLEWPADKVRSTFIDFFCSKNHVNHLSSSVVPHEDPTLLFANAGMNQFKPIFLGTVDPNSDMSKLKRATNTQKC

IRAGGKHNDLDDVGKDVYHHTFFEMLGNWSFGDYFKKDAISFAWELLTKVYRLSEDRLYVTYFGGDPSQNLPEDIEAKQL

WIDIGVPESRILPFGTKDNFWEMGEVGPCGPCSEIHYDRIGNRNAAHLVNLDDPDVLEIWNLVFMQYNRESQTELKPLPN

SHIDTGMGFERLVSVLQNKSSNYDTDVFVPIFNAIQEKSGVREYTGKVGPEDVDGIDMAYRVIADHVRTLLFAITDGAVP

SNEGRGYVVRRILRRGARYARKKMQVELGNFFPSLLDVVVQQMSPTFPEILKKVDFIKQTLQSEEISFARTLDRGEKLFD

ATLNKMRASGQTTVPGAEVWKLYDTFGFPVDLTRVMADENSVFIDEAEFEAEKNKSKELSKKKKGNDSSSETVLLDVHTI

SHLETSLKIQKTNDSFKFSKSDITSSILSIYSNKEFVQSQESSSDGSANAGQLFGILLDKTNFYAEAGGQEWDTGYLLSQ

SQDSQFVVTQVKNYGGYVLHVGYLKNGSLSVGESVLCCYDQDRRRSLCSNHTSTHILNHSLRNVLVSDELDQRGSLVASD

KLRFDFSFNGQISADQIKEIELKCKKVIDDDLEVFSSDVPLQNSKEINGLRAVFGETYPNPVRVVSIGKSVEELVNDPHN

ESWINYSIELCGGTHVKSTKEISDFIITEEASISKGIRRIVAVTGPESEQVLNSYNIAKQMVDKLSTLSGSQLESEIKVV

GKFLDNVNAGVYNKHVLRTKFNQIRSDFIEADKAAKALATKFAIDQVNFAIESNPDAKYHVINLQFDNSLISNSGKALSN

AIAHVKSLNSKAVYLISPDATLGKVAHLCYVPKNLIDSGLNAKQWAESVSSVIGGKCGGKNESAQGSGTNVESVSDAERI

ANDFAKLNIK

>CDO74931.1 hypothetical protein BN946_scf184945.g3 [Trametes cinnabarina]

MASTNSWPAAKVREEFFNYFKSKDHTYVPSSSTIPYEDPTLLFANAGMNQFKSIFLGTVDPQSELARLKRAFNSQKCIRA

GGKHNDLEDVGKDSYHHTFFEMLGNWSFGDYFKKEAIVYSWELLTEVYKLPKEQLYVTYFEGDPKAGLEPDLEARQYWLD

TGVPEDHIIPGNAKDNFWEMGATGPCGPCSEIHFDRIGGRNASHLVNQDDPDVLEIWNNVFIQFNREEDGTLKPLPAKHV

DTGMGFERLVSVVQNKRSNYDTDVFQPLFTTIQELTGVRPYSGKFGNEDENGIDTAYRVIADHVRTLTFALSDGGVPNNV

GRGYVLRRILRRGARFARKKLGVQIGSFFSSLVPVVVEQMGSVFPEITQKTAEIKEILDEEEESFSRTLDRGEKLFEQYA

QKALQSGQKELSGKDVWRLYDTYGFPVDLTRLMAEELGLSINEAEFEAAQAHSKEASKAGVKKGSGEVVALDVHDIAALE

KNDAVPKTDDSFKFQQGNITAKVVAIYSKKAFLQSTEEVPEDTSFGLILDRTSFYAESGGQEHDTGSIIIDGVAEFEVTN

VQVFNGYVLHIGHMKYGKLNVGDEIVSSYDELRRWPLRNNHTATHILNWSLREVLGDHIDQKGSLVAPTKLRFDFSHKAQ

VSGPELTKIESLSQDWIRRNVRVFGKELPLPVAQKIHSLRAVFGESYPDPVRVVSLEYDVEEIAKDPLNHKWRSTSIELC

GGTHVAKTGEIKDFVVTEESGIAKGIRRVVAVTGHEAAEARRLGESLTAQLNRIETMPDAEKDAALKAYTVALGQADIAL

LKKNELRDRLASVRKAFDKHLKDKEAAANKAAVDALQQFFKDKPDAPAYFAILDVNANAKVMQGVVLQAKKLNRSIYIFS

ADAVSGKVAHVNAVSEEAKAKGLDGREWAAAVVALLGGKAGGKPDGAQGVGTEVSKVQEAFDAARKYFESKTGLSV

>KXT07630.1 hypothetical protein AC578_10120 [Mycosphaerella eumusae]

MRRSLIPHLRITSNLLYLSRTTASLSKHTPTTPLSRYSIRTRMASTAGQEPEWTAVKVRKTFIDYFVKHGHTFVPSGSVV

PLADPTLLFTNAGMNQYKSIFLGTVDPDSDFAKLKRAANSQKCIRAGGKHNDLDDVGKDSYHHTFFEMLGNWSFGDYFKK

EAIHFSWELLTKVYGLSPDRLYVTYFEGKKDVGLEPDTEARDFWRAEGVPDDHILTGDMKDNFWEMGDQGPCGPCSEIHY

DRIGGRNASDLVNKDDPNVIEIWNNVFIQYNREADKSLRSLPAKHIDTGMGFERLVSILQNKYSNYDTDVFTPLFARIQE

ITGARGYGGKFGEEDADGIDTAYRVVADHVRLLTFAISDGGIPNNVGRGYVVRRVLRRGARYARKYFNTDIGSFFSKIVP

TLVEQMGEMFPEIKKKQAEVMEILDEEELSFAKTLDRGENMFEKYAQKCKANNAKVLEGDDVWRLYDTYGFPVDLTRLMA

EERGLDIIDEDVAKAQEKAREASKGEKKAGADLVKLDVHDINSLEQMEDVPKTDDSPKFQRDNITATIKAIYHGRKFLQS

TEEVPKGEQFGVFLDRTNFYAEQGGQENDTGRILIDGEAEIDVQNVQVYGGYVMHTGYMNYGTLNVGDQVLSEYDELRRQ

PIRNNHTGTHILNFALRETLGDDVNQKGSLVAPEKLRFDFSHKQGCSDAELEKIEELSTGYIRQNSEVYAKDVPLATAKQ

IEGVRAVFGETYPDPVRVVSIGVPVEDLLEDPKRSDWRNVSVEFCGGTHVKNTTEIKDLIVLEESGIAKGIRRIVAVTGQ

TAHEIQLLGEQWNKKVTCIEGMKYGPEKEAAVKEAQHELGKLEMAALTKTKLRDRVAKVVKENLEHQKAAQKAENKKLLD

AVNEHFDKNKDSKTLVLSFPVSAGSKAIQETMKVIGNKQKDKTVYLLGKTEDGKVVHGCHVSSDAQGKGADAAKWSSEVA

GLVGGKAGGKGATSLGQGTNPDKVDDAVEIARKYLEGLSI

>KRX69891.1 Alanine--tRNA synthetase, cytoplasmic [Trichinella sp. T6]

MTVLLYTTSCCVINNLKRSKMDMLLTANAIRRTFINYFTEKRNHQYVHSSSVIPKGDPSLLFTNAGMNQFKPIFLGTADP

NSQMASWKRVVNSQKCIRAGGKHNDLDDVGKDVYHHTFFEMLGNWSFGDYFKKEICAWAWEVLTKEFKIPKEQLYVTYFG

GSEKYRLQPDVECRQIWLEIGLPSERVLPFGMKDNFWEMGDIGPCGPCSEIHYDRIGNRNAANLINKNDPDVLEIWNLVF

ITYNRDKDGSLHPLPKRHIDCGLGFERLVSVIQGKKSNYDTDLFVPIFNRIAETAKVRPYMGKVGKEDEQQIDMAYRVVA

DHIRTLTVALSDGGRPDNCGRGYVLRRILRRGVRYATEKLNAPSGFFANLVPVVVETLGDTFPELKEDPNSVMEIINEEE

SLFLKTLSRGSRVLRQEIEKASKDKLIPGVVAWRLYASYGFPVDLTQLMAEESGYKVNMEEYEECRQQAQMSSSSKFSQA

NDVVDFNINAVNYLLNGKVPLTDDKFKYAYRLTENGDDDYEFDEVKCEVLALRRNGEFVSEVCSGDSCCLICDKTAFYAE

SGGQIYDEGSMEGEKCEFRVRNVQARSGYVIHFGEVMGTITRGSKLLQNVDQKRRVQVMKNHTATHLLNFALREVLGQVE

QKGSLVLMDRLRFDFTCKAPLTVEQLVRIEQIVANIVNSDVEIYMQEVPLGVAKTIAGLRSSADEVIRRRSTVQFFYTAL

HLQTYPDPVRVVSVGTPVDALLKDPDGPGAKLASVEFCGGT

>XP_024711579.1 alanine-tRNA synthetase [[Candida] pseudohaemulonis]

MTTWPASTVRQTFLDFFKGKQHTFVPSSSVVPHNDPSLLFANAGMNQYKPIFLGTVDTSTDFGSLKRAANSQKCIRAGGK

HNDLDDVGKDSYHHTFFEMLGNWSFGDYFKKEAIEWSWELLTEVFKLEKDRLYVTYFEGDEKNGLQPDLEAKQFWLDVGV

AEDHILPGDASDNFWEMGDQGPCGPCSEIHYDRIGGRNAAHLVNMDDPNVLEVWNVVFIQYNREADSSLRPLPNKHIDTG

MGFERLVSILQSKSSNYDTDVFSPLFDKIRDITGVRPYTGKFGSDDVDGIDTAYRVIADHVRTLTFAITDGGVPNNEGRG

YVLRRILRRGSRYVRKYMNYPIGHFFEQLVDVVIQENSQIFPEIASHKDELKEILVEEELSFAKTLDRGEKLFEQYAIIA

SKTPEQTLSGKDVWRLYDTYGFPVDLTRLMAEEAGLKIDEEAFEKAKEESREASKASGSKTGADLIKLDVHALSELDGND

AVPKTDDHYKFGLENTKSKILAFYDGTSFVDSIDKTGQQFGILLDKTPFYAEQGGQEFDTGSLVIDGSAEFHVDNVQSYA

GYVLHTGTLSEGTLKVGDKVIATYDELRRWPIRNNHTGTHVLNFALREVLGDSVDQKGSLVAAEKLRFDFSNKQALTLKE

VEEVERISNENIKANLEVFAEDVSLTDAKLINGLRAVFGEVYPDPVRVVSIGVPVSKLLSEPENKEWHKYSIELCGGTHV

AKTSEIKDLVIIEESGIAKGTRRIVAVTGHDAHRVQQIARDFGAELDAAANLPFGPDKEQKAKELGVALKKLSISVVDKL

KLNEKFTKLDKSIKDNLKAKQKEESKKTLAVVNDWLKDEEKLKAAFLVAHVPISANAKAITEAFNLIKKENKDKSLYLIT

GTEDKVAHGCYISDEAISKGVDANEVAKVAIAHIGGKAGGRGNIVQGMGDKPEGVEQAVEAVKKALTEKLA

>OJD25819.1 alanine-tRNA synthetase [Blastomyces percursus]

MVSAQTVEQPEWSALRVRNAFLGFFKENGHTFVPSSSVVPLSDPTLLFTNAGMNQYKSIFLGTVDPNSDFATLKRAHNSQ

KCIRAGGKHNDLDDVGKDSYHHTFFEMLGNWSFGDYFKKEAITYSWDLLTKVYGLEPDRLYVTYFEGNPDAGIGPDLEAK

ELWLSVGVAEDHLLSGNMKDNFWEMGEQGPCGPCSEIHYDRIGGRNAAHLVNQDDPNVLEIWNNVFIQYNREPDRSLRPL

PSKHIDTGMGYERLVSILQNKSSNYDTDVFTPLFEKIREITGCRPYTGKFGEEDTDGIDTAYRVVADHIRTLTFAISDGA

SPNNEGRGYVVRRVLRRGARYARKYLNVEIGSFFSKIVPTLVEQMGDMFPEIRRKQTDVMEILDEEEISFAKTLDRGERL

FEEYALQAKQKGLGKLHGADVWRLYDTFGFPVDLTQLMAEERGLTIDNTEFEEARLKAKEASKGQGKQATDVVKLTVHDL

GLLETMDNVPKTNDYAKFGRGNITSQIKAIYHNKKFVDSTKDIPEGEQFGIILDRTNFYAEQGGQENDTGKIIIDGKAEL

AVEDVQVYGGYILHTGFMKYGSFNVDDSVIAEYDELRRWPIRNNHTGTHILNFALRQVLGEGVEQKGSLVAAEKLRFDFS

HKSAVSDSDLEKIERISTDYIRQNCAVYAKDVPLSTAREICGVRAVFGETYPDPVRVVSVGVELEEILQNVHDRRWKDIS

IEFCGGTHVQKTGDIKDLVILEENGIAKGIRRIIAVTGEEAHEVQRIAKEFGERLHRFDKMELGPQKEMEAKLLQVDLNQ

LTISAVEKARFREQFMQIHKKVVEGQKAAQKLESKKALDAITSYFETPKNKDAPHLVLQLPISANPKALSDSLNFVKTKM

QDKSVYVFAADKGAGKVVHGCHVAQSLSAQGATPNEWANTVSSIVGGRAGGKAPVAIGNGTETEKLEDAIKAATEYLVKF

TL

>OEJ84690.1 Alanine--tRNA synthetase, mitochondrial [Hanseniaspora uvarum]

MSDINFGNKSYWTASKVRTTFLDYFKEKHNHTYVKSSPVIPFDDPTLLFANAGMNQYKSIFLGTVDPSNELFYNLKRAVN

SQKCIRAGGKHNDLEDVGKDSYHHTFFEMLGNWSFGDYFKKEAITFSWELLTKVYGIPKECLYVTYFEGDLKNGLEPDLE

AKQIWKDVGVPEDHILPGNAKDNFWEMGDQGPCGPCSEVHYDRLGLKLNRNASSLVNEDDPDVLEIWNNVFIQYNREXNG

ELKPLPAKHVDTGMGFERLVSVLQDVRSNYDTDVFIPLFEKIQEITGCRPYTGKFGAEDVDGIDTAYRVLADHVRTLVFA

LADGGVPNNDGRGYVLRRILRRGARYVRKYMNYEIGSFFSKLAPTLIDQVKDIFPEVEKDTAYLFEILNEEEASFAKTLD

RGEKLFESYAKVAEESAEKTLNGKEVWRLYDTFGFPVDLTRLMAEEKGLKIDEEGFEIAKKESYEASKRGGKNKGTSVLK

LDVHAISEASTVLNLPKTNDSFKYGLEDVQSKVLAIYDGESFANEISQTGKQFGVILDKTPFYAEQGGQEYDTGKLVIDG

ELEFNVTNVQVYGGYVLHTGELVEGTLKVGSEVISSYDELRRFPLRNNHTGTHMLNFALMETLGNEVDQKGSLVSQEKLR

FDFSNKKALSLEELXKVEDIVNKQIKXNIPVYYKEVPLELASKISSVRAVFGETYPDPVRVVSVGRPVEDLIANPESDEW

KNYSVEFCGGTHVAKTGDIKQFVIIEESGIAKGIRRIVAVSGSEAVEVQRVAADYDSKLESAGKMETSAAKEKVLKELGV

ALKTLQISTIDKQNLVAKFNKIEKVMKDELKKKAKEDTKIVLDLVAKHFSENEDSKFLAAHXNIGTNSKAITEAITVLKK

NKQDKVLYLITGNEENKLGHACYVPDSLLETVKPQDVTAKSSEFIGGKTGGKGNIVQGNGMKLSEVNNAIEAVTDLLKSK

LVI

>XP_019019323.1 hypothetical protein PICMEDRAFT_16022 [Pichia membranifaciens NRRL Y-2026]

MSIYTNPATNPKVQEWPASKVRDTFLDFFKQKQHTFVPSSSVIPHDDPTLLFANAGMNQYKPIFLGTVDPSSSFSTLKRA

VNSQKCIRAGGKHNDLEDVGRDSYHHTFFEMLGNWSFGDYFKKEAIQYSWELLTKVYGIPADRLYVTYFEGDEKAGLEPD

IEAKELWSAIGVPDDHILPGNAKDNFWEMGDQGPCGPCSEIHYDRIGGRNAASLVNEDDPDVLEIWNNVFIQFNREPDGS

LKPLPAKHIDTGLGFERLVSLLQDVRSNYDTDVFEPLFLKIQEITGVRPYSGKFGKDDVDGIDTAYRVLADHVRTLTFAL

ADGGVPNNEGRGYVLRRILRRGARYTRKYMNYPIGTFFSQLAPTLIEQDKHIFPELAKDPSSIFEILDEEEASFARTLDR

GEKLFEQYALIASKTPEQTLSGKDVWRLYDTYGFPVDLTRLMAEEAGLKIDEEGFEKARLASKEASKGGASKSAKALLKL

DVHALALLGEQKVPTTDDSAKYDTKDINSQVLAIYTDGGFVDSVTADSFKDKQIGILLDKTPFYAEQGGQEYDTGRIVID

GKSEFHVENVQVYAGYVLHTGFIDEGELKVNDIVISGYDELRRWPLRNNHTGTHILNFALRDVIGDGVEQKGSLVASEKL

RFDFSHKKSVELKELDQVEQIVNQIIKENLKVYAKPVALETAKSINGLRAVFGESYPDPVRVVSVGIPVEDLLADPSNEK

WKKYSIEFCGGTHVAKTDDIKEFVIIEESGIAKGIRRIVAVSGSEAREVQRKAAEFSKILDDVSKLPYGETKERRVKDLG

TELGKLSISVVEKAALKAKFTAIEKSVKDETKLRVKQDTTKVMDAVKSFFAAPDHKSVCAVHVSAGPNAKIISEAINYMK

KEQKDSCLYIVLAEVEGKAAQGVYLSPAVQEKLPAVEVATKGAAFIGGRAGGKGNTCQGMGDKAEGADAAVEEVKTLLEK

LAI

>KKA03260.1 Alanine--tRNA synthetase, mitochondrial [Hanseniaspora uvarum DSM 2768]

MLEMFVSFFLNIFFKLITVQCSNIYQINIPFKVFRLNSLHFYKRHHISVKMSDINFGNKSYWTASKVRTTFLDYFKEKHN

HTYVKSSPVIPFDDPTLLFANAGMNQYKSIFLGTVDPSNELFYNLKRAVNSQKCIRAGGKHNDLEDVGKDSYHHTFFEML

GNWSFGDYFKKEAITFSWELLTKVYGIPKECLYVTYFEGDLKNGLEPDLEAKQIWKDVGVPEDHILPGNAKDNFWEMGDQ

GPCGPCSEVHYDRLGLKLNRNASSLVNEDDPDVLEIWNNVFIQYNREPNGELKPLPAKHVDTGMGFERLVSVLQDVRSNY

DTDVFIPLFEKIQEITGCRPYTGKFGAEDVDGIDTAYRVLADHVRTLVFALADGGVPNNDGRGYVLRRILRRGARYVRKY

MNYEIGSFFSKLAPTLIDQVKDIFPEVEKDTAYLFEILNEEEASFAKTLDRGEKLFESYAKVAEESAEKTLNGKEVWRLY

DTFGFPVDLTRLMAEEKGLKIDEEGFEIAKKESYEASKRGGKNKGTSVLKLDVHAISEASTVLNLPKTNDSFKYGLEDVQ

SKVLAIYDGESFANEISQTGKQFGVILDKTPFYAEQGGQEYDTGKLVIDGELEFNVTNVQVYGGYVLHTGELIEGTLKVG

SEVISSYDELRRFPLRNNHTGTHMLNFALMETLGNEVDQKGSLVSQEKLRFDFFNKKALSLEELIKVENIVNKQIKENIP

VYYKEVPLELASKISSVRAVFGETYPDPVRVVSVGRPVEDLIANPESDEWKNYSVEFCGGTHVAKTGDIKQFVIIEESGI

AKGIRRIVAVSGSEAVEVQRVAADYDSKLESAGKMETSAAKEKVLKELGVALKTLQISTIDKQNLVAKFNKIEKVMKDEL

KKKAKEDTKIVLDLVAKHFSENEDSKFLAAHVNIGTNSKAITEAITVLKKNKQDKVLYLITGNEENKLGHACYVPDSLLE

TVKPQDVTAKSSEFIGGKTGGKGNIAQGNGMKLSEVNNAIEAVTDLLKSKLVI

>KXN90914.1 Alanine--tRNA synthetase [Leucoagaricus sp. SymC.cos]

MSYQSYSGPWTSRKVRDAFFDYFRSKNHTFVPSSSTIPYEDPTLLFANAGMNQYKSIFLGTVDPHSEYAKLKRAFNSQKC

IRAGGKHNDLDDVGKDSYHHTFFEMLGNWSFGDYFKSEAIQYSWELLTEVYQLPRDRLYVTYFEGDPKNGLEPDLEAKEH

WLAQGVPEDHILPGNAKDNFWEMGATGPCGPCSEIHYDRIGGRNAAHLVNQDDPDVLEIWNNVFIQFNREEDGSLKPLPS

KHVDTGMGFERLVSVIQDKRSNYDTDVFLPIFAKTQELTGVRPYEGKFGEDDKDGIDTAYRVIADHVRTLTFALSDGGIP

NNVGRGYVLRRILRRGARYARKKLGVPIGSFFSSLVPVVVETMGPVFPEITKKAAEIKEILDEEEESFSRTLDRGEKLFD

QYTSRAKEQGLKELSGKDVWRLYDTYGFPVDLTLLMAEELSLGINHKEFEEAQAWSKEASKGSGSKGPADVVKLDVHDIA

ALEKNPDVPKTDDSAKFNTGNITAKIKAVFYNKNFLPSTAGVPEHATFGLLLDKTSFYAETGGQEYDTGNIVIDGIADFE

VTNVQVYNGYVLHTGHLKYGTLEVGNEVISCYDELRRWPLRNNHTATHILNFNLREVLGDHIDQKGSLVAPTKLRFDFSH

KSQISPPELAKIENMSVDWICRNVKVYSKELDLQTAHKIPGLRAVFGESYPDPVRVVTLEYDLDEITKDIENPKWRQTSV

EFCGGTHVAKTGDIKDFVITEESGIAKGIRRIVAVTGQEAQDAIRLANSLKDRLDVLERLTGKEKDAGLKAYGVELGQAE

ISILRKAELKDRLATIRKAFDKQVKEREAATNKEAIDRVVKYFEENEKAEAFVGLLDVDGNAKILQSVILQARKLGKTVY

VLSVDSDGGKVAHVNFVSPSYKAKGADARAWATRVSEVLGGKTGGKEESSQGSGVNVSCVQEAIDVAQSYLNSLN

>KIW65631.1 alanine-tRNA synthetase [Phialophora americana]

MRSTRLHIGRAFSAGCQSATALTSRAPRFSKIADLTHAFNTKWPRRFRGLSHSHRVGERSNYTRSNERLSSANSRSRILV

GGPLTALRSSIVRRHTTSTMGSSTPDNTQQWTAPKVRETFLKYFEERGHTFVKSSPVVPLSDPTLLFTNAGMNQFKPIFL

GTVDPTSKEGQYKRVVNSQKCIRAGGKHNDLDDVGKDSYHHTFFEMLGNWSFGDYFKQEAITFSWELLTKVFGLEPDRLY

VTYFEGNEAGGLEPDLEAKEIWKSVGVAEDHILPGNMKDNFWEMGDQGPCGPCSEIHYDRLGGRNAAHLVNQDDPNVLEI

WNNVFIQFNREPDRSLRPLPNKHVDTGMGFERLVSVLQNKLSNYDTDVFTPIFARIQEVTGARPYSGKFGEEDTDGIDTA

YRVVADHVRTLTFAISDGGIPNNEGRGYVVRRVLRRGARYARKYFNVEIGDFFSKIVPTLVEQMGGMFKEIVAKEEEVKE

ILNEEEESFAKTLDRGEKQFEVYAQKSQVQGLKSLHGADVWRLYDTFGFPVDLTRLMAEERGLSIDDNEFEAARLAARDA

SKGQKKATSDLLKLDVHDLGELEKMPGVTKTDDSAKYGRQNIIGTIQAVYHAKKFHPGTKNIPEGDQIGLILDRTCFYAE

QGGQEYDTGRIVIDGQAELDVENVQLYAGYVLHTGYMKYGHFSLGDKVICEYDELRRWPIRNNHTGTHILNFALREVLGL

GVEQRGSLVAQEKLRFDFSHKSGVSDEDLSKIENISTEYIRQNCQVYAKDVPLVIAREIAGVRAVFGETYPDPVRVVSVG

VDVDDILKNVQDKGWEKVSIEFCGGTHVRSTGDIKDLVILEENGIAKGIRRIIAVTGEDAHEVQRVAEEFSGKLEKLEKM

PFGSPKEQEAKNIQLELNNLSISAVRKSQFRDRVSKISKEILEQMKKLQKEQTQKAIDTITGFFQANENAQGAVLKLPVG

GSTKLIPEVIKHVQTKLKDKSVYIVVADEKDPEGKVYHGCFVGQSGTNAGLKSPEWSSTVSNLVGGKAGGKAPVAIGTGT

DQSKVDEALKAATEYLEKFKL

>KDQ33558.1 hypothetical protein PLEOSDRAFT_1091537 [Pleurotus ostreatus PC15]

MAYTGPWTAPVVREAFFEFFRSKDHTFVPSSSTIPYDDPTLLFANAGMNQYKSIFLGTVDPHSDMSKLKRAFNTQKCIRA

GGKHNDLDDVGKDSYHHTFFEMLGNWSFGDYFKKEAIQYSWELLTDVFKLPKDRLYVTYFEGDAKNGLEPDLEAKQYWLD

QGVAEDHILPGNAKDNFWEMGATGPCGPCSEIHYDRIGGRNAAHLVNMDDPDVLEIWNNVFIQFNREEDSSLKPLPSKHV

DTGMGFERLVSVVQDKRSNYDTDVFTPIFDKVQELTGVRPYEGRFGDDDKDGIDTAYRVVADHVRTLTFALSDGGVPNNV

GRGYVLRRILRRGARYARKKLGVNIGSFFSSLMPAVVNDMGHVFPEITKKLDELKEILDEEEESFSRTLDRGEKLFDQYA

ARVKEQGGKELNGKDVWRLYDTFGFPVDLTRLMAEELGLGINEKEFEEAQAWSKEASKGAVKKGNTDLVKLDVHDLASLE

KNNDVPKTDDSAKFNLGNIDATVKAIFHNKQFVPSTSDIPEDTSFGILLDKTSFYAESGGQEYDTGNIVVDGVADFEVTN

VQVYNGYVLHTGHLKYGELKVGDQVVSSYDELRRWPLRNNHTATHILNYCLREVLGDHIDQKGSLVAPTKLRFDFSHKAQ

IALPELQKIETMSLDWIRRNVPVFSKELDLATARVIPGLRAVFGETYPDPVRVVTLEFDVEEIAKDIQNPKWRQTSVEFC

GGTHVAKTGDIKGFVLTEESGIAKGIRRIVAVTGHEAHEVTRVADNLEAKLKSIDQLTGKEKDTALKAFTVELNQADISV

LRKADVKEKLNAIRKAFDKDIKEKETALNKLAIDSIQQYFKENEKADTYIAILDVEGNAKPLSSAVLQAKKLGKSVYLFS

PDKQGGKVAHVNFVSPELKQKGVDARTWAGKVTDVLGGKAGGKEDSAQGVGANVDKVEGAVEAAKQYLSSL

>EER42379.1 alanyl-tRNA synthetase [Histoplasma capsulatum H143]

MASAQTVEQPEWPALRVRNAFLDFFKENGHTFVPSSSVVPLSDPTLLFTNAGMNQYKSIFLGTVDPNSDFATLRRAHNSQ

KCIRAGGKHNDLDDVGKDSYHHTFFEMLGNWSFGDYFKKEAIRYSWDLLTKVYGLNPDRLYVTYFEGNPDAGIGPDLEAK

ELWLSVGVAEDHLLSGNMKDNFWEMGEQGPCGPCSEIHYDRIGARNASHLVNQDDPNVLEIWNNVFIQYNREPDKSLRPL

PNKHVDTGMGYERLVSILQNKSSNYDTDVFTPLFEKISEITGCRPYTGKFHEEDTDGIDTAYRVVADHVRTLTFAISDGA

SPNNEGRGYVVRRVLRRGARYARKYLNVEIGGFFSKIVPTLVDQMGDMFPEIRRKQTDVMEILDEEEISFAKTLDRGERL

FEEYALQAKQKGSGKLHGADVWRLYDTFGFPVDLTRLMAEERGLTIDNAEFEEARLKSKEASKGQGKRTADVVKLTVHDL

GLLETMDDVPKTNDDAKFGRGNITSQIKAIYYNKKFVDSTKDIPQGEQFGIILDRTNFYAEQGGQENDTGKIIIDGKAEL

AVEDVQVYGGYILHTGFMKYGSFNVNDSVIAEYDELRRWPIRNNHTGTHILNFALRQVLGDGIEQKGSLVAAEKLRFDFS

HKSAVSDSDLEKIERISTDYIRQNCAVYAKDVPLATARGISGVRAVFGETYPDPVRVVSVGVELEEILQNVHDPRWKDIS

IEFCGGTHVQKTGDIKDLVILEESGIAKGIRRIIAVTGEEAHEVQRIAKEFGERLRRFEKMELGPQKEIEAKLLQVDLNQ

LSISAVEKARFREQFAQIHKKVLEGQKAAQKLESKKALDAITGYFEAPENKVASHLVMQLPISANPKALSDSLNFVKTKM

QDKSVYVFAADKEAGKVVHGCHVAQSLSEQGATPNDWANAVSSIVGGRAGGKAPISIGNGTEAEKLDEAIKAATEYLEKF

L

>OQN95605.1 Alanine--tRNA synthetase [Rachicladosporium antarcticum]

MVSQAQDEVQWTAQKVRSTFLEFFEKNGHTIVPSGSVVPLSDPTLLFTNAGMNQFKSIFLGTVDPDSDFAKLKRAVDSQK

CIRAGGKHNDLDDVGKDSYHHTFFEMLGNWSFGDYFKKEAIAFSWELLTKVYGLEPDRMYVTYFEGKADVGLEPDLEAKE

LWKSVGVADDHILPGDMKDNFWEMGDQGPCGPCSEIHYDRIGGRNAAHLVNQDDPNVLEIWNNVFIQYNREPDKSLKPLP

SKHVDTGMGFERLVSVLQDKLSNYDTDVFTPLFKAVQDITGAREYSGKFGEDDVDGIDTAYRVVADHVRLLTFAISDGGV

PNNVGRGYVVRRVLRRGARYARKYFKTEIGGFFSKLVPTLVNQMGDMFPEIKKKELDVKEILDEEELSFAKTLDRGEVMF

EKYAEQCKQRDSKELSGADVWRLYDTYGFPVDLTKLMAEERGLSIDDDAVHEAQEKAREASKGEKKSSAELGKFSVHDIN

TIENMEDVPKTDDSSKFLRENVSGTVKAIFHGGKFLQSTADAPEGEQLGIVLDKTCFYAEQGGQEFDMGRILIDGEAEID

VQNVQVYAGYVLHTGFMSYGALKTGDNVLCQYDELRRQPIRNNHTGTHILNFALREVLGEDVNQRGSLVAPEKLRFDFSH

KAGCTDAELEKIEEISTSYIRQNSEVYSTDVPLATARNIEGVRAVFGETYPDPVRVVSIGVPVEELLEDTTRKEWRSVSV

EFCGGTHVKNTSEIKDLVVVEESGIAKGIRRIIAFTGAAAHEVQRIAGEWDEKFTRLEAQAYTPEKEARTKEYQHALNSL

EISALAKTKLRDRVSKIVKANMEHQKTAQKAENKALTDAVTAHFTQHPDAKSFVTSHPVSAGSKAIQETLKLVSSKQKDK

NVYIVGASADGKVVHGCFVSSDAQAKGADAGKWSTTVAGVVGGKAGGKGATSFGQGTEAEKVDEGVEAARQYLEGLGL

>OQE43263.1 hypothetical protein PENCOP_c003G08023 [Penicillium coprophilum]

MTSLGSEWTAQRVRDTFLDYFKQNGHTFVPSSPVAPLSDPTLLFTNAGMNQFKSIFLGTVDASSEFGKMKRATNSQKCLR

AGGKHNDLDDVGKDSYHHTFFEMLGNWSFGDYFKKEAITYSWTLLTEVYGLDPDRLYVTYFEGNEAGGLEPDLEAKELWK

SVGVPESHILPGDMKDNFWEMGDQGPCGPCSEVHYDRIGGRNAAHLVNEDDPNVLEIWNNVFIQYNREPDSSLRPLPNKH

VDTGMGFERLVSILQDKSSNYDTDVFTPIFQAIQDTTGAREYRGKFGADDVDGIDTAYRVIADHVRTLMFAISDGVVPNN

EGRGYVIRRVLRRGARYARKYFQVDIGNFFAKLVPTVVDQLGDMFPELKRKQQDVIEILDEEELSFAKTLDRGERQFEQY

AQQAKVKGTDVLHGADVWRLYDTFGFPVDLTRIMAEERGLRIDDVEFEEARLKAKEASKGQKKAAEDTVKLDVHDLGNLE

KMNDVHKTDDSPKFGRGNITSKVKAIYHGKIFHNSTDDIPEGAQIGVILDTTNFYAEQGGQEYDTGKIIIDGQAELEVGN

VQSYAGYVLHTGFMKYGSFTVGNSVICEYDELRRWPIRNNHTGTHILNFALKEILGDGIDQKGSLVAAEKLRFDFSHKSA

ISDAELEKIEAKATEYIRQNCTVYSQDLPLATAQQISGVRAVFGETYPDPVRVVSVGVELEEILQNVKDPRWKEVSIEFC

GGTHVQKTGDIKDLIILEESGIAKGIRRIIAVTGEDAHAVQRVAEEFGQRLDGLNALPLGSKKEQEAKQIQVDLNQLAIS

AVVKAKFRERFAKINKQVLDGQKAQQKLESKKALDTINSYFEAPENQDKSWLVVELPISANAKAVSESLNHVKSKLQGKS

VYVLAGDSEQGRVSHGCYISQGLSEQGASASEWAAVVSGAVGGKAGGKGPTSIGNGTNPDKVEEAISLAADYLSKFKL

>OCB87358.1 hypothetical protein A7U60_g5498 [Sanghuangporus baumii]

MAYKPYEGDWSSKKVRQTFFDYFKDRGHTYVPSSSTIPYEDPTLLFANAGMNQACFKAIFLGTVDPHSEMAKWKRVHNSQ

KCIRAGGKHNDLDDVGKDSYHHTFFEMLGNWSFGDYFKKEAIEFSWELLTEVYKLPKDRLYVTYFEGDPKNGLEPDNEAR

DFWKAQGLPDSHILTGDAKDNFWEMGATGPCGPCSEIHYDRIGGRNAAHLVNQDDPNVLEIWNNVFIQFNREPDGSLRPL

PAKHVDTGMGFERLVSVIQDRSSNYDTDIFEPLFKRIQELTGVRPYAGKFGKDDEDGIDMAYRVVADHVRTLTFALADGG

VPNNVGRGYVLRRILRRGARYARKKLGVQIGSFFSSLVPSVVEELGDVFPELHKRTDEIKEILDEEEESFARTLDRGEKL

FEGYSSKALQSPSKTLNGEDVWRLYDTFGFPVDLTLLMAEELGLGIDQEGFEKAQARSKEASKAGVKKGDKDLVKLDVHD

IAALDKNDSITKTDDDPKFELGTINATVKAIYHDKSFKDTTEGISEDTTFGILLDRTNFYAEAGGQEYDTGSITIDGAAE

FEVTNVQAYSGYVLHTGILKYGKLAVGDEVIATYDELRRWPLRNNHTATHILNFCLREVLGDHIDQKGSLVAPTKLRFDF

SHKAQISPSELQQIEDMSIDWIKRDVKVYSKELDLKSGYKIPGLRAVFGEAYPDPVRVVSLEYDVDEMKKDLENPKWRKT

SVEFCGGTHVNKTGDIKGFVITEESGIAKGIRRITAVTGHEAADVTRQAKMLIATLDKIEKATGKEKDAMLKTFQIELNQ

ADISVLKKSELRDRLTAIRKAFDKQAKEREQSLQKAATRSLTDYFTTTSRNSSVYISLISVDGNAKILQSILAQARKLDK

AAYLFSIDTEAEGGKGKVVHANYLPEAFRKDEFNAKMWANAVSEVLGGKAGGKDEGAQGVGVNVDKVDEALNVAKELFSK

VGSI

>PGH13139.1 alanyl-tRNA synthetase [Helicocarpus griseus UAMH5409]

MSSSQTQEQPEWPALRVRNAFLDFFKNNGHTFVPSSSVVPLSDPTLLFANAGMNQYKSIFLGTVDPNSDFAQLKRAHNSQ

KCIRAGGKHNDLDDVGKDSYHHTFFEMLGNWSFGDYFKKEAIQYSWDLLTKVYGLDPDRLYVTYFEGNPAAGIDPDLEAK

KLWLSVGVAEDHLLSGNMKDNFWEMGEQGPCGPCSEVHYDRIGGRNAAHLVNQDDPNVLEIWNNVFIQYNREPDRSLRPL

PNKHIDTGLGYERLVSILQNKSSNYDTDVFTPLFEKIREITGCRPYAGKFNEEDPDGIDTAYRVVADHIRTLTFAISDGA

VPNNEGRGYVVRRVLRRGARYARKYLNVEIGSFFSKIVPTLVEQMGDMFPEIRRKQTDVMEILDEEEISFSKTLDRGERL

FEEYAQQAKQKGLDKLHGADVWRLYDTFGFPVDLTQLMAEERGLAIDNNEFEEARLKAKEASKGQGKKAADIVKLTVHDL

GKLDTMDDVPKTDDSAKFGRGNITSQIKAIYHNKAFTDSTKDIPEGEQFGVILDRTNFYAEQGGQENDTGKIIIDGKAEL

AVDDVQVYGGYVLHTGFMKYGTFNVGDSVIAEYDELRRWPIRNNHTGTHILNFALRQVLGDGVEQKGSLVASEKLRFDFS

HKSAVSDADLDKIERLSTDYIRQNCAVYAQDVPLSTAREIAGVRAVFGETYPDPVRVVSVGVELEEILKDVHDPRWKDIS

IEFCGGTHVQKTGDIKDLVILEESGIAKGIRRIIAVTGEEAHEVQRIAKEFGERLQRFEKMELGPKKETEAKLLQVDLNQ

LTISAVEKARFRDVFGQIHKKVVEGQKAAQKAESKKALDAIGEFFKAPENKDKASLVLQLPVSGANAKAVSDSLNFVKTK

MADKSVYVFAADEAGAGAGGKVAHGCYVAQDLSQQGATPNDWASAVSGVVGGRAGGKAPIAIGNGTETEKVDEALKAATE

YLGKFKL

>XP_007317881.1 hypothetical protein SERLADRAFT_361256 [Serpula lacrymans var. lacrymans S7.9]

MSNVYSGPWTAHKVRDEFFNYFRSKGHTFVPSSATIPYEDPTLLFANAGMNQYKSIFLGTVDPNSDMAKLKRAFNSQKCI

RAGGKHNDLDDVGKDSYHHTFFEMLGNWSFGDYFKKEAIEYSWELLTEVYGLPKDRLYVTYFEGDSKNGLQADEEARQYW

RDRGVAEDHILPGNAKDNFWEMGATGPCGPCSEIHFDRIGGRNAAHLVNQDDPDVLEIWNNVFIQFNREEDGSLKSLPSK

HVDTGMGFERLVSVVQDKRSNYDTDVFTPIFDKIQELTGVRPYAGKFGDEDADGIDTAYRVVADHVRTLSFALSDGGVPN

NVGRGYVLRRILRRGSRYARKKLNVPIGSFFSSLMPVVVETMGDVFPELTKKVDEIKEILDEEEESFSRTLDRGEKLFDQ

YASRAQQQSTKELNGKDVWRLYDTYGFPVDLTRLMAEELHLGINDKEFEEAQEASKQASKGTLKKDSHPVIKLDVHDIAA

LEKNDAIPKTDDSAKFQLGNIMAQVKGIYYNKNFFESTSSIPEDTTFGVLLDRTSFYAESGGQEYDTGNIVIDGVSDFEV

TNVQSFNGYVLHIGHLKYGKLDIGDEVVSSYDELRRWPLRSNHTATHILNFGLREVLGDHIDQKGSLVAPTKLRFDFSHK

SQISLPELTKIESMSLDWIKKNVKVYSKELDLKSAQQIPGLRAVFGESYPDPVRVVTLEFDVDDMAKDIENPKWRNTSVE

FCGGTHVAKTGDIKDFVITEESGIAKGIRRITAVTGQEAHEAARQANAFKARLDQLEAMSGKDKDAGLKALTVELGQADI

SVIRKAELKDRLATVRKAFDKQIKEKEAAANKAAIDALVHHFDENKNAEGYFAILDVDGNAKKSLQNVVTQGKKLGKAVY

VFSVDADNTKVAHANHVPEAAKAKGLDARVWASKVTEVLGGKAGGKEDGAQGVGVNCGKAEEALSAAKDFFSQYNK

>EGC43441.1 alanyl-tRNA synthetase [Histoplasma capsulatum H88]

MASAQTVEQPEWPALRVRNAFLDFFKENGHTFVPSSSVVPLSDPTLLFTNAGMNQYKSIFLGTVDPNSDFATLRRAHNSQ

KCIRAGGKHNDLDDVGKDSYHHTFFEMLGNWSFGDYFKKEAIRYSWDLLTKVYGLNPDRLYVTYFEGNPDAGIGPDLEAK

ELWLSVGVAEDHLLSGNMKDNFWEMGEQGPCGPCSEIHYDRIGARNASHLVNQDDPNVLEIWNNVFIQYNREPDKSLRPL

PNKHVDTGMGYERLVSILQNKSSNYDTDVFTPLFEKISEITGCRPYTGKFHEEDTDGIDTAYRVVADHVRTLTFAISDGA

SPNNEGRGYVVRRVLRRGARYARKYLNVEIGGFFSKIVPTLVDQMGDMFPEIRRKQTDVMEILDEEEISFAKTLDRGERL

FEEYALQAKQKGSGKLHGADVWRLYDTFGFPVDLTRLMAEERGLTIDNAEFEEARLKSKEASKGQGKRTADVVKLTVHDL

GLLETMDDVPKTNDDAKFGRGNITSQIKAIYYNKKFVDSTKDIPQGEQFGIILDRTNFYAEQGGQENDTGKIIIDGKAEL

AVEDVQVYGGYILHTGFMKYGSFNVNDSVIAEYDELRRWPIRNNHTGTHILNFALRQVLGDGIEQKGSLVAAEKLRFDFS

HKSAVSDSDLEKIERISTDYIRQNCVVYAKDVPLATARGISGVRAVFGETYPDPVRVVSVGVELEEILQNVHDPRWKDIS

IEFCGGTHVQKTGDIKDLVILEESGIAKGIRRIIAVTGEEAHEVQRIAKEFGERLRRFEKMELGPQKEIEAKLLQVDLNQ

LSISAVEKARFREQFAQIHKKVLEGQKAAQKLESKKALDAITGYFEAPENKVASHLVMQLPISANPKALSDSLNFVKTKM

QDKSVYVFAADKEAGKVVHGCHVAQSLSEQGATPNDWANAVSSIVGGRAGGKAPISIGNGTEAEKLDEAIKAATEYLEKF

L

>EGA60508.1 Ala1p [Saccharomyces cerevisiae FostersO]

MTIGDKQKWTATNVRNTFLDYFKSKEHKFVKSSPVVPFDDPTLLFANAGMNQYKPIFLGTVDPASDFYTLKRAYNSQKCI

RAGGKHNDLEDVGKDSYHHTFFEMLGNWSFGDYFKKEAITYSWTLLTEVYGIPKDRLYVTYFEGDEKLGLEPDTEARELW

KNVGVPDDHILPGNAKDNFWEMGDQGPCGPCSEIHYDRIGGRNAASLVNMDDPDVLEVWNLVFIQFNREQDGSLKPLPAK

HIDTGMGFERLVSVLQDVRSNYDTDVFTPLFERIQEITSVRPYSGNFGENDKDGIDTAYRVLADHVRTLTFALADGGVPN

NEGRGYVLRRILRRGARYARKYMNYPIGNFFSTLAPTLISQVQDIFPELAKDPAFLFEILDEEEASFAKTLDRGERLFEK

YASAASKTESKTLDGKQVWRLYDTYGFPVDLTELMAEEQGLKIDGPGFEKAKQESYEASKRGGKKDQSDLIKLNVHELSE

LNDAKVPKTNDEFKYGSANVEGTILKLHDGTNFVDEITEPGKKYGIILDKTCFYAEQGGQEYDTGKIVIDDAAEFNVENV

QLYNGFVFHTGSLEEGKLSVGDKIIASFDELRRFPIKNNHTGTHILNFALKETLGNDVDQKGSLVAPEKLRFDFSHKKAV

SNEELKKVEDICNEQIKENLQVFYKEIPLDLAKSIDGVRAVFGETYPDPVRVVSVGKPIEELLANPANEEWTKYSIEFCG

GTHVNKTGDIKYFVILEESGIAKGIRRIVAVTGTEAFEAQRLAEQFAADLDAADKLPFSPIKEKKLKELGVKLGQLSISV

ITKNELKQKFNKIEKAVKDEVKSRAKKRKQTNS

>ODV95869.1 hypothetical protein PACTADRAFT_41773 [Pachysolen tannophilus NRRL Y-2460]

MSTSARLELQEKWNASTVRNTFLDYFKERGHTFVPSSSVIPYEDPTLLFANAGMNQYKPIFLGTVEPSSDFASLKRAVNS

QKCIRAGGKHNDLEDVGRDSYHHTFFEMLGNWSFGDYFKKEAIEMSWDLLTGVFGLDKDRLYVTYFGGDEALKLEPDLEA

KEYWLAVGVAEDHILPGSTADNFWEMGDQGPCGPCSEIHYDRIGGRNAASLVNMDDPDVLEIWNNVFIQFNREADGSLRS

LPAKHIDTGMGFERLVSILQDVRSNYDTDVFQPLFHKIQEITGVRPYSGKFGSADIDGIDTTYRVLADHVRTLTFALADG

GVPNNEGRGYVLRRILRRGARYVRKYMNYPIGNFFSQLAPTLMDQVSDIFPELSKNRAEIFEILDEEEASFARTLDRGEK

LFEQYAIIASKTPEQTLAGKDVWRLYDTYGFPVDLTRLMADEASLKIDEEAFEKAKEASREASKGAGKSTGKDILKLDVH

ALGKLDSEGVVPKTDDHFKYGVDNVQAKVVAIYDGKEFLESTKDQTGTVGILLDKTPFYAEQGGQTYDTGKIVIDGIAEF

NVENVQQYAGYVLHTGYMLEGNFSINDDVIATYEELRRWPIRNNHTGTHILNFALREVLGNGVEQKGSLVAPEKLRFDFS

HKQGLTLDEVKKVEDISNQYIKKNTPVYSKEVALPIGKKIYGLRAVFGESYPDPVRVVSIGVPVDEILQNPENPEWENIS

IEFCGGTHVAKTGDIKDFVIVEESGIAKGIRRIVAVSGKDAHAVQRIVSEFDAKLDEIAKMANGPSKEKHLKESNTALKQ

MTISILEKDRLNNKFSKIEKEYKDDLKRKQKEESKKVTEAVKAFFEEHKNSPFYVGHIDVSPNAKAITDAINFVKKENKE

KSVFIITGNNTDSKVAQGCYISDDSTAKVGISASDLTTLATKYIGGKAGGKGNVSQGMGVEVGGIDTAIIEVEKLLKEKL

SI

>XP_015226920.1 PREDICTED: alanine--tRNA synthetase, mitochondrial [Cyprinodon variegatus]

MEFDCACALTQPVVLEADHDSGRFWCADTVIQMSRLLLQKLQRLRRLSPGSERSLSSLPSAQVRKTFLDFFRDKHRHLVV

PSAPVRPRGDPSLLFVNAGMNQFKPIFLGTVDPRSPMASYRRVANSQRCVRAGGKHNDLDDVGRDMYHHTFFEMLGNWSF

GDYFKEEACQMAWSLLTEHYGIPPERLYVSYFGGDAASGLLADEETRNIWLDLGVPPARVLPFGLKDNFWEMGDTGPCGP

CTEIHYDHIGGRDAARLVNADSPEVVEIWNLVFIQYNREADHSLRLLPQFSVDTGMGLERLVSILQGKRSNYDTDLFTPL

LDAIHQRSGVGPYGGRTGEADAGQVDRAYRVVADHIRTLSVCIADGIHPGMSGAELVLRRILRRAVRFSVEVLKAPQGAL

ASLVPTVVHILGDTNPDLTREADRIVDVINENEAHFLSSLQHGSRLIQRTVSRMGAAGSPFPASVAWSLHRDLGFPLDLV

DLMLEERGVQVDRLELDRLISENQKMLLEQQAGVRPRLLPDVLSLAELQRSGVPNTDDTPKYRYQLEDGRYVFPACRATV

LALYDGEALVPEVSEGQRCGVILDRTCFYSEQGGQSHDQGYLTRDGLQDVLYSVEAVTRAGGYVVHQITAADKLRTGDQV

HLHLDQAHRLSCMAKHTATHILNFALRKVLGQSVQQRGSHISADRLRFDFSVKGSLSSSQLQQVETCVNDIISADQVVHS

LEVPLQKARTIQGLRTVDEVYPDPVRVVSLQVPVSELLDGGTERETSVELCCGTHLLQTSTIQDLVIVSEKQLVKGISRI

VAVTGQDAVQARDMGRVLSQEVDSLSARLSDSAPSSLGSAHHFSKEASTLSDSVDNTPIPQWQRRELQARLRAMLRAINT

LIRKLETQQAAKNAEVLFQKNGKKELLVDCVETDSLSVLMKTVNQLSAATPLSHVMLLSHQKDSGKILCACQVPKESSTL

PASDWAVAVCRQLGGSAGGSALVAKGTGSFGDIKEALRWAEDFARQKTKR

>XP_010212319.1 PREDICTED: alanine--tRNA synthetase, mitochondrial, partial [Tinamus guttatus]

VRAAFLRFFEQRHGHRRLPSAPVRPRGDPRLLFVNAGMNQFKPVFLGTVHPRSELAQHRRVVNCQKCVRAGGKHNDLEDV

GRDTSHHTFFEMLGNWSFGDYFKKEACSMAWELLTEVYKIPKDRLYVTYFGGDSSLGLSADEECRDVWLRLGVPASHVLP

FSLKDNFWEMGDTGPCGPCTEIHYDHVGGGRNAAALVNQGSPDVVEIWNLVFMQYNREVEGNLLPLPQHHVDTGMGLERL

VTVLQNKRSNYDTDLFTPILDAIHKGCGGPRYQGLVGRADASGVDTAYRVVADHARTLCVCIADGVYPGLSGAELVLRRI

LRRAVRFCSEVLHAPPGLLASLVPTVVDVLGDAYPELRKNTDQIADIINENEAAFLSSLERGRRVIERTVQHMEPSTDFP

AEVAWSLYGNLGFPLDLIDLMLEEKGIRLDSAAFDELALEDARRKARDPQAGQLEGADWRLDVHSLAQLQSNNVPVTDDS

PKYAYTLGQRGQYEFSPCQATVLMLYRNQSLQKEVGAGQHCGVILDRTNFYAEQGGQASDRGYLMLLGQQDMLFPVKSVH

LCGGYVVHEVTAVEMLRAGDQVQLFVDEAQRLACMTNHTAAHLLNFALRHVLGDNTEQRGSHVTAERLRFDFNTKDPVTI

EQLQQVERVVQEVINRNEVVHMAEVPLALARRVQGLRSVDEEYPDPVRIVSLGVPVESVLIHNSEAAMHTSVELCCGTHL

LRTGVMEDLAIISERQLAKGISRVIAVTGGQAKQAREVGQCLAMEVDSVSIRMKQGSTSIPDVQHLSKEVGQLTQVVANT

AMPQWQRKELQTTLKALQRTANTAIKKLEIQQAAEKAHSLLAKHCNQPLIIDTVPADSASILMKVVNQLCGKSPGTSVLL

LSPQASGEVLCACQVSKNCLPIFSAADWAAAVCTQMEGKAGGSCVVAKGSGNAKGMQRALTTAMEFAQSKLGNKCDVL

>XP_009493046.1 alanyl-tRNA synthetase [Fonticula alba]

MVSTECSQTVWTGDKVRQTFISFFEERGHSFVPSSSTIPHDDPTLLFTNAGMNQFKPIFQGTVDPNSEFSRLKRAANSQK

CIRAGGKHNDLDDVGKDSYHHTFFEMLGNWSFGDYFKVDAINWAWELLTEVFGLDKDRLYVSYFGGDAEANLPADLETRD

LWLAKGVAPERVLPFGMSENFWEMGDTGPCGPCSEIHYDRIGGRTVPELVNADDPDLLEIWNLVFMEFNREADRSLKRLP

SQSVDTGMGLERLVSVLQDRRANYDTDLFSYLFEAIEKGTGARPYSGKYGKDDADGIDTAYRVLADHIRTLAFALSDGGV

PDKDDRGYVLRRVLRRAIRFAHEKLGAKPGFFPNLITAVVDKMGHAFPELKKNPAGVIEILVEEEAQFRRTLDRGIRLFT

QVVENPANTSAGTLSGEVAFKLYDTYGFPKDLTQIMAEERGLTVDLAGYAAAEAEAKARSQSTKSTAGAAVALDVHTIAE

LNQKAVPRTDDSFKYTHSEVEARVLAIFHGGAFVESFDANATHDLFGLILDQTNFYAEQGGQIFDTGLIHLTGGSEGVLE

VENVQVYNGFVLHTVSMSTGVVRVGDAVSCAFDPARRRPIRRNHTATHLLNNALASVLGGEVNQQGSLVAPDRLRFDFSF

GKAVPAEKLREVEALVREQIAADLPVYYDNVPLVDAEKIAGVKAMFGQSYPDPVRVLSVGTPIATLVADPASGSGVASVE

FCGGTHVARSSDIQDFVLLSEEAVSRGVRRVVAVTGPEAVRAGELADQLQQAIADVVKNATGASLVGSLNGLDAQVTEAP

LSLVRKQALRAQIAEAKRAQAEQDKLVRVAQEKAANQRAEELLAKLTPDDKFLVEVFDLGSNAKHLSQLSRQFPKVGVFF

MSVDRDLGRVLHAANVPAALADTLSAKDWSAHVVDGALGGKSGGSKTTAQGAGPEVGAVEKACELATAFAKLKVSSE

>XP_021246040.1 alanine--tRNA synthetase, mitochondrial isoform X1 [Numida meleagris]

MAAAGPLRFPLLAVRRPWSCCSFRGAACPPAGQIRAAFLRFFEEKHGHRCLPSAPVRPRGDPTLLFVNSGMNQFKPVFLG

TAHPRSELAQQRRVVNSQKCVRAGGKHNDLEDVGRDTYHHTFFEMLGNWSFGDYFKEEACSMAWELLTEVYKIPKDRLYV

TYFGGDSSLGLSADEECRDVWLSLGVPGSHVLPFPMKDNFWEMGDTGPCGPCTEIHYDHMGGGRNASALVNQGSPDVVEI

WNLVFMQYNREVDGNLLPLPQRHVDTGMGLERLVTILQNKHSNYDTDLFTPILDAIHKGCGGPRYQGLVGNADVGHVDMA

YRVVADHVRTLCVCITDGIYPGLSGAELVLRRILRRAVRFCSEVLHAPHGLLASLVPTVVEVLGDAYPELRKNADQVMDI

INENEVAFLSSLERGRRIIERTVQQMKPSANFPAEVAWSLYGNLGFPLDLIDLMLEEKGIRLDLAAFNELVLEDAKRKAR

GQQEGRQLGGTDVHLDVHSLAQLQRDNIPATDDSPKYSYKLGQHGQYEFSPCQATILMLYRDQALQKEVGAGQHCGVILD

RTNFYAEQGGQASDQGYLIRLGQQDILFPVESVRLCGGYVIHEVTAVETLRVGDQVQLFVDEVQRLACMTNHTATHLLNF

ALRHILGDSTEQRGSHVTAEQLRFDFNIKGPVTVEQLQQVEQVVQDVIKRNEAVYMAEVPLALARRVQGLRALDEEYPDP

VRIVSLGVPVETVLTSNSQAAMQTSVELCCGTHLLQTGAMEDLAIVSERQLVKGISRVIAVTGRQAKQAREVGQCLAMEV

DSVSIRMKQRGSTSVLEMQNLSKEVGQLTKVVASTAMPQWQRKELQSILKSLQRTANTAIKKLEVQQAEEKAQRLLAKHC

NQPVIIDTVPADSLSILMKVVNQLCDKSPGSSVLLLSPQDSGEVLCACQVSKNCLPVFSAADWAVAVCTQMEGKAGGSPV

VAKGSGNAKGMQGALTTALEFARSKL

>XP_003030369.1 hypothetical protein SCHCODRAFT_82606 [Schizophyllum commune H4-8]

MAAIPHRSSRQVRQDFFDYFQSKGHTFVRSSPTVPYDDPTLLFANAGMNQFKPLFLGTVDPHSDLSKLKRAYNSQKCIRA

GGKHNDLDDVGKDSYHHTFFEMLGNWSFGDYFKKEAIAYSWELLTEVYKLPKDRLYVSYFEGDPKNGLEPDLEAKQYWLD

IGVPEDHILPGNAKDNFWEMGATGPCGPCSEIHYDRIGGRNAAHLVNQDDPDVLEIWNNVFIQFNREDDGSLKPLPSKHV

DTGMGFERLVSVVQDKRSNYDTDVFSPIFQRVQELTGVRPYQGLFGPDDKDGIDTAYRVVADHVRTLTFALSDGGVPNNV

GRGYVLRRILRRGARYARKKLGVTIGSFFSSLMPVVVEHMGDVFPEITKKLPEIKEILDEEEESFARTLDRGEKLFDQYA

KRAKEQNLKELNGKDVWRLYDTYGFPVDLTTLMAEELGLGINEKEFEDAQAWSKEASKATQNKDAATVVKLDVHDIAALE

KGGDVAKTDDSSKYNQGNITATVKKIFHNKAFLDSTSDIPEATSFGIILDRTNFYAESGGQENDTGGIVIDGVAEFEVTD

VQVYNGYVLHVGHLKYGDLEVGNEVVASYDELRRWPLRNNHTATHILNYCLREVLGDHIDQKGSLVAPTKLRFDFSHKAQ

IPLPELTQIESMSVDWIKKNVKVYGKDMDLKSAQKIPGLRAVFGETYPDPVRVVSLEFSVDEISADIENPKWRSTSIEFC

GGTHVAKTGDIKHFVITEESGIAKGIRRIVAVTGHEAAEATRLADALQIKLDQIEKMTGKEADAAIKAFTVTLNQADTSI

IRKSQLKDRLASVRKAFDKQVKEREAALNKDALDQFRTFFEQTPGAPAFVSILHVDGNAKLLQNVVAQARKAGKAVYLFS

PDPEGTKVAHVNFVPPALREKGLDAKTWAAKVTDVLGGKAGGKEDSAQGVGTNVDRVDDALKAAGQYVA

>OQO19401.1 Alanine--tRNA synthetase [Rachicladosporium sp. CCFEE 5018]

MGSHATDEPQWTAQKVRSTFLEFFEKNGHTIVPSGSVVPLSDPTLLFTNAGMNQFKSIFLGTVDPDSDFAKLKRAVDSQK

CIRAGGKHNDLDDVGKDSYHHTFFEMLGNWSFGDYFKKEAIAFSWELLTKVYGLEPDRMYVTYFEGKADVGLEPDLEAKE

LWKSVGVADDHILPGDMKDNFWEMGDQGPCGPCSEIHYDRIGGRNAAHLVNQDDPNVLEIWNNVFIQYNREPDKSLKPLP

SKHVDTGMGFERLVSVLQDKLSNYDTDVFTPLFKAVQDITGAREYSGKFGEDDVDGIDTAYRVVADHVRLLTFAISDGGV

PNNVGRGYVVRRVLRRGARYARKYFKTEIGGFFSKLVPTLVNQMGDMFPEIKKKELDVKEILDEEELSFAKTLDRGEVMF

EKYAEQCKQRDSKELSGADVWRLYDTYGFPVDLTKLMAEERGLSIDDDAVHEAQEKAREASKGEKKSAAELVKFSVHDIN

TIENMEDVTKTDDSSKFLRENVNGTVKAIFHGGKFLQSTADVPKGEQLGIVLDKTCFYAEQGGQEFDMGRILIDGEAEID

VQNVQVYAGYVLHTGFMSYGALKTGDNVLCQYDELRRQPIRNNHTGTHILNFALREVLGEDVNQRGSLVAPEKLRFDFSH

KAGCSDGELEKIEEISTSYIRQNSEVYSTDVSLATARNIEGVRAVFGETYPDPVRVVSIGVPVEELLEDTTRKEWRSVSV

EFCGGTHVKNTSEIKDLVVVEESGIAKGIRRIIAFTGAAAHEVQRIAGEWDEKFTRLEAQAYTPEKEAKTKEYQHALNSL

EISALAKTKLRDRVSKIVKANMEHQKTAQKAENKALTDAVTAHFTQHPDAKSFITSHPVSAGSKAIQETLKLVSSKQKDK

NVYIVGASADGKVVHGCFVSSEAQAKGADAGKWSTTVAGVVGGKAGGKGATSFGQGTEAEKVDEGVEAARKYLEGLGL

>XP_018259138.1 alanine-tRNA synthetase [Kwoniella dejecticola CBS 10117]

MSYADKKPAVPTTAPHPWPSPADWPAAKVRQTYIDYFVKQPGFEHTFWPSSGVIPFDDDTLLFANAGMNQYKPLFLGTAD

PKSDLSKLIRAVNSQKCIRAGGKHNDLDDVGKDTYHHTFFEMLGNWSFGNYFKIGALTMAWDLLTRVYGLPKDRLYVTYF

EGDAKQGLEPDLEAQQIWRDLGVPESHILPGNAKDNFWEMGATGPCGPCSEIHFDRIGGREVPELVNADDPNVLEIWNNV

FIQYNREQSGELRPLPAKHVDTGMGFERLVSVLHNVSSNYDTDVFTPIFAKIQELTGGRPYAGKLGEEDVDGVDTAYRVI

ADHIRTLTIAISDGGVPDKDGRGYVLRRILRRGVRYASNKFNVKIGNFFSSLVPVVVESLSPIFPEVTKKIPELVEILNE

EEASFARTLTRGEALFNKYASTALEEKRDVLGGKDIWRLYDTYGFPVDLTQIMAEERGLKIDQEAFEKARLESLEASKAG

GKDKGVAGTVKLDVHDLGALEANDQVPKTEDSAKYQLDDIKATVKSIYHSSKFYSSTSEVPANAPFGILLDKTNFYAESG

GQEYDTGVLAIDGQAEFKVEDVQVYNGYVLHIGRMEEGEIKVGDEVISTYDELRRWPIRNNHTGTHILNFALREVLGDHI

DQKGSLVAPTKLRFDFSHGKSIGVPELTKIEAISNEWIKKAAPVYAQEMPLAEACKIPGLRAVFGEAYPDPVRVVSLGYP

LEEIAQNIEDSKWRGTSIEFCGGTHVAKTDSIKDFVIIEESSIAKGIRRIVAVTGHEAHDVSRKAAEFEQRLNKIAELQG

KEKEAAMKPYLVELGQSSISLIKKASFKATFEKMQSEIVAATKAKTAADSKVIQDAIKAYFKENPNDNVYVGEFEVAGGN

AKTLSAAVTTAKSLSKAVYVFSTETETGKVAHTNYLPKQILDRKVLDAKTWLGEVSKIVGGKGGGRDDSATGVGSEPSKV

TEAVAAAKSFYLSKVEA

>XP_015468808.1 Alanine--tRNA synthetase [Debaryomyces fabryi]

MSTSSNNWPASRVRSTFLDYFKVKQNHTFVPSSSVVPHNDPTLLFANAGMNQYKPIFLGTVDPASDFASLKRAVNSQKCI

RAGGKHNDLEDVGRDSYHHTFFEMLGNWSFGDYFKKEAISWSWELLTEVYNLDKDRLYVTYFGGDEKQGLQPDLEAKNFW

LSVGVAEDHILPGDVHDNFWEMGDQGPCGPCSEIHYDRIGGRNASSLVNMDDPNVLEVWNIVFMQYNRELDASLRPLPAK

HIDTGLGFERLVSILQNKSSNYDTDVFQPIFNKIREITGVRPYTGKFGAEDRDGIDTAYRVIADHVRTLTFSITDGGVPN

NEGRGYVLRRILRRGARYVRKYMNYPIGSFFQQLVPVVIEQNSEIFPEIVKNQQDLTEILNEEELSFAKTLDRGEKLFEQ

YAIIASKTPEQTLSGKDVWRLYDTYGFPVDLTRLMAEEAGLKIDEEGFEKAKEESREASKATGNKGGVSLIKLDVHALSE

LDGNDKVPKTDDSFKYGLENIKANVVAIYDGSSFVNSISEVGSQAGILLDKTPFYAELGGQEYDTGKLVIDGAAEFSVSN

VQVYGGYVLHTGSLLEGSLKLEDEIIATYDELRRWPIRNNHTGTHVLNFALREVLGDNVDQKGSLVAPEKLRFDFSHKQG

LTPEELLKVETMSNDYIKENKQVYAKDIKLGDAKEINGLRAVFGETYPDPVRVVSIGTSIEDLLKEPKNPEWHKVSIELC

GGTHVAKTGEIKDLVIIEESGIAKGIRRIVAVTGTEAHQVQKIAQDFDKELEHAMALAFGPIKETKAKELGVSLKKLSIS

VLDKRNLNDKFNKLDKSIKDQLKAVQKEESKKVLEVVNEWLNAKDAKDYFVSFVPISANAKAITEAINLLKKQHKSKSLY

LITGSDKVAHGCYVSDEAIAKGVNATDLATAVSSKVGGKAGGKGNVVQGMGDKPQGIDEAIDELKKLFAEKL

>XP_016221338.1 alanine-tRNA synthetase [Exophiala mesophila]

MRHTSCQSQLVEATWRAITSSPRRILIHTSELTHNSRRFTSPTLAQFLQPRRIHSLRPSTTRHTPSDDPNQSISRPCLIS

HSLNQSYLGLKTSSPLLGLRSQRTTATMATTKQWTAQEVRDTFLNFFQERGHAFVKSSPVVPLSDPTLLFTNAGMNQFKP

IFLGTVDPTSKEGQYKRAVNSQKCIRAGGKHNDLDDVGKDSYHHTFFEMLGNWSFGDYFKKEAINWSWELLTKVYGLDPD

RLYVTYFEGYPEGGLEPDLEAKELWKAVGVPDDHILPGDMKDNFWEMGDQGPCGPCSEIHYDRIGGRNAAHLVNQDDPNV

LEIWNNVFIQFNREPDKSLRPLPNKHVDTGMGFERLVSVLQDKLSNYDTDVFTPIFERIQEVTGARPYSGKFGDDDKDGI

DTAYRVIADHVRTLTFAISDGGYPNNEGRGYVVRRVLRRGARYARKYFNVDIGDFFAKIVPNLVQQMGGMFKEIVSKEEE

VKEILKEEELSFAKTLDRGEKQFEVYAQKSQGQGINSIHGADVWRLYDTFGFPVDLTRLMAQERGLSIDDNEFETARLQA

REASKGEKKAASDLVKLDVHDLGELEKMPGVTKTDDSAKYGRSNTTAKIQAIYHAKKFVSDTKSIPTGDQLGVLLDKTCF

YAEQGGQEYDTGRIVIDGQAELDVQNVQLYAGYVLHTGYLKYGQLSVADEVITEYDEFRRSPIRNNHTGTHILNFALREV

LGDGVDQRGSLVAQEKLRFDFSHKSGVTDAELQKIEDICTEYIRQACPVYAKDVPLATALEISGVRAVFGETYPDPVRVV

SVGVEVDDILKNVGDERWKTVSIEFCGGTHVRSTGDIKDLIILEENAIAKGIRRIIAVTGDDAHEVQRVAEEFSERLDRL

EKLPYGPEKEQETKATQSDLDALSISAVKKKQLRDRAEKISKGLLEHQKKMQKEQLKKAVDAVTKYFETNPEAHSAVLRL

PVGGSAKIVPDVTKHVQNKLKDKSVYIVVSDEQDPEGKVYHGCFVSADATKAGVSSAEWSSVVSKLVGGKAGGRAPVAVG

TGTDSSKVDDALKAATEYLEKFKL

>XP_010273668.1 PREDICTED: alanine--tRNA synthetase [Nelumbo nucifera]

MGHWRCSSNSCSAGVNVAAAAVASASLVSSSLSSLVSSNRACSKLLARVRACSGTHLSFFSTASVFPSSLSSSTIAAASA

MPGVQSQTVEWPASRVRDTFLSFFEGKGHVNWNSSPVVPLNDPTLLFANAGMNQFKPIFLGTVDPNTSLSKLTRACNTQK

CIRAGGKHNDLDDVGKDTYHHTFFEMLGNWSFGDYFKREAIGWAWELLTQVYKLPADRIYATYFGGDEKSGLSADDEAKE

LWLKFLPPERVLPFGSKDNFWEMGDTGPCGPCTEIHYDRIGNRDAASLVNNDDPTCIEIWNLVFIQFNRESDGSLKPLPA

KHVDTGMGFERLTSVLQNKLSNYDTDVFLPIFDAIQKATGARPYSGKVGLDDVDKVDMAYRVVADHIRTLSFAIADGSCP

GNEGREYVLRRILRRAVRYGNEVLKAQEGFFSGLVDVVVKVMGDVFPELKQHEVNIRDIIAAEEISFGKTLLKGIEQFKK

ASQDVQGKILSGQDAFVLWDTYGFPLDLTQLMAEERGLTVDVEGFNTAMEEARKRSRSAQNKQAGGAIVMDADATSQLHK

KGISPTDDSSKFIWFQDHESVIKAIYTGAEFVESATAGDEVGVILETTSFYAEQGGQIFDTGSLEGSFGSFQVLNVQIFG

GFVLHIGSIAGGTGRFTVGDKVVCKVDYDRRTLIAPNHTCTHMLNFALREVLGDHVDQKGSIVLPEKLRFDFSHGKPVHP

DDLRKVESIVNKQIRDELNVFAKEVNLADAKRIAGLRAVFGEVYPDPVRVVAIGGKVEDLLANPDNKEWLSISTELCGGT

HISNTREAKAFALLSEEGIAKGVRRITAVTTDCAFKAIESARSLEQEISDAAKLDVSQMEKKVASLKSRVQSAAIPATMK

ADLGAKISQLQDQVRKATKKIAQENIQKATKTAAEMADIAATNGKAFCICRVNVGLDAAAIREAVIKTMDQKGMSVMIFS

TDETTNKAVVCAGVSENGSKSKDLDLLEWFTAAMEPLKGRGGKGSVGKRFYAQGQGTDASQVQQAIEIATAFASMKLK

>AAH26611.2 Aars protein, partial [Mus musculus]

REKMFVDEVVTGQECGVVLDKTCFYAEQGGQIYDEGYLVKVDDSSEDKTEFTVKNAQVRGGYVLHIGTIYGNLKVGDQVR

LFIDEPRRRPVMSNHTATHILNFALRSVLGEADQKGSLVAPDRLRFDFTAKGAMSTQQIKKAEEIVNGMIEAAKPVYTQD

CPLAAAKAIQGLRAVFDETYPDPVRVVSIGVPVSELLDDPCGPAGSLTSVEFCGGTHLRNSSHAGAFVIVTEEAIAKGIR

RIVAVTGAEAQKALRKSETLKKSLSAMEAKVKAQTAPNKDVQREIADLGEALATAVIPQWQKDEQRETLKSLKKVMDDLD

RASKADVQKRVLEKTKQLIDSNPNQPLVILEMESGASAKALNEALKLFKTHSPQTSAMLFTVDNEAGKITCLCQVPQNAA

NRGLKASEWVQQVSGLMDGKGGGKDMSAQATGKNVGCLQEALQLATSFAQLRLGDVKN

>SCU91028.1 LADA_0F07690g1_1 [Lachancea dasiensis CBS 10888]

MTLGDKQKWTAANVRTTFLDYFKERNHKFVASSPVIPYDDPTLLFANAGMNQYKPIFLGTVDPSSEFYHLKSAVNSQKCI

RAGGKHNDLEDVGRDSYHHTFFEMLGNWSFGDYFKKEAIQYGWDLLTKVYGIPADRLYVTYFEGDAKQGLDADVEARDFW

LQVGVLEDHILTGDAKDNFWEMGDQGPCGPCSEIHYDRIGGRNAASLVNMDDPDVLEIWNIVFMQYNREQNGKLRSLPAK

HIDTGMGFERLVSVLQDVRSNYDTDVFQPLFARIQEITGVRHYTGKFGAEDVDGIDTAYRVLADHVRTLMFALADGGVPN

NEGRGYVLRRILRRGARYARKYMNFPIGKFFSTLAPTLIEQTKEMFPEIAKDPSYLFEILDEEELSFAKTLDRGEKLFEK

YAQVASQSENKTLDGVQVWRLYDTYGFPVDLTELMAEEQGLKIDGPGFEKAKQESYEASKKGGKKGASDLIKLDVHELST

LNSENTLKTNDSFKYDSDNLEAVVLKIHDGEKFVDEMTEVGKQYGVILDRTCFYAEQGGQEYDMGTLVIDGVTEFKVENV

QLYNGYVFHTGSLAEGKLSIGDNIIASFDELRRFPIKNNHTGTHILNFALKEVLGNEVDQRGSLVAPEKLRFDFSHKKGL

SIEELQAIENICHEQIKANLPVYYKNVPLELAQKISSVRAVFGESYPDPVRVVSVGQPIDDMLANPSSEEWQKYSVEFCG

GTHVIKTGDIKEFVIVEESGIAKGIRRVVAVSGAEAHEVQRVALGFDNDLTATENLPFSPLKEKKLKELGVKLGQLQVSV

ITKNELKAKFTKIEKTVKDEIRTRAKKEIKQTVDEVKNFFNNNESAHFFVKYIDIPTNPKAITEAVTYIKTSAKDKSIYL

LNGSEKVAHGCYISDYALEKGVDGAQLLEAISNNVGGKAGGKKNVFQGVGDKPEGIAQAVNEVEDIFRSKLMI

>KZV89392.1 hypothetical protein EXIGLDRAFT_838571 [Exidia glandulosa HHB12029]

MAHHTPYTGHWTAANVRKAYFDFFEQNGHTFVRSSSTIPYDDPTLLFANAGMNQYKPIFLGTVDPNSDLSKLKRAYNSQK

CIRAGGKHNDLEDVGKDSYHHTFFEMLGNWSFGDYFKKEAITFSWKLLTEVYGLPKDRLYVTYFEGNPAAGLEPDHEARD

IWLSLGLDPSRVLTGSAKDNFWEMGATGPCGPCTEIHYDRVGGRDAGHLVNNDDPLVLEIWNNVFIQFNREDDGSLKPLP

AKHVDTGMGFERLVSVCQDKLSNYDTDLFAPLFDRIQELTGARPYNGGFGKDDPDGIDTAYRVIADHVRTLTFALSDGGV

PNNVGRGYVLRRILRRGARYARKKFNVEIGSFFSTLAPTLIDQLGDVFPELTKKYDDIKEILDEEEESFARTLDRGEKLF

EKYVAQAREAGKTELGGKDVWRLYDTFGFPVDLTRLMAEELGLGINEAEFEAAQAHSKEASKGAGKQGSKETVKLDVHDI

AKLEADASVPKTNDSAKFRVGKAAELTATVKAIYYNKSLLQSSESIPEGESFGVILDKTNFYAESGGQEYDTGTIVIDGE

AAFDVTNVQLYNGYVLHIGSLKEGTLKVGDAVLPTYDELRRWPLRNNHTATHILNYSLRETLGDHIDQKGSLVAPTKLRF

DFSHKAQIPLPDLQKIEQGSRDWIKKNVKVFSKELDLKSGYKIPGLRAVFGEAYPDPVRVVTLEYDVEDIAKDLENPKWR

NTSVEFCGGTHVAETGTIKDFIITEESGIAKGIRRIIAVTGHEAAEVTRVAAGIEQSLARAESLTGKEKDSALKALTVEL

NNADISVLRKAELKETLTKLRKAFDDDVKAREKAQTKTAVDAVTNHFKESPNSPAFVSVVEGGNAKVLQSVLNQGKALDK

PVYVFTVDDTGDKVMHAAFVPQTQISTSFDARVWASSVTDILGGKAGGKADTAQGVGANVTRLEEAVAAARKRFA

>KIY74247.1 hypothetical protein CYLTODRAFT_427840 [Cylindrobasidium torrendii FP15055 ss-10]

MSTSTYDGPWPSATVRDEFFKFFKSKGHTYVASSPTIPYEDPTLLFANAGMNQYKSIFLGTVDPHSEMAKLKRAFNSQKC

IRAGGKHNDLEDVGKDSYHHTFFEMLGNWSFGDYFKKEAIGYSWELLTEVYKLPKDRLYVTYFEGDAKAGLEPDNEAKQY

WLDVGVAEDHILPGNAKDNFWEMGATGPCGPCSEIHYDRIGGRNAAHLVNQDDPNVLEVWNNVFIQYNREEDSSLRSLPA

KHVDTGMGFERLVSVVQNKMSNYDTDVFSPIFARVQELTGVRPYEGKFGAEDVDGIDTAYRVVADHVRTLTFALSDGGVP

NNVGRGYVLRRILRRGARFARKKLGVTIGSFFSSLLPVVVDTMGHVFPELTTRVDEIKEILDEEEESFSRTLDRGEKLFE

QYAARAQEQGLKELNGKDVWRLYDTYGFPEDLTRLMAEEIGLGINDKEFADAQAASKEASKAQGKAGAAEVVKLDVHDLS

ALKTMQITETNDSAKFKTGNIDAKIVALYHDKKFPKSTKEIPEGAAFGIILDNTSFYAEAGGQEADTGNIVIDGAADFDV

ADVQVYSGYVLHTGRLKYGELSVGDQVVSSYDELRRWPLRNNHTATHILNFGLREVLGDHIDQKGSLVAPTKLRFDFSHK

APISPAELAKIEAIAVGWIKKNVKVFSKDISLSSAYKIPGLRAVFGEKYPDPVRLVTLEYDVEEIAADLENPKWKQTSVE

FCGGTHVAKTGDIKDFVLIEESGIAKGIRRIIAVTGHEANEVTRQAAEFQTRLDQLELLSGKDKDVAMKSLSVELGQSSI

SAVKKADMKDKLAAMRKAFDKAGKEKENALNKAAVDQLQQHFKEGHEKYFVALLDAQGNAKVLQNVLAQGRKLNKPVYVF

TVDTEAGKVAHANFVPAALKSNGVDAKVWAGKVSDILGGKAGGKDDSSQGVGTNVEKVNEAIAAAKEYLSSYV

>EJU04465.1 hypothetical protein DACRYDRAFT_20988 [Dacryopinax primogenitus]

MSTLTPFGGPYGHWPADKVRQTFIDYFKARGHTFWPSSSTIPYEDPTLLFANAGMNQYKAIFLGTVDPQSELAKLKRAVN

SQKCIRAGGKHNDLEDVGKDTYHHTFFEMLGNWSFGDYFKKEAIQWSWELLTEVYKLPKDRLYVTYFEGDSKQGLDADLE

ARQYWLDVGVPEDHILTGDAKDNFWEMGATGPCGPCSEIHFDRLGGRNAAHLVNQDDPNVIEIWNNVFIQFNREEDGSLR

ELPAKHVDTGMGFERLVSCLQDLPSNYDTDVFVPIFAKIQELTGARRYAGKLGAEDVDGIDTAYRVVADHVRTLTFALSD

GGVPNNVGRGYVLRRILRRGARYVRKKFGVPIGSFFSSLAPTVIQQMGHFFPEITRKLSDIKEILDEEEESFSRTLDRGE

KLFEEYVTATKKKGSSSLHGHDVWRLYDTYGFPVDLTRLMAEELHLTINEHEFEEARLRSLEASKASQKKGNEDAVKLDV

HDLAALESNPKVPKTDDRPKFGLGNISATIQAIYFNKSFLESTTAIPEDAAFGILLDRTSFYAESGGQENDTGSIIIDGK

TEFMVQNVQVYNGYVLHSGALKYGQFSVGDEVISSYDELRRWPLRNNHTGTHILNFALREILGDHIDQKGSLVAPTKLRF

DFSHKAQVTLEEMEKIEEICNGWIKRDVKVFSQEVDLHTAQEFAGVRAVFGEAYPDPVRVVSLGYEVQDIIQDPQNPKWR

GTSIEFCGGTHVAKTGDIKALVLTEESGIAKGIRRIIAVTGEEANEVNRLADSFETRLIQIERSAGKEKDAALKAYTFEL

GQADISALRKAALREKFAGIRKTFDDANKAHLASLTKELTKTIDKHFQEKPNESVYLANIDSLEGNTKIMQSIVQHARTL

NKAVYIFSVDVSAGKVAHVNFLPKHAITQSFSAKTWANAVSVILGGKAGGKDDVAQGVGVNVEKVDEALQLARNLYHRTS

>XP_023663476.1 alanine--tRNA synthetase, mitochondrial isoform X1 [Paramormyrops kingsleyae]

MAAVVPLRISRKCISTITCTNGYRLFSTTQKNSVLTSKDVRRMFIDFFKDRHQHRVVSSSPVRPRGDPSLLFVNAGMNQF

KPIFLGSVDPHTEMARYRRVVNSQKCVRAGGKHNDLDDVGRDVYHHTFFEMLGNWSFGDYFKEDSCAMAWQLLTEVYGIP

PDRLYVSYFGGDERLCLKPDRDTREIWLSMGVHSNRILPFGMKDNFWEMGETGPCGPCTEIHYDHVGGRDAACLVNGDSA

DVVEIWNLVFMQYNREADGSLCALPQLSVDTGMGLERLLAVLQDHRSNYDTDLFTPLLASIHQCSKCPEYRGQTGESDVS

GVDMAYRVIADHIRALTVCIADGVYPGMSGAELVLRRILRRAVRFSIEVLQSPPGSLARLVPTVAHILGDAYPEVAKETE

TIMDVINENEAQFLSSLSQGRRVIDRTLWRMDGARLFPGSIAWSLHRNLGFPLDLISLMVEEKGVAMDTETLHRLAMEEE

KLKTEAQASDMQSCAELDVLTLTELQNKGVPFTEDSPKYHYSLGQDGKYAFLPCQATIQALYCGQALVQEVHEGQRCGII

LDQTCFYAVQGGQSHDQGYFTQDSQPDVLFPVEKVQLVGGYVIHQVTAAETLRKGDQLQLFVDEAQRLACMVKHTATHIL

NFTLRQLLGEEVEQRGSHITTERLRFDFSIKKPLSTHQLQDVEKMMQDIIRRDEPVYAEELPLLVARAIPGLRTVDEVYP

DPVRVVSVGVPASDLLYSSNPSTTSVELCCGTHLLRTGYIRDFVIVSERQMVKGISRVVAVTGEEANQAREAGHALAQVV

ESLSARVPAAPPCLSSAHGLSKEAGLLIDAVENTAIPQWQRRELQSQLRALQRVANTMVRKLEIAEAASKVQALLMKHRE

EKLVVDTVDTDSISVLMKAVNQYSNQAPGTLVMLLSHQQSGKVLCACQVPKGCGPLSASDWALAVCTSVGGNAGGSTTVA

KGTGYTTNITEAVHWAKEFAKGNWRS

>OQR71419.1 alanine--tRNA synthetase, partial [Tropilaelaps mercedesae]

MSSAKEIRAKFIDFFRVKYKHTFVPSSCVIPHDDPTLLFANAGMNQFKPIFLGTVDPSSETAKWKRAVNTQKCIRAGGKH

NDLDDVGKDVYHHTFFEMLGNWSFGDFFKREICEWAWELLTKEFGLAEDRLYVTYFGGNKQAGLEADDECKNIWKSLGLP

EERILPGSMKDNFWEMGETGPCGPCSEIHYDRIGGRNAANLVNMDDPDVLEIWNLVFIQFNRETGGGLKLLPKQHVDTGM

GLERLVSVIQNKRANYDTDLFTPIFAAIEKATGARPYAGKVGAEDADGIDMAYRVLADHARTLSIALSDGGHPDNVGRGY

VLRRILRRAVRYATEKMGAKPGMFAGLVPIVVEILGDVFPELRKDPEGVMEIINEEEAQFLKTLSRGRRLLEKSVLQLED

GALLPGDVAWRLYDTYGFPVDLTQLMVNEKGIFIDMASYEKAKQAAQLASQGGGDRVDNTSKLDVHAIAELQDQKKVPAT

DDSPKYIYSSEGTGKTAKYAFEPCNGKVVAIRTNKHFVDEANPGDFCGVVLDRSNFYAEQGGQIYDTGFMQLMGSDDVEF

AVSEVHLQGGYVVHIGRLVGGTLKVGDDLRLQIDESRRLSVMRNHTGTHVLNYALRKVLSADADQRGSLVAPERMRFDFV

SNRPLTAAEVRKTEEAATELIQTNGDVYAKDSSLVRAKAVK

>XP_009547440.1 hypothetical protein HETIRDRAFT_476425 [Heterobasidion irregulare TC 32-1]

MANTTGPSDQKSWTASRIRQEFFDYFQAKEHTFVPSSSTIPYEDPTLLFANAGMNQYKPIFLGTVDPHSDLSKLKRAYNS

QKCIRAGGKHNDLDDVGKDSYHHTFFEMLGNWSFGDYFKKEAVHYSWELLTEVYKLPKDRLYVTYFEGDAKNGLEPDLEV

REYWIKQGVLEDHILPGNAKDNFWEMGSTGPCGPCSEIHYDRIGGRNAAHLVNQDDPDVLEIWNNVFIQFNREDDGSLRP

LPAKHVDTGLGYERLVSVIQDKRSNYDTDVFSPIFAKIQELTGVREYRGTFGAEDADGIDTAYRVVADHVRTLTFALSDG

GVPNNVGRGYVLRRILRRGSRYARKKLGVTIGSFFSSLMPVVVEQLGGVFPELTKKVDDLKEILDEEEESFSRTLDRGEK

LFEQYAANARERGSTQLNGKDVWRLYDTYGFPVDLTRIMAEELGLTVNEEEFIEAQAQSKEASKASLKKGTKDVVRLDVH

DIAALEKNADVPKTDDSPKFSTGNTDATIKAIYYNKAFINSTSGVSSDSTFGVILDRTSFYAEAGGQEYDTGNIVIDGVA

DFEVTNVQVFNGYVLHIGHLKYGQLDVGNAVLSSYDELRRWPLRNNHTATHILNFCLREVLGDHIDQKGSLVAPTKLRFD

FSHKAQIALPELAKIESMSNDWIKKNVKVFSQELDLKTAQKIPGLRAVFGESYPDPVRVVTLEYDVAEMARDIENPKWRG

TSVEFCGGTHVARTGDIKQFVLIEESGIAKGIRRIVAVTGHEAHDVVRQAETFKSRLQQVEQSSGKAKDAGLKELSVEIG

QADISILVKSELKDRLTTLRKAFDKEVKEKEAAASKAAVDGVIRYFEEKPNATGYFAVLDVQGNSKILQAVVLQAKKLGK

AVYVFSVDTDAQKVAHVNHVPEAFRSKGVDARTWASKVTEILGGKAGGKEEGAQGVGSNLDKVQDALTAAEQYFNEIKP

>EPS26059.1 hypothetical protein PDE_00995 [Penicillium oxalicum 114-2]

MASDTSRPEWTAPRVRETFLDYFKQNGHTFVPSSSVAPLSDPTLLFTNAGMNQYKSIFLGTVDPQSDFAKLRRAVNSQKC

IRAGGKHNDLDDVGKDSYHHTFFEMLGNWSFGDYFKKEAITYSWTLLTKVYGLNPDRLYVTYFEGDEKGGLEPDFEARDL

WKSVGVPEDHILPGNMKDNFWEMGDQGPCGPCSEVHYDRIGGRNAASLVNMDDPNVLEIWNNVFIQYNREADRSLRPLPN

KHVDTGMGFERLVSVLQDKSSNYDTDVFTPIFQAIQNATGAREYRGRFGAEDSDGIDTAYRVVADHVRTLMFAISDGVMP

NNEGRGYVIRRVLRRGARYARKYFQVEIGNFFSKLVPTVVEQLGGMFPELVKKQQDVMEVLDEEEISFAKTLDRGERQFE

QYAQHAKVKGDDKLHGADVWRLYDTFGFPVDLTQIMAEERGLRIDDAEFEEARLKAKEASKGQKKTAENLVKLDVHDLGK

LEKMNDVRKTDDSAKFGRENITAHVKAIYHGKNFYESTDKVAEGEQLGVILDCTNFYAEQGGQEADTGRIIIDGKAELEV

GDVQVYAGYVLHTGFMKYGSLSVGDSVICEYDELRRWPIRNNHTGTHILNFALREVLGDGIDQKGSLVSAEKLRFDFSHK

AAVSDADLEKIESKSTEYIRQNCTVYSKELPLATAQQISGVRAVFGETYPDPVRVVSVGVEIEEILRNVKDPRWEQVSIE

FCGGTHVQKTGDIKDLIVLEESGIAKGIRRIIAVTGEEAHEVQRVAKDFEARLDRLEAMPLGPQKERESKQVQVDLNQLV

VSAVRKAQFRTRYAAINKQVLDGQKAQQKLESKMALEAITSYFDAPENKDSTTLVVKLPMPASAKAVSESINHVKSKLQN

KSVYVMAVDPEQTRVAHGCHVAKDLGDKGASASEWASVVSSSVGGKAGGKGATSIGNGTNPDKVDEAVALATEYLSKFKL

>SGZ37965.1 probable alanine--tRNA synthetase [Hanseniaspora guilliermondii]

MSDVNFGNKSYWTASKVRNTFLDYFKEKHNHTYVKSSPVIPFDDPTLLFANAGMNQYKSIFLGTVDPSNELFYNLKRAVN

SQKCIRAGGKHNDLEDVGKDSYHHTFFEMLGNWSFGDYFKKEAITFSWELLTKVYGIPKECLYVTYFEGDLKNGLEPDLE

AKQIWKDVGVPEDHILPGNAKDNFWEMGDQGPCGPCSEIHYDRLGLKLKRNASSLVNEDDPDVLEIWNNVFIQYNREPNG

ELKPLPAKHVDTGMGFERLVSVLQDVRSNYDTDVFIPLFERIQEITGCRPYTGKFGDEDVDGIDTAYRVLADHVRTLVFA

LADGGVPNNDGRGYVLRRILRRGARYVRKYMNYEIGSFFSKLAPTLIDQVKDIFPEVEKDTAYLFEILNEEEVSFAKTLG

RGEKLFESYAKVAEETAEKTLNGKEVWRLYDTFGFPVDLTRLMAEEKGLKIDEEGFEIAKKESYEASKRGGKNKGTSVLK

LDVHAISEASNGLKLPKTDDSFKYGLDDIKSNVLAIYNGESFIEEINENGKQYGIILDKTPFYAEQGGQEYDTGKLVIDG

EVEFNVTNVQVYGGYVLHTGELAEGTLKVGSEVISSYDELRRFPLRNNHTGTHMLNFALMETLGNEVDQKGSLVSQEKLR

FDFSHKKALSLDELVKVEDIVNKQIKENMPVYYKEVPLELASKISSVRAVFGETYPDPVRVVSVGRPVEDLIANPESDEW

KNYSVEFCGGTHVAKTGDIKQFVIIEESGIAKGIRRIVAVSGSEALEVQRIAAEYDSKLDAAGKMETSAAKEKVLKELGV

ALKTLQISTIDKQNLVAKFNKIEKVMKDELKKKAKEDTKIVLDLVAKHFKENEDSKFLAAHVNISTNSKAITEAITTLKK

NKQDKVLYLITGNEENKLGHACYVPDSLLETVKPQDVTAKSSEFIGGKTGGKGNIVQGNGMKLAEVEKAIEAVNELLKSK

LVI

>OCT49259.1 Alanine--tRNA synthetase [Cladophialophora carrionii]

MASSTPEIPQQWTAPKVRETFLKYFEERGHTFVKSSPVVPLSDPTLLFTNAGMNQFKPIFLGTVDPSSKEGQYKRVVNSQ

KCIRAGGKHNDLDDVGKDSYHHTFFEMLGNWSFGDYFKKEAITFSWELLTKVFGLQPDRLYVTYFEGNEAGGLEPDLEAK

EIWKSVGVAEDHILPGNMKDNFWEMGDQGPCGPCSEIHFDRLGGRNAAHLVNQDDPNVLEIWNNVFIQYNREPDRSLRPL

PNKHVDTGMGFERLVSVLQNKLSNYDTDVFTPLFARIQEVTGARPYSGKFGEEDTDGIDTAYRVVADHVRTLTFAISDGG

IPNNEGRGYVVRRVLRRGARYARKYFNVEIGDFFSKIVPTLVEQMGGMFKEIVAKEEEVKEILNEEEESFAKTLDRGEKQ

FEVYAQKSQVQGQKSLHGADVWRLYDTFGFPVDLTRLMAEERGLSIDDNEFEAARLAARDASKGQKKAASDLLKLDVHDL

GELEKMPGVTKTDDSAKHGRQNITGTIQAVYHAKKFHPGTKNIPEGDQIGLILDRTCFYAEQGGQEYDTGRIVIDGQAEL

DVENVQLYAGYVLHTGYMKYGHFSLGDKVICEYDELRRWPIRNNHTGTHILNFALREVLGDGVEQRGSLVAQEKLRFDFS

HKSGVSDEDLARIEDISTDYIRQNCQVYAKDVPLVIAREIAGVRAVFGETYPDPVRVVSVGVDVDDILKNVRDKGWEKVS

IEFCGGTHVRSTGDIKDLVILEESGIAKGIRRIIAVTGEDAHEVQRVAEEFSGKLEKLEKMPFGSPKEQEAKNIQLELNN

LSISAVKKSQFRDRVSKISKEILEQTKKLQKEQTQKAIDTITEYFQKNENAQAAVLRLPTGGSSKLVPEVIKHVQTKLKD

KSVYVVVADEKDPEGKVYHGCFVGQNGTAAGLKSPEWSSTVSNLVGGKAGGKAPVAIGTGTDQSKVDEALKAATDYLEKF

KL

>XP_007308256.1 hypothetical protein STEHIDRAFT_149395 [Stereum hirsutum FP-91666 SS1]

MATLTSNSIPNWTSSKIREEFFNYFRSRDHTFVRSSSTIPYEDPTLLFANAGMNQYKSIFLGTVDPLSDLAKLRRAFNSQ

KCIRAGGKHNDLDDVGKDSYHHTFFEMLGNWSFGDYFKKEAVHYSWELLTDVFKLPKDRLYVTYFEGDPTAGLEPDLEVR

QFWLDQGVADDHILPGNAKDNFWEMGATGPCGPCSEIHYDRIGGRNAAHLVNQDDPDVLEIWNNVFIQFNREDDGSLKSL

PLKHVDTGMGFERLVSVIQDKRSNYDTDVFLPIFARVQELTGVRGYQGKFGEEDDDGIDTAYRVVADHVRTLTFALSDGG

VPNNVGRGYVLRRILRRGARYARKKLGVTIGTFFSSLMPVVVDQLGATFPELTKKLPEIKEILDEEEESFSRTLDRGEKL

FEQYAASARERGVRELNGKDVWRLYDTYGFPVDLTRLMAEELGLTINEREFEAAQASSKEASKASQKKHANSVVKLDVHD

LATLEKNTDVPKTDDSAKFSVGNVEATVKAIYFSKSFFQTTSNVSEDTSFGIILDRTSFYAESGGQEHDTGNIVIDGFAD

FEVTNVQVYNGYVLHIGYLKYGKLSVGDKVVASYDELRRWPLRNNHTATHILNFCLREILGDHIDQRGSLVAPTKLRFDF

SHKAQIPINELVKIESMANEWVKKNVKVYSQELDLKTAQRISGLRAVFGESYPDPVRVVSLEYDVADIAKDVESPKWRST

SIEFCGGTHVARTGDIKQFVVIEESGIAKGTRRIIAVTGHEAQEVTRQAETLRERLHQVEKLAGNTKDTALKTLTVELSQ

ADISLIEKTRMRDHLAALRKTFDKEIKEKDLAANKAAVEDVVKYFEENPNEPAYITVLDVEGNTKILQAVVLQAKKLGKA

VYVFSVDANAGKVAHANHIPESYRARGLDARTWASKVTAVIGGKAGGKEEGAQGVGQEVSKVEDAVIAAKKYLDEVKA

>OSD06527.1 hypothetical protein PYCCODRAFT_1431532 [Trametes coccinea BRFM310]

MASSTSNPWSAAKVREEFFNYFKSKDHTYVPSSSTIPYEDPTLLFANAGMNQFKSIFLGTVDPQSELARLKRAFNSQKCI

RAGGKHNDLEDVGKDSYHHTFFEMLGNWSFGDYFKKEAIAYSWELLTEVYKLPKEQLYVTYFEGDPKAGLEPDLEAKQYW

LQTGVPEDHIIPGNAKDNFWEMGATGPCGPCSEIHYDRIGGRNASHLVNQDDPDVLEIWNNVFIQFNREEDGSLKPLPAK

HVDTGMGFERLVSVVQNKRSNYDTDVFQPLFAKIQELTGVRPYSGKFGEEDENGIDTAYRVIADHVRTLTFALSDGGVPN

NVGRGYVLRRILRRGARFARKKMGVQIGSFFSSLVPVVVEQMGSVFPEITQKTAEIKEILDEEEESFSRTLDRGEKLFEQ

YAQKALQSGQKELSGKDVWRLYDTYGFPVDLTRLMAEELGLSINEAEFEAAQAHSKEASKAGVKKGTGEVVALDVHDIAA

LEKNDAVPKTDDSFKFQQGNITAKVVAIYSKKAFLQTTEEIPEDAPFGLILDRTSFYAESGGQEHDTGSIIIDGVAEFEV

TDVQVYNGYVLHIGHMKYGKLNVGDEIVSSYDELRRWPLRNNHTATHILNWSLREVLGDHIDQKGSLVAPTKLRFDFSHK

AQVSGPELTKIESLSQDWIRRNVRVFGKELPLPDAQKIHSLRAVFGESYPDPVRVVSLEFDVEEIAKDPLNPKWRNTSIE

LCGGTHVAKTGEIKDFVVTEESGIAKGIRRVVAVTGHEAAEARRLGESLTAQLNRIEGMPDAEKDAALKAYTVALGQADI

ALLKKNELRDRLASVRKAFDKHLKDKEAAANKAAVDALQQYFKDKPEAPAYFAILDVNANAKVMQGVVLQAKKLGKSIYV

LSADASTGKVAHVNAVSEEAKAKGLDGREWAAAVVEIVGGKAGGKPDGAQGVGTEVSKVQEAFDAARKFFESKTGLSA

>OJJ31791.1 hypothetical protein ASPWEDRAFT_117659 [Aspergillus wentii DTO 134E9]

MRVSTFMRDGELALTHLFSPFRSRPRTSHLHTFPNPTIFRTKTTLAHSRMITDTNGPEWTAKHVRETFLDYFKKNGHTFV

PSSPVVPHSDPTLLFANAGMNQFKSIFLGTVDPQSDFANLKSAVNSQKCIRAGGKHNDLDDVGKDSYHHTFFEMLGNWSF

GDYFKEEAIRYSWELLTKVYGLDPERLYVTFFEGNEAGNLEPDLEAKELWRSVGVPEDHILPGNMKDNFWEMGDQGPCGP

CSEIHYDRIGGRNAANLVNQDDPNVLEIWNNVFIQYNRESDRSLRPLPNKHVDTGMGFERLVSVLQNKSSNYDTDVFSPL

FQTIKDVTGAREYRGQFGADDPDGIDTAYRVVADHVRTLMFAISDGATPNNEGRGYVVRRVLRRGARYARKYFRVEIGDF

FSKIVPTVVDQLGDMFPELKRKQQDVREILDEEEISFAKTLDRGERQFENYAQQAKIKGADKLHGADVWRLYDTFGFPVD

LTRIMAEERGLRIDDHEFEEARLRAKEASKGQKKSAADLVKLDVHDLGKLEKMSDVHKTDDTAKYEKGNITSQVKAIYHG

NMFHSSTERIPSGEQLGIILDRTNFYAEQGGQENDTGRIVIDGQAELEVGDVQLYAGYVLHTGFIKYGSFSVGDTVISEY

DEIRRWPIRNNHTGTHILNFALREVLGDGIEQRGSLVAAEKLRFDFSHKSAISDHDLEKIEEKSTNYIRQNCAVYAKEIP

LATAREISGVRAVFGETYPDPVRVVSVGVELEEIIENIKDPRWNEVSIEFCGGTHVQKTGDIKDLIILEEGGIAKGIRRI

IAVTGEHAHEVQRMAAEFEKRLDNLDAMTLGPKKEQEAKQIQVELNQLSISAVQKAKFRGRFATINKQVTDGQKAQQKLE

SKKALDTITSFFQAPENKDKKSLVVKLPISANAKAISESLNHVKSKLQDKTVYLLAADSEQGRVAHGCYVSKDMSDRGAL

ASDWAAIVSSAVGGKAGGKGPTSIGNGVNPDKTDEAIELASGYLSRFQL

>KZO95648.1 hypothetical protein CALVIDRAFT_538046 [Calocera viscosa TUFC12733]

MTTATAVSTVGPYGDWPADKVRATFIDYFKQRGHTFWPSSSTIPYEDPTLLFANAGMNQYKAIFLGTVDPHSEMSKLKRA

VNSQKCIRAGGKHNDLEDVGKDTYHHTFFEMLGNWSFGDYFKKDAIKFSWELLTEVYGLPKDRLYVTYFEGDPKQNLEPD

LEARQYWLDVGVPEAQILTGDAKDNFWEMGATGPCGPCSEIHFDRIGGRNASHLVNQDDPNVLEIWNNVFIQFNREESGS

LRELPAKHVDTGMGFERLVSCLQDRPSNYDTDVFHPIFVRIQELTGARPYTGKLGEEDVDGIDTAYRVVADHVRTLTFAL

SDGGVPNNVGRGYVLRRILRRGARYVRKKFGVPIGSFFSSLAPTVSEQMGHFFPEITKKMGDIKEILDEEEESFSRTLDR

GERLFEDYVAAAKEKGSTSLNGRDVWRLYDTYGFPVDLTRLMAEELHFTIDEQEFEEARLQSLEASKASHKKGDKDEVKL

DVHDIASLEKNSLVPKTDDSAKFGLGNITATVKAIYHNKAFHESTSAIPEGAPFGILLDRTSFYAEAGGQEYDTGSIIID

GKAEFDVQNVQVYNGYVLHSGELKYGQLSVGNEVISSYDELRRWPLRNNHTGTHILNFALRDILGDHIDQKGSLVAPTKL

RFDFSHKAQVALDDMEKIEEICNAWIKKDVKVFSQDVDLRTAQEIAGVRAVFGEAYPDPVRVVSLGYEVKDIVRDVQNPK

WRGTSIEFCGGTHVAKTGDIKSLIITEESGIAKGIRRIIAVTGEDANEVTRVADSFEARLNQIERSSGKEKDASLKAYTF

ELGQADISALRKAALKERFAKIRKAFDEANKAQQTALTKEIVENMNKFFQEKPNENIYVASLDTLEGNTKIMQTIAQHAR

TLDKAVYLFSIDGPSGKIAHLNFVPKRNISKSFSAKTWANSIAAILGGKAGGKDDVAQGVGTNVDKLGEALETAEKLYRE

AP

>CDQ01943.1 BMA-AARS-2, isoform i [Brugia malayi]

MKALTCDEVRAAFINFFKDREHTYVHSSSVIPFDDPTLLFANAGMNQPIFLGTVEPSSEMAKLKRAVNTQKCIRAGGKHN

DLDDVGKDVYHHTFFEMLGNWSFGDYFKLEVCNWAWELLTKVLGIPGERLYVSYFGGDEKTGLDADEECRQIWLNIGVPP

SHILSFGMKDNFWEMGDVGPCGPCSEIHYDRVGNRDASHLVNADDPMVVEIWNLVFMQFNREEDGSLKQLPRKHIDCGLG

FERLVAVLQNKLSNYDTDIFTPLFNAIHNGTKVRPYSGKVGADDVDGIDMAYRVIADHIRTLSIALSDGGRPDNTGRGYV

LRRILRRGVRYANEKLSAPPGFLSSLVPTVVNLLGKTFPELHKDPQAVMDIINDEEKQFLKTLSRGRTLFQKAVAGLQGH

NILPGDVAWRLYDTYGFPLDLTQLMAEERGLLVDFEVFENCRQKAAGISAAHANKMRDTIDLDVNAISELKNKGIPATDD

SPKYKYYALSSEDQQTLYNFEKCEGIILALRKDKIFLDTLNSGDFGALILDRTNFYAEQGGQIFDTGVLTEVDKGSEFIV

SNVQIRGGYVVLVGTVEGQLCVGDKVIQAIDEDRRNLIMKNHTGTHVLNFALRKVIGEVEQKGSLVAPDRLRFDLTAKQP

LTYEQIRMVEKESQMLIDTNATVFAKNVPLAEARQINGLRAVFEELTRTQ

>XP_007380880.1 hypothetical protein PUNSTDRAFT_141744 [Punctularia strigosozonata HHB-11173 SS5]

MATSKYTGPWTAPAVRSQFFDYFRSKGHTYVPSSSTIPYEDPTLLFANAGMNQFKAIFLGTVDPHSEMAKWKRVFNSQKC

IRAGGKHNDLDDVGKDSYHHTFFEMLGNWSFGDYFKKEAIQYSWELLTEVYKLPKDRLYVTYFEGDAKNGLVPDEEARQY

WLDQGVSEDHILPGNAKDNFWEMGATGPCGPCSEIHFDRIGGRNAAHLVNQDDPDVLEIWNNVFIQFNREEDGSLKHLPS

KHVDTGMGFERLVSVLQDVRSNYDTDVFLPIFDKVQELTGVRPYGGKFGAEDVDGIDTAYRVVADHVRTLTMALSDGGVP

NNVGRGYVLRRILRRGARYARKKLGVEIGSFFSSLMPVVIEQMGDIFPEVRKRPDEIKEILDEEEESFSRTLDRGEKLFE

QYATRTQQSGEKDLSGKDVWRLYDTYGFPVDLTLLMAEELGLGVNQAEFEEAQAASKEASKASLKKGAADVVKLDVHDIA

ALETNPDVPKTDDSAKFNQGNITAEIKAIYYNKQFLPSSSGITEGTTFGLLLDRTSFYAEAGGQEYDTGNIVIDGVADFE

VTNVQTFNGYVLHIGHLKYGNLEVGNQVVSSYDELRRWPLRNNHTATHILNFALREVLGDHIDQKGSLVAPTKLRFDFSH

KAAIASDELTRIESISKDWIKKNVKVYSKELDLKTAHRIPGLRAVFGESYPDPVRVVTLEYDVEEIAKDIENLKWRSTSV

EFCGGTHVAKTGDIKDFIITEESGIAKGIRRVIAVTGHEAAAANRCAEEMKAKLDRIEGLDGKEKDVALKTFPIELGQMD

MSVVIKGQLRDRHAAIRKTFDKQTKDKEAAASKAAVEELTSHFDKNPSEGSYFANIDVNGNMKILQSVVLQGKKLGKAIY

VFSVDKEGSKVVHVNHSPAEARSKGLDARTWATKVAEVLGGKTGGKEDGAQGVGTETDKVDEAIAVARLHYTSIAQ

>XP_020543256.1 Alanine--tRNA synthetase, partial [Pichia kudriavzevii]

MSIYTNPATNPKIKEWPAAKVRDTFLNYFKSKGHTFVPSSSVIPHDDPTLLFANAGMNQYKPIFLGTVDPSSSFANLKRA

VNSQKCIRAGGKHNDLEDVGRDSYHHTFFEMLGNWSFGDYFKEGAIKYSWELLTEVYGIPKDRLYVTYFGGDEKTGLEPD

LEAKGLWQQVGVADDHILPGNVKDNFWEMGDQGPCGPCSEIHYDRIGGRNAASLVNEDDPDVLEIWNNVFMQYNREPDGS

LVPLPAKHIDTGLGFERLVSLLQDVRSNYDTDVFYPIFQKIQEITGVRPYTGKFGKDDSDGIDTAYRVLADHVRTLTFAL

ADGGVPNNEGRGYVLRRILRRGARYVRKYMNYPIGNFFSQLAPTLIEQVKHIFPELEKDPSTIFEILDEEEASFARTLDR

GEKLFEQYALIASKTPEQTLSGKDVWRLYDTYGFPVDLTRLMAEEAGLKIDEEGFEKARLESKEASKGTGTKTGKTLLKL

DVHALASLNDQSVPKTDDSFKYDTKDIHAKVLSIYSNGQFVDSISQSEQQVGIVLDKTPFYAEQGGQEFDTGKIVIDGKS

EFSVENVQVYGGYVFHTGFVQEGELKVGDDIIAGYDELRRWPLRNNHTGTHILNFALRKVLGDSVDQKGSLVAPEKLRFD

FSHKKAVELKELEQVENITNKLIKDNLKVYAKPVELEIAKGINGLRAVFGETYPDPVRVVSVGVPVDDLLADPSNEKWNN

YSIEFCGGTHVAKTDDIKEFVIIEESGIAKGIRRIVAVSGMEAKEVQRKASEFNGILNEVEKLPYGEVKERRIKELGTEL

GKLSISVIEKAALKDKFSKIEKAVKDETKSRVKKDTAKVMDAVKSYFETPEHSAVCTLKVTAGANAKIVAEAINYMKKEQ

KESCLYLVLGEDQGKVAQGVYLSPAVQDKIAAVEVATKAAEYIGGRAGGKGNSCQGMGDKAENADKAVEEIKTLLEKLSL

GNLSGSSLNTLFSATIDHNIDDEYKSTDIVNSYILAESVPVKDNIPYGSMLGYRFKPNMSAEFTHSYQIIIYLSASLCQL

PVNWNTSISNNGLTLYYTFNETIANNTDFKNMEKVDFQGGYASAIAETEIISTDSDYMLYLMIQPQQCNNCTAEDIWVYE

FAASQRKVMFLYDTEPRISVLDVDYEAAIFQTGDIPFGQNSSYSLYLFKDEYPIPIGLNQSWCAISENQQYDKKISVNES

LVSSLKNAFVVSDLDIAKTYTGVLVITNDIIPYGGGIFEPFVFTMSKTRSCKLAYGLEFCDEVAYAVPIS

>OEJ82573.1 Alanine--tRNA synthetase, mitochondrial [Hanseniaspora opuntiae]

MSDINFGNKSYWTASKVRNTFLEYFKEKHNHTYVKSSPVIPFDDPTLLFANAGMNQYKSIFLGTVDPSNELFYNLKRAVN

SQKCIRAGGKHNDLEDVGKDSYHHTFFEMLGNWSFGDYFKKEAITFSWELLTKVYGIPKECLYVTYFEGDLKNGLEPDLE

AKQIWKDVGVPEDHILPGNAKDNFWEMGDQGPCGPCSEIHYDRLGLKLKRNASSLVNEDDPDVLEIWNNVFIQYNREPSG

ELKPLPAKHVDTGMGFERLVSVLQDVRSNYDTDVFIPLFERIQEITGCRPYSGKFGAEDVDGIDTAYRVLADHVRTLVFX

LADGGVPNNDGRGYVLRRILRRGARYVRKYMNYEIGSFFSKLAPTLIDQVKDIFPEVEKDTAYLFEILNEEEVSFAKTLG

RGEKLFESYAKVAEESAEKTLNGKEVWRLYDTFGFPVDLTRLMAEEKGLKIDEEGFEIAKKESYEASKRGGKNKGTSVLK

LDVHAISEASSGLNLPKTDDSFKYGLEDIQSKVLAIYNGESFVDEINENSKQFGIILDKTPFYAEQGGQEYDTGKLVIDG

EVEFNVTNVQVYGGYVLHTGELVEGTLKVGNEVISSYDELRRFPLRNNHTGTHMLNFALMETLGNEVDQKGSLVSQEKLR

FDFSHKKALSLDELVKVEDIVNKQIKENMPVYYKEVPLELASKISSVRAVFGETYPDPVRVVSVGRPVEDLIANPESDEW

KNYSVEFCGGTHVAKTGDIKQFVIIEESGIAKGIRRIVAVSGSEALEVQRIAAEYDSKLEAAGKMETSAAKEKVLKELGV

ALKTLQISTIDKQNLVAKFNKIEKVMKDELKKKAKEDTKIVLDLVAKHFNENEDSKFLAAHVNISTNSKAITEAITTLKK

NKQDKVLYLITGNEENKLGHACYVPDALLEXVKPQDVTAKSSEFIGGKTGGKGNIVQGNGMKLAEVEKAIEAVNELLKSK

LVI

>KXL51535.1 hypothetical protein FE78DRAFT_135585 [Acidomyces richmondensis]

MGSTAGQAPQWTAPKVRSTFLEYFKDKGHTFVPSSSVVPLSDPTLLFTNAGMNQYKSIFLGTVDPDSDFAKLKRAVNSQK

CIRAGGKHNDLDDVGKDSYHHTFFEMLGNWSFGDYFKKEAIEFSWDLLTNVFGLEPDRLYVTYFEGKPDAGLEPDLEAKE

YWKAVGVAEDHILPGNMKDNFWEMGDQGPCGPCSEIHYDRIGGRNAAHLVNQDDPNVLEIWNNVFIQYNREPDKSLRSLP

SKHVDTGMGFERLVSVLQKKMSNYDTDVFTPLFATIQEVTGAREYRGKFGEEDTDGIDTAYRVVADHVRTLTFAISDGGV

PNNVGRGYVVRRVLRRGARYARKYFNVEIGNFFAKIVPKLVDQMGDMFPEIARKKADVIEILNEEELSFAKTLDRGETMF

EKFAQECKKRQTRDLAGADVWRLYDTYGFPVDLTRLMAEERGLEIDDNAVAEAQEKAREASKGEKKGAADLLKLNVHDIN

TLENMTDVHKTDDSPKYQKDNINATIKAIYCGGKFLQSTEDIADGEQVGIILDKTNFYAEQGGQIFDTGRILIDGEAEIE

VQNVQVYAGYVMHTGYMSYGNLKVGDEVLCQYDELRRQPIRNNHTGTHILNYALREVLGDEVDQRGSLVAPEKLRFDFSH

KAACSDEEINKVEDFCTRYIRQNTDVYAAEVPLATARNIMGVRAVFGETYPDPVRVVSVGVPVEQLLEDVQNPEWRNISV

EFCGGTHVKNTTEIKELVVLEESGIAKGIRRIVALTGTEAQSAQQEAMEWDKKVSQLEKMQYGVEKETKTKEWQHELNKA

EIPAVAKSLLRKRFEKIVKGNLDHQKGAQKAENKMVMDKVNEHFEKNKENKTLVISVPVSSGSKAVQEAIKNISSKQKDK

TVYLIGKSDDKVMHGCYVSAEAQSKGADANKWANEVAVLVGGKAGGKGATSLGQGTQPDKVDDAVEAARKYLQGLNL

>KIV86028.1 alanine-tRNA synthetase [Exophiala sideris]

MQIGGGGAAIWRAARFSSAVHTIVTAQTPRFRNLVLTHNSGVQLFRTCYTVALQAPTTQTGPSKTRNLLFCSITSQSSRN

WEFSSNPKVRLHTTRRMASNTTGEAHQWSAQVVRDTFLKFFEERGHTFVRSSPVVPLSDPTLLFTNAGMNQFKPIFLGTV

DPTSKEGQYKRAVNSQKCIRAGGKHNDLDDVGKDSYHHTFFEMLGNWSFGDYFKEEAITYSWDLLTQKFGLDPDRLYVTY

FEGNEAGGLEPDIEAKELWKSVGVADDHILPGNMKDNFWEMGDQGPCGPCSEIHYDRIGGGRNAAHLVNQDDPNVLEIWN

NVFIQYNREPDKSLRSLPNKHVDTGMGFERLVSVLQNKMSNYDTDVFTPLFERIQEVTGARPYSGKFGEEDADGIDTAYR

VVADHVRTLTFAISDGGIPNNEGRGYVVRRVLRRGARYARKYFNVEIGDFFSKIVPTLVEQMGGMFKEIVAKEDEVKEIL

NEEELSFAKTLDRGERQFEVYAQKSQTQGLKSLHGADVWRLYDTFGFPVDLTRLMAEERGLSIDDNEFEAARLRAKEASK

GEKKAASDLLKLDVHDLGELEKMPGVTKTDDSAKYGRENITGTIQAIYHAKKFHSDTQGLPEGDQIGIILDKTCFYAEQG

GQEYDTGRIVIDGQAELEVENVQLYAGYVLHTGYMKYGHFSVADKAICEYDELRRWPIRNNHTGTHILNFALREVLGDGI

DQRGSLVAQEKLRFDFSHKAAVSDADLQKVEDISTEYIRQNCPVYAKEVPLTVAREISGLRAVFGETYPDPVRVVSVGVE

VDEILKNVKDDRWKTVSIEFCGGTHVKATADIKDLVVLEESGIAKGIRRIIAVTGEDAYQVQRVAEEFSEKLKKLEDMPF

GASKESEAKTMQAELNNLSISAVKKSQFRERMTKISKDVLDQQKKLQKEELKKATDTITQHFEKNKDSKAAVLRLPIGGN

AKIVPDVVKHVQTKLKDKSVYVVVADEKDPEGKVFHGCFVSPDGTKAGITSADWSSQVSQLVGGKAGGKAPVAIGTGTDQ

SKVDDALNAATEYLEKFKL

>KIK48384.1 hypothetical protein CY34DRAFT_71989 [Suillus luteus UH-Slu-Lm8-n1]

MSSSYDGPWTARKVREEFFNYFRSKNHTFVPSSSTVPYDDPTLLFANAGMNQYKSIFLGTVDPNSERGKLKRAFNSQKCI

RAGGKHNDLEDVGKDSYHHTFFEMLGNWSFGDYFKKEAIEFSWDLLTNVYGLPKDRLYVTYYAGDPQNGIPTDEEAKQHW

LSQGISPDHVIASKGNFWEMGATGPCGPCSEIHFDRIGGRNAAHLVDGDDPNVLEIWNNVFIQFNRDDDGSLKPLPNKHV

DTGMGFERLVSVLQNKLSNYDTDVFSPIFTRIQELTGARPYSGKFGNDDVDGIDTAYRVVADHVRTLSFALSDGGVPNNV

GRGYVLRRILRRGSRYVRKKLGAPIGSFFSSLMPVVVETMGDIFPELTKKQDDIKEILDEEEESFSRTLDRGEKLFDQYA

TRTKDQGAKELNGADVWRLYDTYGFPVDLTRLMAEELELGINEKEFEDAQEHSKLASKGSFKKDKTDLVKLDVHDLAALE

KNDAIPKTDDSSKFQLGNITATVKGIYYHKAFISSTSNIPADANFGVLLDRTSFYAEAGGQEYDTGNIVIDGATDFEVTN

VQAYNGYVLHTGSLKYGQLSVGDEVISSYDELRRWPLRSNHTATHILNFCLREVLGDHIDQRGSLVAPTKLRFDFSHKAQ

ISLPELAKIESMSIDWIKKNVKVYGKDLDLTTAHKISGLRAVFGEAYPDPVRVVVLGYDVDEVAKDIDNPKWRSTSVEFC

GGTHVAKTGDIKDFVITEESGIAKGIRRIVAVTGQEAYEASRVANSLKVRLEQLESAEGKEKDSGLKALSVELGQADISV

LLKAELKDRLATLRKALDKAIKEKENAINKAAVNSLVTFFEEKPDSEVYFAILEVDGNAKILQNVVMQGKKLGKTVYVFS

VDAEGGKVAHANYVSESARSRGLDARTWASKVSDILGGKAGGKEDGAQGVGVNVAQVEEALKIARETFSSKYN

>KGL76824.1 hypothetical protein N309_13365, partial [Tinamus guttatus]

VRAAFLRFFEQRHGHRRLPSAPVRPRGDPRLLFVNAGMNQFKPVFLGTVHPRSELAQHRRVVNCQKCVRAGGKHNDLEDV

GRDTSHHTFFEMLGNWSFGDYFKKEACSMAWELLTEVYKIPKDRLYVTYFGGDSSLGLSADEECRDVWLRLGVPASHVLP

FSLKDNFWEMGDTGPCGPCTEIHYDHVGGGRNAAALVNQGSPDVVEIWNLVFMQYNREVEGNLLPLPQHHVDTGMGLERL

VTVLQNKRSNYDTDLFTPILDAIHKGCGGPRYQGLVGRADASGVDTAYRVVADHARTLCVCIADGVYPGLSGAELVLRRI

LRRAVRFCSEVLHAPPGLLASLVPTVVDVLGDAYPELRKNTDQIADIINENEAAFLSSLERGRRVIERTVQHMEPSTDFP

AEVAWSLYGNLGFPLDLIDLMLEEKGIRLDSAAFDELALEDARRKARDPQAGQLEGADWRLDVHSLAQLQSNNVPVTDDS

PKYAYTLGQRGQYEFSPCQATVLMLYRNQSLQKEVGAGQHCGVILDRTNFYAEQGGQASDRGYLMLLGQQDMLFPVKSVH

LCGGYVVHEVTAVEMLRAGDQVQLFVDEAQRLACMTNHTAAHLLNFALRHVLGDNTEQRGSHVTAERLRFDFNTKDPVTI

EQLQQVERVVQEVINRNEVVHMAEVPLALARRVQGLRSVDEEYPDPVRIVSLGVPVESVLIHNSEAAMHTSVELCCGTHL

LRTGVMEDLAIISERQLAKGISRVIAVTGGQAKQAREVGQCLAMEVDSVSIRMKQGSTSIPDVQHLSKEVGQLTQVVANT

AMPQWQRKELQTTLKALQRTANTAIKKLEIQQAAEKAHSLLAKHCNQPLIIDTVPADSASILMKVVNQLCGKSPGTSVLL

LSPQASGEVLCACQVSKNCLPIFSAADWAAAVCTQMEGKAGGSCVVAKGSGNAKGMQRALTTAMEFAQSKL

>KDQ57564.1 hypothetical protein JAAARDRAFT_35260 [Jaapia argillacea MUCL 33604]

MSINAYNGPWSARKVRDEFFNYFRSKDHTFVPSSPTIPYEDPTLLFANAGMNQYKAIFLGTVDPHSEMAKWKRAFNTQKC

IRAGGKHNDLEDVGKDSYHHTFFEMLGNWSFGDYFKKEAIQYSWELLTEVYHLPKDRLYVTYFEGDLQNGLEPDHEAKQY

WLDQGVLEDHILPGNAKDNFWEMGATGPCGPCSEIHFDRIGGRNAAHRVNQDDPDVLEIWNNVFIQFNREDDGSLKPLPA

KHVDTGMGFERLVSVIQDKRSNYDTDVFTPIFSRVQELTGVRPYTGKFGAQDVDGIDTAYRVVADHVRTLTFALSDGGVP

NNVGRGYVLRRILRRGSRYARKKLGVTIGSFFSSLMPVVVESMGDTFPEITTKTEAIKEILDEEEESFSRTLDRGEKLFE

KYASQALQSGAKELNGKDVWRLYDTYGFPEDLTRLMAEELGLGINDEEFQRAQAHSKEASKATSKSGGADVLKLDVHDIA

ALEKDTSVPKTDDSSKYALGNVTAQIKAIYHNKTFVQSTAGIPSDSTFGLILDRTSFYAESGGQEYDTGSIVIDDVAEFE

VTNVQVYNGYVLHSGHLKYGNLNTGSEVVCSYDELRRWPIRNNHTGTHILNFCLREVLGDHIDQKGSLVAPTKLRFDFSH

KAQVSPQDLGKIESMSKAWIKKNVKVFSKELDLKTSQKIPGLRAVFGETYPDPVRVVSLEYDVNEIASDVTNPKWRSTSV

EFCGGTHVAKTGEIKDIVITEESGIAKGIRRITAVTGNEAEEVTRLASQLTARLDHLESLSGKEKDAALKAFSVELGQAD

ISVLIKGDLRDRLGSIRKAFDKQVKEKEATLNKTAVEEMVRYFEGKGDSEAYFALLDVDGNSKILQNLVVNARKLQKPIY

VFSVDHQGGRVVHVNYVPESSRSGGLDARVWASKVADLVGGKAGGKEDTAQGVGVHVDEVDEAIELAKKYYQEVKRS

>XP_008725247.1 alanyl-tRNA synthetase [Cladophialophora carrionii CBS 160.54]

MASSTPEIPQQWTAPKVRETFLKYFEERGHTFVKSSPVVPLSDPTLLFTNAGMNQFKPIFLGTVDPSSKEGQYKRVVNSQ

KCIRAGGKHNDLDDVGKDSYHHTFFEMLGNWSFGDYFKKEAITFSWELLTKVFGLEPDRLYVTYFEGNEAGGLEPDLEAK

EIWKSVGVAEDHILPGNMKDNFWEMGDQGPCGPCSEIHFDRLGGRNAAHLVNQDDPNVLEIWNNVFIQYNREPDRSLRPL

PNKHVDTGMGFERLVSVLQNKLSNYDTDVFTPLFARIQEVTGARPYSGKFGEEDTDGIDTAYRVVADHVRTLTFAISDGG

IPNNEGRGYVVRRVLRRGARFARKYFNVEIGDFFSKIVPTLVEQMGGMFKEIVAKEEEVKEILNEEEESFAKTLDRGEKQ

FEVYAQKSQVQGQKSLHGADVWRLYDTFGFPVDLTRLMAEERGLSIDDNEFEAARLAARDASKGQKKAASDLLKLDVHDL

GELEKMPGVTKTDDSAKHGRQNITGTIQAVYHAKKFHPGTKNIPEGDQIGLILDRTCFYAEQGGQEYDTGRIVIDGQAEL

DVENVQLYAGYVLHTGYMKYGHFSLGDKVICEYDELRRWPIRNNHTGTHILNFALREVLGDGVEQRGSLVAQEKLRFDFS

HKSGVSDEDLAKIEDISTDYIRQNCQVYAKEVPLVIAREIAGVRAVFGETYPDPVRVVSVGVDVDDILKNVRDKGWEKVS

IEFCGGTHVRSTGDIKDLVILEESGIAKGIRRIIAVTGEDAHEVQRVAEEFSGKLEKLEKMPFGSPKEQEAKNIQLELNN

LSISAVKKSQFRDRVSKISKEILEQTKKLQKEQTQKAIDTITEYFQKNENAQAAVLRLPTGGSSKLVPEVIKHVQTKLKD

KSVYIVVADEKDPEGKVYHGCFVGQNGTAAGLKSPEWSSTVSNLVGGKAGGKAPVAIGTGTDQSKVDEALKAATEYLEKF

KL

>KZT56563.1 hypothetical protein CALCODRAFT_524254 [Calocera cornea HHB12733]

MTTISTVGPYGDWPADKVRATFMSYFEERGHTFWPSSSTIPYEDPTLLFANAGMNQYKAIFLGTVDPHSAMAKLKRAVNS

QKCIRAGGKHNDLEDVGKDTYHHTFFEMLGNWSFGDYFKKDACKFSWQLLTEVYGLPKDRLYVTYFEGDPKQNLEPDLEV

KQWWLDVGVPEAQILTGDAKDNFWEMGATGPCGPCSEIHFDRIGGRNASHLVNQDDPNVIEIWNNVFIQFNREEDGSLKE

LPAKHVDTGMGFERLVSCLQDRPSNYDTDVFHPIFLKIQELTGARPYAGKLGAEDVDGIDTAYRVVADHVRTLTFALSDG

GVPNNVGRGYVLRRILRRGARYVRKKFGVPIGSFFSSLAPTVVEQMGHFFPEITKKMDDIKEILDEEEESFSRTLDRGEK

LFEEYVAAAKAKGSTSLSGRDVWRLYDTYGFPVDLTRLMAEELHFTIDDQEFEAARLHSLEASKASTKKGDKEEVKLDVH

DIAALEKNSLVPKTDDSPKFGLGNVTATIKAIYHNKAFHSSTSAIPEGAPFGILLDRTSFYAESGGQEYDTGSIIIDGKA

EFDVQNVQVYNGYVLHSGELKYGELSVGDEIISSYDELRRWPLRNNHTGTHILNFALRDILGDHIDQKGSLVAPTKLRFD

FSHKAQVALDDMVKIEEICNSWIKKDVKVFSQEVDLHTAQEIAGVRAVFGEAYPDPVRVVSLGYEVKDIVQDPQNAKWRG

TSIEFCGGTHVAKTGDIKSLIITEESGIAKGIRRIIAVTGEDAKEVTRLGDSFEARLLQIERSSGKEKDTALKAYTFELG

QADISALRKAALKEKFAKIRKAFDDANKVQQAALTKEIVDSMDKFFEEKPNEDIYVASLDMLEGNTKIMQTIVQHARSVD

KAVYLFSIDGPSGKVAHVNFVPKRIITKSFSAKTWANAIAEILGGKAGGKDDVAQGVGTNVDKLGEALEMAEKLFRDRA

>XP_013287298.1 alanine-tRNA synthetase [Fonsecaea pedrosoi CBS 271.37]

MASTTPTTTTHEWTAPKVRDTFIKYFEDRGHTFVKSSPVVPLSDPTLLFTNAGMNQFKPIFLGTVDPSSKEGQYRRVVNS

QKCIRAGGKHNDLDDVGKDSYHHTFFEMLGNWSFGDYFKKEAILFSWELLTGVFGLEPDRLYVTYFEGNEAGGLEPDLEA

KEIWKSVGVPEDHILPGNMKDNFWEMGDQGPCGPCSEIHYDRIGGRNAASLVNQDDPNVLEIWNNVFIQYNREPDRSLRP

LPNKHVDTGMGFERLVSVLQNKMSNYDTDVFTPLFSRIQEVTGARPYSGKFGEEDVDGIDTAYRVVADHVRTLTFAISDG

GIPNNEGRGYVVRRVLRRGARYARKYFNVEIGDFFSKIVPTLVEQMGGMFKEIVAKEEEVKEILNEEELSFAKTLDRGER

QFEVYAQKSQVQGLKKLHGADVWRLYDTFGFPVDLTRLMAEERNLSIDDNEFEAARLAAKEASKGQKKAASDVLKLDVHD

LGQLEKMPGVTKTDDSAKYGRDNITSTIQAIYHAKQFHSDTKNIPEDDQVGIILDRTCFYAEQGGQEYDTGRIVIDGQAE

LEVENVQLYAGYVLHTGYMKYGHFSVGDKAISEYDELRRWPIRNNHTGTHILNFALREVLGDGVEQKGSLVAQEKLRFDF

SHKSGVSDADLQKIEDISTEYIRQNCPVYAKDVPLVIAREISGVRAVFGETYPDPVRVVSVGVEVDDILNNVKDDKWKTV

SIEFCGGTHVRSTGDIKDLVILEESGIAKGIRRIIAVTGEDAHEVQRVADDFSAKLDKLEKMPSGTPKEHEAKAIQLELN

NLSISAVKKSQFRDRFSKISKEILDQQKKLQKEETKKAIDTITEYFEKSPNAQAAVLRLPIGGNPKIVPDVVKHVQTKMK

DKSVYIMVADEKDPEGKVYHGCFVSQNGTKAGMKSPEWSSTVSKLVGGKAGGKEPVAIGTGTEQSNIDEALKAATDYLEK

FKL

>CEI87424.1 Putative Alanyl-tRNA synthetase [Rhizopus microsporus]

MFLGTLDPNSPLANVKRACNSQKCIRAGGKHNDLDDVGKDTYHHTFFEMLGNWSFGDYFKKEATKLAWEFLTKVISLPED

RLYVTYFGGDEKMGLPADLEAKQLWLDLGVDEKRLIPGNVKDNFWEMGETGPCGPCSELHFDRIGNRDAACLVNMDDPNV

IEIWNLVFMQFNREVDGTLRPLPNQHIDTGLGLERLVSILQNKYSNYDTDVFTPMFDRIKETTGVRSYTGKLGDEDVDGI

DTAYRVVADHIRTLTFAIADGGVPSNEGRGYVLRRILRRGARYVRKYFNVPIGSFFSSLVDTLIAQMHEGYPELAKKAND

IKAILDEEEAAFAKTLDRGEKLFEAYMNKTKESGSNVMSGDDVWRLYDTYGFPVDLTRIMAEENGLQVNEAQFEAAQEAA

KNKSRKARGGALGQDTVALDVHDLGALDNDSSVPKTDDSAKYSRGNIQAKVKAIFFDHKFLKNTSEIPDERNFGLLLDKT

NFYAESGGQEYDIGSIITLDGNTEFVVEDCQLYGGYVLHVGHLKYGQLSVDEDVEVSYDEDRRSPLRHNHTATHILNFAL

REVLSEDIDQKGSLVAPEKFRFDFSYKSPVNVKQLEQVENICNEFITKNQQVYSQEVPLAVAKAIHGLRAVFGETYPDPV

RVVSIGFDIDEIVQDVSNPKWKTSSVEFCGGTHVAKTGDIKHFVIIEESGIAKGIRRIVALTGEAAVQAQREADTFVTKL

DRLEKLSGSQLEAELKVVSKELDNAVISSVSKAAFRERFAQIKKAFDNAEKVRKAEQVKEATEKVKKYFEENPDAPYYIA

ELHVGNNSKALSGAINYAKTSLKDKAVYVFSADRDSGRVAHNCIVGKVYIEKGLKASEWANAVCEKIGGKKGGKDDAAQG

SGDNIDGLPDALKAAEEFAKLKIN

>XP_020542769.1 Alanine--tRNA synthetase [Pichia kudriavzevii]

MSIYTNPATNPKIKEWPAAKVRDTFLNYFKSKGHTFVPSSSVIPHDDPTLLFANAGMNQYKPIFLGTVDPSSSFANLKRA

VNSQKCIRAGGKHNDLEDVGRDSYHHTFFEMLGNWSFGDYFKEGAIKYSWELLTEVYGIPKDRLYVTYFGGDEKTGLEPD

LEAKGLWQQVGVADDHILPGNVKDNFWEMGDQGPCGPCSEIHYDRIGGRNAASLVNEDDPDVLEIWNNVFMQYNREPDGS

LVPLPAKHIDTGLGFERLVSLLQDVRSNYDTDVFYPIFQKIQEITGVRPYTGKFGKDDSDGIDTAYRVLADHVRTLTFAL

ADGGVPNNEGRGYVLRRILRRGARYVRKYMNYPIGNFFSQLAPTLIEQVKHIFPELEKDPSTIFEILDEEEASFARTLDR

GEKLFEQYALIASKTPEQTLSGKDVWRLYDTYGFPVDLTRLMAEEAGLKIDEEGFEKARLESKEASKGTGTKTGKTLLKL

DVHALASLNDQSVPKTDDSFKYDTKDIHAKVLSIYSNGQFVDSISQSEQQVGIVLDKTPFYAEQGGQEFDTGKIVIDGKS

EFSVENVQVYGGYVFHTGFVQEGELKVGDDIIAGYDELRRWPLRNNHTGTHILNFALRKVLGDSVDQKGSLVAPEKLRFD

FSHKKAVELKELEQVENITNKLIKDNLKVYAKPVELEIAKGINGLRAVFGETYPDPVRVVSVGVPVDDLLADPSNEKWNN

YSIEFCGGTHVAKTDDIKEFVIIEESGIAKGIRRIVAVSGMEAKEVQRKASEFNGILNEVEKLPYGEVKERRIKELGTEL

GKLSISVIEKAALKDKFSKIEKAVKDETKSRVKKDTAKVMDAVKSYFETPEHSAVCTLKVTAGANAKIVAEAINYMKKEQ

KESCLYLVLGEDQGKVAQGVYLSPAVQDKIAAVEVATKAAEYIGGRAGGKGNSCQGMGDKAENADKAVEEIKTLLEKLSL

GNLSGSSLNTLFSATIDHNIDDEYKSTDIVNSYILAESVPVKDNIPYGSMLGYRFKPNMSAEFTHSYQIIIYLSASLCQL

PVNWNTSISNNGLTLYYTFNETIANNTDFKNMEKVDFQGGYASAIAETEIISTDSDYMLYLMIQPQQCNNCTAEDIWVYE

FAASQRKVMFLYDTEPRISVLDVDYEAAIFQTGDIPFGQNSSYSLYLFKDEYPIPIGLNQSWCAISENQQYDKKISVNES

LVSSLKNAFVVSDLDIAKTYTGVLVITNDIIPYGGGIFEPFVFTMSKTRSCKLAYGLEFCDEVAYAVPISESYLYGKESW

SEFVSSYDNYSESLYQPFEYALQQVACDTELDARYSPIRTCNDCKYSYKQWLCAVTIPRCVSSLNSGVFNLNYTAEQGRN

EYIAEHIKPPLPYSEILPCLNVCQAIVRDCPADFGFGCPETPELVQLSYGDQELLKGNSDADDTDDDESNAIAELVRETL

SDGSIQTYRLCNYLGVSNIYTSSSSAEATSSAST

>KDR75441.1 hypothetical protein GALMADRAFT_249500 [Galerina marginata CBS 339.88]

MAVAPYTGPWTAARVRQQFFDFFRSKNHVFVPSSPTIPFDDPTLLFANAGMNQYKSIFLGTVDPHSDMSKLKRAFNSQKC

IRAGGKHNDLEDVGRDSYHHTFFEMLGNWSFGDYFKNEAIGYSWELLTQVYKLPADRLYVTYFEGDLKNGLEPDLEAKQF

WLDYGVTEDHIIPGNAKDNFWEMGATGPCGPCSEIHYDRIGGRNAAHLVNQDDPDVLEIWNNVFIQFNREDDGSLRPLPS

KHVDTGMGFERLVSAIQDRRSNYDTDIFLPIFDKVHELTGVRPYQGRFGADDADGIDTAYRVVADHVRTLTFALSDGGVP

NNVGRGYVLRRILRRGARYARKKLGVNIGSFFSSLMPVVIGTMGETFPEITKKVDEIKEILDEEEESFSRTLDRGEKLFD

QFAARANEQGVRELNGKDVWRLYDTFGFPVDLTRLMAEELGLSINDVEFEQAQAHSKEASKVSTKKDALNVVKLDVHDIA

SLEKSDQIPKTDDSAKFNQGNINSIVKAVFYEKSFLQSTDTIPEGATFGILLDRTSFYAESGGQEYDTGNIVIDGVADFE

VVNVQVYNGYVLHTGHLKYGTLNLEDEVVSSYDELRRWPLRNNHTATHILNFSLREILGDHIDQKGSLVAPTKLRFDFSH

KAQISLPELQKIEFLSTDWIKRNVKVFSKDLSLQTASRIPGLRAVFGESYPDPVRVVTLEYDVDEIAGDIENPKWRQTSV

EFCGGTHVAKTGDIKDFVITEESGIAKGIRRIVAVTGHEAQEVARLAQALEEKLKNLDLSTGKDKDTGLKAFSVELGQAD

ISVLRKAHLKDRLAAIRKAHDKQIKDRETQANKQAIEHLQQYFKDNKTSDAYVALIEADGNAKILQSLISQARQLDKSLY

VFSVDTANQKVAHANYVSPSLKSLGADARNWASQVTDVLGGKAGGKEDSAQGVGINIDKIQDALNLASSYLSGIRS

>AGO12026.1 AaceriADR363Cp [Saccharomycetaceae sp. 'Ashbya aceri']

MTIGDKKWTAQNVRSTFIDYFKAKDHKLVPSSPVIPYDDPTLLFANAGMNQFKPIFLGMVDPASDFYTLKRAVNTQKCIR

AGGKHNDLEDVGLDSYHHTFFEMLGNWSFGDYFKKEAITYSWELLTKVYGLPEDQLYVTYFEGDESLGLAADEEARDLWL

SVGVAPDHVIAANVKDNFWEMGDQGPCGPCSEVHFDRIGGRNAAALVNQDDPNVLEIWNNVFIQFNREQDGSLRPLPAKH

VDTGMGFERLVSVLQGVPSNYDTDVFQPLFARIQEITGVRPYSGKFGADDKDGIDTAYRVLADHVRTLTIALADGGVPDN

DGRGYVLRRILRRGARYVRKYMNYPTGKFFSQLAPTLIEQFKDVYPELTKNQSYLFDILDEEEASFAKTLDRGEKLFEKY

AETAKNSGSKILDGKQVWRLYDTYGFPVDLTQLMADEHGLKVDNEGFERAKQESYEASKKNGSKGDVNLIKLNVHEVSQL

RDEGVLRTDDSAKYGTEDIEATIVRIHDGENFVEQMTEVGKQYGVLLDRTCFYAEQGGQEYDQGKIVIDGAAEFNVENVQ

QYSGYVFHTGNLADGKLAVGDKIIASYDELRRYPIRNNHTGTHLLNYALRTILGDNIDQRGSLVTPEKLRFDFSHKKPMS

YEELEQVENICNEQIRENRSVYYKDIPLDVAKSIHSIRAVFGETYPDPVRVVSVGKSVDEILENPSDEEWAKYSIELCGG

THVSKTGDIKEFVIVEESGIAKGIRRIVAVSAHEAYEVQRVAKEFALELDAIDKLPFSMMKEKKIREIGVRLDQLNISVI

TKNELKTKFTKMEKAVKDEVKVKAKKEMKQTLDAVKEYFAANEDATFFVKSLDISANPKAVTEAVQYLKNSARDKTIYLF

TGNDPEGKVAHGCFISDGALAKGIDGAALAKIVSSKIGGKAGGKGNVFQGMGDKPAGIDSAIAEVEASFKEKLIL

>EMD37438.1 hypothetical protein CERSUDRAFT_83193 [Gelatoporia subvermispora B]

MATPATQPWSSRQVREEFFKYFQSKEHTFVPSSSTIPYEDPTLLFANAGMNQYKSIFLGTVDPQSDLARLKRAYNSQKCI

RAGGKHNDLEDVGKDSYHHTFFEMLGNWSFGDYFKKEAIAYSWELLTVVYKLPKEQLYVTYFEGDPANGLEPDLEAKQYW

LDQGVPEDHVLPGNAKDNFWEMGATGPCGPCSEVHFDRIGGRNAAHLVNHDDPDVLEIWNNVFIQFNREDDGSLRLLPSK

HVDTGMGFERLVSVVQGKRSNYDTDVFSPIFSKIQELTGVRPYQGKFGDEDENGIDTAYRVVADHVRTLTFALSDGGVPN

NVGRGYVLRRILRRGCRYARKKLGVNIGSFFSSLLPVVVEQMGDVFPEITKKMAEIKEILDEEEESFSRTLDRGEKLFEQ

YANRTLQQGSKQLSGKDVWRLYDTYGFPVDLTRLMAEELGLVVDEEEFEAAQAHSKEASKASLKKGAKDVVALDVHDIAA

LEKNTNVPKTDDSAKFALGNINAKVVAIYHNKTFPDATSSIPEDAVFGLLLDRTSFYAESGGQEYDTGSIVIDGVAEFEV

TNVQVSNGYVLHIGSMKYGSLSVGDEVVSSYDELRRWPLRNNHTATHILNYCLRETLGDHIDQKGSLVAPTKLRFDFSHK

AGISAPDLAKIEGMSREWIRKNVKVYSKELALKTAQEVPGLRAVFGESYPDPVRVVALEFDINDILKDLHNPKWRSTSIE

LCGGTHVAKTGDIKDFVVVEESGIAKGIRRIVAVTGHEAAEAQRRADALTARLNYLTSASDAEKDSGLKSFTVELGQAEI

SLLRKNELRDQLATVRKAFDKQMKEKEAAANKAAVDSVIEYFQKNENAQAYFAVLDVGANAKVLQSVAQQGRKLGKTIYV

FSADAEGGKVAHINYVSEAAKSNGLDGRTWAGVVAEILGGKAGGKEDSAQGVGINVTKVNEALEAARSYFASKVSS

>XP_007677352.1 hypothetical protein BAUCODRAFT_25335 [Baudoinia panamericana UAMH 10762]

MGSTTGQEPEWTAPKVRSAFIETLRWNTKANHDDSYFKDRGHTFVPSSSVVPLSDPTLLFTNAGMNQYKSIFLGTVDPDS

DFAKLKRAVNSQKCIRAGGKHNDLDDVGKDSYHHTFFEMLGNWSFGDYFKKEAIEMSWELLTKVFGLDPDRLYVTYFEGK

SDAGLEPDEEARKYWKAVGVADDHILTGNMKDNFWEMGDQGPCGPCSEIHYDRIGGRNAAHLVNQDDPNVLEIWNNVFIQ

YNREPDRSLRPLPNKHVDTGLGFERLVSVLQNKMSNYDTDVFTPLFKTIQEVTGAREYRGKFGDEDVDGIDTAYRVIADH

VRTLTFAISDGGVPNNVGRGYVVRRVLRRGARYARKYFDVTIGDFFAKIVPTLVAQMGEMFPEIKAKQSDVVQILDEEEV

SFAKTLDRGESMFERFAQQCKKTDAKELSGADVWRLYDTYGFPVDLTKLMAEERGLDIDDKAVAEAQEKAREASKGEKKA

AADLLKLNVHDINALEQMPDVPKTDDSPKFQRENVHGVIRAIYVGGKFVDSTKEISESDQIGLILDRTSFYAEQGGQEND

TGRILIDGEAEIDVQNVQVYAGYVMHTGFMNYGSFKVGDEVLCDYDELRRQPIRNNHTGTHILNFALREVLGDEVNQRGS

LVAPEKLRFDFSHKAGCSDEELVKIEDLSTSYIRQNSEVYASEVPLATARNIEGVRAVFGETYPDPVRVVSIGVPVEELL

EDVKRKEWRSVSIEFCGGTHVKNTTEIKELVILEESGIAKGIRRIVAVTGAAAHDVQREASEWDKKISKLETMEYGVEKE

ARTKEWQHELGKVEIAAVAKSKLRERVAKVVKENLDHQKTAQKAENKRVLDVVNDHFEQNKDAKTLVLSLPVSAGSKAVQ

ETIKVISSKHKDKTVYLLGKADDKVLHGCFVSEEAQGKGADASKWANEVAGVVGGKAGGKGATSLGQGTNLDKIDEGVEA

ARKYLQGLKLSLIRCRTSEGHGIPKLSLDPLCHCAQGQLATVILNYVAIPKPLSHAANKSSVEFYCPFICMMSFRVAARS

AYSLPRTHATPFSAGIAQARGLVSHPTTNNGFRKAFVQDSHNALRQFSSSPTMATSALASIKSNCRKVMCIGRNYADHVK

ELNNQRPKQPFFFLKPPSSILLPNEGPVLRPAGVNMHFEVELGLVIGKTIKDLNTEDDNEILQYIDSYLLAIDMTGRNVQ

EEAKKRGLPWSIAKGYDTFLPIAGPIAKSKLTDPHNIELYLKVNGETKQEDNTELMLFRLPRQLSDISKVMTLEAGDIVL

TGTPKGWNEDQRQGGGGGAVEGRGEG

>EKC98782.1 alanine-tRNA synthetase [Trichosporon asahii var. asahii CBS 8904]

MSRRESKPTVPTTAAAKWPSPEDWPAAKVRSTFIDYQGVHVADLIMFWVATSHLSPPFWSCLNTPSPFLLLSLDPPKTST

PRGCSISEGCMLFARTVRPLSISSQHLHRTTPFTLRSFAPAGNMSRRESKPTVPTTAAAKWPSPEDWPAAKVRSTFIDYF

VNQPGFEHTFWPSSGSIPFDDDTLLFANAGMNQYKPLFLGTADPKSDLAKLSRAVNSQKCIRAGGKHNDLDDVGKDSYHH

TFFEMLGNWSFGDYFKLGALTMAWDLLTRVYGLPKDRLYVTYFEGDAAQGLEPDLEAKKIWQDLGVPESHILPGNAKDNF

WEMGATGPCGPCSEIHFDRIGGREAAHLVNMDDPDVLEIWNNVFIQYNRENDGSLRPLPATHIDTGMGFERLVSVLHNVS

SNYDTDVFTPIFKRIQELTGARPYEGKLGADDKDGIDTAYRVIADHIRTLTIAISDGGVPDKDGRGYVLRRILRRGVRYA

NSKFGAEMGTFFSSLVDTTVETLKEIFPEVTKKIPELKEILDEEEQSFARTLKRGEILFNKYAEAAAAGDKTLAGRDTWR

LYDTYGFPVDLTQIMAEERGLKIDEEAFEKARLESLEASKAGGKKGAAGGVKFDVHDLGALEANDAVPKTDDDFKYKKGD

ISAAIKAIYHQSKFYESTKDVPEGEPFGILLDKTNFYAESGGQEYDTGVIAIDGQAEFKVEDVQVYNGYVLHVGRLEEGE

LKLNDEVICTIDELRRWPIRNNHTGTHILNFALRDVLGDGIDQKGSLVAPTKLRFDFSHNKAISPADLKKIEDLCNAEIK

KALAVYSKELPLATAQKIPGLRAVFGERYPDPVRVVSIGFPLEDIEKNIEDPKWATTSIEFCGGTHVAKTDDIKDFVIVE

ESSIAKGIRRMVAVTGHEAADVQRKSAEFGRKLDRIANMEPKEREAALKPIPQELGQSGISVLNKLAHQERYNKLTAELA

AAAKAQTAAAQKVVTDDIKQYFAEHPNENVYVASFPSAQGNSKILSAAQTAAKAAKKAVYAFSVDEAGGKVAHLNFLPKD

EVSKAFNAKTWLQDVSKIITGKGGGKDETATGVGMNVDKLDAAMDEAKKAYLASKQ

>PVU98093.1 hypothetical protein BB561_000088 [Smittium simulii]

MATETQEWSNSKVRSTFIDFFKSKGHTFYQSSSVVPHDDPTLLFANAGMNQFKPIFLGITDPNSDMAKLKSACNTQKCIR

AGGKHNDLEDVGKDVYHHTFFEMLGNWSFGSYFKKEAIEYAWELLTDVYSLEKSRLYITYFGGDQAQGLEPDLEAKAFWL

NLGVSESNILPFGTKDNFWEMGEAGPCGPCSEIHYDRIGDRDASSLVNLDDPDVLEIWNLVFMQYNRENANTLKPLPNKH

IDTGMGFERLVSVLQKKSSNYDTDVFVPIFEAIQQKSGVRPYSGHVGNDDIDGIDMAYRVIADHIRTLIFAICDGAIPSN

EGRGYVVRRILRRGARYARKKMNVELGDFFPNLVDTVVAQLGDTFSEISSNTDFIKKTLAEEEKSFSRTLDRGEKLFEGT

LAKMREGGQTVVPGSEVWKLYDTFGFPVDLTRIMAEENNVTIDELEFEKEKLKSKEISRKKKSGNLNADQVTLDVYTLAE

LEKDSSISKTVDNFKFQNPQISSKVVAILVDKSFAQSQDSSLENNSDSAESFGILLDQTNFYAESGGQEFDIGTIISDSS

NFEFIVTNVKNYGGYILHAGYLSKGKVCVGDTATCIYNEERRRLLMSNHSATHILNHSLRNILESPEVDQRGSLVSEDKL

RFDFGYNNQITPEVIRDIQSDTQTTVNSGLKVYTKELDLQVAKSIYGIRAVFGETYPNPVRVVSVGADLESVISDPNNTQ

WSNYSIELCGGTHVNNSKEIDNFVITEESSISKGIRRIIAVTGKVATQVNLNGENFEKELDSISSLKGSQLESALKTIGK

SLDTLYTGVWQKHLLRLKYNDIRKKFIEADKEAKLAASKFVSDQVANAIESNPEKNFHVLKVDFESSNGASNSKALANAI

TFAKSLNSKAVYLLSSDKQTGRVSHLCFVPKDLISKGLKAKDWASSVAKVAGGKSGGKDDTAQGSGTNAEAMDDILDAAK

EFALLSLNK

>ORZ29574.1 tRNA synthetases class II (A)-domain-containing protein, partial [Catenaria anguillulae PL171]

MHASVRIRPWLAAPRSIAATPTRPAAAAPPPAASTAIPLHSIRRRHWTAPKVRSTFINYFASTHNHTHVPSSNTIPHDDP

TLLFANAGMNQFKPIFLGTADPNSPMAKWTRAVNSQKCIRAGGKHNDLEDVGKDTYHHTFFEMLGNWSFGNYFKKEAIEY

AWVLLTEVYGLDKNRLYVTYFGGDEKLGLHADTEARDLWIKLGLPADRVLPYGSKDNFWEMGDQGPCGPCSEIHYDLLNT

GRFVPTLVNADDPTLIEIWNLVFIQFNREPDGSLRRLPTKHVDTGMGFERLVCALQGKLSNYDTDVFTPIFARIQQVTGA

RPYAGKLGTEDTDGIDMAYRVVADHVRTLTIAIADGGMPSNDGRGYVLRRILRRGVRYARSAFGRPADTPLFSSLVDAVV

ESLGDAFPEVKANAETVKQILDEEEVAFAKTLDRGLRLFESIAARSAHQKRISGADAWKLYDTFGFPIDLTRLIAEEKGL

QVDDAGFEAEQAKAKELSRRRKETEGEQVIRLDVHAIKAAEAAGVAKTDDSFKYNTATLDGAKVIAVYKNGEWILDGKHV

AISEGEAAGVLLDRTSFYAESGGQEADKGSLAIDGELEFAVENVHVYAGYVLHTGLLKYGALKVGQSVTASIDTLRRWPL

RNNHTATHVLNHALRSVLGEGVDQKGSLVAPTKLRFDFSAKAPLAHKDLVNIEAKTADTIKRALPVFAKEVPLAHAKAIN

GLRAVFGEVYPDPVRVVSIGVSVEDLVSDPNNAKWPEYSIELCGGTHAGTSLELGSFAVMEESAIAKGVRRIVAVTGPQA

IAAHDEAASFVQELNKLSGLSGFALGEAVKEAAKEVDLRVMPAAAKMHAREVVGAHKKRFDDWNKAQKAAKATAAVEEVK

AYFAGDEANNKPYLVRVLDVGGNNKAVQLAIAAVKATGGEGKAALFITVAEGAKVSYAAYVDKSLHGKVTATQWAEVVAK

AVDGKMGGKEEMAQGAGPRWRLWMRLSGWPRSLPRSSSPKFT

>XP_019005656.1 alanine-tRNA synthetase [Kwoniella mangroviensis CBS 8507]

MSFADKKPTVPTTAPHPWPSPSDWPASKVRQTYIDYFVNQPGFEHTFWPSSGVIPFDDDTLLFANAGMNQYKPLFLGTAD

PKSDLSKLIRAVNSQKCIRAGGKHNDLDDVGKDTYHHTFFEMLGNWSFGNYFKIGALTMAWDLLTRVYGLPKDRLYVTYF

EGDAKQGLEPDTEAQQIWRDLGVPESHILPGNAKDNFWEMGATGPCGPCSEIHFDRIGGREVPELVNADDPNVLEIWNNV

FIQYNREQSGELRSLPAKHVDTGMGFERLVSVLHNVSSNYDTDVFTPIFAEIQRLTGSRPYQGKLGEEDVDGIDTAYRVI

ADHIRTLTIAISDGGVPDKDGRGYVLRRILRRGVRYASNKFNVKIGNFFSSLVPVVVDSLSGIFPEVTKKIPELVEILNE

EEASFARTLTRGEALFNKYASAALEEKRDVLGGKDIWRLYDTYGFPVDLTQIMAEERGLKIDQEAFEKARLESLEASKAG

GKDKGVAGTVKLDVHDLGALEANDQVPKTNDSFKYQLDDIKATVKSIYHSSKFYSSTSELPPNAPFGVLLDKTNFYAESG

GQEYDTGVLAIDGEAEFKVEDVQVYNGYVLHIGQMEEGEIKVGDEVICTYDELRRWPIRNNHTGTHILNFGLREVLGDHI

DQKGSLVAPTKLRFDFSHGKSITTPELVKIEAICNEWIKRAAPVYAKEMPLAEAYKIPGLRAVFGEAYPDPVRVVSLGYP

LEEIAQNIENSKWRGTSIEFCGGTHVAKTDDIKDFVIVEESSIAKGIRRIVAVTGHEAHEVSRKAAEFERRLNTIAELQG

KDKEVAMKPYLVELGQSGISLIKKASLKSTFEKMQSELVAAAKAKTAADSKVIQDAIKTFFKENPNDNVYVGEFEVAGGN

AKTLSAAVTAGKSLSKAIYVFSTDQETGKVAHTNYLPKEVLDKKVIDGKTWLGEVSQIVGGKGGGKDESATGVGSEPTKV

KEAIVLAKNFYLSKVEA

>XP_007759596.1 alanyl-tRNA synthetase [Cladophialophora yegresii CBS 114405]

MASSTPDIPQRWTAPKVRDTFLKYFEERGHTFVKSSPVVPLSDPTLLFTNAGMNQFKPIFLGTVDPTSKEGQYKRVVNSQ

KCIRAGGKHNDLDDVGKDSYHHTFFEMLGNWSFGDYFKKEAITFSWELLTKVFGLEPDRLYVTYFEGNEAGGLEPDVEAK

EIWKSVGVAEDHILPGNMKDNFWEMGDQGPCGPCSEIHFDRLGGRNAAHLVNQDDPNVLEIWNNVFIQYNREPDRSLRPL

PNKHVDTGMGFERLVSVLQNKLSNYDTDVFTPLFARIQEVTGARPYSGKFGEEDADGIDTAYRVVADHVRTLTFAISDGG

IPNNEGRGYVVRRVLRRGARYARKYFNVEIGDFFSKIVPTLVEQMGGMFKEIVAKEEEVKEILNEEEESFAKTLDRGEKQ

FEVYAHKSQVQGQKSLHGADVWRLYDTFGFPVDLTRLMAEERGLSIDDNEFEAARLAARDASKGQKKAASDLLKLDVHDL

GELEKMPGVTKTDDSAKYGRQNITGTIQAVYHAKKFHPGTKNIPEGDQIGLILDRTCFYAEQGGQEYDTGRIVIDGQAEL

DVENVQLYAGYVLHTGYMKYGHFSVGDKVICEYDELRRWPIRNNHTGTHILNFALREVLGDGVEQRGSLVAQEKLRFDFS

HKSGVSDEDLAKIENISTDYIRQNCQVYTKDVPLVIAREIAGVRAVFGETYPDPVRVVSVGVDVDDILKNVQDKGWEKVS

IEFCGGTHVRSTGDIKDLIILEESGIAKGIRRIIAVTGEDAHEVQRLAEEFSGKLEKLEKMPFGLPKEQEAKNIQLELNN

LSISAVRKSQFRDRVSKISKEILEQTKKLQKEQTQKAIDTITEYFQKNEKAQTAVLRLPTGGSSKLVPDVIKHVQTKMKD

KSVYIVVADEKDPEGKVYHGCFVGQSGTTAGLKSPEWSSTVSTLVGGKAGGKAPVAIGTGTDQSKVDEALKAATEYLEKF

KL

>OUM51247.1 hypothetical protein BVG19_g337 [[Candida] boidinii]

MNSFGSKLFKLNIINNSNLHSNPFLKKIPFNTTSNTINTTIIFNQRMSSTTATNNINSSSLTKEQIEWPASKVRNTFLEY

FKENGHTFVPSSSVVPHDDPTLLFANAGMNQYKPIFLGTVDPSSPLATLKRAVNSQKCIRAGGKHNDLEDVGRDSYHHTF

FEMLGNWSFGDYFKKEAIAFSWNLLTKVYALPKDRLYVTYFGGDEKLGLEPDLEAKEYWQQIGVDDDHILPGSAKDNFWE

MGDQGPCGPCSEIHYDRIGGRNASSLVNEDDPNVLEIWNNVFIQFNREADGSLKSLPAKHIDTGMGFERVVSILQNVYSN

YDTDVFEPLFSKIQEITGVRPYSGKFGKDDLDGIDTAYRVLADHVRTLSFAIADGGVPNNEGRGYVLRRILRRGARYARK

YMNYPIGKFFSQLAPTLIEQVKDIFPELSKDPSTIYEILDEEEASFARTLDRGEKLFEQYAIVASKTPEQTLSGKDVWRL

YDTYGFPVDLTRLMAEEAGLKIDEAGFEKAKLAAYEASKGNGKKAGKELVKLDVHALGYLDSNDSIPKTDDSFKYGTKDI

NSKIVAIYNGKEFIDSTDSLEEPTKQQIGILLDKSPFYAEQGGQEFDTGKIVIDGVSEFNVENVQAYAGYVLHTGFLVEG

SLKVNDSVIAGYDELRRWPLRNNHTGTHILNLALKKTLGDGIDQKGSLVAAEKLRFDFSHKSGISLDDLKKIEDICNKQI

EDNLQVFSKPVKLELAKEIKGLRAVFGETYPDPVRVVSVGVPVEDLLSNPSNEEWENYSIEFCGGTHVAKTNDIKKFVIT

EENGIAKGIRRIVALSGTEAFEAQRIANEFNDLLDSIDKLPFGDDKERKLKENGTKLSQLNVSLLDKDQLKKKFNKIDKE

VKNEIKIRQKADSKKTLDTVKNYFIENKDSEFLVAHVPINANAKVITEAINFLKKENKDKSLFLITGKQDESKIAQGVYI

SDAAIAKGLDAAEIANVASKFIGGKAGGKGNVCQGMGTESAGINDAIDAVEKLLKEKLSI

>XP_024342967.1 hypothetical protein POSPLADRAFT_1064626 [Postia placenta MAD-698-R-SB12]

MAHTLPSWSAPRVREEFFAFFRARNHVFVPSSPTVPFDDPTLLFANAGMNQFKSIFLGTVDPQSDLARLSRAFNSQKCIR

AGGKHNDLDDVGKDSYHHTFFEMLGNWSFGDYFKKEAIAYSWELLTEVYKLPKDQLYVTYFEGDRKNGLAADLEARQFWI

AQGVAEDHILPGDAKDNFWEMGATGPCGPCSEIHFDRIGGRNAASLVNHDDPDVLEIWNNVFIQFNREDDGSLKSLPSKH

VDTGMGFERLVSVIQNKRSNYDTDIFTPIFARIQELTGAPPYGGRFGEDDENGVDTAYRVVADHVRTLTFALSDGGVPNN

VGRGYVLRRILRRGCRYARKKLGVNIGSFFSSLVPVVIEQMGDVFPEITKRTQEIKEILDEEEESFSRTLDRGEKLFDQY

AARAKLSGANKMSGKDVWRLYDTYGFPVDLTRLMAEELGLEINDEEFEAAQAHSKQASKANQKKTDKDAVKLDVHDIAAL

EKTRNVPKTDDSAKYSLGNTNARILAIYSNKTFLDSTSTVPEDSMFGLVLDRTSFYAESGGQEYDTGSIVIDDVAEFEVT

NVQVFSGYVLHIGHMKYGSLSLGDEVVSSYDELRRWPLRNNHTATHILNYGLREVLGDHVDQKGSLVAPTKLRFDFSHKA

QIAPQDLTKIEGISRDWIRKNVQVYSKELDLPTAYKISGLRAVFGESYPDPVRVVALEYPVDEITADVENPKWRTTSVEL

CGGTHVAKTGDIKDFIITEESGIAKGIRRIVAITGHEAAEAHRRADALAATLARLESMRGGEKDAGLKSLTVELGQAEIS

VLRKAELRDRLAVVRKAFDKETKEAEAIATKAVVENITEFFKREDTAEVYLTILDSTGNAKVLQAAVQHAKKLGKSIYVF

SPDIASGKVAHVNYVSPTMKAKGLDARSWGSVVTEIVGGKAGGKEDTAQGVGLEPGKVKEALEAAQKYFVQHTNVS

>XP_018696955.1 alanine-tRNA synthetase [Fonsecaea erecta]

MALSTSTHEWSAPKVRDTFLKYFEDRGHTFVKSSPVVPLSDPTLLFTNAGMNQFKPIFLGTVDPSSKEGQYKRVVNSQKC

IRAGGKHNDLDDVGKDSYHHTFFEMLGNWSFGDYFKKEAIMFSWELLTDVFGLAPDRLYVTYFEGNEAGGLEPDLEAKEI

WKSVGVPEDHILPGNMKDNFWEMGDQGPCGPCSEIHYDRIGGRNAASLVNQDDPNVLEIWNNVFIQYNREPDKSLRPLPN

KHVDTGMGFERLVSVLQNKMSNYDTDVFTPIFARIQEVTGARPYSGKFGEEDVDGIDTAYRVVADHVRTLTFAISDGGIP

NNEGRGYVVRRVLRRGARYARKYFNVEIGDFFSKIVPTLVEQMGGMFKEIVAKADEVKEILNEEELSFAKTLDRGERQFE

VYAQKSQVQGLKKLHGADVWRLYDTFGFPVDLTRLMAEERNLSIDDNEFEAARLAAKEASKGQKKAASDVLKLDVHDLGQ

LEKMPGVTKTDDSAKYGRDNITSTIQAIYHAKKFHSDTKNIPQDDQVGIILDQTCFYAEQGGQEFDTGRIVIDGQAELEV

ENVQLYAGYVLHTGYMKYGHFSVGDKAISEYDELRRWPIRNNHTGTHILNFALREVLGDGVEQRGSLVAQEKLRFDFSHK

AGVSDADLQKIEDISTEYIRQNCPVYAKNVPLSIAREIAGVRAVFGETYPDPVRVVSVGVEVDDILKNVKDERWKTVSIE

FCGGTHVRSTGDIKDLIILEESGIAKGIRRIIAVTGEDAHEVQRVAEDFSTKLDKLEKMPFGSPKEQEAKAVQLELNNLS

ISAVRKSQFRERFAKISKEILDQQKKLQKEETKKAIDTITEYFEKNPKAQGAVLRLPIGGNPKIVPDVVKHVQTKMKDKS

VYIIVADEKDPEGKVYHGCFVGQNGAKAGMKSPEWSSTVSKLVGGKAGGKEPVAIGTGTELSKTDEALKAATDYLEKFKL

>XP_022512920.1 alanine-tRNA synthetase [Fonsecaea monophora]

MASTTPTTTTHEWTAPKVRDTFIKYFEDRGHTFVKSSPVVPLSDPTLLFTNAGMNQFKPIFLGTVDPSSKEGQYRRVVNS

QKCIRAGGKHNDLDDVGKDSYHHTFFEMLGNWSFGDYFKKEAILFSWELLTGVFGLEPDRLYVTYFEGNEAGGLEPDLEA

KEIWKSVGVPEDHILPGNMKDNFWEMGDQGPCGPCSEIHYDRIGGRNAASLVNQDDPNVLEIWNNVFIQYNREPDRSLRP

LPNKHVDTGMGFERLVSVLQNKMSNYDTDVFTPLFSRIQEVTGARPYSGKFGEEDVDGIDTAYRVVADHVRTLTFAISDG

GIPNNEGRGYVVRRVLRRGARYARKYFNVEIGDFFSKIVPTLVEQMGGMFKEIVAKEEEVKEILNEEELSFAKTLDRGER

QFEVYAQKSQVQGLKKLHGADVWRLYDTFGFPVDLTRLMAEERNLSIDDNEFEAARLAAKEASKGQKKAASDVLKLDVHD

LGQLEKMPGVTKTDDSAKYGRDNITSTIQAIYHAKQFHSDTKNIPEDDQVGIILDRTCFYAEQGGQEYDTGRIVIDGQAE

LEVENVQLYAGYVLHTGYMKYGHFSVGDKAISEYDELRRWPIRNNHTGTHILNFALREVLGDGVEQKGSLVAQEKLRFDF

SHKSGVSDADLQKIEDISTEYIRQNCPVYAKDVPLVIAREISGVRAVFGETYPDPVRVVSVGVEVDDILNNVKDDKWKTV

SIEFCGGTHVRSTGDIKDLVILEESGIAKGIRRIIAVTGEDAHEVQRVADDFSAKLDKLEKMPSGTPKEHEAKAIQLELN

NLSISAVKKSQFRDRFSKISKEILDQQKKLQKEETKKAIDTITEYFEKSPNAQAAVLRLPIGGNPKLVPDVVKHVQTKMK

DKSVYIMVADEKDPEGKVYHGCFVSQNGTKAGMKSPEWSSTVSKLVGGKAGGKEPVAIGTGTEQSKIDEALKAATDYLEK

FKL

>KKY25989.1 putative alanyl-trna synthetase [Phaeomoniella chlamydospora]

MRAAVRVKNYCALSHEVSGAFLKALPSTLVFTTLAQLRRSHFSGQQRVLRMASTTSGTPEWGATKVRSTFLDYFKENGHT

FVPSSSVVPLSDPTLLFTNAGMNQYKSIFLGTVDPNSDFAHLKRAVNSQKCIRAGGKHNDLDDVGKDSYHHTFFEMLGNW

SFGDYFKKDAITYSWELLTKVYGLQPDRLYVTYFEGAEANGLEPDVEAKELWKSVGVPDDHILPGNMKDNFWEMGDQGPC

GPCSEVHYDRIGGRNASALVNQDDPNVLEIWNNVFIQYNREPDKSLRPLPNKHVDTGMGFERLVSVLQDKLSNYDTDVFT

PLFKRIQEVTGARPYSGKFGEEDTDGIDTAYRVVADHVRTLTFAISDGAAPNNEGRGYVVRRVLRRGARYARKYLNVEIG

SFFSKIVPTLVEQMGDMFPEIKKKETDVMEILDEEELSFAKTLDRGERQFETYATKSKEKGLKSLHGADVWRLYDTFGFP

VDLTRLMAEERRLEIDDNEFEDARLKAKEASKGEKKAASEKIKLDVHDLGTLEKMPGVPKTDDSAKFGRSNINAGIKAIY

HAKKFVDDTKEVPEGDEIGIILDKTNFYAEQGGQENDTGKIVIDGQAELDVENVQVYAGYVLHTGYMKYGSFTVGDNVIC

EYDELRRWPIRNNHTGTHILNFALRETLGDGIDQKGSLVAAEKLRFDFSHKSGISDADLKAIEDKSTKYIHQNCAVYSKE

VPLSIAREISGVRAVFGETYPDPVRVVSVGVEVEEILNNVQDPRWKEISIEFCGGTHVAKTGDIKDLIILEESGIAKGIR

RIIAVTGEEAHEVQRVADKFSQKLSALDKLPLGSQKEAEAKTIGVELNQLSVSAVRKSEFREWFAKITKSISDHAKAQQK

LESKTAVEAVANYFKDEKNKDSSSLIVSLPISGNAKALTDALNHVKKDLPQKNIYIFAKEGETNKVSHGCYLSEPAIKAG

ASPSSWAATVSAIVGGKAGGKSPTAIGNGTQVDKVDEALKAARSYLEKLGL

>XP_014532716.1 Alanyl-tRNA synthetase, putative [Penicillium digitatum Pd1]

MTSLGSEWTAQRVRDTFLDYFKQNGHTFVPSSPVAPLSDPTLLFTNAGMNQFKSIFLGTVDASSDFGKMKRATNSQKCLR

AGGKHNDLDDVGKDSYHHTFFEMLGNWSFGDYFKKEAITYSWTLLTEVYGLDPDRLYVTYFEGNEAGGLEPDLEAKELWK

SVGVPESHILPGNMKDNFWEMGDQGPCGPCSEVHYDRIGGRNAAHLVNEDDPNVLEIWNNVFIQYNREADSSLRSLPNKH

VDTGMGFERLVSILQDKPSNYDTDVFTPIFQAIQNTTGAREYRGHFGADDSDGIDTAYRVVADHVRTLMFAISDGVIPNN

EGRGYVIRRVLRRGARYARKYFQVEIGNFFAKLVPTVVDQLGEMFPELKRKQQDVIEILDEEELSFAKTLDRGERQFEQY

AQQAKIKDTDMLHGADVWRLYDTFGFPVDLTRIMAEERGLRIDDVEFEEARLKAKEASKGQKKAAENTVKLDVHDLGKLE

KMNDVQKTDDSPKFGRGNITSHVKAIYHGKTFHNSTDDVPDGAQLGVILDCTNFYAEQGGQENDTGKIIIDGQAELEVGD

VQSYAGYVLHTGFMKYGSFAVGDSVICEYDELRRWPIRNNHTGTHILNFALKEILGDGVDQKGSLVSAEKLRFDFSHKSA

ISDAELEKIETKATDYIRQNCAVYSQELPLATAQQISGVRAVFGETYPDPVRVVSVGVELEEILQNVKDPRWQEVSIEFC

GGTHVQKTGDIKDLVILEENGIAKGIRRIIAVTGEEAHEVQRAAEEFGHRLDRLNALPLGPEKEQEAKQVQVDLNQLAIS

AVQKAKFRERFAKINKQVLDGQKAQQKLESKKAVDTITSYFDAPENQDKSWLVVQLPISANAKAVSESLNHVKSKMQGKS

VYLLAADSEQGRVSHGCYISQGLSDQGASAADWAAVVSGAVGGKAGGKGPTSIGNGTNADKVEEAISLAADYLNKFKL

>XP_022502491.1 alanine-tRNA synthetase [Fonsecaea nubica]

MASTAPTTTAHEWTAPKVRDTFIKYFEDRGHTFVKSSPVVPLSDPTLLFTNAGMNQFKPIFLGTVDPSSKEGQYRRVVNS

QKCIRAGGKHNDLDDVGKDSYHHTFFEMLGNWSFGDYFKKEAILFSWELLTGVFGLEPDRLYVTYFEGNEAGGLEPDLEA

KEIWKSVGVPEDHILPGNMKDNFWEMGDQGPCGPCSEIHYDRIGGRNAASLVNQDDPNVLEIWNNVFIQYNREPDRSLRP

LPNKHVDTGMGFERLVSVLQNKMSNYDTDVFTPLFSRIQEVTGARPYSGKFGEEDVDGIDTAYRVVADHVRTLTFAISDG

GIPNNEGRGYVVRRVLRRGARYARKYFNVEIGDFFSKIVPTLVEQMGGMFKEIVAKEEEVKEILNEEELSFAKTLDRGER

QFEVYAQKSQVQGLKKLHGADVWRLYDTFGFPVDLTRLMAEERNLSIDDNEFEAARLAAKEASKGQKKAASDVLKLDVHD

LGQLEKMPGVTKTDDSAKYGRDNITSTIQAIYHAKQFHSDTKNIPEDDQVGIILDRTCFYAEQGGQEYDTGRIVIDGQAE

LEVENVQLYAGYVLHTGYMKYGHFSVGDKAISEYDELRRWPIRNNHTGTHILNFALREVLGDGVEQKGSLVAQEKLRFDF

SHKSGVSDADLQKIEDISTEYIRQNCPVYAKDVPLVIAREISGVRAVFGETYPDPVRVVSVGVEVDDILNNVKDDKWKTV

SIEFCGGTHVRSTGDIKDLVILEESGIAKGIRRIIAVTGEDAHEVQRVADDFSVKLDKLEKMPSGTPKEHEAKAIQLELN

NLSISAVKKSQFRDRFSKISKEILDQQKKLQKEETKKAIDTITEYFEKSPSAQAAVLRLPIGGNPKIVPDVVKHVQTKLK

DKSVYIMVADEKDPEGKVYHGCFVSQNGTKAGMKSPEWSSTVSKLVGGKAGGKEPVAIGTGTEQSKIDEALKAATDYLEK

FKL

>PGH02976.1 alanyl-tRNA synthetase [Polytolypa hystricis UAMH7299]

MASTAQQHAPEWPALRVRNTFLDYFKDNGHTFVPSSSVVPLSDPTLLFANAGMNQYKSIFLGTIDPNSDFAQLKRAHNTQ

KCIRAGGKHNDLDDVGKDSYHHTFFEMLGNWSFGDYFKTEAIRYSWELLTKVYGLDPDRLYVSYFEGNPDAGIEPDLEAK

NLWLSCGVAEDHILPGNMKDNFWEMGDQGPCGPCSEIHYDRIGGRNAAHLVNQDDPDVLEIWNNVFIQYNREADKTLLPL

PSKHVDTGLGFERLVSVLQDKRSNYDTDVFTPLFGAIRDITGARPYAGKFGAEDADGVDTAYRVVADHVRTLTFAISDGA

TPNNEGRGYVVRRVLRRGARYARKYFQVEIGSFFSKIVPTLVEQMGDMFPEIKRKQADVMEILDEEEISFSKTLDRGERQ

FENYAEQAKQRNSNKLHGADVWRLYDTFGFPVDLTQIMAEERGLKIDNNEFEEARLKAKEASKGQAKIATETVKLSVHDI

SNLEKMESVPKTDDEAKFGIGNITAQIKAIFDAKEFVSSTEKIPEGKQIGVLLDRTNFYAEQGGQEHDTGKIIIDGKAEL

DVQDVQVYAGYVLHTGSMKYGTLSVGDSVISEYDELRRWPIRNNHTGTHILNFALREVLGDGIEQKGSLVAAEKLRFDFS

HKSGVTDEQLERIERKSTEYIRQNCAVYAKDVPLSVAREISGVRAVFGETYPDPVRVVSVGVELEEILQNVEDPRWKEIS

IEFCGGTHVQKTGDIKDLVILEESGIAKGIRRIVAVTGEDAHEVQRIAKEFGERLARFEKMEIGPKKEQESKIIQVDLNQ

LTISAVEKAKFRDLFAKIHKQLLDGQKAQQKLESKKAVEAVSAYFQDPETKDNPYLVLQLPVSANTKAVSESLNHVKAKL

PSKSVYFFAGDESEGKVIHGCYVSEDLIQQGGAASDWAGVVAGVVGGRAGGKAPVSIGNGTDASKVDEAVKAATEYLSKF

KL

>OWB65405.1 hypothetical protein B5S30_g729 [[Candida] boidinii]

MNSFGSKLFKLNIINNSNLHSNPFLKKIPFNNISNTINTTIIFNQRMSSTTATNNINSSSLTKEQIEWPASKVRNTFLEY

FKENGHTFVPSSSVVPHDDPTLLFANAGMNQYKPIFLGTVDPSSPLATLKRAVNSQKCIRAGGKHNDLEDVGRDSYHHTF

FEMLGNWSFGDYFKKEAITFSWNLLTKVYALPKDRLYVTYFGGDEKLGLEPDLEAKEYWQQIGVDDDHILPGSAKDNFWE

MGDQGPCGPCSEIHYDRIGGRNASSLVNEDDPNVLEIWNNVFIQFNREADGSLKSLPAKHIDTGMGFERVVSILQNVYSN

YDTDVFEPLFSKIQEITGVRPYSGKFGKDDLDGIDTAYRVLADHVRTLSFAIADGGVPNNEGRGYVLRRILRRGARYARK

YMNYPIGKFFSQLAPTLIEQVKDIFPELSKDPSTIYEILDEEEASFARTLDRGEKLFEQYAIVASKTPEQTLSGKDVWRL

YDTYGFPVDLTRLMAEEAGLKIDEAGFEKAKLAAYEASKGNGKKAGKELVKLDVHALGYLDSNDSIPKTDDSFKYGTKDI

NSKIVAIYSGKEFIDSTDSLEEPTKQQIGILLDKSPFYAEQGGQEFDTGKIVIDGVSEFNVENVQAYAGYVLHTGFLVEG

SLKVNDSVIAGYDELRRWPLRNNHTGTHILNLALKKTLGDGIDQKGSLVAAEKLRFDFSHKSGISLDDLKKIEDICNKQI

EDNLQVFSKPVKLELAKEIKGLRAVFGETYPDPVRVVSVGVPVEDLLSNPSNEEWENYSIEFCGGTHVAKTNDIKKFVIT

EENGIAKGIRRIVALSGTEAFEAQRIANEFNDLLDSIDKLPFGDDKEKKLKENGTKLSQLNVSLLDKDQLKNKFNKIDKE

VKNEIKIRQKADSKKTLDTVKNYFIENKDSEFLVAHVPINANAKVITEAINFLKKENKDKSLFLITGKQDESKIAQGVYI

SDAAIAKGLDAAEIANVASKFIGGKAGGKGNVCQGMGTESAGISDAIDAVEKLLNEKLSI

>OCF54527.1 alanine-tRNA synthetase [Kwoniella mangroviensis CBS 10435]

MSFADKKPAVPTTAPHPWPSPSDWPASKVRQTYIDYFVNQPGFEHTFWPSSGVIPFDDDTLLFANAGMNQYKPLFLGTAD

PKSDLSKLIRAVNSQKCIRAGGKHNDLDDVGKDTYHHTFFEMLGNWSFGNYFKIGALTMAWDLLTRVYGLPKDRLYVTYF

EGDAKQGLEPDTEAQQIWRDLGVPESHILPGNAKDNFWEMGATGPCGPCSEIHFDRIGGREVPELVNADDPNVLEIWNNV

FIQYNREQSGELRSLPAKHVDTGMGFERLVSVLHNVSSNYDTDVFTPIFAEIQRLTGSRPYQGKLGEEDVDGIDTAYRVI

ADHIRTLTIAISDGGVPDKDGRGYVLRRILRRGVRYASNKFNVKIGNFFSSLVPVVVDSLSGIFPEVTKKIPELVEILNE

EEASFARTLTRGEALFNKYASAALEEKRDVLGGKDIWRLYDTYGFPVDLTQIMAEERGLKIDQEAFEKARLESLEASKAG

GKDKGVAGTVKLDVHDLGALEANDQVPKTDDSFKYQLDDIKATVKSIYHSSKFYSSTSELPPNAPFGVLLDKTNFYAESG

GQEYDTGVLAIDGEAEFKVEDVQVYNGYVLHIGQMEEGEIKVGDEVICTYDELRRWPIRNNHTGTHILNFGLREVLGDHI

DQKGSLVAPTKLRFDFSHGKSITTPELVKIEAICNEWIKKAAPVYAKEMPLAEAYKIPGLRAVFGEAYPDPVRVVSLGYP

LEEIAQNIENSKWRGTSIEFCGGTHVAKTDDIKDFVIVEESSIAKGIRRIVAVTGHEAHEVSRKAAEFERRLNKIAELQG

KEKEVAMKPYLVELGQSGISLIKKASLKSIFEKMQSELVAAAKAKTAADSKVIQDTIKTFFKENPNDNVYVGEFEVAGGN

AKTLSAAVTAGKSLSKAIYVFSTDQETGKVAHTNYLPKEVLDKKVIDGKTWLGEVSQIVGGKGGGKDESATGVGSEPTKV

KEAIVLAKNFYLSKVEA

>KZT19894.1 hypothetical protein NEOLEDRAFT_1123481 [Neolentinus lepideus HHB14362 ss-1]

MYSVLFRSHRPSFRRLSTMASSSYQGPWTARKVREEFFNYFRSKSHTFVPSSSTIPYEDPTLLFANAGMNQFKNIFLGTV

DPHSEMAKWKRAFNSQKCIRAGGKHNDLEDVGKDSYHHTFFEMLGNWSFGDYFKKEAIQFSWELLTVVYGLPKDRLYVTY

FEGDPKNNLEPDLEAKKYWLDQGVPEDHILPGNAKDNFWEMGATGPCGPCSEIHYDRIGGRNAAHLVNQDDPDVLEIWNN

VFIQFNREDDGSLKSLPSKHVDTGMGFERLVSVIQDKRSNYDTDVFLPVFAKIQDLTGARPYGGKFGADDADGIDTAYRV

VADHVRTLTFALSDGGVPSNVGRGYVLRRILRRGSRYARKKLGVTIGSFFSSLMPVVIETMGDVFPEITKNTDELKSILD

EEEESFSRTLDRGEKLFEQYASRAQQQGLKELNGKDVWRLYDTYGFPEDLTRLMAAELALGINDKQFEEAQAASKEASKA

SAKKGAKEVLKLDVHDIAALEKNTSVSKTDDAAKYATGTITAKVKAVYYNKSFLESTASIPEDATFGLILDRTNFYAESG

GQEYDQGSIVIDGAADFEVEDVQVFNGYVLHAGHLKYGSIGTGAEVICQYDELRRWPIRNNHTATHILNFSLREVLGDHI

DQKGSLVAPTKLRFDFSHKAQISSEDLSKIESISKDWIKKNVGVYSKEVDLKVAQKIPGLRAVFGESYPDPVRVVALEYD

VNDIVKDIENPKWRGTSVEFCGGTHVAKTSDIKDFVIIEESGIAKGIRRITAVTGNEAHDVNRLAAALRSRLDGIDRMNG

KQKDAELKAYSVELGQVDISAVLKAELRDRLAAMRKAFDKQIKEKEATANKAALDIVTQYFQDNESSEVYIGVLDVDGNT

KILQNVVVNARKMSKAVYVFSSDPEGKRVAHANFVSEAAKSRGIDGRKWAAAVSEVLGGKAGGKEDSAQGVGMDIGKLQD

AIKTAQKFYEESKKN

>XP_021004068.1 alanine--tRNA synthetase, cytoplasmic [Parasteatoda tepidariorum]

MIQIYFNRENGSLKPLPKKHVDTGMGLERLVSVIQDKSSNYDTDLFQPIFLSIQQSTGAVPYGGKVGPDDVTGIDMAYRV

LADHARTLTVALADGGRPDNTGRGYVLRRILRRAVRYASEKLSAKPGMFASLVPVVINILGDIFPELSKDPQTIMDIIND

EEAQFLKTLNRGRLLLERAIKKLTSNVLPGDVAWRLYDTYGFPVDLTQLMVEEKGLEVDMIGYEEAKKQAQLMSQAKGEG

VVDEIKLDVHAISDLQSKGVPATDDSPKYSYRSLSDSMDSKYEFDSCLGTVLALRIQKAFTDHVENGQECGIILDRTSFY

AEAGGQIYDTGFMVKENDEDTEFTVKEVQLQGGYVIHLGTLCGSVKVGDKMKLMIDEERRKLVMNNHTGTHILNFALRKA

LATECDQKGSLVAPDRLRFDFTNKGAMTVAQVKEAEIVANQAVRKNGEVFAKDTPLPEAKAIQGLRAVFGEVYPDPVRVI

SIGISVDDLLSDPSGPGGNETSVEFCGGTHLKRSGHIGEFVISSEEAIAKGIRRIVALTGPEAVKANKRSELLQNEVENL

KNLMCDKRASISQKEMVKRIVDLTEEISQSNISYWKKDEMRNNLKNLKKQLDDLDRANKAAVVQTITEEAKHMFETQSEN

KNIPFLVHEFAAGFNAKALDAALKQAKLYSPETAVMFFTRDADAEKVLCMSSVPQNATSKGLKANEWVQQVSQVINGKGG

GKPESAQATGTNTGAVDEAKNIAKKFAEMKLS

>XP_022491916.1 hypothetical protein PENARI_c003G02817 [Penicillium arizonense]

MTSVGSEWTALRVRETFLDYFKQNGHTFVPSSPVAPLSDPTLLFTNAGMNQFKSIFLGTVDPSSDFGKLKRAANSQKCLR

AGGKHNDLDDVGKDSYHHTFFEMLGNWSFGDYFKKEAITYSWTLLTKVYGLNPDRLYVTYFEGNEAGGLEPDLEAKELWK

SVGVPESHILPGDMKDNFWEMGDQGPCGPCSEVHYDRIGGRNAAHLVNQDDPNVLEIWNNVFIQYNREPDSSLRSLPNKH

VDTGMGFERLVSILQDKSSNYDTDVFTPIFQAIQTVTGAREYRGNFGADDADGIDTAYRVVADHVRTLMFAISDGVIPNN

EGRGYVIRRVLRRGARYARKYFQVDIGNFFAKLVPTVVDQLGDMFPELKRKQQDVIEILDEEELSFAKTLDRGERQFEQY

AQQAKVKGTDKLHGADVWRLYDTFGFPVDLTRIMAEERGLRIDDVEFEEARLKAKEASKGQKKAAETTVKLDVHDLGKLE

KMNDVQKTDDSAKFGRGNITAHVKAIYHAKRFYDNTDNVPDGAQLGVILDCTNFYAEQGGQENDTGKIIIDGQAELEVGD

VQSYAGYVLHTGFMKYGSFAVGDSVICEYDELRRWPIRNNHTGTHILNFALKVILGDGVDQKGSLVAAEKLRFDFSHKSA

ISDAELEKIEAKATEYIRQNCSVYSKDIPLATAQEISGVRAVFGETYPDPVRVVSVGVELEEILQNVKDPRWQEVSIEFC

GGTHVQKTGDIKDLIILEESGIAKGIRRIIAVTGEEAHEVQRVAQDFGKRLDRLNALPLGSEKEQEAKQVQVDLNQLSIS

AVEKAKFRERFAKINKQVLDGQKAQQKLESKKAVDTITSYFEAPENKDKSWLVIQLPISANAKAVSESLNHVKSKLQGKS

VYLLAADSEQGRVSHGCYISQDLSTQGASASDWAAVVSGAVGGKAGGKGPTSIGNGTNSDKVEEAIALASDYLSKFKL

>XP_016243378.1 alanine-tRNA synthetase [Cladophialophora immunda]

MRSPSAIWRAAAAGCHRGATITGTTANCTPRSSKIARLTHDSNPLLTKKFSTGPSRHVASRQRRGSTRDDLSARRSLSQS

SSIWCVSTALRPSIVRRHTTSTMASSAPTTTHEWSAPKVRDTFLKYFEDRGHTFVKSSPVVPLSDPTLLFTNAGMNQFKP

IFLGTVDPSSKEGQYKRVVNSQKCIRAGGKHNDLDDVGKDSYHHTFFEMLGNWSFGDYFKKEAIIFSWELLTQVFGLEPD

RLYVTYFEGNEAGGLEPDLEAKEIWKSVGVPEDHILPGNMKDNFWEMGDQGPCGPCSEIHYDRIGGRNAASLVNQDDPNV

LEIWNNVFIQYNREPDKSLRPLPNKHVDTGMGFERLVSVLQDKMSNYDTDVFTPIFSRIQEVTGARPYTGNFGEEDVDGI

DTAYRVVADHVRTLTFAISDGGIPNNEGRGYVVRRVLRRGARYARKYFNVEIGDFFSKIVPTLVEQMGGMFKEIVAKAEE

VKEILNEEELSFAKTLDRGERQFEVYAQKSQVQGLKKLHGADVWRLYDTFGFPVDLTRLMAEERNLSIDDNEFEAARLAA

KEASKGQKKAANDVLKLDVHDLGQLEKMPGVTKTDDSSKYGRENITSTIQAIYHAKKFHSDTKSIPEDDQVGVILDRTCF

YAEQGGQEFDTGRIVIDGQAELEVENVQLYAGYVLHTGYMKYGHFSVGDKAISEYDELRRWPIRNNHTGTHILNFALREV

LGDGVEQKGSLVAQEKLRFDFSHKSGVSDADLQRIEDISTEYIRQNCPVYAKNVPLSIAREIAGVRAVFGETYPDPVRVV

SVGVEVDDILKNVKDERWKTVSIEFCGGTHVRSTGDIKDLIILEESGIAKGIRRIIAVTGEDAHEVQRVAEDFSAKLEKL

EKMPFGSPKEQEAKAIQLELNNLSISAVRKSQFRDRFAKISKEILDQQKKVQKEETKKAIDTITEYFEKNPSAQGAVLRL

PIGGNPKIVPDVVKHVQTKMKDKSVYIIVADEKDPEGKVYHGCFVGQNGTKAGMKSPEWSSTVSKLVGGKAGGKEPVAIG

TGTEQSKIDEALKAATDYLEKFKL

>XP_018606223.1 PREDICTED: alanine--tRNA synthetase, mitochondrial [Scleropages formosus]

MAATVFHFGLWSNVRVKAKRLFFASGVRELRACRWREGSTLTSSAARRMFIEFFKERHGHRVVPSAPVRPRGDPSLLFVN

AGMNQFKPIFLGTVDPRSEMARYRRVVNSQKCVRAGGKHNDLDDVGRDVYHHTFFEMLGNWSFGDYFKEEACAMAWQLLT

GSYGIPRDRLYVSYFGGDRTLGLEADEETREIWLNIGVQPNRILPFGMKDNFWEMGETGPCGPCTEIHYDHVGGRDVAHL

VNSDSSDVVEIWNLVFMQYSREADGSLRALHQHSVDTGMGLERLVAVLQGKRSNYDTDLFTPLFSAIQQHFRCPEYRGQT

GVADKSKVDMAYRVIADHIRALTVCIADGVYPGMSGAQLVLRHILRRAVRFSTEVLKSTPGSLSKLVPTVVHILGDTYPE

VLKETDRIMDIINDNEAQFLSSLKQGRRVIDRTLQNMNGSTVFPGSVAWSLHCHLGFPLDLLRLMVEEKGVAMDTEALDR

LALENKKVRAQTQASEHDSCVDLDVKVLPELQGLGITYTNDLPKYQYSLGQDGRYVFLPCHATIQALICGQGLVPEVEKG

QRCGVIFDKTCFYTEQGGQDHDQGYFTQEILPDVLFPVEQVHLVGGYVVHQVTATERLKRGDQVQLFLDEGQRLACMVKH

TATHILNHCLRQLLGDGVEQRGSHVSADHLRFDFSVKRSLSIQQLQEIEKMIRDIIEGNEPVYIEELPLKVAKGIPGLRT

VDEVYPDPARVVSVGVPIADLLKGHNNKPASVELCCGTHLLMTGEIQDFVVVSERQIAKGVSRIVAVTGAQAKEAREAGR

VLECEVDSLRVRLTAASPSLPGIHRLSKEAGLLIDAVENTAIPQWQRRELSNQLKSLQRSSNTMIRKLEIAEAAKKVQSL

LKKHTNRSLVVDTVDTDSISVVMKTVNQYSDQAPGVMVMLLSHQESGKVLCACQVPKGFSAFSANDWALAVCAHLGGNAG

GSDTVAKGTGSAVDIAEAIQWARNFAEGRMQQR

>KXT18678.1 hypothetical protein AC579_2726 [Pseudocercospora musae]

MRPSLIPHFRITSNFLYLSRSTAGLPTHTPTSRLSRRPIRTMASTTGHEPEWTAVKVRKTFIDYFGKNGHTFVPSGSVVP

LADPTLLFTNAGMNQYKSIFLGTVDPDSDFAKLKRAANSQKCIRAGGKHNDLDDVGKDSYHHTFFEMLGNWSFGDYFKKE

AIHFSWELLTKVYGLSPDRLYVTYFEGKQDAGLEPDTEARDFWRAEGVPDDHILTGDMKDNFWEMGDQGPCGPCSEIHYD

RIGGRNASDLVNKDDPNVIEIWNNVFIQYNREPDKSLRSLPAKHVDTGMGFERLVSILQNKYSNYDTDVFTPLFARIQEI

TGAREYGGKFGEEDADGIDTAYRVVADHVRLLTFAISDGGIPNNVGRGYVVRRVLRRGARYARKYFNTDVGSFFSKIVPT

LVEQMGEMFPEIKKKQAEVIEILDEEELSFAKTLDRGENMFEKYAQKCKANNAKVLEGDDVWRLYDTYGFPVDLTRLMAE

ERGLDIIDEDVAKAQERAREASKGEKKAGADLVKLDVHDINSLEQMTDVPKTDDSPKFQRDNITATIKAIYHGRKFLQST

EEVPKGEQFGVFLDRTNFYAEQGGQENDTGRILIDGEAEIDVQNVQLYGGYVMHTGYMNYGTLRVGDQVLSEYDELRRQP

IRNNHTGTHILNFALRETLGDDVNQKGSLVAPEKLRFDFSHKQGCSDAELEKIEELSTGYIRQNSEVYAKDVPLATAKQI

EGVRAVFGETYPDPVRVVSIGVPVEDLLEDPKRSDWRNVSVEFCGGTHVKNTTEIKDLIVLEESGIAKGIRRIVAVTGQT

AHEIQLLGEQWNKKVTYIESMKYGPEKEAAVKEAQHDLGQVEMAALTKTKLRDRVAKVVKENLDHQKAVQKAENRKFIDA

VNEHFDKNKGSKTLVLSFPVSTGSKAVQETMKVIGNKQKDKTVYLLGKTEDGKVVHGCHVSSDAQGKGADAAKWSSEVAG

VVGGKAGGKGATSLGQGTNPDKVEDAVEIARKYLEGLGI

>KUM57470.1 hypothetical protein ACN42_g9714 [Penicillium freii]

MTSLGSEWTAQRVRDTFLDYFKQNGHTFVPSSPVAPLSDPTLLFTNAGMNQFKSIFLGTVDASSEFGKMKRATNSQKCLR

AGGKHNDLDDVGKDSYHHTFFEMLGNWSFGDYFKKEAITYSWTLLTEVYGLDPDRLYVTYFEGNEAGGLEPDLEAKELWK

SVGVPESHILPGDMKDNFWEMGDQGPCGPCSEVHYDRIGGRNAAHLVNEDDPNVLEIWNNVFIQYNREPDSSLRSLPNKH

VDTGMGFERLVSILQDKPSNYDTDVFTPIFQAIQNTTGAREYRGHFGAEDADGIDTAYRVVADHVRTLMFAISDGVIPNN

EGRGYVIRRVLRRGARYARKYFQVDIGNFFAKLVPTVVDQLGEMFPELKRKQQDVIEILDEEELSFAKTLDRGERQFEQY

AQQAKVKGTDMLHGADVWRLYDTFGFPVDLTRIMAEERGLRIDDVEFEEARLKAKEASKGQKKAAENTVKLDVHDLGKLE

KMNDVQKTDDSPKFGRGNITSHVKAIYHGKTFHNSTDDVPEGAQLGVILDCTNFYAEQGGQENDTGKIIIDGQAELEVGD

VQSYAGYVLHTGFMKYGSFAVGDSVICEYDELRRWPIRNNHTGTHILNFALKEILGDGVDQKGSLVAAEKLRFDFSHKSA

ISDAELEKIEAKATDYIRQNCAVYSQEVPLATAQQISGVRAVFGETYPDPVRVVSVGVELEEILQNVKDPRWQEVSIEFC

GGTHVQKTGDIKDLIILEENGIAKGIRRIIAVTGEDAHEVQRVAEEFGQRLDRLNALPSGSKKEQEAKQVQVDLNQLTIS

AVQKAKFRERFAKINKQVLDGQKAQQKLESKKAVDAITSYFEAPENQDKSWLVVQLPISANAKAVSESLNHVKSKMQGKS

VYLLAADSEQGRVSHGCYISQGLSDQGASASDWAAVVSGAVGGKAGGKGPTSIGNGTNADKVEEAISLAADYLNKFKL

>XP_024457385.1 alanine--tRNA synthetase [Populus trichocarpa]

MKRITSSRPLLSSPLLRLLRQPPISLAAARVSSKQPNFSPFTSLARSFSTMSGGIDSPQPDVDWPANRVRDTFIKFFEEK

NHVDWKSSPVVPVNDPTLLFANAGMNQFKPIFLGTADPNTALSKLSRACNTQKCIRAGGKHNDLDDVGKDTYHHTFFEML

GNWSFGDYFKKEAIEWAWELLTKVYKLPADRIYATYFGGDEKAGLAPDNEARDIWLQFLPPGRVLPFGCKDNFWEMGDTG

PCGPCTEIHYDRVGNRNAAMLVNNDDPTCLEIWNLVFIQFNRESDGSLKSLPAKHVDTGMGFERLTSVLQNKMSNYDTDV

FMPIFDAIQQATGARPYSGKVGADDVDGVDMAYRVVADHIRTLSFAIADGSCPGNEGREYVLRRILRRAVRYGSEVLKAQ

EGFFNGLVNVVVKVMSDVFPELKRNEEHIRETIAAEEASFGKTLLKGIEKFKKAAQEVEGKVLSGQDAFVLWDTYGFPLD

LTQLMAEERGLRVDVEGFNNAMDEARERSRNAQNKQAGGAIVMDADATSALHKKGVSATDDSFKFICFEDHESVIKAIYN

GSEFLESAAAANEVGIVLESTSFYAEQGGQIFDTGVLEGPFGSFQVCNVQIFGGFIVHIGSLTGVSGRFSVGEKVTCKVD

YDRRKLIAPNHTCTHMLNFALREVLGNHVDQKGSIVLPEKLRFDFSHGKPVDPELLRKIESIVNGQIKDELGVFAKEATL

AEAKQINGLRAVFGEVYPDPVRVVSIGRKVEDLLANPENEEWLSISAELCGGTHITNTREAKAFALLSEEGIAKGIRRIT

AVTTDGAFKAMELAHSLEQEVDEASQADGSLLEKKVASLKTRVDSALIPAAKKADIRAKISVLQNKVRKAQKKIAEENMQ

KAVEVATEMAEVASSDGKAFCISHVDVGLDAAAVREAVLKVLERKGISAMVFSTDESTNKVVVCAGVPEKLDKGKGLEVS

EWLTTALGPLKGRCGKGKAGLATGQGTDASHLDEAMGLATTFAQMKLR

>OQV09054.1 hypothetical protein CLAIMM_13234 [Cladophialophora immunda]

MASSAPTTTHEWSAPKVRDTFLKYFEDRGHTFVKSSPVVPLSDPTLLFTNAGMNQFKPIFLGTVDPSSKEGQYKRVVNSQ

KCIRAGGKHNDLDDVGKDSYHHTFFEMLGNWSFGDYFKKEAIIFSWELLTQVFGLEPDRLYVTYFEGNEAGGLEPDLEAK

EIWKSVGVPEDHILPGNMKDNFWEMGDQGPCGPCSEIHYDRIGGRNAASLVNQDDPNVLEIWNNVFIQYNREPDKSLRPL

PNKHVDTGMGFERLVSVLQDKMSNYDTDVFTPIFSRIQEVTGARPYTGNFGEEDVDGIDTAYRVVADHVRTLTFAISDGG

IPNNEGRGYVVRRVLRRGARYARKYFNVEIGDFFSKIVPTLVEQMGGMFKEIVAKAEEVKEILNEEELSFAKTLDRGERQ

FEVYAQKSQVQGLKKLHGADVWRLYDTFGFPVDLTRLMAEERNLSIDDNEFEAARLAAKEASKGQKKAANDVLKLDVHDL

GQLEKMPGVTKTDDSSKYGRENITSTIQAIYHAKKFHSDTKSIPEDDQVGVILDRTCFYAEQGGQEFDTGRIVIDGQAEL

EVENVQLYAGYVLHTGYMKYGHFSVGDKAISEYDELRRWPIRNNHTGTHILNFALREVLGDGVEQKGSLVAQEKLRFDFS

HKSGVSDADLQRIEDISTEYIRQNCPVYAKNVPLSIAREIAGVRAVFGETYPDPVRVVSVGVEVDDILKNVKDERWKTVS

IEFCGGTHVRSTGDIKDLIILEESGIAKGIRRIIAVTGEDAHEVQRVAEDFSAKLEKLEKMPFGSPKEQEAKAIQLELNN

LSISAVRKSQFRDRFAKISKEILDQQKKVQKEETKKAIDTITEYFEKNPSAQGAVLRLPIGGNPKIVPDVVKHVQTKMKD

KSVYIIVADEKDPEGKVYHGCFVGQNGTKAGMKSPEWSSTVSKLVGGKAGGKEPVAIGTGTEQSKIDEALKAATDYLEKF

KL

>OUT22979.1 alanine-tRNA synthetase [Pichia kudriavzevii]

MSIYTNPATNPKIKEWPAAKVRDTFLNYFKSKGHTFVPSSSVIPHDDPTLLFANAGMNQYKPIFLGTVDPSSSFANLKRA

VNSQKCIRAGGKHNDLEDVGRDSYHHTFFEMLGNWSFGDYFKEGAIKYSWELLTEVYGIPKDRLYVTYFGGDEKTGLEPD

LEAKGLWQQVGVADDHILPGNVKDNFWEMGDQGPCGPCSEIHYDRIGGRNAASLVNEDDPDVLEIWNNVFMQYNREPDGS

LVPLPAKHIDTGLGFERLVSLLQDVRSNYDTDVFYPIFQKIQEITGVRPYTGKFGKDDSDGIDTAYRVLADHVRTLTFAL

ADGGVPNNEGRGYVLRRILRRGARYVRKYMNYPIGNFFSQLAPTLIEQVKHIFPELEKDPSTIFEILDEEEASFARTLDR

GEKLFEQYALIASKTPEQTLSGKDVWRLYDTYGFPVDLTRLMAEEAGLKIDEEGFEKARLESKEASKGTGTKTGKTLLKL

DVHALASLNDQSVPKTDDSFKYDTKDIHAKVLSIYSDGQFVDSISQSEQQVGIVLDKTPFYAEQGGQEFDTGKIVIDGKS

EFSVENVQVYGGYVFHTGFVQEGELKVGDDIIAGYDELRRWPLRNNHTGTHILNFALRKVLGDSVDQKGSLVAPEKLRFD

FSHKKAVELKELEQVENITNKLIKDNHKVYAKPVELEIAKGINGLRAVFGETYPDPVRVVSVGVPVDDLLADPSNEKWNN

YSIEFCGGTHVAKTDDIKEFVIIEESGIAKGIRRIVAVSGMEAKEVQRKASEFNDILNEVEKLPYGEVKERRIKELGTEL

GKLSISVIEKAALKDKFSKIEKAVKDETKSRVKKDTAKVMDAVKSYFATPEHSAVCTLKVTAGANAKIVAEAINYMKKEQ

KESCLYLVLGEDQGKVAQGVYLSPAVQDKIAAVDVATKAAEYIGGRAGGKGNSCQGMGDKAENADKAVEEIKTLLEKLFF

TLAVELGNLSGSSLNTLFSATIDHSIDDEYKSTDIVNSYILAESVPVKDNIPYGSMLGYRFKPNMSAEFTHSYQIIIYLS

ASLCQLPVNWNTSISNNGLTLYYTFNETIANNTDFKNMEKVDFQGGYASAIAETKLSPLIVITSEDIWVYEFAASQRKVM

FLYDTEPRISVLDVDYEAAIFQTGDIPFGQNSSYSLYLFKDEYPIPIGLNQSWCAISENQQYDKKISVNESLVASLKNAF

VVSDLDIAKTYTGVLVITNDIIPYGGGIFEPFVFTMSKTRSCKLAYGLEFCDEVAYAVPISESYLYGKESWSEFVSSYDN

YSESLYQPFEYALQQVACDTELDARYSPIRTCNDCKYSYKQWLCAVTIPRCVSSLNSGVFNLNYTAEQGRNEYIAEHIKP

PLPYSEILPCLNVCQAIVRDCPADFGFGCPETPEQVQLSYGDQELLKGNSDADDTDDDESNAIAELVRETLSDGSIQTYR

LCNYLGVSNIYTSSSSAEATSSAST

>CRL28433.1 Alanyl-tRNA synthetase, class IIc [Penicillium camemberti]

MTSPSSEWTAQRVRDTFLDYFKQNGHTFVPSSPVAPLSDPTLLFTNAGMNQFKSIFLGTVDASSEFGKMKRATNSQKCLR

AGGKHNDLDDVGKDSYHHTFFEMLGNWSFGDYFKKEAITYSWTLLTEVYGLDPDRLYVTYFEGNEAGGLEPDLEAKELWK

SVGVPESHILPGDMKDNFWEMGDQGPCGPCSEVHYDRIGGRNAAHLVNEDDPNVLEIWNNVFIQYNREPDSSLRSLPNKH

VDTGMGFERLVSILQDKPSNYDTDVFTPIFQAIQNTTGAREYRGHFGAEDADGIDTAYRVVADHVRTLMFAISDGVIPNN

EGRGYVIRRVLRRGARYARKYFQVDIGNFFAKLVPTVVDQLGEMFPELKRKQQDVIEILDEEELSFAKTLDRGERQFEQY

AQQAKVKGTDMLHGADVWRLYDTFGFPVDLTRIMAEERGLRIDDVEFEEARLKAKEASKGQKKAAENTVKLDVHDLGKLE

KMNDVQKTDDSPKFGRGNITSHVKAIYHGKTFHNSTDDVPEGAQLGVILDCTNFYAEQGGQENDTGKIIIDGQAELEVGD

VQSYAGYVLHTGFMKYGSFAVGDSVICEYDELRRWPIRNNHTGTHILNFALKEILGDGVDQKGSLVAAEKLRFDFSHKSA

ISDAELEKIEAKATEYIRQNCAVYSQELPLATAQQISGVRAVFGETYPDPVRVVSVGVELEEILQNVKDPRWKEVSIEFC

GGTHVQKTGDIKDLIILEENGIAKGIRRIIAVTGEDAHEVQRVAEEFGQRLDRLNALPLGSEKEQEAKQVQVDLNQLAIS

AVQKAKFRERFAKINKQVLDGQKAQQKLESKKALDAITSYFEAPENQDKSWLVVQLPISANAKAVSESLNHVKSKLQGKS

VYLLAADSEQGRVSHGCYISKGLSDQGASASDWAAVVSGAVGGKAGGKGPTSIGNGTNADKVEEAISLAADYLNKFKL

>XP_012065800.1 alanine--tRNA synthetase [Jatropha curcas]

MKRISIVSSSLCPGLHLYTNKKTPLRAPVFLVAAAAAAARVSVNRPLATLFASFSSSSLSRIAIMPDVDPQKVEWPAKKV

RDTFISFFEDKNHVHWKSSPVVPVNDPTLLFANAGMNQFKPIFLGTADPNTALSKLIRACNTQKCIRAGGKHNDLDDVGK

DTYHHTFFEMLGNWSFGDYFKKEAIEWAWELLTKVYKLPTDRIYATYFGGDDKAGLAPDNEARDIWLQFLPPERILPFGC

KDNFWEMGDTGPCGPCTEIHYDRVGNRDAAHLVNNDDPTCIEIWNLVFIQFNRESDGSLKSLPAKHVDTGMGFERLTSVL

QNKMSNYDTDVFLPIFDAIQQATGARPYSGKVGADDVDRIDMAYRVVADHIRTLSFAIADGSRPGNEGREYVLRRILRRA

VRYGSEVLKAQEGFFNGLVNVVVKVMGDVFPELKQNEEHIREIIAEEEASFGKTLLKGIEKFKKAAQEVQGKVLSGQDAF

VLWDTYGFPLDLTQLMAEERGLRVDVEGFNDAMDEARERSRNAQNKQAGGDIVMDADATSALHKKGVPVTDDSYKFTWFQ

DHESVIKAIYTGSEILETFTTGDEVGIVLESTSFYAEQGGQIFDIGLLEGSFGSFQVSNVQIFGGFVVHIGSFTGKTGKI

SVGDKVICKVDYDRRKLIAPNHTCTHMLNFALKEVLGSHVDQKGSIVLPEKLRFDFSHGKPVDPDLLRKIESIVNEQIKA

ELEVFAKEATLSEAKRINGLRAVFGEVYPDPVRVVAIGRKVEDLLADPESKEWLTISAELCGGTHISNTREAKAFALLSE

EGIAKGVRRITAVTTDVAFKALELASSLEQEVHETSKAEGSLLEKKVASLKSRVDSAPIPAAKKADIRAKISVLQNQVRK

AQKKIAEENMQKAVKVATEMAEIAASEGKGFCISRVDVGLDAAAVREAVLKVLEKKGISAMVFSMDETTNKVVVCAGVPE

KKDQGKKLEVSEWLTKALEPLNGRCGKGKGGLATGQGTDGVHVDKAIDLAATFAEMKLR

>OQD60125.1 hypothetical protein PENPOL_c027G06495 [Penicillium polonicum]

MRVSGVVTNGERALTHLFRRNQPRTFSPCFVHSLSTNPPTFNRMTSLGSEWTAQRVRDTFLDYFKQNGHTFVPSSPVAPL

SDPTLLFTNAGMNQFKSIFLGTVDASSEFGKMKRATNSQKCLRAGGKHNDLDDVGKDSYHHTFFEMLGNWSFGDYFKKEA

ITYSWTLLTEVYGLDPDRLYVTYFEGNEAGGLEPDLEAKELWKSVGVPESHILPGDMKDNFWEMGDQGPCGPCSEVHYDR

IGGRNAAHLVNEDDPNVLEIWNNVFIQYNREPDSSLRSLPNKHVDTGMGFERLVSILQDKPSNYDTDVFTPIFQAIQNTT

GAREYRGHFGAEDADGIDTAYRVVADHVRTLMFAISDGVIPNNEGRGYVIRRVLRRGARYARKYFQVDIGNFFAKLVPTV

VDQLGEMFPELKRKQQDVIEILDEEELSFAKTLDRGERQFEQYAQQAKVKGTDMLHGADVWRLYDTFGFPVDLTRIMAEE

RGLRIDDVEFEEARLKAKEASKGQKKAAENTVKLDVHDLGKLEKMNDVQKTDDSPKFGRGNITSHVKAIYHGKTFHNSTD

DVPEGAQLGVILDCTNFYAEQGGQENDTGKIIIDGQAELEVGDVQSYAGYVLHTGFMKYGSFAVGDSVICEYDELRRWPI

RNNHTGTHILNFALKEILGDSIDQKGSLVAAEKLRFDFSHKSAISDAELEKIEAKATDYIRQNCAVYSQELPLATAQQIS

GVRAVFGETYPDPVRVVSVGVELEEILQNVKDPRWQEVSIEFCGGTHVQKTGDIKDLIILEENGIAKGIRRIIAVTGEDA

HEVQRVAEEFGQRLDRLNALPLSSKKEQEAKQVQVDLNQLTISAVQKAKFRERFAKINKQVLDGQKAQQKLESKKAVDAI

TSYFEAPENQDKSWLVVQLPISANAKAVSESLNHVKSKMQGKSVYLLAADSEQGRVSHGCYISQGLSDQGASASDWAAVV

SGAVGGKAGGKGPTSIGNGTNADKVEEAISLAADYLNKFKL

>NP_984459.1 ADR363Cp [Eremothecium gossypii ATCC 10895]

MTIGDKQRWTAQNVRSTFIDYFKAKDHKLVPSSPVIPYDDPTLLFANAGMNQFKPIFLGMVDPASDFYTLKRAVNTQKCI

RAGGKHNDLEDVGLDSYHHTFFEMLGNWSFGDYFKKEAIAYSWELLTKVYGLPEDRLYVTYFEGDESLGLAADEEARDLW

LSVGVAPDHVIAANVKDNFWEMGDQGPCGPCSEVHFDRIGGRNAAALVNQDDPNVIEIWNNVFIQFNREHDGSLRPLPAK

HVDTGMGFERLVSVLQDVPSNYDTDVFQPLFARIQEITGVRPYSGKFGADDKDGIDTAYRVLADHVRTLTIALADGGVPD

NDGRGYVLRRILRRGARYVRKYMNYPTGKFFSQLAPTLIEQFKDVYPELTKNQSYLFDILDEEEASFAKTLDRGEKLFEK

YAETAKNSGSKILDGKQVWRLYDTYGFPVDLTQLMADEHGLKVDNEGFEQAKQESYEASKKNGSKGDANLIKLNVHEVSQ

LSDEGVPRTDDSAKYGTQDIEATILRIHDGENFVEQMTEVGKQYGILLDRTCFYAEQGGQEYDKGKLVIDDAAEFNVDNV

QQYSGYVFHTGNLADGKLCVGDKIIASYDELRRYPIRNNHTGTHLLNHALRTILGDNIDQRGSLVTPEKLRFDFSHKKPM

SYEELEQVETICNQQIRENRSVYYKDIPLDVAKSIHSIRAVFGETYPDPVRVVSVGKSVDEILESPSNEEWAKYSIELCG

GTHVSKTGDIKEFVIIEESGIAKGIRRIVAVSASEAYEVQRVAREFALELDAIDKLPFSMIKEKKIREIGVRLDQLNISV

ITKNELKTKFTKMEKAVKDEVKTKAKKEMKQTLDAVKEYFAANEDATFFVKSLDISANPKAVTEAVQYLKNSARDKTIYL

FTGNDPEGKVAHGCFISDDALAKGIDGPALAKVVSSKIGGKAGGKGNVFQGMGDKPAGIDSAIAEVEASFKEKLIL

>XP_016268586.1 alanine-tRNA synthetase [Exophiala oligosperma]

MSIWRATAASPAAIISSAPRYTNSPLTHNFHILTPARSIRLLWSLTPTTPDQISNRCRAEGPRRQNYRRPYSPALGRVQW

HTTVRMASTTADKPHKWSAPVVRDTFLKYFEDRGHTFVKSSPVVPLSDPTLLFTNAGMNQFKPIFLGTVDPTSKEGQYKR

AVNSQKCIRAGGKHNDLDDVGKDSYHHTFFEMLGNWSFGDYFKKEAITYSWELLTQVFGLEPDRLYVTYFEGDEAGGLEP

DLEAKEIWKSVGVAEDHILPGNMKDNFWEMGEQGPCGPCSEIHYDRIGGRNAAHLVNQDDPNVLEIWNNVFIQYNREPDK

SLRSLPNKHVDTGMGFERLVSVLQNKMSNYDTDVFTPIFARIQEVTGARPYSGKFGEEDKDGIDTAYRVVADHVRTLTFA

ISDGGIPNNEGRGYVVRRVLRRGARYARKYFNVEIGDFFAKLVPSLVEQMGGMFKEIVAKEDEVKEILNEEELSFAKTLD

RGERQFETYAQKSQGQGLKSLHGADVWRLYDTFGFPVDLTRLMAEERNLSIDDNEFEAARLRAKEASKGEKKAALDLLKL

DVHDLGELEKMPGVTKTDDSAKYGLENVTGTIQAIYHAKKFHPDTQSIPEGEQIGIILDKTCFYAEQGGQEYDTGRIVID

GQAELEVENVQLYAGYVLHTGYMKYGHFSVGHKAICEYDELRRWPIRNNHTGTHILNFALREVLGDGIDQRGSLVAQEKL

RFDFSHKSGISDADLQKVEDLSTEYIRQNCPVYAKDVPLVVAREISGVRAVFGETYPDPVRVVSVGVEVDDILKNVKDER

WKTVSIEFCGGTHVKATADIKDLVILEESGIAKGIRRIIAVTGEDAYEVQRVAKEFSERLKKLEEMPFGAQKEQEAKALQ

IELNTLSISAVKKSQLRERMNKVAKEILDQQKKMQKEELKKATDTITEYFEKNKHAKAAVLRLPVGGNPKLVPDVVKHVQ

TKLKDKSVYIVVADASEPDGKVFHGCFVSPDGVKAGITSADWSSEVSKLVGGKAGGKAPTAIGTGTDQGKVDDAIEAARQ

YLEKFTL

>DAA77577.1 TPA_exp: Alanine--tRNA synthetase [Trichophyton benhamiae CBS 112371]

MAASTSSQPPAGQQTVEWPALRVRDTFLDYFKKNGHTFVPSSSVVPLSDPTLLFANAGMNQYKSIFLGTVDPNSDFAQLK

RAHNSQKCIRAGGKHNDLDDVGKDSYHHTFFEMLGNWSFGDYFKKEAIQFSWELLTKVYGLDPDRLYVTYFEGNEAAGVG

PDIEARDIWRSVGVKDDHILTGDMKDNFWEMGDQGPCGPCSEIHYDRIGGRNAAHLVNQDDPDVLEIWNNVFIQYNREPD

RSLRPLPNQHVDTGLGYERLVSILQNKSSNYDTDVFTPLFNAIQNITGARPYTGKFGAEDADGIDTAYRVVADHLRTLTF

AITDGAAPNNEGRGYVVRRVLRRGVRYARKYLQVEIGSFFSKLVPALVEQMGDMFPEIKRKQTDVMEILDEEEVSFAKTL

DRGERQFENYAQQALDSGAGKLHGADVWRLYDTFGFPVDLTRLMAEERQLTIDDAEFEEARLKAKEASKGQAKTASDLLK

LTVHTLSTLEKNGVPKTDDDAKFGKGNIVANIKAICHGKEVIDSTKDIPANEQIGIILDKTNFYAEQGGQEYDTGKIVID

GQAELDVQNVQMYGGYVLHTGFIKYGNFSVNDSVIAEYDELRRWPIRNNHTGTHILNFALREVLGDGIDQKGSLVAAEKL

RFDFSHKSAISDSDLEKIEAKSTDYIRQNCAVYSQEVPLATAREITGVRAVFGETYPDPVRVVSVGVELDEILKNVKDPR

WEGVSIEFCGGTHVQKTGDIKDLVILEESGIAKGIRRIIAVTGEDAHEVQRVAREFGERLNRFEKMPAGPERDAEIKSLQ

VDLNQLTISAVEKTKYRNQFTQIHKKITDAQKAAQKAEVKTAIDAISAYFEENKDSPSLVIKLPITANQKAISESLNHVK

TKLSGKALYVFAADASKVAHGCYVTPDLTTKGASANEWASAVSSIVGGKAGGKAPVAIGNGTEVNKVDEALDEATKYLEK

FKL

>XP_001383420.2 Alanyl-tRNA synthetase, cytoplasmic (Alanine--tRNA synthetase) (AlaRS) [Scheffersomyces stipitis CBS 6054]

MSTTTTSEWTASKVRSTFLDYFTNKRDHKFVPSSSVVPHNDPTLLFANAGMNQYKPIFLGTADPASDLASLKRAANSQKC

IRAGGKHNDLEDVGRDSYHHTFFEMLGNWSFGDYFKPEAITWAWELLTEVYGLDKDRLYVTYFEGDAKQGLEPDLEAKSF

WLKVGVAEDHILPGNAKDNFWEMGDQGPCGPCSEIHYDRIGGRNAASLVNQDDPNVLEVWNIVFIQYNREADSSLKTLPA

KHIDTGMGFERLVSVLQDKSSNYDTDVFLPIFDKIRDITGIRPYGGKFGAEDTDGIDTAYRVIADHVRTLTFAICDGGVP

NNDGAGYVLRRILRRGSRYVRKYMNYPIGSFFQQLVDVVIEQNKEIFPEIVHGAQDLKEILNEEELSFAKTLDRGEKLFE

QYAIIASKTPEQTLSGKDVWRLYDTYGFPSDLTRLMAEEAGLKIDEPAFEKARLESKEASKAIGNKDGVELVKLDVHSLS

ELDSNANVSKTDDSAKYGRENIKATIQAIYSSSGFVDSIDDSSVQYGVLLDKTPFYAEQGGQIYDTGKLVIDGKAEFNVT

NVQVYGGYVLHTGNIVEGSLNVKDDVIATYDELRRWPIRNNHTGTHVLNYALREVLGDSVDQRGSLVAPEKLRFDFSHKQ

ALTPKELAAVESISNEYIKSNKEVFYKDVSLNEAREINGLRAVFGETYPDPVRVVSIGVPVEDLLAEPKKADWHKVSIEF

CGGTHVAKTGDIKDLVVIEESGIAKGIRRIVAVTGHDAHAVQKIASEFEAELDHAAGLPFGSVKETKAKELGVALKKLSI

SVLDKQKLTEKFNKIDKSIKDDLKTKQKAETKQTLDVVNEWLEKKDAGDYLVAHVPINANAKAITEAFNLLKKSHKDKSL

YLITGLTDKVAHGCYIADEAIAKGVNASDLAQAVSAKIGGKAGGKGNIVQGMGDKPEGIKEAVNEVTQLLAEKL

>CDM36177.1 Alanyl-tRNA synthetase [Penicillium roqueforti FM164]

MRVSGVVTNGERALTHLFRRNQPRTFFPCLVHSLSTKHPTFDRMTSLGPEWTAQRVRDTFLDYFKQNGHTFVPSSPVAPL

SDPTLLFTNAGMNQFKSIFLGTVDASSDFGKMKRATNSQKCLRAGGKHNDLDDVGKDSYHHTFFEMLGNWSFGDYFKKEA

ITYSWTLLTEVYGLDPDRLYVTYFEGNEAGGLEPDLEAKEIWKSVGVPESHILPGDMKDNFWEMGDQGPCGPCSEVHYDR

IGGRNAAHLVNEDDPNVLEIWNNVFIQYNREPDSSLRPLPNKHVDTGMGFERLVSILQDKPSNYDTDVFTPIFQAIQNAT

GAREYRGHFGADDADGIDTAYRVIADHVRTLMFAISDGVIPNNEGRGYVIRRVLRRGARYARKYFQVDIGNFFAKLVPTV

VDQLGEMFPELKRKQQDVIEILDEEELSFAKTLDRGERQFEQYAQHAKAKGTDMLHGADVWRLYDTFGFPVDLTRIMAEE

RGLRIDDVEFEEARLKAKEASKGQKKAADNTVKLDVHDLGNLEKMNDVQKTDDSPKFGRGNITSHVKAIYHGKTFHNSTD

DVPEGAQLGVILDRTNFYAEQGGQENDTGKIIIDGQAELEVGDVQSYAGYVLHTGFMKYGSFSVGNSVICEYDELRRWPI

RNNHTGTHILNFALKEILGDGVDQKGSLVAAEKLRFDFSHKSAISDAELEKIEAKATDYIRQNCAVYSQELPLATAQQIS

GVRAVFGETYPDPVRVVSVGVELEEILQNVKDPRWQEVSIEFCGGTHVQKTGDIKDLIILEESGIAKGIRRIIAVTGEDA

HEVQRVAEEFGQRLDRLDALPLGSEKEQEAKQVQVDLNQLAISAVRKAKFRERFAKINKQVLDDQKAQQKLESKKAVDAI

TSYFEAPENQDKSWLVVQLPISANAKAVSESLNHVKSKMQGKSVYLLAADSEQGRVSHGCYISQALSDQGASASDWAAVV

SGAVGGKAGGKGPTSIGNGTNADKVEEAISLAADYLNKFKL

>EME44029.1 hypothetical protein DOTSEDRAFT_44310 [Dothistroma septosporum NZE10]

MASTTGHEPEWTAQKVRSTFIDYFKQHGHTFVPSSSVVPLSDPTLLFTNAGMNQYKSIFLGTVDPNSDFAKLKRAANTQK

CIRAGGKHNDLDDVGKDSYHHTFFEMMGNWSFGDYFKKEAINFSWELLTKVYGLDPNRLYVTYFEGKTDVGLEPDNEARD

LWKSVGVADDHILTGDMKDNFWEMGDQGPCGPCSEIHYDRIGGRNAAHLVNQDDPNVLEIWNNVFIQYNREPDRSLKSLP

AKHVDTGMGYERLVSVLQNKMSNYDTDVFSPLFARIQEVTGVREYQGKFGEDDADGIDTAYRVVADHVRLLTFAISDGAP

PSNVGRGYVVRRVLRRGARYARKYFNVEIGSFFSKIVPTLVEQVGDMFPEIKKKQAEVTETLDEEEVSFAKTLDRGEAMF

EKYAQASKTKGSKDIAGADVFRLYDTYGFPVDLTKLMAEERGLDIDEEAVAEAEKKHQKASKGAKKEGADYPKLTVHDIN

ALENMPDVPKTNDDAKFAKGNISATIKAIYHKGNFLQSTKEVPEGEQFGVVLDKTNFYAEQGGQENDAGSLLIDGEAEID

VQNVQSYAGFVLHTGYMKEGVLNVGSEVLCQYSEERRQPIRNNHTGTHILNFALREVLGDDINQKGSLVAPEKLRFDFSH

KSGCSDAEISQIEEKSTSYIRQNSEVYSKEVPLATARQIEGVRAVFGETYPDPVRVVSIGVPVEDLLEDPPRKDWRSVSV

EFCGGTHVKNTSEIKDLIVVEESGIAKGIRRIIAVTGRTAHKLQRDTEEWGKSISELEKMTFGPEKEQRTKVDQQRLNAE

ELPAVAKNQFRDRLGKVVKDNLDHQKTAQKAENKRVLDAVNEHFEKNKENKTLVLSVPGLSSGTKAVQETIKVISSKQKD

KTVYLLGAVDADGKIVHGCHVSSEAQGKGADAAKWSGEVATVVGGKAGGKGATSLGQGMNVDKVNDAVETARKYLESLSL

>PUU77344.1 tRNA synthetases class II (A)-domain-containing protein [Tuber borchii]

MASPLSTVEWPASLVRKTFIDYFRHNGHTFVPSSSVVPLSDPTLLFANAGMNQYKSIFLGTVDPSSDFASLKRAANSQKC

IRAGGKHNDLDDVGKDSYHHTFFEMLGNWSFGDYFKKEAVEYSWELLTEIYGIDPDRLYVTYFEGNQERGLEPDLETKEL

WKAVGVTDDHILPGNMKDNFWEMGDQGPCGPCSEVHYDHIGGRNASHLVNMDDPNVVEIWNNVFIQYNREPDRSLRPLPN

KHVDTGMGFERLVSVLQNKTSNYDTDVFSPLFQKIQEITKARPYGGKFGANDVDGIDTAYRVIADHVRTLTFAIADGGVP

NNEGRGYVVRRVLRRGARYARRYFQVTIGEFFSKIVPTLVLQLGDVFPEIKKKESDVKEILNEEEESFSRTLDRGERMFE

VFSKKATEQNSRVLNGTDVWRLYDTFGFPVDLTRIMAQENGLEINEEEFERAKAESKEASKARKSSGNVELLRLDVHDLG

ELEQRAEVPKTDDVYKFGRGNITSHIVAIYCDKSFHSSTKDTSPDAQIGIILDKTNFYAEQGGQEYDTGKILIDGKAELD

VQNVQVYGGYVLHTGYMKYGALSVGDDVICEYDELRRWPIRNNHTGTHVLNYALREVLGDGVEQKGSLVAAEKLRFDFSH

KSGVTDAELEKIEKISTDYIRQNLEVYAEDVPLSVAREIEGVRAVFGETYPDPVRVVSVGVPIEDLTRDVKNKEWRKVSI

EFCGGTHVQKTGDIKDLIILEENGIAKGIRRIVAVTGEDAHEVQRIASEFEKRINALGRLPAGPHKEAEIKSVQADLNGL

VISAVEKVQLKDKFGKIHKQLLDDQKAKQKAELRKAVETVTEYFKQNPDMPYFVSKLPISANSKALSDAIKHVQTKAKDK

TIYLFAADDDAEGKVVHGCYVAENAGRQLSAKDLADSISQIVGGKAGGKGATSQGIGNKVDKVDEAVDAAMKALQQLNL

>KIM78966.1 hypothetical protein PILCRDRAFT_824096 [Piloderma croceum F 1598]

MSSSPYTGPWTAHKVRQQFFDYFRSKDHTFVPSSSTIPYDDPTLLFANAGMNQYKSIFLGTVDPHSDMAKWKRAFNSQKC

IRAGGKHNDLEDVGKDSYHHTFFEMLGNWSFGDYFKKEAIGYSWEILTEVYKLPKDRLYVTYFEGDPKNGLVPDEEARQI

WLDRGVAEDHILPGNAKDNFWEMGATGPCGPCSEIHFDRIGNRNAAHLVNNDDPDVLEIWNNVFIQFNREDDGSLRPLPA

KHVDTGMGFERLVSVVQGQRSNYDTDVFTPIFQKVQDISGIRPYQGKFGEEDVDGIDTAYRVVADHIRTLAFSLSDGGVP

NNVGRGYVLRRILRRGSRYARKKLGVPIGSFFSSLLPVVIENMGDVFPELTKKVDDIKEILDEEEESFSRTLDRGEKLFD

QYATKAKQQGAKELSGKDVWRLYDTYGFPVDLTRLMAEELGLGVNEEEFEKAQSLSKEASKASTKKGTSDVIKLDVHDLA

ALEKNSDIPKTDDSAKFLLGNVTAHVKAIYHDKSFVQSTSALPEDIIFGILLDRTSFYAESGGQEYDTGNIVIDGVADFQ

VTNVQVFNGYVLHIGHLKYGQLDVGNEVLSCYDELRRWPLRSNHTATHILNFCLREVLGDHIDQRGSLVAPTKLRFDFSH

KAQVSLPELLKIESMSLDWVKKNVRVFSKDLDLQTAQKIPGLRAVFGESYPDPVRVVTLEYDVQEISQDIENAKWRNTSV

EFCGGTHVAKTGDIKDFVITEESGIAKGIRRIIAVTGREAQEVTRKAIDLKTKLDQLDQFTGKDKDTGLKAFTVELNQSD

ISVLLKAELRDRLGTIRKAFDKQIKDREAATNKIAVDNIIKYFEEDEKSEAYFAILDVEGNAKILANVISQGKKLGKAVY

VFSVDHEDNKVAHGNHVPVASKGMGLDGRTWASKVSEVLGGRVGGKDDGAQGVGTNISKLEEALQVAKDYYWATTQR

>XP_013020657.1 cytoplasmic alanine-tRNA synthetase Ala1 [Schizosaccharomyces octosporus yFS286]

MFEYANRFAVFHIKGFKKFHIQSLHKRKYSGFGFDPSVSQLQKNFRATQTYRLPLHQRTLMTIPTESLKWPASKVRSTFL

KFFEERGHTIVPSSSVVPYDDPSLLFANAGMNQFKPIFLGTVDPSHEYSKLKRAVDSQKCIRAGGKHNDLEDVGRDNYHH

TMFEMLGNWSFGDYFKKEAIQMAWELLTEVYGLEKDQLYVTYFGGHEESGLGPDLEARQLWLDIGIPESRVLPGDLKDNF

WEMGDQGPCGPCSEIHYDRIGNRSVPELVNMDDPNVLEIWNIVFIQYNREKDSTLRPLPNRHVDTGMGFERLVSVIQNKT

SNYDTDVFTPLFEKIQELTGARPYTGKMGEEDVDGIDTAYRVVADHVRTLTFAISDGGVPNNEGRGYVLRRILRRGARYV

RKKFGVPIGSFFSQLSLTVVEQMGEFFPELHRKVADVRELLDEEEVSFSKTLDRGEKMFEQYAAAAKKSSSNVLQGNDVW

RLYETYGFPVDLTHLMAEEAGIKIDEPGFEAAQAHSKEMSKRATKSGSSGGELLVLDVHALGDLAKMDTVPKTNDSYKHH

DATLMSTIKAIYHRDGFQSTTKVFESGEQIGILLDRTNFYAEQGGQENDTGHIVIDGIADFRVTNVQVYAGYVLHTGFLE

YGNLNVGDKVTCEYDEIRRWHLMNNHTVTHVLNLALRNVLGEGIDQRGSLVSQEKMRFDFSYKSSVPIDKLLEVENFCND

QIKKNLPVYSKDVPLKDTKEINGLRAVFGEVYPDPVRLVCIGVDIDTLLKNPKNPEWHNYSIEFCGGTHVYNSGEIKDFV

ILEESGIAKGIRRIVSVTSTEANRVSRVANEFDVRINELEKMPFGAAKEAELKKIGVDLSKLTVSAVRKHNMREHVAKIT

KQFQDQVKAANAAEQKEAVNLVAEYFKENPEAHFVVAKVPISANAKALSFALAHTKKNLKGKSVYLVASDNVKVAHACLV

SNEALQKITPQEWSQKVCLQIGGRSGGKGDTCQGVGDKAENVDAAIEEAIEFFQGKLTL

>EJD37949.1 hypothetical protein AURDEDRAFT_116636 [Auricularia subglabra TFB-10046 SS5]

MSHTPYTGEWSAKNVRKAFFDFFEKNGHTFVRSSSTIPHEDPTLLFANAGMNQYKSIFLGTVDPKSDLAKLKRAYNSQKC

IRAGGKHNDLEDVGKDSYHHTFFEMLGNWSFGDYFKKEAITWSWQLLTEVYKLPKDRLYVTYFEGNPAAGLEPDHEARDI

WLSLGLDPSRVLTGSAKDNFWEMGATGPCGPCSEIHYDRIGGRNAAHLVNQDDPLVLEIWNNVFIQFNREDDGSLKSLPA

KHVDTGMGFERLVSVCQDKHSNYDTDLFAPLFSRIQELTGARPYKGGFGQDDPDGIDTAYRVIADHVRTLTFALSDGGVP

NNVGRGYVLRRILRRGARFARKKFNVEIGSFFSSLAPTLIDQLGDVFPELTKKYDDIKEILDEEEASFARTLDRGEKLFE

RYVAQARESGAKELGGKDVWRLYDTYGFPVDLTRLMAEELGLGINEADFEAARLHSLEASKGAGKQGNKDIVKLDVHDIA

KLEADATVPKTEDSSKFLQGKAAEITAVVKAIYYNKSFVQSTKDITEGESFGVILDRTNFYAEAGGQENDTGSIVIDGEA

AFEVSNVQAFNGYVLHVGELKEGTLKVGDTVLPMYDELRRWPLRNNHTATHILNFCLRETLGDHIDQKGSLVAPTKLRFD

FSHKAQIALPDLQKIEEGSKEWIRRNVKVFSKEVGLKNGFKIPGLRAVFGEAYPDPVRVVALEYDVDEIAKDLENPKWRS

TSIEFCGGTHVAETGTIKDFIITEESGIAKGIRRIIAVTGYEAAEVTRVAAGLEQSIARLDSLHGKEKDTAMKALTLELN

QADISVLRKAELKESLLKVRKAFDDEVKAREKAQTKTAVDTVSQHFKDSPNSPVWVGVVEGGNAKLLQSVMNQGKALNKP

VYVFTRDEAGEKILHAAFVPQSQVSSSFDARVWAASVSEIIGGKAGGKPDAAQGVGTNVEKLEEALAVARKRFV

>OAX38373.1 hypothetical protein K503DRAFT_800484 [Rhizopogon vinicolor AM-OR11-026]

MASSYHGPWTASKVREQFFNYFRSKNHTFVPSSSTVPYDDPTLLFANAGMNQYKSIFLGTVDPNSERGKLKRAFNSQKCI

RAGGKHNDLEDVGKDSYHHTFFEMLGNWSFGDYFKKEAIEFSWELLTKVYGLPKDRLYVTYYAGDPQNGIPTDEEAKQHW

LNQGISPDHVIASMGNFWEMGATGPCGPCSEIHFDRIGGRNAAHLVDGDDPNVLEIWNNVFIQFNREDDGSLKSLPSKHV

DTGMGFERLVSVLQDKLSNYDTDVFLPIFTRIQELTGARPYTGKFGNDDVDGIDTAYRVIADHVRTLSFALSDGGVPNNV

GRGYVLRRILRRGSRYVRKKLGAPIGSFFSSLMPVIVETMGDVFPELTKKQAEIKEILDEEEESFSRTLDRGEKLFDQYA

TRAKEAGVKELNGADVWRLYDTYGFPVDLTRLMAEELGLGINEKEFEEAQEQSKIASKGSFKKDKADLVKLDVHDLAALE

KNETIPKTDDSFKFQLGNITATVKGIYFNREFISSSSDIPKSANFGVLLDRTSFYAESGGQEYDTGSIVIDGATDFEVTN

VQSYSGYVLHTGSLKYGQLSVGDEVISSYDELRRWPLRSNHTATHILNFCLREVLGDHIDQKGSLVAQTKLRFDFSHKAQ

ISLPELAKIESMSIDWIKKNVKVYSKDLDLIAAHKIPGLRAVFGEAYPDPVRVVVLGYDVDEMANDIDNPKWRSTSVEFC

GGTHVAKTGDIKDFVITEESGIAKGIRRIVAVTGEEAHEASRVANALKVRLEQLESTEGKEKDAGLKTLSVELGQADISV

LLKAELKDRLAAVRKALDKQTKEKENAMNKAAVDALVTFFEEKPDSEAYFAILEVEGNAKILQNVVMRGKKLGKAVYVFS

VDAEGGKVAHANYVPESARSRGLDARTWASKVSDIIGGKAGGKEDGAQGVGVNISQVEDALRVAQETFSSK

>OAJ24290.1 hypothetical protein A4X03_g2606 [Tilletia caries]

MTQSTSSQFGDWPAVKVRQTFFDFFEKENKHTFVKSSATIPYDDPTLLFANAGMNQYKPIFLGTVDPNSAFASLKRAVNS

QKCIRAGGKHNDLDDVGKDTYHHTFFEMLGNWSFGDYFKNEAVGMAWELLTKVYGLPADRLYVTYFEGDAKLGLEPDVET

RDIWRKLGVPEDHILTGNAKDNFWEMGATGPCGPCTEIHYDRIGGGRNAAHLVNMDDPDVLEIWNNVFIQFNREADGGLR

PLPAKHVDTGMGFERLVSCLQDKPSNYDTDVFGPLFDKIRELTGARPYAGKLGSEDVDGIDTAYRVVADHVRTLTFAISD

GGVPDKDGRGYVLRRILRRGTRYVRKYFQVPIGSFFSSLVPALVEQMGEMFPEITKKIDDVKAILDEEEESFSRTLDRGE

KLFEQFASKAKQSGAKELNGNDVWRLYDTYGFPVDLTLIMAEELGMGVNEREFEEAQALSKEKSRGQGKKEGSEWPKFDV

HDLGKLESNKDVPKTEDSAKYKLGNVGGVVKAIYQDHAFFNSSSEVDADKPLGILLDRTNFYAEQGGQQGDTGSIVIDGQ

TEFNVEDCQVANGYVLHSGFFKYGELKVGDEVVASYDELRRWPLKNNHTATHILNFALREVLGDHIDQKGSLVAPNKLRF

DFSHKTGITIPELTKISELSMGWVKKNVPVYSKELPLELAYKIAGLRAVFGEAYPDPVRVVSLEYDVDEIAKDLENPKWR

GTSVEFCGGTHVAKTGDIKDFVVIEESGIAKGIRRIVAVTGEEAAQVNRLAANAKLKLETIDAETDRAKKDTALKAFGTE

LNQLEMSVLRKAELNEAFSTIRKKHDAEAKAKQNAETKLVQDAVTSYFSTEQPNATFLVRQFDVGSNTKALQAGILVAKK

LNKAIYLFSEEVLPSEAQPQTEEEGAGTRVKARVAHVNFVPKQDLERGLKAKEWAEVITKSIGGRAGGKDDGVQGVGEDG

GPALGDALIAAERFVVLKTAESKMPMQ

>KLO11725.1 hypothetical protein SCHPADRAFT_998656 [Schizopora paradoxa]

MAYKPYEGEWTSSKVRQTFFDYFQARGHTFMPSSSTIPYEDPTLLFANAGMNQFKSIFLGTTDPNSELSKLKRAFNTQKC

IRAGGKHNDLDDVGKDSYHHTFFEMLGNWSFGDYFKKDAIAYSWELLTKVYKLPSDRLYVTYFEGDSKNGLAPDNEARDF

WRAQGVPDDHILTGNAKDNFWEMGATGPCGPCSEIHFDRIGGRNAASLVNQDDPDVIEIWNNVFIQFNREQDGSLRVLPA

QHVDTGMGFERLVSVVQDRKSNYDTDVFEPLFKRIHELTGVRPYQGLFGAEDKDDIDMAYRVVADHVRTLTFALADGGVP

NKDGRGYVLRRILRRGARYARRKLGVEIGSFFSTLMPTLIDQLGDVFPELRKRTDEIKEILDEEEESFSRTLDRGEKLFE

QYASKVVQSGGKTLNGLDVWRLYDTYGFPLDLTELMAEEKGLLIDKDGVKAAEAKSLEASKAGVKKNAGDVLKLDVHDLA

TLEKNTDVPKTDDSFKYGHDNVEAKVKAIFVNKTFVQQTKDGVEDIYGVLLDRTSFYAESGGQEYDMGNIVIDGVADFEV

TNVQSFGGYVLHIGSMKYGTLSVGDDVVAMYDEIRRWPIRNNHTATHILNYSLREILGDHIDQKGSLVAATKLRFDFSHK

AQVSLPELAKIESLCVDWIKRDVKVYSKDLDLNAGYKIPGLRAVFGEKYPDPVRVVSLEYEVDEIAKDLENPKWRKTSIE

FCGGTHVVKTGDIKGFVITEESGIAKGIRRIIAVTGSDASDVTRQANALSSRLDSIDKLVGKQKDAEMKDFGVRLNTADI

SVLRKSELRDRLAVMKKAFDKETKERETTVQKAEIDRLNEHFKSNPSAEAYFAHLDVDGNMKILQAVVTQAKKMKKAIYV

FSSSLPGSEDGAEGKVVHVNHVPDAFRSKGIDGRTWASKVSEIIGGKAGGKEDGAQGVGTVTDKIQDALLVAQKHFEEFL

K

>XP_016595461.1 Translation elongation/initiation factor/Ribosomal, beta-barrel [Penicillium expansum]

MASLGSEWTAQRVRDTFLDYFKQNGHTFVPSSPVAPLSDPTLLFTNAGMNQFKSIFLGTVDASSDFGKMKRATNSQKCLR

AGGKHNDLDDVGKDSYHHTFFEMLGNWSFGDYFKKEAITYSWTLLTEVYGLDPDRLYVTYFEGNEAGGLEPDLEAKELWK

SVGVPESHILPGDMKDNFWEMGDQGPCGPCSEVHYDRIGGRNAAHLVNEDDPNVLEIWNNVFIQYNREPDSSLRSLPNKH

VDTGMGFERLVSILQDKPSNYDTDVFTPIFQAIQDTTGAREYRGHFGADDADGIDTAYRVVADHVRTLMFAISDGVIPNN

EGRGYVIRRVLRRGARYARKYFQVDIGNFFAKLVPTVVDQLGEMFPELKRKQQDVIEILDEEELSFAKTLDRGERQFEQY

AQQAKVKGTDMLHGADVWRLYDTFGFPVDLTRIMAEERGLRIDDAEFEEARLKAKEASKGQKKAAESTVKLDVHDLGKLE

KMNDVQKTDDSPKFGRGNITSHVKAIYHGKTFHNSTDDVPDGAQLGVILDCTNFYAEQGGQENDTGKIIIDGQAELEVGD

VQSYAGYVLHTGFMKYGSFAVGDSVICEYDELRRWPIRNNHTGTHILNFALKEILGDGVDQKGSLVAAEKLRFDFSHKSA

ISDAELEKIEAKATDYIRQNCAVYSQDVPLATAQQISGVRAVFGETYPDPVRVVSVGVELEEILQNVKDPRWQEVSIEFC

GGTHVQKTGDIKDLVILEESGIAKGIRRIIAVTGEDAHEVQRVAEEFGQRLDRLNALPLGSEKEQEAKQVQVDLNQLTIS

AVQKAKFRERFAKINKQVLDGQKAQQKLESKKAVDTITSYFEAPENQDKSWLVVQLPISANAKAVSESLNHVKSKMQGKS

VYLLAADSEQGRVSHGCYISQGLSDQGASASDWAAVVSGAVGGKAGGKGPTSIGNGTNADKVEEAISLAADYLNKFKL

>XP_007763572.1 hypothetical protein CONPUDRAFT_115791 [Coniophora puteana RWD-64-598 SS2]

MSAPYDGPWTARNVRQAFFDFFTSKDHTFVPSSSTIPYDDPTLLFANAGMNQYKSIFLGTVDPNSERGKLRRAFNSQKCI

RAGGKHNDLEDVGKDSYHHTFFEMLGNWSFGDYFKKEAITYSWEALTKVYGLPKERLYVTYYAGDPKAGIPADEDAKRYW

LEQGVDPSHVLPSTGNFWEMGATGPCGPCSEIHFDRIGGRDVAHLVDGDDPTVIEIWNNVFIQFNREEDGSLRSLPSKHI

DTGMGFERLVSILQDKRSNYDTDVFTPLFDKIQELTGARPYAGKFGDEDADGVDTAYRVVADHVRTLAFALSDGGVPNNV

GRGYVLRRVLRRGSRFVRKKLNAPIGSFFSSLLPVVVETMGDAFPELTKKQDEIKEILDEEEESFSRTLDRGEKLFEQYA

TKAKEQGLKELHGGDIWRLYDTYGFPVDLTRLMAEEHGLSINEKQFEEAQEASKEASKGSFKKDKGAAVKLDVHDLAALD

ANDAIPKTNDSFKFQLGNINAIVKGIYHDKQFLSSTSNIPEGATFGVLLDRTSFYAEAGGQENDTGNIVIDGVADFEVTN

VQSYSGYVLHTGHLKYGQLNAGDDVISSYDELRRWPLRSNHTATHILNYALREVLGDHIDQKGSLVAPTKLRFDFSHKAQ

IGLKELQQIETISIDWIKKNVKVYTKDLTLEEAYKIPGVRAVFGEAYPDPVRVVVLGYDVDEIARNVENPQWRKTSIEFC

GGTHVAKTGDIKEFVIIEESGIAKGIRRIIAVTGQEAQEASRIASSLKTRLDELERSSGKEKDAGLKALTVELGQADISV

LLKAELKDRLASVRKALDKQIKEKETALNKEALNALAKHFEENPNSEGYFAAIEVDGNAKILQNIVMQAKKLNKPAYVFS

VDPERTKVAHANFVPEGARSKGFTAKGWASKIGEVIGGKAGGKDESAQGVGANVDKVAEAVVLAKEVFISSST

>XP_006956391.1 hypothetical protein WALSEDRAFT_31222 [Wallemia mellicola CBS 633.66]

MTEWTAKRVRSTYIDYFKQQHAHTFVPSSSSIPYEDPTLLFANAGMNQYKSIFLGTVDKNSQFGQLKRAVNSQKCVRAGG

KHNDLDDVGKDTYHHTFFEMLGNWSFGDYFKKEAIEMAWKLLVDVYKLPADQLYVTYFEGDPKQGLEPDYEARDIWRSLG

LPDDHILTGDATDNFWEMGATGPCGPCSEIHFDRIGGRNAAHIVNADDPNVIEIWNNVFMQFNREADGSLRPLPAKHIDT

GLGFERLVSILQNKPSNYDTDVFTPIFEKIQQMTKARPYAGTLGAEDVDGIDTAYRVIADHVRTLTITISDGGVPNNVGR

GYVLRRILRRGCRYARKKFGVPIGSFFSTLAPVVIEQMGDMFPEITKKYDDIKEILDEEEESFSRTLDRGEKLFDSYAQK

TLNTPEKQLGGKEVWRLYDTYGFPVDLTVLMAEELGLTINMEEFEKARIESHEASKGTKKKGDVESVKFDVHDLAALEKD

NIAKTDDIYKYTHGTVDAKIISIFQDRQFHKSTKDINTSKPFGILLDKTNFYAESGGQEYDTGMITIDGKADFAVEDVQV

YSGYVLHSGALKYGELNLNDDVIATYDELRRWPIKNNHTGTHLLNFALKEVLGDHIDQKGSLVAPTKLRFDFNHKAGLSV

AELNKVEDLCDQFIKRNVPVFSQEFNLDDARKIPGLRAVFGEVYPDPVRVVSVEFNPQDIKHDIENEKWSKSSIEFCGGT

HVNTTGEIKLFKIVEESSIAKGIRRIIAVTADDAVEVMRKSSELANKLAFIDGLTDMKEKDIALKAYAVEIGQAEISTIR

KIELREQYNKIRKTVDDANKAAQAAQAKYAGDTIKNYFNENEEAKSFVGTLDLGSNTKALGQALSAARQLGKQVYVFSVD

KDASKVAHQCYIPKELLKDGYNAKIWAAKVAEILGGKAGGKDDSAQGVGSEVSKVEEAIGAAKSFI

>XP_015713130.1 PREDICTED: alanine--tRNA synthetase, mitochondrial isoform X1 [Coturnix japonica]

MPLRAPHRVGSRRSSAVPSGPASSEVSVPTSAPANMAAAGPLRLPLLAVRPPWPRCPRRGASCPPSAQIRAAFLRFFQEK

HGHRFLPSAPVRPRGDPTLLFVNSGMNQFKPVFLGTAHPRSELAQQRRVVNSQKCVRAGGKHNDLEDVGRDTYHHTFFEM

LGNWSFGDYFKEEACSMAWELLTEVYKIPKDRLYVTYFGGDSSLGLGADEECRDVWLSLGVPASHVLPFPMKDNFWEMGD

TGPCGPCTEIHYDHVGGGRNAAALVNQGSPDVVEIWNLVFMQYSREVDGNLLPLPQHHVDTGMGLERLVTVLQNKRSNYD

TDLFTPILDAIHKGCGGPRYQGLVGNADVGHVDMAYRVVADHIRTLCVCITDGIYPGPSGAEVVLRRILRRAVRFCSEVL

HAPHGLLASLVPTVVEVLGDAYPELKKNAEQVMDIINENEVAFLSSLERGRRIIERTVQQMEPSANFPTEVAWSLYGNLG

FPLDLIDLMLEEKGIRLDSAAFNELLLEDAKRKARGPQEGQQLEGTVVHLDVHSLAQLQRDNIPATDDSPKYSYRLGQHG

QYEFSPCQATILMLYRDQSLQKEVGAGQHCGVILDRTNFYAEQGGQASDQGYLIRLGQQDILFPIESVRLCGGYVIHEVT

AVETLRVGDQVQLFVDEVQRLACMTNHTATHLLNFALRCVLGDSTEQRGSHVTAEHLRFDFNIKGPVTVEQLQQVEQVVQ

DVIKRNEAVYMAEVPLALARRVQGLRALDEGYPDPVRIVSLGIPVETVLTSNTQAAMQTSVELCCGTHLLQTGAMEDLAI

VNERQLVKGISRVIAVTGRQAKQAREVGQCLAMEVDSVSIRMKQRGSASVLEMQNLSKEVGQLTKVVASTAMPQWQRKEL

QSILKLLQRTANTAIKKLEVQQAEEKAQSLLAKHCNQPVIIDTVPADSLSILMKVVNQLCDKSPGTSVLLLSPQDSGEVL

CACQVSKNCLPMFSAADWAVAVCTQMEGKAGGSPVVAKGSGNAKGMQGALTTALEFAQSKL

>XP_008039052.1 hypothetical protein TRAVEDRAFT_21080 [Trametes versicolor FP-101664 SS1]

MASSTHTDWSASKVREEFFNYFKNREHTFVPSSSTIPYDDPTLLFANAGMNQYKGIFLGTVDPQSELARLKRAFNSQKCI

RAGGKHNDHSQFAALAKIEAAGAWSLATDTYVGKDSYHHTFFEMLGNWSFGDYFKKEAIEYSWELLTEVYGLPKDQLYVT

YFEGDPKAGLEPDLEAKDWWLKTGVAEDHIIPGNAKDNFWEMGATGPCGPCSEIHYDRIGGRNAAGHVNQDDPDVLEIWN

NVFIQFNREDDGSLKPLPAKHVDTGMGFERLVSVVQDKRSNYDTDVFQPIFARIQEVTGVRPYGGKFGDEDENGIDTAYR

VIADHVRTLTFALSDGGVPNNVGRGYVLRRILRRGARFARKKLGVQIGSFFSSLVPVVVEQMGAVFPEITQKSAEIKEIL

DEEEESFSRTLDRGEKLFEQYAQKTLQSGQKELSGKDVWRLYDTYGFPVDLTRLMAEELGLSINEAEFEAAQAHSKEASK

GGVKKGTGEVVALDVHDIAALEKNDGVPKTDDSFKFKQGNITAKVVAIYSKKAFLKSTEEIPEEASFGLLLDRTSFYAES

GGQEYDTGSIVIDGVAEFEVTNVQVYNGYVLHIGHMKYGKLNIGDEIVSSYDELRRWPLRNNHTATHILNYSLREVLGDH

IDQKGSLVAPTKLRFDFSHKAQVSTSELTKIESISQDFIKRNVGVYGKELPLHVAQKINSLRAVFGESYPDPVRVVSLEF

DVDDIAKDLENPKWRKTSIELCGGTHVARTGEIKDFVVTEESGIAKGIRRVVAVTGHEAAEARRLGESLTAQLNRIESMP

DAEKDAALKAYTVHIGQVDIALLKKNELRDRLTSVRKAFDKHLKDKEAAANKASVDALQQYFKDKPDAPAYFAALDVNAN

AKVMQGVVLQAKKLGKSIYIFSADQSAGKVAHVNAVSEEAKGKGLDGREWASAVVEILGGKAGGKPDGAQGVGAEVSKVQ

DALAAAQKFFESKTGLSV

>OCL09326.1 alanyl-tRNA synthetase [Glonium stellatum]

MASSPIMEEKHEWNAPRVRETFLEYFKKNGHTFVPSSSVVPLSDPTLLFTNAGMNQYKSIFLGTVDPASDFASLKRAVNS

QKCIRAGGKHNDLDDVGKDSYHHTFFEMLGNWSFGDYFKKEAIGYSWELLTKVYGLDPERLYVTYFEGADGLEPDLEAKE

LWRSVGVPEDHILPGDMKDNFWEMGDQGPCGPCSEVHYDRIGGRNAAHLVNKDDPNVLEIWNNVFIQYNREPDRSLRSLP

NKHVDTGMGFERLVSVLQDKMSNYDTDVFTPLFKRIQEITGVREYRGKFGDEDPDGIDTAYRVVADHVRTLTFAIADGGV

PNNVGRGYVVRRVLRRGARYARKYLNTEIGDFFSKIVPSLVHQMSSMFPEIAKKESDVKEILDEEEKSFAKSLDRGETMF

EKYAQQCKLLDSNDLPGTDVWRLYDTYGFPVDLTKLMAEERGLIINDKEVEEAQAKAREASKGEKKAAAGLVKLDVHDLG

ALELMKDVPKTDDSPKFGRENITAVIKCIYHNKRFLKSTKEVPEGEQFGVLLDRTNFYAEQGGQEYDTGKLVVDGVAEID

VQNVQVYAGYVLHTGYINYGELSVGDEVICEYDELRRHPIRSNHTGTHILNYALREVLGSEVDQKGSLVAPEKLRFDFSH

KSGISDADLAKIEEISTFYIRQNSEVYALDVPLATAKQIRGVRAVFGETYPDPVRVVSVGVPVEDLLSDVKNEGWEKVSV

EFCGGTHVKQTGEIKELIILEESGIAKGIRRIIAVTGQTAYEVQRIASEFEEGLARLEKAPYSAAKEAEAKKMQVALNEL

SCSALTKSSFRDRFAKIQKSILDQQKAVAKADNKRVLDTIVDFFNKEENEAKKHLVAKLEWSPTVSKAIGEAIKTVSGKG

PMKDKSVYLFSANEEEGKVVHGCYVAEHFSSQGASAVEWSKNVASIIGGKAGGKGATSIGQGTNADKVDEGLEEATRYLE

KFQL

>OAL70047.1 alanyl-tRNA synthetase [Trichophyton violaceum]

MAASTSSQPPAGQQTVEWPALRVRDTFLDYFKKNGHTFVPSSSVVPLSDPTLLFANAGMNQYKSIFLGTVDPNSDFAQLK

RAHNSQKCIRAGGKHNDLDDVGKDSYHHTFFEMLGNWSFGDYFKKEAIQFSWELLTKVYGLDPDRLYVTYFEGNEAAGVG

PDIEARDIWRSVGVKDDHILTGDMKDNFWEMGDQGPCGPCSEIHYDRIGGRNAAHLVNQDDPDVLEIWNNVFIQYNREPD

RSLRPLPNQHVDTGLGYERLVSILQNKSSNYDTDVFTPLFTAIQNITGARPYTGKFGAEDADGIDTAYRVVADHLRTLTF

AITDGAAPNNEGRGYVVRRVLRRGVRYARKYLQVEIGSFFSKLVPALVEQMGDMFPEIRRKQTDVMEILDEEEVSFAKTL

DRGERQFENYAQQALDSGAGKLHGADVWRLYDTFGFPVDLTRLMAEERQLTIDDAEFEEARLKAKEASKGQAKTASDLLK

LTVHTLSTLEKNGVPKTDDDAKFGKGNIVANIKAICHGKEVIDSTKDIPANEQIGIILDKTNFYAEQGGQEYDTGKIVID

GQAELDVQNVQMYGGYVLHTGFIKYGNFSVNDSVIAEYDELRRWPIRNNHTGTHILNFALREVLGDGVDQKGSLVAAEKL

RFDFSHKSAISDSDLEKIEAKSTDYIRQNCAVYSQEVPLATAREITGVRAVFGETYPDPVRVVSVGVELDEILKNVKDPR

WEGVSIEFCGGTHVQKTGDIKDLVILEESGIAKGIRRIIAVTGEDAHEVQRIAREFGERLNRFEKMPAGPERDAEIKSLQ

VDLNQLTISAVEKTKYRNQFTQIHKKITDAQKAAQKAEVKTAIDAISAYFEENKESPSLVIKLPITANQKAISESLNHVK

TKLSGKALYVFAADASKVAHGCYVTPDLTTKGASANEWASAVSSIVGGKAGGKAPVAIGNGTEVNKVDEALDEATKYLEK

FKL

>KOC16279.1 alanyl-tRNA synthetase, cytoplasmic [Aspergillus flavus AF70]

MNSNIQSSPEWTAKSVRDAFIQYFQKNGHTFVASAPVVPHSDPTLLFTNAGMNQFKSIFLGTVDPQSDFANLKSAVNTQK

CIRAGGKHNDLDDVGKDSYHHTFFEMLGNWSFGDYFKKEAINYSWELMTKIYGLDPQRLYVTYFEGSKEGGLSPDLEAKE

LWKAVGVPEDHILPGNMKDNFWEMGDQGPCGPCSEIHYDRIGGRNASNLVNQDDPNVLEIWNNVFIQYNRESDRSLKPLP

NKHVDTGMGFERLVSVLQNKSSNYDTDVFSPLFQTIKDITGARPYQGRFGVEDADGIDTAYRVVADHVRTLTFAISDGVA

PNNEGRGYVIRRVLRRGARYARKYFRVEIGNFFSKIVPTLVDQMGDMFPEVKRKQQDVMEILDEEEISFAKTLDRGERQF

EHYAQQAKAKGDDKLHGADVWRLYDTFGFPVDLTRIMAEERGLRIDDTEFEEARSRAKEASKGQKKAAADTVKLDVHDLG

KLEKMNDVPKTNDSAKFDRGNITAQVKAIYHGKSFHLSTEQIPEGDQLGLILDRTNFYAEQGGQENDTGRIVIDGQAELE

VGDVQLYAGYVLHTGFMKYGSLSVGDEVICEYDELRRWPIRNNHTGTHILNFALKEVLGDGVEQKGSLVAAEKLRFDFSH

KSPVTDKDLERIEDISTEYIRQNCAVYSQEVPLATARQISGVRAVFGETYPDPVRVVSVGVELEEILKNVKDPRWEKVSI

EFCGGTHVQKTGDIKELVILEESGIAKGIRRIIAVTGEDAHEVQRVALGFEKRLDRLEAMALGPEKEQDAKQIQVDLSQL

LISAVQKSRFRERFAHINKQIIDSQKAQQKLESKKALEAITSYFENPENKDKSWLVARLPISANAKAVSESLNHVKSKLQ

DKTVYVLAADSNQGRVVHGCYMSKAISDQGASASDWAAIVSTAVGGKAGGKGPTSIGNGTNPDKTDEAIALASDYLSKFK

L

>XP_025004460.1 alanine--tRNA synthetase, mitochondrial isoform X2 [Gallus gallus]

MAAAGPLRLPLLAVRPPWPRCPRRGAARPPAGQVRAAFLRFFEEKHGHRCLPSAPVRPRGDPTLLFVNSGMNQFKPVFLG

TAHPRSELAQQRRVVNSQKCVRAGGKHNDLEDVGRDTYHHTFFEMLGNWSFGDYFKEEACSMAWELLTEVYKIPKDRLYV

TYFGGDSSLGLNADEECRDVWLSLGVPANHVLPFPMKDNFWEMGDTGPCGPCTEIHYDHVGGGRNASALVNQGSPDVVEI

WNLVFMQYSREVDGNLLPLPQHHVDTGMGLERLVTVLQNKRSNYDTDLFTPILDAIHKGCGGPRYQGLVGNADVGHVDMA

YRVVADHVRTLCVCITDGIYPGLSGAELVLRRILRRAVRFCAEVLHAPRGLLASLVPAVVEVLGDAYPELKKNADQVMDI

INENEVAFLSSLERGRRIIERTVQQMKPSADFPAEVAWSLYGNLGFPLDLIDLMLEEKGIRLDSAAFNELVLEDAKRKAH

GPQEGRQLEGTVVHLDVHSMAQLQRDNIPATDDSPKYSYKLGQHGQYEFSPCQATILMLYRDQSLQKEVGAGQHCGVILD

RTNFYAEQGGQASDRGYLIRLGQQDILFPVESVRLCGGYVIHEVTAVETLRAGDQVQLFVDEVQRLACMTNHTATHLLNF

ALRHVLGDSTEQRGSHVTAEQLRFDFNIKGPVTVEQLQQVEQVVQDVIKRNEAVYMAEVPLALARRVQGLRALDEGYPDP

VRIVSLGVPVETVLTSNSQAAMQTSVELCCGTHLLQTGAMEDLAIVSERQLVKGISRVIAVTGRQAKQAREVGQCLAMEV

DSVSIRMKQKRITSVLEMQNLSKEVGQLTKVVASTAMPQWQRRELQSILKSLQRTANTTIKKLEVQQAEEKAQRLLAKHC

NQPVIIDTVPADSLSILMKVVNQLCDKSPGTSVLLLSPQDSGEVLCACQVSKNCLPMFSAADWAVAVCTQMEGKAGGSPV

VAKGSGNAKGMQGALTTALEFARSKL

>ORZ17358.1 mitochondrial and cytoplasmic alanine-tRNA synthetase Ala1 [Absidia repens]

MTVVWSMNKVRSSFIEYFESKGHSFYASSPTVPHDDPSLLFANAGMNQYKSIFLGTVDPSSPLYNLKKATNSQKCIRAGG

KHNDLDDVGKDTYHHTFFEMLGNWSFGDYFKVEAARYVWEYLTVVLKLPQDRLYVTYFGGDEKMGLEADLEAKQIWIDLG

VKENRLLPGDSKDNFWEMGETGPCGPCSEIHYDRIGGRDATHLVNMDDPNVLEIWNVVFIQFNREQGGKLRPLPNKHIDT

GLGLERLVSILQNKYSNYDTDVFGLLFDRIKALTGARGYTGKLGDEDIDGVDTAYRVVADHIRTLTFAITDGGVPSNDGR

GYVLRRILRRVDTLVEQMGETFPELLTKVDEVKAILDEEEAAFAKTLDRGEKLFEQYLDAAKNRQTKTLSGVDVWRLYDT

YGFPVDLTRVMAEENGVTIDDVELEVSQEAAKEISRRGKADNNGGEAVTLDVHDLAELGKNTQVPKTDDSFKYVGGSTQG

FIKSIYYQHRFLDDTSNIPRGANFGILTDKTNFYAESGGQEFDTGNMMTLDGNAEFIVMDCQLFGGYVLHVGYLKYGQLK

CEDQVELSFDEQRRFSLRNNHTATHLLNYALRQVLDESIDQRGSLVARDKLRFDFSHKSPVSLTQLAHVESICNEFVTND

IPVYSKPVALPIAKTIQGLRAVFGETYPDPVRVVSIGVDVDQVTADVSNPRWATTSVEFCGGTHVVKTGDIQRFAILEET

GIAKGIRRIIAVTGEQAIKADRTAKAFAQRLDQVSTMTTMAELKDMARDLDALVVSAVVKSDLRDRYLKMKKLHDDRDKR

QQKEHAQKVTLTMQRYFEQHPDSKYTVISFMDDDTNNYNKAFIAAITYAKTHLKDKAVYLLSSDASSGRVTHHCMVGPCL

TAHYRFKASDWADVVSDKIGGKKGGNDDSAQGSGDNIAGLDQAIKAAHAFAKSKIGV

>OJA12349.1 hypothetical protein AZE42_02795 [Rhizopogon vesiculosus]

MASSYHGPWTASKVREQFFNYFRSKNHTFVPSSSTVPYDDPTLLFANAGMNQYKSIFLGTVDPNSERGKLKRAFNSQKCI

RAGGKHNDLEDVGKDSYHHTFFEMLGNWSFGDYFKKEAIEFSWELLTKVYGLPKDRLYVTYYAGDPQNGIPTDEEAKQHW

LNQGISPDHVIASMGNFWEMGATGPCGPCSEIHFDRIGGRNAAHLVDGDDPNVLEIWNNVFIQFNREDDGSLKSLPNKHV

DTGMGFERLVSVLQDKLSNYDTDVFLPIFIRIQELTGARPYTGKFGNDDVDGIDTAYRIIADHVRTLSFALSDGGVPNNV

GRGYVLRRILRRGSRYVRKKLGAPIGSFFSSLMPVIVETMGDVFPELTKKQAEIKEILDEEEESFSRTLDRGEKLFDQYA

TRAKEAGVKELNGADVWRLYDTYGFPVDLTRLMAEELGLGINEKEFEEAQEQSKIASKGSFKKDKADLVKLDVHDLAALE

KNEAIPKTDDSFKFQLGNITATVKGIYFNREFISSSSDIPKGENFGVLLNRTSFYAESGGQEYDTGSIVIDGATDFEVTN

VQSYSGYVLHTGSLKYGQLSVGDEVISSYDELRRWPLRSNHTATHILNFCLREVLGDHIDQKGSLVAQTKLRFDFSHKAQ

ISLPELAKIESMSIDWIKKNVKVYSKDLDLIAAHKIPGLRAVFGEAYPDPVRVVVLGYDVDEIANDIDNPKWRGTSVEFC

GGTHVAKTGDIKDFVITEESGIAKGIRRIVAVTGEEAHEASRAASALKVRLEQLESTEGKEKDAGLKTLSVELGQADISV

LLKAKLKDRLAAVRKALDKQTKEKENAMNKAAVDALVTFFEEKPDSEAYFAILEVEGNAKILQNVVMRGKKLGKAVYVFS

VDAEGGKVAHANYVPESARSRGLDARTWASKVSDIIGGKAGGKEDGAQGVGVNINQVEDALRVAQETFSSK

>XP_018983322.1 hypothetical protein BABINDRAFT_163044 [Babjeviella inositovora NRRL Y-12698]

MSTQSYKIPGSSPGYLNRTCPNIHPNLHTLSSNAQARGHSKIYACQNFHAYLSAGRVIKTAQSNTGPFHAPFMLRTLFKR

NMSINPLQQKWPAQKVRQTFLEYFQNKQHTFVPSSSVVPFDDPTLLFANAGMNQYKPIFLGTVDPSSDFSTLKRAVNSQK

CIRAGGKHNDLEDVGRDSYHHTFFEMLGNWSFGDYFKQETIVWSWELLTAVYGLPADRLYVTYFGGDEKLGLPADLEAKQ

MWLDAGVAEDHILPGSAEDNFWEMGDQGPCGPCSEIHFDRIGGRNAAHLVNQDDPDVLEIWNNVFMQFNREADGSLRPLP

AKHIDTGMGFERLVSILQDVRSNYDTDCFQPLFARIQEITGARAYTGHFGAEDVDGIDTAYRVLADHVRTLTFAIADGGV

PNNEGRGYVLRRILRRGARYARKYMGYPIGKFFSQLVPTLVEQVQDIFPEVAKNTAEIFEILDDEEASFAKALDRGEKLF

EQYAATAVASASKTLAGKDVWRLYDTYGFPVDLTRLMAEEAGLEIDEAAFEKARLESLEASKKGGKNSKGGELVKLDVHA

LAELESNTAVPKTDDSSKYVATSTDATIVALYNGKEFVSEISGDAQFGVLLNKTPFYAEQGGQEYDTGKIVIDGASQFEV

ANVQVYAGYVLHTGSLTEGSLKVGDAVIAEFDLERRLSIKNNHTGTHVLNYALRAVLGNAVDQRGSLVSQEKLRFDFTHK

AGLAVSEVEKIEAISNEYITKNLGVFSKDVALEVAKGITGLRAVFGETYPDPVRVVSVGVPVEDLLANPANAEWNNYSIE

FCGGTHVGKTGEIKHMVLIEENGIAKGIRRIVAVSGKEAAEVQRIAAAFDASLDAVSKMVFGEAKQTAVKELGVQLNQLS

ISILDKNQLKTKFAAVDKSIKDEVKAIQKAESKLTLDTVKNHFTVEGTVPEYLVAHIPISANAKALTEAINLMKKDNKEK

SIYLFAGDKATGGKVAHGCYISDAAIAKGVDASKVAQFAAGFIGGRAGGKGNVVQGMGENVAGIDEAVAEVEKLLSQKLT

I

>XP_011022066.1 PREDICTED: alanine--tRNA synthetase [Populus euphratica]

MKRITSSRPLLSSPLLRLLRRPPISLAAARVSSKQPYLSPFTSLARFFSTMSGGIDSPQPDVDWPANRVRDTFIKFFEEK

KHVDWKSSPVVPVNDPTLLFANAGMNQFKPIFLGTADPNTALSKLSRACNTQKCIRAGGKHNDLDDVGKDTYHHTFFEML

GNWSFGDYFKKEAIEWAWELLTKVYKLPADRIYATYFGGDEKAGLAPDNEARDIWLQFLPPGRVLPFGCKDNFWEMGDTG

PCGPCTEIHYDRVGNRNAAMLVNNDDPTCIEIWNLVFIQFNRESDGSLKSLPAKHVDTGMGFERLTSVLQNKMSNYDTDV

FMPIFDAIRQATGARPYSGKVGADDVDGVDMAYRVVADHIRTLSFAIADGSYPGNEGREYVLRRILRRAVRYGSEVLKAQ

EGFFNGLVNVVVKVMSDVFPELKRNEEHIRETIAAEEASFGKTLLKGIEKFKKAAQEVEGKVLSGQDAFVLWDTYGFPLD

LTQLMAEERGLQVDVEGFNNAMDEARERSRNAQNKQAGGAIVMDADATSALHKKGVSATDDSFKYISFEDHESVIKAIYN

GSEFLESAAAANEVGIVLESTSFYAEQGGQIFDTGVLEGPFGSFQVCNVQIFGGFIVHIGSLTGVSGRFSVGEKVTCKVD

YDRRKLIAPNHTCTHMLNFALREVLGNHVDQKGSIVLPEKLRFDFSHGKPVDPELLRKIESIVNGQIKDELGVFAKEATL

AEAKQINGLRAVFGEVYPDPVRVVSIGRKVEDLLANPENEEWLSISAELCGGTHITNTREAKAFALLSEEGIAKGIRRIT

AVTTDGAFKAMELACSLEQEVDEASKADGSLLEKKVASLKTRVDSALIPAAKKADIRAKISVLQNKVRKAQKKIAEENMQ

KAVEVATEMAEVASSDGKAFCISHVDVGLDVAAVREAVLKVLDRKGISAMVFSTDESTNKVVVCAGVPEKLDKGKGLEVS

EWLTTALGPLKGRCGKGKAGLATGQGTDASHLDEAMGLATTFAQMKLR

>KGO65716.1 Translation elongation/initiation factor/Ribosomal, beta-barrel [Penicillium italicum]

MTSLSSDWTAQRVRETFLDYFKQNGHTFVPSSPVAPLSDPTLLFTNAGMNQFKSIFLGTVDASSDFGKMKRATNSQKCLR

AGGKHNDLDDVGKDSYHHTFFEMLGNWSFGDYFKKEAITYSWTLLTEVYGLDPDRLYVTYFEGNEAGGLEPDLEAKELWK

SVGVPESHILPGDMKDNFWEMGDQGPCGPCSEVHYDRIGGRNAAHLVNQDDPNVLEIWNNVFIQYNREPDSSLRSLPNKH

VDTGMGFERLVSILQDKPSNYDTDVFTPIFQAIQNATGAREYRGHFGADDVDGIDTAYRVVADHVRTLMFAISDGVVPNN

EGRGYVIRRVLRRGARYARKYFQVDIGNFFAKLVPTVVDQLGEMFPELKRKQQDVIEILDEEELSFAKTLDRGERQFEQY

AQQAKVKGTEMLHGADVWRLYDTFGFPVDLTRIMAEERGLRIDDVEFEEARLKAKEASKGQKKAAENTVKLDVHDLGKLE

KMNDVQKTDDSPKFGRGNITSHVKAIYHGKTFHRSTDNVPDGAQLGVILDCTNFYAEQGGQENDTGKIIIDGQAELEVGD

VQSYAGYVLHTGFMKYGSFAVGDSVICEYDELRRWPIRNNHTGTHILNFALKEILGDGVDQKGSLVAAEKLRFDFSHKSA

ISDAELEKIEAKATNYIRQNCAVYSQELPLATAQQISGVRAVFGETYPDPVRVVSVGVELEEILQNVKDPRWQEVSIEFC

GGTHVQKTGDIKDLVILEESGIAKGIRRIIAVTGEDAHEVQRVAEEFGQRLDRLNSLPLGSEKEQEAKQVQVDLNQLTIS

AVQKAKFRERFAKINKQVLDGQKAQQKLESKKAVDTITSYFQAPENQDKSWLVVQLPISANAKAVSESLNHVKSKMQGKS

VYLLAADSEQGRVSHGCYISQGLSDQGASASDWAAVVSGAVGGKAGGKGPTSIGNGTNADKVEEAISLAADYLNKFKL

>XP_002565032.1 Pc22g10240 [Penicillium rubens Wisconsin 54-1255]

MASPGSEWTAQRVRDTFLDYFKQNGHTFVPSSPVAPLSDPTLLFTNAGMNQFKSIFLGTVDASSDFGKMKRATNSQKCLR

AGGKHNDLDDVGKDSYHHTFFEMLGNWSFGDYFKKEAITYSWTLLTEVYGLDPDRLYVTYFEGNEAGGLEPDLEAKELWK

SVGVPESHILPGDMKDNFWEMGDQGPCGPCSEVHYDRIGGRNAAHLVNEDDPNVLEIWNNVFIQYNREPDSSLRSLPNKH

VDTGMGFERLVSILQNKPSNYDTDVFTPIFQAIQNTTGAREYRGQFGADDADGIDTAYRVVADHVRTLMFAISDGVIPNN

EGRGYVIRRVLRRGARYARKYFQVDIGNFFAKLVPTVVDQLGEMFPELKRKQQDVVEILDEEELSFAKTLDRGERQFEQY

AQQAKVKGTDMLHGADVWRLYDTFGFPVDLTRIMAEERGLRIDDVEFEEARLKAKEASKGQKKAAENTVKLDVHDLGKLE

KMNDVQKTDDSPKFGRGNITAHVKAIYHGKTFHKSTDDVPEGAQLGIILDCTNFYAEQGGQENDTGKIIIDGQAELEVGD

VQSYAGYVLHTGFMKYGSFTVGNSVICEYDELRRWPIRNNHTGTHILNFALKEVLGDGVEQKGSLVAAEKLRFDFSHKTA

ISDAELENIEAKSTEYIRQNCAVYSQELPLATAQQISGVRAVFGETYPDPVRVVSVGVELEEILKNVKDPRWQEVSIEFC

GGTHVQKTGDIKDLIILEESGIAKGVRRIIAVTGEDAHEVQRVAEEFGQRLDRLNALPLGSEKEQEAKQVQVDLNQLVIS

AVQKAKFRERFAKINKQVLDGQKAQQKLESKKAVEAITSYFETPENQDKSWLVVQLPISANAKAVSESLNHVKSKMQGKS

VYLLAADSEQGRVSHGCYISQGLSDQGASASDWAAVVSGAVGGKAGGKGPTSIGNGTNTDKVEEAISLAADYLNKFKL

>EGA56826.1 Ala1p [Saccharomyces cerevisiae FostersB]

MTIGDKQKWTATNVRNTFLDYFKSKEHKFVKSSPVVPFDDPTLLFANAGMNQYKPIFLGTVDPASDFYTLKRAYNSQKCI

RAGGKHNDLEDVGKDSYHHTFFEMLGNWSFGDYFKKEAITYSWTLLTEVYGIPKDRLYVTYFEGDEKLGLEPDTEARELW

KNVGVPDDHILPGNAKDNFWEMGDQGPCGPCSEIHYDRIGGRNAASLVNMDDPDVLEVWNLVFIQFNREQDGSLKPLPAK

HIDTGMGFERLVSVLQDVRSNYDTDVFTPLFERIQEITSVRPYSGNFGENDKDGIDTAYRVLADHVRTLTFALADGGVPN

NEGRGYVLRRILRRGARYARKYMNYPIGNFFSTLAPTLISQVQDIFPELAKDPAFLFEILDEEEASFAKTLDRGERLFEK

YASAASKTESKTLDGKQVWRLYDTYGFPVDLTELMAEEQGLKIDGPGFEKAKQESYEASKRGGKKDQSDLIKLNVHELSE

LNDAKVPKTNDEFKYGSANVEGTILKLHDGTNFVDEITEPGKKYGIILDKTCFYAEQGGQEYDTGKIVIDDAAEFNVENV

QLYNGFVFHTGSLEEGKLSVGDKIIASFDELRRFPIKNNHTGTHILNFALKETLGNDVDQKGSLVAPEKLRFDFSHKKAV

SNEELKKVEDICNEQIKENLQVFYKEIPLDLAKSIDGVRAVFGETYPDPVRVVSVGKPIEELLANPANEEWTKYSIEFCG

GTHVNKTGDIKYFVILEESGIAKGIRRIVAVTGTEAFEAQRLAEQFAADLDAADKLPFSPIKEKKLKELGVKLGQLSISV

ITKNRTEAXI

>XP_001731546.1 hypothetical protein MGL_1729 [Malassezia globosa CBS 7966]

MSNSSATVVNEWPAAKVRSTFLDFFREKEHTFVPSSSTVPYDDPTLLFANAGMNQYKSIFLGTVDPSSEFSKLRRAVNSQ

KCIRAGGKHNDLDDVGKDTYHHTFFEMLGNWSFGDYFKEEAISFAWKLLTEVYGLPSDRLYVTYFEGDPKQGLEPDVETL

EIWRKVGVPDERILKGNAKDNFWEMGATGPCGPCSEIHFDRVGGRNAASLVNMDDPDVIEVWNNVFIQFNREEDGSLRPL

PARHIDTGMGFERLVSILQDKRSNYDTDVFMPLFSRIQELTGARPYSGKLGAEDVDGLDTAYRVVADHIRTLTFAISDGC

IPDKDGRGYVLRRILRRGTRFVRKYFQVPIGAFFSQLVPTLVEQMGSFFPEITRKTKELQAILNEEEQSFARTLDRGEKL

FEQYANAARTEGRTKLSGADVWRLYDTYGFPVDLTSIMAEEQGLTFDQAEFEKAQAESKEASKGQGKQQEALAVKLDVHD

LGHLDADASVPQTNDVFKYDTPSMQAHVKAIFQEHKFVSDSNSLAKAGEPFGILLDQTNMYAESGGQQADTGSLVIDGKA

EFEVSDVQASNGYVLHIGFLKYGKLSVGDQVVVSYDESRRRPLQNNHTGTHILNFGLREILGDHVDQKGSLVAPSKLRFD

FSHKAPVNVSELQKIEDISNSFIERDVSVYAKDMVLEEAYKIPGLRAVFGESYPNPVRVVALEFDIDQMAKDLTNPRWRQ

TSIEFCGGTHVKKTGEIGRLVVTEESGIAKGIRRIVAVTGDEAVAVTATADEASKRLDEIDQVSDDVAKEAQLKAFSVEL

ARMDISVVRKDELKARFTKIRKALDTRLKARAAADVKVAQEAVQTYFNENPDARVYIAQLDTGANSKALQSGVAVARKLN

KSVYLFARESGSEKTKTLYGNFVPKDELERGLDAVSWNKAVSEKLQGRGGGKPDGAQGQGEGTKADVDEAIKLAQSFFDM

QLK

>APR64342.1 alanyl-tRNA synthetase family protein [Populus tomentosa]

MKRITSSRPLLSSPLLRLLRRPPISLAAARVSSKQPYLSPFTSLARFFSTMSGGIDSPQPDVDWPANRVRDTFIKFFEEK

KHVDWKSSPVVPVNDPTLLFANAGMNQFKPIFLGTADPNTALSKLSRACNTQKCIRAGGKHNDLDDVGKDTYHHTFFEML

GNWSFGDYFKKEAIEWAWELLTKVYKLPADRIYATYFGGDEKAGLAPDNEARDIWLQFLPPGRVLPFGCKDNFWEMGDTG

PCGPCTEIHYDRVGNRNAAMLVNNDDPTCIEIWNLVFIQFNRESDGSLKSLPAKHVDTGMGFERLTSVLQNKMSNYDTDV

FMPIFDAIRQATGARPYSGKVGADDVDGVDMAYRVVADHIRTLSFAIADGSYPGNEGREYVLRRILRRAVRYGSEVLKAQ

EGFFNGLVNVVVKVMSDVFPELKRNEEHIRETIAAEEASFGKTLLKGIEKFKKAAQEVEGKVLSGQDAFVLWDTYGFPLD

LTQLMAEERGLRVDVEGFNNAMDEARERSRNAQTKQAGGAIVMDADATSALHKKGVSATDDSFKFICFEDHESVIKAIYN

GSEFLESAAAANEVGIVLESTSFYAEQGGQIFDTGVLEGPFGSFQVCNVQIFGGFIVHIGSLTGVSGKCSVGEKVTCKVD

YDRRKLIAPNHTCTHMLNFALREVLGNHVDQKGSIVLPEKLRFDFSHGKPVDPELLRKIESIVNGQIKDELGVFAKEATL

AEAKQINGLRAVFGEVYPDPVRVVSIGRKVEDLLANPENEEWLSISAELCGGTHITNTREAKAFALLSEEGIAKGIRRIT

AVTTDGAFKAMELAHSLEQEVDEASKADGSLLEKKVASLKTRVDSALIPAAKKADIRAKISVLQIKVRKAQKKIGEENMQ

KAVEVATEMAEVASSDGKAFCISHVDVGLDAAAVREAVLKVLERKGISAMVFSTDESTNKVVVCAGVPEKLDKGKGLEVS

EWLTTALGPLKGRCGKGKAGLATGQGTDASHLDEAMGLATTFAQMKLR

>XP_022383676.1 alanyl-tRNA synthetase [Aspergillus bombycis]

MNSNIQSSPEWTAKSVRDAFIQYFQKNGHTFVTSAPVVPHSDPTLLFTNAGMNQFKSIFLGTVDPHSDFANLKSAVNTQK

CIRAGGKHNDLDDVGKDSYHHTFFEMLGNWSFGDYFKKEAINYSWELMTKIYGLDPQRLYVTYFEGSEEGGLSPDLEAKE

LWKAVGVPEDHILPGNMKDNFWEMGDQGPCGPCSEIHYDRIGGRNASNLVNQDDPNVLEIWNNVFIQYNRESDRSLKPLP

NKHVDTGMGFERLVSILQNKSSNYDTDVFSPLFQTIKDITGARQYQGRFGVEDPDGIDTAYRVVADHVRTLTFAISDGVT

PNSEGRGYVIRRVLRRGVRYARKYFRVEIGNFFSKIVPTLVDQMGDMFPEVKRKQQDVMEILDEEEISFAKTLDRGEKQF

EHYAQQAKAKGDDKLHGADVWRLYDTFGFPVDLTRIMAEERGLRIDDTEFEEARSRAKEASKGQKKAAADTVKLDVHDLG

KLEKMNGVPKTNDAAKFDRGNITAQVKAIYHEKSFHPSTERIPEGDQIGLILDRTNFYAEQGGQENDTGRIVIDGQAELE

VGDVQLYAGYVLHTGFMKYGSLSVGDEVICEYDELRRWPIRNNHTGTHILNFALKEVLGDGVEQKGSLVASEKLRFDFSH

KSQVTDKDLEKIEDISTEYIRQNCAVYSQEVPLATARQISGVRAVFGETYPDPVRVVSVGVELEEILKNVKDPRWEKVSI

EFCGGTHVQKTGDIKDLVILEESGIAKGIRRIIAVTGEDAHEVQRVAQEFEKRLDSLEAMALGPEKEQDAKQIQVDLSQL

LISAVQKSRFRERFAHINKQIIDSQKAQQKLESKKALEAITSYFQDPENQDKSWLVARLPISANAKAVSESLNHVKSKLQ

DKTVYVLAADSSQGRVVHGCYVSKAVTDQGASASEWAAIVSTAVGGKAGGKGPTSIGNGTNPDKTDEAITLASDYLSKFK

L

>KZN91407.1 Alanine-tRNA synthetase [Penicillium chrysogenum]

MRVSGLVTNGERALTHLFRRNQPRTFSSCFVHSLATNPPTFDRMASPGSEWTAQRVRDTFLDYFKQNGHTFVPSSPVAPL

SDPTLLFTNAGMNQFKSIFLGTVDASSDFGKMKRATNSQKCLRAGGKHNDLDDVGKDSYHHTFFEMLGNWSFGDYFKKEA

ITYSWTLLTEVYGLDPDRLYVTYFEGNEAGGLEPDLEAKELWKSVGVPESHILPGDMKDNFWEMGDQGPCGPCSEVHYDR

IGGRNAAHLVNEDDPNVLEIWNNVFIQYNREPDSSLRSLPNKHVDTGMGFERLVSILQNKPSNYDTDVFTPIFQAIQNTT

GAREYRGQFGADDADGIDTAYRVVADHVRTLMFAISDGVIPNNEGRGYVIRRVLRRGARYARKYFQVDIGNFFAKLVPTV

VDQLGEMFPELKRKQQDVVEILDEEELSFAKTLDRGERQFEQYAQQAKVKGTDMLHGADVWRLYDTFGFPVDLTRIMAEE

RGLRIDDVEFEEARLKAKEASKGQKKAAENTVKLDVHDLGKLEKMNDVQKTDDSPKFGRGNITAHVKAIYHGKTFHKSTD

DVPEGAQLGIILDCTNFYAEQGGQENDTGKIIIDGQAELEVGDVQSYAGYVLHTGFMKYGSFTVGNSVICEYDELRRWPI

RNNHTGTHILNFALKEVLGDGVEQKGSLVAAEKLRFDFSHKTAISDAELENIEAKSTEYIRQNCAVYSQELPLATAQQIS

GVRAVFGETYPDPVRVVSVGVELEEILKNVKDPRWQEVSIEFCGGTHVQKTGDIKDLIILEESGIAKGVRRIIAVTGEDA

HEVQRVAEEFGQRLDRLNALPLGSEKEQEAKQVQVDLNQLVISAVQKAKFRERFAKINKQVLDGQKAQQKLESKKAVEAI

TSYFETPENQDKSWLVVQLPISANAKAVSESLNHVKSKMQGKSVYLLAADSEQGRVSHGCYISQGLSDQGASASDWAAVV

SGAVGGKAGGKGPTSIGNGTNTDKVEEAISLAADYLNKFKL

>EPS99866.1 hypothetical protein FOMPIDRAFT_1024013 [Fomitopsis pinicola FP-58527 SS1]

MSSGTYLNWPAAKVRQEFFNYFQSKGHTLVPSSSTIPHDDPTLLFANAGMNQFKAIFLGTVDPQSDLARLNRAFNTQKCI

RAGGKHNDLDDVGKDSYHHTFFEMLGNWSFGDYFKEEAIAYSWELLTEVYKLPKEQLYVTYFEGDPKNGIAPDTEARQYW

LDQGVAEDHILPGNAKDNFWEMGATGPCGPCSEVHFDRIGGRNAAHLVNQDDPDVLEIWNNVFIQFNREDDGSLKALPSK

HVDTGMGFERLVSVIQNKRSNYDTDVFSPIFAKIQELTGTRPYQGKFGDADPDGIDTAYRVVADHVRTLTFALSDGGVPD

KVGRGYVIRRILRRGCRYARKKLGVQIGSFFSSLVPAVVEQLGDVFPEITRRNDELKEILDEEEESFSRTLDRGEKMFDV

YANKAKEAGTKQLSGRDVWRLYDTYGFPVDLTRLMAEELGLDINDEEFAIAEAESKEASKGTKQKGDKDAVKLDVHDIAN

LEKTLNIPKTDDSAKYKLGNINARILSIYSKKSFVDSTASLSGDGEFGIILDKTSFYAESGGQEYDTGGIIIDGVAEFDV

SNVQVFSGYVLHIGHLKYGTLKVGDEVVASYDELRRWPLRNNHTATHILNFGLREVLGDHIDQKGSLVAPTKLRFDFSHK

AQVAPAELSKIESISKDWISRNVKVFSKEVILKTAYEIPGLRAVFGESYPDPVRVVSLEYSVDDMLQDIHNPKWRATSTE

LCGGTHVVRTGEIKDLVVIEESGIAKGIRRITAVTGHEAAEARRLADAFTARLNQLDSMSGPEKDAGLKSYTVELGQADI

SVLRKAELRDRLASIRKAFDKEIKEKEAKANKAAVDAVQEYFQTNKDAEAYITILDVSGNAKVLQSVVQQGRKLGKAIYV

ISPDPVNGRVAHINFVSEVAKAKGLDANQWAATVTGLIGGKAGGKGDSTQGSGPNTDKAKEALEAAREYFVKQTGLTV

>XP_024670728.1 putative alanyl-tRNA synthetase [Aspergillus candidus]

MRALKVLRDGELALTHLLSPHRLKPRSSYNFHTSPHPTAPHTTTIPAYTKMASDSKGSPDWTAKNVRDTFLDYFKKNGHT

FVPSSSVAPLSDPTLLFTNAGMNQYKSIFLGTVDPNSDFAKLKRAVNSQKCIRAGGKHNDLDDVGKDSYHHTFFEMLGNW

SFGDYFKKDAIRYSWELLTVVYGLDPQRLYVTYFEGDKEGGLEPDLEAKGIWREVGVPEDHILPGNMKDNFWEMGDQGPC

GPCSEIHFDRIGGRNAAKLVNEDDPNVLEIWNNVFIQYNRESDRSLRPLPNKHVDTGMGFERLVSVLQNKPSNYDTDVFT

PLFEAIRTATGAREYQGLFGNDDSDGIDTAYRVVADHVRTLMFAISDDVIPNNEGRGYVVRRVLRRGARYARKYFGVEIG

GFFSKLVPTVVDQLGEMFPELVLKQNDVKEILDEEEISFAKTLDRGERQFETYAQQAKLNGGDKLNGADVWRLYDTFGFP

VDLTRIMAEERGLQIDDLEFEQARLKAKEASKGEKRAATGTVKLDVHDLGKLENMNDVPKTDDSAKFERGNITAQIKALY

HKNAFHTTTEEISDGEQIGIILDRTNFYAEQGGQENDTGRIVIDGKAEIEVGDVQLYAGYVLHTGFMKYGAFAVGDTVIS

EYDELRRWPIRNNHTGTHILNFALREVLGEGIEQKGSLVAPEKLRFDFSHKAAVTDAELEKIESISTDYIRQNCAVYSQE

VSLAVAREISGVRAVFGETYPDPVRVVSVGVELEEILKNVNDPRWKEVSIEFCGGTHVQKTGDIKDLVIIEEAGIAKGIR

RMIAVTGEDAHEVQRLAKDFEQRLERLQSMSPGPEKEQEAKQVQVELSRLTISAVHKSRLRERFAAINKQILDGQKAQQK

LDSKRALETVTSYFQAPENAEKSWLVAELPISANSKVLSETLNYVKSKMQDKTVYILAADPDQGRVAHGCHVSKAFSDQG

ATANDWAAVVSSAVGGKAGGKGATCIGNGNNPVQVEEAISLATEYLGKFKI

>XP_456501.2 DEHA2A03652p [Debaryomyces hansenii CBS767]

MKVAYLLTQWTLWYSEICSIHPNLVNVIETKLTHTRPLKKLSIFLYGVHQRVISSQIKFMLKTLVHKRIMSTSSNNWPAS

RVRSTFLDYFKVKQNHTFVPSSSVVPHNDPTLLFANAGMNQYKPIFLGTVDPASDFSTLKRAVNSQKCIRAGGKHNDLED

VGRDSYHHTFFEMLGNWSFGDYFKKEAISWSWELLTEVYKLEQDRLYVTYFGGDEKQGLQPDLEAKNFWLAVGVAEDHIL

PGNLEDNFWEMGDQGPCGPCSEIHYDRIGGRNASSLVNMDDPNVLEVWNIVFMQYNREPDSSLRSLPAKHIDTGLGFERL

VSILQNKSSNYDTDVFQPIFNKIREITGVRPYTGKFGSEDRDGIDTAYRVIADHVRTLTFAITDGGVPNNEGRGYVLRRI

LRRGSRYVRKYMNYPIGSFFQQLVPVVVEQNSEIFPEILKNQQDLTEILNEEELSFAKTLDRGEKLFEQYAIIASKTPEQ

TLSGKDVWRLYDTYGFPVDLTRLMAEEAGLKIDEEGFEKAKEESREASKATNNKAGVSLIKLDVHALSELDGNDKVPKTD

DSFKYDIENIKANVVAIYNGSSFVDSISEAGSQAGILLDKTPFYAESGGQEYDTGKLVIDGVAEFSVSNVQVYGGYVLHT

GSLLEGSLKLEDEIIATYDELRRWPIRNNHTGTHVLNFALREVLGDNVDQKGSLVAPEKLRFDFSHKQGLTPDELLKVET

ISNDYIKENKQVYAKDIRLNDAKEIDGLRAVFGETYPDPVRVVSIGTSVEDLLKDPKNSEWHKVSIELCGGTHVAKTGDI

KDLVIIEESGIAKGIRRIVAVTGTEAHQVQKVASDFDKELDHTMGLASGPIKETKAKELGVSLKKLSISVLDKRSLNDKF

NKLDKSIKDQLKAVQKEESKKVLEVVNTWLNAEDAKDYLVSFVPISANAKAITEAINLLKKQHKSKSIYLITGSDKVAHG

CYVSDEAIAKGIDATNLATAVSSKVGGKAGGKGNVVQGMGDKPQGIDEAINELKKLFAEKL

>XP_007864521.1 hypothetical protein GLOTRDRAFT_110560 [Gloeophyllum trabeum ATCC 11539]

MLSRFRLHRPSVRRLSTMAANSYQGPWTARKVREEFFNYFRSKDHTFVPSSSTIPYEDPTLLFANAGMNQFKNIFLGTVD

PHSEMAKWKRAFNSQKCIRAGGKHNDLEDVGKDSYHHTFFEMLGNWSFGDYFKKEAIQFSWEVLTVVYGLPKDRLYVTYF

EGDPRNNLEPDLEAKQYWLEQGVPEDHILPGNAKDNFWEMGATGPCGPCSEIHYDRIGGRNAAHLVNQDDPDVLEIWNNV

FIQFNRETDGTLKSLPSKHVDTGMGFERLVSVIQDKRSNYDTDVFLPIFAKIQELTGVRPYAGKFGDEDVDGIDTAYRVV

ADHVRTLTFALSDGGVPSNVGRGYVLRRILRRGSRYARKKLGVTIGSFFSSLIPVVVETMGDVFPEITKNTEETKRILDE

EEESFSRTLDRGEKLFEQYASRARQQGLKELNGKDVWRLYDTYGFPEDLTRLMAAELGLGINDKEFEEAQAASKEASKAS

AKKGTKDVLKLDVHDIAALEKNSSVPKTDDSAKYSLGTISANVKAIYYNKSFVESTADIPEDATFGLILDRTNFYAESGG

QEYDQGNIVIDGAADFEVEDVQVFNGYVLHAGHLKYGAIQTGAEVICSYDELRRWPIRNNHTATHILNFCLREVLGDHID

QKGSLVAPTKLRFDFSHKAQISSQDLAKIESMSKDWIKKNVGVYSKDMDLKVAQKIPGLRAVFGESYPDPVRVVALEYDV

NEIMKDVENPKWRSTSVEFCGGTHVARTGEIKDFVIIEESGIAKGIRRITAVTGHEALEVTQVAAALRTRLDDIDRMTGK

QKDAELKAYSVELGQVDISAVLKAELRDRLAAIRKAFDKQIKEREAAANKAAVDVVTQYFKDNESSEVYVGVLDVDGNAK

ILQNVVANARKLNKAVYVFSRDAEGTRVVHANFVSEAAKSRGIDGRKWATAVSEVIGGKAGGKEDSAQGVGSDASRLEDA

VKAAQKYFEESKKD

>PIG84143.1 alanyl-tRNA synthetase [Aspergillus arachidicola]

MLRHPNHKQPGNELENFSADLMRAPKLLRDGQLALTHLLSPFRSNPRTFYHLHTSPPPPPLRSALSANTISAPFRMNSNI

QSSPEWTAKSVRDAFIQYFQKNGHTFVASAPVVPHSDPTLLFTNAGMNQFKSIFLGTVDPQSDFANLKSAVNTQKCIRAG

GKHNDLDDVGKDSYHHTFFEMLGNWSFGDYFKKEAINYSWELMTKIYGLDPQRLYVTYFEGSKEGGLSPDLEAKELWKAV

GVPEDHILPGNMKDNFWEMGDQGPCGPCSEIHYDRIGGRNASNLVNQDDPNVLEIWNNVFIQYNRESDRSLKPLPNKHVD

TGMGFERLVSVLQNKLSNYDTDVFSPLFQTIKDITGARPYQGRFGVEDSDGIDTAYRVVADHVRTLTFAISDGVAPNNEG

RGYVIRRVLRRGARYARKYFRVEIGNFFSKIVPTLVDQMGDMFPEVKRKQQDVMEILDEEEISFAKTLDRGERQFEHYAQ

QAKAKGDDKLHGADVWRLYDTFGFPVDLTRIMAEERGLRIDDTEFEEARSRAKEASKGQKKAAADTVKLDVHDLGKLEKM

NDVPKTNDSAKFDRGNITAQVKAIYHGKSFHLSTEQIPEGDQLGLILDRTNFYAEQGGQENDTGRIVIDGQAELEVGDVQ

LYAGYVLHTGFMKYGSLSVGDEVICEYDELRRWPIRNNHTGTHILNFALKEVLGDGVEQKGSLVAAEKLRFDFSHKSPVT

DKDLERIEDISTEYIRQNCAVYSQEVPLATARQISGVRAVFGETYPDPVRVVSVGVELEEILKNVKDPRWEKVSIEFCGG

THVQKTGDIKELVILEESGIAKGIRRIIAVTGEDAHEVQRVALEFEKRLDRLEAMALGPEKEQDAKQIQVDLSQLLISAV

QKSRFRERFANINKQIIDSQKAQQKLESKKALEAITSYFQNPENKDKSWLVARLPISANAKAVSESLNHVKSKLQDKTVY

VLAADSNQGRVVHGCYMSKAISDQGASASEWAAIVSTAVGGKAGGKGPTSIGNGTSPDKTDEAIALASDYLSKFKL

>OAL66015.1 alanyl-tRNA synthetase [Trichophyton rubrum]

MAASTSSQPPAGQQTVEWPALRVRDTFLDYFEKNGHTFVPSSSVVPLSDPTLLFANAGMNQYKSIFLGTVDPNSDFAQLK

RAHNSQKCIRAGGKHNDLDDVGKDSYHHTFFEMLGNWSFGDYFKKEAIQFSWELLTKVYGLDPDRLYVTYFEGNEAAGVG

PDIEARDIWRSVGVKDDHILTGDMKDNFWEMGDQGPCGPCSEIHYDRIGGRNAAHLVNQDDPDVLEIWNNVFIQYNREPD

RSLRPLPNQHVDTGLGYERLVSILQNKSSNYDTDVFTPLFTAIQNITGARPYTGKFGAEDADGIDTAYRVVADHLRTLTF

AITDGAAPNNEGRGYVVRRVLRRGVRYARKYLQVEIGSFFSKLVPALVEQMGDMFPEIRRKQTDVMEILDEEEVSFAKTL

DRGERQFENYAQQALDSGAGKLHGADVWRLYDTFGFPVDLTRLMAEERQLTIDDAEFEEARLKAKEASKGQAKTASDLLK

LTVHTLSTLEKNGVPKTDDDAKFGKGNIVANIKAICHGKEVIDSTKDIPANEQIGIILDKTNFYAEQGGQEYDTGKIVID

GQAELDVQNVQMYGGYVLHTGFIKYGNFSVNDSVIAEYDELRRWPIRNNHTGTHILNFALREVLGDGVDQKGSLVAAEKL

RFDFSHKSAISDSDLEKIEAKSTDYIRQNCAVYSQEVPLATAREITGVRAVFGETYPDPVRVVSVGVELDEILKNVKDPR

WEGVSIEFCGGTHVQKTGDIKDLVILEESGIAKGIRRIIAVTGEDAHEVQRIAREFGERLNRFEKMPASPERDAEIKSLQ

VDLNQLTISAVEKTKYRNQFTQIHKKITDAQKAAQKAEVKTAIDAISAYFEENTESPSLVIKLPITANQKAISESLNHVK

TKLSGKALYVFAADASKVAHGCYVTPDLTTKGASANEWASAVSSIVGGKAGGKAPVAIGNGTEVNKVDEALDEATKYLEK

FKL

>OAI99028.1 hypothetical protein A4X13_g7919 [Tilletia indica]

MRLSPTPSALRRFAFFPNHNPFTTNSSSARLAPPTRTLPTAHQLFYRTSMTTSTSSQFGDWPAVKVRQTFFDFFEKQNKH

TFVKSSATIPYDDPTLLFANAGMNQYKPIFLGTVDPNSAFASLKRAVNSQKCIRAGGKHNDLDDVGKDTYHHTFFEMLGN

WSFGDYFKNEAVGFAWELLTKVYKLPADRLYVTYFEGDAKLGLEPDLETRDIWRKLGVPDDHILTGNAKDNFWEMGATGP

CGPCTEIHYDRIGGGRNAAHLVNYDDPDVLEIWNNVFIQFNREADGSLRPLPAKHVDTGMGFERLVSCLQDKRSNYDTDV

FGPLFDKIRELTGARAYEGKLGAEDVDGIDTAYRVVADHVRTLTFAISDGGVPDKDGRGYVLRRILRRGTRYVRKYFQVP

IGSFFSSLVPTLVEQMGEMFPEITKKIDDVKAILDEEEESFSRTLDRGEKLFEQYATKAKQSGAKQLNGNDVWRLYDTYG

FPVDLTLIMAEELGMGVNEREFEEAQALSKEKSKGQGKKEGSEWPKFDVHDLGKLESNKDVPKTEDSAKYQLGNVSGVVK

AIYQDHAFFNSSSEVNSDKPLGILLDRTNFYAESGGQQGDTGSIVIDGQSEFAVEDCQVANGYVLHSGYFKYGELKVGDE

VVASYDELRRWPLKNNHTATHILNYALREVLGDHIDQKGSLVAPTKLRFDFSHKTGITIPELTKISELSTNWVKKNVPVY

SKELSLEVAYKIPGLRAVFGEAYPDPVRVVSLEFDVDDIAKDIENPKWRGTSVEFCGGTHVAKTGDIKDFVIIEESGIAK

GIRRVVAVTGEEAAEVNRVAADAKLRLETIDAESDRAKKDAALKAFSTELGQLEISVLRKAELNETFSTIRKKHDSEAKA

KQTAETKLVQDAVTTYFSTEQPNATFLVRQFDVGSNAKALQAGIAVAKKLNKAVYLFSEEALPADAQPQTKEEGAGTRVK

ARVSHVNFVPKQDLERGLKAKEWADVITQSIGGRAGGKDDGVQGVGEDGGPALKDALIAAERFVILKTGDSKMPLQ

>KOS45537.1 hypothetical protein ACN38_g3554 [Penicillium nordicum]

MTSLSSEWTAQRVRDTFLDYFKQNGHTFVPSSPVAPLSDPTLLFTNAGMNQFKSIFLGTVDASSEFGKMKRATNSQKCLR

AGGKHNDLDDVGKDSYHHTFFEMLGNWSFGDYFKKEAITYSWTLLTEVYGLDPDRLYVTYFEGNEAGGLEPDLEAKELWK

SVGVPESHILPGDMKDNFWEMGDQGPCGPCSEVHYDRIGGRNAAHLVNEDDPNVLEIWNNVFIQYNREPDSSLRSLPNKH

VDTGMGFERLVSILQDKSSNYDTDVFTPIFQAIQNTTGAREYRGHFGAEDADGIDTAYRVVADHVRTLMFAISDGVIPNN

EGRGYVIRRVLRRGARYARKYFQVDIGNFFAKLVPTVVNQLGEMFPELKRKQQDVIEILDEEELSFAKTLDRGERQFEQY

AQQAKVKGTDMLHGADVWRLYDTFGFPVDLTRIMAEERGLRIDDVEFEEARLKAKEASKGQKKAAENTVKLDVHDLGKLE

KMNDVQKTDDSPKFGRGNITSHVKAIYHGKTFHNSTDDVPDGAQLGVILDCTNFYAEQGGQENDTGKIIIDGQAELEVGD

VQSYAGYVLHTGFMKYGSFVVGDSVICEYDELRRWPIRNNHTGTHILNFALKEILGDGVDQKGSLVAAEKLRFDFSHKSA

ISDAELEKIEAKATDYIRQNCAVYSQELPLATAQQISGIRAVFGETYPDPVRVVSVGVELEEILQNIKDPRWQEVSIELC

GGTHVQKTGDIKDLIILEENGIAKGIRRIIAVTGEDAHEVQRVAEEFGQRLDRLNGLPLGSEKEQEAKQVQIDLNQLAIS

AVQKAKFRERFAKINKQVLDGQKAQQKMESKKAVDAITSYFEAPENQDKSWLVVQLPISANAKAVSESLNHVKSKMQGKS

VYLLAADSEQGRVSHGCYISQGLSDQGASASDWAAVVSGAVGGKAGGKGPTSIGNGTNADKVEEAISLAADYLNKFKL

>KIK68832.1 hypothetical protein GYMLUDRAFT_152556 [Gymnopus luxurians FD-317 M1]

MASSVYTGPWTAVNVREAFFTFFRSRNHTFVPSSSTIPYDDPTLLFANAGMNQYKAIFLGTVDPNSDLSKLKRAFNSQKC

IRAGGKHNDLDDVGKDSYHHTFFEMLGNWSFGDYFKKEAIAYSWELLTDVFKLPKDRLYVTYFEGDSSSGLEPDLEAKQF

WLDQGVPEDHILPGNAKDNFWEMGATGPCGPCSEIHCDRIGGRNAAHLVNQDDPDVLEIWNNVFIQFNREDDGSLKSLPS

KHVDTGMGFERLVSVVQDKRSNYDTDVFTPIFSRIQALTGVRPYQGRFGADDTDGIDTAYRVVADHVRTLTFALSDGGVP

NKEGRGYVLRRILRRGARYARKKLGVQIGSFFSSLMPIVVETMGHTFPEITKKTAEIKEILDEEEESFSRTLDRGEKLFD

QYTAQVKQQGLKTLSGKDVWRLYDTYGFPVDLTRLMAEELELEINDAEFDEAQAASKEASKATLKKSAGDVVKLDVHDIA

ALEKDDSVPKTDDSAKYNFGNITAAVKAIFYSKSFLHSTEKIPPESTFGVVLDKTSFYAESGGQEYDTGNIVIDGETDFE

VTNVQVYNGYVLHTGFLKYGQLQVGSQVVASYDELRRWPLRNNHTATHILNYCLREVLGDHIDQRGSLVAPTKLRFDFSH

KAQIGIPDLTKIEAMSVEWIQKNVKVFSKELDLQNAYKIPGLRAVFGESYPDPVRVVALEYDVDEIAKNIENPLWRKTSV

EFCGGTHVAKTGDIKDFVITEESGIAKGIRRIVAVTGHEAQEMIRLAEDFKARIVHISQLIGKDQDTGLKALSVELGHAD

ISVIKKAEMKERLAVMRKAFDKQVKEKEAAKAKLALDQFQQYFKENETAVSFIAVLDVDGNSKILQNVVMQGRKLGKAVY

VFSVDEASGKVAHVNFVPPSIRAKGADARTWATKVTDMIGGKAGGKEESAQGVGDDPSKITDAVNAARSYLSSM

>EZF33517.1 alanyl-tRNA synthetase [Trichophyton interdigitale H6]

MAASTSSQPPAGQETVEWPALRVRDTFLDYFKKNGHTFVPSSSVVPLSDPTLLFANAGMNQYKSIFLGTVDPNSDFAQLK

RAHNSQKCIRAGGKHNDLDDVGKDSYHHTFFEMLGNWSFGDYFKKEAIQFSWELLTKVYGLDPDRLYVTYFEGNEAAGVG

PDIEARDIWRSVGVKDDHILTGDMKDNFWEMGDQGPCGPCSEIHYDRIGGRNAAHLVNQDDPDVLEIWNNVFIQYNREPD

RSLRPLPNQHVDTGLGYERLVSILQNKSSNYDTDVFSPLFTAIQNITGARPYTGKFGAEDADGIDTAYRVVADHLRTLTF

AITDGAAPNNEGRGYVVRRVLRRGVRYARKYLQVEIGSFFSKLVPALVEQMGDMFPEIKRKQTDVMEILDEEEVSFAKTL

DRGERQFENYAQQALDSGAGKLHGADVWRLYDTFGFPVDLTRLMAEERQLTIDDAEFEEARLKAKEASKGQAKTASDLLK

LTVHTLSTLEKNGVPKTDDDAKFGKGNIVANIKAICHGKEVIDSTKDIPANEQIGIILDKTNFYAEQGGQEYDTGKIVID

GQAELDVQNVQMYGGYVLHTGFIKYGNFSVNDSVIAEYDELRRWPIRNNHTGTHILNFALREVLGDGVDQKGSLVAAEKL

RFDFSHKSAISDSDLEKIEAKSTDYIRQNCAVYSQEVPLATAREITGVRAVFGETYPDPVRVVSVGVELDEILKNVKDPR

WEGVSIEFCGGTHVQKTGDIKDLVILEESGIAKGIRRIIAVTGEDAHEVQRIAREFGERLNRFEKMPAGPERDAEIKSLQ

VDLNQLTISAVEKTKYRNQFTQIHKKITDAQKAAQKAEVKTAIDAISAYFEENKDSPSLVIKLPITANQKAISESLNHVK

TKLSGKALYVFAADTSKVAHGCYVTPDLTTKGASANEWASAVSSIVGGKAGGKAPVAIGNGTEVNKVDEALDEATKYLEK

FKL

>XP_023901461.1 alanine--tRNA synthetase-like [Quercus suber]

MASTTGQEPEWTAVKVRSTFIDFYKTRGHTFVPSGSVVPLADPTLLFTNAGMNQFKPIFLGTVDPSSDFATLKRAVDTQK

CIRAGGKHNDLDDVGKDSYHHTFFEMLGNWSFGDYFKEKGIALSWELLTTTFGIDPDRLYVTYFEGKPEAGLEPDHESKE

LWKAIGVADDHILPGNMKDNFWEMGDQGPCGPCSEVHYDRIGGRNAAHLVNMDDPNVLEIWNIVFIQYNREPDRTLKPLP

NKHVDTGLGYERLVSVLQDKLSNYDTDIFAPLFDRIKELTGARAYSGNFGDDDTDGVDTAYRVIADHVRLLTFAISDGAV

PNNAGRGYVVRRVLRRGARYARKYFNVEIGDFFSKIVPTLVSQMGEMFPEIKKKEAEVKDILNEEEISFAKTLDRGEAMF

EKFAAKCKASNSKTLEGADVWRLYDTYGFPEDLTRLMAEERGLEIKDEDVRAAQEKAREASKGEKKAGAELVKLNVHDIN

TLEKMPNVPKTDDSPKYQRENINSKIVAIYRGGEFLNATSEIPEGEQFGVVLDRTNFYAEQGGQENDTGRILIDGEAEID

VQNVQVYGGYVMHTGYMNYGALKVGDTVLCEFDELRRQPIRNNHTGTHILNFALREVLGDDVNQRGSLVAPEKLRFDFSH

KAGCSDAELAKIEELSTSYIRQNSEVYAVDVPLVTAREIEGVRAVFGETYPDPVRVVSIGVPVEELLEDVKRPEWRNVSV

EFCGGTHVKNTAEIKDLVVLEESGIAKGIRRIVAVTGTTAHEVQRIGKEWSDKATQLEKMSYSAEKEAKTKEWQHELGRV

EISALTKTSLRDRFGKVVKDNLEHQKAAQKAENKQVIDAVTQHFESNKDSNTLVLSVPISANSKAVQEAIKTISSKQKDK

SVYLLGKADDKVVHGCFVSSEAQSKGADASKWASEVASLLGGKAGGKGATCVGQGTETEKVDAAVEAARKYLDGLSL

>XP_024688793.1 putative alanyl-tRNA synthetase [Aspergillus campestris IBT 28561]

MRALKVLRDGELALTHLLSPHRLKPRSSYNFHTSPHPTAPHTTTVPAYTRMASDSKGSPDWTAKNVRDTFLDYFKKNGHT

FVPSSSVAPLSDPTLLFTNAGMNQYKSIFLGTVDPNSDFAKLKRAVNSQKCIRAGGKHNDLDDVGKDSYHHTFFEMLGNW

SFGDYFKKDAIRYSWELLTAVYGLDPQRLYVTYFEGDKEGGLEPDLEAKSIWREVGVPEDHILPGNMKDNFWEMGDQGPC

GPCSEIHYDRIGGRNAAKLVNEDDPNVLEIWNNVFIQYNRESDRSLRPLPSKHVDTGMGFERLVSILQNKPSNYDTDVFT

PLFEAIRIATGAREYQGLFGNDDSDGVDTAYRVVADHVRTLMFAISDDVIPNNEGRGYVVRRVLRRGARYARKYFGVEIG

GFFSKLVPTVVDQLGDMFPELVLKQNDVKEILDEEEISFAKTLDRGERQFETYAQQAKLQGGDKLNGADVWRLYDTFGFP

VDLTRIMAEERGLQIDDLEFEQARLKAKEASKGEKRAATGTVKLDVHDLGKLENMNDVPKTDDSAKFERGNITAQIKALY

HKNAFHTTTEEIPDGEQIGIILDRTNFYAEQGGQENDTGRIVIDGKAEIEVGDVQLYAGYVLHTGFMKYGAFAVGDTVIS

EYDELRRWPIRNNHTGTHILNFALREVLGEGIEQKGSLVAPEKLRFDFSYKAAVTDAELEKIESISTDYIRQNCAVYSQE

VSLAVAREISGVRAVFGETYPDPVRVVSVGVELEEILKNVNDPRWKEVSIEFCGGTHVQKTGDIKDLVIIEEAGIAKGIR

RMIAVTGEDAHEVQRLAKDFEQRLDRLQSMSPGPEKEQEAKQVQVELSRLTISAVHKSRLRERFAAINKQILDGQKAQQK

LDSKRALETVTSYFQAPENAEKSWLVAELPISANSKVLSETLNYVKSKMQDKTVYILAADPSQGRVAHGCHVSKAFSDQG

ATANDWAAVVSSAVGGKAGGKGATCIGNGNNPVQVEEAISLATEYLGKFKI

>PCH43709.1 hypothetical protein WOLCODRAFT_26125 [Wolfiporia cocos MD-104 SS10]

MSTQSSAWTSSRVRSEFFSYFQSKDHTFVPSSSTIPYEDPTLLFANAGMNQYKSIFLGTVDPQSDMARLKRAYNSQKCIR

AGGKHNDLEDVGKDSYHHTFFEMLGNWSFGDYFKKEAVAYSWELLTAVYQLPKDRLYVTYFEGDPKNGLEADLEAKQYWL

DQGVPEDHILPGNAKDNFWEMGATGPCGPCSEIHYDRIGGRNAAHLVNNDDPDVLEIWNNVFIQYNREEDGSLRPLPSKH

VDTGMGFERLVSVIQDRRSNYDTDVFTPIFARVQELTGVRPYQGKFGEEDEGGIDTAYRVVADHVRTLTFALSDGGVPNN

VGRGYVLRRILRRGCRYARKKLGVQIGSFFSSLVPVVVEQLGDVFPELTKRTAEIKEILDEEEESFSRTLDRGEKLFDQY

ASRAKAAGAKELSGKDVWRLYDTYGFPVDLTRLMAEELGLGVNETEFEQAQAASKEASKAGLKKGEKEAVKLDVHDIANL

ETNLKLLKTDDSAKYALGNINARVIAIYHEKSFHENALSIPDDALFGLILDRTNFYAESGGQEYDTGSIIVDGEAEFEVT

NVQVFSGYVLHIGRMKYGKLAVGDEVVSSYDELRRWPLRNNHTATHILNYCLRDILGDHIDQRGSLVAPTKLRFDFSHKA

QIPTKELSQIEVSSRDWVKKNVSVYSKELNLRLAQEIPGLRAVFGESYPDPVRVVSLGFSVDDIVQDIHSQKWKTTSIEL

CGGTHVAKTGEIKDFIITEESGIAKGIRRITAVTGNEAAEARRAADALRARLNTLDGLRGSEKDAGIKSLTWDLGQADIS

VLQKAELRERLDSIRKAHDKETKEKEAQANKAAVDAVVAYFESNKDAEAYFAILAEGSNAKVLQSVVQQGKKLGKTVYVF

SADPAGGKATHVNFVSPAAKAKGLDARTWAAAVAEVLGGKAGGKEDSAQGSGVNVLKVDEALQVAREYFTKQSGL

>OQE14417.1 hypothetical protein PENFLA_c038G08665 [Penicillium flavigenum]

MRVSGLVTNGERALTHLFRRNQPRTFSSCFVHSLATNPPTFDRMTSLGSEWTAQRVRDTFLDYFKQNGHTFVPSSPVAPL

SDPTLLFTNAGMNQFKSIFLGTVDASSDFGKMKRATNSQKCLRAGGKHNDLDDVGKDSYHHTFFEMLGNWSFGDYFKKEA

ITYSWTLLTEVYGLDPDRLYVTYFEGNEAGGLEPDLEAKELWKSVGVPESHILPGDMKDNFWEMGDQGPCGPCSEVHYDR

IGGRNAAHLVNEDDPNVLEIWNNVFIQYNREPDSSLRSLPNKHVDTGMGFERLVSILQNKPSNYDTDVFTPIFQAIQNTT

GAREYRGQFGADDADGIDTAYRVVADHVRTLMFAISDGVIPNNEGRGYVIRRVLRRGARYARKYFQVDIGNFFAKLVPTV

VDQLGEMFPELKRKQQDVIEILDEEELSFAKTLDRGERQFEQYAQQAKVKGTDMLHGADVWRLYDTFGFPVDLTRIMAEE

RGLRIDDVEFEEARLKAKEASKGQKKAAENTVKLDVHDLGKLEKMNDVQKTDDSPKFGRGNITAHVKAIYHGKTFHNSTD

DVPEGAQLGIILDCTNFYAEQGGQENDTGKIIIDGQAELEVGDVQSYAGYVLHTGFMKYGSFTVGNSVICEYDELRRWPI

RNNHTGTHILNFALKEVLGDGIEQKGSLVAAEKLRFDLSHKSAISDAELEKIEAKSTDYIRQNCAVYSQELPLATAQQIS

GVRAVFGETYPDPVRVVSVGVELEEILKNVKDPRWQEVSIEFCGGTHVQKTGDIKDLIILEESGIAKGIRRIIAVTGEDA

HAVQRVAEEFGQRLDRLDALPLGSEKEQEAKQVQVDLNQLVISAVQKAKFRERFAKINKQVLDGQKAQQKLESKKAVEAI

TSYFEAPENQDKSWLVVQLPISANAKAVSESLNHVKSKMQGKSVYLLAADSEQGRVSHGCYISQGLSDQGASASDWAAVV

SGAVGGKAGGKGPTSIGNGTNTDKVEEAISLAADYLEKFKL

>OOQ86394.1 Alanine--tRNA synthetase [Penicillium brasilianum]

MASDTSRPEWTAPRVRETFLEYFKQNGHTFVPSSPVAPLSDPTLLFTNAGMNQYKSIFLGTVDPSSDFAKLRRAVNTQKC

IRAGGKHNDLDDVGKDSYHHTFFEMLGNWSFGDYFKKEAITYSWTLLTEVYGLSPDRLYVTYFEGDEAGGLEPDLEAREI

WKSVGVPESHILPGNMKDNFWEMGDQGPCGPCSEVHYDRIGGRNAASLVNQDDPNVLEIWNNVFIQYNREADRSLKSLPN

KHVDTGMGFERLVSILQDKSSNYDTDVFTPIFQAIQNATGAREYRGHFGAEDSDGIDTAYRVVADHVRTLMFAISDGVIP

NNEGRGYVIRRVLRRGARFARKYFQVDIGNFFSKLVPTVVDQLGDMFPELIKKQHDVMEILDEEELSFAKTLDRGERQFE

QYAQHAKLKGSEKLHGADVWRLYDTFGFPVDLTQIMAEERGLRIDDVEFEEARLKAKEASKGQKKAAANVVKLDVHDLGK

LEKMSDVHKTDDSAKFGRGNITAHIKAIYHGKSFYESTDKAPSGEQLGVILDCTNFYAEQGGQEADTGRIVIDGQAELEV

GDVQVYAGYVLHTGFMKYGSLSVGDAVICEYDELRRWPIRNNHTGTHILNFALREVLGDGVEQKGSLVAAEKLRFDFSHK

AAVSDADLETIETKATEYIRQNCTVYSKELPLATAQQIYGVRAVFGETYPDPVRVVSVGVDLEDILRNVKDPQWEKVSIE

FCGGTHVQKTGDIKDLIILEESGIAKGIRRIIAVTGEEAHEVQRIAQEFEARLDKLEAMPLGPEKERESKQVQVDLNQLV

ISAVRKAQFRTRYTAINKQVLDGQKAQQKLESKMALEAITSYFEAPENQNSSSLVVRLPMPASAKAVSESINHVKSKMQN

KNVYVMAVDPENTRVAHGCYVSKELGELGGSASDWAAVVSSSIGGKAGGKGATSIGNGTNPDKVDEAVSLATEYLSKFKL

>OAJ22368.1 hypothetical protein A4X09_g397 [Tilletia walkeri]

MRLSPTPSALRRFAIFPNHNPFTNNSSARLAPPTRTLPTAHQLFYRTSMTTSTSSQFGDWPAVKVRQTFFDFFEKQNKHT

FVKSSATIPYDDPTLLFANAGMNQYKPIFLGTVDPNSAFASLKRAVNSQKCIRAGGKHNDLDDVGKDTYHHTFFEMLGNW

SFGDYFKNEAVGFAWELLTKVYKLPADRLYVTYFEGDAKLGLEPDLETRDIWRKLGVPDDHILTGNAKDNFWEMGATGPC

GPCTEIHYDRIGGGRNAAHLVNYDDPDVLEIWNNVFIQFNREADGSLRPLPAKHVDTGMGFERLVSCLQDKRSNYDTDVF

GPLFDKIRELTGARAYEGKLGAEDVDGIDTAYRVVADHVRTLTFAISDGGVPDKDGRGYVLRRILRRGTRYVRKYFQVPI

GSFFSSLVPTLVEQMGEMFPEITKKIDDVKAILDEEEESFSRTLDRGEKLFEQYATKAKQSGAKQLNGNDVWRLYDTYGF

PVDLTLIMAEELGMGVNEREFEEAQALSKEKSKGQGKKEGSEWPKFDVHDLGKLESNKDVPKTEDSAKYQLGNVSGAVKA

IYQDHAFFNSSSEVNSDKPLGILLDRTNFYAESGGQQGDTGSIVIDGQSEFAVEDCQVANGYVLHSGYFKYGELKVGDEV

VASYDELRRWPLKNNHTATHILNYALREVLGDHIDQKGSLVAPTKLRFDFSHKTGITIPELTKISELSMNWVKKNVPVYS

KELSLEVAYKIPGLRAVFGEAYPDPVRVVSLEFDVDDIAKDIENPKWRGTSVEFCGGTHVAKTGDIKDFVIIEESGIAKG

IRRVVAVTGEEAAEVNRVAADAKLRLETIDAESDRAKKDAALKAFSTELGQLEISVLRKAELNETFSTIRKKHDSEAKAK

QTAETKLVQDAVTTYFSTEQPNATFLVRQFDVGSNAKALQAGIAVAKKLNKAVYLFSEEALPADAQPQTKEEGAGTRVKA

RVSHVNFVPKQDLERGLKAKEWADVITQSIGGRAGGKDDGVQGVGEDGGPALKDALIAAERFVILKTGDSKMPLQ

>XP_013431212.1 alanyl-tRNA synthetase [Aureobasidium namibiae CBS 147.97]

MASQSKDQQWNAVKVRSTFIDYFVKKRGHTFVPSSSVVPHSDPTLLFANAGMNQYKSIFLGTVDPSSDFASLKRATNSQK

CIRAGGKHNDLDDVGKDSYHHTFFEMLGNWSFGDYFKKEAIAWSWELLTNEFGLDPARMYVTYFEGDKAGGLEPDLEAKE

LWKSVGVPEDHILPGNMKDNFWEMGDQGPCGPCSEIHYDRIGGRNAAHLVNMDDPNVLEIWNNVFIQYNRESDKSLRSLP

NKHVDTGMGFERLVSILQDKPSNYDTDVFTPLFDRIQEITGVRKYEGKFGSEDADGVDTAYRVVADHVRLLTFAITDGGI

PNNMGRGYVVRRVLRRGARYARKYLNTDIGDFFSKIVPTLVAQMGDMFPEIKGKEQDVKEILNEEELSFAKTLDRGEAMF

EKYAQQCKDNNTKSLSGSDVWRLYDTYGFPVDLTQLMAEERGLTINEEEVNAAQAKAKEASKGEKKAGADLVKLDVHDLG

KLDTMAEVSKTDDEPKFGRGNINATVKAIYFNKNFLSSTSEVPEGEQFGVILDKTNFYAEQGGQENDTGKILIDGEAELD

VQNVQSYAGYVLHTGYMTYGTFKTGDSVICEYDELRRHPIRNNHTGTHILNFALRAVLGDDINQKGSLVAAEKLRFDFSH

KAALSDKELQEVETKSTSYIRQNSPVYSQEVPLATARNIKGVRAVFGETYPDPVRVVSVGVPVEQLLEDVHNPQWENVSV

EFCGGTHVDKTGEIKELIVLEEGGIAKGIRRIIAVTGQAAHEVQRVAQEFSDDLALLEKMEYSPQKEQKAKALQVQLNQL

TISAVTKTQLKERFAKVSKANLDQQKAFLKAENKTVTDTIAKHFTDNPDSKRLVVRVPISANQKAISEAIKTISTKQKDK

TIYIIAADEKDGKVVHGCHVSAEAKEKGADAPAWATEVAGVLGGKAGGKGATSLGQGTQPENADQAVELATKYLEKLGL

>NP_593640.1 putative alanine--tRNA (Ala) synthetase Ala1 [Schizosaccharomyces pombe]

MTAESEVVNWPANEIRRTFLKYFEDHGHTIVPSSSVIPYDDPTLLFANAGMNQFKPIFLGTVDPSSDFAKLKRACDSQKC

IRAGGKHNDLEDVGKDNYHHTMFEMLGNWSFGDYFKKEAIAMAWDLLTNVYGLKKDQLYVTYFGGHEESGLEPDLEARQL

WLDIGIDESRVIPGSLKDNFWEMGDQGPCGPCSEIHYDRIGNRTVPELVNMDDPNVLEIWNIVFIQFNREKDGSLRPLPN

RHVDTGMGFERLVSVIQNKTSNYDTDVFSPIFAKIQELTNARPYTGKMGDEDVDGIDTAYRVVADHVRTLTFAISDGGVP

NNEGRGYVLRRILRRGARYVRKKFGVPIGNFFSRLSLTVVEQMGDFFPELKRKVDDVRELLDEEEESFSRTLDRGEKMFE

QYAAAAKKTPSKTLQGNDVWRLYETYGFPVDLTHLMAEEAGIKIDEPGFEAAQARSKEISKQASKGGSSGDDLLVLDVHA

LGALSKMDDIPETDDVFKHNSVSLKSVIKGIYHKGGFQKSTEGFNSGEQLGLLLDRTNFYAEQGGQEYDTGHIVIDGVAD

FRVTNVQVYAGYVLHTGFLEYGNLTVNDSVVCEYDEIRRWHLMNNHTVTHILNLALRNTLGDGIDQRGSLVSQEKLRFDF

SYKSSIPIDKLLLVENYCNNVIQDNLSVYSKEVALSKAKEINGLRAVFGEVYPDPVRVVCIGVDIDTLLQEPKKPDWTKY

SIEFCGGTHCDKSGEIKDFVILEENGIAKGIRRIVAVTSTEANRVSRLANEFDARIAKLEKMPFSPAKEAELKKISVDLS

KLVVAAVRKHAMKERIAKITKQVQEHVKAINAAEQKEVVNVVTEYFKENPDMSFVVAKVPISANPKALSFALTYAKKNLK

DKSIYLLASDDTKVAHACLVSPEAMKKLTPQEWSQKVCHSIGGRSGGKGDTCQGVGDKPLSIDVAVEEAIEFFQGKLTI

>EGE01186.1 alanyl-tRNA synthetase [Trichophyton equinum CBS 127.97]

MAASTSSQPPAGQETVEWPALRVRDTFLDYFKKNGHTFVPSSSVVPLSDPTLLFANAGMNQYKSIFLGTVDPNSDFAQLK

RAHNSQKCIRAGGKHNDLDDVGKDSYHHTFFEMLGNWSFGDYFKKEAIQFSWELLTKVYGLDPDRLYVTYFEGNEAAGVG

PDIEARDIWRSVGVKDDHILTGDMKDNFWEMGDQGPCGPCSEIHYDRIGGRNAAHLVNQDDPDVLEIWNNVFIQYNREPD

RSLRPLPNQHVDTGLGYERLVSILQNKSSNYDTDVFSPLFTAIQNITGARPYTGKFGAEDADGIDTAYRVVADHLRTLTF

AITDGAAPNNEGRGYVVRRVLRRGVRYARKYLQVEIGSFFSKLVPALVEQMGDMFPEIKRKQTDVMEILDEEEVSFAKTL

DRGERQFENYAQQALDSGAGKLHGADVWRLYDTFGFPVDLTRLMAEERQLTIDDAEFEEARLKAKEASKGQAKTASDLLK

LTVHTLSTLEKNGVPKTNDDAKFGKGNIVANIKAICHGKEVIDSTKDIPANEQIGIILDKTNFYAEQGGQEYDTGKIVID

GQAELDVQNVQMYGGYVLHTGFIKYGNFSVNDSVIAEYDELRRWPIRNNHTGTHILNFALREVLGDGVDQKGSLVAAEKL

RFDFSHKSAISDSDLEKIEAKSTDYIRQNCAVYSQEVPLATAREITGVRAVFGETYPDPVRVVSVGVELDEILKNVKDPR

WEGVSIEFCGGTHVQKTGDIKDLVILEESGIAKGIRRIIAVTGEDAHEVQRIAREFGERLNRFEKMSAGPERDAEIKSLQ

VDLNQLTISAVEKTKYRNQFTQIHKKITDAQKAAQKAEVKTAIDAISAYFEENKDSPSLVIKLPITANQKAISESLNHVK

TKLSGKALYVFAADTSKVAHGCYVTPDLTTKGASANEWASAVSSIVGGKAGGKAPVAIGNGTEVNKVDEALDEATKYLEK

FKL

>XP_023893788.1 alanine--tRNA synthetase isoform X1 [Quercus suber]

MRKGFVGNSKRSVLLCLLNNKNPYLSHSQSHCYSQLPQPPPLSLSHQHHHHPMVEEWPANRVRDTFIKFFEEKKHTNWKS

SPVVPHNDPTLLFANAGMNQFKPIFLGTADPNTPLSKLNRACNTQKCIRAGGKHNDLDDVGKDTYHHTFFEMLGNWSFGD

YFKADAIQWAWQLLTQVYKLPKDRFYATYFGGDEKLGLAPDAEARDIWLQLLPPERVLPFGCKDNFWEMGDTGPCGPCTE

IHYDRIGHRDAASLVNHDDPTCLEIWNLVFIQFNRESDGSLKPLPAKHVDTGMGFERLTSVLQNKMSNYDTDVFMPIFDA

IQQATGARPYSGKVGQDDLDKVDMAYRVVADHIRTLSFAIADGSCPGNEGREYVLRRILRRAVRYGSEVLKAQEGFFNGL

VNVVVRVMGDVFPELKQNEMRIRDIIAEEEASFGKTLLKGIEKFKKAAQDVQGKILSGQDAFVLWDTYGFPLDLTQLMAE

ERGLLVDVDGFNNAMDEARERSRNAQNKQAGGAIIMDAEATSALHKRGVAATDDSFKFIWFKDHESVIRAIYTGSQFLES

IGSGSEVGIVLESTSFYAEQGGQIFDTGSLEGPFGSFQVGNVQIYGGFILHIGSFSGENGRFSVGEKVICKVDYDRRKLI

APNHTCTHMLNFALREVLGNHVDQKGSIVLPEKLRFDFSHGKPVVPDHLKKIESIVNEQIKAELDVFAKEATLAEAKRIN

GLRAVFGEVYPDPVRVVAIGQKVEDLLADPENDEWLSISAELCGGTHISNTREAKAFALLSEEGIAKGIRRITAVTTDGA

FKAMELAYLLEQEVNDASKIEGSLLEKTVASLKSRVDSTSIPAAKKADIRAKISVLQNQVRKAQKKIAEENIQKAVKITI

EKAEVAASEGKAFCVSHVDVGLDAAAVREAVLKVIEQKGISAMVFSTDETTNKAVVCAGVPEKGDRNKHLEVSEWLTEAL

EPLKGRCGKGKGGLASGQGTDASQVNEAIELATAFALMKLR

>XP_004177771.1 hypothetical protein TBLA_0A04580 [Tetrapisispora blattae CBS 6284]

MTIGDKQKWTAENVRSTFLDYFKSNNHTFVPSSPVVPFDDPTLLFANAGMNQYKPIFLGTVDPSSDFYSLKRAYNSQKCI

RAGGKHNDLEDVGRDSYHHTFFEMLGNWSFGDYFKEGAITFAWNLLTKVYSIPSERLYVTYFEGDASQGLEPDLEAKQLW

LNAGVPEDHILPGDTHDNFWEMGEQGPCGPCSEIHYDRIGGRNASSLVNQDDPDVLEIWNLVFMQFNRESDHSLKKLPAC

HVDTGMGFERLVSVLQDVRSNYDTDVFQPLFTRIQEICNVRPYSGKFGAEDKDGIDTAYRVLADHVRTLTFSITDGGVPN

NEGRGYVLRRILRRGARYARKYMNYPIGNFFSTLAPTLIGQVKVIFPEVTKDPSFLYEILDEEEASFAKTLDRGEKLFEK

YVEAAAQSGTKVLDGKQVWRLYDTYGFPVDLTELMAEEHGLKIDGPGFEKAKQESYEASKRGGKKEADNLIKLNVHTLSQ

LNEAKVPKTDDSAKYGNKDIESTILKIFDGSEFVTEIKEIGKSYGIILDKTCFYAEQGGQEYDTGKLVIDGSSEFNVENV

QLYNGFVFHTGILADGSLSVGDKIIASFDELRRFPIKNNHTGTHILNFALKETLGGEVDQKGSLVAPEKLRFDFSHKKAV

SLDELAKIEAICNKQIEESLAVYYKDVPLEKARTISSVRAVFGETYPDPVRVVSVGVPVEDLMEDPTNEKWTNYSVEFCG

GTHVAKTSDIKFFVIIEESGIAKGIRRIVAVTGTEAFEVQRIANEFNSALDAAEKLPFSPAKEKKLKELGVELTQLSISV

VTKNQLKAKFTKIEKVVKDEIKARAKKETKQTLDAVQTYFTENADATYFVKHINIPTNAKAITEAINYMKSSAKDKSIYL

ITGNDPTGRVAHGCYISNVALDKGVDGAGLAKTVSGIIGGKAGGKGNVFQGMGDKPDSIEKAISEIETALKEKL

>GAT57707.1 predicted protein [Mycena chlorophos]

MSQWTAPRVRQAFFDFFQSKGHTYVPSSSTIPYEDPTLLFANAGMNQYKSIFLGTVDPNSDLSKLKRAYNSQKCIRAGGK

HNDLDDVGKDSYHHTFFEMLGNWSFGDYFKKDAIAYSWELLTEVYKLPKDRLYVTYFEGDAKNKLEPDLEAKEFWLAQGV

PEDHILPGNAKDNFWEMGATGPCGPCSEIHYDRIGGRNAAHLVNQDDPDVLEIWNNVFIQFNREDDGSLKQLPSKHVDTG

MGFERLVSVVQDKRSNYDTDVFSPIFAKIQELTGVRPYEGRFGAEDADGIDTAYRVVADHVRTLTFALSDGGVPNNVGRG

YVLRRILRRGARYARKKLGVTIGSFFSSLMPVLVETMGDTFPEITKKLDEIKEILDEEEESFSRTLDRGERLFDQFASRA

LAQGVKELNGKDVWRLYDTFGFPVDLTQLMAEELGLGINGEEFEAAQAVSKEASKASLKKNTTDAVKLDVHDIAALEKNS

EVPKTDDSAKFALGNTTATVKCIFYNKSFVASTADIPENTSFGVLLDKTSFYAESGGQEHDTGNIVIDGTADFEVTDVQV

FNGYVLHVGHLKYGQLDVGSQVVASYDELRRWPLRNNHTATHILNFSLREVLGDHIDQRGSLVAPTKLRFDFSHKAQIGV

PELQKIEDMSKDWIKRNVKVYSKELDLQTAHKIPGLRAVFGESYPDPVRVVTLEYDVDEIAQDITNPKWRSTSVEFCGGT

HVAKTGDIKDFVITEESGIAKGIRRIIAVTGHEAQEVARIAETFKIRLDQADFLQGAEKDAALKTLNVELGQSDMSVIRK

TELKERLASIRKAFDKQTKEREAAALKESVDALQRYFQENEGAQAYIAAVSVDGNAKMLQTLLTHGRKLDKAVYVFSSDG

SKVAHANYIPPSIKAKGADARTWAAKVTEVLGGKAGGKEDSVQGVGTNVDKVDAAVESGKAYLSAYL

>XP_009557499.1 PREDICTED: alanine--tRNA synthetase, mitochondrial, partial [Cuculus canorus]

RDAFLRFFEERHGHRRLPSAPVRPRGDPRLLFVNAGMNQFKPIFLGTVHPRSELSRHRRVVNSQKCVRAGGKHNDLEDVG

RDTYHHTFFEMLGNWSFGDYFKEEACSMAWELLTEVYEIPKDRLYVTYFGGDSLLGLSADEECRDVWLRLGVPASHVLPF

PLKDNFWEMGDTGPCGPCTEIHYDYVGGGRNAAALVNQGNPDVVEIWNLVFMQYNREVEGNLLPLPQHHVDTGMGLERLV

TVLQNKRSNYDTDLFTPILDAIHKGCRGPRYQGLVGDADVGRVNMAYRVVADHVRTLCVCITDGIYPGLSGAELVLRRIL

RRAVRFSSEVLHAPPGLLASLVPTVVEVLGDAYPELTKDADQIMDIINENEAAFLSSLERGRRVIDQTVQQLEPSTNFPA

EVAWSLYANLGFPLDLIDLMLEEKGISLDSTAFNELVLEDAKRKAGGLQGGPLEHPNTHLDVHSLAQLRSNNVPATDDSP

KYAYTLGQHGQYEFSPCKATVLMLYRDQSLQEEVGAGQRCGVILDRTNFYAEQGGQASDQGYMIRLGQQDVLFPVKLVHI

CGGYVIHEVITVETLRAGDQVQLFVDEAQRLACMTNHTATHLLHFALHHVLGDNTEQRGSHVTAERLRFDFDVKGPVTVE

QLKQVEQVVQGMIKRNEVVHMAEVPLTLARRVQGLRAVDEDYPDPVRIVSLGVPVESVLARDSEAAMQTSVELCCGTHLL

QTGAVEDLAIINERQLVKGISRVIAVTGGQAKQAREVGQSLAMEVDSVSLRMKQRNTSIPEMQNLSKEVGQLTEVVNSTA

IPQWQRKELQTILKALQRTANTAIKKLEVQQAEKKAQSLLAKHSNQPVIIDTVPAESLSILMKVVNQLCDKSPGTSVLLL

SPQASGEVLCACQVSKNYLPVFSAADWAVAVCAQMEGKAGGSPVVAKGSGNAKGIQGALTTALEFAQSKL

>XP_007328463.1 hypothetical protein AGABI1DRAFT_112599, partial [Agaricus bisporus var. burnettii JB137-S8]

MSTQAYTGPWTAPRIRDAFFDYFRARNHSFVPSSPTIPYEDPTLLFANAGMNQYKSIFLGTVDPNSDHAKLRRAFNSQKC

IRAGGKHNDLDDVGKDSYHHTFFEMLGNWSFGDYFKKEAVGYSWEVLTEVYKLPKDRLYVTYFEGDPKTGLEPDLEAKQY

WLDQGVDEDHILPGNAKDNFWEMGATGPCGPCSEIHFDRIGGRNAAHLVNKDDPDVLEIWNNVYIQFNREEDGSLRPLPS

KHVDTGLGFERLVSIIQDKHSNYDTDVFLPLFTRIQELTGVRPYQGKFGEEDTDGIDTAYRVVADHVRTLTFALSDGGVP

NNIGRGYVLRRILRRGSRFARKKLGVPIGSFFSSLVPTVIETMGAVFPEITKKYDEIKEILDEEEESFSRTLDRGEKLFD

QYAKKAKEQGLQLLDGKDVWRLYDTYGFPVDLTLLMAEEMGLGINQKEFDEAQAWSKEASKGPTKKGVSDMVKLDVHDIA

TLEKNPNVPKTNDSAKFGTGNITATIKAIFHNKAFVSSTENIPGYGTFGLLLDKTSFYAEAGGQEYDTGNIVIDGVADFE

VTNVQVYKGYVLHTGYLKYGTLEVGNEVISSYDELRRWPLRNNHTGTHILNFSLREILGDHIDQKGSLVAPSKLRFDFSH

KSQISLSELAKIQEMCTDWIRRNVKVYGKDLDLQTAYKIPGLRAVFGESYPDPVRVVSLEYDVDEIAKDIENPKWRQTSI

EFCGGTHVARTGDIKDIVVVEESGIAKGIRRIVAVTGQEAHDVTQLADTFRDRLNNLERLSGKEKDANLKAFAVELGQAE

ISVTRKADLKERLAAIRKAFDKQVKEKEAAANKEAIDHVMKYFESNEKAEVFIGTVDVEGNAKILQNIVSQARKLGKAVY

VFSLDPSSGKVSHVNFISNSLKEKGADARVWATKVSEILGGKAGGKDDSAQGMGINSDRLEEALEVAQSYLSTL

>EGD95966.1 alanyl-tRNA synthetase [Trichophyton tonsurans CBS 112818]

MAASTSSQPPAGQETVEWPALRVRDTFLDYFKKNGHTFVPSSSVVPLSDPTLLFANAGMNQYKSIFLGTVDPNSDFAQLK

RAHNSQKCIRAGGKHNDLDDVGKDSYHHTFFEMLGNWSFGDYFKKEAIQFSWELLTKVYGLDPDRLYVTYFEGNEAAGVG

PDIEARDIWRSVGVKDDHILTGDMKDNFWEMGDQGPCGPCSEIHYDRIGGRNAAHLVNQDDPDVLEIWNNVFIQYNREPD

RSLRPLPNQHVDTGLGYERLVSILQNKSSNYDTDVFSPLFTAIQNITGARPYTGKFGAEDADGIDTAYRVVADHLRTLTF

AITDGAAPNNEGRGYVVRRVLRRGVRYARKYLQVEIGSFFSKLVPALVEQMGDMFPEIKRKQTDVMEILDEEEVSFAKTL

DRGERQFENYAQQALDSGAGKLHGADVWRLYDTFGFPVDLTRLMAEERQLTIDDAEFEEARLKAKEASKGQAKTASDLLK

LTVHTLSTLEKNGVPKTNDDAKFRKGNIVANIKAICHGKEVIDSTKDIPANEQIGIILDKTNFYAEQGGQEYDTGKIVID

GQAELDVQNVQMYGGYVLHTGFIKYGNFSVNDSVIAEYDELRRWPIRNNHTGTHILNFALREVLGDGVDQKGSLVAAEKL

RFDFSHKSAISDSDLEKIEAKSTDYIRQNCAVYSQEVPLATAREITGVRAVFGETYPDPVRVVSVGVELDEILKNVKDPR

WEGVSIEFCGGTHVQKTGDIKDLVILEESGIAKGIRRIIAVTGEDAHEVQRIAREFGERLNRFEKMSAGPERDAEIKSLQ

VDLNQLTISAVEKTKYRNQFTQIHKKITDAQKAAQKAEVKTAIDAISAYFEENKDSPSLVIKLPITANQKAISESLNHVK

TKLSGKALYVFAADTSKVAHGCYVTPDLTTKGASANEWASAVSSIVGGKAGGKAPVAIGNGTEVNKVDEALDEATKYLEK

FKL

>PPQ89537.1 hypothetical protein CVT25_012209 [Psilocybe cyanescens]

MSVLRPSISSMAVTPYTGPWSAPKVRQQFYDYFRSKNHVFVPSSPTIPFEDPTLLFANAGMNQYKSIFLGTVDPNSEMSK

LKRAFNSQKCIRAGGKHNDLEDVGRDSYHHTFFEMLGNWSFGDYFKTEAIEYSWELLTKVFQLPADRIYVTYFEGDPKAG

LEPDLEAKQFWIDRGVAEDHIIPGNAKDNFWEMGATGPCGPCSEIHYDRIGGRNAAHLVNQDDPNVLEIWNNVFIQFNRE

EDGSLRSLPSKHVDTGMGFERLVSVIQDRTSNYDTDIFLPIFDKVHELTGVRPYEGRFGADDVDGIDTAYRVVADHVRTL

TFALSDGGVPNNVGRGYVLRRILRRGARYARKKLGVNIGSFFSSLMPVVIDTMGEAFPEITKKFDEIKEILDEEEESFSR

TLDRGEKLFDQFATRAKEQGLTQLSGKDVWRLYDTFGFPVDLTRLMAEELGLTINEEEFEAAQAHSKEASKASTKKSSLI

AVKLDVHDIASLEKNDKIPKTDDSAKFNLGNITSTVKAIFHEKSFLESTEDIPEDSTFGIILDRTSFYAESGGQEYDTGN

IVIDGVADFEVSNVQVYNGYVLHTGHLKYGQLNVEDEVVSSYDELRRWPLRNNHTATHILNYSLREILGDHIDQKGSLVA

PTKLRFDFSHKAQIALPELQKIETMSIDWIKRNVRVFSKDLSLETAQRIPGLRAVFGESYPDPVRVVTLEYDVDEISKDI

ENPKWRKTSVEFCGGTHVAKTGDIKDFVITEESGIAKGIRRVVAVTGHEAQEVTRLARSLEEKLDALEASSGKEKDSGLK

TFSVELGQTDISVLKKAQLKDRLALIRKAHDKQIKDRETQANKVAVEQLQQYFKENTTSDVYVASIDVDGNAKILQSLVS

QAKQLDKSLYVFSTDVENNKVAHANFVSSSLKARGGDARTWASKVTDIIGGKAGGKEDSAQGVGINADKADEALVAAKEY

LTGFKSS

>POE49453.1 alanine--trna synthetase [Quercus suber]

MFQRSLPSKLLSTRYTLHSRFWQTARYTSPPYKMASTTGQEPEWTAVKVRSTFIDFYKTRGHTFVPSGSVVPLADPTLLF

TNAGMNQFKPIFLGTVDPSSDFATLKRAVDTQKCIRAGGKHNDLDDVGKDSYHHTFFEMLGNWSFGDYFKEKGIALSWEL

LTTTFGIDPDRLYVTYFEGKPEAGLEPDHESKELWKAIGVADDHILPGNMKDNFWEMGDQGPCGPCSEVHYDRIGGRNAA

HLVNMDDPNVLEIWNIVFIQYNREPDRTLKPLPNKHVDTGLGYERLVSVLQDKLSNYDTDIFAPLFDRIKELTGARAYSG

NFGDDDTDGVDTAYRVIADHVRLLTFAISDGAVPNNAGRGYVVRRVLRRGARYARKYFNVEIGDFFSKIVPTLVSQMGEM

FPEIKKKEAEVKDILNEEEISFAKTLDRGEAMFEKFAAKCKASNSKTLEGADVWRLYDTYGFPEDLTRLMAEERGLEIKD

EDVRAAQEKAREASKGEKKAGAELVKLNVHDINTLEKMPNVPKTDDSPKYQRENINSKIVAIYRGGEFLNATSEIPEGEQ

FGVVLDRTNFYAEQGGQENDTGRILIDGEAEIDVQNVQVYGGYVMHTGYMNYGALKVGDTVLCEFDELRRQPIRNNHTGT

HILNFALREVLGDDVNQRGSLVAPEKLRFDFSHKAGCSDAELAKIEELSTSYIRQNSEVYAVDVPLVTAREIEGVRAVFG

ETYPDPVRVVSIGVPVEELLEDVKRPEWRNVSVEFCGGTHVKNTAEIKDLVVLEESGIAKGIRRIVAVTGTTAHEVQRIG

KEWSDKATQLEKMSYSAEKEAKTKEWQHELGRVEISALTKTSLRDRFGKVVKDNLEHQKAAQKAENKQVIDAVTQHFESN

KDSNTLVLSVPISANSKAVQEAIKTISSKQKDKSVYLLGKADDKVVHGCFVSSEAQSKGADASKWASEVASLLGGKAGGK

GATCVGQGTETEKVDAAVEAARKYLDGLSL

>PIL35624.1 hypothetical protein GSI_02352 [Ganoderma sinense ZZ0214-1]

MRSSRMLLRGLRPSLHRHRFCPPRPLARMGSSQTPHLTAAQVRDQFFNFFKSKEHPYVASSSTIPYEDPTLLFANAGMNQ

FKAIFLGTVDPQSELARLKRAFNSQKCIRAGGKHNDLEDVGKDSYHHTFFEMLGNWSFGDYFKKEAISYSWELLTEVYKL

PKDQLYVTYFEGDLKNGLEPDLEAKQYWLDTGVAEDHIIPGNAKDNFWEMGATGPCGPCSEIHFDRIGGRNAAHLVNQDD

PDVLEIWNNVFIQFNREEDGSLKGLPAKHVDTGMGFERLVSVVQNKRSNYDTDVFAPLFTKIQELTGVRPYAGKFGDEDP

DGIDTAYRVIADHVRTLTFALSDGGVPNNVGRGYVLRRILRRGARFARKKLGVPIGSFFSSLVPVVIEQMGSVFPEITQK

YDEIKEILDEEEESFSRTLDRGEKLFEQYSKKTLDAGQKELNGKDVWRLYDTYGFPVDLTRLMAEELGLSVNEAEFEAAQ

AASKEASKAGVKKGAGETVALDVHDIAALEKNADVPKTNDSFKFQRGSINAKVVAIYNKKAFLRDTEAVPEDTTFGLILD

RTSFYAEAGGQEYDTGSIIIDGVAEFEVTNVQVFNGYVLHIGHMKYGKLNVGDEVVSSYDELRRWPLRNNHTATHILNYA

LRDVLGDHIDQKGSLVAPTKLRFDFSHKAQVTQAELEKIEGSSQEWVRRNVGVYARELALPVAQKIYSLRAVFGESYPDP

VRVVSLEYDVEEIAADLENPKWRKTSIELCGGTHVAKTGEIKDFIVTEESGIAKGIRRVVAVTGHEAAEARRQGESLTAL

LNRIENLPDAEKDAALKAYTVQIGQADISLLKKNELRDRLGAMRKAFDKYLKDKEAAANKAAGEALQQYFKEKPDEAVYF

AVIDVNANAKVLQSVVLQAKKLEKSIYIFSADTANGKVAHVNVVSDAAKAKGLDGREWAATVVEILGGKAGGKPDGAQGV

GTEISKVQDALEAARKYFQTKTSS

>OQE07055.1 hypothetical protein PENVUL_c015G06651 [Penicillium vulpinum]

MRVSGVVTNGERALTHLFRRNQPRPFHPRFVHSRSTNPPKFDRMTSLGAEWTAQRVRDTFLDYFKQNGHTFVPSSPVAPL

SDPTLLFTNAGMNQFKSIFLGTVDASSEFGKMKRATNSQKCLRAGGKHNDLDDVGKDSYHHTFFEMLGNWSFGDYFKKEA

ITYSWTLLTEVYGLDPNRLYVTYFEGNEAGGLEPDLEAKELWKSVGVPESHILPGNMQDNFWEMGDQGPCGPCSEVHYDR

IGGRNAAHLVNQDDPNVLEIWNNVFIQYNREPDSSLRSLPNKHVDTGMGFERLVSILQDKSSNYDTDVFTPIFQAIQDTT

GAREYRGHFGADDVDGIDTAYRVVADHVRTLMFAISDGVIPNNEGRGYVIRRVLRRGARYARKYFQVDIGNFFAKLVPTV

VDQLGEMFPELKRKQQDVIEILDEEELSFAKTLDRGERQFEQYAQQAKLKDTDMLHGADVWRLYDTFGFPVDLTRIMAEE

RGLRIDDVEFEEARLKAKEASKGQKKAAGDTVKLDVHDLGKLEKMNDVQKTDDSPKFGRGNITSHVKAIYHGKTFHSSTN

DIPEGAQLGVILDCTNFYAEQGGQENDTGKIIIDGQAELEVGDVQSYAGYVLHTGFMKYGSFAVGDSVICEYDELRRWPI

RNNHTGTHILNFALKEILGDGVDQKGSLVAAEKLRFDFSHKSAISDAELEKIEAKATEYIRQNCTVYSKELPLATAQQIS

GVRAVFGETYPDPVRVVSVGVELEEILKNVKDPRWQEVSIEFCGGTHVQKTGDIKDLIILEESGIAKGIRRIIAVTGEDA

HEVQRVAEEFGQRLDQLNALPLGPKKEQEAKQVQVDLNQLAISAVQKAKFRERFAKINKQVLDGQKAQQKLESKKALDAI

NSYFEAPENQDKSWLVVQLPISANSKAVSESLNHVKSKLQGKSVYLLAADSEQGRVAHGCFVSQGLSDQGASASEWAAVV

SGAVGGKAGGKGPTSIGNGTNADKVEEAISLAADYLNKFKL

## **Sequences used to build HMM for Methioninyl—tRNA synthetase (MRS)**

>NP_004981.2 methionine--tRNA synthetase, cytoplasmic [Homo sapiens]

MRLFVSDGVPGCLPVLAAAGRARGRAEVLISTVGPEDCVVPFLTRPKVPVLQLDSGNYLFSTSAICRYFFLLSGWEQDDL

TNQWLEWEATELQPALSAALYYLVVQGKKGEDVLGSVRRALTHIDHSLSRQNCPFLAGETESLADIVLWGALYPLLQDPA

YLPEELSALHSWFQTLSTQEPCQRAAETVLKQQGVLALRPYLQKQPQPSPAEGRAVTNEPEEEELATLSEEEIAMAVTAW

EKGLESLPPLRPQQNPVLPVAGERNVLITSALPYVNNVPHLGNIIGCVLSADVFARYSRLRQWNTLYLCGTDEYGTATET

KALEEGLTPQEICDKYHIIHADIYRWFNISFDIFGRTTTPQQTKITQDIFQQLLKRGFVLQDTVEQLRCEHCARFLADRF

VEGVCPFCGYEEARGDQCDKCGKLINAVELKKPQCKVCRSCPVVQSSQHLFLDLPKLEKRLEEWLGRTLPGSDWTPNAQF

ITRSWLRDGLKPRCITRDLKWGTPVPLEGFEDKVFYVWFDATIGYLSITANYTDQWERWWKNPEQVDLYQFMAKDNVPFH

SLVFPCSALGAEDNYTLVSHLIATEYLNYEDGKFSKSRGVGVFGDMAQDTGIPADIWRFYLLYIRPEGQDSAFSWTDLLL

KNNSELLNNLGNFINRAGMFVSKFFGGYVPEMVLTPDDQRLLAHVTLELQHYHQLLEKVRIRDALRSILTISRHGNQYIQ

VNEPWKRIKGSEADRQRAGTVTGLAVNIAALLSVMLQPYMPTVSATIQAQLQLPPPACSILLTNFLCTLPAGHQIGTVSP

LFQKLENDQIESLRQRFGGGQAKTSPKPAVVETVTTAKPQQIQALMDEVTKQGNIVRELKAQKADKNEVAAEVAKLLDLK

KQLAVAEGKPPEAPKGKKKK

>BAF83546.1 unnamed protein product [Homo sapiens]

MRLFVSDGVPGCLPVLAAAGRARGRAEVLISTVGPEDCVVPFLTRPKVPVLQLDSGNYLFSTSAICRYFFLLSGWEQDDL

TNQWLEWEATELQPALSAALYYLVVQGKKGEDVLGSVRRALTHIDHSLSRQNCPFLAGETESLADIVLWGALYPLLQDPA

YLPEELSALHSWFQTLSTQEPCQRAAETVLKQQGVLALRPYLQKQPQPSPAEGRAVTNEPEEEELATLSEEEIAMAVTAW

EKGLESLPPLRPQQNPVLPVAGERNVLITSALPYVNNVPHLGNIIGCVLSADVFARYSRLRQWNTLYLCGTDEYGTATET

KALEEGLTPQEICDKYHIIHADIYRWFNISFDIFGRTTTPQQTKITQDIFQQLLKRGFVLQDTVEQLRCEHCARFLADRF

VEGVCPFCGYEEARGDQCDKCGKLINAVELKKPQCKVCRSCPVVQSSQHLFLDLPKLEKRLEEWLGRTLPGSDWTPNAQF

ITRSWLRDGLKPRCITRDLKWGTPVPLEGFEDKVFYVWFDATIGYLSITANYTDQWERWWKNPEQVDLYQFMAKDNVPFH

SLVFPCSALGAEDNYTLVSHLIATEYLNYEDGKFSKSRGVGVFGDMAQDTGIPADIWRFYLLYIRPEGQDSAFSWTDLLL

KNNSELLNNLGNFINRAGMFVSKFFGGYVPEMVLTPDDQRLLAHVTLELQHYHQLLEKVRIRDALRSILTISRHGNQYIQ

VNEPWKRIKGSEADRQRAGTVTGLAVNIAALLSVMLQPYMPTVSATIQAQLQLPPPACSILLTNFLCTLPAGHQIGTVSP

LFQRLENDQIESLRQRFGGGQAKTSPKPAVVETVTTAKPQQIQALMDEVTKQGNIVRELKAQKADKNEVAAEVAKLLDLK

KQLAVAEGKPPEAPKGKKKK

>BAD96487.1 methionine-tRNA synthetase variant, partial [Homo sapiens]

MRLFVSDGVPGCLPVLAAAGRARGRAEVLISTVGPEDCVVPFLTRPKVPVLQLDSGNYLFSTSAICRYFFLLSGWEQDDL

TNQWLEWEATELQPALSAALYYLVVQGKKGEDVLGSVRRALTHIDHSLSRQNCPFLAGETESLADIVLWGALYPLLQDPA

YLPEELSALHSWFQTLSTQEPCQRAAETVLKQQGVLALRPYLQKQPQPSPAEGRAVTNEPEEEELATLSEEEIAMAVTAW

EKGLESLPPPRPQQNPVLPVAGERNVLITSALPYVNNVPHLGNIIGCVLSADVFARYSRLRQWNTLYLCGTDEYGTATET

KALEEGLTPQEICDKYHIIHADIYRWFNISFDIFGRTTTPQQTKITQDIFQQLLKRGFVLQDTVEQLRCEHCARFLADRF

VEGVCPFCGYEEARGDQCDKCGKLINAVELKKPQCKVCRSCPVVQSSQHLFLDLPKLEKRLEEWLGRTLPGSDWTPNAQF

ITRSWLRDGLKPRCITRDLKWGTPVPLEGFEDKVFYVWFDATIGYLSITANYTDQWERWWKNPEQVDLYQFMAKDNVPFH

SLVFPCSALGAEDNYTLVSHLIATEYLNYEDGKFSKSRGVGVFGDMAQDTGIPADIWRFYLLYIRPEGQDSAFSWTDLLL

KNNSELLNNLGNFINRAGMFVSKFFGGYVPEMVLTPDDQRLLAHVTLELQHYHQLLEKVRIRDALRSILTISRHGNQYIQ

VNEPWKRIKGSEADRQRAGTVTGLAVNIAALLSVMLQPYMPTVSATIQAQLQLPPPACSILLTNFLCTLPAGHQIGTVSP

LFQKLENDQIESLRQRFGGGQAKTSPKPAVVETVTTAKPQQIQALMDEVTKQGNIVRELKAQKADKNEVAAEVAKLLDLK

KQLAVAEGKPPEAPKGKKKK

>AIC54737.1 MARS, partial [synthetic construct]

MRLFVSDGVPGCLPVLAAAGRARGRAEVLISTVGPEDCVVPFLTRPKVPVLQLDSGNYLFSTSAICRYFFLLSGWEQDDL

TNQWLEWEATELQPALSAALYYLVVQGKKGEDVLGSVRRALTHIDHSLSRQNCPFLAGETESLADIVLWGALYPLLQDPA

YLPEELSALHSWFQTLSTQEPCQRAAETVLKQQGVLALRPYLQKQPQPSPAEGRAVTNEPEEEELATLSEEEIAMAVTAW

EKGLESLPPLRPQQNPVLPVAGERNVLITSALPYVNNVPHLGNIIGCVLSADVFARYSRLRQWNTLYLCGTDEYGTATET

KALEEGLTPQEICDKYHIIHADIYRWFNISFDIFGRTTTPQQTKITQDIFQQLLKRGFVLQDTVEQLRCEHCARFLADRF

VEGVCPFCGYEEARGDQYDKCGKLINAVELKKPQCKVCRSCPVVQSSQHLFLDLPKLEKRLEEWLGRTLPGSDWTPNAQF

ITRSWLRDGLKPRCITRDLKWGTPVPLEGFEDKVFYVWFDATIGYLSITANYTDQWERWWKNPEQVDLYQFMAKDNVPFH

SLVFPCSALGAEDNYTLVSHLIATEYLNYEDGKFSKSRGVGVFGDMAQDTGIPADIWRFYLLYIRPEGQDSAFSWTDLLL

KNNSELLNNLGNFINRAGMFVSKFFGGYVPEMVLTPDDQRLLAHVTLELQHYHQLLEKVRIRDALRSILTISRHGNQYIQ

VNEPWKRIKGSEADRQRAGTVTGLAVNIAALLSVMLQPYMPTVSATIQAQLQLPPPACSILLTNFLCTLPAGHQIGTVSP

LFQKLENDQIESLRQRFGGGQAKTSPKPAVVETVTTAKPQQIQALMDEVTKQGNIVRELKAQKADKNEVAAEVAKLLDLK

KQLAVAEGKPPEAPKGKKKK

>XP_009423338.1 methionine--tRNA synthetase, cytoplasmic isoform X2 [Pan troglodytes]

MRLFVSEGVPGCLPVLAAAGRARGRAEVLISTVGPEDCVVPFLTRPKVPVLQLDSGNYLFSTSAICRYFFLLSGWEQDDL

TNQWLEWEATELQPALSAALYYLVVQGKKGEDVLGSVRRALTHIDHSLSRQNCPFLAGETESLADIVLWGALYPLLQDPA

YLPEELSALHSWFQTLSTQEPCQRAAETVLKQQGVLALRPYLQKQPQPSPTEGRAVTNEPEEEELATLSEEEIAMAVTAW

EKGLESLPPLRPQQNPVLPVAGERNVLITSALPYVNNVPHLGNIIGCVLSADVFARYSRLRQWNTLYLCGTDEYGTATET

KALEEGLTPQEICDKYHIIHADIYRWFNISFDIFGRTTTPQQTKITQDIFQQLLKRGFVLQDTVEQLRCEHCARFLADRF

VEGVCPFCGYEEARGDQCDKCGKLINAVELKKPQCKVCRSCPVVQSTQHLFLDLPKLEKRLEEWLGRTLPGSDWTPNAQF

ITRSWLRDGLKPRCITRDLKWGTPVPLEGFEDKVFYVWFDATIGYLSITANYTDQWERWWKNPEQVDLYQFMAKDNVPFH

SLVFPCSALGAEDNYTLVSHLIATEYLNYEDGKFSKSRGVGVFGDMAQDTGIPADIWRFYLLYIRPEGQDSAFSWTDLLL

KNNSELLNNLGNFINRAGMFVSKFFGGYVPEMVLTPDDQRLLAHVTLELQHYHQLLEKVRIRDALRSILTISRHGNQYIQ

VNEPWKRIKGSEADRQRAGTVTGLAVNIAALLSVMLQPYMPTVSATIQAQLQLPPPACSILLTNFLCTLPAGHQIGTVSP

LFQKLENDQIESLRQRFGGGQAKTSPKPAVVETVTTARPQQIQALMDEVTKQGNIVRELKAQKADKNEVAAEVAKLLDLK

KQLAVAEGKPPEAPKGKKKK

>AAH15011.1 MARS protein, partial [Homo sapiens]

RLFVSDGVPGCLPVLAAAGRARGRAEVLISTVGPEDCVVPFLTRPKVPVLQLDSGNYLFSTSAICRYFFLLSGWEQDDLT

NQWLEWEATELQPALSAALYYLVVQGKKGEDVLGSVRRALTHIDHSLSRQNCPFLAGETESLADIVLWGALYPLLQDPAY

LPEELSALHSSFQTLSTQEPCQRAAETVLKQQGVLALRPYLQKQPQPSPAEGRAVTNEPEEEELATLSEEEIAMAVTAWE

KGLESLPPLRPQQNPVLPVAGERNVLITSALPYVNNVPHLGNIIGCVLSADVFARYSRLRQWNTLYLCGTDEYGTATETK

ALEEGLTPQEICDKYHIIHADIYRWFNISFDIFGRTTTPQQTKITQDIFQQLLKRGFVLQDTVEQLRCEHCARFLADRFV

EGVCPFCGYEEARGDQCDKCGKLINAVELKKPQCKVCRSCPVVQSSQHLFLDLPKLEKRLEEWLGRTLPGSDWTPNAQFI

TRSWLRDGLKPRCITRDLKWGTPVPLEGFEDKVFYVWFDATIGYLSITANYTDQWERWWKNPEQVDLYQFMAKDNVPFHS

LVFPCSALGAEDNYTLVSHLIATEYLNYEDGKFSKSRGVGVFGDMAQDTGIPADIWRFYLLYIRPEGQDSAFSWTDLLLK

NNSELLNNLGNFINRAGMFVSKFFGGYVPEMVLTPDDQRLLAHVTLELQHYHQLLEKVRIRDALRSILTISRHGNQYIQV

NEPWKRIKGSEADRQRAGTVTGLAVNIAALLSVMLQPYMPTVSATIQAQLQLPPPACSILLTNFLCTLPAGHQIGTVSPL

FQKLENDQIESLRQRFGGGQAKTSPKPAVVETVTTAKPQQIQALMDEVTKQGNIVRELKAQKADKNEVAAEVAKLLDLKK

QLAVAEGKPPEAPKGKKKK

>CAA64381.1 yeast methionyl-tRNA synthetase homolog [Homo sapiens]

MRLFVSDGVPGCLPVLAAAGRARGRAEVLISTVGPEDCVVPFLTRPKVPVLQVDSGNYLFSTSAICRYFFLLSGWEQDDL

TNQWLEWEATELQPALSAPLYYLVVQGKKGEDVLGSVRRALTHIDHSLSRQNCPFLAGETESLADIVLWGAQYPLLQDPA

YLPEELSALHSWFQTLSTQEPCQRAAETVLKQQGVLALRPYLQKQPQPSPAEGRAVTNEPEEEELATLSEEEIAMAVTAW

EKGLESLPPLRPQQNPVLPVAGERNVLITSALPYVNNVPHLGNIIGCVLSADVFARYSRLRQWNTLYLCGTDEYGTATET

KALEEGLTPQEICDKYHIIHADIYRWFNISFDIFGRTTTPQQTKITQDIFQQLLKRGFVLQDTVEQLRCEHCARFLADRF

VEGVCPFCGYEEARGDQCDKCGKLINAVELKKPQCKVCRSCPVVQSSQHLFLDLPKLEKRLEEWLGRTLPGSDWTPNAQF

ITRSWLRDGLKPRCITRDLKWGTPVPLEGFEDKVFYVWFDATIGYLSITANYTDQWERWWKNPEQVDLYQFMAKDNVPFH

SLVFPCSALGAEDNYTLVSHLIATEYLNYEDGKFSKSRGVGVFGDMAQDTGIPADIWRFYLLYIRPEGQDSAFSWTDLLL

KNNSELLNNLGNFINRAGMFVSKFFGGYVPEMVLTPDDQRLLGHVTLELQHYHQLLEKVRIRDALRSILTISRHGNQYIQ

VNEPWKRIKGSEADRQRAGTVTGLAVNIAALLSVMLQPYMPTVSATIQAQLQLPPPACSILLTNFLCTLPAGHQIGTVSP

LFQKLENDQIESLRQRFGGGQAKTSPKPAVVETVTTAKPQQIQALMDEVTKQGNIVRELKAQKADKNEVAAEVAKLLDLK

KQLAVAEGKPPEAPKGKKKK

>XP_009423337.1 methionine--tRNA synthetase, cytoplasmic isoform X1 [Pan troglodytes]

MRLFVSEGVPGCLPVLAAAGRARGRAEVLISTVGPEDCVVPFLTRPKVPVLQLDSGNYLFSTSAICRYFFLLSGWEQDDL

TNQWLEWEATELQPALSAALYYLVVQGKKGEDVLGSVRRALTHIDHSLSRQNCPFLAGETESLADIVLWGALYPLLQDPA

YLPEELSALHSWFQTLSTQEPCQRAAETVLKQQGVLALRPYLQKQPQPSPTEGRAVTNEPEEEELATLSEEEIAMAVTAW

EKGLESLPPLRPQQNPVLPVAGERNVLITSALPYVNNVPHLGNIIGCVLSADVFARYSRLRQWNTLYLCGTDEYGTATET

KALEEGLTPQEICDKYHIIHADIYRWFNISFDIFGRTTTPQQTKITQDIFQQLLKRGFVLQDTVEQLRCEHCARFLADRF

VEGVCPFCGYEEARGDQCDKCGKLINAVELKKPQCKVCRSCPVVQSTQHLFLDLPKLEKRLEEWLGRTLPGSDWTPNAQF

ITRSWLRDGLKPRCITRDLKWGTPVPLEGFEDKVFYVWFDATIGYLSITANYTDQWERWWKNPEQVDLYQFMAKDNVPFH

SLVFPCSALGAEDNYTLVSHLIATEYLNYEDGKFSKSRGVGVFGDMAQDTGIPADIWRFYLLYIRPEGQDSAFSWTDLLL

KNNSELLNNLGNFINRAGMFVSKFFGGYVPEMVLTPDDQRLLAHVTLELQHYHQLLEKVRIRDALRSILTISRHGNQYIQ

VNEPWKRIKGSEADRQRAGTVTGLAVNIAALLSVMLQPYMPTVSATIQAQLQLPPPACSILLTNFLCTLPAGHQIGTVSP

LFQKLENDQIESLRQRFGGGQLEETFDLKAKTSPKPAVVETVTTARPQQIQALMDEVTKQGNIVRELKAQKADKNEVAAE

VAKLLDLKKQLAVAEGKPPEAPKGKKKK

>XP_008976744.1 LOW QUALITY PROTEIN: methionine--tRNA synthetase, cytoplasmic [Pan paniscus]

MRLFVSEGVPGCLPVLAAAGRARGRAEVLISTVGPEDCVVPFLTRPKVPVLQLDSGNYLFSTSAICRYFFLLSGWEQDDL

TNQWLEWEATELQPALSAALYYLVVQGKXGEDVLGSVRRALTHIDHSLSRQNCPFLAGETESLADIVLWGALYPLLQDPA

YLPEELSALHSWFQTLSTQEPCQRAAETVLKQQGVLALRPYLQKQPQPSPTEGRAVTNEPEEEELATLSEEEIAMAVTAW

EKGLESLPPLRPQQNPVLPVAGERNVLITSALPYVNNVPHLGNIIGCVLSADVFARYSRLRQWNTLYLCGTDEYGTATET

KALEEGLTPQEICDKYHIIHADIYRWFNISFDIFGRTTTPQQTKITQDIFQQLLKRGFVWQDTVEQLRCEHCARFLADRF

VEGMCPFCGYEEARGDQCDKCGKLINAVELKKPQCKVCRSCPVVQSTQHLFLDLPKLEKRLEEWLGRTLPGSDWTPNAQF

ITRSWLRDGLKPRCITRDLKWGTPVPLEGFEDKVFYVWFDATIGYLSITANYTDQWERWWKNPEQVDLYQFMAKDNVPFH

SLVFPCSALGAEDNYTLVSHLIATEYLNYEDGKFSKSRGVGVFGDMAQDTGIPADIWRFYLLYIRPEGQDSAFSWTDLLL

KNNSELLNNLGNFINRAGMFVSKFFGGYVPEMVLTPDDQRLLAHVTLELQHYHQLLEKVRIRDALRSILTISRHGNQYIQ

VNEPWKRIKGSEADRQRAGTVTGLAVNIAALLSVMLQPYMPTVSATIQAQLQLPPPACSILLTNFLCTLPAGHQIGTVSP

LFQKLENDQIESLRQRFGGGQAKTSLKPAVVETVTTARPQQIQALMDEVTKQGNIVRELKAQKADKNEVAAEVAKLLDLK

KQLAVAEGKPPEAPKGKKKK

>XP_018893707.1 PREDICTED: methionine--tRNA synthetase, cytoplasmic isoform X3 [Gorilla gorilla gorilla]

MRLFVSEGVPGCLPVLAAAGRARGRAEVLISTVGPEDCVVPFLTRPKVPVLQLDSGNYLFSTSAICRYFFLLSGWEQDDL

TNQWLEWEATELQPALSAALYYLVVQGKKGEDVLGSVRRALTHIDHSLSRQNCPFLAGETESLADIVLWGALYPLLQDPA

YLPEELSALHSWFQTLSTQEPCQRAAETVLKQQGVLALRPYLQKQPQPSPPEGRAVTNEPEEEELATLSEEEIAMAVTAW

EKGLESLPPLRPQQNPVLPVAGERNVLITSALPYVNNVPHLGNIIGCVLSADVFARYSRLRQWNTLYLCGTDEYGTATET

KALEEGLTPQEICDKYHIIHADIYRWFNISFDIFGRTTTPQQTKITQDIFQRLLKRGFVLQDTVEQLRCEHCARFLADRF

VEGVCPFCGYEEARGDQCDKCGKLINAVELKKPQCKVCRSCPVVQSTQHLFLDLPKLEKRLEEWLGRTLPGSDWTPNARF

ITLSWLRDGLKPRCITRDLKWGTPVPLEGFEDKVFYVWFDATIGYLSITANYTDQWERWWKNPEQVDLYQFMAKDNVPFH

SLVFPCSALGAEDNYTLVSHLIATEYLNYEDGKFSKSRGVGVFGDMAQDTGIPADIWRFYLLYIRPEGQDSAFSWMDLLL

KNNSELLNNLGNFINRAGMFVSKFFGGYVPEMVLTPDDQRLLAHVTLELQHYHQLLEKVRIRDALRSILTISRHGNQYIQ

VNEPWKRIKGSEADRQRAGTVTGLAVNIAALLSVMLQPYMPTVSATIQAQLQLPPPACSILLTNFLCTLPAGHQIGTVSP

LFQKLENDQIESLRQRFGGGQAKTSPKPAVVETVTTARPQQIQALMDEVTKQGNIVRELKAQKADKNEVAAEVVKLLDLK

KQLAVAEGKPPEAPKGKKKK

>XP_018893705.1 PREDICTED: methionine--tRNA synthetase, cytoplasmic isoform X1 [Gorilla gorilla gorilla]

MRLFVSEGVPGCLPVLAAAGRARGRAEVLISTVGPEDCVVPFLTRPKVPVLQLDSGNYLFSTSAICRYFFLLSGWEQDDL

TNQWLEWEATELQPALSAALYYLVVQGKKGEDVLGSVRRALTHIDHSLSRQNCPFLAGETESLADIVLWGALYPLLQDPA

YLPEELSALHSWFQTLSTQEPCQRAAETVLKQQGVLALRPYLQKQPQPSPPEGRAVTNEPEEEELATLSEEEIAMAVTAW

EKGLESLPPLRPQQNPVLPVAGERNVLITSALPYVNNVPHLGNIIGCVLSADVFARYSRLRQWNTLYLCGTDEYGTATET

KALEEGLTPQEICDKYHIIHADIYRWFNISFDIFGRTTTPQQTKITQDIFQRLLKRGFVLQDTVEQLRCEHCARFLADRF

VEGVCPFCGYEEARGDQCDKCGKLINAVELKKPQCKVCRSCPVVQSTQHLFLDLPKLEKRLEEWLGRTLPGSDWTPNARF

ITLSWLRDGLKPRCITRDLKWGTPVPLEGFEDKVFYVWFDATIGYLSITANYTDQWERWWKNPEQVDLYQFMAKDNVPFH

SLVFPCSALGAEDNYTLVSHLIATEYLNYEDGKFSKSRGVGVFGDMAQDTGIPADIWRFYLLYIRPEGQDSAFSWMDLLL

KNNSELLNNLGNFINRAGMFVSKFFGGYVPEMVLTPDDQRLLAHVTLELQHYHQLLEKVRIRDALRSILTISRHGNQYIQ

VNEPWKRIKGSEADRQRAGTVTGLAVNIAALLSVMLQPYMPTVSATIQAQLQLPPPACSILLTNFLCTLPAGHQIGTVSP

LFQKLENDQIESLRQRFGGGQLEETFDLKAKTSPKPAVVETVTTARPQQIQALMDEVTKQGNIVRELKAQKADKNEVAAE

VVKLLDLKKQLAVAEGKPPEAPKGKKKK

>XP_018893708.1 PREDICTED: methionine--tRNA synthetase, cytoplasmic isoform X4 [Gorilla gorilla gorilla]

MRLFVSEGVPGCLPVLAAAGRARGRAEVLISTVGPEDCVVPFLTRPKVPVLQLDSGNYLFSTSAICRYFFLLSGWEQDDL

TNQWLEWEATELQPALSAALYYLVVQGKKGEDVLGSVRRALTHIDHSLSRQNCPFLAGETESLADIVLWGALYPLLQDPA

YLPEELSALHSWFQTLSTQEPCQRAAETVLKQQGVLALRPYLQKQPQPSPPEGRAVTNEPEEEELATLSEEEIAMAVTAW

EKGLESLPPLRPQQNPVLPVAGERNVLITSALPYVNNVPHLGNIIGCVLSADVFARYSRLRQWNTLYLCGTDEYGTATET

KALEEGLTPQEICDKYHIIHADIYRWFNISFDIFGRTTTPQQTKITQDIFQRLLKRGFVLQDTVEQLRCEHCARFLADRF

VEGVCPFCGYEEARGDQCDKCGKLINAVELKKPQCKVCRSCPVVQSTQHLFLDLPKLEKRLEEWLGRTLPGSDWTPNARF

ITLSWLRDGLKPRCITRDLKWGTPVPLEGFEDKVFYVWFDATIGYLSITANYTDQWERWWKNPEQVDLYQFMAKDNVPFH

SLVFPCSALGAEDNYTLVSHLIATEYLNYEDGKFSKSRGVGVFGDMAQDTGIPADIWRFYLLYIRPEGQDSAFSWMDLLL

KNNSELLNNLGNFINRAGMFVSKFFGGYVPEMVLTPDDQRLLAHVTLELQHYHQLLEKVRIRDALRSILTISRHGNQYIQ

VNEPWKRIKGSEADRQRAGTVTGLAVNIAALLSVMLQPYMPTVSATIQAQLQLPPPACSILLTNFLCTLPAGHQIGTVSP

LFQKLENDQIESLRQRFGGGQAKTSPKPAVVETVTTARPQQIQALMDEVTKQGNIVRELKAQKADKNEVAAEVVKLLDLK

KQLAVAEGKPPEAPKGGET

>NP_001253877.1 methionine--tRNA synthetase, cytoplasmic [Macaca mulatta]

MRLFVSEGAPGCLPVLAAAGRARGRAELLISTVGPEDCVVPFLTRPKVPVLQLDSGNYLFSTSAICRYFFLLSGWEQDDL

TNQWLEWEATELQPALSAALYYLVVQGKKGEDVLGSVRRALTHIDHSLSRQNCPFLAGETESLADIVLWGALYPLLQDPA

YLPEELSALHSWFQTLSTQEPCQRAAETVLKQQGVLALRPYLQKQPQPSPAEGRAVTNEPEEEELATLSEEEIAMAVTAW

EKGLESLPPLRPQQNPVLPVTGERNVLITSALPYVNNVPHLGNIIGCVLSADVFARYSRLRQWNTLYLCGTDEYGTATET

KAMEEGLTPQEICDKYHIIHADIYRWFNISFDTFGRTTTPQQTKITQDIFQRLLTRGFVLQDTVEQLRCEHCARFLADRF

VEGVCPFCGYEEARGDQCDKCGKLINAVELKKPQCKVCRSCPVVQSTHHLFLDLPKLEKRLEEWLGRTLPGSDWTPNARF

ITRSWLRDGLKPRCITRDLKWGTPVPLEGFEDKVFYVWFDATIGYLSITANYTDQWERWWKNPEQVDLYQFMAKDNVPFH

GIVFPCSALGAEDNYTLVSHLIATEYLNYEDGKFSKSRGVGVFGDMAQDTGIPADIWRFYLLYIRPEGQDSAFSWTDLLL

KNNSELLNNLGNFINRAGMFVSKFFGGYVPEMVLTPDDQRLLAHVTLELQHYHQLLEKVRIRDALRSILTISRHGNQYIQ

VNEPWKRIKGSEADRQRAGTVTGLAVNIAALLSVMLQPYMPTVSATIQAQLQLPPPACSILLTNFLCTLPAGHQIGTVSP

LFQKLENDQIESLRQRFGGGQAKTSPKPTVIETVTTAGPQQIQALMDEVTKQGNIVRELKAQKADKNQVAAEVAKLLDLK

KQLAVAEGKPPEAPKGKKKK

>XP_009179310.1 methionine--tRNA synthetase, cytoplasmic isoform X2 [Papio anubis]

MRLFVSQGAPGCLPVLAAAGRARGRAELLISTVGPEDCVVPFLTRPKVPVLQLDSGNYLFSTSAICRYFFLLSGWEQDDL

TNQWLEWEATELQPALSAALYYLVVQGKKGEDVLGSVRRALTHIDHSLSRQNCPFLAGETESLADIVLWGALYPLLQDPA

YLPEELSALHSWFQTLSTQEPCQRAAETVLKQQGVLALRPYLQKQPQPSPAEGRAVTNEPEEEELATLSEEEIAMAVTAW

EKGLESLPPLRPQQNPVLPVTGERNVLITSALPYVNNVPHLGNIIGCVLSADVFARYSRLRQWNTLYLCGTDEYGTATET

KAMEEGLTPQEICDKYHIIHADIYRWFNISFDTFGRTTTPQQTKITQDIFQRLLTRGFVLQDTVEQLRCEHCARFLADRF

VEGVCPFCGYEEARGDQCDKCGKLINAVELKKPQCKVCRSCPVVQSTHHLFLDLPKLEKRLEEWLGRTLPGSDWTPNARF

ITRSWLRDGLKPRCITRDLKWGTPVPLEGFEDKVFYVWFDATIGYLSITANYTDQWERWWKNPEQVDLYQFMAKDNVPFH

GIVFPCSALGAEDNYTLVSHLIATEYLNYEDGKFSKSRGVGVFGDMAQDTGIPADIWRFYLLYIRPEGQDSAFSWTDLLL

KNNSELLNNLGNFINRAGMFVSKFFGGYVPEMVLTPDDQRLLAHVTLELQHYHQLLEKVRIRDALRSILTISRHGNQYIQ

VNEPWKRIKGSEADRQRAGTVTGLAVNIAALLSVMLQPYMPTVSATIQAQLQLPPPACSILLTNFLCTLPAGHQIGTVSP

LFQKLENDQIESLRQRFGGGQAKTSPKPTVIETVTTAGPQQIQALMDEVTKQGNIVRELKAQKADKNQVAAEVAKLLDLK

KQLAVAEGKPPEAPKGKKKK

>XP_011918690.1 PREDICTED: methionine--tRNA synthetase, cytoplasmic isoform X2 [Cercocebus atys]

MRLFVSEGAPGCLPVLAAAGRARGRAELLISTVGPEDCVVPFLTRPKVPVLQLDSGNYLFSTSAICRYFFLLSGWEQDDL

TNQWLEWEATELQPALSAALYYLVVQGKKGEDVLGSVRRALTHIDHSLSRQNCPFLAGETESLADIVLWGALYPLLQDPA

YLPEELSALHSWFQTLSTQEPCQRAAETVLKQQGVLALRPYLQKQPQPSPAEGRAVTNEPEEEELATLSEEEIAMAVTAW

EKGLESLPPLRPQQNPVLPVTGERNVLITSALPYVNNVPHLGNIIGCVLSADVFARYSRLRQWNTLYLCGTDEYGTATET

KAMEEGLTPQEICDKYHIIHADIYRWFNISFDTFGRTTTPQQTKITQDIFQRLLTRGFVLQDTVEQLRCEHCARFLADRF

VEGVCPFCGYEEARGDQCDKCGKLINAVELKKPQCKVCRSCPVVQSTHHLFLDLPKLEKRLEEWLGRTLPGSDWTPNARF

ITRSWLRDGLKPRCITRDLKWGTPVPLEGFEDKVFYVWFDATIGYLSITANYTDQWERWWKNPEQVDLYQFMAKDNVPFH

GIVFPCSALGAEDNYTLVSHLIATEYLNYEDGKFSKSRGVGVFGDMAQDTGIPADIWRFYLLYIRPEGQDSAFSWTDLLL

KNNSELLNNLGNFINRAGMFVSKFFGGYVPEMVLTPDDQRLLAHVTLELQHYHQLLEKVRIRDALRSILTISRHGNQYIQ

VNEPWKRIKGSEADRQRAGTVTGLAVNIAALLSVMLQPYMPTVSATIQAQLQLPPPACSILLTNFLCTLPAGHQIGTVSP

LFQKLENDQIESLRQRFGGGQAKTSPKPTVIETVTTAGPQQIQALMDEVTKQGNIVRELKAQKADKNQVAAEVAKLLDLK

KQLAVAEGKPPEASKGKKKK

>XP_005571393.1 PREDICTED: methionine--tRNA synthetase, cytoplasmic isoform X2 [Macaca fascicularis]

MRLFVSEGAPGCLPVLAAAGRARGRAELLISTVGPEDCVVPFLTRPKVPVLQLDSGNYLFSTSAICRYFFLLSGWEQDDL

TNQWLEWEATELQPALSAALYYLVVQGKKGEDVLGSVRRALTHIDHSLSRQNCPFLAGETESLADIVLWGALYPLLQDPA

YLPEELSALHSWFQTLSTQEPCQRAAETVLKQQGVLALRPYLQKQPQPSPAEGRAVTNEPEEEELATLSEEEIAMAVTAW

EKGLESLPPLRPQQNPVLPVTGERNVLITSALPYVNNVPHLGNIIGCVLSADVFARYSRLRQWNTLYLCGTDEYGTATET

KAMEEGLTPQEICDKYHIIHADIYRWFNISFDTFGRTTTAQQTKITQDIFQRLLTRGFVLQDTVEQLRCEHCARFLADRF

VEGVCPFCGYEEARGDQCDKCGKLINAVELKKPQCKVCRSCPVVQSTHHLFLDLPKLEKRLEEWLGRTLPGSDWTPNARF

ITRSWLRDGLKPRCITRDLKWGTPVPLEGFEDKVFYVWFDATIGYLSITANYTDQWERWWKNPEQVDLYQFMAKDNVPFH

GIVFPCSALGAEDNYTLVSHLIATEYLNYEDGKFSKSRGVGVFGDMAQDTGIPADIWRFYLLYIRPEGQDSAFSWTDLLL

KNNSELLNNLGNFINRAGMFVSKFFGGYVPEMVLNPDDQRLLAHVTLELQHYHQLLEKVRIRDALRSILTISRHGNQYIQ

VNEPWKRIKGSEADRQRAGTVTGLAVNIAALLSVMLQPYMPTVSATIQAQLQLPPPACSILLTNFLCTLPAGHQIGTVSP

LFQKLENDQIESLRQRFGGGQAKTSPKPTVIETVTTAGPQQIQALMDEVTKQGNIVRELKAQKADKNQVAAEVAKLLDLK

KQLAVAEGKPPEAPKGKKKK

>XP_002823470.1 methionine--tRNA synthetase, cytoplasmic [Pongo abelii]

MRLFVSEGVPGCLPVLAAAGRARGRAEVLISTVGPEDCVVPFLTRPKVPVLQLDSGNYLFSTNAICRYFFLLSGWEQDDL

TNQWLEWEATQLQPTLSAALYYLVVQGKKGEDVLGSVRRALTHIDHSLSRQNCPFLAGETESLADIVLWGALYPLLQDPA

YLPEELSALHSWFQTLSTQEPCQQAAETVLKQQGVLALRPYLQKQPQPSSPEGRAVTNEPEEEELATLSEEEIAMAVTAW

EKGLESLPPLRPHQNPVLPVAGERNVLITSALPYVNNVPHLGNLIGCVLSADVFARYSRLRQWNTLYLCGTDEYGTATET

KALEEGLTPQEICDKYHIIHADIYRWFNISFDIFGRTTTPQQTKITQDIFQRLLKRGFVLQDTVEQLRCEHCARFLADRF

VEGVCPFCGYEEARGDQCDKCGKLINAIELKKPQCKVCRSCPVVQSSQHLFLDLPKLEKRLEEWLGRTLPGSDWTPNARF

IIRSWLRDGLKPRCITRDLKWGTPVPLEGFEDKVFYVWFDATFGYLSITANYTDQWERWWKNPEQVDLYQFMAKDNVPFH

GLVFPCSALGAEDNYTLVSHLIATEYLNYEDGKFSKSRGVGVFGDMAQDTGIPADIWRFYLLYIRPEGQDSAFSWTDLLL

KNNSELLNNLGNFINRAGMFVSKFFGGYVPEMVLTPDDKRLLAHVTLELQHYHQLLEKVRIRDALRSILTISRHGNQYIQ

VNEPWKRIKGSEADRQRAGTVTGLAVNIAALLSVMLQPYMPTVSATIQAQLQLPPPACSILLTNFLCTLPAGHQIGTVSP

LFQKLENDQIESLRQRFGGGQAKTSPKPAVVETVTTARPQQIQALVDEVTKQGNIVRELKAQKADKNEVAAEVAKLLDLK

KQLAVAEGKPPEAPKGKKKK

>XP_018893706.1 PREDICTED: methionine--tRNA synthetase, cytoplasmic isoform X2 [Gorilla gorilla gorilla]

MRLFVSEGVPGCLPVLAAAGRARGRAEVLISTVGPEDCVVPFLTRPKVPVLQLDSGNYLFSTSAICRYFFLLSGWEQDDL

TNQWLEWEATELQPALSAALYYLVVQGKKGEDVLGSVRRALTHIDHSLSRQNCPFLAGETESLADIVLWGALYPLLQDPA

YLPEELSALHSWFQTLSTQEPCQRAAETVLKQQGVLALRPYLQKQPQPSPPEGRAVTNEPEEEELATLSEEEIAMAVTAW

EKGLESLPPLRPQQNPVLPVAGERNVLITSALPYVNNVPHLGNIIGCVLSADVFARYSRLRQWNTLYLCGTDEYGTATET

KALEEGLTPQEICDKYHIIHADIYRWFNISFDIFGRTTTPQQTKITQDIFQRLLKRGFVLQDTVEQLRCEHCARFLADRF

VEGVCPFCGYEEARGDQCDKCGKLINAVELKKPQCKVCRSCPVVQSTQHLFLDLPKLEKRLEEWLGRTLPGSDWTPNARF

ITLSWLRDGLKPRCITRDLKWGTPVPLEGFEDKVFYVWFDATIGYLSITANYTDQWERWWKNPEQVDLYQFMAKDNVPFH

SLVFPCSALGAEDNYTLVSHLIATEYLNYEDGKFSKSRGVGVFGDMAQDTGIPADIWRFYLLYIRPEGQDSAFSWMDLLL

KNNSELLNNLGNFINRAGMFVSKFFGGYVPEMVLTPDDQRLLAHVTLELQHYHQLLEKVRIRDALRSILTISRHGNQYIQ

VNEPWKRIKGSEADRQRAGTVTGLAVNIAALLSVMLQPYMPTVSATIQAQLQLPPPACSILLTNFLCTLPAGHQIGTVSP

LFQKLENDQIESLRQRFGGGQLEETFDLKAKTSPKPAVVETVTTARPQQIQALMDEVTKQGNIVRELKAQKADKNEVAAE

VVKLLDLKKQLAVAEGKPPEAPKGGET

>XP_015007536.1 PREDICTED: methionine--tRNA synthetase, cytoplasmic isoform X1 [Macaca mulatta]

MRLFVSEGAPGCLPVLAAAGRARGRAELLISTVGPEDCVVPFLTRPKVPVLQLDSGNYLFSTSAICRYFFLLSGWEQDDL

TNQWLEWEATELQPALSAALYYLVVQGKKGEDVLGSVRRALTHIDHSLSRQNCPFLAGETESLADIVLWGALYPLLQDPA

YLPEELSALHSWFQTLSTQEPCQRAAETVLKQQGVLALRPYLQKQPQPSPAEGRAVTNEPEEEELATLSEEEIAMAVTAW

EKGLESLPPLRPQQNPVLPVTGERNVLITSALPYVNNVPHLGNIIGCVLSADVFARYSRLRQWNTLYLCGTDEYGTATET

KAMEEGLTPQEICDKYHIIHADIYRWFNISFDTFGRTTTPQQTKITQDIFQRLLTRGFVLQDTVEQLRCEHCARFLADRF

VEGVCPFCGYEEARGDQCDKCGKLINAVELKKPQCKVCRSCPVVQSTHHLFLDLPKLEKRLEEWLGRTLPGSDWTPNARF

ITRSWLRDGLKPRCITRDLKWGTPVPLEGFEDKVFYVWFDATIGYLSITANYTDQWERWWKNPEQVDLYQFMAKDNVPFH

GIVFPCSALGAEDNYTLVSHLIATEYLNYEDGKFSKSRGVGVFGDMAQDTGIPADIWRFYLLYIRPEGQDSAFSWTDLLL

KNNSELLNNLGNFINRAGMFVSKFFGGYVPEMVLTPDDQRLLAHVTLELQHYHQLLEKVRIRDALRSILTISRHGNQYIQ

VNEPWKRIKGSEADRQRAGTVTGLAVNIAALLSVMLQPYMPTVSATIQAQLQLPPPACSILLTNFLCTLPAGHQIGTVSP

LFQKLENDQIESLRQRFGGGQLEETFDLKAKTSPKPTVIETVTTAGPQQIQALMDEVTKQGNIVRELKAQKADKNQVAAE

VAKLLDLKKQLAVAEGKPPEAPKGKKKK

>XP_021778978.1 methionine--tRNA synthetase, cytoplasmic isoform X1 [Papio anubis]

MRLFVSQGAPGCLPVLAAAGRARGRAELLISTVGPEDCVVPFLTRPKVPVLQLDSGNYLFSTSAICRYFFLLSGWEQDDL

TNQWLEWEATELQPALSAALYYLVVQGKKGEDVLGSVRRALTHIDHSLSRQNCPFLAGETESLADIVLWGALYPLLQDPA

YLPEELSALHSWFQTLSTQEPCQRAAETVLKQQGVLALRPYLQKQPQPSPAEGRAVTNEPEEEELATLSEEEIAMAVTAW

EKGLESLPPLRPQQNPVLPVTGERNVLITSALPYVNNVPHLGNIIGCVLSADVFARYSRLRQWNTLYLCGTDEYGTATET

KAMEEGLTPQEICDKYHIIHADIYRWFNISFDTFGRTTTPQQTKITQDIFQRLLTRGFVLQDTVEQLRCEHCARFLADRF

VEGVCPFCGYEEARGDQCDKCGKLINAVELKKPQCKVCRSCPVVQSTHHLFLDLPKLEKRLEEWLGRTLPGSDWTPNARF

ITRSWLRDGLKPRCITRDLKWGTPVPLEGFEDKVFYVWFDATIGYLSITANYTDQWERWWKNPEQVDLYQFMAKDNVPFH

GIVFPCSALGAEDNYTLVSHLIATEYLNYEDGKFSKSRGVGVFGDMAQDTGIPADIWRFYLLYIRPEGQDSAFSWTDLLL

KNNSELLNNLGNFINRAGMFVSKFFGGYVPEMVLTPDDQRLLAHVTLELQHYHQLLEKVRIRDALRSILTISRHGNQYIQ

VNEPWKRIKGSEADRQRAGTVTGLAVNIAALLSVMLQPYMPTVSATIQAQLQLPPPACSILLTNFLCTLPAGHQIGTVSP

LFQKLENDQIESLRQRFGGGQLEETFDLKAKTSPKPTVIETVTTAGPQQIQALMDEVTKQGNIVRELKAQKADKNQVAAE

VAKLLDLKKQLAVAEGKPPEAPKGKKKK

>XP_011918689.1 PREDICTED: methionine--tRNA synthetase, cytoplasmic isoform X1 [Cercocebus atys]

MRLFVSEGAPGCLPVLAAAGRARGRAELLISTVGPEDCVVPFLTRPKVPVLQLDSGNYLFSTSAICRYFFLLSGWEQDDL

TNQWLEWEATELQPALSAALYYLVVQGKKGEDVLGSVRRALTHIDHSLSRQNCPFLAGETESLADIVLWGALYPLLQDPA

YLPEELSALHSWFQTLSTQEPCQRAAETVLKQQGVLALRPYLQKQPQPSPAEGRAVTNEPEEEELATLSEEEIAMAVTAW

EKGLESLPPLRPQQNPVLPVTGERNVLITSALPYVNNVPHLGNIIGCVLSADVFARYSRLRQWNTLYLCGTDEYGTATET

KAMEEGLTPQEICDKYHIIHADIYRWFNISFDTFGRTTTPQQTKITQDIFQRLLTRGFVLQDTVEQLRCEHCARFLADRF

VEGVCPFCGYEEARGDQCDKCGKLINAVELKKPQCKVCRSCPVVQSTHHLFLDLPKLEKRLEEWLGRTLPGSDWTPNARF

ITRSWLRDGLKPRCITRDLKWGTPVPLEGFEDKVFYVWFDATIGYLSITANYTDQWERWWKNPEQVDLYQFMAKDNVPFH

GIVFPCSALGAEDNYTLVSHLIATEYLNYEDGKFSKSRGVGVFGDMAQDTGIPADIWRFYLLYIRPEGQDSAFSWTDLLL

KNNSELLNNLGNFINRAGMFVSKFFGGYVPEMVLTPDDQRLLAHVTLELQHYHQLLEKVRIRDALRSILTISRHGNQYIQ

VNEPWKRIKGSEADRQRAGTVTGLAVNIAALLSVMLQPYMPTVSATIQAQLQLPPPACSILLTNFLCTLPAGHQIGTVSP

LFQKLENDQIESLRQRFGGGQLEETFDLKAKTSPKPTVIETVTTAGPQQIQALMDEVTKQGNIVRELKAQKADKNQVAAE

VAKLLDLKKQLAVAEGKPPEASKGKKKK

>XP_012365764.1 PREDICTED: methionine--tRNA synthetase, cytoplasmic [Nomascus leucogenys]

MRLFVSEGVPGCLPVLAAARRARGRAELLISTVGPEDCVVPFLTRPKIPVLQLDSGNYLFSTSAICRYFFLLSGWEQDDL

TNQWLEWEATELQPALSAALYYLVVQGKKGEDVLGSVRRALTHIDHSLSRQNCPFLAGETESLADIVLWGALYPLLQDPA

YLPEELSALHSWFQTLSTQEPCQQAAETVLKQQGVLALRPYFQKQPQPSPPEGRAVTNEPEEEELATLSEEEIATAVTAW

EKGLESLPPLRPQQNPVLPVAGERNVLITSALPYVNNVPHLGNIIGCVLSADVFARYSRLRQWNTLYLCGTDEYGTATET

KALEEGLTPQEICDKYHIIHADIYRWFNISFDIFGRTTTPQQTKITQDIFQRLLKRGFVLQDTVEQLRCEHCARFLADRF

VEGVCPFCGYEEARGDQCDKCGKLINAVELKKPQCKVCRSCPVVQSTQHLFLDLPKLEKRLEEWLGRTLPGSDWTPNARF

IIRSWLRDGLKPRCITRDLKWGTPVPLEGFKDKVFYVWFDATIGYLSITANYTDQWERWWKNPEQVDLYQFMAKDNVPFH

GLVFPCSVLGAEDNYTLVSHIIATEYLNYEDGKFSKSRGVGVFGDMAQDTGIPADIWRFYLLYIRPEGQDSAFSWTDLLL

KNNSELLNNLGNFINRAGMFVSKFFGGCVPEMVLTPDDQRLLAHVTLELQHYHQLLEKVRIRDALRSILTVSRHGNQYIQ

VNEPWKRIKGSEADRQRAGTVTGLAVNIAALLSVMLQPYMPTVSATIQAQLQLPPPACSILLTNFLCTLPAGHQIGTVSP

LFQKLENDQIESLRQRFGGGQVKTSPKPAVVETVTTARPQQIQVLMDEVTKQGNVVRELKAQKADKNEVAAEVAKLLDLK

KQLAVAEGKPPEAPKGKKKK

>XP_005571392.1 PREDICTED: methionine--tRNA synthetase, cytoplasmic isoform X1 [Macaca fascicularis]

MRLFVSEGAPGCLPVLAAAGRARGRAELLISTVGPEDCVVPFLTRPKVPVLQLDSGNYLFSTSAICRYFFLLSGWEQDDL

TNQWLEWEATELQPALSAALYYLVVQGKKGEDVLGSVRRALTHIDHSLSRQNCPFLAGETESLADIVLWGALYPLLQDPA

YLPEELSALHSWFQTLSTQEPCQRAAETVLKQQGVLALRPYLQKQPQPSPAEGRAVTNEPEEEELATLSEEEIAMAVTAW

EKGLESLPPLRPQQNPVLPVTGERNVLITSALPYVNNVPHLGNIIGCVLSADVFARYSRLRQWNTLYLCGTDEYGTATET

KAMEEGLTPQEICDKYHIIHADIYRWFNISFDTFGRTTTAQQTKITQDIFQRLLTRGFVLQDTVEQLRCEHCARFLADRF

VEGVCPFCGYEEARGDQCDKCGKLINAVELKKPQCKVCRSCPVVQSTHHLFLDLPKLEKRLEEWLGRTLPGSDWTPNARF

ITRSWLRDGLKPRCITRDLKWGTPVPLEGFEDKVFYVWFDATIGYLSITANYTDQWERWWKNPEQVDLYQFMAKDNVPFH

GIVFPCSALGAEDNYTLVSHLIATEYLNYEDGKFSKSRGVGVFGDMAQDTGIPADIWRFYLLYIRPEGQDSAFSWTDLLL

KNNSELLNNLGNFINRAGMFVSKFFGGYVPEMVLNPDDQRLLAHVTLELQHYHQLLEKVRIRDALRSILTISRHGNQYIQ

VNEPWKRIKGSEADRQRAGTVTGLAVNIAALLSVMLQPYMPTVSATIQAQLQLPPPACSILLTNFLCTLPAGHQIGTVSP

LFQKLENDQIESLRQRFGGGQLEETFDLKAKTSPKPTVIETVTTAGPQQIQALMDEVTKQGNIVRELKAQKADKNQVAAE

VAKLLDLKKQLAVAEGKPPEAPKGKKKK

>XP_011781410.1 PREDICTED: methionine--tRNA synthetase, cytoplasmic [Colobus angolensis palliatus]

MRLFLSEGAPGCLPVLAAAGRARGRAELLISTVGPEDCVVPFLTRPKVPVLQLDSGNYLFSTSAICRYFFLLSGWEQDDL

TNQWLEWEATELQPALSAALYYLVVQGKKGEDVLGSVRRALTHIDHSLSRQNCPFLAGETESLADIVLWGALYPLLQDPA

YLPEELSALHSWFQTLSTQEPCQRAAETVLKQQGVLAFRPYLQKQPQPSPPEGRAVTNEPEEEELATLSEEEIAMAVTAW

EKGLESLPPLRPQQSPVLPVTGERNVLITSALPYVNNVPHLGNIIGCVLSADVFARYSRLRQWNTLYLCGTDEYGTATET

KAMEEGLTPQEICDKYHIIHADIYRWFNISFDTFGRTTTPQQTKITQDIFQRLLTRGFVLQDTVEQLRCEHCARFLADRF

VEGVCPFCGYEEARGDQCDKCGKLINAVELKKPQCKVCRSCPVVQSTHHLFLDLPKLEKRLEEWLGRTLPGSDWTPNARF

ITRSWLRDGLKPRCITRDLKWGTPVPLEGFEDKVFYVWFDATIGYLSITANYTDQWERWWKNPEQVDLYQFMAKDNVPFH

GIVFPCSALGAEDNYTLVSHLIATEYLNYEDGKFSKSRGVGVFGDMAQDTGIPADIWRFYLLYIRPEGQDSAFSWTDLLL

KNNSELLNNLGNFINRAGMFVSKFFGGYVPEMVLTPDDQRLLAHVTLELQHYHQLLEKVRIRDALRSILTISRHGNQYIQ

VNEPWKRIKGSEADRQRAGTVTGLAVNIAALLSVMLQPYMPTVSATIQAQLQLPPPACSILLTNFLCTLPAGHQIGTVSP

LFQKLENDQIESLRQRFGGGQAKTSPKPAVIETVTTAGPQQIQALMDEVTKQGNIVRELKAQKADKNQVAAEVAKLLDLK

KQLAVAEGKPPEAPKGGET

>XP_017391703.1 PREDICTED: methionine--tRNA synthetase, cytoplasmic isoform X3 [Cebus capucinus imitator]

MRLFVSEGAPGSLPVLAAAGRARGRAELLISTVGPEDCVVPFLTRPKVPVLQLDSGNYLFSTSAICRYFFLLSGWEQDDL

TNQWLEWEATELQPALSAALYYLVVQGKKGDDVLGSVRRALTHIDHSLRRQNCPFLAGETESLADIVLWGALYPLLQDPA

YLPEELSALHSWFQTLSTQEPCQRAAETVLKQQGVLALRPYLQKQPQPSPPEGRAVTNEPEEEELATLSEEEIAMAVTAW

EKGLESLPPLRPQQNPVLPVAGERNVLITSALPYVNNVPHLGNIIGCVLSADVFARYSRLRQWNTLYLCGTDEYGTATET

KAMEEGLTPQEICDKYHIIHADIYRWFNISFDVFGRTTTPQQTKITQDIFQRLLARGFVLQDTVEQLRCERCARFLADRF

VEGVCPFCGFEEARGDQCDKCGKLINAVELKKPQCKVCRSCPVVKSSQHLFLDLPKLEKRLEEWLGKTLPGSDWTPNARF

IIRSWLRDGLKPRCITRDLKWGTPVPLEGFEDKVFYVWFDATIGYLSITANYTDQWERWWKNPEQVDLYQFMAKDNVPFH

GLVFPCSALGAEDNYTLVSHLIATEYLNYEDGKFSKSRGVGVFGDMAQDTGIPADIWRFYLLYIRPEGQDSAFSWTDMLL

KNNSELLNNLGNFINRAGMFVSKFFGGYVPEMVLTPDDQRLLAHVTLELRRYHQLLEKVRIRDALRSMLTISRHGNQYIQ

VNEPWKRIKGSEADRQRAGTVTGLAVNIAALLSVMLQPYMPTVSATIQAQLQLPPPACSILLTNFLCTLPAGHQIGTVSP

LFQKLENDQIESLRQRFGGGQAKMSPKPAVAETGTTAAPQQIQALMDEVTKQGNIVRELKAQKADKNQVAAEVAKLLDLK

KQLAVAEGKPPEVPKGKKKK

>XP_012325153.1 methionine--tRNA synthetase, cytoplasmic isoform X2 [Aotus nancymaae]

MRLFVSEGAPGSLPVLAAAGRARGRAELLISTVGPEDCVVPFLTRPKVPVLQLDSGNYLFSTSAICRYFFLLSGWEQDDL

TNQWLEWEATELQPALSAALYYLVVQGKKGEDVLGSVRRALTHIDHSLRRQNCPFLAGETESLADIVLWGALYPLLQDPA

YLPEELSALHSWFQTLSTQEPCQRAAETVLKQQGVLALRPYLQKQPQPSPPEGRAVTNEPEEEELATLSEEEIALAVTAW

EKGLESLPPLQPQQNPVLPVAGERNVLITSALPYVNNVPHLGNIIGCVLSADVFARYSRLRQWNTLYLCGTDEYGTATET

KAMEEGLTPQEICDKYHVIHADIYRWFNISFDVFGRTTTPQQTKITQDIFQRLLARGFVLQDTVEQLRCERCARFLADRF

VEGVCPFCGFEEARGDQCDKCGKLINAVELKKPQCKVCRSCPVVKSSQHLFLDLPKLEKRLEEWLGRTLPGSDWTPNARF

IIRSWLRDGLKPRCITRDLKWGTPVPLEGFEDKVFYVWFDATIGYLSITANYTDQWERWWKNPEQVDLYQFMAKDNVPFH

GLVFPCSALGAEDNYTLVSHLIATEYLNYEDGKFSKSRGVGVFGDMAQDTGIPADIWRFYLLYIRPEGQDSAFSWTDMLL

KNNSELLNNLGNFINRAGMFVSKFFGGYVPEMVLTPDDQRLLAHVTLELRRYHQLLEKVRIRDALRSILTISRHGNQYIQ

VNEPWKRIKGSEADRQRAGTVTGLAVNIAALLSVMLQPYMPTVSATIQAQLQLPPPACSILLTNFLCTLPAGHQIGTVSP

LFQKLENDQIESLRQRFGGGQAKTSPKPAVAETGTTAGPQQIQALMDEVTKQGNIVRELKAQKADKNQVAAEVAKLLDLK

KQLAVAEGKPPEAPKSKKKK

>XP_010387260.1 PREDICTED: LOW QUALITY PROTEIN: methionine--tRNA synthetase, cytoplasmic [Rhinopithecus roxellana]

MRLFVSEGAPGCLPVLAAAGRARGRAELLISTVXWGWGAGPKDCVVPFLTRPKVPVLQLDSGNYLFSTSAICRYFFLLSG

WEQDDLTNQWLEWEATELQPALSAALYYLVVQGKKGEDVLGSVRRALTYIDHSLSRQNCPFLAGETESLADIVLWGALYP

LLQDPAYLPEELSALHSWFQTLSTQEPCQRAAETVLKQQGVLALRPYLQKQPQPSSPEGRAVTNEPEEEELATLSEEEIA

MAVTAWENGLESLPPLRPQQNPVLPVTGERNVLITSALPYVNNVPHLGNIIGCVLSADVFARYSRLRQWNTLYLCGTDEY

GTATETKAMEEGLTPQEICDKYHIIHADIYRWFNISFDTFGRTTTPQQTKITQDIFQRLLTRGFVLQDTVEQLRCERCAR

FLADRFVEGVCPFCGYEEARGDQCDKCGKLINAVELKKPQCKVCRSCPVVQSTHHLFLDLPKLEKRLEEWLGRTLPGSDW

TPNARFITRSWLRDGLKPRCITRDLKWGTPVPLEGFEDKVFYVWFDATIGYLSITANYTDQWERWWKNPEQVDLYQFMAK

DNVPFHGIVFPCSALGAEDNYTLVSHLIATEYLNYEDGKFSKSRGVGVFGDMAQDTGIPADIWRFYLLYIRPEGQDSAFS

WTDLLLKNNSELLNNLGNFINRAGMFVSKFFGGYVPEMVLTPDDQRLLAHVTLELQRYHQLLEKVRIRDALRSILTISRH

GNQYIQVNEPWKRIKGSEADRQRAGTVTGLAVNIAALLSVMLQPYMPTVSATIQAQLQLPPPACSILLTNFLCTLPAGHQ

IGTVSPLFQKLENDQIESLRQRFGGGQAKTSPKPAVIETVTTAGPQQIQALMDEVTKQGNIVRELKAQKADKNQVAAEVA

KLLDLKKQLAVAEGKPPEAPKGKKKK

>XP_017391702.1 PREDICTED: methionine--tRNA synthetase, cytoplasmic isoform X2 [Cebus capucinus imitator]

MRLFVSEGAPGSLPVLAAAGRARGRAELLISTVGPEDCVVPFLTRPKVPVLQLDSGNYLFSTSAICRYFFLLSGWEQDDL

TNQWLEWEATELQPALSAALYYLVVQGKKGDDVLGSVRRALTHIDHSLRRQNCPFLAGETESLADIVLWGALYPLLQDPA

YLPEELSALHSWFQTLSTQEPCQRAAETVLKQQGVLALRPYLQKQPQPSPPEGRAVTNEPEEEELATLSEEEIAMAVTAW

EKGLESLPPLRPQQNPVLPVAGERNVLITSALPYVNNVPHLGNIIGCVLSADVFARYSRLRQWNTLYLCGTDEYGTATET

KAMEEGLTPQEICDKYHIIHADIYRWFNISFDVFGRTTTPQQTKITQDIFQRLLARGFVLQDTVEQLRCERCARFLADRF

VEGVCPFCGFEEARGDQCDKCGKLINAVELKKPQCKVCRSCPVVKSSQHLFLDLPKLEKRLEEWLGKTLPGSDWTPNARF

IIRSWLRDGLKPRCITRDLKWGTPVPLEGFEDKVFYVWFDATIGYLSITANYTDQWERWWKNPEQVDLYQFMAKDNVPFH

GLVFPCSALGAEDNYTLVSHLIATEYLNYEDGKFSKSRGVGVFGDMAQDTGIPADIWRFYLLYIRPEGQDSAFSWTDMLL

KNNSELLNNLGNFINRAGMFVSKFFGGYVPEMVLTPDDQRLLAHVTLELRRYHQLLEKVRIRDALRSMLTISRHGNQYIQ

VNEPWKRIKGSEADRQRAGTVTGLAVNIAALLSVMLQPYMPTVSATIQAQLQLPPPACSILLTNFLCTLPAGHQIGTVSP

LFQKLENDQIESLRQRFGGGQLEETLDLKAKMSPKPAVAETGTTAAPQQIQALMDEVTKQGNIVRELKAQKADKNQVAAE

VAKLLDLKKQLAVAEGKPPEVPKGKKKK

>XP_012325151.1 methionine--tRNA synthetase, cytoplasmic isoform X1 [Aotus nancymaae]

MRLFVSEGAPGSLPVLAAAGRARGRAELLISTVGPEDCVVPFLTRPKVPVLQLDSGNYLFSTSAICRYFFLLSGWEQDDL

TNQWLEWEATELQPALSAALYYLVVQGKKGEDVLGSVRRALTHIDHSLRRQNCPFLAGETESLADIVLWGALYPLLQDPA

YLPEELSALHSWFQTLSTQEPCQRAAETVLKQQGVLALRPYLQKQPQPSPPEGRAVTNEPEEEELATLSEEEIALAVTAW

EKGLESLPPLQPQQNPVLPVAGERNVLITSALPYVNNVPHLGNIIGCVLSADVFARYSRLRQWNTLYLCGTDEYGTATET

KAMEEGLTPQEICDKYHVIHADIYRWFNISFDVFGRTTTPQQTKITQDIFQRLLARGFVLQDTVEQLRCERCARFLADRF

VEGVCPFCGFEEARGDQCDKCGKLINAVELKKPQCKVCRSCPVVKSSQHLFLDLPKLEKRLEEWLGRTLPGSDWTPNARF

IIRSWLRDGLKPRCITRDLKWGTPVPLEGFEDKVFYVWFDATIGYLSITANYTDQWERWWKNPEQVDLYQFMAKDNVPFH

GLVFPCSALGAEDNYTLVSHLIATEYLNYEDGKFSKSRGVGVFGDMAQDTGIPADIWRFYLLYIRPEGQDSAFSWTDMLL

KNNSELLNNLGNFINRAGMFVSKFFGGYVPEMVLTPDDQRLLAHVTLELRRYHQLLEKVRIRDALRSILTISRHGNQYIQ

VNEPWKRIKGSEADRQRAGTVTGLAVNIAALLSVMLQPYMPTVSATIQAQLQLPPPACSILLTNFLCTLPAGHQIGTVSP

LFQKLENDQIESLRQRFGGGQLEETLDLKAKTSPKPAVAETGTTAGPQQIQALMDEVTKQGNIVRELKAQKADKNQVAAE

VAKLLDLKKQLAVAEGKPPEAPKSKKKK

>XP_011726767.1 methionine--tRNA synthetase, cytoplasmic [Macaca nemestrina]

MRLFVSEGAPGCLPVLAAAGRARGRAELLISTVGPEDCVVPFLTRPKVPVLQLDSGNYLFSTSAICRYFFLLSGWEQDDL

TNQWLEWEATELQPALSAALYYLVVQGKKGEDVLGSVRRALTHIDHSLSRQNCPFLAGETESLADIVLWGALYPLLQDPA

YLPEELSALHSWFQTLSTQEPCQRAAETVLKQQGVLALRPYLQKQPQPSPAEGRAVTNEPEEEELATLSEEEIAVAVTAW

EKGLESLPPLRPQQNPVLPVTGERNVLITSALPYVNNVPHLGNIIGCVLSADVFARYSRLRQWNTLYLCGTDEYGTATET

KAMEEGLTPQEICDKYHIIHADIYRWFNISFDTFGRTTTPQQTKITQDIFQRLLTRGFVLQDTVEQLRCEHCARFLADRF

VEGVCPFCGYEEARGDQCDKCGKLINAVELKKPQCKVLDHAPWCSRSHHLFSPLSQLEKRLEEWLGRTLPGSDWTPNARF

ITRSWLRDGLKPRCITRDLKWGTPVPLEGFEDKVFYVWFDATIGYLSITANYTDQWERWWKNPEQVDLYQFMAKDNVPFH

GIVFPCSALGAEDNYTLVSHLIATEYLNYEDGKFSKSRGVGVFGDMAQDTGIPADIWRFYLLYIRPEGQDSAFSWTDLLL

KNNSELLNNLGNFINRAGMFVSKFFGGYVPEMVLTPDDQRLLAHVTLELQHYHQLLEKVRIRDALRSILTISRHGNQYIQ

VNEPWKRIKGSEADRQRAGTVTGLAVNIAALLSVMLQPYMPTVSATIQAQLQLPPPACSILLTNFLCTLPAGHQIGTVSP

LFQKLENDQIESLRQRFGGGQAKTSPKPTVIETVTTAGPQQIQALMDEVTKQGNIVRELKAQKADKNQVAAEVAKLLDLK

KQLAVAEGKPPEAPKGKKKK

>XP_012507164.1 PREDICTED: methionine--tRNA synthetase, cytoplasmic isoform X2 [Propithecus coquereli]

MRLFVSEGAPGNLPVLAAAGRARGRVELLISTVDPEECMVPFLTRPKVPVLQLDSGNYLFSASSICRYFFLLSGWEQDDL

TNQWLEWEATELQPALSAALYYLVVQGKKGEDVLGPVRRALTHIDHSLSRQNCPFLAGETESLADIVLWGALYPLLQDPA

YLPEELGALRSWFQTLSTQEPCQRAAETVLKQQGVLALRPYLQKQPQPSSPDGRAVTNEPQEEELATLSEEEIAMAVTAW

EKGLESLPPLRPPQNPVLPVAGERNVLITSALPYVNNVPHLGNIIGCVLSADVFARYSRLRQWNTLYLCGTDEYGTATET

KAMEEGLTPQEICDKYHIIHADIYRWFNISFDIFGRTTTPQQTKITQDIFQRLLTRGFVLQDTVEQLRCEHCARFLADRF

VEGVCPFCGYEEARGDQCDKCGKLINAIELKKPRCKVCRSCPVVKSSQHLFLDLPKLEKRLEEWLGKTLPGSDWTPNARF

IIRSWLRDGLKPRCITRDLKWGTPVPLEGFEDKVFYVWFDATIGYLSITANYTDQWERWWKNPEQVDLYQFMAKDNVPFH

GLVFPCSALGTEDNYTLVSHLIATEYLNYEDGKFSKSRGVGVFGDMAQDTGIPADIWRFYLLYIRPEGQDSAFSWTDMLL

KNNSELLNNLGNFINRAGMFVSKFFGGYVPEMVLTPDDQRLLAHVTLELRHYHQLLEKVRIRDALRSILTISRHGNQYIQ

VNEPWKRIKGSEADRQRAGTVTGLAVNIAALLSVMLQPYMPTVSATIQAQLHLPPPACSILLKNFLCTLPAGHQIGTVSP

LFQKLENDQIESLKQRFGGGQAKVSPKPEVIETVTTAGSQQIQVLTDEVTKQGNIVRELKAQKADKNQIAAEVAKLLDLK

KQLALAEGKPLETPKGKKKK

>XP_012507156.1 PREDICTED: methionine--tRNA synthetase, cytoplasmic isoform X1 [Propithecus coquereli]

MRLFVSEGAPGNLPVLAAAGRARGRVELLISTVDPEECMVPFLTRPKVPVLQLDSGNYLFSASSICRYFFLLSGWEQDDL

TNQWLEWEATELQPALSAALYYLVVQGKKGEDVLGPVRRALTHIDHSLSRQNCPFLAGETESLADIVLWGALYPLLQDPA

YLPEELGALRSWFQTLSTQEPCQRAAETVLKQQGVLALRPYLQKQPQPSSPDGRAVTNEPQEEELATLSEEEIAMAVTAW

EKGLESLPPLRPPQNPVLPVAGERNVLITSALPYVNNVPHLGNIIGCVLSADVFARYSRLRQWNTLYLCGTDEYGTATET

KAMEEGLTPQEICDKYHIIHADIYRWFNISFDIFGRTTTPQQTKITQDIFQRLLTRGFVLQDTVEQLRCEHCARFLADRF

VEGVCPFCGYEEARGDQCDKCGKLINAIELKKPRCKVCRSCPVVKSSQHLFLDLPKLEKRLEEWLGKTLPGSDWTPNARF

IIRSWLRDGLKPRCITRDLKWGTPVPLEGFEDKVFYVWFDATIGYLSITANYTDQWERWWKNPEQVDLYQFMAKDNVPFH

GLVFPCSALGTEDNYTLVSHLIATEYLNYEDGKFSKSRGVGVFGDMAQDTGIPADIWRFYLLYIRPEGQDSAFSWTDMLL

KNNSELLNNLGNFINRAGMFVSKFFGGYVPEMVLTPDDQRLLAHVTLELRHYHQLLEKVRIRDALRSILTISRHGNQYIQ

VNEPWKRIKGSEADRQRAGTVTGLAVNIAALLSVMLQPYMPTVSATIQAQLHLPPPACSILLKNFLCTLPAGHQIGTVSP

LFQKLENDQIESLKQRFGGGQLEESLELKAKVSPKPEVIETVTTAGSQQIQVLTDEVTKQGNIVRELKAQKADKNQIAAE

VAKLLDLKKQLALAEGKPLETPKGKKKK

>XP_012632541.1 methionine--tRNA synthetase, cytoplasmic isoform X2 [Microcebus murinus]

MRLFVSEGAPGNLPVLAAAGRVRGRAELLISTVGPEECMVPFLTRPKVPVLQLDSGNYLFSTSSICRYFFLLSGWEQDDL

TNQWLEWEATELQPALSAALYCLVVQGKKGEDVLGPVRRALTHIDHSLSRQNCPFLAGETESLADIVLWGALYPLLQDPA

YLPEELGALHSWFQTLSTQEPCQRAAETVLKQQGVLALRPYLQKQPQPSPPDESAVTNEPEEEELATLSEEEIAMAVTAW

ENGLESLPPLRPRQNPVLPVAGERNVLITSALPYVNNVPHLGNIIGCVLSADVFARYSRLRQWNTLYLCGTDEYGTATET

KAMEEGLTPQEICDKYHVIHADIYRWFNISFDIFGRTTTPQQTKITQDIFQRLLTRGFVLRDTVEQLRCEHCARFLADRF

VEGVCPFCGYEEARGDQCDKCGKLINAIELKKPQCKVCRSCPVVKSSQHLFLDLPKLEKRLEEWLGKTLPGSDWTPNARF

IIRSWLRDGLKPRCITRDLKWGTPVPLEGFEDKVFYVWFDATIGYLSITANYTDQWERWWKNPEQVDLYQFMAKDNVPFH

GLVFPCSALGTEDNYTLVSHLIATEYLNYEDGKFSKSRGVGVFGDMAQDTGIPADIWRFYLLYIRPEGQDSAFSWADMLL

KNNSELLNNLGNFINRAGMFVSKFFGGYVPEMVLTPDDQRLLAHVTLELRHYHQLLEKVRIRDALRSILTISRHGNQYIQ

VNEPWKRIKGNEADRQRAGTVTGLAVNIAALLSVMLQPYMPTVSTTIQDQLQLPRPACSILLTNFLCTLPAGHQIGTVSP

LFQKLENDQIESLKQRFGGGQAKVSPKPEVVETVTTAGPQQIQVLMDEVTKQGNTVRELKAQKADKNQIAAEVAKLLELK

KQLALAEGKPLETSKGKKKK

>XP_014928705.1 PREDICTED: methionine--tRNA synthetase, cytoplasmic isoform X2 [Acinonyx jubatus]

MRLFVSEGAPGSLPVLAAAGRAQGREELLISTVGPEECVVPFLTRPKVPVLQLDSGNYLFSTSAICRYFFLLSGWEQDDL

TNQWLEWEATELQPALSAALYYLVVQGKKGEDVLGPVRRALTHIDHSLSRRSCPFLAGETESLADIVLWGALYPLLQDPA

YLPEELGALHSWFQTLSSQEPCQRAAETVLKQQGVLALRPYLQKQPLPSSFEGRAVSNEPEEEELATLSEEEIAMAVTAW

EKGLGSLPPLRPQQNPVLPVAGERNVLITSALPYVNNVPHLGNIIGCVLSADVFARYSRLRQWNTLYLCGTDEYGTATET

KAMEEGLTPQEICDKYHAIHANIYRWFNISFDIFGRTTTPQQTKITQDIFQRLLTRGFVFQDTVEQLRCEHCARFLADRF

VEGVCPFCGYEEARGDQCDKCGKLINAIELKKPQCKVCRSCPVVKSSQHLFLDLPKLEKRLEEWLGKTLPGSDWTPNARF

IIRSWLRDGLKPRCITRDLKWGTPVPLEGFEDKVFYVWFDATIGYVSITANYTDQWERWWKNPEQVNLYQFMAKDNVPFH

GLVFPCSALGAEDNYTLVSHLIATEYLNYEDGKFSKSRGVGVFGDMAQDTGIPADIWRFYLLYIRPEGQDSAFSWTDMLL

KNNSELLNNLGNFINRAGMFVSKFFGGCVPEMVLTPDDRRLLAHVTLELQHYHQLLEKVRIREALRSILTISRHGNQYIQ

VNEPWKRIKGSEADRQRAGTVTGLAVNIAALLSIMLQPYMPTVSATIQAQLQLPAPACSILLTNFLCTLPAGHQIGTVSP

LFQKLENDQIESLRQRFGGGQAKAPPKPAVVEAMATAGPQQIQVLTDEVTKQGNIVRELKAQKADKNQVAAEVAKLLDLK

KQLALAEGKPLETPKGKKKK

>XP_019310441.1 PREDICTED: methionine--tRNA synthetase, cytoplasmic isoform X2 [Panthera pardus]

MRLFVSEGAPGSLPVLAAAGRAQGREELLISTVGPEECVVPFLTRPKVPVLQLDSGNYLFSTSAICRYFFLLSGWEQDDL

TNQWLEWEATELQPALSAALYYLVVQGKKGEDVLGPVRRALTHIDHSLSRRSCPFLAGETESLADIVLWGALYPLLQDPA

YLPEELGALHSWFQTLSSQEPCQRAAETVLKQQGVLALRPYLQKQPLPSSFEGRAVSNEPEEEELATLSEEEIAMAVTAW

EKGLGSLPPLRPQQNPVLPVAGERNVLITSALPYVNNVPHLGNIIGCVLSADVFARYSRLRQWNTLYLCGTDEYGTATET

KAMEEGLTPQEICDKYHAIHANIYRWFNISFDIFGRTTTPQQTKITQDIFQRLLTRGFVFQDTVEQLRCEHCARFLADRF

VEGVCPFCGYEEARGDQCDKCGKLINAIELKKPQCKVCRSCPVVKSSQHLFLDLPKLEKRLEEWLGKTLPGSDWTPNARF

IIRSWLRDGLKPRCITRDLKWGTPVPLEGFEDKVFYVWFDATIGYVSITANYTDQWERWWKNPEQVNLYQFMAKDNVPFH

GLVFPCSALGAEDNYTLVNHLIATEYLNYEDGKFSKSRGVGVFGDMAQDTGIPADIWRFYLLYIRPEGQDSAFSWTDMLL

KNNSELLNNLGNFINRAGMFVSKFFGGCVPEMVLTPDDRRLLAHVTLELQHYHQLLEKVRIREALRSILTISRHGNQYIQ

VNEPWKRIKGSEADRQRAGTVTGLAVNIAALLSVMLQPYMPTVSATIQAQLQLPAPACSILLTNFLCTLPAGHQIGTVSP

LFQKLENDQIESLRQRFGGGQAKAPPKPAVVEAMATAGPQQIQVLTDEVTKQGNIVRELKAQKADKNQVAAEVAKLLDLK

KQLALAEGKPLETPKGKKKK

>EHH66424.1 Methionyl-tRNA synthetase, cytoplasmic, partial [Macaca fascicularis]

SVFASDCVVPFLTRPKVPVLQLDSGNYLFSTSAICRYFFLLSGWEQDDLTNQWLEWEATELQPALSAALYYLVVQGKKGE

DVLGSVRRALTHIDHSLSRQNCPFLAGETESLADIVLWGALYPLLQDPAYLPEELSALHSWFQTLSTQEPCQRAAETVLK

QQGVLALRPYLQKQPQPSPAEGRAVTNEPEEEELATLSEEEIAMAVTAWEKGLESLPPLRPQQNPVLPVTGERNVLITSA

LPYVNNVPHLGNIIGCVLSADVFARYSRLRQWNTLYLCGTDEYGTATETKAMEEGLTPQEICDKYHIIHADIYRWFNISF

DTFGRTTTPQQTKITQDIFQRLLTRGFVLQDTVEQLRCEHCARFLADRFVEGVCPFCGYEEARGDQCDKCGKLINAVELK

KPQCKVCRSCPVVQSTHHLFLDLPKLEKRLEEWLGRTLPGSDWTPNARFITRSWLRDGLKPRCITRDLKWGTPVPLEGFE

DKVFYVWFDATIGYLSITANYTDQWERWWKNPEQVDLYQFMAKDNVPFHGIVFPCSALGAEDNYTLVSHLIATEYLNYED

GKFSKSRGVGVFGDMAQDTGIPADIWRFYLLYIRPEGQDSAFSWTDLLLKNNSELLNNLGNFINRAGMFVSKFFGGYVPE

MVLTPDDQRLLAHVTLELQHYHQLLEKVRIRDALRSILTISRHGNQYIQVNEPWKRIKGSEADRQRAGTVTGLAVNIAAL

LSVMLQPYMPTVSATIQAQLQLPPPACSILLTNFLCTLPAGHQIGTVSPLFQKLENDQIESLRQRFGGGQAKTSPKPTVI

ETVTTAGPQQIQALMDEVTKQGNIVRELKAQKADKNQVAAEVAKLLDLKKQLAVAEGKPPEAPKGKKKK

>XP_007075038.1 PREDICTED: methionine--tRNA synthetase, cytoplasmic isoform X2 [Panthera tigris altaica]

MRLFVSEGAPGSLPVLAAAGRAQGREELLISTVGPEECVVPFLTRPKVPVLQLDSGNYLFSTSAICRYFFLLSGWEQDDL

TNQWLEWEATELQPALSAALYYLVVQGKKGEDVLGPVRRALTHIDHSLSRRSCPFLAGETESLADIVLWGALYPLLQDPA

YLPEELGALHSWFQTLSSQEPCQRAAETVLKQQGVLALRPYLQKQPLPSSFEGRAVSNEPEEEELATLSEEEIAMAVTAW

EKGLGSLPPLRPQQNPVLPVAGERNVLITSALPYVNNVPHLGNIIGCVLSADVFARYSRLRQWNTLYLCGTDEYGTATET

KAMEEGLTPQEICDKYHAIHANIYRWFNISFDIFGRTTTPQQTKITQDIFQRLLTRGFVFQDTVEQLRCEHCARFLADRF

VEGVCPFCGYEEARGDQCDKCGKLINAIELKKPQCKVCRSCPVVKSSQHLFLDLPKLEKRLEEWLGKTLPGSDWTPNARF

IIRSWLRDGLKPRCITRDLKWGTPVPLEGFEDKVFYVWFDATIGYVSITANYTDQWERWWKNPEQVNLYQFMAKDNVPFH

GLVFPCSALGAEDNYTLVNHLIATEYLNYEDGKFSKSRGVGVFGDMAQDTGIPADIWRFYLLYIRPEGQDSAFSWTDMLL

KNNSELLNNLGNFINRAGMFVSKFFGGCVPEMVLTPDDRRLLAHVTLELQHYHQLLEKVRIREALRSILTISRHGNQYIQ

VNEPWKRIKGSEADRQRAGTVTGLAVNIAALLSVMLQPYMPTVSATIQAQLQLPAPACSILLTNFLCTLPAGHQIGTVSP

LFQKLENDQIESLRQRFGGGQAKAPPKPAVVEAMATAGPQQIQVLTDEVTKQGNIVRELKAQKADKNQIAAEVAKLLDLK

KQLALAEGKPLETPKGKKKK

>XP_023112936.1 methionine--tRNA synthetase, cytoplasmic [Felis catus]

MRLFVSEGAPGSLPVLAAAGRAQGREELLISTVGPSECVVPFLTRPKVPVLQLDSGNYLFSTSAICRYFFLLSGWEQDDL

TNQWLEWEATELQPALSAALYYLVVQGKKGEDVLGPVRRALTHIDHSLSRRSCPFLAGETESLADIVLWGALYPLLQDPA

YLPEELGALHSWFQTLSSQEPCQRAAETVLKQQGVLALRPYLQKQPLPSSFEGRAVSNEPEEEELVTLSEEEIAMAVTAW

EKGLGSLPPLRPQQNPVLPVAGERNVLITSALPYVNNVPHLGNIIGCVLSADVFARYSRLRQWNTLYLCGTDEYGTATET

KAMEEGLTPQEICDKYHAIHANIYRWFNISFDIFGRTTTPQQTKITQDIFQRLLTRGFVFQDTVEQLRCEHCARFLADRF

VEGVCPFCGYEEARGDQCDKCGKLINAIELKKPQCKVCRSCPVVKSSQHLFLDLPKLEKRLEEWLGKTLPGSDWTPNARF

IIRSWLRDGLKPRCITRDLKWGTPVPLEGFEDKVFYVWFDATIGYVSITANYTDQWERWWKNPEQVNLYQFMAKDNVPFH

GLVFPCSALGAEDNYTLVNHLIATEYLNYEDGKFSKSRGVGVFGDMAQDTGIPADIWRFYLLYIRPEGQDSAFSWTDMLL

KNNSELLNNLGNFINRAGMFVSKFFGGCVPEMVLTPDDRRLLAHVTLELQHYHQLLEKVRIREALRSILTISRHGNQYIQ

VNEPWKRIKGSEADRQRAGTVTGLAVNIAALLSVMLQPYMPTVSATIQAQLQLPAPACSILLTNFLCTLPAGHQIGTVSP

LFQKLENDQIESLRQRFGGGQAKAPPKPAVVEAVATAGPQQIQVLTDEVTKQGNIVRELKAQKADKNQVAAEVAKLLDLK

KQLALAEGKPLETPKGKKKK

>XP_012632540.1 methionine--tRNA synthetase, cytoplasmic isoform X1 [Microcebus murinus]

MRLFVSEGAPGNLPVLAAAGRVRGRAELLISTVGPEECMVPFLTRPKVPVLQLDSGNYLFSTSSICRYFFLLSGWEQDDL

TNQWLEWEATELQPALSAALYCLVVQGKKGEDVLGPVRRALTHIDHSLSRQNCPFLAGETESLADIVLWGALYPLLQDPA

YLPEELGALHSWFQTLSTQEPCQRAAETVLKQQGVLALRPYLQKQPQPSPPDESAVTNEPEEEELATLSEEEIAMAVTAW

ENGLESLPPLRPRQNPVLPVAGERNVLITSALPYVNNVPHLGNIIGCVLSADVFARYSRLRQWNTLYLCGTDEYGTATET

KAMEEGLTPQEICDKYHVIHADIYRWFNISFDIFGRTTTPQQTKITQDIFQRLLTRGFVLRDTVEQLRCEHCARFLADRF

VEGVCPFCGYEEARGDQCDKCGKLINAIELKKPQCKVCRSCPVVKSSQHLFLDLPKLEKRLEEWLGKTLPGSDWTPNARF

IIRSWLRDGLKPRCITRDLKWGTPVPLEGFEDKVFYVWFDATIGYLSITANYTDQWERWWKNPEQVDLYQFMAKDNVPFH

GLVFPCSALGTEDNYTLVSHLIATEYLNYEDGKFSKSRGVGVFGDMAQDTGIPADIWRFYLLYIRPEGQDSAFSWADMLL

KNNSELLNNLGNFINRAGMFVSKFFGGYVPEMVLTPDDQRLLAHVTLELRHYHQLLEKVRIRDALRSILTISRHGNQYIQ

VNEPWKRIKGNEADRQRAGTVTGLAVNIAALLSVMLQPYMPTVSTTIQDQLQLPRPACSILLTNFLCTLPAGHQIGTVSP

LFQKLENDQIESLKQRFGGGQLEESLELKAKVSPKPEVVETVTTAGPQQIQVLMDEVTKQGNTVRELKAQKADKNQIAAE

VAKLLELKKQLALAEGKPLETSKGKKKK

>XP_007179732.1 PREDICTED: methionine--tRNA synthetase, cytoplasmic isoform X2 [Balaenoptera acutorostrata scammoni]

MMGEIVYFPGVWPGGKGHIRNSISRRASRRSLIPLAGDRFRLHQRGIHGEMRLFVSEGAPGSLPVLAAAGRAQCRAELLI

STVGPEECVVPFLTRPKVPVLQLDSGNYLFSTSAICRYFFLLSGWEQDDLTNQWLEWEATELQPALSAALYYLVVQGKKG

EDVFSPVRRALTHIDHSLSRRSCPFLAGETESLADIVLWGALYPLLQDPAYLPEELGALHSWFQTLSSQEPCQRAAETVL

KQQGVLALRPYLQKQPQPSSLEGRAVSNEPEEEELATLSEEEIAMAVAAWEKGLESLPPVQPQQNPVLPVAGERNVLITS

ALPYVNNVPHLGNIIGCVLSADVFARYSRLRQWNTLYLCGTDEYGTATETKAMEEGLTPQEICDKYHVIHADIYRWFNIS

FDIFGRTTTPQQTKITQDIFQRLVARGFVLQDTVEQLRCEHCARFLADRFVEGVCPFCGYEEARGDQCDKCGKLINAIEL

KKPQCKVCRSCPVVKSSQHLFLDLPKLGKPLEEWLEKTLPGSDWTPNARFIIRSWIRDGLKPRCITRDLKWGTPVPLVGF

EDKVFYVWFDATIGYLSITANYTDQWEKWWKNPEQVSLYQFMAKDNVPFHGLVFPCSALGAEDNYTLVSHLIATEYLNYE

DGKFSKSRGVGVFGDMAQDTGIPADIWRFYLLYIRPEGQDSAFSWTDMLLKNNSELLNNLGNFINRAGMFVSKFFGGFVP

EMVLTSDDRRLLAHVTLELQHYHQLLEKVRIRDALRSILTISRHGNQYIQVNEPWKRIKGSEADRQRAGTVTGLAVNIAA

LLSIMLQPYMPTVSATIQAQLQLPPPACSILLTNFLCTLPAGHQIGTVSPLFQKLENDQIESLRQRFSGGQAKASPKPAA

VETMTTAGPQQIQVLMDEVTKQGNIVRELKAQKADKNQVAAEVAKLLDLKKQLALAEGKPLETPKGKKKK

>XP_014928704.1 PREDICTED: methionine--tRNA synthetase, cytoplasmic isoform X1 [Acinonyx jubatus]

MRLFVSEGAPGSLPVLAAAGRAQGREELLISTVGPEECVVPFLTRPKVPVLQLDSGNYLFSTSAICRYFFLLSGWEQDDL

TNQWLEWEATELQPALSAALYYLVVQGKKGEDVLGPVRRALTHIDHSLSRRSCPFLAGETESLADIVLWGALYPLLQDPA

YLPEELGALHSWFQTLSSQEPCQRAAETVLKQQGVLALRPYLQKQPLPSSFEGRAVSNEPEEEELATLSEEEIAMAVTAW

EKGLGSLPPLRPQQNPVLPVAGERNVLITSALPYVNNVPHLGNIIGCVLSADVFARYSRLRQWNTLYLCGTDEYGTATET

KAMEEGLTPQEICDKYHAIHANIYRWFNISFDIFGRTTTPQQTKITQDIFQRLLTRGFVFQDTVEQLRCEHCARFLADRF

VEGVCPFCGYEEARGDQCDKCGKLINAIELKKPQCKVCRSCPVVKSSQHLFLDLPKLEKRLEEWLGKTLPGSDWTPNARF

IIRSWLRDGLKPRCITRDLKWGTPVPLEGFEDKVFYVWFDATIGYVSITANYTDQWERWWKNPEQVNLYQFMAKDNVPFH

GLVFPCSALGAEDNYTLVSHLIATEYLNYEDGKFSKSRGVGVFGDMAQDTGIPADIWRFYLLYIRPEGQDSAFSWTDMLL

KNNSELLNNLGNFINRAGMFVSKFFGGCVPEMVLTPDDRRLLAHVTLELQHYHQLLEKVRIREALRSILTISRHGNQYIQ

VNEPWKRIKGSEADRQRAGTVTGLAVNIAALLSIMLQPYMPTVSATIQAQLQLPAPACSILLTNFLCTLPAGHQIGTVSP

LFQKLENDQIESLRQRFGGGQLEESLELKAKAPPKPAVVEAMATAGPQQIQVLTDEVTKQGNIVRELKAQKADKNQVAAE

VAKLLDLKKQLALAEGKPLETPKGKKKK

>XP_019310440.1 PREDICTED: methionine--tRNA synthetase, cytoplasmic isoform X1 [Panthera pardus]

MRLFVSEGAPGSLPVLAAAGRAQGREELLISTVGPEECVVPFLTRPKVPVLQLDSGNYLFSTSAICRYFFLLSGWEQDDL

TNQWLEWEATELQPALSAALYYLVVQGKKGEDVLGPVRRALTHIDHSLSRRSCPFLAGETESLADIVLWGALYPLLQDPA

YLPEELGALHSWFQTLSSQEPCQRAAETVLKQQGVLALRPYLQKQPLPSSFEGRAVSNEPEEEELATLSEEEIAMAVTAW

EKGLGSLPPLRPQQNPVLPVAGERNVLITSALPYVNNVPHLGNIIGCVLSADVFARYSRLRQWNTLYLCGTDEYGTATET

KAMEEGLTPQEICDKYHAIHANIYRWFNISFDIFGRTTTPQQTKITQDIFQRLLTRGFVFQDTVEQLRCEHCARFLADRF

VEGVCPFCGYEEARGDQCDKCGKLINAIELKKPQCKVCRSCPVVKSSQHLFLDLPKLEKRLEEWLGKTLPGSDWTPNARF

IIRSWLRDGLKPRCITRDLKWGTPVPLEGFEDKVFYVWFDATIGYVSITANYTDQWERWWKNPEQVNLYQFMAKDNVPFH

GLVFPCSALGAEDNYTLVNHLIATEYLNYEDGKFSKSRGVGVFGDMAQDTGIPADIWRFYLLYIRPEGQDSAFSWTDMLL

KNNSELLNNLGNFINRAGMFVSKFFGGCVPEMVLTPDDRRLLAHVTLELQHYHQLLEKVRIREALRSILTISRHGNQYIQ

VNEPWKRIKGSEADRQRAGTVTGLAVNIAALLSVMLQPYMPTVSATIQAQLQLPAPACSILLTNFLCTLPAGHQIGTVSP

LFQKLENDQIESLRQRFGGGQLEESLELKAKAPPKPAVVEAMATAGPQQIQVLTDEVTKQGNIVRELKAQKADKNQVAAE

VAKLLDLKKQLALAEGKPLETPKGKKKK

>XP_007075037.1 PREDICTED: methionine--tRNA synthetase, cytoplasmic isoform X1 [Panthera tigris altaica]

MRLFVSEGAPGSLPVLAAAGRAQGREELLISTVGPEECVVPFLTRPKVPVLQLDSGNYLFSTSAICRYFFLLSGWEQDDL

TNQWLEWEATELQPALSAALYYLVVQGKKGEDVLGPVRRALTHIDHSLSRRSCPFLAGETESLADIVLWGALYPLLQDPA

YLPEELGALHSWFQTLSSQEPCQRAAETVLKQQGVLALRPYLQKQPLPSSFEGRAVSNEPEEEELATLSEEEIAMAVTAW

EKGLGSLPPLRPQQNPVLPVAGERNVLITSALPYVNNVPHLGNIIGCVLSADVFARYSRLRQWNTLYLCGTDEYGTATET

KAMEEGLTPQEICDKYHAIHANIYRWFNISFDIFGRTTTPQQTKITQDIFQRLLTRGFVFQDTVEQLRCEHCARFLADRF

VEGVCPFCGYEEARGDQCDKCGKLINAIELKKPQCKVCRSCPVVKSSQHLFLDLPKLEKRLEEWLGKTLPGSDWTPNARF

IIRSWLRDGLKPRCITRDLKWGTPVPLEGFEDKVFYVWFDATIGYVSITANYTDQWERWWKNPEQVNLYQFMAKDNVPFH

GLVFPCSALGAEDNYTLVNHLIATEYLNYEDGKFSKSRGVGVFGDMAQDTGIPADIWRFYLLYIRPEGQDSAFSWTDMLL

KNNSELLNNLGNFINRAGMFVSKFFGGCVPEMVLTPDDRRLLAHVTLELQHYHQLLEKVRIREALRSILTISRHGNQYIQ

VNEPWKRIKGSEADRQRAGTVTGLAVNIAALLSVMLQPYMPTVSATIQAQLQLPAPACSILLTNFLCTLPAGHQIGTVSP

LFQKLENDQIESLRQRFGGGQLEESLELKAKAPPKPAVVEAMATAGPQQIQVLTDEVTKQGNIVRELKAQKADKNQIAAE

VAKLLDLKKQLALAEGKPLETPKGKKKK

>XP_020947526.1 methionine--tRNA synthetase, cytoplasmic isoform X2 [Sus scrofa]

MMGEIVYFRNPGPGNSFSRNSISRRAARQSLIPLAGDRFRLHQRGTHGEMRLFVSEGAPGSLPVLAAAGRAQGRVELLIS

TVGPEECVVPFLTRPKVPVLQLDSGNYLFSASAICRYFFLLSGWEQDDLTNQWLEWEATELQPALSAALYYLVVQGKKGE

DVLGPVRRALTHIDHSLSRRSCPFLAGETESLADIVLWGALYPLLQDPAYLPEELGTLHSWFQTLSSQEPCQRAVETVLK

QQGVLALRPYLQKQPQPSFLEGRAVSNEPEEEELATLSEEEIAMAVAAWEKGLGSLPPLRPQQNPVLPVAGERNVLITSA

LPYVNNVPHLGNIIGCVLSADVFARYSRLRQWNTLYLCGTDEYGTATETKAMEEGLTPQEICDKYHAVHADIYRWFNISF

DIFGRTTTPQQTKITQDIFQRLFTRGFVLQDTVEQLRCEHCARFLADRFVEGVCPFCGYEEARGDQCDKCGKLINAIELK

KPQCKVCRSCPVVKSSRHLFLDLPKLEKRLEEWLEKTLPGSDWTPNARFIIRSWLRDGLKPRCITRDLKWGTPVPLEGFE

DKVFYVWFDATIGYLSITANYTDQWERWWKNPEQVSLYQFMAKDNVPFHGLVFPCSALGTEDNYTLVSHLIATEYLNYED

GKFSKSRGVGVFGDMAQNTGIPADIWRFYLLYIRPEGQDTAFSWTDMLLKNNSELLNNLGNFINRAGMFVSKFFGCFVPE

MVLTSDDRRLLAHVTLELQHYHQLLEKARIRDALRSILTISRHGNQYIQVNEPWKRIKGSEADRQRAGTVTGLAVNIAAL

LSVMLQPYMPTVSATIQAQLQLSPPACSILPTNFLCTLPAGHQIGTVSPLFQKLENDQIESLRQRFGGGQAKVSPKPAVV

ETVTTAGPQQVQVLMDEVTKQGNIVRELKAQKADKNQVAAEVAKLLDLKKQLALAEGKPFETPKGKKKR

>XP_007179731.1 PREDICTED: methionine--tRNA synthetase, cytoplasmic isoform X1 [Balaenoptera acutorostrata scammoni]

MMGEIVYFPGVWPGGKGHIRNSISRRASRRSLIPLAGDRFRLHQRGIHGEMRLFVSEGAPGSLPVLAAAGRAQCRAELLI

STVGPEECVVPFLTRPKVPVLQLDSGNYLFSTSAICRYFFLLSGWEQDDLTNQWLEWEATELQPALSAALYYLVVQGKKG

EDVFSPVRRALTHIDHSLSRRSCPFLAGETESLADIVLWGALYPLLQDPAYLPEELGALHSWFQTLSSQEPCQRAAETVL

KQQGVLALRPYLQKQPQPSSLEGRAVSNEPEEEELATLSEEEIAMAVAAWEKGLESLPPVQPQQNPVLPVAGERNVLITS

ALPYVNNVPHLGNIIGCVLSADVFARYSRLRQWNTLYLCGTDEYGTATETKAMEEGLTPQEICDKYHVIHADIYRWFNIS

FDIFGRTTTPQQTKITQDIFQRLVARGFVLQDTVEQLRCEHCARFLADRFVEGVCPFCGYEEARGDQCDKCGKLINAIEL

KKPQCKVCRSCPVVKSSQHLFLDLPKLGKPLEEWLEKTLPGSDWTPNARFIIRSWIRDGLKPRCITRDLKWGTPVPLVGF

EDKVFYVWFDATIGYLSITANYTDQWEKWWKNPEQVSLYQFMAKDNVPFHGLVFPCSALGAEDNYTLVSHLIATEYLNYE

DGKFSKSRGVGVFGDMAQDTGIPADIWRFYLLYIRPEGQDSAFSWTDMLLKNNSELLNNLGNFINRAGMFVSKFFGGFVP

EMVLTSDDRRLLAHVTLELQHYHQLLEKVRIRDALRSILTISRHGNQYIQVNEPWKRIKGSEADRQRAGTVTGLAVNIAA

LLSIMLQPYMPTVSATIQAQLQLPPPACSILLTNFLCTLPAGHQIGTVSPLFQKLENDQIESLRQRFSGGQLEESLSLKA

KASPKPAAVETMTTAGPQQIQVLMDEVTKQGNIVRELKAQKADKNQVAAEVAKLLDLKKQLALAEGKPLETPKGKKKK

>XP_020947525.1 methionine--tRNA synthetase, cytoplasmic isoform X1 [Sus scrofa]

MMGEIVYFRNPGPGNSFSRNSISRRAARQSLIPLAGDRFRLHQRGTHGEMRLFVSEGAPGSLPVLAAAGRAQGRVELLIS

TVGPEECVVPFLTRPKVPVLQLDSGNYLFSASAICRYFFLLSGWEQDDLTNQWLEWEATELQPALSAALYYLVVQGKKGE

DVLGPVRRALTHIDHSLSRRSCPFLAGETESLADIVLWGALYPLLQDPAYLPEELGTLHSWFQTLSSQEPCQRAVETVLK

QQGVLALRPYLQKQPQPSFLEGRAVSNEPEEEELATLSEEEIAMAVAAWEKGLGSLPPLRPQQNPVLPVAGERNVLITSA

LPYVNNVPHLGNIIGCVLSADVFARYSRLRQWNTLYLCGTDEYGTATETKAMEEGLTPQEICDKYHAVHADIYRWFNISF

DIFGRTTTPQQTKITQDIFQRLFTRGFVLQDTVEQLRCEHCARFLADRFVEGVCPFCGYEEARGDQCDKCGKLINAIELK

KPQCKVCRSCPVVKSSRHLFLDLPKLEKRLEEWLEKTLPGSDWTPNARFIIRSWLRDGLKPRCITRDLKWGTPVPLEGFE

DKVFYVWFDATIGYLSITANYTDQWERWWKNPEQVSLYQFMAKDNVPFHGLVFPCSALGTEDNYTLVSHLIATEYLNYED

GKFSKSRGVGVFGDMAQNTGIPADIWRFYLLYIRPEGQDTAFSWTDMLLKNNSELLNNLGNFINRAGMFVSKFFGCFVPE

MVLTSDDRRLLAHVTLELQHYHQLLEKARIRDALRSILTISRHGNQYIQVNEPWKRIKGSEADRQRAGTVTGLAVNIAAL

LSVMLQPYMPTVSATIQAQLQLSPPACSILPTNFLCTLPAGHQIGTVSPLFQKLENDQIESLRQRFGGGQLEESLALKAK

VSPKPAVVETVTTAGPQQVQVLMDEVTKQGNIVRELKAQKADKNQVAAEVAKLLDLKKQLALAEGKPFETPKGKKKR

>XP_007467730.1 PREDICTED: methionine--tRNA synthetase, cytoplasmic [Lipotes vexillifer]

MRLFVSEGAPGSLPVLAAAGRAQGRAELLISTVGPEECVVPFLTRPKVPVLQLDSGNYLFSTSAICRYFFLLSGWEQDDL

TNQWLEWEATELQPALSAALYYLVVQGKKGEDVFSPVRRALTHIDHSLSRRSCPFLAGETESLADLVLWGALYPLLQDPA

YLPEELGALHSWFQTLSSQEPCQRAAETVLKQQGVLALRPYLQKQPQPSSLEGRAVSNEPEEEELATLSEEEIAMAVAAW

EKGLESLPPVQPQQNPVLPVAGERNVLITSALPYVNNVPHLGNIIGCVLSADVFARYSRLRQWNTLYLCGTDEYGTATET

KAMEEGLTPQEICDKYHVIHADIYRWFNISFDIFGRTTTPQQTKITQDIFQRLVARGFVLQDTVEQLRCEHCARFLADRF

VEGMCPFCGYEEARGDQCDKCGKLINAIELKKPQCKVCRSCPVVKSSQHLFLDLPKLGKPLEEWLEKTLPGSDWTPNARF

IIRSWLRDGLKPRCITRDLKWGTPVPLVGFEDKVFYVWFDATIGYLSITANYTDQWEKWWKNPEQVSLYQFMAKDNVPFH

GLVFPCSALGAEDNYTLVSHLIATEYLNYEDGKFSKSRGVGVFGDMAQDTGIPADIWRFYLLYIRPEGQDSAFSWTDMLL

KNNSELLNNLGNFINRAGMFVSKFFGGFVPEMVLTSDDRRLLAHVTLELQHYHQLLEKVRIRDALRSILTISRHGNQYIQ

VNEPWKRIKGNEADRQRAGTVTGLAVNIAALLSIMLQPYMPTVSATIQAQLQLPPPACSILLTNFLCTLPAGHQIGTVSP

LFQKLENDQIESLRQRFSGGQAKASPKPAAVETMTTAGPQQIQVLMDEVTKQGNIVRELKAQKADKNQVAAEVAKLLDLK

KQLALAEGKPLETPKGKKKK

>XP_007103952.1 methionine--tRNA synthetase, cytoplasmic [Physeter catodon]

MRLFVSEGAPGSLPVLAAAGRAQGRAELLISTVGPEECVVPFLTRPKVPVLQLDSGNYLFSTSAICRYFFLLSGWEQDDL

TNQWLEWEATELQPALSAALYYLVVQGKKGEDVFSPVRRALTHIDHSLSRRSCPFLAGETESLADIVLWGALYPLLQDPA

YLPEELGALHSWFQTLSSQEPCQRAAETVLKQQGVLALRPYLQKQPQPSSFEGRAVSNEPEEEELATLSEEEIAMAVAAW

EKGLESLPPVQPQQNPVLPVAGERNVLITSALPYVNNVPHLGNIIGCVLSADVFARYSRLRQWNTLYLCGTDEYGTATET

KAMEEGLTPQEICDKYHVIHADIYRWFNISFDIFGRTTTPQQTKITQDIFQRLVARGFVLQDTVEQLRCEHCARFLADRF

VEGVCPFCGYEEARGDQCDKCGKLINAIELKKPQCKVCRSCPVVKSSQHLFLDLPKLGKPLEEWLEKTLPGSDWTPNARF

IIRSWLRDGLKPRCITRDLKWGTPVPLVGFEDKVFYVWFDATIGYLSITANYTDQWEKWWKNPEQVSLYQFMAKDNVPFH

GLVFPCSALGAEDNYTLVSHLIATEYLNYEDGKFSKSRGVGVFGDMAQDTGIPADIWRFYLLYIRPEGQDSAFSWTDMLL

KNNSELLNNLGNFINRAGMFVSKFFGGFVPEMVLTSDDRRLLAHVTLELQHYHQLLEKVRIRDALRSILTISRHGNQYIQ

VNEPWKRIKGSEADRQRAGTVTGLAVNIAALLSIMLQPYMPTVSATIQDQLQLPPPACSILLTNFLCTLPAGHQIGTVSP

LFQKLENDQIESLRQRFSGGQAKASPKPAAIETMTTAGPQQIQVLMDEVTKQGNIVRELKAQKADKNQVAAEVAKLLDLK

KQLALAEGKPLENPKGKKKK

>XP_022427446.1 methionine--tRNA synthetase, cytoplasmic isoform X1 [Delphinapterus leucas]

MRLFVSEGAPGSLPVLAAAGRAQGRAELLISTVGPEECVVPFLTRPKVPVLQLDSGNYLFSTSAICRYFFLLSGWEQDDL

TNQWLEWEATELQPALSAALYYLVVQGKKGEDVFSPVRRALTHIDHSLSRRSCPFLAGETESLADIVLWGALYPLLQDPA

YLPEELGALHSWFQTLSSQEPCQRAAETVLKQQGVLALRPYLQKQPQPSFLEGRAVSNEPEEEELATLSEEEIAMAVAAW

EKGLESLPPVQPQQNPVLPVAGEKNVLITSALPYVNNVPHLGNIIGCVLSADVFARYSRLRQWNTLYLCGTDEYGTATET

KAMEEGLTPQEICDKYHVIHADIYRWFNISFDIFGRTTTPQQTKITQDIFQRLVARGFVLQDTVEQLRCEHCARFLADRF

VEGMCPFCGYEEARGDQCDKCGKLINAIELKKPQCKVCRSCPVVKSSRHLFLDLPKLGKPLEEWLEKTLPGSDWTPNARF

IIRSWLRDGLKPRCITRDLKWGTPVPLVGFEDKVFYVWFDATIGYLSITANYTDQWEKWWKNPEQVSLYQFMAKDNVPFH

GLVFPCSALGAEDNYTLVSHLIATEYLNYEDGKFSKSRGVGVFGDMAQDTGIPADIWRFYLLYIRPEGQDSAFSWTDMLL

KNNSELLNNLGNFINRAGMFVSKFFGGFVPEMVLTSDDRRLLAHVTLELQHYHQLLEKVRIRDALRSILTISRHGNQYIQ

VNEPWKRIKGSEADRQRAGTVTGLAVNIAALLSIMLQPYMPTVSATIQAQLQLPPPACSILLTNFLCTLPAGHQIGTVSP

LFQKLENDQIESLRQRFSGGQAKASPKPAAVETMTTAGPQQIQVLMDEVTKQGNIVRELKAQKADKNQVAAEVAKLLDLK

KQLALAEGKPLETPKGKKKK

>XP_004276586.1 PREDICTED: methionine--tRNA synthetase, cytoplasmic [Orcinus orca]

MRLFVSEGAPGSLPVLAAAGRAQGRAELLISTVGPEECVVPFLTRPKVPVLQLDSGNYLFSTSAICRYFFLLSGWEQDDL

TNQWLEWEATELQPALSAALYYLVVQGKKGEDVFSPVRRALTHIDHSLSRRSCPFLAGETESLADIVLWGALYPLLQDPA

YLPEELGALHSWFQTLSSQEPCQRAAETVLKQQGALALRPYLQKQPQPSFLEGRAVSNEPEEEELATLSEEEIAMAVAAW

EKGLESLPPVQPQQNPVLPVAGERNVLITSALPYVNNVPHLGNIIGCVLSADVFARYSRLRQWNTLYLCGTDEYGTATET

KAMEEGLTPQEICDKYHVIHADIYRWFNISFDIFGRTTTPQQTKITQDIFQRLVARGFVLQDTVEQLRCEHCARFLADRF

VEGMCPFCGYEEARGDQCDKCGKLINAIELKKPQCKVCRSCPVVKSSRHLFLDLPKLGKPLEEWLEKTLPGSDWTPNARF

IIRSWLRDGLKPRCITRDLKWGTPVPLVGFEDKVFYVWFDATIGYLSITANYTDQWEKWWKNPEQVSLYQFMAKDNVPFH

GLVFPCSALGAEDNYTLVSHLIATEYLNYEDGKFSKSRGVGVFGDMAQDTGIPADIWRFYLLYIRPEGQDSAFSWTDMLL

KNNSELLNNLGNFINRAGMFVSKFFGGFVPEMVLTSDDRRLLAHVTLELQHYHQLLEKVRIRDALRSILTISRHGNQYIQ

VNEPWKRIKGSEADRQRAGTVTGLAVNIAALLSIMLQPYMPTVSATIQAQLQLPPPACSILLTNFLCTLPAGHQIGTVSP

LFQKLENDQIESLRQRFSGGQAKASPKPAAVETMTTAGPQQIQVLMDEVTKQGNIVRELKAQKADKNQVAAEVAKLLDLK

KQLALAEGKPLETPKGKKKK

>XP_010642910.1 PREDICTED: methionine--tRNA synthetase, cytoplasmic isoform X2 [Fukomys damarensis]

MRLFVSEGAPGSLPVLAAAARAQGLAELLISTVGPEECVVPFLTQPKVPVLQLDSGNYLFSTSAICRYFFLLSGWEQDDL

TNQWLEWEATELQPALSAALYYLVVQGKKGEDVLSPLGRALTHIDHSLSRQNCPFLAGETESLADIVLWGALYPLLQDPA

YLPEELGALRSWFQTLSTQEPCQRAAETVLKQRGVLALRPYLQKQPQPSPPEGRGVSNEPEEEELATISEEEIAMAVTAW

EKGPESLPPLRPRQNPVLPVAGERNVLITSALPYVNNVPHLGNIIGCVLSADVFARYSRLRQWNTLYLCGTDEYGTATET

KAMEEGLTPQEICDKYHTIHADIYHWFNISFDIFGRTTTPQQTKITQDIFQRLLTRGFVLQDTVEQLRCEHCARFLADRF

VEGVCPFCGYEEARGDQCDKCGKLINAIELKKPQCKVCRSCPVVKSSQHLFLDLPKLEKRLEEWLGKTLPGSDWTPNARF

IIRSWLRDGLKPRCITRDLKWGTPVPLEGFEDKVFYVWFDATIGYLSITANYTDQWERWWKNPEQVDLYQFMAKDNVPFH

GLVFPCSALGAEDNYTLVKYLIATEYLNYEDGKFSKSRGVGVFGDMAQNTGIPADIWRFYLLYIRPEGQDSAFSWADMLL

KNNSELLNNLGNFINRAGMFVSKFFGGYVPEMVLTPDDQRLLARITLELQNYHQLLEKVRIRDALRSILTVSRHGNQYIQ

VNEPWKQIKGSEADRQRAGTVTGMAVNIAALLSVMLEPYMPSVSATIQAQLQLPLPACSILLTDFVCTLPAGHQIGTVSP

LFQKLENDQIESLKQRFGGGQAKVSPTPAVVETVTTAGPQQIQTLMDEVTKQGNTVRELKAQKADKNQVAAEVAKLLDLK

KQLALAEGKPLETPKGKKKK

>XP_024592963.1 methionine--tRNA synthetase, cytoplasmic isoform X1 [Neophocaena asiaeorientalis asiaeorientalis]

MRLFVSEGAPGSLPVLAAAGRAQGRAELLISTVGPEECVVPFLTRPKVPVLQLDSGNYLFSTSAICRYFFLLSGWEQDDL

TNQWLEWEATELQPALSAALYYLVVQGKKGEDVFSPVRRALTHIDHSLSRRSCPFLAGETESLADIVLWGALYPLLQDPA

YLPEELGALHSWFQTLSSQEPCQRAAETVLKQQGVLALRPYLQKQPQPSFLEGRAVSNEPEEEELATLSEEEIAMAVAAW

EKGLESLPPVQPQQNPVLPVAGEKNVLITSALPYVNNVPHLGNIIGCVLSADVFARYSRLRQWNTLYLCGTDEYGTATET

KAMEEGLTPQEICDKYHVIHADIYRWFNISFDIFGRTTTPQQTKITQDIFQRLVARGFVLQDTVEQLRCEHCARFLADRF

VEGMCPFCGYEEARGDQCDKCGKLINAIELKKPQCKVCRSCPVVKSSRHLFLDLPKLGKPLEEWLEKTLPGSDWTPNARF

IIRSWLRDGLKPRCITRDLKWGTPVPLEGFEDKVFYVWFDATIGYLSITANYTDQWEKWWKNPEQVSLYQFMAKDNVPFH

GLVFPCSALGAEDNYTLVSHLIATEYLNYEDGKFSKSRGVGVFGDMAQDTGIPADIWRFYLLYIRPEGQDSAFSWTDMLL

KNNSELLNNLGNFINRAGMFVSKFFGGFVPEMVLTSDDRRLLAHVTLELQHYHQLLEKVRIRDALRSILTISRHGNQYIQ

VNEPWKRIKGSEADRQRAGTVTGLAVNIAALLSIMLQPYMPTVSATIQAQLQLPPPACSILLTNFLCTLPAGHQIGTVSP

LFQKLENDQIESLRQRFSGGQAKAPPKPAAVETMTTAGPQQIQVLMDEVTKQGNTVRELKAQKADKNQVAAEVAKLLDLK

KQLALAEGKPLETPKGKKKK

>XP_001488941.1 methionine--tRNA synthetase, cytoplasmic isoform X2 [Equus caballus]

MRLFVSEGAPGSLPVLAAARRAQGRAELLISTVGPEECVVPFLTQPKVPVLQLDNGNYLFSTSAICRYFFLLSGWEQDDL

TNQWLEWEATELQPALSAALYYLVVQGKKGEDVLGPVRRALTHIDHSLSRRSCPFLAGETESLADIVLWGALYPLLQDPA

YLPEELGALHSWFQTVSSQEPCQRAAETVLKQQGVLALRPYLQKQPQPSSLQGRAVSNEPEEEELATLSEEEIAMAVTAW

EKGLGNLPPLRPPQNPVLPVAGERNVLITSALPYVNNVPHLGNIIGCVLSADVFARYSRLRQWNTLYLCGTDEYGTATET

KAMEEGLTPQEICDKYHAIHADIYRWFNISFDIFGRTTTPQQTKITQDIFQRLFSRGFVLQDTVEQLRCEQCARFLADRF

VEGVCPFCGYEEARGDQCDKCGKLINATELKKPQCKVCRSCPVVKSSQHLFLDLPKLEKRLEEWLENTLPGSDWTPNARF

IIRSWLRDGLKPRCITRDLKWGTPVPLEGFEDKVFYVWFDATIGYLSITANYTDQWERWWKNPEQVSLYQFMAKDNVPFH

GLVFPCSALGAEDNYTLVSHLIATEYLNYEDGKFSKSRGVGVFGDMAQDTGIPADIWRFYLLYIRPEGQDSAFSWTDMLL

KNNSELLNNLGNFINRAGMFVSKFFGGYVPEMVLTSDDQRLLAHVTLELQHYHQLLEKVRIRDALRSILTISRHGNQYIQ

VNEPWKRIKGSEADRQRAGTVTGLAVNIAALLSVMLQPYMPTVSATIQAQLQLPPPACGILLTNFLCTLPAGHLIGTVSP

LFQKLENDQIESLRQRFGGGQAKVSSKPAVGETVTATGPQQIQVLMDEVTKQGNIVRELKAQKADKNQVAAEVAKLLDLK

KQLALAEGKPLETPKGKKKK

>XP_008575616.1 PREDICTED: methionine--tRNA synthetase, cytoplasmic isoform X2 [Galeopterus variegatus]

MRLFVSEGAPGTLPVLAAAGRTRGRAELLISTVGPEECVVPFLTWPKVPVLQLDNGNYLFSTSAICRYFFLLSGWEQDDL

TNQWLEWEATELQPALSAALYYLVVQGKKGEDVLGPVRRALIHIDHSLSRQNCPFLGGETESLADIVLWGALYPLLQDSA

YLPEELGALCSWFQTLSTQEPCQRAAETVLKQHSVLALRPYLQKQPQPSTPEERAVSNEPEEEDLATLSEEEIAMAVTAW

EKGLGSLPPLRPQQNPVLPVVGERNILITSALPYVNNVPHLGNIIGCVLSADVFARFARLRQWNILYLCGTDEYGTATET

KAMEEGLTPQEICDKYHVIHADIYRWFNISFDFFGRTTTPQQTKITQDIFQRLLARGFVLQDTVEQLRCERCARFLADRF

VEGVCPFCGFEEARGDQCDKCGKLINAIELKKPRCKVCQSCPVVKSSRHLFLDLPKLEKRLQEWLGKTLSGSDWTPNARF

IIRSWLRDGLKPRCITRDLKWGTPVPLEGFEDKVFYVWFDATIGYLSITANYTDQWERWWKNPEQVELYQFMAKDNVPFH

GLVFPCSALGAEDNYTLVSHIIATEYLNYEDGKFSKSRGVGVFGDMAQDTGIPADIWRFYLLYIRPEGQDSAFSWTDMLL

KNNSELLNNLGNFINRAGMFVSKFFGGYVPEMVLTPDDQRLLAHVTLELQHYHQLLEKVRIRDALRSILTISRHGNQYIQ

VNEPWKRIKGSEADRQRAGTVTGLAVNIAALLSVMLQPYMPTVSATIQAQLQLPLPACNILPTNFLCTLPAGHQIGTVSP

LFQKLENDLIESLRQRFGGGQAKASPKPAVVETVTTAGSQKIQVLMDEVTKQGNIVRELKAQKADKNQVAAEVAKLLDLK

KQLALAEGKPLETSKGKKKK

>XP_005611399.1 methionine--tRNA synthetase, cytoplasmic isoform X1 [Equus caballus]

MRLFVSEGAPGSLPVLAAARRAQGRAELLISTVGPEECVVPFLTQPKVPVLQLDNGNYLFSTSAICRYFFLLSGWEQDDL

TNQWLEWEATELQPALSAALYYLVVQGKKGEDVLGPVRRALTHIDHSLSRRSCPFLAGETESLADIVLWGALYPLLQDPA

YLPEELGALHSWFQTVSSQEPCQRAAETVLKQQGVLALRPYLQKQPQPSSLQGRAVSNEPEEEELATLSEEEIAMAVTAW

EKGLGNLPPLRPPQNPVLPVAGERNVLITSALPYVNNVPHLGNIIGCVLSADVFARYSRLRQWNTLYLCGTDEYGTATET

KAMEEGLTPQEICDKYHAIHADIYRWFNISFDIFGRTTTPQQTKITQDIFQRLFSRGFVLQDTVEQLRCEQCARFLADRF

VEGVCPFCGYEEARGDQCDKCGKLINATELKKPQCKVCRSCPVVKSSQHLFLDLPKLEKRLEEWLENTLPGSDWTPNARF

IIRSWLRDGLKPRCITRDLKWGTPVPLEGFEDKVFYVWFDATIGYLSITANYTDQWERWWKNPEQVSLYQFMAKDNVPFH

GLVFPCSALGAEDNYTLVSHLIATEYLNYEDGKFSKSRGVGVFGDMAQDTGIPADIWRFYLLYIRPEGQDSAFSWTDMLL

KNNSELLNNLGNFINRAGMFVSKFFGGYVPEMVLTSDDQRLLAHVTLELQHYHQLLEKVRIRDALRSILTISRHGNQYIQ

VNEPWKRIKGSEADRQRAGTVTGLAVNIAALLSVMLQPYMPTVSATIQAQLQLPPPACGILLTNFLCTLPAGHLIGTVSP

LFQKLENDQIESLRQRFGGGQLEESLELKAKVSSKPAVGETVTATGPQQIQVLMDEVTKQGNIVRELKAQKADKNQVAAE

VAKLLDLKKQLALAEGKPLETPKGKKKK

>XP_010642909.1 PREDICTED: methionine--tRNA synthetase, cytoplasmic isoform X1 [Fukomys damarensis]

MRLFVSEGAPGSLPVLAAAARAQGLAELLISTVGPEECVVPFLTQPKVPVLQLDSGNYLFSTSAICRYFFLLSGWEQDDL

TNQWLEWEATELQPALSAALYYLVVQGKKGEDVLSPLGRALTHIDHSLSRQNCPFLAGETESLADIVLWGALYPLLQDPA

YLPEELGALRSWFQTLSTQEPCQRAAETVLKQRGVLALRPYLQKQPQPSPPEGRGVSNEPEEEELATISEEEIAMAVTAW

EKGPESLPPLRPRQNPVLPVAGERNVLITSALPYVNNVPHLGNIIGCVLSADVFARYSRLRQWNTLYLCGTDEYGTATET

KAMEEGLTPQEICDKYHTIHADIYHWFNISFDIFGRTTTPQQTKITQDIFQRLLTRGFVLQDTVEQLRCEHCARFLADRF

VEGVCPFCGYEEARGDQCDKCGKLINAIELKKPQCKVCRSCPVVKSSQHLFLDLPKLEKRLEEWLGKTLPGSDWTPNARF

IIRSWLRDGLKPRCITRDLKWGTPVPLEGFEDKVFYVWFDATIGYLSITANYTDQWERWWKNPEQVDLYQFMAKDNVPFH

GLVFPCSALGAEDNYTLVKYLIATEYLNYEDGKFSKSRGVGVFGDMAQNTGIPADIWRFYLLYIRPEGQDSAFSWADMLL

KNNSELLNNLGNFINRAGMFVSKFFGGYVPEMVLTPDDQRLLARITLELQNYHQLLEKVRIRDALRSILTVSRHGNQYIQ

VNEPWKQIKGSEADRQRAGTVTGMAVNIAALLSVMLEPYMPSVSATIQAQLQLPLPACSILLTDFVCTLPAGHQIGTVSP

LFQKLENDQIESLKQRFGGGQLEESLELKAKVSPTPAVVETVTTAGPQQIQTLMDEVTKQGNTVRELKAQKADKNQVAAE

VAKLLDLKKQLALAEGKPLETPKGKKKK

>XP_004647061.1 methionine--tRNA synthetase, cytoplasmic isoform X1 [Octodon degus]

MRLFVSEGAPGSLPVLVAAGRVEGRAELLISTVAPEECVVPFLTQPRLPVLQLDSGNYLFSTSAICRYFFLLSGWEPDDL

TNQWLEWEATELQPALSAALHYLVIQGKKGEDVLGPLGRALTHIDHSLSRQNCPFLAGETESLADIVLWGALYPLLQDPA

YLPEELGALRSWFQTLSTQEPCQRAAETVLKKQGVLALRPYLQKQPQPSPPEGRAVSNEPEEEELAVLSEEEIAAAVTAW

EKGLEILPPPRPQQNPVLPVAGERNVLITSALPYVNNVPHLGNIIGCVLSADVFARYSRLRQWNTLYLCGTDEYGTATET

KAMEEGLTPQEICDKYHVIHADIYHWFNISFDIFGRTTTPQQTKITQDIFQRLLTRGFILQDTVEQLRCERCARFLADRF

VEGVCPFCGYEEARGDQCDKCGKLINAIELKKPQCKVCRSCPVVKSSQHLFLDLPKLEKRLEEWLGKTLPGNNWTPNARF

IIRSWLRDGLKPRCITRDLKWGTPVPLEGFEDKVFYVWFDATIGYLSITANYTDQWERWWKNPEQVDLYQFMAKDNVPFH

GLVFPCSALGAEDNYTLVKYLIATEYLNYEDGKFSKSRGVGVFGDMAQDTGIPADIWRFYLLYIRPEGQDSAFSWTDMLL

KNNSELLNNLGNFINRAGMFVSKFFGGSVPEMLLTPDDQRLLAHITLELQHYHQLLEKVRIRDALRAILTVSRHGNQYIQ

VNEPWKRIKGSEADRQRAGTVTGVAVNIAALLSVMLQPYMPTVSATIQAQLQLPLPACSVLPTDFLCTLPAGHQIGTVSP

LFQKLENDQIENLKQRFGGGQAKISPSLAVVETVTTAGPQQIQTLMEEVTKQGNIVRELKAQKADKNQVAAEVAQLLDLK

KQLALAEGKPLETPKGKKKK

>XP_019597798.1 PREDICTED: methionine--tRNA synthetase, cytoplasmic isoform X2 [Rhinolophus sinicus]

MRLFVSEGAPGSLPVLAAAERAQGRAELLISTVGPEDCVVPFLTWPKLPVLQLDSGNYLFSTSAICRYFFLLSGWEQDDL

TNQWLEWEATELQPALSAALYYLVVQGKKGEDVLGPVRRALTHIDHSLSRQSCPFLAGETESLADIVLWGALYPLLQDPA

YVPEELGALHSWFQTLSSQEPCQRAAETVLKQQGILALRPYLQKQPQPSSLERRAVSNEPEEEELATLSEEDIAVAVTAW

EKGLGSLPPLRPKQNPVLPVAGERNVLITSALPYVNNVPHLGNIIGCVLSADVFARYSRLRQWNTLYLCGTDEYGTATET

KAMEEGLTPQEICDKYHVIHADIYRWFNISFDIFGRTTTPQQTEITQDIFQRLWNRGFVLQDTVEQLRCERCARFLADRF

VEGVCPFCAYEEARGDQCDKCGKLINAIELKKPQCKVCRSRPVVKSSRHLFLDLPKLEDQLEGWLGKTLPGSDWTPNARF

IIRSWLRDGLKPRCITRDLKWGTPVPLKGFEDKVFYVWFDATFGYLSITANYTDQWERWWKNPEQVNLYQFMAKDNVPFH

GLVFPCSALGAEDNYTLVSHLIATEYLNYEDGKFSKSRGVGVFGDMAQDTGIPADIWRFYLLYIRPEGQDSAFSWTDMLL

KNNSELLNNLGNFINRAGMFVSKFFGGYVPEMMLTSDDQRLLAHVTLELKHYHQLLEKVRIRDALRSILTISRHGNQYIQ

VNEPWKRIKGNEADRQRAGTVTGLAVNIAALLSIMLQPYMPTVSATIAAQLQLPTAACSILPTDFLCTLPAGHQIGTVSP

LFQKLENDQIESLRQRFGGGQAKASPKPAVVETVTAAGSQQIQVLMDEVTKQGNIVRELKAQKADKNQVAAEVAKLLELK

KQLALAEGKPLDTPKGKKKK

>XP_008575615.1 PREDICTED: methionine--tRNA synthetase, cytoplasmic isoform X1 [Galeopterus variegatus]

MRLFVSEGAPGTLPVLAAAGRTRGRAELLISTVGPEECVVPFLTWPKVPVLQLDNGNYLFSTSAICRYFFLLSGWEQDDL

TNQWLEWEATELQPALSAALYYLVVQGKKGEDVLGPVRRALIHIDHSLSRQNCPFLGGETESLADIVLWGALYPLLQDSA

YLPEELGALCSWFQTLSTQEPCQRAAETVLKQHSVLALRPYLQKQPQPSTPEERAVSNEPEEEDLATLSEEEIAMAVTAW

EKGLGSLPPLRPQQNPVLPVVGERNILITSALPYVNNVPHLGNIIGCVLSADVFARFARLRQWNILYLCGTDEYGTATET

KAMEEGLTPQEICDKYHVIHADIYRWFNISFDFFGRTTTPQQTKITQDIFQRLLARGFVLQDTVEQLRCERCARFLADRF

VEGVCPFCGFEEARGDQCDKCGKLINAIELKKPRCKVCQSCPVVKSSRHLFLDLPKLEKRLQEWLGKTLSGSDWTPNARF

IIRSWLRDGLKPRCITRDLKWGTPVPLEGFEDKVFYVWFDATIGYLSITANYTDQWERWWKNPEQVELYQFMAKDNVPFH

GLVFPCSALGAEDNYTLVSHIIATEYLNYEDGKFSKSRGVGVFGDMAQDTGIPADIWRFYLLYIRPEGQDSAFSWTDMLL

KNNSELLNNLGNFINRAGMFVSKFFGGYVPEMVLTPDDQRLLAHVTLELQHYHQLLEKVRIRDALRSILTISRHGNQYIQ

VNEPWKRIKGSEADRQRAGTVTGLAVNIAALLSVMLQPYMPTVSATIQAQLQLPLPACNILPTNFLCTLPAGHQIGTVSP

LFQKLENDLIESLRQRFGGGQLEESLELKAKASPKPAVVETVTTAGSQKIQVLMDEVTKQGNIVRELKAQKADKNQVAAE

VAKLLDLKKQLALAEGKPLETSKGKKKK

>XP_012876681.1 PREDICTED: methionine--tRNA synthetase, cytoplasmic isoform X4 [Dipodomys ordii]

MRLFVSEGTPGSLPVLAAAGRVRGRIELLINTVGPEECVVPFLTRPKVPVLQLDSGNYLFSTTAICRYFFLLSGWEQDDL

TNQWLEWEATELQPALSAALYYLVVQGKKGEDILGPLRKALTHIDQSLSHQNCPFLAGETESLADIVLWGALYPLLQDPA

YLPEELGALRSWFQTLSTQEPCQRAVETVLKQQGVLALRSYLQKQPQAHPSEGKVVSNEPEEEELATLSEEEIAMAVTAW

EKGLGSLPSLRPQQNPVLPVAGERNVLITSALPYVNNVPHLGNIIGCVLSADVFARYSRLRQWNTLYLCGTDEYGTATET

KAMEEGLTPREICDKYHRIHADIYHWFNISFDIFGRTTTPQQTKITQDIFQRLLTRGFVVQDTVEQLRCEHCARFLADRF

VEGVCPFCGYEEARGDQCDKCGKLINAIELKKPQCKVCRSCPVVKSSKHLFLDLPKLENRLEDWLGKTLPGSDWTPNARF

IIRSWLRDGLKPRCITRDLKWGTPVPLEGFEDKVFYVWFDATIGYLSITANYTEQWERWWKNPEQVDLYQFMAKDNVPFH

GLVFPCSALGAEDNYTLVKHLIATEYLNYEDGKFSKSRGVGVFGDMAQNTGIPADIWRFYLLYIRPEGQDSAFSWTDMLL

KNNSELLNNLGNFVNRAGMFVSKFFGGYVPEMVLTPDDQRLLAHITLELQHYHQLLEKVRIRDALRSILTISRHGNQYIQ

VNEPWKRIKGSEADRQRAGTVTGLAVNIAALLSVMIQPYMPTVSATIQTQLQLPLQVCSVLPIKFLCTLPAGHQIGTVSP

LFQKLENDQIESLRQRFGGGQAKASPKSTVVETVTAGLQQIQVLMDEVTKQGNIVRELKAQKADKNQVAAEVAKLLDLKK

QLAVAEGKPLETPKGKKKK

>XP_022378864.1 methionine--tRNA synthetase, cytoplasmic [Enhydra lutris kenyoni]

MRLFVSEGAPGSLPVLAAAGRAQGRAELLISTVGPEECVVPFLTRPKVPVLQLDSGNYLFSTSAICRYFFLLSGWEQDDL

TNQWLEWEATELQPALSAALYYLVVQGKKGEDVLGPVRRALTHIDQSLSRRSCPFLAGETESLADVVLWGALYPLLQDPA

YLPEELGALHSWFQTLSSQEPCQRAAETVLKQQGVLALRPYLQKQPQPGSFEGRAVSNEPEEEELATLSEEEIAMAVTAW

EKGLESLPPLRPQQNPVLPVAGERNVLITSALPYVNNVPHLGNIIGCVLSADVFARYSRLRQWNTLYLCGTDEYGTATET

KAMEEGLTPQEICDKYHAIHANIYRWFNISFDIFGRTTTPQQTKITQDIFQRLLTRGFVLEDTVEQLRCERCARFLADRF

VEGVCPFCGYEEARGDQCDKCGKLINAIELKKPQCKVCRSCPVVKSSRHLFLDLPKLEKRLEEWLGKTLPGSDWTPNARF

IIRSWLRDGLKPRCITRDLKWGTPVPLEGFEDKVFYVWFDATIGYLSITANYTDQWERWWKNPEQVNLYQFMAKDNVPFH

GLVFPCSALGAEDNYTLVSHLIATEYLNYEDGKFSKSRGVGVFGDMAQDTGIPADIWRFYLLYIRPEGQDSAFSWTDMLL

KNNSELLNNLGNFINRAGMFVSKFFGGYVPEMVLTSDDRRLVAHVTLELQHYHQLLEKVRIREALRSILTISRHGNQYIQ

VNEPWKRIKGSEADRQRAGTVTGLAVNIAALLSVMLQPYMPTVSATIQAQLQLPLPACSILITNFLCTLPAGHQIGTVSP

LFQKLENDQIESLRQRFGGGQARVPPMPAVVEAAATAAPQQVQVLMDEVTRQGNIVRELKAQKADKNQVAAEVAKLLDLK

KQLALAEGKPLETPKGKKKK

>XP_012661013.1 methionine--tRNA synthetase, cytoplasmic isoform X5 [Otolemur garnettii]

MRLFVSEGAPGSLPVLAAAGRARGRAELLISTVGPEECVVPFLTQPKVPVLQLDSGNYLFSTSSICRYFFLLSGWEQDDL

TNQWLEWEATELQPALSAALYYLVVQGKKGEDVLGPVRRALKHIDYSLSRQNCPFLAGETESLADIVLWGALYPLLQDPA

YLPEELGSLCSWFQTLSTQEPCQRAAETVLKQQGVLALRPYLQKQPQPSPPNGRAVTNEPEEELATLSEEEITMAVTAWE

KGLGGLPPLRPPQNPVLPMAGERNVLITSALPYVNNVPHLGNIIGCVLSADVFARYSRLRQWNTLYLCGTDEYGTATETK

AMEEGLTPQEICDKYHAIHADIYRWFNISFDTFGRTTTPQQTKITQDIFRRLLTRGFVLQDTVEQLHCEKCARFLADRFV

EGVCPFCGYEEARGDQCDKCGKLINAIELKKPQCKVCRSCPVVKSSQHLFLDLPKLETRLEEWLGKTLPGSDWTPNSRFI

IRSWLRDGLKPRCITRDLKWGTPVPLEGFEDKVFYVWFDATIGYLSITANYTDQWERWWKNPEQVDLYQFMAKDNVPFHG

LVFPCSALGAEDNYTLVSHLIATEYLNYEDGKFSKSRGVGVFGDMAQDTGIPADIWRFYLLYIRPEGQDSAFSWTDLLLK

NNSELLNNLGNFINRAGMFVSKFFGGYVPEMVLTPDDQRLLAHVTLELRHYHQLLEKVRIRDALRSILTISRHGNQYIQV

NEPWKWIKGTEADRQRAGTVTGLAVNIAALLSVMLQPYMPSVSATIQAQLQLSPPACSVLLTNFLCTLPAGHQIGTVSPL

FQKLENDQIESLRQRFGGGQAKVSPKPAVVDTVAATGPQQIQMLMDEVTKQGNIVRELKAQKADKNQIAAEVAKLLDLKK

QLSLAEGKPPEAPKCKKKK

>XP_004429368.1 PREDICTED: methionine--tRNA synthetase, cytoplasmic isoform X2 [Ceratotherium simum simum]

MRLFVSEGAPGSLPVLAAARRAQGRAELLISTVGPEECVVPFLTQPKIPVLQLDSGNYLFSSSAICRYFFLLSGWEQDDL

TNQWLEWEVTELQPALSAALYCLVVQGKKGEDVLGPVRRALTHIDHSLSHRSCPFLAGETESLADIVLWGALYPLLQDPA

YLPEELGALHSWFQTLSSQEPCQRAAETVLKQQGVLALRPYLQKQPQPSSLQGRAVSNEPEEEELATLSEEEITMAVTAW

EKGLGSLPPLRPQQNPVLPVPGERNVLITSALPYVNNVPHLGNIIGCVLSADVFARYSRLRQWNTLYLCGTDEYGTATET

KAMEEGLTPQEICDKYHAIHADIYRWFNISFDIFGRTTTPQQTKITQDIFQRLFARGFVLQDTMEQLRCEHCARFLADRF

VEGVCPFCGYEEARGDQCDKCGKLINAIELKKPQCKVCRSCPVVKSSQHLFLDLPKLEGRLEEWLEKTLPGSDWTPNAWF

IIRSWLRDGLRPRCITRDLRWGTPVPLEGFEEKVFYVWFDATIGYLSITANYTDQWERWWKNPEQVSLYQFMAKDNVPFH

GLVFPCSALGAEDNYTLVKHLIATEYLNYEDGKFSKSRGVGVFGDMAQDTGIPADIWRFYLLYIRPEGQDSAFSWTDMLH

KNNSELLNNLGNFINRAGMFVSKFFGGYVPEMVLTSDDQRLLAHVTLELQHYHQLLEKVRIRDALRSILTISRHGNQYIQ

VNEPWKRIKGSEADRQRAGTVTGLAVNIAALLSVMLQPYMPTVSATIQAQLQLPPPACSILPTSFLCTLPAGHQIGTVSP

LFQKLENDQIESLRQRFGGGQAKVSSKPAVAETMTTAGPQQIQVLMEEVTKQGNIVRELKAQKADKNQVAAEVAKLLDLK

KQLALAEGKPLETPKGKKKK

>XP_019597797.1 PREDICTED: methionine--tRNA synthetase, cytoplasmic isoform X1 [Rhinolophus sinicus]

MRLFVSEGAPGSLPVLAAAERAQGRAELLISTVGPEDCVVPFLTWPKLPVLQLDSGNYLFSTSAICRYFFLLSGWEQDDL

TNQWLEWEATELQPALSAALYYLVVQGKKGEDVLGPVRRALTHIDHSLSRQSCPFLAGETESLADIVLWGALYPLLQDPA

YVPEELGALHSWFQTLSSQEPCQRAAETVLKQQGILALRPYLQKQPQPSSLERRAVSNEPEEEELATLSEEDIAVAVTAW

EKGLGSLPPLRPKQNPVLPVAGERNVLITSALPYVNNVPHLGNIIGCVLSADVFARYSRLRQWNTLYLCGTDEYGTATET

KAMEEGLTPQEICDKYHVIHADIYRWFNISFDIFGRTTTPQQTEITQDIFQRLWNRGFVLQDTVEQLRCERCARFLADRF

VEGVCPFCAYEEARGDQCDKCGKLINAIELKKPQCKVCRSRPVVKSSRHLFLDLPKLEDQLEGWLGKTLPGSDWTPNARF

IIRSWLRDGLKPRCITRDLKWGTPVPLKGFEDKVFYVWFDATFGYLSITANYTDQWERWWKNPEQVNLYQFMAKDNVPFH

GLVFPCSALGAEDNYTLVSHLIATEYLNYEDGKFSKSRGVGVFGDMAQDTGIPADIWRFYLLYIRPEGQDSAFSWTDMLL

KNNSELLNNLGNFINRAGMFVSKFFGGYVPEMMLTSDDQRLLAHVTLELKHYHQLLEKVRIRDALRSILTISRHGNQYIQ

VNEPWKRIKGNEADRQRAGTVTGLAVNIAALLSIMLQPYMPTVSATIAAQLQLPTAACSILPTDFLCTLPAGHQIGTVSP

LFQKLENDQIESLRQRFGGGQLEESLELKAKASPKPAVVETVTAAGSQQIQVLMDEVTKQGNIVRELKAQKADKNQVAAE

VAKLLELKKQLALAEGKPLDTPKGKKKK

>XP_013376348.1 PREDICTED: methionine--tRNA synthetase, cytoplasmic [Chinchilla lanigera]

MRLFVSEGAPGSLPVLVAAGRAQGRAELQVRAVGPEECVVPFLTQPKLPVLQLDSGNYLFSTSAICRYFFLLSGWEADDL

TNQWLEWEATELQPALSAALYYLVVQGKKGEDVLGPLGRALTHIDHSLSRQNCPFLAGETESLADIVLWGALYPLLQDPA

YLPEELGALRSWFQTLSAQEPCQRAAETVLKQQGVLALRPYLQKQPQPSPPESRAVSNEPEEEELATLSEEEIAAAVTAW

EKGLENLPPLRPQQNPVLPVAGDRNVLITSALPYVNNVPHLGNIIGCVLSADVFARYSRLRQWNTLYLCGTDEYGTATET

KAMEEGLTPQEICDKYHVIHADIYRWFNISFDTFGRTTTAQQTKITQDIFQRLLARGFILQDTVEQLRCERCARFLADRF

VEGVCPFCGYEEARGDQCDKCGKLINAIELKKPQCKVCRSCPVVRSSQHLFLDLPKLEKRLEEWLGKTLPGSNWTPNARF

IIRSWLRDGLKPRCITRDLKWGTPVPLEGFEDKVFYVWFDATIGYLSITANYTDQWERWWKNPEQVDLYQFMAKDNVPFH

GLVFPCSALGAEDNYTLVKYLIATEYLNYEDGKFSKSRGVGVFGDMAQDTEIPADIWRFYLLYIRPEGQDSAFSWTDMLL

KNNSELLNNLGNFINRAGMFVSKFFGGCVPEMVLNPDDQRLLAHITLELRHYHQLLEKVRIRDALRSILTVSRHGNQYIQ

VNEPWKRIKGSEADRQRAGTVTGMAVNIAALLSVMLQPYMPTVSATIQAQLQLPRPACSVLPTDFLCTLPAGHQIGTVSP

LFQKLENDQIESLKQRFGGQAKVSPNPAVVETVTTAGQQQIQTLTDEVAKQGNVVRELKAQKADKNQVAAEVAKLLDLKK

QLALAEGRPLETPKGKKTK

>XP_012876673.1 PREDICTED: methionine--tRNA synthetase, cytoplasmic isoform X3 [Dipodomys ordii]

MRLFVSEGTPGSLPVLAAAGRVRGRIELLINTVGPEGIRASAECVVPFLTRPKVPVLQLDSGNYLFSTTAICRYFFLLSG

WEQDDLTNQWLEWEATELQPALSAALYYLVVQGKKGEDILGPLRKALTHIDQSLSHQNCPFLAGETESLADIVLWGALYP

LLQDPAYLPEELGALRSWFQTLSTQEPCQRAVETVLKQQGVLALRSYLQKQPQAHPSEGKVVSNEPEEEELATLSEEEIA

MAVTAWEKGLGSLPSLRPQQNPVLPVAGERNVLITSALPYVNNVPHLGNIIGCVLSADVFARYSRLRQWNTLYLCGTDEY

GTATETKAMEEGLTPREICDKYHRIHADIYHWFNISFDIFGRTTTPQQTKITQDIFQRLLTRGFVVQDTVEQLRCEHCAR

FLADRFVEGVCPFCGYEEARGDQCDKCGKLINAIELKKPQCKVCRSCPVVKSSKHLFLDLPKLENRLEDWLGKTLPGSDW

TPNARFIIRSWLRDGLKPRCITRDLKWGTPVPLEGFEDKVFYVWFDATIGYLSITANYTEQWERWWKNPEQVDLYQFMAK

DNVPFHGLVFPCSALGAEDNYTLVKHLIATEYLNYEDGKFSKSRGVGVFGDMAQNTGIPADIWRFYLLYIRPEGQDSAFS

WTDMLLKNNSELLNNLGNFVNRAGMFVSKFFGGYVPEMVLTPDDQRLLAHITLELQHYHQLLEKVRIRDALRSILTISRH

GNQYIQVNEPWKRIKGSEADRQRAGTVTGLAVNIAALLSVMIQPYMPTVSATIQTQLQLPLQVCSVLPIKFLCTLPAGHQ

IGTVSPLFQKLENDQIESLRQRFGGGQAKASPKSTVVETVTAGLQQIQVLMDEVTKQGNIVRELKAQKADKNQVAAEVAK

LLDLKKQLAVAEGKPLETPKGKKKK

>XP_023369311.1 methionine--tRNA synthetase, cytoplasmic isoform X3 [Otolemur garnettii]

MRLFVSEGAPGSLPVLAAAGRARGRAELLISTVGPEECVVPFLTQPKVPVLQLDSGNYLFSTSSICRYFFLLSGWEQDDL

TNQWLEWEATELQPALSAALYYLVVQGKKGEDVLGPVRRALKHIDYSLSRQNCPFLAGETESLADIVLWGALYPLLQDPA

YLPEELGSLCSWFQTLSTQEPCQRAAETVLKQQGVLALRPYLQKQPQPSPPNGRAVTNEPEVWNRTKPEGLKWEEELATL

SEEEITMAVTAWEKGLGGLPPLRPPQNPVLPMAGERNVLITSALPYVNNVPHLGNIIGCVLSADVFARYSRLRQWNTLYL

CGTDEYGTATETKAMEEGLTPQEICDKYHAIHADIYRWFNISFDTFGRTTTPQQTKITQDIFRRLLTRGFVLQDTVEQLH

CEKCARFLADRFVEGVCPFCGYEEARGDQCDKCGKLINAIELKKPQCKVCRSCPVVKSSQHLFLDLPKLETRLEEWLGKT

LPGSDWTPNSRFIIRSWLRDGLKPRCITRDLKWGTPVPLEGFEDKVFYVWFDATIGYLSITANYTDQWERWWKNPEQVDL

YQFMAKDNVPFHGLVFPCSALGAEDNYTLVSHLIATEYLNYEDGKFSKSRGVGVFGDMAQDTGIPADIWRFYLLYIRPEG

QDSAFSWTDLLLKNNSELLNNLGNFINRAGMFVSKFFGGYVPEMVLTPDDQRLLAHVTLELRHYHQLLEKVRIRDALRSI

LTISRHGNQYIQVNEPWKWIKGTEADRQRAGTVTGLAVNIAALLSVMLQPYMPSVSATIQAQLQLSPPACSVLLTNFLCT

LPAGHQIGTVSPLFQKLENDQIESLRQRFGGGQAKVSPKPAVVDTVAATGPQQIQMLMDEVTKQGNIVRELKAQKADKNQ

IAAEVAKLLDLKKQLSLAEGKPPEAPKCKKKK

>XP_010335721.1 PREDICTED: methionine--tRNA synthetase, cytoplasmic [Saimiri boliviensis boliviensis]

MRLFLSEGSPGSLPVLAAAGRARGRAELLISTVGPEDCVVPFLTQPKVPVLQLDSGNYLFSTSAICRYFFLLSGWEQDDL

TNQWLEWEATELQPALSAALYYLVVQGKKGDDVLGSVRRALTHIDHSLRRQNCPFLAGETESLADIVLWGALYPLLQDPA

YLPEELSALHSWFQTVSTQEPCQRAAETVLKQQGVLALRPYLQKQPQPSPAEGRTVTNEPEEEELATLSEEEIAMAVTAW

EKGLESLPPLRPQQNPVLPVAGERNVLITSALPYVNNVPHLGNIIGCVLSADVFARYSRLRQWNTLYLCGTDEYGTATET

KAMEEGLTPQEICDKYHIIHADIYRWFNISFDVFGRTTTPQQTKITQDIFQRLLARGFVLQDTVEQLRCERCARFLADRF

VEGVCPFCGFEEARGDQCDKCGKLINAVELKVRQESPSEPGRRHNSSFTLCLSLLSQLEKRLEEWLGRTLPGSDWTPNAR

FIIRSWLRDGLKPRCITRDLKWGTPVPLEGFEDKVFYVWFDATIGYLSITATYTDQWERWWKNPEQVDLYQFMAKDNVPF

HGLVFPCSALGAEDNYTLVSHLIATEYLNYEDGKFSKSRGVGVFGDMAQDTGIPADIWRFYLLYIRPEGQDSAFSWTDML

LKNNSELLNNLGNFINRAGMFVSKFFGGYVPEMVLTPDDQRLLAHVTLELRRYHQLLEKVRIRDALRSILTISRHGNQYI

QVNEPWKRIKGSEADRQRAGTVTGLAVNIAALLSVMLQPYMPTVSATIQAQLQLPPPACSILLTDFLCTLPAGHQIGTVS

PLFQKLENDQIESLRQRFGGGQSKTSPKPAVAETGTTAGPQQIQALMDEVTKQGNIVRELKAQKADKNQVAAEVAKLLDL

KKQLAVAEGKPPEAPKGKKKK

>XP_012876664.1 PREDICTED: methionine--tRNA synthetase, cytoplasmic isoform X2 [Dipodomys ordii]

MRLFVSEGTPGSLPVLAAAGRVRGRIELLINTVGPEECVVPFLTRPKVPVLQLDSGNYLFSTTAICRYFFLLSGWEQDDL

TNQWLEWEATELQPALSAALYYLVVQGKKGEDILGPLRKALTHIDQSLSHQNCPFLAGETESLADIVLWGALYPLLQDPA

YLPEELGALRSWFQTLSTQEPCQRAVETVLKQQGVLALRSYLQKQPQAHPSEGKVVSNEPEEEELATLSEEEIAMAVTAW

EKGLGSLPSLRPQQNPVLPVAGERNVLITSALPYVNNVPHLGNIIGCVLSADVFARYSRLRQWNTLYLCGTDEYGTATET

KAMEEGLTPREICDKYHRIHADIYHWFNISFDIFGRTTTPQQTKITQDIFQRLLTRGFVVQDTVEQLRCEHCARFLADRF

VEGVCPFCGYEEARGDQCDKCGKLINAIELKKPQCKVCRSCPVVKSSKHLFLDLPKLENRLEDWLGKTLPGSDWTPNARF

IIRSWLRDGLKPRCITRDLKWGTPVPLEGFEDKVFYVWFDATIGYLSITANYTEQWERWWKNPEQVDLYQFMAKDNVPFH

GLVFPCSALGAEDNYTLVKHLIATEYLNYEDGKFSKSRGVGVFGDMAQNTGIPADIWRFYLLYIRPEGQDSAFSWTDMLL

KNNSELLNNLGNFVNRAGMFVSKFFGGYVPEMVLTPDDQRLLAHITLELQHYHQLLEKVRIRDALRSILTISRHGNQYIQ

VNEPWKRIKGSEADRQRAGTVTGLAVNIAALLSVMIQPYMPTVSATIQTQLQLPLQVCSVLPIKFLCTLPAGHQIGTVSP

LFQKLENDQIESLRQRFGGGQLEESLEFKAKASPKSTVVETVTAGLQQIQVLMDEVTKQGNIVRELKAQKADKNQVAAEV

AKLLDLKKQLAVAEGKPLETPKGKKKK

>XP_531646.2 methionine--tRNA synthetase, cytoplasmic isoform X1 [Canis lupus familiaris]

MRLFVSEGAPGSLPVLAAAGRAQGRAELLVSTVGPEECVVPFLTRPKIPVLQLDSGNYLFSTSAICRYFFLLSGWEQDDL

TNQWLEWEATELQPALSAALYYLVVQGRKGEDVLGPVRRALTHIDYSLSRRSCPFLAGDTESLADIVLWGALYPLLQDPA

YLPEELGALHSWFQTLSSQEPCQRAAETVLKQQGVLALRPYLQKQPQPGSFEGRAVSNEPEEEELATLSEEEIAMAVTAW

EKGLGSLPPLRPQQNPVLPVAGERNVLITSALPYVNNVPHLGNIIGCVLSADVFARYSRLRQWNTFYLCGTDEYGTATET

KAMEEGLTPQEICDKYHAIHADIYRWFNISFDIFGRTTTPQQTKITQDIFQRLLTRGFVLQDTVEQLRCERCARFLADRF

VEGVCPFCGYEEARGDQCDKCGKLINAIELKKPQCKVCRSCPVVKSSQHLFLDLPKLEKRLEEWLGKTLPGSDWTPNARF

IIRSWLRDGLKPRCITRDLKWGTPVPLEGFEDKVFYVWFDATIGYLSITANYTDQWERWWKNPEQVNLYQFMAKDNVPFH

GLVFPCSALGAEDNYTLVSHLIATEYLNYEDGKFSKSRGVGVFGDMAQDTGIPADIWRFYLLYIRPEGQDSAFSWTDMLL

KNNSELLNNLGNFINRAGMFVSKFFGGYVPEMVLTSDDRRLLAHVTLELQHYHQLLEKVRIREALRSILTISRHGNQYIQ

VNEPWKRIKGSEADRQRAGTVTGLAVNIAALLSVMLQPYMPTVSATIQAQLQVPPPACSILLTNFLCTLPAGHQIGTVSP

LFQKLENDQIESLRQRFGGGQAKTPPTPAEAMAAAAPQQIQVLMDEVTKQGNIVRELKAQKADKNQVAAEVAKLLDLKKQ

LALAEGKPLETPKGKKKK

>XP_024432031.1 methionine--tRNA synthetase, cytoplasmic isoform X2 [Desmodus rotundus]

MRLFVSEGAPGTLPVLAAAGRARGRAELLVITVGPEEHVVPFLTRPKVPVLQLDSGNYLFSTSAICRYFFLLSGWEQDDL

TNQWLEWEATELQPALSAALYYLVVQGKKGEDVLGPVRRALTHIDHSLSRRSCPFLAGETESLADIVLWGALYPLLQDPA

YLPEELGALHSWFQTLSSQEPCQRAAETVLKQQGILALRPYLQKQPQPSLLEGRAVSNEPEEEELVTLSEEEIAMAVTAW

EKGLGSLPPLRPKQNPVLPVAGERNVLITSALPYVNNVPHLGNIIGCVLSADVFARYSRLRQWNTLYVCGTDEYGTATET

KAMEEGLTPQEICDKYHVIHADIYRWFNISFDIFGRTTTPQQTEITQDIFQRLWNRGFVLQDTVEQLRCERCARFLADRF

VEGVCPFCGYEEARGDQCDKCGKLINAIELKKPQCKVCRSCPVVKSSRHLFLDLPKLEDQLEEWLGKTLPGSDWTPNARF

IIRSWLRDGLKPRCITRDLKWGTPVPLEGYEDKVFYVWFDATFGYLSITANYTDQWERWWKNPEQVNLYQFMAKDNVPFH

GLVFPCSALGAEDNYTLVSHLIATEYLNYEDGKFSKSRGIGVFGDMAQDTGIPADIWRFYLLYIRPEGQDSAFSWTDMLL

KNNSELLNNLGNFINRAGMFVSKFFAGSVPEMVLTSDDQRLLAHVTLELQHYHQLLEKVRIRDALRSILTISRHGNQYIQ

VNEPWKRIKGNEADRQRAGTVTGLAVNIAALLSIMLQPYMPTVSSTIAAQLQLPPPACSVLPTDFLCTLPAGHQIGTVSP

LFQKLENDQIETLRQRFGGGQAKVFPKPAAVETVTTAGPQQIQELMDEVTKQGNIVRELKAQKADKNQVAAEVAKLLDLK

KQLALAEGKPLETPKGKKKK

>XP_004773267.1 PREDICTED: methionine--tRNA synthetase, cytoplasmic isoform X1 [Mustela putorius furo]

MRLFVSEGAPGSLPVLAAAGRAQGRAELLISTVGPEECVVPFLTRPKVPVLQLDSGNYLFSTSAICRYFFLLSGWEQDDL

TNQWLEWEATELQPALSAALYYLVVQGKKGEDVLGLVRRALTHIDQSLSRRSCPFLAGETESLADVVLWGALYPLLQDPA

YLPEELGALHSWFQTLSSQEPCQRAAETVLKQQGVLALRPYLQKQPQPGSFEGRAVSNEPEEEELATLSEEEISMAVTAW

EKGLENLPPLRPQQNPVLPVAGERNVLITSALPYVNNVPHLGNIIGCVLSADVFARYSRLRQWNTLYLCGTDEYGTATET

KAMEEGLTPQEICDKYHAIHANIYRWFNISFDIFGRTTTPQQTKITQDIFQRLLTRGFVLEDTVEQLRCERCARFLADRF

VEGVCPFCGYEEARGDQCDKCGKLINAIELKKPQCKVCRSCPVVKSSRHLFLDLPKLEKRLEEWLGKTLPGSDWTPNARF

IIRSWLRDGLKPRCITRDLKWGTPVPLKGFEDKVFYVWFDATIGYLSITANYTDQWERWWKNPEQVNLYQFMAKDNVPFH

GLVFPCSALGAEDNYTLVSHLIATEYLNYEDGKFSKSRGVGVFGDMAQDTGIPADIWRFYLLYIRPEGQDSAFSWTDMLL

KNNSELLNNLGNFINRAGMFVSKFFGGYVPEMVLTSDDRRLVAHVTLELQHYHQLLEKVRIREALRSILTISRHGNQYIQ

VNEPWKRIKGSEADRQRAGTVTGLAVNIAALLSVMLQPYMPTVSATIQAQLQLPLPACSILLTNFLCTLPAGHQIGTVSP

LFQKLENDQIESLRQRFGGGQAQVPPMPVVVEAAATAAPRQVQVLMDEVARQGNIVRELKAQKADKNQVAAEVAKLLDLK

KQLALAEGKPLETPKGKKKK

>XP_003790616.1 methionine--tRNA synthetase, cytoplasmic isoform X4 [Otolemur garnettii]

MRLFVSEGAPGSLPVLAAAGRARGRAELLISTVGPEECVVPFLTQPKVPVLQLDSGNYLFSTSSICRYFFLLSGWEQDDL

TNQWLEWEATELQPALSAALYYLVVQGKKGEDVLGPVRRALKHIDYSLSRQNCPFLAGETESLADIVLWGALYPLLQDPA

YLPEELGSLCSWFQTLSTQEPCQRAAETVLKQQGVLALRPYLQKQPQPSPPNGRAVTNEPEEELATLSEEEITMAVTAWE

KGLGGLPPLRPPQNPVLPMAGERNVLITSALPYVNNVPHLGNIIGCVLSADVFARYSRLRQWNTLYLCGTDEYGTATETK

AMEEGLTPQEICDKYHAIHADIYRWFNISFDTFGRTTTPQQTKITQDIFRRLLTRGFVLQDTVEQLHCEKCARFLADRFV

EGVCPFCGYEEARGDQCDKCGKLINAIELKKPQCKVCRSCPVVKSSQHLFLDLPKLETRLEEWLGKTLPGSDWTPNSRFI

IRSWLRDGLKPRCITRDLKWGTPVPLEGFEDKVFYVWFDATIGYLSITANYTDQWERWWKNPEQVDLYQFMAKDNVPFHG

LVFPCSALGAEDNYTLVSHLIATEYLNYEDGKFSKSRGVGVFGDMAQDTGIPADIWRFYLLYIRPEGQDSAFSWTDLLLK

NNSELLNNLGNFINRAGMFVSKFFGGYVPEMVLTPDDQRLLAHVTLELRHYHQLLEKVRIRDALRSILTISRHGNQYIQV

NEPWKWIKGTEADRQRAGTVTGLAVNIAALLSVMLQPYMPSVSATIQAQLQLSPPACSVLLTNFLCTLPAGHQIGTVSPL

FQKLENDQIESLRQRFGGGQLEESLELKAKVSPKPAVVDTVAATGPQQIQMLMDEVTKQGNIVRELKAQKADKNQIAAEV

AKLLDLKKQLSLAEGKPPEAPKCKKKK

>XP_004861281.1 methionine--tRNA synthetase, cytoplasmic isoform X2 [Heterocephalus glaber]

MRLFVSEGAPGSLPVLAAAARAQGRAELLISTVGPEECVVPFLTEPKVPALQLDSGNYLFSTSAICRYFFLLSGWEQDDL

TNQWLEWEATELQPALSAALYYLVVQGKKSEDVLGPIGRVLTHIDHSLSRQNCPFLAGETESLADIVLWGALYPLLQDPA

YLPEELGDLCSWFQTLSTQEPCQRAAQTVLKQRGVLALRPYLQKQPQPRPPEGRVVSNEPEEEELATLSEEEIAMAVTAW

EKGLESLPPLRPQQNPVLPVAGERNVLITSALPYVNNVPHLGNIIGCVLSADVFARYSRLRQWNTLYLCGTDEYGTATET

KAMEEGLTPQEICDKYHTIHADIYHWFNISFDIFGRTTTPQQTKITQDIFQRLLTRGFILQDSVEQLRCDRCARFLADRF

VEGVCPFCGYEEARGDQCDKCGKLINAIELKKPQCKVCRSSPVVKSSLHLFLDLPKLEKTLEEWLGKTLPGSDWTPNARF

IIRSWLRDGLKPRCITRDLKWGTPVPLEGFEDKVFYVWFDATIGYLSITANYTDQWERWWKNPEQVDLYQFMAKDNVPFH

GLVFPCSALGAEDNYTLVKHLIATEYLNYEDGKFSKSRGVGVFGDMAQDTGIPADIWRFYLLYIRPEGQDSAFSWTDMLL

KNNSELLNNLGNFINRAGMFVSKFFGGFVPEMVLTPDDQRLLAHITLELRHYHQLLEKVRIRDALRSILTVSRHGNQYIQ

VNEPWKRIKGSEGDRQRAGTVTGMAVNIAALLSVMLQPYMPTVSATIQAQLQLPLPVCSVLLTDFVCTLPAGHQIGTVSP

LFQKLENDQIESLKQRFGGGQAKVSPMPAVVETVKTAGAQQIQTLMDEVTKQGNIVRELKAQKADKNQVAAEVAKLLDLK

KQLALAEGKPLETPTGKKKK

>XP_023369310.1 methionine--tRNA synthetase, cytoplasmic isoform X2 [Otolemur garnettii]

MRLFVSEGAPGSLPVLAAAGRARGRAELLISTVGPEECVVPFLTQPKVPVLQLDSGNYLFSTSSICRYFFLLSGWEQDDL

TNQWLEWEATELQPALSAALYYLVVQGKKGEDVLGPVRRALKHIDYSLSRQNCPFLAGETESLADIVLWGALYPLLQDPA

YLPEELGSLCSWFQTLSTQEPCQRAAETVLKQQGVLALRPYLQKQPQPSPPNGRAVTNEPEVWNRTKPEGLKWEEELATL

SEEEITMAVTAWEKGLGGLPPLRPPQNPVLPMAGERNVLITSALPYVNNVPHLGNIIGCVLSADVFARYSRLRQWNTLYL

CGTDEYGTATETKAMEEGLTPQEICDKYHAIHADIYRWFNISFDTFGRTTTPQQTKITQDIFRRLLTRGFVLQDTVEQLH

CEKCARFLADRFVEGVCPFCGYEEARGDQCDKCGKLINAIELKKPQCKVCRSCPVVKSSQHLFLDLPKLETRLEEWLGKT

LPGSDWTPNSRFIIRSWLRDGLKPRCITRDLKWGTPVPLEGFEDKVFYVWFDATIGYLSITANYTDQWERWWKNPEQVDL

YQFMAKDNVPFHGLVFPCSALGAEDNYTLVSHLIATEYLNYEDGKFSKSRGVGVFGDMAQDTGIPADIWRFYLLYIRPEG

QDSAFSWTDLLLKNNSELLNNLGNFINRAGMFVSKFFGGYVPEMVLTPDDQRLLAHVTLELRHYHQLLEKVRIRDALRSI

LTISRHGNQYIQVNEPWKWIKGTEADRQRAGTVTGLAVNIAALLSVMLQPYMPSVSATIQAQLQLSPPACSVLLTNFLCT

LPAGHQIGTVSPLFQKLENDQIESLRQRFGGGQLEESLELKAKVSPKPAVVDTVAATGPQQIQMLMDEVTKQGNIVRELK

AQKADKNQIAAEVAKLLDLKKQLSLAEGKPPEAPKCKKKK

>XP_014642150.1 PREDICTED: methionine--tRNA synthetase, cytoplasmic isoform X1 [Ceratotherium simum simum]

MRLFVSEGAPGSLPVLAAARRAQGRAELLISTVGPEECVVPFLTQPKIPVLQLDSGNYLFSSSAICRYFFLLSGWEQDDL

TNQWLEWEVTELQPALSAALYCLVVQGKKGEDVLGPVRRALTHIDHSLSHRSCPFLAGETESLADIVLWGALYPLLQDPA

YLPEELGALHSWFQTLSSQEPCQRAAETVLKQQGVLALRPYLQKQPQPSSLQGRAVSNEPEEEELATLSEEEITMAVTAW

EKGLGSLPPLRPQQNPVLPVPGERNVLITSALPYVNNVPHLGNIIGCVLSADVFARYSRLRQWNTLYLCGTDEYGTATET

KAMEEGLTPQEICDKYHAIHADIYRWFNISFDIFGRTTTPQQTKITQDIFQRLFARGFVLQDTMEQLRCEHCARFLADRF

VEGVCPFCGYEEARGDQCDKCGKLINAIELKKPQCKVCRSCPVVKSSQHLFLDLPKLEGRLEEWLEKTLPGSDWTPNAWF

IIRSWLRDGLRPRCITRDLRWGTPVPLEGFEEKVFYVWFDATIGYLSITANYTDQWERWWKNPEQVSLYQFMAKDNVPFH

GLVFPCSALGAEDNYTLVKHLIATEYLNYEDGKFSKSRGVGVFGDMAQDTGIPADIWRFYLLYIRPEGQDSAFSWTDMLH

KNNSELLNNLGNFINRAGMFVSKFFGGYVPEMVLTSDDQRLLAHVTLELQHYHQLLEKVRIRDALRSILTISRHGNQYIQ

VNEPWKRIKGSEADRQRAGTVTGLAVNIAALLSVMLQPYMPTVSATIQAQLQLPPPACSILPTSFLCTLPAGHQIGTVSP

LFQKLENDQIESLRQRFGGGQLEEPLELKAKVSSKPAVAETMTTAGPQQIQVLMEEVTKQGNIVRELKAQKADKNQVAAE

VAKLLDLKKQLALAEGKPLETPKGKKKK

>XP_012876655.1 PREDICTED: methionine--tRNA synthetase, cytoplasmic isoform X1 [Dipodomys ordii]

MRLFVSEGTPGSLPVLAAAGRVRGRIELLINTVGPEGIRASAECVVPFLTRPKVPVLQLDSGNYLFSTTAICRYFFLLSG

WEQDDLTNQWLEWEATELQPALSAALYYLVVQGKKGEDILGPLRKALTHIDQSLSHQNCPFLAGETESLADIVLWGALYP

LLQDPAYLPEELGALRSWFQTLSTQEPCQRAVETVLKQQGVLALRSYLQKQPQAHPSEGKVVSNEPEEEELATLSEEEIA

MAVTAWEKGLGSLPSLRPQQNPVLPVAGERNVLITSALPYVNNVPHLGNIIGCVLSADVFARYSRLRQWNTLYLCGTDEY

GTATETKAMEEGLTPREICDKYHRIHADIYHWFNISFDIFGRTTTPQQTKITQDIFQRLLTRGFVVQDTVEQLRCEHCAR

FLADRFVEGVCPFCGYEEARGDQCDKCGKLINAIELKKPQCKVCRSCPVVKSSKHLFLDLPKLENRLEDWLGKTLPGSDW

TPNARFIIRSWLRDGLKPRCITRDLKWGTPVPLEGFEDKVFYVWFDATIGYLSITANYTEQWERWWKNPEQVDLYQFMAK

DNVPFHGLVFPCSALGAEDNYTLVKHLIATEYLNYEDGKFSKSRGVGVFGDMAQNTGIPADIWRFYLLYIRPEGQDSAFS

WTDMLLKNNSELLNNLGNFVNRAGMFVSKFFGGYVPEMVLTPDDQRLLAHITLELQHYHQLLEKVRIRDALRSILTISRH

GNQYIQVNEPWKRIKGSEADRQRAGTVTGLAVNIAALLSVMIQPYMPTVSATIQTQLQLPLQVCSVLPIKFLCTLPAGHQ

IGTVSPLFQKLENDQIESLRQRFGGGQLEESLEFKAKASPKSTVVETVTAGLQQIQVLMDEVTKQGNIVRELKAQKADKN

QVAAEVAKLLDLKKQLAVAEGKPLETPKGKKKK

>XP_024432030.1 methionine--tRNA synthetase, cytoplasmic isoform X1 [Desmodus rotundus]

MRLFVSEGAPGTLPVLAAAGRARGRAELLVITVGPEEHVVPFLTRPKVPVLQLDSGNYLFSTSAICRYFFLLSGWEQDDL

TNQWLEWEATELQPALSAALYYLVVQGKKGEDVLGPVRRALTHIDHSLSRRSCPFLAGETESLADIVLWGALYPLLQDPA

YLPEELGALHSWFQTLSSQEPCQRAAETVLKQQGILALRPYLQKQPQPSLLEGRAVSNEPEEEELVTLSEEEIAMAVTAW

EKGLGSLPPLRPKQNPVLPVAGERNVLITSALPYVNNVPHLGNIIGCVLSADVFARYSRLRQWNTLYVCGTDEYGTATET

KAMEEGLTPQEICDKYHVIHADIYRWFNISFDIFGRTTTPQQTEITQDIFQRLWNRGFVLQDTVEQLRCERCARFLADRF

VEGVCPFCGYEEARGDQCDKCGKLINAIELKKPQCKVCRSCPVVKSSRHLFLDLPKLEDQLEEWLGKTLPGSDWTPNARF

IIRSWLRDGLKPRCITRDLKWGTPVPLEGYEDKVFYVWFDATFGYLSITANYTDQWERWWKNPEQVNLYQFMAKDNVPFH

GLVFPCSALGAEDNYTLVSHLIATEYLNYEDGKFSKSRGIGVFGDMAQDTGIPADIWRFYLLYIRPEGQDSAFSWTDMLL

KNNSELLNNLGNFINRAGMFVSKFFAGSVPEMVLTSDDQRLLAHVTLELQHYHQLLEKVRIRDALRSILTISRHGNQYIQ

VNEPWKRIKGNEADRQRAGTVTGLAVNIAALLSIMLQPYMPTVSSTIAAQLQLPPPACSVLPTDFLCTLPAGHQIGTVSP

LFQKLENDQIETLRQRFGGGQMEESLALKAKVFPKPAAVETVTTAGPQQIQELMDEVTKQGNIVRELKAQKADKNQVAAE

VAKLLDLKKQLALAEGKPLETPKGKKKK

>XP_005901749.1 PREDICTED: methionine--tRNA synthetase, cytoplasmic isoform X2 [Bos mutus]

MRLFVSEGAPGSLPVLAAAGRAQGRAELLISTVGPEECVVPFLTRPKVPVLQLDSGNYLFSTSAICRYFFLLSGWEQDDL

TNQWLEWEATELQPALSAALYYLVVQGKKGEDVLGPVRRALTHIDHSLSRRSCPFLAGETESLADIVLWGALYPLLQDPS

YLPEELGALHSWFQTLSSQEPCQRAAETVLKQQGVLALRPYLQKQPQPSSLEGRLVSNEPEEEELATLSEEEIAVAVAAW

EKGLESLPPLRPQQNPVLPVAGERNVLITSALPYVNNVPHLGNIIGCVLSADVFARYSRLRQWNTLYLCGTDEYGTATET

KAMEEGLTPQEICDKYHVIHADIYRWFNISFDFFGRTTTPQQTKITQDIFQRLLARGFVLQDTVEQLRCEHCARFLADRF

VEGVCPFCGYEEARGDQCDKCGKLINAIELKKPQCKVCRSCPVVKSSQHLFLDLPKLAARVEEWLEKTLPGSDWTANARF

IIRSWLRDGLKPRCITRDLKWGTPVPLEGFEDKVFYVWFDATIGYLSITANYTDQWEKWWKNPEQVNLYQFMAKDNVPFH

GIVFPSSALGAEDNYTLVSHLIATEYLNYEDGKFSKSRGVGVFGDMAQDTGIPADIWRFYLLYIRPEGQDSAFSWTDMLF

KNNSELLNNLGNFINRAGMFVSKFFGGFVPEMVLTSDDQRLLTHITLELQHYHQLLEKVRIRDALRSILTISRHGNQYIQ

VNEPWKRIKGGEADRQRAGTVTGLAVNIAALLSVMLQPYMPTVSATIQAQLQLPPPACSILPTNFLCTLPAGHQIGTVSP

LFQKLENDQIESLKQRFSGGQAKASPKTAAGLTTAGPQQIQALTEEVTKQGNIVRELKAQKADKNQIAAEVAKLLDLKKQ

LALAEGKPLETSKGKKKK

>XP_019513163.1 PREDICTED: methionine--tRNA synthetase, cytoplasmic isoform X2 [Hipposideros armiger]

MRLFVSEGAPGSLPVLAAAERAQGRAELLISTVGPEDCVVPFLTWPKVPVLQLDSGNYLFSTSAICRYFFLLSGWEQDDL

TNQWLEWEATELQPALSAALYYLVVQGKKGEDVLGLVRRALTHIDQSLSRQSCSFLVGETESLADIVLWGALYPLLQDPA

YLPEELGALHSWFQTLSSQEPCQRAAETVLKQQGILALRPYLQKQPQPSSLERRVVSNEPEEEELATLSEEDIATAVTAW

EKGLGSLPPLRPKQIPVLPVAGERNVLITSALPYVNNVPHLGNIIGCVLSADVFARYSRLRQWNTLYLCGTDEYGTATET

KAMEEGLTPQEICDKYHVIHANIYHWFNISFDIFGRTTTPQQTQITQDIFQRLWNRGFVLQDTVEQLRCERCARFLADRF

VEGVCPFCGYEEARGDQCDKCGKLINATELKKPQCKVCRSCPVVKSSQHLFLDLPKLEDRLEEWLGKTLSGSDWTPNARF

IIRSWLRDGLKPRCITRDLKWGTPVPLKGFEDKVFYVWFDATFGYLSITANYTDQWERWWKNPEQVNLYQFMAKDNVPFH

GLVFPCSALGAEDNYTLVSHLIATEYLNYEDGKFSKSRGVGVFGDMAQDTGIPADIWRFYLLYIRPEGQDSAFSWTDLLL

KNNSELLNNLGNFINRAGMFVSKFFGGYVPEMMLTSDDQRLLAHVTLELQHYHQLLEKVRIRDALRSILTISRHGNQYIQ

VNEPWKRIKGSEADRQRAGTVTGLAVNIAALLSIMLQPYMPTVSATIAAQLQLPPPACSILPTDFLCTLPAGHQIGTVSP

LFQKLENDQIESLRQRFGGGQAKASPKPAVVETVTAAGSQQIQVLMDEVTKQGNIVRELKAQKADKNQVAAEVAKLLDLK

KQLALAEGKPLETPKGKKKK

>XP_002720960.1 PREDICTED: methionine--tRNA synthetase, cytoplasmic isoform X2 [Oryctolagus cuniculus]

MRLFVSPGSPGSLPVLAAAGRARGRAELLISTVGPEECVVPFLARPKVPALQLDGGNYLFSTSAICRYFFLLSGWEQDDL

TNQWLEWEATELQPVLSAALYYLVVQGKKGEDVLGPVRRALTHVDQSLSRQNSPFLTGETESLADIVLWGALYPLLQDPA

YLPDELGALRSWFQTLSTQEPCQRAAETVLKQQGILALRPYLQKQPQPSPPEDKAVSNEPEEEELATLSEEEIAVAVTAW

EKGLESLPPRRPQQNPVLPVAGERNVLITSALPYVNNVPHLGNIIGCVLSADVFARYSRLRQWNTLYLCGTDEYGTATET

KAMEEGLTPQEICDKYHIIHADIYRWFNISFDIFGRTTTPQQTKITQDIFQRLLSRGFVLQDTVEQLRCERCARFLADRF

VEGVCPFCGYEEARGDQCDKCGKLINAIELKKPQCKVCRSSPVVKSSQHLFLDLPKLEERLEEWLGKTLPGSDWTPNARF

IIRSWLRDGLKPRCITRDLKWGTPVPLEGFEDKVFYVWFDATIGYLSITANYTDQWERWWKNPEQVDLYQFMAKDNVPFH

GLVFPCSALGAEDNYTLVKHLIATEYLNYEDGKFSKSRGVGVFGDMAQDTGIPADIWRFYLLYIRPEGQDSAFSWTDMLL

KNNSELLNNLGNFINRAGMFVSKFFGGCVPEMVLTSDDRRLLAHVTLELQHYHQLLEKARIRDALRSILTISRHGNQYIQ

VNEPWKRIKGDEADRQRAGTVTGMAVNVAALLSVMLQPYMPTVSASIQAQLQLPPPACSVLPTNFLCTLPAGHQIGTVSP

LFQKLENDQIESLRQRFGGGQAKASPKPAAEETRKTAGPQEIQTLLEEVTKQGNLVRELKAQKADKNQVAAEVTKLLDLK

KQLALAEGKPLETPKGKKKK

>XP_015338597.1 PREDICTED: methionine--tRNA synthetase, cytoplasmic [Marmota marmota marmota]

MRLFVSEGAPGSLPVLAAAGRARGRATELLISTVGPEECVVPFLTRPKVPVLQLDSGNYLFSTTAICRYFFLLSGWEQDD

LTNQWLEWEATELQPALSAALYYLIVQGKKGEDVLGLIRRALTHIDHSLSRQNCPFLAGETESLADIVLWGALYPLLQDP

AYIPEELGALRSWFQTLTTQEPCQRAVETVLKQQGVLALRSYLQKQPQPSLPEERAVSNEPEEEELATLSEEEIAIAVTA

WEKGLESLPPFRPQQNPVLPVAGERNVLITSALPYVNNVPHLGNIIGCVLSADVFARYSRLRQWNTLYLCGTDEYGTATE

TKAMEEGLTPQEICDKYHTIHADIYRWFNISFDIFGRTTTPQQTKITQDIFHRLMARGFVLQDSVEQLRCERCARFLADR

FVEGVCPFCGYEEARGDQCDKCGKLINAIELKKPQCKVCRSCPVVKSSQHLFLDLPKLEKRLEEWLGKTLPGSDWTPNAR

FIIRSWLRDGLKPRCITRDLKWGTPVPLKGFEDKVFYVWFDATIGYLSITANYTDQWEQWWKNPEQVDLYQFMAKDNVPF

HGLVFPCSALGAEDNYTLVKHLIATEYLNYEDGKFSKSRGVGVFGDMAQDTGIPADIWRFYLLYIRPEGQDSAFSWTDML

LKNNSELLNNLGNFINRAGMFVSKFFGGCVPEMVLTPDDQRLLAHITLELRHYHQLLEKVRIRDALRSILTISRHGNQYI

QVNEPWKWIKGSEADRQRAGTVTGLAVNIAALLSTMLQPFMPMVSTTIQAQLQLPLPACSVLPTNFLCTLPAGHQIGTVT

PLFQKLENYQIESLRQRFGGGQAKVFPKPAVIETVTTVGPQQIQMLMDEVTKQGNIVRELKAQKADKNQVAAEVAKLLDL

KKQLALAEGKPLETPKGKKKK

>NP_001033180.1 methionine--tRNA synthetase, cytoplasmic [Bos taurus]

MRLFVSEGAPGSLPVLAAAGRAQGRAELLISTVGPEECVVPFLTRPKVPVLQLDSGNYLFSTSAICRYFFLLSGWEQDDL

TNQWLEWEATELQPALSAALYYLVVQGKKGEDVLGPVRRALTHIDHSLSRRSCPFLAGETESLADIVLWGALYPLLQDPS

YLPEELGALHSWFQTLSSQEPCQRAAETVLKQQGVLALRPYLQKQPQPSSLEGRLVSNEPEEEELATLSEEEIAVAVAAW

EKGLESLPPLRPQQNPVLPVAGERNVLITSALPYVNNVPHLGNIIGCVLSADVFARYSRLRQWNTLYLCGTDEYGTATET

KAMEEGLTPQEICDKYHVIHADIYRWFNISFDFFGRTTTPQQTKITQDIFQRLLARGFVLQDTVEQLRCEHCARFLADRF

VEGVCPFCGYEEARGDQCDKCGKLINAIELKKPQCKVCRSCPVVKSSQHLFLDLPKLAARVEEWLEKTLPGSDWTANARF

IIRSWLRDGLKPRCITRDLKWGTPVPLEGFEDKVFYVWFDATIGYLSITANYTDQWEKWWKNPEQVNLYQFMAKDNVPFH

GIVFPSSALGAEDNYTLVSHLIATEYLNYEDGKFSKSRGVGVFGDMAQDTGIPADIWRFYLLYNRPEGQDSAFSWTDMLF

KNNSELLNNLGNFINRAGMFVSKFFGGFVPEMVLTSDDQRLLTHITLELQHYHQLLEKVRIRDALRSILTISRHGNQYIQ

VNEPWKRIKGGEADRQRAGTVTGLAVNIAALLSVMLQPYMPTVSATIQAQLQLPPPACSILPTNFLCTLPAGHQIGTVSP

LFQKLENDQIESLKQRFSGGQAKASPKTAAGLTTAGPQQIQALTEEVTKQGNIVRELKAQKADKNQIAAEVAKLLDLKKQ

LALAEGKPLETSKGKKKK

>XP_010837628.1 PREDICTED: methionine--tRNA synthetase, cytoplasmic isoform X2 [Bison bison bison]

MRLFVSEGAPGSLPVLAAAGRAQGRAELLISTVGPEECVVPFLTRPKVPVLQLDSGNYLFSTSAICRYFFLLSGWEQDDL

TNQWLEWEATELQPALSAALYYLVVQGKKGEDVLGPVRRALTHIDHSLSRRSCPFLAGETESLADIVLWGALYPLLQDPS

YLPEELGALHSWFQTLSSQEPCQRAAETVLKQQGVLALRPYLQKQPQPSSLEGRLVSNEPEEEELATLSEEEIAVAVAAW

EKGLESLPPLRPQQNPVLPVAGERNVLITSALPYVNNIPHLGNIIGCVLSADVFARYSRLRQWNTLYLCGTDEYGTATET

KAMEEGLTPQEICDKYHVIHADIYRWFNISFDFFGRTTTPQQTKITQDIFQRLLARGFVLQDTVEQLRCEHCARFLADRF

VEGVCPFCGYEEARGDQCDKCGKLINAIELKKPQCKVCRSCPVVKSSQHLFLDLPKLAARVEEWLEKTLPGSDWTANARF

IIRSWLRDGLKPRCITRDLKWGTPVPLEGFEDKVFYVWFDATIGYLSITANYTDQWEKWWKNPEQVNLYQFMAKDNVPFH

GIVFPSSALGAEDNYTLVSHLIATEYLNYEDGKFSKSRGVGVFGDMAQDTGIPADIWRFYLLYIRPEGQDSAFSWTDMLF

KNNSELLNNLGNFINRAGMFVSKFFGGFVPEMVLTSDDQRLLTHITLELQHYHQLLEKVRIRDALRSILTISRHGNQYIQ

VNEPWKRIKGGEADRQRAGTVTGLAVNIAALLSVMLQPYMPTVGATIQAQLQLPPPACSILPTNFLCTLPAGHQIGTVSP

LFQKLENDQIESLKQRFSGGQAKASPKTAAGLTTAGPQQIQALTEEVTKQGNIVRELKAQKADKNQIAAEVAKLLDLKKQ

LALAEGKPLETSKGKKKK

>XP_008842296.1 PREDICTED: methionine--tRNA synthetase, cytoplasmic isoform X5 [Nannospalax galili]

MRLFVCEGAPGSLPVLAAAGRARDRAELLISTVGSEECVVPFLTRPKVPVLQLDSGNYLFSSSAICRYFFLLSGWEQDDL

TNQWLEWEATELQPALSAALYCLVVQGKKGEHVLGPLRRALTHIDYSLSRQNCPFLAGDTESLADIVLWGALYPLLQDPT

YLPEELGALHSWFQTLSTQEPCRRAAETVLKQQGVLALRPYLQKQPQPQSHPPEGRAVSNEPEEEELATLSEEEIAMAVT

AWEKGLGSLPPLRPPQNPVLPVAGERNVLITSALPYVNNVPHLGNIIGCVLSADVFARYSRLRQWNTLYLCGTDEYGTAT

ETKAMEEGLTPQEICDKYHRIHADIYCWFNISFDTFGRTTTAQQTKITQDIFQRLLTRGFVLQDTVEQLRCEQCARFLAD

RFVEGVCPFCGYEEARGDQCDKCGKLINAIELKKPQCKVCRSCPVVKSSQHLFLDLPKLEKRLEEWLGKTLPGSDWTPNA

RFIIRSWLRDGLKPRCITRDLKWGTPVPLEGFEDKVFYVWFDATIGYLSITANYTDQWERWWKNPDQVDLYQFMAKDNVP

FHGLVFPCSALGAEDNYTLVRHLIATEYLNYEDGKFSKSRGVGVFGDMAQDTGIPADIWRFYLLYIRPEGQDSAFSWTDM

LIKNNSELLNNLGNFINRAGMFVSKFFGGCVPKMVLTPDDQRLLAHISLELQHYHQLLEKVRIRDALRSILTISRHGNQY

IQVNEPWKRIKGSEADRQRAGTVTGLAVNIAALLSVMLQPYMPTVSATIQAQLQLPLQACGILLTNFICTLPTGHHIGTV

SPLFQKLENDQIESLRQRFGGGQAKVSPKPAVVETVTTAGPQHIQMLMDEVTKQGNVVRELKAQKADKNQVAAEVAKLLD

LKKQLALAEGKPIETPKGKKKK

>XP_008842294.1 PREDICTED: methionine--tRNA synthetase, cytoplasmic isoform X2 [Nannospalax galili]

MRLFVCEGAPGSLPVLAAAGRARDRAELLISTVGSEECVVPFLTRPKVPVLQLDSGNYLFSSSAICRYFFLLSGWEQDDL

TNQWLEWEATELQPALSAALYCLVVQGKKGEHVLGPLRRALTHIDYSLSRQNCPFLAGDTESLADIVLWGALYPLLQDPT

YLPEELGALHSWFQTLSTQEPCRRAAETVLKQQGVLALRPYLQKQPQPQSHPPEGRAVSNEPEEEELATLSEEEIAMAVT

AWEKGLGSLPPLRPPQNPVLPVAGERNVLITSALPYVNNVPHLGNIIGCVLSADVFARYSRLRQWNTLYLCGTDEYGTAT

ETKAMEEGLTPQEICDKYHRIHADIYCWFNISFDTFGRTTTAQQTKITQDIFQRLLTRGFVLQDTVEQLRCEQCARFLAD

RFVEGVCPFCGYEEARGDQCDKCGKLINAIELKKPQCKVCRSCPVVKSSQHLFLDLPKLEKRLEEWLGKTLPGSDWTPNA

RFIIRSWLRDGLKPRCITRDLKWGTPVPLEGFEDKVFYVWFDATIGYLSITANYTDQWERWWKNPDQVDLYQFMAKDNVP

FHGLVFPCSALGAEDNYTLVRHLIATEYLNYEDGKFSKSRGVGVFGDMAQDTGIPADIWRFYLLYIRPEGQDSAFSWTDM

LIKNNSELLNNLGNFINRAGMFVSKFFGGCVPKMVLTPDDQRLLAHISLELQHYHQLLEKVRIRDALRSILTISRHGNQY

IQVNEPWKRIKGSEADRQRAGTVTGLAVNIAALLSVMLQPYMPTVSATIQAQLQLPLQACGILLTNFICTLPTGHHIGTV

SPLFQKLENDQIESLRQRFGGGQAKVSPKPAVVETVTTAGPQHIQMLMDEVTKQGNVVRELKAQKADKNQVAAEVAKLLD

LKKQLALAEGKPIETPKGKKKKSFLCSKPSSGCFMYIED

>XP_007535016.1 PREDICTED: methionine--tRNA synthetase, cytoplasmic isoform X2 [Erinaceus europaeus]

MRLFVSEGAPGSLPVLAAAGRAQGKAELLISTVGPEECVVPFLTRPKVPVLQLDNGNYLFSTTAICRYFFLLSGWEQDDL

TNQWLEWEATELQPAVSAALYCLVVQGKKGEDVLGSVRRALTHIDHSLSRRSCPFLTGETESLADIVLWGALYPLLQDAA

YLPEELGALHSWFQTLNSQEPCQQAVETVLKQHGILALRPYLQKQPQLSSPEGKLVSNDPEEEELATLSEEDIAMAVSAW

EKGLGSLPTLRPQQNPVLPVAGERNVLITSALPYVNNVPHLGNIIGCVLSADVFARYSRLRQWNTLYLCGTDEYGTATET

KAMEEGLTPQEICDKYYAIHASIYRWFNISFDTFGRTTTPQQTKITQDIFQRLYERGFVLQDTVEQLRCERCARFLADRF

VEGVCPFCGYEEARGDQCDKCGKLINATELKKPQCKVCRSCPVVRSSRHLFLDLPKLEERLERWLEKTVPGSDWTPNARF

IIRSWLRDGLKPRCITRDLKWGTPVPLEGFEDKVFYVWFDATIGYLSITANYTDQWEKWWKNPEQVDLYQFMAKDNVPFH

GLVFPCTALGAEDNYTLVSHLIATEYLNYEDGKFSKSRGVGVFGDMAQDTGIPADIWRFYLLYIRPEGQDSAFSWTDLLL

KNNSELLNNLGNFINRAGMFVSKFFGGCVPEMVLTSDDQRLLTHVTLELQHYHQLLEKARIRDALRSILTISRHGNQYIQ

VNEPWKRIKGNEADRQRAGTVTGMAVNIAALLSVMLQPYMPTVSATIQAQLQLSLPACSVLPTNFLCTLPAGHQIGTVSP

LFQKLENDQIESLRQHFGGGQVKVSPKPAVEEAVTTAGSQQIQMLMDEVTKQGNIVRELKAQKADKNQIAAEVAKLLDLK

KQLALAEGKPLEAPKGKKKK

>XP_004650075.1 PREDICTED: methionine--tRNA synthetase, cytoplasmic isoform X2 [Jaculus jaculus]

MRLFVSEGAPGSLPVLAAAARARGRAELLVSAVGPEECVVPFLTRAKVPVLQLDSGNYLFSTSAICRYFFLLSGWEQDDL

TNQWLEWEATELQPALSAALYCLVVQGKKGDDVLGPLRRALSHIDHSLNRQDRPFLTGETESVADIVLWGALYPLLQDPA

YLPEELGALRSWFQTLSTQEPCQRAAETVLKQQGVLALRPYLQKQPQPQTYFSEGRAVSNEPEEEELVTLSEEEIAKAVT

AWEKGRESLPPLRPQQNPVLPVAGERNVLITSALPYVNNVPHLGNIIGCVLSADVFARYSRLRQWNTLYLCGTDEYGTAT

ETKAMEEGLTPQEICDKYHRIHADIYRWFNISFDTFGRTTTPQQTKITQDIFQRLLTRGFVLQDNVEQLRCEHCARFLAD

RFVEGVCPFCGYEEARGDQCDKCGKLINAIELKKPQCKVCRSCPVVKSSRHLFLDLPKLEKRLEEWLGKTLPGSDWTPNA

RFIIRSWLRDGLKPRCITRDLKWGTPVPLEGFQDKVFYVWFDATIGYLSITANYTDEWERWWKNPEQVDLYQFMAKDNVP

FHGLVFPCSALGAEDNYTLVKHLIATEYLNYEDGKFSKSRGIGVFGDMAQDTGIPADIWRFYLLYIRPEGQDSAFSWTDM

LIKNNSELLNNLGNFINRAGMFVSKFFGGFVPEVVLTPDDQRLLAHITLELQHYHQLLEKVRIRDALRSILTISRHGNQY

IQVNEPWKRIKGSEADRQRAGTVTGLAVNIAALLSVMLQPYMPTVSATIQAQLQLPLAVCSILPTNFICTLPAGHQIGTV

SPLFQKLENDQIESLRQRFGGGQAKVSPKPAVVETGTTAGPQQIQMLMDEVTKQGNTVRELKAQKADKNQIAAEVAKLLD

LKKQLALAEGKPLETPKGKKKK

>XP_003476192.1 methionine--tRNA synthetase, cytoplasmic [Cavia porcellus]

MRLFVSEGAPGSLPVLVAAGRAPGRSELLISTVGPEECVVPFLTQPKFPVLQLDSGNYLFSTSAICRYFFLLSGWEPDDF

TNQWLEWEATELQPVLSAALYYLVIQGKKGEDVLDPLGKALTHIDHSLSRQNCPFLGGETESLADVVLWGALYPLLQDPA

YLPEELGALRSWFQTLSTQEPCQRAAETVLKQKGVLALRPYLQKQPQPSLPEGRAVSNEPEEEELATLSEEEIAAAVTAW

KKGLEGLPVLRPQQNPVLPVAGERNVLITSALPYVNNVPHLGNIIGCVLSADVFARYSRLRQWNTLYLCGTDEYGTATET

KAMEEGLTPQEICDKYHTIHADIYRWFNISFDFFGRTTTPQQTKITQDIFHRLLTRGFILEDTVEQLRCEHCARFLADRF

VEGVCPFCGYEEARGDQCDKCGKLINAIELKKPQCKVCRSCPVVKSSRHLFLDLPKLEKRLEEWLGKTLPGSDWTPNARF

IIRSWLRDGLKPRCITRDLKWGTPVPLEGFEDKVFYVWFDATIGYLSITANYTDQWEKWWKNPEQVDLYQFMAKDNVPFH

GLVFPCSALGTDDNYTLVKYLIATEYLNYEDGKFSKSRGVGVFGDMAQDTGIPADIWRFYLLYIRPEGQDSAFSWTDMLL

KNNSELLNNLGNFINRAGMFVSKFFGGYVPEMVLTPDDQRLLTHITLELQHYHQLLEKVRIRDALRSILTVSRHGNQYIQ

VNEPWKRIKGNEADRQRAGTVTGMAVNIAALLSVMLQPYMPTVSATIQAQLQLPLPACSVLPTDFLCTLPAGHQIGIVSP

LFQKLENDQIESLKQHFGGSQAKVSPNPAAIETVTTPGPQQIQMLMAEVTKQGNIVREMKAQKADKNLVAAEVAKLLDLK

KQLALTEGKPLETLKGKKKK

>XP_015007537.1 PREDICTED: methionine--tRNA synthetase, cytoplasmic isoform X2 [Macaca mulatta]

MRLFVSEGAPGCLPVLAAAGRARGRAELLISTVGPEDCVVPFLTRPKVPVLQLDSGNYLFSTSAICRYFFLLSGWEQDDL

TNQWLEWEATELQETESLADIVLWGALYPLLQDPAYLPEELSALHSWFQTLSTQEPCQRAAETVLKQQGVLALRPYLQKQ

PQPSPAEGRAVTNEPEEEELATLSEEEIAMAVTAWEKGLESLPPLRPQQNPVLPVTGERNVLITSALPYVNNVPHLGNII

GCVLSADVFARYSRLRQWNTLYLCGTDEYGTATETKAMEEGLTPQEICDKYHIIHADIYRWFNISFDTFGRTTTPQQTKI

TQDIFQRLLTRGFVLQDTVEQLRCEHCARFLADRFVEGVCPFCGYEEARGDQCDKCGKLINAVELKKPQCKVCRSCPVVQ

STHHLFLDLPKLEKRLEEWLGRTLPGSDWTPNARFITRSWLRDGLKPRCITRDLKWGTPVPLEGFEDKVFYVWFDATIGY

LSITANYTDQWERWWKNPEQVDLYQFMAKDNVPFHGIVFPCSALGAEDNYTLVSHLIATEYLNYEDGKFSKSRGVGVFGD

MAQDTGIPADIWRFYLLYIRPEGQDSAFSWTDLLLKNNSELLNNLGNFINRAGMFVSKFFGGYVPEMVLTPDDQRLLAHV

TLELQHYHQLLEKVRIRDALRSILTISRHGNQYIQVNEPWKRIKGSEADRQRAGTVTGLAVNIAALLSVMLQPYMPTVSA

TIQAQLQLPPPACSILLTNFLCTLPAGHQIGTVSPLFQKLENDQIESLRQRFGGGQLEETFDLKAKTSPKPTVIETVTTA

GPQQIQALMDEVTKQGNIVRELKAQKADKNQVAAEVAKLLDLKKQLAVAEGKPPEAPKGKKKK

>XP_006859537.1 PREDICTED: methionine--tRNA synthetase, cytoplasmic [Chrysochloris asiatica]

MRLFVSEGVPGILSVLAAASRVRGKAELLISTVGPEECLVPFLTAPKVPVLQLDSGNYLFSTSAICRYFFLLTGWEQDDL

TNQWLEWEAVELQPALSAALYYLVVQGKKGEDVLNPVQRALTHIDHSLSRHSCPFLTGETESLADIVLWGTLYPLLQDPA

YLPEELGALRSWFQMLSTQGPCQQAVETVLKQQGVLALRPYLQKQPQPKLLEGRTVSNEPEEEELATLSEEEIAMAVTAW

EKGLESLPPLRPQKKPVLPVAGEKNVLITSALPYVNNVPHLGNIIGCVLSADVFARYSRLRQWNTLYLCGTDEYGTATET

KAMEEGLSPQEICDKYHTIHANIYRWFNISFDIFGRTTTPQQTKITQDIFQRLLARGFVLQDTVEQLRCERCARFLADRF

VEGVCPFCGYEEARGDQCDKCGKLINAIELKKPQCKVCRSCPVVRSSRHLFLDLPKLESRLEEWLGKILPGSDWTPNARF

IIRSWLRDGLKPRCITRDLKWGTPVPLEGFEDKVFYVWFDATIGYLSITANYTDQWERWWKNPEQVDLYQFMAKDNVPFH

GLVFPCSVLGTDDNYTLVSHIIATEYLNYEDGKFSKSRGVGVFGDMAQDTGIPADIWRFYLLYIRPEGQDSAFSWTDMLL

KNNSELLNNLGNFINRAGMFVSKFFGGSVPKMVLTSDDQRLVAHVTLELQHYHQLLEKVRIRDALRSILTISRHGNQYIQ

VNEPWKRIKGNEADRQRAGTVTGLAVNVAALLSIMIQPYMPTVSTTIQAQLQLPSLACSILPTDFLCTLPAGHQIGTVSP

LFQKLENDQIESLRQRFGGGQVKASPKPVVVETVTTAGPQQIQTLMDEVTKQGNTVRELKAQKADKNQVAAEVTKLLELK

KQLALAEGKPLDPPKGKKKK

>XP_010803461.1 methionine--tRNA synthetase, cytoplasmic isoform X1 [Bos taurus]

MRLFVSEGAPGSLPVLAAAGRAQGRAELLISTVGPEECVVPFLTRPKVPVLQLDSGNYLFSTSAICRYFFLLSGWEQDDL

TNQWLEWEATELQPALSAALYYLVVQGKKGEDVLGPVRRALTHIDHSLSRRSCPFLAGETESLADIVLWGALYPLLQDPS

YLPEELGALHSWFQTLSSQEPCQRAAETVLKQQGVLALRPYLQKQPQPSSLEGRLVSNEPEEEELATLSEEEIAVAVAAW

EKGLESLPPLRPQQNPVLPVAGERNVLITSALPYVNNVPHLGNIIGCVLSADVFARYSRLRQWNTLYLCGTDEYGTATET

KAMEEGLTPQEICDKYHVIHADIYRWFNISFDFFGRTTTPQQTKITQDIFQRLLARGFVLQDTVEQLRCEHCARFLADRF

VEGVCPFCGYEEARGDQCDKCGKLINAIELKKPQCKVCRSCPVVKSSQHLFLDLPKLAARVEEWLEKTLPGSDWTANARF

IIRSWLRDGLKPRCITRDLKWGTPVPLEGFEDKVFYVWFDATIGYLSITANYTDQWEKWWKNPEQVNLYQFMAKDNVPFH

GIVFPSSALGAEDNYTLVSHLIATEYLNYEDGKFSKSRGVGVFGDMAQDTGIPADIWRFYLLYIRPEGQDSAFSWTDMLF

KNNSELLNNLGNFINRAGMFVSKFFGGFVPEMVLTSDDQRLLTHITLELQHYHQLLEKVRIRDALRSILTISRHGNQYIQ

VNEPWKRIKGGEADRQRAGTVTGLAVNIAALLSVMLQPYMPTVSATIQAQLQLPPPACSILPTNFLCTLPAGHQIGTVSP

LFQKLENDQIESLKQRFSGGQLEESLALKAKASPKTAAGLTTAGPQQIQALTEEVTKQGNIVRELKAQKADKNQIAAEVA

KLLDLKKQLALAEGKPLETSKGKKKK

>XP_004861280.1 methionine--tRNA synthetase, cytoplasmic isoform X1 [Heterocephalus glaber]

MRLFVSEGAPGSLPVLAAAARAQGRAELLISTVGPEECVVPFLTEPKVPALQLDSGNYLFSTSAICRYFFLLSGWEQDDL

TNQWLEWEATELQPALSAALYYLVVQGKKSEDVLGPIGRVLTHIDHSLSRQNCPFLAGETESLADIVLWGALYPLLQDPA

YLPEELGDLCSWFQTLSTQEPCQRAAQTVLKQRGVLALRPYLQKQPQPRPPEGRVVSNEPEEEELATLSEEEIAMAVTAW

EKGLESLPPLRPQQNPVLPVAGERNVLITSALPYVNNVPHLGNIIGCVLSADVFARYSRLRQWNTLYLCGTDEYGTATET

KAMEEGLTPQEICDKYHTIHADIYHWFNISFDIFGRTTTPQQTKITQDIFQRLLTRGFILQDSVEQLRCDRCARFLADRF

VEGVCPFCGYEEARGDQCDKCGKLINAIELKKPQCKVCRSSPVVKSSLHLFLDLPKLEKTLEEWLGKTLPGSDWTPNARF

IIRSWLRDGLKPRCITRDLKWGTPVPLEGFEDKVFYVWFDATIGYLSITANYTDQWERWWKNPEQVDLYQFMAKDNVPFH

GLVFPCSALGAEDNYTLVKHLIATEYLNYEDGKFSKSRGVGVFGDMAQDTGIPADIWRFYLLYIRPEGQDSAFSWTDMLL

KNNSELLNNLGNFINRAGMFVSKFFGGFVPEMVLTPDDQRLLAHITLELRHYHQLLEKVRIRDALRSILTVSRHGNQYIQ

VNEPWKRIKGSEGDRQRAGTVTGMAVNIAALLSVMLQPYMPTVSATIQAQLQLPLPVCSVLLTDFVCTLPAGHQIGTVSP

LFQKLENDQIESLKQRFGGGQLEESLELKAKVSPMPAVVETVKTAGAQQIQTLMDEVTKQGNIVRELKAQKADKNQVAAE

VAKLLDLKKQLALAEGKPLETPTGKKKK

>XP_021778979.1 methionine--tRNA synthetase, cytoplasmic isoform X3 [Papio anubis]

MRLFVSQGAPGCLPVLAAAGRARGRAELLISTVGPEDCVVPFLTRPKVPVLQLDSGNYLFSTSAICRYFFLLSGWEQDDL

TNQWLEWEATELQETESLADIVLWGALYPLLQDPAYLPEELSALHSWFQTLSTQEPCQRAAETVLKQQGVLALRPYLQKQ

PQPSPAEGRAVTNEPEEEELATLSEEEIAMAVTAWEKGLESLPPLRPQQNPVLPVTGERNVLITSALPYVNNVPHLGNII

GCVLSADVFARYSRLRQWNTLYLCGTDEYGTATETKAMEEGLTPQEICDKYHIIHADIYRWFNISFDTFGRTTTPQQTKI

TQDIFQRLLTRGFVLQDTVEQLRCEHCARFLADRFVEGVCPFCGYEEARGDQCDKCGKLINAVELKKPQCKVCRSCPVVQ

STHHLFLDLPKLEKRLEEWLGRTLPGSDWTPNARFITRSWLRDGLKPRCITRDLKWGTPVPLEGFEDKVFYVWFDATIGY

LSITANYTDQWERWWKNPEQVDLYQFMAKDNVPFHGIVFPCSALGAEDNYTLVSHLIATEYLNYEDGKFSKSRGVGVFGD

MAQDTGIPADIWRFYLLYIRPEGQDSAFSWTDLLLKNNSELLNNLGNFINRAGMFVSKFFGGYVPEMVLTPDDQRLLAHV

TLELQHYHQLLEKVRIRDALRSILTISRHGNQYIQVNEPWKRIKGSEADRQRAGTVTGLAVNIAALLSVMLQPYMPTVSA

TIQAQLQLPPPACSILLTNFLCTLPAGHQIGTVSPLFQKLENDQIESLRQRFGGGQLEETFDLKAKTSPKPTVIETVTTA

GPQQIQALMDEVTKQGNIVRELKAQKADKNQVAAEVAKLLDLKKQLAVAEGKPPEAPKGKKKK

>XP_002916060.2 PREDICTED: methionine--tRNA synthetase, cytoplasmic isoform X1 [Ailuropoda melanoleuca]

MMGEIVYFPDRCPEKDLVQNSISRRASRRTLHSQAGGRFRLHQRGIHGEMRLFVSEGAPGSLPVLAAAGRAQGRAELLIS

TVGPEECVVPFLTRPKVPVLQLDSGNYLFSTSAICRYFFLLSGWEQDDLTNQWLEWEATELQPALSAALYYLVVQGKKGE

DVLGPVRRALTHIDQSLSRRSCPFLAGETESLADVVLWGALYPLLQDPAYLPEELGALHSWFQTLSSQEPCQRAAETVLK

QQGVLALRPYLQKQPQPGSFEGRAVSNEPEEEGLAALSEEEIAMAVTAWEKGLESLPPLRPQQTPVLPVAGERNVLITSA

LPYVNNVPHLGNIIGCVLSADVFARYSRLRQWNTLYLCGTDEYGTATETKAMEEGLTPQEICDKYHAIHADIYRWFNISF

DTFGRTTTPQQTKITQDIFQRLLTRGFVLQDTVEQLRCERCARFLADRFVEGVCPFCGYEEARGDQCDKCGKLINAVELK

KPQCKVCRSCPVVKSSRHLFLDLPKLEKRLEEWLGKTLPGSDWTPNARFIIRSWLRDGLKPRCITRDLKWGTPVPLEGFE

DKVFYVWFDATIGYLSITANYTDQWERWWKNPEQVNLYQFMAKDNVPFHGLVFPCSALGAEDNYTLVNHLIATEYLNYED

GKFSKSRGVGVFGDMAQDTGIPADIWRFYLLYIRPEGQDSAFSWTDMLLKNNSELLNNLGNFINRAGMFVSKFFGGYVPE

MVLTSDDRRLLAHVTLELQHYHQLLEKVRIREALRSILTISRHGNQYIQVNEPWKRIKGSEADRQRAGTVTGLAVNIAAL

LSVMLQPYMPTVGGTIQAQLQLPPPACRVLLTNFVCTLPAGHQIGTVSPLFQKLENDQIESLRQRFGGGQAKAPAAPAVV

EAVVTAAPQQVQVLMDEVTKQGNIVRELKAQKADKNQVAAEVAKLLDLKKQLALAEGKPPETPKGKKKK

>XP_011918691.1 PREDICTED: methionine--tRNA synthetase, cytoplasmic isoform X3 [Cercocebus atys]

MRLFVSEGAPGCLPVLAAAGRARGRAELLISTVGPEDCVVPFLTRPKVPVLQLDSGNYLFSTSAICRYFFLLSGWEQDDL

TNQWLEWEATELQETESLADIVLWGALYPLLQDPAYLPEELSALHSWFQTLSTQEPCQRAAETVLKQQGVLALRPYLQKQ

PQPSPAEGRAVTNEPEEEELATLSEEEIAMAVTAWEKGLESLPPLRPQQNPVLPVTGERNVLITSALPYVNNVPHLGNII

GCVLSADVFARYSRLRQWNTLYLCGTDEYGTATETKAMEEGLTPQEICDKYHIIHADIYRWFNISFDTFGRTTTPQQTKI

TQDIFQRLLTRGFVLQDTVEQLRCEHCARFLADRFVEGVCPFCGYEEARGDQCDKCGKLINAVELKKPQCKVCRSCPVVQ

STHHLFLDLPKLEKRLEEWLGRTLPGSDWTPNARFITRSWLRDGLKPRCITRDLKWGTPVPLEGFEDKVFYVWFDATIGY

LSITANYTDQWERWWKNPEQVDLYQFMAKDNVPFHGIVFPCSALGAEDNYTLVSHLIATEYLNYEDGKFSKSRGVGVFGD

MAQDTGIPADIWRFYLLYIRPEGQDSAFSWTDLLLKNNSELLNNLGNFINRAGMFVSKFFGGYVPEMVLTPDDQRLLAHV

TLELQHYHQLLEKVRIRDALRSILTISRHGNQYIQVNEPWKRIKGSEADRQRAGTVTGLAVNIAALLSVMLQPYMPTVSA

TIQAQLQLPPPACSILLTNFLCTLPAGHQIGTVSPLFQKLENDQIESLRQRFGGGQLEETFDLKAKTSPKPTVIETVTTA

GPQQIQALMDEVTKQGNIVRELKAQKADKNQVAAEVAKLLDLKKQLAVAEGKPPEASKGKKKK

>XP_004481358.1 methionine--tRNA synthetase, cytoplasmic isoform X2 [Dasypus novemcinctus]

MRLFVSEGAPGSLPVLAAAGRARGSAEVLISTVGPEECVVPFLTRPKIPVLQLDSGNYLFSTSAICRYFFLLSGWEQDDL

TNQWLEWEATELQPALSAAFYYLVVQGKKREDVLDPVRRALTHIDHSLSRRSCPFLAGETESVADIVLWGALYPLLQDPA

YLPEELGALRSWFQTLSIQEPCQQAAETVLKQQGALAFRPYLQKQPQPSPLEGRAVSNEPEEEELATLSEEEIAMAVTAW

ERGLGSLPTLRPQQNPVLPVAGERNVLITSALPYVNNVPHLGNIIGCVLSADVFARYSRLRQWNTLYLCGTDEYGTATET

KAMEEGLTPQQICDKYHIIHADIYRWFNISFDIFGRTTTPQQTLITQDIFQRLLVRGFVLQDTVEQLRCERCARFLADRF

VEGVCPFCGYEEARGDQCDKCGKLINAIELKKPQCKVCRSCPVVKSSKHLFLDLPKLEKRLEEWLEKTLPGSDWTTNAQF

ITRSWLRDGLKPRCITRDLKWGTPVPLEGFEDKVFYVWFDATFGYISITANYTDEWERWWKNPEQVDLYQFMAKDNVPFH

GLVFPCSVLGAEDNYTLVKHLIATEYLNYEDGKFSKSRGVGVFGDMAQDTGIPADIWRFYLLYIRPEGQDSAFSWTDMLL

KNNSELLNNLGNFINRAGMFVSKFFGGRVPEMVLIPDDQRLLAHVTMELRHFHQLLEKVRIRDALRSILTISRHGNQYIQ

VNEPWKRIKGNEADRQRAGTVTGLAVNIAALLSIMLQPYMPAVSATIQAQLQLPPPACNILLTDFLCTLPAGHQIGTVSP

LFQKLENDQMEGLRQRFGGGQAKPSPKPAAAETVTATGQQQIHMLMNEVTKQGNIVRELKAQKADKNQVAAEVAKLLELK

KQLALAEGKPTEPPKGKKKK

>XP_010837626.1 PREDICTED: methionine--tRNA synthetase, cytoplasmic isoform X1 [Bison bison bison]

MRLFVSEGAPGSLPVLAAAGRAQGRAELLISTVGPEECVVPFLTRPKVPVLQLDSGNYLFSTSAICRYFFLLSGWEQDDL

TNQWLEWEATELQPALSAALYYLVVQGKKGEDVLGPVRRALTHIDHSLSRRSCPFLAGETESLADIVLWGALYPLLQDPS

YLPEELGALHSWFQTLSSQEPCQRAAETVLKQQGVLALRPYLQKQPQPSSLEGRLVSNEPEEEELATLSEEEIAVAVAAW

EKGLESLPPLRPQQNPVLPVAGERNVLITSALPYVNNIPHLGNIIGCVLSADVFARYSRLRQWNTLYLCGTDEYGTATET

KAMEEGLTPQEICDKYHVIHADIYRWFNISFDFFGRTTTPQQTKITQDIFQRLLARGFVLQDTVEQLRCEHCARFLADRF

VEGVCPFCGYEEARGDQCDKCGKLINAIELKKPQCKVCRSCPVVKSSQHLFLDLPKLAARVEEWLEKTLPGSDWTANARF

IIRSWLRDGLKPRCITRDLKWGTPVPLEGFEDKVFYVWFDATIGYLSITANYTDQWEKWWKNPEQVNLYQFMAKDNVPFH

GIVFPSSALGAEDNYTLVSHLIATEYLNYEDGKFSKSRGVGVFGDMAQDTGIPADIWRFYLLYIRPEGQDSAFSWTDMLF

KNNSELLNNLGNFINRAGMFVSKFFGGFVPEMVLTSDDQRLLTHITLELQHYHQLLEKVRIRDALRSILTISRHGNQYIQ

VNEPWKRIKGGEADRQRAGTVTGLAVNIAALLSVMLQPYMPTVGATIQAQLQLPPPACSILPTNFLCTLPAGHQIGTVSP

LFQKLENDQIESLKQRFSGGQLEESLALKAKASPKTAAGLTTAGPQQIQALTEEVTKQGNIVRELKAQKADKNQIAAEVA

KLLDLKKQLALAEGKPLETSKGKKKK

>XP_002720959.1 PREDICTED: methionine--tRNA synthetase, cytoplasmic isoform X1 [Oryctolagus cuniculus]

MRLFVSPGSPGSLPVLAAAGRARGRAELLISTVGPEECVVPFLARPKVPALQLDGGNYLFSTSAICRYFFLLSGWEQDDL

TNQWLEWEATELQPVLSAALYYLVVQGKKGEDVLGPVRRALTHVDQSLSRQNSPFLTGETESLADIVLWGALYPLLQDPA

YLPDELGALRSWFQTLSTQEPCQRAAETVLKQQGILALRPYLQKQPQPSPPEDKAVSNEPEEEELATLSEEEIAVAVTAW

EKGLESLPPRRPQQNPVLPVAGERNVLITSALPYVNNVPHLGNIIGCVLSADVFARYSRLRQWNTLYLCGTDEYGTATET

KAMEEGLTPQEICDKYHIIHADIYRWFNISFDIFGRTTTPQQTKITQDIFQRLLSRGFVLQDTVEQLRCERCARFLADRF

VEGVCPFCGYEEARGDQCDKCGKLINAIELKKPQCKVCRSSPVVKSSQHLFLDLPKLEERLEEWLGKTLPGSDWTPNARF

IIRSWLRDGLKPRCITRDLKWGTPVPLEGFEDKVFYVWFDATIGYLSITANYTDQWERWWKNPEQVDLYQFMAKDNVPFH

GLVFPCSALGAEDNYTLVKHLIATEYLNYEDGKFSKSRGVGVFGDMAQDTGIPADIWRFYLLYIRPEGQDSAFSWTDMLL

KNNSELLNNLGNFINRAGMFVSKFFGGCVPEMVLTSDDRRLLAHVTLELQHYHQLLEKARIRDALRSILTISRHGNQYIQ

VNEPWKRIKGDEADRQRAGTVTGMAVNVAALLSVMLQPYMPTVSASIQAQLQLPPPACSVLPTNFLCTLPAGHQIGTVSP

LFQKLENDQIESLRQRFGGGQLEESLELKAKASPKPAAEETRKTAGPQEIQTLLEEVTKQGNLVRELKAQKADKNQVAAE

VTKLLDLKKQLALAEGKPLETPKGKKKK

>XP_006077954.1 PREDICTED: methionine--tRNA synthetase, cytoplasmic isoform X3 [Bubalus bubalis]

MRLFVSEGAPGSLPVLAAAGRAQGRAELLISTVGPEECVVPFLTRPKVPVLQLDSGNYLFSTSAICRYFFLLSGWEQDDL

TNQWLEWEATELQPALSAALYYLVVQGKKGEDVLGPVRRALTHIDHSLSRRSCPFLAGETESLADIVLWGALYPLLQDPS

YLPEELGALHSWFQTLSSQEPCQRAAETVLKQQGVLALRPYLQKQPQPSSLEGRLVSNEPEEEELATLSEEEIAVAVAAW

EKGLESLPSLRPQQNPVLPVAGERNVLITSALPYVNNVPHLGNIIGCVLSADVFARYSRLRQWNTLYLCGTDEYGTATET

KAMEEGLTPQEICDKYHVIHADIYRWFNISFDFFGRTTTPQQTKITQDIFQRLLARGFVLRDTVEQLRCEHCARFLADRF

VEGVCPFCGYEEARGDQCDKCGKLINAIELKKPQCKVCRSCPVVKSSQHLFLDLPKLAARVEAWLEKTLPGSDWTANARF

IIRSWLRDGLKPRCITRDLKWGTPVPLEGFENKVFYVWFDATIGYLSITANYTDQWEKWWKNPEQVNLYQFMAKDNVPFH

GIVFPSSALGAEDNYTLVSHLIATEYLNYEDGKFSKSRGVGVFGDMAQDTGIPADIWRFYLLYIRPEGQDSAFSWTDMLF

KNNSELLNNLGNFINRAGMFVSKFFGGFVPEMVLISDDQRLLTHITLELQHYHQLLEKVRIRDALRSILTISRHGNQYIQ

VNEPWKRIKGGEADRQRAGTVTGLAVNIAALLSVMLQPYMPTVSATIQAQLQLPPPACSILPTNFLCTLPAGHQIGTVSP

LFQKLENDQIENLKQRFSGGQAKASPKTAAELTTAGPQQIQALTEEVTKQGNIVRELKAQKADKNQIAAEVAKLLDLKKQ

LALAEGKPLETPKGKKKK

>XP_004401781.1 PREDICTED: methionine--tRNA synthetase, cytoplasmic [Odobenus rosmarus divergens]

MRLFVSEGAPGSLPVLAAAGRAQGRAELLISTVGPEVCVVPFLTWPKVPVLQLDSGNYLFSTSAICRYFFLLSGWEQDDL

TNQWLEWEATELQPALSAALYYLVVQGKKGEDVLGPVRKALTHIDQSLSRRSCPFLAGETESLADIVLWGALYPLLQDPA

YLPEELGALHSWFQTLSSQEPCQRAAETVLKQQGVLALRPYLQKQPQPGSFEGRAVSNEPEEEELATLSEEEIAMAVTAW

EKGLESLPPLRPQQNPVLPVAGERNVLITSALPYVNNVPHLGNLIGCVLSADVFARYSRLRQWNTLYLCGTDEYGTATET

KAMEEGLTPQEICDKYHAIHADIYRWFNISFDIFGRTTTPQQTKITQDIFQRLLTRGFVLQDTVEQLRCERCARFLADRF

VEGVCPFCGYEEARGDQCDKCGKLINATELKKPQCKVCRSCPVVKSSRHLFLDLPKLEKRLEEWLGKTLPGSDWTPNARF

IIRSWLRDGLKPRCITRDLKWGTPVPLEGFEDKVFYVWFDATIGYLSITANYTDQWERWWKNPEQVNLYQFMAKDNVPFH

GLVFPCSALGAEDNYTLVNHLIATEYLNYEDGKFSKSRGVGVFGDMAQDTGIPADIWRFYLLYIRPEGQDSAFSWTDMLL

KNNSELLNNLGNFINRAGMFVSKFFGGYVPEMVLTSDDRRLLAHVTLELQHYHQLLEKVRIREALRSILTISRHGNQYIQ

VNEPWKRIKGSEADRQRAGTVTGLAVNIAALLSVMLQPYMPTVSATIQAQLQLPPPACSVLLTNFLCTLPAGHQIGTVSP

LFQKLENDQIESLRQRFGGDQARAPPPPAVVGAVAAAAPWQVQVLTDEVTKQGNIVRELKAQKADKNQVAAEVAKLLDLK

KQLALAEGKPLETPKGKKKK

>XP_019513162.1 PREDICTED: methionine--tRNA synthetase, cytoplasmic isoform X1 [Hipposideros armiger]

MRLFVSEGAPGSLPVLAAAERAQGRAELLISTVGPEDCVVPFLTWPKVPVLQLDSGNYLFSTSAICRYFFLLSGWEQDDL

TNQWLEWEATELQPALSAALYYLVVQGKKGEDVLGLVRRALTHIDQSLSRQSCSFLVGETESLADIVLWGALYPLLQDPA

YLPEELGALHSWFQTLSSQEPCQRAAETVLKQQGILALRPYLQKQPQPSSLERRVVSNEPEEEELATLSEEDIATAVTAW

EKGLGSLPPLRPKQIPVLPVAGERNVLITSALPYVNNVPHLGNIIGCVLSADVFARYSRLRQWNTLYLCGTDEYGTATET

KAMEEGLTPQEICDKYHVIHANIYHWFNISFDIFGRTTTPQQTQITQDIFQRLWNRGFVLQDTVEQLRCERCARFLADRF

VEGVCPFCGYEEARGDQCDKCGKLINATELKKPQCKVCRSCPVVKSSQHLFLDLPKLEDRLEEWLGKTLSGSDWTPNARF

IIRSWLRDGLKPRCITRDLKWGTPVPLKGFEDKVFYVWFDATFGYLSITANYTDQWERWWKNPEQVNLYQFMAKDNVPFH

GLVFPCSALGAEDNYTLVSHLIATEYLNYEDGKFSKSRGVGVFGDMAQDTGIPADIWRFYLLYIRPEGQDSAFSWTDLLL

KNNSELLNNLGNFINRAGMFVSKFFGGYVPEMMLTSDDQRLLAHVTLELQHYHQLLEKVRIRDALRSILTISRHGNQYIQ

VNEPWKRIKGSEADRQRAGTVTGLAVNIAALLSIMLQPYMPTVSATIAAQLQLPPPACSILPTDFLCTLPAGHQIGTVSP

LFQKLENDQIESLRQRFGGGQLEESLELKAKASPKPAVVETVTAAGSQQIQVLMDEVTKQGNIVRELKAQKADKNQVAAE

VAKLLDLKKQLALAEGKPLETPKGKKKK

>XP_008842293.1 PREDICTED: methionine--tRNA synthetase, cytoplasmic isoform X1 [Nannospalax galili]

MRLFVCEGAPGSLPVLAAAGRARDRAELLISTVGSEECVVPFLTRPKVPVLQLDSGNYLFSSSAICRYFFLLSGWEQDDL

TNQWLEWEATELQPALSAALYCLVVQGKKGEHVLGPLRRALTHIDYSLSRQNCPFLAGDTESLADIVLWGALYPLLQDPT

YLPEELGALHSWFQTLSTQEPCRRAAETVLKQQGVLALRPYLQKQPQPQSHPPEGRAVSNEPEEEELATLSEEEIAMAVT

AWEKGLGSLPPLRPPQNPVLPVAGERNVLITSALPYVNNVPHLGNIIGCVLSADVFARYSRLRQWNTLYLCGTDEYGTAT

ETKAMEEGLTPQEICDKYHRIHADIYCWFNISFDTFGRTTTAQQTKITQDIFQRLLTRGFVLQDTVEQLRCEQCARFLAD

RFVEGVCPFCGYEEARGDQCDKCGKLINAIELKKPQCKVCRSCPVVKSSQHLFLDLPKLEKRLEEWLGKTLPGSDWTPNA

RFIIRSWLRDGLKPRCITRDLKWGTPVPLEGFEDKVFYVWFDATIGYLSITANYTDQWERWWKNPDQVDLYQFMAKDNVP

FHGLVFPCSALGAEDNYTLVRHLIATEYLNYEDGKFSKSRGVGVFGDMAQDTGIPADIWRFYLLYIRPEGQDSAFSWTDM

LIKNNSELLNNLGNFINRAGMFVSKFFGGCVPKMVLTPDDQRLLAHISLELQHYHQLLEKVRIRDALRSILTISRHGNQY

IQVNEPWKRIKGSEADRQRAGTVTGLAVNIAALLSVMLQPYMPTVSATIQAQLQLPLQACGILLTNFICTLPTGHHIGTV

SPLFQKLENDQIESLRQRFGGGQLEESLELKAKVSPKPAVVETVTTAGPQHIQMLMDEVTKQGNVVRELKAQKADKNQVA

AEVAKLLDLKKQLALAEGKPIETPKGKKKKSFLCSKPSSGCFMYIED

>XP_005680340.2 PREDICTED: methionine--tRNA synthetase, cytoplasmic isoform X2 [Capra hircus]

MRLFVSEGAPGSLPVLAAAGRAQGRAELLISTVGPEECVVPFLTRPKVPVLQLDSGNYLFSTSAICRYFFLLSGWEQDDL

TNQWLEWEATELQPALSAALYYLVVQGKKGEDVLGPVRRALMHIDHSLSRRSCPFLAGETESLADIVLWGALYPLLQDPS

YLPEELGALHSWFQTLSSQEPCQRAAETVLKQQGVLALRPYLQKQPQPGSLEGRLISNEPEEEELATLSEEEIAVAVAAW

EKGLESLPPLRPQQNPVLPVAGERNVLITSALPYVNNVPHLGNIIGCVLSADVFARYSRLRQWNTLYLCGTDEYGTATET

KAMEEGLTPQEICDKYHVIHADIYRWFNISFDFFGRTTTPKQTKITQDIFQRLLARGFVLQDTVEQLRCEHCARFLADRF

VEGVCPFCGYEEARGDQCDKCGKLINAIELKKPQCKVCRSCPVVKSSQHLFLDLPKLGARVEEWLEKTLPGSDWTPNARF

IIRSWLRDGLKPRCITRDLKWGTPVPLEGFEDKVFYVWFDATIGYLSITANYTDQWEKWWKNPEQVNLYQFMAKDNVPFH

GIVFPSSALGAEDNYTLVSHLIATEYLNYEDGKFSKSRGVGVFGDMAQDTGIPADIWRFYLLYIRPEGQDSAFSWTDMLF

KNNSELLNNLGNFINRAGMFVSKFFGGFVPEMVLTSDDQRLLTHVTLELQHYHQLLEKVRIRDALRSILTISRHGNQYIQ

VNEPWKRIKGGEADRQRAGTVTGLAVNIAALLSVMLQPYMPTVSATIQAQLQLPPPACSILPTNFLCTLPAGHQIGMVSP

LFQKLENDQIESLKQRFSGGQAKASPKTAAGPQQIQALTEEVTKQGNIVRELKAQKADKNQIAAEVAKLLDLKKQLAQAE

GKPLETPKGKKKK

>XP_005571394.1 PREDICTED: methionine--tRNA synthetase, cytoplasmic isoform X3 [Macaca fascicularis]

MRLFVSEGAPGCLPVLAAAGRARGRAELLISTVGPEDCVVPFLTRPKVPVLQLDSGNYLFSTSAICRYFFLLSGWEQDDL

TNQWLEWEATELQETESLADIVLWGALYPLLQDPAYLPEELSALHSWFQTLSTQEPCQRAAETVLKQQGVLALRPYLQKQ

PQPSPAEGRAVTNEPEEEELATLSEEEIAMAVTAWEKGLESLPPLRPQQNPVLPVTGERNVLITSALPYVNNVPHLGNII

GCVLSADVFARYSRLRQWNTLYLCGTDEYGTATETKAMEEGLTPQEICDKYHIIHADIYRWFNISFDTFGRTTTAQQTKI

TQDIFQRLLTRGFVLQDTVEQLRCEHCARFLADRFVEGVCPFCGYEEARGDQCDKCGKLINAVELKKPQCKVCRSCPVVQ

STHHLFLDLPKLEKRLEEWLGRTLPGSDWTPNARFITRSWLRDGLKPRCITRDLKWGTPVPLEGFEDKVFYVWFDATIGY

LSITANYTDQWERWWKNPEQVDLYQFMAKDNVPFHGIVFPCSALGAEDNYTLVSHLIATEYLNYEDGKFSKSRGVGVFGD

MAQDTGIPADIWRFYLLYIRPEGQDSAFSWTDLLLKNNSELLNNLGNFINRAGMFVSKFFGGYVPEMVLNPDDQRLLAHV

TLELQHYHQLLEKVRIRDALRSILTISRHGNQYIQVNEPWKRIKGSEADRQRAGTVTGLAVNIAALLSVMLQPYMPTVSA

TIQAQLQLPPPACSILLTNFLCTLPAGHQIGTVSPLFQKLENDQIESLRQRFGGGQLEETFDLKAKTSPKPTVIETVTTA

GPQQIQALMDEVTKQGNIVRELKAQKADKNQVAAEVAKLLDLKKQLAVAEGKPPEAPKGKKKK

>XP_016048856.1 PREDICTED: methionine--tRNA synthetase, cytoplasmic isoform X1 [Erinaceus europaeus]

MRLFVSEGAPGSLPVLAAAGRAQGKAELLISTVGPEECVVPFLTRPKVPVLQLDNGNYLFSTTAICRYFFLLSGWEQDDL

TNQWLEWEATELQPAVSAALYCLVVQGKKGEDVLGSVRRALTHIDHSLSRRSCPFLTGETESLADIVLWGALYPLLQDAA

YLPEELGALHSWFQTLNSQEPCQQAVETVLKQHGILALRPYLQKQPQLSSPEGKLVSNDPEEEELATLSEEDIAMAVSAW

EKGLGSLPTLRPQQNPVLPVAGERNVLITSALPYVNNVPHLGNIIGCVLSADVFARYSRLRQWNTLYLCGTDEYGTATET

KAMEEGLTPQEICDKYYAIHASIYRWFNISFDTFGRTTTPQQTKITQDIFQRLYERGFVLQDTVEQLRCERCARFLADRF

VEGVCPFCGYEEARGDQCDKCGKLINATELKKPQCKVCRSCPVVRSSRHLFLDLPKLEERLERWLEKTVPGSDWTPNARF

IIRSWLRDGLKPRCITRDLKWGTPVPLEGFEDKVFYVWFDATIGYLSITANYTDQWEKWWKNPEQVDLYQFMAKDNVPFH

GLVFPCTALGAEDNYTLVSHLIATEYLNYEDGKFSKSRGVGVFGDMAQDTGIPADIWRFYLLYIRPEGQDSAFSWTDLLL

KNNSELLNNLGNFINRAGMFVSKFFGGCVPEMVLTSDDQRLLTHVTLELQHYHQLLEKARIRDALRSILTISRHGNQYIQ

VNEPWKRIKGNEADRQRAGTVTGMAVNIAALLSVMLQPYMPTVSATIQAQLQLSLPACSVLPTNFLCTLPAGHQIGTVSP

LFQKLENDQIESLRQHFGGGQLEESLAFKVKVSPKPAVEEAVTTAGSQQIQMLMDEVTKQGNIVRELKAQKADKNQIAAE

VAKLLDLKKQLALAEGKPLEAPKGKKKK

>XP_008842295.1 PREDICTED: methionine--tRNA synthetase, cytoplasmic isoform X4 [Nannospalax galili]

MRLFVCEGAPGSLPVLAAAGRARDRAELLISTVGSEECVVPFLTRPKVPVLQLDSGNYLFSSSAICRYFFLLSGWEQDDL

TNQWLEWEATELQPALSAALYCLVVQGKKGEHVLGPLRRALTHIDYSLSRQNCPFLAGDTESLADIVLWGALYPLLQDPT

YLPEELGALHSWFQTLSTQEPCRRAAETVLKQQGVLALRPYLQKQPQPQSHPPEGRAVSNEPEEEELATLSEEEIAMAVT

AWEKGLGSLPPLRPPQNPVLPVAGERNVLITSALPYVNNVPHLGNIIGCVLSADVFARYSRLRQWNTLYLCGTDEYGTAT

ETKAMEEGLTPQEICDKYHRIHADIYCWFNISFDTFGRTTTAQQTKITQDIFQRLLTRGFVLQDTVEQLRCEQCARFLAD

RFVEGVCPFCGYEEARGDQCDKCGKLINAIELKKPQCKVCRSCPVVKSSQHLFLDLPKLEKRLEEWLGKTLPGSDWTPNA

RFIIRSWLRDGLKPRCITRDLKWGTPVPLEGFEDKVFYVWFDATIGYLSITANYTDQWERWWKNPDQVDLYQFMAKDNVP

FHGLVFPCSALGAEDNYTLVRHLIATEYLNYEDGKFSKSRGVGVFGDMAQDTGIPADIWRFYLLYIRPEGQDSAFSWTDM

LIKNNSELLNNLGNFINRAGMFVSKFFGGCVPKMVLTPDDQRLLAHISLELQHYHQLLEKVRIRDALRSILTISRHGNQY

IQVNEPWKRIKGSEADRQRAGTVTGLAVNIAALLSVMLQPYMPTVSATIQAQLQLPLQACGILLTNFICTLPTGHHIGTV

SPLFQKLENDQIESLRQRFGGGQLEESLELKAKVSPKPAVVETVTTAGPQHIQMLMDEVTKQGNVVRELKAQKADKNQVA

AEVAKLLDLKKQLALAEGKPIETPKGKKKK

>XP_012014777.1 PREDICTED: methionine--tRNA synthetase, cytoplasmic isoform X1 [Ovis aries musimon]

MRLFVSEGAPGSLPVLAAAGRAQGRAELLISTVGPEECVVPFLTRPKVPVLQLDSGNYLFSTSAICRYFFLLSGWEQDDL

TNQWLEWEATELQPALSAALYYLVVQGKKGEDVLGPVRRALMHIDHSLSRRSCPFLAGETESLADIVLWGALYPLLQDPS

YLPEELGALHSWFQTLSSQEPCQRAAETVLKQQGVLALRPYLQKQPQPGSLEGRLISNEPEEEELATLSEEEIAVAVAAW

EKGLESLPPLRPQQNPVLPVAGERNVLITSALPYVNNVPHLGNIIGCVLSADVFARYSRLRQWNTLYLCGTDEYGTATET

KAMEEGLTPQEICDKYHVIHADIYRWFNISFDFFGRTTTPQQTKITQDIFQRLLARGFVLQDTVEQLRCEHCARFLADRF

VEGVCPFCGYEEARGDQCDKCGKLINAIELKKPQCKVCRSCPVVKSSQHLFLDLPKLGARVEEWLEKTLPGSDWTPNARF

IIRSWLRDGLKPRCITRDLKWGTPVPLEGFEDKVFYVWFDATIGYLSITANYTDQWEKWWKNPEQVNLYQFMAKDNVPFH

GIVFPSSALGAEDNYTLVSHLIATEYLNYEDGKFSKSRGVGVFGDMAQDTGIPADIWRFYLLYIRPEGQDSAFSWTDMLF

KNNSELLNNLGNFINRAGMFVSKFFGGFVPEMVLTSDDQRLLTHVTLELQHYHQLLEKVRIRDALRSILTISRHGNQYIQ

VNEPWKRIKGGEADRQRAGTVTGLAVNIAALLSVMLQPYMPTVSATIQAQLQLPPPARSILPTNFLCTLPAGHQIGTVSP

LFQKLENDQIESLKQRFSGGQAKASPKTAAGPQQIQALTEEVTKQGNIVRELKAQKADKNQIAAEVAKLLDLKKQLAQAE

GKPLETPKGKKKK

>XP_004650074.1 PREDICTED: methionine--tRNA synthetase, cytoplasmic isoform X1 [Jaculus jaculus]

MRLFVSEGAPGSLPVLAAAARARGRAELLVSAVGPEECVVPFLTRAKVPVLQLDSGNYLFSTSAICRYFFLLSGWEQDDL

TNQWLEWEATELQPALSAALYCLVVQGKKGDDVLGPLRRALSHIDHSLNRQDRPFLTGETESVADIVLWGALYPLLQDPA

YLPEELGALRSWFQTLSTQEPCQRAAETVLKQQGVLALRPYLQKQPQPQTYFSEGRAVSNEPEEEELVTLSEEEIAKAVT

AWEKGRESLPPLRPQQNPVLPVAGERNVLITSALPYVNNVPHLGNIIGCVLSADVFARYSRLRQWNTLYLCGTDEYGTAT

ETKAMEEGLTPQEICDKYHRIHADIYRWFNISFDTFGRTTTPQQTKITQDIFQRLLTRGFVLQDNVEQLRCEHCARFLAD

RFVEGVCPFCGYEEARGDQCDKCGKLINAIELKKPQCKVCRSCPVVKSSRHLFLDLPKLEKRLEEWLGKTLPGSDWTPNA

RFIIRSWLRDGLKPRCITRDLKWGTPVPLEGFQDKVFYVWFDATIGYLSITANYTDEWERWWKNPEQVDLYQFMAKDNVP

FHGLVFPCSALGAEDNYTLVKHLIATEYLNYEDGKFSKSRGIGVFGDMAQDTGIPADIWRFYLLYIRPEGQDSAFSWTDM

LIKNNSELLNNLGNFINRAGMFVSKFFGGFVPEVVLTPDDQRLLAHITLELQHYHQLLEKVRIRDALRSILTISRHGNQY

IQVNEPWKRIKGSEADRQRAGTVTGLAVNIAALLSVMLQPYMPTVSATIQAQLQLPLAVCSILPTNFICTLPAGHQIGTV

SPLFQKLENDQIESLRQRFGGGQLEESLESKAKVSPKPAVVETGTTAGPQQIQMLMDEVTKQGNTVRELKAQKADKNQIA

AEVAKLLDLKKQLALAEGKPLETPKGKKKK

>XP_021542961.1 methionine--tRNA synthetase, cytoplasmic [Neomonachus schauinslandi]

MKLFVSEGAPGSLPVLAAAGRAQGRAELLISTVGPEECVVPFLTRPKVPVLQLDSGNYLFSTSAICRYFFLLSGWEQDDL

SNQWLEWEATELQPALSAALYYLVVQGKKGEDVLGPVRRALTHIDQSLSRRSCPFLAGETESLADVVLWGALYPLLQDPA

YLPEELGALHSWFQTLSSQEPCQRAAETVLKQQGVLALRPYLQKQPQPGSVEGRAVSNEPEEEELATLSEEEIAMAVTAW

EKGLESLPPLRPQQNPVLPVAGERNVLITSALPYVNNVPHLGNLIGCVLSADVFARYSRLRQWNTLYLCGTDEYGTATET

KAMEEGLTPQEICDKYHAIHADIYRWFNISFDIFGRTTTPQQTKITQDIFRRLLTRGFVLQDTVEQLRCERCARFLADRF

VEGVCPFCGYEEARGDQCDKCGKLINATELKKPQCKVCRSCPVVKSSRHLFLDLPKLEERLEEWLGKTLPGSDWTPNARF

IIRSWLRDGLKPRCITRDLKWGTPVPLEGFEDKVFYVWFDATIGYLSITANYTDQWERWWKNPEQVNLYQFMAKDNVPFH

GLVFPCSALGAEDNYTLVNHLIATEYLNYEDGKFSKSRGVGVFGDMAQDTGIPADIWRFYLLYIRPEGQDSAFSWTDMLL

KNNSELLNNLGNFINRAGMFVSKFFGGYVPEMVLTSDDRRLLAHVTLELQHYHQLLEKVRIREALRSILTISRHGNQYIQ

VNEPWKRIKGSEADRQRAGTVTGLAVNIAALLSVMLQPYMPTVSATIQAQVQLPPLACSVLLTNFLCTLPAGHQIGTVSP

LFQKLENDQIESLRQRFGGDQARAPPTPAVVGAVAAAAPQQVQVLMDEVTKQGNTVRELKAQKADKNQVAAEVAKLLDLK

KQLALAEGKPLETPKGKKKK

>XP_019816519.1 PREDICTED: methionine--tRNA synthetase, cytoplasmic isoform X2 [Bos indicus]

MRLFVSEGAPGSLPVLAAAGRAQGRAELLISTVGPEAHSGPTPFQRSYPGPSPECVVPFLTRPKVPVLQLDSGNYLFSTS

AICRYFFLLSGWEQDDLTNQWLEWEATELQPALSAALYYLVVQGKKGEDVLGPVRRALTHIDHSLSRRSCPFLAGETESL

ADIVLWGALYPLLQDPSYLPEELGALHSWFQTLSSQEPCQRAAETVLKQQGVLALRPYLQKQPQPSSLEGRLVSNEPEEE

ELATLSEEEIAVAVAAWEKGLESLPPLRPQQNPVLPVAGERNVLITSALPYVNNVPHLGNIIGCVLSADVFARYSRLRQW

NTLYLCGTDEYGTATETKAMEEGLTPQEICDKYHVIHADIYRWFNISFDFFGRTTTPQQTKITQDIFQRLLARGFVLQDT

VEQLRCEHCARFLADRFVEGVCPFCGYEEARGDQCDKCGKLINAIELKKPQCKVCRSCPVVKSSQHLFLDLPKLAARVEE

WLEKTLPGSDWTANARFIIRSWLRDGLKPRCITRDLKWGTPVPLEGFEDKVFYVWFDATIGYLSITANYTDQWEKWWKNP

EQVNLYQFMAKDNVPFHGIVFPSSALGAEDNYTLVSHLIATEYLNYEDGKFSKSRGVGVFGDMAQDTGIPADIWRFYLLY

IRPEGQDSAFSWTDMLFKNNSELLNNLGNFINRAGMFVSKFFGGFVPEMVLTSDDQRLLTHITLELQHYHQLLEKVRIRD

ALRSILTISRHGNQYIQVNEPWKRIKGGEADRQRAGTVTGLAVNIAALLSVMLQPYMPTVSATIQAQLQLPPPACSILPT

NFLCTLPAGHQIGTVSPLFQKLENDQIESLKQRFSGGQAKASPKTAAGLTTAGPQQIQALTEEVTKQGNIVRELKAQKAD

KNQIAAEVAKLLDLKKQLALAEGKPLETSKGKKKK

>XP_017656356.1 PREDICTED: methionine--tRNA synthetase, cytoplasmic isoform X3 [Nannospalax galili]

MRLFVCEGAPGSLPVLAAAGRARDRAELLISTVGSEECVVPFLTRPKVPVLQLDSGNYLFSSSAICRYFFLLSGWEQDDL

TNQWLEWEATELQPALSAALYCLVVQGKKGEHVLGPLRRALTHIDYSLSRQNCPFLAGDTESLADIVLWGALYPLLQDPT

YLPEELGALHSWFQTLSTQEPCRRAAETVLKQQGVLALRPYLQKQPQPQSHPPEGRAVSNEPEEEELATLSEEEIAMAVT

AWEKGLGSLPPLRPPQNPVLPVAGERNVLITSALPYVNNVPHLGNIIGCVLSADVFARYSRLRQWNTLYLCGTDEYGTAT

ETKAMEEGLTPQEICDKYHRIHADIYCWFNISFDTFGRTTTAQQTKITQDIFQRLLTRGFVLQDTVEQLRCEQCARFLAD

RFVEGVCPFCGYEEARGDQCDKCGKLINAIELKKPQCKVCRSCPVVKSSQHLFLDLPKLEKRLEEWLGKTLPGSDWTPNA

RFIIRSWLRDGLKPRCITRDLKWGTPVPLEGFEDKVFYVWFDATIGYLSITANYTDQWERWWKNPDQVDLYQFMAKDNVP

FHGLVFPCSALGAEDNYTLVRHLIATEYLNYEDGKFSKSRGVGVFGDMAQDTGIPADIWRFYLLYIRPEGQDSAFSWTDM

LIKNNSELLNNLGNFINRAGMFVSKFFGGCVPKMVLTPDDQRLLAHISLELQHYHQLLEKVRIRDALRSILTISRHGNQY

IQVNEPWKRIKGSEADRQRAGTVTGLAVNIAALLSVMLQPYMPTVSATIQAQLQLPLQACGILLTNFICTLPTGHHIGTV

SPLFQKLENDQIESLRQRFGGGQLEESLELKAKVSPKPAVVETVTTAGPQHIQMLMDEVTKQGNVVRELKAQKADKNQVA

AEVAKLLDLKKQLALAEGKPIETPKGKKKNL

>XP_006171959.2 PREDICTED: methionine--tRNA synthetase, cytoplasmic isoform X2 [Tupaia chinensis]

MRLFVSEGAPGSLPVLAAAGRARGRAELLISTVGPEECVVPFLTRPKVPVLQLDSGNYLFSTSAICRYLFLLSGWEQDDL

TNQWLEWEATELQPALSAALYYLVVQGKKGDDVLGPVRRALTHIDHSLSRQNCPFLAGETESLADIVLWGALYPLLQDPA

YFPEELSALHSWFQTLSTQEPCQQAVETVLKQQGVLALRPYLQKQPQPSAPEGKTVSNEPEEEELSTLSEEDIAMAVTAW

EKGLRGLPPPRPQQNPVLPVAGERNVLITSALPYVNNVPHLGNIIGCVLSADVFARYSRLRQWNTLYLCGTDEYGTATET

KAMDEGLTPQEICDKYHVIHADIYRWFNISFDIFGRTTTPQQTKITQDIFQRLLARGFVLQDSVEQLRCERCARFLADRF

VEGVCPFCGYEEARGDQCDKCGKLINAIELKKPLCKVCRSSPVVKSSQHLFLDLPKLEKRVEEWLERTLPGSDWTPNARF

IIRSWLRDGLKPRCITRDLKWGTPVPLEGFESKVFYVWFDATIGYLSITANYTDQWEKWWKNPEQVELYQFMAKDNVPFH

GIVFPCSALGAEDNYTLVSHLIATEYLNYEDGKFSKSRGVGVFGDMAQDTGIPADIWRFYLLYIRPEGQDSAFSWTDMLL

KNNSELLNNLGNFINRAGMFVSKFFGGYVPEMVLTPDDQRLLAHVTQELRHYHQLLEKVRIRDALRSILTISRHGNQYIQ

VNEPWKQIKGSEADRQRAGTVTGVAVNIAALLSVMLQPYMPTVSATIQTQLQLPPPACNILVTDFLCTLPAGHQIGTVSP

LFQKLENDQIESLRQRFGGGQVKPSPKPAVVETVTAAGPQQIQVLMEEVTKQGNIVRELKAQKADKNLVAAEVAKLLDLK

KQLALAEGKPLEAPKGKKKK

>XP_005879087.1 PREDICTED: methionine--tRNA synthetase, cytoplasmic isoform X2 [Myotis brandtii]

MRLFVSEGVPGTLPVLAAAGRAQGRAQLLISTVGPEECVVPFLTRPKIPVLQLDSGNYLFSTNAICRYFFLLSGSEQDDL

TNQWLEWEATELQPALSAAMYYLVVQGKKGEDVLGPIRRALTHIDHSLSRRSCPFLAGETESVADIVLWGALYPLLQDPA

YLPEELGALHSWFQTLSSQEPCQRAAETVLKKQGILALRPYLQKQLQPSFLEGRAVSNEPEEEELATLSEEEIAMAVTAW

EKGLGSLPPLRPKQKPVLPVAGERNVLITSALPYVNNVPHLGNIIGCVLSADVFARYSRLRQWNTLYLCGTDEYGTATET

KAMEEGLTPQEICDKYHVIHADTYRWFNISFDIFGRTTTSQQTEITQDIFQRLWNRGFVLQDTVEQLRCERCARFLADRF

VEGVCPFCGYEEARGDQCDKCGKLINAIELKKPQCKVCRSCPVVRSSRHLFLDLPKLEDRLEEWLGKTVPGSDWTPNARF

IIRSWLRDGLKPRCITRDLKWGTPVPLEGFEDKVFYVWFDATFGYISITANYTDQWEKWWKNPEQVNLYQFMAKDNVPFH

GLVFPCSALGAEDNYTLVSHLIATEYLNYEDGKFSKSRGVGVFGDMAKDTGIPADIWRFYLLYIRPEGQDSAFSWTDMLL

KNNSELLNNLGNFINRAGMFVSKFFGGYVPEMVLTSDDQRLLAHVSLELQHYHQLLEKVRIRDALRSILTISRYGNQYIQ

VNEPWKRIKGSEADRQRAGTVTGLAVNIAALLSTMLQPYMPTVSSMIAAQLQLPPPACSILPTDFLCTLPAGHQIGTVSP

LFQKLENDQIENLRQRFGGGQAKVSPKPAVVETAGPQQIQVLMDEVTKQGNIVRELKAQKADKNQVAAEVAKLLDLKKQL

ALAEGKPLETPKGKKKK

>XP_004006592.1 PREDICTED: methionine--tRNA synthetase, cytoplasmic isoform X1 [Ovis aries]

MRLFVSEGAPGSLPVLAAAGRAQGRAELLISTVGPEECVVPFLTRPKVPVLQLDSGNYLFSTSAICRYFFLLSGWEQDDL

TNQWLEWEATELQPALSAALYYLVVQGKKGEDVLGPVRRALMHIDHSLSRRSCPFLAGETESLADIVLWGALYPLLQDPS

YLPEELGALHSWFQTLSSQEPCQRAAETVLKQQGVLALRPYLQKQPQPGSLEGRLISNEPEEEELATLSEEEIAVAVAAW

EKGLESLPPLRPQQNPVLPVAGERNVLITSALPYVNNVPHLGNIIGCVLSADVFARYSRLRQWNTLYLCGTDEYGTATET

KAMEEGLTPQEICDKYHVIHADIYRWFNISFDFFGRTTTPQQTKITQDIFQRLLARGFVLQDTVEQLRCEHCARFLADRF

VEGVCPFCGYEEARGDQCDKCGKLINAIELKKPQCKVCRSCPVVKSSQHLFLDLPKLGARVEEWLEKTLPGSDWTPNARF

IIRSWLRDGLKPRCITRDLKWGTPVPLEGFEDKVFYVWFDATIGYLSITANYTDQWEKWWKNPEQVNLYQFMAKDNVPFH

GIVFPSSALGAEDNYTLVSHLIATEYLNYEDGKFSKSRGVGVFGDMAQDTGIPADIWRFYLLYIRPEGQDSAFSWTDMLF

KNNSELLNNLGNFINRAGMFVSKFFGGFVPEMVLTSDDQRLLTHVTLELQHYHQLLEKVRIRDALRSILTISRHGNQYIQ

VNEPWKRIKGGEADRQRAGTVTGLAVNIAALLSVMLQPYMPTVSATIQAQLQLPPPARSILPTNFLCTLPAGHQIGTVSP

LFQKLENDQIESLKQRFSGGQARASPKTAAGPQQIQALTEEVTKQGNIVRELKAQKADKNQIAAEVAKLLDLKKQLAQAE

GKPLETPKGKKKK

>XP_006750750.1 PREDICTED: methionine--tRNA synthetase, cytoplasmic [Leptonychotes weddellii]

MKLFVSEGAPGSLPVLAAAGRAQGRAELLISTVGPEECVVPFLTRPKVPVLQLDSGNYLFSTSAICRYFFLLSGWEQDDL

SNQWLEWEATELQPALSAALYYLVVQGKKGEDVLGPVRRALTHIDQSLSRRSCPFLAGETESLADVVLWGALYPLLQDPA

YLPEELGALHSWFQTLSSQEPCQRAAETVLKQQGVLALRPYLQKQPQPGSVEGRAVSNEPEEEELATLSEEEIAMAVTAW

EKGLESLPPLRPQQNPVLPVAGERNVLITSALPYVNNVPHLGNLIGCVLSADVFARYSRLRQWNTLYLCGTDEYGTATET

KAMEEGLTPQEICDKYHAIHADIYRWFNISFDIFGRTTTPQQTKITQDIFRRLLTRGFVLQDTVEQLRCERCARFLADRF

VEGVCPFCGYEEARGDQCDKCGKLINATELKKPKCKVCRSCPVVKSSRHLFLDLPKLEERLEEWLGKTLPGSDWTPNARF

IIRSWLRDGLKPRCITRDLKWGTPVPLEGFEDKVFYVWFDATIGYLSITANYTDQWERWWKNPEQVNLYQFMAKDNVPFH

GLVFPCSALGAEDNYTLVNHLIATEYLNYEDGKFSKSRGVGVFGDMAQDTGIPADIWRFYLLYIRPEGQDSAFSWTDMLL

KNNSELLNNLGNFINRAGMFVSKFFGGYVPEMVLTSDDRRLLAHVTLELQHYHQLLEKVRIREALRSILTISRHGNQYIQ

VNEPWKRIKGSEADRQRAGTVTGLAVNIAALLSVMLQPYMPTVSATIQAQLQLPPLACSVLLTNFLCTLPAGHQIGTVSP

LFQKLENDQIESLRQRFGGDQARAPPTPAVVGAVAAAAPQQVQVLMDEVTKQGNTVRELKAQKADKNQVAAEVAKLLDLK

KQLALAEGKPLETPKGKKKK

>XP_017391701.1 PREDICTED: methionine--tRNA synthetase, cytoplasmic isoform X1 [Cebus capucinus imitator]

MRLFVSEGAPGSLPVLAAAGRARGRAELLISTVGPEDCVVPFLTRPKVPVLQLDSGNYLFSTSAICRYFFLLSGWEQDDL

TNQWLEWEATELQPALSAALYYLVVQGKKGDDVLGSVRRALTHIDHSLRRQNCPFLAGETESLADIVLWGALYPLLQDPA

YLPEELSALHSWFQTLSTQEPCQRAAETVLKQQGVLALRPYLQKQPQPSPPEGRAVTNEPEEEELATLSEEEIAMAVTAW

EKGLESLPPLRPQQNPVYSRLRQWNTLYLCGTDEYGTATETKAMEEGLTPQEICDKYHIIHADIYRWFNISFDVFGRTTT

PQQTKITQDIFQRLLARGFVLQDTVEQLRCERCARFLADRFVEGVCPFCGFEEARGDQCDKCGKLINAVELKKPQCKVCR

SCPVVKSSQHLFLDLPKLEKRLEEWLGKTLPGSDWTPNARFIIRSWLRDGLKPRCITRDLKWGTPVPLEGFEDKVFYVWF

DATIGYLSITANYTDQWERWWKNPEQVDLYQFMAKDNVPFHGLVFPCSALGAEDNYTLVSHLIATEYLNYEDGKFSKSRG

VGVFGDMAQDTGIPADIWRFYLLYIRPEGQDSAFSWTDMLLKNNSELLNNLGNFINRAGMFVSKFFGGYVPEMVLTPDDQ

RLLAHVTLELRRYHQLLEKVRIRDALRSMLTISRHGNQYIQVNEPWKRIKGSEADRQRAGTVTGLAVNIAALLSVMLQPY

MPTVSATIQAQLQLPPPACSILLTNFLCTLPAGHQIGTVSPLFQKLENDQIESLRQRFGGGQLEETLDLKAKMSPKPAVA

ETGTTAAPQQIQALMDEVTKQGNIVRELKAQKADKNQVAAEVAKLLDLKKQLAVAEGKPPEVPKGKKKK

>XP_005970643.1 PREDICTED: methionine--tRNA synthetase, cytoplasmic isoform X2 [Pantholops hodgsonii]

MRLFVSEGAPGSLPVLAAAGRAQGRAELLISTVGPEECVVPFLTRPKVPVLQLDSGNYLFSTSAICRYFFLLSGWEQDDL

TNQWLEWEATELQPALSAALYYLVVQGKKGEDVLGPVRRALTHIDHSLSRRSCPFLAGETESLADIVLWGALYPLLQDPS

YLPEELGALHSWFQTLSSQEPCQRAAETVLKQQGVLALRPYLQKQPQPGSLEDRLISNEPEEEELATLSEEEIAVAVAAW

EKGLESLPPLRPQQNPVLPVAGERNVLITSALPYVNNVPHLGNIIGCVLSADVFARYSRLRQWNTLYLCGTDEYGTATET

KAMEEGLTPQEICDKYHVIHADIYRWFNISFDFFGRTTTPQQTKITQDIFQRLLARGFVLQDIVEQLRCEHCARFLADRF

VEGVCPFCGYEEARGDQCDKCGKLINAIELKKPQCKVCRSCPVVKSSQHLFLDLPKLGARVEEWLEKTLPGSDWTPNARF

IIRSWLRDGLKPRCITRDLKWGTPVPLEGFEDKVFYVWFDATIGYLSITANYTDQWEKWWKNPEQVNLYQFMAKDNVPFH

GIVFPSSALGAEDNYTLVSHLIATEYLNYEDGKFSKSRGVGVFGDMAQDTGIPADIWRFYLLYIRPEGQDSAFSWTDMLF

KNNSELLNNLGNFINRAGMFVSKFFGGFVPEMVLTSDDQRLLTHVTLELQHYHQLLEKVRIRDALRSILTISRHGNQYIQ

VNEPWKRIKGGEADRQRAGTVTGLAVNIAALLSVMLQPYMPTVSATIQAQLQLPPPARSILPTNFLCTLPAGHQIGTVSP

LFQKLENDQIESLKQRFSGGQAKASPKTAAGPQQIQALTEEVTKQGSIVRELKAQKADKNQIAAEVAKLLDLKKQLAQAE

GKPLETPKGKKKK

>XP_006077952.1 PREDICTED: methionine--tRNA synthetase, cytoplasmic isoform X1 [Bubalus bubalis]

MRLFVSEGAPGSLPVLAAAGRAQGRAELLISTVGPEECVVPFLTRPKVPVLQLDSGNYLFSTSAICRYFFLLSGWEQDDL

TNQWLEWEATELQPALSAALYYLVVQGKKGEDVLGPVRRALTHIDHSLSRRSCPFLAGETESLADIVLWGALYPLLQDPS

YLPEELGALHSWFQTLSSQEPCQRAAETVLKQQGVLALRPYLQKQPQPSSLEGRLVSNEPEEEELATLSEEEIAVAVAAW

EKGLESLPSLRPQQNPVLPVAGERNVLITSALPYVNNVPHLGNIIGCVLSADVFARYSRLRQWNTLYLCGTDEYGTATET

KAMEEGLTPQEICDKYHVIHADIYRWFNISFDFFGRTTTPQQTKITQDIFQRLLARGFVLRDTVEQLRCEHCARFLADRF

VEGVCPFCGYEEARGDQCDKCGKLINAIELKKPQCKVCRSCPVVKSSQHLFLDLPKLAARVEAWLEKTLPGSDWTANARF

IIRSWLRDGLKPRCITRDLKWGTPVPLEGFENKVFYVWFDATIGYLSITANYTDQWEKWWKNPEQVNLYQFMAKDNVPFH

GIVFPSSALGAEDNYTLVSHLIATEYLNYEDGKFSKSRGVGVFGDMAQDTGIPADIWRFYLLYIRPEGQDSAFSWTDMLF

KNNSELLNNLGNFINRAGMFVSKFFGGFVPEMVLISDDQRLLTHITLELQHYHQLLEKVRIRDALRSILTISRHGNQYIQ

VNEPWKRIKGGEADRQRAGTVTGLAVNIAALLSVMLQPYMPTVSATIQAQLQLPPPACSILPTNFLCTLPAGHQIGTVSP

LFQKLENDQIENLKQRFSGGQLEESLALKAKASPKTAAELTTAGPQQIQALTEEVTKQGNIVRELKAQKADKNQIAAEVA

KLLDLKKQLALAEGKPLETPKGKKKK

>XP_004481357.1 methionine--tRNA synthetase, cytoplasmic isoform X1 [Dasypus novemcinctus]

MRLFVSEGAPGSLPVLAAAGRARGSAEVLISTVGPEECVVPFLTRPKIPVLQLDSGNYLFSTSAICRYFFLLSGWEQDDL

TNQWLEWEATELQPALSAAFYYLVVQGKKREDVLDPVRRALTHIDHSLSRRSCPFLAGETESVADIVLWGALYPLLQDPA

YLPEELGALRSWFQTLSIQEPCQQAAETVLKQQGALAFRPYLQKQPQPSPLEGRAVSNEPEEEELATLSEEEIAMAVTAW

ERGLGSLPTLRPQQNPVLPVAGERNVLITSALPYVNNVPHLGNIIGCVLSADVFARYSRLRQWNTLYLCGTDEYGTATET

KAMEEGLTPQQICDKYHIIHADIYRWFNISFDIFGRTTTPQQTLITQDIFQRLLVRGFVLQDTVEQLRCERCARFLADRF

VEGVCPFCGYEEARGDQCDKCGKLINAIELKKPQCKVCRSCPVVKSSKHLFLDLPKLEKRLEEWLEKTLPGSDWTTNAQF

ITRSWLRDGLKPRCITRDLKWGTPVPLEGFEDKVFYVWFDATFGYISITANYTDEWERWWKNPEQVDLYQFMAKDNVPFH

GLVFPCSVLGAEDNYTLVKHLIATEYLNYEDGKFSKSRGVGVFGDMAQDTGIPADIWRFYLLYIRPEGQDSAFSWTDMLL

KNNSELLNNLGNFINRAGMFVSKFFGGRVPEMVLIPDDQRLLAHVTMELRHFHQLLEKVRIRDALRSILTISRHGNQYIQ

VNEPWKRIKGNEADRQRAGTVTGLAVNIAALLSIMLQPYMPAVSATIQAQLQLPPPACNILLTDFLCTLPAGHQIGTVSP

LFQKLENDQMEGLRQRFGGGQLEESLELKAKPSPKPAAAETVTATGQQQIHMLMNEVTKQGNIVRELKAQKADKNQVAAE

VAKLLELKKQLALAEGKPTEPPKGKKKK

>XP_007948219.1 PREDICTED: methionine--tRNA synthetase, cytoplasmic [Orycteropus afer afer]

MKLFLSEGAPGSLPVLAAASRVRGRAELLISSIGPEECVVPFLTRPKVPVLQLDNGNYLFSTSAICRYFFLLSGWEQDDL

TNQWLEWEAVELQPVLSAALYCLVVQGKKGEDVLGPVQRALTHIDHSLSRQSCPFLTGETESLADIVLWGALYPLLQDPA

YFPEELGALRSWFQTLSTQEPCQRAAETVLKQQGSLALRPYLQKQPQPRPPEGRAVSNEPEEEELTTLSEEEIAMAVTAW

EKGLENLPPLRPQKNPVLPVAGEKNVLITSALPYVNNVPHLGNIIGCVLSADVFARYSRLRQWNTLYLCGTDEYGTATET

KAMEEGLTPQEICDKYHIIHADIYRWFNISFDIFGRTTTPQQTKITQDIFQRLLSRGFVLEDTVEQLRCEHCARFLADRF

VEGVCPFCGYEEARGDQCDKCGKLINAIELKKPQCKVCRACPVVRSSQHLFLDLPKLENRLEEWLGKMLSGSDWTPNARF

IIRSWLRDGLKPRCITRDLKWGTPVPLEGFEDKVFYVWFDATFGYLSITANYTDQWEKWWKNPEQVDLYQFMAKDNVPFH

GLVFPCSVLGAEDNYTLVSHLIATEYLNYEDGKFSKSRGVGVFGDMAQETGIPADIWRFYLLYIRPEGQDSAFSWTDMLL

KNNSELLNNLGNFINRAGMFVSKFFGGYVPEMVLTSDDQRLLAHVTLELQHYHQLLEKVRIRDALRSILTISRHGNQYIQ

VNEPWKRIKGSEADRQRAGTVTGLAVNIAALLSIMIQPYMPTVSTTIQSQLQLPTLACSILPINFLCTLPAGHQIGTVSP

LFQKLENDQIESLRQRFGGGRVKASPKPAVVETVTTAEPQQIQVLMDEVTKQGNIVRELKAQKADKNHVAAEVAKLLELK

KQLALAEGKLLDLPKGKKKK

>XP_005680339.2 PREDICTED: methionine--tRNA synthetase, cytoplasmic isoform X1 [Capra hircus]

MRLFVSEGAPGSLPVLAAAGRAQGRAELLISTVGPEECVVPFLTRPKVPVLQLDSGNYLFSTSAICRYFFLLSGWEQDDL

TNQWLEWEATELQPALSAALYYLVVQGKKGEDVLGPVRRALMHIDHSLSRRSCPFLAGETESLADIVLWGALYPLLQDPS

YLPEELGALHSWFQTLSSQEPCQRAAETVLKQQGVLALRPYLQKQPQPGSLEGRLISNEPEEEELATLSEEEIAVAVAAW

EKGLESLPPLRPQQNPVLPVAGERNVLITSALPYVNNVPHLGNIIGCVLSADVFARYSRLRQWNTLYLCGTDEYGTATET

KAMEEGLTPQEICDKYHVIHADIYRWFNISFDFFGRTTTPKQTKITQDIFQRLLARGFVLQDTVEQLRCEHCARFLADRF

VEGVCPFCGYEEARGDQCDKCGKLINAIELKKPQCKVCRSCPVVKSSQHLFLDLPKLGARVEEWLEKTLPGSDWTPNARF

IIRSWLRDGLKPRCITRDLKWGTPVPLEGFEDKVFYVWFDATIGYLSITANYTDQWEKWWKNPEQVNLYQFMAKDNVPFH

GIVFPSSALGAEDNYTLVSHLIATEYLNYEDGKFSKSRGVGVFGDMAQDTGIPADIWRFYLLYIRPEGQDSAFSWTDMLF

KNNSELLNNLGNFINRAGMFVSKFFGGFVPEMVLTSDDQRLLTHVTLELQHYHQLLEKVRIRDALRSILTISRHGNQYIQ

VNEPWKRIKGGEADRQRAGTVTGLAVNIAALLSVMLQPYMPTVSATIQAQLQLPPPACSILPTNFLCTLPAGHQIGMVSP

LFQKLENDQIESLKQRFSGGQLEESLALKAKASPKTAAGPQQIQALTEEVTKQGNIVRELKAQKADKNQIAAEVAKLLDL

KKQLAQAEGKPLETPKGKKKK

>XP_016080012.1 PREDICTED: methionine--tRNA synthetase, cytoplasmic isoform X2 [Miniopterus natalensis]

MRLFVSEGAPGTLPVLAAAGRAQGRAELLISTVGPEECVVPFLTRPKAPVLQLDSGNYLFSTNAICRYFFLLSGWEQDDL

TNQWLEWEATELQPALSTALYYLVVQGKKGEDVLGPVRRALAHIDHSLSRRSCPFLAGETESVADVVLWGALYPLLQDPA

YLPEELGALHSWFQTLSSQEPCQRAAETVLKQQGILALRPYLQKQPQPNFLEGRAVSNEPEEEELATLSEDEIAMAVTAW

EKGLGSLPPLRPKQNPVLPVAGERNVLITSALPYVNNVPHLGNIIGCVLSADVFARYSRLRQWNTLYLCGTDEYGTATET

KAMEEGLTPQEICDKYHVIHADVYRWFNISFDIFGRTTTQQQTKITQDIFQRLWNRGFVLQDAVEQLRCERCARFLADRF

VEGVCPFCGYEEARGDQCDKCGKLINAIELKKPQCKVCRSCPVVKSSRHLFLDLPKLEDRLEEWLGKTLPGSDWTPNARF

IIRSWLRDGLKPRCITRDLKWGTPVPLEGFEDKVFYVWFDATFGYLSITANYTDQWERWWKNPEQVSLYQFMAKDNVPFH

GLVFPCSALGAEDNYTLVSHLIATEYLNYEDGKFSKSRGVGVFGDMAQDTGIPADIWRFYLLYIRPEGQDSAFSWTDMLL

KNNSELLNNLGNFINRAGMFVSKFFGGYVPEMVLTSDDQRLLAHVTLELQHYHQLLEKVRIRDALRSILTISRHGNQYIQ

VNEPWKRIKGSEADRQRAGTVTGLAVNIAALLSIMLQPYMPTVSSTIAAQLQLPSPACSILPTDFLCTLPTGHQIGTVSP

LFQKLENEQMESLRQRFSGGQAKVSPKPAVVETVATAGPQQIQMLMDEVTKQGNIVRELKAQKADKNQVAAEVAKLLDLK

KQLALAEGKPLETPKGKKKK

>XP_006897651.1 PREDICTED: methionine--tRNA synthetase, cytoplasmic [Elephantulus edwardii]

MRLFVSEGAPGSLPVLAAAGRARVGAELLVSTVGPEECVVPFLTGPKVPVLQLDSGTYLFSTSAICRYFFLLSGWEQDDL

TNQWLEWEAMELQPALSAALYYLVVQGKKGEDVLAPVQRALTHIDHSLSHHGCPFLTGETESLADIVLWGALYPLLQDPA

YLPEELGALCSWFQTLSTQEPCQRAAETVLKQQGALALRPYLQKQPQPRPPEGRAVSNEPEEEELATLSEEEIAMAVTAW

EKGLEALPLPRPQKNPVLPVTGEKNVLITSALPYVNNVPHLGNIIGCVLSADVFARYCRLRQWNTLYLCGTDEYGTATET

KAMEEGLTPQEICDKYHVIHADIYRWFNISFDIFGRTTTPQQTRITQDIFQRLLARGFVLQDNVEQLRCDHCARFLADRF

VEGICPFCGYEEARGDQCDKCGKLINAMELKKPQCKICRSCPVVRSSQHLFLDLPKLENRLEEWLGEVLPGSDWTPNARF

IIRSWLRDGLKPRCITRDLKWGTPVPLEGFEDKVFYVWFDATIGYLSITANYTDQWEKWWKNPEQVELYQFMAKDNVPFH

GLVFPCTALGAEDNYTLVSHIIATEYLNYEDGKFSKSRGVGVFGDMAKDTGIPADIWRFYLLYIRPEGQDSAFSWTDMLL

KNNSELLNNLGNFINRAGMFVSKFFGGSVPEMVLTSDDQRLLAHITLELRHYHQLLEKVRIRDALRSILTISRHGNQYIQ

VNEPWKRIKGSESDRQRAGTVTGLAVNIAALLSILLQPYMPTVSATIQAQLQLPPPVCSILPTNFLCTLPAGHQIGTVSP

LFQKLENDQIESLRQRFGGGQAKASQKPVAVETITAPEPQQIQVLMDEVTKQGNIVRELKAQKADKNQVAAEVAKLLELK

KQLAVAEGKPLDPPKGKKKK

>XP_008146914.1 PREDICTED: methionine--tRNA synthetase, cytoplasmic [Eptesicus fuscus]

MRLFVSEGAPGTLPVLAAAGRAQGRAQLLISTVGPEECVVPFLTRPKIPVLQLDSGNYLFSTNAICRYFFLLSGSEQDDL

TNQWLEWEATELQPALSAALYYLVVQGKKGEDVLGPIRRALTHIDHSLSRRSCPFLAGETESVADIVLWGALYPLLQDPA

YLPEELGALHSWFQTLSSQEPCQRAAETVLKKQGILALRPYLQKQLQPSFLEGRAVSNEPEEEELATLSEEEIAMAVTAW

EKGLGNLPPLRPKQKPVLPVAGERNVLITSALPYVNNVPHLGNIIGCVLSADVFARYSRLRQWNTLYLCGTDEYGTATET

KAMEEGLTPQEICDKYHAIHADIYRWFNISFDIFGRTTTSQQTEITQDIFQRLWNRGFVLQDTVEQLRCERCSRFLADRF

VEGVCPFCGYEEARGDQCDKCGKLINAIELKKPQCKVCRSCPVVRSSRHLFLDLPKLEDRLEEWLGKTLPGSDWTPNARF

IIRSWLRDGLKPRCITRDLKWGTPVPLEGFEDKVFYVWFDATFGYISITANYTDQWERWWKNPEQVNLYQFMAKDNVPFH

GLVFPCSALGAEDNYTLVSHLIATEYLNYEDGKFSKSRGVGVFGDMAQDTGIPADIWRFYLLYIRPEGQDSAFSWTDMLL

KNNSELLNNLGNFINRAGMFVSKFFGGYVPEMVLTSDDQRLLAHVTLELQHYHQLLEKVRIRDALRSILTISRYGNQYIQ

VNEPWKRIKGNEADRQRAGTVTGLAVNIAALLSTMLQPYMPTVSSTIATQLQLPPPACGILPTDFLCILPAGHQIGTINP

LFQKLENDQIENLRQRFGGGQAKVSPKPAVVETAGPQQIQVLMDEVTKQGNIVRELKAQKADKNQVAAEVAKLLDLKKQL

ALAEGKPLETPKGKKKK

>XP_006093636.1 methionine--tRNA synthetase, cytoplasmic isoform X2 [Myotis lucifugus]

MRLFVSEGAPGTLPVLAAAGRAQGRAQLLISTVGPEECVVPFLTRPKIPVLQLDSGNYLFSTNAICRYFFLLSGSEQDDL

TNQWLEWEATELQPALSAALYYLVVQGKKGEDVLGPIRRALTHIDHSLSRRSCPFLAGETESVADIVLWGALYPLLQDPA

YLPEELGALHSWFQTLSSQEPCQRAAETVLKKQGILALRPYLQKQLQPSFLEGRAVSNEPEEEELATLSEEEIAMAVTAW

EKGLGSLPPLRPKQKPVLPVAGERNVLITSALPYVNNVPHLGNIIGCVLSADVFARYSRLRQWNTLYLCGTDEYGTATET

KAMEEGLTPQEICDKYHVIHADIYRWFNISFDIFGRTTTSQQTEITQDIFQRLWNRGFVLQDTVEQLRCERCARFLADRF

VEGVCPFCGYEEARGDQCDKCGKLINAVELKKPQCKVCRSCPVVRSSRHLFLDLPKLEDRLEEWLGKTVPGSDWTPNARF

IIRSWLRDGLKPRCITRDLKWGTPVPLEGFEDKVFYVWFDATFGYISITANYTDQWEKWWKNPEQVNLYQFMAKDNVPFH

GLVFPCSALGAEDNYTLVSHLIATEYLNYEDGKFSKSRGVGVFGDMAKDTGIPADIWRFYLLYIRPEGQDSAFSWTDMLL

KNNSELLNNLGNFINRAGMFVSKFFGGYVPEMVLTSDDQRLLAHVSLELQHYHQLLEKVRIRDALRSILTISRYGNQYIQ

VNEPWKRIKGSEADRQRAGTVTGLAVNIAALLSTMLQPYMPTVSSMIAAQLQLPSPACSILPTDFLCTLPAGHQIGTVSP

LFQKLENDQIENLRQRFGGGQAKVSPKPAVVEIAGPQQIQVLMDEVTKQGNIVRELKAQKADKNQVAAEVAKLLDLKKQL

ALAEGKPLETPKGKKKK

>XP_019816518.1 PREDICTED: methionine--tRNA synthetase, cytoplasmic isoform X1 [Bos indicus]

MRLFVSEGAPGSLPVLAAAGRAQGRAELLISTVGPEAHSGPTPFQRSYPGPSPECVVPFLTRPKVPVLQLDSGNYLFSTS

AICRYFFLLSGWEQDDLTNQWLEWEATELQPALSAALYYLVVQGKKGEDVLGPVRRALTHIDHSLSRRSCPFLAGETESL

ADIVLWGALYPLLQDPSYLPEELGALHSWFQTLSSQEPCQRAAETVLKQQGVLALRPYLQKQPQPSSLEGRLVSNEPEEE

ELATLSEEEIAVAVAAWEKGLESLPPLRPQQNPVLPVAGERNVLITSALPYVNNVPHLGNIIGCVLSADVFARYSRLRQW

NTLYLCGTDEYGTATETKAMEEGLTPQEICDKYHVIHADIYRWFNISFDFFGRTTTPQQTKITQDIFQRLLARGFVLQDT

VEQLRCEHCARFLADRFVEGVCPFCGYEEARGDQCDKCGKLINAIELKKPQCKVCRSCPVVKSSQHLFLDLPKLAARVEE

WLEKTLPGSDWTANARFIIRSWLRDGLKPRCITRDLKWGTPVPLEGFEDKVFYVWFDATIGYLSITANYTDQWEKWWKNP

EQVNLYQFMAKDNVPFHGIVFPSSALGAEDNYTLVSHLIATEYLNYEDGKFSKSRGVGVFGDMAQDTGIPADIWRFYLLY

IRPEGQDSAFSWTDMLFKNNSELLNNLGNFINRAGMFVSKFFGGFVPEMVLTSDDQRLLTHITLELQHYHQLLEKVRIRD

ALRSILTISRHGNQYIQVNEPWKRIKGGEADRQRAGTVTGLAVNIAALLSVMLQPYMPTVSATIQAQLQLPPPACSILPT

NFLCTLPAGHQIGTVSPLFQKLENDQIESLKQRFSGGQLEESLALKAKASPKTAAGLTTAGPQQIQALTEEVTKQGNIVR

ELKAQKADKNQIAAEVAKLLDLKKQLALAEGKPLETSKGKKKK

>XP_011381165.1 methionine--tRNA synthetase, cytoplasmic [Pteropus vampyrus]

MRLFVSEGSPGSLPVLAAAGRAQGRAELRISTVGPEERVVPFLTQPKVPVLQLDSGNYLFSTSAICRYFFLLSGWEQDDL

TNQWLEWEVTELQPALSAALYYLVVQGKKGEEVLGPVRGALIHIDNSLSRRSCPFLAGETESLADIVLWGALYPLLQDPA

YLPEELGALHSWFQTLSSQEPCQRAAETVLKQQGVLALRPYLQKQLQPSFLEGKAVSNEPEEEELATLSEEEIAVAVTAW

EKGLGSLPPFRPKQTPVLPVAGERNVLITSALPYVNNVPHLGNIIGCVLSADVFARYSRLRQWNTLYLCGTDEYGTATET

KAMEEGLTPQEICDKYHVIHADIYRWFNISFDIFGRTTTPQQTEITQDIFQRLWNRGFVLQDTVEQLRCESCARFLADRF

VEGVCPFCGYEEARGDQCDKCGKLINAIELKKPQCKLCRSCPVVKSSRHLFLDLPKLEDRLEEWLGKTLPGSDWTPNARF

IIRSWLRDGLKPRCITRDLKWGTPVPLEGFEDKVFYVWFDATFGYLSITANYTDQWERWWKNPEQVNLYQFMAKDNVPFH

GLVFPCSALGAEDNYTLVSHLIATEYLNYEDGKFSKSRGVGVFGDMAQDTGIPADIWRFYLLYIRPEGQDSAFSWTDMLL

KNNSELLNNLGNFINRAGMFVSKFFGGYVPEMVLTSDDQRLLAHVALELQHYHQLLEKVRIRDALRSILTISRHGNQYIQ

VNEPWKRIKGSEADRQRAGTVTGLAVNIAALLSIMLQPYMPTVSTTIAAQLQLPPLACSILSTDFLCTLPAGHQIGTVSP

LFQKLENDQIESLKQRFGGGQAKASPKPTDVETVTTVGLQQIKVLMDEVTKQGNIVRELKTQKADKNQVAAEVAKLLDLK

KQLALAEGKPLETPKGKKKK

>XP_005879086.1 PREDICTED: methionine--tRNA synthetase, cytoplasmic isoform X1 [Myotis brandtii]

MRLFVSEGVPGTLPVLAAAGRAQGRAQLLISTVGPEECVVPFLTRPKIPVLQLDSGNYLFSTNAICRYFFLLSGSEQDDL

TNQWLEWEATELQPALSAAMYYLVVQGKKGEDVLGPIRRALTHIDHSLSRRSCPFLAGETESVADIVLWGALYPLLQDPA

YLPEELGALHSWFQTLSSQEPCQRAAETVLKKQGILALRPYLQKQLQPSFLEGRAVSNEPEEEELATLSEEEIAMAVTAW

EKGLGSLPPLRPKQKPVLPVAGERNVLITSALPYVNNVPHLGNIIGCVLSADVFARYSRLRQWNTLYLCGTDEYGTATET

KAMEEGLTPQEICDKYHVIHADTYRWFNISFDIFGRTTTSQQTEITQDIFQRLWNRGFVLQDTVEQLRCERCARFLADRF

VEGVCPFCGYEEARGDQCDKCGKLINAIELKKPQCKVCRSCPVVRSSRHLFLDLPKLEDRLEEWLGKTVPGSDWTPNARF

IIRSWLRDGLKPRCITRDLKWGTPVPLEGFEDKVFYVWFDATFGYISITANYTDQWEKWWKNPEQVNLYQFMAKDNVPFH

GLVFPCSALGAEDNYTLVSHLIATEYLNYEDGKFSKSRGVGVFGDMAKDTGIPADIWRFYLLYIRPEGQDSAFSWTDMLL

KNNSELLNNLGNFINRAGMFVSKFFGGYVPEMVLTSDDQRLLAHVSLELQHYHQLLEKVRIRDALRSILTISRYGNQYIQ

VNEPWKRIKGSEADRQRAGTVTGLAVNIAALLSTMLQPYMPTVSSMIAAQLQLPPPACSILPTDFLCTLPAGHQIGTVSP

LFQKLENDQIENLRQRFGGGQLEESLVLKAKVSPKPAVVETAGPQQIQVLMDEVTKQGNIVRELKAQKADKNQVAAEVAK

LLDLKKQLALAEGKPLETPKGKKKK

>XP_006171958.2 PREDICTED: methionine--tRNA synthetase, cytoplasmic isoform X1 [Tupaia chinensis]

MRLFVSEGAPGSLPVLAAAGRARGRAELLISTVGPEECVVPFLTRPKVPVLQLDSGNYLFSTSAICRYLFLLSGWEQDDL

TNQWLEWEATELQPALSAALYYLVVQGKKGDDVLGPVRRALTHIDHSLSRQNCPFLAGETESLADIVLWGALYPLLQDPA

YFPEELSALHSWFQTLSTQEPCQQAVETVLKQQGVLALRPYLQKQPQPSAPEGKTVSNEPEEEELSTLSEEDIAMAVTAW

EKGLRGLPPPRPQQNPVLPVAGERNVLITSALPYVNNVPHLGNIIGCVLSADVFARYSRLRQWNTLYLCGTDEYGTATET

KAMDEGLTPQEICDKYHVIHADIYRWFNISFDIFGRTTTPQQTKITQDIFQRLLARGFVLQDSVEQLRCERCARFLADRF

VEGVCPFCGYEEARGDQCDKCGKLINAIELKKPLCKVCRSSPVVKSSQHLFLDLPKLEKRVEEWLERTLPGSDWTPNARF

IIRSWLRDGLKPRCITRDLKWGTPVPLEGFESKVFYVWFDATIGYLSITANYTDQWEKWWKNPEQVELYQFMAKDNVPFH

GIVFPCSALGAEDNYTLVSHLIATEYLNYEDGKFSKSRGVGVFGDMAQDTGIPADIWRFYLLYIRPEGQDSAFSWTDMLL

KNNSELLNNLGNFINRAGMFVSKFFGGYVPEMVLTPDDQRLLAHVTQELRHYHQLLEKVRIRDALRSILTISRHGNQYIQ

VNEPWKQIKGSEADRQRAGTVTGVAVNIAALLSVMLQPYMPTVSATIQTQLQLPPPACNILVTDFLCTLPAGHQIGTVSP

LFQKLENDQIESLRQRFGGGQLEESLELKVKPSPKPAVVETVTAAGPQQIQVLMEEVTKQGNIVRELKAQKADKNLVAAE

VAKLLDLKKQLALAEGKPLEAPKGKKKK

>XP_008682242.1 PREDICTED: methionine--tRNA synthetase, cytoplasmic [Ursus maritimus]

MRLFVSEGAPGSLPVLAAAGRAQGRADLLISTVGPEECVVPFLTRPKVPVLQLDSGNYLFSTSAICRYFFLLSGWEQDDL

TNQWLEWEATELQPALSAALYYLVVQGKKGEDVLGPVRRALTHIDQSLSRRSCPFLAGETESLADVVLWGALYPLLQDPA

YLPEELGALHSWFQTLSSQEPCQKAAETVLKQQGVLALRPYLQKQPQPGSFEGRAVSNEPEEEELAALSEEEIAMAVTAW

EKGLESLPPLRPQQTPVLPVAGERNVLITSALPYVNNVPHLGNIIGCVLSADVFARYSRLRQWNTLYLCGTDEYGTATET

KAMEEGLTPQEICDKYHAIHADIYRWFNISFDIFGRTTTPQQTKITQDIFQRLLTRGFVLQDTVEQLRCERCARFLADRF

VEGVCPFCGYEEARGDQCDKCGKLINAIELKKPQCKVCRSCPVVKSSRHLFLDLPKLEKRLEEWLGKTLPGSDWTPNARF

IIRSWLRDGLKPRCITRDLKWGTPVPLEGFEDKVFYVWFDATIGYLSITANYTDQWERWWKNPEQVNLYQFMAKDNVPFH

GLVFPCSALGAEDNYTLVNHLIATEYLNYEDGKFSKSRGVGVFGDMAQDTGIPADIWRFYLLYIRPEGQDSAFSWTDMLL

KNNSELLNNLGNFINRAGMFVSKFFGGYVPEMVLTSDDRRLLAHVTLELQHYHQLLEKVRIREALRSILTISRHGNQYIQ

VNEPWKRIKGSEADRQRAGTVTGLAVNVAALLSVMLQPYMPTVGATIQAQLQLPPPACRVLLTNFVCTLPAGHQIGTVSP

LFQKLENDQIESLRQRFGGGQAKAPATPAVVEAVVTAAPQQVQMLMDEVTKQGNIVRELKAQKADKNQVAAEVAKLLDLK

KQLALAEGKPPETPKGKKKK

>XP_006093635.1 methionine--tRNA synthetase, cytoplasmic isoform X1 [Myotis lucifugus]

MRLFVSEGAPGTLPVLAAAGRAQGRAQLLISTVGPEECVVPFLTRPKIPVLQLDSGNYLFSTNAICRYFFLLSGSEQDDL

TNQWLEWEATELQPALSAALYYLVVQGKKGEDVLGPIRRALTHIDHSLSRRSCPFLAGETESVADIVLWGALYPLLQDPA

YLPEELGALHSWFQTLSSQEPCQRAAETVLKKQGILALRPYLQKQLQPSFLEGRAVSNEPEEEELATLSEEEIAMAVTAW

EKGLGSLPPLRPKQKPVLPVAGERNVLITSALPYVNNVPHLGNIIGCVLSADVFARYSRLRQWNTLYLCGTDEYGTATET

KAMEEGLTPQEICDKYHVIHADIYRWFNISFDIFGRTTTSQQTEITQDIFQRLWNRGFVLQDTVEQLRCERCARFLADRF

VEGVCPFCGYEEARGDQCDKCGKLINAVELKKPQCKVCRSCPVVRSSRHLFLDLPKLEDRLEEWLGKTVPGSDWTPNARF

IIRSWLRDGLKPRCITRDLKWGTPVPLEGFEDKVFYVWFDATFGYISITANYTDQWEKWWKNPEQVNLYQFMAKDNVPFH

GLVFPCSALGAEDNYTLVSHLIATEYLNYEDGKFSKSRGVGVFGDMAKDTGIPADIWRFYLLYIRPEGQDSAFSWTDMLL

KNNSELLNNLGNFINRAGMFVSKFFGGYVPEMVLTSDDQRLLAHVSLELQHYHQLLEKVRIRDALRSILTISRYGNQYIQ

VNEPWKRIKGSEADRQRAGTVTGLAVNIAALLSTMLQPYMPTVSSMIAAQLQLPSPACSILPTDFLCTLPAGHQIGTVSP

LFQKLENDQIENLRQRFGGGQLEESLVLKAKVSPKPAVVEIAGPQQIQVLMDEVTKQGNIVRELKAQKADKNQVAAEVAK

LLDLKKQLALAEGKPLETPKGKKKK

>XP_016080011.1 PREDICTED: methionine--tRNA synthetase, cytoplasmic isoform X1 [Miniopterus natalensis]

MRLFVSEGAPGTLPVLAAAGRAQGRAELLISTVGPEECVVPFLTRPKAPVLQLDSGNYLFSTNAICRYFFLLSGWEQDDL

TNQWLEWEATELQPALSTALYYLVVQGKKGEDVLGPVRRALAHIDHSLSRRSCPFLAGETESVADVVLWGALYPLLQDPA

YLPEELGALHSWFQTLSSQEPCQRAAETVLKQQGILALRPYLQKQPQPNFLEGRAVSNEPEEEELATLSEDEIAMAVTAW

EKGLGSLPPLRPKQNPVLPVAGERNVLITSALPYVNNVPHLGNIIGCVLSADVFARYSRLRQWNTLYLCGTDEYGTATET

KAMEEGLTPQEICDKYHVIHADVYRWFNISFDIFGRTTTQQQTKITQDIFQRLWNRGFVLQDAVEQLRCERCARFLADRF

VEGVCPFCGYEEARGDQCDKCGKLINAIELKKPQCKVCRSCPVVKSSRHLFLDLPKLEDRLEEWLGKTLPGSDWTPNARF

IIRSWLRDGLKPRCITRDLKWGTPVPLEGFEDKVFYVWFDATFGYLSITANYTDQWERWWKNPEQVSLYQFMAKDNVPFH

GLVFPCSALGAEDNYTLVSHLIATEYLNYEDGKFSKSRGVGVFGDMAQDTGIPADIWRFYLLYIRPEGQDSAFSWTDMLL

KNNSELLNNLGNFINRAGMFVSKFFGGYVPEMVLTSDDQRLLAHVTLELQHYHQLLEKVRIRDALRSILTISRHGNQYIQ

VNEPWKRIKGSEADRQRAGTVTGLAVNIAALLSIMLQPYMPTVSSTIAAQLQLPSPACSILPTDFLCTLPTGHQIGTVSP

LFQKLENEQMESLRQRFSGGQLEESFTLKAKVSPKPAVVETVATAGPQQIQMLMDEVTKQGNIVRELKAQKADKNQVAAE

VAKLLDLKKQLALAEGKPLETPKGKKKK

>XP_005970642.1 PREDICTED: methionine--tRNA synthetase, cytoplasmic isoform X1 [Pantholops hodgsonii]

MRLFVSEGAPGSLPVLAAAGRAQGRAELLISTVGPEECVVPFLTRPKVPVLQLDSGNYLFSTSAICRYFFLLSGWEQDDL

TNQWLEWEATELQPALSAALYYLVVQGKKGEDVLGPVRRALTHIDHSLSRRSCPFLAGETESLADIVLWGALYPLLQDPS

YLPEELGALHSWFQTLSSQEPCQRAAETVLKQQGVLALRPYLQKQPQPGSLEDRLISNEPEEEELATLSEEEIAVAVAAW

EKGLESLPPLRPQQNPVLPVAGERNVLITSALPYVNNVPHLGNIIGCVLSADVFARYSRLRQWNTLYLCGTDEYGTATET

KAMEEGLTPQEICDKYHVIHADIYRWFNISFDFFGRTTTPQQTKITQDIFQRLLARGFVLQDIVEQLRCEHCARFLADRF

VEGVCPFCGYEEARGDQCDKCGKLINAIELKKPQCKVCRSCPVVKSSQHLFLDLPKLGARVEEWLEKTLPGSDWTPNARF

IIRSWLRDGLKPRCITRDLKWGTPVPLEGFEDKVFYVWFDATIGYLSITANYTDQWEKWWKNPEQVNLYQFMAKDNVPFH

GIVFPSSALGAEDNYTLVSHLIATEYLNYEDGKFSKSRGVGVFGDMAQDTGIPADIWRFYLLYIRPEGQDSAFSWTDMLF

KNNSELLNNLGNFINRAGMFVSKFFGGFVPEMVLTSDDQRLLTHVTLELQHYHQLLEKVRIRDALRSILTISRHGNQYIQ

VNEPWKRIKGGEADRQRAGTVTGLAVNIAALLSVMLQPYMPTVSATIQAQLQLPPPARSILPTNFLCTLPAGHQIGTVSP

LFQKLENDQIESLKQRFSGGQLEESLALKAKASPKTAAGPQQIQALTEEVTKQGSIVRELKAQKADKNQIAAEVAKLLDL

KKQLAQAEGKPLETPKGKKKK

>XP_006182566.1 PREDICTED: methionine--tRNA synthetase, cytoplasmic isoform X2 [Camelus ferus]

MRLFVSEGAPGTLPVLAAAGRAQGRVELLISTVGPEECVVPFLTRPKVPVLQLDSGNYLFSTSAICRYFFLLSGWEQDDL

TNQWLEWEATELQPALSAALYYLVVQGKKGEDVLGPVRRALTHIDHSLSRRSCPFLAGETESLADIVLWGALYPLLQDPV

YLPEELGALNSWFQTLSSQEPCQRAAETVLKQQGVLALRPYLQKQPQPSSFEGRAVSNEPEEEELAALSEEEIALAVAAW

EKGLGSLPPLQPQQSPVLPVAGERNVLITSALPYVNNVPHLGNIIGCVLSADVFARYSRLRQWNTLYLCGTDEYGTATET

KAMEEGLTPQEICDKYHAIHADIYRWFNISFDIFGRTTTPQQTKITQDIFQRLLARGFVLQDTVEQLRCERCARFLADRF

VEGVCPFCGFEEARGDQCDKCGKLINATELKKPQCKICRSCPVVKSSRHLFLDLPKLEKRLEEWLEKTLPGSDWTPNARF

IIRSWLRDGLKPRCITRDLKWGTPVPLEGFEDKVFYVWFDATIGYVSITANYTDQWERWWKNPEQVDLYQFMAKDNVPFH

GLVFPCSVLGAEDNYTLVKNLIATEYLNYEDGKFSKSRGVGVFGDMAQDTGIPADIWRFYLLYIRPEGQDSAFSWTDMLL

KNNSELLNNLGNFINRAGMFVSKFFGGFVPEMVLTSDDRRLLAHITLELQHYHQLLEKVRIRDALRSILTISRHGNQYIQ

VNEPWKRIKGSEADRQRAGTVTGLAVNIAALLSVMLQPYMPTVSTTIQAQLQLPPPTCSILPTNFLCILPAGHQIGTVSP

LFQKLENDQIESLRQRFSGGQAKAGPKPAAAGTMTTAGPEQIQVLMDEVTKQGNIVRELKAQKADKNQVAAEVAKLLDLK

KQLALAEGKPLETPKGKKKK

>XP_008059027.1 methionine--tRNA synthetase, cytoplasmic isoform X3 [Carlito syrichta]

MKLFVSEGTPGSLPVLAAAERARGRAELLVSTVGPEECVVPFLTQPKVPVLQLDSGNYLFSTSAICRYFFLLSGWEQDDL

TNQWLEWEATELQPALSAALYYLVVQGKKGEEVLGPVRRALTHIDHSLSRQNCPFLAGETESLTDIVLWGALYPLLQDPA

YLPEELSALRSWFQTLSTQEPCQQAAETVLKQQGVLAFRPYLQKQPQPSPPEGKPITNEPEEEELATLSEEEIAMAVAAW

EKGLDSLPPLRPQQNPVLPVDGERNVLITSALPYVNNVPHLGNIIGCVLSADVFARYSRLRQWNTLYLCGTDEYGTATET

KAMEEGLSPQEICDKYHTIHADIYRWFNISFDIFGRTTTPQQTKITQDIFQRLLARGFVLQDTVEQLRCERCARFLADRF

VEGVCPFCRYEEARGDQCDKCGKLINAIELKKPQCKLCRSCPVVKSSRHLFLDLPKLEKRVEEWLGKSLPGSDWTPNARF

ITRSWLRDGLKPRCITRDLKWGTPVPLEGFEDKVFYVWFDATIGYLSITANYTDQWERWWKNPEQVDLYQFMAKDNVPFH

GIVFPCSVLGAEDNYTLVSHLIATEYLNYEDGKFSKSRGVGVFGDMAQDTGIPADIWRFYLLYIRPEGQDSAFSWMDMLL

KNNSELLNNLGNFINRAGMFVSKFFGGCVPEMVLTSDDQRLVAHVTLELRHYHQLLEKVRIRDALRSILTISRHGNQYIQ

VNEPWKQIKGSEADRQRAGTVTGLAVNIAALLSIMLQPYMPMVSATIQAQLQLPPPACSILPTDFLCTLSAGHRIGIVSP

LFQKLENDQIENLRQRFGGGQAKAAPKPAAEEAVPTARPQQIQALMDAVTKQGNIVRELKAQKADKNQIAAEVAKLLDLK

KHLALAEGKPLETPKGKKKK

>XP_010944603.1 PREDICTED: methionine--tRNA synthetase, cytoplasmic [Camelus bactrianus]

MRLFVSEGAPGTLPVLAAAGRAQGRVELLISTVGPEECVVPFLTRPKVPVLQLDSGNYLFSTSAICRYFFLLSGWEQDDL

TNQWLEWEATELQPALSAALYYLVVQGKKGEDVLGPVRRALTHIDHSLSRRSCPFLAGETESLADIVLWGALYPLLQDPV

YLPEELGALNSWFQTLSSQEPCQRAAETVLKQQGVLALRPYLQKQPQPSSFEGRAVSNEPEEEELAALSEEEIALAVAAW

EKGLGSLPPLQPQQSPVLPVAGERNVLITSALPYVNNVPHLGNIIGCVLSADVFARYSRLRQWNTLYLCGTDEYGTATET

KAMEEGLTPQEICDKYHAIHADIYRWFNISFDIFGRTTTPQQTKITQDIFQRLLARGFVLQDTVEQLRCERCARFLADRF

VEGVCPFCGFEEARGDQCDKCGKLINATELKKPQCKICRSCPVVKSSRHLFLDLPKLEKRLEEWLEKTLPGSDWTPNARF

IIRSWLRDGLKPRCITRDLKWGTPVPLEGFEDKVFYVWFDATIGYVSITANYTDQWERWWKNPDQVDLYQFMAKDNVPFH

GLVFPCSVLGAEDNYTLVKNLIATEYLNYEDGKFSKSRGVGVFGDMAQDTGIPADIWRFYLLYIRPEGQDSAFSWTDMLL

KNNSELLNNLGNFINRAGMFVSKFFGGFVPEMVLTSDDRRLLAHITLELQHYHQLLEKVRIRDALRSILTISRHGNQYIQ

VNEPWKRIKGSEADRQRAGTVTGLAVNIAALLSVMLQPYMPTVSTTIQAQLQLPPPTCSILPTNFLCILPAGHQIGTVSP

LFQKLENDQIESLRQRFSGGQAKAGPKPAAAGTMTTAGPEQIQVLMDEVTKQGNIVRELKAQKADKNQVAAEVAKLLDLK

KQLALAEGKPLETPKGKKKK

>XP_005335733.1 methionine--tRNA synthetase, cytoplasmic [Ictidomys tridecemlineatus]

MRLFVSEGAPGSLPVLAAAGRARGRATELLISTVGPEECVVPFLTRPKVPVLQLDSGNYLFSTTAICRYFFLLSGWEQDD

LTNQWLEWEATELQPALSAALYYLIVQGKKGEDVLGLLRRALTHIDHSLSRQNCPFLAGETESLADIVLWGALYPLLQDP

AYLPEELGALRSWFQTLTTQEPCQRAVETVLKQQGVLALRSYLQKQPQPSLPEERAVSNEPEEEELATLTEEEIAMAVTA

WEKGLESLPPFRPQQNPVLPVAEERNVLITSALPYVNNVPHLGNIIGCVLSADVFARYSRLRQWNTLYLCGTDEYGTATE

TKAMEEGLTPQEICDKYHTIHADIYRWFNISFDIFGRTTTPQQTKITQDIFHRLMARGFVLQDSVEQLRCERCARFLADR

FVEGVCPFCGYEEARGDQCDKCGKLINAIELKKPKCKVCRSCPVVKSSQHLFLDLPKLEKRLEEWLGKTLPGSDWTPNAR

FIIRSWLRDGLKPRCITRDLKWGTPVPLKGFEDKVFYVWFDATIGYLSITANYTDQWEQWWKNPEQVDLYQFMAKDNVPF

HGLVFPCSALGAEDNYTLVKHLIATEYLNYEDGKFSKSRGVGVFGDMAQDTGIPADIWRFYLLYIRPEGQDSAFSWTDML

LKNNSELLNNLGNFINRAGMFVSKFFGGCVPEMVLTPDDQRLLAHITLELRHYHQLLEKVRIRDALRSILTISRHGNQYI

QVNEPWKWIKGSEADRQRAGTVTGLAVNIAALLSTMLQPFMPTVSTTIQAQLQLPLPACSVLPMNFLCTLPAGHQIGTVT

PLFQKLENDQIESLRQRFGGGQAKVFPKPAVVETVTTVGPQQIQMLMDEVTKQGNIVRELKAQKADKNQVAAEVAKLLDL

KKQLALAEGKPLETPKGKKKK

>XP_004700579.1 PREDICTED: methionine--tRNA synthetase, cytoplasmic [Echinops telfairi]

MRLFVSPGAPGSLPVLVAASRARGRAELFISTVDPEERVVPFLTRPRVPVLQLDSGNYLFSTSAICRYFFLLSGWEQDDL

TDQWLEWEAVELQPALSAALHYLVVQGKKGEDVLGLMRRALTHIDHSLSRQSCPFLTGEAESLADIVLWGALYPLLQDPA

YLPEELGALRSWFQALSTQEPCQRAAETVLKQQGALALRPYLQKQPLPKPLQDGAVSNEPEEEELATLSEEEIAAAVTAW

EKGLESLPPLRPQEKPVLPVAGEKNVLITSALPYVNNVPHLGNIIGCVLSADVFARYSRLRQWNTLYLCGTDEYGTATET

KAMEEGLTPQEICDKYHAIHANIYRWFNISFDIFGRTTTPQQTKITQDIFQRLWARGFVLQDTVEQLRCERCARFLADRF

VEGVCPFCGYEEARGDQCDKCGKLINAIELKKPQCKVCRSCPVVRTSQHLFLDLPKLESRLEKWLGKVLPGSDWTPNARF

IIRSWLRDGLKPRCITRDLKWGTPVPLKGFEDKVFYVWFDATIGYLSITANYTDQWERWWKNPEQVDLYQFMAKDNVPFH

GLVFPCSALGTDENYTLVSHLIATEYLNYEDGKFSKSRGVGVFGDMAQDTGIPADIWRFYLLYIRPEGQDSAFSWTDMLL

KNNSELLNNLGNFINRAGMFVSKFFGGAVPEMVLTSDDRRLVAHITLEVRHYHQLLEKARIRDALRSILTISRHGNQYIQ

VNEPWKRIKGNEADRQRAGTVTGLAVNIAALLSIMIQPYMPTVSATIQSQLQLPPPACSFLPPAFLCILRAGHQIGTISP

LFQKLENDQIESLKQRFGGGQAKATPKPAVVETVPTAGPQQLQVLVDEVTKQGNIVRELKAQKADKNQVAAEVAKLLELK

KQLALAEGKPLDPPKGKKKK

>XP_008059026.1 methionine--tRNA synthetase, cytoplasmic isoform X1 [Carlito syrichta]

MKLFVSEGTPGSLPVLAAAERARGRAELLVSTVGPEECVVPFLTQPKVPVLQLDSGNYLFSTSAICRYFFLLSGWEQDDL

TNQWLEWEATELQPALSAALYYLVVQGKKGEEVLGPVRRALTHIDHSLSRQNCPFLAGETESLTDIVLWGALYPLLQDPA

YLPEELSALRSWFQTLSTQEPCQQAAETVLKQQGVLAFRPYLQKQPQPSPPEGKPITNEPEEEELATLSEEEIAMAVAAW

EKGLDSLPPLRPQQNPVLPVDGERNVLITSALPYVNNVPHLGNIIGCVLSADVFARYSRLRQWNTLYLCGTDEYGTATET

KAMEEGLSPQEICDKYHTIHADIYRWFNISFDIFGRTTTPQQTKITQDIFQRLLARGFVLQDTVEQLRCERCARFLADRF

VEGVCPFCRYEEARGDQCDKCGKLINAIELKKPQCKLCRSCPVVKSSRHLFLDLPKLEKRVEEWLGKSLPGSDWTPNARF

ITRSWLRDGLKPRCITRDLKWGTPVPLEGFEDKVFYVWFDATIGYLSITANYTDQWERWWKNPEQVDLYQFMAKDNVPFH

GIVFPCSVLGAEDNYTLVSHLIATEYLNYEDGKFSKSRGVGVFGDMAQDTGIPADIWRFYLLYIRPEGQDSAFSWMDMLL

KNNSELLNNLGNFINRAGMFVSKFFGGCVPEMVLTSDDQRLVAHVTLELRHYHQLLEKVRIRDALRSILTISRHGNQYIQ

VNEPWKQIKGSEADRQRAGTVTGLAVNIAALLSIMLQPYMPMVSATIQAQLQLPPPACSILPTDFLCTLSAGHRIGIVSP

LFQKLENDQIENLRQRFGGGQLEESFKLKAKAAPKPAAEEAVPTARPQQIQALMDAVTKQGNIVRELKAQKADKNQIAAE

VAKLLDLKKHLALAEGKPLETPKGKKKK

>XP_007631231.1 PREDICTED: methionine--tRNA synthetase, cytoplasmic isoform X2 [Cricetulus griseus]

MRLFVSEGSPGSLPVLAAAARARGRAELLISTVGPEECVVPFLTRPKVPVLQLDSGNYLFSTSAICRYFFLLSGWEQDDL

TNQWLEWEATELQPVLSAALHCLVVQGKKGEDILGPLREALTHIDHSLSRQHCPFLVGDTESLADIVLWGALYPLLHDPT

YLPEELGALQSWFQTLSTQEPCQRAAETVLKQQGVLALRPYLQKQPQPRPPEGRAVSNEPEEEELATLSEEDILMAVTAW

EKGMGSLPPLQPQQHPVLPVAGERNVLITSALPYVNNVPHLGNIIGCVLSADVFARYSRLRQWNTLYLCGTDEYGTATET

KAMEEGLTPQEICDKYHAIHADVYRWFNISFDTFGRTTTPHQTKITQDIFQRLLTRGFVLQDTVEQLRCEQCARFLADRF

VEGVCPFCGYEEARGDQCDKCGKLINAIELKKPQCKVCRSRPVVRSSQHLFLDLPKLEKRLEDWLGKTLPGSDWTPNARF

ITRSWLRDGLKPRCITRDLKWGTPVPLEGFKDKVFYVWFDATIGYLSITANYTDQWERWWKNPEQVDLYQFMAKDNVPFH

GIVFPCSALGAEDNYTLVKNLIATEYLNYEDGKFSKSRGIGVFGDMAQDTGIPADIWRFYLLYIRPEGQDSAFSWTDMMI

KNNSELLNNLGNFINRAGMFVSKFFGGCVPEMVLTQDDRRLLAHISWELQHYHQLLEKVRIRDALRSILTISRHGNQYIQ

VNEPWKRIKGSEVDRQRAGTVTGVAVNIAALLSVMLQPYMPTVSATIQTQLQLPLLACRVLVTNFICTLPAGHQIGTVSP

LFQKLENDQIENLRQRFGGGQTKGSPKPAVVEEVTTAGPQQIQMLMDEVTKQGNIVRELKAQKADKNQVAAQVTKLLELK

KQLALAEGKPIETPKGKKKK

>OBS58202.1 hypothetical protein A6R68_10642 [Neotoma lepida]

MRLFVSEGSPGSLPVLAAAARARGQAELLISTVGPEECVVPFLTRPKVPVLQLNSGNYLFSTSAICRYFFLLSGWEQDDL

NNQWLEWEATELQPVLSAALHCLVVQGKKGEDVLGPLRGALSHIDHSLSRQNCPFLAGDTESLADIVLWGALYPLLQDPT

YLPEELGALQSWFQTLSTQEPCQRAAETVLKQQGVLALRPYLQKQPQPQARPPDGRAVSNEPEEEELATLSEEDIVVAVT

AWEKGLGSLPPLQPQQHPVLPVAGERNVLITSALPYVNNVPHLGNIIGCVLSADVFARYSRLRQWNTLYLCGTDEYGTAT

ETKAMEEGLTPQEICDKYHAIHADIYRWFNISXDTFGRTTTPQQTKITQDIFQRLLTRGFVLQDTVEQLRCEQCARFLAD

RFVEGVCPFCGYEEARGDQCDKCGKLINAIELKKPQCKVCRSCPVVRSSQHLFLDLPKLEKRLEDWLGKTLPGSDWTPNA

RFIIRSWLRDGLKPRCITRDLKWGTPVPLEGFEDKVFYVWFDATIGYLSITANYTDQWERWWKNPEQVDLYQFMAKDNVP

FHGLVFPCSALGAEDNYTLVKHLIATEYLNYEDGKFSKSRGVGVFGDMAQDTGIPADIWRFYLLYIRPEGQDSAFSWTDM

MIKNNSELLNNLGNFINRAGMFVSKFFGGCVPEMVLAHEDRRLLAHISWELQHYHQLLEKVRIRDALRSILTISRHGNQY

IQVNEPWKRIKGTEVDRQRAGTVTGVAVNIAALLSVMLQPYMPTVSSTIQTQLQLPLPACRILVTNFICTLPAGHRIGTV

SPLFQKLENDQIENLRQRFGGGQLEESLELKAKGSPKPAVVEAVTTAGSQQIQMLMDEVTKQGNIVRELKAQKADKNQVA

AEVTKLLDLKKQLALAEGRPIETPKGKKKK

>XP_003507546.1 PREDICTED: methionine--tRNA synthetase, cytoplasmic isoform X2 [Cricetulus griseus]

MRLFVSEGSPGSLPVLAAAARARGRAELLISTVGPEECVVPFLTRPKVPVLQLDSGNYLFSTSAICRYFFLLSGWEQDDL

TNQWLEWEATELQPVLSAALHCLVVQGKKGEDILGPLREALTHIDHSLSRQHCPFLVGDTESLADIVLWGALYPLLHDPT

YLPEELGALQSWFQTLSTQEPCQRAAETVLKQQGVLALRPYLQKQPQPRPPEGRAVSNEPEEEELATLSEEDILMAVTAW

EKGMGSLPPLQPQQHPVLPVAGERNVLITSALPYVNNVPHLGNIIGCVLSADVFARYSRLRQWNTLYLCGTDEYGTATET

KAMEEGLTPQEICDKYHAIHADVYRWFNISFDTFGRTTTPHQTKITQDIFQRLLTRGFVLQDTVEQLRCEQCARFLADRF

VEGVCPFCGYEEARGDQCDKCGKLINAIELKKPQCKVCRSRPVVRSSQHLFLDLPKLEKRLEDWLGKTLPGSDWTPNARF

ITRSWLRDGLKPRCITRDLKWGTPVPLEGFKDKVFYVWFDATIGYLSITANYTDQWERWWKNPEQVDLYQFMAKDNVPFH

GIVFPCSALGAEDNYTLVKNLIATEYLNYEDGKFSKSRGIGVFGDMAQDTGIPADIWRFYLLYIRPEGQDSAFSWTDMMI

KNNSELLNNLGNFINRAGMFVSKFFGGCVPEMVLTQDDRRLLAHISWELQHYHQLLEKVRIRDALRSILTISRHGNQYIQ

VNEPWKRIKGSEVDRQRAGTVTGVAVNIAALLSVMLQPYMPTVSATIQTQLQLPLLACRVLVTNFICTLTAGHQIGTVSP

LFQKLENDQIENLRQRFGGGQTKGSPKPAVVEEVTTAGPQQIQMLMDEVTKQGNIVRELKAQKADKNQVAAQVTKLLELK

KQLALAEGKPIETPKGKKKK

>XP_014412912.1 PREDICTED: methionine--tRNA synthetase, cytoplasmic isoform X1 [Camelus ferus]

MRLFVSEGAPGTLPVLAAAGRAQGRVELLISTVGPEECVVPFLTRPKVPVLQLDSGNYLFSTSAICRYFFLLSGWEQDDL

TNQWLEWEATELQPALSAALYYLVVQGKKGEDVLGPVRRALTHIDHSLSRRSCPFLAGETESLADIVLWGALYPLLQDPV

YLPEELGALNSWFQTLSSQEPCQRAAETVLKQQGVLALRPYLQKQPQPSSFEGRAVSNEPEEEELAALSEEEIALAVAAW

EKGLGSLPPLQPQQSPVLPVAGERNVLITSALPYVNNVPHLGNIIGCVLSADVFARYSRLRQWNTLYLCGTDEYGTATET

KAMEEGLTPQEICDKYHAIHADIYRWFNISFDIFGRTTTPQQTKITQDIFQRLLARGFVLQDTVEQLRCERCARFLADRF

VEGVCPFCGFEEARGDQCDKCGKLINATELKKPQCKICRSCPVVKSSRHLFLDLPKLEKRLEEWLEKTLPGSDWTPNARF

IIRSWLRDGLKPRCITRDLKWGTPVPLEGFEDKVFYVWFDATIGYVSITANYTDQWERWWKNPEQVDLYQFMAKDNVPFH

GLVFPCSVLGAEDNYTLVKNLIATEYLNYEDGKFSKSRGVGVFGDMAQDTGIPADIWRFYLLYIRPEGQDSAFSWTDMLL

KNNSELLNNLGNFINRAGMFVSKFFGGFVPEMVLTSDDRRLLAHITLELQHYHQLLEKVRIRDALRSILTISRHGNQYIQ

VNEPWKRIKGSEADRQRAGTVTGLAVNIAALLSVMLQPYMPTVSTTIQAQLQLPPPTCSILPTNFLCILPAGHQIGTVSP

LFQKLENDQIESLRQRFSGGQLEESLALKAKAGPKPAAAGTMTTAGPEQIQVLMDEVTKQGNIVRELKAQKADKNQVAAE

VAKLLDLKKQLALAEGKPLETPKGKKKK

>XP_005358044.1 PREDICTED: methionine--tRNA synthetase, cytoplasmic isoform X2 [Microtus ochrogaster]

MRLFLSEGSPGSLPVLAAAARARGRAELLISPVGPEECVVPFLTRPKVPVLQLDSGNYLFSTSAICRYFFLLSGWEQDDL

TNQWLEWEATELQPVLSAALHCLVVQGKKGEEVLGPLRRALTHIDHSLSRQPCPFLAGDTESVADIVLWGALYPLLQDPA

YLPEELGALQSWFQTLSTQEPCQRAAETVLKQQGVLALRPYLQKQPQPRPPEGRAVSNEPEEEELATLSEEDIAMAVTAW

EKGLGSLPPLQPQQHPVLPVAGERNVLITSALPYVNNVPHLGNIIGCVLSADVFARYSRLRQWNTLYLCGTDEYGTATET

KAMEEGLTPQQICDKYHALHADIYRWFNISFDTFGRTTTPQQTKITQDIFQRLLTRGFVLQDTVEQLRCEQCARFLADRF

VEGVCPFCGYEEARGDQCDKCGKLINAIELKKPQCKVCRSCPVVRASRHLFLDLPKLEKRLEDWLGKTLPGSDWTPNARF

IIRSWLRDGLKPRCITRDLKWGTPVPLEGFEDKVFYVWFDATIGYLSITANYTDQWERWWKNPEQVDLYQFMAKDNVPFH

GLVFPCSALGTEDNYTLVKNLIATEYLNYEDGKFSKSRGVGVFGDMAQDTGIPADIWRFYLLYIRPEGQDSAFSWTDMMI

KNNSELLNNLGNFINRAGMFVSKFFGGCVPEMVLTHDDRRLLAHISWELQHYHQLLEKVRIRDALRSILTISRHGNQYIQ

VNEPWKRIKGDEVDRQRAGTVTGVAVNIAALLSVMLQPYMPTVSATIQTQLQLPLPACRILVTNFICTLPAGHRIGTVSP

LFQKLENDQIENLRQRFGGGQAKAPPKSAVAEAVPTAGPQQIQMLMDEVTKQGNIVRELKAQKADKNQVAAEVTKLLDLK

KQLALAEGKPIETPKGKKKK

>XP_010991650.1 PREDICTED: methionine--tRNA synthetase, cytoplasmic isoform X1 [Camelus dromedarius]

MRLFVSEGAPGTLPVLAAAGRAQGRVELLISTVGPEECVVPFLTRPKVPVLQLDSGNYLFSTSAICRYFFLLSGWEQDDL

TNQWLEWEATELQPALSAALYYLVVQGKKGEDVLGPVRRALTHIDHSLSRRSCPFLAGETESLADIVLWGALYPLLQDPV

YLPEELGALNSWFQTLSSQEPCQRAAETVLKQQGVLALRPYLQKQPQPSSFEGRAVSNEPEEEELAALSEEEIALAVAAW

EKGLGSLPPLQPQQSPVLPVAGERNVLITSALPYVNNVPHLGNIIGCVLSADVFARYSRLRQWNTLYLCGTDEYGTATET

KAMEEGLTPQEICDKYHAIHADIYRWFNISFDIFGRTTTPQQTKITQDIFQRLLARGFVLQDTVEQLRCERCARFLADRF

VEGVCPFCGFEEARGDQCDKCGKLINATELKKPQCKICRSCPVVKSSRHLFLDLPKLEKRLEEWLEKTLPGSDWTPNARF

IIRSWLRDGLKPRCITRDLKWGTPVPLEGFEDKVFYVWFDATIGYVSITANYTDQWERWWKNPDQVDLYQFMAKDNVPFH

GLVFPCSVLGAEDNYTLVKNLIATEYLNYEDGKFSKSRGVGVFGDMAQDTGIPADIWRFYLLYIRPEGQDSAFSWTDMLL

KNNSELLNNLGNFINRAGMFVSKFFGGFVPEMVLTSDDRRLLAHITLELQHYHQLLEKVRIRDALRSILTISRHGNQYIQ

VNEPWKRIKGSEADRQRAGTVTGLAVNIAALLSVMLQPYMPTVSTTIQAQLQLPPPTCSILPTNFLCILPAGHQIGTVSP

LFQKLENDQIESLRQRFSGGQLEESLALKAKAGPKPAAAGTMTTAGPEQIQVLMDEVTKQGNIVRELKAQKADKNQVAAE

VAKLLDLKKQLALAEGKPLETPKGKKKK

>XP_007631230.1 PREDICTED: methionine--tRNA synthetase, cytoplasmic isoform X1 [Cricetulus griseus]

MRLFVSEGSPGSLPVLAAAARARGRAELLISTVGPEECVVPFLTRPKVPVLQLDSGNYLFSTSAICRYFFLLSGWEQDDL

TNQWLEWEATELQPVLSAALHCLVVQGKKGEDILGPLREALTHIDHSLSRQHCPFLVGDTESLADIVLWGALYPLLHDPT

YLPEELGALQSWFQTLSTQEPCQRAAETVLKQQGVLALRPYLQKQPQPRPPEGRAVSNEPEEEELATLSEEDILMAVTAW

EKGMGSLPPLQPQQHPVLPVAGERNVLITSALPYVNNVPHLGNIIGCVLSADVFARYSRLRQWNTLYLCGTDEYGTATET

KAMEEGLTPQEICDKYHAIHADVYRWFNISFDTFGRTTTPHQTKITQDIFQRLLTRGFVLQDTVEQLRCEQCARFLADRF

VEGVCPFCGYEEARGDQCDKCGKLINAIELKKPQCKVCRSRPVVRSSQHLFLDLPKLEKRLEDWLGKTLPGSDWTPNARF

ITRSWLRDGLKPRCITRDLKWGTPVPLEGFKDKVFYVWFDATIGYLSITANYTDQWERWWKNPEQVDLYQFMAKDNVPFH

GIVFPCSALGAEDNYTLVKNLIATEYLNYEDGKFSKSRGIGVFGDMAQDTGIPADIWRFYLLYIRPEGQDSAFSWTDMMI

KNNSELLNNLGNFINRAGMFVSKFFGGCVPEMVLTQDDRRLLAHISWELQHYHQLLEKVRIRDALRSILTISRHGNQYIQ

VNEPWKRIKGSEVDRQRAGTVTGVAVNIAALLSVMLQPYMPTVSATIQTQLQLPLLACRVLVTNFICTLPAGHQIGTVSP

LFQKLENDQIENLRQRFGGGQLEESLELKTKGSPKPAVVEEVTTAGPQQIQMLMDEVTKQGNIVRELKAQKADKNQVAAQ

VTKLLELKKQLALAEGKPIETPKGKKKK

>XP_020734092.1 methionine--tRNA synthetase, cytoplasmic isoform X2 [Odocoileus virginianus texanus]

MRLFVSEGAPGSLPVLAAAGRAQGRAELLISTVGPEECVVPFLTRPKVPVLQLDSGNYLFSTSAICRYFFLLSGWEQDDL

TNQWLEWEATELQPALSTALYCLVVQGKKGEDVLGPVRRALMHIDHSLSRRSSPFLAGETESLADIVLWGALYPLLQDPS

YLPEELGALHSWFQTLSSQEPCQRAAETVLKRQGVLALRPYLQKQPQPSSLEGRVVSNEPEEEELATLSEEEIAVAVAAW

EKGLESLPPLRPQQNPVLPVAGERNVLITSALPYVNNVPHLGNIIGCVLSADVFARYSRLRQWNTLYLCGTDEYGTATET

KALEEGLTPQEICDKYHIIHADIYRWFNISFDFFGRTTTPQQTKITQDIFQRLLARGFVLQDTVEQLRCERCARFLADRF

VEGVCPFCGYEEARGDQCDKCGKLINAVELKKPQCKVCRSCPVVRSSRHLFLDLPKLGARVEEWLERTLPGSDWTPNARF

ITRSWLRDGLKPRCITRDLKWGTPVPLEGFEDKVFYVWFDATIGYLSITANYTDQWEKWWKNPEQVNLYQFMAKDNVPFH

GIVFPSSALGAEDNYTLVSHLIATEYLNYEDGKFSKSRGVGVFGDMAQDTGIPADIWRFYLLYIRPEGQDSAFSWTDMLF

KNNSELLNNLGNFINRAGMFVSKFFGGFVPEMVLTSDDQRLLTHVTLELQHYHQLLEKVRIRDALRSILTISRHGNQYIQ

VNEPWKRIRGGEADRQRAGTVTGLAVNIAALLSVMLQPYMPTVSATIQAQLQLPRPACSILPTSFLCTLPAGHQIGTVSP

LFQKLENDQIESLKQRFSGGQAKASPRTAAGPIAAGPQQVQALTEEVAKQGDTVRELKAQKADESRIAAEVARLLDLKKQ

LALAEGKPLGAPKGKKRK

>XP_005358043.1 PREDICTED: methionine--tRNA synthetase, cytoplasmic isoform X1 [Microtus ochrogaster]

MRLFLSEGSPGSLPVLAAAARARGRAELLISPVGPEECVVPFLTRPKVPVLQLDSGNYLFSTSAICRYFFLLSGWEQDDL

TNQWLEWEATELQPVLSAALHCLVVQGKKGEEVLGPLRRALTHIDHSLSRQPCPFLAGDTESVADIVLWGALYPLLQDPA

YLPEELGALQSWFQTLSTQEPCQRAAETVLKQQGVLALRPYLQKQPQPRPPEGRAVSNEPEEEELATLSEEDIAMAVTAW

EKGLGSLPPLQPQQHPVLPVAGERNVLITSALPYVNNVPHLGNIIGCVLSADVFARYSRLRQWNTLYLCGTDEYGTATET

KAMEEGLTPQQICDKYHALHADIYRWFNISFDTFGRTTTPQQTKITQDIFQRLLTRGFVLQDTVEQLRCEQCARFLADRF

VEGVCPFCGYEEARGDQCDKCGKLINAIELKKPQCKVCRSCPVVRASRHLFLDLPKLEKRLEDWLGKTLPGSDWTPNARF

IIRSWLRDGLKPRCITRDLKWGTPVPLEGFEDKVFYVWFDATIGYLSITANYTDQWERWWKNPEQVDLYQFMAKDNVPFH

GLVFPCSALGTEDNYTLVKNLIATEYLNYEDGKFSKSRGVGVFGDMAQDTGIPADIWRFYLLYIRPEGQDSAFSWTDMMI

KNNSELLNNLGNFINRAGMFVSKFFGGCVPEMVLTHDDRRLLAHISWELQHYHQLLEKVRIRDALRSILTISRHGNQYIQ

VNEPWKRIKGDEVDRQRAGTVTGVAVNIAALLSVMLQPYMPTVSATIQTQLQLPLPACRILVTNFICTLPAGHRIGTVSP

LFQKLENDQIENLRQRFGGGQLEESSELKAKAPPKSAVAEAVPTAGPQQIQMLMDEVTKQGNIVRELKAQKADKNQVAAE

VTKLLDLKKQLALAEGKPIETPKGKKKK

>XP_021569486.1 methionine--tRNA synthetase, cytoplasmic isoform X2 [Carlito syrichta]

MKLFVSEGTPGSLPVLAAAERARGRAELLVSTVGPEECVVPFLTQPKVPVLQLDSGNYLFSTSAICRYFFLLSGWEQDDL

TNQWLEWEATELQPALSAALYYLVVQGKKGEEVLGPVRRALTHIDHSLSRQNCPFLAGETESLTDIVLWGALYPLLQDPA

YLPEELSALRSWFQTLSTQEPCQQAAETVLKQQGVLAFRPYLQKQPQPSPPEGKPITNEPEEEELATLSEEEIAMAVAAW

EKGLDSLPPLRPQQNPVLPVDGERNVLITSALPYVNNVPHLGNIIGCVLSADVFARYSRLRQWNTLYLCGTDEYGTATET

KAMEEGLSPQEICDKYHTIHADIYRWFNISFDIFGRTTTPQQTKITQDIFQRLLARGFVLQDTVEQLRCERCARFLADRF

VEGVCPFCRYEEARGDQCDKCGKLINAIELKKPQCKLCRSCPVVKSSRHLFLDLPKLEKRVEEWLGKSLPGSDWTPNARF

ITRSWLRDGLKPRCITRDLKWGTPVPLEGFEDKVFYVWFDATIGYLSITANYTDQWERWWKNPEQVDLYQFMAKDNVPFH

GIVFPCSVLGAEDNYTLVSHLIATEYLNYEDGKFSKSRGVGVFGDMAQDTGIPADIWRFYLLYIRPEGQDSAFSWMDMLL

KNNSELLNNLGNFINRAGMFVSKFFGGCVPEMVLTSDDQRLVAHVTLELRHYHQLLEKVRIRDALRSILTISRHGNQYIQ

VNEPWKQIKGSEADRQRAGTVTGLAVNIAALLSIMLQPYMPMVSATIQAQLQLPPPACSILPTDFLCTLSAGHRIGIVSP

LFQKLENDQIENLRQRFGGGQLEESFKLKAKAAPKPAAEEAVPTARPQQIQALMDAVTKQGNIVRELKAQKADKNQIAAE

VAKLLDLKKHLALAEGKPLETPKGGDI

>XP_003507547.1 PREDICTED: methionine--tRNA synthetase, cytoplasmic isoform X1 [Cricetulus griseus]

MRLFVSEGSPGSLPVLAAAARARGRAELLISTVGPEECVVPFLTRPKVPVLQLDSGNYLFSTSAICRYFFLLSGWEQDDL

TNQWLEWEATELQPVLSAALHCLVVQGKKGEDILGPLREALTHIDHSLSRQHCPFLVGDTESLADIVLWGALYPLLHDPT

YLPEELGALQSWFQTLSTQEPCQRAAETVLKQQGVLALRPYLQKQPQPRPPEGRAVSNEPEEEELATLSEEDILMAVTAW

EKGMGSLPPLQPQQHPVLPVAGERNVLITSALPYVNNVPHLGNIIGCVLSADVFARYSRLRQWNTLYLCGTDEYGTATET

KAMEEGLTPQEICDKYHAIHADVYRWFNISFDTFGRTTTPHQTKITQDIFQRLLTRGFVLQDTVEQLRCEQCARFLADRF

VEGVCPFCGYEEARGDQCDKCGKLINAIELKKPQCKVCRSRPVVRSSQHLFLDLPKLEKRLEDWLGKTLPGSDWTPNARF

ITRSWLRDGLKPRCITRDLKWGTPVPLEGFKDKVFYVWFDATIGYLSITANYTDQWERWWKNPEQVDLYQFMAKDNVPFH

GIVFPCSALGAEDNYTLVKNLIATEYLNYEDGKFSKSRGIGVFGDMAQDTGIPADIWRFYLLYIRPEGQDSAFSWTDMMI

KNNSELLNNLGNFINRAGMFVSKFFGGCVPEMVLTQDDRRLLAHISWELQHYHQLLEKVRIRDALRSILTISRHGNQYIQ

VNEPWKRIKGSEVDRQRAGTVTGVAVNIAALLSVMLQPYMPTVSATIQTQLQLPLLACRVLVTNFICTLTAGHQIGTVSP

LFQKLENDQIENLRQRFGGGQLEESLELKTKGSPKPAVVEEVTTAGPQQIQMLMDEVTKQGNIVRELKAQKADKNQVAAQ

VTKLLELKKQLALAEGKPIETPKGKKKK

>XP_020734090.1 methionine--tRNA synthetase, cytoplasmic isoform X1 [Odocoileus virginianus texanus]

MRLFVSEGAPGSLPVLAAAGRAQGRAELLISTVGPEECVVPFLTRPKVPVLQLDSGNYLFSTSAICRYFFLLSGWEQDDL

TNQWLEWEATELQPALSTALYCLVVQGKKGEDVLGPVRRALMHIDHSLSRRSSPFLAGETESLADIVLWGALYPLLQDPS

YLPEELGALHSWFQTLSSQEPCQRAAETVLKRQGVLALRPYLQKQPQPSSLEGRVVSNEPEEEELATLSEEEIAVAVAAW

EKGLESLPPLRPQQNPVLPVAGERNVLITSALPYVNNVPHLGNIIGCVLSADVFARYSRLRQWNTLYLCGTDEYGTATET

KALEEGLTPQEICDKYHIIHADIYRWFNISFDFFGRTTTPQQTKITQDIFQRLLARGFVLQDTVEQLRCERCARFLADRF

VEGVCPFCGYEEARGDQCDKCGKLINAVELKKPQCKVCRSCPVVRSSRHLFLDLPKLGARVEEWLERTLPGSDWTPNARF

ITRSWLRDGLKPRCITRDLKWGTPVPLEGFEDKVFYVWFDATIGYLSITANYTDQWEKWWKNPEQVNLYQFMAKDNVPFH

GIVFPSSALGAEDNYTLVSHLIATEYLNYEDGKFSKSRGVGVFGDMAQDTGIPADIWRFYLLYIRPEGQDSAFSWTDMLF

KNNSELLNNLGNFINRAGMFVSKFFGGFVPEMVLTSDDQRLLTHVTLELQHYHQLLEKVRIRDALRSILTISRHGNQYIQ

VNEPWKRIRGGEADRQRAGTVTGLAVNIAALLSVMLQPYMPTVSATIQAQLQLPRPACSILPTSFLCTLPAGHQIGTVSP

LFQKLENDQIESLKQRFSGGQLEESLALKAKASPRTAAGPIAAGPQQVQALTEEVAKQGDTVRELKAQKADESRIAAEVA

RLLDLKKQLALAEGKPLGAPKGKKRK

>XP_015983188.1 PREDICTED: methionine--tRNA synthetase, cytoplasmic [Rousettus aegyptiacus]

MRLFVSEGDPGSLPVLAAAGRAQGRAELRISTVGPEERVVPFLTQPKVPVLQLDSGNYLFSTSAICRYFFLLSGWEQDDL

TNQWLEWEVTELQPALSAALYYLVVQGKKGEDVLGPVRRALIHIDNSLSRRSCPFLAGETESLADIVLWGALYPLLQDPT

YLPEELGALHSWFQTLSSQEPCQRAAETVLKQQGVLALRPYLQKQLQPSLLEGKAVSNEPEEEELATLSEEEITIAVTAW

EKGLGSLPPLRPKQTPVLPVAGERNVLITSALPYVNNVPHLGNIIGCVLSADVFARYSRLRQWNTLYLCGTDEYGTATET

KAMEEGLTPQEICDKFHVIHADIYRWFNISFDIFGRTTTPQQTEITQDIFQRLWNRGFVLQDTVEQLRCESCARFLADRF

VEGVCPFCGYEEARGDQCDKCGKLINAIELKNPQCKLCRSCPVVKSSQHLFLDLPKLEDRLEEWLGKTLPGSDWTPNARF

IIRSWLRDGLKPRCITRDLKWGTPVPLEGFENKVFYVWFDATFGYLSITANYTDQWEKWWKNPEQVNLYQFMAKDNVPFH

GLVFPCSALGAEDNYTLVNHLIATEYLNYEDGKFSKSRGVGVFGDMAQDTGIPADIWRFYLLYIRPEGQDSAFSWTDMLL

KNNSELLNNLGNFINRAGMFVSKFFGGYVPEMVLTSDDQRLLAHVTLELHHYHQLLEKVRIRDALRSILTISRHGNQYIQ

VNEPWKRIKGNEADRQRAGTVTGLAVNIAALLSIMLQPYMPTVSTTIAAQLQLPLPACSILSTDFLCTLPAGHQIGTVSP

LFQKLENDQIESLKQRFGGGQAKASRKPTDVETVTTDVETVTTAGPQQIKVLMDEVTKQGNIVRELKAQKADKNQVAAEV

AKLLELKKQLALAEGKPLETPKGKKKK

>XP_006909012.1 methionine--tRNA synthetase, cytoplasmic [Pteropus alecto]

MRLFVSEGSPGSLPVLAAAGRAQGRAELRISTVGPEERVVPFLTQPKVPVLQLDSGNYLFSTSAICRYFFLLSGWEQDDL

TNQWLEWEVTELQPALSAALYYLVVQGKKGEDVLGPVRGALIHIDNSLSRRSCPFLAGETESLADIVLWGALYPLLQDPA

YLPEELGALHSWFQTLSSQEPCQRAAETVLKQQGVLALRPYLQKQLQPSFLEGKAVSNEPEEEELATLSEEEIAVAVTAW

EKGLGSLPPLRPKQTPVLPVAGERNVLITSALPYVNNVPHLGNIIGCVLSADVFARYSRLRQWNTLYLCGTDEYGTATET

KAMEEGLTPQEICDKYHVIHADIYRWFNISFDIFGRTTTPQQTEITQDIFQRLWNRGFVLQDTVEQLRCESCARFLADRF

VEGVCPFCGYEEARGDQCDKCGKLINAIELKKPQCKLCRSCPVVKSSRHLFLDLPKLEDRLEEWLGKTLPGSDWTSNARF

IIRSWLRDGLKPRCITRDLKWGTPVPLEGFEDKVFYVWFDATFGYLSITANYTDQWERWWKNPEQVNLYQFMAKDNVPFH

GLVFPCSALGAEDNYTLVSHLIATEREGHDLSKWFSFILXXXXXXDGKFSKSRGVGVFGDMAQDTGIPADIWRFYLLYIR

PEGQDSAFSWTDMLLKNNSELLNNLGNFINRAGMFVSKFFGGYVPEMVLTSDDQRLLAHVALELQHYHQLLEKVRIRDAL

RSILTISRHGNQYIQVNEPWKRIKGSEADRQRAGTVTGLAVNIAALLSIMLQPYMPTVSTTIAAQLQLPPLACSILSTDF

LCTLPAGHQIGTVSPLFQKLENDQIESLKQRFGGGQAKASPKPTDVETVTTVGLQQIKVLMDEVTKQGNIVRELKTQKAD

KNQVAAEVAKLLDLKKQLALAEGKPLETPKGKKKK

>XP_004379937.2 methionine--tRNA synthetase, cytoplasmic isoform X1 [Trichechus manatus latirostris]

MIVFSGVLPLHDGRNSQLSGSGARNFIQYSISRCALQRPLNSEVGNRFRLHQRGVRGEMRLFVSEGAPGTLSVLAAARRA

RERAELLISTVGPEECVVPFLTGPKVPVLQLDSGNYLFSTSAICRYFFLLSGWEQDDLTNQWLEWEAMELQPALSAALYY

LVIQGKKGEDVLGPVRRALTHIDHSLNHQSCPFLTGETESLADIVLWGALYPLLQDPAYLPEELGALRSWFQTLSTQEPC

QRAAETVLKQQGALALRPYLQKQPQPRPPEGRSVSNEPEEEELTTLSEEEIAMVVTAWEKGLGSLPPLQPQKNPVLPVAG

EKNVLITSALPYVNNVPHLGNIIGCVLSADVFARYSRLRQWNTLYLCGTDEYGTATETKAMEEGLTPKEICDKYHAIHAD

IYRWFNISFDVFGRTTTPQQTKITQAIFQQLLARGFVLQDTVEQLRCEHCSRFLADRFVEGVCPFCGYEEARGDQCDKCG

KLINAIELKKPQCKVCRSCPVVKSSQHLFLDMPKLENRLEEWLGKTLAGSNWTPNARFIIRSWLRDGLKPRCITRDLKWG

TPVPLEGFEDKVFYVWFDATIGYLSITANYTDQWERWWKNPEQVDLYQFMAKDNVPFHGLVFPCTALGTEDSYTLVSHLI

ATEYLNYEDGKFSKSRGVGVFGDMAQDTGIPADIWRFYLLYIRPEGQDSAFSWTDMLLKNNSELLNNLGNFINRAAMFVS

KFFGGCVPEMVLMLDDQRLVAHVTLELQHYHQLLEKVRIRDALRSILTISRHGNQYIQVNEPWKRIKGSEADRQRAGTVT

GLAVNIAALLSVMLQPYMPTVSATIQDQLQLPPPACSILPKNFLCTLPAGHQIGTVSPLFQKLENDQIESLRQRFGGGQA

KTSPKPAVVETVTAAGPQQIQMLMDEVTKQGNIVRELKAQNADNHQVAAEVAKLLELKKQLALAEGKPLDPPKGKKKK

>XP_006757393.1 PREDICTED: methionine--tRNA synthetase, cytoplasmic isoform X2 [Myotis davidii]

MRLFVSEGAPGTLPVLAAAGRAQGRAQLLVSTVGPEECVVPFLTRPKIPVLQLDSGNYLFSTNAICRYFFLLSGSEQDDL

TNQWLEWEATELQPALSAALYYLVVQGKKGEDVLGPIRRALTHIDHSLSRRSCPFLAGETESVADIVLWGALYPLLQDPA

YLPEELGALHSWFQTLSSQEPCQRAAETVLKKQGILALRPYLQKQLQPSFLEGRAVSNEPEEEELATLSEEEIAVAVTAW

EKGLGSLPPLRPKQKPVLPVAGERNVLITSALPYVNNVPHLGNIIGCVLSADVFARYSRLRQWNTLYLCGTDEYGTATET

KAMEEGLTPQEICDKYHVIHADIYRWFNISFDIFGRTTTSQQTEITQDIFQRLWNRGFVLQDTVEQLRCERCARFLADRF

VEGVCPFCGYEEARGDQCDKCGKLINAVELKKPQCKVCRSCPVVRSSRHLFLDLPKLEDRLEEWLGKTVPGSDWTPNARF

IIRSWLRDGLKPRCITRDLKWGTPVPLKGFEDKVFYVWFDATFGYISITANYTDQWEKWWKNPEQVNLYQFMAKDNVPFH

GLVFPCSALGAEDNYTLVSHLIATEYLNYEDGKFSKSRGVGVFVADIWRFYLLYIRPEGQDSAFSWTDMLLKNNSELLNN

LGNFINRAGMFVSKFFGGYVPEMVLTSDDQRLLAHVSLELQHYHQLLEKVRIRDALRSILTISRYGNQYIQVNEPWKRIK

GSEADRQRAGTVTGLAVNIAALLSTMLQPYMPTVSSMIAAQLQLPPPACSILPTDFLCTLPAGHQIGTVSPLFQKLENDQ

IENLRQRFGGGQAKVSPKPAVIETAGPQQIQVLMDEVTKQGNIVRELKAQKADKNQVAAEVAKLLDLKKQLALAEGKPLE

TPKGKKKK

>XP_004583003.1 PREDICTED: methionine--tRNA synthetase, cytoplasmic isoform X2 [Ochotona princeps]

MLEGRFRLHQRGFRGRMRLFVSQGCPGSLPVLAAAGRARGRAELLISTVGPEDCVVPFLTRPKVPVLELDGGNYLFSTSA

ICRYFFLLSGWEQDDLTNQWLEWEATELQPALSAALYYLVVQGKKGEDVLGPVRRALTHLDQSLSRQNSPFLAGETESVA

DIVLWGALCPLLQDPAYLPEELGALRSWFQTLSTQEPCQRAAETVLQQQGAAALRPYLQRQPQPRPTEERPVSNEPEEEE

LATLSEEEIGLAVSAWEKGLESLPPLRPRPNPVLPVAGERNVLITSALPYVNNVPHLGNIIGCVLSADVFARYSRLRQWN

TLYLCGTDEYGTATETKAMEEGLTPQEICDKYHTIHADIYQWFNISFDIFGRTTTPQQTKITQDIFQRLLSRGFVLQDTV

EQLRCENCARFLADRFVEGVCPFCGYEEARGDQCDKCGKLINAVELKKPQCKVCRSSPVVKSSQHLFLDLPKLEERLEAW

LGKTLPGSDWTPNARFIIRSWLRDGLKPRCITRDLKWGTPVPLEGFEDKVFYVWFDATIGYLSITANYTDQWERWWKNPE

QVDLYQFMAKDNVPFHGLVFPCSALGAEDNYTLVKHLIATEYLNYEDGKFSKSRGVGVFGDMAQDTGIPADIWRFYLLYI

RPEGQDSAFSWTDMLLKNNSELLNNLGNFINRAGMFVSKFFGGSVPEMVLTPDDRRLLAHITLELQHYHQLLEKARIRDA

LRSILTISRHGNQYIQVNEPWKLIKGGEADRQRAGTVTGMAVNIAALLSVMLQPYMPTVSATIQTQLQLSSAACSVLPTN

FLCTLPAGHQIGIVSPLFQKLENDQIESLRQRFGGGQAKACSKPAAAETVKTAGPQQIQVLMDEVTKQGNIVRELKAQKA

DKNQVAAEVTKLLDLKKQLALAEGKPLDTPKGKKKK

>EFB28882.1 hypothetical protein PANDA_004122, partial [Ailuropoda melanoleuca]

ECVVPFLTRPKVPVLQLDSGNYLFSTSAICRYFFLLSGWEQDDLTNQWLEWEATELQPALSAALYYLVVQGKKGEDVLGP

VRRALTHIDQSLSRRSCPFLAGETESLADVVLWGALYPLLQDPAYLPEELGALHSWFQTLSSQEPCQRAAETVLKQQGVL

ALRPYLQKQPQPGSFEGRAVSNEPEEEGLAALSEEEIAMAVTAWEKGLESLPPLRPQQTPVLPVAGERNVLITSALPYVN

NVPHLGNIIGCVLSADVFARYSRLRQWNTLYLCGTDEYGTATETKAMEEGLTPQEICDKYHAIHADIYRWFNISFDTFGR

TTTPQQTKITQDIFQRLLTRGFVLQDTVEQLRCERCARFLADRFVEGVCPFCGYEEARGDQCDKCGKLINAVELKKPQCK

VCRSCPVVKSSRHLFLDLPKLEKRLEEWLGKTLPGSDWTPNARFIIRSWLRDGLKPRCITRDLKWGTPVPLEGFEDKVFY

VWFDATIGYLSITANYTDQWERWWKNPEQVNLYQFMAKDNVPFHGLVFPCSALGAEDNYTLVNHLIATEYLNYEDGKFSK

SRGVGVFGDMAQDTGIPADIWRFYLLYIRPEGQDSAFSWTDMLLKNNSELLNNLGNFINRAGMFVSKFFGGYVPEMVLTS

DDRRLLAHVTLELQHYHQLLEKVRIREALRSILTISRHGNQYIQVNEPWKRIKGSEADRQRAGTVTGLAVNIAALLSVML

QPYMPTVGGTIQAQLQLPPPACRVLLTNFVCTLPAGHQIGTVSPLFQKLENDQIESLRQRFGGGQAKAPAAPAVVEAVVT

AAPQQVQVLMDEVTKQGNIVRELKAQKADKNQVAAEVAKLLDLKKQLALAEGKPPETPKGKKKK

>XP_010837629.1 PREDICTED: methionine--tRNA synthetase, cytoplasmic isoform X3 [Bison bison bison]

MKACQGPAGRCVYRECVVPFLTRPKVPVLQLDSGNYLFSTSAICRYFFLLSGWEQDDLTNQWLEWEATELQPALSAALYY

LVVQGKKGEDVLGPVRRALTHIDHSLSRRSCPFLAGETESLADIVLWGALYPLLQDPSYLPEELGALHSWFQTLSSQEPC

QRAAETVLKQQGVLALRPYLQKQPQPSSLEGRLVSNEPEEEELATLSEEEIAVAVAAWEKGLESLPPLRPQQNPVLPVAG

ERNVLITSALPYVNNIPHLGNIIGCVLSADVFARYSRLRQWNTLYLCGTDEYGTATETKAMEEGLTPQEICDKYHVIHAD

IYRWFNISFDFFGRTTTPQQTKITQDIFQRLLARGFVLQDTVEQLRCEHCARFLADRFVEGVCPFCGYEEARGDQCDKCG

KLINAIELKKPQCKVCRSCPVVKSSQHLFLDLPKLAARVEEWLEKTLPGSDWTANARFIIRSWLRDGLKPRCITRDLKWG

TPVPLEGFEDKVFYVWFDATIGYLSITANYTDQWEKWWKNPEQVNLYQFMAKDNVPFHGIVFPSSALGAEDNYTLVSHLI

ATEYLNYEDGKFSKSRGVGVFGDMAQDTGIPADIWRFYLLYIRPEGQDSAFSWTDMLFKNNSELLNNLGNFINRAGMFVS

KFFGGFVPEMVLTSDDQRLLTHITLELQHYHQLLEKVRIRDALRSILTISRHGNQYIQVNEPWKRIKGGEADRQRAGTVT

GLAVNIAALLSVMLQPYMPTVGATIQAQLQLPPPACSILPTNFLCTLPAGHQIGTVSPLFQKLENDQIESLKQRFSGGQL

EESLALKAKASPKTAAGLTTAGPQQIQALTEEVTKQGNIVRELKAQKADKNQIAAEVAKLLDLKKQLALAEGKPLETSKG

KKKK

>XP_017536286.1 PREDICTED: methionine--tRNA synthetase, cytoplasmic isoform X2 [Manis javanica]

MRLFVSEGAPGSLPVLAAAGRAQGRAELLISTVGPEECVVPFLTRPKVPVLQLDSGNYLFSTSAICRYFFLLSGWEQDDL

TNQWLEWEVTELQPALSAALYCLAVQGKKGEDVLGPVRSALTYIDHSLSHHRSCPFLAGETESLADIVLWGALYPLLQDP

AYLPGELGALHSWFQTLSSQEPCRRAAETVLKQQGVVALQPYLQKQPQPNLVERRAVSNEPEEEELASLSEEEIAMALTA

WEKGLGSLPSLKPQQNPVLPVAGEKNVLITSALPYVNNVPHLGNIIGCVLSADVFARYSRLRQWNTLFLCGTDEYGTATE

TKAMEEGLTPQEICDKYHAIHADIYRWFNISFDIFGRTTTPQQTKITQDIFQRLLTRGFVLQDTVEQLRCEHCARFLADR

FVEGVCPFCGYEEARGDQCDKCGKLINATELKKPQCKVCRSRPVVRSSQHLFLDLPKLEKRLEEWLGKTLPNSDWTPNAR

FIIRSWLRDGLKPRCITRDLKWGTPVPLEGFEDKVFYVWFDATIGYLSITANYTDQWEKWWKNPEQVNLYQFMAKDNVPF

HGLVFPCSALGAEDNYTLVNHIIATEYLNYEDGKFSKSRGVGVFGDMAQDTGIPADIWRFYLLYIRPEGQDSAFSWTDML

LKNNSELLNNLGNFINRAGMFVSKFFGGFVPEMVLTSDDQRLLAHVTLELQHYHQLLEKVRIRDALRSILTISRHGNQYI

QVNEPWKRIKGSEADRRRAGTVTGLAVNIAALLSTMLQPYMPTVSATIQAQLQLPAPACSILPTDFPCSLSAGHQIGTVS

PLFQKLENDQIESFRQRFGGSQAKVSPNPAVVETMTTAGPQQIQVLMDEVTRQGNIVRELKAQKADKNQVAVEVAKLLDL

KKQLALAEGKPLDTPKGKKKK

>XP_006757392.1 PREDICTED: methionine--tRNA synthetase, cytoplasmic isoform X1 [Myotis davidii]

MRLFVSEGAPGTLPVLAAAGRAQGRAQLLVSTVGPEECVVPFLTRPKIPVLQLDSGNYLFSTNAICRYFFLLSGSEQDDL

TNQWLEWEATELQPALSAALYYLVVQGKKGEDVLGPIRRALTHIDHSLSRRSCPFLAGETESVADIVLWGALYPLLQDPA

YLPEELGALHSWFQTLSSQEPCQRAAETVLKKQGILALRPYLQKQLQPSFLEGRAVSNEPEEEELATLSEEEIAVAVTAW

EKGLGSLPPLRPKQKPVLPVAGERNVLITSALPYVNNVPHLGNIIGCVLSADVFARYSRLRQWNTLYLCGTDEYGTATET

KAMEEGLTPQEICDKYHVIHADIYRWFNISFDIFGRTTTSQQTEITQDIFQRLWNRGFVLQDTVEQLRCERCARFLADRF

VEGVCPFCGYEEARGDQCDKCGKLINAVELKKPQCKVCRSCPVVRSSRHLFLDLPKLEDRLEEWLGKTVPGSDWTPNARF

IIRSWLRDGLKPRCITRDLKWGTPVPLKGFEDKVFYVWFDATFGYISITANYTDQWEKWWKNPEQVNLYQFMAKDNVPFH

GLVFPCSALGAEDNYTLVSHLIATEYLNYEDGKFSKSRGVGVFVADIWRFYLLYIRPEGQDSAFSWTDMLLKNNSELLNN

LGNFINRAGMFVSKFFGGYVPEMVLTSDDQRLLAHVSLELQHYHQLLEKVRIRDALRSILTISRYGNQYIQVNEPWKRIK

GSEADRQRAGTVTGLAVNIAALLSTMLQPYMPTVSSMIAAQLQLPPPACSILPTDFLCTLPAGHQIGTVSPLFQKLENDQ

IENLRQRFGGGQLEESLVLKAKVSPKPAVIETAGPQQIQVLMDEVTKQGNIVRELKAQKADKNQVAAEVAKLLDLKKQLA

LAEGKPLETPKGKKKK

>XP_004583004.1 PREDICTED: methionine--tRNA synthetase, cytoplasmic isoform X1 [Ochotona princeps]

MRLFVSQGCPGSLPVLAAAGRARGRAELLISTVGPEDCVVPFLTRPKVPVLELDGGNYLFSTSAICRYFFLLSGWEQDDL

TNQWLEWEATELQPALSAALYYLVVQGKKGEDVLGPVRRALTHLDQSLSRQNSPFLAGETESVADIVLWGALCPLLQDPA

YLPEELGALRSWFQTLSTQEPCQRAAETVLQQQGAAALRPYLQRQPQPRPTEERPVSNEPEEEELATLSEEEIGLAVSAW

EKGLESLPPLRPRPNPVLPVAGERNVLITSALPYVNNVPHLGNIIGCVLSADVFARYSRLRQWNTLYLCGTDEYGTATET

KAMEEGLTPQEICDKYHTIHADIYQWFNISFDIFGRTTTPQQTKITQDIFQRLLSRGFVLQDTVEQLRCENCARFLADRF

VEGVCPFCGYEEARGDQCDKCGKLINAVELKKPQCKVCRSSPVVKSSQHLFLDLPKLEERLEAWLGKTLPGSDWTPNARF

IIRSWLRDGLKPRCITRDLKWGTPVPLEGFEDKVFYVWFDATIGYLSITANYTDQWERWWKNPEQVDLYQFMAKDNVPFH

GLVFPCSALGAEDNYTLVKHLIATEYLNYEDGKFSKSRGVGVFGDMAQDTGIPADIWRFYLLYIRPEGQDSAFSWTDMLL

KNNSELLNNLGNFINRAGMFVSKFFGGSVPEMVLTPDDRRLLAHITLELQHYHQLLEKARIRDALRSILTISRHGNQYIQ

VNEPWKLIKGGEADRQRAGTVTGMAVNIAALLSVMLQPYMPTVSATIQTQLQLSSAACSVLPTNFLCTLPAGHQIGIVSP

LFQKLENDQIESLRQRFGGGQLEESLEFKAKACSKPAAAETVKTAGPQQIQVLMDEVTKQGNIVRELKAQKADKNQVAAE

VTKLLDLKKQLALAEGKPLDTPKGKKKK

>XP_023590307.1 methionine--tRNA synthetase, cytoplasmic isoform X2 [Trichechus manatus latirostris]

MRLFVSEGAPGTLSVLAAARRARERAELLISTVGPEECVVPFLTGPKVPVLQLDSGNYLFSTSAICRYFFLLSGWEQDDL

TNQWLEWEAMELQPALSAALYYLVIQGKKGEDVLGPVRRALTHIDHSLNHQSCPFLTGETESLADIVLWGALYPLLQDPA

YLPEELGALRSWFQTLSTQEPCQRAAETVLKQQGALALRPYLQKQPQPRPPEGRSVSNEPEEEELTTLSEEEIAMVVTAW

EKGLGSLPPLQPQKNPVLPVAGEKNVLITSALPYVNNVPHLGNIIGCVLSADVFARYSRLRQWNTLYLCGTDEYGTATET

KAMEEGLTPKEICDKYHAIHADIYRWFNISFDVFGRTTTPQQTKITQAIFQQLLARGFVLQDTVEQLRCEHCSRFLADRF

VEGVCPFCGYEEARGDQCDKCGKLINAIELKKPQCKVCRSCPVVKSSQHLFLDMPKLENRLEEWLGKTLAGSNWTPNARF

IIRSWLRDGLKPRCITRDLKWGTPVPLEGFEDKVFYVWFDATIGYLSITANYTDQWERWWKNPEQVDLYQFMAKDNVPFH

GLVFPCTALGTEDSYTLVSHLIATEYLNYEDGKFSKSRGVGVFGDMAQDTGIPADIWRFYLLYIRPEGQDSAFSWTDMLL

KNNSELLNNLGNFINRAAMFVSKFFGGCVPEMVLMLDDQRLVAHVTLELQHYHQLLEKVRIRDALRSILTISRHGNQYIQ

VNEPWKRIKGSEADRQRAGTVTGLAVNIAALLSVMLQPYMPTVSATIQDQLQLPPPACSILPKNFLCTLPAGHQIGTVSP

LFQKLENDQIESLRQRFGGGQLEEPLELKAKTSPKPAVVETVTAAGPQQIQMLMDEVTKQGNIVRELKAQNADNHQVAAE

VAKLLELKKQLALAEGKPLDPPKGKKKK

>XP_006218009.1 PREDICTED: methionine--tRNA synthetase, cytoplasmic isoform X2 [Vicugna pacos]

MRLFVSEGAPGSLPVLAAAGRAQGRAELLISTVGPEECVVPFLTRPKVPVLQLDSGNYLFSTSAICRYFFLLSGWEQDDL

TNQWLEWEATELQPALSAALYYLVVQGKKGEDVLGPVRRALTHIDHSLSRRSCPFLAGETESLADIVLWGALYPLLQDPA

YLPEELGALNSWFQTLSSQEPCQRAAEMVLKQQGVLALRPYLQKQPQPSLFEGRAVSNEPEEEELAALSEEEIALAVAAW

EKGLGSLPPLQPQQSPVLPVAGERNVLITSALPYVNNVPHLGNIIGCVLSADVFARYSRLRRWNTLYLCGTDEYGTATET

KAMEEGLTPQEICDKYHAIHADIYRWFNISFDIFGRTTTPQQTKITQDIFQRLLARGFVLQDTVEQLRCERCARFLADRF

VEGVCPFCGFEEARGDQCDKCGKLINATELKKPQCKICRSCPVVKSSRHLFLDLPKLEKRLEEWLEKTLPGSDWTPNARF

IIRSWLRDGLKPRCITRDLKWGTPVPLEGFEDKVFYVWFDATIGYVSITANYTDQWERWWKNPEQVDLYQFMAKDNVPFH

GLVFPCSVLGAEDNYTLVKNLIATEYLNYEDGKFSKSRGVGVFGDMAQDTGIPADIWRFYLLYIRPEGQDSAFCWTDMLL

KNNSELLNNLGNFINRAGMFVFKFFGGFVPEMVLTSDDRRLLAHVTLELQHYHQLLEKVRIRDALRSILTISRHGNQYIQ

VNEPWKRIKGSEADRQRAGTVTGLAVNIAALLSVMLQPYMPTVSTTIQAQLQLPPPACSILPTNFLCTLPAGHQIGTVSP

LFQKLENDQIENLRQRFSGGQAKAGPKPAAVETMTTAGPEQIQVLMDEVTKQGNIVRELKAQKADKNQVAAEVAKLLDLK

KQLALAEGKPLETPKGKKKK

>XP_012014778.1 PREDICTED: methionine--tRNA synthetase, cytoplasmic isoform X2 [Ovis aries musimon]

MKACRGPAGRCVCRECVVPFLTRPKVPVLQLDSGNYLFSTSAICRYFFLLSGWEQDDLTNQWLEWEATELQPALSAALYY

LVVQGKKGEDVLGPVRRALMHIDHSLSRRSCPFLAGETESLADIVLWGALYPLLQDPSYLPEELGALHSWFQTLSSQEPC

QRAAETVLKQQGVLALRPYLQKQPQPGSLEGRLISNEPEEEELATLSEEEIAVAVAAWEKGLESLPPLRPQQNPVLPVAG

ERNVLITSALPYVNNVPHLGNIIGCVLSADVFARYSRLRQWNTLYLCGTDEYGTATETKAMEEGLTPQEICDKYHVIHAD

IYRWFNISFDFFGRTTTPQQTKITQDIFQRLLARGFVLQDTVEQLRCEHCARFLADRFVEGVCPFCGYEEARGDQCDKCG

KLINAIELKKPQCKVCRSCPVVKSSQHLFLDLPKLGARVEEWLEKTLPGSDWTPNARFIIRSWLRDGLKPRCITRDLKWG

TPVPLEGFEDKVFYVWFDATIGYLSITANYTDQWEKWWKNPEQVNLYQFMAKDNVPFHGIVFPSSALGAEDNYTLVSHLI

ATEYLNYEDGKFSKSRGVGVFGDMAQDTGIPADIWRFYLLYIRPEGQDSAFSWTDMLFKNNSELLNNLGNFINRAGMFVS

KFFGGFVPEMVLTSDDQRLLTHVTLELQHYHQLLEKVRIRDALRSILTISRHGNQYIQVNEPWKRIKGGEADRQRAGTVT

GLAVNIAALLSVMLQPYMPTVSATIQAQLQLPPPARSILPTNFLCTLPAGHQIGTVSPLFQKLENDQIESLKQRFSGGQA

KASPKTAAGPQQIQALTEEVTKQGNIVRELKAQKADKNQIAAEVAKLLDLKKQLAQAEGKPLETPKGKKKK

>XP_014950268.1 PREDICTED: methionine--tRNA synthetase, cytoplasmic isoform X2 [Ovis aries]

MKACRGPAGRCVCRECVVPFLTRPKVPVLQLDSGNYLFSTSAICRYFFLLSGWEQDDLTNQWLEWEATELQPALSAALYY

LVVQGKKGEDVLGPVRRALMHIDHSLSRRSCPFLAGETESLADIVLWGALYPLLQDPSYLPEELGALHSWFQTLSSQEPC

QRAAETVLKQQGVLALRPYLQKQPQPGSLEGRLISNEPEEEELATLSEEEIAVAVAAWEKGLESLPPLRPQQNPVLPVAG

ERNVLITSALPYVNNVPHLGNIIGCVLSADVFARYSRLRQWNTLYLCGTDEYGTATETKAMEEGLTPQEICDKYHVIHAD

IYRWFNISFDFFGRTTTPQQTKITQDIFQRLLARGFVLQDTVEQLRCEHCARFLADRFVEGVCPFCGYEEARGDQCDKCG

KLINAIELKKPQCKVCRSCPVVKSSQHLFLDLPKLGARVEEWLEKTLPGSDWTPNARFIIRSWLRDGLKPRCITRDLKWG

TPVPLEGFEDKVFYVWFDATIGYLSITANYTDQWEKWWKNPEQVNLYQFMAKDNVPFHGIVFPSSALGAEDNYTLVSHLI

ATEYLNYEDGKFSKSRGVGVFGDMAQDTGIPADIWRFYLLYIRPEGQDSAFSWTDMLFKNNSELLNNLGNFINRAGMFVS

KFFGGFVPEMVLTSDDQRLLTHVTLELQHYHQLLEKVRIRDALRSILTISRHGNQYIQVNEPWKRIKGGEADRQRAGTVT

GLAVNIAALLSVMLQPYMPTVSATIQAQLQLPPPARSILPTNFLCTLPAGHQIGTVSPLFQKLENDQIESLKQRFSGGQA

RASPKTAAGPQQIQALTEEVTKQGNIVRELKAQKADKNQIAAEVAKLLDLKKQLAQAEGKPLETPKGKKKK

>XP_021060827.1 methionine--tRNA synthetase, cytoplasmic isoform X2 [Mus pahari]

MRLFVSEGSPGSLPVLAAAARARGRAELLISTVGPEECVVPFLNRPKVPVLQLDSGNYLFSPSAICRYFFLLCGWEQDDL

TNQWLEWEATELQPVLSAALHCLVVQGKKGEDVLGPLRRVLTHMDHSLSRQNCPFLAGDTESLADIVLWGALYPLLQDPA

YLPEELGALQSWFQTLSTQEPCQRAAETVLKQQGVLALRPYLQKQPQPQPPPPEGRAVSNEPEEEELATLSEEDIVTAVA

AWEKGLESLPPLRLQQHPVLPVPGERNVLITSALPYVNNVPHLGNIIGCVLSADVFARYCRLRQWNTLYLCGTDEYGTAT

ETKAMEEGLTPQEICDKYHAIHADIYRWFSISFDTFGRTTTPQQTKITQDIFQRLLTRGFVLQDTVEQLRCERCARFLAD

RFVEGVCPFCGYEEARGDQCDKCGKLINAIELKKPQCKICRSCPVVKSSRHLFLDLPKLEKRLEDWLGKTVPGSDWTPNA

RFIIRSWLRDGLKPRCITRDLKWGTPVPLEGFEDKVFYVWFDATIGYVSITANYTDQWEKWWKNPEQVDLYQFMAKDNVP

FHGLVFPCSALGAEDNYTLVKHIIATEYLNYEDGKFSKSRGIGVFGDMAQDTGIPADIWRFYLLYIRPEGQDSAFSWTDL

LIKNNSELLNNLGNFINRAGMFVSKFFGGCVPEMALTPDDRRLVAHISWELQRYHQLLEKVRIRDALRSILTISRHGNQY

IQVNEPWKRIKGGEMDRQRAGTVTGMAVNMAALLSVMLQPYMPTVSSTIQTQLQLPPAACRILATSFVCTLPAGHRIGTV

SPLFQKLENDQIENLRQRFGGGQAKGSPKPAAVEAVTTAGSQHIQRLVDEVTKQGNIVRELKAQKADKNQVAAEVAKLLD

LKKQLALAEGKPIETPKGKKKK

>XP_015106412.1 PREDICTED: methionine--tRNA synthetase, cytoplasmic isoform X1 [Vicugna pacos]

MRLFVSEGAPGSLPVLAAAGRAQGRAELLISTVGPEECVVPFLTRPKVPVLQLDSGNYLFSTSAICRYFFLLSGWEQDDL

TNQWLEWEATELQPALSAALYYLVVQGKKGEDVLGPVRRALTHIDHSLSRRSCPFLAGETESLADIVLWGALYPLLQDPA

YLPEELGALNSWFQTLSSQEPCQRAAEMVLKQQGVLALRPYLQKQPQPSLFEGRAVSNEPEEEELAALSEEEIALAVAAW

EKGLGSLPPLQPQQSPVLPVAGERNVLITSALPYVNNVPHLGNIIGCVLSADVFARYSRLRRWNTLYLCGTDEYGTATET

KAMEEGLTPQEICDKYHAIHADIYRWFNISFDIFGRTTTPQQTKITQDIFQRLLARGFVLQDTVEQLRCERCARFLADRF

VEGVCPFCGFEEARGDQCDKCGKLINATELKKPQCKICRSCPVVKSSRHLFLDLPKLEKRLEEWLEKTLPGSDWTPNARF

IIRSWLRDGLKPRCITRDLKWGTPVPLEGFEDKVFYVWFDATIGYVSITANYTDQWERWWKNPEQVDLYQFMAKDNVPFH

GLVFPCSVLGAEDNYTLVKNLIATEYLNYEDGKFSKSRGVGVFGDMAQDTGIPADIWRFYLLYIRPEGQDSAFCWTDMLL

KNNSELLNNLGNFINRAGMFVFKFFGGFVPEMVLTSDDRRLLAHVTLELQHYHQLLEKVRIRDALRSILTISRHGNQYIQ

VNEPWKRIKGSEADRQRAGTVTGLAVNIAALLSVMLQPYMPTVSTTIQAQLQLPPPACSILPTNFLCTLPAGHQIGTVSP

LFQKLENDQIENLRQRFSGGQLEESLALKAKAGPKPAAVETMTTAGPEQIQVLMDEVTKQGNIVRELKAQKADKNQVAAE

VAKLLDLKKQLALAEGKPLETPKGKKKK

>XP_017536285.1 PREDICTED: methionine--tRNA synthetase, cytoplasmic isoform X1 [Manis javanica]

MRLFVSEGAPGSLPVLAAAGRAQGRAELLISTVGPEECVVPFLTRPKVPVLQLDSGNYLFSTSAICRYFFLLSGWEQDDL

TNQWLEWEVTELQPALSAALYCLAVQGKKGEDVLGPVRSALTYIDHSLSHHRSCPFLAGETESLADIVLWGALYPLLQDP

AYLPGELGALHSWFQTLSSQEPCRRAAETVLKQQGVVALQPYLQKQPQPNLVERRAVSNEPEEEELASLSEEEIAMALTA

WEKGLGSLPSLKPQQNPVLPVAGEKNVLITSALPYVNNVPHLGNIIGCVLSADVFARYSRLRQWNTLFLCGTDEYGTATE

TKAMEEGLTPQEICDKYHAIHADIYRWFNISFDIFGRTTTPQQTKITQDIFQRLLTRGFVLQDTVEQLRCEHCARFLADR

FVEGVCPFCGYEEARGDQCDKCGKLINATELKKPQCKVCRSRPVVRSSQHLFLDLPKLEKRLEEWLGKTLPNSDWTPNAR

FIIRSWLRDGLKPRCITRDLKWGTPVPLEGFEDKVFYVWFDATIGYLSITANYTDQWEKWWKNPEQVNLYQFMAKDNVPF

HGLVFPCSALGAEDNYTLVNHIIATEYLNYEDGKFSKSRGVGVFGDMAQDTGIPADIWRFYLLYIRPEGQDSAFSWTDML

LKNNSELLNNLGNFINRAGMFVSKFFGGFVPEMVLTSDDQRLLAHVTLELQHYHQLLEKVRIRDALRSILTISRHGNQYI

QVNEPWKRIKGSEADRRRAGTVTGLAVNIAALLSTMLQPYMPTVSATIQAQLQLPAPACSILPTDFPCSLSAGHQIGTVS

PLFQKLENDQIESFRQRFGGSQLEESSELKAKVSPNPAVVETMTTAGPQQIQVLMDEVTRQGNIVRELKAQKADKNQVAV

EVAKLLDLKKQLALAEGKPLDTPKGKKKK

>XP_021060826.1 methionine--tRNA synthetase, cytoplasmic isoform X1 [Mus pahari]

MRLFVSEGSPGSLPVLAAAARARGRAELLISTVGPEECVVPFLNRPKVPVLQLDSGNYLFSPSAICRYFFLLCGWEQDDL

TNQWLEWEATELQPVLSAALHCLVVQGKKGEDVLGPLRRVLTHMDHSLSRQNCPFLAGDTESLADIVLWGALYPLLQDPA

YLPEELGALQSWFQTLSTQEPCQRAAETVLKQQGVLALRPYLQKQPQPQPPPPEGRAVSNEPEEEELATLSEEDIVTAVA

AWEKGLESLPPLRLQQHPVLPVPGERNVLITSALPYVNNVPHLGNIIGCVLSADVFARYCRLRQWNTLYLCGTDEYGTAT

ETKAMEEGLTPQEICDKYHAIHADIYRWFSISFDTFGRTTTPQQTKITQDIFQRLLTRGFVLQDTVEQLRCERCARFLAD

RFVEGVCPFCGYEEARGDQCDKCGKLINAIELKKPQCKICRSCPVVKSSRHLFLDLPKLEKRLEDWLGKTVPGSDWTPNA

RFIIRSWLRDGLKPRCITRDLKWGTPVPLEGFEDKVFYVWFDATIGYVSITANYTDQWEKWWKNPEQVDLYQFMAKDNVP

FHGLVFPCSALGAEDNYTLVKHIIATEYLNYEDGKFSKSRGIGVFGDMAQDTGIPADIWRFYLLYIRPEGQDSAFSWTDL

LIKNNSELLNNLGNFINRAGMFVSKFFGGCVPEMALTPDDRRLVAHISWELQRYHQLLEKVRIRDALRSILTISRHGNQY

IQVNEPWKRIKGGEMDRQRAGTVTGMAVNMAALLSVMLQPYMPTVSSTIQTQLQLPPAACRILATSFVCTLPAGHRIGTV

SPLFQKLENDQIENLRQRFGGGQLEESLELKAKGSPKPAAVEAVTTAGSQHIQRLVDEVTKQGNIVRELKAQKADKNQVA

AEVAKLLDLKKQLALAEGKPIETPKGKKKK

>NP_001121131.1 methionine--tRNA synthetase, cytoplasmic [Rattus norvegicus]

MRLFVSEGSPGSLPVLAAAARARGRAELLISTVGPEECVVPFLTRPKVPVLQLDSGNYLFSASAICRYFFLLCGWEQDDL

TNQWLEWEATELQPVLSAALYCLVVQGKKGEDVLGPLRRVLTHIDHSLSRQNCPFLAGDTESLADIVLWGALYPLLQDPA

YLPEELGALQSWFQTLSTQEPCQRAAETVLKQQGVLALRPYLQKQPQPQPLPPEGRAVSNEPEEEELATLSEEDIIAAVA

AWEKGLENLPPLQPQQHPVLPVPGERNVLITSALPYVNNVPHLGNIIGCVLSADVFARYSRLRQWNTLYLCGTDEYGTAT

ETKAMEEGLTPQEICDKYHAIHVDIYRWFNISFDTFGRTTTPLQTKITQDIFQRLLTRGFVLQDTVEQLRCEQCARFLAD

RFVEGVCPFCGYEEARGDQCDKCGKLINAIELKRPQCKVCRSHPVVKSSKHLFLDLPKLEKRLEDWLGKTMPGSDWTPNA

RFIIRSWLRDGLKPRCITRDLKWGTPVPLEGFEDKVFYVWFDATIGYLSITANYTDQWERWWKNPEQVDLYQFMAKDNVP

FHGLVFPCSALGAEDNYTLVKNLIATEYLNYEDGKFSKSRGIGVFGDMAQDTGIPADIWRFYLLYIRPEGQDSAFSWTDL

LIKNNSELLNNLGNFINRAGMFVSKFFGGCVPEMVLTHDDRRLVAHVSWELQRYHQLLEKVRIRDALRSILTISRHGNQY

IQVNEPWKRIKGDEMDRQRAGTVTGMAVNIAALLSVMLQPYMPTVSSTIQTQLQLPEAACRILATSFICTLPAGHRIGTV

SPLFQKLENDQIENLRQRFGGGQAKGSPKPAVVEAVTTAGSQDIQVLVDEVTKQGNIVRELKAQKADKNQVAAEVAKLLD

LKKQLALAEGKPIETPKGKKKK

>XP_006973359.1 PREDICTED: methionine--tRNA synthetase, cytoplasmic isoform X2 [Peromyscus maniculatus bairdii]

MRLFVSEGSPGSLPVLAAAARARGQAELLISTVGPEECVVPFLTRPKVPVLQLNSGNYLFSTSAICRYFFLLSGWEQDDL

TNQWLEWEATELQPVLSAALHCLVVQGKRGEDVLGPLRRALTHIDHSLSRQNCPFLAGDTESLADIVLWGALYPLLQDPA

YLPEELGALQSWFQTLSTQEPCRQAAETVLKQQGALALRPYLQKQPQPQPRPPEGRAVSNEPEEEELATLSEEDIVMAVT

AWEEGLGSLPPPQPRPQPVLPVAGERNVLITSALPYVNNVPHLGNIIGCVLSADVFARYSRLRQWNTLYLCGTDEYGTAT

ETKAMEEGLTPQEICDKYHAIHADIYRWFNISFDTFGRTTTPQQTKITQDIFQRLLTRGFVLEDTVEQLRCEQCARFLAD

RFVEGVCPFCGYEEARGDQCDKCGKLINAIELKKPQCKVCRSRPVVRSSQHLFLDLPKLEKRLEDWLGKTLPGSDWTPNA

RFIIRSWLRDGLKPRCITRDLKWGTPVPLEGFEDKVFYVWFDATIGYLSITANYTDQWERWWKNPEQVDLYQFMAKDNVP

FHGLVFPCSALGAEDNYTLVKHLIATEYLNYEDGKFSKSRGVGVFGDMAQDTGIPADIWRFYLLYIRPEGQDSAFSWTDM

MTKNNSELLNNLGNFINRAGMFVSKFFGGCVPEMVLAPEDRRLLAHVSWELQHYHQLLEKVRIRDALRSILTISRHGNQY

IQVNEPWKRIKGSEVDRQRAGTVTGVAVNIAALLSVMLQPYMPTVSATIQTQLQLPRPACRVLLTNFICTLPAGHQIGTV

SPLFQKLENDQIENLKQRFGGGQAKGSPKPAAVEAVTTAGPVQIQMLMEEVTKQGNIVRELKAQKADKNQVAAEVTKLLD

LKKQLALAEGKPVETPKGKKKK

>XP_003405202.1 methionine--tRNA synthetase, cytoplasmic isoform X3 [Loxodonta africana]

MRLFVSEGAPGALPVLAAASRARERAELLISTVGPEECVVPFLSGPKIPVLQLDSGNYLFSTSAICRYFFLLSGWEQDDL

TNQWLEWEAVELQPVLSAALYYLVIQGKKGEDVLCPVRKALTHIDHSLSRQSCPFLTGETESLADIVLWGALYPLLQDPA

YLPEELGALCSWFQMLSTQEPCQQAAETVLKQQGALALRPYLQKQPQPRPREGKSVSNEPEEEELTTLSEEEIAMAVTAW

EKGLGGLPPLRPQKNPVLPVAGEKNVLITSALPYVNNVPHLGNIIGCVLSADVFARYSRLRQWNTLYLCGTDEYGTATET

KAMEEGLTPQEICDKYHVIHADIYRWFNISFDIFGRTTTQQQTEITQDIFQRLLARGFVLQDTVEQLRCEHCARFLADRF

VEGVCPFCGYEEARGDQCDKCGKLINAIELKKPQCKVCRSPPVVKSSQHLFLDLPKLENLLETWLEKVLASSDWTPNARF

IIRSWLRDGLKPRCITRDLKWGTPVPLEGFEDKVFYVWFDATIGYLSITANYTDQWERWWKNPEQVDLYQFMAKDNVPFH

GLVFPCSALGTEDNYTLVSHLIATEYLNYEDGKFSKSRGVGVFGDMAQDTGIPADIWRFYLLYIRPEGQDSAFSWTDMLL

KNNSELLNNLGNFINRAGMFVSKFFGGYVPEMVLTLDDQRLLAHVTLELQHYHQLLEKVRIRDALRSILTISRHGNQYIQ

VNEPWKWIKGSEADRQRAGTVTGLAVNIAALLSVMLQPYMPTVSATIQDQLQLPPPACSVLPTNFLCTLPAGHQIGTVSP

LFQKLENDQIESLRQRFGGGQAKASPKPAVVETVITVGPQQIQVLMDEVTKQGNIVRELKAQKADNNQVAAEVAKLLELK

KQLALAEGKPLDPPKGKKKK

>XP_021513528.1 methionine--tRNA synthetase, cytoplasmic isoform X2 [Meriones unguiculatus]

MRLFVSEGSPGSLPVLAAAAKARGRAELLISNVGPEECVVPFLTQPKVPALQLESGNYLFSASAICRYFFLLSGWDQDDL

TNQWLEWEATELQPVLSAALHCLVVQGKKGEDVLGPLRRALTHIDHSLSRQNCPFLAGDTESLADIVLWGALYPLLQDPA

YLPEELSSLQSWFHTLSTQEPCQRAAETVLKQQGVLALRPYLQKQPQPQARPSEGRPVSNEPEEEELATLSEEDIIIAVN

AWEKGLDSLPPLQPQQYPVLPVAGERNVLITSALPYVNNVPHLGNIIGCVLSADVFARYSRLRQWNTLYVCGTDEYGTAT

ETKAMEEGLTPQEICDKYHAIHADIYRWFNISFDTFGRTTTPQQTKISQDIFQRLLTRGFVLQDTVEQLRCEQCARFLAD

RFVEGVCPFCGYEEARGDQCDKCGKLINAIELKKPQCKVCRSHPVVESSQHLFLDLPKLEKRLEDWLGRTLPGSDWTPNA

RFITRSWLRDGLKARCITRDLKWGTPVPLEGFEDKVFYVWFDAPIGYLSITANYTDQWERWWKNPEQVDLYQFMAKDNVP

FHGIIFPCSALGAEDNYTLVKHLIATEYLNYEDGKFSKSRGVGVFGDMAQDTGIPADIWRFYLLYIRPEGQDSAFSWTDM

LTKNNSELLNNLGNFINRAGMFVSKFFGGCVPDMVLTQDDRRMLAHISWELQHYHQLLDKVRIRDALRRILTISRHGNQY

IQVNEPWKRIKGGEVDRQRAGTVTGVAVNIAALLSVMLHPYMPTVSSTIQTQLQLPLPACRVLLTHFTCTLPAGHRIGTV

SPLFQKLENDQIENLRQRFGGGQAKGPPRPVVVEPVTAPGPLQTQMLMDEVTRQGNIVRELKAQKADKNQVAAEVAKLLD

LKKQLALAEGKPIETSKGKKKK

>XP_006241513.1 PREDICTED: methionine--tRNA synthetase, cytoplasmic isoform X1 [Rattus norvegicus]

MRLFVSEGSPGSLPVLAAAARARGRAELLISTVGPEECVVPFLTRPKVPVLQLDSGNYLFSASAICRYFFLLCGWEQDDL

TNQWLEWEATELQPVLSAALYCLVVQGKKGEDVLGPLRRVLTHIDHSLSRQNCPFLAGDTESLADIVLWGALYPLLQDPA

YLPEELGALQSWFQTLSTQEPCQRAAETVLKQQGVLALRPYLQKQPQPQPLPPEGRAVSNEPEEEELATLSEEDIIAAVA

AWEKGLENLPPLQPQQHPVLPVPGERNVLITSALPYVNNVPHLGNIIGCVLSADVFARYSRLRQWNTLYLCGTDEYGTAT

ETKAMEEGLTPQEICDKYHAIHVDIYRWFNISFDTFGRTTTPLQTKITQDIFQRLLTRGFVLQDTVEQLRCEQCARFLAD

RFVEGVCPFCGYEEARGDQCDKCGKLINAIELKRPQCKVCRSHPVVKSSKHLFLDLPKLEKRLEDWLGKTMPGSDWTPNA

RFIIRSWLRDGLKPRCITRDLKWGTPVPLEGFEDKVFYVWFDATIGYLSITANYTDQWERWWKNPEQVDLYQFMAKDNVP

FHGLVFPCSALGAEDNYTLVKNLIATEYLNYEDGKFSKSRGIGVFGDMAQDTGIPADIWRFYLLYIRPEGQDSAFSWTDL

LIKNNSELLNNLGNFINRAGMFVSKFFGGCVPEMVLTHDDRRLVAHVSWELQRYHQLLEKVRIRDALRSILTISRHGNQY

IQVNEPWKRIKGDEMDRQRAGTVTGMAVNIAALLSVMLQPYMPTVSSTIQTQLQLPEAACRILATSFICTLPAGHRIGTV

SPLFQKLENDQIENLRQRFGGGQLEESLELQAKGSPKPAVVEAVTTAGSQDIQVLVDEVTKQGNIVRELKAQKADKNQVA

AEVAKLLDLKKQLALAEGKPIETPKGKKKK

>XP_006973358.1 PREDICTED: methionine--tRNA synthetase, cytoplasmic isoform X1 [Peromyscus maniculatus bairdii]

MRLFVSEGSPGSLPVLAAAARARGQAELLISTVGPEECVVPFLTRPKVPVLQLNSGNYLFSTSAICRYFFLLSGWEQDDL

TNQWLEWEATELQPVLSAALHCLVVQGKRGEDVLGPLRRALTHIDHSLSRQNCPFLAGDTESLADIVLWGALYPLLQDPA

YLPEELGALQSWFQTLSTQEPCRQAAETVLKQQGALALRPYLQKQPQPQPRPPEGRAVSNEPEEEELATLSEEDIVMAVT

AWEEGLGSLPPPQPRPQPVLPVAGERNVLITSALPYVNNVPHLGNIIGCVLSADVFARYSRLRQWNTLYLCGTDEYGTAT

ETKAMEEGLTPQEICDKYHAIHADIYRWFNISFDTFGRTTTPQQTKITQDIFQRLLTRGFVLEDTVEQLRCEQCARFLAD

RFVEGVCPFCGYEEARGDQCDKCGKLINAIELKKPQCKVCRSRPVVRSSQHLFLDLPKLEKRLEDWLGKTLPGSDWTPNA

RFIIRSWLRDGLKPRCITRDLKWGTPVPLEGFEDKVFYVWFDATIGYLSITANYTDQWERWWKNPEQVDLYQFMAKDNVP

FHGLVFPCSALGAEDNYTLVKHLIATEYLNYEDGKFSKSRGVGVFGDMAQDTGIPADIWRFYLLYIRPEGQDSAFSWTDM

MTKNNSELLNNLGNFINRAGMFVSKFFGGCVPEMVLAPEDRRLLAHVSWELQHYHQLLEKVRIRDALRSILTISRHGNQY

IQVNEPWKRIKGSEVDRQRAGTVTGVAVNIAALLSVMLQPYMPTVSATIQTQLQLPRPACRVLLTNFICTLPAGHQIGTV

SPLFQKLENDQIENLKQRFGGGQLEESLELKAKGSPKPAAVEAVTTAGPVQIQMLMEEVTKQGNIVRELKAQKADKNQVA

AEVTKLLDLKKQLALAEGKPVETPKGKKKK

>XP_010596579.1 methionine--tRNA synthetase, cytoplasmic isoform X2 [Loxodonta africana]

MRLFVSEGAPGALPVLAAASRARERAELLISTVGPEECVVPFLSGPKIPVLQLDSGNYLFSTSAICRYFFLLSGWEQDDL

TNQWLEWEAVELQPVLSAALYYLVIQGKKGEDVLCPVRKALTHIDHSLSRQSCPFLTGETESLADIVLWGALYPLLQDPA

YLPEELGALCSWFQMLSTQEPCQQAAETVLKQQGALALRPYLQKQPQPRPREGKSVSNEPEEEELTTLSEEEIAMAVTAW

EKGLGGLPPLRPQKNPVLPVAGEKNVLITSALPYVNNVPHLGNIIGCVLSADVFARYSRLRQWNTLYLCGTDEYGTATET

KAMEEGLTPQEICDKYHVIHADIYRWFNISFDIFGRTTTQQQTEITQDIFQRLLARGFVLQDTVEQLRCEHCARFLADRF

VEGVCPFCGYEEARGDQCDKCGKLINAIELKKPQCKVCRSPPVVKSSQHLFLDLPKLENLLETWLEKVLASSDWTPNARF

IIRSWLRDGLKPRCITRDLKWGTPVPLEGFEDKVFYVWFDATIGYLSITANYTDQWERWWKNPEQVDLYQFMAKDNVPFH

GLVFPCSALGTEDNYTLVSHLIATEYLNYEDGKFSKSRGVGVFGDMAQDTGIPADIWRFYLLYIRPEGQDSAFSWTDMLL

KNNSELLNNLGNFINRAGMFVSKFFGGYVPEMVLTLDDQRLLAHVTLELQHYHQLLEKVRIRDALRSILTISRHGNQYIQ

VNEPWKWIKGSEADRQRAGTVTGLAVNIAALLSVMLQPYMPTVSATIQDQLQLPPPACSVLPTNFLCTLPAGHQIGTVSP

LFQKLENDQIESLRQRFGGGQLQESLELKAKASPKPAVVETVITVGPQQIQVLMDEVTKQGNIVRELKAQKADNNQVAAE

VAKLLELKKQLALAEGKPLDPPKGKKKK

>XP_021513519.1 methionine--tRNA synthetase, cytoplasmic isoform X1 [Meriones unguiculatus]

MRLFVSEGSPGSLPVLAAAAKARGRAELLISNVGPEECVVPFLTQPKVPALQLESGNYLFSASAICRYFFLLSGWDQDDL

TNQWLEWEATELQPVLSAALHCLVVQGKKGEDVLGPLRRALTHIDHSLSRQNCPFLAGDTESLADIVLWGALYPLLQDPA

YLPEELSSLQSWFHTLSTQEPCQRAAETVLKQQGVLALRPYLQKQPQPQARPSEGRPVSNEPEEEELATLSEEDIIIAVN

AWEKGLDSLPPLQPQQYPVLPVAGERNVLITSALPYVNNVPHLGNIIGCVLSADVFARYSRLRQWNTLYVCGTDEYGTAT

ETKAMEEGLTPQEICDKYHAIHADIYRWFNISFDTFGRTTTPQQTKISQDIFQRLLTRGFVLQDTVEQLRCEQCARFLAD

RFVEGVCPFCGYEEARGDQCDKCGKLINAIELKKPQCKVCRSHPVVESSQHLFLDLPKLEKRLEDWLGRTLPGSDWTPNA

RFITRSWLRDGLKARCITRDLKWGTPVPLEGFEDKVFYVWFDAPIGYLSITANYTDQWERWWKNPEQVDLYQFMAKDNVP

FHGIIFPCSALGAEDNYTLVKHLIATEYLNYEDGKFSKSRGVGVFGDMAQDTGIPADIWRFYLLYIRPEGQDSAFSWTDM

LTKNNSELLNNLGNFINRAGMFVSKFFGGCVPDMVLTQDDRRMLAHISWELQHYHQLLDKVRIRDALRRILTISRHGNQY

IQVNEPWKRIKGGEVDRQRAGTVTGVAVNIAALLSVMLHPYMPTVSSTIQTQLQLPLPACRVLLTHFTCTLPAGHRIGTV

SPLFQKLENDQIENLRQRFGGGQLEETLELKAKGPPRPVVVEPVTAPGPLQTQMLMDEVTRQGNIVRELKAQKADKNQVA

AEVAKLLDLKKQLALAEGKPIETSKGKKKK

>XP_014928706.1 PREDICTED: methionine--tRNA synthetase, cytoplasmic isoform X3 [Acinonyx jubatus]

MRLFVSEGAPGSLPVLAAAGRAQGREELLISTVGPEECVVPFLTRPKVPVLQLDSGNYLFSTSAICRYFFLLSGWEQDDL

TNQWLEWEATELQPALSAALYYLVVQGKKGEDVLGPVRRALTHIDHSLSRRSCPFLAGETESLADIVLWGALYPLLQDPA

YLPEELGALHSWFQTLSSQEPCQRAAETVLKQQGVLALRPYLQKQPLPSSFEGRAVSNEPEEEELATLSEEEIAMAVTAW

EKGLGSLPPLRPQQNPVLPVAGERNVLITSALPYVNNVPHLGNIIGCVLSADVFARYSRLRQWNTLYLCGTDEYGTATET

KAMEEGLTPQEICDKYHAIHANIYRWFNISFDIFGRTTTPQQTKITQDIFQRLLTRGFVFQDTVEQLRCEHCARFLADRF

VEGVCPFCGYEEARGDQCDKCGKLINAIELKKPQCKVCRSCPVVKSSQHLFLDLPKLEKRLEEWLGKTLPGSDWTPNARF

IIRSWLRDGLKPRCITRDLKWGTPVPLEGFEDKVFYVWFDATIGYVSITANYTDQWERWWKNPEQVNLYQFMAKDNVPFH

GLVFPCSALGAEDNYTLVSHLIATEYLNYEDGKFSKSRGVGVFGDMAQDTGIPADIWRFYLLYIRPEGQDSAFSWTDMLL

KNNSELLNNLGNFINRAGMFVSKFFGGCVPEMVLTPDDRRLLAHVTLELQHYHQLLEKVRIREALRSILTISRHGNQYIQ

VNEPWKRIKGSEADRQRAGTVTGLAVNIAALLSIMLQPYMPTVSATIQAQLQLPAPACSILLTNFLCTLPAGHQIGTVSP

LFQKLENDQIESLRQRFGGGQVRKLKTVLSPSKSHGVLSLSPSSPHSLLFPPFDPAASLLIVFLSCLSWKSPWS

>XP_019807880.1 PREDICTED: methionine--tRNA synthetase, cytoplasmic isoform X1 [Tursiops truncatus]

MMGEIVYFPGVWPGERPHSELHFPACFAAVAHPTGGRPVPVASAWDPRRNEVVRERGRPGEPARAGRRREGPGQSGAAYQ

HCRPGRYFFLLSGWEQDDLTNQWLEWEATELQPALSAALYYLVVQGKKGEDVFSPVRRALTHIDHSLSRRSCPFLAGETE

SLADIVLWGALYPLLQDPAYLPEELGALHSWFQTLSSQEPCQRAAETVLKQQGALALRPYLQKQPQPSFLEGRAVSNEPE

EEELATLSEEEIAMAVAAWEKGLESLPPVQPQQNPVLPVAGERNVLITSALPYVNNVPHLGNIIGCVLSADVFARYSRLR

QWNTLYLCGTDEYGTATETKAMEEGLTPQEICDKYHVIHADIYRWFNISFDIFGRTTTPQQTKITQDIFQRLVARGFVLQ

DTVEQLRCEHCARFLADRFVEGMCPFCGYEEARGDQCDKCGKLINAIELKKPQCKVCRSCPVVKSSRHLFLDLPKLGKPL

EEWLEKTLPGSDWTPNARFIIRSWLRDGLKPRCITRDLKWGTPVPLVGFEDKVFYVWFDATIGYLSITANYTDQWEKWWK

NPEQVSLYQFMAKDNVPFHGLVFPCSALGAEDNYTLVSHLIATEYLNYEDGKFSKSRGVGVFGDMAQDTGIPADIWRFYL

LYIRPEGQDSAFSWTDMLLKNNSELLNNLGNFINRAGMFVSKFFGGFVPEMVLTSDDRRLLAHVTLELQHYHQLLEKVRI

RDALRSILTISRHGNQYIQVNEPWKRIKGSEADRQRAGTVTGLAVNIAALLSIMLQPYMPTVSATIQAQLQLPPPACSIL

LTNFLCTLPAGHQIGTVSPLFQKLENDQIESLRQRFSGGQAKASPKPAAVETMTTAGPQQIQVLMDEVTKQGNIVRELKA

QKADKNQVAAEVAKLLDLKKQLALAEGKPLETPKGKKKK

>XP_023413831.1 methionine--tRNA synthetase, cytoplasmic isoform X1 [Loxodonta africana]

MRLFVSEGAPGALPVLAAASRARERAELLISTVGPEECVVPFLSGPKIPVLQLDSGNYLFSTSAICRYFFLLSGWEQDDL

TNQWLEWEAVELQPVLSAALYYLVIQGKKGEDVLCPVRKALTHIDHSLSRQSCPFLTGETESLADIVLWGALYPLLQDPA

YLPEELGALCSWFQMLSTQEPCQQAAETVLKQQGALALRPYLQKQPQPRPREGKSVSNEPEEEELTTLSEEEIAMAVTAW

EKGLGGLPPLRPQKNPVLPVAGEKNVLITSALPYVNNVPHLGNIIGCVLSADVFARYSRLRQWNTLYLCGTDEYGTATET

KAMEEGLTPQEICDKYHVIHADIYRWFNISFDIFGRTTTQQQTEITQDIFQRLLARGFVLQDTVEQLRCEHCARFLADRF

VEGVCPFCGYEEARGDQCDKCGKLINAIELKKPQCKVCRSPPVVKSSQHLFLDLPKLENLLETWLEKVLASSDWTPNARF

IIRSWLRDGLKPRCITRDLKWGTPVPLEGFEDKVFYVWFDATIGYLSITANYTDQWERWWKNPEQVDLYQFMAKDNVPFH

GLVFPCSALGTEDNYTLVSHLIATEYLNYEDGKFSKSRGVGVFGDMAQDTGIPADIWRFYLLYIRPEGQDSAFSWTDMLL

KNNSELLNNLGNFINRAGMFVSKFFGGYVPEMVLTLDDQRLLAHVTLELQHYHQLLEKVRIRDALRSILTISRHGNQYIQ

VNEPWKWIKGSEADRQRAGTVTGLAVNIAALLSVMLQPYMPTVSATIQDQLQLPPPACSVLPTNFLCTLPAGHQIGTVSP

LFQKLENDQIESLRQRFGGGQLQESLELKVIAFSLRCCIKLEVGGYLWQGGDNSLVHLQAKASPKPAVVETVITVGPQQI

QVLMDEVTKQGNIVRELKAQKADNNQVAAEVAKLLELKKQLALAEGKPLDPPKGKKKK

>NP_001003913.1 methionine--tRNA synthetase, cytoplasmic isoform 2 [Mus musculus]

MRLFVSEGSPGSLPVLAAAARARGRAELLISTVGPEECVVPFLTRPKVPVLQLDSGNYLFSASAICRYFFLLCGWEQDDL

TNQWLEWEATELQPVLSAALHCLVVQGKKGEDILGPLRRVLTHIDHSLSRQNCPFLAGDTESLADIVLWGALYPLLQDPA

YLPEELGALQSWFQTLSTQEPCQRAAETVLKQQGVLALRLYLQKQPQPQPPPPEGRTVSNELEEEELATLSEEDIVTAVA

AWEKGLESLPPLKLQQHPVLPVPGERNVLITSALPYVNNVPHLGNIIGCVLSADVFARYCRLRQWNTLYLCGTDEYGTAT

ETKAMEEGLTPREICDKYHAIHADIYRWFGISFDTFGRTTTPQQTKITQDIFQRLLTRGFVLRDTVEQLRCERCARFLAD

RFVEGVCPFCGYEEARGDQCDRCGKLINAIELKKPQCKICRSCPVVRSSQHLFLDLPKLEKRLEDWLGKTVPGSDWTPNA

RFIIRSWLRDGLKPRCITRDLKWGTPVPLEGFEDKVFYVWFDATIGYVSITANYTDQWEKWWKNPEQVDLYQFMAKDNVP

FHGLVFPCSVLGAEDNYTLVKHIIATEYLNYEDGKFSKSRGIGVFGDMAKDTGIPADIWRFYLLYIRPEGQDSAFSWTDL

LIKNNSELLNNLGNFINRAGMFVSKFFGGCVPEMALTPDDRRLVAHVSWELQHYHQLLEKVRIRDALRSILTISRHGNQY

IQVNEPWKRIKGGEMDRQRAGTVTGMAVNMAALLSVMLQPYMPTVSSTIQTQLQLPPAACRILATSFICTLPAGHRIGTV

SPLFQKLENDQIENLRQRFGGGQAKGSPKPAAVEAVTAAGSQHIQTLTDEVTKQGNVVRELKAQKADKNQVAAEVAKLLD

LKKQLALAEGKPIETPKGKKKK

>XP_022279803.1 methionine--tRNA synthetase, cytoplasmic isoform X2 [Canis lupus familiaris]

MRLFVSEGAPGSLPVLAAAGRAQGRAELLVSTVGPEECVVPFLTRPKIPVLQLDSGNYLFSTSAICRYFFLLSGWEQDDL

TNQWLEWEATELQPALSAALYYLVVQGRKGEDVLGPVRRALTHIDYSLSRRSCPFLAGDTESLADIVLWGALYPLLQDPA

YLPEELGALHSWFQTLSSQEPCQRAAETVLKQQGVLALRPYLQKQPQPGSFEGRAVSNEPEEEELATLSEEEIAMAVTAW

EKGLGSLPPLRPQQNPVYSRLRQWNTFYLCGTDEYGTATETKAMEEGLTPQEICDKYHAIHADIYRWFNISFDIFGRTTT

PQQTKITQDIFQRLLTRGFVLQDTVEQLRCERCARFLADRFVEGVCPFCGYEEARGDQCDKCGKLINAIELKKPQCKVCR

SCPVVKSSQHLFLDLPKLEKRLEEWLGKTLPGSDWTPNARFIIRSWLRDGLKPRCITRDLKWGTPVPLEGFEDKVFYVWF

DATIGYLSITANYTDQWERWWKNPEQVNLYQFMAKDNVPFHGLVFPCSALGAEDNYTLVSHLIATEYLNYEDGKFSKSRG

VGVFGDMAQDTGIPADIWRFYLLYIRPEGQDSAFSWTDMLLKNNSELLNNLGNFINRAGMFVSKFFGGYVPEMVLTSDDR

RLLAHVTLELQHYHQLLEKVRIREALRSILTISRHGNQYIQVNEPWKRIKGSEADRQRAGTVTGLAVNIAALLSVMLQPY

MPTVSATIQAQLQVPPPACSILLTNFLCTLPAGHQIGTVSPLFQKLENDQIESLRQRFGGGQAKTPPTPAEAMAAAAPQQ

IQVLMDEVTKQGNIVRELKAQKADKNQVAAEVAKLLDLKKQLALAEGKPLETPKGKKKK

>NP_001165053.1 methionine--tRNA synthetase, cytoplasmic isoform 1 [Mus musculus]

MRLFVSEGSPGSLPVLAAAARARGRAELLISTVGPEECVVPFLTRPKVPVLQLDSGNYLFSASAICRYFFLLCGWEQDDL

TNQWLEWEATELQPVLSAALHCLVVQGKKGEDILGPLRRVLTHIDHSLSRQNCPFLAGDTESLADIVLWGALYPLLQDPA

YLPEELGALQSWFQTLSTQEPCQRAAETVLKQQGVLALRLYLQKQPQPQPPPPEGRTVSNELEEEELATLSEEDIVTAVA

AWEKGLESLPPLKLQQHPVLPVPGERNVLITSALPYVNNVPHLGNIIGCVLSADVFARYCRLRQWNTLYLCGTDEYGTAT

ETKAMEEGLTPREICDKYHAIHADIYRWFGISFDTFGRTTTPQQTKITQDIFQRLLTRGFVLRDTVEQLRCERCARFLAD

RFVEGVCPFCGYEEARGDQCDRCGKLINAIELKKPQCKICRSCPVVRSSQHLFLDLPKLEKRLEDWLGKTVPGSDWTPNA

RFIIRSWLRDGLKPRCITRDLKWGTPVPLEGFEDKVFYVWFDATIGYVSITANYTDQWEKWWKNPEQVDLYQFMAKDNVP

FHGLVFPCSVLGAEDNYTLVKHIIATEYLNYEDGKFSKSRGIGVFGDMAKDTGIPADIWRFYLLYIRPEGQDSAFSWTDL

LIKNNSELLNNLGNFINRAGMFVSKFFGGCVPEMALTPDDRRLVAHVSWELQHYHQLLEKVRIRDALRSILTISRHGNQY

IQVNEPWKRIKGGEMDRQRAGTVTGMAVNMAALLSVMLQPYMPTVSSTIQTQLQLPPAACRILATSFICTLPAGHRIGTV

SPLFQKLENDQIENLRQRFGGGQLEESLELKAKGSPKPAAVEAVTAAGSQHIQTLTDEVTKQGNVVRELKAQKADKNQVA

AEVAKLLDLKKQLALAEGKPIETPKGKKKK

>XP_021029281.1 methionine--tRNA synthetase, cytoplasmic isoform X2 [Mus caroli]

MRLFVSEGSPGSLPVLAAAARARGRAELLISTVGPEECVVPFLTRPKVPVLQLDSGNYLFSASAICRYFFLLCGWEQDDL

TNQWLEWEATELQPVLSAALHCLVVQGKKGEDVLGPLRRVLTHIDHSLSRQNWPFLAGDTESLADIVLWGALYPLLQDPA

YLPEELGALQSWFKTLSTQEPCQRAVETVLKQQGVLALRLYLQKQPQPQPPPPEGRTVSNELEEEELATLSEEDIVTAVA

AWEKGLESLPPLQLQQHPVLPVPGERNVLITSALPYVNNVPHLGNIIGCVLSADVFARYCRLRQWNTLYLCGTDEYGTAT

ETKAMEEGLTPQEICDKYHAIHADIYRWFGISFDTFGRTTTPQQTKITQDIFQRLLTRGFVLRDTVEQLRCEQCARFLAD

RFVEGVCPFCGYEEARGDQCDKCGKLINAIELKKPQCKICRSCPVVRSSQHLFLDLPKLEKRLEDWLGKTVPGSDWTPNA

RFIIRSWLRDGLKPRCITRDLKWGTPVPLEGFEDKVFYVWFDATIGYVSITANYTDQWEKWWKNPEQVDLYQFMAKDNVP

FHGLVFPCSVLGAEDNYTLVKHIIATEYLNYEDGKFSKSRGIGVFGDMAKDTGIPADIWRFYLLYIRPEGQDSAFSWTDL

LIKNNSELLNNLGNFINRAGMFVSKFFGGCVPAMALTPDDRRLVAHVSWELQHYHQLLEKVRIRDALRSILTISRHGNQY

IQVNEPWKRIKGGEMDRQRAGTVTGIAVNMAALLSVMLQPYMPTVSSTIQTQLQLPPAACRVLATSFICTLPAGHRIGTV

SPLFQKLENDQIENLRQRFGGGQAKGSPKPAAVEAVTAAGSQHIQMLTDEVTKQGNIVRELKAQKADKNQVAAEVAKLLD

LKKQLALAEGKPIETPKGKKKK

>EDL24498.1 methionine-tRNA synthetase, isoform CRA_b [Mus musculus]

MRLFVSEGSPGSLPVLAAAARARGRAELLISTVGPEGTGVAAECVVPFLTRPKVPVLQLDSGNYLFSASAICRYFFLLCG

WEQDDLTNQWLEWEATELQPVLSAALHCLVVQGKKGEDILGPLRRVLTHIDHSLSRQNCPFLAGDTESLADIVLWGALYP

LLQDPAYLPEELGALQSWFQTLSTQEPCQRAAETVLKQQGVLALRLYLQKQPQPQPPPPEGRTVSNELEEEELATLSEED

IVTAVAAWEKGLESLPPLKLQQHPVLPVPGERNVLITSALPYVNNVPHLGNIIGCVLSADVFARYCRLRQWNTLYLCGTD

EYGTATETKAMEEGLTPREICDKYHAIHADIYRWFGISFDTFGRTTTPQQTKITQDIFQRLLTRGFVLRDTVEQLRCERC

ARFLADRFVEGVCPFCGYEEARGDQCDRCGKLINAIELKKPQCKICRSCPVVRSSQHLFLDLPKLEKRLEDWLGKTVPGS

DWTPNARFIIRSWLRDGLKPRCITRDLKWGTPVPLEGFEDKVFYVWFDATIGYVSITANYTDQWEKWWKNPEQVDLYQFM

AKDNVPFHGLVFPCSVLGAEDNYTLVKHIIATEYLNYEDGKFSKSRGIGVFGDMAKDTGIPADIWRFYLLYIRPEGQDSA

FSWTDLLIKNNSELLNNLGNFINRAGMFVSKFFGGCVPEMALTPDDRRLVAHVSWELQHYHQLLEKVRIRDALRSILTIS

RHGNQYIQVNEPWKRIKGGEMDRQRAGTVTGMAVNMAALLSVMLQPYMPTVSSTIQTQLQLPPAACRILATSFICTLPAG

HRIGTVSPLFQKLENDQIENLRQRFGGGQLEESLELKAKGSPKPAAVEAVTAAGSQHIQTLTDEVTKQGNVVRELKAQKA

DKNQVAAEVAKLLDLKKQLALAEGKPIETPKGKKKK

>XP_021029280.1 methionine--tRNA synthetase, cytoplasmic isoform X1 [Mus caroli]

MRLFVSEGSPGSLPVLAAAARARGRAELLISTVGPEECVVPFLTRPKVPVLQLDSGNYLFSASAICRYFFLLCGWEQDDL

TNQWLEWEATELQPVLSAALHCLVVQGKKGEDVLGPLRRVLTHIDHSLSRQNWPFLAGDTESLADIVLWGALYPLLQDPA

YLPEELGALQSWFKTLSTQEPCQRAVETVLKQQGVLALRLYLQKQPQPQPPPPEGRTVSNELEEEELATLSEEDIVTAVA

AWEKGLESLPPLQLQQHPVLPVPGERNVLITSALPYVNNVPHLGNIIGCVLSADVFARYCRLRQWNTLYLCGTDEYGTAT

ETKAMEEGLTPQEICDKYHAIHADIYRWFGISFDTFGRTTTPQQTKITQDIFQRLLTRGFVLRDTVEQLRCEQCARFLAD

RFVEGVCPFCGYEEARGDQCDKCGKLINAIELKKPQCKICRSCPVVRSSQHLFLDLPKLEKRLEDWLGKTVPGSDWTPNA

RFIIRSWLRDGLKPRCITRDLKWGTPVPLEGFEDKVFYVWFDATIGYVSITANYTDQWEKWWKNPEQVDLYQFMAKDNVP

FHGLVFPCSVLGAEDNYTLVKHIIATEYLNYEDGKFSKSRGIGVFGDMAKDTGIPADIWRFYLLYIRPEGQDSAFSWTDL

LIKNNSELLNNLGNFINRAGMFVSKFFGGCVPAMALTPDDRRLVAHVSWELQHYHQLLEKVRIRDALRSILTISRHGNQY

IQVNEPWKRIKGGEMDRQRAGTVTGIAVNMAALLSVMLQPYMPTVSSTIQTQLQLPPAACRVLATSFICTLPAGHRIGTV

SPLFQKLENDQIENLRQRFGGGQLEESLELKAKGSPKPAAVEAVTAAGSQHIQMLTDEVTKQGNIVRELKAQKADKNQVA

AEVAKLLDLKKQLALAEGKPIETPKGKKKK

>XP_004601695.1 PREDICTED: methionine--tRNA synthetase, cytoplasmic [Sorex araneus]

MRLFVSEGAPGTLPVLVAAGRAQGRAELLISTVGLEECVVPFLSRPKIPALQLDNGNYLFSTSAICRYFFLLSGWEQDDL

TNQWLEWEATELQPALSAALYYLVVQGKKGEDVLSPVRRALTHIDHSLSQQSWPFLAGETESLADIVLWGALYPLLQDPT

YLPEELGALHTWFQTLSSQEPCQRAVETVLKQQGVLALRPYLQKQPQPSSLEGSTVSNEPEEVLASLSEEQIASAVAAWH

QGLGSLPALRPQQKPVLPVAGERNVLITSALPYVNNVPHLGNIIGCVLSADVFARYSRLRQWNTVYLCGTDEYGTATETK

AMEEGLTPKQICDKYHAIHADIYRWFNISFDFFGRTTTPHQTTITQDIFQRLLARGFVLQDTVEQLYCEHCVRFLADRFV

EGVCPFCSYEEARGDQCDKCGKLINAIELKRPQCKICRSCPVVRSSRHLFLDLPKLEKRLQDWLGKTMPGSDWTPNARFI

TRSWLRDGLKARCITRDLKWGTPVPLEGFEDKVFYVWFDATIGYLSITANYTDQWEKWWKNPEQVDLYQFMAKDNVPFHS

LVFPCSALGAEDNYTLVRNLIATEYLNYEDGKFSKSRGVGVFGDMAQDTGIPADIWRFYLLYIRPEGQDSAFSWTDMMVK

NNSELLNNLGNFINRAGMFVSKFFGGRVPEMVLTSDDRRLVAHVSLELQHYHQLLEKVHIRDALRSILTISRHGNQYIQV

NEPWKRIKGDEADRQRAGTVTGLAVNVAALLAIMLQPYMPTVSATIQTQLQLPPAACSVLATSFVCTLPAGHQIGTVSPL

FQKLENDQIESLKQRFGGGQAKAPPKAAAAATPATAGQQQIQVLMDEVTKQGNIVRELKAQKADKDQIAVEVAKLLDLKK

KLALAEGKPLEAPKGKKKK

>XP_004692670.1 PREDICTED: methionine--tRNA synthetase, cytoplasmic isoform X2 [Condylura cristata]

MRLFVSEGAPGSLPVLAAAGRARGRAELLISTVGPEECVVPFLTRPKVPALQLDSGNYLFTTSAICRYFFLLSGWEQDDL

TNQWLEWEATELQPALSAALYYLVVQGKKGEDVLGPVRRALTHINHSLSHRSCPFLAGETESLADIVLWGALYPLLQDPA

YLPEELGALHDWFQTLSSQEPCQRAAETMLKQQGVMALRPYLQKLPQPGPLEGKAVSNEPEEEEPAALSEEEIAAAVTAW

ESGLGSLPPLRPQQSPVLPVAGERNVLITSALPYVNNVPHLGNIIGCVLSADVFARYSRLRGWNTLYLCGTDEYGTATET

KAMEEGLSPREICDKYHAIHASIYRWFAISFDVFGRTTTPQQTTITQDIFQRLLARGFVLQDTVEQLRCERCARFLADRF

VEGVCPFCSYEEARGDQCDRCGKLINAIELKNPQCKICRSCPVVKSSQHLFLDLPKLEKRVEAWLGATLPGSDWTPNARF

ITRSWLRDGLKPRCITRDLKWGTPVPLEGFEDKVFYVWFDATIGYLSITANYTDQWERWWKNPEHVTLYQFMAKDNVPFH

GIVFPCSALGAEDNYTLVSHLIATEYLNYEDGKFSKSRGVGVFGDMAQDTGIPADIWRFYLLYIRPEGQDSAFSWTDMLL

KNNSELLNNLGNFINRAGMFVSRFFGGSVPAMVLTSDDRRLLAHVSRELQRYHQLLEKVRIRDALRSILTVSRHGNQYIQ

VNEPWKQIKGSEADRQRAGTVTGVAVNIAALLSVMLQPYMPAVSATIQAQLQLPRAACSALPPGFLCTLPAGHRIGTISP

LFQKLENDQIENLRQRFSGGQAKAAPKPAAETVPAAGPQQIQVLMEEVTKQGNVVRELKAQKADKNQVAAEVAKLLDLKR

QLALAEGKPLETAKGKKKK

>XP_012587846.1 PREDICTED: methionine--tRNA synthetase, cytoplasmic isoform X1 [Condylura cristata]

MRLFVSEGAPGSLPVLAAAGRARGRAELLISTVGPEECVVPFLTRPKVPALQLDSGNYLFTTSAICRYFFLLSGWEQDDL

TNQWLEWEATELQPALSAALYYLVVQGKKGEDVLGPVRRALTHINHSLSHRSCPFLAGETESLADIVLWGALYPLLQDPA

YLPEELGALHDWFQTLSSQEPCQRAAETMLKQQGVMALRPYLQKLPQPGPLEGKAVSNEPEEEEPAALSEEEIAAAVTAW

ESGLGSLPPLRPQQSPVLPVAGERNVLITSALPYVNNVPHLGNIIGCVLSADVFARYSRLRGWNTLYLCGTDEYGTATET

KAMEEGLSPREICDKYHAIHASIYRWFAISFDVFGRTTTPQQTTITQDIFQRLLARGFVLQDTVEQLRCERCARFLADRF

VEGVCPFCSYEEARGDQCDRCGKLINAIELKNPQCKICRSCPVVKSSQHLFLDLPKLEKRVEAWLGATLPGSDWTPNARF

ITRSWLRDGLKPRCITRDLKWGTPVPLEGFEDKVFYVWFDATIGYLSITANYTDQWERWWKNPEHVTLYQFMAKDNVPFH

GIVFPCSALGAEDNYTLVSHLIATEYLNYEDGKFSKSRGVGVFGDMAQDTGIPADIWRFYLLYIRPEGQDSAFSWTDMLL

KNNSELLNNLGNFINRAGMFVSRFFGGSVPAMVLTSDDRRLLAHVSRELQRYHQLLEKVRIRDALRSILTVSRHGNQYIQ

VNEPWKQIKGSEADRQRAGTVTGVAVNIAALLSVMLQPYMPAVSATIQAQLQLPRAACSALPPGFLCTLPAGHRIGTISP

LFQKLENDQIENLRQRFSGGQLEEPLKLQAKAAPKPAAETVPAAGPQQIQVLMEEVTKQGNVVRELKAQKADKNQVAAEV

AKLLDLKRQLALAEGKPLETAKGKKKK

>XP_023369309.1 methionine--tRNA synthetase, cytoplasmic isoform X1 [Otolemur garnettii]

MRLFVSEGAPGSLPVLAAAGRARGRAELLISTVGPEECVVPFLTQPKVPVLQLDSGNYLFSTSSICRYFFLLSGWEQDDL

TNQWLEWEATELQPALSAALYYLVVQGKKGEDVLGPVRRALKHIDYSLSRQNCPFLAGETESLADIVLWGALYPLLQDPA

YLPEELGSLCSWFQTLSTQEPCQRAAETVLKQQGVLALRPYLQKQPQPSPPNGRAVTNEPEEELATLSEEEITMAVTAWE

KGLGGLPPLRPPQNPVLPMAGERNVLITSALPYVNNVPHLGNIIGCVLSADVFARYSRLRQWNTLYLCGTDEYGTATETK

AMEEGLTPQEICDKYHAIHADIYRWFNISFDTFGRTTTPQQTKITQDIFRRLLTRGFVLQDTVEQLHCEKCARFLADRFV

EGVCPFCGYEEARGDQCDKCGKLINAIELKKPQCKVCRSCPVVKSSQHLFLDLPKLETRLEEWLGKTLPGSDWTPNSRFI

IRSWLRDGLKPRCITRDLKWGTPVPLEGFEDKVFYVWFDATIGYLSITANYTDQWERWWKNPEQVDLYQFMAKDNVPFHG

LVFPCSALGAEDNYTLVSHLIATEYLNYEDGKFSKSRGVGVFGDMAQDTGIPADIWRFYLLYIRPEGQDSAFSWTDLLLK

NNSELLNNLGNFINRAGMFVSKFFGGYVPEMVLTPDDQRLLAHVTLELRHYHQLLEKVRIRDALRSILTISRHGNQYIQV

NEPWKWIKGTEADRQRAGTVTGLAVNIAALLSVMLQPYMPSVSATIQAQLQLSPPACSVLLTNFLCTLPAGHQIGTVSPL

FQKLENDQIESLRQRFGGGQVRKLKTAVVSLSISHGVTSLVSLSSPDLSLFPSLTLLLLHLQFFFPILARRVLGAKGNHS

PGNIYNWRLGSFMATGSNSVVLFQAKVSPKPAVVDTVAATGPQQIQMLMDEVTKQGNIVRELKAQKADKNQIAAEVAKLL

DLKKQLSLAEGKPPEAPKCKKKK

>XP_012406996.1 methionine--tRNA synthetase, cytoplasmic isoform X3 [Sarcophilus harrisii]

MQLFVSDGAPGNLPVLAAAGRAGARGKLLISTVGPEERVVPFLTRPKLPVLKLDSDRYLFSTSAICRYFFLLSGWEQDDL

TNQWLEWETTELQPALSVALYNLVVQGKKGEDVLSPVRRALIHIDHSLSRRSCPFLVGDSESLADIVLWGALYPLLQDPT

YLPDELAALQTWFRTLSVQEPCQQAVESVLKKQGILAFRSYLQKQPQPSVPLERPVSNEPEEDELPAHTLSEEEIVIAVT

IWEQGLEALPTLRPRQNPVLPVAGERNVLITSALPYVNNVPHLGNIIGCVLSADVFARYSRLRQWNTLYLCGTDEYGTAT

ETKAMEEGLTPQQICDKYHTIHASIYRWFNISFDFFGRTTTPLQTKITQDIFQRLLSRGFLLKDTVEQLRCERCARFLAD

RFVEGICPFCGYEEARGDQCDKCGKLINAIELKKPQCKICRESPVVKSSQHLFLDLPKLEKRLEEWLGRTLASSDWTPNA

RFITRSWLRDGLKPRCITRDLKWGTPVPLEGFQDKVFYVWFDATIGYLSITANYTDQWERWWKNPDQVELYQFMAKDNVP

FHSVVFPCSALGAEDNYTLVSHLIATEYLNYEDGKFSKSRGVGVFGDMAQNTGIPSDIWRFYLLYVRPEGQDSSFSWTDL

MLKNNSELLNNLGNFINRAGMFVCKFFGGCVPKMTLTPDDQRLLAHITLELHQYHQLLEKVRIRDALRCILTISRHGNQY

IQVNEPWKRIKGNEADRLRAGTVTGVAVNMAALLSIMLQPYMPTVSTTIQTQLQLPPAACAVLPTNFPCFLPAGHQIGIV

SPLFQKLENDQIESLKKRFGGGQAKVSKAEAPTEIANPEQIKLLTDAVTKQGNIVRELKAQKADKAQVTAEVAKLLELKQ

QLALAEGKPLEASKGKKKK

>XP_020845771.1 LOW QUALITY PROTEIN: methionine--tRNA synthetase, cytoplasmic [Phascolarctos cinereus]

MRLFVSDGFPGNLPVLAAAWRAGARDKLLISTVGPEECVVPFLTRPKLPALKLDSGHYLFSTTAICRYFFLLSGWEQDDL

TNQWLEWETTELQPVLSIALYNLVVRGKKGEDVLSPVRRALIHIDHSLSRRNYPFLAGDSESLADIVLWGALYPLLQDPT

YLPDELAALQTWFRTLSVQEPCQQAVESVLKNQGALALRVYLQKQPQPSVPLERPVSNEPEEDELSVHTLSEEEIAMAVT

AWEQGRDALPPVRPRQNPVLPVAGERNVLITSALPYVNNVPHLGNIIGCVLSADVFARYSRLRQWNTLYLCGTDEYGTAT

ETKAMEEGLTPQQICDKYHTIHASIYHWFNISFDFFGRTTTPLQTKITQDIFQRLLSRGFLLKDTVEQLRCEQCSRFLAD

RFVEGICPFCGYEEARGDQCDKCGKLINAIELKNPKCKICRESPVVKSSQHLFLDLPKLENRLEEWLSRTLAGSDWTPNA

RFITRSWLRDGLKPRCITRDLKWGTPVPLEGFQDKVFYVWFDATIGYLSIAANYTDQWERWWKNPEQVELYQFMAKDNVP

FHSVVFPCSALGAEDNYTLVSHLIATEYLNYEDGKFSKSRGVGVFGDMAQNTGIPSDIWRFYLLYVRPESQDSSFSWSDL

MLKNNSELLNNLGNFVNRAGMFVCKFFGGCVPKMTLTPDDQRLLAHVTLELRQYHQLLEKVRIRDALRCILTISRHGNQY

IQVNEPWKRIKGNEADRLHAGTVTGVAVNMAALLSIMMQPYMPTVSTTIQSQLQLPPAACAVLPTDFPCFLPAGHQIGIV

SPLFQKLENDQIESLRKRFGGGQLEDSLELKAKVSKAEASTEQADPEQIKLLMDAVTTQGNVVREFKAQKADKAQVAAEV

AKLLELKQQLALAEGKPPEPSKGKKKK

>XP_003772328.1 methionine--tRNA synthetase, cytoplasmic isoform X2 [Sarcophilus harrisii]

MQLFVSDGAPGNLPVLAAAGRAGARGKLLISTVGPEERVVPFLTRPKLPVLKLDSDRYLFSTSAICRYFFLLSGWEQDDL

TNQWLEWETTELQPALSVALYNLVVQGKKGEDVLSPVRRALIHIDHSLSRRSCPFLVGDSESLADIVLWGALYPLLQDPT

YLPDELAALQTWFRTLSVQEPCQQAVESVLKKQGILAFRSYLQKQPQPSVPLERPVSNEPEEDELPAHTLSEEEIVIAVT

IWEQGLEALPTLRPRQNPVLPVAGERNVLITSALPYVNNVPHLGNIIGCVLSADVFARYSRLRQWNTLYLCGTDEYGTAT

ETKAMEEGLTPQQICDKYHTIHASIYRWFNISFDFFGRTTTPLQTKITQDIFQRLLSRGFLLKDTVEQLRCERCARFLAD

RFVEGICPFCGYEEARGDQCDKCGKLINAIELKKPQCKICRESPVVKSSQHLFLDLPKLEKRLEEWLGRTLASSDWTPNA

RFITRSWLRDGLKPRCITRDLKWGTPVPLEGFQDKVFYVWFDATIGYLSITANYTDQWERWWKNPDQVELYQFMAKDNVP

FHSVVFPCSALGAEDNYTLVSHLIATEYLNYEDGKFSKSRGVGVFGDMAQNTGIPSDIWRFYLLYVRPEGQDSSFSWTDL

MLKNNSELLNNLGNFINRAGMFVCKFFGGCVPKMTLTPDDQRLLAHITLELHQYHQLLEKVRIRDALRCILTISRHGNQY

IQVNEPWKRIKGNEADRLRAGTVTGVAVNMAALLSIMLQPYMPTVSTTIQTQLQLPPAACAVLPTNFPCFLPAGHQIGIV

SPLFQKLENDQIESLKKRFGGGQLEDSLELKAKVSKAEAPTEIANPEQIKLLTDAVTKQGNIVRELKAQKADKAQVTAEV

AKLLELKQQLALAEGKPLEASKGKKKK

>XP_011844017.1 PREDICTED: methionine--tRNA synthetase, cytoplasmic [Mandrillus leucophaeus]

MQDYPKLTKHFFSFPFFFFNPFSVFASDCVVPFLTRPKVPVLQLDSGNYLFSTSAICRYFFLLSGWEQDDLTNQWLEWEA

TELQPALSAALYYLVVQGKKGEDVLGSVRRALTHIDHSLSRQNCPFLAGETESLADIVLWGALYPLLQDPAYLPEELSAL

HSWFQTLSTQEPCQRAAETVLKQQGVLALRPYLQKQPQPSPAEGRAVTNEPEEEELATLSEEEIAMAVTAWEKGLESLPP

LRPQQNPVLPVTGERNVLITSALPYVNNVPHLGNIIGCVLSADVFARYSRLRQWNTLYLCGTDEYGTATETKAMEEGLTP

QEICDKYHIIHADIYRWFNISFDTFGRTTTPQQTKSKLTWGQKPQCKVCRSCPVVQSTHHLFLDLPKLEKRLEEWLGRTL

PGSDWTPNARFITRSWLRDGLKPRCITRDLKWGTPVPLGGFEDKVFYVWFDATIGYLSITANYTDQWERWWKNPEQVDLY

QFMAKDNVPFHGIVFPCSALGAEDNYTLVSHLIATEYLNYEDGKFSKSRGVGVFGDMAQDTGIPADIWRFYLLYIRPEGQ

DSAFSWTDLLLKNNSELLNNLGNFINRAGMFVSKFFGGYVPEMVLTPDDQRLLAHVTLELQHYHQLLEKVRIRDALRSIL

TISRHGNQYIQVNEPWKRIKGCEADRQRAGTVTGLAVNIAALLSVMLQPYMPTVSATIQAQLQLPPPACSILLTNFLCTL

PAGHQIGTVSPLFQKLENDQIESLRQRFGGGQAKTSPKPTVIETVTTAGPQQIQALMDEVTKQGNIVRELKAQKADKNQV

AAEVAKLLDLKKQLAVAEGKPPEAPKGKKKK

>XP_012507170.1 PREDICTED: methionine--tRNA synthetase, cytoplasmic isoform X3 [Propithecus coquereli]

MGSNRATVVQGKKGEDVLGPVRRALTHIDHSLSRQNCPFLAGETESLADIVLWGALYPLLQDPAYLPEELGALRSWFQTL

STQEPCQRAAETVLKQQGVLALRPYLQKQPQPSSPDGRAVTNEPQEEELATLSEEEIAMAVTAWEKGLESLPPLRPPQNP

VLPVAGERNVLITSALPYVNNVPHLGNIIGCVLSADVFARYSRLRQWNTLYLCGTDEYGTATETKAMEEGLTPQEICDKY

HIIHADIYRWFNISFDIFGRTTTPQQTKITQDIFQRLLTRGFVLQDTVEQLRCEHCARFLADRFVEGVCPFCGYEEARGD

QCDKCGKLINAIELKKPRCKVCRSCPVVKSSQHLFLDLPKLEKRLEEWLGKTLPGSDWTPNARFIIRSWLRDGLKPRCIT

RDLKWGTPVPLEGFEDKVFYVWFDATIGYLSITANYTDQWERWWKNPEQVDLYQFMAKDNVPFHGLVFPCSALGTEDNYT

LVSHLIATEYLNYEDGKFSKSRGVGVFGDMAQDTGIPADIWRFYLLYIRPEGQDSAFSWTDMLLKNNSELLNNLGNFINR

AGMFVSKFFGGYVPEMVLTPDDQRLLAHVTLELRHYHQLLEKVRIRDALRSILTISRHGNQYIQVNEPWKRIKGSEADRQ

RAGTVTGLAVNIAALLSVMLQPYMPTVSATIQAQLHLPPPACSILLKNFLCTLPAGHQIGTVSPLFQKLENDQIESLKQR

FGGGQLEESLELKAKVSPKPEVIETVTTAGSQQIQVLTDEVTKQGNIVRELKAQKADKNQIAAEVAKLLDLKKQLALAEG

KPLETPKGKKKK

>XP_012406994.1 methionine--tRNA synthetase, cytoplasmic isoform X1 [Sarcophilus harrisii]

MQLFVSDGAPGNLPVLAAAGRAGARGKLLISTVGPEERVVPFLTRPKLPVLKLDSDRYLFSTSAICRYFFLLSGWEQDDL

TNQWLEWETTELQPALSVALYNLVVQGKKGEDVLSPVRRALIHIDHSLSRRSCPFLVGDSESLADIVLWGALYPLLQDPT

YLPDELAALQTWFRTLSVQEPCQQAVESVLKKQGILAFRSYLQKQPQPSVPLERPVSNEPEEDELPAHTLSEEEIVIAVT

IWEQGLEALPTLRPRQNPVLPVAGERNVLITSALPYVNNVPHLGNIIGCVLSADVFARYSRLRQWNTLYLCGTDEYGTAT

ETKAMEEGLTPQQICDKYHTIHASIYRWFNISFDFFGRTTTPLQTKITQDIFQRLLSRGFLLKDTVEQLRCERCARFLAD

RFVEGICPFCGYEEARGDQCDKCGKLINAIELKKPQCKICRESPVVKSSQHLFLDLPKLEKRLEEWLGRTLASSDWTPNA

RFITRSWLRDGLKPRCITRDLKWGTPVPLEGFQDKVFYVWFDATIGYLSITANYTDQWERWWKNPDQVELYQFMAKDNVP

FHSVVFPCSALGAEDNYTLVSHLIATEYLNYEDGKFSKSRGVGVFGDMAQNTGIPSDIWRFYLLYVRPEGQDSSFSWTDL

MLKNNSELLNNLGNFINRAGMFVCKFFGGCVPKMTLTPDDQRLLAHITLELHQYHQLLEKVRIRDALRCILTISRHGNQY

IQVNEPWKRIKGNEADRLRAGTVTGVAVNMAALLSIMLQPYMPTVSTTIQTQLQLPPAACAVLPTNFPCFLPAGHQIGIV

SPLFQKLENDQIESLKKRFGGGQLEDSLELKVIIFPRAVKRDNCKHVSMWGGIIYALGKNDSFILPEAKVSKAEAPTEIA

NPEQIKLLTDAVTKQGNIVRELKAQKADKAQVTAEVAKLLELKQQLALAEGKPLEASKGKKKK

>XP_022427448.1 methionine--tRNA synthetase, cytoplasmic isoform X2 [Delphinapterus leucas]

MGSDRAAVVQGKKGEDVFSPVRRALTHIDHSLSRRSCPFLAGETESLADIVLWGALYPLLQDPAYLPEELGALHSWFQTL

SSQEPCQRAAETVLKQQGVLALRPYLQKQPQPSFLEGRAVSNEPEEEELATLSEEEIAMAVAAWEKGLESLPPVQPQQNP

VLPVAGEKNVLITSALPYVNNVPHLGNIIGCVLSADVFARYSRLRQWNTLYLCGTDEYGTATETKAMEEGLTPQEICDKY

HVIHADIYRWFNISFDIFGRTTTPQQTKITQDIFQRLVARGFVLQDTVEQLRCEHCARFLADRFVEGMCPFCGYEEARGD

QCDKCGKLINAIELKKPQCKVCRSCPVVKSSRHLFLDLPKLGKPLEEWLEKTLPGSDWTPNARFIIRSWLRDGLKPRCIT

RDLKWGTPVPLVGFEDKVFYVWFDATIGYLSITANYTDQWEKWWKNPEQVSLYQFMAKDNVPFHGLVFPCSALGAEDNYT

LVSHLIATEYLNYEDGKFSKSRGVGVFGDMAQDTGIPADIWRFYLLYIRPEGQDSAFSWTDMLLKNNSELLNNLGNFINR

AGMFVSKFFGGFVPEMVLTSDDRRLLAHVTLELQHYHQLLEKVRIRDALRSILTISRHGNQYIQVNEPWKRIKGSEADRQ

RAGTVTGLAVNIAALLSIMLQPYMPTVSATIQAQLQLPPPACSILLTNFLCTLPAGHQIGTVSPLFQKLENDQIESLRQR

FSGGQAKASPKPAAVETMTTAGPQQIQVLMDEVTKQGNIVRELKAQKADKNQVAAEVAKLLDLKKQLALAEGKPLETPKG

KKKK

>XP_019807882.1 PREDICTED: methionine--tRNA synthetase, cytoplasmic isoform X3 [Tursiops truncatus]

MGSDRAAVVQGKKGEDVFSPVRRALTHIDHSLSRRSCPFLAGETESLADIVLWGALYPLLQDPAYLPEELGALHSWFQTL

SSQEPCQRAAETVLKQQGALALRPYLQKQPQPSFLEGRAVSNEPEEEELATLSEEEIAMAVAAWEKGLESLPPVQPQQNP

VLPVAGERNVLITSALPYVNNVPHLGNIIGCVLSADVFARYSRLRQWNTLYLCGTDEYGTATETKAMEEGLTPQEICDKY

HVIHADIYRWFNISFDIFGRTTTPQQTKITQDIFQRLVARGFVLQDTVEQLRCEHCARFLADRFVEGMCPFCGYEEARGD

QCDKCGKLINAIELKKPQCKVCRSCPVVKSSRHLFLDLPKLGKPLEEWLEKTLPGSDWTPNARFIIRSWLRDGLKPRCIT

RDLKWGTPVPLVGFEDKVFYVWFDATIGYLSITANYTDQWEKWWKNPEQVSLYQFMAKDNVPFHGLVFPCSALGAEDNYT

LVSHLIATEYLNYEDGKFSKSRGVGVFGDMAQDTGIPADIWRFYLLYIRPEGQDSAFSWTDMLLKNNSELLNNLGNFINR

AGMFVSKFFGGFVPEMVLTSDDRRLLAHVTLELQHYHQLLEKVRIRDALRSILTISRHGNQYIQVNEPWKRIKGSEADRQ

RAGTVTGLAVNIAALLSIMLQPYMPTVSATIQAQLQLPPPACSILLTNFLCTLPAGHQIGTVSPLFQKLENDQIESLRQR

FSGGQAKASPKPAAVETMTTAGPQQIQVLMDEVTKQGNIVRELKAQKADKNQVAAEVAKLLDLKKQLALAEGKPLETPKG

KKKK

>XP_007179733.1 PREDICTED: methionine--tRNA synthetase, cytoplasmic isoform X3 [Balaenoptera acutorostrata scammoni]

MGSDRAAVVQGKKGEDVFSPVRRALTHIDHSLSRRSCPFLAGETESLADIVLWGALYPLLQDPAYLPEELGALHSWFQTL

SSQEPCQRAAETVLKQQGVLALRPYLQKQPQPSSLEGRAVSNEPEEEELATLSEEEIAMAVAAWEKGLESLPPVQPQQNP

VLPVAGERNVLITSALPYVNNVPHLGNIIGCVLSADVFARYSRLRQWNTLYLCGTDEYGTATETKAMEEGLTPQEICDKY

HVIHADIYRWFNISFDIFGRTTTPQQTKITQDIFQRLVARGFVLQDTVEQLRCEHCARFLADRFVEGVCPFCGYEEARGD

QCDKCGKLINAIELKKPQCKVCRSCPVVKSSQHLFLDLPKLGKPLEEWLEKTLPGSDWTPNARFIIRSWIRDGLKPRCIT

RDLKWGTPVPLVGFEDKVFYVWFDATIGYLSITANYTDQWEKWWKNPEQVSLYQFMAKDNVPFHGLVFPCSALGAEDNYT

LVSHLIATEYLNYEDGKFSKSRGVGVFGDMAQDTGIPADIWRFYLLYIRPEGQDSAFSWTDMLLKNNSELLNNLGNFINR

AGMFVSKFFGGFVPEMVLTSDDRRLLAHVTLELQHYHQLLEKVRIRDALRSILTISRHGNQYIQVNEPWKRIKGSEADRQ

RAGTVTGLAVNIAALLSIMLQPYMPTVSATIQAQLQLPPPACSILLTNFLCTLPAGHQIGTVSPLFQKLENDQIESLRQR

FSGGQLEESLSLKAKASPKPAAVETMTTAGPQQIQVLMDEVTKQGNIVRELKAQKADKNQVAAEVAKLLDLKKQLALAEG

KPLETPKGKKKK

>XP_024592965.1 methionine--tRNA synthetase, cytoplasmic isoform X2 [Neophocaena asiaeorientalis asiaeorientalis]

MGSDRAAVVQGKKGEDVFSPVRRALTHIDHSLSRRSCPFLAGETESLADIVLWGALYPLLQDPAYLPEELGALHSWFQTL

SSQEPCQRAAETVLKQQGVLALRPYLQKQPQPSFLEGRAVSNEPEEEELATLSEEEIAMAVAAWEKGLESLPPVQPQQNP

VLPVAGEKNVLITSALPYVNNVPHLGNIIGCVLSADVFARYSRLRQWNTLYLCGTDEYGTATETKAMEEGLTPQEICDKY

HVIHADIYRWFNISFDIFGRTTTPQQTKITQDIFQRLVARGFVLQDTVEQLRCEHCARFLADRFVEGMCPFCGYEEARGD

QCDKCGKLINAIELKKPQCKVCRSCPVVKSSRHLFLDLPKLGKPLEEWLEKTLPGSDWTPNARFIIRSWLRDGLKPRCIT

RDLKWGTPVPLEGFEDKVFYVWFDATIGYLSITANYTDQWEKWWKNPEQVSLYQFMAKDNVPFHGLVFPCSALGAEDNYT

LVSHLIATEYLNYEDGKFSKSRGVGVFGDMAQDTGIPADIWRFYLLYIRPEGQDSAFSWTDMLLKNNSELLNNLGNFINR

AGMFVSKFFGGFVPEMVLTSDDRRLLAHVTLELQHYHQLLEKVRIRDALRSILTISRHGNQYIQVNEPWKRIKGSEADRQ

RAGTVTGLAVNIAALLSIMLQPYMPTVSATIQAQLQLPPPACSILLTNFLCTLPAGHQIGTVSPLFQKLENDQIESLRQR

FSGGQAKAPPKPAAVETMTTAGPQQIQVLMDEVTKQGNTVRELKAQKADKNQVAAEVAKLLDLKKQLALAEGKPLETPKG

KKKK

>XP_023413848.1 methionine--tRNA synthetase, cytoplasmic isoform X4 [Loxodonta africana]

MRLFVSEGAPGALPVLAAASRARERAELLISTVGPEECVVPFLSGPKIPVLQLDSGNYLFSTSAICRYFFLLSGWEQDDL

TNQWLEWEAVELQPVLSAALYYLVIQGKKGEDVLCPVRKALTHIDHSLSRQSCPFLTGETESLADIVLWGALYPLLQDPA

YLPEELGALCSWFQMLSTQEPCQQAAETVLKQQGALALRPYLQKQPQPRPREGKSVSNEPEEEELTTLSEEEIAMAVTAW

EKGLGGLPPLRPQKNPVLPVAGEKNVLITSALPYVNNVPHLGNIIGCVLSADVFARYSRLRQWNTLYLCGTDEYGTATET

KAMEEGLTPQEICDKYHVIHADIYRWFNISFDIFGRTTTQQQTEITQDIFQRLLARGFVLQDTVEQLRCEHCARFLADRF

VEGVCPFCGYEEARGDQCDKCGKLINAIELKKPQCKVCRSPPVVKSSQHLFLDLPKLENLLETWLEKVLASSDWTPNARF

IIRSWLRDGLKPRCITRDLKWGTPVPLEGFEDKVFYVWFDATIGYLSITANYTDQWERWWKNPEQVDLYQFMAKDNVPFH

GLVFPCSALGTEDNYTLVSHLIATEYLNYEDGKFSKSRGVGVFGDMAQDTGIPADIWRFYLLYIRPEGQDSAFSWTDMLL

KNNSELLNNLGNFINRAGMFVSKFFGGYVPEMVLTLDDQRLLAHVTLELQHYHQLLEKVRIRDALRSILTISRHGNQYIQ

VNEPWKWIKGSEADRQRAGTVTGLAVNIAALLSVMLQPYMPTVSATIQDQLQLPPPACSVLPTNFLCTLPAGHQIGTVSP

LFQKLENDQIESLRQRFGGGQLQESLELKAKASPKPAVVETVITVGPQQIQVLMDEVTKQV

>XP_023563068.1 methionine--tRNA synthetase, cytoplasmic isoform X2 [Octodon degus]

MGGHRVAVIQGKKGEDVLGPLGRALTHIDHSLSRQNCPFLAGETESLADIVLWGALYPLLQDPAYLPEELGALRSWFQTL

STQEPCQRAAETVLKKQGVLALRPYLQKQPQPSPPEGRAVSNEPEEEELAVLSEEEIAAAVTAWEKGLEILPPPRPQQNP

VLPVAGERNVLITSALPYVNNVPHLGNIIGCVLSADVFARYSRLRQWNTLYLCGTDEYGTATETKAMEEGLTPQEICDKY

HVIHADIYHWFNISFDIFGRTTTPQQTKITQDIFQRLLTRGFILQDTVEQLRCERCARFLADRFVEGVCPFCGYEEARGD

QCDKCGKLINAIELKKPQCKVCRSCPVVKSSQHLFLDLPKLEKRLEEWLGKTLPGNNWTPNARFIIRSWLRDGLKPRCIT

RDLKWGTPVPLEGFEDKVFYVWFDATIGYLSITANYTDQWERWWKNPEQVDLYQFMAKDNVPFHGLVFPCSALGAEDNYT

LVKYLIATEYLNYEDGKFSKSRGVGVFGDMAQDTGIPADIWRFYLLYIRPEGQDSAFSWTDMLLKNNSELLNNLGNFINR

AGMFVSKFFGGSVPEMLLTPDDQRLLAHITLELQHYHQLLEKVRIRDALRAILTVSRHGNQYIQVNEPWKRIKGSEADRQ

RAGTVTGVAVNIAALLSVMLQPYMPTVSATIQAQLQLPLPACSVLPTDFLCTLPAGHQIGTVSPLFQKLENDQIENLKQR

FGGGQAKISPSLAVVETVTTAGPQQIQTLMEEVTKQGNIVRELKAQKADKNQVAAEVAQLLDLKKQLALAEGKPLETPKG

KKKK

>OWK16969.1 MARS [Cervus elaphus hippelaphus]

MRLFVSEGAPGSLPVLAAAGRAQGRAELLISTVGPEECVVPFLTRPKVPVLQLDSGNYLFSTSAICRYFFLLSGWEQDDL

TNQWLEWEATELQPALSTALYYLVVQGKKGEDVLGPVRRALVHVDRSLSQELGALHSWFQTLSSQEPCQRAAETVLKQQG

VLALRPYLQKQPQPSSLEGRVVSNEPEEEELATLSEEEIAVAVAAWEKGLEGLPPLRPQQNPVLPVAGERNVLITSALPY

VNNVPHLGNIIGCVLSADVFARCSQLLGDGPEGAVSPREIGLKWGDWKDPGPVEGAELGLVIAAFPSRYSRLRQWNTLYL

CGTDEYGTATETKALEEGLTPQEICDKYHAIHADIYRWFNISFDFFGRTTTPQQTKITQDIFQRLLARGFVLQATVEQLR

CEHCARFLADRFVEGVCPFCGYEEARGDQCDKCGKLINAIELKKPQCKVCRSCPVVRSSQHLFLDLPQLGTRVEEWLERT

LPGSDWTPNARFITRSWLRDGLKPRCITRDLKWGTPVPLEGFEDKVFYVWFDATIGYLSITANYTDHLYQFMAKDNVPFH

GIVFPSSALGAEDNYTLVSHLIATEYLNYEDGKFSKSRGVGVFGDMAQDTGIPADIWRFYLLYIRPEGQDSAFSWTDMLL

KNNSELLNNLGNFINRAGMFVSKFFGGFVPEMVLTSDDQRLLTHVTLELQHYHQLLEKVRIRDALRSILTISRHGNQYIQ

VNEPWKRIKGGEADRQRAGTVTGLAVNIAALLSIMLQPYMPAVSATIRAQLQLPPPACSVLPTSFLCTLPAGHQIGTVSP

LFQKLENDQIESLKQRFSGGQLEESLALKAKASPKTAAGPQQVQALTEEVAKQGDIVRELKAQKADKNQIAAEVAKLLDL

KKQLALAEGKPLETPKGKKKK

>XP_021513533.1 methionine--tRNA synthetase, cytoplasmic isoform X3 [Meriones unguiculatus]

MNKGNPSILTYFLSLGRYFFLLSGWDQDDLTNQWLEWEATELQPVLSAALHCLVVQGKKGEDVLGPLRRALTHIDHSLSR

QNCPFLAGDTESLADIVLWGALYPLLQDPAYLPEELSSLQSWFHTLSTQEPCQRAAETVLKQQGVLALRPYLQKQPQPQA

RPSEGRPVSNEPEEEELATLSEEDIIIAVNAWEKGLDSLPPLQPQQYPVLPVAGERNVLITSALPYVNNVPHLGNIIGCV

LSADVFARYSRLRQWNTLYVCGTDEYGTATETKAMEEGLTPQEICDKYHAIHADIYRWFNISFDTFGRTTTPQQTKISQD

IFQRLLTRGFVLQDTVEQLRCEQCARFLADRFVEGVCPFCGYEEARGDQCDKCGKLINAIELKKPQCKVCRSHPVVESSQ

HLFLDLPKLEKRLEDWLGRTLPGSDWTPNARFITRSWLRDGLKARCITRDLKWGTPVPLEGFEDKVFYVWFDAPIGYLSI

TANYTDQWERWWKNPEQVDLYQFMAKDNVPFHGIIFPCSALGAEDNYTLVKHLIATEYLNYEDGKFSKSRGVGVFGDMAQ

DTGIPADIWRFYLLYIRPEGQDSAFSWTDMLTKNNSELLNNLGNFINRAGMFVSKFFGGCVPDMVLTQDDRRMLAHISWE

LQHYHQLLDKVRIRDALRRILTISRHGNQYIQVNEPWKRIKGGEVDRQRAGTVTGVAVNIAALLSVMLHPYMPTVSSTIQ

TQLQLPLPACRVLLTHFTCTLPAGHRIGTVSPLFQKLENDQIENLRQRFGGGQLEETLELKAKGPPRPVVVEPVTAPGPL

QTQMLMDEVTRQGNIVRELKAQKADKNQVAAEVAKLLDLKKQLALAEGKPIETSKGKKKK

>XP_004773268.1 PREDICTED: methionine--tRNA synthetase, cytoplasmic isoform X2 [Mustela putorius furo]

MGSYRAAVVQGKKGEDVLGLVRRALTHIDQSLSRRSCPFLAGETESLADVVLWGALYPLLQDPAYLPEELGALHSWFQTL

SSQEPCQRAAETVLKQQGVLALRPYLQKQPQPGSFEGRAVSNEPEEEELATLSEEEISMAVTAWEKGLENLPPLRPQQNP

VLPVAGERNVLITSALPYVNNVPHLGNIIGCVLSADVFARYSRLRQWNTLYLCGTDEYGTATETKAMEEGLTPQEICDKY

HAIHANIYRWFNISFDIFGRTTTPQQTKITQDIFQRLLTRGFVLEDTVEQLRCERCARFLADRFVEGVCPFCGYEEARGD

QCDKCGKLINAIELKKPQCKVCRSCPVVKSSRHLFLDLPKLEKRLEEWLGKTLPGSDWTPNARFIIRSWLRDGLKPRCIT

RDLKWGTPVPLKGFEDKVFYVWFDATIGYLSITANYTDQWERWWKNPEQVNLYQFMAKDNVPFHGLVFPCSALGAEDNYT

LVSHLIATEYLNYEDGKFSKSRGVGVFGDMAQDTGIPADIWRFYLLYIRPEGQDSAFSWTDMLLKNNSELLNNLGNFINR

AGMFVSKFFGGYVPEMVLTSDDRRLVAHVTLELQHYHQLLEKVRIREALRSILTISRHGNQYIQVNEPWKRIKGSEADRQ

RAGTVTGLAVNIAALLSVMLQPYMPTVSATIQAQLQLPLPACSILLTNFLCTLPAGHQIGTVSPLFQKLENDQIESLRQR

FGGGQAQVPPMPVVVEAAATAAPRQVQVLMDEVARQGNIVRELKAQKADKNQVAAEVAKLLDLKKQLALAEGKPLETPKG

KKKK

>ELV09236.1 Methionyl-tRNA synthetase, cytoplasmic [Tupaia chinensis]

MAGVSCLLMWHDGRSSPLSGGGAGPSLTQNFISRRGSRRPHNPKTKDRFRLRQRGLHGGMRLFVSEGAPGSLPVLAAAGR

ARGRAELLISTVGPEECVVPFLTRPKVPVLQLDSGNYLFSTSAICRYLFLLSGWEQDDLTNQWLEWEATELQPALSAALY

YLVVQGKKGDDVLGPVRRALTHIDHSLSRQNCPFLAGETESLADIVLWGALYPLLQDPAYFPEELSALHSWFQTLSTQEP

CQQAVETVLKQQGVLALRPYLQKQPQPSAPEGKTVSNEPELPSRAFSSFCLTRLPVAGERNVLITSALPYVNNVPHLGNI

IGCVLSADVFARYSRLRQWNTLYLCGTDEYGTATETKAMDEGLTPQEICDKYHVIHADIYRWFNISFDIFGRTTTPQQTK

ITQDIFQRLLARGFVLQDSVEQLRCERCARFLADRFVEGVCPFCGYEEARGDQCDKCGKLINAIELKKPLCKVCRSSPVV

KSSQHLFLDLPKLEKRVEEWLERTLPGSDWTPNARFIIRSWLRDGLKPRCITRDLKWGTPVPLEGFESKVFYVWFDATIG

YLSITANYTDQWEKWWKNPEQVELYQFMAKDNVPFHGIVFPCSALGAEDNYTLVSHLIATEYLNYEDGKFSKSRGVGVFG

DMAQDTGIPADIWRFYLLYIRPEGQDSAFSWTDMLLKNNSELLNNLGNFINRIRDALRSILTISRHGNQYIQVNEPWKQI

KGSEADRQRAGTVTGVAVNIAALLSVMLQPYMPTVSATIQTQLQLPPPACNILVTDFLCTLPAGHQIGTVSPLFQKLEND

QIESLRQRFGGGQVKPSPKPAVVETVTAAGPQQIQVLMEEVTKQGNIVRELKAQKADKNLVAAEVAKLLDLKKQLALAEG

KPLEAPKGKKKK

>XP_019651771.1 PREDICTED: methionine--tRNA synthetase, cytoplasmic isoform X2 [Ailuropoda melanoleuca]

MGSYGAAVVQGKKGEDVLGPVRRALTHIDQSLSRRSCPFLAGETESLADVVLWGALYPLLQDPAYLPEELGALHSWFQTL

SSQEPCQRAAETVLKQQGVLALRPYLQKQPQPGSFEGRAVSNEPEEEGLAALSEEEIAMAVTAWEKGLESLPPLRPQQTP

VLPVAGERNVLITSALPYVNNVPHLGNIIGCVLSADVFARYSRLRQWNTLYLCGTDEYGTATETKAMEEGLTPQEICDKY

HAIHADIYRWFNISFDTFGRTTTPQQTKITQDIFQRLLTRGFVLQDTVEQLRCERCARFLADRFVEGVCPFCGYEEARGD

QCDKCGKLINAVELKKPQCKVCRSCPVVKSSRHLFLDLPKLEKRLEEWLGKTLPGSDWTPNARFIIRSWLRDGLKPRCIT

RDLKWGTPVPLEGFEDKVFYVWFDATIGYLSITANYTDQWERWWKNPEQVNLYQFMAKDNVPFHGLVFPCSALGAEDNYT

LVNHLIATEYLNYEDGKFSKSRGVGVFGDMAQDTGIPADIWRFYLLYIRPEGQDSAFSWTDMLLKNNSELLNNLGNFINR

AGMFVSKFFGGYVPEMVLTSDDRRLLAHVTLELQHYHQLLEKVRIREALRSILTISRHGNQYIQVNEPWKRIKGSEADRQ

RAGTVTGLAVNIAALLSVMLQPYMPTVGGTIQAQLQLPPPACRVLLTNFVCTLPAGHQIGTVSPLFQKLENDQIESLRQR

FGGGQAKAPAAPAVVEAVVTAAPQQVQVLMDEVTKQGNIVRELKAQKADKNQVAAEVAKLLDLKKQLALAEGKPPETPKG

KKKK

>XP_019597800.1 PREDICTED: methionine--tRNA synthetase, cytoplasmic isoform X3 [Rhinolophus sinicus]

MGSNRTTVVQGKKGEDVLGPVRRALTHIDHSLSRQSCPFLAGETESLADIVLWGALYPLLQDPAYVPEELGALHSWFQTL

SSQEPCQRAAETVLKQQGILALRPYLQKQPQPSSLERRAVSNEPEEEELATLSEEDIAVAVTAWEKGLGSLPPLRPKQNP

VLPVAGERNVLITSALPYVNNVPHLGNIIGCVLSADVFARYSRLRQWNTLYLCGTDEYGTATETKAMEEGLTPQEICDKY

HVIHADIYRWFNISFDIFGRTTTPQQTEITQDIFQRLWNRGFVLQDTVEQLRCERCARFLADRFVEGVCPFCAYEEARGD

QCDKCGKLINAIELKKPQCKVCRSRPVVKSSRHLFLDLPKLEDQLEGWLGKTLPGSDWTPNARFIIRSWLRDGLKPRCIT

RDLKWGTPVPLKGFEDKVFYVWFDATFGYLSITANYTDQWERWWKNPEQVNLYQFMAKDNVPFHGLVFPCSALGAEDNYT

LVSHLIATEYLNYEDGKFSKSRGVGVFGDMAQDTGIPADIWRFYLLYIRPEGQDSAFSWTDMLLKNNSELLNNLGNFINR

AGMFVSKFFGGYVPEMMLTSDDQRLLAHVTLELKHYHQLLEKVRIRDALRSILTISRHGNQYIQVNEPWKRIKGNEADRQ

RAGTVTGLAVNIAALLSIMLQPYMPTVSATIAAQLQLPTAACSILPTDFLCTLPAGHQIGTVSPLFQKLENDQIESLRQR

FGGGQLEESLELKAKASPKPAVVETVTAAGSQQIQVLMDEVTKQGNIVRELKAQKADKNQVAAEVAKLLELKKQLALAEG

KPLDTPKGKKKK

>XP_012014779.1 PREDICTED: methionine--tRNA synthetase, cytoplasmic isoform X3 [Ovis aries musimon]

MGSNRAAVVQGKKGEDVLGPVRRALMHIDHSLSRRSCPFLAGETESLADIVLWGALYPLLQDPSYLPEELGALHSWFQTL

SSQEPCQRAAETVLKQQGVLALRPYLQKQPQPGSLEGRLISNEPEEEELATLSEEEIAVAVAAWEKGLESLPPLRPQQNP

VLPVAGERNVLITSALPYVNNVPHLGNIIGCVLSADVFARYSRLRQWNTLYLCGTDEYGTATETKAMEEGLTPQEICDKY

HVIHADIYRWFNISFDFFGRTTTPQQTKITQDIFQRLLARGFVLQDTVEQLRCEHCARFLADRFVEGVCPFCGYEEARGD

QCDKCGKLINAIELKKPQCKVCRSCPVVKSSQHLFLDLPKLGARVEEWLEKTLPGSDWTPNARFIIRSWLRDGLKPRCIT

RDLKWGTPVPLEGFEDKVFYVWFDATIGYLSITANYTDQWEKWWKNPEQVNLYQFMAKDNVPFHGIVFPSSALGAEDNYT

LVSHLIATEYLNYEDGKFSKSRGVGVFGDMAQDTGIPADIWRFYLLYIRPEGQDSAFSWTDMLFKNNSELLNNLGNFINR

AGMFVSKFFGGFVPEMVLTSDDQRLLTHVTLELQHYHQLLEKVRIRDALRSILTISRHGNQYIQVNEPWKRIKGGEADRQ

RAGTVTGLAVNIAALLSVMLQPYMPTVSATIQAQLQLPPPARSILPTNFLCTLPAGHQIGTVSPLFQKLENDQIESLKQR

FSGGQAKASPKTAAGPQQIQALTEEVTKQGNIVRELKAQKADKNQIAAEVAKLLDLKKQLAQAEGKPLETPKGKKKK

>XP_012030062.1 PREDICTED: methionine--tRNA synthetase, cytoplasmic isoform X3 [Ovis aries]

MGSNRAAVVQGKKGEDVLGPVRRALMHIDHSLSRRSCPFLAGETESLADIVLWGALYPLLQDPSYLPEELGALHSWFQTL

SSQEPCQRAAETVLKQQGVLALRPYLQKQPQPGSLEGRLISNEPEEEELATLSEEEIAVAVAAWEKGLESLPPLRPQQNP

VLPVAGERNVLITSALPYVNNVPHLGNIIGCVLSADVFARYSRLRQWNTLYLCGTDEYGTATETKAMEEGLTPQEICDKY

HVIHADIYRWFNISFDFFGRTTTPQQTKITQDIFQRLLARGFVLQDTVEQLRCEHCARFLADRFVEGVCPFCGYEEARGD

QCDKCGKLINAIELKKPQCKVCRSCPVVKSSQHLFLDLPKLGARVEEWLEKTLPGSDWTPNARFIIRSWLRDGLKPRCIT

RDLKWGTPVPLEGFEDKVFYVWFDATIGYLSITANYTDQWEKWWKNPEQVNLYQFMAKDNVPFHGIVFPSSALGAEDNYT

LVSHLIATEYLNYEDGKFSKSRGVGVFGDMAQDTGIPADIWRFYLLYIRPEGQDSAFSWTDMLFKNNSELLNNLGNFINR

AGMFVSKFFGGFVPEMVLTSDDQRLLTHVTLELQHYHQLLEKVRIRDALRSILTISRHGNQYIQVNEPWKRIKGGEADRQ

RAGTVTGLAVNIAALLSVMLQPYMPTVSATIQAQLQLPPPARSILPTNFLCTLPAGHQIGTVSPLFQKLENDQIESLKQR

FSGGQARASPKTAAGPQQIQALTEEVTKQGNIVRELKAQKADKNQIAAEVAKLLDLKKQLAQAEGKPLETPKGKKKK

>XP_012376203.1 methionine--tRNA synthetase, cytoplasmic isoform X3 [Dasypus novemcinctus]

MGSNRAAVVQGKKREDVLDPVRRALTHIDHSLSRRSCPFLAGETESVADIVLWGALYPLLQDPAYLPEELGALRSWFQTL

SIQEPCQQAAETVLKQQGALAFRPYLQKQPQPSPLEGRAVSNEPEEEELATLSEEEIAMAVTAWERGLGSLPTLRPQQNP

VLPVAGERNVLITSALPYVNNVPHLGNIIGCVLSADVFARYSRLRQWNTLYLCGTDEYGTATETKAMEEGLTPQQICDKY

HIIHADIYRWFNISFDIFGRTTTPQQTLITQDIFQRLLVRGFVLQDTVEQLRCERCARFLADRFVEGVCPFCGYEEARGD

QCDKCGKLINAIELKKPQCKVCRSCPVVKSSKHLFLDLPKLEKRLEEWLEKTLPGSDWTTNAQFITRSWLRDGLKPRCIT

RDLKWGTPVPLEGFEDKVFYVWFDATFGYISITANYTDEWERWWKNPEQVDLYQFMAKDNVPFHGLVFPCSVLGAEDNYT

LVKHLIATEYLNYEDGKFSKSRGVGVFGDMAQDTGIPADIWRFYLLYIRPEGQDSAFSWTDMLLKNNSELLNNLGNFINR

AGMFVSKFFGGRVPEMVLIPDDQRLLAHVTMELRHFHQLLEKVRIRDALRSILTISRHGNQYIQVNEPWKRIKGNEADRQ

RAGTVTGLAVNIAALLSIMLQPYMPAVSATIQAQLQLPPPACNILLTDFLCTLPAGHQIGTVSPLFQKLENDQMEGLRQR

FGGGQLEESLELKAKPSPKPAAAETVTATGQQQIHMLMNEVTKQGNIVRELKAQKADKNQVAAEVAKLLELKKQLALAEG

KPTEPPKGKKKK

>XP_016080013.1 PREDICTED: methionine--tRNA synthetase, cytoplasmic isoform X3 [Miniopterus natalensis]

MGSNRTAVVQGKKGEDVLGPVRRALAHIDHSLSRRSCPFLAGETESVADVVLWGALYPLLQDPAYLPEELGALHSWFQTL

SSQEPCQRAAETVLKQQGILALRPYLQKQPQPNFLEGRAVSNEPEEEELATLSEDEIAMAVTAWEKGLGSLPPLRPKQNP

VLPVAGERNVLITSALPYVNNVPHLGNIIGCVLSADVFARYSRLRQWNTLYLCGTDEYGTATETKAMEEGLTPQEICDKY

HVIHADVYRWFNISFDIFGRTTTQQQTKITQDIFQRLWNRGFVLQDAVEQLRCERCARFLADRFVEGVCPFCGYEEARGD

QCDKCGKLINAIELKKPQCKVCRSCPVVKSSRHLFLDLPKLEDRLEEWLGKTLPGSDWTPNARFIIRSWLRDGLKPRCIT

RDLKWGTPVPLEGFEDKVFYVWFDATFGYLSITANYTDQWERWWKNPEQVSLYQFMAKDNVPFHGLVFPCSALGAEDNYT

LVSHLIATEYLNYEDGKFSKSRGVGVFGDMAQDTGIPADIWRFYLLYIRPEGQDSAFSWTDMLLKNNSELLNNLGNFINR

AGMFVSKFFGGYVPEMVLTSDDQRLLAHVTLELQHYHQLLEKVRIRDALRSILTISRHGNQYIQVNEPWKRIKGSEADRQ

RAGTVTGLAVNIAALLSIMLQPYMPTVSSTIAAQLQLPSPACSILPTDFLCTLPTGHQIGTVSPLFQKLENEQMESLRQR

FSGGQLEESFTLKAKVSPKPAVVETVATAGPQQIQMLMDEVTKQGNIVRELKAQKADKNQVAAEVAKLLDLKKQLALAEG

KPLETPKGKKKK

>XP_021569488.1 methionine--tRNA synthetase, cytoplasmic isoform X4 [Carlito syrichta]

MGSDRAAALSAALYYLVVQGKKGEEVLGPVRRALTHIDHSLSRQNCPFLAGETESLTDIVLWGALYPLLQDPAYLPEELS

ALRSWFQTLSTQEPCQQAAETVLKQQGVLAFRPYLQKQPQPSPPEGKPITNEPEEEELATLSEEEIAMAVAAWEKGLDSL

PPLRPQQNPVLPVDGERNVLITSALPYVNNVPHLGNIIGCVLSADVFARYSRLRQWNTLYLCGTDEYGTATETKAMEEGL

SPQEICDKYHTIHADIYRWFNISFDIFGRTTTPQQTKITQDIFQRLLARGFVLQDTVEQLRCERCARFLADRFVEGVCPF

CRYEEARGDQCDKCGKLINAIELKKPQCKLCRSCPVVKSSRHLFLDLPKLEKRVEEWLGKSLPGSDWTPNARFITRSWLR

DGLKPRCITRDLKWGTPVPLEGFEDKVFYVWFDATIGYLSITANYTDQWERWWKNPEQVDLYQFMAKDNVPFHGIVFPCS

VLGAEDNYTLVSHLIATEYLNYEDGKFSKSRGVGVFGDMAQDTGIPADIWRFYLLYIRPEGQDSAFSWMDMLLKNNSELL

NNLGNFINRAGMFVSKFFGGCVPEMVLTSDDQRLVAHVTLELRHYHQLLEKVRIRDALRSILTISRHGNQYIQVNEPWKQ

IKGSEADRQRAGTVTGLAVNIAALLSIMLQPYMPMVSATIQAQLQLPPPACSILPTDFLCTLSAGHRIGIVSPLFQKLEN

DQIENLRQRFGGGQLEESFKLKAKAAPKPAAEEAVPTARPQQIQALMDAVTKQGNIVRELKAQKADKNQIAAEVAKLLDL

KKHLALAEGKPLETPKGKKKK

>XP_019513164.1 PREDICTED: methionine--tRNA synthetase, cytoplasmic isoform X3 [Hipposideros armiger]

MGSNRTTVVQGKKGEDVLGLVRRALTHIDQSLSRQSCSFLVGETESLADIVLWGALYPLLQDPAYLPEELGALHSWFQTL

SSQEPCQRAAETVLKQQGILALRPYLQKQPQPSSLERRVVSNEPEEEELATLSEEDIATAVTAWEKGLGSLPPLRPKQIP

VLPVAGERNVLITSALPYVNNVPHLGNIIGCVLSADVFARYSRLRQWNTLYLCGTDEYGTATETKAMEEGLTPQEICDKY

HVIHANIYHWFNISFDIFGRTTTPQQTQITQDIFQRLWNRGFVLQDTVEQLRCERCARFLADRFVEGVCPFCGYEEARGD

QCDKCGKLINATELKKPQCKVCRSCPVVKSSQHLFLDLPKLEDRLEEWLGKTLSGSDWTPNARFIIRSWLRDGLKPRCIT

RDLKWGTPVPLKGFEDKVFYVWFDATFGYLSITANYTDQWERWWKNPEQVNLYQFMAKDNVPFHGLVFPCSALGAEDNYT

LVSHLIATEYLNYEDGKFSKSRGVGVFGDMAQDTGIPADIWRFYLLYIRPEGQDSAFSWTDLLLKNNSELLNNLGNFINR

AGMFVSKFFGGYVPEMMLTSDDQRLLAHVTLELQHYHQLLEKVRIRDALRSILTISRHGNQYIQVNEPWKRIKGSEADRQ

RAGTVTGLAVNIAALLSIMLQPYMPTVSATIAAQLQLPPPACSILPTDFLCTLPAGHQIGTVSPLFQKLENDQIESLRQR

FGGGQLEESLELKAKASPKPAVVETVTAAGSQQIQVLMDEVTKQGNIVRELKAQKADKNQVAAEVAKLLDLKKQLALAEG

KPLETPKGKKKK

>EDL24497.1 methionine-tRNA synthetase, isoform CRA_a, partial [Mus musculus]

RGTMRLFVSEGSPGSLPVLAAAARARGRAELLISTVGPEECVVPFLTRPKVPVLQLDSGNYLFSASAICRYFFLLCGWEQ

DDLTNQWLEWEATELQPVLSAALHCLVVQGKKGEDILGPLRRVLTHIDHSLSRQNCPFLAGDTESLADIVLWGALYPLLQ

DPAYLPEELGALQSWFQTLSTQEPCQRAAETVLKQQGVLALRLYLQKQPQPQPPPPEGRTVSNELEEEELATLSEEDIVT

AVAAWEKGLESLPPLKLQQHPVLPVPGERNVLITSALPYVNNVPHLGNIIGCVLSADVFARYCRLRQWNTLYLCGTDEYG

TATETKAMEEGLTPREICDKYHAIHADIYRWFGISFDTFGRTTTPQQTKITQDIFQRLLTRGFVLRDTVEQLRCERCARF

LADRFVEGVCPFCGYEEARGDQCDRCGKLINAIELKKPQCKICRSCPVVRSSQHLFLDLPKLEKRLEDWLGKTVPGSDWT

PNARFIIRSWLRDGLKPRCITRDLKWGTPVPLEGFEDKVFYVWFDATIGYVSITANYTDQWEKWWKNPEQVDLYQFMAKD

NVPFHGLVFPCSVLGAEDNYTLVKHIIATEYLNYEDGKFSKSRGIGVFGDMAKDTGIPADIWRFYLLYIRPEGQDSAFSW

TDLLIKNNSELLNNLGNFINRAGMFVSKFFGGCVPEMALTPDDRRLVAHVSWELQHYHQLLEKVRIRDALRSILTISRHG

NQYIQVNEPWKRIKGGEMDRQRAGTVTGMAVNMAALLSVMLQPYMPTVSSTIQTQLQLPPAACRILATSFICTLPAGHRI

GTVSPLFQKLENDQIENLRQRFGGGQVSKLDCALPQQKPWQCPPISPSILTLSFHL

>EHH20895.1 Methionyl-tRNA synthetase, cytoplasmic [Macaca mulatta]

MGWSGRLHSHVWHLEELSALHSWFQTLSTQEPCQRAAETVLKQQGVLALRPYLQKQPQPSPAEGRAVTNEPEEEELATLS

EEEIAMAVTAWEKGLESLPPLRPQQNPVLPVTGERNVLITSALPYVNNVPHLGNIIGCVLSADVFARYSRLRQWNTLYLC

GTDEYGTATETKAVEEGLTPQEICDKYHVIHADIYRWFNISFDTFGRTTTPQQTKITQDIFQRLLTRGFVLQDTVEQLRC

EHCARFLADRFVEGVCPFCGYEEARGDQCDKCGKLINAVELKKPQCKVCRSCPVVQSTHHLFLDLPKLEKRLEEWLGRTL

PGSDWTPNARFITRSWLRDGLKPRCITRDLKWGTPVPLEGFEDKVFYVWFDATIGYLSITANYTDQWERWWKNPEQVDLY

QFMAKDNVPFHGIVFPCSALGAEDNYTLVSHLIATEYLNYEDGKFSKSRGVGVFGDMAQDTGIPADIWRFYLLYIRPEGQ

DSAFSWTDLLLKNNSELLNNLGNFINRAGMFVSKFFGGYVPEMVLTPDDQRLLAHVTLELQHYHQLLEKVRIRDALRSIL

TISRHGNQYIQVNEPWKRIKGSEADRQRAGTVTGLAVNIAALLSVMLQPYMPTVSATIQAQLQLPPPACSILLTNFLCTL

PAGHQIGTVSPLFQKLENDQIESLRQRFGGGQLEETFDLKAKTSPKPAVIETVTTAGPQQIQALMDEVTKQGNIVRELKA

QKADKNQVAAEVAKLLDLKKQLAVAEGKPPEAPKGKKKK

>KFO24536.1 Methionyl-tRNA synthetase, cytoplasmic [Fukomys damarensis]

MRLFVSEGAPGSLPVLAAAARAQGLAELLISTVGPEEELGALRSWFQTLSTQEPCQRAAETVLKQRGVLALRPYLQKQPQ

PSPPEGRGVSNEPEEEELATISEEEIAMAVTAWEKGPESLPPLRPRQNPVLPVAGERNVLITSALPYVNNVPHLGNIIGC

VLSADVFARYSRLRQWNTLYLCGTDEYGTATETKAMEEGLTPQEICDKYHTIHADIYHWFNISFDIFGRTTTPQQTKITQ

DIFQRLLTRGFVLQDTVEQLRCEHCARFLADRFVEGVCPFCGYEEARGDQCDKCGKLINAIELKKPQCKVCRSCPVVKSS

QHLFLDLPKLEKRLEEWLGKTLPGSDWTPNARFIIRSWLRDGLKPRCITRDLKWGTPVPLEGFEDKVFYVWFDATIGYLS

ITANYTDQWERWWKNPEQVDLYQFMAKDNVPFHGLVFPCSALGAEDNYTLVKYLIATEYLNYEDGKFSKSRGVGVFGDMA

QNTGIPADIWRFYLLYIRPEGQDSAFSWADMLLKNNSELLNNLGNFINRAGMFVSKFFGGYVPEMVLTPDDQRLLARITL

ELQNYHQLLEKVRIRDALRSILTVSRHGNQYIQVNEPWKQIKGSEADRQRAGTVTGMAVNIAALLSVMLEPYMPSVSATI

QAQLQLPLPACSILLTDFVCTLPAGHQIGTVSPLFQKLENDQIESLKQRFGGGQLEESLELKAKVSPTPAVVETVTTAGP

QQIQTLMDEVTKQGNTVRELKAQKADKNQVAAEVAKLLDLKKQLALAEGKPLETPKGKKKK

>XP_006719461.1 methionine--tRNA synthetase, cytoplasmic isoform X1 [Homo sapiens]

MAVTAWEKGLESLPPLRPQQNPVLPVAGERNVLITSALPYVNNVPHLGNIIGCVLSADVFARYSRLRQWNTLYLCGTDEY

GTATETKALEEGLTPQEICDKYHIIHADIYRWFNISFDIFGRTTTPQQTKITQDIFQQLLKRGFVLQDTVEQLRCEHCAR

FLADRFVEGVCPFCGYEEARGDQCDKCGKLINAVELKKPQCKVCRSCPVVQSSQHLFLDLPKLEKRLEEWLGRTLPGSDW

TPNAQFITRSWLRDGLKPRCITRDLKWGTPVPLEGFEDKVFYVWFDATIGYLSITANYTDQWERWWKNPEQVDLYQFMAK

DNVPFHSLVFPCSALGAEDNYTLVSHLIATEYLNYEDGKFSKSRGVGVFGDMAQDTGIPADIWRFYLLYIRPEGQDSAFS

WTDLLLKNNSELLNNLGNFINRAGMFVSKFFGGYVPEMVLTPDDQRLLAHVTLELQHYHQLLEKVRIRDALRSILTISRH

GNQYIQVNEPWKRIKGSEADRQRAGTVTGLAVNIAALLSVMLQPYMPTVSATIQAQLQLPPPACSILLTNFLCTLPAGHQ

IGTVSPLFQKLENDQIESLRQRFGGGQAKTSPKPAVVETVTTAKPQQIQALMDEVTKQGNIVRELKAQKADKNEVAAEVA

KLLDLKKQLAVAEGKPPEAPKGKKKK

>EPY82940.1 methionine-tRNA synthetase isoform 3-like protein [Camelus ferus]

MRLFVSEGAPGTLPVLAAAGRAQGRVELLISTVGPEGFDTLFIPMPTEELGALNSWFQTLSSQEPCQRAAETVLKQQGVL

ALRPYLQKQPQPSSFEGRAVSNEPEEEELAALSEEEIALAVAAWEKGLGSLPPLQPQQSPVLPVAGERNVLITSALPYVN

NVPHLGNIIGCVLSADVFARYSRLRQWNTLYLCGTDEYGTATETKAMEEGLTPQEICDKYHAIHADIYRWFNISFDIFGR

TTTPQQTKITQDIFQRLLARGFVLQDTVEQLRCERCARFLADRFVEGVCPFCGFEEARGDQCDKCGKLINATELKKPQCK

ICRSCPVVKSSRHLFLDLPKLEKRLEEWLEKTLPGSDWTPNARFIIRSWLRDGLKPRCITRDLKWGTPVPLEGFEDKVFY

VWFDATIGYVSITANYTDQWERWWKNPEQVDLYQFMAKDNVPFHGLVFPCSVLGAEDNYTLVKNLIATEYLNYEDGKFSK

SRGVGVFGDMAQDTGIPADIWRFYLLYIRPEGQDSAFSWTDMLLKNNSELLNNLGNFINRAGMFVSKFFGGFVPEMVLTS

DDRRLLAHITLELQHYHQLLEKVRIRDALRSILTISRHGNQYIQVNEPWKRIKGSEADRQRAGTVTGLAVNIAALLSVML

QPYMPTVSTTIQAQLQLPPPTCSILPTNFLCILPAGHQIGTVSPLFQKLENDQIESLRQRFSGGQLEESLALKAKAGPKP

AAAGTMTTAGPEQIQVLMDEVTKQGNIVRELKAQKADKNQVAAEVAKLLDLKKQLALAEGKPLETPKGKKKK

>XP_017714340.1 PREDICTED: methionine--tRNA synthetase, cytoplasmic [Rhinopithecus bieti]

MAVTAWENGLESLPPLRPQQNPVLPVTGERNVLITSALPYVNNVPHLGNIIGCVLSADVFARYSRLRQWNTLYLCGTDEY

GTATETKAMEEGLTPQEICDKYHIIHADIYRWFNISFDTFGRTTTPQQTKITQDIFQRLLTRGFVLQDTVEQLRCEHCAR

FLADRFVEGVCPFCGYEEARGDQCDKCGKLINAVELKKPQCKVCRSCPVVQSTHHLFLDLPKLEKRLEEWLGRTLPGSDW

TPNARFITRSWLRDGLKPRCITRDLKWGTPVPLEGFEDKVFYVWFDATIGYLSITANYTDQWERWWKNPEQVDLYQFMAK

DNVPFHGIVFPCSALGAEDNYTLVSHLIATEYLNYEDGKFSKSRGVGVFGDMAQDTGIPADIWRFYLLYIRPEGQDSAFS

WTDLLLKNNSELLNNLGNFINRAGMFVSKFFGGYVPEMVLTPDDQRLLAHVTLELQRYHQLLEKVRIRDALRSILTISRH

GNQYIQVNEPWKRIKGSEADRQRAGTVTGLAVNIAALLSVMLQPYMPTVSATIQAQLQLPPPACSILLTNFLCTLPAGHQ

IGTVSPLFQKLENDQIESLRQRFGGGQLEETFDLKAKTSPKPAVIETVTTAGPQQIQALMDEVTKQGNIVRELKAQKADK

NQVAAEVAKLLDLKKQLAVAEGKPPEAPKGKKKK

>EHB13062.1 Methionyl-tRNA synthetase, cytoplasmic [Heterocephalus glaber]

MTVYTLQLEKHFRKTVDLESLPVLAAASRAQGRAELLLSTVGPEECVVPFLTKPKVPVLQLDSGNYLFSTSAICRYFFLL

SGWEQDDLTNQWLEWEATELQPALSTALYYSVVQGKKGEDVLGPIGRALTHTDHSLSHQNCPFLAGETESLADIVLWGAL

YPLLQDPAYLPEELGALRSWFQTLSTQEPCQRAAQTVLKQRGVLALRPYLQKQTQPSPPEGTAWEKGPESLPLLWPQQNP

VLPVAGERNVLITSGLPYVNHVPHLGNIIGCVLSAEVFARCSHLCQWNTLYLCGADEYGPATETKAVEEGLTPQGICDKY

HTIHADIYHWFNISFDIFGRTTTPQQTQITQDIFQRLLARGFVFQDSVEQLQCECCACFLADHFVEGVGPFCGYEEARGD

QCDKCGKLINATELKKPQCKLEKTLEEWLGKTLPGSDWTLNAWFIIQSWLRDGLKPHCITRDLKWGTPVPLEGFEDKVFY

AWFDATIGYLSITANYTDQWERWWKNPEQVDLYQFMAKDNVPFHGLVFPCSALGAEDNYTLVKHLIATDMLLKNNSELLN

NFINRAGMFVSKFFGGYVPEMVLTPDDQRLLAHITLELRHYHQLLQKVRIQDALCSILTVSRHGNQYIQVNEPWKRIKGS

EDDRQRAGTVTGMAVNIAALLSVMLQPYMPAVSATVQAQLQLPLPVFSVLLTDFMCTLPAGHQIGTVSPLFQKLENDQIE

SLQQCFGGGQEKVSPMPAVLETVETAGAQQIQTLMDEVTKQDNIVRELKAQKADKNQVASEVAKLLDPKKQLALARRNPL

KLLKARRKSEKPFIKSQKSL

>PNJ44590.1 MARS isoform 13, partial [Pongo abelii]

MRLFVSEGVPGCLPVLAAAGRARGRAEVLISTVGPEDCVVPFLTRPKVPVLQLDSGNYLFSTNAICRYFFLLSGWEQDDL

TNQWLEWEATQLQPTLSAALYYLVVQGKKGEDVLGSVRRALTHIDHSLSRQNCPFLAGETESLADIVLWGALYPLLQDPA

YLPEELSALHSWFQTLSTQEPCQQAAETVLKQQGVLALRPYLQKQPQPSSPEGRAVTNEPEEEELATLSEEEIAMAVTAW

EKGLESLPPLRPHQNPVLPVAGERNVLITSALPYVNNVPHLGNLIGCVLSADVFARYSRLRQWNTLYLCGTDEYGTATET

KALEEGLTPQEICDKYHIIHADIYRWFNISFDIFGRTTTPQQTKITQDIFQRLLKRGFVLQDTVEQLRCEHCARFLADRF

VEGVCPFCGYEEARGDQCDKCGKLINAIELKKPQCKVCRSCPVVQSSQHLFLDLPKLEKRLEEWLGRTLPGSDWTPNARF

IIRSWLRDGLKPRCITRDLKWGTPVPLEGFEDKVFYVWFDATFGYLSITANYTDQWERWWKNPEQVDLYQFMAKDNVPFH

GLVFPCSALGAEDNYTLVSHLIATEYLNYEDGKFSKSRGVGVFGDMAQDTGIPADIWRFYLLYIRPEGQDSAFSWTDLLL

KNNSELLNNLGNFIN

>XP_019807884.1 PREDICTED: methionine--tRNA synthetase, cytoplasmic isoform X4 [Tursiops truncatus]

MMGEIVYFPGVWPGERPHSELHFPACFAAVAHPTGGRPVPVASAWDPRRNEVVRERGRPGEPARAGRRREGPGQSGAAYQ

HCRPGRYFFLLSGWEQDDLTNQWLEWEATELQPALSAALYYLVVQGKKGEDVFSPVRRALTHIDHSLSRRSCPFLAGETE

SLADIVLWGALYPLLQDPAYLPEELGALHSWFQTLSSQEPCQRAAETVLKQQGALALRPYLQKQPQPSFLEGRAVSNEPE

EEELATLSEEEIAMAVAAWEKGLESLPPVQPQQNPVLPVAGERNVLITSALPYVNNVPHLGNIIGCVLSADVFARYSRLR

QWNTLYLCGTDEYGTATETKAMEEGLTPQEICDKYHVIHADIYRWFNISFDIFGRTTTPQQTKITQDIFQRLVARGFVLQ

DTVEQLRCEHCARFLADRFVEGMCPFCGYEEARGDQCDKCGKLINAIELKKPQCKVCRSCPVVKSSRHLFLDLPKLGKPL

EEWLEKTLPGSDWTPNARFIIRSWLRDGLKPRCITRDLKWGTPVPLVGFEDKVFYVWFDATIGYLSITANYTDQWEKWWK

NPEQVSLYQFMAKDNVPFHGLVFPCSALGAEDNYTLVSHLIATEYLNYEDGKFSKSRGVGVFGDMAQDTGIPADIWRFYL

LYIRPEGQDSAFSWTDMLLKNNSELLNNLGNFINRAGMFVSKFFGGFVPEMVLTSDDRRLLAHVTLELQHYHQLLEKVRI

RDALRSILTISRHGNQYIQVNEPWKRIKGSEADSGQGQ

>XP_021088467.1 methionine--tRNA synthetase, cytoplasmic [Mesocricetus auratus]

MNASTVYFSLSPTGHRISADIVLWGALYPLLQDPNYLPEELSALQSWFQTLSTQEPCQRAAETVLKQQGVLALRPYLQKQ

PQPCLPEGRAISNEPEEEELATLSEEDILMAVTAWEKGVGSLPPLQPQQHPVLPVAGERNVLITSALPYVNNVPHLGNII

GCVLSADVFARYCRLRQWNTLYLCGTDEYGTATETKAMEEGLTPQEICDKYHAIHADIYRWFGISFDTFGRTTTPHQTKI

TQDIFQRLLTRGFVLQDTVEQLRCEQCARFLADRFVEGVCPFCGYEEARGDQCDKCGKLINAIELKKPQCKVCRSRPVVR

SSRHLFLDLPKLEKRLEDWLGKTLPGSDWTPNARFIIRSWLRDGLKPRCITRDLKWGTPVPLEGFEDKVFYVWFDATIGY

LSITANYTDQWERWWKNPEQVDLYQFMAKDNVPFHGLVFPCSALGAEDNYTLVKNLIATEYLNYEDGKFSKSRGVGVFGD

MAQDTGIPADIWRFYLLYIRPEGQDSAFSWTDMMIKNNSELLNNLGNFINRAGMFVSKFFGGCVPEMVLTQDDRRLLAHI

SWELQHYHQLLEKVRIRDALRSILTISRHGNQYIQVNEPWKRIKGSGVDRQRAGTVTGVAVNIAALLSVMLQPYMPTVSA

TIQTQLQLPPPACRILATNFICTLPAGHQIGTVSPLFQKLENDQIESLRQRFGGGQVRKLDCAFPANMSAPP

>OBS59155.1 hypothetical protein A6R68_09720, partial [Neotoma lepida]

GQAELLISTVGPEECVVPFLTRPKVPVLQLNSGNYLFSTSAICRYFFLLSGSEQDDLTNQWLEWEATELQPVLSAALHCL

VVQGKKGEDVXGPLRGALNXVDHSLSRQNCPFLARDTESLADIVLWGALYPLMQDSTYLPEELGALQSWFQTLSTQEPCQ

LAAETVLKQQGVLALHPYLQKQPQPQAGPPDGRAVSNEPEEKELATLSEEDIVVAVTVWEKDLGSLPPLQPQQHPVLPVA

XERNVLITSALPYVNNVPHLGNIIGCVLSADVFARYSRLXQWNTLXLCVTDEYDTATETKAVEEGLTPQEICDKYHAIHT

DIYRWFNISFDSFDTVEQLRCEQCARFLADRFVEGVCPFCGYEEAGGDQCDKCGKLINAIELKKPQCKVCQSCPVVRSSQ

HLFLDLRFEDKVFYVWFDATIGYLSITANYTDXWERWXKNPEQVDLYQFMAKDNVPFHGLVFPCSALGAEDNYTLVKHLI

ATEYLNYEDGKFSKSPGVGVFGDMAQDTGIPANIWRFYLLYIRPEGQDSAFSWTDMMIKNNSELLNNLGNFINRAGMFVS

KFFGGCVPEIVLAHEDRCLLAHISWELQHYHQLLEKVRIWDALCSILTISRHGNQYIQVNEPWKWIKGTEVDRQRAGIVT

GVAVNIAALLSVILQPYMPPVSSTIQTQLQLXLPTCRILVTNFICTLPASHRIGTVSPLFQKLENDQIENLRQRFGGGQA

KGSPKPAVVEAVXTAGSQQIQMLMDEVTKQGNIDRELKAQKADKNQVAAEVIKLLDLKKQLALAEGRPIETPKGKKKM

>XP_013816343.1 PREDICTED: methionine--tRNA synthetase, cytoplasmic [Apteryx australis mantelli]

MATLSPLSRPWVPTLELDSGTVLFSPNAICQYFFLASGEEPTDLTNQWLEWEATELQPAVLAALYAQLVQGKKGAEAVAA

LGKSLTYIDQSLSRRGTAFLAGDAKSVADVVVWGALFPVLQDESSLPDELKALRTWFQSMSLSEACRKAADSVLVPKGLL

EFKSYLQKQPPPCLAVERPTSNEPEEEEASERALTEEEIGAAADAWARGTAALPKPWQPQKPVLPVEGMRNVLITSALPY

VNNVPHLGNIIGCVLSADTFARYCRLRNWNTLYVCGTDEYGTATETKAVEEGLTPQEICDKYNAIHADIYRWFDISFDYF

GRTTTPHQTTIAQDIFQRLLARGFLLQDTVEQLRCEGCQRFLADRFVEGICPFCSYEEARGDQCDKCGKLINAVELKRPQ

CKLCRGIPVVRPTQHLFLDLPKLEERLEPWLEQSWTKGDWTANARYITRSWIRDGLKPRCITRDLKWGTPVPLDSFRDKV

FYVWFDAPIGYLSITANYTDQWERWWKNPQQVELYNFMAKDNVPFHSVVFPCSLLGADDNYTLVNHLIATEYLNYEDGKF

SKSRGVGVFGDMAKDTGIPADIWRFYLLYLRPEGQDSAFSWSDLMLKNNSELLNNLGNFINRAGMFVCKFFGGTVPAMVL

TPDDKRLLARVTLELHQYHQLLEKVRIRDALRCILSISRHGNQYIQVNEPWKRIKGDEEDRQRAGTVTGVAVNMASLLAV

MVQPYMPSVSAAIQAQLCIPPECFVLGHDFTCTLPAGHRVGTVSPLFQKLEHDQIEALRKRFGGGQPEDLTLVPQAKPTP

PASGSPPAPASGTPPAPGDPQLIQELTEQVAKQGNHVRQLKASKAEKVLVDAEVAKLLELKKQLVLAEGKSPEVPVPKGK

RKK

>XP_010569810.1 PREDICTED: methionine--tRNA synthetase, cytoplasmic [Haliaeetus leucocephalus]

MAAPAPVIPTPRRGPPPGLTGLLPPPPPADRPLAPLSPPWAPALELDSGTVLFSPNAICQFFFLARGEEPTDLTNQWLEW

EATELQPAASAALYAQLVHGRKGLEAVEVLGKLLAHIEQNLSRRGTAYLAGDTKSVADVVVWGTLFPVLQDETSLPSELQ

VLRTWFQSMTLSEACRKAADSVLMPKALLEFKSYLQKQPPPCLAMERATSNEPEEEEGSEPALTEEEITAAVDAWARGVA

ALPKPWQPQKPVLPVEGMRNVLITSALPYVNNVPHLGNIIGCVLSADTFARYCRLRNWNTLYVCGTDEYGTATETKAVEE

GLTPQEICNKYNTIHADIYRWFDISFDYFGRTTTPYQTMIAQDIFQRLLARGFLLQDTVEQLQCEDCQRFLADRFVEGTC

PFCSYEEARGDQCDKCGKLINAVELKNPQCKLCRGVPVVKPTQHLFLDLPKLEEQLEPWLEQSWATGDWTANARYITRSW

IRDGLKPRCITRDLKWGTPVPLDGFRDKVFYVWFDAPIGYLSITANYTDQWERWWKNPQQVELYNFMAKDNVPFHSVIFP

CSLLGADDNYTLVNHLIATEYLNYEDGKFSKSRGVGVFGDMAKDTGIPADIWRFYLLYLRPEGQDSTFSWTDLMLKNNSE

LLNNLGNFINRAGMFVCKFFGGTVPDMVLTPDDKRLLARVTLELHQYHQLLEKVRIRDALRCILSISRHGNQYIQVNEPW

KRIKGDEKDRQRAGTVTGVAVNMASMLAVMLQPYMPSVSLAIQGQLCIPPDCFILSHNFTCTLPPGHRVGTVSPLFQKLE

HDQIEALRKRFGGGQAKQTPPASVTLTAPGDPQLIQELTEEVAKQGNHVRQLKANKAEKAQVDAEVAKLLELKKQLALAE

GKSPEVLVPKGKRKK

>OPJ90009.1 methionine--tRNA synthetase, cytoplasmic isoform A [Patagioenas fasciata monilis]

MRLRVAPGGPAALKVLAAAGAAAAPVRLVWGDPLERPLASLSPPWVPALELDSGTVLFSPNAICQFFFLARGEEPTDLTN

QWLEWEATELQPAASAALYAQLVHGKKGLEAVEALGQLLDHIEQNLTRRGTAYLAGDTKSVADVVVWGALFPVLQDETSL

PSELQALRTWFQNMTLSEACRKAADSVLMPKALLEFKSYLQKHPPPCVATERATSNEPEEEDGSERALTEEEITAAVDAW

ARGVAALPKPWKPQKPVLPVEGMRNVLITSALPYVNNVPHLGNIIGCVLSADTFARYCRLRNWNTLYVCGTDEYGTATET

KAMEEGLTPQEICNKYNAIHADIYRWFDISFDYFGRTTTPHQTTIAQDIFQRLLARGFLLQDTVEQLRCEDCQRFLADRF

VEGVCPFCNYEEARGDQCDKCGKLINAVELKNPQCKLCRGVPVVKTTQHLFLDLPKLEERLEPWLEQSWATGDWTANARY

ITRSWIRDGLKPRCITRDLKWGTPVPLDGFQDKVFYVWFDAPIGYLSITANYTDQWERWWKNPQQVELYNFMAKDNVPFH

SVIFPCSLLGADDNYTLVNHLIATEYLNYEDGKFSKSRGVGVFGDMAKDTGIPADIWRFYLLYLRPEGQDSAFSWSDLML

KNNSELLNNLGNFINRAGMFVCKFFGGVVPDMVLTLDDKRLLARVTLELRQYHQLLEKVRIRDALRCILSISRHGNQYIQ

VNEPWKRIKGDEKDRQRAGTVTGVAVNMASMLAIMLQPYMPSVSLAIQGQLRIPPDCFILSHDFTCTLPAGHCVGTVSPL

FQKLENDQVESLRKRFGGGQTEDVAVKPQAEQTHPAPATLGAPGDPQLIQELTEKVAKQGNYVRQLKANKAEKGEVDTEV

AKLLELKKQLLLAEGKSPEVPVPKGKRKK

>OPJ90010.1 methionine--tRNA synthetase, cytoplasmic isoform B [Patagioenas fasciata monilis]

MRLRVAPGGPAALKVLAAAGAAAAPVRLVWGDPLERPLASLSPPWVPALELDSGTVLFSPNAICQFFFLARGEEPTDLTN

QWLEWEATELQPAASAALYAQLVHGKKGLEAVEALGQLLDHIEQNLTRRGTAYLAGDTKSVADVVVWGALFPVLQDETSL

PSELQALRTWFQNMTLSEACRKAADSVLMPKALLEFKSYLQKHPPPCVATERATSNEPEQEEDGSERALTEEEITAAVDA

WARGVAALPKPWKPQKPVLPVEGMRNVLITSALPYVNNVPHLGNIIGCVLSADTFARYCRLRNWNTLYVCGTDEYGTATE

TKAMEEGLTPQEICNKYNAIHADIYRWFDISFDYFGRTTTPHQTTIAQDIFQRLLARGFLLQDTVEQLRCEDCQRFLADR

FVEGVCPFCNYEEARGDQCDKCGKLINAVELKNPQCKLCRGVPVVKTTQHLFLDLPKLEERLEPWLEQSWATGDWTANAR

YITRSWIRDGLKPRCITRDLKWGTPVPLDGFQDKVFYVWFDAPIGYLSITANYTDQWERWWKNPQQVELYNFMAKDNVPF

HSVIFPCSLLGADDNYTLVNHLIATEYLNYEDGKFSKSRGVGVFGDMAKDTGIPADIWRFYLLYLRPEGQDSAFSWSDLM

LKNNSELLNNLGNFINRAGMFVCKFFGGVVPDMVLTLDDKRLLARVTLELRQYHQLLEKVRIRDALRCILSISRHGNQYI

QVNEPWKRIKGDEKDRQRAGTVTGVAVNMASMLAIMLQPYMPSVSLAIQGQLRIPPDCFILSHDFTCTLPAGHCVGTVSP

LFQKLENDQVESLRKRFGGGQTEDVAVKPQAEQTHPAPATLGAPGDPQLIQELTEKVAKQGNYVRQLKANKAEKGEVDTE

VAKLLELKKQLLLAEGKSPEVPVPKGKRKK

>XP_017832047.1 PREDICTED: methionine--tRNA synthetase, cytoplasmic, partial [Callithrix jacchus]

SPRHALFSSSFLTRLPVAGERNVLITSALPYVNNVPHLGNIIGCVLSADVFARYSRLRQWNTLYLCGTDEYGTATETKAM

EEGLTPQEICDKYHIIHADIYRWFNISFDVFGRTTTPQQTKITQDIFQRLLARGFVLQDTVEQLRCERCARFLADRFVEG

VCPFCGFEEARGDQCDKCGKLINAVELKKPQCKVCRSCPVVKSSQHLFLDLPKLEKRLEEWLGRTLPGSDWTPNAWFITR

SWLRDGLKPRCITRDLKWGTPVPLEGFEDKVFYVWFDATIGYLSITANYTDQWERWWKNPEQVDLYQFMAKDNVPFHGIV

FPCSALGAEDNYTLVSHLIATEYLNYEDGKFSKSRGVGVFGDMAQDTGIPADIWRFYLLYMRPEGQDSTFSWTDMLLKNN

SELLNNLGNFINRAGMFVSKFFGGYVPEMVLTPDDQRLLAHVTLELRHYHQLLEKVRIRDALRSILTISRHGNQYIQVNE

PWKQIKGSEADRQRAGTVTGLAVNIAALLSVMLQPYMPTVSATIQAQLQLPPPACSILLTNFLCTLPAGHQIGTVSPLFQ

KLENDQIESLRQRFGGGQLEETLDLKAKTSLKPTVAETGTTAGPQQIQALMDEVTKQGNIVRELKAQKADKNQVAAEVAK

LLDLKKQLAVAEGKPPEAPKGKKKK

>XP_014815388.1 PREDICTED: methionine--tRNA synthetase, cytoplasmic [Calidris pugnax]

MEVQVGDPVVLRAPGWPSTHGDLFPPAERPLAPLSPLWAPALELESGTVLFSPNAICQFFFLARGEEPTDLTNQWLEWEA

TELQPAASAALYAQLVHGKKGLEAVDPLGKLLAHVEQNLSRRGTAYLAGDTKSVADVVVWGTLFPVLQDETSLPIELQAL

RTWFQSMTLSEACRKAADSVLVPTALLEFKSYLQKQPPPCLAMERAPSNEPEDEEGFERALTEDEISAAAEAWARGAAVL

PKPWKPQKPVLPVEGMRNVLITSALPYVNNVPHLGNIIGCVLSADTFARYCRLRNWNTLFVCGTDEYGTATETKAVEEGL

TPQEICDKYNAIHADIYRWFDISFDYFGRTTTPHQTTIAQDIFQRLLARGFLLQDTVEQLRCENCQRFLADRFVEGTCPF

CSYEEARGDQCDKCGKLINAVELKNPQCKLCRGVPVVKPTQHLFLDLPKLEERLEPWLEQSWATGDWTANARYITRSWIR

DGLKPRCITRDLKWGTPVPLDGFRDKVFYVWFDAPIGYLSITANYTDQWERWWKNPQQVELYNFMAKDNVPFHSVIFPCS

LLGAEDNYTLVNHLIATEYLNYEDGKFSKSRGVGVFGDMAKDTGIPADIWRFYLLYLRPEGQDSAFSWSDLMLKNNSELL

NNLGNFINRAGMFVCKFFSGTVPDMVLTLDDKRLLARVTVELHQYHQLLEKVRIRDALRCILTISRHGNQYIQVNEPWKR

IKGDEKDRQRAGTVTGVAVNMASLLAVLIQPYMPSVSSAIQRQLCIPPDCCVLSHDFTCTLPPGHRVGTVSPLFQKLEND

QVEALRKRFGGGQAKQPSPAPAVPVTPGDSQLIQELTEEVAKQGNHVRQLKANKAEKAQIDAEVAKLLELKKQLAIAEGK

SPEVPVPKGKRKK

>XP_011599589.1 PREDICTED: methionine--tRNA synthetase, cytoplasmic [Aquila chrysaetos canadensis]

MAIPHSNPVQKQWRDHVLAQFLVLGKPMPSPGVLAAAGAAAAPVRLRLGRPLRDPLVPAPVIPTPRRGPPGLTGLLPPAE

QPLAPLSPPWTPALELDSGTVLFSPNAICQFFFLARGEEPTDLTNQWLEWEATELQPAASAALYAQLVHGRKGLEAVEVL

GKLLAHIELNLSRRGTAYLAGDTKSVADVVVWGTLFPVLQDETSLPSELQVLRTWFQSMTLSEACRKAADSVLMPKALLE

FKSYLQKQPPPCLAMERATSNEPEEEEGSEPALTEEEITAAVDAWARGVAALPKPWQPQKPVLPVEGMRNVLITSALPYV

NNVPHLGNIIGCVLSADTFARYCRLRNWNTLYVCGTDEYGTATETKAVEEGLTPQEICNKYNAIHTDIYRWFDISFDYFG

RTTTPYQTMIAQDIFQRLLARGFLLQDTVEQLQCEDCQRFLADRFVEGTCPFCSYEEARGDQCDKCGKLINAVELKNPQC

KLCRGVPVVKPTQHLFLDLPKLEEQLEPWLEQSWATGDWTANARYITRSWIRDGLKPRCITRDLKWGTPVPLDGFRDKVF

YVWFDAPIGYLSITANYTDQWERWWKNPQQVELYNFMAKDNVPFHSVIFPCSLLGADDNYTLVNHLIATEYLNYEDGKFS

KSRGVGVFGDMAKDTGIPADIWRFYLLYLRPEGQDSAFSWSDLMLKNNSELLNNLGNFINRAGMFVCKFFGGTVPDMVLT

PDDKRLLARVTLELHQYHQLLEKVRIRDALRCILSISRHGNQYIQVNEPWKRIKGDEKDRQRAGTVTGVAVNMASMLAVM

LQPYMPSVSLAIQGQLCIPPDCFVLSHNFTCTLPPGHRVGTVSPLFQKLEHDQIEALRKRFGGGQPEDLAVEPQAKQTPP

ASVTLMAPGDPELIQELTEEVAKQGNHVRQLKANKAEKAQVDTEVAKLLELKKQLALAEGKSPEVLVPKGKRKK

>XP_019346961.1 PREDICTED: methionine--tRNA synthetase, cytoplasmic [Alligator mississippiensis]

MPAGGGLPVNPALPEMRLHVGAGPHGAKVLAAAAWGPPGLAWAPPGEHVVPFLTQPCLPALQLDSGAVLFSPNAICRYFF

LLSGKEQDDVANQWLEWEATELQPAVSAALYLQLVQGKPGEEALAPVREALAHVDRGLAQRGTTYLTGDAESVADVVVWG

ALFPLFQDGSCLPDNLGLLGSWFRSMSQLAPCRAAAAAVLTPEGARELRAHLQRQPVPSPASKRPATNEPEEEESAEHAL

SEEDIAAAADAWAQGTAALPEPWQPQHPVLPVAGRKNVLITSALPYVNNVPHLGNIIGCVLSADVFARYCRLRNWNTLYV

CGTDEYGTATETKAMEEGLTPRQICDKYHAIHARVYSWFGISFDYFGRTTTPQQTTIAQDIFGRLLERGFLRSDMVEQLR

CERCQRFLADRFVEGVCPFCAYEEARGDQCDKCGRLINAIELKKPQCKICHDSPVVKSSQHLFLDLPKLEQRLEQWLEQS

WATGDWTANARFITRSWLRDGLRPRCITRDLAWGTPVPLDGFRDKVFYVWFDAPIGYLSITANYTDQWERWWKNPEQVEL

YNFMAKDNVPFHSVVFPCSLLGAEDGYTLVNHLVATEYLNYEDGKFSKSRGVGVFGDMAKDTGIPADVWRFYLLSLRPEG

QDSAFSWADLLLRNNSELLNNLGNFVNRAGMFVCKFFGGCVPEMELSPDDKRLLARVGLELRHYTQLLDKVRIRDALRCV

LNISRHGNQYIQVNEPWKRIKGNDADRGRASTVTGVAVNMACLLSIMLQPYMPSISASIQDQLRAPPACCVLPSAFVCTL

PPGHRIGTVTPLFQKLENDQVEALRKRFGGGQAKAAPPKPPTPGVDLQKVKELSEEVAKQGTHVRELKAAKVEKGQIDTE

VAKLLELKRQLVLVEGRPLEPPAPKGKRKK

>KYO34317.1 methionine--tRNA synthetase, cytoplasmic [Alligator mississippiensis]

MRLHVGAGPHGAKVLAAAAWGPPGLAWAPPGEHVVPFLTQPCLPALQLDSGAVLFSPNAICRYFFLLSGKEQDDVANQWL

EWEATELQPAVSAALYLQLVQGKPGEEALAPVREALAHVDRGLAQRGTTYLTGDAESVADVVVWGALFPLFQDGSCLPDN

LGLLGSWFRSMSQLAPCRAAAAAVLTPEGARELRAHLQRQPVPSPASKRPATNEPEEEESAEHALSEEDIAAAADAWAQG

TAALPEPWQPQHPVLPVAGRKNVLITSALPYVNNVPHLGNIIGCVLSADVFARYCRLRNWNTLYVCGTDEYGTATETKAM

EEGLTPRQICDKYHAIHARVYSWFGISFDYFGRTTTPQQTTIAQDIFGRLLERGFLRSDMVEQLRCERCQRFLADRFVEG

VCPFCAYEEARGDQCDKCGRLINAIELKKPQCKICHDSPVVKSSQHLFLDLPKLEQRLEQWLEQSWATGDWTANARFITR

SWLRDGLRPRCITRDLAWGTPVPLDGFRDKVFYVWFDAPIGYLSITANYTDQWERWWKNPEQVELYNFMAKDNVPFHSVV

FPCSLLGAEDGYTLVNHLVATEYLNYEDGKFSKSRGVGVFGDMAKDTGIPADVWRFYLLSLRPEGQDSAFSWADLLLRNN

SELLNNLGNFVNRAGMFVCKFFGGCVPEMELSPDDKRLLARVGLELRHYTQLLDKVRIRDALRCVLNISRHGNQYIQVNE

PWKRIKGNDADRGRASTVTGVAVNMACLLSIMLQPYMPSISASIQDQLRAPPACCVLPSAFVCTLPPGHRIGTVTPLFQK

LENDQVEALRKRFGGGQAKAAPPKPPTPGVDLQKVKELSEEVAKQGTHVRELKAAKVEKGQIDTEVAKLLELKRQLVLVE

GRPLEPPAPKGKRKK

>XP_005986254.1 PREDICTED: methionine--tRNA synthetase, cytoplasmic [Latimeria chalumnae]

MKLYLGEGSPGSLKALAAVEVTGVRVQLQFVSLEEHVVPYLTQPKVPALHLDGGNVLFSANAICQYLFTLTGKDQCDLTN

QWLEWEATELEPAVLAVLYSAMVQGKSKEEGLGVLRKLLIHVNQSLLKTSNPYLAGNSVSAADIVLWGSLYPLLSASVSL

TEEFKTLNCWLQNLSSSEPCQKAADAALKDAHLLKVYLQKLPVPAPLSQKPTCNEPEEEASTEHAFSEEEVAAAFQAWLK

GREASPKPRQFQHPILPEKNQKNILITSALPYVNNVPHLGNIIGCVLSADVFARYCRLRNWNILYICGTDEYGTATETKA

MEEGLTPQQICDKYNAIHSQIYQWFNISFDYFGRTTTHQQTEIAQDIFRRLLDRNFLLNDVVEQLKCEACNRFLADRFVE

GVCPLCNYEEARGDQCDKCGKLINAIELKKPVCKICKATPVVQSSQHLFLDLPKLEGTLEKWLEQSWATGDWTANARYIT

RSWIRDGLKPRCITRDLKWGTPVPLKEFEDKVFYVWFDAPIGYLSITACYTDQWEKWWKNPEQVQLYQFMAKDNVPFHSV

VFPCSQLGAEDNYTLVNHLIATEYLNYEDGKFSKSRGVGVFGDMAKDTGIPADIWRFYLLFIRPEGQDSAFSWNDLLLKN

NSELLNNLGNFINRAGMFVCKFFEGRVPEMELNAEDKRLIAQVTWELQQSNQFLEKVRIRDALKCILNISRHGNQYIQVN

EPWKRIKGDDADQKRAGTVTGVAVNVACLLSVMLQPYMPSVSATIQEQLQAPQDCNVLTNQFLCTLPAGHRIGMVSPLFQ

KLENDQIESLRRRFSGQQPKDEKKATEVKPGAAEVEKGGDLERSKELTQEVEKQGNLIRELKMQKAEKALIDAEVEKLLE

LKKQLTLAEGKTPEAPAQKGKKKK

>5GL7_A Chain A, Crystal Structure Of A Truncated Human Cytosolic Methionyl-trna Synthetase

MSEEEELATLSEEEIAMAVTAWEKGLESLPPLRPQQNPVLPVAGERNVLITSALPYVNNVPHLGNIIGCVLSADVFARYS

RLRQWNTLYLCGTDEYGTATETKALEEGLTPQEICDKYHIIHADIYRWFNISFDIFGRTTTPQQTKITQDIFQQLLKRGF

VLQDTVEQLRCEHCARFLADRFVEGVCPFCGYEEARGDQCDKCGKLINAVELKKPQCKVCRSCPVVQSSQHLFLDLPKLE

KRLEEWLGRTLPGSDWTPNAQFITRSWLRDGLKPRCITRDLKWGTPVPLEGFEDKVFYVWFDATIGYLSITANYTDQWER

WWKNPEQVDLYQFMAKDNVPFHSLVFPCSALGAEDNYTLVSHLIATEYLNYEDGKFSKSRGVGVFGDMAQDTGIPADIWR

FYLLYIRPEGQDSAFSWTDLLLKNNSELLNNLGNFINRAGMFVSKFFGGYVPEMVLTPDDQRLLAHVTLELQHYHQLLEK

VRIRDALRSILTISRHGNQYIQVNEPWKRIKGSEADRQRAGTVTGLAVNIAALLSVMLQPYMPTVSATIQAQLQLPPPAC

SILLTNFLCTLPAGHQIGTVSPLFQKLENDQIESLRQRFGGGQAKTSPKPAVVETVLEHHHHHH

>XP_014381585.1 PREDICTED: methionine--tRNA synthetase, cytoplasmic [Alligator sinensis]

MVTSRCSDVSQTRRGVTYRAPPRFGRPGDGGCFRLAPLPVTRCMPAGGGLPVNPALPEMRLHVEHVVPFLTQPCLPALQL

DSGAVLFSPNAICRYFFLLSGKEQDDVANQWLEWEATELQPAVSAALYLQLVQGKSGEEALAPVREALAHVDQGLAQRGT

TYLTGDAESVADVVVWGALFPLFQDGSCLPDNLGLLGSWFRSMSQLAPCRAAAAAVLTPEGARELRAHLQRQPVPSPAFK

RPATNEPEEEESAEHALSEEDIAAAADAWAQGTAALPEPWQPQHPVLPVAGRKNVLITSALPYVNNVPHLGNIIGCVLSA

DVFARYCRLRNWNTLYVCGTDEYGTATETKAMEEGLTPRQICDKYHAIHARVYSWFGISFDYFGRTTTPQQTKIAQDIFG

RLLERGFLRSDMVEQLRCERCQRFLADRFVEGICPFCAYEEARGDQCDKCGRLINAIELKRPQCKICHDSPVVKSSQHLF

LDLPKLEQRLEQWLEQSWATGDWTANARFITRSWLRDGLRPRCITRDLAWGTPVPLDGFRDKVFYVWFDAPIGYLSITAN

YTDQWERWWKNPEQVELYNFMAKDNVPFHSVVFPCSLLGAEDGYTLVNHLVATEYLNYEDGKFSKSRGVGVFGDMAEDTG

IPADVWRFYLLSLRPEGQDSAFSWADLLLRNNSELLNNLGNFVNRAGMFVCKFFGGCVPEMELSSDDKRLLARVGLELRR

YTQLLDKVRIRDALRCVLNISRHGNQYIQVNEPWKRIKGNDADRGRASTVTGVAVNMACLLSIMLQPYMPSISASIQDQL

RAPPACCVLPSAFVCTLPPGHRIGTVTPLFQKLENDQVEALRKRFGGGQPEDDALEPQAKAAPPKPPPGVDLQKVKELSE

QVAKQGTHVRELKAAKVEKGQIDREVAKLLELKRQLALVEGRPPEPPAPKGKRKK

>NP_001079984.1 methionine--tRNA synthetase, cytoplasmic [Xenopus laevis]

MKLFVGEGNPQGVKVLAAAALWAQHVQIDRLQQEEKIVPFMSQPRLPVLDLENGNYLFLSNAICRYFYLSSGHDMCDLSN

QWLEWEAAELQPALSAALYAHVVQGKKKEDVMATISASLKHLDQSLAGKSSPYLIKDALTVVDIVVWGSIYPLIVDASNL

PEEMASLKRWFQNVSQLEQCQKAASSLLKDKGSSVFKPFLQKQPAPITPPGKSVCKEQEGEDMPSLSEEDIQAAAEAWAK

GLTGASKPKQRPHPILPVEGEKNVLITSALPYVNNVPHLGNIIGSVLSADVFARYCRLRNWNTLYICGTDEYGTATETKA

MEEGLTPQQICDKYNAIHTAIYQWFNISFDYFGRTTTQHQTTISQDIFHRLLEREFLLTDTVEQLRCEKCQRFLADRFVE

GICPFCNYEEARGDQCDKCGKLINAVELKKPQCKICKQSPVIKSSKHLFLDLPKLEKRLEQWLEQSFSTGDWTSNARFIT

RSWIRDGLKPRCITRDLKWGTPVPLDGFRDKVFYVWFDAPIGYISITANYTDQWEKWWKSPQQVQLYNFMAKDNVPFHSV

VFPSCLLGAEDNYTLVNHLVATEYLNYEDGKFSKSRGVGVFGDMAKDTGIPADIWRFYLLYVRPEGQDSAFSWSDLMLKN

NSELLNNLGNFVNRAGMFVQKFFNGCVPEMELLSEDKRLLAQVAAELQQYNLLLEKVRIRDALRCILNISRHGNQYIQVN

EPWKCIKGNQQEQKRAGTVTGVAVNMAALLSIMLHPYMPTISSVIQEQLLMPQESKVLTTDFCCCLQSGHQIGNVSPLFQ

KLENDQIESLRKRFGGGQVKTESKVSPSQEAPEQQAPKASGPERVKELMQELEKQGNHVRELKGKKAEKSVIDPEVQKLL

ALKKELALAEGKSPDPPTQKGKKKK

>OCT92865.1 hypothetical protein XELAEV_18015931mg [Xenopus laevis]

MKLFVGEGNPQGVKVLAAAALWAQHVQIDRLQQEEKIVPFMSQPRLPVLDLENGNYLFLSNAICRYFYLSSGHDMCDLSN

QWLEWEAAELQPALSAALYAHVVQGKKKEDVMATISASLKHLDQSLAGKSSPYLIKDALTVVDIVVWGSIYPLIVDASNL

PEEMASLKRWFQNVSQLEQCQKAASSLLKDKGSSVFKPFLQKQPAPITPPGKSVCKEQEGEDMPSLSEEDIQAAAEAWAK

GLTGASKPKQRPHPILPVEGEKNVLITSALPYVNNVPHLGNIIGSVLSADVFARYCRLRNWNTLYICGTDEYGTATETKA

MEEGLTPQQICDKYNAIHTAIYQWFNISFDYFGRTTTQHQTTISQDIFHRLFEREFLLTDTVEQLRCEKCQRFLADRFVE

GICPFCNYEEARGDQCDKCGKLINAVELKKPQCKICKQSPVIKSSKHLFLDLPKLEKRLEQWLEQSFSTGDWTSNARFIT

RSWIRDGLKPRCITRDLKWGTPVPLDGFRDKVFYVWFDAPIGYISITANYTDQWEKWWKSPQQVQLYNFMAKDNVPFHSV

VFPSCLLGAEDNYTLVNHLVATEYLNYEDGKFSKSRGVGVFGDMAKDTGIPADIWRFYLLYVRPEGQDSAFSWSDLMLKN

NSELLNNLGNFVNRAGMFVQKFFNGCVPEMELLSEDKRLLAQVAAELQQYNLLLEKVRIRDALRCILNISRHGNQYIQVN

EPWKCIKGNQQEQKRAGTVTGVAVNMAALLSIMLHPYMPTISSVIQEQLLMPQESKVLTTDFCCCLQSGHQIGNVSPLFQ

KLENDQIESLRKRFGGGQVKTESKVSPSQEAPEQQAPKASGPERVKELMQELEKQGNHVRELKGKKAEKSVIDPEVQKLL

ALKKELELAEGKSPDPPTQKGKKKK

>KFV81551.1 Methionine--tRNA synthetase, cytoplasmic, partial [Struthio camelus australis]

GTVLFSPNAICQYFFLVSGEEPTDLTNQWLEWEATELQPAVLAALYAQLVQGKKGAEAVGALVKSLTYIDQSLSRRGTAY

LARDVKSVADVVVWGALFPVLQDESSLPDELKVLRTWFQSMSLLEACRKAADSVLVPKGLLEFKCYLQKQPPPCLAVERP

TSNEPEEEEASERALTEEEVAAPGAAWARGAAALPKPWQPQKPVLPVEGMRNVLITSALPYVNNVPHLGNIIGCVLSADT

FARYCRLRNWNTLYVCGTDEYGTATETKAVEEGLTPQEICDKYNAIHADIYRWFNISFDYFGRTTTPHQTTIAQDIFQRL

LAHGFLLQDTVEQLRCEGCQRFLADRFVEGTCPFCSYEEARGDQCDKCGKLINAVELKRPQCKICRGIPVVKPTQHLFLD

LPKASLEEQLEPWLEQSWTKGDWTANARYITRSWIRDGLKPRCITRDLKWGTPVPLDGFCDKVFYVWFDAPIGYLSITAN

YTDQWERWWKNPQQVELYNFMAKDNVPFHSVIFPCSLLGADDNYTLVNHLIATEYLNYEDGKFSKSRGVGVFGDMAKDTG

IPADIWRFYLLYLRPEGQDSAFSWSDLMLKNNSELLNNLGNFINRAGMFVCKFFGGTVPDMVLTPDDKRLLARVTLELRQ

YHQLLEKVRIRDALRCILSISRHGNQYIQVNEPWKRIKGEEEDRQRAGTVTGIAVNMASLLAVMVQPYMPSISVAIQAQL

CIPPDCFVLGHDFTCTLPPGHRVGTVSPLFQKLEHDQVEALRKQFGGGQVRSLPAPASGTPPAPGDPQLIQELMEQEAKQ

GTHVRQLKASKVEKVLVDAEVAKLLELKKQLALAEGKSPEVPVPKGKRKK

>XP_009673664.1 PREDICTED: methionine--tRNA synthetase, cytoplasmic, partial [Struthio camelus australis]

GTVLFSPNAICQYFFLVSGEEPTDLTNQWLEWEATELQPAVLAALYAQLVQGKKGAEAVGALVKSLTYIDQSLSRRGTAY

LARDVKSVADVVVWGALFPVLQDESSLPDELKVLRTWFQSMSLLEACRKAADSVLVPKGLLEFKCYLQKQPPPCLAVERP

TSNEPEEEEASERALTEEEVAAPGAAWARGAAALPKPWQPQKPVLPVEGMRNVLITSALPYVNNVPHLGNIIGCVLSADT

FARYCRLRNWNTLYVCGTDEYGTATETKAVEEGLTPQEICDKYNAIHADIYRWFNISFDYFGRTTTPHQTTIAQDIFQRL

LAHGFLLQDTVEQLRCEGCQRFLADRFVEGTCPFCSYEEARGDQCDKCGKLINAVELKRPQCKICRGIPVVKPTQHLFLD

LPKLEEQLEPWLEQSWTKGDWTANARYITRSWIRDGLKPRCITRDLKWGTPVPLDGFCDKVFYVWFDAPIGYLSITANYT

DQWERWWKNPQQVELYNFMAKDNVPFHSVIFPCSLLGADDNYTLVNHLIATEYLNYEDGKFSKSRGVGVFGDMAKDTGIP

ADIWRFYLLYLRPEGQDSAFSWSDLMLKNNSELLNNLGNFINRAGMFVCKFFGGTVPDMVLTPDDKRLLARVTLELRQYH

QLLEKVRIRDALRCILSISRHGNQYIQVNEPWKRIKGEEEDRQRAGTVTGIAVNMASLLAVMVQPYMPSISVAIQAQLCI

PPDCFVLGHDFTCTLPPGHRVGTVSPLFQKLEHDQVEALRKQFGGGQPEELTVVPQAKPTPPASGSLPAPASGTPPAPGD

PQLIQELMEQEAKQGTHVRQLKASKVEKVLVDAEVAKLLELKKQLALAEGKSPEVPVPKGKRKK

>XP_020634197.1 methionine--tRNA synthetase, cytoplasmic isoform X2 [Pogona vitticeps]

MKLHLSEGSPGGLKVLAAAKATGAPVELQWLPQDAPVVPFLTRPCLPVLQLENGSFLFSTNAICQYFFRLSGKETSDFSQ

QQSDFANHLFEWEATELEPALSAALYLHVVQGKKGEEIWGIIKKPLNYIDQTLNKKGTSYFVGDAESAVDVALWGSLFPL

LRDESFLPDELKALRRWFQNMNLKEYCKKAVEAVWTSKGLLELKAYLQKHSTPNLAFEKPATNEAKEEELAQHQLSEEEI

AAIADVWSQGLASLPKPWKPQHPIMPVEGMKNVLITSALPYVNNVPHLGNIIGCVLSADTFARYCRLRNWNTLYLCGTDE

YGTATETKAMEEGLTPKQICDKYYAVHDHIYQWFEISFDYFGRTTTPQQTTIAQDIFQRLLEHDYLLRETVDQLKCEKCD

RFLADRFVEGTCPFCNYEEARGDQCDKCGKLINATELKKPVCKVCQGTPVVKSSQHLFLDLPKLEEKLEKWLSFSQSTGD

WTSNARYIARSWIRDGLKPRCITRDLKWGTPVPLDGFREKVFYVWFDAPIGYLSITANYTDQWEKWWKNPEQVQLYNFMA

KDNVPFHSVVFPCSLLGTGEQYTLVNHLIATEYLNYEDGKFSKSRGVGVFGDMAKDTGIPADIWRFYLLYLRPESQDSAF

SWNDLLIKNNSELLNTLGNFINRAGTFVCKFFGGHVPAMELSQDDKRLLAHITLELRQYNRLLEKVKIRDALRIILNIAR

HGNQYLQVNEPWKHIKGSDVDKKRAGTVIGVAVNVASLLSVILQPYMPTVSAAIQEQLCVPANCNVVTDNFVCTLPAGHQ

IGTVTPLFQKLETEQIESLRKRFGGGQLEDLPQQPQQKAATNSGAPMGAKELQEEVTNQDRYVQQLKAQKAEKQQIDVAT

ARLVELQKQLSLATGKGKKKK

>XP_020634191.1 methionine--tRNA synthetase, cytoplasmic isoform X1 [Pogona vitticeps]

MKLHLSEGSPGGLKVLAAAKATGAPVELQWLPQDAPVVPFLTRPCLPVLQLENGSFLFSTNAICQYFFRLSGKETSDFSQ

QQSDFANHLFEWEATELEPALSAALYLHVVQGKKGEEIWGIIKKPLNYIDQTLNKKGTSYFVGDAESAVDVALWGSLFPL

LRDESFLPDELKALRRWFQNMNLKEYCKKAVEAVWTSKGLLELKAYLQKHSTPNLAFEKPATNEAKEEELAQHQLSEEEI

AAIADVWSQGLASLPKPWKPQHPIMPVEGMKNVLITSALPYVNNVPHLGNIIGCVLSADTFARYCRLRNWNTLYLCGTDE

YGTATETKAMEEGLTPKQICDKYYAVHDHIYQWFEISFDYFGRTTTPQQTTIAQDIFQRLLEHDYLLRETVDQLKCEKCD

RFLADRFVEGTCPFCNYEEARGDQCDKCGKLINATELKKPVCKVCQGTPVVKSSQHLFLDLPKLEEKLEKWLSFSQSTGD

WTSNARYIARSWIRDGLKPRCITRDLKWGTPVPLDGFREKVFYVWFDAPIGYLSITANYTDQWEKWWKNPEQVQLYNFMA

KDNVPFHSVVFPCSLLGTGEQYTLVNHLIATEYLNYEDGKFSKSRGVGVFGDMAKDTGIPADIWRFYLLYLRPESQDSAF

SWNDLLIKNNSELLNTLGNFINRAGTFVCKFFGGHVPAMELSQDDKRLLAHITLELRQYNRLLEKVKIRDALRIILNIAR

HGNQYLQVNEPWKHIKGSDVDKKRAGTVIGVAVNVASLLSVILQPYMPTVSAAIQEQLCVPANCNVVTDNFVCTLPAGHQ

IGTVTPLFQKLETEQIESLRKRFGGGQQKAATNSGAPMGAKELQEEVTNQDRYVQQLKAQKAEKQQIDVATARLVELQKQ

LSLATGKGKKKK

>NP_001086723.1 methionyl-tRNA synthetase L homeolog [Xenopus laevis]

MKLFVAEGNPHGVKVLAAAALWGQHVQINRLQQEEKIVPFMSQPRLPALDLENGNFLFLSNAICRYFYLSCGHDMCDQSN

QWLEWEAVELQPALSAALYAHVVQGKKKEDVLVTVSASLRHLDQSLAGKSSPYLVKDVLTVVDIVVWGSIYPLIVDASSL

PEEMTSLKRWFQNLNQLEQCQKAASSLFKDEGSSVFKPYLQKQPAAITPSGKPICNEQEGEDIESLSEEDIQAAAEAWAK

GLSGAPKPKLQPHPILPVEGEKNILITSALPYVNNVPHLGNIIGSVLSADVFARYCRLRNWNTLYICGTDEYGTATETKA

MEEGLTPQQICDKYNAVHTAIYQWFNISFDYFGRTTTQQQTTIAQDIFHRLLERDFLLTDTVEQLRCETCKRFLADRFVE

GICPFCNYEEARGDQCDKCGKLINAVELKKPQCKVCKQSPVIKSSKHLFLDLPKLEKRLEQWLEQSFSSGDWTSNARFIT

RSWIRDGLKPRCITRDLKWGTPVPLDGFRDKVFYVWFDAPIGYISITANYTDQWEMWWKSPQQVQLYNFMAKDNVPFHSV

IFPSCLLGAEDNYTLVNHLVATEYLNYEDGKFSKSRGVGVFGDMAQETGIPADIWRFYLLYVRPEGQDSAFSWSDLMLKN

NSELLNNLGNFVNRAAMFVQKFFNGCVPEMELLSEDKRLLAQVAAELQQFNQLLEKVRIRDALRCILNISRHGNQYIQVN

EPWKCIKGNQEEQKRAGTVTGVAVNMAALLSVMLHPYMPTISGVIQEQLLMPQESKVLTTDFCCRLQSGHRIGNVSPLFQ

KLENDQIESLRKRFGGGQVKTESKVPPSQEAPLQKTPKASGPERVKELMQEVEKQGNHVRELKGKKAEKSTIDSEVQKLL

ALKKELALAEGKSPDPPAQKGKKKK

>NP_001017000.2 methionine--tRNA synthetase, cytoplasmic [Xenopus tropicalis]

MKLFVGEGNPHGVKVLAAAALWAEHVHINRLQQEEKIVPFMSQPRLPALDLENGNYLFLSNAVCRYFYLSSGHDVCNLSN

QWLEWEAVELQPAFSAALYAHVVQGRKKEEVIASLSASLSHLDQSLAGKPSPYLVKDALTVADIVVWGSLYPLIEDASSL

PEEMKLLKHWFQTVSQLEQCQKAVSFLMKDGGSSVFKPYLQKQPVPITPSGKPTCNEHEGEDMASLSEEDIQAAANAWAK

GLTGAQKPKQRLHPILPVEGEKNVLITSALPYVNNVPHLGNIIGSVLSADVFARYCRLRNWNTLYICGTDEYGTATETKA

MEEGLTPQQICDKYNAVHTAIYQWFDISFDFFGRTTTQHQTTIAQDIFHRLLKRDFLLTDTVEQLRCETCQRFLADRFVE

GTCPFCNYEEARGDQCDKCGKLINAVELKKPQCKVCKKSPVKKSSKHLFLDLPKLEKRLEQWLEKSFSSGDWTSNARFIT

RSWIRDGLKPRCITRDLKWGTPVPLDGFRDKVFYVWFDAPIGYISITANYTDQWEKWWKSTQQVQLYHFMAKDNVPFHSV

IFPSCLLGAEDNYTLVNHIIATEYLNYEDGKFSKSRGVGVFGDMAKDTGIPADIWRFYLLYVRPEGQDSAFSWSDLMLKN

NSELLNNLGNFVNRAGMFVQKFFNGCVPEMELLSEDKRLLAQVAAELQQYNQHLEKVRIRDALRCILNISRHGNQYIQVN

EPWKCIKGNQQEQKRAGTVTGVAVNMAALLSVMLHPYMPTVSGLIQEQLLMPQESKVLTTDFCCRLQSGHQIGNVSPLFQ

KLENDQIESLRKRFGGGQVKTESKAPPCQEAPLKEAPKASGPERVKELMQEVEKQGNLVRELKGKKAEKSTIDTEVQKLL

ALKKELALAEGKSPDPPAQKGKKKK

>XP_018425241.1 PREDICTED: methionine--tRNA synthetase, cytoplasmic isoform X2 [Nanorana parkeri]

MKLFVSEGNPHGVKVLAAAGLWAAQVEIQRLQQEEKVVPFLSQPKLPALELDDGNFLFTSNAICRCFSLSAEKEPSDLIN

QWLEWEATELQPSISAALYAHVVQGKKKEEVLQTVNKSLIYIAQKLTKKAYLAGDALSVADIVVWGAIYPLIVDGASLPE

ELSSLKSWFKNVTELDQCLKAAESLLKGQGPSVFKAFLQKLPAPVLVSEKSLSNEQEGEDTPARLLSEEDIAAAAEAWKK

GLAEAPKPKPQQHPILPIEGEKNLLITSALPYVNNVPHLGNIIGSVLSADVFARYCRLRNWNTLYVCGTDEYGTATETKA

MEEGLTPQQICDKYNAVHTAIYKWFNISFDYFGRTTTSHQTKIAQEIFHRLLERGFLLKDTVEQLRCETCQRFLADRFVE

GTCPLCNYEEARGDQCDKCGKLINAVELKRPQCKVCKQSPVIKSTEHLFLDLPKLEERLEKWLDQSLATGDWTANARYIT

RSWLRDGLKPRCITRDLKWGTPVPLDGFRDKVFYVWFDAPIGYISITANYTDEWEKWWKNPEQVQLYNFMAKDNVPFHSV

IFPSCLLGVEDNYTVVNHLVATEYLNYEDGKFSKSRGVGVFGDMAQDTGIPADIWRFYLLYVRPEGQDSAFSWSDLMLKN

NSELLNNLGNFVNRAGMFVQKFFNGQVPEIELLPEDKKLLAQVTAELQQYNQLLEKVRIRDALRAILNISRHGNQYIQVN

EPWKCIKGTQQEQKRAGTVTGLAVNMAALLSNMLQPYMPTISSVIREQLLMPEEANVLTSDFRCILQSGHRIGNVSPLFQ

KLETDQIESLRKRFGGGQVKAPAPTPEPAKETPKVGDPQRIQELTQAVEKQGYHVRELKSKAAEKSLIDSEVQKLLKLKK

DLALAEGKSPDPPAQKGKKKK

>XP_018425248.1 PREDICTED: methionine--tRNA synthetase, cytoplasmic isoform X3 [Nanorana parkeri]

MKLFVSEGNPHGVKVLAAAGLWAAQVEIQRLQQEEKVVPFLSQPKLPALELDDGNFLFTSNAICRCFSLSAEKEPSDLIN

QWLEWEATELQPSISAALYAHVVQGKKKEEVLQTVNKSLIYIAQKLTKKAYLAGDALSVADIVVWGAIYPLIVDGASLPE

ELSSLKSWFKNVTELDQCLKAAESLLKGQGPSVFKAFLQKLPAPVLVSEKSLSNEQEGEDTPARLLSEEDIAAAAEAWKK

GLAEAPKPKPQQHPILPIEGEKNLLITSALPYVNNVPHLGNIIGSVLSADVFARYCRLRNWNTLYVCGTDEYGTATETKA

MEEGLTPQQICDKYNAVHTAIYKWFNISFDYFGRTTTSHQTKIAQEIFHRLLERGFLLKDTVEQLRCETCQRFLADRFVE

GTCPLCNYEEARGDQCDKCGKLINAVELKRPQCKVCKQSPVIKSTEHLFLDLPKLEERLEKWLDQSLATGDWTANARYIT

RSWLRDGLKPRCITRDLKWGTPVPLDGFRDKVFYVWFDAPIGYISITANYTDEWEKWWKNPEQVQLYNFMAKDNVPFHSV

IFPSCLLGVEDNYTVVNHLVATEYLNYEDGKFSKSRGVGVFGDMAQDTGIPADIWRFYLLYVRPEGQDSAFSWSDLMLKN

NSELLNNLGNFVNRAGMFVQKFFNGQVPEIELLPEDKKLLAQVTAELQQYNQLLEKVRIRDALRAILNISRHGNQYIQVN

EPWKCIKGTQQEQKRAGTVTGLAVNMAALLSNMLQPYMPTISSVIREQLLMPEEANVLTSDFRCILQSGHRIGNVSPLFQ

KLETDQIESLRKRFGGGQVRFVVKAPAPTPEPAKETPKVGDPQRIQELTQAVEKQGYHVRELKSKAAEKSLIDSEVQKLL

KLKKDLALAEGKSPDPPAQKGKKKK

>XP_018425257.1 PREDICTED: methionine--tRNA synthetase, cytoplasmic isoform X4 [Nanorana parkeri]

MKLFVSEGNPHGVKVLAAAGLWAAQVEIQRLQQEEKVVPFLSQPKLPALELDDGNFLFTSNAICRCFSLSAEKEPSDLIN

QWLEWEATELQPSISAALYAHVVQGKKKEEVLQTVNKSLIYIAQKLTKKAYLAGDALSVADIVVWGAIYPLIVDGASLPE

ELSSLKSWFKNVTELDQCLKAAESLLKGQGPSVFKAFLQKLPAPVLVSEKSLSNEQEGEDTPARLLSEEDIAAAAEAWKK

GLAEAPKPKPQQHPILPIEGEKNLLITSALPYVNNVPHLGNIIGSVLSADVFARYCRLRNWNTLYVCGTDEYGTATETKA

MEEGLTPQQICDKYNAVHTAIYKWFNISFDYFGRTTTSHQTKIAQEIFHRLLERGFLLKDTVEQLRCETCQRFLADRFVE

GTCPLCNYEEARGDQCDKCGKLINAVELKRPQCKVCKQSPVIKSTEHLFLDLPKLEERLEKWLDQSLATGDWTANARYIT

RSWLRDGLKPRCITRDLKWGTPVPLDGFRDKVFYVWFDAPIGYISITANYTDEWEKWWKNPEQVQLYNFMAKDNVPFHSV

IFPSCLLGVEDNYTVVNHLVATEYLNYEDGKFSKSRGVGVFGDMAQDTGIPADIWRFYLLYVRPEGQDSAFSWSDLMLKN

NSELLNNLGNFVNRAGMFVQKFFNGQVPEIELLPEDKKLLAQVTAELQQYNQLLEKVRIRDALRAILNISRHGNQYIQVN

EPWKCIKGTQQEQKRAGTVTGLAVNMAALLSNMLQPYMPTISSVIREQLLMPEEANVLTSDFRCILQSGHRIGNVSPLFQ

KLETDQIESLRKRFGGGQVRFVKAPAPTPEPAKETPKVGDPQRIQELTQAVEKQGYHVRELKSKAAEKSLIDSEVQKLLK

LKKDLALAEGKSPDPPAQKGKKKK

>XP_018425231.1 PREDICTED: methionine--tRNA synthetase, cytoplasmic isoform X1 [Nanorana parkeri]

MKLFVSEGNPHGVKVLAAAGLWAAQVEIQRLQQEEKVVPFLSQPKLPALELDDGNFLFTSNAICRCFSLSAEKEPSDLIN

QWLEWEATELQPSISAALYAHVVQGKKKEEVLQTVNKSLIYIAQKLTKKAYLAGDALSVADIVVWGAIYPLIVDGASLPE

ELSSLKSWFKNVTELDQCLKAAESLLKGQGPSVFKAFLQKLPAPVLVSEKSLSNEQEGEDTPARLLSEEDIAAAAEAWKK

GLAEAPKPKPQQHPILPIEGEKNLLITSALPYVNNVPHLGNIIGSVLSADVFARYCRLRNWNTLYVCGTDEYGTATETKA

MEEGLTPQQICDKYNAVHTAIYKWFNISFDYFGRTTTSHQTKIAQEIFHRLLERGFLLKDTVEQLRCETCQRFLADRFVE

GTCPLCNYEEARGDQCDKCGKLINAVELKRPQCKVCKQSPVIKSTEHLFLDLPKLEERLEKWLDQSLATGDWTANARYIT

RSWLRDGLKPRCITRDLKWGTPVPLDGFRDKVFYVWFDAPIGYISITANYTDEWEKWWKNPEQVQLYNFMAKDNVPFHSV

IFPSCLLGVEDNYTVVNHLVATEYLNYEDGKFSKSRGVGVFGDMAQDTGIPADIWRFYLLYVRPEGQDSAFSWSDLMLKN

NSELLNNLGNFVNRAGMFVQKFFNGQVPEIELLPEDKKLLAQVTAELQQYNQLLEKVRIRDALRAILNISRHGNQYIQVN

EPWKCIKGTQQEQKRAGTVTGLAVNMAALLSNMLQPYMPTISSVIREQLLMPEEANVLTSDFRCILQSGHRIGNVSPLFQ

KLETDQIESLRKRFGGGQLDADSKETQVKAPAPTPEPAKETPKVGDPQRIQELTQAVEKQGYHVRELKSKAAEKSLIDSE

VQKLLKLKKDLALAEGKSPDPPAQKGKKKK

>BAG57322.1 unnamed protein product [Homo sapiens]

MAVTAWEKGLESLPPLRPQQNPVLPVAGERNVLITSALPYVSNVPHLGNIIGCVLSADVFARYSRLRQWNTLYLCGTDEY

GTATETKALEEGLTPQEICDKYHIIHADIYRWFNISFDIFGRTTTPQQTKITQDIFQQLLKRGFVLQDTVEQLRCEHCAR

FLADRFVEGVCPFCGYEEARGDQCDKCGKLINAVELKKPQCKVCRSCPVVQSSQHLFLDLPKLEKRLEEWLGRTLPGSDW

TPNAQFITRSWLRDGLKPRCITRDLKWGTPVPLEGFEDKVFYVWFDATIGYLSITANYTDQWERWWKNPEQVDLYQFMAK

DNVPFHSLVFPCSALGAEDNYTLVSHLIATEYLNYEDGKFSKSRGVGVFGDMAQDTGIPADIWRFYLLYIRPEGQDSAFS

WTDLLLKNNSELLNNLGNFINRAGMFVSKFFGGYVPEMVLTPDDQRLLAHVTLELQHYHQLLEKVRIRDALRSILTISRH

GNQYIQVNEPWKRIKGSEADRQRAGTVTGLAVNIAALLSVMLQPYMPTVSATIQAQLQLPPPACSILLTNFLCTLPAGHQ

IGTVSPLFQKLENDQIESLRQRFGGGQVRKLKAVPSLHNSHSITSLISLSSPHPSLFPSFGPAASSLTVFLSCLN

>XP_008113280.1 PREDICTED: methionine--tRNA synthetase, cytoplasmic isoform X1 [Anolis carolinensis]

MKLHLSEGSPAGLKGLAAANATESPVELQWVSLEAHVVPFLTHPQSTVLQLEDGTFLFSTNTICQYFFRLSGKEMSDFGQ

EQSGFTNQLFEWEATELEPALSAALYLLLVQGKKGEEILGIIKKPLNYIEQILTKKSTPYLTGETESAADIVLWGSLFPL

LKDESYLPDELQALRKWFQTMSLKEYCKKAVEAIWTPKGLLALKAYLQKHPAPNLTFEKPATNEPKEEDSAQRHLSEEEI

AVIAEVWSRGSASLPKPWKPQHPILPVEGMKNVLITSALPYVNNVPHLGNIIGCVLSADTFARYCRLRNWNTLYLCGTDE

YGTATETKAMEEGLTPQQICDKYYAVHDQIYKWFNISFDFFGRTTTTQQTTIAQDIFHCLLKRNFLLTETVDQLKCEKCE

RFLADRFVEGTCPFCNYEEARGDQCDKCGKLINATELKKPVCKVCQGTPVVRSSQHLFLDLPKLEESLEKWLALSQSTGD

WTPNARYIARSWIRDGLKPRCITRDLKWGTPVPLDGFRDKVFYVWFDAPIGYLSIAANYTDQWEKWWKNPEQVQLYNFMA

KDNVPFHSVVFPCTLLGTGDKYTLVNHLIATEYLNYEDGKFSKSRGVGVFGDMAKDTGIPSDIWRFYLLYLRPESQDSAF

SWTDLMTKNNSELLNTLGNFINRAGSFVCKFFGGHVPAMELNQDDKRLLAHITLELRQYNYLLEKVKIREALRSILNMAR

HGNHYLQVNEPWKHIKGSEADKKRAGTVIGVAVNIASLLSVVLQPYMPNVSATIQQQLSIPADYNVVSNDFVCTLPAGHQ

IGTVTPLFQKLEAEQIENLRKRFGGGQLEDFPLKPKQKAAANCDATLGSKTLQEEVAKQDNYVQQLKAQKAEKQQIDAAV

SKLGELQKQLSLAGGQEKKKK

>XP_008113281.1 PREDICTED: methionine--tRNA synthetase, cytoplasmic isoform X2 [Anolis carolinensis]

MKLHLSEGSPAGLKGLAAANATESPVELQWVSLEAHVVPFLTHPQSTVLQLEDGTFLFSTNTICQYFFRLSGKEMSDFGQ

EQSGFTNQLFEWEATELEPALSAALYLLLVQGKKGEEILGIIKKPLNYIEQILTKKSTPYLTGETESAADIVLWGSLFPL

LKDESYLPDELQALRKWFQTMSLKEYCKKAVEAIWTPKGLLALKAYLQKHPAPNLTFEKPATNEPKEEDSAQRHLSEEEI

AVIAEVWSRGSASLPKPWKPQHPILPVEGMKNVLITSALPYVNNVPHLGNIIGCVLSADTFARYCRLRNWNTLYLCGTDE

YGTATETKAMEEGLTPQQICDKYYAVHDQIYKWFNISFDFFGRTTTTQQTTIAQDIFHCLLKRNFLLTETVDQLKCEKCE

RFLADRFVEGTCPFCNYEEARGDQCDKCGKLINATELKKPVCKVCQGTPVVRSSQHLFLDLPKLEESLEKWLALSQSTGD

WTPNARYIARSWIRDGLKPRCITRDLKWGTPVPLDGFRDKVFYVWFDAPIGYLSIAANYTDQWEKWWKNPEQVQLYNFMA

KDNVPFHSVVFPCTLLGTGDKYTLVNHLIATEYLNYEDGKFSKSRGVGVFGDMAKDTGIPSDIWRFYLLYLRPESQDSAF

SWTDLMTKNNSELLNTLGNFINRAGSFVCKFFGGHVPAMELNQDDKRLLAHITLELRQYNYLLEKVKIREALRSILNMAR

HGNHYLQVNEPWKHIKGSEADKKRAGTVIGVAVNIASLLSVVLQPYMPNVSATIQQQLSIPADYNVVSNDFVCTLPAGHQ

IGTVTPLFQKLEAEQIENLRKRFGGGQQKAAANCDATLGSKTLQEEVAKQDNYVQQLKAQKAEKQQIDAAVSKLGELQKQ

LSLAGGQEKKKK

>XP_005532122.1 PREDICTED: methionine--tRNA synthetase, cytoplasmic [Pseudopodoces humilis]

MRLRVPPGAPAALKVLAAAGAAPAPVRPHLGGPAERPRSPPSPPRGPGLELELDNGTVLFSANAICQYFYLCRGEEPTDL

TTQWLEWEATELQPAVAAALYLRLVQGRPGPDTLGVLWHLLAHLEQHLARSGTTHLAGDAASVADVVVWGSLFPVLQDET

SLPSELQVLRSWFRAMSLWEPCRDAAALLPEALRELRTHLAQHPPPLLAPRHEPQDEEGPERTLTDDDIAAAVDIWAQGP

AALPPPRQPRTPVLPVEGERNVLITSALPYVNNVPHLGNIIGCVLSADTFARYCRLRNWNTLFVCGTDEYGTATETRALE

EGLSPQELCDRYHAVHADVYAWFRISFDHFGRTTTPQQTRIAQDIFQRLLARGFLLQDTLEQLRCESCGRYLADRFVEGT

CPFCGYAEARGDQCDKCGKLINAVELKSPQCKLCRGTPVVTPTQHLFLDLPKLEGQLEAWLERTWASGDWTANARHITRT

WLRDGLKPRCITRDLTWGTPVPLDGFRDKVFYVWFDAPIGYLSITANYTEQWERWWKNPQQVELYNFMAKDNVPFHSVVF

PCSLLGADDNYTLVKHLIATEYLNYEDGKFSKSRGVGVFGDMAKDTGIPADVWRFYLLYLRPEGQDSAFSWADLLLKNNS

ELLNNLGNFINRAGMFVCKFFAGTVPDMVLTAEDQRLLARLTLEIRQYHQLMEKVRIRDALRCVLSISRHGNQYIQVNEP

WKRIKGDERDRQRAGTVTGVAVNVAAALAALLQPFVPGVSAAIQGQLRIPPERCGLGPGLTCTLPAGHRVGTVSPLFQKL

EPEQVEALRQRFGGTQAKEPPPASVPTPAPNTSAAPGDPKLIQELTEQVAKQGNLVRQLKAAQAEKAQVDAQVAKLLELK

QQLALAEGRSPEVPKGKRKK

>XP_005297306.1 LOW QUALITY PROTEIN: methionine--tRNA synthetase, cytoplasmic [Chrysemys picta bellii]

MRLYLSEGSAQGLKVVAAAGASGVRVRLERVPPDGRVVPFLTHPRVPALHLDSGTFLFSPNAICQYFFLLAGKEPNDLTN

QWLEWEATELQPAVSTALYCHLVQGKKGGEVLGAIRKTLTYIDQSLTSKATPYLTGEAESVADIVLWATLFPVLRDEFCL

PDELEALRSWFQTMNLSEPCRSAVASVLTPKGAQEFKGYLQKQPGPSLHWEKPAANEPEEEELAERSLSEEEIAAAAEAW

ARGPAALPERWQPASPVLPVEGKRNILITSALPYVNNVPHLGNIIGCVLSADVFARYCRLRNWNTLYVCGTDEYGTATET

KALEEGLTPQQICDKYNAIHAQIYRWFDISFDYFGRTTTAHQTTIAQDIFRRLLERDMLLTDTVDQLRCERCQRFLADRF

VEGTCPFCNYEEARGDQCDKCGKLINAVELKKPQCKVCKDSPVVKSSQHLFLDLPKLEGLLDKWLEQSWATGDWTANSRF

ITRSWIRDGLKPRCITRDLKWGTPVPLDGFRDKVFYVWFDAPIGYLSITANYTDQWERWWKNPQQVQLYNFMAKDNVPFH

SVVFPCTLLGAEDNYTLVNHLIATEYLNYEDGKFSKSRGMGVFGDMAQDTGIPADIWRFYLLFLRPEGQDSAFSWTDLML

KNNSELLNNLGNFVNRAGMFVCKFFGGRVPPMELGADDKRLLAHVTLELHQYNQLLEKVRLPMVLILAINFAAGSQADTP

QNTVIARVCAPLXRGGVPFSRTRAGTVTGVAVNIASLISVMLQPYMPSISATIQRQLCVPQACNVLTDTFLCLLPPGHQI

GTVTPLFQKLESEQIEALRKRFGGGQPEDSTLELKAHAARMASADMTPPGPGGGAVLAGDPQKVKELAEEVAKQADRVRQ

LKASKAEKGQIDAEVAKLLELKKQLALAEGKTPEPPAPKGKKRK

>EOA95490.1 Methionyl-tRNA synthetase, cytoplasmic, partial [Anas platyrhynchos]

QPVVAAALYAQLVHGKKGLEAAGHLGKLLAYIEQSLSGRGTAYLAGDTQSVADVVVWGALFPVLQDEASLSNELQALRTW

FQTMTLSEACRKAADSVLMPKGLLEFKSYLQKQPPPCLAMERATSNEPEEEEASERALTEEEIRAAADAWARGAAALPKP

WCPQKPVLPVEGMRNVLITSALPYVNNVPHLGNIIGCVLSADTFARYCRLRNWNTLYVCGTDEYGTATETKAVEEGLTPR

EICDKYNAIHADIYRWFHISFDYFGRTTTPHQTTIAQDIFQRLLARGFLLQDTVEQLQCEGCQRFLADRFVEGICPFCGY

EEARGDQCDKCGKLINAVELKRPQCKLCRGVPVVKPTQHLFLDLPKLEEQLEPWLEHSWSSGDWTANARYITRSWIRDGL

KPRCITRDLKWGTPVPLDGFRDKVFYVWFDAPIGYLSITANYTDQWERWWKNPQQVELYNFMAKDNVPFHSVIFPCSLLG

ADDNYTLVNHLIATEYLNYEDGKFSKSRGVGVFGDMAKDTGIPADIWRFYLLYLRPEGQDSAFSWSDLMLKNNSELLNNL

GNFINRAGMFVCKFFGGVVPDMVLTADDKRLLARVTLELRQYHQLLEKVRIRDALRCILSISRHGNQYIQVNEPWKRIKG

NEEDRQRAGTVTGVAVNMASLLAVMVQPYMPSVSLAIQGQLCIPPECFVLSHIFTCTLPPGHRVGTVSPLFQKLENDQIE

ALRKRFGGGQVTPVPAPASGPVVAPGDPQLIQELTEQVAKQGNHVRQLKANKAEKAEIDAEVAKLLELKKQLALAEGKSP

EVLVPKGKRKK

>XP_014747287.1 PREDICTED: methionine--tRNA synthetase, cytoplasmic [Sturnus vulgaris]

MRLRVPPGAPAALKVLAAAGAAPAPVRPHLDGPAERPELELDNGTVLFSANAICQYFYLYRGEQPTDLTTQWLEWEATEL

QPAVASALYLRLVQSRPGPDALGVLQHLLAHLEQHLARSGTSHLAGDAASVADVVVWGSLFPVLQDETSLPSELQVLRSW

FRAMSQWEPCRAAAALLPEALRELRSHLAQHPPPLLGPRHEPQDEEGLERMLTDDDIAAAVDIWAQGPAALPPPRQPQTP

VLPVEGERNVLITSALPYVNNVPHLGNIIGCVLSADTFARYCRLRRWNTLFVCGTDEYGTATETRALEEGLSPQQLCDRY

HALHADIYTWFRISFDHFGRTTTPQQTRIAQDIFQRLLARGFLLQDTLEQLRCEKCGRYLADRFVEGTCPFCGYAEARGD

QCEKCGKLINAVELKNPQCKICRGTPVVTPTQHLFLDLPKLEGQLEAWLERTLAAGDWTANARHITRTWLRDGLKPRCIT

RDLTWGTPVPLDGFRDKVFYVWFDAPIGYLSITANYTDQWERWWKNPQQVELYNFMAKDNVPFHSVVFPCSLLGADDNYT

LVNHLIATEYLNYEDGKFSKSRGVGVFGDMAKDTGIPADVWRFYLLYVRPEGQDSAFSWADLLLKNNSELLNNLGNFINR

AGMFVSKFFGGAVPDMVLTAEDQRLLARVTLEVRQYHQLMEKVRIRDALRCVLSISRHGNQYIQVNEPWKRIKGDERDRQ

RAGTVTGVAVNVAAALAALLQPFVPGVSAAIQEQLQIPPERCGFGPGLTCTLPAGHRIGTVSPLFQKLEPEQVEALRQRF

GGGQDKQPPPSSASAPPPAPNTSAAPGDPKLIQELTEQVAKQGNLVRQLKAAQVEKAQVDAQVAKLLELKQQLALAEGRS

PEVPKGKRKK

>KFR05377.1 Methionine--tRNA synthetase, cytoplasmic, partial [Nipponia nippon]

QPAASAALYAQLVQGKKGLEAVEALGKLLAHIEQNLSRRGTAYLAGDTKSVADMVVWGTLFPVFQDETSLPSELQALRTW

FQSMTLLDACRKAADSVLMPKALLEFKSYLQKQPPPCLAMERATSNEPEEEEGSERALTEEEITAAADAWAHGAAALPKP

WQPQKPVLPVEGMRNVLITSALPYVNNVPHLGNIIGCVLSADTFARYCRLRNWNTLYVCGTDEYGTATETKAVEEGLTPQ

EICNKYNAIHADIYRWFDISFDYFGRTTTPYQTTIAQDIFQRLLARGFLLQDTVEQLRCEDCQRFLADRFVEGTCPFCSY

EEARGDQCDKCGKLINAVELKNPQCKLCRGVPVVKPTQHLFLDLPKLEEQLEPWLEQSWATGDWTANARYITRSWIRDGL

KPRCITRDLKWGTPVPLDGFRDKVFYVWFDAPIGYLSITANYTDQWERWWKNPQQVDLYNFMAKDNVPFHSVIFPCSLLG

ADDNYTLVNHLIATEYLNYEDGKFSKSRGVGVFGDMAKDTGIPADIWRFYLLYVRPEGQDSAFSWSDLMLKNNSELLNNL

GNFINRAGMFVCKFFGGTVPNMVLTLDDKRFLARVTLELRQYHQLLEKVRLRDALRCILSISRHGNQYIQVNEPWKRIKG

DEKDRQCAGTVTGVAVNMASMLAVLLQPYMPGVSLAIQRQLCIPPECFVLSHDFTCTLPPGHRVGTVSPLFQKLENDQVE

ALRKRFGGGQVTSEPSPAPATVVAPGDPQLIQELTEEVAKQGNHVRQLKANKAEKAQVDAEVAKLLELKKQLALAEGKSP

EVPVPKGKRKK

>XP_007422123.1 methionine--tRNA synthetase, cytoplasmic isoform X1 [Python bivittatus]

MSDPFPTRRRAGEGVHGESALISRPASLHQLGTKRGWEAEGGRSMKLCLSEGSLGGLKVLAAAKATGAPVELQWLPQEAH

VVPFLTHPQLPVLQLENGSFLFSTNAISQYFFRLSGKEMSGFKNEQSDFANQLFEWDVTELEPALSAALYLHVVQEKKGE

DVLNIIRKPLDYLDQILTKKGTSYLTGDVESAADVVLWGSLFPLLRDESFLPNELKALRSWFQNMNLKEHCRKAVESIWT

PKGLLELKAYLQKHPAPSMAFEKSATNEAKAEESIQQHLSDVEIEAIAEVWSRGPASLPSPWKPQHPIVPVEGMKNVLIT

SALPYVNNVPHLGNIIGCVLSADTFARYCRLRNWNTLYVCGTDEYGTATETKAMEEGLTPQEICDKYYSVHAQIYQWFNI

SFDYFGRTTTAQQTTIAQGIFQQLLERNYLLTETVDQLKCEKCVRFLADRFVEGICPFCNYEEARGDQCDKCGKLINATE

LKKPVCKICQGTPVVKSSEHLFLDLPKLEDSLEKWLAVSQSSGDWTPNSRYITRSWIRDGLKPRCITRDLKWGTPVPLDG

FRDKVFYVWFDAPIGYLSITANYTDQWEKWWKNPEQIQLYNFMAKDNVPFHSVVFPCSLLGTGDNYTLVNHLIATEYLNY

EDGKFSKSRGVGVFGDMAKDTGIPADIWRFYLLYLRPESQDSAFSWNDLMIKNNSELLNTLGNFINRAGTFVCKFFGGCV

PPMNLNQDDKRLLAHITLELRQYNQLLEKVKIRDALRSILNIARHGNQYLQVNEPWKCIKGSDADKKRASTVIGVAVNIA

SLLSVVLQPYMPSVSATIQDQLCIPSSFNVVTNDFVCTLPVGHQIGTVTPLFQRLEAEQIENLRKRFSGGQFEDFPLKPK

QKAAANVQKTLGVKAHQEGAAKQGSYLKHTKAQGAAKEQVDTMVSELVELQKQLSVVEGNGKKKK

>XP_009470683.1 PREDICTED: methionine--tRNA synthetase, cytoplasmic [Nipponia nippon]

MPPVLSTPAASAALYAQLVQGKKGLEAVEALGKLLAHIEQNLSRRGTAYLAGDTKSVADMVVWGTLFPVFQDETSLPSEL

QALRTWFQSMTLLDACRKAADSVLMPKALLEFKSYLQKQPPPCLAMERATSNEPEEEEGSERALTEEEITAAADAWAHGA

AALPKPWQPQKPVLPVEGMRNVLITSALPYVNNVPHLGNIIGCVLSADTFARYCRLRNWNTLYVCGTDEYGTATETKAVE

EGLTPQEICNKYNAIHADIYRWFDISFDYFGRTTTPYQTTIAQDIFQRLLARGFLLQDTVEQLRCEDCQRFLADRFVEGT

CPFCSYEEARGDQCDKCGKLINAVELKNPQCKLCRGVPVVKPTQHLFLDLPKLEEQLEPWLEQSWATGDWTANARYITRS

WIRDGLKPRCITRDLKWGTPVPLDGFRDKVFYVWFDAPIGYLSITANYTDQWERWWKNPQQVDLYNFMAKDNVPFHSVIF

PCSLLGADDNYTLVNHLIATEYLNYEDGKFSKSRGVGVFGDMAKDTGIPADIWRFYLLYVRPEGQDSAFSWSDLMLKNNS

ELLNNLGNFINRAGMFVCKFFGGTVPNMVLTLDDKRFLARVTLELRQYHQLLEKVRLRDALRCILSISRHGNQYIQVNEP

WKRIKGDEKDRQCAGTVTGVAVNMASMLAVLLQPYMPGVSLAIQRQLCIPPECFVLSHDFTCTLPPGHRVGTVSPLFQKL

ENDQVEALRKRFGGGQVKPSPAPATVVAPGDPQLIQELTEEVAKQGNHVRQLKANKAEKAQVDAEVAKLLELKKQLALAE

GKSPEVPVPKGKRKK

>XP_007422124.1 methionine--tRNA synthetase, cytoplasmic isoform X2 [Python bivittatus]

MSDPFPTRRRAGEGVHGESALISRPASLHQLGTKRGWEAEGGRSMKLCLSEGSLGGLKVLAAAKATGAPVELQWLPQEAH

VVPFLTHPQLPVLQLENGSFLFSTNAISQYFFRLSGKEMSGFKNEQSDFANQLFEWDVTELEPALSAALYLHVVQEKKGE

DVLNIIRKPLDYLDQILTKKGTSYLTGDVESAADVVLWGSLFPLLRDESFLPNELKALRSWFQNMNLKEHCRKAVESIWT

PKGLLELKAYLQKHPAPSMAFEKSATNEAKAEESIQQHLSDVEIEAIAEVWSRGPASLPSPWKPQHPIVPVEGMKNVLIT

SALPYVNNVPHLGNIIGCVLSADTFARYCRLRNWNTLYVCGTDEYGTATETKAMEEGLTPQEICDKYYSVHAQIYQWFNI

SFDYFGRTTTAQQTTIAQGIFQQLLERNYLLTETVDQLKCEKCVRFLADRFVEGICPFCNYEEARGDQCDKCGKLINATE

LKKPVCKICQGTPVVKSSEHLFLDLPKLEDSLEKWLAVSQSSGDWTPNSRYITRSWIRDGLKPRCITRDLKWGTPVPLDG

FRDKVFYVWFDAPIGYLSITANYTDQWEKWWKNPEQIQLYNFMAKDNVPFHSVVFPCSLLGTGDNYTLVNHLIATEYLNY

EDGKFSKSRGVGVFGDMAKDTGIPADIWRFYLLYLRPESQDSAFSWNDLMIKNNSELLNTLGNFINRAGTFVCKFFGGCV

PPMNLNQDDKRLLAHITLELRQYNQLLEKVKIRDALRSILNIARHGNQYLQVNEPWKCIKGSDADKKRASTVIGVAVNIA

SLLSVVLQPYMPSVSATIQDQLCIPSSFNVVTNDFVCTLPVGHQIGTVTPLFQRLEAEQIENLRKRFSGGQQKAAANVQK

TLGVKAHQEGAAKQGSYLKHTKAQGAAKEQVDTMVSELVELQKQLSVVEGNGKKKK

>XP_015678102.1 PREDICTED: methionine--tRNA synthetase, cytoplasmic [Protobothrops mucrosquamatus]

MSGPASLHQLGTKRGWEAEGGRNMKLCLSEGSLGGLKVLAAAKATGAPVELRWLPQEAHVVPFLTRPQLPALQLENGSFL

FSTNVISQYFFRLSGKEMSSFSNEHSDFANQFFEWDITELEPALSAALYLHVVQEKKGEDILGIIRKPLDYLDQIFTKKG

TSYLTGDVESAADIVLWGSLFPLLRDDSFLPNELKALRSWFQNMNLKEHCRKAVESVWTPKGLLELKAYLQKHPAPSLAF

EQSATNEAKEEESVQQHLSDVEIEAIAEIWSRGPASLPSPWKPQHPVLPVEGMKNVLITSALPYVNNVPHLGNIIGCVLS

ADTFARYCRLRNWNTLYICGTDEYGTATETKAMEEGLTPQQICDKYYSIHAQIYQWFNISFDYFGRTTTAQQTTIAQSIF

QQLLERKYLLTETVDQLKCEKCARFLADRFVEGICPFCNYEEARGDQCDKCGKLINATELKKPMCKICQGTPVVKSSQHL

FLDLPKLEESLEKWLAVSQSSGDWTPNSRYITRSWIRDGLKPRCITRDLKWGTSVPLDGFREKVFYVWFDAPIGYLSITA

NYTDQWEKWWKNPEQVQLYNFMAKDNVPFHSVVFPCSLLGTGDNYTLVNHLIATEYLNYEDGKFSKSRGVGVFGDMAKDT

GIPADIWRFYLLYLRPESQDSAFSWNDLMIKNNSELLNTLGNFINRAGTFVCKFFGGCVPAMDLTQDDKRLLAHITLELR

QYNQLLEKVKIRDALRSILNIARHGNQYLQVNEPWKCIKGSDADKKRASTVIGLAVNIASLLSVVLQPYMPTVSAAIQDQ

LCIASSFNVVTNDFVCTLPAGHQIGTVTPLFQKLEAEQIENLRKRFSGGQQKAAANIQKTLGGKEHQDGDKRGNKVKHTK

AQGAGKEQVDAVVSELVELQKQHSVVEGKGKKKK

>XP_009706285.1 PREDICTED: methionine--tRNA synthetase, cytoplasmic, partial [Cariama cristata]

LEAVEVLGKLLAHIEQNLSRRGTAYLAGDTKSVADVVVWGTLFPVLQDETSLPSELQALRTWFQSMTLSEACRKAADSVL

MPKALLEFKSYLQKQPPPCLAMERPTSNEPEEEEGSEHALTEEEITAAVDAWARGAAALPKPWQPQKPVLPVEGMRNVLI

TSALPYVNNVPHLGNIIGCVLSADTFARYCRLRNWNTLYVCGTDEYGTATETKAVEEGLTPQEICNKYNAIHADIYRWFN

ISFDYFGRTTTPYQTTIAQDIFQRLLAHGFLLQDTVEQLRCEDCQRFLADRFVEGTCPFCSYEEARGDQCDKCGKLINAV

ELKNPQCKLCRGVPVVKPTQHLFLDLPKLEEQLEAWLEQSWATGDWTANARYITRSWIRDGLKPRCITRDLKWGTPVPLD

GFRDKVFYVWFDAPIGYLSITANYTDQWERWWKNPQQVELYNFMAKDNVPFHSVIFPCSLLGADDNYTLVNHLIATEYLN

YEDGKFSKSRGVGVFGDMAKDTGIPADIWRFYLLYLRPEGQDSAFSWSDLMLKNNSELLNNLGNFINRAGMFVCKFFGGT

VPNMALTPDDKRVLARVTLELRQYHQLLEKVRIRDALRCILSISRHGNQYIQVNEPWKRIKGDEKDRQRAGTVTGVAVNM

ASMLAIMLQPYMPSVSLAIQGQLCIPPDCFVLSHDFTCTLPAGHHVGTVSPLFQKLENDQVEALRKRFGGGQAKQTPPPS

DHPPASATVMVPGDPQLIQELTEEVAKQGNHIRQLKTNKAEKAQVNMEVAKLLELKKQLALAEGKSPEVPVPKGKRKK

>KFM09454.1 Methionine--tRNA synthetase, cytoplasmic, partial [Aptenodytes forsteri]

QPAASAALYAQLVHGRKGLEAVEALGKLLAHIEQNLSRRGTAYLAGDTKSVADVVVWGTLFPVLQDETSLPSELQALRIW

FQSMTLSEACRKAADSVLMPKALLEFKSYLQKQPPPCLAMERATSNEPEEEEGSERALTEEEITAAADAWARGAAALPKP

WQPQKPVLPVEGMRNVLITSALPYVNNVPHLGNIIGCVLSADTFARYCRLRNWNTLYVCGTDEYGTATETKAVEEGLTPQ

EICNKYNAIHADIYRWFDISFDYFGRTTTPYQTTIAQDIFQRLLAHGFLLQDTVEQLRCEDCQRFLADRFVEGTCPFCNY

EEARGDQCDKCGKLINAVELKNPQCKLCRGVPVVKPTQHLFLDLPKLEERLEPWLEQSWATGDWTANARYITRSWIRDGL

KPRCITRDLKWGTPVPLDGFRDKVFYVWFDAPIGYLSITANYTDQWERWWKNPQQVELYNFMAKDNVPFHSVIFPCSLLG

ADDNYTLVNHLIATEYLNYEDGKFSKSRGVGVFGDMAKDTGIPADIWRFYLLYLRPEGQDSAFSWSDLMLKNNSELLNNL

GNFINRAGMFVCKFFGGTVPNMVLTPDDKRLLARVTLELRQYHQLLEKVRIRDALRCILSISRQGNQYIQVNEPWKRIKG

DEKDRQRAGTVTGVAVNMASMLAILLQPYMPSVSLAIQGQLCIPLDCFILSHDFTCILPPGHRVGTVSPLFQKLENDQVE

ALRKRFGGGQVISEPPPAPVTLMAPGDPQLIQELTEEVAKQGNHVRQLKANKAEKAQVDMEVAKLLELKKQLALAEGKSP

EDPVPKGKRK

>KFP61317.1 Methionine--tRNA synthetase, cytoplasmic, partial [Cariama cristata]

KLLAHIEQNLSRRGTAYLAGDTKSVADVVVWGTLFPVLQDETSLPSELQALRTWFQSMTLSEACRKAADSVLMPKALLEF

KSYLQKQPPPCLAMERPTSNEPEEEEGSEHALTEEEITAAVDAWARGAAALPKPWQPQKPVLPVEGMRNVLITSALPYVN

NVPHLGNIIGCVLSADTFARYCRLRNWNTLYVCGTDEYGTATETKAVEEGLTPQEICNKYNAIHADIYRWFNISFDYFGR

TTTPYQTTIAQDIFQRLLAHGFLLQDTVEQLRCEDCQRFLADRFVEGTCPFCSYEEARGDQCDKCGKLINAVELKNPQCK

LCRGVPVVKPTQHLFLDLPKLEEQLEAWLEQSWATGDWTANARYITRSWIRDGLKPRCITRDLKWGTPVPLDGFRDKVFY

VWFDAPIGYLSITANYTDQWERWWKNPQQVELYNFMAKDNVPFHSVIFPCSLLGADDNYTLVNHLIATEYLNYEDGKFSK

SRGVGVFGDMAKDTGIPADIWRFYLLYLRPEGQDSAFSWSDLMLKNNSELLNNLGNFINRAGMFVCKFFGGTVPNMALTP

DDKRVLARVTLELRQYHQLLEKVRIRDALRCILSISRHGNQYIQVNEPWKRIKGDEKDRQRAGTVTGVAVNMASMLAIML

QPYMPSVSLAIQGQLCIPPDCFVLSHDFTCTLPAGHHVGTVSPLFQKLENDQVEALRKRFGGGQAKQTPPPSDHPPASAT

VMVPGDPQLIQELTEEVAKQGNHIRQLKTNKAEKAQVNMEVAKLLELKKQLALAEGKSPEVPVPKGKRKK

>XP_007907515.1 PREDICTED: methionine--tRNA synthetase, cytoplasmic [Callorhinchus milii]

MKVYLNSGNPGGLKVVAAAELSGVGPEFQPVSLEERVVPFLSQSKLPALQLPTGSFIFSTNAICQYLFTLSGKEPSDATN

QWLEWEAARLQPAVNVWLHTSVVQGKKNEQAASAVSKLLAHINQSLIKTATPYLTSAAVTVADVVLWGALYPILVDPTHL

SEELLMVCEWFERMNMLDSCRKASESVLQGKGVQAFKAYLQKQPVSTLLSEKPASNGQEEGEAPGQTFTEEEIQAAEDAW

RHGRVQMSKPKLVQHPILPKKNERNILITSALPYVNNVPHLGNIIGCVLSADVFARYCRMRNWNTLYICGTDEYGTATET

KAMEEGLTPQQICDKYNAIHSQIYQWFHISFDYFGRTTTEHQTRIAQDIFNRLLQRDFLVNDTVEQLRCNRCERFLADRF

VEGSCPFCNYEEARGDQCDKCGKLINAVELKNPQCKICKDTPVIKSSKHLFIDLPKLQERLEEWFDKSVATGDWTTNARL

ITRSWIRDGLKPRCITRDLKWGTPVPLEEFKDKVFYVWFDAPIGYISITADYTEHWEKWWKNPEEVQLYNFMAKDNVPFH

SVIFPCSLLGAEDNYTLVNSLIATEYLNYEDGKFSKSRGVGVFGNMAKDTGIPADIWRFYLLFIRPEGQDSSFSWNDFLL

KNNSELLNNLGNFINRAGMFVTKFFDRLVPEMELNLEDKQLLAQVTWELQQYHQHLEKARIRDALKCILNISRCGNQYIQ

VNEPWKRIQGSDADKKRAGTVTGVAVNVSALLALMLQPYMPTVTVTIQAQLQTPEDYSVLTTHFVCLLPAGHRIGTVSPL

FQKLETEQIEALKKKYGSGQQAAASTKSTAAPNQPGQPIAPTQPGQPATPEQIKEMTEAVAKQGNCVRELKAQKAEKLLI

DKEVALLLALKKQLVVAEGKPIETPSSGGKKK

>XP_012962606.1 methionine--tRNA synthetase, cytoplasmic, partial [Anas platyrhynchos]

GSGSWPRLPDPPLSRSALCSFQPVVAAALYAQLVHGKKGLEAAGHLGKLLAYIEQSLSGRGTAYLAGDTQSVADVVVWGA

LFPVLQDEASLSNELQALRTWFQTMTLSEACRKAADSVLMPKGLLEFKSYLQKQPPPCLAMERATSNEPEEEEASERALT

EEEIRAAADAWARGAAALPKPWCPQKPVLPVEGMRNVLITSALPYVNNVPHLGNIIGCVLSADTFARYCRLRNWNTLYVC

GTDEYGTATETKAVEEGLTPREICDKYNAIHADIYRWFHISFDYFGRTTTPHQTTIAQDIFQRLLARGFLLQDTVEQLQC

EGCQRFLADRFVEGICPFCGYEEARGDQCDKCGKLINAVELKRPQCKLCRGVPVVKPTQHLFLDLPKLEEQLEPWLEHSW

SSGDWTANARYITRSWIRDGLKPRCITRDLKWGTPVPLDGFRDKVFYVWFDAPIGYLSITANYTDQWERWWKNPQQVELY

NFMAKDNVPFHSVIFPCSLLGADDNYTLVNHLIATEYLNYEDGKFSKSRGVGVFGDMAKDTGIPADIWRFYLLYLRPEGQ

DSAFSWSDLMLKNNSELLNNLGNFINRAGMFVCKFFGGVVPDMVLTADDKRLLARVTLELRQYHQLLEKVRIRDALRCIL

SISRHGNQYIQVNEPWKRIKGNEEDRQRAGTVTGVAVNMASLLAVMVQPYMPSVSLAIQGQLCIPPECFVLSHIFTCTLP

PGHRVGTVSPLFQKLENDQIEALRKRFGGGQPEDLTVVRQAKPTPPASAPVPAPASGPVVAPGDPQLIQELTEQVAKQGN

HVRQLKANKAEKAEIDAEVAKLLELKKQLALAEGKSPEVLVPKGKRKK

>XP_025001055.1 methionine--tRNA synthetase, cytoplasmic isoform X1 [Gallus gallus]

MRLRVAAGSPAALKVVAAAHCAGAPVQLCWAEPGEPPPSPLCPPWAPALQLESGAVLFSPNAICQYFFLRRGEQPTDLTT

QWLEWEATELQPAALAALYAHLVHGRTGVEALGEPLGHIERSLVGRGTAYLAGDTESVADVVVWSTLFPVLRDEAIPAAE

PLQSWFRSMTFSEACRNAADTVLLPRHLQEFTACLQKLPLPRPPPDTAPGCEEDDVSERALTEEDMAAAVDAWTRGAAAL

PKPWRPPKPVLPVEGMRNVLITSALPYVNNVPHLGNIIGCVLSADAFARYCRLRNWNTLYVCGTDEYGTATETKAVEEGL

TPREICDKYHAVHADIYRWFDISFDYFGRTTTPQQTVISQDIFQRLQAHGFVLQDTVQQLHCASCQRFLADRFVEGICPF

CSYQEARGDQCDKCGKLINAVELKTPRCKLCSGSPTVRPTQHLFLDLPKLEERLQPWLENCWATGNWTANARYITRSWIR

DGLRPRCITRDLQWGTPVPLDGFRDKVFYVWFDAPIGYLSITANYTEHWERWWKNPQQVELYNFMAKDNVPFHSVVFPCS

LLGTDDNYTLVNHLIATEYLNYEDGKFSKSRGVGVFGDMAKDTGIAADVWRFYLLYVRPEGQDSAFSWGDLMLKNNSELL

NNLGNFINRAGMFVCKFFGSAVPPMVLTPDDHRLLARVTVELRQYHQLLERVRIRDALRCVLNISRHGNQYIQVNEPWKR

IKGGEEDRQRAGTVTGVAVNVAALLAVLVQPYMPTVSAAIQAQLCVPPECSVLSHIFTCTLPPGHRVGTVSPLFQKLESE

RIDALRKRFGGGQVKPPIPPPQLDPPSVPGKTPPDPSRIQELTEQVAKQGNCVRQLKASGSDRARIDSEVGKLLALKREL

ALAQNMSPDGPRGKGKK

>KGL98605.1 Methionine--tRNA synthetase, cytoplasmic, partial [Charadrius vociferus]

QPAAWAALYAQLVHGKKGLEAVEALGKLLAHIEQNLSRRGTAYLAGETKSVADVVVWATLFPVFQDETSLPIELQALRTW

FQSMTLLEACRKAADFVLMPKALLEFKSYLQKQPPPCLAMERAPSNEPEEEEGSERALTEEEITAAADAWAHGAAALPKP

WQPQKPVLPVEGMRNVLITSALPYVNNVPHLGNIIGCVLSADTFARYCRLRNWNTLYVCGTDEYGTATETKAVEEGLTPQ

EICNKYNAIHADIYRWFDISFDYFGRTTTPYQTTIAQDIFQRLLARGFLLQDTVEQLRCENCQRFLADRFVEGTCPFCSY

KEARGDQCDKCGKLINAVELKNPQCKLCRGVPVVKPTQHLFLDLPKLEERLEPWLEQSWATGDWTANARYITRSWIRDGL

KPRCITRDLKWGTPVPLDGFHDKVFYVWFDAPIGYLSITANYTDQWERWWKNPQQVELYNFMAKDNVPFHSVIFPCSLLG

AEDNYTLVNHLIATEYLNYEDGKFSKSRGVGVFGDMAKDTGIPADIWRFYLLYLRPEGQDSAFSWSDLMLKNNSELLNNL

GNFINRAGMFVCKFFGGTVPNMVLTPDDKRLLARVTLELHQYHQLLEKVRIRDALRCILSISRHGNQYIQVNEPWRRIKG

DENDRERAGTVTGVAVNMASMLAVLLQPYMPSVSLAIQGQLCIPPDCFVLSHYFTCTLPPGHRVGTVSPLFQKLENDQVE

ALRKRFGGGQARQTPPASHPSPAPGDPQLIQQLTEEVAKQGNHVRQLKANKAEKAQVDAEVAKLLELKQQLAVAEGKSPE

VPVPKGKRKK

>KFZ57349.1 Methionine--tRNA synthetase, cytoplasmic, partial [Podiceps cristatus]

QPAAAAALYAQLVHGKKGLEALRKLLAHIEQNLSRRGSAYLAGWLVGAADVVVWGTLFPVLQDETSLPNELQALRTWFQS

MTLSEACRKAADSILMPKALMEFKSYLQKQPPPCLAMERATSNEPEEEEGSERALTEEEITAAADAWAHGAVALPKPWQP

QKPVLPVEGMKNVLITSALPYVNNVPHLGNIIGCVLSADTFARYCRLRNWNTLYVCGTDEYGTATETKAVEEGLTPQEIC

NKYNAIHADIYRWFDISFDYFGRTTTPYQTTIAQDIFQRLLDRGFLLQDTVEQLRCENCQRFLADRFVEGTCPFCSYEEA

RGDQCDKCGKLINAVELKNPQCKLCRGIPVVKPTQHLFLDLPKLEERLEPWLQQSWATGDWTVNARYITRSWIRDGLKPR

CITRDLKWGTPVPLDGFRDKVFYVWFDAPIGYLSITANYTDQWERWWKNPQQVELYNFMAKDNVPFHSVIFPCSLLGADD

NYTLVNHLIATEYLNYEDGKFSKSRGVGVFGDMAKDTGIPADIWRFYLLYLRPEGQDSAFSWSDLMLKNNSELLNNLGNF

INRAGMFVCKFFSGVVPNMVLTPDDKRLLARVTLELRQYHQLLEKVRIRDALRCILSISRHGNQYIQVNEPWKRIKGDEK

DRQRAGTVTGVAVNMASLLAVLLQPYMPSVSSAIQGQLCIPADCFVLSHNFTCTLPPGHCVGTVSPLFQKLENDQVEALR

KRFGGGQARQTPPAPATLVATGAPHLIQELTEEVAKQGNHIRQLKANKAEKAQIDTEVAKLLELKKQLALAEGKNPEVPV

SKGKRNK

>XP_009280454.1 PREDICTED: methionine--tRNA synthetase, cytoplasmic [Aptenodytes forsteri]

MRPMARMCPTARLCPMAMDSPPTMAMPHSNPVRKQWWDHVLPQFLVPGEPMPDPGPAASAALYAQLVHGRKGLEAVEALG

KLLAHIEQNLSRRGTAYLAGDTKSVADVVVWGTLFPVLQDETSLPSELQALRIWFQSMTLSEACRKAADSVLMPKALLEF

KSYLQKQPPPCLAMERATSNEPEEEEGSERALTEEEITAAADAWARGAAALPKPWQPQKPVLPVEGMRNVLITSALPYVN

NVPHLGNIIGCVLSADTFARYCRLRNWNTLYVCGTDEYGTATETKAVEEGLTPQEICNKYNAIHADIYRWFDISFDYFGR

TTTPYQTTIAQDIFQRLLAHGFLLQDTVEQLRCEDCQRFLADRFVEGTCPFCNYEEARGDQCDKCGKLINAVELKNPQCK

LCRGVPVVKPTQHLFLDLPKLEERLEPWLEQSWATGDWTANARYITRSWIRDGLKPRCITRDLKWGTPVPLDGFRDKVFY

VWFDAPIGYLSITANYTDQWERWWKNPQQVELYNFMAKDNVPFHSVIFPCSLLGADDNYTLVNHLIATEYLNYEDGKFSK

SRGVGVFGDMAKDTGIPADIWRFYLLYLRPEGQDSAFSWSDLMLKNNSELLNNLGNFINRAGMFVCKFFGGTVPNMVLTP

DDKRLLARVTLELRQYHQLLEKVRIRDALRCILSISRQGNQYIQVNEPWKRIKGDEKDRQRAGTVTGVAVNMASMLAILL

QPYMPSVSLAIQGQLCIPLDCFILSHDFTCILPPGHRVGTVSPLFQKLENDQVEALRKRFGGGQAKQTPPVSEPPPAPVT

LMAPGDPQLIQELTEEVAKQGNHVRQLKANKAEKAQVDMEVAKLLELKKQLALAEGKSPEDPVPKGKRK

>XP_008318853.1 methionine--tRNA synthetase, cytoplasmic isoform X2 [Cynoglossus semilaevis]

MKLFVSEGNPHCLKVLAALEETGAKCGVDYINHEENVVPFLSRPAIPALLLPSGSHLFSSNAICQFLFEVNGQETPPLCN

QWLEWEATELQPALFQALYMAVVQGKGSEVSKVLQAPLNYLDQGLTKGNLPYLTGNAVTVADVVLWAALYPILSDSSLQL

GEHKSVRSWFDRVNARQSCQSATQKVLQGKGLQGMKSYLQRQPAPQVNQPRDSQPSNSPSAECEEGERVVSEEEMEAAAL

TWSKGLSTGPVLKERQQPILPEKDKRNVLVTSALPYVNNVPHLGNIIGCVLSADVFSRYGRMRGWNLLYVCGTDEYGTAT

ENKAREEGLTPQQICDKYHAIHANIYKWFQIEFDFFGRTTTEKQTEIAQNIFWRLHEHGFLMEDTVEQLRCEKCQRFLAD

RFVEGICPHCRYPEARGDQCDKCGRLINAVELGEPQCKVCKQTPVIRSSKHLFLDLPKLEAQLEQWLETSTGTGDWTTNA

KQITRSWLRDGLKPRCITRDLQWGTPVPHPDFKEKVFYVWFDAPIGYLSITANYTDQWEKWWKNPEQVELYNFMAKDNVP

FHSVVFPCSLLGAQDNYTLVNHLVATEYLNYEDTKFSKSRGVGVFGDMAKDTGIPSDVWRFYLLYVRPEGQDSAFSWADM

ALKNNSELLNNLGNFINRAGMFVTKFFDGCVPVMDLQQEDKKLLAMVSWELQHYIQLMDKVKIRDALKHILNISRHGNQY

IQVNEPWKKIKGGDADRQRAGTVTGVSVNIACLLSAMLQPYMPTVSQTIRDQLNAPPTCVHTMVQGSGTFACSLSAGHRI

GTVSPLFQKLEADQIEALKKRFGGQQATAQKAVSPAAAAVPVAAAAAPGAEPATANGVDPEKAKQLSQAVTEQGEKVRAL

KAQKADKAAITAEVAKLLDLKKQLAVAEGKNPEPAPQKGI

>XP_009889994.1 PREDICTED: methionine--tRNA synthetase, cytoplasmic [Charadrius vociferus]

MGLAPAAWAALYAQLVHGKKGLEAVEALGKLLAHIEQNLSRRGTAYLAGETKSVADVVVWATLFPVFQDETSLPIELQAL

RTWFQSMTLLEACRKAADFVLMPKALLEFKSYLQKQPPPCLAMERAPSNEPEEEEGSERALTEEEITAAADAWAHGAAAL

PKPWQPQKPVLPVEGMRNVLITSALPYVNNVPHLGNIIGCVLSADTFARYCRLRNWNTLYVCGTDEYGTATETKAVEEGL

TPQEICNKYNAIHADIYRWFDISFDYFGRTTTPYQTTIAQDIFQRLLARGFLLQDTVEQLRCENCQRFLADRFVEGTCPF

CSYKEARGDQCDKCGKLINAVELKNPQCKLCRGVPVVKPTQHLFLDLPKLEERLEPWLEQSWATGDWTANARYITRSWIR

DGLKPRCITRDLKWGTPVPLDGFHDKVFYVWFDAPIGYLSITANYTDQWERWWKNPQQVELYNFMAKDNVPFHSVIFPCS

LLGAEDNYTLVNHLIATEYLNYEDGKFSKSRGVGVFGDMAKDTGIPADIWRFYLLYLRPEGQDSAFSWSDLMLKNNSELL

NNLGNFINRAGMFVCKFFGGTVPNMVLTPDDKRLLARVTLELHQYHQLLEKVRIRDALRCILSISRHGNQYIQVNEPWRR

IKGDENDRERAGTVTGVAVNMASMLAVLLQPYMPSVSLAIQGQLCIPPDCFVLSHYFTCTLPPGHRVGTVSPLFQKLEND

QVEALRKRFGGGQARQTPPASHPSPAPGDPQLIQQLTEEVAKQGNHVRQLKANKAEKAQVDAEVAKLLELKQQLAVAEGK

SPEVPVPKGKRKK

>XP_021406293.1 methionine--tRNA synthetase, cytoplasmic [Lonchura striata domestica]

MRLRVPPGAPAALKVLAAAAAAPAPGPPRPGGPAERPSAPPAPGQEQQLQLELDSGTVLFSANAICQYFYLCRGEEPTDL

TTQWLEWEATELQPAVAAALYLRLVQGRPGPEALGVLQPLLAHLEQHLARSGTSHLAGDAASVADVVVWGSLFPVLQDET

SVPSELQVLRSWFGAVSAWEPCRAAAALLPEALRELRAHLARCPPALPDPKHLPQDEESPERLLSDADIAAAAELWARGP

AALPPPRQPATPVLPVEGERNVLITSALPYVNNVPHLGNIIGCVLSADTFARYCRLRGWNTLFVCGTDEYGTATETRALQ

EGLSPRQLCDRYHALHADVYAWFRISFDHFGRTTTPQQTRIAQDIFQRLLARGFLLQDTLQQLRCESCGRYLADRFVEGT

CPGCGYAEARGDQCDKCGKLINAVELQSPQCKLCRGTPVVTPTQHLFLDLPKLEGRLQAWLERSWAAGHWTANARHITRT

WLRDGLKPRCITRDLAWGTPVPLDGFRDKVFYVWFDAPIGYLSITANYTEHWERWWKNPQQVELYNFMAKDNVPFHSVVF

PCSLLGADDNYTLVNHLIATEYLNYEDGKFSKSRGVGVFGDMAKDTGIPADVWRFYLLYLRPEGQDSAFSWADLMLKNNS

ELLNNLGNFINRAGMFVCKFFGGAVPAMVLTAEDRRLLARVTLEMRQYHQLMEKVHIRDALRCVLSISRHGNQYIQVNEP

WKRIKGDEGDRQRAGTVTGVAVNVAAALAALLQPFVPGVSAAIQAQLRIPPERCGLGPGLTCTLPTGHRVGTVSPLFQKL

EPEQVEALRQRFGGGQAKQPPPASAPPPAPNTSAAPGDPKVIQELTEQVAKQGNLVRQLKAAQVEKAQVDAQVAKLLELK

QQLALAEGRSPEVPKGKRKK

>XP_014191448.1 PREDICTED: methionine--tRNA synthetase, cytoplasmic isoform X1 [Haplochromis burtoni]

MNLFVSEGNPHCLKALAALEATGVQCGVQFVNHDERVVPFLSRPTLPALALPSGQYLFSSNAICRYLFEVNGQECSELCN

QWLEWEATVLQPALQQALHMAVLQGKSSEVSKALQGPINVLEQSLSKRNTPYLTGDSVSAADLVLWAGFFPVLSDSSLTL

GEHKSLKAWFDRVAAMNACQSAAQKVLQGKGLQGMKSYMQKQPAPQSSQCRETQPCNSNSAEEEGGERVVSEEEMEAAAL

TWSKGLNTCPPATKKQHPILPQEGKRNVLVTSALPYVNNVPHLGNIIGCVLSADVFSRYGHLRGWNMLFVCGTDEYGTAT

ENKAREEGLTPQQICDKYHAVHSSIYKWFQIDFDYFGRTTTEKQTEIAQDIFWRLHERGFLIEDTVEQLRCENCQRFLAD

RFVEGICPHCSYPEARGDQCDKCGRLINAVDLREPQCKVCRQTPVIRSSKHLFLDLPKLESQLEQWLEKSTSTGDWTANA

KQITRSWLRDGLKPRCITRDLQWGTPVPHADFKEKVFYVWFDAPIGYLSITANYTSEWEKWWKNPHQVELYNFMAKDNVP

FHSVVFPCSLLGAQDNYTLVNHLIATEYLNYEDTKFSKSRGVGVFGDMAKDTGIPSDVWRFYLLYVRPEGQDSAFSWADM

ALKNNSELLNNLGNFINRAGMFVTKFFEGCVPVMELQEEDKKLLALVSWELQQYIQLMDKIKIRDALKHILNISRHGNQY

IQVNEPWKKIKGGDSERQRAGTVTGVSVNIACLLSVMLSPYMPAVSQTIRDQLNAPPSCINTMLEDNGTFVCSLSAGHRI

GAVSPLFQKLEADQIEALKKKFGGQQPEDEPPKKKTTVRNAASAQSAAVPAPAVAVEVGTVNQVDPEKAKQLTQAVAEQG

EKVRALKAQKAEKAVITAEVAKLLDLKKQLALAEGKNPEPAPQKGKKK

>XP_005740101.1 PREDICTED: methionine--tRNA synthetase, cytoplasmic isoform X2 [Pundamilia nyererei]

MNLFVSEGNPHCLKALAALEATGVQCGVQFVNQDERVVPFLSRPTLPALALPSGQYLFSSNAICRYLFEVNGQECSELCD

QWLEWEATVLQPALQQALHMAVLQGKSSEVSKALQGPINVLEQSLSKRNAPYLTGDSVSAADLVLWAGFFPVLSDSSLTL

GEHKSLKAWFDRVAAMTACQSAAQKVLQGKGLQGMKSYMQKQPAPQSSQCRETQPCNSNSAEEEGGERVVSEEEMEAAAL

TWSKGLNTCPPATKKQHPILPQEGKRNVLVTSALPYVNNVPHLGNIIGCVLSADVFSRYGHLRGWNMLFVCGTDEYGTAT

ENKAREEGLTPQQICDKYHAVHSSIYKWFQIDFDYFGRTTTEKQTEIAQDIFWRLHERGFLIEDTVEQLRCENCQRFLAD

RFVEGICPHCSYPEARGDQCDKCGRLINAVDLREPQCKVCRQTPVIRSSKHLFLDLPKLESQLEQWLEKSTSTGDWTANA

KQITRSWLRDGLKPRCITRDLQWGTPVPHADFKEKVFYVWFDAPIGYLSITANYTSKWEKWWKNPHQVELYNFMAKDNVP

FHSVVFPCSLLGAQDNYTLVNHLIATEYLNYEDTKFSKSRGVGVFGDMAKDTGIPSDVWRFYLLYVRPEGQDSAFSWADM

ALKNNSELLNNLGNFINRAGMFVTKFFEGCVPVMELQEEDKKLLALVSWELQQYIQLMDKIKIRDALKHILNISRHGNQY

IQVNEPWKKIKGGDSERQRAGTVTGVSVNIACLLSVMLSPYMPAVSQTIRDQLNAPPSCINTMLEDNGTFVCSLSAGHRI

GAVSPLFQKLEADQIETLKKKFGGQQTTVRNAASAQSAAVPAPAVAVEVGTVNQVDPEKAKQLTQAVAEQGEKVRALKAQ

KAEKAVITAEVTKLLDLKKQLALAEGKNPEPAPQKGKKK

>XP_008318852.1 methionine--tRNA synthetase, cytoplasmic isoform X1 [Cynoglossus semilaevis]

MKLFVSEGNPHCLKVLAALEETGAKCGVDYINHEENVVPFLSRPAIPALLLPSGSHLFSSNAICQFLFEVNGQETPPLCN

QWLEWEATELQPALFQALYMAVVQGKGSEVSKVLQAPLNYLDQGLTKGNLPYLTGNAVTVADVVLWAALYPILSDSSLQL

GEHKSVRSWFDRVNARQSCQSATQKVLQGKGLQGMKSYLQRQPAPQVNQPRDSQPSNSPSAECEEGERVVSEEEMEAAAL

TWSKGLSTGPVLKERQQPILPEKDKRNVLVTSALPYVNNVPHLGNIIGCVLSADVFSRYGRMRGWNLLYVCGTDEYGTAT

ENKAREEGLTPQQICDKYHAIHANIYKWFQIEFDFFGRTTTEKQTEIAQNIFWRLHEHGFLMEDTVEQLRCEKCQRFLAD

RFVEGICPHCRYPEARGDQCDKCGRLINAVELGEPQCKVCKQTPVIRSSKHLFLDLPKLEAQLEQWLETSTGTGDWTTNA

KQITRSWLRDGLKPRCITRDLQWGTPVPHPDFKEKVFYVWFDAPIGYLSITANYTDQWEKWWKNPEQVELYNFMAKDNVP

FHSVVFPCSLLGAQDNYTLVNHLVATEYLNYEDTKFSKSRGVGVFGDMAKDTGIPSDVWRFYLLYVRPEGQDSAFSWADM

ALKNNSELLNNLGNFINRAGMFVTKFFDGCVPVMDLQQEDKKLLAMVSWELQHYIQLMDKVKIRDALKHILNISRHGNQY

IQVNEPWKKIKGGDADRQRAGTVTGVSVNIACLLSAMLQPYMPTVSQTIRDQLNAPPTCVHTMVQGSGTFACSLSAGHRI

GTVSPLFQKLEADQIEALKKRFGGQQPEDELPMNKATAQKAVSPAAAAVPVAAAAAPGAEPATANGVDPEKAKQLSQAVT

EQGEKVRALKAQKADKAAITAEVAKLLDLKKQLAVAEGKNPEPAPQKGI

>XP_004547749.1 methionine--tRNA synthetase, cytoplasmic isoform X2 [Maylandia zebra]

MNLFVSEGNPHCLKALAALEATGVQCGVQFVNHDERVVPFLSRPTLPALALPSGQYLFSSNAICRYLFEVNGQECSELCD

QWLEWEATVLQPALQQALHMAVLQGKSSEVSKVLQGPINVLEQSLSKRNAPYLTGDSVSAADLVLWAGFFPVLSDSSLTL

GEHKSLKAWFDRVAAMNACQSAAQKVLQGKGLQGMKSYMQKQPAPQSSQCRETQPCNSNSAEEEGGERVVSEEEMEAAAL

TWSKGLNTCPPATKKQHPILPQEGKRNVLVTSALPYVNNVPHLGNIIGCVLSADVFSRYGHLRGWNMLFVCGTDEYGTAT

ENKAREEGLTPQQICDKYHAVHSSIYKWFQIDFDYFGRTTTEKQTEIAQDIFWRLHERGFLIEDTVEQLRCENCQQFLAD

RFVEGICPHCSYPEARGDQCDKCGRLINAVDLREPQCKVCRQTPVIRSSKHLFLDLPKLESQLEQWLEKSTSTGDWTANA

KQITRSWLRDGLKPRCITRDLQWGTPVPHADFKEKVFYVWFDAPIGYLSITANYTNEWEKWWKNPHQVELYNFMAKDNVP

FHSVVFPCSLLGAQDNYTLVNHLIATEYLNYEDTKFSKSRGVGVFGDMAKDTGIPSDVWRFYLLYVRPEGQDSAFSWADM

ALKNNSELLNNLGNFINRAGMFVTKFFEGCVPVMELQEEDKKLLALVSWELQQYIQLMDKIKIRDALKHILNISRHGNQY

IQVNEPWKKIKGGDSERQRAGTVTGVSVNIACLLSVMLSPYMPAVSQTIRDQLNAPPSCINTMLEGNGTFVCSLSAGHRI

GAVSPLFQKLEADQIEALKKKFGGQQTTVRNAASAQSAAVPAPAVAVEVGTVNQVDPEKAKQLTQAVAEQGEKVRALKAQ

KAEKAVITAEVAKLLDLKKQLALAEGKNPEPAPQKGKKK

>AAH57463.1 Mars protein [Danio rerio]

MKLFIGEGNPHCLKVLAALELSGVRCETQLVKHEEKVVPYLTHPVLPILQLPSGQHLFSPNSICQYLFDISGQKATDATN

QWLEWEATNLQPAVLQSLQLVALQGKRVESAAVMKEPLSWLEQSLSKRKTSFLTDEVVSVADVVLWAALYPLLSDSAFEP

GDLQAVRGWFERVCSVSACQSAALRVLQGKGAEALKSFLQKQPVTHTPRRDSPSNSTEAEDSERELSPEEIEEAAQVYSE

GLKDFTVVTERKHPVLPQEGKRNVLITSALPYVNNVPHLGNIIGCVLSADVFARYGRLRGWNLLYICGTDEYGTATENKA

REEGLTPQQICDKYHCIHASIYQWFQIDFDFFGRTTTQHQTEIAQDIFWRLHERGFLLEDTVEQLRCEGCQRFLADRFVE

GECPHCRYPEARGDQCDKCGRLINAVELKNPQCKVCKETPVIRSSKHLFLNLPKLEQDLEQWLQTSTAAGDWTTNARHIT

RSWLRDGLKPRCITRDLKWGTPVPHPDYKEKVFYVWFDAPIGYLSITANYTDQWERWWKNPQQVELYNFMAKDNVPFHSV

VFPCSLLGAQDNYTLVNNLIATEYLNYEDTKFSKSRGVGVFGDMAKDTGIPSDVWRFYLLYLRPEGQDSAFSWTDMALKN

NSELLNNLGNFINRAGMFVSKFFEGCVPEMLLNDDDKRLIAQVCWELKQYIQLLDKVRIRDALKCILNMSRHGNQYIQLN

EPWKKIKGGAEDRCRAGTVTGVSVNVACLLAAVLEPFMPTVSLSIRSQLQAPESSSRAMLSGPGAFICTLPAGHRIGTVS

PLFQKLENEQIEALRKRFGGLQTPSNSSVAVESKPNAGAAQHTPASVTADPERAKQLTALVAEQGEKVRALKAQKAEKSA

IGVEVAKLLDLKNQLCLAEGKTPEPPAQKTKKK

>XP_005740100.1 PREDICTED: methionine--tRNA synthetase, cytoplasmic isoform X1 [Pundamilia nyererei]

MNLFVSEGNPHCLKALAALEATGVQCGVQFVNQDERVVPFLSRPTLPALALPSGQYLFSSNAICRYLFEVNGQECSELCD

QWLEWEATVLQPALQQALHMAVLQGKSSEVSKALQGPINVLEQSLSKRNAPYLTGDSVSAADLVLWAGFFPVLSDSSLTL

GEHKSLKAWFDRVAAMTACQSAAQKVLQGKGLQGMKSYMQKQPAPQSSQCRETQPCNSNSAEEEGGERVVSEEEMEAAAL

TWSKGLNTCPPATKKQHPILPQEGKRNVLVTSALPYVNNVPHLGNIIGCVLSADVFSRYGHLRGWNMLFVCGTDEYGTAT

ENKAREEGLTPQQICDKYHAVHSSIYKWFQIDFDYFGRTTTEKQTEIAQDIFWRLHERGFLIEDTVEQLRCENCQRFLAD

RFVEGICPHCSYPEARGDQCDKCGRLINAVDLREPQCKVCRQTPVIRSSKHLFLDLPKLESQLEQWLEKSTSTGDWTANA

KQITRSWLRDGLKPRCITRDLQWGTPVPHADFKEKVFYVWFDAPIGYLSITANYTSKWEKWWKNPHQVELYNFMAKDNVP

FHSVVFPCSLLGAQDNYTLVNHLIATEYLNYEDTKFSKSRGVGVFGDMAKDTGIPSDVWRFYLLYVRPEGQDSAFSWADM

ALKNNSELLNNLGNFINRAGMFVTKFFEGCVPVMELQEEDKKLLALVSWELQQYIQLMDKIKIRDALKHILNISRHGNQY

IQVNEPWKKIKGGDSERQRAGTVTGVSVNIACLLSVMLSPYMPAVSQTIRDQLNAPPSCINTMLEDNGTFVCSLSAGHRI

GAVSPLFQKLEADQIETLKKKFGGQQPEDEPPKKKTTVRNAASAQSAAVPAPAVAVEVGTVNQVDPEKAKQLTQAVAEQG

EKVRALKAQKAEKAVITAEVTKLLDLKKQLALAEGKNPEPAPQKGKKK

>XP_004547748.1 methionine--tRNA synthetase, cytoplasmic isoform X1 [Maylandia zebra]

MNLFVSEGNPHCLKALAALEATGVQCGVQFVNHDERVVPFLSRPTLPALALPSGQYLFSSNAICRYLFEVNGQECSELCD

QWLEWEATVLQPALQQALHMAVLQGKSSEVSKVLQGPINVLEQSLSKRNAPYLTGDSVSAADLVLWAGFFPVLSDSSLTL

GEHKSLKAWFDRVAAMNACQSAAQKVLQGKGLQGMKSYMQKQPAPQSSQCRETQPCNSNSAEEEGGERVVSEEEMEAAAL

TWSKGLNTCPPATKKQHPILPQEGKRNVLVTSALPYVNNVPHLGNIIGCVLSADVFSRYGHLRGWNMLFVCGTDEYGTAT

ENKAREEGLTPQQICDKYHAVHSSIYKWFQIDFDYFGRTTTEKQTEIAQDIFWRLHERGFLIEDTVEQLRCENCQQFLAD

RFVEGICPHCSYPEARGDQCDKCGRLINAVDLREPQCKVCRQTPVIRSSKHLFLDLPKLESQLEQWLEKSTSTGDWTANA

KQITRSWLRDGLKPRCITRDLQWGTPVPHADFKEKVFYVWFDAPIGYLSITANYTNEWEKWWKNPHQVELYNFMAKDNVP

FHSVVFPCSLLGAQDNYTLVNHLIATEYLNYEDTKFSKSRGVGVFGDMAKDTGIPSDVWRFYLLYVRPEGQDSAFSWADM

ALKNNSELLNNLGNFINRAGMFVTKFFEGCVPVMELQEEDKKLLALVSWELQQYIQLMDKIKIRDALKHILNISRHGNQY

IQVNEPWKKIKGGDSERQRAGTVTGVSVNIACLLSVMLSPYMPAVSQTIRDQLNAPPSCINTMLEGNGTFVCSLSAGHRI

GAVSPLFQKLEADQIEALKKKFGGQQLEDEPPKKKTTVRNAASAQSAAVPAPAVAVEVGTVNQVDPEKAKQLTQAVAEQG

EKVRALKAQKAEKAVITAEVAKLLDLKKQLALAEGKNPEPAPQKGKKK

>XP_013859608.1 PREDICTED: methionine--tRNA synthetase, cytoplasmic isoform X1 [Austrofundulus limnaeus]

MKLFVSEGNPHCLKVLAALEVTGLQCGVQYVNHEEKVVPFLNRPSLPALLLPSGQYLFSSSSICRYFFEANGQECNDLCN

QWLEWEITVLQPALLQTLHMAVLQGKGSEVSRVLQSPLNVLDQSLSKGTMPFLTGEAVSVADVVIWGALHPVLSDSSLAL

SEHVSVRAWFDRVAVMHSCQSAAQKVLQGKGLQAMKSYMQKQPAPQSSQCRDTQPCNSSPAENEEGERVVSEEEMEAAVL

TWRKSLNTSSPATDKQLPVLPKEDKRNVLVTSALPYVNNVPHLGNIIGCVLSGDVFSRYGRLRGWNMLYVCGTDEYGTAT

ENKAREEGLTPQQICDKYHMVHSSIYKWFQIDFDFFGRTTTEKQTEIAQDIFWRLHKNQYLVEDTVEQLRCEKCQRFLAD

RFVEGTCPHCSYPEARGDQCDKCGRLINAVELREPQCKVCRQTPVIRSSKHLFLDLPKLESQLEQWLDKSTSSGDWTANA

KQITRSWIRDGLKPRCITRDLKWGTPVPHPDFKEKVFYVWFDAPIGYLSITANYTKEWEKWWKNPHQVELYNFMAKDNVP

FHSVVFPCSLLGAQDNYTLVNHLIATEYLNYEDTKFSKSRGVGVFGDMAKDTGIPSDVWRFYLLYVRPEGQDSAFSWADM

ALKNNSELLNNLGNFINRAGMFVSRFFEGCVPVMDLQREDKKLLALVGWELQQYIQLLDKVRIRDALKHILNISRHGNQY

IQVNEPWKKIKGGELERRRAGTVTGVSVNISCLLSVMLLPYMPEVSQTIRDQLNAPQSCINTMLQGTGTFVCALSAGHRI

GTVSPLFQKLEADQIEALKKRFGGQQPEEEPSNKKKPSENITTAQPAAAAAPSDGVKPTAVVGVDPEKAKQLTKAVAEQG

EKVRALKAQKAEKDVITAEVSKLLDLKKQLALAEGKNPEPQKGKKK

>XP_003451873.2 PREDICTED: methionine--tRNA synthetase, cytoplasmic isoform X2 [Oreochromis niloticus]

MKLFVSEGNPHCLKALAALEATGVQCGVQFVNHDERVVPFLSRPTLPALALPSGQYLFSSNAICRYLFEVNGQECSELCD

QWLEWEATVLQPALQQALHMAVLQGKSSEVSKVLQGPINVLEQSLSKRNTPYLTGDSVSAADLVLWAGFFPVLSDSSLTL

GEHKSLKAWFDRVAAMNACQSAAQKVLQGKGLQGMKSYMQRQPAPQSSQCRETQPCNSNSAEEEGGERVVSEEEMEAAAL

TWSKGLNTCPPATKKQHPILPQEDKRNVLVTSALPYVNNVPHLGNIIGCVLSADVFSRYGRLRGWNMLFVCGTDEYGTAT

ENKAREEGLTPQQICDKYHAVHSSIYKWFQIDFDYFGRTTTEKQTEIAQDIFWRLHERDFLIEDTVEQLRCENCQRFLAD

RFVEGICPHCSYPEARGDQCDKCGRLINAVDLREPQCKVCRQTPVIRSSKHLFLDLPKLESQLEQWLEKSTSTGDWTANA

KQITRSWLRDGLKPRCITRDLKWGTPVPHADFKEKVFYVWFDAPIGYLSITANYTSEWEKWWKNPHQVELYNFMAKDNVP

FHSVVFPCSLLGAQDNYTLVNHLIATEYLNYEDTKFSKSRGVGVFGDMAKDTGIPSDVWRFYLLYVRPEGQDSAFSWADM

ALKNNSELLNNLGNFINRAGMFVTKFFEGCVPVMELQEEDKKLLALVSWELQQYIQLMDKIKIRDALKHILNISRHGNQY

IQVNEPWKKIKGGDSERQRAGTVTGVSVNIACLLSVMLFPYMPAVSQTIRDQLNAPPSCINTMLEGNGTFVCSLSAGHRI

GAVSPLFQKLEADQIEALKKKFGGQQTPVRNAASAQSAAVPAPAAAVEVGTVNQVDPEKAKQLTQAVAEQGEKVRALKAQ

KAEKAVITAEVAKLLDLKKQLALAEGKNPEPAPQKGKKK

>XP_013859609.1 PREDICTED: methionine--tRNA synthetase, cytoplasmic isoform X2 [Austrofundulus limnaeus]

MKLFVSEGNPHCLKVLAALEVTGLQCGVQYVNHEEKVVPFLNRPSLPALLLPSGQYLFSSSSICRYFFEANGQECNDLCN

QWLEWEITVLQPALLQTLHMAVLQGKGSEVSRVLQSPLNVLDQSLSKGTMPFLTGEAVSVADVVIWGALHPVLSDSSLAL

SEHVSVRAWFDRVAVMHSCQSAAQKVLQGKGLQAMKSYMQKQPAPQSSQCRDTQPCNSSPAENEEGERVVSEEEMEAAVL

TWRKSLNTSSPATDKQLPVLPKEDKRNVLVTSALPYVNNVPHLGNIIGCVLSGDVFSRYGRLRGWNMLYVCGTDEYGTAT

ENKAREEGLTPQQICDKYHMVHSSIYKWFQIDFDFFGRTTTEKQTEIAQDIFWRLHKNQYLVEDTVEQLRCEKCQRFLAD

RFVEGTCPHCSYPEARGDQCDKCGRLINAVELREPQCKVCRQTPVIRSSKHLFLDLPKLESQLEQWLDKSTSSGDWTANA

KQITRSWIRDGLKPRCITRDLKWGTPVPHPDFKEKVFYVWFDAPIGYLSITANYTKEWEKWWKNPHQVELYNFMAKDNVP

FHSVVFPCSLLGAQDNYTLVNHLIATEYLNYEDTKFSKSRGVGVFGDMAKDTGIPSDVWRFYLLYVRPEGQDSAFSWADM

ALKNNSELLNNLGNFINRAGMFVSRFFEGCVPVMDLQREDKKLLALVGWELQQYIQLLDKVRIRDALKHILNISRHGNQY

IQVNEPWKKIKGGELERRRAGTVTGVSVNISCLLSVMLLPYMPEVSQTIRDQLNAPQSCINTMLQGTGTFVCALSAGHRI

GTVSPLFQKLEADQIEALKKRFGGQQKPSENITTAQPAAAAAPSDGVKPTAVVGVDPEKAKQLTKAVAEQGEKVRALKAQ

KAEKDVITAEVSKLLDLKKQLALAEGKNPEPQKGKKK

>XP_012696681.1 PREDICTED: methionine--tRNA synthetase, cytoplasmic isoform X2 [Clupea harengus]

MQKMKLFVSEGNPHCLKALAVLELTGVKCDVQHVNHEEKVVPFLSRPTLPVLHLPSGQYLFSSNAICQYLFDVSKKGSSK

QSHQWLEWEATELQPALMYALHLVVVQGKTADSAKGLEAPLSFLEQSLSKRTTAFLTAETVGVADIVLWAALYPAFLENG

LKTSELKSTRTWFERVGVLPACQASVQTVFKGKGVEALKGYLQKQPAPHGQHTQPRNTHTSSNGSPTETEDSDHNTTEEE

IEAAAQIWNQQIDCQSKPERKHPILPEEGKRNVLITSALPYVNNVPHLGNIIGCVLSADVFARYGRQRGWNLLYICGTDE

YGTATENKAREEGLTPQEICDKYHAIHTDIYKWFQIDFDFFGRTTTQKQTEIAQNIFWRLHERGFLIEDIVEQLRCESCQ

RFLADRFVEGVCPFCQYPEARGDQCDKCGRLINAVELKEPQCKVCKQTPVIRSSKHLFLDLPKLEANLEQWLDRSVSTGD

WTTNARNITRSWLRDGLKPRCITRDLRWGTPVPHPDFSGKVFYVWFDAPIGYLSITANYTDQWEKWWKNPEQVELYNFMA

KDNVPFHSVVFPCSLLGAQDNYTLVNNLVATEYLNYEDTKFSKSRGVGVFGDMAKDTGIPSDVWRFYLLYLRPEGQDAAF

SWADMALKNNSELLNNLGNFINRAGMFVGKFFEGCVPQVVLQEEDKRLLAQVGWELKQYIQSLDKVRIRDALKCILNISR

HGNQYIQVNEPWKKIKGGDADRQRAGTVTAVSVNMSVLLSAMLEPYMPTVSETIRSQLQAPPASAHFMLQGEGNFVCSLP

AGHRIGTVSPLFQKLEAEQIETLKKKFGGQQPQTITQNNAATKASAPVAASETSVKADPELVKQLVAAIAEQGDKVRALK

GQKAEKAVIGAEVAKLLEFKKQLALAEGKSPETAPQKGKKK

>XP_010796028.1 PREDICTED: methionine--tRNA synthetase, cytoplasmic isoform X2 [Notothenia coriiceps]

MKLFVSEGNPHCLKVLAAVEVSGVQCDVQYVNHEERVVPFLSRPALPALLLPSGLHLFSSNAICRYLFEVSGEDSSELCN

QWLEWEATDLQPALLQALHLAVVQGKVSEVSKVLQGPLNYLEQSLSKGNTPYLTGEAVSVADVVLWAALYPVLSDASLAL

GERASIKAWFDRGAVMHSCQSAAQKVLQGKGLQGMKSYMQRQPAPPSSQCRDSQPCNGNPAESEESERAVSAEEMETAAL

IWSKGLNTCPMATSRQHPILPQEGKRNILLTSALPYVNNVPHLGNIIGCVLSTDVFSRYGRMRGWNMLFICGTDEYGTAT

ENKAREEGLTPQQICDKYHAIHSNIYKWFQIDFDFFGRTTTEKQTEIAQNIFWRIHKHGFLMEDTVEQLRCEKCQRFLAD

RFVEGICPHCSYPEARGDQCDKCGKLINAVELREPQCKVCKETPSIRSSKHLFLDLPKLEGQLEQWLDKSTSTGDWTANA

KQITRSWLRDGLKPRCITRDLRWGTPVPHPDFKEKVFYVWFDAPIGYLSITANYTDQWEKWWKNPHQVELYNFMAKDNVP

FHSVVFPCSLLGAQDNYTLVNHLIATEYLNYEDTKFSKSRGVGVFGDMAKDTGIPSDVWRFYLLYVRPEGQDSAFSWADM

ALKNNSELLNNLGNFINRAGMFVTKFFEGCVPAMELQQEDKRLLALVGWELQQYIQLLDKVKIRDSLRHILNISRHGNQY

IQLNEPWKKIKGGDTERQRAGTVTGVSVNIACLLSVMLLPYMPTVSQTIRDQFNAPQSCVDTMLQGTGTFVCSLSAGHRI

GTVSPLFQKLEVDQIEALKKKFGGQQKTAQSAGSPPPAAGPAAAAEVVAGIGADPEKAKQLTQAVAEQGEKVRELKAQKA

EKAAITAEVAKLLDLKKQLALAEGKSSDPAPQKGKKK

>XP_008278954.1 PREDICTED: methionine--tRNA synthetase, cytoplasmic isoform X2 [Stegastes partitus]

MKLFVSEGNPHCLKVLAALEVTGVQCSVQYVNHEEKVVPFLSRPVLPALLLPSGLHLFSSNAICRYLFEVSGQESSELCN

QWLEWEATDLQPALLQALHMAVLQGKGSEVSKVLQGPLKFLDQSLTKGGKPYLNGDTVSLADLVLWAALYPVLSDSSLAL

GEHKSVNSWFDRVASIHSCLSAAQKVLQGKGLQGMKSYMQRQPAPQSNQCRDTQPCNSNPAEGEEGERDFSEEEMEAAAL

TWSKGLSTSPVVTDRQHPILPQEGKRNILVTSALPYVNNVPHLGNIIGCVLSADVFSRYGRLRDWNLLFVCGTDEYGTAT

ENKAREEGLTPQLICDKYHAVHSSIYKWFQIDFDFFGRTTTEKQTEIAQNIFWRLHKNGFLVEDTVEQLRCENCQRFLAD

RFVEGICPHCKYPEARGDQCDKCGRLINAVELREPQCKVCRQTPVIRSSKHLFLDLPKLETQLEQWLEKSTSTGDWTANA

KQITRSWLRDGLKPRCITRDLLWGTPVPHPDFKEKVFYVWFDAPIGYLSITANYTDKWEKWWKNPHQVELYNFMAKDNVP

FHSVVFPCSLLGAQDNYTLVNHLVATEYLNYEDTKFSKSRGVGVFGDMAKDTGIPADVWRFYLLYVRPEGQDSAFSWADM

ALKNNSELLNNLGNFINRAGMFVAKFFEGCVPVMELQQEDKRLLALVGWELQQYIQLMDKVKIRDALKHILNISRHGNQY

IQVNEPWKKIKGGDTERQRAGTVTGVSVNIACLLSVMLMPYMPAVSQTIRDQLNAPQSCVNTMLQGTGTFVCSLCAGHRI

GTVSPLFQKLEADQIEALKKRFGGQQKTPQSTASPQPAAVPATAAADEVAMVNGVDPEKAKQLTQAVAEQGDKVRALKAQ

KAEKAVITAEVAKLLDLKKQLALAEGKSPEPAPQKGKKK

>XP_018534426.1 PREDICTED: LOW QUALITY PROTEIN: methionine--tRNA synthetase, cytoplasmic [Lates calcarifer]

MKLFVSEGNPHCLKVLAALGVTGVQCDIQYVNHEETVVPFLSRPALPALLLPSGLHLFSSNAICQYLFEVNGQESSELCY

QWLEWEATDLQPALLEALHMAVLQGKGSEVSKVLQGPLNYLDQSLSKGNMPYLTGEAVSVADVVLWAALYPVLSESSLAL

GERKSVKSWFDRVAAMHSCQSAAQKVLQGKGLQGMKSYMQRQPAPRSSHCRDTQPCNSNPAECEEGERAVSEEEMEAAVL

TWSKDLNAGPLAADRQHPILPQEGKRNILVTSALPYVNNVPHLGNIIGCVLSADVFSRYGRLRGWNLLFVCGTDEYGTAT

ENKAREEGLTPQQICDKYHAVHSSIYKWFQIDFDFFGRTTTEKQTEIAQNIFWRLHEHGFLVEDTVEQLRCENCQRFLAD

RFVEGTCPHCSYPEARGDQCDKCGRLINAVELREPQCKVCRQTPVIRSSKHLFLDLPKLENQLEQWLEKSTSTGDWTVNA

KQITRSWLRDGLKPRCITRDLQWGTPVPHPDFKEKVFYVWFDAPIGYLSITANYTDKWEKWWKNPQQVELYNFMAKDNVP

FHSVVFPCSLLGAQDNYTLVSHLVATEYLNYEDTKFSKSRGVGVFGDMAKDTGIPSDVWRFYLLYVRPEGQDSAFSWADM

ALKNNSELLNNLGNFINRAGMFVTKFFEGCVPAMELQQEDKKLLAVVGWELQQYIQLMDKVKIRDALKHILNISRHGNQY

IQVNEPWKKIKGGDTERQRAGTVTGVSVNIACLLSVMLFPYMPSVSHTIRDQLNAPQSCINTMLQGSGSFVCTLRAGHRI

GTVSPLFQKLEVDQIEALKKRFGGQQPEDEPPKKKTTAQSAASAQPAAVPAPAAPAEVAAVNGVDPEKAKQLAQAVAEQG

DKVRALKAQKAEKAVITAEVAKLLDLKKQLAAAEGKSPEPAPQKSKKK

>KFQ67321.1 Methionine--tRNA synthetase, cytoplasmic, partial [Phaethon lepturus]

QPAASAALYAQLVHGQKGVEALGKLLAHIEQNLSRRGTAYLTGVTKSVADVVVWGTLFPVVQDETTLPSELQALRTWFQN

MTLSKACRKAADSVLVPKALLEFKSYLQKQPPPCLARERVTSNEPEEEEGFECALTEEDITAAADAWARGAAALPKPWQP

QKPVLPMAGMRNVLITSALPYVNNVPHLGNIIGCVLSADTFARYCRLRNWNTLYICGTDEYGTATETKAMEEGLTPQEIC

NKYNAIHADIYRWFNISFDYFGRTTTPYQTTIAQDIFQRLLARGFLLQDTVEQLQCEDCQRFLADRFVEGTCPFCSYEEA

RGDQCDKCGKLINAVELKNPQCKLCRGVPVIKSTQHLFLDLPKLEEKLEPWLEQSWATGDWTANARYITRSWIRDGLKPR

CITRDLKWGTPVPLDGFHEKVFYVWFDAPIGYLSITANYTDQWERWWKNPQQVELYNFMAKDNVPFHSVIFPCSLLGADD

NYTLVNHLIATEYLNYEDGKFSKSRGVGVFGDMAKDTGIPADIWRFYLLYLRPEGQDSAFSWSDLMLKNNSELLNNLGNF

INRAGMFVCKFFGGTVPNMVLTPDDRRLLARVTLELRQYHQLLEKVRIRDALRCILSISRHGNQYIQVNEPWKRIKGDEK

DRQHAGTVTGVAVNMASILAVLIQPYIFRRRLATPPDCFVLSHEFTCTLPPGHRVGTVSPLFQKLENDQIEALRKRFGGG

QAKQTSPVPVALMAPGDPQRMQELTEEVAKQGNHVQQLKASKAEKAQVDMEVTKLLELKKQLALAEGGSPEVPVLKGKRK

>XP_022603987.1 methionine--tRNA synthetase, cytoplasmic isoform X2 [Seriola dumerili]

MKLFVSEGNPHCLKVLAALEVTGVQCDVQYVNHEETVVPFLSRPALPAMFLPSGLYLFSANAICQYLFEVNGQESSELCS

QWLEWEATDLQPALLLALHMAVLQGKGSQVSNVLQGPLNYLDQSLSKGNTPYLTGEAVSVADVVLWAALYPVLSDSSLAL

GERKSVKPWFDRVAAMHSCQSAVQKVLRGKGLQGMKSYMQRQPAPQSSQCRDTQPCNSNPAECEEGERVVSEEEMEAAVL

TWSKDLNGGPLATERQHPILPQEGKRNILLTSALPYVNNVPHLGNIIGCVLSADVFSRYGRLRGWNLLFVCGTDEYGTAT

ENKAREEGLTPQQICDKYHAIHSSIYKWFQIDFDFFGRTTTEKQTEIAQNIFWRLHKHEFLVEDTVEQLRCENCQRFLAD

RFVEGICPHCSYPEARGDQCDKCGRLINAVELREPQCKVCRQTPVIRSSKHLFLDLPKLETQLEQWLDKSTSTGDWTVNA

KQITRSWLRDGLKPRCITRDLQWGTPVPHPDFKEKVFYVWFDAPIGYLSITANYTDKWEKWWKNPQQVELYNFMAKDNVP

FHSVVFPCSLLGAQDNYTLVNHLVATEYLNYEDTKFSKSRGVGVFGDMAKDTGIPADVWRFYLLYVRPEGQDSAFSWADM

ALKNNSELLNNLGNFINRAGMFVTKFFEGCVPAMELQQEDKKLLALVGWELQQYIQLMDKVKIRDALKHILNISRHGNQY

IQVNEPWKKIKGGDTERQRAGTVTGVSVNIACLLSVMLLPYMPTVSQTIRDQLNAPQSCINTMLQGTGTFVCTLRAGHRI

GTVSPLFQKLEVDQIEALKKRFGGQQTTAQSAASAQPAAVPAPAAPAAEVATVNGVDPEKAKQLTQAVAEQGDKVRALKA

QKAEKAVITAEVAKLLDLKKQLAVAEGKNPEPAPQKSKKK

>XP_010285364.1 PREDICTED: methionine--tRNA synthetase, cytoplasmic, partial [Phaethon lepturus]

SVCSQPAASAALYAQLVHGQKGVEALGKLLAHIEQNLSRRGTAYLTGVTKSVADVVVWGTLFPVVQDETTLPSELQALRT

WFQNMTLSKACRKAADSVLVPKALLEFKSYLQKQPPPCLARERVTSNEPEEEEGFECALTEEDITAAADAWARGAAALPK

PWQPQKPVLPMAGMRNVLITSALPYVNNVPHLGNIIGCVLSADTFARYCRLRNWNTLYICGTDEYGTATETKAMEEGLTP

QEICNKYNAIHADIYRWFNISFDYFGRTTTPYQTTIAQDIFQRLLARGFLLQDTVEQLQCEDCQRFLADRFVEGTCPFCS

YEEARGDQCDKCGKLINAVELKNPQCKLCRGVPVIKSTQHLFLDLPKLEEKLEPWLEQSWATGDWTANARYITRSWIRDG

LKPRCITRDLKWGTPVPLDGFHEKVFYVWFDAPIGYLSITANYTDQWERWWKNPQQVELYNFMAKDNVPFHSVIFPCSLL

GADDNYTLVNHLIATEYLNYEDGKFSKSRGVGVFGDMAKDTGIPADIWRFYLLYLRPEGQDSAFSWSDLMLKNNSELLNN

LGNFINRAGMFVCKFFGGTVPNMVLTPDDRRLLARVTLELRQYHQLLEKVRIRDALRCILSISRHGNQYIQVNEPWKRIK

GDEKDRQHAGTVTGVAVNMASILAVLIQPYIFRRRLATPPDCFVLSHEFTCTLPPGHRVGTVSPLFQKLENDQIEALRKR

FGGGQAKQTSPVPVALMAPGDPQRMQELTEEVAKQGNHVQQLKASKAEKAQVDMEVTKLLELKKQLALAEGGSPEVPVLK

GKRK

>XP_021425783.1 methionine--tRNA synthetase, cytoplasmic [Oncorhynchus mykiss]

MKLFVSDGNPHCVKVLAVLEFTGVKCDVKHVNHEERVVPYLSRPALPALLLPSGTHLFSPNSICQYLIELNGQESSDIVN

QWLEWEATELQPAVMQALHMVVLQGKGAETPRVLQGPLSYLDQSLSQRTTLFLTAEMVSVADVVLWAALYPILSDSSLVS

GNQTLQLWFERVGMLAACVSAAQTVLQGKGLKAESLKTYLQRQPTTHHGQGQVQGRGTQPCNSPEGDDDDDDCVLSEEEI

QSAAVTWGKGLTDLPMVPERQHPILPQEGKRNVLVTSALPYVNNVPHLGNIIGCVLSADVFARYGRLRGWNVLYVCGTDE

YGTATENKAREEGLTAQQICDKYHAVHASIYQWFQVDFDFFGRTTTKKQTEIAQNIFWRLHERGFLLEDIVEQLRCEACQ

RFLADRFVEGTCPFCSYPEARGDQCDKCGKLINAVELREPTCKVCSQTPVVQSSKHLFLDLPKLEAELEQWLERSTGSGD

WTANAKQITRSWVRDGLKPRCITRDLKWGTPVPHPDFSDKVFYVWFDAPIGYLSITANYTDQWEKWWKNPQQVELYNFMA

KDNVPFHSVVFPCSLLGAQDNYTLVNHLVATEYLNYEDTKFSKSRGVGVFGDMAKDTGIPSDVWRFYLLYLRPEGQDSAF

SWVDMATKNNSELLNNLGNFINRAGMFVSKFFESCVPAMALQQEDKRLLAQVGWELKQYINLMDRVKIRDALKCILNISR

HGNQYIQVNEPWKKIKGDETDRQRSGTVTGVSVNVACLLSVMLQPYMPTVSHTIRQQLNAPPSVTGTLLQGTGNFVCSLP

AGHHIGTVSPLFQKLESEQIESLKKRFGGQQPKTTAQPDVNAQPATTPASDPAAQTAVNGADAERARELTLAVAEQGEKV

RTLKGQKAEKTVIGAEVTKLLELKKQLSLAEGKDPEPVLTKGKKNAQPATTPASDPAAQTAVNGADAERARELTLAVAEQ

GEKVRTLKGQKAEKTVIGAEVTKLLELKKQLSLAEGKNPEPVLTKGKKK

>XP_023138692.1 methionine--tRNA synthetase, cytoplasmic isoform X2 [Amphiprion ocellaris]

MELFVSEGNPHCLKVLAALEVTGVQCSVQYVNHEERVVPFLSRPTLPALLLPTGLHLFSSNAICRYLFEVSGQECSEFCN

QWLEWEATDLQPALLQALYMAVLQGKGSEVSTVLQGPLKFLDQSLSKGGMPYLTGEAVSVADVVLWAALYPVLSDSSQAL

GEHKSVNGWFDRVASIHSCLSAAQKVLQGKGLQGMKSYMQRQPAPQSSQGRNTQPCNCNPIEGEEGERAFSEEELEAAIL

NWSKGLSTSPLATDRQHPILPQEGKRNILVTSALPYVNNVPHLGNIIGCVLSADVFSRYGRLRGWNLLFVCGTDEYGTAT

ENKAREEGLTPQLICDKYHAVHSSIYKWFQIDFDFFGRTTTEKQTEIAQDIFWRLHKNGFLVEDTVEQLRCEQCQRFLAD

RFVEGICPHCKYPEARGDQCDKCGRLINAVELREPQCKVCRQTPVIRSSKHLFLDLPKLETQLEQWLEKSTSTGDWTTNA

KQITRSWLRDGLKPRCITRDLLWGTPVPHPDFKEKVFYVWFDAPIGYLSITANYTDEWEKWWKNPHQVELYNFMAKDNVP

FHSVVFPCSLLGAQDNYTLVSHLVATEYLNYEDTKFSKSRGVGVFGDMAKDTGIPADVWRFYLLYVRPEGQDSAFSWADM

ALKNNSELLNNLGNFINRAGMFVTKFFEGCVPVMELQQEDKKLLALVGWELQQYIQLMDKVKIRDALKHILNISRHGNQY

IQVNEPWKNIKGGDTERQRAGTVTGVSVNIACLLSVMLMPYMPEVSQTIRNQLNAPQSCVSTMLQGNGTFVCSLCAGHRI

GTVSPLFQKLEADQIEALKKRFGGQQTPQSAASTQPAAVPAPAAAVEVVTVNGVDPEKAKQLTQAVAEQGDKVRALKAQN

AEKAVITAEVAKLLDLKKQLALAEGKSPEPAPQKGKKKK

>XP_022603986.1 methionine--tRNA synthetase, cytoplasmic isoform X1 [Seriola dumerili]

MKLFVSEGNPHCLKVLAALEVTGVQCDVQYVNHEETVVPFLSRPALPAMFLPSGLYLFSANAICQYLFEVNGQESSELCS

QWLEWEATDLQPALLLALHMAVLQGKGSQVSNVLQGPLNYLDQSLSKGNTPYLTGEAVSVADVVLWAALYPVLSDSSLAL

GERKSVKPWFDRVAAMHSCQSAVQKVLRGKGLQGMKSYMQRQPAPQSSQCRDTQPCNSNPAECEEGERVVSEEEMEAAVL

TWSKDLNGGPLATERQHPILPQEGKRNILLTSALPYVNNVPHLGNIIGCVLSADVFSRYGRLRGWNLLFVCGTDEYGTAT

ENKAREEGLTPQQICDKYHAIHSSIYKWFQIDFDFFGRTTTEKQTEIAQNIFWRLHKHEFLVEDTVEQLRCENCQRFLAD

RFVEGICPHCSYPEARGDQCDKCGRLINAVELREPQCKVCRQTPVIRSSKHLFLDLPKLETQLEQWLDKSTSTGDWTVNA

KQITRSWLRDGLKPRCITRDLQWGTPVPHPDFKEKVFYVWFDAPIGYLSITANYTDKWEKWWKNPQQVELYNFMAKDNVP

FHSVVFPCSLLGAQDNYTLVNHLVATEYLNYEDTKFSKSRGVGVFGDMAKDTGIPADVWRFYLLYVRPEGQDSAFSWADM

ALKNNSELLNNLGNFINRAGMFVTKFFEGCVPAMELQQEDKKLLALVGWELQQYIQLMDKVKIRDALKHILNISRHGNQY

IQVNEPWKKIKGGDTERQRAGTVTGVSVNIACLLSVMLLPYMPTVSQTIRDQLNAPQSCINTMLQGTGTFVCTLRAGHRI

GTVSPLFQKLEVDQIEALKKRFGGQQPEDEPPKKKTTAQSAASAQPAAVPAPAAPAAEVATVNGVDPEKAKQLTQAVAEQ

GDKVRALKAQKAEKAVITAEVAKLLDLKKQLAVAEGKNPEPAPQKSKKK

>XP_022061489.1 methionine--tRNA synthetase, cytoplasmic isoform X2 [Acanthochromis polyacanthus]

MELFVSEGNPHCLKVLAALEVTGVQCSVQYVNHEERVVPFLSRPTLPALILPGGLHLFSSNAICRYLFEVSGQESSELCN

QWLEWEATDLQPALLQALYMAVLQGKGSEVSTVLQGPLNFLDQSLSKGGTPYLTGEAVSVADVVLWAALYPVLSDSSQAL

GEHKSVNGWFDRVASVHSCLSAAQKVLQGKGLQGMKSYMQRQPAPQSSQGRNTQPCNCNPAEGEEGERAFSEEELEAAIL

NWSKGLSTSPLATDRQHPILPQEGKRNILLTSALPYVNNVPHLGNIIGCVLSADVFSRYGRLRGWNLLFVCGTDEYGTAT

ENKAREEGLTPQLICDKYHAVHASIYKWFQIDFDFFGRTTTEKQTEIAQNIFWRLHNNGFLVEDTVEQLRCEHCQRFLAD

RFVEGICPHCKYPEARGDQCDKCGRLINAVELREPQCKVCRQTPVICSSKHLFLDLPKLETQLEQWLEKSTSSGDWTTNA

KQITRSWLRDGLKPRCITRDLLWGTPVPHPDFTDKVFYVWFDAPIGYLSITANYTDEWEKWWKNPHQVELYNFMAKDNVP

FHSVVFPCTLLGAQDNYTLVSHLVATEYLNYEDTKFSKSRGVGVFGDMAKDTGIPADVWRFYLLYVRPEGQDSAFSWADM

ALKNNSELLNNLGNFINRAGMFVTKFFEGCVPVMELQQEDKKLLALVGWELQQYIQLMDKVKIRDALKHILNISRHGNQY

IQVNEPWKNIKGGDTERQRAGTVTGVSVNIACLLSVMLSPYMPEVSQTIRDQLNAPQSCVNTMLQGNGTFVCSLCAGHRI

GTVSPLFQKLEADQIEALKKRFGGQQTPQSAASSQPAAVPAPAAAVEVVTVNGVDPEKAKQLTQAVAEQGDKVRALKAQN

AEKAVITAEVAKLLDLKKQLALAEGKSPEPAPQKGKKKK

>XP_012696680.1 PREDICTED: methionine--tRNA synthetase, cytoplasmic isoform X1 [Clupea harengus]

MQKMKLFVSEGNPHCLKALAVLELTGVKCDVQHVNHEEKVVPFLSRPTLPVLHLPSGQYLFSSNAICQYLFDVSKKGSSK

QSHQWLEWEATELQPALMYALHLVVVQGKTADSAKGLEAPLSFLEQSLSKRTTAFLTAETVGVADIVLWAALYPAFLENG

LKTSELKSTRTWFERVGVLPACQASVQTVFKGKGVEALKGYLQKQPAPHGQHTQPRNTHTSSNGSPTETEDSDHNTTEEE

IEAAAQIWNQQIDCQSKPERKHPILPEEGKRNVLITSALPYVNNVPHLGNIIGCVLSADVFARYGRQRGWNLLYICGTDE

YGTATENKAREEGLTPQEICDKYHAIHTDIYKWFQIDFDFFGRTTTQKQTEIAQNIFWRLHERGFLIEDIVEQLRCESCQ

RFLADRFVEGVCPFCQYPEARGDQCDKCGRLINAVELKEPQCKVCKQTPVIRSSKHLFLDLPKLEANLEQWLDRSVSTGD

WTTNARNITRSWLRDGLKPRCITRDLRWGTPVPHPDFSGKVFYVWFDAPIGYLSITANYTDQWEKWWKNPEQVELYNFMA

KDNVPFHSVVFPCSLLGAQDNYTLVNNLVATEYLNYEDTKFSKSRGVGVFGDMAKDTGIPSDVWRFYLLYLRPEGQDAAF

SWADMALKNNSELLNNLGNFINRAGMFVGKFFEGCVPQVVLQEEDKRLLAQVGWELKQYIQSLDKVRIRDALKCILNISR

HGNQYIQVNEPWKKIKGGDADRQRAGTVTAVSVNMSVLLSAMLEPYMPTVSETIRSQLQAPPASAHFMLQGEGNFVCSLP

AGHRIGTVSPLFQKLEAEQIETLKKKFGGQQPEEEASKKQPQTITQNNAATKASAPVAASETSVKADPELVKQLVAAIAE

QGDKVRALKGQKAEKAVIGAEVAKLLEFKKQLALAEGKSPETAPQKGKKK

>XP_005456440.1 PREDICTED: methionine--tRNA synthetase, cytoplasmic isoform X1 [Oreochromis niloticus]

MKLFVSEGNPHCLKALAALEATGVQCGVQFVNHDERVVPFLSRPTLPALALPSGQYLFSSNAICRYLFEVNGQECSELCD

QWLEWEATVLQPALQQALHMAVLQGKSSEVSKVLQGPINVLEQSLSKRNTPYLTGDSVSAADLVLWAGFFPVLSDSSLTL

GEHKSLKAWFDRVAAMNACQSAAQKVLQGKGLQGMKSYMQRQPAPQSSQCRETQPCNSNSAEEEGGERVVSEEEMEAAAL

TWSKGLNTCPPATKKQHPILPQEDKRNVLVTSALPYVNNVPHLGNIIGCVLSADVFSRYGRLRGWNMLFVCGTDEYGTAT

ENKAREEGLTPQQICDKYHAVHSSIYKWFQIDFDYFGRTTTEKQTEIAQDIFWRLHERDFLIEDTVEQLRCENCQRFLAD

RFVEGICPHCSYPEARGDQCDKCGRLINAVDLREPQCKVCRQTPVIRSSKHLFLDLPKLESQLEQWLEKSTSTGDWTANA

KQITRSWLRDGLKPRCITRDLKWGTPVPHADFKEKVFYVWFDAPIGYLSITANYTSEWEKWWKNPHQVELYNFMAKDNVP

FHSVVFPCSLLGAQDNYTLVNHLIATEYLNYEDTKFSKSRGVGVFGDMAKDTGIPSDVWRFYLLYVRPEGQDSAFSWADM

ALKNNSELLNNLGNFINRAGMFVTKFFEGCVPVMELQEEDKKLLALVSWELQQYIQLMDKIKIRDALKHILNISRHGNQY

IQVNEPWKKIKGGDSERQRAGTVTGVSVNIACLLSVMLFPYMPAVSQTIRDQLNAPPSCINTMLEGNGTFVCSLSAGHRI

GAVSPLFQKLEADQIEALKKKFGGQQPEDEPPKKKTPVRNAASAQSAAVPAPAAAVEVGTVNQVDPEKAKQLTQAVAEQG

EKVRALKAQKAEKAVITAEVAKLLDLKKQLALAEGKNPEPAPQKGKKK

>XP_008278953.1 PREDICTED: methionine--tRNA synthetase, cytoplasmic isoform X1 [Stegastes partitus]

MKLFVSEGNPHCLKVLAALEVTGVQCSVQYVNHEEKVVPFLSRPVLPALLLPSGLHLFSSNAICRYLFEVSGQESSELCN

QWLEWEATDLQPALLQALHMAVLQGKGSEVSKVLQGPLKFLDQSLTKGGKPYLNGDTVSLADLVLWAALYPVLSDSSLAL

GEHKSVNSWFDRVASIHSCLSAAQKVLQGKGLQGMKSYMQRQPAPQSNQCRDTQPCNSNPAEGEEGERDFSEEEMEAAAL

TWSKGLSTSPVVTDRQHPILPQEGKRNILVTSALPYVNNVPHLGNIIGCVLSADVFSRYGRLRDWNLLFVCGTDEYGTAT

ENKAREEGLTPQLICDKYHAVHSSIYKWFQIDFDFFGRTTTEKQTEIAQNIFWRLHKNGFLVEDTVEQLRCENCQRFLAD

RFVEGICPHCKYPEARGDQCDKCGRLINAVELREPQCKVCRQTPVIRSSKHLFLDLPKLETQLEQWLEKSTSTGDWTANA

KQITRSWLRDGLKPRCITRDLLWGTPVPHPDFKEKVFYVWFDAPIGYLSITANYTDKWEKWWKNPHQVELYNFMAKDNVP

FHSVVFPCSLLGAQDNYTLVNHLVATEYLNYEDTKFSKSRGVGVFGDMAKDTGIPADVWRFYLLYVRPEGQDSAFSWADM

ALKNNSELLNNLGNFINRAGMFVAKFFEGCVPVMELQQEDKRLLALVGWELQQYIQLMDKVKIRDALKHILNISRHGNQY

IQVNEPWKKIKGGDTERQRAGTVTGVSVNIACLLSVMLMPYMPAVSQTIRDQLNAPQSCVNTMLQGTGTFVCSLCAGHRI

GTVSPLFQKLEADQIEALKKRFGGQQPEDEPSKEKKTPQSTASPQPAAVPATAAADEVAMVNGVDPEKAKQLTQAVAEQG

DKVRALKAQKAEKAVITAEVAKLLDLKKQLALAEGKSPEPAPQKGKKK

>XP_010796027.1 PREDICTED: methionine--tRNA synthetase, cytoplasmic isoform X1 [Notothenia coriiceps]

MKLFVSEGNPHCLKVLAAVEVSGVQCDVQYVNHEERVVPFLSRPALPALLLPSGLHLFSSNAICRYLFEVSGEDSSELCN

QWLEWEATDLQPALLQALHLAVVQGKVSEVSKVLQGPLNYLEQSLSKGNTPYLTGEAVSVADVVLWAALYPVLSDASLAL

GERASIKAWFDRGAVMHSCQSAAQKVLQGKGLQGMKSYMQRQPAPPSSQCRDSQPCNGNPAESEESERAVSAEEMETAAL

IWSKGLNTCPMATSRQHPILPQEGKRNILLTSALPYVNNVPHLGNIIGCVLSTDVFSRYGRMRGWNMLFICGTDEYGTAT

ENKAREEGLTPQQICDKYHAIHSNIYKWFQIDFDFFGRTTTEKQTEIAQNIFWRIHKHGFLMEDTVEQLRCEKCQRFLAD

RFVEGICPHCSYPEARGDQCDKCGKLINAVELREPQCKVCKETPSIRSSKHLFLDLPKLEGQLEQWLDKSTSTGDWTANA

KQITRSWLRDGLKPRCITRDLRWGTPVPHPDFKEKVFYVWFDAPIGYLSITANYTDQWEKWWKNPHQVELYNFMAKDNVP

FHSVVFPCSLLGAQDNYTLVNHLIATEYLNYEDTKFSKSRGVGVFGDMAKDTGIPSDVWRFYLLYVRPEGQDSAFSWADM

ALKNNSELLNNLGNFINRAGMFVTKFFEGCVPAMELQQEDKRLLALVGWELQQYIQLLDKVKIRDSLRHILNISRHGNQY

IQLNEPWKKIKGGDTERQRAGTVTGVSVNIACLLSVMLLPYMPTVSQTIRDQFNAPQSCVDTMLQGTGTFVCSLSAGHRI

GTVSPLFQKLEVDQIEALKKKFGGQQPDDEPPKKKKTAQSAGSPPPAAGPAAAAEVVAGIGADPEKAKQLTQAVAEQGEK

VRELKAQKAEKAAITAEVAKLLDLKKQLALAEGKSSDPAPQKGKKK

>AAT68164.1 methionine-tRNA synthetase [Danio rerio]

MKLFIGEGNPHCLKVLAALELSGVRCETQLVKHEEKVVPYLTHPVLPILQLPSGQHLFSPNSICQYLFDISGQKATDATN

QWLEWEATNLQPAVLQSLQLVALQGKRVESAAVMKEPLSWLEQSLSKRKTSFLTDEVVSVADVVLWAALYPLLSDSAFEP

GDLQAVRGWFERVCSVSACQSAALRVLQGKGAEALKSFLQKQPVTHTPRRDSPSNSTEAEDSERELSPEEIEEAAQVYSE

GLKDFTVVTERKHPVLPQEGKRNVLITSALPYVNNVPHLGNIIGCVLSADVFARYGRLRGWNLLYICGTDEYGTATENKA

REEGLTPQQICDKYRCIHASIYQWFQIDFDFFGRTTTQHQTEIAQDIFWRLHERGFLLEDTVEQLRCEGCQRFLADRFVE

GECPHCRYPEARGDQCDKCGRPINAVELKNPQCKVCKETPVIRSSKHLFLNLPKLEQDLEQWLQTSTAAGDWTTNARHIT

RSWLRDGLKPRCITRDLKWGTPVPHPDYKEKVFYVWFDAPIGYLSITANYTDQWERWWKNPQQVELYNFMAKDNVPFHSV

VFPCSLLGAQDNYTLVNNLIATEYLNYEDTKFSKSRGVGVFGDMAKDTGIPSDVWRFYLLYLRPEGQDSAFSWTDMALKN

NSELLNNLGNFINRAGMFVSKFFEGCVPEMLLNDDDKRLIAQVCWELKQYIQLLDKVRIRDALKCILNMSRHGNQYIQLN

EPWKKIKGGAEDRCRAGTVTGVSVNVACLLAAVLEPFMPTVSLSIRSQLQAPESSSRAMLSGPGAFICTLPAGHRIGTVS

PLFQKLENEQIEALRKRFGGLQTPSNSSVAVESKPNAGAAQHTPASVTADPERAKQLTALVAEQGEKVRALKAQKAEKSA

IGVEVAKLLDLKNQLCLAEGKTPEPPAQKTKKK

>NP_956370.1 methionine--tRNA synthetase, cytoplasmic [Danio rerio]

MKLFIGEGNPHCLKVLAALELSGVRCETQLVKHEEKVVPYLTHPVLPILQLPSGQHLFSPNSICQYLFDVSGQKATDATN

QWLEWEATNLQPAVLQSLQLVALQGKRVEAAAVMKEPLSWLEQSLSKRKTSFLTDEVVSVADVVLWAALYPLLSDSAFEP

GDLQAVRGWFERVCSVSACQSAALRVLQGKGAEALKSFLQKQPVTHTPRRDSPSNSTEAEDSERELSPEEIEEAAQVYSE

GLKDFTVVTERKHPVLPQEGKRNVLITSALPYVNNVPHLGNIIGCVLSADVFARYGRLRGWNLLYICGTDEYGTATENKA

REEGLTPQQICDKYHCIHASIYQWFQIDFDFFGRTTTQHQTEIAQDIFWRLHERGFLLEDTVEQLRCEGCQRFLADRFVE

GECPHCRFPEARGDQCDKCGRLINAVELKNPQCKVCKETPVIRSSKHLFLNLPKLEQDLEQWLQTSTAAGDWTTNARHIT

RSWLRDGLKPRCITRDLKWGTPVPHPDYKEKVFYVWFDAPIGYLSITANYTDQWERWWKNPQQVELYNFMAKDNVPFHSV

VFPCSLLGAQDNYTLVNNLIATEYLNYEDTKFSKSRGVGVFGDMAKDTGIPSDVWRFYLLYLRPEGQDSAFSWTDMALKN

NSELLNNLGNFINRAGMFVSKFFDGCVPEMLLNDDDKRLIAQVCWELKQYIQLLDKVRIRDALKCILNMSRHGNQYIQLN

EPWKKIKGGAEDRCRAGTVTGVSVNVACLLAAVLEPFMPTVSLSIRSQLQAPESSSRAMLSGPGAFICTLPAGHRIGTVS

PLFQKLENEQIEALRKRFGGLQPEEESAGLKTPSNSSVAVESKPNAGAAQHTPASVTADPERAKQLTALVAEQGEKVRAL

KAQKAEKSAIGVEVAKLLDLKNQLCLAEGKTPEPPAQKTKKK

>XP_023268609.1 methionine--tRNA synthetase, cytoplasmic isoform X1 [Seriola lalandi dorsalis]

MKLFVSEGNPHCLKVLAALEVTGVQCDVQYVNHEETVVPFLSRPALPAMLLPSGLYLFSANAICQYLFEVNGQESSELCS

QWLEWEATDLQPALLLALHMAVLQGKGSQVSNVLQGPLNYLDQSLSKGNTPYLTGEAVSVADVVLWAALYPVLSDSSLAL

GEHKSVKPWFDRVAAMHSCQSAVQKVLRGKGLQGMKSYMQRQPAPQSSQCKDTQPCNSNPAECEEGERVVSEEEMEAAVL

TWSKDLNGGPLATERQHPILPQEGKRNILLTSALPYVNNVPHLGNIIGCVLSADVFSRYGRLRGWNLLFVCGTDEYGTAT

ENKAREEGLTPQQICDKYHAIHSSIYKWFQIDFDFFGRTTTEKQTEIAQNIFWRLHKHEFLVEDTVEQLRCENCQRFLAD

RFVEGICPHCSYPEARGDQCDKCGRLINAVELREPQCKVCRQTPVIRSSKHLFLDLPKLETQLEQWLDKSTSTGDWTVNA

KQITRSWLRDGLKPRCITRDLQWGTPVPHPDFKEKVFYVWFDAPIGYLSITANYTDKWEKWWKNPQQVELYNFMAKDNVP

FHSVVFPCSLLGAQDNYTLVNHLVATEYLNYEDTKFSKSRGVGVFGDMAKDTGIPADVWRFYLLYVRPEGQDSAFSWADM

ALKNNSELLNNLGNFINRAGMFVTKFFEGCVPAMELQQEDKKLLALVGWELQQYIQLMDKVKIRDALKHILNISRHGNQY

IQVNEPWKKIKGGDTERQRAGTVTGVSVNIACLLSVMLLPYMPTVSQTIRDQLNAPQSCINTMLQGTGTFVCTLRAGHRI

GTVSPLFQKLEVDQIEALKKRFGGQQPEDEPPKKKTTAQSAASAQPAAVPAPAAPAAEVLTVNGVDPEKAKQLTQAVAEQ

GDKVRALKTQKAEKAVITAEVAKLLDLKKQLAVAEGKNPEPAPQKSKKK

>XP_023268610.1 methionine--tRNA synthetase, cytoplasmic isoform X2 [Seriola lalandi dorsalis]

MKLFVSEGNPHCLKVLAALEVTGVQCDVQYVNHEETVVPFLSRPALPAMLLPSGLYLFSANAICQYLFEVNGQESSELCS

QWLEWEATDLQPALLLALHMAVLQGKGSQVSNVLQGPLNYLDQSLSKGNTPYLTGEAVSVADVVLWAALYPVLSDSSLAL

GEHKSVKPWFDRVAAMHSCQSAVQKVLRGKGLQGMKSYMQRQPAPQSSQCKDTQPCNSNPAECEEGERVVSEEEMEAAVL

TWSKDLNGGPLATERQHPILPQEGKRNILLTSALPYVNNVPHLGNIIGCVLSADVFSRYGRLRGWNLLFVCGTDEYGTAT

ENKAREEGLTPQQICDKYHAIHSSIYKWFQIDFDFFGRTTTEKQTEIAQNIFWRLHKHEFLVEDTVEQLRCENCQRFLAD

RFVEGICPHCSYPEARGDQCDKCGRLINAVELREPQCKVCRQTPVIRSSKHLFLDLPKLETQLEQWLDKSTSTGDWTVNA

KQITRSWLRDGLKPRCITRDLQWGTPVPHPDFKEKVFYVWFDAPIGYLSITANYTDKWEKWWKNPQQVELYNFMAKDNVP

FHSVVFPCSLLGAQDNYTLVNHLVATEYLNYEDTKFSKSRGVGVFGDMAKDTGIPADVWRFYLLYVRPEGQDSAFSWADM

ALKNNSELLNNLGNFINRAGMFVTKFFEGCVPAMELQQEDKKLLALVGWELQQYIQLMDKVKIRDALKHILNISRHGNQY

IQVNEPWKKIKGGDTERQRAGTVTGVSVNIACLLSVMLLPYMPTVSQTIRDQLNAPQSCINTMLQGTGTFVCTLRAGHRI

GTVSPLFQKLEVDQIEALKKRFGGQQTTAQSAASAQPAAVPAPAAPAAEVLTVNGVDPEKAKQLTQAVAEQGDKVRALKT

QKAEKAVITAEVAKLLDLKKQLAVAEGKNPEPAPQKSKKK

>XP_022061424.1 methionine--tRNA synthetase, cytoplasmic isoform X1 [Acanthochromis polyacanthus]

MELFVSEGNPHCLKVLAALEVTGVQCSVQYVNHEERVVPFLSRPTLPALILPGGLHLFSSNAICRYLFEVSGQESSELCN

QWLEWEATDLQPALLQALYMAVLQGKGSEVSTVLQGPLNFLDQSLSKGGTPYLTGEAVSVADVVLWAALYPVLSDSSQAL

GEHKSVNGWFDRVASVHSCLSAAQKVLQGKGLQGMKSYMQRQPAPQSSQGRNTQPCNCNPAEGEEGERAFSEEELEAAIL

NWSKGLSTSPLATDRQHPILPQEGKRNILLTSALPYVNNVPHLGNIIGCVLSADVFSRYGRLRGWNLLFVCGTDEYGTAT

ENKAREEGLTPQLICDKYHAVHASIYKWFQIDFDFFGRTTTEKQTEIAQNIFWRLHNNGFLVEDTVEQLRCEHCQRFLAD

RFVEGICPHCKYPEARGDQCDKCGRLINAVELREPQCKVCRQTPVICSSKHLFLDLPKLETQLEQWLEKSTSSGDWTTNA

KQITRSWLRDGLKPRCITRDLLWGTPVPHPDFTDKVFYVWFDAPIGYLSITANYTDEWEKWWKNPHQVELYNFMAKDNVP

FHSVVFPCTLLGAQDNYTLVSHLVATEYLNYEDTKFSKSRGVGVFGDMAKDTGIPADVWRFYLLYVRPEGQDSAFSWADM

ALKNNSELLNNLGNFINRAGMFVTKFFEGCVPVMELQQEDKKLLALVGWELQQYIQLMDKVKIRDALKHILNISRHGNQY

IQVNEPWKNIKGGDTERQRAGTVTGVSVNIACLLSVMLSPYMPEVSQTIRDQLNAPQSCVNTMLQGNGTFVCSLCAGHRI

GTVSPLFQKLEADQIEALKKRFGGQQPEDEPSKEKTPQSAASSQPAAVPAPAAAVEVVTVNGVDPEKAKQLTQAVAEQGD

KVRALKAQNAEKAVITAEVAKLLDLKKQLALAEGKSPEPAPQKGKKKK

>XP_023138691.1 methionine--tRNA synthetase, cytoplasmic isoform X1 [Amphiprion ocellaris]

MELFVSEGNPHCLKVLAALEVTGVQCSVQYVNHEERVVPFLSRPTLPALLLPTGLHLFSSNAICRYLFEVSGQECSEFCN

QWLEWEATDLQPALLQALYMAVLQGKGSEVSTVLQGPLKFLDQSLSKGGMPYLTGEAVSVADVVLWAALYPVLSDSSQAL

GEHKSVNGWFDRVASIHSCLSAAQKVLQGKGLQGMKSYMQRQPAPQSSQGRNTQPCNCNPIEGEEGERAFSEEELEAAIL

NWSKGLSTSPLATDRQHPILPQEGKRNILVTSALPYVNNVPHLGNIIGCVLSADVFSRYGRLRGWNLLFVCGTDEYGTAT

ENKAREEGLTPQLICDKYHAVHSSIYKWFQIDFDFFGRTTTEKQTEIAQDIFWRLHKNGFLVEDTVEQLRCEQCQRFLAD

RFVEGICPHCKYPEARGDQCDKCGRLINAVELREPQCKVCRQTPVIRSSKHLFLDLPKLETQLEQWLEKSTSTGDWTTNA

KQITRSWLRDGLKPRCITRDLLWGTPVPHPDFKEKVFYVWFDAPIGYLSITANYTDEWEKWWKNPHQVELYNFMAKDNVP

FHSVVFPCSLLGAQDNYTLVSHLVATEYLNYEDTKFSKSRGVGVFGDMAKDTGIPADVWRFYLLYVRPEGQDSAFSWADM

ALKNNSELLNNLGNFINRAGMFVTKFFEGCVPVMELQQEDKKLLALVGWELQQYIQLMDKVKIRDALKHILNISRHGNQY

IQVNEPWKNIKGGDTERQRAGTVTGVSVNIACLLSVMLMPYMPEVSQTIRNQLNAPQSCVSTMLQGNGTFVCSLCAGHRI

GTVSPLFQKLEADQIEALKKRFGGQQPEDEPSKEKTPQSAASTQPAAVPAPAAAVEVVTVNGVDPEKAKQLTQAVAEQGD

KVRALKAQNAEKAVITAEVAKLLDLKKQLALAEGKSPEPAPQKGKKKK

>XP_010159195.1 PREDICTED: methionine--tRNA synthetase, cytoplasmic, partial [Eurypyga helias]

HIEQNLTRRGTAYLAGDTKSVADVVVWGTLFPVLQNETSLPSELQALRSWFQSMTLSEACRKAVDSILMPKALLEFKSYL

QKEPPPCLAVERVTSNEPEEEESAERTLTEEEIAAAADAWARGAAALPKPWQPQNPVLPVEGMRNVLITSALPYVNNVPH

LGNIIGCVLSADTFARYCRLRNWNTLYVCGTDEYGTATETKAVEEGLTPQEICNKYHAIHADVYRWFNISFDYFGRTTTS

YQTTIAQDIFQRLLARGFLLQDTVEQLRCEDCQRFLADRFVEGTCPFCSYEEARGDQCDKCGKLINAVELKNPQCKLCRG

APVVKPTQHLFLDLPKLEERLEPWLEQSWATGDWTANARYITRSWIRDGLKPRCITRDLKWGTPVPLDGFRDKVFYVWFD

APIGYLSITANYTEQWERWWKNPQQVELYNFMAKDNVPFHSVIFPCSLLGADDNYTLVNHLIATEYLNYEDGKFSKSRGV

GVFGDMAKDTGIPADIWRFYLLYLRPEGQDSAFSWSDLMLKNNSELLNNLGNFINRAGMFVCKFFGGTVPNMVLTLDDKR

LLARVTLELRQYHQLLEKVRIRDALRCILSISRHGNQYIQVNEPWKRIKGDEKDRQRAGTVTGVAVNMASMLAVMLQPYM

PSVSSAIQGQLCIPPDCLVLSHDFTCTLPPGHRVGTVSPLFQKLENDQVEALRKRFGGGQAKPTPPASAPPPAPGDPQLV

MQLTEEVAKQGNHIRQLKANKAEKAQVAVEVAKLLELKKQLALAEGRSPEVLVPKGKQKK

>XP_023651134.1 methionine--tRNA synthetase, cytoplasmic isoform X2 [Paramormyrops kingsleyae]

MKLYLSEGNPQCMKVLAAVELTGAKCDIHHAHPEERIVPTLSRPVLPVLQPSVGVHLFMPNAICRYLFELSGQEASSQTD

QWLEWEATELQPALMQALHLSVLQAKRTEAAEALEGPLRFLEQSLSKKPTAFLTAETATVADIVLWGALYPILSDASFLP

GDSQHVAAWFERVTALPACQAAAGSILNEKGGKAALRGFVQRQPVPHTQRRDAQPCSQPEENSSEHEMTEEEIQAAEETW

RRQVADFPAPAERQHPILPQEGHQNVLITSALPYVNNVPHLGNIIGCVLSADVFARYGRLRGWNVLFVCGTDEYGTATEN

KAREEGLTPREICDKYHKVHADIYSWFQIDFDYFGRTTTDQQTEIAQDIFWRLHQHGFLVSDMVDQLRCENCERFLADRF

VEGVCPHCRYPEARGDQCDKCGRLINAVELKDPQCKVCRQTPVVRSSKHLFLDLPKLESQLDQWLDQSMSSGNWTSNARH

ITRSWLRDGLKPRCITRDLQWGTPVPHPDFSGKVFYVWFDAPIGYLSITANYTDQWEKWWKNPKQVELYNFMAKDNVPFH

SVVFPCSLLGAQDNYTLVNNLIATEYLNYEDTKFSKSRGVGVFGDMAKDTGIPADVWRFYLLYLRPEGQDSAFSWSDMAL

KNNSELLNNLGNFVNRAGMFVTKFFDGCVPEMVLQQEDLRLLAQVGWELRQYIQLLDRVRIRDALKCILNISRHGNQYIQ

MNEPWKKIKGGSQDRQRASTVTGVSVNVACLLSAMLQPYMPTVSATICAQLRAPPNCADAMLRGAGAFICTLPCGHRIGT

ISPLFQKLEADQIEALRKEFGGQQAQAPPKASDTPPAPTGSVGNGTDPERAKRLEKEVAEQGDKVRVLKTQKAEKNVITV

EVTKLLELKKQLAVAEGRDPEPVPQKGKKK

>XP_019900678.1 PREDICTED: methionine--tRNA synthetase, cytoplasmic isoform X3 [Esox lucius]

MKLFVSEGNPHCLKVLAVLKLTGVKCDVQHVNHEEKVVPHLSRPTLPALLLPSGMHLFSANSICRYLGEVSGQPSSDLNN

QWLEWEASELQPAVAQALHLAVLQGKGSEASSALQGQLSYLEQSLCKKTTPFLTAEIVSVADVVLWAALYPILSDHGLVL

GDQGKVQNWFERVGMLAACESAAQEVLQGKGLKSEALKAYMQKQPAPPHSQAQKRDNQPCNSPEQVADGDRVMSEEEILS

ATVTWAKGLADRPMISDRQHPILPKEGKRNILVTSALPYVNNVPHLGNIIGCVLSADVYARYGRLRGWNVLYVCGTDEYG

TATENKAREEGLTPQQICDKYHTVHASIYQWFQVDFDYFGRTTTEKQTQIAQNIFWRLHERGFLLEDTVEQLRCEMCQRF

LADRFVEGTCPYCAYPEARGDQCDKCGKLINAVELQEPKCKVCNQTPVVRSSKHLFLDLPKLEAPLEQWLEKSMGSGDWT

ANAKQITRSWLRDGLKPRCITRDLKWGTPVPHPDFSDKVFYVWFDAPIGYLSITANYTDQWEKWWKNPEQVELYNFMAKD

NVPFHSVVFPCSLLGAQDNYSLVNHLVATEYLNYEDTKFSKSRGVGVFGDMAKDTGIPSDVWRFYLLYLRPEGQDSAFSW

ADMAIKNNSELLNNLGNFINRAGMFVSKFFENCVPAMDLQPEDKRLLALVGWELKQYISLMDKVKIRDSLKCILNISRLG

NQYIQVNEPWKKIKGGDTDRQRAGTVTGVSVNIACLLSVMLLPYMPTVCQTIREQLNAPPTVTATMLQGTGNFVCCLPAG

HHIGVVSPLFQKLEADQIEDLKKRFGGQQPKASTQADVVTQPATTSAGQTVKNGADPERAKELTLAVAGQGEKVRALKGQ

KAEKAVIAVEVDKLLDLKKQLALAEGKNQEPDQTKGTKNTQHAPCPATTAQAVINKADPERAKELTLAVAEQGEKVRMLK

GQKAEKAAITTEVDKLLDLKKQLALAEGKNPEPTLTKGKKK

>XP_015742471.1 PREDICTED: methionine--tRNA synthetase, cytoplasmic [Coturnix japonica]

MRLRVLPGSPAALKVVAAARSAAAAVQLCWARPGEPPPSPLCAPWAPALQLESGAVLFSANAICQFFFLQRGEEPNELST

QWLEWEATELQPAALQALYARLVLGRTEAAAALGEALGHVERSLAGSGTAFLAGDKPSVADVVVWATLFPLLRDEAVPAA

EPLQNWFRNMTLSEACRSAADTVLLPQHLQEFTACLQKLPRPLLDAASGCKEDDVLEQALTEEDVAAAADAWTRGAAALP

KPWRPPKPVLPVEGMRNVLITSALPYVNNVPHLGNIIGCVLSADAFARYSRLRNWNTLYVCGTDEYGTATETKAVEEGLT

PREICDKYHAVHADIYRWFDISFDYFGRTTTPQQTVISQDIFQRLQARGFVLQDTVQQLHCAACQRFLADRFVEGICPFC

TYQEARGDQCDKCGKLINAVELKAPRCKLCGGSPTVRPTRHLFLDLPKLEERLQPWLENCWATGNWTANARYITRSWIRD

GLRPRCITRDLQWGTPVPLDGFRDKVFYVWFDAPIGYLSITANYTEHWERWWKNPQQVELYNFMAKDNVPFHSVVFPCSL

LGAEDNYTLVNHLIATEYLNYEDGKFSKSRGVGVFGDMAKDTGIAADVWRFYLLYVRPEGQDSAFSWGDLMLKNNSELLN

NLGNFINRAGMFVCKFFDGAVPPMVLTPGDHRLLARVTMELRQYHQLLERVRIRDALRCVLNISRHGNQYIQVNEPWKRI

KGGEEDRQRAGTVTGVAVNVAALLAVLVQPYMPAVSAAIQAQLCVPPECSVLSHVFTCTLPPGHRVGTVSPLFQKLESEH

IDALRKRFGGGQAKPPNPPAQSDPPSVPGLAPTDPNRIQELTEQVAEQGDCVRRLKASGADRARIDSEVGKLLALKRELA

LAQNKSPDTPRGKGKK

>PNJ44589.1 MARS isoform 9, partial [Pongo abelii]

EEFCLFLLYRVLPVAGERNVLITSALPYVNNVPHLGNLIGCVLSADVFARYSRLRQWNTLYLCGTDEYGTATETKALEEG

LTPQEICDKYHIIHADIYRWFNISFDIFGRTTTPQQTKITQDIFQRLLKRGFVLQDTVEQLRCEHCARFLADRFVEGVCP

FCGYEEARGDQCDKCGKLINAIELKKPQCKVCRSCPVVQSSQHLFLDLPKLEKRLEEWLGRTLPGSDWTPNARFIIRSWL

RDGLKPRCITRDLKWGTPVPLEGFEDKVFYVWFDATFGYLSITANYTDQWERWWKNPEQVDLYQFMAKDNVPFHGLVFPC

SALGAEDNYTLVSHLIATEYLNYEDGKFSKSRGVGVFGDMAQDTGIPADIWRFYLLYIRPEGQDSAFSWTDLLLKNNSEL

LNNLGNFINRAGMFVSKFFGGYVPEMVLTPDDKRLLAHVTLELQHYHQLLEKVRIRDALRSILTISRHGNQYIQVNEPWK

RIKGSEADRQRAGTVTGLAVNIAALLSVMLQPYMPTVSATIQAQLQLPPPACSILLTNFLCTLPAGHQIGTVSPLFQKLE

NDQIESLRQRFGGGQVRKLKAVPSLHNSHSITSLISLNSPHPSLFPSFGPAASSLSFSFLS

>XP_015257726.1 PREDICTED: methionine--tRNA synthetase, cytoplasmic isoform X2 [Cyprinodon variegatus]

MMELFVSEGNPHCLKVLAALEVTGVQCGVHFINHEEKVVPFLSRPALPALLLPSKQHIFSSNTICRYLFELNGPESSDQC

NQWLEWEATVLQPALLRALHMAVLQGKGSEVSKVLQSPLNVLDQSLGKGNMSYLTGKDVSVADIVLWGALHPVLFNSSLA

LSDHKFVKAWFDRVAAMSGCQSAAQKVLQGKGLEAMKSYMQRQPAPQSRDTQPCNSSPVESEEGERVVSEEEMEAAALTW

RKDINACSQTTEKQHPIMPKEGMRNVLITSALPYVNNVPHLGNIIGCVLSADVFSRYGRLRGWNVLFVCGTDEYGTATEN

KAREEGLTPKQICDKYHAVHSSIYRWFQIDFDFFGRTTTEKQTEIAQDIFWRLQKNGFLVEDTVEQLRCENCQRFLADRF

VEGICPHCSYPEARGDQCDKCGRLINAVELREPQCKVCKQTPVIRSSKHLFLDLPKLETQLEQWLEKSTSSGDWTANAKQ

ITRSWLRDGLKPRCITRDLQWGTPVPHPDFKEKVFYVWFDAPIGYLSITANYTSDWEKWWKNPHQVELYNFMAKDNVPFH

SVVFPCSLLGAQDNYTLVNHLVATEYLNYEDTKFSKSRGVGVFGDMAKDTGIPSDVWRFYLLYVRPEGQDSAFSWADMAL

KNNSELLNNLGNFINRAGMFVTKFFGGCVPEMELQQEDKKLLAVVGWELQQYIQLMDKVKIRDALKHILNISRHGNQYIQ

VNEPWKKIKGGESERRRAGTVTGVSVNISCLLSVMLLPYMPAVSQTIRDQLNAPQSCINTMLLGPGSFVCALRAGHRIGT

VSPLFQKLEADQIEALKKRFGGQQITASSQQPAVVPAPPAVAEVKTGVGADPEKAKQLAKAVAEQGDKVRALKSQKAEKD

VIAAEVSKLLDLKKQLALAEGKSPEPAAQKGKKK

>KFO99679.1 Methionine--tRNA synthetase, cytoplasmic, partial [Calypte anna]

FLAQDTKSVADVVVWGTLFPVLQDETSLPSELPALKTWFQSMTLSEACRKAADSVLIPRALQEFTSYLQKQPPPCLPVQR

TTSNEPEEEEGSERALTEEEITAAASAWARGAAALPKPWQPQKPVLPVEGMRNILITSALPYVNNVPHLGNIIGCVLSAD

TFARYCRLRNWNTLYVCGTDEYGTATETKALEEGMTPQEICNKYHAIHADIYRWFDISFDYFGRTTTPHQTRIAQDIFKR

LLARGFLLEDTVEQLCCESCQRFLADRFVEGTCPFCSYREARGDQCDKCGKLINAVELKSPQCKLCRSVPVVKATRHLFL

DLPKLEEQLEPWLEQSLATGDWTPNARYITRSWIRDGLKPRCITRDLKWGTPVPLDGFQDKVFYVWFDAPIGYLSITANY

TQHWERWWKNPQQVELYNFMAKDNVPFHSVVFPCSLLGAEDNYTLVNHLIATEYLNYEDGKFSKSRGVGVFGDMAKDTGI

PADIWRFYLLYLRPEGQDSAFSWSDLMLKNNSELLNNLGNFINRAGMFVCKFFGGTVPSMVLTLDDKRLLARVTLELRQY

HQLLEKVRIRDALRCVLSISRHGNQYIQVNEPWKRIKGDEKDRQRAGTVTGVAVNMAALLAVMVQPYMPSVSSAIQGQLH

IPPDCFVLSHDFTCTLPPGHHVGTVSPLFQKLENDQVEALRRRFGGGQNSPASGSPPAPATQVTPGDPQLIQLLTEEVAK

QGNHVRQLKANKAEKAQVDAEVAKLLELKKQLALAEGKSPEIPVPKGKSKK

>XP_013057410.1 PREDICTED: methionine--tRNA synthetase, cytoplasmic, partial [Anser cygnoides domesticus]

GDSGAALFPGELCSPPVFPEAEPRARAGAVSGLDAAFLGQDTKSVADVVVWGALFPVLQDEASLSNELQALRTWFQSMTL

SEACRKAADSVLMPERLLEFKSYLQKQPPPCLAMERATSNEPEEEEASERALTEEEITAAADAWAHGAAALPKPWHPQKP

VLPVEGMRNVLITSALPYVNNVPHLGNIIGCVLSADTFARYCRLRNWNTLYVCGTDEYGTATETKAVEEGLTPREICDKY

NAIHADIYRWFHISFDYFGRTTTPHQTAIAQDIFQRLLARGFLLQDTVEQLQCEGCQRFLADRFVEGICPFCSYEEARGD

QCDKCGKLINAVELKKPQCKLCRGVPVVKPTQHLFLDLPKLEEQLEPWLERSWTTGDWTANARYITRSWIRDGLKPRCIT

RDLKWGTPVPLDGFRDKVFYVWFDAPIGYLSITANYTDQWERWWKNPQQVELYNFMAKDNVPFHSVIFPCSLLGADDNYT

LVNHLIATEYLNYEDGKFSKSRGVGVFGDMAKDTGIPADIWRFYLLYLRPEGQDSAFSWSDLMLKNNSELLNNLGNFINR

AGMFVCKFFGGIVPDMVLTADDKRLLARVTLELRQYHQLLEKVRIRDALRCILSISRHGNQYIQVNEPWKRIKGNEEDRQ

RAGTVTGVAVNMASLLAVMVQPYMPSVSVAIQGQLCIPPECFVLSPVFTCTLPPGHRVGAVSPLFQKLENDQIEALRKRF

GGGQPEDLNVVRQAKPTPPASDPVPAPASGSVVAPADPQLIQELTEQVAKQGNHVRQLKANKAEKAQVDSEVAKLLELKK

QLALAEGKSPEILVPKGKRKK

>XP_013989462.1 PREDICTED: methionine--tRNA synthetase, cytoplasmic [Salmo salar]

MKLFVSDGNPHCVKVLAVLEFTGVKCDVKHVNHEERVVPYLSRPALPALLLPSGTYLFSPNSICQYLIEVNGQESSDIVN

QWLEWEATELQPAVMQALHMVVLQGKGAQTTRVLQGPLSYLDQSLSKRTTLFLTAEMVSVADVVLWAALYPILSDSSLVS

GNQTELQHWFERVGMLAACGSAAQTALHGKGLKAESLKTYLQRQPSTHHGQGQVQGRGSQPCNSPEGDDDGDCVLSEEEI

QSFAVTWGKGLADLPMVPERQHPILPVEGKRNVLVTSALPYVNNVPHLGNIIGCVLSADVFARYGRLRGWNVLYVCGTDE

YGTATENKAREEGLTAQQICDKYHAVHASIYQWFQVDFDFFGRTTTKKQTEIAQNIFWRLHERGFLLEDIVEQLRCEACQ

RFLADRFVEGTCPYCSYPEARGDQCDKCGKLINAVELREPTCKVCSQTPVVQSSKHLFLDLPKLEAELEQWLEKSTGSGD

WTANAKQITRSWVRDGLKPRCITRDLKWGTPVPHPDFSDKVFYVWFDAPIGYLSITANYTDQWEMWWKNPQQVELYNFMA

KDNVPFHSVVFPCSLLGAQDNYTLVNHLVATEYLNYEDTKFSKSRGVGVFGDMAKDTGIPSDVWRFYLLYLRPEGQDSAF

SWVDMATKNNSELLNNLGNFINRAGMFVSKFFESYVPAMDLQQEDKRLLAQVGWELKQYMNLMDRVKIRDALKCILNISR

HGNQYIQVNEPWKKIKGDETDRQRSGTVTGVSVNVACLLSVMLQPYMPTVSQTICQQLNAPPTVTGTMLQGTGNFVCSLP

AGHHIGTVSPLFQKLEAEQIESLKKRFGGQQPEEEEEEAKPKKPKTAAQTDVNAQPAPSPASHPAAQTAVNGADAERARE

LTLAVAEQGEKVRTLKGQKAEKAVIGAEVTKLLELKKQLSLAEGKHPEPVLIKGKKNAQPAPSPASHPAAQTAVNGADAE

RARELTLAVAEQGEKVRTLKGQKAEKAVIGAEVTKLLELKKQLSLAEGKHPEPVLIKGKKK

>XP_017264024.1 methionine--tRNA synthetase, cytoplasmic isoform X1 [Kryptolebias marmoratus]

MKLFVSEGNPHCLKVLAALEVTGVQCGVQTVSHEEKVVPFLSRPALPALLLPSGQYLFSSNAICRYFFEANGQECNNLCN

QWLEWETTVLQPALLQTLHMAVLQGKGSEASKVLQSPLNVLDQSLSKGNMSYLTGEAVSVADVVLWGTLHPVLSDSPSAS

SEHESVRTWFDRVAALHSCQSAAQKVLQGKGLQAMKSYVQKQPAPPSSQCRDAQPCNSSPAESEEGERVVSEEEMEAAVN

TWSKGLNSTSLAADKQHPILPQEGKRNVLVTSALPYVNNVPHLGNIIGCVLSADVFSRYGRLRGWNVLFVCGTDEYGTAT

ENKAREEGLTPQQICDKYHAVHASIYKWFQIDFDFFGRTTTEKQTEIAQDIFWRLHQNEYLVEDTVEQLRCEKCQRFLAD

RFVEGMCPHCSYPEARGDQCDKCGRLINAVELREPQCKVCRQTPVIRSSKHLFLDLPKLESQLEQWLDKSTSSGDWTLNA

KQITRSWIRDGLKPRCITRDLQWGTPVPHPDFREKVFYVWFDAPIGYLSITANYTKEWEKWWKNPHQVELYNFMAKDNVP

FHSVVFPCSLLGAQDNYTLVNHLVATEYLNYEDTKFSKSRGVGVFGDMAKDTGIPSDVWRFYLLYVRPEGQDSAFSWADM

ALKNNSELLNNLGNFINRAGMFVTKFFEGCVPVMDLQREDKKLLALVGWELQQYIQLMDKVKIRDALKHILNISRHGNQY

IQVNEPWKKIKEGDPERQRAGTVTGVSVNIACLLSVMLFPYMPAVSQTIRDQLNAPQSCIDTMLQGTGTFVCSLSAGHRI

GTVSPLFQKLEADQIEALKKRFGGQQPEDEPSNKKKTLQNAANAQPAVAAAAAVGVKATTSGGVDPEKAKQLTKAVAEQG

DKVRALKAQKAEKDVVAAEVSKLLDLKKQLALAEGKNPDPAPQKGKKK

>XP_017264025.1 methionine--tRNA synthetase, cytoplasmic isoform X2 [Kryptolebias marmoratus]

MKLFVSEGNPHCLKVLAALEVTGVQCGVQTVSHEEKVVPFLSRPALPALLLPSGQYLFSSNAICRYFFEANGQECNNLCN

QWLEWETTVLQPALLQTLHMAVLQGKGSEASKVLQSPLNVLDQSLSKGNMSYLTGEAVSVADVVLWGTLHPVLSDSPSAS

SEHESVRTWFDRVAALHSCQSAAQKVLQGKGLQAMKSYVQKQPAPPSSQCRDAQPCNSSPAESEEGERVVSEEEMEAAVN

TWSKGLNSTSLAADKQHPILPQEGKRNVLVTSALPYVNNVPHLGNIIGCVLSADVFSRYGRLRGWNVLFVCGTDEYGTAT

ENKAREEGLTPQQICDKYHAVHASIYKWFQIDFDFFGRTTTEKQTEIAQDIFWRLHQNEYLVEDTVEQLRCEKCQRFLAD

RFVEGMCPHCSYPEARGDQCDKCGRLINAVELREPQCKVCRQTPVIRSSKHLFLDLPKLESQLEQWLDKSTSSGDWTLNA

KQITRSWIRDGLKPRCITRDLQWGTPVPHPDFREKVFYVWFDAPIGYLSITANYTKEWEKWWKNPHQVELYNFMAKDNVP

FHSVVFPCSLLGAQDNYTLVNHLVATEYLNYEDTKFSKSRGVGVFGDMAKDTGIPSDVWRFYLLYVRPEGQDSAFSWADM

ALKNNSELLNNLGNFINRAGMFVTKFFEGCVPVMDLQREDKKLLALVGWELQQYIQLMDKVKIRDALKHILNISRHGNQY

IQVNEPWKKIKEGDPERQRAGTVTGVSVNIACLLSVMLFPYMPAVSQTIRDQLNAPQSCIDTMLQGTGTFVCSLSAGHRI

GTVSPLFQKLEADQIEALKKRFGGQQKTLQNAANAQPAVAAAAAVGVKATTSGGVDPEKAKQLTKAVAEQGDKVRALKAQ

KAEKDVVAAEVSKLLDLKKQLALAEGKNPDPAPQKGKKK

>XP_008490507.1 PREDICTED: methionine--tRNA synthetase, cytoplasmic [Calypte anna]

MNDCIVLQSEFHSVPAAAFLAQDTKSVADVVVWGTLFPVLQDETSLPSELPALKTWFQSMTLSEACRKAADSVLIPRALQ

EFTSYLQKQPPPCLPVQRTTSNEPEEEEGSERALTEEEITAAASAWARGAAALPKPWQPQKPVLPVEGMRNILITSALPY

VNNVPHLGNIIGCVLSADTFARYCRLRNWNTLYVCGTDEYGTATETKALEEGMTPQEICNKYHAIHADIYRWFDISFDYF

GRTTTPHQTRIAQDIFKRLLARGFLLEDTVEQLCCESCQRFLADRFVEGTCPFCSYREARGDQCDKCGKLINAVELKSPQ

CKLCRSVPVVKATRHLFLDLPKLEEQLEPWLEQSLATGDWTPNARYITRSWIRDGLKPRCITRDLKWGTPVPLDGFQDKV

FYVWFDAPIGYLSITANYTQHWERWWKNPQQVELYNFMAKDNVPFHSVVFPCSLLGAEDNYTLVNHLIATEYLNYEDGKF

SKSRGVGVFGDMAKDTGIPADIWRFYLLYLRPEGQDSAFSWSDLMLKNNSELLNNLGNFINRAGMFVCKFFGGTVPSMVL

TLDDKRLLARVTLELRQYHQLLEKVRIRDALRCVLSISRHGNQYIQVNEPWKRIKGDEKDRQRAGTVTGVAVNMAALLAV

MVQPYMPSVSSAIQGQLHIPPDCFVLSHDFTCTLPPGHHVGTVSPLFQKLENDQVEALRRRFGGGQPKGFVEPQAKQNSP

ASGSPPAPATQVTPGDPQLIQLLTEEVAKQGNHVRQLKANKAEKAQVDAEVAKLLELKKQLALAEGKSPEIPVPKGKSKK

>XP_015257718.1 PREDICTED: methionine--tRNA synthetase, cytoplasmic isoform X1 [Cyprinodon variegatus]

MMELFVSEGNPHCLKVLAALEVTGVQCGVHFINHEEKVVPFLSRPALPALLLPSKQHIFSSNTICRYLFELNGPESSDQC

NQWLEWEATVLQPALLRALHMAVLQGKGSEVSKVLQSPLNVLDQSLGKGNMSYLTGKDVSVADIVLWGALHPVLFNSSLA

LSDHKFVKAWFDRVAAMSGCQSAAQKVLQGKGLEAMKSYMQRQPAPQSRDTQPCNSSPVESEEGERVVSEEEMEAAALTW

RKDINACSQTTEKQHPIMPKEGMRNVLITSALPYVNNVPHLGNIIGCVLSADVFSRYGRLRGWNVLFVCGTDEYGTATEN

KAREEGLTPKQICDKYHAVHSSIYRWFQIDFDFFGRTTTEKQTEIAQDIFWRLQKNGFLVEDTVEQLRCENCQRFLADRF

VEGICPHCSYPEARGDQCDKCGRLINAVELREPQCKVCKQTPVIRSSKHLFLDLPKLETQLEQWLEKSTSSGDWTANAKQ

ITRSWLRDGLKPRCITRDLQWGTPVPHPDFKEKVFYVWFDAPIGYLSITANYTSDWEKWWKNPHQVELYNFMAKDNVPFH

SVVFPCSLLGAQDNYTLVNHLVATEYLNYEDTKFSKSRGVGVFGDMAKDTGIPSDVWRFYLLYVRPEGQDSAFSWADMAL

KNNSELLNNLGNFINRAGMFVTKFFGGCVPEMELQQEDKKLLAVVGWELQQYIQLMDKVKIRDALKHILNISRHGNQYIQ

VNEPWKKIKGGESERRRAGTVTGVSVNISCLLSVMLLPYMPAVSQTIRDQLNAPQSCINTMLLGPGSFVCALRAGHRIGT

VSPLFQKLEADQIEALKKRFGGQQPEDEPSNKKITASSQQPAVVPAPPAVAEVKTGVGADPEKAKQLAKAVAEQGDKVRA

LKSQKAEKDVIAAEVSKLLDLKKQLALAEGKSPEPAAQKGKKK

>XP_019900677.1 PREDICTED: methionine--tRNA synthetase, cytoplasmic isoform X2 [Esox lucius]

MKLFVSEGNPHCLKVLAVLKLTGVKCDVQHVNHEEKVVPHLSRPTLPALLLPSGMHLFSANSICRYLGEVSGQPSSDLNN

QWLEWEASELQPAVAQALHLAVLQGKGSEASSALQGQLSYLEQSLCKKTTPFLTAEIVSVADVVLWAALYPILSDHGLVL

GDQGKVQNWFERVGMLAACESAAQEVLQGKGLKSEALKAYMQKQPAPPHSQAQKRDNQPCNSPEVADGDRVMSEEEILSA

TVTWAKGLADRPMISDRQHPILPKEGKRNILVTSALPYVNNVPHLGNIIGCVLSADVYARYGRLRGWNVLYVCGTDEYGT

ATENKAREEGLTPQQICDKYHTVHASIYQWFQVDFDYFGRTTTEKQTQIAQNIFWRLHERGFLLEDTVEQLRCEMCQRFL

ADRFVEGTCPYCAYPEARGDQCDKCGKLINAVELQEPKCKVCNQTPVVRSSKHLFLDLPKLEAPLEQWLEKSMGSGDWTA

NAKQITRSWLRDGLKPRCITRDLKWGTPVPHPDFSDKVFYVWFDAPIGYLSITANYTDQWEKWWKNPEQVELYNFMAKDN

VPFHSVVFPCSLLGAQDNYSLVNHLVATEYLNYEDTKFSKSRGVGVFGDMAKDTGIPSDVWRFYLLYLRPEGQDSAFSWA

DMAIKNNSELLNNLGNFINRAGMFVSKFFENCVPAMDLQPEDKRLLALVGWELKQYISLMDKVKIRDSLKCILNISRLGN

QYIQVNEPWKKIKGGDTDRQRAGTVTGVSVNIACLLSVMLLPYMPTVCQTIREQLNAPPTVTATMLQGTGNFVCCLPAGH

HIGVVSPLFQKLEADQIEDLKKRFGGQQTEEEETIWNKPKASTQADVVTQPATTSAGQTVKNGADPERAKELTLAVAGQG

EKVRALKGQKAEKAVIAVEVDKLLDLKKQLALAEGKNQEPDQTKGTKNTQHAPCPATTAQAVINKADPERAKELTLAVAE

QGEKVRMLKGQKAEKAAITTEVDKLLDLKKQLALAEGKNPEPTLTKGKKK

>XP_019900676.1 PREDICTED: methionine--tRNA synthetase, cytoplasmic isoform X1 [Esox lucius]

MKLFVSEGNPHCLKVLAVLKLTGVKCDVQHVNHEEKVVPHLSRPTLPALLLPSGMHLFSANSICRYLGEVSGQPSSDLNN

QWLEWEASELQPAVAQALHLAVLQGKGSEASSALQGQLSYLEQSLCKKTTPFLTAEIVSVADVVLWAALYPILSDHGLVL

GDQGKVQNWFERVGMLAACESAAQEVLQGKGLKSEALKAYMQKQPAPPHSQAQKRDNQPCNSPEQVADGDRVMSEEEILS

ATVTWAKGLADRPMISDRQHPILPKEGKRNILVTSALPYVNNVPHLGNIIGCVLSADVYARYGRLRGWNVLYVCGTDEYG

TATENKAREEGLTPQQICDKYHTVHASIYQWFQVDFDYFGRTTTEKQTQIAQNIFWRLHERGFLLEDTVEQLRCEMCQRF

LADRFVEGTCPYCAYPEARGDQCDKCGKLINAVELQEPKCKVCNQTPVVRSSKHLFLDLPKLEAPLEQWLEKSMGSGDWT

ANAKQITRSWLRDGLKPRCITRDLKWGTPVPHPDFSDKVFYVWFDAPIGYLSITANYTDQWEKWWKNPEQVELYNFMAKD

NVPFHSVVFPCSLLGAQDNYSLVNHLVATEYLNYEDTKFSKSRGVGVFGDMAKDTGIPSDVWRFYLLYLRPEGQDSAFSW

ADMAIKNNSELLNNLGNFINRAGMFVSKFFENCVPAMDLQPEDKRLLALVGWELKQYISLMDKVKIRDSLKCILNISRLG

NQYIQVNEPWKKIKGGDTDRQRAGTVTGVSVNIACLLSVMLLPYMPTVCQTIREQLNAPPTVTATMLQGTGNFVCCLPAG

HHIGVVSPLFQKLEADQIEDLKKRFGGQQTEEEETIWNKPKASTQADVVTQPATTSAGQTVKNGADPERAKELTLAVAGQ

GEKVRALKGQKAEKAVIAVEVDKLLDLKKQLALAEGKNQEPDQTKGTKNTQHAPCPATTAQAVINKADPERAKELTLAVA

EQGEKVRMLKGQKAEKAAITTEVDKLLDLKKQLALAEGKNPEPTLTKGKKK

>XP_023651133.1 methionine--tRNA synthetase, cytoplasmic isoform X1 [Paramormyrops kingsleyae]

MKLYLSEGNPQCMKVLAAVELTGAKCDIHHAHPEERIVPTLSRPVLPVLQPSVGVHLFMPNAICRYLFELSGQEASSQTD

QWLEWEATELQPALMQALHLSVLQAKRTEAAEALEGPLRFLEQSLSKKPTAFLTAETATVADIVLWGALYPILSDASFLP

GDSQHVAAWFERVTALPACQAAAGSILNEKGGKAALRGFVQRQPVPHTQRRDAQPCSQPEENSSEHEMTEEEIQAAEETW

RRQVADFPAPAERQHPILPQEGHQNVLITSALPYVNNVPHLGNIIGCVLSADVFARYGRLRGWNVLFVCGTDEYGTATEN

KAREEGLTPREICDKYHKVHADIYSWFQIDFDYFGRTTTDQQTEIAQDIFWRLHQHGFLVSDMVDQLRCENCERFLADRF

VEGVCPHCRYPEARGDQCDKCGRLINAVELKDPQCKVCRQTPVVRSSKHLFLDLPKLESQLDQWLDQSMSSGNWTSNARH

ITRSWLRDGLKPRCITRDLQWGTPVPHPDFSGKVFYVWFDAPIGYLSITANYTDQWEKWWKNPKQVELYNFMAKDNVPFH

SVVFPCSLLGAQDNYTLVNNLIATEYLNYEDTKFSKSRGVGVFGDMAKDTGIPADVWRFYLLYLRPEGQDSAFSWSDMAL

KNNSELLNNLGNFVNRAGMFVTKFFDGCVPEMVLQQEDLRLLAQVGWELRQYIQLLDRVRIRDALKCILNISRHGNQYIQ

MNEPWKKIKGGSQDRQRASTVTGVSVNVACLLSAMLQPYMPTVSATICAQLRAPPNCADAMLRGAGAFICTLPCGHRIGT

ISPLFQKLEADQIEALRKEFGGQQPEDEEPKNQAQAPPKASDTPPAPTGSVGNGTDPERAKRLEKEVAEQGDKVRVLKTQ

KAEKNVITVEVTKLLELKKQLAVAEGRDPEPVPQKGKKK

>XP_015814627.1 PREDICTED: methionine--tRNA synthetase, cytoplasmic isoform X2 [Nothobranchius furzeri]

MKLFVSEGNPHCLKVLAALEVTGVHCGVQYISHEEKVVPFLSRPSFPALLLPSGQHLFSSNAICRYFFEVNGQESNDVSN

QWLEWETIVLQPVLHQALHMAVVQGKGSEVSRVLQSPLNFLDQSLSKGSMPHLTGESVSVADVILWAALYPVLSDSSLAL

GEHKFVRAWFDHLAAMHSCQSAAQKVLQGKAMKNYMQKQSVPQSFQCRDTQPSNSSPAESEEGERAVSEEEMEAATLTWR

KGLTSCSLATERQHPLLPEEGKRNILLTSALPYVNNVPHLGNIIGCVLSADVFSRYGRLRGWNLLFVCGTDEYGTATENK

AREEGLTPQQICDKYHAVHSSIYKWFQIDFDFFGRTTTEKQTEIAQDIFWRLHKNGFLVEDTVEQLRCENCQRFLADRFV

EGTCPHCSYPEARGDQCDKCGRLINAVELREPQCKVCRQTPIIRSSKHLFLDLPKLERQLEQWLEKSTSTGDWTANAKQI

TRSWIRDGLKPRCITRDLQWGTPVPHPDFKEKVFYVWFDAPIGYLSITASYTNEWDKWWKNPQQVELYNFMAKDNVPFHS

VVFPCSLLGAQDNYTLVNHLVATEYLNYEDTKFSKSRGVGVFGDMAKDTGIPSDVWRFYLLYVRPEGQDSAFSWADMALK

NNSELLNNLGNFINRAGMFVTKFFEGCVPEMDLQREEKKLLAVVGWELQQYIQLMDKVRIRDALKHILNISRHGNQYIQV

NEPWKKIKGGESDRRRAGTVTGVSVNIACLLSVMLFPYMPLVSQTIRDQLNAPQSCIGTMLQGTGTFVCALSAGHRIGTV

SPLFQKLETDQIEALKKRFGGQQKPAQSSVSAPAAPGAEVKVVGGVDPEKAEQLTKAVAEQGEKVRALKAQKAEKDVIAA

EVSKLLELKKHLALAEGKNAAPAPQTGKKKK

>XP_016402089.1 PREDICTED: methionine--tRNA synthetase, cytoplasmic [Sinocyclocheilus rhinocerous]

MKLYIADGTPQCVKVLAALELTGVKCEIEAVSHEERVVPHLSLLESPVLLLPDGQHLFSPNSICQYLFDISGQKATDATN

EWLEWEATSLQPAVLQSLHMVALQGKRVEAAAVMKEPLKWLEQSLSKKNSSFLTAEAVSVADVVLWAALYPLLSDSSFDV

GELRSVRGWFERVGDLAACQAAANRVLHGKGVEALKTFLQKHPATQTQRRDSPTNATEAEVAEHVITVEEIEEAAQIWSE

GLKSSPAVTERKHPVLPQEGKRNVLLPSALPYVNNVPHLGNLIGCVLSADVFARYGRLRGWNLLYICGTDEYGTATENKA

REEGLTPQEICDKYHAIHADIYQWFQIDFDFFGRTTTKQQTEIAQDIFWRLHERGFLLEDTVEQLRCESCQRFLADRFVE

GECPHCRYPEARGDQCDKCGRLINAVELRNPQCKVCKETPVICSSKHLFLNLPKLEDELEQWLDKSTSSGDWTLNARHIT

RSWIRDGLKPRCITRDLQWGTPVPHEDYKEKVFYVWFDAPIGYLSITANYTDQWEKWWKNPQQVELYNFMAKDNVPFHSV

VFPCSLLGAQDNYTLVNHLIATEYLNYEDTKFSKSRGVGVFGDMAKDTGIPSDVWRFYLLYLRPEGQDSAFSWSDMALKN

NSELLNNLGNFINRAGMFVSKFFDGCVPEMLLNEDDKRLIAQVRWELKQYIQLLDKVRIRDALKCILNISRHGNQYMQLN

EPWKKIKGGDGDRQRAGTVTGVSVNVACLLSAVLEPYMPSVSHTIRQQLQAPHTCVSAMLQNTGTFICTLPAGHRIGAVS

PLFQKLENEQIEVLRKRFGGQQAPSSAAETKPSAGAVHDASVNVNADPERAKQLTALVAEQGNKLRGLKAQKADKSVIDD

EVEKLLQLKKQLSLAEGKNPEPAAQKGKKK

>XP_023180936.1 methionine--tRNA synthetase, cytoplasmic isoform X2 [Xiphophorus maculatus]

MELFVSEGNPHCLKVLAALEVTGVQCGVQFVNHEGKVVPFLPRPAVPTLFLPSGQHLFSSNTICRYLFELNGPESSDQCN

QWLEWEATILQPALLRALHLAVLQGKGSEVPKVLQGPLNVLDQSLVKGSMAYLTGEAVSVADVVLWGALHPVLSVSSLEL

SDHKSAKAWFDRVAAVRGCQSAAQKVLQGKGLEAMKSYMQKQPAPQSRDTQPCNSSPAENDEGERTVSEEEMEAAALTWS

KDLNACSYPAEKQHPVMPQEGNRNVLITSALPYVNNVPHLGNIIGCVLSADVFSRYGQLRGWNVLFVCGTDEYGTATENK

AREEGLTPQQICDKYHAVHSSIYKWFQIDFDFFGRTTTEKQTEIAQDIFWRLHKNGFLVEDTVEQLRCENCQRFLADRFV

EGMCPHCSYPEARGDQCDKCGRLINAVELREPQCKVCRQTPEIRSSKHLFLDLPKLETQLENWLERSTSSGDWTANAKQI

TRSWLRDGLKPRCITRDLQWGTPVPHPDFKEKVFYVWFDAPIGYLSITANYTNEWEKWWKNPHQVELYNFMAKDNVPFHS

VVFPCSLLGAQDNYTLVSHLIATEYLNYEDTKFSKSRGVGVFGDMAKDTGIPSDVWRFYLLYVRPEGQDSAFSWADMALK

NNSELLNNLGNFINRGGMFVTKFFGGCVPAMELQQEDKKLLAVVGWELQQYIQLMDKVKIRDALKHILNISRHGNQYIQV

NEPWKKIKGGESERQRAGTVTGVSVNIACLLSVMLLPYMPAVSQTIRDQLNAPQTCINTMLQGPGTFVCALRAGHRIGTV

SPLFQKLEADQIEALKKRFGGQQITASTQPAAVPAPPVVAEMKVVDGSDPEKAKQLAEAVAEQGDKVRALKSQKAEKAII

TAEVSKLLDLKKQLALAEGKSPEPAPQKGKKK

>XP_015814626.1 PREDICTED: methionine--tRNA synthetase, cytoplasmic isoform X1 [Nothobranchius furzeri]

MKLFVSEGNPHCLKVLAALEVTGVHCGVQYISHEEKVVPFLSRPSFPALLLPSGQHLFSSNAICRYFFEVNGQESNDVSN

QWLEWETIVLQPVLHQALHMAVVQGKGSEVSRVLQSPLNFLDQSLSKGSMPHLTGESVSVADVILWAALYPVLSDSSLAL

GEHKFVRAWFDHLAAMHSCQSAAQKVLQGKAMKNYMQKQSVPQSFQCRDTQPSNSSPAESEEGERAVSEEEMEAATLTWR

KGLTSCSLATERQHPLLPEEGKRNILLTSALPYVNNVPHLGNIIGCVLSADVFSRYGRLRGWNLLFVCGTDEYGTATENK

AREEGLTPQQICDKYHAVHSSIYKWFQIDFDFFGRTTTEKQTEIAQDIFWRLHKNGFLVEDTVEQLRCENCQRFLADRFV

EGTCPHCSYPEARGDQCDKCGRLINAVELREPQCKVCRQTPIIRSSKHLFLDLPKLERQLEQWLEKSTSTGDWTANAKQI

TRSWIRDGLKPRCITRDLQWGTPVPHPDFKEKVFYVWFDAPIGYLSITASYTNEWDKWWKNPQQVELYNFMAKDNVPFHS

VVFPCSLLGAQDNYTLVNHLVATEYLNYEDTKFSKSRGVGVFGDMAKDTGIPSDVWRFYLLYVRPEGQDSAFSWADMALK

NNSELLNNLGNFINRAGMFVTKFFEGCVPEMDLQREEKKLLAVVGWELQQYIQLMDKVRIRDALKHILNISRHGNQYIQV

NEPWKKIKGGESDRRRAGTVTGVSVNIACLLSVMLFPYMPLVSQTIRDQLNAPQSCIGTMLQGTGTFVCALSAGHRIGTV

SPLFQKLETDQIEALKKRFGGQQPEDEASNEKKPAQSSVSAPAAPGAEVKVVGGVDPEKAEQLTKAVAEQGEKVRALKAQ

KAEKDVIAAEVSKLLELKKHLALAEGKNAAPAPQTGKKKK

>XP_020492407.1 methionine--tRNA synthetase, cytoplasmic isoform X2 [Labrus bergylta]

MKLYVSEGNPHCLKVLAALDVTGVQCDVHYVNHEERVVPYLSRPALPALLLPSGLHLFSSNAICRYLFEVSGKDCTELCG

QWLEWEATDLQPALLQALHMAVLQGKGSDVSKVLQGPLNYLEQSLSKGNLPYLTGDAVSAADVVLWAALYPVLSDSSQAL

GERKFVKTWFDRGAVMHSLQSAAQKVLQGKGLQGTKSYMQKQPAPQSNQCRDTQPCNSSPAEGEESERLVSEEEMEAAAL

TWSKGLNSCQSDTDRQHPILPKEGKRNLLLTSALPYVNNVPHLGNIIGCVLSTDVFSRYGRLRDWNVLFVCGTDEYGTAT

ENKAREEGLTPQQICDKFHTIHASIYKWFQIDFDFFGRTTTEKQTEIAQDIFWRLHKQGFLVEDTVEQLRCESCQRFLAD

RFVEGICPHCSYPEARGDQCDKCGRLINAVELSEPQCKVCRQTPVIRSSKHLFLDLPKLESQLEQWLDKSTSTGDWTANA

KQITRSWLRDGLKPRCITRDLKWGTPVPHPDFKEKVFYVWFDAPIGYLSITANYTDQWEKWWKNPNQVELYNFMAKDNVP

FHSVVFPCSLLGAQDNYTLVNHLIATEYLNYEDSKFSKSRGVGVFGDMAKDTGIPSDVWRFYLLYVRPEGQDSVFSWADM

ATKNNSELLNNLGNFINRAGMFVAKFFEGCVPAMDLQQEDKKLLALVGWELQQYIQLMDKVKIRDALKHILNISRHGNQY

IQVNEPWKKIKGGDTERQRSGTVTGVSVNIACLLSVMLHPYMPTVSQTIRDQLNAPQSCINTMLKGTGTFVCALCPGHRI

GTVSPLFQKLELDQIEALKKRFGGQQTAAQKAAGTQPAAVPVPAAAEVASVNGVDPEKAKQLTQAVAEQGEKVRALKAQK

AEKAAITAEVAKLLDLKKQLALAEGKSPEPAPQKGKKK

>XP_020468787.1 methionine--tRNA synthetase, cytoplasmic isoform X4 [Monopterus albus]

MKLFVSEGNPQCLKVLCALEVTGVQCDVHYVNHEERVVPFLSRPALPALLLPSGRHLFSPNAICRYIFEVNGQESSDLCN

QWLEWEATHLQPALAEALHMAVLQGKGSEVSQVLQGPLNFLDQSLNNGNTPYLIGEAVSVADVVLWAALCPVLSDSSLVL

GKHKSVKAWFDHVAAMHSCQSAAQKVLQGKGLQGMKSYMQRQPAPQSSQCRDTQPCNSSPAECGEEEGERVVSEEDMNAA

ALKWSKGLNADPLSKEKQHPILPQEGKRNILVTSALPYVNNVPHLGNIIGCVLSADVFSRYGRLRGWNLLYVCGTDEYGT

ATENKAREEGMTPQQICDKYHVIHSSIYKWFQIDFDFFGRTTTEKQTEIAQDIFWRLHNHGFLVEDTVEQLRCENCQRFL

ADRFVEGTCPHCSYPEARGDQCDKCGRLINAVELREPQCKVCRQIPVIRSSKHLFLDLPKLETQLKQWLDTSTSTGHWTA

NAKQITRSWLRDGLKPRCITRDLQWGTPVPHPDFKEKVFYVWFDAPIGYLSITAKYTDKWEKWWKNPQQVELYNFMAKDN

VPFHSVVFPCSLLGAQDNYTLVSHLVATEYLNYEDTKFSKSRGVGVFGDMAMDTDIPSDVWRFYLLYVRPEGQDSAFSWA

DMAMKNNSELLNNLGNFINRAAMFVTKFFEGCVPAVELQQEDKKLLALVGWELQQYIQLMDKVKIRDALKHILNISRHGN

QYIQVNEPWKKIKGVGTERQRAGTVTGVSVNIACLLSVMLLPYMPTVSQTIRDQLHAPQSCVSTMLQGTGTFVCALKAGH

RIGTVTPLFQKLETDQIEALKKRFGGQQTAQNAATVPAPAAPAAEVAMVNGVDPEKAKRLTQAVAEQGDKVRALKAQKAE

KAVVTAEVAKLLDLKKQLALAEGKSPEPAPQKGKKK

>XP_010730412.2 PREDICTED: methionine--tRNA synthetase, cytoplasmic isoform X1 [Larimichthys crocea]

MKLFVSEGNPHCLKVLAALEVTGAKCDVQYVNHEERVVPFLSRPALPALLLPSGLHLFSSNAICRYLFEVNGQDSSELCN

QWLEWEATELQPALLQALHMAVLQGKGSEVSKVLQGPLNYLDQSLSKKNTPYLTGEAVSVADVVLWAALYPILSDSSLAL

GECKSVKIWFDHGAAMHSLQCAAQKVLQGKGLQGMKSYMQKQPAPQSSQCRDTQPCNSSPAESEEGERLVSEEEIEAAAL

TWSKSLNACPLVANRQHPILPQEGKRNLLVTSALPYVNNVPHLGNIIGCVLSADVFSRYGRLRDWNVLFVCGTDEYGTAT

ENKAREEGLTPQQICDKYHAVHSSIYKWFQIDFDYFGRTTTEKQTEIAQNIFWRLHEHGFLVEDTVEQLRCESCQRFLAD

RFVEGTCPHCSYPEARGDQCDKCGRLINAVELREPQCKVCRQTPVIRSSKHLFLDLPKLETQLEQWLDKSTSTGDWTANA

KQITRSWLRDGLKPRCITRDLKWGTPVPHPDFKEKVFYVWFDAPIGYLSITANYTDKWETWWKNPHQVELYNFMAKDNVP

FHSVVFPCSLLGAQDNYTLVNHLVATEYLNYEDTKFSKSRGVGVFGDMAKDTGILSDVWRFYLLYVRPEGQDSAFSWADM

ATKNNSELLNNLGNFINRAGMFVTKFFEGCVPPMELQHEDKKLLALVGWELQQYIQLMDKVKIRDALKHILNISRHGNQY

IQVNEPWKKIKGGDTERQRAGTVTGVSVNISCLLSVMLFPYMPSVSQTIRDQLNAPQSCINTMLQGTGAFVCALSAGHRI

GTVSPLFQKLEVDQIEALKKRFGGQQPEDEPPKKKTAAQAAASAQPATVPAPAAPAAAAEVVTVSGVDPEKAKQLTQAVA

EQGEKVRALKAQKAEKAVITAEVAKLLDLKKQLALAEGKSPEPAPQKGKKK

>XP_022531356.1 methionine--tRNA synthetase, cytoplasmic isoform X2 [Astyanax mexicanus]

MKLFVSEGNPHCVKALAALELSGLKCDVQLVNHEERVVPFLNRPVIPVLLLPSGQHLFSANAICQLLLEMAGKSTDESKQ

LLEWEATELQPALLQALHKQALQGKSSEAAAVLKGPLGWLDQYLSKRTTSFLTEEILADIVLGAALYPVLSDPAFEVGDL

PSLQAWFNHVRELPACQSAAQKVLNGKGLEALKGFLQKQPVPHLQRRDSPPSASEMEESEPVMTQEEIEAAAQAWRKGLP

RCSATPQKKHPILPEEGKRNVLLTSALPYVNNVPHLGNIIGCVLSADVFARYGRLRGWNLLYVCGTDEYGTATENKAREE

GLTPQEICDKYHAVHCAIYKWFQISFDYFGRTTTERQTEISQNIFWRLHERGFLLEDTVEQLHCENCQRFLADRFVEGVC

PHCRYPEARGDQCDKCGRLINAVELKEPQCKVCQQTPVVRSSKHLFLDLPKLESELEQWLDKSTSSGDWTNNAKNITRSW

LRDGLKPRCITRDLKWGTPVPHPDYSDKVFYVWFDAPIGYLSITANYTSEWEKWWKNPQQVELYNFMAKDNVPFHSVVFP

CSLLGAQDNYTLVNNLVATEYLNYEDTKFSKSRGVGVFGDMAKDTGIPSDVWRFYLLYLRPEGQDSAFSWADMALKNNSE

LLNNLGNFINRAGMFVSKFFEGHVPEMVLQDEDKRLIAQVCWELKQYIQLLDKVRIRDALKCILNISRHGNQYIQVNEPW

KKIKGGDDDRQRAGTVTGVSVNVACLLSAMLEPYMPTVSQTIRDQLQAPAHCSSATLGTDGGFLCTLSAGHPIGTVSPLF

QKLESEQIEALKKRFGGQQVPSSMVNSAQPKPPAAVEPTVNASVSAADPERAKQLAAAVAEQGDKVRALKSQKAEKSVIT

AEVTKLLELKKELAVAEGKNPEPSPQKGKKK

>XP_010307252.1 PREDICTED: LOW QUALITY PROTEIN: methionine--tRNA synthetase, cytoplasmic, partial [Balearica regulorum gibbericeps]

AAEALGKLLAHIEQNLSRRGTAYLTGDTKSVADVVVWGTLFPVLQDQTSLPSELQALRTWFQSMTLLEVCRKAADSVLTP

KALLEFKSYLQKQPPPSLAMERATSNEPEEEEGSERALTEEEITAAADAWAHGAAVLPKPWQPQKPVLPVEGMRNILITS

ALPYVNNVPHLGNIIGCVLSADTFARYCRLRNWNTLYVCGTDEYGTATETKAVEEGLTPQEICNKYNAIHADIYRWFNIS

FDYFGRTTTPYQTMIAQDIFQRLLARGFLLEDTVEQLRCEDCQRFLADRFVEGTCPFCSYEEARGDQCDKCGKLINAVEL

KNPQCKLCRGVPVVKPTKHLFLDLPKLEEQLEPWLQQSWATGNWTANARYITRSWIRDGLKPRCITRDLKWGTPVPLDGF

RDKVFYVWFDAPIGYLSITANYTDQWERWWKNPQQVELYNFMAKDNVPFHSVIFPCSLLGADDNYTLVNHLIATEYLNYE

DGKFSKSRGVGVFGDMAKDTGIPADIWRFYLLYLRPEGQDSAFSWNDLMLKNNSELLNNLGNFINRAGMFVCKFFGGTVP

NMVLTPDDKRLLARVTLELHQYHQLLEKVRIRDALRCILSISRHGNQYIQVNEPWKQIKGDEKDRQRAGTVTGVAVNMAS

MLAVMLQPYMPSVSLAIQGQLCIPLDCFILRHDFTXKNPPGHRVGTVSPLFQKLENDQIEGLRKRFGGGQPEDLAVEPQV

TPQYCLRGTGSNLPLSSAKQSPPASELPPAPATLMAPSDPQLIQELSEKVAKQGNHVRQLKANKAEKTQVDMEVAKLLEL

KKQLALAEGKSPEVPAPKGKRKK

>XP_020468701.1 methionine--tRNA synthetase, cytoplasmic isoform X3 [Monopterus albus]

MKLFVSEGNPQCLKVLCALEVTGVQCDVHYVNHEERVVPFLSRPALPALLLPSGRHLFSPNAICRYIFEVNGQESSDLCN

QWLEWEATHLQPALAEALHMAVLQGKGSEVSQVLQGPLNFLDQSLNNGNTPYLIGEAVSVADVVLWAALCPVLSDSSLVL

GKHKSVKAWFDHVAAMHSCQSAAQKVLQGKGLQGMKSYMQRQPAPQSSQCRDTQPCNSSPAECGEEEGERVVSEEDMNAA

ALKWSKGLNADPLSKEKQHPILPQEGKRNILVTSALPYVNNVPHLGNIIGCVLSADVFSRYGRLRGWNLLYVCGTDEYGT

ATENKAREEGMTPQQICDKYHVIHSSIYKWFQIDFDFFGRTTTEKQTEIAQDIFWRLHNHGFLVEDTVEQLRCENCQRFL

ADRFVEGTCPHCSYPEARGDQCDKCGRLINAVELREPQCKVCRQIPVIRSSKHLFLDLPKLETQLKQWLDTSTSTGHWTA

NAKQITRSWLRDGLKPRCITRDLQWGTPVPHPDFKEKVFYVWFDAPIGYLSITAKYTDKWEKWWKNPQQVELYNFMAKDN

VPFHSVVFPCSLLGAQDNYTLVSHLVATEYLNYEDTKFSKSRGVGVFGDMAMDTDIPSDVWRFYLLYVRPEGQDSAFSWA

DMAMKNNSELLNNLGNFINRAAMFVTKFFEGCVPAVELQQEDKKLLALVGWELQQYIQLMDKVKIRDALKHILNISRHGN

QYIQVNEPWKKIKGVGTERQRAGTVTGVSVNIACLLSVMLLPYMPTVSQTIRDQLHAPQSCVSTMLQGTGTFVCALKAGH

RIGTVTPLFQKLETDQIEALKKRFGGQQKTAQNAATVPAPAAPAAEVAMVNGVDPEKAKRLTQAVAEQGDKVRALKAQKA

EKAVVTAEVAKLLDLKKQLALAEGKSPEPAPQKGKKK

>XP_010730413.2 PREDICTED: methionine--tRNA synthetase, cytoplasmic isoform X2 [Larimichthys crocea]

MKLFVSEGNPHCLKVLAALEVTGAKCDVQYVNHEERVVPFLSRPALPALLLPSGLHLFSSNAICRYLFEVNGQDSSELCN

QWLEWEATELQPALLQALHMAVLQGKGSEVSKVLQGPLNYLDQSLSKKNTPYLTGEAVSVADVVLWAALYPILSDSSLAL

GECKSVKIWFDHGAAMHSLQCAAQKVLQGKGLQGMKSYMQKQPAPQSSQCRDTQPCNSSPAESEEGERLVSEEEIEAAAL

TWSKSLNACPLVANRQHPILPQEGKRNLLVTSALPYVNNVPHLGNIIGCVLSADVFSRYGRLRDWNVLFVCGTDEYGTAT

ENKAREEGLTPQQICDKYHAVHSSIYKWFQIDFDYFGRTTTEKQTEIAQNIFWRLHEHGFLVEDTVEQLRCESCQRFLAD

RFVEGTCPHCSYPEARGDQCDKCGRLINAVELREPQCKVCRQTPVIRSSKHLFLDLPKLETQLEQWLDKSTSTGDWTANA

KQITRSWLRDGLKPRCITRDLKWGTPVPHPDFKEKVFYVWFDAPIGYLSITANYTDKWETWWKNPHQVELYNFMAKDNVP

FHSVVFPCSLLGAQDNYTLVNHLVATEYLNYEDTKFSKSRGVGVFGDMAKDTGILSDVWRFYLLYVRPEGQDSAFSWADM

ATKNNSELLNNLGNFINRAGMFVTKFFEGCVPPMELQHEDKKLLALVGWELQQYIQLMDKVKIRDALKHILNISRHGNQY

IQVNEPWKKIKGGDTERQRAGTVTGVSVNISCLLSVMLFPYMPSVSQTIRDQLNAPQSCINTMLQGTGAFVCALSAGHRI

GTVSPLFQKLEVDQIEALKKRFGGQQTAAQAAASAQPATVPAPAAPAAAAEVVTVSGVDPEKAKQLTQAVAEQGEKVRAL

KAQKAEKAVITAEVAKLLDLKKQLALAEGKSPEPAPQKGKKK

>XP_020468536.1 methionine--tRNA synthetase, cytoplasmic isoform X1 [Monopterus albus]

MKLFVSEGNPQCLKVLCALEVTGVQCDVHYVNHEERVVPFLSRPALPALLLPSGRHLFSPNAICRYIFEVNGQESSDLCN

QWLEWEATHLQPALAEALHMAVLQGKGSEVSQVLQGPLNFLDQSLNNGNTPYLIGEAVSVADVVLWAALCPVLSDSSLVL

GKHKSVKAWFDHVAAMHSCQSAAQKVLQGKGLQGMKSYMQRQPAPQSSQCRDTQPCNSSPAECGEEEGERVVSEEDMNAA

ALKWSKGLNADPLSKEKQHPILPQEGKRNILVTSALPYVNNVPHLGNIIGCVLSADVFSRYGRLRGWNLLYVCGTDEYGT

ATENKAREEGMTPQQICDKYHVIHSSIYKWFQIDFDFFGRTTTEKQTEIAQDIFWRLHNHGFLVEDTVEQLRCENCQRFL

ADRFVEGTCPHCSYPEARGDQCDKCGRLINAVELREPQCKVCRQIPVIRSSKHLFLDLPKLETQLKQWLDTSTSTGHWTA

NAKQITRSWLRDGLKPRCITRDLQWGTPVPHPDFKEKVFYVWFDAPIGYLSITAKYTDKWEKWWKNPQQVELYNFMAKDN

VPFHSVVFPCSLLGAQDNYTLVSHLVATEYLNYEDTKFSKSRGVGVFGDMAMDTDIPSDVWRFYLLYVRPEGQDSAFSWA

DMAMKNNSELLNNLGNFINRAAMFVTKFFEGCVPAVELQQEDKKLLALVGWELQQYIQLMDKVKIRDALKHILNISRHGN

QYIQVNEPWKKIKGVGTERQRAGTVTGVSVNIACLLSVMLLPYMPTVSQTIRDQLHAPQSCVSTMLQGTGTFVCALKAGH

RIGTVTPLFQKLETDQIEALKKRFGGQQYEDEPPKEKKTAQNAATVPAPAAPAAEVAMVNGVDPEKAKRLTQAVAEQGDK

VRALKAQKAEKAVVTAEVAKLLDLKKQLALAEGKSPEPAPQKGKKK

>XP_020468619.1 methionine--tRNA synthetase, cytoplasmic isoform X2 [Monopterus albus]

MKLFVSEGNPQCLKVLCALEVTGVQCDVHYVNHEERVVPFLSRPALPALLLPSGRHLFSPNAICRYIFEVNGQESSDLCN

QWLEWEATHLQPALAEALHMAVLQGKGSEVSQVLQGPLNFLDQSLNNGNTPYLIGEAVSVADVVLWAALCPVLSDSSLVL

GKHKSVKAWFDHVAAMHSCQSAAQKVLQGKGLQGMKSYMQRQPAPQSSQCRDTQPCNSSPAECGEEEGERVVSEEDMNAA

ALKWSKGLNADPLSKEKQHPILPQEGKRNILVTSALPYVNNVPHLGNIIGCVLSADVFSRYGRLRGWNLLYVCGTDEYGT

ATENKAREEGMTPQQICDKYHVIHSSIYKWFQIDFDFFGRTTTEKQTEIAQDIFWRLHNHGFLVEDTVEQLRCENCQRFL

ADRFVEGTCPHCSYPEARGDQCDKCGRLINAVELREPQCKVCRQIPVIRSSKHLFLDLPKLETQLKQWLDTSTSTGHWTA

NAKQITRSWLRDGLKPRCITRDLQWGTPVPHPDFKEKVFYVWFDAPIGYLSITAKYTDKWEKWWKNPQQVELYNFMAKDN

VPFHSVVFPCSLLGAQDNYTLVSHLVATEYLNYEDTKFSKSRGVGVFGDMAMDTDIPSDVWRFYLLYVRPEGQDSAFSWA

DMAMKNNSELLNNLGNFINRAAMFVTKFFEGCVPAVELQQEDKKLLALVGWELQQYIQLMDKVKIRDALKHILNISRHGN

QYIQVNEPWKKIKGVGTERQRAGTVTGVSVNIACLLSVMLLPYMPTVSQTIRDQLHAPQSCVSTMLQGTGTFVCALKAGH

RIGTVTPLFQKLETDQIEALKKRFGGQQYEDEPPKEKTAQNAATVPAPAAPAAEVAMVNGVDPEKAKRLTQAVAEQGDKV

RALKAQKAEKAVVTAEVAKLLDLKKQLALAEGKSPEPAPQKGKKK

>XP_023180935.1 methionine--tRNA synthetase, cytoplasmic isoform X1 [Xiphophorus maculatus]

MELFVSEGNPHCLKVLAALEVTGVQCGVQFVNHEGKVVPFLPRPAVPTLFLPSGQHLFSSNTICRYLFELNGPESSDQCN

QWLEWEATILQPALLRALHLAVLQGKGSEVPKVLQGPLNVLDQSLVKGSMAYLTGEAVSVADVVLWGALHPVLSVSSLEL

SDHKSAKAWFDRVAAVRGCQSAAQKVLQGKGLEAMKSYMQKQPAPQSRDTQPCNSSPAENDEGERTVSEEEMEAAALTWS

KDLNACSYPAEKQHPVMPQEGNRNVLITSALPYVNNVPHLGNIIGCVLSADVFSRYGQLRGWNVLFVCGTDEYGTATENK

AREEGLTPQQICDKYHAVHSSIYKWFQIDFDFFGRTTTEKQTEIAQDIFWRLHKNGFLVEDTVEQLRCENCQRFLADRFV

EGMCPHCSYPEARGDQCDKCGRLINAVELREPQCKVCRQTPEIRSSKHLFLDLPKLETQLENWLERSTSSGDWTANAKQI

TRSWLRDGLKPRCITRDLQWGTPVPHPDFKEKVFYVWFDAPIGYLSITANYTNEWEKWWKNPHQVELYNFMAKDNVPFHS

VVFPCSLLGAQDNYTLVSHLIATEYLNYEDTKFSKSRGVGVFGDMAKDTGIPSDVWRFYLLYVRPEGQDSAFSWADMALK

NNSELLNNLGNFINRGGMFVTKFFGGCVPAMELQQEDKKLLAVVGWELQQYIQLMDKVKIRDALKHILNISRHGNQYIQV

NEPWKKIKGGESERQRAGTVTGVSVNIACLLSVMLLPYMPAVSQTIRDQLNAPQTCINTMLQGPGTFVCALRAGHRIGTV

SPLFQKLEADQIEALKKRFGGQQPEDEPSNKKITASTQPAAVPAPPVVAEMKVVDGSDPEKAKQLAEAVAEQGDKVRALK

SQKAEKAIITAEVSKLLDLKKQLALAEGKSPEPAPQKGKKK

>KFV99218.1 Methionine--tRNA synthetase, cytoplasmic, partial [Eurypyga helias]

VSRPDSASLAQDTKSVADVVVWGTLFPVLQNETSLPSELQALRSWFQSMTLSEACRKAVDSILMPKALLEFKSYLQKEPP

PCLAVERVTSNEPEEEESAERTLTEEEIAAAADAWARGAAALPKPWQPQNPVLPVEGMRNVLITSALPYVNNVPHLGNII

GCVLSADTFARYCRLRNWNTLYVCGTDEYGTATETKAVEEGLTPQEICNKYHAIHADVYRWFNISFDYFGRTTTSYQTTI

AQDIFQRLLARGFLLQDTVEQLRCEDCQRFLADRFVEGTCPFCSYEEARGDQCDKCGKLINAVELKNPQCKLCRGAPVVK

PTQHLFLDLPKLEERLEPWLEQSWATGDWTANARYITRSWIRDGLKPRCITRDLKWGTPVPLDGFRDKVFYVWFDAPIGY

LSITANYTEQWERWWKNPQQVELYNFMAKDNVPFHSVIFPCSLLGADDNYTLVNHLIATEYLNYEDGKFSKSRGVGVFGD

MAKDTGIPADIWRFYLLYLRPEGQDSAFSWSDLMLKNNSELLNNLGNFINRAGMFVCKFFGGTVPNMVLTLDDKRLLARV

TLELRQYHQLLEKVRIRDALRCILSISRHGNQYIQVNEPWKRIKGDEKDRQRAGTVTGVAVNMASMLAVMLQPYMPSVSS

AIQGQLCIPPDCLVLSHDFTCTLPPGHRVGTVSPLFQKLENDQVEALRKRFGGGQAKPTPPASAPPPAPGDPQLVMQLTE

EVAKQGNHIRQLKANKAEKAQVAVEVAKLLELKKQLALAEGRSPEVLVPKGKQKK

>XP_020492406.1 methionine--tRNA synthetase, cytoplasmic isoform X1 [Labrus bergylta]

MKLYVSEGNPHCLKVLAALDVTGVQCDVHYVNHEERVVPYLSRPALPALLLPSGLHLFSSNAICRYLFEVSGKDCTELCG

QWLEWEATDLQPALLQALHMAVLQGKGSDVSKVLQGPLNYLEQSLSKGNLPYLTGDAVSAADVVLWAALYPVLSDSSQAL

GERKFVKTWFDRGAVMHSLQSAAQKVLQGKGLQGTKSYMQKQPAPQSNQCRDTQPCNSSPAEGEESERLVSEEEMEAAAL

TWSKGLNSCQSDTDRQHPILPKEGKRNLLLTSALPYVNNVPHLGNIIGCVLSTDVFSRYGRLRDWNVLFVCGTDEYGTAT

ENKAREEGLTPQQICDKFHTIHASIYKWFQIDFDFFGRTTTEKQTEIAQDIFWRLHKQGFLVEDTVEQLRCESCQRFLAD

RFVEGICPHCSYPEARGDQCDKCGRLINAVELSEPQCKVCRQTPVIRSSKHLFLDLPKLESQLEQWLDKSTSTGDWTANA

KQITRSWLRDGLKPRCITRDLKWGTPVPHPDFKEKVFYVWFDAPIGYLSITANYTDQWEKWWKNPNQVELYNFMAKDNVP

FHSVVFPCSLLGAQDNYTLVNHLIATEYLNYEDSKFSKSRGVGVFGDMAKDTGIPSDVWRFYLLYVRPEGQDSVFSWADM

ATKNNSELLNNLGNFINRAGMFVAKFFEGCVPAMDLQQEDKKLLALVGWELQQYIQLMDKVKIRDALKHILNISRHGNQY

IQVNEPWKKIKGGDTERQRSGTVTGVSVNIACLLSVMLHPYMPTVSQTIRDQLNAPQSCINTMLKGTGTFVCALCPGHRI

GTVSPLFQKLELDQIEALKKRFGGQQPEDEPPKKKTAAQKAAGTQPAAVPVPAAAEVASVNGVDPEKAKQLTQAVAEQGE

KVRALKAQKAEKAAITAEVAKLLDLKKQLALAEGKSPEPAPQKGKKK

>XP_014902958.1 PREDICTED: methionine--tRNA synthetase, cytoplasmic [Poecilia latipinna]

MELFVSEGNPHCLKVLAALEVTGVQCGVQFVNHEGKVVPFLPRPAVPTLFLPSGQHLFSSNTICRYLFELNGPESSDQCN

QWLEWEATILQPALLRALHLAVLQGKGSEVPKVLQGPLNVLDQSLAKGSTAYLTGEAVSVADVVLWGALHPVLSVSSLAL

SDHKSVKAWFDRVAAVRGCQSAAQKVLQGKGLEAMKSYMQKQPAPQSRDTQPCNGSPAETDEGERAVSEEEMEAAALTWS

KDLNACSHPAEKQHPVMPQEGNRNVLITSALPYVNNVPHLGNIIGCVLSADVFSRYGQLRGWNVLFVCGTDEYGTATENK

AREEGLTPQQICDKYHAVHSSIYKWFQIDFDFFGRTTTEKQTEIAQDIFWRLHKNGFLVEDTVEQLRCENCQRFLADRFV

EGTCPHCSYPEARGDQCDKCGRLINAVELREPQCKVCRRTPEIRSSKHLFLDLPKLETQLENWLERSTSSGDWTANAKQI

TRSWLRDGLKPRCITRDLQWGTPVPHPDFKEKVFYVWFDAPIGYLSITANYTNEWEKWWKNPHQVELYNFMAKDNVPFHS

VVFPCSLLGAQDNYTLVSHLIATEYLNYEDTKFSKSRGVGVFGDMAKDTGIPSDVWRFYLLYVRPEGQDSAFSWADMALK

NNSELLNNLGNFINRGGMFVTKFFGGCVPAMELQQEDKKLLAVVGWELQQYIQLMDKVKIRDALKHILNISRHGNQYIQV

NEPWKKIKGGQSERQRAGTVTGVSVNIACLLSVMLLPYMPAVSQTIRDQLNAPQTCINTMLQGPGTFVCALRAGHRIGTV

SPLFQKLEADQIEALKKRFGGQQITASTQPAAVPAPPVVAEVKVVDGSDPEKAKQLAEAVAEQGDKVRALKSEKAEKAVI

TAEVSKLLDLKKQLALAEGKSPEPAPQKGKKK

>XP_007548781.1 PREDICTED: methionine--tRNA synthetase, cytoplasmic isoform X2 [Poecilia formosa]

MELFVSEGNPHCLKVLAALKVTGVQCGVQFVNHEGKVVPFLPRPAVPTLFLPSGQHLFSSNTICRYLFELNGPESSDQCN

QWLEWEATILQPALLRALHLAVLQGKGSEVPKVLQGPLNVLDQSLAKGSTAYLTGEAVSVADVVLWGALHPVLSVSSLVL

SDHKSVKAWFDRVAAVRGCQSAAQKVLQGKGLEAMKSYMQKQPAPQSRDTQPCNGSPAETDEGERAVSEEEMEAAALIWS

KDLNACSHPAEKQHPVMPQEGNRNVLITSALPYVNNVPHLGNIIGCVLSADVFSRYGQLRGWNVLFVCGTDEYGTATENK

AREEGLTPQQICDKYHAVHSSIYKWFQIDFDFFGRTTTEKQTEIAQDIFWRLHKNGFLVEDTVEQLRCENCQRFLADRFV

EGTCPHCSYPEARGDQCDKCGRLINAVELREPQCKVCRQTPEIRSSKHLFLDLPKLETQLENWLERSTSSGDWTANAKQI

TRSWLRDGLKPRCITRDLQWGTPVPHPDFKEKVFYVWFDAPIGYLSITANYTNEWEKWWKNPHQVELYNFMAKDNVPFHS

VVFPCSLLGAQDNYTLVSHLIATEYLNYEDTKFSKSRGVGVFGDMAKDTGIPSDVWRFYLLYVRPEGQDSAFSWADMALK

NNSELLNNLGNFINRGGMFVTKFFGGCVPAMELQQEDKKLLAVVGWELQQYIQLMDKVKIRDALKHILNISRHGNQYIQV

NEPWKKIKGGESERQRAGTVTGVSVNIACLLSVMLLPYMPAVSQTIRDQLNAPQTCINTMLQGPGTFVCALRAGHRIGTV

SPLFQKLEADQIEALKKRFGGQQITASTQPAAVPAPPVVAEVKVVDGSDPEKAKQLAEAVAEQGDKVRALKSEKAEKAVI

TAEVSKLLDLKKQLALAEGKSPEPAPQKGKKK

>XP_016525580.1 PREDICTED: methionine--tRNA synthetase, cytoplasmic isoform X1 [Poecilia formosa]

MELFVSEGNPHCLKVLAALKVTGVQCGVQFVNHEGKVVPFLPRPAVPTLFLPSGQHLFSSNTICRYLFELNGPESSDQCN

QWLEWEATILQPALLRALHLAVLQGKGSEVPKVLQGPLNVLDQSLAKGSTAYLTGEAVSVADVVLWGALHPVLSVSSLVL

SDHKSVKAWFDRVAAVRGCQSAAQKVLQGKGLEAMKSYMQKQPAPQSRDTQPCNGSPAETDEGERAVSEEEMEAAALIWS

KDLNACSHPAEKQHPVMPQEGNRNVLITSALPYVNNVPHLGNIIGCVLSADVFSRYGQLRGWNVLFVCGTDEYGTATENK

AREEGLTPQQICDKYHAVHSSIYKWFQIDFDFFGRTTTEKQTEIAQDIFWRLHKNGFLVEDTVEQLRCENCQRFLADRFV

EGTCPHCSYPEARGDQCDKCGRLINAVELREPQCKVCRQTPEIRSSKHLFLDLPKLETQLENWLERSTSSGDWTANAKQI

TRSWLRDGLKPRCITRDLQWGTPVPHPDFKEKVFYVWFDAPIGYLSITANYTNEWEKWWKNPHQVELYNFMAKDNVPFHS

VVFPCSLLGAQDNYTLVSHLIATEYLNYEDTKFSKSRGVGVFGDMAKDTGIPSDVWRFYLLYVRPEGQDSAFSWADMALK

NNSELLNNLGNFINRGGMFVTKFFGGCVPAMELQQEDKKLLAVVGWELQQYIQLMDKVKIRDALKHILNISRHGNQYIQV

NEPWKKIKGGESERQRAGTVTGVSVNIACLLSVMLLPYMPAVSQTIRDQLNAPQTCINTMLQGPGTFVCALRAGHRIGTV

SPLFQKLEADQIEALKKRFGGQQPEDEPSNKKITASTQPAAVPAPPVVAEVKVVDGSDPEKAKQLAEAVAEQGDKVRALK

SEKAEKAVITAEVSKLLDLKKQLALAEGKSPEPAPQKGKKK

>KFO06072.1 Methionine--tRNA synthetase, cytoplasmic, partial [Balearica regulorum gibbericeps]

KLLAHIEQNLSRRGTAYLTGDTKSVADVVVWGTLFPVLQDQTSLPSELQALRTWFQSMTLLEVCRKAADSVLTPKALLEF

KSYLQKQPPPSLAMERATSNEPEEEEGSERALTEEEITAAADAWAHGAAVLPKPWQPQKPVLPVEGMRNILITSALPYVN

NVPHLGNIIGCVLSADTFARYCRLRNWNTLYVCGTDEYGTATETKAVEEGLTPQEICNKYNAIHADIYRWFNISFDYFGR

TTTPYQTMIAQDIFQRLLARGFLLEDTVEQLRCEDCQRFLADRFVEGTCPFCSYEEARGDQCDKCGKLINAVELKNPQCK

LCRGVPVVKPTKHLFLDLPKLEEQLEPWLQQSWATGNWTANARYITRSWIRDGLKPRCITRDLKWGTPVPLDGFRDKVFY

VWFDAPIGYLSITANYTDQWERWWKNPQQVELYNFMAKDNVPFHSVIFPCSLLGADDNYTLVNHLIATEYLNYEDGKFSK

SRGVGVFGDMAKDTGIPADIWRFYLLYLRPEGQDSAFSWNDLMLKNNSELLNNLGNFINRAGMFVCKFFGGTVPNMVLTP

DDKRLLARVTLELHQYHQLLEKVRIRDALRCILSISRHGNQYIQVNEPWKQIKGDEKDRQRAGTVTGVAVNMASMLAVML

QPYMPSVSLAIQGQLCIPLDCFILRHDFTKKPPWPSCGHCRGNPSSSWLQVSPLFQKLENDQIEGLRKRFGGGQAKQSPP

ASELPPAPATLMAPSDPQLIQELSEKVAKQGNHVRQLKANKAEKTQVDMEVAKLLELKKQLALAEGKSPEVPAPKGKRKK

>XP_014860068.1 PREDICTED: methionine--tRNA synthetase, cytoplasmic isoform X2 [Poecilia mexicana]

MELFVSEGNPHCLKVLAALKVTGVQCGVQFVNHEGKVVPFLPCPAVPTLFLPSGQHLFSSNTICRYLFELNGPESSDQCN

QWLEWEATILQPALLRALHLAVLQGKGSEVPKVLQGPLNVLDQSLAKGSTAYLTGEAVSVADVVLWGALHPVLSVSSLAL

SDHKSVKAWSDRVAAVRGCQSAAQKVLQGKGLEAMKSYMQKQPAPQSRDTQPCNGSPAETDEGERAVSEEEMEAAALIWS

KDLNACSHPAEKQHPVMPQEGNRNVLITSALPYVNNVPHLGNIIGCVLSADVFSRYGQLRGWNVLFVCGTDEYGTATENK

AREEGLTPQQICDKYHAVHSSIYKWFQIDFDFFGRTTTEKQTEIAQDIFWRLHKNGFLVEDTVEQLRCENCQRFLADRFV

EGTCPHCSYPEARGDQCDKCGRLINAVELREPQCKVCRQTPEIRSSKHLFLDLPKLETQLENWLERSTSSGDWTANAKQI

TRSWLRDGLKPRCITRDLQWGTPVPHPDFKEKVFYVWFDAPIGYLSITANYTNEWEKWWKNPHQVELYNFMAKDNVPFHS

VVFPCSLLGAQDNYTLVSHLIATEYLNYEDTKFSKSRGVGVFGDMAKDTGIPSDVWRFYLLYVRPEGQDSAFSWADMALK

NNSELLNNLGNFINRGGMFVTKFFGGCVPAMELQQEDKKLLAVVGWELQQYIQLMDKVKIRDALKHILNISRHGNQYIQV

NEPWKKIKGGESERQRAGTVTGVSVNIACLLSVMLLPYMPAVSQTIRDQLNAPQTCINTMLQGPGTFVCALRAGHRIGTV

SPLFQKLEADQIEALKKRFGGQQITASTQPAAVPAPPVVAEVKVVDGSDPEKAKQLAEAVAEQGDKVRALKSQKAEKAVI

TAEVSKLLDLKKQLALAEGKSPEPAPQKGKKK

>XP_012724786.1 methionine--tRNA synthetase, cytoplasmic isoform X2 [Fundulus heteroclitus]

MELFVGEGNPHCLKVLAALEVTGVQCGVQFINHEEKVVPFLSRPALPALLLPSRQHLFSPNTICRYMFELNGPESSNQCN

QWLEWEATVLQPVLFRALHMAVLQGKGSEVAKVLQGPLNVLDQSLGKGSTTYLTGEALSVADVVLWGALHPVLSDSSLAL

SAHQSVKAWFDRVAAVRGCQSAAQKVLQGKGLEAMRSYMQKQPAPQSSQGRDAQPCNSSPAESEEGDRVVSEEEMEAAVL

TWGKDLNACSQATEKQHPIMPQEGKRNVLITSALPYVNNVPHLGNIIGCVLSADVFSRYGRLRGWNTLFVCGTDEYGTAT

ENKAREEGLTPKQICDKYHAVHSSIYKWFQIDFDFFGRTTTEKQTEIAQDIFWRLQKNGFLVEDTVEQLRCESCQRFLAD

RFVEGTCPHCSYPEARGDQCDKCGRLINAVELREPQCKVCRQTPVIRSSKHLFLDLPKLESQLEQWLEKSTSSGDWTANA

KQITRSWLRDGLKPRCITRDLLWGTPVPHPDFKEKVFYVWFDAPIGYLSITANYTNQWEKWWKNPHQVELYNFMAKDNVP

FHSVVFPCSLLGAQDNYTLVNHLVATEYLNYEDTKFSKSRGIGVFGDMAKDTGIPSDVWRFYLLYVRPEGQDSAFSWADM

ALKNNSELLNNLGNFINRAGMFVTKFFEGCVPVMELQQEDKKLLAVVGWELQQYIQLMDKVKIRDALKHILNISRHGNQY

IQVNEPWKKIKGGESERRRAGTVTGVSVNIACLLSVMLLPYMPAVSQTIREQLNAPQSCVNAMLQGAGTFACALRAGHRI

GAVSPLFQKLEADQIEALKKRFGGQQVQIGASTQPAAVPAPAAVAQVQAAVGSDPEKAKQLAKAVAEQGDKVRALKSQKA

EKAVIAGEVAKLLDLKKQLALAEGKNPEPAPQKGKKK

>XP_012724785.1 methionine--tRNA synthetase, cytoplasmic isoform X1 [Fundulus heteroclitus]

MELFVGEGNPHCLKVLAALEVTGVQCGVQFINHEEKVVPFLSRPALPALLLPSRQHLFSPNTICRYMFELNGPESSNQCN

QWLEWEATVLQPVLFRALHMAVLQGKGSEVAKVLQGPLNVLDQSLGKGSTTYLTGEALSVADVVLWGALHPVLSDSSLAL

SAHQSVKAWFDRVAAVRGCQSAAQKVLQGKGLEAMRSYMQKQPAPQSSQGRDAQPCNSSPAESEEGDRVVSEEEMEAAVL

TWGKDLNACSQATEKQHPIMPQEGKRNVLITSALPYVNNVPHLGNIIGCVLSADVFSRYGRLRGWNTLFVCGTDEYGTAT

ENKAREEGLTPKQICDKYHAVHSSIYKWFQIDFDFFGRTTTEKQTEIAQDIFWRLQKNGFLVEDTVEQLRCESCQRFLAD

RFVEGTCPHCSYPEARGDQCDKCGRLINAVELREPQCKVCRQTPVIRSSKHLFLDLPKLESQLEQWLEKSTSSGDWTANA

KQITRSWLRDGLKPRCITRDLLWGTPVPHPDFKEKVFYVWFDAPIGYLSITANYTNQWEKWWKNPHQVELYNFMAKDNVP

FHSVVFPCSLLGAQDNYTLVNHLVATEYLNYEDTKFSKSRGIGVFGDMAKDTGIPSDVWRFYLLYVRPEGQDSAFSWADM

ALKNNSELLNNLGNFINRAGMFVTKFFEGCVPVMELQQEDKKLLAVVGWELQQYIQLMDKVKIRDALKHILNISRHGNQY

IQVNEPWKKIKGGESERRRAGTVTGVSVNIACLLSVMLLPYMPAVSQTIREQLNAPQSCVNAMLQGAGTFACALRAGHRI

GAVSPLFQKLEADQIEALKKRFGGQQPEDEPSNKKVQIGASTQPAAVPAPAAVAQVQAAVGSDPEKAKQLAKAVAEQGDK

VRALKSQKAEKAVIAGEVAKLLDLKKQLALAEGKNPEPAPQKGKKK

>XP_022531355.1 methionine--tRNA synthetase, cytoplasmic isoform X1 [Astyanax mexicanus]

MKLFVSEGNPHCVKALAALELSGLKCDVQLVNHEERVVPFLNRPVIPVLLLPSGQHLFSANAICQLLLEMAGKSTDESKQ

LLEWEATELQPALLQALHKQALQGKSSEAAAVLKGPLGWLDQYLSKRTTSFLTEEILADIVLGAALYPVLSDPAFEVGDL

PSLQAWFNHVRELPACQSAAQKVLNGKGLEALKGFLQKQPVPHLQRRDSPPSASEMEESEPVMTQEEIEAAAQAWRKGLP

RCSATPQKKHPILPEEGKRNVLLTSALPYVNNVPHLGNIIGCVLSADVFARYGRLRGWNLLYVCGTDEYGTATENKAREE

GLTPQEICDKYHAVHCAIYKWFQISFDYFGRTTTERQTEISQNIFWRLHERGFLLEDTVEQLHCENCQRFLADRFVEGVC

PHCRYPEARGDQCDKCGRLINAVELKEPQCKVCQQTPVVRSSKHLFLDLPKLESELEQWLDKSTSSGDWTNNAKNITRSW

LRDGLKPRCITRDLKWGTPVPHPDYSDKVFYVWFDAPIGYLSITANYTSEWEKWWKNPQQVELYNFMAKDNVPFHSVVFP

CSLLGAQDNYTLVNNLVATEYLNYEDTKFSKSRGVGVFGDMAKDTGIPSDVWRFYLLYLRPEGQDSAFSWADMALKNNSE

LLNNLGNFINRAGMFVSKFFEGHVPEMVLQDEDKRLIAQVCWELKQYIQLLDKVRIRDALKCILNISRHGNQYIQVNEPW

KKIKGGDDDRQRAGTVTGVSVNVACLLSAMLEPYMPTVSQTIRDQLQAPAHCSSATLGTDGGFLCTLSAGHPIGTVSPLF

QKLESEQIEALKKRFGGQQPEDETPAGEVPSSMVNSAQPKPPAAVEPTVNASVSAADPERAKQLAAAVAEQGDKVRALKS

QKAEKSVITAEVTKLLELKKELAVAEGKNPEPSPQKGKKK

>XP_019952461.1 PREDICTED: methionine--tRNA synthetase, cytoplasmic [Paralichthys olivaceus]

MKLLLSEGNPHCLKVLAALQVTGVQCDVQYVNHEETVVPFLSRPTLPALLLPSGLHLFSSNAICQYLYEVKGQESTDLCN

QWLEWEATDLQPALLQALHMALVQGKASEVSKVLQAPLNYLDQGLTKGNTPYLTGEVVSVADVVVWAALYPLLSDSSLAL

GDRKSVKGWFDRMTEMHGCQSAAQKVLQGMKSYMQKQPARQINHCSSPAECEEGERVVSQEEMEAAALTWSKGLKDSPLV

TDRQHPILPQEGKRNILVTSALPYVNNVPHLGNIIGCVLSADVFSRYARLRGWNLLFVCGTDEYGTATENKAREEGLTPQ

QICDKYHAVHASIYKWFQIDFDFFGRTTTEKQTEIAQNIFLRLHKHGFLAEDTVEQLRCENCQRFLADRFVEGICPHCNY

PEARGDQCDKCGKLINAVELGEPQCKVCRQTPVVRSSKHLFLDLPKLEAQLEQWLDKSMSAGDWTPNAKQITRSWLRDGL

KPRCITRDLKWGIPVPHPDFKDKVFYVWFDAPIGYLSVTANYTDQWEKWWKNPRQVELYNFMAKDNVPFHSVVFPCSLLG

AQDNYTLVSHLVATEYLNYEDTKFSKSRGTGVFGDMAKDTGIPSDVWRFYLLFVRPEGQDSAFSWADMALKNNSELLNNL

GNFINRAGMFVTKFFEGCVPAMELQQEDKKLLALVSWELQQYIQLMDKVKIRDALKHILNISRHGNQYIQVNQPWKNIKG

EDTERQRAGTVTGISVNIACLLSVMLLPYMPTVSQTIRDQLNAPQSGLHTMFQGTGNFVCSLRAGHRIGTVSPLFQKLEG

DQIEALKKRFGGQQMTAQSAASSQPAAVPAPPAAGEVAATTVNGADPEKAKLLTQAVTEQGEKVRALKTQKAEKAVITAE

VAKLLDLKKQLAVAEGKNPEPAPQKSKKK

>XP_008406691.1 PREDICTED: methionine--tRNA synthetase, cytoplasmic isoform X2 [Poecilia reticulata]

MELFVSEGNPHCLKVLAALEVTGVQCGVQFVHHEGKVVPFLPRPAVPTLFFPSGQHLFSSNTICRYLFELNGPESSDQCN

QWLEWEATILQPAVLRALHLAVLQGKGSEVPKVLQGPLNVLDQSLTKGSTAYLTGEAVSVADVVLWGALHPXLSVSSLAL

SDHKSVKAWFDRVAAVRGCQSAAQKVLQGKGLEAMKSYMQKQPAPQSRDTQPCNGSPAETDEGERAVSEEEMEAAALTWS

KDLNACSHPAEKQHPVMPQEGNRNVLITSALPYVNNVPHLGNIIGCVLSADVFSRYGQLRGWNVLFVCGTDEYGTATENK

AREEGLTPQQICDKYHAVHSSIYKWFQIDFDFFGRTTTEKQTEIAQDIFWRLHKNGFLVEDTVEQLRCENCQRFLADRFV

EGTCPHCSYPEARGDQCDKCGRLINAVELREPQCKVCRQTPEIRSSKHLFLDLPKLETQLENWLESSTSSGDWTANAKQI

TRSWLRDGLKPRCITRDLQWGTPVPHPDFKEKVFYVWFDAPIGYLSITANYTNEWEKWWKNPHQVELYNFMAKDNVPFHS

VVFPCSLLGAQDNYTLVSNLIATEYLNYEDTKFSKSRGVGVFGDMAKDTGIPSDVWRFYLLYVRPEGQDSAFSWADMALK

NNSELLNNLGNFINRGGMFVTKFFGGCVPAMELQQEDKTLLAVVGWELQQYIQLMDKVKIRDALKHILNISRHGNQYIQV

NEPWKKIKGGESERQRAGTVTGVSVNIACLLSVMLLPYMPAVSQTIRDQLNAPQTCINTMLQGPGTFVCALRAGHRIGTV

SPLFQKLEADQIEALKKKFGGQQITASTQPAAVPAPLVVAEVKVADGSDPEKAKQLAEAVAEQGDKVRALKSQKAEKAVI

TAEVSKLLDLKKQLALAEGKSPEPAPQKGKKK

>XP_023853547.1 LOW QUALITY PROTEIN: methionine--tRNA synthetase, cytoplasmic, partial [Salvelinus alpinus]

MKLFVSDGNPHCVKVLAVLGFTGVKCDVKHVNHEERVVPYLTXPALPALLLPSGTYLFXPNSICQYLIEVNGQESSDIVN

QWLEWEATELQPAVMQALHMVVLQGKEXRTTRVLQGPLSYLDQGLSKRTTLFLTAEKVSVADVVCXGALYPILSDSSLVS

GSNQTLQHWFERVGMLAACGSAAQTALYGKGLKAESLKTYLQRQPATHQGQGQGRGTQPCNSPEGDDDGDCVLSEEEIQS

AAVTWGKGLADLPMVPERQHPILPKEGKRNVLVTSALPYVNNVPHLGNIIGCVLSADVFARYGRLRGWNVLYVCGTDEYG

TATENKAREEGLTAQQICDKYHAVHASIYQWFQVDFDFFGRTTTKKQTEIAQNIFWRLHERGFLLEDIVEQLRCEACQRF

LADRFVEGTCPFCSYPEARGDQCDKCGKLINAVELREPTCKVCSQTPVVQSSKHLFLDLPKLEPELEQWLERSTGSGDWT

ANAKQITRSWVRDGLKPRCITRDLKWGTPVPHPDFSDKVFYVWFDAPIGYLSITANYTDQWEKWWKNPQQVELYNFMAKD

NVPFHSVVFPCSLLGAQDNYTLVNHLVATEYLNYEDTKFSKSRGVGVFGDMAKDTGIPSDVWRFYLLYLRPEGQDSAFSW

VDMATKNNSELLNNLGNFINRAGMFVSKFFENCVPAMALQQEDKRLLAQVGWELKQYINLMDRVKIRDALKCILNISRHG

NQYIQVNEPWKKIKGDETDRQRSGTVTGVSVNVACLLSVMLQPYMPTVSQTIRQQLNAPPTVTGTMLQGTGNFVCSLPAG

HHIGTVSPLFQKLESDQIESLKKRFGGQQPKTTAQTDVNAQPAPTPASDPAAQTAVNGADAERARDLTLAVAEQGEKVRT

LKGQKAEKAVIGAEVTKLLELKKQLSLAEGKHPEPVLTKGKKSAQPAPTPASDPAAQTAVNGADAERARDLTLAVAEQG

>XP_014860059.1 PREDICTED: methionine--tRNA synthetase, cytoplasmic isoform X1 [Poecilia mexicana]

MELFVSEGNPHCLKVLAALKVTGVQCGVQFVNHEGKVVPFLPCPAVPTLFLPSGQHLFSSNTICRYLFELNGPESSDQCN

QWLEWEATILQPALLRALHLAVLQGKGSEVPKVLQGPLNVLDQSLAKGSTAYLTGEAVSVADVVLWGALHPVLSVSSLAL

SDHKSVKAWSDRVAAVRGCQSAAQKVLQGKGLEAMKSYMQKQPAPQSRDTQPCNGSPAETDEGERAVSEEEMEAAALIWS

KDLNACSHPAEKQHPVMPQEGNRNVLITSALPYVNNVPHLGNIIGCVLSADVFSRYGQLRGWNVLFVCGTDEYGTATENK

AREEGLTPQQICDKYHAVHSSIYKWFQIDFDFFGRTTTEKQTEIAQDIFWRLHKNGFLVEDTVEQLRCENCQRFLADRFV

EGTCPHCSYPEARGDQCDKCGRLINAVELREPQCKVCRQTPEIRSSKHLFLDLPKLETQLENWLERSTSSGDWTANAKQI

TRSWLRDGLKPRCITRDLQWGTPVPHPDFKEKVFYVWFDAPIGYLSITANYTNEWEKWWKNPHQVELYNFMAKDNVPFHS

VVFPCSLLGAQDNYTLVSHLIATEYLNYEDTKFSKSRGVGVFGDMAKDTGIPSDVWRFYLLYVRPEGQDSAFSWADMALK

NNSELLNNLGNFINRGGMFVTKFFGGCVPAMELQQEDKKLLAVVGWELQQYIQLMDKVKIRDALKHILNISRHGNQYIQV

NEPWKKIKGGESERQRAGTVTGVSVNIACLLSVMLLPYMPAVSQTIRDQLNAPQTCINTMLQGPGTFVCALRAGHRIGTV

SPLFQKLEADQIEALKKRFGGQQPEDEPSNKKITASTQPAAVPAPPVVAEVKVVDGSDPEKAKQLAEAVAEQGDKVRALK

SQKAEKAVITAEVSKLLDLKKQLALAEGKSPEPAPQKGKKK

>XP_021260791.1 methionine--tRNA synthetase, cytoplasmic isoform X1 [Numida meleagris]

MRLRVAAGSPAALKVVAAAHRAAAPVQLCWAEPGEPPPSPLCPSWAPALQLESGAVLYSPNAICQYFFLRRGELPTELTT

QWLEWEATELQPAALAALYARLVHGQTGVEAAAALGGLLGHIERSLAGRGTAYLAGDTESVADVVVWGTLFPVLRDEVIP

SAEPLRSWFQSMTLSEACRSAADAVLLPQRLQEFTACLQKLPPLRPRPDMAPGSEEDDTLERALTEEDVAAAVDAWARGA

AALPKPWRPPKPVLPVEGMRNVLITSALPYVNNVPHLGNIIGCVLSADTFARYCRLRNWNTLYVCGTDEYGTATETKAVE

EGLTPQEICDKYHAVHADIYRWFNISFDYFGRTTTPQQTIISQDIFQRLQARGFVLQDTVQQLHCASCQRFLADRFVEGT

CPFCAYQEARGDQCDKCGKLINAVELKTPRCKLCSSTPTVQPTRHLFLDLPKLEERLQPWLKHCWATGNWTANARYITRS

WIRDGLRPRCITRDLQWGTPVPLDGFRNKVFYVWFDAPIGYLSITANYTEHWERWWKNPQQVELYNFMAKDNVPFHSVMF

PCSLLGADDNYTLVNHLIATEYLNYEDGKFSKSRGVGVFGDMAKDTGIAADVWRFYLLYVRPEGQDSAFSWGDLMLKNNS

ELLNNLGNFINRAGMFVCKFFGGAVPAMALTLDDHRLLARITVELRQYHRLLERVCIRDALRCVLNISRHGNQYIQVNEP

WKRIKGDEEDRQRAGTVTGVAVNVAALLAVLVQPYMPAVSAAIQAQLCMPLESLVLGHVFTCTLPPGHRVGTVSPLFQKL

ESERINALRKRFGAGQAKPPAPPTQPDSPLVPSVAAGDPSRIQELTEQVAEQGDCVRRLKASGADRACIDSEVGKLLALK

RELALAQNVSPDGPRAQGKK

>XP_020774228.1 methionine--tRNA synthetase, cytoplasmic [Boleophthalmus pectinirostris]

MKLFLSEGNPQCLKALAALEVTRAKCDVQYVNHEDKVVPFLSRPVLPALLLPSGQHLFSPNAICRYMFEVSGQDSGALCD

QWLEWEATNLQPALVQALHLAVLQGKGSEVPKIIQGSLNYLDQNLSKGKTTYLTGESISAADIVLWAALFPLFSDSTVPT

GEHKTVTLWFNHVNGLNACQSSAQKVLQGKGLQGMKSYMQKQPAPPSSHSRDVQPSTSSPAEGDDGERLVSEEEMEAAAL

SYSKGLSIFKDTERKHPILPQEGKRNILLTSALPYVNNVPHLGNIIGCVLSADVFSRYGRLRGWNLLYVCGTDEYGTATE

NKAREEGLTPQQICDKYHAVHASIYKWFQIDFDFFGRTTTSKQTEIAQDIFWHLHKHDFLVEDTVEQLRCENCQRFLADR

FVEGICPFCNYPEARGDQCDKCGRLINAVELKQPQCKVCKQTPVIQTSKHLFLNLPKLETQLEQWLDKSTSTGDWTANAK

QITRSWLRDGLKPRCITRDLQWGTPVPHPDFKEKVFYVWFDAPIGYLSITANYTDQWEKWWKNPQQVELYNFMAKDNVPF

HSVVFPCSLLGAQDNYTLVSHLVATEYLNYEDTKFSKSRGVGVFGDMAKDTGIPSDVWRFYLLYVRPEGQDSAFSWADMA

MKNNSELLNNLGNFINRAGMFVTKFFEGCVPVMDLQPEDKRVLALVGWELQQYIQLMDKVKLRDALKHILNISRHGNQYI

QVNEPWKKIKGGDSERQRAGTVTGVSVNISCLLSVMLLPYMPTVSQTIRDQLNAPPSCISSMLQGSGTFVCALNAGHRIG

TVSPLFQKLEAEHIETLKKKFGGQQEDEPPKIQPKSTASSAASPQPAVVPAPATEAIVAGGVDPEKAKLLTQAVAEQGEK

VRALKTQKADKAVITAEVAKLLDLKKHCIQSDI

>XP_024125643.1 methionine--tRNA synthetase, cytoplasmic [Oryzias melastigma]

MKLFVSEGNPHCLKVLAALELTAVKCDVQYVNHEDKVVQFLSHPALPALLLPSGQQLFSANAICRYLFEVSGQNCNDHCN

QWLEWEVTVLQPALLRALRSAVLQGKGSDVSQILQSPLNFLDQSLLKGGKPYLTGEAISVADVVLWAALHPILSDSSFTL

GEHPSVKTWFDQVAAVHSCQSAVQKVLQGKGLQAMKSYMQRQPAPPNSQCRDAQPCNNNPAESEERQHSVSEEECEAAAL

TWSKGLSSCPPTDKQHPILPQEGKRNVLVTSALPYVNNVPHLGNIIGCVLSADVFSRYGRLRGWNMLFVCGTDEYGTATE

NKAREEGLTPQQICDKYHAVHSSIYSWFQIDFDFFGRTTTEKQTEIAQNIFWRLHEHGYLVEDTVEQLRCEKCQRFLADR

FVEGTCPHCSYPEARGDQCDKCGRLINAVELRDPQCKVCRQTPVIRSSKHLFLDLPKLESQLEQWLEKSTSTGDWTTNAK

QITHSWIRDGLKPRCITRDLHWGTPVPHPDFKEKVFYVWFDAPIGYLSITANYTNEWEKWWKNPQQVELYNFMAKDNVPF

HSVVFPCSLLGAQDNYTLVNHLVATEYLNYEDTKFSKSRGVGVFGDMAKDTGIPSDVWRFYLLYVRPEGQDSAFSWADMA

MKNNSELLNNLGNFINRAGMFVTKFFEGCVPVMELQREDKELLAVVGWELQQYIQLMDKVKIRDALKHILNISRHGNQYI

QVNQPWKKIKGDESERQRAGTVTGVAVNIACLLSVMLSPYMPLVSQTIRDQLNAPQSCVYTMLQGGGTFVCSLSAGHRIG

TVSPLFQKLEAEQIEALKKRFGGQQAPFQSTSSTPAAAPAAAIKVAAVEGGDSEKAEQLAKAVAEQGDKVRALKAQKAEK

DVITAEVSKLLELKTQLAVAEGKNPEPTSKKGKKK

>XP_008406690.1 PREDICTED: methionine--tRNA synthetase, cytoplasmic isoform X1 [Poecilia reticulata]

MELFVSEGNPHCLKVLAALEVTGVQCGVQFVHHEGKVVPFLPRPAVPTLFFPSGQHLFSSNTICRYLFELNGPESSDQCN

QWLEWEATILQPAVLRALHLAVLQGKGSEVPKVLQGPLNVLDQSLTKGSTAYLTGEAVSVADVVLWGALHPXLSVSSLAL

SDHKSVKAWFDRVAAVRGCQSAAQKVLQGKGLEAMKSYMQKQPAPQSRDTQPCNGSPAETDEGERAVSEEEMEAAALTWS

KDLNACSHPAEKQHPVMPQEGNRNVLITSALPYVNNVPHLGNIIGCVLSADVFSRYGQLRGWNVLFVCGTDEYGTATENK

AREEGLTPQQICDKYHAVHSSIYKWFQIDFDFFGRTTTEKQTEIAQDIFWRLHKNGFLVEDTVEQLRCENCQRFLADRFV

EGTCPHCSYPEARGDQCDKCGRLINAVELREPQCKVCRQTPEIRSSKHLFLDLPKLETQLENWLESSTSSGDWTANAKQI

TRSWLRDGLKPRCITRDLQWGTPVPHPDFKEKVFYVWFDAPIGYLSITANYTNEWEKWWKNPHQVELYNFMAKDNVPFHS

VVFPCSLLGAQDNYTLVSNLIATEYLNYEDTKFSKSRGVGVFGDMAKDTGIPSDVWRFYLLYVRPEGQDSAFSWADMALK

NNSELLNNLGNFINRGGMFVTKFFGGCVPAMELQQEDKTLLAVVGWELQQYIQLMDKVKIRDALKHILNISRHGNQYIQV

NEPWKKIKGGESERQRAGTVTGVSVNIACLLSVMLLPYMPAVSQTIRDQLNAPQTCINTMLQGPGTFVCALRAGHRIGTV

SPLFQKLEADQIEALKKKFGGQQPEDEPSNKKITASTQPAAVPAPLVVAEVKVADGSDPEKAKQLAEAVAEQGDKVRALK

SQKAEKAVITAEVSKLLDLKKQLALAEGKSPEPAPQKGKKK

>XP_017560692.1 PREDICTED: methionine--tRNA synthetase, cytoplasmic isoform X2 [Pygocentrus nattereri]

MKLFVSEGNPHCVKVLAGLEVTGVKCDVQLVSHEERVVPFLNRPVLPVLLLPSGQHLFSPNAICQYLFEVVGKEASDDGN

QLLEWEATELQPAVLQALHMLMLQGKSGEAGTVLKGPLGWLEQYLSKRTTPFLTEETVAVADIVLWAALYPILSNSTFEA

VVGELPSVRGWFKRVSQVPACEAAVQRVLQGKGLDALKNFLQKQPAPHIQRRDSPSSANATEMEDSELMMTEEEVEVAAQ

AWSKGLPDCSDSTQKKHPILPQEGKRNVLVTSALPYVNNVPHLGNIIGCVLSADVFARYGRLRGWNLLYVCGTDEYGTAT

ENKAREEGLTPQEICDKYHAVHSSIYKWFQIDFDYFGRTTTEQQTEIAQDIFWRLHERGYLLEDVVEQLRCENCQRFLAD

RFVEGVCPHCKYPEARGDQCDKCGRLINAVELKEPQCKVCQQTPEIRSSKHLFLDLPKLEGELEQWLEKSMGSGDWTVNA

KHITRSWLRDGLKPRCITRDLQWGTPVPHPDFSDKVFYVWFDAPIGYLSITANYTSEWEKWWKNPQQVELYNFMAKDNVP

FHSVVFPCSLLGAQDNYTLVNNLVATEYLNYEDTKFSKSRGVGVFGDMAKDTGIPSDVWRFYLLYLRPEGQDSAFSWADM

ALKNNSELLNNLGNFINRAGMFVSKFFEGHVPEMVLQDEDKRLIAQVCWELKQYIQLLDKVRIRDALKCILNISRHGNQY

IQVNEPWKKIKGGDDDRKRAGTVTGVSVNVACLLSAMLEPYMPTVSQTIRDQLQAPAHCARAMLYADRGFLCSLSAGHRI

GTVSPLFQKLESEQIEALRKRFGGQQVPSSTVNSAQPKPPAAAAPAVNASVSAADPEKAKQLAAAVAKQGDKVRALKAQK

AEKSVVTAEVTKLLELKKELALAEGKNPEPSLQKGKKK

>XP_018597871.1 PREDICTED: methionine--tRNA synthetase, cytoplasmic [Scleropages formosus]

MSEMKLFVSDGNPHCLKVLAVAELTGVKCDICHASDDERLVPFLSHPVRPVLLLPCGQHLFKANPICLYFLEVSGRDVCD

LTSQWLEWEAAELQPALMQALYQCVLLGKMTEAANTLEGHLTYMEQRLSKGATPFLAAEEATVVDIVLWAALYPLLSNSS

FLPDKLQCVARWFEQVNALPVCQTVAQRVQQQKSLESLKRFLQKQPLPPARCRNAPLCNRAEEEDAEREMTDEEIQAAGA

TWSRGLADRPTPAERRHPILPQEGKRNVLITSALPYVNNVPHLGNIIGCVLSADVFARYGRLRGWNALFVCGTDEYGTAT

ENKAREEGLSPQEICDKYHAVHASIYKWFQIDFDHFGRTTTEKQTEIAQDIFWRLYDRGFLIPDTVDQLHCENCQRFLAD

RFVEGICPNCRYPEARGDQCDKCGRLINAVELKDPQCKVCKRTPVVRSSAHLFLDLPKLESQLEQWLEQSMSTGNWTSNA

RHITRSWLRDGLKPRCITRDLRWGTPVPHPDFSGKVFYVWFDAPIGYLSITASYTDEWEKWWKNPEKVELYNFMAKDNVP

FHSVVFPCSLLGAQDNYTLVSNLIATEYLNYEDTKFSKSRGVGVFGDMAKDTDIPSDVWRFYLLYLRPEGQDSAFSWADM

ALKNNSELLNNLGNFINRAGMFASKFFEGRVPQMELQQEDKRLLAQVGFELRQYIQLLDRVRIRDALKCILNISRHGNQY

IQVNEPWKKIKGGDEDRLRAGTVTGVSVNVACLLSAMLRPYMPAVSATIREQLCAPANCADAMFQGAGSFVCTLPAGHRI

GTISPLFQKLEAEQIESLKRRFGGQQIQSAAPAAATVPASPSATIETSANPERAKQLLQEVAEQGDRVRALKAQKAEKTL

ITAEVAKLLQLKKQLAAAEGKNPEPSPQKGKKK

>NP_001244301.1 methionine--tRNA synthetase, cytoplasmic [Gallus gallus]

MRLRVAAGSPAALKVVAAAHCAGAPVQLCWAEPGEPPPSPLCPPWAPALQLESGAVLFSPNAICQYFFLRRGEQPTDLTT

QWLEWEATELQPAALAALYAHLVHGRTGVEALGEPLGHIERSLVGRGTAYLAGDTESVADVVVWSTLFPVLRDEAIPAAE

PLQSWFRSMTFSEACRNAADTVLLPRHLQEFTACLQKLPLPRPPPDTAPGCEEDDVSERALTEEDMAAAVDAWTRGAAAL

PKPWRPPKPVLPVEGMRNVLITSALPYVNNVPHLGNIIGCVLSADAFARYCRLRNWNTLYVCGTDEYGTATETKAVEEGL

TPREICDKYHAVHADIYRWFDISFDYFGRTTTPQQTVISQDIFQRLQAHGFVLQDTVQQLHCASCQRFLADRFVEGICPF

CSYQEARGDQCDKCGKLINAVELKTPRCKLCSGSPTVRPTQHLFLDLPKLEERLQPWLENCWATGNWTANARYITRSWIR

DGLRPRCITRDLQWGTPVPLDGFRDKVFYVWFDAPIGYLSITANYTEHWERWWKNPQQVELYNFMAKDNVPFHSVVFPCS

LLGTDDNYTLVNHLIATEYLNYEDGKFSKSRGVGVFGDMAKDTGIAADVWRFYLLYVRPEGQDSAFSWGDLMLKNNSELL

NNLGNFINRAGMFVCKFFGSAVPPMVLTPDDHRLLARVTVELRQYHQLLERVRIRDALRCVLNISRHGNQYIQVNEPWKR

IKGGEEDRQRAGTVTGVAVNVAALLAVLVQPYMPTVSAAIQAQLCVPPECSVLSHIFTCTLPPGHRVGTVWGQHRGWREE

GGGGGETPHPPPQLDPPSVPGKTPPDPSRIQELTEQVAKQGNCVRQLKASGSDRARIDSEVGKLLALKRELALAQNMSPD

GPRGKGKK

>XP_011473173.1 methionine--tRNA synthetase, cytoplasmic isoform X2 [Oryzias latipes]

MKLFVSEGNPHCLKVLAALELTGVKCDVQYVNHEEKVVQFLNHPALPALILPAGQQLFSANAICRYLFEVSGQNYNDHCN

QWLEWEVTVLQPALLRALRTAVLQGKGSDVSQILQSPLNFLDQSLLKGSKPYLTGEAVSVADIVLWGALHPILSDSSFTL

GEHNSVKTWFDQVAAVHSCQSAVQKVLQGKGLQAMKSYMQRQPAPQNSQCRDAQPCNNNPAESEERQHFVSEEECEAAVL

AWNTGLSSCPAPDRQHPILPQEGKRNVLVTSALPYVNNVPHLGNIIGCVLSADVFSRYGRLRRWNMLFVCGTDEYGTATE

NKAREEGLTPKQICDKYHAVHSSIYSWFQIDFDFFGRTTTEKQTEIAQNIFWRLHKHGYLVEDTVEQLRCENCQRFLADR

FVEGTCPHCSYPEARGDQCDKCGRLINAVELRDPQCKVCRQTPVIRSSKHLFLDLPKLESQLEQWLEKSTSTGDWTTNAK

QITHSWIRDGLKPRCITRDLHWGTPVPHPDFKEKVFYVWFDAPIGYLSITANYTSEWERWWKNPQQVELYNFMAKDNVPF

HSVVFPCSLLGAQDNYTLVNHLVATEYLNYEDTKFSKSRGTGVFGDMAKDTGIPSDVWRFYLLYVRPEGQDSAFSWADMA

MKNNSELLNNLGNFINRAGMFVTKFFEGCVPVMELQREDKELLAVVGWELQQYIQLMDKVKIRDALKHILNISRHGNQYI

QVNQPWKKIKGEESERLRAGTVTGVAVNIACLLSVMLLPYMPLVSQTIRDQLNAPQSCVYTMLQGSGTFVCSLSAGHRIG

TVSPLFQKLEAEQIEALKKRFGGQQAPLQSTPSTPAAAPAAAIKVAAVEGLDSEKAEQLVKAVAEQGDKVRALKAQKAEK

DVIAAEVSKLLELKTQLALAEGKNLEPTSKKGKKK

>XP_019716615.1 PREDICTED: methionine--tRNA synthetase, cytoplasmic isoform X4 [Hippocampus comes]

MKLFISEGNPHCLKVLAALEEAGVQCDVQFVNHEENLVPFLSRPSLPALLLPNGQHLFSPNAICRYMFEVSGQECSEACD

QWLEWEATDLQPALLQALHMAVLQGKELEATKVLAGPLSYLDQSLTKANTPYLTGDVVSVADLVVCAALHPILSAPSLAS

GDHKSVRLWFDRVAASQSCVSASHKVLQGKGLQAMKSFMQRQPAPQSGQSRGGTHPCNSGPAEDEERERAVSEEEMEAAA

LTWNKGFNASAAATERLHPILPQEGKRNILLTSALPYVNNVPHLGNIIGCVLSADVFSRYSRLRGWNSLFVCGTDEYGTA

TENKAREEGLTPQQICDKYHAIHASIYEWFQIDFDFFGRTTTEKQTEIAQNIFWRLHENGFLLEDVVEQLRCEQCQRFLA

DRFVEGICPHCNYPDARGDQCDKCGRLINAVELKAPHCKVCRQTPVIRSSKHLFLDLPKLEGQLEQWLNKSTSSGDWTAN

AKQITRSWLRDGLKPRCITRDLRWGTPVPHPDFQEKVFYVWFDAPIGYVSITANYTDKWEQWWKNPGQVEMYNFMAKDNV

PFHSVVFPCSLLGAQDNYTLVNHLIATEYLNYEDTKFSKSRGIGVFGDMAKDTGIPSDVWRFYLLYVRPEGQDSAFSWAD

MALKNNSELLNNLGNFINRAGMFVSKFFDGCVPAMELLQEDKKLLALVGWELQQYIQLMDKVKIRDALKHILNISRHGNQ

YIQVNEPWKKIKGGDAERQRAGTVTGVSINVACLLSVMLLPYMPAMSQIIRDQLNAPYSCVSAMLHGSGAFTRVLDPGHR

IGTVSPLFQKLEVEQIEALKKRFGGQQTATQNAANPQSAATLPTPAIEVNAGADPEKARQLTQAVAEQGEKVRALKAQKA

DKATIMAEVSKLLELKKQLALAEGTSPEPTPVKGKKK

>XP_019716614.1 PREDICTED: methionine--tRNA synthetase, cytoplasmic isoform X3 [Hippocampus comes]

MKLFISEGNPHCLKVLAALEEAGVQCDVQFVNHEENLVPFLSRPSLPALLLPNGQHLFSPNAICRYMFEVSGQECSEACD

QWLEWEATDLQPALLQALHMAVLQGKELEATKVLAGPLSYLDQSLTKANTPYLTGDVVSVADLVVCAALHPILSAPSLAS

GDHKSVRLWFDRVAASQSCVSASHKVLQGKGLQAMKSFMQRQPAPQSGQSRGGTHPCNSGPAEDEERERAVSEEEMEAAA

LTWNKGFNASAAATERLHPILPQEGKRNILLTSALPYVNNVPHLGNIIGCVLSADVFSRYSRLRGWNSLFVCGTDEYGTA

TENKAREEGLTPQQICDKYHAIHASIYEWFQIDFDFFGRTTTEKQTEIAQNIFWRLHENGFLLEDVVEQLRCEQCQRFLA

DRFVEGICPHCNYPDARGDQCDKCGRLINAVELKAPHCKVCRQTPVIRSSKHLFLDLPKLEGQLEQWLNKSTSSGDWTAN

AKQITRSWLRDGLKPRCITRDLRWGTPVPHPDFQEKVFYVWFDAPIGYVSITANYTDKWEQWWKNPGQVEMYNFMAKDNV

PFHSVVFPCSLLGAQDNYTLVNHLIATEYLNYEDTKFSKSRGIGVFGDMAKDTGIPSDVWRFYLLYVRPEGQDSAFSWAD

MALKNNSELLNNLGNFINRAGMFVSKFFDGCVPAMELLQEDKKLLALVGWELQQYIQLMDKVKIRDALKHILNISRHGNQ

YIQVNEPWKKIKGGDAERQRAGTVTGVSINVACLLSVMLLPYMPAMSQIIRDQLNAPYSCVSAMLHGSGAFTRVLDPGHR

IGTVSPLFQKLEVEQIEALKKRFGGQQPQTATQNAANPQSAATLPTPAIEVNAGADPEKARQLTQAVAEQGEKVRALKAQ

KADKATIMAEVSKLLELKKQLALAEGTSPEPTPVKGKKK

>XP_017560691.1 PREDICTED: methionine--tRNA synthetase, cytoplasmic isoform X1 [Pygocentrus nattereri]

MKLFVSEGNPHCVKVLAGLEVTGVKCDVQLVSHEERVVPFLNRPVLPVLLLPSGQHLFSPNAICQYLFEVVGKEASDDGN

QLLEWEATELQPAVLQALHMLMLQGKSGEAGTVLKGPLGWLEQYLSKRTTPFLTEETVAVADIVLWAALYPILSNSTFEA

VVGELPSVRGWFKRVSQVPACEAAVQRVLQGKGLDALKNFLQKQPAPHIQRRDSPSSANATEMEDSELMMTEEEVEVAAQ

AWSKGLPDCSDSTQKKHPILPQEGKRNVLVTSALPYVNNVPHLGNIIGCVLSADVFARYGRLRGWNLLYVCGTDEYGTAT

ENKAREEGLTPQEICDKYHAVHSSIYKWFQIDFDYFGRTTTEQQTEIAQDIFWRLHERGYLLEDVVEQLRCENCQRFLAD

RFVEGVCPHCKYPEARGDQCDKCGRLINAVELKEPQCKVCQQTPEIRSSKHLFLDLPKLEGELEQWLEKSMGSGDWTVNA

KHITRSWLRDGLKPRCITRDLQWGTPVPHPDFSDKVFYVWFDAPIGYLSITANYTSEWEKWWKNPQQVELYNFMAKDNVP

FHSVVFPCSLLGAQDNYTLVNNLVATEYLNYEDTKFSKSRGVGVFGDMAKDTGIPSDVWRFYLLYLRPEGQDSAFSWADM

ALKNNSELLNNLGNFINRAGMFVSKFFEGHVPEMVLQDEDKRLIAQVCWELKQYIQLLDKVRIRDALKCILNISRHGNQY

IQVNEPWKKIKGGDDDRKRAGTVTGVSVNVACLLSAMLEPYMPTVSQTIRDQLQAPAHCARAMLYADRGFLCSLSAGHRI

GTVSPLFQKLESEQIEALRKRFGGQQPDEETPTGKVPSSTVNSAQPKPPAAAAPAVNASVSAADPEKAKQLAAAVAKQGD

KVRALKAQKAEKSVVTAEVTKLLELKKELALAEGKNPEPSLQKGKKK

>XP_016097259.1 PREDICTED: methionine--tRNA synthetase, cytoplasmic, partial [Sinocyclocheilus grahami]

MKLYISDGNPHCIKVLAALELTGGTCDTEVVSHEEKVVPHFTRPVSPALLLPDGQHLFSPNSICQYLFDISGQKATDATN

QWLEWEATNLQPAVLQSLHMVALQGKHVEAAAVMKEPLNWLEQSLSKKNTPFLTAEAVSVADVVLWAALFPLLSDSGFDA

GELRSVRGWFQQVGGLEACQTAARRVLQGKGVEALKTFLQKHPATQTQRRDSPTNATEAEDGEHVMTVEEIEEAAQIWSE

GLKASPAVTERKHPVLPQEGKRNVLLTSALPYVNNVPHLGNIIGCVLSADVFARYGRLRGWNLLFICGTDEYGTATENKA

REEGLTPQEICDKYHAVHADIYQWFQIDFDFFGRTTTERQTEIAQDIFWRLHERGFLLEDTVEQLRCESCQRFLADRFVE

GECPHCRYPEARGDQCDKCGRLINAVELRNPQCKVCKETPVIRSSKHLFLNLPKLEDELEQWLDKSTGSGDWTANARHIT

HSWLRDGLKPRCITRDLRWGTPVPHEDYKEKVFYVWFDAPIGYLSITANYTDQWEKWWKNPQQVELYNFMAKDNVPFHSV

VFPCSLLGAQDNYTLVNHLIATEYLNYEDTKFSKSRGVGVFGDMAKDTGIPSDVWRFYLLYLRPEGQDSAFSWSDMALKN

NSELLNNLGNFINRAGMFVSKFFDGCVPEMLLNEDDKRLIAQVRWELKQYIQLLDKVRIRDALKCILNISRHGNQYIQVN

EPWKKIKGGDGDRQRAGTVTGLSVNVACLLSVVLEPYMPSVSYTIRHQLQAPPTCVSAMLQNTGTFICTLPAGHSIGTVS

PLFQKLENEQIEVLRKRFGGQQPEEESAKVKTRVETKPSAGAALNASVNVNADPERAKQLTALVAEQVRTHTLTL

>XP_004068814.1 methionine--tRNA synthetase, cytoplasmic isoform X1 [Oryzias latipes]

MKLFVSEGNPHCLKVLAALELTGVKCDVQYVNHEEKVVQFLNHPALPALILPAGQQLFSANAICRYLFEVSGQNYNDHCN

QWLEWEVTVLQPALLRALRTAVLQGKGSDVSQILQSPLNFLDQSLLKGSKPYLTGEAVSVADIVLWGALHPILSDSSFTL

GEHNSVKTWFDQVAAVHSCQSAVQKVLQGKGLQAMKSYMQRQPAPQNSQCRDAQPCNNNPAESEERQHFVSEEECEAAVL

AWNTGLSSCPAPDRQHPILPQEGKRNVLVTSALPYVNNVPHLGNIIGCVLSADVFSRYGRLRRWNMLFVCGTDEYGTATE

NKAREEGLTPKQICDKYHAVHSSIYSWFQIDFDFFGRTTTEKQTEIAQNIFWRLHKHGYLVEDTVEQLRCENCQRFLADR

FVEGTCPHCSYPEARGDQCDKCGRLINAVELRDPQCKVCRQTPVIRSSKHLFLDLPKLESQLEQWLEKSTSTGDWTTNAK

QITHSWIRDGLKPRCITRDLHWGTPVPHPDFKEKVFYVWFDAPIGYLSITANYTSEWERWWKNPQQVELYNFMAKDNVPF

HSVVFPCSLLGAQDNYTLVNHLVATEYLNYEDTKFSKSRGTGVFGDMAKDTGIPSDVWRFYLLYVRPEGQDSAFSWADMA

MKNNSELLNNLGNFINRAGMFVTKFFEGCVPVMELQREDKELLAVVGWELQQYIQLMDKVKIRDALKHILNISRHGNQYI

QVNQPWKKIKGEESERLRAGTVTGVAVNIACLLSVMLLPYMPLVSQTIRDQLNAPQSCVYTMLQGSGTFVCSLSAGHRIG

TVSPLFQKLEAEQIEALKKRFGGQQPEDEPSNLKAPLQSTPSTPAAAPAAAIKVAAVEGLDSEKAEQLVKAVAEQGDKVR

ALKAQKAEKDVIAAEVSKLLELKTQLALAEGKNLEPTSKKGKKK

>XP_019716613.1 PREDICTED: methionine--tRNA synthetase, cytoplasmic isoform X2 [Hippocampus comes]

MKLFISEGNPHCLKVLAALEEAGVQCDVQFVNHEENLVPFLSRPSLPALLLPNGQHLFSPNAICRYMFEVSGQECSEACD

QWLEWEATDLQPALLQALHMAVLQGKELEATKVLAGPLSYLDQSLTKANTPYLTGDVVSVADLVVCAALHPILSAPSLAS

GDHKSVRLWFDRVAASQSCVSASHKVLQGKGLQAMKSFMQRQPAPQSGQSRGGTHPCNSGPAEDEERERAVSEEEMEAAA

LTWNKGFNASAAATERLHPILPQEGKRNILLTSALPYVNNVPHLGNIIGCVLSADVFSRYSRLRGWNSLFVCGTDEYGTA

TENKAREEGLTPQQICDKYHAIHASIYEWFQIDFDFFGRTTTEKQTEIAQNIFWRLHENGFLLEDVVEQLRCEQCQRFLA

DRFVEGICPHCNYPDARGDQCDKCGRLINAVELKAPHCKVCRQTPVIRSSKHLFLDLPKLEGQLEQWLNKSTSSGDWTAN

AKQITRSWLRDGLKPRCITRDLRWGTPVPHPDFQEKVFYVWFDAPIGYVSITANYTDKWEQWWKNPGQVEMYNFMAKDNV

PFHSVVFPCSLLGAQDNYTLVNHLIATEYLNYEDTKFSKSRGIGVFGDMAKDTGIPSDVWRFYLLYVRPEGQDSAFSWAD

MALKNNSELLNNLGNFINRAGMFVSKFFDGCVPAMELLQEDKKLLALVGWELQQYIQLMDKVKIRDALKHILNISRHGNQ

YIQVNEPWKKIKGGDAERQRAGTVTGVSINVACLLSVMLLPYMPAMSQIIRDQLNAPYSCVSAMLHGSGAFTRVLDPGHR

IGTVSPLFQKLEVEQIEALKKRFGGQQPEDESPNKKTATQNAANPQSAATLPTPAIEVNAGADPEKARQLTQAVAEQGEK

VRALKAQKADKATIMAEVSKLLELKKQLALAEGTSPEPTPVKGKKK

>XP_019716611.1 PREDICTED: methionine--tRNA synthetase, cytoplasmic isoform X1 [Hippocampus comes]

MKLFISEGNPHCLKVLAALEEAGVQCDVQFVNHEENLVPFLSRPSLPALLLPNGQHLFSPNAICRYMFEVSGQECSEACD

QWLEWEATDLQPALLQALHMAVLQGKELEATKVLAGPLSYLDQSLTKANTPYLTGDVVSVADLVVCAALHPILSAPSLAS

GDHKSVRLWFDRVAASQSCVSASHKVLQGKGLQAMKSFMQRQPAPQSGQSRGGTHPCNSGPAEDEERERAVSEEEMEAAA

LTWNKGFNASAAATERLHPILPQEGKRNILLTSALPYVNNVPHLGNIIGCVLSADVFSRYSRLRGWNSLFVCGTDEYGTA

TENKAREEGLTPQQICDKYHAIHASIYEWFQIDFDFFGRTTTEKQTEIAQNIFWRLHENGFLLEDVVEQLRCEQCQRFLA

DRFVEGICPHCNYPDARGDQCDKCGRLINAVELKAPHCKVCRQTPVIRSSKHLFLDLPKLEGQLEQWLNKSTSSGDWTAN

AKQITRSWLRDGLKPRCITRDLRWGTPVPHPDFQEKVFYVWFDAPIGYVSITANYTDKWEQWWKNPGQVEMYNFMAKDNV

PFHSVVFPCSLLGAQDNYTLVNHLIATEYLNYEDTKFSKSRGIGVFGDMAKDTGIPSDVWRFYLLYVRPEGQDSAFSWAD

MALKNNSELLNNLGNFINRAGMFVSKFFDGCVPAMELLQEDKKLLALVGWELQQYIQLMDKVKIRDALKHILNISRHGNQ

YIQVNEPWKKIKGGDAERQRAGTVTGVSINVACLLSVMLLPYMPAMSQIIRDQLNAPYSCVSAMLHGSGAFTRVLDPGHR

IGTVSPLFQKLEVEQIEALKKRFGGQQPEDESPNKKPQTATQNAANPQSAATLPTPAIEVNAGADPEKARQLTQAVAEQG

EKVRALKAQKADKATIMAEVSKLLELKKQLALAEGTSPEPTPVKGKKK

>KFQ21348.1 Methionine--tRNA synthetase, cytoplasmic, partial [Mesitornis unicolor]

LFPVLQDETSLPSELQMLRTWFQSMTLSEACRKAADSVLVPKALLEFKSYLQKQPPPCLAMERVTSNEPEEEEGSERALT

EEEIAAAADAWARGAAVLPKRWDPQKPVLPVEGRRNILITSALPYVNNVPHLGNIIGCVLSADTFARYCRLRGWNTLYVC

GTDEYGTATETKAVEEGLTPQEICDKYNAIHADIYRWFNISFDYFGRTTTPHQTIIAQDIFQRLLARGFLLQDTVEQLRC

EKCQRFLADRFVEGTCPFCSYEEARGDQCDKCGKLINAVELKNPQCKLCRSVPVVKPTQHLFLDLPKLEERLETWLEQSW

ATGDWTANARYITRSWIRDGLKPRCITRDLKWGTPVPLDGFRDKVFYVWFDAPVGYLSITANYTDQWERWWKNPQQVELY

NFMAKDNVPFHSVIFPCSLLGADDNYTLVNHLIATEYLNYEDGKFSKSRGVGVFGDMAKDTGIPADIWRFYLLYLRPEGQ

DSAFSWSDLMLKNNSELLNNLGNFINRAGMFVCKFFGGTVPNMVLTADDKRLLARVTLELRQYHQLLEKVRIRDALRCIL

SISRHGNQYIQVNEPWKRIKGNDKDRERAGTVTGVAVNMASLLAVLLQPYMPSISLTIQGQLCIPPDCFILSHSFTCTLP

PGHRVGTVSPLFQKLENDQVEALRKRFGGGQPKQAPPAPVTTVTPGDHQLIQELTEKVAKQGNHVRQLKASKAERAQVDT

EVAKLLELKKQLALAEGKSPDVPMPKGKRKK

>XP_025001056.1 methionine--tRNA synthetase, cytoplasmic isoform X2 [Gallus gallus]

MRLRVAAGSPAALKVVAAAHCAGAPVQLCWAEPGEPPPSPLCPPWAPALQLESGAVLFSPNAICQYFFLRRGEQPTDLTT

QWLEWEATELQPAALAALYAHLVHGRTGVEALGEPLGHIERSLVGRGTAYLAGDTESVADVVVWSTLFPVLRDEAIPAAE

PLQSWFRSMTFSEACRNAADTVLLPRHLQEFTACLQKLPLPRPPPDTAPGCEEDDVSERALTEEDMAAAVDAWTRGAAAL

PKPWRPPKPVLPVEGMRNVLITSALPYVNNVPHLGNIIGCVLSADAFARYCRLRNWNTLYVCGTDEYGTATETKAVEEGL

TPREICDKYHAVHADIYRWFDISFDYFGRTTTPQQTVISQDIFQRLQAHGFVLQDTVQQLHCASCQRFLADRFVEGICPF

CSYQEARGDQCDKCGKLINAVELKTPRCKLCSGSPTVRPTQHLFLDLPKLEERLQPWLENCWATGNWTANARYITRSWIR

DGLRPRCITRDLQWGTPVPLDGFRDKVFYVWFDAPIGYLSITANYTEHWERWWKNPQQVELYNFMAKDNVPFHSVVFPCS

LLGTDDNYTLVNHLIATEYLNYEDGKFSKSRGVGVFGDMAKDTGIAADVWRFYLLYVRPEGQDSAFSWGDLMLKNNSELL

NNLGNFINRAGMFVCKFFGSAVPPMVLTPDDHRLLARVTVELRQYHQLLERVRIRDALRCVLNISRHGNQYIQVNEPWKR

IKGGEEDRQRAGTVTGVAVNVAALLAVLVQPYMPTVSAAIQAQLCVPPECSVLSHIFTCTLPPGHRVGTVSPLFQKLESE

RIDALRKRFGGGQPQAPPVVLIHHGTTFIRRHRTHTSLSLSPSSGETPHPPPPVGPPLSARQDSP

>XP_003973541.1 PREDICTED: methionine--tRNA synthetase, cytoplasmic [Takifugu rubripes]

MKLFVSEGNPHCLKVLAALEETGVKCDVQYVSHEERVVPFISRPVLPALLPPTGPHLFSSNAICSYLFEVGGKDCSDLCN

QWLEWETTELQPMLLQALHMAVVLEKGSDVSKVLQGPLNYLDQSLSKQNTSYLTGETLSVADIVMWAALYPILSGSSTIL

GERNSVKAWFERGAKLQSIQSAAQKVLQGKGLQGMKTFLQRQPNPQSKEIQTCTSSPAEAEESERVVSEEEMEAAALTWS

KELQDWPLPEDRQHPILPQEDKKNFLVTSALPYVNNVPHLGNIIGCVLSADVFSRYSRLRGWNVLFVCGTDEYGTATENK

AIEEGLTPQEICDKYHAIHSAIYKWFQIDFDFFGRTTTEKQTVIAQDIFWRLHKNNFLLEDTVEQLRCENCQRFLADRFV

EGICPHCAYPEARGDQCDKCGRLINAVELKEPQCKICRKTPIICSSKHLFLDLPKLEIQLEQWLDKSTSTGDWTANAKQI

TRSWLRDGLKPRCITRDLKWGTPVPHPDFKEKVFYVWFDAPIGYLSITANYTDKWEKWWKNPPQVELYNFMAKDNVPFHS

VVFPCSLLGAQDNYTLVNHLVATEYLNYEDTKFSKSRGVGVFGDMAKETDIPCDVWRFYLLYVRPEGQDSSFSWADLATK

NNSELLNNLGNFINRAGMFVTKFFEGCVPAMVLQQEDKKLLALVAWELQQYIQLMDKVKIRDSLKHILNISRHGNQYIQV

NEPWKNIKAGDAERQRAGTVTGVSVNIACLLSVMLYPYMPTTSQTIRDQLNCPQSVIGTMLRGNGNFVCALGAGHRVGAV

SPLFQKLEGDQIEALKKRFSGQQTTAQKVASSEAATAPAPAAEATAAVADPERAKQLSQAVVEQGEKVRNLKAQKAEKAL

ITAEVTLLLDLKKQLVLAEGKTPEPAPQKGKKK

>XP_010178800.1 PREDICTED: methionine--tRNA synthetase, cytoplasmic, partial [Mesitornis unicolor]

PVLQDETSLPSELQMLRTWFQSMTLSEACRKAADSVLVPKALLEFKSYLQKQPPPCLAMEREEEGSERALTEEEIAAAAD

AWARGAAVLPKRWDPQKPVLPVEGRRNILITSALPYVNNVPHLGNIIGCVLSADTFARYCRLRGWNTLYVCGTDEYGTAT

ETKAVEEGLTPQEICDKYNAIHADIYRWFNISFDYFGRTTTPHQTIIAQDIFQRLLARGFLLQDTVEQLRCEKCQRFLAD

RFVEGTCPFCSYEEARGDQCDKCGKLINAVELKNPQCKLCRSVPVVKPTQHLFLDLPKLEERLETWLEQSWATGDWTANA

RYITRSWIRDGLKPRCITRDLKWGTPVPLDGFRDKVFYVWFDAPVGYLSITANYTDQWERWWKNPQQVELYNFMAKDNVP

FHSVIFPCSLLGADDNYTLVNHLIATEYLNYEDGKFSKSRGVGVFGDMAKDTGIPADIWRFYLLYLRPEGQDSAFSWSDL

MLKNNSELLNNLGNFINRAGMFVCKFFGGTVPNMVLTADDKRLLARVTLELRQYHQLLEKVRIRDALRCILSISRHGNQY

IQVNEPWKRIKGNDKDRERAGTVTGVAVNMASLLAVLLQPYMPSISLTIQGQLCIPPDCFILSHSFTCTLPPGHRVGTVS

PLFQKLENDQVEALRKRFGGGQPKQAPPAPVTTVTPGDHQLIQELTEKVAKQGNHVRQLKASKAERAQVDTEVAKLLELK

KQLALAEGKSPDVPMPKGKRKK

>XP_015276844.1 PREDICTED: LOW QUALITY PROTEIN: methionine--tRNA synthetase, cytoplasmic [Gekko japonicus]

MRLFVSEGSPGGLKVLAAAKATDTPVELQWVPHDASVVPFLTLPRVPALLLEGGAFMFSPNAICLYFFFSSGKEASNFTQ

AQRNFSIQLLEWEATELQPAVSVALYHHVVQGKKGEDVLGIVRQPLNYIDQTLTKKNTPYLNGDAESTVDIVFWGALFPL

LQDGTVLPDDLKVLRNWFQNMSLKEHCKKTVETVWTRKGVQELKAHLQKQSGPSLAFEKPATDEPKEEEPLQRLSEEEIA

AVAEVWSRGTAALPKPWKPQQPVVPVEGMRNVLITSALPYVNNVPHLGNIIGCVLSADTFARYSRLRNWNTLYLCGTDEY

GTATETKAMEEGLSPQQICDKYHVVHAQIYQWFNISFDYFGRTTTPQQTRIAQGIFQQLLEHNYLLTETVDQLKCEHCDR

FLADRFVEGVCPFCNYEEARGDQCDKCGKLINATELKKPLCKVCQRTPVVKPSQHLFLDLPRLEDRLEKWLTLSQSTGDW

TPNAKFISRAWIRDGLKPRCITRDLKWGTPVPLDGFHDKVELYNFMRRVPLLVAGHRGKLYLEYLNYEDGKFSKSRGIGV

FGDMAKDTGIPADIWRFYLLFVRPESQDSAFSWNDLMLKNNSELLNNLGNFVNRGNRKQGICWAVLWSVVFPCSLLGTGE

NYTLVSHLIATEYLNYEDGKFSKSRGVGVFGDMAKDTGIPADIWRFYLLFVRPESQDSAFSWNDLMLKNNSELLNNLGNF

VNRAGSFVCKFFGGRVPDMELNQDDKRLLAHITLELRQYNQLLEKVRIRDALRSILSISRHGNQYLQVNEPWKRIKGNDA

ERKRAGTVIGVAVNIAALLSVMVQPYMPSVSTAIQGQFCIPXHNVVTNDFVCTLPAGHQIGTVTPLFQKLESEQIENLRK

RFGGGQLEDFLLKPKQKNVTVAEKGVKVLQEEVTKQDNYVRQLKAQKAEKKQIDAAVSKLTELQKQLCSADGKNPAPQAP

KGKGKK

>XP_009633824.1 PREDICTED: methionine--tRNA synthetase, cytoplasmic [Egretta garzetta]

MDQPQGMAWAGPPGLGPPQPDSASLAQDTKSVADVEVWGTLFPVLRDETSLPNDLQALRTWFQSMTLLEACRKAADSVLM

PKALLEFKSYLQKQPPPCLAVERATTNEPEEEEDSEHALTEEEITAAADAWARGAAALPKPWKQQKPVLPVEGMRNVLIT

SALPYVNNVPHLGNIIGCVLSADTFARYCRLRNWNTLYVCGTDEYGTATETKAMEEGLTPQEICNKYNAIHADIYRWFDI

SFDYFGRTTTPQQTMIAQDIFQRLLARGFLLQDTVEQLCCEGCQRFLADRFVEGTCPFCSYEEARGDQCDKCGKLINAVE

LKNPQCKLCRGVPVVKPTQHLFLDLPKLEERLEPWLEQSWATGDWTANARYITRSWIRDGLKPRCITRDLKWGTPVPLDG

FRDKVFYVWFDAPIGYLSITANYTDQWERWWKNPQQVELYNFMAKDNVPFHSVIFPCSLLGADDNYTLVNHLIATEYLNY

EDGKFSKSRGVGVFGDMAKETGIPADIWRFYLLYLRPEGQDTAFSWSDLMLKNNSELLNNLGNFINRAGMFVCKFFGGIV

PDMVLTQDDKRLLARVTLELCQYHQLLEKVRIRDALRCILSISRHGNQYIQVNEPWKRIKGDETDRQRAGTVTGVAVNMA

SMLAVLLQPYMPSVSLAIQGQLCIPPDCFVLSHCFTCTLPPGHRVGTVSPLFQKLENSQIEALRKRFGGGQGNHVRQLKA

NKAEKAQVDMEVAKLLELKKQLALAEGKSPEVPVPKGKRKK

>XP_008937665.1 PREDICTED: methionine--tRNA synthetase, cytoplasmic, partial [Merops nubicus]

EAVEALGNLLAHLEQNLSERGTAYLAGDTESVADGADSLLMPNALLELKSYLQKQPPPCLAMERTTSNEPEEEEGSEHSL

TEEEITAAVDAWARGAAALPKPWQPQKPVLPVEGMRNVLITSALPYVNNVPHLGNIIGCVLSADTFARYCRLRNWNTLYI

CGTDEYGTATETKAVEEGLTPQEICNKYNAIHADIYRWFNISFDYFGRTTTPHQTTIAQDIFQRLLAHGFLLQDTVEQLR

CEDCQQFLADRFVEGTCPFCSYEEARGDQCDKCGKLINAVELKNPQCKLCRGVPVVKPTQHLFLDLPKLEERLEPWLEQS

WATGDWTANARYITRSWIRDGLKPRCITRDLKWGTPVPLDGFRDKVFYVWFDAPIGYLSITANYTDQWERWWKNPQQVEL

YNFMAKDNVPFHSVIFPCSLLGADDNYTLVNHLIATEYLNYEDGKFSKSRGVGVFGDMAKDTGIPSDIWRFYLLYLRPEG

QDSAFSWSDLMLKNNSELLNNLGNFINRAGMFVCKFFSGTVPNMVLTPDDKRLLARVTLELHQYHQLLEKVRIRDALRCI

LSISRHGNQYIQVNEPWKRIKGDEKDRQRAGTVTGVAVNMASLLAVLLQPYMPSVSLAIQAQLCIPPDCFVLSHDFTCTL

PSGHRVGTVSPLFQKLENDQVEALRRRFGGGQVSWAAVAPQLWASPPPEIPVAPGDPQLIQELTEKVAKQGNHIRQLKAN

KAEKAQVDTEVAKLLELKKQLALAEGKTPEVLVPKGKRKK

>KFQ28870.1 Methionine--tRNA synthetase, cytoplasmic, partial [Merops nubicus]

KSADSLLMPNALLELKSYLQKQPPPCLAMERTTSNEPEEEEGSEHSLTEEEITAAVDAWARGAAALPKPWQPQKPVLPVE

GMRNVLITSALPYVNNVPHLGNIIGCVLSADTFARYCRLRNWNTLYICGTDEYGTATETKAVEEGLTPQEICNKYNAIHA

DIYRWFNISFDYFGRTTTPHQTTIAQDIFQRLLAHGFLLQDTVEQLRCEDCQQFLADRFVEGTCPFCSYEEARGDQCDKC

GKLINAVELKNPQCKLCRGVPVVKPTQHLFLDLPKLEERLEPWLEQSWATGDWTANARYITRSWIRDGLKPRCITRDLKW

GTPVPLDGFRDKVFYVWFDAPIGYLSITANYTDQWERWWKNPQQVELYNFMAKDNVPFHSVIFPCSLLGADDNYTLVNHL

IATEYLNYEDGKFSKSRGVGVFGDMAKDTGIPSDIWRFYLLYLRPEGQDSAFSWSDLMLKNNSELLNNLGNFINRAGMFV

CKFFSGTVPNMVLTPDDKRLLARVTLELHQYHQLLEKVRIRDALRCILSISRHGNQYIQVNEPWKRIKGDEKDRQRAGTV

TGVAVNMASLLAVLLQPYMPSVSLAIQAQLCIPPDCFVLSHDFTCTLPSGHRVGTVSPLFQKLENDQVEALRRRFGGGQA

KQASPPPEIPVAPGDPQLIQELTEKVAKQGNHIRQLKANKAEKAQVDTEVAKLLELKKQLALAEGKTPEVLVPKGKRKK

>BAG53819.1 unnamed protein product [Homo sapiens]

MRLFVSDGVPGCLPVLAAAGRARGRAEVLISTVGPEDCVVPFLTRPKVPVLQLDSGNYLFSTSAICRYFFLLSGWEQDDL

TNQWLEWEATELQPALSAALYYLVVQGKKGEDVLGSVRRALTHIDHSLSRQNCPFLAGETESLADIVLWGALYPLLQDPA

YLPEELSALHSWFQTLSTQEPCQRAAETVLKQQGVLALRPYLQKQPQPSPAEGRAVTNEPEEEELATLSEEEIAMAVTAW

EKGLESLPPLRPQQNPVLPVAGERNVLITSALPYVNNVPHLGNIIGCVLSADVFARYSRLRQWNTLYLCGTDEYGTATET

KALEEGLTPQEICDKYHIIHADIYRWFNISFDIFGRTTTPQQTKITQDIFQQLLKRGFVLQDTVEQLRCEHCARFLADRF

VEGVCPFCGYEEARGDQCDKCGKLINAVELKKPQCKVCRSCPVVQSSQHLFLDLPKLEKRLEEWLGRTLPGSDWTPNAQF

ITRSWLRDGLKPRCITRDLKWGTPVPLEGFEDKVFYVWFDATIGYLSITANYTDQWERWWKNPEQP

>XP_016333003.1 PREDICTED: methionine--tRNA synthetase, cytoplasmic-like [Sinocyclocheilus anshuiensis]

MKLYIAEGNPQCVKVLAALELTGVKCEIEAVSHEEKVVPPEPVSPVWRHNGLFACLHRYLFDISGQKATDATNEWLEWEA

TSLQPAVLQSLHMVALQGKRVEAAAVMKEPLKWLEQSLSKINSSFLTAEAVSVADVVLWAALYPLLSDSCFDVGELRSVR

GWFERVGDLAACQAAANRVLHGKGVEALKTFLQKHPATQTQRRDSPTNATEAEVAEHVITVEEIEEAAQIWSEGLKGSPA

VTERKHPVLPQEGKRNMLLTSALPYVNNVPHLGNIIGCVLSADVFARYGRLRGWNLLYICGTDEYGTATENKAREEGLTP

QEICDKYHAIHADIYKWFQIDFDFFGRTTTKQQTEIAQDIFWRLHERGFLLEDTVEQLRCESCQRFLADRFVEGECPHCR

YPEARGDQCDKCGRLINAVELRNPQCKVCKETPVIRSSKHLFLNLPKLEDELEQWLDKSTSSGDWTVNARHITRSWIRDG

LKPRCITRDLQWGTPVPHEDYKEKVFYVWFDAPIGYLSITANYTDQWEKWWKNPQQVELYNFMAKDNVPFHSVVFPCSLL

GAQDNYTLVNHLIATEYLNYEDTKFSKSRGVGVFGDMAKDTGIPSDVWRFYLLYLRPEGQDSAFSWSDMALKNNSELLNN

LGNFINRAGMFVSKFFDGCVPEMLLNEDDKRLIAQVRWELKQYIQLLDKVRIRDALKCILSISRHGNQYMQLNEPWKKIK

GGDGDRQRAGTVTGVSVNVACLLSAVLEPYMPSVSHTIRQQLQAPHTCVSAILQNTGTFICTLPAGHRISAVSPLFQKLE

NEQIEVLRKRFGGQQAPSSAAETKPSTGAVHDASVNVNADPERAKQLTALVAEQGNKLRGLKAQKADKSVIDDEVEKLLQ

LKKQLSLAEGKNPEPAAQKGKKK

>XP_025001058.1 methionine--tRNA synthetase, cytoplasmic isoform X4 [Gallus gallus]

MAGVGGHRAPAALAALYAHLVHGRTGVEALGEPLGHIERSLVGRGTAYLAGDTESVADVVVWSTLFPVLRDEAIPAAEPL

QSWFRSMTFSEACRNAADTVLLPRHLQEFTACLQKLPLPRPPPDTAPGCEEDDVSERALTEEDMAAAVDAWTRGAAALPK

PWRPPKPVLPVEGMRNVLITSALPYVNNVPHLGNIIGCVLSADAFARYCRLRNWNTLYVCGTDEYGTATETKAVEEGLTP

REICDKYHAVHADIYRWFDISFDYFGRTTTPQQTVISQDIFQRLQAHGFVLQDTVQQLHCASCQRFLADRFVEGICPFCS

YQEARGDQCDKCGKLINAVELKTPRCKLCSGSPTVRPTQHLFLDLPKLEERLQPWLENCWATGNWTANARYITRSWIRDG

LRPRCITRDLQWGTPVPLDGFRDKVFYVWFDAPIGYLSITANYTEHWERWWKNPQQVELYNFMAKDNVPFHSVVFPCSLL

GTDDNYTLVNHLIATEYLNYEDGKFSKSRGVGVFGDMAKDTGIAADVWRFYLLYVRPEGQDSAFSWGDLMLKNNSELLNN

LGNFINRAGMFVCKFFGSAVPPMVLTPDDHRLLARVTVELRQYHQLLERVRIRDALRCVLNISRHGNQYIQVNEPWKRIK

GGEEDRQRAGTVTGVAVNVAALLAVLVQPYMPTVSAAIQAQLCVPPECSVLSHIFTCTLPPGHRVGTVSPLFQKLESERI

DALRKRFGGGQVKPPIPPPQLDPPSVPGKTPPDPSRIQELTEQVAKQGNCVRQLKASGSDRARIDSEVGKLLALKRELAL

AQNMSPDGPRGKGKK

>PNI39251.1 MARS isoform 1 [Pan troglodytes]

MRLFVSEGVPGCLPVLAAAGRARGRAEVLISTVGPEDCVVPFLTRPKVPVLQLDSGNYLFSTSAICRYFFLLSGWEQDDL

TNQWLEWEATELQPALSAALYYLVVQGKKGEDVLGSVRRALTHIDHSLSRQNCPFLAGETESLADIVLWGALYPLLQDPA

YLPEELSALHSWFQTLSTQEPCQRAAETVLKQQGVLALRPYLQKQPQPSPTEGRAVTNEPEEEELATLSEEEIAMAVTAW

EKGLESLPPLRPQQNPVLPVAGERNVLITSALPYVNNVPHLGNIIGCVLSADVFARYSRLRQWNTLYLCGTDEYGTATET

KALEEGLTPQEICDKYHIIHADIYRWFNISFDIFGRTTTPQQTKITQDIFQQLLKRGFVLQDTVEQLRCEHCARFLADRF

VEGVCPFCGYEEARGDQCDKCGKLINAVELKKPQCKVCRSCPVVQSTQHLFLDLPKLEKRLEEWLGRTLPGSDWTPNAQF

ITRSWLRDGLKPRCITRDLKWGTPVPLEGFEDKVFYVWFDATIGYLSITANYTDQWERWWKNPEQP

>XP_006783493.1 PREDICTED: LOW QUALITY PROTEIN: methionine--tRNA synthetase, cytoplasmic-like [Neolamprologus brichardi]

MNLFVSEGNPHCLKALAALEATGVQCGVQFVNHDERVVPFLSRPTLPALALPSGQYLFSSNAICRYLFEVNGQECSELCD

QWLEWEATVLQDSVSAADLVLWAGFFPVLSDSSLTLGEHKSLKAWFDRVAAMNACQSAAQKVLQGKGLQGMKSYMQKQPA

PQSSQCRETQPCNSNSAEEEAGERVVSEEEMEAAALTWSKGLNTCPPATKKQHPILPQEGKRNVLVTSALPYVNNVPHLG

NIIGCVLSADVFSRYGHLRGWNMLFVCGTDEYGTATENKAREEGLTPQQICDKYHAVHSSIYKWFQIDFDYFGRTTTEKQ

TEXKXXXXXXXDIFWRLHERGFLIEDTVEQLRCENCQRFLADRFVEGICPHCSYPEARGDQCDKCGRLINAVDLREPQCK

VCRQTPVIRSSKHLFLDLPKLESQLEQWLEKSTSTGDWTANAKQITRSWLRDGLKPRCITRDLQWGTPVPHADFKEKVFY

VWFDAPIGYLSITANYTSEWEKWWKNPHQVELYNFMAKDNVPFHSVVFPCSLLGAQDNYTLVNHLIATEYLNYEDTKFSK

SRGVGVFGDMAQDTGIPSDVWRFYLLYVRPEGQDSAFSWADMALKNNSELLNNLGNFINRAGMFVTKFFEGCVPVMELQE

EDKKLLALVSWELQQYIQLMDKIKIRDALKHILNISRHGNQYIQVNEPWKKIKGGDSERQRAGTVTGVSVNIACLLSVML

SPYMPAVSQTIRDQLNAPPSCINTMLEGNCTFVCSLSAGHRIGAVSPLFQKLEADQIEALKKKFGGQQPEDEPPKKKTTV

RNAASAQSAAVAAPAVAVEVGTVNQVDPEKAEQLTQAVAEQGEKVRALKDQKAEKAVITAEVTKLLDLKKQLALAEGKNP

EPAPQKGKKK

>CAF96510.1 unnamed protein product [Tetraodon nigroviridis]

MKLFVSEGNPHCLKVLAALEDTGVKCDVQFVNHEVXXXXXXXXXXXXXXXXXXXXXXXXXXXXXXXXXXXXXXXXXXXXX

XXXXXXXXXXXXXXXXXXXXXXXXXXXXXXXXXXXXXXXXXXXXXXXXXXXXXXXXXXXXXXXXXXXXXXXXXXXXXXXX

XXXXXXXXXXXXXXXXXXXXXXXXXXXXXXXXXXXXXXXXXXXXXXXXXXXXXXXXXXXXXXXXXXXXXXXXXXXXXXXX

XXXXXXXXXXXXXXXXXXXXXXXXXXXXXXXXXXXXXXXXXXXXXXXXXXXXXXXXXXXXXXXXXXXXXXXXXXXXXXXX

XXXXXXXXXXXXXXXXXXXXXXXXXXXXXXXXXXXXXXXXXXXXYLNIFLPVYLSICVCVYIYIYGYFFFSFPPIYTPRY

LFEVSGKGCTDLCNQWLEWETADLQPALQHALHVAVVLEKGSEVSKVLQGPLNHLEQSLTKASTAYLTGESVSIADVVLW

AALYPVLSDPSIPLGERKTVKAWFERGAKLRSFQTAAQKVLQGKGLQGMKTYLQKQPIHLSTQCKEPCTNNPFEGEELER

AVTEEEMEAAAITWSKKLPDCPSAEDRQHPILPQKDKKNVLVTSALPYVNNVPHLGNIIGCVLSADVFSRYSRLRGWNVL

YLCGTDEYGTATETKALEEGLTPLEICDKYHAVHAAIYKWFQIDFDFFGRTTTEKQTEIAQDIFWRLHKNDFLLEDTVEQ

LRCDNCERFLADRFVEGICPHCAFPEARGDQCDQCGRLINAIELKEPQCKVCRKTPAIRSSKHLFLDLPKLETELEEWLD

KSISTGDWTTNSKQITRSWLRDGLKPRCITRDLKWGTPVPHEDFKDKVFYVWFDAPIGYLSITANYTDEWEKWWKNPTQV

ELYNFMAKDNVPFHSVVFPCSLLGARDNYTLVNHLVATEYLNYEDTKFSKSRGVGVFGDMAKDTDIPCDVWRFYLLYVRP

EGQDSSFSWTDLATKNNSELLNNLGNFINRAGMFVTKFFEGCVPAMVLQQEDKKLLALVGWELQQYIQLMDRVKIRDALK

HILNISRHGNQYIQINEPWKKIKGADADRQRAGTVTGVSVNIACLLSVMLSPYMPIVSQTIRDQLNCPPNVLSAMLVGSG

NFVCTLSAGHRVGTVSPLFQKLEMDQIEALKKRFSGAQTEPNAASGQAAAVPTTAAAADPERAKQLSQAVVKQGEKVRDL

KAQKAEETLITAEVNKLLDLKHQLSLAEGKGPEPAPQKGKKK

>XP_023651135.1 methionine--tRNA synthetase, cytoplasmic isoform X3 [Paramormyrops kingsleyae]

MKLYLSEGNPQCMKVLAAVELTGAKCDIHHAHPEERIVPTLSRPVLPVLQPSVGVHLFMPNAICRYLFELSGQEASSQTD

QWLEWEATELQPALMQALHLSVLQAKRTEAAEALEGPLRFLEQSLSKKPTAFLTAETATVADIVLWGALYPILSDASFLP

GDSQHVAAWFERVTALPACQAAAGSILNEKGGKAALRGFVQRQPVPHTQRRDAQPCSQPEENSSEHEMTEEEIQAAEETW

RRQVADFPAPAERQHPILPQEGHQNVLITSALPYVNNVPHLGNIIGCVLSADVFARYGRLRGWNVLFVCGTDEYGTATEN

KAREEGLTPREICDKYHKVHADIYSWFQIDFDYFGRTTTDQQTEIAQDIFWRLHQHGFLVSDMVDQLRCENCERFLADRF

VEGVCPHCRYPEARGDQCDKCGRLINAVELKDPQCKVCRQTPVVRSSKHLFLDLPKLESQLDQWLDQSMSSGNWTSNARH

ITRSWLRDGLKPRCITRDLQWGTPVPHPDFSGKVFYVWFDAPIGYLSITANYTDQWEKWWKNPKQVELYNFMAKDNVPFH

SVVFPCSLLGAQDNYTLVNNLIATEYLNYEDTKFSKSRGVGVFGDMAKDTGIPADVWRFYLLYLRPEGQDSAFSWSDMAL

KNNSELLNNLGNFVNRAGMFVTKFFDGCVPEMVLQQEDLRLLAQVGWELRQYIQLLDRVRIRDALKCILNISRHGNQYIQ

MNEPWKKIKGGSQDRQRASTVTGVSVNVACLLSAMLQPYMPTVSATICAQLRAPPNCADAMLRGAGAFICTLPCGHRIGT

ISPLFQKLEADQIEALRKEFGGQQFPNSPPLSLSLSQLARGRGAQKSGTGSAQSQRHTTCSHGFRGERHRS

>XP_016335172.1 PREDICTED: methionine--tRNA synthetase, cytoplasmic-like [Sinocyclocheilus anshuiensis]

MKLYIADGNPHCIKVLAALELTGGTCDTAVVSHEEKVVPHFTRPVSPALLLPDGQHLFSPNSICQYLFDISGQKATDATN

QWLEWEATNLQPAVLQSLHMVALQGKHVEAAAVMKEPLNWLEQSLSKKNTPFLTAVSPVHHKHTHLCLCEEPAVLQSLHM

VALQGKHVEAAAVMKEPLNWLEQSLSKKNTPFLTAAEDGEHVMTVEEIEEAAQIWSEGLKGSPAVTERKHPVLPQEGKRN

VLLTSALPYVNNVPHLGNIIGCVLSADVFARYGRLRGWNLLFICGTDEYGTATENKAREEGLTPQEICDKYHAVHADIYQ

WFQIDFDFFGRTTTERQTEIAQDIFWRLHGRGFLLEDTVEQLRCESCQRFLADRFVEGECPHCRYPEARGDQCDKCGRLI

NAVELRNPQCKVCKETPVIRSSKHLFLNLPKLEDELEQWLDKSTGSGDWTANARHITHSWLRDGLKPRCITRDLRWGTPV

PHEEYKEKVFYVWFDAPIGYLSITANYTDQWEKWWKNPQQVELYNFMAKDNVPFHSVVFPCSLLGAQDNYTLVNHLIATE

YLNYEDTKFSKSRGVGVFGDMAKDTGIPSDVWRFYLLYLRPEGQDSAFSWSDMALKNNSELLNNLGNFINRAGMFVSKFF

DGCVPEMLLNEDDNRLIAQVRWELKQYIQLLDKVRIRDALKCILNISRHGNQYIQVNEPWKKIKGGDGDRQRAGTVTGVS

VNVACLLSVVLEPYMPSVSHTIRQQLQAPPTCVSAMLQNTGTFICTLPAGHCIGTVSPLFQKLENEQIEVLRKRFGGQQP

EEESAKVKARVETKPSAGAAQDASVNVNADPERAKQLTALVAEQGNKVRGLKAQKAEQSVIDEAVETLLDLKKQLSLAEG

KNPEPAAQKRKKK

>KQK84961.1 methionine--tRNA synthetase, cytoplasmic [Amazona aestiva]

MRCCLCLLPPPTEELTSTSVEHLIINPNAAYEKFRDKRLGTEGVDFSDRITKSRTTGYESGEYEMMAELEKRLAQLEATV

RCEPDSQNPLLVGLKGSSLMETVQVLQAKVNILDVAVLDQVEARLQSVLGKVNEIAKHKSTVQDADTQSKVGEDLGAKET

PQQRYQRLQYEVQELAREVEQIQSTVKEVAAEEELTPMALARQVEGLKQQLVSSHLEKLLGPTAAIDFADPDGALAKAAA

DTVLVPEALLEFTSCLQKQPPPCPAVQRATSNEPEEEEGSERALTEDEIAAAADAWARGAAALPRPWQPQEPVLPVEGMR

NVLITSALPYVNNVPHLGNIIGCVLSADTFARYCRLRNWNTLYVCGTDEYGTATETKAVEEGLTPQEICDKYNAIHADIY

RWFGISFDCFGRTTTPSQTTIAQDIFQRLLARGFLLQDTVEQLRCEGCQRFLADRFVEGTCPFCGYEEARGDQCDKCGKL

INAVELKSPHCKLCRGVPIVKPTQHLFLDLPKLEQRLEAWLEQSWATGDWTANARYITRSWIRDGLKPRCITRDLKWGTP

VPLDGFRDKVFYVWFDAPIGYLSITANYTPEWERWWKNPQQVELYNFMAKDNVPFHSVIFPCSLLGADDNYTLVNHLIAT

EYLNYEDGKFSKSRGVGVFGDMAKDTGIPADVWRFYLLYLRPEGQDSAFSWSDLMLKNNSELLNNLGNFINRAGMFVCKF

FGGAVPSMVLTPDDKRLLARVTLELRRYHQLLEKVRIRDALRCILSISRHGNQYIQVNEPWKRIKGDEQDRQRAGTVTGV

AVNMASLLAVMLQPYMPSISVAIQGQLRIPAECFVLSXDFTCTLPPGHRVGTVSPLFQKLENDRVEALRRRFGGAQPEDL

AVEPQAKQTPPAPETPVAPTDPQRLQELTQEVARQGNHVRQLKAHKAEKAQIAVEVAKLLELKQQLALAEGKSPQVPAPK

GKQK

>XP_010168847.1 PREDICTED: methionine--tRNA synthetase, cytoplasmic, partial [Antrostomus carolinensis]

ETLGKLLAHIEQNLYRRGTAYLAGDTKSVADVVVWSTLFPVLQDETSLPSELQALRTWFQRMTLSEACRKAADSVLMPKA

LLEFKSYLQKQPPPCLAMERVTSNEPEEEEGSERALTEEEITAAADAWARGAEALPKPWQPQKPVLPVEGMRNVLITSAL

PYVNNVPHLGNIIGCVLSADTFARYCRLRNWNTLYVCGTDEYGTATETKAVEEGLTPQEICDKYNAIHTDIYHWFNISFD

YFGRTTTPYQTTIAQDIFQRLLARGFLLQDTVEQLQSFCSYEEARGDQCDKCGKLINAVELKNPQCKLCRGVPVVKPTQH

LFLDLPKLEKQLEPWLEQSWATGDWTANARYITRSWIRDGLKPRCITRDLKWGTPVPLDGFRDKVFYVWFDAPIGYLSIT

ANYTDQWERWWKNPQQVELYNFMAKDNVPFHSVIFPCSLLGADDNYTLVNHLIATEYLNYENGKFSKSRGVGVFGDMAKD

TGIPADIWRFYLLYLRPEGQDSAFSWSDLMIKNNSELLNNLGNFINRAGMFVCKFFGGTVPNMVLTLDDKRLLARVTLEL

RQYHQLLEKVRIRDALRCILSISRHGNQYIQVNEPWKRIKGDEKDRQRAGTVTGVAVNMAFLPAGMFQPLIXXXXXXXXX

XYMPTVSSAIQGQLCIPSDCFVLSHDFTCTLPPGHRVGTVSPLFQKLENDQIETLRKRFGGGQPDDLAVEPQAKQTPPTP

ATPITPGDPQLIQELSEEVAKQGNYVRQLKANKAEKAQIDTEVAKLLELKRQLALAEGKSPDVPVPKGKQKK

>PNJ44581.1 MARS isoform 1 [Pongo abelii]

MRLFVSEGVPGCLPVLAAAGRARGRAEVLISTVGPEDCVVPFLTRPKVPVLQLDSGNYLFSTNAICRYFFLLSGWEQDDL

TNQWLEWEATQLQPTLSAALYYLVVQGKKGEDVLGSVRRALTHIDHSLSRQNCPFLAGETESLADIVLWGALYPLLQDPA

YLPEELSALHSWFQTLSTQEPCQQAAETVLKQQGVLALRPYLQKQPQPSSPEGRAVTNEPEEEELATLSEEEIAMAVTAW

EKGLESLPPLRPHQNPVLPVAGERNVLITSALPYVNNVPHLGNLIGCVLSADVFARYSRLRQWNTLYLCGTDEYGTATET

KALEEGLTPQEICDKYHIIHADIYRWFNISFDIFGRTTTPQQTKITQDIFQRLLKRGFVLQDTVEQLRCEHCARFLADRF

VEGVCPFCGYEEARGDQCDKCGKLINAIELKKPQCKVCRSCPVVQSSQHLFLDLPKLEKRLEEWLGRTLPGSDWTPNARF

IIRSWLRDGLKPRCITRDLKWGTPVPLEGFEDKVFYVWFDATFGYLSITANYTDQWERWWKNPEQP

>XP_016431335.1 PREDICTED: methionine--tRNA synthetase, cytoplasmic-like [Sinocyclocheilus rhinocerous]

MKLYIADGNPHCIKVLAALELTGGKCDTEVVSHEEKVVPHFTHPVSPALLLPDGQHLFSPNSICQYLFDISGQKATDATN

QWLEWEATNLQPAVLQSLHMVALQGKHVEAAAVMKEPLNWLEQSLSKNNTPFLTAEAVSVADVVLWAALYPLLSDSGFDA

GELRSVRGWFQRVGGLEACQTAARRVLQGKGVEALKTFLQKHPATQTPRRDSPTNATEAEDGEHVMTVEEIEEAAQIWSE

GLKASPAVTERKHPVLPQEGKRNVLLTSALPYVNNVPHLGNIIGCVLSADVFARYGRLRGWNLLYICGTDEYGTATENKA

REEGLMPQEICDKYHAVHADIYQWFQIDFDFFGRTTTERQTEIAQDIFWRLHERGFLLEDTVEQLRCESCQRFLADRFVE

GECPHCRYPEARGDQCDKCGRLINAVELRNPQCKVCKETPVIRSSKHLFLNLPKLEDELEQWLDKSTGSGDWTANARHIT

HSWLRDGLKPRCITRDLRWGTPVPHEEYKEKVFYVWFDAPIGYLSITANYTDQWEKWWKNPQQVELYNFMAKDNVPFHSV

VFPCSLLGAQDNYTLVNHLIATEYLNYEDTKFSKSRGVGVFGDMAKDTGIPSDVWHFNQFATFSSHQSCCWSLLRAGMFV

SKFFDGCVPEMLLNEDDKRLIAEVHWELKQYIQLLDKVRIRDALKCILNISRHGNQYIQVNEPWKKIKGGDGDRQRAGTV

TGVSVNVACLLSVVLEPYMPSVSRTIRQQLQAPPTCVSAMLQNTGTFICTLPAGHCIGTVSPLFQKLENEQIEVLRKRFG

GQQPEEESAKVKTRVETKPSAGAAQNASVNVNADPERAKQLTALVAEQGNKVRGLKAQKAEQSVIDEAVEALLDLKKQLS

LAEGKNPEPAAQKRKKK

>XP_013926569.1 PREDICTED: methionine--tRNA synthetase, cytoplasmic [Thamnophis sirtalis]

MKAHSPSTNVISQYFFRLSGKEISSFSNEQSDFANQFFEWDITELQPALSAALYLHVVQEKKGEDVLGIIRKPLDYLDQI

LTKKGTSYLTGDVESAADIVLWGSLFPLLRDDSFLPNELKALRSWFQNMNLKEYCRKAVESVWTPKGLLELKAYLQKHPT

PSLSFEKSATNEAKEEESAQQHLSDVEIEAIAQIWTRGPASLPSPWKPQHPILPVEGMKNVLITSALPYVNNVPHLGNII

GCVLSADTFARYCRLRNWNTLYICGTDEYGTATETKAMEEGLTPQQICDKYYSIHAQIYQWFNISFDYFGRTTTAHQTTI

AQTIFQQLLERNYLLRETVDQLKCEKCARFLADRFVEGICPFCNYEEARGDQCDKCGKLINATELKKPVCKICQGTPVVK

SSQHLFLDLPKLEESLEKWLAVSQSSGDWTPNSRYIARSWIRDGLKPRCITRDLKWGTPVPLDGFREKVRNGTEGDIFSV

IECHTIIDIKYKGTPHQIQLYNFMAKDNVPFHSVVFPCTLLGTGDNYTLVNHLIATEYLNYEDGKFSKSRGVGVFGDMAK

DTGIPADIWRFYLLYLRPESQDSAFSWNDLMTKNNSELLNTLGNFINRAGTFVCKFFGGCVPTMDLTQDDKRLLAHITLE

LRQYNQLLEKVKIRDALRSILNIARHGNQYLQVNEPWKCIKGSNADKKRASTVIGVAINIASLLSVVLQPYMPNVSATIQ

DQLCIASSFNVVTNDFVCTLPAGHQIGTVTPLFQKLEAEQIENLRKRFGGGQFEDFPLKPKQKASANIQKTLGGKEHQEG

ERGNHVKNAKAQSARKEQVEVMVSDLVELQKQHHVVEGKGKKKK

>KFP58191.1 Methionine--tRNA synthetase, cytoplasmic, partial [Cathartes aura]

QPAASAALYAQLVHGKKGLEAVEALGKLLAHIEQNLSRRGTAYLAGGEHSLCSCGCKNHAALCLSLAPQGELQVLRTWFQ

SMTLSEACRKAADSVLMPKALLEFKSYLQKQPPPCLAMERATSNEPEEEEGSEPALTEEEITAAADAWGRGAAALPKPWQ

PQKPVLPVEGMRNILITSALPYVNNVPHLGNIIGCVLSADTFARYCRLRNWNTLYVCGTDEYGTATETKAVEEGLTPQEI

CNKYNAIHADIYRWFNISFDFFGRTTTPYQTMIAQDIFQRLLARGFLLQDTMEQLRCEDCQRFLADRFVEGTCPFCSYKE

ARGDQCDKCGKLINAVELKNPQCKLCRGIPVVKPTQHLFLDLPKLQEQLEPWLEQSWATGDWTANARYITRSWIRDGLKP

RCITRDLKWGTPVPLDGFRDKVFYVWFDAPIGYLSITANYTDQWERWWKNPQQVELYNFMAKDNVPFHSVIFPCSLLGAD

DNYTLVNHLIATEYLNYEDGKFSKSRGVGVFGDMAKDTGIPADIWRFYLLYLRPEGQDSAFSWSDLMLKNNSELLNNLGN

FINRAGMFVCKFFGGTVPNMVLTPDDKRLLARVTLELRQYHQLLEKVRIRDALRCILNISRHGNQYIQVNEPWKRIKGDE

KDRQRAGTVTGVAVNIASMLAIMLQPYMPAVSLAIQRQLCIPPDCFVLSHDFTCTLPPGHCVGTVSPLFQKLENDQVEAL

RKRFGGGQ

>KFO72658.1 Methionine--tRNA synthetase, cytoplasmic, partial [Cuculus canorus]

QPAASAALYAQLVRGKKGPEAVEALGPLLAHLEQSLRRRGAAYLAGDTESVADVVVWGTLFPVLQDETSLPGELQALRTW

FQRMTFSEACRKAADSILTPEALLEFKSYLQKQPPPCSAMEKAASNEVEEEEGSERALTEEEITAATDAWDRGVAALPKP

WQPQKPVLPVEGMRNVLITSALPYVNNVPHLGNIIGCVLSADTFARYCRLRNWNTLYVCGTDEYGTATETKAVEEGLTPQ

EICDKYNAIHAEIYRWFDISFDFFGRTTTPCQTRIAQDIFQRLLARGFLLEDTVEQLRCENCQRFLADRFVEGTCPFCGY

EEARGDQCDKCGKLINAVELKKPQCKLCKSVPVVKPTRHLFLDLPKLEERLESWLEQSWATGDWTANARYITRSWIRDGL

KPRCITRDLKWGTPVPLDGFCDKVFYVWFDAPIGYLSITANYTEHWEKWWKNPKQVELYNFMAKDNVPFHSVIFPCSLLG

TEDDYTLVNHLVATEYLNYEDGKFSKSRGVGVFGDMAKDTGIPADIWRFYLLSVRPEGQDSAFSWSDLMLKNNSELLNNL

GNFINRAGMFVCKFFGGTVPDMILTPDDKRLLARVTLELRQYHQLLEKVRIRDALRCVLSISRHGNQYIQVNEPWKRIKG

DEEDRRRAGTVTGVAVNMASLLGVLVQPYMPSVSRAIQGQLRIPPECFVLGYDFTCTLPAGHRVGTV

>XP_017335273.1 PREDICTED: methionine--tRNA synthetase, cytoplasmic isoform X2 [Ictalurus punctatus]

MKLFVSEGSPQCVKVLAAREHTGQQCDVQLLGHEERVVSFLSRPVLPAALLPSGKQLFSANAICQYLFEVGGKEITDTSR

QLLEWEATELQPVLLQALHVAVIQGKSSEAGTLLKKPLSWLEQYLSGKSTQFLTADTVEVADIVLWAALYPVLSNPALEA

GELRSVRGWFDRVGQLPACTKAVQKILPGKDLNALKNFLQKQPAPHSQRRENLPNETDDAEHVITEEEIEVAAQAWSKGL

AECHDIMERQHPILPKEGKRNVLVTSALPYVNNVPHLGNIIGCVLSADVFSRYGRLRGWNLLYLCGTDEYGTATENKALE

EGLTPQEICDKYFVVHDAIYKWFQIDFDFFGRTTTPQQTEISQNIFFRLLERGFLNEDTVEQLRCEKCQRFLADRFVEGG

CPHCAYPEARGDQCDKCSRLINAVELKNPQCKVCGETPVIRSSKHLFLDLPKLEGELEKWLEQSLNTGDWTANARQITRS

WIRDGLKPRCITRDLKWGTPVPLPDYSEKVFYVWFDAPIGYLSITANYTSEWEKWWKNPQQVELYNFMAKDNVPFHSVVF

PCSLLGAQDNYTLVNHLVATEYLNYEDTKFSKSRGVGVFGDMAKDTGIPSDVWRFYLTYVRPESQDSSFSWADLALKNNS

ELLNNLGNFINRAGMFVSKFFNSCVPEIVLQDEDKRLIAQVSWELKQYIQLLDKVRIRDALKSILNISRHGNQYIQSSEP

WKKIKGDDEDRKRAGTVTGLSVNVACLLSAMLEPYMPTVSQTIRTQLKAPDNCAFAMLGAEGCFLRSLPTGHQIGTVSPL

FQKLEPEQIEILRKKFGGQQPEEESPTGKTEDTEHVITPKEIEVAAQAWSKGLAECHDIIERKHPILPKEGKRNVLVTSA

LPYVNNVPHLGNIIGCVLSADVFSRYGRLRGWNLLYLCGTDEYGTATENKALEEGLTPQEICDKYFVVHDAIYKWFQIDF

DFFGRTTTPQQTEISQNIFFRLLERGFLNEDTVEQLRCEKCQRFLADRFVEGGCPHCAYPEARGDQCDKCSRLINAVELK

NPQCKLCGETPVIRSSKHLFLDLPKLEGELEKWLEQSLNTGDWTANARQITRSWIRDGLKPRCITRDLKWGTPVPLPDYS

EKVFYVWFDAPIGYLSITANYTSEWEKWWKNPQQVELYNFMAKDNVPFHSVVFPCSLLGAQDNYTLVNHLVATEYLNYED

TKFSKSRGVGVFGDMAKDTGIPSDVWRFYLTYVRPESQDSSFSWADLALKNNSELLNNLGNFINRAGMFVSKFFNSCVPE

IVLQDEDKRLIAQVSWELKQYIQLLDKVRIRDALKSILNISRHGNQYIQSSEPWKKIKGDDEDRKRAGTVTGLSVNVACL

LSAMLEPYMPTVSQTIRTQLKAPDNCAFAMLGAEGCFLRSLPTGHQIGTVSPLFQKLEPEQIEILRKKFGGQQGPSDKST

AETKPPTAAAPTASQGKK

>XP_017335272.1 PREDICTED: methionine--tRNA synthetase, cytoplasmic isoform X1 [Ictalurus punctatus]

MKLFVSEGSPQCVKVLAAREHTGQQCDVQLLGHEERVVSFLSRPVLPAALLPSGKQLFSANAICQYLFEVGGKEITDTSR

QLLEWEATELQPVLLQALHVAVIQGKSSEAGTLLKKPLSWLEQYLSGKSTQFLTADTVEVADIVLWAALYPVLSNPALEA

GELRSVRGWFDRVGQLPACTKAVQKILPGKDLNALKNFLQKQPAPHSQRRENLPNETDDAEHVITEEEIEVAAQAWSKGL

AECHDIMERQHPILPKEGKRNVLVTSALPYVNNVPHLGNIIGCVLSADVFSRYGRLRGWNLLYLCGTDEYGTATENKALE

EGLTPQEICDKYFVVHDAIYKWFQIDFDFFGRTTTPQQTEISQNIFFRLLERGFLNEDTVEQLRCEKCQRFLADRFVEGG

CPHCAYPEARGDQCDKCSRLINAVELKNPQCKVCGETPVIRSSKHLFLDLPKLEGELEKWLEQSLNTGDWTANARQITRS

WIRDGLKPRCITRDLKWGTPVPLPDYSEKVFYVWFDAPIGYLSITANYTSEWEKWWKNPQQVELYNFMAKDNVPFHSVVF

PCSLLGAQDNYTLVNHLVATEYLNYEDTKFSKSRGVGVFGDMAKDTGIPSDVWRFYLTYVRPESQDSSFSWADLALKNNS

ELLNNLGNFINRAGMFVSKFFNSCVPEIVLQDEDKRLIAQVSWELKQYIQLLDKVRIRDALKSILNISRHGNQYIQSSEP

WKKIKGDDEDRKRAGTVTGLSVNVACLLSAMLEPYMPTVSQTIRTQLKAPDNCAFAMLGAEGCFLRSLPTGHQIGTVSPL

FQKLEPEQIEILRKKFGGQQPEEESPTGKTEDTEHVITPKEIEVAAQAWSKGLAECHDIIERKHPILPKEGKRNVLVTSA

LPYVNNVPHLGNIIGCVLSADVFSRYGRLRGWNLLYLCGTDEYGTATENKALEEGLTPQEICDKYFVVHDAIYKWFQIDF

DFFGRTTTPQQTEISQNIFFRLLERGFLNEDTVEQLRCEKCQRFLADRFVEGGCPHCAYPEARGDQCDKCSRLINAVELK

NPQCKLCGETPVIRSSKHLFLDLPKLEGELEKWLEQSLNTGDWTANARQITRSWIRDGLKPRCITRDLKWGTPVPLPDYS

EKVFYVWFDAPIGYLSITANYTSEWEKWWKNPQQVELYNFMAKDNVPFHSVVFPCSLLGAQDNYTLVNHLVATEYLNYED

TKFSKSRGVGVFGDMAKDTGIPSDVWRFYLTYVRPESQDSSFSWADLALKNNSELLNNLGNFINRAGMFVSKFFNSCVPE

IVLQDEDKRLIAQVSWELKQYIQLLDKVRIRDALKSILNISRHGNQYIQSSEPWKKIKGDDEDRKRAGTVTGLSVNVACL

LSAMLEPYMPTVSQTIRTQLKAPDNCAFAMLGAEGCFLRSLPTGHQIGTVSPLFQKLEPEQIEILRKKFGGQQPEEESPT

GKGPSDKSTAETKPPTAAAPTASQGKK

>XP_009554499.1 PREDICTED: methionine--tRNA synthetase, cytoplasmic, partial [Cuculus canorus]

MTGPELNSGFKPAASAALYAQLVRGKKGPEAVEALGPLLAHLEQSLRRRGAAYLAGDTESVADVVVWGTLFPVLQDETSL

PGELQALRTWFQRMTFSEACRKAADSILTPEALLEFKSYLQKQPPPCSAMEKAASNEVEEEEGSERALTEEEITAATDAW

DRGVAALPKPWQPQKPVLPVEGMRNVLITSALPYVNNVPHLGNIIGCVLSADTFARYCRLRNWNTLYVCGTDEYGTATET

KAVEEGLTPQEICDKYNAIHAEIYRWFDISFDFFGRTTTPCQTRIAQDIFQRLLARGFLLEDTVEQLRCENCQRFLADRF

VEGTCPFCGYEEARGDQCDKCGKLINAVELKKPQCKLCKSVPVVKPTRHLFLDLPKLEERLESWLEQSWATGDWTANARY

ITRSWIRDGLKPRCITRDLKWGTPVPLDGFCDKVFYVWFDAPIGYLSITANYTEHWEKWWKNPKQVELYNFMAKDNVPFH

SVIFPCSLLGTEDDYTLVNHLVATEYLNYEDGKFSKSRGVGVFGDMAKDTGIPADIWRFYLLSVRPEGQDSAFSWSDLML

KNNSELLNNLGNFINRAGMFVCKFFGGTVPDMILTPDDKRLLARVTLELRQYHQLLEKVRIRDALRCVLSISRHGNQYIQ

VNEPWKRIKGDEEDRRRAGTVTGVAVNMASLLGVLVQPYMPSVSRAIQGQLRIPPECFVLGYDFTCTLPAGHRVGT

>XP_017335275.1 PREDICTED: methionine--tRNA synthetase, cytoplasmic isoform X3 [Ictalurus punctatus]

MKLFVSEGSPQCVKVLAAREHTGQQCDVQLLGHEERVVSFLSRPVLPAALLPSGKQLFSANAICQYLFEVGGKEITDTSR

QLLEWEATELQPVLLQALHVAVIQGKSSEAGTLLKKPLSWLEQYLSGKSTQFLTADTVEVADIVLWAALYPVLSNPALEA

GELRSVRGWFDRVGQLPACTKAVQKILPGKDLNALKNFLQKQPAPHSQRRENLPNETDDAEHVITEEEIEVAAQAWSKGL

AECHDIMERQHPILPKEGKRNVLVTSALPYVNNVPHLGNIIGCVLSADVFSRYGRLRGWNLLYLCGTDEYGTATENKALE

EGLTPQEICDKYFVVHDAIYKWFQIDFDFFGRTTTPQQTEISQNIFFRLLERGFLNEDTVEQLRCEKCQRFLADRFVEGG

CPHCAYPEARGDQCDKCSRLINAVELKNPQCKVCGETPVIRSSKHLFLDLPKLEGELEKWLEQSLNTGDWTANARQITRS

WIRDGLKPRCITRDLKWGTPVPLPDYSEKVFYVWFDAPIGYLSITANYTSEWEKWWKNPQQVELYNFMAKDNVPFHSVVF

PCSLLGAQDNYTLVNHLVATEYLNYEDTKFSKSRGVGVFGDMAKDTGIPSDVWRFYLTYVRPESQDSSFSWADLALKNNS

ELLNNLGNFINRAGMFVSKFFNSCVPEIVLQDEDKRLIAQVSWELKQYIQLLDKVRIRDALKSILNISRHGNQYIQSSEP

WKKIKGDDEDRKRAGTVTGLSVNVACLLSAMLEPYMPTVSQTIRTQLKAPDNCAFAMLGAEGCFLRSLPTGHQIGTVSPL

FQKLEPEQIEILRKKFGGQQTEDTEHVITPKEIEVAAQAWSKGLAECHDIIERKHPILPKEGKRNVLVTSALPYVNNVPH

LGNIIGCVLSADVFSRYGRLRGWNLLYLCGTDEYGTATENKALEEGLTPQEICDKYFVVHDAIYKWFQIDFDFFGRTTTP

QQTEISQNIFFRLLERGFLNEDTVEQLRCEKCQRFLADRFVEGGCPHCAYPEARGDQCDKCSRLINAVELKNPQCKLCGE

TPVIRSSKHLFLDLPKLEGELEKWLEQSLNTGDWTANARQITRSWIRDGLKPRCITRDLKWGTPVPLPDYSEKVFYVWFD

APIGYLSITANYTSEWEKWWKNPQQVELYNFMAKDNVPFHSVVFPCSLLGAQDNYTLVNHLVATEYLNYEDTKFSKSRGV

GVFGDMAKDTGIPSDVWRFYLTYVRPESQDSSFSWADLALKNNSELLNNLGNFINRAGMFVSKFFNSCVPEIVLQDEDKR

LIAQVSWELKQYIQLLDKVRIRDALKSILNISRHGNQYIQSSEPWKKIKGDDEDRKRAGTVTGLSVNVACLLSAMLEPYM

PTVSQTIRTQLKAPDNCAFAMLGAEGCFLRSLPTGHQIGTVSPLFQKLEPEQIEILRKKFGGQQPEEESPTGKGPSDKST

AETKPPTAAAPTASQGKK

>XP_006513631.1 PREDICTED: methionine--tRNA synthetase, cytoplasmic isoform X1 [Mus musculus]

MEEGLTPREICDKYHAIHADIYRWFGISFDTFGRTTTPQQTKITQDIFQRLLTRGFVLRDTVEQLRCERCARFLADRFVE

GVCPFCGYEEARGDQCDRCGKLINAIELKKPQCKICRSCPVVRSSQHLFLDLPKLEKRLEDWLGKTVPGSDWTPNARFII

RSWLRDGLKPRCITRDLKWGTPVPLEGFEDKVFYVWFDATIGYVSITANYTDQWEKWWKNPEQVDLYQFMAKDNVPFHGL

VFPCSVLGAEDNYTLVKHIIATEYLNYEDGKFSKSRGIGVFGDMAKDTGIPADIWRFYLLYIRPEGQDSAFSWTDLLIKN

NSELLNNLGNFINRAGMFVSKFFGGCVPEMALTPDDRRLVAHVSWELQHYHQLLEKVRIRDALRSILTISRHGNQYIQVN

EPWKRIKGGEMDRQRAGTVTGMAVNMAALLSVMLQPYMPTVSSTIQTQLQLPPAACRILATSFICTLPAGHRIGTVSPLF

QKLENDQIENLRQRFGGGQLEESLELKAKGSPKPAAVEAVTAAGSQHIQTLTDEVTKQGNVVRELKAQKADKNQVAAEVA

KLLDLKKQLALAEGKPIETPKGKKKK

>OXB53369.1 hypothetical protein ASZ78_007560, partial [Callipepla squamata]

PAALLALHALLVRGRTAAEAAAALGEALGHIGQSLAGRGTAFLAGDTQSVADVVVWGTLFPVLRDDAVPLAEPLQSWFQS

MTLSDACRSAADAVLLPQHLQELTACLQQLPPPHPSPGTALGTEEDDVSERALTEEDVAAAVEAWGCGAAVLPKPWRPPE

PVLPVEGMRNVLITSALPYVNNVPHLGNIIGCVLSADAFARYCRLRNWNTLYVCGTDEYGTATETKAVEEGLTPREICDK

YHAVHADIYRWFHISFDYFGRTTTAQQTAICQDIFQRLLAHGFVLQDTVQQLHCAGCGRFLADRFVEGTCPFCAFQEARG

DQCDKCGKLINAVELKAPRCKLCGHSPTARPTQHLFLDLPKLEERLQPWLERCWATGNWTANARHITRSWIRDGLRPRCI

TRDLQWGTPVPLDGFRDKVFYVWFDAPIGYLSITANYTEQWERWWKNPQQVGLGGSGQDNVPFHSVVFPCSLLGANDNYT

LVNHLIATEYLNYEDGKFSKSRGVGVFGDMAKETGIAADVWRFYLLYVRPEGQDSAFSWGDLMLKNNSELLNNLGNFINR

AGMFVCKFFGGVVPSMVLTPDDHRLLARVTLELRQYHRLLERVRIRDALRCVLNISRHGNQYIQVNEPWKRIKGSEEDRQ

RAGTVTGVAVNVAALLAVLVQPYMPTVSAAIQAQLCLPPECSVLGHVFTCTLLPGHRVGTVSPLFQKLESERIDALRKRF

GGGQTKPPAPPPPADPSSEPCTAPADPSRIQELTARVAEQGDCVRRLKASGAERARIDSEVGKLLALKRELGQAQNAGSG

GQRNK

>KFZ60734.1 Methionine--tRNA synthetase, cytoplasmic, partial [Antrostomus carolinensis]

VSRLNCACLAQDTKSVADVVVWSTLFPVLQDETSLPSELQALRTWFQRMTLSEACRKAADSVLMPKALLEFKSYLQKQPP

PCLAMERVTSNEPEEEEGSERALTEEEITAAADAWARGAEALPKPWQPQKPVLPVEGMRNVLITSALPYVNNVPHLGNII

GCVLSADTFARYCRLRNWNTLYVCGTDEYGTATETKAVEEGLTPQEICDKYNAIHTDIYHWFNISFDYFGRTTTPYQTTI

AQDIFQRLLARGFLLQDTVEQLHYEEARGDQCDKCGKLINAVELKNPQCKLCRGVPVVKPTQHLFLDLPKLEKQLEPWLE

QSWATGDWTANARYITRSWIRDGLKPRCITRDLKWGTPVPLDGFRDKVFYVWFDAPIGYLSITANYTDQWERWWKNPQQV

ELYNFMAKDNVPFHSVIFPCSLLGADDNYTLVNHLIATEYLNYENGKFSKSRGVGVFGDMAKDTGIPADIWRFYLLYLRP

EGQDSAFSWSDLMIKNNSELLNNLGNFINRAGMFVCKFFGGTVPNMVLTLDDKRLLARVTLELRQYHQLLEKVRIRDALR

CILSISRHGNQYIQVNEPWKRIKGDEKDRQRAGTVTGVAVNMAFLPAGISAIQGQLCIPSDCFVLSHDFTCTLPPGHRVG

TVSPLFQKLENDQIETLRKRFGGGQAKQTPPTPATPITPGDPQLIQELSEEVAKQGNYVRQLKANKAEKAQIDTEVAKLL

ELKRQLALAEGKSPDVPVPKGKQKK

>XP_019214900.1 PREDICTED: methionine--tRNA synthetase, cytoplasmic isoform X3 [Oreochromis niloticus]

MAVLQGKSSEVSKVLQGPINVLEQSLSKRNTPYLTGDSVSAADLVLWAGFFPVLSDSSLTLGEHKSLKAWFDRVAAMNAC

QSAAQKVLQGKGLQGMKSYMQRQPAPQSSQCRETQPCNSNSAEEEGGERVVSEEEMEAAALTWSKGLNTCPPATKKQHPI

LPQEDKRNVLVTSALPYVNNVPHLGNIIGCVLSADVFSRYGRLRGWNMLFVCGTDEYGTATENKAREEGLTPQQICDKYH

AVHSSIYKWFQIDFDYFGRTTTEKQTEIAQDIFWRLHERDFLIEDTVEQLRCENCQRFLADRFVEGICPHCSYPEARGDQ

CDKCGRLINAVDLREPQCKVCRQTPVIRSSKHLFLDLPKLESQLEQWLEKSTSTGDWTANAKQITRSWLRDGLKPRCITR

DLKWGTPVPHADFKEKVFYVWFDAPIGYLSITANYTSEWEKWWKNPHQVELYNFMAKDNVPFHSVVFPCSLLGAQDNYTL

VNHLIATEYLNYEDTKFSKSRGVGVFGDMAKDTGIPSDVWRFYLLYVRPEGQDSAFSWADMALKNNSELLNNLGNFINRA

GMFVTKFFEGCVPVMELQEEDKKLLALVSWELQQYIQLMDKIKIRDALKHILNISRHGNQYIQVNEPWKKIKGGDSERQR

AGTVTGVSVNIACLLSVMLFPYMPAVSQTIRDQLNAPPSCINTMLEGNGTFVCSLSAGHRIGAVSPLFQKLEADQIEALK

KKFGGQQPEDEPPKKKTPVRNAASAQSAAVPAPAAAVEVGTVNQVDPEKAKQLTQAVAEQGEKVRALKAQKAEKAVITAE

VAKLLDLKKQLALAEGKNPEPAPQKGKKK

>EMP41996.1 Methionyl-tRNA synthetase, cytoplasmic [Chelonia mydas]

MPSAFPSAGRVVPFLTHPRVPALHLDSGTFLFSPNAICQYFFLLAGKEPDDLTNQWLEWEATELQEEELAERSLSEEEIA

AAAEAWPRGLAALPERWQPPSPVLPVEGRRNILITSALPYVNNVPHLGNIIGCVLSADVFARYCRLRNWNTLYVCGTDEY

GTATETKALEEGLTPQQICDKYNAIHAQIYRWFDISFDYFGRTTTPHQTTIAQDIFQRLLERDMLRTDTVDQLRCERCQR

FLADRFVEGTCPFCNYEEARGDQCDKCGKLINAVELKKPQCKVCKDSPVVKSSQHLFLDLPKLEGRLEKWLEQSWATGDW

TANSRFITRSWIRDGLKPRCITRDLKWGTPVPLDGFRDKVFYVWFDAPIGYLSITANYTDQWEKWWKNPQQVQLYNFMAK

DNVPFHSVVFPCTLLGAEDNYTLVNHLVATEYLNYEDGKFSKSRGVGVFGDMAQDTGIPADIWRFYLLFLRPEGQDSTFS

WTDLMLKNNSELLNNLGNFVNRAGMFVCKFFGGRVPHMELGTDDKRLLAHVTLELRQYNQLLEKVRIRDALRCILSVSRY

GNQYIQVNEPWKRIKGHDADRTRAGTVTGVAVNIASLISVMLQPYMPSISATIQRQLCVPKACNVLTDTFLCLLPPGHQI

GTVSPLFQKLESEQIEALRKRFGEGQLWPCGSDSPPTGARASPALHGAGGSQSSELTAALCAQADHVRQLKASKAEKGQI

DAEVAKLLELKKQLALAEGKTPEPPAPKGKKRK

>XP_007422126.1 methionine--tRNA synthetase, cytoplasmic isoform X3 [Python bivittatus]

MSDPFPTRRRAGEGVHGESALISRPASLHQLGTKRGWEAEGGRSMKLCLSEGSLGGLKVLAAAKATGAPVELQWLPQEAH

VVPFLTHPQLPVLQLENGSFLFSTNAISQYFFRLSGKEMSGFKNEQSDFANQLFEWDVTELEPALSAALYLHVVQEKKGE

DVLNIIRKPLDYLDQILTKKGTSYLTGDVESAADVVLWGSLFPLLRDESFLPNELKALRSWFQNMNLKEHCRKAVESIWT

PKGLLELKAYLQKHPAPSMAFEKSATNEAKAEESIQQHLSDVEIEAIAEVWSRGPASLPSPWKPQHPIVPVEGMKNVLIT

SALPYVNNVPHLGNIIGCVLSADTFARYCRLRNWNTLYVCGTDEYGTATETKAMEEGLTPQEICDKYYSVHAQIYQWFNI

SFDYFGRTTTAQQTTIAQGIFQQLLERNYLLTETVDQLKCEKCVRFLADRFVEGICPFCNYEEARGDQCDKCGKLINATE

LKKPVCKICQGTPVVKSSEHLFLDLPKLEDSLEKWLAVSQSSGDWTPNSRYITRSWIRDGLKPRCITRDLKWGTPVPLDG

FRDKVFYVWFDAPIGYLSITANYTDQWEKWWKNPEQIQLYNFMAKDNVPFHSVVFPCSLLGTGDNYTLVNHLIATEYLNY

EDGKFSKSRGVGVFGDMAKDTGIPADIWRFYLLYLRPESQDSAFSWNDLMIKNNSELLNTLGNFINRAGTFVCKFFGGCV

PPMNLNQDDKRLLAHITLELRQYNQLLEKVKIRDALRSILNIARHGNQYLQVNEPWKCIKGSDADK

>XP_009506493.1 PREDICTED: LOW QUALITY PROTEIN: methionine--tRNA synthetase, cytoplasmic, partial [Phalacrocorax carbo]

QAELQQLRMWFRSMTLSEACRKAADSVLMPRALLEFKSYLQKQPPPSLAMERATSNEPEEEEGSERALTEEEITAAADAW

AHGAAGLPKPWQPQKPVLPVEGMRNVLITSALPYVNNVPHLGNIIGCVLSADTFARYCRLRNWNTLYVCGTDEYGTATET

XEGLTPQEICNKYNAIHADIYRWFDISFDYFGRTTTPYQTMIAQDIFQRLLARGFLLQDTVEQLRCEDCQRFLADLFVEG

PCPFCSYEEARGDQCDKCGKLINAVELKNPQCKLCKGIPVVKPTQHLFLDLPKLEERLEPWLEQSWATGDWTANARYITR

SWFRDGLKPRCITRDLRWGTPVPLDGFRDKVFYVWFDAPIGYLSITANYTDQWERWWKNPQQVELYNFMAKDNVPFHSVI

FPCSLLGAEDNYTLVNHLIATEYLNYEDGKFSKSRGVGVFGDMAKDTGIPADIWRFYLLYVRPEGQDSAFSWSDLMLKNN

SELLNNLGNFINRAGMFVCKFFGGAVPNMILTLDDKRLLAHVTLELRQYHQLLEKVRIRDALRCILSISRHGNQYIQANE

PWKRIKGDEKDRQRASTVTGIAVNMASMLAVLLQPYMPGVSLAIQEQLCIPPDCFVMSHNFTCTLPPGHRIGTVSPLFQK

LENDQVEALRKHFGGGQAKQTPPASEPAPGDPQLIQELTEEVAKQ

>XP_009996174.1 PREDICTED: methionine--tRNA synthetase, cytoplasmic, partial [Chaetura pelagica]

LPKPWQPQKPVLPVEGMRNVLITSALPYVNNVPHLGNIIGCVLSADTFARYCRLRNWNTLFVCGTDEYGTATETKAVEEG

LTPQQICDKYHALHADIYRWFHISFDYFGRTTTPHQTTIAQDIFQRLLARGFLLQDSLQQLQCETCQRFLADRFVEGTCP

FCSYGEARGDQCDKCGKLINAVELKNPQCKLCRGVPVVKPTQHLFLDLPKLEEQLEPWLEQTWARGDWTANARYITRSWL

RDGLKPRCITRDLKWGTPVPLDGFRDKVFYVWFDAPIGYLSITANYTEHWERWWKNPQQVELYNFMAKDNVPFHSVIFPS

SLLGADDNYTLVNHLIATEYLNYEDGKFSKSRGVGVFGDMAKDTGIPADIWRFYLLFLRPEGQDSAFSWSDLLLKNNSEL

LNNLGNFINRAGMFVCKFFGGTVPEMVLTLDDKRLLARVTLELHQYHQLLEKVRIRDALRCVLSISRHGNQYIQVNEPWK

RIKGDEQDRQRAGTVTGVAVTMAALLAVLLQPYMPSVSAAIQGQLCIPPDCFVLSHSLTCTLPPGHRVGTVSPLFQKLEN

DQIEALRKRFGGGQPEDFVVESQAKQAPPASACPAATPPTPGDPQLIQVLTEEVAKQGNHVRQLKANKAEKAQVDVEVAK

LLELKKQLALAEGKTPEVSVPKGKRKK

>XP_022109155.1 methionine--tRNA synthetase, cytoplasmic-like isoform X2 [Acanthaster planci]

MKLLTDKGNQHCLKAVIAACLAGVKLDVEEVENTDHVVPYLRKNRLPVLNLGNGDFIFSANSICRYLLAQGALDVDDTQV

DEWLEWEATQLQPVLGSFLALLYGSGKANADCLKPLLTHLNSSLQGNRYLVGNGLSVADISVWSALYPAQATRSDLLPAY

PHVESWFRGLSGQKAFEASVAVVTHSQGAGSFKASILAQPIVQMPVGDARTAKDEESADTEDVTSEEIERARQAWLLGKA

DAPTPRARNHPLLPEKDRRNVLITSALPYVNNVPHLGNIIGCVLSADVFSRFCRLRNYNCLYICGTDEYGTATETKALAE

GLTPQQICDKYYRIHYDVYTWFSIDFDYFGRTTTKQQTEIAQDIFLRLHQRGFLLKDTVEQLLCVKCNRFLADRFVEGTC

PLCYYPDARGDQCDKCGKLINATELKKPRCKVCKKSPIIKNSEHLFLDLPKLQPQVQAYFERVAAAGDWTANAKYVTSSW

LRDGLKPRCITRDLKWGTPVPLDGFRDKVFYVWFDAPIGYPSITANYTDQWERWWKNPEEVELYNFMAKDNIPFHSVVFP

CTLIGAEDNYTIVNHLIAAEYLNYEDGKFSKSRGIGVFGDMAKDTGIPSDIWRFYLLYVRPETMDSTFSWADLMVKNNSE

LLNNLGNFINRSLTFLEKNFGSVVPEMTLEEDDERLLALVTRELTSYKQLLEKSKIRDALRHILNISRLGNQHIQANQPW

VLVKGGDKERARAGGVIGLCTNISCLVSTMLLPYMPLTSRTIQEQLNAPPECNVITEKFVCYLETGHKIGTPKPLFGKLE

PSLIESLKKKFSGQQKQVGDSGDPKAAPVSSVPVDGPAAPAGDASEVKRLTDEVAKQGNIVRKLKSEKADKSEIEKEVKK

LLALKQELALTSGQDPTPVESSKGKRKKGGKKKEEKSKNSE

>XP_022109154.1 methionine--tRNA synthetase, cytoplasmic-like isoform X1 [Acanthaster planci]

MKLLTDKGNQHCLKAVIAACLAGVKLDVEEVENTDHVVPYLRKNRLPVLNLGNGDFIFSANSICRYLLAQGALDVDDTQV

DEWLEWEATQLQPVLGSFLALLYGSGKANADCLKPLLTHLNSSLQGNRYLVGNGLSVADISVWSALYPAQATRSDLLPAY

PHVESWFRGLSGQKAFEASVAVVTHSQGAGSFKASILAQPIVQMPVGDARTAKDEESADTEQDVTSEEIERARQAWLLGK

ADAPTPRARNHPLLPEKDRRNVLITSALPYVNNVPHLGNIIGCVLSADVFSRFCRLRNYNCLYICGTDEYGTATETKALA

EGLTPQQICDKYYRIHYDVYTWFSIDFDYFGRTTTKQQTEIAQDIFLRLHQRGFLLKDTVEQLLCVKCNRFLADRFVEGT

CPLCYYPDARGDQCDKCGKLINATELKKPRCKVCKKSPIIKNSEHLFLDLPKLQPQVQAYFERVAAAGDWTANAKYVTSS

WLRDGLKPRCITRDLKWGTPVPLDGFRDKVFYVWFDAPIGYPSITANYTDQWERWWKNPEEVELYNFMAKDNIPFHSVVF

PCTLIGAEDNYTIVNHLIAAEYLNYEDGKFSKSRGIGVFGDMAKDTGIPSDIWRFYLLYVRPETMDSTFSWADLMVKNNS

ELLNNLGNFINRSLTFLEKNFGSVVPEMTLEEDDERLLALVTRELTSYKQLLEKSKIRDALRHILNISRLGNQHIQANQP

WVLVKGGDKERARAGGVIGLCTNISCLVSTMLLPYMPLTSRTIQEQLNAPPECNVITEKFVCYLETGHKIGTPKPLFGKL

EPSLIESLKKKFSGQQKQVGDSGDPKAAPVSSVPVDGPAAPAGDASEVKRLTDEVAKQGNIVRKLKSEKADKSEIEKEVK

KLLALKQELALTSGQDPTPVESSKGKRKKGGKKKEEKSKNSE

>XP_021260792.1 methionine--tRNA synthetase, cytoplasmic isoform X2 [Numida meleagris]

MTLSEACRSAADAVLLPQRLQEFTACLQKLPPLRPRPDMAPGSEEDDTLERALTEEDVAAAVDAWARGAAALPKPWRPPK

PVLPVEGMRNVLITSALPYVNNVPHLGNIIGCVLSADTFARYCRLRNWNTLYVCGTDEYGTATETKAVEEGLTPQEICDK

YHAVHADIYRWFNISFDYFGRTTTPQQTIISQDIFQRLQARGFVLQDTVQQLHCASCQRFLADRFVEGTCPFCAYQEARG

DQCDKCGKLINAVELKTPRCKLCSSTPTVQPTRHLFLDLPKLEERLQPWLKHCWATGNWTANARYITRSWIRDGLRPRCI

TRDLQWGTPVPLDGFRNKVFYVWFDAPIGYLSITANYTEHWERWWKNPQQVELYNFMAKDNVPFHSVMFPCSLLGADDNY

TLVNHLIATEYLNYEDGKFSKSRGVGVFGDMAKDTGIAADVWRFYLLYVRPEGQDSAFSWGDLMLKNNSELLNNLGNFIN

RAGMFVCKFFGGAVPAMALTLDDHRLLARITVELRQYHRLLERVCIRDALRCVLNISRHGNQYIQVNEPWKRIKGDEEDR

QRAGTVTGVAVNVAALLAVLVQPYMPAVSAAIQAQLCMPLESLVLGHVFTCTLPPGHRVGTVSPLFQKLESERINALRKR

FGAGQAKPPAPPTQPDSPLVPSVAAGDPSRIQELTEQVAEQGDCVRRLKASGADRACIDSEVGKLLALKRELALAQNVSP

DGPRAQGKK

>ELK15312.1 Methionyl-tRNA synthetase, cytoplasmic [Pteropus alecto]

MRLFVSEGSPGSLPVLAAAGRAQGRAELRISTVGPEERVVPFLTQPKVPVLQLDSGNYLFSTSAICRYFFLLSGWEQDDL

TNQWLEWEVTELQPALSAALYYLVVQGKKGEDVLGPVRGALIHIDNSLSRRSCPFLAGSADYLFSPMPTEELGALHSWFQ

TLSSQEPCQRAAETVLKQQGVLALRPYLQKQLQPSFLEGKAVSNEPEEEELATLSEEEIAVAVTAWEKGLGSLPPLRPKQ

TPVLPVAGERNVLITSALPYVNNVPHLGNIIGCVLSADVFARYSRLRQWNTLYLCGTDEYGTATETKAMEEGLTPQEICD

KYHVIHADIYRWFNISFDIFGRTTTPQQTEITQDIFQRLWNRGFVLQDTVEQLRCESCARFLADRFVEGVCPFCGYEEAR

GDQCDKCGKLINAIELKKPQCKLCRSCPVVKSSRHLFLDLPKLEDRLEEWLGKTLPGSDWTSNARFIIRSWLRDGLKPRC

ITRDLKWGTPVPLEGFEDKVFYVWFDATFGYLSITANYTDQWERWWKNPEQVNLYQFMAKDNVPFHGLVFPCSALGAEDN

YTLVSHLIATGTFEDLTGIVMGNEG

>XP_014191449.1 PREDICTED: methionine--tRNA synthetase, cytoplasmic isoform X2 [Haplochromis burtoni]

MNACQSAAQKVLQGKGLQGMKSYMQKQPAPQSSQCRETQPCNSNSAEEEGGERVVSEEEMEAAALTWSKGLNTCPPATKK

QHPILPQEGKRNVLVTSALPYVNNVPHLGNIIGCVLSADVFSRYGHLRGWNMLFVCGTDEYGTATENKAREEGLTPQQIC

DKYHAVHSSIYKWFQIDFDYFGRTTTEKQTEIAQDIFWRLHERGFLIEDTVEQLRCENCQRFLADRFVEGICPHCSYPEA

RGDQCDKCGRLINAVDLREPQCKVCRQTPVIRSSKHLFLDLPKLESQLEQWLEKSTSTGDWTANAKQITRSWLRDGLKPR

CITRDLQWGTPVPHADFKEKVFYVWFDAPIGYLSITANYTSEWEKWWKNPHQVELYNFMAKDNVPFHSVVFPCSLLGAQD

NYTLVNHLIATEYLNYEDTKFSKSRGVGVFGDMAKDTGIPSDVWRFYLLYVRPEGQDSAFSWADMALKNNSELLNNLGNF

INRAGMFVTKFFEGCVPVMELQEEDKKLLALVSWELQQYIQLMDKIKIRDALKHILNISRHGNQYIQVNEPWKKIKGGDS

ERQRAGTVTGVSVNIACLLSVMLSPYMPAVSQTIRDQLNAPPSCINTMLEDNGTFVCSLSAGHRIGAVSPLFQKLEADQI

EALKKKFGGQQPEDEPPKKKTTVRNAASAQSAAVPAPAVAVEVGTVNQVDPEKAKQLTQAVAEQGEKVRALKAQKAEKAV

ITAEVAKLLDLKKQLALAEGKNPEPAPQKGKKK

>XP_023010852.1 methionine--tRNA synthetase, cytoplasmic isoform X3 [Maylandia zebra]

MNACQSAAQKVLQGKGLQGMKSYMQKQPAPQSSQCRETQPCNSNSAEEEGGERVVSEEEMEAAALTWSKGLNTCPPATKK

QHPILPQEGKRNVLVTSALPYVNNVPHLGNIIGCVLSADVFSRYGHLRGWNMLFVCGTDEYGTATENKAREEGLTPQQIC

DKYHAVHSSIYKWFQIDFDYFGRTTTEKQTEIAQDIFWRLHERGFLIEDTVEQLRCENCQQFLADRFVEGICPHCSYPEA

RGDQCDKCGRLINAVDLREPQCKVCRQTPVIRSSKHLFLDLPKLESQLEQWLEKSTSTGDWTANAKQITRSWLRDGLKPR

CITRDLQWGTPVPHADFKEKVFYVWFDAPIGYLSITANYTNEWEKWWKNPHQVELYNFMAKDNVPFHSVVFPCSLLGAQD

NYTLVNHLIATEYLNYEDTKFSKSRGVGVFGDMAKDTGIPSDVWRFYLLYVRPEGQDSAFSWADMALKNNSELLNNLGNF

INRAGMFVTKFFEGCVPVMELQEEDKKLLALVSWELQQYIQLMDKIKIRDALKHILNISRHGNQYIQVNEPWKKIKGGDS

ERQRAGTVTGVSVNIACLLSVMLSPYMPAVSQTIRDQLNAPPSCINTMLEGNGTFVCSLSAGHRIGAVSPLFQKLEADQI

EALKKKFGGQQLEDEPPKKKTTVRNAASAQSAAVPAPAVAVEVGTVNQVDPEKAKQLTQAVAEQGEKVRALKAQKAEKAV

ITAEVAKLLDLKKQLALAEGKNPEPAPQKGKKK

>KFU89763.1 Methionine--tRNA synthetase, cytoplasmic, partial [Chaetura pelagica]

LPVEGMRNVLITSALPYVNNVPHLGNIIGCVLSADTFARYCRLRNWNTLFVCGTDEYGTATETKAVEEGLTPQQICDKYH

ALHADIYRWFHISFDYFGRTTTPHQTTIAQDIFQRLLARGFLLQDSLQQLQCETCQRFLADRFVEGTCPFCSYGEARGDQ

CDKCGKLINAVELKNPQCKLCRGVPVVKPTQHLFLDLPKASFVAGGGDGAPFPHSPPMGVPLQLEEQLEPWLEQTWARGD

WTANARYITRSWLRDGLKPRCITRDLKWGTPVPLDGFRDKVFYVWFDAPIGYLSITANYTEHWERWWKNPQQVELYNFMA

KDNVPFHSVIFPSSLLGADDNYTLVNHLIATEYLNYEDGKFSKSRGVGVFGDMAKDTGIPADIWRFYLLFLRPEGQDSAF

SWSDLLLKNNSELLNNLGNFINRAGMFVCKFFGGTVPEMVLTLDDKRLLARVTLELHQYHQLLEKVRIRDALRCVLSISR

HGNQYIQVNEPWKRIKGDEQDRQRAGTVTGVAVTMAALLAVLLQPYMPSVSAAIQGQLCIPPDCFVLSHSLTCTLPPGHR

VGTVSPLFQKLENDQIEALRKRFGGGQAKQAPPASACPAATPPTPGDPQLIQVLTEEVAKQGNHVRQLKANKAEKAQVDV

EVAKLLELKKQLALAEGKTPEVSVPKGKRKK

>XP_005740102.1 PREDICTED: methionine--tRNA synthetase, cytoplasmic isoform X3 [Pundamilia nyererei]

MTACQSAAQKVLQGKGLQGMKSYMQKQPAPQSSQCRETQPCNSNSAEEEGGERVVSEEEMEAAALTWSKGLNTCPPATKK

QHPILPQEGKRNVLVTSALPYVNNVPHLGNIIGCVLSADVFSRYGHLRGWNMLFVCGTDEYGTATENKAREEGLTPQQIC

DKYHAVHSSIYKWFQIDFDYFGRTTTEKQTEIAQDIFWRLHERGFLIEDTVEQLRCENCQRFLADRFVEGICPHCSYPEA

RGDQCDKCGRLINAVDLREPQCKVCRQTPVIRSSKHLFLDLPKLESQLEQWLEKSTSTGDWTANAKQITRSWLRDGLKPR

CITRDLQWGTPVPHADFKEKVFYVWFDAPIGYLSITANYTSKWEKWWKNPHQVELYNFMAKDNVPFHSVVFPCSLLGAQD

NYTLVNHLIATEYLNYEDTKFSKSRGVGVFGDMAKDTGIPSDVWRFYLLYVRPEGQDSAFSWADMALKNNSELLNNLGNF

INRAGMFVTKFFEGCVPVMELQEEDKKLLALVSWELQQYIQLMDKIKIRDALKHILNISRHGNQYIQVNEPWKKIKGGDS

ERQRAGTVTGVSVNIACLLSVMLSPYMPAVSQTIRDQLNAPPSCINTMLEDNGTFVCSLSAGHRIGAVSPLFQKLEADQI

ETLKKKFGGQQPEDEPPKKKTTVRNAASAQSAAVPAPAVAVEVGTVNQVDPEKAKQLTQAVAEQGEKVRALKAQKAEKAV

ITAEVTKLLDLKKQLALAEGKNPEPAPQKGKKK

>XP_009577199.1 PREDICTED: methionine--tRNA synthetase, cytoplasmic, partial [Fulmarus glacialis]

PDSASLAQDTKSVADVVVWGTLFPVLRDETSLPSELQVLRTWFQSMTLSEACRKAADSILTPKALLEFKSYLQKQPPPCL

AMERATSNEPEEEEGSEHALTEEEITAAEDAWSCGVAALPKLWQPRKPVLPVEGRRNVLITSALPYVNNVPHLGNIIGCV

LSADTFARYCRLRNWNTLYVCGTDEYGTATETKAVEEGLTPQEICNKYNAIHADIYRWFNISFDYFGRTTTPYQTTIAQD

IFQRLLTHGFLQQDTVEQLRCEDCQRFLADRFVEGTCPFCSYEEARGDQCDKCGKLINAVELKNPQCKLCRGIPVVKPTQ

HLFLDLPKLEERLERWLEQSWATGDWTANARYITRSWIRDGLKPRCITRDLKWGTPVPLDGFRDKVFYVWFDAPIGYLSI

TANYTDQWERWWKNPQQVELYNFMAKDNVPFHSVIFPCSLLGADDNYTLVNHLIATEYLNYEDGKFSKSRGVGVFGDMAK

DTGIPADIWRFYLLYLRPEGQDSAFSWSDLMLKNNSELLNNLGNFINRAGMFVCKFFGGTVPNMVLTLDDKRLLARVTLE

LRQYHQLLEKVRIRDALRCILSISRHGNQYIQVNEPWKRIKGDEKDRQRAGTVTGVAVNVASMPVSPLFQKLENDQVEAL

RRRFGGGQ

>XP_021139174.1 methionine--tRNA synthetase, cytoplasmic, partial [Columba livia]

RPEPCVCVRQPAAPAALYAQLVHGQKGLEALEPLGQLLEHIEQDLSSRGTAYLAGDTQSVADVVVWGALFPVLQDETGLP

SELPALRTWFQNMTLSEACRKAAGSVLMPEALLEFKSYLQKHPPPCLATGRTTSNAPEAQPGRALSEEEIAAAVAAWARG

ARSDCSVRARLPVHGRRNVLITSALPYVNNVPHLGNIIGCVLSADTFARYCRLRNWNTLYVCGTDEYGTATETKAMEEGL

TPQEICDKYNAIHADIYRWFDISFDYFGRTTTPHQTRIAQDIFQRLLARGFLLQDTVEQLRCEGCQRFLADRFVEGTCPF

CNYEEARGDQCDKCGKLINAVELKNPQCKLCGGVPVVKTTQHLFLDLPKLQERLEPWLEQSLATGDWTANARYITRSWIR

DGLKPRCITRDLKWGTPVPLDGFQDKVFYVWFDAPIGYLSITANYTEQWERWWKNPQQVELYNFMAKDNVPFHSVIFPCS

LLGADDNYTLVNHLIATEYLNYEDGKFSKSRGVGVFGDMAKDTGIPADIWRFYLLSVRPEGQDSAFSWGDLMLKNNSELL

NNLGNFINRLRDTLGNQYIQVNEPWKRIKGDEKDRQRAGTVTGVAVNVASALAVMLQPYMPSVSVAIQGQLRIPVSPLFQ

KLENDQVEALRKRFGGGQPEDLAVKPQAKQTHPAPAAPVAPGDPQLIQELTEKVAEQGNYVRQLKANKAEKAEVDTEVAK

LLELKKQLLQAEGKSPEVPVPKGKRKK

>KFQ77160.1 Methionine--tRNA synthetase, cytoplasmic, partial [Phoenicopterus ruber ruber]

QPAASAALYAQLVYGKKGLEAVEALGKLLAHIEQNLSRRGTAYLAGDTKSVADVVVWGTLFPVLQDETSLPNELQALRTW

FQSMTLSEACRKAADSVLMPKALLEFKSYLQKQPPPCLAMERATSNEPEEEEGSERALTEEEITAAVDAWAHGAAALPKP

WQPQKPVLPVEGMRNVLVTSALPYVNNVPHLGNIIGCVLSADTFARYCRLRNWNTLYVCGTDEYGTATETKAVEEGLTPQ

EICNKYNAIHADIYRWFNISFDYFGRTTTPYQTTIAQDIFQRLLARGFLLQDTVEQLRCENCQRFLADRFVEGTCPFCSY

EEARGDQCDKCGKLINAVELKNPQCKLCRGNPVVKPTQHLFLDLPKLEERLGQSWATGDWTANARYITRSWIRDGLKPRC

ITRDLKWGTPVPLDGFHDKVFYVWFDAPIGYLSITANYTDQWERWWKNPQQVELYNFMAKDNVPFHSVIFPCSLLGADDN

YTLVNHLIATEYLNYEDGKFSKSRGVGVFGDMAKDTGIPADIWRFYLLYLRPEGQDSAFSWSDLMLKNNSELLNNLGNFI

NRAGMFVCKFFGGIVPSMVLTPDDKRLLARVTLELRQYHQLLEKVRIRDALRCILSISRHGNQYIQVNEPWKRIKGDETD

R

>XP_025001057.1 methionine--tRNA synthetase, cytoplasmic isoform X3 [Gallus gallus]

MRLRVAAGSPAALKVVAAAHCAGAPVQLCWAEPGEPPPSPLCPPWAPALQLESGAVLFSPNAICQYFFLRRGEQPTDLTT

QWLEWEATELQPAALAALYAHLVHGRTGVEALGEPLGHIERSLVGRGTAYLAGDTESVADVVVWSTLFPVLRDEAIPAAE

PLQSWFRSMTFSEACRNAADTVLLPRHLQEFTACLQKLPLPRPPPDTAPGCEEDDVSERALTEEDMAAAVDAWTRGAAAL

PKPWRPPKPVLPVEGMRNVLITSALPYVNNVPHLGNIIGCVLSADAFARYCRLRNWNTLYVCGTDEYGTATETKAVEEGL

TPREICDKYHAVHADIYRWFDISFDYFGRTTTPQQTVISQDIFQRLQAHGFVLQDTVQQLHCASCQRFLADRFVEGICPF

CSYQEARGDQCDKCGKLINAVELKTPRCKLCSGSPTVRPTQHLFLDLPKLEERLQPWLENCWATGNWTANARYITRSWIR

DGLRPRCITRDLQWGTPVPLDGFRDKVFYVWFDAPIGYLSITANYTEHWERWWKNPQQVELYNFMAKDNVPFHSVVFPCS

LLGTDDNYTLVNHLIATEYLNYEDGKFSKSRGVGVFGDMAKDTGIAADVWRFYLLYVRPEGQDSAFSWGDLMLKNNSELL

NNLGNFINRAGMFVCKFFGSAVPPMVLTPDDHRLLARVTVELRQYHQLLERVRIRDALRCVLNISRHGNQYIQVNEPWKR

IKGGEEDR

>XP_007053260.1 PREDICTED: methionine--tRNA synthetase, cytoplasmic [Chelonia mydas]

MRGVAVRGSSPAVAVHGSRRMMAMCGPSPAVALGTCMGCPGRVVPFLTHPRVPALHLDSGTFLFSPNAICQYFFLLAGKE

PDDLTNQWLEWEATELQPAVSTALYCHLVQGKKEEVLGAIRKPLTYIDQSLTSKATPYLAGEAESVADVVLWEEELAERS

LSEEEIAAAAEAWPRGLAALPERWQPPSPVLPVEGRRNILITSALPYVNNVPHLGNIIGCVLSADVFARYCRLRNWNTLY

VCGTDEYGTATETKALEEGLTPQQICDKYNAIHAQIYRWFDISFDYFGRTTTPHQTTIAQDIFQRLLERDMLRTDTVDQL

RCERCQRFLADRFVEGTCPFCNYEEARGDQCDKCGKLINAVELKKPQCKVCKDSPVVKSSQHLFLDLPKLEGRLEKWLEQ

SWATGDWTANSRFITRSWIRDGLKPRCITRDLKWGTPVPLDGFRDKVFYVWFDAPIGYLSITANYTDQWEKWWKNPQQVQ

LYNFMAKDNVPFHSVVFPCTLLGAEDNYTLVNHLVATEYLNYEDGKFSKSRGVGVFGDMAQDTGIPADIWRFYLLFLRPE

GQDSTFSWTDLMLKNNSELLNNLGNFVNRAGMFVCKFFGGRVPHMELGTDDKRLLAHVTLELRQYNQLLEKVRIRDALRC

ILSVSRYGNQYIQVNEPWKRIKGHDADRTRAGTVTGVAVNIASLISVMLQPYMPSISATIQRQLCVPKACNVLTDTFLCL

LPPGHQIGTVSPLFQKLESEQIEALRKRFGEGQVRKGEPQSKAQPHLLCGWGLPRLADWKEAPAPQGSRGGAQKSPWFLS

DMTLGGDPGQQGLEDSTLELKAHAAYVASADVTPPGPGGGVVLAGDPQKAEELAEELAKQADHVRQLKASKAEKGQIDAE

VAKLLELKKQLALAEGKTPEPPAPKGKKRK

>PKK16793.1 hypothetical protein A306_00000167, partial [Columba livia]

SAPSRPEPCVCVRQPAAPAALYAQLVHGQKGLEALEPLGQLLEHIEQDLSSRGTAYLAGDTQSVADVVVWGALFPVLQDE

TGLPSELPALRTWFQNMTLSEACRKAAGSVLMPEALLEFKLPVHGRRNVLITSALPYVNNVPHLGNIIGCVLSADTFARY

CRLRNWNTLYVCGTDEYGTATETKAMEEGLTPQEICDKYNAIHADIYRWFDISFDYFGRTTTPHQTRIAQDIFQRLLARG

FLLQDTVEQLRCEGCQRFLADRFVEGTCPFCNYEEARGDQCDKCGKLINAVELKNPQCKLCGGVPVVKTTQHLFLDLPKL

QERLEPWLEQSLATGDWTANARYITRSWIRDGLKPRCITRDLKWGTPVPLDGFQDKVFYVWFDAPIGYLSITANYTEQWE

RWWKNPQQVELYNFMAKDNVPFHSVIFPCSLLGADDNYTLVNHLIATEYLNYEDGKFSKSRGVGVFGDMAKDTGIPADIW

RFYLLSVRPEGQDSAFSWGDLMLKNNSELLNNLGNFINRAGMFVCKFFGGVVPDMVLTPDDERLLARVTLELRQYHQLLE

RVNEPWKRIKGDEKDRQRAGTVTGVAVNVASALAVMLQPYMPSVSVSPLFQKLENDQVEALRKRFGGGQELTEKVAEQVS

GGATPALSWLYSPSPQPPRLPQGNYVRQLKANKAEKAEVDTEVAKLLELKKQLLQAEGKSPEVPVPKGKRKK

>AAI59044.1 Unknown (protein for IMAGE:7540772), partial [Xenopus tropicalis]

RLHPILPVEGEKNVLITSALPYVNNVPHLGNIIGSVLSADVFARYCRLRNWNTLYICGTDEYGTATETKAMEEGLTPQQI

CDKYNAVHTAIYQWFDISFDFFGRTTTQHQTTIAQDIFHRLLKRDFLLTDTVEQLRCETCQRFLADRFVEGTCPFCNYEE

ARGDQCDKCGKLINAVELKKPQCKVCKKSPVKKSSKHLFLDLPKLEKRLEQWLEKSFSSGDWTSNARFITRSWIRDGLKP

RCITRDLKWGTPVPLDGFRDKVFYVWFDAPIGYISITANYTDQWEKWWKSTQQVQLYHFMAKDNVPFHSVIFPSCLLGAE

DNYTLVNHIIATEYLNYEDGKFSKSRGVGVFGDMAKDTGIPADIWRFYLLYVRPEGQDSAFSWSDLMLKNNSELLNNLGN

FVNRAGMFVQKFFNGCVPEMELLSEDKRLLAQVAAELQQYNQHLEKVRIRDALRCILNISRHGNQYIQVNEPWKCIKGNQ

QEQKRAGTVTGVAVNMAALLSVMLHPYMPTVSGLIQEQLLMPQESKVLTTDFCCRLQSGHQIGNVSPLFQKLENDQIESL

RKRFGGGQVKTESKAPPCQEAPLKEAPKASGPERVKELMQEVEKQGNLVRELKGKKAEKSTIDTEVQKLLALKKELALAE

GKSPDPPAQKGKKKK

>XP_007658064.1 PREDICTED: methionine--tRNA synthetase, cytoplasmic, partial [Ornithorhynchus anatinus]

MRLFVSEGAPGNLPVLAAAGRAGHTGLQVCTVGPDERVVPFLSRPRVPALELDGGGFLFSTNAICRYFFLLSGWEQDDLT

QQWLEWETTELQPALSASLSALVLQGCQGEKVLQPMRRALAHIDHSLSRRSCPFLSGDEESLADIVLWGTLYPLLQDPAY

LPAQVPGPSGGDVATHSLSEEEIAVAAAAWEKGVDALPQPRPPQHPVLPVPGERNVLITSALPYVNNVPHLGNIIGCVLS

ADVFARYARLRQWNTLYVCGTDEYGTATETKAMEEGLTPQQICDKYHALHATIYRWFAISFDHFGRTTTPQQTTIAQDIF

QRLLARGFLHTATVAQLRCERCARFLADRFVEGVCPFCGYEEARGDQCDKCGKLINAVELKKPQCKICRSTPEVKSSRHL

FLDLPQLEKRLEPWLTKTLAASDWTPNARFITRSWLRDGLKPRCITRDLQWGTPVPLEDFQEKVFYVWFDAPIGYLSITA

TYTDQWERWWKNPEQVELYQFMAKDNVPFHSVVFPCSLLGAEDNYTLVNHLIATEYLNYEDGKFSKSRGVGVFGDMAQGT

GIPADIWRFYLLFVRPEGQDSAFSWADLQLKNNSELLNNLGNFINRAGSFGIH

>OCT95752.1 hypothetical protein XELAEV_18013439mg [Xenopus laevis]

MSFYSLPVEGEKNILITSALPYVNNVPHLGNIIGSVLSADVFARYCRLRNWNTLYICGTDEYGTATETKAMEEGLTPQQI

CDKYNAVHTAIYQWFNISFDYFGRTTTQQQTTIAQDIFHRLLERDFLLTDTVEQLRCETCKRFLADRFVEGICPFCNYEE

ARGDQCDKCGKLINAVELKKPQCKVCKQSPVIKSSKHLFLDLPKLEKHLEQWLEQSFSSGDWTSNARFITRSWIRDGLKP

RCITRDLKWGTPVPLDGFRDKVFYVWFDAPIGYISITANYTDQWEMWWKSPQQVQLYNFMAKDNVPFHSVIFPSCLLGAE

DNYTLVNHLVATEYLNYEDGKFSKSRGVGVFGDMAQETGIPADIWRFYLLYVRPEGQDSAFSWSDLMLKNNSELLNNLGN

FVNRAAMFVQKFFNGCVPEMELLSEDKRLLAQVAAELQQFNQLLEKVRIRDALRCILNISRHGNQYIQVNEPWKCIKGNQ

EEQKRAGTVTGVAVNMAALLSVMLHPYMPTISGVIQEQLLMPQESKVLTTDFCCRLQSGHRIGNVSPLFQKLENDQIESL

RKRFGGGQVKTESKVPPSQEAPLQKTPKASGPERVKELMQEVEKQGNHVRELKGKKAEKSTIDSEVQKLLALKKELALAE

GKSPDPPAQKGKKKK

>XP_010010076.1 PREDICTED: methionine--tRNA synthetase, cytoplasmic, partial [Nestor notabilis]

LPVEGMRNILITSALPYVNNVPHLGNIIGCVLSADTFARYCRLRNWNTLYLCGTDEYGTATETKAVEEGLTPQEICNKYH

AIHADIYRWFGISFDHFGRTTTPSQTTIAQDIFQRLLARGFLLQDTVEQLRCEGCQRFLADRFVEGTCPFCSYEEARGDQ

CDKCGKLINAVELKNPQCKLCRGVPIVKPTQHLFLDLPKVSLRLEAWLEQSWATGDWTANARYITRSWIRDGLKPRCITR

DLKWGTPVPLDGFRDKVFYVWFDAPIGYLSITANYTDQWERWWKNPQQVELYNFMAKDNVPFHSVIFPCSLLGADDNYTL

VNHLIATEYLNYEDGKFSKSRGVGVFGDMAKDTGIPADVWRFYLLYLRPEGQDSAFSWSDLMLKNNSELLNNLGNFINRA

GMFVCKFFGGTVPDMVLTPDDKRLLARVTLELRQYHQLLEKVRIREALRCILSISRHGNQYIQVNEPWKRIKGDEKDRQH

AGTVTGVAVNMASLLAVMLQPYMPSISLAIQGQLHIPPDCFVLSHDFTCTLPPGHRVGTVSPLFQKLENDRVEALRRRFG

GGQPEDLTVEPQ

>XP_023930473.1 methionine--tRNA synthetase, cytoplasmic [Lingula anatina]

MKLISDAGNIHTLKIAIASELTGTHLQYEEVKHDEKVSGALTQNKLPILEVDTGLTLFSANAACRYILCKAGQDVDSLMV

DQWLEWEAAELQPVLWPYLVSSVGQGKVESKQVQAVKGLLAHLDDALHLKELIVRDQISAADIVLWSALFPAFFDIAALQ

DWSSQYLNISRWFNKLSQHPAFQKGFTMVTSGKGVSAFKASLLAQPVPPPAIWDTKKLKMMLTGSGDKENTEPPSEEKSV

SVTAEETKAAVAAWKQMTTPTPVQRTHPILPQEGKRNILITSALPYVNNVPHLGNIIGCVLSADVYARFCRLRNYNILYI

CGTDEYGTATETKALEEGLTPQQICDKYNKLHSEIYEWFNIDFDYFGRTSTTQQTEISQDIFWKLYEKGFVITDTVEQLH

CAKCDRFLADRFVEGTCPLCAYEDARGDQCDKCGKLINATELKNPKCKVCKTTPEIKTSEHLFLDLPKLEPTLSTYLDKQ

FEDGIWTQNAKVITKSWIRDGLKPRCITRDLKWGTPVPLEGFTDKVFYVWFDAPIGYISIMANYTDKWKQWWKNPQQVQL

YNFMAKDNVPFHSVIFPCTLLGADDNFTIVNHLSACEYLNYEDGKFSKSRGVGVFGNNAQETGVPADIWRFYLLFIRPES

QDSTFAWEDFVLKNNSELLNNLGNFINRALMFVANSFGGQVQEITLTSEDEELLALITRDLKAYVDCLEKIKLREGITHI

LNISRLGNQYMQANKPWELVKGDATQKSRAGTVVSLSANIACLLSVLLQPYMPQVSATIQMQVNAPKECSVITENFVCLL

KPGHKIGTPQPLFKKMDPKEAEAHKKKFAGQGDDSKKATSGGKGKGPAAAVAANPAEAERLTAEVAKQGDAVRQLKASKA

DKTTIDAAVAVLLDLKKQLSAAQGQNSHGHSNTADPAAAERLTAEVIKQGDVVRQLKASKADKSSIDEAVAVLLDLKKQL

ALAQGQNPTDVQGSGKKKGKKK

>XP_024260904.1 methionine--tRNA synthetase, cytoplasmic-like, partial [Oncorhynchus tshawytscha]

MVPERQHPILPQEGKRNVLVTSALPYVNNVPHLGNIIGCVLSADVFARYGRLRGWNVLYVCGTDEYGTATENKAREEGLT

AQQICDKYHAVHASIYQWFQVDFDFFGRTTTKKQTEIAQNIFWRLHERGFLLEDIVEQLRCEACQRFLADRFVEGTCPFC

SYPEARGDQCDKCGKLINAVELREPTCKVCSQTPVVQSSKHLFLDLPKLEAELEQWLERSTGSGDWTANAKQITRSWVRD

GLKPRCITRDLKWGTPVPHPDFSDKVFYVWFDAPIGYLSITANYTDQWEKWWKNPQQVELYNFMAKDNVPFHSVVFPCSL

LGAQDNYTLVNHLVATEYLNYEDTKFSKSRGVGVFGDMAKDTGVPSDVWRFYLLYLRPEGQDSAFSWVDMATKNNSELLN

NLGNFINRAGMFVSKFFESCVPAMALQQEDKRLLAQVGWELKQYINLMDRVKIRDALKCILNISRHGNQYIQVNEPWKKI

KGDETDRQRSGTVTGVSVNVACLLSVMLQPYMPTVSHTIRQQLNAPPSVTGTLLQGTGNFVCSLPVGHHIGTVSPLFQKL

ESEQIESLKKRFGGQQPKTTAQPATTPASDPAAQTAVNGADAERARELTLAVAEQGEKVRTLKGQKAEKTVIGAEVTKLL

ELKKQLSLAEGKDPEPVLTKGKTTAQPATTPASDPAAQTAVNGADAERARELTLAVAEQGEKVRTLKGQKAEKTVIGAEV

TKLLELKKQLSLAEGKDPEPVLTKGK

>XP_013384289.1 methionine--tRNA synthetase, cytoplasmic [Lingula anatina]

MKLISDAGNIHTLKIAIASELTGTHLQYEEVKHDEKVAGALTQNKLPILEVDTGLTLFSANAACRYILCKAGQDVDSLMV

DQWLEWEAAELQPVLWPYLVSSVGQGKVESKQVQAVKGLLAHLDDALHLKELIVRDQISAADIVLWSALFPAFFDIAALQ

DWSSQYLNISRWFNKLSQHPAFQKGFTMVTSGKGVSAFKASLLAQPVPLPAIWDTKKLKMTLTGSGDKEKTEPPSEEKSV

SVTAEETKAAVAAWKQMTTPTPVQRTHPILPQEGKRNILITSALPYVNNVPHLGNIIGCVLSADVYARFCRLRNYNILYI

SGTDEYGTATETKALEEGLTPQQICDKYNKLHSEIYEWFNIDFDYFGRTSTTQQTEISQDIFWKLYEKGFVITDTVEQLH

CAKCDRFLADRFVEGTCPLCAYEDARGDQCDKCGKLINATELKNPKCKVCKTTPEIKTSEHLFLDLPKLEPTLSSYLDKQ

FEDGIWTQNAKVITKSWIRDGLKPRCITRDLKWGTPVPLEGFTDKVFYVWFDAPIGYISIMANYTDKWKQWWKNPQQVQL

YNFMAKDNVPFHSVIFPCTLLGADDNFTIVNHLSACEYLNYEDGKFSKSRGIGVFGNNAQETGVPADIWRFYLLFIRPES

QDSTFAWEDFVLKNNSELLNNLGNFINRALMFVANSFGGQVQEITLTSEDEELLALITRDLKAYVDCLEKIKLREGITHI

LNISRLGNQYMQANKPWELVKGDAAQKSRAGTVVSLSANIACLLSVLLQPYMPQVSATIQMQVNAPKECNVITEKFVCLL

KPGHKIGTPQPLFKKMDPKEAEAHKKKFAGQGDDSKKATSGGKGKGPAAAVAANPAEAERLTAEVAKQGDAVRQLKASKA

DKTTIDAAVAVLLDLKKQLSAAQGQNSHGHSNTADPAAAERLTAEVTKQGDVVRQLKASKADKSSIDEAVAVLLDLKKQL

ALAQGQNPTDVQGSGKKKGKKK

>KFQ47148.1 Methionine--tRNA synthetase, cytoplasmic, partial [Nestor notabilis]

LPVEGMRNILITSALPYVNNVPHLGNIIGCVLSADTFARYCRLRNWNTLYLCGTDEYGTATETKAVEEGLTPQEICNKYH

AIHADIYRWFGISFDHFGRTTTPSQTTIAQDIFQRLLARGFLLQDTVEQLRCEGCQRFLADRFVEGTCPFCSYEEARGDQ

CDKCGKLINAVELKNPQCKLCRGVPIVKPTQHLFLDLPKVSLIPGEWDWTANARYITRSWIRDGLKPRCITRDLKWGTPV

PLDGFRDKVFYVWFDAPIGYLSITANYTDQWERWWKNPQQVELYNFMAKDNVPFHSVIFPCSLLGADDNYTLVNHLIATE

YLNYEDGKFSKSRGVGVFGDMAKDTGIPADVWRFYLLYLRPEGQDSAFSWSDLMLKNNSELLNNLGNFINRAGMFVCKFF

GGTVPDMVLTPDDKRLLARVTLELRQYHQLLEKVRIREALRCILSISRHGNQYIQVNEPWKRIKGDEKDRQHAGTVTGVA

VNMASLLAVMLQPYMPSISLAIQGQLHIPPDCFVLSHDFTCTLPPGHRVGTVSPLFQKLENDRVEALRRRFGGGQ

>XP_021373988.1 methionine--tRNA synthetase, cytoplasmic-like [Mizuhopecten yessoensis]

MHLHVQNAEVPTRLRNNLDLNEYLWSLLSNLAIAVPVSAANLRNIEIQCKIHAVFTKHFTSGKQRHLFIRVKIALYNNLN

VLTRVANHLHTFYIQYNRVFVCQNISYLIFLNGVCAYTFSSNKTKVLTSGPLSQEVSYRPLHTCVIFTLPAILLTFHINR

GNFGMKLHTNPWNFNSLKLLAAAELSGIKLEVVERKHDDKVVPYLTRNTLPVLEVFPNKFLFSVGAATRYLLNQQSLSPE

DDSTVDRWLEWDSNQLQPSLVPYLVSVIGQNKPDAFLLSQIQTLLKLLNTELKGRQTLTKTGITAGDITLWGSLYPIFKP

GKASIKGWEGLYPNIKAWFCRVEEEPAVQKSISVVVGGKDTGVFKDSLLAQPVPLSPPTPSSLKPDKVVADSNKKQQSKG

KPEKMEADPAESQILAARPVTPEELAAAVKAWNSGKGSSPKVRERVHPVLPKDGERNIMITSALPYVNNVPHLGNIIGCV

LSADVYSRYCRLRNYNVFYLCGTDEYGTATETKAIEEGLTPQQICDKYNKIHSDIYEWFNIDFDYFGRTTTTQQTKIAQD

IFWKLYNQGHILKDSMEQLQCVKCNRFLADRFVEGTCPLCNFDDARGDQCDACGKLINATELKSPKCKLCKSTPIVKTSD

HLFLDLPKMEPFLQKHLDSAYSTGTWSNNAKNITNSWIRDGLKPRCISRDLKWGTQVPLEGYTDKVFYVWFDAPIGYISI

TANYTDQWEKWWKNPKQVEMYNFLGKDNVPFHSVIFPCTLLGADDNYTIVNHMVATEYLNYEDGKFSKSRGTGVFGNNAR

DTGIPADIYRFYLLYVRPETQDSSFSWDDFLLKNNSELLNNLGNFINRALMFVSNNFDGTIQEMILTDDDNQLLVQVTRE

LRTYIENMEQAKIRDSIRNILSISRLGNQFMQANQPWVLVKGSDADKKRAGSVVSLAANISTLLSVLLQPYMPATSETIQ

KQLNCPKEANVVHPEFLCHLPPGHKIGQPSPLFEKIAASKMDDLKKKFAGEPAPEKKETKSSKRTGGDAAAAASGPCDPV

EVARLTDEVAKQGLVVRDLKTKKADKSEIDAQVAKLLDLKKRLALAQGQNSVPAAGGNKKKDKKSAKQPASGQGSAPPAS

VAPPASSNAPGSSAEVDRLTEEVSKQGIMVRDLKSKKAEKSQIDGEVAKLLDLKKQLALAQGQDPNAAVGGGKKKGKKK

>OWF40602.1 Methionine--tRNA synthetase, cytoplasmic [Mizuhopecten yessoensis]

MKLHTNPWNFNSLKLLAAAELSGIKLEVVERKHDDKVVPYLTRNTLPVLEVFPNKFLFSVGAATRYLLNQQSLSPEDDST

VDRWLEWDSNQLQPSLVPYLVSVIGQNKPDAFLLSQIQTLLKLLNTELKGRQTLTKTGITAGDITLWGSLYPIFKPGKAS

IKGWEGLYPNIKAWFCRVEEEPAVQKSISVVVGGKDTGVFKDSLLAQPVPLSPPTPSSLKPDKVVADSNKKQQSKGKPEK

MEADPAESQILAARPVTPEELAAAVKAWNSGKGSSPKVRERVHPVLPKDGERNIMITSALPYVNNVPHLGNIIGCVLSAD

VYSRYCRLRNYNVFYLCGTDEYGTATETKAIEEGLTPQQICDKYNKIHSDIYEWFNIDFDYFGRTTTTQQTKIAQDIFWK

LYNQGHILKDSMEQLQCVKCNRFLADRFVEGTCPLCNFDDARGDQCDACGKLINATELKSPKCKLCKSTPIVKTSDHLFL

DLPKMEPFLQKHLDSAYSTGTWSNNAKNITNSWIRDGLKPRCISRDLKWGTQVPLEGYTDKVFYVWFDAPIGYISITANY

TDQWEKWWKNPKQVEMYNFLGKDNVPFHSVIFPCTLLGADDNYTIVNHMVATEYLNYEDGKFSKSRGTGVFGNNARDTGI

PADIYRFYLLYVRPETQDSSFSWDDFLLKNNSELLNNLGNFINRALMFVSNNFDGTIQEMILTDDDNQLLVQVTRELRTY

IENMEQAKIRDSIRNILSISRLGNQFMQANQPWVLVKGSDADKKRAGSVVSLAANISTLLSVLLQPYMPATSETIQKQLN

CPKEANVVHPEFLCHLPPGHKIGQPSPLFEKIAASKMDDLKKKFAGEPAPEKKETKSSKRTGGDAAAAASGPCDPVEVAR

LTDEVAKQGLVVRDLKTKKADKSEIDAQVAKLLDLKKRLALAQGQNSVPAAGGNKKKDKKSAKQPASGQGSAPPASVAPP

ASSNAPGSSAEVDRLTEEVSKQGIMVRDLKSKKAEKSQIDGEVAKLLDLKKQLALAQGQDPNAAVGGGKKKGKKK

>CAA89153.1 orf, partial [Homo sapiens]

KLINAVELKKPQCKVCRSCPVVQSSQHLFLDLPKLEKRLEEWLGRTLPGSDWTPNAQFITRSWLRDGLKPRCITRDLKWG

TPVPLEGFEDKVFYVWFDATIGYLSITANYTDQWERWWKNPEQVDLYQFMAKDNVPFHSLVFPCSALGAEDNYTLVSHLI

ATEYLNYEDGKFSKSRGVGVFGDMAQDTGIPADIWRFYLLYIRPEGQDSAFSWTDLLLKNNSELLNNLGNFINRAGMFVS

KFFGGYVPEMVLTPDDQRLLGHVTLELQHYHQLLEKVRIRDALRSILTISRHGNQYIQVNEPWKRIKGSEADRQRAGTVT

GLAVNIAALLSVMLQPYMPTVSATIQAQLQLPPPACSILLTNFLCTLPAGHQIGTVSPLFQKLENDQIESLRQRFGGGQA

KTSPKPAVVETVTTAKPQQIQALMDEVTKQGNIVRELKAQKADKNEVAAEVAKLLDLKKQLAVAEGKPPEAPKGKKKK

>PIO34351.1 hypothetical protein AB205_0158050 [Rana catesbeiana]

MRINPDVLPIEGEKNLLITSALPYVNNVPHLGNIIGSVLSADVFARYCRLRNWNTLFVCGTDEYGTATETKAMEEGLTPQ

QICDKYNAVHTAIYKWFNISFDYFGRTTTSHQTKIAQDIFHRLLERGFLLEDTVEQLRCETCQRFLADRFVEGICPFCNY

EEARGDQCDKCGKLINAVELKRPQCKVCKQSPVIKSSRHLFLDLPKLEERLEKWLDESLATGDWTTNARYITRSWIRDGL

KPRCITRDLKWGTPVPLDGFRDKVFYVWFDAPIGYISITANYTAQWEKWWKNPKQVQLYNFMAKDNVPFHSVIFPSCLLG

AEDNYTVVNHLIATEYLNYEDGKFSKSRGVGVFGDMAQDTGIPADIWRFYLLYVRPEGQDSAFSWGDLMLKNNSELLNNL

GNFVNRAGMFVQKFFNGQVPEIVLLPEDKRLLAHVTAELQQYNQLLEKVRIRDALRCILNISRHGNQYIQVNEPWKCIKG

TQEEQKRAGTVTGLAVNMAALLSIMLQPYMPTISSVIREQLLMPEEANVLTNDFPCILQSGHHIGNVSPLFQKLENDQIE

SLRKRFGGGQLDADSKEPEVFLYALLKIAWQLFHSSISYS

>XP_022343888.1 LOW QUALITY PROTEIN: methionine--tRNA synthetase, cytoplasmic-like [Crassostrea virginica]

MKLYTDRGNFNSLKVIAALEFTALPYSVTECGLEDRVVPYLSAPKLPVLEVXPQRFLFSPNSATRYILNKKSEEDDPNID

KWLEWESVQLQPYVVRYLVSVIVXKKPDLESLSRIQNLLKFLNAELKSKSLTKIKDCAADFVLWGSLFPLFRGTAELKGW

ESQYSNIKSWFNGVDSLPCCQTAQSKVIPQSDTTCLKASLNAQPLPLLETETRSEEPAQKPKPRKXKMEKPSELPPAEES

LLAKEATPEEIQAAKESWRAGLSKCPKPRQRKHPILPKSGERNLLITSALPYVNNVPHLGNIIGCVLSADVFSRYCQLRN

YNCLYVSGTDEYGTATETKAIEEGLTPQEICDKYNKIHTEIYQWFNISFDKFSRTSIPAHTKIAQDIFVKLEKNGYISKD

GLEQLQCQQCLRFLADRFVEGTCPLCGFDDARGDQCDGCGKLLNAVDLLNPKCKVCNGIPQIKSSNHLFLDLPKVENKLK

SHLDAQFKKGIWTNNAKVITNAWIRDGLKPRCISXDLKWGIPIPLEGYQDKVFYVWYDAPIGYISATASYTDQWEQWWKN

PKQVELFNFLGKDNVPFHSVIFPSTLLGADDNFTVVNHMSATEYLNYEDTKFSKSRGTGVFGHQARETGIPADIFRFYLL

YVRPESQDSSFSWDDLQLKNNSELLNNLGNFINRALMFVSNSFGGEIQQMKLTEEDFHLLALVNRELITYIENMEQIKLR

DSIRNILSISRLGNQYMQANQPWVLVKGSEQDRQRAGTVVSLCANIATLLSVLVQPYMPAVSATIQHQLKTPPDCNVIHK

ELVCRLPPGHKIGKPSPLFEKIETXKDSEMKKKFEGKPAPQKATANASSGNPQEVQKLTDEVAAQGNIVRELKAQKAEKA

VINEEVAKLLALKKRLTAMTGEPVPAGGKQNSKQAKQENKTPVKVNNGKEDSQEVERLTEAVNKQGTLVRDLKSQKAEKS

RIDXEVAVLLDLKRQLAVAQGQDPEALTGGKGKKGKKK

>XP_020331439.1 methionine--tRNA synthetase, cytoplasmic-like, partial [Oncorhynchus kisutch]

MLASCVSAAQTVLQGKGLKAESLKTYLQRQPTTHHGQGQVQGRGTQPCNSPEGGDDDDCVLSEEEIQSAAVTWGKGLTDL

PMVPERQHPILPQEGKRNVLVTSALPYVNNVPHLGNIIGCVLSADVFARYGRLRGWNVLYVCGTDEYGTATENKAREEGL

TAQQICDKYHAVHASIYQWFQVDFDFFGRTTTKKQTEIAQNIFWRLHERGFLLEDIVEQLRCEACQRFLADRFVEGTCPF

CSYPEARGDQCDKCGKLINAVELREPTCKVCSQTPVVQSSKHLFLDLPKLEAELEQWLERSTGSGDWTANAKQITRSWVR

DGLKPRCITRDLKWGTPVPHPDFSDKVFYVWFDAPIGYLSITANYTDQWEKWWKNPQQVELYNFMAKDNVPFHSVVFPCS

LLGAQDNYTLVNHLVATEYLNYEDTKFSKSRGVGVFGDMAKDTGIPSDVWRFYLLYLRPEGQDSAFSWVDMATKNNSELL

NNLGNFINRAGMFVSKFFESCVPAMALQQEDKRLLAQVGWELKQYINLMDRVKIRDALKCILNISRHGNQYIQVNEPWKK

IKGDETDRQRSGTVTGVSVNVACLLSVMLQPYMPTVSQTIRRQLNAPPSVTGTLLQGTGNFVCSLPAGHHIGTVSPLFQK

LESEQIESLKKRFGGQQPEEEEEAKLKPKTRAQPDVNAQPATTP

>PVD37251.1 hypothetical protein C0Q70_04247 [Pomacea canaliculata]

MKLYTDFGNFQTLKVLSAVKIAGIAIEKTDVDHGAQVVPYLKKSRLPVLEVSPNKFLFSANSIVSYLFSSQQQQTLGDEE

QQWLEWESVVLQPVALPYLTSALGHEKPEKDLLKSFPPLLQELDNGLRDRQYLVGEKPSAADVVLFGTVFPLLQDKLFND

ILHQFPAVRTWINTIQSQKAVQEAINEVTRGTGAKAFKQSLLTQPVPSVPVSAVANVPVPISKKQTTNANNIPQFSASDV

PEVEKVSAHEAEAAFTAWAADSKHFPCPLPRKHPILPVDGGRNILITSALPYVNNVPHLGNIIGCVLSADAFSRFCRLRN

YNVLYICGTDEYGTATETKALEEGLTPQEICDKYNKLHTEIYQWFNIDFDYFGRTTTVQQTEIAQDIFWRLHERGFILSD

SIEQLHCSHCDKFLADRYVEGTCPLCGFDDARGDQCDGCGRLINATELKNPRCKLCQKKPSPRVSQHLFLDLPQLEPQLK

SHLDKVFSSGTWTTNSRTITSSWIRDGLKPRCISRDLKWGTPVPLEGYTDKVFYVWFDAPIGYLSITANYTDHWKKWWKN

PEKVELYNFLGKDNVPFHSVIFPSCLLGADDNYTVVNHMVATEYLNYEDTKFSKSRGTGVFGDQAKDTGIPADIYRFYLL

YVRPESQDSSFSWDDLLLKNNSELLNNLGNFINRALMFLSNNFGGVVQEMIIEDEDKQLLALINRELATYITNMDHGRLR

DGIRNILTISRLGNQYMQSNKPWVHVKGSAEEKKRAGTVVSLSANVASLLSVLLIPYMPDTSATIQKQLNYPPDAIIISD

TFLCRLPPGHALGKPSPLFQKIEASTIAELKTKFAGKPSPPNPATPSAKGDAKSASDPAEVSRLTQEVSNQGNRVREMKA

AKADKKDIETEVAKLLELKRILALAQGDDTSSSATGVSKKKGKKK

>XP_019922394.1 PREDICTED: methionine--tRNA synthetase, cytoplasmic [Crassostrea gigas]

MKLYTDRGNFKSLKVIAALEFTGLSYSLTECGLEERVVPYLSGTSLPVLEVSPQRFLFSPNTATRYILNKKSDEDDPNID

KWLEWESVQLQPYLVRYLVSVSVHKKPDAESLNRLQQLLKFLNAELKSKSLTKTKDCAADFVLWASLFPVFKGAAEIKGW

EGQYSNIKSWFNTINSLPCCQTALDKVIPGGDTTCLKASLNTQPVPLLETETRQEEPVQKSKPQKLKMEKPSETPTPEES

LLTKEASPEEIQDAKESWLTGKSKFPKPRERKHPILPKSGERNLLITSALPYVNNVPHLGNIIGCVLSADVFSRYCRLRN

YNCLYICGTDEYGTATETKAIEEGVTPREICDKYNKIHAEIYDWFNISFDKFSRTSIPEQTKIAQDIFWKLEKNGFISRD

SVEQLQCQQCLRFLADRFVEGTCPLCGFDDARGDQCDGCGKLLNAIELKDPKCKVCKGIPQVKSSNHLFLDLPKIEDKLK

AHLNAQFDKGIWTNNARVITNSWIRDGLKPRCISRDLKWGIPVPLEGYQDKVFYVWFDAPIGYISATASYTDRWEQWWKN

PKQVELYNFLGKDNVPFHSVIFPCSLLGADDNFTVVNHMSATEYLNYEDTKFSKSRGTGVFGHQAKDTGIPADIFRFYLL

FVRPESQDSSFSWDDFQLKNNSELLNNLGNFINRALMFVSNSFGGQIQEMTLTPADFHLLALVNRELASYIDNMEQIKLR

DSIRNILSISRLGNQYMQANKPWVLVKGSESDKRRAGTVVSLSANLATLLSVLVQPYMPAVSDTIQGQLKTPPQCNVIFK

EFVCQLPPGHKIGKPSPLFEKIETPKIQELKKKFEGKPAPQKNASKASAGNPEETQKLTDEVAAQGNLVRELKAKKAEKA

VVTEEVAKLLALKKKLADLMGEPAPAGGKQNSKQAKKESKKPEVVSSGTTANGVVDSQEVERLTEAVSKQGLLVRDLKSQ

KAEKSRIDSEVAVLLDLKRKLAVAQGQDPEALTGGKGKKGKKK

>XP_022787444.1 methionine--tRNA synthetase, cytoplasmic-like [Stylophora pistillata]

MFRTCSGFSSGALLRSVILTRLLHCRRSLSSQISEIMKLYTDRGNPFLLSILAAKNLAGVPLTVQYISHEDGSKLNCPGD

RKLPVLEAEHGVLLYSPNSICRYLLDLKSNLEETPDPNREAIVDQWVEWESRSLQPVLFRHLVSSIGLGRQKSSHVDDLM

NLLTHADQALSAYKFFSGDQLGLADVILWGSLHPLFIADSSFSDPQINNIRKWYGQLMSQDVFHKSASEVTGDKGASAFK

DSLLAMSGTQVPPSSKPQEGTKSKQSVKGSQPPQQEKGNGISQDEVGQVRVASSEEIAEAVKNWNQGSKNRPKRRRLMDP

ILPIEGEKNILITSALPYVNNVPHLGNIIGCVLSADVFARYCRLRQHNTLYICGTDEYGTATETKALEEGLTPQQICDKY

FKIHKEVYEWFQIKFDFFGRTTTQQQTEIAQDIFWRLHKQDHLVEDTLEQLQCVDCKRFLADRFVEGTCPFCNFEDARGD

QCDSCGKLINAIELKKPRCKVCGGTPKVRSSKHLFLNLAKLEPSLKSWVDKSSSEGTWTSNARHITRGWVQEGLRPRCIT

RDLKWGTPVPLEGYTDKVFYVWFDAPIGYLSITANYTKEWEKWWKNPQQVTLHQFMAKDNVPFHTVVFPCTLIGADDNYS

LLNSISATEYLNYEDDKFSKSRGLGVFGNDAADTGIPADIWRFYLLYVRPENQDSAFSWTDFVSRNNTELLNNFGNFINR

ALMFVQNFFGGAIPSIDLTDEDKNFLALINKELKTYIDDLEKMRLREGLRGVLNISKLGNQYFQSNQPWVLVKGSESDRS

RAGTVISIAANLSWQLSVLIHPYMPGVSEEIQRQLQVSDDGNVVLDNFIPYLQPGHKIGTPKPLFQKIDASAADQFRAKY

GGNKQKQQTAGDKAANTPNGNAADMDVATLQAEITKQGDKVRKLKTEKAEKEKVDAEVAILLALKKNLAVAEGKDPNEQE

GSSKGKKKGKKK

>KXJ18562.1 Methionine--tRNA synthetase, cytoplasmic [Exaiptasia pallida]

MKLYSDKGBPNLLRILVAKNLSGQXVDLKYLSQEEKQKLPLPGIHTKLPVLELDDGHFLFSPNAICRYFMEGIAKKKLNA

EQDAIDNHWIEWENTHLQPAILSCLQSNNTKQSKGNLSVFLSHLELALKXQRHISGEKPGLADVVVWSSLYPLVCSEPQI

FGAQYENIRKWNSTIAELKAFSDGVEEGNFSCATFKEFFQSHSNSXTAASDSQPSDESQRQPKAIKNNKSKGQQKPXATK

VNGTAEENTGITITKIQASASEISDSFNAWVASNHTTAKKRDHPILPIEGEKNILISSALPYVNNVPHLGNIIGCVLSAD

VFARYCRLRGYNTLYICGTDEYGTATETKALEEGLTPKEICDKYYKIHFDVYSWFNIKFDYFGRTTTEQQTKIAQDIFWR

LHKRGHLLEDSIEQLQCTACKRFLADRFVEGTCPFCNYEDARGDQCDACGKLINAIELKNPKCKVCGGKPEAKSSKHLFL

DLAELEPNLKAWVEXSSSEGNWTVNSRQITRSWIQEGLKPRCITRDLKWGTPVPLDGYTDKVFYVWFDAPIGYLSITANY

TEEWERWWKNPQQVTLYQFMAKDNVPFHTVVFPSTLLGADDNYTLLNSISATEYLNYEDDKFSKSRGVGVFGDNATDSGI

PADIWRFYLLYMRPEGQDSNFSWSDFVLKNNSELLNNLGNFINRALMFLNNNFNGVVPNMELQDEDKNFIALVNRELKSF

LDNMEKIRLREGLKVVLNISKYGNQYVQANKPWVLVKGDDNERKRAGTVIGLASNLSFLLSVLIHPYMPSISAEIRSQLQ

VESDFNVLTEYFIPILKPXHKIGQPKPLFQKIEPSLADXLRKKYAGQKKTTPQKPAPSPSKPPPAAANGNQDIERLQNEV

TAQGDKVRNLKTNKAEKSVIDAEVARLLDLKKQLALAQGKDPAENTASTKSKKKNKKK

>XP_020892873.1 methionine--tRNA synthetase, cytoplasmic-like [Exaiptasia pallida]

MFTAIKPVAGLFRHPLHFKKIFSGFPTRLRYYYKISCDMKLYSDKGBPNLLRILVAKNLSGQXVDLKYLSQEEKQKLPLP

GIHTKLPVLELDDGHFLFSPNAICRYFMEGIAKKKLNAEQDAIDNHWIEWENTHLQPAILSCLQSNNTKQSKGNLSVFLS

HLELALKXQRHISGEKPGLADVVVWSSLYPLVCSEPQIFGAQYENIRKWNSTIAELKAFSDGVEEGNFSCATFKEFFQSH

SNSXTAASDSQPSDESQRQPKAIKNNKSKGQQKPXATKVNGTAEENTGITITKIQASASEISDSFNAWVASNHTTAKKRD

HPILPIEGEKNILISSALPYVNNVPHLGNIIGCVLSADVFARYCRLRGYNTLYICGTDEYGTATETKALEEGLTPKEICD

KYYKIHFDVYSWFNIKFDYFGRTTTEQQTKIAQDIFWRLHKRGHLLEDSIEQLQCTACKRFLADRFVEGTCPFCNYEDAR

GDQCDACGKLINAIELKNPKCKVCGGKPEAKSSKHLFLDLAELEPNLKAWVEXSSSEGNWTVNSRQITRSWIQEGLKPRC

ITRDLKWGTPVPLDGYTDKVFYVWFDAPIGYLSITANYTEEWERWWKNPQQVTLYQFMAKDNVPFHTVVFPSTLLGADDN

YTLLNSISATEYLNYEDDKFSKSRGVGVFGDNATDSGIPADIWRFYLLYMRPEGQDSNFSWSDFVLKNNSELLNNLGNFI

NRALMFLNNNFNGVVPNMELQDEDKNFIALVNRELKSFLDNMEKIRLREGLKVVLNISKYGNQYVQANKPWVLVKGDDNE

RKRAGTVIGLASNLSFLLSVLIHPYMPSISAEIRSQLQVESDFNVLTEYFIPILKPXHKIGQPKPLFQKIEPSLADXLRK

KYAGQKKTTPQKPAPSPSKPPPAAANGNQDIERLQNEVTAQGDKVRNLKTNKAEKSVIDAEVARLLDLKKQLALAQGKDP

AENTASTKSKKKNKKK

>XP_005098473.2 PREDICTED: methionine--tRNA synthetase, cytoplasmic-like [Aplysia californica]

MASFISDSVVPYLSKQKLPVLEIEDGKFIFSSNSAAKYLFLKGKIPTEEASMDEILSWESEVLLPVIAPFLVFLVGQAKR

DSKLIEKVEKALQFVDKKLGASKFLTGDTFGVADVCVFCTIYPLFNLKNSSLADKFTNLRRFYDEVSSKAVVQGVLSEEW

KENNQQDAFKASLLAQRFPPIPGASASVSPAAAKPSASVSLSVSSEDQVPVEKVSQEEADAALKAWGKDPKDCPKPRVRQ

HPVLPDPKARNIMVTSALPYVNNVPHLGNIIGCVLSADVYSRFARLRNYNVLYVCGTDEYGTATETKAMEEGVTPKEICD

KYNKLHTQIYEWFNIDFDYFGRTTTELQTEIAQDIFWKLYQEGYILKDSIEQLYCTKCNKFLADRFVEGTCPMCGFDDAR

GDQCDGCGKLINAVELINPKCKMCKSSPVVKSTQHLFVDLPKLEPKLKDHLNRVYESGTWTNNARVITNSWIRDGLKPRC

ISRDLKWGTPIPLEGYTDKVFYVWFDAPIGYISITANYTDKWREWWKNDEKVEMYNFLGKDNVPFHSVIFPSCLLGANDK

YTVVNHMIATEYLNYEDTKFSKSRGSGVFGDQARDTGIPADVYRFYLLYLRPESQDTAFSWDDFLLKNNSELLNNLGNFI

NRALMFLNNNFAGKVQEMQVNEEDTQLLALISREIQGYVGNLEACRLRDGIRNILTISRLGNQYMQNNKPWVLAKGNAEE

KLRAGCVVSLCANVSCLLSVLLQPYMPSISATIQGQLQAPSTVNVITGEFVAMLKPGHQIGKPSPLFQKIEASTMADLKA

RFEGKPAEKPAPKKGGGAPAVAVTKASPEEVERLTKAVSDQGNKVRELKTAKAEKSIVDAEVATLLKLKQELAAAQGVDP

NAAAGGKKKGGKSGGGGGGGGKSGGGGGGGGKKEKAGGGGSGAAPAVASPAVNGGAGDSPSSPEVARLTQAVADQGNKVR

DLKAAKAEKSAIDTEVASLLQLKRELAVAQGQDPDAAVGGGSSKKKGKKK

>XP_009819363.1 PREDICTED: methionine--tRNA synthetase, cytoplasmic, partial [Gavia stellata]

PAASAALYAQLVHGKKGLEAAEALGKLLAHIEQNLSRRGTAYLAGNTESVAGVXXXGTLFPVLRDETSLPSELQALRTWF

WSMTLSEACRKAADSVLMPKALLEFKAYLQKQPPPCLAVEKATSNEPEEEEGSERALTEEEITAAVDAWARGAAALPKPW

QSQKPVLPVEGMRNVLITSALPYVNNVPHLGNIIGCVLSADTFARYCRLRNWNTLYVCGTDEYGTATETKAVEEGLTPQE

ICNKYNAIHADIYRWFDISFDYFGRTTTPYQTTIAQDIFQRLLARGFLLQDTVEQLRCEDCQRFLADRFVEGTCPFCSYE

EARGDQCDKCGKLINAVELKNPQCKLCRGVPVVKPTQHLFLDLPKLEERLEPWLEQSWATGDWTANARYITRSWIRDGLK

PRCITRDLKWGTPVPLDGFRDKVFYVWFDAPIGYLSITANYTDQWERWWKNPQQVELYNFMAKDNVPFHSVIFPCSLLGA

DDNYTLVNHLIATEYLNYEDGKFSKSWGVGVFGDMAKDTGIPADIWRFYLLYLRPEGQDSAFSWSDLMLKNNSELLNNLG

HFINRAGMFVCKFFGGTVPNMVLTADDKRLLARVTLELRQYHQLLEKVR

>PNI39261.1 MARS isoform 18, partial [Pan troglodytes]

MRLFVSEGVPGCLPVLAAAGRARGRAEVLISTVGPEDCVVPFLTRPKVPVLQLDSGNYLFSTSAICRYFFLLSGWEQDDL

TNQWLEWEATELQPALSAALYYLVVQGKKGEDVLGSVRRALTHIDHSLSRQNCPFLAGETESLADIVLWGALYPLLQDPA

YLPEELSALHSWFQTLSTQEPCQRAAETVLKQQGVLALRPYLQKQPQPSPTEGRAVTNEPEEEELATLSEEEIAMAVTAW

EKGLESLPPLRPQQNPVLPVAGERNVLITSALPYVNNVPHLGNIIGCVLSADVFARYSRLRQWNTLYLCGTDEYGTATET

KALEEGLTPQEICDKYHIIHADIYRWFNISFDIFGRTTTPQQTKITQDIFQQLLKRGFVLQDTVEQLRCEHCARFLADRF

VEGVCPFCGYEEARGDQCDKCGKLINAVELKKPQCKVCRSCPVVQSTQHLFLDLPK

>KFM59101.1 Methionine--tRNA synthetase, cytoplasmic, partial [Stegodyphus mimosarum]

MKLYIDDNCSISSLKILAISKFYCINVNVSTSSGVRNSRVPIFEASDGSSYFSANASCRYLVEVSGNPVNYETDAWLEWE

SSTLQPSVGLLLKTGKGETMPADFKRNLIYLNAVLGKQNYFSGEDSLNVSDIVIWANLYPLFKSSLLSDVSDLQNIQRWI

LSLKSQDSLNKAFLIIFGDAKSENLKKKLAVQPVHYIQPCSFMPIEVPGISLETLNIAEVASKENEESVKEPEVTSEEIT

TAYEKWLKGRTMLLSIKKEVPVLPKKGEKNVLITSALPYVNNVPHLGNIIGSVLSADVFARYCRLRGYNTLYICGTDEYG

TATETKAIEKGVTPREICDEYNKLHSEIYEWFNISFDYFGRTTTDKQTEIAQDIFKKLHSQNLLLTDTVEQLYCESCKRF

LADRFVEGTCPLCSYEDARGDQCDKCGKLINAPDLKNPQCKVCRSTPHLCSSKHLFLDLPKLEASIKRHLDKATSTGHWS

NTAQVISYSWLKDGLKPRCITRDLKWGTPVPLEDFKDKVFYVWFDAPIGYLSITANYTEHWEKWWKQPDLVQLYQFMAKD

NVPFHSIIFPASQIGTKDNYTVVNHLAAVEYLNYEDVKFSKSRGIGVFGDDAKVTGIPSDIWRFYLLYLRPEAQDTYFSW

NDLMLKVNSELLNNLGNFINRALSFISNNFGGVIPELLLGDSEKLLIAQVDRELKSYISCMDKTRLRDAIKYILNISRIG

NQYIQSNKPWELVKGSAAEKARAGTILGLSANICCLLCTLLEPYMPDTFKNLKTQLNTEQSHHILINDFVCLLPQNHKIG

KPVPLFKKIEQDLINNLKTKYGGRQSPDKDSSSPKRLPEKTVNKEAANNLPNGLDAEEVASLESLVTVQGNKVRELKAKK

ESKTEIDEAVAVLLELKKRLAVAKGENPDQFQGKSKSKKKR

>OCA46517.1 hypothetical protein XENTR_v90006328mg, partial [Xenopus tropicalis]

KFIRPFLLPCTRQQLVDHACFSRYCRLRNWNTLYICGTDEYGTATETKAMEEGLTPQQICDKYNAVHTAIYQWFDISFDF

FGRTTTQHQTTIAQDIFHRLLKRDFLLTDTVEQLRCETCQRFLADRFVEGTCPFCNYEEARGDQCDKCGKLINAVELKKP

QCKVCKKSPVKKSSKHLFLDLPKLEKRLEQWLEKSFSSGDWTSNARFITRSWIRDGLKPRCITRDLKWGTPVPLDGFRDK

VFYVWFDAPIGYISITANYTDQWEKWWKSTQQVQLYHFMAKDNVPFHSVIFPSCLLGAEDNYTLVNHIIATEYLNYEDGK

FSKSRGVGVFGDMAKDTGIPADIWRFYLLYVRPEGQDSAFSWSDLMLKNNSELLNNLGNFVNRAGMFVQKFFNGCVPEME

LLSEDKRLLAQVAAELQQYNQHLEKVRIRDALRCILNISRHGNQYIQVNEPWKCIKGNQQEQKRAGTVTGVAVNMAALLS

VMLHPYMPTVSGLIQEQLLMPQESKVLTTDFCCRLQSGHQIGNVSPLFQKLENDQIESLRKRFGGGQVKTESKAPPCQEA

PLKEAPKASGPERVKELMQEVEKQGNLVRELKGKKAEKSTIDTEVQKLLALKKELALAEGKSPDPPAQKGKKKK

>XP_019636662.1 PREDICTED: methionine--tRNA synthetase, cytoplasmic-like [Branchiostoma belcheri]

MKLFTDPGNPHALKAVIASNIAGTVLQIQEVDRTRWTGNLPILELPSGRAIFSANAACRYLLGNKNTDDITVDEWLEWES

SQLQPFLAPYIVACVGQGKKDNALFAAMFPLLEHLDKALKDRQYIVGTSVTAADIVLWSALFPLYGDSTKITDELTRYSG

IVDWFQNLSTQEAFKSAIATVTKSKGVSACKASLLAQPLPLKPVIQSSPTKAQSEAGSTSSELSVSAEEVTAAISAFKDG

KSPKPRTRTHPMFCRLRNWNTLYICGTDEYGTATETKALEEGLSPQQICDKYNRIHTEIYKWFEIDFDYFGRTTTPQQTV

ISQDIFWRLYKRNFLLTETVEQLKCQQCDKFLADRFVEGTCPLCNYDDARGDQCDKCGKLINATELKKPRCKLCNKTPVV

QTSEHLFLDLPKLEPALSAFLNTIYPTDDWSNNAKVITRSWVRDGLKPRCITRDLKWGTPVPLEGFTEKVFYVWFDAPIG

YLSITANYTDQWEKWWKNPEQVTLYNFMAKDNVPFHSVVFPCSLLGAEDNYTLVNHLQATEYLNYEDGKFSKSRGVGVFG

NNAKDTGIPADIYRFYLLFVRPESQDSTFSWEDLMTKNNSELLNNLGNFVNRALMFVSNFFQGVMPAVEVTEEDGQLLAQ

INRELQAFLQCLEKVRLREGLVRILNISRLGNQYIQAQKPWVLVKGSPAEKARAGTVIGLAANVSCLLSVLVQPYMPATS

STIQQQLQAPPEVNILTDTFVCQLPAGHKIGKPSPLFQKLEASTMAELKKKFAGTQMERKEQEQKSKTPAQPVQNTPPVK

VDQAEVDRLTAEVQKQGEAVRQLKTDKAEKKLVDAEVQKLLALKAQLAAAKGENPAEIIGGKGKKNKKGKK

>XP_002116645.1 hypothetical protein TRIADDRAFT_31114, partial [Trichoplax adhaerens]

EEISLADVIIWGTLYPFVDSSPASVNAKKYKSVHTWYQRLSSMDDFKTAVNEATGSKGVTAFKNYFQQAPIPSVSLSTSQ

SVTEDSENSVTQDEIDEAYHAWTSGRENYPKREKRPHPLLPQEGKRNILLTSALPYVNNVPHLGNIVGCVLSADAFARYC

RARNYNILYICGTDEYGTATETKAVEEGVTPQQICDKYHALHAEVYKWFDISFDHFGRTTTKEQTEIAQDIFWRLQDRNY

VTEENVDQLFCENCQRFLADRFVEGTCPFCTFPDARGDQCDKCGKLINAIELKAPRCKVCKSQPVIKSSKHLFLDLPKIE

PQLHKYYGRPDSLTDWSPAARNITQTWLRDGVKSRCITRDLKWGTPVPLEGFTEKVFYVWYDAPIGYISITAAYTDQWEK

WWKNPQNVEMYNFMAKDNVPFHSIIFPACLLAADDNYTVVNKLSATEYLNYEDGKFSKSRGIGVFGNDAKATGIPVDIWR

FYLLYVRPETQDSSFSWSDLEAKNNSELLANLGNFVNRAMLFVSNFFNGEIPEMDLIEEDKVLLAQITREIRTYNNNLER

TRIRDGIRNILSVSRLGNQYMQSHTPWKLVKQSEADKKRAGTVVGVCANITCLLSILLQPYMPTTSKEILSQAKLVDDYS

VITDKFVCLLPAGHRIGKPKHLFEKIEPSKTEEFKTKYGGRTNATAVNFKKTETTPVANDVKELEEQVSKQGDLVRQLKA

DKAEKFVIQSEVQKLLELKKHLAIAKGEPVPSKDSKKKKKK

>OCA46518.1 hypothetical protein XENTR_v90006328mg, partial [Xenopus tropicalis]

KFIRPFLLPCTRQQLVDHACFSRYCRLRNWNTLYICGTDEYGTATETKAMEEGLTPQQICDKYNAVHTAIYQWFDISFDF

FGRTTTQHQTTIAQDIFHRLLKRDFLLTDTVEQLRCETCQRFLADRFVEGTCPFCNYEEARGDQCDKCGKLINAVELKKP

QCKVCKKSPVKKSSKHLFLDLPKLEKRLEQWLEKSFSSGDWTSNARFITRSWIRDGLKPRCITRDLKWGTPVPLDGFRDK

VFYVWFDAPIGYISITANYTDQWEKWWKSTQQVQLYHFMAKDNVPFHSVIFPSCLLGAEDNYTLVNHIIATEYLNYEDGK

FSKSRGVGVFGDMAKDTGIPADIWRFYLLYVRPEGQDSAFSWSDLMLKNNSELLNNLGNFVNRAGMFVQKFFNGCVPEME

LLSEDKRLLAQVAAELQQYNQHLEKVRIRDALRCILNISRHGNQYIQVNEPWKCIKGNQQEQKRAGTVTGVAVNMAALLS

VMLHPYMPTVSGLIQEQLLMPQESKVLTTDFCCRLQSGHQIGNVSPLFQKLENDQIESLRKRFGGGQLNGDNTEPQVKTE

SKAPPCQEAPLKEAPKASGPERVKELMQEVEKQGNLVRELKGKKAEKSTIDTEVQKLLALKKELALAEGKSPDPPAQKGK

KKK

>CBN80834.1 Methionyl-tRNA synthetase, cytoplasmic [Dicentrarchus labrax]

MKLFVSEGNPHCLKVLAALGVTGVQCDVQYVNHEERVVPFLSRPTLPALLLPSGLHLFSSNAICRYLFEVSGKDSSERCN

QWLEWEATDLQPALLQALHMAVLQGKGSEVSKVLQGPLNYLDQSLSKGNTPYLTGEAVSVADVVLWAALYPILSDSSLAL

GECKSVKTWFDRGAAVMCSFQAAAQKVLQGKGLQGMKSYMQRQPAPQSSQCRDTQSCNSNPAEGEEGERVVSEEEMEAAA

VTFSKGLNACPLATNRQHPILPQEGKRNLLVTSALPYVNNVPHLGNIIGCVLSADVFSRYGRLRGWNMLFVCGTDEYGTA

TENKAREEGLTPQQICDKYHAIHSSIYKWFQIDFDFFGRTTTEKQTEIAQNIFWRLHKHDFLVEDTVEQLRCESCQRFLA

DRFVEGTCPHCSYPEARGDQCDKCGRLINAVELREPQCKVCRQTPVIRSSKHLFLDLPKVFYVWFDAPIGYLSITANYTD

KWEKWWKNPHQVELYNFMAKDNVPFHSVVFPCSLLGAQDNYTLVNHLVATEYLNYEDTKFSKSRGVGVFGDMAKDTGIPS

DVWRFYLLYVRPEGQDSAFSWADMATKNNSELLNNLGNFINRAGMFVTKFFEGCVPAIELQQEDKKLLALVGWELQQYIQ

LMDKVKIRDALKHILNISRHGNQYIQVNEPWKKIKGGDIERQRAGTVTGLSVNIACLLSVMLFPYMPTVSQTIRDQLNAP

QSCINAMLQGTGTFVCALSAGHRVGTVSPLFQKLEVDQIEALKKKFGGQQKTAQTAASTQPAAVPAPLAEVAMVNGVDPE

KAKQLTLAVTEQGEKVRALKAQKAEKAVITAEVTKLLDLKKQLALAEGKSPEPAPQKGKKK

>XP_014774996.1 PREDICTED: methionine--tRNA synthetase, cytoplasmic-like isoform X1 [Octopus bimaculoides]

MALPTLHTDVANNPETLKLFIAVNLSNRNINVVHRKKDERIVPYLSKTQLPVLELAKDDFLFSTNSATRYLLSGDVVMND

ANIDTQVDMWLQWEACQLQSTVKDIISIASGKNVSKEDNGANLALLHSHLKYMEDKLKKNDNNLVKGQMTAADVTVWSTL

YHVFNQTDVIKDWEKDYPKVSSWFKCLSGKKDFQDGVNQLTDSQRTAALTESVCKSNQKSSKATWESKSGSKLQDDQKPK

SLTSKLPVKPVKQPENQEAANDVLVNSNEISKSVAAWKLPCTTNATKREHPILPVPGARNILITSALPYVNNVPHLGNII

GSTLSADIFSRYSQLRGYNCLYICGTDEYGTATETKAMEEGLTPQQICDKYNVIHEEIYKWFNISFDYFGRTTTKLQTEI

AQDIFWKLHKKGYISEDTVDQLKCEKCDRFLADRFVEGICPLCGFDDARGDQCDKCGKLINAIDLVKPRCKTCSNTPIVK

SSRHLFLDLPKLEPDLKKHLDHVFPKGIWTNNAKVITNSWLRDGLKPRCISRDLKWGTPVPLKGFTDKVFYVWFDAPIGY

ISITANYTEKWEQWWKNSSQVQMYNFLGKDNVPFHSVIFPATLLGADPNYTLVDSVIATEYLNYEDGKFSKSRGVGVFGN

NAKDTGIPADIFRFYLVYMRPESQDTAFSWDDFQLKNNCELLNNIGNFINRALMFVSNSFQQTIQAMDLTEGDEKLIAQI

NKLLHRYIENMECTKMRDSLRILLEMSTLGNQYMQMHKPWVLVKGTNEEKQRAGTIVSLSSNIAALLSVLLQPFMPQTSA

TIQLQMQFPPEKNVILNNFSCYLSAGHKIGKPKPLFQKLEYDFIQELKKKFSSTLTTAASNKTNQTAAAATLGSEADVES

LNQLVTEQGLKVRELKANKAEKSVIDGEVAELLELKRQLALKQGVDPASLGVGNKKKNKKK

>PIK60223.1 putative methionine--tRNA synthetase, cytoplasmic isoform X2 [Apostichopus japonicus]

MKLFTDKGNQHSLKILVAAELAGISLEIEHVDSAAIVVPFLKRNVLPVLEVETNEYLFSANAITRYILATVGKDSEDITV

STWLEWESTDLQAALSSYLPVLYGGGKADSQKLQPHLSKLDNSLGGAQFIVQDQLSAADVCLFAALYPLYANSKSCFENF

SNLNAWFTKLHAEPPFGASVAKVTEGKGSSSLKASILAQPLVNTLPVARSVSNEKGKSSKDKENSTTEEVVAKEFSLEEV

RAAKETFLTGKKVAPKPVQRNHPLLPRKDAKNVLITSALPYVNNIPHLGNIVGCVLSADVFSRFCRIRNYNVLYICGTDE

YGTATETKALEEGLTPQQICDKYFKLHDHVYRWFNIDCDHFGRTTTPQQTKIAQDIFWGLHKEKHLLEQKVEQLKCEDCD

RFLADRFVEGTCPLCNYDDARGDQCDKCGKLINATDLKNPRCKICNRTPVVKSSDHLFLDLPRLESRLQSFLDKSTTEGD

WTSNAKMITSSWIRDGLKPRCITRDLKWGTPVPLEPYTDKVFYVWFDAPIGYPSITANYTPDWEKWWKNPDEVQLYQFMA

KDNVPFHSVVFPCSLLGCGEKWTLVNHLIATEYLNYEDSKFSKSRGVGVFGNHAEQTGIPADIWRFYLLFIRPESQDSYF

NWDDFVLKNNSELLNNLGNFINRGLMFLEKNFGGLVQELKLEQLDCQLLAAVSQELKQYVSLLEKVRIKEALRHVLNISR

LGNQHIQVTQPWVLVKGSPEEKSRAGTVTSLCVNISCLLSVMLHPYMPDTSKTIQEQLNASADVNVLTGDFVCYLEPGHK

IGTPKPLFAKIEQAQADQLRNQYAGKND

>XP_013788881.1 methionine--tRNA synthetase, cytoplasmic-like [Limulus polyphemus]

MKLNIILKNLCSLKTVIAAGITDAQVEIVKIPPKASCSGFCNNVSTSSPISRPPVLELPSSECLFSANAICWYLFHQRES

GNNTTFDPITEEWLEWESNCLQPYVTTFIANFFENNKLNDALFSTLMNHLQYLDSFLTSTPHFPRSSASGTLPGIGDVVI

WSTVYPVITDEKLGADTVKKFSSLCRWFHEIGLNESFKEAVRYVFGEKKPSACKETLIEKDPLLHNFALSTQRGKLVPQV

INSSDVEMNTSQVEEDVISQEEMFVVFKNWLKGKSFMPKKSEEKFPILPQEGERNILVTSALPYVNNVPHLGNIIGAVLS

ADVFARYCRNRNYVTLYICGTDEYGTATETKALEEGVTPWEICNKYHRLHKEVYQWFNISFDYFGRTTTDWQKQIAQDIF

LKLYAKNYLFEDTVEQLFCEKCHRFLADRFVLGVCPFCLYDDARGDQCDKCGRLINAVELKEPKCKVCSSTPILKTSRHL

FLDLPKLQPDLHTWLDQSCESGYWTNTAKVITYSWLKEGLKPRCLTRDLKWGIPVPVKGYEGKVFYVWFDAPIGYLSITA

SYTDQWENWWKQPEKVQLYQFMAKDNVPFHSIIFPASIIGTKEKFTMVNHLIAVEYLNYENDKFSKSRGIGVFGTDAKET

GIPSDVWRFYLLSLRPESQDSSFSWNDLLLKNNSELLNNFGNFVNRALMFLANNFSCVIPEMKINEEDKMLLVRVNRELQ

SYIELMEKVKLRDAIRPVFSISRLGNQHIQSNKPWEHVKGSNEQRERAGTVMGLCANIACLLATLIEPYMPETSTKLKQQ

LNTTPDCHVITSFFTCCLPSGHKIDYPKPLFKKLETSFIEDLKKKFAGDEQPKEKSPSKKMSSKGGKSTSVVEPSTQIHD

TVDQASLDHLTDLVAKQGNLVRDLKSRKAEKAEIEREVQQLLELKRQLALASGKDPNEVSGQQKKKNRKK

>KFV48345.1 Methionine--tRNA synthetase, cytoplasmic, partial [Gavia stellata]

QPAASAALYAQLVHGKKGLEAAEALGKLLAHIEQNLSRRGTAYLAGNTESVAGVESCCLLPLTPQGELQALRTWFWSMTL

SEACRKAADSVLMPKALLEFKAYLQKQPPPCLAVEKATSNEPEEEEGSERALTEEEITAAVDAWARGAAALPKPWQSQKP

VLPVEGMRNVLITSALPYVNNVPHLGNIIGCVLSADTFARYCRLRNWNTLYVCGTDEYGTATETKAVEEGLTPQEICNKY

NAIHADIYRWFDISFDYFGRTTTPYQTTIAQDIFQRLLARGFLLQDTVEQLRCEDCQRFLADRFVEGTCPFCSYEEARGD

QCDKCGKLINAVELKNPQCKLCRGVPVVKPTQHLFLDLPKLEERLEPWLEQSWATGDWTANARYITRSWIRDGLKPRCIT

RDLKWGTPVPLDGFRDKVFYVWFDAPIGYLSITANYTDQWERWWKNPQQVELYNFMAKDNVPFHSVIFPCSLLGADDNYT

LVNHLIATEYLNYEDGKFSKSWGVGVFGDMAKDTGIPADIWRFYLLYLRPEGQDSAFSWSDLMLKNNSELLNNLGHFINR

AGMFVCKFFGGTVPNMVLTADDKRLLARVTLELRQYHQLLEKVR

>XP_009919581.1 PREDICTED: LOW QUALITY PROTEIN: methionine--tRNA synthetase, cytoplasmic-like [Haliaeetus albicilla]

MTLSEACRKAADSVLMPKALLEFKSYLQQQPQPCLAMQRATSNEPEEEEGSEPALTEEEITAAVDAWARGVAALPKPWQP

QKPVLPVEGMRNVLITSALPYVNNVPHLGNIIGCVLSADTFARYCRLRNWNTLYVCGTDEYGTATETKAVEEGLTPQEIC

NKYNTIHADIYRWFDISFDYFGRTTTPYQTMIAQDIFQRLLARGFLLQDTVEQLQCETNCERVRPGRCLEGPCPFCSYEE

ARGDQCDKCGKLINAVELKNPQCKLCRGVPVVKPTQHLFLDLPKLEEQLEPWLEQSWATGDWTANARYITRSWIRDGLKP

RCITRDLKWGTPVPLDGFRDKVFYVWFDAPIGYLSITANYTDQWERWWKNPQQVELYNFMAKDNVPFHSVIFPCSLLGAD

DNYTLVNHLIATEYLNYEDGKFSKSRGVGVFGDMAKDTGIPADIWRFYLLYLRPEGQDSTFSWTDLMLKNNSELLNNLGN

FINRAGMFVCKFFGGTVPDMVLTPDDKRLLARVTLELHQYHQLLEKVRIRDALRCILSISRHGNQYIQVNEPWKRIKGDE

KDR

>XP_022792356.1 methionine--tRNA synthetase, cytoplasmic-like [Stylophora pistillata]

MSPVFGRVSSVVFLRTVKFTARLNYRKLASSLISEVMKLYSNRGNPSLLRILAAKKLADVSMTIQYITNGDRAKFTPQGS

SKLPVLETDGGKLLHSPNIICRYLLDLGGSSDVAPEPYEKAIVDQWVEWESTSLQPVIFNHLVRSIGQGRSREHSGLVDD

VVNLLRHADEGLTSKKYFSGDHLGLADVILWGSLHPLFMEDPKLSDPCISNLIRWHDLLMSQELFIKTESEVAGGKGFSA

FKDALLAMLDAHEETTKGSPSSQQEQERDSATEERILAKVLSREEIDLAFEHWNSGSKNRPKTRRLVDPILPIEGERNIL

ITSALPYVNNVPHLGNIIGCVLSADVFARFCRLRQYNTLYICGTDEYGTATETKALEEGLTPQQICDKYFKIHKEVYEWF

QIKFDFFGRTTTQQQTEIAQDIFWRLHKQDHLVEDTLEQLQCVECKRFLADRFVEGTCPFCNYEGARGDQCDSCGKLINA

IELKKPRCKVCGGTSKVRSSKHLFLNLPKLELSLQTWIDKSSSEGTWTLNARQITQSWIKEGLKPRCITRDLKWGTPVPL

EGYTDKVFYVWFDAPIGYLSITANYTNEWEKWWKNPQQVSLHQFMAKDNVPFHTVVFPCTLIGADDNYSLLNSISATEYL

NYEDDKFSKSRGVGVFGDHAADTGIAADVWRFYLLFVRPESQDSAFSWSDFVSRNNTELLNNFGNFINRALMFVQNNFDS

CIPAMALTDEDKKFIALINKELKTYIDNLEKMRLREGLKDVLNISKLGNQYIHSNQPWVLFKGSESERSRAGTVIGITAN

LSWLLSVLVHPYMPGVSEEIQRQLQVSDEGNVVLDNFIPYLETGHKIGTPKPLFHKIDASVADQFRAKYGGYKQKQPAAD

KKTKEASKG

>KPP64476.1 methionine--tRNA synthetase, cytoplasmic-like [Scleropages formosus]

MTPALMQALYQCVLLGKMTEAANTLEGHLTYMEQRLSKGATPFLAAVRRPSTDCHELTRSISTVAQRVQQQKSLESLKRF

LQKQPLPPARCRNAPLCNRAEEEDAEREMTDEEIQAAGATWSRGLADRPTPAERRHPILPQEGKRNVLITSALPYVNNVP

HLGNIIGCVLSADRPLPAPISVASPVFRDPSVAAAAIEDAELPLRSSARKLASAPGHVVGAFGVEAAASGPPLTRVCSSV

RYGRLRGWNALFVCGTDEYGTATENKAREEGLSPQEICDKYHAVHASIYKWFQIDFDHFGRTTTEKQTEIAQDIFWRLYD

RGFLIPDTVDQLHCENCQRFLADRFVEGICPNCRYPEARGDQCDKCGRLINAVELKDPQCKVCKRTPVVRSSAHLFLDLP

KLESQLEQWLEQSMSTGNWTSNARHITRSWLRDGLKPRCITRDLRWGTPVPHPDFSGKVFYVWFDAPIGYLSITASYTDE

WEKWWKNPEKVELYNFMAKDNVPFHSVVFPCSLLGAQDNYTLVSNLIATEYLNYEDTKFSKSRGVGVFGDMAKDTDIPSD

VWRFYLLYLRPEGQDSAFSWADMALKNNSELLNNLGNFINRAGMFASKFFEGRVPQMELQQEDKRLLAQVGFELRQYIQL

LDRVRIRDALKCILNISRHGNQYIQVNEPWKKIKGGDEDRLRAGTVTGVSVNVACLLSAMLRPYMPAVSATIREQLCAPA

NCADAMFQGAGSFVCTLPAGHRIGTISPLFQKLEAEQIESLKRRFGGQQIQSAAPAAATVPASPSATIETSANPERAKQL

LQEVAEQGDRVRALKAQKAEKTLITAEVAKLLQLKKQLAAAEGKNPEPSPQKGKKK

>XP_021000482.1 methionine--tRNA synthetase, cytoplasmic-like [Parasteatoda tepidariorum]

MKLSIDDKCNLNSLKILVVSEFCGVKLNIEKCSDVKKQVRLPLLEIKDQCTFFSSNASCRYLWSVSGQPLYPDIDQWLEW

ECAILQPSISALLKASKDDSLPLSFKRNLNYLNALLGSRSFLSGKDAPDLSDLVLWANIYPLYKASLLPDVSNLANLQKW

LNTLKDLGSLNTAFTHIGGKEKSDLKKNLLCEVSFTGDYFFMPIHVADFVPSVETDDSNAEQSKEPEITAEEVIRSYSCW

VKGKASIPQPKENKVPVLPKKGERNILVTSALPYVNNIPHLGNIIGSVLSADVFARYCRSRGYNTLYICGTDEYGTATET

KAIASKMTPREICDKYNKLHSDIYKWFNIAFDHFGRTTTEKQTEIAQDIFHKLYAENLILTDSVEQLFCESCQRFLADRF

VEGICPLCAYEDARGDQCDKCGKLLNAADLKSPQCKLCKKTPHLRSSRHLFLDLPKLEAPLKKYLDNSTPNSQWSNTAKV

IAYSWLKEGLKPRCITRDLKWGTPVPLEGFTDKVFYVWYDAPIGYLSITANYTDKWEQWWKQPENVQLYQFMAKDNVPFH

GIIFPACQIGTKDTYTIVNHLAATEYLNYEDVKFSKSRGVGVFGDDAKETGLPSDVWRFYLLYIRPEAQDTYFSWNDLML

KINSELLNNLGNFINRALTFVSNNFGGIIPDMQVGDGEKQLIAQVDRELKQYINCMEKTRMRDAIKYLLNISRLGNFYIQ

SNKPWELVKKSPAEKALAGTVLGLSANICCLLCTLMEPYMPDTCANLKSQLNTERIHHVLIDDFVCLLPSGHKIGKPSPL

FKKIEQDVINELKSKYAGRQSQEKETVPPSKETKAAVPASNMTNGVDLQALEASVTEQGEKVRSLKVAKADKASIDKEVA

LLLQLKREVMIAKGENPDQQPAKSKNKKKR

>XP_020620896.1 methionine--tRNA synthetase, cytoplasmic-like [Orbicella faveolata]

MPCLGSQASHQENKSAVPLEEASQTKVCSKEEVDEAVESWNNGCKSRPKTRRLADPVLPIEGEKNILITSALPYVNNVPH

LGNIIGCVLSADVFARFCRLRQYNTLYICGTDEYGTATETKALEEGLTPQQICDKYFKLHKEVYEWFEIKFDYFGRTTTE

EQTEIAQDIFWRLHKRDHLVEDTLEQLQCVDCKRFLADRFVEGTCPFCNFEDARGDQCDACGKLINAIELKKPRCKVCGG

TPKVRSSKHLFLNLTKLEPSLRQWVEKSSSEGTWTSNARHITRGWIQEGLRPRCITRDLKWGTPVPLEGYTDKVFYVWFD

APIGYLSITANYTKEWEKWWKNPQQVTLYQFMAKDNVPFHTVVFPCTLIGADDNYSLLNSISATEYLNYEDDKFSKSRGV

GVFGNDAANTGIPADIWRFYLLYVRPENQDSAFSWSDFIQKNNTELLNNFGNFINRALMFVHNNFGGAVPAMDLTEEDKN

FIALINKELKTYIDDLEKMRLREGLKGVLNMSKFGNQYIQSNQPWVLVKGSESDRSRAGTVLGVATNLSWLLSVLIHPYM

PGVSEEIQRQLQVTDEGNVVLDNFVPYLQPGHKIGTPSPLFQKIDASVADQLRAKYGGNKPKQQPEGDKSTNAVTENTAD

TDVAVLQAEITKQGDKVRQLKAEKAEKEKVEAEVAILLALKKKLAVAEGKDPNEQDATSKGKKKGKKK

>XP_014774997.1 PREDICTED: methionine--tRNA synthetase, cytoplasmic-like isoform X2 [Octopus bimaculoides]

MNDANIDTQVDMWLQWEACQLQSTVKDIISIASGKNVSKEDNGANLALLHSHLKYMEDKLKKNDNNLVKGQMTAADVTVW

STLYHVFNQTDVIKDWEKDYPKVSSWFKCLSGKKDFQDGVNQLTDSQRTAALTESVCKSNQKSSKATWESKSGSKLQDDQ

KPKSLTSKLPVKPVKQPENQEAANDVLVNSNEISKSVAAWKLPCTTNATKREHPILPVPGARNILITSALPYVNNVPHLG

NIIGSTLSADIFSRYSQLRGYNCLYICGTDEYGTATETKAMEEGLTPQQICDKYNVIHEEIYKWFNISFDYFGRTTTKLQ

TEIAQDIFWKLHKKGYISEDTVDQLKCEKCDRFLADRFVEGICPLCGFDDARGDQCDKCGKLINAIDLVKPRCKTCSNTP

IVKSSRHLFLDLPKLEPDLKKHLDHVFPKGIWTNNAKVITNSWLRDGLKPRCISRDLKWGTPVPLKGFTDKVFYVWFDAP

IGYISITANYTEKWEQWWKNSSQVQMYNFLGKDNVPFHSVIFPATLLGADPNYTLVDSVIATEYLNYEDGKFSKSRGVGV

FGNNAKDTGIPADIFRFYLVYMRPESQDTAFSWDDFQLKNNCELLNNIGNFINRALMFVSNSFQQTIQAMDLTEGDEKLI

AQINKLLHRYIENMECTKMRDSLRILLEMSTLGNQYMQMHKPWVLVKGTNEEKQRAGTIVSLSSNIAALLSVLLQPFMPQ

TSATIQLQMQFPPEKNVILNNFSCYLSAGHKIGKPKPLFQKLEYDFIQELKKKFSSTLTTAASNKTNQTAAAATLGSEAD

VESLNQLVTEQGLKVRELKANKAEKSVIDGEVAELLELKRQLALKQGVDPASLGVGNKKKNKKK

>XP_023066379.1 methionine--tRNA synthetase, cytoplasmic [Piliocolobus tephrosceles]

MRLFLSEGAPGCLPVLAAAGRARGRAELLISTVGPEDCVVPFLTRPKVPVLQLDSGNYLFSTSAICRYFFLLSGWEQDDL

TNQWLEWEATELQPALSAALYYLVVQGKKGEDVLGSVRRALTHIDHSLSRQNCPFLAGETESLADIVLWGALYPLLQDPA

YLPEELSALHSWFQTLSTQEPCQRAAETVLKQQGVLALRPYLQKQPQPSPPEGRAVTNEPEEEELATLSEEEIAVAVTAW

EKGLESLPPLRPQQNPTFLNQPSCLSPLSQLEKRLEEWLGRTLPGSDWTPNARFITRSWLRDGLKPRCITRDLKWGTPVP

LEGFEDKVFYVWFDATIGYLSITANYTDQWERWWKNPEQVDLYQFMAKDNVPFHGIVFPCSALGAEDNYTLVSHLIATEY

LNYEDGKFSKSRGVGVFGDMAQDTGIPADIWRFYLLYIRPEGQDSAFSWTDLLLKNNSELLNNLGNFINRAGMFVSKFFG

GYVPEMVLTPDDQRLLAHVTLELQHYHQLLEKVRIRDALRSILTISRHGNQYIQVNEPWKRIKGSEADRQRAGTVTGLAV

NIAALLSVMLQPYMPTVSATIQAQLQLPPPACSILLTNFLCTLPAGHQIGTVSPLFQKLENDQIESLRQRFGGGQLEETF

DLKAKTSPKPAVIETVTTAGPQQIQALMDEVTKQGNIVRELKAQKADKNQVAAEVAKLLDLKKQLAVAEGNPPEAPKGKK

KK

>AIC62429.1 hypothetical protein, partial [synthetic construct]

MRLFVSDGVPGCLPVLAAAGRARGRAEVLISTVGPEDCVVPFLTRPKVPVLQLDSGNYLFSTSAICRYFFLLSGWEQDDL

TNQWLEWEATELQPALSAALYYLVVQGKKGEDVLGSVRRALTHIDHSLSRQNCPFLAGETESLADIVLWGALYPLLQDPA

YLPEELSALHSWFQTLSTQEPCQRAAETVLKQQGVLALRPYLQKQPQPSPAEGRAVTNEPEEEELATLSEEEIAMAVTAW

EKGLESLPPLRPQQNPVLPVAGERNVLITSALPYVNNVPHLGNIIGCVLSADVFARYSRLRQWNTLYLCGTDEYGTATET

KALEEGLTPQEICDKYHIIHADIYRWFNISFDIFGRTTTPQQTKITQDIFQQLLKRGFVLQDTVEQLRCEHCARFLADRF

VEGVCPFCGYEEARGDQCDKCGKLINAVELKKMELALVPVSAHGNFYEGDCYVILSTRRVASLLSQDIHFWIGKDSSQDE

QSCAAIYTTQLDDYLGGSPVQHREVQYHESDTFRGYFKQGIIYKQGGVASGMKHVETNTYDVKRLLHVKGKRNIRATEVE

MSWDSFNRGDVFLLDLGKVIIQWNGPESNSGERLKAMLLAKDIRDRERGGRAEIGVIEGDKEAASPELMKVLQDTLGRRS

IIKPTVPDEIIDQKQKSTIMLYHISDSAGQLAVTEVATRPLVQDLLNHDDCYILDQSGTKIYVWKGKGATKAEKQAAMSK

ALGFIKMKSYPSSTNVETVNDGAESAMFKQLFQKWSVKDQTMGLGKTFSIGKIAKVFQDKFDVTLLHTKPEVAAQERMVD

DGNGKVEVWRIENLELVPVEYQWYGFFYGGDCYLVLYTYEVNGKPHHILYIWQGRHASQDELAASAYQAVEVDRQFDGAA

VQVRVRMGTEPRHFMAIFKGKLVIFEGGTSRKGNAEPDPPVRLFQIHGNDKSNTKAVEVPAFASSLNSNDVFLLRTQAEH

YLWYGKVGWLGPGSDQPLGAQTCTPLLLSARSXDLE

>XP_002161815.2 PREDICTED: methionine--tRNA synthetase, cytoplasmic-like [Hydra vulgaris]

MICTRFLRQVLESNRIVSLIVATQYRTFTDSATLRLALCIKKRNMNTISSKNSLKLITDAGNPSALKIIASSLYFEVPID

IQVSQKKETKSCIYNQLPVLECGDNHYLFNANSASCYLYNISKVKHEFSTENKEKQDEILEWESFKLQAAVQPYLLSLFS

GKKDSNTIIMESVFQSINSYLGQNPYLLGSFITASDIVVWASLYAVMQEKDIQDISFKLPAVKNWFNKLSISDNTLKAVD

LITQSKGISAFKVFFENYSLPSHLSKNIKHIKDAAEELQQTEETEGPSDADIKNAKSFWLKGRDLVAKPRKLTHPILPKD

GEKNILITSALPYVNNVPHLGNIIGCTLSADVYARYCRLRNLNVLYICGTDEYGTATETKAFSEGLTPQQICDKYNKIHS

EIYTWFNIEFDCFGRTTTEHQTKITQDIFWKIEKNGYLCKDAVDQLKCEQCERFLADRFVEGICPFCSYEDARGDQCDAC

GKLINATELKKPRCKQCGSSPVIKSSDHIFLDLPKIEPMLKDWIMKSEKANWTVNAKNITRGWIAEGLKPRCITRDLKWG

TPVPMEGYTDKVFYVWFDAPIGYISITANYTDQWEKWWKNPEQVTLVQFMAKDNVPFHTVVFPSSLLAAKDNYTILNEIS

ATEYLNYENDKFSKSRGVGVFGDHAESTGIPADMFRFYLLYIRPESQDSFFQWDDFITKNNSELLNNLGNFVNRALMFLK

NSFNSTLPNLQLQCEDYKLIAGINRDLHQYIEHLDAIKLKDGLRCILNMSRLGNQYMQAHKPWVLIKGDEQEQMRAATVV

GLAVNVSALIAVLILPYMPLVSKQLQLQLDLPSDFLIVPDNFVPMLPEGHKIGTPAPLFQKIEVSLGDELKKRFSGIKDA

TVLPTNPVKSESSGEKIVVSDQVIETLTVAELEEEITKQGNIVRKVKAEKANKDIIDGEVKKLLDLKKALASTQGKSNES

SKEKVCKKTKKK

>XP_014132857.1 PREDICTED: methionine--tRNA synthetase, cytoplasmic [Falco cherrug]

MRNIVRKLKQSDVRGVPPHALPCLSPACRLQPAASAALYAQLVQGKKGAEAVEALGKPLAHLEQNLSRRGSAYLAGATRS

VADVVVWGTLFPVLQDEASLPSELRALRTWFQSMTLSEACKKAASSVLMPEALLEFKSYLQKQPPPRLAMERATSNEPEE

EEGAERALTEEEITAAADAWARGTAVLPQPWQPQQPVLPVEGRRNVLITSALPYVNNVPHLGNIIGCVLSADTFARYCRL

RNWNTLYVCGTDEYGTATETKAAEEGLTPQEICDKYNAIHADIYRWFDISFDYFGRTTTPHQTRIAQDIFQRLLARGFLL

QDTVEQLRCEGCQRFLADRFVEGTCPFCSYEEARGDQCDKCGKLINAVELKNPQCKLCRGVPVVKPTQHLFLDLPKLEGR

LEPWLEQSWATGDWTANARYITRSWIRDGLKPRCITRDLKPIGYLSITANYTDQWEKWWKNPQQVELYNFMAKDNVPFHS

VIFPCSLLGAEDNYTLVNHLIATEYLNYEDGKFSKSRGVGVFGDMAKETGIPADIWRFYLLSVRPEGQDSAFSWGDLMLK

NNSELLNNLGNFINRAGMFVCKFFGGAVPDMVLTPDDKRLLAPVTPELHQDPGNHVRQLKATKAEKAQVDAEVAKLLELK

RRLALAGGETPDVPVPKGRRKK

>XP_019851278.1 PREDICTED: methionine--tRNA synthetase, cytoplasmic-like isoform X1 [Amphimedon queenslandica]

MASNSESVDGVSGCYGDPDVLKVAVLKELNPTIKVTFRKCTDENSKKSVSLLLSNGFCVTAANEICRYLVKKEGGAVVSL

RDWALIDYWLEWEAISLKPAILSSVINNSPSDQLLSCLTQLESHLTSNQKIVSCNEFSLADVVVWSTLLVIQSPESKLSS

NIARDYPKIAQWYRSLARDSRFSSAVWAASNDKGFEGFYDFIIPSGSGKSKSTTTGGKGQSSKAEKERTQSASGLSPSSS

TGDMIGQADIDKAMAHWNEGPDAAPKPRLHSHPILPIPGERNVLVTSALPYVNNVPHLGNIIGCVLSGDVFVRYARLRNY

NTLYVCGTDEYGTATEVKAVKEGLTPQQICDKYYALHSEIYEWFNVDFDIFGRTSTPQQTQIAQDIFWHLYDRDLIKKDT

VDQLLCTSCDKYLADRFVEGICPFCAYPDARGDQCDKCGKLINAVELKSPRCSICSSTPVVKQSNHLFLDLEKLQSPVFD

WAQKSISEGVWSQNAQFITKSWLRDGLKPRCITRDLKWGTPVPLEGYTDKVFYVWFDAPIGYISITACYTDQWEKWWKNP

EQVQLYQFMAKDNVPFHTVVFPSTLIGTGVDYSLLNHISSTEYLNYEDGKFSKSRGVGVFGNNAVETGIPADIYRFYLLY

VRPESQDSAFSWSDFVMKNNSELLNNLGNFVNRTLSFIKSSFDSCIPVMTLSHDDSVIVVKVTQELQGYIECLERHKQRD

GLKHILAISRLGNGHIQAHKPWELVKGDKNQKLLAGSLLGLCANICCLISVLLQPYMPSISKEIQNQLQAPSECNIIWDY

FVPYLPTGHKIGQPLPLFKKIDDATANELKKQFSGQKQDTGASSAMGESSKSKLEAELAKQAEVVRSLKASGSATKETLD

AEIAKLLELKKQLGISGASVGSKKKGKKTK

>XP_008001972.1 PREDICTED: methionine--tRNA synthetase, cytoplasmic [Chlorocebus sabaeus]

MRLFVSEGAPGCLPVLAAAGRARGRAELLISTVGPEDCVVPFLTRPKVPVLQLDSGNYLFSTSAICRYFFLLSGWEQDDL

TNQWLEWEATELQPALSAALYYLVVQGKKGEDVLGSVRRALTHIDHSLSRQNCPFLAGETESLADIVLWGALYPLLQDPA

YLPEELSALHSWFQTLSTQEPCQRAAETVLKQQGVLALQPYLQKQPQPSPAEGRAVTNEPEEEELATLSEEEIAMAVTAW

EKGLESLPPLRPQQNPVLPVTGERNVLITSALPYVNNVPHLGNIIGCVLSADVFARYSRLRQWNTLYLCGTDEYGTATET

KAMEEGLTPQEICDKYHIIHADIYRWFNISFDTFGRTTTPQQTKITQDIFQRLLTRGFVLQDTVEQLRCEHCARFLADRF

VEGVCPFCGYEEARGDQCDKCGKLINAVELKVRGGSPWEPGSRQSLFLRDSLLVPFRIFILAF

>PNF41623.1 Methionine--tRNA synthetase, cytoplasmic, partial [Cryptotermes secundus]

MKLVTNENNPLSLKVLISANFAQKEILCEIADVDDPRFQSPRRLPVLELDTGEFLFSANSASRLLFPPHQSTSVVVDKWL

EWESSELQPVLAYYLSATSKPDTGVSRQLKEYLTTLNLALSGKKFLTGEGVSVADVAVWSILFLLATGAKHLQELLSEQS

HVQRWFSHLSLQPEVQVAVDQLCPKVGIAAYHSLANATWYPVNAVLSYVEKVPHSRIPSVNSLDGMSKESPLSEAEINAA

AEAWQSGAARRPKPKPQVSPVLPVPGERNVLISSALPYVNNIPHLGTIIGCVLSGDVFARFSRLCNNNTLYICGTDEYGT

ATETKALEEGLTPQQICDKYFKIHDAVYKWFNISFDHFGRTTTAEQTEISQDLFLQLYENGFLSKESVEQLLCENCNRYL

ADRFVEGTCPRCAYEDARGDQCDGCGHLVNATELVSPRCKICQKRPVVTKSDQFFLELPKTEPLLRNWLIRVIDGWSHNA

RVITQAWLKDGLKPRCVTRDLKWGVPVPLDGYRNKVFYVWFDAPIGYMSITKTYTDQWEKWWRPERNTTVTYYNFIGKDN

VPFHSVMFPSCLLGANRNYTLVSHIMATEFLNYEDEKFSKSRGVGVFGTDAKETGIPSDVWRFYLLYVRPESQDSNFSWV

DLATKNNSELLNNLGNFINRSLVFAEKNFSRQVPDMLPGVQEWTLLALITRELKAYMGALENAKLRDGIRHILNISRHGN

QYMQLLQPWVLLKGNDEERAHAGTVIGICCNIACLLAVLLKPYMPVTADILKEQLEAPDDVWIIIPEFTVLLPSGHKLGK

PSPLFSKIEPSRVDQLKKQFSGRQKSQSPEVNTTSSKPATSTVNVVTTQVQGDPSVIAKLSAAVAQQADLVRKLKGSGAA

PSIWQPHVAVLLELKRHLASAQSINGNVNEASTATLNGLQVPNPAEVARLEAGIAKQV

>XP_013158052.1 PREDICTED: LOW QUALITY PROTEIN: methionine--tRNA synthetase, cytoplasmic [Falco peregrinus]

MRNIVRKLKQSDVRGVPPHALPCLSPACRLQPAASAALYAQLVQGKKGAEAVEALGKLLAHLEQNLSRRGSAYLAGATRS

VADVVVWGTLFPVLQDEASLPSELRALRTWFQSMTLSEACKKAASSVLMPEALLEFKSYLQKQPPPRLAMERTTSNEPEE

EEGAERALTEEEITAAADAWARGTAALPQPWQPQQPVLPVEGRRNVLITSALPYVNNVPHLGNIIGCVLSADTFARYCRL

RNWNTLYVCGTDEYGTATETKAAEEGLTPQEICDKYNAIHADIYRWFDISFDYFGRTTTPHQTRIAQDIFQRLLARGFLL

QDTVEQLRCEGCQRFLADRFVEGTCPFCSYEEARGDQCDKCGKLINAVELKNPQCKLCRGLPVVKPTQHLFLDLPKLEGR

LEPWLEQSWATGDWTANARYITRSWIRDGLKPRCITRDLKWGTPVPLDGFRDKDARVHPCVSPCPPPPLPTACQVELYNF

MAKDNVPFHSVIFPCSLLGAEDNYTLVNHLIATEYLNYEDGKFSKSRGVGVFGDMAKETGIPADIWRFYLLSVRPEGQDS

AFSWGDLMLKNNSELLNNLGNFINRAGMFVCKFFGGAVPDMAAPDRVLPPDDKRLLARVTLELHQYHQLLEKVRSGLDSG

GWGVWGGQSGGRDPRCPCAQGEAEEIAGAAPPSPQTKLGCTGQHLELCNKINCTTVYGGGV

>XP_017768839.1 PREDICTED: methionine--tRNA synthetase, cytoplasmic [Nicrophorus vespilloides]

MPVIYTNESNPAGLKLIISAKFGSVNYQVKIIESADKTVPAPRVLPYVETDRETVIFSSNAGAQYFYPNKEVDSTNSVQQ

WLDFEVYELAQSLALVIGGSSKVQKIKDILSSSLKLLNDKLKEGAPLNGKTIEVCDIAVWSALYPLHNDKKMSKEYLEPY

PDILNWMTHFTENTFVKEALAEFKILDALTSYQALLIGAKYTAVNIKSSTEVATANPDVVSPEELKSAEEAFKKTLNNLP

KPKKEVHPILPVAGEKNVLITSALPYVNNVPHLGNIIGCVLSSDVFARFSRLCNWNTLLIGGTDEYGTATETKALEEGLT

PQQICDKYFEIHNNIYRWFNIGFDYFGRTTTPEQTRLVQDMFITLNNNGFMQTQPVEQLLCEKCNRYLADRFVEGGCPHP

GCNYEDARGDQCDGCGKLVNSTELKNPRCKVCRTAPVKRTSKQFFLDLPKLEPLLHHWMEKSSQGWTNNAKVISKSWMKE

GLKPRCITRDLKWGVPVPLDGYRDKVFYVWFDAPLGYVSMTSKYTKDWEKWWRPAKETDITLFQFMAKDNVPFHSVMFPA

TLLGANKGYTLVSHIMATEYLNYEDGKFSKSRGVGVFGTDAQETGIPSDVWRFYLLYVRPEGQDSSFSWVDLATKNNSEL

LNNLGNFVNRALVFTEKFFGSTVPAMNLTEEDLTVLALCTRELKSYVASLEKAKLRDGIRYILAISKHGNQYMQSTQPWV

LLKRTDDDKQRAGTVVGVCVNIACLLATLMLPYMPDSSRALKAQLNAPPETNILTPDNPEIVMFLPEGHKIGKPAPLFSK

IEPAQVEEMKKKFGGQQSDKPSAVSGDVSALEKAIQEQGDKVRVLKSSGSPKTTWQPEVAILLDLKKKLEAAQKAVPASA

PTSVSGDVATLEKAVQEQGEKVRSLKSSGVPKATWQPEVTILLDLKKKLEAAQKAAPASAPSSGSDDVASLEKAVQEQGE

KVRSLKSSGVPKATWQPEVTILLDLKKKLEAAQKSTVTASPSTNGVAIADVNEIQCAIDKQALLVRQLKEGGAAKSEWSP

QVTILLDLKAKLAAATGQPSTGGKKSKK

>XP_009063905.1 hypothetical protein LOTGIDRAFT_229481 [Lottia gigantea]

MKVITDRGNFQTVKILIAAKAQTAGSGSGTDLKVTEVKHGEKVVPYLTESKLPVLEVKPEKFLFSVNSAVRLLLTSTGIE

SDSKQIDEWLEWESSQLLPYVFPVLVNHIGHNKTDKQSDSLLQPLLTYLNTSVKNKTYLVGDSLTSADVCVFGTVYPLTS

TPILKGFPELTSWIEKLSLIDAFKEAISIVTQNKGPVVFKESLLSQPAMPSPDSLTSKSTSKSSSVSSQPTESSEVEKQL

TEEELNAAEKCWRDGVAKLPELRERKHPILPIKGEKNILICSALPYVNNVPHLGNIIGCVLSADVYSRYCRLRNYNSVYL

CGTDEYGTATENKALEEGLTPQEICDKYNKLHTEIYDWFNISFDHFGRTTTTEQTKIVQDIFWKVYNNGHVIKDSIDQLH

CDKCNRFLADRFLADRFLAGRFLADRFVSGICPMCSYDDARGDQCDGCGKLINAVELINPKCKSCTSTPTVKTTQHLFLD

LPKLEPELRQHLDKAFDNGLWTHNSKVITNSWIRDGLKPRCITRDLKWGIPVPLEGYTDKVFYVWFDAPIGYISITATYT

KDWEKWWKNPNEVELFNFLGKDNVPFHSVIFPCTLLGANDNFTLVKHMSATEYLNYEDTKFSKSRGVGVFGNNAKDTGIP

ADVFRFYLLFVRPESQDTAFSWDDFLLKNNSELLNNLGNFINRLRDSIRNILSISRLGNQMMQANKPWVLVKRTPDEKAR

AGSVVSLAANIAWLIGVLLQPYMPSTSEIICTQLNGNPKNMVIPTQFIPVLKTGHKIGKPSPLFQKIEASLIEEMKKKFQ

GKQEKSVPAKPEVKSAPANSVLPKASDSEIASITDQVTKQGEVVRELKTQKAEKSKIDKEVALLLDLKKKLSLAQGIDPA

TASSNKKKGKKK

>XP_023721049.1 methionine--tRNA synthetase, cytoplasmic [Cryptotermes secundus]

MKLVTNENNPLSLKVLISANFAQKEILCEIADVDDPRFQSPRRLPVLELDTGEFLFSANSASRLLFPPHQSTSVVVDKWL

EWESSELQPVLAYYLSATSKPDTGVSRQLKEYLTTLNLALSGKKFLTGEGVSVADVAVWSILFLLATGAKHLQELLSEQS

HVQRWFSHLSLQPEVQVAVDQLCPKVGIAAYHSLANATWYPVNAVLSYVEKVPHSRIPSVNSLDGMSKESPLSEAEINAA

AEAWQSGAARRPKPKPQVSPVLPVPGERNVLISSALPYVNNIPHLGTIIGCVLSGDVFARFSRLCNNNTLYICGTDEYGT

ATETKALEEGLTPQQICDKYFKIHDAVYKWFNISFDHFGRTTTAEQTEISQDLFLQLYENGFLSKESVEQLLCENCNRYL

ADRFVEGTCPRCAYEDARGDQCDGCGHLVNATELVSPRCKICQKRPVVTKSDQFFLELPKTEPLLRNWLIRVIDGWSHNA

RVITQAWLKDGLKPRCVTRDLKWGVPVPLDGYRNKVFYVWFDAPIGYMSITKTYTDQWEKWWRPERNTTVTYYNFIGKDN

VPFHSVMFPSCLLGANRNYTLVSHIMATEFLNYEDEKFSKSRGVGVFGTDAKETGIPSDVWRFYLLYVRPESQDSNFSWV

DLATKNNSELLNNLGNFINRSLVFAEKNFSRQVPDMLPGVQEWTLLALITRELKAYMGALENAKLRDGIRHILNISRHGN

QYMQLLQPWVLLKGNDEERAHAGTVIGICCNIACLLAVLLKPYMPVTADILKEQLEAPDDVWIIIPEFTVLLPSGHKLGK

HPSPLFSKIEPSRVDQLKKQFSGRQKSQSPEVNTTSSKPATSTVNVVTTQVQGDPSVIAKLSAAVAQQADLVRKLKGSGA

APSIWQPHVAVLLELKRHLASAQSINGNVNEASTATLNGLQVPNPAEVARLEAGIAKQPESYNIHLVFPENCN

>KDR14813.1 Methionyl-tRNA synthetase, cytoplasmic [Zootermopsis nevadensis]

MKLYTNENNPLSLKVLISANFAQKDLRPEITNVDDPRFQSPRRLPVLELDTGEFLFSTNSASRLLFPPLQSSAVAVDQWL

EWESSQLQPVLAFYLSATSKPDTGVISHLKELLTTLNLALSGKEFLTGQGVTAADVAVWSILLPLATGTKQQKELLSEHS

QVQHWFSRLGAHPEVKAAVDLLSPKAGMDAYHSLATATWYPVNAVVSQTEKVSHVRIPSVNSLDGMSKESPLSEAEINAA

AEAWQNGAARRPKPKPQVCPVLPVPGEHNVLISSALPYVNNIPHLGTIIGCVLSGDVFARFSRLCNNNTLYICGTDEYGT

ATETKALEEDLTPQQICDKYYKIHDAVYKWFNISFDHFGRTTTPEQTEISQDLFLQLYENGFLTMESVEQLLCENCDRYL

ADRFVEGTCPRCAYLDARGDQCDGCGHLVNATELISPRCKICQKKPVVAKSDQFFLELPKTEPLLRKWLSGVIGGWSNNA

RVITQAWLKDGLKPRCVTRDLKWGVPVPLDGYRNKVFYVWFDAPIGYMSITKTYTDQWEKWWRPESTTSVSYYNFIGKDN

VPFHSVMFPSCLLGTYRNYTLVSYIMATEFLNYEDEKFSKSRGVGVFGTDAKETGIPSDVWRFYLLYVRPESQDSNFSWV

DLATKNNSELLNNLGNFVNRALVFVEKNFSCQVPKMSPGTQEWTLLAFITREVKAYMVALENAKLRDGIRHILNISRHGN

QYMQLLQPWVLIKGSDADRARAGTVIGICCNIACLLAVLLKPYMPVTADTLKEQLQAPDDVWIIIPEFTVLLPAGHKLGK

PSPLFSKIEPARVDRLKQQFSGRQKSRSPDQNTAAKSAPPPVNVATNQMQADASVISKLSAAVAQQAELVHKMKASGAAR

SIWQPHVAVLMELKQQLASAQIFNGGLNEVGAATSNGLQATNLADVARLEAEIVKQGDLIRQMKLRGVSKSEWQPELNVL

LSLKSELAAAQGSPLPPKGKSKGXXXXXXXXXXXXXRFPPRSTLYTMMRSNCMI

>XP_014259531.1 methionine--tRNA synthetase, cytoplasmic isoform X2 [Cimex lectularius]

MKLYSNPGNPHALKILACANLAGQEVEVELVPITDKRFQCPKKLPVLELPSGYSIFSSNVACSYLFRPPKKLEVATTEWL

TWDTVKLQPLIPFIGTKTEPTDKIEFVLTELNNTLNIKQYLVSDQLTSADICVCFTLYPLLFDEKLGKQFLLNRQNILKW

SKHVLTLDQVKSASKKFEVKSGEEALLSVTNSSWFPSPTSENIVLPDFDSDEHEQEVVLSQNHILQATKSWESGLDTRSN

LVLPSQPVLPVKDKKNVLITSALPYVNNVPHLGNIIGCVLSADVFARYSRFRGHNTFFVCGTDEYGTATETKALEEGLTP

QQICDKYFEIHDNVYKWFNISFDYFGRTTTAEQKEVVHEMFKSCYNNGYTSTSSVDQLLCENCNRYLADRFVEGICPDCK

YEDARGDQCDGCGHLINAVELIDPKCKLCKNTPVIKQSKQIFIELPKVEEKLKKWVAETSKGWSHNARVITEAWLKGGLK

PRCITRDLKWGVPVPLEGFEQKVFYVWFDAPIGYMSMTKRYTDKWLKWWQPSSTEKVVLYQFMAKDNVPFHSIMFPATLM

ASNGNYTIVNHLMATEYLNYENGKFSKSRGIGVFGNDAKDTGIPSDIFRFYLLYIRPESQDTNFSWADLALKNNTELLNN

LGNFIMRTLSFVDKCYNSIIPLIKLKTEEQELLGNISLELNSYVRSMDKSKFRDGIKYILNISRHGNQYMQNTKPWVLIK

GSESDKEKSATAVALLSNVVCLIASLLQPFMPETVQTIATQVNLPVEMFTITDTFYPYLKTGHVIGKVSPLFQKIEQDFM

ENLKAKYAGKQNSPSPTFVTVPSVVVDSTVMDRAKAVASLEEAVAKQGDLVRSMKAAGKSKDELKPMINTLLDLKKQLAA

LKENVGQDKMGSQVTNESDRVTEPAKINDVATLEELIAKQGDLVRAMKAASKSKEEVKPQIDKLLDLKKQLALLKGEPEE

SKKLKNGPVAKTQQSISGKKKNKKK

>XP_014259530.1 methionine--tRNA synthetase, cytoplasmic isoform X1 [Cimex lectularius]

MKLYSNPGNPHALKILACANLAGQEVEVELVPITDKRFQCPKKLPVLELPSGYSIFSSNVACSYLFRPPKKLEVATTEWL

TWDTVKLQPLIPFIGTKTEPTDKIEFVLTELNNTLNIKQYLVSDQLTSADICVCFTLYPLLFDEKLGKQFLLNRQNILKW

SKHVLTLDQVKSASKKFEVKSGEEALLSVTNSSWFPSPTSENIVLPDFDSDQEHEQEVVLSQNHILQATKSWESGLDTRS

NLVLPSQPVLPVKDKKNVLITSALPYVNNVPHLGNIIGCVLSADVFARYSRFRGHNTFFVCGTDEYGTATETKALEEGLT

PQQICDKYFEIHDNVYKWFNISFDYFGRTTTAEQKEVVHEMFKSCYNNGYTSTSSVDQLLCENCNRYLADRFVEGICPDC

KYEDARGDQCDGCGHLINAVELIDPKCKLCKNTPVIKQSKQIFIELPKVEEKLKKWVAETSKGWSHNARVITEAWLKGGL

KPRCITRDLKWGVPVPLEGFEQKVFYVWFDAPIGYMSMTKRYTDKWLKWWQPSSTEKVVLYQFMAKDNVPFHSIMFPATL

MASNGNYTIVNHLMATEYLNYENGKFSKSRGIGVFGNDAKDTGIPSDIFRFYLLYIRPESQDTNFSWADLALKNNTELLN

NLGNFIMRTLSFVDKCYNSIIPLIKLKTEEQELLGNISLELNSYVRSMDKSKFRDGIKYILNISRHGNQYMQNTKPWVLI

KGSESDKEKSATAVALLSNVVCLIASLLQPFMPETVQTIATQVNLPVEMFTITDTFYPYLKTGHVIGKVSPLFQKIEQDF

MENLKAKYAGKQNSPSPTFVTVPSVVVDSTVMDRAKAVASLEEAVAKQGDLVRSMKAAGKSKDELKPMINTLLDLKKQLA

ALKENVGQDKMGSQVTNESDRVTEPAKINDVATLEELIAKQGDLVRAMKAASKSKEEVKPQIDKLLDLKKQLALLKGEPE

ESKKLKNGPVAKTQQSISGKKKNKKK

>XP_023230927.1 methionine--tRNA synthetase, cytoplasmic-like [Centruroides sculpturatus]

MDSFSKAVKTVTNDTNANVFKESLLTSFKHLHFQTKYLCDNEVISVSNQDEEEKVKITEEEKEKAYNYWLEGVKCIPAVK

PLIDPILPVKGEKNILVTSALPYVNNVPHLGNIIGAVLSADVFARYCRLQQYNTLYICGTDEYGTATETKALEEGVTPRE

ICDKYFRLHSEIYSWFNISFNYFGRTTTEWQTKIAQEIFWKLHEKEYLIDDKMEQLFCQQCQRFLADRFVEGICPFCSYE

DARGDQCDKCSKLINAAELKSPQCKVCKATPTMRTSNHLFLNLPKLEPLLKDWLDNSTKCGEWSQTAKVICNSWLKDGLK

PRCITRDLKWGTPVPLEEYKNKVFYVWYDAPIGYPSITANYTEKWEEWWKQPNDVQLYQFMAKDNVPFHGIIFPSCLLGT

GDPYTKVNHLAAVEYLNYEDTKFSKSRGIGVFGNDAKETNIPPDIWRFYLLYIRPEGQDTSFNWVDLMTKNNSELLNNLG

NFINRALTFLTKNFDNSIPDMNLVEQDKILIAEINKELHQYINLMEKIKLRDGIRPILTISRLGNQNIQANKPWELIKGT

EEQKKRAGTVMGLSANIACLLANMLQPYMPDTSDILRKQLNMPSNCAVLSSFFTCFLPPGHKLGTNFPLFKKIEQSQIEE

LKKKYSGKQTTPERKIIINKDNINISNMENNDKIGDPQEIARYTNLVAQQGDKVRQLKLQKAEKDLIDKEIALLLELKKQ

LSIINKQDSLNIGTVSQKNKKKGK

>XP_002128793.1 PREDICTED: methionine--tRNA synthetase, cytoplasmic [Ciona intestinalis]

MMEINGQPSPEKLKVLLACFETGLHKIKVNSKPGSALSVRLDNEIEFTSSNAAVRYTYETQGVKVDVETLHWFEWEVTIL

RKLVGSYFSLSDKKILNINEFPNLISSLQYLENSINSGKLECDFSTKISAANCVIFCTISPLFCNPGIEGEFPVLRKHFA

IFMNRGSFRSAWKEVTGGEGRLAFQAYFAATKAQWKETKPIISTETSKPTEPNNSSKEVVSEDEIEAARKSWDSCNVQTA

KPRVHPVLPKEGEKNILITSALPYVNNVPHLGNIIGCVLSADVFSRYCRLRGWNVLYICGTDEYGTATETKAAQEGVTPK

EICDKYHKIHSEVYEWFSIGFDYFGRTTTECQTKIAQDIFWHLHRNNHLIKQTVEQLYCGRCERFLADRFVEGTCPLCAF

EDARGDQCDKCGKLINAVELKSPKCKMCSDTPAIRTSEHLFVDLSKLEPKLRRCLQESFKGEGWSNNAKTITETWLRDGL

KPRCITRDLKWGTPVPLEGFTDKVFYVWFDAPIGYLSITANYTDKWELWWKNPKNVQLYNFMAKDNVPFHSVVFPSCLLG

TDDEYTIVNHLNATEYLNYEEGKFSKSRGVGVFGDHASKTNIDSDIWRFYLLSMRPEGQDTHFSWSDFLLKNNSELLNNL

GNFINRSLKFVSNAYGGVVPDPVLNNTDLELLALVNRELKDYVTDMDKLKIRDNLRRILSISRLGNQYLQENQPWKLIKG

SNDDKSRASTVTSIAANLSCLLCILIHPYMPQTSSIIQDQLNLRGGYLIPDAITRMLPSGHKINKPSPLFRKLEEAEIEE

YKAKYRGREDGKKEVAKVEVGDCKGLEEEVLKQGNLVRELKTAKAEKLEIEREVSKLLELKKKLSLLKGEEPVQKKKGKK

KKK

>XP_023323108.1 methionine--tRNA synthetase, cytoplasmic-like [Eurytemora affinis]

MRLFTNSSNINSLKVQVAAELAGTNLQVVGVAPNDSRVGLRNILPVLEVEGSRFFLSAPAAIFILQNAGVLSDKEVEAYS

GLIEWESGTLYPTCLSFLQSAAINKNPDTSLKTQLNSALTLLAGGCEKGFVRGDKLTILDILVWCDLYPLATDKIFRKEL

EKFPSLVSWFDKLSALDCFKKPSEKFGRGLDGCKGSVSSLVGFTKQAVGATVPSEQESETKPKENPVSEEEIATAKAGWS

HTAHEARPVPGAGPVLPEDGKRNIMITSALPYVNNVPHLGNIVGCVLSADIYARFARLRGYNVLFICGTDEYGTSTETKA

VEEGLTPRQICDKYNGLHTEIYEWFSISFDKFGRTTTERQTEIAQQIFWDLHKEGKTSEASVEQLHCEPCDRFLADRFVE

GECPLCGFSDARGDQCDACGKLINAIELKSPRCKLCSKCPTIRTSNHLFLDLPQLEKSLELWLDKSSEKWSNNARVIAKS

WLKGGLQPRCITRDLKWGTPVPLAGYESKVFYVWFDAPIGYISITAEYTSEWELWWKNPDNVEYWQFMAKDNVPFHSVVF

PSTLLGTGKEWTLVNRLMSTEYLNYEDAKFSKSRGVGVFGNDAKDTGIPADVWRFYLMYTRPENQDSTFKWEDLMLKNNS

ELLANLGNFVNRATKFTKDNFDYTVPEMSLNAEDWEIVCLVNREVTHYIQLLEDAREREAISAVFSISRLGNQLMQHNTP

WKLVKGDDKEKVRAGTVIGISVNLACLLSVLIQPYMPNLSRELQNQLGIQEGSLNYIPEKFSVLLPPGHRIGEPSPLVAE

IKQALISDLKVRYAGKQNTRISAESKPEPSAEIKPTGVDPAAAALLEVQVADQGIKVRDLKARKAEKDEIAGAVATLLDL

KKRLATAQGVEVHVPDKKGKKKK

>XP_018897879.1 PREDICTED: methionine--tRNA synthetase, cytoplasmic isoform X1 [Bemisia tabaci]

MKLITNENNPNALKLFICGNLTCKTIDCEKVDSKDTKLHHSRYPALILDNGKQLFSSNAAALYLFPSFPTDIVDQWLEWE

ASHLVPSVLKSLSKEGSTTLTNNIKILDTVLSKTKFLHEGFITVADITVWSTLFGVITEPKLREKYCSSAANVIRWFEAI

HLLPEVQAAMKSWNGKSGEAVLTSLQSSSSCLAVTEASSTSTITAKLAKTSISNAPASETTPVKDVVSEDEISACIEAWE

KKSATRPKLKPHVHPILPKEGENNVLITSALPYVNNVPHLGNIIGCVLSADVFARYCRLRNRNTLFVSGTDEYGTATETK

ALEEKLTPQQICDKYFVIHKDIYEWFNIKFDQFGRTTTQKQTEIAQSIFKLVHNNGFTSTASVEQLLCEKCDRFLADRFV

EGTCPKCKYEDARGDQCDGCGSLINATELINPRCKVCGNTPVIRHSEQLFLELPKLEADLKKWVDNTSSGWSHNARVIAK

SWLKDGLKPRCITRDLKWGVQVPLQGFEKKVFYVWFDAPIGYMSISAAYTDEWEKWWLPSKSTQITLYQFMAKDNVPFHS

IMFPAIEMATQKNYTKVSHIMATEYLNYEDGKFSKSRGVGVFGNDARDTGISADVFRFYLLYIRPEAQDSSFSWADMAAK

TNTELLNNLGNFTNRALTFCEKFFGSVIPDMKLNKEDFQLLALITRELRQFMAVLDKAKFRDGIKHILCISKYCNQLMQA

EKPWVLMKGSEEEKSRAGTVIGLLSNLACLLSIMIRPYMPDTSKIMAEQLNAPESLYVLTEDVTMLLPPGHKIGHPSPIF

SLITPKTVEELKARFAGKQNDSAATKPTKTEAKSTDKSKSAPSQPASVPANAETIEKLEAAVAAQGLVVRKLKSETKDKA

VWEPEVKKLLDLKQQLASAKGEQVGNAATKKKK

>XP_023026965.1 methionine--tRNA synthetase, cytoplasmic [Leptinotarsa decemlineata]

MVIIYSNEFNPATLKLIVAKNFAKVAVDIKIVDPHDQIPQPKHLPCIEIDSDTTLFSSNAALCYLLPPGEDSVEVYNWLE

WEAATLSPSLTLLFGTSSKNDKLKSNLLGALNTLEKEFNNKKFLVGNNITNADIAIWSTLYPFLTCPAATKEYITPLSNL

SNWFQTLEDNPHFKDAISQFKIELNSSYNALLNGAKYPAPTAEYFHNVEKSSHIEETVTSEEIKLAQKAWQSDISKLPKL

KEQKDCVLPVPGERNILITSALPYVNNVPHLGNIIGCVLSADVFARYSRLCNNNTLYIGGTDEYGTATETKALEEGLTCQ

EICDKYFHIHNEAYKWFNISFNHFGRTTYPEQTEICQEMFLQLNDNGFMFTESVEQLHCENCNRFLADRFVEGGCPNLGC

TYEDARGDQCDKCGKLVNAVELKNPKCKICGKTPKLKSSNQFFIDLPKLEPLIHHWIKFSAPGWSNNAQIIAKSWLKEGL

KPRCITRDLKWGVPVPIDGFRDKVFYVWFDALIGYLSISKAYTKEFEQWWRPAKGTDVTLYNFMAKDNVPFHAILFPSML

MGTNKGYITVSHIMATEYLNYEDGKFSKSRGVGVFGTDAKSTGIPSDVWRFYLLYVRPESQDSSFSWNDLVIKNNSELLN

NLGNFINRALMFVKNNFDKTIPEMNPNNEDYTLLALCTKELKGYIASLEKAKFRDGIRHILNISRHGNHYMQSNQPWVLL

KGTDEEKARARTVIGICCNLSCLLATLLQPYMPDTSDNLKSQMNCSNVVITASNPQIVKLLIAGHKIGEPFPLFAKIEQA

TADELKKKFAGTQSERAGSENAEIKKLEEEVAKQGDKVRLLKSSGAAKTVWQPEVTILQELKKKLEVAQKTAVVQKKTVQ

IITMDAEKVKKLEQEVAKQALEVRKLKEGGANKSLWAPEVEKLLALKSRLAEATGSTLKDIQGDKKGKK

>XP_020381842.1 methionine--tRNA synthetase, cytoplasmic, partial [Rhincodon typus]

MTVDTAPMPVPLYSIGRYLFTLXGEEPNDITNQWLEWDATQLQTAVSAALRATVLQKRKVEDSLGTLRRQLSHLNQALLD

ATSPCLTSGTVTVADVVLWGDLYPLLQDVSCLPGEFEALRGWLQQLSQTRACRRAAAAVLDGGGLEVFRSHLQKLPAPAP

LSERPPSNDCEEGDAPDCQLTEEEVQAADEAWHSGLSALPKPGKVQHPVLPKPSERNILITSALPYVNNIPHLGNIIGCV

LSADVFARFCRLRGWNTLYICGTDEYGTATETKAVEEGLTPRQICDKYNAIHSDIYSWFQISFDYFGRTTTEHQTRIAQD

IFRRLLERGFLRSDTVDQLRCEACERFLADRFVEGHCPFCGYEEARGDQCDLCGKLINAVELKNPQCKICRATPVIRTSK

HLFLELPKLEEKLETWLQRSIETGDWTANARFITHSWIRDGLKARCITRDLKWGTPVPLEEFKDKVFYVWFDAPIGYISI

TADYTEHWEKWWKNPEEVKLYNFMAKDNVPFHSVVFPCSLLGADDNYTLVNSLIATEYLNYEDGKFSKSRGVGVFGDMAK

ETGIPVDIWRFYLLLVRPEGQDSSFSWHDLMLRNNAELLNNLGNFVN

>XP_018575973.1 methionine--tRNA synthetase, cytoplasmic [Anoplophora glabripennis]

MVKLYTNRSNPAALKLIIANNFSSAKVKLDITIVNLDGRTISEPKFLPTLEINNVQSIFMPNAGASYLFPASESNFFPVN

ELLEWEATILSPCLAYTLGNSAKNEKIRNNLLIALRKLNTVLENKKNLVGDSVTVADVAIWSCLYPLVKCDKLSKDYLSD

LPNINGWFLRLENEVQFKDAVAEFKIEIGQTAYTALLAGARYLAPPLLASDAPIKEKESPQHHIEAVTSEELLAAKKAWK

KNITQLPALKDQKNIVVPTPGEKNILITSALPYVNNVPHLGNIIGCVLSADVFARFSRLCNYNTLYICGTDEYGTATETK

ALEEGLSCKAICDKYFSIHDNIYKWFNISFNYFGRTSTPQQTEICQDMFLQLNSNGFMFTQCVEQLHCEKCDRYLADRFV

EGGCPNIGCNYQDARGDQCDGCGKLVNAVELKNPRCKICGQTPKLKTSNQFFIDLPKLEPLLHHWIKISSPGWSNNAQVI

ARSWIKEGLKPRCITRDLKWGVPVPLDGFRDKVFYVWFDAPIGYLSISKAYTKDFLKWWIPERGTEVALYQFMAKDNVPF

HSVLFPCMLLGANRGYTTVSHIMATEYLNYESGKFSKSRGVGVFGTDAQDTGIPSDVWRFYLLYIRPESQDSSFSWDDLA

TKNNSELLNNLGNFINRALVFAKNNFNRTVPEIKPTEEDYTLLALCTRELKGYVAALEKAKLRDGIRHILSISRHGNQYM

QANQPWVLLKGSDDEKIRAGAVIGICCNLACLLATLLQPYMPDTSASLHKQLNSSDILITPSNLEIVTYLPAGHKLGEPF

PLFAKIELTRVEELKKKFAGKQEEKENGKVDVKVIEAEVAKQAEKVRALKAKTTDKLVWKPEIDILLSLKKKLEAAQKNG

PVVNGGQSPVVKNGVSPDEIKKLEEEVAKQGLIVRKLKEGGSDKSIWQPEVQKLLSLKNQLQLAAGESPSPQKNKKGKK

>XP_019851279.1 PREDICTED: methionine--tRNA synthetase, cytoplasmic-like isoform X2 [Amphimedon queenslandica]

MGSYFLETILSSVINNSPSDQLLSCLTQLESHLTSNQKIVSCNEFSLADVVVWSTLLVIQSPESKLSSNIARDYPKIAQW

YRSLARDSRFSSAVWAASNDKGFEGFYDFIIPSGSGKSKSTTTGGKGQSSKAEKERTQSASGLSPSSSTGDMIGQADIDK

AMAHWNEGPDAAPKPRLHSHPILPIPGERNVLVTSALPYVNNVPHLGNIIGCVLSGDVFVRYARLRNYNTLYVCGTDEYG

TATEVKAVKEGLTPQQICDKYYALHSEIYEWFNVDFDIFGRTSTPQQTQIAQDIFWHLYDRDLIKKDTVDQLLCTSCDKY

LADRFVEGICPFCAYPDARGDQCDKCGKLINAVELKSPRCSICSSTPVVKQSNHLFLDLEKLQSPVFDWAQKSISEGVWS

QNAQFITKSWLRDGLKPRCITRDLKWGTPVPLEGYTDKVFYVWFDAPIGYISITACYTDQWEKWWKNPEQVQLYQFMAKD

NVPFHTVVFPSTLIGTGVDYSLLNHISSTEYLNYEDGKFSKSRGVGVFGNNAVETGIPADIYRFYLLYVRPESQDSAFSW

SDFVMKNNSELLNNLGNFVNRTLSFIKSSFDSCIPVMTLSHDDSVIVVKVTQELQGYIECLERHKQRDGLKHILAISRLG

NGHIQAHKPWELVKGDKNQKLLAGSLLGLCANICCLISVLLQPYMPSISKEIQNQLQAPSECNIIWDYFVPYLPTGHKIG

QPLPLFKKIDDATANELKKQFSGQKQDTGASSAMGESSKSKLEAELAKQAEVVRSLKASGSATKETLDAEIAKLLELKKQ

LGISGASVGSKKKGKKTK

>XP_019851280.1 PREDICTED: methionine--tRNA synthetase, cytoplasmic-like isoform X3 [Amphimedon queenslandica]

MGSYFLENQLLSCLTQLESHLTSNQKIVSCNEFSLADVVVWSTLLVIQSPESKLSSNIARDYPKIAQWYRSLARDSRFSS

AVWAASNDKGFEGFYDFIIPSGSGKSKSTTTGGKGQSSKAEKERTQSASGLSPSSSTGDMIGQADIDKAMAHWNEGPDAA

PKPRLHSHPILPIPGERNVLVTSALPYVNNVPHLGNIIGCVLSGDVFVRYARLRNYNTLYVCGTDEYGTATEVKAVKEGL

TPQQICDKYYALHSEIYEWFNVDFDIFGRTSTPQQTQIAQDIFWHLYDRDLIKKDTVDQLLCTSCDKYLADRFVEGICPF

CAYPDARGDQCDKCGKLINAVELKSPRCSICSSTPVVKQSNHLFLDLEKLQSPVFDWAQKSISEGVWSQNAQFITKSWLR

DGLKPRCITRDLKWGTPVPLEGYTDKVFYVWFDAPIGYISITACYTDQWEKWWKNPEQVQLYQFMAKDNVPFHTVVFPST

LIGTGVDYSLLNHISSTEYLNYEDGKFSKSRGVGVFGNNAVETGIPADIYRFYLLYVRPESQDSAFSWSDFVMKNNSELL

NNLGNFVNRTLSFIKSSFDSCIPVMTLSHDDSVIVVKVTQELQGYIECLERHKQRDGLKHILAISRLGNGHIQAHKPWEL

VKGDKNQKLLAGSLLGLCANICCLISVLLQPYMPSISKEIQNQLQAPSECNIIWDYFVPYLPTGHKIGQPLPLFKKIDDA

TANELKKQFSGQKQDTGASSAMGESSKSKLEAELAKQAEVVRSLKASGSATKETLDAEIAKLLELKKQLGISGASVGSKK

KGKKTK

>XP_014289891.1 methionine--tRNA synthetase, cytoplasmic [Halyomorpha halys]

MKLYTNDSNTISLKILISCEIVGKNVEIEIVQVDDKRFQGPKRLPVLLLDNGINLFSSNTAVSVLVHPSEKDIQAVNKWL

SWEAEKLQPVLVLYHNKTEFISIVEEFFNELDSTLSSSTHLVGDKITAADICVWAALYPLYSEDKFKTSLFDSRKGILKW

FENLYQEKYFKDAEKKLNAKKGLVALHSIASACWYPLSGSASDDVSASSKEKSDELVSAEEVNLAVSAWEKGPSSRVKIK

QQKKPILPVKGEKNILITSSLPYVNNVPHLGNIIGCVLSADVFARYCRARSWNTLYISGTDEYGTATETKALEEGVTPQE

ICDKYFAIHSDIYSWFNISFDHFGRTTTVDQTEVAQCIFTACHENGFTSTAEMDQLFCEKCERFLADRFVEGTCPDCKFE

DARGDQCDGCGHLMNTVDLISPRCKLCQNTPVVRQSQQLFLELPKIESWLEDWVNRTSDGWSHNAKVITKSWLNKGLKPR

CITRDLKWGVPVPLKNFGTKVFYVWFDAPIGYMSMTKVYTKDWLKWWKVDPQFDVKLYQFMAKDNVPFHGIMFPATLYAC

KGSHVMVSHLMATEFLNYEDGKFSKSRGIGVFGNDAKETGIPSDIFRFYLLYIRPESQDSNFSWSDFALKVNTELLNNVG

NFINRSLMFADKFYAGCIPPICVTEEEKKLLGLVRMELKSYISALDKAKLRDGIKYILNISRHGNLYIQSHKPWELVKGS

DDEKSRAGTIVGIACNLVCLITSLLQPFMPDTVKTISQQLNLPIESFSIQPDFIPYLDAGHKIGKPFPLFTKIEPALAES

LKAKYAGRQKSGSPESATPSIPSNLNNLANANDYLKAIKSLEEAVTKQGDLVRCMKAAGKSKDELQPMIGVLLDLKKKHT

EMKSAYEKQTNGLPLPEKEKGSNPDIISSLEKEVANQGEKVRSLKSSGADKSVWQPEVNILLDLKKKLAEATGTPVVQPQ

PAGKKKKSKK

>EFX80629.1 hypothetical protein DAPPUDRAFT_318233 [Daphnia pulex]

MNFKIKETHTEQAEEEEAAISEEEIEEAVKYWAQSNIPKPKTIIHPVLPVEGERNILITSALPYVNNFPHLGNIIGCVLS

ADVFARYCRLRNYNTLYICGTDEYGTATETKALEEGLTPQQICDKYFKLHDEVYKWFDISFDHFGRTTTPNQTKIAQDIF

LKLHRNNYLVEDTMEQLLCQHCKIFLADRFVEGTCPLCGYEDARGDQCDKCGKLVNAVELKLPRCKMCRHTPVIKTSKHL

FLDLPKIEDKLTSWMESVTAGWTHNARVIANSWIKEGLKPRCITRDLKWGTPVPLEGYTDKVFYVWFDAPIGYLSITACY

TEKWERWWKNREQVVHYEFMAKDNVPFHSVVFPSSQLGTHDPYTLVNHLIATEYLNYEDGKFSKSRGVGVFGNNAQETGI

PADVWRFYLLYVRPESQDSTFSWADLMTKNNTELLNNLGNFANRALSFLEKNYDSCVPSMTLGQEENMLLVRINRELKQY

ISLLENAKLRDAIRPIFSISRLGNQLMQAAQPWVLIKSTKDEEKKRAGTVIGLCANIICQLSVMLLPYMPNLSGKLQEQL

GVTSDVNHLIPEFTCRLPAGHRIGKPAPLFQKIEQSLIDELKQKFSGAQVGSAAPPPGNPAATPVAKEAASDSLETPENL

VLTVAQQGDKVRQLKAAKAEKSIVTAAVAELLDLKKRLATAEEAAKQIHQAPPPAATENPTPSSSDLENLTKQVAEQGDK

VRGLKTAKADKASIDAAVAALLDLKKQLALAEGKDPAPANPAASKGKKKGQQK

>XP_021928713.1 methionine--tRNA synthetase, cytoplasmic, partial [Zootermopsis nevadensis]

MTRTAIHLADPQLKNNGLHAANCSMKQDPRFQSPRRLPVLELDTGEFLFSTNSASRLLFPPLQSSAVAVDQWLEWESSQL

QPVLAFYLSATSKPDTGVISHLKELLTTLNLALSGKEFLTGQGVTAADVAVWSILLPLATGTKQQKELLSEHSQVQHWFS

RLGAHPEVKAAVDLLSPKAGMDAYHSLATATWYPVNAVVSQTEKVSHVRIPSVNSLDGMSKESPLSEAEINAAAEAWQNG

AARRPKPKPQVCPVLPVPGEHNVLISSALPYVNNIPHLGTIIGCVLSGDVFARFSRLCNNNTLYICGTDEYGTATETKAL

EEDLTPQQICDKYYKIHDAVYKWFNISFDHFGRTTTPEQTEISQDLFLQLYENGFLTMESVEQLLCENCDRYLADRFVEG

TCPRCAYLDARGDQCDGCGHLVNATELISPRCKICQKKPVVAKSDQFFLELPKTEPLLRKWLSGVIGGWSNNARVITQAW

LKDGLKPRCVTRDLKWGVPVPLDGYRNKVFYVWFDAPIGYMSITKTYTDQWEKWWRPESTTSVSYYNFIGKDNVPFHSVM

FPSCLLGTYRNYTLVSYIMATEFLNYEDEKFSKSRGVGVFGTDAKETGIPSDVWRFYLLYVRPESQDSNFSWVDLATKNN

SELLNNLGNFVNRALVFVEKNFSCQVPKMSPGTQEWTLLAFITREVKAYMVALENAKLRDGIRHILNISRHGNQYMQLLQ

PWVLIKGSDADRARAGTVIGICCNIACLLAVLLKPYMPVTADTLKEQLQAPDDVWIIIPEFTVLLPAGHKLGKHPSPLFS

KIEPARVDRLKQQFSGRQKSRSPDQNTAAKSAPPPVNVATNQMQADASVISKLSAAVAQQAELVHKMKASGAARSIWQPH

VAVLMELKQQLASAQIFNGGLNEVGAATSNGLQATNLADVARLEAEIVKQGDLIRQMKLRGVSKSEWQPELNVLLSLKSE

LAAAQGSPLPPKGKSK

>XP_022196447.1 methionine--tRNA synthetase, cytoplasmic isoform X2 [Nilaparvata lugens]

MKLFTNPGNSASLKILISSVFGKNEIVIEVISINDASFQAPKRLPVLQVDDVSIFSSNAAAQLLYPPKDSTLSAVQKWLD

WEAVSLQPALVKSSKIENCSIVGELVGKLNSTLANQQHLIKDILTVSDIVVWSKLFPIFSDDQLEPIFSAKYESVAKWFK

NLANLPEFESGVEKFANAGGVSCIQSVGAAGYFPAVSTVTTASESSKKTKPHKDPLPSESSESSDALPKEPPITDAELQS

AQKAWKSGPTATPKPTVTPVLPVAGERNVLITSALPYVNNVPHLGNIIGCVLSADVFARYSRLKNWNTLFVCGTDEYGTA

TETKAIEEGLTPEQICNKYFEIHKEIYSWFNIQFDKFGRTTTKEQTEVVHEMFRSVHSNGFTSISAMEQLLCEKCDKYLA

DRFVEGTCPKCSYEDARGDQCDGCGNLINAVELINPRCKLCQTRPVVRLSDQLFLELPKVEKQVEQWVESTSDGWSNNAK

VISKAWLKDGLKPRCITRDLKWGIPVPLPGFEKKVFYVWFDAPIGYLSITKTYTDKWRSWWEKDKSGKVKVSLYQFMAKD

NVPFHSIMFPSTLFAAGGHFNLVNHLMATEYLNYEDGKFSKSRGVGVFGTDAKETGIPSDIFRFYLLYVRPEGQDSSFSW

HDLVTKNNSELLNNLGNFINRALMFVEKFFDYLIPEINLNEDDFTYVVYINRLLSEYSSALENGRLRFGLRHILGISRYG

NLYFQTNQPWALMKGSPEDKKRAGSVIGLSCNIACLLAILLKPYMPQTCDVIAKQLNAPASVFVLPSQLAQLLPPGHKIG

KPAPLFTKIEPSTVDELKKRFAGKQQPVANAGPIGATNKKKQASAPSPSSSSPTSAAIKAMEEAVAKQGNLVREMKGSGS

PKSEWQSQVTVLLDLKKQLAELQMNTPVANSESATMDVETLKKKVAEQGDLVRKIKSSGVPKSEWQPHVDVLLSLKKQLA

EAEKQSPSSNSSVADPKQAIKDIEEKIAKQGDLVRKMKGSGVARSEWQPQVDVLLDLKKQLVAVQKS

>XP_019618782.1 PREDICTED: methionine--tRNA synthetase, cytoplasmic-like [Branchiostoma belcheri]

MADTAQGTPTEGPVTPEERTEALASFTEGKSACPKPVIREHPILPKPGEKNVLITSALPYVNNVPHLGNIIGCVLSADVF

SRFCRLRGWNSLYICGTDEYGTATETRALEEGLTPQQICDKYHALHADIYRWFNIDFDFFGRTTTPQQTEIAQDIFWRLH

QRDHLLTDTVDQLLCEKCDRFLADRFVEGTCPLCNYEDARGDQCDKCGKLIVATELKKPRCKICSASPVVKTSTHLFLNL

PQIEPKLNGWLEEILPNDSWSSNARVITRTWLRDGLKPRCITRDLHWGTPVPLKGFTEKVFYVWFDAPIGYLSITANYTE

RWDKWWKNPDEVILYNFMAKDNVPFHSVVFPCSLLGAEDNYTLVNHLQATEFLNYEDGKFSKSRGIGVFGDDAKDTSIPA

DIWRFYLLYVRPESQDTTFSWEDLMVKHNSELLNNLGNFIYRALTFVVNNFHGTIPAMELGESDTEMLAQVNRELQLYNF

NMEKVRLREGLTTILNISRLGNQYIQANKPWVLLKGTEQDRARAGTVVGLCANVACLLSVLLQPYMPDISTQIQEHLAAP

AGVNVLVDKLVCHLEPGHKIGQPKPLFEKLEASLVQDLKKKFAGRQKE

>XP_020018748.1 methionine--tRNA synthetase, cytoplasmic [Castor canadensis]

MRLFVSEGAPGSLPVLAAAGRARGRAELLISTVGPEECVVPFFTRPKIPVLQLDSGNYLFSTSAICRYFFLLSGWEQDDL

TNQWLEWEATELQPALSIALYYLVVQGKKGEDVLGPLRRALTFIDHSLSRQNCPFLAGETESLADIVLWGALYPLLQDPA

YLPEELGALRSWFQTLSIQEPCQQAAETVLKQRGVLALRPYLQKQPQPQPQPNPPEGRVVSNEPEEEELATLSEEEIAMA

VTAWEKGLESLPSLRPQQKPVLPVAGERNVLITSALPYVNNVPHLGNIIGCVLSADVFARYSRLRQWNTLYLCGTDEYGT

ATETKAMEEGLTPQEICDKYHTIHADIYRWFNISFDIFGRTTTPQQTKITQDIFQRLLARGFVLKDTVEQLRCEHCARFL

ADRFVEGVCPFCGYEEARGDQCDKCGKLINAIELKKPQCKVCRSSPVVKSSQHLFLDLPKRDKDEESVDQRGERCDLSRW

FFLLFCSEYLNYEDGKFSKSRGVGVFGDMAQDTGIPADIWRFYLLYIRPEGQDSAFSWTDMLLKNNSELLNNLGNFINRA

GMFVSKFFGGYVPEMVLTLDDQRLLAHITLELQHYHQLLEKVRIRDALRSILTISRHGNQYIQVNEPWKRIKGSEAERQR

AGTVTGLAVNIAALLSVMLQPFMPTVSATIQAQLQLPLPACRILLTNFLCTLPAGHQIGTVSPLFQKLENDQIESLRQQF

GGGQLEESLELKAKVSPKPAVVETVTTAGPQQIQILMDEVAKQGNIVRELKAQKADKNQVAAEVAKLLDLKKQLALAEGK

PLETSKGKKKK

>XP_015518469.1 PREDICTED: methionine--tRNA synthetase, cytoplasmic [Neodiprion lecontei]

MIILTNDNNPNALKLIIGLKLAQQSVEINTVSCRDAVLGYLPSLQLPSGSSLFSTSAALQFLIPTPEESSVLINKWLEWD

ISQLQPALVSYGGSGKLDTSHKPRIWSLLKEFNRCLKSKERLIETNTVLSVADVSIWVSLWSTIAAVEVSKELTKEFPHV

QEWLNNLESLPVVQESLKEFKFERGMNAIGSIQAASWFPLNSYTGGTLNKQLSNEVNTPKEKEVASISEEEILNVKKNWT

SKDYPNLKEPSYPVLPKKGEKNILITSALPYVNNVPHLGNIIGCVLSGDIFARYCRQRNYNTLYISGTDEYGTATEAKAV

EENMTPQQICDKFFSIHNDIYRWFSIGFDYFGRTTTPEQTEIVQSLFLRLKDKGFINMETIDQLLCEKCDRFLADRFVEG

TCPGCKYEDARGDQCDGCGHLVNATELLNPRCKVCNNSPVIKPSVQFFLDLPKIEPILKKWAAKTEGEWSPTARVVAKPW

LRDGLKPRCITRDLKWGIPVPIEEYKKKVFYVWFDAPVGYISITKRYTKDYEQWWKPKDHDVSLYQFMAKDNVPFHAIMF

PASLLAADNGHTLVNHIMAIEYLNYEDTKFSKSRGIGVFGTDARDTGIPADVWRFYLAYIRPESQDSNFNWVDLATKNNS

ELLNNLGNFVNRALTFAEKNFGSKVPPMELREDDLIVLALAQRELSQYVAALEKAKMRDALRHILAISKHGNQYMQSQEP

WVKIKGSDDDKKKAGTVIGVCSNLACLLSALLGPYMPSTSRELRAQLGLDKISYGYIPDTIMTFLPAGHKIGKPSPLFSK

IEDQQVELLRKKYAGKQDSPSRELKSVIPAAATDTAALEAAIVKQGLLVRDLKAKGDKTIWQPQVQILLQLKKQLAELTS

STAKINSQSTPVNAKKSVSAATSELNGDGGQDVATLEAAIAKQGLLVRDLKTKGDKSVWQPEVEILLNLKKQLASITGAP

VSAANDKKSKKKK

>XP_022196446.1 methionine--tRNA synthetase, cytoplasmic isoform X1 [Nilaparvata lugens]

MKLFTNPGNSASLKILISSVFGKNEIVIEVISINVFSDASFQAPKRLPVLQVDDVSIFSSNAAAQLLYPPKDSTLSAVQK

WLDWEAVSLQPALVKSSKIENCSIVGELVGKLNSTLANQQHLIKDILTVSDIVVWSKLFPIFSDDQLEPIFSAKYESVAK

WFKNLANLPEFESGVEKFANAGGVSCIQSVGAAGYFPAVSTVTTASESSKKTKPHKDPLPSESSESSDALPKEPPITDAE

LQSAQKAWKSGPTATPKPTVTPVLPVAGERNVLITSALPYVNNVPHLGNIIGCVLSADVFARYSRLKNWNTLFVCGTDEY

GTATETKAIEEGLTPEQICNKYFEIHKEIYSWFNIQFDKFGRTTTKEQTEVVHEMFRSVHSNGFTSISAMEQLLCEKCDK

YLADRFVEGTCPKCSYEDARGDQCDGCGNLINAVELINPRCKLCQTRPVVRLSDQLFLELPKVEKQVEQWVESTSDGWSN

NAKVISKAWLKDGLKPRCITRDLKWGIPVPLPGFEKKVFYVWFDAPIGYLSITKTYTDKWRSWWEKDKSGKVKVSLYQFM

AKDNVPFHSIMFPSTLFAAGGHFNLVNHLMATEYLNYEDGKFSKSRGVGVFGTDAKETGIPSDIFRFYLLYVRPEGQDSS

FSWHDLVTKNNSELLNNLGNFINRALMFVEKFFDYLIPEINLNEDDFTYVVYINRLLSEYSSALENGRLRFGLRHILGIS

RYGNLYFQTNQPWALMKGSPEDKKRAGSVIGLSCNIACLLAILLKPYMPQTCDVIAKQLNAPASVFVLPSQLAQLLPPGH

KIGKPAPLFTKIEPSTVDELKKRFAGKQQPVANAGPIGATNKKKQASAPSPSSSSPTSAAIKAMEEAVAKQGNLVREMKG

SGSPKSEWQSQVTVLLDLKKQLAELQMNTPVANSESATMDVETLKKKVAEQGDLVRKIKSSGVPKSEWQPHVDVLLSLKK

QLAEAEKQSPSSNSSVADPKQAIKDIEEKIAKQGDLVRKMKGSGVARSEWQPQVDVLLDLKKQLVAVQKS

>XP_018329162.1 PREDICTED: methionine--tRNA synthetase, cytoplasmic [Agrilus planipennis]

MVLTVYTNETNPSTLKILVAANFSKAEVNLKIVSSQERELQQPRHLPYLELEDSCNLFIPNAAAKFLFPSKDNEEVFVNE

WLEWESSVLSPNIALLFGFPVKIEDRKEGLKRHLHYLNDAIRDFSTSQKQIIVSDVVIWSTLYPFYKDTDLSKEYLPELD

DLNTWIKKLEQMEEFQKAVNGYEVKPGQPSYQVLLNSSKYLSPVNLAICPPCPSRPSIQTQTSIESVSVDDISKAKESWL

KGSANLSKPKSDIIPVLPKPGEKNVLITSALPYVNNVPHLGNIIGCVLSADVFARYCRLCNYNTLFICGTDEYGTATETK

ALEEGLTPQQICDKYFEVHNSIYRWFNIGFDYFGRTTTPQQTILVQEMFQILNKNGFMFTETVDQLLCQKCNRYLADRFV

EGGCPHLGCLYEDARGDQCDGCGKLVNATELRNPRCKVCGTTPVIRSSNQFFFDLPKLEPLLLCWMKQSSTGWSSNARII

GNSWLKEGLKPRCITRDLKWGVPVPLEGFRDKVFYVWFDAVLGYISITQRYTKEWAKWWKPEKGTEVKLYQFMAKDNVPF

HAVIFPAILKGVNDGFITVSHIMATEYLNYEDGKFSKSRGIGVFGNDAQNTGIPSDVWRFYLLYVRPESQDSSFSWADLV

TKNNSELLNNLGNFINRALVFAEKFFDSHIPNMNLLEDDFILLAQCTKELQAYVVSLENGKLRDGIRHILSISRYGNQYM

QSMQPWVLVKGTSEDKERAGTVVGVCCNLVCLLATLLFPYMPETGRTIRNQLNAPNETFALTSLHPEIVQLLPSGHKLGK

PVPLFAKIELSRVEELKTQFSGKQADSNGASKKEDNLSVTELEEAIKKQGDKVRNLKASGVEKAVWQPEVNILLDLKKKL

EIAQKGTNIKQSSVKILNENGVLPCASPEEIKKLEERVQKQGDHVRQLKSSGSPKSEWQPQVNILLQLKARLAAALGAPP

AKPDGKGKKSKN

>XP_001626448.1 predicted protein [Nematostella vectensis]

LPKEGEKNILITSALPYVNNVPHLGNIIGCVLSGDVFARYCRIRGFNTLYISGTDEYGTATETKALEEGLTPQQVCDKYH

KLHCSVYDWFNIQFDFFGRTTTENQTKIAQDIFWKLHQQNHLVEDTVEQLQCTGCQRFLADRFVEGICPFCNYEDARGDQ

CDACGKLINATELKSPKCKVCGGTPEKKTSRHLFLNLTESSQDISCTSPGNWSPNARQITRSWIQEGLKPRCITRDLKWG

TPVPLEGYTDKVFYVWFDAPIGYLSITANYTDQWEKWWKNPKQVTLYQFMAKDNVPFHTVVFPSTLIGANDNYTLLNNIS

ATEYLNYEDDKFSKSRGVGVFGDNAADSGIPADIWRFYLLYMRPEGQDSAFSWSDFAIKNNSELLNNLGNFINRALMFLQ

NNFAGKIPDLELMDEDTNFIALVNRELKVYYENMDKLRLREGLKVVLNISKYGNQYVQANKPWVLVKGSDAERCRAGTVI

GLACNLSFMLSLLIHPYMPTVSKEIQTQLKVRAQEDCGILTEHFVPFLKPGHVIGTPKPLFQKIDPAVCEKLKAKYAGKK

APSKAKPSATPSSTPAPGAASPATIEALQAEVTQQGEKVRSLKTNKAEKAVIDAEVTRLLELKKKLSLAEGKDPNQPSTG

GKSKKK

>XP_008003572.1 PREDICTED: methionine--tRNA synthetase, cytoplasmic-like [Chlorocebus sabaeus]

MLTIYETFLNQPSCLSPLSQLEKRLEEWLGRTLPGSDWTPNARFITRSWLRDGLKPRCITRDLKWGTPVPLEGFEDKVFY

VWFDATIGYLSITANYTDQWERWWKNPEQVDLYQFMAKDNVPFHGIVFPCSALGAEDNYTLVSHLIATEYLNYEDGKFSK

SRGVGVFGDMAQDTGIPADIWRFYLLYIRPEGQDSAFSWTDLLLKNNSELLNNLGNFINRAGMFVSKFFGGYVPEMVLTP

DDQRLLAHVTLELQHYHQLLEKVRIRDALRSILTISRHGNQYIQVNEPWKRIKGSEADRQRAGTVTGLAVNIAALLSVML

QPYMPTVSATIQAQLQLPPPACSILLTNFLCTLPAGHQIGTVSPLFQKLENDQIESLRQRFGGGQLEETFDLKVITFPLR

CCIKLEGNIVRELKAQKADKNHVAAEVVKLLDLKKQLAVAEGKPPEAPKGKKKK

>XP_017051723.1 PREDICTED: methionine--tRNA synthetase, cytoplasmic [Drosophila ficusphila]

MIIYTNEGNPLGLQLLMLAKFAKRPVQVQLVNLNDAKYKDLLVLPTLELDNGLRLFSPAAIAKYLLVDEGHQRRDEWLEW

SATLLAPALAHHMGVGHKADANALPVLNGLVKKLDDNLKSSAFLAGDKLGAADIAVWSLLAPDGTLKGAQNVDSLRDWYG

RIKALPEVQEVLTEQPLKDLSFNALQQSNRYGGLHHVPLKRSSLADAGKLLVDTTPTVADTVTDEEISAAQAAFTYTTPK

QIKEERTVLPKAGERNVLITSALPYVNNVPHLGNIIGCVLSADIYARYSRSAGYNTLLICGTDEYGTATENKALAENLTP

REICDKYFELHNAIYRWFGIGFDYFGRTTTQEQTDIVQEAFKDVLKEGYIITESVEQLLCQKCDRFLADRFVEGTCPHPG

CGYEDARGDQCDKCGKLVNATELIRPRCKVCNSAPVLRSSDQLFIDLPKAEPKLKEWVDKSEGGWTHNAKVITRAWLKEG

LKPRCITRDLKWGIPVPHEGFENKVFYVWFDAPFGYVSMTKRYTKEYQQWWQPAKGTDVELFQFMAKDNVPFHSVVWPSV

LLAINKGHTLVSHIMATEYLNYEDGKFSKSRGIGVFGNDAQETGIPADVWRFYLASARPEGQDSSFSWNDLAARNNSELL

NNLGNFVNRALVFCEKNFNGTVPEVAVTQDELVLLALINRELRGYINSLEKAKLRDGIRHLLAISRHGNGYMQTQQPWVL

LKGSEDQKKRASTIIGLCVNIACLLANLLFPYMPTTARTLFDQLNAKQTPLNAEKPLATLLLPAGHKIGKPAPLFAKLEQ

AFIDELKGRYGGAQDSKTPAQSQTSAADLEQAVQAQADKVRQLKASTKDKSVWQPEVTKLLDLKKQLEEAKKTPAAAAAT

PPSNSSPSVQDLEKAIQEQGDKVRKLKGSTKDKSVWQPEVNVLLDLKKQLESAQKAAKAAPPATVPVPSPVSSADAAQVK

ALEDKIAQQAEKVRTLKASGDASVWKPEVDILLALKNELATLTGTPAAGGQGKGKKKK

>XP_018779641.1 PREDICTED: LOW QUALITY PROTEIN: methionine--tRNA synthetase, cytoplasmic, partial [Serinus canaria]

WEATELQPAVAAALYLQLVQGRAGPDALGVLQPLLAHLEQHLARSGTSHLAGDAASVADVVVWGSLFPALQDDPLLARLP

VEGERNVLITSALPYVNNVPHLGNIIGCVLSADTFARYCRLRRWNTLFVCGTDEYGTATETRALQEGLSPQELCDRYHAL

HADXXXXXHLHWRYLWLMXPPFPGRIAQDIFQRLLARGFLLQDTLEQLRCESCGRYLADRFVEGTCPFCGYAEARGDQCD

KCGKLINAVELQNPQCKLCRGTPVVTPTQHLFLDLPKLEGRLEAWLERTWAAGEWTANARHITRTWLRDGLKPRCITRDL

TWGTPVPLDGFRDQGPALPQVFYVWFDAPIGYLSITANYTDQWERWWKNPQQVELYNFMAKDNVPFHSVVFPCSLLGADD

NYTLVNHLIATEYLNYEDGKFSKSRGVGVFGDMAKDTGIPADVWRFYLLYLRPEGQDSAFSWADLLLKNNSELLNNLGNF

INRAGMFVCKFFGGTVPAMALTDEDQRLLARVTLEMRQYHQLMEKVRLRDALRCVLGISRHGNQYIQVNEPWKHIKGDER

DRQRAGTVTGGTSPHLFLLQAKQPPPASAPPPAPNTSAAPGDPKLIQELTEQVAKQGNLVRQLKAAKVEKAQVAAQVAKL

LELKQQLALAEGRSPEVPKGKRKK

>XP_019868561.1 PREDICTED: LOW QUALITY PROTEIN: methionine--tRNA synthetase, cytoplasmic [Aethina tumida]

MSVIYTNENNPAGLKLIIAKSQSKKEEVAVKIVNVHERSLEQPRHLPYLEIDNSLVLFSSNAALFYLCPLPSKVDAQAAN

EWLEWESTVLAPSLALLIGSNLKSDYLKSTLNTALKKLNTFLKNKKFILSDQLTIVDISIWCTLYPLYVFGKTEEHFKEY

HANLTDIDAWFKNLENDATFQESLQSFTLKGCHPSYNSLLNGGKYLVSASQDASTPKTEQTPEVTVTEEELEAAKNAFLG

SETFPPLKPIEKTVLPVSGEKNVFITSALPYVNNVPHLGNIIGCVLSSDVFARFSRLCNQNTLFISGTDEYGTATETKAL

EEGLSCQAICDKYHKIHRETYEWFNISFNYFGRTSTPHQTEICQDMFLKLNDNGFMFTQSVEQLHCDNCKKFLADRFVEG

GCPNAGCTYEDARGDQCDACGKLVNAIELKNPRCKICSQSPKIKTSNQFFIDLPKLEPLLHHWMKKSSSNWSNNAQVIAR

TWLREGLKPRCITRDLKWGVPVPLEGFRDKVFYVWFDAPIGYMSILKAYTDKFEKWWIPEKDADIDLYQFMAKDNVPFHS

VLFPACLLGANRGYLTVSHIMATEYLNYEDGKFSKSRGVGVFGTDVQKTGIPSDVWRFYLLYVRPESQDSSFSWVDLATK

NNSELLNNLGNFINRSLMFAKNCFDSVVPAINQLSNEDLTLLALCTKEFKQYVNALEKCKLRDGIRHILAISKLGNQFMQ

SNQPWVLVKGTDEQKLRAGTVIGLCCNIVALLELLLRPYMPITSDTIKKQINGKDLAINLANVCIVQLLPSGHKLGEPSP

LFAKIEPARVDELKKMFAGKQGDRVPVEVQQLELEVQKQADKVRALKSSKADKKIWQPEVAILLDLKKKLEECQKNKAKA

TAGGDAPVQGTPEEVKALESEIAKQGDKVRSLKASGVEKAVWQPEVNILLSLKKKLEELQKFISSKSSAPQNGVNPDEIK

RLEEEVNKQALKVRKLKEGKAEKSVWQPEVDILLKLKAELVAAGGAPAAAPQKGKKGK

>PSN51504.1 hypothetical protein C0J52_00742 [Blattella germanica]

MKLFTNENNSQSLKLKISANFSEKDVKAEITHIEDPRFQCPRRLPVLELDNGEHIFMPNAASRYLFPPHQRCSRLVDHWL

EWEAYWLQPVVALYLSSTSKLDAGVGERLKGLLATLNSALHSKFLAGDEVTVADISIWVTLFPLATEPKYKQDLLSGLTR

VQEWFASVQNMPQVKISVEQLCPKGGSAAAQSAADATWFPINAVGFQSEKSSRAQLQSVSSVDSTSKEPPITEAELKAAA

EAWQNGSTRRPTPKRQVAPVLPVPGERNVLITSALPYVNNIPHLGTIIGCVLSADVFARFSRLCNNNTLYICGTDEYGTA

TETKALEEGLTPQEICNKYFQIHDAVYKWFNISFDNFGRTTTPEQTEICQDIFLQLNENGFMTTESVEQLLCESCQRYLA

DRFVEGTCPRCAYEDARGDQCDGCGHLVNATDLIKPRCKVCQKTPVIRKSEQFFLELPKIEPLLRKWLEGVISGWSNNAR

VITKSWLKDGLKPRCITRDLKWGIPVPLEGFRKKVFYVWFDAPIGYMSITKKYTDKWEKWWRPDEHTEVTLYNFIGKDNV

PFHSVMFPSSLLGANRNYTLVSYIMATDYRERLSLQDEVTVADISIWVTLFPLATEPKYKQDLLSGLTRVQEWFASVQNM

PQVKVRLAKAFQVLLQISVEQLCPKGGSAAAQSAADATWIPINAVGFQSEKSSRAQLQSVSSVDSTSKEPPITEAELKAA

AEAWQNGSTRRPTPKRQVAPVLPVPGERNVLITSALPYVNNIPHLGTIIGCVLSADVFARFSRLCNNNTLYICGTDEYGT

ATETKALEEGLTPQEICNKYFQIHDAVYKWFNISFDNFGRTTTPEQTEICQDIFLQLNENGFMTTESVEQLLCESCQRYL

ADRFVEGTCPRCAYEDARGDQCDGCGHLVNATDLIKPRCKVCQKTPVIRKSEQFFLELPKIEPLLRKWLEGVISGWSNNA

RVITKSWLKDGLKPRCITRDLKWGIPVPLEGFRKKVFYVWFDAPIGYMSITKKYTDKWEKWWRPDEHTEVTLYNFIGKDN

VPFHSVMFPSSLLGANRNYTLVSYIMATEFLNYEDEKFSKSRGVGVFGTDAKETGIPSDVWRFYLLYVRPESQDSNFSWV

DLATKNNSELLNNLGNFVNRALVFAEKNFSGVVPDMVPDATEWTLLAAITRELKAYMAALESSKLRDGIRHILNISRHGN

QYMQMQQPWVLLKGSDADRVRAGTVIGTCCNIACLLAILLKPYMPETSDVMKDQLQAPDDVFIITPEFTLLLPPGHKLGK

PSPLFSKIEPDRVEQLKQQFSGRQKSRSPEANPVEGDSIAKLTAAVAQQAELVRKMKSSGAARSDWQPHVAVLLELKQQL

AAAQNIEVASANSNGLQVTNAAEVARLEAEIAQQGDLVRKLKSSGVPKSEWQPQVNVLLGLKSQLAELQGSSTPAKNKKG

KKMSVSFIRIQKKKPRKVSACRSELLDRVNSEEREVQRMERCFDCLSLMDECESETNRSLDGMIAEMIADNETNAASEYV

VDMVRSLRPELKLEANRILLLLEEAEMLKDRLEWERKVLKEEGRAVRDDMTQYEDYICNEQYYNFKNATWDALKNVGRDE

LQRRRSKLQKMRNDLNGKYQMLCQKIRVYQFLLSKLNEEEIQREQRCHALSLHGGSSKMQIHRDLIEYVRTEIDRTQVTE

KFITNKLKQDIIQVKETWSQNILNVQRLGCTFDFVLQHRVNKGGSRGYYDAVVGIFEKEIPVFHADAICEKSMILMKQVF

GRQRTRLEKEMDIYLWMTNAKPSRVPGASVLEQPKPDLTPQKPEFEKDAPKQGEMPKVQENASKCADKQDVEHCNQRIDE

KRGKTKKQTKKLEEKTDELAKSMSEVKEVNKAILKKLNSLIPKIHQSKRLFPCEAKSLIRHKEIDETKALEDKNTLQETT

EALSAFVTRGEDLENKRCSFQPADLSIEEQKRLASLQILKKLHSFLKRNEETRAFAESEEMSLNVIPRQLLSISIISQFL

NDSVLKLRCLEISEMKRMETQDFQLRDVHEALMYLTNNEVITWADEDLCDEFCSICEERDSC

>OQV15980.1 Methionine--tRNA synthetase, cytoplasmic [Hypsibius dujardini]

MADSTKPLTETSTRTFSPEEVSTCQLQWRTGKTSLTGPKARIHPILPEAGARNILITSALPYVNNVPHLGNIIGCVLSAD

AFSRYCRLRGYNSLFVCGTDEYGTATETKALEEGVTPREICDKYNAIHEEVYRWFNIDFDVFGRTSTEHQTKVTQDIFWK

LQKRGYMLEESVEQLHCSRCNRFLADRYVEGTCPLCGYPDCRGDQCDKCGKLVNATELIQPKCKICGTAPAIRASNHLFL

DLPKLQPDLEKYLEEAFRTGTWSHNAKVITQSWCRDGLKPRCVTRDLVWGTPVPLEGFTDKVFYVWFDACIGYISITAAY

TSEWEKWWKNPEQVDLYNFLGKDNVPFHSVVFPISLLGTQDGYTMVKHMSATEYLNYEDDKFSKSRGVGVFGTDAQSTGI

EPDIYRFYLLFVRPEDQDTKFSWSDLVLKVNTELLNNVGNFINRALSFLDKTYGGVVPAMQLDAEDLCLLANIHADIAEY

LEHMEKIHLREALRSILSVSRLGNQMMQAAKPWALVKGTDEEKARAGSVIGLAANICLQLSVMLLPFMPHYSKVIQDQLN

APDRCYFLQEYLVCQLEAGHKIGTPAPLFRKIENAEAEDLKKRFAGKKPVVAAATTAGGAAVGGESAAELNEQITKQGEV

VRDLKAKKAAKEEIDREVLKLLDLKKRLTLSGGGGDAAAAPADKAGKKEKGGKKTGDTKKPVVVASDGEAKPADKP

>XP_017471733.1 PREDICTED: methionine--tRNA synthetase, cytoplasmic [Rhagoletis zephyria]

MKIYTNEGNPFGLKLQLLAKFAKQPVEVVNVTLNDTECKDFLVLPTLVLENGVKLFSTDAVAKYLFPDEGALRDEWLEWS

TVKLAPALAQHLAVGHRANPDALPVLNMLVRKLDDRLAESPFLVGDKITSADIAVWSLLAPDGTLKGALNIEHLKAWYRK

ISALAAVQDVLTETPLAKLTFTSLQNSNRYGGLNHVPVHLHTSSESEKLLAETSGNLADTVTEEELNTAKRNFVQVAVPQ

RIEPRTVLPKSGERNVLITSALPYVNNVPHLGNIIGCVLSADIFARYSRAAGYNTLYICGTDEYGTATENKALAENLTPK

QICDKYFDLHNSIYRWFGIGFDFFGRTSSLQQTEIVQEAFNDVYKNGYIFTESVEQLLCVKCDRFLADRFVEGTCPHSGC

GYEDARGDQCDKCGKLVNATELIRPRCKICNTAPILRSSDQLFLDLPKVEPQLRAWMEGSEMGWTNNARVIARSWLREGL

RPRCITRDLKWGIPVPLAGFEKKVFYVWFDAPYGYISMTKHYTSEYKQWWIPSNDVKVELFQFMAKDNVPFHSVIFPSSL

LATNKGHTMVTNIMATEYLNYEDGKFSKSRGIGVFGNDAQETGIPADIWRFYLACARPEGQDSSFSWNDLAARNNSDLLN

NLGNFVNRALVFCEKNFDGVIQAMTLTKDELVLLALINREVRAYVNSLEKAKLRDGIRHLLSISRQGNGYMQAQQPWVLL

KGTDEQKARAATIIGVCANIAAILANLLFPYMPATARTLLTQLNAKQTQLNPRQPFINILLPTGHKIGKPAPLFTKLEQS

FIDELKQKFGGVQDQNIDKKATLVANTSKPDTAKASLITNGTQTIAQLEEAVRLQGEKVRQLKASTKDKSVWQPEVNILL

DLKKQLTVAQAAPPVTKAAVVPQASDAKVKELEQRITQQGEKVRQLKAAGDASVWKPEVDVLLQMKKELATLTGAPQPTV

QGKNKKKK

>XP_017149708.1 PREDICTED: methionine--tRNA synthetase, cytoplasmic [Drosophila miranda]

MIIYTNEGNPLGLQLLMLAKFAKRSVQLVNLNLNDAKFKDFLILPTLELDNGLRLFSPAAIAKYLFVDEGQLRDEWLEWS

TTLLAPALSHHMAVGHRADPNALAVLNALVKKLDDCLKATPYLAGDKLSAADIAVWSLLAPDGTLKGAQNLESLHGWYNR

VKALPEVQQVLAEQPLKDLSFNALQQSNRYGGLHHVPLKRLSLADAGKLLAETASTVADTVTDEELAAAGASFTYTAPKD

VQEQRTVLPKAGERNVLITSALPYVNNVPHLGNIIGCVLSADIFARYSRSAGYNTLLICGTDEYGTATENKALAENMTPR

EICDKYFELHNSIYRWFGIGFDYFGRTTTQEQTDIVQEAFKDVLSAGYIITESVEQLLCQKCDRFLADRFVEGTCPHPGC

GYADARGDQCDKCGKLVNATELIRPRCKVCNTAPILRSSDQLFIDLPKAEPRLREWVDKAENGWTHNAKVITRAWLREGL

KPRCITRDLKWGIPVPHSGFEKKVFYVWFDAPFGYVSMTKRYTKEYQQWWQPAKGTDVELFQFMAKDNVPFHSVVWPSVL

LAINKGHTLVSHIMATEYLNYEDGKFSKSRGIGVFGNDAQATGIPADVWRFYLASARPEGQDSSFSWNDLAARNNSELLN

NLGNFVNRALVFCEKNFNSIVPEVVATEEELILLALVNRELRGYINSLEKAKLRDGIRHLLAISRHGNGYMQTQQPWVLL

KGSDEQKKRAATIIGLCANIACLLANLLFPYMPSTARTLFAQLNAKQTPLNAEKPLATLLLPAGHKIGKPAPLFAKLEQS

FIDELKAKYGGSQDTKAAVQSQSSTADLEQAVQAQADKVRQLKTSTKDKTVWQPEVSKLLDLKKQLEEAKKTAVAAPASA

PALSNGGQSVQDLEKAVQAQGEKVRNLKGSTKDKTVWQPEVNVLLDLKKQLDAAQKAAKAAAAAPAATTAAPPAASADAG

QVKALEDKLAQQAEKVRTLKAKGDASVWKPEVDILLALKNQLAALTGAPAAGGQGKDKKKK

>XP_018897880.1 PREDICTED: methionine--tRNA synthetase, cytoplasmic isoform X2 [Bemisia tabaci]

MKLITNENNPNALKLFICGNLTCKTIDCEKVDSKDTKLHHSRYPALILDNGKQLFSSNAAALYLFPSFPTDIVDQWLEWE

ASHLVPSVLKSLSKEGSTTLTNNIKILDTVLSKTKFLHEGFITVADITVWSTLFGVITEPKLREKYCSSAANVIRWFEAI

HLLPEVQAAMKSWNGKSGEAVLTSLQSSSSCLAVTEASSTSTITAKLAKTSISNAPASETTPVKDVVSEDEISACIEAWE

KKSATRPKLKPHVHPILPKEGENNVLITSALPYVNNVPHLGNIIGCVLSADVFARYCRLRNRNTLFVSGTDEYGTATETK

ALEEKLTPQQICDKYFVIHKDIYEWFNIKFDQFGRTTTQKQTEIAQSIFKLVHNNGFTSTASVEQLLCEKCDRFLADRFV

EGTCPKCKYEDARGDQCDGCGSLINATELINPRCKVCGNTPVIRHSEQLFLELPKLEADLKKWVDNTSSGWSHNARVIAK

SWLKDGLKPRCITRDLKWGVQVPLQGFEKKVFYVWFDAPIGYMSISAAYTDEWEKWWLPSKSTQITLYQFMAKDNVPFHS

IMFPAIEMATQKNYTKVSHIMATEYLNYEDGKFSKSRGVGVFGNDARDTGISADVFRFYLLYIRPEAQDSSFSWADMAAK

TNTELLNNLGNFTNRALTFCEKFFGSVIPDMKLNKEDFQLLALITRELRQFMAVLDKAKFRDGIKHILCISKYCNQLMQA

EKPWVLMKGSEEEKSRAGTVIGLLSNLACLLSIMIRPYMPDTSKIMAEQLNAPESLYVLTEDVTMLLPPGHKIGHPSPIF

SLITPKTVEELKARFAGKQNDSAATKPTKTEAKSTDKSKSAPSQPASVPANAETIEKLEAAVAAQESVMGFLRKSCTSAN

TIICHEAALAELKLQVDSMKKLAHCM

>XP_017106922.1 PREDICTED: methionine--tRNA synthetase, cytoplasmic [Drosophila bipectinata]

MIIYTNEGNPLGLQLLMLAKFAKQVVKVQLVNLNDAKYKDLLVLPTLELDNGLRLFSPSAIAKYLLVDEGQLRDEWLEWS

ATLLAPALAHHMAVGHKADANALPVLNALVKKLDDSLKATPYLAGDKLTAADIAVWSLLAPDGTLKGAQNVESLRDWYGR

VKALPEVQEVLSEQPLKDLSFNALQQSNRYGGLHHVPLKRLSLADAGKLLVDTTPTVADTVTDEEISAAQAAFTYTAPKE

VKEERTILPKAGQRNVLITSALPYVNNVPHLGNIIGCVLSADIFARYSRSAGYNTLLICGTDEYGTATENKALAENLTPR

EICDKYFELHNSIYRWFGIGFDYFGRTTTQEQTDIVQEAFKDVLSAGYIITESVEQLLCQKCDRFLADRFVEGTCPHPGC

GYEDARGDQCDKCGKLVNATELIRPRCKVCNSAPVLRSSDQLFIDLPKAEPQLKDWVDKSEGGWTNNAKVITRAWLKEGL

KPRCITRDLKWGIPVPHKGFEKKVFYVWFDAPFGYVSMTKRYTKEYQQWWQPAKGTDVELFQFMAKDNVPFHSVVWPSVL

LAINKGHTLVSHIMATEYLNYEDGKFSKSRGIGVFGNDAQETGIPADVWRFYLASARPEGQDSSFSWNDLAARNNSELLN

NLGNFVNRALVFCEKNFNSTVPEVALTQDELILLALINRELKGYVNSLEKAKLRDGIRHLLAISRHGNGYMQTQQPWVLL

KGSDEQKKRAATIIGLSANIACLLANLLFPYMPTTARTLFSQLNAKQTPLNAEKPLATLLLPAGHKIGKPAPLFAKLEQA

FIDELKGKYGGAQETKASPKTQTSAADLEQAVAAQADKVRHLKASTKDKAQWQPEVNKLLDLKKQLEEAKKTAPASIATP

ASASTSGSQSVKDLEKAIQEQGDKVRKLKGSTKDKSVWQPEVDILLSLKKQLEAAQKAAKAAPVASAPAPSSAANPAQVK

ALEDKIAQQGEKVRTLKASGDASVWKPEVDILLGLKNELAALTGAPAAGGQGKGKKKK

>ODM96096.1 Methionine--tRNA synthetase, cytoplasmic, partial [Orchesella cincta]

TLEGKQRPRLPILVVDEVTRLFDTNAIVTYLMETGKVQLDPKLLGLMNFDAHRLSPAILAYLSEKGDADFGVTKELTAAL

IEIEKKINPYGFLSEERIPKYEDAIIWSTVYPLLASNDKVGSKFPKLMEWFSNTCRYAAFAEALAAIKPLSGKEGAKTIF

ICNPAAVISTFCERFARAEVSSTVKTEDMEEQSGTVVNVSNEEVAAARQAWKGQIPHSAVKGKRSGQILPLAGQRNYLIT

SALPYVNNVPHLGNIIGCVLSADVFARYCRVRGRNILYVSGTDEYGTATETKALEEGLTPQQICDKYHALHSEIYKWFDI

GFDYFGRTTTKEQTQIGQDIFMRLYQNEYLKEMTVLQLFCEPCKRFLSDRFVEGTCPSCGYEDARGDQCDKCSRLTNSTD

LKNPRCKVCGSAQVITKNSEQVFLDLPALEQKERLAEWLEKASEQWTPNARHIAKSWMKEGLKERCITRDLKWGTPVPLD

KFKEKVFYVWFDAPIGYISMTANYTSNWKQWWINSSKDVEIEYYQFMAKDNVPFHSILFPASLKGTKEPWTLVRHLIATE

YLNYEDGKFSKSRGVGVFGTDVMETGIPADIWRFYLIYVRPESQDSAFSWDDFMTKNNSELLNNLGNFVNRSLTFLEKFF

HSEVPTIVMSEDDYRVVAEVNREIVEYINLLELCKLRDALRQILAISKIGNVYFQSQKPWTLTKNAADRQRCGTVCGVGA

QIVFLLMTLLDPFMPGVARTIREQLNVSKPIFSLSLLPFLSPGHKIGKVSPLFQKLELDMIEKLRKKYAGNQAERKEKEA

KKLKSPSKSSSVVSTDSGNNNANNASDTASTGGGAAADPAEVAKLEAEIAKQGNLVRDLKTKKADKTEIDKEVQALLKLK

FQLGLLSGNETPVNDSKSKKKGTNKKK

>XP_020808130.1 methionine--tRNA synthetase, cytoplasmic [Drosophila serrata]

MIIYTNEGNPLGLQLLMLVKHAKREVQVQLVNLNDPNYKDLLVLPTLQLDNGLSLFSPAAIAKYLLVDEGQLRDEWLEWS

ATLLAPALAHHMAVGHKADANALPVLNALVKKLDDSLKTSPFLAGDKLTAADIAVWSLLAPEGTLKGAQNVDRLRDWYGR

IKVLPEVQEVLAEQPLKDLSFNALQQSNRYGGLYHIPLKRLSVADAGKLLVDTTPTVADTVTDDEIRAAQAAFTYTAPKD

IKEERTVLPKAGQRNVLITSALPYVNNVPHLGNIIGCVLSADIFARYSRSAGYNTLLICGTDEYGTATENKALAENLTPR

EICDKYFELHNAIYRWFGIGFDYFGRTTTQEQTDIVQEAFQDVLKAGYIITESVEQLLCQKCDRFLADRFVEGTCPHPGC

GYEDARGDQCDKCGKLVNATELIRPRCKVCNSAPVLRSSDQLFIDLPKAEPNLKEWVDKSEGGWTHNAKVITRAWLKEGL

KPRCITRDLKWGIPVPHAGFEKKVFYVWFDAPFGYVSMTKRYTKEYQQWWQPAKGTDVELFQFMAKDNVPFHSVVWPSVL

LAINKGHTLVSHIMATEYLNYEDGKFSKSRGIGVFGNDAQETGIPADVWRFYLASARPEGQDSSFSWNDLAARNNSELLN

NLGNFVNRALVFCEKNFNSTVPEVVATQDELILLALINRELRGYINSLEKAKLRDGIRHLLAISRHGNGYMQTQQPWVLL

KGSEDQKKRAATIIGLCANIACLLANLLFPYMPATARTLFSQLNAKQTALNADKPLATLLLPAGHKIGKPAPLFAKLEQS

FIDELKTKYGGAQTAAVHSQGSAAELEKAVQDQADKVRQLKASTKDKTVWQPEVNKLLDLKKQLEEAKKAAPVATLAPAN

GSQSVQDLEKAIQEQGDKVRKLKGSTKDKSVWQPEVNILLDLKKQLDAAQKAAKAAPSTVAATTSSVDAAQVKALEDKIA

QQGEKVRTLKAAGNADVWKPEVATLLALKNELSALTGAPAAGGQAKGKKK

>XP_023304852.1 methionine--tRNA synthetase, cytoplasmic [Lucilia cuprina]

MKIYTNEGNPLGLKLQILAKFAKREVSVEKVNLNDIKSKDLLVLPTLELENGLRLFSTDAVAKYLFPNEGQLRDEWLEWS

STLLAPALVHHMGHGHRANPNALPVLNALVKKLEDNLSKTPFLAGDKLTAADLSVWSLLAPDGTLKGAQQIEHLLVWYRK

ISAMPEIKSVLQTQPLKDLTFNALQNSNLYGGLYHVPLKMPASLSEAGNLLADTGSTLADTITDEELNLASTNFVYVPMA

ERKEPRTVLPKAGERNVLVTSALPYVNNVPHLGNIIGCVLSADIFARYSRAAGYNTLYISGTDEYGTATENKALAENLTP

REICDKYFELHNAIYRWFGIGFDYFGRTTTQEQTEIVQEAFKDVYDNGYILTESVEQLLCQKCDRFLADRFVEGTCPHPG

CGYEDARGDQCDKCGKLVNATDLIRPRCKVCNTAPVVRSSDQLFLDLPKIENKLREWVDHSENGWTNNARVITRAWLREG

LKPRCITRDLKWGIPVPHKGFENKVFYVWFDAPFGYVSMTKRYSKEFKQWWQPEKGVDVELFQFMAKDNVPFHSVVWPSA

LLAINKGNTLVSHIMATEYLNYEDGKFSKSRGIGVFGNDAQDTGIPADVWRFYLASARPEGQDSSFSWNDLAARNNSELL

NNLGNFVNRALVFCEKNFNGVVPAMNLTNDEHVLLALINRELRGYVNSLEKARLRDGIRHMLSISRHGNGYMQAQQPWVL

LKGNDQQKSRAATIIGLCCNIACLLANLIFPYMPTTARTMFEQLNAKQSQLNVEKPFTSILLPSGHKIGKPAPLFAKLEQ

SFIDEMKKKYAGPQVLGSSDAQTSSANIADLEKAIQEQGDKVRQLKASTKDKAVWQPEVNKLLDLKKQLTEAQSKQSAET

KQNGAAPKSVKDLEAAIQAQGDKVRQLKASTKDKAVWQPEVNKLLDLKKQLAEAQASITSAATTTTSSPASVDPKKVKEV

EEKIAKQAEKVRTLKASADASVWKPEVEILLALKKELTTLTGAPEPNAQGKNKKKK

>XP_016958619.1 PREDICTED: methionine--tRNA synthetase, cytoplasmic [Drosophila biarmipes]

MIIYTNEGNPLGLQLLMLAKFAKRPVQVQLVNLNDAKYKDLLVLPTLELDNGLRLFSPAAIAKYLSVDEGQRRDEWLEWS

ATLLAPALAHHMAVGHKADANALPVLNALVKKLDDNLKSSPFLAGNQLTAADIAVWSLLAPDGTLKGAQNVDSLRDWYGR

VKALPEVQEVLNAQPLKDLSFNALQQSNRYGGLHHVPLKRLSLADAGKLLVDTTPTVADTVTDEEISAAQAAFTYTTPKE

LKEERTVLPKAGERNVLITSALPYVNNVPHLGNIIGCVLSADIYARYSRSAGYNTLLICGTDEYGTATENKALAENLTPR

EICDKYFELHNAIYRWFGIGFDYFGRTTTQEQTDIVQEAFQDVLKAGYIITESVEQLLCQKCDRFLADRFVEGTCPHPGC

GYEDARGDQCDKCGKLVNATELIRPRCKVCNSAPVLRSSDQLFIDLPKAEPQLKEWVDKSEGGWTHNAKVITRAWLKEGL

KPRCITRDLKWGIPVPHEGFEKKVFYVWFDAPFGYVSMTKRYTKEYQQWWQPAKGTDVELFQFMAKDNVPFHSVVWPSVL

LAINKGHTLVSHIMATEYLNYEDGKFSKSRGIGVFGNDAQETGIPADVWRFYLASARPEGQDSSFSWNDLAARNNSELLN

NLGNFVNRALVFCEKNFGSTVPEVATTQDDLVLLALINRELRGYINSLEKAKLRDGIRHLLAISRHGNGYMQTQQPWVLL

KGTDDQKKRASTIIGLCVNIACLLANLLFPYMPTTARTLFGQLNAKQTSLNAEKPLATLLLPAGHKIGKPAPLFAKLEQS

FIDELKSKYGGAQTAAQSQTSAADLEQAVQAQADKVRQLKASTKDKAVWQPEVTKLLDLKKQLEEAKKTAPAAAPAAATA

PSNGSQSVQDLEKAIQEQGDKVRKLKGSTKDKAVWQPEVNVLLDLKKQLEAAQKAAKAAPAAAAPVPSPAASADPAQVKV

LEDKIAQQAEKVRTLKASGDATVWKPEVDILLGLKTQLAALTGTPAAGGQGKGKKKK

>KNC29254.1 hypothetical protein FF38_02414, partial [Lucilia cuprina]

DQHKAKMKIYTNEGNPLGLKLQILAKFAKREVSVEKVNLNDIKSKDLLVLPTLELENGLRLFSTDAVAKYLFPNEGQLRD

EWLEWSSTLLAPALVHHMGHGHRANPNALPVLNALVKKLEDNLSKTPFLAGDKLTAADLSVWSLLAPDGTLKGAQQIEHL

LVWYRKISAMPEIKSVLQTQPLKDLTFNALQNSNLYGGLYHVPLKMPASLSEAGNLLADTGSTLADTITDEELNLASTNF

VYVPMAERKEPRTVLPKAGERNVLVTSALPYVNNVPHLGNIIGCVLSADIFARYSRAAGYNTLYISGTDEYGTATENKAL

AENLTPREICDKYFELHNAIYRWFGIGFDYFGRTTTQEQTEIVQEAFKDVYDNGYILTESVEQLLCQKCDRFLADRFVEG

TCPHPGCGYEDARGDQCDKCGKLVNATDLIRPRCKVCNTAPVVRSSDQLFLDLPKIENKLREWVDHSENGWTNNARVITR

AWLREGLKPRCITRDLKWGIPVPHKGFENKVFYVWFDAPFGYVSMTKRYSKEFKQWWQPEKGVDVELFQFMAKDNVPFHS

VVWPSALLAINKGNTLVSHIMATEYLNYEDGKFSKSRGIGVFGNDAQDTGIPADVWRFYLASARPEGQDSSFSWNDLAAR

NNSELLNNLGNFVNRALVFCEKNFNGVVPAMNLTNDEHVLLALINRELRGYVNSLEKARLRDGIRHMLSISRHGNGYMQA

QQPWVLLKGNDQQKSRAATIIGLCCNIACLLANLIFPYMPTTARTMFEQLNAKQSQLNVEKPFTSILLPSGHKIGKPAPL

FAKLEQSFIDEMKKKYAGPQVLGSSDAQTSSANIADLEKAIQEQGDKVRQLKASTKDKAVWQPEVNKLLDLKKQLTEAQS

KQSAETKQNGAAPKSVKDLEAAIQAQGDKVRQLKASTKDKAVWQPEVNKLLDLKKQLAEAQASITSAATTTTSSPASVDP

KKVKEVEEKIAKQAEKVRTLKASADASVWKPEVEILLALKKELTTLTGAPEPNAQGKNKKKK

>XP_017839759.1 PREDICTED: methionine--tRNA synthetase, cytoplasmic [Drosophila busckii]

MIIYTNEGNPFGLQLLMLAKFAKRQVQLQLVNLNDAKYKDLLVLPTLELDNGLRLFSLAAIAKYMFGDGEGESQQRDEWL

EWCTTLLAPALAHHMGVGHRADPNALPVLNGLVKKLNDTLQSKPYLAGDKLSSADIAVWSLLAPEGTLKGAQQVENLRSW

YDKIKALPEVQQALAEQPLQQLSFSALQQSNRYGGLYHVPLKRDSISNVTGKLLVDTTPTVADTVTDEELNAARAAFIYT

QSTEAVVPRTVLPKAGERNVLITSALPYVNNVPHLGNIIGCVLSADIFARYSRAAGYNTLLICGTDEYGTATENKALAEN

MTPREICDKYFELHNGIYRWFGIGFDYFGRTTTQEQTDIVQEAFHDVYQAGYVLTESVEQLLCQKCDRFLADRFVEGTCP

HPGCGYEDARGDQCDKCGKLVNATELVRPRCKVCNTAPVLRNSDQLFIDLPKAEPQLREWVERSEQGWTHNARVITRAWL

REGLKPRCITRDLKWGIPVPHKGFEKKVFYVWFDAPFGYVSMTKRYTKEYQQWWQPAKGTDVELFQFMAKDNVPFHSVVW

PSILLGINKGHTLVSHIMATEYLNYEDGKFSKSRGIGVFGNDAQETGIPADVWRFYLASARPEGQDSSFSWNDLAARNNS

ELLNNLGNFVNRALVFCEKNFDSCVPEVQLTQDELLLLALINRELRGYVQSMEKAKLRDGIRHLLAISRHGNGYMQTQQP

WVLLKGTNEQKTRAASIIGLSANIACLLANLLFPYMPTTARTLFAQLNAKQTPINADKPLATLLLPAGHKIGKPAPLFAK

LEQTYIDELKGKYGGAQQSQTQPAQASAAELEAAVQAQADKVRQLKASTKDKTVWQPEVSKLLELKKQLEAAQKSAPAVP

QKGVQTVADLEKAVQAQGEKVRQLKGSTKDKAVWQPEVNVLLDLKKQLEAAQKSAKEAPTAPAAAAAAAPVAASDDKVKA

LEAQITQQGEKVRSLKASGDAAAWKPEVEILLNLKKELSTLTGVPVTAASQGKQKKKK

>NP_611382.1 Methionyl-tRNA synthetase [Drosophila melanogaster]

MIIYTNDGNPLGLQLLMLAKFAKQPVQVQLVNLNDAKYKDLLVLPTLELDNGLRLFSPAAIAKYLFVGKGQQRDEWLEWS

ATLLAPALAHHMAVGHKADANALPVLNALVKKLDDKLKATPYLAGDKPSAADIAIWSLLAPDGTLKGAQNVDNLRDWYGR

VKALPEVQEVLAEQPLKDLSFNALQQSNRYGGLHHVPLKRLSLADASKLLVDTTPTVADTVTNEEISAAKAAFTYTAPKE

IKEERTVLPKPGERNVLITSALPYVNNVPHLGNIIGCVLSADIYARYSRSAGYNTLLICGTDEYGTATENKALAENLTPR

EICDKYFELHNAIYRWFGIGFDYFGRTTTQEQTDIVQEAFKDVLKAGYIITESVEQLLCQKCDRFLADRFVEGTCPHPGC

GYEDARGDQCDKCGKLVNATELIRPRCKVCNSAPVLRSSDQLFIDLPKAEPQLKEWVDKSEGGWTHNAKVITRAWLKEGL

KPRCITRDLKWGIPVPHEGFEKKVFYVWFDAPFGYVSMTKRYTKEYQQWWQPAKGTDVELFQFMAKDNVPFHSVVWPSVL

LAINKGHTLVSHIMATEYLNYEDGKFSKSRGIGVFGNDAQETGIPADVWRFYLASARPEGQDSSFSWNDLAARNNSELLN

NLGNFVNRALVFCEKNFSSTVPGVITTQDELVLLALINRELRGYINSMEKAKLRDGVRHLLAISRHGNGYMQSQQPWVLL

KGTDDQKTRASTIIGLCVNIACLLANLLFPYMPTTARTLFGQLNAKQTPLNAEKPLVTLLLPAGHQIGKPAPLFAKLEQS

FIDELKGKYGGAQATNDAAHSQISAADLEKAVQAQADKVRELKASTKDKAIWQPEVTKLLDLKKQLEEAKKKTATAAAPA

ATPAPSNGSQSVQDLEKAIQEQGDKVRKLKGSTKDKTVWQPEVNILLDLKKQLEAVQKAAKAAPAANAAPAASPAATADA

AKVKALEDKIAQQAEKVRTLKATGDAAVWKPEVDILLSLKNELAALTGTPVAGGQGKGKKKK

>XP_018788470.1 PREDICTED: methionine--tRNA synthetase, cytoplasmic [Bactrocera latifrons]

MKIYTNEGNPFGLKLQLLAKFANKPVEVIKVTLNDPASKDFLVLPILELESGLKLFSTDAVARYLFPDEGALRDEWLEWS

TVKLAPALSQHLSVGHRANPDALPVLNLLVRKLDDRLADSAFLTGDKVSTADIAVWSLLAPDGTMKGAQNIQHLKAWYRK

ITALPEVQSVLAGTPLADLSFTSLQNSNRYGGLNHVPVRVHTASESENLLADTTGNLADTVTEDELANAQNSFVQVSSPQ

RVEPRTVLPKAGERNVLITSALPYVNNVPHLGNIIGCVLSADIFARYSRAVGNNTLYICGTDEYGTATENKALAENLTPK

QICDKYFDLHNSIYRWFGIGFDFFGRTSSQHQTEIVQEAFQDVYKNGYIFTESVDQLHCVKCDRFLADRFVEGTCPHPGC

GYEDARGDQCDKCGKLVNATELIRPRCKICNSAPVIRSSDQLFLDLPKIEPQLRVWLEGSEMGWTNNARVITRSWLREGL

KPRCITRDLKWGIPVPLAGFEKKVFYVWFDAPYGYISMTKHYTSEYKQWWIPSKDVKVELFHFMAKDNVPFHSVVFPSSL

LATNKGHTMVTNIMATEYLNYEDGKFSKSRGIGVFGNDAQQTGISADIWRFYLASARPEGQDSNFSWNDLAARNNSDLLN

NLGNFVNRALVFCEKNFNCVVPTMTLTKNELILLALINREVRGYIHSLEKAKLRDGIRHLLSISRHGNGYMQSQQPWVLL

KGSTEQKARAATIIGICANIAALLANLMLPYMPTIARTLFSQLNAKPTLLNPEKPFINILLPTGHNIGKPAPLFTKLEQN

FIEELKKKFGGVQDQTNDSQPSSATTNTNSATAKPSALPSSTLSVAQLEEAVCLQGEKVRQLKASTKDKAVWQPEVNILL

DLKKQLTAAQAVSTPEASDEKVKDLEQRITQQGEKVRQLKASGDVSVWKPEVEKLLQLKKDLATLSGAPQPAAQGKNKKK

K

>ERL92272.1 hypothetical protein D910_09589 [Dendroctonus ponderosae]

MGTIEGNENNPATLKLVLAGKLAKTQYAVKIRGHNEEGQYLPYLALSSEDNIFVPNAAIWFILPPAEELSDKVREVLEME

STVLSPSLALLLGTNLKSEKVKSSVLSALQSLNSTLEGSQFLVQDAITVADVAVFSTLFPIFKAAKLLKEYLGSFPSISK

YLNQLELLPEVKDSVAIVFHQKCNIGPVSYQVLFQSSKYLVPQIQSDPKTVNRLDSVEKESSKHEVETVSSEELEAAEKA

WKKSPGSLPKLKTPNKVVLPKTGEKNVFITSALPYVNNVPHLGNIIGCVLSADVFARFSRLCNNNTLYICGTDEYGTATE

TKALEEGLSCQAICDKYFKIHQEIYQWFNISFDSFGRTSTPLQTELAQDLFLQLNHNGFMSTQAVDQLLCEKCNRYLADR

FVEGGCPNVGCTYEDARGDQCDGCGKLVNAIELKNPRCKICGSTPKVKSSSQFFIDLPKLEPLLLHWVKNSSPGWSHNAE

VIAKAWLKEGLKPRCITRDLHWGVPVPLDGFRNKVFYVWFDAPIGYMSISKAYTKEYEKWWRPAKDTEVKLYQFMAKDNV

PFHAILFPAILLGANREYVTVSHIMATEYLNYEEGKFSKSRGIGVFGNDARDTKIPSDVWRFYLLYVRPEAQDASFSWND

LATKNNSELLNNLGNFINRALIFVKNNFNSTIPKLELTSEDYTLLALCTRELKGYIAALDKCKLRDGIRHILAISRHGNQ

YMQLNQPWVKIKQGEEGKLRGGTVVGVCANLSCLLATLLQPYLPETSEKLKAQLNCEYFVLNPENIEIINLLPTGHSIGE

PQPLFTKIEPEVIEEYKKKFAGSQESRKTPPKEIVKPDLCNGDAETLEEAIAKQGLVVRKLKEGGFDKTVWAPEVAKLVA

LKARLAELTGVAPDKKSSSKKSNATTNNNVQKVANNMPAKSKKKPVNNSN

>XP_969201.1 PREDICTED: methionine--tRNA synthetase, cytoplasmic [Tribolium castaneum]

MLIYTNANNPAALKLLIAAKFAQKSVDIKVVDVNDGSLEQPRHLPYVKTGDNETIFVPNAGVWWLWPPAQQAAKAASEYL

EWEAAVLAPTLAHVVGGGSKQENLKACLNSALNKLNKVLESQPYLSEKKLNASDIVVWCTLYPLALDDTLAKEYLSNNLH

VLKWMKSVESDKEVAEVLKSFKISSGIAAYQALLAGAKFIISNISTDNNVQSPQSVPEIVTSDELEAAQKAWLKDIKDIP

LMKTQPKVVLPKSTERNILITSALPYVNNVPHLGNIIGCVLSSDVFARFCRICNYNTLFIGGTDEYGTATETKALEEGLS

CQAICDKYYAIHKDIYNWFNISFDYFGRSSSPQQTELCQDMFLQLNNNGFMFTEAVEQLHCEKCDRYLADRFVEGGCPHP

GCNYEDARGDQCDGCGKLVNAVELKNPRCKICRSTPKLKTSNQFFIDLPKLEPLLLHWMKQSAPGWSSNAQVIGKAWIRE

GLKPRCITRDLKWGVPVPLKDFKDKVFYVWFDAPIGYMSINKAYTEKWEEWWRPKPEHKITLYQFMAKDNVPFHAVMFPA

CLLGANRGYITVSHIMATEYLNYEDGKFSKSRGVGVFGNDARDTGIPSDVWRFYLLYVRPESQDSSFSWSDLATKNNSEL

LNNLGNFINRALVFAKNNFNCTVPEMQLTKDDFTLLVLCTRELKGYVNCLEKCKLRDGIRHILSISRLGNQYMQFNQPWV

LLKGTPEEKVRAATVVGLCCNIAALLATLLHPYMPDTSNTLKKQLNCANTALTPESIEIRQLLPPGHKLGNPAPLFAKLE

PAKVDELKKKFAGAETKPRQMNGELKMTDLTKSIAEIEKRLKTLKSGKADKKKIKEEEAILADLRSKLNSQVQNNKDDIK

SIEDQIAVQGSLVRKMKESGASKPEWQPEVAKLLELKAKLASLQPR

>XP_003425875.1 PREDICTED: methionine--tRNA synthetase, cytoplasmic [Nasonia vitripennis]

MKAASNPNNPNLLKLIVAAEIAGKELTLDDKTEPKQSSLNRLPVLKLESSDELFSVSAAVQYLAPVSEENIVAANRWLEW

DASQLQPAIVSYGGSGKLDVSHKPRIWSLLKELNTALKDAQYLIKDKDNLTVVDASIWVTLWSTVVVTEIGEVITKEFAN

VDKWLKDIQSLPAIQKSLKKIKLERGVQAIISINAASWFPLNSYTGAGKSQSAPTGDGSPSEEKEDKAITQEEMDSVKNN

WTSITYSSIKEASYPVLPQKGEKNVLITSALPYVNNVPHLGNIIGCVLSADIFARYCRQRNYNTLYISGTDEYGTATEAK

ALEEKLTPQQICDKFFDIHNDIYRWFSIGFDYFGRTTTPEQTEIVQAMFLRIKEQGYILPKTVDQLLCEKCERFLADRFV

EGTCPRCKYEDARGDQCDGCGHLVNATELINPRCKVCSTKPVIKASTQFFLDLPKIQEGLKQWATEAEGGWSSVARAVTK

PWLRDGLKPRCITRDLKWGIPVPLDGYRDKVFYVWFDAPLGYLSITKKYTKEFEKWWKPNGVDVSLYQFMAKDNVPFHAI

MFPATLLAADQGHTLVRHIMATEYLNYEDTKFSKSRGIGVFGTDARDTGIPADVWRFYLAYIRPETMDSNFNWVDLATKN

NSELLNNLGNFVNRALMFAEKNFNSKIPEMELLEDDLHVLALVQRELTSYITLLEQARLRDSLRHILAISKHGNQYMQSQ

QPWVKVKGSEEDKKKGATVIGVCCNLACLLSALLAPFMPNTARELRAQLGLPKQNYGYIPEVLSIMLPAGHSIGKPSPLF

TKIEDKQVEELRKKYAGRQESTPPRETVAVPTDVASLEAAVAKQALLVRDLKAKQDKSVWQPQVQILLDLKKKLAEAQSK

GNQAGAAKDNKAQSATAPQQNGNVTAEIAELEAAVLKQGNLVRDLKGKGDKSVWQPQVEILLQLKQKLSALTGAPAAAPA

DKKSKKKK

>XP_017865171.1 PREDICTED: methionine--tRNA synthetase, cytoplasmic isoform X2 [Drosophila arizonae]

MILFTNEGNPIGLQLLVLAKYLKRPMFLQQITITDAKYKDLILLPTVELDNGLRLFSTAAIYKYLLMHEGYPRNEWLEWG

ATLLTPALEHYMATGHRADSRAGPVLNALVKKLDDTLSVSPFIAGDKLTSADLFIWSLLAPDGTLKGAQNVEHLHSWYNK

IKALPEVQEALKELPINTLSFNALQQGNRYGGLAHVQLRRGISQDADKVLADSGPTVADTVSEEEINTAREAFIYTEPKE

LTEPRTVLPKPGERNVLITSALPYVNNVPHLGNIIGCVLSADIFARYSRAAGYNTLLICGTDEYGTATENKALAENMTPR

EICDKYFELHNSIYRWFGIGFDYFGRTTTPEQTEIVQEAFKDVYEAGYILTESVEQLLCQKCDRFLADRFVEGTCPHPGC

GYEDARGDQCDKCGKLVNATELVRPRCKVCNTAPVLRSSDQLFLDLPKAEPKLREWVDRAEQGWTHNARVITRAWLREGL

KPRCITRDLKWGIPVPHKNFEQKVFYVWFDAPFGYVSITKRYTKDYLQWWQPAPGTDVELFQFMAKDNVPFHSVVWPSVL

LAINKGNTLVSHIMATEYLNYEDGKFSKSRGIGVFGNDAQETGIPADVWRFYLASARPEGQDSSFSWTDLAARNNSELLN

NLGNFVNRALSFCERNFDSKLPQMDLNHNDFLLLALINRELRGYINSLEKAKMRDGIRHLLSISRHGNGYIQYQQPWALL

KGSDDQKSRAGTIIGLSINIVCLLANLLFPFMPTTARAMFAQLNTKQTPLDANKPFATQLLRPGHKIGTPSPLFDKLDHA

FIESLKAKFGGSQADKEAVAAAGAAAAQPQSSTVELEAAVKAQGEKVRQLKASTKDKAVWQPEVNKLLELKKQLEAAQKA

APVSTGAVAAAAAAAAAAPVPSASPSAQSVQSLEQAVQAQGDKVRQLKSTTKDKAVWQPEVNKLLDLKKQLEEAKAASGA

TSASVSPAPKGIDAAAVKALQEKIAQQGEKVRNLKATGDASVWKPEVEILLNLKKELAALDGSAAAAPQTKNKKKK

>XP_017865170.1 PREDICTED: methionine--tRNA synthetase, cytoplasmic isoform X1 [Drosophila arizonae]

MILFTNEGNPIGLQLLVLAKYLKRPMFLQQITITDAKYKDLILLPTVELDNGLRLFSTAAIYKYLLMHEGYPRNEWLEWG

ATLLTPALEHYMATGHRADSRAGPVLNALVKKLDDTLSVSPFIAGDKLTSADLFIWSLLAPDGTLKGAQNVEHLHSWYNK

IKALPEVQEALKELPINTLSFNALQQGNRYGGLAHVQLRRGISQDADKVLADSGPTVADTVSEEEINTAREAFIYTEPKE

LTEPRTVLPKPGERNVLITSALPYVNNVPHLGNIIGCVLSADIFARYSRAAGYNTLLICGTDEYGTATENKALAENMTPR

EICDKYFELHNSIYRWFGIGFDYFGRTTTPEQTEIVQEAFKDVYEAGYILTESVEQLLCQKCDRFLADRFVEGTCPHPGC

GYEDARGDQCDKCGKLVNATELVRPRCKVCNTAPVLRSSDQLFLDLPKAEPKLREWVDRAEQGWTHNARVITRAWLREGL

KPRCITRDLKWGIPVPHKNFEQKVFYVWFDAPFGYVSITKRYTKDYLQWWQPAPGTDVELFQFMAKDNVPFHSVVWPSVL

LAINKGNTLVSHIMATEYLNYEDGKFSKSRGIGVFGNDAQETGIPADVWRFYLASARPEGQDSSFSWTDLAARNNSELLN

NLGNFVNRALSFCERNFDSKLPQMDLNHNDFLLLALINRELRGYINSLEKAKMRDGIRHLLSISRHGNGYIQYQQPWALL

KGSDDQKSRAGTIIGLSINIVCLLANLLFPFMPTTARAMFAQLNTKQTPLDANKPFATQLLRPGHKIGTPSPLFDKLDHA

FIESLKAKFGGSQADKEAVAAAGAAAAQPQSSTVELEAAVKAQGEKVRQLKASTKDKAVWQPEVNKLLELKKQLEAAQKA

GAGLTAAAGESPAPGGQSVQALEQAVQAQGDKVRQLKASTKDKAVWQPEVNKLLELKKQLEAAQKAAPVSTGAVAAAAAA

AAAAPVPSASPSAQSVQSLEQAVQAQGDKVRQLKSTTKDKAVWQPEVNKLLDLKKQLEEAKAASGATSASVSPAPKGIDA

AAVKALQEKIAQQGEKVRNLKATGDASVWKPEVEILLNLKKELAALDGSAAAAPQTKNKKKK

>XP_017014484.1 PREDICTED: methionine--tRNA synthetase, cytoplasmic [Drosophila takahashii]

MIIYTNEGNPLGLQLLMLAKFAKRPVQVQLVNLNDAKFKDLLVLPTLELDNGLRLFSPAAIAKYLLVDEGQRRDEWLEWS

ATLLAPALAHHMAVGHKADANALPVLNALVKKLDDSLKSSPFLAGNAPSAADIAVWSLLAPEGTLKGAQNVDSLRDWYAR

VKELPEVQQVLSEQPLKDLSFNALQQSNRYGGLHHVPLKRLSLADAGKLLVDTTPTVADTVTEEEISAAQAAFTYTAPKE

LKQERTVLPKAGERNVLITSALPYVNNVPHLGNIIGCVLSADIYARYSRSAGYNTLLICGTDEYGTATENKALAENLTPR

EICDKYFELHNAIYRWFGIGFDYFGRTTTQEQTDIVQEAFQDVLKSGYIITESVEQLLCQKCDRFLADRFVEGTCPHPGC

GYEDARGDQCDKCGKLVNATELIRPRCKVCNSAPVLRSSDQLFIDLPKAEPQLREWVDKSEGGWTHNAKVITRAWLKEGL

KPRCITRDLKWGIPVPHEGFEKKVFYVWFDAPFGYVSMTKRYTKEYQQWWQPAKGTDVELFQFMAKDNVPFHSVVWPSVL

LAINKGHTLVSHIMATEYLNYEDGKFSKSRGIGVFGNDAQETGIPADVWRFYLASARPEGQDSSFSWNDLAARNNSELLN

NLGNFVNRALVFCEKNFDSTVPEVATTQDELVLLALINRELRGYINSLEKAKLRDGIRHLLAISRHGNGYMQTQQPWVLL

KGSDEQKKRAATIIGLCVNVACLLANLLFPYMPTTARTLFGQLNAKQTPLNAEKPFATLLIPADHKIGKPAPLFAKLEQS

FIDELKSKYGGAQETKTADQTSAADLEQAVQAQADKVRQLKASTKDKAVWQPEVNKLLELKKQLEEAKKTATAAPAAPAP

SNGSQSVQDLEKAIQEQGDKVRKLKGSTKDKAVWQPEVNVLLDLKKQLEAAQKAAKAAPAAAAPAPSAAVSADAAQVKAL

EDKIAQQAEKVRTLKATGDAAVWKPQVEVLLGLKNELAALTGTPAAGGQGKGKKKK

>XP_001961196.1 uncharacterized protein Dana_GF13746 [Drosophila ananassae]

MIIYTNEGNPLGLQLLMLAKFAKQAVKVQLVNLNDAKFKDLLVLPTLELDNGLRLFSPSAIAKYLLVDEGQLRDEWLEWS

ATLLAPALAHHMAVGHKADANALPVLNALVKKLDDNLKATPYLAGNKLTAADIAVWSLLAPEGTLKGAQNVESLRDWYGR

VKALPEVQEVLSEQPLKDLSFNALQQSNRYGGLHHVPLKRLSLADAGKLLVDTTPTVADTVTDEEISAAQAAFTYTAPKE

VKEERTILPKPGQRNVLITSALPYVNNVPHLGNIIGCVLSADIFARYSRSAGYNTLLICGTDEYGTATENKALAENLTPR

EICDKYFELHNSIYRWFGIGFDYFGRTTTQEQTDIVQEAFKDVLSAGYIIKESVEQLLCQKCDRFLADRFVEGTCPHPGC

GYEDARGDQCDKCGKLVNATELIRPRCKVCNSAPVIRSSDQLFIDLPKAEPQLKDWVDKSEGGWTNNAKVITRAWLKEGL

KPRCITRDLKWGIPVPHKGFEKKVFYVWFDAPFGYVSMTKRYTKEYQQWWQPAKGTDVELFQFMAKDNVPFHSVVWPSIL

LAVNKGHTLVSHIMATEYLNYEDGKFSKSRGIGVFGNDAQETGIPADVWRFYLASARPEGQDSSFSWNDLAARNNSELLN

NLGNFVNRALVFCEKNFNSTVPEVALTQDELILLALINRELKGYINSMEKAKLRDGIRHLLAVSRHGNGYMQTQQPWVLL

KGSDEQKKRAATIIGLSANIACLLANLLFPYMPTTARTLFSQLNAKQTPLNAEKPLATLLLPAGHKIGKPAPLFAKLEQA

FIDELKGKYGGAQESKASPNTQTSAADLEKAVAAQADKVRQLKASTKDKAQWQPEVNKLLDLKKQLEEAKKTAPASTAAP

ASAPTNGSQSVKDLEKAIQEQGDKVRKLKGSTKDKSVWQPEVDVLLGLKKQLEAAQKAAKEAPASAPPAPAPSSAANPAQ

VKALEDKIALQGEKVRTLKASGDASVWKPEVDILLALKNELAALTGAPAAGGQGKGKKKK

>XP_016991501.1 PREDICTED: methionine--tRNA synthetase, cytoplasmic [Drosophila rhopaloa]

MIIYTNEGNPLGLQLLMLAKFAKRPVQVQLVNLNDVKYKDLLALPTLELDSGLRLFSPAAIAKYLLVDEGQLRDEWLEWS

ATLLAPALAHHMAVGHKADANALPVLNALVKKLDDSLKSSPFLAGDKLSAADIAVWSLLAPDGTLKGAQNVDSLRDWYGR

IKALPEVQQVLAEQPLKDLTFNALQQSNRYGGLHHVPLKRLSLADAGKLLVDTTPTVADTVTDEEISAAKAAFTYTAPKE

IKEQKTVLPKAGERNVLITSALPYVNNVPHLGNIIGCVLSADIYARYSRSAGYNTLLICGTDEYGTATENKALAENLTPR

EICDKYFELHNAIYRWFGIGFDYFGRTTTQEQTDIVQEAFKDVLKSGYIITESVEQLLCQKCDRFLADRFVEGTCPHLGC

GYEDARGDQCDKCGKLVNATELIRPRCKVCNSAPVLRSSDQLFIDLPKAEPQLKEWVDKSEGGWTHNAKVITRAWLKEGL

KPRCITRDLKWGIPVPHEGFEKKVFYVWFDAPFGYVSMTKRYTKEYQQWWQPAKGTDVELFQFMAKDNVPFHSVVWPSVL

LAINKGHTLVSHIMATEYLNYEDGKFSKSRGIGVFGNDAQETGIPADVWRFYLASARPEGQDSSFSWNDLAARNNSELLN

NLGNFVNRALVFCEKNFNSTVPEVATTQDELVLLALINRELKGYINSLEKAKLRDGIRHLLAISRHGNGYMQTQQPWVLL

KGSDDQKKRASTIIALCVNIACLLANLLFPYMPTTARTLFGQLNAKQTPLNAEKPLATLLLPAGHKIGKPAPLFAKLEQS

FIDELKGKYGGAQETKAAAQSQPSAADLEQAVQAQADKVRQLKASTKDKAVWQPEVTKLLELKKQLEGAKKTAPNPAPAP

APTSSNGVQSVQDLEKAIQEQGDKVRRLKGSTKDKAVWQPEVNILLDLKKKLEAAQKAAKAGPAAAVPAPSAAPSADAGQ

VKALEDKIAQQAEKVRTLKASGDASVWKPEVDALLALKNQLTALTGIPAAGSQGKSKKKK

>XP_017125904.1 PREDICTED: methionine--tRNA synthetase, cytoplasmic [Drosophila elegans]

MIIYTNEGNPLGLQLLMLAKFAKRPLQMQLVNLNDAKYKDLLVLPTLELDNGLRLFSPAAIAKYLLVDEGQLRDEWLEWS

ATLLAPALAHHMAVGHKADANALPVLNALVKKLDDSLKSSPFLAGDKPSAADIAVWSLLAPDGTLKGAQNVDSLRDWYGR

IKALPEVQQVLVEQPLKDLTFNALQQSNRYGGLHHVPLKRLSLADAGKLLVDTTPTVADTVTDEEISAAQAAFTYTAPKE

IKEERTVLPKAGERNVLITSALPYVNNVPHLGNIIGCVLSADIYARYSRSAGYNTLLICGTDEYGTATENKALAENLTPR

EICDKYFELHNAIYRWFGIGFDYFGRTTTQEQTDIVQEAFKDVLKAGYIITESVEQLLCQKCDRFLADRFVEGTCPHPGC

GYEDARGDQCDKCGKLVNATELIRPRCKVCNSAPVLRSSDQLFIDLPKSEAQLKEWVDKSEGGWTHNAKVITRAWLKEGL

KPRCITRDLKWGIPVPHEGFEKKVFYVWFDAPFGYVSMTKRYTKEYQQWWQPAKGTDVELFQFMAKDNVPFHSVVWPSVL

LAINKGHTLVSHIMATEYLNYEDGKFSKSRGIGVFGNDAQETGIPADVWRFYLASARPEGQDSSFSWNDLAARNNSELLN

NLGNFVNRALVFCEKNFNSTVPEVAATQDELVLLALINRELRGYINSLEKTKLRDGIRHLLAISRHGNGYMQAQQPWVLL

KGSDDQKKRAATIIGLCANIACLLANLLFPYMPSTARTLFGQLNAKQTPLNAEKPLATLLLPAGHKIGKPAPLFAKLEQS

FIDELKSKYGGAQETKTAVQGQTSAADLEQAVQAQADKVRQLKASTKDKAVWQPEVTKLLELKKQLEQAKKSAPASAPAT

ARTPTNGPQSVQDLEKAIQEQGDKVRKLKGSTKDKAVWQPEVNILLDLKKKLEAAQKAAKAAPVAAAPAPAASPSADAAQ

VKALEDKIAQQGEKVRTLKASGDASVWKPEVDTLVALKNQLAALTGVPVAGGQSKGKKKK

>XP_011196457.1 PREDICTED: methionine--tRNA synthetase, cytoplasmic [Zeugodacus cucurbitae]

MKIFTNEGNPFGLKLQLLAKFANKQVEVVKVTLNDPACKDYLVLPTVELENGLKLFSTDAVAHFLFPDEGALRDEWLEWS

TVKLAPALAQHLSVGHRANPDALPVLNILVRKLDDRLAESAFLTGDKVSAADIAVWSLLAPDGTLKGAQNIEHLKAWYRK

MTALPEIQAVLAETPLAGLTFTSLQNSNRYGGLNHVPVRVHTESESEKLLAEATGSLADTVTEDELANAQNSFVPVSLPQ

RVEPRTVLPKAGERNILITSALPYVNNVPHLGNIIGCVLSADIFARYSRAAGYNTLLICGTDEYGTATENKALAENLTPK

QICDKYFELHNSIYRWFGIGFDFFGRTSSQHQTEIVQEAFHDVYNNGYIFTESVEQLLCVKCDRFLADRFVEGTCPHPGC

GYEDARGDQCDKCGKLVNATELIRPRCKICNSAPVLRSSDQLFLDLPKVESQLRVWMEGSEMGWTNNARVIARSWLREGL

KPRCITRDLKWGIPVPLAGFEKKVFYVWFDAPYGYISMTKNYTSEYKQWWIPSKDVKVELFQFMAKDNVPFHSVVFPSAL

LATNKGHTMVTNIMATEYLNYEDGKFSKSRGIGVFGNDAQETGIPADIWRFYLASARPEGQDSSFSWNDLGARNNSDLLN

NLGNFVNRALVFCEKNFNCVVPTMTLTKDDLILLALINREVRGYINSLEKAKLRDGIRHLLSISRHGNGYMQSQQPWVLL

KGTDEQKARAATIIGVCANIAALLANLILPYMPTIARTLFAQLNAKPTQLNPEKPCINILLPSGHKIGKPAPLFTKLEQS

FIEELKMKFGGVQDQTNGNQAATATSDTSKPSPLANGTQSVAQLEEAVRLQGEKVRQLKASTKDKAVWQPEVNVLLDLKK

QLAAAQAAPPTTAAAAATPVASDGKVKELEQRITQQGEKVRQLKASGDASVWKPEVEVLLQLKKELATLTGAPQPAAQGK

NKKKK

>XP_023317993.1 methionine--tRNA synthetase, cytoplasmic [Trichogramma pretiosum]

MKVLSNPNNPNSLKLVIAASISGKKLKIDYTAESKQSSLHRLPILKDEEQGIELFSLSAAVQYLAPLSTNNSVIANRWLE

WEACQLLPAITAYGGTGKIDLSHKPRVWSLLKELDSTLKNSQYLIQEQSDINVVDSSIWVTLWSTLIPTKIGEVIPQEFP

NVNKWLLLIENLPIIQQSLKKVTIERGLPAIVAVNAVSWYPINSYSGSEKPQSKTSKISEVTCNEEKEDKAVSQEELNSV

KNNWTSVPFLDYKESAYPVLPKKDERNVLITSALPYVNNVPHLGNIIGCVLSADIFARYCRQRNYNTLYISGTDEYGTAT

EAKALEEKLTPQQICDKYFNIHNDIYRWFSIGFDYFGRTTTPEQTEIVQAMFLRIKETGYVISNSVDQLLCEKCDRFLAD

RFVEGVCPGCKYEDARGDQCDGCGHLVNATELINPRCKVCSTTPIIKASTQFFLDLPKIQNNLKEWANKVEDGWTPVARA

VTKPWLRDGLKPRCITRDLKWGIPVPLDGYRNKVFYVWFDAPIGYLSITKKYTKEYEKWWKPKDSKVDLYQFMAKDNVPF

HAIMFPATLMAANEGHTLVKHIMATEYLNYEDTKFSKSRGIGVFGTDARDTGIPADVWRFYLAYIRPETMDSNFNWIDLA

TKNNSELLNNLGNFINRALSFADKTFNGTVPAIDLKEDDLNFLALAQRELTSYVNLLEQARLRDALKHVLMISKHGNQYM

QYQQPWVKVKGNDEDKKKGATVIGLCCNIACLLASLIGPFMPNTARELRSQLGLSVQTYGYIPNVISLMIPCGHKLGKPK

PLFAKIEDKQVEELRQKYAGKQNSETIKKNNLALDNAALDKVALEAAIEKQGNLVRELKAKGDKSVWQPEVNILLDLKKK

LANLASSSKAQTNAASDTDASALEAAIQKQGNLVRELKAKGDKSVWQPEVNILLDLKKKLANLVASSPKAQTNPASDTNV

SALEAAIQKQGNLVRELKAKGDKSVWQPQVDILLDLKKQLEAFKPSSAATSSNSKKSKNKK

>XP_016941202.1 PREDICTED: methionine--tRNA synthetase, cytoplasmic [Drosophila suzukii]

MIIYTNEGNPLGLQLLMLAKFAKRPVQVQLVNLNDAKFKDLLVLPTLELDNGLRLFSPAAIAKYLLVDEGQRRDEWLEWS

ATLLAPALAHHMAVGHKADPNALPVLNALVKKLDDSLKSSPFLAGDKLTSADIAVWSLLAPDGTLKGAQNVDSLRDWYGR

VKALPEVQQVLNEQPLKDLTFNALQQSNRYGGLHHVPLKRLSLADAGKLLVDTTPTVADTVTDEEISAAQAAFTYTAPKE

LKEERTVLPKAGERNVLITSALPYVNNVPHLGNIIGCVLSADIYARYSRSAGYNTLLICGTDEYGTATENKALAENLTPR

EICDKYFELHNAIYRWFGIGFDYFGRTTTQEQTDIVQEAFQDVLKAGYIITESVEQLLCQKCDRFLADRFVEGTCPHPGC

GYEDARGDQCDKCGKLVNATELIRPRCKVCNSAPVLRSSDQLFIDLPKAEAQLKEWVDKSEGGWTHNAKVITRAWLKEGL

KPRCITRDLKWGIPVPHEGFEKKVFYVWFDAPFGYVSMTKRYTKEYQQWWQPAKGTDVELFQFMAKDNVPFHSVVWPSVL

LAINKGHTLVSHIMATEYLNYEDGKFSKSRGIGVFGNDAQETGIPADVWRFYLASARPEGQDSSFSWNDLAARNNSELLN

NLGNFVNRALVFCEKNFESTVPEVATTQDELVLLALINRELRGYINSLEKAKLRDGIRHLLAISRHGNGYMQTQQPWVLL

KGTDDQKKRASTIIGLCVNIACLLANLLFPYMPSTARTLFGQLNAKQTPLNAEKPLATLLLRAGHKIGKPAPLFAKLEQS

FIDELKSKYGGAQETKSAAQSQTSAAELEQAVQAQADKVRELKASTKDKAVWQPEVTKLLALKKQLEEAKKTAPAATPAA

APAPSNGSQSVQDLEKAIQEQGDKVRKLKGSTKDKAVWQPEVNVLLDLKKQLEAAQKAAKAAPAAAASVPSPAASADAAQ

VKALEDKIAQQAEKVRTLKASGDAALWKPEVDILLGLKNQLAALTGIPAAGGQGKGKKKK

>XP_001360316.1 uncharacterized protein Dpse_GA13491 [Drosophila pseudoobscura pseudoobscura]

MIIYTNEGNPLGLQLLMLAKFAKRSVQLVNLNLNDAKFKDFLILPTLELDNGLRLFSPAAIAKYLFVDEGQLRDEWLEWS

TTLLAPALAHHMAVGHRADPNALPVLNALVKKLDDCLKATPYLAGDKLSAADIAAWSLLAPDGTLKGAQNLESLHGWYNR

VKALPEVQHVLAEQPLKDLSFNALQQSNRYGGLHHVPLKRLSLADAGKLLAETASTVADTVTDEELAAAGASFTYTAPKD

VQEQRTVLPKAGERNVLITSALPYVNNVPHLGNIIGCVLSADIFARYSRCAGYNTLLICGTDEYGTATENKALAENMTPR

EICDKYFELHNSIYRWFGIGFDYFGRTTTQEQTDIVQEAFKDVHSAGYIITESVEQLLCQKCDRFLADRFVEGTCPHPGC

GYADARGDQCDKCGKLVNATELIRPRCKVCNTAPILRSSDQLFIDLPKAEPRLREWVDKAENGWTHNAKVITRAWLREGL

KPRCITRDLKWGIPVPHSGFEKKVFYVWFDAPFGYVSMTKRYTKEYQKWWQPAKGTDVELFQFMAKDNVPFHSVVWPSVL

LAINKGHTLVSHIMATEYLNYEDGKFSKSRGIGVFGNDAQATGIPADVWRFYLASARPEGQDSSFSWNDLAARNNSELLN

NLGNFVNRALVFCEKNFNSIVPEVVATEEELILLALVNRELRGYINSLEKAKLRDGIRHLLAISRHGNGYMQTQQPWVLL

KGSDEQKKRAATIIGLCANIACLLANLLFPYMPSTARTLFAQLNAKQTPLNAEKPLATLLLPAGHKIGKPAPLFAKLEQS

FIDELKTKYGGSQETKASVQSQSSTADLEQAVQAQADKVRQLKTSTKDKTVWQPEVSKLLDLKKQLEEAKKTAVAAPASA

PAPSNGGQSVQDLEKAVQAQGEKVRNLKGSTKDKTVWQPEVNVLLDLKKQLEAAQKAAKAAAAAPAATTAAPPAASADAG

QVKALEDKLAQQAEKVRTLKAKGDASVWKPEVDILLALKNQLAALTGAPAAGGQGKGKKKK

>XP_017033519.1 PREDICTED: methionine--tRNA synthetase, cytoplasmic [Drosophila kikkawai]

MIIYTNEGNPLGLQLLMLVKHAKRQVQVQLVNLNDPKYKDLLVLPTLQLDNGLSLFSPAAIAKYLLVDEGKQRDEWLEWS

ATLLAPALAHHMAVGHKADANALPVLNALVKKLDDSLQSSPFLAGDKLTAADIAVWSLLAPEGTLKGAQNVDRLRDWYGR

IKVLPEVQEVLAEQPLKDLSFNALQQSNRYGGLYHIPLKRLSVADAGKLLVDTTPTVADTVTDDEIKAAQAAFTYTAPKE

IKEERTVLPKAGQRNVLITSALPYVNNVPHLGNIIGCVLSADIFARYSRSAGYNTLLICGTDEYGTATENKALAEKLTPR

EICDKYFELHNGIYRWFGIGFDYFGRTTTQEQTDIVQEAFKDVLKAGYIITESVEQLLCQKCDRFLADRFVEGTCPHPGC

GYEDARGDQCDKCGKLVNATELIRPRCKVCNSAPVLRSSDQLFIDLPKAEPYLKDWVDKSEGGWTHNAKVITRAWLKEGL

KPRCITRDLKWGIPVPHPGFEKKVFYVWFDAPFGYVSMTKRYTKEYQQWWQPVKGTDVELFQFMAKDNVPFHSVVWPSVL

LAINKGHTLVSHIMATEYLNYEDGKFSKSRGIGVFGNDAQETGIPADVWRFYLASARPEGQDSSFSWNDLAARNNSELLN

NLGNFVNRALVFCEKNFNSTVPEVVATQDDLILLALVNRELRGYINSLEKAKLRDGIRHLLAISRHGNGYMQTQQPWVLL

KGSADQKTRAATIIGLCANIACLLANLLFPYMPATARTLFGQLNAKQTPLNADKPLATLLLPAGHKIGKPAPLFAKLEQS

FIDELKSKYGGDQVAAVQSPGNAAELEKAVQDQADKVRQLKASTKDKAVWQPEVNKLLDLKKQLEEAKKATPAASATPAP

ASSNGKQSVQDLEKAIQEQGDKVRKLKGSTKDKAVWQPEVNVLLELKKQLEAAQKAAKAAPAAVPAPTPAPAATSSVDPT

QVKALEDKIAQQGEKVRTLKAAGNADVWKPEVATLLALKNELAALTGAPAAGGQNKGKKKK

>XP_002004875.2 uncharacterized protein Dmoj_GI20156, isoform D [Drosophila mojavensis]

MILFTNEGNPIGLQLLVLAKYLKRPMFLQQITITDAKYKDLILLPTVELDNGLRLFSTAAIYKYLLMHEGYPRNEWLEWG

ATLLTPALEHYMATGHRADSRAGPVLNALVKKLDDTLSVSPFIAGDKLTSADLFIWSLLAPDGTLKGAQNVEHLHSWYNK

IKALPEVQEALKELPINTLSFNALQQGNRYGGLAHVQLRRGISQDADKVLADSGPTVADTVSEEEINTAREAFIYTEPKE

LVEPRTVLPKPGERNVLITSALPYVNNVPHLGNIIGCVLSADIFARYSRAAGYNTLLICGTDEYGTATENKALAENMTPR

EICDKYFELHNSIYRWFGIGFDYFGRTTTPEQTEIVQEAFKDVYEAGYILTESVEQLLCQKCDRFLADRFVEGTCPHPGC

GYEDARGDQCDKCGKLVNATELVRPRCKVCNTAPVLRSSDQLFLDLPKAEPKLREWVDRAEQGWTHNARVITRAWLREGL

KPRCITRDLKWGIPVPHKNFEQKVFYVWFDAPFGYVSITKRYTKDYLQWWQPAPGTDVELFQFMAKDNVPFHSVVWPSVL

LAINKGNTLVSHIMATEYLNYEDGKFSKSRGIGVFGNDAQETGIPADVWRFYLASARPEGQDSSFSWTDLAARNNSELLN

NLGNFVNRALSFCERNFDSKLPQIDLNHNDFLLLALINRELRGYINSLEKAKMRDGIRHLLSISRHGNGYIQYQQPWALL

KGSDDQKTRAGTIIGLSINIVCLLANLLFPFMPTTARAMFAQLNTKQTPLDANKPFATLLLRPGHKIGTPSPLFNKLDHS

FIESLKAKFGGSQADKEAVAAAGAAAALPQLSTVELEAAVKAQGEKVRQLKASTKDKAVWQPEVNKLLELKKQLEAAQKA

TPVSTGAVAAAAAAPVPSASPSAQSVQSLEQAVQAQGDKVRQLKSTTKDKAVWQPEVNKLLDLKKQLEEAKTASGAASAT

DSPAPKGIDAAAVKALQEKITQQGEKVRNLKATGDASVWKPEVEILLNLKKELAALDGSAAAAPQTKNKKKK

>XP_013104836.1 PREDICTED: methionine--tRNA synthetase, cytoplasmic [Stomoxys calcitrans]

MKVYTNDGNPFGLKLQMLAKFAKREVVVEKVNLNDAKGKELMLLPTLELDGGIQLFSTDAVAKYFFPNEGPLRDEWLEWS

TTILAPALVHSMGVGHRANPNAMPVLNALVKKLEVTLSKSPYLAGDKLSAADISVWSLLAPDGTLKGAQQIDNIMAWYRK

INALPEVQAVLASEPLKDLTFNALQNSNRFGGLYHVPLKVSASSTDAGKLLADTGSNLADTLTDDELSSARANFVFTPPN

ERKEPRTVLPKAGERNVLITSALPYVNNVPHLGNIIGCVLSADIFARYSRAAGYNTLLVCGTDEYGTATENKALAENLTP

REICDKYFELHNAIYCWFGIGFDYFGRTTTQEQTDIVQEAFLDVYKNGYIITESVEQLLCQKCDRFLADRFVEGTCPHAG

CGYEDARGDQCDKCGKLVNATELIRPRCKVCNTAPVVRSSEQLFLDLPKAEAQLREWVDRSEQGWTNNARVITRAWLREG

LKPRCITRDLKWGIPVPHKGFENKVFYVWFDAPFGYVSITKRYTKEYKQWWQPEKGVDVELFQFMAKDNVPFHSVVWPSA

LLAINKGNTMVSHIMATEYLNYEDGKFSKSRGIGVFGNDAQDTGIPADVWRFYLASARPEGQDSSFSWNDLAARNNSELL

NNLGNFVNRALVFCEKNFKSTVPVMNLTKDEEVLLALINRELRGYINSLEKARLRDGIRHLLSISRHGNGYMQSQQPWVL

LKGNDEQKARAATIIGLCTNIACLLANLIFPYMPTTARTMFEQLNTKQGSINAEKPFIGILLPSGHKIGKPAPLFAKLEQ

SFIDEMKKKYAGPQVLSEENSSPAGPSVADLEKAIQAQGDKVRQLKSSTKDKAVLQPEINILLDLKKQLTEAQSRVQPSA

ASESKQDGLSVKELEAAVQAQGEKVRQLKAQTKDKAVWQPEVNKLLELKKHLAAAQSSTSPATANTASVGSSQVKDLEEQ

IAKQGEKVRTLKAGGDPSIWKPEVEILLSLKKQLSSITGAPDTNTNQGKNKKKK

>XP_001974747.1 uncharacterized protein Dere_GG20938 [Drosophila erecta]

MIIYTNEGNPLGLQLLMLARFAKRPVQVQLVNLNDAKYRDLLVLPTLELDNGLRLFSPAAIAKYLLVDEGQRRDEWLEWS

ATLLAPALAHHMAVGHKADANALPVLNALVKKLDDSLKGTPFLAGDKPSAADIAVWSLLAPDGTLKGAQNVDSLRDWYGR

VKALPEVQEVLAEQPLKDLTFNALQQSNRYGGLHHVPLKRLSLADASKLLVDTTPTVADTVTDEEISAAQAAFTYTAPKE

FKEERTVLPKPGERNVLITSALPYVNNVPHLGNIIGCVLSADIYARYSRSAGYNTLLICGTDEYGTATENKALAEKLTPR

EICDKYFELHNAIYRWFGIGFDYFGRTTTQEQTDIVQEAFKDVLKAGYIITESVEQLLCQKCDRFLADRFVEGTCPHPGC

GYEDARGDQCDKCGKLVNATELIRPRCKVCNSAPVLRSSDQLFIDLPKAEPQLKEWVDKSEGGWTHNAKVITRAWLKEGL

KPRCITRDLKWGIPVPHEGFEKKVFYVWFDAPFGYVSMTKRYTKEYQQWWQPAEGTDVELFQFMAKDNVPFHSVVWPSVL

LAINKRHTLVSHIMATEYLNYEDGKFSKSRGIGVFGNDAQETGIPADVWRFYLASARPEGQDSSFSWNDLAARNNSELLN

NLGNFVNRALVFCEKNFNSTVPEVVTTQDELVLLALINRELRGYINSMEKAKLRDGIRHLLSISRHCNGYMQSQQPWVLL

KGNDDQKARASTIIGLCVNIACLLANLLFPYMPTTARTLFGQLNAKQTPLNAEKPLVTLLLRAGHKIGKPAPLFAKLEQS

FIDELKGKYGGAQDTKSAAQSQTSAADLEKAVQAQADKVRQLKATTKDKAVWQPEVTKLLDLKKQLEEAKKTAPSAVPAT

APAPSNGSQSVQDLDKAIQEQGDKVRKLKGSTKDKAVWQPEVNVLLDLKKQLEAAQKAAKAAPAANVAPAPSTTAPADPV

QVKALEDKIAQQAERVRTLKASGDAAVWKPEVDILLSLKNQMAALTGTPAAGGQGKGKKKK

>XP_002061477.1 methionine--tRNA synthetase, cytoplasmic [Drosophila willistoni]

MIIYTNEGNPLGLQLQILAKFAKREVQVQLVNLNDSKYKHLLVLPTLELDNGLQLFSTAAIAKYLLADEGQQRDEWLEWS

TTLLAPALAHHMGVGHKADPNALPVLNGLVKKLDDHLKSSPYLAGDKITSADLAVWSLLAPDGTLKGAQNVDHLRDWYGR

LKVLPEVQQVLQQQPLKDLSFNALQQSNRYGGLHHITLKRPSLVDAGKLLAETGNTLADTITDEEIEAARTAFTYVEPKK

LLEPRTVLPKAGERNVLITSALPYVNNVPHLGNIIGCVLSADIFARYSRSAGYNTLLICGTDEYGTATENKALAENLTPR

EICDKYFELHNAIYRWFGIGFDYFGRTTHQEQTEIVQEAFKDVHSAGYIITESVEQLLCQKCDRFLADRFVEGTCPHPGC

GYEDARGDQCDKCGKLVNATELIRPRCKVCNSAPVLRSSDQLFIDLPKAEPKLREWVDKADNGWTHNARVITRAWLREGL

KPRCITRDLKWGIPVPHAGFEKKVFYVWFDAPFGYVSMTKRYTKDYQQWWQPAKGTDVELFQFMAKDNVPFHSVVWPSVL

LAINKGHTLVSHIMATEYLNYEDGKFSKSRGIGVFGNDAQETGIPADVWRFYLASARPEGQDSSFSWNDLAARNNSELLN

NLGNFVNRALVFCEKNFNSLVPEVVLTQDELILLALINRELRGYINSLEKAKLRDGIRHLLAISRHGNGYMQTQQPWVLL

KGDETQKKRAATIIGLSANIACLLANLLFPYMPSTARTLFDQLNSKQTTLNASKPLATLLLPAGHKIGKPAPLFAKLEQA

FIDELKGKYGGVQAQTESAKSDQLSVADLEQAIQAQGNKVRELKASTKDKSVWQPEVNKLLELKKQLEAAQKAAPSAVQA

PTKSGTPSVQDLEKAVQAQGEKVRQLKSSTKDKSVWQPEVNVLLDLKKQLEAAQKTASAAPVPAASAPATPNGEQVKALE

NKIAQQGEKVRTLKASGDATVWKPEVEILLSLKKELATLTGVPAAAPAQGKSKKKK

>XP_002050955.1 uncharacterized protein Dvir_GJ22433 [Drosophila virilis]

MIIYTNEGNPLGLQLLMLAKFAKRAVQVQLVNLNDAKYKDLLVLPTLELDTGLRLFSTAAIAKYLLGDEGQQRDEWLEWS

TTLLAPALAHHMAVGHRADPNALPVLNGLVKKLDDCLRNAPFLAGDKLSAADIAIWCLLAPDGTLKGAQHVDNLRSWYDK

IKALPEVQQVLGEQPLKELSFNALQQANRYGGLHHVPLRRSSSVDAGKLLAETAPTVADTVTDEEINAARAAFTYTKPKE

LAEARTVLPKAGERNVLITSALPYVNNVPHLGNIIGCVLSADIFARYSRSAGYNTLLICGTDEYGTATENKALAENMTPR

EICDKYFELHNSIYRWFGIGFDYFGRTTTQEQTDIVQEAFHDVYKAGYILTESVEQLLCQKCDRFLADRFVEGTCPHPGC

GYEDARGDQCDKCGKLVNATELVRPRCKVCNSAPVLRSSDQLFLDLPKAEPQLREWVDRVEQGWTHNARVITRAWLREGL

KPRCITRDLKWGIPVPHKNFEKKVFYVWFDAPFGYVSMTKRYTKEYLQWWQPAAGTNVELFQFMAKDNVPFHSVVWPSVL

LAINKGNTLVSHIMATEYLNYEDGKFSKSRGIGVFGNDAQETGIPADVWRFYLASARPEGQDSSFSWNDLAARNNSELLN

NLGNFVNRALVFCEKNFNSTVPQIVLNQDELVLLALINRELRGYINSLEKAKLRDGIRHLLAISRHGNGYMQTQQPWVLL

KGNDEQKSRAATIIGLCANIACLLANLLFPYMPSTARNLFAQLNAKQTPLDASKPLATILLPAGHKIGKPSPLFTKLEQA

FIDELKGKYGGTQETRAEEPQQAAELEAAVQAQSDKVRQLKASTKDKTVWQPEVNKLLELKKQLEAAQKTTPAAAAPAAA

TQSVQDLENAVQAQGEKVRQLKSSTKDKAVWQPEVNVLLALKKQLDAAKAAPAAAAPAAAVPTAAAPANVGADAGKIKAL

EDKIAQQGEKVRSLKASGDATIWKPELDVLLNLKKELAALTGAPAAGTQSKNKKKK

>XP_019764461.1 PREDICTED: methionine--tRNA synthetase, cytoplasmic [Dendroctonus ponderosae]

MGTIEGNENNPATLKLVLAGKLAKTQYAVKIRGHNEEGQYLPYLALSSEDNIFVPNAAIWFILPPAEELSAKVREVLEME

STVLSPSLALLLGTNLKSEKVKSSVLSALQSLNSTLEGSQFLVQDAITVADVAVFSTLFPIFKAAKLLKEYLGSFPSISK

YLNQLELLPEVKDSVAIVFHQKCNIGPVSYQVLFQSSKYLVPQIQSDPKTVNRVDSVEKESPKHEVETVSSEELEAAEKA

WKKSPGSLPKLKTPNKVVLPKTGEKNVFITSALPYVNNVPHLGNIIGCVLSADVFARFSRLCNNNTLYICGTDEYGTATE

TKALEEGLSCQAICDKYFKIHQEIYQWFNISFDSFGRTSTPLQTELAQDLFLQLNHNGFMSTQAVDQLLCEKCNRYLADR

FVEGGCPNVGCTYEDARGDQCDGCGKLVNAIELKNPRCKICGSTPKVKSSSQFFIDLPKLEPLLLHWVKNSSPGWSHNAE

VIAKAWLKEGLKPRCITRDLHWGVPVPLDGFRNKVFYVWFDAPIGYMSISKAYTKEYEKWWRPAKDTEVKLYQFMAKDNV

PFHAILFPAILLGANREYVTVSHIMATEYLNYEEGKFSKSRGIGVFGNDARDTKIPSDVWRFYLLYVRPEAQDASFSWND

LATKNNSELLNNLGNFINRALIFVKNNFNSTIPKLELTSEDYTLLALCTRELKGYIAALDKCKLRDGIRHILAISRHGNQ

YMQLNQPWVKIKQGEEGKLRGGTVVGVCANLSCLLATLLQPYLPETSEKLKAQLNCEYFVLNPENIEIINLLPTGHSIGE

PQPLFTKIEPEVIEEYKKKFAGSQESRKTPPKEIVKPDLCNGDAETLEEAIAKQGLVVRKLKEGGFDKTVWAPEVAKLVA

LKARLAELTGVVPDKKSSSKKSNATTNNNVQKVANNMPAKSKKKPVNNSN

>XP_014085549.1 PREDICTED: methionine--tRNA synthetase, cytoplasmic [Bactrocera oleae]

MIIYTNEGNPFGLKLQLLAKFANKPVDVIKVTLNDPACKEFLVLPILELESGLKLFSTDAVARYLFPDEGALRDEWLEWS

TVKLAPALSQHLAVGHRANPDALPVLNLLVRKLDDRLADSAFITGDKISAADIAVWSLLAPDGTLKGAQNIEHLKAWYRK

ITALAEVQAVLAETPLAGLTFTSLQNSNRYGGLSHVPIRVHTTSESEKLLADTTGNLADTVTEDELGNAQKSFVHVSLPQ

RIEARTVLPKAGERNILITSALPYVNNVPHLGNIIGCVLSADIFARYSRAAGYNTLYICGTDEYGTATENKALAENLSPK

QICDKYFDLHNSIYRWFGIGFDFFGRTSSQQQTEIVQEAFHDVYNNGYIFTESVEQLLCVKCDRFLADRFVEGTCPHPGC

GYEDARGDQCDKCGKLVNATELIRPRCKICNSAPVLRSSDQLFLDLPKIEPQLRVWLEGSEMGWTNNARVITRSWLREGL

KPRCITRDLKWGIPVPLAGFEKKVFYVWFDAPYGYISMTKHYTSEYKQWWIPSKDVKVELFQFMAKDNVPFHSVVFPSSL

LATNKGHTMVTNIMATEYLNYEDGKFSKSRGIGVFGNDAQQTGISADIWRFYLASARPEGQDSSFSWNDLAARNNSDLLN

NLGNFVNRALVFCEKNFNCVVPTMTLTKDELILLALINREVRGYINSLEKAKLRDGIRHLLSISRHGNGYMQSQQPWVLL

KGTDVQKARAATIIGICANIAALLANLMLPYMPTIARTLFSQLNAKPTPLNPEKPFINMLLPTGHNIDKPAPLFTKLEQS

FIEELKKKFGGVQDQSNGSQPPSTTTNTNSTTAKPSALPNGTQSVAQLEEAVRLQGEKVRQLKATTKDKAVWQPEVNVLL

DLKKQLTAAQAAPPIATAAMATPVASSEKVKELEQRITQQGEKVRQLKASGDASVWKPEVEILLQLKKDLAALTGAPQPA

TQAKNKKKK

>XP_002015762.1 GL10845 [Drosophila persimilis]

MIIYTNEGNPLGLQLLMLAKFAKRSVQLVNLNLNDAKFKDFLILPTLELDNGLRLFSPAAIAKYLFVDEGQLRDEWLEWS

TMLLAPALAHHMAVGHRADPNALPVLNALVKKLDDCLKATPYLAGDKLSAADIAVWSLLAPDGTLKGAQNLESLHGWYNR

VKALPEVQQVLAEQPLKDLSFNALQQSNRYGGLHHVPLKRLSLADAGKLLAETASTVADTVTDEELAAAGASFTYTAPKD

VQEQRTVLPKAGERNVLITSALPYVNNVPHLGNIIGCVLSADIFARYSRCAGYNTLLICGTDEYGTATENKALAENMTPR

EICDKYFELHNSIYRWFGIGFDYFGRTTTQEQTDIVQEAFKDVHSAGYIITESVEQLLCQKCDRFLADRFVEGTCPHPGC

GYADARGDQCDKCGKLVNATELIRPRCKVCNTAPILRSSDQLFIDLPKAEPRLREWVDKAENGWTHNAKVITRAWLREGL

KPRCITRDLKWGIPVPHSGFEKKVFYVWFDAPFGYVSMTKRYTKEYQKWWQPAKGTDVELFQFMAKDNVPFHSVVWPSVL

LAINKGHTLVSHIMATEYLNYEDGKFSKSRGIGVFGNDAQATGIPADVWRFYLASARPEGQDSSFSWNDLAARNNSELLN

NLGNFVNRALVFCEKNFNSIVPEVVATEEELILLALVNRELRGYINSLEKAKLRDGIRHLLAISRHGNGYMQTQQPWVLL

KGNDEQKKRAATIIGLCANIACLLANLLFPYMPSTARTLFAQLNAKQTPLNAEKPLATLLLPAGHKIGKPAPLFAKLEQS

FIDELKTKYGGSQETKASVQSQSSTADLEQAVQAQADKVRQLKTSTKDKTVWQPEVSKLLDLKKQLEEAKKTAVAAPASA

PAPSNGGQSVQDLEKAVQAQGEKVRNLKGSTKDKTVWQPEVNVLLDLKKQLEAAQKAAKAAAAAPAATTAAPPAASADAG

QVKALEDKLAQQAEKVRTLKAKGDASVWKPEVDILLALKNQLAALTGAPAAGGQGKGKKKK

>XP_015018887.1 uncharacterized protein Dmoj_GI20156, isoform C [Drosophila mojavensis]

MILFTNEGNPIGLQLLVLAKYLKRPMFLQQITITDAKYKDLILLPTVELDNGLRLFSTAAIYKYLLMHEGYPRNEWLEWG

ATLLTPALEHYMATGHRADSRAGPVLNALVKKLDDTLSVSPFIAGDKLTSADLFIWSLLAPDGTLKGAQNVEHLHSWYNK

IKALPEVQEALKELPINTLSFNALQQGNRYGGLAHVQLRRGISQDADKVLADSGPTVADTVSEEEINTAREAFIYTEPKE

LVEPRTVLPKPGERNVLITSALPYVNNVPHLGNIIGCVLSADIFARYSRAAGYNTLLICGTDEYGTATENKALAENMTPR

EICDKYFELHNSIYRWFGIGFDYFGRTTTPEQTEIVQEAFKDVYEAGYILTESVEQLLCQKCDRFLADRFVEGTCPHPGC

GYEDARGDQCDKCGKLVNATELVRPRCKVCNTAPVLRSSDQLFLDLPKAEPKLREWVDRAEQGWTHNARVITRAWLREGL

KPRCITRDLKWGIPVPHKNFEQKVFYVWFDAPFGYVSITKRYTKDYLQWWQPAPGTDVELFQFMAKDNVPFHSVVWPSVL

LAINKGNTLVSHIMATEYLNYEDGKFSKSRGIGVFGNDAQETGIPADVWRFYLASARPEGQDSSFSWTDLAARNNSELLN

NLGNFVNRALSFCERNFDSKLPQIDLNHNDFLLLALINRELRGYINSLEKAKMRDGIRHLLSISRHGNGYIQYQQPWALL

KGSDDQKTRAGTIIGLSINIVCLLANLLFPFMPTTARAMFAQLNTKQTPLDANKPFATLLLRPGHKIGTPSPLFNKLDHS

FIESLKAKFGGSQADKEAVAAAGAAAALPQLSTVELEAAVKAQGEKVRQLKASTKDKAVWQPEVNKLLELKKQLEAAQKA

GAGVTAAAGESPAPAGQSVQALEQAVQAQGDKVRQLKASTKDKAVWQPEVNKLLELKKQLEAAQKATPVSTGAVAAAAAA

PVPSASPSAQSVQSLEQAVQAQGDKVRQLKSTTKDKAVWQPEVNKLLDLKKQLEEAKTASGAASATDSPAPKGIDAAAVK

ALQEKITQQGEKVRNLKATGDASVWKPEVEILLNLKKELAALDGSAAAAPQTKNKKKK

>XP_002596718.1 hypothetical protein BRAFLDRAFT_78382 [Branchiostoma floridae]

MLYIFVGGNFVVDTLKTKFPPRTYSLPKPGEKNVLITSALPYVNNVPHLGNIIGCVLSADVFSRYCRLRGWNALYICGTD

EYGTATETRALEEGLTPQQICDKYHALHADIYRWFNIDFDFFGRTTTPQQTEIAQDIFWRLHQRDHLLTDTVDQLLCEKC

DRFLADRFVEGTCPLCNYEDARGDQCDKCGKLIVATELKKPRCKVCSASPVVKTSTHLFLNLPQIEPKLSGWLEEILPSD

SWSSNARVITRTWLRDGLKPRCITRDLQWGTPVPLKGFTEKVFYVWFDAPIGYLSITANYTDQWEKWWKNPDEVTLYNFM

AKDNVPFHSVVFPCSLLGAEDNYTLVNHLQATEFLNYEDGKFSKSRGIGVFGDDAKDTSIPADIWRFYLLYVRPENQDTT

FSWEDLMVKHNSELLNNLGNFIYSTIPTMDLDESDTEMLAQVNRELQMYNFNMERVRLREGLTTILNISRLGNQYIQASK

PWVLLKGTEQERARAGTVVGLCANIACVLSVLLHPYMPDISTQIQAQLAAPVDVNVVGDRLVCHLAPGHKIGQPKPLFEK

LEAAVVQDLKNKFAGRQKE

>XP_017959206.1 PREDICTED: methionine--tRNA synthetase, cytoplasmic [Drosophila navojoa]

MILFTNEGNPIGLQLLVLAKYLKRPIFLQQITLTDAKFKDLILLPTVELDNGLRLFSTAAIYKYLLMYEGYPRNEWLEWG

ATLLTPALEHYMATGHRADSRAGPVLNALVKKLDDTLSVSPFIAGDKLTSADLFIWSLLAPDGTLKGAQNVEHLHSWYNK

IKALPEVQEALKELPISSLSFNALQQGNRYGGLAHVQLRRGISQDTDKVLADSAPTVADTVSDEEINAAREAFIYTEPKE

LVEPRTVLPKPGERNVLITSALPYVNNVPHLGNIIGCVLSADIFARYSRAAGYNTLLICGTDEYGTATENKALAENMTPR

EICDKYFELHNSIYRWFGIGFDYFGRTTTPEQTEIVQEAFKDVYDAGYILTESVEQLLCQKCDRFLADRFVEGTCPHPGC

GYEDARGDQCDKCGKLVNATELVRPRCKVCNTAPVLRSSDQLFLDLPKAEPKLREWVDRAEQGWTHNARVITRAWLREGL

KPRCITRDLKWGIPVPHKNFEQKVFYVWFDAPFGYVSITKRYTKDYLQWWQPAPGTDVELFQFMAKDNVPFHSVVWPSVL

LAINKGNTLVSHIMATEYLNYEDGKFSKSRGIGVFGNDAQETGIPADVWRFYLASARPEGQDSSFSWTDLAARNNSELLN

NLGNFVNRALSFCERNFDSKLPQMDLNHNDYLLLALINRELRGYINSLEKAKMRDGIRHLLSISRHGNGYIQYQQPWALL

KGSDDQKSRAGTIIGLSINIVCLLANLLFPFMPTTARAVFAQLNAKQTPLDANKPFATLLLRPGHKIGTPSPLFDKLDVS

FIEGLKARFGGSQADKEAVAAAAQPQSSTVELEAAVKAQGEKVRQLKASTKDKAVWQPEVNKLLELKKQLEAAQKAGAGV

TAAAGGSPAPAGQSVQALEQAVQAQGDKVRQLKASTKDKAVWQPEVNKLLELKKQLEAAKKEAPASQGAAAPVPSPSPQS

VQSLEQAVQAQGDKVRQLKSTTKDKAVWQPEVNKLLDLKKQLEAAKTASGAASAPTAKGIDAAAVKALQEKIAQQGEKVR

TLKASGDASVWKPEVEILLNLKKELAALDGSAAAAPQTKNKKKK

>XP_005182375.1 PREDICTED: methionine--tRNA synthetase, cytoplasmic [Musca domestica]

MKIYTNEGNPFGLKLQLLAKFAKREVVVEKVNLNDANGKDFLLLPTYELENGIKLFSTDAVAKYLFPEEGLQRDEWLEWS

TTLLAPALVHKMGVGHRANPNASPVLNALVKKLEDTLSKTPFLIGDKLSAADISVWSLLAPDGTLKGAQQIDNLLVWYRK

ICAMPEVQAVLATEPLKDLTFNALQNSNRFGGLYHVPLKVSASSADAGKFLADAGSNIADTVTEEEITSARNNFVYKKPA

ERQEPRTVLPKAGERNVLITSALPYVNNVPHLGNIIGCVLSADIFARYSRSVGYNTLLICGTDEYGTATENKALAEGLTP

REICDKYFELHNGIYRWFGIGFDYFGRTTTQEQTEIVQEAFHDVYNNGYILTESVEQLLCQKCDRFLADRFVEGTCPHPG

CGYEDARGDQCDKCGKLVNATELIRPRCKVCNTAPVIRSSDQLFLDLPKVEPQLREWIERAEHGWTNNARVITRAWLREG

LKPRCITRDLKWGIPVPHKGFENKVFYVWFDAPFGYVSMTKRYTKDFKQWWQPEKGVDVELYQFMAKDNVPFHSVVWPSA

LLAINKGNTMVSHIMATEYLNYEDGKFSKSRGIGVFGNDAQDTGIPADVWRFYLACARPEGQDSSFSWNDLAARNNSELL

NNLGNFVNRALVFCEKNFNGTVPAMTLTNDEEVLLALINRELRGYISSLEKARLRDGIRHLLSISRHGNGYLQSQQPWVL

LKGSEEQKARAATIIGICVNIACWLANLIFPYMPSTARTMFEQLNTKQILLNAEKPYVSILLQTGHKIGKPAPLFAKLEQ

QFIDEMKKKYSGPQVTTSSGSGLSVADLEKAVQEQGEKLRQLKSTTKDKAVLQPEIDKLLDLKKQLSEAKAAGENKQNGT

SSVKDLEAAVQAQGEKVRQLKAQTKDKSVWQPEVNKLLDLKKQLAAAQSGTSTPAPTNPAPASDANQVKELEDKIAKQGE

KVRTLKAAGDASVWKPEVEILLALKKQLSALTGAPDANAQGKNKKKK

>ABM68611.1 AAEL014709-PA [Aedes aegypti]

MIIYTNAGNPFGLKLLICAKIAKKEVQVKTISLNDAELKEMKHLPILQLESGVQLFSTDVAVKYLLQGETPVIQRDEWLE

WSTTRLAPALTHNMAVGNRQDPNAKPILNSLVKFLDDCLSKNTFLTGEKLTSADIAVWSLLAPDGTLKGAQNIDNLLRWY

RAINAMPEVKAALEQLPLKELSFASLKHSNRFEGLHHIVLDPEIIDFEGQVLVDTSDNIAATVQKEEIQAAKDSFVVAQE

RAVPEEKVVLPKVGQKNNLITSALPYVNNVPHLGNIIGCVLSADVFARYSRLCGYNTLYICGTDEYGTATETKALAENLT

PKQICDKYFEIHNSIYRWFGIGFDYFGRTTTAEQTQIVQDMFKDLYAGGFIETRAVDQLLCQKCDRYLADRFVEGTCPYP

GCGYEDARGDQCDACGKLINAIELVRPRCKVCNTSPIIRQSSQFFLDLPKIEPKLREWVDKAEHGWTHNARVITRAWLKE

GLKSRCITRDLKWGIPVPLEGYEDKVFYVWFDAPIGYISMTSRYSKEWKQWWQPSAETKVDLYQFMAKDNVPFHSVMFPA

SLIGIDKGYTLVSHIMATEYLNYEDGKFSKSRGVGVFGNDAHDTGIPADVWRFYLCSSRPEGQDSSFSWNDLAARNNNEL

LKNLGNFVNRALVFCEKYFDSIIPPMVLNEEDFTLLALINRDLKGYVHQMEKARLRDGIRHLLGISRYGNQLMQAEEPWV

LVKGTDDQKTRAGTVIGLCCNLACVLATLIFPFMPSTARNLYAQLNVSSGQIESEKPQMRIFLPSGHKIGKPAVLFNKID

QAKIDELKKKYAGAQQQAPKATPAKPVSNKLFTSLVEAQKAVDEQAAKVRKIKSTGADKSVWQPEVNILLELKKQLQTLS

QPRTSESTAQPSQAAPSDPEPETVQSLEAEIAKQGEKVRTLKGNGSEKSVWQPEVSVLLALKEKLAKLTGVVAPAPGGGG

KKKNKNKKVIEKNKIGVFCFVSLLPSFRLACFARDLEYLAGIDVQCEETVNLLLINFAN

>EAT33248.1 AAEL014494-PA [Aedes aegypti]

MIIYTNAGNPFGLKLLVCAKIAKKEVQVKTISLNDAELKDMKHLPILQLESGVQLFSTDVAVKYLLQGETPVIQRDEWLE

WSTTRLAPALTHNMAVGNRQDPNAKPILNSLVKFLDDCLSKNTFLTGEKLTSADIAVWSLLAPDGTLKGAQNIDNLLRWY

RAINAMPEVKAALEQLPLKELSFASLKHSNRFEGLHHIVLDPEIIDFEGQVLVDTSDNIAATVQKEEIQAAKDSFVVAQE

RAVPEEKVVLPKVGQKNNLITSALPYVNNVPHLGNIIGCVLSADVFARYSRLCGYNTLYICGTDEYGTATETKALAENLT

PKQICDKYFEIHNSIYRWFGIGFDYFGRTTTAEQTQIVQDMFKDLYAGGFIETRAVDQLLCQKCDRYLADRFVEGTCPYP

GCGYEDARGDQCDACGKLINAIELVRPRCKVCNTSPIIRQSSQFFLDLPKIEPKLREWVDKAEHGWTHNARVITRAWLKE

GLKSRCITRDLKWGIPVPLEGYEDKVFYVWFDAPIGYISMTSRYSKEWKQWWQPSAETKVDLYQFMAKDNVPFHSVMFPA

SLIGIDKGYTLVSHIMATEYLNYEDGKFSKSRGVGVFGNDAHDTGIPADVWRFYLCSSRPEGQDSSFSWNDLAARNNNEL

LKNLGNFVNRALVFCEKYFDSIIPPMVLNEEDFTLLALINRDLKGYVHQMEKARLRDGIRHLLGISRYGNQLMQAEEPWV

LVKGTDDQKTRAGTVIGLCCNLACVLATLIFPFMPSTARNLYAQLNVSSGQIESEKPQMRIFLPSGHKIDKPAVLFNKID

QAKIDELKKKYAGAQQQAPKATSAKPASNKLFTSLVEAQKAVDEQAAKVRKIKSTGADKSVWQPEVNILLELKKQLQTLS

QPRTSESTAQPSQSAPSDPEPETVQSLEAEIAKQGEKVRTLKGNGSEKSVWQPEVSVLLALKEKLAKLTGVVAPAPGGGG

KKKNKK

>EAT33034.1 AAEL014709-PA [Aedes aegypti]

MIIYTNAGNPFGLKLLICAKIAKKEVQVKTISLNDAELKEMKHLPILQLESGVQLFSTDVAVKYLLQGETPVIQRDEWLE

WSTTRLAPALTHNMAVGNRQDPNAKPILNSLVKFLDDCLSKNTFLTGEKLTSADIAVWSLLAPDGTLKGAQNIDNLLRWY

RAINAMPEVKAALEQLPLKELSFASLKHSNRFEGLHHIVLDPEIIDFEGQVLVDTSDNIAATVQKEEIQAAKDSFVVAQE

RAVPEEKVVLPKVGQKNNLITSALPYVNNVPHLGNIIGCVLSADVFARYSRLCGYNTLYICGTDEYGTATETKALAENLT

PKQICDKYFEIHNSIYRWFGIGFDYFGRTTTAEQTQIVQDMFKDLYAGGFIETRAVDQLLCQKCDRYLADRFVEGTCPYP

GCGYEDARGDQCDACGKLINAIELVRPRCKVCNTSPIIRQSSQFFLDLPKIEPKLREWVDKAEHGWTHNARVITRAWLKE

GLKSRCITRDLKWGIPVPLEGYEDKVFYVWFDAPIGYISMTSRYSKEWKQWWQPSAETKVDLYQFMAKDNVPFHSVMFPA

SLIGIDKGYTLVSHIMATEYLNYEDGKFSKSRGVGVFGNDAHDTGIPADVWRFYLCSSRPEGQDSSFSWNDLAARNNNEL

LKNLGNFVNRALVFCEKYFDSIIPPMVLNEEDFTLLALINRDLKGYVHQMEKARLRDGIRHLLGISRYGNQLMQAEEPWV

LVKGTDDQKTRAGTVIGLCCNLACVLATLIFPFMPSTARNLYAQLNVSSGQIESEKPQMRIFLPSGHKIGKPAVLFNKID

QAKIDELKKKYAGAQQQAPKATPAKPVSNKLFTSLVEAQKAVDEQAAKVRKIKSTGADKSVWQPEVNILLELKKQLQTLS

QPRTSESTAQPSQAAPSDPEPETVQSLEAEIAKQGEKVRTLKGNGSEKSVWQPEVSVLLALKEKLAKLTGVVAPAPGGGG

KKKNKK

>XP_001649418.2 methionine--tRNA synthetase, cytoplasmic [Aedes aegypti]

MIIYTNAGNPFGLKLLVCAKIAKKEVQVKTISLNDAELKEMKHLPILQLESGVQLFSTDVAVKYLLQGETPVIQRDEWLE

WSTTRLAPALTHNMAVGNRQDPNAKPILNSLVKFLDDCLSKNTFLTGEKLTSADIAVWSLLAPDGTLKGAQNIDNLLRWY

RAINAMPEVKAALEQLPLKELSFASLKHSNRFEGLHHIVLDPEIIDFEGQVLVDTSDNIAATVQKEEIQAAKDSFVVAQE

RAVPEEKVVLPKVGQKNNLITSALPYVNNVPHLGNIIGCVLSADVFARYSRLCGYNTLYICGTDEYGTATETKALAENLT

PKQICDKYFEIHNSIYRWFGIGFDYFGRTTTAEQTQIVQDMFKDLYAGGFIETRAVDQLLCQKCDRYLADRFVEGTCPYP

GCGYEDARGDQCDACGKLINAIELVRPRCKVCNTSPIIRQSSQFFLDLPKIEPKLREWVDKAEHGWTHNARVITRAWLKE

GLKSRCITRDLKWGIPVPLEGYEDKVFYVWFDAPIGYISMTSRYSKEWKQWWQPSAETKVDLYQFMAKDNVPFHSVMFPA

SLIGIDKGYTLVSHIMATEYLNYEDGKFSKSRGVGVFGNDAHDTGIPADVWRFYLCSSRPEGQDSSFSWNDLAARNNNEL

LKNLGNFVNRALVFCEKYFDSIIPPMVLNEEDFTLLALINRDLKGYVHQMEKARLRDGIRHLLGISRYGNQLMQAEEPWV

LVKGTDDQKTRAGTVIGLCCNLACVLATLIFPFMPSTARNLYAQLNVSSGQIESEKPQMRIFLPSGHKIGKPAVLFNKID

QAKIDELKKKYAGAQQQAPKATPAKPASNKLFTSLVEAQKAVDEQAAKVRKIKSTGADKSVWQPEVNILLELKKQLQTLS

QPRTSESTAQPSQAAPSDPEPETVQSLEAEIAKQGEKVRTLKGNGSEKSVWQPEVSVLLALKEKLAKLTGVVAPAPGGGG

KKKNKK

>XP_002091851.1 uncharacterized protein Dyak_GE13875 [Drosophila yakuba]

MIIYTNEGNPLGLQLLMLAKFAKRPVQVQLVNLNDAKYKDLLVLPTLELDNGLRLFSPAAIAKYLLVDEGQRRDEWLEWS

ATLLAPALAHHMAVGHKADANALPVLNALVKKLDDSLKVTPYLAGDKPSAADIAVWSLLAPDGTLKGAQNVESLRDWYGR

VKALPEVQEVLAEQPLKDLTFNALQQSNRYGGLHHVPLKRLSLADASKLLVDTTPTVADTVTDEEISAAQAAFTYTAPKE

FKEERTVLPKPGERNVLITSALPYVNNVPHLGNIIGCVLSADIYARYSRSAGYNTLLICGTDEYGTATENKALAENLTPR

EICDKYFELHNAIYRWFGIGFDYFGRTTTQEQTDIVQEAFKDVLKAGYIITESVEQLLCQKCDRFLADRFVEGTCPHPGC

GYEDARGDQCDKCGKLVNATELIRPRCKVCNSAPVLRSSDQLFIDLPKAEPQLKDWVDKSEGGWTHNAKVITRAWLKEGL

KPRCITRDLKWGIPVPHEGFEKKVFYVWFDAPFGYVSMTKRYTKEYQQWWQPAKGTDVELFQFMAKDNVPFHSVVWPSVL

LAINKGHTLVSHIMATEYLNYEDGKFSKSRGIGVFGNDAQETGIPADVWRFYLASARPEGQDSSFSWNDLAARNNSELLN

NLGNFVNRALVFCEKNFNSTIPEVITTQDELVLLALINRELRGYINSMEKAKLRDGIRHLLAISRHGNGYMQSQQPWVLL

KGTDDQKKRASTIIGLCVNIACLLANLLFPYMPSTARTLFGQLNAKQTPLNAEKPLVTLLLPSGHKIGKPAPLFAKLEQP

FIDELKGKYGGAQDTNGAAKSQTSAADLEKAVQAQADKVRQLKASTKDKAVWQPEVTKLLDLKKQLEEAKKTAPAAAPAT

AAAPSGGSQSVQNLEKAIQEQGDKVRKLKGSTKDKAVWQPEVNILLDLKKQLEAAQKAAKAAPAANATPASTSAAPADAG

QVKALEEKIAQQAEKVRTLKTSGDAAVWKPEVDILLSLKNQLAALTGTPAAGGQGKGKKKK

>XP_002034518.1 GM19865 [Drosophila sechellia]

MIIYTNEGNPLGLQLLMLAKFAKRPVQVQLVNLNDAKYKDLLVLPTLELDNGLRLFSPAAIAKYLLVGEGQQRDEWLEWS

ATLLAPALAHHMAVGHKADANALPVLNALVKKLDDKLKATPYLTGDKPSAADIAIWSLLAPDGTLKGAQNVDNLRDWYGR

VKAIPEVQEVLAEQPLKDLSFNALQQSNRYGGLHHVPLKRLSLADASKLLVDTTPTVADTVTNEEISAAKAAFTYTAPKE

IKEERTVLPKPGERNVLITSALPYVNNVPHLGNIIGCVLSADIYARYSRSAGYNTLFICGTDEYGTATENKALAENLTPR

EICDKYFELHNAIYRWFGIGFDYFGRTTTQEQTDIVQEAFKDVLKAGYIITESVEQLLCQKCDRFLADRFVEGTCPHPGC

GYEDARGDQCDKCGKLVNATELIRPRCKVCNTAPVLRSSDQLFIDLPKAEPQLKDWVDKSEGGWTHNAKVITRAWLKEGL

KPRCITRDLKWGIPVPHEGFEKKVFYVWFDAPFGYVSMTKRYTKEYQQWWQPAKGTDVELFQFMAKDNVPFHSVVWPSVL

LAINKGHTLVSHIMATEYLNYEDGKFSKSRGIGVFGNDAQETGIPADVWRFYLASARPEGQDSSFSWNDLAVRNNSELLN

NLGNFVNRALVFCEKNFSSTVPEVITTQDELVLLALINRELRGYINSMEKAKLRDGVRHLLAISRHGNGYMQSQQPWNLL

KGTDDQKKRASTIIGLCVNIACLLANLLFPYMPTTARTLFGQLNAKQTPLNAEKPFVTLLLPAGHIIGKPSPLFAKLEQS

FIDELKGKYGGAQDTNAVDQSQISAADLEKAVQAQADKVRELKASTKDKAVWQPEVTKLLDLKKQLEEAKKKTAAAAAPT

ATPAPSNGSQSVQALEKAIQEQGDKVRKLKGSTKDKAVWQPEVNILLDLKKQLEAAQKAAKAAPAANAAPAASPAAPAAA

AQVKALEDKIAQQAEKVRTLKATGDAALWKPEVDILLSFKNELAALTGTPAAGGQGKGKKKK

>XP_011209646.1 PREDICTED: methionine--tRNA synthetase, cytoplasmic [Bactrocera dorsalis]

MKIYTNEGNPFGLKLQLLAKFANKPVEVIKVTLNDPASKDFLVLPILELESGLKLFSTDAVARYLFPDEGALRDEWLEWS

TTKLAPALSQHLSVGHRANPDALPVLNLLVRTLDDRLADSAFLTGDKVSAADIAVWSLLAPDGTLKGAQNIEHLKAWYRK

ITALPEVQAVLAETPLAGLTFTSLQNSNRYGGLSHVPVRVHTASESENLLADTTGNLADTVTEDELANAQKSFVHVSLPQ

RVEPRTVLPKAGERNVLITSALPYVNNVPHLGNIIGCVLSADIFARYSRAAGYNTLYICGTDEYGTATENKALAENLTPK

QICDKYFDLHNSIYRWFGIGFDFFGRTSSQQQTEIVQEAFHDVYNNGYIFTESVDQLLCVKCDRFLADRFVEGTCPHPGC

GYEDARGDQCDKCGKLVNATELIRPRCKICNSAPVIRSSDQLFLDLPKIEPQLRVWLEGSEMGWTNNARVITRSWLREGL

KPRCITRDLKWGIPVPLAGFEKKVFYVWFDAPYGYISMTKHYTSEYKQWWIPSKDVKVELFQFMAKDNVPFHSVVFPSSL

LATNKGHTMVTNIMATEYLNYEDGKFSKSRGIGVFGNDAQQTGISADIWRFYLASARPEGQDSSFSWNDLAARNNSDLLN

NLGNFVNRALVFCEKNFNCVVPTMTLTKDELILLALINREVRGYIHSLEKAKLRDGIRHLLSISRHGNGYMQSQQPWVLL

KGTDEQKARAATIIGICANIAALLANLMLPYMPTIARTLFSQLNAKPTPLNPEKPFINILLPTGHNIGKPAPLFTKLEQS

FIEELKKKFGGVQDQSNGSQPSSTTTNTNSATAKPSALPNGTQSVAQLEEAVRLQGEKVRQLKASTKDKAVWQPEVNVLL

DLKKQLTAAQAAPPTAPAVEATPVASDEKVKELEQRITQQGEKVRQLKASGDASVWKPEVEILLQLKKDLATLTGTPQPA

AQGKNKKKK

>EDX07747.1 GD25352 [Drosophila simulans]

MIIYTNEGNPLGLQLLMLAKFAKRPVKVQLVNLNDSKYKDLLVLPTLELDNGLRLFSPAAIAKYLLVGEGQQRDEWLEWS

ATLLAPALAHHMAVGHKADANALPVLNALVKKLDDKLKATPYLAGAKPSAADIAIWSLLAPDGTLKGAQNVDNLRDWYGR

VKALPEVQEVLAEQPLKDLSFNALQQSNRYGGLHHVPLKRLSLADASKLLVDTTPTVADTVTSEEISAAKAAFTYTAPKE

IKEERTVLPKPGERNVLITSALPYVNNVPHLGNIIGCVLSADIYARYSRSAGYNTLLICGTDEYGTATENKALAENLTPR

EICDKYFELHNAIYRWFGIGFDYFGRTTTQEQTDIVQEAFKDVLKAGYIITESVEQLLCQKCDRFLADRFVEGTCPHPGC

GYEDARGDQCDKCGKLVNATELIRPRCKVCNTAPVLRSSDQLFIDLPKAEPQLKDWVDKSEGGWTHNAKVITRAWLKEGL

KPRCITRDLKWGIPVPHEGFEKKVFYVWFDAPFGYVSMTKRYTKEYQQWWQPAKGTDVELFQFMAKDNVPFHSVVWPSVL

LAINKGHTLVSHIMATEYLNYEDGKFSKSRGIGVFGNDAQETGIPADVWRFYLASARPEGQDSSFSWNDLAARNNSELLN

NLGNFVNRALVFCEKNFSSTVPEVITTQDELVLLALINRELRGYINSMEKAKLRDGVRHLLAISRHGNGYMQSQQPWSLL

KGTDDQKKRASTIIGLCVNIACLLANLLFPYMPTTARTLFGQLNAKQTPLNAEKPLVTLLLPAGHIIGKPSPLFAKLEQS

FIDELKGKYGGAQDTNGAAQRQISAADLEKAVQAQADKVRELKASTKDKAVWQPEVTKLLDLKKQLEEAKKKTAAAAAPA

ATPAPSSGSQSVQDLEKAIQEQGDKVRKLKGSTKDKAVWQPEVNILLDLKKQLEAAQKAAKAPPAANAAPAASPAAPAAA

AQVKALEDKIAQQAEKVRTLKATGDAAVWKPEVDILLSLKNELAALTGTPAAGGQGKGKKKK

>XP_018897881.1 PREDICTED: methionine--tRNA synthetase, cytoplasmic isoform X3 [Bemisia tabaci]

MKSWNGKSGEAVLTSLQSSSSCLAVTEASSTSTITAKLAKTSISNAPASETTPVKDVVSEDEISACIEAWEKKSATRPKL

KPHVHPILPKEGENNVLITSALPYVNNVPHLGNIIGCVLSADVFARYCRLRNRNTLFVSGTDEYGTATETKALEEKLTPQ

QICDKYFVIHKDIYEWFNIKFDQFGRTTTQKQTEIAQSIFKLVHNNGFTSTASVEQLLCEKCDRFLADRFVEGTCPKCKY

EDARGDQCDGCGSLINATELINPRCKVCGNTPVIRHSEQLFLELPKLEADLKKWVDNTSSGWSHNARVIAKSWLKDGLKP

RCITRDLKWGVQVPLQGFEKKVFYVWFDAPIGYMSISAAYTDEWEKWWLPSKSTQITLYQFMAKDNVPFHSIMFPAIEMA

TQKNYTKVSHIMATEYLNYEDGKFSKSRGVGVFGNDARDTGISADVFRFYLLYIRPEAQDSSFSWADMAAKTNTELLNNL

GNFTNRALTFCEKFFGSVIPDMKLNKEDFQLLALITRELRQFMAVLDKAKFRDGIKHILCISKYCNQLMQAEKPWVLMKG

SEEEKSRAGTVIGLLSNLACLLSIMIRPYMPDTSKIMAEQLNAPESLYVLTEDVTMLLPPGHKIGHPSPIFSLITPKTVE

ELKARFAGKQNDSAATKPTKTEAKSTDKSKSAPSQPASVPANAETIEKLEAAVAAQGLVVRKLKSETKDKAVWEPEVKKL

LDLKQQLASAKGEQVGNAATKKKK

>XP_002082162.2 uncharacterized protein Dsimw501_GD25352 [Drosophila simulans]

MIIYTNEGNPLGLQLLMLAKFAKRPVKVQLVNLNDSKYKDLLVLPTLELDNGLRLFSPAAIAKYLLVGEGQQRDEWLEWS

ATLLAPALAHHMAVGHKADANALPVLNALVKKLDDKLKATPYLAGAKPSAADIAIWSLLAPDGTLKGAQNVDNLRDWYGR

VKALPEVQEVLAEQPLKDLSFNALQQSNRYGGLHHVPLKRLSLADASKLLVDTTPTVADTVTNEEISAAKAAFTYTAPKE

IKEERTVLPKPGERNVLITSALPYVNNVPHLGNIIGCVLSADIYARYSRSAGYNTLLICGTDEYGTATENKALAENLTPR

EICDKYFELHNAIYRWFGIGFDYFGRTTTQEQTDIVQEAFKDVLKAGYIITESVEQLLCQKCDRFLADRFVEGTCPHPGC

GYEDARGDQCDKCGKLVNATELIRPRCKVCNTAPVLRSSDQLFIDLPKAEPQLKDWVDKSEGGWTHNAKVITRAWLKEGL

KPRCITRDLKWGIPVPHEGFEKKVFYVWFDAPFGYVSMTKRYTKEYQQWWQPAKGTDVELFQFMAKDNVPFHSVVWPSVL

LAINKGHTLVSHIMATEYLNYEDGKFSKSRGIGVFGNDAQETGIPADVWRFYLASARPEGQDSSFSWNDLAARNNSELLN

NLGNFVNRALVFCEKNFSSTVPEVITTQDELVLLALINRELRGYINSMEKAKLRDGVRHLLAISRHGNGYMQSQQPWNLL

KGTDDQKKRASTIIGLCVNIACLLANLLFPYMPTTARTLFGQLNAKQTPLNAEKPFVTLLLPAGHIIGKPSPLFAKLEQS

FIDELKGKYGGAQDTNGAAQRQISAADLEKAVQAQADKVRELKASTKDKAVWQPEVTKLLDLKKQLEEAKKKTAAAAAPV

ATPAPSNGSQSVQDLEKAIQEQGDKVRKLKGSTKDKAVWQPEVNILLDLKKQLEAAQKAAKAAPAANAAPAASPAAPAAA

AQVKALEGKIAQQAEKVRTLKATGDAAVWKPEVDILLSLKNELAALTGTPAAGGQGKGKKKK

>XP_004521981.1 methionine--tRNA synthetase, cytoplasmic [Ceratitis capitata]

MLDKIPNMKIYTNEGNPFGFKLQLLAKFANKPVEVFKVALNDPICKDFLVLPTVELKNGLKLFSTDAVAKYLFPDEGALR

DEWLEWSTVNLAPALAQHLASGHRANPDALPVLNLLVRKLDDRLAESPFLTGDKLTAADIAVWSLLAPDGTLKGAQNIEY

LKAWYRKVTALPEVQAVLAETPLAGLTFTSLLNSNRYGGLSLVPVHVHSVSESEKLLAETAGSLSDTVTEDELNNARNSF

VQVSVPQRIEPRTVLPKVGERNVLITSALPYVNNVPHLGNIIGCVLSADIFARYSRAAGYNTLFICGTDEYGTATENKAL

AENLTPKQICDKYFDLHNSIYRWFGIGFDFFGRTSSSQQTEIVQEAFNDVYKNGYIFTESVEQLMCVKCDRFLADRFVEG

TCPHLGCGYEDARGDQCDKCGKLVNATELIRPRCKTCSSAPVVRSTEQLFLDLPKIEPQLRAWMESSEMGWTNNARVIAR

SWLREGLKARCITRDLKWGIPVPLAGFENKVFYVWFDAPYGYISMTKHYTSEYKQWWIPAKDVKVELFQFMAKDNVPFHS

VVFPSSLLATNKGHTMVTNIMATEYLNYEDGKFSKSRGIGVFGNDAQETGIPADIWRFYLASARPEGQDSSFSWNDLAAR

NNSDLLNNLGNFVNRALVFCEKNFNSIVPTMTLTKDELVLLALINREVRGYINSLEKAKLRDGIRHMLSISRHGNGYMQA

QQPWVLLKGTEEQKARAATIIGVCANIAALLADLMLPYMPTIARTLFAQLNTKQTQLNPDKPFINIFLPTGHKIGKPAPL

FTKLEQSFIDELKKKFGGVQDQSNGNKATPANAPQSNTITAKPAASLPNGTQTVAQLEEAVRLQGEKVRQLKASTKDKAV

WQPEVNVLLDLKKQLAAAQAAPPAAVASAPAVVDNAKAKELEERITQQGEKVRQLKAAGDASVWKPEVEVLLKLKGELAT

LTGAPQGKNKKKK

>XP_022911829.1 methionine--tRNA synthetase, cytoplasmic [Onthophagus taurus]

MIIITNPKNPAVLKILIAGKCAGKDIEVKIMNSQDSLVGTRLLPYLKLDGLECIFLPNPAGKYLFPIKKDDAKLEEWLSW

ESSTLTPLLAQFSSIIKNEKKKGLLVNYLKYLNDNLTQKYLCGEDLLFCDISIWSVLYPLFKDQSLSGEYFGEFKNLLNW

FKEIEQESFVKSILEKYTINPAIEAYNALGEGARYITNNLPELSTQSSLENDDVASKQELDNAKKSFLEGTVRFVEPRND

KNPILPIPGEKNILITSALPYVNNVPHLGNIIGCVLSADVFARFCRLCNYNTLYICGTDEYGTATETKALEEGLTPQQIC

DKYFEIHNSVYRWFNIGFDYFGRTTTPEQTEIVQDLFHAVNKNGFVLTQSVDQLLCEKCNRYLADRFVEGGCPHPGCNYE

DARGDQCDGCGKLINAVELKNPRCKVCKGTPVLRSSNQFFLDLPKLEPLLLHWIEKSSSGWSNNARVIAKSWMKEGLKPR

CITRDLKWGVPVPIDGFRDKVFYVWFDAPIGYISMTQRYCKDYEKWWRPNSDTKVTLYQFMGKDNVPFHSVMFPGMLLGA

NRNFELVSHIMATEYVNYEDGKFSKSRGVGVFGTDAQQTGISSDVWRFYLLYIRPEGQDSNFSWVDLATKCNSELLNNFG

NFVNRALVFTEKFFKSSIPNLKPTDDDFNLLALCNREIKGYISALNQGRLRDGIRYILNISRHGNQYLQSKQPWVLLKGT

DEQKEQAGTVIGLCCNITLMLTSLLFPYMPDTSRTILKQLNAENLQPMINPNKPEIVVYLKPGHKIGKPVPLFTKLESEK

IDELKLKFAGKQQSPQKEIINNDVAKKVENNNSGLQLSNEESIKELTQKIETQGALVRKLKESGAPKADWQPQVEILLKL

KGQLSVMTGTPPAAPGKGKKGKK

>XP_014220044.1 methionine--tRNA synthetase, cytoplasmic [Copidosoma floridanum]

MQASSNSNNPNVLKLIIAAELAGCRLDLDDLKREPKQSFANRLPVLKISSTGDELFSLSSAVQYIAPVGQEHNVDANRWL

EWDATQLLPAIVAYGGSGKLDTSHKPRIWSLLKELNTALKDKEYLIKCKNVTVVDTTIWVTLWSTLVTTEIGEIISKEFS

NLSKWLKSIESLPEITKSLEKVQLERGIQAIVAISAAAWYPLNLYTGAEKLSNAQAPAAHEKNPVDEVKVISEEELNAVK

NNWSSVAYSCVKDSQYPILPKKGEKNILITSALPYVNNVPHLGNIIGCVLSADIFARYCRQRNYNTLYISGTDEYGTATE

AKALEEKMTPQEICNKYFDIHNDIYRWFNIGFDYFGRTSTPEQTEVTQEIFLRVLEQGYVISNSVDQLLCGNCDKFLADR

FVEGTCPGCKYEDARGDQCDGCGHLINAIELINPRCKVCNSRPTIKASTQLFLDLPKIEKRLQEWAPTVTNGWSAVARSV

MKPWLRDGLKQRCITRDLKWGIPVPLDGYRDKVFYVWFDAPIGYISITKKYTKEYEQWWKPKDVDVDLYQFMAKDNVPFH

SIMFPATLFAANQGHTIIKYLMATEYLNYEDTKFSKSRGVGVFGSDARETGIPADVWRFYLAYIRPENMDSNFNWVDLAT

KNNSELLNNFGNFVNRALMFVEKNFDSKVPEINLQADDLRVLALIQRELTSYVTLLEQAKLRDGLRHVLAISKHGNQYMQ

YQQPWVKVKGDEEEKKKGATVIGVCCNIACLLSALIAPYMPNTARELRAQLGLTKQNYGYIPEVVTLMLSTGHSIGKPSP

LFTKIEDSRVEELRKSYAGRQEPTPPPKGTSAPHADVATLEASIAKQGDIVRQLKATEEKSVWLPQVEILKKLKQKLADL

QSKSKPEISPSIAQTASPNLTTPATQHNSVPADVAELESAIAKQGNLVRELKAQGDKSVWEPQVKVLLQMKQKLSELTGS

TTAAPSHKKSKKKK

>XP_022226071.1 methionine--tRNA synthetase, cytoplasmic [Drosophila obscura]

MIIYTNEGNPLGLQLLMLAKFAKRSVQVQLVNLNDAKFKDLLILPTLELDDGLRLFSSAAIAKYLLVDEGQLRDEWLEWA

STLLAPALAHHMAVGHRADPNSFPVLNALVKKLDDCLKGSPYLAGDKLSAADIAVWALLAPDGTLKGAQNLESLHGWYNR

VKALPEVQQVLAEQPLKELTFNALQQSNRYGGLHHVPLKRLSLADAGKLLAEATSTVADTVTAEELAAAGAAFTYTAPKD

VQQQRIVLPKAGERNVLITSALPYVNNVPHLGNIIGCVLSADIYARYSRSAGYNTLLICGTDEYGTATENKALAENLTPR

EICDKYFELHNSIYRWFGIGFDYFGRTTTQEQTDIVQEAFRDVNSAGYILTESVEQLLCQKCDRFLADRFVEGTCPHPGC

GYADARGDQCDKCGKLVNATELIRPRCKVCNTAPILRSSDQLFIDLPKAEPRLREWVDKAENGWTNNAKVITRAWLREGL

KPRCITRDLKWGIPVPHSGFEKKVFYVWFDAPFGYVSMTKRYTKEYQQWWQPAKGTDVELFQFMAKDNVPFHSVVWPSVL

LAINKGHTLVSHIMATEYLNYEDGKFSKSRGIGVFGNDAQDTGIPADVWRFYLASARPEGQDSSFSWNDLAARNNSELLN

NLGNFVNRALVFCEKNFNSTVPEVVATEEELILLALVNRELRGYINSLEKAKLRDGIRHLLAISRHGNGYMQTQQPWVLL

KGSDDQKQRAATIIALCANIACLLANLLFPYMPSTARTLFAQLNANQTPLNAEKPLATLLLPAGHKIGKPAPLFAKLEQS

FIDELKAKYGGSQDTRGAAAVQSQPSTADLEQAVQAQADKVRQLKTSTKDKSVWQPEVSKLLDLKKQLEEAKKTAASAPA

SAPAPSIGAQSVQELEKAVQAQGEKVRNLKGSTKDKSAWQPEVNVLLDLKKQLEAAQKVAKVTPAAPAAAAATPVASASA

DAGQVKALEDKLAQQAEKVRTLKSHGDASVWKPEVDILLALKNQLAALTGAPAAGGQGKGKKKK

>KXJ68752.1 hypothetical protein RP20_CCG001876 [Aedes albopictus]

MIIYTNAGNPFGLKLLICAKFAKKDVQVKTIALNDTELKDMKHLPILQLESGVQLFSTDVAVKYLLQGETPVIQRDEWLE

WSTTRLAPALTHNMAVGSRQDPNTKPILNSLVKFLDDCLSRNTFLTGDKLTSADISVWSLLAPDGTLKGAQNIDNLLRWY

RAINAMPEVKAALEVLPLKELSFASLKHSNRFEGLHHIVLDPEIIDFEGQVLVDTSDNIAATVQKEEIQAARDSFVAVQE

RSVPDEKIVLPKVGQKNNLITSALPYVNNVPHLGNIIGCVLSADVFARYSRLCGYNTLYICGTDEYGTATETKALAEKLT

PKQICDKYFEIHNSIYRWFGIGFDYFGRTTTAEQTQIVQEMFKDLYAGGFIETRAVDQLLCQKCDRYLADRFVEGTCPYP

GCGYEDARGDQCDACGKLINAIELVRPRCKVCNTSPIIRQSSQFFLDLPKIEPRLRDWVDKAEHGWTHNARVITRAWLKE

GLKSRCITRDLKWGIPVPLEGYEDKVFYVWFDAPIGYISMTSRYSKEWKQWWQPSPETKVDLYQFMAKDNVPFHSVMFPA

SLIGIDKGYTLVSHIMATEYLNYEDGKFSKSRGIGVFGNDAHDTGIPADVWRFYLCSSRPEGQDSSFSWNDLAARNNNEL

LKNVGNFVNRALVFCEKCFDSTIPPLVLNDEDYTLLALINRELKGYVHQMEKARLRDGIRHLLAISRYGNQLMQAEEPWV

LVKGTDDQKTRAGTVIGLCCNLACLLATLIFPFMPSTARNLYTQLNVQSCHLDADKPQMRIFLPSGHKIGKPAVLFNKID

QAKIDELKKKYAGAQQQPESKPAAAVKPASNKLFTSLVEAQKAVDEQAAKVRKIKSTGADKSVWQPEVNILLELKKQLQT

LSQPRTSESTAATPATPTSAPTPETLQSLEAEIAKQGEKVRTLKAGGSEKAVWQPEVTVLLALKEKLAKLTGAPQAAAPA

SGKKKNKK

>AAM50099.1 AT05114p [Drosophila melanogaster]

MIIYTNDGNPLGLQLLMLAKFAKQPVQVQLVNLNDAKYKDLLVLPTLELDNGLRLFSPAAIAKYLFVGEGQQRDEWLEWS

ATLLAPALAHHMAVGHKADANALPVLNALVKKLDDKLKATPYLAGDKPSAADIAIWSLLAPDGTLKGAQNVDNLRDWYGR

VKALPEVQEVLAEQPLKDLSFNALQQSNRYGGLHHVPLKRLSLADASKLLVDTTPTVADTVTNEEISAAKATFTYTAPKE

IKEERTVLPKPGERNVLITSALPYVNNVPHLGNIIGCVLSADIYARYSRSAGYNTLLICGTDEYGTATENKALAENLTPR

EICDKYFELHNAIYRWFGIGFDYFGRTTTQEQTDIVQEAFKDVLKAGYIITESVEQLLCQKCDRFLADRFVEGTCPHPGC

GYEDARGDQCDKCGKLVNATELIRPRCKVCNSAPVLRSSDQLFIDLPKAEPQLKEWVDKSEGGWTHNAKVITRAWLKEGL

KPRCITRDLKWGIPVPHEGFEKKVFYVWFDAPFGYVSMTKRYTKEYQQWWQPAKGTDVELFQFMAKDNVPFHSVVWPSVL

LAINKGHTLVSHIMATEYLNYEDGKFSKSRGIGVFGNDAQETGIPADVWRFYLASARPEGQDSSFSWNDLAARNNSELLN

NLGNFVNRALVFCEKNFSSTVPGVITTQDELVLLALINRELRGYINSMEKAKLRDGVRHLLAISRHGNGYMQSQQPWVLL

KGTDDQKTRASTIIGLCVNIACLLANLLFPYMPTTARTLFGQLNAKQTPLNAEKPLVTLLLPAGHQIGKPAPLFAKLEQS

FIDELKGKYGGAQATNDAAQAVQAQADKVRELKASTKDKAIWQPEVTKLLDLKKQLEEAKKKTATAAAPAATPAPSNGSQ

SVQDLEKAIQEQGDKVRKLKGSTKDKTVWQPEVNILLDLKKQLEAVQKAAKAAPAANAAPAASPAATADAAKVKALEDKI

AQQAEKVRTLKATGDAAVWKPEVDILLSLKNELAALTGTPVAGGQGKGKKKK

>XP_003702412.1 PREDICTED: methionine--tRNA synthetase, cytoplasmic [Megachile rotundata]

MIISTNEDNPNALKLIIAAKIAQQSVTVKTVQPKASFLGRLPTVELPSGLVLFSTNAALQLLVPPSKDSNILINKWLEWD

ISKLQPAVVLYGNTGTYKTTSKSCLWSLLGELDKELKEKKYLIKDQNDLSLADTCIWVTVWSTVLVTEIEKELSKEYVCL

KNWLINIELLPIIQDSIKEYKFERGVKAILSIQAASWFPVISSLNSQSTKPLPSDVSPTKEKEMEAVSQEEIHNIASNWS

SKSYPDMKNSPYPVLPKKGERNILITSALPYVNNVPHLGNIIGCVLSADIFARYCRQRNYNTLFICGTDEYGTATEAKAL

EEKTTPQAICDKFFDIHNDIYRWFSIGFDYFGRTTTPEQTEIVQSFFLKIKSQGYILSETVDQLLCESCNRFLADRFVEG

ICPRCKYEDARGDQCDGCGHLVNATDLISPRCKVCNNRPVVKKSEQFFLDLPKLESKLKEWSPTVEKGWSSVARVVAKPW

LRDGLKPRCITRDLKWGIPVPVKGFENKVFYVWFDAPFGYISITKRYTKEYEQWWKPKDVAVDLYQFMAKDNVPFHAIMF

PAYLLAANDGYILMKHLMATEYLNYEDTKFSKSRGIGVFGNDARETGIPADVWRFYLAYVRPETQDSNFNWIDLACKNNN

ELLNNFGNFVNRALVFAEKYFESYIPPMTLQEEDLILLVLAQRELSSYIHALEQAKLRDGLKHVLAISKHGNQYMQFQEP

WVKIKGTDSDKKRAGTIIGICCNLACLLSALLAPFMPNAARELRTQLGLDASNYGYIPDIITNILPAGHKIGKPSPLFKK

IEDKDVEALRQKYAGKQESQNNTQKNNNNDVKSLEAAIAEQGNKVRELKAKHDKTIWQPEVQILLNLKKKLTNLISGEKE

SKSTESQPKNKKPEVVFVPEQNGDALTDVASLESAIAKQGNLVRELKAKEQKSVWQPEVEKLLTFKKQLADLTGTSPAPT

DKKSKKKK

>XP_012242290.1 methionine--tRNA synthetase, cytoplasmic isoform X1 [Bombus impatiens]

MKMNISTNEGNPNTLKLIIAAKVAQHAVTVKTVQPKVGFLSRLPSIELSSGLVLFSTNAAMQLIVPSSSQESKLLNNKWL

EWEISRLQPAIAFYGNTGIYKTTQKSCLWSLLRELNNALEDKQYLIKDNNDLSLADICIWVTLWSTVMVTEIGNELNKEY

LSIKNWISSIELLPVIQDSIEEYKFERGSKAIFSIQAVSWFPVNTNSNPKPPKPADLQLSPTKEKEMEAVNQEEIQNISS

NWSSKSYPDVKNFPYPVLPKKGEKNILITSALPYVNNVPHLGNIIGCVLSADIFARYCRQRNYNTLFISGTDEYGTATEA

KALEEKTTPQNICDKFFDIHNDVYRWFNIGFDYFGRTTTPEQTEIVQSFFLKIKSEGYVLSETVDQLLCESCDRFLADRF

VEGTCPRCKYEDARGDQCDGCGHLVNATDLISPRCKVCSNRPIVKKSQQFFLDLPKLENKLKEWSSTVEKGWSSVAKVVA

KPWLRDGLKPRCITRDLKWGIPVPIKGFENKVFYVWFDAPFGYISITKRYTKEYQQWWKPTDVQVDLYQFMAKDNVPFHA

IMFPAYLLAANEGYTLMKHLMATEYLNYEDTKFSKSRGIGVFGTDARDTGIPADVWRFYLAYVRPETQDSNFNWVDLATK

NNSELLNNFGNFVNRALVFAEKYFDSKIPPIELQEDDLILLALAQRELSSYIHALEQAKLRDGLKNVLAISKHGNQYMQF

QEPWVKIKGTDNDKKRAGTIIGICCNLACLLSALLAPFMPCTARELRSQLGLQTNNYGYIPDVITSILPTGHKISKPSPL

FKKIEDKDVEILRRKYAGKQETTSSSSGDVKSLDSAITELDNKVKKLKAKFDKSGWPSSQIQIFSALKKKLPDLINEKNK

SPESKSKKPETVSVPEQNGDVAIDVASLEAAIAKQGDLVRQLKAKEERSVWQPEVEKLLKLKKQLGDLTGTAAAPTDKKS

KKKK

>XP_023160349.1 methionine--tRNA synthetase, cytoplasmic [Drosophila hydei]

MIIYTNEGNPLGLQLLVLAKFAKKTAQVQLVNLNDAKHKDLLVLPTLELDNGLRLFSGAAIAKYLLGDEGQRRDEWLEWS

TTLLAPALEHHMAVGHRADPNALPVLNGLVKKLNDCLSTSPFLAGDKLSAADLSIWCLLAPDGTLKGAQNVDQLRSWYDK

IKAMPEVQQGLKELPIKDLTFNALQQSNRYGGLQHVQLKRSSSVDAGKLLAEAASTVADTVYDEEINAARAAFIYTKAEE

LSQPRTVLPKPGQRNVLITSALPYVNNVPHLGNIIGCVLSADIFARYSRAAGYNTLLICGTDEYGTATENKALAENMTPR

EICDKYFELHNSIYRWFGIGFDYFGRTTTQEQTDIVQEAFKDVYNAGYILTESVEQLLCQKCDRFLADRFVEGTCPHPGC

GYEDARGDQCDKCGKLVNATELVRPRCKVCNTAPVLRSSDQLFLDLPKSEPQLREWVDRAEHGWTNNARVITRAWLREGL

KPRCITRDLKWGIPVPHKNFEKKVFYVWFDAPFGYVSITKRYTKEYLQWWQPAAGTDVELFQFMAKDNVPFHSVVWPSVL

LAINKGNTLVSHIMATEYLNYEDGKFSKSRGIGVFGNDAQETGIPADVWRFYLASARPEGQDSSFSWNDLAARNNSELLN

NLGNFVNRALVFCEKNFDSTVPQIVLNQDDLVLLALINRELRGYISSLEKARLRDGIRHLLAVSRHGNGYMQTQQPWVLL

KGSNDQKARAATIIGLCTNIACLLANLLFPYMPTTARTMFAQLNTKQTPLDANKPLATMLLPTGHKIGKPSPLFTKLEQS

FIDELKGKYGGTQESQAAAAQEQSSSADLEAAVQAQANKVRELKASTKDKSVWQPEVDKLLDLKNQLKKAASAPAPAAAA

PAPTGQSVEALEQAVQAQGEKVRQLKSTTKDKAVWQPEVNKLLDLKKQFEAAKGAPAAAPATPTAATDVAAVKALEDKIT

QQGEKVRNLKATGDASVWKPEVETLLNLKKELATLTGAPVAATQTKNKKKK

>XP_001987835.1 GH19733 [Drosophila grimshawi]

MIIYTNEGNPLGLQLLMLAKFAKRTAQVQLVNLNDAKYKDLLVLPTMELDNGLRLFSGAAIAKYFLGDESQQRDEWLEWS

TTLLAPALAHHMAVGHRADPNALPVLNNLVKKLDDHLKNSSYLAGDKVTAADIAVWSLLAPDGTLKGAQNVDNLRSWYGK

IKALSEVQQVLGEQSLKDLSFNALQQANRYGGLHHVPLKRSISTDAGKLLAETASTVADTITDEEISAARAAFIYNKPQE

FAEPRTVLPKAGERNVLITSALPYVNNVPHLGNIIGCVLSADIFARYSRAAGYNTLLICGTDEYGTATENKALAENMTPR

EICDKYFELHNSIYRWFGIGFDYFGRTTTQEQTDIVQEAFKDVYNAGYILTESVEQLLCQKCDRFLADRFVEGTCPHPGC

GYEDARGDQCDKCGKLVNATELVRPRCKVCNTAPVLRSSEQLFLDLPKAEPQLREWVDRVENGWTHNARVITRAWLREGL

KPRCITRDLKWGIPVPHKNFEKKVFYVWFDAPFGYVSMTKRYTKDYEQWWRPDANTDVQLFQFMAKDNVPFHSVVWPSVL

LAVNKGNTLVSHIMATEYLNYEDGKFSKSRGIGVFGNDAQETGIPADVWRFYLASARPEGQDSSFSWNDLAARNNSELLN

NLGNFVNRALVFCEKNFSSTVPHVVLAQDEVVLLALINRELRGYINSMEKAKLRDGIRHLLAISRHGNGYMQTQQPWVLL

KGNADQKTHAATIIGLCINIACLLANLLFPYMPSTARQLFAQLNAKQTPLKADKPLTTLLLPAGHKIGKPSPLFSKLEQQ

FINELKAKYGGVQDTQTATSQQSNVADLEAAVQAQADQVRQLKASTKDKTVWQPEVSKLLELKKQLEAAQKTATTATPAA

AVAAAIPQKSTQSSSVQDLEKMVQAQGEKVRQLKSSTKDKAVWQPEVNVLLDLKKQLEAAKAEPPAPRAAATSTQAAPDA

ATQVKDLEDKITKQGEKVRTLKASGDATIWKPEVDILLNLKKELSSLTGAPVAGAQGKNKKKK

>XP_012281975.1 methionine--tRNA synthetase, cytoplasmic [Orussus abietinus]

MIVLTNEGNPNTLKLIIAAKLGQQSVTVKTVTPKDTALGRLPILNLPSGVSLFSTSAAVQLLVPSTEEFSILANRWLEWD

ISQLQPAIVAYGGTGILDTSHKPRISSLLKELNIVLKATKYLIKNKDDLTVADISVWVTLWSTLSVLEVSKELFKEFPYV

DKWIKSIENLPIVRESIQEYKLERGLKAMAAIQAVSWFPVNSYSVVSSPKSSPNNIDANKEKEIDSVSQDEISTVKNNWM

TQSYPEVNKASYPVLPKKGEKNVLITSALPYVNNVPHLGNIIGCVLSADIFARYCRQRNYNTLYISGTDEYGTATEAKAL

EEKMTPQEICDKYFSIHNDVYRWFGIGFDYFGRTTTTEQTEIVQSFFLRIKSQGYIITESVDQLLCESCQRFLADRFVEG

TCPGCKYEDARGDQCDGCGHLVNATELINPRCKVCNSSPIVKPSTQFFLDLPRLEEKLKEWAVTVENEWSTVARVVAKPW

LRDGLKPRCITRDLKWGIPVPVEGFRDKVFYVWFDAPLGYISITKRYTKEYEQWWKPQASEVSLYQFMAKDNVPFHAIMF

PAALLAADQGHTLVRHIMAIEYLNYEDTKFSKSRGIGVFGTDARDTGIPADVWRFYLAYIRPENQDSNFNWVDLATKNNS

ELLNNLGNFVNRALTFVEKNFASKVPPMELQEDDLTVLALAQRELTLYIIALEQAKLRDGIKHMLAISKHGNQYMQSQEP

WAKIKGTDDDKKKAGTVVGICCNLACLLSALLAPYMPNTARKLRSQLGLSSEDYGFIPESIATMLPAGHLIGKPSPLFSK

IEDQKIEELRRKYGENQEASTQGNSHENGAVYKDVASLEAAITKQGQLVRELKSKQEKSVWQPQVDVLLQLKKQLAEMKS

NAGKTEAGRSVNDKGQSAVTPQQNGTAADIAALEETVAKQGNLVRELKAKGDKSVWQPQVDILLKFKKQLADLTGTVSSI

SADKKSKKKK

>XP_012242291.1 methionine--tRNA synthetase, cytoplasmic isoform X2 [Bombus impatiens]

MKMNISTNEGNPNTLKLIIAAKVAQHAVTVKTVQPKVGFLSRLPSIELSSGLVLFSTNAAMQLIVPSSSQESKLLNNKWL

EWEISRLQPAIAFYGNTGIYKTTQKSCLWSLLRELNNALEDKQYLIKDNNDLSLADICIWVTLWSTVMVTEIGNELNKEY

LSIKNWISSIELLPVIQDSIEEYKFERGSKAIFSIQAVSWFPVNTNSNPKPPKPADLSPTKEKEMEAVNQEEIQNISSNW

SSKSYPDVKNFPYPVLPKKGEKNILITSALPYVNNVPHLGNIIGCVLSADIFARYCRQRNYNTLFISGTDEYGTATEAKA

LEEKTTPQNICDKFFDIHNDVYRWFNIGFDYFGRTTTPEQTEIVQSFFLKIKSEGYVLSETVDQLLCESCDRFLADRFVE

GTCPRCKYEDARGDQCDGCGHLVNATDLISPRCKVCSNRPIVKKSQQFFLDLPKLENKLKEWSSTVEKGWSSVAKVVAKP

WLRDGLKPRCITRDLKWGIPVPIKGFENKVFYVWFDAPFGYISITKRYTKEYQQWWKPTDVQVDLYQFMAKDNVPFHAIM

FPAYLLAANEGYTLMKHLMATEYLNYEDTKFSKSRGIGVFGTDARDTGIPADVWRFYLAYVRPETQDSNFNWVDLATKNN

SELLNNFGNFVNRALVFAEKYFDSKIPPIELQEDDLILLALAQRELSSYIHALEQAKLRDGLKNVLAISKHGNQYMQFQE

PWVKIKGTDNDKKRAGTIIGICCNLACLLSALLAPFMPCTARELRSQLGLQTNNYGYIPDVITSILPTGHKISKPSPLFK

KIEDKDVEILRRKYAGKQETTSSSSGDVKSLDSAITELDNKVKKLKAKFDKSGWPSSQIQIFSALKKKLPDLINEKNKSP

ESKSKKPETVSVPEQNGDVAIDVASLEAAIAKQGDLVRQLKAKEERSVWQPEVEKLLKLKKQLGDLTGTAAAPTDKKSKK

KK

>PFX24554.1 Methionine--tRNA synthetase, cytoplasmic [Stylophora pistillata]

MHNQTEASLPVLETDGGKLLHSPNIICRYLLDLGGSSDVAPEPYEKAIVDQWVEWESTSLQPVIFNHLVRSIGQGRSREH

SGLVDDVVNLLRHADEGLTSKKYFSGDALLAMLDAHEETTKGSPSSQQEQERDSATEERILAKVLSREEIDLAFEHWNSG

SKNRPKTRRLVDPILPIEGERNILITSALPYVNNVPHLGNIIGCVLSADVFARFCRLRQYNTLYICGTDEYGTATETKAL

EEGLTPQQICDKYFKIHKEVYEWFQIKFDFFGRTTTQQQTEIAQDIFWRLHKQDHLVEDTLEQLQCVECKRFLADRFVEG

TCPFCNYEGARGDQCDSCGKLINAIELKLELSLQTWIDKSSSEGTWTLNARQITQSWIKEGLKPRCITRDLKWGTPVPLE

GYTDKVFYVWFDAPIGYLSITANYTNEWEKWWKNPQQVSLHQFMAKDNVPFHTVVFPCTLIGADDNYSLLNSISATEYLN

YEDDKFSKSRGVGVFGDHAADTGIAADVWRFYLLFVRPESQDSAFSWSDFVSRNNTELLNNFGNFINRALMFVQNNFDSC

IPAMALTDEDKKFIALINKELKTYIDNLEKMRLREGLKDVLNISKLGNQYIHSNQPWVLFKGSESERSRAGTVIGITANL

SWLLSVLVHPYMPGVSEEIQRQLQVSDEGNVVLDNFIPYLETGHKIGTPKPLFHKIDASVADQFRAKYGGYKQKQPAADK

KTKEASKG

>XP_017077122.1 PREDICTED: methionine--tRNA synthetase, cytoplasmic [Drosophila eugracilis]

MIIYTNEGNPLGLQLLMLAKFAKRPVQVQLVNLNDAKYRDLLILPTLELDNGLRLFSPAAIAKYLLVDEGQRRDEWLEWS

ATLLAPALSHHMAVGHKADANALPVLNALVKKLNDSLKSSPFLAGDKLTAADIAVWSLLAPEGTLKGAQNVDSLRDWYGR

VKSLPEVEEVLAEQPLKNLSFNALQQSNRYGGLHHVPLKRLSLADAGKLLVDTTPTVADTVTDEEISAARAAFVYTAPKE

IKEERTVLPKAGERNVLVTSALPYVNNVPHLGNIIGCVLSADIYARYSRSAGYNTLLISGTDEYGTATENKALAENLTPR

EICDKYFELHNAIYRWFGIGFDYFGRTTAQEQTDIVQEAFKDVLKNGYIITESVEQLLCQKCDRFLADRFVEGTCPHPGC

GYEDARGDQCDKCGKLVNATELIRPRCKVCNSAPVLRSSDQLFIDLPKAEPQLKEWVDKSEGGWTHNAKVITRAWLKEGL

KPRCITRDLKWGIPVPHEGFENKVFYVWFDAPFGYVSMTKHYTKEYQQWWQPDKGTEVELFQFMAKDNVPFHSVVWPSVL

LAINKGHTLVSHIMATEYLNYEDGKFSKSRGIGVFGNDAQETGIPADVWRFYLASARPEGQDSSFSWNDLAARNNSELLN

NLGNFVNRALVFCEKNFDSTVPEVVITQDELVLLALINRELRGYINSLEKAKLRDGIRHLLAISRHGNGYMQIQQPWVLL

KGTEDQKKRASTIIGLCVNIACLLANLLFPFMPTTARTLFGQLNAKQTPLNAEKPLATLLLTAGHKLGKPVPLFAKLEQS

FIDELKGKYGGAQNTKAAAQSQTSSADMEQAVQAQADKVRELKASTKDKTVWQPEVTKLLELKKKLEEAKKTATNVTPIA

APAASNGSQSVKDLEKAIQEQGDKVRKLKGSTKDKTIWQPEVNVLLDLKKQLEAAQKAAKTAPAAGAPASSSTSPADASQ

VKALEDKIAQQAEKVRTLKASGDASVWKPEVDILLSLKNELASLTGQPAAGGQGKSKKKK

>XP_020718979.1 LOW QUALITY PROTEIN: methionine--tRNA synthetase, cytoplasmic [Bombus terrestris]

MNISTNEGNPNTLKLVIAAKVAQHAVTVKTVQPKVGFLSRLPSIELSSGLVLFSTNAAMQLIVPSSSQESKILNNKWLEW

EISQLQPAIAFYGNTGIYKTTQKSCLWSLLRELNNALEDKQYLIKDNNDLSLADICIWVTLWSTVMATEIGNELNKEYLS

IKNWISSIELLPVIQDSIEEYKFERGSKAIFSIQAVSWFPVNANSNPKPSKPADLQLSPTKEKEMEAVSQEEIQNISSNW

SSRSYPDVKNFPYPVLPKKGEKNILITSALPYVNNVPHLGNIIGCVLSADIFARYCRQRNYNTLFISGTDEYGTATEAKA

LEEKTTPQNICDKFFDIHNDVYRWFNIGFDYFGRTTTPEQTEIVQSFFLKIKSEGYVLSETVDQLLCESCDRFLADRFVE

GTCPRCKYEDARGDQCDGCGHLVNATDLVSPRCKVCSNRPIVKKSQQFFLDLPKLENKLKEWSSTVEKGWSSVAKVVAKP

WLRDGLKPRCITRDLKWGIPVPVKGFENKVFYVWFDAPFGYISITKRYTKEYRQWWKPTDVQVDLYQFMAKDNVPFHAIM

FPAYLLAANEGYTLMKHLMATEYLNYEDTKFSKSRGIGVFGTDARDTGIPADVWRFYLAYVRPETQDSNFNWVDLATKNN

SELLNNFGNFVNRALVFAEKYFDSKIPPIELQEDDLILLVLAQRELSSYIHALEQAKLRDGLKNVLAISKHGNQYMQFQE

PWVKIKGTDNDKKRAGTIIGICCNLACLLSALLAPFMPCTARELRSQLGLQTSNYGYIPDVITSILPVGHKIGKPSPLFK

KIEDKDVEILRKKYAGKQETTSSSGHGDVKSLDGAIAELDNKVKXLKAKFDKSGWPSSQIQIFSALKKLPDLINEKNKSP

ESQSKKPETVLVPEQNGDVAMDVASLEAAIAKQGDLVRQLKAKEERSVWQPEVEKLLKLKKQLGDLTGTAAAPTDKKSKK

KK

>XP_014475976.1 PREDICTED: methionine--tRNA synthetase, cytoplasmic isoform X1 [Dinoponera quadriceps]

MVTMNVSTNQGNPNALKLIIAAKLAQELVTVKIVEPKDAFLGRLPTLTTPSGLSLFSVSSALRLLLPVAAELEILNNKWL

EWEISQLQPAILSYASGKTSSKDLLSLFKDLNSELKNKQYLIETSSYLSPADISIWVSLWSTLLFAECKDISAQLLNIVH

WLTNMEQQPVIQESIKEFDLVRGAKAIASIEAASWFPVNAVTNAQVRQESLLSDISPTKEKEMETVSQEEVRSVMNIWFT

RRYPEVKESSYPILPKKGERNVLVTSALPYVNNVPHLGNIIGCVLSADIFARYCRQRNYNTLYISGTDEYGTATEAKALQ

EKTTPQIICDKFFDIHNDVYRWFGIGFDYFGRTTTPEQTEIVQSFFLKIKSQGYILNESVEQLHCEFCDRFLADRFVEGT

CPRCKYEDARGDQCDGCSHLVNAVELIEPRCKMCNTKPVVRNSVQFFLDLPKLEDKLKQWSATVNQGWSSVAKVVSKSWL

RDGLKPRCITRDLKWGIPVPVNGFENKVFYVWFDAPFGYVSITKRYTKEYEKWWKPQDTKVDLCQFMAKDNVPFHAIMFP

ACLLAANQNNTMIKYLMATEYLNYEDKKFSKSRGIGVFGTDARDTGIPADVWRFYLAYIRPETQDSNFNWIDLATKNNSE

LLNNLGNFVNRALMFSEKFFDSNVPPMKLEEADWIVLALAQRELSSYISAMEQAKLRDGLKYILAISKHGNQYMQFQQPW

VKIKGTDDDKNRAGTVVAICCNLACLLSALLAPFMPTTAKQLRSQLGLSNKSYGYIPETITNMLPTWHKIGKPSPLFTKI

EEQQVEMLRKKYAGQQEINGRTAATNVASDESVDSLETAIMVQGNLVRELKAKHEKSVWQPQVEILLSLKKKLADLKVSA

PKAPEKNSPVKNKKVETNVNKQHARLYEQKISDLKALHSAINERRLKESKKDAVGNAATDVATLESAIAKQSNLVRELKA

KGDANVWEPQVEMLLKLKEQLMGLTEATPVAVDKKSKKKK

>XP_011328992.1 PREDICTED: methionine--tRNA synthetase, cytoplasmic [Ooceraea biroi]

MIISTNEGNPNALKLIIAAKLAREPFTVQIVNPANAFLGRLPTLKASAELSLFSVSSALRFLLSVPKESEILNDRWLEWE

ITQLQPAILSHANMRTPKNLQKSDLWSLLKDLNSELKDKQYLIKEHSHLSPADICIWVSLWSTMLLEDVKDNVAKNLPNL

YNWLANIEKQSIIQESIKEFNLERGAKAVASIEAASWFPVNAVSNSRVSESVSSDVNPKEKDAEAISEEELRNVAHSWTT

GQYLEVKESSYPILPKKSERNVLITSALPYVNNVPHLGNIIGCVLSADIFARYCRQRNYNTLYISGTDEYGTATEAKALQ

EKTTPQAICDKFFDIHNDVYRWFGIGFDYFGRTSTPEQTEIVQAFFLKIKSRGYVRSETVEQLHCELCDRFLADRFVEGT

CPGCKYEDARGDQCDGCGHLVNAIELINPRCKMCNSTPVVKHSVQFFLDLPKIEEKLKAYNATVEKGWSSVARVVSKSWL

RDGLKPRCITRDLKWGIPVPVEGFENKVFYVWFDAPFGYISITKRYTKEYQKWWQPPQDVKVDLCQFMAKDNVPFHTVMF

PACLLAVNQNYTLMKYLMATEYLNYEDKKFSKSRGIGVFGTDARDTGISADVWRFYLAYIRPETQDSNFNWVDLATKNNS

ELLNNLGNFVNRALVFAEKFFNSSIPPIELAEEADWSVLALAQRELSSYINAMEHAKLRDGLKHILAISKHGNQYMQFQQ

PWVKIKGTEDDKKRAGTVVAVCCNLACLLSALLAPFMPTTSKQLRSQLGLSNKSYGYIPETITNILPTWHKIGKPSPLFT

KIEDQSVEVLRKKYAGQQEVNGEVSTTNKAPDDGIASLETAITKQGTLVRELKAKHDKSVWQPQVAILLDLKKKLADLKG

NVNVPEKKLPAKKTPAKNKKAEPTQVLEQNGDALTDVAALESAIAKQGNLVRELKAKEEASVWKPQVEILLKLKQRLMGL

TGTTTPNATDKKSKKKK

>KXJ70857.1 hypothetical protein RP20_CCG022269 [Aedes albopictus]

MIIYTNAGNPFGLKLLICAKFAKKDVQVKTIALNDTELKDMKHLPILQLESGVQLFSTDVAVKYLLQGETPVIQRDEWLE

WSTTRLAPALTHNMAVGSRQDPNTKPILNSLVKFLDDCLSRNTFLTGDKLTSADISVWSLLAPDGTLKGAQNIDNLLRWY

RAINAMPEVKAALEVLPLKELSFASLKYSNRFEGLHHIVLDPEIIDFEGQVLVDTSDNIAATVQKEEIQAARDSFVAVQE

RNVPDEKIVLPKVGQKNNLITSALPYVNNVPHLGNIIGCVLSADVFARYSRLCGYNTLYICGTDEYGTATETKALAEKLT

PKQICDKYFEIHNSIYRWFGIGFDYFGRTTTAEQTQIVQEMFKDLYAGGFIETRAVDQLLCQKCDRYLADRFVEGTCPYP

GCGYEDARGDQCDACGKLINAIELVRPRCKVCNTSPIIRQSSQFFLDLPKIEPRLRDWVDKAEHGWTHNARVITRAWLKE

GLKSRCITRDLKWGIPVPLEGYEDKVFYVWFDAPIGYISMTSRYSKEWKQWWQPSPETKVDLYQFMAKDNVPFHSVMFPA

SLIGIDKGYTLVSHIMATEYLNYEDGKFSKSRGIGVFGNDAHDTGIPADVWRFYLCSSRPEGQDSSFSWNDLAARNNNEL

LKNVGNFVNRALVFCEKCFDSTIPPLVLNDEDYTLLALINRELKGYVHQMEKARLRDGIRHLLAISRYGNQLMQAEEPWV

LVKGTDDQKTRAGTVIGLCCNLACLLATLIFPFMPSTARNLYTQLNVQSCHLDADKPQMRIFLPSGHKIGKPAVLFNKID

QAKIDELKKKYAGAQQQPESKPAAAAKPASNKLFTSLVEAQKAVDEQAAKVRKVKSTGADKSVWQPEVNILLELKKQLQT

LSQPRTSESTAATPATPTSAPAPETLQSLEAEIAKQGEKVRTLKAGGSEKAVWQPEVTVLLALKEKLAKLTGAPQAAAPA

SGKKKNKK

>XP_015367374.1 PREDICTED: methionine--tRNA synthetase, cytoplasmic [Diuraphis noxia]

MSLSTVEVGEFELLNAKNSWISKSCLKDINKIECQPVLPVKGEKNVLVTSALPYVNNVPHLGNIIGCVLSADVFARYCRL

RGWNTLYISGTDEYGTATETKALEEKLTPQQICDKFFKIHNEIYKWFNISFDYFGRTTSNKQTEIVQEIFKEVHSNGYTS

TASMEQLLCENCDRFLADRFVEGTCPLCNYEDARGDQCDGCGHLINATELLKPRCKVCQKTPVIRSSQQLFLNLPKVEAK

LNDWIDKSGDDWSYNAKVITKSWLKDGLKERCITRDLKWGIPVPLEDFKSKVFYVWFDAPIGYMSMSAVYTPEWRKWWQP

EDDTEVTYYQFMAKDNVPFHSVMFPACLFATERNYTMVNHIMATEYLNYEDGKFSKSRGIGIFGNDAQDTGLPADVFRFY

LLFVRPESQDSSFSWADLATKNNSELLNNLGNFCNRALSFLEKFFDCVIPEIKLRDDELTLLALVTRDLKEYTSMLDKAK

LRDGLRLILSISRHGNQYMQFQKPWVLIKGSSEEKIRAGTVIGLSCNLVCLLAVMLQPYMPQTVDVIGKQLNVDISTFLL

NNFVSVFLQPGHKIGKPAPLFEKIDVSVVNQLKXRFAGKQESQEKELPVASGLDLKQIISVEQIEEAISKQGDKVRKLKE

AKVEKSELQPEINLLIELKSKLCISKGIPVENGQKKKQKPNKK

>PCG80048.1 hypothetical protein B5V51_11904 [Heliothis virescens]

MKIYTNQNNTSTLKLLIAANLAGKKVDLVSASFEDDTFAGPRQLPLLEVDDNLSFFSSNAAAQYLFPVVDVTDHGECQQT

QEWEATRLHPAVSAVLTSKTVASDLKQVLQALLSTVNGTLEKHKYILGDKLSPADISIWSTLYPLYHKDELKQAYIADCA

HILRWFQDLASAKAVQEAVKQWGGSATGPFGAASALGIPQVPSLSSPVGSPDEGAAVELGPTPEELEAAKDNWINGLKKL

QAPLVPGKLVLPVKGRKNILITSALPYVNNVPHLGNIIGCVLSADIFARYCRLCDYNTLYISGTDEYGTATETKALEEGI

TPREICDKYFDIHNSVYRWFNIGFDYFGRTSTPQQTEIAQRMFLKLRDNGFVSSQSVDQLLCTNCDRFLADRFVEGTCPY

PGCGYEGARGDQCDKCGKLITATELLQPRCKVCGHAPTVRPSQQLFIELGQLEPSLRTWISSTEKLWSPPARAVSRAWMR

EPLRSRAVTRDLKWGVPVPVEGFTNKVFYVWFDAPIGYLSITQNATKEFEKWWKPDKEYDVKLYQFMAKDNVPFHCIMFP

ATLMGVNEGHVLVSHLYATEYLNYEDGKFSKSRGVGVFGTDAQDTGIPSDVWRFYLASIRPETSDSSFSWTELGTRNNSE

LLNNLGNFCHRSLSFCANSYKGRVPDLELTQADYELIVLVNREVIAYVQHMEKGRLREALRHVLSVSRLGNQHMQAQQPW

VLLKGSDDDKKRGGTAIGLCCQLVALLCALLSPYLPETVRKLREQLNLKQESLRINPENPSLLQYLPSGHVIGKPEPLFA

KIEPELLDKLRAKYAGTQSERAGKENGTAPGSVAELEEAVKQQGEKVRQLKASTKDKTVWQPEVAKLLDLKKQLEAAQKT

QAAQTKGAQGDVSSLEKAVAAQADKVRELKASTKDKTVWQPEVNKLLDLKKQLAAAQSAPAVAQTAPAVTQSASANSTDL

SALEKELAEQADKVRKLKASTKDKSVWQPEVNKLLAIKKQLADLQINK

>XP_019526200.1 PREDICTED: methionine--tRNA synthetase, cytoplasmic-like [Aedes albopictus]

MIIFTNAGNPFGLKLLICAKFAKKDVQVKTIALNDTELKDMKHLPILQLESGVQLFSTDVAVKYLLQGETPVIQRDEWLE

WSTTRLAPALTHNMAVGSRQDPNTKPILNSLVKFLDDCLSRNTFLTGDKLTSADISVWSLLAPDGTLKGAQNIDNLLRWY

RAINAMPEVKAALEVLPLKELSFASLKHSNRFEGLHHIVLDPEIIDFEGQVLVDTSDNIAATVQKEEIQAARDSFVAVQE

RSVPDEKVVLPKVGQKNNLITSALPYVNNVPHLGNIIGCVLSADVFARYSRLCGYNTLYICGTDEYGTATETKALAEKLT

PKQICDKYFEIHNSIYRWFGIGFDYFGRTTTAEQTQIVQEMFKDLYAGGFIETRAVDQLLCQKCDRYLADRFVEGTCPYP

GCGYEDARGDQCDACGKLINAIELVRPRCKVCNTSPIIRQSSQFFLDLPKIEPRLRDWVDKAEHGWTHNARVITRAWLKE

GLKSRCITRDLKWGIPVPLEGYEDKVFYVWFDAPIGYISMTSRYSKEWKQWWQPSPETKVDLYQFMAKDNVPFHSVMFPA

SLIGIDKGYTLVSHIMATEYLNYEDGKFSKSRGIGVFGNDAHDTGIPADVWRFYLCSSRPEGQDSSFSWNDLAARNNNEL

LKNVGNFVNRALVFCEKCFDSTIPPLVLNDEDYTLLALINRELKGYVHQMEKARLRDGIRHLLAISRYGNQLMQAEEPWV

LVKGTDDQKTRAGTVIGLCCNLACLLATLIFPFMPSTARNLYTQLNVQSCHLDADKPQMRIFVPSGHKIGKPAVLFNKID

QAKIDELKKKYAGAQQQPESKPAAAAAKPASNKLFTSLVEAQKAVDEQAAKVRKVKSTGADKSVWQPEVNILLELKKQLQ

TLSQPRTSESTAATPATPTSAPTPETLQSLEAEIAKQGEKVRTLKAGGSEKAVWQPEVTVLLALKEKLAKLTGAPQAAAP

ASGKKKNKK

>XP_006618853.1 PREDICTED: methionine--tRNA synthetase, cytoplasmic-like [Apis dorsata]

MNILTNEDNPNTLKLIIAAKVVQQSVTVKKVEPKGSFLGRLPSIELSSGLVLFSTNAAMQLIIPPSEESKIINSKWLEWE

ISQLQSAIVFYGSTGIYKTTHKSDLWSLLEELNNVLKDKKYLIKDNNDLSLADICIWVTLWSTVLVTEIGNELCREYQHI

NNWISNIESHPIIQDSIKEYKFERGLKAIFSIQAVSWFPINTIQNFRSPKSADIQLSPTKEKEIEAVSQEEMHIISSNWS

SILYPDVKNFPYPVLPKKDEKNILITSALPYVNNVPHLGNIIGCVLSADIFARYCRQRNYNTLFISGTDEYGTATEAKAL

EEKTTPQSICDKFFDIHNDVYRWFNIGFDYFGRTTTPEQTEIVQTFFLRIKSQGYILSETVDQLLCESCDRFLADRFVEG

TCPRCKYEDARGDQCDGCGHLINATDLISPRCKVCSNRPIIKKSEQFFLDLPKLENKLKEWSSTVEKGWSGVARVVAKPW

LRDGLKPRCITRDLKWGIPVPVKGFENKVFYVWFDAPFGYISITKRYTKEYEQWWKPKDIKVDLYQFMAKDNVPFHAIMF

PASLLAANEDYILLKHLMATEYLNYEDTKFSKSRGIGVFGTDARDTGIPSDVWRFYLAYVRPETQDSNFNWVDLATKNNS

ELLNNFGNFVNRALVFVERYFESKIPPIELQEDDLILLALAQRELSSYIYAMEQAKLRDGLKHILAISKHGNQYMQFQEP

WVKIKGTDNDKKRAGTIIGISCNMACLLSALLAPFMPSTARELRSQLGLATNSYGYIPDLITNILPTAHKIGKPSPLFKK

IEDKNIETLRKKYAGKQETTNEHSDVKSLDNAIAELDTKVKELKAKHDKSGWQSSQVQIFSDLKKKLSDLISEKNKSSDS

KNKKPEIVLVPEQNGDVSMDIASLESAIAMQGNLVRTLKAKEEKSVWQPEVEKLLKLKKQLADLTGTAPTSNEKKSKKKK

>XP_317601.4 AGAP007891-PA [Anopheles gambiae str. PEST]

MIIYTNTGNPFGLKLLICANLAKTNVEVKHVTLNDPALGDMKHLPILQLNSGVQLFSTDVAAKYLLPGDDASKVVQRDEW

LEWSSKRLAPALTHNMAVGSRADPNTKPILNTLVKMLDDCLSNSTFLTGDKISSADVAVWSLLAPDGTLKGAVNIDNVLR

WYRQITALPEVQSALAALPLKELSFASLQHSNRFGGLHHIVLNPEIADFEGELLADTSANIAETVQREEIEAARAAFGET

DDRAVRDEPRIVLPKPGQKNNLITSALPYVNNVPHLGNIVGCVLSADIFARYSRLCGYNTLYIGGTDEYGTATETKALAE

NMTPRQICDKYFDIHNSIYRWFGIGFDYFGRTTTAEQTRIVQDIFKELYASGYIETRSVEQLLCQKCDRYLADRFVEGTC

PHPGCGYEDARGDQCDGCGKLVNAVELLRPRCKMCNTSPVIRESSQFFLDLPKIEPTLREWVDRSEGGWTHNARVITRAW

LKEGLKSRCITRDLKWGIPVPLEGYEKKVFYVWFDAPIGYISITSRYCKEWKQWWQPDTANTGVKVDLYQFMAKDNVPFH

SVMFPASLLAVDKGYTLVSHVMATEYLNYEDGKFSKSRGIGVFGNDAQDTGIPADVWRFYLGAARPEGQDSSFSWSDLQA

RNNSELLKNLGNFFNRALVFCEKFFDSTIPPMTLLEEDYTLLALINRELKGYVNQMEKARMRDGIRHLLQISRYGNQLMQ

SEEPWVKVKGTDDQKARAGTVIGLCCNFACLLATLIFPFMPTTARNMYSQLNVRGGFINSDKPLIRTLLPTGHRIGKPAV

LFTKIEDARIEELKQKYGGVQQDKDKSPAPVKAPQGFTSLKDAQKAVDEQAAKVRKIKSSGADRTVWQPEVNILLELKKQ

LQTLSQPRTSESAATAAPGEKPAKQAPKQAPKPATALEEEIAQQGNKVRKLKEANSDKSVWQPEVSLLLSLKQKLASLTG

VPVAAPAGGGKKKGNKK

>XP_397235.3 PREDICTED: methionine--tRNA synthetase, cytoplasmic isoform X1 [Apis mellifera]

MNILTNEDNPNTLKLIIAAKVVQQSVTVKKVEPKESFLGRLPSIELSSGLVLFSTNAAMQLIIPPSEELKIINSKWLEWE

ISQLQSAITFYGSTGIYKTTHKSGLWSLLEELNNVLKDKKYLIKDNNDLSLADICIWVTLWSTVLVTEIGNELCREYQHI

NNWISNIESHPIIQDSIKEYKFERGLKAISSIQAVSWFPINTIQNFRSPKSADIQLSPTKEKEMEAVSQEEMHIISSNWS

SILYPDVKNFPYPVLPKKDEKNVLITSALPYVNNVPHLGNIIGCVLSADIFARYCRQRNYNTLFISGTDEYGTATEAKAL

EEKTTPQSICDKFFDIHNDIYRWFNIGFDYFGRTTTPEQTEIVQKFFLRIKSQGYILSETVDQLLCESCDRFLADRFVEG

TCPRCKYEDARGDQCDGCSHLINATDLISPRCKVCSNRPIIKKSEQFFLDLPKLENKLKEWLSTVENGWSGVARVVAKPW

LRDGLKPRCITRDLKWGIPVPIKGFENKVFYVWFDAPFGYMSITKRYTKEYEQWWKPKDIKVDLYQFMAKDNVPFHAIMF

PASLLAANEDYILLKHLMATEYLNYEDTKFSKSRGIGVFGTDARDTGIPSDVWRFYLAYVRPETQDSNFNWVDLATKNNS

ELLNNFGNFVNRALVFVERYFESKIPPIELQEDDLIVLALAQRELSSYICAMEQAKLRDGLKHILAISKHGNQYMQFQEP

WVKIKGTDNDKKRAGTIIGISCNMACLLSALLAPFMPSTARELRSQLGLATNSYGYIPDLITNILPTAHKIGKPSPLFKK

IEDKSIETLRKKYAGKQETTNEHSDVKSLDNAIAELDTKVKELKAKHDKSGWQSSQVQIFSDLKKKLSDLISEKNKSSDS

KNKKPEIVLVPEQNGDVSMDVASLESAIAMQGNLVRTLKSKEEKSVWQPEVEKLLKLKKQLADLTGIAPTSNEKKSKKKK

>XP_019561333.1 PREDICTED: methionine--tRNA synthetase, cytoplasmic-like [Aedes albopictus]

MIIYTNAGNPFGLKLLICAKFAKKDVQVKTIALNDTELKDMKHLPILQLESGVQLFSTDVAVKYLLQGETPVIQRDEWLE

WSTTRLAPALTHNMAVGSRQDPNTKPILNSLVKFLDDCLSRNTFLTGDKLTSADISVWSLLAPDGTLKGAQNIDNLLRWY

RAINAMPEVKAALEVLPLKELSFASLKHSNRFEGLHHIVLDPEIIDFEGQVLVDTSDNIAATVQKEEIQAARDSFVAVQE

RSVPDEKVVLPKVGQKNNLITSALPYVNNVPHLGNIIGCVLSADVFARYSRLCGYNTLYICGTDEYGTATETKALAEKLT

PKQICDKYFEIHNSIYRWFGIGFDYFGRTTTAEQTQIVQEMFKDLYAGGFIETRAVDQLLCQKCDRYLADRFVEGTCPYP

GCGYEDARGDQCDACGKLINAIELVRPRCKVCNTSPIIRQSSQFFLDLPKIEPRLRDWVDKAEHGWTHNARVITRAWLKE

GLKSRCITRDLKWGIPVPLEGYEDKVFYVWFDAPIGYISMTSRYSKEWKQWWQPSPETKVDLYQFMAKDNVPFHSVMFPA

SLIGIDKGYTLVSHIMATEYLNYEDGKFSKSRGIGVFGNDAHDTGIPADVWRFYLCSSRPEGQDSSFSWNDLAARNNNEL

LKNVGNFVNRALVFCEKCFDSTIPPLVLNDEDYTLLALINRELKGYVHQMEKARLRDGIRHLLAISRYGNQLMQAEEPWV

LVKGTDDQKTRAGTVIGFCCNLACLLATLIFPFMPSTARNLYTQLNVQSCHLDADKPQMRIFLPSGHKIGKPAVLFNKID

QAKIDELKKKYAGAQQQPESKPAAAAAKPASNKLFTSLVEAQKAVDEQAAKVRKVKSTGADKSVWQPEVNILLELKKQLQ

TLSQPRTSESTAATPATSTSAPAAETLQSLEAEIAKQGEKVRTLKAGGSEKAVWQPEVTVLLALKEKLAKLTGAPQAAAP

GSSKKKNKK

>XP_012338968.1 PREDICTED: methionine--tRNA synthetase, cytoplasmic [Apis florea]

MNILTNEDNPNTLKLVIAAKIVQQSVTVKKVEPKGSFLGRLPSIELSSGLVLFSTNAAMQLIISPSEESKIINNKWLEWE

ISQLQSAIIFYGNTGIYKTTHKSCLWSLLEELNNVLKDKKYLIKDNNDLSLADICIWVTLWSTVLVTEIGNELCKEYQHI

NNWISNIESHPIIQDSIKEYKFERGLKAIFSIQTVSWFPINTIQNFRSPKSADIQLSPTKEKEVEAVSQEEMHIISSNWS

SISYPDIKNFPYPVLPKKDERNILITSALPYVNNVPHLGNIIGCVLSADIFARYCRQRNYNTLFISGTDEYGTATEAKAL

EEKTTPQSICDKFFDIHNDVYRWFNIGFDYFGRTTTPEQTEIVQKFFLRIKSQGYILSETVDQLLCETCDRFLADRFVEG

TCPRCKYEDARGDQCDGCGHLINATDLISPRCKVCSNRPIVKKSEQFFLDLPKLENKLKEWSSTVEKGWSGVARVVAKPW

LRDGLKPRCITRDLKWGIPVPVKGFENKVFYVWFDAPFGYISITKRYTKEYEQWWKPKDIKVDLYQFMAKDNVPFHAIMF

PASLLAANEDYILLKHLMATEYLNYEDTKFSKSRGIGVFGTDARDTGIPSDVWRFYLAYVRPETQDSNFNWVDLATKNNS

ELLNNFGNFVNRALVFVERYFESKIPPIELQEDDLILLALAQRELSSYIHAMEQAKLRDGLKHILAISKHGNQYMQFQEP

WVKIKGTDNDKKRAGTIIGISCNVACLLSALLAPFMPSIARELRSQLGLTTNSYGYIPDFITNILPTAHKIGKPSPLFKK

IEDKNIETLRKKYAGKQETTNEHSDVKSLDNAIAELDTKVKELKAKHDKNGWQSSQVQIFSDLKKKLSDLISEKNKSFDS

KNKKPEVVLVPEQNGDVSMDVATLESAIAMQGNLVRTLKAKEEKSVWQPEVEKLLKLKKQLADLTGTAPTSNEKKSKKKN

K

>XP_014475977.1 PREDICTED: methionine--tRNA synthetase, cytoplasmic isoform X2 [Dinoponera quadriceps]

MVTMNVSTNQGNPNALKLIIAAKLAQELVTVKIVEPKGRLPTLTTPSGLSLFSVSSALRLLLPVAAELEILNNKWLEWEI

SQLQPAILSYASGKTSSKDLLSLFKDLNSELKNKQYLIETSSYLSPADISIWVSLWSTLLFAECKDISAQLLNIVHWLTN

MEQQPVIQESIKEFDLVRGAKAIASIEAASWFPVNAVTNAQVRQESLLSDISPTKEKEMETVSQEEVRSVMNIWFTRRYP

EVKESSYPILPKKGERNVLVTSALPYVNNVPHLGNIIGCVLSADIFARYCRQRNYNTLYISGTDEYGTATEAKALQEKTT

PQIICDKFFDIHNDVYRWFGIGFDYFGRTTTPEQTEIVQSFFLKIKSQGYILNESVEQLHCEFCDRFLADRFVEGTCPRC

KYEDARGDQCDGCSHLVNAVELIEPRCKMCNTKPVVRNSVQFFLDLPKLEDKLKQWSATVNQGWSSVAKVVSKSWLRDGL

KPRCITRDLKWGIPVPVNGFENKVFYVWFDAPFGYVSITKRYTKEYEKWWKPQDTKVDLCQFMAKDNVPFHAIMFPACLL

AANQNNTMIKYLMATEYLNYEDKKFSKSRGIGVFGTDARDTGIPADVWRFYLAYIRPETQDSNFNWIDLATKNNSELLNN

LGNFVNRALMFSEKFFDSNVPPMKLEEADWIVLALAQRELSSYISAMEQAKLRDGLKYILAISKHGNQYMQFQQPWVKIK

GTDDDKNRAGTVVAICCNLACLLSALLAPFMPTTAKQLRSQLGLSNKSYGYIPETITNMLPTWHKIGKPSPLFTKIEEQQ

VEMLRKKYAGQQEINGRTAATNVASDESVDSLETAIMVQGNLVRELKAKHEKSVWQPQVEILLSLKKKLADLKVSAPKAP

EKNSPVKNKKVETNVNKQHARLYEQKISDLKALHSAINERRLKESKKDAVGNAATDVATLESAIAKQSNLVRELKAKGDA

NVWEPQVEMLLKLKEQLMGLTEATPVAVDKKSKKKK

>XP_015438440.1 PREDICTED: methionine--tRNA synthetase, cytoplasmic [Dufourea novaeangliae]

MSISTNEGNPNTLKLIVAAKMAQQSVTVKTVQPNVSLLGRLPTIELSNGSVLFSTSAAMQLILPPSEEFKDVNNKWLEWD

ISQLQPAIVCYGNTGTTNKSCLWPLLKELNSALKNRKYLIKDKNDLSLADVCIWVTIWSTVLVTDVGQKLCKEYSSIKDW

LLNIELLPVIQDSLKEYNFERGIKAILNIQAASWFPVNTVMNSRSSKPVLSDISPTKEKEKEVEAVSQEEMQNIASNWSS

KSYPAVKNFPYPVLPKKGEKNILITSALPYVNNVPHLGNIIGCVLSADIFARYCRQRNYNTLFISGTDEYGTATEAKAIE

EKTTPQAICDKFFDIHNDVYRWFSIGFDYFGRTTTPEQTEIVQSFFLRIKSEGYVLSETVDQLLCESCDRFLADRFVEGT

CPRCKYEDARGDQCDGCGHLVNATDLISPRCKVCSNRPVVKKSVQFFLDLPKAKNKLKQWSQTVEKGWSSVARVVAKPWL

RDGLKPRCITRDLKWGIPVPVKGFENKVFYVWFDAPFGYISITKRYTKEYEQWWKPKDVNVDLYQFMAKDNVPFHAIMFP

ACLLAANKGHTLMKHLMATEYLNYEDTKFSKSRGIGVFGTDARDTGIPADVWRFYLAYVRPETQDSNFNWVDLATKNNSE

LLNNLGNFVNRALMFAEKYFDSKLPQMELLEDDLVLLALAQRELSSYIQALEQAKLRDGLKHVLAISKHGNQFMQFQEPW

VKIKGTDDDKKRAGTIIGICCNLACLLSALLAPFMPNTSRELRSQLGLNSINHGYIPDIITNILPTGHKIGIPSPLFKKI

EVKDVETLRSKYGGKQDGANKIEKKIVTSSDVKSLETAITTQGNKVRELKAKHDKSVWQPEVQILVDLKQKLADLIGGGN

KSSDLAKKNKPETVFVPEQNGDASMDAASLEAAVAKQGNLVRELKSKAEKSVWQPEVEKLLKLKKQLTDLTGTSPSTTNK

KSKKKK

>XP_015117336.1 PREDICTED: methionine--tRNA synthetase, cytoplasmic [Diachasma alloeum]

MIVSTNKSNPNALKLLIGGEISGNSVDVRIVPTNDSPLGRLPTLKLPNGITLISSNAALQYLVPGDEQQDVLVNKWLEWD

STQLQPAVVSYGSSGSLDTSHKPRIWSLLTQLNTSLKSQKYVITASPTSADIAIFSTLWCTITVTEISKELSTEFPHVDR

WLKDLEALPAMQSPLSKLPLLDLERGLKSISSLHTSTWFPLNSYTGVRSQKPPQDVNTPSSPTKEPETAITKHELETSRN

NWSSPAHPSIKPPTYPVLPKQNERNILITSALPYVNNVPHLGNIIGCVLSADVFARYCRQRDYNTLYISGTDEYGTATEA

KALEENMTPQEICDKYFGIHNDIYRWFNIGFDYFGRTTTPEQTEIVQESFLRIKSQGYIITESVDQLLCEHCDRFLADRF

VEGTCPGCKYEDARGDQCDGCGHLVNATDLINPRCKVCSKTPVIKASNQFFLDLPKIQPNLKEWASKAEKFWSNVARAVT

RPWLRDGLKQRCITRDLKWGIPVPLEGFRDKVFYVWFDAPLGYISITKRYTQEYERWWKPREDTKVDLYQFMAKDNVPFH

AIMFPATLLAADQGHTLVKHIMATEYLNYEDTKFSKSRGIGVFGTDARETGIPADVWRFYLTYIRPESQDSNFNWVDLAT

KNNSELLNNFGNFVNRALTFAVKNFKGVVPEMEIREEDLQVLALVQRELSQYIVAMEQGKFRDGVRHLLAISKHGNQYMQ

SQQPWVLIKEKGKDRVRAAGTVIGVCCNISCLLSALLGPFMPETSRELRKQLGLDGSTYGYIPDTVSILLPTGHQLGEAS

PLFSKIEESVVEELRKKYAGKQENGNNGTRAGESVEELQALIAAQGVKVRELKAKTDKSVWQPEVQILLQLKGKLAKMTE

SSEGTVNTATIGVEKQVNGPTQNGKNEYDIAALEAAIARQALFVRDLKASADRSVWQPEVNTLLKLKKELSDATGVITPN

PGDKKSKKKK

>XP_015586892.1 methionine--tRNA synthetase, cytoplasmic isoform X2 [Cephus cinctus]

MNIVTNDGNPNALKLVLAAKLGQQHATITTVSTDYAELGRLPVLELPSGIRIFSISAAVQFLVPMGEEFSIHINKWLEWD

ISQLQPALVSYGGLGKLDTSHKPWIWSLLKELNAALKDKKYLIKDKDNPSIVDLSIWITLWSTITPLDISKDIAVEFPTV

NKWLKAIEVIPIVQESIKEFNIEKGLKAIASIQAVSWFPVNSYTDSSSSISALSDEQEIETVSEEELNNVAKNWIVTCYS

TVKEPPYPVLPIKGERNIMITSALPYANNVPHLGNIIGCVLSADIFARYCRQRNYNVLFICGTDEYGTATEAKAVEEKTT

PQEICDKFFGIHNDIYRWFSIGFDYFGRTTTPEQTEIVQAIFLRIQSQGYIFTENVDQLLCESCQRFLADRFVEGTCPRC

SYTDARGDQCDGCGHLVNAIELINPRCKVCNSKPMIKVSTQFFLDLPKIEPRLQEWAKSAEVNWSSVARAVSKPWFRDGL

KPRCITRDLKWGIPVPLEGYRDKVFYVWFDAPLGYVSITKRYTKEYEQWWKPKYVDVSLYQFMAKDNVPFHSVMFPASLL

AADQGHTLVKHIMATEYLNYEDTKFSKSRGIGVFGTDAKETGILPDVWRFYLAYVRPESQDSNFNWVDLATKNNSELLNN

LGNFINRALMFVERNFASKVPPMEPQEDDLTVLALSQRELSSYVAALEQAKLRDALRHILAISKHGNQYMQSQEPWVKVK

GTDDDKKKAGTVIGICCNLVALLSALLAPYMPNTSREVRSQLGLDKSSYGYIPDTITTILPAGHTIGKPSPLFTKIDDQK

VEELRKKYGGKQECPIQKVSSGTIEKCNDAASLEAAIAKQGLLVRELKAKQDKSVWQPQVQILLQLKKQLADLTSNTGKT

SDQAKEKHNSTPGVVLEQNGDGINGAADLEAAIAKQGVLVREMKAKAEKSVWQPQVEILLKMKKQLADLTGAIIPESSNK

KSKKKK

>XP_015586891.1 methionine--tRNA synthetase, cytoplasmic isoform X1 [Cephus cinctus]

MNIVTNDGNPNALKLVLAAKLGQQHATITTVSTDYAELGRLPVLELPSGIRIFSISAAVQFLVPMGEEFSIHINKWLEWD

ISQLQPALVSYGGLGKLDTSHKPWIWSLLKELNAALKDKKYLIKDKDNPSIVDLSIWITLWSTITPLDISKDIAVEFPTV

NKWLKAIEVIPIVQESIKEFNIEKGLKAIASIQAVSWFPVNSYTDSSSSISALSDVNVLKEQEIETVSEEELNNVAKNWI

VTCYSTVKEPPYPVLPIKGERNIMITSALPYANNVPHLGNIIGCVLSADIFARYCRQRNYNVLFICGTDEYGTATEAKAV

EEKTTPQEICDKFFGIHNDIYRWFSIGFDYFGRTTTPEQTEIVQAIFLRIQSQGYIFTENVDQLLCESCQRFLADRFVEG

TCPRCSYTDARGDQCDGCGHLVNAIELINPRCKVCNSKPMIKVSTQFFLDLPKIEPRLQEWAKSAEVNWSSVARAVSKPW

FRDGLKPRCITRDLKWGIPVPLEGYRDKVFYVWFDAPLGYVSITKRYTKEYEQWWKPKYVDVSLYQFMAKDNVPFHSVMF

PASLLAADQGHTLVKHIMATEYLNYEDTKFSKSRGIGVFGTDAKETGILPDVWRFYLAYVRPESQDSNFNWVDLATKNNS

ELLNNLGNFINRALMFVERNFASKVPPMEPQEDDLTVLALSQRELSSYVAALEQAKLRDALRHILAISKHGNQYMQSQEP

WVKVKGTDDDKKKAGTVIGICCNLVALLSALLAPYMPNTSREVRSQLGLDKSSYGYIPDTITTILPAGHTIGKPSPLFTK

IDDQKVEELRKKYGGKQECPIQKVSSGTIEKCNDAASLEAAIAKQGLLVRELKAKQDKSVWQPQVQILLQLKKQLADLTS

NTGKTSDQAKEKHNSTPGVVLEQNGDGINGAADLEAAIAKQGVLVREMKAKAEKSVWQPQVEILLKMKKQLADLTGAIIP

ESSNKKSKKKK

>XP_003246352.1 PREDICTED: methionine--tRNA synthetase, cytoplasmic [Acyrthosiphon pisum]

MSLTTIEVDEFELLNAKNSWISKSCLKDINRVECQPVLPVKDEKNILVTSALPYVNNVPHLGNIIGCVLSADVFARYCRL

RGWNTLYISGTDEYGTATETKALEEKLTPQQICDKFFKIHNEIYKWFNISFDHFGRTTSQKQTEIVQEIFKEVHSNGYTS

TASMEQLLCENCDRFLADRFVEGTCPLCNYEDARGDQCDGCGHLINATELIKPRCKVCQKTPVIRSSQQLFLNLPKVEAK

LNDWVDKSGDDWSYNAKVITKSWLKDGLKERCITRDLKWGIPVPLEDFKSKVFYVWFDAPIGYMSMSAAYTPDWRKWWQP

EDDTKVTYYQFMAKDNVPFHSVMFPACLFATERNYTMVNHIMATEYLNYEDGKFSKSRGVGVFGNDAQDTGLPADVFRFY

LLFVRPESQDSSFSWADLATKNNSELLNNLGNFCNRALSFLEKFFDCVIPEIQLGNDELTLLALVTRDLKEYTSMLDKAK

LRDGLRLILSISRHGNQYMQFQKPWVLIKGSSEEKIRAGTVIGLSCNLACLLAVMLQPYMPQTVNIIGKQLNVDISTFLL

NNFVSVFLPPGHKIGKPAPLFEKIDPSVVNQLKSRFAGKQKSQEKELSVASGLDLKQITSVEQMEEAISKQGDMVRQLKQ

SGAQKSEWEPYVKALLEMKKSLEQMKLESNPISIDCLEEEISKQGDKVRKLKEAKVEKSELQPEIDLLLQLKSKLSISKG

IPVENGQKKKQKPIKK

>KMQ99403.1 methionyl-trna cytoplasmic [Lasius niger]

MIISTNENNPNALKLIIAAKLAQDTFIVKVVKPGDAVLGRLPTLTLPSGTSLFSVSSALRLLLPVPKELEVLNNRWLEWE

ISQLQPTILSHTSARTSKIPQKSDLWSLLKDLNSELKDKQYLIEKHSNLSPADISIWVSLWSTILLADMKDNISKDLPNV

SNWLVNIEKQPVIQESIKEFNLERGTKAIASIEAASWFPVNTTSSSSQRCESVSSDISPTKEKEIEAVSQEELNNIASSW

TTGQYSEVKESSYPILPKKGERNVLVTSALPYVNNVPHLGNIIGCVLSADIFARYCRQRNYNTLYISGTDEYGTATEAKA

LQEKTTPQAICDKFFDIHNDVYRWFGIGFDYFGRTTTPEQTEIVQDFFLKIKSQGYILTEIVEQLHCESCDRFLADRFVE

GICPRCKYEDARGDQCDGCGHLMNAVELINPRCKMCNTRPAIKNSTQFFLDLPKIEERLKEYSATVEKGWSNVARVVSKA

WLRDGLKPRCITRDLKWGIPVPVEGFENKVFYVWFDAPFGYISITKRYTKEYKKWWQPPQDTKVDLCQFMAKDNVPFHAI

MFPAYLLAANQNYTLIKYLMATEYLNYEDKKFSKSRGIGVFGTDARDTGIPADVWRFYLAYIRPETQDSNFNWVDLATKN

NSELLNNLGNFVNRALAFSEKFFNSTVPPIERPEEADWTVLALAQRELSCYINAMEQAKLRDGLKYILAISKHGNQYMQF

QQPWVKIKGTDDDKKRAGTIIAICCNLACLLSALLAPFMPSTAKELRSQLGLSNKSYGYIPETITNMLPTWHKIGKPSPI

FTKIEDQKVEMLRKKYAGQQETNNEMSENKADESVASLEAAITKQGNLVRELKAKHDKSTWQPQVEILLDLKKKINDLKN

NAPEKKLPTKNKKAEPTHVPEQNGDALTDVVALEMAIAKQGNLVRELKAKEDGSVWKPQVEILLKLKQRLIDLTGTAPVA

VDKKSKKKK

>XP_011500338.1 PREDICTED: methionine--tRNA synthetase, cytoplasmic [Ceratosolen solmsi marchali]

MNVTSNPNNPNALKLIVAAEISKKELAIEFTSDPKQCSLNRLPTLKIDSSRKELFSVNAAVQYISPISNKDVIVANRWLE

WDVSQLQPAIVSYGGSGKLDVSHKPRIWSLIKELNASLKNKEYLIKDKDNITIVDASIWVTLWSTVVVTDMGEVIRKEFP

NVDRWLKHIESLPIIKKCLEKMNLERGIQAIMSMNAASWFPLNSYTGVANSQIISAEKNNNNLEDKEIKAITDEEINLVK

NSWTSVVYPSVKEIPYPVLPKKGEKNILVTSALPYVNNVPHLGNIIGCVLSADIFARYCRQRNYNTLYISGTDEYGTATE

AKALEEKVTPQQICDKFFDIHNDIYRWFSIGFDYFGRTTTSEQTEIVQAMFLRIKDKGYVLTNIIDQLLCENCDRFLADR

FVEGTCPRCKYEDARGDQCDGCGHLVNATELINPRCKVCSNKPVIKASTQFFLDLPKIQENLKQWASKVEDGWSLVAKTV

TKPWLRDGLKARCITRDLKWGIPVPLDGYRDKVFYVWFDAPLGYLSITKKYTKEYEKWWKPNNVDVNLYQFMAKDNVPFH

AIMFPATLLAANEDHILVKHIMATEYLNYEDTKFSKSRGIGVFGTDARETGISADVWRFYLAYIRPESMDSNFNWVDLAT

KNNSELLNNLGNFINRALMFVEKNFNSKIPQMELSDEDMHVLALVQRELTNYISFLEQAKLRDGLKHVLAISKHGNQYMQ

YQQPWVKIKGTEEEKKKGATVIGVCCNISCLISALLAPFMPNTAREIRVQLGLSKQNYGYIPEILSIMLPAGHSIGKPSP

LFSKIEEKQVEELRHKYGGKQSVSVENSQDSTNLASLEKAVANQAQLVRDLKAKQGKDVWQPQVQILLDLKKKLAEFQVN

SNVSGQATAKSKSSSTAAAPEQNGNVTAEITELEAAVLKQGNLVRELKAKNEKSVWQPQVEVLLQLKQKLSALTGAVHSP

PPGDKKSKKKKINS

>XP_024892470.1 methionine--tRNA synthetase, cytoplasmic [Temnothorax curvispinosus]

MIVSTNGGNPNALKLIVAAKLAQETVSVRILKPSDAALGRLPTLTTPSGVSLFSVSSALRFLLPVPKESEVLNDRWLEWE

ISQLQPTILSHASTRTSKTPQKSDLWSLLKDLNNALKEKEYLIEKCSNLSPADISIWVSLWSTILVTDIANNIAKDLPNL

DSWLANIQKQSVIQQSIKESIEMFNLERGAKAIASIEAASWFPVNTTSNSQVHESVPSDISPTKEKEVEIVSQEELRNVA

KSWTTDQYPEVKESSYPILPKKGERNVLITSALPYVNNVPHLGNIIGCVLSADIFARYCRQRNYNTLYISGTDEYGTATE

AKALQEKTTPQAICDKFFDIHNDVYRWFGIGFDYFGRTTTPEQTEIVQAFFLKIKSQGYIFTETVEQLHCESCDRFLADR

FVEGICPGCKYEDARGDQCDGCGHLVNAVELINPRCKMCNNRPVVKNSVQFFLDLPKLEQELKKYSATVEKGWSNVARVV

SKAWLRDGLKPRCITRDLKWGIPVPVEGFENKVFYVWFDAPFGYISITKRYTKEYKKWWQPPQDTKIDLCQFMAKDNVPF

HVIMFPACLLAADQNYTLMKYIMATEYLNYEDKKFSKSRGIGVFGTDARDTGIPADVWRFYLAFIRPETQDSTFNWVDLA

TKNNSELLNNLGNFVNRALVFSEKFFDSNVPPIEPEEADWTLLALAQRELSSYVNAMEQAKFRDGLRHILAISKHGNQYM

QFQQPWVKIKGNDDDKKRAGTVVAICCNLACLLSALLAPFMPSTTKQLRSQLGLSNKTYGYIPETITNMLPTWHKIGKPS

PLFTKIEDQKVEMLRKKYAGQQENNGQAAAKSSDEIVASLEAAITKQGNLVRELKAKHDKSVWQPQVEILLDLKKKLIDL

KSNTNAPEKKSPPKNKKAEPKHVPEQNGEQNAPTDVAALESAIAKQGNLVRELKAKEDGSVWKPQVEILVKLKQRLADLT

GTAPVVVDKKSKKKK

>EZA60354.1 Methionyl-tRNA synthetase, cytoplasmic [Ooceraea biroi]

MQQQWEKALERNRKNAFLGRLPTLKASAELSLFSVSSALRFLLSVPKESEILNDRWLEWEITQLQPAILSHANMRTPKNL

QKSDLWSLLKDLNSELKDKQYLIKEHSHLSPADICIWVSLWSTMLLEDVKDNVAKNLPNLYNWLANIEKQSIIQESIKEF

NLERGAKAVASIEAASWFPVNAVSNSRVSESVSSDVNPKEKDAEAISEEELRNVAHSWTTGQYLEVKESSYPILPKKSER

NVLITSALPYVNNVPHLGNIIGCVLSADIFARYCRQRNYNTLYISGTDEYGTATEAKALQEKTTPQAICDKFFDIHNDVY

RWFGIGFDYFGRTSTPEQTEIVQAFFLKIKSRGYVRSETVEQLHCELCDRFLADRFVEGTCPGCKYEDARGDQCDGCGHL

VNAIELINPRCKMCNSTPVVKHSVQFFLDLPKIEEKLKAYNATVEKGWSSVARVVSKSWLRDGLKPRCITRDLKWGIPVP

VEGFENKVFYVWFDAPFGYISITKRYTKEYQKWWQPPQDVKVDLCQFMAKDNVPFHTVMFPACLLAVNQNYTLMKYLMAT

EYLNYEDKKFSKSRGIGVFGTDARDTGISADVWRFYLAYIRPETQDSNFNWVDLATKNNSELLNNLGNFVNRALVFAEKF

FNSSIPPIELAEEADWSVLALAQRELSSYINAMEHAKLRDGLKHILAISKHGNQYMQFQQPWVKIKGTEDDKKRAGTVVA

VCCNLACLLSALLAPFMPTTSKQLRSQLGLSNKSYGYIPETITNILPTWHKIGKPSPLFTKIEDQSVEVLRKKYAGQQEV

NGEVSTTNKAPDDGIASLETAITKQGTLVRELKAKHDKSVWQPQVAILLDLKKKLADLKGNVNVPEKKLPAKKTPAKNKK

AEPTQVLEQNGDALTDVAALESAIAKQGNLVRELKAKEEASVWKPQVEILLKLKQRLMGLTGTTTPNATDKKSKKKK

>XP_020708517.1 methionine--tRNA synthetase, cytoplasmic isoform X2 [Athalia rosae]

MIVKMFVSTNKNNPNVLKLIIAAKLARQSVEIKTIPPQDVALGRLPLLQIPSGTNLFGTSAALQFLVPTEDSSSILINKW

LEWDISQLQPALVSYGGSGKLDMSHKPRIWSLLKQLNTSLQNKKYLIEAKNYPSVADFSIWVSLWSTIAATEVNVEIIKE

FPLVEGWSNNIECLPTVQESLKEFKIVRGLRAIADIQAASWFPLNSYTGGASHKASPNDVIVNSVKEKEIILVSEEELKF

VTECWTVKNYPSLKDPAYPVLPKKGERNILITSALPYVNNVPHLGNIIGCVLSADIFARYCRQRSYNTLYISGTDEYGTA

TEAKAIEENMTPQQICDKFFSIHNDIYRWFSIGFDYFGRTTTLEQTKIVQDLFLRLKHKGFINAESVDQLLCQTCDRFLA

DRFVEGICPGCKYEDARGDQCDGCGHLVNATELINPRCKMCNNSPIIKPSTQFFLDLPKIEPTLKKWAENVESEWSPTAR

VVAKPWLRDGLKPRCITRDLKWGIPVPLDNYRNKVFYVWFDAPLGYISITKRYTKEYEQWWLPKDIPVDLYQFMAKDNVP

FHAVMFPASLLAADQGHTLVKHIMAIEYLNYENTKFSKSRGIGVFGTDARDTGIPADVWRFYLAYVRPESQDSNFNWVDL

ATKNNSELLSNLGNFINRSLMFAEKNFGSKIPPMDLQKDDLIVLALAQRELTSYVAAMERAKLRDALRHILAISKHGNQY

MQSQAPWVKMKGTDDDKNKAGTVIGVCCNLACLISALLGPYMPNISRDLRAQLGLDKTMYGYIPDTIIMLLPAGHKIGKP

SPLFTKILDEQVELLRQKYSGKQDFHACGVSSSP

>XP_012534645.1 PREDICTED: methionine--tRNA synthetase, cytoplasmic [Monomorium pharaonis]

MMMIVSTNEGNPNALKLIIAAKLAQKVVTVKILEPRDAVLGRLPTLSTPSGLSLFSVSAALRLILPEPEDLEASIDRWLE

WEISQLQPAILSYAKTSQKTQLWSFLQDLNNELKNKQYLIEKCSDLSTADISIWVSLWSTVLITDTDMINNIAQDLPNVN

SWLTNIYKQSVIQQSIEESVKMFKFERGAKAIASIEAASWFPVNSALNFQVRESVPADINLTKEKEEIVNQEEICNVAKN

WTTEKYLEVKESSYPILPKKGERNILITSALPYVNNVPHLGNIIGCVLSADIFARYCRQRNYNTLYISGTDEYGTATEAK

ALQEKTTPQAICDKFFDIHNDVYRWFSIGFDYFGRTTTPEQTEIVQEFFLKIKSKGYILTETVEQLHCEFCNRFLADRFV

EGMCPRCKYEDARGDQCDGCGHLVNAVELINPRCKICNTSPIMKNSVQFFLDLPKLEKKLKEYSAPLEKGWSNVARVITK

AWLRDGLKPRCITRDLKWGIPVPVEGFEKKVFYVWFDAPFGYISITKRYTKEYKKWWQPAQDTKVDLCQFMAKDNVPFHT

IMFPAYLLAVNQNYTLMRYIMATEYLNYEDKKFSKSRGIGVFGTDARDTGIPADVWRFYLAYIRPETQDSNFNWVDLATK

NNSELLNNLGNFVNRALVFCEKFFDSNVPPLEAEEADWTVLALAQRELLSYINAMEQAKLRDGLKHILTISKHGNQYMQF

QQPWVKIKGNDDDKKKAGTIVAICCNLACLLSALLAPFMPNTAKQLRSQLGLSNKNYGYIPETITNMLPTWHKIGKPSPL

FTKIEDHKVEMLRKKYAGQQETNGEVTNKNESIVSLEATITKQGNLVRELKGKHDKSVWQPQVEILLDLKKKLADLKGNV

NVPAKKSPTKNKKVEPTHVPEQNGDALTDVAALESAIAKQGNLVRELKAKEDGSVWKPQVEILLKLKQRLSDLTGTVPVA

IDKKSKKKK

>XP_011175606.1 PREDICTED: methionine--tRNA synthetase, cytoplasmic [Solenopsis invicta]

MMIVSTNEGNPNALKLIIAAKLAQEAVTVKILKPRDAVLGRLPTLTTPSGLSLFSVSAALRLLLPEPKDLEVSNDRWLEW

EISQLQPTILSHAKTSQKSRLWSFLQDLNNELKDKKYLIEKCSNLSPADISIWVSLWSTILVTDIDMANDITQNLPNVNS

WLTNIYNQSVIQQSIEESTKMFKFERGGKAIASIEVASWFPVNSVSSSQVHESVSADISPTKEKEVEIVNQEELCNVAKS

WTSEHYLEVKESSYPILPKKGERNILITSALPYVNNVPHLGNIIGCVLSADIFARYCRQRNYNTLYISGTDEYGTATEAK

ALQEKTTPQAICDKFFDIHNDVYRWFGIGFDYFGRTTTPEQTEIVQAFFLKIKSQGYVLTETVEQLHCEFCDRFLADRFV

EGTCPGCKYEDARGDQCDGCGHLVNAVELINPRCKICNTRPVVKNSVQFFLDLPKLEEKLKEYSATVYKGWSNVAKVVSK

TWLRDGLKPRCITRDLKWGIPVPVEGFENKVFYVWFDAPFGYISITKRYTKEYKKWWQPPQDTKVDLCQFMAKDNVPFHA

IMFPACLLAVNQNYTLTRNILATEYLNYEDKKFSKSRGIGVFGTDARDTGIPADVWRFYLAYIRPETQDSTFNWVDLATK

NNSELLNNLGNFVNRALVFCEKFFDSNVPPLEAEEADWTVLALAQRELSSYINAMEQAKLRDGLRHILAISKHGNQYMQF

QQPWVKIKGNDDDKKKAGTVVAICCNLACLLSALLAPFMPNVAKQLRSQLGLSNKNYGYIPEKITNMLPTWHKIGKPSPL

FTKIEDQKIEMLRKKYAGQQETNGEVINKSSDESIASLEAAITKQGNLVRELKAKHDKNVWQPQVEILLDLKKKLVDLKG

NVNTPTKKSPAKNKKAEPMHVPEQNGDALTDVAALESAIAKQGNLVRELKAKEDGSVWKPQVEILLKMKQRLSDLTGTAP

VTVDKKSKKKK

>XP_002607629.1 hypothetical protein BRAFLDRAFT_123956 [Branchiostoma floridae]

MKLLTDSGNQHALKAVIAANITGTELQIEEVDQASCSGQLPILELPSGKALFSANLTCRYLLGNKDADDFQVDEWLEWES

SQLQPFLAPYLVTCIGQGKKDSALLAAVVPLLEHLDKALKDRQYIVGSSVTSADVVVWSALFPLYGDSSKLADELTRYSG

IVDWFQNLSTHEAFKSAIATVTKGKGVSACKAGSTSSELSVSAEEVTAAISAFKDGKSPKPWIRTHPMFCRLRNWNTLYI

CGTDEYGTATETKALEEGLTPQQICDKYNKIHSEIYQWFEIGFDFFGRTTTPQQTEFLADRFVEGTCPLCNYDDARGDQC

DKCGKLINATELKLEPALSAFLNTIYPTDDWSNNAKVITRSWVRDGLKPRCITRDLKWGTPVPLEGFTEKVFYVWFDAPI

GYLSITANYTDQWEKWWKNPQQVTLYNFMAKDNVPFHSVVFPCSLLGAEDNYTLVNHLQATEYLNYEDGKFSKSRGVGVF

GNNAKDTGIPADIYRFYLLFVRPESQDSTFSWEDFMTKNNSELLNNLGNFVNRALMFVSNFFQGVIPAVDVTEDDGQLLA

QVNRELQAFIQCLEKVSWGGVGGGNQYIQGQKPWVLVKGNPGEKARAGTVIGLAANVACLLSVMVQPYMPATSNTIQQQL

QAPAEVNILTDTFVCRLQAGHKIGKPSPLFQKIEASTMAELKKKFAGTQMERKEKGQKSKTAAPPVQNAAPVKVDQAEVD

RLTAEVQKQGDAVRQLKTDKAEKKLVDAEVQKLIALKQQLAAAKGENPAEIIGGKGKKNKKGKK

>BAG64411.1 unnamed protein product [Homo sapiens]

MRLFVSDGVPGCLPVLAAAGRARGRAEVLISTVGPEEELSALHSWFQTLSTQEPCQRAAETVLKQQGVLALRPYLQKQPQ

PSPAEGRAVTNEPEEEELATLSEEEIAMAVTAWEKGLESLPPLRPQQNPVLPVAGERNVLITSALPYVNNVPHLGNIIGC

VLSADVFARYSRLRQWNTLYLCGTDEYGTATETKALEEGLTPQEICDKYHIIHADIYRWFNISFDIFGRTTTPQQTKITQ

DIFQQLLKRGFVLQDTVEQLRCEHCARFLADRFVEGVCPFCGYEEARGDQCDKCGKLINAVELKKPQCEVCRSCPVVQSS

QHLFLDLPKLEKRLEEWLGRTLPGSDWTPNAQFITRSWLRDGLKPRCITRDLKWGTPVPLEGFEDKVFYVWFDATIGYLS

ITANYTDQWERWWKNPEQVSSSWLGCAWGG

>OQR78339.1 methionine--tRNA synthetase [Tropilaelaps mercedesae]

MKLYIGEGSVQVCKLDAILHVTKSKVNRIQCTVKESPLKRLPTFELPDGDYLFSANAICRYLWHEAGNTVNSEDEHWLSY

ESLVLLPTVAGILQGKKASLRLQDFNTPPRQASLGYLVTCSTLSALSTNKNYTSILSSIPFYMKFATDFGSKIPKSIASE

SLQGLYIDHVQANSQADTIDEQNLKLTSEFIEKVTATWNEKIGGTPLLQKNTTILPEKGKRNILVTAALPYINNVPHLGN

IVGSVLSADVFARFCRMRGYNTLYIGGTDEYGTATETKALEMECTPKELCDKFHDIHRDIYKWFNIEFDYFGRTSTPKQT

EVAQTIFRKLHANGFLNEDSVEQLYCEKCPRFLADRFVEGICPICAYEDARGDQCDKCSKLINAVELKNPKCKLCKTTPV

VRTSRHLFLDLPKIEPAYRKWQEQSVSTGQWTQSAKVITNSWLRDGLRPRCITRDLKWGIPVPVKGFEGKVFYVWFDAPI

GYMSITASYTDHWEKWWKNPDEVELYQYMGKDNVLFHSLIFPMCLIGTGENFTKVSNVVATEYLNYEDTKFSKSRGIGVF

GTDAKETGIAPDVWRFYMMYLRPESQDTAFSWIDLQTKNNSELLNNLGNFINRCLTFTGNNFIGKIPPFIGDLLKDDKIV

IAQVSRFYHDFLHNLEHNHIRDALRSLLMISKVGNQYIQSTKPWELVKAADTKARCGSILYVGVQICALIAGCLEPFMPD

TAKELRRQLNIPQLQLENQFRPLIPSGHKIKNSAPLFKKIESDAIEKFRKKFGGHQQQPIQSTTNPAELEAKVKKQGDKV

RALKSSKANKETITREVNALLDLKIQLEAALKVATTAAVDPTASVSPIEPEAKIKEQGGKFRALKNSKADKVAVTIGRNC

ISAQETT

>XP_017889841.1 PREDICTED: methionine--tRNA synthetase, cytoplasmic [Ceratina calcarata]

MNVSTNEGNPNVLKLVIAAKMARKSVTVKIIQPKDSSLDRLPSVELSSGLVLFSTNVAMQLLAPPPEDLKTDNDKWLEWE

ITQLQPAIVFYGSTGTCKAIHKSCLWSLLKKLNNALKDKKYLIEDKNGLSLADACIWVTIWSTILVTEVGSELCKEYSSI

NEWLSNIESLSLIQDSIKEYEFERGSKAILSIQAVSWFPVNSTLSSQSLKPSTAHLSPTKEKEVEAVGQEEMQNISSNWS

SKSYPDMKNLSYPVLPKKGEKNILITSALPYVNNVPHLGNIIGCVLSADIFARYCRQRNYNTLFISGTDEYGTATEAKAL

EEKTTPQAICDKFFDIHNDVYRWFSIGFDHFGRTTTPEQTEIVQSFFLKIKSQGYILSETVDQLLCETCDRFLADRFVEG

TCPRCKYEDARGDQCDGCGHLVNATDLISPRCKVCNNRPVVKISQQFFLDLPKLEDKLKEWSGTVEKGWSNVARVVAKPW

LRDGLKPRCITRDLKWGIPVPVKGFENKVFYVWFDAPFGYISITKRYTKEYEQWWKPSDVQVDLYQFMAKDNVPFHAIMF

PACLLAANQGYTLMKHLMATEYLNYEDTKFSKSRGIGVFGTDARDTGIPADVWRFYLAYVRPESQDSNFNWEDLATKNNS

ELLNNFGNFVNRALVFAERYFESKIPSIELQEDDLVLLALAQRELSSYVSALEQAKLRDGLKHVLGISKHGNQYMQSQEP

WVKIKGTDSXRKRAGTIIGICCNLACLLSALLAPFMPNTTRELRLQLGLDSSNYGYIPDYISNILPTGHKIGKPSPLFKK

IEDKDVEALRKKYSGKQDTTNNGERRDAKSLDNAIAELDKKVKELKDKHDKSGWQANQVQIFSDLKKKLSDLISEKNKSP

ESKNKKAEVVSVAEQNGDISMDVANLESAIAKQGNLVRELKAKGDKSVWQPEVDKLLKLKKQLVDITGSVATPTDKKSKK

KK

>XP_011688583.1 PREDICTED: methionine--tRNA synthetase, cytoplasmic [Wasmannia auropunctata]

MIISTNEGNPNALKLIVAAKLAQEAVSVRILKPSDATLGRLPTLTTPTGLSLFSVSSALRLLLSVPKELEVLNDRWLEWE

ISQLQPVILSHAGAKTSKTPQKSDLWSLLKDLNSELKDKQYLIEKCSNLSPADISIWVSLWSTILVTDIANDITKDLPNV

NSWLANIQKQSVIQQSIKEFTEMLKLERGAKAIASIEAASWFPVNTSNIQVRESVPGDISPTKEKEVEIKEELNQEELCN

VAKSWSTEQYPEVKESSYPILPKKGERNVLITSALPYVNNVPHLGNIIGCVLSADIFARYCRQRNYNTLYICGTDEYGTA

TEAKALQEKTTPQAICDKFFDIHNDVYRWFGIGFDYFGRTTTPEQTEIVQAFFLKIKSQGYVLSETVEQLHCESCDRFLA

DRFVEGTCPGCKYEDARGDQCDGCGHLVNAVELINPRCKMCNTRPVVKNSIQFFLDLPKLEEKLKEYTATVEKGWSNVAR

VVLKAWLRDGLKPRCITRDLKWGIPVPVEGFENKVFYVWFDAPFGYISITKRYTKEYKKWWQPPQDMKIDLYQFMAKDNV

PFHAIMFPACLLAANQNNTLLRCLMATEYLNYEDKKFSKSRGIGVFGTDARDTGIPADVWRFYLAYIRPETQDSNFNWVD

LATKNNSELLNNLGNFVNRALVFCEKFFDSNVPPIEPEEADWTVLALAQRELTSYINVMEQARLRDGLKHILAISKHGNQ

YMQFQQPWVKIKGNDDDKKKAGTVVAICCNLACLLSALLAPFMPTTAKQLRSQLGLSNKSYGYIPDTITNMLPTWHKIGK

PSPLFTKIEDQKVEMLRKKYAGQQDTNGDVANKSSQESIASLEAAVTKQGNLVRELKAKNDKSVWQPQVEILLDLKKKLN

DLKSSANAPAKKSPAKNKKAEPTHVPEQNGDALTDVAALESAIAKQGNLVRELKAKEDGSVWKPQVEILLKLKQRLSDLT

GTAPVAVDKKSKKKK

>XP_022817111.1 methionine--tRNA synthetase, cytoplasmic isoform X1 [Spodoptera litura]

MKIYTNENNTSTLKLLIAANLAGKKVELVSASFEDDVFAGPRQLPLLEVDDNLTFFSSNAAASYLFPVLDLTDDGQSEQM

QEWEATRLHPAVAAVLTAKSVASDLKQALQALLSTVDSNLGKHKYILGAKLSAADISIWSTLYPIYYNPELKSGYLAQCS

HISRWIEELASAKAIQEAVKQWGGSAAGPFGSSSALGIPQVASLSSPVGSPDEGAAVELGPTAEEIQAAKDNWLNGLKNL

QKPLIPGKLVLPVKNRKNVLITSALPYVNNVPHLGNIIGCVLSADIFARYCRLCDYNTLYISGTDEYGTATETKALEEGI

SPREICDKYFDIHNSVYRWFNIGFDYFGRTSTPQQTEIAQRMFLKLKDNGFVSSQSVDQLLCTNCDRFLADRFVEGTCPY

PGCGYEGARGDQCDKCGKLITATELVTPRCKVCGTTPSVTPSQQLFIELGQLEPSIRTWITSTEKQWSPPARAVSRAWMR

EPLRARAVTRDLKWGVPVPVEGFTNKVFYVWFDAPIGYLSITQNATKEFEKWWKPDKEYNVKLYQFMAKDNVPFHCIMFP

ATLMGVNEGHVLVSHLYATEYLNYEDGKFSKSRGVGVFGTDAQDTGIPSDVWRFYLASIRPETSDSSFSWIELGTRNNSE

LLNNLGNFCHRSLSFCANTFKGRVPDVAFTHADYELIALVNREVVAYVQHMEKGRLREALRHVLSISRLGNQHMQFQQPW

VLLKGSDDDKKCGATAIGLCCQLVALLCALLTPYLPETTRKLREQLNLKPEKLRINPEKPSLLQYLPAGHVIGKPEPLFT

KIETELLYKLRAKYAGTQSERANNSKENGSAGSVAELEEAVKQQGEKVRQLKSSTKDKSVWQPEVTKLLELKKQLDAAVK

LQATPAPPGVTSVEKLEKDVADQGEKVRQLKASTKDKSVWQPEVAKLLELKKQLEDAKLQTALQATMAKGAGDVSAVEKA

VAEQADKVRKLKASTKDKAVWQPEVAKLLDLKKQLADLQIKK

>XP_022817112.1 methionine--tRNA synthetase, cytoplasmic isoform X2 [Spodoptera litura]

MKIYTNENNTSTLKLLIAANLAGKKVELVSASFEGPRQLPLLEVDDNLTFFSSNAAASYLFPVLDLTDDGQSEQMQEWEA

TRLHPAVAAVLTAKSVASDLKQALQALLSTVDSNLGKHKYILGAKLSAADISIWSTLYPIYYNPELKSGYLAQCSHISRW

IEELASAKAIQEAVKQWGGSAAGPFGSSSALGIPQVASLSSPVGSPDEGAAVELGPTAEEIQAAKDNWLNGLKNLQKPLI

PGKLVLPVKNRKNVLITSALPYVNNVPHLGNIIGCVLSADIFARYCRLCDYNTLYISGTDEYGTATETKALEEGISPREI

CDKYFDIHNSVYRWFNIGFDYFGRTSTPQQTEIAQRMFLKLKDNGFVSSQSVDQLLCTNCDRFLADRFVEGTCPYPGCGY

EGARGDQCDKCGKLITATELVTPRCKVCGTTPSVTPSQQLFIELGQLEPSIRTWITSTEKQWSPPARAVSRAWMREPLRA

RAVTRDLKWGVPVPVEGFTNKVFYVWFDAPIGYLSITQNATKEFEKWWKPDKEYNVKLYQFMAKDNVPFHCIMFPATLMG

VNEGHVLVSHLYATEYLNYEDGKFSKSRGVGVFGTDAQDTGIPSDVWRFYLASIRPETSDSSFSWIELGTRNNSELLNNL

GNFCHRSLSFCANTFKGRVPDVAFTHADYELIALVNREVVAYVQHMEKGRLREALRHVLSISRLGNQHMQFQQPWVLLKG

SDDDKKCGATAIGLCCQLVALLCALLTPYLPETTRKLREQLNLKPEKLRINPEKPSLLQYLPAGHVIGKPEPLFTKIETE

LLYKLRAKYAGTQSERANNSKENGSAGSVAELEEAVKQQGEKVRQLKSSTKDKSVWQPEVTKLLELKKQLDAAVKLQATP

APPGVTSVEKLEKDVADQGEKVRQLKASTKDKSVWQPEVAKLLELKKQLEDAKLQTALQATMAKGAGDVSAVEKAVAEQA

DKVRKLKASTKDKAVWQPEVAKLLDLKKQLADLQIKK

>XP_018395032.1 PREDICTED: methionine--tRNA synthetase, cytoplasmic [Cyphomyrmex costatus]

MIIFTNEGNPNALKLVVAAKLAQETMSVRILKPSDATLGRLPILTIPTGLSLFSVSSALRFLLSVPKDLEVLNDRWLEWE

ISQLQPAILSHTSTKTSKTLQKSDLWSLLKDLNSELKDKQYLIEKCNNLSPADISISVSLWSTILVTDIGNDIAKDLPSI

DSWLANIQKQPVIQQSIKESIEMFKLERGLKAIASIEATSWFPGNAASNPQIHESVPSDISPTKEREVEIVNQEELNNVA

KSWTEQYPEVKESSYPILPKKGERNMLITSALPYVNNVPHLGNIIGCVLSADIFARYCRQRNYNTLYICGTDEYGTATEA

KALQEKTTPQAICDKFFDIHNDVYRWFGIGFDYFGRTTTPEQTEIVQAFFLKIKSQDYVLTETVEQLHCESCDRFLADRF

VEGTCPECKYEDARGDQCDGCGHLVNAVELINPRCKICNTRPVVKNSVQFFLDLPKLEEKLKEYSTARDKGWSNIARVVS

RAWLRDGLQPRCITRDLKWGIPVPVEGFENKVFYVWFDAPFGYISITKRYTKEYKQWWQPPQDTKIDLYQFMAKDNIPFH

TIMFPACLLAANQNYTLMKCTMATEYLNYEDKKFSKSRGIGVFGTDARDTGIPADVWRFYLAYIRPETQDSNFNWVDLAT

KNNSELLNNLGNFVNRALVFSEKFFDSNIPPIEPEEADWTVLALAQRELSAYINAMEQGKLKDGLKRILAISKHGNQYMQ

FQQPWVKIKGNDDDKKKAGTVVAICCNLACLLSALLAPFMPSTAKQLRSQLGLSNKSYGYIPETITNMLPTWHKIGKPSP

LFTKIEDQKVEMLRKKYAGQQETNGDGMNKSSDESIVLLEAAITKQGNLVRELKAKNDKSVWQPQVEILLDLKKKLTDLK

SNTNVPAKKSPAKNKKTEPTHVLEQNGDALTNVTALESAIAKQGNLVRELKAKEDGSVWKPQVEILLKLKQRLSDLTGTA

PVAVDKKSKKKK

>XP_011664075.1 PREDICTED: methionine--tRNA synthetase, cytoplasmic [Strongylocentrotus purpuratus]

MKIYSDPGNQHTLKAVVAAALANLKLEIQFTSITDSKVPYLKRGKLPVLEISPNEYIFSANNICRYILSNGSEQTWELKV

EEWVEWESTHLQALLAPYLTAVFGGGKKNAEPFKLPFCKLNDVLANKQYLVGVSILYGASAFKEAILSQHVKVPLSDAGR

ERGQQAAISQASDGQDESKKDIPPEEVAAAKAAFTRGRKSLPKLTKRVHPVLPQSSGKNILITSALPYVNNVPHLGTIIG

CVLSGDVFSRFCRLRNYNTLYICGTDEYGTATETKALEEGLTPQQICDKYHALHRDIYKWFNIDFDHFGRTTTQKQTEIA

QDIFHRLHAAGHILEDTVEQLQCQKCNRFLADRFVEGICPLCSYEDARGDQCDKCGKLINATDLKKPRCKVCSNTPVIKT

SRHLFLDLPKLAPALEVFLQKSTSEGNWSANAKLITNSWVRDGLKPRCITRDLKWGTPVPLKGYTDKVFYVWYDAPIGYP

SITANYTDQWEKWWKNPQQVQLYNFMAKDNVPFHSVIFPSCLIGANDNYTMVNHLIATEYLNYEDTKFSKSRGIGVFGDN

AQSTGIPADIWRFYLLYIRPETQDSAFSWADFVWKNNSELLNNLGNFINRGLTFLENSFGSVIPEIRLESEDERLIAEIN

RELKSYIALQEKVKIRDALKHILNISRLGNQHIQACKPWVLVKGNPQEK

>XP_011315424.1 PREDICTED: methionine--tRNA synthetase, cytoplasmic [Fopius arisanus]

MILSTNKNNPNALKLVIGGEISGNSVEIRIITTNESTLSRLPTLQLPTGITLISSNAALQYLLPGNEQDLENKWLEWDST

QLQPAIASYGNSGALDTSHKPRIWSLLSQLNSELKTKKYLITPDTHTSADIAIFCTLWCTICSTEVSAEITKEFPHVDKW

LENISILPHVQQSLSKFPLENGFKAISSLNSSSWFPMNSYTGIRQKTAALEPSSPTKEPEAITKQELETSKKNWSTPLHP

SIKPPTYPVLPRKNERNILITSALPYVNNVPHLGNIIGCVLSADVFARYCRQRDYNTLYISGTDEYGTATEAKALEENMT

PQEICDKFFDIHNDIYRWFNIGFDYFGRTTTPEQTEIVQESFLRIKSQGYIITESVDQLLCQNCNRFLADRFVEGTCPGC

KYEDARGDQCDGCGHLVNATELINPRCKVCSKTPVIKASTQFFLDLPKIQPKLKEWASKAEKNWSNVARAVTRPWLRDGL

KPRCITRDLKWGIPVPIDEFKDKVFYVWFDAPLGYVSITKRYTQEYEKWWKPSEGTDVNLYQFMAKDNVPFHAIMFPATL

LAANEGHTLVKHIMATEYLNYEDTKFSKSRGIGVFGTDARETGIPADVWRFYLTYIRPENQDSNFNWVDLATKNNSELLN

NFGNFVNRALTFAVKNFEGKVPEMELREEDHQVLVLVQRELSHYIVAMEQGKFRDGVRHLLGISKHGNQYMQAQQPWVMV

KGKEVKTAGTVIGVCCNISCLLSALLGPFMPDTSRELRKQLGLDGSTYGYIPEKVSILLSPGHKLGEPTPLFSKIEEAVV

EELRSKYGGKQDNGTNGGKTEGSVEELQGQIAAQGLKVRELKSKTDKSVWQPEVQILLKLKEQLAKITETSIITVNTATI

GEKNKENGPVQNGKIDIPALEAAIAKQGLVVRELKANSEKSVWQPELNILLKLKKELSDATGVITPNAVDKKSKKKK

>XP_009063904.1 hypothetical protein LOTGIDRAFT_107815, partial [Lottia gigantea]

SVTEEELSAAKKCWRDGVAKLPELRERKHPILPVPGERNIMICSAEPYVNNVPHLGNIIGCVLSGDAFSRYCRLRNYNSV

YLCGTDEYGTATENKALEDGVTPQEICDKYYKLHTEIYDWFNISFDRFSRTTSTEHTEIVQDIFWKLYNNGYIIKDWVDQ

LHCDNCNRILADRFVSGICPLCSYDDARGDQCDGCGKLINAVELINPKCKSCTNIPKVKATQHLFLDLPKLESEFRQHID

KVFDNGIWSQNAKVITNSWIRDGLKPRCITRDLKWGIPVPLEGFTDKVFYVWFEITIGYVSIAATYTKDWEKWWKNPNEV

ELIHFLGKDNVPFHSVIFPCTLLGANDNFTLISHMSATEFLNYEDTKFSKSRGVGVFGDNAQDTGIPADIFRFYLLFVRP

EAQDTAFSWDDFLLKNNSELLNNLGNFINRGLAFVSSSFNGTIQEINLTSEDLQTVALISRELTIYIQNMEQAKLRDSIR

NVLSISRLGNEMMQVNKPWVLVEGTPDEKARAGSVVSLAANIAWLIGVLLQPYMPSTSEIICTQLNGNPKNMVIPTQFIP

VLKTGHKIGKPTPLFQKIEASLIEKMKKKFQGKQEKVGRNDFLSETLCVKY

>XP_012257625.1 methionine--tRNA synthetase, cytoplasmic isoform X1 [Athalia rosae]

MIVKMFVSTNKNNPNVLKLIIAAKLARQSVEIKTIPPQADVALGRLPLLQIPSGTNLFGTSAALQFLVPTEDSSSILINK

WLEWDISQLQPALVSYGGSGKLDMSHKPRIWSLLKQLNTSLQNKKYLIEAKNYPSVADFSIWVSLWSTIAATEVNVEIIK

EFPLVEGWSNNIECLPTVQESLKEFKIVRGLRAIADIQAASWFPLNSYTGGASHKASPNDVIVNSVKEKEIILVSEEELK

FVTECWTVKNYPSLKDPAYPVLPKKGERNILITSALPYVNNVPHLGNIIGCVLSADIFARYCRQRSYNTLYISGTDEYGT

ATEAKAIEENMTPQQICDKFFSIHNDIYRWFSIGFDYFGRTTTLEQTKIVQDLFLRLKHKGFINAESVDQLLCQTCDRFL

ADRFVEGICPGCKYEDARGDQCDGCGHLVNATELINPRCKMCNNSPIIKPSTQFFLDLPKIEPTLKKWAENVESEWSPTA

RVVAKPWLRDGLKPRCITRDLKWGIPVPLDNYRNKVFYVWFDAPLGYISITKRYTKEYEQWWLPKDIPVDLYQFMAKDNV

PFHAVMFPASLLAADQGHTLVKHIMAIEYLNYENTKFSKSRGIGVFGTDARDTGIPADVWRFYLAYVRPESQDSNFNWVD

LATKNNSELLSNLGNFINRSLMFAEKNFGSKIPPMDLQKDDLIVLALAQRELTSYVAAMERAKLRDALRHILAISKHGNQ

YMQSQAPWVKMKGTDDDKNKAGTVIGVCCNLACLISALLGPYMPNISRDLRAQLGLDKTMYGYIPDTIIMLLPAGHKIGK

PSPLFTKILDEQVELLRQKYSGKQDFHACGVSSSP

>GAV02250.1 hypothetical protein RvY_12841 [Ramazzottius varieornatus]

MSSGVESGEEKRTTQGQQFSAEELATTQEQWLSGAAQSPPVQRSHPVLPKEGARNILISSALPYVNNVPHLGNIIGCVLS

ADVFSRFCRLRGYNSLYVCGTDEYGTATETKALEEGLTPKQICDKYNAIHKEIYQWFNIDFDTFGRTSTEDQTKICQDIF

WKLQKKGLMLEESVKQLFCDSCSRFLADRYVEGMCPYCGADGCRGDQCDKCGKLINAIELITPKCQICKSSPTEKTSNHL

FLDLPTLQPSLEAHLKSVFEKGIWTNNAKGIVQAWLRDGLKPRCISRDLTWGVPVPLEGYTSKVFYVWFDAPIGYISITA

GYTPEHWEKWWKHPKQVEYFNFLGKDNVTFHAAVFPSMLLGADDNFTVVTSISATEYLNYEDDKFSKSRGVGVFGNDAQT

TGIEADIYRFYLLFVRPENQDTVFSWTDLVLKNNTELLNNLGNFVNRALSFLDKNFGGVVPEMKLNQEDLYFLSSVHTEI

TEYLDHMEHVRLREALRSVLSVSRLGNQMMQSAMPWVLVKGSAEEKVRGGTIIGLATNLCLQLSIMLKPFMPHYSKVIQE

QLNASDECYVLHEYVICRLQSGHKIGTPAPLFRKIEDAEAEELRKRFAGRGKKPAQEGAAAAVVEDLEKLKLEHDQQGEI

VRELKANKAAKEEIDRAVAALLDIKKLLKLAGGDTDAVGKKDKKDTGGKKKAI

>XP_022166274.1 methionine--tRNA synthetase, cytoplasmic, partial [Myzus persicae]

MIRGTPSSYVLVNANQLITDFITSIFPPVKCTLTLTGNFYYRLPVKGEKNILVTSALPYVNNVPHLGNIIGCVLSADVFA

RYCRLRGWNTLYISGTDEYGTATETKALEEKLTPQQICDKFFKIHNEIYKWFNISFDHFGRTTSHKQTEIVQEIFKEVHS

NGYTSTASMEQLLCENCDRFLADRFVEGTCPLCNYEDARGDQCDGCGHLINATELLKPRCKVCQKTPVVRSSQQLFLNLP

KVEAKLNDWVDKSGDDWSYNAKVITKSWLKDGLKERCITRDLKWGIPVPLDEFKSKVFYVWFDAPIGYMSMSAVYTPEWR

KWWQPEDDTKVTYYQFMAKDNVPFHSVMFPACLFATERNYTMVNHIMATEYLNYEDGKFSKSRGIGVFGNDAQDTGLPAD

VFRFYLLFVRPESQDSSFSWADLATKNNSELLNNLGNFCNRALSFLEKFFDCVIPEIQLGNDELTLLALVTRDLKEYTSM

LDKAKLRDGLRLILSISRHGNQYMQFQKPWVLIKGSSEEKIRAGTVIGLSCNLACLLAVMLQPYMPQTVNIIAKQLNVDI

STFLLNNFVSVFLQPGHKIGKPAPLFEKIDPSVVNQLKSRFSGKQKSQEKELAVASGLDLKQITSVEQMEEAISKQGDMV

RQLKQSGAQKSDWEPYVKALLEMKKSLEQMKLASNPISIDDLEEEISKQGDKVRKLKEAKVEKSELQPEIDLLIQLKT

>XP_017483024.1 PREDICTED: methionine--tRNA synthetase, cytoplasmic-like [Rhagoletis zephyria]

MLTEAEIDDAFHQFTRTDIPLWKDLRVGDPENVPVPGEQNILITSALPYVNNVPHLGNIVGSVLSADVFARYCRLKGYNV

LYICGTDEYGTATENKALEEKLTPKEICDKYHQIHADIYTWFNISFDKFGRTTTDVQTEIAQDIFWKIYNNGYVTEQEIE

QLFCLQCEKFLADRFVEGVCPYCAYEDARGDQCDKCGKLINAVELKSPRCKTCRNTPEVKISKHLFIDLPKIQPQLEKWI

FGALEKEGNNWSQTAKVITHSWIKGGLQPRCITRDLKWGTPVPLKGYENKVFYVWFDAPIGYLSITAGFTPHWEKWWKNP

DHVELYNFLGKDNVAFHAVIFPSTELSTGENWTMVSHIPAVEYLNYEDTKFSKSRGVGVFGDHAKETGIPSDIWRFYLLY

MRPENQDTTFSWSDLMYKNNSELLNSLGNFVNRSLAFLSNSFGGVVPELQLNETDKNLLVQLNRELASYFEYMDKVQLKD

GLKPILWGISRLGNQYMQNEKPWVLVKNEDTKPRAATVIGLAANISAMLSILLQPYMPDTCVQLQEQMNIKCNQVPSNRK

FICILKTGHQIGKPQPLFKKLDPKEVEGFRTKFSGSSTDATVKAAADEAKLTELVESLALKNELEIAQLVTAQGDKVRQL

KAAKADKAVIAQEVKILLELKKSQTAKAAGAK

>XP_011058454.1 PREDICTED: methionine--tRNA synthetase, cytoplasmic [Acromyrmex echinatior]

MIVSTNEGNPNALKLIIATKLAQETVSVRILKPSDAILGRLPTLTTPTGLSLFSVSSALRFLLSVPKELEVLNDRWLEWE

ISQLQPAILSHANASKTLQKSNLWSLLKDLNSELKDKQYLIEKCSNLSPADISIWVSLWSTILITDIANDIAKDLPSVNS

WLANIQKQPIIQQSIKESNEMFKLERGPKAIASIETASWFPINTTSNSQVHESVPSDISPTKEKEMDIVNPEELRNVAKN

WTTEQYPEVKENSYPILPKKGERNMLITSALPYVNNVPHLGNIIGCVLSADIFARYCRQRNYNTLYICGTDEYGTATEAK

ALQEKMTPQAICDKFFDIHNDIYRWFGIGFDYFGRTSTPEQTEIVQAFFLKIKSQGYVLTETVEQLHCEFCDRFLADRFV

EGTCPGCKYEDARGDQCDGCGHLVNAVELINPRCKICNIKPVVKNSVQFFLDLPKLEEKLKEYSTTRDKGWSNVARVVSK

AWLRDGLQPRCITRDLKWGIPVPVEGFEHKVFYVWFDAPFGYISITKRYTKEYKQWWQPPSQDIKIDLYQFMAKDNIPFH

TIMFPACLLAANQNYTLMKCILATEYLNYEDKKFSKSRGIGVFGTDARDTGIPADVWRFYLAYIRPETQDSNFNWVDLAT

KNNSELLNNLGNFVNRALVFCEKFFDSNIPPIEPEEADWTVLALVQRELSAYINAMEQGKLKDGLKRILAISKHGNQYMQ

FQQPWVKIKGNDDNKKKAGTVVAICCNLACLLSALLAPFMPNIAKQLRSQLGLSNKNYGYIPETITNMLPTWHKIGKPSP

LFTKIEDQKVEMLRKKYAGQQETNGDGTNKSSDESIALLETAITKQGNLVRELKAKNDKSVWHPQIEILLDLKKKLTDLK

SNANAPAKKSPAKKSPAKNKKAEPMQVPEQNGDALTDVAALESAIAKQGNLVRELKAKEDGNVWKPQVEILLKLKQRLSD

LTGTTPVIVDKKSKKKK

>XP_023799032.1 methionine--tRNA synthetase, cytoplasmic [Cyanistes caeruleus]

MSLPPPVIPAHSWWALCIQVVLGRSLQVCTGVPWMQCHLALPYLIPELSVGSLPIPVPTPGVCCRYCRLRNWNTLFVCGT

DEYGTATETRALEEGLSPQELCDRYHAVHADVYAWFRISFDHFGRTTTPQQTRIAQDIFQRLLARGFLLQDTLEQLRCES

CGRYLADRFVEGTCPFCGYAEARGDQCDKCGKLINAVELKNPQCKLCRGTPVVTPTQHLFLDLPKLEGQLEAWLERSWAS

GDWTANARHITRTWLRDGLKPRCITRDLTWGTPVPLDGFRDKVFYVWFDAPIGYLSITANYTEQWERWWKNPQQVELYNF

MAKDNVPFHSVVFPCSLLGADDNYTLVNHLIATEYLNYEDGKFSKSRGVGVFGDMAKDTGIPADVWRFYLLYLRPEGQDS

AFSWADLLLKNNSELLNNLGNFINRAGMFVCKFFAGTVPDMVLTAEDQRLLARLTLEICQYHQLMEKVRIRDALRCVLSI

SRHGNQYIQVNEPWKRIKGDERDR

>ETN60175.1 methionine-tRNA synthetase [Anopheles darlingi]

MIIYTNTGNPFGLKLLICAKLAKREVEVKHVTLNDPSLDDWKHLPVLELDSGKRLFSTDLAVKYLLSDEDASSTVVQRDE

WLEWSTKRLAPALTHNMAVGSRSDPNTKPILNTLVKMLEDCLSKSTFLTGDKLTSADVSVWSLIAPDGTLKGAVNIDNLL

RWYRQISTLPEVQAALGVLPLKELSFASLQHSNRFGGLHHIVLNPEIADFEGELLADTAANIAETVQPEEIEAARVAFAD

VDNRAIRDEPRIVLPKAGQKNNLITSALPYVNNVPHLGNIVGCVLSADIFARYSRLCGYNTLYIGGTDEYGTATETKALA

ENMTPRQICDKYFDIHNSIYRWFGIGFDYFGRTTTTEQTRIVQELFGELYAKGYIETRAVEQLLCTKCDRYLADRFVEGT

CPHPGCGYEDARGDQCDGCGKLVNAIELLRPRCKVCNSSPIMRESNQFFLDLPKIEPELRAWIDRSDPGWTHNARVITRS

WLKEGLKSRCITRDLKWGIPVPVEGYEKKVFYVWFDAPIGYISITSRYCKEWKQWWQPDTANTGVKVDLYQFMAKDNVPF

HSVMFPASLLAVNQGHTLVSHIMATEYLNYEDGKFSKSRGIGVFGNDAQDTGIPADVWRFYLGASRPEGQDSSFSWSDLQ

ARNNNELLKNLGNFVNRALVFCEKFFDSTIPPMTLIEDDYTLLALANRELKGYVHQMEKARMRDGIRHMLQISRYGNQFM

QNEEPWVKVKGTDEQKARAGTVIGLCCNFACLLATLIFPFMPATARNLYSQLNVRGGYINADKPLLRTVLPSGHRIGKPA

VLFSKIEDSRLAELKQKYGGQQQSSASETAKPAASAKSAPNSLKEAQKAVDEQAAKVRKVKSSGADRSVWQPEVNILLEL

KKQLQALGLPRTSESAPQAPGEPQQQQPSAAPASPAATTADPQAVKTLEEDIAKQGEKVRQLKASKSDKSVWQPEVSILL

SLKEKLASLTGVVAPAPSSGSGKKKGNKK

>KFB39744.1 AGAP007891-PA-like protein [Anopheles sinensis]

MIIYTNTGNPFGLKLLICANLAKKDVEVKNVTLNDSELGDMKHLPTLQLDSGVQLFSTDVAAKYLLKGENEDTVVLRDEW

LEWSSKRLAPALTHNMAVGSRADPNTKPILNTLVKMLDDCLSSRTFLTGEKISSADVAVWSLLAPDGTLKGAVNIDNLLR

WYRQISNLPEVQAALAKMPLKELSFASLQHSNRFGGLHHIVLNPEIADFEGELLADTSANIAETVQKEEIEAARVAFNEV

DERAVRDEPRIVLPKPGQKNNLITSALPYVNNVPHLGNIVGCVLSGDMFARYSRLCGYNTLYIGGTDEYGTATETKALAE

NLTPKQICDKYFDVHNSIYRWFGIGFDYFGRTTTNEQTRIVQELFKDLYATGYIETRSVEQLLCQKCDRFLADRFVEGTC

PHPGCGFEDARGDQCDGCGKLVNAIELVRPRCKVCNTSPVIRDSNQFFLDLPRIEPTLREWVERVDHGWTHNARVITRAW

LKEGLKSRCITRDLKWGIPVPVEGYESKVFYVWFDAPIGYISITSRYCKEWQQWWRPDTANTGVKVDLYQFMAKDNVPFH

SVMFPASLLAVDKGYTLVSHIMATEYLNYEDGKFSKSRGIGVFGNDAQETGIPADVWRFYLGASRPEGQDSTFSWADLQA

RNNNELLKNLGNFVNRALVFCEKFFDSTIPVMTLTEEDLTLLALINRELKGYVNQMEKARMRDGIRHLLQISRYGNQFMQ

SEEPWVKVKGTDDQKARAGTVVGLCCNFACLLATLIFPFMPTTARNLYGQLNVRGGFINSEKPLLRTLLPSGHRIGKPAV

LFSKIEDSRIEELKKRYAGQQQEKDKSPAPGAKLSAVAEEKKLPSLKEAQKAVDEQAAKVRKIKSSGADRTVWQPEVNLL

LELKKQLQSLSQPRTSESAPPSPLEGKPTKGVPTASKEQVNPQPAGVNNQPVADSNAVKALEEEIAQQGEKVRKLKAASS

DKSAWQPEVTLLLSLKQKLASLTGIAPAAPASSGKKKGKK

>XP_022690176.1 probable methionine--tRNA synthetase, cytoplasmic [Varroa jacobsoni]

MKLYICEDSVQTCKLDAVLQETKYQVERVQCKAKESPLKRLPAFELADGDHLFSTNAICRYLWAKAGKTVDTEDEYWLSY

ESSVLLPSVAGIVLGKKIALRLEDFNPPPKQASLGYLVICSALSALSTDENCAALLSSISFYAKFASDFANIIPKSIALE

SLLNLYTEQIQVTKQSQSCSDEQHIKLTHEFIDKAVTLWKDKVGGNPLTEKTAPILPEKGKRNMLITAALPYINNVPHLG

NIVSSVLSADVFARFCRMRGYNTLFIGGTDEYGTATETKALELKCTPRELCDKYHTIHKDIYKWFNIEFDYFGRTSTSKQ

TEMAQAVFNKLHENGYLSADSVEQLYCEECPRFLADRFVEGACPFCAYENARGDQCDKCSKLINAIELKHPKCKLCKSTP

IIKASQHLFLDMPKIDSAFREWLDVSIKTGQWTQTAKVITNSWLREGLRPRCITRDLKWGVPVPIQGYENKVFYVWFDAP

IGYLSITSSYTDQWEKWWKNPEEVELSQFMGKDNVPFHAIIFPMCLIGTGENFTKVNNVVATEYLNYENTKFSKSRGVGV

FGTDAKETGIVSDVWRFYMMYLRPESQDTAFSWADLQTKNNSELLNNLGNFVNRCLTFIGNNFAGQVPSFVGDLLEEDKM

VIAQVAQFYHDFLQKLEHNHIRDALRSLLMISKMGNQYIQSTRPWELVKTADTRARCGNTLYIGVQICALIAGCLEPFMP

DTAKELRHQLNITKLQLDDQFRPLVAAGHKINKATPLFKKIDTDVIEEFRKKYAGDQQGRTQLVKDSTELMTKLKKQEDK

LRTLKAAKADRQLIIRETNVLFDMKKLLEVSQIAEASSVIGSDQALFLTAAEFETRVKEQGDKVRALKTNKAEKEIISKE

VDVLLSLKKQLEAARILDGFQAPNYKGDAISSPEEIQKKVTAQGDKVRRLKADNAEKVVIDKEVTILLELKRQLAMAKGE

SPTTSRTTTTNNNNKDSKEK

>XP_018306644.1 PREDICTED: methionine--tRNA synthetase, cytoplasmic isoform X1 [Trachymyrmex zeteki]

MIVSTNEDNPNALKLVIAAKLAQETVSIRILKPSDATLGRLPTLTTPTGLSLFSVSSALRFLLSVPKELEVFNDKWLEWE

ISQLQPAILSHASAKTSKTLQKSDLWSLLKDLNSELKDKQYLIEKCSNLSPADISIWVSLWSTILITDIANNIAKDLPSV

DSWLANIQKQPIIQQSIKESIEMFKLERGSKAIASIEAASWFPVNTASNPQVHESVPSDISPTNKEKEIDVVNQEELRNV

AKSWTTEQYPEVKESSYPILPKKSERNILITSALPYVNNVPHLGNIIGCVLSADIFARYCRQRNYNTLYICGTDEYGTAT

EAKALQEKTTPQAICDKFFNIHNDIYRWFGIGFDYFGRTSTLEQTEIVQAFFLKIKSQGYVLTETVEQLHCESCDRFLAD

RFVEGMCPGCKYEDARGDQCDGCGHLVNAVELINPRCKICNTRPVVKNSVQFFLDLPKLEEKLKEYSTTRDKGWSNVARV

VTKAWLRDGLQPRCITRDLKWGISVPVEGFENKVFYVWFDAPFGYISITKRYTKEYKQWWQPPQDTKIDLYQFMAKDNIP

FHAIMFPACLLAANQNYTLMKCIMATEYLNYEDKKFSKSRGIGVFGTDARDTGIPADVWRFYLAYIRPETQDSTFNWVDL

ATKNNSELLNNLGNFVNRALVFCEKFFDSNIPPIEPEEADWTVLALAQRELSAYINAMEQGKLKDGLKRILAISKHGNQY

MQFQQPWVKIKGNDDDKKKAGTVVAICCNLACLLSALLAPFMPSTAKQLRSQLGLSNKSYGYIPETITNTLPTWHKIGKP

SPLFTKIEDQKVEMLRKKYAGQQESNGDGTNKSSNESIASLEAAITKQGNLVRELKAKNDKSIWQPQVEILLDLKKKLTD

LKSNANAPAKKSPAKNKKAEPIHVPEQNGDALTDVAALESAIAKQGNLVRELKAKEDGSVWKPQVEILLKLKQRLSDLTG

TAPVAVDKKSKKKK

>XP_011882967.1 PREDICTED: methionine--tRNA synthetase, cytoplasmic [Vollenhovia emeryi]

MIVSTNEGNPNALKLIVAAKLAQRTVSVRILKPSDAALGRLPTLTAPSGLSLFSVSSALRFLLPVPKETEVLNDRWLEWE

ISQLQPAILRDHANAGASKGLQQSDLWSLLKDLNSELKDKEYLIEKCSNLSPADISIWVSLWSTILVTDIASDIAKDLPN

VDRWSANIQKQSVVQQSIKESIEMFNLERGAKAVASIQAASWFPVNSAPNFQVRESVPSXISPTKEKEEIVSKEELCKVT

KNWPTEKYPEVKESSYPILPKKDERNILITSALPYVNNVPHLGNIIGCVLSADIFARYCRQRNYNTLYICGTDEYGTATE

AKALQEKTTPQAICDKFFDIHNDVYRWFGIGFDYFGRTTTPEQTEIVQDFFLKIKSQGYVLSESVEQLHCESCDRFLADR

FVEGTCPGCKYEDARGDQCDGCGHLVNAVELINPRCKICSTRPVVKNSVQFFLDLPKLEDRLKKYSVAREKGWSNVARVV

SRAWLRDGLKPRCITRDLKWGIPVPVEGFENKVFYVWFDAPFGYISITKRYTKDYKEWWQPPQDTKIDLCQFMAKDNVPF

HVIMFPACLIAANQNYTLMRYIMATEYLNYEDKKFSKSRGIGVFGTDARDTGIPADVWRFYLAYIRPETQDSNFNWVDLA

TKNNSELLNNLGNFVNRALVFAEKFFDSNVPPIEAEEADWTVLALAQRELSSYINAMEQAKLRDGLKHILAISKHGNQYM

QFQQPWVKVKGNDDDKKKAGTVIAICCNLACLLSSLLAPFMPSTAKELRLQLGLSNKSYGYIPETITNMLPTWHKLGKPS

PLFTKIEDQKIEMLRKKYAGQQENNGEGANKSSNESIASLEAAITKQGNQVRELKAKHDKSVWQPQVEILLDLKKKLNDF

KSNASAPGKKSPAKNKKAGPTHVPEQNGDALTNVAALESAIAKQGNLVRELKAKEDGSVWKPQVEILLKLKQRLTDLTGT

TPVAVDKKSKKKK

>XP_015586893.1 methionine--tRNA synthetase, cytoplasmic isoform X3 [Cephus cinctus]

MPLLQLCPRIVNAELGRLPVLELPSGIRIFSISAAVQFLVPMGEEFSIHINKWLEWDISQLQPALVSYGGLGKLDTSHKP

WIWSLLKELNAALKDKKYLIKDKDNPSIVDLSIWITLWSTITPLDISKDIAVEFPTVNKWLKAIEVIPIVQESIKEFNIE

KGLKAIASIQAVSWFPVNSYTDSSSSISALSDVNVLKEQEIETVSEEELNNVAKNWIVTCYSTVKEPPYPVLPIKGERNI

MITSALPYANNVPHLGNIIGCVLSADIFARYCRQRNYNVLFICGTDEYGTATEAKAVEEKTTPQEICDKFFGIHNDIYRW

FSIGFDYFGRTTTPEQTEIVQAIFLRIQSQGYIFTENVDQLLCESCQRFLADRFVEGTCPRCSYTDARGDQCDGCGHLVN

AIELINPRCKVCNSKPMIKVSTQFFLDLPKIEPRLQEWAKSAEVNWSSVARAVSKPWFRDGLKPRCITRDLKWGIPVPLE

GYRDKVFYVWFDAPLGYVSITKRYTKEYEQWWKPKYVDVSLYQFMAKDNVPFHSVMFPASLLAADQGHTLVKHIMATEYL

NYEDTKFSKSRGIGVFGTDAKETGILPDVWRFYLAYVRPESQDSNFNWVDLATKNNSELLNNLGNFINRALMFVERNFAS

KVPPMEPQEDDLTVLALSQRELSSYVAALEQAKLRDALRHILAISKHGNQYMQSQEPWVKVKGTDDDKKKAGTVIGICCN

LVALLSALLAPYMPNTSREVRSQLGLDKSSYGYIPDTITTILPAGHTIGKPSPLFTKIDDQKVEELRKKYGGKQECPIQK

VSSGTIEKCNDAASLEAAIAKQGLLVRELKAKQDKSVWQPQVQILLQLKKQLADLTSNTGKTSDQAKEKHNSTPGVVLEQ

NGDGINGAADLEAAIAKQGVLVREMKAKAEKSVWQPQVEILLKMKKQLADLTGAIIPESSNKKSKKKK

>XP_018365133.1 PREDICTED: methionine--tRNA synthetase, cytoplasmic isoform X1 [Trachymyrmex cornetzi]

MIVSTNEGNPNALKLIIATKLAQETVSVRILKPSDATLGRLPTLATPTGLSLFSVSSALRFLLSVPKELEVLNDRWLEWE

ISQLQPAILSHASAKTSKALIKQKSNLWSLLKDLNSELKDKQYLIEKCSNLSPADISIWVSLWSTILITDIANDIAKDLS

SVNSWLANIQKQPIIQQSIKESIEMFKLERGPKAIASIEAVSWFPINTVSNPQVHESVSSDISPTKEKEMDIVNQKELCN

VAKSWATEQYPEVKESSYPILPKKDERNMLITSALPYVNNVPHLGNIIGCVLSADIFARYCRQRNYNTLYICGTDEYGTA

TEAKALQEKTTPQAICDKFFDIHNDVYRWFGIGFDYFGRTSTPEQTEIVQAFFLKIKSQGYVLTETVEQLHCESCDRFLA

DRFVEGTCPGCKYEDARGDQCDGCGHLVNAVELINPRCKICNTKPVVKNSVQFFLDLPKLEEKLKEYSTTRDKGWSNVAR

VVSKAWLRDGLQPRCITRDLKWGIPVPVKGFENKVFYVWFDAPFGYISITKRYTKEYKQWWQPPQDTKINLYQFMAKDNI

PFHTIMFPACLLAANQNYTLMKCILATEYLNYEDKKFSKSRGIGVFGTDARDTGIPADVWRFYLAYIRPETQDSNFNWVD

LATKNNSELLNNLGNFVNRALAFSEKFFDSNIPPIEPEEADWTVLALAQRELSAYINAMEQGKLKDGLKRILAISKHGNQ

YMQFQQPWMKIKGNNDDKKKAGTVVAICCNLACLLSALLAPFMPSTAKQLRSQLGLSNKSYGYIPETITNMLPTWHKIGK

PNPLFTKIEDQKVEMLRKKYAGQQETNGDGTNKSSNESIALLEAAITKQGNLVRELKAKNDKSVWQPQVEILLDLKKKLT

DLKSNANAPAKKSPAKNKKAEPMHVPEQNGDTLTDVATLESAIAKQGNLVRELKAKEDGSVWKPQVEILLKLKQRLSDLT

GTAPVVVDKKSKKKK

>XP_015191643.1 PREDICTED: methionine--tRNA synthetase, cytoplasmic [Polistes dominula]

MIVSTNESNPNALKLLIAAKLEKKTINIKITVPNETVLNRLPFLELPNGSKIFSTSAGMQFLAAPSEEYEIITNKWLEWD

ASQLQPAITYYGTTGMYKTDSKSCLWSLLSEINNALKDKMYLIKDKNDISLADASIWVSIWSTVMVTEIGNEILKEYVYL

SDWLKNIELLPIIQDSLKVFSLERGLKAINSIQAGSWFPVNSTKSCKSTTNELNLNKEKDEKDEVVSQEELDKAATNWNS

CNCPNVKEPSYPVLPKKGERNILITSALPYVNNVPHLGNIVGCVLSADIFARYCRQRNYNTLYISGTDEYGTATEAKALE

EKTTPQEICDKFFSIHNDVYRWFGIGFDYFGRTTTAEQTEIVQSFFLKIKSQGYVLMDTVDQLLCESCDRFLADRFVEGI

CPRCKYEDARGDQCDGCGHLVNATELINPRCKICSNRPIIKKSRQFFLDLPKLEERLKVWSAKVEKGWSAVARVVTKPWL

RDGLKPRCITRDLKWGIPVPAEGFENKVFYVWFDAPFGYISITKRYTKEYEQWWKPNGTDISLYQFMAKDNVPFHTIMFP

ASLLATNEDYTLLKHLMAIEYLNYEDTKFSKSRGIGVFGTDARETGIPADVWRFYLAYIRPESQDSNFNWADLATKNNNE

LLNNLGNFINRALVFTEKFFESKVPPMEPQEDDLTLLALAQRELTSYIIALEQAKLRDGIKYVLAISKHGNQYMQSQQPW

AKVKGTDDDKKKAGTVIGICCNLTCLLSALLAPYMPSTSREIRGQLGLDKTRYGYIPDTIMNMLPSGHKIGKASPLFTKI

EDQLIEELRKKYSGKQESNGTQSKMNNGTEQLVKEVICNNDKNVQQPNVDVPVKMQNKQADLKQDTDTANNKKVKPLHTP

EQNGDTSTDINSLEAAIAKQGTLVRELKAKEEKSVWQPQVDILLKLKNQLASLTGTKIPDKKSKKKK

>XP_022655681.1 probable methionine--tRNA synthetase, cytoplasmic [Varroa destructor]

MKLYICEDSVQTCKLDAVLQETKYQVERVQCKAKESPLKRLPAFELADGDHLFSTNAICRYLWAKAGKTVDTEDEYWLSY

ESSVLLPSVAGIVLGKKIALRLEDFNPPPKQASLGYLVICSALSALSTDENCAALLSSISFYAKFASDFANIIPKSIALE

SLLNLYTEQIQVTKQSQSCSDEQHIKLTHEFIDKAVTLWKDKVGGNPLTEKTAPILPEKGKRNMLITAALPYINNVPHLG

NIVSSVLSADVFARFCRMRGYNTLFIGGTDEYGTATETKALELKCTPRELCDKYHTIHKDIYKWFNIEFDYFGRTSTLKQ

TEMAQAVFNKLHENGYLSADSVEQLYCEECPRFLADRFVEGACPFCAYEDARGDQCDKCSKLINAIELKHPKCKLCKSTP

IIKASQHLFLDMPKIDSAFREWLDVSIKTGQWTQTAKVITNSWLREGLRPRCITRDLKWGVPVPIQGYENKVFYVWFDAP

IGYLSITSSYTDQWEKWWKNPEEVELSQFMGKDNVPFHAIIFPMCLIGTGENFTKVNNVVATEYLNYENTKFSKSRGVGV

FGTDAKETGIVSDVWRFYMMYLRPESQDTAFSWADLQTKNNSELLNNLGNFVNRCLTFIGNNFAGQVPSFVGDLLEEDKM

VIAQVAQFYHDFLQKLEHNHIRDALRSLLMISKMGNQYIQSTRPWELVKTADTRARCGNTLYIGVQICALIAGCLEPFMP

DTAKELRHQLNITKLQLDDQFRPLVAAGHKINKATPLFKKIDTDVIEEFRKKYAGDQQGRTQLVKDSTELKTKLKKQEDK

LRTLKAAKADRQLIIRETNVLFDMKKLLEVSQIAEASSVIGSDQALFLTAAEFETRVKEQGDKVRALKTNKAEKEIISKE

VDVLLSLKKQLEAARILDGFQAPNYKGDAISSPEEIQKKVTAQGDKVRRLKADNAEKVVIDKEVTILLELKRQLAMAKGE

SPTTSRTTTTNNNNKDSKEK

>XP_019884194.1 PREDICTED: methionine--tRNA synthetase, cytoplasmic isoform X1 [Camponotus floridanus]

MIISTNENNPNALKLIIAAKLIQKTFIVKVVKSGACIALCTDAVLGRLPTLTVSSGLSLFSVSSALRLLLPISKELEVLN

NRWLEWEISQLQPTILSYTSARTPKILQKSDLWSLLKDLNSELKNKQYLIEEHSNLSPAFILSPADISIWVSLWSTILLP

DIRNDISKDLSNVNNWLINIEKQPVIQESIKEFNLERGARAIASIEAVSWFPVNATSNNSQLCKPVSSDISPTKEKEMET

VSQEELHNVEKSWTTGQYPEVKENSYPILPKKGERNILVTSALPYVNNVPHLGNIIGCVLSADIFARYCRQRNYNTLYIS

GTDEYGTATEAKALQEKTTPQVICDKFFDIHNDVYRWFGIGFDYFGRTTTPEQTEIVQAFFLRIKSQGYVSSETVEQLHC

ESCDRFLADRFVEGTCPRCKYEDARGDQCDGCSHLVNAVELINPRCKMCNTRPVIKNSIQFFLDLPKIEKRLKEYSITVE

KEWSSVARAVSKSWLRDGLKPRCITRDLKWGIPVPIEGFENKVFYVWFDAPFGYISITKRYTKEYKKWWQPLQDTKIDLC

QFMAKDNVPFHAIMFPAYLLAANQNYTLIKYLMATEYLNYEDKKFSKSRGIGVFGTDARDTGIPADVWRFYLAYIRPETQ

DSNFNWVDLATKNNSELLNNLGNFVNRALVFSEKFFNSTVPPIEAEEADWTVLALAQRELSCYINAMEQAKLRDGLKYIL

AISKHGNQYMQFQQPWVKVKGTDDNKKRAGTVIAICCNLACLLSALLAPFMPSTAKQLRSQLGLSNKSYGYIPETITNML

PTWHKIGKPNPLFTKIEDQKIEMLRKKYDGQQETNNGISANKVSDESITLEAAITKQGNLVRELKAKRDKSIWQPQVEIL

LDLKKKLNDLKNNVNTSEKKLPAKNKKVEPIHAPEQNGDALTDVMALEMAIAKQGNLVRELKAKEDGSIWKPQVEILLEM

KQRLMDLTGTAPVVVDKKSKKKK

>XP_011262093.1 PREDICTED: methionine--tRNA synthetase, cytoplasmic isoform X2 [Camponotus floridanus]

MIISTNENNPNALKLIIAAKLIQKTFIVKVVKSGDAVLGRLPTLTVSSGLSLFSVSSALRLLLPISKELEVLNNRWLEWE

ISQLQPTILSYTSARTPKILQKSDLWSLLKDLNSELKNKQYLIEEHSNLSPAFILSPADISIWVSLWSTILLPDIRNDIS

KDLSNVNNWLINIEKQPVIQESIKEFNLERGARAIASIEAVSWFPVNATSNNSQLCKPVSSDISPTKEKEMETVSQEELH

NVEKSWTTGQYPEVKENSYPILPKKGERNILVTSALPYVNNVPHLGNIIGCVLSADIFARYCRQRNYNTLYISGTDEYGT

ATEAKALQEKTTPQVICDKFFDIHNDVYRWFGIGFDYFGRTTTPEQTEIVQAFFLRIKSQGYVSSETVEQLHCESCDRFL

ADRFVEGTCPRCKYEDARGDQCDGCSHLVNAVELINPRCKMCNTRPVIKNSIQFFLDLPKIEKRLKEYSITVEKEWSSVA

RAVSKSWLRDGLKPRCITRDLKWGIPVPIEGFENKVFYVWFDAPFGYISITKRYTKEYKKWWQPLQDTKIDLCQFMAKDN

VPFHAIMFPAYLLAANQNYTLIKYLMATEYLNYEDKKFSKSRGIGVFGTDARDTGIPADVWRFYLAYIRPETQDSNFNWV

DLATKNNSELLNNLGNFVNRALVFSEKFFNSTVPPIEAEEADWTVLALAQRELSCYINAMEQAKLRDGLKYILAISKHGN

QYMQFQQPWVKVKGTDDNKKRAGTVIAICCNLACLLSALLAPFMPSTAKQLRSQLGLSNKSYGYIPETITNMLPTWHKIG

KPNPLFTKIEDQKIEMLRKKYDGQQETNNGISANKVSDESITLEAAITKQGNLVRELKAKRDKSIWQPQVEILLDLKKKL

NDLKNNVNTSEKKLPAKNKKVEPIHAPEQNGDALTDVMALEMAIAKQGNLVRELKAKEDGSIWKPQVEILLEMKQRLMDL

TGTAPVVVDKKSKKKK

>XP_013136733.1 PREDICTED: methionine--tRNA synthetase, cytoplasmic isoform X2 [Papilio polytes]

MKVYTNHGNTGTLKVLIAAQLAGKRVEKCLASFEGPQALPLLEVDDLLTFFSSNAAVQYLFPTLDLSENGQCQQMLEWEA

TRLQPAVMALLSGKAVAADLRQVLQASLQMLDKLLQKNKYILGEKLSAADISIWSTLYPVCFDESHKQTYITGCDNIVRW

NTNLASAKAIQEAVQQWGWSPMSPYGVSSALGIPQIASIVSPIGSPDEDPAAISVSPEEIEAAKDGWINGVKNMQAPIQH

EKVVLPVKDRKNVLITSALPYVNNVPHLGNIIGCVLSADVFARYCRQCDYNTLYICGTDEYGTATETKALEEGITPREIC

DKYFDIHNAVYRWFDIGFDYFGRTSTKEQTEISQKLFLKLIDNGFVSTQSVDQLYCEKCERFLADRFVEGVCPHPGCLYG

GARGDQCDKCGKLINAIELVEPRCKVCSHRPTVRPSEQLFIELGQLEPSLRTWIGRAESGWSAPARAVTRAWLRDSLRAR

AVTRDLKWGVPVPIDKHAHKVFYVWFDAPIGYMSITQCLTKHYEKWWKPDKDVDVKLYQFMAKDNVPFHVIMFPATVIGV

NEGYKLVDHIFATEYLNYEEGKFSKSRGVGVFGTDAQETGIPSDVWRFYLTSIRPETSDSSFSWVELGTRNNSELLNNLG

NFCHRSLSFCANSFKGAVPDVALTAADLELIALVNREVRSYVENMEKGRLREALRHVLSVSRLGNQHMQAEQPWVLLKGS

QHDKVRGATAVGVCCQLAALVCALLAPFMPHTARRLREQLNLPEVALRINPQNPSLVQYLPPGHVIGKPEPLFSKIEPEL

LEKLRAKYAGTQSDRAKQNGDTKSSPSKSVADLEAAVEKQGNKVREIKASTKDKTVWQPEVNKLLDLKKQLEAAKVAHKN

TSTTSSDSVASLEKAIAEQGEKVRKLKASTKDKSVWQPEVNKLLQLKKQLTAAQAQANTASAASVASTESKDDVASLEKA

ITEQGDKVRKLKASTKDKAVWQPEVNVLLDLKKRLAALQVK

>XP_022121588.1 methionine--tRNA synthetase, cytoplasmic isoform X1 [Pieris rapae]

MKIYTNDNNTSTLKLLIAANLAGKKLELQKSTFEDQFSGPKCLPLLEVDEELTFFSSNAAVQYLFPILDLSKNGQCQQMQ

EWEAIRLQPAVANVLSGKTVPADVKQVLQSLVLVLNELLKKHKYIIGDKLSAADISIWSTLYPLLYNEELKSYLSECSHI

LQWAEELNKAKAFQEAAKQWGGNPKGPFSSISALGTPQITNLPTPTGSPDDSVPISVSQEEADLAQENWLNGLKNLQAPI

KQEKLVLPVKDRKNVLITSALPYVNNIPHLGNIIGCVLSADIFARYCRLCDYNTLYISGTDEYGTATETKALEEGVTPRQ

ICDKYYEIHNSVYRWFNIGFDYFGRTSTPEQTEIVQKLFLKVLDNGFMSTQSVEQLFCEKCERFLADRFVEGKCPHPGCA

YDGARGDQCDKCGKLINAIELIEPKCKVCAHRPTVRPSEQLFIELGQLEPSIRSWMSSVEGNWSGPARAVSRAWLREGLR

ARAVTRDLKWGVPVPLDKYKHKVFYVWFDAPVGYTSITQCATKQYEKWWKPDKDYDVKLYQFMAKDNVPFHAIMFPASVI

GANEGHKLVDYIFATEYLNYEEGKFSKSRGVGVFGTDAQETGIPSDVWRFYLASIRPETSDSSFNWTELCTRNNSELLNN

LGNFCHRSLSFCANTFKGKIPEMSCSNTDYVLFALVNREVVAYVQNMEKGRLREALRHVMSVSRLGNQHMQSEQPWVLLK

GSPEEKIRGSTAIAVCCQLVALLCTLIAPYMPDTSRKLKAQLNLPDTSFRINPTNPALFQYIPKGHVIGKPEPLFAKIEQ

DLVDAMRAKYAGTQAERAKQNGDVKTGASTNVADLEKAIAAQGEKVRQLKASTKDKTVWQPEVNKLLELKKQLTAAQSQP

VMDVASLEKAIAEQGEKVRKLKASTKDKTVWQPEVNKLLELKKQMANAQSSIATPANVANLEKAIAEQGDKVRKLKASTK

DKSVWQQEVNVLLDLKKQLAAAQTK

>XP_022121589.1 methionine--tRNA synthetase, cytoplasmic isoform X2 [Pieris rapae]

MKIYTNDNNTSTLKLLIAANLAGKKLELQKSTFEGPKCLPLLEVDEELTFFSSNAAVQYLFPILDLSKNGQCQQMQEWEA

IRLQPAVANVLSGKTVPADVKQVLQSLVLVLNELLKKHKYIIGDKLSAADISIWSTLYPLLYNEELKSYLSECSHILQWA

EELNKAKAFQEAAKQWGGNPKGPFSSISALGTPQITNLPTPTGSPDDSVPISVSQEEADLAQENWLNGLKNLQAPIKQEK

LVLPVKDRKNVLITSALPYVNNIPHLGNIIGCVLSADIFARYCRLCDYNTLYISGTDEYGTATETKALEEGVTPRQICDK

YYEIHNSVYRWFNIGFDYFGRTSTPEQTEIVQKLFLKVLDNGFMSTQSVEQLFCEKCERFLADRFVEGKCPHPGCAYDGA

RGDQCDKCGKLINAIELIEPKCKVCAHRPTVRPSEQLFIELGQLEPSIRSWMSSVEGNWSGPARAVSRAWLREGLRARAV

TRDLKWGVPVPLDKYKHKVFYVWFDAPVGYTSITQCATKQYEKWWKPDKDYDVKLYQFMAKDNVPFHAIMFPASVIGANE

GHKLVDYIFATEYLNYEEGKFSKSRGVGVFGTDAQETGIPSDVWRFYLASIRPETSDSSFNWTELCTRNNSELLNNLGNF

CHRSLSFCANTFKGKIPEMSCSNTDYVLFALVNREVVAYVQNMEKGRLREALRHVMSVSRLGNQHMQSEQPWVLLKGSPE

EKIRGSTAIAVCCQLVALLCTLIAPYMPDTSRKLKAQLNLPDTSFRINPTNPALFQYIPKGHVIGKPEPLFAKIEQDLVD

AMRAKYAGTQAERAKQNGDVKTGASTNVADLEKAIAAQGEKVRQLKASTKDKTVWQPEVNKLLELKKQLTAAQSQPVMDV

ASLEKAIAEQGEKVRKLKASTKDKTVWQPEVNKLLELKKQMANAQSSIATPANVANLEKAIAEQGDKVRKLKASTKDKSV

WQQEVNVLLDLKKQLAAAQTK

>XP_013136732.1 PREDICTED: methionine--tRNA synthetase, cytoplasmic isoform X1 [Papilio polytes]

MKVYTNHGNTGTLKVLIAAQLAGKRVEKCLASFEDGIFTGPQALPLLEVDDLLTFFSSNAAVQYLFPTLDLSENGQCQQM

LEWEATRLQPAVMALLSGKAVAADLRQVLQASLQMLDKLLQKNKYILGEKLSAADISIWSTLYPVCFDESHKQTYITGCD

NIVRWNTNLASAKAIQEAVQQWGWSPMSPYGVSSALGIPQIASIVSPIGSPDEDPAAISVSPEEIEAAKDGWINGVKNMQ

APIQHEKVVLPVKDRKNVLITSALPYVNNVPHLGNIIGCVLSADVFARYCRQCDYNTLYICGTDEYGTATETKALEEGIT

PREICDKYFDIHNAVYRWFDIGFDYFGRTSTKEQTEISQKLFLKLIDNGFVSTQSVDQLYCEKCERFLADRFVEGVCPHP

GCLYGGARGDQCDKCGKLINAIELVEPRCKVCSHRPTVRPSEQLFIELGQLEPSLRTWIGRAESGWSAPARAVTRAWLRD

SLRARAVTRDLKWGVPVPIDKHAHKVFYVWFDAPIGYMSITQCLTKHYEKWWKPDKDVDVKLYQFMAKDNVPFHVIMFPA

TVIGVNEGYKLVDHIFATEYLNYEEGKFSKSRGVGVFGTDAQETGIPSDVWRFYLTSIRPETSDSSFSWVELGTRNNSEL

LNNLGNFCHRSLSFCANSFKGAVPDVALTAADLELIALVNREVRSYVENMEKGRLREALRHVLSVSRLGNQHMQAEQPWV

LLKGSQHDKVRGATAVGVCCQLAALVCALLAPFMPHTARRLREQLNLPEVALRINPQNPSLVQYLPPGHVIGKPEPLFSK

IEPELLEKLRAKYAGTQSDRAKQNGDTKSSPSKSVADLEAAVEKQGNKVREIKASTKDKTVWQPEVNKLLDLKKQLEAAK

VAHKNTSTTSSDSVASLEKAIAEQGEKVRKLKASTKDKSVWQPEVNKLLQLKKQLTAAQAQANTASAASVASTESKDDVA

SLEKAITEQGDKVRKLKASTKDKAVWQPEVNVLLDLKKRLAALQVK

>XP_011646980.1 PREDICTED: methionine--tRNA synthetase, cytoplasmic [Pogonomyrmex barbatus]

MMMIVTTNEGNPNALKLIFALHLAQKDFTVKIVKPGDVVLNRLPALTTPSGLYLFSVSAALRFLLPVPKELEVFNDRWLE

WEISQLQPAILRYVSAKSSKTPQKSDLWFLIKDLNNELNNKKYLIEKCSNLSPADISIWVSLWSIDEDIINEFSNVKTWL

TDIHQSFYVQEHISSSFCDKSKITIASIEAASWFPVNTISNSQTHESIPNDISSIKEKETEIISQEELHNVTKNWFGQYP

EVKESSYPILPKKDERNVLITSALPYVNNVPHLGNIIGCVLSADIFARYCRQRNYNTLYICGTDEYGTATEAKALQEKTT

PQAICDKFFDIHNDVYRWFGIGFDYFGRTTTPEQTEIVQSFFLKIKSQGYVLTETVEQLHCESCDRFLADRFVEGTCPGC

KYEDARGDQCDGCGHLVNAVELINPRCKICNTKPIVKNSTQFFLDLPKLEEKVKEYSAVVEKGWSNVARVVSKAWLRDGL

KPRCITRDLKWGIPVPVEGFENKVFYVWFDAPFGYISITKRYTKEYKKWWQPSQDTKIDLWQFMAKDNVPFHVIMFPACL

LAVNENYTLMKHLMATEYLNYEDKKFSKSRGIGVFGTDARDTGIPADVWRFYLAYIRPETQDSNFNWVDLATKNNSELLN

NLGNFVNRALVFSEKFFNSNIPPIEQPEEADWTVLALAQRELTSYLNAMEQAKLRDGLKHILAISKHGNQYMQSQQPWVK

IKGNEDDKKRAGTIVAICCNLACLLSALLAPFMPATAKQLRSQLGLSNKSYGYIPETITSMLPTWHKIGKPTPLFTKIED

QNIEMLREKYAGQQETNGTVQLSTNKYIDKIMSLNAAIKKQNDLVHELKTKNDESVWVPQYNILLDLQKELVEILNKEKS

MCEKKSPVKNKKTEPMHVPEQNGDALTDVTALELAISKQGNLVRELKAKEDASVWKPQVDILLKLKHRLADLTGTTPVSV

DKKSKKKKKLKKTFFSAAIFILLCANEYSESFIFWRGSFNLADTIAAE

>KPI96460.1 Methionyl-tRNA synthetase, cytoplasmic [Papilio xuthus]

MKVYTNHGNTGTLKVLIAAHLAGKKVEKCLATFEDGIFTGPQSLPLLEVDDLLTFFSSNAAVQYLFPTLDLSENGLCQQM

LEWEATRLQPAVAAIVSGKSVASDIRQALQSSLQMLDKLLQKNKYILGDKLSAADISIWSTLYPVCFDEPLKQTYISGCD

NIIRWNTNLASANAIKQAVEQWGWSPKGPYGASSALGIPQIASIVSPIGSPDEDPAAIGVSPEEVEAAKEGWFNGVKNMQ

APIQHEKVVLPLKDRKNVLITSALPYVNNVPHLGNIIGCVLSADIFARYCRQCDYNTLYICGTDEYGTATETKALEEGIT

PREICDKYFDIHNAVYRWFDIGFDYFGRTSTKEQTEIAQKLFLKLIDNGFVSTQSVEQLYCEKCERFLADRFVEGVCPHP

GCLYDSARGDQCDKCGKLINAIELVEPRCKVCAHKPTVRPSEQLFIELGQLEPSLRTWISSAEQGWSAPARAVSRAWLRD

TLRARAVTRDLKWGVPVPIEKHAHKVFYVWFDAPIGYMSITQCLTKHYEKWWKPDKDVDVKLYQFMAKDNVPFHVIMFPA

TVIGVNEGHKLVDHIFATEYLNYEEGKFSKSRGVGVFGTDAQETGIPSDVWRFYLTSIRPETSDSSFSWVELGTRNNSEL

LNNLGNFCHRSLSFCANSFKGKVPDVTLTPADLELIALVNREVISYVQNMEKGRLREALRHVLSVSRLGNQHMQAEQPWV

LLKGSEQDKVRGGTAIGLCCQLVALLCALLAPFVPHTARRLREQLNVDAVALRINPQNPSLVQYLPAGHVIGKPEPLFSK

IEPELLEKLRAKYAGTQSDRAKQNGDSKSSPSKGSVADLEAAVEEQGNKVRQLKASTKDKAVWQPEVNKLLDLKKQLEAA

KATPKETTATPSESVASLEKAIAEQGDKVRKLKASTKDKGVWQPEVDKLLQLKKQLTAAQAQTKAASTDSKDDVTSLEKA

IVEQGDKVRKLKASTKDKAVWQPEVNVLLDLKKRLAALQIK

>XP_013177114.1 PREDICTED: methionine--tRNA synthetase, cytoplasmic isoform X2 [Papilio xuthus]

MKVYTNHGNTGTLKVLIAAHLAGKKVEKCLASFEGPQSLPLLEVDDLLTFFSSNAAVQYLFPTLDLSENGLCQQMLEWEA

TRLQPAVAAIVSGKSVASDIRQALQSSLQMLDKLLQKNKYILGEKLSAADISIWSTLYPVCFDEPLKQTYISGCDNIIRW

NTNLASANAIKQAVEQWGWSPKGPYGASSALGIPQIASIVSPIGSPDEDPAAIGVSPEEVEAAKEGWFNGVKNMQAPIQH

EKVVLPLKDRKNVLITSALPYVNNVPHLGNIIGCVLSADIFARYCRQCDYNTLYICGTDEYGTATETKALEEGITPREIC

DKYFDIHNAVYRWFDIGFDYFGRTSTKEQTEIAQKLFLKLIDNGFVSTQSVEQLYCEKCERFLADRFVEGVCPHPGCLYD

SARGDQCDKCGKLINAIELVEPRCKVCAHKPTVRPSEQLFIELGQLEPSLRTWISSAEQGWSAPARAVSRAWLRDTLRAR

AVTRDLKWGVPVPIEKHAHKVFYVWFDAPIGYMSITQCLTKHYEKWWKPDKDVDVKLYQFMAKDNVPFHVIMFPATVIGV

NEGHKLVDHIFATEYLNYEEGKFSKSRGVGVFGTDAQETGIPSDVWRFYLTSIRPETSDSSFSWVELGTRNNSELLNNLG

NFCHRSLSFCANSFKGKVPDVSLTPADLELIALVNREVISYVQNMEKGRLREALRHVLSVSRLGNQHMQAEQPWVLLKGS

EQDKVRGGTAIGLCCQLVALLCALLAPFVPHTARRLREQLNVDAVALRINPQNPSLVQYLPAGHVIGKPEPLFSKIEPEL

LEKLRAKYAGTQSDRAKQNGDSKSSPSKGSVADLEAAVEEQGNKVRQLKASTKDKAVWQPEVNKLLDLKKQLEAAKATPK

ETTTPSESVASLEKAIAEQGDKVRKLKASTKDKGVWQPEVDKLLQLKKQLTAAQAQTKAASTDNKDDVASLEKAIVEQGD

KVRKLKASTKDKAVWQPEVNVLLDLKKRLAALQIK

>XP_013177113.1 PREDICTED: methionine--tRNA synthetase, cytoplasmic isoform X1 [Papilio xuthus]

MKVYTNHGNTGTLKVLIAAHLAGKKVEKCLASFEDGIFTGPQSLPLLEVDDLLTFFSSNAAVQYLFPTLDLSENGLCQQM

LEWEATRLQPAVAAIVSGKSVASDIRQALQSSLQMLDKLLQKNKYILGEKLSAADISIWSTLYPVCFDEPLKQTYISGCD

NIIRWNTNLASANAIKQAVEQWGWSPKGPYGASSALGIPQIASIVSPIGSPDEDPAAIGVSPEEVEAAKEGWFNGVKNMQ

APIQHEKVVLPLKDRKNVLITSALPYVNNVPHLGNIIGCVLSADIFARYCRQCDYNTLYICGTDEYGTATETKALEEGIT

PREICDKYFDIHNAVYRWFDIGFDYFGRTSTKEQTEIAQKLFLKLIDNGFVSTQSVEQLYCEKCERFLADRFVEGVCPHP

GCLYDSARGDQCDKCGKLINAIELVEPRCKVCAHKPTVRPSEQLFIELGQLEPSLRTWISSAEQGWSAPARAVSRAWLRD

TLRARAVTRDLKWGVPVPIEKHAHKVFYVWFDAPIGYMSITQCLTKHYEKWWKPDKDVDVKLYQFMAKDNVPFHVIMFPA

TVIGVNEGHKLVDHIFATEYLNYEEGKFSKSRGVGVFGTDAQETGIPSDVWRFYLTSIRPETSDSSFSWVELGTRNNSEL

LNNLGNFCHRSLSFCANSFKGKVPDVSLTPADLELIALVNREVISYVQNMEKGRLREALRHVLSVSRLGNQHMQAEQPWV

LLKGSEQDKVRGGTAIGLCCQLVALLCALLAPFVPHTARRLREQLNVDAVALRINPQNPSLVQYLPAGHVIGKPEPLFSK

IEPELLEKLRAKYAGTQSDRAKQNGDSKSSPSKGSVADLEAAVEEQGNKVRQLKASTKDKAVWQPEVNKLLDLKKQLEAA

KATPKETTTPSESVASLEKAIAEQGDKVRKLKASTKDKGVWQPEVDKLLQLKKQLTAAQAQTKAASTDNKDDVASLEKAI

VEQGDKVRKLKASTKDKAVWQPEVNVLLDLKKRLAALQIK

>XP_012242292.1 methionine--tRNA synthetase, cytoplasmic isoform X3 [Bombus impatiens]

MQLIVPSSSQESKLLNNKWLEWEISRLQPAIAFYGNTGIYKTTQKSCLWSLLRELNNALEDKQYLIKDNNDLSLADICIW

VTLWSTVMVTEIGNELNKEYLSIKNWISSIELLPVIQDSIEEYKFERGSKAIFSIQAVSWFPVNTNSNPKPPKPADLQLS

PTKEKEMEAVNQEEIQNISSNWSSKSYPDVKNFPYPVLPKKGEKNILITSALPYVNNVPHLGNIIGCVLSADIFARYCRQ

RNYNTLFISGTDEYGTATEAKALEEKTTPQNICDKFFDIHNDVYRWFNIGFDYFGRTTTPEQTEIVQSFFLKIKSEGYVL

SETVDQLLCESCDRFLADRFVEGTCPRCKYEDARGDQCDGCGHLVNATDLISPRCKVCSNRPIVKKSQQFFLDLPKLENK

LKEWSSTVEKGWSSVAKVVAKPWLRDGLKPRCITRDLKWGIPVPIKGFENKVFYVWFDAPFGYISITKRYTKEYQQWWKP

TDVQVDLYQFMAKDNVPFHAIMFPAYLLAANEGYTLMKHLMATEYLNYEDTKFSKSRGIGVFGTDARDTGIPADVWRFYL

AYVRPETQDSNFNWVDLATKNNSELLNNFGNFVNRALVFAEKYFDSKIPPIELQEDDLILLALAQRELSSYIHALEQAKL

RDGLKNVLAISKHGNQYMQFQEPWVKIKGTDNDKKRAGTIIGICCNLACLLSALLAPFMPCTARELRSQLGLQTNNYGYI

PDVITSILPTGHKISKPSPLFKKIEDKDVEILRRKYAGKQETTSSSSGDVKSLDSAITELDNKVKKLKAKFDKSGWPSSQ

IQIFSALKKKLPDLINEKNKSPESKSKKPETVSVPEQNGDVAIDVASLEAAIAKQGDLVRQLKAKEERSVWQPEVEKLLK

LKKQLGDLTGTAAAPTDKKSKKKK

>XP_018339699.1 PREDICTED: methionine--tRNA synthetase, cytoplasmic isoform X1 [Trachymyrmex septentrionalis]

MIVSTNEGNPNALKLVIATKLAQETVSIRILKPSNATLGRLPTLTTSTGLSLFSISSALRFLLSVPKEVEVFNDRWLEWE

ISQLQPAILSHTSAKTCKTLQKSYLWSLLKDLNNELRDKQYLIEKCSNLSPADISIWVSLWSTILITDIANDIAKDLPSV

NSWLANIQKQPIIQQSIKESIEMFKLERGSKAIASIEAASWFPINTASNPQVHESVPSDISPTKEKEMDIVNQEELCNMA

KSWTTEQYPEVKESSYPILPKKDERNMLITSALPYVNNVPHLGNIIGCVLSADIFARYCRQRNYNTLYICGTDEYGTATE

AKALQEKTTPQAICDKFFDIHNDVYRWFGIGFDYFGRTSTPEQTEIVQGFFLNIKSQGYVLTETVEQLHCESCDRFLADR

FVEGTCPGCKYEDARGDQCDGCGHLVNAVELINPRCKICNTKPVVKNSIQFFLDLPKLEEKLKEYSTTRDKGWSNIARVV

SKAWLRDGLQPRCITRDLKWGIPVPVEGFENKVFYVWFDAPFGYISITKRYTKEYKQWWQPSAQDIKIDLYQFMAKDNIP

FHTIMFPACLLAANQNYTLMKCILATEYLNYEDKKFSKSRGIGVFGTDARDTGIPADVWRFYLAYIRPETQDSNFNWVDL

ATKNNSELLNNLGNFVNRALVFSEKFFNSNIPPIEPEEADWTVLALAQRELSAYINAMEQGKLKDGLKRILAISKHGNQY

MQFQQPWVKIKGNDNDKKKAGTVVAICCNLACLLSALLAPFMPSTAKQLRSQLGLNNKSYGYIPEIITNMLPTWHKIGKP

SPLFTKIEDQKVEMLRKKYAGQQETNGDGTNKSSDESIAILEAAITKQGNLVRELKAKNDKSIWQPQVEILLDLKKKLTD

LKSNANAPAKKSPAKNKKAEPMHVPEQNGNALTDVAALESAIAKQGNLVRELKTKEDGSVWKPQVEILLKLKQRLSDLTG

TTPVVVDKKSKKKK

>XP_012056005.1 PREDICTED: methionine--tRNA synthetase, cytoplasmic [Atta cephalotes]

MIVSTNEGNPNALKLVIATKLAQETVSVRILKPSDATLGRLPTLTISTGLSLFSVSSALRFLLSVPKELEVLNDRWLEWE

ISQLQPAILSHASVKTSKTLQKSNLWSLLKDLNSDLRDKQYLIEKCSNLSPADISIWVSLWSTILVTDIINDIAKDLPSV

NSWLANIQKQPIIQQSIKESIEMFKLERGPKAIASIESASWFPINTASNSQVHESVPSDISPIKEKEMDIVNQEELRNMA

KSWTTEQYLEVKESSYPILPKKGERNILITSALPYVNNVPHLGNIIGCVLSADIFARYCRQRNYNTLYICGTDEYGTATE

AKALQEKTTPQAICDKFFDIHNDVYRWFGIGFDYFGRTSTPEQTEIVQAFFLKIKSQGYVLTETVEQLHCESCDRFLADR

FVEGMCPGCKYEDARGDQCDGCGHLVNAVELINPRCKICNTKPVVKNSVQFFLDLPKLEEKLKEYSTTHDKGWSNVARVV

SKAWLRDGLQPRCITRDLKWGIPVPVEGFENKVFYVWFDAPFGYISITKRYTKEYKQWWQPPSQDIKIDLYQFMAKDNIP

FHTIMFPACLLAANQNYTLMKCILATEYLNYEDKKFSKSRGIGVFGTDARDTGIPADVWRFYLAYIRPETQDSNFNWVDL

ATKNNSELLNNLGNFVNRALAFSEKFFDSNIPPIEPEETDWTVLALAQRELSAYINAMEQGKLKDGLKRILAISKHGNQY

MQFQQPWVKIKGNDDDKKKAGTVVAICCNLACLLSALLAPFMPNTAKQLRSQLGLSNKNYGYIPETITNMLPTWHKIGKP

SPLFTKIEDQKVEMLRKKYAGQQETNGDGMNKSSESIALLEAAIIKQGNLVRELKAKNDKGVWQPQVEILLDLKKKLTDL

KSNANAPAKKSPAKNKKAEPMHMPEQNGDALTDVATLESAIAKQGNLVRELKAKEDGSVWKPQVEILLKLKQRLSDLTGT

TPVIVDKKSKKKK

>KYM92310.1 Methionyl-tRNA synthetase, cytoplasmic [Atta colombica]

MIVSTNEGNPNALKLVIATKLAQETVSVRILKPSDATLGRLPTLTISTGLSLFSVSSALRFLLSVPKELEVLNDRWLEWE

ISQLQPAILSHASVKTSKTLQKSNLWSLLKDLNSDLRDKQYLIEKCSNLSPADISIWVSLWSTILVTDIINDIAKDLPNV

NSWLANIQKQPIIQQSIKESIEMFKLERGPKAIASIESASWFPINTASNSQVHESVPSDISPIKEKEMDIVNQEELRNMA

KSWTTEQYLEVKESSYPILPKKGERNILITSALPYVNNVPHLGNIIGCVLSADIFARYCRQRNYNTLYICGTDEYGTATE

AKALQEKTTPQAICNKFFDIHNDVYRWFGIGFDYFGRTSTPEQTEIVQAFFLKIKSQGYVLTETVEQLHCESCDRFLADR

FVEGMCPGCKYEDARGDQCDGCGHLVNAVELINPRCKICNTKPVVKNSVQFFLDLPKLEEKLKEYSTTHDKGWSNVARVV

SKAWLRDGLQPRCITRDLKWGIPVPVEGFENKVFYVWFDAPFGYISITKRYTKEYKQWWQPPSQDIKIDLYQFMAKDNIP

FHTIMFPACLLAANQNYTLMKCILATEYLNYEDKKFSKSRGIGVFGTDARDTGIPADVWRFYLAYIRPETQDSNFNWVDL

ATKNNSELLNNLGNFVNRALAFSEKFFDSNIPPIEPEETDWTVLALAQRELSAYINAMEQGKLKDGLKRILAISKHGNQY

MQFQQPWVKIKGNDDDKKKAGTVVAICCNLACLLSALLAPFMPNTAKQLRSQLGLSNKNYGYIPETITNMLPTWHKIGKP

SPLFTKIEDQKVEILRKKYAGQQETNGDGTNKSSESIALLEAAIIKQGNLVRELKAKNDKGVWQPQVEILLDLKKKLTDL

KSNANAPAKKSPAKNKKAEPMHMPEQNGDALTDVATLESAIAKQGNLVRELKAKEDGSVWKPQVEILLKLKQRLSDLTGT

TPVIVDKKSKKKK

>XP_017797626.1 PREDICTED: methionine--tRNA synthetase, cytoplasmic [Habropoda laboriosa]

MNISTNEGNPNVLKLVIAAKMAQQSFTVKTVQPKVSFLGRLPFIELSSGLVLFSTNAAMQLLVSSPEDSKVVTNKWLEWE

ASQLQPSIAVYGSTGTYKTANKSCLWSLLKELNIALKDKKYLIEDNNDLSLADACIWVTIWSTILVTDIGNELCKEYVSI

KNWLSNIELLPIIQNSLKEYKFQRGLKAILSVQAVSWFPANTAVISRSAPNSKPTPSDVSPTKEKEVETISQEEIQNITS

NWSSTSYPEMKNSSYPVLPKKGEKNILITSALPYVNNVPHLGNIIGCVLSADIFARYCRQRNYNTPCYLLNFTITIIYYI

SGTDEYGTATEAKALEEKTTPQAICNKFFDIHNDIYRWFRIGFDYFGRTTTPEQTEIVQSFFLRIKSQGYILNETVDQLL

CESCDRFLADRFVEGTCPRCKYEDARGDQCDGCGHLVNATDLISPRCKVCSSRPIVKKSVQFFLDLPKLEDKLKEWSPTV

EKGWSSVARVVAKPWLRDGLKPRCITRDLKWGIPVPVEGYENKVFYVWFDAPFGYISITKRYTQEYEQWWKPKDVQVDLY

QFMAKDNVPFHAIMFPACLIAANEGHTLIKHLMATEYLNYEDTKFSKSRGIGVFGTDARDTGIPADVWRFYLAYVRPETQ

DSNFNWVDLATKNNSELLNNFGNFVNRALVFAEKYFDSKVPLIELQEDDLVLLVLAQRELSSYVHSMKQAKLRDGLKHVL

AISKHANVYMQFQEPWVKIKGTDNDKKRAGTIIGICCNLACLLSALLAPFMPNTARELRSQLGLDNNHYGYISDVITNIL

PTGHKIGKPSPLFKKIEDKDVEALRKKYAGKQETENNGENSDIKSLESEVAELGNKVKELVAKHEGQIFSNLKKMFTDII

GEKSKSPESKSKKSDLVFVPEQNGDASMDVASLEAAIAKQANLVRELKAKEEKSVWRPEVEKLLNLKKQLADLNGTAPAP

TDKKSKKKK

>EFN64410.1 Methionyl-tRNA synthetase, cytoplasmic [Camponotus floridanus]

MIISTNENNPNALKLIIAAKLIQKTFIVKVVKSGDAVLGRLPTLTVSSGLSLFSVSSALRLLLPISKELEVLNNRWLEWE

ISQLQPTILSYTSARTPKILQKSDLWSLLKDLNSELKNKQYLIEEHSNLSPAFILSPADISIWVSLWSTILLPDIRNDIS

KDLSNVNNWLINIEKQPVIQISPTKEKEMETVSQEELHNVEKSWTTGQYPEVKENSYPILPKKGERNILVTSALPYVNNV

PHLGNIIGCVLSADIFARYCRQRNYNTLYISGTDEYGTATEAKALQEKTTPQVICDKFFDIHNDVYRWFGIGFDYFGRTT

TPEQTEIVQAFFLRIKSQGYVSSETVEQLHCESCDRFLADRFVEGTCPRCKYEDARGDQCDGCSHLVNAVELINPRCKMC

NTRPVIKNSIQFFLDLPKIEKRLKEYSITVEKEWSSVARAVSKSWLRDGLKPRCITRDLKWGIPVPIEGFENKVFYVWFD

APFGYISITKRYTKEYKKWWQPLQDTKIDLCQFMAKDNVPFHAIMFPAYLLAANQNYTLIKYLMATEYLNYEDKKFSKSR

GIGVFGTDARDTGIPADVWRFYLAYIRPETQDSNFNWVDLATKNNSELLNNLGNFVNRALVFSEKFFNSTVPPIEAEEAD

WTVLALAQRELSCYINAMEQAKLRDGLKYILAISKHGNQYMQFQQPWVKVKGTDDNKKRAGTVIAICCNLACLLSALLAP

FMPSTAKQLRSQLGLSNKSYGYIPETITNMLPTWHKIGKPNPLFTKIEDQKIEMLRKKYDGQQETNNGISANKVSDESIT

LEAAITKQGNLVRELKAKRDKSIWQPQVEILLDLKKKLNDLKNNVNTSEKKLPAKNKKVEPIHAPEQNGDALTDVMALEM

AIAKQGNLVRELKAKEDGSIWKPQVEILLEMKQRLMDLTGTAPVVVDKKSKKKK

>XP_018054220.1 PREDICTED: methionine--tRNA synthetase, cytoplasmic [Atta colombica]

MTMIVSTNEGNPNALKLVIATKLAQETVSVRILKPSDATLGRLPTLTISTGLSLFSVSSALRFLLSVPKELEVLNDRWLE

WEISQLQPAILSHASVKTSKTLQKSNLWSLLKDLNSDLRDKQYLIEKCSNLSPADISIWVSLWSTILVTDIINDIAKDLP

NVNSWLANIQKQPIIQQSIKESIEMFKLERGPKAIASIESASWFPINTASNSQVHESVPSDISPIKEKEMDIVNQEELRN

MAKSWTTEQYLEVKESSYPILPKKGERNILITSALPYVNNVPHLGNIIGCVLSADIFARYCRQRNYNTLYICGTDEYGTA

TEAKALQEKTTPQAICNKFFDIHNDVYRWFGIGFDYFGRTSTPEQTEIVQAFFLKIKSQGYVLTETVEQLHCESCDRFLA

DRFVEGMCPGCKYEDARGDQCDGCGHLVNAVELINPRCKICNTKPVVKNSVQFFLDLPKLEEKLKEYSTTHDKGWSNVAR

VVSKAWLRDGLQPRCITRDLKWGIPVPVEGFENKVFYVWFDAPFGYISITKRYTKEYKQWWQPPSQDIKIDLYQFMAKDN

IPFHTIMFPACLLAANQNYTLMKCILATEYLNYEDKKFSKSRGIGVFGTDARDTGIPADVWRFYLAYIRPETQDSNFNWV

DLATKNNSELLNNLGNFVNRALAFSEKFFDSNIPPIEPEETDWTVLALAQRELSAYINAMEQGKLKDGLKRILAISKHGN

QYMQFQQPWVKIKGNDDDKKKAGTVVAICCNLACLLSALLAPFMPNTAKQLRSQLGLSNKNYGYIPETITNMLPTWHKIG

KPSPLFTKIEDQKVEILRKKYAGQQETNGDGTNKSSESIALLEAAIIKQGNLVRELKAKNDKGVWQPQVEILLDLKKKLT

DLKSNANAPAKKSPAKNKKAEPMHMPEQNGDALTDVATLESAIAKQGNLVRELKAKEDGSVWKPQVEILLKLKQRLSDLT

GTTPVIVDKKSKKKK

>NP_001296503.1 methionine--tRNA synthetase, cytoplasmic [Bombyx mori]

MKIYTNKNNTGTLKLLIAANLAAKKVEIVEAEFEDDVFAGPRQLPLLEVDPELRFFSSNAAVQYLFPILDMSENGQCQQL

QEWEATRLQPSLSAALGKSPASDVKQVLKSSLEHVNGLLGGHQYVLGDKISAADISIWSTLYPLTHNDDLREQYLKELGN

IRRWSDAIGGAKSVQEAVRQWGGSPTGPFGQISALGLPQVANLVSPAGSPDDDAEVQEGPTPEEIEAAKEGWLNGLSKLQ

PPLKQEKIVLPVKGRKNVLITSALPYVNNVPHLGNIIGCVLSADIFARYCRLCDYNTLYICGTDEYGTATETKALEEGVT

PKQICDKYFEIHNNVYRWFNIGFDYFGRTSTEEQAEITQRLFLKLNENGFVSSQTVDQLFCEKCDRFLADRFVEGTCPHP

GCGYEGARGDQCDKCGRLVNAPELLQPRCKVCAHPPVLKPSEQLFIELGQLEPSLRSWMSRWEHLWSAPARAVGRAWLRE

PLRARALTRDLRWGVPVPLDGYRHKVFYVWFDAPIGYMSITQCATKQYEAWWKPDKEYDVSLYQFMAKDNVPFHVVMFPA

TVLGVNEGHLLVKHLFATEYLNYEEGKFSKSRGVGVFGNDAQDTGIPSDVWRFYLASIRPETSDSSFSWTELGTRNNSEL

LNNLGNFCHRSLSFCSNAFKGLIPEPNPRRSDYELMALVNREIAAYVQNMEKGRLREALRHVLSISRLGNQHMQSEQPWV

LLKGSEDDKQRGATAIGLCCQLVALLCALLAPYVPDTTRKLRGQLNVDAEKLRLNPENPRLLRYLPPGHIIGKPEPLFVK

IEQERLEELRARYAGTQSQRADKKNSKTLDIAELEKAIAEQGDAVRKLKSTTKDKSVWQPEVEKLLELKKRLTEAQKQAE

TRPAANQAAGDVASLEKAIAEQGDAVRKLKSTTKDKSVWQPEVEKLLELKKRLTEAQKQAETRPAANQAAGDVASLEKAI

AEQGDKVRKLKASTKDKAVWQPEVNVLLDLKKRLQALQVKK

>XP_014616658.1 PREDICTED: methionine--tRNA synthetase, cytoplasmic [Polistes canadensis]

MIVLTNESNSNALKLIIAAKLDKKTINVKIALPNETFLNRLPILELPNGSKIFSTSAGMQFLVAPSEEYEIITNKWLEWD

ASQLQPAITYYGTTGIYKTDSKSCLWSLLNEINNALKDKMYLIKDKNDISLADASIWVSIWSTVMVTEIGNEILKEYVYL

SDWLKNIELLPIIQDSLKVFSLERGLKAINSIQAGSWFPVNSTKSCKSTTNELNLVKEKDIEVVSQEELDKAATNWNSCN

CPNVKESSYPVLPKKGERNILITSALPYVNNVPHLGNIVGCVLSADIFARYCRQRNYNTLYISGTDEYGTATEAKALEEK

TTPQEICDKFFSIHNDIYRWFGIGFDYFGRTTTAEQTEIVQSFFLKIKSQGYVLMDTVDQLLCESCNRFLADRFVEGICP

RCKYEDARGDQCDGCGHLVNATELINARCKICSNRPIIKKSRQFFLDLPKLEDRLKIWSAKVEKGWSAVARGVTKPWLRD

GLKPRCITRDLKWGIPVPAEGFENKVFYVWFDAPFGYISITKRYTKEYEQWWKPNGTDISLYQFMAKDNVPFHTIMFPAY

LLATNEDYTLLKHLMAIEYLNYEDTKFSKSRGIGVFGTDARETGIPADVWRFYLAYIRPESQDSNFNWADLATKNNNELL

NNLGNFINRALVFTEKFFESKVPPMEPQEDDLTLLALAQRELTSYLIALEQAKLRDGIKYVLAISKHGNQYMQSQQPWVK

VKGTDDDKKKAGTVIGICCNLTCLLSALLAPYMPSTSREIRGQLGLDKTRYGYIPDTIMNMLPSGHKIGKASPLFTKIED

QLIEELRQKYSGKQESNGTQSKTNNITDQLVKEVIYSNDKNVQQPNVDVPVKMQNKQADLKEDTDKCDTANNKKVKPLYT

PEQNGDTSTDINSLEAAIAKQGTLVRKLKAKEEKSVWQPQVDILLKLKNQLASLTGTKIPDKKSKKKK

>XP_021955150.1 methionine--tRNA synthetase, cytoplasmic-like [Folsomia candida]

MDHQNQTIKIITNTGNPASLKPLIAASLYGFKVALESVSVSPDGKQRPRLPVLVANEKTKLFDTNVMVTYLLELAGIPHP

SANLLNLLNFEAHSLFPLVLSYLLEKADTDFGVGKELLNVLVELERRLKGESFFSGNVKPDIEDAIVWATLYPLFSANDK

ITKKCPLLLKWYILVESADLTKAAVNLLQPVSGREAAKQILVCNPTASISLYGEKIKMEKLKLDDLPGGDATSTAVSSEE

IQLARESWVRPTPTSAVKSKRAGRILPLAGQKNYFITSALPYVNNVPHLGTIIGCVLSADVFARYCRIRGRNTLYICGTD

EYGTATETKALEENLTPQQICDKYHKLHKEIYDWFDISFDYFGRTTTKEQTEVGQEIFLKLWNNGFLKEMVVQQLFCNPC

KRFLSDRFVEGTCPACAYEDARGDQCDKCGRLTNSIELKNPRCKVCGSKDVTTKNSDQVFLDLPKLEEKVGLEEWLNRSS

EEWTSNARHIAKSWIREGLKERCITRDLKWGTPVPIEKYKDKVFYVWFDAPIGYISMTKCYTKDYWKAWWMNPDPEIDIE

YYQFMAKDNVPFHSILFPASLRGTKDPWTLVRHIMATEYLNYEDGKFSKSRGVGVFGNDAIQTGIPADIWRFYLIFVRPE

NQDSAFSWDDFMLKNNSELLNNLGNFINRSLTFLAKFFNSKIPPMNLNDDDFRVVAEINKEVSEYVNFLETCKLKDGLRK

ILAVSRIGNVYFQTQQPWTLNKNDQDLERCGTICGLGAQIVFLLMTLMEPFLPGVSRQIREQLNVPKPIFSKQLVPFLSV

GHELGKVSPLFQKLDADFIAGLRKKYVGQQQPVEASPKKGTAKAAPADSPLKPESNGVPGSPEEIAALEAAIALQGGVVR

DLKAAKADKAALQPEIEKLLKLKSDLSVKQGVDPKAAAAPAASSKSKGKSKKK

>XP_020291650.1 methionine--tRNA synthetase, cytoplasmic isoform X1 [Pseudomyrmex gracilis]

MHVLTNNGNPNALKLIVASKLAGESVTVKIVEPNKALLGRLPTLTTSSGSSLFSVSSALQLLLPVPKDLEVSNNKWLEWE

ISQLQPTILSYASEKTLKISQKTDLLSLLKDLNSELKDKQYLIETHSNLSPADISIWVSLWSTVFVYDIEDLSRTLPNVT

SWLANIEKQLVVQESIKMCVKEFKLERGTKAVASIEAASWFPSNTNSSCRVRNLASSDLSPTKEKEKEIEVISQEELLNV

ENNWTTGQYLEVKENSYPILPKKGERNVLVTSALPYVNNVPHLGNIIGCVLSADIFARYCRQRNYNTLYISGTDEYGTAT

EAKALQEKTTPQAICDKFFDIHNDVYRWFGIGFDYFGRTTTPEQTEIVQAFFLKIKSQGYVLSQTVEQLHCEFCDRFLAD

RFVEGTCPGCKYEDARGDQCDGCGHLVNAVELINPRCKICSNRPVIKNSVQFFLDLPKVEQKLKEYSSTVEKGWSSVARV

VSRAWLRDGLKPRCITRDLKWGIPVPVKGYENKVFYVWFDAPFGYISITKRYTKEYMQWWQPSQDVKVDLCQFMAKDNVP

FHAIMFPACLLATNQNYTLIKYLMATEYLNYEDKKFSKSRGIGVFGTDARDTGIPADVWRFYLAYIRPETQDSNFNWIDL

ATKNNSELLNNLGNFVNRALMFCQKFFDSNIPPIEQPEEADWTVLAEAQRELTSYIIAMEQAKLRDGLKHILAISKHGNQ

YMQFQQPWVKIKGTDDDKKKAGTVVAICCNLACLLSALLGPFMPTTAKQLRSQLGLSNKNYGYIPETITNMLPTWHKIGK

PNPLFTRIEDQTVELLRKKYAGQQETNGKMPSETSKESAKTSEKSKESAKISDKDCPNNVAVLEAAITKQANLVRELKSK

HEKSIWQPQVEILLDLKKKLAALKGVANTSEKNAKNKKTPVSQQNGWVSQQGGALPFKNILFFLLIIIVSCFVTLGAHFE

FVA

>XP_020291660.1 methionine--tRNA synthetase, cytoplasmic isoform X2 [Pseudomyrmex gracilis]

MHVLTNNGNPNALKLIVASKLAGESVTVKIVEPNKALLGRLPTLTTSSGSSLFSVSSALQLLLPVPKDLEVSNNKWLEWE

ISQLQPTILSYASEKTLKISQKTDLLSLLKDLNSELKDKQYLIETHSNLSPADISIWVSLWSTVFVYDIEDLSRTLPNVT

SWLANIEKQLVVQESIKMCVKEFKLERGTKAVASIEAASWFPSNTNSSCRVRNLASSDLSPTKEKEKEIEVISQEELLNV

ENNWTTGQYLEVKENSYPILPKKGERNVLVTSALPYVNNVPHLGNIIGCVLSADIFARYCRQRNYNTLYISGTDEYGTAT

EAKALQEKTTPQAICDKFFDIHNDVYRWFGIGFDYFGRTTTPEQTEIVQAFFLKIKSQGYVLSQTVEQLHCEFCDRFLAD

RFVEGTCPGCKYEDARGDQCDGCGHLVNAVELINPRCKICSNRPVIKNSVQFFLDLPKVEQKLKEYSSTVEKGWSSVARV

VSRAWLRDGLKPRCITRDLKWGIPVPVKGYENKVFYVWFDAPFGYISITKRYTKEYMQWWQPSQDVKVDLCQFMAKDNVP

FHAIMFPACLLATNQNYTLIKYLMATEYLNYEDKKFSKSRGIGVFGTDARDTGIPADVWRFYLAYIRPETQDSNFNWIDL

ATKNNSELLNNLGNFVNRALMFCQKFFDSNIPPIEQPEEADWTVLAEAQRELTSYIIAMEQAKLRDGLKHILAISKHGNQ

YMQFQQPWVKIKGTDDDKKKAGTVVAICCNLACLLSALLGPFMPTTAKQLRSQLGLSNKNYGYIPETITNMLPTWHKIGK

PNPLFTRIEDQTVELLRKKYAGQQETNGKMPSETSKESAKTSEKSKESAKISDKDCPNNVAVLEAAITKQANLVRELKSK

HEKSIWQPQVEILLDLKKKLAALKGVANTSEKNAKNKKTPVSQQNGWVSQQG

>XP_004365688.2 methionyl-tRNA synthetase [Capsaspora owczarzaki ATCC 30864]

MSLTVAVLDGKSLAALKVEHALALRSTTSSSSTTTVAIATASEAKQLADRAAAVAPATTAAVVLALDERRTLVNPNAIAV

TVLGQCTADAATWIQLDESRLASQLAVVLAPPKVPALVTASKAAVLAALKTIASAITAAKGQPLSDAAHVVLGATLFPAF

HATGAFALEAAIRSQVASVEQYFQKNVFSTEAFRQALAKTGFADDSALAAHVAALSATAAAVLPSKASAVASAAAPAPAA

AVTHHQPAPDAKTGIVDLDIMEKATPKELEDALSNWTKDALPTGKVGSDPTAPIPGQVLLPKPDQRNILITSALPYVNNV

PHLGNIIGCVLSADVYARFCRERGYNTLYICGTDEYGTATETKALEEGLTPQQICDKYHKLHREIYEYFQISFDHFGRTT

TPQQTEISQDIFLRALKNDTLLEESVEQLLCETCNRYLADRFVEGICPSCKYDDARGDQCDKCGKLLNAVELVEPRCKLC

RSRPVVRPSRHMFLNLPMLDGQIKEWYAGSSVKGDWTASAKHITSSWLKEGVKPRCITRDLKWGVPVPLDGFREKVFYVW

FDAPIGYISITAAYTAEWEKWWKNPSQVQLYQFMAKDNVPFHSIIFPASQLAAKDNYTLVHHISAVEFLNYEDGKFSKSR

GVGVFGNHARDTGLAIDIFRFYLLGIRPEYTDSVFSWTDLASKVNNELLANLGNLVQRSLSFIKANFAGKVPKVVLNVDT

DAQSCNFVALVNRYLREYIDLLEQVHLRDALRLVLKISSIANQYFQTVQPWKTIKLTDDLSKQRTATCLGLCANFVHLLS

TLLFPFMPVTGKKILEQLNAAPGSLRADHWGLTLPEGHEIGTPSVLFEKIEPAVVETLRAKYGGKQEGAAAAGPVSVTES

AALKKSKTQQASSPQPAAGTAAAGSSSTGDVAALTAKVDAQGNLVRKLKGEKADKAKIDAEVKVLLDLKSQLERATKAAA

AAAPVAAPSAAPAASASAAPSATTKALEDQIAVQGNKVRDLKSQKADKAVVTAEVNILLALKQQLASLSK

>XP_012544873.1 methionine--tRNA synthetase, cytoplasmic isoform X2 [Bombyx mori]

MKIYTNKNNTGTLKLLIAANLAAKKVEIVEAEFEDDVFAGPRQLPLLEVDPELRFFSSNAAVQYLFPILDMSENGQCQQL

QEWEATRLQPSLSAALGKSPASDVKQVLKSSLEHVNGLLGGHQYVLGDKISAADISIWSTLYPLTHNDDLREQYLKELGN

IRRWSDAIGGAKSVQEAVRQWGGSPTGPFGQISALGLPQVANLVSPAGSPDDDAEVQEGPTPEEIEAAKEGWLNGLSKLQ

PPLKQEKIVLPVKGRKNVLITSALPYVNNVPHLGNIIGCVLSADIFARYCRLCDYNTLYICGTDEYGTATETKALEEGVT

PKQICDKYFEIHNNVYRWFNIGFDYFGRTSTEEQAEITQRLFLKLNENGFVSSQTVDQLFCEKCDRFLADRFVEGTCPHP

GCGYEGARGDQCDKCGRLVNAPELLQPRCKVCAHPPVLKPSEQLFIELGQLEPSLRSWMSRWEHLWSAPARAVGRAWLRE

PLRARALTRDLRWGVPVPLDGYRHKVFYVWFDAPIGYMSITQCATKQYEAWWKPDKEYDVSLYQFMAKDNVPFHVVMFPA

TVLGVNEGHLLVKHLFATEYLNYEEGKFSKSRGVGVFGNDAQDTGIPSDVWRFYLASIRPETSDSSFSWTELGTRNNSEL

LNNLGNFCHRSLSFCSNAFKGLIPEPNPRRSDYELMALVNREIAAYVQNMEKGRLREALRHVLSISRLGNQHMQSEQPWV

LLKGSEDDKQRGATAIGLCCQLVALLCALLAPYVPDTTRKLRGQLNVDAEKLRLNPENPRLLRYLPPGHIIGKPEPLFVK

IEQERLEELRARYAGTQSQRADKKNSKTLDIAELEKAIAEQGDAVRKLKSTTKDKSVWQPEVEKLLELKKRLTEAQKQAE

TRPAANQAAGDVASLEKAIAEQGDKVRKLKASTKDKAVWQPEVNVLLDLKKRLQALQVKK

>XP_012544872.1 methionine--tRNA synthetase, cytoplasmic isoform X1 [Bombyx mori]

MKIYTNKNNTGTLKLLIAANLAAKKVEIVEAEFEGPRQLPLLEVDPELRFFSSNAAVQYLFPILDMSENGQCQQLQEWEA

TRLQPSLSAALGKSPASDVKQVLKSSLEHVNGLLGGHQYVLGDKISAADISIWSTLYPLTHNDDLREQYLKELGNIRRWS

DAIGGAKSVQEAVRQWGGSPTGPFGQISALGLPQVANLVSPAGSPDDDAEVQEGPTPEEIEAAKEGWLNGLSKLQPPLKQ

EKIVLPVKGRKNVLITSALPYVNNVPHLGNIIGCVLSADIFARYCRLCDYNTLYICGTDEYGTATETKALEEGVTPKQIC

DKYFEIHNNVYRWFNIGFDYFGRTSTEEQAEITQRLFLKLNENGFVSSQTVDQLFCEKCDRFLADRFVEGTCPHPGCGYE

GARGDQCDKCGRLVNAPELLQPRCKVCAHPPVLKPSEQLFIELGQLEPSLRSWMSRWEHLWSAPARAVGRAWLREPLRAR

ALTRDLRWGVPVPLDGYRHKVFYVWFDAPIGYMSITQCATKQYEAWWKPDKEYDVSLYQFMAKDNVPFHVVMFPATVLGV

NEGHLLVKHLFATEYLNYEEGKFSKSRGVGVFGNDAQDTGIPSDVWRFYLASIRPETSDSSFSWTELGTRNNSELLNNLG

NFCHRSLSFCSNAFKGLIPEPNPRRSDYELMALVNREIAAYVQNMEKGRLREALRHVLSISRLGNQHMQSEQPWVLLKGS

EDDKQRGATAIGLCCQLVALLCALLAPYVPDTTRKLRGQLNVDAEKLRLNPENPRLLRYLPPGHIIGKPEPLFVKIEQER

LEELRARYAGTQSQRADKKNSKTLDIAELEKAIAEQGDAVRQLKSTTKDKSVWQPEVEKLLELKKRLTEAQKQAETRPAA

NQAAGDVASLEKAMAEQGDAVRKLKSTTKDKSVWQPEVEKLLELKKRLTEAQKQAETRPAANQAAGDVASLEKAIAEQGD

KVRKLKASTKDKAVWQPEVNVLLDLKKRLQALQVKK

>KJE89321.1 hypothetical protein CAOG_009344 [Capsaspora owczarzaki ATCC 30864]

MGSTLSKLWFFNLQTLSILQVEGFVFIAHSSFSSFFISYFYFSPMSLTVAVLDGKSLAALKVEHALALRSTTSSSSTTTV

AIATASEAKQLADRAAAVAPATTAAVVLALDERRTLVNPNAIAVTVLGQCTADAATWIQLDESRLASQLAVVLAPPKVPA

LVTASKAAVLAALKTIASAITAAKGQPLSDAAHVVLGATLFPAFHATGAFALEAAIRSQVASVEQYFQKNVFSTEAFRQA

LAKTGFADDSALAAHVAALSATAAAVLPSKASAVASAAAPAPAAAVTHHQPAPDAKTGIVDLDIMEKATPKELEDALSNW

TKDALPTGKVGSDPTAPIPGQVLLPKPDQRNILITSALPYVNNVPHLGNIIGCVLSADVYARFCRERGYNTLYICGTDEY

GTATETKALEEGLTPQQICDKYHKLHREIYEYFQISFDHFGRTTTPQQTEISQDIFLRALKNDTLLEESVEQLLCETCNR

YLADRFVEGICPSCKYDDARGDQCDKCGKLLNAVELVEPRCKLCRSRPVVRPSRHMFLNLPMLDGQIKEWYAGSSVKGDW

TASAKHITSSWLKEGVKPRCITRDLKWGVPVPLDGFREKVFYVWFDAPIGYISITAAYTAEWEKWWKNPSQVQLYQFMAK

DNVPFHSIIFPASQLAAKDNYTLVHHISAVEFLNYEDGKFSKSRGVGVFGNHARDTGLAIDIFRFYLLGIRPEYTDSVFS

WTDLASKVNNELLANLGNLVQRSLSFIKANFAGKVPKVVLNVDTDAQSCNFVALVNRYLREYIDLLEQVHLRDALRLVLK

ISSIANQYFQTVQPWKTIKLTDDLSKQRTATCLGLCANFVHLLSTLLFPFMPVTGKKILEQLNAAPGSLRADHWGLTLPE

GHEIGTPSVLFEKIEPAVVETLRAKYGGKQEGAAAAGPVSVTESAALKKSKTQQASSPQPAAGTAAAGSSSTGDVAALTA

KVDAQGNLVRKLKGEKADKAKIDAEVKVLLDLKSQLERATKAAAAAAPVAAPSAAPAASASAAPSATTKALEDQIAVQGN

KVRDLKSQKADKAVVTAEVNILLALKQQLASLSNPSIAATHYSNADALSSPSSSSSSSSSSSHCPTVTDTYSDTTSSNAH

RIRTIASSAAAAAAMASVSPSAAPRRLPVIYVSHGSPMSMTQTKEPFYQAFRKVRNVLGLPNADAIKDAVRAIVVVSAHW

ESHSNGVRVTGGANPGLIYDFGGFPRELYNWKYNAPGAPDVAEQVAQLLNKSGVSELACTVDNSRGWDHGAWVPLDIMFP

EATVPVVQLSLNGNLSPDTHIKMGRALAPLRDQGILIIGSGSVTHNLGDFDPSGKSAKVAEWAASFEQFVDTIVTSPDLT

QAERESQLSAFEKHPNGRHAHPRTEHFVPIFVALGANLPDDTTNKQSPPAVPFFRGFVFRTLSMTSYVFGAPQP

>KOC69527.1 Methionine--tRNA synthetase, cytoplasmic [Habropoda laboriosa]

MNISTNEGNPNVLKLVIAAKMAQQSFTVKTVQPKVSFLGRLPFIELSSGLVLFSTNAAMQLLVSSPEDSKVVTNKWLEWE

ASQLQPSIAVYGSTGTYKTANKSCLWSLLKELNIALKDKKYLIEDNNDLSLADACIWVTIWSTILVTDIGNELCKEYVSI

KNWLSNIELLPIIQNSLKEYKFQRGLKAILSVQAVSWFPANTAVISRSAPNSKPTPSDVSPTKEKEVETISQEEIQNITS

NWSSTSYPEMKNSSYPVLPKKGEKNILITSALPYVNNVPHLGNIIGCVLSADIFARYCRQRNYNTPCYLLNFTITIIYYI

SGTDEYGTATEAKALEEKTTPQAICNKFFDIHNDIYRWFRIGFDYFGRTTTPEQTEIVQSFFLRIKSQGYILNETVDQLL

CESCDRFLADRFVEGTCPRCKYEDARGDQCDGCGHLVNATDLISPRCKVCSSRPIVKKSVQFFLDLPKAKDKLKEWSPTV

EKGWSSVARVVAKPWLRDGLKPRCITRDLKWGIPVPVEGYENKVFYVWFDAPFGYISITKRYTQEYEQWWKPKDVQVDLY

QFMAKDNVPFHAIMFPACLIAANEGHTLIKHLMATEYLNYEDTKFSKSRGIGVFGTDARDTGIPADVWRFYLAYVRPETQ

DSNFNWVDLATKNNSELLNNFGNFVNRALVFAEKYFDSKVPLIELQEDDLVLLVLAQRELSSYVHSMKQAKLRDGLKHVL

AISKHANVYMQFQEPWVKIKGTDNDKKRAGTIIGICCNLACLLSALLAPFMPNTARELRSQLGLDNNHYGYISDVITNIL

PTGHKIGKPSPLFKKIEDKDVEALRKKYAGKQETENNGENSDIKSLESEVAELGNKVKELVAKHEGQIFSNLKKMFTDII

GEKSKSPESKSKKSDLVFVPEQNGDASMDVASLEAAIAKQANLVRELKAKEEKSVWRPEVEKLLNLKKQLADLNGTAPAP

TDKKSKKKK

>PAA56250.1 hypothetical protein BOX15_Mlig003940g1, partial [Macrostomum lignano]

TLPVKVEMCEQLKVKLASLLAEGCNSSQSASRQQLRSSNAIVCQTLGERPLKGSLAELLELEACHLRPALHQHLLQHQHL

LQHQHLLQHQQQQPSKALQSALTALESYLEKQQQKQKKQQQQAQIFLSGSCDKPGAADVVLWSSLSPLLLPPASPLSAAL

TKSFPRLAGWFGHVARSHPGFFSLGGDTTTASAAEEARTPGLWVNACAEPAFGCLREPAPLPPTSTTAAAAGQQQQQKQP

QKQKSKQQQQQQQKQPDEQQDELPYPEATQEELVLCRDAFLTDNPLRPFPAKGGRANPVLPVAGRRNLLVTSALPYVNNV

PHLGNIIGCVLSADVYARFHRQRGVNVLYISGTDEYGTATETKALAEGVTPQQICDKYHRIHADIYRWFGIDFDYFGRTT

TEQQTSIAQDIFWRLHRAGHVIRDSVSQLYCSKCEKFLADRFVEGTCPLCQYADCRGDQCDKCGRLINAVELINPRCKVC

SAAPTVRSSEHLFLDLPRLQPQLLAHLESAMDAPDSQWTDNAKHITRSWIKTGLQPRCISRDLKWGTPVPLEGYTDKVFY

VWFDAPIGYVSITANYTKDWEQWWKRPDDVTLVQFMAKDNVPFHSVIFPSCCLGAADNYTMVRHLVATEYLNYEDGKFSK

SRGVGVFGNDVQATGIEADVWRFYLLYVRPESQDSSFSWEDLMLKNNSELLNNLGNFINRPLQFLKKYFDAKVPEISLSQ

EDLRFLHAVNHELQIYIDSLEKAKLRDGLRRVLNISRYGNQYMQLNKPWAQLSNKDTAARAASVVALAANVACLLGALLQ

PYMPSVAIRVAQQCGCLPTKSAQQPQAEFGDRPPLLPIQHPMRRLLPAGHRIGEPSPLFAKVQRDQVDAMRAKFGGNGAR

GE

>XP_017752456.1 PREDICTED: methionine--tRNA synthetase, cytoplasmic [Eufriesea mexicana]

MNISTNEGNPNTLKLVIAAKLTQQSVNIKTVQAKVSFLGRLPSIELSSGLVLFSTNAAMQLIAPSSEELTFINNKWLEWE

ISQLQPAIAFYGSTGIYKTTQKSCLWSLLEKLNNELKDSKYLIKDDLSLADVCIWVTLWSTILVTEIGNELCKEYLSIKD

WLSNIEILPVIQDAIKEYKFERGLKAIFSIQAVSWFPINNIIPNFQRSKLTDLSPTKEKEAETVSQEEIQNISSNWSSNS

YPDAKSFPYPVLPKKGEKNILITSALPYVNNVPHLGNIIGCVLSADIFARYCRQRNYNTLFISGTDEYGTATEAKALEEK

TTPQAICDKFFDIHNDIYRWFSIGFDHFGRTTTPEQTEIVQSFFLRIKSQGYVLSETVDQLLCEFCDRFLADRFVEGTCP

RCKYEDARGDQCDGCGHLVNATDLISPRCKVCSNRPIKKKSEQFFLDLPKLQNKLNEWSSVVEKEWSNVARVVAKPWLRD

GLKPRCITRDLKWGIPVPVKGFENKVFYVWFDAPFGYISITKRYTKEYEQWWKPKDVEVNLYQFMAKDNVPFHAIMFPAC

LLAVDEGYILMKHLMATEYLNYEDTKFSKSRGIGVFGTDARDTGIPADVWRFYLAYVRPETQDSNFNWVDLATKNNSELL

NNFGNFVNRALVFAERYFESKVPPIELQEDDLVLLALAQRELSFYIHALEQAKLRDGIKHVLAISKHGNQYMQFQEPWVK

IKGTDNDKKRAGTIIGICCNLACLLSALLAPFMPSTARELRSQLGLHINSYGYIPDVIMNILPTGHKIGKPSPLFKKIED

KDVEILRKKYAGKQEATNNGEHGDIKSLDNAVTELGKKVEELKAKHDKCAWQSNQIQIFSDLKKKLCNLVSEKSSEPTNK

KPQVVLTPEQNGDVLSDIEILKEAIAKQGDIVRKLKANEEKSVWKPEVEKLLKFKKQLADLTGTPLVPTDKKSKKKK

>XP_002424206.1 methionine-tRNA synthetase, putative [Pediculus humanus corporis]

MKVLTNENNINSLKIVISSKITKKSIHVLTNSIEKLNLKSPKTIPQLVLDSGVHIFSSNASSKFLFPPSVEHNSCVNYYL

DVEATVIQPLIATFSGKVSDKNKKSFEEILNQCNNDLISKKFLCGNEITVSDVSIWCALYPIFTDTKFNDILLNKDSLKK

WFEFIFKLPEVQESLKLCNIKQGLSSIENLACVQKSTSVKPGSSSSQKFSIPSTPEKTIKDNSLTVTNEEKKIAAAAWKE

NLPVAGETNILITSALPYVNNVPHLGNIIGCVLSGDVFARFCRLRGYNTLYISGTDEYGTATETKALEEGLTPREICDKY

YKIHDAIYKWFGISFDYFGRTTTPQQTEICQLIFKKINENNYIISDVVEQLLCEKCDRYLADRFVEGVCPRCSYEDARGD

QCDGCGHLINAPELIKPRCKMCKQTPVLRQSKQFFLDLPKIEPLLKEWIQQRWLDWSHNAREISKAWLNDGLKPRCITRD

LKWGIPVPLEGFESKVFYVWFDAPIGYISITKGYTSEWKKWWMPDKNTKVNLYQFMAKDNVPFHSVMFPASLIASKENYV

TVSNIFATEYLNYEDGKFSKSRGIGVFGNDAQETGIPADVWRFYLLYVRPESQDSSFSWSDLALKNNSELLSVLGNFINR

ALMFCEKTFDSKIPNMNLTDENYELVALVNRELHEYISVIEKGKLREGIRHLLSIARLGNQFMQSSQPWVLCKGSEDDKK

PFPLFNKIEDDLINKLKAKYAGVQSANSHGKGGDDGKKTGGHIGNDVSSLEAAVVAQGDVVRKMKAAGKTKAEWGPEVTI

LLDLKKKLAEAQANNQNNSATNPVTPNQKSITEMEEKVKKQGDHVRKLKESGADKSQWQPEVNVLLDLKKQLAELKGEPL

PAPSGKKNKKK

>XP_011141268.1 PREDICTED: methionine--tRNA synthetase, cytoplasmic [Harpegnathos saltator]

MIVSTNQGNPNALKLIIAAKLAQETVTIKIVKPKDAFLGRLPMLITPSGLSLFSVSSALRFLLPIAAELEILNNRWLEWE

ISQLQPAILSLASGKISSKDLLSLLQDLNSELKDKQYLIETNNYLSPADISIWVSLWSTLLLAECKDISAHLPNIINWLT

NMEQQPVIQESIKEFNSVRGPKAIASIEATSWFPVNTVLNTHAHESVSNDISPTREKEVEAVSQEELRNVTNSWFTGQYL

EVKEGSYPILPKKGERNVLITSALPYVNNVPHLGNIIGCVLSADIFARYCRQRNYNTLYICGTDEYGTATEAKALQEKTT

PQAICDKFFDIHNDVYRWFGIGFDYFGRTTTPEQTEIVQSFFLKIKSQGYILNETVEQLHCELCDRFLADRFVEGTCPGC

KYEDARGDQCDGCGHLMNAVELVEPRCKICNTKPVVKNSVQFFLDLPKIEDRLKQWSDTVSKGWSSVAKVVSKSWLRDGL

KPRCITRDLKWGIPVPVNGFENKVFYVWFDAPFGYISITKRYTKEYEKWWKPQDTKVDLCQFMAKDNVPFHAIMFPACLL

AANQNNTLIKYLMATEYLNYEDKKFSKSRSVGVFGTDARDTGIPADVWRFYLAYIRPETQDSNFNWIDLATKNNSELLNN

LGNFVNRALMFSEKFFDCNVPPTKLEEADWTVLALAQRELTSYINAMEQAKLRDGLKHILAISKHGNQYMQFQQPWVKIK

GTDDDKNRAGTVVAICCNLACLLSALLAPFMPATAKQLRSQLGLSNKNYGYIPEMITNMLPTWHKIGKPSPLFTKIKDQQ

VEALRKKYAGQQEINGHAPAHIGANTSFSGH

>PAA91399.1 hypothetical protein BOX15_Mlig033525g2, partial [Macrostomum lignano]

TLPVKVEMCEQLKVKLASLLAEGCNSSQSGSRQQLRSSNAIVCQTLGERPLKGSLAELLELEACHLRPALHQHLLQHQQQ

QPSKALQSALTALESYLEKQQQKQKKQQQQAQIFLSGSCDKPGAADVVLWSSLSPLLLPPASPLSAALTKSFPRLAGWFG

HVASSRPDFVALAGETATASAAEEARTPGLWVNACAEPAFGCLREPAPLPPTSTTAAAAGQQQQQKQPQKQKSNQQKQQQ

QKQPDEQQDELPYPEATQEELMLCRDAFLTDNPLRPFPAKGGRASPVLPVAGQRNLLVTSALPYVNNVPHLGNIIGCVLS

ADVYARFHRQRGVNVLYISGTDEYGTATETKALAESVTPQQICDKYHRIHADIYRWFGIDFDYFGRTTTEQQTSIAQDIF

WRLHRAGHVMRDSVSQLYCAKCEKFLADRFVEGTCPLCQYADCRGDQCDKCGRLINAVELVNPRCKVCSAAPTVRSSEHL

FLDLPRLQPQLLAHLESVMDAPDSQWTDNAKHITRSWIKIGLQPRCISRDLKWGTPVPLEGYTDKVFYVWFDAPIGYVSI

TANYTKDWEQWWKRPDEVTLVQFMAKDNVPFHSVIFPSCCLGAADNYTMVRHLVATEYLNYEDGKFSKSRGVGVFGNDVQ

ATGIEADVWRFYLLYVRPESQDSSFSWEDLMLKNNSELLNNLGNFINRPLQFLKKYFDAKVPEISLSQEDLRFLHAVNHE

LQIYIDSLEKAKLRDGLRRVLNISRYGNQYMQLNKPWAQLSNKDTAARAASVVALAANVACLLGALLQPYMPSVAIRVAQ

QCGCLPTKSAQQPQAEFGDRPPLLPIQHPMRRLLPAGHRIGEPSPLFAKVQRDQVDAMRAKFGGNGARGE

>PAA93937.1 hypothetical protein BOX15_Mlig012846g1, partial [Macrostomum lignano]

TLPVKVEMCEQLKVKLASLLAEGCNSSQSGSRQQLRSSNAIVCQTLGERPLKGSLAELLELEACHLRPALHQHLLQHQQQ

QPSKALQSALTALESYLEKQQQKQKKQQQQAQIFLSGSCDKPGAADVVLWSSLSPLLLPPASPLSAALTKSFPRLAGWFG

HVASSRPDFVALAGETATASAAEEARTPGLWVNACAEPAFGCLREPAPLPPTSTTAAAAGQQQQQKQPQKQKSNQQKQQQ

QKQPDEQQDELPYPEATQEELMLCRDAFLTDNPLRPFPARGGRASPVLPVAGQRNLLVTSALPYVNNVPHLGNIIGCVLS

ADVYARFHRQRGVNVLYISGTDEYGTATETKALAEGVTPQQICDKYHRIHADIYRWFGIDFDYFGRTTTEQQTSIAQDIF

WRLHRAGHVMRDSVSQLYCAKCEKFLADRFVEGTCPLCQYADCRGDQCDKCGRLINAVELVNPRCKVCSAAPTVRSSEHL

FLDLPRLQPQLLAHLESVMDAPDSQWTDNAKHITRSWIKIGLQPRCISRDLKWGTPVPLEGYTDKVFYVWFDAPIGYVSI

TANYTKDWEQWWKRPDEVTLVQFMAKDNVPFHSVIFPSCCLGAADNYTMVRHLVATEYLNYEDGKFSKSRGVGVFGNDVQ

ATSIEADVWRFYLLYVRPESQDSSFSWEDLMLKNNSELLNNLGNFINRPLQFLKKYFDAKVPEISLSQEDLRFLHAVNHE

LQIYIDSLDKARLRDGLRRVLNISRYGNQYMQLNKPWAQLSNKDTAARAASVVALAANVACLLGALLQPYMPRVAIRVAQ

QCGCLPTKSAQQPQAEFGDRPPLLPIQHPMRRLLPAGHRIGEPSPLFAKVQRDQVDAMRAKFGGNGARGD

>XP_017300143.1 PREDICTED: LOW QUALITY PROTEIN: methionine--tRNA synthetase, cytoplasmic [Diaphorina citri]

MKCLSNRNNVQTLKLLITAEFSTTKLDVELVDVADARLLLSKTVPVLELDDGSQLFSANQSCLYLSKFDTKNESAINQWL

EWESVELFPSVLSFFDNPKVLPDNLKTKLNVLNEAISNSFLIENSTTVADICIWTCVYPLLSDSKVRPLLSSYTNITPWY

DRISKLPQVQKALKTWTEVLKGSANDVGSLSEALFSLSIASQSGNTVLNSLNKLAPLCKKSNKQQPESKAKPAAVVEDDL

SSKSKDVTSEEIEQTKVAWTQGSPVPLRTRTAPVLPQPNENNILITSALPYVNNVPHLGNIIGCVLSADVFSRFCRLVNR

NTLYVSGTDEYGTATETKALEEGLTPRQICDKYFAIHREVYEWFEIDFDYFGRTSTPQQTEIVQHLFDRVHKAGFTSSAS

VEQLHCAQCDRFLADRFVEGVCPKCRYEDARGDQCDGCGQLINAIELIQPRCKQCSQAPTVKSSTQLFLELPKIEDKLQS

WVSETSDQWSSNARVICKAWLKDGLKPRCITRDLKWGIPVPLPGFENKVSNTPVLVVWLAXSRGFQCVIVQYEATPQSKI

TLYQFMAKDNVPFHSIMFPATLVAAGDQYTKVNHIMATEYLNYEDGKFSKSRGTGVFGNNAKDTGLPADIFRFYLLYVRP

ENQDTSFSWQDLATKNNSELLNNLGNFINRALSFIEKNYGSTIPEISPIHEDFLLLALINRELSAYYSVLDKAKLRDGIR

YILNISRYCNQYMQTQQPWVLFKGSPEQKSRASTVLGILCQVSYLLSLLLSPYMPSTASTIASQLGASHLKYSLSSSPSL

VQFLPTGHKIGTPAPLFRKIEQAQVDEWKLKYGGQQNKESSNNKKEKKQAEITFSSVEELEAAVATQGNLVRQVKSSGVE

KSEWQPHVDKLLSLKKQLEAMKSQQRAATDANQTSATPSASDNSADVKALEAEIEKQAEVVRTLKASAGGDKSVFAADLA

KLLSLKQQLSALTGTTGTTNNTKCNNSSKNSSSKKKQKK

>OAD60119.1 Methionine--tRNA synthetase, cytoplasmic [Eufriesea mexicana]

MNISTNEGNPNTLKLVIAAKLTQQSVNIKTVQAKVSFLGRLPSIELSSGLVLFSTNAAMQLIAPSSEELTFINNKWLEWE

ISQLQPAIAFYGSTGIYKTTQKSCLWSLLEKLNNELKDSKYLIKDDLSLADVCIWVTLWSTILVTEIGNELCKEYLSIKD

WLSNIEILPVIQDAIKEYKFERGLKAIFSIQAVSWFPINNIIPNFQRSKLTDLSPTKEKEAETVSQEEIQNISSNWSSNS

YPDAKSFPYPVLPKKGEKNILITSALPYVNNVPHLGNIIGCVLSADIFARYCRQRNYNTLFISGTDEYGTATEAKALEEK

TTPQAICDKFFDIHNDIYRWFSIGFDHFGRTTTPEQTEIVQSFFLRIKSQGYVLSETVDQLLCEFCDRFLADRFVEGTCP

RCKYEDARGDQCDGCGHLVNATDLISPRCKVCSNRPIKKKSEQFFLDLPKLNEWSSVVEKEWSNVARVVAKPWLRDGLKP

RCITRDLKWGIPVPVKGFENKVFYVWFDAPFGYISITKRYTKEYEQWWKPKDVEVNLYQFMAKDNVPFHAIMFPACLLAV

DEGYILMKHLMATEYLNYEDTKFSKSRGIGVFGTDARDTGIPADVWRFYLAYVRPETQDSNFNWVDLATKNNSELLNNFG

NFVNRALVFAERYFESKVPPIELQEDDLVLLALAQRELSFYIHALEQAKLRDGIKHVLAISKHGNQYMQFQEPWVKIKGT

DNDKKRAGTIIGICCNLACLLSALLAPFMPSTARELRSQLGLHINSYGYIPDVIMNILPTGHKIGKPSPLFKKIEDKDVE

ILRKKYAGKQEATNNGEHGDIKSLDNAVTELGKKVEELKAKHDKCAWQSNQIQIFSDLKKKLCNLVSEKSSEPTNKKPQV

VLTPEQNGDVLSDIEILKEAIAKQGDIVRKLKANEEKSVWKPEVEKLLKFKKQLADLTGTPLVPTDKKSKKKK

>XP_011569068.1 PREDICTED: methionine--tRNA synthetase, cytoplasmic [Plutella xylostella]

MKLTVKGEPLFAGPRSLPLLEVDEELLFFSSNAAVQYLVPVLDLSQNGQCQQMQEWEATRLLPATLSAVTGKPAPELRQL

LTSLLTDIDAVLTKKKYILGDKLSAADISIWSTLYPLYYSATHKSTYLEATKNIQKWFEDIAAAKAVQEAVKQWGGSAQG

PFGAPSALGLPQVPSLVATATSPEEPVEVGPTPEEIELAEENWKHGLERLQPKLEQGKVILPIKGRKNILITSALPYVNN

VPHLGNIIGCVLSADIFARYCRLCDYNTLYISGTDEYGTATETKALEEGVTPREICDKYFAIHNNVYRWFNIGFDHFGRT

STAEQTEITQKMFLKLQDNGFISKKSVDQLYCQQCSRFLADRFVEGVCHHPGCLYPDARGDQCDKCGKLINATELVNPRC

KVCGDSPIIRASEQLFIELNQLEPSIRAWSDKASAGWAGGARAVLRAWLKETLRPRAVTRDLKWGVPVPNYPDKVFYVWF

DAPLGYMSITQSATKDYEKWWRPDKECDVKLYQFMAKDNVPFHALMFPAALLGVNEGHLLVHHIYSTEYLNYEEGKFSKS

RGVGVFGTDAQDTGIPADVWRFYLASIRPESSDSSFSWAELGTKNNSELLNNLGNFCHRSLSFCYNSFKGAVPDVNPGPE

EYELMALVNRELIAYVTEMSQGRLRFALRRVLSISRLGNQRMQSAQPWILLKGTDEDKIRGATTIGLCCQIAALLCAVLS

PYMPVTARQLAKQLNVDMGTLRINPDNPSLLQYLPRGHVINQPSPLFSKLEPDSLETLRTKYAGTQSEREGKKNDAASVA

ALEEAIAKQGEKVRQLQASTKDKSVWQPEVNILLDLKKQLTAAQAQAKAAPKPSAAAGTLSVAHLEDAIAKQGEKVRQLK

ASTKDKAVWTPEVNILLDLKKQLTAAQAQAKTQTAPAAAPAQGASVSQLEEAVAKQGEKVRQLKASTKDKAVWTPEVNIL

LDLKKQLTAAQAQAKSSAPVAAAAVNGASAAGLEAAIAKQGDKVRQLKASTKDKSVWQPEVNVLLDLKKQLAALQVK

>KYN41264.1 Methionyl-tRNA synthetase, cytoplasmic [Trachymyrmex septentrionalis]

MIVSTNEGNPNALKLVIATKLAQETVSIRILKPSNATLGRLPTLTTSTGLSLFSISSALRFLLSVPKEVEVFNDRWLEWE

ISQLQPAILSHTSAKTCKTLQKSYLWSLLKDLNNELRDKQYLIEKCSNLSPADISIWVSLWSTILITDIANDIAKDLPSV

NSWLANIQKQPIIQQSIKESIEMFKLERGSKAIASIEAASWFPINTASNPQVHESVPSDISPTKEKEMDIVNQEELCNMA

KSWTTEQYPEVKESSYPILPKKDERNMLITSALPYVNNVPHLGNIIGCVLSADIFARYCRQRNYNTLYICGTDEYGTATE

AKALQEKTTPQAICDKFFDIHNDVYRWFGIGFDYFGRTSTPEQTEIVQGFFLNIKSQGYVLTETVEQLHCESCDRFLADR

FVEGTCPGCKYEDARGDQCDGCGHLVNAVELINPRCKICNTKPVVKNSIQFFLDLPKLEEKLKEYSTTRDKGWSNIARVV

SKAWLRDGLQPRCITRDLKWGIPVPVEGFENKVFYVWFDAPFGYISITKRYTKEYKQWWQPSAQDIKIDLYQFMAKDNIP

FHTIMFPACLLAANQNYTLMKCILATEYLNYEDKKFSKSRGIGVFGTDARDTGIPADVWRFYLAYIRPETQDSNFNWVDL

ATKNNSELLNNLGNFVNRALVFSEKFFNSNIPPIEPEEADWTVLALAQRELSAYINAMEQGKLKDGLKRILAISKHGNQY

MQFQQPWVKIKGNDNDKKKAGTVVAICCNLACLLSALLAPFMPSTAKQLRSQLGLNNKSYGYIPEIITNMLPTWHKIGKP

SPLFTKIEDQKVEMLRKKYAGQQETNGDGTNKSSDESIAILEAAITKQFFSFLIIQESSCIFYVFGNLVRELKTKEDGSV

WKPQVEILLKLKQRLSDLTGTTPVVVDKKSKKKK

>XP_002407128.1 methionyl-tRNA synthetase, putative [Ixodes scapularis]

LPKKGERNVLITSALPYVNNVPHLGNIVGSVLSADVFARYCRLRNWNTLYVCGTDEYGTATETKALEMGITPREICDRFN

KLHADIYKWFNISFDHFGRTTTEQQTKIAQDIFLKLHKNQYMREEVVEQLFCNRCERFLADRFVEGTCPFCAYEDARGDQ

CDLCSKLINPTELKQARCKLCKEPPVLKTSRHLFLDLPKLEPAFGEWYRKKTEGDEWTQTAKVITHAWVRDGLKPRCITR

DLKWGTPVPLHDYKDKVTSYAAFLTWAKFPQINLDVEKSAQVFQHKQDFVTLYQFMAKDNVPFHGIVFPMTQLGTHEHYT

TVDHLVAVEYLNYEDSKFSKSRGIGVFGNDAQDTGIPADVWRFYLMYQRPEHQDTSFCWNDLLMKNNSELLNNLGNFINR

ALTFLSNNFGGIVPELQLLEEDKVVIAKVASLVEHYVSLMDKVRLRDSIRCILSVSKVGNQYIQFNKPWELVKGTPAQKL

RSATVIGLSANIACLLSGLLQPFMPETSEALRHQLNIPLFQLEPHFAPLLKAGHQINKPVPLFKKIEQSTLDTLKKRFEG

KSPTPEKKNGEVNHENANKASGDVSQTPEELARCVQNQGNKVRELKAAKADKATVEKEVACLLELKKQLALSLGQEPEEP

KRQPQKKKK

>XP_018306654.1 PREDICTED: methionine--tRNA synthetase, cytoplasmic isoform X2 [Trachymyrmex zeteki]

MPAILSHASAKTSKTLQKSDLWSLLKDLNSELKDKQYLIEKCSNLSPADISIWVSLWSTILITDIANNIAKDLPSVDSWL

ANIQKQPIIQQSIKESIEMFKLERGSKAIASIEAASWFPVNTASNPQVHESVPSDISPTNKEKEIDVVNQEELRNVAKSW

TTEQYPEVKESSYPILPKKSERNILITSALPYVNNVPHLGNIIGCVLSADIFARYCRQRNYNTLYICGTDEYGTATEAKA

LQEKTTPQAICDKFFNIHNDIYRWFGIGFDYFGRTSTLEQTEIVQAFFLKIKSQGYVLTETVEQLHCESCDRFLADRFVE

GMCPGCKYEDARGDQCDGCGHLVNAVELINPRCKICNTRPVVKNSVQFFLDLPKLEEKLKEYSTTRDKGWSNVARVVTKA

WLRDGLQPRCITRDLKWGISVPVEGFENKVFYVWFDAPFGYISITKRYTKEYKQWWQPPQDTKIDLYQFMAKDNIPFHAI

MFPACLLAANQNYTLMKCIMATEYLNYEDKKFSKSRGIGVFGTDARDTGIPADVWRFYLAYIRPETQDSTFNWVDLATKN

NSELLNNLGNFVNRALVFCEKFFDSNIPPIEPEEADWTVLALAQRELSAYINAMEQGKLKDGLKRILAISKHGNQYMQFQ

QPWVKIKGNDDDKKKAGTVVAICCNLACLLSALLAPFMPSTAKQLRSQLGLSNKSYGYIPETITNTLPTWHKIGKPSPLF

TKIEDQKVEMLRKKYAGQQESNGDGTNKSSNESIASLEAAITKQGNLVRELKAKNDKSIWQPQVEILLDLKKKLTDLKSN

ANAPAKKSPAKNKKAEPIHVPEQNGDALTDVAALESAIAKQGNLVRELKAKEDGSVWKPQVEILLKLKQRLSDLTGTAPV

AVDKKSKKKK

>XP_018365134.1 PREDICTED: methionine--tRNA synthetase, cytoplasmic isoform X2 [Trachymyrmex cornetzi]

MPAILSHASAKTSKALIKQKSNLWSLLKDLNSELKDKQYLIEKCSNLSPADISIWVSLWSTILITDIANDIAKDLSSVNS

WLANIQKQPIIQQSIKESIEMFKLERGPKAIASIEAVSWFPINTVSNPQVHESVSSDISPTKEKEMDIVNQKELCNVAKS

WATEQYPEVKESSYPILPKKDERNMLITSALPYVNNVPHLGNIIGCVLSADIFARYCRQRNYNTLYICGTDEYGTATEAK

ALQEKTTPQAICDKFFDIHNDVYRWFGIGFDYFGRTSTPEQTEIVQAFFLKIKSQGYVLTETVEQLHCESCDRFLADRFV

EGTCPGCKYEDARGDQCDGCGHLVNAVELINPRCKICNTKPVVKNSVQFFLDLPKLEEKLKEYSTTRDKGWSNVARVVSK

AWLRDGLQPRCITRDLKWGIPVPVKGFENKVFYVWFDAPFGYISITKRYTKEYKQWWQPPQDTKINLYQFMAKDNIPFHT

IMFPACLLAANQNYTLMKCILATEYLNYEDKKFSKSRGIGVFGTDARDTGIPADVWRFYLAYIRPETQDSNFNWVDLATK

NNSELLNNLGNFVNRALAFSEKFFDSNIPPIEPEEADWTVLALAQRELSAYINAMEQGKLKDGLKRILAISKHGNQYMQF

QQPWMKIKGNNDDKKKAGTVVAICCNLACLLSALLAPFMPSTAKQLRSQLGLSNKSYGYIPETITNMLPTWHKIGKPNPL

FTKIEDQKVEMLRKKYAGQQETNGDGTNKSSNESIALLEAAITKQGNLVRELKAKNDKSVWQPQVEILLDLKKKLTDLKS

NANAPAKKSPAKNKKAEPMHVPEQNGDTLTDVATLESAIAKQGNLVRELKAKEDGSVWKPQVEILLKLKQRLSDLTGTAP

VVVDKKSKKKK

>XP_013185674.1 PREDICTED: methionine--tRNA synthetase, cytoplasmic [Amyelois transitella]

MKIYTNENNTSTLKLLIAANLAGKKVELLNANFGDDIFAGPRQLPILEVDDQLRFFSSNAAAQYLFPILDLSENGQCQQM

QEWEATRLQPAVAAVVSARPSPELKQALTSLLHLLEQMLTKHQYILGDKLSAADISIWSTVYPVYYNNDLKQQYMSEFGN

LKRWVEELAGAKAIQEAVKQWNGSVTGPFAPSSALGIPQIPSLSSPVGSPDEADIAITADEIESAKDNWLNGAAKLDPPL

KQEKIVLPIKGRKNVLITSALPYVNNVPHLGNIIGCVLSADIFARYCRLCGYNTLYISGTDEYGTATETKALEEGVTPRQ

ICDKYFEIHNAVYRWFNIGFDYFGRTSTDEQTKICQSLFLKLKDNGFISTQSVDQLLCEKCDRFLADRFVEGTCPHPGCG

YDNARGDQCDKCGKLINATELIHARCKICGTTPVVRASEQMFIELPQLEPSLRSWISRHESQWSAPARAVCRSWLREALR

ARAVTRDLRWGVKVPVAGFENKVFYVWFDAPIGYMSITELATRQYETWWKPDKEYDVKLYQFMAKDNVPFHVIMFPSTLL

GINEGHLMVSHVYATEYLNYEEGKFSKSRGVGVFGTDAQETGIPSDVWRFYLASIRPETSDSSFSWAELGTRNNSELLNN

LGNFCHRSLSFCANSFSGIVPAVELTRSDYELIALVNREVVAYVHNMEKGRLREGLRHVLSVSKLGNQHMQSRQPWVLLR

GSGDDKVLGATAIGLCCQLVALLCALLAPFVPDAQRRLREQLNVPQELLRISPTNPSFVQYLPPGHKIGKPEPLFAKIEP

EVLEKLRAKYAGTQSERAGKQNGVVGSVAELEAAIAVQGDKVRQLKASTKDKSVWQPEVERLLQLKKQLAEAQARSSAGE

TDSVASLERAIAEQGDKVRKLKASTKDKNVWTPEVNVLLDLKKRLAALQVK

>XP_018306662.1 PREDICTED: methionine--tRNA synthetase, cytoplasmic isoform X3 [Trachymyrmex zeteki]

MVGMGNFSITNIANNIAKDLPSVDSWLANIQKQPIIQQSIKESIEMFKLERGSKAIASIEAASWFPVNTASNPQVHESVP

SDISPTNKEKEIDVVNQEELRNVAKSWTTEQYPEVKESSYPILPKKSERNILITSALPYVNNVPHLGNIIGCVLSADIFA

RYCRQRNYNTLYICGTDEYGTATEAKALQEKTTPQAICDKFFNIHNDIYRWFGIGFDYFGRTSTLEQTEIVQAFFLKIKS

QGYVLTETVEQLHCESCDRFLADRFVEGMCPGCKYEDARGDQCDGCGHLVNAVELINPRCKICNTRPVVKNSVQFFLDLP

KLEEKLKEYSTTRDKGWSNVARVVTKAWLRDGLQPRCITRDLKWGISVPVEGFENKVFYVWFDAPFGYISITKRYTKEYK

QWWQPPQDTKIDLYQFMAKDNIPFHAIMFPACLLAANQNYTLMKCIMATEYLNYEDKKFSKSRGIGVFGTDARDTGIPAD

VWRFYLAYIRPETQDSTFNWVDLATKNNSELLNNLGNFVNRALVFCEKFFDSNIPPIEPEEADWTVLALAQRELSAYINA

MEQGKLKDGLKRILAISKHGNQYMQFQQPWVKIKGNDDDKKKAGTVVAICCNLACLLSALLAPFMPSTAKQLRSQLGLSN

KSYGYIPETITNTLPTWHKIGKPSPLFTKIEDQKVEMLRKKYAGQQESNGDGTNKSSNESIASLEAAITKQGNLVRELKA

KNDKSIWQPQVEILLDLKKKLTDLKSNANAPAKKSPAKNKKAEPIHVPEQNGDALTDVAALESAIAKQGNLVRELKAKED

GSVWKPQVEILLKLKQRLSDLTGTAPVAVDKKSKKKK

>XP_015789737.1 methionine--tRNA synthetase, cytoplasmic [Tetranychus urticae]

MEGATIDELTEAVKYFSIHQDIPEAVETVEPVEPSVVLPGENCRNILVTSALPYVNNVPHLGNIVGSVLSADVFARYCRL

RGYRTIYICGTDEYGTATETKAMEEKCTPAELCEKYYNCHKEVYEWFNIHFDHFGRTSTTKQTEICHNIFWALHKNGFIL

EDETEQLYCEACERFLADRFVEGTCPFCQYEDARGDQCDKCGKLMNAPDLVNSRCKVCSAHPIIRSSKHLFLDLPKIESS

TNDWFNKTVEDSGKWTQTARVVAGTWLKDGLKPRCITRDLKWGTPVPLEGYQSKVFYVWFDAPIGYLSISSSYTKDWEKW

WKNPDQVELFQFMAKDNVPFHAVIFPSCLLGTKEPYTKVNHLSGIEYLNYENTKFSKSRSIGIFGNDAKDTGIDSDIWRF

YLMYIRPETQDTSFSWVDLMTKNNSELLNNLGNFINRSLMFIAKNFGSKIPAIELDQSDKELIAAINRELKLYVELLESI

KLRDGLRQIFNIGRLGNQHIQVNTPWNLVKGSEEEKKRAGSIMALCANITALIALLLAPYTPKTSEKLRDQLNFKGTTLE

NDHLICFLKEGHSIGTPSPLFRKIDQAEIDALKKKFSGEIEVPKPAKGGKKNTKKPGQSKEPKQPKEPKQPKEPKQPKEP

KEPKQPKEAEQPKQPVEVKD

>XP_018339700.1 PREDICTED: methionine--tRNA synthetase, cytoplasmic isoform X2 [Trachymyrmex septentrionalis]

MVGMGNFSITNIANDIAKDLPSVNSWLANIQKQPIIQQSIKESIEMFKLERGSKAIASIEAASWFPINTASNPQVHESVP

SDISPTKEKEMDIVNQEELCNMAKSWTTEQYPEVKESSYPILPKKDERNMLITSALPYVNNVPHLGNIIGCVLSADIFAR

YCRQRNYNTLYICGTDEYGTATEAKALQEKTTPQAICDKFFDIHNDVYRWFGIGFDYFGRTSTPEQTEIVQGFFLNIKSQ

GYVLTETVEQLHCESCDRFLADRFVEGTCPGCKYEDARGDQCDGCGHLVNAVELINPRCKICNTKPVVKNSIQFFLDLPK

LEEKLKEYSTTRDKGWSNIARVVSKAWLRDGLQPRCITRDLKWGIPVPVEGFENKVFYVWFDAPFGYISITKRYTKEYKQ

WWQPSAQDIKIDLYQFMAKDNIPFHTIMFPACLLAANQNYTLMKCILATEYLNYEDKKFSKSRGIGVFGTDARDTGIPAD

VWRFYLAYIRPETQDSNFNWVDLATKNNSELLNNLGNFVNRALVFSEKFFNSNIPPIEPEEADWTVLALAQRELSAYINA

MEQGKLKDGLKRILAISKHGNQYMQFQQPWVKIKGNDNDKKKAGTVVAICCNLACLLSALLAPFMPSTAKQLRSQLGLNN

KSYGYIPEIITNMLPTWHKIGKPSPLFTKIEDQKVEMLRKKYAGQQETNGDGTNKSSDESIAILEAAITKQGNLVRELKA

KNDKSIWQPQVEILLDLKKKLTDLKSNANAPAKKSPAKNKKAEPMHVPEQNGNALTDVAALESAIAKQGNLVRELKTKED

GSVWKPQVEILLKLKQRLSDLTGTTPVVVDKKSKKKK

>XP_018365135.1 PREDICTED: methionine--tRNA synthetase, cytoplasmic isoform X3 [Trachymyrmex cornetzi]

MVGMGNFSITNIANDIAKDLSSVNSWLANIQKQPIIQQSIKESIEMFKLERGPKAIASIEAVSWFPINTVSNPQVHESVS

SDISPTKEKEMDIVNQKELCNVAKSWATEQYPEVKESSYPILPKKDERNMLITSALPYVNNVPHLGNIIGCVLSADIFAR

YCRQRNYNTLYICGTDEYGTATEAKALQEKTTPQAICDKFFDIHNDVYRWFGIGFDYFGRTSTPEQTEIVQAFFLKIKSQ

GYVLTETVEQLHCESCDRFLADRFVEGTCPGCKYEDARGDQCDGCGHLVNAVELINPRCKICNTKPVVKNSVQFFLDLPK

LEEKLKEYSTTRDKGWSNVARVVSKAWLRDGLQPRCITRDLKWGIPVPVKGFENKVFYVWFDAPFGYISITKRYTKEYKQ

WWQPPQDTKINLYQFMAKDNIPFHTIMFPACLLAANQNYTLMKCILATEYLNYEDKKFSKSRGIGVFGTDARDTGIPADV

WRFYLAYIRPETQDSNFNWVDLATKNNSELLNNLGNFVNRALAFSEKFFDSNIPPIEPEEADWTVLALAQRELSAYINAM

EQGKLKDGLKRILAISKHGNQYMQFQQPWMKIKGNNDDKKKAGTVVAICCNLACLLSALLAPFMPSTAKQLRSQLGLSNK

SYGYIPETITNMLPTWHKIGKPNPLFTKIEDQKVEMLRKKYAGQQETNGDGTNKSSNESIALLEAAITKQGNLVRELKAK

NDKSVWQPQVEILLDLKKKLTDLKSNANAPAKKSPAKNKKAEPMHVPEQNGDTLTDVATLESAIAKQGNLVRELKAKEDG

SVWKPQVEILLKLKQRLSDLTGTAPVVVDKKSKKKK

>XP_014363860.1 PREDICTED: methionine--tRNA synthetase, cytoplasmic [Papilio machaon]

MKVYTNIGNTGTLKVLIAAHLAGKSVEKCLATFEDGIFTGPQALPLLEVDDLLTFFSSNAAVQYLFPTLDLSENGQCQQM

LEWEATRLQPAVAAIVSGKSVASDIRQALQSSLQMLDKLLQKNKYILGEKLSAADISIWSTLYPVCFDDALKQTYISGCD

NIVRWNTNLASAKAIQQAVQQWGWSPKDQYGASSALGIPQIASIVSPIGLPLKDRKNVLITSALPYVNNVPHLGNIIGCV

LSADIFARYCRQCDYNTLFICGTDEYGTATETKALEEGITPREICDKYFDIHNAVYRWFDIGFDYFGRTSTKEQTEIAQK

LFLKLIDNGFVSTQSVEQLYCEKCERFLADRFVEGVCPHPGCLYDSARGDQCDKCGKLINAIELVEPRCKVCSHKPTVRP

SEQLFIELGQLEPSLRTWISSAEQGWSAPARAVSRAWLRDSLRARAVTRDLKWGVPVPIDKHAHKVFYVWFDAPIGYMSI

TQCLTKHYEKLWKPDKDVDVKLYQFMAKDNVPFHVIMFPATVIGVNEGHKLVDHIFATEYLNYEEGKFSKSRGVGVFGTD

AQETGIPSDVWRFYLTSIRPETSDSSFSWVELGTRNNSELLNNLGNFCHRSLSFCANSFKGKVPDVTLTPADLELIALVN

REVISYVQNMEKGRLRESLRHVLSVSRLGNQHMQAEQPWVLLKGSEQDKVRGATAIGLCCQLVALLCALLAPFMPHTARR

LREQLNIDAVALRINPQNPSLVQYLPAGHIIGKPEPLFSKIEPELLEKLRAKYAGTQSDRAKQNGDAKSSPSKGSVAELE

AAVEEQGNKVRQLKASTKDKAVWQPEVNKLLDLKKQLETAKAAPKETTATPSESVASLEKAIAEQGDKVRKLKASTKDKG

VWQPEVDKLLQLKKQLTAAQAQTNAASTNNKDDIASLEKAITEQGDKVRKLKASTKDKAVWQPEVNVLLDLKKRLAALQI

K

>PBC29010.1 Methionyl-tRNA synthetase, cytoplasmic [Apis cerana cerana]

MNILTNEDNPNTLKLIIAAKVVQQSVTVKKVEPKGSFLGRLPSIELSSGLVLFSTNAAMQLIIPPSEESKIINSKWLEWE

ISQLQINKALSYIVQSAITFYGSTGIYKTTHKSGLWSLLEELNNVLKDKKYLIKDNNDLSLADICIWVTLWSTVLVTEIG

NELCREYQHINNWISNIESHPIIQDSIKEYKFERGLKAIFSIQAVSWFPINTIQNFRSPKSADVQLSPTKEKEMEAVSQE

EMHVISSNWSSILYPDVKNFPYPVLPKKDEKNVLITSALPYVNNVPHLGNIIGCVLSADIFARYCRQRNYNTLFISGTDE

YGTATEAKALEEKTTPQSICDKFFDIHNDVYRWFNIGFDYFGRTTTPEQTEIVQTFFLRIKSQGYILSETVDQLLCESCD

RFLADRFVEGTCPRCKYEDARGDQCDGCSHLINATDLISPRCKVCSNRPIIKKSEQFFLDLPKLENKLKEWSSTVEKGWS

GVARVVAKPWLRDGLKPRCITRDLKWGIPVPVKGFENKVFYVWFDAPFGYISITKRYTKEYEQWWKPKDIKVDLYQFMAK

DNVPFHAIMFPASLLAANEDYILLKHLMATEYLNYEDTKFSKSRGIGVFGTDARDTGIPSDVWRFYLAYVRPETQDSNFN

WVDLATKNNSELLNNFGNFVNRALVFVERYFESKIPPIELQEDDLILLALAQRELSSYIYAMEQAKLRDGLKHILAISKH

GNQYMQFQEPWDLKHCMPYRLENLYFLGTLVQCGLLLHEKRAGTIIGISCNMACLLSALLAPFMPSTARELRSQLGLATN

SYGYIPDLITNILPTAHKIETLRKKYAGKQETTNEHSDVKSLDNAIAELDTKVKELKAKHDKSGWQSSQVQIFSDLKKKL

SDLISEKNKSSDSKNKKPEIVLVPEQNGDILMDVASLESAIAMQGNLVRTLKAKEEKSVWQPEVEKLLKLKKQLADLTGT

APTSNEKKSKKKK

>EKC18185.1 Methionyl-tRNA synthetase, cytoplasmic [Crassostrea gigas]

MKLYTDRGNFKSLKVIAALEFTGLSYSLTECGLEERVVPYLSGTSLPVLEVSPQRFLFSPNTATRYILNKKSDEDDPNID

KWLEWESVQLQPYLVRYLVSVSVHKKPDAESLNRLQQLLKFLNAELKSKSLTKTKDCAADFVLWASLFPVFKGAAEIKGW

EGQYSNIKSWFNTINSLPCCQTALDKVIPGGDTTCLKASLNTQPVPLLETETRQEEPVQKSKPQKLKMEKPSETPTPEES

LLTKEASPEEIQDAKESWLTGKSKFPKPRERKHPILPKSGERNLLITSALPYVNNVPHLGNIIGCVLSADVFSRYCRLRN

YNCLYICGTDEYGTATETKAIEEGVTPREICDKYNKIHAEIYDWFNISFDKFSRTSIPEQTKIAQDIFWKLEKNGFISRD

SVEQLQCQQCLRFLADRFVEGTCPLCGFDDARGDQCDGCGKLLNAIELKDPKCKVCKGIPQVKSSNHLFLDLPKIEDKLK

AHLNAQFDKGIWTNNARVITNSWIRDGLKPRCISRDLKWGIPVPLEGYQDKVFYVWFDAPIGYISATASYTDRWEQWWKN

PKQVELYNFLGKDNVPFHSVIFPCSLLGADDNFTVVNHMSATEYLNYEDTKFSKSRGTGVFGHQAKDTGIPADIFRFYLL

FVRPESQDSSFSWDDFQLKNNSELLNNLGNFINRQVI

>OWR49585.1 hypothetical protein KGM_210187 [Danaus plexippus plexippus]

MKIYTNENNTATLKLLIAAKLAGKDVELLKGTHEESPGPAVLPRLEVHEELSFFSSNAAVQYLFPVLDLSQNGQCLQMLE

WEATRLYPVVSTVLTSKTVSSELKEALNTSLLIADNLLAKHQYILGDKLSPVDVSIFSTLYPLCCTDLKDTYLKEYSHVL

RWSGDIGNSEAVQEAVKQWGGSPNSPPSASSLLGTPQVVIQTPTGSPDEVPEKLSAEELEMARDNFLNGINKLQPPLKRE

GVVLPDKDRRNVLITSALPYVNNVPHLGNIIGCVLSADIFSRYCRLCGFNTLFVCGTDEYGTATETKALEEGVTPRQICD

KYFAIHDAVYRWFNIDFDYFGRTSTEQQTRIAQDLFKKLNANGFVSKQTVEQLYCEKCDRFLADRFVEGTCPHPGCLYDN

ARGDQCDKCGKLINAVELREARCKVCSSSPAVRNSDQLFIELPQLEPSLRSWASRAEAGWSGPARAVLRAWMRDKLRCRA

VTRDLKWGVPVPITGFENKVFYVWFDAPIGYLSITECATGNYEKWWKRSPDYDVKLYQFMAKDNVPFHVIMFPATVIGVN

EGHLLVDHIYATEYLNYEDTKFSKSRGVGVFGTDARDTGIPSDVWRFYLAMIRPETSDSSFSWADLATRNNSELLNNLGN

FCHRSLSFCYSSFSAAVPDTQLTPTDLEIIAGVNRDVVAYVQHLERGRLRDALRHVLRVSRAGNLYMQDTQPWALLKGGT

QDRVKAATTIGVCCELVALLAALLAPYMPDTSKRLCTQLNIDQSELRINPTEPCMVRFLGPGHTINKPEPLFTKIEQQTV

DELRRKYAGTQADRRKSNGDCKKLSAAELEAAISAQGEKVRKLKSSTKDKAVWQPEVDVLLALKKQLTLAHTHADQQTGS

AAELERAVAEQGDKVRKLKASTKDKTVWQPEVNKLLALKKQLAEHTDRQTGNHSPGSVEQLEKAIAEQGDKVRKLKASTK

DKSVWQPEVNVLLDLKKQLTALQANK

>EAW97019.1 methionine-tRNA synthetase, isoform CRA_a [Homo sapiens]

MRLFVSDGVPGCLPVLAAAGRARGRAEVLISTVGPEDCVVPFLTRPKVPVLQLDSGNYLFSTSAICRYFFLLSGWEQDDL

TNQWLEWEATELQPALSAALYYLVVQGKKGEDVLGSVRRALTHIDHSLSRQNCPFLAGETESLADIVLWGALYPLLQDPA

YLPEELSALHSWFQTLSTQEPCQRAAETVLKQQGVLALRPYLQKQPQPSPAEGRAVTNEPEEEELATLSEEEIAMAVTAW

EKGLESLPPLRPQQNPVLPVAGERNVLITSALPYVNNVPHLGNIIGCVLSADVFARYSRLRQWNTLYLCGTDEYGTATET

KALEEGLTPQEICDKYHIIHADIYRWFNISFDIFGRTTTPQQTKSLSVKSADHALWCSRASTCFWTCLSWRSDWRSGWGG

HCLAVTGHPMPSLSPVLGFGMASSHAA

>XP_003090777.1 hypothetical protein CRE_09872 [Caenorhabditis remanei]

MGYDLAEVKKKFAAKTVNYVEKKDPKRFAESHKNNLFLFSSILPQAGKRNILITAALPYVNNVPHLGNIIGCVLSADVFA

RYCNLRGHQTFYVGGTDEYGTATETKALQEKCTPRELCDKYHTIHKGIYEWFGIDFTHFGRTTTDHQTEICQDMFMKLHQ

NGYTSTQSVDQLFCNNCDKFLADRFVTGTCPYCAYDDARGDQCDGCGKLINAVELKNAKCHMCKSAPEVKQSTHIFLSLD

KLQEKTAKYLDVVMNDNPDSRWSANAVGITNSWTKLGLEPRCITRDLKWGTPVPLEGFEKKVFYVWFDAPIGYLSITKCV

LGDDWTKWWKNPKNVELYNFVGKDNVAFHAVMFPCSQLGANDNYTVVNHLCATEYLNYEDTKFSKSRGTGIFGDAAQGTG

IPADIWRFYLLYMRPESQDTAFSWDDFVLKVNSELLNNLGNFINRALSFVSNFFGGVIPEIQLTDADAEFLEEVNKECEQ

WDKQFDRVHLKDAVKSILNVSRLGNQYMQAQTPWVLMKGSDEEKKRAGTIIGVAANIAYHLAVILYPVMPSISATIREQC

GLPKLPIFSPFPINYLKSGHKIGKPSPLFQKLDPVQIAEFKAKFGGSQDAQNASPAAEKPKQQKKQAPTKDKKGGDKKKM

ASTAAFVELEQGAKMISQLLAQNMKKFEQAKALFARNQLQKLEEENKQLILDAKSLQHHLIELETAAGIKQIPKPVVSCT

PTPTSTPASGIIAETPKSAEPAPAPAAEPKKAKEQKKGKGGGAAAAPVDDSIDVGRLDMRVGRIIKCEKHPDADALYVEQ

IDVGEASPRTVVSGLVRHVPLDQMQNRLVVVLCNLKPAKMRGVESRAMVMCASSPDKVEIMEVSPDSVPGTPVVCPPYTH

RPDEQLNPKKKIWETVAEDLKVSADGFAEWKGHPLLVGNATKMTAPTLRGVHVK

>OZF92252.1 hypothetical protein FL83_21336, partial [Caenorhabditis latens]

MGYDLSEVKKKFAAKTVNYIEKKDPKSILPQEGKRNILITAALPYVNNVPHLGNIIGCVLSADVFARYCNLRGHQTFYVG

GTDEYGTATETKALQEKCTPRELCDKYHTIHKGIYEWFGIDFTHFGRTTTDHQTEICQDMFMKLHQNGYTSTQSVDQLFC

DKCDKFLADRFVTGTCPFCAYDDARGDQCDGCGKLINAVELKNAKCHMCKSAPEVKQSTHIFLSLDKLQEKTAKYLDVVM

NENPDSRWSANAVGITNSWTKLGLEPRCITRDLKWGTPVPLEGFEKKVFYVWFDAPIGYLSITKCVLGDDWTKWWKNPKN

VELYNFVGKDNVAFHAVMFPCSQLGANDNYTVVNHLCATEYLNYEDTKFSKSRGTGIFGDAAKETGIPADIWRFYLLYMR

PESQDTAFSWDDFVLKVNSELLNNLGNFINRALSFVGNFFGGVIPEIQLTDADAEFLEEVNKECEQWDKQFDRVHLKDAV

KSILNVSRLGNQYMQAQTPWVLMKGSDEDKKRAGTIIGVAANIAYHLAVILYPVMPSISATIREQCGLPKLPLFSPFPIN

YLQSGHKIGKPSPLFQKLDPVQIAEFKAKFGGSQDAQNASPAAEKPKQQKKQAPTKDKKGGDKKKMASTAAFVELEQGAK

MISQLLAQNMKKFEQAKALFARNQLQKLEEENKQLILDVKSLQHHLIELETAAGIKQIPKPVVSCTPTPTSTPASGIIAE

TPKIAETAPAPAAEPKKAKEQKKGKGGGAAAAPVDDSIDVGRLDMRVGRIIKCEKHPDADALYVEQIDVGEASPRTVVSG

LVRHVPLDQMQNRLVVVLCNLKPAKMRGVESRAMVMCASSPDKVEIMEVSPDSVPGTPVVCPPYTHRPDEQLNPKKKIWE

TVAEDLKVSADGFAEWKGQPLLVGNATKMTAPTLRGVHVK

>XP_003107904.1 CRE-MARS-1 protein [Caenorhabditis remanei]

MGYDLAEVKKKFAAKTVNYVEKKDPKSILPQAGKRNILITAALPYVNNVPHLGNIIGCVLSADVFARYCNLRGHQTFYVG

GTDEYGTATETKALQEKCTPRELCDKYHTIHKGIYEWFGIDFTHFGRTTTDHQTEICQDMFMKLHQNGYTSTQSVDQLFC

NNCDKFLADRFVTGTCPYCAYDDARGDQCDGCGKLINAVELKNAKCHMCKSAPEVKQSTHIFLSLDKLQEKTAKYLDVVM

NDNPDSRWSANAVGITNSWTKLGLEPRCITRDLKWGTPVPLEGFEKKVFYVWFDAPIGYLSITKCVLGDDWTKWWKNPKN

VELYNFVGKDNVAFHAVMFPCSQLGANDNYTVVNHLCATEYLNYEDTKFSKSRGTGIFGDAAQGTGIPADIWRFYLLYMR

PESQDTAFSWDDFVLKVNSELLNNLGNFINRALSFVSNFFGGVIPEIQLTDADAEFLEEVNKECEQWDKQFDRVHLKDAV

KSILNVSRLGNQYMQAQTPWVLMKGSDEEKKRAGTIIGVAANIAYHLAVILYPVMPSISATIREQCGLPRLPIFSPFPIN

YLKSGHKIGKPSPLFQKLDPVQIAEFKAKFGGSQDAQNASPVAEKPKQQKKQAPTKDKKGGDKKKMASTAAFVELEQGAK

MISQLLAQNMKKFEQAKALFARNQLQKLEEENKQLILDAKSLQHHLIELETAAGIKQIPKPVVSCTPTPTSTPASGIIAE

TPKSAEPAPAPAAEPKKAKEQKKGKGGGAAAAPVDDSIDVGRLDMRVGRIIKCEKHPDADALYVEQIDVGEASPRTVVSG

LVRHVPLDQMQNRLVVVLCNLKPAKMRGVESRAMVMCASSPDKVEIMEVSPDSVPGTPVVCPPYTHRPDEQLNPKKKIWE

TVAEDLKVSADGFAEWKGQPLLVGNATKMTAPTLRGVHVK

>OZF97997.1 hypothetical protein FL82_03861, partial [Caenorhabditis remanei]

MGYDLAEVKKKFAAKTVNYVEKKDPKSILPQAGKRNILITAALPYVNNVPHLGNIIGCVLSADVFARYCNLRGHQTFYVG

GTDEYGTATETKALQEKCTPRELCDKYHTIHKGIYEWFGIDFTHFGRTTTDHQTEICQDMFMKLHQNGYTSTQSVDQLFC

NNCDKFLADRFVTGTCPYCAYDDARGDQCDGCGKLINAVELKNAKCHMCKSAPEVKQSTHIFLSLDKLQEKTAKYLDVVM

NDNPNSRWSANAVGITNSWTKLGLEPRCITRDLKWGTPVPLEGFEKKVFYVWFDAPIGYLSITKCVLGDDWTKWWKNPKN

VELYNFVGKDNVAFHAVMFPCSQLGANDNYTVVNHLCATEYLNYEDTKFSKSRGTGIFGDAAQGTGIPADIWRFYLLYMR

PESQDTAFSWDDFVLKVNSELLNNLGNFINRALSFVSNFFGGVIPEIQLTDADAEFLEEVNKECEQWDKQFDRVHLKDAV

KSILNVSRLGNQYMQAQTPWVLMKGSDEEKKRAGTIIGVAANIAYHLAVILYPVMPSISATIREQCGLPRLPIFSPFPIN

YLKSGHKIGKPSPLFQKLDPVQIAEFKAKFGGSQDAQNGSPVAEKPKQQKKQAPTKDKKGGDKKKMASTAAFVELEQGAK

MISQLLAQNMKKFEQAKALFARNQLQKLEEENKQLILDAKSLQHHLIELETAAGIKQIPKPVVSCTPTPTSTPASGIIAE

TPKSAEPAPAPAAEPKKAKEQKKGKGGGAAAAPVDDSIDVGRLDMRVGRIIKCEKHPDADALYVEQIDVGEASPRTVVSG

LVRHVPLDQMQNRLVVVLCNLKPAKMRGVESRAMVMCASSPDKVEIMEVSPDSVPGTPVVCPPYTHRPDEQLNPKKKIWE

TVAEDLKVSADGFAEWKGQPLLVGNATKMTAPTLRGVHVK

>KPM07843.1 methionine-tRNA synthetase, cytoplasmic-like protein [Sarcoptes scabiei]

MSSTISEEEISKAKLEFNRSGLKFWVDRSARINLIPREDTRNILIASALPYVNNEPHLGNIIGCTLSADVFARFCRLRGA

NVLYVCGTDEYGTATENKAAEENMSPREICDKYHQVHSNVYKWFNIEFDEFGRTSTEIQTRIVQEIFKKTYENGFILEEE

IEQLFCENCQKFLADRFVEGVCPFCDYEDARGDQCDRCSKLINAIELKNPRCKTCSKSPVKRTSKHLFLDLPKIQPKLEK

WLESKLSVENNWSQTAKVITNSWLKEGLKARCITRDLKWGIPVPLEGYKDKVFYVWFDAPIGYVSITASICPDWEKWWKN

PENVELYHFLGKDNVAFHSIIFPSTQLASNDKWTMCTNISATEYLNYEDSKFSKSRGTGVFGESAKNTGIPADIWRFYLL

YIRPENQDTNFDWDDFMAKNNSELLNNLGNYINRTLSFLSNSFDSTVGHIQLNDDDFKLLAKINRELQYYIDLMENLKLR

DGIKAILNISRLGNQYLQSEKPWKLIKDEATKNRAISVISLSTNLCLLLSILIEPYMPETSKQIQSQIDYKCNVLPKNNN

FTRILPENHKINKPEIIFKQITIDQIKKFREQFGSDPKKEMESKLKNELRSKSLSEIEALISQQGDKIHDVPDTAELSSI

IRGVPRMPTGMEKEEECEHHLQRAISAQQAYGRTIDHVIPTPEVYIVENEHYDQLYTANFTMPRQLIHIQLSLFEQEQPE

YDIDSEDEEWLIQMKPLLNISDTKFEEMIECLEKNSAVHKVIELPEAKPLLRGVDESVQISVYDYWLNKKLGVKKSLIPS

VKTEKRDGISGNNPYIAFRRRTEKMQTRKNRKNDEHSYEKMLKLRKDFDSIRNILSLVRRRDITKREILKVDLEIWDTSR

TVNEFNEDFLTSEDDNSNDVVYQNNHINNIDDDADNPDGIFSFKRKKGCFYHAPLEKLSGWQWESSEGLNNEKLKINLQE

CYPDYFTGYFRRRIGRGGRLRIARNGDIVDDDDSDSEEM

>XP_024224067.1 methionine--tRNA synthetase, cytoplasmic isoform X4 [Bombus impatiens]

MKMNISTNEGNPNTLKLIIAAKVAQHAVTVKTVQPKVGFLSRLPSIELSSGLVLFSTNAAMQLIVPSSSQESKLLNNKWL

EWEISRLQPAIAFYGNTGIYKTTQKSCLWSLLRELNNALEDKQYLIKDNNDLSLADICIWVTLWSTVMVTEIGNELNKEY

LSIKNWISSIELLPVIQDSIEEYKFERGSKAIFSIQAVSWFPVNTNSNPKPPKPADLQLSPTKEKEMEAVNQEEIQNISS

NWSSKSYPDVKNFPYPVLPKKGEKNILITSALPYVNNVPHLGNIIGCVLSADIFARYCRQRNYNTLFISGTDEYGTATEA

KALEEKTTPQNICDKFFDIHNDVYRWFNIGFDYFGRTTTPEQTEIVQSFFLKIKSEGYVLSETVDQLLCESCDRFLADRF

VEGTCPRCKYEDARGDQCDGCGHLVNATDLISPRCKVCSNRPIVKKSQQFFLDLPKLENKLKEWSSTVEKGWSSVAKVVA

KPWLRDGLKPRCITRDLKWGIPVPIKGFENKVFYVWFDAPFGYISITKRYTKEYQQWWKPTDVQVDLYQFMAKDNVPFHA

IMFPAYLLAANEGYTLMKHLMATEYLNYEDTKFSKSRGIGVFGTDARDTGIPADVWRFYLAYVRPETQDSNFNWVDLATK

NNSELLNNFGNFVNRALVFAEKYFDSKIPPIELQEDDLILLALAQRELSSYIHALEQAKLRDGLKNVLAISKHGNQYMQF

QEPWVKIKGTDNDKYPCSVRIT

>CDS17000.1 methionyl tRNA synthetase cytoplasmic [Echinococcus granulosus]

MWRLFIDTHNRGCLAALAAAYKTKKAEILKYGAERISDGVVLETESGLISSVNAIVSFILGVDALTPKLNAFLLWESTVL

KPGLNRITGSNTGDATDLLIDLQNKLRNLEKEMGEMDGLVANLIIWADISHLLPSIVTLDHLEYLHNLLRTRNTEEYCSH

ALRDLSLKVHSQESSTITPKMPQRKQGQNKFAKANEKSVQNDEDELRRLHVEVSTDQMLKAQWTFSSTVLPATSIHSEKR

TILPLKKTEGVRNILISSALPYVNNVPHLGNLIGSLLSANIFALYCQTAGYNVLSICGTDEYGTATEARAQAEGLTPQQI

TDKYHKLHCQVYDWFDIHFDYFGRTSTPVHTEITQEIFWDLYNNGFVTEASVEQLFCEKCEKFLADRFVEGVCPFCKAED

ARGDQCDTCGKLINAVELIKPQCITCRSYPTVRSSNHLFLDLPKLEEKVDVFFTESVEDPNCKWTQVAQSIARTWLRDGF

KKRCITRDLKWGVPVPLLNFKNKVFYVWFDAPIGYISITANYTRDWRQWWLPSEDVAPVEYFQFMAKDNVLFHSAIFPAC

LLGSGKKFTLVKHIIATEYLNYEGKKFSKSRGVGVFGNNAEDSGIESNIWRFYLAYIRPETQDSTFSWEDFALKNNSELL

NNLGNFINRPLMFVMKFFNSTVPSMKSLQPDDVTFLARVNSILHEYISHMENCRLREGIRSVLGISRLGNGYLQVQRPWV

LYKSSDTIQRAGVVTGISSNIAALLGILLRPFMPTIGEEIFAQCKLPSERRSLAPMVTNGGHIICLLPEGHVIGEPKPLF

KKIEAHDVQRLKKLYYPTS

>NP_001255570.1 Methionine--tRNA synthetase, cytoplasmic [Caenorhabditis elegans]

MGHDLADIKKSFEASLPGYVEKKDPKSILPQPGKRNILITAALPYVNNVPHLGNIIGCVLSADVFARYCNLRGHQTFYVG

GTDEYGTATETKALQEGCTPRELCDKYHAIHKGIYEWFGIDFSHFGRTTTDHQTEICQDMFLKLHKNGYTSSQSVDQLYC

NQCEKFLADRFVTGTCPMCAYDDARGDQCDGCGKLINAVDLKDAKCHMCKATPEVKQSTHIFLSLDKLQQKTTEHLDREL

AKEDNRWSSNAVGITKAWMKLGLDPRCITRDLKWGTAVPLDGFEKKVFYVWFDAPIGYLSITKCVLGDNWTKWWKNPENV

ELFNFVGKDNVAFHAVMFPCSQLGANDNYTVVNNLCATEYLNYEDTKFSKSRGTGIFGDAAQGTEIPADIWRFYLLYMRP

ESQDTAFSWDDFVLKVNSELLNNLGNFINRALSFVANSFGGVVPEMNLTNDDAEVLSEIHNECMQWDKQFDGVHLKDAVK

TILNVSRLGNQYMQAQTPWVLMKKDEEGKKRAGTIIGVAANIAYHVSVLLYPIMPTISATIREQCGLPALPLFTPFPICY

LKAGHKIGQPSPLFQKLDPAQIAEFKAKFGGSQDAQSSAPKTAEKPKQQKKQAPTKDKKGDKKMASTAAFVELEQGAKVI

SQLIAQNLKKFDQAKALFTRNQLQRLDGENKQLTIDVKTLQHQLIELETAAGIKQVPKPVVSCTPTPTSTPASGIITEAP

KKEAPSTPAPSEPKKAKEQKKGKGGAAAAPVDDTIDVGRLDMRVGRIIKCEKHPDADALYVEQIDVGESAPRTVVSGLVR

HVPLDQMQNRLVVVLCNLKPAKMRGVESRAMVMCASSPDKVEIMEVPADSKPGTPVVCPPYTHRPDEQLNPKKKIWETVA

EDLKVSAEGFAEWKGQPLLIGSESKMTAPTLRGVHVK

>EFZ21518.1 hypothetical protein SINV_15365, partial [Solenopsis invicta]

MMIVSTNEGNPNALKLIIAAKLAQEAVTVKILKPRDAVLGRLPTLTTPSGLSLFSVSAALRLLLPEPKDLEVSNDRWLEW

EISQLQPTILSHAKTSQKSRLWSFLQDLNNELKDKKYLIEKCSNLSPADISIWVSLWSTILVTDIDMANDITQNLPNVNS

WLTNIYNQSVIQQSIEESTKMFKFERGGKAIASIEVASWFPVNSVSSSQVHESVSADISPTKEKEVEIVNQEELCNVAKS

WTSEHYLEVKESSYPILPKKGERNILITSALPYVNNVPHLGNIIGCVLSADIFASGTDEYGTATEAKALQEKTTPQAICD

KFFDIHNDVYRWFGIGFDYFGRTTTPEQTEIVQAFFLKIKSQGYVLTETVEQLHCEFCDRFLADRFVEGTCPGCKYEDAR

GDQCDGCGHLVNAVELINPRCKICNTRPVVKNSVQFFLDLPKLEEKLKEYSATVYKGWSNVAKVVSKTWLRDGLKPRCIT

RDLKWGIPVPVEGFENKVFYVWFDAPFGYISITKRYTKEYKKWWQPPQDTKVDLCQFMAKDNVPFHAIMFPACLLAVNQN

YTLTRNILATGTISKECKSLGCIYFMTVFVSYFLCHLFRNISEYLNYEDKKFSKSRGIGVFGTDARDTGIPADVWRFYLA

YIRPETQDSTFNWVDLATKNNSELLNNLGNFVNRFVVSQKLVMSRALVFCEKFFDSNVPPLEAEEADWTVLALAQRELSS

YINAMEQAKLRDGLRHILAISKHGNQYMQFQQPWVKIKGNDDDKKKAGTVVAICCNLACLLSALLAPFMPNVAKQLRSQL

GLSNKNYGYIPEKITNMLPTWHKIGKPSPLFTKIEDQKIEMLRKKYAGQQETNGEVINKSSDESIASLEAAITKQVSRAY

FFFSSCHDNHGNLVRELKAKHDKNVWQPQVEILLDLKKKLVDLKGNVNTPTKKSPAKNKKAEPMHVPEQNGDALTDVAAL

ESAIAKQGNLVRELKAKEDGSVWKPQVEILLKMKQRLSDLTGTAPVTVDKKSKKKK

>PAV56255.1 hypothetical protein WR25_15695 isoform A [Diploscapter pachys]

MASITEAFKTSPQFLEPVPKEKLLPVPGKKNVLITAALPYVNNVPHLGNLIGCVLSADVFARYCRMKGYQTLYICGTDEY

GTATETKALQEGVTPKALCDKYHEIHKAIYEWFNIEFDNFGRTTTQNHTEVTQDIFMQLHKNGFTTADSIDQLFCETCNM

FLADRFVSGICNLCGFDDARGDQCDACGKLINAVELKQPKCHMCKKEPVVRKSTHIFLQLDKLQDEIQAYVEKEISKSDT

KWSANAIAIVKGWFKNGLERRCITRDLKWGIPVPIKEYDDKVFYVWFDAPIGYFSITKSLLKDDWLKWWKNPDDVQLYQF

VGKDNVVFHGVMFPCTLLGTKNGYTVVNHLCATEYLNYEDTKFSKSRGTGVFGDGAKGTGIEADVWRFYLIYMRPENQDS

AFAWDDFAFKVNSELLANLGNFVNRALTFVTNFFESHIPEMHLENDDVQFLKDVHNETVLWDETFDKVKLRDSIKQVLAI

TRHGNAYLQVHQPWVLVKGDEAQRKRAGTIIGVAANVSYHVAVLLHPVMPEISKRIREQCGLPELPLFSPYVINYLKPGH

KIQKIAPLFKKIEPDQVKALREKFGGGQEKGPAKPGEKPDKKAQKKEKPQTPQDPSKLAEKEALKEAKKEAKRMEASRIF

PNLAKNQTAIQQLLRGNWAKFEKARDLFVARKLAEFEKERVRLEKEVESLKTKLFDAETAAGVKQYEVPKREKTPICATS

APGKPEKPADGKEEKGKPKKDEQKEGKQASGAQQNGAAADAEVHVGRLDLRVGRIVKCEKHPDADSLYVEQIDLGEGKPR

TVVSGLVKHVPLNQV

>PAV56256.1 hypothetical protein WR25_15695 isoform B [Diploscapter pachys]

MASITEAFKTSPQFLEPVPKEKLLPVPGKKNVLITAALPYVNNVPHLGNLIGCVLSADVFARYCRMKGYQTLYICGTDEY

GTATETKALQEGVTPKALCDKYHEIHKAIYEWFNIEFDNFGRTTTQNHTEVTQDIFMQLHKNGFTTADSIDQLFCETCNM

FLADRFVSGICNLCGFDDARGDQCDACGKLINAVELKQPKCHMCKKEPVVRKSTHIFLQLDKLQDEIQAYVEKEISKSDT

KWSANAIAIVKGWFKNGLERRCITRDLKWGIPVPIKEYDDKVFYVWFDAPIGYFSITKSLLKDDWLKWWKNPDDVQLYQF

VGKDNVVFHGVMFPCTLLGTKNGYTVVNHLCATEYLNYEDTKFSKSRGTGVFGDGAKGTGIEADVWRFYLIYMRPENQDS

AFAWDDFAFKVNSELLANLGNFVNRALTFVTNFFESHIPEMHLENDDVQFLKDVHNETVLWDETFDKVKLRDSIKQVLAI

TRHGNAYLQVHQPWVLVKGDEAQRKRAGTIIGVAANVSYHVAVLLHPVMPEISKRIREQCGLPELPLFSPYVINYLKPGH

KIQKIAPLFKKIEPDQVKALREKFGGGQEKGPAKPGEKPDKKAQKKEKPQTPQDPSKLAEKEALKEAKKEAKRMEASRIF

PNLAKNQTAIQQLLRGNWAKFEKARDLFVARKLAEFEKERVRLEKEVESLKTKLFDAETAAGGDFIFKN

>XP_002633364.1 C. briggsae CBR-MRS-1 protein [Caenorhabditis briggsae]

MGVDLNEIKKNFNVNTSAYVEKKDPKSILPQPGKRNILITAALPYVNNVPHLGNIIGCVLSADVFARYCNLRGHQTFYVG

GTDEYGTATETKALQEGCTPRELCDKYHAIHKGIYEWFGIDFTHFGRTTTDHQTEICQDMFLKLHKNGFTTTQTVDQLFC

NQCDKFLADRFVTGTCPNCAYDDARGDQCDGCGKLINAVELKDAKCHMCKSTPEVKQSTHIFLSLDKLQESTMDLIMKQM

DLPGSKWSANAKGITKSWENQGLEPRCITRDLKWGTPVPLEGFEKKVFYVWFDAPIGYLSITKCVLGDDWTKWWKNPSNV

ELYNFVGKDNVAFHGVMFPCSQVGANDNYTVVNHLCATEYLNYEDTKFSKSRGTGIFGDAAQRTGIPADIWRFYLLYMRP

ESQDTAFSWDDFVLKVNSELLNNLGNFINRALSFVANFFGGVIPNMDLTEADATILEEIHQECLQWDTQFDGVRLKDAVK

SILNVSRIGNQYMQSQTPWVLMKGDDAAKKRAGTIIGVAANIAYHLAVILYPVMPSVSETIRQQCDLSKLPLFSPFPRNY

LKPGHKIGKPSPLFQKLDPIQIAEFKAKFGGGQNPQNAAVAAAAGNQKQQKKTPPTKEKKGGDKKKMASTAAFAELEQGA

KLISQLLTTNLKKFEQAKALFARNKLQKLEEENRQLTLDAKSLQQQLVELEKAAGIKQIPKPVASCTPTPTSTPASGIIN

QQKNATPAPQPTAEPKKAKEQKKGKGGGAAAAAPVDDAIDIGRLDMRVGRIIKCEKHPDADALYVEQIDVGEESPRTVVS

GLVRHVPLDQMQNRLVVVLCNLKPAKMRGVESRAMVMCASTPDKVEIMEVAADSKPGTPVVCPPLTHRPDEQLNPKKKIW

ETVAEDLKVSAEGFAEWKGHPLLVGNATKMTAPTLRGCHVK

>OTF70519.1 methionine-tRNA synthetase, cytoplasmic-like protein [Euroglyphus maynei]

MCFLLYIAFHPNHFHALYTSTSNTFRFLFQVITSCNNNKKKMSFEVSNDELTEAIREFQRDDIVYWKEPNLSNPNKVPIP

GERNTLITSALPYVNNVPHLGNIIGCTLSGDVFARYCRIRGDNVLYICGTDEYGTATENKAQEEKLSPREICDKYHALHD

EVYKWFNIEFDRFGRTSTEIQTQIVQDIFKKVYSNGYIIKEEIEQLYCEKCSKFLADRFVEGACPYCAYEDARGDQCDKC

GKLINAIELKMPRCKTCSSAPVVKSSRHLFIDLPKLQPKLEIWLDETLKKENYWSQTAKVITNSWLKEGLKPRCITRDLK

WGIPVPLEGYTDKVFYVWFDAPIGYISITAALCPQWEKWWKDPDNVESYNFLAKDNVTFHAIIFPSTLLATHEKWSMVSH

IPAVEYLNYEDSKFSKSRSIGVFGDSVRETGIPSDIWRFYLLYVRPENQDTTFCWNDLMAKNNTELLNNLGNFLNRVLTF

LSNNFAATIGDIKLTSSDQELLVQLNRELLIFIDSMNRVKLRDGIKIILNISRIGNQYIQSEKPWTLVKDLSTRERAHSV

LSLGANICLLLSILMEPYMPDTTRQLQEQINFRCNLLPKNHRFVCLLKPGHKIGKPSPLFKKLEQKEIDIFRKKFAGSNA

DREKETTNAVQLAKFFESISLKTDKELEQMIADQGDKVRRLKADKAEKKIINDEVQILLKLKSTLAEKK

>NP_001255572.1 Methionyl Amino-acyl tRNA Synthetase [Caenorhabditis elegans]

MGHDLADIKKSFEASLPGYVEKKDPKSILPQPGKRNILITAALPYVNNVPHLGNIIGCVLSADVFARYCNLRGHQTFYVG

GTDEYGTATETKALQEGCTPRELCDKYHAIHKGIYEWFGIDFSHFGRTTTDHQTEICQDMFLKLHKNGYTSSQSVDQLYC

NQCEKFLADRFVTGTCPMCAYDDARGDQCDGCGKLINAVDLKDAKCHMCKATPEVKQSTHIFLSLDKLQQKTTEHLDREL

AKEDNRWSSNAVGITKAWMKLGLDPRCITRDLKWGTAVPLDGFEKKVFYVWFDAPIGYLSITKCVLGDNWTKWWKNPENV

ELFNFVGKDNVAFHAVMFPCSQLGANDNYTVVNNLCATEYLNYEDTKFSKSRGTGIFGDAAQGTEIPADIWRFYLLYMRP

ESQDTAFSWDDFVLKVNSELLNNLGNFINRALSFVANSFGGVVPEMNLTNDDAEVLSEIHNECMQWDKQFDGVHLKDAVK

TILNVSRLGNQYMQAQTPWVLMKKDEEGKKRAGTIIGVAANIAYHVSVLLYPIMPTISATIREQCGLPALPLFTPFPICY

LKAGHKIGQPSPLFQKLDPAQIAEFKAKFGGSQDAQSSAPKTAEKPKQQKKQAPTKDKKGGEWWSRRAEKYTESCTQCIQ

LRMDCMPKFYYFLLFQLSLSLLPVQFINLPAQSSAS

>CDS42025.1 methionyl tRNA synthetase cytoplasmic [Echinococcus multilocularis]

MWRLFIDTHNRGCLAALAAVYKTKKAEILKYGAERVSDGVVLETESGLISSVNAIVSFILGVDALAPKLNAFLLWESAVL

KPGLNRITGSNTRDATDLLIDLQSRLRNLKKEMGEMDGLVANLIIWADISHLLPSIVTLDHLEYLHNLLRTCNTEEHCSH

ALRDLSLKVHSQESSTITPKMSQRRQGQNRFAKANEKSVQNDEDELRRLHVEVSTDQMLKAQWTFSSTVLPATSIHSEKR

TILPLKKTEGVRNILISSALPYVNNVPHLGNLIGSLLSANIFALYCQTAGYNVLSICGTDEYGTATEARAQAEGLTPQQI

TDKYHKLHCQVYDWFDIHFDYFGRTSTPAHTEITQEIFWDLYNNGFVTEASVEQLFCEKCEKFLADRFVEGVCPSCKAED

ARGDQCDTCGKLINAVELIRPQCITCRSYPTVRSSNHLFLDLPKLEEKVDVFFTESVEDPNCKWTQVAQSIARTWLRDGF

KKRCITRDLKWGVPVPLLNFKNKVFYVWFDAPIGYISITANYTKDWRQWWLPSEDVAPVEYFQFMAKDNVLFHSAIFPAC

LLGSGKKFTLVKHIIATEYLNYEGKKFSKSRGVGVFGNNAEDSGIESNIWRFYLAYIRPETQDSTFSWEDFALKNNSELL

NNLGNFINRPLMFVMKFFNSTVPSMKSLQPDDVTFLARVNSILREYISHMENCRLREGIRSVLGISRLGNGYLQVQRPWV

LYKSSDTIQRAGVVTGVSSNIAALLGILLRPFMPTVGEEIFAQCKLPPERRSLAPMVTNGGHIICLLPEGHVIGEPKPLF

KKIEAHDVQRLKKLYYPTS

>PIC34356.1 hypothetical protein B9Z55_014033 [Caenorhabditis nigoni]

MGVDLNEIKKNFDVKTSAYVEKKDPKSILPQPGKRNILITAALPYVNNVPHLGNIIGCVLSADVFARYCNLRGHQTFYVG

GTDEYGTATETKALQEGCTPRELCDKYHAIHKGIYEWFGIDFTHFGRTTTDHQTEICQDMFLKLHKNGFTTTQTVDQLFC

NQCDKFLADRFVTGTCPNCAYDDARGDQCDGCGKLINAVELKDAKCHMCKSTPEVKQSTHIFLSLDKLQESTMKLIVKQM

GRPDSKWSANAKGITKSWQDQGLEPRCITRDLKWGTPVPLEGFEKKVFYVWFDAPIGYLSITKCVLGDDWTKWWKNPENV

ELYNFVGKDNVAFHGVMFPCSQVGANDNYTVVNNLCATEYLNYEDTKFSKSRGTGIFGDAAQGTGIPADIWRFYLLYMRP

ESQDTAFSWDDFVLKVNSELLNNLGNFINRALSFVANFFGGVIPNMDLTEADATILEEIHQECLQWDTQFDGVRLKDAVK

TILNVSRIGNQYMQSQTPWVLMKGDDAAKKRAGTIIGVAANIAYHLAVILYPVMPSVSETIRQQCDLPKLPLFSPFPRNY

LKPGHKIGKPSPLFQKLDPVQIAEFKAKFGGGQNPQNDAVAAAAGNQKQQKKTAPTKEKKGGDKKKMASTAAFAELEQGA

KLISQLLTNNLKKFEQAKALFARNQLQKLEEENRQLTLDAKSLQQQLVELEKAAGIKQIPKPVASCTPTPTSTPASGIIN

QQKSATPAPQSTAEPKKAKEQKKGKGGGAAAAAPVDDAIDIGRLDMRVGRIIKCEKHPDADALYVEQIDVGEESPRTVVS

GLVRHVPLDQMQNRLVVVLCNLKPAKMRGVESRAMVMCASTPDKVEIMEVAPDSKPGTPVVCPPLTHRPDEQLNPKKKIW

ETVAEDLKVSAEGFAEWKGHPLLVGNATKMTAPTLRGCHVK

>XP_023941495.1 methionine--tRNA synthetase, cytoplasmic, partial [Bicyclus anynana]

FYALQDKISSADISVWSTLYPIAFNKTLRQAYLNEFTNITKWAEEFASIKAVEDALSQWDGTVEGPFVPNSALGVPQITN

MVSPIGSPDESPELSVTAEEREAAKENFLNGVKSLPEPLKSEKPILPVKGRKNVLITSALPYVNNVPHLGNIIGCVLSAD

IFARYCRLCGYNTLFVSGTDEYGTATETKALEEGITPREICDKYFAIHDAVYRWFEIGFDCFGRTTTAQQTEIAQRMFLK

LMDNGFVTNQSVSQLFCENCQRFLADRFVEGTCPHPGCLYDGARGDQCDKCGKLINAIELIEPRCKVCAHRPTVRPSEHL

FIELGQLEPSIRTWFNTVEGGWSPPARAVTRAWLRDSLRARALTRDLKWGVPVPVPGYTNKVFYVWFDAPIGYLSITQCF

TQNYEKWWKKDKDYDVKLYQFMAKDNVPFHGIMFPATIIGVNEGLTLVDHIFATEYLNYEEGKFSKSRGVGVFGTDAQET

GIPADVWRFYLASIRPETSDSSFSWTELGTRNNSELLNNLGNFCHRSLSFCASTFKGVVPDMTLTDADYELIALVNREIA

AYVSTIERGRLREGLRHVLSVSRLGNQHMQAAQPWVLLKGSDDDKVKGATAIALCCQLVALLCALLAPYVPATCRRLRDQ

LNIHEEALRINPLKPSLVRYLPAGHTIGKPEPLFAKLEPELIDKLRAKYAGSQSDRAKQNGEVPQTAAELEAAIAAQGEK

VRKLKSSTKDKSIWQPEVDKLLALKKQLSSLPAQQPAVNNTTSSDSVADLEKAVAEQGDKVRKLKSTTKDKAVWQPEVDK

LLSLKKQLTEAQTKQKSSAANVASNSSSAADLEKAIAEQGDKVRKLKASTKDKTIWQPEVNTLLDLKKQLAALQVNNK

>PDM70957.1 mars-1, partial [Pristionchus pacificus]

MGNWQWWIRSQARRFRESNVAQRMRYFFNITNPCTCLDSTFHKNRLVIIYSLFLKCNLHRFLFRRHIQTRRRLSLLLCLI

ILISLWIFFPRTSIKESVSQTCLENKLREYDGQLKNFSVARDEESIRFVGNGYIGVGEDGELRAARGNSLINSVLTGFRP

FVHLKLEGISDTPSTAILSHFIKGKLIKVQCFSIDNECLCATTTSFVHRTKKNIMMEEVKVMNPTKSVIIIEMYREESPT

WVSEQVIPAAQERNFINSPLLVRENLGAGGRSVGCIICSSLVPHYEIQPRREETFRLTCSVDFREKIGEEWLKDSREAAV

ASFVEATRKVVNLDKEHEFAWEKLNEVTFHLSHSFAPNVLNGVLINGTRYAIMSNVRAPILEDGVSPQEKMILESKGSRR

DLCYTGHSTMLYPSRLWTKAKNSEELESLVDTWLVTLEKRGCANVLSMGATGVAQAFVQSLSAATFHDGHLEFGMDPADM

HREISVSGLELNDASKSKLYFRVGIHKDNRPFLVVSSSSEVYACDGGCRSDPIPISPSGTKIAVMLTKPLTSILYIAPNR

KHLTQLRSAIHVSEIDVAPAHDDDVISAHKGEDGGLPTIIWAILGVILIAFHVFLIKLLYSEWQKGDSTPYNSFLRKMDE

RRSLRHQWTTQSPSYIKKTAAKDIRPIKGEKNVLITSALPYVNNVPHLGNIIGCVLSADVFARFARMKGYNTLYVCGTDE

YGTATETKAIQEGLSPKAVCDKYHAIHKKVYEWFNIGFDHFGRTTTQHQTEITQDIFNKIDTNGFTSILTVDQLHCNNCS

KFLADRFVSGECPMCGYDDARGDQCDACGKLVNAVELKDPKCHLCKKTPVIKQSSHIFLELDKLQEKVGDYLEKKLSEDN

HWSANAISIVKGWIKGGLEKRCITRDLMWGTPVPREEFNNKVFYVWFDAPIGYLSITKDLIGKDWTKWWKNPDDVQLYQF

IGKDNVAFHGVMFPCTLMAANDGYTIVNHVCATEYLNYEDTKFSKSRGTGVFGDMAMETGIEADIWRFYLLYMRPENQDT

AFSWDDFSLKVNSELLANIGNFINRALSFCSTNFEGVIPEMELTDDDVDFLLDIQKEMFEWENCLEHVRLKDAVRSILSL

SRRANQYMQAQAPWVSIKGDEQQKKRAGSVIGIVANVAYLIAILLSPIMPEISRRIREQCGLEELPAFSHSIICYLKKGH

KIGNPKPLFTKMEKGKVDEWKAKFGGSATTEVSSKKEVRGGKKKGRKEEEKLTKDEVMASRHFPQLAGNKKQITSLLSII

SPKFEQARQAFVQSTRSNLEKENEKLEMDIICLKERLVKAEVSAGIPQYAIPSKKVEVTMSNGVTTTPVVNTLLTTSTAD

KKKDGKGTKKGGESCVGSKNGSNGDDCIDIGRLDLRVGRIIKCDKHPDADALYVEQIDVGEESPRTVVSGLVKHVPIDQM

QNRLVVVLCNLKPAKMRGVESRAMVMCASSPDKVEIMEVNGCSIPGTRVECPPFVNRPDAQLNPKKKIWETVAEDLKVSA

EGYATWKGEALLVGGKTKMTAPTLRGIMKGAEGLSDSRFTHLKSDPKFGGLKRKERKVVVGDRFASALTDARFTRAPLVD

KRGRKVKRTGDATLHRLYEIEKKGAKEEDEEEEVESEVNEDESIENEDNEHDHLKYDLARGDGEYVSSSSEEDDEEEDED

WKLEKADVDHNWGNLDKEVRRVEWASRRLAICNLDWDSLSSQDLFLLVSSFKPLGGHIESLSIYLSDLGKEKLEREEKEG

PQLIMNENGKEDSDEEDDGGLDEETLKHHDKKREAIRRYQIERLKYFYAVLVCDSEQTASAVYETCDGVEYENSGLRLDL

RFVPDDMTFDESRLKERVTTDDVDNGKYKPREISNMAAASTKVKLTWDESDLNRKKKMRGAFDVEADLDEVSRDLIAPSS

GEDSGEEDHTFDMDDRMITKKERLAILLGGGDKDDGEMEVEWEKYDDDEKEEVTIKKKSKSSWEEYLEKRKAKRKEKKNV

VKEERRKAREEREKDEDEKRMEKKKVLKKEVMKEKDDRSVTVDARFNALFTHSAFSIDTSSNLNKSKGLADKQNAVRRKR

KTDVETNSSAQEGHHSLVDKLKSNTNELDGIEGIRENVSRIPLFTRRSTPNTEEHAIMKM

>CDJ82188.1 unnamed protein product [Haemonchus contortus]

MDPQRAAQVRAAWSQSPTFLQLTPKERRVPLKGKRNILITSALPYVNNVPHLGNIIGCVLSADVFSRYCRMRGHETLYVC

GTDEYGTATETKALQEGLTPKEVCDKYHVIHKKIYEWFNIDFDIFGRTTTLQQTEIAQDIFLKLHKNGYTSSCSIDQLHC

QNCDKFLADRFVTGICPFCAFDDARGDQCDSCGRLINAVELKSPKCHICKQEPKVRQSTHIFLHLDALQDEVRQYVENEI

AKPNMRWSSNAIAIVKGWIKGGLEKRCITRDLKWGTAVPLAGYENKVFYVWYDAPIGYLSITKCLVGDNWTKWWKNPDEV

ELFNFIGKDNVAFHGVMFPCTQLGARDNYTIVNHVCATEYLNYEDTKFSKSRNTGVFGDNAKDTGIEADVWRFYLLYMRP

ETQDTSFCWDDFALKVNSELLANLGNFINRALSFLSNSFGGVVPEMHLNDTDVELLAGIEEDSAEWDQLMDAVRQKDAVK

AVLSMSRRGNQYMQSQQPWVLIKGSEEERKRAGTVIGIAANISYHLAIVLHPIMPEISARIRSQCALEELPSFSTYPISY

LKPGHKIGTPKPLFTKMESVKVTEWKAKFGGSKVVDNMQKKQKDKTKQAPKAEAGEKSAKKSERHKSDMATKGILAYPHL

AKNQQQIKQLLDASLKKFSQARELFVKQKGAELAANISKLEKEVEEVKRQLIEAETAAGIKQVAVPKSAKAVVKEEPITN

APVEKPSKKEKPEVEKKQSASGSGKKAGNATVDDQIDVGRLDLRVGRIVKCEKHPDADALYVEQIDVGESSPRTVVSGLV

RHVPLDQMQNRLVVVLCNLKPAKMRGIESRAMVMCASSPEKVEIMEVDPSAQPGTPVKCPPYVHRPDAQLNPKKKVWETV

AEDLKVSAEGYAVWKDCPLLVGGSTKMTAPTLRGVFIK

>CDJ83374.1 Aminoacyl-tRNA synthetase and tRNA-binding region domain containing protein [Haemonchus contortus]

MDPQRAAQVRAVWSQSPTFLQLTPKERRVPQKGKRNILITSALPYVNNVPHLGNIIGCVLSADVFSRYCRMRGHETLYVC

GTDEYGTATETKALQEGLTPKEVCDKYHVIHKKIYEWFNIDFDIFGRTTTPQQTEIAQDIFLKLHENGYTSSCSIDQLHC

QNCDKFLADRFVTGICPFCAFDDARGDQCDSCGRLINAVELKSPKCHICKQEPKVRQSTHIFLHLDALQDEVRQYVENEI

AKPNMRWSSNAIAIVKGWIKGGLEKRCITRDLKWGTAVPLAGYENKVFYVWYDAPIGYLSITKCLVGDNWTKWWKNPNEV

ELFNFIGKDNVAFHGVMFPCTQLGARDNYTIVNHVCATEYLNYEDTKFSKSRNTGVFGDNAKDTGIEADVWRFYLLYMRP

ETQDTSFCWDDFALKVNSELLANLGNFINRALSFLSNSFGGVVPEMHLNDTDVELLAGIEEDSAEWDQLMDAVRQKDAVK

VVLSMSRRGNQYMQSQQPWVLIKGSEEERKRAGTVIGIAANISYHLAIVLHPIMPEISARIRSQCALEQLPSFSTYPLSY

LKPGHKIGTPKPLFTKMESVKVTEWKAKFGGSKVVDNTQKKQKDKTKQAPKAEAGEKSAKKSEKHKSDMATKGILAYPHL

AKNQQQIKQLLDASLKKFSQARELFVRQKGAELAANISKLEKEVEEVKRQLIEAETAARIKQVAVPKLAKAVEKEEPITK

APVEKSSKKEKTDVEKKQSASGSGKKAGSTTVDDQIDVGRLDLRVGRIVKCEKHPDADALYVEQIDVGEPAPRTVVSGLV

RHVPLDQMQNRLVVVLCNLKPAKMRGIESRAMVMCASSPEKVEIMEVDPSAQPGTPVKCPPYVHRPDAQLNPKKKIWETV

AEDLKVSAEGYAVWKDCPLLVGGSTKMTAPTLRGVFIK

>XP_008543768.1 PREDICTED: methionine--tRNA synthetase, cytoplasmic [Microplitis demolitor]

MSTPTIESNQKQCKISQENNLKDKESEEMLITEDELNAIKNNWLTKTQLTSNKLEYPVLPKKEARNILITSALPYVNNIP

HLGNIIGCVLSADIFARYCRQRNYNTLYIGGTDEYGTATEAKVLEEKMSCKEICDKYYNIHDEVYRWFNIAFDRFGRTTT

KEQTEVVQDFFLRVKDEGFIFTDTVDQLRCENCEKFLADRFVEGTCPDCKYEDARGDQCDGCGHLINAPELINPRCKLCN

QTPVIKSSEHFFLDLPKIEPKVKEWVAEAESKWSSVARAIAKPWLRDGLKSRCITRDIKWGIPVPLEGYEDKVFYVWFDA

PLGYLSISKNYHKDYEQWWKPEKEMDVSLYQFMAKDNVPFHAIMFPASLLAANQGYILVKHIMAVEYLNYEDTKFSKSRG

VGVFGTDARDTGIPADVWRFYLAYVRPEGQDTNFNWTDLATKTNSELLNNFGNFVNRSLAFAEKNYDGVIQEMELRDEEY

NLLAHVQRELTQYNLLMEQAKLRDSLKLILSISKLGNQYLQAQQPWAKIKGNDDDKKIAGTVSGICCNLSCLLSSLIGPF

MPMISLALRSQLGLESHYGLIPDTVTILLEPGHKIGKSFPLFTRITDKEIQAYRDKFGGVKEEKVKKNKKTQKQSKQNKN

PVAELEKSMADLKN

>KHN77202.1 Methionine--tRNA synthetase, cytoplasmic [Toxocara canis]

MTQICCKACEYRKVKVKSEQIKLLADNVSALRTDLLEPWLTMGIESLWNTAPKFVEKVDSSGKVPVKGEKNVLITSALPY

VNNVPHLGNIIGCVLSADVFSRYCLLKGWRHLFVCGTDEYGTATETKALQEGVTPQQICDKYFNIHKSIYDWFNIDFDRF

GRTTNPHQIELTQDLFLKLHSNGFISSSTIDQLHCEKCQRFLADRFVHGECPFCHFEDARGDQCDACSKLINAVELIRPR

CHVCSGTPVVKPSTHLFVQLDKLSAEVEEHMNKQISASANHWSANAVSIAKGWIKGGLEKRCITRDLKWGVPVPLPEFAD

KVFYVWFDAPVGYMSITKDLVGDAWTSWWKNPAQVELYNFVGKDNVAFHAVMFPASQIGARDNYTVVNHLCATEYLNYED

TKFSKSRGTGVFGDMARDTGIEPDVWRFYLLYIRPETQDTSFAWDDFALKVNSELLSNLGNFINRALSFVAASFGGMIPE

MELGEVETELLEGVAHDLSEYDSLLTAVKLRDGISKILQISRRGNQYMQSMEPWLLVKGDEQQKKRAGTVIGVSANIAYL

IAVLMHPYMPQVSRRIREQCGLLDLPLMPSSPIAFLQTGHKIGKPKPIFVKMEKALVEECKKKFGGSGQREPSRASGKVG

SKKNKSAAANADHLQKTNTKQRQLTDAGRMNESEDRSECASPEKILIRDGNQDRRAQKSESAGVKEQGAMPSLASYSKLV

LNQQTVDKLLKVATSKLNQKKDLFVKAKLAELNEENGILKDEVQKLLTRLNEVETAGGVKQYKIAAPISSDKATTAVKES

SANRHSQPAEAATGGRSKSPKAEAKKAKGESKKQSKKEGATAGGADDSVDVGRLDLRVGRILEAARHPDADTLYVEKIDL

GEAQPRTVVSGLVRHVPVEAMQNKLVMCLCNLKPMKMRGVESQAMVMCASTPERVEILEVDPSSKPGQVVTCPPFVHRPD

AVLNPKKKVWETVAVDLAVSADGRAAYKGYPLLVDGQVPVTAPSLRGVPVK

>XP_018013844.1 PREDICTED: methionine--tRNA synthetase, cytoplasmic-like [Hyalella azteca]

MFSFRPGKISSGCVLSADCYARFCRLRGDNVAYICGTDEYGTATETKAIGEGLTPQQICDKYHKIHSDVYKDFNISFDYF

GRTTTPSQTEIVHELFWDLEKEGKISRESMEQLFCEHCSRFLADRFAEGTCPHVGCGYEDARGDQCDGCGKLVNAVDLVN

PRCKLCSKTPKIKSSNHLFIDLPKIEGKLREWFVESSKNWSSNASIICNTWLRDGLLARCITRDLKWGVPVPLEGYDDKV

FYVWFDAPIGYLSITKEYTKDWEMWWKNPDQVNLYQFMAKDNVPFHGIIFPCTLTGSPKPWTRVTHLMATEYLNYEDGKF

SKSRGVGVFGDQAMKTGIPSDVWRFYLLYIRPESQDTVFSWADLQMKNNTELLNNLGNFVNRSLKFVSQFYDGLVPEAKP

NEDDFKVMAAVNHELFNYHNALSNVRLRDGLRHILAMTRIGNQYIQDNEPYKLIKPDRSTEDKERGASVISVSANLVFLV

AVCLQPYMPDTSAKILQIMNLPDATHWRLPTALPHAVPSGCKVTKPEPLIKKITDEELQQWQLQFKGTDDSAAKCQVKKN

SAVVLPAIVNVDIESLKKMIDEQGDKVRALKAGGASKADISADVATLLQLKNQLAQAQGAPAVPPAKDKKQKKGEAVRTL

KSSGASKDAVAAEVTKLLQLKSELAVALGVDVSSLSAPSKSSKKKK

>EYC29887.1 hypothetical protein Y032_0005g2319 [Ancylostoma ceylanicum]

MSAPSAAEIRKAWDGSPRFVEHTPKEKLVPIKGKRNILITSALPYVNNVPHLGNIIGCVLSADVFARYCRMRGYETLYVC

GTDEYGTATETKALQEGKTPREVCDKYHAIHKSIYQWFNIDFDIFGRTTTPQQTEIAQDIFLKLHKNGFTSSASIDQLHC

QNCDKFLADRFVTGICPFCSFDDARGDQCDGCGRLINAVELRSPKCHICKQEPKVRQSTHIFLHLDALQDEVRDYVENEI

AKPDTRWSSNAIAIVKGWIKGGLEKRCITRDLKWGTAVPLAGYEDKVFYVWFDAPIGYLSITKCLVGNNWTKWWKNPREV

ELYNFIGKDNVAFHGVMFPCTQLGTRDNYTVVNHVCATEYLNYEDSKFSKSRNTGVFGDSAKETGIEADVWRFYLLYMRP

ESQDTSFSWDDFALKVNSELLANLGNFINRALSFLSNNFGGAVPEMELTDIDLELLMGVQADSVEWDQLMNAVRQKDAVR

TVLAMSRRGNQYMQSQQPWVLMKGNEGDKKRAATVIGVAANISYHLAVVLHPIMPEVSARIRAQCGAERLPTFSTFPISY

LKPGHKIGTPKPLFTKMESAKISEWKAKFGGGVAVAEEPSRKGNGRRIFLLLENF

>EYC29888.1 hypothetical protein Y032_0005g2319 [Ancylostoma ceylanicum]

MTRHFLCYCNMSAPSAAEIRKAWDGSPRFVEHTPKEKLVPIKGKRNILITSALPYVNNVPHLGNIIGCVLSADVFARYCR

MRGYETLYVCGTDEYGTATETKALQEGKTPREVCDKYHAIHKSIYQWFNIDFDIFGRTTTPQQTEIAQDIFLKLHKNGFT

SSASIDQLHCQNCDKFLADRFVTGICPFCSFDDARGDQCDGCGRLINAVELRSPKCHICKQEPKVRQSTHIFLHLDALQD

EVRDYVENEIAKPDTRWSSNAIAIVKGWIKGGLEKRCITRDLKWGTAVPLAGYEDKVFYVWFDAPIGYLSITKCLVGNNW

TKWWKNPREVELYNFIGKDNVAFHGVMFPCTQLGTRDNYTVVNHVCATEYLNYEDSKFSKSRNTGVFGDSAKETGIEADV

WRFYLLYMRPESQDTSFSWDDFALKVNSELLANLGNFINRALSFLSNNFGGAVPEMELTDIDLELLMGVQADSVEWDQLM

NAVRQKDAVRTVLAMSRRGNQYMQSQQPWVLMKGNEGDKKRAATVIGVAANISYHLAVVLHPIMPEVSARIRAQCGAERL

PTFSTFPISYLKPGHKIGTPKPLFTKMESAKISEWKAKFGGGVAVAEEPSRKGNGRRIFLLLENF

>EGT37651.1 hypothetical protein CAEBREN_11795 [Caenorhabditis brenneri]

MGEDLGGITKQFEAEATGYVEWKDPKTILPAKGKRNILITAALPYVNNVPHLGNIIGCVLSADVFARYCNLRGHQTFYVG

GTDEYGTATETKALQEGCTPRELCDKYHTIHKAIYDWFGIEFTHFGRTTTEHQTEICQDMFMKLHNNGYTSTQSVDQLFC

VKCDKFLADRFVTGTCPMCNYDDARGDQCDGCGKLINAVDLIDAKCHMCKSTPEVRQSTHIFLSLDKLQEKTNEYLKVEM

SRESSRWSSNAIGITNAWMKQGLDPRCITRDLKWGTPVPLEGFEKKVFYVWFDAPIGYLSITKCVLEDNWTKWWKNPENV

ELYNFVGKDNVAFHAVMFPCSQLGANDNYTVVNNLCATEYLNYENTKFSKSRGTGIFGDAAQGTGIPSDIWRFYLLYMRP

ESQDTAFSWDDFVLKVNSELLNNLGNFINRALSFVANSFAGVIPDMDLTEEDAAVLSEIHKECVQWDEQFDKVHLKDAVK

TTLNVSRLGNQYMQAQKPWVLIKGNDADKRRAGTIIGVAANIAYHLAVLLYPVMPTISATIRDQCGLPKLPLFTPFPRRY

LRSGHKIATPAPLFQKLDPVVIAEFKAKFGGSQEAQNTTPKTTEKPKKQAPTKDKKGGDKKKMASTAAYVELEAGARAIS

QLLAGNFKKFEQAKALFTRNQLQKLEEENRKLILDAKSLQQQLIELETAAGIKQIAKPIASCTPTPTSTPASGIITEAPK

TATPSAASATEPKKAKEQKKGKGGGAVAAPVDDTIDVGRLDMRVGRIIKCEKHPDADALYVEQIDVGEAAPRTVVSGLVR

HVPLDQMQNRLVVVLCNLKPAKMRGIESRAMVMCASSPEKVEIMEVDPNSKPGTPVVCPPYTHRPDDVMNPKKKIWETVA

EDLKVSADGFAEWKGQPLLVGNATKMTAPTLRGVHVK

>EGT58248.1 hypothetical protein CAEBREN_25557 [Caenorhabditis brenneri]

MGEDLGGITKQFEAEATGYVEWKDPKTILPAKGKRNILITAALPYVNNVPHLGNIIGCVLSADVFARYCNLRGHQTFYVG

GTDEYGTATETKALQEGCTPRELCDKYHTIHKAIYDWFGIEFTHFGRTTTEHQTEICQDMFMKLHNNGYTSTQSVDQLFC

VKCDKFLADRFVTGTCPMCNYDDARGDQCDGCGKLINAVDLIDAKCHMCKSTPEVRQSTHIFLSLDKLQGKTNEYLKVEM

SRESSRWSSNAIGITNAWMKQGLDPRCITRDLKWGTPVPLEGFEKKVFYVWFDAPIGYLSITKCVLEDNWTKWWKNPENV

ELYNFVGKDNVAFHAVMFPCSQLGANDNYTVVNNLCATEYLNYENTKFSKSRGTGIFGDAAQGTGIPSDIWRFYLLYMRP

ESQDTAFSWDDFVLKVNSELLNNLGNFINRALSFVANSFAGVIPDMNLTEEDAAVLSEIHKECVQWDEQFDKVHLKDAVK

TTLNVSRLGNQYMQAQKPWVLIKGNDADKRRAGTIIGVAANIAYHLAVLLYPVMPTISATIRDQCGLPKLPLFTPFPRKY

LRSGHKIATPAPLFQKLDPVVIAEFKAKFGGSQEAQNTTPKTTEKPKKQAPTKDKKGGDKKKMASTAAYVELEAGARAIS

QLLAGNFKKFEQAKALFTRNQLQKLEEENRKLILDAKSLQQQLIELETAAGIKQIAKPIASCTPTPTSTPASGIITEAPK

TATPSAASATEPKKAKEQKKGKGGGAVAAPVDDTIDVGRLDMRVGRIIKCEKHPDADALYVEQIDVGEAAPRTVVSGLVR

HVPLDQMQNRLVVVLCNLKPAKMRGIESRAMVMCASSPEKVEIMEVDPNSKPGTPVVCPPYTHRPDDVMNPKKKIWETVA

EDLKVSADGFAEWKGQPLLVGNATKMTAPTLRGVHVK

>CRZ24874.1 BMA-MARS-1 [Brugia malayi]

MDFEKTWFKNPEYVQSVDKSKKLPKPGEKNILITSALPYVNNVPHLGNIVGCVLSADVFSRYCYLKGWRRLFICGTDEYG

TATETKAIQEGLTPRQICDKYHAIHAKIYKWFNIDFDHFGRTTTQHQTELTQEIFLKLHKNGFTSTDTVDQLHCDSCSRF

LADRFVCGECPHCHFDDARGDQCDACGKLINAVELINPRCHLCKATPIVKPSNHIFLHLDKLADKVEKHLNEQLNLGKNQ

WSPNAISIAKSWLKGGLEKRCITRDLKWGVSVPLESFSNKVFYVWFDAPIGYLSITKEYLGEDWSQWWKNPSIVDLYNFV

GKVAYFFVPFHDNVAFHAVMFPATQIGTGDNYTVVNRLCATEYLNYESMKFSKSRGTGVFGDMAAETGIDADIWRFYLLY

IRPETQDTSFSWDDFSLKVNTELLNNLGNFVNRALIFLSKYFDGVVPDMHLQEQDKALLAQVSSDLSDFDLCLSSVKLRD

GIVKVLSISRHGNLYIQSTQPWVLIKGSENDRKRAGTVIGIVANLAYLIAVMLYPYMPSISAKIREHCGHLALASLPSTP

IAFLKPGHKIGEPKPLFVKMEKSAIDDFKKKFGSASESIINEQVKKKKIINEKKKTKNEEKDNNERLIKDVASSSVYSTL

LTNQKKIDSLLKVASMKLEQRRAQFEKVKLQEENEQALQLRAEIERLKAKLIELETMGGIKQVSGIPVFEKSFLVKTNND

LTPSVQMEKSNEQNVNEVRILRKEVKDRNESAKSKKEGSKNEEVDEVDIGRLDIRVGRILKVEKHADADSLYVEQMDVGE

DKPRTVVSGLVRHVPIEQMQNRLVICVCNLKPVKMRGIESQGMVLCASTPEKVELIEFDESCIPGQPVTCEGYKRRPDPV

LNPKKKIWEGVAPDLKVNFEGRVVYKGQPLLVNGLSPLLAPSLRNVPVK

>XP_011403559.1 PREDICTED: methionine--tRNA synthetase, cytoplasmic-like [Amphimedon queenslandica]

MDRESKDILLKGPVAAAWNDASYVVKCFRAKPSEPILPVSGERNVLVTSALPYVNNVPHLGNLIGCVLSGDVFARYCRQR

NYNVLYVCGTDEYGTATETKAIEEGLTPKEICDKYFEIHDKVYKWFNIDFDIFGRTSTAHQTRIAQEIFWDLYHNGFIVK

DNVDQLLCQTCDRFLADRFVEGICPFCSFDGARGDQCDKCGHLINAIELKSPKCKLCSSEPCIKTSNHLFLDLAKLQDPV

NELFMKSSDQGVWSNNAKIITSTWLRDGLNRRCISRDLKWGTPVPLDGYTNKVFYVWFDAPIGYISITAGYIDEWEKWWK

NPDEVKLYQFMAKDNVPFHTVVFPASLIGTKKPYTFLDSISSTEFLNYEDGKFSKSRGVGVFGNDAIDTGIPADVFRFYL

LLVRPETQDSVFSWSDFAIKVNAELVNNLGNFINRTLTFLGKNYELVMPPVFPNDGDIHFLEQINVELQNYIENMEKCKL

RDGLKCILSISKLGNGYLQAGEPWKLLKLNNNADKLRADSIIGISTNVVYLLCILLYPFMPSAGSDILKQLNVMENCIAI

PEAMAPFLAVGHKINEPFPLFVKLEDSQVLGLKKKYSGNDTC

>OAF66437.1 hypothetical protein A3Q56_05845 [Intoshia linei]

MLNLPWVKHNLKIRAKALNPVLPIKGQNNVLITSALPYVNNIPHLGNIIGSVLSGDVFSKYCKLRGHNTLYICGTDEFGT

ATQTAALKSNVTPKEICDKYYKIHYDVYKWFNIEFDNFGRTTCKEQTPISQYIFNSLYKKGYISSKTVEQMYCQNCKMFL

ADRFIEGICYLCNFPDARGDQCDKCGKLMNAIELINSKCKMCKSDPVRKTSDHLFIDLDKLQPLLEDHVSKSNFSKNWTE

NAVGITKGWLKEKLKARCITRDLHWGTPVPLEGYKDKVFYVWFDAPIGYISFTAEYTKQWEKWWKNPDEVEYYQFIGKDN

VPFHAVMFPCSLLGTGEAWTYVTHLCATEYLNYEGQKFSKSRGVGLFGDEVQKTGIPSDIWRFYLLAVRPESNDSTFSWE

DLVHKTNFELLNNIGNFVHRSCSFLKNFFGGKISHVNLTDDSIDLISKIDIETVRYVEKMEKTEFREALKSVLSISRLGN

QFMQLNKPWETVKTSNTKQLALETCSLCVNVVAHISVLLSPFMPDTNKTIKKFLKLDDYILSEPFGLKQYLSVGHEISKP

VILFEKLDMEKIEKFISNKQVVPNVIKLTEQQVQQIEQDIQTQGDLIRKLKLEKSTKSQLKPHIDKLIDMKQKLKIGK

>XP_010115034.1 PREDICTED: methionine--tRNA synthetase, cytoplasmic, partial [Chlamydotis macqueenii]

LEEQLEPWLEQSWATGDWTANARYITRSWIRDGLKPRCITRDLKWGTPVPLDGFRDKVFYVWFDAPIGYLSITANYTDQW

ERWWKNPQQVELYNFMAKDNIPFHSVIFPCSLLGADDNYTLVNHLIATEYLNYEDGKFSKSRGVGVFGDMAKDTGIPADI

WRFYLLYLRPEGQDSAFSWSDLMLKNNSELLNNLGNFINRAGMFVCKFFGGTVPNMVLTSDDKRLLARITLELRQYHQLL

EKVRIRDALRCILSISRHGNQYIQVNEPWKRIKGDEKDRQRAGTVTGVAVNMASMLAVMLQPYMPSISLAIQGQLLIPSS

CFVLSQDFICTLPPGHCVGTVSPLFQKLENDQVEALRKRFGGGQPKPHHPASETLVSSGDPQLIQELTEEVAKQGNHVRQ

LKANKAEKAQVDMEVAKLLELKKQLSLAEGKSPEGPVPKGKRKK

>XP_003738939.2 PREDICTED: methionine--tRNA synthetase, cytoplasmic [Galendromus occidentalis]

MYIDDKSLERAALGDVSKILQSVNSTEQNAEEQKYNQDSEAFFKLIELWKTKIGGVPVHEHKKPALPSENRRNVLITTAL

PYVNNVPHLGNIVGSVLSADVFCRFCRMRDYNCVYVGGTDEYGTATETKALEVKCTPRELCDKFNTQHSEIYRWFDIDFD

IFGRTSTEKQTEIVQNLFLKLHGNGNFSEDTVDQLYCGSCPRFLADRFVEGTCPFCGYEDARGDQCDKCSKLINAIELKD

PRCKICKSSPTVKTSRHLFLDMPKLDSQFREWQKKSSDSGKWTQSAKVITNSWLRDGLKPRCITRDMKWGVPVPLAGYES

KVFYVWFDACIGYMSITATYTDQWEKWWRNPEKVELFQFMGKDNVPFHAIVFPMCQIGSGEKFTKVNHVVATEYLNYEDK

KFSKSRGVGVFGDQAAETGIPSDVWRFYMMYMRPENQDTSFSWVDLQTKNNSELLNNLGNFIFRCLSFACARFENRVPES

EGELEDTDKNVLAQVARYYQEFLENFENLHIRDALRAVLMISKAGNQYVQASKPWEKVKNKETIGRSKTIVYIGIQICAL

VAGCLEPFMPKTAAELRRQLNIERLVLEDEFRPLVAGNHPLNEPKPLFRKIETEQVVEFQKKFAGQPVAEASSAELCAEE

FSSAAATGEKAGGGGGRRGDFRRSDRPTARRGKVSRIQNKST

>KRZ70580.1 Methionine--tRNA synthetase, cytoplasmic, partial [Trichinella papuae]

LKSIEMTAKEKIIFPVPGERNILITAALPYVNNYPHLGTIIGCVLSADVFSRYCRLRGYNTLYICGTDEYGTATETKAIE

EGVTPIQLCDKYYELHREVYEWFNIKFDYFGRTTTEQQTEIAQHIFKRIYDAGFTTTKNVDQLYCEKCERFLADRFVEGE

CPFCGYEEARGDQCDKCGHLINAPELKLPKCKLCSHPPIIRTSEHLFLDLPKLQLNLEKYLKEQWASSDCCWSHNAIAIT

KKWLKEGLKERCITRDLKWGTPVPLENFSDKVFYVWFDAPIGYLSITANYTPEHWKKWWMDDKHVELYHFVGKDNVPFHS

IIFPATLIATNEQYCKVKNISATEYLNYESSKFSKSRGVGVFGNNARETGIPADVWRFYLLYTRPESQDAAFNWDDFALK

VNSELLSNLGNFVNRALSFVERRFHGVIPEVDQSLLDKDRLQAFIDNVNETLNNYIDCLDNLKLRDSVKCILNISRLGNQ

YMQSERPWELIKQNERQAGSVLAIVANVVAILAALVHPIMPEISTTLCKQLQLEKLIFCSSFQFVLKAGHHIGQVTTLFV

KFEKETVQQFRKMFAGFQANSEVKNVKKTQSAKNVCKSVKGSQKYCEIFAEIEETRHRISALKLFLTQRAKRRLAAENEE

LQKHLEKLKLDYQILMDRRELYSKLGLNTVSKEYVSLENSNEDEKFATTKHVIKDKKEKPKKKVAEKTDKVTAVCSSDQK

NAVNISSLDIRVGLIKKAVKHPQADTLYVEEVDVGEDAVRTVVSGLVNFVPLEKMQNRFAVLLCNLKPAKMRGVESQAMV

LCASTKEAVEILEPPEGAVPGDRIFCPGYEGMPEAQLNPKKKIWESVQPLLLVNADGQACYANKPLRIVGKEGCLTAPTL

RSVPIK

>XP_009490023.1 PREDICTED: LOW QUALITY PROTEIN: methionine--tRNA synthetase, cytoplasmic-like, partial [Pelecanus crispus]

NPQCKLCRGVPVVKPTQHLFLDLPKLEERXWVEQSWATGDWTANARYITRSWIRDGLKPRCITRDLKWGTPVPLDGFRDK

VFYVWFDAPIGYLSITANYTDQWERWWKNPQQVELYNFMAKDNVPFHSVIFPCSLLGAEDNYTLVNHLIATEYLNYEDGK

FSKSRGVGVFGDMAKDTGIPADIWRFYLLYLRPEGQDSAFSWSDLMLKNNSELLNNLGNFINRAGMFVCKFFGGTVPDMV

LTPDDKRLLARVTLELRQYHQLLEKVRIRDALRCILSISRHGNQYIQVNEPWKRIKGDEKDRQRAGTVTGVAVNMASLLA

VLLQPYMPSVSSAIQGQLCIPPDCFVLSHDFTCTLPPGHHVGTVSPLFQKLENNQIEALRKRFGGRQPEDLAVEPQAKQS

PPASEPPPAPATLGPPGDPQLIQELTEEVAKQGNYVRQLKANKAEKAQVDMEVAKLLQLKKQLALAEGKSPEIPVPKGKR

KK

>KRZ06501.1 Methionine--tRNA synthetase, cytoplasmic, partial [Trichinella zimbabwensis]

LKSIEMTAKDKIIFPVPGERNILITAALPYVNNYPHLGTIIGCVLSADVFSRYCRLRGYNTLYICGTDEYGTATETKAIE

EGVTPIQLCDKYYELHREVYEWFNIKFDYFGRTTTEQQTEIAQHIFKKIYDAGFTTTKNVDQLYCEKCERFLADRFVEGE

CPFCGYEEARGDQCDKCGHLINAPELKLPKCKLCSHPPIIRTSEHLFLDLPKLQLNLEKYLKEQWASSDCCWSHNAIAIT

KKWLKEGLKERCITRDLKWGTPVPLENFSDKVFYVWFDAPIGYLSITANYTPEHWKKWWMDDKHVELYHFVGKDNVPFHS

IIFPATLIATNEQYCKVKNISATEYLNYESSKFSKSRGVGVFGNNARETGIPADVWRFYLLYTRPESQDAAFNWDDFALK

VNSELLSNLGNFVNRALSFLERRFHGVIPEVDQNLLDKDRLQAFIDNVNETLNNYIDCLDNLKLRDSVKCILNISRLGNQ

YMQSERPWELIKQNERQAGSVLAIVANVVAILAALVHPIMPEISTTICKQLQLEKLIFCSSFQFVLKAGHRIGQVTTLFV

KFEKETVQQFRKMFAGFQANSEVKNVKKTQSAKNVCKSVKGSQKYCEIFAEIEETRHRISALKLFLTQRAKRRLAAENEE

LQKHLEKLKLDYQILMDRRELYSKLGLNTVSKEYVSLENSNEDEKFATTKHVIKDKKEKPKKKVAEKTDKVTAVCSSDQK

NAVNISSLDIRVGLIKKAVKHPQADTLYVEEVDVGEDAVRTVVSGLVNFIPLEKMQNRFAVLLCNLKPAKMRGVESQAMV

LCASTKEAVEILEPPEGAIPGDRIFCPGYEGMPEAQLNPKKKIWESVQPLLLVNADGQACYANKPLRIIGKEGCLTAPTL

RSVPIK

>KRZ19806.1 Methionine--tRNA synthetase, cytoplasmic [Trichinella pseudospiralis]

MKAVYPIEAIGSCSLKSLEMIDKDKIIFPVPGERNILITSALPYVNNYPHLGTIIGCVLSADVFSRYCRLRGYNTLYICG

TDEYGTATETKAIEEGVTPIQLCDKYYELHREVYEWFNIKFDYFGRTTTEQQTEIAQHIFKKIYDAGFTTTKNVDQLYCE

KCERFLADRFVEGECPFCGYEEARGDQCDKCGHLINAPELKLPKCKLCSHPPIIRTSEHLFLDLPKLQLNLEKYLKEQWA

SSDCCWSHNAIAITKKWLKEGLKERCITRDLKWGTPVPLENFSDKVFYVWFDAPIGYLSITANYTPEHWKKWWMDDKHVE

LYHFVGKDNVPFHSIIFPATLIATNEQYCKVKNISATEYLNYENSKFSKSRGVGVFGNNARETGIPADVWRFYLLYTRPE

SQDAAFNWDDFALKVNSELLSNLGNFVNRALSFVERRFDGVIPEVDQSLLDKDRLQAFIDNVNETLNNYIDCLDNLKLRD

SVKCILNVSRLGNQYMQSERPWELIKQNERQAGSVLAIVANVVAILAALVHPIMPEISMTICKQLQLEKLIFCSSFQFVL

KPGHRIGQVTTLFVKFEKETVQQFRKMFAGFQANSEVENVKKIPSAKNVCKSVKGSQKYCEIFAEIKETRHRISALKLFL

TQRAKRRLAAENEELQKHLEKLKLDYQILMDRRELYSKLGLNTVSKEYVSLENSNEDETFPTAKHVIKDKKEKPKKKVAE

KTDKVTAVCTSDQKNAVNISSLDIRVGFIKKAVKHPQADTLYVEEVDVGEDAVRTVVSGLVNFIPLEKMQNRFAVLLCNL

KPAKMRGVESQAMVLCASTKEAVEILEPPDGAVPGDRIFCPGHEGTPEAQLNPKKKIWESVQPLLLVNADGQACYANEPL

RIVGKEGCLTAPTLRSVPIK

>KRY84004.1 Methionine--tRNA synthetase, cytoplasmic [Trichinella pseudospiralis]

MKAVYPIEAIGSCSLKSIEMIDKDKIIFPVPGERNILITSALPYVNNYPHLGTIIGCVLSADVFSRYCRLRGYNTLYICG

TDEYGTATETKAIEEGVTPIQLCDKYYELHREVYEWFNIKFDYFGRTTTEQQTEIAQHIFKKIYDAGFTTTKNVDQLYCE

KCERFLADRFVEGECPFCGYEEARGDQCDKCGHLINAPELKLPKCKLCSHPPIIRTSEHLFLDLPKLQLNLEKYLKEQWA

SSDCCWSHNAIAITKNWLKEGLKERCITRDLKWGTPVPLENFSDKVFYVWFDAPIGYLSITANYTPEHWKKWWMDDKHVE

LYHFVGKDNVPFHSIIFPATLIATNEQYCKVKNISATEYLNYENSKFSKSRGVGVFGNNARETGIPADVWRFYLLYTRPE

SQDAAFNWDDFALKVNSELLSNLGNFVNRALSFVERRFDGVIPEVDQSLLDKDRLQAFIDNVNETLNNYIDCLDNLKLRD

SVKCILNVSRLGNQYMQSERPWELIKQNERQAGSVLAIVANVVAILAALVHPIMPEISMTICKQLQLEKLIFCSSFQFVL

KPGHRIGQVTTLFVKFEKETVQQFRKMFAGFQANSEVENVKKIPSAKNVCKSVKGSQKYCEIFAEIKETRHRISALKLFL

TQRAKRRLAAENEELQKHLEKLKLDYQILMDRRELYSKLGLNTVSKEYVSLENSNEDETFPTAKHVIKDKKEKPKKKVAE

KTDKVTAVCTSDQKNAVNISSLDIRVGFIKKAVKHPQADTLYVEEVDVGEDAVRTVVSGLVNFIPLEKMQNRFAVLLCNL

KPAKMRGVESQAMVLCASTKEAVEILEPPDGAVPGDRIFCPGHEGTPEAQLNPKKKIWESVQPLLLVNADGQACYANEPL

RIVGKEGCLTAPTLRSVPIK

>XP_016768374.1 PREDICTED: methionine--tRNA synthetase, cytoplasmic isoform X2 [Apis mellifera]

MNILTNEDNPNTLKLIIAAKVVQQSVTVKKVEPKESFLGRLPSIELSSGLVLFSTNAAMQLIIPPSEELKIINSKWLEWE

ISQLQSAITFYGSTGIYKTTHKSGLWSLLEELNNVLKDKKYLIKDNNDLSLADICIWVTLWSTVLVTEIGNELCREYQHI

NNWISNIESHPIIQDSIKEYKFERGLKAISSIQAVSWFPINTIQNFRSPKSADIQLSPTKEKEMEAVSQEEMHIISSNWS

SILYPDVKNFPYPVLPKKDEKNVLITSALPYVNNVPHLGNIIGCVLSADIFARYCRQRNYNTLFISGTDEYGTATEAKAL

EEKTTPQSICDKFFDIHNDIYRWFNIGFDYFGRTTTPEQTEIVQKFFLRIKSQGYILSETVDQLLCESCDRFLADRFVEG

TCPRCKYEDARGDQCDGCSHLINATDLISPRCKVCSNRPIIKKSEQFFLDLPKLENKLKEWLSTVENGWSGVARVVAKPW

LRDGLKPRCITRDLKWGIPVPIKGFENKVFYVWFDAPFGYMSITKRYTKEYEQWWKPKDIKVDLYQFMAKDNVPFHAIMF

PASLLAANEDYILLKHLMATEYLNYEDTKFSKSRGIGVFGTDARDTGIPSDVWRFYLAYVRPETQDSNFNWVDLATKNNS

ELLNNFGNFVNRALVFVERYFESKIPPIELQEDDLIVLALAQRELSSYICAMEQAKLRDGLKHILAISKHGNQYMQFQEP

WVKIKGTDNDKYPSMWIAT

>KRZ32149.1 Methionine--tRNA synthetase, cytoplasmic, partial [Trichinella pseudospiralis]

LKSLEMIDKDKIIFPVPGERNILITSALPYVNNYPHLGTIIGCVLSADVFSRYCRLRGYNTLYICGTDEYGTATETKAIE

EGVTPIQLCDKYYELHREVYEWFNIKFDYFGRTTTEQQTEIAQHIFKKIYDAGFTTTKNVDQLYCEKCERFLADRFVEGE

CPFCGYEEARGDQCDKCGHLINAPELKLPKCKLCSHPPIIRTSEHLFLDLPKLQLNLEKYLKEQWASSDCCWSHNAIAIT

KKWLKEGLKERCITRDLKWGTPVPLENFSDKVFYVWFDAPIGYLSITANYTPEHWKKWWMDDKHVELYHFVGKDNVPFHS

IIFPATLIATNEQYCKVKNISATEYLNYENSKFSKSRGVGVFGNNARETGIPADVWRFYLLYTRPESQDAAFNWDDFALK

VNSELLSNLGNFVNRALSFVERRFDGVIPEVDQSLLDKDRLQAFIDNVNETLNNYIDCLDNLKLRDSVKCILNVSRLGNQ

YMQSERPWELIKQNERQAGSVLAIVANVVAILAALVHPIMPEISMTICKQLQLEKLIFCSSFQFVLKPGHRIGQVTTLFV

KFEKETVQQFRKMFAGFQANSEVENVKKIPSAKNVCKSVKGSQKYCEIFAEIKETRHRISALKLFLTQRAKRRLAAENEE

LQKHLEKLKLDYQILMDRRELYSKLGLNTVSKEYVSLENSNEDETFPTAKHVIKDKKEKPKKKVAEKTDKVTAVCTSDQK

NAVNISSLDIRVGFIKKAVKHPQADTLYVEEVDVGEDAVRTVVSGLVNFIPLEKMQNRFAVLLCNLKPAKMRGVESQAMV

LCASTKEAVEILEPPDGAVPGDRIFCPGHEGTPEAQLNPKKKIWESVQPLLLVNADGQACYANEPLRIVGKEGCLTAPTL

RSVPIK

>KRY00378.1 Methionine--tRNA synthetase, cytoplasmic, partial [Trichinella pseudospiralis]

LKSIEMIDKDKIIFPVPGERNILITSALPYVNNYPHLGTIIGCVLSADVFSRYCRLRGYNTLYICGTDEYGTATETKAIE

EGVTPIQLCDKYYELHREVYEWFNIKFDYFGRTTTEQQTEIAQHIFKKIYDAGFTTTKNVDQLYCEKCERFLADRFVEGE

CPFCGYEEARGDQCDKCGHLINAPELKLPKCKLCSHPPIIRTSEHLFLDLPKLQLNLEKYLKEQWASSDCCWSHNAIAIT

KKWLKEGLKERCITRDLKWGTPVPLENFSDKVFYVWFDAPIGYLSITANYTPEHWKKWWMDDKHVELYHFVGKDNVPFHS

IIFPATLIATNEQYCKVKNISATEYLNYENSKFSKSRGVGVFGNNARETGIPADVWRFYLLYTRPESQDAAFNWDDFALK

VNSELLSNLGNFVNRALSFVERRFDGVIPEVDQSLLDKDRLQAFIDNVNETLNNYIDCLDNLKLRDSVKCILNVSRLGNQ

YMQSERPWELIKQNERQAGSVLAIVANVVAILAALVHPIMPEISMTICKQLQLEKLIFCSSFQFVLKPGHRIGQVTTLFV

KFEKETVQQFRKMFAGFQANSEVENVKKIPSAKNVCKSVKGSQKYCEIFAEIKETRHRISALKLFLTQRAKRRLAAENEE

LQKHLEKLKLDYQILMDRRELYSKLGLNTVSKEYVSLENSNEDETFPTAKHVIKDKKEKPKKKVAEKTDKVTAVCTSDQK

NAVNISSLDIRVGFIKKAVKHPQADTLYVEEVDVGEDAVRTVVSGLVNFIPLEKMQNRFAVLLCNLKPAKMRGVESQAMV

LCASTKEAVEILEPPDGAVPGDRIFCPGHEGTPEAQLNPKKKIWESVQPLLLVNADGQACYANEPLRIVGKEGCLTAPTL

RSVPIK

>XP_020303052.1 methionyl-tRNA synthetase [Loa loa]

MDFEKTWNQNPEYVQSFDKSKKLPKPGEKNILITSALPYVNNVPHLGNIIGCVLSADAFSRYCYLKGWRRLFICGTDEYG

TATERKAVQEGLTPRQICDKYHAIHAKIYKWFNIDFDRFGRTTTQHQTELTQEIFLKLHKNGFTSTDVVDQLHCDNCNRF

LADRFVFGECPYCHFDDARGDQCDACGKLINAVEIINPRCDLCKATPVVKPSSHIFLHLDKLAGEVEKHLNKQLSSGKNH

WSANAISIAKSWLKGGLERRCITRDLKWGVPVPIDSFSNKVFYVWFDAPIGYLSITKEYLGEDWSQWWKNPSIVDLYNFV

GKTIFPFHDNVAFHAVMFPATQIGTGDNYTMVNRLCATEYLNYESTKFSKSRGTGVFGDMAAETGIDADIWRFYLLYIRP

ETQDTSFSWDDFSLKVNTELLNNLGNFVNRALMFLSKYFNGVVPDMYLEEEDKALLAQVASDLNDFDSCLSSVKLRDGIV

KVLSISRHGNLYMQSTQPWVLLKGNENDRRRAGTVIGVVANLTYLIAVMIYPYMPSISEKIREHCGHPTLALLPRTPIAF

LKPGHKIGEPKPLFVKMEKSTIDDFKKRFGGVDESVVNEQMKKRN

>ETE63822.1 Methionyl-tRNA synthetase, cytoplasmic [Ophiophagus hannah]

MKLCLSEGSLGGLKVLAAAKATGAPVEVQWLPQEAHVVPFLTRPQLPALQLENGSFLFSTNVISQYFFRLSGKEISSFNN

EQSDFANQFFEWDITELEPALSAALYLHVVQEKKGEDVLGIIRKPLDYLDQILTKKGTSYLTGDVESAADIVLWGSLFPL

LRDDSFLPNELKALRSWFQNMNLKEYCRKAVESVWTPKGLLELKAYLQKHPAPSLAFEKSATNEAKEEESAQQHLLPVEG

MKNVLITSALPYVNNVPHLGNIIGCVLSADTFARYCRLRNWNTLYICGTDEYGTATETKAMEEGLTPQQICDKYYAIHAE

IYQWFNISFDYFGRTTTAQQTTIAQGIFQQLLERSYLLTETVDQLKCEKCARFLADRFVEGICPFCNYEEARGDQCDKCG

KLINATELKKPVCKVCQGTPVVKSSQHLFLDLPKLEESLEKWLAVSQSSGDWTPNSRYIARSWIRDGLKPRCITRDLKWG

TPVPLDGFREKVFYVWFDAPIGYLSITANYTDQWEKWWKNPEQVS

>XP_001893903.1 methionyl-tRNA synthetase [Brugia malayi]

MDFEKTWFKNPEYVQSVDKSKKLPKPGEKNILITSALPYVNNVPHLGNIVGCVLSADVFSRYCYLKGWRRLFICGTDEYG

TATETKAIQEGLTPRQICDKYHAIHAKIYKWFNIDFDHFGRTTTQHQTELTQEIFLKLHKNGFTSTDTVDQLHCDSCSRF

LADRFVCGECPHCHFDDARGDQCDACGKLINAVELINPRCHLCKATPIVKPSNHIFLHLDKLADKVEKHLNEQLNLGKNQ

WSPNAISIAKSWLKGGLEKRCITRDLKWGVSVPLESFSNKVFYVWFDAPIGYLSITKEYLGEDWSQWWKNPSIVDLYNFV

GKVAYFFVPFHDNVAFHAVMFPATQIGTGDNYTVVNRLCATEYLNYESMKFSKHGFAFLFDIFQSRGTGVFGDMAAETGI

DADIWRFYLLYIRPETQDTSFSWDDFSLKVNTELLNNLGNFVNRALIFLSKYFDGVVPDMHLQEQDKALLAQVSSDLSDF

DLCLSSVKLRDGIVKVLSISRHGNLYIQSTQPWVLIKGSENDRKRAGTVIGIVANLAYLIAVMLYPYMPSISAKIREHCG

HLALASLPSTPIAFLKPGHKIGEPKPLFVKMEKSAIDDFKKKFGSASESIINEQVKKKKIINEKKKTKNEEKDNNERLIK

DVASSSVYSTLLTNQKKIDSLLKVASMKLEQRRAQFEKVKLQEENEQALQLRAEIERLKAKLIELETMGGIKQVSGIPVF

EKSFLVKTNNDLTPSVQMEKSNEQNVNEVRILRKEVKDRNESAKSKKXGSKNEEVDEVDIGRLDIRVGRILKVEKHADAD

SLYVEQMDVGEDKPRTVVSGLVRHVPIEQMQNRLVICVCNLKPVKMRGIESQGMVLCASTPEKVELIEFDESCIPGQPVT

CEGYKRRPDPVLNPKKKIWEGVAPDLKVNFEGXVVYKGQPLLVNGLSPLLAPSLRNVPVK

>KRY00377.1 Methionine--tRNA synthetase, cytoplasmic, partial [Trichinella pseudospiralis]

LKSIEMIDKDKIIFPVPGERNILITSALPYVNNYPHLGTIIGCVLSADVFSRYCRLRGYNTLYICGTDEYGTATETKAIE

EGVTPIQLCDKYYELHREVYEWFNIKFDYFGRTTTEQQTEIAQHIFKKIYDAGFTTTKNVDQLYCEKCERFLADRFVEGE

CPFCGYEEARGDQCDKCGHLINAPELKLPKCKLCSHPPIIRTSEHLFLDLPKLQLNLEKYLKEQWASSDCCWSHNAIAIT

KKWLKEGLKERCITRDLKWGTPVPLENFSDKVFYVWFDAPIGYLSITANYTPEHWKKWWMDDKHVELYHFVGKDNVPFHS

IIFPATLIATNEQYCKVKNISATEYLNYENSKFSKSRGVGVFGNNARETGIPADVWRFYLLYTRPESQDAAFNWDDFALK

VNSELLSNLGNFVNRALSFVERRFDGVIPEVDQSLLDKDRLQAFIDNVNETLNNYIDCLDNLKLRDSVKCILNVSRLGNQ

YMQSERPWELIKQNERQLNDIFYLLIYRAGSVLAIVANVVAILAALVHPIMPEISMTICKQLQLEKLIFCSSFQFVLKPG

HRIGQVTTLFVKFEKETVQQFRKMFAGFQANSEVENVKKIPSAKNVCKSVKGSQKYCEIFAEIKETRHRISALKLFLTQR

AKRRLAAENEELQKHLEKLKLDYQILMDRRELYSKLGLNTVSKEYVSLENSNEDETFPTAKHVIKDKKEKPKKKVAEKTD

KVTAVCTSDQKNAVNISSLDIRVGFIKKAVKHPQADTLYVEEVDVGEDAVRTVVSGLVNFIPLEKMQNRFAVLLCNLKPA

KMRGVESQAMVLCASTKEAVEILEPPDGAVPGDRIFCPGHEGTPEAQLNPKKKIWESVQPLLLVNADGQACYANEPLRIV

GKEGCLTAPTLRSVPIK

>XP_016905471.1 PREDICTED: methionine--tRNA synthetase, cytoplasmic [Apis cerana]

MNILTNEDNPNTLKLIIAAKVVQQSVTVKKVEPKGSFLGRLPSIELSSGLVLFSTNAAMQLIIPPSEESKIINSKWLEWE

ISQLQSAITFYGSTGIYKTTHKSGLWSLLEELNNVLKDKKYLIKDNNDLSLADICIWVTLWSTVLVTEIGNELCREYQHI

NNWISNIESHPIIQDSIKEYKFERGLKAIFSIQAVSWFPINTIQNFRSPKSADVQLSPTKEKEMEAVSQEEMHVISSNWS

SILYPDVKNFPYPVLPKKDEKNVLITSALPYVNNVPHLGNIIGCVLSADIFARYCRQRNYNTLFISGTDEYGTATEAKAL

EEKTTPQSICDKFFDIHNDVYRWFNIGFDYFGRTTTPEQTEIVQTFFLRIKSQGYILSETVDQLLCESCDRFLADRFVEG

TCPRCKYEDARGDQCDGCSHLINATDLISPRCKVCSNRPIIKKSEQFFLDLPKLENKLKEWSSTVEKGWSGVARVVAKPW

LRDGLKPRCITRDLKWGIPVPVKGFENKVFYVWFDAPFGYISITKRYTKEYEQWWKPKDIKVDLYQFMAKDNVPFHAIMF

PASLLAANEDYILLKHLMATEYLNYEDTKFSKSRGIGVFGTDARDTGIPSDVWRFYLAYVRPETQDSNFNWVDLATKNNS

ELLNNFGNFVNRALVFVERYFESKIPPIELQEDDLILLALAQRELSSYIYAMEQAKLRDGLKHILAISKHGNQYMQFQEP

WVKIKGTDNDKYPCSVWIAVT

>KRZ32151.1 Methionine--tRNA synthetase, cytoplasmic, partial [Trichinella pseudospiralis]

LKSLEMIDKDKIIFPVPGERNILITSALPYVNNYPHLGTIIGCVLSADVFSRYCRLRGYNTLYICGTDEYGTATETKAIE

EGVTPIQLCDKYYELHREVYEWFNIKFDYFGRTTTEQQTEIAQHIFKKIYDAGFTTTKNVDQLYCEKCERFLADRFVEGE

CPFCGYEEARGDQCDKCGHLINAPELKLPKCKLCSHPPIIRTSEHLFLDLPKLQLNLEKYLKEQWASSDCCWSHNAIAIT

KKWLKEGLKERCITRDLKWGTPVPLENFSDKVFYVWFDAPIGYLSITANYTPEHWKKWWMDDKHVELYHFVGKDNVPFHS

IIFPATLIATNEQYCKVKNISATEYLNYENSKFSKSRGVGVFGNNARETGIPADVWRFYLLYTRPESQDAAFNWDDFALK

VNSELLSNLGNFVNRALSFVERRFDGVIPEVDQSLLDKDRLQAFIDNVNETLNNYIDCLDNLKLRDSVKCILNVSRLGNQ

YMQSERPWELIKQNERQLVYTDLAGSVLAIVANVVAILAALVHPIMPEISMTICKQLQLEKLIFCSSFQFVLKPGHRIGQ

VTTLFVKFEKETVQQFRKMFAGFQANSEVENVKKIPSAKNVCKSVKGSQKYCEIFAEIKETRHRISALKLFLTQRAKRRL

AAENEELQKHLEKLKLDYQILMDRRELYSKLGLNTVSKEYVSLENSNEDETFPTAKHVIKDKKEKPKKKVAEKTDKVTAV

CTSDQKNAVNISSLDIRVGFIKKAVKHPQADTLYVEEVDVGEDAVRTVVSGLVNFIPLEKMQNRFAVLLCNLKPAKMRGV

ESQAMVLCASTKEAVEILEPPDGAVPGDRIFCPGHEGTPEAQLNPKKKIWESVQPLLLVNADGQACYANEPLRIVGKEGC

LTAPTLRSVPIK

>KRX70434.1 Methionine--tRNA synthetase, cytoplasmic, partial [Trichinella sp. T6]

LKPIEMAGNDKIIYNFEFSFPVPGERNILITSALPYVNNYPHLGTIIGCVLSADVFSRYCRLRGYNTLYICGTDEYGTAT

ETKAIEEGVTPIQLCDKYYELHREVYEWFNIKFDYFGRTTTKQQTEIAQHIFKKIYDAGFTTTKNVDQLYCEKCQRFLAD

RFVEGECPFCGYEEARGDQCDKCGHLINAPELKLPKCKLCSHPPIIRTSEHLFLDLPKLQLNLEKYLEEQWASSDCCWSH

NAIAITKKWLKEGLKERCITRDLKWGTPVPLENFSNKVFYVWFDAPIGYLSITANYSPEHWKKWWMDDKHVELYHFVGKD

NVPFHSIIFPATLIATNEQYCKVKNISATEYLNYESSKFSKSRGVGVFGNNARETGIPADVWRFYLLYTRPESQDAAFNW

DDFALKVNSELLSNLGNFVNRALSFVERRFHGVIPEVDESLLDKDRLQVFIHNVNETLNNYIDCFDNLKLRDSVKCILNI

SRLGNQYMQSERPWELIKQNERQAGSVLAIVANVVAILAALVHPIMPEISTTICKQLQLEKLVFCSSFQFLLKPGHRIGQ

VTTLFVKFEKETVEQFRKMFAGFQTNSEVANVKKSPSSGKNICKAVKGSQKYCEIFAEIEETRHRISALKLFLTQRAKRR

LAAENAELEKHLEKLKLDYQILMDRRELYSKLGLNTVSNENISLENSNEKFTTTKDVVKDKKEKPKKKVAEKMDKVTASD

QKNAVNISSLDIRVGLIKKAVKHPQADTLYVEEVDVGEDGVRTVVSGLVNFIPLEKMQNRFAVLLCNLKPAKMRGVESQA

MVLCASTKDAVEILEPPEGAVPGDRIFCPGYEGTPEAQLNPKKKIWESVQPLLLVNADGQACYANKPLRIVGKEGCLTAP

TLRSVPIK

>KRZ50693.1 Methionine--tRNA synthetase, cytoplasmic, partial [Trichinella nativa]

LKPIEMTGNDKIIFPVPGERNILITSALPYVNNYPHLGTIIGCVLSADVFSRYCRLRGYNTLYICGTDEYGTATETKAIE

EGVTPIQLCDKYYELHREVYEWFNIKFDYFGRTTTKQQTEIAQHIFKKIYDAGFTTTKNVDQLYCEKCQRFLADRFVEGE

CPFCGYEEARGDQCDKCGHLINAPELKLPKCKLCSHPPIIRTSEHLFLDLPKLQLNLEKYLEEQWASSDCCWSHNAIAIT

KKWLKEGLKERCITRDLKWGTPVPLENFSNKVFYVWFDAPIGYLSITANYSPEHWKKWWMDDKHVELYHFVGKDNVPFHS

IIFPATLIATNEQYCKVKNISATEYLNYESSKFSKSRGVGVFGNNARETGIPADVWRFYLLYTRPESQDAAFNWDDFALK

VNSELLSNLGNFVNRALSFVERRFHGVIPEVDESLLDKDRLQAFIHNVNETLNNYIDCFDNLKLRDSVKCILNISRLGNQ

YMQSERPWELIKQNEKQAGSVLAIVANVVAILAALVHPIMPEISTTICKQLQLEKLVFCSSFQFLLKPGHRIGQVTTLFV

KFEKETVEQFRKMFAGFQTNSEVANVKKSPSSGKNICKTVKGSQKYCEMFAEIEETRHRISALKLFLTQRAKRRLAAENE

ELEKHLEKLKLDYQILMDRRELYSKLGLNTVSNENISLENSNEKFTTTKGVVKDKKEKPKKKVAEKMDKVTASDQKNAVN

ISSLDIRVGLIKKAVKHPQADTLYVEEVDVGEDGVRTVVSGLVNFIPLEKMQNRFAVLLCNLKPAKMRGVESQAMVLCAS

TKDAVEILEPPEGAVPGDRIFCPGYEGTPEAQLNPKKKIWESVQPLLLVNADGQACYANKPLRIVGKEGCLTAPTLRSVP

IK

>KRY16136.1 Methionine--tRNA synthetase, cytoplasmic, partial [Trichinella patagoniensis]

LKSIEMTGNDKIIFPVPGERNILITSALPYVNNYPHLGTIIGCVLSADVFSRYCRLRGYNTLYICGTDEYGTATETKAIE

EGVTPIQLCDKYYELHREVYEWFNIKFDYFGRTTTKQQTEIAQHIFKKIYDAGFTTTKNVDQLYCEKCQRFLADRFVEGE

CPFCGYEEARGDQCDKCGHLINAPELKLPKCKLCSHPPIIRTSEHLFLDLPKLQLNLEKYLEEQWASSDCCWSHNAIAIT

KKWLKEGLKERCITRDLKWGTPVPLENFSNKVFYVWFDAPIGYLSITANYSPEHWKKWWMDDKHVELYHFVGKDNVPFHS

IIFPATLIATNEQYCKVKNISATEYLNYESSKFSKSRGVGVFGNNARETGIPADVWRFYLLYTRPESQDAAFNWDDFALK

VNSELLSNLGNFVNRALSFVERRFHGIIPEVDESLLDKDRLQAFIHNVNETLNNYIDCFDNLKLRDSVKCILNISRLGNQ

YMQSERPWELIKQNERQAGSVLAIVANVVAILAALVHPIMPEISTTICKQLQLEKLVFCSSFQFLLKPGHRIGQVTTLFV

KFEKETVEQFRKMFAGFQTNSEVANVKKSPSSGKNICKTVKGSQKYCEMFAEIEETRHRISALKLFLTQRAKRRLAAENE

ELQKHLEKLKLDYQILMDRRELYSKRGLNTVSNENISLENSNEKFTTTKDVVKDKKEKSKKKVAEKMDKVTASDQKNAVN

ISSLDIRVGLIKKAVKHPQADTLYVEEVDVGEDGVRTVVSGLVNFIPLEKMQNRFAVLLCNLKPAKMRGVESQAMVLCAS

TKDAVEILEPPERAVPGDRIFCPGYEGTPEAQLNPKKKIWESVQPLLLVNADGQACYANKPLRIVGKEGCLTAPTLRSVP

IK

>XP_003376438.1 methionyl-tRNA synthetase, cytoplasmic [Trichinella spiralis]

MTANDKIIFPVPGERNILITSALPYVNNYPHLGTIIGCVLSADVFSRYCRLRGYNTLYICGTDEYGTATETKAIEEGVTP

IQLCDKYYELHRDVYEWFNIKFDYFGRTTTKQQTEIAQHIFKKIYDAGFTTTKNVDQLYCEKCERFLADRFVEGECPFCG

YEEARGDQCDKCGHLINAPELKLPKCKLCSHPPIIRTSEHLFLDLPKLQLNLEKYLEEQWASSDCCWSHNAIAITKKWLK

EGLKERCITRDLKWGTPVPLENFSNKVFYVWFDAPIGYLSITANYTPEHWKKWWMDDKHVELYHFVGKDNVPFHSIIFPA

TLIATNEQYCKVKNISATEYLNYESSKFSKSRGVGVFGNNARETGIPADVWRFYLLYTRPESQDAAFNWDDFALKVNSEL

LSNLGNFVNRALSFVERRFHGVIPEVDESLLDEDRLQAFIHNVNETLNNYIDCFDNLKLRDSVKCILNISRLGNQYMQSE

RPWELIKQNERQAGSVLAIVANVVAILAALVHPIMPEISTKICKQLQLEKLVFCSSFQFLLKPGHRIGQVTTLFVKFEKE

TVEQFRKMFAGFQTNSEVANVKKSPSSGKNICKTVKGSQKYCEMFAEIEETRHRISALKLFLTQRAKRRLAAENEELQKH

LEKLKLDYQILMDRRELYSKLGLNTVSNENISLGNSNEKFATTKDVVKDKKEKPKKKVAEKMDKVAASDQKNAVNISSLD

IRVGLIKKAVKHPQADTLYVEEVDVGEDGVRTVVSGLVNFIPLEKMQNRFAVLLCNLKPAKMRGVESQAMVLCASTKDSV

EILEPPEGAVPGDRIFCPGYEGTPEAQLNPKKKIWESVQPLLLVNADGQACYANKPLRIVGKEGCLTAPTLRTNNSRIWG

KNNGQLKLGVGIRNLRYFKLAVVGDCPEAGSEAAAVSASALVQPSLPNCLLSDESNRRAISLCVDTKSRFVLRFLGELFP

VQFAWFELVAQLQLD

>KRY42828.1 Methionine--tRNA synthetase, cytoplasmic [Trichinella spiralis]

MTANDKIIFPVPGERNILITSALPYVNNYPHLGTIIGCVLSADVFSRYCRLRGYNTLYICGTDEYGTATETKAIEEGVTP

IQLCDKYYELHRDVYEWFNIKFDYFGRTTTKQQTEIAQHIFKKIYDAGFTTTKNVDQLYCEKCERFLADRFVEGECPFCG

YEEARGDQCDKCGHLINAPELKLPKCKLCSHPPIIRTSEHLFLDLPKLQLNLEKYLEEQWASSDCCWSHNAIAITKKWLK

EGLKERCITRDLKWGTPVPLENFSNKVFYVWFDAPIGYLSITANYTPEHWKKWWMDDKHVELYHFVGKDNVPFHSIIFPA

TLIATNEQYCKVKNISATEYLNYESSKFSKSRGVGVFGNNARETGIPADVWRFYLLYTRPESQDAAFNWDDFALKVNSEL

LSNLGNFVNRALSFVERRFHGVIPEVDESLLDEDRLQAFIHNVNETLNNYIDCFDNLKLRDSVKCILNISRLGNQYMQSE

RPWELIKQNERQAGSVLAIVANVVAILAALVHPIMPEISTKICKQLQLEKLVFCSSFQFLLKPGHRIGQVTTLFVKFEKE

TVEQFRKMFAGFQTNSEVANVKKSPSSGKNICKTVKGSQKYCEMFAEIEETRHRISALKLFLTQRAKRRLAAENEELLKH

LEKLKLDYQILMDRRELYSKLGLNTVSNENISLGNSNEKFATTKDVVKDKKEKPKKKVAEKMDKVAASDQKNAVNISSLD

IRVGLIKKAVKHPQADTLYVEEVDVGEDGVRTVVSGLVNFIPLEKMQNRFAVLLCNLKPAKMRGVESQAMVLCASTKDSV

EILEPPEGAVPGDRIFCPGYEGTPEAQLNPKKKIWESVQPLLLVNADGQACYANKPLRIVGKEGCLTAPTLRSVPIK

>KRY16135.1 Methionine--tRNA synthetase, cytoplasmic, partial [Trichinella patagoniensis]

LKSIEMTGNDKIIYNFEFSFPVPGERNILITSALPYVNNYPHLGTIIGCVLSADVFSRYCRLRGYNTLYICGTDEYGTAT

ETKAIEEGVTPIQLCDKYYELHREVYEWFNIKFDYFGRTTTKQQTEIAQHIFKKIYDAGFTTTKNVDQLYCEKCQRFLAD

RFVEGECPFCGYEEARGDQCDKCGHLINAPELKLPKCKLCSHPPIIRTSEHLFLDLPKLQLNLEKYLEEQWASSDCCWSH

NAIAITKKWLKEGLKERCITRDLKWGTPVPLENFSNKVFYVWFDAPIGYLSITANYSPEHWKKWWMDDKHVELYHFVGKD

NVPFHSIIFPATLIATNEQYCKVKNISATEYLNYESSKFSKSRGVGVFGNNARETGIPADVWRFYLLYTRPESQDAAFNW

DDFALKVNSELLSNLGNFVNRALSFVERRFHGIIPEVDESLLDKDRLQAFIHNVNETLNNYIDCFDNLKLRDSVKCILNI

SRLGNQYMQSERPWELIKQNERQAGSVLAIVANVVAILAALVHPIMPEISTTICKQLQLEKLVFCSSFQFLLKPGHRIGQ

VTTLFVKFEKETVEQFRKMFAGFQTNSEVANVKKSPSSGKNICKTVKGSQKYCEMFAEIEETRHRISALKLFLTQRAKRR

LAAENEELQKHLEKLKLDYQILMDRRELYSKRGLNTVSNENISLENSNEKFTTTKDVVKDKKEKSKKKVAEKMDKVTASD

QKNAVNISSLDIRVGLIKKAVKHPQADTLYVEEVDVGEDGVRTVVSGLVNFIPLEKMQNRFAVLLCNLKPAKMRGVESQA

MVLCASTKDAVEILEPPERAVPGDRIFCPGYEGTPEAQLNPKKKIWESVQPLLLVNADGQACYANKPLRIVGKEGCLTAP

TLRSVPIK

>KRZ50690.1 Methionine--tRNA synthetase, cytoplasmic, partial [Trichinella nativa]

LKPIEMTGNDKIIYNFEFSFPVPGERNILITSALPYVNNYPHLGTIIGCVLSADVFSRYCRLRGYNTLYICGTDEYGTAT

ETKAIEEGVTPIQLCDKYYELHREVYEWFNIKFDYFGRTTTKQQTEIAQHIFKKIYDAGFTTTKNVDQLYCEKCQRFLAD

RFVEGECPFCGYEEARGDQCDKCGHLINAPELKLPKCKLCSHPPIIRTSEHLFLDLPKLQLNLEKYLEEQWASSDCCWSH

NAIAITKKWLKEGLKERCITRDLKWGTPVPLENFSNKVFYVWFDAPIGYLSITANYSPEHWKKWWMDDKHVELYHFVGKD

NVPFHSIIFPATLIATNEQYCKVKNISATEYLNYESSKFSKSRGVGVFGNNARETGIPADVWRFYLLYTRPESQDAAFNW

DDFALKVNSELLSNLGNFVNRALSFVERRFHGVIPEVDESLLDKDRLQAFIHNVNETLNNYIDCFDNLKLRDSVKCILNI

SRLGNQYMQSERPWELIKQNEKQAGSVLAIVANVVAILAALVHPIMPEISTTICKQLQLEKLVFCSSFQFLLKPGHRIGQ

VTTLFVKFEKETVEQFRKMFAGFQTNSEVANVKKSPSSGKNICKTVKGSQKYCEMFAEIEETRHRISALKLFLTQRAKRR

LAAENEELEKHLEKLKLDYQILMDRRELYSKLGLNTVSNENISLENSNEKFTTTKGVVKDKKEKPKKKVAEKMDKVTASD

QKNAVNISSLDIRVGLIKKAVKHPQADTLYVEEVDVGEDGVRTVVSGLVNFIPLEKMQNRFAVLLCNLKPAKMRGVESQA

MVLCASTKDAVEILEPPEGAVPGDRIFCPGYEGTPEAQLNPKKKIWESVQPLLLVNADGQACYANKPLRIVGKEGCLTAP

TLRSVPIK

>KRY51152.1 Methionine--tRNA synthetase, cytoplasmic, partial [Trichinella britovi]

LKPIEMTGNDKIIFPVPGERNILITSALPYVNNYPHLGTIIGCVLSADVFSRYCRLRGYNTLYICGTDEYGTATETKAIE

EGVTPIQLCDKYYELHREVYEWFNIKFDYFGRTTTKQQTEIAQHIFKKIYDAGFTTTKNVDQLYCEKCQRFLADRFVEGE

CPFCGYEEARGDQCDKCGHLINAPELKLPKCKLCSHPPIIRTSEHLFLDLPKLQLNLEKYLEEQWASSDCCWSHNAIAIT

KKWLKEGLKERCITRDLKWGTPVPLENFSNKVFYVWFDAPIGYLSITANYSPEHWKKWWMDDKHVELYHFVGKDNVPFHS

IIFPATLIATNEQYCKVKNISATEYLNYESSKFSKSRGVGVFGNNARETGIPADVWRFYLLYTRPESQDAAFNWDDFALK

VNSELLSNLGNFVNRALSFVERRFHGVIPEVDESLLDKDRLQAFIHNVNETLNNYIDCFDNLKLRDSVKCILNISRLGNQ

YMQSERPWELIKQNERQAGSVLAIVANVVAILAALVHPIMPEISTTICKQLQLEKLVFCSSFQFLLKPGHRIGQVTTLFV

KFEKETVEQFRKMFAGFQTNSEVANVKKSPSSGKNICKAVKGSQKYCEMFAEIEETRHRISALKLFLTQRAKRRLAAENE

ELQKHLEKLKLDYQILMDRRELYSKLGLNTVSNENISLENSNEKFTTTKDVVKDKKEKLKKKVAEKMDKVTASDQKNAVN

ISSLDIRVGLIKKAVKHPQANTLYVEEVDVGEDGVRTVVSGLVNFIPLEKMQNRFAVLLCNLKPAKMRGVESQAMVLCAS

TKDAVEILEPPEGAVPGDRIFCPGYEGTPEAQLNPKKKIWESVQPLLLVNADGQACYANKPLRIVGKEGCLTAPTLRSVP

IK

>KRY51151.1 Methionine--tRNA synthetase, cytoplasmic, partial [Trichinella britovi]

LKPIEMTGNDKIIFPVPGERNILITSALPYVNNYPHLGTIIGCVLSADVFSRYCRLRGYNTLYICGTDEYGTATETKAIE

EGVTPIQLCDKYYELHREVYEWFNIKFDYFGRTTTKQQTEIAQHIFKKIYDAGFTTTKNVDQLYCEKCQRFLADRFVEGE

CPFCGYEEARGDQCDKCGHLINAPELKLPKCKLCSHPPIIRTSEHLFLDLPKLQLNLEKYLEEQWASSDCCWSHNAIAIT

KKWLKEGLKERCITRDLKWGTPVPLENFSNKVFYVWFDAPIGYLSITANYSPEHWKKWWMDDKHVELYHFVGKDNVPFHS

IIFPATLIATNEQYCKVKNISATEYLNYESSKFSKSRGVGVFGNNARETGIPADVWRFYLLYTRPESQDAAFNWDDFALK

VNSELLSNLGNFVNRALSFVERRFHGVIPEVDESLLDKDRLQAFIHNVNETLNNYIDCFDNLKLRDSVKCILNISRLGNQ

YMQSERPWELIKQNERQAGSVLAIVANVVAILAALVHPIMPEISTTICKQLQLEKLVFCSSFQFLLKPGHRIGQVTTLFV

KFEKETVEQFRKMFAGFQTNSEVANVKKSPSSGKNICKAVKGSQKYCEMFAEIEETRHRISALKLFLTQRAKRRLAAENE

ELQKHLEKLKLDYQILMDRRELYSKLGLNTVSNENISLENSNEKFTTTKDVVKDKKEKLKKKVAEKMDKVTASDQKNAVN

ISSLDIRVGLIKKAVKHPQANTLYVEEVDVGEDGVRTVVSGLVNFIPLEKMQNRFAVLLCNLKPAKMRGVESQAMVLCAS

TKDAVEILEPPEGAVPGDRIFCPGYEGTPEAQLNPKKKIWESVQPLLLVNADGQACYANKPLRIVGKEGCLTAPTLRSVP

INERGKESRKEFFIFLKMILSKQQPHMG

>KRX68196.1 Methionine--tRNA synthetase, cytoplasmic, partial [Trichinella sp. T9]

LKSIEMTGNDKIIFPVPGERNILITSALPYVNNYPHLGTIIGCVLSADVFSRYCRLRGYNTLYICGTDEYGTATETKAIE

EGVTPIQLCDKYYELHREVYEWFNIKFDYFGRTTTKQQTEIAQHIFKKIYDAGFTTTKNVDQLYCEKCQRFLADRFVEGE

CPFCGYEEARGDQCDKCGHLINAPELKLPKCKLCSHPPIIRTSEHLFLDLPKLQLNLEKYLEEQWASSDCCWSHNAIAIS

KKWLKEGLKERCITRDLKWGTPVPLENFSNKVFYVWFDAPIGYLSITANYSPEHWKKWWMDDKHVELYHFVGKDNVPFHS

IIFPATLIATNEQYCKVKNISATEYLNYESSKFSKSRGVGVFGNNARETGIPADVWRFYLLYTRPESQDAAFNWDDFALK

VNSELLSNLGNFVNRALSFVERRFHGVIPEVDESLLDKDRLQAFIHNVNETLNNYIDCFDNLKLRDSVKCILNISRLGNQ

YMQSERPWELIKQNERQAGSVLAIVANVVAILAALVHPIMPEISTTICKQLQLEKLVFCSSFQFLLKPGHRIGQVTTLFV

KFEKETVEQFRKMFAGFKTNSEVANVKKSPSSGKNICKTVKGSQKYCEMFAEIEETRHRISALKLFLTQRAKRRLAAENE

ELQKHLEKLKLDYQILMDRRELYSKLGLNTVSNENISLENSNEKFTTTKDVVKDKKEKPKKKVAEKMDKVTASDQKNAVN

ISSLDIRVGLIKKAVKHPQADTLYVEEVDVGENGVRTVVSGLVNFIPLEKMQNRFAVLLCNLKPAKMRGVESQAMVLCAS

TKDAVEILEPPEGAVPGDRIFCPGYEGTPEAQLNPKKKIWESVQPLLLVNADGQACYANKPLRIVGKEGCLTAPTLRSVP

IK

>KRZ83683.1 Methionine--tRNA synthetase, cytoplasmic [Trichinella sp. T8]

MTGNDKIIFPVPGERNILITSALPYVNNYPHLGTIIGCVLSADVFSRYCRLRGYNTLYICGTDEYGTATETKAIEEGVTP

IQLCDKYYELHREVYEWFNIKFDYFGRTTTKQQTEIAQHIFKKIYDAGFTTTKNVDQLYCEKCQRFLADRFVEGECPFCG

YEEARGDQCDKCGHLINAPELKLPKCKLCSHPPIIRTSEHLFLDLPKLQLNLEKYLEEQWASSDCCWSHNAIAITKKWLK

EGLKERCITRDLKWGTPVPLENFSNKVFYVWFDAPIGYLSITANYSPEHWKKWWMDDKHVELYHFVGKDNVPFHSIIFPA

TLIATNEQYCKVKNISATEYLNYESSKFSKSRGVGVFGNNARETGIPADVWRFYLLYTRPESQDAAFNWDDFALKVNSEL

LSNLGNFVNRALSFVERRFHGVIPEVDESLLDKDRLQAFIHNVNETLNNYIDCFDNLKLRDSVKCILNISRLGNQYMQSE

RPWELIKQNERQAGSVLAIVANVVAILAALVHPIMPEISTTICKQLQLEKLVFCSSFQFLLKPGHRIGQVTTLFVKFEKE

TVEQFRKMFAGFQTNSEVANVKKSPSSGKNICKTVKGSEKYCEMFAEIEETRHRISALKLFLTQRAKRRLAAENEELLKH

LEKLKLDYQILMDRRELYSKLGLNTVSNENISLENSNEKFTTTKDVVKDKKEKPKKKVAEKMDKVTASDQKNAVNISSLD

IRVGLIKKAVKHPQADTLYVEEVDVGEDGVRTVVSGLVNFIPLEKMQNRFAVLLCNLKPAKMRGVESQAMVLCASTKDAV

EILEPPEGAVPGDRIFCPGYEGTPEAQLNPKKKIWESVQPLLLVNADGQACYANKPLRIVGKEGCLTAPTLRSVPIK

>KRY51150.1 Methionine--tRNA synthetase, cytoplasmic, partial [Trichinella britovi]

LKPIEMTGNDKIICNFEFSFPVPGERNILITSALPYVNNYPHLGTIIGCVLSADVFSRYCRLRGYNTLYICGTDEYGTAT

ETKAIEEGVTPIQLCDKYYELHREVYEWFNIKFDYFGRTTTKQQTEIAQHIFKKIYDAGFTTTKNVDQLYCEKCQRFLAD

RFVEGECPFCGYEEARGDQCDKCGHLINAPELKLPKCKLCSHPPIIRTSEHLFLDLPKLQLNLEKYLEEQWASSDCCWSH

NAIAITKKWLKEGLKERCITRDLKWGTPVPLENFSNKVFYVWFDAPIGYLSITANYSPEHWKKWWMDDKHVELYHFVGKD

NVPFHSIIFPATLIATNEQYCKVKNISATEYLNYESSKFSKSRGVGVFGNNARETGIPADVWRFYLLYTRPESQDAAFNW

DDFALKVNSELLSNLGNFVNRALSFVERRFHGVIPEVDESLLDKDRLQAFIHNVNETLNNYIDCFDNLKLRDSVKCILNI

SRLGNQYMQSERPWELIKQNERQAGSVLAIVANVVAILAALVHPIMPEISTTICKQLQLEKLVFCSSFQFLLKPGHRIGQ

VTTLFVKFEKETVEQFRKMFAGFQTNSEVANVKKSPSSGKNICKAVKGSQKYCEMFAEIEETRHRISALKLFLTQRAKRR

LAAENEELQKHLEKLKLDYQILMDRRELYSKLGLNTVSNENISLENSNEKFTTTKDVVKDKKEKLKKKVAEKMDKVTASD

QKNAVNISSLDIRVGLIKKAVKHPQANTLYVEEVDVGEDGVRTVVSGLVNFIPLEKMQNRFAVLLCNLKPAKMRGVESQA

MVLCASTKDAVEILEPPEGAVPGDRIFCPGYEGTPEAQLNPKKKIWESVQPLLLVNADGQACYANKPLRIVGKEGCLTAP

TLRSVPIK

>KRX68197.1 Methionine--tRNA synthetase, cytoplasmic, partial [Trichinella sp. T9]

LKSIEMTGNDKIIYNFEFSFPVPGERNILITSALPYVNNYPHLGTIIGCVLSADVFSRYCRLRGYNTLYICGTDEYGTAT

ETKAIEEGVTPIQLCDKYYELHREVYEWFNIKFDYFGRTTTKQQTEIAQHIFKKIYDAGFTTTKNVDQLYCEKCQRFLAD

RFVEGECPFCGYEEARGDQCDKCGHLINAPELKLPKCKLCSHPPIIRTSEHLFLDLPKLQLNLEKYLEEQWASSDCCWSH

NAIAISKKWLKEGLKERCITRDLKWGTPVPLENFSNKVFYVWFDAPIGYLSITANYSPEHWKKWWMDDKHVELYHFVGKD

NVPFHSIIFPATLIATNEQYCKVKNISATEYLNYESSKFSKSRGVGVFGNNARETGIPADVWRFYLLYTRPESQDAAFNW

DDFALKVNSELLSNLGNFVNRALSFVERRFHGVIPEVDESLLDKDRLQAFIHNVNETLNNYIDCFDNLKLRDSVKCILNI

SRLGNQYMQSERPWELIKQNERQAGSVLAIVANVVAILAALVHPIMPEISTTICKQLQLEKLVFCSSFQFLLKPGHRIGQ

VTTLFVKFEKETVEQFRKMFAGFKTNSEVANVKKSPSSGKNICKTVKGSQKYCEMFAEIEETRHRISALKLFLTQRAKRR

LAAENEELQKHLEKLKLDYQILMDRRELYSKLGLNTVSNENISLENSNEKFTTTKDVVKDKKEKPKKKVAEKMDKVTASD

QKNAVNISSLDIRVGLIKKAVKHPQADTLYVEEVDVGENGVRTVVSGLVNFIPLEKMQNRFAVLLCNLKPAKMRGVESQA

MVLCASTKDAVEILEPPEGAVPGDRIFCPGYEGTPEAQLNPKKKIWESVQPLLLVNADGQACYANKPLRIVGKEGCLTAP

TLRSVPIK

>KRZ50692.1 Methionine--tRNA synthetase, cytoplasmic, partial [Trichinella nativa]

LKPIEMTGNDKIIYNFEFSFPVPGERNILITSALPYVNNYPHLGTIIGCVLSADVFSRYCRLRGYNTLYICGTDEYGTAT

ETKAIEEGVTPIQLCDKYYELHREVYEWFNIKFDYFGRTTTKQQTEIAQHIFKKIYDAGFTTTKNVDQLYCEKCQRFLAD

RFVEGECPFCGYEEARGDQCDKCGHLINAPELKLPKCKLCSHPPIIRTSEHLFLDLPKLQLNLEKYLEEQWASSDCCWSH

NAIAITKKWLKEGLKERCITRDLKWGTPVPLENFSNKVFYVWFDAPIGYLSITANYSPEHWKKWWMDDKHVELYHFVGKD

NVPFHSIIFPATLIATNEQYCKVKNISATEYLNYESSKFSKSRGVGVFGNNARETGIPADVWRFYLLYTRPESQDAAFNW

DDFALKVNSELLSNLGNFVNRALSFVERRFHGVIPEVDESLLDKDRLQAFIHNVNETLNNYIDCFDNLKLRDSVKCILNI

SRLGNQYMQSERPWELIKQNEKQLVYANLAGSVLAIVANVVAILAALVHPIMPEISTTICKQLQLEKLVFCSSFQFLLKP

GHRIGQVTTLFVKFEKETVEQFRKMFAGFQTNSEVANVKKSPSSGKNICKTVKGSQKYCEMFAEIEETRHRISALKLFLT

QRAKRRLAAENEELEKHLEKLKLDYQILMDRRELYSKLGLNTVSNENISLENSNEKFTTTKGVVKDKKEKPKKKVAEKMD

KVTASDQKNAVNISSLDIRVGLIKKAVKHPQADTLYVEEVDVGEDGVRTVVSGLVNFIPLEKMQNRFAVLLCNLKPAKMR

GVESQAMVLCASTKDAVEILEPPEGAVPGDRIFCPGYEGTPEAQLNPKKKIWESVQPLLLVNADGQACYANKPLRIVGKE

GCLTAPTLRSVPIK

>KRZ83682.1 Methionine--tRNA synthetase, cytoplasmic [Trichinella sp. T8]

MTGNDKIICNFEFSFPVPGERNILITSALPYVNNYPHLGTIIGCVLSADVFSRYCRLRGYNTLYICGTDEYGTATETKAI

EEGVTPIQLCDKYYELHREVYEWFNIKFDYFGRTTTKQQTEIAQHIFKKIYDAGFTTTKNVDQLYCEKCQRFLADRFVEG

ECPFCGYEEARGDQCDKCGHLINAPELKLPKCKLCSHPPIIRTSEHLFLDLPKLQLNLEKYLEEQWASSDCCWSHNAIAI

TKKWLKEGLKERCITRDLKWGTPVPLENFSNKVFYVWFDAPIGYLSITANYSPEHWKKWWMDDKHVELYHFVGKDNVPFH

SIIFPATLIATNEQYCKVKNISATEYLNYESSKFSKSRGVGVFGNNARETGIPADVWRFYLLYTRPESQDAAFNWDDFAL

KVNSELLSNLGNFVNRALSFVERRFHGVIPEVDESLLDKDRLQAFIHNVNETLNNYIDCFDNLKLRDSVKCILNISRLGN

QYMQSERPWELIKQNERQAGSVLAIVANVVAILAALVHPIMPEISTTICKQLQLEKLVFCSSFQFLLKPGHRIGQVTTLF

VKFEKETVEQFRKMFAGFQTNSEVANVKKSPSSGKNICKTVKGSEKYCEMFAEIEETRHRISALKLFLTQRAKRRLAAEN

EELLKHLEKLKLDYQILMDRRELYSKLGLNTVSNENISLENSNEKFTTTKDVVKDKKEKPKKKVAEKMDKVTASDQKNAV

NISSLDIRVGLIKKAVKHPQADTLYVEEVDVGEDGVRTVVSGLVNFIPLEKMQNRFAVLLCNLKPAKMRGVESQAMVLCA

STKDAVEILEPPEGAVPGDRIFCPGYEGTPEAQLNPKKKIWESVQPLLLVNADGQACYANKPLRIVGKEGCLTAPTLRSV

PIK

>XP_011568041.1 PREDICTED: methionine--tRNA synthetase, cytoplasmic-like [Plutella xylostella]

MKIYTNENNTSTLKLLIAANLAGKKVELKNANFGEPLFAGPRSLPLLEVDEELLFFSSNAAAQYLMPVLDLSQNGRCQQM

QEWEATRLLPATLSAVTGKPAPELRQLLTSLLTDIDAVLTKKKYILGDKLSAADISIWSTLYPLYYNATHKTTYLDATKN

IQKWFEDIAAAKAVQEAVKQWGGSAQGPFGAPSALGLPQVPSLVATAQSPEEPVEVGPTPEEIELAEENWKHGLERLQPK

LEQGKVILPIKGRKNILITSALPYVNNVPHLGNIIGCVLSADIFARYCRLCDYNTLYISGTDEYGTATETKALEEGVTPR

EICDKYFAIHNSVYRWFNIGFDHFGRTSTAEQTEITQKMFLKLQDNGFISKKSVDQLYCQQCSRFLADRFVEGVCHHPGC

LYPDARGDQCDKCGKLINATELVNPRCKVCGDSPIIRASEQLFIELNQLEPSIRAWSDKASAGWAGGARAVLRAWLKETL

RPRAVTRDLKWGVPVPNYPDKVFYVWFDAPLGYMSITQSATKDYEKWWRPDKECDVKLYQFMAKDNVPFHALMFPAALLG

VNEGHLLVHHIYSTEYLNYEEGKFSKSRGVGVFGTDAQDTGIPADVWRFYLASIRPESSDSSFSWAELGTKNNSELLNNL

GNFCHRSLSFCYNSFKGAVPDVNPGPEEYELMALVNRELIAYVTEMSQGRLRFALRRVLSISRLGNQRMQAAQPWILLKG

TDEDKIRGATTIGLCCQIAALLCADELLSTSGLGKLPGTFKIQLEKNASPTVCPVRKIPIGVRDKLLDALNNMVKLGVIR

KVSHPTAWVNGIVLAPKKSGELRVCLDPRPLNRVIRRAHYPLPTLTDIATKLQGARYFSKLDAPELNPEGSIPEVQG

>OON20474.1 methionine--tRNA synthetase, partial [Opisthorchis viverrini]

CAEALKSVAAASITSGVELREDANATGLYKLLNEFSIPVHPATLTTRSLTPAIKRYINSHNSVSSSTGVSTSELDSVLKD

LERRVSVSESLFRLDPLSPCSLIVWSALLPLFYPESPFGECAHCSHIILPTEAVSFHEELARFFEMVDSLLRSEQILRLF

KDDLKTLWSSAPSIAQPNSCTSRPETKACAPKQGSAKPMAKTSQQKAVYSIKQPPANDSATKSVPDLLDDGSSLPTTLPV

TEDDLHQATACFKPLGSFKRPVYPVPKLPQSKSSGVRNILITSALPYVNNVPHLGNMIGSTLSASVFALYCDVAGYNVLS

ICGTDEYGTATEAKALSEHMTPRQICDKYHKLHCDIYSWFDIQFDYFGRTTTDKHQEIVQDLFQKLWENQYITEDTLEQL

FCENCSKFLADRFVEGTCPFCKYDDARGDQCDKCGRLMNAIELKQPRCKTCQHSPVVRSSKHLFLDLPKIESKLAAFVEQ

RAYNPTSLWTTNARTISSTWIRDGLRPRCITRDLNWGVPVPLEEYKNKVFYVWFDAPIGYLSITANYTPEWRHWWQPTPK

QISEMGPIELFQFMAKDNVPFHAIIFPACLLAANAGHTLVQHLLSTEYMNYEGTKFSKSRGIGVFGDGAMSSGIHSNVWR

FYLLYRRPETQDSAFVWDDFMLVTNCELLNNLGNFINRALAFVARFFNGKIPNMDGLDPSDVEFLAQVAQLIRSYAMHME

AGRLREGLRQILAISRLGNVYFQVNQPWVAIKKEETKPRAGVVIGVAANVACLLGMLLYPFMPTIGRQIWTEQCNLSTDV

LSLLSLSRQDYRLAQLLPAGHQIGRPTPLFRKMEPEDINKLSKLYGSGMTASSDSPSGETSCSGLSLSHSDLLFSDEFEI

DVIL

>XP_012759792.1 hypothetical protein SAMD00019534_034460 [Acytostelium subglobosum LB1]

MSNPPNHVVLPEQGRRNILITSALPYVNNVPHLGNIIGCVLSADVYARYCRLRDYNCIYICGTDEYGTATETKALQEKCT

PQQICDRYHAIHRDIYQWFNIGFDKFGRTSTQSQTEITQDIFLKLYERQLTFSEDVEQLYCEKCPMFLADRYVEGICPHC

QFEDARGDQCDRCTKMLNPTELISPRCKLDGTTPILRTTKHIFLDLPKIQQRLDAFVDKTSKIWTENSQKITNSWVKGEL

KPRCITRDLKWGTPVPLEEFKEKVFYVWFDAPIGYLSITKEYTPEWEKWWKNPENVQLVQFMGKDNVPFHTVIFPSSLIG

SDDPYTLLHNLSTTEYLNYETGKFSKSRNTGVFGDNAQGTGVPADVWRFYLLSNRPENQDSTFSWDDFGFKTSELLNNFG

NFVNRVLKSVVSSFDCRIPEIQLQESDEVFIKEVDNLYQQYLEALEKVNIKDGLKLAMTISKLGNQYMQENKPWALEKTD

KARCGTILAVLVNVIKLLATVFEPYLPAITGKIHEQLNCAPSKYTPCFEFLIKAGHEISKNIEPLIQKIEKKDLDVYREK

FAGTVQGEFPIDLQVAVIREVEEHPKADHLYILKAEIGKGVQRKVVAGLKATHTKEQLIGKKIALLLNLKPAKFKDVVSE

GMVVVADNGDKDAPVVSLLLINNDVELGTQLNARGMKVNPKPAMDYVKEFKKLDLKVKDGVLTYKDEPITAGNQSIIFEA

PIANGTLR

>KRY42829.1 Methionine--tRNA synthetase, cytoplasmic, partial [Trichinella spiralis]

LITSALPYVNNYPHLGTIIGCVLSADVFSRYCRLRGYNTLYICGTDEYGTATETKAIEEGVTPIQLCDKYYELHRDVYEW

FNIKFDYFGRTTTKQQTEIAQHIFKKIYDAGFTTTKNVDQLYCEKCERFLADRFVEGECPFCGYEEARGDQCDKCGHLIN

APELKLPKCKLCSHPPIIRTSEHLFLDLPKLQLNLEKYLEEQWASSDCCWSHNAIAITKKWLKEGLKERCITRDLKWGTP

VPLENFSNKVFYVWFDAPIGYLSITANYTPEHWKKWWMDDKHVELYHFVGKDNVPFHSIIFPATLIATNEQYCKVKNISA

TEYLNYESSKFSKSRGVGVFGNNARETGIPADVWRFYLLYTRPESQDAAFNWDDFALKVNSELLSNLGNFVNRALSFVER

RFHGVIPEVDESLLDEDRLQAFIHNVNETLNNYIDCFDNLKLRDSVKCILNISRLGNQYMQSERPWELIKQNERQAGSVL

AIVANVVAILAALVHPIMPEISTKICKQLQLEKLVFCSSFQFLLKPGHRIGQVTTLFVKFEKETVEQFRKMFAGFQTNSE

VANVKKSPSSGKNICKTVKGSQKYCEMFAEIEETRHRISALKLFLTQRAKRRLAAENEELLKHLEKLKLDYQILMDRREL

YSKLGLNTVSNENISLGNSNEKFATTKDVVKDKKEKPKKKVAEKMDKVAASDQKNAVNISSLDIRVGLIKKAVKHPQADT

LYVEEVDVGEDGVRTVVSGLVNFIPLEKMQNRFAVLLCNLKPAKMRGVESQAMVLCASTKDSVEILEPPEGAVPGDRIFC

PGYEGTPEAQLNPKKKIWESVQPLLLVNADGQACYANKPLRIVGKEGCLTAPTLRSVPIK

>XP_012797861.1 Methionine--tRNA synthetase, cytoplasmic [Schistosoma haematobium]

MIDLTEEITEPSSIHITDEDQRRAGDIFLDYSVFTKPVYETPKKPQPKQTGIRNIMITSALPYVNNVPHLGNMIGSTLSA

SVFALYCDLAGYNVLSICGTDEYGTATEAKAVSENMTPRQICDKFHKLHCDIYKWFQIEFDYFGRTTTDQHTEIVQELFK

RLWDKGFITEDSVEQLYCEKCPKFLADRFVEGVCPLCKYDDARGDQCDKCGRLMNAVELIEPRCKICCQRPVVRSSRHLF

LDLPKIESKLSTFVEQRINDSSCIWTSNACTISNSWLRDGLKPRCITRDLHWGVPVPLEAYKDKVFYVWFDAPIGYISIT

ANYTKHWKEWWQPSDDSEIELFQFMAKDNVPFHAIVFPSCLLAADAGYTLVKHLLSTEYMNYENTKFSKSRGIGVFGDDA

MKSGVISDVWRFYLLYRRPESQDSAFIWDDFMLVNNSELLNNLGNFVNRSLSFVSRFFNSIIPSITKLEPVDTEFLARIN

KLFASYMRNMELCHLRDAIRNVLTIARLGNGYFQANQPWVAVKKPETKQRSGVVISVAANVACLLGAMIYPYMPTIGKQI

WIEQCDLPESQMSFVQIIKNEFTVKRLLPDGHRIGKPVPLFRKIDPDEIKRLSQVYSGSSAASVTNGNEPK

>KRY42830.1 Methionine--tRNA synthetase, cytoplasmic, partial [Trichinella spiralis]

LITSALPYVNNYPHLGTIIGCVLSADVFSRYCRLRGYNTLYICGTDEYGTATETKAIEEGVTPIQLCDKYYELHRDVYEW

FNIKFDYFGRTTTKQQTEIAQHIFKKIYDAGFTTTKNVDQLYCEKCERFLADRFVEGECPFCGYEEARGDQCDKCGHLIN

APELKLPKCKLCSHPPIIRTSEHLFLDLPKLQLNLEKYLEEQWASSDCCWSHNAIAITKKWLKEGLKERCITRDLKWGTP

VPLENFSNKVFYVWFDAPIGYLSITANYTPEHWKKWWMDDKHVELYHFVGKDNVPFHSIIFPATLIATNEQYCKVKNISA

TEYLNYESSKFSKSRGVGVFGNNARETGIPADVWRFYLLYTRPESQDAAFNWDDFALKVNSELLSNLGNFVNRALSFVER

RFHGVIPEVDESLLDEDRLQAFIHNVNETLNNYIDCFDNLKLRDSVKCILNISRLGNQYMQSERPWELIKQNERQAGSVL

AIVANVVAILAALVHPIMPEISTKICKQLQLEKLVFCSSFQFLLKPGHRIGQVTTLFVKFEKETVEQFRKMFAGFQTNSE

VANVKKSPSSGKNICKTVKGSQKYCEMFAEIEETRHRISALKLFLTQRAKRRLAAENEELLKHLEKLKLDYQILMDRREL

YSKLGLNTVSNENISLGNSNEKFATTKDVVKDKKEKPKKKVAEKMDKVAASDQKNAVNISSLDIRVGLIKKAVKHPQADT

LYVEEVDVGEDGVRTVVSGLVNFIPLEKMQNRFAVLLCNLKPAKMRGVESQAMVLCASTKDSVEILEPPEGAVPGDRIFC

PGYEGNLRTLRNLSILGTPEAQLNPKKKIWESVQPLLLVNADGQACYANKPLRIVGKEGCLTAPTLRSVPIK

>XP_004350883.1 methionyl-tRNA synthetase [Cavenderia fasciculata]

MSENIYLRSNPPKDVEVLPIEGQRNILITSALPYVNNVPHLGNIIGCVLSGDVYSRYCRLKGHNCIYICGTDEYGTATET

KALQEKCTPKEICDRYHAIHKEIYEWFNIGFDKFGRTSTQSQTEITQDIFMKLHQNGLTFSDTVEQLFCEQCKIFLADRY

VEGICPHCQYEDARGDQCDKCTKLLNPTDLIQPRCKVCSTTPIIKSTKHIFLDLPKVQERLDTFVQGSSGKWSENSQNIT

NSWVKGELKPRCITRDLKWGTPVPLEEFKEKVFYVWFDAPIGYLSITKEYTKEWEKWWKNPENVELVQFMGKDNVPFHTV

IFPSSLIGTEDPYTMLHNLSTTEYLNYETGKFSKSRNTGVFGDGARDTKIPADIWRFYLLSNRPEVSDSVFSWDDFSAKT

LELLNNFGNLVNRVLKFVSVNFDGKIPKIDLNDSDKKFVQEVNDNVKLYHEALEAVRIKEGLRLAMAISRLGNQYQQENK

PWEVIKTDKARCGASVALLVNTIKLLATLFEPYLPLISQQIHEQLNFESTKYTVGFEFLIPEGHEISKNITPLIQAIPDV

DLKSFRTKFAGAVADEFPLDLRVAKIVEVEDHPAADSLYIIKCDIGGGVTKRAVAGLTTAFTKSQLVGQKLAVLLNLKPS

KFKGVQSEAMIVVADDKSPIGVSLLLSDAEIGSKIGARDSKIVPKSNIDYQKEFIHMDLRVIDGVPCYKEKPLLVNGKPL

DLQKKEIISAKLR

>KRX22800.1 Methionine--tRNA synthetase, cytoplasmic, partial [Trichinella nelsoni]

LITSALPYVNNYPHLGTIIGCVLSADVFSRYCRLRGYNTLYICGTDEYGTATETKAIEEGVTPIQLCDKYYELHREVYEW

FNIKFDNFGRTTTKQQTEIAQHIFKKIYDAGFTTTKNVDQLYCEKCERFLADRFVEGECPFCGYEEARGDQCDKCGHLIN

APELKLPKCKLCSHPPIIRTSEHLFLDLPKLQLNLEKYLEEQWASSDCCWSHNAIAITKKWLKEGLKERCITRDLKWGTP

VPLENFSNKVFYVWFDAPIGYLSITANYSPEHWKKWWMDDKHVELYHFVGKDNVPFHSIIFPATLIATNEQYCKVKNISA

TEYLNYESSKFSKSRGVGVFGNNARETGIPADVWRFYLLYTRPESQDAAFNWDDFALKVNSELLSNLGNFVNRALSFVER

RFHGVIPEMDESLLDKDRLQAFIHNVNETLNNYIDCFDNLKLRDSVKCILNISRLGNQYMQSERPWELIKQNERQAGSVL

AIVANVVAILAALVHPIMPEISTKICKQLQLEKLVFCSSFQFLLKPGHRIGQVTTLFVKFEKETVEQFRKMFAGFQTNSE

VANVKKSPSSGKNICKTVKGSQKYCKMFAEIEETRHRISALKLFLIQRAKRRLAAENEELQKHLEKLKLDYQILMDRREL

YSKLGLNTVSNENISLENSNEKIATTKDVVKDKKEKPKKKVAEKMDKITASDQKNAVNISSLDIRVGLIKKAVKHPQADT

LYVEEVDVGEDGMRTVVSGLVNFIPLEKMQNRFAVLLCNLKPAKMRGVESQAMVLCASTKDAVEILEPPEGAVPGDRIFC

PGYEGTPEAQLNPKKKIWESVQPLLLVNADGQACYANNPLRIVGKEGCLTAPTLRSVPIK

>XP_024057555.1 methionine--tRNA synthetase, cytoplasmic [Terrapene mexicana triunguis]

MASEVLWQPRPGTASCWAERYFFLLAGKEPNDLTNQWLEWEATELQPAVSTALYCHLVQGKKGGEVLGAIRKTLTYIDQS

LTSKATPYLAGEAESVADIVLWATLFPVLRDEFCLPDELEALRSWFQTMNLSEPCRSAVASVLTPQGAQEFKAYLQKQPG

PSLHWEKPAANEPEEEELAERSLSEEEIAAAAEAWARGPAALPERWQPASPVLPVEGRRNVLITSALPYVNNVPHLGNII

GCVLSADVFARYCRLRNWNTLYVCGTDEYGTATETKALEEGLTPQQICDKYNAIHTQIYRWFDISFDYFGRTTTAHQTTI

AQDIFRRLLERDMLLTDTVDQLRCERCQRFLADRFVEGTCPFCNYEEARGDQCDKCGKLINAVELKKPQCKVCKDSPVVK

SSQHLFLDLPKLEGLLEKWLEQSWATGDWTANSRFITRSWIRDGLKPRCITRDLKWGTPVPLDGFRDKVFYVWFDAPIGY

LSITANYTDQWERWWKNPQQEML

>PNW86475.1 hypothetical protein CHLRE_02g087950v5 [Chlamydomonas reinhardtii]

MAALHARKGDVQALKVDAAAHLYGKKLNFTAAASVNDVISKGSNPFATNTILFSSGELQLSEPSAAATFLAGGRASLPLS

VLKWVDWAEAVLRPATYLGGDALVAAEVELEAALTAGELNAAAAGLAEVFVFATLLPLGLRKKLPQQLDSFVGAVAAAPA

VQSAVADVGCDDGAAVVGVWHDDAAAYTAARPKRPIPGRRNILITSALPYVNNVPHLGNIIGCVLSADVYARYCRARGHN

CIYVCGTDEYGTATETKALEEGLTCQQICDKYHAIHAGIYQWFDIAFDRFGRTPTRAQTKIAQDIFKSLQSRGQLVQQEM

QQLYSEAAGKFLADRFVTGTCPKCGYEDARGDQCDGCGNLLNPTELIRPKCKLTGTTPVMRSTRHVFLDLPQLSPQLQEY

ITTTSAQGGWSANCVTLTNAWMRDGLKLRCITRDLKWGTPVPLEGFEEKVFYVWFDAPIGYISITANYTPDWEAWWKSPK

DVELVQFMGKDNVPFHTVIFPATLLGTGGDWTMMRNISVTEYLNYEGGKFSKSRGTGVFGNQAQETGIPVEVWRYYLLAN

RPEQADTDFKWSDLAAKNNSELLANLGNFINRALMFVSKFFDGVVPGATAAGQAEAEELGKTVGPKVDEYIAAMERIKLK

EGLRLVMSISADGNKFLQDTKPWVAVKEDKDKCATLVATSVGLVRLLSALMAPYMPSLSAKILQQLNLPYESSVALSDEL

VAGCRQPQSLVPAGHAIGTPGPLISTISDETVAELRARFGGNQADDEAAAAATAGGAKPGDKKAAGGKGGADKQGGAAAA

GGEKKAAREGTPDVSWLDVRVGVITKAWKHPDAESLYVEEVDVGEPQPRQVVSGLVKYIPTAEALVGRRVVLLCNLKPAA

MRGVQSQAMVLAASSADGAQLELLEPPAGAKVGERISWPGFPGEPEEQLNPRKKVFEAIQPDFTTDAGCVAVYKGAAFTT

SAGPVKVATIVGASIK

>CDS25457.2 methionyl tRNA synthetase cytoplasmic [Hymenolepis microstoma]

MVRLFADPKNRNCLAALVVAHKSKEVEILKYGADNSPDGAILETESGLISSVNSIVSFIQGVDLDSQVIVDFLRWESSTL

SPALKQAGTDENVTDILVELQIKLQALERSLTESDKSAIHIIVWADLSQLSLSGASKNLDHLHKIVQTLSADEHCIQALK

DLSGKALAKGSVVAAGKNLPRKQLQSSTPKCDVTPQISEIDYELKRLQIEVSTDQMLKAQWTFFSPYPSVKTHNPKIDGK

VIPAKKDEGVRNILITSALPYVNNVPHLGNLIGSLLSANIFALYCKTAGYNVLSICGTDEYGTATETKALAEGLTPQQIT

DKYHAIHRQVYDWFGIHFDYFGRTSTPAHTEITQEVFKQLYNNGYISEASVEQLYCGHCEKFLADRFVEGNCPYCKSDDA

RGDQCDSCGKLINAVELIKPRCITCLKSPVIRSSDHLFLNLPQLEEKVGAFFKERTEAKDCKWTHVAQSIARTWLRDGFK

KRCITRDLKWGVPVPLTNFKDKVFYVWFDAPMGYMSITANYTKDWRQWWQPSEDLAPVEYFQFMAKDNVPFHACIFPASL

LGSSKKYTLVKHIIATDYLNYEGKKFSKSKGVGVFGNNAKDSGIESNIWRFYLCYIRPETQDSTFSWEDFALKNNTELLN

NLGNFINRPLMFVAKFFKSRVPAMKFLQPDDLTFLARVNSILAGYIDHMENCRLREGIRSVLSISRLGNGYVQAQKPWVL

YKSPDTMNGQVWLVVCNIAALLGILLRPFMPAFSDEIFSQCQLLPEHQTLAPIIANGGYLPCLLPEGHVIGEVSLFHL

>XP_008222906.1 PREDICTED: probable methionine--tRNA synthetase isoform X2 [Prunus mume]

MGDLTMTQKAPKLPIPGKRNILITSALPYVNNVPHLGNIIGCVLSADVFARYCRLRGYNTVYICGTDEYGTATETKAMEE

KCSPQEICDKYHAIHREVYEWFNISFEKFGRTSTPQQTEVCQDIFKKLLENGWLSENTMQQLYCDTCKRFLADRLVEGTC

PTQGCDYGSARGDQCENCGKLLNPTELKDPKCKVCKTTPRIRDTNHLFLELPSLKDKLEEYINRTSVAGSWSQNAIQATN

AWLKEGLKQRCITRDLKWGVPVPHDNFKDKVFYVWFDAPIGYVSITKCYTDDWEQWWKNPKNVELYQFMGKDNVPFHTVM

FPSTLLGTGENWTLMKTISVTEYLNYEAGKFSKSKGIGVFGNNAKDTNIPVEVWRYYLLTNRPEVSDTLFTWADLQAKLN

SELLNNLGNFINRVLSFVAKPSGQGYGSIIPDAPSAESDPLTEKLAEKVGKYVEQYIESMEKVKLKQGLKTAMSISSEGN

AYLQESQFWKLYKEDQPRCAVVMRTSVGLVHLLACLLEPFMPSFSLEVFKQLDLPPETHISLYDDKGDIASARRPWEIVP

IGHKIGKPEPLFKELKDEEVESLRKKFAGSQADRKEREEAEAVKVAAQLKKMKVSDNSGKKKQQATKSAAEAEISISRLD

IRVGLITKAQKHPDADSLYVEEIDVGEEQTRTVVSGLVKYIPIEEMQNRKVCVLCNLKPATMRGIKSQAMVLAASNSDHT

TVELVYPPKAAQVGERVTFPGFVGEPDEVLNPKKKVWETLQVDLHTNTDLVACYRDIPLTTSAGVCTVSSIRGGSIR

>XP_007221944.2 probable methionine--tRNA synthetase isoform X1 [Prunus persica]

MIFDDDANANACHCTRELDMGDLTMTFKEDQKAPKLPIPGKRNILITSALPYVNNVPHLGNIIGCVLSADVFARYCRLRG

YNTVYICGTDEYGTATETKAMEEKCSPQEICDKYHAIHREVYEWFNISFEKFGRTSTPQQTEVCQEIFKKLLENGWLSEN

TMQQLYCDTCKRFLADRLVEGTCPTQGCDYGSARGDQCENCGKLLNPTELKDPKCKVCKTTPRIRDTNHLFLELPSLKDK

LEEYINRTSVAGSWSQNAIQATNAWLKEGLKQRCITRDLKWGVPVPHDNFKDKVFYVWFDAPIGYVSITKCYTDDWEQWW

KNPENVELYQFMGKDNVPFHTVMFPSTLLGTGENWTLMKTISVTEYLNYEAGKFSKSKGIGVFGNNAKDTNIPVEVWRYY

LLTNRPEVSDTLFTWADLQAKLNSELLNNLGNFINRVLSFVAKPSGQGYGSIIPDAPSAESDPLTEKLAEKVGKYVEQYI

ESMEKVKLKQGLKTAMSISSEGNAYLQESQFWKLYKEDQPRCAVVMRTSVGLVHLLACLLEPFMPSFSLEVFKQLDLPPE

THISLYDDKGDIASARRPWEIVPIGHKIGKPEPLFKELKDEEVESLRKKFAGSQADRKEREEAEAVKVAAQLKKMKVSDN

SGKKKQQATKSAAEAEISISRLDIRVGLITKAQKHPDADSLYVEEIDVGEGQTRTVVSGLVKYIPIEEMQNRKVCVLCNL

KPATMRGIKSQAMVLAASNSDHTTVELVDPPKAAQVGERVTFPGFVGEPDEVLNPKKKVWETLQVDLHTNTDLVACHRDI

PLTTSAGVCTVSSIRGGSIR

>XP_020409818.1 probable methionine--tRNA synthetase isoform X2 [Prunus persica]

MGDLTMTFKEDQKAPKLPIPGKRNILITSALPYVNNVPHLGNIIGCVLSADVFARYCRLRGYNTVYICGTDEYGTATETK

AMEEKCSPQEICDKYHAIHREVYEWFNISFEKFGRTSTPQQTEVCQEIFKKLLENGWLSENTMQQLYCDTCKRFLADRLV

EGTCPTQGCDYGSARGDQCENCGKLLNPTELKDPKCKVCKTTPRIRDTNHLFLELPSLKDKLEEYINRTSVAGSWSQNAI

QATNAWLKEGLKQRCITRDLKWGVPVPHDNFKDKVFYVWFDAPIGYVSITKCYTDDWEQWWKNPENVELYQFMGKDNVPF

HTVMFPSTLLGTGENWTLMKTISVTEYLNYEAGKFSKSKGIGVFGNNAKDTNIPVEVWRYYLLTNRPEVSDTLFTWADLQ

AKLNSELLNNLGNFINRVLSFVAKPSGQGYGSIIPDAPSAESDPLTEKLAEKVGKYVEQYIESMEKVKLKQGLKTAMSIS

SEGNAYLQESQFWKLYKEDQPRCAVVMRTSVGLVHLLACLLEPFMPSFSLEVFKQLDLPPETHISLYDDKGDIASARRPW

EIVPIGHKIGKPEPLFKELKDEEVESLRKKFAGSQADRKEREEAEAVKVAAQLKKMKVSDNSGKKKQQATKSAAEAEISI

SRLDIRVGLITKAQKHPDADSLYVEEIDVGEGQTRTVVSGLVKYIPIEEMQNRKVCVLCNLKPATMRGIKSQAMVLAASN

SDHTTVELVDPPKAAQVGERVTFPGFVGEPDEVLNPKKKVWETLQVDLHTNTDLVACHRDIPLTTSAGVCTVSSIRGGSI

R

>XP_008222905.1 PREDICTED: probable methionine--tRNA synthetase isoform X1 [Prunus mume]

MDVEGGGRHVMIFDDDANACHCTRELDMGDLTMTQKAPKLPIPGKRNILITSALPYVNNVPHLGNIIGCVLSADVFARYC

RLRGYNTVYICGTDEYGTATETKAMEEKCSPQEICDKYHAIHREVYEWFNISFEKFGRTSTPQQTEVCQDIFKKLLENGW

LSENTMQQLYCDTCKRFLADRLVEGTCPTQGCDYGSARGDQCENCGKLLNPTELKDPKCKVCKTTPRIRDTNHLFLELPS

LKDKLEEYINRTSVAGSWSQNAIQATNAWLKEGLKQRCITRDLKWGVPVPHDNFKDKVFYVWFDAPIGYVSITKCYTDDW

EQWWKNPKNVELYQFMGKDNVPFHTVMFPSTLLGTGENWTLMKTISVTEYLNYEAGKFSKSKGIGVFGNNAKDTNIPVEV

WRYYLLTNRPEVSDTLFTWADLQAKLNSELLNNLGNFINRVLSFVAKPSGQGYGSIIPDAPSAESDPLTEKLAEKVGKYV

EQYIESMEKVKLKQGLKTAMSISSEGNAYLQESQFWKLYKEDQPRCAVVMRTSVGLVHLLACLLEPFMPSFSLEVFKQLD

LPPETHISLYDDKGDIASARRPWEIVPIGHKIGKPEPLFKELKDEEVESLRKKFAGSQADRKEREEAEAVKVAAQLKKMK

VSDNSGKKKQQATKSAAEAEISISRLDIRVGLITKAQKHPDADSLYVEEIDVGEEQTRTVVSGLVKYIPIEEMQNRKVCV

LCNLKPATMRGIKSQAMVLAASNSDHTTVELVYPPKAAQVGERVTFPGFVGEPDEVLNPKKKVWETLQVDLHTNTDLVAC

YRDIPLTTSAGVCTVSSIRGGSIR

>XP_020433732.1 methionyl-tRNA synthetase [Heterostelium album PN500]

MQIMSAPPSEVVLPVEGQRNILITSALPYVNNVPHLGNIIGCVLSGDVYARYCRMRGYNTIYICGTDEYGTATETKALQE

GVTPKEICDRYHVIHRDIYKWFNIGFDKFGRTSTDSQTEIAQDIFMKLYKNELTTSEEIEQLYCERCNMFLADRYVEGTC

PHCQFEDARGDQCDNCTKMLNPTDLIRPRCKLDSTTPVLKSTKHIFLSLPKIQQRLDDFVTQSSPIWSENSQRITNSWVK

GELKSRCITRDLKWGTPVPLEEFKEKVFYVWFDAPIGYLSITKEYTEQWEQWWKNPKNVELVQFMGKDNVPFHTVIFPSS

LIGSGDQYTLLHNLSTTEYLNYESGKFSKSRNTGVFGDNAMGTGVPADIWRYYLLSNRPEKIDSTFSWADFGLKTNTELL

SNFGNLVNRVLKQVSSDLNGVIPKIELNDEDRQFIADVDKLYIEYLNDLEHVHIKDGLRTAMSMSKLGNQYMQSNKPWEL

KEKDPLRFGTILTVLVNTIKLLATVFEPYLPEITAKVHEQLNMQPTKYSVNFEFLITEGHAISKNIVPLINKIENTDLEK

FRSKFSGPVLEEFPLDLQVAQIKEVADHPTAESLYVLKAELAGGAVRTVVAGLKATHTKEQLLNKKIALLLNLKPAKFQG

VASEGMVVVADNENKESPVVSLLLVADNTELGTKINVKGMKLAPKPGMDYVKGFKKLDLKVKDGEILFKGAPIHINNQPL

TLEKPISTGILR

>XP_024353617.1 Methionyl-tRNA synthetase, cytoplasmic [Echinococcus granulosus]

MPQRKQGQNKFAKANEKSVQNDEDELRRLHVEVSTDQMLKAQWTFSSTVLPATLPLKKTEGVRNILISSALPYVNNVPHL

GNLIGSLLSANIFALYCQTAGYNVLSICGTDEYGTATEARAQAEGLTPQQITDKYHKLHCQVYDWFDIHFDYFGRTSTPV

HTEITQEIFWDLYNNGFVTEASVEQLFCEKCEKFLADRFVEGVCPFCKAEDARGDQCDTCGKLINAVELIKPQCITCRSY

PTVRSSNHLFLDLPKLEEKVDVFFTESVEDPNCKWTQVAQSIARTWLRDGFKKRCITRDLKWGVPVPLLNFKNKVFYVWF

DAPIGYISITANYTRDWRQWWLPSEDVAPVEYFQFMAKDNVLFHSAIFPACLLGSGKKFTLVKHIIATEYLNYEGKKFSK

SRGVGVFGNNAEDSGIESNIWRFYLAYIRPETQDSTFSWEDFALKNNSELLNNLGNFINRPLMFVMKFFNSTVPSMKSLQ

PDDVTFLARVNSILHEYISHMENCRLREGIRSVLGISRLGNGYLQVQRPWVLYKSSDTIQRAGVVTGISSNIAALLGILL

RPFMPTIGEEIFAQCKLPSERRSLAPMVTNGGHIICLLPEGHVIGELFVECRRLQKRTNLLAEVETPQTELLPLQVQALR

RSIGILKRRLSSLCHTCGCIKPIRTKHCSLCNRCVKVMDHHCPVTDNCVGQDNRVWFLLTSIMVTIVSSWIGWLAVRYWQ

KADSTSLYEIMTLVLLSIGWMFGLNACFMTISSAIFNMTTNELTNWRRYEHFGTAKTGFRNPFNQGYWSNVVEFFRPRHY

ETERELYKHSATDGGRYPFVV

>KRY42831.1 Methionine--tRNA synthetase, cytoplasmic, partial [Trichinella spiralis]

LITSALPYVNNYPHLGTIIGCVLSADVFSRYKFKKLIYFRYCRLRGYNTLYICGTDEYGTATETKAIEEGVTPIQLCDKY

YELHRDVYEWFNIKFDYFGRTTTKQQTEIAQHIFKKIYDAGFTTTKNVDQLYCEKCERFLADRFVEGECPFCGYEEARGD

QCDKCGHLINAPELKLPKCKLCSHPPIIRTSEHLFLDLPKLQLNLEKYLEEQWASSDCCWSHNAIAITKKWLKEGLKERC

ITRDLKWGTPVPLENFSNKVFYVWFDAPIGYLSITANYTPEHWKKWWMDDKHVELYHFVGKDNVPFHSIIFPATLIATNE

QYCKVKNISATEYLNYESSKFSKSRGVGVFGNNARETGIPADVWRFYLLYTRPESQDAAFNWDDFALKVNSELLSNLGNF

VNRALSFVERRFHGVIPEVDESLLDEDRLQAFIHNVNETLNNYIDCFDNLKLRDSVKCILNISRLGNQYMQSERPWELIK

QNERQAGSVLAIVANVVAILAALVHPIMPEISTKICKQLQLEKLVFCSSFQFLLKPGHRIGQVTTLFVKFEKETVEQFRK

MFAGFQTNSEVANVKKSPSSGKNICKTVKGSQKYCEMFAEIEETRHRISALKLFLTQRAKRRLAAENEELLKHLEKLKLD

YQILMDRRELYSKLGLNTVSNENISLGNSNEKFATTKDVVKDKKEKPKKKVAEKMDKVAASDQKNAVNISSLDIRVGLIK

KAVKHPQADTLYVEEVDVGEDGVRTVVSGLVNFIPLEKMQNRFAVLLCNLKPAKMRGVESQAMVLCASTKDSVEILEPPE

GAVPGDRIFCPGYEGTPEAQLNPKKKIWESVQPLLLVNADGQACYANKPLRIVGKEGCLTAPTLRSVPIK

>KHJ44292.1 methionine--tRNA synthetase [Trichuris suis]

MLLSRGRSIDVARNTSGAVEGGFIDETDMLVTTWYSSASRASLSKSPSSVWAKQKETCSGSATVPMNDARRHLVRAVSSL

GTDNAVVVGCLKQGEKLDILGVPIRNMENKKNDCSARNGHRPVVPEVGKRNVLITSALPYVNNVPHLGTIIGCVLSADVF

SRYCRLRGFNTLFICGTDEYGTATETKAIEEGVTPLEICNKYYKLHKEVYEWFNIEFDYFGRTSTEKQTEFFFIYLELLE

KFFSINQYLQDQLNVLTKFPSFKIVQDIFNRLYLNGYTSTKCVDQLHCSTCSRFLADRFVEGECPFCSYHDARGDQCDLC

GHLINAPELKNPKCKLCHQTPTLRSSEHLFLDLAKLQPQVEAYLLKLWSNGAVWSSNAIAITKGWLSDGLKERCITRDLK

WGTPVPLEQFADKVFYVWFDATIGYMSITANYLGDNWKDWWMGKEKVLFLLRDVRIFKVRFSKVELYNFVGKDNVLFHSI

VFPATLLATGQSYTMVSHISATEYLTYEDTKFSKSRGIGVFGTDAQDTSIPADIWRFYLLYIRPETQDSSFNWTDFALKV

NSELSNNLGNFINRTLSFLAKNFGGIVASVSAEDHRKSKRPFDCYYFANTLIRFRDSIRCILNISRLCNHFIQSSQPWVL

VKGSASERTEAAKLISLAVNAIAILEPLLQPFMPETSKTIRSQLNVQSSELHDCFKVVLKPGHVIGEVSPLFSKFENDLV

EAYRERFRGDAHVDVGAGDKNAVQSKKVQAAPTIKRKKPLETDLKNAGLFSRKVLEANADVKDSLNDSIPNDVIFETSEY

EKIATMLQSFEQLKVQLNERTDEMKRLKSSIQSLESKLFSKIERELEQDHEMLRKKLAVKQAEYFNVTGKKNFITGAQLG

STAVEVESGKKNESAELRQQVLASSKGEVTPSEEKTKGKPSKAKQHVVEEAAPDFSRLDVRVGLIKTARKHPDADSLYVE

EVDVGEDKPRTVISGLVNFIPVDQMQERFAVLLCNLKPVKMRGIESQAMVLCASTKEKVEILEPPAGSVPGDPVICPGYE

DNRGCFSAYPKGSLCCGPRKYSDCPWTPGRRPVSILLTNALKGLSFKHNTISRRWAVGSDDFVFPALTEAFVRSIWFIVI

ICVYAIRSPETCEYAHSLKLYVVFILIHNAVLIAHSLYAAYVSSRGTIFNAIPRRLVPKLLYFRCIFFIFDVVLAVVGAS

LASRAVSSGTCNHFFLLSIQILVVASWLVVAGTLLGVWLIFDPLGRHHHDDIRVAKMAPSLWKARLQRALCCFSSSDRSK

MAIEEAAEVFTFLFEDVDLVFTDIFSGLLLLADSRENQLLHLGAKCEDQDTAPSWMNTRLAERFMEISLGVYGWPYYVCS

SPWFCRPLFKLLSHLRCCYQCRCDSVLVIRDNCCLCNSVAFMLHTSLAEADLFYVNYHNEIWETPFIACVDHSTRNIVVA

IRGSFSLRDALTDLCIREDCLELIAEGSYLNNDMPTKCYVHRGILKSARYVCDKLNGENVLGDLFVLHPDYGLVVTGHSL

GASVASVVSLLLHSTYPSVHCFAFSPLGCVVDENTSHYMKKFVCTFVLGDDFACRLSYHSVLRLKALIREALERCSRPKY

ELLVRGCFHWLFTSRDQRYNEIVDDEASICESQESSRQKRPSNLTDRLMAKSKPSYSASDTVSNGQQCRIKLCCPGRIYH

LVRTCPWNGGFTIKNCEAADFEEIHLSSSMISDHFPQSMMKALRSLSLVSHL

>XP_024500968.1 tRNA-binding domain and Aminoacyl-tRNA synthetase, class 1a, anticodon-binding domain and Nucleic acid-binding, OB-fold domain and Rossmann-like alpha/beta/alpha sandwich fold domain and Methionyl-tRNA synthetase family and Methionyl/Leucyl tRNA synthetase family and Methionine-tRNA synthetase, type 1 family-containing protein [Strongyloides ratti]

MESIEKTFFSEPTFYHDTVTSALPYVNNVPHLGNIIGCVLSADVFVRFCNITGINCLYISGTDEYGTATEVKALQEKMSP

SEICSKYHEIHKKIYDWFNIKFDAFGRTSTEAQTEITQDVFNKLYANGFIESQDVDQLHCSSCNIFLADRYVNGICPLCT

YDDARGDQCDGCGKLINAIELKEPKCLLCKKTPSLKKSKHLFLCLDKLTTDVYENLENILKNENNHWSSNAISIAKSWLK

TGLEKRCITRDLKWGVKVPIEGFQDKVFYVWFDAPIGYISITKQLLGDDYVKWWKNSKDVELYNFLGKDNVAFHSIMFPA

TLFGTKDNYTIVKHINATEYFNYEDQKFSKSRGIGVFGDAASETGIPADVWRFYLLYMRPESSDTYFSWDDFANKINSEL

LSNLGNFCNRSLSFLSKNYNGIIPNIIVNDEDKELFIGINKDLEEYHKCLTEVKLRDGLLKILSISRKGNQYLQNNAPWK

LIKGNDEEKHRAETVIGISANLVYLLSVVLYPYMPKVSEEIRKQCNKDELTMLPKNICCVLKSGHKIGTPATLFTKLESE

DIALFKAKFGTSSKTETKVDKKPDNKKDQKKKPQDSNKKMETKIIESNFFYPKAQANIKNIDNVMASLKESTEKAKSLFE

KEQLIKLSEDKKKLSVEIEKLLKQLKEEEIKGGIKQIDVPINVNETMNLLEESNIKKEEIVIEKKKKESVLLKNEKKEKS

TKGSEKTSNLSDDSVDIGRLDLRVGKIIECKRHPDADALYLETIDLGEEKPRTVISGLVKFVPIEEMQNRLVICLCNLKP

AKMRGIESQAMVMCASTPEKVEIMEVDPSCTPGMRITCGSFVSRPDSVLNPKKKVWEQVAPDLKVNNEGKATYKGELLLV

NSQTFVVAPTLRDVNIK

>KFD65297.1 hypothetical protein M514_00606 [Trichuris suis]

MNDARRHLVRAVSSLGTDNAVVVGCLKQGEKLDILGVPIRNMENKKNDCSARNGHRPVVPEVGKRNVLITSALPYVNNVP

HLGTIIGCVLSADVFSRYCRLRGFNTLFICGTDEYGTATETKAIEEGVTPLEICNKYYKLHKEVYEWFNIEFDYFGRTST

EKQTEIVQDIFNRLYLNGYTSTKCVDQLHCSTCSRFLADRFVEGECPFCSYHDARGDQCDLCGHLINAPELKNPKCKLCH

QTPTLRSSEHLFLDLAKLQPQVEAYLLKLWSNGAVWSSNAIAITKGWLSDGLKERCITRDLKWGTPVPLEQFADKVFYVW

FDATIGYMSITANYLGDNWKDWWMGKEKVELYNFVGKDNVLFHSIVFPATLLATGQSYTMVSHISATEYLTYEDTKFSKS

RGIGVFGTDAQDTSIPADIWRFYLLYIRPETQDSSFNWTDFALKVNSELSNNLGNFINRFRDSIRCILNISRLCNHFIQS

SQPWVLVKGSASERTEAAKLISLAVNAIAILEPLLQPFMPETSKTIRSQLNVQSSELHDCFKVVLKPGHVIGEVSPLFSK

FENDLVEAYRERFRGDAHVDVGAGDKNAVQSKKVQAAPTIKRKKPLETDLKNAGLFSRKVLEANADVKDSLNDSIPNDVI

FETSEYEKIATMLQSFEQLKVQLNERTDEMKRLKSSIQSLESKLFSKIERELEQDHEMLRKKLAVKQAEYFNVTGKKNFI

TGAQLGSTAVEVESGKKNESAELRQQVLASSKGEVTPSEEKTKGKPSKAKQHVVEEAAPDFSRLDVRVGLIKTARKHPDA

DSLYVEEVDVGEDKPRTVISGLVNFIPVDQMQERFAVLLCNLKPVKMRGIESQAMVLCASTKEKVEILEPPAGSVPGDPV

ICPGYEGLPEKQLNPKKKVWEAVQPSLLVDSNGRACFKCTPLQVAGKGEILAPSLREVLGLLCSQPVTMPSMRMFNRRWA

VGSDDFVFPALTEAFVRSIWLAFIVIICVYAIRSPETCEYAHSLKLYVVFILIHNAVLIAHSLYAAYVSSRGTIFNAIPR

RLVPKLLYFRCIFFIFDVVLAVVGASLASRAVSSGTCNHFFLLSIQILVVASWLVVAGTLLGVWLIFDPLGRHHHDDIRV

AKMAPSLWKARLQRALCCFSSSDRSKMAIEEAAEVFTFLFEDVDLVFTDIFSGLLLLADSRENQLLHLGAKCEDQDTAPS

WMNTRLAERFMEISLGVYGWPYYVCSSPWFCRPLFKLLSHLRCCYQCRCDSVLVIRDNCCLCNSVAFMLHTSLAEADLFY

VNYHNEIWETPFIACVDHSTRNIVVAIRGSFSLRDALTDLCIREDCLELIAEGSYLNNDMPTKCYVHRGILKSARYVCDK

LNGENVLGDLFVLHPDYGLVVTGHSLGASVASVVSLLLHSTYPSVHCFAFSPLGCVVDENTSHYMKKFVCTFVLGDDFAC

RLSYHSVLRLKALIREALERCSRPKYELLVRGCFHWLFTSRDQRYNEIVDDEASICESQESSRQKRPSNLTDRLMAKSKP

SYSASDTVSNGQQCRIKLCCPGRIYHLVRTCPWNGGFTIKNCEAADFEEIHLSSSMISDHFPQSMMKALRSLSLVSHL

>XP_641872.1 methionyl-tRNA synthetase [Dictyostelium discoideum AX4]

MSKPSNTPPLPKDGERNILITSALPYVNNVPHLGNIIGCVLSADVYARYCRLKNYNCIYICGTDEYGTATETKALSEGCT

PKEICDKYHEIHKEIYEWFNISFDKFGRTSTNSQTEIAQDIFNKIKDNGYTLTQEIEQLYCEQTCKMFLADRFVEGTCPH

CKFEDARGDQCDGCSKLLNPTELINPRCKVCSKPPVIKSTKHIFIDLPQLQQQVDQFVETNSKGGNWSENSIAITNTWVK

GELKPRCITRDLKWGTPVPMEEFKDKVFYVWFDAPIGYISITAEYTNEWEKWWKNPENVKLVQFMGKDNVPFHTVIFPAS

LIGSKDNYTLLNNLSTTEFLNYETGKFSKSRNTGVFGDGAKATGIPSEVWRFYLLNNRPESSDSIFSWDDFNFKNNELLN

NFGNLVNRVLKMLNTNAAFNGVVPKIGELNEVDKKLVQEVDEHLVQYFQKLEEISLKEGLKIAMSISKLGNTYMQDNKPW

DLSGKDNERCGQVLAILINLIKLLSTLLEPYIPSLTDKVHQQLNVEPTKYSTHFDINAIPAGHEISKEILPLVKKIEADD

LKKWRTKFSGIGPEFPIDMKIATVLEVNDHPSAENLYVIKLSLGGDQTKTAVSGIKANFEKSQLIGKKLAVVLNLKPSKF

KGVLSEAMILVADDGQATKESLSFLVPSNPQSIEAGSKIAGKGMSIKPKPTIDYQKEFLHYDLTCIDKIISYNKSQLFIN

QNEPLVSEKIGNGKVR

>OAJ42670.1 methionine-tRNA synthetase [Batrachochytrium dendrobatidis JEL423]

MSATKSIITATSQYQLLVDSNHSDAIRSSSALKLILALEIAGFPYSVVTASTPGNRIYQLIFTPFQAIASISWLTTIILS

YSALPFHSKVSVLTPSGLVLGNPNTAVQHILLKFKPEDDVASATLDSIVEWDLHVLGAVITCSDLTTKAAHSALAAANIH

LSNLANFQENSAASVVFFGTLFPMWSKLVALEEFVTKYSDLNKWFSTMQDCPQYKSAISAWKNATILGVDGKNTKEALSN

SNLPAPKVNADVVIALDHHSRKISPKKNERNVLITSALPYVNNIPHLGNLIGSVLSADVYARYSRIRGENVLFICGTDEY

GTATETKAIEEKLSCKQLCDKYFKFHDETYKWFQIDFDHFGRTITEQQTIITQDIYLKLEKNGHVMEDTVTQLYCNQHES

FLADRFVEGTCPLCGYEDARGDQCDSCGKLLNAIELKNPRCKLDNAAPVARDSKHLFLNLTGQQEKLTEWFKKASSEGCW

SANSQTITNSWLKEGLKPRCITRDLKWGTPVPKEGYENKVFYVWFDAPIGYFSITANYTKDWELWWKNPKDVKLYQFMGK

DNVPFHTVIFPSTLLGTNENWTLLHHLSTTEYLQYESGKFSKSRGVGVFGNNVVDSGIPVEVWRYYLLSIRPESGDSYFS

WNSFIAANNTELLANLGNFVNRLVKFINAKYDGVIPAYKTTGEPETKLISSVNTLLHQFIEALNDVKIRTGLRLFMDISA

RGNLYLQENRIDNSLFANSRERCDNVVAVAANLAYLISAVVYPYMPSTSEGILRQLNLPMRRITDTWNAEDITAGHKIGA

AEYLFKRLDESLEEKLRQKYSGKQDPAAKTGTDSGTATGGSKSKKSKSAKAAVPASNVVKTPEMIAVETRIAEQGGIVRD

LKTAKADPNVVKEAVDALLALKAQLAALTTA

>KFD58380.1 hypothetical protein M513_00606 [Trichuris suis]

MNDARRHLVRAVSSLGTDNAVVVGCLKQGEKLDILGVPIRNMENKKNDCSARNGHRPVVPEVGKRNVLITSALPYVNNVP

HLGTIIGCVLSADVFSRYCRLRGFNTLFICGTDEYGTATETKAIEEGVTPLEICNKYYKLHKEVYEWFNIEFDYFGRTST

EKQTEIVQDIFNRLYLNGYTSTKCVDQLHCSTCSRFLADRFVEGECPFCSYHDARGDQCDLCGHLINAPELKNPKCKLCH

QTPTLRSSEHLFLDLAKLQPQVEAYLLKLWSNGAVWSSNAIAITKGWLSDGLKERCITRDLKWGTPVPLEQFADKVFYVW

FDATIGYMSITANYLGDNWKDWWMGKEKVELYNFVGKDNVLFHSIVFPATLLATGQSYTMVSHISATEYLTYEDTKFSKS

RGIGVFGTDAQDTSIPADIWRFYLLYIRPETQDSSFNWTDFALKVNSELSNNLGNFINRFRDSIRCILNISRLCNHFIQS

SQPWVLVKGSASERTEAAKLISLAINAIAILEPLLQPFMPETSKTIRSQLNVQSSELHDCFKVVLKPGHVIGEVSPLFSK

FENDLVEAYRERFRGDAHVDVGAGDKNAVQSKKVQAAPTIKRKKPLETDLKNAGLFSRKVLEANADVKDSLNDSIPNDVI

FETSEYEKIATMLQSFEQLKVQLNERTDEMKRLKSSIQSLESKLFSKIERELEQDHEMLRKKLAVKQAEYFNVTGKKNFI

TGAQLGSTAVEVESGKKNESAELRQQVLASSKGEVTPSEEKTKGKPSKAKQHVVEEAAPDFSRLDVRVGLIKTARKHPDA

DSLYVEEVDVGEDKPRTVISGLVNFIPVDQMQERFAVLLCNLKPVKMRGIESQAMVLCASTKEKVEILEPPAGSVPGDPV

ICPGYEGLPEKQLNPKKKVWEAVQPSLLVDSNGRACFKCTPLQVAGKGEILAPSLREIIVVAFRPIRKVVYAVVRGSTRV

LGLLCSQPVTMPSMRMFNRRWAVGSDDFVFPALTEAFVRSIWLAFIVIICVYAIRSPETCEYAHSLKLYVVFILIHNAVL

IAHSLYAAYVSSRGTIFNAIPRRLVPKLLYFRCIFFIFDVVLAVVGASLASRAVSSGTCNHFFLLSIQILVVASWLVVAG

TLLGVWLIFDPLGRHHHDDIRVAKMAPSLWKARLQRALCCFSSSDRSKMAIEEAAEVFTFLFEDVDLVFTDIFSGLLLLA

DSRENQLLHLGAKCEDQDTAPSWMNTRLAERFMEISLGVYGWPYYVCSSPWFCRPLFKLLSHLRCCYQCRCDSVLVIRDN

CCLCNSVAFMLHTSLAEADLFYVNYHNEIWETPFIACVDHSTRNIVVAIRGSFSLRDALTDLCIREDCLELIAEGSYLNN

DMPTKCYVHRGILKSARYVCDKLNGENVLGDLFVLHPDYGLVVTGHSLGASVASVVSLLLHSTYPSVHCFAFSPLGCVVD

ENTSHYMKKFVCTFVLGDDFACRLSYHSVLRLKALIREALERCSRPKYELLVRGCFHWLFTSRDQRYNEIVDDEASICES

QESSRQKRPSNLTDRLMAKSKPSYSASDTVSNGQQCRIKLCCPGRIYHLVRTCPWNGGFTIKNCEAADFEEIHLSSSMIS

DHFPQSMMKALRSLSLVSHL

>XP_008232512.2 PREDICTED: probable methionine--tRNA synthetase [Prunus mume]

MGDLTVTSKEEQTAAKLPIPGKRNILITSALPYVNNVPHLGNIIGCVLSADVFARYCRLRGYNTVYVCGTDEYGTATETK

AMEEKCSPQQICDKYHAIHKEVYKWFNISFDIFGRTSAPQQTEVCQAIFKKLLENEWLSENTMQQLYCDTCKRFLADRLV

EGTCPTQGCDYASARGDQCENCGKLLNPTELKDPKCKVCKTTPRIRDTNHLFLELPLLKDKLEEYINKTSVDGSWSQNAI

QATNAWLKEGLKQRCITRDLKWGVPVPHDNFKEKVFYVWFDAPIGYVSITKCYTDEWEKWWKNPENVELYQFMGKDNVPF

HTVMFPSTLLGTGENWTLMKTISVTEYLNYEAGKFSKSKGIGVFGNDAKDTNIPVEVWRYYLLTNRPEVSDTLFTWADLQ

AKLNSELLNNLGNFINRVLSFVAKPSGQGYGSIIPDAPRAESDLLTEKLAEKVGKYVEQYIEAMEKVKLKQGLKTAMSIS

SEGNAYLQESQFWKLYKEDQPRCAVVIRTSVGLVHLLACLLEPFMPSFSLEVFKQLNLPPEKHISLCDDKGDIDRARRPW

EIVPVGHKIGKPEPLFKELKDEEVESLRKKFAGSQADRKEREEAEAVKVAAQLKKMKVSDNSGKKKQQATKSVAEAEISI

SRLDIRVGLITKAQKHPDADSLYIQEIDVGEGQTRTVVSGLVKYIPIEEMQNRKVCVLCNLKPATMRGIKSQAMVLAASN

SDHATVELVNPPKAAQVGERVTFPGFAGEPDEVLNPKKKVWESLQVDLHTNTDLVACYRDIPLTTSAGVCTVSSICGGSV

R

>XP_007204253.2 probable methionine--tRNA synthetase [Prunus persica]

MGDLAVTSKEEQTAAKLPIPGKRNILITSALPYVNNVPHLGNIIGCVLSADVFARYCRLRGYNTVYVCGTDEYGTATETK

AMEEKCSPQQICDKYHAIHREVYKWFNISFDKFGRTSAPQQTEVRQAIFKKLLENEWLSENTMQQLYCDTCKRFLADRLV

EGTCPSQGCDYASARGDQCENCGKLLNPTELKDPKCKVCKTTPQIHDTNHLFLELPLLKDKLEEYINKTSVVGSWSQNAI

QATNAWLKEGLKQRCITRDLKWGVPVPHDNFKDKVFYVWFDAPIGYVSITKCYTDEWEKWWKNPENVELYQFMGKDNVPF

HTVMFPSTLLGTGENWTLMKTISVTEYLNYEAGKFSKSKGIGVFGNDAKDTNIPVEVWRYYLLTNRPEVSDTLFTWADLQ

AKLNSELLNNLGNFINRVLSFVSKPSGQGYGSIIPDAPRAESDLLTEKLAEKVGKYVEQYIEAMEKVKLKQGLKTAMSIS

SEGNAYLQESQFWKLYKEDQPRCAFVIRTSVGLVHLLACLLEPFMPSFSLEVFKQLNLPPEKHISLCDDKGDIDRARRPW

EIVPVGHKIGKPEPLFKELKDEEVESLRKKFAGSQADRKEREEAEAVKVAAQLKKMKVSDNSGKKKQQATKSAAEAEISI

SRLDIRVGLITKAQKHPDADSLYIQEIDVGEGQARTVVSGLVKYIPIEDMQNRKVCVLCNLKPATMRGIKSQAMVLVASN

SDHATVELVNPPKAAQVGERVTFSGFAGEPDEVLNPKKKVWESLQVDLHTNTDLVACYRDIPLTTSAGVCTVSSICGGSI

R

>ONI22450.1 hypothetical protein PRUPE_2G130000 [Prunus persica]

MDMGDLAVTSKEEQTAAKLPIPGKRNILITSALPYVNNVPHLGNIIGCVLSADVFARYCRLRGYNTVYVCGTDEYGTATE

TKAMEEKCSPQQICDKYHAIHREVYKWFNISFDKFGRTSAPQQTEVRQAIFKKLLENEWLSENTMQQLYCDTCKRFLADR

LVEGTCPSQGCDYASARGDQCENCGKLLNPTELKDPKCKVCKTTPQIHDTNHLFLELPLLKDKLEEYINKTSVVGSWSQN

AIQATNAWLKEGLKQRCITRDLKWGVPVPHDNFKDKVFYVWFDAPIGYVSITKCYTDEWEKWWKNPENVELYQFMGKDNV

PFHTVMFPSTLLGTGENWTLMKTISVTEYLNYEAGKFSKSKGIGVFGNDAKDTNIPVEVWRYYLLTNRPEVSDTLFTWAD

LQAKLNSELLNNLGNFINRVLSFVSKPSGQGYGSIIPDAPRAESDLLTEKLAEKVGKYVEQYIEAMEKVKLKQGLKTAMS

ISSEGNAYLQESQFWKLYKEDQPRCAFVIRTSVGLVHLLACLLEPFMPSFSLEVFKQLNLPPEKHISLCDDKGDIDRARR

PWEIVPVGHKIGKPEPLFKELKDEEVESLRKKFAGSQADRKEREEAEAVKVAAQLKKMKVSDNSGKKKQQATKSAAEAEI

SISRLDIRVGLITKAQKHPDADSLYIQEIDVGEGQARTVVSGLVKYIPIEDMQNRKVCVLCNLKPATMRGIKC

>GAX73155.1 hypothetical protein CEUSTIGMA_g608.t1 [Chlamydomonas eustigma]

MATLYFRAGDAQALKPVIASSLTKSQLHCSPVADISSLRNKLSNMFNSNNLGFVSESLQLTDPNAMCKYLGGPDLYPPHL

EDNINTWTAWEETILRPSILKTGGIDAEVMQHLEQSLIPNQLIVGDSLTLADVVLFSTLLPLRQQLPTQTRLYIEKLAAL

PIITQAIEKAGAPNSDAMTWLLKEDSCKYKEQLPKLPAPGQRNILITSALPYVNNVPHLGNIIGCVLSADCYARFCRSRG

YNTVFICGTDEYGTATETKAMEEGMTPQQICDKYHAIHKQIYEWFDIDFDRFGRTPSRHQTAICQSVFKSLQDKNCLVEQ

SMEQLYSEALGKFLADRFVVGTCPKCKYEDARGDQCDACGNLLNPTELINPKCKFSGTKPVIRSTRHIFLDLPQLSEKLQ

EYITTTSQLGGWSSNCVQVTGAWMRDGLKNRCITRDLKWGTPVPMEGYEDKVFYVWFDAPIGYISITAGYTPDWEKWWKN

PEDVEMVQFMGKDNVPFHTVIFPATLLGTGEKWTMMRNISVTEYLNYEGGKFSKSRGTGVFGNDAKDTGIPVEVWRYYLL

SNRPEQQDTDFKWSDLAAKNNNELIKNLGNFVNRVLPFVTKNFEGRVPGVVPGKMEGALSELGNAVGPKVAEYVSAMEKI

KLREGIQFAMAISSDGNKFLQDNKPWDVIKVDKEACASLVTAAVGMVRLLAVLLQPYTPSLSDRLQQQLNLPASSLMLTD

ELMKASYLPHTLVEAGHSIGVATPLLSAISDETIEDLRARFGGNQAERDAKAVAAAGTPPSKGDQAAAAAGSKKQAAEGG

KQAPAAAASAGKGAPVKKEAEGPVDVSRLNIRVGIIRKAWRHPDAESLYVEEIDVGEDQPRQVVSGLVKYIPESEMQGRR

VLVLCNLKPANMRGVTSHAMVLAATSVDGDKVELVEPPEGTLVGERVCFDGYSGEPDEQLNPKKKIFEQVQPDFKVGENL

VAQYKDIPFMTSQGPCKVKTAVGGSIR

>OON10605.1 methionine-tRNA synthetase [Batrachochytrium salamandrivorans]

MQNQPEPTAEATNIAAENVTDPIQSTSTLGGKGQVKMNLPEPLVDSTVVITLDHHSRKITPKEGQRNILITSALPYVNNI

PHLGNLIGSVLSADVFARYSRLRSANVLFICGTDEYGTATETKAIEEKMSCKQLCDKYFKFHDETYKWFQIDFDHFGRTT

TDEQTVITQDIFLSLEKNGHVMEDTITQLYCTQHSSFLADRFVEGTCPLCGYGDARGDQCDGCGKLLNAIELVNPRCKLD

GATPIARDSKHLFLNLTDQQEKLEKWFLQASTEGMWSANSQTITNSWLKEGLKPRCITRDLKWGTPVPREGYENKVFYVW

FDAPIGYLSITATYTNDWEKWWKNPKDVKLYQFMGKDNVPFHTVIFPSTLQGTDQDWTLLHHLSTTEYLQYESGKFSKSR

GVGVFGNNVVDSGIPVEVWRYYLLSIRPESGDSYFSWNAFITANNTELLANLGNYVNRLVKFINAKYDGVIPTYQTSDEP

EKKLTADVAVLLRQYIDALDGVKIRQGLRLFMDISARGNLYLQENRIDNSLFANSRERCNNVVAVAANLAYLLSALVYPY

LPSTSEGIRRQLNLPMRRISDTWDGTDIVAGHKIGAAEYLFKKLDESLEKELRIKYSGKQDPAADTGANATGSQSGKKRS

NKKSTSTTLPSSVPASDVVKTPEMTALEDQIAQQAGTVRSLKADKAAPSDVKTAVDELLALKSQLAALYIA

>OON10606.1 methionine-tRNA synthetase, variant 1 [Batrachochytrium salamandrivorans]

MQNQPEPTAEATSTSTLGGKGQVKMNLPEPLVDSTVVITLDHHSRKITPKEGQRNILITSALPYVNNIPHLGNLIGSVLS

ADVFARYSRLRSANVLFICGTDEYGTATETKAIEEKMSCKQLCDKYFKFHDETYKWFQIDFDHFGRTTTDEQTVITQDIF

LSLEKNGHVMEDTITQLYCTQHSSFLADRFVEGTCPLCGYGDARGDQCDGCGKLLNAIELVNPRCKLDGATPIARDSKHL

FLNLTDQQEKLEKWFLQASTEGMWSANSQTITNSWLKEGLKPRCITRDLKWGTPVPREGYENKVFYVWFDAPIGYLSITA

TYTNDWEKWWKNPKDVKLYQFMGKDNVPFHTVIFPSTLQGTDQDWTLLHHLSTTEYLQYESGKFSKSRGVGVFGNNVVDS

GIPVEVWRYYLLSIRPESGDSYFSWNAFITANNTELLANLGNYVNRLVKFINAKYDGVIPTYQTSDEPEKKLTADVAVLL

RQYIDALDGVKIRQGLRLFMDISARGNLYLQENRIDNSLFANSRERCNNVVAVAANLAYLLSALVYPYLPSTSEGIRRQL

NLPMRRISDTWDGTDIVAGHKIGAAEYLFKKLDESLEKELRIKYSGKQDPAADTGANATGSQSGKKRSNKKSTSTTLPSS

VPASDVVKTPEMTALEDQIAQQAGTVRSLKADKAAPSDVKTAVDELLALKSQLAALYIA

>SAM00476.1 hypothetical protein [Absidia glauca]

MVRPLNVTVPKLVPSQRHYANTLKLLIAAETAGIKINQLPGDNLTVVLDDVTILDPNVAVRYILDATKFDLFESLAIEKE

STSFGPLINKKKYDAVLKDINQFMEEYPVFANTDKLTAVDIIYFGSLYEALSEVDAAKYPKVAAWVHLTSQAPAVKAAVE

TIGQQVQAKAPKKKHQATEKKVTEVTPLAELNQATQKLNGEAFYKPKIQTGKLLPVEGERNVLVTSALPYVNNIPHLGNI

VGSTLSADVYARYCRVRGYNTLYICGTDEYGTATETKALEEGVSCQALCDKYHTIHASVYKWFDLSFDHFGRTTTEKQTQ

ITQDIFKKVNENGYVVQDTMTQLFCEQCQRFLADRYVEGICPNCLYDDARGDQCDACGRLLNATELVKPRCKLDGNSPIT

KDSRHLFLDLGKLQGQIEAFNTKSHAEGKWSANGINITGSWLKEGLRPRCITRDLKWGTPVPLEGFEDKVFYVWFDACIG

YPSITATYTDDWEKWWKNPNNVKLYQFMGKDNVPFHSVIFPGTELATKEDWTLVHHISTTEYLNYEGGKFSKSRNVGVFG

TNAEETGIPPSVWRYYLLSGRPESSDSMFTWNEFITKNNTELLNNLGNFVNRAIKFVLAKYDGVLPPASETPLDGALEKG

LVKDVNELLAQYVDQLEQVKLRAGLATAMAISARGNLYLQESNLSNSLYNDQRAQCNAVVTTAINLIYILSSLISPYMPA

TSESISRQINAPLRLIPNAFTYDILAGHKLNGSEYLFTRIDEKMEDVWKAKYGGNDKK

>XP_008232482.1 PREDICTED: probable methionine--tRNA synthetase [Prunus mume]

MGDLTVTSSEDQKAAKLPIPGKRNILITSALPYVNNVPHLGNIIGCVLSADVFARYCRIRGYNTVYICGTDEYGTATETK

AIEEQCSPQEICDKYHAIHREVYQWFNISFEKFGRTSTPQQTEVCQAIFKKLLENDWLSENTMQQLYCDTCKRFLADRLV

EGTCPTQGCDYASARGDQCENCGKLLNPTELKDPKCKVCKTTPRIRDTNHMFLELPLLRDKLEEYINKTSVAGSWSQNAI

QATNAWLKEGLKQRCITRDLKWGVPVPHDNFKDKVFYVWFDAPIGYVSITKCYTDDWEKWWKNPENVELYQFMGKDNVPF

HTVMFPSTLLGTGENWTLMKTISVTEYLNYEAGKFSKSKGIGVFGNDAKDTNIPVEVWRYYLLTNRPEVSDTLFTWADLQ

AKLNSELLNNLGNFINRVLSFVAKPSGQGYGSIIPDAPSADSDLLTEKLAEKVGKCVEQYIEAMEKVKLKQGLKTAMSIS

SEGNAYLQESQFWKLYKEDQPRCAVVMRTSVGLVYLLACLLEPFMPSFSLEVFKQLNLPPETHISLCDDKGDIDRARRPW

EIVPIGHKIGKPEPLFKELKDEEVESLRKKFAGSQADRKEREEAEAVKVAAQLKKMKVSDNSGKKKQQATKSAAEAEISI

SRLDIRVGLITKAQKHPDADSLYVEEIDVGEGQTRTVVSGLVKYIPIEEMQNRKVCVLCNLKPATMRGIKSQAMVLAAST

SDHATVELVDPPKAAQVGERVTFPGFVGEPDEVLNPKKKVWETLQVDLHTNTDLVACYRDIPLTTSAGVCTVSSICGGSI

R

>OON10607.1 methionine-tRNA synthetase, variant 2 [Batrachochytrium salamandrivorans]

MNLPEPLVDSTVVITLDHHSRKITPKEGQRNILITSALPYVNNIPHLGNLIGSVLSADVFARYSRLRSANVLFICGTDEY

GTATETKAIEEKMSCKQLCDKYFKFHDETYKWFQIDFDHFGRTTTDEQTVITQDIFLSLEKNGHVMEDTITQLYCTQHSS

FLADRFVEGTCPLCGYGDARGDQCDGCGKLLNAIELVNPRCKLDGATPIARDSKHLFLNLTDQQEKLEKWFLQASTEGMW

SANSQTITNSWLKEGLKPRCITRDLKWGTPVPREGYENKVFYVWFDAPIGYLSITATYTNDWEKWWKNPKDVKLYQFMGK

DNVPFHTVIFPSTLQGTDQDWTLLHHLSTTEYLQYESGKFSKSRGVGVFGNNVVDSGIPVEVWRYYLLSIRPESGDSYFS

WNAFITANNTELLANLGNYVNRLVKFINAKYDGVIPTYQTSDEPEKKLTADVAVLLRQYIDALDGVKIRQGLRLFMDISA

RGNLYLQENRIDNSLFANSRERCNNVVAVAANLAYLLSALVYPYLPSTSEGIRRQLNLPMRRISDTWDGTDIVAGHKIGA

AEYLFKKLDESLEKELRIKYSGKQDPAADTGANATGSQSGKKRSNKKSTSTTLPSSVPASDVVKTPEMTALEDQIAQQAG

TVRSLKADKAAPSDVKTAVDELLALKSQLAALYIA

>XP_005707241.1 methionyl-tRNA synthetase isoform 1 [Galdieria sulphuraria]

METRRVVNPQSKNILITSALPYVNNVPHLGNLVGCVLSADVYARFVRQRKENVLYICGTDEYGTATETKALAEGVTPREI

CDKFFKLHKDIYDWFDIRFDYFGRTSTSLQTEICQQIFWDIFHNGYVTEESLEQLFCESCGRFLADRYITGTCPMCGFED

ARGDQCDNCGRLLNPTDLLTARCSLCKNSPVVRKSDHLFLDLPALKDQLEKWIEKSSVKGDWSYNAIHSTYKWLNEGLKK

RCITRDLKWGTPVPLEKYKDKVFYVWFDAPIGYLSITANYTSGWKDWWQNPENVTLFQFMGKDNVPFHTVIFPATLIGTQ

RKWTMLHHISVTDYLNYEDGKFSKSRGVGVFGNDAKDTGIPTEVWRYYLLVNRPENADTVFSWDDFAAKNNNELLANLGN

FVNRTIAFLNSYFEARVPEYELQELDERYIQQVDEELQNYLQTMERVSLKNGLRIAMSISRLGNMYLQETAPWKWMNSDK

NRAATVLVVAVNICRLLAVVLEPFMGAVFSRKIFAQLNIPHTDETNYIPDKFHLHLVEPGHVVGKPFLLFSKITTEQASQ

LRKRFAGGQLKESSPIQTHGEEALAIEMRVGRIVQVEEHVDSNHLYICQVDLGEWGGIKQVVAGIRNYFTKDELLGRTVI

VVCNLQPACLAGVESQGMILTAEKKGKVHLLSCNGSGSPGDEDHNYHDVENKHDEGTTESPKVGTLVTINNTTMPMVPLP

ILDRKAFQNASKLLKIDAQGHVVYNKKYKLIAGTQFVHGSGAQENAKVR

>XP_002953607.1 hypothetical protein VOLCADRAFT_82392 [Volvox carteri f. nagariensis]

MGATLPSPAAETPSSTLAVFDEDAAAYRASRPRLPVPGRRNVLITSALPYVNNVPHLGNIIGCVLSADCYARYCRARGHN

CIYMCGTDEYGTATETKALEEGLTCQQICDKYHAIHAEIYRWFDIAFDRFGRTPTRAQTAIAQDIFKSLHANGQLVQQEM

QQLYSEAAGKFLADRFVYGTCPKCGYDDARGDQCDGCGNLLNPTELINPRCKLTGTTPVLRSTRHVFLDLPQLSPALQEY

ITTTSTQGGWSANCVQLTNAWMRDGLKLRCITRDLKWGTPVPLEGYEDKVFYVWFDAPIGYISITAGYTPDWEAWWKSPK

DVELVQFMGKDNVPFHTVIFPATQLGTQQPWTMMKSISVTEYLNYEGGKFSKSRGTGVFGNQAKDTGIPVEVWRYYLLAN

RPEQADTDFKWSDLAAKNNSELLANLGNFINRSLMFVAKFFEGVIPGATEAGLAEAEELGKTVEEYISALERIKLKEGLR

LVMSISADGNKFLQDTKPWVAVKEDKDKCATLVATSMGLVRLLAAVMAPYMPSLSSKILSQMALPLESSIALTDELVAGS

YRPQTLVPAGHPIGTPGPLISQITEEQVEALRARFGGNQAADEAAAAAAASTSKPAAAGKGKAAAAAALGGDKKAGGGGG

GGAADDGPIDVSRLDVRVGRILKAWRHPDADSLYVEEVDVGEEKPRQVVSGLVRFIPSAEDLAGRRVVLLCNLKPAAMRG

VQSQAMVLAATSQDGAKLELLEPPEGAPLGERVTWPGYGAEGRQPDEVLNPRKKIFETVQPDFTTDAQCVAIYKGAPFTT

SAGPVKVATIVGATIK

>XP_013297879.1 methionine--tRNA synthetase [Necator americanus]

MTAPSAAEIRKAWNEYPSFVGQTPKEKLVPVKGKRNILITSALPYVNNVPHLGNIIGCVLSADVFARYCRMRGYETLYVC

GTDEYGTATETKALQESMTPREVCDKYHVIHRDIYKWFNVEFDIFGRTTTPQQTEFLADRFVTGICPFCAYDDARGDQCD

ACGRLINAVELKSPKCHICKLEPKVRQSTHIFLHLDALQDEVRQYVEKEIAKPDSRWSANAIAIVKGWIKGGLEKRCITR

DLKWGTAVPLVFYVWYDAPIGYLSITKCLVGDNWTKWWKNPGEVELFNFIGKDNVAFHGVMFPCTQLGARDNYTIVNHVC

ATEYLNYEDSKFSKSRNTGVFGDSAKETGIEADVWRFYLLYMRPESQDTSFSWDDFALKLSILTVNSELLANLGNFINRA

LSFLANSFGGVVSDMELNDTDIELIAGVQADSMEWDQFMDAVREKDAVRAVLSMSRRGNQYMQSQQPWVLMKGSEEDKKR

AGTVIGVAANISYHLAVVLHPIMPEISARIREQCGFERLPAFSTYPISYLKPGHKIGTPKPLFTKMESSKIAEWKVKFGG

TDASPREPQTKGKDKTKHSKKASEAGNSSKKCENVKLKKNMAAANCVFPHLAKNQQQIKQLFEATLKKFSQARDQFVKQK

GAELAADVAKLEKEIEALQQQLVEAETAAGIKQIPVPKVVKSVEKSNPPAAASASPVKQAKTATETPSKEKKGAEKKAAP

GGNKKTENAVGGDTIDIGRLDLRVGRIIKCEKHPDADALYVEQIDVGESTPRTVVSGLVRHVPLDQMQNRLVVVLCNLKP

AKMRGVESRAMVMCASSPEKVEIIEVDQLSQPGTPVVCPPYTHRPDAQLNPKKKIWETVAEDLKVSPDGYAVWKDCPLLV

GGKTKMTAPTLRGNELFEDDVVDNSRMNCYIFILYFRMPKKKGSERLGLEDERFAHLRSDPKFSGLSNKEKKVTIGKRFA

AVVTDPRFSAKARVDKYGRPVRKTGGDLLDLYEVEDSDEKEEVKTPEKKVKVSERAGEKRKTEKKVEEENDTDEKEPSDS

DDDRPIKIDLARGEGNVESSSDDDDSDSESEWQADEELQMEIDLAHLDDDAEQVEWATNRIAACNMDWNVVHCEDLMILG

KSFIPPGGSIKRITIYLSDFGAERLAEEDKHGPRLQLKKPLGEYDGDKIDDETKLAMRKYQVDQLRYYYAVIECDSTETA

VAIYDQCDGYQFEASNIKMDLRFVPEGMEFDKDRIKEELSEDDVNVGKYKSKEKIISAVAQTSARIGWDENEELRRKKFE

EAFNSDDDAGADLIADSDSDDEEREKSRQALMSLLGNKEEVDGLQVDWDNQQVEVKVTSNLIKNTYQAYLERRKQKKAER

RQKIKELKEKERDALKAVGDQAKAKQKENRAALKAAKQREEKEGVLVEAVAGDERFKALFTDSAFAIDQSSRNYKGSKLI

EKQVSVKRKANEQCQQSGDTNEPDLISKLKNKSAKWKKQK

>XP_005707242.1 methionyl-tRNA synthetase isoform 2 [Galdieria sulphuraria]

METRRVVNPQSKNILITSALPYVNNVPHLGNLVGCVLSADVYARFVRQRKENVLYICGTDEYGTATETKALAEGVTPREI

CDKFFKLHKDIYDWFDIRFDYFGRTSTSLQTEICQQIFWDIFHNGYVTEESLEQLFCESCGRFLADRYITGTCPMCGFED

ARGDQCDNCGRLLNPTDLLTARCSLCKNSPVVRKSDHLFLDLPALKDQLEKWIEKSSVKGDWSYNAIHSTYKWLNEGLKK

RCITRDLKWGTPVPLEKYKDKVFYVWFDAPIGYLSITANYTSGWKDWWQNPENVTLFQFMGKDNVPFHTVIFPATLIGTQ

RKWTMLHHISVTDYLNYEDGKFSKSRGVGVFGNDAKDTGIPTEVWRYYLLVNRPENADTVFSWDDFAAKNNNELLANLGN

FVNRTIAFLNSYFEARVPEYELQELDERYIQQVDEELQNYLQTMERVSLKNGLRIAMSISRLGNMYLQETAPWKWMNSDK

NRAATVLVVAVNICRLLAVVLEPFMGAVFSRKIFAQLNIPHTDETNYIPDKFHLHLVEPGHVVGKPFLLFSKITTEQASQ

LRKRFAGGQLKESSPIQTHGEEALAIEMRVGRIVQVEEHVDSNHLYICQVDLGEWGGIKQVVAGIRNYFTKDELLGRTVI

VVCNLQPACLAGVESQGMILTAEKKGKVHLLSCNGSGSPGDEDHNYHDVENKHDEERYGIGTTESPKVGTLVTINNTTMP

MVPLPILDRKAFQNASKLLKIDAQGHVVYNKKYKLIAGTQFVHGSGAQENAKVR

>XP_024195324.1 probable methionine--tRNA synthetase [Rosa chinensis]

MGDLAVTSKEGPKLPIPGQRNVLITSALPYVNNVPHLGNIIGCVLSADVFARYCRLRGYNTVYICGTDEYGTATETKALE

EKCSPKEICDKYHAIHRQVYEWFNISFDKFGRTSMPEQTEVCQAIFNRLLENNWLSENTMQQLYCDTCTRFLADRLVEGT

CPTQGCDYTSARGDQCENCGKLLNPTELKDPKCKVCKSTPRIRDTNHLFLELPLLKDRLEEYINKMSVEGSWSQNAIQAT

NAWLREGLKQRCITRDLKWGVPVPHDNFRDKVFYVWFDAPIGYVSITKCYTPDWEKWWKNPEDVELYQFMGKDNVPFHTV

MFPSTLLGTGENWTLMKTISVTEYLNYEAGKFSKSKGIGVFGNDARDTNIPVEVWRYYLLTNRPEVSDTLFTWADLQAKL

NSELLNNLGNFINRVLSFVAKPSGQGYDSIIPDAQDAISDPLTEKIAEKVGKCVEQYVEAMEKVKLKQGLKLAMSISSEG

NAYLQESQFWKLYKEDQPRCSIVMRTAVGLVHLLACLLEPFMPSFSVEVFKQLNLSPETHISLCDDKGDTDRARRPWEIV

PRGHKIGKPEPLFKELNDDEVDSLRKRFAGSQADRKEREEAEAVKLAEQLKKSKVSDKSGKKKQQATKSATEAEISISRL

DIRVGLITKAQKHPDADALYVEEIDVGEGQPRTVVSGLVRFIPLEEMQNRKVCVLCNLKPATMRGIKSQAMVLAASNSDH

TTVELVDPPKSAQVGERITFPGFQGEPDDVLNPKKKVWETLQVDLHSNPELVACYKDIPLTTSAGVCTVKSIRGGSIR

>XP_010264123.1 PREDICTED: probable methionine--tRNA synthetase [Nelumbo nucifera]

MGDVSDTAGHRKVPKLPIEGRRNILITSALPYVNNVPHLGNIIGCVLSADVFARYCRLRGYNTIYICGTDEYGTATETKA

MEENCTPQQICDKYHAIHREVYKWFDISFDEFGRTSTPQQTEICQAIFKKLLENNWLSENTMQQLYCNTCKRFLADRFVE

GTCPTPDCNYESARGDQCEKCGKMLNPTELKDPKCKICRTTPHIRDTDHMFLELPLLKHKLEEYINNMSVAGSWSQNAVQ

ATNAWLKEGLKPRCITRDLKWGVPVPHDKFKDKVFYVWFDAPIGYVSITKCYTDEWEKWWKNPENVELFQFMGKDNVPFH

TVMFPSTLLGTGENWTLMKTISVTEYLNYEAGKFSKSKGVGVFGNDAKDTKIPAEVWRYYLLTNRPEVSDTLFTWADLQA

KLNSELLNNLGNFINRVLSFIVKPQGLGYASIIPDAPDAETHELTKILAEKVGKLVEQYVESMEKVKLKQGLKIAMSISS

EGNSYLQDSQFWKLYKDDQCSCSIVMRTSVGLVYLLASLLEPFMPSFSVEVLKQLNWPKTQLSLCDEKGDLDNARRPWEI

LPAGHEIGVPSPLFKELKDEEVEFFREKFAGSQADRVLKAEAEANKVAEQLKKTKVSDGDDKKRPNKSTVVAKLKATAEV

VSISRLDIRVGLIKKVQKHPNADSLYVEEIDVGEGSPRTVVSGLVKYIPLEEMQNRRVCVLCNLKPVTMRGVKSQAMVLA

ASNNDHTKVELVDPPQSAPVGERITFPGFHGEPDDVLNPKKKVWETIQVDLHTDTELVAHYKNVPFTTSAGICKVASIAG

GTIQ

>XP_007218925.1 probable methionine--tRNA synthetase [Prunus persica]

MGDLTVTSNEDQKAAKLPIPGKRNILITSALPYVNNVPHLGNIIGCVLSADVFARYCRLRGYNTVYICGTDEYGTATETK

AIEEQCSPQEICDKYHAIHREVYQWFNISFEKFGRTSTPQQTEVCQAIFNKLLENDWLSENTMQQPYCDTCKRFLADRLV

EGTCPTQGCDYASARGDQCENCGKLLNPTELKDPKCKVCKTTPRIRDTNHMFLELPLLRDKLEEYINKTSVAGSWSQNAI

QATNAWLKEGLKQRCITRDLKWGVPVPHDNFKDKVFYVWFDAPIGYVSITKCYTDDWEKWWKNPENVELYQFMGKDNVPF

HTVMFPSTLLGTGENWTLMKTISVTEYLNYEAGKFSKSKGIGVFGNDAKDTNIPVEVWRYYLLTNRPEVSDTLFTWADLQ

AKLNSELLNNLGNFINRVLSFVAKPSGQGYGSIIPDAPSADSDLLTEKLAEKVGKCVEQYIEAMEKVKLKQGLKTAMSIS

SEGNAYLQESQFWKLYKEDQPRCAVVMRTSVGLVHLLACLLEPFMPSFSLEVFKQLNLPPETHISLCDDKGDIDRARRPW

EIVPIGHKIGKPEPLFKELKDEEVESLRKKFAGSQADRKEREEAEAVKVAAQLKKMKVSDNSGKKKQQATKSAAEAEISI

SRLDIRVGLITKAQKHPDADSLYVEEIDVGEGQTRTVVSGLVKYIPIEEMQNRKVCVLCNLKPATMRGIKSQAMVLAAST

SDHATVELVDPPKAAQVGEKVTFPGFVGEPDEVLNPKKKVWETLQVDLHTNTDLVACYRDIPLTTSAGVCTVSSICGGSI

R

>XP_008380519.1 PREDICTED: probable methionine--tRNA synthetase isoform X1 [Malus domestica]

MGDLKANSKGDQKVAKLPIPGKRNILITSALPYVNNVPHLGNIIGCVLSADVYARYCRLRGYNTLYICGTDEYGTATETK

ALEEKCSPQEICDKYHAIHRDVYEWFNISFDKFGRTSSPEQTEVCQAIFKKLLENDWLTENTMQQLYCDTCERFLADRLV

EGTCPTQGCGYASARGDQCENCGKLLNPTELKDPKCKVCKSTPRIRDTNHLFLELPLLKDKLEQYINEMSVAGSWSQNAI

QATNAWLKEGLRQRCITRDLKWGVPVPHDNFRDKVFYVWFDAPIGYVSITKCYTDDWEKWWKNPENVELYQFMGKDNVPF

HTVMFPSTLLGTGENWTLMKTISVTEYLNYESGKFSKSKGIGVFGNNAKDTKIPVEVWRYYLLTNRPEVSDTLFTWADLQ

AKLNSELLNNLGNFINRVLSFVAKPSGQGYGSIIPDAPQAEADPLTEKLAEKVGKYAEQYIEAMEKVKLKQGLKTAMAIS

SEGNAYLQESQFWKLYKEDQPRCAIVMRTSVGLVHLLACLLEPFMPSFSLEVFKQLNLPPETHLSLCDDEGDIDRARRPW

EIVPIGHKIGKPEPLFKELKDEEVEFWRQKFAGSQADRKEREEAEAIKVAAQLKKTKVSADSTGKKKQQPTKSAAEAEIS

ISRLDIRVGLITKAQKHPDADSLYVEDIDVGEGQTRTVVSGLVKYIPLEKMQNRKVCVLCNLKPANMRGIKSEAMVLAAS

NSDHTTVELVEPPEGAQVGERVTFPGFVGEPDEVLNPKKKVWETLQVDLHTNTELVACYKDVPLTTSAGVCTVSSIAGGS

IK

>ORZ20370.1 tRNA synthetases class I (M)-domain-containing protein [Absidia repens]

MVRPINVTVPKLVPSQRHYANTLKLLIAAEAAGVKINQIPGDTLTILLDDMTIHDPNVAVRYILSATTFDLYQSLAIEKE

TTVFGPLINKKKYDVVLQDINQFMNDNSAFGNTDKLTATDIIYFGSIYEALSDIDAAKYPKLAAWSHIISQASAVKTALE

IIGQQVQVKAPKKKHQATEKKAAEVTPLAELNPATQKLNDKVYYVPKPEGEKQTPVEGERNILITSALPYVNNIPHLGNI

VGSTLSADVYARYCRIRGYNTLYICGTDEYGTATETKALEEGVSCQALCDKYNAIHASVYKWFGLSFDYFGRTTTEKQTQ

ITQDIFHKVNENGFVVQDTMTQLFCEQCQRFLADRYVEGVCPNCQYEDARGDQCDSCGRLLNATELIAPRCKLDGNAPIT

KDSRHLFLDLGKLQDQIESFNKKSSVEGKWSANGVNITGSWLKEGLRPRCITRDLKWGTPVPLEGFENKVFYVWFDACIG

YPSITANYTDDWEKWWKNPKNVKLYQFMGKDNVPFHSVIFPGTELATKEDWTLVNHISTTEYLNYEGGKFSKSRNVGVFG

TNAEETGIPPSVWRYYLLSGRPESSDSMFTWNEFITKNNTELLNNLGNFVNRAIKFVLAKYDGILPPASETPLDGANEKS

LVKDVNELLAQYVDQLEQVKIRAGLATAMAISARGNLYLQESNLSNGLYNDQRPQCNAVVTTAINLIYILSSLISPYMPT

TCDSISRQLNAPLRLIPDTFSYDIRGGHKLNGAEYLFTRIDEKMEDVWKAKYGGNSKK

>XP_004296962.1 PREDICTED: probable methionine--tRNA synthetase [Fragaria vesca subsp. vesca]

MGDLAAAPKLPIPGQRNVLITSALPYVNNVPHLGNIIGCVLSADVFARYCRLRGYNTVYICGTDEYGTATETKALEEKCS

PQEICDKYHAIHKQVYEWFNISFDKFGRTSTPEQTEVCQGIFNRLLENNWLSENTMQQLYCDTCTRFLADRLVEGTCPTQ

GCDYASARGDQCENCGKLLNPTELKDPKCKVCKSTPRIRDTNHLFLELPLLKDRLKEYIDKMSVEGSWSQNAIQATNAWL

KEGLRQRCITRDLKWGVPVPHDNFRDKVFYVWFDAPIGYVSITKCYTPDWEKWWKNPEDVELYQFMGKDNVPFHTVMFPS

TLLGTGENWTLMKTISVTEYLNYEAGKFSKSKGIGVFGNDAKDTNIPVEVWRYYLLTNRPEVSDTLFTWADLQAKLNSEL

LNNLGNFINRVLSFVAKPSGQGYDSIVPDAPDAVSDPLTEKIGEKVGKCVEEYVEAMEKVKLKQGLKLAMSISSEGNAYL

QESQFWKLYKQDRPRCSVVMRTAVGLVHLLACLLEPFMPSFSVEVFKQLNLPAETHISLCDDKGDIDRARKPWEIVPIGH

KIGKPEPLFKELNDDEVDFLRKKFAGSQADRKEREEAEAVKLAEQLKKSKLSDKTGKKKQQATKSATEAEISISRLDIRV

GLITKAQKHPDADSLYVEEIDVGEGQPRTVVSGLVKFIPLEEMQNRKVCVLCNLKPATMRGIKSQAMVLAASNSDHTAVE

LVDPPLSAQVGERITFPGFEGEPDDVLNPKKKVWETLQVDLQSNNELVACYKDIPLATSAGVCTVKSIRGGAIR

>GAO48471.1 hypothetical protein G7K_2644-t1 [Saitoella complicata NRRL Y-17804]

MAAVKITIPSAPKESPLFTQAIKVCVAAEAYKSKNVALAGGAAQKTVTLELAKEFKTLIEANAIVRYLFTHAFPESQISI

LEAALIEWEESFLTPAVQDKAADLDRICASVSSVIEKAGFTEAQPIPAEIIIFATLYNKLSTADLSKFPVLEKWFKDFTG

KEYVKKGVELAAQHTSAGKVSVPQDTTPAGPAARKVKAGIEMKTLYEKEILPRKGEKNILITSALPYVNNVPHLGNIVGS

VLSADVFARYNKARNFNTLFICGTDEYGTATETKALEDGVTPKELCDKYHAIHADVYRWFEIGFDHFGRTTTPKQTEIAQ

DIFMRLYEKGYLQEDTMTQLWCEKHQGFLADRYVEGTCPKCLYEDARGDQCDKCGQLLNASELIEPRCKLDGNKPVLRDS

KHIFLALDKLQEKVEAWSKESAEKGKWSANGRAITESWLKEGLNPRCITRDLKWGTPVPLEGFTDKVLYVWFDATIGYVS

ITANYTDKWQEWWQNPEDVKLYQFMGKDNVPFHTVVFPATLLGTGQNWTMLHHINTTDYLNYEGGKFSKSRNVGVFGNNA

QQTGLSPSVWRYYLIMARPETSDTQFTWKEFVSRNNTELLANLGNFVNRVIKFVNAKYGGVIAEYSSDLAEDDESKIAQL

KENVNELLAQYNKDLEASRLRAGLQTVMAISARGNVFLQDNKLDNTLFAEHPARCANVLAAALNLIYLISALAYPYMPST

SSSIAEQLNAPLRAIPDVWSNDDLQTGHKLGKAKYLFGRVDEKMEEEWRAKFGGGSGTPVVDEKKEAKKAQKEAKKAKKA

AEKAASAEGEKKEEEVKARACELFGKYDSASARVHFTFNIDKKMRGSHGVYYVDDSTHSLSHPRIVARERSGGPSDDATF

APSQGKQAGSYLQAIRAIDASDMAMVEDSTPTTAQSVTSDPETVDASKHPIDPNDASQDDLQPGETYRIPENFAMISPGL

YRSSLPKPPSFPYLKKLGLKTIIRLNQEEYPEENVAFCARNGIRTYHFGVPGNKEPFVKVPEEEITRALAVILDLANHPI

LIHCRSGRHRTGCLVGCVRRIQNWALTVIFDEYRSFANPKSRSLDQQFIELYDLGRVWRRIGTKGIERLPWRLEVGEEAR

AQAMIGEEKECDGEGGEIDDEGYGKSLPNGKNSNNVEIGRLEYGNRVWQLKPERGNRITEQGTRSRRQESPTGGVLHAWI

RLLGTYNLQDYNDGIYNQYYIYA

>XP_001382405.1 methionyl tRNA synthetase [Scheffersomyces stipitis CBS 6054]

MSALYASKKSTLEALATNLKLSIASSAFQKQVSVSVNEDADSTALLVDKKAGFELSEPNAVVKYLSSDFTDASGAIAFEE

SQLYPALKANSADKISQIATPAYFEKAEITAAQIILFSALYPALKDSKSTSETTEIAKWFQDFASIEVVQSGIKHALSIT

TLERPKAVNTGAQKVKTGHLVKPQAERIVPKDNERNILITSALPYVNNVPHLGNIIGSVLSADIYARYAKNRNYNAVYVC

GTDEYGTATETKALEEKVTPQQLCDKYHKIHKEVYDWFDIGFDYFGRTTTPLQTEIAQDIFTRLHKNNYLEERTTEQLYC

EEHKSFLADRFVEGTCPHCQYEDARGDQCDKCGNLLDPLQLIKPRCKVDGATPVVRESTHIYLKLNELEGELAEWVDHAS

SHGEWSKNAKTITASWIKRGLEPRCITRDLSWGTPVPLEGYEEKVLYVWFDATIGYVSITANYFKDHNGDDWRKWWKNPE

NVDLYQFMGKDNVPFHTVVFPASQIGTRDVWTKLHHLSTTEYLQYENGKFSKSRGVGVFGNSAKDTGIPASVWRYYLASV

RPETSDSQFSWEEFVTKNNSELLANLGNFVNRIVKFVIAKYNGVIPKFDTKNVPDYEKFETEVNQLLKSYVDTMEAVSLR

KGLELAMAISSRGNQFLQDNKLDNSLYANLPDKSDAVVGVALNLVYLVSAIIFPFMPETTKQINEILNAPALSITDKFEL

VLEAGHNIGKAQYLFKRIDEKKIAEWRSLYGGQQEKK

>XP_008380520.1 PREDICTED: probable methionine--tRNA synthetase isoform X2 [Malus domestica]

MGDLKANSKGDQKVAKLPIPGKRNILITSALPYVNNVPHLGNIIGCVLSADVYARYCRLRGYNTLYICGTDEYGTATETK

ALEEKCSPQEICDKYHAIHRDVYEWFNISFDKFGRTSSPEQTEVCQAIFKKLLENDWLTENTMQQLYCDTCERFLADRLV

EGTCPTQGCGYASARGDQCENCGKLLNPTELKDPKCKVCKSTPRIRDTNHLFLELPLLKDKLEQYINEMSVAGSWSQNAI

QATNAWLKEGLRQRCITRDLKWGVPVPHDNFRDKVFYVWFDAPIGYVSITKCYTDDWEKWWKNPENVELYQFMGKDNVPF

HTVMFPSTLLGTGENWTLMKTISVTEYLNYESGKFSKSKGIGVFGNNAKDTKIPVEVWRYYLLTNRPEVSDTLFTWADLQ

AKLNSELLNNLGNFINRVLSFVAKPSGQGYGSIIPDAPQAEADPLTEKLAEKVGKYAEQYIEAMEKVKLKQGLKTAMAIS

SEGNAYLQESQFWKLYKEDQPRCAIVMRTSVGLVHLLACLLEPFMPSFSLEVFKQLNLPPETHLSLCDDEGDIDRARRPW

EIVPIGHKIGKPEPLFKELKDEEVEFWRQKFAGSQADRKEREEAEAIKVAAQLKKTKVSDSTGKKKQQPTKSAAEAEISI

SRLDIRVGLITKAQKHPDADSLYVEDIDVGEGQTRTVVSGLVKYIPLEKMQNRKVCVLCNLKPANMRGIKSEAMVLAASN

SDHTTVELVEPPEGAQVGERVTFPGFVGEPDEVLNPKKKVWETLQVDLHTNTELVACYKDVPLTTSAGVCTVSSIAGGSI

K

>XP_006679786.1 hypothetical protein BATDEDRAFT_12267, partial [Batrachochytrium dendrobatidis JAM81]

PKKNERNVLITSALPYVNNIPHLGNLIGSVLSADVYARYSRIRGENVLFICGTDEYGTATETKAIEEKLSCKQLCDKYFK

FHDETYKWFQIDFDHFGRTITEQQTIITQDIYLKLEKNGHVMEDTVTQLYCNQHESFLADRFVEGTCPLCGYEDARGDQC

DSCGKLLNAIELKNPRCKLDNAAPVARDSKHLFLNLTGQQEKLTEWFKKASSEGCWSANSQTITNSWLKEGLKPRCITRD

LKWGTPVPKEGYENKVFYVWFDAPIGYFSITANYTKDWELWWKNPKDVKLYQFMGKDNVPFHTVIFPSTLLGTNENWTLL

HHLSTTEYLQYESGKFSKSRGVGVFGNNVVDSGIPVEVWRYYLLSIRPESGDSYFSWNSFIAANNTELLANLGNFVNRLV

KFINAKYDGVIPAYKTTGEPETKLISSVNTLLHQFIEALNDVKIRTGLRLFMDISARGNLYLQENRIDNSLFANSRERCD

NVVAVAANLAYLISAVVYPYMPSTSEGILRQLNLPMRRITDTWNAEDITAGHKIGAAEYLFKRLDESLEEKLRQKYSGKQ

DPAAKTGTDSGTATGGSKSKKSKSAKAAVPASNVVKTPEMIAVETRIAEQGGIVRDLKTAKADPNVVKEAVDALLALKAQ

LAALTTA

>PON36032.1 Methionyl-tRNA synthetase [Parasponia andersonii]

MADHSGGRGGVVKNPKLPIPGKRNILVTSALPYVNNVPHLGNIIGCVLSADVFARYCRLRGYNAIYICGTDEYGTATETK

AMEENSTPQQICDKYHAIHKEVYDWFNISFDEFGRTSTPQQTEICQAIFTRLMENNWLSDNTMQQLYCEKCNRFLADRLV

EGTCPMLDCNYHSARGDQCEKCGKLLNPTELKDPKCKVCRTTPQIRDTDHLFLELPLLKDKLEEYISNMSVAGSWSQNAI

QATYAWLKEGLKPRCITRDLKWGVPVPHEKFKDKVFYVWFDAPIGYVSITSCYTSDWEKWWKNPDNVELYQFMGKDNVPF

HTVMFPSTLLGTGENWTMMKSISVTEYLNYESGKFSKSKGIGVFGNEAKDTNIPSEVWRYYLLTNRPEVSDSLFTWVDLQ

AKLNSELLNNLGNFINRVLSFITKPQGIGYGSIIPDSPGAELHPLTRTLAEKISKFVDQYIEAMEKVKLKQALKIGMSIS

SEGNAYLQESQFWKLYKQDQDSCNIVIRTSVGLVFLLACLLEPFMPSFSLKVLKQLDIPPENQLSLSNESGDLERARKPW

EFISAGHKIGTPEPLFKELKDEDVELFRTKFAGSQADRIVKLEEANEASKMAEQLAKTKI

>XP_021806686.1 LOW QUALITY PROTEIN: probable methionine--tRNA synthetase [Prunus avium]

MGDLTITSNEDQKAAKLPIPGKRNILITSALPYVNNVPHLGNIIGCVLSADVFARYCHLRGYNAVYICGTDEYGTATETK

AMEEKCSPQEICDKYHAIHREVYQWFNISFEKFGRTSTPQQTEVCQAIFKKLLENDWLSENTMQQLYCDTCKRFLADRLV

EGTCPTQGCDYASARGDQCENCGKLLNPTELKDPKCKVCKRTPRIRDTNHMFLELPLLRDKLEEYINNTSVAGSWSQNAI

QATNAWLKEGLKQRCITRDLKWGVPVPHDNFKDKVFYVWFDAPIGYVSITKCYTDDWEKWWKNPENVELYQFMGKDNVPF

HTVMFPSTLLGTGENWTLMKTISVTEYLNYESGKFSKSKGIGVFGNDAKDTNIPVEVWRYYLLTNRPEVSDTLFTWADLQ

AKLNSELLNNLGNFINRVLSFVAKPSGQGYGSIIPDAPSADSDLLTEKLAEKVGKCVEQYIEAMEKVKLKQGLKTAMSIS

SEGNAYLQESQFWKLYKEDQPRCAVVMRTSVGLVHLLACLLEPFMPSFSLEVFKQLNLPPETHISLCDDKGDIDRARRPW

EIVPVGHKIGKPEPLFKELKDEEVESLRKKFAGSQADRKEREEAEAVKVAAQLKKMKVSDNSGKKKQQATKSAAEAEISI

SRLRTKSAAELEISISRLDIRVGLITKAQKHPDADSLYVEEIDVGEGQTRTVVSGLVKYIPIEEMQNRKVCVLCNLKPAT

MRGIKSQAMVLAASTSDHATVELVDPPKAAQVGERVTFPGFVGEPDEVLNPKKKVWETLQVDLHTNTDLVACYRDIPLTT

SAGVCTVSSICGGSIR

>ORX44635.1 methionyl-tRNA synthetase [Piromyces finnis]

MGFPAEQKVDTKNSIILNHHERVILPVEGKRNILVTSALPYVNNVPHLGNIIGAMLSADVFSRYCRMRDYNIIFINGTDE

YGTATETKALEEKCTCQALCDKYHALHKEVYKWFGIDFDYFGRTTTPKQTEIAQDIFLKLNKNGYLKEDTVTQLFCNTCK

LFLADRFVEGECPFCHYDDARGDQCDKCGKLINAVELIKPRCKVCNSTPETKTSNHIFIDLPKLQDKCAEFVKKNSVEGD

WSSNSISITNGWLKEGLKPRCITRDLKWGTPVPLEHMKDKVFYVWFDAPIGYISMTANYTDEWKKWWMNPEQVQLYQFMG

KDNIPFHTVIFPCSEIGTGDNYTLLNRLSTTEYLNYENGKFSKSRNVGVFGNNAVDTGVPVEVWRYYLLINRPETNDSSF

TWNDFIARNNNELLNNIGNFVNRLLKFVAAKYDSVIPEYDLSGPSEQKLIEDVNKLLKDYLRNMEACKLRAGLKTAMDIS

QVGNAYLQENKIDNKLFTEKRQVCNNVVAASVNLIYLLSSLIYPYMPTTTDSILKQINAPLRRITDVWEANEILPGHTIG

KPAYLFKRIDPEMENVWREKYGAAKKQKKAPKENKKAAPAAAAAAETGPDPSKLDIRVGHIIEAHKHEQADSLYVETIDL

GEPEPR

>XP_002868338.1 methionine--tRNA synthetase, cytoplasmic [Arabidopsis lyrata subsp. lyrata]

MEDDGKSNPKLPIPGKRNILITSALPYVNNVPHLGNIIGCVLSADVYARYCRLRGYNAIYICGTDEYGTATETKALEENC

TPKEICDKYHAIHKEVYDWFDISFDKFGRTSTPEQTEVCQAIFKKLWDNKWLSENTMQQLYCDTCKKFLADRLVEGSCPF

EGCNYDSARGDQCEKCGKLLNPTELKDPKCKVCQNTPRIRDTDHLFIELPLLKDRLEKYINETSVAGSWSQNAIQTTNAW

LRDGLRQRCITRDLKWGVPVPHEKYKEKVFYVWFDAPIGYVSITSCYTSEWEKWWKNPENVELYQFMGKDNVPFHTVMFP

STQLGTGENWTLMKTISVTEYLNYEDGKFSKSKGVGVFGNDVKDTNIPVEVWRYYLLTNRPEVSDTSFSWTDLQAKLNGE

LLNNLGNFVNRVLSFIAKPNNAGYGSVIPDAHDAESHPLTKSLAEKVEKFVAEYVEAMEKVKLKQGLKTAMLISSEGNFY

LQTSQFWKLYKEDKPLCAIVIRTAAGLVHLLAQLLEPFMPSFSCEVFKQLNLPPQFSLSDERGEVLLASKPWEILPPSHR

IGTAQPLFKELENDEVTQYREKFAGSQADRRARDEAANLTDQLNKTKLSDAKKQKASSSKGGGKPKPQPAADREITMARL

DIRVGKIVKAERHPKADTLYVEEIDVGGGEIRTVVSGLVKYIPLEEMQNRMVCVLCNLKPAKMRDIVSQAMVLAASSSDG

SKVELVEPPESANIGERVTFPGFEGEPDDVLNPKKKVWETLLVDLNTNENLVACYKDVPFTTSAGVCKVSSISNGTIR

>XP_009371840.1 PREDICTED: probable methionine--tRNA synthetase [Pyrus x bretschneideri]

MDDQSDGGEGNSQRNPKLPVPGKRNILITSALPYVNNIPHLGTIIGCVLSADVYARYCRLRGYNAIYICGTDEYGTATET

KAREENCTPQQICDKYYEIHKQVYEWFDISFDKFGRTSTPQQTEICQHIFKKLMENGYFSENTIQQLFCETCDRFLADRL

VEGTCPTLDCGYDSARGDQCDKCGKLLNPTELKDPRCKACQARPCIRETNHLFLELPLLKDKVEEFINNLSVEGSWSPNA

IQTTYAWLREGLKVRCMTRDLKWGVPVPLEKFEDKVFYVWFDAPIGYISITSCYTPDWEKWWKNPGNVELYQFMGKDNVP

FHTVMFPCTLLGTGENWTLMKSLSVTEYLMYETGKFSKSRGIGIFGNDVQETNIPSEVWRYYLLANRPEVSDTVFTWADL

QAKLNAELLNNLGNFINRVLIFIAKPQGQGYGSIIPEASGAESHSLTKRLAERIGIYVDQYIEAMEKVKLKQALKIAMSI

SSEGNAYLQENQFWKLYKQDQASCCIVIKTSAGLVYLLACVLEPFMPSFSRKVLKQLNLPPENQFSLCNEDVERARKPWE

ILPDGHKIGTPEPLFKELKDEDVEFFRSQFSGSQAERVAKGEAEAKKMAEKLEQTKI

>XP_024018664.1 probable methionine--tRNA synthetase [Morus notabilis]

MGDVSNSESNLADRKVPKLPIPGQRNILITSALPYVNNVPHLGNIIGCVLSADVFARYCRLRGYNAIYICGTDEYGTATE

TKAMEEKCSTKEICDKYHAIHRDVYKWFNISFDEFGRTSAPQQTEVCQAIFKKLLDNNWLSENTMQQLYCDTCKRFLADR

LVEGSCPTPGCGYDSARGDQCENCGKLLNPTELTDPRCKVCGTTPHIRDTNHLFLELPLLKDKLVEYINKTSVAGSWSQN

AIQATNAWLKEGLKQRCITRDLKWGVPVPHDKFRDKVFYVWFDAPIGYVSITSCYTPEWEKWWKNPEEVELFQFMGKDNV

PFHTVMFPSTLLGTGENWTLMKTISVTEYLNYEAGKFSKSKGIGVFGNDAKDTNIPVEVWRYYLLTNRPEVSDTLFTWAD

LQAKLNSELLNNLGNFINRVLSFIAKPSGQGYDSTIPDAPNDVSDPVTEKLAERVGKYVEQYVEAMEKVKLKQGLKTAMS

ISSEGNAYLQESQFWKLFKEDKPRCSIVMKTSVGLVHILACLLEPFIPSFSLEVFKQLNLSPEQLSLADGKGDIDRAKRP

WLIVPSGHKIGKPEPLFKELKDEEVESFRKRFAGSQADRIERAEAEAEKVAEQLKNIKVSDNSGKKQKSTKSEKSIKSEK

SAKSATEPEISISRLDIRVGLITRAQKHPDADSLYVEEIDVGEGQPRTVVSGLVKYIPLEEMQNRKVCVLCNLKPATMRG

IKSQAMVLAASNSDHTKVELVEPPKSAYVGERVSFPGFAGQPDDVLNPKKKVWETLQVDLHTNSHLVACYKDIPFTTLAG

VCTVSTIPEGSIR

>ORX91886.1 methionyl-tRNA synthetase [Basidiobolus meristosporus CBS 931.73]

MTDTTEKIVRPVADESNRKVREGIALNLNKDVKLPVKGERNILITSALPYVNNVPHLGNIIGAVLSADVFARFTRSRGYN

VLYVCGTDEYGTATETKALEEGVSCQELCDKYNALHAQVYEWFGVDFDYFGRTTTNQQTEIGQDIFLKLHKNGYLIEDTM

TQLFCEKCQRFLADRYVEGTCPKCGYVDARGDQCDQCQQLLNATELISPRCKLDGNAPITRDSTHMFLDLPKLQQKCEEF

VEESSQKGSWANNGKIITQSWLKEGLKARCITRDLKWGTPVPLEHMKDKVFYVWFDAPIGYPSITANYTSEWEKWWKNPE

DVKLYQFMGKDNIPFHTVIFPCSLIGTEDPWTMLHHISTTEYLNYENGKFSKSRNVGVFGNNVMDTGIPVAVWRYYLLSN

RPETNDSVFTWKDFITRNNSELLANVGNFCNRVIKFVDSAKYNGTLPAFSLEGENEKQLIEDVNKLLDAYIDALENVKLR

SGLEIAMKISQRGNQYLQESKLDNTLYNNERAQCDTVVTVAINLIYLLSAVFYPYMPTTSESICRQLNAPLPRIPETFQM

NILEGHVIGKPEYLFKRIDGKMEEVYKQKYGGGAKEEPAKKKKSKKSKKPAEPKPQADGEKAEEAAPPAEDAKTGESA

>XP_019252161.1 PREDICTED: probable methionine--tRNA synthetase [Nicotiana attenuata]

MGENCKTFPKLPIPGKRNILITSALPYVNNVPHLGNIIGCVLSADVFARYCRLRGYNAIYMCGTDEYGTATETKALEENC

TPKEICDEYHAIHKEVYKWFDISFDEFGRTSTPQQTEVCQAIFKKLFENNWLSENTMQQLYCDTCKKFLADRLVEGNCPT

PGCNYDSARGDQCEKCGKLLNPTELMEPKCKVCRNTPCIRDTDHLFLELPLLKDRLEAYVNNMSVAGGWSQNAIYTTHAW

LREGLKPRCITRDLKWGVPVPHEKYKDKVFYVWFDAPIGYVSITSCYTTEWEKWWKNPDDVELYQFMGKDNVPFHTVIFP

STLLGTGENWTLMKTISVTEYLNYEAGKFSKSKGIGVFGNDAKDTNIPVEVWRYYLLTNRPEVSDTLFTWADLQAKLNSE

LLSNLGNFVNRVLSFIAKDSASGYGSIIPDVEGAESHPLTKALGEKVGNYVEQYIEAMEKVKLKQGLKVAMSVSGEGNGY

LQESQFWKLYKEDRRSCSIVMRTACGLVYLLACLLEPFMPSFSREVLKQLNLPPETQLSLADEKGDVANSKRPWNIIPAG

HKIGTPAPLFKELKDEEVEFYREKFAGSQADRADRALKAETDAKKMAEQLNKTKISDGNKKERAKKSSETKSKAPATVEG

EISISRLDIRVGLITKAQKHPNADSLYVEEIDVGEALPRTVVSGLVNYIPLEEMQNRKVCVLCNLKPVSMRGIKSQAMVL

AASNSDHTKVELVEPPKDAAIGERVTFPGFDGKPDDVLNSKKKIWETVQLDLHTNKELVACYKDLPFTTSSGICKVASIS

EGSIR

>XP_010247959.1 PREDICTED: probable methionine--tRNA synthetase [Nelumbo nucifera]

MADVSDAAGDRKAPKLPIEGHRNILITSALPYVNNVPHLGNIIGCVLSADVFARYCRLRGYNAIYICGTDEYGTATETKA

MEENCTPQQICDKYHAIHREVYKWFDISFDEFGRTSTPQQTEVCQAIFKKLLENNWLSENTMQQLYCDTCKRFLADRFVE

GICPTPACNYESARGDQCEKCGKMLNPTELKDPRCKLCQTTPRIRDTDHLFLELPLLKDKLEEYINKMSVAGSWSQNAVQ

ATHAWLKEGLKQRCITRDLKWGVPVPHEKFKDKVFYVWFDAPIGYVSISKCYTNEWEKWWKNPENVELYQFMGKDNVPFH

TVMFPSTLLGTGENWTLMKTISVTEYLNYEAGKFSKSKGVGVFGNDAKDTNIPVEVWRYYLLTNRPEVSDTLFTWVDLQA

KLNSELLNNLGNFINRVLSFVAKPQGLGYASVIPDAPGAECHQLTNTLAEKVGKLVEQYVEAMEKVKLKQGLKIAMSISS

EGNAYLQDSQFWKLYKDDQASCSIVMKTSIGLVYLLASLLEPFMPSFSAEVLKQLNCPQTQLSLCDEKGDVERAKKPWEI

LPAGHEIGVPAPLFKELKDEEVQYFRERFAGSQADRVLKAAEAEAKKVAEQLKKTKISDGNGKKKPSKSTTETKPKAAAE

AEISISRLDIRVGIITKVQKHPDADSLYVEEIDVGEGSXRTVVSGLVKYIPLEEMQNRKVCVLCNLKPATMRGVKSQAMV

LAASNSDHTKVELVDPPQSAPVGERLTFPGYEGEADGVLNPKKKVWETVQVDLHTDNELVARYKDVAFTTSAGVCKVASI

SGGSIR

>XP_006842391.1 probable methionine--tRNA synthetase [Amborella trichopoda]

MGDATGNGGPKLPIPGMRNIMITSALPYVNNVPHLGNIIGCVLSADVFARYSRLRGYNTIYMCGTDEYGTATETKALEEN

CTPQEICDKYYAIHNEIYEWFDISFDKFGRTSCPDNTEISHAIFHKLLENNWLSENTMQQLYCDTCQRFLADRLVEGACP

TPGCGYEDARGDQCEKCGKLLNPNELIDPRCKVCRSSPRSRDTNHLFLELPLLKDKLEEYVKTTSVAGSWSQNAIQTTNS

WLKEGLKPRCITRDLKWGVPVPHEKFKDKVFYVWFDAPIGYVAITSSYTSEWAKWWKDPENVELFQFMGKDNVPFHTVMF

PSMLFGTGENWTLMKTISVTEYLNYEAGKFSKSKGVGVFGNDAKDTKIPVEVWRYYLLTNRPEVSDTLFTWTDLQAKLNN

ELLKNLGNFIHRVLSFIAKPEGEGYGSVIPDAPNADSHLLTKKLAETVGNLVEQFVEAMEKVKLKQGLKIAMSVSSEGNA

YLQDNQFWKLHKTDPTSCATVMKTSVGLVHLLASLLEPFMPSFSRKVVAQLNLPCESLSICKQKGDAARERRPWDIVPAG

HKIGKPEPLFFEMKDDEVNDYRERFAGSQSDRAVKAEAEAQKIAEHLKKTTVSDGTTKKKGQSKLPAAEKEKLKEVEISV

ARLDIRVGLVTKVQKHPDADSLYVEEIDVGEESPRTVVSGLVKYIPLEKMQNRKVCVLCNLKPATMRGIKSQAMVLAAST

EDHTIVELVDPPENARVGERVTFPGFSGEPDEVLNPKKKVWETVQVDLRTTEDLVASYKGVPFTTSGGVCKVSSIACGSI

R

>GAQ88771.1 Methionyl-tRNA synthetase [Klebsormidium nitens]

MDQLEQGAKGSEGKELTLADVAVFWRLIPVLGGADARPSKVAEWFGTLVASKHVASAIKSILGDQNPQTLADQSRKAISA

SVTAASAKPKPSKSQTPAEKPAASKPSKPAPANASDSKIAAKAGSKGPVKAEAAVKPVVITDPGSPYLEVTGPPRAPVKG

KRNVLVTSALPYVNNVPHLGNIIGSVLSADVYARYCRRRGYNVIYMCGTDEYGTATENKALEEGLTPREICDKYHVIHRD

IYKWFDISFDHFGRTSTPQQTDIAQAIFRSLLDQNRLIEDSMEQLYCDLDQKFLADRFVEGTCPNYYKYKEARDADKCRK

LLNPTDLINPRCKICGTTPRIRSTEHLFLDLPTLKPQLEDYINATSGVGEWSANSIATTGSWIRDGLKPRCITRDLKWGV

PVPLEKFKDKVFYVWFDAPIGYISITANYTDSWKEWWHSPKDVELVQFMGKDNVPFHTVIFPSTLLGTAEPWTLMKTISV

TEYLNYEDGKFSKSRGVGVFGNDAQSTGVPSEVWRYYLLVNRPEVSDTVFMWSDLQAKTNGELLANLGNFVNRALKFLAD

RLDATIPEAKAAASNAAVVALGKEVQDNVREYVSKLEKAKLKDGLKIAMNISALGNKFFQDNKPWVLLKEDKPACEDIVA

TAVGLVRLLATLLEPYMPAFAHKVLKQLNLPPSSLSLTDADVELTSELWKLVPAGHKIGAPEPLFREMSNEEVAANKLKF

AGSQADRKAAESAATASAASGASSASTSSSGPATPAANNGKPKKEKDKGAKEPKGGSSRGAKEAEKPVDVSRLDIRVGKI

VSVKKHPDADSLYVEEIDVGEAEPRTVVSGLVKYIPLEGMQNQVILTVCNLKPVAMRGVKSHAMVLCASDAEKTKIEFVR

PPEGAKVGERITFPAYPGEPDEQLNPKKKVWETVQPGFVTTESLVATWKGDPFTTSAGVCTVASIPGGSIS

>CBI17224.3 unnamed protein product, partial [Vitis vinifera]

MTDPSNDAAHGRTAPKLPIQGKRNILITSALPYVNNVPHLGNIIGCVLSADVFARYCRLRGYNAIYICGTDEYGTATETK

AMEEKCTPQQICDKYHAIHKEVYKWFNISFDEFGRTSSPQQTEVCQAIFKKLMENNWLSENVMQQLYCDKCQKFLADRLV

EGICPTQDCNYDSARGDQCEKCGKLLNPIELKDPRCKFCHSTPHIRDTNHLFLELPLLKDKLEEYINLMSVAGSWSQNAI

QATHAWLKEGLKPRCITRDLKWGVPVPHEKFKDKVFYVWFDAPIGYVSITSCYTPDWEKWWKNPENVELYQFMGKDNVPF

HTVMFPSTLLGTSENWTLMKTISVTEYLNYEAGKFSKSKGVGVFGNDAKDTNIPVEVWRYYLLTNRPEVSDTLFTWVDLQ

AKLNSELLNNLGNFINRVLSFIAKPAGLGYGSVIPDAPGAELHQLTKTLAEKVGRFVEQYVEAMEKVKLKQGLKTAMSIS

SEGNAYLQESQFWKLYKEDQPSCSIVVKTSLGLVHLLACLLEPFMPSFSLEVLKQLNMPPETSFLLCDEKGDIERAKRPW

EIVPAGHRIGTPEPLFKELKDEDVEFFREKFAGSQADRIVKAEAEAKKIAEQLKKTKVSALCTSNYYLLHVGRFKYLKST

SDNQSVSEIATALHHVKASLSMSSFMLCNHNANKYLASNLLRLSIISLDIERFQWTKIYQCHCHHLQCLTIVIKAEPSLA

CMTYCVDMGVLGEVEEFRVSCVLAHTFFVLIMLVQSISVSFYGNGKKQRPTKSAEAKPKAAAEAEISISRLDIRVGLITK

VQKHPDADSLYVEEIDVGEGSTRTVVSGLVKFIPLEEMQNRKVCVLCNLKPATMRGIKSQAMVLAASNSEHTKVELVEPP

QSAAVGERVTFPGFEGEPDDVLNPKKKVWETLQVDLRTDSELVALYKDVPFTTTAGICKVSSICDGSIR

>XP_016160621.1 PREDICTED: methionine--tRNA synthetase, cytoplasmic [Ficedula albicollis]

MCPRCARPVTPDVTSLCPQPAVAAALYLRQDAASVADVVVWGSLFPVLQDETSLPSVTGVRLPGTGGWAVYRCYRCAPPW

DWGLGCAQVLQVCHTAVIGVHAQVLQVPCRCASCPCRCARCARCPCQVCQVPLQVCQVPCRCARCPCRRARHTCQVRQVW

QAAPPRRYCRLRGWNTLFVCGTDEYGTATETRALEEGLSPQQLCDRYHALHADVYRWFRISFDHFGRTTTPQQTRIAQDI

FQRLLARGFLLQDTLEQLRCESCGRYLADRFVEGTCPFCGYAEARGDQCEKCGKLINAVELKNPQCKLCRGTPVVTPTQH

LFLDLPKLEGPLEAWLERSWAAGDWTPNARHITRTWLRDGLKPRCITRDLTWGTPVPLDGFRDKVFYVWFDAPIGYLSIT

ANYTDQWERWWKNPQQVELYNFMAKDNVPFHSVVFPCSLLGADDNYTLVNHLIATEYLNYEDGKFSKSRGVGVFGDMAKD

TGIPADVWRFYLLYLRPEGQDSAFSWADLLLKNNSELLNNLGNFINRSGDAAGSRAAAPWLRDGLKPRCITRDLTWGTPV

PLDGFRAPAPAPNTSAAPGDPKLIQELTEQVAKQGNLVRQLKAAGGGVRQLKAAQVEKAQVDAQVAKLLELKQQLALAEG

RSPEVPKGKRKK

>XP_009779530.1 PREDICTED: probable methionine--tRNA synthetase [Nicotiana sylvestris]

MGENCKTIPKLPIPGKRNILITSALPYVNNVPHLGNIIGCVLSADVFARYCRLRGYNAIYMCGTDEYGTATETKALEENC

TPKQICDKYHAIHKEVYKWFDISFDEFGRTSMPQQTEVCQAIFKKLFENNWLSENTMQQLYCDTCKKFLADRLVEGNCPT

PGCNYDSARGDQCEKCGKLLNPTELMEPKCKVCRKTPCIRDTDHLFLELPLLKDRLEAYVNNMSVAGGWSQNAIYTTHAW

LREGLKPRCITRDLKWGVPVPHEKYKDKVFYVWFDAPIGYVSITSCYTTEWEKWWKNPDDVELYQFMGKDNVPFHTVIFP

STLLGTGENWTLMKTISVTEYLNYEAGKFSKSKGIGVFGNDAKDTNIPVEVWRYYLLTNRPEVSDTLFTWADLQAKLNSE

LLSNLGNFVNRVLSFIAKDSASGYGSIIPDVEGAESHPLTKALGEKVGNYVEQYIEAMEKVKLKQGLKVAMSVSGEGNGY

LQESQFWKLYKEDRRSCSIVMRTACGLVYLLACLLEPFMPSFSREVLKQLNLPPEMQLSLADEKGDVANSKRPWNIIPAG

HKIGTPAPLFKELKDEEVEFYREKFAGSQADRADRALKAETDTKKMAEQLNKTKISDGNKKERAKKSSETKSKAPATDEG

EISISRLDIRVGLITKAQKHPNADSLYVEEIDVGEALPRTVVSGLVNYIPLEEMQNRKVCILCNLKPVSMRGIKSQAMVL

AASNSDHTKVELVEPPKDAAIGERITFPGFDGEPDDVLNNKKKIWETVQLDLHTNKELVACYKDLPFTTSSGICKVASIS

EGSIR

>XP_008372495.1 PREDICTED: probable methionine--tRNA synthetase [Malus domestica]

MDDQSDGGEGNSQRNPKLPVPGKRNILITSALPYVNNIPHLGTIIGCVLSADVYARYCRLRGYNAIYICGTDEYGTATET

KAREENCTPQQICDKYYEIHKQVYEWFDISFDKFGRTSTPQQTEICQHIFKKLLENGYFSENTIQQLFCETCDRFLADRL

VEGTCPTLDCGYDSARGDQCDKCGKLLNTTELKDPRCKACQARPCIRETNHLFLELPLLKDKVEEFVNNLSVEGSWSPNA

IQTTYAWLREGLKVRCMTRDLKWGVPVPLEKFEDKVFYVWFDAPIGYISITSCYTPDWEKWWKNPKNVELYQFMGKDNVP

FHTVMFPSTLLGTGENWTLMKSLSVTEYLMYETGKFSKSRGIGIFGNDVQETNIPSEVWRYYLLANRPEVSDTVFTWADL

QAKLNTELLNNLGNFINRVLSFIAKPQGXGYGSIIPDASXAESHSLTKRLAERIGIYVDQYIEAMEKVKLKQALKIAMSI

SSEGNAYLQENQFWKLYKQDQASCCIVIKTSAGLVYLLACVLEPFMPSFSLKVLKQLNLPPENQFSLCNEDVERARKPWE

ILPDGHKIGTPEPLFKELKDEDVEFFRSKFSGSQAERVAKGEAEAKKMAEKLEQTKI

>XP_002269672.1 PREDICTED: probable methionine--tRNA synthetase [Vitis vinifera]

MTDPSNDAAHGRTAPKLPIQGKRNILITSALPYVNNVPHLGNIIGCVLSADVFARYCRLRGYNAIYICGTDEYGTATETK

AMEEKCTPQQICDKYHAIHKEVYKWFNISFDEFGRTSSPQQTEVCQAIFKKLMENNWLSENVMQQLYCDKCQKFLADRLV

EGICPTQDCNYDSARGDQCEKCGKLLNPIELKDPRCKFCHSTPHIRDTNHLFLELPLLKDKLEEYINLMSVAGSWSQNAI

QATHAWLKEGLKPRCITRDLKWGVPVPHEKFKDKVFYVWFDAPIGYVSITSCYTPDWEKWWKNPENVELYQFMGKDNVPF

HTVMFPSTLLGTSENWTLMKTISVTEYLNYEAGKFSKSKGVGVFGNDAKDTNIPVEVWRYYLLTNRPEVSDTLFTWVDLQ

AKLNSELLNNLGNFINRVLSFIAKPAGLGYGSVIPDAPGAELHQLTKTLAEKVGRFVEQYVEAMEKVKLKQGLKTAMSIS

SEGNAYLQESQFWKLYKEDQPSCSIVVKTSLGLVHLLACLLEPFMPSFSLEVLKQLNMPPETSFLLCDEKGDIERAKRPW

EIVPAGHRIGTPEPLFKELKDEDVEFFREKFAGSQADRIVKAEAEAKKIAEQLKKTKVSDGNGKKQRPTKSAEAKPKAAA

EAEISISRLDIRVGLITKVQKHPDADSLYVEEIDVGEGSTRTVVSGLVKFIPLEEMQNRKVCVLCNLKPATMRGIKSQAM

VLAASNSEHTKVELVEPPQSAAVGERVTFPGFEGEPDDVLNPKKKVWETLQVDLRTDSELVALYKDVPFTTTAGICKVSS

ICDGSIR

>XP_020677328.1 probable methionine--tRNA synthetase [Dendrobium catenatum]

MGEIPGKKNAKLPIPGKRNILITSALPYVNNVPHLGNIIGCVLSADVFARYCRLRGYNTIYICGTDEYGTSTETKAMEEN

CTPKEICDKYHAIHNEVYKWFDISFDEFGRTSSPQQTEICQAIFQKLLENKWLSENTMQQLYCDTCKRFLADRLVEGTCP

RSNCNYESARGDQCEKCGNLLNPTELQDPKCKVCRNTPRIRDTDHLFLELPLLKDKLEEYVNLTSVVGGWSQNAVQATHA

WLKEGLKPRCITRDLKWGVPVPHEKYRDKVFYVWFDAPIGYVSITSCYTPDWEKWWKNPDNVDLFQFMGKDNVPFHTVMF

PSTLLGTGENWTLLKTISVTEYLNYESGKFSKSKGIGIFGNDAKDTNIPTEVWRYYLLTNRPEVSDTLFTWTDLQAKLNT

ELLNNLGNFINRVLSFVAKPSGSGYNSIIPDAPDAESHHLTKALAEKVSKLVEQYLDAMEKVKLKQGLKTAMSISSEGNS

YLQESQFWKLYKDDPSSCSIVMKTSTGLVLLLACLLEPFMPSFSSEVLKQLNLPPETHLSLCDDKGDIDKAKQPWNFLPS

GHKIGKPEPLFRELKDEEVESLRQRYAGSQAERIEKAEADAKKIAEQLKSTKISGVSAKKQPAKSTVNTKPKAPEAEISV

SRLDIRVGQIRKVQKHPDADSLYVEEIDVGEESTRTVVSGLVKFIPLEEMQNRKVCVLCNLKPATMRGIKSHAMVFAASN

DDHTKVELVDPPASAVVGERITFPGYSGEPDGLLNAKSKVWEKVQVDLHTDAELVAHYKDSPFTTSAGICKVLSIASGAI

R

>ORY58964.1 methionyl-tRNA synthetase [Neocallimastix californiae]

MGFPAEQKVDNKNSLVLNHHERIILPVDDKKNILVTCALSYVNNVPHLGNIIGSMLSADCYVRYCRMREYNVLFINGTDE

YGTATETKALEEKCTCQELCDKYHAIHDQIYQWFGIDFDYFGRTTTPQQTEIAQDIFLKLNKNGYLLEDTVTQLYCETCK

LFLADRFVEGECPFCHYEDARGDQCDKCGKLINAVELIKPRCKVCNNTPETKTSNHIFIDLPKLQDKCEAFVKKNSAEGE

WSSNSISITNGWLKEGLKPRCITRDLKWGTPVPLEHMKDKVFYVWFDAPIGYLSITANYTPEWKQWWMNPDKVQLIQFMG

KDNIPFHTVIFPCSEIGTGDNYTLLNRLSTTEYLNYENGKFSKSRNVGVFGNNVVDTGVPVEVWRYYLLINRPESNDSAF

TWNDFIARNNNELLNNIGNFVNRLLKFVASKYDSIIPEYDLSGPTEQKLIADVNKLLKEYINNMEAIKLRAGLKTAMDIS

QVGNAYLQENKIDNKLFTEKRQVCNNVVAASVNLIYLLSSLIYPMMPTTTESILKQINAPLRKISDVWEANEILPGHTIG

KPTYLFKRIDPEMEHVWREKYGGNAPKEAKPADKKNKKASKDKKAQKK

>XP_020584174.1 probable methionine--tRNA synthetase [Phalaenopsis equestris]

MGEVAVKKTAKLPIPGKRNILITSALPYVNNVPHLGNIIGCVLSADVFARYCRLRGYNTVYICGTDEYGTSTETKAMEEN

CTPQEICDKYHAIHKDVYKWFDISFDEFGRTSTPQQTEVCQEIFQALLENNWLSENTMQQLYCDTCERFLADRLVEGTCP

RTNCNYESARGDQCEKCGNLLNPTELQDPKCKVCRNTPRVRDTDHLFLELPLLKDKLEEYINRTSVTGGWSQNAIQATHA

WLKEGLKPRCITRDLKWGVPVPHEKYKDKVFYVWFDAPIGYISITSCYTPDWEKWWKNPDNVELFQFMGKDNVPFHTVMF

PSTLLGTGENWTLLKTISVTEYLNYESGKFSKSKGIGIFGNDAEDTEIPTEVWRYYLLTNRPEVSDTLFTWTDLQAKLNS

ELLNNLGNFVNRVLSFIAKPSESGYNSTIPDAVDAESHHLTKTLAEKVFKLVEQYIDAMEKVKLKQGLKTAMSISSEGNA

YLQESQFWKLYKKDPLSCSIVIKTSAGLVLLLACLLEPFMPSFSVKVLQQLNLSPETYLSLFSEGGDIDKAKQPWNLLPS

GHKIGKPEPLFTELEDEKVESLRQRYAGSQAERVEKAEADAKKIAEQLKSTKISETSSKKQQAKPTNTKLKAPEAEISVS

RLDIRVGQITKVQKHPDADSLYVEEIDLGEESRTVVSGLVNYIPIEEMENRKVCVLCNLKPANMRGIKSYAMVFAASNDD

HTKVELVDPPASAVVGERITFPGYSGQPDALLNAKAKVWEKVQVDLHTDAELIAHYKNSPFTTSAGICKVLSIANGAIR

>XP_016478698.1 PREDICTED: probable methionine--tRNA synthetase [Nicotiana tabacum]

MGENCKTIPKLPIPGKRNILITSALPYVNNVPHLGNIIGCVLSADVFARYCRLRGYNAIYMCGTDEYGTATETKALEENC

TPKQICDKYHAIHKEVYKWFDISFDEFGRTSMPQQTEVCQAIFKKLFENNWLSENTMQQLYCDTCKKFLADRLVEGNCPT

PGCNYDSARGDQCEKCGKLLNPTELMEPKCKVCRKTPCIRDTDHLFLELPLLKDRLEAYVNNMSVAGGWSQNAIYTTHAW

LREGLKPRCITRDLKWGVPVPHEKYKDKVFYVWFDAPIGYVSITSCYTTEWEKWWKNPDDVELYQFMGKDNVPFHTVIFP

STLLGTGENWTLMKTISVTEYLNYEAGKFSKSKGIGVFGNDAKDTNIPVEVWRYYLLTNRPEVSDTLFTWADLQAKLNSE

LLSNLGNFVNRVLSFIAKDSASGYGSIIPDVEGAESHPLTKALGEKVGNYVEQYIEAMEKVKLKQGLKVAMSVSGEGNGY

LQESQFWKLYKEDRRSCSIVMRTACGLVYLLACLLEPFMPSFSREVLKQLNLPPEMQLSLADEKGDVANSKRPWNIIPAG

HKIGTPAPLFKELKDEEVEFYREKFAGSQSDRADRALKAETDTKKMAEQLNKTKISDGNKKERAKKSSETKSKAPATDEG

EISISRLDIRVGLITKAQKHPNADSLYVEEIDVGEALPRTVVSGLVNYIPLEEMQNRKVCILCNLKPVSMRGIKSQAMVL

AASNSDHTKVELVEPPKDAAIGERITFPGFDGEPDDVLNNKKKIWETVQLDLHTNKELVACYKDLPFTTSSGICKVASIS

EGSIR

>XP_001744032.1 hypothetical protein [Monosiga brevicollis MX1]

MPKVHVRPPAPILPKAGARNILVTSALPYVNNVPHLGNIIGCVLSADVYARFARNQGHTVLFVCGTDEYGTATETKALAE

GMTPREICDKYHAIHKGVYEWFNISFDHFGRTSTPLQTEICQDIFKKCYANGFVQQKDEQQWYCTSCETFLADRFVEGIC

PDCGYDDARGDQCDECGHTFDAIKLKSPRCRYRKDCNKAPELRTSRHLYLQLDKLQPLTEKWYAESNDKHAWSKIAQDIS

RNWFNMGLEARAITRDLKWGIPVPLEGFTEKVFYVWFDAPIGYISITANYTDQWEQWWKNPENVELFQFMAKDNVPFHSI

VFPSALLASQDNWTMLNHLNAVQYLNYEGTQFSKSRGVGVFGDSARDSGLAADVYRFYLLYMRPEASDANFDWEDLVAKH

NNELLNNLGNFANRALSFCKRFFDGKVPKIALDDTAVEAVAQVNTELRAYTEGMESQVRIRDAIRGILSVSRIGNQYLQS

TMPWKLVKNPETAAQAGSIVGVVLNLTYLIALMLEPIMPDTAQELLQQIGAERRFLPSAFAPLLDEGHVIGEPKPLFEKI

DPDMAKEWRSRYGGRQAKAPEAPKVELLPTDRKRKTWSDRCGNRFWHPISFDSLLR

>XP_009619836.1 PREDICTED: probable methionine--tRNA synthetase [Nicotiana tomentosiformis]

MGENCKTIPKLPIPGKRNILITSALPYVNNVPHLGNIIGCVLSADVFARYCRLRGYNAIYMCGTDEYGTATETKALEENC

TPKQICDKYHAIHKEVYKWFDISFDEFGRTSTPQQTEVCQAIFKNLFENNWLSENTMQQLYCDTCKKFLADRLVEGNCPT

PGCNYDSARGDQCEKCGKLLNPTELMEPKCKVCHNTPCIRDTDHLFLELPLLKDRLEAYVNNMSVAGGWSQNAIYTTHAW

LREGLKPRCITRDLKWGVPVPHEKYKDKVFYVWFDAPIGYVSITSCYTTEWEKWWKNPDDVELYQFMGKDNVPFHTVIFP

STLLGTGENWTLMKTISVTEYLNYEAGKFSKSKGIGVFGNDAKDTNIPVEVWRYYLLTNRPEASDTLFTWADLQAKLNSE

LLSNLGNFVNRVLSFIAKDSASGYGSIIPDVEEAESHPLTKALGEKVGNYVEQYIEAMEKVKLKQGLKVAMSISGEGNGY

LQESQFWKLYKEDRRSCSIVMRTACGLVYLLACLLEPFMPPFSHEVLKQLNLPPETQLSLADEKGDVANSKRPWNIIPAG

HKIGTPAPLFKELKDEEVEFYREKFAGSQSDRADRALKAETDAKKMAEQLNKTKISDGNKKKERAKKSSETKSKAPATVE

GEVSISRLDIRVGLITKAQKHPNADSLYVEEIDVGEALPRTVVSGLVNFIPLEDMQNRKVCVLCNLKPVSMRGIKSLAMV

LAASNSDHTKVELVEPPKDAAIGERVTFPGFDGKPDDVLNNKKKVWETVQLDLHTNMELVACYKDLPFTTSSGICKVASI

SEGSIG

>EPZ30937.1 Methionyl/Leucyl tRNA synthetase domain-containing protein [Rozella allomycis CSF55]

MRKVLEGEITEMDSLIGRDGDVKSVLSILYFARMSFCEIEDESVKKWMEVARKEYGCVVESLPAKYLKEVKARKVVVSEG

IKVERRKDKEILPIEGERNILITSALPYVNNVPHLGNIIGCVLSADVYSRFCRLKGYNSLFICGTDEYGTATETKALEEG

ISCQELCDRFHKIHSDVYKWFDIDFDSFGRTTTPSQTEIAQDIFLKLNKNGHLQKETVSQLYSESCKMFLADRYVEGTCP

LCGYNDARGDQCDSCGKLLNAMELKNPRCKIDGSTPIIKESTHVFIKLNDLQQKCEDFVKESSLKGQWSHNSVSVTNAWF

KEGLKPRCITRDLKWGTPVPLEELKEKVFYVWFDAPIGYLSITAEYTKNWEKWWKNPDNVQLYQFMGKDNVPFHTVIFPC

SLLGTGDSFTMLHHLSTTEYLNYETGKFSKSRNVGVFGHNVVDSGIPSSIWRYYLLSNRPEQDDSTFLWDDFAAKTNSEL

LANLGNFVSRTTKFLSAKYNGIVPNATLGDLENKFIEKINEELQVYNQNMEAVKIKAGLKSAMAISALGNLYLTEAKIDN

NLFLNHRERCDTVLAFSLNIVYILSSLLYPFIPAACDSILEQLNAPLRFIPTAFSIDLLGGHQIGVPTHLFKKIEEAKID

ELKKLYSGSA

>XP_002268996.1 PREDICTED: probable methionine--tRNA synthetase [Vitis vinifera]

MGDENEKGGRKKVPVEGKRNILITSALPYVNNVPHLGNIIGCVLSADVFARYCRLRGYNVLYICGTDEYGTATETKAMEE

NCSPQQICDKYHKIHKEVYDWFGISFDEFGRTSTPQHTEVCQAIFKKLMENNWLSENRMQQLYCETCQRFLADRLVEGTC

PTLDCNYDSARGDQCEKCGKLLNPTELKDPRCKVCQSSPQIRDTDHLFLELPLLKDKLEEYINNMSIAGCWSQNAIQATY

AWLKEGLRSRCITRDLKWGVPVPHEKYKDKVFYVWFDAPIGYVSITACYTPEWEKWWKNPENVDLYQFMGKDNVPFHTVM

FPSTLIGTGENWTLMKSISVTEYLNYEAGKFSKSKGVGVFGNDVKDTKIPAEVWRYYLLTNRPEVSDSLFTWVDLQAKLN

TELLNNLGNFINRVLSFIAKAPGLGYGSSIPDAPGAESHYLTKALAEKVGAYLEQYIEAMEKVKLKQGLKIGMSISSEGN

GYLQESQFWKLYKEDRASCSIVMRTSVGLVYLLSCLLEPFMPSFSLEVLKQLNLPPETRLSLCDENGDLERARKPWELLP

AGHKMGTPEPLFNELRDEDVEFFREKFAGSQADRVVKAEAEAKKIVEQLKEAKISDGNGKKQKSTKSGSEVQIKKSCKSE

VSISRLDIRVGLIKEAQKHPDADSLYVKEIDVGEVATRTVVSGLVKHVPLEEMLNRKVCVLCNLKPATMRGVNSQAMVLT

ASDKNGTKVELVEPPHSASVGERVTFPGFEGKPDRVLIPKKKIWETLQSDLHTDVQLFAFYKDVPFTTSAGVCKVKSISN

GVIS

>NP_193114.1 methionine-tRNA synthetase, putative / methionyl-tRNA synthetase, putative / MetRS [Arabidopsis thaliana]

MEDDGKSSPKLPIPGKRNILITSALPYVNNVPHLGNIIGCVLSADVYARYCRLRGYNAIYICGTDEYGTATETKALEENC

TPKEICDKYHAIHKEVYDWFGISFDKFGRTSTPEQTEVCQAIFNKLWDNKWLSENTMQQLYCDTCKKFLADRLVEGSCPF

EGCNYDSARGDQCEKCGKLLNPTELKDPKCKVCQNTPRIRDTDHLFIELPLLKDRLEAYIKKTSVTGSWSQNAIQTTNAW

LRDGLRQRCITRDLKWGVPVPHEKYKDKVFYVWFDAPIGYVSITSCYTSEWEKWWKNPENVELYQFMGKDNVPFHTVMFP

STQLGTEENWTLMKTISVTEYLNYEDGKFSKSKGVGVFGNDVKDTNIPVEVWRYYLLTNRPEVSDTSFSWTDLQAKLNGE

LLSNLGNFVNRVLSFIAKPDNAGYGSVIPDAHDAESHSLTKSLAEKVEKFVAEYVEAMEKVKLKQGLKTAMLISSEGNYY

LQASQFWKLYKEDKPLCAVVIRTAAGLVHLLAQLLEPFMPSFSCEVFKQLNLPPQFSLSDERGEVLLASRPWDILPPSHR

IGTPQPLFKELENDEVARYREKFAGSQSDRRARDEAANLADQLNKTKLSDAKKQKASSKGGGKPKPQPAADREITMARLD

IRVGKIVKAEKHPKADALYVEEIDVGGGEIRTVVSGLVKYIPLEEMQNRMVCVLCNLKPAKMRDIVSQAMVLAASSSDGS

KVELVEPPKTANIGERVTFPGFEGEPDDVLNPKKKVWETLLVDLNTKENLVACYKDVPFTTSAGVCKVSSISNGTIR

>OAP00931.1 hypothetical protein AXX17_AT4G15730 [Arabidopsis thaliana]

MEDDGKSSPKLPIPGKRNILITSALPYVNNVPHLGNIIGCVLSADVYARYCRLRGYNAIYICGTDEYGTATETKALEENC

TPKEICDKYHAIHKEVYDWFGISFDKFGRTSTPEQTEVCQAIFNKLWDNKWLSENTMQQLYCDTCKKFLADRLVEGSCPF

EGCNYDSARGDQCEKCGKLLNPTELKDPKCKVCQNTPRIRDTDHLFIELPLLKDRLEAYIKKTSVTGSWSQNAIQTTNAW

LRDGLRQRCITRDLKWGVPVPHEKYKDKVFYVWFDAPIGYVSITSCYTSEWEKWWKNPENVELYQFMGKDNVPFHTVMFP

STQLGTEENWTLMKTISVTEYLNYEDGKFSKSKGVGVFGNDVKDTNIPVEVWRYYLLTNRPEVSDTSFSWTDLQAKLNGE

LLSNLGNFVNRVLSFIAKPDNAGYGSVIPDAHDAESHSLTKSLAEKVEKFVAEYVEAMEKVKLKQGLKTAMLISSEGNYY

LQASQFWKLYKEDKPLCAVVIRTAAGLVHLLAQLLEPFMPSFSCEVFKQLNLPPQFSLSDERGEVLLASRPWDILPPSHR

IGTPQPLFKELENDEVARYREKFAGSQSDRRARDEAANLADQLNKTKLSDAKKQKASSKGGGKPKPQPAADREITMARLD

IRVGKIVKAEKHPKADALYVEEIDVGGGEIRTVVSGLVKYIPLEEMQNRMVCVLCNLKPAKMRDIVSQAMVLAASSSDGS

KVELVEPPKTANIGERVTFPGFEGEPDDVLYPKKKVWETLLVDLNTKENLVACYKDVPFTTSAGVCKVSSIRNGTIR

>XP_016503502.1 PREDICTED: probable methionine--tRNA synthetase [Nicotiana tabacum]

MGENCKTIPKLPIPGKRNILITSALPYVNNVPHLGNIIGCVLSADVFARYCRLRGYNAIYMCGTDEYGTATETKALEENC

TPKQICDKYHAIHKEVYKWFDISFDEFGRTSTPQQTEVCQAIFKNLFENNWLSENTMQELYCDTCKKFLADRLVEGNCPT

PGCNYDSARGDQCEKCGKLLNPTELMEPKCKVCHNTPCIRDTDHLFLELPLLKDRLEAYVNNMSVAGGWSQNAIYTTHAW

LREGLKPRCITRDLKWGVPVPHEKYKDKVFYVWFDAPIGYVSITSCYTTEWEKWWKNPDDVELYQFMGKDNVPFHTVIFP

STLLGTGENWTLMKTISVTEYLNYEAGKFSKSKGIGVFGNDAKDTNIPVEVWRYYLLTNRPEASDTLFTWADLQAKLNSE

LLSNLGNFVNRVLSFIAKDSASGYGSIIPDVEEAESHPLTKALGEKVGNYVEQYIEAMEKVKLKQGLKVAMSISGEGNGY

LQESQFWKLYKEDRRSCSIVMRTACGLVYLLACLLEPFMPPFSHEVLKQLNLPPETQLSLADEKGDVANSKRPWNIIPAG

HKIGTPAPLFKELKDEEVEFYREKFAGSQSDRADRALKAETDAKKMAEQLNKTKISDGNKKKERAKKSSETKSKAPATVE

GEVSISRLDIRVGLITKAQKHPNADSLYVEEIDVGEALPRTVVSGLVNFIPLEDMQNRKVCVLCNLKPVSMRGIKSLAMV

LAASNSDHTKVELVEPPKDAAIGERVTFPGFDGKPDDVLNNKKKVWETVQLDLHTNMELVACYKDLPFTTSSGICKVASI

SEGSIG

>XP_011101554.1 probable methionine--tRNA synthetase [Sesamum indicum]

MGDSSAPAATKLPVPGKRNILITSALPYVNNVPHLGNIIGCVLSADVFARYCRMRGYNAIYICGTDEYGTATETKAMEEG

CSPKEICDKYHAIHKDVYQWFNISFDEFGRTSSPQQTEVCQAIFKKLLENNWLSENTMQQLYCDTCKRFLADRLVEGNCP

TPGCNYDSARGDQCEKCGKLLNPTELLDPKCKVCRNTPRIRDTNHLFLELPLLKDRLEEYIRTTSVAGGWSQNAIQATHA

WLREGLKPRCITRDLKWGVPVPHEKFKDKVFYVWFDAPIGYVSITSCHTPEWEKWWKNPENVELFQFMGKDNVPFHTVMF

PSTLLGTGENWTFMKTISVTEYLNYETGKFSKSKGVGVFGNDAKDTNIPVEVWRYYLLTNRPEVSDTLFTWVDLQAKLNS

ELLSNLGNFINRVLSFIAKDPASGYGSIIPDAQGADSHPLTKALGEKVGSYVEQYIEAMEKVKLKQGLKLAMTVSGEGNA

YLQESQFWKLYKEDRPSCSVVMKTSAGLVYLLACLLEPFMPSFSLGVLKQLNLPLETQLSLSDEKGDVERAKRPWDILPA

GHKIGTPAPLFRELKDEEVEFYREKFAGSQADRIVKAQAESEAKKLAGKLKETKVSDGNNAKKDRPKKSADSKPKASAPA

ESEISVSRLDIRVGLITKAQKHPDADSLYVEEIDVGEPQPRTVVSGLVKYIPLEDMQNRKVCVLCNLKPASMRGIKSQAM

VLAASNSDHTKVELVEPPQGAAVGERVTFPGFDGLPDDVLNPKKKVWETVQVDLHTDKELVARYKDLPFTTSAGVCRVAS

ITEGSIR

>CDP11143.1 unnamed protein product [Coffea canephora]

MGDLENSGKNNPPQNVKLPIPGKRNILITSALPYVNNVPHLGNIIGCVLSADVFARYCRLRGYNVIYICGTDEYGTATEN

KALEEGVTPQEICDKYHAIHKSIYEWFDISFDKFGRTSSPEQTKVCQAIFKKLLENNWLSENTMQQLFCDTCQRFLADRF

VEGSCPHCNYDSARGDQCENCGKLLNPTELLDPKCKVCKSAPHIRDTDHLFLELPLLKDKLEAYINEMSVAGGWSQNAIQ

ATNAWLREGLKPRCITRDLKWGVPVPHEKYKDKVFYVWFDAPIGYVSITACYTTEWEKWWKNPEEVELFQFMGKDNVPFH

TVMFPSTLLGTGENWTLMKTISVTEYLNYESGKFSKSKGIGVFGNDAKDTNIPIEVWRYYLLTNRPEVSDALFTWDDLRA

KLNNELLKNLGNFINRVLNFIAKAPGLGYGSILPDAQDAESHPSTKALGDKVGELVEQYVEAMEKVKLKQGLKIAMAIST

EGNGYLQENEFWRLYKDDQPTCSIVMRTSAGLVYLIASLLEPFMPSFSIEVLKQLNLPLEQISLCDEKGDIERAKRPWEI

LSAGHRIGTPKPLFKELKEDEAEMYRGKFSGSQADRADRKLKEEAEAKISEQLNKAKLSVVLECFPSVYLDILSLSKYVR

QPFLLYATLLGFSKVEFDLLFRTILLHFLQLTNIRFNGASFCCILFPWLFSLISNGWMSPVQSSTSEVNSQASAVLSLFD

TFISISASVVSYRIQHQLSLIEFSILNVVFCFVTPLTIVNGNAKKGKPKKSAQGASKTASLAEAEISVSRLDIRVGLIKK

AEKHSSADSLYVEEIDVGEPQPRKVVSGLVKYIPLEKMQNRKVCVLCNLKPANMRGVKSEAMVLCASNSEDTKVELVEPP

EGAVIGERVKFPGCEGSPDDQLNPKKKVWETVQPDLRTDEELVACYKGIPFTTSAGVCKVSSLNEAKIR

>PRQ56114.1 putative methionine--tRNA synthetase [Rosa chinensis]

MGGLKLPIPGQRNVLITSALPYVNNVPHLGNIIGCVLSADVFARYCRLRGYNTVYICGTDEYGTATETKAMEDKCSPKEI

CDKYHAIHRQVYDWFNISFDEFGRTSTPEQTEVCQAIFNRLLENNWLSDNTMQQLYCDTCTRFLADRLVEGTCPTQGCDY

TSARGDQCENCGKLLNPTELKDPKCKVCKSTPRIRDTNHLFLELPLLKDRLEEYINKMSVEGSWSQNAIQATNAWLREGL

KQRCITRDLKWGVPVPHDNFRDKVFYVWFDAPIGYVSITKCYTPDWEKWWKNPEDVELYQFMGKDNVPFHTVMFPSTLLG

TGENWTLMKTISVTEYLNYEAGKFSKSKGIGVFGNDAKDTNIPVEVWRYYLLTNRPEVSDTLFTWADLQAKLNSELLNNL

GNFINRVLSFVAKPSGQGYDSIIPDGQDAISDPLTEKIAEKVGKYVEQYVEAMEKVKLKQGLKLAMSISSEGNAYLQESR

FWKLYKEDQPRCSIVMRTAVGLVHLLACLLEPFMPSFSVKVFKQLNLPPETHISLCDDKGDTDRARRPWEIVPRGHKIGK

PEPLFKELNNDEVDSLRKKFAGSQADRKEREEAEAVKLAEQLKKSKVSESEGMRPLQPEASDHTAY

>XP_018822921.1 PREDICTED: methionine--tRNA synthetase, cytoplasmic [Juglans regia]

MGDVSGHAAGDRKVPKLPIEGKRNILITSALPYVNNVPHLGNIIGCVLSADVFARYCRLRSYNAIYMCGTDEYGTATETK

AMEENSTPKEICDKYYAIHKEVYEWFNISFDKFGRTSAPQQTEVCQAIFKKLLENNWLSENTMQQLYCDTCKRFLADRLV

EGTCPKEGCDYKSARGDQCEKCSKLLNPTELKDPRCKVCQKTPRIRDTNHLFLELPLLKDRLEEYINKMSVAGSWSQNAI

QATYAWLKEGLKSRCITRDLKWGVPVPHERYSDKVFYVWFDAPIGYVSITSCYTPEWEKWWKNPENVELYQFMGKDNVPF

HTVMFPSTLLGTEENWTLMKTISVTEYLNYEAGKFSKSKGIGVFGNDAKDTNIPVEVWRYYLLNNRPEVSDTLFTWADLQ

AKLNSELLNNLGNFINRVLSFIAKPPGQGYGSVIPKPSDSDPDPLTHEPTKKLVCSIVECVEQYVEAMEKVKLKQGLKTA

MSISNEGNGYLQESQFWKLYKDDQPSCSLVIRTSAGLVYLLACLLEPFMPSFSLEVLKQLNFPPETPFSLEKGDIDRASR

PWDILPAGHKIGTPEPLFKELKDEEVEFFRTKFAGSQADRIVRAEAEAEKMAEQLKKTKVSESSGKKQQNKKPASEAKGR

ASAEPEISIARLDIRVGLITSAQKHPDADSLYVEEIDVGEGQTRTVVSGLVKYIPLEEMQNRKVCVLCNLKPATMRGIKS

QAMVLAASNSDHTKVELVEPPKSAQVGERVTIPGFDGEPDDVLNPKKKVWETLQVDLHTNTELVACYKDIPFTTSAGVCT

VSSITGGSIR

>XP_009414754.1 PREDICTED: probable methionine--tRNA synthetase [Musa acuminata subsp. malaccensis]

MADSSAKKAPKLPIPGRRNILITSALPYVNNVPHLGNIIGCVLSADVFARYCRLRGYNTIYMCGTDEYGTATETKAMEEN

CSPKEICDKYHAIHRDVYEWFDISFDKFGRTSAPQQTEVCQAIFHKLLDNKWLSENTMQQLYCDTCQRFLADRLVEGTCP

TLDCNYDSARGDQCEKCGKLLNPTELKDPKCKVCRTAPRIRDTDHLFLELPLLREKLEEYINTMSVAGSWSQNAIQATNA

WLKEGLRPRCITRDLKWGVPVPHEKYKDKVFYVWFDAPIGYISITACYTSEWEQWWKNPDNVELYQFMGKDNVPFHTVMF

PSTLLGTGERWTMMKTISVTEYLNYESGKFSKSKGIGVFGNDAKDTKIPTEVWRYYLLTNRPEVSDTLFTWADLQAKLNT

ELLNNLGNFINRVLSFIAKPEGAGYGSIIPDASNPQSQQLTKYLGEKVGKLVDQYLDAMEKVKLKQGLKIAMSISSEGNA

YLQESQFWKLFKEDPSSCAIVMKTSAGLVYLLACLLEPFMPSFSVEVLRQLNLPLEANLSFCDENGETDKAKRPWDFLPS

GHHIGKPEPLFKELKDEDVESFRVRFAGSQAERIVKAETDANKVAEQLKATKITGNAKKQQNKPSGNTKAKTAETEISVS

KLDVRVGLIKKVQKHPDADSLYVEEIDVGEESPRTVVSGLVKYIPLEEMQNRKVCVLCNLKPVTMRGIKSHAMVLAASND

DHTKVELVDPPSSAAVGERVTFPGYSGEPDGILSAKSKVWEKVQVDLQSNSELVACYKDVPFTTSAGVCKVLSIANGAIR

>AFY06992.1 methionyl tRNA synthetase [Ditylenchus destructor]

MADWSSSEPEYFDSSFKKRLPIEGQRNILITSALPYVNNVPHLGNIIGSVLSADVFSRYCQMRGYQSLFICGTDEYGTAT

EMKALKDGASCQEICDKFHRLHKEIYDWFNIDFDYFGRTTTQHQTEICQDIFKNAHSNKYTSTSSLEQLHCETCDKFLAD

RFVFGICPHCKFTDARGDQCDGCGKLLDGVDLVEPKCHICQNTPKPKKSEHIFLNLDDLAGEVEKYVEKVINAEDSHWSS

NAIAIVRSWFKQGLEKRCITRDLKWGTPVPLEGFDSKVFYVWFDAPIGYLSMTKTLVGEDWVKWWKNPNQVELYQFLGKD

NVAFHGVIFPATQIATGENYTKVRHVCATEYLNYEDKKFSKSRNTGVFGDTVSTIGIPSDIWRFYLIYMRPENQDTAFCW

DDFMLKVNSELLANLGNFILRALSFLFTNFNGEQPEIHLTSAENELIAAIDNELAEFTRNMENVRLREALIKILAISRLG

NQYIQSHTPWVLVKGSPEEKQRAQTIIGVAANISILLSTLLYPFMPAVSKQIREECNIKQALKLPSSFMQMIKPGHKISK

PSALFTKLEPAKVTEWKLRFGASDEASAVQATAKSNKNQKQNKQKAMSKSSDVIVEREPTNFPAIATAQETIDSLLNSVG

QKLDLAKKLFTSNEVEKIETVNAKLRQEVESLKLQLGGNASILNVTGSSTNDDIKNDGSVKTTQANELDSSTQATKNSKE

PTKKTPQKDQVKAAPSEKSAKGTPSANVNDEVVDVGRLDLRVGRIIEAKKHPDADSLYVEQIDLGEGQPRTVVSGLVRFV

SLEQMQNRLVVCCCNLKPAKMRGIESQAMVLCASTPEKVEIMEVPAECQPGDVVLCDGFTRRPDAVLNPKKKVWETVAVD

LKVSDDGVAVYKGKPLKIGGSHPMTAPTLRNVPVK

>XP_024156288.1 probable methionine--tRNA synthetase [Rosa chinensis]

MGGLKLPIPGQRNVLITSALPYVNNVPHLGNIIGCVLSADVFARYCRLRGYNTVYICGTDEYGTATETKAMEDKCSPKEI

CDKYHAIHRQVYDWFNISFDEFGRTSTPEQTEVCQAIFNRLLENNWLSDNTMQQLYCDTCTRFLADRLVEGTCPTQGCDY

TSARGDQCENCGKLLNPTELKDPKCKVCKSTPRIRDTNHLFLELPLLKDRLEEYINKMSVEGSWSQNAIQATNAWLREGL

KQRCITRDLKWGVPVPHDNFRDKVFYVWFDAPIGYVSITKCYTPDWEKWWKNPEDVELYQFMGKDNVPFHTVMFPSTLLG

TGENWTLMKTISVTEYLNYEAGKFSKSKGIGVFGNDAKDTNIPVEVWRYYLLTNRPEVSDTLFTWADLQAKLNSELLNNL

GNFINRVLSFVAKPSGQGYDSIIPDGQDAISDPLTEKIAEKVGKYVEQYVEAMEKVKLKQGLKLAMSISSEGNAYLQESR

FWKLYKEDQPRCSIVMRTAVGLVHLLACLLEPFMPSFSVKVFKQLNLPPETHISLCDDKGDTDRARRPWEIVPRGHKIGK

PEPLFKELNNDEVDSLRKKFAGSQADRKEREEAEAVKLAEQLKKSKVSDKSGISMSFLGLVSNLCVCWSGTESEGMRPLQ

PEASDHTAY

>XP_010907221.1 PREDICTED: probable methionine--tRNA synthetase [Elaeis guineensis]

MGDSPAKKSPTLPVPGRRNILITSALPYVNNVPHLGNIIGCVLSADVFARYCRLRSYNTVYICGTDEYGTATETKAMEEN

CSPKEICDKYHAIHRDIYKWFDISFDEFGRTSTPQQTEVCQSIFQKLMENKWLSENTMQQLYCDTCQRFLADRLVEGTCP

TLDCNYESARGDQCEKCGKLLNPTELKDPKCKVCQTTPQIRDTDHLFLELPLLRDKLEEYINSMSVAGAWSQNAIQATYA

WLKEGLKPRCITRDLKWGVPVPHEKYKDKVFYVWFDAPIGYISITACYTMDWEKWWKNPENVELYQFMGKDNVPFHTVMF

PSTLLGTGEEWTMMKTISVTEYLNYESGKFSKSKGIGVFGNDAKDTRIPPEVWRYYLLTNRPEVSDTLFTWADLQAKLNS

ELLNNLGNFINRVLSFIAKPPGAGYDYVIPDAPDAESHQLTKSLAEKIGKLVDQYLDAMEKVKLKQGLRTAMSISSEGNA

YLQDSQFWKLYKEDPSSCAIVMKTSVGLVYLLASLLEPFMPSFSVEALRQLNIAPETHLSFCDEKGETEKAKKPWDFLPS

GHKIGKPEPLFKELKDEDVEFFRMKFAGSQSDRKVKAEAEEKKIAEQLKSTKIREGNAKKQQTKPVGSSKSKASETEISI

SKLDIRVGMIKKVQKHPDADSLYVEEIDVGEDSTRTVVSGLVKYISLEEMQNRKVCVLCNLKPATMRGIKSHAMVLAASN

EDNSKVELVDPPESAVVGERVSFPGYSGEPDGVLNAKSKVWERVQADLHTDAELVACYKDAPFTTSVGVCKVLSIASGAI

R

>PKC08871.1 methionyl-tRNA synthetase [Rhizophagus irregularis]

MSTNSVSERKINSGVALEIEKEKNKEPIKGERNILITSALPYVNNIPHLGNIIGSVLSADVFARYSRARRYNTLFVCGTD

EYGTATETKALEEGTTCQELCDKYNAIHKQVYEWFELDFDYFGRTTTRQQTEIAQDIFMKLYKNGYLSEDAMIQLFCEKC

QRFLADRYVEGTCPKCNYEDARGDQCDSCGQLLNATELVNPRCKLEGSTPIMRESKHMFLDLDTLQKTVEDWVIKTSVEG

KWSTNSKTITNSWLKEGLKPRCITRDLKWGTPVPLEEMKEKVFYVWFDAPIGYLSITANYTPHWEKWWKNPDNVKLYQFM

GKDNVPFHTVIFPSSLFGTGEDWTLLYHINTTEYLNYENTKFSKSRNIGVFGTDAKDTGINVSVWRYYLIASRPETNDSV

FTWKEFIAKNNNELLANLGNFVNRVIKFSASKYNSIVPEFSYDKIEEKEFIENIDSLLKNYIETLENVKLRAGLEIAMEI

SRLGNGYLQESKLDNSLFQNNIEKCSTVIGIAINLIYLLSALFFPYMPSTTESICRQLNAPLRDIPDTWTIDILPGHKLG

KAEYLFKKIDDKKEEEYLNRYGGGDGKVAETKKKAKKGKNVNTVTNNKSSKIVNG

>XP_004242831.1 PREDICTED: methionine--tRNA synthetase, cytoplasmic [Solanum lycopersicum]

MGDSSTAGNGDNLSPASPKLPIPGKRNILITSALPYVNNVPHLGNIIGSVLSADVFARYCRLRDYNIIYMCGTDEYGTAT

ETKALEENCTPKQICDKYHAIHREVYKWFNISFDHFGRTSTPQQTEVCQAIFKKLWENNWLSENTMQQPYCETCKKFLAD

RLVEGNCPTPGCNYDSARGDQCEKCGKLLNPTELKDPRCKVCRNTPCIRDTDHLFLELPLLKDELEAYVNDLSVAGGWSQ

NAIHTTHAWLREGLKPRCITRDLKWGVPVPHEKYKDKVFYVWFDAPIGYVSITSCYTTEWEKWWKNPENVELYQFMGKDN

VPFHTVIFPSTLLGTRENWTLMKTISVTEYLNYEAGKFSKSKGVGVFGNDAKETNIPVEVWRYYLLTNRPEVSDTLFSWV

DLQAKLNSELLNNLGNFVNRVLSFIAKDPASGYGSIIPNPEGAESHPLTKALGEKVGNYVEEYIEAMEKVKLKQGLKIAM

SISGEGNGYLQESQFWRLYKEDRPSCSIVMSTASGLVYLLACLLEPFMPSFSREVLKQLNFPPETKPSLSDDRGDIEKSK

RPWNILPAGHKIGIPTPLFKELKDEEVEFYREKFAGSQADRNLKAETEARKITDQLHKAKISDANKKKERATKSSEAKAK

GSASVEAEISISRLDIRVGLITRAQKHPDADSLYVEEIDVGEAQPRTVVSGLVKYIPLEEMQNRKVCVLCNLKPASMRGI

KSQAMVLAASNSDHTKVELVEPPKDAAIGERVMFPGLDGKADDVLNPKKKVWETLQVDLHTNKELVACYKDLPLTTSVGV

CKVTSISEGSIR

>PKY55601.1 methionyl-tRNA synthetase [Rhizophagus irregularis]

MSTNSVSERKINSGVALEIEKEKNKEPIKGERNILITSALPYVNNIPHLGNIIGSVLSADVFARYSRARRYNTLFVCGTD

EYGTATETKALEEGTTCQELCDKYNAIHKQVYEWFELDFDYFGRTTTQQQTEIAQDIFMKLYKNGYLSEDAMIQLFCEKC

QRFLADRYVEGTCPKCNYEDARGDQCDSCGQLLNATELVNPRCKIEGSTPIMRESKHMFLDLATLQKTVEDWVIKTSVEG

KWSTNSKTITNSWLKEGLKPRCITRDLKWGTPVPLEEMKEKVFYVWFDAPIGYLSITANYTPHWEKWWKNPDDVKLYQFM

GKDNVPFHTVIFPSSLFGTGEDWTLLYHINTTEYLNYENTKFSKSRNIGVFGTDAKDTGINVSVWRYYLIASRPETNDSV

FTWKEFIAKNNNELLANLGNFVNRVIKFSASKYNSIVPEFSYDKIEEKEFIENIDSLLKNYIETLENVKLRAGLEIAMEI

SRLGNGYLQESKLDNSLFQNNIEKCSTVIGIAINLIYLLSALFFPYMPSTTESICRQLNAPLRDIPDTWTIDILPGHKLG

KAEYLFKKIDDKKEEEYLNKYGGGDGKVAETKKKAKKGKNVNTVTNNKSSKIVNG

>XP_015082271.1 PREDICTED: methionine--tRNA synthetase, cytoplasmic [Solanum pennellii]

MGDSSTAGNGDNHSPASPKLPIPGKRNILITSALPYVNNVPHLGNIIGSVLSADVFARYCRLRDYNIIYMCGTDEYGTAT

ETKALEENCTPKQICDKYHAIHREVYKWFNISFDHFGRTSTPQQTEVCQAIFKKLWENNWLSENTMQQPYCETCKKFLAD

RLVEGNCPTPGCNYDSARGDQCEKCGKLLNPTELKDPRCKVCRNTPCIRDTDHLFLELPLLKDELEAYVNDLSVAGGWSQ

NAIHTTHAWLREGLKPRCITRDLKWGVPVPHEKYKDKVFYVWFDAPIGYVSITSCYTTEWEKWWKNPENVELYQFMGKDN

VPFHTVIFPSTLLGTRENWTLMKTISVTEYLNYEAGKFSKSKGVGVFGNDAKETNIPVEVWRYYLLTNRPEVSDTLFSWV

DLQSKLNSELLNNLGNFVNRVLSFIAKDLASGYGSIIPNPEGAESHPLTKALGEKVGNYVEEYIEAMEKVKLKQGLKIAM

SISGEGNGYLQESQFWRLYKEDRPSCSIVMSTASGLVYLLACLLEPFMPSFSREVLKQLNFPPETKPSLSDDRGDIEKSK

RPWHILPAGHKIGIPTPLFKELKDEEVEFYREKFAGSQADRNLKAETEARKITDQLHKAKISDANKKKERATKSSEAKAK

GSASVEAEISISRLDIRVGLITRAQKHPDADSLYVEEIDVGEAQPRTVVSGLVKYIPLEEMQNRKVCVLCNLKPASMRGI

KSQAMVLAASNSDHTKVELVEPPKDAAIGERVTFPGFDGKADDVLNPKKKVWETLQVDLHTNKELVACYKDLPLTTSVGV

CKVTSISEGSIR

>EXX67532.1 methionine--tRNA synthetase MES1 [Rhizophagus irregularis DAOM 197198w]

MSTNSVSERKINSGVALEIEKEKNKEPIKGERNILITSALPYVNNIPHLGNIIGSVLSADVFARYSRARRYNTLFVCGTD

EYGTATETKALEEGTTCQELCDKYNAIHKQVYEWFELDFDYFGRTTTRQQTEIAQDIFMKLYKNGYLSEDAMIQLFCEKC

QRFLADRYVEGTCPKCNYEDARGDQCDSCGQLLNATELVNPRCKLEGSTPIMRESKHMFLDLDTLQKTVEDWVIKTSVEG

KWSTNSKTITNSWLKEGLKPRCITRDLKWGTPVPLEEMKEKVFYVWFDAPIGYLSITANYTPHWEKWWKNPDDVKLYQFM

GKDNVPFHTVIFPSSLFGTGEDWTLLYHINTTEYLNYENTKFSKSRNIGVFGTDAKDTGINVSVWRYYLIASRPETNDSV

FTWKEFIAKNNNELLANLGNFVNRVIKFSASKYNSIVPEFSYDKIEEKEFIENIDSLLKNYIETLENVKLRAGLEIAMEI

SRLGNGYLQESKLDNSLFQNNIEKCSTVIGIAINLIYLLSALFFPYMPSTTESICRQLNAPLRDIPDTWTIDILPGHKLG

KAEYLFKKIDDKKEEEYLNRYGGGDGKVAETKKKAKKGKNVNTVTNNKSSKIVNG

>KMZ68773.1 Methionine--tRNA synthetase [Zostera marina]

MSTAAADAPKLPIPGKRNILITSALPYVNNVPHLGNIIGCVLSADVFARYCRLRGYNAIYMCGTDEYGTATETKALEEKC

TPKEICDKYHTIHKQVYDWFDISFDHFGRTSTPQQTEVCQTIFQKLLDGKWLTENTMHQLYCDTCTKFLADRLVEGICPT

LGCGYESARGDQCEKCGKLLNPTDLKDPQCKVCHNPPRIRDTDHLFLELPLLREVLEKYINKMSVAGGWSQNAIQATNAW

LKEGLKQRCITRDLKWGVPVPHEKYKDKVFYVWFDAPIGYLSITSCYTPEWEKWWKDPENVELYQFMGKDNVPFHTVMFP

STLLGTGQNWTLMKTISVTEYLNYEAGKFSKSKGIGVFGNDAKDTKIPSEVWRYYLLTNRPEVSDTMFTWTDLQAKLNSE

LLSNLGNFINRVLSFMAKPQGQGYNSIVPDAPNAESHLLTKSFAEKVSKYVEQYADAMEKVKLKQGLKIAMSISSEGNAY

LQESQFWKLYKEDPTSCSVVIKTSAGIVYILAALLEPFMPSFSAEVTKQLNLSPGAKFSLINEKGELILGKQPWNILPCG

HKIGTPEPLFKELKDEEIEFFREKFAGSQADRALKAETEAKIIAERVKNINVSDKKKKIKSTSETKAKPVENVISVSRLD

IRVGVIKKVQKHPDADSLYVEEIDCGEDLPRTVVSGLVKYIPIEEMQNRKVCVLCNLKPASMRGIKSHAMVLAASNEDHT

RVELVEPPVDAPIGERIIFPGFLGEPDGVLNPKKKVWEAIQPDLSTDGDLIARYKDKPFTTSTGICKVSSIGCGSIK

>CEP01556.1 hypothetical protein PBRA_002162 [Plasmodiophora brassicae]

MMQQLGGVRSADMAPSASVGEAQRRPVLPKDGARNILITSALPYVNNVPHLGNIIGCVLSADVYARFCRLRGHNAIYVCG

TDEYGTATEQKAIQEGLTPREICDKFHAIHAQVYEWFGISFDIFGRTSTPLQTEIVQRIFKECETNGIVHEDTVEQLFCT

KCDMFLADRYVRGTCPDCGHVGAHGDQCDKCGHLMTATLLKDPYCASDKSTPVIRKTEHLFLDLPLMSERLEKWIDQRSE

KGFWSQNSIQVTKAWIATGLKSRSITRDLKWGVPVPKDGFRDKVFYVWFDVSSAPIGYMSITAAYTPEWERWWRNPEQVE

LVQFMGKDNIPFHTVIWPSVLLGADQGYTMLHHINTTEYLQYESGKFSKSMGVGVFGDSVQLTGVSPSVWRYYLLVNRPE

TNDTVFTWDDFMQKNNGELLKNVGNFVNRALVFIKNNFSSVVPECTLTPDDESFIKDMNEHAKTYVALLEKVEIKDGLKQ

AMQASHRCNLYLQDNAPWHLIKDDKARAGSVINVSVNAVVLLATMLEPYMPELVDRIFKQLNTDRSVLSLKGDGPYFRHS

IRSGHVIGEPQVLFTKISEEQITTWRRLYAGQQPVVEDFPLNLVTGVIRSVSEHETAEHLFVLQVDIGSETRQIVSGLRA

HYSSNELLNKKVVVLTNLKASNFRGVRSAGMLLCGVKPDGSLGLLLSPGNAGDRVGPKGYNSLTSSIEKKHFSKVSPHLR

VGDDSLAYFHETPIVVADTGNQVVIDRIQGPAQIS

>XP_006361630.1 PREDICTED: methionine--tRNA synthetase, cytoplasmic-like [Solanum tuberosum]

MGDFSTAGNGDNLPPASPKLPIPGKRNILITSALPYVNNVPHLGNIIGSVLSADVFARYCRLRDYNTIYMCGTDEYGTAT

ETKALEENCTPKQICDKYHVIHREVYKWFDISFDHFGRTSTPQQTEVCQAIFKKLWENNWLSENTMQQPYCETCKKFLAD

RLVEGSCPTPGCNYDSARGDQCEKCGKLLNPTELKDPRCKVCRNTPCIRDTDHLFLELPLLKDELEAYVNDLSVAGGWSQ

NAIHTTYAWLREGLKPRCITRDLKWGVPVPHEKYKDKVFYVWFDAPIGYVSITSCYTTEWEKWWKNPENVELYQFMGKDN

VPFHTVIFPSTLLGTRENWTLMKTISVTEYLNYEAGKFSKSKGVGVFGNDAKETNIPVEVWRYYLLTNRPEVSDTLFSWV

DLQAKLNSELLNNLGNFVNRVLSFIAKDPASGYGSIIPNPEGAESHPLTKALGEKVGNYVEQYIEAMEKVKLKQGLKIAM

SISGEGNGYLQESQFWRLYKEDKPSCSIVMSTASGLVYLLACLLEPFIPSFSREVLKQLNFPPETQLSLSDERGDIEKSK

RPWHILPAGHKIGIPTPLFKELKDEEVEFYREKFAGSQAERNLKAETEARKITDQLNKAKISDANKKKERATKSSEAKAK

GSASVEAEISISRLDIRVGLITRAEKHPDADSLYVEEIDVGEAQPRTVVSGLVKYIPLEEMQNRKVCVLCNLKPASMRGI

KSQAMVLAASNSDHTKVELVEPPKDAAIGERVTFPGFDGKADDVLNPKKKVWETLQVDLHTNEELVACYKDLPLTTSVGV

CKVTSISEGSIR

>XP_024180156.1 probable methionine--tRNA synthetase [Rosa chinensis]

MGDLAKQDQSVPKLPIPGERNILITSALPYVNNVPHLGNIIGCVLSADVFARYCRLRGYNTVYISGTDEYGTATETKAME

EKCSPQEICDKYHKIHREVYEWFNISFDQFGRTSTPQQTEVCQQIFKKLLDSDRLAENTIQQLHCDSCKRFLADRFVEGT

CPTQGCYYDSARGDQCDKCGKLLNPTELKDPKCKVCKETPRIRKTNHLFLELPLLKDKLEDYINKASVEGCWSQNAVQIT

NAWLKEGLKQRCITRDLKWGVPVPHDGFRDKVFYVWFDAPIGYISITKCHTPDHWEKWWKNPEDVELYQFMGKDNVPFHT

VMFPSALLGTGENWTLMKNISVTEYLNYEEGKFSKSRGIGVFGNDAKGTGIPVEVWRYYLLTNRPEVSDTLFTWADLQAK

LKSELVGNLGNFINRVLSFVAKPSAKGGYGDTIPDAPHADSDALTLELAEIVGRYVEQYIEAMEKLKLKQGLKIAMSISS

EGNAYLQESKFWKLYKEDRPRCSIVIKTSVGLVHLLACLLEPYMPSFSARVLEQLNLPPESIKSLCDVNKAKRPWEIVPD

GHKIGQPKTLFTTLTGEEVESLRKKFSGSQADREKRTQ

>PON59393.1 Methionyl-tRNA synthetase [Trema orientalis]

MADHSGGRGVKNPNLPIPGKRNILVTSALPYVNNVPHLGNIIGSVLSADVFARYCRLRGYNVIYICGTDEYGTATETKAM

EEKSTPQQICDKYHAIHKEVYDWFNISFDEFGRTSTPQQTEICQAIFTRLMENNWLSDNTMQQLYCEKCNRFLADRLVEG

TCPMLDCNYDSARGDQCEKCGKLLNPTELKDPKCKVCQTTPQIRDTDHLFLELPLLKDKLEEYISNMSVAGSWSQNAIQA

TYAWLKEGLKARCITRDLKWGVPVPHEKFKDKVFYVWFDAPIGYVSITSCYTSDWEKWWKNPENVELYQFMGKDNVPFHT

VMFPSTLLGTGENWTMMKSISVTEYLNYESGKFSKSKGIGVFGNDAKDTNIPSEVWRYYLLTNRPEVSDTLFTWVDLQAK

LNSELLNNLGNFINRVLSFITKPQGIGYGSIIPDSPGAEVHPLTKTLAEKISKFVDQYIEAMEKVKLKQALKIGMSISSE

GNAYLQESQFWKLYKQDQDSCNIVIRTSVGLVFLLACLLEPFMPSFSLKVLKQLDIPPENQLSLSNESGDLERARKPWEF

ISAGHKIGTPEPLFKELKDEAVELFRTKFAGSQAERIVKLEEANEASKMAEQLAKTKI

>XP_019158384.1 PREDICTED: methionine--tRNA synthetase, cytoplasmic [Ipomoea nil]

MGEISEKAPKVPIPGKRNILITSALPYVNNVPHLGNIIGCVLSADVFARYCRLRGYNAIYICGTDEYGTATETKALEENS

SPQQICDKYHEIHKQVYQWFDISFDKFGRTSTPQQTEVCQSIFKKLLDNNWLTENTMQQPYCDTCKKFLADRLVEGTCPT

PGCNYDSARGDQCEKCGKLLNPTELKEPRCKVCRNTPCIKDTDHLFLELPLLKDKLESYVNSMSVAGGWSQNAIHTTHAW

LREGLKSRCITRDLKWGVPVPHEKYKDKVFYVWFDAPIGYVSITSCYTADWEKWWKNPENVELYQFMGKDNVPFHTVIFP

STLFGTGENWTFMKTISVTEYLNYESGKFSKSKGIGVFGNDVKDTTIPVEVWRYYLLANRPEVSDTLFTWTDLQAKLNSE

LLSNLGNFINRVLSFIAKDPASGYGSVIPDAHGVESHSSTKALAEKVGSYLDQYIEAMEKVKLKQGLKIAMSLSGEGNAY

LQESQFWKLYKEDKPSCSVVMRTAAGLVYLLACLLEPFMPSFSREVFKQLNLPESRLSLSDENGDIERAKKPWDILPAGH

KIGTPVPLFKELKDEEVEMYREKFAGSQADRTLRVEAEAKKITEQLNKAKISNEKTKKERSTKSAQAKSKAGASVSAEAE

VSISRLDIRVGLIKKAQKHPDADSLYVEEIDVGEAQPRTVVSGLVKYIPLEEMQNRKVCVLCNLKPATMRGIKSQAMVLA

ASNNDHTKVELVEPPQDAGVGERVTFLGFEGKADDVLNPKKKVWETLQVDFHTNKELVACYKDIPFTTSAGVCKVSSISE

GSIR

>XP_010905659.1 PREDICTED: probable methionine--tRNA synthetase isoform X1 [Elaeis guineensis]

MANPPAKNAPKLPVPGRRNILITSALPYVNNVPHLGNIIGSVLSADVFARYCRLRGYNAIYICGTDEYGTATETKAMEEN

CSPKEICDKYHAIHRDIYKWFDISFDEFGRTSTPQQTEVCQSIFHKLMENNWLSENTMQQLYCDTCKRFLADRLVEGTCP

TLDCNYESARGDQCEKCGKLLNPTELKDPKCKVCRATPRIRDTDHLFLELPLLRDKLEEYINNMSVAGSWSQNAIQATHA

WLKEGLKPRCITRDLKWGVPVPHEKYKDKVFYVWFDAPIGYISITACYTMDWEKWWKNPENVELYQFMGKDNVPFHTVMF

PSTLLGTSERWTMMKTISVTEYLNYESGKFSKSKGTGVFGNDAKDTKIPAEVWRYYLLTNRPEASDTLFMWADLQAKLNS

ELLNNLGNFINRVLSFIAKPPGAGYDSVIPDAPDAESHQLTKSLAEKVGKLVDQYLEAMEKVKLKQGLKTAMNISSEGNA

YLQESQFWKLYKEDPSSCAIVMKTSVGLVYLLASLLEPFMPSFSVEVLRQLNIAPETHLSFCDEKGETEKAKKLWAFLSS

GHKIGKPEPLFKELKDEDVEFFRVKFAGSQADRKERAEADEKKIAEQLKRTKIAEGNAKKQQAKPVGSSKSKVSETEISI

SKLDIRVGLIKKVQKHPDADSLYVEEIDVGEDSTRTVVSGLVKYIPLEEMQNRKVLVLCNLKPATMRGIKSHAMVLAASN

EDHSKVELVDPPESAVVGERVSFRGYSGEPDGILNAKSKVWEKVQADLHTDAELVACYKDVPFTTSAGVCKVLSIASGAI

R

>XP_008812681.1 PREDICTED: probable methionine--tRNA synthetase [Phoenix dactylifera]

MGDSPAKKPPKLPVPGRRNILITSALPYVNNVPHLGNIIGCVLSADVFARYCRLRGYNTIYICGTDEYGTATETKAMEEN

CSPKEICDKYHAIHRDIYKWFDISFDEFGRTSTPQQTEVCQSIFQKLMENKWLSENTMQQLFCETCPRFLADRLVEGTCP

TLDCNYESARGDQCEKCGKLLNPTELKDPKCKVCQTTPRIRDTDHLFLELPLLRDKLEEYINNMSVAGAWSQNAIQATYA

WLKEGLKPRCITRDLKWGVPVPHEKYKDKVFYVWFDAPIGYISITACYTMDWEKWWKNPENVELYQFMGKDNVPFHTVMF

PSTLLGTGEEWTMMKTISVTEYLNYESGKFSKSKGIGVFGNDAKDTKIPAEVWRYYLLTNRPEVSDTLFTWADLQAKLNS

ELLNNLGNFINRVLSFIAKPQGAGYDSVIPGALDSESHQLTKSLAEKVGKLVGQYLDAMEKVKLKQGLRTAMSISSEGNA

YLQDSQFWKLYKEDPSSCAIVMKTSVGLVYLLASLLEPFMPSFSVEVLRQLNIAPETHLSFCDEKGETEKAKKPWDFLPS

GHKIGKPEPPFKELKDEDVEFFRVKFAGSQADRKVKAEAEEKKIAEQLKNTKIREGNAKKQQTKPAGSSKSKASETEISI

SKLDIRVGMVKKVQKHPDADSLYVEEIDVGDNSTRTVVSGLVKYISLEEMQNRKVCVLCNLKPATMRGIKSHAMVLAASN

EDHSKVELVDPPESAVVGERVSFPGYSGEPDGVLNAKSKIWEKVQADLHTDAELVACYKNAPFTTSAGVCKVLSIASGAI

R

>XP_023919164.1 probable methionine--tRNA synthetase [Quercus suber]

MGDEKKDKKVETGKKVPIAGKRNILITSALPYVNNVPHLGNIIGSVLSADVFARYCRLRSYNVIYICGTDEYGTTTETKA

LEENCTPKQICDKYYAIHKEVYNWFNISFDEFGRTSTPQQTDICQSIFKKLMENNWLSENMIQQLYCDTCDRFLADRFVE

GTCPFPTCNYDSARGDQCEKCGELLNTTELKDPRCKVCRKTPQIRDTNHLFLELPLLKDKLEVYINDMSVAGSWSQNAIQ

ATHGWLKGGLKPRCITRDLKWGVPVPHEKFKDKVFYVWFDAPIGYVSITACYTPDWEKWWKNPENVELYQFMGKDNVPFH

TVMFPSALLGTGENWTLMKSISVTEYLNYEAGKFSKSKGIGVFGNDVKDTNIPSEVWRYYLLTNRPEVSDTLFTWADLQA

KLNTELLNNLGNFINRVLSFIAKPPGLGYGSVIPDAPGVESNLLTKTLSEKIGNYVEQYVEAMEKVKLKQALKIAMSISS

EGNAYLQESQFWKLYKQDQASCSLVVRTSVGLVYLLTCLLEPFMPSFSIKVLKQLNLPPEKISLCDETGDLDRAKKPWES

LPAGHKIGTPEPLFKELKDEDVEFFREKFAGSQADRDVKDRSRKEITEQLGKTKLT

>XP_021745553.1 probable methionine--tRNA synthetase [Chenopodium quinoa]

MADLHKNGDAATENRHILPIPGKRNVLITSALPYVNNVPHLGNIIGCVLSADVFARYCRLRGYNTIYICGTDEYGTATET

KAMEEKCTPKEICDKYHAIHKEVYEWFNISFDKFGRTSSPEQTEACQAIFKKLMDNNWLTENTMQQPYCDTCEKFLADRL

VEGTCPKPNCNYDSARGDQCEKCGNLLNPTELKDPKCKTCQSTPRIRDTNHLFLELPLLQDKLEEYINKMSEAGSWSQNA

IQVTNGWLKEGLRSRCITRDLKWGVPVPHEKFKDKVFYVWYDAPIGYISITACYTPEWEKWWKNPEDVELYQFMGKDNVP

FHTVMFPSTLLGTGENWTLMKSISVTEYLNYEAGKFSKSKGIGVFGNDAKDTSIPVEVWRYYLLTNRPEVSDTLFTWADL

QAKLNSELLSNLGNFVNRVLSFIAKPSGQGYGSVIPDAPQAEAHSLTKALAEKVGKHVEQYVEAMEKVKLKQGLKTAMAV

SSEGNAYLQESQFWKLYKEDQSACAVVMRTAAGLVYLLACLLEPFMPSFSLEVLKQLNMPQLQVSLSDENGNLEKIRKLW

DILPAGHSIGKPEPLFKELKDEEVEFYRDKFAGSQADRALKAAAEKTAEQLKKTKISDKSEKKKAAKPAAGSKTKSSPEA

EITIARLDIRVGLITKVQKHPDADSLYVEEIDVGEATPRTVVSGLVNYIPIEDMQNRKVCVLCNLKPASMRGIKSHAMVL

AASNSEHTKVELVEPPKEAPVGERVKFSGFDGEPDEVLNPKKKVWETLQVDLNTDSNLVARFKDLPFTTSAGICKVSSIS

NGTIK

>EIE81944.1 hypothetical protein RO3G_06649 [Rhizopus delemar RA 99-880]

MSKVSITLPKLAPNTKPFANALKVTIASQVKGIKATEVPGDLVSLVVSNEVSLTDANAAVNPFIVKRRLDQAFQTVEELV

SKYGKLFAKESAQVGFVDVIFYGTLVEAFASFKEPSKYPLLAAWFDIVSKNSKVSAAVEHIKEQVFTERKKKLPKMTHID

VYVQQNSKGSKTKKQGNHAEKKVTKDIPKPAFNAATQKVNADVASKPVDKNIKVLPKDNVRNIMITSALPYVNNVPHLGN

IIGSTLSADVFARYCRARGYNTAYFCGTDEYGTATETKALEEGVSCQELCDKYNAVHSEVYKWFDLSFDHFGRTTTERQT

EIVQDMFWKNYKNGNITKESMIQLYCENCQRFLADRYVEGICPLCQYNDARGDQCDACGKLLNATELVEPRCKLDGNRPI

IKESNHLFLDLSKLQPVIEEGFKKSSTEGGWSANGINITQSWLKEGLKPRCITRDLKWGVPVPLEGYEDKVFYVWFDAPV

GYPSITANYTDEWEKWWKNPEQVKLYQFMGKDNVPFHSVIFPGNQMGTGENWTMVNHISTCEYLNYEGGKFSKSRNVGVF

GNNAKDTGIPPSVWRYYLLSVRPETSDSMFTWNEFITKNNSELLNNFGNFVNRAIRFIFAKYDGVIPKADFSGPLETDLF

KDVNGLLKQYVEQMEAVKLRAGLGTLMSISARGNQYLQEANLSNALFDETRAKCDAVVASAVNLIYLLSGLVSPFMPAVA

DSMCRQLNAPARLIPEEFSVDILPGHVLGGAEYLFTKIDEKMEDVWKNKYGTGA

>PUU83014.1 tRNA synthetases class I (M)-domain-containing protein [Tuber borchii]

MASLKVNIPIPAPSGSSAFTQTLKVCLAASAYGVPVQFAQVSGQTNISLEVPRENKILIEANAIVRYFATTGSRPWLKSG

EVALKDSALIEFEESGQGIKAADVAESVISKANFPTDPALPPSPAEIILFSNLWDLASTSTSDDRPTLSSWLIRIIGTEP

TLSSWLIKIIGTEWAKVGVEQATAHTADKPVTKSTGKGVGKGVGKGVGKGAGKGAGKNTSIPVDDTPREMIQKIKEGTNM

KIRDPKEPILPVPGERNVLITSALPYVNNVPHLGNIVGSVLSADAFSRYAKARNYNTLYICGTDEYGTATETKALEEGVT

PRALCDKYNALHAGVYKWFDIGFDYFGRTTTDAQTEIAQDIFLKLQKNGFLVEKTMTQLFCEKHQGFLADRFVEGTCPKC

DYEDARGDQCDTCGSLLDPLDLINPRCKLDGTGPIKRDSKHIFIQLDELQSEIEEWSKKTSEEGQWTKNGRIITESWLKT

GLKPRCITRDLTWGTPVPVPGFENKVMYVWFDACIGYVSITANYTKEWEKWWKNKNEVKLYQFMGKDNVPFHTVVFPGTQ

IGTRENWTMLHHISTTEYLQYEGGKFSKSRGVGVFGNNAQDTGIPPDIWRYYLLSSRPETSDTQFVWQDFATKNNSELLA

NLGNFVNRLIKFVNAKYAGVVPDYTAGINDLSFNQYKEAVNALLKSYVEELEEVHIRAGLEKAMAISGEGNRFLQDNKLD

SALFTNSPEKAAAVVGFGLNLIYLLSALIYPYMPATSESIVKQLNAPPRSIPDVWDADGLLPGHVIGRAEYLFSRIEENQ

VQEWKSRYGGKQEAAPRDTKKEKKKGKSAARQQNAKEGAASLGPAPTSTANVEVRLIV

>XP_019168455.1 PREDICTED: methionine--tRNA synthetase, cytoplasmic-like isoform X1 [Ipomoea nil]

MGEVSEKVPKVPIPGKRNILITSALPYVNNVPHLGNIIGCVLSADVFARYCRLRGYNAIYICGTDEYGTATETKALEENS

SPQQICDKYHEIHKQVYQWFDISFDKFGRTSTPQQTEVCQSIFKKLLDNNWLTENTIQQPYCDTCKKFLADRLVEGTCPT

PGCNYDSARGDQCEKCGKLLNPTELKEPRCKVCRNTPCIKDTDHLFLELPLLKDKLESYVNSMSVAGGWSQNAIHTTHAW

LREGLKSRCITRDLKWGVPVPHEKYKDKVFYVWFDAPIGYVSITSCYTAEWEKWWKNPENVELYQFMGKDNVPFHTVIFP

STLFGTGENWTFMKTISVTEYLNYESGKFSKSKGIGVFGNDVKDTTIPVEVWRYYLLANRPEVSDTLFTWTDLQAKLNSE

LLSNLGNFINRVLSFIAKDPASGYGSVIPDAHGVESHSSTKALAEKVGNYLDQYIEAMEKVKLKQALKIAMSLSGEGNAY

LQESQFWKLYKEDKPSCSVVMRTAAGLVYLLAFLLEPFMPSFSREVFKQLNLPESWLSLSDENGDIERAKKPWDILPAGH

KIGTPVPLFKELKDEEVEMYREKFAGSQADRTLRVEAEAKKITEQLNKAKISNEKIKKERSTKSAEAKAKAGASVSAEAE

VSISRLDIRVGLIKKAQKHPDVDSLYVEEIDVGEAQPRTVVSGLVKYIPLEEMQNCKVCVLCNLKPATMRGIKSHAMVLA

ASNNDHTKVCGDLLLGLKEFIILSF

>XP_017700174.1 PREDICTED: LOW QUALITY PROTEIN: probable methionine--tRNA synthetase [Phoenix dactylifera]

MANSPAKNALKLPVLGRRNILITSALPYVNNVPHLGNIIGCVLSADVFARYCRLRGYNAIYICGTDEYGTATETKAMEEN

CSPKEICDKYHAIHRDIYKWFDISFDKFGRTSTPQQTDVCQSIFHKLMENSWLSENTMQQLYCDSCERFLADRLVEGTCP

TLDCNYESARGDQCEKCGKLLNPTELKDPKCKVCQTTPRIRNTDHLFLELPLLRDKLEEYINNMSVAGSWSQNAIQATHA

WLKEGLKPRCITRDLKWGVPVPHEEYKDKVFYVWFDAPIGYISITACYTTDWEKWWKNPENVELYQFMGKDNVPFHTVMF

PSTLLGTGEKWTMMKTISVTEYLNYESGKFSKSKGTGVFGNDAKDTKIPVEVWRYYLLTNRPEASDTLFMWADLQAKLNS

ELLNNLGNFINRVLSFIAKPPGAGYDSVIPDAPDAESHQLTKSLAEKVGKLVDQYLEAMEKVKLKQGLKTAMNISSEGNA

YLQESQFWKLYKEDPSSCAIVMKTSVGLVYLLASLLEPFMPSFSVEVLRQLNIAPETHLSFCDEKGETEKAKKPWDFLPS

GHKIGKPGPLFKELKDEDVEFFRVKFAGSQADRKVKAEADDKKIAEQLKRTKITGIAKEQQSKPARSSKSKASETEISIS

KLDIRVGLIKKVEKHPDADSLYVEEIDVGEDSTRTVVSGLVKYIPLEEMQNRKVCVLCNLKPATMRGIKSHAMVLAASSE

DHSKVELVDPPESAAVGERVSFPGYSGEADGVLNAKSKVWEKVQADLHTDAERVACYKDVPFTTSAGVCKVLSVASGAIR

>XP_018296318.1 hypothetical protein PHYBLDRAFT_130614 [Phycomyces blakesleeanus NRRL 1555(-)]

MKTNEQTPLAELNPETQKVNRKVFMKKHDTSVKPVPVEGERNILITSALPYVNNIPHLGNVVGSTLSADVYARYCRARGY

NIIYICGTDEYGTATETKALEEGVSCQALCDKYNAVHRSVYKWFDISFDYFGRTTTPKQTEIAQDMFLKAKANGYLLEQT

MTQLYCEHCKRFLADRYVEGVCPVCKYEDARGDQCDACGRLLNASELVKPRCKLDGHPPITRDSNHMFLDLSKLQDKIEK

FNAKVNVEGKWSSNGINITQSWLKEGLKPRCITRDLKWGTPVPLAGFEDKVFYVWFDAPIGYPSITANYTDEWEKWWKNP

ANVKLYQFMGKDNVPFHSVIFPGSEMATGDDWTLVHHISTTEYLNYEGGKFSKSRNIGVFGTSAEETGIPPSVWRYYLLS

SRPETSDSMFTWNEFITKNNSELLNNLGNFVNRAIKFVLAKYDGVIPQANLSGESEVTMIKDVNVLLAQYNDQLENVKIR

GALATAMAISARGNQYLQDSGLSNSTFAEQRGKCDAVVNVAVNLIYLLSAILFPYMPEVSENISRQLNAPLRRIPDEFSL

DILPGHKLNGSAYLFTQIDEKMEDIWKQKYGTSAK

>PON95468.1 Methionyl-tRNA synthetase [Trema orientalis]

MGDVSNPDAGSGDGKAPKLPIPGRRNILITSALPYVNNVPHLGNIIGCVLSADVFARYCRLRGYNAIYICGTDEYGTATE

TKALEEKCSPKEICDKYHAIHRDVYKWFNISFDEFGRTSSPQQTEVCQAIFKKLLENNWLSENTMQQPYCDTCKKFLADR

LVEGTCPTPGCDYDSARGDQCEKCGKLLNPTELKNPRCKVCGTTPHIRDTNHLFLELPLLRDKLEKYINEMSVAGSWSQN

AIQATNAWLKEGLKPRCITRDLKWGVPVPHDNFRDKVFYVWFDAPIGYVSITSCYTSDWEQWWKNPENVELYQFMGKDNV

PFHTVMFPSTLLGTGEAWTMMKTISVTEYLNYEAGKFSKSKGIGVFGNDAKDTGIPVEVWRYYLLTNRPEVSDTLFTWAD

LQAKLNSELLNNLGNFINRVLSFIAKPSGQGYDSIIPDAPHDVSDALTVKLADKVGTFVEDYVAAMEKVKLKQGLKTAMS

ISSEGNAYLQESQFWKLYKEDQPRCSIVMKTSVGLVHILARLLEPFMPSFSLEVFKQLNLEPEQLSLADERGDIDRARRP

WDIVPSGHRIGKPEPLFKELKDEEVESFRKRFAGSQADRVERAEAEAEKVAQQLKKTKITENSGKKQKSAKPATEVEISI

SRLDIRVGLITKAQKHPDADSLYVEEIDVGEGQPRTVVSGLVKYIPLEEMQNRKVCVLCNLKPAAMRGIKSQAMVLAASN

SDHTKVELVEPPKSAQVGERVSFPGFEGQPDDVLNPKKKVWETLQVDLHTNTDLVACYKDIPLTTSAGVCTVSTIREGSI

R

>PKK78255.1 methionyl-tRNA synthetase [Rhizophagus irregularis]

MSTNSVSERKINSGVALEIEKEKNKEPIKGERNILITSALPYVNNIPHLGNIIGSVLSADVFARYSRARRYNTLFVCGTD

EYGTATETKALEEGTTCQELCDKYNAIHKQVYEWFELDFDYFGRTTTRQQTEIAQDIFMKLYKNGYLSEDAMIQLFCEKC

QRFLADRYVEGTCPKCNYEDARGDQCDSCGQLLNATELVNPRCKLEGSTPIMRESKHMFLDLDTLQKTVEDWVIKTSVEG

KWSTNSKTITNSWLKEGLKPRCITRDLKWGTPVPLEEMKEKIFYVWFDAPIGYLSITANYTPHWEKWWKNPDDVKLYQFM

GKDNVPFHTVIFPSSLFGTGEDWTLLYHINTTEYLNYENTKFSKSRNIGVFGTDAKDTGINVSVWRYYLIASRPETNDSV

FTWKEFIAKNNNELLANLGNFVNRVIKFSASKYNSIVPEFSYDKIEEKEFIENIDSLLKNYIETLENVKLRAGLEIAMEI

SRLGNGYLQESKLDNSLFQNNIEKCSTVIGIAINLIYLLSALFFPYIPSTTESICRQLNAPLRDIPDTWTIDILPGHKLG

KAEYLFKKIDDKKEEEYLNRYGGGDGKVAETKKKAKKGKNVNTVTNNKSSKIVNG

>PKC60057.1 methionyl-tRNA synthetase [Rhizophagus irregularis]

MSTNSVSERKINSGVALEIEKEKNKEPIKGERNILITSALPYVNNIPHLGNIIGSVLSADVFARYSRARRYNTLFVCGTD

EYGTATETKALEEGTTCQELCDKYNAIHKQVYEWFELDFDYFGRTTTRQQTEIAQDIFMKLYKNGYLSEDAMIQLFCEKC

QRFLADRYVEGTCPKCNYEDARGDQCDSCGQLLNATELVNPRCKLEGSTPIMRESKHMFLDLDTLQKTVEDWVIKTSVEG

KWSTNSKTITNSWLKEGLKPRCITRDLKWGTPVPLEEMKEKVFYVWFDAPIGYLSITANYTPHWEKWWKNPDNVKLYQFM

GKDNVPFHTVIFPSSLFGTGEDWTLLYHINTTEYLNYENTKFSKSRNIGVFGTDAKDTGINVSVWRYYLIASRPETNDSV

FTWKEFIAYNNNELLANLGNFVNRVIKFSASKYNSIVPEFSYDKIEEKEFIENIDSLLKNYIETLENVKLRAGLEIAMEI

SRLGNGYLQESKLDNSLFQNNIEKCSTVIGIAINLIYLLSALFFPYMPSTTESICRQLNAPLRDIPDTWTIDILPGHKLG

KAEYLFKKIDDKKEEEYLNRYGGGDGKVAETKKKAKKGKNVNTVTNNKSSKIVNG

>PHU18272.1 putative methionine--tRNA synthetase [Capsicum chinense]

MGDNCKSTLKVPIAGKRNILITSALPYVNNVPHLGNIIGCVLSADVFARYCRIRGYNAIYMCGTDEYGTATETKALEENC

TPKQICDKYHAIHKDVYKWFDISFDEFGRTSTPQQTEVCQAIFKKLWDNNWLSENTMQQLYCDTCKKFLADRLVEGNCPT

PGCNYDSARGDQCEKCGKLLNPTELLEPRCKVCRNTPCIRDTDHLFLELPLLKDKLEAYINEMSVSGGWSQNAIYTTHAW

LREGLKSRCITRDLKWGVPVPHEKYKDKVFYVWFDAPIGYVSITSCYTTEWEKWWKNPDNVELYQFMGKDNVPFHTVIFP

STLLGTGERWTLMKTISVTEYLNYEAGKFSKSKGIGVFGNDVKDTNIPVEVWRYYLLTNRPEVSDTLFTWADLQAKLNSE

LLSNLGNFINRVLSFIAKDQASGYGSIIPDAEGAESHSLTKALGERVGSCVQQYIEAMEKVKLKQGLKVAMSISSEGNGY

LQESQFWKLYKEDRPSCSIVMRTACGLVYLLACLLEPFMPSFSREVLKQLNLPPEMLSLSDEGGDIENSKRPWNILPAGH

RIGTPAPLFKELKDEEVEFYRAKFAGSQADRADRALKAETEAKNVAEKLNKVKISDGNKKKERATKSSETKSKAPPSVEA

EISISRLDIRVGLITKAQKHPDADSLYVEEIDVGESQPRTVVSGLVKYIPLEEMQNRKVCVLCNLKPASMRGIKSQAMVL

AASNSDHTKVELVEPPKDAVVGERVTFPGFDGKPDDILNPKKKVWETVQVDLQTNKKLVACYKDVPLTTSVGTCKVASIS

EGSIR

>XP_019024018.1 methionyl-tRNA synthetase [Saitoella complicata NRRL Y-17804]

MKTLYEKEILPRKGEKNILITSALPYVNNVPHLGNIVGSVLSADVFARYNKARNFNTLFICGTDEYGTATETKALEDGVT

PKELCDKYHAIHADVYRWFEIGFDHFGRTTTPKQTEIAQDIFMRLYEKGYLQEDTMTQLWCEKHQGFLADRYVEGTCPKC

LYEDARGDQCDKCGQLLNASELIEPRCKLDGNKPVLRDSKHIFLALDKLQEKVEAWSKESAEKGKWSANGRAITESWLKE

GLNPRCITRDLKWGTPVPLEGFTDKVLYVWFDATIGYVSITANYTDKWQEWWQNPEDVKLYQFMGKDNVPFHTVVFPATL

LGTGQNWTMLHHINTTDYLNYEGGKFSKSRNVGVFGNNAQQTGLSPSVWRYYLIMARPETSDTQFTWKEFVSRNNTELLA

NLGNFVNRVIKFVNAKYGGVIAEYSSDLAEDDESKIAQLKENVNELLAQYNKDLEASRLRAGLQTVMAISARGNVFLQDN

KLDNTLFAEHPARCANVLAAALNLIYLISALAYPYMPSTSSSIAEQLNAPLRAIPDVWSNDDLQTGHKLGKAKYLFGRVD

EKMEEEWRAKFGGGSGTPVVDEKKEAKKAQKEAKKAKKAAEKAASAEGEKKEEEVKA

>XP_021594539.1 probable methionine--tRNA synthetase [Manihot esculenta]

MGDLSSTNSSGDGRTAPKVPIPGKRNILITSALPYVNNVPHLGNIIGCVLSADVFARYCRLRGYNAIYICGTDEYGTATE

TKALEENCTPKEICDKYHAIHREVYKWFNISFDEFGRTSSPQQTEVCQAIFKKLLENNWLSENAMQQLYCDTCKRFLADR

LVEGNCPTEGCNYDSARGDQCEKCGKLLNPTELKDPRCKVCRNTPHIRETNHLFLELPLLRDKLGKYIESMSIAGSWSQN

AIQATNAWLKEGLKPRCITRDLKWGVPVPHEKFMDKVFYVWFDAPIGYVSITACYTPDWEKWWKNPENVELYQFMGKDNV

PFHTVMFPSTLLGTGENWTLMKTISVTEYLNYEAGKFSKSKGIGVFGNDVKDTKIPVEVWRYYLLTNRPEVSDTLFTWSD

LQAKLNSELLNNLGNFINRVLAFISKPPGAGYGSIIPDAPNADSHPLTKKLAEEVHKYVEQYVEAMEKVKLKQGLKTAMS

ISSEGNAYLQESQFWKLYKEDQSSCNIVMRTAVGLVYLLACLLEPFMPSFSLEVFKQLNLPPEKASLCDEKGDIDRAKKP

WEYLSTNHKIGTPEPLFKELKDEEVDFFRQKFAGSQAERAEAEAAKIAEQLKKTKVSDVSGKKQQTKKPSAEAKPKGTVE

PEISITRLDIRVGLIKKAQKHPDADSLYVEEIDVGGGETRTVVSGLVKYIPLEEMQNRKVCVLCNLKPATMRGIKSQAMV

LAASNSDHTKVELVDPPESAAVGERVTFPGFEGQPDDVLNPKKKVWETLQVDLHSNTDLVACYKDIPFTTSAGVCKVASI

QSGSIR

>XP_016573621.1 PREDICTED: methionine--tRNA synthetase, cytoplasmic [Capsicum annuum]

MGDNCKSTLKVPIAGKRNILITSALPYVNNVPHLGNIIGCVLSADVFARYCRIRGYNAIYMCGTDEYGTATETKALEENC

TPKQICDKYHAIHKDVYKWFDISFDEFGRTSTPQQTEVCQAIFKKLWDNNWLSENTMQQLYCDTCKKFLADRLVEGNCPT

PGCNYDSARGDQCEKCGKLLNPTELLEPRCKVCRNTPCIRDTDHLFLELPLLKDKLEAYINEMSVSGGWSQNAIYTTHAW

LREGLKSRCITRDLKWGVPVPHEKYKDKVFYVWFDAPIGYVSITSCYTTEWEKWWKNPDNVELYQFMGKDNVPFHTVIFP

STLLGTGERWTLMKTISVTEYLNYEAGKFSKSKGIGVFGNDVKDTNIPVEVWRYYLLTNRPEVSDTLFTWADLQAKLNSE

LLSNLGNFINRVLSFIAKDQASGYGSIIPDAEGAESHSLTKALGERVGSCVQQYIEAMEKVKLKQGLKVAMSISSEGNGY

LQESQFWKLYKEDRPSCSIVMRTACGLVYLLACLLEPFMPSFSREVLKQLNLPPEMLSLSDEGGDIENSKRPWNILPAGH

RIGTPAPLFKELKDEEVEFYRAKFAGSQADRADRALKAETEAKNVAEKLNKVKISDGNKKKERATKSSETKSKAPPSVEA

EISISRLDIRVGLITKAQKHPDADSLYVEEIDVGESQPRTVVSGLVKYIPLEEMQNRKVCVLCNLKPASMRGIKSQAMVL

AASNSDHTKVELVEPPKDAVVGERVTFPGFDGKPDDILNPKKKVWETVQVDLHTNKELVACYKDVPLTTSVGTCKVASIS

EGSIR

>XP_010905660.1 PREDICTED: probable methionine--tRNA synthetase isoform X2 [Elaeis guineensis]

MANPPAKNAPKLPVPGRRNILITSALPYVNNVPHLGNIIGSVLSADVFARYCRLRGYNAIYICGTDEYGTATETKAMEEN

CSPKEICDKYHAIHRDIYKWFDISFDEFGRTSTPQQTEVCQSIFHKLMENNWLSENTMQQLYCDTCKRFLADRLVEGTCP

TLDCNYESARGDQCEKCGKLLNPTELKDPKCKVCRATPRIRDTDHLFLELPLLRDKLEEYINNMSVAGSWSQNAIQATHA

WLKEGLKPRCITRDLKWGVPVPHEKYKDKVFYVWFDAPIGYISITACYTMDWEKWWKNPENVELYQFMGKDNVPFHTVMF

PSTLLGTSERWTMMKTISVTEYLNYESGKFSKSKGTGVFGNDAKDTKIPAEVWRYYLLTNRPEASDTLFMWADLQAKLNS

ELLNNLGNFINRVLSFIAKPPGAGYDSVIPDAPDAESHQLTKSLAEKVGKLVDQYLEAMEKVKLKQGLKTAMNISSEGNA

YLQESQFWKLYKEDPSSCAIVMKTSVGLVYLLASLLEPFMPSFSVEVLRQLNIAPETHLSFCDEKGETEKAKKLWAFLSS

GHKIGKPEPLFKELKDEDVEFFRVKFAGSQADRKERAEADEKKIAEQLKRTKIAGNAKKQQAKPVGSSKSKVSETEISIS

KLDIRVGLIKKVQKHPDADSLYVEEIDVGEDSTRTVVSGLVKYIPLEEMQNRKVLVLCNLKPATMRGIKSHAMVLAASNE

DHSKVELVDPPESAVVGERVSFRGYSGEPDGILNAKSKVWEKVQADLHTDAELVACYKDVPFTTSAGVCKVLSIASGAIR

>XP_006373073.1 probable methionine--tRNA synthetase [Populus trichocarpa]

MGDLDRPVPKLPIPGKRNILITSALPYVNNVPHLGNIIGCVLSADVFARYCRLRGYNAIYICGTDEYGTATETKAMEENS

TPKQICDKYHAIHREVYKWFNISFDEFGRTSSPQQTEVCQAIFKKLLDNNWLSENTMKQLYCDTCERFLADRLVEGTCPT

EGCNYDSARGDQCENCGKLLNPTELINPRCKQCKNTPRIRDTNHLFLELPQLKDKLVSYIESMSVAGSWSQNAIQTTNAW

LKEGLKPRCITRDLKWGVPVPLDEFKDKVFYVWFDAPIGYVSITSCYTPDWEKWWKNPDNVELYQFMGKDNVPFHTVMFP

STLIGTGENWTMMKTISVTEYLNYEAGKFSKSKGVGVFGNDAKDTNIPVEVWRYYLLTNRPEVSDTLFTWSDLQAKLNSE

LLNNLGNFINRVLSFLAKPPGLGYGSIIPDAPGADSHPLTKKLAGEVGKYVEQYLEAMEKVKLKQGLKTAMSISSEGNAY

LQESQFWKLYKEDQPSCSIVIKTSLGLVYLLACLLEPFIPSFSVEVFKQLNLPLEQASLCDEKGDMDRAKRPWEILPAGH

KIGTPEPLFKELKDEEVEDYRRRFAGSQADRLERDEAEKAAKLAEQLKKKASVGGGKKQQAKKPASEAKSKGSVEREISI

TRLDIRVGLIKKVQKHPDADSLYVEEIDLGEGDCRTVVSGLVKYIPLEEMQNRKVCVLCNLKPASMRGIKSQAMVLAASN

SDHTKVELVDPPPSAVVGERVTFQGFEGEPDDVLNPKKKVWETLQVDLQTNSDLIACYKDIPLTTSAGVCKVASISCGSI

R

>XP_023921000.1 methionine--tRNA synthetase, cytoplasmic [Quercus suber]

MAAKVPIKGKRNILITSALPYVNNVPHLGNIIGCVLSADVFARYCRLRGYNALYICGTDEYGTATETKALEENSTPKQIC

DKYHAIHKEVYDWFNISFDKFGRTSTPEQTEICQAIFKKILENNWLSENSMQQLYCDTCKRFLADRLVEGTCPKEGCNYP

SARGDQCENCGSLLNPTELKDPRCKVCQTTPHIRDTNHLFLELPLLKDKLEEYINKMSMAGSWSQNAIQTTNAWLKEGLK

SRCITRDLKWGVPVPLERFSDKVFYVWFDAPIGYVSITACHTPEWEKWWKNPEDVELYQFMGKDNVPFHTVMFPSTLLGT

GENWTLMKTISVTEYLNYEAGKFSKSKGIGVFGNDAKDTNIPVEVWRYYLLTNRPEVSDTLFTWADLQAKLNSELLNNLG

NFINRVLSFIVKPPGQGYGSIIPQPSDADPDPLQHEPTRKLVGSIVDYVEQYVEAMEKVKLKQGLKIAMSISSEGNGYLQ

ESRFWKLYNDDRPSCSLVIRTSAGLVYLLACLLEPFMPSFSLEVLKQLNLPPDTPFLLEKGDIDRVSRPWEILPAGHKIG

TPEPLFKELRDEEVEFFREKFAGSQADRIVRAEAEAEKIAEKLKKTKVSDSSGKKKRPVKPATESKAKANAETEISITRL

DIRVGLITSVQKHPDADSLYVEEIDVGEGQTRTVVSGLVKYIPLEEMQNRKVCVLCNLKPATMRGIKSQAMVLAASDSDH

TKVELVDPPKSATVGERVTFPGFNGEPDDVLNPKKKVWETLQVDLNTNTELVASYKDIPFTTSAGVCTVSSIASGSIR

>PHT48749.1 putative methionine--tRNA synthetase [Capsicum baccatum]

MGDNCKSTLKVPIAGKRNILITSALPYVNNVPHLGNIIGCVLSADVFARYCRIRGYNAIYMCGTDEYGTATETKALEESC

TPKQICDKYHAIHKDVYKWFDISFDEFGRTSTPQQTEVCQAIFKKLWDNNWLSENTMQQLYCDTCKKFLADRLVEGNCPT

PGCNYDSARGDQCEKCGKLLNPTELLEPRCKVCRNTPCIRDTDHLFLELPLLRDKLEAYINEMSVSGGWSQNAIYTTHAW

LREGLKSRCITRDLKWGVPVPHEKYKDKVFYVWFDAPIGYVSITSCYTTEWEKWWKNPDNVELYQFMGKDNVPFHTVIFP

STLLGTGERWTLMKTISVTEYLNYEAGKFSKSKGIGVFGNDVKDTNIPVEVWRYYLLTNRPEVSDTLFTWADLQAKLNSE

LLSNLGNFINRVLSFIAKDQASGYGSIIPDAEGAESHSLTKALGERVGSCVQQYIEAMEKVKLKQGLKVAMSISSEGNGY

LQESQFWKLYKEDRPSCSIVMRTACGLVYLLACLLEPFMPSFSREVLKQLNLPPEMLSLSDEGGDIENSKRPWNILPAGH

RIGTPAPLFKELKDEEVEFYRAKFAGSQADRADRALKAETEAKNVAEKLNKVKISDGNKKKERATKSSETKSKAPPSVEA

EISISRLDIRVGLITKAQKHPDADSLYVEEIDVGESQPRTVVSGLVKYIPLEEMQNRKVCVLCNLKPASMRGIKSQAMVL

AASNSDHTKVELVEPPKDAVVGERVTFPGFDGKPDDILNPKKKVWETVQVDLHTNKELVACYKDVPLTTSVGTCKVATIS

EGSIR

>KZM85945.1 hypothetical protein DCAR_026633 [Daucus carota subsp. sativus]

MSNLSAAAAAGDDRKPIKLPISGKRNVLITSALPYVNNVPHLGNIIGCVLSADVFARYCRLRGYNAIYVCGTDEYGTATE

TKALEENCTPKEICDKYHAIHKKVYEWFDIKFDEFGRTSTPQQTEVCQAIFTKLMENNWLSENTMQQLYCDTCKRFLADR

LVEGSCPTQGCNYDSARGDQCEKCGKLLNPTELIDPKCKVCQNTPRIRDTDHLFLELPLLKDKLVEYINNMSVAGGWTQN

AIQATNAWLKEGLRQRCITRDLKWGVPVPHEKYKDKVFYVWFDAPIGYVSITSCYTAEWEKWWKNPENVELYQFMGKDNV

PFHTVMFPSTLLGTGENWTLMKTISVTEYLNYEAGKFSKSKGIGVFGNDAKDTNIPVEVWRYYLLTNRPEVSDTLFTWAD

LQAKLNGELLNNLGNFVNRVLSFIAKDQASGYGSIIPDAPGAESHLLTKNLGDKVGSCVEQYIEAMEKVKLKQGLKLAMS

ISGEGNAYLQESQFWKLYKEDKASCSIVLRTAAGLVYLLACLLEPFMPSFSAEVLKQLNMPLETQVTLTDEKGDIERSKR

PWELLPAGHKIGTPAPLFKELSDKDVESFRQKFAGSQADRIVKAEAEAKQITEQLKKAKVSDGNVKKERTKKSASENKPK

AAEQEISISRLDIRVGLITKVQKHPDADSLYVEEIDVGEGQPRTVVSGLVKFIPLEEMQNRKVCVLCNLKPATMRGIKSH

AMVLAVSNADHSKVELVDPPQSAPVGERLTFSGFEGDPDDVLNPKKKVWETIQPDLHTNKELIACYKDLPFTTSVGVCKS

SSEHYASFDGNVKKERTKKSASENKPKAAEQEISISRLDIRVGLITKVQKHPDADSLYVEEIDVGEGQPRTVVSGLVKFI

PLEEMQNRKVCVLCNLKPATMRGIKSHAMVLAVSNADHSKVELVDPPQSAPVGERLTFSGFEGDPDDVLNPKKKVWETIQ

PDLHTNKELIACYKDLPFTTSVGVCKVSSISDGSIR

>XP_010069016.1 PREDICTED: methionine--tRNA synthetase, cytoplasmic [Eucalyptus grandis]

MGSSGGTKLPIPGKRNVLITSALPYVNNVPHLGNIIGCVLSADVFARYCRLRGYNAVYICGTDEYGTATETKAMEEKCTP

QQICDKYHAVHKQVYDWFDISFDKFGRTSTPQQTEVCQAIFKKLYDNNWLTENTMQQLYCDNCPRFLADRLVEGVCPTPG

CNYDSARGDQCEKCGKLLNPTELIDPRCKVCQTTPRIRDTNHLFLELPQLKDKLVEYINEMSEAGSWSQNAIQTTHAWLR

EGLKPRCITRDLKWGVPVPHEKFEDKVFYVWFDAPIGYVSITSCYTPEWEQWWKNPENVELFQFMGKDNVPFHTVIFPST

LLGTGEKWTLMKTISVTEYLNYEAGKFSKSKGIGVFGNDVKDTNIPVEVWRYYLLTNRPEVSDTLFTWVDLQAKLNSELL

NNLGNFVNRVLSFVAKPTGLGYGSVIPDAQGADSHSLTVELAEKVGKLVEQYVEAMEKVKLKQGLKTAMSISSEGNAYLQ

ASQFWKLYKEDKPSCSIVMRTSVGLVHLLACLLEPFMPSFSIEVFKQLNLPPETQVSLQDENGEVERAKRPWEIIPAGHK

IGVPEPLFKELKDEEVEFYRTKFAGSQADRIVRAEAEAAKVAEQLRKTKVSDGSKKRSQPAKSSAGAKSKVPAAEAEISI

TRLDIRVGLITKVQKHPDADSLYVEEIDVGEGQNRTVVSGLVKHIPLEEMQNRKVCVLCNLKPATMRGIKSQAMVLCASN

KENTKVELIEPPESAKVGERVTFPGFEGAPDDVLNPKKKVWETLQPDLHTDSGLVAHFKNIPFTTSAGVCKASSIQDGSI

R

>XP_006592017.1 PREDICTED: probable methionine--tRNA synthetase isoform X2 [Glycine max]

MAENLIAPKLPVEGKRNILITSALPYVNNVPHLGNIIGCVLSADVFARYCRLRGYNAIYICGTDEYGTATETKAMEENCS

PKEICDKYHAIHKEVYNWFNISFDEFGRTSSPEQTEVCQAIFRKIFENKWLSENTMQQLYCNTCERFLADRLVEGTCPTP

GCEYDSARGDQCEKCGKLLNPTELKNPRCKVCQKTPHIRDTDHLFLELPLLKDRLEKYISEMSVVGGWSQNAIQTTNSWL

REGLKPRCITRDLKWGVPVPHEKYSDKVFYVWFDAPIGYVSITSCYTHEWEKWWKNPENVELYQFMGKDNVPFHTVMFPS

TLLGTSENWTLMKTISVTEYLNYEAGKFSKSKGIGVFGNDAKDTNIPVEVWRYYLLTNRPEVSDTLFTWPDLQAKLNSEL

LNNLGNFINRVLSFIAKPAGQGYDSIIPSVPDNVSGDSHGPTKKLADKVAAYIDQYIEAMEKVKLKQGLKTAMSISSEGN

AYLQEAEFWRLYKENKSLCSLVMKTAAGIVYLLACLLEPFIPSFTLEVFKQLNLPADTHVSLSDDKGDVDRVKRPWDIIS

AGHKIGTPKPLFRELKDEEVEFYRKKFAGSQADRIVRAEAEAQNVAEQLKKTKVSDGNGKKKPGKSSNEAKNKAAAEPDI

TITRLDIRVGLIIKAQKHPDADALYVEEIDVGEEQTRTVVSGLVKFIPLDEMQNRKVCVLCNLKPVTMRGIKSQAMVLAA

SDGDHTKVELVEPPSSAQPGERITFPGYEGNPDELLNPKKKVWETLQVDLHTNEELVACYKNVPLTTSAGVCKVSSISCG

SIR

>KHN46449.1 Putative methionine--tRNA synthetase [Glycine soja]

MTENLKAPKLPVEGKRNILITSALPYVNNVPHLGNIIGCVLSADVFARYCRLRGYNAIYICGTDEYGTATETKAMEENCS

PKEICDKYHAIHKEVYNWFNISFDEFGRTSSPEQTEVCQAIFRKIFENKWLSENTMQQLYCNTCERFLADRLVEGTCPTP

GCEYDSARGDQCEKCGKLLNPTELKNPRCKVCQKTPHIRDTDHLFLELPLLKDRLEKYISEMSVVGGWSQNAIQTTNSWL

REGLKPRCITRDLKWGVPVPHEKYSDKVFYVWFDAPIGYVSITSCYTREWEKWWKNPENVELYQFMGKDNVPFHTVMFPS

TLLGTSENWTLMKTISVTEYLNYEAGKFSKSKGIGVFGNDAKDTNIPVEVWRYYLLTNRPEVSDTLFTWPDLQAKLNSEL

LNNLGNFINRVLSFIAKPAGQGYDSIIPSVPDNVSGDSHGPTKKLADKVAAYIDQYIEAMEKVKLKQGLKTAMSISSEGN

AYLQEAEFWRLYKENKSLCSLVMKTAAGIVYLLACLLEPFIPSFTLEVFKQLNLPADTHVSLSDDKGDVDRVKRPWDIIS

AGHKIGTPKPLFRELKDEEVEFYRKKFAGSQADRIVRAEAEAQNVAEQLKKTKVSDGNGKKKPGKSSNEAKNKAAAEPDI

TITRLDIRVGLIIKAQKHPDADALYVEEIDVGEEQTRTVVSGLVKFIPLDEMQNRKVCVLCNLKPVTMRGIKSQAMVLAA

SDGDHTKVELVEPPSSAQPGERITFPGYEGNPDELLNPKKKVWETLQVDLHTNEELVACYKNVPLTTSAGVCKVSSISCG

SIR

>XP_012071110.1 probable methionine--tRNA synthetase [Jatropha curcas]

MGDLSSNNTTGEVRTAPKVPIAGKRNILITSALPYVNNVPHLGNIIGCVLSADVFARYCRLRGYNAIYVCGTDEYGTATE

TKAMEENCTPKEICDKYHAIHKEVYKWFNISFDEFGRTSTPQQTEVCQTIFKKLLENNWLSENTMQQLYCDTCKRFLADR

LVEGTCPTEGCNYDSARGDQCEKCGKLLNPTELKDPKCKVCRNTPRIRDTNHLFLELPLLKDKLVQYIENMSVAGSWTQN

AIQATNAWLKEGLKSRCITRDLKWGVPVPHEKFKDKVFYVWFDAPIGYVSITKCYTPDWEKWWKNPENVELYQFMGKDNV

PFHTVMFPSTLLGTGENWTLMKTISVTEYLNYEAGKFSKSKGIGVFGNDAKDTNIPVEVWRYYLLTNRPEASDTLFTWSD

LQAKLNSELLNNLGNFINRVLSFIAKPSGTGYGSIIPESPNADSHPLTKKLAQEVGKYVEQYVEAMEKVKLKQGLKTAMS

ISSEGNAYLQESQFWKLYKEDQPSCNIVMRTSVGLVYLLACLLEPFIPSFSLEVFKQLNLSPEQASLSDEKGDIDKAKRP

WEFVPPNHKIGPPEPLFKELKDEEVDFFRKKFAGSQAERAEAEAAKVAEQLKKTKVSDSGKKQQTKKPAAQAKSKSTVEP

EISITRLDIRVGLITKAQKHPDADSLYVEEIDLGGETRTVVSGLVKYIPLEEMQNRKVCVLCNLKPATMRGIKSQAMVLA

ASNSDHTKVELGEPPASAAVGERVTFPGFEGEPDDVLNPKKKVWETLQVDLQTNADLVACYKDTPLTTSAGVCKVASIGS

GSIR

>PVV03752.1 hypothetical protein BB560_001759 [Smittium megazygosporum]

MASNVSNPITIFVGNAKPTTGTPAAISLLKVFLACKISNVPFVIKKDPQVVGKLPFLLELNSGCRFVDPNAFIRYSCQLS

SFGISARLEQEFLLEWEEKFLFNFIENNLKTSSELLSLVDSRIKNEWLKDGFSAAEIAIFSDVFNLFSILKDSEKSAYPN

LCSWFGKFKMSEHVQSALSVYNENTKELLVRQVPTAENVKVSTEALFSYDPSQKVQVKHGERNILITSALPYVNNVPHLG

NIIGSVLSADAFARYARARNISTLFVCGTDEYGTATETKALAENITCQELCDKYHKVHDEVYKWFDISFDIFGRTTTPKQ

TSITQDMFMKCHNNGYTISDTMQQLYCEQCKRFLADRFVEGTCPKCGYDDCRGDQCDNCGQLINAIELVSPRCKLDGNAP

IVKSSTHLFLDLTKLQPECEKFVARSYENGKWSSNGYTITKSWLSEGLKPRCITRDLKWGVPVPLPGFEDKVFYVWFDAC

IGYVSITANYTDEWETWWKNPDHVQLYQFMGKDNVPFHTVVFPSTQVATGDPSTMLHHISTTEYLNYESGKFSKSRGIGV

FGNNAKDTGVAADVWRYYLLSNRPESSDTLFTWNEFIARNNNELLANFGNFCNRMLKFTDAASKYAGVVPNPSLELLTDP

NLTHASLLSDINALLAKYNSSMESVHLKAGLRIAMEISARGNQYLQESKFDNSLFMNNKKDCDTVVFVALNLIYLLSAVF

SPFMPSTSSSICQQLNAPPRTIPDTFEFDIKPGHVIGYPKHLFSPIDEKMADFWREKYGGGKQVE

>OUM66810.1 hypothetical protein PIROE2DRAFT_68070 [Piromyces sp. E2]

MGFPAEQKVDTKNSIVLNHHERVILPVEGKKNILVTSALPYVNNVPHLGNIIGAMLSADVFSRYCRMRDYNIIFINGTDE

YGTATETKALEEKCTCQALCDKYHALHKEVYKWFGIDFDYFGRTTTPQQTEIAQDIFLKLHKNGYLMEDTVTQLYCNTCK

LFLADRFVEGECPFCHYDDARGDQCDKCGKLINAVELIKPRCKVCNSTPETKTSNHIFIDLPKLQDKCADFVKKNSVEGE

WSSNSISITNGWLKEGLKPRCITRDLKWGTPVPLEHMKDKVFYVWFDAPIGYISMTACYTPEWKKWWMNPEQVQLYQFMG

KDNIPFHTVIFPCSEIGTGDNYTLLNRLSTTEYLNYENGKFSKSRNVGVFGNNAVDTGVPVEVWRYYLLINRPETNDSSF

TWNDFIARNNNELLNNIGNFVNRLLKFVAAKYDSIIPQYDLSGPVEQKLIEDVNKLLKDYITNMEACKLRAGLKTAMDIS

QVGNAYLQENKIDNKLFIEKRQVCDNVVSASVNLIYLLSSLIYPYMPTTSESILKQINAPLRKITETWEANEILPGHTIG

KPAYLFKRIDPEMENVWREKFGAAKKQKKAPKENKKAAVAPAAAETGPDPSKLDIRVGHIIEAHKHEQADSLYVETIDLG

EPEPR

>XP_021641466.1 probable methionine--tRNA synthetase isoform X2 [Hevea brasiliensis]

MGDLSSSNSSGEGQTAPKLPIPGKRNILITSALPYVNNVPHLGNIIGSVLSADVFARYCRLRGYNTIYMCGTDEYGTATE

TKAMEENCSPKKICDKYHAIHREVYKWFNISFDEFGRTSTPQQTEVCQAIFKKLLENKWLSESTMQQLFCDTCQRFLADR

LVEGTCPTEGCNYDSARGDQCEKCGKLLNPTELKDPRCKVCQTTPRVRDTNHLFLELPLLKDKLTKYIESMSIAGSWSQN

AIQATNAWLKEGLKPRCITRDLKWGVPVPHERFMDKVFYVWFDAPIGYVSITACYTPDWEKWWKNPENVELYQFMGKDNV

PFHTVMFPSTLLGTGESWTMMKTISVTEYLNYEAGKFSKSKGIGVFGNDAKDTNIPVEVWRYYLLTNRPEVSDTLFAWSD

LQAKLNSELLNNLGNFINRVLSFIAKPSGTGYGSIVPDAPDADSHPLTKKLAEEVGKYVEQYVEAMEKVKLKQGLKIAMS

ISSEGNAYLQESQFWKLYKEDQSSCNIVMRTSVGLVCILACLLEPFMPSFSVEVFKQLNLPPEKASLCDEKGDIDRAKRP

WEFLPPNHKIGTPEPLFKELKDEEVDFFRKKFAGSQAERAEAEAARIAEQLKKTKVSDGSGKKQQTKKPSAATKSKSTVE

PEISITRLDIRVGLIKKAQKHPDADSLYVEEIDVGEVETRTVVSGLVKYIPLEQMQNRKVCVLCNLKPATMRGIKSQAMV

LTASNSDHTKFELVDPPESAAVGERVAFPGFEGQPDDVLNPKKKVWETLQVDLQTNTDLVACYKDIPFTTSAGVCKVASI

QSGSIS

>OVA01635.1 tRNA-binding domain [Macleaya cordata]

MGDIEGTEGDGKSPKLPIPGRRNIMITSALPYVNNVPHLGNIIGCVLSADVFARYCRLRGYNAIYMCGTDEYGTATETKA

MEEKCSPKEICDKYHAIHKEVYKWFDISFDEFGRTSSPQQTEVCQAIFKKLMANNWLSENTMQQLYCDTCKRFLADRLVE

GTCPRPGCNYESARGDQCEKCGNLLNPTELQDPRCKVCQTTPRIRDTDHLFLELPLLKDKLEEYINNMSVAGSWSQNAIQ

ATNAWLKEGLKQRCITRDLKWGVPVPHEKYKDKVFYVWFDAPIGYVSITSCYTSDWEKWWKNPENVELYQFMGKDNVPFH

TVMFPSTLLGTGENWTLMKTISVTEYLNYEAGKFSKSKGIGVFGNDAKDTKIPSEVWRYYLLTNRPEVSDTLFTWKDLQA

KLNGELLSNLGNFINRVLSFIAKPSGLGYGSVIPDAPNAGSHPLTKTLAEKVGKYVEQYVEAMEKVKLKQGLKTAMSISS

EGNAYLQDSQFWKLYKEDPASCSIVMKTSVGLVYLLASLLEPFMPSFSVEVVKQLNLPPEIQLSLCDEKGNIDRARRPWE

IIPTGHKIGIPEPLFKELKDEEVEFFREKFAGSQAERIVKAEADAKKMADQLKKTKLSDGNVKKQKPTKSAAGASTNEAE

VSITRLDIRVGLIAKVQKHPDADSLYVEEIDVGEAAPRTVVSGLVKFIPLEEMQNRKVCVLCNLKPATMRGIKSHAMVLA

TSNEDHTKVELVDPPQSAVVGERVTFPGFQGEPDEVLNPKKKIWEALQVDLKTDAELVARYKDVPLTTSAGVCKVSSIAG

GPIR

>OBZ83797.1 putative methionine--tRNA synthetase, cytoplasmic [Choanephora cucurbitarum]

MPAAIKITLPKLAANTKPYANALKLSIAAKVTGAKLVETPGEVVTFIASDNLVLTDANAALKFIFDATAFNALQSIVIEK

EETLVSPLIIKRRIDQAFQNTEDILSKHGNAFAKESDKVTVVDAIYYGTLFETLTSAKDLSKFPILSSWFATVSKNEKMA

AGVDFVKELTNKGTKPKNKNAHAEKKVTKEIPKPVYNKATQKVKADIYAKTVDPSVKVLPKDNERNILITSALPYVNNVP

HLGNIIGSTLSADVYARYCRARGYNTVYFCGTDEYGTATETKALEEGVTCQALCDKYNAIHSAVYKWFDLSFDHFGRTTT

EKQTEIAQEIFLKNYQNGNVVKESMTQLYCEKCSRFLADRYVEGICPHCKYDDARGDQCDACGKLLNATELIEPRCKLDN

NKPITKESNHLFLDLAKLQPEIEKVFKKSAEEGKWSANGVTITQSWLKEGLKPRCITRDLKWGTPVPLEGYEDKVFYVWF

DAPIGYPSIAANYTDEWQKWFKNPENVKLYQFMGKDNVPFHSVIFPGSQIGTGEDWTIVNHISTCEYLNYEGGKFSKSRN

IGVFGNNAEDTGIPASVWRYYLLSVRPETSDSMFTWNEFITKNNSELLNNLGNFVNRGIRFMFAKYDGVIPKADFSGPLE

TALIKDVNALLKQYVDTMEAVKLRAGLGLAMTISARGNQYLQEANLSNALFDESRAKCDAVVAVAANLIYLLSALISPYM

PASAESICRQLNAPLRTIPDEFTMDILEGHVLGGAEYLFTRIDEKMEDVWKAKYGTCSK

>XP_017222896.1 PREDICTED: probable methionine--tRNA synthetase [Daucus carota subsp. sativus]

MSNLSAAAAAGDDRKPIKLPISGKRNVLITSALPYVNNVPHLGNIIGCVLSADVFARYCRLRGYNAIYVCGTDEYGTATE

TKALEENCTPKEICDKYHAIHKKVYEWFDIKFDEFGRTSTPQQTEVCQAIFTKLMENNWLSENTMQQLYCDTCKRFLADR

LVEGSCPTQGCNYDSARGDQCEKCGKLLNPTELIDPKCKVCQNTPRIRDTDHLFLELPLLKDKLVEYINNMSVAGGWTQN

AIQATNAWLKEGLRQRCITRDLKWGVPVPHEKYKDKVFYVWFDAPIGYVSITSCYTAEWEKWWKNPENVELYQFMGKDNV

PFHTVMFPSTLLGTGENWTLMKTISVTEYLNYEAGKFSKSKGIGVFGNDAKDTNIPVEVWRYYLLTNRPEVSDTLFTWAD

LQAKLNGELLNNLGNFVNRVLSFIAKDQASGYGSIIPDAPGAESHLLTKNLGDKVGSCVEQYIEAMEKVKLKQGLKLAMS

ISGEGNAYLQESQFWKLYKEDKASCSIVLRTAAGLVYLLACLLEPFMPSFSAEVLKQLNMPLETQVTLTDEKGDIERSKR

PWELLPAGHKIGTPAPLFKELSDKDVESFRQKFAGSQADRIVKAEAEAKQITEQLKKAKVSDGNVKKERTKKSASENKPK

AAEQEISISRLDIRVGLITKVQKHPDADSLYVEEIDVGEGQPRTVVSGLVKFIPLEEMQNRKVCVLCNLKPATMRGIKSH

AMVLAVSNADHSKVELVDPPQSAPVGERLTFSGFEGDPDDVLNPKKKVWETIQPDLHTNKELIACYKDLPFTTSVGVCKV

SSISDGSIR

>XP_021863544.1 probable methionine--tRNA synthetase [Spinacia oleracea]

MADLNNHGVAAAILPIPGKRNVLITSALPYVNNVPHLGNIIGCVLSADVFARYCRLRGYNTVYICGTDEYGTATETKAME

EKCTPKQICDKYHAIHKEVYEWFNISFDKFGRTSSPEQTEVCQSVFKKLMENNWLTENTMQQPYCDTCEKFLADRLVEGI

CPTPGCNYDSARGDQCEKCGKLLNPTDLKDPKCKACQSTPRIRDTDHLFLELPLLQDKLEEYINKMSEAGSWSQNAIQVT

NGWLKEGLRSRCITRDLKWGVPVPHEKFKDKVFYVWYDAPIGYISITACYTPDWEKWWKNPEDVELYQFMGKDNVPFHTV

MFPSTLLGTGESWTLMKSISVTEYLNYEAGKFSKSKGIGVFGNDAKDTSIPVEVWRYYLLTNRPEVSDTLFTWADLQAKL

NSELLSNLGNYVNRVLSFIAKPSDQGYGSVIPDAPQAEAHSLTKALAEKVGKHVEQYVEAMEKVKLKQGLKTAMAISSEG

NAYLQESQFWKLYKEDQPACAVVMRTAAGLVYLLACLLEPFMPSFSLEVLKQLNMPRLQVSLSDENGDLEKIRILWDILP

AGHKIGKPEPLFKELKDEEMEFYREKFAGSQADRAVKAAAEKTAEQLKKTKISDKSEKKKAAKPAGGSKTKSTPEPEVTI

ARLDIRVGLITKVEKHPDADSLYVEEIDVGEAAPRTVVSGLVKYIPIEEMQNRKVCVLCNLKPASMRGIKSHAMVLAASN

SEHTKVELVEPPKDASIGERVKFSGFDGEPDDVLNPKKKVWETLQVDLSTDSNLVARFKDLPFTTSAGICKVSSIANGTI

K

>KIH68843.1 methionine--tRNA synthetase [Ancylostoma duodenale]

MSAASAAEVRKAWDASPSFIEHTPKEKYVNRLYEGLGSQADLVLVICSLLQIHKKGVRNLNYPDFRYCRMRGYETLYVCG

TDEYGTATETKALQEGKTPREVCDKYHAIHESIYQWFNIEFDIFGRTTTPQQTEIAQDIFLKLHQNGFTSSSSIDQLHCQ

NCDKFLADRFVTGTCPFCSFDDARGDQCDGCGRLINAVELKSPKCHICKQEPKVRQSTHIFLHLDALQDEVRQYVENEIA

KPDTRWSPNAIAIVKGWIKGGLEKRCITRDLKWGTAVPLAGYEDKVFYVWFDAPIGYLSITKCLVGDNWTKWWKNPKEVE

LFNFIGKDNVAFHGVMFPCTQLGTRDNYTIVNHVCATEYLNYEDSKFSKSRNTGVFGDNAKETGIEADVWRFYLLYMRPE

SQDTSFSWDDFALKVNSELLANLGNFINRALSFLSNNFGGIVPEMQLTDIDLELLMGVQADSVEWDQLMNAVRQKDAVRT

VLAMSRRGNQYMQSQQPWVLMKGSESEKKRAATVIGVAANISYHLAVVLHPIMPEVSARIRAQCGAERLPAFSTYPISYL

KPGHKIGTVGSETFILAPTPLFTKMESAKITEWKTKFGGAAAVLEEPRKKAKGDLFVKQKSAEIGAIMAKLEKEIETLQR

QLVEAEEAAGIKQVPVSKTVKSVEKSASPQVASAPPTKQAKVEKPSKNKESGGEKKAVAGGGGGKKTENVSADDGIDVGR

LDLRVGRIIKCEKHPDADALYVEQIDVGEPTPRTVVSGLVRHVPLDQVEIMEVDQSSQPGTPVLCPPYIHRPDAQLNPKK

KVKDLLFLRNCLVMFVV

>PSC71729.1 putative methionine-tRNA synthetase [Micractinium conductrix]

MATLVCRSGDPQALKPAAAAAATGSQLALLPLEQATAWRKLLSDASEAPSAQLFLMLPDGTAVADATAAARCVGASLAPQ

LDLNVESWVEWEEAVLRPAVYAGDTAALAAAVARLAAALAGGRAFLAGSTLSLADICVYATLSPVAGGEAVAPLAGYLSG

LAAQPAIAAAAEQVGAGAPGAGPSGAFLADCAAAKATKPKLPIPGQRNILITSALPYVNNVPHLGNIIGCVLSADVYARF

CRARGYNCVYVCGTDEYGTATETKALEEGLTCQQICDKYHAIHKAIYEWFDIGFDKFGRTPTRPQTQIGQDIFRTLRERG

CLVEQTIEQLYSEPLGKFLADRFVVGTCPKCKYEDARGDQCDACGSLLNPTELINPKCKLTGSTPVVRQTKHIFLDLPTL

SPELQAYIDRTSTLGGWSSNCVQVTKAWMEQGLKVRCITRDLRWGTPVPLEGYEDKVFYVWFDAPIGYISITANYCDNWE

AWWKNPKDVELVQFMGKDNVPFHTVIFPATLIGTQEPWTMMKNISVTEYLNYEDGKFSKSRGVGVFGNDAKDTGIPVEVW

RYYLLAVRPETSDSAFQWDDFAAKNNAELNDNLGNFINRTLKFIYARFDKRVPGAAASGAGMEAVQGLGEKVSALVEQYI

EALEGLRMKEALKAAMLVSKAGNAFFQETEIWVAYKQDKEAAAAYISACAGVVLLLATLLQPFMPSFTAKLLAQLGASET

ARLSDELVKAATNPAALLPAGHPIGTAEPTPLFRKIADDEVEQLRARYAGSQADRAAAPAAASSSSAASAAPAGGKGGKG

KPAAAAAAAAAASGKAGKKGAAAADGNAADSNAAAADGKAAAGGKKGGGGGGGKAPERPVDVGRLDLRVGFITKAWRHPD

AESLYVEEVDVGEGAPRTVVSGLVKFIPEAEMQQRRVVLVCNLKPANMRGIQSQAMVLAATSPDGAKVELVEPPAGAAVG

ERLSVEGFDCSAPDELLAPKKKIFEAVQPDLASNEQCVACYRGVPLLTSAGPCTVKSIQGGSIK

>KXN70372.1 methionyl-tRNA synthetase [Conidiobolus coronatus NRRL 28638]

MAKLNKLLLSKKTEVNSIEYINLIKLVLSTLIWNNKYQQVILDTIEEGIQLPSYECSKEKISLFEPSSLVIYLLNNNVTY

KVWQLIEFETSKLKPLVKANLGNSQVYEQIASQLEDLVAKYESEESLKQVLQYLVIGNLYAFVNTEFDKLSQYPKVSQIY

QTVQQLENFQGLVNDLANGVVATVNEEKKQEIEVIVGDESHRKVDSSAVLPETGNQVKQPIPGEKNILITSALPYVNNVP

HLGNLIGSTLSADVYARFCKIRSTNYLFICGTDEYGTATETKALELGVTCQELCDKYHKVHKEIYEWFDIDFDLFGRTTT

PQQTEIAQDIFLRCKDNGYLLEDSITQLFCEHCQRFLADRYVEGECPKCHYIDARGDQCDQCGGLLNAHELINPRCKLDN

NTPVLRDSTHVFLDLPKLQPACEEFVAKHSEEGKWTNNGKVITQSWLKEGLRPRCITRDLKWGTPVPLEHLKDKVFYVWF

DAPIGYLSITANYTKDWEAWWKNPDNVKLYQFMGKDNVPFHTVIFPCSELATGEDWTLLHHINTTEYLNYEDGKFSKSRN

VGVFGNNAQDTGIPASVWRYYLISNRPESNDSVFSWNEFITRNNSELLNNLGNFCNRVLKFLAAGSKYAGVVPKFDLNDD

LSKQLVENVNQLLERYIADMELGKFRSGLRLMMEISQLGNQYLQNVKIDNTLFNDHREVCDRAVGLSVNLIYLIAALTFP

FMPETTKQICAQLNAPIPKLQDTWNTAAILEGHRIGKPTHLFTRIEEKLISVFRSKFGGNGGPATPPTN

>KYP68017.1 putative methionyl-tRNA synthetase [Cajanus cajan]

MAENRKAAKLPVEGKRNILITSALPYVNNVPHLGNIIGCVLSADVFARYCRLRGYNAIYICGTDEYGTATETKAMEEKCS

PKEICDKYHAVHKEVYDWFNISFDEFGRTSCPEQTEVCQAIFKKIFDNKWLSENTMQQLYCDTCEKFLADRLVEGTCPTL

GCDYDSARGDQCEKCGKLLNPTELKSPRCKVCQKAPRIRDTDHLFLELPLLKDRLEKYISEMSVAGGWSQNAIQTTNSWL

REGLKPRCITRDLKWGVPVPHEKYSDKVFYVWFDAPIGYVSITSCYTRDWEKWWKNPENVELYQFMGKDNVPFHTVMFPS

TLLGTGENWTLMKTISVTEYLNYEAGKFSKSKGIGVFGNDVKDTNIPVEVWRYYLLTNRPEVSDTLFTWPDLQAKLNSEL

LNNLGNFINRVLSFIAKPAGQGYDSIIPSVPDDVSGDSHNLTKKLADKVSAYIDQYIEAMEKVKLKQGLKTAMSISSEGN

AYLQEAEFWRLYKENRSLCSLVMKTAAGIVYLLACLLEPFIPSFTLEVFKQLNLPAEIHVSLCDEKGDVNRVKKPWDIIS

AGHRIGTPKPLFKELKDEEVEFYRKKFAGSQADRIVRAEAEAEKVAEQLKKTKVSDANGKKKSAKSSNEAKNKAAAEXII

TITRLDIRVGLIKKAQKHPDADSLYVEEIDVGEEQTRTVVSGLVKYIPLDEMQNRKVCVLCNLKPATMRGIKSQAMVLAA

SNDDHTKVELVEPPSSAQVGERITFPGYEGSPDELLNPKKKVWETLQVDLHSNEELVACYKDVPLTTSAGICKVSSIRSG

SIR

>PSS09655.1 Methionine--tRNA synthetase [Actinidia chinensis var. chinensis]

MGDQATEEDERVPKLPIQGKRNILITSALPYVNNVPHLGNIIGSVLSADVFARYCRLRGYNVLYICGTDEYGTATETKAM

EENCSPQQICDKYHAIHKEVYKWFNISFDEFGRTSTPQQTEICQSIFKKLLENNWLSENTMQQLYCDACERFLADRLVEG

TCPTPDCNYDSARGDQCEKCGKLLNPTELREPRCKVCHATPQIRNTEHLFLELPLLKDKLEEYINEMSVVGSWSQNAIQA

TYAWLKEGLRARCITRDLKWGVPVPHEKFKDKVFYVWFDAPIGYVSITSCHTPEWEKWWKNPENVELYQFMGKDNVPFHT

VMFPSTLIGTGENWTLMKSISVTEYLNYEAGKFSKSKGVGVFGNDAKDTNIPAEVWRYYLLTNRPEVSDTLFAWVDLQAK

LNTELLNNLGNFINRVLSFIAKPLGLGYGSIIPDTPGAESHLLTKTLAEKVTKYVQQYVEAMEKVKIKQGLRIAMSMSSE

GNTYLQDSKFWKLYKEDKASCSIVVRTSAGLIYLLACLLEPFMPSVSLEVLKQLNLPPETQLSICDEYGDHEKARKPWEI

LPAGHTIGAPYSLFKALKDEDVDFFREKFAGSQADRQRS

>XP_021641465.1 probable methionine--tRNA synthetase isoform X1 [Hevea brasiliensis]

MGDLSSSNSSGEGQTAPKLPIPGKRNILITSALPYVNNVPHLGNIIGSVLSADVFARYCRLRGYNTIYMCGTDEYGTATE

TKAMEENCSPKKICDKYHAIHREVYKWFNISFDEFGRTSTPQQTEVCQAIFKKLLENKWLSESTMQQLFCDTCQRFLADR

LVEGTCPTEGCNYDSARGDQCEKCGKLLNPTELKDPRCKVCQTTPRVRDTNHLFLELPLLKDKLTKYIESMSIAGSWSQN

AIQATNAWLKEGLKPRCITRDLKWGVPVPHERFMDKVFYVWFDAPIGYVSITACYTPDWEKWWKNPENVELYQFMGKDNV

PFHTVMFPSTLLGTGESWTMMKTISVTEYLNYEAGKFSKSKGIGVFGNDAKDTNIPVEVWRYYLLTNRPEVSDTLFAWSD

LQAKLNSELLNNLGNFINRVLSFIAKPSGTGYGSIVPDAPDADSHPLTKKLAEEVGKYVEQYVEAMEKVKLKQGLKIAMS

ISSEGNAYLQESQFWKLYKEDQSSCNIVMRTSVGLVCILACLLEPFMPSFSVEVFKQLNLPPEKASLCDEKGDIDRAKRP

WEFLPPNHKIGTPEPLFKELKDEEVDFFRKKFAGSQAERAEAEAARIAEQLKKTKVSVAYTSRTFFFLGWDCDLTDGSGK

KQQTKKPSAATKSKSTVEPEISITRLDIRVGLIKKAQKHPDADSLYVEEIDVGEVETRTVVSGLVKYIPLEQMQNRKVCV

LCNLKPATMRGIKSQAMVLTASNSDHTKFELVDPPESAAVGERVAFPGFEGQPDDVLNPKKKVWETLQVDLQTNTDLVAC

YKDIPFTTSAGVCKVASIQSGSIS

>XP_024931511.1 probable methionine--tRNA synthetase [Ziziphus jujuba]

MGDQKNSEDRKVPKLPIPSRRNILITSALPYVNNVPHLGNIIGCVLSADVFARYCRLRDYNAIYICGTDEYGTATETKAL

EERCSPKEICDKYYAIHKEVYKWFNISFDEFGRTSTPQQTEVCQAIFKRLLDNNWLTENTMQQLYCDTCKRFLADRLVEG

TCPTPGCDYDSARGDQCEKCGKLLNPTELKDPKCKQVCGTTPHIRDTNHLFLELPLLRDKLEEYINKTSVAGSWSQNAIQ

ATNAWLKEGLKQRCITRDLKWGVPVPHDKFQDKVFYVWFDAPIGYVSITSCYTPDWEKWWKNPENVELFQFMGKDNVPFH

TVMFPSTLIGTGENWTLMKTISVTEYLNYEAGKFSKSKGVGVFGNDAKDTNIPVEVWRYYLLTNRPEVSDTLFTWADLQA

KLNSELLNNLGNFINRVLSFIAKPSGQGYGSIIPDAPPGESDSLTEKLAEKVGKYVEQYVEAMEKVKLKQGLKTAMNISS

EGNAYLQESQFWKLYKEDQPRCSIVVRTSVGLVHILACLLEPFMPSFSLEVFKQLNLSPEAQLSLSDEKGDIDRARRPWD

IIPSGHKIGKPEPLFKELKDEEVEAFRQRFAGSQADRIERAEAEAEKIAAQLKKTKVSDNSGKKKPTKSASEAEITISRL

DIRVGLITKAQKHPDADSLYVEEIDVGEGQPRTVVSGLVKYIPLEEMQNRKVCVLCNLKPAALKGIKSQAMVLAASNTDH

TLVELVEPPKSANVGERVTFAGFEGEPDNVLNPKKKVWETLQVDLHTNADLVACYKDLPFTTSAGVCTVSTIRDGSIR

>XP_021879983.1 tRNA synthetases class I (M)-domain-containing protein [Lobosporangium transversale]

MSSPTTIKAGIALPPKNPHKVPIPGQRNVLITSALPYVNNVPHLGNIIGSTLSADVYARYQRVRGINALYICGTDEYGTA

TETKALSDGVTCQELCDKYHPIHKQCYEWMEIDFDKFGRTTTEEQTKIAQDIFMRLYERDLLVEDSMPQLYCSQCDRYLA

DRFVEGTCPRCNYDDARGDQCDQCGNLLNAIDLINPRCKLDGNRPVQKHSKHLFLDLGKLQARCEAWAEKAAVEGKWSAN

GRIITQAWFKEGLRLRCITRDVKWGTPVPLEGWEGKVFYVWFDAPIGYPSITATYTKEWEKWWKNPKDVKLYQFMGKDNV

PFHTVIFPSSLIGTGEEWTLLNTLSTTEYLQYETGKFSKSRGVGIFGNNVQETGVPPSVWRYYLLSNRPETNDSQYLWKD

FIARNNNELLANVGNFVNRVIKFVNAKYDNVVPSLETAAAPTESDEKLIREVNEKLKLYIEALDDVKIREALRLAMEISA

LGNVYLQENKIDNTLFAEHKDRCDRVVITALNLIYLLSAILFPYMPSTSDSIVRQLNAPLRLLPDEFRIDAILAGHVINK

AEYLFKRIDEKMEDIWRNKYGGNSSVPEPDDKKKKKKAAAKAAAPVFQGEKPAEAIALEKQIEEQGAKVRELKTAKADNA

VVQNEVQTLLALKKDLADLFAKLTV

>XP_020214766.1 probable methionine--tRNA synthetase [Cajanus cajan]

MAENRKAAKLPVEGKRNILITSALPYVNNVPHLGNIIGCVLSADVFARYCRLRGYNAIYICGTDEYGTATETKAMEEKCS

PKEICDKYHAVHKEVYDWFNISFDEFGRTSCPEQTEVCQAIFKKIFDNKWLSENTMQQLYCDTCEKFLADRLVEGTCPTL

GCDYDSARGDQCEKCGKLLNPTELKSPRCKVCQKAPRIRDTDHLFLELPLLKDRLEKYISEMSVAGGWSQNAIQTTNSWL

REGLKPRCITRDLKWGVPVPHEKYSDKVFYVWFDAPIGYVSITSCYTRDWEKWWKNPENVELYQFMGKDNVPFHTVMFPS

TLLGTGENWTLMKTISVTEYLNYEAGKFSKSKGIGVFGNDVKDTNIPVEVWRYYLLTNRPEVSDTLFTWPDLQAKLNSEL

LNNLGNFINRVLSFIAKPAGQGYDSIIPSVPDDVSGDSHNLTKKLADKVSAYIDQYIEAMEKVKLKQGLKTAMSISSEGN

AYLQEAEFWRLYKENRSLCSLVMKTAAGIVYLLACLLEPFIPSFTLEVFKQLNLPAEIHVSLCDEKGDVNRVKKPWDIIS

AGHRIGTPKPLFKELKDEEVEFYRKKFAGSQADRIVRAEAEAEKVAEQLKKTKVSDANGKKKSAKSSNEAKNKAAAEPDI

ITITRLDIRVGLIKKAQKHPDADSLYVEEIDVGEEQTRTVVSGLVKYIPLDEMQNRKVCVLCNLKPATMRGIKSQAMVLA

ASNDDHTKVELVEPPSSAQVGERITFPGYEGSPDELLNPKKKVWETLQVDLHSNEELVACYKDVPLTTSAGICKVSSIRS

GSIR

>XP_011041943.1 PREDICTED: probable methionine--tRNA synthetase [Populus euphratica]

MGDLDRPVPKLPIPGKRNILITSALPYVNNVPHLGNIIGCVLSADVFARYCRLRGYNAIYICGTDEYGTATETKAMEENS

TPKQICDKYHAIHREVYKWFNISFDEFGRTSSPQQTEVCQAIFKKLLDNNWLSENTMKQLYCDTCERFLADRLVEGTCPT

EGCNYDSARGDQCENCGKLLNPTELINPRCKQCKNTPRIRDTNHLFLELPQLKDKLVSYIESMSVAGSWSQNAIQTTNAW

LKEGLKPRCITRDLKWGVPVPLDEFKDKVFYVWFDAPIGYVSITSCYTPDWEKWWKNPDNVELYQFMGKDNVPFHTVMFP

STLIGTGENWTMMKTISVTEYLNYEAGKFSKSKGVGVFGNDAKDTNIPVEVWRYYLLTNRPEVSDTLFTWSDLQAKLNSE

LLNNLGNFINRVLSFLAKPPGSGYGSIIPDAPGADSHPLTKKLAGEVGKYVEQYLEAMEKVKLKQGLKTAMSISSEGNAY

LQESQFWKLYKEDQLSCSIVIKTSLGLVYLLACLLEPFIPSFSVEVFKQLNLPLEQASLCDEKGDMDRAKRPWEILPAGH

KIGTPEPLFKELKDEEVEDYRRRFAGSQADRLERDEAEKAAKLAEQLKKKASVGGGKKQQAKKPASEAKSKGSVEPEISI

TRLDIRVGLIKKAQKHPDADSLYVEEIDLGEGDCRTVVSGLVKYIPLEEMQNRKVCVLCNLKPASMRGIKSQAMVLAASN

SDHTKVELVDPPPSAVVGERVTFQGFEGEPDDVLNPKKKVWETLQVDLQTNSELVACYKDIPLTTSAGVCKVASISCGSI

R

>XP_010673414.1 PREDICTED: probable methionine--tRNA synthetase [Beta vulgaris subsp. vulgaris]

MADLNNGEKRHILPVAGKRNVLITSALPYVNNVPHLGNIIGCVLSADVFARYCRLRGYNTLYICGTDEYGTATETKAMEE

KCTPKQICDKYHAIHKQVYEWFNISFDHFGRTSTPQQTDVCQSIFKKLMDNNWLTENTMQQPYCDTCEKFLADRLVEGIC

PTPGCNYDSARGDQCEKCGKLLNPTDLKDPKCKACQCAPRIRDTNHLFLELPLLKDKLEEYINKMSEAGSWSQNAIQVTN

GWLKEGLRSRCITRDLKWGVPVPHEKFKDKVFYVWYDAPIGYISITACHTPDWEKWWKNPENVELYQFMGKDNVPFHTVM

FPSTLLGTGENWTLMKSISVTEYLNYEAGKFSKSKGIGVFGNDAKDTDIPVEVWRYYLLTNRPEVSDTLFTWVDLQAKLN

GELLSNLGNFVNRVLSFIAKPSGQGYGSIIPDAPQAELHSLTKTLAEKVGKHVEQYVEAMEKVKLKQGLKTAMAISSEGN

AYLQESQFWKLYKEDQSACAVVMRTSAGLVYLLACLLEPFMPSFSLEVLKQLNMPQLQVSLSDENGDLEKIKILWDVLPA

GHKLGKPEPLFKELKDEQMEFYRDKFAGSQADRAEKAAAEKTAEQLKKTKISDKSEKKKPAKSAAGGKAKSTPEPETTIA

RLDIRVGLIIKVQKHPDADSLYVEEIDVGEATPRTVVSGLVNHIPIDEMQNRKVCVLCNLKPATMRGIKSHAMVLAASDS

EHTKVELVEPPKDAVIGERVKFSGFDGEPDDVLNPKKKVWETLQVDLNTDSDLVARFKDLPFTTSAGICKVSSIANGSIR

>XP_017249975.1 PREDICTED: probable methionine--tRNA synthetase isoform X4 [Daucus carota subsp. sativus]

MEFLVVEDMKKVPIPGKRNMLITSALPYVNNVPHLGNIIGCVLSADVFARFCRLRGYNVLYICGTDEYGTATETKAMEEG

LTPKEICEKYHAIHKDIYNWFDISFDEFGRTSTPQQTGICQAIFTKLFKNRWISENTMQQPFCDTCQRFLADRFVEGTCP

KPDCSYDSARGDQCEKCGKLLNPAELKNPRCKVCRATPRIRDTDHLFLELPLLKDILVKYINKMSVEGSWSENALKATQA

WLKEGLKPRCITRDLKWGVPVPHEKYEDKVFYVWYDAPIGYISITSCHTSEWEKWWKNPENVELYQFMGKDNVPFHTVMF

PSTLLGTGENWTLMKSISVTEYLNYESGKFSKSKGVGVFGNDAKDTKIPSEVWRYYLLTNRPEASDTLFTWKDLQAKLNS

ELLNNLGNFVNRVLSFIAKPKGRGYGSIVPDAPGAETHCPTKALAERVGKYVEQYMDDMEKIKLKQGLKTGMSISSEGNA

YLQNTEFWKLYKEDESSCAIVIRTSVGLVYLIACLLEPFMPSFTMEVFKQLNLPPKKVFSLSDKDGDLGQVSRPWEFLPA

GHVIGTPAPLFKELTNEEVEHFSRKFAGSQATRAAT

>XP_010532767.1 PREDICTED: methionine--tRNA synthetase, cytoplasmic-like [Tarenaya hassleriana]

MAEETSAAKLPIPGKRNILITSALPYVNNVPHLGNIIGCVLSADVYARYCRLRGYNAIYICGTDEYGTATETKALEENCS

PKEICDKYHAIHKEVYEWFDILFDKFGRTSTPQQTEVCQAIFKKLLENNWLSENTMQQLYCETCKKFLADRLVEGTCPFE

GCNDGSARGDQCDTCGKLLNPTELKDPKCKVCRATPHIRDTDHLFLELPLLKDKLEEYINKTSVAGSWSQNAIQITNSWL

KEGLKQRCITRDLKWGVPVPLEKYKDKVFYVWFDAPIGYVSITSCYTSEWEKWWKNPENVELYQFMGKDNVPFHTVMFPS

TQLGTGENWTLMKTISVTEYLNYEAGKFSKSKGVGVFGNDAKDTNIPVEVWRYYLLTNRPEVSDTQFTWDDLQAKLNGEL

LNNLGNFINRVLSFIAKPAGQGYGSVIPDAPGAESHPLTISLAEKVGKLVDQYVEAMEKVKLKQGLKTAMSISSEGNAYL

QESQFWKLYKQDQPSCAIVMRTAAGLVNILARLLEPFMPSFSREVFKQLNVPPQFSLSDENGDMLKASKPWEILPPNHKM

GTPQPLFKELKDEEVQQYREKFAGSQDDRRVREAEAANMAEQLKKTKISDGKKQKPSKASGEAKAKAKPKPTEEPEITIT

RLDIRVGKIVKAQKHPDADSLYVEEIDVGEEKPRTVVSGLVKHIPLEEMQNRMVCVLCNLKPAKMRGIQSQAMVLAASNS

DDSKVELVEPPESSKIGERVTFPGFEGEPDDVLNPKKKVWETLQPDLRTDDNLVARFKDLAFTTSAGICKVSSIKSGKIR

>CDS13740.1 hypothetical protein LRAMOSA05914 [Lichtheimia ramosa]

MVLKVVLPKLTPQTKPFANALKVVLATLVAGVKFQQIPGTAVTVETNNGTTLTDANVAVKYILDVLTFDGSETALLQSAA

IEQEEAISRLVLQKSTHEAFKIVDGYISKYGSKMFSSGDKLNAVDVIYFGTLYEFLNNNEIISQYPAMNTWFTNVSKLPA

IASALETVDKLISNAAKKKMAGADRKSANKQTALAELNPETQKLKTEKFITVPDGKTKILPKDGERNILITSALPYVNNI

PHLGNIIGSTLSADCYARYCRARGYNTLYICGTDEYGTATETKALEEGVSCRALCDKYNAIHSNVYKWFQLSFDHFGRTT

TDEQTEIAQDIFNKVHANGFTSEAVMTQLFCEKCQRFLADRYVEGVCPNCKYNDARGDQCDACGRLLNATELEEPRCKLD

GTRPITKESKHIFLDLDRLQGDIEKFNARVNVEGKWSANGVHITQSWLKEGLRPRCITRDLKWGTPVPVPGFENKVFYVW

FDAPIGYPSITACYTKEWEKWWKNPDNVKLYQFMGKDNVPFHSVIFPGSQLGTKDNWTMVHHISTTEYLNYEGGKFSKSR

NIGVFGTNAEESGISPSVWRYYLLSSRPETSDSMFTWNEFITKNNSELLNNLGNFVNRVIKFAVAKYDGVVPEADLSGDA

EKALIKDVNELLAQYVDAMENVKIRQGLNIAMSISARGNVYLQESNLSNTLFTETRGKCDAVIHLALNLIYLLSSLLYPY

MPATSESISRQLNAPLGAIPDTFSCNILAGHKLNGAAYLFSKIDEKMEQVWKAKYGGSNNA

>XP_017249973.1 PREDICTED: probable methionine--tRNA synthetase isoform X2 [Daucus carota subsp. sativus]

MKFSRCKGNHIPFGIGRVDRLCLKKEALMVSGNSTCMLMLTQTLSVLCFRNMKKVPIPGKRNMLITSALPYVNNVPHLGN

IIGCVLSADVFARFCRLRGYNVLYICGTDEYGTATETKAMEEGLTPKEICEKYHAIHKDIYNWFDISFDEFGRTSTPQQT

GICQAIFTKLFKNRWISENTMQQPFCDTCQRFLADRFVEGTCPKPDCSYDSARGDQCEKCGKLLNPAELKNPRCKVCRAT

PRIRDTDHLFLELPLLKDILVKYINKMSVEGSWSENALKATQAWLKEGLKPRCITRDLKWGVPVPHEKYEDKVFYVWYDA

PIGYISITSCHTSEWEKWWKNPENVELYQFMGKDNVPFHTVMFPSTLLGTGENWTLMKSISVTEYLNYESGKFSKSKGVG

VFGNDAKDTKIPSEVWRYYLLTNRPEASDTLFTWKDLQAKLNSELLNNLGNFVNRVLSFIAKPKGRGYGSIVPDAPGAET

HCPTKALAERVGKYVEQYMDDMEKIKLKQGLKTGMSISSEGNAYLQNTEFWKLYKEDESSCAIVIRTSVGLVYLIACLLE

PFMPSFTMEVFKQLNLPPKKVFSLSDKDGDLGQVSRPWEFLPAGHVIGTPAPLFKELTNEEVEHFSRKFAGSQATRAAT

>XP_017249972.1 PREDICTED: probable methionine--tRNA synthetase isoform X1 [Daucus carota subsp. sativus]

MKFSRCKGNHIPFGIGRVDRLCLKKEALMVSGNSTCMLMLTQTLSVLCFRKDMKKVPIPGKRNMLITSALPYVNNVPHLG

NIIGCVLSADVFARFCRLRGYNVLYICGTDEYGTATETKAMEEGLTPKEICEKYHAIHKDIYNWFDISFDEFGRTSTPQQ

TGICQAIFTKLFKNRWISENTMQQPFCDTCQRFLADRFVEGTCPKPDCSYDSARGDQCEKCGKLLNPAELKNPRCKVCRA

TPRIRDTDHLFLELPLLKDILVKYINKMSVEGSWSENALKATQAWLKEGLKPRCITRDLKWGVPVPHEKYEDKVFYVWYD

APIGYISITSCHTSEWEKWWKNPENVELYQFMGKDNVPFHTVMFPSTLLGTGENWTLMKSISVTEYLNYESGKFSKSKGV

GVFGNDAKDTKIPSEVWRYYLLTNRPEASDTLFTWKDLQAKLNSELLNNLGNFVNRVLSFIAKPKGRGYGSIVPDAPGAE

THCPTKALAERVGKYVEQYMDDMEKIKLKQGLKTGMSISSEGNAYLQNTEFWKLYKEDESSCAIVIRTSVGLVYLIACLL

EPFMPSFTMEVFKQLNLPPKKVFSLSDKDGDLGQVSRPWEFLPAGHVIGTPAPLFKELTNEEVEHFSRKFAGSQATRAAT

>XP_008679089.1 probable methionine--tRNA synthetase [Zea mays]

MASPSSSPPPKLPIPGRRNILITSALPYVNNVPHLGNIIGCVLSADVFARYCRLRGYNALYICGTDEYGTATETKAMEEE

CSPKEICDKYHAIHSEVYKWFDIEFDKFGRTSSPQQTTICQAIFHKLMENNWLSENTMQQLYCDTCQRFLADRLVEGACP

TEGCTNKAARGDQCDNCSHMLNPTELIDPKCKVCKNTPHIRETDHLFLDLPRIEDKLVNYINDTSVAGMWSQNAIQATNA

WLKEGLKSRCITRDLKWGVPVPIEKYKDKVFYVWFDAPIGYVSITSSYTPEWERWWKNPDNVELFQFMGKDNVPFHTIMF

PSTLLGTGENWTMMKTICVTEYLNYEGGKFSKSKGIGVFGNDAKDTNIPPEVWRYYLLMNRPEASDTLFTWTDLQAKLNS

ELLNNLGNFINRVLSFIAKPAGAGYDSIIPDAPGAESHPSTSVIAEKVSKSVEQYLDAMEKVKLKQGLKSAMGISTDGNR

YLQDSEFWKLYKDNPASCAIVMKTSVGLVYLLACLLEPFMPSFSKEVLRQLNLSPEENISFSEENGESVKAKPPWDLVPA

GHKIGRPVPLFEELKDEKVSEYREKYAGSQAERNSKAVADAEATKIANQTQSIALSGGSKKEQKKQSGNSKSKVAEAELS

VAKLEIRVGLIRKAEKHPDADSLYVEEIDVGEEAPRTVVSGLVKYIPLEEIQNRKVCVLCNLKPVVMRGIKSDAMVLAAS

NEDHTRVELVEPPADAAVGERVTFAGHSGETEASLSGKSKVWEKLAADLRSNGDLVACYQDVPFMTSAGVCKVQTIANGE

IR

>XP_021749273.1 methionine--tRNA synthetase, cytoplasmic-like [Chenopodium quinoa]

MADLHKNGGAVTENRHILPIPGKRNVLITSALPYVNNVPHLGNIIGCVLSADVFARYCRLRGYNTIYICGTDEYGTATET

KAMEEKCTPKEICDKYHAIHKEVYEWFNISFDKFGRTSSPEQTEACQAIFKKLMDNNWLTENTMQQPYCDTCEKFLADRL

VEGTCPKPNCNYDSARGDQCEKCGNLLNPTELKDPKCKTCQSTPRIRDTNHLFLELPLLQDKLEEYINKMSEAGSWSQNA

IQVTNGWLKEGLRSRCITRDLKWGVPVPHEKFKDKVFYVWYDAPIGYISITACYTPEWEKWWKNPEDVELYQFMGKDNVP

FHTVMFPSTLLGTGENWTLMKSISVTEYLNYEAGKFSKSKGIGVFGNDAKDTSIPVEVWRYYLLTNRPEVSDTLFTWADL

QAKLNSELLSNLGNFVNRVLSFIAKPSGQGYGSVIPDAPQAEAHSLTMALAEKVGKHVEQYVEAMEKVKLKQGLKTAMAV

SSEGNAYLQESQFWKLYKEDQPACAVVMRTAAGLVYLLACLLEPFMPSFSVEVLKQLNMPQLQVTLSDENGDLEKIRKLW

DILPDGHCIGKPEPLFKELKDEEVEFYRDKFAGSQADRALKAAAEKTAEQLKKTKISDKSEKKKAAKPAAGSKTKSSPEA

EITISRLDIRVGLITKVQKHPDADSLYVEEIDVGEATPRTVVSGLVKYIPIEEMQNRKVCVLCNLKPASMRGIKSHAMVL

AASNSEHTKVELVEPPKEASVGERVKFSGFDGEPDEVLNPKKKVWETLQVDLNTDSNLVARFKDLPFTTSAGICKVSSIS

NGTIK

>CDH54678.1 methionyl-trna synthetase [Lichtheimia corymbifera JMRC:FSU:9682]

MVLKVVLPKLTPQTKPFANALKVVLAALVAGVKFQQVAGDAVTVETNNGTTLTDANVAVKYILDVLTFDGSETALLQSAA

IEQEEAISRLVLQKSTHEAYKILDGYMTKYGSKMFSSGDKLNAVDVIYFGTLYEFLNNNEDSSKYPALSTWFTNVSKLPA

IASALETVDKLISKAAKKKMAGADRKSANKQTELAALNPETQKLKTEKFITVPDGKTKILPKDGERNVLITSALPYVNNI

PHLGNIIGSTLSADCYARYCRARGYNTLYICGTDEYGTATETKALEEGVSCRALCDKYNAIHSNVYKWFDLSFDHFGRTT

TDEQTEIAQDIFNKVHANGFTSEAVMTQLFCEKCQRFLADRYVEGVCPNCKYNDARGDQCDSCGRLLNATELEEPRCKLD

GTRPITKDSKHIFLDLDRLQGDIEKFNARVNVEGKWSSNGVHITQSWLKEGLRPRCITRDLKWGTPVPMPGFENKVFYVW

FDAPIGYPSITACYTKEWEKWWKNPDNVKLYQFMGKDNVPFHSVIFPGSQLGTKDNWTMVHHISTTEYLNYEGGKFSKSR

NVGVFGTNAEESGISPSVWRYYLLSSRPETSDSMFTWNEFITKNNSELLNNLGNFVNRVIKFALAKYDGVVPEADLSGDA

EQALIKDVNELLGQYVDTMENVKLRQGLSIAMSISARGNVYLQESNLSNTLFTETRGKCDAVINLALNLIYLLSSLLYPY

MPATSESISRQLNAPLGAIPDTFVCNILAGHKLNGAAYLFSKIDEKMEQVWKDKYGGSSNA

>KCW57236.1 hypothetical protein EUGRSUZ_H00043 [Eucalyptus grandis]

MGSSGGTKLPIPGKRNVLITSALPYVNNVPHLGNIIGCVLSADVFARYCRLRGYNAVYICGTDEYGTATETKAMEEKCTP

QQICDKYHAVHKQVYDWFDISFDKFGRTSTPQQTEVCQAIFKKLYDNNWLTENTMQQLYCDNCPRFLADRLVEGVCPTPG

CNYDSARGDQCEKCGKLLNPTELIDPRCKVCQTTPRIRDTNHLFLELPQLKDKLVEYINEMSEAGSWSQNAIQTTHAWLR

EGLKPRCITRDLKWGVPVPHEKFEDKVFYVWFDAPIGYVSITSCYTPEWEQWWKNPENVELFQFMGKDNVPFHTVIFPST

LLGTGEKWTLMKTISVTEYLNYEAGKFSKSKGIGVFGNDVKDTNIPVEVWRYYLLTNRPEVSDTLFTWVDLQAKLNSELL

NNLGNFVNRVLSFVAKPTGLGYGSVIPDAQGADSHSLTVELAEKVGKLVEQYVEAMEKVKLKQGLKTAMSISSEGNAYLQ

ASQFWKLYKEDKPSCSIVMRTSVGLVHLLACLLEPFMPSFSIEVFKQLNLPPETQVSLQDENGEVERAKRPWEIIPAGHK

IGVPEPLFKELKDEEVEFYRTKFAGSQADRIVRAEAEAAKVAEQLRKTKVSGGG

>PTQ39331.1 hypothetical protein MARPO_0045s0011 [Marchantia polymorpha]

MDATVSGLKSLVLDSNTEGGSRGSGLQTRVKREIRVPIPGKRNVMVTSALPYVNNVPHLGNIIGCVLSADVYARYCRIRG

YNVIYMCGTDEYGTATETKAMEEGISPKEICDKYHAIHKEIYEWFDIDFDMFGRTSTPEQTVICQAIFQKLLEKDRLLED

KMEQPYCNTCLRFLADRFVEGICPHATCRYEDARGDQCDKCGKLLNAADLINPRCKRCGNPPQIRETEHMFLDLPQLKDR

LEAYVTNTSVSGGWSQNSVQTTNAWIRDGLKPRCITRDLKWGVPVPLEKYKEKVFYVWFDAPIGYMSITANYTSDWEKWW

KSPDDVELVQFMGKDNVPFHTVIFPSTLLGTDDPWTLMKTISVTEYLNYESGKFSKSRGVGVFGNDAKTTDIPVEVWRYY

LLTNRPEMSDTLFTWADLQAKQNNELLKNLGNFVHRSLSFLVKPAGEGYAGVIPEAPGAESHSLTVTLGEKVGELVKDYV

EVMEKVKLKQGLRIAMNVSSLGNLYLQDTKFWKLYKEDPPACAIVLRTSLGLVHLLATLLEPFMPSFSHKVLAQLNLPPS

SLSLSDEKGDLEKAFKPWELVPANHHIGSPTPLFAEMKDEEMERYRAKFAGSQADRAAADATAAATTGTPAVSKGDTKKN

VKSSKGGEAKSKKSATEVKDAPPAARLDIRVGLITKVQKHPDADSLYVEEIDVGEAAPRTVVSGLVKFIPIEQMQDRRVC

VLCNLKPASMRGIKSQAMVLAASNADHTKVELVTPPEGAQIGERVTFPGYEGEPDEVLNPKKKIWETVQPELATTEELVA

VYKDVPFTTSTGVCKVASITKGSIK

>ORX77082.1 methionyl-tRNA synthetase [Anaeromyces robustus]

MGFPAEQKVDTKNSITLNHHDRIILPVNDKKNILVTSALPYVNNVPHLGNIVGAMLSADCFTRYGRLRDYNIIFINGTDE

YGTATETKALEEKCTCQELCDKYHALHKEIYQWFGIDFDYFGRTTTEKQTEVAQDIFLKLHKNGYLLEDTVTQLYCETCK

LFLADRFVEGECPFCHYDDARGDQCDKCGKLINAVELIKPRCKVCNNTPKTKTSEHIFIDLPKLQDKCEAFVKKNSVEGE

WSSNSISITNGWLKEGLKPRCITRDLKWGTPVPLEKYKDKVFYVWFDAPIGYISMTANYTPEWKQWWMNPDNVQLYQFMG

KDNIPFHTVIFPCSEIGTGDNYTLLNRLSTTEYLNYENGKFSKSRNVGVFGNNVVETGVPVEVWRYYLLINRPESNDSAF

TWNDFISRNNNELLANIGNFVNRLLKFVAAKYDSIIPEYDLSGPIEQKLIEDVNKLLKEYIHNMEVCKLRAGLKTAMDIS

QVGNAYLQENKIDNKLFTEKREICNNVVAASVNLIYLLSSLVYPFMPTTTESILKQINAPLRKITDVWEANEILPGHTIG

KPAYLFKRIDPEMENIWREKYGGNAPKESKPAAKKQKKASKENKTSKN

>OMJ91070.1 hypothetical protein SteCoe_6427 [Stentor coeruleus]

MKLVYESAKEPAKACLLALQYTGIPAKKQIEPVPVVTLELGDQDKTGNFIELMGELSITRYLMKQCSWQVPDNLHLDEWV

LYHLKPIVSNTPKSQVKSNQDLLSSIGYLEQQISEQFICGQKPGSTDILSFTYLYQYFSGNPWMVKTFPKLANSYKQISE

VYNVVQEELGEKVKVPAKVGKAVEEFAPYATILRDKNVEKVPVEGKRNILITSALPYVNNVPHLGNIIGCVLSADVFSRY

CRLRGYNSIYICGTDEYGTATETKAIQEGLTPQEICTKYHEIHKKIYDWFDIDFDYFGRTTTNQQTEIAQNIFLKLKENG

ELNEDNVEQSFCEPCNRFLADRFILGTCPSCNYLDARGDQCDKCGKLLNPEELIDAKCFLCSSSPKMRISKHIFLNLPNI

RERLENWVSKSSSDGRWSANCLKITSAWIRDGLKPRCITRDLKWGTPVPLPEFSEKVFYVWFDAPIGYISITANYTSQWE

KWWKNTNNVELIQFMGKDNIPFHTVIFPSSLLGTGENWTLLSNISSTEYLNYEDGKFSKSRGVGVFGDNIIETNIPVEIF

RYYLLSNRPETSDTVFKWTDLAEKNNNELLPNLGNLFNRLLKYLAKSNKPIPNASTITEFDENTLENIYILVKRYLDEME

ELKLKNALKTAMEVSALGNKYFQDTEFWTLEKKDVERFKTVINLAANVARLVALLLEPFMPSFSAKVYNQFGIQRDAREE

SLLEVLAASTSSRVILTLLKEGTKVGNVMPIFRIITPEQVNTFKARYAGKKDN

>XP_019168456.1 PREDICTED: methionine--tRNA synthetase, cytoplasmic-like isoform X2 [Ipomoea nil]

MGEVSEKVPKVPIPGKRNILITSALPYVNNVPHLGNIIGCVLSADVFARYCRLRGYNAIYICGTDEYGTATETKALEENS

SPQQICDKYHEIHKQVYQWFDISFDKFGRTSTPQQTEVCQSIFKKLLDNNWLTENTIQQPYCDTCKKFLADRLVEGTCPT

PGCNYDSARGDQCEKCGKLLNPTELKEPRCKVCRNTPCIKDTDHLFLELPLLKDKLESYVNSMSVAGGWSQNAIHTTHAW

LREGLKSRCITRDLKWGVPVPHEKYKDKVFYVWFDAPIGYVSITSCYTAEWEKWWKNPENVELYQFMGKDNVPFHTVIFP

STLFGTGENWTFMKTISVTEYLNYESGKFSKSKGIGVFGNDVKDTTIPVEVWRYYLLANRPEVSDTLFTWTDLQAKLNSE

LLSNLGNFINRVLSFIAKDPASGYGSVIPDAHGVESHSSTKALAEKVGNYLDQYIEAMEKVKLKQALKIAMSLSGEGNAY

LQESQFWKLYKEDKPSCSVVMRTAAGLVYLLAFLLEPFMPSFSREVFKQLNLPESWLSLSDENGDIERAKKPWDILPAGH

KIGTPVPLFKELKDEEVEMYREKFAGSQADRTLRVEAEAKKITEQLNKAKISIGYLQLMWLSFL

>XP_010527469.1 PREDICTED: methionine--tRNA synthetase, cytoplasmic-like isoform X1 [Tarenaya hassleriana]

MAEEKSGPKLPIPGKRNILITSALPYVNNFPHLGNIIGCVLSADVFARYCRIRGYNSIYICGTDEYGTATETKALEENCS

PKEICDKYHAIHKEVYDWFDILFDKFGRTSTPQQTEVCQAIFKKLLKNNWLSENTMQQLYCETCKKFLADRLVEGTCPFE

ICNNDSARGDQCDKCGKLLNPTELKDPRCKVCGTSPQIRDTDHLFLELPLLKDKLDEYINKTSVAGSWSQNAIQITNSWL

KEGLKQRCITRDLKWGVPVPLEKYKDKVFYVWFDAPIGYVSITSCYTSEWEKWWKNPENVELYQFMGKDNVPFHTVMFPS

TQLGTEENWTLMKTISVTEYLNYEGGKFSKTKGVGVFGNDAKETNIPVEVWRYYLLTNRPEVSDTQFTWDDLQAKLNGEL

LNNLGNFVNRVLSFIAKPAGQGYGSVIPDAPGAESHPLTNSMAEKVGKLVEQYVEAMEKVKLKQGLRIAMSISSEGNAYL

QESQFWKLYKQDEPSCSIVMRTACGIVNILARLLEPFMPSFSHEVFKQLNLPLQFSLSDENGDIFKASRPWEILPPNHKI

GTPQPLFKELKDEEVQQYQEKFAGSQGDRRAREAEAANMVEQLKKTQLSDGKKQKPSKASEAAKAKPKPAAEPEITITRL

DIRVGKIVKVQKHPDADSLYIEEIDVGEEKPRTVVSGLVKHIPLDEMQNRMVCVLCNLKPVKMRGIQSQAMVLAASNSDD

SKVELVEPPESAKIGERVKFLGLEGDADDVLNPKKKVWETLQPDLRTNDNLVAHFKALPFTTSAGFCKVSSIKSGKIR

>XP_023000645.1 probable methionine--tRNA synthetase [Cucurbita maxima]

MGDVSGDAAAGGTKATILPIPGKRNILITSALPYVNNVPHLGNIIGCVLSADVFARYCRLRGYNTIYICGTDEYGTATET

KAMEEKCSPKEICDKYHAIHKEVYNWFGISFDEFGRTSSPQQTEVCQAIFGKLLENNWLSENTMQQLYCDTCERFLADRL

VEGICPTPGCEYDSARGDQCEKCGKLLNPTELKDPRCKVCQTTPRIRDTNHMFLELPLLRNKLEEYINKTSVAGSWSQNA

IQATNAWLKEGLKPRCITRDLKWGVPVPLERFKDKVFYVWFDAPIGYVSITSCYTNEWEKWWKNPENVELFQFMGKDNVP

FHTVMFPSTLLGTGEDWTLMKTISVTEYLNYEAGKFSKSKGVGVFGNDAKDTNIPVEVWRYYLLANRPEVSDTLFTWADL

QAKLNGELLNNLGNFIHRVLSFIAKPLGQGYGSIIPDVSDADSHGLTLGLAEKVGKYVDQYVESMEKVKLKQGLKSAMAI

STEGNIYLQTAQFWNLYKEDKPSCNLVMRTSAGLVYLLACLLEPFMPSFSQEVFKQLNIPVERHMSLCEEKGDIERAKQP

WKILPSGHKIGTPEPLFKELKDEEVELYRDKFAGSQAQRIVRAEAEAEKLAAQLKSTNVSGSGKKQQAKSTGSKSKAAVE

PEITITRLDIRVGLITKAQKHPDADSLYVEEIDVGESQPRTVVSGLVKYIPLEEMQNRKVCVLCNLKPAAMRGIKSQAMV

LAASNSDHTKVELVEPPKDAIVGERIKFSGFEGEPDSVLNPKKKVWETLQVDLATNADLVACFKDIPFTTSAGICKVSSI

SSGSIR

>PIN17951.1 Methionyl-tRNA synthetase [Handroanthus impetiginosus]

MGDRSAAAAPATKLPVPGKRNILITSALPYVNNVPHLGNIIGSVLSADVFARYCRIRGYNALYICGTDEYGTATETKALE

DGCSPKEICDKYHAIHSEVYKWFNISFDEFGRTSSPQQTEVCQAIFKRLLDNNWLSENTMQQLYCDTCKRFLADRLVEGN

CPTPGCLYDSARGDQCEKCGKLLNPTELLEPKCKVCRNTPHIRDTNHLFLELPLLKDKLEEYINNMSVAGGWSQNAIQTT

NAWLREGLKARCITRDLKWGVPVPLEKFKDKVFYVWFDAPIGYVSITACHTPEWEKWWKNPENVELYQFMGKDNVPFHTV

MFPSTLLGTGESWTLMKTISVTEYLNYEAGKFSKSKGVGVFGNDAKDTNIPVEVWRYYLLTNRPEVSDTLFTWVDLQAKL

NGELLSNVGNFINRVLSFIAKDPGSGYGSVIPDAQGADSHPLTKALGDKVGNYVEQYTEAMEKVKLKQGLKIAMTISGEG

NGYLQESQFWKLYKEDRPSCSIVMKTSAGLVYLLACLLEPFMPSFSLEVLKQLNLPLETISLSDEKGDIERAKRPWDFLP

VGHKIGTPAPLFKELKDEEVEFYRQKFAGSQADRIVRAEAEAEAKKLADKLKETKVSDGKTANKDRPKKCAEAKPKASAR

ANEEISISRLDIRVGLITKAQKHPDADALYVEEIDVGEPQPRTVVSGLVKYIPLEEMQNRKVCVLCNLKPATMRGIKSQA

MVLAASNNDHTKVELVEPPEGAVVGERVTFPGFEGLPDDVLNPKKKVWETLQVDLHTDKELVAHYKDLPFTTSAGVCKVS

SISEGSIR

>XP_023514495.1 probable methionine--tRNA synthetase [Cucurbita pepo subsp. pepo]

MGDVSGDAAAGGTKAIILPIPGKRNILITSALPYVNNVPHLGNIIGCVLSADVFARYCRLRGYNTIYICGTDEYGTATET

KAMEEKCSPKEICDKYHAIHKEVYNWFGISFDEFGRTSSPQQTEVCQAIFGKLLENNWLSENTMQQLYCDTCERFLADRL

VEGICPTPGCEYDSARGDQCEKCGKLLNPTELKDPRCKVCQTTPRIRDTNHMFLELPLLRNKLEEYINKTSVAGSWSQNA

IQATNAWLKEGLKPRCITRDLKWGVPVPLERFKDKVFYVWFDAPIGYVSITSCYTNEWEKWWKNPENVELFQFMGKDNVP

FHTVMFPSTLLGTGEDWTLMKTISVTEYLNYEAGKFSKSKGVGVFGNDAKDTNIPVEVWRYYLLANRPEVSDTLFTWADL

QAKLNGELLNNLGNFIHRVLSFIAKPLGQGYGSIIPDVSDADSHGLTLGLAEKVGKYVDQYVESMEKVKLKQGLKSAMAI

STEGNIYLQTAQFWNLYKEDKPSCNLVMRTSAGLVYLLACLLEPFMPSFSQEVFKQLNVPVERHMSLCEEKGDIERAKQP

WKILPSGHKIGTPEPLFKELKDEEVELYRDKFAGSQAQRIVRAEAEAEKLAAQLKSTNVSGGGKKQQAKSTGSKSKAAVE

PEITITRLDIRVGLITKAQKHPDADSLYVEEIDVGESQPRTVVSGLVKYIPLEEMQNRKVCVLCNLKPAAMRGIKSQAMV

LAASNSDHTKVELVEPPKDAIVGERIKFSGFEGEPDSVLNPKKKVWETLQVDLATNGDLVACFKDIPFTTSAGICKVSSI

SSGSIR

>XP_022871486.1 probable methionine--tRNA synthetase [Olea europaea var. sylvestris]

MGDQAPSAAAKKLPIPGKRNILITSALPYVNNVPHLGNIIGCVLSADVFARYCRNRGYNAVYICGTDEYGTATETKALEE

KCSPKEICDKYHAIHKEVYNWFNISFDEFGRTSSPQQTEVCQAIFKKLLENNWLSENTMQQLYCGSCKRFLADRLVEGNC

PTPGCNYDSARGDQCEKCGKLLNPTELLEPRCKVCGNTPRIQDTNHLFLELPLLKDKLEEYINNMSVAGGWSQNAIQATH

AWLREGLKQRCITRDLKWGVPVPHEKFMDKVFYVWFDAPIGYVSITSCYTPEWEKWWKNPENVELYQFMGKDNVPFHTVM

FPSTLIGTAENWTLMKTISVTEYLNYEAGKFSKSKGVGVFGNDAKDTNIPVEVWRYYLLTNRPEVSDTLFTWTDLQAKLN

SELLSNLGNFINRVLSFIAKDPGSGYGSIIPDAQGAESHPLTKVLGEKVSSYVEQYIEAMEKVKLKQGLKIAMLISGEGN

AYLQESQFWKLYKEDRPSCSVVMKTSVGIVYLLACLLEPFMPSFSLEVLKQLNLPLEMQISLSDEKGDVERAKRPWEIIP

AGHKIGTPAPLFRELTDKEVEFFREKFAGSQADRIVKAEAEAKKLTEKLKGTNISDGSTSKKDRPKKSAEAKPQVSAPAE

AGISISRLDIRVGLITKAQKHPDADSLYVEEIDVGEAQPRTVVSGLVKYIPLEEMQNRKVCVLCNLKPAAMRGIKSQAMV

LAASNSDHTKVELVEPPQGAVVGERVTFPGFEGTPDEVLNPKKKVWETLQVDLHSDKELVACYKDVPFTTSAGVCKVSSV

PDGSIR

>OAE23403.1 hypothetical protein AXG93_1660s1620 [Marchantia polymorpha subsp. ruderalis]

MDATVSGLKSLVLDSNTEGGSRGSGLQTRVKREIRVPIPGKRNVMVTSALPYVNNVPHLGNIIGCVLSADVYARYCRIRG

YNVIYMCGTDEYGTATETKAMEEGISPKEICDKYHAIHKEIYEWFDIDFDMFGRTSTPEQTVICQAIFQKLLEKDRLLED

KMEQPYCNTCLRFLADRFVEGICPHATCRYEDARGDQCDKCGKLLNAADLINPRCKRCGNPPQIRETEHMFLDLPQLKDR

LEAYVTNTSVSGGWSQNSVQTTNAWIRDGLKPRCITRDLKWGVPVPLEKYKEKVFYVWFDAPIGYMSITANYTSDWEKWW

KSPDDVELVQFMGKDNVPFHTVIFPSTLLGTDDPWTLMKTISVTEYLNYESGKFSKSRGVGVFGNDAKTTDIPVEVWRYY

LLTNRPEMSDTLFTWADLQAKQNNELLKNLGNFVHRSLSFLVKPAGEGYAGVIPEAPGAESHSLTVTLGEKVGELVKDYV

EVMEKVKLKQGLRIAMNVSSLGNLYLQDTKFWKLYKEDPPACAIVLRTSLGLVHLLATLLEPFMPSFSHKVLAQLNLPPS

SLSLSDEKGDLEKAFKPWELVPANHHIGSPTPLFAEMKDEEMERYRAKFAGSQADRAAADATAAATTGTPAVSKGDTKKN

VKSSKGGEAKSKKSATEVKDAPPAARLDIRVGLITKVQKHPDADSLYVEEIDVGEAAPRTVVSGLVKFIPIEQMQDRRVC

VLCNLKPASMRGIKSQAMVLAASNADHTKVELVTPPEGAQIGERVTFPGYEGEPDEVLNPKKKIWETVQPELATTEELVA

VYKDVPFTTSTGLGTTSAGIKDP

>XP_022964435.1 probable methionine--tRNA synthetase [Cucurbita moschata]

MGDVSGDAAAGGTKATILPIPGRRNILITSALPYVNNVPHLGNIIGCVLSADVFARYCRLRGYNTIYICGTDEYGTATET

KAMEEKCSPKEICDKYHAIHKEVYNWFGISFDEFGRTSSPQQTEVCQAIFGKLLVNNWLSENTMQQLYCDTCERFLADRL

VEGICPTPGCEYDSARGDQCEKCGKLLNPTELKDPRCKVCQTTPRIRDTNHMFLELPLLRNKLEEYINKTSVAGSWSQNA

IQATNAWLKEGLKPRCITRDLKWGVPVPLERFKDKVFYVWFDAPIGYVSITSCYTNEWEKWWKNPENVELFQFMGKDNVP

FHTVMFPSTLLGTGEDWTLMKTISVTEYLNYEAGKFSKSKGVGVFGNDAKDTNIPVEVWRYYLLANRPEVSDTLFTWADL

QAKLNGELLNNLGNFIHRVLSFIAKPLGQGYGSIIPDVSDADSHGLTLGLAEKVGKYVDQYMESMEKVKLKQGLKSAMAI

STEGNIYLQTAQFWNLYKEDKPSCNLVMRTSAGLVYLLACLLEPFMPSFSQEVFKQLNIPVERHMSLCEEKGDIERAKQP

WKILPSGHKIGTPEPLFKELKDEEVELYRDKFAGSQAQRIVRAEAEAEKLAAQLKSTNVSGSGKKQQAKSTGSKSKAAVE

PEITITRLDIRVGLITKAQKHPDADSLYVEEIDVGESQPRTVVSGLVKYIPLEEMQNRKVCVLCNLKPAAMRGIKSQAMV

LAASNSDHTKVELVEPPKDAIVGERIKFSGFEGEPDSVLNPKKKVWETLQVDLATNADLVACFKDIPFTTSAGICKVSSI

SSGSIR

>XP_019454325.1 PREDICTED: probable methionine--tRNA synthetase [Lupinus angustifolius]

MTEENPKLTKLPIEGKRNILITSALPYVNNVPHLGNIIGCVLSADVFARYCRLRGYNAIYICGTDEYGTATETKALEENC

SPKQICDKYHAIHKEVYNWFNISFDEFGRTSSPQQTEVCQAIFKRLYDNKWLSENTMQQLYCDTCEKFLADRLVEGDCPT

PGCEYDSARGDQCEKCGKLLNPTELKNPRCKVCRKSPRIRDTDHLFLELPLLKDRLEKYINEMSVVGLWSQNAIQTTNSW

LREGLKQRCITRDLKWGVPVPHEKYSDKVFYVWFDAPIGYVSITSCYTPEWEKWWKNPENVELYQFMGKDNVPFHTVMFP

STLLGTHENWTLMKTISVTEYLNYEAGKFSKSKGVGVFGNDAKDTGIPVEVWRYYLLTNRPEVSDTLFTWSDLQAKLNSE

LLNNLGNFINRVLSFIAKPAGQGYDSIIPAIPDDVSDDSHELTKKLANEVSANLEQYIDAMEKVKLKQGLKIAMSISSEG

NRYLQEAEFWHLYKDNKSLCSLVMKTAVGIVYLLACLLEPFIPSFTLEVFKQLNLSPETHLSLCDEKGDIDRVKRPWDII

SAGHKIGKPQPLFRELKDEEVEFYREKFAGSQADRIVRAEAEKVAEQLKKTKVSDGSGKKKSSAKSSKEAKNKAVAEPDI

SITRLDIRVGLIKKAEKHPAADTLYVEEIDVGEEQPRTVVSGLVNYIPLDEMQNRKVCVLCNLKPVTMRGIKSHAMVLAA

SNDSKVELVEPPTDARVGERVTFPGYEGNPDEILNPKKKVWETLQVDLHSNDELVACYKDVPLTTSAGICKVSSIPGGSI

K

>XP_018484569.1 PREDICTED: methionine--tRNA synthetase, cytoplasmic [Raphanus sativus]

MEDGESCPKLPIPGKRNILITSALPYVNNVPHLGNIIGCVLSADVYARYCRLRGYNAIYICGTDEYGTATETKALEENCT

PKEICDKYHAIHKEVYDWFDISFDKFGRTSTPEQTQVCQAIFNKLFENNFLSENTMQQLYCDTCQKFLADRLVEGSCPFP

ECNYDSARGDQCEKCGKLLNPTELKDPRCKVCRTTPRIRDTDHLFIELPLLKDRLEKYIDDTSVTGSWSQNAIQTTKAWL

KEGLRQRCITRDLKWGVPVPHEKYKEKVFYVWFDAPIGYVSITSCYTSEWEKWWKNPENVELYQFMGKDNVPFHTVMFPS

TQLGTGENWTLMKTISVTEYLNYESGKFSKSKGVGVFGNDVKDTNIPVEVWRYYLLTNRPEVSDTLFLWGDLQAKLTGEL

LNNLGNFVNRVLSFIAKPEPAGYGSIIPDAPGAESHPLTKSLAEKVGKLVEEYVQAMEKVKLKQGLKTAMTISNEGNWYL

QETQFWKLFKEDKPSCAIVIRTAAGLVYLLAQILEPFMPSFSREVFKQLNLPLHFSLSDEGIASRLWEILPPGHKIGTPK

PLFKELTDEEVQQYKDKFAGSQADRRDRDVEAANMAEQLKKTKLSDAKKQKASKGAGTSKSQPAATLEITMARLDIRVGK

ILKAEKHPNADSLYVEQIDVGGGEIRTVVSGLVKYIPLEEMQNRMVCVLCNLKPSKMRDIMSQAMVLAASSSDGSKVELV

EPPESAMIGERVRFAGHEGEPDVVLNPKKKVWETLLVDLNTNENLVACYKDLPFTTDAGVCKVSSISNGTIR

>XP_017216907.1 PREDICTED: probable methionine--tRNA synthetase [Daucus carota subsp. sativus]

MISFLRTDILTKLTSHSHKLTKLSYITFKTLIKPQMADLAAGDRKTIKLPIPGQRNILITSALPYVNNVPHLGNIIGCVL

SADVFARYCRLRGYNAIYVCGTDEYGTATETKAMEENCTPKEICDKYHAIHKEVYEWFNIKFDEFGRTSTPQQTEVCQAI

FSKLMENHWLSENTMQQLYCDTCKRFLADRLVEGTCPTQGCNYDSARGDQCENCGKLLNPTELIDPKCKVCQNTPHIRDT

NHLFLELPLLKDKLVEYINSMSVTGSWTQNAIQATNAWLKEGLRQRCITRDLKWGVPVPHEKFKDKVFYVWFDAPIGYVS

ITSCYTSEWEKWWKNPENVELYQFMGKDNVPFHTVMFPSTLLGTGENWTLMKTISVTEYLNYEAGKFSKSKGVGVFGNDA

KDTKIPVEVWRYYLLTNRPEVSDTLFTWVDLQAKLNTELLNNLGNFINRVLSFIAKDQASGYGFIIPDAPGAESHILTKK

LGDKVGNYVEQYIEAMEKVKLKQGLKLAMSISGEGNAYLQETQFWKLYKEDKASCSIVMKTSAGLVYLLACLLEPFMPSF

SVEVLKQLNMPPETQISLADEKGDIDRSKRPWEFLPAGHKIGTPVPLFKELSDKDVEFFRQKFAGSQADRIVKAEAEAKQ

IAEQLKRAKVSDGNVKKERSKKSSENKPKSTAEQEISISRLDIRVGLITKVQKHPDADSLYVEEIDVGEGQPRIVVSGLV

KYIPLEEMQNRKVCVLCNLKPVTMRGIKSYAMVLAVSNDDHTKVELVDPPQSAPVGERVTFSGFEGSPDDVLNPKKKVWE

TIQPELHTSNELIACYKDMPFSTSVGLCKVKSISDGSIR

>NP_595586.1 putative methionine--tRNA (Met) synthetase Mrs1 [Schizosaccharomyces pombe]

MATYKVQIPASFKTSYSFAESLKVSIAISAFSVKVPCEGAANCNSVRLVNSKEPEKYVSDANAIVSFLYWKQEEDLFNSF

ISSKLSILDWEALQFTPKAYTAKTKEDFAYLLSQLETIFKENEILNEFTPVEVALASDIYFCVLNGAPVREYPLLSAWYL

KIEKQKPFVQALKLTFEKTLGQPAVTSTEKIPVSETTRNVNSQHLMRERVPGEKILPKSNERNILITSALPYVNNVPHLG

NIVGSTLSADVFARYHRARNHNTLYICGTDEYGTATETKALEEGVSPKELCDKYHALHKEVYDWFEIDFDHFGRTTTPKQ

TGIAQHIFTKLYNNDYMAIDTMTQLYCEVHQGYLADRYVEGTCPKCGYDDARGDQCDGCGGLLNAFELIDPKCKLDRATP

VKRETKHVFLSLDKLQPAVESWAMQSAVEGKWSNNGRSITESWLKEGLRPRCITRDLKWGTPVPLEEFKGKVLYVWFDAT

IGYISITANYTDEWEKWWRNPEQVKLYQFMGKDNVPFHTVIFPSSLLGTGEKWTMLHHINTTDYLNYETGKFSKSRGVGV

FGNTAQDIGLSPSVWRYYLLSSRPETSDTMFTWKEFITRHNSELLANLGNFVNRTLKFTTAKYNGLVPHYLTDPSVGAGK

LKADFVKDVNALLAKYNAALEASKLREGLRLAMEISARGNQYLQDNRIDNKCYLYERQKCADAIGYALNLIYLLAAIFYP

YMPSTSTSIYKQLNAPAAAIPDTWELCLLPGHRIGEPEYLFTRIDESMEEEWRSKYGGNGSN

>XP_020082578.1 probable methionine--tRNA synthetase [Ananas comosus]

MGEQTSPRKLPVLGRRNILITSALPYVNNVPHLGNIIGCVLSADVFARYCRLREYNTVYICGTDEYGTATETKAMEENCS

PKEICDKYHAIHKEVYNWFNISFDEFGRTSTPQQTEICQSIFQRLMENKCLSENTMQQLYCETCQRFLADRFVEGTCPIA

GCNYESARGDQCEKCGKLLNPTELNDPKCKVCRNTPCIRVTDHIFLDLPLLSDKVEKYVNEMSVVGSWSQNAIQLTYALL

KEGLKPRCITRDLKWGVPVPHDKYKDKVFYVWFDAPIGYISITACYTPDWEKWWKDPDNVELYQFMGKDNVQFHTVMFPS

TLIGTGENWTMMKTISVTQYLNYETGKFSKSKGIGVFGNDAKDTNIPADVWRYYLLTNRPEVSDTLFTWVDLQAKLNSEL

LSNLGNFINRVLSFVAKPPGIGYDLVIPDASNAESHQLTKVLAENVAKLIDQYLDAMEKVKLKQGLKTAMSISTEGNGYL

QESQFWRLYKKDPSSCAIVMKTSVGLVYLLACLLEPFMPSFSSEVLGQLNLSPEVHLSFNDEKCDIEKAKRPWDLLPSGH

KIGKPEPLFKELKDEEVEFFRQKFAGSQADRITKAEADAQRIAEQLETTKITG

>KFH70174.1 methionyl-tRNA synthetase [Mortierella verticillata NRRL 6337]

MSTPATIKAGIAMPPKNEHKLPIPGKRNVLITSALPYVNNVPHLGNIIGSTLSADVYARYCRVRDVNALYICGTDEYGTA

TETKALSDGVTCQELCDKYYPIHKQCYEWMEIDFDKFGRTTTEEQTKIAQDIFLRLHERDLLVQQAMLQLYCAHCDRYLA

DRFVEGTCPKCNYDDARGDQCDQCGSLLNATELINPRCKLDGNRPVQKESQHLFLDLGKLQPQCEAWFEKASVEGKWSSN

GKVITKSWFKEGLQLRCITRDLKWGTPVPLPGWEGKVFYVWFDAPIGYPSITATYTKEWEKWWKNPEDVKLYQFMGKDNV

PFHTVIFPSTLIGTEEKWTLLNTLSTTEYLQYETGKFSKSRGVGIFGNNVQETGIPPSVWRYYLLSNRPESNDSQYLWKD

FIARNNNELLANAGNFVNRVIKFVNAKYDSNVPSLETAAAPTESDEKLIREVNEKLKAYIEASENVEIRAALRLAMEISA

LGNVYLQENKIDNTLFSEHRNRCDRVVITALNLIYLLSALFFPFMPSTSESIARQLNAPVRLLPDEFTVDSILPGHVINK

AEYLFKRIDEKMEEVWRAKYGGNGSEPAEDKKKKKRGKTAAPAAPIFEGEKPAEVIALEKQIEEQGVKVRDLKAAKADPA

EVTTEVQALLGLKKDLTNLFAKLTV

>XP_019255703.1 PREDICTED: methionine--tRNA synthetase, cytoplasmic-like [Nicotiana attenuata]

MGDSSTAGNGNYLPPTTPKLPIPGKRNILITSALPYVNNVPHLGNIIGSVLSADVFARYCRLRDYNAIYMCGTDEYGTAT

ETKALEENSTPKQICDKYHAIHREVYKWFDISFDQFGRTSTPQQTEVCQAIFNKLWENNWLSENTMQQPYCDTCKKFLAD

RLVEGNCPTPGCNYDSARGDQCEKCGKLLNPTELKDPRCKVCRNTPCIRDTDHLFLELPLLKDKLETYVNNMSVSGGWSQ

NAVHTTNAWLREGLKQRCITRDLKWGVPVPHEKYKEKVFYVWFDAPIGYVSITSCYTTEWEKWWKNPENVELYQFMGKDN

VPFHTVIFPSTLLGTGENWTLMKTISVTEYLNYEAGKFSKSKGIGVFGNDAKDTNIPVEVWRYYLLTNRPEVSDTLFSWA

DLQAKLNSELLSNLGNFVNRVLSFIAKDPVSGYGSIIPNPEGAESHPLTKALGEKVGNYVEQYIEAMEKVKLKQGLKIAM

SISGEGNGYLQESQFWKLYKEDKPSCSIVMSTAAGLVYLLACLLEPFMPSFSREVLKQLNFPPETQVSLADERGDIEKSK

RPWHILPAGHKIGIPAPLFKELKDEEVEFYREKFAGSQADRNLKAEAEAKKITDQLNKAKISDANKKKERATKPSVAKSK

GSASVEAEISISRLDIRVGLITRAQKHPDADSLYVEEIDVGEAQPRTVVSGLVNYIPLEEMQNRKVCVLCNLKPASMRGI

KSQAMVLAASSSDHTRVELVEPPKDAAIGERVTFPGFDGTPDDVLNPKKKVWETLLVDLHSNKELVACYKDLPFTTSAGV

CKVASISEGSIR

>XP_013723180.1 methionine--tRNA synthetase, cytoplasmic [Brassica napus]

MEDGESCPKLPIPGKRNILITSALPYVNNVPHLGNIIGCVLSADVYARYCRLRGYNAIYICGTDEYGTATETKALEENCT

PKEICDKYHAIHKEVYDWFDISFDKFGRTSTPEQTQVCQAIFKKLFENNFLSENTMQQLYCDTCQKFLADRLVEGSCPFP

ECNYDSARGDQCEKCGKLLNPTELKDPRCKVCQTTPRIRDTDHLFIELPLLKDRLEKYIDDTSVTGSWSQNAIQTTKAWL

KEGLRQRCITRDLKWGVPVPHEKYKEKVFYVWFDAPIGYVSITSCYTSEWEKWWKNPENVELYQFMGKDNVPFHTVMFPS

TQLGTGENWTLMKTISVTEYLNYETGKFSKSKGVGVFGNDVKDTNIPVEVWRYYLLTNRPEVSDTLFTWGDLQAKLTGEL

LNNLGNFVNRVLSFIAKPEPSGYGSVIPDAPGAESHPLTQSLAEKVGKFVKEYVEAMEKVKLKQGLKIAMTISNEGNAYL

QEAQFWKLFKEDKPSCAIVIRTAAGLVYLLAQLLEPFMPSFSREVFKQLNLPLQFSLSDEGIASRLWEILPPGHRIGTPQ

PLFKELKDEEVQQYKDKFAGNQADRRARDVEAANMAEQLKKTKLSDAKKQKASKGAGTSKPQPAATLEITMARLDIRVGK

ILKAEKHPNADSLYVEQIDVGGGEIRTVVSGLVKYIPLEEMQNRMVCVLCNLKPSKMRDIMSQAMVLAASSSDGSKVELV

EPPESATIGERVRFAGHEGEPDVVLNPKKKVWETLLVDLNTNENLVACYKDIPFTTDAGVCKVSSISNGTIR

>XP_013637095.1 PREDICTED: probable methionine--tRNA synthetase [Brassica oleracea var. oleracea]

MEDGESCPKLPIPGKRNILITSALPYVNNVPHLGNIIGCVLSADVYARYCRLRGYNAIYICGTDEYGTATETKALEENCT

PKEICDKYHAIHKEVYDWFDISFDKFGRTSTPEQTEVCQAIFKKLFENNFLSENTMQQLYCDTCQKFLADRLVEGSCPFP

ECNYDSARGDQCEKCGKLLNPTELKDPRCKVCQTTPRIRDTDHLFIELPLLKDRLEKYIDDTSVTGSWSQNAIQTTKAWL

KEGLRQRCITRDLKWGVPVPHEKYKEKVFYVWFDAPIGYVSITSCYTSEWEKWWKNPENVELYQFMGKDNVPFHTVMFPS

TQLGTGENWTLMKTISVTEYLNYETGKFSKSKGVGVFGNDVKDTNIPVEVWRYYLLTNRPEVSDTLFTWGDLQAKLTGEL

LNNLGNFVNRVLSFIAKPEPSGYGSVIPDAPGAESHPLTQSLAEKVGKFVKEYVEAMEKVKLKQGLKIAMTISNEGNAYL

QEAQFWKLFKEDKPSCAIVIRTAAGLVYLLAQLLEPFMPSFSREVFKQLNLPLQFSLSDEGIASRLWEILPPGHRIGTPQ

PLFKELKDEEVQQYKDKFAGNQADRRARDVEAANMAEQLKKTKLSDAKKQKASKGAGTSKPQPAATLEITMARLDIRVGK

ILKAEKHPNADSLYVEQIDVGGGEIRTVVSGLVKYIPLEEMQNRMVCVLCNLKPSKMRDIMSQAMVLAASSSDGSKVELV

EPPESATIGERVRFAGHEGEPDVVLNPKKKVWETLLVDLNTNENLVACYKDIPFTTDAGVCKVSSISNGTIR

>EPB81657.1 methionine-tRNA synthetase (EC:6.1.) [Mucor circinelloides f. circinelloides 1006PhL]

MPAAIKITVPKLSANTKPYANALKLSIAAQVTGAKLVETPGDALTFVVSNDVSLTDANAALKYIFDATAFNALQSLVIEK

EETSVSPLVIKRRIDQAFQNAEEIFVKHADAFAKEASNVTLVDVIYYGTLFETLSSAKDLSKYPTLSAWFNNISKNEKFT

AGVEHVKEQTNKGAKPKNKAVFAEKKVAKEIPKPVFNKTTQKVNNVAAKGLDQSVKVLPKENERNILITSALPYVNNVPH

LGNIVGSTLSADVYARYCRARGYNTAFFCGTDEYGTATETKALEEGVSCQALCDKYNAIHNAVYEWFDLSFDLFGRTTTE

KQTEIAQDIFLKNYKNGNIIKETMTQLFCEKCSRFLADRYVEGTCPHCKYDDARGDQCDACGKLLNATDLVNPRCKLDGN

APITKDSNHLFLDLAKLQPEIEAAFKQSAVDGKWSANGVNITQSWLKEGLRPRCITRDLKWGTPVPLEGYEDKVFYVWFD

APIGYPSITANYTDEWEKWWKNPENVKLYQFMGKDNVPFHSVIFPGCEIGTGEKWTVVNHISTCEYLNYEGGKFSKSRNV

GVFGNNAEATGIPPSVWRYYLMSVRPETSDSMFTWNEFITKNNSELLNNLGNFVNRAIRFIFAKYDGVIPKADFSGKLET

DLIKDVNELLKQYIDAMENVKLRSGLGIAMNISARGNQYLQEANLSNALFDESRPKCDAVVATAANLIYLLSAVLSPYMP

AASESMCRQLNAPLRTIPDELAMDIKEGHVLGGAEYLFTRIDEKMEEVWKGKYGTGSS

>XP_004335113.1 methionine-tRNA synthetase [Acanthamoeba castellanii str. Neff]

MECEKTKEQFAEDLKVLEDGQAVFQKEFVELKKAKADKAKIDEVLNRLKDGKEKIAALQKEFKALYPEDTLGGKAKAKPK

EKKEPVVKKEKFEVEVATYTLPEELLPGVGVESTNPILPVDDKRNILITSSLPYVNNVPHLGNIIGCVLSADVFARYCRL

RGHNVIYICGTDEYGTATETKALQEGMTPIEVCNKYYKVHREIYEWFGCQFDKFGRTTTEQQTTIAQQIFLDIQKNDYLL

TQTLEQLYCPTCAKFLADRFVEGTCPKEGCGYVDARGDQCDQCGSLLNPIELVNPRCKTCGTTPEPRFSEHLFLDLPRLE

GQLNEWVDKSISQGTWSQNSISTTKDKWLKEGLQPRCITRDLKWGTPVPLDKYKEKVFYVWFDAPIGYISITANYTEEWE

KWWKNPKNVELHQFMGKDNVPFHCVIFPASLMATGGDWTLLHSISTTEYLNYENTQFSKSRGTGVFGDAAKNTGIPAEVW

RYYLLANRPETSDSDFNWEDFGAKNNNELLANLGNFVNRCLKFTKDRFASTVPALGQLTEDDQTLIKEVDEQLEVYVKEL

DLAKMKASLQAAMHISRLGNYYLQKQEPWVLFKTDPQRCATVVHVGLNLVKLLTALLEPFMPHFSEKVLRQMQLDHSQIP

DRWTFEVAAGHVISDAVPIFRQIKEQELKEFARRFAGKQEQEGKAEVVKPKAVPAASSSSA

>PIN19081.1 Methionyl-tRNA synthetase [Handroanthus impetiginosus]

MGDRSAAASASAAKLPVPEKRNILITSALPYVNNVPHLGNIIGSVLSADVFARYCRIRGYNALYICGTDEYGTATETKAL

EDGCSPKEICDKYHAIHSEVYKWFNISFDEFGRTSSPQQTEVCQAIFKKLLDNNWLSENTMQQLYCDTCKRFLADRLVEG

NCPTPGCLYDSARGDQCEKCGKLLNPTELLEPKCKVCRNTPHIRDTNHLFLELPLLKDKLEEYINNMSVAGGWSQNAIQT

TNAWLREGLKARCITRDLKWGVPVPLEKFKDKVFYVWFDAPIGYVSITACHTPEWEKWWKNPENVELYQFMGKDNVPFHT

VMFPSTLLGTGESWTLMKTISVTEYLNYEAGKFSKSKGVGVFGNDAKDTNIPVEVWRYYLLTNRPEVSDTLFTWVDLQAK

LNGELLSNLGNFINRVLSFIAKDPGSGYGSIIPDAQGADSHPLTKALGDKVGNYVEQYIEAMEKVKLKQGLKIAMTISGE

GNGYLQESQFWKLYKEDRPSCSIVMKTSAGLVYLLACLLEPFMPSFSFEVLKQLNLPLETISLSDEKGDIERAKRPWDFL

PAGHKIGTPAPLFKELKDEEVEFYRQKFAGSQADRIVRAEAEAEAKKLADKLKETKVSDGKTAKKDWPKKSAEAKPKASA

RANEEISISRLDIRVGLITKAQKHPDADALYVEEIDVGEPQPRTVVSGLVKYIPLEEMQNRKVCVLCNLKPATMRGIKSQ

AMVLAASNNDHTKIELVEPPEGAVVGERVTFPGFEGLPDDILNPKKKVWETLQVDLHTDKELVARYKDLPFTTSAGVCKV

SSISEGSIR

>ORY94849.1 tRNA synthetases class I (M)-domain-containing protein [Syncephalastrum racemosum]

MVATISLTKLTPGTKPYINAFKLVLAGIVSDAKVNQQPGDLVSLIVNKDLLLTDANAAVKYLVGSGFDGSDAALKESAAI

EKEEALVAPLVAQKKTSEALEAAEKVLAAQKKPADKLTAVDVIYFASLYDVLIASSDIAKYPTIAAWFKTLQENKNTAAA

LESIDQQISKAAKKKQLGAQDKKSAGSKANVLADLDPATQKLDEQVFMPKRGEEKVLPKDGHRNVLITSALPYVNNIPHL

GNIIGSTLSADCFARYCRARGYNTLLICGTDEYGTATETKALEEGVSCQALCDKYSAIHEKVYDWFGLSFDHFGRTSTPE

QTQISQDIFNKVHANGYTSENVMTQLFCEKCQRFLADRYVEGTCPLCNYEDARGDQCDACGKLLNATELVNPRCKLDGTA

PITKDSRHLFLDLDRLQPDIDAFNARVNTEGKWSSNGVHITQSWLKEGLRPRCITRDLKWGTPVPLPGFESKVFYVWFDA

PIGYPSITARYTADWEKWWKNPDNVKLYQFMGKDNVPFHSVIFPGTQIATKEAWTMVHHISTTEYLNYEGGKFSKSRNVG

VFGTNAEETGIPPSVWRYYLLSSRPETSDSMFTWNEFITKNNSELLNNLGNFVNRVIKFAMAKYDGVVPEADLTGQSEAD

LTNDINKLLQEYIDNLENTKLRQGLAVAMAISARGNLYLQDSNVSNTLFTEQRSKCDAVVNMAVNLIYLLSALIYPYMPA

TSESISRQLNAPLRLIPDTFTFDIKGGHKMNGAAYLFSRIDEKMEEVWKSKYGSSGTK

>ORE02919.1 methionyl-tRNA synthetase [Rhizopus microsporus var. microsporus]

MSKLNLTLPKLAPNTKPFANALKIHIAAKVKDIKLNEVPGEHVSLVLSDEISLTDANAVVNPFIIKRRLDQAFQIVEETL

AKHRNLFAKVSDEVGFLDIIYYGALVEAFASFKEPSKYPLLAAWFDIVSKNTKVAAAVEYVKEQVIRMCYDSFVTQNSKG

TKSKKQGHAEKKVTKEIPKPTVNPATQKVNTNVATKLVDKNVKVLPKEGARNIMITSALPYVNNVPHLGNIIGSTLSADV

FARYCRARGYNTAYFCGTDEYGTATETKALEEGVSCKELCDKYNAIHTEVYKWFDLSFDLFGRTTTDRQTEIVQDMFKKN

YANGNVVKESMTQLYCEQCHRFLADRYVEGICPLCQYNDARGDQCDACGKLLNATELVEPRCKLDGNRPILKESNHLFLD

LTKLQPAIEETFKQSSTEGGWSANGIHITESWLKEGLKPRCITRDLKWGVPVPLEGYEDKVFYVWFDAPIGYPSITANYT

DEWEKWWKNPEQVKLYQFMGKDNVPFHSVIFPGVQIGTGEKWTKVNHISTCEYLNYEGGKFSKSRNVGVFGNNAKETGIP

PSVWRYYLLSVRPESSDSMFTWSEFITKNNSELLNNFGNFVNRAIRFIFAKYDGVIPKADFNGQLETDLFKDVNALLKQF

VEQMEAVKLRAALGTVMALSARGNQYLQEANLSNALFDESRPKCDAVVASAANLIYLLSALVSPFMPATSDSMCRQLNAP

ARNIPDQFTLDILPGHVLGGAEYLFTRIDEKMEQVWKSKYGTGA

>KZM86736.1 hypothetical protein DCAR_023870 [Daucus carota subsp. sativus]

MADLAAGDRKTIKLPIPGQRNILITSALPYVNNVPHLGNIIGCVLSADVFARYCRLRGYNAIYVCGTDEYGTATETKAME

ENCTPKEICDKYHAIHKEVYEWFNIKFDEFGRTSTPQQTEVCQAIFSKLMENHWLSENTMQQLYCDTCKRFLADRLVEGT

CPTQGCNYDSARGDQCENCGKLLNPTELIDPKCKVCQNTPHIRDTNHLFLELPLLKDKLVEYINSMSVTGSWTQNAIQAT

NAWLKEGLRQRCITRDLKWGVPVPHEKFKDKVFYVWFDAPIGYVSITSCYTSEWEKWWKNPENVELYQFMGKDNVPFHTV

MFPSTLLGTGENWTLMKTISVTEYLNYEAGKFSKSKGVGVFGNDAKDTKIPVEVWRYYLLTNRPEVSDTLFTWVDLQAKL

NTELLNNLGNFINRVLSFIAKDQASGYGFIIPDAPGAESHILTKKLGDKVGNYVEQYIEAMEKVKLKQGLKLAMSISGEG

NAYLQETQFWKLYKEDKASCSIVMKTSAGLVYLLACLLEPFMPSFSVEVLKQLNMPPETQISLADEKGDIDRSKRPWEFL

PAGHKIGTPVPLFKELSDKDVEFFRQKFAGSQADRIVKAEAEAKQIAEQLKRAKVSDGNVKKERSKKSSENKPKSTAEQE

ISISRLDIRVGLITKVQKHPDADSLYVEEIDVGEGQPRIVVSGLVKYIPLEEMQNRKVCVLCNLKPVTMRGIKSYAMVLA

VSNDDHTKVELVDPPQSAPVGERVTFSGFEGSPDDVLNPKKKVWETIQPELHTSNELIACYKDMPFSTSVGLCKVKSISD

GSIR

>XP_014619901.1 PREDICTED: probable methionine--tRNA synthetase isoform X1 [Glycine max]

MAENLIAPKLPVEGKRNILITSALPYVNNVPHLGNIIGCVLSADVFARYCRLRGYNAIYICGTDEYGTATETKAMEENCS

PKEICDKYHAIHKEVYNWFNISFDEFGRTSSPEQTEVCQAIFRKIFENKWLSENTMQQLYCNTCERFLADRLVEGTCPTP

GCEYDSARGDQCEKCGKLLNPTELKNPRCKVCQKTPHIRDTDHLFLELPLLKDRLEKYISEMSVVGGWSQNAIQTTNSWL

REGLKPRCITRDLKWGVPVPHEKYSDKVFYVWFDAPIGYVSITSCYTHEWEKWWKNPENVELYQFMGKDNVPFHTVMFPS

TLLGTSENWTLMKTISVTEYLNYEAGKFSKSKGIGVFGNDAKDTNIPVEVWRYYLLTNRPEVSDTLFTWPDLQAKLNSEL

LNNLGNFINRVLSFIAKPAGWSNSFIILVKLFITVFGTWQGYDSIIPSVPDNVSGDSHGPTKKLADKVAAYIDQYIEAME

KVKLKQGLKTAMSISSEGNAYLQEAEFWRLYKENKSLCSLVMKTAAGIVYLLACLLEPFIPSFTLEVFKQLNLPADTHVS

LSDDKGDVDRVKRPWDIISAGHKIGTPKPLFRELKDEEVEFYRKKFAGSQADRIVRAEAEAQNVAEQLKKTKVSDGNGKK

KPGKSSNEAKNKAAAEPDITITRLDIRVGLIIKAQKHPDADALYVEEIDVGEEQTRTVVSGLVKFIPLDEMQNRKVCVLC

NLKPVTMRGIKSQAMVLAASDGDHTKVELVEPPSSAQPGERITFPGYEGNPDELLNPKKKVWETLQVDLHTNEELVACYK

NVPLTTSAGVCKVSSISCGSIR

>XP_009797160.1 PREDICTED: probable methionine--tRNA synthetase [Nicotiana sylvestris]

MGDSSTAGNGDYLPPTTPKLPIPGKRNILITSALPYVNNVPHLGNIIGSVLSADVFARYCRLRNYNAIYMCGTDEYGTAT

ETKALEENSTPKQICDKYHAIHREVYKWFDISFDQFGRTSTPQQTEVCQAIFNKLWENNWLSENTMQQPYCDTCKKFLAD

RLVEGNCPTPGCNYDSARGDQCEKCGKLLNPTDLKDPRCKVCRNTPCIRDTDHLFLELPLLKDKLETYVNNMSVAGGWSQ

NAVHTTNAWLREGLKQRCITRDLKWGVPVPHEKYKEKVFYVWFDAPIGYVSITSCYTTEWEKWWKNPENVELYQFMGKDN

VPFHTVIFPSTLLGTGENWTLMKTISVTEYLNYEAGKFSKSKGVGVFGNDAKDTNIPVEVWRYYLLTNRPEVSDTLFSWA

DLQAKLNSELLSNLGNFVNRVLSFIAKDPVSGYGSIIPNHEGAESHPLTKALGEKVGNYVEQYIEAMEKVKLKQGLKIAM

SISGEGNGYLQESQFWKLYKEDKPSCSIVMSTAAGLVYLLACLLEPFMPSFSREVLKQLNFPPETQVSLADERGDIEKSK

RPWHILPAGHKIGIPAPLFKELKDEEVEFYREKFAGSQADRNLKAEAEAKKITDQLNKAKISDANKKKERATKPSAAKSK

GSASVEAEISISRLDIRVGLITRAQKHPDADSLYVEEIDVGEAQPRTVVSGLVNYIPLEEMQNRKVCVLCNLKPASMRGI

KSQAMVLAASSSDHTRVELVKPPKDAAIGERVTFPGFDGTPDDVLNPKKKVWETLLVDLHSNKELVACYKDLPFTTSAGV

CKVASISEGSIR

>XP_009406026.1 PREDICTED: probable methionine--tRNA synthetase isoform X1 [Musa acuminata subsp. malaccensis]

MEDTPAENSPKLPVPGRRNILITSALPYVNNVPHLGNIIGCVLSADVFARYCRLRGYNTIYICGTDEYGTATETKAMEEN

CSPKEICDKYHEIHKEVYKWFDISFDEFGRTSSPQQTEICQSIFHKLLENNWLSEKTMLQLYCDTCQRFLADRLVEGTCP

TLDCNYESARGDQCEKCGKLLNPTELIDPKCKVCQRTPHICDTDHLFLELPLLRKKLEDYIDSMSITGSWSQNAIQATQA

WLKEGLKPRCITRDLKWGVPVPHEKYKDKVFYVWFDAPIGYISITACYTLEWEKWWKNPENVELYQFMGKDNVPFHTVMF

PSTLIGTGEKWTMMKTISVTEYLNYETGKFSKSKGVGVFGNDAKDTNIPTEVWRYYLLTNRPEVSDTLFTWSDLQAKLNS

ELLNNLGNFVNRVLSFIAKPEGAGYNSIVPDAPDAESHNLTKDLGEKVGKLVDQYLDAMEKVKLKQGLRTAMSISSEGNA

YLQESQFWKLYKEDPSSCAIVMKTSAGLVYLLATLLEPFMPSFSIEVLRQLNLSPKTSLSFCNEKGETDDKKKPWNFLPS

GHSISKPEPLFKELKDEDVEYFRMRFAGSQADRKVKAEADAKKITEQLKSTNITEANAKKQQCKPEGSPKAKPTETEIPI

TKLDIRVGLIKKVQKHPDADALYVEEIDVGEESTRTVVSGLVKYIPLEEMQNRKVCVLCNLKPATMRGIKSHAMVLAASS

DDHSKVEMVDPPASAAVGERVTFSGYSGEPDNIISAKSKVWEKVQLDMHTDAELVACYKDVPFMTSAGVCKVSSIANGII

S

>XP_008457156.1 PREDICTED: methionine--tRNA synthetase, cytoplasmic [Cucumis melo]

MGDDSGKAAGTKATKLPIPGKRNILITSALPYVNNVPHLGNIIGCVLSADVFARYCRLRGYNAIYICGTDEYGTATETKA

MEEKCSPKDICDKYHAIHKEVYNWFGISFDEFGRTSSPQQTEVCQAIFGKLLENNWLSENTMQQLYCDTCERFLADRLVE

GICPTPGCEYDSARGDQCEKCGKLLNPTELQDPRCKVCQTTPRIRDTNHMFLELPLLREKLEEYINKMSVAGSWSQNAIQ

ATHAWLKEGLKPRCITRDLKWGVPVPLERFKDKVFYVWFDAPIGYVSITSCYTNEWEKWWKNPENVELYQFMGKDNVPFH

TVMFPSTLLGTGEDWTLMKTISVTEYLNYEAGKFSKSKGVGVFGNDAKDTNIPVEVWRYYLLANRPEVSDTLFTWADLQA

KLNGELLNNLGNFIHRVLSFIAKPLGQGYGSIIPDASDVVSHELTLSLADKVGKYVDQYVESMEKVKLKQGLKAAMAISS

EGNIYLQTAQFWNLYKADKPSCNIVMRTSAGLVYLLACLLEPFMPSFSLEVFRQLDLPLERHISLCEEKGDIETVKQPWK

ILPSGHKIGTPEPLFKELKDEEVELYRDKFAGSQAQRIVRAEAEAEKLAAQLKKTNVSGGGKKQQAKSTGGKPKAAVEQE

ITITRLDIRVGLITKAQKHPDADSLYVEEIDVGESQPRTVVSGLVKYIPIEEMQNRKVCVLCNLKPASMRGIKSQAMVLA

ASNSDHTKVELVEPPKEALVGERIKFAGFEGEADDVLNPKKKVWETLQVDLSTNGDLVACFKDIPFTTTAGVCKVSSISC

GSIR

>XP_012855641.1 PREDICTED: probable methionine--tRNA synthetase [Erythranthe guttata]

MGDCSAAAAAAAATSAKLPIPGKRNILITSALPYVNNIPHLGNIIGCVLSADVFARYCRLRGYNAIYICGTDEYGTATET

KALEEGCSPKEICDKYHAIHKEVYKWFNISFDEFGRTSSPQQTEVCQDIFKKLLDNNWLSENTMQQLYCDTCDKFLADRL

VDGNCPTPGCNYDSARGDQCEKCGKLLNPTELLEPKCKFCRKTPRIRDTNHLFLELPLLKDKLEEYISTTSVSGGWSQNA

IQTTNAWLREGLKPRCITRDLKWGVPVPHENFKEKVFYVWFDAPIGYVSITSCHTKEWEKWWKNPEDVELYQFMGKDNVP

FHTVMFPSTLLGTGESWTLMKTISVTEYLNYESGKFSKTKGVGVFGNDAKDTNIPVEVWRYYLLTNRPEVSDTLFTWPDL

QAKLNSELLSNLGNFINRVLSFIAKDPATGYGSIIPEAEFVDSHPLTKALGDKIGNYVEQYVEAMEKVKLKQGLKIAMAI

SGEGNAYLQESQFWKLYREDPPSCAVVMKTSAGLVYLLACLLEPFMPSFSLEVLKQLNLPAETQFSLSDDKGEVERAKRP

WEILPAGHKIGTPAPLFRELKDEEVAFYREKFAGSQADRKVKADAAAKKLTDKLQEVEISDGKKAKKEKAKKSAETKTKV

SAPAEAEISISRLDIRVGLITKAQKHPDADSLYVEEIDVGEAQPRTVVSGLVKYIPLEEMQNRKVCVLCNLKPAAMRGIK

SQAMVLAASNNDHTKVELVEPPQGAVVGERVTFQGFEGLPDDVLNPKKKVWETLQVDLLTDKELVACYKDLPFTTSAGVC

KVSSISEGTIR

>ESR56818.1 hypothetical protein CICLE_v10024095mg, partial [Citrus clementina]

MGDSEQEPDGERNHRVAKLPVPGKRNILITSALPYVNNVPHLGNIIGCVLSADVFARYCRLRGYNALYICGTDEYGTATE

TKAMEENCTPQQICDKYYAIHKAVYNWFNISFDEFGRTSTPQQTEICQAIFKKLLENNWLSEYTTQQLYCDTCKRFLADR

LVEGSCPTLGCNYDSARGDQCEKCGKLLNPTELKDPRCKVCQATPKIRDTDHLFLELPLIKDKLEEYISKMSVSGSWSQN

AIQGTYAWLKDGLKPRCITRDLKWGVPVPHEKFKDKVFYVWFDAPIGYISITSCYTPDWEEWWKNPENVALYQFMGKDNA

PFHTVMFPSTLLGTEENWTLMKTISVTEYLNYEAGKFSKSKGVGVFGNDVKDTNIPAEVWRYYLITNRPEVSDTLFTWED

FQAKLNTELLNNLGNFVNRVLSFIAKPPGRGYGSIIPDSPKADLHPSTKMLAEKISNHIDQYIEAMEKVKLKQGLRIGMS

ISSEGNAYLQESQFWKLYNEDQGSCSIVIRTSVGLVYLLACLLEPFMPSFSLEVLKQLNLPTETKFSLSDENGDLERARK

PWEILPAGHRIGMPSPLFKELKDEDVRFFREKFAGSQADRAVQAEAEAKRLAEHLKKTKIS

>XP_022149372.1 methionine--tRNA synthetase, cytoplasmic-like [Momordica charantia]

MGGVSGDTVAAGRTKATKLPIAGRRNVLITSALPYVNNVPHLGNIIGCVLSADVFARYCRIRGYNAIYVCGTDEYGTATE

TKAMEEKCTPQEICDKYHAIHKEVYNWFGISFDEFGRTSSPQQTEVCQAIFGKLLENNWLSENTMQQLYCDTCERFLADR

LVEGICPTPGCDHDSARGDQCEKCGKLLNPTELKDPRCKVCQTSPRIRDTNHMFLELPLLRDRLEEYINKMSVAGSWSQN

AIQATHAWLKEGLKPRCITRDLKWGVPVPVERFKDKVFYVWFDXPIGYVSITSCYTNEWEKWWKNPENVELFQFMGKDNV

PFHTVMFPSTLLGTGESWTLMKTINVTEYLNYEAGKFSKSKGVGVFGNDAKDTNIPVEVWRYYLLANRPEVSDTLFTWAD

LQAKLNGELLNNLGNFIHRVLSFIAKPLGQGYGSIIPDAPDAESHGLTTGLAEKVGKYVDQYVESMEKVKLKQGLKAAMA

ISSEGNTYLQTAQFWNLYKEDKPSCNVVMRTSAGLVHLLACLLEPFMPSFSLEVFKQLNLPGEWHASLCEEKGDIEKAKQ

PWKILPSGHKIGTPEPLFKELKDEEVEIYRDKFAGSQAQRIVRAEAEAEKLAAQLKKTNVSGGGKKQQAKSTGSKPKAAV

EPEITITRLDIRVGLITKAQKHPDADSLYVEEIDVGEQQPRTVVSGLVKYIPLEEMQNRKVCVLCNLKPASMRGIKSQAM

VLAASNSDHTKVELVEPPKDANIGERVKFPGFEGEPDDVLNPKKKIWETLQVDLATNTELVACFKDIPFTTSAGICKVSS

ISSGSIR

>ORX60728.1 methionyl-tRNA synthetase [Hesseltinella vesiculosa]

MVQSITLTVPKLVPSQRHFANTLKVFIAAKVVDVKVTQQQGDSFSVEVDGVALQDPNVVVRCILGDLDFDLSRSYCVEKE

SSQLGPLITKKKYDAALKEINQLMEQHKVFGNTDKLTSVDAIYYGSLYEALGSVDAAKYPTLAAWTHLTGQSEVVKAAVD

LIQQQIQTKAPKKKHQANEKKAVQSVQITQLNPADQKLNKNVLYVAPTATDAKVTPKDGEHNILITSALPYVNNIPHLGN

IIGSTLSADCYARYCRLRGYNTLYICGTDEYGTATETKALEEGVSCQALCDKYNAVHSKVYEWFGISFDYFGRTTTEKQT

QITQDIFKKVNDNGYIIQDTMTQLYCEKCSRFLADRYVEGECPNCKYEDARGDQCDACGRLLNATELVKPRCKLDGNTPI

TRDSRHLFLNLEKLQSGIEAFVEKSTVEGKWSSNGVNITHSWLKEGLHPRCITRDLKWGTPVPLAGFEDKVFYVWFDACI

GYPSITANYTDDWEKWWKNPDQVKLYQFMGKDNVPFHSVIFPGTELATQERWTMVNHISTTEYLNYEGGKFSKSRNVGVF

GTNAEETGISPSVWRYYLLSGRPESNDSMFTWNEFITKNNTELLNNLGNFVNRAIKFVVAKYDGMLPADAPLGELETKLA

KDVNELLAQYVDQMEKVKLRAGLATAMAVSARGNQYLQESNLSNQLFNDQRAQCDAVVTTAVNLIHLLASLISPFMPATA

DSISLQINAPLRQIPDTFALDIHGGHKLNGADYLFTRIDEKMEDVWKSKYGGKK

>XP_001524999.1 methionyl-tRNA synthetase [Lodderomyces elongisporus NRRL YB-4239]

MSLELFGAKRSSLNNFVKNLKLSIAAAVYKPDLKVELSPETEIKDVLLRDKKNPAFQLTEPNAIVKYLADDFTNEELIKF

EETVLYKALISNKRESLNDIELKLPESVKLKPAQIILFASVYPRFEGDEKSAPKWFTEFAQDEKVASGIKHALSISKLER

RKETNTGAQNLVKDHLVKSQADRILPKADERNILITSALPYVNNVPHLGNIVGSVLSADIYARYAKNRNYNAIFICGTDE

YGTATETKALEDGVTPQQLCDKYHKIHKQVYDWFDIGFDYFGRTTTPLQTEIAQDIFKKLDKGEYLEEKTTEQLYCEHHH

SFLADRFVEGICPKCGYEDARGDQCDKCGQLLDPLELIEPRCKVDGTKPIVKPSTHIYLKLNELETPLKEWVDISSEKGA

WSKNSKTITNSWIKQGLEPRCITRDLKWGTPVPRPGYEDKVLYVWFDATIGYVSITANYFKSIDESSVDNWQKWWKNPEN

VDLYQFMGKDNVPFHTVVFPASQIGTGDNWTKLHHLSTTEYLQYEGGKFSKSRGVGVFGNNAEETGVPASVWRYYLASSR

PETSDAHFSWDDFVTKNNSELLANLGNFVNRIVKFAIAKYNGVLPQYDTKNIPNYDQFLTDINTLLGTYIKNMEAVNLRK

GLETAMLISSRGNQFLQENKLDNTLYNEYPDKSDAVVAIGLNLVYLVSAVISPFMPDTTKKINEILNAPALSITNTFIPI

LSPGHHIGQAQYLFTRIDEKKIAEWRALYGGQQKK

>XP_009614577.1 PREDICTED: methionine--tRNA synthetase, cytoplasmic-like [Nicotiana tomentosiformis]

MGDSSTAGNGDYLPPTTPKLPIPGKRNILITSALPYVNNVPHLGNIIGSVLSADVFARYCRLRDYNAVYMCGTDEYGTAT

ETKALEENSTPKQICDKYHAIHRDVYKWFDISFDQFGRTSTPQQTEVCQAIFNKLWENNWLSENTMQQPYCDTCKKFLAD

RLVEGNCPTPGCNYDSARGDQCEKCGKLLNPTELKDPRCKVCRNTPCIRDTDHLFLELPLLKDKLETYVNNMSVAGGWSQ

NAVHTTNAWLREGLKQRCITRDLKWGVPVPHEKYKEKVFYVWFDAPIGYVSITSCYTTEWEKWWKNPENVELYQFMGKDN

VPFHTVIFPSTLLGTGENWTLMKTISVTEYLNYEAGKFSKSKGIGVFGNDAKDTNIPVEVWRYYLLTNRPEVSDTLFSWA

DLQAKLNSELLSNLGNFVNRVLSFIAKDLVSGYGSIIPNPEGAESHPLTKALGEKVGNYVEQYIEAMEKVKLKQGLKIAM

SISGEGNGYLQESQFWKLYKEDKPSCSIVMSTAAGLVYLLACLLEPFIPSFSREVLKQLNFPPETQVSLADEKGDVEKSK

RPWHILPAGHKIGIPAPLFKELKDEEVEFYREKFAGSQADRNLKAEAEAKKITDQLNKAKISDANKKKERATKPSVAKSK

GSASVEAEISISRLDIRVGLITRAQKHPDADSLYVEEIDVGEAQPRTVVSGLVNYIPLEEMQNRKVCVLCNLKPASMRGI

KSQAMVLAASSSDHTRVELVEPPKDAAIGERVTFPGFDGTPDDVLNPKKKVWETLLVDLHSNKELVACYKDLPFTTSAGV

CKVASISEGSIR

>XP_006479256.1 probable methionine--tRNA synthetase [Citrus sinensis]

MGDSEQEPDGERNHRVAKLPVPGKRNILITSALPYVNNVPHLGNIIGCVLSADVFARYCRLRGYNALYICGTDEYGTATE

TKAMEENCTPQQICDKYYAIHKAVYNWFNISFDEFGRTSTPQQTEICQAIFKKLLENNWLSEYTTQQLYCDTCKRFLADR

LVEGSCPTLGCNYDSARGDQCEKCGKLLNPTELKDPRCKVCQATPKIRDTDHLFLELPLIKDKLEEYISKMSVSGSWSQN

AIQGTYAWLKDGLKPRCITRDLKWGVPVPHEKFKDKVFYVWFDAPIGYISITSCYTPDWEEWWKNPENVALYQFMGKDNA

PFHTVMFPSTLLGTEENWTLMKTISVTEYLNYEAGKFSKSKGVGVFGNDVKDTNIPAEVWRYYLITNRPEVSDTLFTWED

FQAKLNTELLNNLGNFVNRVLSFIAKPPGRGYGSIIPDSPKADLHPSTKMLAEKISNHIDQYIEAMEKVKLKQGLRIGMS

ISSEGNAYLQESQFWKLYNEDQGSCSIVIRTSVGLVYLLACLLEPFMPSFSLEVLKQLNLPTETKFSLSDENGDLERARK

PWEILPAGHRIGMPSPLFKELKDEDVRFFREKFAGSQADRAVQAEAEAKRLAEHLKKTKISVPLN

>XP_007131531.1 hypothetical protein PHAVU_011G020800g [Phaseolus vulgaris]

MAQNGKPPKLPVEGKRNILITSALPYVNNVPHLGNIIGCVLSADVFARYCRIRGYNTIYICGTDEYGTATETKAMEEKCS

PKEICDKYYAIHKEVYNWFNISFDEFGRTSCPQQTEVCQEIFKKIFDNKWLSENTMQQLYCDTCEKFLADRLVEGTCPTP

GCEYDSARGDQCEKCGKLLNPTELKNPRCKVCKKTPRIRDTDHLFLELPLLKDRLEKYITEMSVVGGWSQNAIQTTNSWL

REGLKPRCITRDLKWGVPVPHEKYKDKVFYVWFDAPIGYVSITSCYTHEWEKWWKNPENVELYQFMGKDNVPFHTVMFPS

TLLGTGENWTLMKTISVTEYLNYESGKFSKSKGIGVFGNDVKDTEIPVEVWRYYLLTNRPEVSDTLFTWPDLQAKLNSEL

LNNLGNFINRVLSFIAKPEGQGYDSIIPSIPDDVSADSHNLTKKLADKVAAYIDQYIEAMEKVKLKQGLKTAMSISSEGN

AYLQEAQFWRLYKENKSLCALVMKTAVGVVYLLACLLEPFIPSFTLEVFKQLNLPAEIHVSLCDDKGDVDRVKRLWDIVS

SGHKLGTPKPLFKELKDEEVDFYRKKFAGSQADRIVRAEVEADKVAEQLKKTKVSDGNGKKKPAKSSNEAKNKAAPEPDI

TITRLDIRVGLITKAQKHPDADALYVEEIDVGEEQTRTVVSGLVKYIPLDEMQNRKVCVLCNLKPASMRGIKSQAMVLAA

SNDDHSKVELVEPPSSATVGERITFPGNEGNPDELLNPKKKVWETLQVDLHTNEDLVACYKDVPLTTSAGVCKVSSISCG

TIK

>XP_006283141.1 methionine--tRNA synthetase, cytoplasmic [Capsella rubella]

MEDGGKSDVPKLPIPGKRNILITSALPYVNNVPHLGNIIGCVLSADVYARYCRLRGYNAIYICGTDEYGTATETKALEEN

CTPKEICDKYHAIHKEVYDWFDISFDKFGRTSTPEQTEVCQAIFNKLWENKWLSENTMQQLYCDTCKKFLADRLVEGSCP

FEGCNYDSARGDQCEKCGKLLNPTELKDPKCKVCQNTPRIRDTDHLFIELPLLKDKLEEYIKETSVAGSWSQNAIQTTNA

WLRDGLRQRCITRDLKWGVPVPHEKYKEKVFYVWFDAPIGYVSITSCYTSEWEKWWKNPENVELYQFMGKDNVPFHTVMF

PSTQLGTGENWTLMKTISVTEYLNYEDGKFSKSKGVGVFGNDVKDTNIPVEVWRYYLLTNRPEVSDTSFSWKDLQAKLNG

ELLSNIGNFVNRVLSFIAKPENAGYGSVIPDAPGAESHPLTISLAEKVEKLVAEYVEAMEKVKLKQGLKTAMLISSEGNF

YLQANQFWKLYKDDKPSCAIVIRTAAGLVHLLAQLLEPFMPSFSREVFKQLNLPLHFSLSDERGEVLLASRPWEILPPSH

RIGTPQPLFKELVDEEVQQYREKFAGSQADRRARDEAANLTDQLNKTKLSDAKKQKAASSKGGGKPKPQPAAEREITMAR

LDIRVGKILKAEKHPKADALYVEEIDVGGGEIRTVVSGLVKYIPLEEMQNRMVCVLCNLKPAKMRDIVSQAMVLAASSSD

GSKVELVEPPKSATIGERVTFPGFEGEPDDVLNPKKKVWETLLVDLNTNENLVACYKDVPFTTSAGECKVSSISNGTIR

>XP_018847750.1 PREDICTED: probable methionine--tRNA synthetase [Juglans regia]

MGDRKEDIRGEEEGCKKIPKLPIPGKRNILITSALPYVNNVPHLGNIIGCVLSADVFARYCRLRGYNVIYICGTDEYGTA

TETKAMEENCTPQEICDKYHAIHKEVYNWFDISFDEFGRTSTPQQTDVCQDIFKKLMENNWLSENTIQQFYCDNCNRFLA

DRLVEGTCPFPNCNYDSARGDQCEKCCEMLNSTELKDPRCKVCQTTPQIRDTNHLFLELPLLKDKLEEYINAMSVAGSWS

QNAIQATYAWLTGGLKPRCITRDLKWGVPVPHEKFKDKVFYVWFDAPIGYISITACYTPDWEKWWKNPENVELYQFMGKD

NVPFHTVMFPSTLLGTGESWTLMKSISVTEYLNYEAGKFSKSKGTGVFGNDAKGTNIPPEVWRYYLLTNRPEVSDTLFSW

TDLQTKLNTELLNNLGNFINRVLSFIVKPPGQGYGSIIPDAQGAESHLLTKTLAEKVGNNVDQYVEAMEKVKLKQALKIG

MSISSEGNAYLQESQFWKLYKQDQASCSVVMRTSVGLVYLLAYLLEPFMPSFSVKVLKQLNLPPDRTLSLSDEAGDLGKA

RKPWEVIPAGHKIGTPDPLFKEMKDEDVAFFQMKFSGRQADRAV

>XP_014522689.1 methionine--tRNA synthetase, cytoplasmic [Vigna radiata var. radiata]

MAQNGKPPKLPVEGKRNILITSALPYVNNVPHLGNIIGCVLSADVFARYCRIRGYNTIYICGTDEYGTATETKAMEEKCS

PKEICDKYYAIHKEVYNWFNISFDEFGRTSCPQQTEVCQEIFKKIFENKWLSENTMQQLYCDTCERFLADRLVEGTCPTP

GCEYDSARGDQCEKCGKLLNPTELKNPRCKVCQKTPRIRDTDHLFLELPLLKDRLEKYITEMSVAGGWSQNAIQTTNSWL

REGLKPRCITRDLKWGVPVPHEKYKDKVFYVWFDAPIGYVSITSCYTRDWEKWWKNPENVELYQFMGKDNVPFHTVMFPS

TLLGTGENWTLMKTISVTEYLNYEAGKFSKSKGIGVFGNDVKDTEIPVEVWRYYLLTNRPEVSDTLFTWPDLQAKLNSEL

LNNLGNFINRVLSFIAKPAGQGYDSIIPSIPDDVSGDSHNLTKKLADKVAAYIDQYIEAMEKVKLKQGLKTAMSISSEGN

AYLQEAQFWRLYKENKSLCALVMKTAVGIVYLLACLLEPFIPSFTLEVFNQLNLPAEIHVSLCDDKGDVDRVKRPWDILS

SGHKIGTPKPLFKELKDEEVDFYRKKFAGSQADRIVRAEAEAENVAEKLRKTKVSDGNGKKKPAKSSNEAKNKAVPEPDI

TITRLDIRVGLITKAQKHPDADALYVEEIDVGEEQTRTVVSGLVKYIPLDEMQNRKVCVLCNLKPASMRGIKSQAMVLAA

SNDDHSKVELVEPPSSAKVGERITFPGSEGNPDELLNPKKKVWETLQVDLHTNEDLVACYKNVPLTTSAGICKVSSIRGG

SIK

>KNE59603.1 methionine-tRNA synthetase [Allomyces macrogynus ATCC 38327]

MSTTKRATPFVRSKAVPLPVPGQRNVLVTSALPYVNNEPHLGNIIGCVLSADAYARFARLRGYNTLYVCGTDEYGTATET

KALEEGVSCQALCDKYHALHKSIYELFEIDFDLFGRTTTPQQTTIAQDIFNKVEANGFVTRDTLTQLYCETCQRFLADRY

VEGTCPHCKFTDARGDQCDACQKLLNAADLIQPRCKLDGSKPVTRETTHLFLDLPKLTPQLTEFVDTKSAAGKWSANSIA

ITRGWLKEGLKPRCITRDLKWGTPVPLEGFTDKVFYVWFDAPIGYPSITANLTPEWERWWKNPKDVQLYQFMGKDNIPFH

TVIFPSSLLATRENWTLLHHVSTTEYLNYEGGKFSKSRKTGVFGSNVRDTGIPVSVWRYYLFANRPETSDSDFSWKDFIA

RNNNELLNNLGNFVNRVVKFVASPKYASVLPAFEWAVHPVDIQLVADIDTLLTQYLDALEHVKIRHGLQLAMAVSARGNQ

YLQDNKLDNALHAADPKRCAQVLGHAANVAYLLAALFYPYMPATAASIARQLNAPQPKYPASFAAGPLLAAESSHAIGKP

EYLFKRIDEKLEGEFRAKYGGQTAGGSAADAAPVVDKKAAKKAAAAKKAADPVKLVEKMDPAKITPAARELQAKVAAQGE

KVKTLKTAKADATTVANEVDVLLKFKADLLAEVQAIAGGK

>XP_006414857.1 methionine--tRNA synthetase, cytoplasmic [Eutrema salsugineum]

MEDGDGNAPRLPIPGMRNILITSALPYVNNVPHLGNIIGCVLSADVFARFCRLLGYNVIYICGTDEYGTATETKALEENC

TPKEICDKYHAIHKEVYDWFEISFDKFGRTSTPEQTEVSQAIFKKLFENNFLSENTMQQLYCDTCQKFLADRLVEGSCPH

EACNYDSARGDQCEKCGKLLNPTELKDPRCKVCRTTPRIRDTDHLFIELPLLEDKLEEYINETSVAGSWSQNAIQTTKAW

LKEGLKQRCITRDLKWGVPVPHEKYNEKVFYVWFDAPIGYVSITSCYTSEWEKWWKNPENVELYQFMGKDNVPFHTVMFP

STQLGTGENWTMMKTISVTEYLNYEAGKFSKSKGVGVFGNDVKDTNIPVEVWRYYLLTNRPEVSDTLFTWDDLQAKLTGE

LLNNLGNFVNRVLSFIAKPEPAGYGSVIPDAPGAESHHLTKVLAEKVGNFVNQYIEAMEKVKLKQGLKIAMSISSEGNAY

LQGNQFWKLYKEDKPSCDIVLRTAAGLVHLLAQLLEPFMPSFSREVFKQLNLPPHFSLSAEKVSLASKPWEILPPGHKIG

TPQPLFKELKDEEVQQYREKFAGSQADRRARDAEAANMAEQLKKTKLSDAKKQKASKGAGKSQPQPAADREITMARLDIR

VGKILKAEKHPNADALYVEEIDVGGEIRTVVSGLVKYIPLEEMQNRMVCVLCNLKPAKMRDIMSQAMVLAATSSDGSKVE

LVEPPKSSKIGERVRFPGFEGEPDDVLNPKKKIWETLVVDLHTDENLVACYKDLPFTTDAGVCKVSSISNGTIR

>OAQ28875.1 methionyl-tRNA synthetase [Mortierella elongata AG-77]

MSTPATIKAGIAMPPKNPNKVPIPGERNVLITSALPYVNNVPHLGNIIGSTLSADVYARYQRVRNINALYICGTDEYGTA

TETKALADGVTCQELCDKYHPIHKQCYEWMEIDFDKFGRTTTEEQTKIAQDIFMRLYERDLLVEDSMLQLYCSQCDRYLA

DRFVEGTCPKCNYDDARGDQCDQCGSLLNATDLVNPRCKLDGNRPVQKESKHLFLDLGKLQPECETWYEKASVEGSWSNN

GKVITQSWFKEGLRLRCITRDLKWGTPVPLPGWEGKVFYVWFDAPIGYPSITATYTKDWEKWWKNPEDVKLYQFMGKDNV

PFHTVIFPSSLIGTGEKWTLLNTLSTTEYLQYETGKFSKSRGVGIFGNNVQETGVPPSVWRYYLLSNRPETNDSQYLWKD

FIARNNNELLANLGNFVNRVIKFVNAKYDSHVPTLDAAAAPATESDEKLVREVNERLRLYVEALDDVKIREGLRLAMEIS

ALGNVYLQENKIDNTLFADHRARCDRVVVTALNLIYLLSAILFPFMPSTSESIARQLNAPVRLLPDSFTVDSILPGHVIN

KAEYLFKRIDEKQEDIWRAKYGGNSSEPAADEKKKKKKPTKAAPAPVFKGEKPAEVVELEKKIEEQGAKVRELKTNKADA

AAVQTEVSALLGLKKDLTDLFAKLAV

>PIA51040.1 hypothetical protein AQUCO_01100100v1 [Aquilegia coerulea]

MEEDNGFVTKKKVPKLPIPGQRNIMITSALPYVNNVPHLGNIIGCVLSADVFARYCRNRGYNTIYMCGTDEYGTATETKA

LEENCSPQQICDKYHKIHREVYEWFDISFDEFGRTSAPQQTEVCQAIFKKLMENNWLSENTMQQLYCNTCQRFLADRLVE

GTCPTPGCNYESARGDQCEHCGKLLNPTELKDPRCKVCRNPPQIRDTDHLFLELPLLKDQLEAYINKTSVAGSWSQNAIQ

VTNAWLKEGLKSRCITRDLKWGVHVPHEKYKDKVFYVWFDAPIGYISITAAYTSDWEKWWKNPKDVELYQFMGKDNVPFH

TVMFPSTLLGTGEEWTFMKTISVTEYLNYEDGKFSKSKGVGVFGNDAKVTGIPTEVWRYYLLTNRPEVSDTLFTWADLQA

KLNGELLNNLGNFINRVLSFIAKDPGSGYGSVLPDAPDADSHSLTKMLAEKVGSYVENYIEAMEKVKLKQGLKIAMNISS

EGNAYLQESQFWKLYKEDHTLCSIVMKTSAGLVYLLASLLEPFMPSFSVEVLKQLNLQPGTQFSLDKGDIDKARTPWDIL

PTGHKIGTPKPLFRELKDEDVESFRKKFAGSQAERIVKAEADAKIAAEQLKRTKISDTRQKGKPAKAKTKTNANVEPSVT

ALDVRVGLITKVQKHPDADSLYVEEIDVGDGSPRTIVSGLVKFIPMEEMLNRKVCVLCNLKPAKMRGVMSDGMVLAASND

DHTKVELVEPPISACVGERVTFPGFEGDPNDSYINKIFEKVQPDLHTDTELVACYRDVPFTTSAGVCKVSSISSGTIR

>XP_020184849.1 probable methionine--tRNA synthetase [Aegilops tauschii subsp. tauschii]

MSSGKGKAKVPVAGRRNVLVTSALPYVNNVPHLGNIIGCVLSADVFARYCRLRGHNVLYVCGTDEYGTATENKALEEGCS

PLEICDKYHAIHKEVYEWFNISFDHFGRTSTPQQTEICQSIFSKLLDNNWLSENTMQQLYCESCQRFLADRFVVGSCPVE

GCGNDTARGDQCDRCGRLLNSTELIDPRCKVCEGIPIVRDTDHLFLELPLLKEQLEKYIDEASATGSWSQNAVRITDAWL

KEGLRPRCITRDLKWGVPVPHEKYKDKVFYVWFDAPIGYISITACYTPEWEKWWKNPENVELYQFMGKDNVPFHTVMFPS

ALLGTGENWTLMKNISVTEYLNYESGKFSKTKGIGVFGNDAKATNIPADVWRYYLLSNRPEASDAVFTWGGLQIKHNNEL

LKNLGNFIYRVLSFIAKPEGAGYGSIIPEAPNADIHPLSQSLAETVGNLIQQYIDAMDKVKLKQGLKIAMAISSEGNAYL

QESQFWKLYKQDPASCTTVMKTSVGVVYLLACLLEPFMPSFSKDVLQQLNLSPNEHISFADEKGESDKAKRPWDLIPSNH

RIGEIVPLFTELKDDAVASFRETFAGSQAERNARAN

>XP_016187834.1 probable methionine--tRNA synthetase [Arachis ipaensis]

MIQGFSACKLTVTSHTLAASLHSLSLSSLTLSPSLSRFFWRRSCSISPAAASHLRFQSPEMAANDRKTPKLPVKGKRNIL

ITSALPYVNNVPHLGNIIGCVLSADVYARYCRLRGYNAIYICGTDEYGTATETKAMEENCSPKEICDKYHAIHKDIYEWF

DISFDEFGRTSAPQQTEVCQAIFKRLLDNKWLSENTMQQLYCDTCKRFLADRLVEGICPTPGCEYDSARGDQCEKCGKLL

NPTELKTPRCKVCQNTPRIRDTDHLFLELPLLREKLEKYIEEVKGSWSSNAKQTTASWLREGLKPRCITRDLKWGVPVPH

EKYSDKVFYVWFDAPIGYVSITACYTTEWEKWWKNPENVELYQFMGKDNVPFHTVMFPSTLLGTGENWTLMKTISVTEYL

NYEAGKFSKSKGIGVFGNDAKDTNIPVEVWRYYLLTNRPEVSDTLFTWPDLQAKLNSELLNNLGNFINRVLSFIAKPEGQ

GYDSAIPALADGLSSESHDPTKKLAERISAYVDQYIEEMEKVRLKQGLKIAMAISTEGNGYLQECEFWRLYKENKPLCSL

VMKTAVGVVYILACLLEPFMPSLTQEVFKQLNLSTELHLSLCDEKGDVNKAKIPWEILNAGHKIGKPQPLFKELKDEDVE

FYRKKFAGSQADRILRAEAEAGKVAEELKKTKISDGGKKKSSSKSSNDGKNKAAPEPEITISRLDIRVGLIKKAQKHPDA

DSLYVEEIDVGEEQPRTVVSGLVKYIPLEEMQNRKVCVLCNLKPATMRGIKSQAMVLAASNSDHTKVELVEPPSSAKVGE

RVTFPGFEGNPDELLNPKKKVWETLQVDLQSNGELVACYKDVPLTTSAGVCKVSSIQNGSIR

>GAN01114.1 methionyl-tRNA synthetase [Mucor ambiguus]

MPAAIKITVPKLSANTKPYANALKLSIAAQVTGAKLVETPGDALTFVVSNDVSLTDANAALKYIFNATAFNALQSVVIEK

EETSVSPLVIKRRIDQAFQNAEEILTKHADAFAKEASSVTLVDVIYYGTLFETLSSAKDLSKYPTLSAWFNNVSKNEKFT

AGVEHVKEQTNKGAKPKNKAVFAEKKVAKEIPKPVFNKATQKVNNVAAKGLDQSVKVLPKDNERNVLITSALPYVNNVPH

LGNIVGSTLSADVYARYCRARGYNTAFFCGTDEYGTATETKALEEGVSCKALCDKYNAIHNAVYEWFDLSFDLFGRTTTD

KQTEIAQDIFLKNYKNGNIIKETMTQLFCEKCSRFLADRYVEGTCPHCKYDDARGDQCDACGKLLNATDLVNPRCKLDGN

APITKDSNHLFLDLAKLQPEIEAAFKQSAVDGKWSANGVNITQSWLKEGLRPRCITRDLKWGTPVPLEGYEDKVFYVWFD

APIGYPSITANYTNEWEKWWKNPENVKLYQFMGKDNVPFHSVIFPGCEIGTGEKWTVVNHISTCEYLNYEGGKFSKSRNV

GVFGNNAEATGIPPSVWRYYLMSVRPETSDSMFTWNEFITKNNSELLNNLGNFVNRAIRFIFAKYDGVIPKADFSGKLET

DLIKDVNELLKQYIDTMENVKLRSGLGIAMNISARGNQYLQEANLSNALFDESRPKCDAVVATAANLIYLLSAVLSPYMP

AASESMCRQLNAPLRTIPDEFAMDIKESHVLGGAEYLFTRIDEKMEEVWKGKYGTGSS

>XP_009139597.1 PREDICTED: methionine--tRNA synthetase, cytoplasmic [Brassica rapa]

MEDGESCPKLPIPGKRNILITSALPYVNNVPHLGNIIGCVLSADVYARYCRLRGYNAIYICGTDEYGTATETKALEENCT

PKEICDKYHAIHKEVYDWFDISFDKFGRTSTPEQTEVCQAIFKKLFENNFLSENTMQQLYCDTCQKFLADRLVEGSCPFP

ECNYDSARGDQCEKCGKLLNPTELKDPRCKVCQTTPRIRDTDHLFIELPLLKDRLEKYIDDTSVTGSWSQNAIQTTKAWL

KEGLRQRCITRDLKWGVPVPHEKYKEKVFYVWFDAPIGYVSITSCYTSEWEKWWKNPENVELYQFMGKDNVPFHTVMFPS

TQLGTGENWTLMKTISVTEYLNYETGKFSKSKGVGVFGNDVKDTNIPVEVWRYYLLTNRPEVSDTLFTWGDLQAKLTGEL

LNNLGNFVNRVLSFIAKPEPSGYGSVIPDAPGAESHPLTQSLAEKVGKFVKEYVEAMEKVKLKQGLKIAMAISNEGNAYL

QEAQFWKLFKEDKPSCAIVIRTAAGLVYLLAQLLEPFMPSFSREVFKQLNLPLHFSLSDEGIASRLWEMLPPGHRIGTPQ

PLFKELKDEEVQQYKDKFAGNQADRRARDVEAANMAEQLKKTKLSDAKKQKASKGAGTSKPQPAATLEITMARLDIRVGK

ILKAEKHPNADSLYVEQIDVGGGEIRTVVSGLVKYIPLEEMQNRMVCVLCNLKPSKMRDIMSQAMVLAASSSDGSKVELV

EPPESATIGERVRFAGHEGEPDVVLNPKKKVWETLLVDLNTNENLVACYKGIPFTTDAGVCKVSSISNGTIR

>XP_007034286.1 PREDICTED: methionine--tRNA synthetase, cytoplasmic [Theobroma cacao]

MEDQNQTAPKLPIPGKRNVLITSALPYVNNVPHLGNIIGCVLSADVFARFCRLRGYNAIYICGTDEYGTTTETKAMEENC

TPQEICDKYHALHREVYDWFDISFDKFGRTSSPDQTEVCQAIFKKLWENNWLSENTMQQLFCNTCQRFLADRLVEGSCPS

EGCNYDSARGDQCEKCGKLLNPTELKDPRCKACQNTPHVRDTKHLFLELPKLEGKLVEYIDKMSVAGSWSQNAINTTQAW

IKEGLKARCITRDLKWGVPVPHENFKDKVFYVWFDAPIGYVSITSCYTPDWEKWWKNPENVELFQFMGKDNVPFHTVMFP

STLLGTGENWTLMKSISVTEYLNYESGKFSKSKGIGVFGNDVKDTKIPVEVWRYYLLTNRPEVSDTLFTWDDLQAKLNNE

LLSNLGNFINRVLSFIAKPPGQGYDFIIPDTPDAESHPLTKALSEKVGNLVEQYIEAMEKVKLKQGLKTAMSISSEGNAY

LQESEFWKLYKEDRPSCSIVMRTAVGLVHILACLLEPFIPSFSVEVFKQLNLPQEQISLSDEKGDVDNARRLWEIVPAGH

KIGDPKPLFEELKSERVEELREQYAGSQADRRARAEADVAKTAEQLKKTEISEGKKQKRTTSTAGSKAKPTTTEPEITIT

RLDIRVGKIVKAQKHPDADSLYVEEIDVGEAHPRTVVSGLVKYIPLEEMQNRLVCVLCNLKPATMRGVKSHAMVLAASDS

DHTKVELVEPPKSATVGERVTFPGFTGEPDDVLNPKKKVWETLQVDLHTDANRVACYKDIPLTTSAGVCKVSSISSGSIE

>OMJ12633.1 putative methionine-tRNA synthetase, cytoplasmic [Smittium culicis]

MSTQNIGSITLVINSSEPATGSLPAIAILKIALAFSLNDLEFNISKNSEVKSKFPFWLVLPSGSKLYEPNSVIRFAFNFG

ALNPSKRFELESLLEWEEKSITSVLETKGEDRLKLISLLNNEVTLRKIGKISNPASIAIFSSVYDTFSNLDNDSKAQFPL

FQDWFQGISSLPNFSKAIEIYENNTKKLLARPTPTLKNRIFNKDIEFNYDPSIKVNVKNGERNILITSALPYVNNIPHLG

NIIGSVLSADVYARYCRSRGINTLYVCGTDEYGTATETKAIEENMSCQDLCDKYHKIHSDVYKWFNISFDFFGRTTTPLQ

TEIVQDMFIKCHNNGYTKTESMVQLYCEMCKRFLADRFVEGTCPKCGYDGARGDQCDKCGQLINAIELISPRCKLDGNTP

IVRESTHLFLDLEALQPKCEDFVKKSSEKGVWSSNGLTITRSWLNEGLKPRCITRDLKWGVPVPLPGFEEKVFYVWFDAC

IGYISLTANYTDKWEEWWKNPENVQLYQFMGKDNVPFHTVVFPSTQIATGDSSTMLHHISTTEYLNYESGKFSKSRGVGV

FGNSAEETGVAPDIWRYFLLSNRPESSDTQFTWNEFISRNNNELLANMGNFCNRILRFVDASSKYAGILPTPSQEILSAE

GSSSHSMISDVNKLLEKYNSFLSSVHIRAALRTAMEISARGNQYLQESKFDNSLFTNSRAECDTVIYVAVNLIYLLSSLF

SPYMPVVCDSICEQLNAPTRLIPDTFGMDLLPGHVIGKPAHLFTTIDEGMAEKWRSKYGGSQKEAESK

>OLY82357.1 putative methionine-tRNA synthetase, cytoplasmic [Smittium mucronatum]

MDSSQAPLPISLVINTATPATNSLPAIGILKIVLALSLNSVSFNLAKPSLQSKFPLWVSLPSGNNLYEPNSVIRFAYNCG

SFGPAERFELDLLLEWEEKIITGLLETKDADRPQLISMLNSGTILNRIGLPNSPASIAVFSSIYDTFSSMTDETRASFPS

VYTWFKAVSDLPNFSSAIKIYTENTKKLAARPAPSPENRKFNRKAEFFRDLSKVNVKPGEKNILITSALPYVNNIPHLGN

IIGSVLSADVYARYCRARGLNTLYICGTDEYGTATETKAIEEKTTCQELCDKYHKIHAQVYNWFDISFDIFGRTTTPLQT

EIVQDMFTKCYDQGYISTESMTQLFCPHHNKFLADRYVEGTCPKCGYEDARGDQCDGCGQLMNAAELIDPRCKLDGHTPI

IKESVHLFLDLEKLQPDCEKFVSKSSKDGGWSPNGLTITRSWLNEGLRPRCITRDLEWGVPVPLPGFQDKVFYVWFDACI

GYISITANYTDRWEDWWKNPDDVDLYQFMGKDNVPFHTVVFPSTQIATGEKCTMLHHISTTEYLNYESGKFSKSRGVGVF

GNSAQETGIAPDVWRYFLLSNRPESSDTQFTWSEFIARNNNELLANLGNFCNRILRFVDATSKYAGALPYPNPESLTTRG

SLDQIMISDINEILGRYNDLLSKVHIRSALRTAMEISARGNSYLQESKFDNALFTNNRAQCDTVIYVAVNLIYLLSAVVS

PYMPQVSQSITIQLNAPARLIPDTFDLDLLPGHVIGKPSHLFSNIDESMADVWRAKYGGSQSHTVAE

>SGZ49964.1 CIC11C00000001628 [[Candida] intermedia]

MSFSLAADKGKTNLALVNNLKVSVAAAKFQPKLEITVSDDSGLLLKDKKSAFELVEANAIVKYLSGDFTASDLFSVDEVA

VYDALKSGSSQKAVEALDSVTVPSNNDFSAANIVLFGAAYPFLKDLKVLATWVQSFAALPEVSRGIKNAQAVTTVERQVA

SATGAVNVKTGFLVKPQDDSILPKDGERNILITSALPYVNNVPHLGNIIGSVLSADIYARYAKNRNYNTLFVCGTDEYGT

ATETKALEEKVTPQQLCDKYHVLHKEVYDWFDIDFDYFGRTTTPLQTEIAQDIFMKLYNNGYLEEQTTEQLYCETHKSFL

ADRFVEGTCPKCGYDDARGDQCDKCGNLLDPLELINPRCKLDDAKPVPKNSTHVYLKLGDLEPELQQWFIEASEKGAWSK

NSKTITNSWLKQGLDSRCITRDLVWGTPVPLEKFKDKVLYVWFDATIGYVSITANYFKDSKDIAGWEKWWRDPENVQLYQ

FMGKDNVPFHTVVFPATQIGTKDPWTKLHHLNTTEYLQYEGGKFSKSRGVGVFGNNAKDTGISPSVWRYYLASVRPESSD

SHFSWKEFVTKNNSELLANLGNFVNRLVKFVIAKYNGVIPAYNPEQIPNYAAFELEVNGLLASYIESMEAVSLRRGLELA

MAISSRGNQFLQDNKLDNSLFNNSPDLSDAVVGVGLNLIYLVSAVISPFMPETRTQISEILNAPPLSITDKFELVLEAGH

NIGKAQYLFKRIDEKKIEEWKALYGGAQKK

>XP_015952821.1 probable methionine--tRNA synthetase [Arachis duranensis]

MIQGFSACKLPVTSHTLAASLHSLSLSSLTLSPSLSRFFWRRSCSISPAAASHLRFRSPEMAANDRKTPKLPVKGKRNIL

ITSALPYVNNVPHLGNIIGCVLSADVYARYCRLRGYNAIYICGTDEYGTATETKAMEENCSPKEICDKYHAIHKDIYEWF

DISFDEFGRTSAPQQTEVCQAIFKRLLDNKWLSENTMQQLYCDTCKRFLADRLVEGICPTPGCEYDSARGDQCEKCGKLL

NPTELKTPRCKVCQNTPRIRDTDHLFLELPLLREKLEKYIEEVKGSWSSNAKQTTASWLREGLKPRCITRDLKWGVPVPH

EKYSDKVFYVWFDAPIGYVSITACYTTEWEKWWKNPENVELYQFMGKDNVPFHTVMFPSTLLGTGENWTLMKTISVTEYL

NYEAGKFSKSKGIGVFGNDAKDTNIPVEVWRYYLLTNRPEVSDTLFTWPDLQAKLNSELLNNLGNFINRVLSFIAKPEGQ

GYDSAIPALADGLSSESHDPTKKLAERISAYVDQYIEEMEKVRLKQGLKIAMAISTEGNGYLQECEFWRLYKENKPLCSL

VMKTAVGVVYILACLLEPFMPSLTQEVFKQLNLSTELHLSLCDEKGDVNKAKIPWEILNAGHKIGKPQPLFKELKDEDVE

FYRKKFAGSQADRILRAEAEAGKVAEELKKTKISDGGKKKSSSKSSNDGKNKAAPEPEITISRLDIRVGLIKKAQKHPDA

DSLYVEEIDVGEEQPRTVVSGLVKYIPLEEMQNRKVCVLCNLKPATMRGIKSQAMVLAASNSDHTKVELVEPPSSAKVGE

RVTFPGFEGNPDELLNPKKKVWETLQVDLQSNGELVACYKDVPLTTSAGVCKVSSIQNGSIR

>EOY05213.1 Methionine--tRNA synthetase / methionyl-tRNA synthetase / MetRS, putative isoform 2 [Theobroma cacao]

MEDQNQTAPKLPIPGKRNVLITSALPYVNNVPHLGNIIGCVLSADVFARFCRLRGYNAIYICGTDEYGTTTETKAMEENC

TPQEICDKYHALHREVYDWFDISFDKFGRTSSPDQTEVCQAIFKKLWENNWLSENTMQQLFCNTCQRFLADRLVEGSCPS

EGCNYDSARGDQCEKCGKLLNPTELKDPRCKACQNTPHVRDTKHLFLELPKLEGKLVEYIDKMSVAGSWSQNAINTTQAW

IKEGLKARCITRDLKWGVPVPHENFKDKVFYVWFDAPIGYVSITSCYTPDWEKWWKNPENVELFQFMGKDNVPFHTVMFP

STLLGTGENWTLMKSISVTEYLNYESGKFSKSKGIGVFGNDVKDTKIPVEVWRYYLLTNRPEVSDTLFTWDDLQAKLNNE

LLSNLGNFINRVLSFIAKPPGQGYDFIIPDTPDAESHPLTKALSEKVGNLVEQYIEAMEKVKLKQGLKTAMSISSEGNAY

LQESEFWKLYKEDRPSCSIVMRTAVGLVHILACLLEPFIPSFSVEVFKQLNLPQEQISLSDEKGDVDNARRLWEIVPAGH

KIGDPKPLFEELKSERVEELREQYAGSQADRRARAEADVAKTAEQLKKTEISEGKKQKRTTSTAGSKAKPTTTEPEITIT

RLDIRVGKIVKAQKHPDADSLYVEEIDVGEAHPRTVVSGLNRLVCVLCNLKPATMRGVKSHAMVLAASDSDHTKVELVEP

PKSATVGERVTFPGFTGEPDDVLNPKKKVWETLQVDLHTDANRVACYKDIPLTTSAGVCKVSSISSGSIE

>GAY40085.1 hypothetical protein CUMW_049300 [Citrus unshiu]

MGDSEQEPDGERNHRVAKLPVPGKRNILITSALPYVNNVPHLGNIIGCVLSADVFARYCRLRGYNALYICGTDEYGTATE

TKAMEENCTTQQICDKYYAIHKAVYNWFNISFDEFGRTSTPQQTEICQAIFKKLLENNWLSEYTTRQLYCDTCKRFLADR

LVEGSCPTLGCNYDSARGDQCEKCGKLLNPTELKDPRCKVCQATPKIRDTDHLFLELPLIKDKLEEYINKMSVSGSWSQN

AIQGTYAWLKDGLKPRCITRDLKWGVPVPHKKFKDKVFYVWFDAPIGYISITSCYTPDWEEWWKNPENVELYQFMGKDNV

PFHTVMFPSTLLGTEENWTLMKTISVTEYLNYEAGKFSKSKGVGVFGNDVKDTNIPAEVWRYYLITNRPEVSDTLFTWED

FQAKLNTELLNNLGNFVNRVLSFIAKPPGRGYGSIIPDSPKADLHPSTKMLAEKISNHIDQYIEAMEKVKLKQGLRIGMS

ISSEGNAYLQESQFWKLYNEDQGSCSIVIRTSVGLVYLLACLLEPFMPSFSLEVLKQLNLPTETKFSLSDENGDLERARK

PWEILPAGHRIGMSSPLFKELKDEDVRFFREKFAGSQADRAVQAEAEAKRLAEHLKKTKISVPLN

>SGZ46950.1 CIC11C00000000596 [[Candida] intermedia]

MSFSLAADKGKTNLALVNNLKVSVAAAKFQPKLEITVSDDSGLLLKDKKSAFELVEANAIVKYLSGDFAASDLFSVDEVA

VYDALKSGSSQKAVEALDSVTVPSNNDFSAANIVLFGAAYPFLKDLKALATWVQSFAALPEVSQGIKNAQAVTTVERQVA

SATGAVNVKTGFLVKPQDDSILPKDGERNILITSALPYVNNVPHLGNIIGSVLSADIYARYAKNRNYNTLFVCGTDEYGT

ATETKALEEKVTPQQLCDKYHVLHKEVYDWFDIDFDYFGRTTTPLQTEIAQDIFMKLYDNGYLEEQTTEQLYCETHKSFL

ADRFVEGTCPKCGYDDARGDQCDKCGNLLDPLELINPRCKLDDAKPVPKNSTHVYLKLGDLEPELQQWFIEASEKGAWSK

NSKTITNSWLKQGLDSRCITRDLVWGTPVPLEKFKDKVLYVWFDATIGYVSITANYFKDSKDIAGWEKWWRDPENVQLYQ

FMGKDNVPFHTVVFPATQIGTKDPWTKLHHLNTTEYLQYEGGKFSKSRGVGVFGNNAKDTGISPSVWRYYLASVRPESSD

SHFSWKEFVTKNNSELLANLGNFVNRLVKFVIAKYNGVIPAYNPEQIPNYSAFELEVNGLLASYIESMEAVSLRRGLELA

MAISSRGNQFLQDNKLDNSLFNNSPDLSDAVVGVGLNLIYLVSAVISPFMPETRTQISEILNAPPLSITDKFELVLEAGH

NIGKAQYLFKRIDEKKIEEWKALYGGAQKK

>KHN46450.1 Putative methionine--tRNA synthetase [Glycine soja]

MTENLKAPKLPVEGKRNILITSALPYVNNVPHLGNIIGCVLSADVFARYCRLRGYNAIYICGTDEYGTATETKAMEENCS

PKEICDKYHAIHKEVYNWFNISFDEFGRTSSPEQTEVCQAIFKKIFENKWLSENTMQQLYCNTCERFLADRLVEGTCPTP

GCEYDSARGDQCEKCGKLLNPTELKNPGCKVCQKTPRIRDTDHLFLELPLLKDRLEKYISEMSVVGGWSQNAIQTTNSWL

REGLKPRCITRDLKWGVPVPHEKYSDKVFYVWFDAPIGYVSITSSYTRDWEKWWKNPENVELYQFMGKDNVPFHTVMFPS

TLLGTSENWTLMKTISVTEYLNYEAGKFSKSKGIGVFGNDAKDTNIPVEVWRYYLLTNRPEVSDTLFTWSDLQAKLNSEL

LNNLGNFINRVLSFIAKPAGQGYDSIIPSVPDDVSGDSHGPTKKLADKVAAYIDQYIEAMEKVKLKQGLKVAMSISSEGN

AYLQEAEFWRLYKENKSLCSLVMKTAAGIVYLLACLLEPFIPSFTLEVFKQLNLPVDTHVSLCDDKGDIDRVKRPWDIIS

ASHKIGTPKPLFRELKDEEVKFYRKKFAGSQADRIVRAEAEAQNVAEQLKKTKVSGVFPGSSISNKFKEVGRKSSR

>XP_010495831.1 PREDICTED: methionine--tRNA synthetase, cytoplasmic [Camelina sativa]

MEDGGKSDGPKLPIPGKRNILITSALPYVNNVPHLGNIIGCVLSADVYARYCRLRGYNAIYICGTDEYGTATETKALEEN

CTPKEICDKYHAIHKEVYDWFDISFDKFGRTSTPEQTEVCQAIFKKLWDNKWLSENTMQQLYCDTCKKFLADRLVEGSCP

FKGCNYDSARGDQCEKCGKLLNPTELKDPKCKVCQNTPRIRDTDHLFIELPLLKDKLEEYIKETSVAGSWSQNAIQTTNA

WLRDGLRQRCITRDLKWGVPVPHEKYKEKVFYVWFDAPIGYVSITSCYTSEWEKWWKNPENVELYQFMGKDNVPFHTVMF

PSTQLGTGENWTLMKTISVTEYLNYEDGKFSKSKGVGVFGNDVKDTNIPVEVWRYYLLTNRPEVSDTSFSWKDLQAKLNG

ELLSNLGNFVNRVLSFIAKPESSGYGSVIPDAPGAESHPLTISLAEKVEKLVAEYVEAMEKVKLKQGLKTAMLISNEGNF

YLQANQFWKLYKDDKPSCAIVIRTAAGLVHLIAQLLEPFMPSFSCEVFKQLNLPLHFSLSDERGEVLLASRPWDILPPSH

RIGTPQPLFKELVDEEVQQYREKFAGSQADRRARDEAANLTDQLNKTKLSDAKKQKASSSKSGGKPKPQPAADREITMAR

LDIRVGKIVKAERHPRADALYVEEIDVGGGEIRTVVSGLVNYIPLEEMQNRMVCVLCNLKPAKMRDIVSQAMVLAASSSD

GSKVELVEPPESAIIGERVIFPGFEGEPDDLLNPKKKIFETLAVDLHTNENLVACYKDVPFTTSAGVCKVSSISNGPVR

>OSS51959.1 hypothetical protein B5807_03923 [Epicoccum nigrum]

MSDTKKIVLPVEGERNVLITSALPYVNNVPHLGNIIGSVLSADVFARFSRARGYNTLYVCGTDEYGTATETKAIEEKCTP

QELCDKYYALHADVYKWFNISFDKFGRTPTQQQTDIAQDIFTKLHKNGYLEEKTTTQPYCETHKSFLADRFVEGTCPLCN

YEDARGDQCDKCGHLLEPLELKNPRCKLDGATPIPRETKHVYLCLDKLQPLEEEWFKKSSTEGNWSSNGVQITASWLKEG

LRARGITRDLKWGTAVPLEGYEDKVMYVWFDACIGYVSITANYTDEWEKWWKNPENVKLYQFMGKDNVPFHTVVFPCSQI

GTKDKWTMLNTISTTEYLNYEKGKFSKSRNIGVFGNNAKDTGIPSDVWRYYLLSHRPETGDSEFEWDSFISGNNNELLKN

LGNFVNRVIKFVNSKVYDSVVPDYTKFEDDYFVQHKKKVNDVLNKYIEELEDVKIRAALSTALHLSSLGNQLLQDNKLDN

NLATTEPDRCAAVVGLALSHIHLLASLIEPYMPDTTKNILSQLNAEFLVIPDNWEAKSLPVGHKIGKPAYLFSQIKPEKE

QEWREQFGGEEARKAKEEKAKKAAAKKADKERKKAKKAAGKAEGVETAEKGKDGPADKKAEEALSSGVEQVTLQTS

>CUS22476.1 LAQU0S05e06260g1_1 [Lachancea quebecensis]

MAFNLSFDRSKDHTYALQLANNIKLAFAQELAGKEIKFSVNDESAKPELTVAGTDFRLFNTNAILRYALNDFEGLESPET

HFAVSSLEAMLYHPSAPQEHVKETVSKALENYLKEFQEPLSATSLITFANAYALSPETLESKFQKLPASLSKALSAAKSY

TPRLSSSFKRTGAVKINNNFAVKKQDAEILPIEGERNVLITSALPYVNNVPHLGNIVGSVLSADIYARYCKARNYNTLFV

CGTDEYGTATETKAIEEGCTPRELCDKYHKIHSDVYKWFQIGFDYFGRTTTEKQTEIAQDIFNKLNDNGYLEEHSMKQLY

CEEHKAYLADRYVEGECPKCHYEDARGDQCDKCGGLLDPFELINPRCKLDNSKPIPRNSDHIFISLDKLEPRIKEWVEKS

SREGGWSKNSRTITQSWLKEGLQPRCITRDLVWGTPVPLEKYKDKVLYVWFDATIGYVSITANYTDGWKKWWKDPENVQL

YQFMGKDNVPFHSVIFPGSQLGTEEDWTMLHHLSTTEYLQYEGGKFSKSRGVGVFGNNAKDTGVSPSVWRYYLASVRPES

SDSHFSWSDFVARNNSELLANLGNFVNRLVKFVNAKYNGVVPEYDQKQLPDFDKLEQDVNSILSNYVVEMEQSHERRGLE

IAMSLSARGNLFLQENKLDNSLFSDFPAKSDAVVGVGLNIVYSVASVIYPFMPEAAQTIYKMLNAPPKKIDEKFYLCIEG

GHNINKAEYLFKRIDEKEIENWRSKYGGQQK

>CDY12710.1 BnaC04g29310D [Brassica napus]

MEDGESCPKLPIPGKRNILITSALPYVNNVPHLGNIIGCVLSADVYARYCRLRGYNAIYICGTDEYGTATETKALEENCT

PKEICDKYHAIHKEVYDWFDISFDKFGRTSTPEQTEVCQAIFKKLFENNFLSENTMQQLYCDTCQKFLADRLVEGSCPFP

ECNYDSARGDQCEKCGKLLNPTELKDPRCKVCQTTPRIRDTDHLFIELPLLKDRLEKYIDDTSVTGSWSQNAIQTTKAWL

KEGLRQRCITRDLKWGVPVPHEKYKEKVFYVWFDAPIGYVSITSCYTSEWEKWWKNPENVELYQFMGKDNVPFHTVMFPS

TQLGTGENWTLMKTISVTEYLNYETGKFSKSKGVGVFGNDVKDTNIPVEVWRYYLLTNRPEVSDTLFTWGDLQAKLTGEL

LNNLGNFVNRVLSFIAKPEPSGYGSVIPDAPGAESHPLTQSLAEKVGKFVKEYVEAMEKVKLKQGLKIAMTISNEGNAYL

QEAQFWKLFKEDKPSCAIVIRTAAGLVYLLAQLLEPFMPSFSREVFKQLNLSLQFSLSDEGIASRLWEILPPGHRIGTPQ

PLFKELKDEEVQQYKDKFAGNQADRRARDVEAANMAEQLKKTKLSDAKKQKASKGAGTSKPQPAATLEITMARLDIRVGK

ILKAEKHPNADSLYVEQIDVGGGEIRTVVSGLVKYIPLEEMQNRMVCVLCNLKPSKMRDIMSQAMVLAASSSDGSKVELV

EPPESATIGERVRFAGHEGEPDVVLNPKKKVWETLLVDLNTNENLVACYKDIPFTTDAGVCKVSSISNGTIR

>ORE14650.1 methionyl-tRNA synthetase [Rhizopus microsporus]

MSKLNLTLPKLAPNTKPFANALKIHIAAKVKDIKLNEVPGEHVSLVLSDEISLTDANAAVNPFIIKRRLDQAFQIVEETL

AKYRNLFAKVSDDVGFLDVIYYGALVEAFASFKEPSKYPLLAAWFDIVSKNTKVAAAVEYVKEQVLRMCYDSFVTQNSKG

TKSKKQGHAEKKVTKEIPKPTVNPATQKVNTNVAIKPVDKNVKVLPKEGARNIMITSALPYVNNVPHLGNIIGSTLSADV

FARYCRARGYNTAYFCGTDEYGTATETKALEEGVSCKELCDKYNAIHTEVYKWFDLSFDLFGRTTTERQTEIVQDMFKKN

YANGNVVKESMIQLYCEQCHRFLADRYVEGICPLCQYNDARGDQCDACGKLLNATELVEPRCKLDGNRPILKESNHLFLD

LTKLQPAIEETFKQSSTEGGWSANGIHITESWLKEGLKPRCITRDLKWGVPVPLEGYEDKVFYVWFDAPIGYPSITANYT

DEWEKWWKNPEQVKLYQFMGKDNVPFHSVIFPGVQIGTGEKWTKVNHISTCEYLNYEGGKFSKSRNVGVFGNNAKETGIP

PSVWRYYLLSVRPESSDSMFTWSEFITKNNSELLNNFGNFVNRAIRFIFAKYDGVIPKADFNGQLETDLFKDVNALLKQF

VEQMEAVKLRAALGTVMALSARGNQYLQEANLSNALFDESRPKCDAVVASAANLIYLLSALVSPFMPATSDSMCRQLNAP

ARNIPDEFTLDILPGHVLGGAEYLFTRIDEKMEQVWKSKYGTGA

>XP_023548158.1 methionine--tRNA synthetase, cytoplasmic-like [Cucurbita pepo subsp. pepo]

MADVLGDAGGGGTKATKLPIPGKRNILITSALPYVNNVPHLGNIIGCVLSADVFARYCRLRGYNAIYICGTDEYGTATET

KAMEEKCSPKEICDKYHKIHKEVYDWFGISFDEFGRTSSSQQTEVCQEIFGKLLENNWLSENTMQQLYCDTCERFLADRL

VEGICPTPGCEYDSARGDQCEKCGKLLNPTELKDPRCKVCQTSPRIRDTNHMFLELPLLRDKLEEYINKMSVAGSWSQNA

IQATQAWLKEGLKPRCITRDLKWGVPVPLERFKDKVFYVWFDAPIGYVSITSCYTNEWEKWWKNPENVELFQFMGKDNVP

FHTVMFPSTLLGTGEDWTLMKTISVTEYLNYEAGKFSKSKGIGVFGNDAKDTNISAEVWRYYLLANRPEVSDTLFTWADL

QAKLNGELLNNLGNFIHRVLSFIAKPLGQGYGSIIPDAPDADSHGLTLGLAEKVGKYVDQYVESMEKVKLKQGLKAAMAI

SSEGNTYLQTSQFWNLYKADKPSCNIVMRTSTGLVHLLACLLEPFMPSFSHEVFRQLDLPLERRISLCEEKGDIERAKQP

WKTLPSGHKIGTPHPLFKELKDEEVEQYRDKFTGSQAQRIVKAEAEKLAAAHLEKTNVSGGEKKQQAKSSTGRKPKAAGG

GGAVEAGITITRLDIRVGLITKAQKHPDADSLYVEEIDVGEAQPRTVVSGLVNYIPLQEMQNRKVCVLCNLKPASMRGIK

SQAMVLAASNSDHTKVELVEPPKDANVGERIKFPGFEGKPDDVLNPKKKVWETLQVDLAINTDGVACFKDIPFNTSAGIC

KVSSISGGSIW

>XP_022138592.1 probable methionine--tRNA synthetase [Momordica charantia]

MGDVSGDAGGGRTKVTKLPVAGKRNILITSALPYVNNVPHLGNIIGCVLSADVFARYCRIRGYNAIYICGTDEYGTATET

KAMEEKCTPKEICDKYYAIHKEVYNWFGISFDEFGRTSSPQQTEVCQEIFGKLLENNWLSENTMQQLYCDTCKRFLADRL

VEGICPTPGCDYDSARGDQCEKCGKLLNPTELKDPRCKVCQTTPRIRDTDHMFLELPLLRAKLEEYINKTSVAGSWSQNA

IQATNAWLKEGLKPRCITRDLKWGVPVPLEKFKDKVFYVWFDAPIGYVSITSCYTNEWEKWWKNPENVELFQFMGKDNVP

FHTVMFPSTLLGTGESWTLMKTISVTEYLNYEAGKFSKSKGIGVFGNDAKDTNIPVEVWRYYLLANRPEVSDTLFTWADL

QAKLNGELLNNLGNFIHRVLSFIAKPLGQGYGSIIPDAPDAESHGLTMGLAEKVGKYVDQYVESMEKVKLKQGLKAAMAI

SSEGNTYLQTAQFWNLYKEDRPSCNVVMRTSAGLVHLLACLLEPFMPSFSLEVFKKLNLPVERHISLCEEKGDIERAKQP

WKILPSGLKIGIPEPLFKELKDEEVELYRDKFAGSQAQRIVRAEAEAEKLAAQLKKTNVAGGGKKQQTKSTGSKPKAAVE

PEITITRLDIRVGLITKAQKHPDADSLYVEEIDVGEQQPRTVVSGLVKYIPLEEMQNRKVCVLCNLKPASMRGIKSQAMV

LAASNSDHTKVELVEPPQDAIVGERVKFPGFEGEPDDVLNPKKKVWETLQVDLGTNAELVACFKDIPFTTSAGICKVSSI

SSGSIR

>OMO88585.1 hypothetical protein COLO4_20192 [Corchorus olitorius]

MGDPNQTAPKLPIPGKRNVLITSALPYVNNVPHLGNIIGCVLSADVFARYCRLRGYNVIYVCGTDEYGTTTETKAMEENC

TPQEICDKYHALHKEVYDWFDISFDKFGRTSTPEQTEVCQAIFQKLWDNNWLSEDTMQQLFCDTCQRFLADRLVEGTCPH

EGCNYDSARGDQCEKCGKLLNPTELKDPRCKACQNAPRIRDTKHLFLELPKLEGQLREYIDKKSVAGSWSQNAINTTQGW

LKDGLKARCITRDLKWGVPVPHENYKDKVFYVWFDAPIGYVSITACYTPEWEKWWKNPENVELFQFMGKDNVPFHTVMFP

STLLGTGENWTLMKSISVTEYLNYEAGKFSKSKGIGVFGNDVKDTNIPVEVWRYYLLTNRPEVSDTLFTWDDLQAKLNSE

LLGNLGNFINRVLTFIAKPSGEGYGSIIPDAPGAESHSLTKDSSERVYKLVEQYIEAMEKVKLKQGLKTAMGISTEGNWY

LQESKFWRLYKEDQPSCSIVMRYAVGLVHILACLLEPFIPSFSVEVFRQLNLHPQAQLSLCDEKRDLDWARRPWEIVPAG

HKIGNPKPMFEELKSERVQELRERYAGSQADRRARAEAEAAKTADQLKKTKISDGKKQKPTKSAADSKAKPTTEPEISIT

RLDIRVGKILKAQKHPDADSLYVEEIDVGEPQPRTVVSGLVKFIPLEEMQNRLVCVLCNLKPATMRGIKSHAMVLAASNS

DHTKVELVEPPKSAIVGERVTFPGFEGEPDDVLNPKKKVWETLQVDLHSDANLVACYKDIPLTTSAGVCKVASISNGSIR

>XP_018682359.1 PREDICTED: probable methionine--tRNA synthetase isoform X2 [Musa acuminata subsp. malaccensis]

MEDTPAENSPKLPVPGRRNILITSALPYVNNVPHLGNIIGCVLSADVFARYCRLRGYNTIYICGTDEYGTATETKAMEEN

CSPKEICDKYHEIHKEVYKWFDISFDEFGRTSSPQQTEICQSIFHKLLENNWLSEKTMLQLYCDTCQRFLADRLVEGTCP

TLDCNYESARGDQCEKCGKLLNPTELIDPKCKVCQRTPHICDTDHLFLELPLLRKKLEDYIDSMSITGSWSQNAIQATQA

WLKEGLKPRCITRDLKWGVPVPHEKYKDKVFYVWFDAPIGYISITACYTLEWEKWWKNPENVELYQFMGKDNVPFHTVMF

PSTLIGTGEKWTMMKTISVTEYLNYETGKFSKSKGVGVFGNDAKDTNIPTEVWRYYLLTNRPEVSDTLFTWSDLQAKLNS

ELLNNLGNFVNRVLSFIAKPEGAGYNSIVPDAPDAESHNLTKDLGEKVGKLVDQYLDAMEKVKLKQGLRTAMSISSEGNA

YLQESQFWKLYKEDPSSCAIVMKTSAGLVYLLATLLEPFMPSFSIEVLRQLNLSPKTSLSFCNEKGETDDKKKPWNFLPS

GHSISKPEPLFKELKDEDVEYFRMRFAGSQADRKVKAEADAKKITEQLKSTNITGLIYLNESFE

>XP_010450162.1 PREDICTED: methionine--tRNA synthetase, cytoplasmic [Camelina sativa]

MEDGGKSDVPKLPIPGKRNILITSALPYVNNVPHLGNIIGCVLSADVYARYCRLRGYNAIYICGTDEYGTATETKALEEN

CTPKEICDKYHAIHKEVYDWFDISFDKFGRTSTPEQTEVCQAIFKKLWENKWLSENTMQQLYCDTCKKFLADRLVEGSCP

FDACNYGSARGDQCENCGKLLNPTELKDPKCKVCQNTPRIRDTDHLFIELPLLKDKLEEYIKETSVAGSWSQNAIQTTNA

WLRDGLRQRCITRDLKWGVPVPHEKYKEKVFYVWFDAPIGYVSITSCYTSEWEKWWKNPENVELYQFMGKDNVPFHTVMF

PSTQLGTGENWTLMKTISVTEYLNYEDGKFSKSKGVGVFGNDVKDTNIPVEVWRYYLLTNRPEVSDTSFSWKDLQAKLNG

ELLSNLGNFVNRVLSFIAKPESSGYGSVIPDAPGAESHPLTISLAEKVEKLVAEYVEAMEKVKLKQGLKTAMLISNEGNF

YLQANQFWKLYKDDKPSCAIVIRTAAGLVHLIAQLLEPFIPSFSCEVFKQLNLPPHFSLSDERGEVLLASRPWEILPPSH

MIGTPQPLFKELVDEEVQQYREKFAGSQADRRARDEAANLRDQLNKTKLSDAKKQKASSSKSGGKPKPQPAADREVTMAR

LDIRVGKILKAERHPRADALYVEEIDVGGGEIRTVVSGLVNYIPLEEMQNRMVCVLCNLKPAKMRDIVSQAMVLAASSSD

GSKVELVEPPESAIIGERVTFPGFEGEPDDLLNPKKKIFETLAVDLHTNENLVACYKDVPFTTSAGVCKVSSISNGPVR

>KDO65962.1 hypothetical protein CISIN_1g006917mg [Citrus sinensis]

MGDSEQEPDGERNHRVAKLPVPGKRNILITSALPYVNNVPHLGNIIGCVLSADVFARYCRLRGYNALYICGTDEYGTATE

TKAMEENCTTQQICDKYYAIHKAVYNWFNISFDEFGRTSTPQQTEICQAIFKKLLENNWLSEYTTQQLYCDTCKRFLADR

LVEGSCPTLGCNYDSARGDQCEKCGKLLNPTELKDPRCKVCQATPKIRDTDHLFLELPLIKDKLEEYINKMSVSGSWSQN

AIQGTYAWLKDGLKPRCITRDLKWGVPVPHEKFKDKVFYVWFDAPIGYISITSCYTPDWEEWWKNPENVALYQFMGKDNA

PFHTVMFPSTLLGTEENWTLMKTISVTEYLNYEAGKFSKSKGVGVFGNDVKDTNIPAEVWRYYLITNRPEVSDTLFTWED

FQAKLNTELLNNLGNFVNRVLSFIAKPPGRGYGSIIPDSPKADLHPSTKMLAEKISNHIDQYIEAMEKVKLKQGLRIGMS

ISSEGNAYLQESQFWKLYNEDQGSCSIVIRTSVGLVYLLACLLEPFMPSFSLEVLKQLNVPTETKFSLSDENGDLERARK

PWEILPAGHRIGMSSPLFKELKDEDVRFFREKFAGSQADRAVQAEAEAKRLAEHLKKTKISVPLN

>XP_022673999.1 methionyl-tRNA synthetase [Kluyveromyces marxianus DMKU3-1042]

MASQISFDQSKLHTATLQLANNIKLAYALLSAPASLKLEENSEVSVSQLIVGSSGFRLFDANAILRYVLNDFESEESDEY

SFAVSSLEALASHKDQIKEHVTESVKKSIETYLTSVEEPLSATRIVTFANSYALDSAAVEAKFPLASVPEKLQKAVALAK

SKAVRSSTDYKHTGAVNVSQEYLMKSQGSEILPKDGERNILITSALPYVNNVPHLGNIVGSVLSADIYARYTKGRNYNTL

FVCGTDEYGTATETKALEDGVTPRELCDKYHKIHADVYKWFQIGFDYFGRTTTEKQTEIAQDIFLKLNDRGYLEEQSMKQ

LYCPVHKGYLADRYVEGTCPKCGYEDARGDQCDKCGALLNPFELIDPRCKLDEGKPEPRNSDHIFLSLDKLEPQIKEWNE

KASSKGEWSKNSKTITQSWLKEGLHPRCITRDLVWGTPVPLEKYQDKVLYVWFDACIGYVSITANYTDKWENWWKNPDNV

DLYQFMGKDNVPFHTVIFPGSQLGTGEDWTMLHHLNTTEYLQYEGGKFSKSRSIGVFGNNAQEIGLSPSVWRYYLASVRP

ETSDSQFSWSDFAARNNSELLANLGNFVNRVVKFVNAKYNGVVPEYKVENLPGYDQLKKDLDEILANYVNTMEHVHLRRG

LEIAMSLSSRGNQFLQENKLDNSLFAQQPDKSDAVVGVALNIIYTVASIMAPFIPETSETIYRMLNAPPLRIDDHFNLPI

RAGHNINKAEYLFTRIDEKDVEEWRKRYAGKQV

>XP_022012726.1 probable methionine--tRNA synthetase [Helianthus annuus]

MGDVPISGGADRKPVKLPIPGKRNILITSALPYVNNVPHLGNIIGCVLSADVYARFCRSRGYNAIYICGTDEYGTATETK

AMEENCTPKEICDKYHAIHKDVYEWFNISFDEFGRTSTPQQTEVCQAIFNKLLDNKWLSENTMQQLYCDTCSKFLADRLV

EGTCPTPGCNYDSARGDQCEKCGKLLNPTELINPRCKVCATSPRIRDTDHLFLELPLLEGKLREYISTMSVAGSWSQNAI

HATNAWLKEGLRQRCITRDLKWGVPVPLEKFKDKVFYVWFDAPIGYVSITNCYTQEWEKWWKNPDHVELYQFMGKDNVPF

HTVMFPSTLIGTGENWTMMKTISVTEYLNYEAGKFSKSKGIGVFGNDAKDTNIPVEVWRYYLLTNRPEVSDTLFTWADLQ

AKLNSELLNNLGNFINRVLSFVAKDPDSGSGKGSGYNSIIPDAPGAESHPLTKALAEKVGNYVDQYVDAMEKVKLKQGLK

IAMSISGEGNAYLQESQFWKLYKEDLPSCSIVMKTSVGLVYLLACLLEPFMPSFSLEVLKQLNLPPQVSLSDEKGDIVKA

KSPWNIIPAGHKIGTPVPLFKELKDEEVEFFRNKFAGSQADRADRAVKEEAEAKKLTEKLKNTKIADKSGKKERPAKSAG

EPKAKAAAVEKEVTIDRLDIRVGVIKEVQKHPDADSLYVEKIDVGEEQPRTVVSGLVKYIPLEEMQNRKVCVLCNLKPAT

MRGIKSQAMVLCASTIDKVELVEPPEGAAVGERVTFAGFVGEPDDVLNPKKKVWETLQVDLHSDKNLVACYKDLPFTTSA

GVCKVSSISDGTIR

>KRH24042.1 hypothetical protein GLYMA_12G018300 [Glycine max]

MTENLKAPKLPVEGKRNILITSALPYVNNVPHLGNIIGCVLSADVFARYCRLRGYNAIYICGTDEYGTATETKAMEENCS

PKEICDKYHAIHKEVYNWFNISFDEFGRTSSPEQTEVCQAIFRKIFENKWLSENTMQQLYCNTCERFLADRLVEGTCPTP

GCEYDSARGDQCEKCGKLLNPTELKNPGCKVCQKTPRIRDTDHLFLELPLLKDRLEKYISEMSVVGGWSQNAIQTTNSWL

REGLKPRCITRDLKWGVPVPHEKYSDKVFYVWFDAPIGYVSITSCYTRDWEKWWKNPENVELYQFMGKDNVPFHTVMFPS

TLLGTSENWTLMKTISVTEYLNYEAGKFSKSKGIGVFGNDAKDTNIPVEVWRYYLLTNRPEVSDTLFTWSDLQAKLNSEL

LNNLGNFINRVLSFIAKPAGQGYDSIIPSVPDDVSGDSHGPTKKLADKVAAYIDQYIEAMEKVKLKQGLKVAMSISSEGN

AYLQEAEFWRLYKENKSLCSLVMKTAAGIVYLLACLLEPFIPSFTLEVFKQLNLPVDTHVSLCDDKGDIDRVKRPWDIIS

ASHKIGTPKPLFRELKDEEVKFYRKKFAGSQADRIVRAEAEAQNVAEQLKKTKVSGVFPGSSISNKFKEVGRKSSR

>BAP69674.1 methionyl-tRNA synthetase [Kluyveromyces marxianus]

MASQISFDQSKLHTATLQLANNIKLAYALLSAPASLKLEENSEVSVSQLIVGSSGFRLFDANAILRYVLNDFESEESDEY

SFAVSSLEALASHKDQIKEHVTESVKKSIDTYLTSVDEPLSATRIVTFANTYALDSAAVEAKFPLASVPEKLQKAVALAK

SKAVRSSTDYKHTGAVNVSQEYLMKSQGSEILPKDGERNILITSALPYVNNVPHLGNIVGSVLSADIYARYTKGRNYNTL

FVCGTDEYGTATETKALEDGVTPRELCDKYHKIHADVYKWFQIGFDYFGRTTTEKQTEIAQDIFLKLNDRGYLEEQSMKQ

LYCPVHKGYLADRYVEGTCPKCGYEDARGDQCDKCGALLNPFELIDPRCKLDEGKPEPRNSDHIFLSLDKLEPQIKEWNE

KASSKGEWSKNSKTITQSWLKEGLHPRCITRDLVWGTPVPLEKYQDKVLYVWFDACIGYVSITANYTDKWENWWKNPDNV

DLYQFMGKDNVPFHTVIFPGSQLGTGEDWTMLHHLNTTEYLQYEGGKFSKSRSIGVFGNNAQEIGLSPSVWRYYLASVRP

ETSDSQFSWSDFAARNNSELLANLGNFVNRVVKFVNAKYNGVVPEYKVENLPGYDQLKKDLDEILANYVNTMEHVHLRRG

LEIAMSLSSRGNQFLQENKLDNSLFAQQPDKSDAVVGVALNIIYTVASIMAPFIPETSETIYRMLNAPPLRIDDHFNLPI

RAGHNINKAEYLFTRIDEKDVEEWRKRYAGKQV

>XP_006592016.1 PREDICTED: probable methionine--tRNA synthetase [Glycine max]

MTENLKAPKLPVEGKRNILITSALPYVNNVPHLGNIIGCVLSADVFARYCRLRGYNAIYICGTDEYGTATETKAMEENCS

PKEICDKYHAIHKEVYNWFNISFDEFGRTSSPEQTEVCQAIFRKIFENKWLSENTMQQLYCNTCERFLADRLVEGTCPTP

GCEYDSARGDQCEKCGKLLNPTELKNPGCKVCQKTPRIRDTDHLFLELPLLKDRLEKYISEMSVVGGWSQNAIQTTNSWL

REGLKPRCITRDLKWGVPVPHEKYSDKVFYVWFDAPIGYVSITSCYTRDWEKWWKNPENVELYQFMGKDNVPFHTVMFPS

TLLGTSENWTLMKTISVTEYLNYEAGKFSKSKGIGVFGNDAKDTNIPVEVWRYYLLTNRPEVSDTLFTWSDLQAKLNSEL

LNNLGNFINRVLSFIAKPAGQGYDSIIPSVPDDVSGDSHGPTKKLADKVAAYIDQYIEAMEKVKLKQGLKVAMSISSEGN

AYLQEAEFWRLYKENKSLCSLVMKTAAGIVYLLACLLEPFIPSFTLEVFKQLNLPVDTHVSLCDDKGDIDRVKRPWDIIS

ASHKIGTPKPLFRELKDEEVKFYRKKFAGSQADRIVRAEAEAQNVAEQLKKTKVSDGNVKKKPAKSLNEVKNKAESF

>XP_022958743.1 probable methionine--tRNA synthetase [Cucurbita moschata]

MADVLGDAGGGGTKATKLPIPGKRNILITSALPYVNNVPHLGNIIGCVLSADVFARYCRLRGYNAIYICGTDEYGTATET

KAMEEKCSPKEICDKYHKIHKEVYDWFGISFDEFGRTSSSQQTEVCQEIFGKLLENNWLSENTMLQLYCDTCERFLADRL

VEGICPTPGCEYDSARGDQCEKCGKLLNPTELKDPRCKVCQTTPRIRDTNHMFLELPLLRDKLEEYINKMSVAGSWSQNA

IQATQAWLKEGLKPRCITRDLKWGVPVPLERFRDKVFYVWFDAPIGYVSITSCYTNEWEKWWKNPENVELFQFMGKDNVP

FHTVMFPSTLLGTGEDWTLMKTISVTEYLNYEAGKFSKSKGIGVFGNDAKDTNISAEVWRYYLLANRPEVSDTLFTWADL

QAKLNGELLNNLGNFIHRVLSFVAKPLGQGYGSIIPDAPNADSHGLTLVLAEKVGKYVDQYVESMEKVKLKQGLKAAMAI

SNEGNTYLQTSQFWNLYKADKPSCNIVMRTSTGLVHLLACLLEPFMPSFSDEVFRQLDLPLERRISLCEEKGDIERAKQP

WKTLPSGHKIGTPQPLFKELKDEEVELYRDKFAGSQAQRIVKAEAEKLAAHLKKTNVSGSEKKQQAKSSTGRKPKAAAGG

GAVEAGITITRLDIRVGLITKAQKHPDADSLYVEEIDVGEAQPRTVVSGLVNYIPLQEMQNRKVCVLCNLKPASMRGIKS

QAMVLAASNSDHTKVELVEPPKDANVGERIKFPGFEGEPEDVLNPKKKVWETLQVDLAINTEGVACFKDIPFNTSAGICK

VPSITSGSIW

>XP_002172414.1 cytoplasmic methionine-tRNA synthetase Mrs1 [Schizosaccharomyces japonicus yFS275]

MATFKLQIPAVSKESFGFHEALKAAIAVSAFAPKTKVEGAAPCNSVRLTDAKDASKYISDANAIVSYLYWKNTNLTAEKF

LSVEHKLLDWDSLNLSPLLIAADAPEKRQIVLTLLDNAIQNFKLDASVTPANVIIFADVYSLINGPEELEPFETLKSFYS

KFAAHPFFQKSLNLAIEKTKGQTAEISSEQIPISEETRKLRPKIELMQERKKSGVLPKPNSRNILITSALPYVNNVPHLG

NIIGSTLSADVFARYHRARNNTTLYICGTDEYGTATETKALEEGVSPKELCDKYHKLHKEVYDWFQLDFDFFGRTTTPKQ

TEISQHIFNRLYERDYMEVDSMTQLYCEEHKGFLADRYVEGTCPKCGFDDARGDQCDGCGQLLNAFELINPTCKLDKTTP

VKRETRHIFLSLDKLQPQIQEWFQKSSKEGHWSNNGVAITDSWLKEGLRPRCITRDLKWGTPVPLEEFKDKVLYVWFDAT

IGYISITANYTDEWKQWWKNPENVKLYQFMGKDNVPFHTVIFPGSLLGTDEKWTMLHHINTTDYLNYESGKFSKSRGIGV

FGNNAKELGLSPSVWRYYLLASRPETSDTMFTWKEFITRHNSELLANIGNLVNRVVKFTTAKYNGLVPYYLEDASVGSGK

LKTELVKDVNAILRKYNTALEAVKLREGLRIAMELSARGNQFLQDNRVDNKLFTNERQKCADAIGYTLNLVYLLASILSP

FIPATSDSIYKQLNAPALAIPDEWTLDLLPGHRIGNAEYLFTRIDESMEEVWRGKYGGSQK

>XP_023006265.1 probable methionine--tRNA synthetase [Cucurbita maxima]

MADVLGDAGGGGTKATKLPIPGKRNILITSALPYVNNVPHLGNIIGCVLSADVFARYCRLRGYNAIYICGTDEYGTATET

KAMEEKCSPKEICDKYHKIHKEVYDWFGISFDEFGRTSSSQQTEVCQEIFGKLLENNWLSENTMQQLYCDTCERFLADRL

VEGICPTPGCEYDSARGDQCEKCGKLLNPTELKDPRCKVCQTSPRIRDTNHMFLELPLLRDKLEEYINKMSVAGSWSQNA

IQATQAWLKEGLKPRCITRDLKWGVPVPLERFKDKVFYVWFDAPIGYVSITSCYTNEWEKWWKNPENVELFQFMGKDNVP

FHTVMFPSTLLGTGEDWTLMKTISVTEYLNYEAGKFSKSKGIGVFGNDAKDTNISAEVWRYYLLGNRPEVSDTLFTWADL

QAKLNGELLNNLGNFIHRVLSFIAKPLGQGYGSIIPDAPDADSHGLTLGLAEKVDKYVDQYVESMEKVKLKQGLKAAMAI

SSEGNTYLQSSQFWNLYKADKPSCNIVMRTSSGLVHLLACLLEPFMPSFSDEVFRQLDLPLERRISLCEEKGDIERAKQP

WKTLPSGHKIGTPHPLFKELKDEEVELYRDKFAGSQAQRIVKAEAEAEAEKLAAHLKKTNVSGSEKKQQAKSSTGRKPKA

AGGGGAVEAGITITRLDIRVGLITKAQKHPDADSLYVEEIDVGEAQPRTVVSGLVNYIPLQEMQNRKVCVLCNLKPASMR

GIKSQAMVLAASNSDHTKVELVEPPKDANVGERIKFPGFEGEPDDVLNPKKKVWETLQVDLAINTEGVACFKDIPFNTSA

GICKVSSISSGSIW

>XP_021658924.1 probable methionine--tRNA synthetase [Hevea brasiliensis]

MGDLSSSNSSGEGRTVPKVPIPGKRNILITSALPYVNNVPHLGNIIGSVLSADVFARYCRLRGYNASYICGTDEYGTATE

TKAMEENCTPKEICDKYHAIHREVYKWFNISFDEFGRTSTPQQTEVCQAIFKLLLENNWLSENTIQQLYCDTCKRFLADR

LVEGICPTEGCNYDSARGDQCEKCGKLLNPTELKDPRCKVCCNTPHIRDTNHLFLELPLLKDKLVKYIESMSVAGSWSQN

AIQATYAWLKEGLKQRCITRDLKWGVPVPHEKFMDKVFYVWFDAPIGYVSITSCYTPDWEKWWKNPENVELYQFMGKDNV

PFHTVMFPSTLLGTGENWTLMKTISVTEYLNYEAGKFSKSKGIGVFGNDAKDTNIPVEVWRYYLLTNRPEVSDTLFTWSD

LQAKLNSELLNNLGNFINRVLSFIAKPPGTGYGSIIPDAPNADSHLLTKKLAEEVGKYVEQYVEAMEKVKLKQGLKTAMS

ISSEGNAYLQESQFWKLYKEDQSSCNIVMRTSVGLVYLLACLLEPFMPSFSFEVFKQLNFPPEKASLCDEKGDIDRAKRP

WEFLPPNHKIGTPEPLFKELKDEEVDFFRKKFAGSQAERAEAEAAKVAEQLKKTKVSDGNGKKQQIKKPSAKAKSKSTVE

PEISITRLDIRVGLIKKAQKHPDADSLYVEEIDVGGGETRKVVSGLVNYIPLEEMQDRKVCVLCNLKPATMRGIKSQAMV

LAASNSDHTKVELVDPPESAAVGERVTFPGFEGEPDDVLNPKKKVWETLQVDLQTNTDLVACYKDIPFTTSAGVCKVASI

QSGSVR

>XP_010496964.1 PREDICTED: methionine--tRNA synthetase, cytoplasmic [Camelina sativa]

MEDGGKSDVPKLPIPGKRNILITSALPYVNNVPHLGNIIGCVLSADVYARYCRLRDYNAIYICGTDEYGTATETKAVQEN

CTPKEICDKFHAIHKEVYDWFDISFDKFGRTSSPQHTQVCQAIFSKLCENNRLSEGTMQQLYCDTCKKFLADRLLEGSCP

FEGCNYDSARGDQCENCGKLLNPTELKDPRCKVCRTTPRVRQTDHLFIELPSLKDKLEEYINETSVNGSWSQNAIHTTNA

WLREGLKQRCITRDLKWGVPVPHEKYKDKVFYVWFDAPIGYVSITSCYTSEWEKWWKNPENVELYQFMGKDNVPFHTVMF

PSTQLGTGENWTLMKTISVTEYLNYEDGKFSKSKGVGVFGNDVKDTNIPVEVWRYYLLTNRPEVSDTSFSWKDLQAKLNG

ELLSNLGNFVNRVLSFIAKPESSGYGSVIPDAPGAESHPLTISLAEKVEKLVGEYVEAMEKVKLKQGLKTAMLISNEGNF

YLQANQFWKLYKDDKPSCAIVIRTAAGLVHLIAQLLEPFIPSFSCEVFKQLNLPPHFSLSDERGEVLLASRPWEILPPSH

MIGTPQPLFKELVDEEVQQYREKFAGSQADRRARDEAANLRDQLNKTKLSDAKKQKASSSKSGGKPKPQPAADREVTMAR

LDIRVGKILKAERHPRADALYVEEIDVGGGEIRTVVSGLVNYIPLEEMQNRMVCVLCNLKPAKMRDIVSQAMVLAASSSD

GSKVELVEPPESAIIGERVTFPGFEGEPDDLLNPKKKIFETLAVDLHTNENLVACYKDVPFTTSAGVCKVSSISNGPVR

>OWM86530.1 hypothetical protein CDL15_Pgr026422 [Punica granatum]

MGDITGNGGGTKLPIPGKRNILITSALPYVNNVPHLGNIIGSVLSADVFARYCRLRGYNAIYICGTDEYGTATETKAMEE

KCTPQEICDKYHAIHRQVYDWFDISFDKFGRTSTPQQTEVCQTIFKKLLENNWLSENTMQQLYCDTCQRFLADRLVEGSC

PFPDCNYDSARGDQCDKCQKLLNPTELKDPRCKVCKTTPRIRDTDHLFLELPLLKDKLEEYINEMSVAGSWSQNAIQATN

AWLREGLRQRCITRDLKWGVPVPHEKYKDKVFYVWFDAPIGYISITACYTSEWEKWWKNPKNVELYQFMGKDNVPFHTVM

FPSTLLGTGESWTLMKTISVTEYLNYEGDVFSTGKFSKSHGIGVFGNDARDTNLPVEVWRYYLLINRPEVSDTQFTWTDL

QAKLNTELLNNLGNFINRVLSFIAKPPGLGYGSVVPDAPGAESHALTANLAEKVGKFVDQYVEAMEKVKLKQGLKTAMSI

SSEGNAYLQESQFWKLYKEDQPSCSIVMRTSVGLVHLLACLLEPFMPSFSFEVFKQINLSPTGISLGDEAGDVDRAKRPW

EIIPAGHKIGKPEPLFKELKDEEVEFLRKKYAGSQADRINRAEAEARKVAEAEAQKVAEQLKTTKVSDSKGKKQQPARSA

SQAKAKATPDAEISISRVNIRIGLIKKVKKHPDADSLYVEEIDVGEAQCRTVVSGLVNYIPLEEMLNRKVCVICNLKPAT

MRGIKSQAMVLAASNSDHTEIELVDPPEAANIGERVSFPGFEGEPDDVLNPKKKVWETVQPDLSTDSELVARYKGVPFTT

SAGVCKVATIQNGSIRNIVLSQACARSQVLDRVWRIPSLIFQ

>OMO71007.1 hypothetical protein CCACVL1_18518 [Corchorus capsularis]

MGDPNQTAPKLPIPGKRNVLITSALPYVNNVPHLGNIIGCVLSADVFARYCRLRGYNVVYVCGTDEYGTTTETKAIEENC

TPQEICDKYHALHKEVYDWFDISFDKFGRTSTPEQTEVCQAIFQKLWDNNWLSEDTMQQLFCDTCQRFLADRLVEGTCPH

EGCNYDSARGDQCEKCGKLLNPTELKDPRCKACQNTPRIRDTKHLFLELPKLEGQLREYIDKMSVAGSWSQNAINTTQGW

LKDGLKARCITRDLKWGVPVPHENYKDKVFYVWFDAPIGYVSITACYTPEWEKWWKNPENVELFQFMGKDNVPFHTVMFP
[truncated: 17,582,782 more chars]
